# Comparative genome-wide analysis of extracellular small RNAs from the mucormycosis pathogen *Rhizopus delemar*

**DOI:** 10.1038/s41598-018-23611-z

**Published:** 2018-03-27

**Authors:** Muxing Liu, Gillian O. Bruni, Christopher M. Taylor, Zhengguang Zhang, Ping Wang

**Affiliations:** 1 Department of Plant Pathology, College of Plant Protection, Nanjing Agricultural University, Nanjing, China; 2 Department of Microbiology, Immunology, and Parasitology, Louisiana State University Health Sciences Center, New Orleans, LA 70112 USA; 3 Department of Pediatrics, Louisiana State University Health Sciences Center, New Orleans, LA 70118 USA

## Abstract

*Rhizopus delemar* is an emerging fungal pathogen causing devastating mucormycosis in immunocompromised individuals. The organism remains understudied and there are urgent needs for new methods of rapid disease diagnosis for timely therapy. Extracellular vesicles with encapsulated RNAs have recently been discovered to have great potential applications for disease diagnoses and treatments. To explore the utilization of ex-RNA in studies of mucormycosis, we have performed RNA-Seq of ex-sRNAs from two clinical strains of *R*. *delemar*. Approximately 3.3 and 3.2 million clean reads were obtained from FGSC-9543 and CDC-8219 strains, respectively. The median sequence length of the sRNAs was 22 nts, with a minimum of 18 and a maximum of 30 nts. Further annotation identified 560 and 526 miRNAs from FGSC-9543 and CDC-8219 strains, respectively. miRNA target prediction and analysis of GO and KEGG pathways have revealed that the regulation of metabolism, secondary metabolite biosynthesis, and two-component system signaling are important during growth. We have also validated RNA-Seq by qRT-PCR and Northern blotting analysis of randomly selected miRNAs. Our results show that *R*. *delemar* has a rich reservoir of secreted ex-sRNAs and our studies could facilitate the development of improved diagnostic methods as well as elucidating virulence mechanisms for *R*. *delemar* infection.

## Introduction

Mucormycosis is a highly invasive and life-threatening fungal infection that occurs in patients with immune deficiency due to hematologic diseases, diabetic ketoacidosis, or organ transplantation. The infection is caused by zygomycetous fungi, including *Rhizopus*, *Mucor*, and *Rhizomucor*^1^. *R*. *delemar* (also known as *R*. *oryzae*) is the most common etiologic agent of mucormycosis, responsible for ~70% of clinical cases^2,3^. Because of the increasing prevalence of diabetes mellitus, cancer, and organ transplantation, the number of patients at risk is dramatically increasing. Despite potentially disfiguring surgical debridement and adjunctive antifungal therapy, the overall mortality rate is formidably high, ranging from 50–100% in those individuals with disseminated infection or persistent neutropenia^3^. Significantly, multiple incidences of nosocomial mucormycosis outbreaks have been reported in recent years. During 2007–2009, six fatalities among young children occurred in a pediatric hospital in New Orleans, Louisiana. The deceased victims ranged from premature infants to 13 years^4^. More recently, hospital-acquired mucormycosis outbreaks occurred in the Queen Mary Hospital in Hong Kong and two university hospitals in Pittsburgh, Pennsylvania among organ transplant recipients, resulting in multiple fatalities^5–7^. Although individual cases occur more frequently, these nosocomial outbreaks highlight the clinical importance of mucormycosis and the urgency of developing rapid and effective diagnosis methods that will improve treatment strategy and patient outcomes.

The signs and symptoms of mucormycosis are non-specific and definitive diagnosis requires direct examination of specimens and culturing of the organism. These processes are time-consuming and limited in sensitivity^8,9^. The resulting delay in diagnosis and implementation of effective treatment during a critical window of time contributes to the high rate of tragic mucormycosis mortalities and underscores the urgent need for a rapid, sensitive diagnostic method. Recent progress in the study of extracellular vesicles (EVs) and encapsulated exRNAs have provided promise for the development of such a method. EVs, including exosomes and microvesicles, are secreted membrane derived vesicles often containing nucleic acids, lipids, and various proteins or other macromolecules^10,11^. In recent years, EVs and exRNAs have emerged as important mediators of intercellular communication that is involved in the regulation of a diverse range of biological processes. They are recognized in diseases, including cancer, neurodegenerative disorders, and infectious diseases, as they carry signals that not only identify themselves but are also capable of altering the function of targeted cells (reviewed in^11,12^).

EV-derived exRNAs have great potentials as biomarkers for diagnosis of *R*. *delemar* infection. Previous studies have demonstrated that other fungal pathogens such as *Candida albicans* and *Cryptococcus neoformans* produce EVs that contain at least 2,000 and 1,244 exRNAs, respectively^13,14^. To examine exRNAs produced by *R*. *delemar*, and to investigate the feasibility of using these exRNA signatures as biomarkers for mucormycosis, we isolated EVs from two clinical strains of *R*. *delemar*: FGSC-9543 and CDC-8219, and extracted exRNAs. The isolated exRNA was subjected to RNA deep sequencing (RNA-Seq). We have found that *R*. *delemar* produces abundant exRNAs, including sRNA, mRNA, and lncRNA, and that the FGSC-9543 and CDC-8219 strains each exhibit shared and distinct exRNA profiles. Our studies provide a preliminary, yet important step in the development of exRNA signature for use in a biomarker-based diagnosis method for mucormycosis. These exRNA profiles may also illuminate molecular mechanisms associated with *R*. *delemar* pathogenesis within the host.

## Results

### EV extraction and exRNA characterization

Previous studies have shown that there are two types of EVs: exosomes and microvesicles. Exosomes originate from the fusion of the multivesicular membrane and are 40–120 nm in size, whereas microvesicles are formed from outward budding of the surface cell membrane and are larger in sizes (50–1000 nm) (reviewed in^11^). Both vesicles contain miRNA, many non-coding RNA (ncRNA), and mRNA, as well as cytoplasmic and membrane proteins such as receptors and phospholipids. Even though the numbers of exRNAs identified from *C*. *albicans* and *C*. *neoformans* were relatively low (2,000 and 1,244, respectively)^13,14^, the results still suggested that *R*. *delemar* may also produce EVs with encapsulated exRNAs.

In our ongoing research effort to develop an efficient genetic transformation method for *R*. *delemar*, we were also interested in the identification of type III RNA polymerase (Pol III) promoter sequences involved in the synthesis of a large variety of small non-coding RNA (ncRNA). Since a significant amount of ncRNA is encapsulated in EVs, systemic characterization of ex-sRNAs may also lead to the identification of such sequences. For this purpose and for exploring exRNA as a biomarker for mucormycosis, we have isolated membrane vesicles from *R*. *delemar* FGSC-9543 and CDC-8219 strains through ultrafiltration and ultracentrifugation. For increased representation, EV preparations from three batches were pooled for exRNA extraction and were found to be of high quality (grade A) suitable for RNA-Seq. cDNA was then synthesized and size fractionated through a PAGE gel. Approximately 100 ng cDNA was subjected to deep sequencing using the BGISEQ-500 desktop sequencer (BGI, Shen Zhen, China).

### sRNA profiling

**Figure 1:**
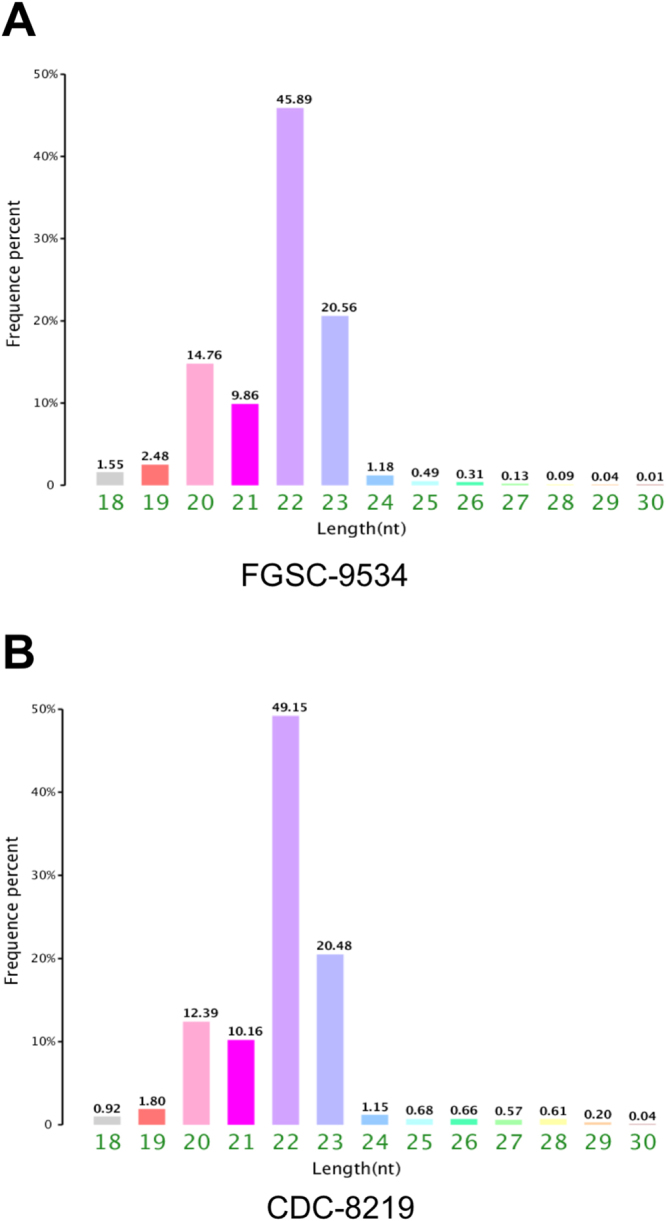
Rhizopus delemar FGSC-9543 and CDC-8219 strains share similar length distribution of ex-sRNAs. The majority of the ex-sRNA was in the range between 20 nt and 23 nt in length, with 22 nt as the major size group. The x-axis indicates tag lengths and the y-axis indicates the percentage of the tags.

**Figure 2:**
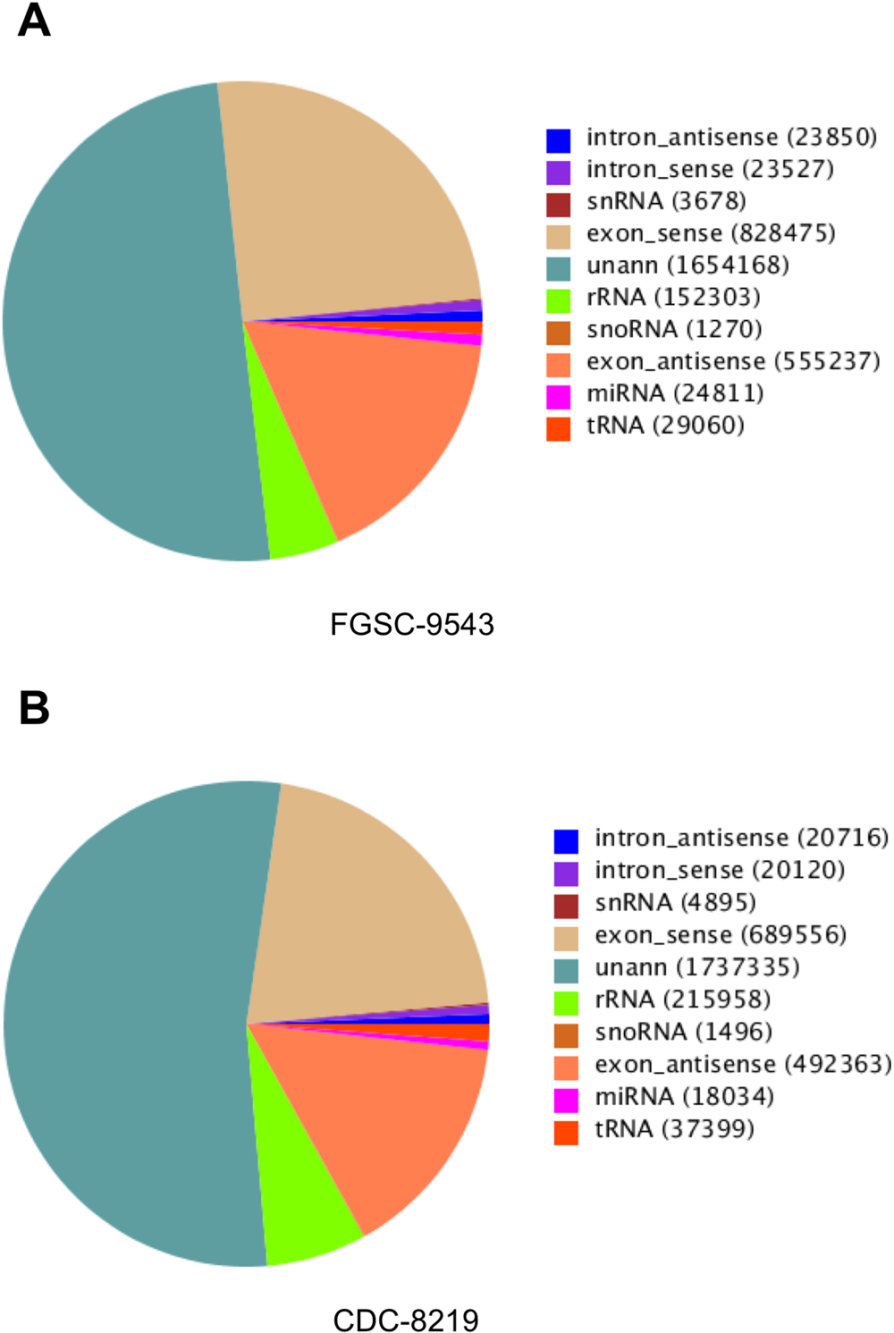
Summary distribution of unique reads in *R*. *delemar* strains. Pie charts depict the category and percentage of unique sRNA reads from FGSC-9543 (**A**) and CDC-8219 (**B**) strains.

The *R*. *delemar* FGSC-9543 strain is a clinical strain isolated in 1999 from a brain abscess of a diabetic patient who developed fatal rhinocerebral mucormycosis (www.broadinstitute.org). This is a type culture strain also known as RA99–880 or ATCC MYA-4621. The CDC-8219 strain was recovered from a deceased pediatric patient in a New Orleans hospital in 2008^4^. Initial characterizations of the basic traits and genetic information, including DNA sequences of *PyrF*, *PyrG*, (RO3G_14508:EIE89797), *CNA1*, *CNA2*, *CNA3*, and *CNB1* (RO3G_16532:EIE91821), suggested that CDC-8219 is genetically similar to FGSC-9543^4^ (Bruni and Wang, unpublished observations). Approximately 3.3 and 3.2 million clean reads were obtained from FGSC-9543 and CDC8219 strains, respectively. 86.1% (FGSC-9543 strain) and 69.1% (CDC-8219 strain) of these reads were mapped to the genome by Short Oligonucleotide Alignment Program (SOAP)^15^. The median sequence length of the sRNA was 22 nt (45.9% and 49.2% for FGSC-9543 and CDC-8219 strains, respectively), with a minimum of 18 nt (1.6% and 0.92% for FGSC-9543 and CDC-8219 strains, respectively) and a maximum of 30 nt (0.01% and 0.04% for FGSC-9543 and CDC-8219 strains, respectively) (Fig. 1A,B). The sRNA reads were aligned to Genbank and Rfam database using BLAST^16^ for identification and removal of ribosomal RNA (rRNA), transfer RNA (tRNA), small nuclear RNA (snRNA), and small nucleolar RNA (snoRNA) associated reads (Supple Fig. 1A–D). Introns and exons were also mapped and removed (Supple Fig. 1E and F). The distribution of unique ex-sRNAs from both strains is summarized in Fig. 2A,B.

### miRNA annotation and differential expression

miRNA sequences were identified using the miRBase database. Approximately 393 and 362 known miRNAs were identified from FGSC9543 and CDC8219 strains, respectively (ST1). In addition, the reads that were mapped to the antisense exon, intron, or intergenic region of the genome, but not to any other RNAs, were also analyzed using MIREAP (http://sourceforge.net/projects/mireap/) to identify 167 and 164 novel miRNAs from FGSC9543 and CDC8219, respectively (ST2).

**Figure 3:**
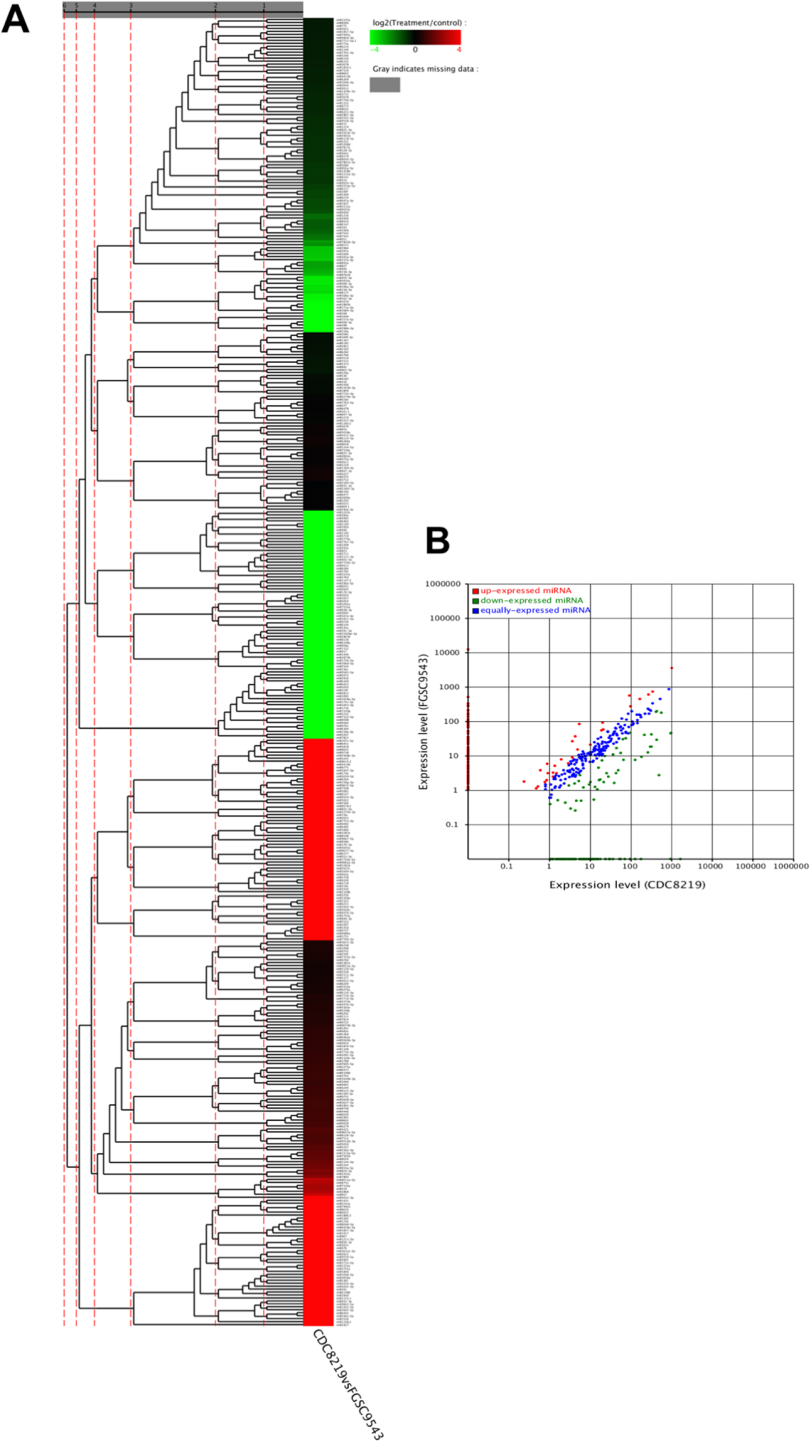
Differential expression of miRNA between FGSC-9543 and CDC-8219 strains. Heat-map diagram of a two-way hierarchical clustering analysis for the differentially expressed miRNAs (**A**) and the scatter plot of differential expressed miRNA between FGSC-9543 and CDC-8219 strains (**B**). Red color represents up-regulated miRNA, green color represents down-regulated miRNA, and blue represents unchanged expressions, p-value < 0.05.

The FGSC9543 and CDC8219 strains are distinct clinical isolates from different sources. Differential expression of known miRNA between the two strains was compiled and presented in a hierarchical clustering heat map (Fig. 3A), and a scatter plot was also generated using the Expdiff method (p value < 0.05) (Fig. 3B). Most of shared miRNA had similar expression profiles, but the expression appeared to be more depressed in CDC-8219 than in FGSC-9543 (Fig. 3B). Certain miRNA exhibited distinctive expression patterns. For example, miR1028a-3p and miR1043–3p were highly expressed in CDC-8219 but not in FGS-9543, while miR1044–3p, miR1047–3p and miR1048–3p were exclusively expressed in FGS-9543 (ST1).

### miRNA target prediction

**Figure 4:**
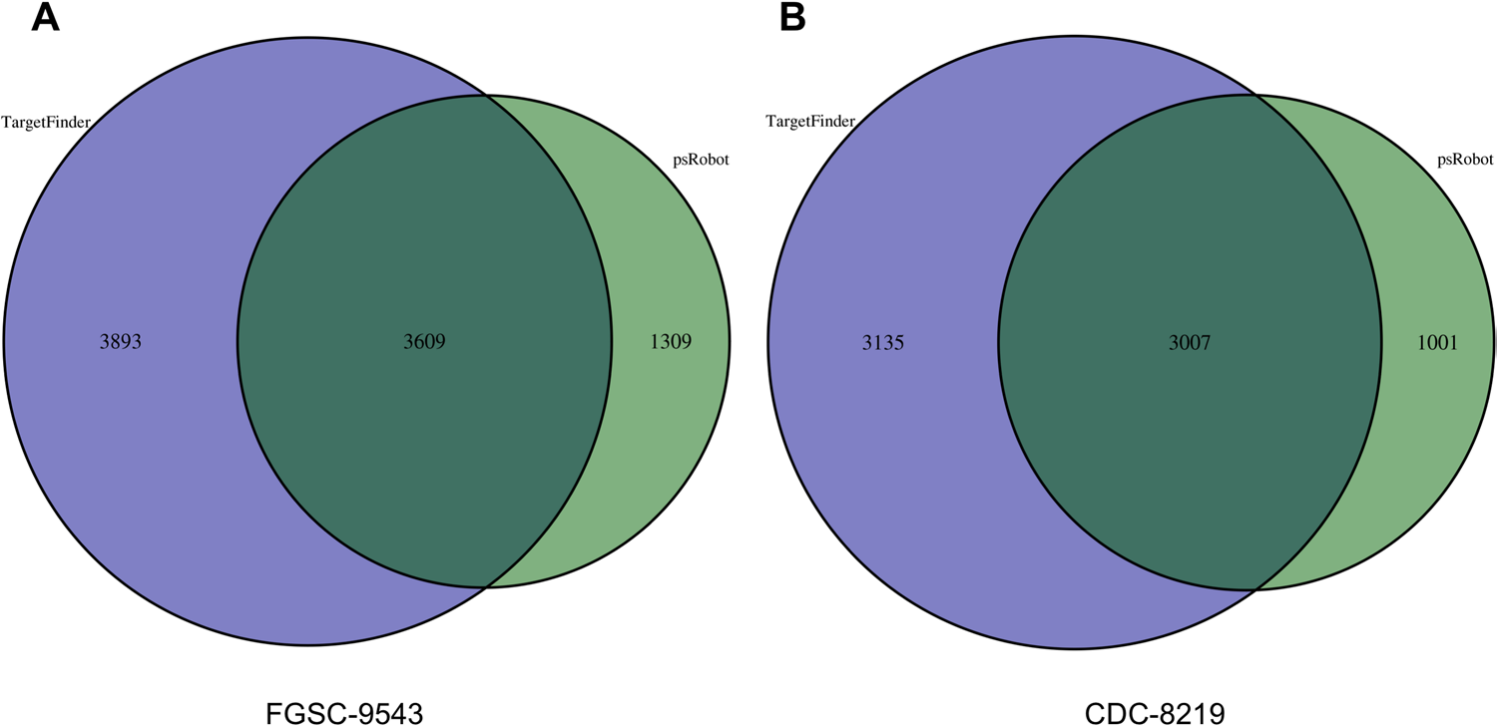
Summary of miRNA target prediction. Venn diagram of miRNA targets predicated by TargetScan and psRobot algorithms, as indicated, of FGSC-9543 (**A**) and CDC-8219 (**B**) strains.

**Figure 5:**
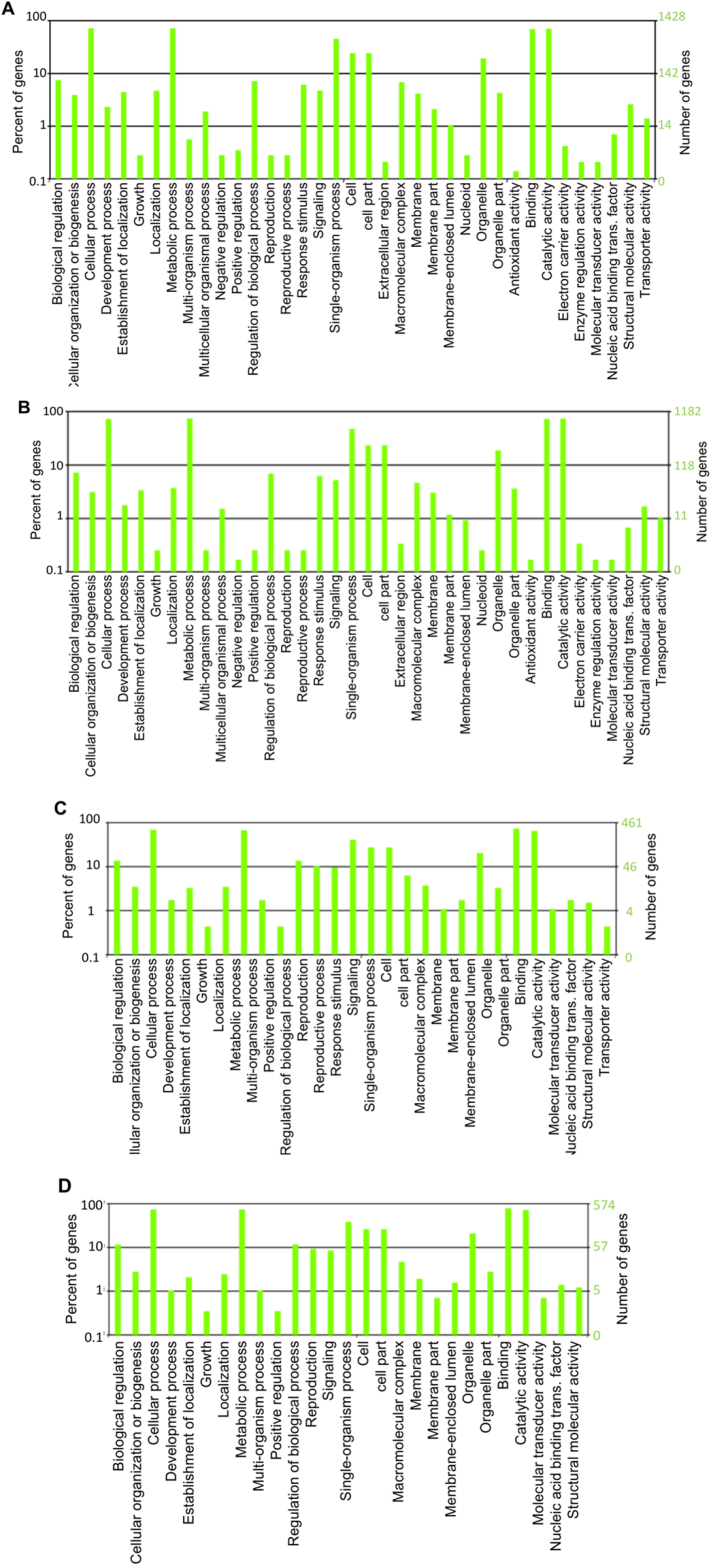
Gene Ontology analysis of miRNA target genes. (**A**) and (**B**) Represent known miRNA target GO terms of FGSC-9543 and CDC-8219 strains, respectively. (**C**) and (**D**) Represent novel miRNA target GO terms of FGSC-9543 and CDC-8219 strains, also respectively. The horizontal coordinates refer to the class of GO, the left vertical coordinates are the percentage of genes, and the right vertical coordinates are numbers of the gene.

To predict the target of miRNA in *R*. *delemar*, several databases including miRanda, targetscan, and PITA were searched using psRobot and TargetFinder software. For FGSC-9543, psRobot predicted 2897 target genes for 315 miRNAs, while TargetFinder predicted 4183 targets for 368 miRNAs (Fig. 4A) (ST3). For CDC-8219, psRobot predicted 2548 target genes for 282 known miRNAs, whereas TargetFinder predicted 3580 targets for 333 miRNAs (Fig. 4B) (ST4). The target genes were similar between two strains and could be categorized into 37 functional groups based on gene ontology (GO) annotation (Fig. 5A and B). For novel miRNA, TargetFinder predicted 1424 targets for 112 miRNAs, whereas psRobot predicted 1231 target genes for 91 miRNAs (ST5) for FGSC-9543. Again, for CDC-8219, TargetFinder predicted 1529 targets for 116 miRNAs, whereas psRobot predicted 1215 target genes for 88 novel miRNAs, respectively (ST6).

### GO terms and KEGG pathway annotation of miRNA targets

GO enrichment and function analysis was performed using the Web Gene Ontology Annotation Plot (WEGO, wego.genomics.org.cn). GO term annotation revealed 37 GO functional categories for known miRNA targets in both FGSC-9543 and CDC-8219 strains. Cellular and metabolic processes and single organism process (biological process), cell and cell component (cellular component), and binding and catalytic activity (molecular function) processes were the most significantly enriched GO terms (Fig. 5A,B). For novel miRNA, the target genes remained similar and they could be grouped into 28 (FGSC-9543) and 27 (CDC-8219) GO terms (Fig. 5C,D).

**Figure 6:**
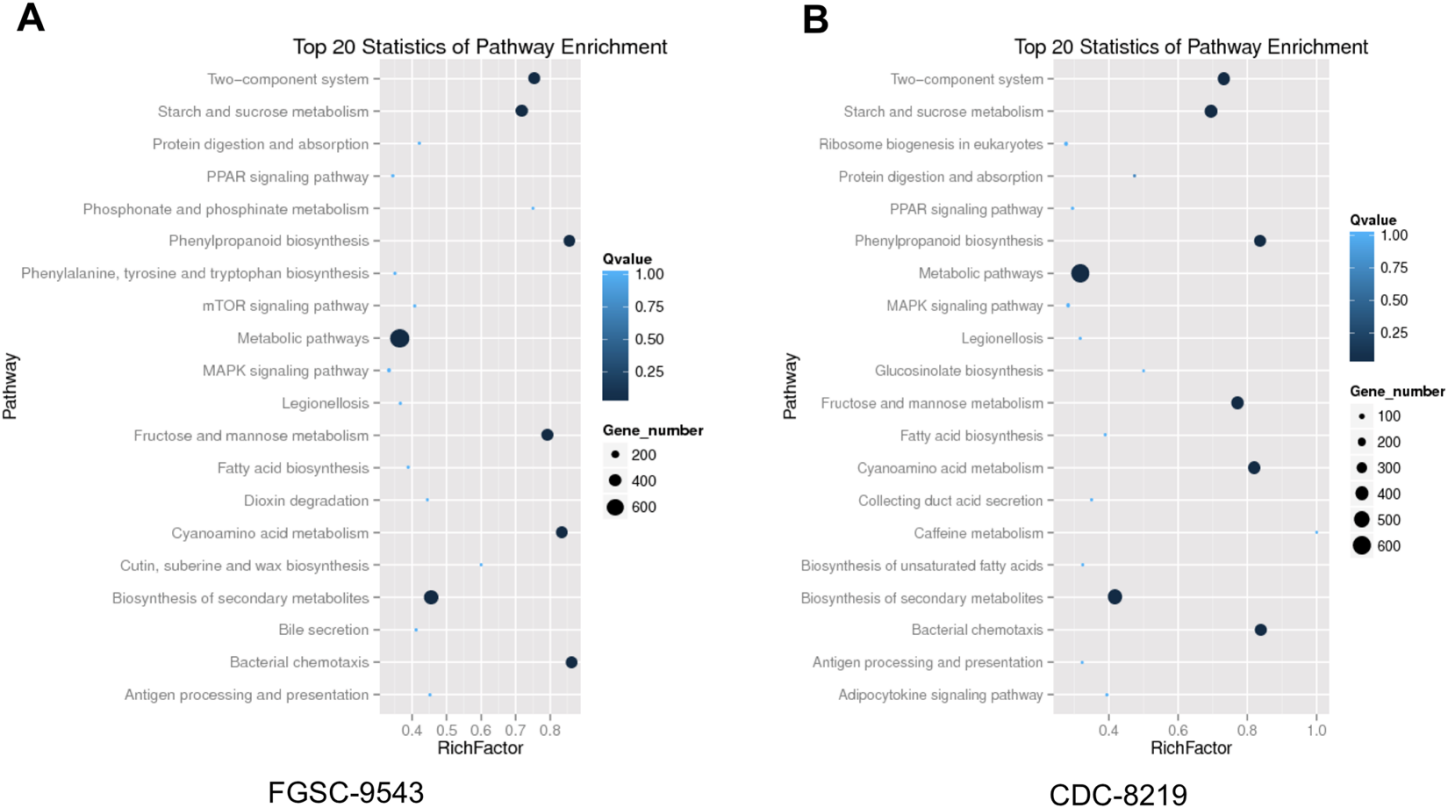
KEGG pathway analysis of miRNA target genes. (**A**) and (**B**) Represent known miRNA target KEGG terms of FGSC-9543 and CDC-8219, respectively. (**A**) and (**B**) Represent novel miRNA target KEGG terms of FGSC-9543 and CDC-8219, respectively. The horizontal coordinates refer to rich factors. The larger the rich factor the higher the enrichment. The right vertical coordinates are pathways. “Rich factor” means that the ratio of the differential expressed gene (DEG) numbers and the number of genes have been annotated in this pathway. The greater of the Rich factor, the greater the degree of enrichment.

The KEGG pathway annotation was performed based on scoring and visualization of the pathways collected in the KEGG database. In the analysis of known miRNA targets, 20 pathways were over-represented in both strains, suggesting that these pathways are significantly regulated during growth of the fungus (Fig. 6A,B). Interestingly, most of the pathways have been shown to be involved in the metabolic pathway and biosynthesis of secondary metabolites. The two-component regulatory system that serves as a basic stimulus-response coupling mechanism to allow organisms to sense and respond to changes in many different environmental conditions was found to be the fourth most significantly regulated. Collectively, these pathway analysis results illustrate the potential roles and mechanisms of miRNAs in regulating the general growth and development of *R*. *delemar*.

### Validation of expression

**Figure 7:**
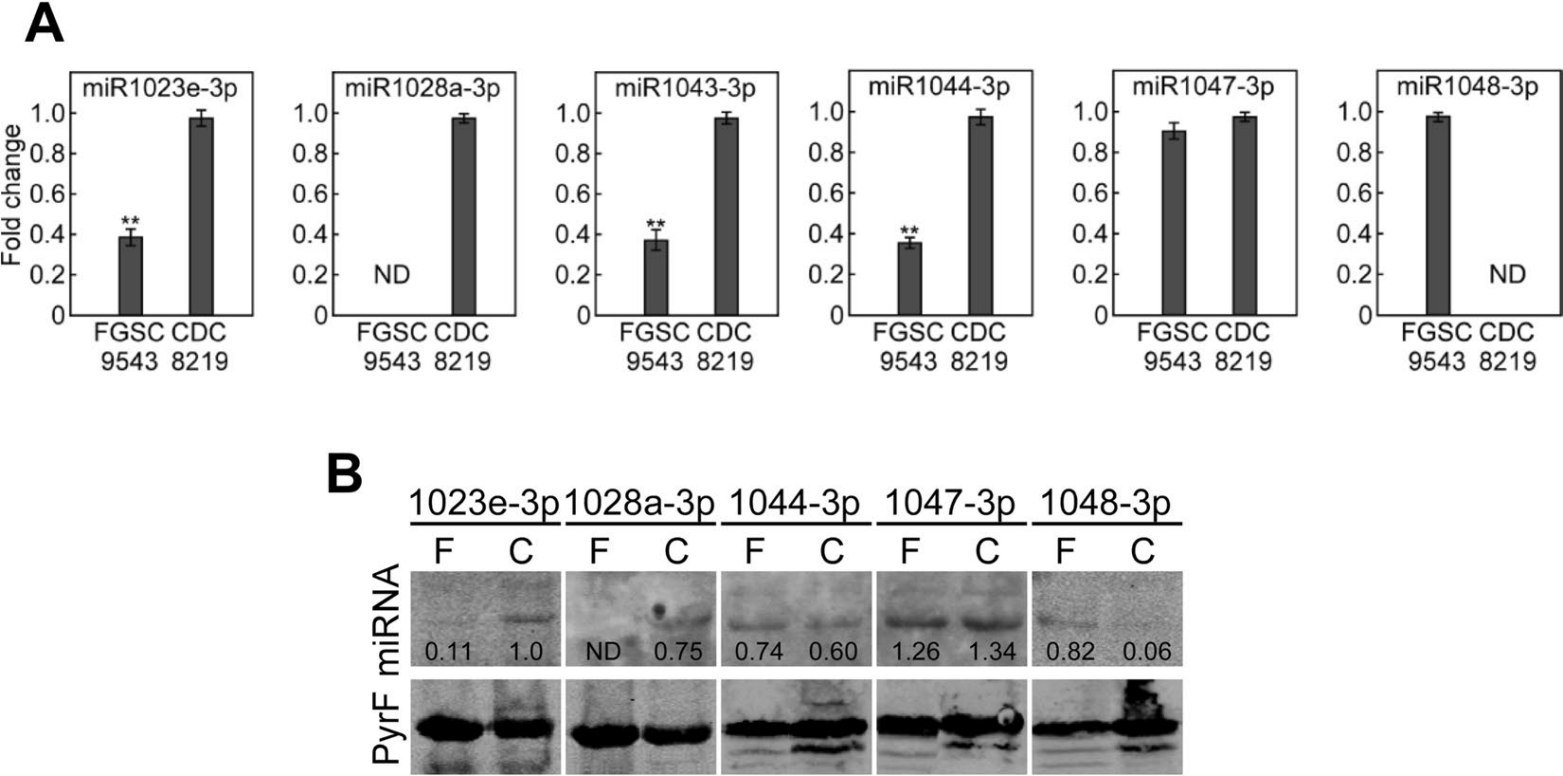
miRNA expression validation by qRT-PCR and Northern blot analysis. (**A**) The expression of six miRNA3 was quantified by quantitative real-time PCR (qRT-PCR) in reference to the expression of the constitutively expressed *PyrF* gene. Error bars show standard deviation (n = 3). Asterisks indicate a statistically significant correlation (p < 0.05). (**B**) Northern blot analysis of five miRNAs. Expression was normalized to the transcript of the *PyrF* gene and the relative value was inserted in the picture beneath the miRNA band. The experiments were repeated once that showed similar results. C denotes the CDC-8219 strain while F indicates the FGSC-9543 strain.

In order to validate the expression profile of exRNAs, we randomly selected six of the known miRNAs and performed quantitative real-time PCR (qRT-PCR) and Northern blot analyses. We extracted total RNA from both strains for cDNA synthesis as we opted to use the expression of the *PyrF* gene, a putative constitutively expressed gene encoding orotate phophoribosyltransferase, as an internal control. Expression of miR1048–3p in FGSC-9543 and miR1028a-3p in CDC-8219 only was consistent with the data from RNA-Seq. Also consistent was miR1023e-3p whose expression could be found in both strains (Fig. 7A). The expression of the remaining miRNAs (miR1043, miR1044, and miR1047) showed slight inconsistency that could be due to variations in fungal growth or during RNA sample preparations (Fig. 7A). For sample preparation, we used a PEG8000/NaCl method to first separate small RNA from total RNA prior to denaturing polyacrylamide gel electrophoresis separation. Similar to the qRT-PCR results, the expression of five miRNAs detected by Northern blot analysis was largely consistent. The only exception was that the expression of 1048–3p was detected in CDC-8219 (Fig. 7B). However, the expression is very low (0.06 in relevance to the expression of the *pyrF* gene control) in comparison to FGSC-9543 (0.82) (Fig. 7B). Taken together, these findings are mostly agreeable with the data obtained from RNA-Seq.

## Discussion

Zygomycetous fungi such as *Rhizopus*, *Mucor*, and *Rhizomucor* are emerging as significant fungal pathogens. Multiple outbreaks of nosocomial mucormycosis, caused by *R*. *delemar*, have occurred in recent years, thereby underscoring the importance of improving diagnostic methods as well as furthering our understanding of how these fungi cause disease. However, this group of fungi is poorly studied and therefore lacks many available genetic research tools and resources that have been developed for studying other medically important fungi, including *Candida*, *C*. *neoformans*, and *A*. *fumigatus*. In order to improve molecular resources for studying *R*. *delemar*, we have performed RNA-Seq analysis of ex-sRNAs from two clinical strains of *R*. *delemar*: FGSC-9543 and CDC-8219. In addition to sRNAs, we have also identified a combined total of 17,884 extracellular mRNAs and extracellular 1,932 lncRNAs from these two strains. We present herein the comparative analysis of secreted sRNAs from two clinical strains of *R*. *delemar*. Since this appears to be the first study in which RNA profiling of ex-sRNAs from a zygomycete was extensively performed, it is unknown if these molecular sequences are unique to *R*. *delemar* or whether some exRNA molecular signatures might be similarly conserved with other zyogmycetes such as *Mucor circinelloides*. Further studies are needed to ascertain the uniqueness of *R*. *delemar* exRNA signatures among other zygomycetes; however, it should be noted that the optimal treatment of infections caused by each of the zyogmycetes is the same.

Previous studies by others have revealed that exRNA is produced in relatively high abundance in response to environmental stress and during infection of the host. Circulating pathogen-derived exRNAs are readily detectable in bodily fluids, including blood, bronchoalveolar lavage, and urine^17–21^. These circulating, pathogen-derived exRNAs can be easily isolated from an infected host in a non-invasive manner and hold great potential as molecular biomarkers of infection. The development of a sensitive and rapid detection method is especially important for diagnosis of mucormycosis, since the symptoms are often non-specific. Furthermore, traditional methods of identification, either by direct examination or culture of the organism, may delay diagnosis and implementation of effective treatment during a critical window of time. This delay in diagnosis and administration of the appropriate antifungal therapy contributes to the tragically high mortality rate of 50–100%^3^. Therefore, in order to explore the potential application of ex-sRNA as a molecular biomarker of mucormycosis, we sought to profile ex-sRNAs in this preliminary study. We included two clinical strains, the type culture FGSC-9543/RA 99–880 isolated from a brain abscess of a diabetic patient and the CDC-8219 strain isolated from a pediatric patient in New Orleans with fatal mucormycosis. Preliminary characterization of the basic physiological traits revealed that these strains are very similar in growth and morphology (unpublished observations), indicating that our selection of strains is representative of the species. In addition, we were able to obtain several million clean RNA-Seq reads from each strain that also suggests that the reads far exceed the number needed to saturate the transcriptome(s) of the strain grown in monocultures.

Small RNA, including miRNAs, small interfering RNAs (siRNAs) and piwi-interacting RNAs (piRNAs) play a critical role in the regulation of cellular growth and development by regulating the expression of target genes by RNA cleavage or transcriptional silencing^22^. miRNAs have been reported as vital participants in post-transcriptional gene regulation by affecting mRNA stability and translation efficiency in organisms, including humans, fruit flies and nematodes (reviewed in^23,24^). miRNAs regulate gene expression through targeting the 3′ untranslated region (UTR) of target mRNAs in a seemingly complex pattern, as a single miRNA can regulate hundreds of target genes. Conversely, a single gene can be targeted simultaneously by multiple miRNAs. In contrast to other model organisms, the functions of sRNAs, especially ex-sRNAs, have been relatively unexplored for zygomycetous fungi such as *Rhizopus* and *Mucor spp* until now. Here, our studies revealed that the FGSC-9543 strain has 393 known miRNAs (373 targeting 4,810 host genes) and the CDC-8219 strain has 362 miRNAs (338 targeting 4,082 host genes) in the secreted sRNA profiles, respectively. In addition, along with known miRNA and targets, 116 novel miRNAs with 1,848 targets and 118 novel miRNAs with 1,936 targets were also predicted for FGSC-9543 and CDC-8219 strains, respectively. While the miRNA profiles of each strain are very similar, strain-specific miRNAs were also present. Interestingly, statistically significant differential expression patterns were observed for more than 50% of the known miRNAs produced by the two strains. Furthermore, we validated the expression of six known miRNAs by qRT-PCR and five miRNAs by Northern blot analysis and found that the expression levels are very similar to the results obtained from RNA-Seq. Further exploration of miRNA expression and characterization of target genes will expand our understanding of the role individual miRNAs play in the growth, development, and even virulence of *R*. *delemar*, and are, thereby, warranted.

There are three ontologies in Gene Ontology (GO): molecular function, cellular component and biological process. Through GO and KEGG pathway analysis of putative targets of miRNA, we found that most of the biological functions are related to carbohydrate utilization and metabolism, and secondary metabolite biosynthesis, pathways that are critical for fungal growth. Interestingly, the two-component system represented one of the major pathways targeted by miRNA in our analysis. This is not surprising since sensing and responding to changes in the environment is central to fungal growth. Based on the GO and KEGG analysis results, it is highly intriguing to consider whether exRNA profile studies involving clinical or *in vivo* samples would reveal pathways that are important during infection, but not expressed *in vitro*.

Future studies will be aimed at profiling additional exRNAs including mRNAs and lncRNAs that could uncover novel molecular mechanisms governing growth, virulence mechanisms, and the response to antifungal treatment. Taken together, our analysis of ex-sRNAs in this study indicates the feasibility of exploring exRNA as both a biomarker for mucomycosis and for further investigating virulence mechanisms in *R*. *delemar*.

## Materials and Methods

### Extracellular vesicle isolation

*R*. *delemar* FGSC-9543 and CDC-8219 strains were grown in potato dextrose broth (PDB) medium at 30 °C for 5 days with shaking at 250 rpm. Hyphae and cell debris were first filtered through Miracloth (EMD Millipore) and the cell-free culture fluids were recovered by centrifugation first at 4,000 × g for 15 min at 4 °C and then at 15,000 × g for 15 min. The latter step was necessary for removing smaller debris or particles. The liquid was filtered through an ultrafiltration filter with a cutoff of 100-kDa (Millipore). The remaining liquid was precipitated by ultracentrifugation (Beckman) at 100, 000 × g for 1 hour and at 4 °C, and the precipitate containing EVs was re-suspended in phosphate-buffered saline (PBS). The vesicle pellets were washed twice in PBS and the final pellets were suspended in PBS and lyophilized for RNA extraction. EV preparations from three repeats were pooled.

### Cloning of ex-sRNAs

RNA was extracted with Trizol (Sigma-Aldrich) and separated on a PAGE gel. The small RNA band at approximately 18–30 nt in size were recovered and cloned with the Small RNA Cloning Kit (TaKaRa). Briefly, sRNA was detected by an Agilent 2100 Bioanalyzer (Agilent Technologies) and 8 μg purified sRNA was ligated with the 3′ and 5′ RNA adaptors included in the kit. cDNA was subsequently synthesized and amplified with the supplemented oligo primers. cDNA was cloned into T-vector pMD20 provided with the kit to generate a sRNA library. The cDNA libraries were loaded onto the flow cell channels of an BGISEQ-500 platform for paired-end 90 bp x 2 sequencing at the Beijing Genomics Institute (BGI), Shenzhen, China.

### Identification of miRNAs via bioinformatics analysis

The ligating adapters with unique synthetic oligonucleotide sequences (sequence tags) were subjected to data cleaning analysis to obtain clean tags/reads. The clean tags were annotated into different categories and tRNA, rRNA, and other impurities were removed. The sequences were mapped to the *R*. *delemar* genome at ftp.ncbi.nlm.nih.gov/genomes/all/GCA/000/149/305/GCA_000149305.1_RO3/GCA_ 000149305.1_RO3_genomic.fna.gz. Known miRNAs were identified by searching against miRBase (www.mirbase.org). Since the miRNA hairpins are mostly located in intergenic regions, introns or reverse repeat sequence of coding sequence, sRNAs that were mapped to these regions, but not to any other RNA, were predicted to be novel miRNAs. The Rfam database is at http://rfam.janella.org. Expression levels of sRNA were calculated by using Transcript Per Million (TPM)^25^.

### miRNA target identification and functional annotation by GO and KEGG analyses

Once miRNA results were obtained, target prediction was performed using psRobot and TargetFinder software^26,27^. Gene ontology (GO) and Kyoto Encyclopedia of Genes and Genomes (KEGG) pathway enrichment analyses were also performed. GO provides a common descriptive framework and functional annotation and classification for analyzing the gene set data (www.geneontology.org), whereas the KEGG pathway database is a recognized and comprehensive database including all kinds of biochemical pathways (www.annotation.jp/KEGG/ko.html)^28,29^.

### Quantitative RT-PCR

For quantitative real time RT-PCR (qRT-PCR), 5 μg of total RNA was reverse transcribed into first-strand cDNA using the oligo(dT) or random primers and M-MLV Reverse Transcriptase (Invitrogen). The qRT-PCR reactions were performed following previously established procedures^30,31^. Primer pairs used in this section are listed in Table I. The expression of the *PyrF* gene that encodes orotate phophoribosyltransferase, a putative constitutively expressed gene, was used as a control. DNA primers are listed in Supple Table 7.

### Northern blot Analysis of miRNAs

Northern blot analysis of miRNA was performed following the protocols described previously^32,33^. Briefly, small RNAs were isolated from total RNA by precipitation in 5% PEG8000 and 0.5 M NaCl. Small RNAs were resolved using 14% denaturing polyacrylamide gel electrophoresis, transferred to the nylon membrane (GE healthcare life sciences) using a semi-dry transfer apparatus (Trans-blot semi-dry transfer cell, Bio-Rad), and detected by a non-radioactive biotin-labeling method (Chemiluminescent nuclei acid detection module kit, NEB). Both miRNA and *PyrF* probes were labeled with biotin at both 5′ terminus and 3′ terminus. Quantitation of the blots was performed using ImageJ and the relative signals were calculated by normalizing to the *PyrF* control band.

## Electronic supplementary material

Supplemental information

## References

[1] Hibbett DS (2007). A higher-level phylogenetic classification of the Fungi. Mycol Res.

[2] Gomes MZ, Lewis RE, Kontoyiannis DP (2011). Mucormycosis caused by unusual mucormycetes, non*-Rhizopu*s, *-Muco*r, and *-Lichtheimi*a species. Clin Microbiol Rev.

[3] Spellberg B, Edwards J, Ibrahim A (2005). Novel perspectives on mucormycosis: pathophysiology, presentation, and management. Clin Microbiol Rev.

[4] Duffy J (2014). Mucormycosis outbreak associated with hospital linens. Pediatr Infect Dis J.

[5] Cheng VCC (2016). Hospital Outbreak of Pulmonary and Cutaneous Zygomycosis due to Contaminated Linen Items From Substandard Laundry. Clin Infect Dis.

[6] Novosad SA (2016). Notes from the Field: Probable Mucormycosis Among Adult Solid Organ Transplant Recipients at an Acute CareHospital — Pennsylvania, 2014–2015. Morbidity and Mortality Weekly Report.

[7] Valle, L. D. Mold at two Pittsburgh hospitals linked to 5 deaths., http://www.cnn.com/2017/2001/2028/health/moldy-hospital-bed-linen-deaths/ (CNN Health, 2017).

[8] Ibrahim AS, Spellberg B, Walsh TJ, Kontoyiannis DP (2012). Pathogenesis of mucormycosis. Clin Infect Dis.

[9] Jeong SJ, Lee JU, Song YG, Lee KH, Lee MJ (2015). Delaying diagnostic procedure significantly increases mortality in patients with invasive mucormycosis. Mycoses.

[10] Ibrahim A, Marban E (2016). Exosomes: Fundamental Biology and Roles in Cardiovascular Physiology. Annual review of physiology.

[11] Quesenberry PJ, Aliotta J, Deregibus MC, Camussi G (2015). Role of extracellular RNA-carrying vesicles in cell differentiation and reprogramming. Stem cell research & therapy.

[12] El Andaloussi S, Lakhal S, Mager I, Wood MJ (2013). Exosomes for targeted siRNA delivery across biological barriers. Advanced drug delivery reviews.

[13] Peres da Silva R (2015). Extracellular vesicle-mediated export of fungal RNA. Scientific reports.

[14] Jiang N, Yang Y, Janbon G, Pan J, Zhu X (2012). Identification and functional demonstration of miRNAs in the fungus Cryptococcus neoformans. PloS one.

[15] Li R, Li Y, Kristiansen K, Wang J (2008). SOAP: short oligonucleotide alignment program. Bioinformatics.

[16] Nawrocki EP (2015). Rfam 12.0: updates to the RNA families database. Nucleic Acids Res.

[17] Arscott WT, Camphausen KA (2011). Analysis of urinary exosomes to identify new markers of non-small-cell lung cancer. Biomarkers in medicine.

[18] Cazzoli R (2013). microRNAs derived from circulating exosomes as noninvasive biomarkers for screening and diagnosing lung cancer. Journal of thoracic oncology: official publication of the International Association for the Study of Lung Cancer.

[19] Dijkstra S (2014). Prostate cancer biomarker profiles in urinary sediments and exosomes. The Journal of urology.

[20] Lau C (2013). Role of pancreatic cancer-derived exosomes in salivary biomarker development. The Journal of biological chemistry.

[21] Li Q (2015). Plasma long noncoding RNA protected by exosomes as a potential stable biomarker for gastric cancer. Tumour biology: the journal of the International Society for Oncodevelopmental Biology and Medicine.

[22] He L, Hannon GJ (2004). MicroRNAs: small RNAs with a big role in gene regulation. Nat Rev Genet.

[23] Bartel DP (2004). MicroRNAs: genomics, biogenesis, mechanism, and function. Cell.

[24] Fabian MR, Sonenberg N, Filipowicz W (2010). Regulation of mRNA translation and stability by microRNAs. Annu Rev Biochem.

[25] t Hoen PA (2008). Deep sequencing-based expression analysis shows major advances in robustness, resolution and inter-lab portability over five microarray platforms. Nucleic Acids Res.

[26] Wu HJ, Ma YK, Chen T, Wang M, Wang XJ (2012). PsRobot: a web-based plant small RNA meta-analysis toolbox. Nucleic Acids Res.

[27] Kielbasa SM, Bluthgen N, Fahling M, Mrowka R (2010). Targetfinder.org: a resource for systematic discovery of transcription factor target genes. Nucleic Acids Res.

[28] Kanehisa M, Goto S (2000). KEGG: kyoto encyclopedia of genes and genomes. Nucleic Acids Res.

[29] Altermann E, Klaenhammer TR (2005). PathwayVoyager: pathway mapping using the Kyoto Encyclopedia of Genes and Genomes (KEGG) database. BMC Genomics.

[30] Varkonyi-Gasic E, Wu R, Wood M, Walton EF, Hellens RP (2007). Protocol: a highly sensitive RT-PCR method for detection and quantification of microRNAs. Plant Methods.

[31] Gong J, Grodsky JD, Zhang Z, Wang P (2014). A Ric8/synembryn homolog promotes Gpa1 and Gpa2 activation to respectively regulate cyclic AMP and pheromone signaling in *Cryptococcus neoforman*s. Eukaryot Cell.

[32] Huang Q, Mao Z, Li S, Hu J, Zhu Y (2014). A non-radioactive method for small RNA detection by northern blotting. Rice (N Y).

[33] Hu J, Zhu Y (2017). Nonradioactive Plant Small RNA Detection Using Biotin-Labeled Probes. Methods Mol Biol.

