# Supplementary material for: Comparative genome-wide analysis of extracellular small RNAs from the mucormycosis pathogen Rhizopus delemar
Source: Sci Rep. 2018 Mar 27;8:5243. doi: 10.1038/s41598-018-23611-z (PMC5869740; doi:10.1038/s41598-018-23611-z)
Supplement: Supplementary file 1 — Supplemental information [file 41598_2018_23611_MOESM1_ESM.pdf]

Supplemental information for

"*Comparative genome-wide analysis of extracellular small RNAs from the mucormycosis pathogen Rhizopus delemar*" by MX Liu, GO Bruni, CM Taylor, ZG Zhang, and P Wang

Supplemental Table 1: FGSC-9543 miRNA
miR419 28551 TGAGGAATGATGACGTGAG
miR9552b-3p 31309 TGGCGAGAGACCGATAGC
miR6478 17010 CCGGCCTTAGCTCAGTTGGTAG
miR2867-3p 10352 TCAGGACTTTGGGATGGCCA
miR6202 5732 TGAGGATTTAAGCTTTGAAG
miR398 2397 TGTGTTCTCAGGTCACCCCT
miR3949 5724 TGAGGTATGAGCAAAAAGTGTAG
miR1870-5p 4321 TTGCTGAATGGACCAGTGGCAT
miR167d 1479 TGAAGCTGCCAGCATGATCT
miR5163b-3p 11881 TAGATACTTTGGTTGTGGGA
miR5632-3p 4262 TTGGATTTAAGTCTGGATACT
miR5506 7838 TAGATCGCTTCGTTGAGG
miR1216 9034 TGATGGGTTAGCGCTTGTAC
miR166h-3p 929 TCGGACCAGGCTTCATTCCC
miR1167 22718 TGGGCTGTGGATGACTTGAAAC
miR847-3p 2321 TGACTCCTCTTCTTCTGGTCG
miR168 937 TCGCTTGGTGCAGGTCGGGA
miR1222a 3731 TATGAAGAAGTTCATTGTTGATA
miR408-5p 646 ACAGGGAACAAGCAGAGCAT
miR3476-5p 3139 TGAACTGGGTGCTGCTGCTGC
miR9674b-3p 3274 TGAATATGCCAGAGCATCAG
miR5185l-3p 44848 TTGGACGGTTGACTTAGAAC
miR2111 1146 TAAACTGTGTTTGGGTTTGG
miR3703 10101 TAGATTCATCAGCCATTGCC
miR159a 480 TTTGGATTGAAGGGAGCTCT
miR7697-3p 10380 TCCAATAACAATGATCGGATA
miR1513a-5p 14033 TGAACAAAGCCATGGACTTGC
miR5177 631 TGAGGTTGGAAAACGAGCAG
miR161.1 1229 TGAAAGTGGCTCATCAAGGGGT
miR7541 552 TGCAATGTCTCTTTTGCTGGCAC
miR2609a 421 TGGAAGTAATGGCTCGTCACTT
miR1431 2700 TTTGGAGTTGGCCACCGCTTGAC
miR4413b 577 TAAGAGCAAGTATAAGTCACCT
miR171n-5p 3394 TATTGGTGGGTTCAAGTACGG
miR8044-5p 514 TTGTCAAAATGGTTGGAGATTT
miR8123-3p 8419 TTGTCGCCATTGGTCCAAGTC
miR832-3p 8023 TTGATCGAATCCAAGCCAAG
miR8179 2349 TTGATCTGCATTCACTTGATCG
miR2275a 1746 TTTGGTTTCCTCCGGTTCTCAT
miR5718 715 TCAGAACCAAGACCAGAGCCAAG
miR5821 1391 TGGACGGAGACGATGGTAGAG
miR6023 2605 TTGATGACAGTTTTTTGGAT
miR169d 393 TTAGCAAGGATGAGATTACCGG
miR171a-3p 387 TGATTGAGCCGCGCCAATAT

miR5562-3p 22759 ATGGGACGCAAGGCTGAAAC
miR1146 4395 TTGGGTCCGACGGGAAGTCTT
miR7536a 7919 TGAAGCATGCTCGAGACTG
miR5679 1709 TTGGTGCAGAAGAAGTTAGA
miR3440b-3p 2585 TGGATCCGGTCAAGGGAAAGCT
miR6221-5p 571 TCTGTCTTCTGGCCCGCTGTCT
miR7761-3p 6731 TCTCGAATCAAGGACGCTCTGG
miR408-3p 193 ATGCACTGCCTCTTCCCTGG
miR826b 731 TGGTCTTGACATCGATGAAAAT
miR403a 754 TTGACGATTCACGCCAATCTTG
miR1098 601 TATGCGTTTTTGTCTGAAAT
miR3435-5p 179 TGCATCAACTCTGTCAATTTCC
miR7763-5p 3196 AACTTCATCGTGCTGGGGATAG
miR9491 2539 TGCTATCTTCCTCTGACTCAG
miR1435a 258 TTTCTTAAAGTGCAAGTTTTTC
miR160b 394 TGCCTGGCTCCCTGTATGCC
miR168a-3p 161 CCCGCCTTGCATCAACTGAA
miR7803b-3p 2047 TGACACGTCGTCAATCATGTACT
miR5293 691 TGATGAAGAAGTGGCGCAGAAGA
miR156i 394 TGGAACAGAAGAAGACAGCAG
miR5741a 3771 TAGGAGCAAATTGATGTGTTT
miR165a-3p 105 TCGGACCAGGCTTCATCCCC
miR394a-3p 195 TGTTGTCATCTCTTTTGTAA
miR2111-3p 511 GAAGTCAGGTGGGATTACC
miR397a 158 TCATTGAGTGCAGCGTTGATG
miR774b-5p 339 TGAAGATGCATATTTGGAGTGAC
miR8034 2833 TATGACAAGCCTGCACAAACT
miR8748 688 TGATGGTGATGATAATAATGAAT
miR5678 2543 TTCCATGCATAAGACTTTCC
miR951 231 TGCTCTTGACGATCTGGCTACG
miR482e 589 TTGCCTATTCCTCTGCCAAG
miR5144-3p 787 TACTCACTGCACAGCACAAGAAAG
miR9558-3p 1616 TTGCAAGGCCAGCTTTTCCTCT
miR5252 1152 TTGTAAAGCTACTGAAGTCTGC
miR4401b 4514 TCAAAGACGGTTGTGAGCAA
miR403-3p 87 TTAGATTCACGCACAAACTC
miR164b 84 TGGAGAAGCAGGGCACGTGC
miR9559-5p 3549 TTTGATTTTGGTTCATTTTG
miR6279 38067 TAGACAAGACTTCCAGCCAGACG
miR6118-5p 139 TGGAATGGGTGCTTGGGAAA
miR9409 499 TTTTGTCATAGTTCATTTTC
miR7512 88 TACTACTTGTAGCTTATGCCAT
miR166b 651 TCGGCCAGGCATTCCTTCCAA
miR6214 8518 TGACGACGAGAGCGACGATCA
miR9657a-3p 455 TGGTGCTTCCTGTCGTACAGGT

miR825-3p 725 TGGTCTCGATAAGGTGCATTAAC
miR529a 989 TTGTCGCTGCTCTCTTCTTC
miR833a-5p 181673 TGGCTTTGTTTGTACCCGGTCTGT
miR8141 1274 TGCATACTAGTTGGTGGT
miR7539 130 TGAGAGAGAAGAGCTATGAGG
miR3627-3p 346 TGCTCGCTGTCCTCGTGACAAG
miR167c-3p 178 TGTATGACTTGGTAGTTTCACC
miR5219 220 TCTGGACTCTCAGCTGCTGCGT
miR6255 1033 TGGGAAAGAATGGACGATGAGT
miR391 5270 TACGCAGAGGGAGACGTCG
miR7817a 8659 TTTGGTTGAACTTGTCTCGAGAC
miR162-3p 54 TCGATAAACCTCTGCATCCA
miR6117 2282 GGGGCTGATCCGGGTTGAAGAC
miR164b-3p 664 GCGCCCAGCTTCTCCACC
miR7719-5p 146 TCCTCCATAAGCATGTTTCTTC
miR7502b 3000 TTGTTAATAAGTTCATGCAT
miR477 2210 TTGGAGTATCTTTGGAGGAGC
miR396b 101 TTCCACAGCTTTCTTGAACT
miR156f-3p 368 TGCTTCACTGCTCTTTTCAGAT
miR6282 941 TTGATCATGTGGGGATGTATAA
miR6277 427 TGTGTGTGAAAGAGACGGAG
miR158-5p 126 CTTTGCCTATCGTTTGGAAAA
miR7716-5p 330 TAGTTGATAAGTAAGAACGAGAC
miR6194 101 TAGTAGGGATCTTGACACACGAG
miR6209 404 TGAGCATCAGAAGCTGTGCG
miR2905 127 TACTGTAGCCGACAAAGGCA
miR7695-5p 196 TTGCCATTGAGCACGCCAGTGAA
miR1425-5p 10962 TACTGATCATCCTTGCTGCT
miR775 3502 TTGCGATGTCTTGCAGTCAA
miR4374b 977 TCTTCAAAGACGGTTGTGAG
miR399f 1564 TGCCAACGCGATTTGTCCTG
miR2611 774 TATTTTCAGTGTTGTGGTAGAA
miR854 213 TGATGAGGATATTGAGAAGGG
miR5565e 423 TATGGTCTAGATGTTGTCGGA
miR319c 734 TTGGATGAAAGGGGCATGCT
miR3712 3414 TGCTGATCAAGATCAAGACCA
miR9727 53 TGTGTTAACTCTGAGAACTGAG
miR1061-3p 1620 TGTTAGCATGGAATAATG
miR1857-5p 10996 TGGATTCTTTTGGAGATGCGG
miR6114-5p 179 TCGAAAGCACCATGAACGTGA
miR1152 714 TAAAAGGAGCGCGTCTTGAA
miR7725a-3p 189 TGCAAACAACTCATTTCGTCAAG
miR835-3p 535 TGAAGAGATACGCCAGGAG
miR416 1269 TGTTAGTCGTACCACTGTTCAG
miR536f 4260 TTCCGTCCAAGCTGTGTTGA

miR5512a 9697 TAGGATCATTGTCATGCTAAAAT
miR773a 993 TTTGCTGCCACTTTTGTCACG
miR1120c-5p 563 TAATATAAAATGCTTTGTTGAC
miR5788 122 TGGATAGTTATCAGACTCTAGTA
miR5561-5p 15108 CATTTGGTGAGAGACATGGACAA
miR1068 371 TAGCCAGTTTTGCTTGCAGGTC
miR8554 80 TGCTTATGACTTTGATTTGGGA
miR5373 793 TCTCTTGTATGCTGATGATGTT
miR475a-3p 917 TTAATGCCCATTGACTTCAG
miR2644 129 TCTCTTCAGTTGATGGTTGT
miR5632-5p 154 TGTCATTCTTTATCCAACTG
miR6248 502 TATTGAGGTGGAGGTTAGTG
miR398b-3p 2330 ATGTGTTCTCAGGTCACCCCT
miR169f-3p 2005 TGATGTCTTCCTTTGGTTACT
miR9760 54 TGATGATGTAGTCTTGATTGGT
miR7120a 192 TGTTATTTGTCAGATTGATTCT
miR1023e-3p 1630 TGGGAAACGGGTTGGAGTGCAT
miR5766 1300 TTAGGCAGAGGAATAGGCAAG
miR1160.1 368 TTCCCTCTTTTTAAGCATGGCC
miR6269 4205 TGCTGCAAGTGATTAGCCATGG
miR4227 1933 TCTGGTACCAATCTTTCA
miR4412-5p 652 TGTTGCGGATATTCTTTGCACT
miR5241a 2663 TGATGATGGAAGAGGGAAT
miR6182 2789 TGGTCTGTGATGGATGCGTT
miR158-3p 74 TTTCCAAATGTAGACAAAGC
miR6266a 192 TATATGCAGGCGGCAAATAT
miR5176-5p 767 TATTCCATGTGTCACATCTC
miR1886.2 2242 TGGATGATATTGTGATTGG
miR6232a-3p 35 TGGATGCTAGCAAAAAAGAAAGT
miR902h-3p 563 TAGAAGGGTCTCCACACAAAC
miR6185 775 TCTGGCAGCACGGGTAACAGTA
miR5526 16789 GAGTTGAGTCAGGTATGAG
miR8581 25 TAGAGAATTTTGTAGACAACTG
miR845a 61 CGGCTCTGATACCAATTGAT
miR5239 1139 TGGGAGAAAACATAGGTG
miR6025e 1670 TGCCATTATAGATCTGACATGC
miR4397-3p 37 TTCAAAGATGTGGAGAAAGACT
miR9752 1252 TGCTTCTTGCTGTTCCATGATTT
miR6485 650 TGGATGTACCAGAGCATCAA
miR869.1 8028 TGGGTTCAAGTCTGGTGATTG
miR6464 138 TTGATTGTTTGTTGGTATTATT
miR5387a 3592 TAACACGCCGGTCTACAGTATC
miR415 189 AAAGGAACAGAAGCAGAAGCAG
miR7807a-3p 368 TGAGGATTTGCATACATAC
miR7542 93 TGTTGCGTTCATAGATGGTA

miR1076-5p 32 TATCACAAGCGCGACTAATTGT
miR7814 106 TAATATGTCTTTGATGCTTTGA
miR2105 59 TTGTGATTTGTGAATGGTTGAT
miR414 126 TACCATCTTCTTCATATCGTCA
miR172k 1138 TGAACTGGATGCTGCTGCT
miR447a-3p 10180 TTGCGGACGAGAATTTTGTG
miR482d-3p 139 TTGGCCGACCCTACCTATGCCA
miR1535a 154 TTCTGTTTGTGCGTGATGTCTT
miR5261 383 TCATGTCAGATGCTTTGGTCT
miR2595 873 TACATTTCTACTTCTCTGTCT
miR8043 171 TGATCATACTATGGATTTGGCC
miR5658 310 TGATGATGATGATGTTGCAGAA
miR2082 108 TGTTGTTCCTTCTTCTTCTCT
miR535d 54 TGAACGACGATAGAGAGCAC
miR7495a 664 TTCTTGTAGTGCCTCCTTCA
miR6108d 75 TAAGGAGATTAAGCTGAAAAAGT
miR8741 1342 TGGCCTGAATGAATGATGATGG
miR1072 42 TATTGTTGTTATTTGAGCTTGG
miR9742 284 TGTATTGTTTGTTTCTGTGA
miR5751 60 TTGATTTGCAGATGCCTTTT
miR1428b 31 TATAAGAAATGCCATGAATTCT
miR5743a 124 TGAGAAGTTTTCCCACCATT
miR6462e 474 TTTTTGCCTTTTTGTCTACT
miR827 22 TTAGATGACCATCAACAAAT
miR5654a 25 ATAAATCCCAAGCATCATCC
miR7508 237 TAAAAAACAAGTCGGGAGG
miR6443 13985 GTAAGGTCAAGATGATGAG
miR863-5p 823 TTATGGCTTGTTGTATATCAT
miR6473 138 TCCACAATGCCAAAGAGTT
miR5144-5p 2073 TTCTTGTGCTGTGCAGTGA
miR7837 70 TGGGTGTAGGAGTGGTAGCTG
miR8148 682 TGATTGGGTCTGCATGCCGTGGT
miR894 87 TGTTTCACTCCGGTTCACAC
miR4244 396 TAGTTGTTCCTGGAGATGTG
miR3623-3p 74 TGGTGCTGTGCAAGAATTTGCT
miR5065 3371 TAGGCTTACTTATACATT
miR444b 542 TGCCGTTGCTGTTTCCAAGTT
miR855 26 TAGACAAGCTAAGGACAAAGGAA
miR5015 565 TTGGTGATGTAGTAGTCTAC
miR8571 624 TTCATCGTCAAGATCCTGTTCT
miR9667-5p 599 AAATTGGCAAACATGGTTG
miR1097 114 TGCCATTGTTTGTTGTTGGAAG
miR857 1867 TTTGAATGAGTGTATGGTGGTAT
miR827-3p 424 TAATATGATCCATCCAACAAACG
miR4348c 464 TGTTAAACCTTGGAAGAGACCA

miR157a-5p 27 TTGACAGAAGATAGAGAGCA
miR8773 38 TTGGATGCAATCGGTGGTTTAT
miR4221 66 TAAGAGGTTCAAAAGGTAGGAAA
miR8602 1063 TCAGGAGAGTATGCTGGCCA
miR902a-5p 336 TACTGCATGCACGCTTCTTCA
miR6270 2366 TTCGCTATTGGAATCTTCATCT
miR3510 539 TTAACCATCTTTCGGGACTGAA
miR1515 207 TATTTTAGCATGACAATGATCC
miR913-5p 422 TGACATCTTGCGAGACCGAGG
miR6116-3p 1325 TCATTTGTCATCCAAGCATTAG
miR7709-3p 65 TGACCTAGTCATATTTGAT
miR163 854 TTGAAGAGCAGCATGGAATTCGT
miR6030 158 TTCAACCATACCAAAGACCC
miR2100-5p 169 TGCTCTCAAGTCTGCCAAACGA
miR8183 52 TTGTAGTTGACGGACAATTGGC
miR9653a-3p 2508 TTGGGACTTTGGGCTGGCC
miR478a 21 TGGGTCGTCTATTTTTAGGAGA
miR3440 843 TGGATTCGATCAAGGGAAAGC
miR9560b-3p 66 TCACTCTTAGTTCTACTCGTGCT
miR7800 4712 TATTTTTGTCTCGTCTGGTC
miR157a-3p 25 GCTCTCTAGTCTTCTGTCAT
miR847-5p 754 TATTGATGAGGAGGATGGA
miR8175 64 CGATCCCCGGCAACGGCGCC
miR5021 123 TGACGATAGAAGAAGGAAAA
miR5499 140 TACAGGAAGATCGTTTGGAA
miR1108 464 TAAGCTGGAAACATAACGCCC
miR5557-3p 1610 TGCTTGCATTATCTTGTTGA
miR8016 2557 TTTTTGAATGAAGGGCCTGTGG
miR9672-5p 228 CCTAAGGCAGTCAGTGGTGTC
miR8775 143 TTGCAGATTGAACCGATGG
miR5648-5p 6143 TTTAAAAATTTGGCCTTGACT
miR3946 26 TGTAGAGAAAAGAGAGAGCATG
miR2868 301 TTGATTTTGTTGTAGTAGCA
miR5246 3951 TTGAGACAGCTTTGACTT
miR5532 108 TGGAATATATCACACAAGTTGG
miR9554-3p 244 TCATAGCTCAAGTCATCATTCAT
miR5554a-5p 515 TGTTGCAGATCTTGAACAATGTT
miR8011a-5p 91 TTGGGTTGAGCTTTCTTTTTTTC
miR5281b 560 TTTTAATAAAAGGAACGGAG
miR1857-3p 1199 TCATGGCTCCAACAAAACAAG
miR7753-3p 659 TTGAGCAATGGAGAAGAAGG
miR8762d 2137 TTGTAAACTTTGATCGATGTCT
miR2633 403 TCGACATTTGCACCAGATTCT
miR822-5p 324 TGCGAAAGCATTTGCCAAG
miR5786 27 TTCGCAGGATAGAGGTCAAGCT

miR1047-3p 141 TGATCAGCATACTTCAGTTG
miR530 298 TGATTATGCACCTGGCTCTC
miR4240 722 TCGACTGACTCGTAACATTTC
miR8658 2180 TTGACAAAGTACAGAGACCAT
miR5208d 161 TTTAGTCATAGTTTGTAGGCAA
miR8632 848 AGAGCTCAAAGTTGGAACC
miR7734-3p 65 TGGAACCAGTCCACATTCAACG
miR5641 31 TGGAGGAAGATAGTAAAGAATTA
miR5534a 2639 TACGTAGCATCAGCTAGAATGG
miR8610.2 206 TTCTTGAACAAATTGAAGGGC
miR1850.1 70 TTGAAAGTTTGGGAGTTGGG
miR9722 131 TAAAGATGGGAAGATGAAGAA
miR170-3p 454 TATTGGCCGTGTAATATT
miR9662a-3p 556 TTAACATCCACAGAGCCAAG
miR1169-3p 365 TGTGGATGTTGCTTTCTGTGACC
miR7711-5p.1 5545 TACTTAGCTGTTTGACTTCTTG
miR159g-3p 221 TTTGGAGTGAAGCGATTTGA
miR6148b 6040 TCTGTTATGTTTGTTCCA
miR5038a 801 TGGGAATCTTGGCCTTTTCCA
miR7716-3p 117 TTCTTCATCCTCCAGTAATGCC
miR840-3p 120 TGGTTTATGTCCTTAGTTTC
miR5244 158 TTCTCTTGAAGTAGTTGTTGGT
miR5369 107 TCAAGAAAAGGAGGATGTCCAT
miR823-5p 44 TTTGTATTGATCACTGACCATT
miR842 273 TATGGTCCCAGATCCGTCAACC
miR1044-3p 94 TGTGGTGACATATTTGTTTG
miR6155 173 TGAGTTGCCTGTGCCTTGCA
miR3631a-3p 3482 TAATTGGATTATGTCTTAACAA
miR2612 967 TGATAGTGGCAGTAGTATCAG
miR2602a 3971 TTCCAGTGATTGCCAGCAT
miR4414b 143 TGTGAATATGCGAGAGCCAAG
miR6459a-3p 1094 TTGATTTGGGCTTGAATTGA
miR5758 762 TAAGTGAAGAATGTCCTTG
miR9486a 56 TCTTTCAAGAGTTAGAGGTTC
miR5568e-3p 183 TATCTAGAAATGGTCAAAACG
miR5020c 99 TGGCATGCAAGAGGTCGAC
miR5566 268 TACATCACCTCCCTGTCTGT
miR6451 179 ATGTAGATCATGTTGATT
miR2927 625925 TGTCTGTCGTGATCAGCCCATG
miR2091-5p 274 TCTACGAGCCAGCAGGAGGAGG
miR8126-3p 100 TTCAGTATATTTGTCATCAGAG
miR867 1458 TTGAACAGGATTATTAGAGAG
miR5037c 777 AGTTGAACTTTGGGCCCG
miR8137 249 TATCGCAACCAGTTCACAAAAT
miR2275b-3p 342 TTCAAGTTTCCATCGTAAATCTA

miR5298d 51 TAGAGATGAAGAAGCTGAAAGAA
miR7492n 2574 TAAGATCAGGGCTTTGAGCTGG
miR9759 12 TAGTAAGCAAGTACGAGTTTAT
miR6147 20 TGACATGCATCAAAACCCCACTG
miR5169a 78 TTTGTCCGATGTGTTGTAGAACA
miR8048-3p 39 TAGATGGAATGCATAATGGACA
miR2654 300 TATTCAGGGACCGAAGTGTGC
miR1885b 13 TACATCTTCTCCGCGGAAGCT
miR476 1845 TGGATCCTTCTTTCAAGGTC
miR396a-3p 15 GTTCAATAAAGCTGTGGGAA
miR3434-3p 4324 TCAGGATCAGCCAAGTTGA
miR5228 574 TAGTGGTGATAGCAACTTGATGGA
miR2651 1721 TGTTGATTGATATGCTGATT
miR3449-5p 782 TCCAATCGCTTTCGTCTGT
miR8135 44 AGGATTTATGCTTGGTGAT
miR824-5p 400 TAGACCATTCTTGAGAAGA
miR6156 2303 TTGAAGAAGTTCTTTGTTCGT
miR9476-5p 123 TCTAGTCTTGCCTACTTTTTTTT
miR1048-3p 4501 TGTCTACGCTTATGTTCCTAG
miR6476a 185 TCGGTAGGTGATGAACATGA
miR1214 219 TACTTAGAGTCTCGCGGCAC
miR1917 1307 ATTGATCAAGGTCTAAAGT
miR1849 4999 TAGCGTATCATAGGTTGGT
miR2678 515 TGAATTGTTGGGATTGTACTT
miR7729a-5p 491 TGTTTTCAAGTAGGACAGTGAGC
miR1512a-3p 580 TGCTTTAAGAATGTCAGTTTTG
miR1115-5p 50 TGAGGTACTGGCCTTTGGTGG
miR3711 61 TAGCCAAGAAGGAGGTGCCT
miR528-5p 31 TGGAATGGGGATGAGAGGAGG
miR1052 14 TTCCTATTTTATTGACTGTGTTA
miR858b 619 TTCGTTCTGTTCGTCGCTTG
miR5243 185 TGGGCCAGAATCGTGGGC
miR9492 665 TACTTCTCTGTCTTGGTAT
miR4387a 29 AACAAGCCGGTGTGGGTGACACT
miR5818 169 TCCAACAGAAGGGCCACTT
miR7543 37 TTCATATACATGTTTGACAA
miR4385 13 TAATGTTGTAAAAAGGATTGGT
miR5022 284 TAATGGGGTATGTCGAAG
miR9677-5p 429 TTTCCTCTCCAACAGCCAAG
miR8603 143 TGAGCTTGTGGTGAGCATTGT
miR6029 35 TGTGGTTGATGATGTTCAGGCT
miR1320-5p 15 TGGAAGGAGTGTAAGTTTATAG
miR8578.2 325 TATGAGTGTATTAGCAATG
miR6477 1266 TGAACAGTAGAGGATGAT
miR5639-5p 163 TTGTTCCACGTGTCTAAGGC

miR6267a 30 TGAGAGAGGTGGTAGAGTGTG
miR1522 91 TTTATTGTTAAGCATGAAAG
miR5227 165 TGAAGAAGAAGAAGTTGAGGAT
miR5645a 454 TTTGCAGTCACGGCGTTAAG
miR9553-3p 45 TAATCATCCCAGCTATGAACAT
miR6253 89 GAGGAAAGTCGGGGAGGTGGGT
miR1428e-3p 80 TAAGAAATGCCATGAACTCTTT
miR8048-5p 1124 CATCATTACATTCCATCTG
miR1211-3p 1626 TCACACCGTCTCTTCATGC
miR5287a 647 TTGCAATATTATGACGGAGGAT
miR9663-5p 8859 AACGTAGTCGACCGATTCT
miR6274b-3p 785 TTGTGGTAGACCGAAAATAG
miR915 42 TGGCAATAAGGACAACGTCGTC
miR2108b 920 TTAATGTATGTGTTTGTAGT
miR5260 1135 TGTTGTATTGATGACTGGCTAT
miR5256 81 TAATGGCTTGTAAGAATTAA
miR5726 136 TAAAGAGTTGCTTGAATCAGG
miR9738 174 TGAAACATATTGGGCTCGTC
miR856-3p 1583 TGAATGTGATTGTGGTTAAA
miR5042-3p 4880 TGGGCTTGAGTACAAGAAGG
miR5080 423 AAAAGGATCATGACGGTACATG
miR950 11 TCTGGTCAGCTGGTTTGAT
miR2656a 4528 TAAGTTGCATATTGGACGTTGG
miR3441-5p.2 11 TCAAGAAAATGAAGTCGCTTTT
miR2622 3447 TTTGAGGCCATCGTGAAACATTA
miR1148.2 25788 TGGAGATCCGCCTGTGCCGGG
miR6264 218 ATTCTTGGACCGTGTCAA
miR8743a 42 TATCGCAAAAGTATAAAATGGTC
miR5809 4005 TCGGCTGGCGACCAAAGC
miR5059 58 CGGCCTGGGCAGTACTACCAAA
miR1514a 50 TATCAATTTGAAACTAGGCATG
miR1172.1 7037 AGGATTTGCAGCAGCAAAGGAG
miR5081 251 TGAATTTGTAAAATTGATACT
miR5570 30 TAAAAAACAAATTAGCATGTGCA
CDC-8219 miRNA
miR398 40402 TGTGTTCTCAGGTCACCCCT
miR167d 17966 TGAAGCTGCCAGCATGATCT
miR166h-3p 12554 TCGGACCAGGCTTCATTCCC
miR168 13383 TCGCTTGGTGCAGGTCGGGA
miR408-5p 10814 ACAGGGAACAAGCAGAGCAT
miR159a 7576 TTTGGATTGAAGGGAGCTCT
miR171a-3p 5290 TGATTGAGCCGCGCCAATAT
miR160b 2733 TGCCTGGCTCCCTGTATGCC
miR2867-3p 12873 TCAGGACTTTGGGATGGCCA
miR6478 14384 CCGGCCTTAGCTCAGTTGGTAG

miR1216 23238 TGATGGGTTAGCGCTTGTAC
miR408-3p 2025 ATGCACTGCCTCTTCCCTGG
miR164b 1616 TGGAGAAGCAGGGCACGTGC
miR171b 1283 TGATTGAGCCGCGTCAATAT
miR6202 3214 TGAGGATTTAAGCTTTGAAG
miR1167 21590 TGGGCTGTGGATGACTTGAAAC
miR9552b-3p 11157 TGGCGAGAGACCGATAGC
miR168a-3p 1821 CCCGCCTTGCATCAACTGAA
miR7541 1507 TGCAATGTCTCTTTTGCTGGCAC
miR397a 1056 TCATTGAGTGCAGCGTTGATG
miR403-3p 893 TTAGATTCACGCACAAACTC
miR158-5p 1167 CTTTGCCTATCGTTTGGAAAA
miR1870-5p 2076 TTGCTGAATGGACCAGTGGCAT
miR165a-3p 736 TCGGACCAGGCTTCATCCCC
miR396b 690 TTCCACAGCTTTCTTGAACT
miR162-3p 622 TCGATAAACCTCTGCATCCA
miR5163b-3p 10861 TAGATACTTTGGTTGTGGGA
miR5506 7191 TAGATCGCTTCGTTGAGG
miR8141 1860 TGCATACTAGTTGGTGGTGT
miR419 3817 TGCGTGAAGCTGAAGATGTTG
miR8179 3178 TGATCTGCATTCACTTGATCGT
miR951 661 TGCTCTTGACGATCTGGCTACG
miR8044-5p 698 TTGTCAAAATGGTTGGAGATTT
miR398b-3p 39848 ATGTGTTCTCAGGTCACCCCT
miR847-3p 1575 TGACTCCTCTTCTTCTGGTCG
miR4413b 652 TAAGAGCAAGTATAAGTCACCT
miR161.1 1043 TGAAAGTGGCTCATCAAGGGGT
miR3703 4410 TAGATTCATCAGCCATTGCC
miR167a-3p 451 GATCATGTTCGCAGTTTCAC
miR1513a-5p 4045 TGAGAGAGATAGCATGACTAC
miR2111 643 TAAACTGTGTTTGGGTTTGG
miR6214 9325 TGACGACGAGAGCCACGATCA
miR157a-5p 509 TTGACAGAAGATAGAGAGCA
miR5185l-3p 36102 TTGGACGGTTGACTTAGAAC
miR158-3p 398 TTTCCAAATGTAGACAAAGC
miR5654a 252 ATAAATCCCAAGCATCATCC
miR9674b-3p 1737 TGAATATGCCAGAGCATCAG
miR845a 287 CGGCTCTGATACCAATTGAT
miR5177 408 TGAGGTTGGAAAACGAGCAG
miR1068 824 TAGCCAGTTTTGCTTGCAGGTC
miR2609a 324 TGGAAGTAATGGCTCGTCACTT
miR156c 275 TTGACAGAAGAGACCGAGCA
miR826b 783 TGGTCTTGACATCGATGAAAAT
miR3435-5p 230 TGCATCAACTCTGTCAATTTCC
miR1146 4822 TTGGGTCCGACGGGAAGTCTT

miR4401b 6013 TCAAAGACGGTTGTGAGCAA
miR5821 1343 TGGACGGAGACGATGGTAGAG
miR894 425 GTTTCACGTCGGGTTCACC
miR9409 954 TTTTGTCATAGTTCATTTTC
miR6221-5p 690 TCTGTCTTCTGGCCCGCTGTCT
miR3476-5p 1781 TGAACTGGGTGCTGCTGCTGC
miR7803b-3p 8932 TGACACGTCGTCAATCATGTACT
miR8123-3p 3600 TTGTCGCCATTGGTCCAAGTC
miR399f 2630 TGCCAACGCGATTTGTCCTG
miR9558-3p 2087 TTGCAAGGCCAGCTTTTCCTCT
miR774b-5p 415 TGAAGATGCATATTTGGAGTGAC
miR1885b 178 TACATCTTCTCCGCGGAAGCT
miR7536a 5802 TGAAGCATGCTCGAGACTG
miR391-5p 153 TTCGCAGGAGAGATAGCGCC
miR529a 1022 TTGTCGCTGCTCTCTTCTTC
miR2275a 773 TTTGGTTTCCTCCGGTTCTCAT
miR827 101 TTAGATGACCATCAACAAAT
miR172b-5p 240 GCAGCACCATTAAGATTCAC
miR157a-3p 190 GCTCTCTAGCCTTCTGTCAT
miR4374b 576 TCTTCAAAGACGGTTGTGAG
miR5679 1203 TTGGTGCAGAAGAAGTTAGA
miR166d-5p 237 GGAATGTTGTCTGGCTCGAG
miR5293 535 TGATGAAGAAGTGGCGCAGAAGA
miR169d 176 TTAGCAAGGATGAGATTACCGG
miR403-5p 100 TGTTTTGTGCGTGAATCTAAT
miR858a 162 TTCGTTGTCTGTTCGACCTT
miR6269 4731 TGCTGCAAGTGATTAGCCATGG
miR5252 1548 TTGTAAAGCTACTGAAGTCTGC
miR8175 546 CGATCCCCGGCAACGGCGCC
miR5562-3p 7003 ATGGGACGCAAGGCTGAAAC
miR156f-3p 158 TGCTCACGGCTCTTTCTGTCAG
miR163 1843 TTGAAGAGCAGCATGGAATTCGT
miR6117 3550 GGGGCTGATCCGGGTTGAAGAC
miR400 84 TATGAGAGTATTATAAGTCA
miR394a-3p 161 TGTTGTCATCTCTTTTGTAA
miR396a-3p 140 GTTCAATAAAGCTGTGGGAA
miR7823 39095 TTGCATGCTGATCTTAGAT
miR7761-3p 7399 TCTCGAATCAAGGACGCTCTGG
miR6255 690 TGGGAAAGAATGGACGATGAGT
miR5711 94 TGTTCTGTGGGTTTTTACCG
miR6030 63 TCCACCCATACCATACAGACC
miR1023e-3p 2150 TGGGAAACGGGTTGGAGTGCAT
miR6118-5p 187 TGGAATGGGTGCTTGGGAAA
miR8748 279 TGATGGTGATGATAATAATGAAT
miR1511-3p 90 AGCCTGGCTCTGATACCATGAAG

miR7763-5p 2699 AACTTCATCGTGCTGGGGATAG
miR6209 257 TGAGCATCAGAAGCTGTGCG
miR394a 80 TTGGCATTCTGTCCACCTC
miR5678 3106 TTCCATGCATAAGACTTTCC
miR164b-3p 756 GCGCCCAGCTTCTCCACC
miR6182 2384 TGGTCTGTGATGGATGCGTT
miR8554 172 TGCTTATGACTTTGATTTGGGA
miR9491 1144 TGCTATCTTCCTCTGACTCAG
miR7817a 11605 TTTGGTTGAACTTGTCTCGAGAA
miR5373 812 TCTCTTGTATGCTGATGATGTT
miR825-3p 948 TGGTCTCGAGAAGGTGCATTATC
miR416 1167 TGTTAGTCGTACCACTGTTCAG
miR390a-5p 48 AAGCTCAGGAGGGATAGCGC
miR6282 914 TTGATCATGTGGGGATGTATAA
miR395b 83 CTGAAGTGTTTGGGGGGACT
miR1857-5p 11899 TGGATTCTTTTGGAGATGCGG
miR857 178 TTTTGTATGTTGAAGGTGTG
miR869.1 6716 TGGGTTCAAGTCTGGTGATTG
miR5719 86 TTGTGATGATAATCCGACTT
miR3946 67 TGTAGAGAAAAGAGAGAGCATG
miR1098 364 TATGCGTTTTTGTCTGAAAT
miR2111-3p 330 GAAGTCAGGTGGGATTACC
miR5219 190 TCTGGACTCTCAGCTGCTGC
miR169f-3p 1898 TGATGTCTTCCTTTGGTTACT
miR1435a 56 TTTCTTAGGGTGCAAACTTTTC
miR775 3724 TTGCGATGTCTTGCAGTCAA
miR7539 150 TGAGAGAGAAGAGCTATGAGG
miR414 178 TACCATCTTCTTCATATCGTCA
miR8034 793 TATGACAAGCCTGCACAAAC
miR170-5p 43 TATTGGCCTGGTTCACTCAG
miR1535a 165 TTCTGTTTGTGCGTGATGTCTT
miR393a 64 TCCAAAGGGATCGCATTGATC
miR7719-5p 85 TCCTCCATAAGCATGTTTCTTC
miR159a-3p 7469 TTGGATTGAAGGGAGCTCT
miR8571 2341 TTCATCGTCAAGATCCTGTTCT
miR7725a-3p 116 TGCAAACAACTCATTTCGTCAA
miR2611 575 TATTTTCAGTGTTGTGGTAGAA
miR6025e 3089 TGCCATTATAGATCTGACATGC
miR7716-5p 192 TAGTTGATAAGTAAGAACGAGAC
miR9657a-3p 152 TGGTGCTTCCTGTCGTACAGGT
miR7502b 866 TTGTTAATAAGTTCATGCAT
miR6028 30 TGGAGAGTAAATGACATTCAG
miR5632-5p 100 TGTCATTCTTTATCCAACTG
miR319f 500 TTGGATGAAAGGGGCATGCTT
miR4227 1308 TCTGGTACCAATCTTTCA

miR1140 86 ACAGCCTAAACCAATCGGAG
miR447a-3p 18239 TTGCGGACGAGAATTTTGTG
miR7512 32 TACTACTTGTAGCTTATGCCAT
miR1520g 1217 CAACTCAGAACTGGTACGGACAA
miR7837 126 TGGGTGTAGGAGTGGTAGCTG
miR6270 3936 TTCGCTATTGGAATCTTCATCT
miR5512a 17250 TAGGATCATTGTCATGCTAAAAT
miR415 246 AAAGGAACAGAAGCAGAAGCAG
miR773a 1082 TTTGCTGCCACTTTTGTCACG
miR477 1867 TTGGAGTATCTTTGGAGGAGC
miR6114-5p 124 TCGAAAGCACCATGAACGTGA
miR6185 741 TCTGGCAGCACGGGTAACAGTA
miR482d-3p 150 TTGGCCGACCCTACCTATGCCA
miR6464 20325 TGTTTGCTTGTTGATGTTGAT
miR5387a 2357 TAACACGCCGGTCTACAGTATC
miR6147 43 TGACATGCATCAAAACCCCACT
miR390-3p 29 CGCTGTCCATCCTGAGTTTC
miR1152 870 TAAAAGGAGCGCGTCTTGAA
miR5246 4345 TTGAGACAGCTTTGACTT
miR5788 57 TGGATAGTTATCAGACTCTAGTA
miR9408-3p 20 TTTCATCTTAGAGAATGTTGT
miR902h-3p 794 TAGAAGGGTCTCCACACAAAC
miR6462e 235 TTTTTGCCTTTTTGTCTACT
miR2105 57 TTGTGATTTGTGAATGGTTGAT
miR475a-3p 691 TTAATGCCCATTGACTTCAG
miR6194 78 TAGTAGGGATCTTGACACACGAG
miR1160.1 327 TTCCCTCTTTTTAAGCATGGCC
miR902a-5p 482 TACTGCATGCACGCTTCTTCA
miR842 278 TATGGTCCCAGATCCGTCAACC
miR3712 2295 TGCTGATCAAGATCAAGACCA
miR6279 14466 TAGACAAGACTTCCAGCCAGACG
miR1120c-5p 261 TAATATAAAATGCTTTGTTGAC
miR6029 28 TGGGGTTGTGAATGTTCAGCTA
miR827-3p 318 TAATATGATCCATCCAACAAACG
miR1428e-3p 95 TAAGAAATGCCATGAACTCTTT
miR835-3p 429 TGAAGAGATACGCCAGGAG
miR8598 2152 TTTAGTGCACGATGCGGAGGTGA
miR854 150 TGATGAGGATATTGAGAAGGG
miR7800 839 TATTTTTGTCTCGTCTGGTC
miR7695-5p 92 TTGCCATTGAGCACGCCAGTGAA
miR1072 32 TATTGTTGTTATTTGAGCTTGG
miR9565-5p 638 TCCGTTCTGCTCGTTTCTCT
miR5565e 240 TATGGTCTAGATGTTGTCGGA
miR2863b 144 TGTTTATTTGGAGCTAGATGT
miR5207 68748 TTAAGGAGGTTTTGGATGGCGA

miR5813 517 AACAGCCTCTGGTCGATGGA
miR1446 177 TTCTGAAATCTCTGCGCATCAT
miR6248 305 TATTGAGGTGGAGGTTAGTG
miR5786 35 TTCGCAGGATAGAGGTCAAGCT
miR4348c 266 TGTTAAACCTTGGAAGAGACCA
miR7716-3p 57 TTCTTCATCCTCCAGTAATGCC
miR5658 121 TGATGATGATGATGTTGCAGAA
miR2082 102 TGTTGTTCCTTCTTCTTCTCTT
miR5632-3p 223 TGGATTATATAGTTGATGAG
miR2100-5p 135 TGCTCTCAAGTCTGCCAAACGA
miR2592bp-3p 154 TGGCCGAACTCCATGCATTGCGA
miR3623-3p 45 TGGTGCTGTGCAAGAATTTGCT
miR1061-3p 658 TGTGTTAGCATGGAATAATG
miR9742 172 TGTATTGTTTGTTTCTGTGAGTA
miR7807a-3p 503 TGAGGATTTGCATACATAC
miR8773 46 TTGGATGCAATCGGTGGTTTAT
miR4244 169 TAGTTGTTCCTGGAGATGTG
miR5648-5p 2487 TTTAAAAATTTGGCCTTGACT
miR9760 33 TGATGATGTAGTCTTGATTGGT
miR393b-3p 35 ATCATGCGATCTCATTGGAT
miR1169-3p 262 TGTGGATGTTGCTTTCTGTGACC
miR5015 432 TTGGTGATGTAGTAGTCTAC
miR5641 42 TGGAGGAAGATAGTAAAGAATTA
miR1428b 45 TAAGAAATGCCATGAACTCT
miR6266a 133 TATATGCAGGCGGCAAATAT
miR2595 535 TACATTTCTACTTCTCTGTCT
miR6116-3p 829 TCATTTGTCATCCAAGCATTAG
miR7767-5p 61 CCCCAAGATGAGTGCTCTCC
miR824-5p 81 TAGACCATTTGTGAGAAGGG
miR478a 15 TGGGTCGTCTATTTTTAGGAGA
miR5291a 323 TGTTTGTAGATGGATGGATGAT
miR6232a-3p 22 TGGATGCTAGCAAAAAAGAAAGT
miR847-5p 652 TATTGATGAGGAGGATGGA
miR9557-3p 14 GCTGAGTTGGAACGCAAAAT
miR1515 212 TATTTTAGCATGACAATGATCC
miR5239 819 TGGGAGAAAACATAGGTG
miR5499 237 TACAGGAAGATCGTTTGGAA
miR2644 151 TCTTCAGTTGATGGTTGTGT
miR824-3p 27 CCTTCTCATCGATGGTCTAG
miR8762d 13170 TTGTAAACTTTGATCGATGTCT
miR5038a 569 TGGGAATCTTGGCCTTTTCCA
miR1222e 78 TTAAAGGAAGCTTCAGTGGACA
miR5554a-5p 805 TGTTGCAGATCTTGAACAATGTT
miR5225a 134 TCTTGTTGAAGGTAAATGGTGC
miR8632 1027 AGAGCTCAAAGTTGGAACC

miR9752 528 TGCTTCTTGCTGTTCCATGATTT
miR1850.1 78 TTGAAAGTTTGGGAGTTGGGA
miR7814 58 TAATATGTCTTTGATGCTTTGA
miR9560b-3p 33 TCACTCTTAGTTCTACTCGTGCT
miR5176-5p 506 TATTCCATGTGTCACATCTCT
miR6155 192 TGAGTTGCCTGTGCCTTGCA
miR7532a 353 GAACAGCCTCTGGTCGATGG
miR5785 120 TAGTGTTGTCCGTGCGAATCAGA
miR5080 611 AAAAGGATCATGACGGTACATG
miR2612 1139 TGATAGTGGCAGTAGTATCAG
miR4385 13 TAATGTTGTAAAAAGGATTGGT
miR4412-5p 465 TGTTGCGGATATTCTTTGCACT
miR5534a 1679 TACGTAGCATCAGCTAGAATGG
miR4995 73 TAGGCATGGCTTGGTCAAGGA
miR5766 1270 TTAGGCAGAGGAATAGGCAAG
miR6169 606 TCGATATCGTCTTTTCTCTCT
miR1512a-3p 844 TGCTTTAAGAATGTCAGTTTTG
miR5261 199 TCATGTCAGATGCTTTGGTCT
miR8741 153 TGGCCTGAATGAATGATGATGG
miR9726 428 TACAGCGTTATTTTTTCTTC
miR6138 20 TACTTTGAGCATTGAAGAATAAA
miR5244 38 TTCTCTTGAAGTAGTTGTTGGT
miR823 68 TGTGGTGATCAAATGAAGAT
miR528-5p 42 TGGAATGGGGATGAGAGGAGG
miR5021 131 TGAGAAGATGAAGAAGATACAG
miR482e 298 TTGCCTATTCCTCTGCCTAA
miR8048-3p 24 TAGATGGAATGCATAATGGACA
miR1108 224 TAAGCTGGAAACATAACGCCC
miR4397-3p 12 TTCAAAGATGTGGAGAAAGACT
miR7122-5p 2363 TTATCAGAGAATACTACGCGTCG
miR833a-5p 42691 TGGCTTTGTTTGTACCCGGTCTGT
miR9761 4000 TGAAGGATCAGGATATGCTTGT
miR5208d 217 TTTAGTCATAGTTTGTAGGCAA
miR5228 379 TAGTGGTGATAGCAACTTGATGGA
miR5144-5p 1517 TTCTATCTGCTGCTGATGAGC
miR2602a 2981 TTCCAGTGATTGCCAGCAT
miR3440b-3p 1127 TGGATTCGATCAAGGGAAAGCCT
miR3695 379 TCGTCGTCTTGGTCGTTTGGG
miR5072 644 CGTTCCCCAGCAGAGTCGCC
miR7699-3p 25 TGTAAATACCATCATTAAATGC
miR8603 165 TGAGCTTGTGGTGAGCATTGT
miR6477 976 TGAACAGTAGAGGATGAT
miR6108d 33 TAAGGAGATTAAGCTGAAAAAGT
miR6274b-3p 673 TTGTGGTAGACCGAAAATAG
miR6148a 158 TGCGGTCGATCGCTTGTTCTT

miR5498 63 TGAGCGCAACCTTGAAGAA
miR530 276 TGATTATGCACCTGGCTCTC
miR160b-3p 12 CGTACAGAGTAGTCAAGCAT
miR3510 541 TTAACCATCTTTCGGGACTGAA
miR3711 73 TAGCCAAGAAGGAGGTGCCT
miR838-3p 389 TTTATACATCTTTTCTTGCACA
miR5369 54 TCAAGAAAAGGAGGATGTCCAT
miR2199 83 TGATAACCAGAACGGATCGAC
miR2654 144 TATTCAGGGACCGAAGTGTGCT
miR9557-5p 9 TTTTGCGTTTCAACTCGGTC
miR8011a-5p 10 TGTGTGATGGTTTTCATTGTTTC
miR5232 1501 TGCATGTCCTCTACCTGAA
miR2905 51 TACTGTAGCCGACAAAGGCA
miR7734-3p 59 TGGAACCAGTCCACATTCAACG
miR5650 291 TCGTTTTGGCCTTAGATACA
miR535a 418 TGGCAACGAAGAAGAGAGCAGC
miR6144 438 TGCGCCACTTCTTCATCATC
miR7729a-3p 97 TAGCAATGGGGTTGTTGGAGGA
miR8602 420 TCAGGAGAGTATGCTGGC
miR2873b 224 TTGGATTGGATTTGGTTATT
miR1214 287 TACTTAGAGTCTCGCGGCAC
miR7495a 718 TTCTTGTAGTGCCTCCTTCA
miR472-3p 18 TTTTGCCTACTCCTCCCATAC
miR7120a 25 TGTTTGTTGTCATGATTGTCAT
miR9484 2970 TAGGCAAGGGAAGTCGGC
miR1028a-3p 733 TGACATTGTAGATGTAGTT
miR5054 307 GTTCCCCACAGTCGGCGCC
miR863-5p 858 TTATGGCTTGTTGTATATCAT
miR9759 12 TAGTAAGCAAGTACGAGTTTAT
miR1043-3p 979 TTGCGCGTTGAGATTTGAAGGA
miR917 184 TCGTTCACTGCTATGTTCGAAG
miR3627-3p 140 TGCTCGCTGTCCTCGTGACAAG
miR5050 481 TGAGGTCGATTCACCAGCAT
miR9411 107 TAGGACGACTTACACGTAA
miR6284 108 TATGACCAGGATGAAGCTT
miR1059 14 TGAAAGCTATCTTCACAACAAC
miR1090 17 TGAAAGCCATACTACAATCAAAC
miR7542 222 TAGCTTCAGCTTAAGATGGTG
miR844-3p 39 CCATCTTACTCGTCTGTTCATT
miR8183 48 TTGTAGTTGACGGACAATTGGC
miR5544 256 AAAACACGCATAGGAAGTTGGT
miR1122 167 TGATATCATCGGCATTTGGA
miR1093 566 TGGATGTTCTTGCCAACGA
miR6300 5277 GGTGGCTGTAGTATAGTGG
miR5059 20 CGATCCTGGGCAGCACCACC

miR8126-3p 36 TTCAGTATATTTGTCATCAGAG
miR444b 222 TGCCGTTGCTGTTTCCAAGTT
miR7711-5p.1 5991 TACTTAGCTGTTTGACTTCTTG
miR1147.2 48 GCTCGGCAAGTCTGGAAAGA
miR1849 4514 TAGCGTATCATAGGTTGGT
miR2678 576 TGAATTGTTGGGATTGTACTT
miR2868 37 TTGGTTTCTGTGTGTAAAAT
miR6422 487 TGTAGATAATGAATGGATAAGGT
miR2108a 22 TTAATTGTTCTTTTTGTCGGG
miR172c-3p 977 AGATATCTTTGACAATGCTGC
miR3707 21 TTGATCTGCCATTGATTCTTGAA
miR1130 13 TCTGTAAGTTATATCAAGAG
miR912 26 TGGATTGATCACCATCCCTGGC
miR8658 1510 TTGACAAAGTACAGAGACCAT
miR1051-3p 464 TTCCCGTACTTGAACCCAC
miR6473 61 TCCACAATGCCAAAGAGTT
miR2645 52 TTTCTTGAGATGAGCATTTT
miR5144-3p 203 TACTCACTGCACAGCACAAGAAAG
miR5557-3p 1395 TGCTTGCATTATCTTGTTGA
miR6156 2541 TTGAAGAAGTTCTTTGTTCGT
miR5798 13 TCTGGACTCAAGAACCCTGGAT
miR6476a 117 TCGGTAGGTGATGAACATGA
miR6031 51 AAGAGGTTCGGAGCGGTTTGAAG
miR8138 147 TAAAGAGGCAACAAAGGAAG
miR4221 22 TAAGAGGTTCAAAAGGTAGGAAA
miR165a-5p 16 GGAATGTTGTCTGGATCGAG
miR9722 72 TAAAGATGGGAAGATGAAGAA
miR1057 292 TTCTAGGTTTAGCTGGGTGGT
miR9653a-3p 1645 TTGGGACTTTGGGCTGGCC
miR5769 134 TGGGGAACTGGAAGACGCGA
miR2091-5p 127 TCTACGAGCCAGCAGGAGGAGG
miR5227 51 TGAAGAAGAAGAAGTTGAGGAT
miR400-3p 9 GACTTATAATGATCTCATGA
miR8581 9 TAGAGAATTTTGTAGACAACTG
miR9553-3p 27 TAATCATCCCAGCTATGAACAT
miR5774a 61 CTGGCGTCGAACTCGTGAGCAT
miR2916 622 TAGGGGATCGAAGATGATCAGAT

Supplemental Table 2: FGSC-9543 Novel miRNA
>novel_mir_128 14333
TGAGCACCGCACACTTCAGAGG
>novel_mir_39 4333
TCTTCGGTAGTATAGTGGTGAG
>novel_mir_33 3401
GGAGGGATGGCCGAGTTGGT
>novel_mir_79 1617
TGACAATATGGGAGAACTGGCT
>novel_mir_101 2370
TACCACAATGTCGAGACCATAAG
>novel_mir_22 1144
TAGCGCCTATTTTCTTTCCTCA
>novel_mir_53 1111
TGCATCGGCTACGTTCTTTTCG
>novel_mir_31 970
TGTTCTGGGTTCATGCATAAAG
>novel_mir_119 983
GGTCTTGTGGTGTAATGGTTAAA
>novel_mir_112 597
TAGTGCTCAGGCCAAAAAGGGA
>novel_mir_37 606
TGGGAACTCGGGCTTTGGTAGG
>novel_mir_71 391
TAAAAGAGGACAAGAGAGGGTG
>novel_mir_70 382
TGGTGGGTTGAAAAGGCTTTGG
>novel_mir_41 231
TACTTGATAATTGACTGATGTT
>novel_mir_24 259
TGCGGGTAGCTCTACAGTGGCC
>novel_mir_116 102
TTGTCCGACGGTCTCAGGTAATT
>novel_mir_108 159
TATTGCCCGGGTCAGATCAA
>novel_mir_58 43
TCTACTTTACAAGGTCTGGTGG
>novel_mir_80 79
TGTAGCTTTTCCTTTGCCTTTG
>novel_mir_78 99
TAGCGTAAAAGATCGAAAGA
>novel_mir_103 71
TGTATCGAAGAGGAGGTCGG
>novel_mir_64 60
TACAAGCAGTCTTAGCAGAAAAG

>novel_mir_113 41
TTAGGGTGGTGTTATAAGTCAA
>novel_mir_16 54
TAGGCACGGTAGTATACCAGTAC
>novel_mir_127 18
TGCTTGATAAGTAGTGACCACC
>novel_mir_102 46
TAATACGTTGTATCTTAGCAGG
>novel_mir_117 27
TAATGGCAGGGATGATAGAAG
>novel_mir_43 17
TCTTTTCGTTTCTATTTGCTTT
>novel_mir_97 14
TGCCAAAGTTTGCTGCATTGCG
>novel_mir_121 15
TGGAGCGGAGAAATTAATGTAT
>novel_mir_42 20
TTGGTTGCTCTTTGTAAGTC
>novel_mir_18 11
TGGAGCCAACTGAGATTGAT
>novel_mir_95 18
TGATGAAGGATAGTGGCATGAT
>novel_mir_13 14
TACGTTCAGGTAAGAGAGGA
>novel_mir_19 18
TGGTCTGTAGGATCGAGTACTA
>novel_mir_28 11
TAGTGATTGCCTTGTAAGTTGT
>novel_mir_124 8
TGCTCTTGTTGCTGTTGTTG
>novel_mir_93 9
TATACAGTGCCTCACAAAACCCT
>novel_mir_69 13
TGATCCTAAACATCGATGAAAGT
>novel_mir_90 15
TAGTAGCCGCTCTATTTAAGG
>novel_mir_88 13
TATCACGCGCTCATTGACAAAGT
>novel_mir_52 7
TGGGGAAGGAGAAGGGGAGGA
>novel_mir_15 10
TCTCCTTTTTCTTTTTCTTTTC
>novel_mir_67 9
TACAGCGGCTTCTTTCAGACTT
>novel_mir_122 9

TGCATCTGCTGCTGCTGTTG
>novel_mir_27 9
TTGCCGAATTCGGATGAGATTG
>novel_mir_5 7
TACCACTGTTCAGAAGCATCGCT
>novel_mir_114 5
TCGTCTGACAGTTGGATTGCTC
>novel_mir_104 6
CAGGGGGGGAAAGGGGGGAA
>novel_mir_208 362492
TCTCCATGTGGTCATCCGCCAT
>novel_mir_141 5715
TAGACCGAGGCAAGGTGTTGGC
>novel_mir_160 751
TTTCAAGATCCATTCTATC
>novel_mir_171 345
TCATTTGGAAAAAAGAGGCG
>novel_mir_199 309
TATTGGTGCGCATGTTTCGATG
>novel_mir_155 239
TCAAGGGCCAGACATCTATCGAA
>novel_mir_152 222
TGAGGAAGTCTAAGGCTGCA
>novel_mir_166 158
TTCCAAAGTTTCTTCTTGATCAG
>novel_mir_140 116
TATGCTTGCTAAATTCACGATG
>novel_mir_192 89
TATGGTATTGCTTGAGGTTATTC
>novel_mir_168 76
TAGTGACCTTTCATGTGCGG
>novel_mir_183 76
TACTACAGAACTAGGCCATTGT
>novel_mir_151 69
ATGCCGTCAAAACTGGTCGCATG
>novel_mir_144 59
TTACGGTCAACAGTGTCATAAG
>novel_mir_175 58
TAATAGAAGACTGTAGGAGA
>novel_mir_200 50
TATGACAACCGTGCTGCCAACG
>novel_mir_203 48
TGATGCTACGTATGCACTTTTGT
>novel_mir_153 47
TGTTGCGTTTCACTTTACTTTTG

>novel_mir_159 39
TTAAACGGTTTGTGGCAGAGAC
>novel_mir_193 36
TGTATGATTTGAAGTCAAGT
>novel_mir_150 28
TTTGACGGCCACTGTATATTAG
>novel_mir_142 28
TTCTGCGGTTTATGCTTTCTCA
>novel_mir_167 23
TAATTGTAAGGCCATATTCTAG
>novel_mir_154 22
TAGGTTCATCTGTTGTAGAGAT
>novel_mir_164 21
TCCAACTGTCAGACGAGCTTTAC
>novel_mir_178 20
TAATGATATAAAGAAGAGCAAG
>novel_mir_188 19
TACGCCATTTCCTATAGTAG
>novel_mir_185 19
TTAAAGCTCAATAAAAAAATCCC
>novel_mir_205 17
TTGAGCTGCAACTTCAATGGAT
>novel_mir_176 15
TTTCCGGAAATAGATTGCACAA
>novel_mir_202 13
TAGGCTTCGCCGGGCGAAGGAA
>novel_mir_179 13
TTCATACGTTAGACTAGCTGTT
>novel_mir_129 13
TTCGAGCCACGCTTTATTTTCA
>novel_mir_195 12
TAGACATCATTCGCTCAAAGTG
>novel_mir_161 12
TATCGTTCCTGTTGTATCCACA
>novel_mir_207 12
TACTGTGACGCAGTTGGTGTAA
>novel_mir_206 12
TCGTTGTTATACTCTAGATCAA
>novel_mir_177 12
TTTAATAAGGAAAATACGACATA
>novel_mir_148 12
TTTTTTTTTGTGTGTGTGTG
>novel_mir_157 11
TTATGTGCTAATGAAAGGTGT
>novel_mir_131 11

TCATAACGTTGGCCTTTGTTCAT
>novel_mir_209 11
AGAATCTGGGCCTAAAGGTC
>novel_mir_173 11
TGATCTTGATCGTCCAAATCAAC
>novel_mir_135 10
TGGTAGATGAAGAATGTTTTTC
>novel_mir_143 10
TTTAAGTGAACTCATCCGACAG
>novel_mir_156 9
TAAAAACAGTAGGAAGTCAATAG
>novel_mir_137 9
TTGTGAAGGACTGGTCAGGGT
>novel_mir_197 9
TTGTCCTCTGGTCTAAATAGTAA
>novel_mir_186 8
TAAAAAAAAAATAAAGGGATCAG
>novel_mir_172 8
TTTCCAGTATCAGGATGTCT
>novel_mir_165 8
TGGTGTAGATGTACGTGGTACA
>novel_mir_182 8
TTAAGGAGAGGTACGACTGGGTG
>novel_mir_190 8
TGATTTGACATGTATAAGGAA
>novel_mir_184 8
TTTGACTTTTCTTTAGGGTG
>novel_mir_149 7
TCGATGCTTTGCTGGTTGCT
>novel_mir_133 7
TAAACTGATAGAGCTATACTGG
>novel_mir_163 7
TTGAAAACGGGAGACAGCATGG
>novel_mir_180 7
CTACAAGGCTGGTTAAATTAT
>novel_mir_198 7
TGGGAGAGGGGAGGAGGGGG
>novel_mir_130 7
TTATTTGACTGGCCAATGACAA
>novel_mir_196 7
TTGATAGACCCTTAGCAACAAGA
>novel_mir_194 7
TTGTGAGGCACTGTATTGTAGAT
>novel_mir_139 7
TATATTAACGATTGGCCACCTAA

>novel_mir_191 7
TGGGTGCTTGGGAGAGCAAGGT
>novel_mir_146 6
TGACACAATAGGCCTCGATCAA
>novel_mir_136 6
TTGATATGTGCTATGTTGGTCA
>novel_mir_169 6
TGCTTAAACGGCTGTCAGAGGA
>novel_mir_158 6
TACACAGGATTTAGTGAACCAG
>novel_mir_145 6
TTCGTGCGCTGTCCCAGGAAAT
>novel_mir_132 6
TGGATAGAATGGGACTTTGTAAG
>novel_mir_170 6
TGACAGTACAAGCTAGGAACAG
>novel_mir_174 5
TGAAGTGGACTTGATGTTGAAAA
>novel_mir_204 5
TAGGCTTCACTGGGCGAAGGAA
>novel_mir_189 5
TCGGATAGCTGAGAAACAGTGG
>novel_mir_201 5
TAACCGATAAGCTTAGTGAAGAG
>novel_mir_138 5
TACCACATTTTCAGCCTTCGC
>novel_mir_181 5
TGGCGCTTTTCTTTTGCAATT
>novel_mir_147 5
TGAGCTAGATCCAAGGTAGGAC
>novel_mir_162 5
TAAACATAGTTGGGGAAAACAC
>novel_mir_187 5
TGACGTAGTTTGGAGGCTGAGG
>novel_mir_134 5
TAGAGCACTTGGTTTAATTGAC
CDC−8219 novel miRNA
>novel_mir_128 10596
TGAGCACCGCACACTTCAGAGG
>novel_mir_39 5349
TCTTCGGTAGTATAGTGGTGAG
>novel_mir_33 1687
GGAGGGATGGCCGAGTTGGT
>novel_mir_79 2039
TGACAATATGGGAGAACTGGCT

>novel_mir_101 1264
TACCACAATGTCGAGACCATAAG
>novel_mir_22 1227
TAGCGCCTATTTTCTTTCCTCA
>novel_mir_53 748
TGCATCGGCTACGTTCTTTTCG
>novel_mir_31 517
TGTTCTGGGTTCATGCATAAAG
>novel_mir_119 276
GGTCTTGTGGTGTAATGGTTAAA
>novel_mir_112 591
TAGTGCTCAGGCCAAAAAGGGA
>novel_mir_37 387
TGGGAACTCGGGCTTTGGTAGG
>novel_mir_71 471
TAAAAGAGGACAAGAGAGGGTG
>novel_mir_70 317
TGGTGGGTTGAAAAGGCTTTGG
>novel_mir_41 290
TACTTGATAATTGACTGATGTT
>novel_mir_24 148
TGCGGGTAGCTCTACAGTGGCC
>novel_mir_116 104
TTGTCCGACGGTCTCAGGTAATT
>novel_mir_108 32
TATTGCCCGGGTCAGATCAA
>novel_mir_58 116
TCTACTTTACAAGGTCTGGTGG
>novel_mir_80 65
TGTAGCTTTTCCTTTGCCTTTG
>novel_mir_78 35
TAGCGTAAAAGATCGAAAGA
>novel_mir_103 49
TGTATCGAAGAGGAGGTCGG
>novel_mir_64 48
TACAAGCAGTCTTAGCAGAAAAG
>novel_mir_113 63
TTAGGGTGGTGTTATAAGTCAA
>novel_mir_16 40
TAGGCACGGTAGTATACCAGTAC
>novel_mir_127 62
TGCTTGATAAGTAGTGACCACC
>novel_mir_102 24
TAATACGTTGTATCTTAGCAGG
>novel_mir_117 21

TAATGGCAGGGATGATAGAAG
>novel_mir_43 29
TCTTTTCGTTTCTATTTGCTTT
>novel_mir_97 27
TGCCAAAGTTTGCTGCATTGCG
>novel_mir_121 20
TGGAGCGGAGAAATTAATGTAT
>novel_mir_42 13
TTGGTTGCTCTTTGTAAGTC
>novel_mir_18 19
TGGAGCCAACTGAGATTGAT
>novel_mir_95 11
TGATGAAGGATAGTGGCATGAT
>novel_mir_13 14
TACGTTCAGGTAAGAGAGGA
>novel_mir_19 9
TGGTCTGTAGGATCGAGTACTA
>novel_mir_28 15
TAGTGATTGCCTTGTAAGTTGT
>novel_mir_124 15
TGCTCTTGTTGCTGTTGTTG
>novel_mir_93 12
TATACAGTGCCTCACAAAACCCT
>novel_mir_69 7
TGATCCTAAACATCGATGAAAGT
>novel_mir_90 5
TAGTAGCCGCTCTATTTAAGG
>novel_mir_88 6
TATCACGCGCTCATTGACAAAGT
>novel_mir_52 11
TGGGGAAGGAGAAGGGGAGGA
>novel_mir_15 6
TCTCCTTTTTCTTTTTCTTTTC
>novel_mir_67 7
TACAGCGGCTTCTTTCAGACTT
>novel_mir_122 6
TGCATCTGCTGCTGCTGTTG
>novel_mir_27 5
TTGCCGAATTCGGATGAGATTG
>novel_mir_5 6
TACCACTGTTCAGAAGCATCGCT
>novel_mir_114 6
TCGTCTGACAGTTGGATTGCTC
>novel_mir_104 5
CAGGGGGGGAAAGGGGGGAA

>novel_mir_85 3409
TGGATCCGATGTGGTTTGAGGC
>novel_mir_40 829
TCATTTGTCGGGTATCGCTGAT
>novel_mir_107 462
TGGACCTGAGTATGCTTGTTGGA
>novel_mir_62 370
TGATGTAGCCTAATGATTGA
>novel_mir_68 344
TAGAAGGAGCTGACCATGTGGA
>novel_mir_12 269
TGCACCGCTTGTTTTTGATTCCC
>novel_mir_3 196
TGAAGTCCCCTGCTATGAAGAGA
>novel_mir_47 195
TCAAGGGCCAGACATCTATCGA
>novel_mir_110 141
TGATAGGGTACTTGGATGATGT
>novel_mir_46 99
TTGTGCTGAGTGCTGATTGGAG
>novel_mir_54 88
TGTGAATGAATGTTAACCGA
>novel_mir_38 87
TGTTATTGATGTCCAGTCTACG
>novel_mir_21 65
TGGTCTAAATTGCGTCTTCCGC
>novel_mir_51 61
TAAACGGTTTGTGGCAGAGACA
>novel_mir_73 60
TAGTGACCTTTCATGTGCGGAT
>novel_mir_105 55
TGGGCAAGACTGGATGATGAGT
>novel_mir_56 51
TTGGAGGCCGGAGTGAAGTCAA
>novel_mir_17 44
TACGGTCAACAGTGTCATAAGC
>novel_mir_32 39
TGACGACGATTTGCTTGATGAT
>novel_mir_2 30
TTTCAGACTCTTCTTCGCCAAC
>novel_mir_118 29
TAGAAATGAGTTGAGGCCGTGA
>novel_mir_9 27
TATAGGGTCAGCTATTGCTTCA
>novel_mir_96 26

```
TGGTTGTTCTATATTCTTGAAG
>novel_mir_125 26
TCTCTCTTGGGAGCAAGTCCGC
>novel_mir_25 25
TAATACGTTACTCATTTCAATTC
>novel_mir_14 19
TAATAAAAAAGAACACGTGTACT
>novel_mir_123 18
TGAGCTGCAACTTCAATGGAT
>novel_mir_83 18
TGAAGAAGAAGATAGCGCGTATA
>novel_mir_8 18
TTTATAACGCGACATTACTTTC
>novel_mir_72 17
TAGCTAGAAGATGATTGTGAAG
>novel_mir_111 17
TATTGAGTGAGTGGTTTGATAT
>novel_mir_44 16
TGAGCCAATGTTTGTTTACGCC
>novel_mir_84 15
TCACTTTCTGCGGTTTATGCTT
>novel_mir_77 14
TTAAGTGAACTCATCCGACAGG
>novel_mir_23 14
TAGAGGACCATTGTTCGTAGTAA
>novel_mir_30 14
TAAGTGAACTCATCCGACAGGTG
>novel_mir_98 12
TGACAAAGGATAGTTACGACCG
>novel_mir_74 12
TAGTGGACGAGTGTAGACGATA
>novel_mir_87 12
TGGTTCGTATATAACAAGGTGCA
>novel_mir_29 11
TATCCCAGATCAGTTAAAG
>novel_mir_7 11
TGAGGATGTTTTTGGGGAGGAG
>novel_mir_86 11
TTTATTGTGGCACAGACAGTCAA
>novel_mir_36 10
TGTTTAAAGTATGTTGGAAT
>novel_mir_10 10
TACCACATTTTCAGCCTTCG
>novel_mir_100 10
TTGAACGAGGAAGTTTTGTTG
```

>novel_mir_65 10
TATTGTTGAGTCTTCCGCTGAAA
>novel_mir_126 10
TTACTGTGACGCAGTTGGTGTAA
>novel_mir_49 10
TAGTCGTTGGTGCATGTGTAGG
>novel_mir_120 10
TATATGGAAGACTACAGCATTTG
>novel_mir_106 9
TGAATTAGGTTCAACATTATGTT
>novel_mir_99 9
TTCGTCGATTATTGGATATGGG
>novel_mir_115 9
TACAACACTCGCGACATGTACA
>novel_mir_94 9
TGAAGGAACTTGCTAAATTT
>novel_mir_66 8
TATTGGAATATTCTGGAGTGGA
>novel_mir_55 8
TAGAGAGAGCAGCACAGAAG
>novel_mir_81 8
TATCGCTTTGGTCATTTAATACA
>novel_mir_82 8
TATATACAGTAGGTCACAAAAC
>novel_mir_61 8
TAGTGGCCTTTCATATTCGG
>novel_mir_4 8
TAATAAAAAAGTTAAGAGCAAG
>novel_mir_63 7
TAGTGGAGAAAAGTGTATTCG
>novel_mir_45 7
TGGCTGATTGTATTTTTTAAATT
>novel_mir_91 7
TGCGCCTTTGGACAAATTATGTT
>novel_mir_48 7
TGAAGAAGATAATTAGAGAGGAT
>novel_mir_1 7
TGCGGCCGATAAATATTGTCAT
>novel_mir_109 7
TATAGTCAACACCATACCATGCT
>novel_mir_75 6
TGGCTTATGAATGTATTGGATT
>novel_mir_20 6
TCCTGGCCTTCTTAGATATCAAA
>novel_mir_92 6

TGCTTGGAAAATTGCATCGACA
>novel_mir_34 6
TATACAGTGACCCACAAAATTGT
>novel_mir_11 6
TGTTGTTCTTTAACTGATCTGGG
>novel_mir_6 6
TAGAAACAAAGATCGAGAAACA
>novel_mir_60 6
TGTCTGGTGCAGATGTTGACGA
>novel_mir_89 6
TGATGTGGGGGTGCATGACGTT
>novel_mir_35 6
TGAAAAACTAGTGAATGAGTCT
>novel_mir_26 6
TGCGAGCTGTGAACATGAGTTT
>novel_mir_50 5
TTGCCATGTAGGAAGATCATT
>novel_mir_57 5
TTATCTATTTGTCATCAGCTAG
>novel_mir_59 5
TCGTTCCTAAATCAATCTCA
>novel_mir_76 5
TTGCTCAATTGGATTCAGTT

Supplemental Table 3: FGSC-9543 known miRNA Target (TargetFinder)
#miRNA mRNA Score Match_pos Strand Pairing target and
miRNA
query=miR1160.1, target=RO3G_17276, score=0, range=918-939, stran
target 5' GGCCAUGCUUAAAAAGAGGGAA 3'
::::::::::::::::::::::
query 3' CCGGUACGAAUUUUUCUCCCUU 5'
query=miR1160.1, target=RO3G_13270, score=0, range=828-849, stran
target 5' GGCCAUGCUUAAAAAGAGGGAA 3'
::::::::::::::::::::::
query 3' CCGGUACGAAUUUUUCUCCCUU 5'
query=miR1160.1, target=RO3G_10774, score=0, range=795-816, stran
target 5' GGCCAUGCUUAAAAAGAGGGAA 3'
::::::::::::::::::::::
query 3' CCGGUACGAAUUUUUCUCCCUU 5'
query=miR1160.1, target=RO3G_13863, score=0, range=528-549, stran
target 5' GGCCAUGCUUAAAAAGAGGGAA 3'
::::::::::::::::::::::
query 3' CCGGUACGAAUUUUUCUCCCUU 5'
query=miR1160.1, target=RO3G_05935, score=0, range=234-255, stran
target 5' GGCCAUGCUUAAAAAGAGGGAA 3'
::::::::::::::::::::::
query 3' CCGGUACGAAUUUUUCUCCCUU 5'
query=miR1160.1, target=RO3G_03626, score=0, range=234-255, stran
target 5' GGCCAUGCUUAAAAAGAGGGAA 3'
::::::::::::::::::::::
query 3' CCGGUACGAAUUUUUCUCCCUU 5'
query=miR1160.1, target=RO3G_03593, score=0, range=234-255, stran
target 5' GGCCAUGCUUAAAAAGAGGGAA 3'
::::::::::::::::::::::
query 3' CCGGUACGAAUUUUUCUCCCUU 5'
query=miR1160.1, target=RO3G_04790, score=0, range=234-255, stran
target 5' GGCCAUGCUUAAAAAGAGGGAA 3'
::::::::::::::::::::::
query 3' CCGGUACGAAUUUUUCUCCCUU 5'
query=miR1160.1, target=RO3G_00678, score=0, range=198-219, stran
target 5' GGCCAUGCUUAAAAAGAGGGAA 3'
::::::::::::::::::::::
query 3' CCGGUACGAAUUUUUCUCCCUU 5'
query=miR1160.1, target=RO3G_11527, score=0, range=234-255, stran
target 5' GGCCAUGCUUAAAAAGAGGGAA 3'
::::::::::::::::::::::
query 3' CCGGUACGAAUUUUUCUCCCUU 5'
query=miR1160.1, target=RO3G_10869, score=0, range=234-255, stran
target 5' GGCCAUGCUUAAAAAGAGGGAA 3'

```
:::::::::::::::::::::::
query 3' CCGGUACGAAUUUUUCUCCCUU 5'
query=miR1160.1, target=RO3G_01589, score=0.5, range=234-255, str
target 5' GGUCAUGCUUAAAAAGAGGGAA 3'
::.::::::::::::::::::::
query 3' CCGGUACGAAUUUUUCUCCCUU 5'
query=miR1160.1, target=RO3G_17249, score=1, range=753-774, stran
target 5' GGCCAUGCUUAAAAAGAGGGGA 3'
:::::::::::::::::::::.:
query 3' CCGGUACGAAUUUUUCUCCCUU 5'
query=miR1160.1, target=RO3G_14363, score=1, range=786-807, stran
target 5' GACCAUGCUUAAAAAGAGGGAA 3'
: :::::::::::::::::::::
query 3' CCGGUACGAAUUUUUCUCCCUU 5'
query=miR1160.1, target=RO3G_14547, score=1, range=234-255, stran
target 5' GACCAUGCUUAAAAAGAGGGAA 3'
: :::::::::::::::::::::
query 3' CCGGUACGAAUUUUUCUCCCUU 5'
query=miR1160.1, target=RO3G_10734, score=2, range=234-255, stran
target 5' GGCCAUGCUUAAAAAGAUGGAA 3'
:::::::::::::::::: ::::
query 3' CCGGUACGAAUUUUUCUCCCUU 5'
query=miR1160.1, target=RO3G_17459, score=2, range=234-255, stran
target 5' GGCCAUGCUUAAAAAGAUGGAA 3'
:::::::::::::::::: ::::
query 3' CCGGUACGAAUUUUUCUCCCUU 5'
query=miR1160.1, target=RO3G_05134, score=3.5, range=1120-1141,
strand=-1
target 5' UGCCAUCCUUAAAGAGAGGGAG 3'
::::: ::::::.:::::::.
query 3' CCGGUACGAAUUUUUCUCCCUU 5'
query=miR1160.1, target=RO3G_05127, score=3.5, range=1120-1141,
strand=-1
target 5' UGCCAUCCUUAAAGAGAGGGAG 3'
::::: ::::::.:::::::.
query 3' CCGGUACGAAUUUUUCUCCCUU 5'
query=miR5281b, target=RO3G_17117, score=2.5, range=665-684, stra
target 5' CUUCAGUCCUUUUAUUAAAA 3'
::.: ::::::::::::::
query 3' GAGGCAAGGAAAAUAAUUUU 5'
query=miR5281b, target=RO3G_08187, score=3, range=1247-1267, stra
target 5' CUCCCGAUCCUUUUAUUGAAA 3'
:: ::: ::::::::::.:::
query 3' GA-GGCAAGGAAAAUAAUUUU 5'
query=miR5281b, target=RO3G_08154, score=3, range=18-37, strand=-
```

```
target 5' AUCCUUUUCUUUUAUUAAAA 3'
::: ::.::::::::::::
query 3' GAGGCAAGGAAAAUAAUUUU 5'
query=miR5281b, target=RO3G_04365, score=3, range=431-450, strand
target 5' AUCCAUUUCUUUUAUUAAAA 3'
::: ::.::::::::::::
query 3' GAGGCAAGGAAAAUAAUUUU 5'
query=miR5281b, target=RO3G_08298, score=3, range=999-1017, stran
target 5' CUCC-UUCUUUUUAUUAAAC 3'
:::: :::.::::::::::
query 3' GAGGCAAGGAAAAUAAUUUU 5'
query=miR5281b, target=RO3G_00916, score=4, range=858-876, strand
target 5' CUUU-UUUCUUUUAUUGAAA 3'
::.. ::.::::::::.:::
query 3' GAGGCAAGGAAAAUAAUUUU 5'
query=miR5281b, target=RO3G_10068, score=4, range=246-265, strand
target 5' CUUAGUUCCUUUUAUUAAUG 3'
::. :::::::::::::: .
query 3' GAGGCAAGGAAAAUAAUUUU 5'
query=miR5281b, target=RO3G_05529, score=4, range=160-180, strand
target 5' AUCCGAUUCCUUUUACUAAAA 3'
:::: ::::::::: :::::
query 3' GAGGC-AAGGAAAAUAAUUUU 5'
query=miR5281b, target=RO3G_13391, score=4, range=444-463, strand
target 5' GUCCUUUCUUUUUAUUGAAA 3'
::: :::.:::::::.:::
query 3' GAGGCAAGGAAAAUAAUUUU 5'
query=miR5281b, target=RO3G_05662, score=4, range=78-97, strand=-
target 5' UUGUGUUUCUUUUAUUAAAU 3'
.: .:::.:::::::::::
query 3' GAGGCAAGGAAAAUAAUUUU 5'
query=miR5281b, target=RO3G_14434, score=4, range=365-384, strand
target 5' UUCAGUUCCUUCUAUUAAAG 3'
.:: ::::::: :::::::.
query 3' GAGGCAAGGAAAAUAAUUUU 5'
query=miR5281b, target=RO3G_12911, score=4, range=1322-1342, stra
target 5' AUCCGAUUCCUUUUACUAAAA 3'
:::: ::::::::: :::::
query 3' GAGGC-AAGGAAAAUAAUUUU 5'
query=miR5281b, target=RO3G_12128, score=4, range=998-1017, stran
target 5' CUCCUCUUCUUUUAUUAAAU 3'
:::: :.:::::::::::
query 3' GAGGCAAGGAAAAUAAUUUU 5'
query=miR5042-3p, target=RO3G_16914, score=2, range=3215-3234,
strand=-1
```

target 5' CCUUCUUGUACUCAAGUCCU 3'
::::::::::::::::.::
query 3' GGAAGAACAUGAGUUCGGGU 5'
query=miR5042-3p, target=RO3G_15166, score=3, range=484-503, stra
target 5' CCUUCUUGUACCCAAGUCCA 3'
::::::::::: ::::.:::
query 3' GGAAGAACAUGAGUUCGGGU 5'
query=miR5042-3p, target=RO3G_02218, score=4, range=377-395, stra
target 5' CCAUC-UGUACUCAAGCCUU 3'
:: :: :::::::::::.
query 3' GGAAGAACAUGAGUUCGGGU 5'
query=miR8632, target=RO3G_19000, score=0, range=450-468, strand=
target 5' GGUUCCAACUUUGAGCUCU 3'
:::::::::::::::::::
query 3' CCAAGGUUGAAACUCGAGA 5'
query=miR8632, target=RO3G_19002, score=0, range=450-468, strand=
target 5' GGUUCCAACUUUGAGCUCU 3'
:::::::::::::::::::
query 3' CCAAGGUUGAAACUCGAGA 5'
query=miR8632, target=RO3G_15016, score=3, range=2706-2723, stran
target 5' GGUUCCAA-UUUGAGUUCU 3'
:::::::: ::::::.:::
query 3' CCAAGGUUGAAACUCGAGA 5'
query=miR8632, target=RO3G_04352, score=3, range=1925-1943, stran
target 5' GAUUCAAACUUUGAGCUCG 3'
: ::: ::::::::::::
query 3' CCAAGGUUGAAACUCGAGA 5'
query=miR8632, target=RO3G_15392, score=3.5, range=2639-2657,
strand=-1
target 5' GGUUCUAACUUUAAGCUCA 3'
::::.:::::: :::::
query 3' CCAAGGUUGAAACUCGAGA 5'
query=miR8632, target=RO3G_03452, score=4, range=757-774, strand=
target 5' GAUUCCAACUUUGA-CUUU 3'
: :::::::::::: ::.:
query 3' CCAAGGUUGAAACUCGAGA 5'
query=miR8632, target=RO3G_12046, score=4, range=775-792, strand=
target 5' GGUUCCAG-UUUGAGUUCU 3'
:::::::. ::::::.:::
query 3' CCAAGGUUGAAACUCGAGA 5'
query=miR8632, target=RO3G_00895, score=4, range=1416-1433, stran
target 5' AGUUCCAA-UUUGAGCUUU 3'
::::::: :::::::::.:
query 3' CCAAGGUUGAAACUCGAGA 5'
query=miR8632, target=RO3G_06934, score=4, range=1429-1448, stran

```
target 5' UGUACACGACUUUGAGCUCU 3'
:: : :.:::::::::::
query 3' CCAAG-GUUGAAACUCGAGA 5'
query=miR5565e, target=RO3G_16107, score=0, range=527-547, strand
target 5' UCCGACAACAUCUAGACCAUA 3'
::::::::::::::::::::
query 3' AGGCUGUUGUAGAUCUGGUAU 5'
query=miR5565e, target=RO3G_09773, score=4, range=571-590, strand
target 5' UCCGAUAAC-UCUGGACCAUG 3'
::::.::: :::.:::::.
query 3' AGGCUGUUGUAGAUCUGGUAU 5'
query=miR5565e, target=RO3G_14812, score=4, range=115-134, strand
target 5' UCC-ACAACAUCUAAAUCAUA 3'
::: ::::::::: :.:::
query 3' AGGCUGUUGUAGAUCUGGUAU 5'
query=miR3711, target=RO3G_09910, score=3, range=704-724, strand=
target 5' GGGCAGCUUCCUUCUUGGCUU 3'
.:::: :.:::::::::::
query 3' UCCGU-GGAGGAAGAACCGAU 5'
query=miR3711, target=RO3G_01286, score=3.5, range=121-140, stran
target 5' AUGCGCAUCCUUCUUGGCUU 3'
: ::.: :::::::::::
query 3' UCCGUGGAGGAAGAACCGAU 5'
query=miR3711, target=RO3G_13432, score=4, range=548-567, strand=
target 5' AGGCAUCAUCUUCUUGGCUG 3'
:::::.: .:::::::::.
query 3' UCCGUGGAGGAAGAACCGAU 5'
query=miR3711, target=RO3G_11375, score=4, range=1394-1413, stran
target 5' GGGCAUCUGCUUCUUGGUUA 3'
.::::.:: ::::::::.::
query 3' UCCGUGGAGGAAGAACCGAU 5'
query=miR3711, target=RO3G_11895, score=4, range=983-1003, strand
target 5' AUGCAACUUCCUUUUUGGCUG 3'
: :: ::.::::.:::::.
query 3' UCCG-UGGAGGAAGAACCGAU 5'
query=miR3711, target=RO3G_03385, score=4, range=1172-1192, stran
target 5' AGGCGUCUUCUUCUUGAGCUA 3'
::::..::.::::::: ::::
query 3' UCCGUGGAGGAAGAAC-CGAU 5'
query=miR3711, target=RO3G_11900, score=4, range=1400-1420, stran
target 5' AGGCGGCUUCCUUCUUGGCAA 3'
:::: .:.::::::::::: :
query 3' UCCG-UGGAGGAAGAACCGAU 5'
query=miR3711, target=RO3G_15319, score=4, range=1145-1165, stran
target 5' AGUUAUCUCCUUCUUUGGCUA 3'
```

```
:: .:.:::::::: :::::::
query 3' UCCGUGGAGGAAG-AACCGAU 5'
query=miR3711, target=RO3G_04341, score=4, range=643-661, strand=
target 5' AUGU-CCUCCUUCUUGGUUG 3'
: :. ::::::::::::.:.
query 3' UCCGUGGAGGAAGAACCGAU 5'
query=miR170-3p, target=RO3G_11045, score=3.5, range=302-319, str
target 5' AAUUUUAUAUGGCCAAUG 3'
::: :::.:.:::::::.
query 3' UUAUAAUGUGCCGGUUAU 5'
query=miR170-3p, target=RO3G_10275, score=3.5, range=207-224, str
target 5' AGCAUUAUACGGCCAAUC 3'
:. ::::.::::::::
query 3' UUAUAAUGUGCCGGUUAU 5'
query=miR170-3p, target=RO3G_10916, score=3.5, range=355-371, str
target 5' GAUAUU-CACGGUCAAUA 3'
.::::: :::::.:::::
query 3' UUAUAAUGUGCCGGUUAU 5'
query=miR170-3p, target=RO3G_16278, score=3.5, range=151-167, str
target 5' GAUAUU-CACGGCCAAUU 3'
.::::: ::::::::::
query 3' UUAUAAUGUGCCGGUUAU 5'
query=miR170-3p, target=RO3G_13560, score=3.5, range=81-98, stran
target 5' AAUGUUGUACGGCCAGUA 3'
:::.::..:::::::.::
query 3' UUAUAAUGUGCCGGUUAU 5'
query=miR170-3p, target=RO3G_08726, score=3.5, range=148-164,
strand=-1
target 5' AAUG-UAUAUGGCCAAUA 3'
:::. ::.:.::::::::
query 3' UUAUAAUGUGCCGGUUAU 5'
query=miR170-3p, target=RO3G_05589, score=3.5, range=3347-3364,
strand=-1
target 5' AGCGAUACACGGCCAAUG 3'
:. . :::::::::::.
query 3' UUAUAAUGUGCCGGUUAU 5'
query=miR170-3p, target=RO3G_02520, score=4, range=710-726, stran
target 5' AAUAUUACAUGG-CAGUA 3'
:::::::::.:: ::.::
query 3' UUAUAAUGUGCCGGUUAU 5'
query=miR170-3p, target=RO3G_11165, score=4, range=4930-4946, str
target 5' AAU-UUACCUGGCCAAUA 3'
::: :::: .::::::::
query 3' UUAUAAUGUGCCGGUUAU 5'
query=miR170-3p, target=RO3G_12187, score=4, range=148-164, stran
```

target 5' CAU-UUACACGGCCAACA 3'
:: ::::::::::: :
query 3' UUAUAAUGUGCCGGUUAU 5'
query=miR170-3p, target=RO3G_14986, score=4, range=148-164, stran
target 5' CAU-UUACACGGCCAACA 3'
:: ::::::::::: :
query 3' UUAUAAUGUGCCGGUUAU 5'
query=miR170-3p, target=RO3G_13154, score=4, range=148-164, stran
target 5' CAU-UUACACGGCCAACA 3'
:: ::::::::::: :
query 3' UUAUAAUGUGCCGGUUAU 5'
query=miR170-3p, target=RO3G_04209, score=4, range=148-164, stran
target 5' CAU-UUACACGGCCAACA 3'
:: ::::::::::: :
query 3' UUAUAAUGUGCCGGUUAU 5'
query=miR170-3p, target=RO3G_02823, score=4, range=148-164, stran
target 5' CAU-UUACACGGCCAACA 3'
:: ::::::::::: :
query 3' UUAUAAUGUGCCGGUUAU 5'
query=miR170-3p, target=RO3G_04693, score=4, range=148-164, stran
target 5' CAU-UUACACGGCCAACA 3'
:: ::::::::::: :
query 3' UUAUAAUGUGCCGGUUAU 5'
query=miR170-3p, target=RO3G_03610, score=4, range=148-164, stran
target 5' CAU-UUACACGGCCAACA 3'
:: ::::::::::: :
query 3' UUAUAAUGUGCCGGUUAU 5'
query=miR170-3p, target=RO3G_14766, score=4, range=148-164, stran
target 5' CAU-UUACACGGCCAACA 3'
:: ::::::::::: :
query 3' UUAUAAUGUGCCGGUUAU 5'
query=miR170-3p, target=RO3G_13188, score=4, range=33-50, strand=
target 5' AAUACAAUACGGCCAAUA 3'
:::: :.::::::::::
query 3' UUAUAAUGUGCCGGUUAU 5'
query=miR170-3p, target=RO3G_10288, score=4, range=4156-4172,
strand=-1
target 5' ACUAUUACACGGUC-AUA 3'
: :::::::::.: :::
query 3' UUAUAAUGUGCCGGUUAU 5'
query=miR170-3p, target=RO3G_01343, score=4, range=723-741, stran
target 5' AAUCUUACCACGGUCAAUA 3'
::: ::: :::::.:::::
query 3' UUAUAAU-GUGCCGGUUAU 5'
query=miR2275b-3p, target=RO3G_06738, score=0, range=92-114, stra

```
target 5' UAGAUUUACGAUGGAAACUUGAA 3'
:::::::::::::::::::::::
query 3' AUCUAAAUGCUACCUUUGAACUU 5'
query=miR2275b-3p, target=RO3G_08840, score=1, range=248-270,
strand=-1
target 5' UAAAUUUACGAUGGAAACUUGAA 3'
:: ::::::::::::::::::::
query 3' AUCUAAAUGCUACCUUUGAACUU 5'
query=miR2275b-3p, target=RO3G_17208, score=1, range=242-264,
strand=-1
target 5' UAAAUUUACGAUGGAAACUUGAA 3'
:: ::::::::::::::::::::
query 3' AUCUAAAUGCUACCUUUGAACUU 5'
query=miR2275b-3p, target=RO3G_00297, score=1, range=92-114, stra
target 5' UAAAUUUACGAUGGAAACUUGAA 3'
:: ::::::::::::::::::::
query 3' AUCUAAAUGCUACCUUUGAACUU 5'
query=miR2275b-3p, target=RO3G_05518, score=1, range=11-33, stran
target 5' UAAAUUUACGAUGGAAACUUGAA 3'
:: ::::::::::::::::::::
query 3' AUCUAAAUGCUACCUUUGAACUU 5'
query=miR2275b-3p, target=RO3G_16695, score=1, range=20-42, stran
target 5' UAAAUUUACGAUGGAAACUUGAA 3'
:: ::::::::::::::::::::
query 3' AUCUAAAUGCUACCUUUGAACUU 5'
query=miR2275b-3p, target=RO3G_09543, score=1, range=149-171,
strand=-1
target 5' UAAAUUUACGAUGGAAACUUGAA 3'
:: ::::::::::::::::::::
query 3' AUCUAAAUGCUACCUUUGAACUU 5'
query=miR2275b-3p, target=RO3G_16701, score=1.5, range=284-306,
strand=-1
target 5' UAAGUUUACGAUGGAAACUUGAA 3'
:: .:::::::::::::::::::
query 3' AUCUAAAUGCUACCUUUGAACUU 5'
query=miR2275b-3p, target=RO3G_07703, score=1.5, range=248-270,
strand=-1
target 5' UAAGUUUACGAUGGAAACUUGAA 3'
:: .:::::::::::::::::::
query 3' AUCUAAAUGCUACCUUUGAACUU 5'
query=miR2275b-3p, target=RO3G_11277, score=2, range=92-114, stra
target 5' UAAAUUUACGAUGGAAGCUUGAA 3'
:: :::::::::::::.::::::
query 3' AUCUAAAUGCUACCUUUGAACUU 5'
query=miR2275b-3p, target=RO3G_13759, score=2, range=92-114, stra
```

```
target 5' UAAAUUUACGAUGGAAGCUUGAA 3'
:: :::::::::::::.::::::
query 3' AUCUAAAUGCUACCUUUGAACUU 5'
query=miR2275b-3p, target=RO3G_17364, score=3.5, range=248-270,
strand=-1
target 5' UACAUUUGCGAUGGAAACUUGUA 3'
:: ::::.:::::::::::::: :
query 3' AUCUAAAUGCUACCUUUGAACUU 5'
query=miR2275b-3p, target=RO3G_10037, score=4, range=233-255,
strand=-1
target 5' UAGAUUUACGAUGGAAAGUUGUA 3'
:::::::::::::::: ::: :
query 3' AUCUAAAUGCUACCUUUGAACUU 5'
query=miR2275b-3p, target=RO3G_14999, score=4, range=92-114, stra
target 5' UAGAUUUACGAUGGAAAGUUGUA 3'
:::::::::::::::: ::: :
query 3' AUCUAAAUGCUACCUUUGAACUU 5'
query=miR2275b-3p, target=RO3G_14360, score=4, range=92-114, stra
target 5' UAGAUUUACGAUGGAAAGUUGUA 3'
:::::::::::::::: ::: :
query 3' AUCUAAAUGCUACCUUUGAACUU 5'
query=miR2275b-3p, target=RO3G_07680, score=4, range=92-114, stra
target 5' UAGAUUUACGAUGGAAAGUUGUA 3'
:::::::::::::::: ::: :
query 3' AUCUAAAUGCUACCUUUGAACUU 5'
query=miR2275b-3p, target=RO3G_15156, score=4, range=92-114, stra
target 5' UAGAUUUACGAUGGAAAGUUGUA 3'
:::::::::::::::: ::: :
query 3' AUCUAAAUGCUACCUUUGAACUU 5'
query=miR2275b-3p, target=RO3G_01202, score=4, range=92-114, stra
target 5' UAGAUUUACGAUGGAAAGUUGUA 3'
:::::::::::::::: ::: :
query 3' AUCUAAAUGCUACCUUUGAACUU 5'
query=miR2275b-3p, target=RO3G_14973, score=4, range=92-114, stra
target 5' UAGAUUUACGAUGGAAAGUUGUA 3'
:::::::::::::::: ::: :
query 3' AUCUAAAUGCUACCUUUGAACUU 5'
query=miR2275b-3p, target=RO3G_04687, score=4, range=92-114, stra
target 5' UAGAUUUACGAUGGAAAGUUGUA 3'
:::::::::::::::: ::: :
query 3' AUCUAAAUGCUACCUUUGAACUU 5'
query=miR2275b-3p, target=RO3G_14985, score=4, range=92-114, stra
target 5' UAGAUUUACGAUGGAAAGUUGUA 3'
:::::::::::::::: ::: :
query 3' AUCUAAAUGCUACCUUUGAACUU 5'
```

```
query=miR2275b-3p, target=RO3G_17237, score=4, range=92-114, stra
target 5' UAGAUUUACGAUGGAAAGUUGUA 3'
:::::::::::::::: ::: :
query 3' AUCUAAAUGCUACCUUUGAACUU 5'
query=miR5743a, target=RO3G_08617, score=0, range=281-300, strand
target 5' AAUGGUGGGAAAACUUCUCA 3'
::::::::::::::::::::
query 3' UUACCACCCUUUUGAAGAGU 5'
query=miR5743a, target=RO3G_03516, score=1, range=1616-1635, stra
target 5' AAUGGUGGGAAAAUUUCUCA 3'
:::::::::::::.::::::
query 3' UUACCACCCUUUUGAAGAGU 5'
query=miR159g-3p, target=RO3G_10142, score=1, range=579-598, stra
target 5' UCAAAUCGCUUCACUUCAAA 3'
::::::::::::::::.::::
query 3' AGUUUAGCGAAGUGAGGUUU 5'
query=miR159g-3p, target=RO3G_03107, score=1, range=579-598, stra
target 5' UCAAAUCGCUUCACUUCAAA 3'
::::::::::::::::.::::
query 3' AGUUUAGCGAAGUGAGGUUU 5'
query=miR159g-3p, target=RO3G_06108, score=3, range=731-751, stra
target 5' UCAAAACGCUUCACAUCCAAA 3'
::::: :::::::: ::::::
query 3' AGUUUAGCGAAGUG-AGGUUU 5'
query=miR159g-3p, target=RO3G_03964, score=3, range=7600-7620,
strand=-1
target 5' UCCAAUCAGCUUCACUUCAAA 3'
:: :::: ::::::::.::::
query 3' AGUUUAG-CGAAGUGAGGUUU 5'
query=miR159g-3p, target=RO3G_01135, score=3.5, range=1733-1753,
strand=1
target 5' CCAAGUCUGCUUCACUUCAAA 3'
:::.:: ::::::::.::::
query 3' AGUUUAG-CGAAGUGAGGUUU 5'
query=miR159g-3p, target=RO3G_04971, score=3.5, range=605-625,
strand=1
target 5' UCAAGUCGCUUUCACUCCAAU 3'
::::.:::: ::::::::::
query 3' AGUUUAGCG-AAGUGAGGUUU 5'
query=miR159g-3p, target=RO3G_08468, score=3.5, range=493-512,
strand=-1
target 5' ACAGGUUGCUUCAUUCCAAA 3'
::..:.::::::.::::::
query 3' AGUUUAGCGAAGUGAGGUUU 5'
query=miR159g-3p, target=RO3G_02881, score=4, range=3815-3834,
```

```
strand=1
target 5' UCAAAUCUCUUUAUUCCAAA 3'
::::::: :::.:.::::::
query 3' AGUUUAGCGAAGUGAGGUUU 5'
query=miR159g-3p, target=RO3G_02867, score=4, range=507-526, stra
target 5' UCAAAUCGCUUCCUUUCAAA 3'
:::::::::::: .:.::::
query 3' AGUUUAGCGAAGUGAGGUUU 5'
query=miR159g-3p, target=RO3G_15320, score=4, range=218-238, stra
target 5' UUGAAUCUGCUUCCCUCCAAA 3'
:..:::: ::::: :::::::
query 3' AGUUUAG-CGAAGUGAGGUUU 5'
query=miR159g-3p, target=RO3G_15485, score=4, range=3574-3592,
strand=1
target 5' UC-GAUUGCUUCACUUCAAU 3'
:: .::.:::::::.:::
query 3' AGUUUAGCGAAGUGAGGUUU 5'
query=miR159g-3p, target=RO3G_08165, score=4, range=556-575, stra
target 5' UCAGAUUGCUUCAUACCAAA 3'
:::.::.:::::. :::::
query 3' AGUUUAGCGAAGUGAGGUUU 5'
query=miR159g-3p, target=RO3G_07951, score=4, range=376-395, stra
target 5' UCCAAUCGCUUCUCUCUAAA 3'
:: ::::::::: :::.:::
query 3' AGUUUAGCGAAGUGAGGUUU 5'
query=miR159g-3p, target=RO3G_15766, score=4, range=788-806, stra
target 5' UCAAAU-GCUUCAUUCCAGC 3'
:::::: ::::::.::::.
query 3' AGUUUAGCGAAGUGAGGUUU 5'
query=miR159g-3p, target=RO3G_08004, score=4, range=2147-2165,
strand=-1
target 5' CUAAGU-GCUUCACUCCAAU 3'
.::.: :::::::::::
query 3' AGUUUAGCGAAGUGAGGUUU 5'
query=miR397a, target=RO3G_06442, score=4, range=5174-5194, stran
target 5' CAUCAUCCCUGUAUUCAAUGA 3'
::::: : :::.:.:::::::
query 3' GUAGUUGCGACGUGAGUUACU 5'
query=miR397a, target=RO3G_02691, score=4, range=8006-8026, stran
target 5' CAUCAUCCCUGUAUUCAAUGA 3'
::::: : :::.:.:::::::
query 3' GUAGUUGCGACGUGAGUUACU 5'
query=miR397a, target=RO3G_09965, score=4, range=4517-4537, stran
target 5' CAUCAUCCCUGUAUUCAAUGA 3'
::::: : :::.:.:::::::
```

```
query 3' GUAGUUGCGACGUGAGUUACU 5'
query=miR397a, target=RO3G_13920, score=4, range=4517-4537, stran
target 5' CAUCAUCCCUGUAUUCAAUGA 3'
::::: : :::.:.:::::::
query 3' GUAGUUGCGACGUGAGUUACU 5'
query=miR397a, target=RO3G_03547, score=4, range=4517-4537, stran
target 5' CAUCAUCCCUGUAUUCAAUGA 3'
::::: : :::.:.:::::::
query 3' GUAGUUGCGACGUGAGUUACU 5'
query=miR397a, target=RO3G_13867, score=4, range=4433-4453, stran
target 5' CAUCAUCCCUGUAUUCAAUGA 3'
::::: : :::.:.:::::::
query 3' GUAGUUGCGACGUGAGUUACU 5'
query=miR397a, target=RO3G_02959, score=4, range=4517-4537, stran
target 5' CAUCAUCCCUGUAUUCAAUGA 3'
::::: : :::.:.:::::::
query 3' GUAGUUGCGACGUGAGUUACU 5'
query=miR397a, target=RO3G_15250, score=4, range=4472-4492, stran
target 5' CAUCAUCCCUGUAUUCAAUGA 3'
::::: : :::.:.:::::::
query 3' GUAGUUGCGACGUGAGUUACU 5'
query=miR397a, target=RO3G_17371, score=4, range=4472-4492, stran
target 5' CAUCAUCCCUGUAUUCAAUGA 3'
::::: : :::.:.:::::::
query 3' GUAGUUGCGACGUGAGUUACU 5'
query=miR397a, target=RO3G_06427, score=4, range=4109-4129, stran
target 5' CAUCAUCCCUGUAUUCAAUGA 3'
::::: : :::.:.:::::::
query 3' GUAGUUGCGACGUGAGUUACU 5'
query=miR397a, target=RO3G_17326, score=4, range=4349-4369, stran
target 5' CAUCAUCCCUGUAUUCAAUGA 3'
::::: : :::.:.:::::::
query 3' GUAGUUGCGACGUGAGUUACU 5'
query=miR397a, target=RO3G_06428, score=4, range=3611-3631, stran
target 5' CAUCAUCCCUGUAUUCAAUGA 3'
::::: : :::.:.:::::::
query 3' GUAGUUGCGACGUGAGUUACU 5'
query=miR397a, target=RO3G_13749, score=4, range=3026-3046, stran
target 5' CAUCAUCCCUGUAUUCAAUGA 3'
::::: : :::.:.:::::::
query 3' GUAGUUGCGACGUGAGUUACU 5'
query=miR397a, target=RO3G_05619, score=4, range=2660-2680, stran
target 5' CAUCAUCCCUGUAUUCAAUGA 3'
::::: : :::.:.:::::::
query 3' GUAGUUGCGACGUGAGUUACU 5'
```

```
query=miR397a, target=RO3G_14355, score=4, range=2723-2743, stran
target 5' CAUCAUCCCUGUAUUCAAUGA 3'
::::: : :::.:.:::::::
query 3' GUAGUUGCGACGUGAGUUACU 5'
query=miR397a, target=RO3G_01330, score=4, range=1751-1771, stran
target 5' CAUCAUCCCUGUAUUCAAUGA 3'
::::: : :::.:.:::::::
query 3' GUAGUUGCGACGUGAGUUACU 5'
query=miR397a, target=RO3G_11776, score=4, range=1607-1627, stran
target 5' CAUCAUCCCUGUAUUCAAUGA 3'
::::: : :::.:.:::::::
query 3' GUAGUUGCGACGUGAGUUACU 5'
query=miR397a, target=RO3G_02222, score=4, range=1571-1591, stran
target 5' CAUCAUCCCUGUAUUCAAUGA 3'
::::: : :::.:.:::::::
query 3' GUAGUUGCGACGUGAGUUACU 5'
query=miR397a, target=RO3G_04115, score=4, range=1460-1480, stran
target 5' CAUCAUCCCUGUAUUCAAUGA 3'
::::: : :::.:.:::::::
query 3' GUAGUUGCGACGUGAGUUACU 5'
query=miR397a, target=RO3G_17404, score=4, range=1145-1165, stran
target 5' CAUCAUCCCUGUAUUCAAUGA 3'
::::: : :::.:.:::::::
query 3' GUAGUUGCGACGUGAGUUACU 5'
query=miR397a, target=RO3G_07668, score=4, range=920-940, strand=
target 5' CAUCAUCCCUGUAUUCAAUGA 3'
::::: : :::.:.:::::::
query 3' GUAGUUGCGACGUGAGUUACU 5'
query=miR397a, target=RO3G_05265, score=4, range=329-349, strand=
target 5' CAUCAUCCCUGUAUUCAAUGA 3'
::::: : :::.:.:::::::
query 3' GUAGUUGCGACGUGAGUUACU 5'
query=miR397a, target=RO3G_05903, score=4, range=329-349, strand=
target 5' CAUCAUCCCUGUAUUCAAUGA 3'
::::: : :::.:.:::::::
query 3' GUAGUUGCGACGUGAGUUACU 5'
query=miR5786, target=RO3G_05613, score=4, range=678-698, strand=
target 5' AGCUUUACGU-UAUCCUGCGAA 3'
::::: :: : ::::::::::
query 3' UCGAACUGGAGAUAGGACGCUU 5'
query=miR8775, target=RO3G_09964, score=0, range=343-361, strand=
target 5' CCAUCGGUUCAAUCUGCAA 3'
:::::::::::::::::::
query 3' GGUAGCCAAGUUAGACGUU 5'
query=miR8775, target=RO3G_01558, score=4, range=1532-1549, stran
```

```
target 5' CUGU-GGUUCGAUUUGCAA 3'
:..: :::::.::.:::::
query 3' GGUAGCCAAGUUAGACGUU 5'
query=miR8775, target=RO3G_14802, score=4, range=3105-3123, stran
target 5' CUACUGGUUCAAUCUGAAA 3'
:.: .:::::::::: ::
query 3' GGUAGCCAAGUUAGACGUU 5'
query=miR8775, target=RO3G_14706, score=4, range=3243-3261, stran
target 5' CUACUGGUUCAAUCUGAAA 3'
:.: .:::::::::: ::
query 3' GGUAGCCAAGUUAGACGUU 5'
query=miR8775, target=RO3G_06905, score=4, range=12-31, strand=1
target 5' UUAUGGUGUUCAAUUUGCAA 3'
..:: : :::::::.:::::
query 3' GGUAGC-CAAGUUAGACGUU 5'
query=miR8775, target=RO3G_06335, score=4, range=484-501, strand=
target 5' UCAUUUGUUCAAUCU-CAA 3'
.:::. ::::::::: :::
query 3' GGUAGCCAAGUUAGACGUU 5'
query=miR8775, target=RO3G_13976, score=4, range=287-305, strand=
target 5' CCUUCUGUUUGAUCUGCAA 3'
:: :: :::..::::::::
query 3' GGUAGCCAAGUUAGACGUU 5'
query=miR3946, target=RO3G_17023, score=1, range=1225-1246, stran
target 5' CAUGCUCCCUCUUUUCUCUACA 3'
::::::: ::::::::::::::
query 3' GUACGAGAGAGAAAAGAGAUGU 5'
query=miR3946, target=RO3G_10379, score=3, range=319-340, strand=
target 5' CUUGUUCUUUCUUUUCUUUACA 3'
: ::.:::.::::::::.::::
query 3' GUACGAGAGAGAAAAGAGAUGU 5'
query=miR3946, target=RO3G_02013, score=4, range=1467-1489, stran
target 5' CAUCUUCUUUCUUUUCUUCUACA 3'
::: .:::.:::::::: ::::::
query 3' GUACGAGAGAGAAAAG-AGAUGU 5'
query=miR8748, target=RO3G_04592, score=0, range=1361-1383, stran
target 5' AUUCAUUAUUAUCAUCACCAUCA 3'
:::::::::::::::::::::::
query 3' UAAGUAAUAAUAGUAGUGGUAGU 5'
query=miR8748, target=RO3G_10217, score=1, range=2189-2211, stran
target 5' AUUCAUUAUCAUCAUCACCAUCA 3'
::::::::: :::::::::::::
query 3' UAAGUAAUAAUAGUAGUGGUAGU 5'
query=miR8748, target=RO3G_16749, score=1, range=2189-2211, stran
target 5' AUUCAUUAUCAUCAUCACCAUCA 3'
```

```
::::::::: ::::::::::::
query 3' UAAGUAAUAAUAGUAGUGGUAGU 5'
query=miR8748, target=RO3G_13273, score=1, range=2189-2211, stran
target 5' AUUCAUUAUCAUCAUCACCAUCA 3'
::::::::: ::::::::::::
query 3' UAAGUAAUAAUAGUAGUGGUAGU 5'
query=miR8748, target=RO3G_13145, score=1, range=2189-2211, stran
target 5' AUUCAUUAUCAUCAUCACCAUCA 3'
::::::::: ::::::::::::
query 3' UAAGUAAUAAUAGUAGUGGUAGU 5'
query=miR8748, target=RO3G_05424, score=1, range=2105-2127, stran
target 5' AUUCAUUAUCAUCAUCACCAUCA 3'
::::::::: ::::::::::::
query 3' UAAGUAAUAAUAGUAGUGGUAGU 5'
query=miR8748, target=RO3G_17257, score=1, range=2105-2127, stran
target 5' AUUCAUUAUCAUCAUCACCAUCA 3'
::::::::: ::::::::::::
query 3' UAAGUAAUAAUAGUAGUGGUAGU 5'
query=miR8748, target=RO3G_16136, score=1, range=2105-2127, stran
target 5' AUUCAUUAUCAUCAUCACCAUCA 3'
::::::::: ::::::::::::
query 3' UAAGUAAUAAUAGUAGUGGUAGU 5'
query=miR8748, target=RO3G_15889, score=1, range=2105-2127, stran
target 5' AUUCAUUAUCAUCAUCACCAUCA 3'
::::::::: ::::::::::::
query 3' UAAGUAAUAAUAGUAGUGGUAGU 5'
query=miR8748, target=RO3G_10735, score=1, range=1634-1656, stran
target 5' AUUCAUUAUCAUCAUCACCAUCA 3'
::::::::: ::::::::::::
query 3' UAAGUAAUAAUAGUAGUGGUAGU 5'
query=miR8748, target=RO3G_09183, score=1, range=1322-1344, stran
target 5' AUUCAUUAUCAUCAUCACCAUCA 3'
::::::::: ::::::::::::
query 3' UAAGUAAUAAUAGUAGUGGUAGU 5'
query=miR8748, target=RO3G_17351, score=1, range=917-939, strand=
target 5' AUUCAUUAUCAUCAUCACCAUCA 3'
::::::::: ::::::::::::
query 3' UAAGUAAUAAUAGUAGUGGUAGU 5'
query=miR8748, target=RO3G_04681, score=1, range=779-801, strand=
target 5' AUUCAUUAUCAUCAUCACCAUCA 3'
::::::::: ::::::::::::
query 3' UAAGUAAUAAUAGUAGUGGUAGU 5'
query=miR8748, target=RO3G_07697, score=1, range=515-537, strand=
target 5' AUUCAUUAUCAUCAUCACCAUCA 3'
::::::::: ::::::::::::
```

```
query 3' UAAGUAAUAAUAGUAGUGGUAGU 5'
query=miR8748, target=RO3G_10040, score=1, range=779-801, strand=
target 5' AUUCAUUAUCAUCAUCACCAUCA 3'
::::::::: :::::::::::::
query 3' UAAGUAAUAAUAGUAGUGGUAGU 5'
query=miR8748, target=RO3G_00422, score=2.5, range=36-57, strand=
target 5' AUUC-UUGUUAUCAUCACUAUCA 3'
:::: ::.::::::::::.::::
query 3' UAAGUAAUAAUAGUAGUGGUAGU 5'
query=miR8748, target=RO3G_00289, score=3, range=917-939, strand=
target 5' AUUCAUUAUCAUUAUCAUCAUCA 3'
::::::::: ::.::::.:::::
query 3' UAAGUAAUAAUAGUAGUGGUAGU 5'
query=miR8748, target=RO3G_13923, score=3, range=228-250, strand=
target 5' GCUUAUUAUUAUCAUCAUCAUCA 3'
. :.::::::::::::::.::::
query 3' UAAGUAAUAAUAGUAGUGGUAGU 5'
query=miR8748, target=RO3G_01836, score=3, range=258-280, strand=
target 5' AUUCAUCAUAAUCAUCAUCAUCA 3'
:::::: :: ::::::::.::::
query 3' UAAGUAAUAAUAGUAGUGGUAGU 5'
query=miR8748, target=RO3G_09516, score=3.5, range=240-262, stran
target 5' UAUUAUUAUUAUCAUCAUCAUCA 3'
:.::::::::::::::.::::
query 3' UAAGUAAUAAUAGUAGUGGUAGU 5'
query=miR8748, target=RO3G_03951, score=3.5, range=672-694, stran
target 5' CUUCAUUAUCAUCAUCAUCAUCG 3'
:::::::: :::::::.::::.
query 3' UAAGUAAUAAUAGUAGUGGUAGU 5'
query=miR8748, target=RO3G_10591, score=3.5, range=999-1021, stra
target 5' AAUCGUCAUUAUCAUCAUCAUCA 3'
: ::.: ::::::::::.:::::
query 3' UAAGUAAUAAUAGUAGUGGUAGU 5'
query=miR8748, target=RO3G_14401, score=4, range=776-799, strand=
target 5' AAUUGUUAUUAUCAUCACCCAUCA 3'
: :..:::::::::::: ::::::
query 3' UAAGUAAUAAUAGUAGU-GGUAGU 5'
query=miR8748, target=RO3G_03827, score=4, range=1063-1085, stran
target 5' AAUCAUCAUCAUCAUCAUCAUCA 3'
: :::: :: :::::::.:::::
query 3' UAAGUAAUAAUAGUAGUGGUAGU 5'
query=miR8748, target=RO3G_00729, score=4, range=1048-1070, stran
target 5' AAUCAUCAUCAUCAUCAUCAUCA 3'
: :::: :: :::::::.:::::
query 3' UAAGUAAUAAUAGUAGUGGUAGU 5'
```

```
query=miR8748, target=RO3G_07191, score=4, range=281-303, strand=
target 5' AUAGAGCAUUAUCAUCACCAUCA 3'
:: : ::::::::::::::::
query 3' UAAGUAAUAAUAGUAGUGGUAGU 5'
query=miR8748, target=RO3G_10973, score=4, range=576-598, strand=
target 5' GGUCGUUAUUAUCAUCGUCAUCA 3'
. ::.:::::::::::..:::::
query 3' UAAGUAAUAAUAGUAGUGGUAGU 5'
query=miR8748, target=RO3G_14835, score=4, range=450-473, strand=
target 5' GUUCUGUUAUUAUCAUCAUCGUCA 3'
.::: .::::::::::::.:.:::
query 3' UAAG-UAAUAAUAGUAGUGGUAGU 5'
query=miR8748, target=RO3G_09517, score=4, range=507-529, strand=
target 5' GUUCAUUGUUAUCAUCAACAUCC 3'
.::::::.::::::::: ::::
query 3' UAAGUAAUAAUAGUAGUGGUAGU 5'
query=miR8748, target=RO3G_13781, score=4, range=210-232, strand=
target 5' AUUUGUCAUCAUCAUCAUCAUCA 3'
:::..: :: :::::::.:::::
query 3' UAAGUAAUAAUAGUAGUGGUAGU 5'
query=miR8748, target=RO3G_11361, score=4, range=192-214, strand=
target 5' AUUCAUCGAUAUCAUCGCCAUCG 3'
:::::: . :::::::.:::::.
query 3' UAAGUAAUAAUAGUAGUGGUAGU 5'
query=miR8748, target=RO3G_00068, score=4, range=933-955, strand=
target 5' CUUCAUCAUCAUCAUCAUCAUCA 3'
::::: :: :::::::.:::::
query 3' UAAGUAAUAAUAGUAGUGGUAGU 5'
query=miR8748, target=RO3G_15427, score=4, range=630-652, strand=
target 5' CUUCAUCAUCAUCAUCAUCAUCA 3'
::::: :: :::::::.:::::
query 3' UAAGUAAUAAUAGUAGUGGUAGU 5'
query=miR8748, target=RO3G_10691, score=4, range=522-544, strand=
target 5' AGUCAUCAUCAUCAUCAUCAUCA 3'
: :::: :: :::::::.:::::
query 3' UAAGUAAUAAUAGUAGUGGUAGU 5'
query=miR8748, target=RO3G_15993, score=4, range=723-746, strand=
target 5' AUGUCAUCAUCAUCAUCAUCAUCA 3'
:: :::: :: :::::::.:::::
query 3' UA-AGUAAUAAUAGUAGUGGUAGU 5'
query=miR8748, target=RO3G_15201, score=4, range=492-514, strand=
target 5' AAUCAUCAUCAUCAUCAUCAUCA 3'
: :::: :: :::::::.:::::
query 3' UAAGUAAUAAUAGUAGUGGUAGU 5'
query=miR8748, target=RO3G_04948, score=4, range=402-424, strand=
```

```
target 5' CAUCAUCAUCAUCAUCACCAUCA 3'
:::: :: ::::::::::::
query 3' UAAGUAAUAAUAGUAGUGGUAGU 5'
query=miR8748, target=RO3G_06092, score=4, range=4407-4428, stran
target 5' CUUCA-ACUUAUCAUCACCAUCA 3'
:::: ::::::::::::::
query 3' UAAGUAAUAAUAGUAGUGGUAGU 5'
query=miR408-3p, target=RO3G_11207, score=4, range=2798-2816, str
target 5' CGAUGGAAGAGGCAG-GCAU 3'
: : :::::::::: ::::
query 3' GGUCCCUUCUCCGUCACGUA 5'
query=miR408-3p, target=RO3G_04583, score=4, range=378-396, stran
target 5' CCAAGGAAGAGG-AGUGUAU 3'
::: :::::::: ::::.::
query 3' GGUCCCUUCUCCGUCACGUA 5'
query=miR9663-5p, target=RO3G_12194, score=0, range=927-945, stra
target 5' AGAAUCGGUCGACUACGUU 3'
:::::::::::::::::::
query 3' UCUUAGCCAGCUGAUGCAA 5'
query=miR9663-5p, target=RO3G_05630, score=0, range=927-945, stra
target 5' AGAAUCGGUCGACUACGUU 3'
:::::::::::::::::::
query 3' UCUUAGCCAGCUGAUGCAA 5'
query=miR9663-5p, target=RO3G_12951, score=0, range=927-945, stra
target 5' AGAAUCGGUCGACUACGUU 3'
:::::::::::::::::::
query 3' UCUUAGCCAGCUGAUGCAA 5'
query=miR9663-5p, target=RO3G_03988, score=0, range=927-945, stra
target 5' AGAAUCGGUCGACUACGUU 3'
:::::::::::::::::::
query 3' UCUUAGCCAGCUGAUGCAA 5'
query=miR9663-5p, target=RO3G_09376, score=0, range=927-945, stra
target 5' AGAAUCGGUCGACUACGUU 3'
:::::::::::::::::::
query 3' UCUUAGCCAGCUGAUGCAA 5'
query=miR9663-5p, target=RO3G_13152, score=0, range=927-945, stra
target 5' AGAAUCGGUCGACUACGUU 3'
:::::::::::::::::::
query 3' UCUUAGCCAGCUGAUGCAA 5'
query=miR9663-5p, target=RO3G_02235, score=0, range=270-288, stra
target 5' AGAAUCGGUCGACUACGUU 3'
:::::::::::::::::::
query 3' UCUUAGCCAGCUGAUGCAA 5'
query=miR9663-5p, target=RO3G_11970, score=0, range=927-945, stra
target 5' AGAAUCGGUCGACUACGUU 3'
```

```
::::::::::::::::::::
query 3' UCUUAGCCAGCUGAUGCAA 5'
query=miR9663-5p, target=RO3G_12979, score=0, range=927-945, stra
target 5' AGAAUCGGUCGACUACGUU 3'
::::::::::::::::::::
query 3' UCUUAGCCAGCUGAUGCAA 5'
query=miR9663-5p, target=RO3G_12087, score=0, range=927-945, stra
target 5' AGAAUCGGUCGACUACGUU 3'
::::::::::::::::::::
query 3' UCUUAGCCAGCUGAUGCAA 5'
query=miR9663-5p, target=RO3G_10555, score=0, range=927-945, stra
target 5' AGAAUCGGUCGACUACGUU 3'
::::::::::::::::::::
query 3' UCUUAGCCAGCUGAUGCAA 5'
query=miR9663-5p, target=RO3G_03103, score=0, range=927-945, stra
target 5' AGAAUCGGUCGACUACGUU 3'
::::::::::::::::::::
query 3' UCUUAGCCAGCUGAUGCAA 5'
query=miR9663-5p, target=RO3G_08612, score=0, range=927-945, stra
target 5' AGAAUCGGUCGACUACGUU 3'
::::::::::::::::::::
query 3' UCUUAGCCAGCUGAUGCAA 5'
query=miR9663-5p, target=RO3G_07305, score=0, range=927-945, stra
target 5' AGAAUCGGUCGACUACGUU 3'
::::::::::::::::::::
query 3' UCUUAGCCAGCUGAUGCAA 5'
query=miR9663-5p, target=RO3G_05280, score=0, range=927-945, stra
target 5' AGAAUCGGUCGACUACGUU 3'
::::::::::::::::::::
query 3' UCUUAGCCAGCUGAUGCAA 5'
query=miR9663-5p, target=RO3G_16686, score=0, range=927-945, stra
target 5' AGAAUCGGUCGACUACGUU 3'
::::::::::::::::::::
query 3' UCUUAGCCAGCUGAUGCAA 5'
query=miR9663-5p, target=RO3G_14704, score=0, range=927-945, stra
target 5' AGAAUCGGUCGACUACGUU 3'
::::::::::::::::::::
query 3' UCUUAGCCAGCUGAUGCAA 5'
query=miR9663-5p, target=RO3G_13859, score=0, range=552-570, stra
target 5' AGAAUCGGUCGACUACGUU 3'
::::::::::::::::::::
query 3' UCUUAGCCAGCUGAUGCAA 5'
query=miR9663-5p, target=RO3G_01203, score=0, range=927-945, stra
target 5' AGAAUCGGUCGACUACGUU 3'
::::::::::::::::::::
```

```
query 3' UCUUAGCCAGCUGAUGCAA 5'
query=miR9663-5p, target=RO3G_01718, score=0, range=927-945, stra
target 5' AGAAUCGGUCGACUACGUU 3'
:::::::::::::::::::
query 3' UCUUAGCCAGCUGAUGCAA 5'
query=miR9663-5p, target=RO3G_15869, score=0, range=927-945, stra
target 5' AGAAUCGGUCGACUACGUU 3'
:::::::::::::::::::
query 3' UCUUAGCCAGCUGAUGCAA 5'
query=miR9663-5p, target=RO3G_11403, score=0, range=927-945, stra
target 5' AGAAUCGGUCGACUACGUU 3'
:::::::::::::::::::
query 3' UCUUAGCCAGCUGAUGCAA 5'
query=miR9663-5p, target=RO3G_06416, score=0, range=927-945, stra
target 5' AGAAUCGGUCGACUACGUU 3'
:::::::::::::::::::
query 3' UCUUAGCCAGCUGAUGCAA 5'
query=miR9663-5p, target=RO3G_11275, score=0, range=927-945, stra
target 5' AGAAUCGGUCGACUACGUU 3'
:::::::::::::::::::
query 3' UCUUAGCCAGCUGAUGCAA 5'
query=miR9663-5p, target=RO3G_16744, score=0, range=927-945, stra
target 5' AGAAUCGGUCGACUACGUU 3'
:::::::::::::::::::
query 3' UCUUAGCCAGCUGAUGCAA 5'
query=miR9663-5p, target=RO3G_10560, score=0, range=927-945, stra
target 5' AGAAUCGGUCGACUACGUU 3'
:::::::::::::::::::
query 3' UCUUAGCCAGCUGAUGCAA 5'
query=miR9663-5p, target=RO3G_07104, score=0, range=927-945, stra
target 5' AGAAUCGGUCGACUACGUU 3'
:::::::::::::::::::
query 3' UCUUAGCCAGCUGAUGCAA 5'
query=miR9663-5p, target=RO3G_04610, score=3.5, range=319-337,
strand=-1
target 5' CGGAUCGGUCAACUACGUU 3'
:.::::::: ::::::::
query 3' UCUUAGCCAGCUGAUGCAA 5'
query=miR9663-5p, target=RO3G_00313, score=4, range=2798-2817,
strand=1
target 5' AGAGUUGCGUCGACUGCGUU 3'
:::.:.: ::::::.::::
query 3' UCUUAGC-CAGCUGAUGCAA 5'
query=miR9663-5p, target=RO3G_13398, score=4, range=326-344, stra
target 5' AGAAUAGGUCGAUUGUGUU 3'
```

```
::::: ::::::.:..:::
query 3' UCUUAGCCAGCUGAUGCAA 5'
query=miR9663-5p, target=RO3G_13700, score=4, range=14-32, strand
target 5' CGUAUCAGUCGACUACGUU 3'
: ::: :::::::::::
query 3' UCUUAGCCAGCUGAUGCAA 5'
query=miR9663-5p, target=RO3G_06018, score=4, range=17-35, strand
target 5' UGAACGGGUCGACUGCGUU 3'
::: ::::::::.::::
query 3' UCUUAGCCAGCUGAUGCAA 5'
query=miR9554-3p, target=RO3G_12520, score=0, range=1561-1583,
strand=1
target 5' AUGAAUGAUGACUUGAGCUAUGA 3'
:::::::::::::::::::::::
query 3' UACUUACUACUGAACUCGAUACU 5'
query=miR9554-3p, target=RO3G_00797, score=0, range=1561-1583,
strand=1
target 5' AUGAAUGAUGACUUGAGCUAUGA 3'
:::::::::::::::::::::::
query 3' UACUUACUACUGAACUCGAUACU 5'
query=miR9554-3p, target=RO3G_02103, score=2, range=88-110, stran
target 5' AUGAACGACGACUUGAGCUAUGA 3'
::::: :: ::::::::::::::
query 3' UACUUACUACUGAACUCGAUACU 5'
query=miR9554-3p, target=RO3G_01994, score=4, range=422-443, stra
target 5' AUG-AUGAUGACUUGAGUUAUAA 3'
::: :::::::::::::.::: :
query 3' UACUUACUACUGAACUCGAUACU 5'
query=miR9554-3p, target=RO3G_05597, score=4, range=54-77, strand
target 5' AUUAAUCGAAGACUUGGGCUAUGA 3'
:: ::: :: :::::.:::::::
query 3' UACUUA-CUACUGAACUCGAUACU 5'
query=miR858b, target=RO3G_16744, score=0, range=1773-1792, stran
target 5' CAAGCGACGAACAGAACGAA 3'
::::::::::::::::::::
query 3' GUUCGCUGCUUGUCUUGCUU 5'
query=miR858b, target=RO3G_01203, score=0, range=1773-1792, stran
target 5' CAAGCGACGAACAGAACGAA 3'
::::::::::::::::::::
query 3' GUUCGCUGCUUGUCUUGCUU 5'
query=miR858b, target=RO3G_11403, score=0, range=1773-1792, stran
target 5' CAAGCGACGAACAGAACGAA 3'
::::::::::::::::::::
query 3' GUUCGCUGCUUGUCUUGCUU 5'
query=miR858b, target=RO3G_06416, score=0, range=1773-1792, stran
```

```
target 5' CAAGCGACGAACAGAACGAA 3'
::::::::::::::::::::
query 3' GUUCGCUGCUUGUCUUGCUU 5'
query=miR858b, target=RO3G_01718, score=0, range=1773-1792, stran
target 5' CAAGCGACGAACAGAACGAA 3'
::::::::::::::::::::
query 3' GUUCGCUGCUUGUCUUGCUU 5'
query=miR858b, target=RO3G_15869, score=0, range=1773-1792, stran
target 5' CAAGCGACGAACAGAACGAA 3'
::::::::::::::::::::
query 3' GUUCGCUGCUUGUCUUGCUU 5'
query=miR858b, target=RO3G_10560, score=0, range=1773-1792, stran
target 5' CAAGCGACGAACAGAACGAA 3'
::::::::::::::::::::
query 3' GUUCGCUGCUUGUCUUGCUU 5'
query=miR858b, target=RO3G_11275, score=0, range=1773-1792, stran
target 5' CAAGCGACGAACAGAACGAA 3'
::::::::::::::::::::
query 3' GUUCGCUGCUUGUCUUGCUU 5'
query=miR858b, target=RO3G_13859, score=0, range=1398-1417, stran
target 5' CAAGCGACGAACAGAACGAA 3'
::::::::::::::::::::
query 3' GUUCGCUGCUUGUCUUGCUU 5'
query=miR858b, target=RO3G_12194, score=0, range=1773-1792, stran
target 5' CAAGCGACGAACAGAACGAA 3'
::::::::::::::::::::
query 3' GUUCGCUGCUUGUCUUGCUU 5'
query=miR858b, target=RO3G_09376, score=0, range=1773-1792, stran
target 5' CAAGCGACGAACAGAACGAA 3'
::::::::::::::::::::
query 3' GUUCGCUGCUUGUCUUGCUU 5'
query=miR858b, target=RO3G_07735, score=3, range=2831-2850, stran
target 5' AGAGCGACGAGCAGAACGAG 3'
.:::::::.::::::::.
query 3' GUUCGCUGCUUGUCUUGCUU 5'
query=miR858b, target=RO3G_10543, score=3, range=2384-2403, stran
target 5' AGAGCGACGAGCAGAACGAG 3'
.:::::::.::::::::.
query 3' GUUCGCUGCUUGUCUUGCUU 5'
query=miR858b, target=RO3G_10744, score=3, range=2930-2949, stran
target 5' AGAGCGACGAGCAGAACGAG 3'
.:::::::.::::::::.
query 3' GUUCGCUGCUUGUCUUGCUU 5'
query=miR858b, target=RO3G_14563, score=3, range=494-513, strand=
target 5' AGAGCGACGAGCAGAACGAG 3'
```

```
.::::::::.::::::::.
query 3' GUUCGCUGCUUGUCUUGCUU 5'
query=miR858b, target=RO3G_10745, score=3, range=1898-1917, stran
target 5' AGAGCGACGAGCAGAACGAG 3'
.::::::::.::::::::.
query 3' GUUCGCUGCUUGUCUUGCUU 5'
query=miR858b, target=RO3G_07862, score=3, range=3041-3060, stran
target 5' AGAGCGACGAGCAGAACGAG 3'
.::::::::.::::::::.
query 3' GUUCGCUGCUUGUCUUGCUU 5'
query=miR858b, target=RO3G_17255, score=3, range=2882-2901, stran
target 5' AGAGCGACGAGCAGAACGAG 3'
.::::::::.::::::::.
query 3' GUUCGCUGCUUGUCUUGCUU 5'
query=miR858b, target=RO3G_17156, score=3, range=1184-1203, stran
target 5' AGAGCGACGAGCAGAACGAG 3'
.::::::::.::::::::.
query 3' GUUCGCUGCUUGUCUUGCUU 5'
query=miR858b, target=RO3G_16919, score=3, range=506-525, strand=
target 5' AGAGCGACGAGCAGAACGAG 3'
.::::::::.::::::::.
query 3' GUUCGCUGCUUGUCUUGCUU 5'
query=miR858b, target=RO3G_13224, score=3.5, range=1649-1669,
strand=-1
target 5' CUGGUAGGCGAACAGAACGAA 3'
: .:. :.:::::::::::::
query 3' GUUCG-CUGCUUGUCUUGCUU 5'
query=miR858b, target=RO3G_13221, score=3.5, range=1649-1669,
strand=-1
target 5' CUGGUAGGCGAACAGAACGAA 3'
: .:. :.:::::::::::::
query 3' GUUCG-CUGCUUGUCUUGCUU 5'
query=miR858b, target=RO3G_04120, score=3.5, range=1649-1669,
strand=-1
target 5' CUGGUAGGCGAACAGAACGAA 3'
: .:. :.:::::::::::::
query 3' GUUCG-CUGCUUGUCUUGCUU 5'
query=miR858b, target=RO3G_04203, score=3.5, range=593-613, stran
target 5' CUGGCAAACGAACAGAACGAA 3'
: .:: ::::::::::::::
query 3' GUUCG-CUGCUUGUCUUGCUU 5'
query=miR858b, target=RO3G_16122, score=3.5, range=62-82, strand=
target 5' CUGGCAAACGAACAGAACGAA 3'
: .:: ::::::::::::::
query 3' GUUCG-CUGCUUGUCUUGCUU 5'
```

```
query=miR858b, target=RO3G_11693, score=4, range=134-153, strand=
target 5' CAAACGACGAAAAGAAUGAA 3'
::: ::::::: ::::.:::
query 3' GUUCGCUGCUUGUCUUGCUU 5'
query=miR858b, target=RO3G_17150, score=4, range=2048-2068, stran
target 5' UAAGCUGACGAGCGGAACGAG 3'
.:::: :::::.:.::::::.
query 3' GUUCG-CUGCUUGUCUUGCUU 5'
query=miR858b, target=RO3G_11767, score=4, range=1202-1222, stran
target 5' UAAGCUGACGAGCGGAACGAG 3'
.:::: :::::.:.::::::.
query 3' GUUCG-CUGCUUGUCUUGCUU 5'
query=miR858b, target=RO3G_12316, score=4, range=53-72, strand=-1
target 5' CAAGUUACGAACAGCACGAG 3'
::::. :::::::: ::::.
query 3' GUUCGCUGCUUGUCUUGCUU 5'
query=miR858b, target=RO3G_16290, score=4, range=821-839, strand=
target 5' CAAGC-ACGAAUAGAAUGAU 3'
::::: :::::.::::.::
query 3' GUUCGCUGCUUGUCUUGCUU 5'
query=miR858b, target=RO3G_00335, score=4, range=11-30, strand=-1
target 5' CUUGCGACCAACAGAACGAA 3'
: ::::: ::::::::::
query 3' GUUCGCUGCUUGUCUUGCUU 5'
query=miR827, target=RO3G_16161, score=3, range=675-694, strand=1
target 5' AUUUAAUGAUGGUCAUUUAA 3'
:::: ::::::::::.:::
query 3' UAAACAACUACCAGUAGAUU 5'
query=miR827, target=RO3G_15356, score=4, range=687-706, strand=1
target 5' AUUUGGUGGUUGUCAUCUAA 3'
::::: ::.: :::::::::
query 3' UAAACAACUACCAGUAGAUU 5'
query=miR827, target=RO3G_00714, score=4, range=616-635, strand=-
target 5' AUUUGUUGAUGGUUUUUUAA 3'
:::::::::::::. :.:::
query 3' UAAACAACUACCAGUAGAUU 5'
query=miR827, target=RO3G_16787, score=4, range=2140-2160, strand
target 5' CCUUGUUGAUGGUUCAUCUAA 3'
:::::::::: ::::::::
query 3' UAAACAACUACC-AGUAGAUU 5'
query=miR171a-3p, target=RO3G_16852, score=4, range=398-416, stra
target 5' AUAUUUUCG-GGCUCAAUCA 3'
::::: :: ::::::::::
query 3' UAUAACCGCGCCGAGUUAGU 5'
query=miR2656a, target=RO3G_02845, score=0, range=214-235, strand
```

```
target 5' CCAACGUCCAAUAUGCAACUUA 3'
::::::::::::::::::::::
query 3' GGUUGCAGGUUAUACGUUGAAU 5'
query=miR2656a, target=RO3G_13919, score=2, range=181-202, strand
target 5' CCAACGUCCAAUAUGCAACCUA 3'
::::::::::::::::::: ::
query 3' GGUUGCAGGUUAUACGUUGAAU 5'
query=miR2656a, target=RO3G_00679, score=2, range=181-202, strand
target 5' CCAACGUCCAAUAUGCAACCUA 3'
::::::::::::::::::: ::
query 3' GGUUGCAGGUUAUACGUUGAAU 5'
query=miR2656a, target=RO3G_13286, score=2, range=181-202, strand
target 5' CCAACGUCCAAUAUGCAACCUA 3'
::::::::::::::::::: ::
query 3' GGUUGCAGGUUAUACGUUGAAU 5'
query=miR2656a, target=RO3G_00284, score=2, range=181-202, strand
target 5' CCAACGUCCAAUAUGCAACCUA 3'
::::::::::::::::::: ::
query 3' GGUUGCAGGUUAUACGUUGAAU 5'
query=miR2656a, target=RO3G_12678, score=2, range=181-202, strand
target 5' CCAACGUCCAAUAUGCAACCUA 3'
::::::::::::::::::: ::
query 3' GGUUGCAGGUUAUACGUUGAAU 5'
query=miR2656a, target=RO3G_03602, score=2, range=181-202, strand
target 5' CCAACGUCCAAUAUGCAACCUA 3'
::::::::::::::::::: ::
query 3' GGUUGCAGGUUAUACGUUGAAU 5'
query=miR2656a, target=RO3G_02706, score=2, range=181-202, strand
target 5' CCAACGUCCAAUAUGCAACCUA 3'
::::::::::::::::::: ::
query 3' GGUUGCAGGUUAUACGUUGAAU 5'
query=miR2656a, target=RO3G_01960, score=2, range=181-202, strand
target 5' CCAACGUCCAAUAUGCAACCUA 3'
::::::::::::::::::: ::
query 3' GGUUGCAGGUUAUACGUUGAAU 5'
query=miR2656a, target=RO3G_13454, score=2, range=181-202, strand
target 5' CCAACGUCCAAUAUGCAACCUA 3'
::::::::::::::::::: ::
query 3' GGUUGCAGGUUAUACGUUGAAU 5'
query=miR2656a, target=RO3G_17222, score=3, range=181-202, strand
target 5' CCAUCGUCCAAUAUGCAACCUA 3'
::: ::::::::::::::: ::
query 3' GGUUGCAGGUUAUACGUUGAAU 5'
query=miR2656a, target=RO3G_07707, score=4, range=181-202, strand
target 5' CCAACGUCCAAUAUGCAAACUA 3'
```

```
:::::::::::::::::: ::
query 3' GGUUGCAGGUUAUACGUUGAAU 5'
query=miR8123-3p, target=RO3G_16904, score=0, range=483-503, stra
target 5' GACUUGGACCAAUGGCGACAA 3'
:::::::::::::::::::::
query 3' CUGAACCUGGUUACCGCUGUU 5'
query=miR8123-3p, target=RO3G_16829, score=0, range=483-503, stra
target 5' GACUUGGACCAAUGGCGACAA 3'
:::::::::::::::::::::
query 3' CUGAACCUGGUUACCGCUGUU 5'
query=miR8123-3p, target=RO3G_16428, score=0, range=120-140, stra
target 5' GACUUGGACCAAUGGCGACAA 3'
:::::::::::::::::::::
query 3' CUGAACCUGGUUACCGCUGUU 5'
query=miR8123-3p, target=RO3G_16721, score=1, range=441-461, stra
target 5' GACUUGGACCAAUGGCGAUAA 3'
::::::::::::::::::.::
query 3' CUGAACCUGGUUACCGCUGUU 5'
query=miR8123-3p, target=RO3G_11411, score=1, range=120-140, stra
target 5' GACUUGGACCAAUGGCGAUAA 3'
::::::::::::::::::.::
query 3' CUGAACCUGGUUACCGCUGUU 5'
query=miR8123-3p, target=RO3G_05945, score=1, range=120-140, stra
target 5' GACUUGGACCAAUGGCGAUAA 3'
::::::::::::::::::.::
query 3' CUGAACCUGGUUACCGCUGUU 5'
query=miR8123-3p, target=RO3G_16703, score=1.5, range=483-503,
strand=1
target 5' GGCUUGGACCAAUGGCGAUAA 3'
:.::::::::::::::::.::
query 3' CUGAACCUGGUUACCGCUGUU 5'
query=miR8123-3p, target=RO3G_13734, score=2, range=483-503, stra
target 5' GACUUGGACUAAUGGCGAUAA 3'
:::::::::.::::::::.::
query 3' CUGAACCUGGUUACCGCUGUU 5'
query=miR8123-3p, target=RO3G_17154, score=2, range=120-140, stra
target 5' GACUUGGACUAAUGGCGAUAA 3'
:::::::::.::::::::.::
query 3' CUGAACCUGGUUACCGCUGUU 5'
query=miR8123-3p, target=RO3G_02224, score=2, range=120-140, stra
target 5' GACUUGGACUAAUGGCGAUAA 3'
:::::::::.::::::::.::
query 3' CUGAACCUGGUUACCGCUGUU 5'
query=miR8123-3p, target=RO3G_12060, score=2, range=120-140, stra
target 5' GACUUGGACUAAUGGCGAUAA 3'
```

```
::::::::.::::::::.::
query 3' CUGAACCUGGUUACCGCUGUU 5'
query=miR8123-3p, target=RO3G_17202, score=3, range=483-503, stra
target 5' GACUUGGACCAAUGGCAAUAA 3'
:::::::::::::::: :.::
query 3' CUGAACCUGGUUACCGCUGUU 5'
query=miR8123-3p, target=RO3G_00676, score=3, range=483-503, stra
target 5' GACUUGGAGCAAUGGCGAUAA 3'
:::::::: :::::::::.::
query 3' CUGAACCUGGUUACCGCUGUU 5'
query=miR8123-3p, target=RO3G_03594, score=3, range=120-140, stra
target 5' GACUUGGACCAAUGGCAAUAA 3'
:::::::::::::::: :.::
query 3' CUGAACCUGGUUACCGCUGUU 5'
query=miR8123-3p, target=RO3G_14688, score=3, range=1604-1624,
strand=-1
target 5' UAUUUUGGCCAAUGGCGACAA 3'
:.:: :.:::::::::::::
query 3' CUGAACCUGGUUACCGCUGUU 5'
query=miR2622, target=RO3G_06442, score=0, range=1126-1148, stran
target 5' UAAUGUUUCACGAUGGCCUCAAA 3'
:::::::::::::::::::::::
query 3' AUUACAAAGUGCUACCGGAGUUU 5'
query=miR2622, target=RO3G_02691, score=0, range=4318-4340, stran
target 5' UAAUGUUUCACGAUGGCCUCAAA 3'
:::::::::::::::::::::::
query 3' AUUACAAAGUGCUACCGGAGUUU 5'
query=miR2622, target=RO3G_09965, score=0, range=829-851, strand=
target 5' UAAUGUUUCACGAUGGCCUCAAA 3'
:::::::::::::::::::::::
query 3' AUUACAAAGUGCUACCGGAGUUU 5'
query=miR2622, target=RO3G_13920, score=0, range=829-851, strand=
target 5' UAAUGUUUCACGAUGGCCUCAAA 3'
:::::::::::::::::::::::
query 3' AUUACAAAGUGCUACCGGAGUUU 5'
query=miR2622, target=RO3G_03547, score=0, range=829-851, strand=
target 5' UAAUGUUUCACGAUGGCCUCAAA 3'
:::::::::::::::::::::::
query 3' AUUACAAAGUGCUACCGGAGUUU 5'
query=miR2622, target=RO3G_02959, score=0, range=829-851, strand=
target 5' UAAUGUUUCACGAUGGCCUCAAA 3'
:::::::::::::::::::::::
query 3' AUUACAAAGUGCUACCGGAGUUU 5'
query=miR2622, target=RO3G_15250, score=0, range=829-851, strand=
target 5' UAAUGUUUCACGAUGGCCUCAAA 3'
```

```
:::::::::::::::::::::::
query 3' AUUACAAAGUGCUACCGGAGUUU 5'
query=miR2622, target=RO3G_17371, score=0, range=829-851, strand=
target 5' UAAUGUUUCACGAUGGCCUCAAA 3'
:::::::::::::::::::::::
query 3' AUUACAAAGUGCUACCGGAGUUU 5'
query=miR2622, target=RO3G_17326, score=0, range=499-521, strand=
target 5' UAAUGUUUCACGAUGGCCUCAAA 3'
:::::::::::::::::::::::
query 3' AUUACAAAGUGCUACCGGAGUUU 5'
query=miR2622, target=RO3G_15585, score=0, range=829-851, strand=
target 5' UAAUGUUUCACGAUGGCCUCAAA 3'
:::::::::::::::::::::::
query 3' AUUACAAAGUGCUACCGGAGUUU 5'
query=miR2622, target=RO3G_04114, score=0, range=829-851, strand=
target 5' UAAUGUUUCACGAUGGCCUCAAA 3'
:::::::::::::::::::::::
query 3' AUUACAAAGUGCUACCGGAGUUU 5'
query=miR2622, target=RO3G_05272, score=0, range=829-851, strand=
target 5' UAAUGUUUCACGAUGGCCUCAAA 3'
:::::::::::::::::::::::
query 3' AUUACAAAGUGCUACCGGAGUUU 5'
query=miR2622, target=RO3G_08608, score=0, range=1345-1367, stran
target 5' UAAUGUUUCACGAUGGCCUCAAA 3'
:::::::::::::::::::::::
query 3' AUUACAAAGUGCUACCGGAGUUU 5'
query=miR2622, target=RO3G_05617, score=0, range=829-851, strand=
target 5' UAAUGUUUCACGAUGGCCUCAAA 3'
:::::::::::::::::::::::
query 3' AUUACAAAGUGCUACCGGAGUUU 5'
query=miR2622, target=RO3G_00307, score=0, range=1261-1283, stran
target 5' UAAUGUUUCACGAUGGCCUCAAA 3'
:::::::::::::::::::::::
query 3' AUUACAAAGUGCUACCGGAGUUU 5'
query=miR2622, target=RO3G_14356, score=0, range=829-851, strand=
target 5' UAAUGUUUCACGAUGGCCUCAAA 3'
:::::::::::::::::::::::
query 3' AUUACAAAGUGCUACCGGAGUUU 5'
query=miR2622, target=RO3G_16696, score=0, range=820-842, strand=
target 5' UAAUGUUUCACGAUGGCCUCAAA 3'
:::::::::::::::::::::::
query 3' AUUACAAAGUGCUACCGGAGUUU 5'
query=miR2622, target=RO3G_11783, score=0, range=829-851, strand=
target 5' UAAUGUUUCACGAUGGCCUCAAA 3'
:::::::::::::::::::::::
```

```
query 3' AUUACAAAGUGCUACCGGAGUUU 5'
query=miR2622, target=RO3G_01615, score=0, range=382-404, strand=
target 5' UAAUGUUUCACGAUGGCCUCAAA 3'
:::::::::::::::::::::::
query 3' AUUACAAAGUGCUACCGGAGUUU 5'
query=miR2622, target=RO3G_06442, score=0, range=1306-1328, stran
target 5' UAAUGUUUCACGAUGGCCUCAAA 3'
:::::::::::::::::::::::
query 3' AUUACAAAGUGCUACCGGAGUUU 5'
query=miR2622, target=RO3G_17326, score=0, range=679-701, strand=
target 5' UAAUGUUUCACGAUGGCCUCAAA 3'
:::::::::::::::::::::::
query 3' AUUACAAAGUGCUACCGGAGUUU 5'
query=miR2622, target=RO3G_01947, score=1, range=499-521, strand=
target 5' UAAUGUUUCACGAUGGCUUCAAA 3'
:::::::::::::::::.:::::
query 3' AUUACAAAGUGCUACCGGAGUUU 5'
query=miR2622, target=RO3G_17158, score=3, range=733-755, strand=
target 5' UAAUGCUUCACGAUUGCCUCAAA 3'
::::: :::::::: ::::::::
query 3' AUUACAAAGUGCUACCGGAGUUU 5'
query=miR2622, target=RO3G_14266, score=4, range=1009-1032, stran
target 5' UAUGUGAUUUAUGAUGGCCUCAAA 3'
:: .:: ::.:.:::::::::::::
query 3' AU-UACAAAGUGCUACCGGAGUUU 5'
query=miR7495a, target=RO3G_15523, score=0, range=150-169, strand
target 5' UGAAGGAGGCACUACAAGAA 3'
::::::::::::::::::::
query 3' ACUUCCUCCGUGAUGUUCUU 5'
query=miR7495a, target=RO3G_09419, score=3, range=776-795, strand
target 5' UGAAGGUGGCUCUACAAGAA 3'
:::::: ::: :::::::::
query 3' ACUUCCUCCGUGAUGUUCUU 5'
query=miR7495a, target=RO3G_04309, score=3.5, range=1169-1189,
strand=1
target 5' UCAAGGCAGGCACUGCAAGAG 3'
: :::: :::::::.:::::.
query 3' ACUUCC-UCCGUGAUGUUCUU 5'
query=miR7495a, target=RO3G_00977, score=3.5, range=488-508, stra
target 5' UCAAGGCAGGCACUGCAAGAG 3'
: :::: :::::::.:::::.
query 3' ACUUCC-UCCGUGAUGUUCUU 5'
query=miR7495a, target=RO3G_05161, score=4, range=2076-2095, stra
target 5' GGAAGGAGGCGAUACAAGAA 3'
:::::::::. ::::::::
```

```
query 3' ACUUCCUCCGUGAUGUUCUU 5'
query=miR7495a, target=RO3G_02132, score=4, range=156-175, strand
target 5' UGAAGGAGGCACGAAAAGAA 3'
:::::::::::: : :::::
query 3' ACUUCCUCCGUGAUGUUCUU 5'
query=miR7495a, target=RO3G_07788, score=4, range=905-924, strand
target 5' UGAAGGUGGCUCUACGAGAA 3'
:::::: ::: ::::.::::
query 3' ACUUCCUCCGUGAUGUUCUU 5'
query=miR7495a, target=RO3G_01923, score=4, range=905-924, strand
target 5' UGAAGGUGGCUCUACGAGAA 3'
:::::: ::: ::::.::::
query 3' ACUUCCUCCGUGAUGUUCUU 5'
query=miR7495a, target=RO3G_14467, score=4, range=485-504, strand
target 5' UGAAGGUGGCUCUACGAGAA 3'
:::::: ::: ::::.::::
query 3' ACUUCCUCCGUGAUGUUCUU 5'
query=miR7495a, target=RO3G_11567, score=4, range=1613-1632, stra
target 5' UGAAGGUGGCUCUACGAGAA 3'
:::::: ::: ::::.::::
query 3' ACUUCCUCCGUGAUGUUCUU 5'
query=miR7495a, target=RO3G_11486, score=4, range=665-684, strand
target 5' UGAAGGUGGCUCUACGAGAA 3'
:::::: ::: ::::.::::
query 3' ACUUCCUCCGUGAUGUUCUU 5'
query=miR7495a, target=RO3G_02354, score=4, range=956-975, strand
target 5' UGAAGGUGGCUCUACGAGAA 3'
:::::: ::: ::::.::::
query 3' ACUUCCUCCGUGAUGUUCUU 5'
query=miR7495a, target=RO3G_08906, score=4, range=956-975, strand
target 5' UGAAGGUGGCUCUACGAGAA 3'
:::::: ::: ::::.::::
query 3' ACUUCCUCCGUGAUGUUCUU 5'
query=miR7495a, target=RO3G_05303, score=4, range=956-975, strand
target 5' UGAAGGUGGCUCUACGAGAA 3'
:::::: ::: ::::.::::
query 3' ACUUCCUCCGUGAUGUUCUU 5'
query=miR7495a, target=RO3G_13903, score=4, range=956-975, strand
target 5' UGAAGGUGGCUCUACGAGAA 3'
:::::: ::: ::::.::::
query 3' ACUUCCUCCGUGAUGUUCUU 5'
query=miR7495a, target=RO3G_13672, score=4, range=956-975, strand
target 5' UGAAGGUGGCUCUACGAGAA 3'
:::::: ::: ::::.::::
query 3' ACUUCCUCCGUGAUGUUCUU 5'
```

```
query=miR7495a, target=RO3G_14722, score=4, range=956-975, strand
target 5' UGAAGGUGGCUCUACGAGAA 3'
:::::: ::: ::::.::::
query 3' ACUUCCUCCGUGAUGUUCUU 5'
query=miR7495a, target=RO3G_13664, score=4, range=956-975, strand
target 5' UGAAGGUGGCUCUACGAGAA 3'
:::::: ::: ::::.::::
query 3' ACUUCCUCCGUGAUGUUCUU 5'
query=miR7495a, target=RO3G_05181, score=4, range=956-975, strand
target 5' UGAAGGUGGCUCUACGAGAA 3'
:::::: ::: ::::.::::
query 3' ACUUCCUCCGUGAUGUUCUU 5'
query=miR7495a, target=RO3G_05194, score=4, range=956-975, strand
target 5' UGAAGGUGGCUCUACGAGAA 3'
:::::: ::: ::::.::::
query 3' ACUUCCUCCGUGAUGUUCUU 5'
query=miR7495a, target=RO3G_01528, score=4, range=524-543, strand
target 5' UGAAGGUGGCUCUACGAGAA 3'
:::::: ::: ::::.::::
query 3' ACUUCCUCCGUGAUGUUCUU 5'
query=miR7495a, target=RO3G_08375, score=4, range=995-1014, stran
target 5' UAAAGGAGGCAUUACAAGUA 3'
: ::::::::.:::::: :
query 3' ACUUCCUCCGUGAUGUUCUU 5'
query=miR7495a, target=RO3G_04110, score=4, range=653-672, strand
target 5' UGAAGGUGGCUCUACGAGAA 3'
:::::: ::: ::::.::::
query 3' ACUUCCUCCGUGAUGUUCUU 5'
query=miR7495a, target=RO3G_04104, score=4, range=653-672, strand
target 5' UGAAGGCGGCUCUACGAGAA 3'
:::::: ::: ::::.::::
query 3' ACUUCCUCCGUGAUGUUCUU 5'
query=miR7495a, target=RO3G_12139, score=4, range=329-348, strand
target 5' UGAAGGUGGCUCUACGAGAA 3'
:::::: ::: ::::.::::
query 3' ACUUCCUCCGUGAUGUUCUU 5'
query=miR7495a, target=RO3G_01518, score=4, range=329-348, strand
target 5' UGAAGGUGGCUCUACGAGAA 3'
:::::: ::: ::::.::::
query 3' ACUUCCUCCGUGAUGUUCUU 5'
query=miR7495a, target=RO3G_05535, score=4, range=341-360, strand
target 5' UGAAGGUGGCUCUACGAGAA 3'
:::::: ::: ::::.::::
query 3' ACUUCCUCCGUGAUGUUCUU 5'
query=miR7495a, target=RO3G_10644, score=4, range=341-360, strand
```

target 5' UGAAGGUGGCUCUACGAGAA 3'
:::::: ::: ::::.::::
query 3' ACUUCCUCCGUGAUGUUCUU 5'
query=miR7495a, target=RO3G_02412, score=4, range=342-360, strand
target 5' AGAA-GAGGCACUACAAGCA 3'
::: ::::::::::::: :
query 3' ACUUCCUCCGUGAUGUUCUU 5'
query=miR7495a, target=RO3G_02795, score=4, range=555-575, strand
target 5' UGAAGAAGGCUAUUACAAGAA 3'
::::: :::: :.::::::::
query 3' ACUUCCUCCG-UGAUGUUCUU 5'
query=miR7495a, target=RO3G_06278, score=4, range=165-183, strand
target 5' UGAAGGAGGC-UUACGAGAA 3'
:::::::::: .:::.::::
query 3' ACUUCCUCCGUGAUGUUCUU 5'
query=miR9742, target=RO3G_06442, score=0, range=3354-3376, stran
target 5' UACUCACAGAAACAAACAAUACA 3'
:::::::::::::::::::::::
query 3' AUGAGUGUCUUUGUUUGUUAUGU 5'
query=miR9742, target=RO3G_02691, score=0, range=6186-6208, stran
target 5' UACUCACAGAAACAAACAAUACA 3'
:::::::::::::::::::::::
query 3' AUGAGUGUCUUUGUUUGUUAUGU 5'
query=miR9742, target=RO3G_09965, score=0, range=2697-2719, stran
target 5' UACUCACAGAAACAAACAAUACA 3'
:::::::::::::::::::::::
query 3' AUGAGUGUCUUUGUUUGUUAUGU 5'
query=miR9742, target=RO3G_13920, score=0, range=2697-2719, stran
target 5' UACUCACAGAAACAAACAAUACA 3'
:::::::::::::::::::::::
query 3' AUGAGUGUCUUUGUUUGUUAUGU 5'
query=miR9742, target=RO3G_03547, score=0, range=2697-2719, stran
target 5' UACUCACAGAAACAAACAAUACA 3'
:::::::::::::::::::::::
query 3' AUGAGUGUCUUUGUUUGUUAUGU 5'
query=miR9742, target=RO3G_13867, score=0, range=2613-2635, stran
target 5' UACUCACAGAAACAAACAAUACA 3'
:::::::::::::::::::::::
query 3' AUGAGUGUCUUUGUUUGUUAUGU 5'
query=miR9742, target=RO3G_02959, score=0, range=2697-2719, stran
target 5' UACUCACAGAAACAAACAAUACA 3'
:::::::::::::::::::::::
query 3' AUGAGUGUCUUUGUUUGUUAUGU 5'
query=miR9742, target=RO3G_15250, score=0, range=2652-2674, stran
target 5' UACUCACAGAAACAAACAAUACA 3'

```
::::::::::::::::::::::
query 3' AUGAGUGUCUUUGUUUGUUAUGU 5'
query=miR9742, target=RO3G_17371, score=0, range=2652-2674, stran
target 5' UACUCACAGAAACAAACAAUACA 3'
::::::::::::::::::::::
query 3' AUGAGUGUCUUUGUUUGUUAUGU 5'
query=miR9742, target=RO3G_06427, score=0, range=2289-2311, stran
target 5' UACUCACAGAAACAAACAAUACA 3'
::::::::::::::::::::::
query 3' AUGAGUGUCUUUGUUUGUUAUGU 5'
query=miR9742, target=RO3G_17326, score=0, range=2529-2551, stran
target 5' UACUCACAGAAACAAACAAUACA 3'
::::::::::::::::::::::
query 3' AUGAGUGUCUUUGUUUGUUAUGU 5'
query=miR9742, target=RO3G_06428, score=0, range=1791-1813, stran
target 5' UACUCACAGAAACAAACAAUACA 3'
::::::::::::::::::::::
query 3' AUGAGUGUCUUUGUUUGUUAUGU 5'
query=miR9742, target=RO3G_13749, score=0, range=1260-1282, stran
target 5' UACUCACAGAAACAAACAAUACA 3'
::::::::::::::::::::::
query 3' AUGAGUGUCUUUGUUUGUUAUGU 5'
query=miR9742, target=RO3G_05619, score=0, range=840-862, strand=
target 5' UACUCACAGAAACAAACAAUACA 3'
::::::::::::::::::::::
query 3' AUGAGUGUCUUUGUUUGUUAUGU 5'
query=miR9742, target=RO3G_14355, score=0, range=903-925, strand=
target 5' UACUCACAGAAACAAACAAUACA 3'
::::::::::::::::::::::
query 3' AUGAGUGUCUUUGUUUGUUAUGU 5'
query=miR9742, target=RO3G_15585, score=0, range=2652-2674, stran
target 5' UACUCACAGAAACAAACAAUACA 3'
::::::::::::::::::::::
query 3' AUGAGUGUCUUUGUUUGUUAUGU 5'
query=miR9742, target=RO3G_04114, score=0, range=2697-2719, stran
target 5' UACUCACAGAAACAAACAAUACA 3'
::::::::::::::::::::::
query 3' AUGAGUGUCUUUGUUUGUUAUGU 5'
query=miR9742, target=RO3G_02221, score=0, range=2619-2641, stran
target 5' UACUCACAGAAACAAACAAUACA 3'
::::::::::::::::::::::
query 3' AUGAGUGUCUUUGUUUGUUAUGU 5'
query=miR9742, target=RO3G_05272, score=0, range=2697-2719, stran
target 5' UACUCACAGAAACAAACAAUACA 3'
::::::::::::::::::::::
```

```
query 3' AUGAGUGUCUUUGUUUGUUAUGU 5'
query=miR9742, target=RO3G_01613, score=0, range=948-970, strand=
target 5' UACUCACAGAAACAAACAAUACA 3'
:::::::::::::::::::::::
query 3' AUGAGUGUCUUUGUUUGUUAUGU 5'
query=miR9742, target=RO3G_00306, score=0, range=1146-1168, stran
target 5' UACUCACAGAAACAAACAAUACA 3'
:::::::::::::::::::::::
query 3' AUGAGUGUCUUUGUUUGUUAUGU 5'
query=miR9742, target=RO3G_10558, score=0, range=450-472, strand=
target 5' UACUCACAGAAACAAACAAUACA 3'
:::::::::::::::::::::::
query 3' AUGAGUGUCUUUGUUUGUUAUGU 5'
query=miR9742, target=RO3G_05368, score=3, range=1917-1939, stran
target 5' UAAUAACAUAAACAAACAAUACA 3'
:: : ::: ::::::::::::::
query 3' AUGAGUGUCUUUGUUUGUUAUGU 5'
query=miR9742, target=RO3G_02099, score=4, range=1832-1855, stran
target 5' UACCUGACAGAAAUAAGCAAUACA 3'
:: :: :::::::.::.:::::::
query 3' AU-GAGUGUCUUUGUUUGUUAUGU 5'
query=miR3434-3p, target=RO3G_09174, score=0, range=665-683, stra
target 5' UCAACUUGGCUGAUCCUGA 3'
:::::::::::::::::::
query 3' AGUUGAACCGACUAGGACU 5'
query=miR3434-3p, target=RO3G_06414, score=0, range=665-683, stra
target 5' UCAACUUGGCUGAUCCUGA 3'
:::::::::::::::::::
query 3' AGUUGAACCGACUAGGACU 5'
query=miR3434-3p, target=RO3G_10730, score=0, range=668-686, stra
target 5' UCAACUUGGCUGAUCCUGA 3'
:::::::::::::::::::
query 3' AGUUGAACCGACUAGGACU 5'
query=miR3434-3p, target=RO3G_17201, score=0, range=665-683, stra
target 5' UCAACUUGGCUGAUCCUGA 3'
:::::::::::::::::::
query 3' AGUUGAACCGACUAGGACU 5'
query=miR3434-3p, target=RO3G_07736, score=0, range=668-686, stra
target 5' UCAACUUGGCUGAUCCUGA 3'
:::::::::::::::::::
query 3' AGUUGAACCGACUAGGACU 5'
query=miR3434-3p, target=RO3G_12967, score=0, range=668-686, stra
target 5' UCAACUUGGCUGAUCCUGA 3'
:::::::::::::::::::
query 3' AGUUGAACCGACUAGGACU 5'
```

```
query=miR3434-3p, target=RO3G_13449, score=0, range=668-686, stra
target 5' UCAACUUGGCUGAUCCUGA 3'
:::::::::::::::::::
query 3' AGUUGAACCGACUAGGACU 5'
query=miR3434-3p, target=RO3G_10533, score=0, range=668-686, stra
target 5' UCAACUUGGCUGAUCCUGA 3'
:::::::::::::::::::
query 3' AGUUGAACCGACUAGGACU 5'
query=miR3434-3p, target=RO3G_10748, score=0, range=668-686, stra
target 5' UCAACUUGGCUGAUCCUGA 3'
:::::::::::::::::::
query 3' AGUUGAACCGACUAGGACU 5'
query=miR3434-3p, target=RO3G_17254, score=0, range=668-686, stra
target 5' UCAACUUGGCUGAUCCUGA 3'
:::::::::::::::::::
query 3' AGUUGAACCGACUAGGACU 5'
query=miR3434-3p, target=RO3G_07861, score=0, range=668-686, stra
target 5' UCAACUUGGCUGAUCCUGA 3'
:::::::::::::::::::
query 3' AGUUGAACCGACUAGGACU 5'
query=miR3434-3p, target=RO3G_07863, score=0, range=668-686, stra
target 5' UCAACUUGGCUGAUCCUGA 3'
:::::::::::::::::::
query 3' AGUUGAACCGACUAGGACU 5'
query=miR3434-3p, target=RO3G_11978, score=0, range=521-539, stra
target 5' UCAACUUGGCUGAUCCUGA 3'
:::::::::::::::::::
query 3' AGUUGAACCGACUAGGACU 5'
query=miR3434-3p, target=RO3G_11821, score=2, range=2204-2222,
strand=1
target 5' UCAACUUGGCUGGUCUUGA 3'
:::::::::::.::.:::
query 3' AGUUGAACCGACUAGGACU 5'
query=miR3434-3p, target=RO3G_14562, score=2, range=605-623, stra
target 5' UCAACUUGGCGGAUCCUGA 3'
:::::::::: ::::::::
query 3' AGUUGAACCGACUAGGACU 5'
query=miR3434-3p, target=RO3G_17220, score=2, range=674-692, stra
target 5' UCAACUUGGCGGAUCCUGA 3'
:::::::::: ::::::::
query 3' AGUUGAACCGACUAGGACU 5'
query=miR3434-3p, target=RO3G_16903, score=2, range=602-620, stra
target 5' UCAACUUGGCGGAUCCUGA 3'
:::::::::: ::::::::
query 3' AGUUGAACCGACUAGGACU 5'
```

```
query=miR3434-3p, target=RO3G_17250, score=2, range=668-686, stra
target 5' UCAACUUGGCGGAUCCUGA 3'
:::::::::: ::::::::
query 3' AGUUGAACCGACUAGGACU 5'
query=miR3434-3p, target=RO3G_16365, score=2, range=668-686, stra
target 5' UCAACUUGGCGGAUCCUGA 3'
:::::::::: ::::::::
query 3' AGUUGAACCGACUAGGACU 5'
query=miR3434-3p, target=RO3G_10538, score=2, range=626-644, stra
target 5' UCAACUUGGCGGAUCCUGA 3'
:::::::::: ::::::::
query 3' AGUUGAACCGACUAGGACU 5'
query=miR3434-3p, target=RO3G_16358, score=2, range=497-515, stra
target 5' UCAACUUGGCUGACCCUGA 3'
::::::::::::: :::::
query 3' AGUUGAACCGACUAGGACU 5'
query=miR3434-3p, target=RO3G_04192, score=2.5, range=611-629,
strand=1
target 5' UCGACUUGGCUGGUUCUGA 3'
::.:::::::::.:.::::
query 3' AGUUGAACCGACUAGGACU 5'
query=miR3434-3p, target=RO3G_15938, score=3, range=590-608, stra
target 5' UCGAUUUGGCUGGUUCUGA 3'
::.:.::::::::.:.::::
query 3' AGUUGAACCGACUAGGACU 5'
query=miR3434-3p, target=RO3G_03248, score=3, range=173-191, stra
target 5' UCGGCUUGGUUGAUCCUGC 3'
::..:::::.::::::::
query 3' AGUUGAACCGACUAGGACU 5'
query=miR3434-3p, target=RO3G_17108, score=3, range=884-902, stra
target 5' GGAACUUGGCUGAUUCUGA 3'
:::::::::::::.::::
query 3' AGUUGAACCGACUAGGACU 5'
query=miR3434-3p, target=RO3G_11790, score=3, range=1238-1256,
strand=1
target 5' UCAACUUGACUGAUCCUGU 3'
:::::::: :::::::::
query 3' AGUUGAACCGACUAGGACU 5'
query=miR3434-3p, target=RO3G_12418, score=3, range=1178-1195,
strand=1
target 5' UCAACUUGGCUGAUUC-GA 3'
::::::::::::::.: ::
query 3' AGUUGAACCGACUAGGACU 5'
query=miR3434-3p, target=RO3G_16276, score=3, range=121-139, stra
target 5' UCAAGUUGGCUGCUCCUGA 3'
```

```
:::: ::::::: ::::::
query 3' AGUUGAACCGACUAGGACU 5'
query=miR3434-3p, target=RO3G_06992, score=3.5, range=443-461,
strand=1
target 5' UCAACUUUGUUGAUCCUGG 3'
::::::: :.:::::::.
query 3' AGUUGAACCGACUAGGACU 5'
query=miR3434-3p, target=RO3G_05895, score=3.5, range=293-311,
strand=1
target 5' UCAAAUUGGCUGAUUUUGG 3'
:::: ::::::::..::.
query 3' AGUUGAACCGACUAGGACU 5'
query=miR3434-3p, target=RO3G_06674, score=3.5, range=245-263,
strand=1
target 5' UUGCCUUGGUUGAUCCUGG 3'
:.. ::::.::::::::.
query 3' AGUUGAACCGACUAGGACU 5'
query=miR3434-3p, target=RO3G_14694, score=3.5, range=962-980,
strand=1
target 5' UCGUCUGGGCUGAUCCUGA 3'
::. :: :::::::::::
query 3' AGUUGAACCGACUAGGACU 5'
query=miR3434-3p, target=RO3G_11190, score=3.5, range=1274-1292,
strand=-1
target 5' UCCGCUUGGCUGAUCUUGC 3'
:: .:::::::::::.::
query 3' AGUUGAACCGACUAGGACU 5'
query=miR3434-3p, target=RO3G_13248, score=3.5, range=443-460,
strand=-1
target 5' UCGACUUGGCUGAU-UUGA 3'
::.:::::::::: .:::
query 3' AGUUGAACCGACUAGGACU 5'
query=miR3434-3p, target=RO3G_04594, score=3.5, range=778-796,
strand=-1
target 5' UAGACUUGCCUGAUCCUGA 3'
: .::::: :::::::::
query 3' AGUUGAACCGACUAGGACU 5'
query=miR3434-3p, target=RO3G_13067, score=4, range=665-683, stra
target 5' AUGACUUGGCUGGUCUUGA 3'
..::::::::.::.:::
query 3' AGUUGAACCGACUAGGACU 5'
query=miR3434-3p, target=RO3G_06633, score=4, range=206-224, stra
target 5' UUCAUUUGGUUGAUUCUGA 3'
:. :.::::.::::.::::
query 3' AGUUGAACCGACUAGGACU 5'
```

query=miR3434-3p, target=RO3G_05448, score=4, range=262-281, stra
target 5' UUGACUGUGGCUGAUUUUGA 3'
:..::: :::::::..:::
query 3' AGUUGA-ACCGACUAGGACU 5'
query=miR3434-3p, target=RO3G_05324, score=4, range=260-278, stra
target 5' UUCAUUUGGUUGAUCCUGU 3'
:. :.::::.:::::::
query 3' AGUUGAACCGACUAGGACU 5'
query=miR3434-3p, target=RO3G_16217, score=4, range=683-701, stra
target 5' UGAACUUGGCUGUUUCUGA 3'
: :::::::::: :.:::
query 3' AGUUGAACCGACUAGGACU 5'
query=miR3434-3p, target=RO3G_02336, score=4, range=1985-2002,
strand=1
target 5' UCAACUUGGCUCAU-CUGA 3'
:::::::::: :: ::::
query 3' AGUUGAACCGACUAGGACU 5'
query=miR3434-3p, target=RO3G_09123, score=4, range=1149-1166,
strand=1
target 5' UAAAC-UGGUUGAUCUUGA 3'
: ::: :::.:::::.:::
query 3' AGUUGAACCGACUAGGACU 5'
query=miR3434-3p, target=RO3G_14740, score=4, range=603-620, stra
target 5' UCAG-UUGGCUGAUCGUGG 3'
:::. :::::::::: ::.
query 3' AGUUGAACCGACUAGGACU 5'
query=miR3434-3p, target=RO3G_03676, score=4, range=156-173, stra
target 5' UGGAUUU-GCUGAUCCUGA 3'
: .:.:: ::::::::::
query 3' AGUUGAACCGACUAGGACU 5'
query=miR3434-3p, target=RO3G_07431, score=4, range=14-31, strand
target 5' UUAACUUGGCU-AUCUUGG 3'
:.::::::::: :::.::.
query 3' AGUUGAACCGACUAGGACU 5'
query=miR3434-3p, target=RO3G_00826, score=4, range=422-440, stra
target 5' UCACCGUGACUGAUCCUGA 3'
::: : :: ::::::::::
query 3' AGUUGAACCGACUAGGACU 5'
query=miR3434-3p, target=RO3G_15224, score=4, range=423-440, stra
target 5' GCAA-UUGGAUGAUCCUGA 3'
::: :::: :::::::::
query 3' AGUUGAACCGACUAGGACU 5'
query=miR3434-3p, target=RO3G_12160, score=4, range=77-94, strand
target 5' UCGGC-UGGCUGAUCCUUA 3'
::..: :::::::::: :

```
query 3' AGUUGAACCGACUAGGACU 5'
query=miR3434-3p, target=RO3G_09400, score=4, range=432-449, stra
target 5' UCAGC-UGGCUGAUCGUGG 3'
:::.: ::::::::: ::.
query 3' AGUUGAACCGACUAGGACU 5'
query=miR3434-3p, target=RO3G_09422, score=4, range=1690-1708,
strand=-1
target 5' UCAAAUUGGCUGAUUUUGU 3'
:::: :::::::::..::
query 3' AGUUGAACCGACUAGGACU 5'
query=miR3434-3p, target=RO3G_10613, score=4, range=1568-1586,
strand=-1
target 5' UUGACUUGGCUGAUCAUGU 3'
:..:::::::::::: ::
query 3' AGUUGAACCGACUAGGACU 5'
query=miR3434-3p, target=RO3G_10979, score=4, range=1133-1151,
strand=-1
target 5' UCGCUUUGGUUGAUCCUGU 3'
::. .::::.::::::::
query 3' AGUUGAACCGACUAGGACU 5'
query=miR3434-3p, target=RO3G_10566, score=4, range=93-112, stran
target 5' UUGACAUUGGCUGAUCUUGC 3'
:..:: ::::::::::.::
query 3' AGUUG-AACCGACUAGGACU 5'
query=miR3434-3p, target=RO3G_08260, score=4, range=345-362, stra
target 5' UCAAC-UGGCUUAUCCUGU 3'
::::: ::::: ::::::
query 3' AGUUGAACCGACUAGGACU 5'
query=miR1115-5p, target=RO3G_13445, score=4, range=524-545, stra
target 5' AUACAAAUAGGCCAGUACCUCG 3'
.:: :: :::::::::::::.
query 3' GGUGGUU-UCCGGUCAUGGAGU 5'
query=miR1115-5p, target=RO3G_00733, score=4, range=242-261, stra
target 5' UAACCGAAGGCCA-UACCUCA 3'
. :::.::::::: :::::::
query 3' GGUGGUUUCCGGUCAUGGAGU 5'
query=miR4348c, target=RO3G_16361, score=0, range=869-890, strand
target 5' UGGUCUCUUCCAAGGUUUAACA 3'
::::::::::::::::::::::
query 3' ACCAGAGAAGGUUCCAAAUUGU 5'
query=miR4348c, target=RO3G_04126, score=0, range=869-890, strand
target 5' UGGUCUCUUCCAAGGUUUAACA 3'
::::::::::::::::::::::
query 3' ACCAGAGAAGGUUCCAAAUUGU 5'
query=miR4348c, target=RO3G_17422, score=0, range=170-191, strand
```

```
target 5' UGGUCUCUUCCAAGGUUUAACA 3'
::::::::::::::::::::::
query 3' ACCAGAGAAGGUUCCAAAUUGU 5'
query=miR4348c, target=RO3G_13722, score=3, range=872-893, strand
target 5' UGAUCUCGUCCAAGGUUUAGCA 3'
:: :::: ::::::::::.::
query 3' ACCAGAGAAGGUUCCAAAUUGU 5'
query=miR4348c, target=RO3G_11064, score=4, range=1257-1278, stra
target 5' UGCUCCCUUCCAAGGUUUAAUC 3'
:: :: :::::::::::::.
query 3' ACCAGAGAAGGUUCCAAAUUGU 5'
query=miR4348c, target=RO3G_14347, score=4, range=875-896, strand
target 5' UGAUCUCGACCAAGGUUUAGCA 3'
:: :::: :::::::::.::
query 3' ACCAGAGAAGGUUCCAAAUUGU 5'
query=miR4348c, target=RO3G_09547, score=4, range=875-896, strand
target 5' UGAUCUCGACCAAGGUUUAGCA 3'
:: :::: :::::::::.::
query 3' ACCAGAGAAGGUUCCAAAUUGU 5'
query=miR4348c, target=RO3G_06425, score=4, range=875-896, strand
target 5' UGAUCUCGACCAAGGUUUAGCA 3'
:: :::: :::::::::.::
query 3' ACCAGAGAAGGUUCCAAAUUGU 5'
query=miR4348c, target=RO3G_17021, score=4, range=875-896, strand
target 5' UGAUCUCGACCAAGGUUUAGCA 3'
:: :::: :::::::::.::
query 3' ACCAGAGAAGGUUCCAAAUUGU 5'
query=miR4348c, target=RO3G_12677, score=4, range=875-896, strand
target 5' UGAUCUCGACCAAGGUUUAGCA 3'
:: :::: :::::::::.::
query 3' ACCAGAGAAGGUUCCAAAUUGU 5'
query=miR4348c, target=RO3G_04933, score=4, range=875-896, strand
target 5' UGAUCUCGACCAAGGUUUAGCA 3'
:: :::: :::::::::.::
query 3' ACCAGAGAAGGUUCCAAAUUGU 5'
query=miR4348c, target=RO3G_05330, score=4, range=875-896, strand
target 5' UGAUCUCGACCAAGGUUUAGCA 3'
:: :::: :::::::::.::
query 3' ACCAGAGAAGGUUCCAAAUUGU 5'
query=miR4348c, target=RO3G_02704, score=4, range=875-896, strand
target 5' UGAUCUCGACCAAGGUUUAGCA 3'
:: :::: :::::::::.::
query 3' ACCAGAGAAGGUUCCAAAUUGU 5'
query=miR4348c, target=RO3G_05344, score=4, range=875-896, strand
target 5' UGAUCUCGACCAAGGUUUAGCA 3'
```

```
:: :::: ::::::::::.::
query 3' ACCAGAGAAGGUUCCAAAUUGU 5'
query=miR1870-5p, target=RO3G_15268, score=0, range=1663-1684,
strand=1
target 5' AUGCCACUGGUCCAUUCAGCAA 3'
::::::::::::::::::::::
query 3' UACGGUGACCAGGUAAGUCGUU 5'
query=miR1870-5p, target=RO3G_04121, score=0, range=1663-1684,
strand=1
target 5' AUGCCACUGGUCCAUUCAGCAA 3'
::::::::::::::::::::::
query 3' UACGGUGACCAGGUAAGUCGUU 5'
query=miR1870-5p, target=RO3G_13217, score=0, range=1663-1684,
strand=1
target 5' AUGCCACUGGUCCAUUCAGCAA 3'
::::::::::::::::::::::
query 3' UACGGUGACCAGGUAAGUCGUU 5'
query=miR1870-5p, target=RO3G_15265, score=0, range=1663-1684,
strand=1
target 5' AUGCCACUGGUCCAUUCAGCAA 3'
::::::::::::::::::::::
query 3' UACGGUGACCAGGUAAGUCGUU 5'
query=miR1870-5p, target=RO3G_14352, score=0, range=1663-1684,
strand=1
target 5' AUGCCACUGGUCCAUUCAGCAA 3'
::::::::::::::::::::::
query 3' UACGGUGACCAGGUAAGUCGUU 5'
query=miR1870-5p, target=RO3G_13222, score=0, range=1657-1678,
strand=1
target 5' AUGCCACUGGUCCAUUCAGCAA 3'
::::::::::::::::::::::
query 3' UACGGUGACCAGGUAAGUCGUU 5'
query=miR1870-5p, target=RO3G_13219, score=0, range=1663-1684,
strand=1
target 5' AUGCCACUGGUCCAUUCAGCAA 3'
::::::::::::::::::::::
query 3' UACGGUGACCAGGUAAGUCGUU 5'
query=miR1870-5p, target=RO3G_16818, score=0, range=1405-1426,
strand=1
target 5' AUGCCACUGGUCCAUUCAGCAA 3'
::::::::::::::::::::::
query 3' UACGGUGACCAGGUAAGUCGUU 5'
query=miR1870-5p, target=RO3G_05640, score=0, range=1411-1432,
strand=1
target 5' AUGCCACUGGUCCAUUCAGCAA 3'
```

```
:::::::::::::::::::::
query 3' UACGGUGACCAGGUAAGUCGUU 5'
query=miR1870-5p, target=RO3G_15266, score=0, range=1663-1684,
strand=1
target 5' AUGCCACUGGUCCAUUCAGCAA 3'
:::::::::::::::::::::
query 3' UACGGUGACCAGGUAAGUCGUU 5'
query=miR1870-5p, target=RO3G_03992, score=0, range=394-415, stra
target 5' AUGCCACUGGUCCAUUCAGCAA 3'
:::::::::::::::::::::
query 3' UACGGUGACCAGGUAAGUCGUU 5'
query=miR1870-5p, target=RO3G_16797, score=0, range=664-685, stra
target 5' AUGCCACUGGUCCAUUCAGCAA 3'
:::::::::::::::::::::
query 3' UACGGUGACCAGGUAAGUCGUU 5'
query=miR1870-5p, target=RO3G_11967, score=2, range=1675-1696,
strand=1
target 5' AUGCCACUGGUUCGUUCAGCAA 3'
:::::::::::.:.::::::::
query 3' UACGGUGACCAGGUAAGUCGUU 5'
query=miR1870-5p, target=RO3G_13225, score=2, range=1675-1696,
strand=1
target 5' AUGCCACUGGUUCGUUCAGCAA 3'
:::::::::::.:.::::::::
query 3' UACGGUGACCAGGUAAGUCGUU 5'
query=miR1870-5p, target=RO3G_16800, score=4, range=1663-1684,
strand=1
target 5' AUGCCACUGGUUCGCUCAGCAA 3'
:::::::::::.:. :::::::
query 3' UACGGUGACCAGGUAAGUCGUU 5'
query=miR1870-5p, target=RO3G_08855, score=4, range=914-935, stra
target 5' ACGCCACAGGUCCAUUCAGUGA 3'
: ::::: ::::::::::..:
query 3' UACGGUGACCAGGUAAGUCGUU 5'
query=miR2905, target=RO3G_15711, score=0, range=2375-2394, stran
target 5' UGCCUUUGUCGGCUACAGUA 3'
:::::::::::::::::::
query 3' ACGGAAACAGCCGAUGUCAU 5'
query=miR2905, target=RO3G_13796, score=0, range=2375-2394, stran
target 5' UGCCUUUGUCGGCUACAGUA 3'
:::::::::::::::::::
query 3' ACGGAAACAGCCGAUGUCAU 5'
query=miR2905, target=RO3G_09539, score=0, range=2375-2394, stran
target 5' UGCCUUUGUCGGCUACAGUA 3'
:::::::::::::::::::
```

```
query 3' ACGGAAACAGCCGAUGUCAU 5'
query=miR2905, target=RO3G_11521, score=0, range=1337-1356, stran
target 5' UGCCUUUGUCGGCUACAGUA 3'
::::::::::::::::::::
query 3' ACGGAAACAGCCGAUGUCAU 5'
query=miR2905, target=RO3G_03627, score=2.5, range=392-411, stran
target 5' UUCCAUUGUCGGCUACAGUG 3'
: :: :::::::::::::.
query 3' ACGGAAACAGCCGAUGUCAU 5'
query=miR2905, target=RO3G_01708, score=2.5, range=392-411, stran
target 5' UUCCAUUGUCGGCUACAGUG 3'
: :: :::::::::::::.
query 3' ACGGAAACAGCCGAUGUCAU 5'
query=miR2905, target=RO3G_11026, score=3.5, range=657-676, stran
target 5' UGCUUUUGUUGGCUAUGGUA 3'
:::.::::.:::::..:::
query 3' ACGGAAACAGCCGAUGUCAU 5'
query=miR5557-3p, target=RO3G_14978, score=0, range=111-130, stra
target 5' UCAACAAGAUAAUGCAAGCA 3'
::::::::::::::::::::
query 3' AGUUGUUCUAUUACGUUCGU 5'
query=miR5557-3p, target=RO3G_17249, score=0, range=630-649, stra
target 5' UCAACAAGAUAAUGCAAGCA 3'
::::::::::::::::::::
query 3' AGUUGUUCUAUUACGUUCGU 5'
query=miR5557-3p, target=RO3G_11527, score=0, range=111-130, stra
target 5' UCAACAAGAUAAUGCAAGCA 3'
::::::::::::::::::::
query 3' AGUUGUUCUAUUACGUUCGU 5'
query=miR5557-3p, target=RO3G_10774, score=0, range=672-691, stra
target 5' UCAACAAGAUAAUGCAAGCA 3'
::::::::::::::::::::
query 3' AGUUGUUCUAUUACGUUCGU 5'
query=miR5557-3p, target=RO3G_13270, score=0, range=705-724, stra
target 5' UCAACAAGAUAAUGCAAGCA 3'
::::::::::::::::::::
query 3' AGUUGUUCUAUUACGUUCGU 5'
query=miR5557-3p, target=RO3G_17276, score=0, range=795-814, stra
target 5' UCAACAAGAUAAUGCAAGCA 3'
::::::::::::::::::::
query 3' AGUUGUUCUAUUACGUUCGU 5'
query=miR5557-3p, target=RO3G_03626, score=0, range=111-130, stra
target 5' UCAACAAGAUAAUGCAAGCA 3'
::::::::::::::::::::
query 3' AGUUGUUCUAUUACGUUCGU 5'
```

```
query=miR5557-3p, target=RO3G_05935, score=0, range=111-130, stra
target 5' UCAACAAGAUAAUGCAAGCA 3'
::::::::::::::::::::
query 3' AGUUGUUCUAUUACGUUCGU 5'
query=miR5557-3p, target=RO3G_01589, score=0, range=111-130, stra
target 5' UCAACAAGAUAAUGCAAGCA 3'
::::::::::::::::::::
query 3' AGUUGUUCUAUUACGUUCGU 5'
query=miR5557-3p, target=RO3G_14547, score=0, range=111-130, stra
target 5' UCAACAAGAUAAUGCAAGCA 3'
::::::::::::::::::::
query 3' AGUUGUUCUAUUACGUUCGU 5'
query=miR5557-3p, target=RO3G_00678, score=0, range=75-94, strand
target 5' UCAACAAGAUAAUGCAAGCA 3'
::::::::::::::::::::
query 3' AGUUGUUCUAUUACGUUCGU 5'
query=miR5557-3p, target=RO3G_04790, score=0, range=111-130, stra
target 5' UCAACAAGAUAAUGCAAGCA 3'
::::::::::::::::::::
query 3' AGUUGUUCUAUUACGUUCGU 5'
query=miR5557-3p, target=RO3G_13863, score=0, range=405-424, stra
target 5' UCAACAAGAUAAUGCAAGCA 3'
::::::::::::::::::::
query 3' AGUUGUUCUAUUACGUUCGU 5'
query=miR5557-3p, target=RO3G_14363, score=0, range=663-682, stra
target 5' UCAACAAGAUAAUGCAAGCA 3'
::::::::::::::::::::
query 3' AGUUGUUCUAUUACGUUCGU 5'
query=miR5557-3p, target=RO3G_10869, score=1, range=111-130, stra
target 5' UCAACAUGAUAAUGCAAGCA 3'
:::::: :::::::::::::
query 3' AGUUGUUCUAUUACGUUCGU 5'
query=miR5557-3p, target=RO3G_03593, score=2, range=111-130, stra
target 5' UCAACAAGAUAGUGCAGGCA 3'
:::::::::::.::::.:::
query 3' AGUUGUUCUAUUACGUUCGU 5'
query=miR5557-3p, target=RO3G_17259, score=2, range=111-130, stra
target 5' UCAACAAGAUAAUGAAAGCA 3'
:::::::::::::: :::::
query 3' AGUUGUUCUAUUACGUUCGU 5'
query=miR5557-3p, target=RO3G_13921, score=2, range=111-130, stra
target 5' UCAACAAGAUAAUGGAAGCA 3'
:::::::::::::: :::::
query 3' AGUUGUUCUAUUACGUUCGU 5'
query=miR5557-3p, target=RO3G_10734, score=2, range=111-130, stra
```

```
target 5' UCAACAACAUAAUGCAAGCA 3'
::::::: :::::::::::: 
query 3' AGUUGUUCUAUUACGUUCGU 5'
query=miR5557-3p, target=RO3G_17459, score=2, range=111-130, stra
target 5' UCAACAACAUAAUGCAAGCA 3'
::::::: :::::::::::: 
query 3' AGUUGUUCUAUUACGUUCGU 5'
query=miR5557-3p, target=RO3G_05423, score=2, range=111-130, stra
target 5' UCAACUAGAUAAUGUAAGCA 3'
::::: ::::::::.:::::
query 3' AGUUGUUCUAUUACGUUCGU 5'
query=miR5557-3p, target=RO3G_10645, score=2, range=2-21, strand=
target 5' ACAAAAAGAUAAUGCAAGCA 3'
::: :::::::::::::::
query 3' AGUUGUUCUAUUACGUUCGU 5'
query=miR5557-3p, target=RO3G_13954, score=2.5, range=449-468,
strand=1
target 5' UUGAGAGGAUAAUGCAAGCA 3'
:..: :.:::::::::::::
query 3' AGUUGUUCUAUUACGUUCGU 5'
query=miR5557-3p, target=RO3G_06186, score=3, range=10-29, strand
target 5' UCAAUGAGGUAAUGCAAGUA 3'
::::..::.::::::::::.:
query 3' AGUUGUUCUAUUACGUUCGU 5'
query=miR5557-3p, target=RO3G_01707, score=3.5, range=158-177,
strand=-1
target 5' ACAACAGGAUAAUGCAAGGA 3'
::::.:::::::::::: :
query 3' AGUUGUUCUAUUACGUUCGU 5'
query=miR5557-3p, target=RO3G_10608, score=4, range=8928-8947,
strand=1
target 5' GCAAGAAGGUGAUGCAAGCA 3'
::: :::.:.:::::::::
query 3' AGUUGUUCUAUUACGUUCGU 5'
query=miR5557-3p, target=RO3G_06444, score=4, range=326-344, stra
target 5' UCAAUAAGGUAAUG-AAGCG 3'
::::.:::.::::: ::::.
query 3' AGUUGUUCUAUUACGUUCGU 5'
query=miR5557-3p, target=RO3G_05820, score=4, range=488-507, stra
target 5' UGAAGAAGAUGAUGCAAGCC 3'
: :: :::::.:::::::::
query 3' AGUUGUUCUAUUACGUUCGU 5'
query=miR5557-3p, target=RO3G_00743, score=4, range=2349-2367,
strand=1
target 5' UCAACAUGAUAAUGC-AGUA 3'
```

```
:::::: :::::::: ::.:
query 3' AGUUGUUCUAUUACGUUCGU 5'
query=miR5557-3p, target=RO3G_03883, score=4, range=586-604, stra
target 5' UCAGC-AGAUAAUGCAAGAG 3'
:::.: ::::::::::: .
query 3' AGUUGUUCUAUUACGUUCGU 5'
query=miR5557-3p, target=RO3G_04024, score=4, range=209-227, stra
target 5' UGAACAAGAUGAU-CAAGCA 3'
: ::::::::.:: ::::::
query 3' AGUUGUUCUAUUACGUUCGU 5'
query=miR5557-3p, target=RO3G_12622, score=4, range=1143-1162,
strand=-1
target 5' UGAACAAGAUGAUGUAAGUA 3'
: :::::::.:::.:::.:
query 3' AGUUGUUCUAUUACGUUCGU 5'
query=miR5557-3p, target=RO3G_02506, score=4, range=2209-2228,
strand=-1
target 5' UCAACAAGAGAAUGCAAGUU 3'
::::::::: ::::::::.
query 3' AGUUGUUCUAUUACGUUCGU 5'
query=miR5557-3p, target=RO3G_01224, score=4, range=369-388, stra
target 5' UCUGCUGGAUAAUGCAGGCA 3'
:: .: .:::::::::.:::
query 3' AGUUGUUCUAUUACGUUCGU 5'
query=miR5557-3p, target=RO3G_01221, score=4, range=369-388, stra
target 5' UCUGCUGGAUAAUGCAGGCA 3'
:: .: .:::::::::.:::
query 3' AGUUGUUCUAUUACGUUCGU 5'
query=miR5557-3p, target=RO3G_02795, score=4, range=491-509, stra
target 5' CGAACAAGAUAAUG-AAGCA 3'
::::::::::: :::::
query 3' AGUUGUUCUAUUACGUUCGU 5'
query=miR5557-3p, target=RO3G_02641, score=4, range=1145-1163,
strand=-1
target 5' UCUGC-AGAUAGUGCAAGCG 3'
:: .: ::::.:::::::.
query 3' AGUUGUUCUAUUACGUUCGU 5'
query=miR8135, target=RO3G_12402, score=0, range=438-456, strand=
target 5' AUCACCAAGCAUAAAUCCU 3'
:::::::::::::::::::
query 3' UAGUGGUUCGUAUUUAGGA 5'
query=miR8135, target=RO3G_15434, score=2, range=3444-3462, stran
target 5' AUUUCUAAGCAUAAAUCCU 3'
::. :.:::::::::::
query 3' UAGUGGUUCGUAUUUAGGA 5'
```

query=miR8135, target=RO3G_13681, score=2.5, range=262-280, stran
target 5' AUCGGCAAGCAUAAAUCCG 3'
:::. ::::::::::::
query 3' UAGUGGUUCGUAUUUAGGA 5'
query=miR8135, target=RO3G_05681, score=3.5, range=1158-1176,
strand=-1
target 5' AUUACCACGCAUAAAUUCU 3'
::.:::: ::::::::.::
query 3' UAGUGGUUCGUAUUUAGGA 5'
query=miR8135, target=RO3G_11062, score=3.5, range=308-327, stran
target 5' AUGCAUCAAGUAUAAAUCCA 3'
:: ::.::::.::::::::
query 3' UA-GUGGUUCGUAUUUAGGA 5'
query=miR8135, target=RO3G_10706, score=3.5, range=219-236, stran
target 5' AUCAAUAAGCAU-AAUCCU 3'
:::: .:::::: ::::::
query 3' UAGUGGUUCGUAUUUAGGA 5'
query=miR8135, target=RO3G_11749, score=4, range=1572-1590, stran
target 5' AUUAUCAGGCAUAAAUUCA 3'
::.:.::.::::::::.:
query 3' UAGUGGUUCGUAUUUAGGA 5'
query=miR8135, target=RO3G_05601, score=4, range=1113-1131, stran
target 5' UUCACCAGGCAUAAACCCU 3'
::::::.:::::: :::
query 3' UAGUGGUUCGUAUUUAGGA 5'
query=miR8135, target=RO3G_15222, score=4, range=2086-2103, stran
target 5' AUC-CCAAGCGUAAAUUUU 3'
::: ::::::.:::::..:
query 3' UAGUGGUUCGUAUUUAGGA 5'
query=miR8135, target=RO3G_05297, score=4, range=2603-2621, stran
target 5' AACACCAAGCAGAAAUCUU 3'
: ::::::::: :::::.:
query 3' UAGUGGUUCGUAUUUAGGA 5'
query=miR8135, target=RO3G_00647, score=4, range=4399-4416, stran
target 5' AUC-UUAAGCAUGGAUCCU 3'
::: ..:::::::..:::::
query 3' UAGUGGUUCGUAUUUAGGA 5'
query=miR8135, target=RO3G_10979, score=4, range=298-315, strand=
target 5' AUU-UCAAGCAUGAAUUCU 3'
::. .::::::::.::::.::
query 3' UAGUGGUUCGUAUUUAGGA 5'
query=miR8135, target=RO3G_15836, score=4, range=75-94, strand=1
target 5' ACCAUCGAGCAUAAAUACCU 3'
: ::.:.::::::::: :::
query 3' UAGUGGUUCGUAUUUA-GGA 5'

```
query=miR8135, target=RO3G_07416, score=4, range=10-27, strand=1
target 5' AUUAUCAAGCAU-AAUCCA 3'
::.:.:::::: :::::
query 3' UAGUGGUUCGUAUUUAGGA 5'
query=miR8135, target=RO3G_03230, score=4, range=197-216, strand=
target 5' CUCAAGCAAGCAUAAAUCCA 3'
:: : :::::::::::
query 3' UAG-UGGUUCGUAUUUAGGA 5'
query=miR8135, target=RO3G_16550, score=4, range=848-865, strand=
target 5' AUC-CGAAGCAUAAGUCCA 3'
::: : ::::::::.:::
query 3' UAGUGGUUCGUAUUUAGGA 5'
query=miR8135, target=RO3G_12166, score=4, range=737-754, strand=
target 5' AUC-CGAAGCAUAAGUCCA 3'
::: : ::::::::.:::
query 3' UAGUGGUUCGUAUUUAGGA 5'
query=miR8135, target=RO3G_08847, score=4, range=737-754, strand=
target 5' AUC-CGAAGCAUAAGUCCA 3'
::: : ::::::::.:::
query 3' UAGUGGUUCGUAUUUAGGA 5'
query=miR8135, target=RO3G_16751, score=4, range=326-343, strand=
target 5' AUC-CGAAGCAUAAGUCCA 3'
::: : ::::::::.:::
query 3' UAGUGGUUCGUAUUUAGGA 5'
query=miR8135, target=RO3G_03603, score=4, range=176-193, strand=
target 5' AUC-CGAAGCAUAAGUCCA 3'
::: : ::::::::.:::
query 3' UAGUGGUUCGUAUUUAGGA 5'
query=miR8135, target=RO3G_12240, score=4, range=573-591, strand=
target 5' AUCACCAGGUAAAAAUCCU 3'
:::::::.:.: :::::::
query 3' UAGUGGUUCGUAUUUAGGA 5'
query=miR8135, target=RO3G_13465, score=4, range=1551-1569, stran
target 5' UUCACCAAGCAUGAAUUCA 3'
:::::::::::.:::.:
query 3' UAGUGGUUCGUAUUUAGGA 5'
query=miR8135, target=RO3G_11318, score=4, range=128-147, strand=
target 5' UUUAUCAGAGCAUAAAUCCU 3'
:.:.:: :::::::::::
query 3' UAGUGGU-UCGUAUUUAGGA 5'
query=miR8135, target=RO3G_14614, score=4, range=343-361, strand=
target 5' UUUGCAAAGCAUAAAUCUU 3'
:..: :::::::::::.:
query 3' UAGUGGUUCGUAUUUAGGA 5'
query=miR8135, target=RO3G_11170, score=4, range=158-175, strand=
```

```
target 5' AUUGCCAAGCAU-GAUCCU 3'
::..::::::::: .:::::
query 3' UAGUGGUUCGUAUUUAGGA 5'
query=miR8135, target=RO3G_11114, score=4, range=3850-3869, stran
target 5' AACACCAAAGCAUAAUUCCU 3'
: :::: :::::::: ::::
query 3' UAGUGG-UUCGUAUUUAGGA 5'
query=miR8135, target=RO3G_12314, score=4, range=1227-1245, stran
target 5' AGCAACAAGCAUCAAUCCU 3'
: :: ::::::: ::::::
query 3' UAGUGGUUCGUAUUUAGGA 5'
query=miR8135, target=RO3G_03692, score=4, range=622-639, strand=
target 5' CUCCCCAAGCAUAAA-CCU 3'
:: :::::::::: :::
query 3' UAGUGGUUCGUAUUUAGGA 5'
query=miR8135, target=RO3G_09208, score=4, range=831-850, strand=
target 5' ACCUCCAAGCAUCAAAUCCU 3'
: : :::::::: :::::::
query 3' UAGUGGUUCGUA-UUUAGGA 5'
query=miR5241a, target=RO3G_11557, score=2, range=519-537, strand
target 5' AUUCACUCUUCCAUCAUCC 3'
:::: ::::::::::::
query 3' UAAGGGAGAAGGUAGUAGU 5'
query=miR5241a, target=RO3G_17296, score=2.5, range=34-52, strand
target 5' GAACCCUCUUCCAUCAUCA 3'
. :::::::::::::::
query 3' UAAGGGAGAAGGUAGUAGU 5'
query=miR5241a, target=RO3G_07498, score=2.5, range=750-767, stra
target 5' AUUCCUUCUU-CAUCAUCA 3'
:::::.:::: ::::::::
query 3' UAAGGGAGAAGGUAGUAGU 5'
query=miR5241a, target=RO3G_05858, score=3, range=676-694, strand
target 5' GUUAUCUCUUCCAUUAUCA 3'
.:: .:::::::::.::::
query 3' UAAGGGAGAAGGUAGUAGU 5'
query=miR5241a, target=RO3G_16310, score=3, range=1230-1248, stra
target 5' AAGUUCUCUUCCAUCAUCA 3'
: ..::::::::::::::
query 3' UAAGGGAGAAGGUAGUAGU 5'
query=miR5241a, target=RO3G_13602, score=3, range=128-146, strand
target 5' AUGCUUUCUUCCAUCGUCA 3'
:: :..:::::::::.:::
query 3' UAAGGGAGAAGGUAGUAGU 5'
query=miR5241a, target=RO3G_04050, score=3.5, range=1258-1276,
strand=1
```

target 5' UUUCCUUCUUCUAUCGUCA 3'
::::.::::::.:::.:::
query 3' UAAGGGAGAAGGUAGUAGU 5'
query=miR5241a, target=RO3G_00301, score=3.5, range=2542-2560,
strand=1
target 5' UUUCCUUCUUCUAUCGUCA 3'
::::.::::::.:::.:::
query 3' UAAGGGAGAAGGUAGUAGU 5'
query=miR5241a, target=RO3G_12949, score=3.5, range=703-721, stra
target 5' UUUCCUUCUUCUAUCGUCA 3'
::::.::::::.:::.:::
query 3' UAAGGGAGAAGGUAGUAGU 5'
query=miR5241a, target=RO3G_15056, score=3.5, range=427-445, stra
target 5' GUUCCCUCUUUCAUCGUCC 3'
.:::::::::.::::.::
query 3' UAAGGGAGAAGGUAGUAGU 5'
query=miR5241a, target=RO3G_09951, score=3.5, range=108-126, stra
target 5' AUUCUCUCCUUCAUCAUCA 3'
::::.::: :.::::::::
query 3' UAAGGGAGAAGGUAGUAGU 5'
query=miR5241a, target=RO3G_01868, score=3.5, range=196-214, stra
target 5' UCUCCCUUUUCCAUCAUCG 3'
:::::.:::::::::::.
query 3' UAAGGGAGAAGGUAGUAGU 5'
query=miR5241a, target=RO3G_17322, score=3.5, range=839-857, stra
target 5' UUUCCUUGUUCCAUCAUCA 3'
::::.: ::::::::::
query 3' UAAGGGAGAAGGUAGUAGU 5'
query=miR5241a, target=RO3G_07919, score=3.5, range=839-857, stra
target 5' UUUCCUUGUUCCAUCAUCA 3'
::::.: ::::::::::
query 3' UAAGGGAGAAGGUAGUAGU 5'
query=miR5241a, target=RO3G_05129, score=3.5, range=839-857, stra
target 5' UUUCCUUGUUCCAUCAUCA 3'
::::.: ::::::::::
query 3' UAAGGGAGAAGGUAGUAGU 5'
query=miR5241a, target=RO3G_11990, score=3.5, range=839-857, stra
target 5' UUUCCUUGUUCCAUCAUCA 3'
::::.: ::::::::::
query 3' UAAGGGAGAAGGUAGUAGU 5'
query=miR5241a, target=RO3G_00002, score=3.5, range=839-857, stra
target 5' UUUCCUUGUUCCAUCAUCA 3'
::::.: ::::::::::
query 3' UAAGGGAGAAGGUAGUAGU 5'
query=miR5241a, target=RO3G_12352, score=3.5, range=839-857, stra

target 5' UUUCCUUGUUCCAUCAUCA 3'
::::.: ::::::::::
query 3' UAAGGGAGAAGGUAGUAGU 5'
query=miR5241a, target=RO3G_10082, score=3.5, range=839-857, stra
target 5' UUUCCUUGUUCCAUCAUCA 3'
::::.: ::::::::::
query 3' UAAGGGAGAAGGUAGUAGU 5'
query=miR5241a, target=RO3G_13707, score=3.5, range=839-857, stra
target 5' UUUCCUUGUUCCAUCAUCA 3'
::::.: ::::::::::
query 3' UAAGGGAGAAGGUAGUAGU 5'
query=miR5241a, target=RO3G_01112, score=3.5, range=839-857, stra
target 5' UUUCCUUGUUCCAUCAUCA 3'
::::.: ::::::::::
query 3' UAAGGGAGAAGGUAGUAGU 5'
query=miR5241a, target=RO3G_07318, score=3.5, range=839-857, stra
target 5' UUUCCUUGUUCCAUCAUCA 3'
::::.: ::::::::::
query 3' UAAGGGAGAAGGUAGUAGU 5'
query=miR5241a, target=RO3G_16076, score=3.5, range=839-857, stra
target 5' UUUCCUUGUUCCAUCAUCA 3'
::::.: ::::::::::
query 3' UAAGGGAGAAGGUAGUAGU 5'
query=miR5241a, target=RO3G_05128, score=3.5, range=839-857, stra
target 5' UUUCCUUGUUCCAUCAUCA 3'
::::.: ::::::::::
query 3' UAAGGGAGAAGGUAGUAGU 5'
query=miR5241a, target=RO3G_05091, score=3.5, range=839-857, stra
target 5' UUUCCUUGUUCCAUCAUCA 3'
::::.: ::::::::::
query 3' UAAGGGAGAAGGUAGUAGU 5'
query=miR5241a, target=RO3G_05692, score=3.5, range=839-857, stra
target 5' UUUCCUUGUUCCAUCAUCA 3'
::::.: ::::::::::
query 3' UAAGGGAGAAGGUAGUAGU 5'
query=miR5241a, target=RO3G_17348, score=3.5, range=839-857, stra
target 5' UUUCCUUGUUCCAUCAUCA 3'
::::.: ::::::::::
query 3' UAAGGGAGAAGGUAGUAGU 5'
query=miR5241a, target=RO3G_02948, score=3.5, range=839-857, stra
target 5' UUUCCUUGUUCCAUCAUCA 3'
::::.: ::::::::::
query 3' UAAGGGAGAAGGUAGUAGU 5'
query=miR5241a, target=RO3G_08729, score=3.5, range=839-857, stra
target 5' UUUCCUUGUUCCAUCAUCA 3'

```
::::.: ::::::::::
query 3' UAAGGGAGAAGGUAGUAGU 5'
query=miR5241a, target=RO3G_03722, score=3.5, range=839-857, stra
target 5' UUUCCUUGUUCCAUCAUCA 3'
::::.: ::::::::::
query 3' UAAGGGAGAAGGUAGUAGU 5'
query=miR5241a, target=RO3G_14908, score=3.5, range=839-857, stra
target 5' UUUCCUUGUUCCAUCAUCA 3'
::::.: ::::::::::
query 3' UAAGGGAGAAGGUAGUAGU 5'
query=miR5241a, target=RO3G_17221, score=3.5, range=839-857, stra
target 5' UUUCCUUGUUCCAUCAUCA 3'
::::.: ::::::::::
query 3' UAAGGGAGAAGGUAGUAGU 5'
query=miR5241a, target=RO3G_17389, score=3.5, range=839-857, stra
target 5' UUUCCUUGUUCCAUCAUCA 3'
::::.: ::::::::::
query 3' UAAGGGAGAAGGUAGUAGU 5'
query=miR5241a, target=RO3G_04035, score=3.5, range=839-857, stra
target 5' UUUCCUUGUUCCAUCAUCA 3'
::::.: ::::::::::
query 3' UAAGGGAGAAGGUAGUAGU 5'
query=miR5241a, target=RO3G_03986, score=3.5, range=839-857, stra
target 5' UUUCCUUGUUCCAUCAUCA 3'
::::.: ::::::::::
query 3' UAAGGGAGAAGGUAGUAGU 5'
query=miR5241a, target=RO3G_04027, score=3.5, range=839-857, stra
target 5' UUUCCUUGUUCCAUCAUCA 3'
::::.: ::::::::::
query 3' UAAGGGAGAAGGUAGUAGU 5'
query=miR5241a, target=RO3G_14748, score=3.5, range=839-857, stra
target 5' UUUCCUUGUUCCAUCAUCA 3'
::::.: ::::::::::
query 3' UAAGGGAGAAGGUAGUAGU 5'
query=miR5241a, target=RO3G_00400, score=3.5, range=839-857, stra
target 5' UUUCCUUGUUCCAUCAUCA 3'
::::.: ::::::::::
query 3' UAAGGGAGAAGGUAGUAGU 5'
query=miR5241a, target=RO3G_13483, score=3.5, range=839-857, stra
target 5' UUUCCUUGUUCCAUCAUCA 3'
::::.: ::::::::::
query 3' UAAGGGAGAAGGUAGUAGU 5'
query=miR5241a, target=RO3G_06439, score=3.5, range=836-854, stra
target 5' UUUCCUUGUUCCAUCAUCA 3'
::::.: ::::::::::
```

query 3' UAAGGGAGAAGGUAGUAGU 5'
query=miR5241a, target=RO3G_05090, score=3.5, range=839-857, stra
target 5' UUUCCUUGUUCCAUCAUCA 3'
::::.: ::::::::::
query 3' UAAGGGAGAAGGUAGUAGU 5'
query=miR5241a, target=RO3G_13265, score=3.5, range=839-857, stra
target 5' UUUCCUUGUUCCAUCAUCA 3'
::::.: ::::::::::
query 3' UAAGGGAGAAGGUAGUAGU 5'
query=miR5241a, target=RO3G_14037, score=3.5, range=195-213, stra
target 5' UUUCUCUCGUCCAUCAUCA 3'
:::.::: :::::::::
query 3' UAAGGGAGAAGGUAGUAGU 5'
query=miR5241a, target=RO3G_02172, score=3.5, range=992-1009, str
target 5' GUUUUC-CUUCCAUCAUCA 3'
.::..: :::::::::::
query 3' UAAGGGAGAAGGUAGUAGU 5'
query=miR5241a, target=RO3G_01269, score=3.5, range=184-202, stra
target 5' UAUCAUUCUUCCAUCAUCA 3'
:: .::::::::::::
query 3' UAAGGGAGAAGGUAGUAGU 5'
query=miR5241a, target=RO3G_14004, score=3.5, range=815-832, stra
target 5' AUUUAC-CUUCCAUCAUCA 3'
:::. : :::::::::::
query 3' UAAGGGAGAAGGUAGUAGU 5'
query=miR5241a, target=RO3G_13481, score=3.5, range=750-768, stra
target 5' UUUCUCUUUUCCAUUAUCA 3'
:::.::.:::::::.::::
query 3' UAAGGGAGAAGGUAGUAGU 5'
query=miR5241a, target=RO3G_09440, score=3.5, range=768-786, stra
target 5' AUUUCCUUUUCCAUCGUCU 3'
:::.:::.:::::::.::
query 3' UAAGGGAGAAGGUAGUAGU 5'
query=miR5241a, target=RO3G_06881, score=3.5, range=30-48, strand
target 5' CUUUCCUCUUCCGUCAUCC 3'
::.::::::::.:::::
query 3' UAAGGGAGAAGGUAGUAGU 5'
query=miR5241a, target=RO3G_02114, score=3.5, range=480-498, stra
target 5' UGUCUCUCUUUCAUCAUCA 3'
::.:::::.::::::::
query 3' UAAGGGAGAAGGUAGUAGU 5'
query=miR5241a, target=RO3G_09478, score=3.5, range=2519-2537,
strand=-1
target 5' UUUCCGUCUUCCAUCAUUG 3'
:::: :::::::::::..

query 3' UAAGGGAGAAGGUAGUAGU 5'
query=miR5241a, target=RO3G_05842, score=3.5, range=1616-1634, strand=-1
target 5' UCUUCCUCUUCCAUCAUCU 3'
:.:::::::::::::
query 3' UAAGGGAGAAGGUAGUAGU 5'
query=miR5241a, target=RO3G_02881, score=3.5, range=7667-7685, strand=-1
target 5' AUUUGCUCUUCCUUCAUCA 3'
:::. ::::::: ::::::
query 3' UAAGGGAGAAGGUAGUAGU 5'
query=miR5241a, target=RO3G_12763, score=3.5, range=1478-1497, strand=-1
target 5' AUCUUCCUCUUCCAUCACCA 3'
:: :.:::::::::::: ::
query 3' UA-AGGGAGAAGGUAGUAGU 5'
query=miR5241a, target=RO3G_01141, score=3.5, range=81-98, strand
target 5' AUUCCUUCUUCC-UCAUCC 3'
::::.:::::: :::::
query 3' UAAGGGAGAAGGUAGUAGU 5'
query=miR5241a, target=RO3G_01137, score=3.5, range=1046-1064, strand=-1
target 5' ACUUGCUCUUCCAUCAUCU 3'
: :. ::::::::::::
query 3' UAAGGGAGAAGGUAGUAGU 5'
query=miR5241a, target=RO3G_01422, score=3.5, range=564-581, stra
target 5' ACUCCUUCUU-CAUCAUCA 3'
: :::.:::: ::::::::
query 3' UAAGGGAGAAGGUAGUAGU 5'
query=miR5241a, target=RO3G_07756, score=3.5, range=1227-1244, strand=-1
target 5' AUAUCCUCUU-CAUCAUCA 3'
:: .:::::: ::::::::
query 3' UAAGGGAGAAGGUAGUAGU 5'
query=miR5241a, target=RO3G_01230, score=4, range=424-442, strand
target 5' UUUUUAUUUUCCAUCAUCA 3'
::.. :.:::::::::
query 3' UAAGGGAGAAGGUAGUAGU 5'
query=miR5241a, target=RO3G_00344, score=4, range=1373-1392, stra
target 5' CUUCUUCUCUUCCAUCAUUG 3'
::: .::::::::::::..
query 3' UAAG-GGAGAAGGUAGUAGU 5'
query=miR5241a, target=RO3G_07775, score=4, range=1405-1423, stra
target 5' UCUUCUUCUUCCAUCAUCU 3'
:.:.::::::::::::

```
query 3' UAAGGGAGAAGGUAGUAGU 5'
query=miR5241a, target=RO3G_06122, score=4, range=964-982, strand
target 5' AUUCACUCAUCUAUCAUCA 3'
:::: ::: ::.:::::::
query 3' UAAGGGAGAAGGUAGUAGU 5'
query=miR5241a, target=RO3G_16590, score=4, range=1572-1591, stra
target 5' AUCUCCUUCUUCCAUCCUCG 3'
:: :::.::::::::: ::.
query 3' UA-AGGGAGAAGGUAGUAGU 5'
query=miR5241a, target=RO3G_06182, score=4, range=403-421, strand
target 5' CCUACCUCUUCUAUCAUCA 3'
: ::::::.:::::::
query 3' UAAGGGAGAAGGUAGUAGU 5'
query=miR5241a, target=RO3G_12564, score=4, range=265-282, strand
target 5' GUUUCCUCUUCC-UCAUCU 3'
.::.:::::::: :::::
query 3' UAAGGGAGAAGGUAGUAGU 5'
query=miR5241a, target=RO3G_14159, score=4, range=431-448, strand
target 5' AUUUAUUCUU-CAUCAUCA 3'
:::. .:::: ::::::::
query 3' UAAGGGAGAAGGUAGUAGU 5'
query=miR5241a, target=RO3G_17338, score=4, range=597-615, strand
target 5' AUUUUCUUUUUCAUCAUCU 3'
:::..::.::.:::::::
query 3' UAAGGGAGAAGGUAGUAGU 5'
query=miR5241a, target=RO3G_03732, score=4, range=356-374, strand
target 5' CUUCUCUCUUCCAUCCUCG 3'
:::.:::::::::: ::.
query 3' UAAGGGAGAAGGUAGUAGU 5'
query=miR5241a, target=RO3G_00543, score=4, range=681-700, strand
target 5' CUUCCUCUUUUUCAUCAUCA 3'
:::: ::.::.::::::::
query 3' UAAGG-GAGAAGGUAGUAGU 5'
query=miR5241a, target=RO3G_06842, score=4, range=3092-3111, stra
target 5' AUCUUCCUCUUCCAUCAUGG 3'
:: :.:::::::::::: .
query 3' UA-AGGGAGAAGGUAGUAGU 5'
query=miR5241a, target=RO3G_07550, score=4, range=1409-1427, stra
target 5' AUUUGUCCUUCCAUCAUCA 3'
:::. . :::::::::::
query 3' UAAGGGAGAAGGUAGUAGU 5'
query=miR5241a, target=RO3G_06592, score=4, range=1403-1421, stra
target 5' AUUUGUCCUUCCAUCAUCA 3'
:::. . :::::::::::
query 3' UAAGGGAGAAGGUAGUAGU 5'
```

```
query=miR5241a, target=RO3G_12732, score=4, range=1217-1235, stra
target 5' AAUUCCUCUUCCAUCAUGG 3'
: :.:::::::::::: .
query 3' UAAGGGAGAAGGUAGUAGU 5'
query=miR5241a, target=RO3G_06133, score=4, range=11-29, strand=-
target 5' AUAUCUUCUUCCAUGAUCA 3'
:: .:.:::::::: ::::
query 3' UAAGGGAGAAGGUAGUAGU 5'
query=miR5241a, target=RO3G_10705, score=4, range=692-710, strand
target 5' AUAUCUUCUUCCAACAUCA 3'
:: .:.::::::: :::::
query 3' UAAGGGAGAAGGUAGUAGU 5'
query=miR5241a, target=RO3G_03189, score=4, range=365-384, strand
target 5' AUUCACAUCUUCUAUCGUCA 3'
:::: : :::::.:::.:::
query 3' UAAG-GGAGAAGGUAGUAGU 5'
query=miR5241a, target=RO3G_15176, score=4, range=453-471, strand
target 5' AUUAGUUCUUCCAUUAUCG 3'
::: .::::::::.:::.
query 3' UAAGGGAGAAGGUAGUAGU 5'
query=miR5241a, target=RO3G_09067, score=4, range=1872-1889, stra
target 5' AUUCUUUCUU-CAUCAUCU 3'
::::..:::: :::::::
query 3' UAAGGGAGAAGGUAGUAGU 5'
query=miR5241a, target=RO3G_10851, score=4, range=969-986, strand
target 5' GUUCCUUCUU-CAUCAUCU 3'
.::::.:::: :::::::
query 3' UAAGGGAGAAGGUAGUAGU 5'
query=miR5241a, target=RO3G_13254, score=4, range=192-209, strand
target 5' CUUCCUUCUUCCAUC-UCG 3'
::::.:::::::::: ::.
query 3' UAAGGGAGAAGGUAGUAGU 5'
query=miR5241a, target=RO3G_16011, score=4, range=549-567, strand
target 5' AUAGCCUUUUCCAUCAUCU 3'
:: :::.::::::::::
query 3' UAAGGGAGAAGGUAGUAGU 5'
query=miR5241a, target=RO3G_11592, score=4, range=539-556, strand
target 5' CAUCCCU-UUCCAUCAUCA 3'
::::: :::::::::::
query 3' UAAGGGAGAAGGUAGUAGU 5'
query=miR5241a, target=RO3G_03088, score=4, range=807-824, strand
target 5' ACUUCUUCUU-CAUCAUCA 3'
: :.:.:::: ::::::::
query 3' UAAGGGAGAAGGUAGUAGU 5'
query=miR5241a, target=RO3G_02482, score=4, range=102-120, strand
```

```
target 5' AUGACGUCUUUCAUCAUCA 3'
:: : ::::.::::::::
query 3' UAAGGGAGAAGGUAGUAGU 5'
query=miR5241a, target=RO3G_08714, score=4, range=347-366, strand
target 5' GUACGCGUCUUCCAUCAUCG 3'
.: : : :::::::::::.
query 3' UAAG-GGAGAAGGUAGUAGU 5'
query=miR5241a, target=RO3G_13397, score=4, range=261-278, strand
target 5' AUAUCCUCUU-CAUCAUCG 3'
:: .:::::: :::::::.
query 3' UAAGGGAGAAGGUAGUAGU 5'
query=miR5241a, target=RO3G_07178, score=4, range=975-992, strand
target 5' AUUUCUUCUU-CAUCGUCA 3'
:::.:.:::: ::::.:::
query 3' UAAGGGAGAAGGUAGUAGU 5'
query=miR1068, target=RO3G_06442, score=0, range=6121-6142, stran
target 5' GACCUGCAAGCAAAACUGGCUA 3'
::::::::::::::::::::::
query 3' CUGGACGUUCGUUUUGACCGAU 5'
query=miR1068, target=RO3G_16557, score=0, range=397-418, strand=
target 5' GACCUGCAAGCAAAACUGGCUA 3'
::::::::::::::::::::::
query 3' CUGGACGUUCGUUUUGACCGAU 5'
query=miR1068, target=RO3G_07098, score=0, range=397-418, strand=
target 5' GACCUGCAAGCAAAACUGGCUA 3'
::::::::::::::::::::::
query 3' CUGGACGUUCGUUUUGACCGAU 5'
query=miR1068, target=RO3G_01745, score=0, range=397-418, strand=
target 5' GACCUGCAAGCAAAACUGGCUA 3'
::::::::::::::::::::::
query 3' CUGGACGUUCGUUUUGACCGAU 5'
query=miR1068, target=RO3G_05271, score=0, range=397-418, strand=
target 5' GACCUGCAAGCAAAACUGGCUA 3'
::::::::::::::::::::::
query 3' CUGGACGUUCGUUUUGACCGAU 5'
query=miR1068, target=RO3G_00290, score=0, range=397-418, strand=
target 5' GACCUGCAAGCAAAACUGGCUA 3'
::::::::::::::::::::::
query 3' CUGGACGUUCGUUUUGACCGAU 5'
query=miR1068, target=RO3G_15134, score=0, range=439-460, strand=
target 5' GACCUGCAAGCAAAACUGGCUA 3'
::::::::::::::::::::::
query 3' CUGGACGUUCGUUUUGACCGAU 5'
query=miR1068, target=RO3G_12470, score=0, range=397-418, strand=
target 5' GACCUGCAAGCAAAACUGGCUA 3'
```

```
:::::::::::::::::::::
query 3' CUGGACGUUCGUUUUGACCGAU 5'
query=miR1068, target=RO3G_16132, score=0, range=397-418, strand=
target 5' GACCUGCAAGCAAAACUGGCUA 3'
:::::::::::::::::::::
query 3' CUGGACGUUCGUUUUGACCGAU 5'
query=miR1068, target=RO3G_01928, score=0, range=397-418, strand=
target 5' GACCUGCAAGCAAAACUGGCUA 3'
:::::::::::::::::::::
query 3' CUGGACGUUCGUUUUGACCGAU 5'
query=miR1068, target=RO3G_11525, score=0, range=397-418, strand=
target 5' GACCUGCAAGCAAAACUGGCUA 3'
:::::::::::::::::::::
query 3' CUGGACGUUCGUUUUGACCGAU 5'
query=miR1068, target=RO3G_00485, score=0, range=397-418, strand=
target 5' GACCUGCAAGCAAAACUGGCUA 3'
:::::::::::::::::::::
query 3' CUGGACGUUCGUUUUGACCGAU 5'
query=miR1068, target=RO3G_09479, score=0, range=397-418, strand=
target 5' GACCUGCAAGCAAAACUGGCUA 3'
:::::::::::::::::::::
query 3' CUGGACGUUCGUUUUGACCGAU 5'
query=miR1068, target=RO3G_09541, score=0, range=397-418, strand=
target 5' GACCUGCAAGCAAAACUGGCUA 3'
:::::::::::::::::::::
query 3' CUGGACGUUCGUUUUGACCGAU 5'
query=miR1068, target=RO3G_14995, score=0.5, range=397-418, stran
target 5' GGCCUGCAAGCAAAACUGGCUA 3'
:.:::::::::::::::::::
query 3' CUGGACGUUCGUUUUGACCGAU 5'
query=miR1068, target=RO3G_02714, score=3.5, range=397-418, stran
target 5' GACUUACAAGCAAAACUAGCUA 3'
:::.: :::::::::: ::::
query 3' CUGGACGUUCGUUUUGACCGAU 5'
query=miR1068, target=RO3G_02962, score=3.5, range=182-202, stran
target 5' AACUU-CAAGCAAAACUGGCUU 3'
::.: ::::::::::::::
query 3' CUGGACGUUCGUUUUGACCGAU 5'
query=miR1068, target=RO3G_06842, score=4, range=5290-5311, stran
target 5' GACUUGCAAGCAAAACUGAUUG 3'
:::.:::::::::::::: .:.
query 3' CUGGACGUUCGUUUUGACCGAU 5'
query=miR1068, target=RO3G_04655, score=4, range=1790-1811, stran
target 5' GACAUGAAAGUAAAACUGGCUU 3'
::: :: :::.:::::::::
```

```
query 3' CUGGACGUUCGUUUUGACCGAU 5'
query=miR1068, target=RO3G_14692, score=4, range=524-546, strand=
target 5' GACCUGCUGGGCAAAACUCGCUA 3'
::::::: ..:::::::: ::::
query 3' CUGGACG-UUCGUUUUGACCGAU 5'
query=miR9491, target=RO3G_16744, score=0, range=278-298, strand=
target 5' CUGAGUCAGAGGAAGAUAGCA 3'
:::::::::::::::::::::
query 3' GACUCAGUCUCCUUCUAUCGU 5'
query=miR9491, target=RO3G_01203, score=0, range=278-298, strand=
target 5' CUGAGUCAGAGGAAGAUAGCA 3'
:::::::::::::::::::::
query 3' GACUCAGUCUCCUUCUAUCGU 5'
query=miR9491, target=RO3G_11403, score=0, range=278-298, strand=
target 5' CUGAGUCAGAGGAAGAUAGCA 3'
:::::::::::::::::::::
query 3' GACUCAGUCUCCUUCUAUCGU 5'
query=miR9491, target=RO3G_06416, score=0, range=278-298, strand=
target 5' CUGAGUCAGAGGAAGAUAGCA 3'
:::::::::::::::::::::
query 3' GACUCAGUCUCCUUCUAUCGU 5'
query=miR9491, target=RO3G_01718, score=0, range=278-298, strand=
target 5' CUGAGUCAGAGGAAGAUAGCA 3'
:::::::::::::::::::::
query 3' GACUCAGUCUCCUUCUAUCGU 5'
query=miR9491, target=RO3G_15869, score=0, range=278-298, strand=
target 5' CUGAGUCAGAGGAAGAUAGCA 3'
:::::::::::::::::::::
query 3' GACUCAGUCUCCUUCUAUCGU 5'
query=miR9491, target=RO3G_10560, score=0, range=278-298, strand=
target 5' CUGAGUCAGAGGAAGAUAGCA 3'
:::::::::::::::::::::
query 3' GACUCAGUCUCCUUCUAUCGU 5'
query=miR9491, target=RO3G_11275, score=0, range=278-298, strand=
target 5' CUGAGUCAGAGGAAGAUAGCA 3'
:::::::::::::::::::::
query 3' GACUCAGUCUCCUUCUAUCGU 5'
query=miR9491, target=RO3G_14704, score=0, range=278-298, strand=
target 5' CUGAGUCAGAGGAAGAUAGCA 3'
:::::::::::::::::::::
query 3' GACUCAGUCUCCUUCUAUCGU 5'
query=miR9491, target=RO3G_12979, score=0, range=278-298, strand=
target 5' CUGAGUCAGAGGAAGAUAGCA 3'
:::::::::::::::::::::
query 3' GACUCAGUCUCCUUCUAUCGU 5'
```

```
query=miR9491, target=RO3G_10555, score=0, range=278-298, strand=
target 5' CUGAGUCAGAGGAAGAUAGCA 3'
:::::::::::::::::::::
query 3' GACUCAGUCUCCUUCUAUCGU 5'
query=miR9491, target=RO3G_05280, score=0, range=278-298, strand=
target 5' CUGAGUCAGAGGAAGAUAGCA 3'
:::::::::::::::::::::
query 3' GACUCAGUCUCCUUCUAUCGU 5'
query=miR9491, target=RO3G_16686, score=0, range=278-298, strand=
target 5' CUGAGUCAGAGGAAGAUAGCA 3'
:::::::::::::::::::::
query 3' GACUCAGUCUCCUUCUAUCGU 5'
query=miR9491, target=RO3G_07305, score=0, range=278-298, strand=
target 5' CUGAGUCAGAGGAAGAUAGCA 3'
:::::::::::::::::::::
query 3' GACUCAGUCUCCUUCUAUCGU 5'
query=miR9491, target=RO3G_03103, score=0, range=278-298, strand=
target 5' CUGAGUCAGAGGAAGAUAGCA 3'
:::::::::::::::::::::
query 3' GACUCAGUCUCCUUCUAUCGU 5'
query=miR9491, target=RO3G_12087, score=0, range=278-298, strand=
target 5' CUGAGUCAGAGGAAGAUAGCA 3'
:::::::::::::::::::::
query 3' GACUCAGUCUCCUUCUAUCGU 5'
query=miR9491, target=RO3G_08612, score=0, range=278-298, strand=
target 5' CUGAGUCAGAGGAAGAUAGCA 3'
:::::::::::::::::::::
query 3' GACUCAGUCUCCUUCUAUCGU 5'
query=miR9491, target=RO3G_05630, score=0, range=278-298, strand=
target 5' CUGAGUCAGAGGAAGAUAGCA 3'
:::::::::::::::::::::
query 3' GACUCAGUCUCCUUCUAUCGU 5'
query=miR9491, target=RO3G_13450, score=0, range=278-298, strand=
target 5' CUGAGUCAGAGGAAGAUAGCA 3'
:::::::::::::::::::::
query 3' GACUCAGUCUCCUUCUAUCGU 5'
query=miR9491, target=RO3G_11970, score=0, range=278-298, strand=
target 5' CUGAGUCAGAGGAAGAUAGCA 3'
:::::::::::::::::::::
query 3' GACUCAGUCUCCUUCUAUCGU 5'
query=miR9491, target=RO3G_13152, score=0, range=278-298, strand=
target 5' CUGAGUCAGAGGAAGAUAGCA 3'
:::::::::::::::::::::
query 3' GACUCAGUCUCCUUCUAUCGU 5'
query=miR9491, target=RO3G_03988, score=0, range=278-298, strand=
```

```
target 5' CUGAGUCAGAGGAAGAUAGCA 3'
:::::::::::::::::::::
query 3' GACUCAGUCUCCUUCUAUCGU 5'
query=miR9491, target=RO3G_12951, score=0, range=278-298, strand=
target 5' CUGAGUCAGAGGAAGAUAGCA 3'
:::::::::::::::::::::
query 3' GACUCAGUCUCCUUCUAUCGU 5'
query=miR9491, target=RO3G_09376, score=0, range=278-298, strand=
target 5' CUGAGUCAGAGGAAGAUAGCA 3'
:::::::::::::::::::::
query 3' GACUCAGUCUCCUUCUAUCGU 5'
query=miR9491, target=RO3G_12194, score=0, range=278-298, strand=
target 5' CUGAGUCAGAGGAAGAUAGCA 3'
:::::::::::::::::::::
query 3' GACUCAGUCUCCUUCUAUCGU 5'
query=miR9491, target=RO3G_07104, score=0, range=278-298, strand=
target 5' CUGAGUCAGAGGAAGAUAGCA 3'
:::::::::::::::::::::
query 3' GACUCAGUCUCCUUCUAUCGU 5'
query=miR9491, target=RO3G_13861, score=0, range=278-298, strand=
target 5' CUGAGUCAGAGGAAGAUAGCA 3'
:::::::::::::::::::::
query 3' GACUCAGUCUCCUUCUAUCGU 5'
query=miR9491, target=RO3G_08632, score=3, range=503-523, strand=
target 5' GUGAAUCAGAGGAAGAUAGCU 3'
::: ::::::::::::::::
query 3' GACUCAGUCUCCUUCUAUCGU 5'
query=miR9491, target=RO3G_12301, score=3.5, range=3578-3598, str
target 5' UUGACUUGGAGGAAGAUAGUA 3'
.::: :..::::::::::.:
query 3' GACUCAGUCUCCUUCUAUCGU 5'
query=miR2602a, target=RO3G_13914, score=0, range=737-755, strand
target 5' AUGCUGGCAAUCACUGGAA 3'
:::::::::::::::::::
query 3' UACGACCGUUAGUGACCUU 5'
query=miR2602a, target=RO3G_12058, score=0, range=737-755, strand
target 5' AUGCUGGCAAUCACUGGAA 3'
:::::::::::::::::::
query 3' UACGACCGUUAGUGACCUU 5'
query=miR2602a, target=RO3G_10801, score=0, range=542-560, strand
target 5' AUGCUGGCAAUCACUGGAA 3'
:::::::::::::::::::
query 3' UACGACCGUUAGUGACCUU 5'
query=miR2602a, target=RO3G_09614, score=0, range=68-86, strand=1
target 5' AUGCUGGCAAUCACUGGAA 3'
```

```
:::::::::::::::::::
query 3' UACGACCGUUAGUGACCUU 5'
query=miR2602a, target=RO3G_17462, score=0, range=869-887, strand
target 5' AUGCUGGCAAUCACUGGAA 3'
:::::::::::::::::::
query 3' UACGACCGUUAGUGACCUU 5'
query=miR2602a, target=RO3G_02233, score=0, range=266-284, strand
target 5' AUGCUGGCAAUCACUGGAA 3'
:::::::::::::::::::
query 3' UACGACCGUUAGUGACCUU 5'
query=miR2602a, target=RO3G_14983, score=1, range=542-560, strand
target 5' AUGCUGGCAAUUACUGGAA 3'
::::::::::.::::::::
query 3' UACGACCGUUAGUGACCUU 5'
query=miR2602a, target=RO3G_03583, score=1, range=542-560, strand
target 5' ACGCUGGCAAUCACUGGAA 3'
: :::::::::::::::::
query 3' UACGACCGUUAGUGACCUU 5'
query=miR2602a, target=RO3G_03556, score=2, range=542-560, strand
target 5' AUGCUGACAAUCACUGGAA 3'
:::::: ::::::::::::
query 3' UACGACCGUUAGUGACCUU 5'
query=miR2602a, target=RO3G_13096, score=3, range=269-287, strand
target 5' AUGGUGGCAGUCGCUGGAA 3'
::: :::::.::.::::::
query 3' UACGACCGUUAGUGACCUU 5'
query=miR2602a, target=RO3G_12495, score=3, range=320-339, strand
target 5' AUGCUUAGCAAUCACUGGAU 3'
:::: : ::::::::::::
query 3' UACG-ACCGUUAGUGACCUU 5'
query=miR2602a, target=RO3G_06752, score=3.5, range=179-197, stra
target 5' AUGAUGGUAAUUACUGGAG 3'
::: :::.:::.::::::.
query 3' UACGACCGUUAGUGACCUU 5'
query=miR2602a, target=RO3G_10535, score=4, range=2123-2141, stra
target 5' GUGUUGGCAAUGACUGGGA 3'
.::.:::::: :::::.:
query 3' UACGACCGUUAGUGACCUU 5'
query=miR2602a, target=RO3G_12181, score=4, range=1292-1310, stra
target 5' GUGUUGGCAAUGACUGGGA 3'
.::.:::::: :::::.:
query 3' UACGACCGUUAGUGACCUU 5'
query=miR2602a, target=RO3G_15136, score=4, range=185-203, strand
target 5' GUGUUGGCAAUGACUGGGA 3'
.::.:::::: :::::.:
```

```
query 3' UACGACCGUUAGUGACCUU 5'
query=miR2602a, target=RO3G_16143, score=4, range=4475-4493, stra
target 5' GUGUUGGCAAUGACUGGGA 3'
.::.::::::: :::::.:
query 3' UACGACCGUUAGUGACCUU 5'
query=miR2602a, target=RO3G_02691, score=4, range=2924-2942, stra
target 5' GUGUUGGCAAUGACUGGGA 3'
.::.::::::: :::::.:
query 3' UACGACCGUUAGUGACCUU 5'
query=miR2602a, target=RO3G_17318, score=4, range=4478-4496, stra
target 5' GUGUUGGCAAUGACUGGGA 3'
.::.::::::: :::::.:
query 3' UACGACCGUUAGUGACCUU 5'
query=miR2602a, target=RO3G_05918, score=4, range=998-1016, stran
target 5' GUGUUGGCAAUGACUGGGA 3'
.::.::::::: :::::.:
query 3' UACGACCGUUAGUGACCUU 5'
query=miR2602a, target=RO3G_12533, score=4, range=3818-3836, stra
target 5' GUGUUGGCAAUGACUGGGA 3'
.::.::::::: :::::.:
query 3' UACGACCGUUAGUGACCUU 5'
query=miR2602a, target=RO3G_07028, score=4, range=3461-3479, stra
target 5' GUGUUGGCAAUGACUGGGA 3'
.::.::::::: :::::.:
query 3' UACGACCGUUAGUGACCUU 5'
query=miR2602a, target=RO3G_05520, score=4, range=1145-1163, stra
target 5' GUGUUGGCAAUGACUGGGA 3'
.::.::::::: :::::.:
query 3' UACGACCGUUAGUGACCUU 5'
query=miR2602a, target=RO3G_17383, score=4, range=977-995, strand
target 5' GUGUUGGCAAUGACUGGGA 3'
.::.::::::: :::::.:
query 3' UACGACCGUUAGUGACCUU 5'
query=miR2602a, target=RO3G_17329, score=4, range=3815-3833, stra
target 5' GUGUUGGCAAUGACUGGGA 3'
.::.::::::: :::::.:
query 3' UACGACCGUUAGUGACCUU 5'
query=miR2602a, target=RO3G_17274, score=4, range=4301-4319, stra
target 5' GUGUUGGCAAUGACUGGGA 3'
.::.::::::: :::::.:
query 3' UACGACCGUUAGUGACCUU 5'
query=miR2602a, target=RO3G_17076, score=4, range=796-814, strand
target 5' ACGUUGGCAAUUACUGGGG 3'
: :.:::::::.:::::..
query 3' UACGACCGUUAGUGACCUU 5'
```

```
query=miR2602a, target=RO3G_03950, score=4, range=202-220, strand
target 5' AUGCUGGCAAGCAUUGGAC 3'
:::::::::: ::.::::
query 3' UACGACCGUUAGUGACCUU 5'
query=miR2602a, target=RO3G_14739, score=4, range=749-766, strand
target 5' AUGCUGGCGAU-AUUGGAA 3'
::::::::.:: :.:::::
query 3' UACGACCGUUAGUGACCUU 5'
query=miR2602a, target=RO3G_15498, score=4, range=1311-1328, stra
target 5' GU-UUGGUAAUUACUGGAA 3'
.: .:::.:::.:::::::
query 3' UACGACCGUUAGUGACCUU 5'
query=miR2602a, target=RO3G_08685, score=4, range=503-521, strand
target 5' AUACUUGCAAUUAUUGGAA 3'
:: :: :::::.:.:::::
query 3' UACGACCGUUAGUGACCUU 5'
query=miR2602a, target=RO3G_12519, score=4, range=285-302, strand
target 5' AUGGU-GCAAUCACUGGGC 3'
::: : :::::::::::.
query 3' UACGACCGUUAGUGACCUU 5'
query=miR2602a, target=RO3G_06658, score=4, range=1991-2009, stra
target 5' AAGAUGGCAAUCGCUGGGA 3'
: : ::::::::.::::.:
query 3' UACGACCGUUAGUGACCUU 5'
query=miR2602a, target=RO3G_16999, score=4, range=4591-4608, stra
target 5' AU-CUAGCAAUUGCUGGAA 3'
:: :: :::::..::::::
query 3' UACGACCGUUAGUGACCUU 5'
query=miR2602a, target=RO3G_00024, score=4, range=470-488, strand
target 5' GCUUUAGCAAUCACUGGAA 3'
. .: ::::::::::::
query 3' UACGACCGUUAGUGACCUU 5'
query=miR2602a, target=RO3G_02639, score=4, range=416-435, strand
target 5' AUCCGGAGCAAUCACUGGAU 3'
:: : : :::::::::::
query 3' UACGAC-CGUUAGUGACCUU 5'
query=miR2602a, target=RO3G_10099, score=4, range=137-154, strand
target 5' CUGCUGGCAAU-GCUGGAA 3'
:::::::::: .::::::
query 3' UACGACCGUUAGUGACCUU 5'
query=miR6147, target=RO3G_05963, score=0, range=387-408, strand=
target 5' AGUGGGGUUUUGAUGCAUGUCA 3'
::::::::::::::::::::::
query 3' UCACCCCAAAACUACGUACAGU 5'
query=miR6147, target=RO3G_08106, score=0, range=387-408, strand=
```

```
target 5' AGUGGGGUUUUGAUGCAUGUCA 3'
::::::::::::::::::::::
query 3' UCACCCCAAAACUACGUACAGU 5'
query=miR6147, target=RO3G_17163, score=0, range=387-408, strand=
target 5' AGUGGGGUUUUGAUGCAUGUCA 3'
::::::::::::::::::::::
query 3' UCACCCCAAAACUACGUACAGU 5'
query=miR6147, target=RO3G_15896, score=0, range=387-408, strand=
target 5' AGUGGGGUUUUGAUGCAUGUCA 3'
::::::::::::::::::::::
query 3' UCACCCCAAAACUACGUACAGU 5'
query=miR6147, target=RO3G_14346, score=0, range=387-408, strand=
target 5' AGUGGGGUUUUGAUGCAUGUCA 3'
::::::::::::::::::::::
query 3' UCACCCCAAAACUACGUACAGU 5'
query=miR6147, target=RO3G_02711, score=0, range=387-408, strand=
target 5' AGUGGGGUUUUGAUGCAUGUCA 3'
::::::::::::::::::::::
query 3' UCACCCCAAAACUACGUACAGU 5'
query=miR396b, target=RO3G_03508, score=2, range=780-800, strand=
target 5' UGUUCAAAGAAAGCUGUGGAA 3'
:::: :::::::::::::::
query 3' UCAAG-UUCUUUCGACACCUU 5'
query=miR396b, target=RO3G_02790, score=4, range=740-759, strand=
target 5' CGCUCAAGAAAGCUAUGGAA 3'
: ::::::::::: :::::
query 3' UCAAGUUCUUUCGACACCUU 5'
query=miR396b, target=RO3G_16340, score=4, range=605-624, strand=
target 5' AGUUGAUGGAAGCUGUGGAU 3'
:::: : :.::::::::::
query 3' UCAAGUUCUUUCGACACCUU 5'
query=miR396b, target=RO3G_07651, score=4, range=4206-4225, stran
target 5' AAUUAAAGAAAGCUGUGAAA 3'
: :: :::::::::::: ::
query 3' UCAAGUUCUUUCGACACCUU 5'
query=miR396b, target=RO3G_08307, score=4, range=804-822, strand=
target 5' AGUUGAAGAAAGCU-UGGAU 3'
:::: ::::::::: ::::
query 3' UCAAGUUCUUUCGACACCUU 5'
query=miR396b, target=RO3G_06158, score=4, range=1190-1209, stran
target 5' UGCGCAUGAAAGCUGUGGAA 3'
: :: :::::::::::::
query 3' UCAAGUUCUUUCGACACCUU 5'
query=miR7711-5p.1, target=RO3G_12954, score=2, range=54-75, stra
target 5' CAAGAAGUCAAACAGCAAAGUA 3'
```

```
:::::::::::::::: :::::
query 3' GUUCUUCAGUUUGUCGAUUCAU 5'
query=miR7711-5p.1, target=RO3G_10762, score=2, range=54-75, stra
target 5' CAAGAAGUCAAACAGCAAAGUA 3'
:::::::::::::::: :::::
query 3' GUUCUUCAGUUUGUCGAUUCAU 5'
query=miR7711-5p.1, target=RO3G_10770, score=2, range=54-75, stra
target 5' CAAGAAGUCAAACAGCAAAGUA 3'
:::::::::::::::: :::::
query 3' GUUCUUCAGUUUGUCGAUUCAU 5'
query=miR7711-5p.1, target=RO3G_00304, score=2, range=54-75, stra
target 5' CAAGAAGUCAAACAGCAAAGUA 3'
:::::::::::::::: :::::
query 3' GUUCUUCAGUUUGUCGAUUCAU 5'
query=miR7711-5p.1, target=RO3G_04690, score=4, range=54-75, stra
target 5' CAAGAAGUCAAACAGCGAAAUA 3'
:::::::::::::::: :: ::
query 3' GUUCUUCAGUUUGUCGAUUCAU 5'
query=miR7711-5p.1, target=RO3G_05431, score=4, range=54-75, stra
target 5' CAAGAAGUCAAACAGCAAAAUA 3'
:::::::::::::::: :: ::
query 3' GUUCUUCAGUUUGUCGAUUCAU 5'
query=miR7711-5p.1, target=RO3G_02822, score=4, range=54-75, stra
target 5' CAAGAAGUCAAACAGCGAAAUA 3'
:::::::::::::::: :: ::
query 3' GUUCUUCAGUUUGUCGAUUCAU 5'
query=miR7711-5p.1, target=RO3G_09168, score=4, range=54-75, stra
target 5' CAAGAAGUCAAACAGCAAAAUA 3'
:::::::::::::::: :: ::
query 3' GUUCUUCAGUUUGUCGAUUCAU 5'
query=miR7711-5p.1, target=RO3G_15141, score=4, range=54-75, stra
target 5' CAAGAAGUCAAACAGCAAAAUA 3'
:::::::::::::::: :: ::
query 3' GUUCUUCAGUUUGUCGAUUCAU 5'
query=miR7711-5p.1, target=RO3G_04787, score=4, range=54-75, stra
target 5' CAAGAAGUCAAACAGCAAAAUA 3'
:::::::::::::::: :: ::
query 3' GUUCUUCAGUUUGUCGAUUCAU 5'
query=miR5373, target=RO3G_13221, score=0, range=1731-1752, stran
target 5' AACAUCAUCAGCAUACAAGAGA 3'
::::::::::::::::::::::
query 3' UUGUAGUAGUCGUAUGUUCUCU 5'
query=miR5373, target=RO3G_13224, score=0, range=1731-1752, stran
target 5' AACAUCAUCAGCAUACAAGAGA 3'
::::::::::::::::::::::
```

```
query 3' UUGUAGUAGUCGUAUGUUCUCU 5'
query=miR5373, target=RO3G_04123, score=0, range=1731-1752, stran
target 5' AACAUCAUCAGCAUACAAGAGA 3'
::::::::::::::::::::::
query 3' UUGUAGUAGUCGUAUGUUCUCU 5'
query=miR5373, target=RO3G_04120, score=0, range=1731-1752, stran
target 5' AACAUCAUCAGCAUACAAGAGA 3'
::::::::::::::::::::::
query 3' UUGUAGUAGUCGUAUGUUCUCU 5'
query=miR5373, target=RO3G_04203, score=0, range=675-696, strand=
target 5' AACAUCAUCAGCAUACAAGAGA 3'
::::::::::::::::::::::
query 3' UUGUAGUAGUCGUAUGUUCUCU 5'
query=miR5373, target=RO3G_13218, score=0, range=144-165, strand=
target 5' AACAUCAUCAGCAUACAAGAGA 3'
::::::::::::::::::::::
query 3' UUGUAGUAGUCGUAUGUUCUCU 5'
query=miR5373, target=RO3G_16122, score=0, range=144-165, strand=
target 5' AACAUCAUCAGCAUACAAGAGA 3'
::::::::::::::::::::::
query 3' UUGUAGUAGUCGUAUGUUCUCU 5'
query=miR5373, target=RO3G_14836, score=1, range=1482-1503, stran
target 5' AACGUCAUCAGCAUACAAGAGG 3'
:::.:::::::::::::::::.
query 3' UUGUAGUAGUCGUAUGUUCUCU 5'
query=miR5373, target=RO3G_06780, score=1, range=759-780, strand=
target 5' CACAUCAUCAGCAUACAAGAGA 3'
:::::::::::::::::::::
query 3' UUGUAGUAGUCGUAUGUUCUCU 5'
query=miR5373, target=RO3G_00484, score=1.5, range=32-53, strand=
target 5' AAUGUCAUCAGCAUACAAGAGG 3'
::..:::::::::::::::::.
query 3' UUGUAGUAGUCGUAUGUUCUCU 5'
query=miR5373, target=RO3G_16800, score=2, range=3363-3384, stran
target 5' CACAUCAUCAGCAUACAGGAGA 3'
:::::::::::::::::.::::
query 3' UUGUAGUAGUCGUAUGUUCUCU 5'
query=miR5373, target=RO3G_15267, score=2, range=1125-1146, stran
target 5' CACAUCAUCAGCAUACAGGAGA 3'
:::::::::::::::::.::::
query 3' UUGUAGUAGUCGUAUGUUCUCU 5'
query=miR5373, target=RO3G_17246, score=2, range=564-585, strand=
target 5' CACAUCAUCAGCAUACAGGAGA 3'
:::::::::::::::::.::::
query 3' UUGUAGUAGUCGUAUGUUCUCU 5'
```

```
query=miR5373, target=RO3G_16819, score=2, range=135-156, strand=
target 5' CACAUCAUCAGCAUACAGGAGA 3'
::::::::::::::::.::::
query 3' UUGUAGUAGUCGUAUGUUCUCU 5'
query=miR5373, target=RO3G_12339, score=3.5, range=1656-1677,
strand=-1
target 5' GACUUCAUCAGCAUUCAAGAGA 3'
.:: :::::::::: :::::::
query 3' UUGUAGUAGUCGUAUGUUCUCU 5'
query=miR5373, target=RO3G_14120, score=4, range=145-166, strand=
target 5' UACAUCAUCGGCAUACAAAAGA 3'
:::::::.:::::::: :::
query 3' UUGUAGUAGUCGUAUGUUCUCU 5'
query=miR5373, target=RO3G_15307, score=4, range=1483-1504, stran
target 5' CACAUCAACAGCAUACAAGACA 3'
:::::: ::::::::::: :
query 3' UUGUAGUAGUCGUAUGUUCUCU 5'
query=miR5373, target=RO3G_14105, score=4, range=162-183, strand=
target 5' UACAUCAUCGGCAUACAAAAGA 3'
:::::::.:::::::: :::
query 3' UUGUAGUAGUCGUAUGUUCUCU 5'
query=miR5373, target=RO3G_13384, score=4, range=1656-1677, stran
target 5' GACUUCGUCAGCAUUCAAGAGA 3'
.:: ::.::::::: :::::::
query 3' UUGUAGUAGUCGUAUGUUCUCU 5'
query=miR832-3p, target=RO3G_15146, score=1.5, range=363-382, str
target 5' CUGGGCUUGGAUUCGAUCAG 3'
:: :::::::::::::::.
query 3' GAACCGAACCUAAGCUAGUU 5'
query=miR832-3p, target=RO3G_12664, score=2.5, range=210-229, str
target 5' CUGGGCUUGGAUUCGAUCGG 3'
:: ::::::::::::::..
query 3' GAACCGAACCUAAGCUAGUU 5'
query=miR832-3p, target=RO3G_07112, score=2.5, range=306-325, str
target 5' CUGGGCUUGGAUUCGAUCGG 3'
:: ::::::::::::::..
query 3' GAACCGAACCUAAGCUAGUU 5'
query=miR832-3p, target=RO3G_08663, score=2.5, range=483-502, str
target 5' CUGGGCUUGGAUUCGAUCGG 3'
:: ::::::::::::::..
query 3' GAACCGAACCUAAGCUAGUU 5'
query=miR832-3p, target=RO3G_07120, score=2.5, range=306-325, str
target 5' CUGGGCUUGGAUUCGAUCGG 3'
:: ::::::::::::::..
query 3' GAACCGAACCUAAGCUAGUU 5'
```

query=miR832-3p, target=RO3G_15001, score=2.5, range=306-325, str
target 5' CUGGGCUUGGAUUCGAUCGG 3'
:: :::::::::::::::..
query 3' GAACCGAACCUAAGCUAGUU 5'
query=miR832-3p, target=RO3G_15575, score=2.5, range=306-325, str
target 5' CUGGGCUUGGAUUCGAUCGG 3'
:: :::::::::::::::..
query 3' GAACCGAACCUAAGCUAGUU 5'
query=miR832-3p, target=RO3G_11240, score=2.5, range=306-325, str
target 5' CUGGGCUUGGAUUCGAUCGG 3'
:: :::::::::::::::..
query 3' GAACCGAACCUAAGCUAGUU 5'
query=miR832-3p, target=RO3G_08794, score=3, range=138-158, stran
target 5' CUUGCCUAUGGAUUUGAUCAA 3'
:::: :: ::::::.::::::
query 3' GAACCGA-ACCUAAGCUAGUU 5'
query=miR832-3p, target=RO3G_12847, score=3, range=293-312, stran
target 5' CCUCGAUUGGAUUCGAUCAA 3'
: : : ::::::::::::::
query 3' GAACCGAACCUAAGCUAGUU 5'
query=miR832-3p, target=RO3G_16594, score=3, range=515-534, stran
target 5' CUUGGAUUGGAUUCGAUUGA 3'
::::: :::::::::::..:
query 3' GAACCGAACCUAAGCUAGUU 5'
query=miR832-3p, target=RO3G_03727, score=3.5, range=1611-1630, strand=1
target 5' CUUGAUUUGGAUUCAAUCAA 3'
:::: .:::::::: :::::
query 3' GAACCGAACCUAAGCUAGUU 5'
query=miR832-3p, target=RO3G_13277, score=3.5, range=483-502, str
target 5' CUGGGCUUGGAUUCGAUCCG 3'
:: ::::::::::::::: .
query 3' GAACCGAACCUAAGCUAGUU 5'
query=miR832-3p, target=RO3G_14621, score=3.5, range=141-160, str
target 5' CUGGGCUUGGAUCCGAUCAG 3'
:: ::::::::: ::::::.
query 3' GAACCGAACCUAAGCUAGUU 5'
query=miR832-3p, target=RO3G_10800, score=3.5, range=141-160, str
target 5' CUGGGCUUGGAUCCGAUCAG 3'
:: ::::::::: ::::::.
query 3' GAACCGAACCUAAGCUAGUU 5'
query=miR832-3p, target=RO3G_16545, score=3.5, range=483-502, str
target 5' CUGGGCUUGGAUCCGAUCAG 3'
:: ::::::::: ::::::.
query 3' GAACCGAACCUAAGCUAGUU 5'

```
query=miR832-3p, target=RO3G_07031, score=3.5, range=483-502, str
target 5' CUGGGCUUGGAUUCGAUCCG 3'
:: :::::::::::::: .
query 3' GAACCGAACCUAAGCUAGUU 5'
query=miR832-3p, target=RO3G_00255, score=3.5, range=483-502, str
target 5' CUGGGCUUGGAUCCGAUCAG 3'
:: ::::::::: ::::::.
query 3' GAACCGAACCUAAGCUAGUU 5'
query=miR832-3p, target=RO3G_16359, score=3.5, range=483-502, str
target 5' CUGGGCUUGGAUCCGAUCAG 3'
:: ::::::::: ::::::.
query 3' GAACCGAACCUAAGCUAGUU 5'
query=miR832-3p, target=RO3G_09612, score=3.5, range=483-502, str
target 5' CUGGGCUUGGAUCCGAUCAG 3'
:: ::::::::: ::::::.
query 3' GAACCGAACCUAAGCUAGUU 5'
query=miR832-3p, target=RO3G_16554, score=3.5, range=483-502, str
target 5' CUGGGCUUGGAUUCGAUCCG 3'
:: :::::::::::::: .
query 3' GAACCGAACCUAAGCUAGUU 5'
query=miR832-3p, target=RO3G_13271, score=3.5, range=483-502, str
target 5' CUGGGCUUGGAUCCGAUCAG 3'
:: ::::::::: ::::::.
query 3' GAACCGAACCUAAGCUAGUU 5'
query=miR832-3p, target=RO3G_03684, score=3.5, range=408-428, str
target 5' CUUGGUUUUGGAUUGGAUCAA 3'
::::: .::::::: ::::::
query 3' GAACC-GAACCUAAGCUAGUU 5'
query=miR832-3p, target=RO3G_03803, score=3.5, range=306-325, str
target 5' CUGGACUUGGAUUCGAUCGG 3'
:: : ::::::::::::..
query 3' GAACCGAACCUAAGCUAGUU 5'
query=miR832-3p, target=RO3G_04019, score=3.5, range=415-434, str
target 5' UUUUCUUUGGAUUCGAUCAG 3'
.:: .:::::::::::::.
query 3' GAACCGAACCUAAGCUAGUU 5'
query=miR832-3p, target=RO3G_05301, score=4, range=435-454, stran
target 5' UUUGGAUUGGAUUUGGUCAG 3'
.:::: :::::::.:.:::.
query 3' GAACCGAACCUAAGCUAGUU 5'
query=miR832-3p, target=RO3G_11266, score=4, range=306-325, stran
target 5' CUGGGCUUGGAUCCGGUCAA 3'
:: ::::::::: ::.::::
query 3' GAACCGAACCUAAGCUAGUU 5'
query=miR832-3p, target=RO3G_01902, score=4, range=3924-3944, str
```

```
target 5' CUAUGGCUUGGAUUUCAUCAA 3'
:: :::::::::::. :::::
query 3' GA-ACCGAACCUAAGCUAGUU 5'
query=miR832-3p, target=RO3G_15416, score=4, range=343-363, stran
target 5' CCUGGCUUUGGAUUGGAUCAA 3'
: :::: ::::::: ::::::
query 3' GAACCG-AACCUAAGCUAGUU 5'
query=miR832-3p, target=RO3G_03634, score=4, range=2610-2630, str
target 5' CUUGCCAAUGGAUUUGAUCAA 3'
:::: : ::::::.::::::
query 3' GAACCG-AACCUAAGCUAGUU 5'
query=miR832-3p, target=RO3G_10693, score=4, range=571-590, stran
target 5' CGUGUGAUGGAUUCGAUCAA 3'
: :: :::::::::::::
query 3' GAACCGAACCUAAGCUAGUU 5'
query=miR832-3p, target=RO3G_01659, score=4, range=1171-1189, str
target 5' CUUGGC-UCGAUUCGAUUAA 3'
:::::: : ::::::::.::
query 3' GAACCGAACCUAAGCUAGUU 5'
query=miR832-3p, target=RO3G_12122, score=4, range=263-282, stran
target 5' CUUGCCUUGGAUUUCAUCAA 3'
:::: ::::::::. :::::
query 3' GAACCGAACCUAAGCUAGUU 5'
query=miR832-3p, target=RO3G_03345, score=4, range=263-282, stran
target 5' CUUGCCUUGGAUUUCAUCAA 3'
:::: ::::::::. :::::
query 3' GAACCGAACCUAAGCUAGUU 5'
query=miR832-3p, target=RO3G_15816, score=4, range=710-729, stran
target 5' GAUGGCCUGGAUUUGAUCAA 3'
:::: :::::.::::::
query 3' GAACCGAACCUAAGCUAGUU 5'
query=miR172k, target=RO3G_17256, score=0, range=356-374, strand=
target 5' AGCAGCAGCAUCCAGUUCA 3'
:::::::::::::::::::
query 3' UCGUCGUCGUAGGUCAAGU 5'
query=miR172k, target=RO3G_15890, score=0, range=359-377, strand=
target 5' AGCAGCAGCAUCCAGUUCA 3'
:::::::::::::::::::
query 3' UCGUCGUCGUAGGUCAAGU 5'
query=miR172k, target=RO3G_15147, score=0, range=323-341, strand=
target 5' AGCAGCAGCAUCCAGUUCA 3'
:::::::::::::::::::
query 3' UCGUCGUCGUAGGUCAAGU 5'
query=miR172k, target=RO3G_13146, score=0, range=359-377, strand=
target 5' AGCAGCAGCAUCCAGUUCA 3'
```

```
:::::::::::::::::::
query 3' UCGUCGUCGUAGGUCAAGU 5'
query=miR172k, target=RO3G_09182, score=0, range=335-353, strand=
target 5' AGCAGCAGCAUCCAGUUCA 3'
:::::::::::::::::::
query 3' UCGUCGUCGUAGGUCAAGU 5'
query=miR172k, target=RO3G_10041, score=0, range=359-377, strand=
target 5' AGCAGCAGCAUCCAGUUCA 3'
:::::::::::::::::::
query 3' UCGUCGUCGUAGGUCAAGU 5'
query=miR172k, target=RO3G_00288, score=0, range=350-368, strand=
target 5' AGCAGCAGCAUCCAGUUCA 3'
:::::::::::::::::::
query 3' UCGUCGUCGUAGGUCAAGU 5'
query=miR172k, target=RO3G_17248, score=0, range=359-377, strand=
target 5' AGCAGCAGCAUCCAGUUCA 3'
:::::::::::::::::::
query 3' UCGUCGUCGUAGGUCAAGU 5'
query=miR172k, target=RO3G_17350, score=0, range=353-371, strand=
target 5' AGCAGCAGCAUCCAGUUCA 3'
:::::::::::::::::::
query 3' UCGUCGUCGUAGGUCAAGU 5'
query=miR172k, target=RO3G_16137, score=0, range=353-371, strand=
target 5' AGCAGCAGCAUCCAGUUCA 3'
:::::::::::::::::::
query 3' UCGUCGUCGUAGGUCAAGU 5'
query=miR172k, target=RO3G_03584, score=0, range=287-305, strand=
target 5' AGCAGCAGCAUCCAGUUCA 3'
:::::::::::::::::::
query 3' UCGUCGUCGUAGGUCAAGU 5'
query=miR172k, target=RO3G_05425, score=1, range=356-374, strand=
target 5' AGCAGAAGCAUCCAGUUCA 3'
::::: :::::::::::::
query 3' UCGUCGUCGUAGGUCAAGU 5'
query=miR172k, target=RO3G_09497, score=1, range=68-86, strand=1
target 5' AACAGCAGCAUCCAGUUCA 3'
: :::::::::::::::::
query 3' UCGUCGUCGUAGGUCAAGU 5'
query=miR172k, target=RO3G_09603, score=2, range=371-389, strand=
target 5' AGCAGCAGCACCCAGUUCA 3'
:::::::::: ::::::::
query 3' UCGUCGUCGUAGGUCAAGU 5'
query=miR172k, target=RO3G_10218, score=2, range=350-368, strand=
target 5' AGCAGCAGCACCCAGUUCA 3'
:::::::::: ::::::::
```

```
query 3' UCGUCGUCGUAGGUCAAGU 5'
query=miR172k, target=RO3G_11537, score=2, range=371-389, strand=
target 5' AGCAGCAGCACCCAGUUCA 3'
:::::::::: ::::::::
query 3' UCGUCGUCGUAGGUCAAGU 5'
query=miR172k, target=RO3G_11236, score=2, range=371-389, strand=
target 5' AGCAGCAGCACCCAGUUCA 3'
:::::::::: ::::::::
query 3' UCGUCGUCGUAGGUCAAGU 5'
query=miR172k, target=RO3G_07738, score=2, range=380-398, strand=
target 5' AGCAGCAGCACCCAGUUCA 3'
:::::::::: ::::::::
query 3' UCGUCGUCGUAGGUCAAGU 5'
query=miR172k, target=RO3G_02228, score=2, range=329-347, strand=
target 5' AGCAGCAGCACCCAGUUCA 3'
:::::::::: ::::::::
query 3' UCGUCGUCGUAGGUCAAGU 5'
query=miR172k, target=RO3G_07981, score=2, range=329-347, strand=
target 5' AGCAGCAGCACCCAGUUCA 3'
:::::::::: ::::::::
query 3' UCGUCGUCGUAGGUCAAGU 5'
query=miR172k, target=RO3G_07720, score=2, range=329-347, strand=
target 5' AGCAGCAGCACCCAGUUCA 3'
:::::::::: ::::::::
query 3' UCGUCGUCGUAGGUCAAGU 5'
query=miR172k, target=RO3G_15897, score=2, range=329-347, strand=
target 5' AGCAGCAGCACCCAGUUCA 3'
:::::::::: ::::::::
query 3' UCGUCGUCGUAGGUCAAGU 5'
query=miR172k, target=RO3G_09602, score=2, range=329-347, strand=
target 5' AGCAGCAGCACCCAGUUCA 3'
:::::::::: ::::::::
query 3' UCGUCGUCGUAGGUCAAGU 5'
query=miR172k, target=RO3G_04211, score=2, range=329-347, strand=
target 5' AGCAGCAGCACCCAGUUCA 3'
:::::::::: ::::::::
query 3' UCGUCGUCGUAGGUCAAGU 5'
query=miR172k, target=RO3G_07696, score=2, range=329-347, strand=
target 5' AGCAGCAGCACCCAGUUCA 3'
:::::::::: ::::::::
query 3' UCGUCGUCGUAGGUCAAGU 5'
query=miR172k, target=RO3G_07306, score=2, range=329-347, strand=
target 5' AGCAGCAGCACCCAGUUCA 3'
:::::::::: ::::::::
query 3' UCGUCGUCGUAGGUCAAGU 5'
```

```
query=miR172k, target=RO3G_09929, score=2, range=329-347, strand=
target 5' AGCAGCAGCACCCAGUUCA 3'
:::::::::: ::::::::
query 3' UCGUCGUCGUAGGUCAAGU 5'
query=miR172k, target=RO3G_14551, score=2, range=329-347, strand=
target 5' AGCAGCAGCACCCAGUUCA 3'
:::::::::: ::::::::
query 3' UCGUCGUCGUAGGUCAAGU 5'
query=miR172k, target=RO3G_04679, score=2, range=299-317, strand=
target 5' AGCAGCAGCACCCAGUUCA 3'
:::::::::: ::::::::
query 3' UCGUCGUCGUAGGUCAAGU 5'
query=miR172k, target=RO3G_06730, score=2, range=329-347, strand=
target 5' AGCAGCAGCACCCAGUUCA 3'
:::::::::: ::::::::
query 3' UCGUCGUCGUAGGUCAAGU 5'
query=miR172k, target=RO3G_16748, score=2, range=329-347, strand=
target 5' AGCAGCAGCACCCAGUUCA 3'
:::::::::: ::::::::
query 3' UCGUCGUCGUAGGUCAAGU 5'
query=miR172k, target=RO3G_01726, score=2, range=374-392, strand=
target 5' AGCAGCAGCACCCAGUUCA 3'
:::::::::: ::::::::
query 3' UCGUCGUCGUAGGUCAAGU 5'
query=miR172k, target=RO3G_11977, score=2, range=293-311, strand=
target 5' AGCAGCAGCACCCAGUUCA 3'
:::::::::: ::::::::
query 3' UCGUCGUCGUAGGUCAAGU 5'
query=miR172k, target=RO3G_10775, score=2, range=357-375, strand=
target 5' AACAGAAGCAUCCAGUUCA 3'
: ::: :::::::::::::
query 3' UCGUCGUCGUAGGUCAAGU 5'
query=miR172k, target=RO3G_02907, score=3, range=1081-1098, stran
target 5' AGCAGU-GCAUCCAGUUCG 3'
:::::. :::::::::::.
query 3' UCGUCGUCGUAGGUCAAGU 5'
query=miR172k, target=RO3G_01612, score=3.5, range=185-203, stran
target 5' AGCAGCAGCAUCUCGUUCG 3'
::::::::::::. ::::.
query 3' UCGUCGUCGUAGGUCAAGU 5'
query=miR172k, target=RO3G_16555, score=3.5, range=185-203, stran
target 5' AGCAGCAGCAUCUCGUUCG 3'
::::::::::::. ::::.
query 3' UCGUCGUCGUAGGUCAAGU 5'
query=miR172k, target=RO3G_16551, score=3.5, range=185-203, stran
```

```
target 5' AGCAGCAGCAUCUCGUUCG 3'
:::::::::::. ::::.
query 3' UCGUCGUCGUAGGUCAAGU 5'
query=miR172k, target=RO3G_08846, score=3.5, range=185-203, stran
target 5' AGCAGCAGCAUCUCGUUCG 3'
:::::::::::. ::::.
query 3' UCGUCGUCGUAGGUCAAGU 5'
query=miR172k, target=RO3G_09483, score=3.5, range=185-203, stran
target 5' AGCAGCAGCAUCUCGUUCG 3'
:::::::::::. ::::.
query 3' UCGUCGUCGUAGGUCAAGU 5'
query=miR172k, target=RO3G_11773, score=3.5, range=185-203, stran
target 5' AGCAGCAGCAUCUCGUUCG 3'
:::::::::::. ::::.
query 3' UCGUCGUCGUAGGUCAAGU 5'
query=miR172k, target=RO3G_07704, score=3.5, range=185-203, stran
target 5' AGCAGCAGCAUCUCGUUCG 3'
:::::::::::. ::::.
query 3' UCGUCGUCGUAGGUCAAGU 5'
query=miR172k, target=RO3G_13726, score=3.5, range=185-203, stran
target 5' AGCAGCAGCAUCUCGUUCG 3'
:::::::::::. ::::.
query 3' UCGUCGUCGUAGGUCAAGU 5'
query=miR172k, target=RO3G_03615, score=3.5, range=185-203, stran
target 5' AGCAGCAGCAUCUCGUUCG 3'
:::::::::::. ::::.
query 3' UCGUCGUCGUAGGUCAAGU 5'
query=miR172k, target=RO3G_09607, score=3.5, range=185-203, stran
target 5' AGCAGCAGCAUCUCGUUCG 3'
:::::::::::. ::::.
query 3' UCGUCGUCGUAGGUCAAGU 5'
query=miR172k, target=RO3G_13742, score=3.5, range=185-203, stran
target 5' AGCAGCAGCAUCUCGUUCG 3'
:::::::::::. ::::.
query 3' UCGUCGUCGUAGGUCAAGU 5'
query=miR172k, target=RO3G_17440, score=3.5, range=1027-1045, str
target 5' AAUAGCAGCAUCCAGUUUC 3'
: .:::::::::::::.
query 3' UCGUCGUCGUAGGUCAAGU 5'
query=miR172k, target=RO3G_13150, score=3.5, range=748-766, stran
target 5' AAUAGCAGCAUCCAGUUUC 3'
: .:::::::::::::.
query 3' UCGUCGUCGUAGGUCAAGU 5'
query=miR172k, target=RO3G_01737, score=3.5, range=541-559, stran
target 5' AAUAGCAGCAUCCAGUUUC 3'
```

```
: .:::::::::::::::.
query 3' UCGUCGUCGUAGGUCAAGU 5'
query=miR172k, target=RO3G_02685, score=3.5, range=748-766, stran
target 5' AAUAGCAGCAUCCAGUUUC 3'
: .:::::::::::::::.
query 3' UCGUCGUCGUAGGUCAAGU 5'
query=miR172k, target=RO3G_10520, score=3.5, range=688-706, stran
target 5' AAUAGCAGCAUCCAGUUUC 3'
: .:::::::::::::::.
query 3' UCGUCGUCGUAGGUCAAGU 5'
query=miR172k, target=RO3G_04258, score=3.5, range=1680-1698,
strand=-1
target 5' AGCAGCAGCAGCCAGUUUG 3'
:::::::::: ::::::..
query 3' UCGUCGUCGUAGGUCAAGU 5'
query=miR172k, target=RO3G_05064, score=3.5, range=117-135, stran
target 5' GUCAGCAGCGUCCGGUUCA 3'
. :::::::.:::.:::::
query 3' UCGUCGUCGUAGGUCAAGU 5'
query=miR172k, target=RO3G_12749, score=3.5, range=2151-2169,
strand=-1
target 5' UGUAUCAGCAUCCAGUUCC 3'
:.: :::::::::::::
query 3' UCGUCGUCGUAGGUCAAGU 5'
query=miR172k, target=RO3G_02393, score=3.5, range=3345-3362,
strand=-1
target 5' AGU-GCAGCAUCCACUUCA 3'
::. :::::::::: ::::
query 3' UCGUCGUCGUAGGUCAAGU 5'
query=miR172k, target=RO3G_01369, score=4, range=326-344, strand=
target 5' AGCAGCAGCAUUCAGUCCU 3'
:::::::::::.:::: :
query 3' UCGUCGUCGUAGGUCAAGU 5'
query=miR172k, target=RO3G_05635, score=4, range=185-203, strand=
target 5' AGCAGCAGCAUCUCGUUCC 3'
::::::::::::. ::::
query 3' UCGUCGUCGUAGGUCAAGU 5'
query=miR172k, target=RO3G_04559, score=4, range=369-387, strand=
target 5' UAAAGCAGCAUCUAGUUCA 3'
:::::::::.::::::
query 3' UCGUCGUCGUAGGUCAAGU 5'
query=miR172k, target=RO3G_03439, score=4, range=519-537, strand=
target 5' AGAAGCAGUACCCAGUUCA 3'
:: :::::.: ::::::::
query 3' UCGUCGUCGUAGGUCAAGU 5'
```

```
query=miR172k, target=RO3G_01016, score=4, range=15-33, strand=1
target 5' AACAGGAGCAUCCGGUUUA 3'
: ::: :::::::.:::.:
query 3' UCGUCGUCGUAGGUCAAGU 5'
query=miR172k, target=RO3G_06536, score=4, range=431-448, strand=
target 5' AGCAGCAGCAGCCA-UUCA 3'
:::::::::: ::: ::::
query 3' UCGUCGUCGUAGGUCAAGU 5'
query=miR172k, target=RO3G_03482, score=4, range=101-118, strand=
target 5' AGCAGCAGCAACCA-UUCA 3'
:::::::::: ::: ::::
query 3' UCGUCGUCGUAGGUCAAGU 5'
query=miR172k, target=RO3G_05735, score=4, range=1565-1582, stran
target 5' AGGAG-AGCAUUCAGUUUA 3'
:: :: :::::.:::::.:
query 3' UCGUCGUCGUAGGUCAAGU 5'
query=miR172k, target=RO3G_09470, score=4, range=1278-1296, stran
target 5' AGCAGCAGCAUCCGCUUCU 3'
:::::::::::::. :::
query 3' UCGUCGUCGUAGGUCAAGU 5'
query=miR172k, target=RO3G_01272, score=4, range=4977-4995, stran
target 5' AGCAGCAGCAUCCUUUUCA 3'
::::::::::::: ::::
query 3' UCGUCGUCGUAGGUCAAGU 5'
query=miR172k, target=RO3G_11148, score=4, range=234-252, strand=
target 5' AGCAGCAGCAUCCUGUACA 3'
::::::::::::: :: ::
query 3' UCGUCGUCGUAGGUCAAGU 5'
query=miR172k, target=RO3G_16173, score=4, range=147-166, strand=
target 5' AGUCAGCAGCAUUCAGUACA 3'
:: :::::::::.:::: ::
query 3' UC-GUCGUCGUAGGUCAAGU 5'
query=miR172k, target=RO3G_06092, score=4, range=3708-3726, stran
target 5' GGCAACAGCAUCCAUUUCG 3'
.::: ::::::::: :::.
query 3' UCGUCGUCGUAGGUCAAGU 5'
query=miR172k, target=RO3G_12555, score=4, range=1934-1953, stran
target 5' AGUACGUUGCAUCCAGUUCA 3'
::.: :. :::::::::::
query 3' UCGU-CGUCGUAGGUCAAGU 5'
query=miR172k, target=RO3G_09685, score=4, range=630-648, strand=
target 5' ACCGAUAGCAUUCAGUUCA 3'
: :. .::::.:::::::
query 3' UCGUCGUCGUAGGUCAAGU 5'
query=miR172k, target=RO3G_06770, score=4, range=102-119, strand=
```

```
target 5' UGCAG-AGCAUCUGGUUCA 3'
:::: ::::::..:::::
query 3' UCGUCGUCGUAGGUCAAGU 5'
query=miR172k, target=RO3G_03308, score=4, range=9-27, strand=-1
target 5' AUCAGAAGCAUUCAGUUCC 3'
: ::: :::::.::::::
query 3' UCGUCGUCGUAGGUCAAGU 5'
query=miR172k, target=RO3G_06887, score=4, range=768-785, strand=
target 5' AG-AGCAGUAUCCAAUUCA 3'
:: :::::::::: ::::
query 3' UCGUCGUCGUAGGUCAAGU 5'
query=miR172k, target=RO3G_16320, score=4, range=441-459, strand=
target 5' AUUUGUAGCAUCCAGUUCU 3'
: . :.::::::::::::
query 3' UCGUCGUCGUAGGUCAAGU 5'
query=miR7837, target=RO3G_11243, score=0, range=158-178, strand=
target 5' CAGCUACCACUCCUACACCCA 3'
:::::::::::::::::::::
query 3' GUCGAUGGUGAGGAUGUGGGU 5'
query=miR7837, target=RO3G_00539, score=3, range=299-319, strand=
target 5' CAGCUCCCACUCCUUCACCCA 3'
::::: :::::::: ::::::
query 3' GUCGAUGGUGAGGAUGUGGGU 5'
query=miR7837, target=RO3G_01660, score=4, range=834-853, strand=
target 5' UA-CUACCACUCCUGCACCUG 3'
.: ::::::::::::.::::..
query 3' GUCGAUGGUGAGGAUGUGGGU 5'
query=miR7837, target=RO3G_14336, score=4, range=1193-1213, stran
target 5' CAGUAUCUACUCCUACAUCCA 3'
:::. :.:::::::::.:::
query 3' GUCGAUGGUGAGGAUGUGGGU 5'
query=miR8016, target=RO3G_13451, score=1, range=1741-1762, stran
target 5' CCACAAGCCCUUCAUUCAAAAA 3'
::::: ::::::::::::::::
query 3' GGUGUCCGGGAAGUAAGUUUUU 5'
query=miR8016, target=RO3G_01193, score=1, range=1495-1516, stran
target 5' CCACAAGCCCUUCAUUCAAAAA 3'
::::: ::::::::::::::::
query 3' GGUGUCCGGGAAGUAAGUUUUU 5'
query=miR8016, target=RO3G_05342, score=2, range=1906-1927, stran
target 5' CCACAAGCCCUUCAUUCAGAAA 3'
::::: ::::::::::::.:::
query 3' GGUGUCCGGGAAGUAAGUUUUU 5'
query=miR8016, target=RO3G_14552, score=2, range=1906-1927, stran
target 5' CCACAAGCCCUUCAUUCAGAAA 3'
```

```
::::: ::::::::::::.:::
query 3' GGUGUCCGGGAAGUAAGUUUUU 5'
query=miR8016, target=RO3G_16368, score=2, range=1906-1927, stran
target 5' CCACAAGCCCUUCAUUCAGAAA 3'
::::: ::::::::::::.:::
query 3' GGUGUCCGGGAAGUAAGUUUUU 5'
query=miR8016, target=RO3G_11542, score=2, range=1741-1762, stran
target 5' CCACAAGCCCUUCAUUCAGAAA 3'
::::: ::::::::::::.:::
query 3' GGUGUCCGGGAAGUAAGUUUUU 5'
query=miR8016, target=RO3G_13708, score=2, range=1741-1762, stran
target 5' CCACAAGCCCUUCAUUCAGAAA 3'
::::: ::::::::::::.:::
query 3' GGUGUCCGGGAAGUAAGUUUUU 5'
query=miR8016, target=RO3G_12646, score=2, range=1741-1762, stran
target 5' CCACAAGCCCUUCAUUCAGAAA 3'
::::: ::::::::::::.:::
query 3' GGUGUCCGGGAAGUAAGUUUUU 5'
query=miR8016, target=RO3G_15886, score=2, range=1741-1762, stran
target 5' CCACAAGCCCUUCAUUCAGAAA 3'
::::: ::::::::::::.:::
query 3' GGUGUCCGGGAAGUAAGUUUUU 5'
query=miR8016, target=RO3G_05908, score=2, range=1741-1762, stran
target 5' CCACAAGCCCUUCAUUCAGAAA 3'
::::: ::::::::::::.:::
query 3' GGUGUCCGGGAAGUAAGUUUUU 5'
query=miR8016, target=RO3G_01324, score=2, range=592-613, strand=
target 5' CCACAAGCCCUUCAUUCAGAAA 3'
::::: ::::::::::::.:::
query 3' GGUGUCCGGGAAGUAAGUUUUU 5'
query=miR8016, target=RO3G_02818, score=2.5, range=241-262, stran
target 5' CCGCAAGCCCUUCAUUCAGAAA 3'
::.:: ::::::::::::.:::
query 3' GGUGUCCGGGAAGUAAGUUUUU 5'
query=miR8016, target=RO3G_12968, score=3, range=1822-1843, stran
target 5' CCACAAGCCCUUCCUUCAAAAA 3'
::::: ::::::: ::::::::
query 3' GGUGUCCGGGAAGUAAGUUUUU 5'
query=miR8016, target=RO3G_05954, score=3, range=1822-1843, stran
target 5' CCACAAGCCCUUCCUUCAAAAA 3'
::::: ::::::: ::::::::
query 3' GGUGUCCGGGAAGUAAGUUUUU 5'
query=miR8016, target=RO3G_05911, score=3, range=1675-1696, stran
target 5' CCACAAGCCCUUCCUUCAAAAA 3'
::::: ::::::: ::::::::
```

```
query 3' GGUGUCCGGGAAGUAAGUUUUU 5'
query=miR8016, target=RO3G_05427, score=3, range=1102-1123, stran
target 5' CCACAAGCCCUUCCUUCAAAAA 3'
::::: ::::::: ::::::::
query 3' GGUGUCCGGGAAGUAAGUUUUU 5'
query=miR8016, target=RO3G_15258, score=3, range=592-613, strand=
target 5' CCACAAGCCCUUCCUUCAAAAA 3'
::::: ::::::: ::::::::
query 3' GGUGUCCGGGAAGUAAGUUUUU 5'
query=miR8016, target=RO3G_05785, score=3.5, range=166-188, stran
target 5' UCACAGGCGCUUUUAUUCAAAAA 3'
.::::::: :.::.:::::::::
query 3' GGUGUCCG-GGAAGUAAGUUUUU 5'
query=miR1515, target=RO3G_17349, score=0.5, range=15-36, strand=
target 5' GGGUCAUUGUCAUGCUAAAAUA 3'
::.:::::::::::::::::::
query 3' CCUAGUAACAGUACGAUUUUAU 5'
query=miR1515, target=RO3G_16894, score=0.5, range=15-36, strand=
target 5' GGGUCAUUGUCAUGCUAAAAUA 3'
::.:::::::::::::::::::
query 3' CCUAGUAACAGUACGAUUUUAU 5'
query=miR1515, target=RO3G_13852, score=1, range=461-482, strand=
target 5' GGAUCAUUGUCAUGCUGAAAUA 3'
::::::::::::::::.:::::
query 3' CCUAGUAACAGUACGAUUUUAU 5'
query=miR1515, target=RO3G_15271, score=2, range=710-731, strand=
target 5' GGAUCAUUGUCGUGCUGAAAUA 3'
:::::::::::.::::.:::::
query 3' CCUAGUAACAGUACGAUUUUAU 5'
query=miR1515, target=RO3G_10771, score=2, range=461-482, strand=
target 5' GGAUCAUUGUCAUGUUGAAAUA 3'
::::::::::::::.:.:::::
query 3' CCUAGUAACAGUACGAUUUUAU 5'
query=miR1515, target=RO3G_16725, score=2, range=452-473, strand=
target 5' GGAUCAUUGUCGUGCUGAAAUA 3'
:::::::::::.::::.:::::
query 3' CCUAGUAACAGUACGAUUUUAU 5'
query=miR1515, target=RO3G_07739, score=2, range=461-482, strand=
target 5' GGAUCAUUCUCAUGCUGAAAUA 3'
:::::::: :::::::.:::::
query 3' CCUAGUAACAGUACGAUUUUAU 5'
query=miR1515, target=RO3G_13731, score=2.5, range=203-225, stran
target 5' GGGUCAUUGUCAUGCUAAAAGUA 3'
::.:::::::::::::::::: ::
query 3' CCUAGUAACAGUACGAUUUU-AU 5'
```

query=miR1515, target=RO3G_10527, score=2.5, range=189-210, stran
target 5' GGGUCAUUGUCAUGCUAAAACA 3'
::.:::::::::::::::::: :
query 3' CCUAGUAACAGUACGAUUUUAU 5'
query=miR1515, target=RO3G_09968, score=2.5, range=15-36, strand=
target 5' GGGUCAUUGUCAUGCUAAAACA 3'
::.:::::::::::::::::: :
query 3' CCUAGUAACAGUACGAUUUUAU 5'
query=miR1515, target=RO3G_09962, score=2.5, range=15-36, strand=
target 5' GGGUCAUUGUCAUGCUAAAACA 3'
::.:::::::::::::::::: :
query 3' CCUAGUAACAGUACGAUUUUAU 5'
query=miR1515, target=RO3G_00745, score=3, range=431-452, strand=
target 5' GGAUCAUUGUCGUGUUGAAAUA 3'
:::::::::::.::.:.:::::
query 3' CCUAGUAACAGUACGAUUUUAU 5'
query=miR1515, target=RO3G_05640, score=3, range=605-626, strand=
target 5' GGAUCAUUAUCGUGCUGAAAUA 3'
:::::::: ::.::::.:::::
query 3' CCUAGUAACAGUACGAUUUUAU 5'
query=miR1515, target=RO3G_10866, score=3, range=719-740, strand=
target 5' GGAUCAUUGUCAUGCUGAAAAA 3'
::::::::::::::::.:::: :
query 3' CCUAGUAACAGUACGAUUUUAU 5'
query=miR1515, target=RO3G_00255, score=3, range=719-740, strand=
target 5' GGAUCAUUGUCAUGCUGAAAAA 3'
::::::::::::::::.:::: :
query 3' CCUAGUAACAGUACGAUUUUAU 5'
query=miR1515, target=RO3G_09612, score=3, range=719-740, strand=
target 5' GGAUCAUUGUCAUGCUGAAAAA 3'
::::::::::::::::.:::: :
query 3' CCUAGUAACAGUACGAUUUUAU 5'
query=miR1515, target=RO3G_13271, score=3, range=719-740, strand=
target 5' GGAUCAUUGUCAUGCUGAAAAA 3'
::::::::::::::::.:::: :
query 3' CCUAGUAACAGUACGAUUUUAU 5'
query=miR1515, target=RO3G_07112, score=3, range=542-563, strand=
target 5' GGAUCAUUGUCAUGCUGAAAAA 3'
::::::::::::::::.:::: :
query 3' CCUAGUAACAGUACGAUUUUAU 5'
query=miR1515, target=RO3G_13266, score=3, range=497-518, strand=
target 5' GGAUCAUUGUCAUGCUGAAAAA 3'
::::::::::::::::.:::: :
query 3' CCUAGUAACAGUACGAUUUUAU 5'
query=miR1515, target=RO3G_15166, score=3, range=707-728, strand=

target 5' GGAUCAUUAUCAUGUUGAAAUA 3'
:::::::: :::::.:.:::::
query 3' CCUAGUAACAGUACGAUUUUAU 5'
query=miR1515, target=RO3G_13714, score=3, range=710-731, strand=
target 5' GGAUCAUUAUCGUGCUGAAAUA 3'
:::::::: ::.::::.:::::
query 3' CCUAGUAACAGUACGAUUUUAU 5'
query=miR1515, target=RO3G_16742, score=3, range=710-731, strand=
target 5' GGAUCAUUAUCGUGCUGAAAUA 3'
:::::::: ::.::::.:::::
query 3' CCUAGUAACAGUACGAUUUUAU 5'
query=miR1515, target=RO3G_17416, score=3, range=710-731, strand=
target 5' GGAUCAUUAUCGUGCUGAAAUA 3'
:::::::: ::.::::.:::::
query 3' CCUAGUAACAGUACGAUUUUAU 5'
query=miR1515, target=RO3G_17291, score=3, range=710-731, strand=
target 5' GGAUCAUUAUCGUGCUGAAAUA 3'
:::::::: ::.::::.:::::
query 3' CCUAGUAACAGUACGAUUUUAU 5'
query=miR1515, target=RO3G_02824, score=3, range=710-731, strand=
target 5' GGAUCAUUAUCGUGCUGAAAUA 3'
:::::::: ::.::::.:::::
query 3' CCUAGUAACAGUACGAUUUUAU 5'
query=miR1515, target=RO3G_16372, score=3, range=710-731, strand=
target 5' GGAUCAUUAUCGUGCUGAAAUA 3'
:::::::: ::.::::.:::::
query 3' CCUAGUAACAGUACGAUUUUAU 5'
query=miR1515, target=RO3G_07705, score=3, range=710-731, strand=
target 5' GGAUCAUUAUCGUGCUGAAAUA 3'
:::::::: ::.::::.:::::
query 3' CCUAGUAACAGUACGAUUUUAU 5'
query=miR1515, target=RO3G_16123, score=3, range=710-731, strand=
target 5' GGAUCAUUAUCGUGCUGAAAUA 3'
:::::::: ::.::::.:::::
query 3' CCUAGUAACAGUACGAUUUUAU 5'
query=miR1515, target=RO3G_17387, score=3, range=710-731, strand=
target 5' GGAUCAUUAUCGUGCUGAAAUA 3'
:::::::: ::.::::.:::::
query 3' CCUAGUAACAGUACGAUUUUAU 5'
query=miR1515, target=RO3G_06717, score=3, range=710-731, strand=
target 5' GGAUCAUUAUCGUGCUGAAAUA 3'
:::::::: ::.::::.:::::
query 3' CCUAGUAACAGUACGAUUUUAU 5'
query=miR1515, target=RO3G_17399, score=3, range=710-731, strand=
target 5' GGAUCAUUAUCGUGCUGAAAUA 3'

```
:::::::: ::.::::.::::
query 3' CCUAGUAACAGUACGAUUUUAU 5'
query=miR1515, target=RO3G_01328, score=3, range=710-731, strand=
target 5' GGAUCAUUAUCGUGCUGAAAUA 3'
:::::::: ::.::::.::::
query 3' CCUAGUAACAGUACGAUUUUAU 5'
query=miR1515, target=RO3G_07137, score=3, range=710-731, strand=
target 5' GGAUCAUUAUCGUGCUGAAAUA 3'
:::::::: ::.::::.::::
query 3' CCUAGUAACAGUACGAUUUUAU 5'
query=miR1515, target=RO3G_05268, score=3, range=710-731, strand=
target 5' GGAUCAUUAUCGUGCUGAAAUA 3'
:::::::: ::.::::.::::
query 3' CCUAGUAACAGUACGAUUUUAU 5'
query=miR1515, target=RO3G_16911, score=3, range=305-326, strand=
target 5' GGAUCAUUGUCAUGCUGAAAAA 3'
::::::::::::::::.::: :
query 3' CCUAGUAACAGUACGAUUUUAU 5'
query=miR1515, target=RO3G_12663, score=3, range=545-566, strand=
target 5' GGAUCAUUAUCGUGCUGAAAUA 3'
:::::::: ::.::::.::::
query 3' CCUAGUAACAGUACGAUUUUAU 5'
query=miR1515, target=RO3G_17355, score=3, range=545-566, strand=
target 5' GGAUCAUUAUCGUGCUGAAAUA 3'
:::::::: ::.::::.::::
query 3' CCUAGUAACAGUACGAUUUUAU 5'
query=miR1515, target=RO3G_04674, score=3, range=461-482, strand=
target 5' GGAUCAUUAUCGUGCUGAAAUA 3'
:::::::: ::.::::.::::
query 3' CCUAGUAACAGUACGAUUUUAU 5'
query=miR1515, target=RO3G_01962, score=3, range=461-482, strand=
target 5' GGAUCAUUAUCGUGCUGAAAUA 3'
:::::::: ::.::::.::::
query 3' CCUAGUAACAGUACGAUUUUAU 5'
query=miR1515, target=RO3G_16706, score=3, range=434-455, strand=
target 5' GGAUCAUUAUCGUGCUGAAAUA 3'
:::::::: ::.::::.::::
query 3' CCUAGUAACAGUACGAUUUUAU 5'
query=miR1515, target=RO3G_17223, score=3, range=434-455, strand=
target 5' GGAUCAUUAUCGUGCUGAAAUA 3'
:::::::: ::.::::.::::
query 3' CCUAGUAACAGUACGAUUUUAU 5'
query=miR1515, target=RO3G_09193, score=3, range=434-455, strand=
target 5' GGAUCAUUAUCGUGCUGAAAUA 3'
:::::::: ::.::::.::::
```

```
query 3' CCUAGUAACAGUACGAUUUUAU 5'
query=miR1515, target=RO3G_14770, score=3, range=710-731, strand=
target 5' GGAUCAUUAUCGUGCUGAAAUA 3'
:::::::: ::.::::.:::::
query 3' CCUAGUAACAGUACGAUUUUAU 5'
query=miR1515, target=RO3G_11781, score=3, range=605-626, strand=
target 5' GGAUCAUUAUCGUGCUGAAAUA 3'
:::::::: ::.::::.:::::
query 3' CCUAGUAACAGUACGAUUUUAU 5'
query=miR1515, target=RO3G_16420, score=3, range=461-482, strand=
target 5' GGAUCAUUAUCGUGCUGAAAUA 3'
:::::::: ::.::::.:::::
query 3' CCUAGUAACAGUACGAUUUUAU 5'
query=miR1515, target=RO3G_07702, score=3, range=461-482, strand=
target 5' GGAUCAUUAUCGUGCUGAAAUA 3'
:::::::: ::.::::.:::::
query 3' CCUAGUAACAGUACGAUUUUAU 5'
query=miR1515, target=RO3G_10800, score=3, range=377-398, strand=
target 5' GGAUCAUUGUCAUGCUGAAAAA 3'
:::::::::::::::::.::: :
query 3' CCUAGUAACAGUACGAUUUUAU 5'
query=miR1515, target=RO3G_14621, score=3, range=377-398, strand=
target 5' GGAUCAUUGUCAUGCUGAAAAA 3'
:::::::::::::::::.::: :
query 3' CCUAGUAACAGUACGAUUUUAU 5'
query=miR1515, target=RO3G_15282, score=3, range=434-455, strand=
target 5' GGAUCAUUAUCGUGCUGAAAUA 3'
:::::::: ::.::::.:::::
query 3' CCUAGUAACAGUACGAUUUUAU 5'
query=miR1515, target=RO3G_05634, score=3, range=188-209, strand=
target 5' GGAUCAUUGUCAUGCUGAAAAA 3'
:::::::::::::::::.::: :
query 3' CCUAGUAACAGUACGAUUUUAU 5'
query=miR1515, target=RO3G_12069, score=3, range=188-209, strand=
target 5' GGAUCAUUGUCAUGCUGAAAAA 3'
:::::::::::::::::.::: :
query 3' CCUAGUAACAGUACGAUUUUAU 5'
query=miR1515, target=RO3G_04695, score=3, range=500-521, strand=
target 5' GGAUCAUUAUCGUGCUGAAAUA 3'
:::::::: ::.::::.:::::
query 3' CCUAGUAACAGUACGAUUUUAU 5'
query=miR1515, target=RO3G_07867, score=3, range=461-482, strand=
target 5' GGAUCAUUAUCGUGCUGAAAUA 3'
:::::::: ::.::::.:::::
query 3' CCUAGUAACAGUACGAUUUUAU 5'
```

query=miR1515, target=RO3G_10793, score=3, range=188-209, strand=
target 5' GGAUCAUUGUCAUGCUGAAAAA 3'
:::::::::::::::::.::: :
query 3' CCUAGUAACAGUACGAUUUUAU 5'
query=miR1515, target=RO3G_10553, score=3.5, range=189-210, stran
target 5' GGGUCAUUGUCAUGCUAAAAAU 3'
::.:::::::::::::::::
query 3' CCUAGUAACAGUACGAUUUUAU 5'
query=miR1515, target=RO3G_10559, score=3.5, range=189-210, stran
target 5' GGGUCAUUGUCAUGCUAAAAAU 3'
::.:::::::::::::::::
query 3' CCUAGUAACAGUACGAUUUUAU 5'
query=miR1515, target=RO3G_07031, score=3.5, range=719-740, stran
target 5' GGGUCAUUGUCAUGCUGAAAAA 3'
::.::::::::::::::.::: :
query 3' CCUAGUAACAGUACGAUUUUAU 5'
query=miR1515, target=RO3G_16554, score=3.5, range=719-740, stran
target 5' GGGUCAUUGUCAUGCUGAAAAA 3'
::.::::::::::::::.::: :
query 3' CCUAGUAACAGUACGAUUUUAU 5'
query=miR1515, target=RO3G_00237, score=3.5, range=176-197, stran
target 5' GGAUCGUUAUCAUGUUGAAAUA 3'
:::::.:: :::::.:.:::::
query 3' CCUAGUAACAGUACGAUUUUAU 5'
query=miR1515, target=RO3G_04692, score=3.5, range=59-80, strand=
target 5' GGAUCGUUAUCAUGUUGAAAUA 3'
:::::.:: :::::.:.:::::
query 3' CCUAGUAACAGUACGAUUUUAU 5'
query=miR1515, target=RO3G_17207, score=3.5, range=59-80, strand=
target 5' GGAUCGUUAUCAUGUUGAAAUA 3'
:::::.:: :::::.:.:::::
query 3' CCUAGUAACAGUACGAUUUUAU 5'
query=miR1515, target=RO3G_15895, score=3.5, range=20-41, strand=
target 5' GGAUCGUUAUCAUGUUGAAAUA 3'
:::::.:: :::::.:.:::::
query 3' CCUAGUAACAGUACGAUUUUAU 5'
query=miR1515, target=RO3G_16536, score=3.5, range=20-41, strand=
target 5' GGAUCGUUAUCAUGUUGAAAUA 3'
:::::.:: :::::.:.:::::
query 3' CCUAGUAACAGUACGAUUUUAU 5'
query=miR1515, target=RO3G_05953, score=4, range=710-731, strand=
target 5' GGGUCGUUGUCAUGCUGAAAAA 3'
::.::.:::::::::.::: :
query 3' CCUAGUAACAGUACGAUUUUAU 5'
query=miR1515, target=RO3G_07688, score=4, range=719-740, strand=

target 5' GGAUCAUUGUCAUGUUGAAAAA 3'
::::::::::::::.:.::: :
query 3' CCUAGUAACAGUACGAUUUUAU 5'
query=miR1515, target=RO3G_16359, score=4, range=719-740, strand=
target 5' GGAUCAUUGUCAUGUUGAAAAA 3'
::::::::::::::.:.::: :
query 3' CCUAGUAACAGUACGAUUUUAU 5'
query=miR1515, target=RO3G_16545, score=4, range=719-740, strand=
target 5' GGAUCAUUGUCAUGUUGAAAAA 3'
::::::::::::::.:.::: :
query 3' CCUAGUAACAGUACGAUUUUAU 5'
query=miR1515, target=RO3G_03772, score=4, range=719-740, strand=
target 5' GGAUCAUUGUCAUGCUGGAAGA 3'
::::::::::::::::..:: :
query 3' CCUAGUAACAGUACGAUUUUAU 5'
query=miR1515, target=RO3G_01618, score=4, range=719-740, strand=
target 5' GGAUCAUUGUCAUGCUGGAAGA 3'
::::::::::::::::..:: :
query 3' CCUAGUAACAGUACGAUUUUAU 5'
query=miR1515, target=RO3G_08842, score=4, range=719-740, strand=
target 5' GGAUCAUUGUCAUGCUGGAAGA 3'
::::::::::::::::..:: :
query 3' CCUAGUAACAGUACGAUUUUAU 5'
query=miR1515, target=RO3G_02844, score=4, range=497-518, strand=
target 5' GGAUCAUUGUCAUGCUGGAAGA 3'
::::::::::::::::..:: :
query 3' CCUAGUAACAGUACGAUUUUAU 5'
query=miR1515, target=RO3G_03766, score=4, range=497-518, strand=
target 5' GGAUCAUUGUCAUGCUGGAAGA 3'
::::::::::::::::..:: :
query 3' CCUAGUAACAGUACGAUUUUAU 5'
query=miR1515, target=RO3G_13137, score=4, range=617-638, strand=
target 5' GGAUCAUUGUCAUGCUGGAAGA 3'
::::::::::::::::..:: :
query 3' CCUAGUAACAGUACGAUUUUAU 5'
query=miR1515, target=RO3G_03554, score=4, range=611-632, strand=
target 5' GGAUCAUUGUCAUGCUGGAAGA 3'
::::::::::::::::..:: :
query 3' CCUAGUAACAGUACGAUUUUAU 5'
query=miR1515, target=RO3G_08657, score=4, range=611-632, strand=
target 5' GGAUCAUUGUCAUGCUGGAAGA 3'
::::::::::::::::..:: :
query 3' CCUAGUAACAGUACGAUUUUAU 5'
query=miR1515, target=RO3G_10772, score=4, range=608-629, strand=
target 5' GGAUCAUUGUCAUGCUGGAAGA 3'

```
::::::::::::::::..:: :
query 3' CCUAGUAACAGUACGAUUUUAU 5'
query=miR1515, target=RO3G_10780, score=4, range=710-731, strand=
target 5' GGGUCGUUGUCAUGCUGAAAAA 3'
::.::.::::::::::.::: :
query 3' CCUAGUAACAGUACGAUUUUAU 5'
query=miR1515, target=RO3G_00256, score=4, range=710-731, strand=
target 5' GGGUCGUUGUCAUGCUGAAAAA 3'
::.::.::::::::::.::: :
query 3' CCUAGUAACAGUACGAUUUUAU 5'
query=miR1515, target=RO3G_14361, score=4, range=710-731, strand=
target 5' GGGUCGUUGUCAUGCUGAAAAA 3'
::.::.::::::::::.::: :
query 3' CCUAGUAACAGUACGAUUUUAU 5'
query=miR1515, target=RO3G_05267, score=4, range=710-731, strand=
target 5' GGGUCGUUGUCAUGCUGAAAAA 3'
::.::.::::::::::.::: :
query 3' CCUAGUAACAGUACGAUUUUAU 5'
query=miR1515, target=RO3G_09967, score=4, range=710-731, strand=
target 5' GGGUCGUUGUCAUGCUGAAAAA 3'
::.::.::::::::::.::: :
query 3' CCUAGUAACAGUACGAUUUUAU 5'
query=miR1515, target=RO3G_04207, score=4, range=710-731, strand=
target 5' GGGUCGUUGUCAUGCUGAAAAA 3'
::.::.::::::::::.::: :
query 3' CCUAGUAACAGUACGAUUUUAU 5'
query=miR1515, target=RO3G_04210, score=4, range=710-731, strand=
target 5' GGGUCGUUGUCAUGCUGAAAAA 3'
::.::.::::::::::.::: :
query 3' CCUAGUAACAGUACGAUUUUAU 5'
query=miR1515, target=RO3G_07860, score=4, range=710-731, strand=
target 5' GGGUCGUUGUCAUGCUGAAAAA 3'
::.::.::::::::::.::: :
query 3' CCUAGUAACAGUACGAUUUUAU 5'
query=miR1515, target=RO3G_09542, score=4, range=710-731, strand=
target 5' GGGUCGUUGUCAUGCUGAAAAA 3'
::.::.::::::::::.::: :
query 3' CCUAGUAACAGUACGAUUUUAU 5'
query=miR1515, target=RO3G_10773, score=4, range=710-731, strand=
target 5' GGGUCGUUGUCAUGCUGAAAAA 3'
::.::.::::::::::.::: :
query 3' CCUAGUAACAGUACGAUUUUAU 5'
query=miR1515, target=RO3G_04127, score=4, range=671-692, strand=
target 5' GGAUCAUUGUCAUGCUGGAAGA 3'
::::::::::::::::..:: :
```

```
query 3' CCUAGUAACAGUACGAUUUUAU 5'
query=miR1515, target=RO3G_00244, score=4, range=671-692, strand=
target 5' GGAUCAUUGUCAUGCUGGAAGA 3'
::::::::::::::::..:: :
query 3' CCUAGUAACAGUACGAUUUUAU 5'
query=miR1515, target=RO3G_05959, score=4, range=710-731, strand=
target 5' GGGUCGUUGUCAUGCUGAAAAA 3'
::.::.::::::::::.::: :
query 3' CCUAGUAACAGUACGAUUUUAU 5'
query=miR1515, target=RO3G_13147, score=4, range=497-518, strand=
target 5' GGAUCAUUGUCAUGCUGGAAGA 3'
::::::::::::::::..:: :
query 3' CCUAGUAACAGUACGAUUUUAU 5'
query=miR1515, target=RO3G_09194, score=4, range=710-731, strand=
target 5' GGGUCGUUGUCAUGCUGAAAAA 3'
::.::.::::::::::.::: :
query 3' CCUAGUAACAGUACGAUUUUAU 5'
query=miR1515, target=RO3G_11966, score=4, range=710-731, strand=
target 5' GGGUCGUUGUCAUGCUGAAAAA 3'
::.::.::::::::::.::: :
query 3' CCUAGUAACAGUACGAUUUUAU 5'
query=miR1515, target=RO3G_07124, score=4, range=710-731, strand=
target 5' GGGUCGUUGUCAUGCUGAAAAA 3'
::.::.::::::::::.::: :
query 3' CCUAGUAACAGUACGAUUUUAU 5'
query=miR1515, target=RO3G_04205, score=4, range=419-440, strand=
target 5' GGAUCAUUGUCAUGCUGGAAGA 3'
::::::::::::::::..:: :
query 3' CCUAGUAACAGUACGAUUUUAU 5'
query=miR1515, target=RO3G_09945, score=4, range=434-455, strand=
target 5' GGGUCGUUGUCAUGCUGAAAAA 3'
::.::.::::::::::.::: :
query 3' CCUAGUAACAGUACGAUUUUAU 5'
query=miR1515, target=RO3G_00287, score=4, range=599-620, strand=
target 5' GGAUUGUUAUCAUGUUGAAAUA 3'
::::..:: :::::.:.:::::
query 3' CCUAGUAACAGUACGAUUUUAU 5'
query=miR1515, target=RO3G_16378, score=4, range=188-209, strand=
target 5' GGAUCAUUGUCAUGUUGAAAAA 3'
::::::::::::::.:.::: :
query 3' CCUAGUAACAGUACGAUUUUAU 5'
query=miR1515, target=RO3G_12649, score=4, range=98-119, strand=-
target 5' GGAUCAUUGUCAUGCUGGAAGA 3'
::::::::::::::::..:: :
query 3' CCUAGUAACAGUACGAUUUUAU 5'
```

```
query=miR1515, target=RO3G_16704, score=4, range=128-149, strand=
target 5' GGAUCAUUGUCAUGCUGGAAGA 3'
::::::::::::::::..:: :
query 3' CCUAGUAACAGUACGAUUUUAU 5'
query=miR1515, target=RO3G_12198, score=4, range=107-128, strand=
target 5' GGAUUGUUAUCAUGUUGAAAUA 3'
::::..:: :::::.:.:::::
query 3' CCUAGUAACAGUACGAUUUUAU 5'
query=miR1515, target=RO3G_07725, score=4, range=98-119, strand=-
target 5' GGAUUGUUAUCAUGUUGAAAUA 3'
::::..:: :::::.:.:::::
query 3' CCUAGUAACAGUACGAUUUUAU 5'
query=miR1515, target=RO3G_16747, score=4, range=98-119, strand=-
target 5' GGAUUGUUAUCAUGUUGAAAUA 3'
::::..:: :::::.:.:::::
query 3' CCUAGUAACAGUACGAUUUUAU 5'
query=miR1515, target=RO3G_07577, score=4, range=59-80, strand=-1
target 5' GGAUUGUUAUCAUGUUGAAAUA 3'
::::..:: :::::.:.:::::
query 3' CCUAGUAACAGUACGAUUUUAU 5'
query=miR1515, target=RO3G_03553, score=4, range=59-80, strand=-1
target 5' GGAUUGUUAUCAUGUUGAAAUA 3'
::::..:: :::::.:.:::::
query 3' CCUAGUAACAGUACGAUUUUAU 5'
query=miR1515, target=RO3G_10562, score=4, range=59-80, strand=-1
target 5' GGAUUGUUAUCAUGUUGAAAUA 3'
::::..:: :::::.:.:::::
query 3' CCUAGUAACAGUACGAUUUUAU 5'
query=miR1515, target=RO3G_16809, score=4, range=59-80, strand=-1
target 5' GGAUUGUUAUCAUGUUGAAAUA 3'
::::..:: :::::.:.:::::
query 3' CCUAGUAACAGUACGAUUUUAU 5'
query=miR1515, target=RO3G_17167, score=4, range=59-80, strand=-1
target 5' GGAUUGUUAUCAUGUUGAAAUA 3'
::::..:: :::::.:.:::::
query 3' CCUAGUAACAGUACGAUUUUAU 5'
query=miR1515, target=RO3G_01952, score=4, range=59-80, strand=-1
target 5' GGAUUGUUAUCAUGUUGAAAUA 3'
::::..:: :::::.:.:::::
query 3' CCUAGUAACAGUACGAUUUUAU 5'
query=miR1515, target=RO3G_07301, score=4, range=59-80, strand=-1
target 5' GGAUUGUUAUCAUGUUGAAAUA 3'
::::..:: :::::.:.:::::
query 3' CCUAGUAACAGUACGAUUUUAU 5'
query=miR1515, target=RO3G_04678, score=4, range=59-80, strand=-1
```

```
target 5' GGAUUGUUAUCAUGUUGAAAUA 3'
::::..:: ::::.:.:::::
query 3' CCUAGUAACAGUACGAUUUUAU 5'
query=miR1515, target=RO3G_07681, score=4, range=59-80, strand=-1
target 5' GGAUUGUUAUCAUGUUGAAAUA 3'
::::..:: ::::.:.:::::
query 3' CCUAGUAACAGUACGAUUUUAU 5'
query=miR1515, target=RO3G_06723, score=4, range=59-80, strand=-1
target 5' GGAUUGUUAUCAUGUUGAAAUA 3'
::::..:: ::::.:.:::::
query 3' CCUAGUAACAGUACGAUUUUAU 5'
query=miR1515, target=RO3G_02720, score=4, range=59-80, strand=-1
target 5' GGAUUGUUAUCAUGUUGAAAUA 3'
::::..:: ::::.:.:::::
query 3' CCUAGUAACAGUACGAUUUUAU 5'
query=miR1515, target=RO3G_12676, score=4, range=20-41, strand=-1
target 5' GGAUUGUUAUCAUGUUGAAAUA 3'
::::..:: ::::.:.:::::
query 3' CCUAGUAACAGUACGAUUUUAU 5'
query=miR1515, target=RO3G_03112, score=4, range=709-731, strand=
target 5' GGAUCAUUAUCAUGCUGAAAGUA 3'
:::::::: ::::::: :::.::
query 3' CCUAGUAACAGUACGA-UUUUAU 5'
query=miR1515, target=RO3G_05522, score=4, range=709-731, strand=
target 5' GGAUCAUUAUCAUGCUGAAAGUA 3'
:::::::: ::::::: :::.::
query 3' CCUAGUAACAGUACGA-UUUUAU 5'
query=miR1515, target=RO3G_00240, score=4, range=652-674, strand=
target 5' GGAUCAUUAUCAUGCUGAAAGUA 3'
:::::::: ::::::: :::.::
query 3' CCUAGUAACAGUACGA-UUUUAU 5'
query=miR1515, target=RO3G_02227, score=4, range=709-731, strand=
target 5' GGAUCAUUAUCAUGCUGAAAGUA 3'
:::::::: ::::::: :::.::
query 3' CCUAGUAACAGUACGA-UUUUAU 5'
query=miR1515, target=RO3G_10797, score=4, range=343-365, strand=
target 5' GGAUCAUUAUCAUGCUGAAAGUA 3'
:::::::: ::::::: :::.::
query 3' CCUAGUAACAGUACGA-UUUUAU 5'
query=miR1515, target=RO3G_17423, score=4, range=343-365, strand=
target 5' GGAUCAUUAUCAUGCUGAAAGUA 3'
:::::::: ::::::: :::.::
query 3' CCUAGUAACAGUACGA-UUUUAU 5'
query=miR1515, target=RO3G_14784, score=4, range=343-365, strand=
target 5' GGAUCAUUAUCAUGCUGAAAGUA 3'
```

```
:::::::: ::::::: :::.::
query 3' CCUAGUAACAGUACGA-UUUUAU 5'
query=miR1515, target=RO3G_16737, score=4, range=343-365, strand=
target 5' GGAUCAUUAUCAUGCUGAAAGUA 3'
:::::::: ::::::: :::.::
query 3' CCUAGUAACAGUACGA-UUUUAU 5'
query=miR1515, target=RO3G_01725, score=4, range=343-365, strand=
target 5' GGAUCAUUAUCAUGCUGAAAGUA 3'
:::::::: ::::::: :::.::
query 3' CCUAGUAACAGUACGA-UUUUAU 5'
query=miR1515, target=RO3G_07723, score=4, range=343-365, strand=
target 5' GGAUCAUUAUCAUGCUGAAAGUA 3'
:::::::: ::::::: :::.::
query 3' CCUAGUAACAGUACGA-UUUUAU 5'
query=miR1515, target=RO3G_17092, score=4, range=343-365, strand=
target 5' GGAUCAUUAUCAUGCUGAAAGUA 3'
:::::::: ::::::: :::.::
query 3' CCUAGUAACAGUACGA-UUUUAU 5'
query=miR1515, target=RO3G_10050, score=4, range=343-365, strand=
target 5' GGAUCAUUAUCAUGCUGAAAGUA 3'
:::::::: ::::::: :::.::
query 3' CCUAGUAACAGUACGA-UUUUAU 5'
query=miR1515, target=RO3G_14981, score=4, range=343-365, strand=
target 5' GGAUCAUUAUCAUGCUGAAAGUA 3'
:::::::: ::::::: :::.::
query 3' CCUAGUAACAGUACGA-UUUUAU 5'
query=miR1515, target=RO3G_12950, score=4, range=343-365, strand=
target 5' GGAUCAUUAUCAUGCUGAAAGUA 3'
:::::::: ::::::: :::.::
query 3' CCUAGUAACAGUACGA-UUUUAU 5'
query=miR1515, target=RO3G_15145, score=4, range=343-365, strand=
target 5' GGAUCAUUAUCAUGCUGAAAGUA 3'
:::::::: ::::::: :::.::
query 3' CCUAGUAACAGUACGA-UUUUAU 5'
query=miR1515, target=RO3G_03791, score=4, range=343-365, strand=
target 5' GGAUCAUUAUCAUGCUGAAAGUA 3'
:::::::: ::::::: :::.::
query 3' CCUAGUAACAGUACGA-UUUUAU 5'
query=miR1515, target=RO3G_09926, score=4, range=343-365, strand=
target 5' GGAUCAUUAUCAUGCUGAAAGUA 3'
:::::::: ::::::: :::.::
query 3' CCUAGUAACAGUACGA-UUUUAU 5'
query=miR1515, target=RO3G_11536, score=4, range=343-365, strand=
target 5' GGAUCAUUAUCAUGCUGAAAGUA 3'
:::::::: ::::::: :::.::
```

```
query 3' CCUAGUAACAGUACGA-UUUUAU 5'
query=miR1515, target=RO3G_05516, score=4, range=343-365, strand=
target 5' GGAUCAUUAUCAUGCUGAAAGUA 3'
:::::::: ::::::: :::.::
query 3' CCUAGUAACAGUACGA-UUUUAU 5'
query=miR1515, target=RO3G_17328, score=4, range=343-365, strand=
target 5' GGAUCAUUAUCAUGCUGAAAGUA 3'
:::::::: ::::::: :::.::
query 3' CCUAGUAACAGUACGA-UUUUAU 5'
query=miR1515, target=RO3G_15170, score=4, range=343-365, strand=
target 5' GGAUCAUUAUCAUGCUGAAAGUA 3'
:::::::: ::::::: :::.::
query 3' CCUAGUAACAGUACGA-UUUUAU 5'
query=miR1515, target=RO3G_16541, score=4, range=343-365, strand=
target 5' GGAUCAUUAUCAUGCUGAAAGUA 3'
:::::::: ::::::: :::.::
query 3' CCUAGUAACAGUACGA-UUUUAU 5'
query=miR1515, target=RO3G_16124, score=4, range=343-365, strand=
target 5' GGAUCAUUAUCAUGCUGAAAGUA 3'
:::::::: ::::::: :::.::
query 3' CCUAGUAACAGUACGA-UUUUAU 5'
query=miR1515, target=RO3G_15286, score=4, range=343-365, strand=
target 5' GGAUCAUUAUCAUGCUGAAAGUA 3'
:::::::: ::::::: :::.::
query 3' CCUAGUAACAGUACGA-UUUUAU 5'
query=miR1515, target=RO3G_13752, score=4, range=343-365, strand=
target 5' GGAUCAUUAUCAUGCUGAAAGUA 3'
:::::::: ::::::: :::.::
query 3' CCUAGUAACAGUACGA-UUUUAU 5'
query=miR1515, target=RO3G_03560, score=4, range=343-365, strand=
target 5' GGAUCAUUAUCAUGCUGAAAGUA 3'
:::::::: ::::::: :::.::
query 3' CCUAGUAACAGUACGA-UUUUAU 5'
query=miR1515, target=RO3G_16141, score=4, range=343-365, strand=
target 5' GGAUCAUUAUCAUGCUGAAAGUA 3'
:::::::: ::::::: :::.::
query 3' CCUAGUAACAGUACGA-UUUUAU 5'
query=miR1515, target=RO3G_07136, score=4, range=34-56, strand=-1
target 5' GGAUCAUUAUCAUGCUGAAAGUA 3'
:::::::: ::::::: :::.::
query 3' CCUAGUAACAGUACGA-UUUUAU 5'
query=miR1515, target=RO3G_11533, score=4, range=343-365, strand=
target 5' GGAUCAUUAUCAUGCUGAAAGUA 3'
:::::::: ::::::: :::.::
query 3' CCUAGUAACAGUACGA-UUUUAU 5'
```

query=miR1515, target=RO3G_10785, score=4, range=343-365, strand=
target 5' GGAUCAUUAUCAUGCUGAAAGUA 3'
:::::::: ::::::: :::.::
query 3' CCUAGUAACAGUACGA-UUUUAU 5'
query=miR1515, target=RO3G_00250, score=4, range=343-365, strand=
target 5' GGAUCAUUAUCAUGCUGAAAGUA 3'
:::::::: ::::::: :::.::
query 3' CCUAGUAACAGUACGA-UUUUAU 5'
query=miR1515, target=RO3G_13267, score=4, range=343-365, strand=
target 5' GGAUCAUUAUCAUGCUGAAAGUA 3'
:::::::: ::::::: :::.::
query 3' CCUAGUAACAGUACGA-UUUUAU 5'
query=miR1515, target=RO3G_12084, score=4, range=343-365, strand=
target 5' GGAUCAUUAUCAUGCUGAAAGUA 3'
:::::::: ::::::: :::.::
query 3' CCUAGUAACAGUACGA-UUUUAU 5'
query=miR1515, target=RO3G_03797, score=4, range=208-230, strand=
target 5' GGAUCACUGUCGUGCUGAAAAUA 3'
:::::: ::::.:::: ::::::
query 3' CCUAGUAACAGUACGA-UUUUAU 5'
query=miR5561-5p, target=RO3G_13288, score=0, range=1229-1251,
strand=-1
target 5' UUGUCCAUGUCUCUCACCAAAUG 3'
:::::::::::::::::::::::
query 3' AACAGGUACAGAGAGUGGUUUAC 5'
query=miR5561-5p, target=RO3G_14787, score=0, range=1229-1251,
strand=-1
target 5' UUGUCCAUGUCUCUCACCAAAUG 3'
:::::::::::::::::::::::
query 3' AACAGGUACAGAGAGUGGUUUAC 5'
query=miR5561-5p, target=RO3G_14800, score=0, range=1229-1251,
strand=-1
target 5' UUGUCCAUGUCUCUCACCAAAUG 3'
:::::::::::::::::::::::
query 3' AACAGGUACAGAGAGUGGUUUAC 5'
query=miR5561-5p, target=RO3G_15875, score=0, range=1229-1251,
strand=-1
target 5' UUGUCCAUGUCUCUCACCAAAUG 3'
:::::::::::::::::::::::
query 3' AACAGGUACAGAGAGUGGUUUAC 5'
query=miR5561-5p, target=RO3G_14793, score=0, range=1229-1251,
strand=-1
target 5' UUGUCCAUGUCUCUCACCAAAUG 3'
:::::::::::::::::::::::
query 3' AACAGGUACAGAGAGUGGUUUAC 5'

```
query=miR5561-5p, target=RO3G_17411, score=0, range=671-693, stra
target 5' UUGUCCAUGUCUCUCACCAAAUG 3'
:::::::::::::::::::::::
query 3' AACAGGUACAGAGAGUGGUUUAC 5'
query=miR5561-5p, target=RO3G_02440, score=4, range=449-471, stra
target 5' UUGAUUAUGGCUCUCACCAGAUG 3'
::: ..:::: :::::::::.:::
query 3' AACAGGUACAGAGAGUGGUUUAC 5'
query=miR7502b, target=RO3G_05880, score=3, range=272-290, strand
target 5' AUG-AUAAACUUAUUGACAA 3'
::: :: ::::::::.::::
query 3' UACGUACUUGAAUAAUUGUU 5'
query=miR7502b, target=RO3G_10183, score=3.5, range=260-278, stra
target 5' UUGC-UGAACUUAUUGACAG 3'
::: :::::::::::.:::.
query 3' UACGUACUUGAAUAAUUGUU 5'
query=miR7502b, target=RO3G_16944, score=3.5, range=191-210, stra
target 5' AUCUGUCAACUUAUUAACAG 3'
:: ..: ::::::::::::.
query 3' UACGUACUUGAAUAAUUGUU 5'
query=miR7502b, target=RO3G_12923, score=4, range=583-602, strand
target 5' UUGCAUGAAUUUAUUAAUGA 3'
::::::::.:::::::..:
query 3' UACGUACUUGAAUAAUUGUU 5'
query=miR7502b, target=RO3G_16530, score=4, range=1012-1031, stra
target 5' UUGAAUGAAUUUAUUAAUAA 3'
:: :::::.:::::::.::
query 3' UACGUACUUGAAUAAUUGUU 5'
query=miR7502b, target=RO3G_02695, score=4, range=3805-3825, stra
target 5' AUGAAUGAAUUUAUUGAACAA 3'
::: :::::.::::: :::::
query 3' UACGUACUUGAAUAA-UUGUU 5'
query=miR7502b, target=RO3G_03550, score=4, range=3805-3825, stra
target 5' AUGAAUGAAUUUAUUGAACAA 3'
::: :::::.::::: :::::
query 3' UACGUACUUGAAUAA-UUGUU 5'
query=miR7502b, target=RO3G_09175, score=4, range=3805-3825, stra
target 5' AUGAAUGAAUUUAUUGAACAA 3'
::: :::::.::::: :::::
query 3' UACGUACUUGAAUAA-UUGUU 5'
query=miR7502b, target=RO3G_11541, score=4, range=1195-1215, stra
target 5' AUGAAUGAAUUUAUUGAACAA 3'
::: :::::.::::: :::::
query 3' UACGUACUUGAAUAA-UUGUU 5'
query=miR7502b, target=RO3G_08532, score=4, range=1304-1322, stra
```

```
target 5' AUGCA-GAACUUAUUAAAGA 3'
::::: ::::::::::: .:
query 3' UACGUACUUGAAUAAUUGUU 5'
query=miR7502b, target=RO3G_11910, score=4, range=24-43, strand=1
target 5' AUCCUUAAAUUUAUUAACAA 3'
:: : : ::.::::::::::
query 3' UACGUACUUGAAUAAUUGUU 5'
query=miR7502b, target=RO3G_08587, score=4, range=2021-2039, stra
target 5' AU-CAAGAACUUAUUGACAU 3'
:: :: :::::::::.:::
query 3' UACGUACUUGAAUAAUUGUU 5'
query=miR7502b, target=RO3G_02564, score=4, range=443-461, strand
target 5' UUACA-GAAUUUAUUAACAA 3'
: :: :::.::::::::::
query 3' UACGUACUUGAAUAAUUGUU 5'
query=miR7502b, target=RO3G_09426, score=4, range=1132-1151, stra
target 5' AAGCACAAACUUAUUAACAU 3'
: ::: ::::::::::::
query 3' UACGUACUUGAAUAAUUGUU 5'
query=miR7502b, target=RO3G_08891, score=4, range=109-127, strand
target 5' AUGCUCGAA-UUAUUAACAA 3'
:::: ::: ::::::::::
query 3' UACGUACUUGAAUAAUUGUU 5'
query=miR7502b, target=RO3G_06589, score=4, range=316-335, strand
target 5' GUGCUUGAGCUUAUUAAUAG 3'
.::: :::.::::::::.:.
query 3' UACGUACUUGAAUAAUUGUU 5'
query=miR7502b, target=RO3G_09234, score=4, range=83-101, strand=
target 5' AU-CAUGGGCUUAUUAGCAA 3'
:: ::::..:::::::.:::
query 3' UACGUACUUGAAUAAUUGUU 5'
query=miR7502b, target=RO3G_02236, score=4, range=308-327, strand
target 5' AUUCUUGAACUUAAUAACAA 3'
:: : :::::::: ::::::
query 3' UACGUACUUGAAUAAUUGUU 5'
query=miR7502b, target=RO3G_08139, score=4, range=407-427, strand
target 5' AUGACAUUAACUUAUUAUCAA 3'
::: ::: ::::::::: :::
query 3' UAC-GUACUUGAAUAAUUGUU 5'
query=miR7502b, target=RO3G_08587, score=4, range=620-639, strand
target 5' UUGCUUUAACUUAUUAACAU 3'
::: : ::::::::::::
query 3' UACGUACUUGAAUAAUUGUU 5'
query=miR6451, target=RO3G_15326, score=0, range=476-493, strand=
target 5' AAUCAACAUGAUCUACAU 3'
```

```
:::::::::::::::::
query 3' UUAGUUGUACUAGAUGUA 5'
query=miR6451, target=RO3G_15197, score=3, range=118-134, strand=
target 5' GAUU-ACAUGGUCUACAU 3'
.::. ::::.:::::::
query 3' UUAGUUGUACUAGAUGUA 5'
query=miR6451, target=RO3G_10684, score=3, range=987-1004, strand
target 5' UAACAACAUGAUCUACAA 3'
: :::::::::::::
query 3' UUAGUUGUACUAGAUGUA 5'
query=miR6451, target=RO3G_10673, score=3, range=987-1004, strand
target 5' UAACAACAUGAUCUACAA 3'
: :::::::::::::
query 3' UUAGUUGUACUAGAUGUA 5'
query=miR6451, target=RO3G_10669, score=3, range=987-1004, strand
target 5' UAACAACAUGAUCUACAA 3'
: :::::::::::::
query 3' UUAGUUGUACUAGAUGUA 5'
query=miR6451, target=RO3G_10667, score=3, range=114-131, strand=
target 5' UAACAACAUGAUCUACAA 3'
: :::::::::::::
query 3' UUAGUUGUACUAGAUGUA 5'
query=miR6451, target=RO3G_10128, score=3, range=524-541, strand=
target 5' AAUCAACAUGGUCUAUGU 3'
:::::::::.::::..:
query 3' UUAGUUGUACUAGAUGUA 5'
query=miR6451, target=RO3G_05236, score=3, range=734-751, strand=
target 5' UAUCAAUAUGAUCUACAA 3'
:::::.:::::::::
query 3' UUAGUUGUACUAGAUGUA 5'
query=miR6451, target=RO3G_10142, score=3, range=806-823, strand=
target 5' CAUCAACAUGAUCAACAU 3'
:::::::::::: ::::
query 3' UUAGUUGUACUAGAUGUA 5'
query=miR6451, target=RO3G_03107, score=3, range=806-823, strand=
target 5' CAUCAACAUGAUCAACAU 3'
:::::::::::: ::::
query 3' UUAGUUGUACUAGAUGUA 5'
query=miR6451, target=RO3G_14682, score=3, range=527-544, strand=
target 5' AAUCAACAUGAUCUUCAG 3'
::::::::::::: ::
query 3' UUAGUUGUACUAGAUGUA 5'
query=miR6451, target=RO3G_14381, score=3, range=278-296, strand=
target 5' AAUCCAAUAUGAUCUACAA 3'
::: :::.::::::::::
```

```
query 3' UUA-GUUGUACUAGAUGUA 5'
query=miR6451, target=RO3G_06628, score=3, range=562-580, strand=
target 5' ACUCAACAUGAUCUACCAU 3'
: ::::::::::::: :::
query 3' UUAGUUGUACUAGAU-GUA 5'
query=miR6451, target=RO3G_00012, score=3, range=485-502, strand=
target 5' AAGGAACAUGAUCUACAA 3'
:: ::::::::::::
query 3' UUAGUUGUACUAGAUGUA 5'
query=miR6451, target=RO3G_15271, score=3.5, range=771-788, stran
target 5' UAUUAGCAUGAUUUACAU 3'
::.:.:::::.:::::
query 3' UUAGUUGUACUAGAUGUA 5'
query=miR6451, target=RO3G_16725, score=3.5, range=513-530, stran
target 5' UAUUAGCAUGAUUUACAU 3'
::.:.:::::.:::::
query 3' UUAGUUGUACUAGAUGUA 5'
query=miR6451, target=RO3G_04929, score=3.5, range=262-279, stran
target 5' AAUUUAUAUGAUUUACAU 3'
:::. :.:::::.:::::
query 3' UUAGUUGUACUAGAUGUA 5'
query=miR6451, target=RO3G_06872, score=3.5, range=901-918, stran
target 5' AAUCGACCUGAUCUACAA 3'
::::.:: :::::::::
query 3' UUAGUUGUACUAGAUGUA 5'
query=miR6451, target=RO3G_02555, score=3.5, range=80-97, strand=
target 5' AAUCGACAUGCUCUACAC 3'
::::.::::: ::::::
query 3' UUAGUUGUACUAGAUGUA 5'
query=miR6451, target=RO3G_13970, score=3.5, range=345-362, stran
target 5' AAUGGGCAUGAUCUACAG 3'
::: ..:::::::::::
query 3' UUAGUUGUACUAGAUGUA 5'
query=miR6451, target=RO3G_08163, score=3.5, range=109-126, stran
target 5' GAACAACAUUAUCUACAU 3'
.: :::::: ::::::::
query 3' UUAGUUGUACUAGAUGUA 5'
query=miR6451, target=RO3G_05137, score=3.5, range=781-799, stran
target 5' GAUCAACAUGAUCAUACAA 3'
.:::::::::::: ::::
query 3' UUAGUUGUACUAG-AUGUA 5'
query=miR6451, target=RO3G_01516, score=3.5, range=2393-2410,
strand=-1
target 5' AAGUAACUUGAUCUACAU 3'
:: .::: :::::::::
```

```
query 3' UUAGUUGUACUAGAUGUA 5'
query=miR6451, target=RO3G_11852, score=3.5, range=542-559, stran
target 5' AGUCCACAUGAUCAACAU 3'
:.:: :::::::: ::::
query 3' UUAGUUGUACUAGAUGUA 5'
query=miR6451, target=RO3G_07782, score=3.5, range=362-379, stran
target 5' AGACAACAUGAUCAACAU 3'
:. ::::::::: ::::
query 3' UUAGUUGUACUAGAUGUA 5'
query=miR6451, target=RO3G_10993, score=3.5, range=1-19, strand=-
target 5' ACUCAGCAUGAUCUAUCAU 3'
: :::.::::::::: :::
query 3' UUAGUUGUACUAGAU-GUA 5'
query=miR6451, target=RO3G_00841, score=3.5, range=1016-1032,
strand=-1
target 5' GAU-AACAUGAUCUAAAU 3'
.:: :::::::::: ::
query 3' UUAGUUGUACUAGAUGUA 5'
query=miR6451, target=RO3G_14199, score=4, range=3412-3429, stran
target 5' AAUCAACAUGGUUUGCAG 3'
:::::::::.:.:.::
query 3' UUAGUUGUACUAGAUGUA 5'
query=miR6451, target=RO3G_02771, score=4, range=1093-1110, stran
target 5' GAUCGGCAUGAUUUACAA 3'
.:::..::::::.::::
query 3' UUAGUUGUACUAGAUGUA 5'
query=miR6451, target=RO3G_08859, score=4, range=100-118, strand=
target 5' AAUCAACAUGAUCUCGUAU 3'
::::::::::::: ..::
query 3' UUAGUUGUACUAGA-UGUA 5'
query=miR6451, target=RO3G_01352, score=4, range=1393-1410, stran
target 5' GAUUUACAUGGUUUACAU 3'
.::. :::::.:.:::::
query 3' UUAGUUGUACUAGAUGUA 5'
query=miR6451, target=RO3G_15840, score=4, range=7813-7830, stran
target 5' AAUCAACAUGGUGUACAA 3'
::::::::::.: ::::
query 3' UUAGUUGUACUAGAUGUA 5'
query=miR6451, target=RO3G_00403, score=4, range=7825-7842, stran
target 5' AAUCAACCUGAUUUACAC 3'
::::::: ::::.::::
query 3' UUAGUUGUACUAGAUGUA 5'
query=miR6451, target=RO3G_05460, score=4, range=273-290, strand=
target 5' CAUCAACGUGAUCUUCAU 3'
::::::.:::::: :::
```

```
query 3' UUAGUUGUACUAGAUGUA 5'
query=miR6451, target=RO3G_07629, score=4, range=252-270, strand=
target 5' CAUCAACAUGAUUUAUCAU 3'
:::::::::::.:: :::
query 3' UUAGUUGUACUAGAU-GUA 5'
query=miR6451, target=RO3G_00880, score=4, range=1080-1097, stran
target 5' UAUUGUCAUGAUCUACAU 3'
::.. :::::::::::
query 3' UUAGUUGUACUAGAUGUA 5'
query=miR6451, target=RO3G_00324, score=4, range=817-834, strand=
target 5' AAUCAACAUCAUCAACAU 3'
::::::::: ::: ::::
query 3' UUAGUUGUACUAGAUGUA 5'
query=miR6451, target=RO3G_05836, score=4, range=118-134, strand=
target 5' GAUU-ACAUGGUUUACAU 3'
.::. :::::.:.:::::
query 3' UUAGUUGUACUAGAUGUA 5'
query=miR6451, target=RO3G_15359, score=4, range=106-123, strand=
target 5' CUUCAACAUGAUCUAGAU 3'
::::::::::::: ::
query 3' UUAGUUGUACUAGAUGUA 5'
query=miR6451, target=RO3G_15507, score=4, range=5107-5124, stran
target 5' AUUCAACAUGAUCCACAA 3'
: ::::::::::: :::
query 3' UUAGUUGUACUAGAUGUA 5'
query=miR6451, target=RO3G_11284, score=4, range=1720-1736, stran
target 5' AAUAAACAUGGUCU-CAU 3'
::: ::::::.::: :::
query 3' UUAGUUGUACUAGAUGUA 5'
query=miR6451, target=RO3G_13853, score=4, range=766-782, strand=
target 5' AAUAAACAUGGUCU-CAU 3'
::: ::::::.::: :::
query 3' UUAGUUGUACUAGAUGUA 5'
query=miR6451, target=RO3G_00705, score=4, range=1720-1736, stran
target 5' AAUAAACAUGGUCU-CAU 3'
::: ::::::.::: :::
query 3' UUAGUUGUACUAGAUGUA 5'
query=miR6451, target=RO3G_03562, score=4, range=1450-1466, stran
target 5' AAUAAACAUGGUCU-CAU 3'
::: ::::::.::: :::
query 3' UUAGUUGUACUAGAUGUA 5'
query=miR6451, target=RO3G_10802, score=4, range=838-854, strand=
target 5' AAUAAACAUGGUCU-CAU 3'
::: ::::::.::: :::
query 3' UUAGUUGUACUAGAUGUA 5'
```

```
query=miR6451, target=RO3G_06473, score=4, range=283-299, strand=
target 5' AAU-AACAUGAUCAACAC 3'
::: ::::::::: :::
query 3' UUAGUUGUACUAGAUGUA 5'
query=miR6451, target=RO3G_04493, score=4, range=258-274, strand=
target 5' AAUACACAUGAUCU-CAU 3'
::: ::::::::: :::
query 3' UUAGUUGUACUAGAUGUA 5'
query=miR6451, target=RO3G_01109, score=4, range=26-43, strand=-1
target 5' AAUUGACAUGGUCUGCAG 3'
:::..::::.:::.::
query 3' UUAGUUGUACUAGAUGUA 5'
query=miR6451, target=RO3G_08530, score=4, range=1979-1996, stran
target 5' AAUCAGCAUGAUUUUCAU 3'
::::.::::::.: :::
query 3' UUAGUUGUACUAGAUGUA 5'
query=miR6451, target=RO3G_12313, score=4, range=647-664, strand=
target 5' AAUCAACAUCAUCUGUAU 3'
::::::::: ::::..::
query 3' UUAGUUGUACUAGAUGUA 5'
query=miR6451, target=RO3G_06338, score=4, range=176-193, strand=
target 5' AAUCAACAUGACUUGCAU 3'
:::::::::: .:.:::
query 3' UUAGUUGUACUAGAUGUA 5'
query=miR6451, target=RO3G_11327, score=4, range=322-339, strand=
target 5' GGUCAACAUGAUCAACGU 3'
..:::::::::: ::.:
query 3' UUAGUUGUACUAGAUGUA 5'
query=miR6451, target=RO3G_15840, score=4, range=5083-5100, stran
target 5' AAUCAAAAUGAUCUGCAG 3'
:::::: :::::::.::
query 3' UUAGUUGUACUAGAUGUA 5'
query=miR6451, target=RO3G_12170, score=4, range=668-685, strand=
target 5' UAUCAAUAAGAUCUACAU 3'
:::::.: ::::::::
query 3' UUAGUUGUACUAGAUGUA 5'
query=miR6451, target=RO3G_01715, score=4, range=668-685, strand=
target 5' UAUCAAUAAGAUCUACAU 3'
:::::.: ::::::::
query 3' UUAGUUGUACUAGAUGUA 5'
query=miR6451, target=RO3G_03739, score=4, range=626-644, strand=
target 5' AAUGCAACAUGGUCUGCAG 3'
::: :::::::.:::.::
query 3' UUA-GUUGUACUAGAUGUA 5'
query=miR6451, target=RO3G_13376, score=4, range=72-88, strand=-1
```

```
target 5' AAUCAACAUGGUCUA-GU 3'
::::::::::.:::: .:
query 3' UUAGUUGUACUAGAUGUA 5'
query=miR6451, target=RO3G_14681, score=4, range=1223-1241, stran
target 5' UGUUGAACAUGGUCUACAU 3'
.:. :::::.:::::::
query 3' UUAG-UUGUACUAGAUGUA 5'
query=miR6451, target=RO3G_15110, score=4, range=1079-1096, stran
target 5' AAUCACCAUGAUCUUCAU 3'
::::: :::::::: :::
query 3' UUAGUUGUACUAGAUGUA 5'
query=miR6451, target=RO3G_04172, score=4, range=1502-1519, stran
target 5' AAUGAACAUGAUUUUCAU 3'
::: ::::::::.: :::
query 3' UUAGUUGUACUAGAUGUA 5'
query=miR6451, target=RO3G_14952, score=4, range=437-454, strand=
target 5' AAACAACAAGGUCUACAU 3'
:: ::::: :.:::::::
query 3' UUAGUUGUACUAGAUGUA 5'
query=miR6451, target=RO3G_06602, score=4, range=200-217, strand=
target 5' AUUCAACAUCAUCUGCAU 3'
: ::::::: ::::.:::
query 3' UUAGUUGUACUAGAUGUA 5'
query=miR6451, target=RO3G_00014, score=4, range=745-762, strand=
target 5' GAGUAACAUGAUGUACAU 3'
.: .:::::::: :::::
query 3' UUAGUUGUACUAGAUGUA 5'
query=miR6451, target=RO3G_14511, score=4, range=203-220, strand=
target 5' AAGCUGCGUGAUCUACAU 3'
:: : .:.::::::::::
query 3' UUAGUUGUACUAGAUGUA 5'
query=miR6451, target=RO3G_16094, score=4, range=185-201, strand=
target 5' AAUCAACAUGAUC-AUAG 3'
:::::::::::::: :.:
query 3' UUAGUUGUACUAGAUGUA 5'
query=miR6451, target=RO3G_06402, score=4, range=728-745, strand=
target 5' AAUCUACAAGAUCUACAC 3'
:::: ::: ::::::::
query 3' UUAGUUGUACUAGAUGUA 5'
query=miR6451, target=RO3G_08950, score=4, range=170-187, strand=
target 5' AAUAAACAGGAUCUACAG 3'
::: :::: ::::::::
query 3' UUAGUUGUACUAGAUGUA 5'
query=miR6451, target=RO3G_12257, score=4, range=462-478, strand=
target 5' GAUU-AUAUGAUCUACAA 3'
```

```
.::. :.:::::::::
query 3' UUAGUUGUACUAGAUGUA 5'
query=miR6451, target=RO3G_09820, score=4, range=149-166, strand=
target 5' AAGCAACAUUAUCUACAC 3'
:: :::::: :::::::
query 3' UUAGUUGUACUAGAUGUA 5'
query=miR6451, target=RO3G_00155, score=4, range=341-359, strand=
target 5' UAUCCAACAUGAUCAACAU 3'
:: :::::::::: ::::
query 3' UUA-GUUGUACUAGAUGUA 5'
query=miR6451, target=RO3G_06617, score=4, range=431-447, strand=
target 5' AAUCAACAUGA-AUACAU 3'
:::::::::: :::::
query 3' UUAGUUGUACUAGAUGUA 5'
query=miR6451, target=RO3G_02490, score=4, range=926-943, strand=
target 5' CACCUACAUGAUCUGCAU 3'
: : :::::::::.:::
query 3' UUAGUUGUACUAGAUGUA 5'
query=miR6451, target=RO3G_02518, score=4, range=452-468, strand=
target 5' AAU-AGCAUGAUCUAAAU 3'
::: :.:::::::: ::
query 3' UUAGUUGUACUAGAUGUA 5'
query=miR6451, target=RO3G_05989, score=4, range=261-277, strand=
target 5' AUUCAGCAUGAU-UACAU 3'
: :::.:::::: :::::
query 3' UUAGUUGUACUAGAUGUA 5'
query=miR6451, target=RO3G_06636, score=4, range=1004-1021, stran
target 5' CCUCUACAUGAUCUACAG 3'
:: :::::::::::
query 3' UUAGUUGUACUAGAUGUA 5'
query=miR6451, target=RO3G_02449, score=4, range=416-433, strand=
target 5' AAAGAACAUUAUCUACAU 3'
:: ::::: ::::::::
query 3' UUAGUUGUACUAGAUGUA 5'
query=miR6451, target=RO3G_15432, score=4, range=731-747, strand=
target 5' AAUCAA-ACGAUCUACAU 3'
:::::: : :::::::::
query 3' UUAGUUGUACUAGAUGUA 5'
query=miR951, target=RO3G_04122, score=0, range=196-217, strand=1
target 5' CGUAGCCAGAUCGUCAAGAGCA 3'
::::::::::::::::::::::
query 3' GCAUCGGUCUAGCAGUUCUCGU 5'
query=miR951, target=RO3G_13220, score=0, range=196-217, strand=1
target 5' CGUAGCCAGAUCGUCAAGAGCA 3'
::::::::::::::::::::::
```

```
query 3' GCAUCGGUCUAGCAGUUCUCGU 5'
query=miR951, target=RO3G_04124, score=0, range=196-217, strand=1
target 5' CGUAGCCAGAUCGUCAAGAGCA 3'
::::::::::::::::::::::
query 3' GCAUCGGUCUAGCAGUUCUCGU 5'
query=miR951, target=RO3G_13223, score=0.5, range=196-217, strand
target 5' CGUAGUCAGAUCGUCAAGAGCA 3'
:::::.::::::::::::::::
query 3' GCAUCGGUCUAGCAGUUCUCGU 5'
query=miR951, target=RO3G_04119, score=2, range=196-217, strand=1
target 5' CGUAGCCAGAUGGUCAAGAGCA 3'
::::::::::: ::::::::::
query 3' GCAUCGGUCUAGCAGUUCUCGU 5'
query=miR7716-3p, target=RO3G_17338, score=0, range=3-24, strand=
target 5' GGCAUUACUGGAGGAUGAAGAA 3'
::::::::::::::::::::::
query 3' CCGUAAUGACCUCCUACUUCUU 5'
query=miR7716-3p, target=RO3G_07958, score=3.5, range=3070-3090,
strand=1
target 5' GACG-UGUUGGAGGAUGAAGAA 3'
: :. :..::::::::::::::
query 3' CCGUAAUGACCUCCUACUUCUU 5'
query=miR7716-3p, target=RO3G_12741, score=4, range=1170-1191,
strand=1
target 5' GAUGGUGCUGGAGGAUGAAGAG 3'
: .. :.::::::::::::::.
query 3' CCGUAAUGACCUCCUACUUCUU 5'
query=miR7716-3p, target=RO3G_14666, score=4, range=840-860, stra
target 5' AGCAUUAAUGGA-GAUGAAGAA 3'
:::::: :::: :::::::::
query 3' CCGUAAUGACCUCCUACUUCUU 5'
query=miR8741, target=RO3G_13273, score=0, range=2206-2227, stran
target 5' CCAUCAUCAUUCAUUCAGGCCA 3'
::::::::::::::::::::::
query 3' GGUAGUAGUAAGUAAGUCCGGU 5'
query=miR8741, target=RO3G_10217, score=0, range=2206-2227, stran
target 5' CCAUCAUCAUUCAUUCAGGCCA 3'
::::::::::::::::::::::
query 3' GGUAGUAGUAAGUAAGUCCGGU 5'
query=miR8741, target=RO3G_16749, score=0, range=2206-2227, stran
target 5' CCAUCAUCAUUCAUUCAGGCCA 3'
::::::::::::::::::::::
query 3' GGUAGUAGUAAGUAAGUCCGGU 5'
query=miR8741, target=RO3G_13145, score=0, range=2206-2227, stran
target 5' CCAUCAUCAUUCAUUCAGGCCA 3'
```

```
::::::::::::::::::::::
query 3' GGUAGUAGUAAGUAAGUCCGGU 5'
query=miR8741, target=RO3G_17257, score=0, range=2122-2143, stran
target 5' CCAUCAUCAUUCAUUCAGGCCA 3'
::::::::::::::::::::::
query 3' GGUAGUAGUAAGUAAGUCCGGU 5'
query=miR8741, target=RO3G_05424, score=0, range=2122-2143, stran
target 5' CCAUCAUCAUUCAUUCAGGCCA 3'
::::::::::::::::::::::
query 3' GGUAGUAGUAAGUAAGUCCGGU 5'
query=miR8741, target=RO3G_15889, score=0, range=2122-2143, stran
target 5' CCAUCAUCAUUCAUUCAGGCCA 3'
::::::::::::::::::::::
query 3' GGUAGUAGUAAGUAAGUCCGGU 5'
query=miR8741, target=RO3G_16136, score=0, range=2122-2143, stran
target 5' CCAUCAUCAUUCAUUCAGGCCA 3'
::::::::::::::::::::::
query 3' GGUAGUAGUAAGUAAGUCCGGU 5'
query=miR8741, target=RO3G_04592, score=0, range=1378-1399, stran
target 5' CCAUCAUCAUUCAUUCAGGCCA 3'
::::::::::::::::::::::
query 3' GGUAGUAGUAAGUAAGUCCGGU 5'
query=miR8741, target=RO3G_09183, score=0, range=1339-1360, stran
target 5' CCAUCAUCAUUCAUUCAGGCCA 3'
::::::::::::::::::::::
query 3' GGUAGUAGUAAGUAAGUCCGGU 5'
query=miR8741, target=RO3G_00289, score=0, range=940-961, strand=
target 5' CCAUCAUCAUUCAUUCAGGCCA 3'
::::::::::::::::::::::
query 3' GGUAGUAGUAAGUAAGUCCGGU 5'
query=miR8741, target=RO3G_17351, score=0, range=934-955, strand=
target 5' CCAUCAUCAUUCAUUCAGGCCA 3'
::::::::::::::::::::::
query 3' GGUAGUAGUAAGUAAGUCCGGU 5'
query=miR8741, target=RO3G_04681, score=0, range=796-817, strand=
target 5' CCAUCAUCAUUCAUUCAGGCCA 3'
::::::::::::::::::::::
query 3' GGUAGUAGUAAGUAAGUCCGGU 5'
query=miR8741, target=RO3G_07697, score=0, range=532-553, strand=
target 5' CCAUCAUCAUUCAUUCAGGCCA 3'
::::::::::::::::::::::
query 3' GGUAGUAGUAAGUAAGUCCGGU 5'
query=miR8741, target=RO3G_10040, score=0, range=796-817, strand=
target 5' CCAUCAUCAUUCAUUCAGGCCA 3'
::::::::::::::::::::::
```

```
query 3' GGUAGUAGUAAGUAAGUCCGGU 5'
query=miR8741, target=RO3G_10735, score=2.5, range=1651-1672, str
target 5' CCAUCAUCAUUCAUUCAGGCGG 3'
:::::::::::::::::::: .
query 3' GGUAGUAGUAAGUAAGUCCGGU 5'
query=miR913-5p, target=RO3G_15268, score=0, range=472-492, stran
target 5' CCUCGGUCUCGCAAGAUGUCA 3'
:::::::::::::::::::::
query 3' GGAGCCAGAGCGUUCUACAGU 5'
query=miR913-5p, target=RO3G_04121, score=0, range=472-492, stran
target 5' CCUCGGUCUCGCAAGAUGUCA 3'
:::::::::::::::::::::
query 3' GGAGCCAGAGCGUUCUACAGU 5'
query=miR913-5p, target=RO3G_16800, score=0, range=472-492, stran
target 5' CCUCGGUCUCGCAAGAUGUCA 3'
:::::::::::::::::::::
query 3' GGAGCCAGAGCGUUCUACAGU 5'
query=miR913-5p, target=RO3G_15265, score=0, range=472-492, stran
target 5' CCUCGGUCUCGCAAGAUGUCA 3'
:::::::::::::::::::::
query 3' GGAGCCAGAGCGUUCUACAGU 5'
query=miR913-5p, target=RO3G_13219, score=0, range=472-492, stran
target 5' CCUCGGUCUCGCAAGAUGUCA 3'
:::::::::::::::::::::
query 3' GGAGCCAGAGCGUUCUACAGU 5'
query=miR913-5p, target=RO3G_13217, score=4, range=472-492, stran
target 5' CCUCGGUCCCGUAAGAUGUCU 3'
:::::::: ::.:::::::
query 3' GGAGCCAGAGCGUUCUACAGU 5'
query=miR913-5p, target=RO3G_14352, score=4, range=472-492, stran
target 5' CCUCGGUCCCGUAAGAUGUCU 3'
:::::::: ::.:::::::
query 3' GGAGCCAGAGCGUUCUACAGU 5'
query=miR913-5p, target=RO3G_15266, score=4, range=472-492, stran
target 5' CCUCGGUCCCGUAAGAUGUCU 3'
:::::::: ::.:::::::
query 3' GGAGCCAGAGCGUUCUACAGU 5'
query=miR8603, target=RO3G_05069, score=3.5, range=1125-1145,
strand=-1
target 5' ACUAUAUUCAUCACAAGCUCA 3'
:: :: .:::.::::::::::
query 3' UGUUACGAGUGGUGUUCGAGU 5'
query=miR8603, target=RO3G_05236, score=4, range=15-36, strand=1
target 5' ACAAUUGCUCAUCACAAGUUUA 3'
:::: ::::::.::::::.:.:
```

query 3' UGUU-ACGAGUGGUGUUCGAGU 5'
query=miR5641, target=RO3G_17273, score=2.5, range=1754-1776, strand=-1
target 5' UAGUUCUUUACUGUCUUCUUCCA 3'
::.:::::::::.:::::.::::
query 3' AUUAAGAAAUGAUAGAAGGAGGU 5'
query=miR5641, target=RO3G_15160, score=2.5, range=1652-1674, strand=-1
target 5' UAGUUCUUUACUGUCUUCUUCCA 3'
::.:::::::::.:::::.::::
query 3' AUUAAGAAAUGAUAGAAGGAGGU 5'
query=miR5641, target=RO3G_03100, score=4, range=3206-3228, stran
target 5' UAGUUCUUUGCUAUCCUCUUCCA 3'
::.:::::::.::::: ::.::::
query 3' AUUAAGAAAUGAUAGAAGGAGGU 5'
query=miR5641, target=RO3G_05340, score=4, range=1589-1611, stran
target 5' UAGUUCUUUGCUAUCCUCUUCCA 3'
::.:::::::.::::: ::.::::
query 3' AUUAAGAAAUGAUAGAAGGAGGU 5'
query=miR4401b, target=RO3G_17451, score=0, range=70-89, strand=1
target 5' UUGCUCACAACCGUCUUUGA 3'
::::::::::::::::::::
query 3' AACGAGUGUUGGCAGAAACU 5'
query=miR4401b, target=RO3G_13914, score=0, range=829-848, strand
target 5' UUGCUCACAACCGUCUUUGA 3'
::::::::::::::::::::
query 3' AACGAGUGUUGGCAGAAACU 5'
query=miR4401b, target=RO3G_12058, score=0, range=829-848, strand
target 5' UUGCUCACAACCGUCUUUGA 3'
::::::::::::::::::::
query 3' AACGAGUGUUGGCAGAAACU 5'
query=miR4401b, target=RO3G_14983, score=0, range=634-653, strand
target 5' UUGCUCACAACCGUCUUUGA 3'
::::::::::::::::::::
query 3' AACGAGUGUUGGCAGAAACU 5'
query=miR4401b, target=RO3G_10801, score=0, range=634-653, strand
target 5' UUGCUCACAACCGUCUUUGA 3'
::::::::::::::::::::
query 3' AACGAGUGUUGGCAGAAACU 5'
query=miR4401b, target=RO3G_09614, score=0, range=160-179, strand
target 5' UUGCUCACAACCGUCUUUGA 3'
::::::::::::::::::::
query 3' AACGAGUGUUGGCAGAAACU 5'
query=miR4401b, target=RO3G_17462, score=0, range=961-980, strand
target 5' UUGCUCACAACCGUCUUUGA 3'

```
::::::::::::::::::::
query 3' AACGAGUGUUGGCAGAAACU 5'
query=miR4401b, target=RO3G_02233, score=0, range=358-377, strand
target 5' UUGCUCACAACCGUCUUUGA 3'
::::::::::::::::::::
query 3' AACGAGUGUUGGCAGAAACU 5'
query=miR4401b, target=RO3G_12667, score=2, range=199-218, strand
target 5' UUGCUCACAACCGUCUUGGA 3'
::::::::::::::::: ::
query 3' AACGAGUGUUGGCAGAAACU 5'
query=miR4401b, target=RO3G_03583, score=2, range=634-653, strand
target 5' UUGCUCACAACAGUCUUUGA 3'
::::::::::: ::::::::
query 3' AACGAGUGUUGGCAGAAACU 5'
query=miR4401b, target=RO3G_16714, score=3.5, range=187-206, stra
target 5' UUGUUGACAACCGUCUUAGA 3'
:::.: ::::::::::: ::
query 3' AACGAGUGUUGGCAGAAACU 5'
query=miR4401b, target=RO3G_04116, score=3.5, range=194-213, stra
target 5' UUGUUGACAACCGUCUUAGA 3'
:::.: ::::::::::: ::
query 3' AACGAGUGUUGGCAGAAACU 5'
query=miR4401b, target=RO3G_01079, score=3.5, range=466-486, stra
target 5' UGGCUCACCAACCGUUUUUGG 3'
: ::::: ::::::.::::.
query 3' AACGAGU-GUUGGCAGAAACU 5'
query=miR4401b, target=RO3G_01425, score=3.5, range=1419-1437,
strand=-1
target 5' UUUCUU-CAACCGUUUUUGA 3'
:: ::. :::::::.:::::
query 3' AACGAGUGUUGGCAGAAACU 5'
query=miR4401b, target=RO3G_03120, score=3.5, range=1419-1437,
strand=-1
target 5' UUUCUU-CAACCGUUUUUGA 3'
:: ::. :::::::.:::::
query 3' AACGAGUGUUGGCAGAAACU 5'
query=miR4401b, target=RO3G_16566, score=3.5, range=1419-1437,
strand=-1
target 5' UUUCUU-CAACCGUUUUUGA 3'
:: ::. :::::::.:::::
query 3' AACGAGUGUUGGCAGAAACU 5'
query=miR4401b, target=RO3G_13806, score=3.5, range=1419-1437,
strand=-1
target 5' UUUCUU-CAACCGUUUUUGA 3'
:: ::. :::::::.:::::
```

```
query 3' AACGAGUGUUGGCAGAAACU 5'
query=miR4401b, target=RO3G_14533, score=3.5, range=1224-1242,
strand=-1
target 5' UUUCUU-CAACCGUUUUUGA 3'
:: ::. :::::::.:::::
query 3' AACGAGUGUUGGCAGAAACU 5'
query=miR4401b, target=RO3G_08383, score=4, range=281-300, strand
target 5' UUGCUCGCAGCCGUCUUGGG 3'
::::::.::.::::::: :.
query 3' AACGAGUGUUGGCAGAAACU 5'
query=miR4401b, target=RO3G_12286, score=4, range=242-260, strand
target 5' UUUCUGA-AACCGUCUUUGA 3'
:: :: : ::::::::::::
query 3' AACGAGUGUUGGCAGAAACU 5'
query=miR4401b, target=RO3G_07324, score=4, range=567-586, strand
target 5' UGGCUGCCAAUCGUCUUUGA 3'
: ::: :::.:::::::::
query 3' AACGAGUGUUGGCAGAAACU 5'
query=miR4401b, target=RO3G_08428, score=4, range=401-419, strand
target 5' CUUCUCAC-ACCGUCUUUGA 3'
: ::::: :::::::::::
query 3' AACGAGUGUUGGCAGAAACU 5'
query=miR4401b, target=RO3G_11133, score=4, range=95-113, strand=
target 5' UUGCUC-CAAUAGUCUUUGA 3'
:::::: :::. ::::::::
query 3' AACGAGUGUUGGCAGAAACU 5'
query=miR171n-5p, target=RO3G_09180, score=0, range=1134-1154,
strand=-1
target 5' CCGUACUUGAACCCACCAAUA 3'
:::::::::::::::::::::
query 3' GGCAUGAACUUGGGUGGUUAU 5'
query=miR171n-5p, target=RO3G_16812, score=0, range=1134-1154,
strand=-1
target 5' CCGUACUUGAACCCACCAAUA 3'
:::::::::::::::::::::
query 3' GGCAUGAACUUGGGUGGUUAU 5'
query=miR171n-5p, target=RO3G_09189, score=0, range=1134-1154,
strand=-1
target 5' CCGUACUUGAACCCACCAAUA 3'
:::::::::::::::::::::
query 3' GGCAUGAACUUGGGUGGUUAU 5'
query=miR171n-5p, target=RO3G_14798, score=0, range=1134-1154,
strand=-1
target 5' CCGUACUUGAACCCACCAAUA 3'
:::::::::::::::::::::
```

```
query 3' GGCAUGAACUUGGGUGGUUAU 5'
query=miR171n-5p, target=RO3G_14791, score=0, range=1134-1154,
strand=-1
target 5' CCGUACUUGAACCCACCAAUA 3'
:::::::::::::::::::::
query 3' GGCAUGAACUUGGGUGGUUAU 5'
query=miR171n-5p, target=RO3G_16733, score=0, range=1134-1154,
strand=-1
target 5' CCGUACUUGAACCCACCAAUA 3'
:::::::::::::::::::::
query 3' GGCAUGAACUUGGGUGGUUAU 5'
query=miR171n-5p, target=RO3G_15270, score=0, range=183-203, stra
target 5' CCGUACUUGAACCCACCAAUA 3'
:::::::::::::::::::::
query 3' GGCAUGAACUUGGGUGGUUAU 5'
query=miR171n-5p, target=RO3G_06734, score=0, range=438-458, stra
target 5' CCGUACUUGAACCCACCAAUA 3'
:::::::::::::::::::::
query 3' GGCAUGAACUUGGGUGGUUAU 5'
query=miR171n-5p, target=RO3G_17260, score=1, range=1013-1033,
strand=-1
target 5' CCAUACUUGAACCCACCAAUA 3'
:: ::::::::::::::::::
query 3' GGCAUGAACUUGGGUGGUUAU 5'
query=miR171n-5p, target=RO3G_01819, score=4, range=1809-1828,
strand=1
target 5' UUUUA-UUGAACCUACCAAUA 3'
.. :: ::::::.:::::::
query 3' GGCAUGAACUUGGGUGGUUAU 5'
query=miR1431, target=RO3G_11979, score=4, range=619-641, strand=
target 5' GUCAAGCUGUGGCCAAUGCCAAA 3'
::::::: ::::::::. :::::
query 3' CAGUUCGCCACCGGUUGAGGUUU 5'
query=miR1431, target=RO3G_17253, score=4, range=635-657, strand=
target 5' GUCAAGCUGUGGCCAAUGCCAAA 3'
::::::: ::::::::. :::::
query 3' CAGUUCGCCACCGGUUGAGGUUU 5'
query=miR158-3p, target=RO3G_02126, score=2, range=520-540, stran
target 5' GCAUUUGUCUACGUUUGGAAA 3'
:: :::::::::.::::::::
query 3' CG-AAACAGAUGUAAACCUUU 5'
query=miR158-3p, target=RO3G_05865, score=4, range=2734-2753, str
target 5' GCUUUGUCUACAUCUGAAAA 3'
:::::::::::: :: :::
query 3' CGAAACAGAUGUAAACCUUU 5'
```

```
query=miR158-3p, target=RO3G_02707, score=4, range=631-650, stran
target 5' GCUUUGUCUACAUCUGAAAA 3'
:::::::::::: :: :::
query 3' CGAAACAGAUGUAAACCUUU 5'
query=miR158-3p, target=RO3G_17446, score=4, range=3082-3101, str
target 5' GCUUUGUCUACAUCUGAAAA 3'
:::::::::::: :: :::
query 3' CGAAACAGAUGUAAACCUUU 5'
query=miR158-3p, target=RO3G_15863, score=4, range=10-29, strand=
target 5' GCUUUGUCUACAUCUGAAAA 3'
:::::::::::: :: :::
query 3' CGAAACAGAUGUAAACCUUU 5'
query=miR158-3p, target=RO3G_01733, score=4, range=2884-2903, str
target 5' GCUUUGUCUACAUCUGAAAA 3'
:::::::::::: :: :::
query 3' CGAAACAGAUGUAAACCUUU 5'
query=miR158-3p, target=RO3G_11945, score=4, range=10-29, strand=
target 5' GCUUUGUCUACAUCUGAAAA 3'
:::::::::::: :: :::
query 3' CGAAACAGAUGUAAACCUUU 5'
query=miR158-3p, target=RO3G_10420, score=4, range=3082-3101, str
target 5' GCUUUGUCUACAUCUGAAAA 3'
:::::::::::: :: :::
query 3' CGAAACAGAUGUAAACCUUU 5'
query=miR158-3p, target=RO3G_02966, score=4, range=2353-2372, str
target 5' GCUUUGUCUACAUCUGAAAA 3'
:::::::::::: :: :::
query 3' CGAAACAGAUGUAAACCUUU 5'
query=miR158-3p, target=RO3G_10991, score=4, range=3082-3101, str
target 5' GCUUUGUCUACAUCUGAAAA 3'
:::::::::::: :: :::
query 3' CGAAACAGAUGUAAACCUUU 5'
query=miR158-3p, target=RO3G_01944, score=4, range=3082-3101, str
target 5' GCUUUGUCUACAUCUGAAAA 3'
:::::::::::: :: :::
query 3' CGAAACAGAUGUAAACCUUU 5'
query=miR158-3p, target=RO3G_13849, score=4, range=3082-3101, str
target 5' GCUUUGUCUACAUCUGAAAA 3'
:::::::::::: :: :::
query 3' CGAAACAGAUGUAAACCUUU 5'
query=miR158-3p, target=RO3G_06502, score=4, range=2959-2978, str
target 5' GCUUUGUCUACAUCUGAAAA 3'
:::::::::::: :: :::
query 3' CGAAACAGAUGUAAACCUUU 5'
query=miR158-3p, target=RO3G_17287, score=4, range=3082-3101, str
```

```
target 5' GCUUUGUCUACAUCUGAAAA 3'
:::::::::::: :: :::
query 3' CGAAACAGAUGUAAACCUUU 5'
query=miR158-3p, target=RO3G_03749, score=4, range=3082-3101, str
target 5' GCUUUGUCUACAUCUGAAAA 3'
:::::::::::: :: :::
query 3' CGAAACAGAUGUAAACCUUU 5'
query=miR158-3p, target=RO3G_15573, score=4, range=3082-3101, str
target 5' GCUUUGUCUACAUCUGAAAA 3'
:::::::::::: :: :::
query 3' CGAAACAGAUGUAAACCUUU 5'
query=miR158-3p, target=RO3G_07325, score=4, range=1306-1325, str
target 5' GCUUUGGUUACAUUUGAAAA 3'
:::::: .:::::::: :::
query 3' CGAAACAGAUGUAAACCUUU 5'
query=miR158-3p, target=RO3G_09758, score=4, range=3112-3131, str
target 5' GCUUUUGCUACAUUAGGAAA 3'
::::: ::::::: :::::
query 3' CGAAACAGAUGUAAACCUUU 5'
query=miR158-3p, target=RO3G_06983, score=4, range=895-914, stran
target 5' GCUUGGUCUACAUCUGGAAU 3'
:::: :::::::: :::::
query 3' CGAAACAGAUGUAAACCUUU 5'
query=miR158-3p, target=RO3G_08644, score=4, range=829-847, stran
target 5' GUGUUG-CUAUAUUUGGAAG 3'
:. ::: :::.:::::::.
query 3' CGAAACAGAUGUAAACCUUU 5'
query=miR158-3p, target=RO3G_17196, score=4, range=215-233, stran
target 5' GCAUUG-CUACAUUUGAAAA 3'
:: ::: ::::::::: :::
query 3' CGAAACAGAUGUAAACCUUU 5'
query=miR1146, target=RO3G_06423, score=0, range=518-538, strand=
target 5' AAGACUUCCCGUCGGACCCAA 3'
:::::::::::::::::::::
query 3' UUCUGAAGGGCAGCCUGGGUU 5'
query=miR1146, target=RO3G_10235, score=0, range=464-484, strand=
target 5' AAGACUUCCCGUCGGACCCAA 3'
:::::::::::::::::::::
query 3' UUCUGAAGGGCAGCCUGGGUU 5'
query=miR1146, target=RO3G_09483, score=0, range=464-484, strand=
target 5' AAGACUUCCCGUCGGACCCAA 3'
:::::::::::::::::::::
query 3' UUCUGAAGGGCAGCCUGGGUU 5'
query=miR1146, target=RO3G_06441, score=0, range=464-484, strand=
target 5' AAGACUUCCCGUCGGACCCAA 3'
```

```
:::::::::::::::::::::
query 3' UUCUGAAGGGCAGCCUGGGUU 5'
query=miR1146, target=RO3G_09499, score=0, range=464-484, strand=
target 5' AAGACUUCCCGUCGGACCCAA 3'
:::::::::::::::::::::
query 3' UUCUGAAGGGCAGCCUGGGUU 5'
query=miR1146, target=RO3G_15675, score=0, range=464-484, strand=
target 5' AAGACUUCCCGUCGGACCCAA 3'
:::::::::::::::::::::
query 3' UUCUGAAGGGCAGCCUGGGUU 5'
query=miR1146, target=RO3G_11401, score=0, range=464-484, strand=
target 5' AAGACUUCCCGUCGGACCCAA 3'
:::::::::::::::::::::
query 3' UUCUGAAGGGCAGCCUGGGUU 5'
query=miR1146, target=RO3G_07875, score=0, range=464-484, strand=
target 5' AAGACUUCCCGUCGGACCCAA 3'
:::::::::::::::::::::
query 3' UUCUGAAGGGCAGCCUGGGUU 5'
query=miR1146, target=RO3G_07303, score=0, range=464-484, strand=
target 5' AAGACUUCCCGUCGGACCCAA 3'
:::::::::::::::::::::
query 3' UUCUGAAGGGCAGCCUGGGUU 5'
query=miR1146, target=RO3G_14359, score=0, range=464-484, strand=
target 5' AAGACUUCCCGUCGGACCCAA 3'
:::::::::::::::::::::
query 3' UUCUGAAGGGCAGCCUGGGUU 5'
query=miR1146, target=RO3G_00681, score=0, range=464-484, strand=
target 5' AAGACUUCCCGUCGGACCCAA 3'
:::::::::::::::::::::
query 3' UUCUGAAGGGCAGCCUGGGUU 5'
query=miR1146, target=RO3G_05336, score=0, range=464-484, strand=
target 5' AAGACUUCCCGUCGGACCCAA 3'
:::::::::::::::::::::
query 3' UUCUGAAGGGCAGCCUGGGUU 5'
query=miR1146, target=RO3G_09544, score=0, range=464-484, strand=
target 5' AAGACUUCCCGUCGGACCCAA 3'
:::::::::::::::::::::
query 3' UUCUGAAGGGCAGCCUGGGUU 5'
query=miR1146, target=RO3G_10732, score=0, range=464-484, strand=
target 5' AAGACUUCCCGUCGGACCCAA 3'
:::::::::::::::::::::
query 3' UUCUGAAGGGCAGCCUGGGUU 5'
query=miR1146, target=RO3G_16807, score=0, range=464-484, strand=
target 5' AAGACUUCCCGUCGGACCCAA 3'
:::::::::::::::::::::
```

```
query 3' UUCUGAAGGGCAGCCUGGGUU 5'
query=miR1146, target=RO3G_17382, score=0, range=464-484, strand=
target 5' AAGACUUCCCGUCGGACCCAA 3'
:::::::::::::::::::::
query 3' UUCUGAAGGGCAGCCUGGGUU 5'
query=miR1146, target=RO3G_02689, score=0, range=464-484, strand=
target 5' AAGACUUCCCGUCGGACCCAA 3'
:::::::::::::::::::::
query 3' UUCUGAAGGGCAGCCUGGGUU 5'
query=miR1146, target=RO3G_01322, score=0, range=350-370, strand=
target 5' AAGACUUCCCGUCGGACCCAA 3'
:::::::::::::::::::::
query 3' UUCUGAAGGGCAGCCUGGGUU 5'
query=miR1146, target=RO3G_17400, score=0, range=338-358, strand=
target 5' AAGACUUCCCGUCGGACCCAA 3'
:::::::::::::::::::::
query 3' UUCUGAAGGGCAGCCUGGGUU 5'
query=miR1146, target=RO3G_05915, score=0, range=464-484, strand=
target 5' AAGACUUCCCGUCGGACCCAA 3'
:::::::::::::::::::::
query 3' UUCUGAAGGGCAGCCUGGGUU 5'
query=miR1146, target=RO3G_01730, score=0, range=464-484, strand=
target 5' AAGACUUCCCGUCGGACCCAA 3'
:::::::::::::::::::::
query 3' UUCUGAAGGGCAGCCUGGGUU 5'
query=miR1146, target=RO3G_13720, score=0, range=341-361, strand=
target 5' AAGACUUCCCGUCGGACCCAA 3'
:::::::::::::::::::::
query 3' UUCUGAAGGGCAGCCUGGGUU 5'
query=miR1146, target=RO3G_16897, score=0, range=464-484, strand=
target 5' AAGACUUCCCGUCGGACCCAA 3'
:::::::::::::::::::::
query 3' UUCUGAAGGGCAGCCUGGGUU 5'
query=miR1146, target=RO3G_17285, score=0.5, range=464-484, stran
target 5' AAGGCUUCCCGUCGGACCCAA 3'
:::.:::::::::::::::::
query 3' UUCUGAAGGGCAGCCUGGGUU 5'
query=miR1146, target=RO3G_01652, score=0.5, range=464-484, stran
target 5' AAGACUUUCCGUCGGACCCAA 3'
:::::::.:::::::::::::
query 3' UUCUGAAGGGCAGCCUGGGUU 5'
query=miR1146, target=RO3G_01956, score=0.5, range=464-484, stran
target 5' AAGGCUUCCCGUCGGACCCAA 3'
:::.:::::::::::::::::
query 3' UUCUGAAGGGCAGCCUGGGUU 5'
```

query=miR1146, target=RO3G_08846, score=0.5, range=464-484, stran
target 5' AAGACUUUCCGUCGGACCCAA 3'
:::::::.:::::::::::::
query 3' UUCUGAAGGGCAGCCUGGGUU 5'
query=miR1146, target=RO3G_03571, score=0.5, range=464-484, stran
target 5' AAGACUUUCCGUCGGACCCAA 3'
:::::::.:::::::::::::
query 3' UUCUGAAGGGCAGCCUGGGUU 5'
query=miR1146, target=RO3G_05635, score=0.5, range=464-484, stran
target 5' AAGACUUUCCGUCGGACCCAA 3'
:::::::.:::::::::::::
query 3' UUCUGAAGGGCAGCCUGGGUU 5'
query=miR1146, target=RO3G_13848, score=0.5, range=464-484, stran
target 5' AAGACUUUCCGUCGGACCCAA 3'
:::::::.:::::::::::::
query 3' UUCUGAAGGGCAGCCUGGGUU 5'
query=miR1146, target=RO3G_09607, score=0.5, range=464-484, stran
target 5' AAGACUUUCCGUCGGACCCAA 3'
:::::::.:::::::::::::
query 3' UUCUGAAGGGCAGCCUGGGUU 5'
query=miR1146, target=RO3G_05622, score=0.5, range=464-484, stran
target 5' AAGACUUUCCGUCGGACCCAA 3'
:::::::.:::::::::::::
query 3' UUCUGAAGGGCAGCCUGGGUU 5'
query=miR1146, target=RO3G_13742, score=0.5, range=464-484, stran
target 5' AAGACUUUCCGUCGGACCCAA 3'
:::::::.:::::::::::::
query 3' UUCUGAAGGGCAGCCUGGGUU 5'
query=miR1146, target=RO3G_07704, score=0.5, range=464-484, stran
target 5' AAGACUUUCCGUCGGACCCAA 3'
:::::::.:::::::::::::
query 3' UUCUGAAGGGCAGCCUGGGUU 5'
query=miR1146, target=RO3G_13151, score=1, range=464-484, strand=
target 5' AAGACUUCCUGUCGGACCCAA 3'
:::::::::.:::::::::::
query 3' UUCUGAAGGGCAGCCUGGGUU 5'
query=miR1146, target=RO3G_11773, score=1, range=464-484, strand=
target 5' AAGAUUUUCCGUCGGACCCAA 3'
::::.::.:::::::::::::
query 3' UUCUGAAGGGCAGCCUGGGUU 5'
query=miR1146, target=RO3G_16551, score=1, range=464-484, strand=
target 5' AAGAUUUUCCGUCGGACCCAA 3'
::::.::.:::::::::::::
query 3' UUCUGAAGGGCAGCCUGGGUU 5'
query=miR1146, target=RO3G_16555, score=1, range=464-484, strand=

```
target 5' AAGAUUUUCCGUCGGACCCAA 3'
::::.::.:::::::::::::
query 3' UUCUGAAGGGCAGCCUGGGUU 5'
query=miR1146, target=RO3G_03615, score=1, range=464-484, strand=
target 5' AAGUCUUCCCGUCGGACCCAA 3'
::: :::::::::::::::::
query 3' UUCUGAAGGGCAGCCUGGGUU 5'
query=miR1146, target=RO3G_13726, score=1.5, range=464-484, stran
target 5' UAGACUUUCCGUCGGACCCAA 3'
::::::.:::::::::::::
query 3' UUCUGAAGGGCAGCCUGGGUU 5'
query=miR1146, target=RO3G_08661, score=2, range=464-484, strand=
target 5' AAGGCUUUUCGUCGGACCCAA 3'
:::.:::..::::::::::::
query 3' UUCUGAAGGGCAGCCUGGGUU 5'
query=miR1146, target=RO3G_05699, score=4, range=81-101, strand=1
target 5' AAGACUUGUUGUUGGACCCAA 3'
::::::: ..::.::::::::
query 3' UUCUGAAGGGCAGCCUGGGUU 5'
query=miR4414b, target=RO3G_17150, score=2, range=1385-1405, stra
target 5' ACUGGCUCUCGCAUAUUCACA 3'
:::::::::::::::::::
query 3' GAACCGAGAGCGUAUAAGUGU 5'
query=miR4414b, target=RO3G_11767, score=2, range=539-559, strand
target 5' ACUGGCUCUCGCAUAUUCACA 3'
:::::::::::::::::::
query 3' GAACCGAGAGCGUAUAAGUGU 5'
query=miR5219, target=RO3G_04102, score=0, range=292-313, strand=
target 5' ACGCAGCAGCUGAGAGUCCAGA 3'
::::::::::::::::::::::
query 3' UGCGUCGUCGACUCUCAGGUCU 5'
query=miR5219, target=RO3G_17126, score=0, range=320-341, strand=
target 5' ACGCAGCAGCUGAGAGUCCAGA 3'
::::::::::::::::::::::
query 3' UGCGUCGUCGACUCUCAGGUCU 5'
query=miR5219, target=RO3G_09090, score=0, range=290-311, strand=
target 5' ACGCAGCAGCUGAGAGUCCAGA 3'
::::::::::::::::::::::
query 3' UGCGUCGUCGACUCUCAGGUCU 5'
query=miR5219, target=RO3G_07568, score=0, range=193-214, strand=
target 5' ACGCAGCAGCUGAGAGUCCAGA 3'
::::::::::::::::::::::
query 3' UGCGUCGUCGACUCUCAGGUCU 5'
query=miR5219, target=RO3G_02387, score=0.5, range=235-256, stran
target 5' AUGCAGCAGCUGAGAGUCCAGA 3'
```

```
:.:::::::::::::::::::
query 3' UGCGUCGUCGACUCUCAGGUCU 5'
query=miR5219, target=RO3G_15231, score=1, range=533-554, strand=
target 5' ACACAGCAGCUGAGAGUCCAGA 3'
:: ::::::::::::::::::
query 3' UGCGUCGUCGACUCUCAGGUCU 5'
query=miR5219, target=RO3G_10585, score=1, range=134-155, strand=
target 5' ACACAGCAGCUGAGAGUCCAGA 3'
:: ::::::::::::::::::
query 3' UGCGUCGUCGACUCUCAGGUCU 5'
query=miR5219, target=RO3G_08723, score=1, range=197-218, strand=
target 5' ACGCAGCAGCUGAGAGUCCGGA 3'
::::::::::::::::::.::
query 3' UGCGUCGUCGACUCUCAGGUCU 5'
query=miR5219, target=RO3G_08544, score=1.5, range=139-160, stran
target 5' ACGUAUCAGCUGAGAGUCCAGA 3'
:::.: :::::::::::::::
query 3' UGCGUCGUCGACUCUCAGGUCU 5'
query=miR5219, target=RO3G_10900, score=2, range=89-110, strand=1
target 5' ACGCAGCAGCUGAGAAUCCAGA 3'
:::::::::::::: ::::::
query 3' UGCGUCGUCGACUCUCAGGUCU 5'
query=miR5219, target=RO3G_12762, score=2, range=290-311, strand=
target 5' ACGCAGCAGCUGAGAGUCGAGA 3'
::::::::::::::::: :::
query 3' UGCGUCGUCGACUCUCAGGUCU 5'
query=miR5219, target=RO3G_10892, score=2, range=131-152, strand=
target 5' ACGCAGCAGCUGAGAAUCCAGA 3'
:::::::::::::: ::::::
query 3' UGCGUCGUCGACUCUCAGGUCU 5'
query=miR5219, target=RO3G_09875, score=2, range=193-214, strand=
target 5' ACACAGCAGUUGAGAGUCCAGA 3'
:: :::::.::::::::::::
query 3' UGCGUCGUCGACUCUCAGGUCU 5'
query=miR5219, target=RO3G_03449, score=2, range=116-137, strand=
target 5' GCACAGCAGCUGAGAGUCCAGG 3'
.: :::::::::::::::::.
query 3' UGCGUCGUCGACUCUCAGGUCU 5'
query=miR5219, target=RO3G_06453, score=2.5, range=136-157, stran
target 5' ACAUAGCAGCUGAGGGUCCAGA 3'
:: .:::::::::.:::::::
query 3' UGCGUCGUCGACUCUCAGGUCU 5'
query=miR5219, target=RO3G_01907, score=3.5, range=103-124, stran
target 5' GCAUAGUAGCUGAGAGUUCAGA 3'
.: .::.:::::::::.::::
```

```
query 3' UGCGUCGUCGACUCUCAGGUCU 5'
query=miR8762d, target=RO3G_10771, score=3, range=484-505, strand
target 5' AAACAUCGAUCAAAGUUCACAA 3'
: :::::::::::::: ::::
query 3' UCUGUAGCUAGUUUCAAAUGUU 5'
query=miR8762d, target=RO3G_07739, score=3, range=484-505, strand
target 5' AAACAUCGAUCAAAGUUCACAA 3'
: :::::::::::::: ::::
query 3' UCUGUAGCUAGUUUCAAAUGUU 5'
query=miR8762d, target=RO3G_13852, score=3, range=484-505, strand
target 5' AAACAUCGAUCAAAGUUCACAA 3'
: :::::::::::::: ::::
query 3' UCUGUAGCUAGUUUCAAAUGUU 5'
query=miR8762d, target=RO3G_05953, score=4, range=733-754, strand
target 5' AAACACCGAUCAAAGUCUACAA 3'
: ::: ::::::::: :::::
query 3' UCUGUAGCUAGUUUCAAAUGUU 5'
query=miR8762d, target=RO3G_17449, score=4, range=733-754, strand
target 5' AAACACCGAUCAAAGUCUACAA 3'
: ::: ::::::::: :::::
query 3' UCUGUAGCUAGUUUCAAAUGUU 5'
query=miR8762d, target=RO3G_05921, score=4, range=733-754, strand
target 5' AAACACCGAUCAAAGUCUACAA 3'
: ::: ::::::::: :::::
query 3' UCUGUAGCUAGUUUCAAAUGUU 5'
query=miR8762d, target=RO3G_10729, score=4, range=733-754, strand
target 5' AAACACCGAUCAAAGUCUACAA 3'
: ::: ::::::::: :::::
query 3' UCUGUAGCUAGUUUCAAAUGUU 5'
query=miR8762d, target=RO3G_11966, score=4, range=733-754, strand
target 5' AAACACCGAUCAAAGUCUACAA 3'
: ::: ::::::::: :::::
query 3' UCUGUAGCUAGUUUCAAAUGUU 5'
query=miR8762d, target=RO3G_05939, score=4, range=733-754, strand
target 5' AAACACCGAUCAAAGUCUACAA 3'
: ::: ::::::::: :::::
query 3' UCUGUAGCUAGUUUCAAAUGUU 5'
query=miR1216, target=RO3G_14801, score=0, range=97-116, strand=1
target 5' GUACAAGCGCUAACCCAUCA 3'
::::::::::::::::::::
query 3' CAUGUUCGCGAUUGGGUAGU 5'
query=miR1216, target=RO3G_14788, score=0, range=97-116, strand=1
target 5' GUACAAGCGCUAACCCAUCA 3'
::::::::::::::::::::
query 3' CAUGUUCGCGAUUGGGUAGU 5'
```

```
query=miR1216, target=RO3G_17261, score=0, range=164-183, strand=
target 5' GUACAAGCGCUAACCCAUCA 3'
::::::::::::::::::::
query 3' CAUGUUCGCGAUUGGGUAGU 5'
query=miR1216, target=RO3G_09190, score=0, range=164-183, strand=
target 5' GUACAAGCGCUAACCCAUCA 3'
::::::::::::::::::::
query 3' CAUGUUCGCGAUUGGGUAGU 5'
query=miR1216, target=RO3G_09181, score=0, range=164-183, strand=
target 5' GUACAAGCGCUAACCCAUCA 3'
::::::::::::::::::::
query 3' CAUGUUCGCGAUUGGGUAGU 5'
query=miR1216, target=RO3G_16732, score=0, range=164-183, strand=
target 5' GUACAAGCGCUAACCCAUCA 3'
::::::::::::::::::::
query 3' CAUGUUCGCGAUUGGGUAGU 5'
query=miR1216, target=RO3G_14792, score=0, range=164-183, strand=
target 5' GUACAAGCGCUAACCCAUCA 3'
::::::::::::::::::::
query 3' CAUGUUCGCGAUUGGGUAGU 5'
query=miR1216, target=RO3G_14799, score=0, range=164-183, strand=
target 5' GUACAAGCGCUAACCCAUCA 3'
::::::::::::::::::::
query 3' CAUGUUCGCGAUUGGGUAGU 5'
query=miR1216, target=RO3G_16813, score=0, range=164-183, strand=
target 5' GUACAAGCGCUAACCCAUCA 3'
::::::::::::::::::::
query 3' CAUGUUCGCGAUUGGGUAGU 5'
query=miR1216, target=RO3G_09818, score=4, range=432-452, strand=
target 5' GUACGAGUGGCUAACCUAUCA 3'
::::.::. :::::::.::::
query 3' CAUGUUCG-CGAUUGGGUAGU 5'
query=miR1216, target=RO3G_13574, score=4, range=219-237, strand=
target 5' GUAAAAGCGC-AACUCAUCA 3'
::: :::::: :::.:::::
query 3' CAUGUUCGCGAUUGGGUAGU 5'
query=miR2612, target=RO3G_13221, score=0, range=767-787, strand=
target 5' CUGAUACUACUGCCACUAUCA 3'
:::::::::::::::::::::
query 3' GACUAUGAUGACGGUGAUAGU 5'
query=miR2612, target=RO3G_04123, score=1, range=767-787, strand=
target 5' CUGAUACCACUGCCACUAUCA 3'
::::::: :::::::::::::
query 3' GACUAUGAUGACGGUGAUAGU 5'
query=miR2612, target=RO3G_04120, score=1, range=767-787, strand=
```

```
target 5' CUGAUACCACUGCCACUAUCA 3'
::::::: :::::::::::::
query 3' GACUAUGAUGACGGUGAUAGU 5'
query=miR2612, target=RO3G_13224, score=1, range=767-787, strand=
target 5' CUGAUACCACUGCCACUAUCA 3'
::::::: :::::::::::::
query 3' GACUAUGAUGACGGUGAUAGU 5'
query=miR2612, target=RO3G_17093, score=1, range=527-547, strand=
target 5' CUGAUACCACUGCCACUAUCA 3'
::::::: :::::::::::::
query 3' GACUAUGAUGACGGUGAUAGU 5'
query=miR2612, target=RO3G_11597, score=2.5, range=895-916, stran
target 5' UUGAUCACUACUGCCAUUAUCA 3'
.:::: ::::::::::.:::::
query 3' GACUA-UGAUGACGGUGAUAGU 5'
query=miR2612, target=RO3G_01516, score=3, range=2025-2045, stran
target 5' UUGAUGCUGCUGCUACUAUCA 3'
.::::.::.::::.:::::::
query 3' GACUAUGAUGACGGUGAUAGU 5'
query=miR2612, target=RO3G_02911, score=3.5, range=113-133, stran
target 5' CUGAUGAUACUGCUGCUAUCA 3'
:::::. ::::::..::::::
query 3' GACUAUGAUGACGGUGAUAGU 5'
query=miR2612, target=RO3G_03005, score=3.5, range=107-127, stran
target 5' UUGUUAUUACUGCCACUAUUG 3'
.:: ::.::::::::::::..
query 3' GACUAUGAUGACGGUGAUAGU 5'
query=miR2612, target=RO3G_08542, score=3.5, range=237-256, stran
target 5' CUG-UGCUACUUCCACUAUCA 3'
::: :.::::: :::::::::
query 3' GACUAUGAUGACGGUGAUAGU 5'
query=miR2612, target=RO3G_02624, score=3.5, range=315-334, stran
target 5' UUGAUAC-ACUGCCGUUAUCA 3'
.:::::: ::::::..:::::
query 3' GACUAUGAUGACGGUGAUAGU 5'
query=miR2612, target=RO3G_08172, score=4, range=605-625, strand=
target 5' AUGAUACUAUUGCUGCUAUCA 3'
::::::::.:::..::::::
query 3' GACUAUGAUGACGGUGAUAGU 5'
query=miR2612, target=RO3G_02915, score=4, range=901-922, strand=
target 5' GUGAUAACUACUGCCAUUGUCA 3'
:::: ::::::::::.:.:::
query 3' GACUA-UGAUGACGGUGAUAGU 5'
query=miR2612, target=RO3G_01852, score=4, range=164-183, strand=
target 5' CUGCUACUACUGCUACU-UCA 3'
```

```
::: ::::::::.::: :::
query 3' GACUAUGAUGACGGUGAUAGU 5'
query=miR2612, target=RO3G_04617, score=4, range=675-694, strand=
target 5' CUUUUACUACUGCCAC-AUCA 3'
:: ::::::::::: ::::
query 3' GACUAUGAUGACGGUGAUAGU 5'
query=miR1148.2, target=RO3G_17296, score=2, range=120-140, stran
target 5' CCCGGCACCGGCGGAUCUCCA 3'
:::::::: :::::::::::
query 3' GGGCCGUGUCCGCCUAGAGGU 5'
query=miR6476a, target=RO3G_03666, score=4, range=110-128, strand
target 5' UCAU-UUCAUUACUUGCCGA 3'
:::: :::::.::.:.::::
query 3' AGUACAAGUAGUGGAUGGCU 5'
query=miR6476a, target=RO3G_04527, score=4, range=780-799, strand
target 5' UGAUGAUCAUCAUCUACUGA 3'
: ::: ::::::.::::.::
query 3' AGUACAAGUAGUGGAUGGCU 5'
query=miR6476a, target=RO3G_05448, score=4, range=1076-1094, stra
target 5' UCAUGGUCAUCACCU-CUGA 3'
::::: ::::::::: :.::
query 3' AGUACAAGUAGUGGAUGGCU 5'
query=miR6476a, target=RO3G_10979, score=4, range=962-980, strand
target 5' UCAAG-GCAUCACCUACUGA 3'
::: : ::::::::::.::
query 3' AGUACAAGUAGUGGAUGGCU 5'
query=miR6476a, target=RO3G_00932, score=4, range=124-142, strand
target 5' UCGUG-UCAUCAUCUACUGG 3'
::.:: :::::.::::.:.
query 3' AGUACAAGUAGUGGAUGGCU 5'
query=miR3949, target=RO3G_16083, score=4, range=660-682, strand=
target 5' UGACAUUUUUUGCUUGUACCUCA 3'
. :::.::::::::..:::::::
query 3' GAUGUGAAAAACGAGUAUGGAGU 5'
query=miR5021, target=RO3G_00301, score=0, range=2541-2560, stran
target 5' UUUUCCUUCUUCUAUCGUCA 3'
::::::::::::::::::::
query 3' AAAAGGAAGAAGAUAGCAGU 5'
query=miR5021, target=RO3G_04050, score=0, range=1257-1276, stran
target 5' UUUUCCUUCUUCUAUCGUCA 3'
::::::::::::::::::::
query 3' AAAAGGAAGAAGAUAGCAGU 5'
query=miR5021, target=RO3G_12949, score=0, range=702-721, strand=
target 5' UUUUCCUUCUUCUAUCGUCA 3'
::::::::::::::::::::
```

query 3' AAAAGGAAGAAGAUAGCAGU 5'
query=miR5021, target=RO3G_06744, score=3, range=972-992, strand=
target 5' UUUUCCUUCUUUUCAUCGUCA 3'
:::::::::::.: :::::::
query 3' AAAAGGAAGAAGA-UAGCAGU 5'
query=miR5021, target=RO3G_14233, score=3.5, range=1104-1124, str
target 5' UUUUCCUUCUUCUAUUGAUCG 3'
::::::::::::::.: ::.
query 3' AAAAGGAAGAAGAUAGC-AGU 5'
query=miR5021, target=RO3G_00801, score=3.5, range=857-876, stran
target 5' UUUUCUUUCUUCAGUCGUCA 3'
::::.:::::: .::::::
query 3' AAAAGGAAGAAGAUAGCAGU 5'
query=miR5021, target=RO3G_09998, score=3.5, range=132-151, stran
target 5' UUUUUCUUCUUCUGUCUUCA 3'
::::.::::::::.:: :::
query 3' AAAAGGAAGAAGAUAGCAGU 5'
query=miR5021, target=RO3G_15993, score=3.5, range=162-181, stran
target 5' UAUUCUUUCUUUUAUUGUCA 3'
: :::.::::.:::.::::
query 3' AAAAGGAAGAAGAUAGCAGU 5'
query=miR5021, target=RO3G_06767, score=3.5, range=2738-2757,
strand=-1
target 5' UUCUUCUUCUUCUAUUGUCU 3'
:: :.::::::::::.:::
query 3' AAAAGGAAGAAGAUAGCAGU 5'
query=miR5021, target=RO3G_16410, score=3.5, range=890-909, stran
target 5' UUCUUCUUCUUUUAUCGUCU 3'
:: :.::::::.:::::::
query 3' AAAAGGAAGAAGAUAGCAGU 5'
query=miR5021, target=RO3G_03413, score=3.5, range=590-609, stran
target 5' UCUUUCUUCUUCUAUCGUUU 3'
: ::.:::::::::::::.
query 3' AAAAGGAAGAAGAUAGCAGU 5'
query=miR5021, target=RO3G_04712, score=3.5, range=74-93, strand=
target 5' UAUAUCUUCUUCUGUCGUCA 3'
: : .::::::::.::::::
query 3' AAAAGGAAGAAGAUAGCAGU 5'
query=miR5021, target=RO3G_08238, score=3.5, range=992-1011, stra
target 5' UUGCUCUUCUUCUGUCGUCA 3'
:: .::::::::.::::::
query 3' AAAAGGAAGAAGAUAGCAGU 5'
query=miR5021, target=RO3G_14008, score=3.5, range=792-810, stran
target 5' CUUUUCUUCUUC-AUCGUCA 3'
:::.:::::::: :::::::

```
query 3' AAAAGGAAGAAGAUAGCAGU 5'
query=miR5021, target=RO3G_13112, score=3.5, range=1851-1869,
strand=-1
target 5' UUCUUCUUCUUCUA-CGUCA 3'
:: :.:::::::: :::::
query 3' AAAAGGAAGAAGAUAGCAGU 5'
query=miR5021, target=RO3G_10608, score=3.5, range=9639-9659,
strand=-1
target 5' AUUUCUUUCUUCUUAUCGUCA 3'
::::.:::::: ::::::::
query 3' AAAAGGAAGAAG-AUAGCAGU 5'
query=miR5021, target=RO3G_06189, score=3.5, range=87-105, strand
target 5' UUGUUCUUCUUC-AUCGUCA 3'
:: :.:::::: :::::::
query 3' AAAAGGAAGAAGAUAGCAGU 5'
query=miR5021, target=RO3G_10250, score=4, range=237-256, strand=
target 5' UUUUCCUUCUUCCAUAGUCA 3'
::::::::::: :: ::::
query 3' AAAAGGAAGAAGAUAGCAGU 5'
query=miR5021, target=RO3G_03019, score=4, range=209-228, strand=
target 5' AUUUUUUUCUUCUGUUGUCA 3'
:::..::::::.:.::::
query 3' AAAAGGAAGAAGAUAGCAGU 5'
query=miR5021, target=RO3G_12821, score=4, range=812-831, strand=
target 5' UCUUACUUUUUCUGUCGUCA 3'
: :: :::.:::.::::::
query 3' AAAAGGAAGAAGAUAGCAGU 5'
query=miR5021, target=RO3G_09002, score=4, range=881-900, strand=
target 5' AUUACCUUCUUCAAUCGUCA 3'
:: :::::::: :::::::
query 3' AAAAGGAAGAAGAUAGCAGU 5'
query=miR5021, target=RO3G_13930, score=4, range=338-356, strand=
target 5' UCUUCUUUCUU-UAUCGUCG 3'
: :::.::::: :::::::.
query 3' AAAAGGAAGAAGAUAGCAGU 5'
query=miR5021, target=RO3G_07645, score=4, range=494-513, strand=
target 5' CUUGACUUCUUCUAUCGUCU 3'
:: :::::::::::::
query 3' AAAAGGAAGAAGAUAGCAGU 5'
query=miR5021, target=RO3G_03189, score=4, range=365-385, strand=
target 5' AAUUCACAUCUUCUAUCGUCA 3'
::: : ::::::::::::
query 3' AAAAG-GAAGAAGAUAGCAGU 5'
query=miR5021, target=RO3G_09319, score=4, range=558-576, strand=
target 5' UUCUACUUCUUCUAU-GUCA 3'
```

```
:: : :::::::::: ::::
query 3' AAAAGGAAGAAGAUAGCAGU 5'
query=miR6025e, target=RO3G_16742, score=0, range=601-622, strand
target 5' GCAUGUCAGAUCUAUAAUGGCA 3'
::::::::::::::::::::::
query 3' CGUACAGUCUAGAUAUUACCGU 5'
query=miR6025e, target=RO3G_02824, score=0, range=601-622, strand
target 5' GCAUGUCAGAUCUAUAAUGGCA 3'
::::::::::::::::::::::
query 3' CGUACAGUCUAGAUAUUACCGU 5'
query=miR6025e, target=RO3G_17416, score=0, range=601-622, strand
target 5' GCAUGUCAGAUCUAUAAUGGCA 3'
::::::::::::::::::::::
query 3' CGUACAGUCUAGAUAUUACCGU 5'
query=miR6025e, target=RO3G_17291, score=0, range=601-622, strand
target 5' GCAUGUCAGAUCUAUAAUGGCA 3'
::::::::::::::::::::::
query 3' CGUACAGUCUAGAUAUUACCGU 5'
query=miR6025e, target=RO3G_13714, score=0, range=601-622, strand
target 5' GCAUGUCAGAUCUAUAAUGGCA 3'
::::::::::::::::::::::
query 3' CGUACAGUCUAGAUAUUACCGU 5'
query=miR6025e, target=RO3G_16372, score=0, range=601-622, strand
target 5' GCAUGUCAGAUCUAUAAUGGCA 3'
::::::::::::::::::::::
query 3' CGUACAGUCUAGAUAUUACCGU 5'
query=miR6025e, target=RO3G_17355, score=0, range=436-457, strand
target 5' GCAUGUCAGAUCUAUAAUGGCA 3'
::::::::::::::::::::::
query 3' CGUACAGUCUAGAUAUUACCGU 5'
query=miR6025e, target=RO3G_01962, score=0, range=352-373, strand
target 5' GCAUGUCAGAUCUAUAAUGGCA 3'
::::::::::::::::::::::
query 3' CGUACAGUCUAGAUAUUACCGU 5'
query=miR6025e, target=RO3G_16706, score=0, range=325-346, strand
target 5' GCAUGUCAGAUCUAUAAUGGCA 3'
::::::::::::::::::::::
query 3' CGUACAGUCUAGAUAUUACCGU 5'
query=miR6025e, target=RO3G_09193, score=0, range=325-346, strand
target 5' GCAUGUCAGAUCUAUAAUGGCA 3'
::::::::::::::::::::::
query 3' CGUACAGUCUAGAUAUUACCGU 5'
query=miR6025e, target=RO3G_17223, score=0, range=325-346, strand
target 5' GCAUGUCAGAUCUAUAAUGGCA 3'
::::::::::::::::::::::
```

```
query 3' CGUACAGUCUAGAUAUUACCGU 5'
query=miR6025e, target=RO3G_04682, score=0, range=601-622, strand
target 5' GCAUGUCAGAUCUAUAAUGGCA 3'
::::::::::::::::::::::
query 3' CGUACAGUCUAGAUAUUACCGU 5'
query=miR6025e, target=RO3G_05640, score=1, range=496-517, strand
target 5' GCAUGUCAGAUCUAUGAUGGCA 3'
:::::::::::::::.::::::
query 3' CGUACAGUCUAGAUAUUACCGU 5'
query=miR6025e, target=RO3G_07705, score=1, range=601-622, strand
target 5' GCAUGUCAGAUCUAUAAUGGUA 3'
::::::::::::::::::::.:
query 3' CGUACAGUCUAGAUAUUACCGU 5'
query=miR6025e, target=RO3G_17387, score=1, range=601-622, strand
target 5' GCAUGUCAGAUCUAUGAUGGCA 3'
:::::::::::::::.::::::
query 3' CGUACAGUCUAGAUAUUACCGU 5'
query=miR6025e, target=RO3G_16123, score=1, range=601-622, strand
target 5' GCAUGUCAGAUCUAUGAUGGCA 3'
:::::::::::::::.::::::
query 3' CGUACAGUCUAGAUAUUACCGU 5'
query=miR6025e, target=RO3G_06717, score=1, range=601-622, strand
target 5' GCAUGUCAGAUCUAUGAUGGCA 3'
:::::::::::::::.::::::
query 3' CGUACAGUCUAGAUAUUACCGU 5'
query=miR6025e, target=RO3G_07137, score=1, range=601-622, strand
target 5' GCAUGUCAGAUCUAUGAUGGCA 3'
:::::::::::::::.::::::
query 3' CGUACAGUCUAGAUAUUACCGU 5'
query=miR6025e, target=RO3G_01328, score=1, range=601-622, strand
target 5' GCAUGUCAGAUCUAUGAUGGCA 3'
:::::::::::::::.::::::
query 3' CGUACAGUCUAGAUAUUACCGU 5'
query=miR6025e, target=RO3G_05268, score=1, range=601-622, strand
target 5' GCAUGUCAGAUCUAUGAUGGCA 3'
:::::::::::::::.::::::
query 3' CGUACAGUCUAGAUAUUACCGU 5'
query=miR6025e, target=RO3G_04674, score=1, range=352-373, strand
target 5' GCAUGUCAGAUCUAUGAUGGCA 3'
:::::::::::::::.::::::
query 3' CGUACAGUCUAGAUAUUACCGU 5'
query=miR6025e, target=RO3G_14770, score=1, range=601-622, strand
target 5' GCAUGUCAGAUCUAUAAUGGUA 3'
::::::::::::::::::::.:
query 3' CGUACAGUCUAGAUAUUACCGU 5'
```

query=miR6025e, target=RO3G_11781, score=1, range=496-517, strand
target 5' GCAUGUCAGAUCUAUAAUGGUA 3'
::::::::::::::::::::.:
query 3' CGUACAGUCUAGAUAUUACCGU 5'
query=miR6025e, target=RO3G_07702, score=1, range=352-373, strand
target 5' GCAUGUCAGAUCUAUAAUGGUA 3'
::::::::::::::::::::.:
query 3' CGUACAGUCUAGAUAUUACCGU 5'
query=miR6025e, target=RO3G_16420, score=1, range=352-373, strand
target 5' GCAUGUCAGAUCUAUAAUGGUA 3'
::::::::::::::::::::.:
query 3' CGUACAGUCUAGAUAUUACCGU 5'
query=miR6025e, target=RO3G_15282, score=1, range=325-346, strand
target 5' GCAUGUCAGAUCUAUAAUGGUA 3'
::::::::::::::::::::.:
query 3' CGUACAGUCUAGAUAUUACCGU 5'
query=miR6025e, target=RO3G_15261, score=1, range=601-622, strand
target 5' GCAUGUCAGAUCUAUGAUGGCA 3'
::::::::::::::::.:::::
query 3' CGUACAGUCUAGAUAUUACCGU 5'
query=miR6025e, target=RO3G_10795, score=1, range=601-622, strand
target 5' GCAUGUCAGAUCUAUAAUGGUA 3'
::::::::::::::::::::.:
query 3' CGUACAGUCUAGAUAUUACCGU 5'
query=miR6025e, target=RO3G_17017, score=1, range=601-622, strand
target 5' GCAUGUCAGAUCUAUAAUGGUA 3'
::::::::::::::::::::.:
query 3' CGUACAGUCUAGAUAUUACCGU 5'
query=miR6025e, target=RO3G_04695, score=1, range=391-412, strand
target 5' GCAUGUCAGAUCUAUGAUGGCA 3'
::::::::::::::::.:::::
query 3' CGUACAGUCUAGAUAUUACCGU 5'
query=miR6025e, target=RO3G_07867, score=1, range=352-373, strand
target 5' GCAUGUCAGAUCUAUGAUGGCA 3'
::::::::::::::::.:::::
query 3' CGUACAGUCUAGAUAUUACCGU 5'
query=miR6025e, target=RO3G_11235, score=1, range=496-517, strand
target 5' GCAUGUCAGAUCUAUAAUGGUA 3'
::::::::::::::::::::.:
query 3' CGUACAGUCUAGAUAUUACCGU 5'
query=miR6025e, target=RO3G_06735, score=1, range=325-346, strand
target 5' GCAUGUCAGAUCUAUAAUGGUA 3'
::::::::::::::::::::.:
query 3' CGUACAGUCUAGAUAUUACCGU 5'
query=miR6025e, target=RO3G_14555, score=1, range=229-250, strand

```
target 5' GCAUGUCAGAUCUAUAAUGGUA 3'
::::::::::::::::::::.:
query 3' CGUACAGUCUAGAUAUUACCGU 5'
query=miR6025e, target=RO3G_06417, score=1, range=229-250, strand
target 5' GCAUGUCAGAUCUAUAAUGGUA 3'
::::::::::::::::::::.:
query 3' CGUACAGUCUAGAUAUUACCGU 5'
query=miR6025e, target=RO3G_14786, score=1, range=229-250, strand
target 5' GCAUGUCAGAUCUAUAAUGGUA 3'
::::::::::::::::::::.:
query 3' CGUACAGUCUAGAUAUUACCGU 5'
query=miR6025e, target=RO3G_15271, score=2, range=601-622, strand
target 5' GCAUGUCAGAUCUAUGAUGGUA 3'
:::::::::::::::.::::.:
query 3' CGUACAGUCUAGAUAUUACCGU 5'
query=miR6025e, target=RO3G_07739, score=2, range=352-373, strand
target 5' GCAUGUCAGAUCUAUGAUGGUA 3'
:::::::::::::::.::::.:
query 3' CGUACAGUCUAGAUAUUACCGU 5'
query=miR6025e, target=RO3G_16725, score=2, range=343-364, strand
target 5' GCAUGUCAGAUCUAUGAUGGUA 3'
:::::::::::::::.::::.:
query 3' CGUACAGUCUAGAUAUUACCGU 5'
query=miR6025e, target=RO3G_06433, score=2, range=325-346, strand
target 5' GCAUGUCAGAUUUAUAAUGGUA 3'
:::::::::::.::::::::.:
query 3' CGUACAGUCUAGAUAUUACCGU 5'
query=miR6025e, target=RO3G_10779, score=2, range=217-238, strand
target 5' GCAUGUCAGAUUUAUAAUGGUA 3'
:::::::::::.::::::::.:
query 3' CGUACAGUCUAGAUAUUACCGU 5'
query=miR6025e, target=RO3G_17277, score=2, range=85-106, strand=
target 5' GCAUGUCAGAUUUAUAAUGGUA 3'
:::::::::::.::::::::.:
query 3' CGUACAGUCUAGAUAUUACCGU 5'
query=miR6025e, target=RO3G_05632, score=2, range=286-307, strand
target 5' GCAUGUCAGAUCUAUAAUGGGA 3'
:::::::::::::::::::: :
query 3' CGUACAGUCUAGAUAUUACCGU 5'
query=miR6025e, target=RO3G_03591, score=2.5, range=217-238, stra
target 5' GCAUGUCGGAUUUAUAAUGGUA 3'
:::::::.:::.::::::::.:
query 3' CGUACAGUCUAGAUAUUACCGU 5'
query=miR6025e, target=RO3G_10771, score=3, range=352-373, strand
target 5' ACAUGUCAGAUCUAUGAUGGUA 3'
```

```
::::::::::::::.::::.:
query 3' CGUACAGUCUAGAUAUUACCGU 5'
query=miR6025e, target=RO3G_13852, score=3, range=352-373, strand
target 5' ACAUGUCAGAUCUAUGAUGGUA 3'
::::::::::::::.::::.:
query 3' CGUACAGUCUAGAUAUUACCGU 5'
query=miR6025e, target=RO3G_04207, score=3, range=601-622, strand
target 5' GCAUGUCAGAUAUAUGAUGGCA 3'
::::::::::: :::.::::::
query 3' CGUACAGUCUAGAUAUUACCGU 5'
query=miR6025e, target=RO3G_17449, score=3, range=601-622, strand
target 5' GCAUGUCAGAUAUAUGAUGGCA 3'
::::::::::: :::.::::::
query 3' CGUACAGUCUAGAUAUUACCGU 5'
query=miR6025e, target=RO3G_05921, score=3, range=601-622, strand
target 5' GCAUGUCAGAUAUAUGAUGGCA 3'
::::::::::: :::.::::::
query 3' CGUACAGUCUAGAUAUUACCGU 5'
query=miR6025e, target=RO3G_14361, score=3, range=601-622, strand
target 5' GCAUGUCAGAUAUAUGAUGGCA 3'
::::::::::: :::.::::::
query 3' CGUACAGUCUAGAUAUUACCGU 5'
query=miR6025e, target=RO3G_01942, score=3, range=598-619, strand
target 5' GCAUGUCAGAUAUAUGAUGGCA 3'
::::::::::: :::.::::::
query 3' CGUACAGUCUAGAUAUUACCGU 5'
query=miR6025e, target=RO3G_17399, score=3, range=601-622, strand
target 5' GCAUGUCAGAUCUAUGAUGACA 3'
:::::::::::::::.::: ::
query 3' CGUACAGUCUAGAUAUUACCGU 5'
query=miR6025e, target=RO3G_05939, score=3, range=601-622, strand
target 5' GCAUGUCAGAUGUAUGAUGGCA 3'
::::::::::: :::.::::::
query 3' CGUACAGUCUAGAUAUUACCGU 5'
query=miR6025e, target=RO3G_07124, score=3, range=601-622, strand
target 5' GCAUGUCAGAUGUAUGAUGGCA 3'
::::::::::: :::.::::::
query 3' CGUACAGUCUAGAUAUUACCGU 5'
query=miR6025e, target=RO3G_09945, score=3, range=325-346, strand
target 5' GCAUGUCAGAUAUAUGAUGGCA 3'
::::::::::: :::.::::::
query 3' CGUACAGUCUAGAUAUUACCGU 5'
query=miR6025e, target=RO3G_11264, score=3, range=601-622, strand
target 5' GCAUGUCAGAUAUAUGAUGGCA 3'
::::::::::: :::.::::::
```

```
query 3' CGUACAGUCUAGAUAUUACCGU 5'
query=miR6025e, target=RO3G_16689, score=3, range=601-622, strand
target 5' GCAUGUCAGAUAUAUGAUGGCA 3'
:::::::::: :::.::::::
query 3' CGUACAGUCUAGAUAUUACCGU 5'
query=miR6025e, target=RO3G_11962, score=3, range=325-346, strand
target 5' GCAUGUCAGAUCUCUAAUGGUA 3'
::::::::::::: ::::::.:
query 3' CGUACAGUCUAGAUAUUACCGU 5'
query=miR6025e, target=RO3G_09542, score=4, range=601-622, strand
target 5' GCAUGUCAGGUAUAUGAUGGCA 3'
:::::::::.: :::.::::::
query 3' CGUACAGUCUAGAUAUUACCGU 5'
query=miR6025e, target=RO3G_04210, score=4, range=601-622, strand
target 5' GCAUGUCAGGUGUAUGAUGGCA 3'
:::::::::.: :::.::::::
query 3' CGUACAGUCUAGAUAUUACCGU 5'
query=miR6025e, target=RO3G_10773, score=4, range=601-622, strand
target 5' GCAUGUCAGGUGUAUGAUGGCA 3'
:::::::::.: :::.::::::
query 3' CGUACAGUCUAGAUAUUACCGU 5'
query=miR6025e, target=RO3G_10780, score=4, range=601-622, strand
target 5' GCAUGUCAGGUGUAUGAUGGCA 3'
:::::::::.: :::.::::::
query 3' CGUACAGUCUAGAUAUUACCGU 5'
query=miR6025e, target=RO3G_14568, score=4, range=601-622, strand
target 5' GCAUGUCAGGUAUAUGAUGGCA 3'
:::::::::.: :::.::::::
query 3' CGUACAGUCUAGAUAUUACCGU 5'
query=miR6025e, target=RO3G_10729, score=4, range=601-622, strand
target 5' GCAUGUCAGGUAUAUGAUGGCA 3'
:::::::::.: :::.::::::
query 3' CGUACAGUCUAGAUAUUACCGU 5'
query=miR6025e, target=RO3G_12663, score=4, range=436-457, strand
target 5' GCAUGUCAGACUUAUGAUGGCA 3'
:::::::::: .:::.::::::
query 3' CGUACAGUCUAGAUAUUACCGU 5'
query=miR6025e, target=RO3G_10798, score=4, range=601-622, strand
target 5' GCAUGUCAGGUAUAUGAUGGCA 3'
:::::::::.: :::.::::::
query 3' CGUACAGUCUAGAUAUUACCGU 5'
query=miR6025e, target=RO3G_17214, score=4, range=601-622, strand
target 5' GCAUGUCAGGUGUAUGAUGGCA 3'
:::::::::.: :::.::::::
query 3' CGUACAGUCUAGAUAUUACCGU 5'
```

```
query=miR6025e, target=RO3G_04117, score=4, range=496-517, strand
target 5' GCAUGUCAGACCUAUGAUGGUA 3'
:::::::::: ::::.::::.:
query 3' CGUACAGUCUAGAUAUUACCGU 5'
query=miR6025e, target=RO3G_15866, score=4, range=325-346, strand
target 5' GCAUGUCAGGUGUAUGAUGGCA 3'
:::::::::.: :::.::::::
query 3' CGUACAGUCUAGAUAUUACCGU 5'
query=miR6025e, target=RO3G_05267, score=4, range=601-622, strand
target 5' GCCUGUCAGAUAUAUGAUGGCA 3'
:: :::::::: :::.::::::
query 3' CGUACAGUCUAGAUAUUACCGU 5'
query=miR6025e, target=RO3G_09967, score=4, range=601-622, strand
target 5' GCCUGUCAGAUAUAUGAUGGCA 3'
:: :::::::: :::.::::::
query 3' CGUACAGUCUAGAUAUUACCGU 5'
query=miR6025e, target=RO3G_07860, score=4, range=601-622, strand
target 5' GCCUGUCAGAUAUAUGAUGGCA 3'
:: :::::::: :::.::::::
query 3' CGUACAGUCUAGAUAUUACCGU 5'
query=miR6025e, target=RO3G_00256, score=4, range=601-622, strand
target 5' GCCUGUCAGAUAUAUGAUGGCA 3'
:: :::::::: :::.::::::
query 3' CGUACAGUCUAGAUAUUACCGU 5'
query=miR6025e, target=RO3G_05959, score=4, range=601-622, strand
target 5' GCCUGUCAGAUAUAUGAUGGCA 3'
:: :::::::: :::.::::::
query 3' CGUACAGUCUAGAUAUUACCGU 5'
query=miR6025e, target=RO3G_09194, score=4, range=601-622, strand
target 5' GCCUGUCAGAUAUAUGAUGGCA 3'
:: :::::::: :::.::::::
query 3' CGUACAGUCUAGAUAUUACCGU 5'
query=miR6025e, target=RO3G_11966, score=4, range=601-622, strand
target 5' GCCUGUCAGAUAUAUGAUGGCA 3'
:: :::::::: :::.::::::
query 3' CGUACAGUCUAGAUAUUACCGU 5'
query=miR6277, target=RO3G_05710, score=3, range=1338-1356, stran
target 5' CUCUGUUUC-UUCACACACA 3'
:::.::.:: ::::::::::
query 3' GAGGCAGAGAAAGUGUGUGU 5'
query=miR6277, target=RO3G_09135, score=4, range=2937-2956, stran
target 5' CAUUGGCUCUUUCACAUACA 3'
: ..: :::::::::::.:::
query 3' GAGGCAGAGAAAGUGUGUGU 5'
query=miR9553-3p, target=RO3G_02695, score=0, range=3665-3686,
```

strand=1
target 5' AUGUUCAUAGCUGGGAUGAUUA 3'
::::::::::::::::::::::
query 3' UACAAGUAUCGACCCUACUAAU 5'
query=miR9553-3p, target=RO3G_09175, score=0, range=3665-3686,
strand=1
target 5' AUGUUCAUAGCUGGGAUGAUUA 3'
::::::::::::::::::::::
query 3' UACAAGUAUCGACCCUACUAAU 5'
query=miR9553-3p, target=RO3G_03550, score=0, range=3665-3686,
strand=1
target 5' AUGUUCAUAGCUGGGAUGAUUA 3'
::::::::::::::::::::::
query 3' UACAAGUAUCGACCCUACUAAU 5'
query=miR9553-3p, target=RO3G_11541, score=0, range=1055-1076,
strand=1
target 5' AUGUUCAUAGCUGGGAUGAUUA 3'
::::::::::::::::::::::
query 3' UACAAGUAUCGACCCUACUAAU 5'
query=miR9553-3p, target=RO3G_00959, score=3.5, range=36-56, stra
target 5' GUGUUUGU-GUUGGGAUGAUUA 3'
.::::..: :.:::::::::::
query 3' UACAAGUAUCGACCCUACUAAU 5'
query=miR2868, target=RO3G_14217, score=0, range=599-618, strand=
target 5' UGCUACUACAACAAAAUCAA 3'
::::::::::::::::::::
query 3' ACGAUGAUGUUGUUUUAGUU 5'
query=miR2868, target=RO3G_13590, score=0, range=599-618, strand=
target 5' UGCUACUACAACAAAAUCAA 3'
::::::::::::::::::::
query 3' ACGAUGAUGUUGUUUUAGUU 5'
query=miR2868, target=RO3G_04048, score=0.5, range=1508-1527, str
target 5' UGCUGCUACAACAAAAUCAA 3'
::::.:::::::::::::::
query 3' ACGAUGAUGUUGUUUUAGUU 5'
query=miR2868, target=RO3G_03617, score=0.5, range=1568-1587, str
target 5' UGCUGCUACAACAAAAUCAA 3'
::::.:::::::::::::::
query 3' ACGAUGAUGUUGUUUUAGUU 5'
query=miR2868, target=RO3G_11515, score=0.5, range=332-351, stran
target 5' UGCUACUACAACAAAAUCAG 3'
:::::::::::::::::::.
query 3' ACGAUGAUGUUGUUUUAGUU 5'
query=miR2868, target=RO3G_03026, score=2.5, range=19-37, strand=
target 5' UCUUA-UACAACAAAAUCAA 3'

```
: .:: :::::::::::::
query 3' ACGAUGAUGUUGUUUUAGUU 5'
query=miR2868, target=RO3G_01909, score=3, range=795-814, strand=
target 5' AGCUGCUACAGCAAAAUCAG 3'
:::.::::.::::::::.
query 3' ACGAUGAUGUUGUUUUAGUU 5'
query=miR2868, target=RO3G_03130, score=3, range=909-928, strand=
target 5' UGCUUCUACAAUGAAAUCAA 3'
:::: ::::::..:::::::
query 3' ACGAUGAUGUUGUUUUAGUU 5'
query=miR2868, target=RO3G_11701, score=3, range=1224-1242, stran
target 5' UGCUACUGCAACAAAA-CAA 3'
:::::::.:::::::: :::
query 3' ACGAUGAUGUUGUUUUAGUU 5'
query=miR2868, target=RO3G_15707, score=3, range=2952-2970, stran
target 5' UGC-AGAACAACAAAAUCAA 3'
::: : ::::::::::::
query 3' ACGAUGAUGUUGUUUUAGUU 5'
query=miR2868, target=RO3G_10856, score=3.5, range=641-660, stran
target 5' UGCUAUUACAGCAAAAUGAA 3'
:::::.::::.:::::: ::
query 3' ACGAUGAUGUUGUUUUAGUU 5'
query=miR2868, target=RO3G_00028, score=3.5, range=771-791, stran
target 5' UGCUUGCUACAACAAAAUCCA 3'
::: :.::::::::::::: :
query 3' ACG-AUGAUGUUGUUUUAGUU 5'
query=miR2868, target=RO3G_01651, score=3.5, range=239-258, stran
target 5' AACUCUUACAACAAAAUCAA 3'
:: .::::::::::::::
query 3' ACGAUGAUGUUGUUUUAGUU 5'
query=miR2868, target=RO3G_16465, score=3.5, range=2071-2090, str
target 5' AGUCUCUACAACAAAAUCAA 3'
:. ::::::::::::::
query 3' ACGAUGAUGUUGUUUUAGUU 5'
query=miR2868, target=RO3G_08992, score=3.5, range=668-686, stran
target 5' UGC-AUUACAACAAAAACAA 3'
::: :.:::::::::: :::
query 3' ACGAUGAUGUUGUUUUAGUU 5'
query=miR2868, target=RO3G_08603, score=3.5, range=438-456, stran
target 5' UGC-AGUACAACGAAAUCAG 3'
::: : ::::::.::::::.
query 3' ACGAUGAUGUUGUUUUAGUU 5'
query=miR2868, target=RO3G_12444, score=3.5, range=498-518, stran
target 5' UGCAGCAGACAACAAAAUCAA 3'
::: .: ::::::::::::
```

```
query 3' ACGAUG-AUGUUGUUUUAGUU 5'
query=miR2868, target=RO3G_03905, score=3.5, range=1874-1892,
strand=-1
target 5' UG-UGCUACAACAAGAUCGA 3'
:: :.:::::::::.:::.:
query 3' ACGAUGAUGUUGUUUUAGUU 5'
query=miR2868, target=RO3G_11177, score=3.5, range=1073-1091,
strand=-1
target 5' UGC-AUUAUAAUAAAAUCAA 3'
::: :.::.::.::::::::
query 3' ACGAUGAUGUUGUUUUAGUU 5'
query=miR2868, target=RO3G_04878, score=3.5, range=1004-1023,
strand=-1
target 5' UUCUUUUACAGCAAAAUCAA 3'
: :: .::::.::::::::
query 3' ACGAUGAUGUUGUUUUAGUU 5'
query=miR2868, target=RO3G_07279, score=4, range=3150-3169, stran
target 5' UGCUACUAUAACAGAAACAA 3'
:::::::::.::::.:: :::
query 3' ACGAUGAUGUUGUUUUAGUU 5'
query=miR2868, target=RO3G_04479, score=4, range=530-549, strand=
target 5' UGUUAUUACACCAGAAUCAA 3'
::.::.:::: ::.::::::
query 3' ACGAUGAUGUUGUUUUAGUU 5'
query=miR2868, target=RO3G_00451, score=4, range=279-298, strand=
target 5' AGCUACAACAAUAAGAUCAA 3'
::::: ::::.::.:::::
query 3' ACGAUGAUGUUGUUUUAGUU 5'
query=miR2868, target=RO3G_11324, score=4, range=132-151, strand=
target 5' UGCUGUUACAACAAAACCAC 3'
::::..:::::::::: ::
query 3' ACGAUGAUGUUGUUUUAGUU 5'
query=miR2868, target=RO3G_04175, score=4, range=3378-3397, stran
target 5' UGUUGCCACAACAGAAUCAU 3'
::.:.: ::::::.:::::
query 3' ACGAUGAUGUUGUUUUAGUU 5'
query=miR2868, target=RO3G_01836, score=4, range=803-822, strand=
target 5' UGUUGCAACAACAAGAUCAU 3'
::.:.: ::::::::.::::
query 3' ACGAUGAUGUUGUUUUAGUU 5'
query=miR2868, target=RO3G_08583, score=4, range=1788-1806, stran
target 5' UGUUAUUACAG-AAAAUCAA 3'
::.::.::::. ::::::::
query 3' ACGAUGAUGUUGUUUUAGUU 5'
query=miR2868, target=RO3G_00460, score=4, range=291-310, strand=
```

```
target 5' UCCUACUAAAACAAAGUCAA 3'
: :::::: ::::::.::::
query 3' ACGAUGAUGUUGUUUUAGUU 5'
query=miR2868, target=RO3G_06532, score=4, range=650-669, strand=
target 5' AGCAACUACAGCAAAAUCAU 3'
:: ::::::.::::::::
query 3' ACGAUGAUGUUGUUUUAGUU 5'
query=miR2868, target=RO3G_09230, score=4, range=195-214, strand=
target 5' UACUACUACAACAAAAGCAC 3'
: :::::::::::::: ::
query 3' ACGAUGAUGUUGUUUUAGUU 5'
query=miR2868, target=RO3G_00896, score=4, range=359-378, strand=
target 5' AGAUAAUGCAACAAAAUCAA 3'
: :: :.::::::::::::
query 3' ACGAUGAUGUUGUUUUAGUU 5'
query=miR2868, target=RO3G_13441, score=4, range=375-394, strand=
target 5' AGCUUUAACAACAAAAUCAG 3'
::: . ::::::::::::.
query 3' ACGAUGAUGUUGUUUUAGUU 5'
query=miR2868, target=RO3G_14147, score=4, range=1136-1154, stran
target 5' UG-UACAAUAAUAAAAUCAA 3'
:: ::: :.::.::::::::
query 3' ACGAUGAUGUUGUUUUAGUU 5'
query=miR2868, target=RO3G_13385, score=4, range=92-111, strand=1
target 5' AUCAACAACAACAAAAUCAA 3'
: :: :::::::::::::
query 3' ACGAUGAUGUUGUUUUAGUU 5'
query=miR2868, target=RO3G_04779, score=4, range=461-479, strand=
target 5' CGC-AAUACAACAAAGUCAA 3'
:: : :::::::::.::::
query 3' ACGAUGAUGUUGUUUUAGUU 5'
query=miR2868, target=RO3G_08932, score=4, range=432-450, strand=
target 5' CGUU-CAACAACAAAAUCAG 3'
:.: : ::::::::::::.
query 3' ACGAUGAUGUUGUUUUAGUU 5'
query=miR2868, target=RO3G_03029, score=4, range=1583-1602, stran
target 5' UUCAACAACAACAAAAUCAC 3'
: : :: ::::::::::::
query 3' ACGAUGAUGUUGUUUUAGUU 5'
query=miR2868, target=RO3G_03922, score=4, range=3036-3054, stran
target 5' UGC-AGAGCAACAAAAUCAA 3'
::: : .::::::::::::
query 3' ACGAUGAUGUUGUUUUAGUU 5'
query=miR2868, target=RO3G_15451, score=4, range=1122-1140, stran
target 5' UACUAC-ACAACGAAAUCAC 3'
```

```
: :::: :::::.::::::
query 3' ACGAUGAUGUUGUUUUAGUU 5'
query=miR2868, target=RO3G_02573, score=4, range=295-315, strand=
target 5' GGUUAUUAUCAACAAAAUCAA 3'
:.::.:: ::::::::::::
query 3' ACGAUGAU-GUUGUUUUAGUU 5'
query=miR2868, target=RO3G_03208, score=4, range=18-36, strand=1
target 5' UGCAAC-ACAACAAAAUUAU 3'
::: :: ::::::::::.:
query 3' ACGAUGAUGUUGUUUUAGUU 5'
query=miR2868, target=RO3G_07160, score=4, range=996-1014, strand
target 5' UGCUGUUA-GACAAAAUCAA 3'
::::..:: .::::::::::
query 3' ACGAUGAUGUUGUUUUAGUU 5'
query=miR2868, target=RO3G_08051, score=4, range=599-618, strand=
target 5' UGUUUUUACAACAAAAUAAA 3'
::.: .:::::::::: ::
query 3' ACGAUGAUGUUGUUUUAGUU 5'
query=miR2868, target=RO3G_17116, score=4, range=230-249, strand=
target 5' AACUGGUACAACAAAAUCAG 3'
::. :::::::::::::.
query 3' ACGAUGAUGUUGUUUUAGUU 5'
query=miR2868, target=RO3G_16533, score=4, range=284-304, strand=
target 5' GGUUGCAAACAACAAAAUCAA 3'
:.:.: ::::::::::::
query 3' ACGAUG-AUGUUGUUUUAGUU 5'
query=miR2868, target=RO3G_03727, score=4, range=1259-1278, stran
target 5' UGCCUUGACAACAAAAUCAG 3'
::: . ::::::::::::.
query 3' ACGAUGAUGUUGUUUUAGUU 5'
query=miR5639-5p, target=RO3G_16544, score=0, range=563-582, stra
target 5' GCCUUAGACACGUGGAACAA 3'
::::::::::::::::::::
query 3' CGGAAUCUGUGCACCUUGUU 5'
query=miR5639-5p, target=RO3G_15468, score=4, range=1555-1574,
strand=1
target 5' GCCUUGGGCACAUGGAACAG 3'
:::::.:.::: :::::::.
query 3' CGGAAUCUGUGCACCUUGUU 5'
query=miR5639-5p, target=RO3G_02557, score=4, range=368-388, stra
target 5' UCCUUAAAACAUGUGGAACAA 3'
:::: : :::.:::::::::
query 3' CGGAA-UCUGUGCACCUUGUU 5'
query=miR2651, target=RO3G_16804, score=0, range=159-178, strand=
target 5' AAUCAGCAUAUCAAUCAACA 3'
```

```
::::::::::::::::::::
query 3' UUAGUCGUAUAGUUAGUUGU 5'
query=miR2651, target=RO3G_13293, score=0, range=159-178, strand=
target 5' AAUCAGCAUAUCAAUCAACA 3'
::::::::::::::::::::
query 3' UUAGUCGUAUAGUUAGUUGU 5'
query=miR2651, target=RO3G_03867, score=1, range=159-178, strand=
target 5' AAUCAGCAUGUCAAUCAACA 3'
:::::::::.::::::::::
query 3' UUAGUCGUAUAGUUAGUUGU 5'
query=miR2651, target=RO3G_01004, score=1, range=159-178, strand=
target 5' AAUCAGCAUGUCAAUCAACA 3'
:::::::::.::::::::::
query 3' UUAGUCGUAUAGUUAGUUGU 5'
query=miR2651, target=RO3G_15131, score=1, range=159-178, strand=
target 5' AAUCAGCAUGUCAAUCAACA 3'
:::::::::.::::::::::
query 3' UUAGUCGUAUAGUUAGUUGU 5'
query=miR2651, target=RO3G_01475, score=1, range=159-178, strand=
target 5' AAUCAGCAUGUCAAUCAACA 3'
:::::::::.::::::::::
query 3' UUAGUCGUAUAGUUAGUUGU 5'
query=miR2651, target=RO3G_13439, score=1, range=159-178, strand=
target 5' AAUCAGCAUGUCAAUCAACA 3'
:::::::::.::::::::::
query 3' UUAGUCGUAUAGUUAGUUGU 5'
query=miR2651, target=RO3G_15002, score=1, range=159-178, strand=
target 5' AAUCAGCAUGUCAAUCAACA 3'
:::::::::.::::::::::
query 3' UUAGUCGUAUAGUUAGUUGU 5'
query=miR2651, target=RO3G_09044, score=1, range=21-40, strand=1
target 5' AAUCAGCAUGUCAAUCAACA 3'
:::::::::.::::::::::
query 3' UUAGUCGUAUAGUUAGUUGU 5'
query=miR2651, target=RO3G_12536, score=1, range=159-178, strand=
target 5' AAUCAGCAUGUCAAUCAACA 3'
:::::::::.::::::::::
query 3' UUAGUCGUAUAGUUAGUUGU 5'
query=miR2651, target=RO3G_10715, score=1, range=159-178, strand=
target 5' AAUCAGCAUGUCAAUCAACA 3'
:::::::::.::::::::::
query 3' UUAGUCGUAUAGUUAGUUGU 5'
query=miR2651, target=RO3G_04309, score=1, range=159-178, strand=
target 5' AAUCAGCAUGUCAAUCAACA 3'
:::::::::.::::::::::
```

```
query 3' UUAGUCGUAUAGUUAGUUGU 5'
query=miR2651, target=RO3G_09718, score=1, range=159-178, strand=
target 5' AAUCAGCAUGUCAAUCAACA 3'
:::::::::.::::::::::
query 3' UUAGUCGUAUAGUUAGUUGU 5'
query=miR2651, target=RO3G_12007, score=1, range=159-178, strand=
target 5' AAUCAGCAUGUCAAUCAACA 3'
:::::::::.::::::::::
query 3' UUAGUCGUAUAGUUAGUUGU 5'
query=miR2651, target=RO3G_10177, score=1, range=159-178, strand=
target 5' AAUCAGCAUGUCAAUCAACA 3'
:::::::::.::::::::::
query 3' UUAGUCGUAUAGUUAGUUGU 5'
query=miR2651, target=RO3G_03524, score=2.5, range=1112-1131,
strand=-1
target 5' GAUCAGCGUGUCAAUCAACA 3'
.::::::.:.::::::::::
query 3' UUAGUCGUAUAGUUAGUUGU 5'
query=miR2651, target=RO3G_11598, score=3, range=1072-1091, stran
target 5' CAUCAGCAUUUCAAUCAACA 3'
:::::::: ::::::::::
query 3' UUAGUCGUAUAGUUAGUUGU 5'
query=miR2651, target=RO3G_13050, score=3, range=2507-2526, stran
target 5' ACUCAGCAUCUCAAUCAACA 3'
: ::::::: ::::::::::
query 3' UUAGUCGUAUAGUUAGUUGU 5'
query=miR2651, target=RO3G_13112, score=4, range=1618-1637, stran
target 5' CGUCAGCGUAUCAAUCAGCG 3'
.:::::.:::::::::.:.
query 3' UUAGUCGUAUAGUUAGUUGU 5'
query=miR2651, target=RO3G_12637, score=4, range=499-518, strand=
target 5' AAUUGGCGUAACAAUCAACA 3'
:::..::.:: :::::::::
query 3' UUAGUCGUAUAGUUAGUUGU 5'
query=miR2651, target=RO3G_15178, score=4, range=1729-1748, stran
target 5' CGUCAACGUAUCAAUCAACG 3'
.::: :.:::::::::::.
query 3' UUAGUCGUAUAGUUAGUUGU 5'
query=miR2651, target=RO3G_07853, score=4, range=959-978, strand=
target 5' AAUCAUCAUAUAAAUCAAUA 3'
::::: ::::: ::::::.:
query 3' UUAGUCGUAUAGUUAGUUGU 5'
query=miR2651, target=RO3G_17129, score=4, range=2074-2093, stran
target 5' AAGCAGAAUGUCGAUCAACA 3'
:: ::: ::.::.:::::::
```

```
query 3' UUAGUCGUAUAGUUAGUUGU 5'
query=miR2651, target=RO3G_08603, score=4, range=284-302, strand=
target 5' AAUUGG-AUAUCAAUCAAUC 3'
:::..: :::::::::::.
query 3' UUAGUCGUAUAGUUAGUUGU 5'
query=miR2651, target=RO3G_10000, score=4, range=47-65, strand=1
target 5' GAUCA-UAUAUCAAUCGACU 3'
.:::: .:::::::::.::
query 3' UUAGUCGUAUAGUUAGUUGU 5'
query=miR2651, target=RO3G_12971, score=4, range=1111-1130, stran
target 5' AACCAACAUAACAAUCAACA 3'
:: :: :::: :::::::::
query 3' UUAGUCGUAUAGUUAGUUGU 5'
query=miR2651, target=RO3G_03799, score=4, range=1069-1088, stran
target 5' AACCAACAUAACAAUCAACA 3'
:: :: :::: :::::::::
query 3' UUAGUCGUAUAGUUAGUUGU 5'
query=miR2651, target=RO3G_09489, score=4, range=1069-1088, stran
target 5' AACCAACAUAACAAUCAACA 3'
:: :: :::: :::::::::
query 3' UUAGUCGUAUAGUUAGUUGU 5'
query=miR2651, target=RO3G_10225, score=4, range=1069-1088, stran
target 5' AACCAACAUAACAAUCAACA 3'
:: :: :::: :::::::::
query 3' UUAGUCGUAUAGUUAGUUGU 5'
query=miR2651, target=RO3G_02696, score=4, range=553-572, strand=
target 5' AACCAACAUAACAAUCAACA 3'
:: :: :::: :::::::::
query 3' UUAGUCGUAUAGUUAGUUGU 5'
query=miR2651, target=RO3G_03095, score=4, range=247-267, strand=
target 5' AAUCAACCAUAUCAAACAACA 3'
:::: : :::::::: :::::
query 3' UUAG-UCGUAUAGUUAGUUGU 5'
query=miR2651, target=RO3G_07453, score=4, range=551-570, strand=
target 5' AAAAAGAAUAUCAAUCGACA 3'
:: :: :::::::::.:::
query 3' UUAGUCGUAUAGUUAGUUGU 5'
query=miR2651, target=RO3G_16091, score=4, range=629-649, strand=
target 5' UAUUAGUGAUGUCAAUCAACA 3'
::.::. ::.::::::::::
query 3' UUAGUCG-UAUAGUUAGUUGU 5'
query=miR2651, target=RO3G_00059, score=4, range=630-649, strand=
target 5' UUUCAGCAUAUAAAUCAACA 3'
::::::::: ::::::::
query 3' UUAGUCGUAUAGUUAGUUGU 5'
```

query=miR2651, target=RO3G_09248, score=4, range=497-516, strand=
target 5' AAUACGCAUAUUAAUCAAUA 3'
::: :::::.::::::.:
query 3' UUAGUCGUAUAGUUAGUUGU 5'
query=miR2651, target=RO3G_16462, score=4, range=816-834, strand=
target 5' AAU-AGCAUAUCCAUUAACA 3'
::: :::::::: ::.::::
query 3' UUAGUCGUAUAGUUAGUUGU 5'
query=miR2651, target=RO3G_00502, score=4, range=50-68, strand=-1
target 5' GGUC-GCAUAUCAAUCAUCA 3'
..:: ::::::::::: ::
query 3' UUAGUCGUAUAGUUAGUUGU 5'
query=miR2651, target=RO3G_04482, score=4, range=525-543, strand=
target 5' AUUCAGCAUAUC-AUCAACC 3'
: :::::::::: ::::::
query 3' UUAGUCGUAUAGUUAGUUGU 5'
query=miR5562-3p, target=RO3G_19004, score=0, range=1117-1136, strand=-1
target 5' GUUUCAGCCUUGCGUCCCAU 3'
::::::::::::::::::::
query 3' CAAAGUCGGAACGCAGGGUA 5'
query=miR5562-3p, target=RO3G_19001, score=0, range=821-840, stra
target 5' GUUUCAGCCUUGCGUCCCAU 3'
::::::::::::::::::::
query 3' CAAAGUCGGAACGCAGGGUA 5'
query=miR8011a-5p, target=RO3G_06442, score=0, range=5896-5918, strand=1
target 5' GAAAAAAAGAAAGCUCAACCCAA 3'
:::::::::::::::::::::::
query 3' CUUUUUUUCUUUCGAGUUGGGUU 5'
query=miR8011a-5p, target=RO3G_02691, score=0, range=8728-8750, strand=1
target 5' GAAAAAAAGAAAGCUCAACCCAA 3'
:::::::::::::::::::::::
query 3' CUUUUUUUCUUUCGAGUUGGGUU 5'
query=miR8011a-5p, target=RO3G_09965, score=0, range=5239-5261, strand=1
target 5' GAAAAAAAGAAAGCUCAACCCAA 3'
:::::::::::::::::::::::
query 3' CUUUUUUUCUUUCGAGUUGGGUU 5'
query=miR8011a-5p, target=RO3G_13920, score=0, range=5239-5261, strand=1
target 5' GAAAAAAAGAAAGCUCAACCCAA 3'
:::::::::::::::::::::::
query 3' CUUUUUUUCUUUCGAGUUGGGUU 5'

query=miR8011a-5p, target=RO3G_03547, score=0, range=5239-5261, strand=1
target 5' GAAAAAAAGAAAGCUCAACCCAA 3'
:::::::::::::::::::::::
query 3' CUUUUUUUCUUUCGAGUUGGGUU 5'
query=miR8011a-5p, target=RO3G_02959, score=0, range=5079-5101, strand=1
target 5' GAAAAAAAGAAAGCUCAACCCAA 3'
:::::::::::::::::::::::
query 3' CUUUUUUUCUUUCGAGUUGGGUU 5'
query=miR8011a-5p, target=RO3G_06427, score=0, range=4831-4853, strand=1
target 5' GAAAAAAAGAAAGCUCAACCCAA 3'
:::::::::::::::::::::::
query 3' CUUUUUUUCUUUCGAGUUGGGUU 5'
query=miR8011a-5p, target=RO3G_06428, score=0, range=4333-4355, strand=1
target 5' GAAAAAAAGAAAGCUCAACCCAA 3'
:::::::::::::::::::::::
query 3' CUUUUUUUCUUUCGAGUUGGGUU 5'
query=miR8011a-5p, target=RO3G_05619, score=0, range=3382-3404, strand=1
target 5' GAAAAAAAGAAAGCUCAACCCAA 3'
:::::::::::::::::::::::
query 3' CUUUUUUUCUUUCGAGUUGGGUU 5'
query=miR8011a-5p, target=RO3G_17404, score=1, range=1716-1738, strand=1
target 5' AAAAAAAAGAAAGCUCAACCCAA 3'
::::::::::::::::::::::
query 3' CUUUUUUUCUUUCGAGUUGGGUU 5'
query=miR8011a-5p, target=RO3G_04115, score=2, range=2031-2053, strand=1
target 5' AAAAAAAAGAAAGCUCAAUCCAA 3'
:::::::::::::::::.::::
query 3' CUUUUUUUCUUUCGAGUUGGGUU 5'
query=miR8011a-5p, target=RO3G_08253, score=3, range=345-367, str
target 5' UAAAAAAAGAAAGCUCAAUCCAC 3'
:::::::::::::::::.:::
query 3' CUUUUUUUCUUUCGAGUUGGGUU 5'
query=miR8011a-5p, target=RO3G_16065, score=4, range=407-429, strand=-1
target 5' GGAAAUAAGAAAACUCAACCCAG 3'
:.::: :::::: :::::::::.
query 3' CUUUUUUUCUUUCGAGUUGGGUU 5'
query=miR1152, target=RO3G_13297, score=0, range=719-738, strand=

```
target 5' UUCAAGACGCGCUCCUUUUA 3'
::::::::::::::::::::
query 3' AAGUUCUGCGCGAGGAAAAU 5'
query=miR1152, target=RO3G_09480, score=0, range=4958-4977, stran
target 5' UUCAAGACGCGCUCCUUUUA 3'
::::::::::::::::::::
query 3' AAGUUCUGCGCGAGGAAAAU 5'
query=miR1152, target=RO3G_10676, score=0, range=4100-4119, stran
target 5' UUCAAGACGCGCUCCUUUUA 3'
::::::::::::::::::::
query 3' AAGUUCUGCGCGAGGAAAAU 5'
query=miR1152, target=RO3G_11284, score=3.5, range=2536-2554, str
target 5' UUCAA-ACGAGCUCCUUUUG 3'
::::: ::: :::::::::.
query 3' AAGUUCUGCGCGAGGAAAAU 5'
query=miR1152, target=RO3G_13853, score=3.5, range=1582-1600, str
target 5' UUCAA-ACGAGCUCCUUUUG 3'
::::: ::: :::::::::.
query 3' AAGUUCUGCGCGAGGAAAAU 5'
query=miR1152, target=RO3G_13301, score=3.5, range=637-655, stran
target 5' UUCAA-ACGAGCUCCUUUUG 3'
::::: ::: :::::::::.
query 3' AAGUUCUGCGCGAGGAAAAU 5'
query=miR1152, target=RO3G_00705, score=3.5, range=2536-2554, str
target 5' UUCAA-ACGAGCUCCUUUUG 3'
::::: ::: :::::::::.
query 3' AAGUUCUGCGCGAGGAAAAU 5'
query=miR1152, target=RO3G_11478, score=3.5, range=121-139, stran
target 5' UUCAA-ACGAGCUCCUUUUG 3'
::::: ::: :::::::::.
query 3' AAGUUCUGCGCGAGGAAAAU 5'
query=miR1152, target=RO3G_10802, score=4, range=1654-1672, stran
target 5' UUUAA-ACGAGCUCCUUUUG 3'
::.:: ::: :::::::::.
query 3' AAGUUCUGCGCGAGGAAAAU 5'
query=miR1152, target=RO3G_01906, score=4, range=1174-1193, stran
target 5' CUUAAGAUGUGCUCCUUUUG 3'
:.::::.:.:::::::::.
query 3' AAGUUCUGCGCGAGGAAAAU 5'
query=miR1152, target=RO3G_10958, score=4, range=796-815, strand=
target 5' CUCAGGAUGCGUUCCUUUUG 3'
:::.::.:::.:::::::.
query 3' AAGUUCUGCGCGAGGAAAAU 5'
query=miR1152, target=RO3G_05195, score=4, range=95-114, strand=-
target 5' UUCUUGACGCGCUUUUUUUA 3'
```

```
::: :::::::..:::::
query 3' AAGUUCUGCGCGAGGAAAAU 5'
query=miR854, target=RO3G_06418, score=0, range=381-401, strand=-
target 5' CCCUUCUCAAUAUCCUCAUCA 3'
:::::::::::::::::::::
query 3' GGGAAGAGUUAUAGGAGUAGU 5'
query=miR854, target=RO3G_11879, score=2.5, range=2138-2158, stra
target 5' CACUUCUUAAUAUCCUUAUCA 3'
: ::::.::::::::.::::
query 3' GGGAAGAGUUAUAGGAGUAGU 5'
query=miR854, target=RO3G_10853, score=3, range=141-161, strand=-
target 5' CUCUUUUGAAUAUCUUCAUCA 3'
:.:::.: :::::.:::::
query 3' GGGAAGAGUUAUAGGAGUAGU 5'
query=miR854, target=RO3G_09011, score=3.5, range=3740-3760, stra
target 5' AUCUCGUCAAUAUCCUCAUCA 3'
.:: ::::::::::::::
query 3' GGGAAGAGUUAUAGGAGUAGU 5'
query=miR854, target=RO3G_12902, score=3.5, range=189-209, strand
target 5' CUCUUUUCACUAUCCUCAUCG 3'
:.:::.::: ::::::::::.
query 3' GGGAAGAGUUAUAGGAGUAGU 5'
query=miR854, target=RO3G_06151, score=3.5, range=1314-1335, stra
target 5' CUCUUCUUCCAUAUCCUCAUCA 3'
:.:::: :: :::::::::::
query 3' GGGAAG-AGUUAUAGGAGUAGU 5'
query=miR854, target=RO3G_04712, score=3.5, range=2535-2555, stra
target 5' UACUCUUCAAUAUCCUCAUCG 3'
. :: .::::::::::::::.
query 3' GGGAAGAGUUAUAGGAGUAGU 5'
query=miR854, target=RO3G_05685, score=3.5, range=678-698, strand
target 5' UCCUCCUCAAUAUCCUCCUCA 3'
.::: ::::::::::: :::
query 3' GGGAAGAGUUAUAGGAGUAGU 5'
query=miR854, target=RO3G_06208, score=3.5, range=213-232, strand
target 5' UCUUUUUCAAU-UCCUCAUCA 3'
.:.::.::::: :::::::::
query 3' GGGAAGAGUUAUAGGAGUAGU 5'
query=miR854, target=RO3G_10188, score=3.5, range=174-195, strand
target 5' CCCUUCUUUAUAUCCUUCAUCA 3'
:::::::. :::::: ::::::
query 3' GGGAAGAGUUAUAGG-AGUAGU 5'
query=miR854, target=RO3G_15519, score=3.5, range=453-473, strand
target 5' UCUUUAAUAAUAUCCUCAUCA 3'
.:.:: .::::::::::::
```

```
query 3' GGGAAGAGUUAUAGGAGUAGU 5'
query=miR854, target=RO3G_13317, score=4, range=466-486, strand=1
target 5' ACCUCCUCAAUAUCUUCAUCC 3'
::: :::::::::.:::::
query 3' GGGAAGAGUUAUAGGAGUAGU 5'
query=miR854, target=RO3G_09338, score=4, range=705-726, strand=1
target 5' AUCAUCAUUAAUAUCCUCAUCA 3'
.: :: :.:::::::::::::
query 3' GGGAAG-AGUUAUAGGAGUAGU 5'
query=miR854, target=RO3G_09517, score=4, range=81-101, strand=-1
target 5' UAUUUAUCAAUAUCUUCAUCA 3'
. .:: ::::::::.::::::
query 3' GGGAAGAGUUAUAGGAGUAGU 5'
query=miR854, target=RO3G_09393, score=4, range=762-783, strand=-
target 5' CUCUUCUUCAUCAUCCUCAUCA 3'
:.:::::. :: ::::::::::
query 3' GGGAAGAGUUA-UAGGAGUAGU 5'
query=miR854, target=RO3G_15786, score=4, range=36-55, strand=-1
target 5' GCCUUCUCAAUA-CCUCAUUA 3'
::::::::::: ::::::.:
query 3' GGGAAGAGUUAUAGGAGUAGU 5'
query=miR3449-5p, target=RO3G_10769, score=0, range=242-260, stra
target 5' ACAGACGAAAGCGAUUGGA 3'
:::::::::::::::::::
query 3' UGUCUGCUUUCGCUAACCU 5'
query=miR3449-5p, target=RO3G_02845, score=0, range=590-608, stra
target 5' ACAGACGAAAGCGAUUGGA 3'
:::::::::::::::::::
query 3' UGUCUGCUUUCGCUAACCU 5'
query=miR3449-5p, target=RO3G_16727, score=0, range=365-383, stra
target 5' ACAGACGAAAGCGAUUGGA 3'
:::::::::::::::::::
query 3' UGUCUGCUUUCGCUAACCU 5'
query=miR3449-5p, target=RO3G_16719, score=0, range=365-383, stra
target 5' ACAGACGAAAGCGAUUGGA 3'
:::::::::::::::::::
query 3' UGUCUGCUUUCGCUAACCU 5'
query=miR3449-5p, target=RO3G_17408, score=0, range=365-383, stra
target 5' ACAGACGAAAGCGAUUGGA 3'
:::::::::::::::::::
query 3' UGUCUGCUUUCGCUAACCU 5'
query=miR3449-5p, target=RO3G_10550, score=0, range=365-383, stra
target 5' ACAGACGAAAGCGAUUGGA 3'
:::::::::::::::::::
query 3' UGUCUGCUUUCGCUAACCU 5'
```

```
query=miR3449-5p, target=RO3G_07690, score=0, range=365-383, stra
target 5' ACAGACGAAAGCGAUUGGA 3'
:::::::::::::::::::
query 3' UGUCUGCUUUCGCUAACCU 5'
query=miR3449-5p, target=RO3G_03624, score=0, range=365-383, stra
target 5' ACAGACGAAAGCGAUUGGA 3'
:::::::::::::::::::
query 3' UGUCUGCUUUCGCUAACCU 5'
query=miR3449-5p, target=RO3G_08847, score=0, range=365-383, stra
target 5' ACAGACGAAAGCGAUUGGA 3'
:::::::::::::::::::
query 3' UGUCUGCUUUCGCUAACCU 5'
query=miR3449-5p, target=RO3G_02706, score=0, range=557-575, stra
target 5' ACAGACGAAAGCGAUUGGA 3'
:::::::::::::::::::
query 3' UGUCUGCUUUCGCUAACCU 5'
query=miR3449-5p, target=RO3G_16550, score=0, range=476-494, stra
target 5' ACAGACGAAAGCGAUUGGA 3'
:::::::::::::::::::
query 3' UGUCUGCUUUCGCUAACCU 5'
query=miR3449-5p, target=RO3G_13286, score=0, range=557-575, stra
target 5' ACAGACGAAAGCGAUUGGA 3'
:::::::::::::::::::
query 3' UGUCUGCUUUCGCUAACCU 5'
query=miR3449-5p, target=RO3G_07707, score=0, range=557-575, stra
target 5' ACAGACGAAAGCGAUUGGA 3'
:::::::::::::::::::
query 3' UGUCUGCUUUCGCUAACCU 5'
query=miR3449-5p, target=RO3G_00679, score=0, range=557-575, stra
target 5' ACAGACGAAAGCGAUUGGA 3'
:::::::::::::::::::
query 3' UGUCUGCUUUCGCUAACCU 5'
query=miR3449-5p, target=RO3G_13919, score=0, range=557-575, stra
target 5' ACAGACGAAAGCGAUUGGA 3'
:::::::::::::::::::
query 3' UGUCUGCUUUCGCUAACCU 5'
query=miR3449-5p, target=RO3G_13743, score=0, range=365-383, stra
target 5' ACAGACGAAAGCGAUUGGA 3'
:::::::::::::::::::
query 3' UGUCUGCUUUCGCUAACCU 5'
query=miR3449-5p, target=RO3G_00284, score=0, range=374-392, stra
target 5' ACAGACGAAAGCGAUUGGA 3'
:::::::::::::::::::
query 3' UGUCUGCUUUCGCUAACCU 5'
query=miR3449-5p, target=RO3G_10741, score=0, range=365-383, stra
```

```
target 5' ACAGACGAAAGCGAUUGGA 3'
:::::::::::::::::::
query 3' UGUCUGCUUUCGCUAACCU 5'
query=miR3449-5p, target=RO3G_10763, score=0, range=365-383, stra
target 5' ACAGACGAAAGCGAUUGGA 3'
:::::::::::::::::::
query 3' UGUCUGCUUUCGCUAACCU 5'
query=miR3449-5p, target=RO3G_17222, score=0, range=557-575, stra
target 5' ACAGACGAAAGCGAUUGGA 3'
:::::::::::::::::::
query 3' UGUCUGCUUUCGCUAACCU 5'
query=miR3449-5p, target=RO3G_12678, score=0, range=557-575, stra
target 5' ACAGACGAAAGCGAUUGGA 3'
:::::::::::::::::::
query 3' UGUCUGCUUUCGCUAACCU 5'
query=miR3449-5p, target=RO3G_00688, score=0, range=365-383, stra
target 5' ACAGACGAAAGCGAUUGGA 3'
:::::::::::::::::::
query 3' UGUCUGCUUUCGCUAACCU 5'
query=miR3449-5p, target=RO3G_10787, score=0.5, range=365-383,
strand=1
target 5' ACAGACGAAAGCGAUUGGG 3'
::::::::::::::::::.
query 3' UGUCUGCUUUCGCUAACCU 5'
query=miR3449-5p, target=RO3G_12166, score=0.5, range=365-383,
strand=1
target 5' ACAGACGAAAGCGAUUGGG 3'
::::::::::::::::::.
query 3' UGUCUGCUUUCGCUAACCU 5'
query=miR3449-5p, target=RO3G_12658, score=0.5, range=365-383,
strand=1
target 5' ACAGACGAAAGCGAUUGGG 3'
::::::::::::::::::.
query 3' UGUCUGCUUUCGCUAACCU 5'
query=miR3449-5p, target=RO3G_03602, score=1, range=506-524, stra
target 5' ACAUACGAAAGCGAUUGGA 3'
::: :::::::::::::::
query 3' UGUCUGCUUUCGCUAACCU 5'
query=miR3449-5p, target=RO3G_03588, score=1, range=242-260, stra
target 5' ACAUACGAAAGCGAUUGGA 3'
::: :::::::::::::::
query 3' UGUCUGCUUUCGCUAACCU 5'
query=miR3449-5p, target=RO3G_01960, score=1, range=557-575, stra
target 5' ACAUACGAAAGCGAUUGGA 3'
::: :::::::::::::::
```

query 3' UGUCUGCUUUCGCUAACCU 5'
query=miR3449-5p, target=RO3G_11281, score=2, range=320-338, stra
target 5' ACAGACGAAAGCAAUUGGA 3'
:::::::::::: ::::::
query 3' UGUCUGCUUUCGCUAACCU 5'
query=miR3449-5p, target=RO3G_13064, score=2, range=23-41, strand
target 5' ACAGACAAAAGCGAUUGGA 3'
:::::: ::::::::::::
query 3' UGUCUGCUUUCGCUAACCU 5'
query=miR3449-5p, target=RO3G_11807, score=3, range=2492-2510,
strand=1
target 5' ACAUACGAAAGCGAUUUGA 3'
::: :::::::::::: ::
query 3' UGUCUGCUUUCGCUAACCU 5'
query=miR3449-5p, target=RO3G_00463, score=3, range=1279-1296,
strand=1
target 5' ACAGAC-AAAGCGAUUGGU 3'
:::::: :::::::::::
query 3' UGUCUGCUUUCGCUAACCU 5'
query=miR3449-5p, target=RO3G_10056, score=3.5, range=2202-2220,
strand=1
target 5' GCAGACGAGAUCGAUUGGA 3'
.:::::::.: ::::::::
query 3' UGUCUGCUUUCGCUAACCU 5'
query=miR3449-5p, target=RO3G_09548, score=3.5, range=232-249,
strand=1
target 5' ACAGGCG-AAGUGAUUGGA 3'
::::.:: :::.::::::
query 3' UGUCUGCUUUCGCUAACCU 5'
query=miR3449-5p, target=RO3G_04776, score=3.5, range=126-144,
strand=1
target 5' ACAAAUGAAAGAGAUUGGA 3'
::: :.::::: :::::::
query 3' UGUCUGCUUUCGCUAACCU 5'
query=miR3449-5p, target=RO3G_07587, score=3.5, range=763-782,
strand=1
target 5' GCAGAACAAAAGCGAUUGGA 3'
.::: :: ::::::::::::
query 3' UGUC-UGCUUUCGCUAACCU 5'
query=miR3449-5p, target=RO3G_10913, score=3.5, range=1153-1170,
strand=1
target 5' GCA-ACGAAAGCGAUCGGA 3'
.:: ::::::::::: :::
query 3' UGUCUGCUUUCGCUAACCU 5'
query=miR3449-5p, target=RO3G_12800, score=3.5, range=128-145,

```
strand=1
target 5' ACA-ACCAAAGCGAUUGGG 3'
::: :: :::::::::::.
query 3' UGUCUGCUUUCGCUAACCU 5'
query=miR3449-5p, target=RO3G_16413, score=3.5, range=944-962,
strand=1
target 5' AGGAAAGAAAGCGAUUGGA 3'
: . : ::::::::::::
query 3' UGUCUGCUUUCGCUAACCU 5'
query=miR3449-5p, target=RO3G_01501, score=3.5, range=1054-1071,
strand=1
target 5' UCAGAUGAAAGC-AUUGGA 3'
::::.:::::: ::::::
query 3' UGUCUGCUUUCGCUAACCU 5'
query=miR3449-5p, target=RO3G_00586, score=3.5, range=423-441,
strand=-1
target 5' ACUGAUGAUAGCGAUUGGA 3'
:: ::.:: :::::::::
query 3' UGUCUGCUUUCGCUAACCU 5'
query=miR3449-5p, target=RO3G_15766, score=3.5, range=815-833,
strand=-1
target 5' GAAGAAGAAAGCGAUUGGU 3'
. ::: ::::::::::::
query 3' UGUCUGCUUUCGCUAACCU 5'
query=miR3449-5p, target=RO3G_08529, score=4, range=775-793, stra
target 5' ACGGAUGAGAGAGAUUGGA 3'
::.::.::.:: :::::::
query 3' UGUCUGCUUUCGCUAACCU 5'
query=miR3449-5p, target=RO3G_04768, score=4, range=144-163, stra
target 5' GCAGGAUGAGAGCGAUUGGC 3'
.:: ::.::.:::::::::
query 3' UGU-CUGCUUUCGCUAACCU 5'
query=miR3449-5p, target=RO3G_00079, score=4, range=1117-1135,
strand=1
target 5' ACAGAGGAUAGUGAUUGGA 3'
::::: :: ::.:::::::
query 3' UGUCUGCUUUCGCUAACCU 5'
query=miR3449-5p, target=RO3G_00099, score=4, range=877-895, stra
target 5' ACAGAGAAAAGCGGUUGGA 3'
::::: ::::::.:::::
query 3' UGUCUGCUUUCGCUAACCU 5'
query=miR3449-5p, target=RO3G_07526, score=4, range=565-583, stra
target 5' GCAGCUGAAAGCUAUUGGA 3'
.::: .:::::: ::::::
query 3' UGUCUGCUUUCGCUAACCU 5'
```

query=miR3449-5p, target=RO3G_02792, score=4, range=2022-2040, strand=1
target 5' GCACUUGAAAGUGAUUGGA 3'
.:: .:::::.:::::::
query 3' UGUCUGCUUUCGCUAACCU 5'
query=miR3449-5p, target=RO3G_03414, score=4, range=601-618, stra
target 5' ACAG-CAAAAGUGAUUGGA 3'
:::: : ::::.:::::::
query 3' UGUCUGCUUUCGCUAACCU 5'
query=miR3449-5p, target=RO3G_09135, score=4, range=4368-4386, strand=1
target 5' GUUGAAGAAAGCGAUUGGU 3'
.. :: :::::::::::
query 3' UGUCUGCUUUCGCUAACCU 5'
query=miR3449-5p, target=RO3G_06541, score=4, range=1555-1573, strand=1
target 5' GCAUUUGAAAGCGAUUGGC 3'
.:: .:::::::::::
query 3' UGUCUGCUUUCGCUAACCU 5'
query=miR3449-5p, target=RO3G_13413, score=4, range=377-395, stra
target 5' ACAGCGGAAAGCCAUUGGA 3'
:::: :::::: ::::::
query 3' UGUCUGCUUUCGCUAACCU 5'
query=miR3449-5p, target=RO3G_10607, score=4, range=6827-6844, strand=1
target 5' ACA-CCGAAAACGAUUGGA 3'
::: ::::: ::::::::
query 3' UGUCUGCUUUCGCUAACCU 5'
query=miR3449-5p, target=RO3G_14286, score=4, range=155-174, stra
target 5' ACGGGCGAGAGCGAUUAGGA 3'
::.:.:::.::::::: :::
query 3' UGUCUGCUUUCGCUAA-CCU 5'
query=miR3449-5p, target=RO3G_08856, score=4, range=2948-2967, strand=-1
target 5' AUGGAACGAAAGCGAGUGGA 3'
:..: :::::::::: ::::
query 3' UGUC-UGCUUUCGCUAACCU 5'
query=miR3449-5p, target=RO3G_01473, score=4, range=1213-1230, strand=-1
target 5' AC-GACGAGAGCGUUUGGA 3'
:: :::::.:::: :::::
query 3' UGUCUGCUUUCGCUAACCU 5'
query=miR3449-5p, target=RO3G_12930, score=4, range=1885-1902, strand=-1
target 5' AC-GACGAGAGCGUUUGGA 3'

```
:: ::::.:::: :::::
query 3' UGUCUGCUUUCGCUAACCU 5'
query=miR3449-5p, target=RO3G_13428, score=4, range=61-78, strand
target 5' AC-GACGAGAGCGUUUGGA 3'
:: ::::.:::: :::::
query 3' UGUCUGCUUUCGCUAACCU 5'
query=miR3449-5p, target=RO3G_12221, score=4, range=49-66, strand
target 5' CCA-AGGAAAGCGAUUGGU 3'
:: : ::::::::::::
query 3' UGUCUGCUUUCGCUAACCU 5'
query=miR3449-5p, target=RO3G_10153, score=4, range=219-236, stra
target 5' ACAGA-GAAAGUGAUUCGA 3'
::::: ::::.:::: ::
query 3' UGUCUGCUUUCGCUAACCU 5'
query=miR3449-5p, target=RO3G_01032, score=4, range=67-84, strand
target 5' ACGCAUGAAA-CGAUUGGA 3'
::. :.:::: ::::::::
query 3' UGUCUGCUUUCGCUAACCU 5'
query=miR1435a, target=RO3G_11285, score=0, range=200-221, strand
target 5' GAAAAACUUGCACUUUAAGAAA 3'
::::::::::::::::::::::
query 3' CUUUUUGAACGUGAAAUUCUUU 5'
query=miR1435a, target=RO3G_13710, score=0, range=200-221, strand
target 5' GAAAAACUUGCACUUUAAGAAA 3'
::::::::::::::::::::::
query 3' CUUUUUGAACGUGAAAUUCUUU 5'
query=miR1435a, target=RO3G_17245, score=1, range=200-221, strand
target 5' GAAGAAUUUGCACUUUAAGAAA 3'
:::.::.:::::::::::::::
query 3' CUUUUUGAACGUGAAAUUCUUU 5'
query=miR1435a, target=RO3G_12006, score=1, range=200-221, strand
target 5' GAAGAAUUUGCACUUUAAGAAA 3'
:::.::.:::::::::::::::
query 3' CUUUUUGAACGUGAAAUUCUUU 5'
query=miR1435a, target=RO3G_14707, score=1, range=200-221, strand
target 5' GAAGAAUUUGCACUUUAAGAAA 3'
:::.::.:::::::::::::::
query 3' CUUUUUGAACGUGAAAUUCUUU 5'
query=miR1435a, target=RO3G_16910, score=1, range=200-221, strand
target 5' GAAGAAUUUGCACUUUAAGAAA 3'
:::.::.:::::::::::::::
query 3' CUUUUUGAACGUGAAAUUCUUU 5'
query=miR1435a, target=RO3G_17365, score=1, range=200-221, strand
target 5' GAAGAAUUUGCACUUUAAGAAA 3'
:::.::.:::::::::::::::
```

```
query 3' CUUUUUGAACGUGAAAUUCUUU 5'
query=miR1435a, target=RO3G_01196, score=1, range=200-221, strand
target 5' GAAGAAUUUGCACUUUAAGAAA 3'
:::.::.:::::::::::::::
query 3' CUUUUUGAACGUGAAAUUCUUU 5'
query=miR1435a, target=RO3G_13738, score=1, range=200-221, strand
target 5' GAAGAAUUUGCACUUUAAGAAA 3'
:::.::.:::::::::::::::
query 3' CUUUUUGAACGUGAAAUUCUUU 5'
query=miR6023, target=RO3G_10323, score=2.5, range=92-111, strand
target 5' AUCUAAGGAACUGUCAUCAG 3'
:::.::..:::::::::::.
query 3' UAGGUUUUUUGACAGUAGUU 5'
query=miR6023, target=RO3G_05182, score=3, range=224-243, strand=
target 5' GUUCAAAGAACUGUUAUCAA 3'
.:.::::.::::::.:::::
query 3' UAGGUUUUUUGACAGUAGUU 5'
query=miR6023, target=RO3G_04478, score=3.5, range=1665-1684, str
target 5' GAUGAAGAAACUGUCAUCAA 3'
. . ::.:::::::::::::
query 3' UAGGUUUUUUGACAGUAGUU 5'
query=miR6023, target=RO3G_03634, score=4, range=49-68, strand=1
target 5' AUCCAAAAGACUGUCAACAU 3'
::::::::.::::::: ::
query 3' UAGGUUUUUUGACAGUAGUU 5'
query=miR6023, target=RO3G_04306, score=4, range=821-839, strand=
target 5' AUCC-AAGAAUUGUCAUCAU 3'
:::: ::.::.::::::::
query 3' UAGGUUUUUUGACAGUAGUU 5'
query=miR6023, target=RO3G_04006, score=4, range=202-220, strand=
target 5' AUCAAAAAGACUGU-AUCAA 3'
::: ::::.::::: :::::
query 3' UAGGUUUUUUGACAGUAGUU 5'
query=miR6023, target=RO3G_13613, score=4, range=344-362, strand=
target 5' AUCC-ACAAGCUGUCAUUAA 3'
:::: : ::.:::::::.::
query 3' UAGGUUUUUUGACAGUAGUU 5'
query=miR6023, target=RO3G_05723, score=4, range=1057-1075, stran
target 5' AUCCAAAAAGCU-UUAUCAA 3'
:::::::::.:: :.:::::
query 3' UAGGUUUUUUGACAGUAGUU 5'
query=miR6023, target=RO3G_13387, score=4, range=583-601, strand=
target 5' AUCCAAAAAGCU-UUAUCAA 3'
:::::::::.:: :.:::::
query 3' UAGGUUUUUUGACAGUAGUU 5'
```

```
query=miR6023, target=RO3G_08960, score=4, range=151-169, strand=
target 5' AUCCAAAGAG-UGUCAUCAA 3'
:::::::.:. :::::::::
query 3' UAGGUUUUUUGACAGUAGUU 5'
query=miR6023, target=RO3G_08422, score=4, range=218-236, strand=
target 5' AUUUGAGAAACUGUC-UCAA 3'
::...:.:::::::: ::::
query 3' UAGGUUUUUUGACAGUAGUU 5'
query=miR6023, target=RO3G_16998, score=4, range=808-828, strand=
target 5' AUCCAAAUAAAGUGUCAUUAA 3'
::::::: ::: ::::::.::
query 3' UAGGUUU-UUUGACAGUAGUU 5'
query=miR6023, target=RO3G_09746, score=4, range=215-234, strand=
target 5' AGUCAAGAAACUUUCAUCAA 3'
: .:::.::::: :::::::
query 3' UAGGUUUUUUGACAGUAGUU 5'
query=miR6023, target=RO3G_09283, score=4, range=335-355, strand=
target 5' GUUGAAUAAAAUUGUCAUCAA 3'
.:. :: ::::.:::::::::
query 3' UAGGUU-UUUUGACAGUAGUU 5'
query=miR9722, target=RO3G_11557, score=3, range=261-280, strand=
target 5' UUCUUCCUC-UCCCAUCUUUA 3'
:::::: :: :::::::::::
query 3' AAGAAGUAGAAGGGUAGAAAU 5'
query=miR9722, target=RO3G_06617, score=3.5, range=798-818, stran
target 5' UUCUUUAUCUUUCCAUCCUUA 3'
:::::.:::::.::::: :::
query 3' AAGAAGUAGAAGGGUAGAAAU 5'
query=miR9722, target=RO3G_09632, score=3.5, range=156-177, stran
target 5' UUCUUUCUCCUUUCCAUCUUUA 3'
:::::. : :::.:::::::::
query 3' AAGAAGUA-GAAGGGUAGAAAU 5'
query=miR9722, target=RO3G_03357, score=4, range=63-84, strand=1
target 5' UUCUUCUUGUUCCUCAUCUUUA 3'
:::::: : :::: ::::::::
query 3' AAGAAGUAGAAGG-GUAGAAAU 5'
query=miR9722, target=RO3G_01662, score=4, range=3-24, strand=-1
target 5' UUCUUCAUCUUCUCCGUCUUUC 3'
:::::::::::: ::.:::::
query 3' AAGAAGUAGAAG-GGUAGAAAU 5'
query=miR9722, target=RO3G_03488, score=4, range=1626-1646, stran
target 5' UUCUUCUUCUUCUCAUCUUCA 3'
:::::: :::::.:::::: :
query 3' AAGAAGUAGAAGGGUAGAAAU 5'
query=miR9722, target=RO3G_16288, score=4, range=175-195, strand=
```

```
target 5' UUCUUCCUUUUCCCAUCUUAA 3'
:::::: :.:::::::::: :
query 3' AAGAAGUAGAAGGGUAGAAAU 5'
query=miR9722, target=RO3G_02442, score=4, range=1080-1099, stran
target 5' UUC-UCAUCUUUCCAUGUUUA 3'
::: ::::::.:::: ::::
query 3' AAGAAGUAGAAGGGUAGAAAU 5'
query=miR9722, target=RO3G_00181, score=4, range=122-141, strand=
target 5' GUCUUCA-CUUCCUAUUUUUA 3'
:::::: :::::.::.::::
query 3' AAGAAGUAGAAGGGUAGAAAU 5'
query=miR9722, target=RO3G_15026, score=4, range=320-339, strand=
target 5' UUCAUCAUCUU-CCAUUUUUA 3'
::: ::::::: ::::.::::
query 3' AAGAAGUAGAAGGGUAGAAAU 5'
query=miR9722, target=RO3G_03004, score=4, range=378-399, strand=
target 5' UUCUUCUUCUUCAUCAUCUUUA 3'
:::::: ::::: .::::::::
query 3' AAGAAGUAGAAG-GGUAGAAAU 5'
query=miR2654, target=RO3G_15134, score=0, range=179-200, strand=
target 5' AGCACACUUCGGUCCCUGAAUA 3'
::::::::::::::::::::::
query 3' UCGUGUGAAGCCAGGGACUUAU 5'
query=miR2654, target=RO3G_03099, score=0, range=179-200, strand=
target 5' AGCACACUUCGGUCCCUGAAUA 3'
::::::::::::::::::::::
query 3' UCGUGUGAAGCCAGGGACUUAU 5'
query=miR2654, target=RO3G_03546, score=2, range=659-680, strand=
target 5' AACUCACUUCGGUCCCUGAAUA 3'
: : ::::::::::::::::::
query 3' UCGUGUGAAGCCAGGGACUUAU 5'
query=miR2654, target=RO3G_08065, score=2, range=245-266, strand=
target 5' AACUCACUUCGGUCCCUGAAUA 3'
: : ::::::::::::::::::
query 3' UCGUGUGAAGCCAGGGACUUAU 5'
query=miR2654, target=RO3G_15293, score=3, range=771-791, strand=
target 5' AGUAC-UUUCGGUCCUUGAAUA 3'
::.:: .::::::::.::::::
query 3' UCGUGUGAAGCCAGGGACUUAU 5'
query=miR2654, target=RO3G_02968, score=4, range=1235-1256, stran
target 5' AGCAUGCUGCGGUCCAUGAAUA 3'
::::..:: :::::: ::::::
query 3' UCGUGUGAAGCCAGGGACUUAU 5'
query=miR7492n, target=RO3G_11534, score=0, range=799-820, strand
target 5' CCAGCUCAAAGCCCUGAUCUUA 3'
```

```
::::::::::::::::::::::
query 3' GGUCGAGUUUCGGGACUAGAAU 5'
query=miR7492n, target=RO3G_08848, score=0, range=109-130, strand
target 5' CCAGCUCAAAGCCCUGAUCUUA 3'
::::::::::::::::::::::
query 3' GGUCGAGUUUCGGGACUAGAAU 5'
query=miR7492n, target=RO3G_10720, score=1, range=799-820, strand
target 5' CCAGCUCAAAGUCCUGAUCUUA 3'
:::::::::::.::::::::::
query 3' GGUCGAGUUUCGGGACUAGAAU 5'
query=miR7492n, target=RO3G_01951, score=1, range=799-820, strand
target 5' CCAGCUCAAAGUCCUGAUCUUA 3'
:::::::::::.::::::::::
query 3' GGUCGAGUUUCGGGACUAGAAU 5'
query=miR7492n, target=RO3G_15154, score=1, range=799-820, strand
target 5' CCAGCUCAAAGUCCUGAUCUUA 3'
:::::::::::.::::::::::
query 3' GGUCGAGUUUCGGGACUAGAAU 5'
query=miR7492n, target=RO3G_03987, score=1, range=799-820, strand
target 5' CCAGCUCAAAGUCCUGAUCUUA 3'
:::::::::::.::::::::::
query 3' GGUCGAGUUUCGGGACUAGAAU 5'
query=miR7492n, target=RO3G_17409, score=1, range=799-820, strand
target 5' CCAGCUCAAAGUCCUGAUCUUA 3'
:::::::::::.::::::::::
query 3' GGUCGAGUUUCGGGACUAGAAU 5'
query=miR7492n, target=RO3G_10044, score=1, range=799-820, strand
target 5' CCAGCUCAAAGUCCUGAUCUUA 3'
:::::::::::.::::::::::
query 3' GGUCGAGUUUCGGGACUAGAAU 5'
query=miR7492n, target=RO3G_11244, score=1, range=799-820, strand
target 5' CCAGCUCAAAGUCCUGAUCUUA 3'
:::::::::::.::::::::::
query 3' GGUCGAGUUUCGGGACUAGAAU 5'
query=miR7492n, target=RO3G_01313, score=1, range=799-820, strand
target 5' CCAGCUCAAAGUCCUGAUCUUA 3'
:::::::::::.::::::::::
query 3' GGUCGAGUUUCGGGACUAGAAU 5'
query=miR7492n, target=RO3G_15873, score=1, range=799-820, strand
target 5' CCAGCUCAAAGUCCUGAUCUUA 3'
:::::::::::.::::::::::
query 3' GGUCGAGUUUCGGGACUAGAAU 5'
query=miR7492n, target=RO3G_03558, score=1, range=799-820, strand
target 5' CCAGCUCAAAGUCCUGAUCUUA 3'
:::::::::::.::::::::::
```

```
query 3' GGUCGAGUUUCGGGACUAGAAU 5'
query=miR7492n, target=RO3G_07994, score=1, range=799-820, strand
target 5' CCAGCUCAAAGUCCUGAUCUUA 3'
::::::::::.::::::::::
query 3' GGUCGAGUUUCGGGACUAGAAU 5'
query=miR7492n, target=RO3G_01194, score=1, range=799-820, strand
target 5' CCAGCUCAAAGUCCUGAUCUUA 3'
::::::::::.::::::::::
query 3' GGUCGAGUUUCGGGACUAGAAU 5'
query=miR7492n, target=RO3G_07683, score=1, range=799-820, strand
target 5' CCAGCUCAAAGUCCUGAUCUUA 3'
::::::::::.::::::::::
query 3' GGUCGAGUUUCGGGACUAGAAU 5'
query=miR7492n, target=RO3G_13447, score=1, range=799-820, strand
target 5' CCAGCUCAAAGUCCUGAUCUUA 3'
::::::::::.::::::::::
query 3' GGUCGAGUUUCGGGACUAGAAU 5'
query=miR7492n, target=RO3G_12526, score=1, range=799-820, strand
target 5' CCAGCUCAAAGUCCUGAUCUUA 3'
::::::::::.::::::::::
query 3' GGUCGAGUUUCGGGACUAGAAU 5'
query=miR7492n, target=RO3G_13706, score=1, range=757-778, strand
target 5' CCAGCUCAAAGUCCUGAUCUUA 3'
::::::::::.::::::::::
query 3' GGUCGAGUUUCGGGACUAGAAU 5'
query=miR7492n, target=RO3G_01735, score=1, range=799-820, strand
target 5' CCAGCUCAAAGUCCUGAUCUUA 3'
::::::::::.::::::::::
query 3' GGUCGAGUUUCGGGACUAGAAU 5'
query=miR7492n, target=RO3G_17211, score=1, range=799-820, strand
target 5' CCAGCUCAAAGUCCUGAUCUUA 3'
::::::::::.::::::::::
query 3' GGUCGAGUUUCGGGACUAGAAU 5'
query=miR7492n, target=RO3G_07119, score=1, range=124-145, strand
target 5' CCAGCUCAAAGUCCUGAUCUUA 3'
::::::::::.::::::::::
query 3' GGUCGAGUUUCGGGACUAGAAU 5'
query=miR7492n, target=RO3G_12013, score=1, range=124-145, strand
target 5' CCAGCUCAAAGUCCUGAUCUUA 3'
::::::::::.::::::::::
query 3' GGUCGAGUUUCGGGACUAGAAU 5'
query=miR7492n, target=RO3G_10742, score=1, range=124-145, strand
target 5' CCAGCUCAAAGUCCUGAUCUUA 3'
::::::::::.::::::::::
query 3' GGUCGAGUUUCGGGACUAGAAU 5'
```

```
query=miR7492n, target=RO3G_10528, score=1, range=124-145, strand
target 5' CCAGCUCAAAGUCCUGAUCUUA 3'
:::::::::::.::::::::::
query 3' GGUCGAGUUUCGGGACUAGAAU 5'
query=miR7492n, target=RO3G_11263, score=1, range=124-145, strand
target 5' CCAGCUCAAAGUCCUGAUCUUA 3'
:::::::::::.::::::::::
query 3' GGUCGAGUUUCGGGACUAGAAU 5'
query=miR7492n, target=RO3G_16118, score=1, range=67-88, strand=1
target 5' CCAGCUCAAAGUCCUGAUCUUA 3'
:::::::::::.::::::::::
query 3' GGUCGAGUUUCGGGACUAGAAU 5'
query=miR7492n, target=RO3G_17213, score=1, range=124-145, strand
target 5' CCAGCUCAAAGUCCUGAUCUUA 3'
:::::::::::.::::::::::
query 3' GGUCGAGUUUCGGGACUAGAAU 5'
query=miR7492n, target=RO3G_12174, score=1, range=124-145, strand
target 5' CCAGCUCAAAGUCCUGAUCUUA 3'
:::::::::::.::::::::::
query 3' GGUCGAGUUUCGGGACUAGAAU 5'
query=miR7492n, target=RO3G_03617, score=3, range=799-820, strand
target 5' CCAGCUCAAAGUCCUGACCUUA 3'
:::::::::::.::::: ::::
query 3' GGUCGAGUUUCGGGACUAGAAU 5'
query=miR7492n, target=RO3G_11948, score=3, range=799-820, strand
target 5' CCAGCUCAAAGUCCUGACCUUA 3'
:::::::::::.::::: ::::
query 3' GGUCGAGUUUCGGGACUAGAAU 5'
query=miR7492n, target=RO3G_09924, score=3, range=799-820, strand
target 5' CCAGCUCAAAGUCCUGACCUUA 3'
:::::::::::.::::: ::::
query 3' GGUCGAGUUUCGGGACUAGAAU 5'
query=miR7492n, target=RO3G_01950, score=3, range=799-820, strand
target 5' CCAGCUCAAAGUCCUGACCUUA 3'
:::::::::::.::::: ::::
query 3' GGUCGAGUUUCGGGACUAGAAU 5'
query=miR7492n, target=RO3G_09199, score=3, range=799-820, strand
target 5' CCAGCUCAAAGUCCUGACCUUA 3'
:::::::::::.::::: ::::
query 3' GGUCGAGUUUCGGGACUAGAAU 5'
query=miR7492n, target=RO3G_05916, score=3, range=799-820, strand
target 5' CCAGCUCAAAGUCCUGACCUUA 3'
:::::::::::.::::: ::::
query 3' GGUCGAGUUUCGGGACUAGAAU 5'
query=miR7492n, target=RO3G_07295, score=3, range=631-652, strand
```

```
target 5' CCAGCUCAAAGUCCUGACCUUA 3'
:::::::::::.::::: ::::
query 3' GGUCGAGUUUCGGGACUAGAAU 5'
query=miR7492n, target=RO3G_10537, score=3, range=622-643, strand
target 5' CCAGCUCAAAGUCCUGACCUUA 3'
:::::::::::.::::: ::::
query 3' GGUCGAGUUUCGGGACUAGAAU 5'
query=miR7492n, target=RO3G_16548, score=3, range=454-475, strand
target 5' CCAGCUCAAAGUCCUGACCUUA 3'
:::::::::::.::::: ::::
query 3' GGUCGAGUUUCGGGACUAGAAU 5'
query=miR7492n, target=RO3G_11528, score=3, range=124-145, strand
target 5' CCAGCUCAAAGUCCUGACCUUA 3'
:::::::::::.::::: ::::
query 3' GGUCGAGUUUCGGGACUAGAAU 5'
query=miR7492n, target=RO3G_11402, score=3, range=424-445, strand
target 5' CCAGCACAAAGCCCUGAUCUCA 3'
::::: ::::::::::::: :
query 3' GGUCGAGUUUCGGGACUAGAAU 5'
query=miR7492n, target=RO3G_09997, score=4, range=1057-1078, stra
target 5' UUAGAUCAAAGUCCUGAUUUUA 3'
..:: :::::.::::::.:::
query 3' GGUCGAGUUUCGGGACUAGAAU 5'
query=miR7492n, target=RO3G_13276, score=4, range=181-202, strand
target 5' CCAGCUCAAAGUCCUGAUUUGA 3'
:::::::::::.::::::.: :
query 3' GGUCGAGUUUCGGGACUAGAAU 5'
query=miR7492n, target=RO3G_16377, score=4, range=124-145, strand
target 5' CCAGCUCAAAGUUCUGACCUUA 3'
:::::::::::..:::: ::::
query 3' GGUCGAGUUUCGGGACUAGAAU 5'
query=miR7492n, target=RO3G_07788, score=4, range=805-826, strand
target 5' CCUGCUCAAAGCCCUGAUUUGA 3'
:: ::::::::::::::.: :
query 3' GGUCGAGUUUCGGGACUAGAAU 5'
query=miR7492n, target=RO3G_11567, score=4, range=1513-1534, stra
target 5' CCUGCUCAAAGCCCUGAUUUGA 3'
:: ::::::::::::::.: :
query 3' GGUCGAGUUUCGGGACUAGAAU 5'
query=miR7492n, target=RO3G_08835, score=4, range=109-130, strand
target 5' CCAGCUCAAAGUCCUGACCUUC 3'
:::::::::::.::::: :::
query 3' GGUCGAGUUUCGGGACUAGAAU 5'
query=miR7492n, target=RO3G_05303, score=4, range=856-877, strand
target 5' CCUGCUCAAAGCCCUGAUUUGA 3'
```

```
:: ::::::::::::::.: :
query 3' GGUCGAGUUUCGGGACUAGAAU 5'
query=miR7492n, target=RO3G_13903, score=4, range=856-877, strand
target 5' CCUGCUCAAAGCCCUGAUUUGA 3'
:: ::::::::::::::.: :
query 3' GGUCGAGUUUCGGGACUAGAAU 5'
query=miR7492n, target=RO3G_08906, score=4, range=856-877, strand
target 5' CCUGCUCAAAGCCCUGAUUUGA 3'
:: ::::::::::::::.: :
query 3' GGUCGAGUUUCGGGACUAGAAU 5'
query=miR7492n, target=RO3G_05194, score=4, range=856-877, strand
target 5' CCUGCUCAAAGCCCUGAUUUGA 3'
:: ::::::::::::::.: :
query 3' GGUCGAGUUUCGGGACUAGAAU 5'
query=miR7492n, target=RO3G_02354, score=4, range=856-877, strand
target 5' CCUGCUCAAAGCCCUGAUUUGA 3'
:: ::::::::::::::.: :
query 3' GGUCGAGUUUCGGGACUAGAAU 5'
query=miR7492n, target=RO3G_05181, score=4, range=856-877, strand
target 5' CCUGCUCAAAGCCCUGAUUUGA 3'
:: ::::::::::::::.: :
query 3' GGUCGAGUUUCGGGACUAGAAU 5'
query=miR7492n, target=RO3G_13664, score=4, range=856-877, strand
target 5' CCUGCUCAAAGCCCUGAUUUGA 3'
:: ::::::::::::::.: :
query 3' GGUCGAGUUUCGGGACUAGAAU 5'
query=miR7492n, target=RO3G_13672, score=4, range=856-877, strand
target 5' CCUGCUCAAAGCCCUGAUUUGA 3'
:: ::::::::::::::.: :
query 3' GGUCGAGUUUCGGGACUAGAAU 5'
query=miR7492n, target=RO3G_14722, score=4, range=856-877, strand
target 5' CCUGCUCAAAGCCCUGAUUUGA 3'
:: ::::::::::::::.: :
query 3' GGUCGAGUUUCGGGACUAGAAU 5'
query=miR7492n, target=RO3G_01923, score=4, range=805-826, strand
target 5' CCUGCUCAAAGCCCUGAUUUGA 3'
:: ::::::::::::::.: :
query 3' GGUCGAGUUUCGGGACUAGAAU 5'
query=miR7492n, target=RO3G_11486, score=4, range=565-586, strand
target 5' CCUGCUCAAAGCCCUGAUUUGA 3'
:: ::::::::::::::.: :
query 3' GGUCGAGUUUCGGGACUAGAAU 5'
query=miR7492n, target=RO3G_04110, score=4, range=553-574, strand
target 5' CCUGCUCAAAGCCCUGAUUUGA 3'
:: ::::::::::::::.: :
```

```
query 3' GGUCGAGUUUCGGGACUAGAAU 5'
query=miR7492n, target=RO3G_01528, score=4, range=424-445, strand
target 5' CCUGCUCAAAGCCCUGAUUUAA 3'
:: ::::::::::::::.: :
query 3' GGUCGAGUUUCGGGACUAGAAU 5'
query=miR7492n, target=RO3G_15496, score=4, range=498-519, strand
target 5' CCUGCUCAAAGCCCUGAUUUGA 3'
:: ::::::::::::::.: :
query 3' GGUCGAGUUUCGGGACUAGAAU 5'
query=miR7492n, target=RO3G_05535, score=4, range=241-262, strand
target 5' CCUGCUCAAAGCCCUGAUUUGA 3'
:: ::::::::::::::.: :
query 3' GGUCGAGUUUCGGGACUAGAAU 5'
query=miR7492n, target=RO3G_10644, score=4, range=241-262, strand
target 5' CCUGCUCAAAGCCCUGAUUUGA 3'
:: ::::::::::::::.: :
query 3' GGUCGAGUUUCGGGACUAGAAU 5'
query=miR7492n, target=RO3G_12139, score=4, range=229-250, strand
target 5' CCUGCUCAAAGCCCUGAUUUGA 3'
:: ::::::::::::::.: :
query 3' GGUCGAGUUUCGGGACUAGAAU 5'
query=miR7492n, target=RO3G_01518, score=4, range=229-250, strand
target 5' CCUGCUCAAAGCCCUGAUUUAA 3'
:: ::::::::::::::.: :
query 3' GGUCGAGUUUCGGGACUAGAAU 5'
query=miR7492n, target=RO3G_05616, score=4, range=145-166, strand
target 5' CCUGCUCAAAGCCCUGAUUUGA 3'
:: ::::::::::::::.: :
query 3' GGUCGAGUUUCGGGACUAGAAU 5'
query=miR7492n, target=RO3G_16893, score=4, range=241-262, strand
target 5' CCUGCUCAAAGCCCUGAUUUGA 3'
:: ::::::::::::::.: :
query 3' GGUCGAGUUUCGGGACUAGAAU 5'
query=miR7492n, target=RO3G_05603, score=4, range=115-136, strand
target 5' CCUGCUCAAAGCCCUGAUUUGA 3'
:: ::::::::::::::.: :
query 3' GGUCGAGUUUCGGGACUAGAAU 5'
query=miR319c, target=RO3G_04030, score=1, range=1107-1126, stran
target 5' AGCAUGCCCUUUUCAUCCAA 3'
::::::::.::::::::::
query 3' UCGUACGGGGAAAGUAGGUU 5'
query=miR319c, target=RO3G_12114, score=2, range=169-189, strand=
target 5' AGCAUGUCCUCUUUCAUCCAA 3'
:::::: ::.::::::::::
query 3' UCGUAC-GGGGAAAGUAGGUU 5'
```

```
query=miR319c, target=RO3G_12923, score=3.5, range=822-842, stran
target 5' AGUAUGGCCCUUGUCAUCCAA 3'
::.::: ::::: ::::::::
query 3' UCGUACGGGGAA-AGUAGGUU 5'
query=miR319c, target=RO3G_11333, score=3.5, range=1003-1022,
strand=-1
target 5' GCCUUGCCCUUUUCAUCCAA 3'
. : ::::.::::::::::
query 3' UCGUACGGGGAAAGUAGGUU 5'
query=miR319c, target=RO3G_13957, score=3.5, range=394-414, stran
target 5' AGCAAUCUCUCUUUCAUCCAA 3'
::: :: .:.::::::::::
query 3' UCG-UACGGGGAAAGUAGGUU 5'
query=miR319c, target=RO3G_01412, score=4, range=80-98, strand=1
target 5' AGGAU-CCCUUUUCAUCCGA 3'
:: :: :::.::::::::.:
query 3' UCGUACGGGGAAAGUAGGUU 5'
query=miR319c, target=RO3G_09067, score=4, range=784-802, strand=
target 5' AACAUG-CCCUUGCAUCCAA 3'
: :::: ::::: :::::::
query 3' UCGUACGGGGAAAGUAGGUU 5'
query=miR319c, target=RO3G_12773, score=4, range=259-278, strand=
target 5' GGCCUGUCCUUUUCAUCCAC 3'
.:: ::.::.::::::::
query 3' UCGUACGGGGAAAGUAGGUU 5'
query=miR319c, target=RO3G_15690, score=4, range=745-765, strand=
target 5' AGCAUUUCUCUUUUCAUCCAA 3'
:::: : :.:.::::::::::
query 3' UCGU-ACGGGGAAAGUAGGUU 5'
query=miR2609a, target=RO3G_07862, score=0, range=905-926, strand
target 5' AAGUGACGAGCCAUUACUUCCA 3'
::::::::::::::::::::::
query 3' UUCACUGCUCGGUAAUGAAGGU 5'
query=miR2609a, target=RO3G_10744, score=0, range=791-812, strand
target 5' AAGUGACGAGCCAUUACUUCCA 3'
::::::::::::::::::::::
query 3' UUCACUGCUCGGUAAUGAAGGU 5'
query=miR2609a, target=RO3G_16366, score=0, range=791-812, strand
target 5' AAGUGACGAGCCAUUACUUCCA 3'
::::::::::::::::::::::
query 3' UUCACUGCUCGGUAAUGAAGGU 5'
query=miR2609a, target=RO3G_07735, score=0, range=710-731, strand
target 5' AAGUGACGAGCCAUUACUUCCA 3'
::::::::::::::::::::::
query 3' UUCACUGCUCGGUAAUGAAGGU 5'
```

```
query=miR2609a, target=RO3G_10543, score=0, range=263-284, strand
target 5' AAGUGACGAGCCAUUACUUCCA 3'
::::::::::::::::::::::
query 3' UUCACUGCUCGGUAAUGAAGGU 5'
query=miR2609a, target=RO3G_10747, score=0, range=905-926, strand
target 5' AAGUGACGAGCCAUUACUUCCA 3'
::::::::::::::::::::::
query 3' UUCACUGCUCGGUAAUGAAGGU 5'
query=miR2609a, target=RO3G_09173, score=0, range=905-926, strand
target 5' AAGUGACGAGCCAUUACUUCCA 3'
::::::::::::::::::::::
query 3' UUCACUGCUCGGUAAUGAAGGU 5'
query=miR2609a, target=RO3G_17218, score=0, range=791-812, strand
target 5' AAGUGACGAGCCAUUACUUCCA 3'
::::::::::::::::::::::
query 3' UUCACUGCUCGGUAAUGAAGGU 5'
query=miR2609a, target=RO3G_02741, score=4, range=1688-1709, stra
target 5' AUGUAACCAGCCAUUACUUCCU 3'
: :: :: :::::::::::::
query 3' UUCACUGCUCGGUAAUGAAGGU 5'
query=miR1169-3p, target=RO3G_11964, score=0, range=25-47, strand
target 5' GGUCACAGAAAGCAACAUCCACA 3'
:::::::::::::::::::::::
query 3' CCAGUGUCUUUCGUUGUAGGUGU 5'
query=miR1169-3p, target=RO3G_13001, score=1, range=1581-1603,
strand=1
target 5' GGUCACAGAAAGUAACAUCCACA 3'
:::::::::::.:::::::::::
query 3' CCAGUGUCUUUCGUUGUAGGUGU 5'
query=miR1169-3p, target=RO3G_06596, score=1, range=1581-1603,
strand=1
target 5' GGUCACAGAAAGUAACAUCCACA 3'
:::::::::::.:::::::::::
query 3' CCAGUGUCUUUCGUUGUAGGUGU 5'
query=miR1169-3p, target=RO3G_03002, score=1, range=1581-1603,
strand=1
target 5' GGUCACAGAAAGUAACAUCCACA 3'
:::::::::::.:::::::::::
query 3' CCAGUGUCUUUCGUUGUAGGUGU 5'
query=miR1169-3p, target=RO3G_13809, score=2, range=59-81, strand
target 5' GGUCACAGAAAGCAACAUACACA 3'
::::::::::::::::::: ::::
query 3' CCAGUGUCUUUCGUUGUAGGUGU 5'
query=miR1169-3p, target=RO3G_02146, score=2, range=2-24, strand=
target 5' UGUCACAGAAAGUAACAUCCACA 3'
```

```
::::::::::.:::::::::
query 3' CCAGUGUCUUUCGUUGUAGGUGU 5'
query=miR1169-3p, target=RO3G_08215, score=4, range=231-252, stra
target 5' GGUCA-AGAAAGCAGCGUCUACA 3'
::::: :::::::.:.::.:::
query 3' CCAGUGUCUUUCGUUGUAGGUGU 5'
query=miR157a-5p, target=RO3G_04595, score=3, range=974-992, stra
target 5' UGCUCU-UGUCUUCUGUCAU 3'
:::::: :.::::::::::
query 3' ACGAGAGAUAGAAGACAGUU 5'
query=miR157a-5p, target=RO3G_16965, score=3, range=1504-1523,
strand=-1
target 5' UGCUCUCCAUCUUCUGUCAU 3'
::::::: ::::::::::
query 3' ACGAGAGAUAGAAGACAGUU 5'
query=miR157a-5p, target=RO3G_11173, score=3.5, range=2754-2774,
strand=1
target 5' UGCUUUCUAUCUUCUCGUUAA 3'
::::.:::::::::: ::.::
query 3' ACGAGAGAUAGAAGA-CAGUU 5'
query=miR157a-5p, target=RO3G_12125, score=3.5, range=41-59, stra
target 5' AGUUC-CUAUCUUCUGUUAA 3'
:.:: :::::::::::.::
query 3' ACGAGAGAUAGAAGACAGUU 5'
query=miR157a-5p, target=RO3G_02069, score=3.5, range=974-992,
strand=1
target 5' UGCU-UUUGUCUUCUGUCAU 3'
:::: :.:.::::::::::
query 3' ACGAGAGAUAGAAGACAGUU 5'
query=miR157a-5p, target=RO3G_07431, score=3.5, range=135-153,
strand=1
target 5' UGCUC-CUAUCUUCUGUGAG 3'
::::: :::::::::: :.
query 3' ACGAGAGAUAGAAGACAGUU 5'
query=miR157a-5p, target=RO3G_12149, score=3.5, range=1069-1089,
strand=-1
target 5' AGCUCUAUUAUCUUCUGUCGA 3'
::::: .::::::::::::.:
query 3' ACGAGA-GAUAGAAGACAGUU 5'
query=miR157a-5p, target=RO3G_00816, score=3.5, range=335-354,
strand=-1
target 5' UGCUUUGUAUCUUCUGUCUA 3'
::::.: :::::::::: :
query 3' ACGAGAGAUAGAAGACAGUU 5'
query=miR157a-5p, target=RO3G_14433, score=3.5, range=5263-5281,
```

strand=-1
target 5' UGAUCUUU-UCUUCUGUCAA 3'
:: :::.: ::::::::::
query 3' ACGAGAGAUAGAAGACAGUU 5'
query=miR157a-5p, target=RO3G_03674, score=4, range=356-375, stra
target 5' UGAUCUCUGUCUUGUGUCAA 3'
:: :::::.:::: ::::::
query 3' ACGAGAGAUAGAAGACAGUU 5'
query=miR157a-5p, target=RO3G_02944, score=4, range=2526-2544,
strand=1
target 5' UGCUCACUAUUUUCU-UCAA 3'
::::: ::::.:::: ::::
query 3' ACGAGAGAUAGAAGACAGUU 5'
query=miR157a-5p, target=RO3G_12366, score=4, range=4974-4992,
strand=1
target 5' AGCACU-UAUCUUUUGUCAA 3'
:: :: ::::::.::::::
query 3' ACGAGAGAUAGAAGACAGUU 5'
query=miR157a-5p, target=RO3G_04854, score=4, range=614-632, stra
target 5' UGCUUUUUAUCUUCU-UUAA 3'
::::.:.:::::::: :.::
query 3' ACGAGAGAUAGAAGACAGUU 5'
query=miR157a-5p, target=RO3G_11943, score=4, range=892-910, stra
target 5' UGUCUUCU-UCUUCUGUCAA 3'
::. .::: ::::::::::
query 3' ACGAGAGAUAGAAGACAGUU 5'
query=miR157a-5p, target=RO3G_13979, score=4, range=842-861, stra
target 5' UUAUCUGUAUCUUCUGUCAU 3'
: ::: :::::::::::
query 3' ACGAGAGAUAGAAGACAGUU 5'
query=miR157a-5p, target=RO3G_08269, score=4, range=235-253, stra
target 5' UCUUUUCU-UCUUCUGUCAA 3'
: .:.::: ::::::::::
query 3' ACGAGAGAUAGAAGACAGUU 5'
query=miR1514a, target=RO3G_15904, score=0, range=657-678, strand
target 5' CAUGCCUAGUUUCAAAUUGAUA 3'
::::::::::::::::::::::
query 3' GUACGGAUCAAAGUUUAACUAU 5'
query=miR1514a, target=RO3G_16782, score=1, range=723-744, strand
target 5' CAUGCCUCGUUUCAAAUUGAUA 3'
::::::: ::::::::::::::
query 3' GUACGGAUCAAAGUUUAACUAU 5'
query=miR1514a, target=RO3G_16780, score=1, range=723-744, strand
target 5' CAUGCCUCGUUUCAAAUUGAUA 3'
::::::: ::::::::::::::

```
query 3' GUACGGAUCAAAGUUUAACUAU 5'
query=miR1514a, target=RO3G_16853, score=1, range=723-744, strand
target 5' CAUGCCUCGUUUCAAAUUGAUA 3'
::::::: ::::::::::::::
query 3' GUACGGAUCAAAGUUUAACUAU 5'
query=miR1514a, target=RO3G_16855, score=1, range=723-744, strand
target 5' CAUGCCUCGUUUCAAAUUGAUA 3'
::::::: ::::::::::::::
query 3' GUACGGAUCAAAGUUUAACUAU 5'
query=miR1514a, target=RO3G_07244, score=2, range=747-768, strand
target 5' CAUACCUCGUUUCAAAUUGAUA 3'
::: ::: ::::::::::::::
query 3' GUACGGAUCAAAGUUUAACUAU 5'
query=miR1514a, target=RO3G_06815, score=2, range=654-675, strand
target 5' CAUACCUCGUUUCAAAUUGAUA 3'
::: ::: ::::::::::::::
query 3' GUACGGAUCAAAGUUUAACUAU 5'
query=miR1514a, target=RO3G_07241, score=2, range=654-675, strand
target 5' CAUACCUCGUUUCAAAUUGAUA 3'
::: ::: ::::::::::::::
query 3' GUACGGAUCAAAGUUUAACUAU 5'
query=miR1514a, target=RO3G_09518, score=3, range=510-531, strand
target 5' CAUGGUUGGUUUCAGAUUGAUA 3'
:::: .:.:::::.:::::::
query 3' GUACGGAUCAAAGUUUAACUAU 5'
query=miR1514a, target=RO3G_06812, score=3, range=720-742, strand
target 5' UAUGAUCUCGUUUCAAAUUGAUA 3'
.::: .:: ::::::::::::::
query 3' GUAC-GGAUCAAAGUUUAACUAU 5'
query=miR1514a, target=RO3G_11033, score=4, range=1146-1167, stra
target 5' CAUGUCUUGUUUCAAAUAGAUG 3'
::::.:: ::::::::: :::.
query 3' GUACGGAUCAAAGUUUAACUAU 5'
query=miR5228, target=RO3G_16693, score=0, range=1470-1493, stran
target 5' UCCAUCAAGUUGCUAUCACCACUA 3'
::::::::::::::::::::::::
query 3' AGGUAGUUCAACGAUAGUGGUGAU 5'
query=miR5228, target=RO3G_12527, score=0, range=1470-1493, stran
target 5' UCCAUCAAGUUGCUAUCACCACUA 3'
::::::::::::::::::::::::
query 3' AGGUAGUUCAACGAUAGUGGUGAU 5'
query=miR5228, target=RO3G_14347, score=0, range=1464-1487, stran
target 5' UCCAUCAAGUUGCUAUCACCACUA 3'
::::::::::::::::::::::::
query 3' AGGUAGUUCAACGAUAGUGGUGAU 5'
```

query=miR5228, target=RO3G_16539, score=0, range=1362-1385, stran
target 5' UCCAUCAAGUUGCUAUCACCACUA 3'
::::::::::::::::::::::::
query 3' AGGUAGUUCAACGAUAGUGGUGAU 5'
query=miR5228, target=RO3G_12538, score=0, range=1362-1385, stran
target 5' UCCAUCAAGUUGCUAUCACCACUA 3'
::::::::::::::::::::::::
query 3' AGGUAGUUCAACGAUAGUGGUGAU 5'
query=miR5228, target=RO3G_06425, score=0, range=1464-1487, stran
target 5' UCCAUCAAGUUGCUAUCACCACUA 3'
::::::::::::::::::::::::
query 3' AGGUAGUUCAACGAUAGUGGUGAU 5'
query=miR5228, target=RO3G_09547, score=0, range=1464-1487, stran
target 5' UCCAUCAAGUUGCUAUCACCACUA 3'
::::::::::::::::::::::::
query 3' AGGUAGUUCAACGAUAGUGGUGAU 5'
query=miR5228, target=RO3G_16361, score=0, range=1464-1487, stran
target 5' UCCAUCAAGUUGCUAUCACCACUA 3'
::::::::::::::::::::::::
query 3' AGGUAGUUCAACGAUAGUGGUGAU 5'
query=miR5228, target=RO3G_05330, score=0, range=1464-1487, stran
target 5' UCCAUCAAGUUGCUAUCACCACUA 3'
::::::::::::::::::::::::
query 3' AGGUAGUUCAACGAUAGUGGUGAU 5'
query=miR5228, target=RO3G_17021, score=0, range=1464-1487, stran
target 5' UCCAUCAAGUUGCUAUCACCACUA 3'
::::::::::::::::::::::::
query 3' AGGUAGUUCAACGAUAGUGGUGAU 5'
query=miR5228, target=RO3G_12677, score=0, range=1464-1487, stran
target 5' UCCAUCAAGUUGCUAUCACCACUA 3'
::::::::::::::::::::::::
query 3' AGGUAGUUCAACGAUAGUGGUGAU 5'
query=miR5228, target=RO3G_04933, score=0, range=1464-1487, stran
target 5' UCCAUCAAGUUGCUAUCACCACUA 3'
::::::::::::::::::::::::
query 3' AGGUAGUUCAACGAUAGUGGUGAU 5'
query=miR5228, target=RO3G_02704, score=0, range=1464-1487, stran
target 5' UCCAUCAAGUUGCUAUCACCACUA 3'
::::::::::::::::::::::::
query 3' AGGUAGUUCAACGAUAGUGGUGAU 5'
query=miR5228, target=RO3G_05344, score=0, range=1464-1487, stran
target 5' UCCAUCAAGUUGCUAUCACCACUA 3'
::::::::::::::::::::::::
query 3' AGGUAGUUCAACGAUAGUGGUGAU 5'
query=miR5228, target=RO3G_03778, score=0, range=1422-1445, stran

target 5' UCCAUCAAGUUGCUAUCACCACUA 3'
::::::::::::::::::::::::
query 3' AGGUAGUUCAACGAUAGUGGUGAU 5'
query=miR5228, target=RO3G_17422, score=0, range=765-788, strand=
target 5' UCCAUCAAGUUGCUAUCACCACUA 3'
::::::::::::::::::::::::
query 3' AGGUAGUUCAACGAUAGUGGUGAU 5'
query=miR5228, target=RO3G_12532, score=0, range=1470-1493, stran
target 5' UCCAUCAAGUUGCUAUCACCACUA 3'
::::::::::::::::::::::::
query 3' AGGUAGUUCAACGAUAGUGGUGAU 5'
query=miR5228, target=RO3G_01936, score=0, range=1470-1493, stran
target 5' UCCAUCAAGUUGCUAUCACCACUA 3'
::::::::::::::::::::::::
query 3' AGGUAGUUCAACGAUAGUGGUGAU 5'
query=miR5228, target=RO3G_09493, score=0, range=255-278, strand=
target 5' UCCAUCAAGUUGCUAUCACCACUA 3'
::::::::::::::::::::::::
query 3' AGGUAGUUCAACGAUAGUGGUGAU 5'
query=miR5228, target=RO3G_00242, score=0, range=1470-1493, stran
target 5' UCCAUCAAGUUGCUAUCACCACUA 3'
::::::::::::::::::::::::
query 3' AGGUAGUUCAACGAUAGUGGUGAU 5'
query=miR5228, target=RO3G_11414, score=0, range=1362-1385, stran
target 5' UCCAUCAAGUUGCUAUCACCACUA 3'
::::::::::::::::::::::::
query 3' AGGUAGUUCAACGAUAGUGGUGAU 5'
query=miR5228, target=RO3G_08105, score=0, range=219-242, strand=
target 5' UCCAUCAAGUUGCUAUCACCACUA 3'
::::::::::::::::::::::::
query 3' AGGUAGUUCAACGAUAGUGGUGAU 5'
query=miR5228, target=RO3G_13918, score=1, range=1470-1493, stran
target 5' UCCAUCAAGUUGCUAUCACCGCUA 3'
:::::::::::::::::::::.:::
query 3' AGGUAGUUCAACGAUAGUGGUGAU 5'
query=miR5228, target=RO3G_10804, score=1, range=1470-1493, stran
target 5' UCCAUCAAGUUGCUAUCACCGCUA 3'
:::::::::::::::::::::.:::
query 3' AGGUAGUUCAACGAUAGUGGUGAU 5'
query=miR5228, target=RO3G_07311, score=1, range=1470-1493, stran
target 5' UCCAUCAAGUUGCUAUCACCGCUA 3'
:::::::::::::::::::::.:::
query 3' AGGUAGUUCAACGAUAGUGGUGAU 5'
query=miR5228, target=RO3G_01614, score=1, range=1470-1493, stran
target 5' UCCAUCAAGUUGCUAUCACCGCUA 3'

```
::::::::::::::::::::.:::
query 3' AGGUAGUUCAACGAUAGUGGUGAU 5'
query=miR5228, target=RO3G_04126, score=1, range=1464-1487, stran
target 5' UCCAUCAAGUUGCUAUCACCGCUA 3'
::::::::::::::::::::.:::
query 3' AGGUAGUUCAACGAUAGUGGUGAU 5'
query=miR5228, target=RO3G_12673, score=1, range=765-788, strand=
target 5' UCCAUCAAGUUGCUAUCACCGCUA 3'
::::::::::::::::::::.:::
query 3' AGGUAGUUCAACGAUAGUGGUGAU 5'
query=miR5228, target=RO3G_03619, score=1, range=1470-1493, stran
target 5' UCCAUCAAGUUGCUAUCACCGCUA 3'
::::::::::::::::::::.:::
query 3' AGGUAGUUCAACGAUAGUGGUGAU 5'
query=miR5228, target=RO3G_02821, score=1, range=1470-1493, stran
target 5' UCCAUCAAGUUGCUAUCACCGCUA 3'
::::::::::::::::::::.:::
query 3' AGGUAGUUCAACGAUAGUGGUGAU 5'
query=miR5228, target=RO3G_05515, score=1, range=459-482, strand=
target 5' UCCAUCAAGUUGCUAUCACCGCUA 3'
::::::::::::::::::::.:::
query 3' AGGUAGUUCAACGAUAGUGGUGAU 5'
query=miR5228, target=RO3G_00295, score=1, range=1308-1331, stran
target 5' UCCAUCAAGUUGCUAUCACCGCUA 3'
::::::::::::::::::::.:::
query 3' AGGUAGUUCAACGAUAGUGGUGAU 5'
query=miR5228, target=RO3G_13722, score=1, range=1461-1484, stran
target 5' UCCAUCAAUUUGCUAUCACCACUA 3'
:::::::: ::::::::::::::
query 3' AGGUAGUUCAACGAUAGUGGUGAU 5'
query=miR1428e-3p, target=RO3G_13882, score=0, range=631-652, str
target 5' AAAGAGUUCAUGGCAUUUCUUA 3'
::::::::::::::::::::::
query 3' UUUCUCAAGUACCGUAAAGAAU 5'
query=miR1428e-3p, target=RO3G_05325, score=0, range=631-652, str
target 5' AAAGAGUUCAUGGCAUUUCUUA 3'
::::::::::::::::::::::
query 3' UUUCUCAAGUACCGUAAAGAAU 5'
query=miR1428e-3p, target=RO3G_00809, score=0, range=631-652, str
target 5' AAAGAGUUCAUGGCAUUUCUUA 3'
::::::::::::::::::::::
query 3' UUUCUCAAGUACCGUAAAGAAU 5'
query=miR1428e-3p, target=RO3G_13730, score=0, range=394-415, str
target 5' AAAGAGUUCAUGGCAUUUCUUA 3'
::::::::::::::::::::::
```

query 3' UUUCUCAAGUACCGUAAAGAAU 5'
query=miR1428e-3p, target=RO3G_03721, score=1, range=631-652, str
target 5' AAAGAAUUCAUGGCAUUUCUUA 3'
::::: ::::::::::::::::
query 3' UUUCUCAAGUACCGUAAAGAAU 5'
query=miR1428e-3p, target=RO3G_00141, score=2, range=631-652, str
target 5' AAAGAAUUCAUGGUAUUUCUUA 3'
::::: :::::::.::::::::
query 3' UUUCUCAAGUACCGUAAAGAAU 5'
query=miR1428e-3p, target=RO3G_02269, score=2.5, range=107-128,
strand=-1
target 5' AAGAAAUUCAUGGCAUUUCUUA 3'
::. : ::::::::::::::::
query 3' UUUCUCAAGUACCGUAAAGAAU 5'
query=miR1850.1, target=RO3G_13985, score=1, range=15-35, strand=
target 5' UCCCAACUCCCAAACUUUCAU 3'
:::::::::::::::::::::
query 3' AGGGUUGAGGGUUUGAAAGUU 5'
query=miR1850.1, target=RO3G_10032, score=2, range=9-29, strand=1
target 5' UCCCAACUCCCAAACUUUCCA 3'
::::::::::::::::::: :
query 3' AGGGUUGAGGGUUUGAAAGUU 5'
query=miR1850.1, target=RO3G_14387, score=3, range=1228-1248, str
target 5' UUUCAAAUCCCAGACUUUCAA 3'
:..::: :::::.::::::::
query 3' AGGGUUGAGGGUUUGAAAGUU 5'
query=miR1850.1, target=RO3G_07512, score=3, range=77-96, strand=
target 5' UCUUAA-UCUCAAACUUUCAA 3'
::..:: ::.:::::::::::
query 3' AGGGUUGAGGGUUUGAAAGUU 5'
query=miR1850.1, target=RO3G_07363, score=4, range=2-22, strand=1
target 5' UGCCACCACCCAAACUUUCAC 3'
: ::: : ::::::::::::
query 3' AGGGUUGAGGGUUUGAAAGUU 5'
query=miR1850.1, target=RO3G_06331, score=4, range=1133-1153,
strand=-1
target 5' GUUCAAGUUCCAAACUUUCAA 3'
..:::: :.::::::::::::
query 3' AGGGUUGAGGGUUUGAAAGUU 5'
query=miR1850.1, target=RO3G_06931, score=4, range=404-424, stran
target 5' UCUUAUCGCCUAAACUUUCAA 3'
::..: : ::.::::::::::
query 3' AGGGUUGAGGGUUUGAAAGUU 5'
query=miR1850.1, target=RO3G_08585, score=4, range=893-912, stran
target 5' UGUUAA-UCCUAAACUUUCAA 3'

```
: ..:: :::.::::::::::
query 3' AGGGUUGAGGGUUUGAAAGUU 5'
query=miR835-3p, target=RO3G_14509, score=0, range=838-856, stran
target 5' CUCCUGGCGUAUCUCUUCA 3'
:::::::::::::::::::
query 3' GAGGACCGCAUAGAGAAGU 5'
query=miR835-3p, target=RO3G_17407, score=3, range=810-828, stran
target 5' CUCAGGGCGUAUUUCUUCA 3'
::: ::::::.::::::
query 3' GAGGACCGCAUAGAGAAGU 5'
query=miR835-3p, target=RO3G_12688, score=3.5, range=500-517, str
target 5' CUUCUGGCG-AUCUCUUCU 3'
::.:::::: ::::::::
query 3' GAGGACCGCAUAGAGAAGU 5'
query=miR835-3p, target=RO3G_15730, score=3.5, range=2547-2566,
strand=-1
target 5' CUACUUGGCGCAUCUCUUCA 3'
:: :.::::: :::::::::
query 3' GA-GGACCGCAUAGAGAAGU 5'
query=miR835-3p, target=RO3G_14020, score=3.5, range=3683-3700,
strand=-1
target 5' UUGCUGGCGUAUCU-UUCA 3'
.: :::::::::: ::::
query 3' GAGGACCGCAUAGAGAAGU 5'
query=miR835-3p, target=RO3G_12347, score=4, range=438-456, stran
target 5' UUCUUGGGGUAUCUCUUCU 3'
.::.::: :::::::::
query 3' GAGGACCGCAUAGAGAAGU 5'
query=miR835-3p, target=RO3G_13123, score=4, range=2850-2867,
strand=-1
target 5' CUUUU-GCAUAUCUCUUCA 3'
::..: :: ::::::::::
query 3' GAGGACCGCAUAGAGAAGU 5'
query=miR7536a, target=RO3G_01539, score=3.5, range=2028-2045,
strand=-1
target 5' UAUUCU-GAGCAUGCUUCA 3'
.: ::: :::::::::::
query 3' GUCAGAGCUCGUACGAAGU 5'
query=miR7536a, target=RO3G_11116, score=3.5, range=266-283, stra
target 5' AAGUUUC-AGCAUGCUUCA 3'
:::.:: ::::::::::
query 3' GUCAGAGCUCGUACGAAGU 5'
query=miR7536a, target=RO3G_01272, score=4, range=3251-3269, stra
target 5' CGGUCUGGAGUAUGCUUCG 3'
:.:::: :::.:::::::.
```

```
query 3' GUCAGAGCUCGUACGAAGU 5'
query=miR7536a, target=RO3G_13195, score=4, range=1963-1982, stra
target 5' CAGCUCGAGAGCAUGCUUCA 3'
::: :: :::::::::::
query 3' GUC-AGAGCUCGUACGAAGU 5'
query=miR7536a, target=RO3G_09900, score=4, range=655-672, strand
target 5' CAGUAU-GAGCAUGCUUCU 3'
:::: : ::::::::::
query 3' GUCAGAGCUCGUACGAAGU 5'
query=miR7536a, target=RO3G_10956, score=4, range=1451-1468, stra
target 5' CA-UCUUGAGCGUGUUUCA 3'
:: :::.::::.::.::::
query 3' GUCAGAGCUCGUACGAAGU 5'
query=miR7536a, target=RO3G_04849, score=4, range=468-486, strand
target 5' CUGUCUCGAGCAUGCAUCC 3'
: ::::::::::::: ::
query 3' GUCAGAGCUCGUACGAAGU 5'
query=miR7536a, target=RO3G_12591, score=4, range=516-533, strand
target 5' CAUUCUUGAGCAUG-UUCA 3'
:: :::.:::::::: ::::
query 3' GUCAGAGCUCGUACGAAGU 5'
query=miR7536a, target=RO3G_15479, score=4, range=4203-4220, stra
target 5' UGGU-GCGAGCAUGUUUCA 3'
..:: :::::::::.::::
query 3' GUCAGAGCUCGUACGAAGU 5'
query=miR7536a, target=RO3G_00227, score=4, range=26-44, strand=-
target 5' CCAUAACGAGCAUGCUUCA 3'
: : ::::::::::::
query 3' GUCAGAGCUCGUACGAAGU 5'
query=miR7536a, target=RO3G_01978, score=4, range=1356-1373, stra
target 5' CUGCCUCGAGCAUG-UUCA 3'
: : :::::::::: ::::
query 3' GUCAGAGCUCGUACGAAGU 5'
query=miR856-3p, target=RO3G_02165, score=2, range=2-21, strand=-
target 5' AAUAACCACAAUCACAUUCA 3'
:::::::::::::::::::
query 3' AAAUUGGUGUUAGUGUAAGU 5'
query=miR856-3p, target=RO3G_08586, score=3, range=206-225, stran
target 5' UUAAAAAACAAUCACAUUCA 3'
:: :: :::::::::::::
query 3' AAAUUGGUGUUAGUGUAAGU 5'
query=miR856-3p, target=RO3G_05389, score=3.5, range=3773-3791,
strand=-1
target 5' UUUAA-UAUAGUCACAUUCA 3'
::::: .:.:.::::::::
```

```
query 3' AAAUUGGUGUUAGUGUAAGU 5'
query=miR856-3p, target=RO3G_14809, score=4, range=135-154, stran
target 5' UCUUACUACAAUCACAUUUG 3'
: : ::.::::::::::..
query 3' AAAUUGGUGUUAGUGUAAGU 5'
query=miR856-3p, target=RO3G_03718, score=4, range=69-89, strand=
target 5' GUUCAUUAACAAUCACAUUCA 3'
:: :.. ::::::::::::
query 3' AAAUUGG-UGUUAGUGUAAGU 5'
query=miR856-3p, target=RO3G_13946, score=4, range=348-368, stran
target 5' UUUACACCACGGUCAUAUUCA 3'
:::: :::::..:::.:::::
query 3' AAAU-UGGUGUUAGUGUAAGU 5'
query=miR856-3p, target=RO3G_02487, score=4, range=5511-5530,
strand=-1
target 5' CAUAAUUAUAAUCACAUUCA 3'
:::..:.:::::::::::
query 3' AAAUUGGUGUUAGUGUAAGU 5'
query=miR856-3p, target=RO3G_02020, score=4, range=195-214, stran
target 5' UUGAACAAUAAUCACAUUUA 3'
:: ::: :.:::::::::.:
query 3' AAAUUGGUGUUAGUGUAAGU 5'
query=miR856-3p, target=RO3G_01403, score=4, range=1716-1734,
strand=-1
target 5' UUUAAGCACAAUCAC-UUUA 3'
::::: ::::::::: ::.:
query 3' AAAUUGGUGUUAGUGUAAGU 5'
query=miR856-3p, target=RO3G_00933, score=4, range=2679-2697,
strand=-1
target 5' UUUAA-GACAAUCAUAUUUA 3'
::::: ::::::.:::.:
query 3' AAAUUGGUGUUAGUGUAAGU 5'
query=miR6030, target=RO3G_02123, score=3.5, range=1708-1728, str
target 5' GGUAUGGGUGGUAUGGGUGGU 3'
::: :: .::::::::::::
query 3' CCAGACAUACCAUACCCACCU 5'
query=miR6030, target=RO3G_02086, score=4, range=2452-2472, stran
target 5' GAACUUAAUGGUAUGGGUGGA 3'
: :: :::::::::::::
query 3' CCAGACAUACCAUACCCACCU 5'
query=miR6030, target=RO3G_06021, score=4, range=713-732, strand=
target 5' GUUCUCU-UGGUAUGGGUGGU 3'
: ::: : :::::::::::
query 3' CCAGACAUACCAUACCCACCU 5'
query=miR773a, target=RO3G_04126, score=0, range=900-920, strand=
```

```
target 5' CGUGACAAAAGUGGCAGCAAA 3'
:::::::::::::::::::::
query 3' GCACUGUUUUCACCGUCGUUU 5'
query=miR773a, target=RO3G_16782, score=2, range=651-670, strand=
target 5' GGUG-CAAAAGUGGCAGCAAA 3'
::: ::::::::::::::::
query 3' GCACUGUUUUCACCGUCGUUU 5'
query=miR773a, target=RO3G_16780, score=2, range=651-670, strand=
target 5' GGUG-CAAAAGUGGCAGCAAA 3'
::: ::::::::::::::::
query 3' GCACUGUUUUCACCGUCGUUU 5'
query=miR773a, target=RO3G_16853, score=2, range=651-670, strand=
target 5' GGUG-CAAAAGUGGCAGCAAA 3'
::: ::::::::::::::::
query 3' GCACUGUUUUCACCGUCGUUU 5'
query=miR773a, target=RO3G_16855, score=2, range=651-670, strand=
target 5' GGUG-CAAAAGUGGCAGCAAA 3'
::: ::::::::::::::::
query 3' GCACUGUUUUCACCGUCGUUU 5'
query=miR773a, target=RO3G_15904, score=2, range=585-604, strand=
target 5' GGUG-CAAAAGUGGCAGCAAA 3'
::: ::::::::::::::::
query 3' GCACUGUUUUCACCGUCGUUU 5'
query=miR773a, target=RO3G_16361, score=3, range=900-920, strand=
target 5' CGUGACAAAAGUUGCGGCAAA 3'
:::::::::::: ::.:::::
query 3' GCACUGUUUUCACCGUCGUUU 5'
query=miR773a, target=RO3G_17422, score=3, range=201-221, strand=
target 5' CGUGACAAAAGUUGCGGCAAA 3'
:::::::::::: ::.:::::
query 3' GCACUGUUUUCACCGUCGUUU 5'
query=miR773a, target=RO3G_07244, score=4, range=674-694, strand=
target 5' GGGUCCAAAAGUGGCAGCAAA 3'
: :::::::::::::::::
query 3' GCACUGUUUUCACCGUCGUUU 5'
query=miR773a, target=RO3G_06815, score=4, range=581-601, strand=
target 5' GGGUCCAAAAGUGGCAGCAAA 3'
: :::::::::::::::::
query 3' GCACUGUUUUCACCGUCGUUU 5'
query=miR773a, target=RO3G_12228, score=4, range=647-667, strand=
target 5' GGGUCCAAAAGUGGCAGCAAA 3'
: :::::::::::::::::
query 3' GCACUGUUUUCACCGUCGUUU 5'
query=miR773a, target=RO3G_12986, score=4, range=647-667, strand=
target 5' GGGUCCAAAAGUGGCAGCAAA 3'
```

```
: :::::::::::::::
query 3' GCACUGUUUUCACCGUCGUUU 5'
query=miR773a, target=RO3G_12985, score=4, range=647-667, strand=
target 5' GGGUCCAAAAGUGGCAGCAAA 3'
: :::::::::::::::
query 3' GCACUGUUUUCACCGUCGUUU 5'
query=miR773a, target=RO3G_12229, score=4, range=647-667, strand=
target 5' GGGUCCAAAAGUGGCAGCAAA 3'
: :::::::::::::::
query 3' GCACUGUUUUCACCGUCGUUU 5'
query=miR773a, target=RO3G_07241, score=4, range=581-601, strand=
target 5' GGGUCCAAAAGUGGCAGCAAA 3'
: :::::::::::::::
query 3' GCACUGUUUUCACCGUCGUUU 5'
query=miR773a, target=RO3G_00573, score=4, range=419-439, strand=
target 5' GGGUUCAAAAGUGGCAGCAAA 3'
: :::::::::::::::
query 3' GCACUGUUUUCACCGUCGUUU 5'
query=miR773a, target=RO3G_06812, score=4, range=647-667, strand=
target 5' GGGUCCAAAAGUGGCAGCAAA 3'
: :::::::::::::::
query 3' GCACUGUUUUCACCGUCGUUU 5'
query=miR773a, target=RO3G_15475, score=4, range=467-487, strand=
target 5' GGGUCCAAAAGUGGCAGCAAA 3'
: :::::::::::::::
query 3' GCACUGUUUUCACCGUCGUUU 5'
query=miR773a, target=RO3G_01424, score=4, range=347-366, strand=
target 5' UGUUGC-GAAGUGGCAGCAAG 3'
.:: .: .::::::::::::.
query 3' GCACUGUUUUCACCGUCGUUU 5'
query=miR5287a, target=RO3G_16747, score=0, range=30-51, strand=-
target 5' AUCCUCCGUCAUAAUAUUGCAA 3'
::::::::::::::::::::::
query 3' UAGGAGGCAGUAUUAUAACGUU 5'
query=miR5287a, target=RO3G_07725, score=0, range=30-51, strand=-
target 5' AUCCUCCGUCAUAAUAUUGCAA 3'
::::::::::::::::::::::
query 3' UAGGAGGCAGUAUUAUAACGUU 5'
query=miR5287a, target=RO3G_15166, score=2, range=639-660, strand
target 5' AUCCUCCGUCAUAAUUUUGCAA 3'
:::::::::::::: ::::::
query 3' UAGGAGGCAGUAUUAUAACGUU 5'
query=miR5287a, target=RO3G_00287, score=2, range=531-552, strand
target 5' AUCCUCCGUCAUAAUCUUGCAA 3'
:::::::::::::: ::::::
```

query 3' UAGGAGGCAGUAUUAUAACGUU 5'
query=miR5287a, target=RO3G_05940, score=2, range=348-369, strand
target 5' AUCCUCCGUCAUAAUCUUGCAA 3'
::::::::::::::: ::::::
query 3' UAGGAGGCAGUAUUAUAACGUU 5'
query=miR5287a, target=RO3G_12198, score=3, range=39-60, strand=-
target 5' AUCCUCCAUCAUAAUCUUGCAA 3'
::::::: ::::::: ::::::
query 3' UAGGAGGCAGUAUUAUAACGUU 5'
query=miR5287a, target=RO3G_00237, score=4, range=108-129, strand
target 5' AUCCUCCGUCAUAACCUUGCAA 3'
:::::::::::::: ::::::
query 3' UAGGAGGCAGUAUUAUAACGUU 5'
query=miR5654a, target=RO3G_10995, score=3.5, range=161-180, stra
target 5' GGAUGGUGUUUUGGAUUUAU 3'
:::::.::.:: ::::::::
query 3' CCUACUACGAACCCUAAAUA 5'
query=miR5654a, target=RO3G_02461, score=3.5, range=1316-1335,
strand=-1
target 5' AAAUUGUGCUUGGGAUUUAU 3'
:: .::::::::::::::
query 3' CCUACUACGAACCCUAAAUA 5'
query=miR5654a, target=RO3G_12701, score=4, range=125-145, strand
target 5' UUUUGACUGCUUGGGAUUUAU 3'
::: ::::::::::::::
query 3' CCUACU-ACGAACCCUAAAUA 5'
query=miR9672-5p, target=RO3G_10363, score=0, range=1486-1506,
strand=-1
target 5' GACACCACUGACUGCCUUAGG 3'
:::::::::::::::::::::
query 3' CUGUGGUGACUGACGGAAUCC 5'
query=miR9672-5p, target=RO3G_01142, score=4, range=259-278, stra
target 5' GGCAUC-CUGACUGCCUUGGC 3'
:.::.: :::::::::::.:
query 3' CUGUGGUGACUGACGGAAUCC 5'
query=miR9672-5p, target=RO3G_08461, score=4, range=691-711, stra
target 5' GAUAUCAGUGAUUGCUUUAGG 3'
::.:.:: :::.:::.:::::
query 3' CUGUGGUGACUGACGGAAUCC 5'
query=miR9672-5p, target=RO3G_14127, score=4, range=162-181, stra
target 5' GA-ACUGCUGACUGCUUUAGC 3'
:: ::..:::::::::.::::
query 3' CUGUGGUGACUGACGGAAUCC 5'
query=miR1512a-3p, target=RO3G_12005, score=1, range=1189-1210,
strand=-1

target 5' CAAAACUGACAUUCUUAAGGCA 3'
::::::::::::::::::.:::
query 3' GUUUUGACUGUAAGAAUUUCGU 5'
query=miR1512a-3p, target=RO3G_04676, score=1, range=1189-1210, strand=-1
target 5' CAAAACUGACAUUCUUAAGGCA 3'
::::::::::::::::::.:::
query 3' GUUUUGACUGUAAGAAUUUCGU 5'
query=miR1512a-3p, target=RO3G_05928, score=1, range=961-982, strand=-1
target 5' CAAAACUGACAUUCUUAAGGCA 3'
::::::::::::::::::.:::
query 3' GUUUUGACUGUAAGAAUUUCGU 5'
query=miR1512a-3p, target=RO3G_12080, score=1, range=772-793, strand=-1
target 5' CAAAACUGACAUUCUUAAGGCA 3'
::::::::::::::::::.:::
query 3' GUUUUGACUGUAAGAAUUUCGU 5'
query=miR1512a-3p, target=RO3G_11407, score=1, range=772-793, strand=-1
target 5' CAAAACUGACAUUCUUAAGGCA 3'
::::::::::::::::::.:::
query 3' GUUUUGACUGUAAGAAUUUCGU 5'
query=miR1512a-3p, target=RO3G_14636, score=1, range=565-586, strand=-1
target 5' CAAAACUGACAUUCUUAAGGCA 3'
::::::::::::::::::.:::
query 3' GUUUUGACUGUAAGAAUUUCGU 5'
query=miR1512a-3p, target=RO3G_12687, score=1.5, range=565-586, strand=-1
target 5' CAAAGCUGACAUUCUUAAGGCA 3'
::::.:::::::::::::.:::
query 3' GUUUUGACUGUAAGAAUUUCGU 5'
query=miR1512a-3p, target=RO3G_13717, score=3, range=564-585, strand=-1
target 5' CAAAACUGAAAUUCUUAAGGCA 3'
::::::::: ::::::::.:::
query 3' GUUUUGACUGUAAGAAUUUCGU 5'
query=miR6264, target=RO3G_00009, score=0, range=312-329, strand=
target 5' UUGACACGGUCCAAGAAU 3'
::::::::::::::::::
query 3' AACUGUGCCAGGUUCUUA 5'
query=miR6264, target=RO3G_15055, score=0, range=312-329, strand=
target 5' UUGACACGGUCCAAGAAU 3'
::::::::::::::::::

```
query 3' AACUGUGCCAGGUUCUUA 5'
query=miR6264, target=RO3G_08282, score=0, range=312-329, strand=
target 5' UUGACACGGUCCAAGAAU 3'
::::::::::::::::::
query 3' AACUGUGCCAGGUUCUUA 5'
query=miR6264, target=RO3G_08159, score=1, range=312-329, strand=
target 5' UUAACACGGUCCAAGAAU 3'
:: :::::::::::::::
query 3' AACUGUGCCAGGUUCUUA 5'
query=miR6264, target=RO3G_03474, score=3, range=538-555, strand=
target 5' GUAACAUGGUCCAAGAAU 3'
: :::.:::::::::::
query 3' AACUGUGCCAGGUUCUUA 5'
query=miR6264, target=RO3G_12260, score=3, range=288-305, strand=
target 5' CUGGUACGGUUCAAGAAU 3'
::..:::::.:::::::
query 3' AACUGUGCCAGGUUCUUA 5'
query=miR6264, target=RO3G_07359, score=3, range=797-815, strand=
target 5' UUGAGCACUGUCCAAGAAU 3'
:::: ::: ::::::::::
query 3' AACU-GUGCCAGGUUCUUA 5'
query=miR6264, target=RO3G_13521, score=3.5, range=513-530, stran
target 5' UUGGCACGGUCCGGGAAG 3'
:::.:::::::::..:::
query 3' AACUGUGCCAGGUUCUUA 5'
query=miR6264, target=RO3G_15623, score=3.5, range=261-278, stran
target 5' UUGGCACGAUCCAAGAAA 3'
:::.:::: ::::::::
query 3' AACUGUGCCAGGUUCUUA 5'
query=miR6264, target=RO3G_17127, score=3.5, range=1739-1756,
strand=-1
target 5' UUGUUACGGUCAAAGAAU 3'
::: .:::::: ::::::
query 3' AACUGUGCCAGGUUCUUA 5'
query=miR6264, target=RO3G_14386, score=4, range=286-303, strand=
target 5' UUGACACGGGCCAAGGAG 3'
::::::::: :::::.:
query 3' AACUGUGCCAGGUUCUUA 5'
query=miR6264, target=RO3G_02400, score=4, range=422-439, strand=
target 5' UAUACAUGGUUCAAGAAU 3'
: :::.:::.:::::::
query 3' AACUGUGCCAGGUUCUUA 5'
query=miR6264, target=RO3G_17159, score=4, range=383-400, strand=
target 5' UUGAAAUGGUACAAGAAU 3'
:::: :.::: :::::::
```

```
query 3' AACUGUGCCAGGUUCUUA 5'
query=miR6264, target=RO3G_02698, score=4, range=263-280, strand=
target 5' UUGAAAUGGUACAAGAAU 3'
:::: :.::: :::::::
query 3' AACUGUGCCAGGUUCUUA 5'
query=miR6264, target=RO3G_08564, score=4, range=403-421, strand=
target 5' UUGACAAUGGUACAAGAAU 3'
::::: :.::: :::::::
query 3' AACUG-UGCCAGGUUCUUA 5'
query=miR6264, target=RO3G_14331, score=4, range=4663-4680, stran
target 5' AUGAAACGGUUCAAGAAA 3'
::: ::::.::::::
query 3' AACUGUGCCAGGUUCUUA 5'
query=miR6264, target=RO3G_02636, score=4, range=455-471, strand=
target 5' UUGA-AUGGUUCAAGAAC 3'
:::: :.:::.::::::
query 3' AACUGUGCCAGGUUCUUA 5'
query=miR6264, target=RO3G_04361, score=4, range=455-471, strand=
target 5' UUGA-AUGGUUCAAGAAC 3'
:::: :.:::.::::::
query 3' AACUGUGCCAGGUUCUUA 5'
query=miR6264, target=RO3G_09340, score=4, range=455-471, strand=
target 5' UUGA-AUGGUUCAAGAAC 3'
:::: :.:::.::::::
query 3' AACUGUGCCAGGUUCUUA 5'
query=miR6264, target=RO3G_00174, score=4, range=455-471, strand=
target 5' UUGA-AUGGUUCAAGAAC 3'
:::: :.:::.::::::
query 3' AACUGUGCCAGGUUCUUA 5'
query=miR6264, target=RO3G_08147, score=4, range=434-450, strand=
target 5' UUGA-AUGGUUCAAGAAC 3'
:::: :.:::.::::::
query 3' AACUGUGCCAGGUUCUUA 5'
query=miR6264, target=RO3G_05475, score=4, range=58-74, strand=1
target 5' UGGAC-CGGUUCAAGAAU 3'
: ::: ::::.:::::::
query 3' AACUGUGCCAGGUUCUUA 5'
query=miR6264, target=RO3G_06915, score=4, range=916-933, strand=
target 5' UCAAGACGGUCCAAGAGU 3'
: : :::::::::::.:
query 3' AACUGUGCCAGGUUCUUA 5'
query=miR6264, target=RO3G_06635, score=4, range=3200-3216, stran
target 5' UUG-CACGAUCCAAGAAA 3'
::: :::: ::::::::
query 3' AACUGUGCCAGGUUCUUA 5'
```

```
query=miR6264, target=RO3G_00491, score=4, range=640-656, strand=
target 5' UUGACAC-GUCUAGGAAU 3'
::::::: :::.:.::::
query 3' AACUGUGCCAGGUUCUUA 5'
query=miR6264, target=RO3G_00494, score=4, range=640-656, strand=
target 5' UUGACAC-GUCUAGGAAU 3'
::::::: :::.:.::::
query 3' AACUGUGCCAGGUUCUUA 5'
query=miR6264, target=RO3G_10833, score=4, range=252-269, strand=
target 5' GUGGUGCGGUCCAAGAGU 3'
::...::::::::::.:
query 3' AACUGUGCCAGGUUCUUA 5'
query=miR6264, target=RO3G_11562, score=4, range=1260-1277, stran
target 5' UUGACAGGUUCCAAGAAU 3'
:::::: : :::::::::
query 3' AACUGUGCCAGGUUCUUA 5'
query=miR6264, target=RO3G_04559, score=4, range=575-593, strand=
target 5' UUGAUCACUGUCCAAGAGU 3'
:::: ::: ::::::::.:
query 3' AACU-GUGCCAGGUUCUUA 5'
query=miR6264, target=RO3G_08751, score=4, range=1253-1270, stran
target 5' UCGCCAUGGUCCAAGGAU 3'
: : ::.::::::::.::
query 3' AACUGUGCCAGGUUCUUA 5'
query=miR6264, target=RO3G_00574, score=4, range=306-323, strand=
target 5' UUAGUACAGUCCAAGAAU 3'
:: ..:: ::::::::::
query 3' AACUGUGCCAGGUUCUUA 5'
query=miR6264, target=RO3G_10152, score=4, range=2664-2682, stran
target 5' CUGAUCACGGUCCAAGAGA 3'
::: ::::::::::::.
query 3' AACU-GUGCCAGGUUCUUA 5'
query=miR6264, target=RO3G_07538, score=4, range=1495-1511, stran
target 5' UUGACACGGU-CAAGAGC 3'
:::::::::: :::::.
query 3' AACUGUGCCAGGUUCUUA 5'
query=miR6264, target=RO3G_01480, score=4, range=203-219, strand=
target 5' UUCCCAC-GUCCAAGAAU 3'
:: ::: ::::::::::
query 3' AACUGUGCCAGGUUCUUA 5'
query=miR6264, target=RO3G_04131, score=4, range=1013-1029, stran
target 5' UUGACA-GGUCCAGGAAA 3'
:::::: ::::::.:::
query 3' AACUGUGCCAGGUUCUUA 5'
query=miR9552b-3p, target=RO3G_19000, score=1, range=676-693,
```

strand=-1
target 5' GCUAUCGGUUUCUCGCCA 3'
::::::::.::::::::
query 3' CGAUAGCCAGAGAGCGGU 5'
query=miR9552b-3p, target=RO3G_19002, score=1, range=676-693,
strand=-1
target 5' GCUAUCGGUUUCUCGCCA 3'
::::::::.::::::::
query 3' CGAUAGCCAGAGAGCGGU 5'
query=miR9552b-3p, target=RO3G_01749, score=3, range=187-204, str
target 5' GCCAUCGGUCUCUCGCUC 3'
:: ::::::::::::.
query 3' CGAUAGCCAGAGAGCGGU 5'
query=miR9552b-3p, target=RO3G_08147, score=3, range=380-398, str
target 5' GUUAUCGUGUCUCUCGCCG 3'
:.::::: :::::::::.
query 3' CGAUAGC-CAGAGAGCGGU 5'
query=miR9552b-3p, target=RO3G_15177, score=3, range=174-191,
strand=-1
target 5' GCCUUCGGUUUCUCGCCA 3'
:: ::::.::::::::
query 3' CGAUAGCCAGAGAGCGGU 5'
query=miR9552b-3p, target=RO3G_09077, score=3.5, range=1057-1074,
strand=1
target 5' GGUGUUGGUCUCUUGCCA 3'
: :.:.:::::::.::::
query 3' CGAUAGCCAGAGAGCGGU 5'
query=miR9552b-3p, target=RO3G_09074, score=3.5, range=1057-1074,
strand=1
target 5' GGUGUUGGUCUCUUGCCA 3'
: :.:.:::::::.::::
query 3' CGAUAGCCAGAGAGCGGU 5'
query=miR9552b-3p, target=RO3G_03948, score=4, range=67-84, stran
target 5' GCUAUCGGUCUCUCAUUA 3'
:::::::::::::: ..:
query 3' CGAUAGCCAGAGAGCGGU 5'
query=miR9552b-3p, target=RO3G_02584, score=4, range=148-165, str
target 5' GCUACCGGUCUCUUGCUC 3'
:::: ::::::::.::.
query 3' CGAUAGCCAGAGAGCGGU 5'
query=miR9552b-3p, target=RO3G_07084, score=4, range=148-165, str
target 5' GCUACCGGUCUCUUGCUU 3'
:::: ::::::::.::.
query 3' CGAUAGCCAGAGAGCGGU 5'
query=miR9552b-3p, target=RO3G_00058, score=4, range=1521-1539,

```
strand=1
target 5' UCUGAUCGGUCUCUUGCCC 3'
:: ::::::::::.:::
query 3' CGA-UAGCCAGAGAGCGGU 5'
query=miR9552b-3p, target=RO3G_14836, score=4, range=85-102, stra
target 5' CCAACCGGUCUUUCGCCA 3'
: : ::::::.::::::
query 3' CGAUAGCCAGAGAGCGGU 5'
query=miR9552b-3p, target=RO3G_00174, score=4, range=380-398, str
target 5' GUUACCGUGUCUCUCGCCG 3'
:.:: :: ::::::::::.
query 3' CGAUAGC-CAGAGAGCGGU 5'
query=miR9552b-3p, target=RO3G_12433, score=4, range=3236-3252,
strand=-1
target 5' ACUAUCGGUCUC-CGUCA 3'
:::::::::: ::.::
query 3' CGAUAGCCAGAGAGCGGU 5'
query=miR9552b-3p, target=RO3G_05760, score=4, range=745-761,
strand=-1
target 5' GC-AUCUGUCUCUUGCCA 3'
:: ::: ::::::.::::
query 3' CGAUAGCCAGAGAGCGGU 5'
query=miR8141, target=RO3G_03764, score=0.5, range=502-521, stran
target 5' GCACCACCAACUAGUAUGCA 3'
.:::::::::::::::::::
query 3' UGUGGUGGUUGAUCAUACGU 5'
query=miR8141, target=RO3G_05431, score=0.5, range=829-848, stran
target 5' GCACCACCAACUAGUAUGCA 3'
.:::::::::::::::::::
query 3' UGUGGUGGUUGAUCAUACGU 5'
query=miR8141, target=RO3G_05421, score=0.5, range=541-560, stran
target 5' GCACCACCAACUAGUAUGCA 3'
.:::::::::::::::::::
query 3' UGUGGUGGUUGAUCAUACGU 5'
query=miR8141, target=RO3G_12666, score=0.5, range=502-521, stran
target 5' GCACCACCAACUAGUAUGCA 3'
.:::::::::::::::::::
query 3' UGUGGUGGUUGAUCAUACGU 5'
query=miR8141, target=RO3G_12954, score=0.5, range=871-890, stran
target 5' GCACCACCAACUAGUAUGCA 3'
.:::::::::::::::::::
query 3' UGUGGUGGUUGAUCAUACGU 5'
query=miR8141, target=RO3G_12071, score=0.5, range=502-521, stran
target 5' GCACCACCAACUAGUAUGCA 3'
.:::::::::::::::::::
```

```
query 3' UGUGGUGGUUGAUCAUACGU 5'
query=miR8141, target=RO3G_03776, score=0.5, range=331-350, stran
target 5' GCACCACCAACUAGUAUGCA 3'
.:::::::::::::::::::
query 3' UGUGGUGGUUGAUCAUACGU 5'
query=miR8141, target=RO3G_14560, score=0.5, range=739-758, stran
target 5' GCACCACCAACUAGUAUGCA 3'
.:::::::::::::::::::
query 3' UGUGGUGGUUGAUCAUACGU 5'
query=miR8141, target=RO3G_13858, score=0.5, range=502-521, stran
target 5' GCACCACCAACUAGUAUGCA 3'
.:::::::::::::::::::
query 3' UGUGGUGGUUGAUCAUACGU 5'
query=miR8141, target=RO3G_16729, score=2, range=739-758, strand=
target 5' ACACCACCAAGUAGUAUGCA 3'
:::::::::: :::::::::
query 3' UGUGGUGGUUGAUCAUACGU 5'
query=miR8141, target=RO3G_02713, score=2.5, range=601-620, stran
target 5' GCACCACCAAGUAGUAUGCA 3'
.::::::::: :::::::::
query 3' UGUGGUGGUUGAUCAUACGU 5'
query=miR8141, target=RO3G_08115, score=2.5, range=574-593, stran
target 5' GCACCACCAAGUAGUAUGCA 3'
.::::::::: :::::::::
query 3' UGUGGUGGUUGAUCAUACGU 5'
query=miR8141, target=RO3G_04690, score=2.5, range=871-890, stran
target 5' GCACCACCAAGUAGUAUGCA 3'
.::::::::: :::::::::
query 3' UGUGGUGGUUGAUCAUACGU 5'
query=miR8141, target=RO3G_15135, score=2.5, range=4077-4096,
strand=-1
target 5' GCACCACCAAGUAGUAUGCA 3'
.::::::::: :::::::::
query 3' UGUGGUGGUUGAUCAUACGU 5'
query=miR8141, target=RO3G_07774, score=4, range=247-266, strand=
target 5' GCACCAUCAGCUGGUAUGCC 3'
.:::::.::.::.::::::
query 3' UGUGGUGGUUGAUCAUACGU 5'
query=miR8141, target=RO3G_09734, score=4, range=219-239, strand=
target 5' GCACCAUGCAACUAGUAUGAA 3'
.:::::. ::::::::::: :
query 3' UGUGGUG-GUUGAUCAUACGU 5'
query=miR8141, target=RO3G_14180, score=4, range=642-662, strand=
target 5' ACAUCAUCAAUUAGUGAUGCA 3'
:::.::.:::.:::: :::::
```

```
query 3' UGUGGUGGUUGAUCA-UACGU 5'
query=miR902a-5p, target=RO3G_12957, score=0.5, range=327-347,
strand=1
target 5' UGAAGAAGCGUGCAUGCAGUG 3'
:::::::::::::::::::.
query 3' ACUUCUUCGCACGUACGUCAU 5'
query=miR902a-5p, target=RO3G_13735, score=1, range=327-347, stra
target 5' UGAAGAAGCGUGUAUGCAGUA 3'
::::::::::::.::::::::
query 3' ACUUCUUCGCACGUACGUCAU 5'
query=miR902a-5p, target=RO3G_12179, score=1, range=327-347, stra
target 5' UGAAGAAGCGUGUAUGCAGUA 3'
::::::::::::.::::::::
query 3' ACUUCUUCGCACGUACGUCAU 5'
query=miR902a-5p, target=RO3G_07110, score=1, range=327-347, stra
target 5' UGAAGAAGCGUGCAUGCAGUU 3'
::::::::::::::::::::
query 3' ACUUCUUCGCACGUACGUCAU 5'
query=miR902a-5p, target=RO3G_07678, score=1, range=327-347, stra
target 5' UGAAGAAGCGUGCAUGCAGUU 3'
::::::::::::::::::::
query 3' ACUUCUUCGCACGUACGUCAU 5'
query=miR902a-5p, target=RO3G_07676, score=1, range=327-347, stra
target 5' UGAAGAAGCGUGCAUGCAGUU 3'
::::::::::::::::::::
query 3' ACUUCUUCGCACGUACGUCAU 5'
query=miR902a-5p, target=RO3G_01949, score=1, range=327-347, stra
target 5' UGAAGAAGCGUGCAUGCAGUU 3'
::::::::::::::::::::
query 3' ACUUCUUCGCACGUACGUCAU 5'
query=miR902a-5p, target=RO3G_05523, score=1, range=327-347, stra
target 5' UGAAGAAGCGUGCAUGCAGUU 3'
::::::::::::::::::::
query 3' ACUUCUUCGCACGUACGUCAU 5'
query=miR902a-5p, target=RO3G_03767, score=1, range=327-347, stra
target 5' UGAAGAAGCGUGCAUGCAGUU 3'
::::::::::::::::::::
query 3' ACUUCUUCGCACGUACGUCAU 5'
query=miR902a-5p, target=RO3G_01968, score=1, range=327-347, stra
target 5' UGAAGAAGCGUGCAUGCAGUU 3'
::::::::::::::::::::
query 3' ACUUCUUCGCACGUACGUCAU 5'
query=miR902a-5p, target=RO3G_17380, score=1, range=327-347, stra
target 5' UGAAGAAGCGUGCAUGCAGUU 3'
::::::::::::::::::::
```

```
query 3' ACUUCUUCGCACGUACGUCAU 5'
query=miR902a-5p, target=RO3G_10525, score=1, range=327-347, stra
target 5' UGAAGAAGCGUGCAUGCAGUU 3'
:::::::::::::::::::::
query 3' ACUUCUUCGCACGUACGUCAU 5'
query=miR902a-5p, target=RO3G_10531, score=1, range=327-347, stra
target 5' UGAAGAAGCGUGCAUGCAGUU 3'
:::::::::::::::::::::
query 3' ACUUCUUCGCACGUACGUCAU 5'
query=miR902a-5p, target=RO3G_11953, score=1, range=204-224, stra
target 5' UGAAGAAGCGUGCAUGCAGUU 3'
:::::::::::::::::::::
query 3' ACUUCUUCGCACGUACGUCAU 5'
query=miR902a-5p, target=RO3G_07868, score=1, range=327-347, stra
target 5' UGAAGAAGCGUGCAUGCAGUU 3'
:::::::::::::::::::::
query 3' ACUUCUUCGCACGUACGUCAU 5'
query=miR902a-5p, target=RO3G_10545, score=1, range=327-347, stra
target 5' UGAAGAAGCGUGCAUGCAGUU 3'
:::::::::::::::::::::
query 3' ACUUCUUCGCACGUACGUCAU 5'
query=miR902a-5p, target=RO3G_04676, score=2, range=327-347, stra
target 5' UGAAGAAGCGUGUAUGCAGUU 3'
::::::::::::.::::::::
query 3' ACUUCUUCGCACGUACGUCAU 5'
query=miR902a-5p, target=RO3G_12005, score=2, range=327-347, stra
target 5' UGAAGAAGCGUGUAUGCAGUU 3'
::::::::::::.::::::::
query 3' ACUUCUUCGCACGUACGUCAU 5'
query=miR902a-5p, target=RO3G_15157, score=2, range=327-347, stra
target 5' UGAAGAAGCGUGUAUGCAGUU 3'
::::::::::::.::::::::
query 3' ACUUCUUCGCACGUACGUCAU 5'
query=miR902a-5p, target=RO3G_14637, score=2, range=327-347, stra
target 5' UGAAGAAGCGUGUAUGCAGUU 3'
::::::::::::.::::::::
query 3' ACUUCUUCGCACGUACGUCAU 5'
query=miR774b-5p, target=RO3G_14626, score=0, range=1870-1892,
strand=1
target 5' GUCACUCCAAAUAUGCAUCUUCA 3'
:::::::::::::::::::::::
query 3' CAGUGAGGUUUAUACGUAGAAGU 5'
query=miR774b-5p, target=RO3G_16138, score=0, range=1870-1892,
strand=1
target 5' GUCACUCCAAAUAUGCAUCUUCA 3'
```

```
::::::::::::::::::::::
query 3' CAGUGAGGUUUAUACGUAGAAGU 5'
query=miR774b-5p, target=RO3G_12072, score=0, range=1870-1892,
strand=1
target 5' GUCACUCCAAAUAUGCAUCUUCA 3'
::::::::::::::::::::::
query 3' CAGUGAGGUUUAUACGUAGAAGU 5'
query=miR774b-5p, target=RO3G_01740, score=0, range=1834-1856,
strand=1
target 5' GUCACUCCAAAUAUGCAUCUUCA 3'
::::::::::::::::::::::
query 3' CAGUGAGGUUUAUACGUAGAAGU 5'
query=miR774b-5p, target=RO3G_05958, score=0, range=1834-1856,
strand=1
target 5' GUCACUCCAAAUAUGCAUCUUCA 3'
::::::::::::::::::::::
query 3' CAGUGAGGUUUAUACGUAGAAGU 5'
query=miR774b-5p, target=RO3G_17441, score=0, range=1597-1619,
strand=1
target 5' GUCACUCCAAAUAUGCAUCUUCA 3'
::::::::::::::::::::::
query 3' CAGUGAGGUUUAUACGUAGAAGU 5'
query=miR774b-5p, target=RO3G_10522, score=0, range=1834-1856,
strand=1
target 5' GUCACUCCAAAUAUGCAUCUUCA 3'
::::::::::::::::::::::
query 3' CAGUGAGGUUUAUACGUAGAAGU 5'
query=miR774b-5p, target=RO3G_07294, score=0, range=1471-1493,
strand=1
target 5' GUCACUCCAAAUAUGCAUCUUCA 3'
::::::::::::::::::::::
query 3' CAGUGAGGUUUAUACGUAGAAGU 5'
query=miR774b-5p, target=RO3G_04785, score=0, range=1834-1856,
strand=1
target 5' GUCACUCCAAAUAUGCAUCUUCA 3'
::::::::::::::::::::::
query 3' CAGUGAGGUUUAUACGUAGAAGU 5'
query=miR774b-5p, target=RO3G_01954, score=0, range=820-842, stra
target 5' GUCACUCCAAAUAUGCAUCUUCA 3'
::::::::::::::::::::::
query 3' CAGUGAGGUUUAUACGUAGAAGU 5'
query=miR774b-5p, target=RO3G_13149, score=0, range=67-89, strand
target 5' GUCACUCCAAAUAUGCAUCUUCA 3'
::::::::::::::::::::::
query 3' CAGUGAGGUUUAUACGUAGAAGU 5'
```

query=miR774b-5p, target=RO3G_17385, score=0.5, range=1870-1892, strand=1
target 5' GUUACUCCAAAUAUGCAUCUUCA 3'
::.::::::::::::::::::::
query 3' CAGUGAGGUUUAUACGUAGAAGU 5'
query=miR774b-5p, target=RO3G_07686, score=0.5, range=1870-1892, strand=1
target 5' GUUACUCCAAAUAUGCAUCUUCA 3'
::.::::::::::::::::::::
query 3' CAGUGAGGUUUAUACGUAGAAGU 5'
query=miR774b-5p, target=RO3G_12009, score=0.5, range=1870-1892, strand=1
target 5' GUUACUCCAAAUAUGCAUCUUCA 3'
::.::::::::::::::::::::
query 3' CAGUGAGGUUUAUACGUAGAAGU 5'
query=miR774b-5p, target=RO3G_11771, score=0.5, range=1870-1892, strand=1
target 5' GUUACUCCAAAUAUGCAUCUUCA 3'
::.::::::::::::::::::::
query 3' CAGUGAGGUUUAUACGUAGAAGU 5'
query=miR774b-5p, target=RO3G_17358, score=0.5, range=1870-1892, strand=1
target 5' GUUACUCCAAAUAUGCAUCUUCA 3'
::.::::::::::::::::::::
query 3' CAGUGAGGUUUAUACGUAGAAGU 5'
query=miR774b-5p, target=RO3G_09940, score=0.5, range=1870-1892, strand=1
target 5' GUUACUCCAAAUAUGCAUCUUCA 3'
::.::::::::::::::::::::
query 3' CAGUGAGGUUUAUACGUAGAAGU 5'
query=miR774b-5p, target=RO3G_12169, score=0.5, range=1870-1892, strand=1
target 5' GUUACUCCAAAUAUGCAUCUUCA 3'
::.::::::::::::::::::::
query 3' CAGUGAGGUUUAUACGUAGAAGU 5'
query=miR774b-5p, target=RO3G_14710, score=0.5, range=1870-1892, strand=1
target 5' GUUACUCCAAAUAUGCAUCUUCA 3'
::.::::::::::::::::::::
query 3' CAGUGAGGUUUAUACGUAGAAGU 5'
query=miR774b-5p, target=RO3G_07976, score=0.5, range=1867-1889, strand=1
target 5' GUUACUCCAAAUAUGCAUCUUCA 3'
::.::::::::::::::::::::
query 3' CAGUGAGGUUUAUACGUAGAAGU 5'

query=miR774b-5p, target=RO3G_10725, score=0.5, range=1834-1856,
strand=1
target 5' GUUACUCCAAAUAUGCAUCUUCA 3'
::.::::::::::::::::::::
query 3' CAGUGAGGUUUAUACGUAGAAGU 5'
query=miR774b-5p, target=RO3G_01309, score=0.5, range=1819-1841,
strand=1
target 5' GUUACUCCAAAUAUGCAUCUUCA 3'
::.::::::::::::::::::::
query 3' CAGUGAGGUUUAUACGUAGAAGU 5'
query=miR774b-5p, target=RO3G_13747, score=0.5, range=1870-1892,
strand=1
target 5' GUUACUCCAAAUAUGCAUCUUCA 3'
::.::::::::::::::::::::
query 3' CAGUGAGGUUUAUACGUAGAAGU 5'
query=miR774b-5p, target=RO3G_11404, score=0.5, range=1870-1892,
strand=1
target 5' GUUACUCCAAAUAUGCAUCUUCA 3'
::.::::::::::::::::::::
query 3' CAGUGAGGUUUAUACGUAGAAGU 5'
query=miR774b-5p, target=RO3G_16736, score=0.5, range=4-26, stran
target 5' GUUACUCCAAAUAUGCAUCUUCA 3'
::.::::::::::::::::::::
query 3' CAGUGAGGUUUAUACGUAGAAGU 5'
query=miR774b-5p, target=RO3G_03552, score=0.5, range=4-26, stran
target 5' GUUACUCCAAAUAUGCAUCUUCA 3'
::.::::::::::::::::::::
query 3' CAGUGAGGUUUAUACGUAGAAGU 5'
query=miR774b-5p, target=RO3G_07128, score=0.5, range=388-410,
strand=1
target 5' GUUACUCCAAAUAUGCAUCUUCA 3'
::.::::::::::::::::::::
query 3' CAGUGAGGUUUAUACGUAGAAGU 5'
query=miR774b-5p, target=RO3G_03580, score=1, range=1795-1817,
strand=1
target 5' AUCACUCCAAAUAUGCAUCUUCA 3'
::::::::::::::::::::::
query 3' CAGUGAGGUUUAUACGUAGAAGU 5'
query=miR774b-5p, target=RO3G_11531, score=1, range=1363-1385,
strand=1
target 5' AUCACUCCAAAUAUGCAUCUUCA 3'
::::::::::::::::::::::
query 3' CAGUGAGGUUUAUACGUAGAAGU 5'
query=miR774b-5p, target=RO3G_03589, score=1, range=1795-1817,
strand=1

```
target 5' AUCACUCCAAAUAUGCAUCUUCA 3'
::::::::::::::::::::::
query 3' CAGUGAGGUUUAUACGUAGAAGU 5'
query=miR774b-5p, target=RO3G_15580, score=1, range=1834-1856,
strand=1
target 5' AUCACUCCAAAUAUGCAUCUUCA 3'
::::::::::::::::::::::
query 3' CAGUGAGGUUUAUACGUAGAAGU 5'
query=miR774b-5p, target=RO3G_06430, score=1.5, range=1834-1856,
strand=1
target 5' GUUACUCCAAAUAUGCAUUUUCA 3'
::.:::::::::::::::.::::
query 3' CAGUGAGGUUUAUACGUAGAAGU 5'
query=miR774b-5p, target=RO3G_01207, score=2.5, range=1687-1709,
strand=1
target 5' GGUACUCCAAAUAUGCAUUUUCA 3'
: .:::::::::::::::.::::
query 3' CAGUGAGGUUUAUACGUAGAAGU 5'
query=miR774b-5p, target=RO3G_08815, score=4, range=1695-1716,
strand=-1
target 5' GUUA-UUGAUAUAUGCAUCUUCA 3'
::.: :. : :::::::::::::
query 3' CAGUGAGGUUUAUACGUAGAAGU 5'
query=miR5741a, target=RO3G_07101, score=3.5, range=52-72, strand
target 5' AAGUUGAUCAAUUUGCUCCUG 3'
::.. ::::::::::::::.
query 3' UUUGUGUAGUUAAACGAGGAU 5'
query=miR5741a, target=RO3G_07788, score=4, range=1520-1540, stra
target 5' AAACACUUCAAUUUGCUCUCA 3'
:::::: :::::::::::. :
query 3' UUUGUGUAGUUAAACGAGGAU 5'
query=miR5741a, target=RO3G_05444, score=4, range=107-127, strand
target 5' AAAUGCUUCAAUUUGCUGCUA 3'
:::..: :::::::::: :::
query 3' UUUGUGUAGUUAAACGAGGAU 5'
query=miR9653a-3p, target=RO3G_10862, score=0, range=329-347, str
target 5' GGCCAGCCCAAAGUCCCAA 3'
:::::::::::::::::::
query 3' CCGGUCGGGUUUCAGGGUU 5'
query=miR9653a-3p, target=RO3G_00677, score=1, range=359-377, str
target 5' GGCCAGCCCAAAGUCCCGA 3'
:::::::::::::::::.:
query 3' CCGGUCGGGUUUCAGGGUU 5'
query=miR9653a-3p, target=RO3G_03605, score=1, range=1259-1277,
strand=1
```

```
target 5' GGCCAGCCCAAAGUCCCGA 3'
:::::::::::::::::.:
query 3' CCGGUCGGGUUUCAGGGUU 5'
query=miR9653a-3p, target=RO3G_05950, score=1, range=275-293, str
target 5' GGCCAGCCCAAAGUCCCGA 3'
:::::::::::::::::.:
query 3' CCGGUCGGGUUUCAGGGUU 5'
query=miR9653a-3p, target=RO3G_16715, score=1, range=257-275, str
target 5' GGCCAGCCCAAAGUCCCGA 3'
:::::::::::::::::.:
query 3' CCGGUCGGGUUUCAGGGUU 5'
query=miR9653a-3p, target=RO3G_17406, score=1, range=158-176, str
target 5' GGCCAGCCCAAAGUCCCGA 3'
:::::::::::::::::.:
query 3' CCGGUCGGGUUUCAGGGUU 5'
query=miR9653a-3p, target=RO3G_01588, score=1, range=137-155, str
target 5' GGCCAGCCCAAAGUCCCGA 3'
:::::::::::::::::.:
query 3' CCGGUCGGGUUUCAGGGUU 5'
query=miR9653a-3p, target=RO3G_00690, score=2, range=746-764, str
target 5' GGCCAGCUCAAAGUCCCGA 3'
:::::::.:::::::::.:
query 3' CCGGUCGGGUUUCAGGGUU 5'
query=miR9653a-3p, target=RO3G_01925, score=2, range=806-824, str
target 5' GGCCAGCUCAAAGUCCCGA 3'
:::::::.:::::::::.:
query 3' CCGGUCGGGUUUCAGGGUU 5'
query=miR9653a-3p, target=RO3G_07024, score=2, range=806-824, str
target 5' GGCCAGCUCAAAGUCCCGA 3'
:::::::.:::::::::.:
query 3' CCGGUCGGGUUUCAGGGUU 5'
query=miR9653a-3p, target=RO3G_07121, score=2, range=806-824, str
target 5' GGCCAGCUCAAAGUCCCGA 3'
:::::::.:::::::::.:
query 3' CCGGUCGGGUUUCAGGGUU 5'
query=miR9653a-3p, target=RO3G_12525, score=2, range=728-746, str
target 5' GGCCAGCUCAAAGUCCCGA 3'
:::::::.:::::::::.:
query 3' CCGGUCGGGUUUCAGGGUU 5'
query=miR9653a-3p, target=RO3G_04786, score=2, range=374-392, str
target 5' GGCCAGCUCAAAGUCCCGA 3'
:::::::.:::::::::.:
query 3' CCGGUCGGGUUUCAGGGUU 5'
query=miR9653a-3p, target=RO3G_09955, score=2, range=491-509, str
target 5' GGCCAGCUCAAAGUCCCGA 3'
```

```
:::::::.:::::::::.:
query 3' CCGGUCGGGUUUCAGGGUU 5'
query=miR9653a-3p, target=RO3G_02700, score=2, range=491-509, str
target 5' GGCCAGCUCAAAGUCCCGA 3'
:::::::.:::::::::.:
query 3' CCGGUCGGGUUUCAGGGUU 5'
query=miR9653a-3p, target=RO3G_03570, score=2, range=491-509, str
target 5' GGCCAGCUCAAAGUCCCGA 3'
:::::::.:::::::::.:
query 3' CCGGUCGGGUUUCAGGGUU 5'
query=miR9653a-3p, target=RO3G_11273, score=2, range=137-155, str
target 5' GGCCAGCUCAAAGUCCCGA 3'
:::::::.:::::::::.:
query 3' CCGGUCGGGUUUCAGGGUU 5'
query=miR9653a-3p, target=RO3G_16905, score=2, range=614-632, str
target 5' GGCCAGCUCAAAGUCCCGA 3'
:::::::.:::::::::.:
query 3' CCGGUCGGGUUUCAGGGUU 5'
query=miR9653a-3p, target=RO3G_04661, score=2, range=437-455, str
target 5' GGCCUGCCCAAAGUCCCGA 3'
:::: ::::::::::::.:
query 3' CCGGUCGGGUUUCAGGGUU 5'
query=miR9653a-3p, target=RO3G_13276, score=3, range=179-197, str
target 5' GGCCAGCUCAAAGUCCUGA 3'
:::::::.::::::::..:
query 3' CCGGUCGGGUUUCAGGGUU 5'
query=miR9653a-3p, target=RO3G_16118, score=3, range=65-83, stran
target 5' GGCCAGCUCAAAGUCCUGA 3'
:::::::.::::::::..:
query 3' CCGGUCGGGUUUCAGGGUU 5'
query=miR9653a-3p, target=RO3G_10537, score=3, range=620-638, str
target 5' GGCCAGCUCAAAGUCCUGA 3'
:::::::.::::::::..:
query 3' CCGGUCGGGUUUCAGGGUU 5'
query=miR9653a-3p, target=RO3G_07295, score=3, range=629-647, str
target 5' GGCCAGCUCAAAGUCCUGA 3'
:::::::.::::::::..:
query 3' CCGGUCGGGUUUCAGGGUU 5'
query=miR9653a-3p, target=RO3G_12174, score=3, range=122-140, str
target 5' GGCCAGCUCAAAGUCCUGA 3'
:::::::.::::::::..:
query 3' CCGGUCGGGUUUCAGGGUU 5'
query=miR9653a-3p, target=RO3G_17213, score=3, range=122-140, str
target 5' GGCCAGCUCAAAGUCCUGA 3'
:::::::.::::::::..:
```

```
query 3' CCGGUCGGGUUUCAGGGUU 5'
query=miR9653a-3p, target=RO3G_08835, score=3, range=107-125, str
target 5' GGCCAGCUCAAAGUCCUGA 3'
:::::::.::::::::..:
query 3' CCGGUCGGGUUUCAGGGUU 5'
query=miR9653a-3p, target=RO3G_10528, score=3, range=122-140, str
target 5' GGCCAGCUCAAAGUCCUGA 3'
:::::::.::::::::..:
query 3' CCGGUCGGGUUUCAGGGUU 5'
query=miR9653a-3p, target=RO3G_11263, score=3, range=122-140, str
target 5' GGCCAGCUCAAAGUCCUGA 3'
:::::::.::::::::..:
query 3' CCGGUCGGGUUUCAGGGUU 5'
query=miR9653a-3p, target=RO3G_12013, score=3, range=122-140, str
target 5' GGCCAGCUCAAAGUCCUGA 3'
:::::::.::::::::..:
query 3' CCGGUCGGGUUUCAGGGUU 5'
query=miR9653a-3p, target=RO3G_07119, score=3, range=122-140, str
target 5' GGCCAGCUCAAAGUCCUGA 3'
:::::::.::::::::..:
query 3' CCGGUCGGGUUUCAGGGUU 5'
query=miR9653a-3p, target=RO3G_10742, score=3, range=122-140, str
target 5' GGCCAGCUCAAAGUCCUGA 3'
:::::::.::::::::..:
query 3' CCGGUCGGGUUUCAGGGUU 5'
query=miR9653a-3p, target=RO3G_11528, score=3, range=122-140, str
target 5' GGCCAGCUCAAAGUCCUGA 3'
:::::::.::::::::..:
query 3' CCGGUCGGGUUUCAGGGUU 5'
query=miR9653a-3p, target=RO3G_17409, score=3, range=797-815, str
target 5' GGCCAGCUCAAAGUCCUGA 3'
:::::::.::::::::..:
query 3' CCGGUCGGGUUUCAGGGUU 5'
query=miR9653a-3p, target=RO3G_01951, score=3, range=797-815, str
target 5' GGCCAGCUCAAAGUCCUGA 3'
:::::::.::::::::..:
query 3' CCGGUCGGGUUUCAGGGUU 5'
query=miR9653a-3p, target=RO3G_01194, score=3, range=797-815, str
target 5' GGCCAGCUCAAAGUCCUGA 3'
:::::::.::::::::..:
query 3' CCGGUCGGGUUUCAGGGUU 5'
query=miR9653a-3p, target=RO3G_09199, score=3, range=797-815, str
target 5' GGCCAGCUCAAAGUCCUGA 3'
:::::::.::::::::..:
query 3' CCGGUCGGGUUUCAGGGUU 5'
```

query=miR9653a-3p, target=RO3G_05916, score=3, range=797-815, str
target 5' GGCCAGCUCAAAGUCCUGA 3'
:::::::.:::::::::..:
query 3' CCGGUCGGGUUUCAGGGUU 5'
query=miR9653a-3p, target=RO3G_15873, score=3, range=797-815, str
target 5' GGCCAGCUCAAAGUCCUGA 3'
:::::::.:::::::::..:
query 3' CCGGUCGGGUUUCAGGGUU 5'
query=miR9653a-3p, target=RO3G_12526, score=3, range=797-815, str
target 5' GGCCAGCUCAAAGUCCUGA 3'
:::::::.:::::::::..:
query 3' CCGGUCGGGUUUCAGGGUU 5'
query=miR9653a-3p, target=RO3G_13447, score=3, range=797-815, str
target 5' GGCCAGCUCAAAGUCCUGA 3'
:::::::.:::::::::..:
query 3' CCGGUCGGGUUUCAGGGUU 5'
query=miR9653a-3p, target=RO3G_03987, score=3, range=797-815, str
target 5' GGCCAGCUCAAAGUCCUGA 3'
:::::::.:::::::::..:
query 3' CCGGUCGGGUUUCAGGGUU 5'
query=miR9653a-3p, target=RO3G_11244, score=3, range=797-815, str
target 5' GGCCAGCUCAAAGUCCUGA 3'
:::::::.:::::::::..:
query 3' CCGGUCGGGUUUCAGGGUU 5'
query=miR9653a-3p, target=RO3G_10044, score=3, range=797-815, str
target 5' GGCCAGCUCAAAGUCCUGA 3'
:::::::.:::::::::..:
query 3' CCGGUCGGGUUUCAGGGUU 5'
query=miR9653a-3p, target=RO3G_01313, score=3, range=797-815, str
target 5' GGCCAGCUCAAAGUCCUGA 3'
:::::::.:::::::::..:
query 3' CCGGUCGGGUUUCAGGGUU 5'
query=miR9653a-3p, target=RO3G_10720, score=3, range=797-815, str
target 5' GGCCAGCUCAAAGUCCUGA 3'
:::::::.:::::::::..:
query 3' CCGGUCGGGUUUCAGGGUU 5'
query=miR9653a-3p, target=RO3G_01950, score=3, range=797-815, str
target 5' GGCCAGCUCAAAGUCCUGA 3'
:::::::.:::::::::..:
query 3' CCGGUCGGGUUUCAGGGUU 5'
query=miR9653a-3p, target=RO3G_15154, score=3, range=797-815, str
target 5' GGCCAGCUCAAAGUCCUGA 3'
:::::::.:::::::::..:
query 3' CCGGUCGGGUUUCAGGGUU 5'
query=miR9653a-3p, target=RO3G_03558, score=3, range=797-815, str

target 5' GGCCAGCUCAAAGUCCUGA 3'
:::::::.::::::::..:
query 3' CCGGUCGGGUUUCAGGGUU 5'
query=miR9653a-3p, target=RO3G_07994, score=3, range=797-815, str
target 5' GGCCAGCUCAAAGUCCUGA 3'
:::::::.::::::::..:
query 3' CCGGUCGGGUUUCAGGGUU 5'
query=miR9653a-3p, target=RO3G_07683, score=3, range=797-815, str
target 5' GGCCAGCUCAAAGUCCUGA 3'
:::::::.::::::::..:
query 3' CCGGUCGGGUUUCAGGGUU 5'
query=miR9653a-3p, target=RO3G_09924, score=3, range=797-815, str
target 5' GGCCAGCUCAAAGUCCUGA 3'
:::::::.::::::::..:
query 3' CCGGUCGGGUUUCAGGGUU 5'
query=miR9653a-3p, target=RO3G_11948, score=3, range=797-815, str
target 5' GGCCAGCUCAAAGUCCUGA 3'
:::::::.::::::::..:
query 3' CCGGUCGGGUUUCAGGGUU 5'
query=miR9653a-3p, target=RO3G_01735, score=3, range=797-815, str
target 5' GGCCAGCUCAAAGUCCUGA 3'
:::::::.::::::::..:
query 3' CCGGUCGGGUUUCAGGGUU 5'
query=miR9653a-3p, target=RO3G_13706, score=3, range=755-773, str
target 5' GGCCAGCUCAAAGUCCUGA 3'
:::::::.::::::::..:
query 3' CCGGUCGGGUUUCAGGGUU 5'
query=miR9653a-3p, target=RO3G_17211, score=3, range=797-815, str
target 5' GGCCAGCUCAAAGUCCUGA 3'
:::::::.::::::::..:
query 3' CCGGUCGGGUUUCAGGGUU 5'
query=miR9653a-3p, target=RO3G_03617, score=3, range=797-815, str
target 5' GGCCAGCUCAAAGUCCUGA 3'
:::::::.::::::::..:
query 3' CCGGUCGGGUUUCAGGGUU 5'
query=miR9653a-3p, target=RO3G_10805, score=3, range=806-824, str
target 5' GGCCAGCCCAAAUUCCCGA 3'
:::::::::::: ::::.:
query 3' CCGGUCGGGUUUCAGGGUU 5'
query=miR9653a-3p, target=RO3G_01587, score=3, range=623-641, str
target 5' GGCCUGCUCAAAGUCCCGA 3'
:::: ::.:::::::::.:
query 3' CCGGUCGGGUUUCAGGGUU 5'
query=miR9653a-3p, target=RO3G_17361, score=3, range=374-392, str
target 5' GGCCUGCUCAAAGUCCCGA 3'

```
:::: ::.::::::::.:
query 3' CCGGUCGGGUUUCAGGGUU 5'
query=miR9653a-3p, target=RO3G_08658, score=3, range=614-632, str
target 5' GGCCUGCUCAAAGUCCCGA 3'
:::: ::.::::::::.:
query 3' CCGGUCGGGUUUCAGGGUU 5'
query=miR9653a-3p, target=RO3G_14989, score=3, range=653-671, str
target 5' GGCCUGCUCAAAGUCCCGA 3'
:::: ::.::::::::.:
query 3' CCGGUCGGGUUUCAGGGUU 5'
query=miR9653a-3p, target=RO3G_11966, score=3, range=797-815, str
target 5' GGCCAUCCCAAAGUCCUGA 3'
::::: ::::::::..:
query 3' CCGGUCGGGUUUCAGGGUU 5'
query=miR9653a-3p, target=RO3G_13857, score=3, range=437-455, str
target 5' GGCCUGCUCAAAGUCCCGA 3'
:::: ::.::::::::.:
query 3' CCGGUCGGGUUUCAGGGUU 5'
query=miR9653a-3p, target=RO3G_05959, score=3, range=797-815, str
target 5' GGCCAUCCCAAAGUCCUGA 3'
::::: ::::::::..:
query 3' CCGGUCGGGUUUCAGGGUU 5'
query=miR9653a-3p, target=RO3G_05953, score=3, range=797-815, str
target 5' GGCCAUCCCAAAGUCCUGA 3'
::::: ::::::::..:
query 3' CCGGUCGGGUUUCAGGGUU 5'
query=miR9653a-3p, target=RO3G_17449, score=3, range=797-815, str
target 5' GGCCAUCCCAAAGUCCUGA 3'
::::: ::::::::..:
query 3' CCGGUCGGGUUUCAGGGUU 5'
query=miR9653a-3p, target=RO3G_05921, score=3, range=797-815, str
target 5' GGCCAUCCCAAAGUCCUGA 3'
::::: ::::::::..:
query 3' CCGGUCGGGUUUCAGGGUU 5'
query=miR9653a-3p, target=RO3G_00256, score=3, range=797-815, str
target 5' GGCCAUCCCAAAGUCCUGA 3'
::::: ::::::::..:
query 3' CCGGUCGGGUUUCAGGGUU 5'
query=miR9653a-3p, target=RO3G_05267, score=3, range=797-815, str
target 5' GGCCAUCCCAAAGUCCUGA 3'
::::: ::::::::..:
query 3' CCGGUCGGGUUUCAGGGUU 5'
query=miR9653a-3p, target=RO3G_14361, score=3, range=797-815, str
target 5' GGCCAUCCCAAAGUCCUGA 3'
::::: ::::::::..:
```

query 3' CCGGUCGGGUUUCAGGGUU 5'
query=miR9653a-3p, target=RO3G_09967, score=3, range=797-815, str
target 5' GGCCAUCCCAAAGUCCUGA 3'
::::: ::::::::::..:
query 3' CCGGUCGGGUUUCAGGGUU 5'
query=miR9653a-3p, target=RO3G_07860, score=3, range=797-815, str
target 5' GGCCAUCCCAAAGUCCUGA 3'
::::: ::::::::::..:
query 3' CCGGUCGGGUUUCAGGGUU 5'
query=miR9653a-3p, target=RO3G_17069, score=3, range=662-680, str
target 5' GGCCAUCCCAAAGUCCUGA 3'
::::: ::::::::::..:
query 3' CCGGUCGGGUUUCAGGGUU 5'
query=miR9653a-3p, target=RO3G_14568, score=3, range=797-815, str
target 5' GGCCAUCCCAAAGUCCUGA 3'
::::: ::::::::::..:
query 3' CCGGUCGGGUUUCAGGGUU 5'
query=miR9653a-3p, target=RO3G_12377, score=3, range=812-830, strand=-1
target 5' GAUCGGCCCGAAGUCCCAA 3'
: .:.::::.:::::::::
query 3' CCGGUCGGGUUUCAGGGUU 5'
query=miR9653a-3p, target=RO3G_10729, score=3.5, range=797-815, strand=1
target 5' GGCUAUCCCAAAGUCCUGA 3'
:::.: ::::::::::..:
query 3' CCGGUCGGGUUUCAGGGUU 5'
query=miR9653a-3p, target=RO3G_07651, score=3.5, range=5193-5211, strand=1
target 5' UGGUAGCUCAAAGUCCCAA 3'
: .:::.:::::::::::
query 3' CCGGUCGGGUUUCAGGGUU 5'
query=miR9653a-3p, target=RO3G_05737, score=3.5, range=390-407, strand=-1
target 5' GGAUAG-CCAAAGUCCCAA 3'
:: .:: :::::::::::
query 3' CCGGUCGGGUUUCAGGGUU 5'
query=miR9653a-3p, target=RO3G_16377, score=4, range=122-140, str
target 5' GGCCAGCUCAAAGUUCUGA 3'
::::::.::::::.:..:
query 3' CCGGUCGGGUUUCAGGGUU 5'
query=miR9653a-3p, target=RO3G_16548, score=4, range=452-470, str
target 5' UGCCAGCUCAAAGUCCUGA 3'
:::::.::::::::..:
query 3' CCGGUCGGGUUUCAGGGUU 5'

query=miR9653a-3p, target=RO3G_12186, score=4, range=806-824, str
target 5' GGCCAGCUCAAAGUCCAGA 3'
:::::::.:::::::: .:
query 3' CCGGUCGGGUUUCAGGGUU 5'
query=miR9653a-3p, target=RO3G_03599, score=4, range=806-824, str
target 5' GGCCAGCUCAAAAUCCCGA 3'
:::::::.:::: ::::.:
query 3' CCGGUCGGGUUUCAGGGUU 5'
query=miR9653a-3p, target=RO3G_12196, score=4, range=728-746, str
target 5' GGCCAGCUCAAAGUCCAGA 3'
:::::::.:::::::: .:
query 3' CCGGUCGGGUUUCAGGGUU 5'
query=miR9653a-3p, target=RO3G_14620, score=4, range=242-260, str
target 5' GGCCUGCUCAAAGUCCUGA 3'
:::: ::.::::::::..:
query 3' CCGGUCGGGUUUCAGGGUU 5'
query=miR9653a-3p, target=RO3G_14987, score=4, range=461-479, str
target 5' GGCCUGCUCAAAGUCCUGA 3'
:::: ::.::::::::..:
query 3' CCGGUCGGGUUUCAGGGUU 5'
query=miR9653a-3p, target=RO3G_06721, score=4, range=611-629, str
target 5' GGCCUGCUCAAAGUCCUGA 3'
:::: ::.::::::::..:
query 3' CCGGUCGGGUUUCAGGGUU 5'
query=miR9653a-3p, target=RO3G_03797, score=4, range=302-320, str
target 5' GGCCUGCUCAAAGUCCUGA 3'
:::: ::.::::::::..:
query 3' CCGGUCGGGUUUCAGGGUU 5'
query=miR9653a-3p, target=RO3G_17293, score=4, range=614-632, str
target 5' GGCCUGCUCAAAGUCCUGA 3'
:::: ::.::::::::..:
query 3' CCGGUCGGGUUUCAGGGUU 5'
query=miR9653a-3p, target=RO3G_04200, score=4, range=614-632, str
target 5' GGCCUGCUCAAAGUCCUGA 3'
:::: ::.::::::::..:
query 3' CCGGUCGGGUUUCAGGGUU 5'
query=miR9653a-3p, target=RO3G_17436, score=4, range=614-632, str
target 5' GGCCUGCUCAAAGUCCUGA 3'
:::: ::.::::::::..:
query 3' CCGGUCGGGUUUCAGGGUU 5'
query=miR9653a-3p, target=RO3G_04654, score=4, range=614-632, str
target 5' GGCCUGCUCAAAGUCCUGA 3'
:::: ::.::::::::..:
query 3' CCGGUCGGGUUUCAGGGUU 5'
query=miR9653a-3p, target=RO3G_15672, score=4, range=614-632, str

target 5' GGCCUGCUCAAAGUCCUGA 3'
:::: ::.:::::::..:
query 3' CCGGUCGGGUUUCAGGGUU 5'
query=miR9653a-3p, target=RO3G_11405, score=4, range=614-632, str
target 5' GGCCUGCUCAAAGUCCUGA 3'
:::: ::.:::::::..:
query 3' CCGGUCGGGUUUCAGGGUU 5'
query=miR9653a-3p, target=RO3G_07689, score=4, range=614-632, str
target 5' GGCCUGCUCAAAGUCCUGA 3'
:::: ::.:::::::..:
query 3' CCGGUCGGGUUUCAGGGUU 5'
query=miR9653a-3p, target=RO3G_07302, score=4, range=614-632, str
target 5' GGCCUGCUCAAAGUCCUGA 3'
:::: ::.:::::::..:
query 3' CCGGUCGGGUUUCAGGGUU 5'
query=miR9653a-3p, target=RO3G_10224, score=4, range=614-632, str
target 5' GGCCUGCUCAAAGUCCUGA 3'
:::: ::.:::::::..:
query 3' CCGGUCGGGUUUCAGGGUU 5'
query=miR9653a-3p, target=RO3G_00247, score=4, range=614-632, str
target 5' GGCCUGCUCAAAGUCCUGA 3'
:::: ::.:::::::..:
query 3' CCGGUCGGGUUUCAGGGUU 5'
query=miR9653a-3p, target=RO3G_12670, score=4, range=614-632, str
target 5' GGCCUGCUCAAAGUCCUGA 3'
:::: ::.:::::::..:
query 3' CCGGUCGGGUUUCAGGGUU 5'
query=miR9653a-3p, target=RO3G_09944, score=4, range=614-632, str
target 5' GGCCUGCUCAAAGUCCUGA 3'
:::: ::.:::::::..:
query 3' CCGGUCGGGUUUCAGGGUU 5'
query=miR9653a-3p, target=RO3G_03785, score=4, range=419-437, str
target 5' GGCCUGCUCAAAGUCCUGA 3'
:::: ::.:::::::..:
query 3' CCGGUCGGGUUUCAGGGUU 5'
query=miR9653a-3p, target=RO3G_17204, score=4, range=677-695, str
target 5' GGCCUGCUCAAAGUCCUGA 3'
:::: ::.:::::::..:
query 3' CCGGUCGGGUUUCAGGGUU 5'
query=miR9653a-3p, target=RO3G_08649, score=4, range=559-577, str
target 5' GGCCUGCUCAAAGUCCUGA 3'
:::: ::.:::::::..:
query 3' CCGGUCGGGUUUCAGGGUU 5'
query=miR9653a-3p, target=RO3G_04207, score=4, range=797-815, str
target 5' GGCCAUCCCAAAGUUCUGA 3'

```
::::: ::::::::.:..:
query 3' CCGGUCGGGUUUCAGGGUU 5'
query=miR9653a-3p, target=RO3G_10226, score=4, range=614-632, str
target 5' GGCCUGCUCAAAGUCCUGA 3'
:::: ::.::::::::..:
query 3' CCGGUCGGGUUUCAGGGUU 5'
query=miR9653a-3p, target=RO3G_12082, score=4, range=692-710, str
target 5' GGCCUGCUCAAAGUCCUGA 3'
:::: ::.::::::::..:
query 3' CCGGUCGGGUUUCAGGGUU 5'
query=miR9653a-3p, target=RO3G_14772, score=4, range=692-710, str
target 5' GGCCUGCUCAAAGUCCUGA 3'
:::: ::.::::::::..:
query 3' CCGGUCGGGUUUCAGGGUU 5'
query=miR9653a-3p, target=RO3G_14972, score=4, range=728-746, str
target 5' GGCCACCACAAAGUCCCGA 3'
::::: : :::::::::.:
query 3' CCGGUCGGGUUUCAGGGUU 5'
query=miR9653a-3p, target=RO3G_01942, score=4, range=794-812, str
target 5' GGACAUCCCAAAGUCCUGA 3'
:: :: ::::::::::..:
query 3' CCGGUCGGGUUUCAGGGUU 5'
query=miR9653a-3p, target=RO3G_09945, score=4, range=521-539, str
target 5' GGACAUCCCAAAGUCCUGA 3'
:: :: ::::::::::..:
query 3' CCGGUCGGGUUUCAGGGUU 5'
query=miR9653a-3p, target=RO3G_09194, score=4, range=797-815, str
target 5' GGACAUCCCAAAGUCCUGA 3'
:: :: ::::::::::..:
query 3' CCGGUCGGGUUUCAGGGUU 5'
query=miR9653a-3p, target=RO3G_03612, score=4, range=284-302, str
target 5' GGACUGCUCAAAGUCCCGA 3'
:: : ::.::::::::.:
query 3' CCGGUCGGGUUUCAGGGUU 5'
query=miR9653a-3p, target=RO3G_02961, score=4, range=284-302, str
target 5' GGACUGCUCAAAGUCCCGA 3'
:: : ::.::::::::.:
query 3' CCGGUCGGGUUUCAGGGUU 5'
query=miR9653a-3p, target=RO3G_10457, score=4, range=148-165, str
target 5' GAACAGCCCAAAG-CCCAA 3'
: ::::::::: :::::
query 3' CCGGUCGGGUUUCAGGGUU 5'
query=miR9653a-3p, target=RO3G_00639, score=4, range=174-193,
strand=-1
target 5' UGCCUGCCCAAAAGUCCCAA 3'
```

```
::: :::: ::::::::::
query 3' CCGGUCGGG-UUUCAGGGUU 5'
query=miR419, target=RO3G_17296, score=0, range=174-192, strand=1
target 5' CUCACGUCAUCAUUCCUCA 3'
:::::::::::::::::::
query 3' GAGUGCAGUAGUAAGGAGU 5'
query=miR419, target=RO3G_13948, score=2.5, range=1809-1827, stra
target 5' CUCACCUCAUCAUUCUUCG 3'
::::: :::::::::.::.
query 3' GAGUGCAGUAGUAAGGAGU 5'
query=miR419, target=RO3G_12214, score=3, range=947-966, strand=1
target 5' CUCAACAUCAUCAUUUCUCA 3'
::: :: ::::::::.::::
query 3' GAG-UGCAGUAGUAAGGAGU 5'
query=miR419, target=RO3G_16237, score=3, range=1366-1385, strand
target 5' CUUAUAGUCAUCAUUCCUCC 3'
::.:. :::::::::::::
query 3' GAGUG-CAGUAGUAAGGAGU 5'
query=miR419, target=RO3G_04424, score=3, range=425-443, strand=-
target 5' CUGCCGUCGUCAUUCCUCA 3'
:: ::::.::::::::::
query 3' GAGUGCAGUAGUAAGGAGU 5'
query=miR419, target=RO3G_02333, score=3.5, range=120-138, strand
target 5' UUCACAUCAUCGUUCUUCA 3'
.:::: :::::.:::.:::
query 3' GAGUGCAGUAGUAAGGAGU 5'
query=miR419, target=RO3G_17067, score=3.5, range=1021-1040, stra
target 5' UUCAAGUUCAUUAUUCCUCA 3'
.::: : ::::.::::::::
query 3' GAGUGC-AGUAGUAAGGAGU 5'
query=miR419, target=RO3G_13039, score=3.5, range=1728-1745, stra
target 5' CUC-UGUCGUCAUUCCUCC 3'
::: .:::.:::::::::
query 3' GAGUGCAGUAGUAAGGAGU 5'
query=miR419, target=RO3G_01820, score=3.5, range=1471-1488, stra
target 5' UUCA-GUCAUCAUUCCCCA 3'
.::: :::::::::: ::
query 3' GAGUGCAGUAGUAAGGAGU 5'
query=miR419, target=RO3G_03632, score=3.5, range=1466-1483, stra
target 5' CUCUCGUCAUCAUU-CUCG 3'
::: :::::::::: :::.
query 3' GAGUGCAGUAGUAAGGAGU 5'
query=miR419, target=RO3G_15326, score=3.5, range=68-85, strand=1
target 5' CUUACAUCAUCA-UCCUCA 3'
::.:: :::::: ::::::
```

```
query 3' GAGUGCAGUAGUAAGGAGU 5'
query=miR419, target=RO3G_13329, score=3.5, range=276-293, strand
target 5' CUC-UGUGAUCAUUCCUCA 3'
::: .:: ::::::::::
query 3' GAGUGCAGUAGUAAGGAGU 5'
query=miR419, target=RO3G_05065, score=4, range=500-518, strand=1
target 5' AACACGUCAUUAUUCUUCA 3'
::::::::.::::.:::
query 3' GAGUGCAGUAGUAAGGAGU 5'
query=miR419, target=RO3G_17196, score=4, range=500-518, strand=1
target 5' AACACGUCAUUAUUCUUCA 3'
::::::::.::::.:::
query 3' GAGUGCAGUAGUAAGGAGU 5'
query=miR419, target=RO3G_14928, score=4, range=29-47, strand=1
target 5' CUCGUGUCAUCAUUACUCU 3'
:::..::::::::: :::
query 3' GAGUGCAGUAGUAAGGAGU 5'
query=miR419, target=RO3G_14934, score=4, range=29-47, strand=1
target 5' CUCGUGUCAUCAUUACUCU 3'
:::..::::::::: :::
query 3' GAGUGCAGUAGUAAGGAGU 5'
query=miR419, target=RO3G_02621, score=4, range=423-440, strand=1
target 5' CUCA-GUCAUCGUUUUUCA 3'
:::: :::::::.::..:::
query 3' GAGUGCAGUAGUAAGGAGU 5'
query=miR419, target=RO3G_16590, score=4, range=368-386, strand=1
target 5' UUCCUGCCAUCAUUCCUCA 3'
.:: .: :::::::::::
query 3' GAGUGCAGUAGUAAGGAGU 5'
query=miR419, target=RO3G_06771, score=4, range=276-293, strand=1
target 5' CUCACGUCGU-AUUCCUCC 3'
::::::::.: :::::::
query 3' GAGUGCAGUAGUAAGGAGU 5'
query=miR419, target=RO3G_02782, score=4, range=392-411, strand=1
target 5' UAUACCGUCAUCAUUCCUUA 3'
. .: :::::::::::::.:
query 3' GAGU-GCAGUAGUAAGGAGU 5'
query=miR419, target=RO3G_17114, score=4, range=473-491, strand=1
target 5' AUCAAGUCAUCAUUCAUCA 3'
::: :::::::::: :::
query 3' GAGUGCAGUAGUAAGGAGU 5'
query=miR419, target=RO3G_12716, score=4, range=269-287, strand=1
target 5' CUCAACUCAUUAUUCCUCC 3'
:::: ::::.:::::::
query 3' GAGUGCAGUAGUAAGGAGU 5'
```

```
query=miR419, target=RO3G_13887, score=4, range=465-483, strand=1
target 5' CUCUCAUCAUCAUUCCCCA 3'
::: : :::::::::: ::
query 3' GAGUGCAGUAGUAAGGAGU 5'
query=miR419, target=RO3G_04821, score=4, range=339-356, strand=1
target 5' ACCAC-UCAUCAUUCCUCC 3'
::: ::::::::::::
query 3' GAGUGCAGUAGUAAGGAGU 5'
query=miR419, target=RO3G_14305, score=4, range=452-470, strand=-
target 5' GUCACGUCAUCAUUCUUUU 3'
::::::::::::::.:.
query 3' GAGUGCAGUAGUAAGGAGU 5'
query=miR419, target=RO3G_00234, score=4, range=1121-1139, strand
target 5' UCCAUAUCAUCAUUCUUCA 3'
. ::. :::::::::.:::
query 3' GAGUGCAGUAGUAAGGAGU 5'
query=miR419, target=RO3G_12448, score=4, range=416-434, strand=-
target 5' CUGAAGUCAUCAUUCUUCU 3'
:: : ::::::::::.::
query 3' GAGUGCAGUAGUAAGGAGU 5'
query=miR419, target=RO3G_01815, score=4, range=678-695, strand=-
target 5' CUCAAGUCAUCA-UCUUCA 3'
:::: ::::::: ::.:::
query 3' GAGUGCAGUAGUAAGGAGU 5'
query=miR419, target=RO3G_10948, score=4, range=747-764, strand=-
target 5' UCCAUGUCAU-AUUCCUCA 3'
. ::.::::: ::::::::
query 3' GAGUGCAGUAGUAAGGAGU 5'
query=miR6477, target=RO3G_16897, score=0, range=93-110, strand=1
target 5' AUCAUCCUCUACUGUUCA 3'
::::::::::::::::::
query 3' UAGUAGGAGAUGACAAGU 5'
query=miR6477, target=RO3G_17215, score=0, range=93-110, strand=1
target 5' AUCAUCCUCUACUGUUCA 3'
::::::::::::::::::
query 3' UAGUAGGAGAUGACAAGU 5'
query=miR6477, target=RO3G_03109, score=0, range=93-110, strand=1
target 5' AUCAUCCUCUACUGUUCA 3'
::::::::::::::::::
query 3' UAGUAGGAGAUGACAAGU 5'
query=miR6477, target=RO3G_10235, score=0, range=93-110, strand=1
target 5' AUCAUCCUCUACUGUUCA 3'
::::::::::::::::::
query 3' UAGUAGGAGAUGACAAGU 5'
query=miR6477, target=RO3G_03615, score=0, range=93-110, strand=1
```

```
target 5' AUCAUCCUCUACUGUUCA 3'
::::::::::::::::::
query 3' UAGUAGGAGAUGACAAGU 5'
query=miR6477, target=RO3G_01322, score=0, range=93-110, strand=1
target 5' AUCAUCCUCUACUGUUCA 3'
::::::::::::::::::
query 3' UAGUAGGAGAUGACAAGU 5'
query=miR6477, target=RO3G_06423, score=0, range=93-110, strand=1
target 5' AUCAUCCUCUACUGUUCA 3'
::::::::::::::::::
query 3' UAGUAGGAGAUGACAAGU 5'
query=miR6477, target=RO3G_09499, score=0, range=93-110, strand=1
target 5' AUCAUCCUCUACUGUUCA 3'
::::::::::::::::::
query 3' UAGUAGGAGAUGACAAGU 5'
query=miR6477, target=RO3G_06441, score=0, range=93-110, strand=1
target 5' AUCAUCCUCUACUGUUCA 3'
::::::::::::::::::
query 3' UAGUAGGAGAUGACAAGU 5'
query=miR6477, target=RO3G_01956, score=0, range=93-110, strand=1
target 5' AUCAUCCUCUACUGUUCA 3'
::::::::::::::::::
query 3' UAGUAGGAGAUGACAAGU 5'
query=miR6477, target=RO3G_05336, score=0, range=93-110, strand=1
target 5' AUCAUCCUCUACUGUUCA 3'
::::::::::::::::::
query 3' UAGUAGGAGAUGACAAGU 5'
query=miR6477, target=RO3G_07303, score=0, range=93-110, strand=1
target 5' AUCAUCCUCUACUGUUCA 3'
::::::::::::::::::
query 3' UAGUAGGAGAUGACAAGU 5'
query=miR6477, target=RO3G_14359, score=0, range=93-110, strand=1
target 5' AUCAUCCUCUACUGUUCA 3'
::::::::::::::::::
query 3' UAGUAGGAGAUGACAAGU 5'
query=miR6477, target=RO3G_00681, score=0, range=93-110, strand=1
target 5' AUCAUCCUCUACUGUUCA 3'
::::::::::::::::::
query 3' UAGUAGGAGAUGACAAGU 5'
query=miR6477, target=RO3G_17285, score=0, range=93-110, strand=1
target 5' AUCAUCCUCUACUGUUCA 3'
::::::::::::::::::
query 3' UAGUAGGAGAUGACAAGU 5'
query=miR6477, target=RO3G_09483, score=0, range=93-110, strand=1
target 5' AUCAUCCUCUACUGUUCA 3'
```

```
::::::::::::::::::
query 3' UAGUAGGAGAUGACAAGU 5'
query=miR6477, target=RO3G_05635, score=0, range=93-110, strand=1
target 5' AUCAUCCUCUACUGUUCA 3'
::::::::::::::::::
query 3' UAGUAGGAGAUGACAAGU 5'
query=miR6477, target=RO3G_16807, score=0, range=93-110, strand=1
target 5' AUCAUCCUCUACUGUUCA 3'
::::::::::::::::::
query 3' UAGUAGGAGAUGACAAGU 5'
query=miR6477, target=RO3G_09544, score=0, range=93-110, strand=1
target 5' AUCAUCCUCUACUGUUCA 3'
::::::::::::::::::
query 3' UAGUAGGAGAUGACAAGU 5'
query=miR6477, target=RO3G_17382, score=0, range=93-110, strand=1
target 5' AUCAUCCUCUACUGUUCA 3'
::::::::::::::::::
query 3' UAGUAGGAGAUGACAAGU 5'
query=miR6477, target=RO3G_07875, score=0, range=93-110, strand=1
target 5' AUCAUCCUCUACUGUUCA 3'
::::::::::::::::::
query 3' UAGUAGGAGAUGACAAGU 5'
query=miR6477, target=RO3G_02689, score=0, range=93-110, strand=1
target 5' AUCAUCCUCUACUGUUCA 3'
::::::::::::::::::
query 3' UAGUAGGAGAUGACAAGU 5'
query=miR6477, target=RO3G_01652, score=0, range=93-110, strand=1
target 5' AUCAUCCUCUACUGUUCA 3'
::::::::::::::::::
query 3' UAGUAGGAGAUGACAAGU 5'
query=miR6477, target=RO3G_10732, score=0, range=93-110, strand=1
target 5' AUCAUCCUCUACUGUUCA 3'
::::::::::::::::::
query 3' UAGUAGGAGAUGACAAGU 5'
query=miR6477, target=RO3G_15675, score=0, range=93-110, strand=1
target 5' AUCAUCCUCUACUGUUCA 3'
::::::::::::::::::
query 3' UAGUAGGAGAUGACAAGU 5'
query=miR6477, target=RO3G_13151, score=0.5, range=93-110, strand
target 5' GUCAUCCUCUACUGUUCA 3'
.:::::::::::::::::
query 3' UAGUAGGAGAUGACAAGU 5'
query=miR6477, target=RO3G_07210, score=1, range=903-920, strand=
target 5' AUUGUCCUCUACUGUUCA 3'
::..::::::::::::::
```

```
query 3' UAGUAGGAGAUGACAAGU 5'
query=miR6477, target=RO3G_00999, score=1, range=1935-1952, stran
target 5' AUUGUCCUCUACUGUUCA 3'
::..::::::::::::::
query 3' UAGUAGGAGAUGACAAGU 5'
query=miR6477, target=RO3G_05622, score=2, range=93-110, strand=1
target 5' AUCAUCCUCCACUGUUCA 3'
::::::::: ::::::::
query 3' UAGUAGGAGAUGACAAGU 5'
query=miR6477, target=RO3G_01612, score=2, range=93-110, strand=1
target 5' AUCAUCCUCCACUGUUCA 3'
::::::::: ::::::::
query 3' UAGUAGGAGAUGACAAGU 5'
query=miR6477, target=RO3G_13742, score=2, range=93-110, strand=1
target 5' AUCAUCCUCCACUGUUCA 3'
::::::::: ::::::::
query 3' UAGUAGGAGAUGACAAGU 5'
query=miR6477, target=RO3G_13726, score=2, range=93-110, strand=1
target 5' AUCAUCCUCCACUGUUCA 3'
::::::::: ::::::::
query 3' UAGUAGGAGAUGACAAGU 5'
query=miR6477, target=RO3G_07704, score=2, range=93-110, strand=1
target 5' AUCAUCCUCCACUGUUCA 3'
::::::::: ::::::::
query 3' UAGUAGGAGAUGACAAGU 5'
query=miR6477, target=RO3G_09607, score=2, range=93-110, strand=1
target 5' AUCAUCCUCCACUGUUCA 3'
::::::::: ::::::::
query 3' UAGUAGGAGAUGACAAGU 5'
query=miR6477, target=RO3G_05915, score=2, range=93-110, strand=1
target 5' AUCAUCCUCUACUGUGCA 3'
::::::::::::::: ::
query 3' UAGUAGGAGAUGACAAGU 5'
query=miR6477, target=RO3G_01730, score=2, range=93-110, strand=1
target 5' AUCAUCCUCUACUGUGCA 3'
::::::::::::::: ::
query 3' UAGUAGGAGAUGACAAGU 5'
query=miR6477, target=RO3G_16551, score=2, range=93-110, strand=1
target 5' AUCAUCCUCCACUGUUCA 3'
::::::::: ::::::::
query 3' UAGUAGGAGAUGACAAGU 5'
query=miR6477, target=RO3G_08846, score=2, range=93-110, strand=1
target 5' AUCAUCCUCCACUGUUCA 3'
::::::::: ::::::::
query 3' UAGUAGGAGAUGACAAGU 5'
```

```
query=miR6477, target=RO3G_11773, score=2, range=93-110, strand=1
target 5' AUCAUCCUCCACUGUUCA 3'
::::::::: ::::::::
query 3' UAGUAGGAGAUGACAAGU 5'
query=miR6477, target=RO3G_11401, score=2, range=93-110, strand=1
target 5' AUCAUCCUCCACUGUUCA 3'
::::::::: ::::::::
query 3' UAGUAGGAGAUGACAAGU 5'
query=miR6477, target=RO3G_16555, score=2, range=93-110, strand=1
target 5' AUCAUCCUCCACUGUUCA 3'
::::::::: ::::::::
query 3' UAGUAGGAGAUGACAAGU 5'
query=miR6477, target=RO3G_03571, score=2, range=93-110, strand=1
target 5' AUCAUCCUCUACUGUACA 3'
:::::::::::::::: ::
query 3' UAGUAGGAGAUGACAAGU 5'
query=miR6477, target=RO3G_13848, score=2, range=93-110, strand=1
target 5' AUCAUCCUCCACUGUUCA 3'
::::::::: ::::::::
query 3' UAGUAGGAGAUGACAAGU 5'
query=miR6477, target=RO3G_01902, score=2.5, range=354-371, stran
target 5' GUCUUCUUCUACUGUUCA 3'
.:: ::.:::::::::::
query 3' UAGUAGGAGAUGACAAGU 5'
query=miR6477, target=RO3G_16099, score=3, range=747-764, strand=
target 5' AUCAUCUUCUACUGAUCA 3'
::::::.::::::: :::
query 3' UAGUAGGAGAUGACAAGU 5'
query=miR6477, target=RO3G_08661, score=3, range=93-110, strand=1
target 5' AUCAUCCUCCACUGUUUA 3'
::::::::: :::::::.:
query 3' UAGUAGGAGAUGACAAGU 5'
query=miR6477, target=RO3G_13605, score=3, range=1012-1028, stran
target 5' AUC-UCUUCUGCUGUUCA 3'
::: ::.:::.:::::::
query 3' UAGUAGGAGAUGACAAGU 5'
query=miR6477, target=RO3G_02290, score=3, range=76-92, strand=1
target 5' AUC-UCUUCUGCUGUUCA 3'
::: ::.:::.:::::::
query 3' UAGUAGGAGAUGACAAGU 5'
query=miR6477, target=RO3G_10445, score=3, range=474-490, strand=
target 5' UUCAUCCUCUACUG-UCA 3'
::::::::::::: :::
query 3' UAGUAGGAGAUGACAAGU 5'
query=miR6477, target=RO3G_09054, score=3, range=1282-1299, stran
```

target 5' AUCAUCCUCUGCUGUUUC 3'
:::::::::.:::::.
query 3' UAGUAGGAGAUGACAAGU 5'
query=miR6477, target=RO3G_04004, score=3, range=334-351, strand=
target 5' AUCAUCUUUUACUGUUCU 3'
::::::.:.::::::::
query 3' UAGUAGGAGAUGACAAGU 5'
query=miR6477, target=RO3G_10565, score=3, range=624-642, strand=
target 5' AUCAUCCUCUGCUGUAUCA 3'
:::::::::.:::: :::
query 3' UAGUAGGAGAUGACA-AGU 5'
query=miR6477, target=RO3G_02067, score=3, range=414-432, strand=
target 5' AUCAUCCUCUGUCUGUUCA 3'
::::::::::. :::::::
query 3' UAGUAGGAGAU-GACAAGU 5'
query=miR6477, target=RO3G_13558, score=3, range=222-240, strand=
target 5' AUACAUCUUCUGCUGUUCA 3'
:: ::::.:::.:::::::
query 3' UA-GUAGGAGAUGACAAGU 5'
query=miR6477, target=RO3G_08586, score=3, range=1000-1017, stran
target 5' AUCAGCCUCUACUGUUUU 3'
:::: :::::::::::.
query 3' UAGUAGGAGAUGACAAGU 5'
query=miR6477, target=RO3G_11740, score=3, range=3743-3759, stran
target 5' AUCAUCCUCUGCUG-UCA 3'
::::::::::.::: :::
query 3' UAGUAGGAGAUGACAAGU 5'
query=miR6477, target=RO3G_15525, score=3, range=986-1002, strand
target 5' CUC-UCCUCUGCUGUUCA 3'
:: ::::::.:::::::
query 3' UAGUAGGAGAUGACAAGU 5'
query=miR6477, target=RO3G_13618, score=3, range=30-46, strand=-1
target 5' ACCAUCCUCUACU-UUCA 3'
: ::::::::::: ::::
query 3' UAGUAGGAGAUGACAAGU 5'
query=miR6477, target=RO3G_02427, score=3, range=803-819, strand=
target 5' ACCA-CCUCUAUUGUUCA 3'
: :: ::::::.::::::
query 3' UAGUAGGAGAUGACAAGU 5'
query=miR6477, target=RO3G_08852, score=3.5, range=561-578, stran
target 5' CUCGUCCUCUGCUGUUCC 3'
::.::::::.::::::
query 3' UAGUAGGAGAUGACAAGU 5'
query=miR6477, target=RO3G_00513, score=3.5, range=5592-5609, str
target 5' GUUCUCCUUUACUGUUCG 3'

```
.:. ::::.:::::::.
query 3' UAGUAGGAGAUGACAAGU 5'
query=miR6477, target=RO3G_04535, score=3.5, range=2019-2036, str
target 5' CUCAUCCUCUACUAUUCG 3'
::::::::::: :::.
query 3' UAGUAGGAGAUGACAAGU 5'
query=miR6477, target=RO3G_14091, score=3.5, range=175-192, stran
target 5' ACCGUCCUUUACUGUUCC 3'
: :.::::.:::::::
query 3' UAGUAGGAGAUGACAAGU 5'
query=miR6477, target=RO3G_11057, score=3.5, range=53-71, strand=
target 5' GUCAUGCCUCUACUGUUUC 3'
.:::: ::::::::::.
query 3' UAGUA-GGAGAUGACAAGU 5'
query=miR6477, target=RO3G_08425, score=3.5, range=577-593, stran
target 5' AUU-UUCUCUAUUGUUCA 3'
::. :.:::::.::::::
query 3' UAGUAGGAGAUGACAAGU 5'
query=miR6477, target=RO3G_11337, score=3.5, range=217-234, stran
target 5' AAGGUCCUCUAUUGUUCA 3'
: .:::::::.::::::
query 3' UAGUAGGAGAUGACAAGU 5'
query=miR6477, target=RO3G_15299, score=3.5, range=911-929, stran
target 5' GUCAUACCUCUCCUGUUCA 3'
.:::: ::::: :::::::
query 3' UAGUA-GGAGAUGACAAGU 5'
query=miR6477, target=RO3G_00043, score=3.5, range=46-63, strand=
target 5' AAUAACCUCUACUGUUCU 3'
: .: :::::::::::
query 3' UAGUAGGAGAUGACAAGU 5'
query=miR6477, target=RO3G_09501, score=3.5, range=988-1004, stra
target 5' AUC-UCCUCUUCUGUUCG 3'
::: :::::: ::::::.
query 3' UAGUAGGAGAUGACAAGU 5'
query=miR6477, target=RO3G_13886, score=3.5, range=749-767, stran
target 5' GUCAUCUUGCUACUGUUCA 3'
.:::::.: :::::::::
query 3' UAGUAGGA-GAUGACAAGU 5'
query=miR6477, target=RO3G_08005, score=3.5, range=400-416, stran
target 5' AUCAUCUUCU-CUGUUCG 3'
::::::.::: ::::::.
query 3' UAGUAGGAGAUGACAAGU 5'
query=miR6477, target=RO3G_14023, score=3.5, range=598-615, stran
target 5' AUCGUCUUCUACUGUUUC 3'
:::.::.::::::::.
```

query 3' UAGUAGGAGAUGACAAGU 5'
query=miR6477, target=RO3G_08882, score=3.5, range=3155-3173,
strand=-1
target 5' CUCAUCCCUCUACUGUUUG 3'
:::: :::::::::::..
query 3' UAGUA-GGAGAUGACAAGU 5'
query=miR6477, target=RO3G_13132, score=3.5, range=889-905, stran
target 5' AUU-UCCUCUGCUGUUCC 3'
::. :::::.:::::
query 3' UAGUAGGAGAUGACAAGU 5'
query=miR6477, target=RO3G_09535, score=3.5, range=1014-1032,
strand=-1
target 5' AUUAUGCUCAUACUGUUCA 3'
::.:: ::: :::::::::
query 3' UAGUAGGAG-AUGACAAGU 5'
query=miR6477, target=RO3G_06479, score=4, range=4041-4058, stran
target 5' AUCGUCGUCUGCUGUUCG 3'
:::.:: :::.:::::.
query 3' UAGUAGGAGAUGACAAGU 5'
query=miR6477, target=RO3G_11450, score=4, range=297-315, strand=
target 5' UUCAUCCUCUAUUGUAUCA 3'
::::::::.::: :::
query 3' UAGUAGGAGAUGACA-AGU 5'
query=miR6477, target=RO3G_09842, score=4, range=2061-2078, stran
target 5' UUCAACUUCUAUUGUUCA 3'
::: :.::::.::::::
query 3' UAGUAGGAGAUGACAAGU 5'
query=miR6477, target=RO3G_16482, score=4, range=105-122, strand=
target 5' AUCAUCCUCUACUUUACA 3'
:::::::::::: : ::
query 3' UAGUAGGAGAUGACAAGU 5'
query=miR6477, target=RO3G_02069, score=4, range=1033-1049, stran
target 5' AUC-UCUUCUGCUGUUUA 3'
::: ::.:::.:::::.:
query 3' UAGUAGGAGAUGACAAGU 5'
query=miR6477, target=RO3G_11156, score=4, range=181-197, strand=
target 5' AUU-UCCUUUACUGUUUG 3'
::. ::::.:::::::..
query 3' UAGUAGGAGAUGACAAGU 5'
query=miR6477, target=RO3G_02593, score=4, range=175-192, strand=
target 5' UGCCUUCUCUACUGUUCA 3'
: :.:::::::::::
query 3' UAGUAGGAGAUGACAAGU 5'
query=miR6477, target=RO3G_06092, score=4, range=1884-1900, stran
target 5' AUCUUCCUCUGCUG-UCA 3'

```
::: ::::::.::: :::
query 3' UAGUAGGAGAUGACAAGU 5'
query=miR6477, target=RO3G_09997, score=4, range=1102-1119, stran
target 5' AUAUUCCUUUACUGUUCU 3'
:: ::::.::::::::
query 3' UAGUAGGAGAUGACAAGU 5'
query=miR6477, target=RO3G_16107, score=4, range=1572-1588, stran
target 5' AUCA-CCACUGCUGUUCA 3'
:::: :: ::.:::::::
query 3' UAGUAGGAGAUGACAAGU 5'
query=miR6477, target=RO3G_01072, score=4, range=2479-2495, stran
target 5' UACA-CUUCUACUGUUCA 3'
:: :.::::::::::
query 3' UAGUAGGAGAUGACAAGU 5'
query=miR6477, target=RO3G_10142, score=4, range=1542-1558, stran
target 5' CUC-UCCUCUCCUGUUCA 3'
:: :::::: :::::::
query 3' UAGUAGGAGAUGACAAGU 5'
query=miR6477, target=RO3G_03107, score=4, range=1544-1560, stran
target 5' CUC-UCCUCUCCUGUUCA 3'
:: :::::: :::::::
query 3' UAGUAGGAGAUGACAAGU 5'
query=miR6477, target=RO3G_04143, score=4, range=706-722, strand=
target 5' AGCAU-CUCUACUGUUCC 3'
: ::: ::::::::::
query 3' UAGUAGGAGAUGACAAGU 5'
query=miR6477, target=RO3G_00987, score=4, range=177-194, strand=
target 5' CUCAUCUUUUGCUGUUCA 3'
:::::.:.:.:::::::
query 3' UAGUAGGAGAUGACAAGU 5'
query=miR6477, target=RO3G_08255, score=4, range=87-105, strand=-
target 5' AUCAUCUUUUACUGUCUCA 3'
::::::.:.:::::: :::
query 3' UAGUAGGAGAUGACA-AGU 5'
query=miR6477, target=RO3G_01827, score=4, range=105-123, strand=
target 5' AUCAUCUUCUACUUGUUCU 3'
::::::.::::: :::::
query 3' UAGUAGGAGAUG-ACAAGU 5'
query=miR6477, target=RO3G_14674, score=4, range=284-300, strand=
target 5' AUCAUCCUUUACU-UUUA 3'
:::::::::.:::: ::.:
query 3' UAGUAGGAGAUGACAAGU 5'
query=miR6477, target=RO3G_01665, score=4, range=428-444, strand=
target 5' AUCAUUCUCUGCU-UUCA 3'
:::::.::::.:: ::::
```

```
query 3' UAGUAGGAGAUGACAAGU 5'
query=miR6477, target=RO3G_00507, score=4, range=481-498, strand=
target 5' AUCAUCCUCUUCUGUUAA 3'
:::::::::: ::::: :
query 3' UAGUAGGAGAUGACAAGU 5'
query=miR6477, target=RO3G_14152, score=4, range=1442-1459, stran
target 5' AUAAUCUUCUACGGUUCA 3'
:: :::.::::: :::::
query 3' UAGUAGGAGAUGACAAGU 5'
query=miR6477, target=RO3G_12763, score=4, range=3051-3069, stran
target 5' AUCCAUCCACUGCUGUUCA 3'
:: ::::: ::.:::::::
query 3' UA-GUAGGAGAUGACAAGU 5'
query=miR6477, target=RO3G_09843, score=4, range=1781-1798, stran
target 5' AGCAAUCUUUACUGUUCA 3'
: :: .::.:::::::::
query 3' UAGUAGGAGAUGACAAGU 5'
query=miR6477, target=RO3G_12344, score=4, range=904-921, strand=
target 5' AUAACCCUCUACUGUUUU 3'
:: : ::::::::::.
query 3' UAGUAGGAGAUGACAAGU 5'
query=miR6477, target=RO3G_17111, score=4, range=691-707, strand=
target 5' GUU-UCCUCUACUGUGCA 3'
.:. :::::::::: ::
query 3' UAGUAGGAGAUGACAAGU 5'
query=miR6477, target=RO3G_13627, score=4, range=434-450, strand=
target 5' ACCAUCCUCUACU-UUCU 3'
: :::::::::: :::
query 3' UAGUAGGAGAUGACAAGU 5'
query=miR7734-3p, target=RO3G_13273, score=0, range=647-668, stra
target 5' CGUUGAAUGUGGACUGGUUCCA 3'
::::::::::::::::::::::
query 3' GCAACUUACACCUGACCAAGGU 5'
query=miR7734-3p, target=RO3G_10217, score=0, range=647-668, stra
target 5' CGUUGAAUGUGGACUGGUUCCA 3'
::::::::::::::::::::::
query 3' GCAACUUACACCUGACCAAGGU 5'
query=miR7734-3p, target=RO3G_16749, score=0, range=647-668, stra
target 5' CGUUGAAUGUGGACUGGUUCCA 3'
::::::::::::::::::::::
query 3' GCAACUUACACCUGACCAAGGU 5'
query=miR7734-3p, target=RO3G_13145, score=0, range=647-668, stra
target 5' CGUUGAAUGUGGACUGGUUCCA 3'
::::::::::::::::::::::
query 3' GCAACUUACACCUGACCAAGGU 5'
```

```
query=miR7734-3p, target=RO3G_11969, score=0, range=647-668, stra
target 5' CGUUGAAUGUGGACUGGUUCCA 3'
::::::::::::::::::::::
query 3' GCAACUUACACCUGACCAAGGU 5'
query=miR7734-3p, target=RO3G_07698, score=0, range=647-668, stra
target 5' CGUUGAAUGUGGACUGGUUCCA 3'
::::::::::::::::::::::
query 3' GCAACUUACACCUGACCAAGGU 5'
query=miR7734-3p, target=RO3G_11237, score=0, range=647-668, stra
target 5' CGUUGAAUGUGGACUGGUUCCA 3'
::::::::::::::::::::::
query 3' GCAACUUACACCUGACCAAGGU 5'
query=miR7734-3p, target=RO3G_04683, score=0, range=647-668, stra
target 5' CGUUGAAUGUGGACUGGUUCCA 3'
::::::::::::::::::::::
query 3' GCAACUUACACCUGACCAAGGU 5'
query=miR7734-3p, target=RO3G_11409, score=0, range=647-668, stra
target 5' CGUUGAAUGUGGACUGGUUCCA 3'
::::::::::::::::::::::
query 3' GCAACUUACACCUGACCAAGGU 5'
query=miR7734-3p, target=RO3G_04212, score=0, range=647-668, stra
target 5' CGUUGAAUGUGGACUGGUUCCA 3'
::::::::::::::::::::::
query 3' GCAACUUACACCUGACCAAGGU 5'
query=miR7734-3p, target=RO3G_14550, score=0, range=647-668, stra
target 5' CGUUGAAUGUGGACUGGUUCCA 3'
::::::::::::::::::::::
query 3' GCAACUUACACCUGACCAAGGU 5'
query=miR7734-3p, target=RO3G_15898, score=0, range=647-668, stra
target 5' CGUUGAAUGUGGACUGGUUCCA 3'
::::::::::::::::::::::
query 3' GCAACUUACACCUGACCAAGGU 5'
query=miR7734-3p, target=RO3G_09930, score=0, range=647-668, stra
target 5' CGUUGAAUGUGGACUGGUUCCA 3'
::::::::::::::::::::::
query 3' GCAACUUACACCUGACCAAGGU 5'
query=miR7734-3p, target=RO3G_06731, score=0, range=647-668, stra
target 5' CGUUGAAUGUGGACUGGUUCCA 3'
::::::::::::::::::::::
query 3' GCAACUUACACCUGACCAAGGU 5'
query=miR7734-3p, target=RO3G_07307, score=0, range=647-668, stra
target 5' CGUUGAAUGUGGACUGGUUCCA 3'
::::::::::::::::::::::
query 3' GCAACUUACACCUGACCAAGGU 5'
query=miR7734-3p, target=RO3G_09601, score=0, range=647-668, stra
```

```
target 5' CGUUGAAUGUGGACUGGUUCCA 3'
::::::::::::::::::::::
query 3' GCAACUUACACCUGACCAAGGU 5'
query=miR7734-3p, target=RO3G_11538, score=0, range=647-668, stra
target 5' CGUUGAAUGUGGACUGGUUCCA 3'
::::::::::::::::::::::
query 3' GCAACUUACACCUGACCAAGGU 5'
query=miR7734-3p, target=RO3G_07721, score=0, range=647-668, stra
target 5' CGUUGAAUGUGGACUGGUUCCA 3'
::::::::::::::::::::::
query 3' GCAACUUACACCUGACCAAGGU 5'
query=miR7734-3p, target=RO3G_01728, score=0, range=605-626, stra
target 5' CGUUGAAUGUGGACUGGUUCCA 3'
::::::::::::::::::::::
query 3' GCAACUUACACCUGACCAAGGU 5'
query=miR7734-3p, target=RO3G_16827, score=0, range=647-668, stra
target 5' CGUUGAAUGUGGACUGGUUCCA 3'
::::::::::::::::::::::
query 3' GCAACUUACACCUGACCAAGGU 5'
query=miR7734-3p, target=RO3G_07980, score=0, range=35-56, strand
target 5' CGUUGAAUGUGGACUGGUUCCA 3'
::::::::::::::::::::::
query 3' GCAACUUACACCUGACCAAGGU 5'
query=miR7734-3p, target=RO3G_07737, score=0, range=8-29, strand=
target 5' CGUUGAAUGUGGACUGGUUCCA 3'
::::::::::::::::::::::
query 3' GCAACUUACACCUGACCAAGGU 5'
query=miR7734-3p, target=RO3G_17257, score=2, range=563-584, stra
target 5' CAUUGAAUGUGGAUUGGUUCCA 3'
: :::::::::::.::::::::
query 3' GCAACUUACACCUGACCAAGGU 5'
query=miR7734-3p, target=RO3G_16136, score=2, range=563-584, stra
target 5' CAUUGAAUGUGGAUUGGUUCCA 3'
: :::::::::::.::::::::
query 3' GCAACUUACACCUGACCAAGGU 5'
query=miR7734-3p, target=RO3G_05424, score=2, range=563-584, stra
target 5' CAUUGAAUGUGGAUUGGUUCCA 3'
: :::::::::::.::::::::
query 3' GCAACUUACACCUGACCAAGGU 5'
query=miR7734-3p, target=RO3G_15889, score=2, range=563-584, stra
target 5' CAUUGAAUGUGGAUUGGUUCCA 3'
: :::::::::::.::::::::
query 3' GCAACUUACACCUGACCAAGGU 5'
query=miR7734-3p, target=RO3G_10735, score=2, range=92-113, stran
target 5' CAUUGAAUGUGGAUUGGUUCCA 3'
```

```
: ::::::::::.:::::::: 
query 3' GCAACUUACACCUGACCAAGGU 5'
query=miR7734-3p, target=RO3G_10039, score=2, range=563-584, stra
target 5' CAUUGAAUGUGGAUUGGUUCCA 3'
: ::::::::::.:::::::: 
query 3' GCAACUUACACCUGACCAAGGU 5'
query=miR7734-3p, target=RO3G_02828, score=2, range=563-584, stra
target 5' CAUUGAAUGUGGAUUGGUUCCA 3'
: ::::::::::.:::::::: 
query 3' GCAACUUACACCUGACCAAGGU 5'
query=miR7734-3p, target=RO3G_11949, score=2, range=8-29, strand=
target 5' CAUUGAAUGUGGAUUGGUUCCA 3'
: ::::::::::.:::::::: 
query 3' GCAACUUACACCUGACCAAGGU 5'
query=miR7734-3p, target=RO3G_00291, score=2, range=563-584, stra
target 5' CAUUGAAUGUGGAUUGGUUCCA 3'
: ::::::::::.:::::::: 
query 3' GCAACUUACACCUGACCAAGGU 5'
query=miR7734-3p, target=RO3G_14991, score=2, range=8-29, strand=
target 5' CAUUGAAUGUGGAUUGGUUCCA 3'
: ::::::::::.:::::::: 
query 3' GCAACUUACACCUGACCAAGGU 5'
query=miR7734-3p, target=RO3G_12072, score=4, range=866-887, stra
target 5' CGUUGAAUGUCGAUUGGUUUCA 3'
:::::::::: ::.:::::.:: 
query 3' GCAACUUACACCUGACCAAGGU 5'
query=miR7734-3p, target=RO3G_17358, score=4, range=866-887, stra
target 5' CGUUGAAUGUCGAUUGGUUUCA 3'
:::::::::: ::.:::::.:: 
query 3' GCAACUUACACCUGACCAAGGU 5'
query=miR7734-3p, target=RO3G_07686, score=4, range=866-887, stra
target 5' CGUUGAAUGUCGAUUGGUUUCA 3'
:::::::::: ::.:::::.:: 
query 3' GCAACUUACACCUGACCAAGGU 5'
query=miR7734-3p, target=RO3G_14710, score=4, range=866-887, stra
target 5' CGUUGAAUGUCGAUUGGUUUCA 3'
:::::::::: ::.:::::.:: 
query 3' GCAACUUACACCUGACCAAGGU 5'
query=miR7734-3p, target=RO3G_17385, score=4, range=866-887, stra
target 5' CGUUGAAUGUCGAUUGGUUUCA 3'
:::::::::: ::.:::::.:: 
query 3' GCAACUUACACCUGACCAAGGU 5'
query=miR7734-3p, target=RO3G_12009, score=4, range=866-887, stra
target 5' CGUUGAAUGUCGAUUGGUUUCA 3'
:::::::::: ::.:::::.:: 
```

```
query 3' GCAACUUACACCUGACCAAGGU 5'
query=miR7734-3p, target=RO3G_09940, score=4, range=866-887, stra
target 5' CGUUGAAUGUCGAUUGGUUUCA 3'
:::::::::: ::.:::::.::
query 3' GCAACUUACACCUGACCAAGGU 5'
query=miR7734-3p, target=RO3G_07976, score=4, range=863-884, stra
target 5' CGUUGAAUGUCGAUUGGUUUCA 3'
:::::::::: ::.:::::.::
query 3' GCAACUUACACCUGACCAAGGU 5'
query=miR7734-3p, target=RO3G_10725, score=4, range=830-851, stra
target 5' CGUUGAAUGUCGAUUGGUUUCA 3'
:::::::::: ::.:::::.::
query 3' GCAACUUACACCUGACCAAGGU 5'
query=miR7734-3p, target=RO3G_03580, score=4, range=791-812, stra
target 5' CGUUGAAUGUCGAUUGGUUUCA 3'
:::::::::: ::.:::::.::
query 3' GCAACUUACACCUGACCAAGGU 5'
query=miR7734-3p, target=RO3G_17441, score=4, range=593-614, stra
target 5' CGUUGAAUGUCGAUUGGUUUCA 3'
:::::::::: ::.:::::.::
query 3' GCAACUUACACCUGACCAAGGU 5'
query=miR7734-3p, target=RO3G_13747, score=4, range=866-887, stra
target 5' CGUUGAAUGUCGAUUGGUUUCA 3'
:::::::::: ::.:::::.::
query 3' GCAACUUACACCUGACCAAGGU 5'
query=miR7734-3p, target=RO3G_08042, score=4, range=398-419, stra
target 5' CGUUGAAUGUCGAUUGGUUUCA 3'
:::::::::: ::.:::::.::
query 3' GCAACUUACACCUGACCAAGGU 5'
query=miR7734-3p, target=RO3G_07294, score=4, range=467-488, stra
target 5' CGUUGAAUGUCGAUUGGUUUCA 3'
:::::::::: ::.:::::.::
query 3' GCAACUUACACCUGACCAAGGU 5'
query=miR7734-3p, target=RO3G_05631, score=4, range=866-887, stra
target 5' CGUUGAAUGUCGAUUGGUUUCA 3'
:::::::::: ::.:::::.::
query 3' GCAACUUACACCUGACCAAGGU 5'
query=miR7734-3p, target=RO3G_07130, score=4, range=770-791, stra
target 5' CGUUGAAUGUCGAUUGGUUUCA 3'
:::::::::: ::.:::::.::
query 3' GCAACUUACACCUGACCAAGGU 5'
query=miR7734-3p, target=RO3G_16739, score=4, range=866-887, stra
target 5' CGUUGAAUGUCGAUUGGUUUCA 3'
:::::::::: ::.:::::.::
query 3' GCAACUUACACCUGACCAAGGU 5'
```

```
query=miR7734-3p, target=RO3G_09192, score=4, range=866-887, stra
target 5' CGUUGAAUGUCGAUUGGUUUCA 3'
:::::::::: ::.:::::.::
query 3' GCAACUUACACCUGACCAAGGU 5'
query=miR7734-3p, target=RO3G_00693, score=4, range=344-365, stra
target 5' CGUUGAAUGUCGAUUGGUUUCA 3'
:::::::::: ::.:::::.::
query 3' GCAACUUACACCUGACCAAGGU 5'
query=miR9476-5p, target=RO3G_09279, score=3.5, range=505-527,
strand=1
target 5' CAAAAAGAGUGGGCAAGACUGGA 3'
:::::.:::.:::::::::.::
query 3' UUUUUUUUCAUCCGUUCUGAUCU 5'
query=miR9476-5p, target=RO3G_11610, score=4, range=2233-2254,
strand=-1
target 5' AAAAUAAAGUAGGUAAGA-UAGA 3'
:::: ::::::::.:::: ::::
query 3' UUUUUUUUCAUCCGUUCUGAUCU 5'
query=miR3476-5p, target=RO3G_07738, score=0, range=378-398, stra
target 5' GCAGCAGCAGCACCCAGUUCA 3'
:::::::::::::::::::::
query 3' CGUCGUCGUCGUGGGUCAAGU 5'
query=miR3476-5p, target=RO3G_01726, score=0, range=372-392, stra
target 5' GCAGCAGCAGCACCCAGUUCA 3'
:::::::::::::::::::::
query 3' CGUCGUCGUCGUGGGUCAAGU 5'
query=miR3476-5p, target=RO3G_11537, score=0, range=369-389, stra
target 5' GCAGCAGCAGCACCCAGUUCA 3'
:::::::::::::::::::::
query 3' CGUCGUCGUCGUGGGUCAAGU 5'
query=miR3476-5p, target=RO3G_11236, score=0, range=369-389, stra
target 5' GCAGCAGCAGCACCCAGUUCA 3'
:::::::::::::::::::::
query 3' CGUCGUCGUCGUGGGUCAAGU 5'
query=miR3476-5p, target=RO3G_07981, score=0, range=327-347, stra
target 5' GCAGCAGCAGCACCCAGUUCA 3'
:::::::::::::::::::::
query 3' CGUCGUCGUCGUGGGUCAAGU 5'
query=miR3476-5p, target=RO3G_07696, score=0, range=327-347, stra
target 5' GCAGCAGCAGCACCCAGUUCA 3'
:::::::::::::::::::::
query 3' CGUCGUCGUCGUGGGUCAAGU 5'
query=miR3476-5p, target=RO3G_06730, score=0, range=327-347, stra
target 5' GCAGCAGCAGCACCCAGUUCA 3'
:::::::::::::::::::::
```

```
query 3' CGUCGUCGUCGUGGGUCAAGU 5'
query=miR3476-5p, target=RO3G_07720, score=0, range=327-347, stra
target 5' GCAGCAGCAGCACCCAGUUCA 3'
:::::::::::::::::::::
query 3' CGUCGUCGUCGUGGGUCAAGU 5'
query=miR3476-5p, target=RO3G_04211, score=0, range=327-347, stra
target 5' GCAGCAGCAGCACCCAGUUCA 3'
:::::::::::::::::::::
query 3' CGUCGUCGUCGUGGGUCAAGU 5'
query=miR3476-5p, target=RO3G_09602, score=0, range=327-347, stra
target 5' GCAGCAGCAGCACCCAGUUCA 3'
:::::::::::::::::::::
query 3' CGUCGUCGUCGUGGGUCAAGU 5'
query=miR3476-5p, target=RO3G_07306, score=0, range=327-347, stra
target 5' GCAGCAGCAGCACCCAGUUCA 3'
:::::::::::::::::::::
query 3' CGUCGUCGUCGUGGGUCAAGU 5'
query=miR3476-5p, target=RO3G_14551, score=0, range=327-347, stra
target 5' GCAGCAGCAGCACCCAGUUCA 3'
:::::::::::::::::::::
query 3' CGUCGUCGUCGUGGGUCAAGU 5'
query=miR3476-5p, target=RO3G_09929, score=0, range=327-347, stra
target 5' GCAGCAGCAGCACCCAGUUCA 3'
:::::::::::::::::::::
query 3' CGUCGUCGUCGUGGGUCAAGU 5'
query=miR3476-5p, target=RO3G_15897, score=0, range=327-347, stra
target 5' GCAGCAGCAGCACCCAGUUCA 3'
:::::::::::::::::::::
query 3' CGUCGUCGUCGUGGGUCAAGU 5'
query=miR3476-5p, target=RO3G_09603, score=0, range=369-389, stra
target 5' GCAGCAGCAGCACCCAGUUCA 3'
:::::::::::::::::::::
query 3' CGUCGUCGUCGUGGGUCAAGU 5'
query=miR3476-5p, target=RO3G_10218, score=0, range=348-368, stra
target 5' GCAGCAGCAGCACCCAGUUCA 3'
:::::::::::::::::::::
query 3' CGUCGUCGUCGUGGGUCAAGU 5'
query=miR3476-5p, target=RO3G_16748, score=0, range=327-347, stra
target 5' GCAGCAGCAGCACCCAGUUCA 3'
:::::::::::::::::::::
query 3' CGUCGUCGUCGUGGGUCAAGU 5'
query=miR3476-5p, target=RO3G_04679, score=0, range=297-317, stra
target 5' GCAGCAGCAGCACCCAGUUCA 3'
:::::::::::::::::::::
query 3' CGUCGUCGUCGUGGGUCAAGU 5'
```

```
query=miR3476-5p, target=RO3G_11977, score=0, range=291-311, stra
target 5' GCAGCAGCAGCACCCAGUUCA 3'
:::::::::::::::::::::
query 3' CGUCGUCGUCGUGGGUCAAGU 5'
query=miR3476-5p, target=RO3G_02228, score=0, range=327-347, stra
target 5' GCAGCAGCAGCACCCAGUUCA 3'
:::::::::::::::::::::
query 3' CGUCGUCGUCGUGGGUCAAGU 5'
query=miR3476-5p, target=RO3G_10041, score=1, range=357-377, stra
target 5' GCAGCAGCAGCAUCCAGUUCA 3'
::::::::::::.::::::::
query 3' CGUCGUCGUCGUGGGUCAAGU 5'
query=miR3476-5p, target=RO3G_17248, score=1, range=357-377, stra
target 5' GCAGCAGCAGCAUCCAGUUCA 3'
::::::::::::.::::::::
query 3' CGUCGUCGUCGUGGGUCAAGU 5'
query=miR3476-5p, target=RO3G_15147, score=1, range=321-341, stra
target 5' GCAGCAGCAGCAUCCAGUUCA 3'
::::::::::::.::::::::
query 3' CGUCGUCGUCGUGGGUCAAGU 5'
query=miR3476-5p, target=RO3G_03584, score=1, range=285-305, stra
target 5' GCAGCAGCAGCAUCCAGUUCA 3'
::::::::::::.::::::::
query 3' CGUCGUCGUCGUGGGUCAAGU 5'
query=miR3476-5p, target=RO3G_15890, score=1, range=357-377, stra
target 5' GCAGCAGCAGCAUCCAGUUCA 3'
::::::::::::.::::::::
query 3' CGUCGUCGUCGUGGGUCAAGU 5'
query=miR3476-5p, target=RO3G_13146, score=1, range=357-377, stra
target 5' GCAGCAGCAGCAUCCAGUUCA 3'
::::::::::::.::::::::
query 3' CGUCGUCGUCGUGGGUCAAGU 5'
query=miR3476-5p, target=RO3G_17256, score=1, range=354-374, stra
target 5' GCAGCAGCAGCAUCCAGUUCA 3'
::::::::::::.::::::::
query 3' CGUCGUCGUCGUGGGUCAAGU 5'
query=miR3476-5p, target=RO3G_16137, score=1, range=351-371, stra
target 5' GCAGCAGCAGCAUCCAGUUCA 3'
::::::::::::.::::::::
query 3' CGUCGUCGUCGUGGGUCAAGU 5'
query=miR3476-5p, target=RO3G_17350, score=1, range=351-371, stra
target 5' GCAGCAGCAGCAUCCAGUUCA 3'
::::::::::::.::::::::
query 3' CGUCGUCGUCGUGGGUCAAGU 5'
query=miR3476-5p, target=RO3G_00288, score=1, range=348-368, stra
```

```
target 5' GCAGCAGCAGCAUCCAGUUCA 3'
:::::::::::.::::::::
query 3' CGUCGUCGUCGUGGGUCAAGU 5'
query=miR3476-5p, target=RO3G_09182, score=1, range=333-353, stra
target 5' GCAGCAGCAGCAUCCAGUUCA 3'
:::::::::::.::::::::
query 3' CGUCGUCGUCGUGGGUCAAGU 5'
query=miR3476-5p, target=RO3G_05425, score=2, range=354-374, stra
target 5' GCAGCAGAAGCAUCCAGUUCA 3'
::::::: ::::.::::::::
query 3' CGUCGUCGUCGUGGGUCAAGU 5'
query=miR3476-5p, target=RO3G_09497, score=2, range=66-86, strand
target 5' GCAACAGCAGCAUCCAGUUCA 3'
::: ::::::::.::::::::
query 3' CGUCGUCGUCGUGGGUCAAGU 5'
query=miR3476-5p, target=RO3G_10775, score=3, range=355-375, stra
target 5' GCAACAGAAGCAUCCAGUUCA 3'
::: ::: ::::.::::::::
query 3' CGUCGUCGUCGUGGGUCAAGU 5'
query=miR3476-5p, target=RO3G_04258, score=3.5, range=1680-1700,
strand=-1
target 5' GCAGCAGCAGCAGCCAGUUUG 3'
:::::::::::: :::::::..
query 3' CGUCGUCGUCGUGGGUCAAGU 5'
query=miR3476-5p, target=RO3G_03734, score=4, range=266-286, stra
target 5' GGGGCAGUAGCACCCAGAUCA 3'
: .::::.::::::::: :::
query 3' CGUCGUCGUCGUGGGUCAAGU 5'
query=miR3476-5p, target=RO3G_03439, score=4, range=517-537, stra
target 5' AAAGAAGCAGUACCCAGUUCA 3'
:: ::::.::::::::::
query 3' CGUCGUCGUCGUGGGUCAAGU 5'
query=miR3476-5p, target=RO3G_06536, score=4, range=429-448, stra
target 5' GCAGCAGCAGCAGCCA-UUCA 3'
:::::::::::: ::: ::::
query 3' CGUCGUCGUCGUGGGUCAAGU 5'
query=miR3476-5p, target=RO3G_01044, score=4, range=1359-1379,
strand=-1
target 5' CCAGCAGCAGCACCUUGUUCA 3'
:::::::::::::. :::::
query 3' CGUCGUCGUCGUGGGUCAAGU 5'
query=miR8137, target=RO3G_03585, score=0, range=77-98, strand=1
target 5' AUUUUGUGAACUGGUUGCGAUA 3'
::::::::::::::::::::::
query 3' UAAAACACUUGACCAACGCUAU 5'
```

query=miR8137, target=RO3G_17257, score=1, range=1403-1424, stran
target 5' AUUUUGUGAACUGGUUGCGGUA 3'
::::::::::::::::::::.::
query 3' UAAAACACUUGACCAACGCUAU 5'
query=miR8137, target=RO3G_05424, score=1, range=1403-1424, stran
target 5' AUUUUGUGAACUGGUUGCGGUA 3'
::::::::::::::::::::.::
query 3' UAAAACACUUGACCAACGCUAU 5'
query=miR8137, target=RO3G_15889, score=1, range=1403-1424, stran
target 5' AUUUUGUGAACUGGUUGCGGUA 3'
::::::::::::::::::::.::
query 3' UAAAACACUUGACCAACGCUAU 5'
query=miR8137, target=RO3G_16136, score=1, range=1403-1424, stran
target 5' AUUUUGUGAACUGGUUGCGGUA 3'
::::::::::::::::::::.::
query 3' UAAAACACUUGACCAACGCUAU 5'
query=miR8137, target=RO3G_10735, score=1, range=932-953, strand=
target 5' AUUUUGUGAACUGGUUGCGGUA 3'
::::::::::::::::::::.::
query 3' UAAAACACUUGACCAACGCUAU 5'
query=miR8137, target=RO3G_09183, score=1, range=620-641, strand=
target 5' AUUUUGUGAACUGGUUGCGGUA 3'
::::::::::::::::::::.::
query 3' UAAAACACUUGACCAACGCUAU 5'
query=miR8137, target=RO3G_00289, score=1, range=215-236, strand=
target 5' AUUUUGUGAACUGGUUGCGGUA 3'
::::::::::::::::::::.::
query 3' UAAAACACUUGACCAACGCUAU 5'
query=miR8137, target=RO3G_17351, score=1, range=215-236, strand=
target 5' AUUUUGUGAACUGGUUGCGGUA 3'
::::::::::::::::::::.::
query 3' UAAAACACUUGACCAACGCUAU 5'
query=miR8137, target=RO3G_11949, score=1, range=848-869, strand=
target 5' AUUUUGUGAACUGGUUGCGGUA 3'
::::::::::::::::::::.::
query 3' UAAAACACUUGACCAACGCUAU 5'
query=miR8137, target=RO3G_10040, score=1, range=77-98, strand=1
target 5' AUUUUGUGAACUGGUUGCGGUA 3'
::::::::::::::::::::.::
query 3' UAAAACACUUGACCAACGCUAU 5'
query=miR8571, target=RO3G_06442, score=0, range=7167-7188, stran
target 5' AGAACAGGAUCUUGACGAUGAA 3'
::::::::::::::::::::::
query 3' UCUUGUCCUAGAACUGCUACUU 5'
query=miR8571, target=RO3G_16557, score=0, range=1443-1464, stran

```
target 5' AGAACAGGAUCUUGACGAUGAA 3'
::::::::::::::::::::::
query 3' UCUUGUCCUAGAACUGCUACUU 5'
query=miR8571, target=RO3G_07098, score=0, range=1443-1464, stran
target 5' AGAACAGGAUCUUGACGAUGAA 3'
::::::::::::::::::::::
query 3' UCUUGUCCUAGAACUGCUACUU 5'
query=miR8571, target=RO3G_01745, score=0, range=1443-1464, stran
target 5' AGAACAGGAUCUUGACGAUGAA 3'
::::::::::::::::::::::
query 3' UCUUGUCCUAGAACUGCUACUU 5'
query=miR8571, target=RO3G_17438, score=0, range=975-996, strand=
target 5' AGAACAGGAUCUUGACGAUGAA 3'
::::::::::::::::::::::
query 3' UCUUGUCCUAGAACUGCUACUU 5'
query=miR8571, target=RO3G_14995, score=0, range=1443-1464, stran
target 5' AGAACAGGAUCUUGACGAUGAA 3'
::::::::::::::::::::::
query 3' UCUUGUCCUAGAACUGCUACUU 5'
query=miR8571, target=RO3G_09540, score=0, range=651-672, strand=
target 5' AGAACAGGAUCUUGACGAUGAA 3'
::::::::::::::::::::::
query 3' UCUUGUCCUAGAACUGCUACUU 5'
query=miR8571, target=RO3G_05271, score=0, range=1443-1464, stran
target 5' AGAACAGGAUCUUGACGAUGAA 3'
::::::::::::::::::::::
query 3' UCUUGUCCUAGAACUGCUACUU 5'
query=miR8571, target=RO3G_03098, score=0, range=651-672, strand=
target 5' AGAACAGGAUCUUGACGAUGAA 3'
::::::::::::::::::::::
query 3' UCUUGUCCUAGAACUGCUACUU 5'
query=miR8571, target=RO3G_00290, score=0, range=1443-1464, stran
target 5' AGAACAGGAUCUUGACGAUGAA 3'
::::::::::::::::::::::
query 3' UCUUGUCCUAGAACUGCUACUU 5'
query=miR8571, target=RO3G_15134, score=0, range=1485-1506, stran
target 5' AGAACAGGAUCUUGACGAUGAA 3'
::::::::::::::::::::::
query 3' UCUUGUCCUAGAACUGCUACUU 5'
query=miR8571, target=RO3G_00486, score=0, range=240-261, strand=
target 5' AGAACAGGAUCUUGACGAUGAA 3'
::::::::::::::::::::::
query 3' UCUUGUCCUAGAACUGCUACUU 5'
query=miR8571, target=RO3G_12470, score=0, range=1443-1464, stran
target 5' AGAACAGGAUCUUGACGAUGAA 3'
```

```
::::::::::::::::::::::
query 3' UCUUGUCCUAGAACUGCUACUU 5'
query=miR8571, target=RO3G_16132, score=0, range=1443-1464, stran
target 5' AGAACAGGAUCUUGACGAUGAA 3'
::::::::::::::::::::::
query 3' UCUUGUCCUAGAACUGCUACUU 5'
query=miR8571, target=RO3G_01928, score=0, range=1443-1464, stran
target 5' AGAACAGGAUCUUGACGAUGAA 3'
::::::::::::::::::::::
query 3' UCUUGUCCUAGAACUGCUACUU 5'
query=miR8571, target=RO3G_11524, score=0, range=510-531, strand=
target 5' AGAACAGGAUCUUGACGAUGAA 3'
::::::::::::::::::::::
query 3' UCUUGUCCUAGAACUGCUACUU 5'
query=miR8571, target=RO3G_16560, score=0, range=513-534, strand=
target 5' AGAACAGGAUCUUGACGAUGAA 3'
::::::::::::::::::::::
query 3' UCUUGUCCUAGAACUGCUACUU 5'
query=miR8571, target=RO3G_02699, score=0, range=240-261, strand=
target 5' AGAACAGGAUCUUGACGAUGAA 3'
::::::::::::::::::::::
query 3' UCUUGUCCUAGAACUGCUACUU 5'
query=miR8571, target=RO3G_14926, score=3, range=31-51, strand=1
target 5' AGAA-AGGAUUUUGAUGAUGAA 3'
:::: :::::.::::.::::::
query 3' UCUUGUCCUAGAACUGCUACUU 5'
query=miR8571, target=RO3G_12504, score=4, range=935-956, strand=
target 5' AGAAAAGGAUCUUGCUGAUGAA 3'
:::: ::::::::: .::::::
query 3' UCUUGUCCUAGAACUGCUACUU 5'
query=miR7719-5p, target=RO3G_13270, score=0, range=525-546, stra
target 5' GAAGAAACAUGCUUAUGGAGGA 3'
::::::::::::::::::::::
query 3' CUUCUUUGUACGAAUACCUCCU 5'
query=miR7719-5p, target=RO3G_14363, score=0, range=483-504, stra
target 5' GAAGAAACAUGCUUAUGGAGGA 3'
::::::::::::::::::::::
query 3' CUUCUUUGUACGAAUACCUCCU 5'
query=miR7719-5p, target=RO3G_10774, score=0, range=492-513, stra
target 5' GAAGAAACAUGCUUAUGGAGGA 3'
::::::::::::::::::::::
query 3' CUUCUUUGUACGAAUACCUCCU 5'
query=miR7719-5p, target=RO3G_13863, score=0, range=225-246, stra
target 5' GAAGAAACAUGCUUAUGGAGGA 3'
::::::::::::::::::::::
```

```
query 3' CUUCUUUGUACGAAUACCUCCU 5'
query=miR7719-5p, target=RO3G_13755, score=0, range=525-546, stra
target 5' GAAGAAACAUGCUUAUGGAGGA 3'
::::::::::::::::::::::
query 3' CUUCUUUGUACGAAUACCUCCU 5'
query=miR7719-5p, target=RO3G_08849, score=0, range=525-546, stra
target 5' GAAGAAACAUGCUUAUGGAGGA 3'
::::::::::::::::::::::
query 3' CUUCUUUGUACGAAUACCUCCU 5'
query=miR7719-5p, target=RO3G_14979, score=0, range=525-546, stra
target 5' GAAGAAACAUGCUUAUGGAGGA 3'
::::::::::::::::::::::
query 3' CUUCUUUGUACGAAUACCUCCU 5'
query=miR7719-5p, target=RO3G_17276, score=0.5, range=615-636,
strand=1
target 5' GAGGAAACAUGCUUAUGGAGGA 3'
::.:::::::::::::::::::
query 3' CUUCUUUGUACGAAUACCUCCU 5'
query=miR7719-5p, target=RO3G_13741, score=0.5, range=525-546,
strand=1
target 5' GAAGAAACGUGCUUAUGGAGGA 3'
::::::::.:::::::::::::
query 3' CUUCUUUGUACGAAUACCUCCU 5'
query=miR7719-5p, target=RO3G_17249, score=1, range=525-546, stra
target 5' AAAGAAACAUGCUUAUGGAGGA 3'
:::::::::::::::::::::
query 3' CUUCUUUGUACGAAUACCUCCU 5'
query=miR7719-5p, target=RO3G_05936, score=2, range=525-546, stra
target 5' GAAGAAACAUGCUUAUGGAGCA 3'
:::::::::::::::::::: :
query 3' CUUCUUUGUACGAAUACCUCCU 5'
query=miR7719-5p, target=RO3G_05420, score=2, range=386-407, stra
target 5' GAAGAAACAUGCUUAUGGAGCA 3'
:::::::::::::::::::: :
query 3' CUUCUUUGUACGAAUACCUCCU 5'
query=miR5177, target=RO3G_17015, score=0, range=1208-1227, stran
target 5' CUGCUCGUUUUCCAACCUCA 3'
::::::::::::::::::::
query 3' GACGAGCAAAAGGUUGGAGU 5'
query=miR5177, target=RO3G_11637, score=2, range=791-809, strand=
target 5' AUGCUC-UUUUCCAACCUCA 3'
::::: :::::::::::::
query 3' GACGAGCAAAAGGUUGGAGU 5'
query=miR5177, target=RO3G_12627, score=3, range=1118-1137, stran
target 5' CUGCUCGUCUUCCAACCUCC 3'
```

```
:::::::: ::::::::::
query 3' GACGAGCAAAAGGUUGGAGU 5'
query=miR5177, target=RO3G_00488, score=3, range=383-403, strand=
target 5' CUGCUCGAUUUUUCAACCUCC 3'
::::::: ::::.::::::
query 3' GACGAGC-AAAAGGUUGGAGU 5'
query=miR5177, target=RO3G_13129, score=4, range=346-365, strand=
target 5' CUGAUCGGUUUCCAACCUUA 3'
::: ::: ::::::::::.:
query 3' GACGAGCAAAAGGUUGGAGU 5'
query=miR5177, target=RO3G_01141, score=4, range=499-517, strand=
target 5' CU-CUCGCUUUCCAAUCUCA 3'
:: :::: :::::::.::::
query 3' GACGAGCAAAAGGUUGGAGU 5'
query=miR5177, target=RO3G_10655, score=4, range=542-560, strand=
target 5' CUGCUC-UUUUCCGUCCUCA 3'
:::::: ::::::. :::::
query 3' GACGAGCAAAAGGUUGGAGU 5'
query=miR5177, target=RO3G_04265, score=4, range=1244-1264, stran
target 5' CUGCUAGAUUUCCAUACCUCA 3'
::::: : :::::: ::::::
query 3' GACGAGCAAAAGGU-UGGAGU 5'
query=miR5177, target=RO3G_14671, score=4, range=288-308, strand=
target 5' UUGCUUGAUUUUUCAACCUUA 3'
.::::.: ::::.::::::.:
query 3' GACGAGC-AAAAGGUUGGAGU 5'
query=miR5177, target=RO3G_01404, score=4, range=720-739, strand=
target 5' CUUUUUGUUUUUCAACCUCU 3'
:: .:.::::.:::::::
query 3' GACGAGCAAAAGGUUGGAGU 5'
query=miR5177, target=RO3G_07652, score=4, range=3246-3265, stran
target 5' UUGUUCCUUUUACAACCUCA 3'
.::.:: :::: ::::::::
query 3' GACGAGCAAAAGGUUGGAGU 5'
query=miR5177, target=RO3G_08766, score=4, range=900-919, strand=
target 5' CUGCUAGUUUGCCAACCUCC 3'
::::: :::: ::::::::
query 3' GACGAGCAAAAGGUUGGAGU 5'
query=miR6248, target=RO3G_04050, score=0, range=1302-1321, stran
target 5' CACUAACCUCCACCUCAAUA 3'
::::::::::::::::::::
query 3' GUGAUUGGAGGUGGAGUUAU 5'
query=miR6248, target=RO3G_12949, score=0, range=747-766, strand=
target 5' CACUAACCUCCACCUCAAUA 3'
::::::::::::::::::::
```

```
query 3' GUGAUUGGAGGUGGAGUUAU 5'
query=miR6248, target=RO3G_00301, score=0, range=2586-2605, stran
target 5' CACUAACCUCCACCUCAAUA 3'
::::::::::::::::::::
query 3' GUGAUUGGAGGUGGAGUUAU 5'
query=miR6248, target=RO3G_09585, score=2.5, range=141-160, stran
target 5' CUUUCACCUCCACCUCAAUA 3'
: .: ::::::::::::::
query 3' GUGAUUGGAGGUGGAGUUAU 5'
query=miR6248, target=RO3G_04538, score=3, range=1576-1595, stran
target 5' AACCAACCUCCACCUCAAUC 3'
:: ::::::::::::::
query 3' GUGAUUGGAGGUGGAGUUAU 5'
query=miR6248, target=RO3G_14139, score=3.5, range=242-260, stran
target 5' CAUUGACCUCCACCUC-AUG 3'
::.:.:::::::::: ::.
query 3' GUGAUUGGAGGUGGAGUUAU 5'
query=miR6248, target=RO3G_10206, score=4, range=405-425, strand=
target 5' CAUCUGGCCACCACCUCAAUA 3'
:: ::..:: ::::::::::
query 3' GU-GAUUGGAGGUGGAGUUAU 5'
query=miR6248, target=RO3G_00949, score=4, range=2027-2045, stran
target 5' CAC-AACCUCCACCACAAUC 3'
::: :::::::::: ::::
query 3' GUGAUUGGAGGUGGAGUUAU 5'
query=miR6248, target=RO3G_00796, score=4, range=419-437, strand=
target 5' CACU-UCCUCCACCUCGAUC 3'
:::: ::::::::::.::
query 3' GUGAUUGGAGGUGGAGUUAU 5'
query=miR6248, target=RO3G_01339, score=4, range=419-437, strand=
target 5' CACU-UCCUCCACCUCGAUC 3'
:::: ::::::::::.::
query 3' GUGAUUGGAGGUGGAGUUAU 5'
query=miR6248, target=RO3G_00107, score=4, range=345-364, strand=
target 5' CAUUGACCUCUACUUCAAUU 3'
::.:.:::::.::.:::::
query 3' GUGAUUGGAGGUGGAGUUAU 5'
query=miR6248, target=RO3G_07692, score=4, range=876-895, strand=
target 5' CAGGGAACUCCACCUCAAUG 3'
:: .: :::::::::::.
query 3' GUGAUUGGAGGUGGAGUUAU 5'
query=miR6221-5p, target=RO3G_04120, score=0, range=747-768, stra
target 5' AGACAGCGGGCCAGAAGACAGA 3'
::::::::::::::::::::::
query 3' UCUGUCGCCCGGUCUUCUGUCU 5'
```

```
query=miR6221-5p, target=RO3G_13224, score=0, range=747-768, stra
target 5' AGACAGCGGGCCAGAAGACAGA 3'
::::::::::::::::::::::
query 3' UCUGUCGCCCGGUCUUCUGUCU 5'
query=miR6221-5p, target=RO3G_04123, score=0, range=747-768, stra
target 5' AGACAGCGGGCCAGAAGACAGA 3'
::::::::::::::::::::::
query 3' UCUGUCGCCCGGUCUUCUGUCU 5'
query=miR6221-5p, target=RO3G_17093, score=0, range=507-528, stra
target 5' AGACAGCGGGCCAGAAGACAGA 3'
::::::::::::::::::::::
query 3' UCUGUCGCCCGGUCUUCUGUCU 5'
query=miR6221-5p, target=RO3G_13221, score=2, range=747-768, stra
target 5' AGACAGCGGACCAGAAGACAGA 3'
::::::::: ::::::::::::
query 3' UCUGUCGCCCGGUCUUCUGUCU 5'
query=miR6221-5p, target=RO3G_01231, score=4, range=889-909, stra
target 5' UGAGAGCGGGCCA-AAGACAGA 3'
:: ::::::::: ::::::::
query 3' UCUGUCGCCCGGUCUUCUGUCU 5'
query=miR6221-5p, target=RO3G_01237, score=4, range=544-564, stra
target 5' UGAGAGCGGGCCA-AAGACAGA 3'
:: ::::::::: ::::::::
query 3' UCUGUCGCCCGGUCUUCUGUCU 5'
query=miR1849, target=RO3G_15157, score=0, range=555-573, strand=
target 5' ACCAACCUAUGAUACGCUA 3'
:::::::::::::::::::
query 3' UGGUUGGAUACUAUGCGAU 5'
query=miR1849, target=RO3G_16810, score=0, range=198-216, strand=
target 5' ACCAACCUAUGAUACGCUA 3'
:::::::::::::::::::
query 3' UGGUUGGAUACUAUGCGAU 5'
query=miR1849, target=RO3G_04676, score=0, range=555-573, strand=
target 5' ACCAACCUAUGAUACGCUA 3'
:::::::::::::::::::
query 3' UGGUUGGAUACUAUGCGAU 5'
query=miR1849, target=RO3G_12005, score=0, range=555-573, strand=
target 5' ACCAACCUAUGAUACGCUA 3'
:::::::::::::::::::
query 3' UGGUUGGAUACUAUGCGAU 5'
query=miR1849, target=RO3G_11407, score=0, range=138-156, strand=
target 5' ACCAACCUAUGAUACGCUA 3'
:::::::::::::::::::
query 3' UGGUUGGAUACUAUGCGAU 5'
query=miR1849, target=RO3G_12080, score=0, range=138-156, strand=
```

```
target 5' ACCAACCUAUGAUACGCUA 3'
:::::::::::::::::::
query 3' UGGUUGGAUACUAUGCGAU 5'
query=miR1849, target=RO3G_05928, score=0, range=327-345, strand=
target 5' ACCAACCUAUGAUACGCUA 3'
:::::::::::::::::::
query 3' UGGUUGGAUACUAUGCGAU 5'
query=miR1849, target=RO3G_11953, score=2.5, range=432-450, stran
target 5' ACCAGCCUAUGAUGCGCUC 3'
::::.::::::::.::::
query 3' UGGUUGGAUACUAUGCGAU 5'
query=miR1849, target=RO3G_07678, score=2.5, range=555-573, stran
target 5' ACCAGCCUAUGAUGCGCUC 3'
::::.::::::::.::::
query 3' UGGUUGGAUACUAUGCGAU 5'
query=miR1849, target=RO3G_07110, score=2.5, range=555-573, stran
target 5' ACCAGCCUAUGAUGCGCUC 3'
::::.::::::::.::::
query 3' UGGUUGGAUACUAUGCGAU 5'
query=miR1849, target=RO3G_10525, score=2.5, range=555-573, stran
target 5' ACCAGCCUAUGAUGCGCUC 3'
::::.::::::::.::::
query 3' UGGUUGGAUACUAUGCGAU 5'
query=miR1849, target=RO3G_07676, score=2.5, range=555-573, stran
target 5' ACCAGCCUAUGAUGCGCUC 3'
::::.::::::::.::::
query 3' UGGUUGGAUACUAUGCGAU 5'
query=miR1849, target=RO3G_01968, score=2.5, range=555-573, stran
target 5' ACCAGCCUAUGAUGCGCUC 3'
::::.::::::::.::::
query 3' UGGUUGGAUACUAUGCGAU 5'
query=miR1849, target=RO3G_10531, score=2.5, range=555-573, stran
target 5' ACCAGCCUAUGAUGCGCUC 3'
::::.::::::::.::::
query 3' UGGUUGGAUACUAUGCGAU 5'
query=miR1849, target=RO3G_17380, score=2.5, range=555-573, stran
target 5' ACCAGCCUAUGAUGCGCUC 3'
::::.::::::::.::::
query 3' UGGUUGGAUACUAUGCGAU 5'
query=miR1849, target=RO3G_05523, score=2.5, range=555-573, stran
target 5' ACCAGCCUAUGAUGCGCUC 3'
::::.::::::::.::::
query 3' UGGUUGGAUACUAUGCGAU 5'
query=miR1849, target=RO3G_03767, score=2.5, range=555-573, stran
target 5' ACCAGCCUAUGAUGCGCUC 3'
```

```
::::.:::::::::.::::
query 3' UGGUUGGAUACUAUGCGAU 5'
query=miR1849, target=RO3G_01949, score=2.5, range=555-573, stran
target 5' ACCAGCCUAUGAUGCGCUC 3'
::::.:::::::::.::::
query 3' UGGUUGGAUACUAUGCGAU 5'
query=miR1849, target=RO3G_10545, score=2.5, range=555-573, stran
target 5' ACCAGCCUAUGAUGCGCUC 3'
::::.:::::::::.::::
query 3' UGGUUGGAUACUAUGCGAU 5'
query=miR1849, target=RO3G_12960, score=2.5, range=216-234, stran
target 5' ACCAGCCUAUGAUGCGCUC 3'
::::.:::::::::.::::
query 3' UGGUUGGAUACUAUGCGAU 5'
query=miR1849, target=RO3G_12974, score=2.5, range=216-234, stran
target 5' ACCAGCCUAUGAUGCGCUC 3'
::::.:::::::::.::::
query 3' UGGUUGGAUACUAUGCGAU 5'
query=miR1849, target=RO3G_04668, score=3.5, range=63-82, strand=
target 5' AUCAACCUAUGAUAUUGCUA 3'
:.:::::::::::: .::::
query 3' UGGUUGGAUACUAU-GCGAU 5'
query=miR1849, target=RO3G_00874, score=3.5, range=1654-1671, str
target 5' AUCAACCU-UGAUACGCUU 3'
:.:::::: ::::::::
query 3' UGGUUGGAUACUAUGCGAU 5'
query=miR1849, target=RO3G_14802, score=3.5, range=1841-1859,
strand=-1
target 5' ACUAUCUUAUGAUAUGCUA 3'
::.: :.:::::::.::::
query 3' UGGUUGGAUACUAUGCGAU 5'
query=miR1849, target=RO3G_09606, score=3.5, range=1169-1187,
strand=-1
target 5' ACUAUCUUAUGAUAUGCUA 3'
::.: :.:::::::.::::
query 3' UGGUUGGAUACUAUGCGAU 5'
query=miR1849, target=RO3G_14706, score=3.5, range=1799-1817,
strand=-1
target 5' ACUAUCUUAUGAUAUGCUA 3'
::.: :.:::::::.::::
query 3' UGGUUGGAUACUAUGCGAU 5'
query=miR1849, target=RO3G_02953, score=3.5, range=1913-1931,
strand=-1
target 5' ACUAUCUUAUGAUAUGCUA 3'
::.: :.:::::::.::::
```

query 3' UGGUUGGAUACUAUGCGAU 5'
query=miR1849, target=RO3G_01303, score=3.5, range=2012-2030, strand=-1
target 5' ACUAUCUUAUGAUAUGCUA 3'
::.: :.:::::::.::::
query 3' UGGUUGGAUACUAUGCGAU 5'
query=miR1849, target=RO3G_05338, score=3.5, range=347-365, stran
target 5' ACUAUCUUAUGAUAUGCUA 3'
::.: :.:::::::.::::
query 3' UGGUUGGAUACUAUGCGAU 5'
query=miR1849, target=RO3G_10561, score=3.5, range=347-365, stran
target 5' ACUAUCUUAUGAUAUGCUA 3'
::.: :.:::::::.::::
query 3' UGGUUGGAUACUAUGCGAU 5'
query=miR1849, target=RO3G_12463, score=3.5, range=191-209, stran
target 5' ACUAUCUUAUGAUAUGCUA 3'
::.: :.:::::::.::::
query 3' UGGUUGGAUACUAUGCGAU 5'
query=miR1849, target=RO3G_17398, score=3.5, range=2012-2030, strand=-1
target 5' ACUAUCUUAUGAUAUGCUA 3'
::.: :.:::::::.::::
query 3' UGGUUGGAUACUAUGCGAU 5'
query=miR1849, target=RO3G_04802, score=3.5, range=1253-1271, strand=-1
target 5' ACUAUCUUAUGAUAUGCUA 3'
::.: :.:::::::.::::
query 3' UGGUUGGAUACUAUGCGAU 5'
query=miR1849, target=RO3G_12489, score=3.5, range=46-64, strand=
target 5' ACAAGCCUAUGAUGCGCUC 3'
:: :.:::::::.::::
query 3' UGGUUGGAUACUAUGCGAU 5'
query=miR1849, target=RO3G_07870, score=4, range=1473-1492, stran
target 5' UCCAUCCUAUGAUAUCGCUA 3'
::: ::::::::: :::::
query 3' UGGUUGGAUACUAU-GCGAU 5'
query=miR1849, target=RO3G_15867, score=4, range=1473-1492, stran
target 5' UCCAUCCUAUGAUAUCGCUA 3'
::: ::::::::: :::::
query 3' UGGUUGGAUACUAU-GCGAU 5'
query=miR1849, target=RO3G_05417, score=4, range=825-844, strand=
target 5' UCCAUCCUAUGAUAUCGCUA 3'
::: ::::::::: :::::
query 3' UGGUUGGAUACUAU-GCGAU 5'
query=miR1849, target=RO3G_17442, score=4, range=1500-1519, stran

target 5' UCCAUCCUAUGAUAUCGCUA 3'
::: ::::::::: :::::
query 3' UGGUUGGAUACUAU-GCGAU 5'
query=miR3627-3p, target=RO3G_12527, score=0, range=1323-1344, strand=-1
target 5' CUUGUCACGAGGACAGCGAGCA 3'
::::::::::::::::::::::
query 3' GAACAGUGCUCCUGUCGCUCGU 5'
query=miR3627-3p, target=RO3G_14347, score=0, range=1317-1338, strand=-1
target 5' CUUGUCACGAGGACAGCGAGCA 3'
::::::::::::::::::::::
query 3' GAACAGUGCUCCUGUCGCUCGU 5'
query=miR3627-3p, target=RO3G_16361, score=0, range=1317-1338, strand=-1
target 5' CUUGUCACGAGGACAGCGAGCA 3'
::::::::::::::::::::::
query 3' GAACAGUGCUCCUGUCGCUCGU 5'
query=miR3627-3p, target=RO3G_09547, score=0, range=1317-1338, strand=-1
target 5' CUUGUCACGAGGACAGCGAGCA 3'
::::::::::::::::::::::
query 3' GAACAGUGCUCCUGUCGCUCGU 5'
query=miR3627-3p, target=RO3G_06425, score=0, range=1317-1338, strand=-1
target 5' CUUGUCACGAGGACAGCGAGCA 3'
::::::::::::::::::::::
query 3' GAACAGUGCUCCUGUCGCUCGU 5'
query=miR3627-3p, target=RO3G_12677, score=0, range=1317-1338, strand=-1
target 5' CUUGUCACGAGGACAGCGAGCA 3'
::::::::::::::::::::::
query 3' GAACAGUGCUCCUGUCGCUCGU 5'
query=miR3627-3p, target=RO3G_17021, score=0, range=1317-1338, strand=-1
target 5' CUUGUCACGAGGACAGCGAGCA 3'
::::::::::::::::::::::
query 3' GAACAGUGCUCCUGUCGCUCGU 5'
query=miR3627-3p, target=RO3G_02704, score=0, range=1317-1338, strand=-1
target 5' CUUGUCACGAGGACAGCGAGCA 3'
::::::::::::::::::::::
query 3' GAACAGUGCUCCUGUCGCUCGU 5'
query=miR3627-3p, target=RO3G_05330, score=0, range=1317-1338, strand=-1

target 5' CUUGUCACGAGGACAGCGAGCA 3'
::::::::::::::::::::::
query 3' GAACAGUGCUCCUGUCGCUCGU 5'
query=miR3627-3p, target=RO3G_04933, score=0, range=1317-1338, strand=-1
target 5' CUUGUCACGAGGACAGCGAGCA 3'
::::::::::::::::::::::
query 3' GAACAGUGCUCCUGUCGCUCGU 5'
query=miR3627-3p, target=RO3G_04126, score=0, range=1317-1338, strand=-1
target 5' CUUGUCACGAGGACAGCGAGCA 3'
::::::::::::::::::::::
query 3' GAACAGUGCUCCUGUCGCUCGU 5'
query=miR3627-3p, target=RO3G_05344, score=0, range=1317-1338, strand=-1
target 5' CUUGUCACGAGGACAGCGAGCA 3'
::::::::::::::::::::::
query 3' GAACAGUGCUCCUGUCGCUCGU 5'
query=miR3627-3p, target=RO3G_03778, score=0, range=1275-1296, strand=-1
target 5' CUUGUCACGAGGACAGCGAGCA 3'
::::::::::::::::::::::
query 3' GAACAGUGCUCCUGUCGCUCGU 5'
query=miR3627-3p, target=RO3G_17422, score=0, range=618-639, stra
target 5' CUUGUCACGAGGACAGCGAGCA 3'
::::::::::::::::::::::
query 3' GAACAGUGCUCCUGUCGCUCGU 5'
query=miR3627-3p, target=RO3G_12532, score=0, range=1323-1344, strand=-1
target 5' CUUGUCACGAGGACAGCGAGCA 3'
::::::::::::::::::::::
query 3' GAACAGUGCUCCUGUCGCUCGU 5'
query=miR3627-3p, target=RO3G_13722, score=0, range=1314-1335, strand=-1
target 5' CUUGUCACGAGGACAGCGAGCA 3'
::::::::::::::::::::::
query 3' GAACAGUGCUCCUGUCGCUCGU 5'
query=miR3627-3p, target=RO3G_11414, score=0, range=1215-1236, strand=-1
target 5' CUUGUCACGAGGACAGCGAGCA 3'
::::::::::::::::::::::
query 3' GAACAGUGCUCCUGUCGCUCGU 5'
query=miR3627-3p, target=RO3G_16693, score=2, range=1323-1344, strand=-1
target 5' CUUGUCACGAGGACAGCGAACA 3'

```
:::::::::::::::::: ::
query 3' GAACAGUGCUCCUGUCGCUCGU 5'
query=miR3627-3p, target=RO3G_16539, score=2, range=1215-1236,
strand=-1
target 5' CUUGUCACGAGGACAGCGAACA 3'
:::::::::::::::::: ::
query 3' GAACAGUGCUCCUGUCGCUCGU 5'
query=miR3627-3p, target=RO3G_12538, score=2, range=1215-1236,
strand=-1
target 5' CUUGUCACGAGGACAGCGAACA 3'
:::::::::::::::::: ::
query 3' GAACAGUGCUCCUGUCGCUCGU 5'
query=miR3627-3p, target=RO3G_09493, score=2, range=108-129, stra
target 5' CUUGUCACGAGGACAGCGAACA 3'
:::::::::::::::::: ::
query 3' GAACAGUGCUCCUGUCGCUCGU 5'
query=miR3627-3p, target=RO3G_02082, score=4, range=186-206, stra
target 5' AUUGUCA-GAGGACAGCGAGAA 3'
:::::: ::::::::::: :
query 3' GAACAGUGCUCCUGUCGCUCGU 5'
query=miR3627-3p, target=RO3G_03629, score=4, range=53-73, strand
target 5' CUUGAAAC-AGGACAGCGAGUA 3'
:::: :: ::::::::::.:
query 3' GAACAGUGCUCCUGUCGCUCGU 5'
query=miR9560b-3p, target=RO3G_11767, score=0, range=1250-1272,
strand=-1
target 5' AGCACGAGUAGAACUAAGAGUGA 3'
:::::::::::::::::::::::
query 3' UCGUGCUCAUCUUGAUUCUCACU 5'
query=miR9560b-3p, target=RO3G_17150, score=1, range=2096-2118,
strand=-1
target 5' AUCACGAGUAGAACUAAGAGUGA 3'
: :::::::::::::::::::::
query 3' UCGUGCUCAUCUUGAUUCUCACU 5'
query=miR9560b-3p, target=RO3G_06111, score=4, range=34-56, stran
target 5' AGCAAGCACAGAACUAAGAGUGA 3'
:::: : ::::::::::::::
query 3' UCGUGCUCAUCUUGAUUCUCACU 5'
query=miR1047-3p, target=RO3G_15127, score=0, range=1264-1283,
strand=1
target 5' CAACUGAAGUAUGCUGAUCA 3'
::::::::::::::::::::
query 3' GUUGACUUCAUACGACUAGU 5'
query=miR1047-3p, target=RO3G_00249, score=3, range=73-92, strand
target 5' CAGCUGGAGUACGCUGAUCA 3'
```

```
::.:::.:::: ::::::::
query 3' GUUGACUUCAUACGACUAGU 5'
query=miR1047-3p, target=RO3G_02142, score=3, range=1780-1799,
strand=1
target 5' CAACUGAAGUAUCCUGAUCC 3'
:::::::::::: ::::::
query 3' GUUGACUUCAUACGACUAGU 5'
query=miR1047-3p, target=RO3G_13898, score=3.5, range=351-370,
strand=1
target 5' CAUCUUGGGUAUGCUGAUCA 3'
:: :: ..::::::::::::
query 3' GUUGACUUCAUACGACUAGU 5'
query=miR1047-3p, target=RO3G_13579, score=3.5, range=1014-1033,
strand=1
target 5' UCACAAAAGUAUGCUGAUCA 3'
. :: ::::::::::::::
query 3' GUUGACUUCAUACGACUAGU 5'
query=miR1047-3p, target=RO3G_07101, score=4, range=138-157, stra
target 5' UAACUUGAGUAUGUUGGUCA 3'
.:::: .::::::.::.:::
query 3' GUUGACUUCAUACGACUAGU 5'
query=miR1047-3p, target=RO3G_14254, score=4, range=108-127, stra
target 5' CAACUGAAGUAUGCUGGGCC 3'
::::::::::::::::. :
query 3' GUUGACUUCAUACGACUAGU 5'
query=miR1047-3p, target=RO3G_06153, score=4, range=1467-1486,
strand=-1
target 5' CAUCUGAAGCAUGUUGAUCA 3'
:: :::::: :::.::::::
query 3' GUUGACUUCAUACGACUAGU 5'
query=miR1047-3p, target=RO3G_08744, score=4, range=108-127, stra
target 5' CAACCGAAGUAUGCUGAGCC 3'
:::: :::::::::::: :
query 3' GUUGACUUCAUACGACUAGU 5'
query=miR5176-5p, target=RO3G_10860, score=0, range=576-595, stra
target 5' GAGAUGUGACACAUGGAAUA 3'
::::::::::::::::::::
query 3' CUCUACACUGUGUACCUUAU 5'
query=miR5176-5p, target=RO3G_17289, score=0, range=576-595, stra
target 5' GAGAUGUGACACAUGGAAUA 3'
::::::::::::::::::::
query 3' CUCUACACUGUGUACCUUAU 5'
query=miR5176-5p, target=RO3G_07729, score=0, range=345-364, stra
target 5' GAGAUGUGACACAUGGAAUA 3'
::::::::::::::::::::
```

query 3' CUCUACACUGUGUACCUUAU 5'
query=miR5176-5p, target=RO3G_08111, score=0, range=576-595, stra
target 5' GAGAUGUGACACAUGGAAUA 3'
::::::::::::::::::::
query 3' CUCUACACUGUGUACCUUAU 5'
query=miR5176-5p, target=RO3G_02682, score=0, range=576-595, stra
target 5' GAGAUGUGACACAUGGAAUA 3'
::::::::::::::::::::
query 3' CUCUACACUGUGUACCUUAU 5'
query=miR5176-5p, target=RO3G_12182, score=0, range=576-595, stra
target 5' GAGAUGUGACACAUGGAAUA 3'
::::::::::::::::::::
query 3' CUCUACACUGUGUACCUUAU 5'
query=miR5176-5p, target=RO3G_11784, score=0.5, range=576-595,
strand=1
target 5' GAGAUGUGACACAUGGAAUG 3'
:::::::::::::::::::.
query 3' CUCUACACUGUGUACCUUAU 5'
query=miR5176-5p, target=RO3G_12541, score=0.5, range=576-595,
strand=1
target 5' GAGAUGUGACACAUGGAAUG 3'
:::::::::::::::::::.
query 3' CUCUACACUGUGUACCUUAU 5'
query=miR5176-5p, target=RO3G_09496, score=0.5, range=576-595,
strand=1
target 5' GAGAUGUGACACAUGGAAUG 3'
:::::::::::::::::::.
query 3' CUCUACACUGUGUACCUUAU 5'
query=miR5176-5p, target=RO3G_11272, score=0.5, range=576-595,
strand=1
target 5' GAGAUGUGACACAUGGAAUG 3'
:::::::::::::::::::.
query 3' CUCUACACUGUGUACCUUAU 5'
query=miR5176-5p, target=RO3G_15862, score=0.5, range=576-595,
strand=1
target 5' GAGAUGUGACACAUGGAAUG 3'
:::::::::::::::::::.
query 3' CUCUACACUGUGUACCUUAU 5'
query=miR5176-5p, target=RO3G_13916, score=0.5, range=576-595,
strand=1
target 5' GAGAUGUGACACAUGGAAUG 3'
:::::::::::::::::::.
query 3' CUCUACACUGUGUACCUUAU 5'
query=miR5176-5p, target=RO3G_15274, score=0.5, range=576-595,
strand=1

```
target 5' GAGAUGUGACACAUGGAAUG 3'
::::::::::::::::::::.
query 3' CUCUACACUGUGUACCUUAU 5'
query=miR5176-5p, target=RO3G_15288, score=0.5, range=576-595,
strand=1
target 5' GAGAUGUGACACAUGGAAUG 3'
::::::::::::::::::::.
query 3' CUCUACACUGUGUACCUUAU 5'
query=miR5176-5p, target=RO3G_02988, score=4, range=1327-1347,
strand=1
target 5' GAUGAUGUGAAACAUGGAAUU 3'
:: ::::::: :::::::::
query 3' CU-CUACACUGUGUACCUUAU 5'
query=miR5176-5p, target=RO3G_15907, score=4, range=1116-1134,
strand=1
target 5' GAGACAU-ACACAUGGAAUA 3'
:::: : ::::::::::::
query 3' CUCUACACUGUGUACCUUAU 5'
query=miR5176-5p, target=RO3G_06014, score=4, range=1633-1651,
strand=-1
target 5' GAGA-GGGAUGCAUGGAAUA 3'
:::: : ::..:::::::::
query 3' CUCUACACUGUGUACCUUAU 5'
query=miR5176-5p, target=RO3G_16929, score=4, range=1519-1539,
strand=-1
target 5' GAGCUUUUGACACAUGGAAUC 3'
::: : :::::::::::::
query 3' CUC-UACACUGUGUACCUUAU 5'
query=miR5726, target=RO3G_13448, score=0, range=955-975, strand=
target 5' CCUGAUUCAAGCAACUCUUUA 3'
:::::::::::::::::::::
query 3' GGACUAAGUUCGUUGAGAAAU 5'
query=miR5726, target=RO3G_12329, score=3, range=298-317, strand=
target 5' CUUGAUUUAAGC-ACUCUUUA 3'
:.:::::.:::: ::::::::
query 3' GGACUAAGUUCGUUGAGAAAU 5'
query=miR5726, target=RO3G_04368, score=4, range=2289-2309, stran
target 5' ACUGAAUAAAGCAACUUUUUA 3'
:::: : ::::::::.::::
query 3' GGACUAAGUUCGUUGAGAAAU 5'
query=miR5726, target=RO3G_01838, score=4, range=774-794, strand=
target 5' CCCAACUCAAGCAACUCUUUU 3'
:: : :::::::::::::
query 3' GGACUAAGUUCGUUGAGAAAU 5'
query=miR5243, target=RO3G_17159, score=0, range=592-609, strand=
```

```
target 5' GCCCACGAUUCUGGCCCA 3'
::::::::::::::::::
query 3' CGGGUGCUAAGACCGGGU 5'
query=miR5243, target=RO3G_02698, score=0, range=472-489, strand=
target 5' GCCCACGAUUCUGGCCCA 3'
::::::::::::::::::
query 3' CGGGUGCUAAGACCGGGU 5'
query=miR5243, target=RO3G_09923, score=3, range=680-698, strand=
target 5' GCCGCACGGUUCUGGCCUA 3'
::: ::::.:::::::.:
query 3' CGG-GUGCUAAGACCGGGU 5'
query=miR5243, target=RO3G_06009, score=4, range=1331-1347, stran
target 5' AGCCA-GAUUCUGGCCCA 3'
::: :::::::::::
query 3' CGGGUGCUAAGACCGGGU 5'
query=miR5243, target=RO3G_10081, score=4, range=129-145, strand=
target 5' GACCUCG-UUCUGGCCCA 3'
: :: :: :::::::::
query 3' CGGGUGCUAAGACCGGGU 5'
query=miR8602, target=RO3G_17276, score=0, range=874-893, strand=
target 5' UGGCCAGCAUACUCUCCUGA 3'
::::::::::::::::::::
query 3' ACCGGUCGUAUGAGAGGACU 5'
query=miR8602, target=RO3G_17259, score=0, range=190-209, strand=
target 5' UGGCCAGCAUACUCUCCUGA 3'
::::::::::::::::::::
query 3' ACCGGUCGUAUGAGAGGACU 5'
query=miR8602, target=RO3G_10869, score=0, range=190-209, strand=
target 5' UGGCCAGCAUACUCUCCUGA 3'
::::::::::::::::::::
query 3' ACCGGUCGUAUGAGAGGACU 5'
query=miR8602, target=RO3G_14363, score=0, range=742-761, strand=
target 5' UGGCCAGCAUACUCUCCUGA 3'
::::::::::::::::::::
query 3' ACCGGUCGUAUGAGAGGACU 5'
query=miR8602, target=RO3G_17249, score=0, range=709-728, strand=
target 5' UGGCCAGCAUACUCUCCUGA 3'
::::::::::::::::::::
query 3' ACCGGUCGUAUGAGAGGACU 5'
query=miR8602, target=RO3G_13270, score=0, range=784-803, strand=
target 5' UGGCCAGCAUACUCUCCUGA 3'
::::::::::::::::::::
query 3' ACCGGUCGUAUGAGAGGACU 5'
query=miR8602, target=RO3G_10774, score=0, range=751-770, strand=
target 5' UGGCCAGCAUACUCUCCUGA 3'
```

```
::::::::::::::::::::
query 3' ACCGGUCGUAUGAGAGGACU 5'
query=miR8602, target=RO3G_04790, score=0, range=190-209, strand=
target 5' UGGCCAGCAUACUCUCCUGA 3'
::::::::::::::::::::
query 3' ACCGGUCGUAUGAGAGGACU 5'
query=miR8602, target=RO3G_05935, score=0, range=190-209, strand=
target 5' UGGCCAGCAUACUCUCCUGA 3'
::::::::::::::::::::
query 3' ACCGGUCGUAUGAGAGGACU 5'
query=miR8602, target=RO3G_03593, score=0, range=190-209, strand=
target 5' UGGCCAGCAUACUCUCCUGA 3'
::::::::::::::::::::
query 3' ACCGGUCGUAUGAGAGGACU 5'
query=miR8602, target=RO3G_01589, score=0, range=190-209, strand=
target 5' UGGCCAGCAUACUCUCCUGA 3'
::::::::::::::::::::
query 3' ACCGGUCGUAUGAGAGGACU 5'
query=miR8602, target=RO3G_03626, score=0, range=190-209, strand=
target 5' UGGCCAGCAUACUCUCCUGA 3'
::::::::::::::::::::
query 3' ACCGGUCGUAUGAGAGGACU 5'
query=miR8602, target=RO3G_13863, score=0, range=484-503, strand=
target 5' UGGCCAGCAUACUCUCCUGA 3'
::::::::::::::::::::
query 3' ACCGGUCGUAUGAGAGGACU 5'
query=miR8602, target=RO3G_11527, score=0, range=190-209, strand=
target 5' UGGCCAGCAUACUCUCCUGA 3'
::::::::::::::::::::
query 3' ACCGGUCGUAUGAGAGGACU 5'
query=miR8602, target=RO3G_00678, score=0, range=154-173, strand=
target 5' UGGCCAGCAUACUCUCCUGA 3'
::::::::::::::::::::
query 3' ACCGGUCGUAUGAGAGGACU 5'
query=miR8602, target=RO3G_14547, score=0.5, range=190-209, stran
target 5' UGGCUAGCAUACUCUCCUGA 3'
::::.:::::::::::::::
query 3' ACCGGUCGUAUGAGAGGACU 5'
query=miR8602, target=RO3G_13921, score=1, range=190-209, strand=
target 5' UGGCCAGCAUACUCUCUUGA 3'
ː::::::::::::::::.:::
query 3' ACCGGUCGUAUGAGAGGACU 5'
query=miR8602, target=RO3G_17459, score=1, range=190-209, strand=
target 5' UGGUCAGCAUACUCUCCUGG 3'
:::.:::::::::::::::.
```

```
query 3' ACCGGUCGUAUGAGAGGACU 5'
query=miR8602, target=RO3G_10734, score=1, range=190-209, strand=
target 5' UGGUCAGCAUACUCUCCUGG 3'
:::.:::::::::::::::.
query 3' ACCGGUCGUAUGAGAGGACU 5'
query=miR8602, target=RO3G_15166, score=3, range=790-809, strand=
target 5' UGGCCAGCAUAUUCACCUGA 3'
:::::::::::.:: :::::
query 3' ACCGGUCGUAUGAGAGGACU 5'
query=miR8602, target=RO3G_07577, score=3, range=142-161, strand=
target 5' UGGCCAGCAUAUUCACCUGA 3'
:::::::::::.:: :::::
query 3' ACCGGUCGUAUGAGAGGACU 5'
query=miR8602, target=RO3G_05940, score=3, range=499-518, strand=
target 5' UGGCCAGCAUAUUCACCUGA 3'
:::::::::::.:: :::::
query 3' ACCGGUCGUAUGAGAGGACU 5'
query=miR8602, target=RO3G_16536, score=3, range=103-122, strand=
target 5' UGGCCAGCAUAUUCGCCUGA 3'
:::::::::::.:: :::::
query 3' ACCGGUCGUAUGAGAGGACU 5'
query=miR8602, target=RO3G_00237, score=3, range=259-278, strand=
target 5' UGGCCAGCAUAUUCACCUGA 3'
:::::::::::.:: :::::
query 3' ACCGGUCGUAUGAGAGGACU 5'
query=miR8602, target=RO3G_15895, score=4, range=103-122, strand=
target 5' UGGCCAGUAUAUUCACCUGA 3'
:::::::.:::.:: :::::
query 3' ACCGGUCGUAUGAGAGGACU 5'
query=miR8602, target=RO3G_04692, score=4, range=142-161, strand=
target 5' UGGCCAGUAUAUUCACCUGA 3'
:::::::.:::.:: :::::
query 3' ACCGGUCGUAUGAGAGGACU 5'
query=miR8602, target=RO3G_17207, score=4, range=142-161, strand=
target 5' UGGCCAGUAUAUUCACCUGA 3'
:::::::.:::.:: :::::
query 3' ACCGGUCGUAUGAGAGGACU 5'
query=miR8602, target=RO3G_07783, score=4, range=658-677, strand=
target 5' UGCACAGCAUUCUCUCCUGA 3'
:: :::::: :::::::::
query 3' ACCGGUCGUAUGAGAGGACU 5'
query=miR8602, target=RO3G_01430, score=4, range=922-941, strand=
target 5' UUGCCCCCAUAUUCUCCUGA 3'
: ::: ::::.::::::::
query 3' ACCGGUCGUAUGAGAGGACU 5'
```

query=miR8602, target=RO3G_16443, score=4, range=124-143, strand=
target 5' UUGCCUCCAUAUUCUCCUGA 3'
: ::: ::::.::::::::
query 3' ACCGGUCGUAUGAGAGGACU 5'
query=miR7814, target=RO3G_06442, score=0, range=3852-3873, stran
target 5' UCAAAGCAUCAAAGACAUAUUA 3'
::::::::::::::::::::::
query 3' AGUUUCGUAGUUUCUGUAUAAU 5'
query=miR7814, target=RO3G_09965, score=0, range=3195-3216, stran
target 5' UCAAAGCAUCAAAGACAUAUUA 3'
::::::::::::::::::::::
query 3' AGUUUCGUAGUUUCUGUAUAAU 5'
query=miR7814, target=RO3G_13867, score=0, range=3111-3132, stran
target 5' UCAAAGCAUCAAAGACAUAUUA 3'
::::::::::::::::::::::
query 3' AGUUUCGUAGUUUCUGUAUAAU 5'
query=miR7814, target=RO3G_02959, score=0, range=3195-3216, stran
target 5' UCAAAGCAUCAAAGACAUAUUA 3'
::::::::::::::::::::::
query 3' AGUUUCGUAGUUUCUGUAUAAU 5'
query=miR7814, target=RO3G_15250, score=0, range=3150-3171, stran
target 5' UCAAAGCAUCAAAGACAUAUUA 3'
::::::::::::::::::::::
query 3' AGUUUCGUAGUUUCUGUAUAAU 5'
query=miR7814, target=RO3G_17371, score=0, range=3150-3171, stran
target 5' UCAAAGCAUCAAAGACAUAUUA 3'
::::::::::::::::::::::
query 3' AGUUUCGUAGUUUCUGUAUAAU 5'
query=miR7814, target=RO3G_06427, score=0, range=2787-2808, stran
target 5' UCAAAGCAUCAAAGACAUAUUA 3'
::::::::::::::::::::::
query 3' AGUUUCGUAGUUUCUGUAUAAU 5'
query=miR7814, target=RO3G_17326, score=0, range=3027-3048, stran
target 5' UCAAAGCAUCAAAGACAUAUUA 3'
::::::::::::::::::::::
query 3' AGUUUCGUAGUUUCUGUAUAAU 5'
query=miR7814, target=RO3G_06428, score=0, range=2289-2310, stran
target 5' UCAAAGCAUCAAAGACAUAUUA 3'
::::::::::::::::::::::
query 3' AGUUUCGUAGUUUCUGUAUAAU 5'
query=miR7814, target=RO3G_13749, score=0, range=1758-1779, stran
target 5' UCAAAGCAUCAAAGACAUAUUA 3'
::::::::::::::::::::::
query 3' AGUUUCGUAGUUUCUGUAUAAU 5'
query=miR7814, target=RO3G_05619, score=0, range=1338-1359, stran

```
target 5' UCAAAGCAUCAAAGACAUAUUA 3'
::::::::::::::::::::::
query 3' AGUUUCGUAGUUUCUGUAUAAU 5'
query=miR7814, target=RO3G_14355, score=0, range=1401-1422, stran
target 5' UCAAAGCAUCAAAGACAUAUUA 3'
::::::::::::::::::::::
query 3' AGUUUCGUAGUUUCUGUAUAAU 5'
query=miR7814, target=RO3G_15585, score=0, range=2997-3018, stran
target 5' UCAAAGCAUCAAAGACAUAUUA 3'
::::::::::::::::::::::
query 3' AGUUUCGUAGUUUCUGUAUAAU 5'
query=miR7814, target=RO3G_11776, score=0, range=285-306, strand=
target 5' UCAAAGCAUCAAAGACAUAUUA 3'
::::::::::::::::::::::
query 3' AGUUUCGUAGUUUCUGUAUAAU 5'
query=miR7814, target=RO3G_02222, score=0, range=249-270, strand=
target 5' UCAAAGCAUCAAAGACAUAUUA 3'
::::::::::::::::::::::
query 3' AGUUUCGUAGUUUCUGUAUAAU 5'
query=miR7814, target=RO3G_10558, score=0, range=948-969, strand=
target 5' UCAAAGCAUCAAAGACAUAUUA 3'
::::::::::::::::::::::
query 3' AGUUUCGUAGUUUCUGUAUAAU 5'
query=miR7814, target=RO3G_04115, score=0, range=138-159, strand=
target 5' UCAAAGCAUCAAAGACAUAUUA 3'
::::::::::::::::::::::
query 3' AGUUUCGUAGUUUCUGUAUAAU 5'
query=miR7814, target=RO3G_05279, score=0, range=141-162, strand=
target 5' UCAAAGCAUCAAAGACAUAUUA 3'
::::::::::::::::::::::
query 3' AGUUUCGUAGUUUCUGUAUAAU 5'
query=miR7814, target=RO3G_02691, score=1, range=6684-6705, stran
target 5' UCAAAGCAUCAAAGAUAUAUUA 3'
:::::::::::::::.::::::
query 3' AGUUUCGUAGUUUCUGUAUAAU 5'
query=miR7814, target=RO3G_03547, score=1, range=3195-3216, stran
target 5' UCAAAGCAUCAAAGAUAUAUUA 3'
:::::::::::::::.::::::
query 3' AGUUUCGUAGUUUCUGUAUAAU 5'
query=miR7814, target=RO3G_13920, score=1, range=3195-3216, stran
target 5' UCAAAGCAUCAAAGAUAUAUUA 3'
:::::::::::::::.::::::
query 3' AGUUUCGUAGUUUCUGUAUAAU 5'
query=miR7814, target=RO3G_01613, score=1, range=1446-1467, stran
target 5' UCAAAGCAUCAAAGGCAUAUUA 3'
```

```
::::::::::::::.:::::::
query 3' AGUUUCGUAGUUUCUGUAUAAU 5'
query=miR7814, target=RO3G_00306, score=2, range=1491-1512, stran
target 5' UCAAAGCAUCAAAGACAUAUCA 3'
:::::::::::::::::::: :
query 3' AGUUUCGUAGUUUCUGUAUAAU 5'
query=miR7814, target=RO3G_01330, score=2, range=429-450, strand=
target 5' UCAAAGCAUCAAAGACAUACUA 3'
::::::::::::::::::: ::
query 3' AGUUUCGUAGUUUCUGUAUAAU 5'
query=miR6116-3p, target=RO3G_12001, score=0, range=785-806, stra
target 5' CUAAUGCUUGGAUGACAAAUGA 3'
::::::::::::::::::::::
query 3' GAUUACGAACCUACUGUUUACU 5'
query=miR6116-3p, target=RO3G_10556, score=0, range=785-806, stra
target 5' CUAAUGCUUGGAUGACAAAUGA 3'
::::::::::::::::::::::
query 3' GAUUACGAACCUACUGUUUACU 5'
query=miR6116-3p, target=RO3G_17369, score=0, range=785-806, stra
target 5' CUAAUGCUUGGAUGACAAAUGA 3'
::::::::::::::::::::::
query 3' GAUUACGAACCUACUGUUUACU 5'
query=miR6116-3p, target=RO3G_15681, score=0, range=44-65, strand
target 5' CUAAUGCUUGGAUGACAAAUGA 3'
::::::::::::::::::::::
query 3' GAUUACGAACCUACUGUUUACU 5'
query=miR6116-3p, target=RO3G_13488, score=0, range=44-65, strand
target 5' CUAAUGCUUGGAUGACAAAUGA 3'
::::::::::::::::::::::
query 3' GAUUACGAACCUACUGUUUACU 5'
query=miR6116-3p, target=RO3G_13491, score=0, range=44-65, strand
target 5' CUAAUGCUUGGAUGACAAAUGA 3'
::::::::::::::::::::::
query 3' GAUUACGAACCUACUGUUUACU 5'
query=miR6116-3p, target=RO3G_05824, score=0, range=44-65, strand
target 5' CUAAUGCUUGGAUGACAAAUGA 3'
::::::::::::::::::::::
query 3' GAUUACGAACCUACUGUUUACU 5'
query=miR6116-3p, target=RO3G_03517, score=0, range=44-65, strand
target 5' CUAAUGCUUGGAUGACAAAUGA 3'
::::::::::::::::::::::
query 3' GAUUACGAACCUACUGUUUACU 5'
query=miR6116-3p, target=RO3G_03518, score=0, range=44-65, strand
target 5' CUAAUGCUUGGAUGACAAAUGA 3'
::::::::::::::::::::::
```

```
query 3' GAUUACGAACCUACUGUUUACU 5'
query=miR6116-3p, target=RO3G_01302, score=0, range=356-377, stra
target 5' CUAAUGCUUGGAUGACAAAUGA 3'
::::::::::::::::::::::
query 3' GAUUACGAACCUACUGUUUACU 5'
query=miR5065, target=RO3G_10854, score=3, range=130-147, strand=
target 5' AAUGCGUAAGUAAGCCUU 3'
:::: .:::::::::::
query 3' UUACAUAUUCAUUCGGAU 5'
query=miR5065, target=RO3G_11814, score=3, range=603-620, strand=
target 5' ACUGAAUAAGUAGGCCUA 3'
: :: :::::::.:::::
query 3' UUACAUAUUCAUUCGGAU 5'
query=miR5065, target=RO3G_16755, score=3.5, range=628-644, stran
target 5' GAUG-AUAAGUGAGCCUU 3'
.::: ::::::.:::::
query 3' UUACAUAUUCAUUCGGAU 5'
query=miR5065, target=RO3G_08665, score=4, range=1577-1594, stran
target 5' AAUUUGUAAGUGAGCUUA 3'
::: :.:::::.:::.::
query 3' UUACAUAUUCAUUCGGAU 5'
query=miR5065, target=RO3G_05981, score=4, range=5104-5122, stran
target 5' GAUGUGAUCAGUAAGCCUG 3'
.:::: :: :::::::::.
query 3' UUACA-UAUUCAUUCGGAU 5'
query=miR5065, target=RO3G_03388, score=4, range=1241-1258, stran
target 5' AAAAUAUAAGUAAGCCAA 3'
:: :::::::::::: :
query 3' UUACAUAUUCAUUCGGAU 5'
query=miR5065, target=RO3G_15240, score=4, range=284-301, strand=
target 5' AAUGUAUAAGGAGGCUUA 3'
:::::::::: :.::.::
query 3' UUACAUAUUCAUUCGGAU 5'
query=miR5065, target=RO3G_02470, score=4, range=452-469, strand=
target 5' AAUGUAUAAGGAGGCUUA 3'
:::::::::: :.::.::
query 3' UUACAUAUUCAUUCGGAU 5'
query=miR5065, target=RO3G_03391, score=4, range=134-152, strand=
target 5' GGUGUAUAAGUAACGCUUA 3'
..:::::::::: ::.::
query 3' UUACAUAUUCAUU-CGGAU 5'
query=miR5065, target=RO3G_03158, score=4, range=189-206, strand=
target 5' ACUGUGUAAGUAUGCCUA 3'
: :::.::::::: :::::
query 3' UUACAUAUUCAUUCGGAU 5'
```

```
query=miR5065, target=RO3G_11357, score=4, range=26-44, strand=-1
target 5' AAGGUAUAAGUAAGACCUC 3'
:: ::::::::::: :::
query 3' UUACAUAUUCAUUC-GGAU 5'
query=miR5645a, target=RO3G_17150, score=0, range=89-108, strand=
target 5' CUUAACGCCGUGACUGCAAA 3'
::::::::::::::::::::
query 3' GAAUUGCGGCACUGACGUUU 5'
query=miR6473, target=RO3G_10651, score=0, range=614-632, strand=
target 5' AACUCUUUGGCAUUGUGGA 3'
:::::::::::::::::::
query 3' UUGAGAAACCGUAACACCU 5'
query=miR6473, target=RO3G_13601, score=3, range=879-897, strand=
target 5' AAUUUUUUGGCAUUGAGGA 3'
::.:.:::::::::: :::
query 3' UUGAGAAACCGUAACACCU 5'
query=miR6473, target=RO3G_03503, score=3, range=617-635, strand=
target 5' AGUUCUUUGGCAUUGUGAA 3'
:..:::::::::::::: :
query 3' UUGAGAAACCGUAACACCU 5'
query=miR6473, target=RO3G_11680, score=3, range=972-989, strand=
target 5' AAAUCUUUGGCAUU-UGGA 3'
:: ::::::::::: ::::
query 3' UUGAGAAACCGUAACACCU 5'
query=miR6473, target=RO3G_05738, score=3.5, range=388-406, stran
target 5' AUCUUUUUGGCAUUUUGGA 3'
: ::.::::::::: ::::
query 3' UUGAGAAACCGUAACACCU 5'
query=miR6473, target=RO3G_05727, score=3.5, range=388-406, stran
target 5' AUCUUUUUGGCAUUUUGGA 3'
: ::.::::::::: ::::
query 3' UUGAGAAACCGUAACACCU 5'
query=miR6473, target=RO3G_05229, score=3.5, range=423-440, stran
target 5' AUCUC-UUGGUAUUGUGGG 3'
: ::: ::::.:::::::.
query 3' UUGAGAAACCGUAACACCU 5'
query=miR6473, target=RO3G_01384, score=3.5, range=179-196, stran
target 5' AACUC-UUGGAAUUGUGGG 3'
::::: :::: :::::::.
query 3' UUGAGAAACCGUAACACCU 5'
query=miR6473, target=RO3G_04420, score=3.5, range=74-92, strand=
target 5' CAUUCUUUGGCAUUGAGGA 3'
:.:::::::::::: :::
query 3' UUGAGAAACCGUAACACCU 5'
query=miR6473, target=RO3G_01452, score=3.5, range=477-495, stran
```

```
target 5' AGCACAUUGGUAUUGUGGA 3'
:.: : ::::.::::::::
query 3' UUGAGAAACCGUAACACCU 5'
query=miR6473, target=RO3G_16362, score=3.5, range=2592-2610,
strand=-1
target 5' AGCCAAUUGGCAUUGUGGA 3'
:.: :::::::::::::
query 3' UUGAGAAACCGUAACACCU 5'
query=miR6473, target=RO3G_11493, score=4, range=682-699, strand=
target 5' CAUUUUUUGGC-UUGUGGA 3'
:.:.:::::: :::::::
query 3' UUGAGAAACCGUAACACCU 5'
query=miR6473, target=RO3G_01660, score=4, range=986-1004, strand
target 5' GACUGUUUGGCAUUGUUGG 3'
.::: ::::::::::: :.
query 3' UUGAGAAACCGUAACACCU 5'
query=miR6473, target=RO3G_02589, score=4, range=951-969, strand=
target 5' AAGUCGUUGGUAUUGUGGU 3'
:: :: ::::.:::::::
query 3' UUGAGAAACCGUAACACCU 5'
query=miR6473, target=RO3G_16173, score=4, range=222-239, strand=
target 5' AAGUUUUUGGCAUU-UGGG 3'
:: :.::::::::: :::.
query 3' UUGAGAAACCGUAACACCU 5'
query=miR6473, target=RO3G_10811, score=4, range=137-154, strand=
target 5' AAC-AUUUGGCAUUGUGAA 3'
::: :::::::::::: :
query 3' UUGAGAAACCGUAACACCU 5'
query=miR855, target=RO3G_07786, score=3.5, range=879-900, strand
target 5' CUUCUCU-UCCUUAGCUUGUCUA 3'
:.:: : :::::::::::::::
query 3' AAGGAAACAGGAAUCGAACAGAU 5'
query=miR3712, target=RO3G_16744, score=0, range=558-578, strand=
target 5' UGGUCUUGAUCUUGAUCAGCA 3'
:::::::::::::::::::::
query 3' ACCAGAACUAGAACUAGUCGU 5'
query=miR3712, target=RO3G_01203, score=0, range=558-578, strand=
target 5' UGGUCUUGAUCUUGAUCAGCA 3'
:::::::::::::::::::::
query 3' ACCAGAACUAGAACUAGUCGU 5'
query=miR3712, target=RO3G_11403, score=0, range=558-578, strand=
target 5' UGGUCUUGAUCUUGAUCAGCA 3'
:::::::::::::::::::::
query 3' ACCAGAACUAGAACUAGUCGU 5'
query=miR3712, target=RO3G_06416, score=0, range=558-578, strand=
```

```
target 5' UGGUCUUGAUCUUGAUCAGCA 3'
:::::::::::::::::::::
query 3' ACCAGAACUAGAACUAGUCGU 5'
query=miR3712, target=RO3G_01718, score=0, range=558-578, strand=
target 5' UGGUCUUGAUCUUGAUCAGCA 3'
:::::::::::::::::::::
query 3' ACCAGAACUAGAACUAGUCGU 5'
query=miR3712, target=RO3G_15869, score=0, range=558-578, strand=
target 5' UGGUCUUGAUCUUGAUCAGCA 3'
:::::::::::::::::::::
query 3' ACCAGAACUAGAACUAGUCGU 5'
query=miR3712, target=RO3G_10560, score=0, range=558-578, strand=
target 5' UGGUCUUGAUCUUGAUCAGCA 3'
:::::::::::::::::::::
query 3' ACCAGAACUAGAACUAGUCGU 5'
query=miR3712, target=RO3G_11275, score=0, range=558-578, strand=
target 5' UGGUCUUGAUCUUGAUCAGCA 3'
:::::::::::::::::::::
query 3' ACCAGAACUAGAACUAGUCGU 5'
query=miR3712, target=RO3G_14704, score=0, range=558-578, strand=
target 5' UGGUCUUGAUCUUGAUCAGCA 3'
:::::::::::::::::::::
query 3' ACCAGAACUAGAACUAGUCGU 5'
query=miR3712, target=RO3G_12979, score=0, range=558-578, strand=
target 5' UGGUCUUGAUCUUGAUCAGCA 3'
:::::::::::::::::::::
query 3' ACCAGAACUAGAACUAGUCGU 5'
query=miR3712, target=RO3G_10555, score=0, range=558-578, strand=
target 5' UGGUCUUGAUCUUGAUCAGCA 3'
:::::::::::::::::::::
query 3' ACCAGAACUAGAACUAGUCGU 5'
query=miR3712, target=RO3G_05280, score=0, range=558-578, strand=
target 5' UGGUCUUGAUCUUGAUCAGCA 3'
:::::::::::::::::::::
query 3' ACCAGAACUAGAACUAGUCGU 5'
query=miR3712, target=RO3G_16686, score=0, range=558-578, strand=
target 5' UGGUCUUGAUCUUGAUCAGCA 3'
:::::::::::::::::::::
query 3' ACCAGAACUAGAACUAGUCGU 5'
query=miR3712, target=RO3G_07305, score=0, range=558-578, strand=
target 5' UGGUCUUGAUCUUGAUCAGCA 3'
:::::::::::::::::::::
query 3' ACCAGAACUAGAACUAGUCGU 5'
query=miR3712, target=RO3G_12087, score=0, range=558-578, strand=
target 5' UGGUCUUGAUCUUGAUCAGCA 3'
```

```
:::::::::::::::::::::
query 3' ACCAGAACUAGAACUAGUCGU 5'
query=miR3712, target=RO3G_08612, score=0, range=558-578, strand=
target 5' UGGUCUUGAUCUUGAUCAGCA 3'
:::::::::::::::::::::
query 3' ACCAGAACUAGAACUAGUCGU 5'
query=miR3712, target=RO3G_05630, score=0, range=558-578, strand=
target 5' UGGUCUUGAUCUUGAUCAGCA 3'
:::::::::::::::::::::
query 3' ACCAGAACUAGAACUAGUCGU 5'
query=miR3712, target=RO3G_13450, score=0, range=513-533, strand=
target 5' UGGUCUUGAUCUUGAUCAGCA 3'
:::::::::::::::::::::
query 3' ACCAGAACUAGAACUAGUCGU 5'
query=miR3712, target=RO3G_13859, score=0, range=183-203, strand=
target 5' UGGUCUUGAUCUUGAUCAGCA 3'
:::::::::::::::::::::
query 3' ACCAGAACUAGAACUAGUCGU 5'
query=miR3712, target=RO3G_11970, score=0, range=558-578, strand=
target 5' UGGUCUUGAUCUUGAUCAGCA 3'
:::::::::::::::::::::
query 3' ACCAGAACUAGAACUAGUCGU 5'
query=miR3712, target=RO3G_13152, score=0, range=558-578, strand=
target 5' UGGUCUUGAUCUUGAUCAGCA 3'
:::::::::::::::::::::
query 3' ACCAGAACUAGAACUAGUCGU 5'
query=miR3712, target=RO3G_03988, score=0, range=558-578, strand=
target 5' UGGUCUUGAUCUUGAUCAGCA 3'
:::::::::::::::::::::
query 3' ACCAGAACUAGAACUAGUCGU 5'
query=miR3712, target=RO3G_09376, score=0, range=558-578, strand=
target 5' UGGUCUUGAUCUUGAUCAGCA 3'
:::::::::::::::::::::
query 3' ACCAGAACUAGAACUAGUCGU 5'
query=miR3712, target=RO3G_12194, score=0, range=558-578, strand=
target 5' UGGUCUUGAUCUUGAUCAGCA 3'
:::::::::::::::::::::
query 3' ACCAGAACUAGAACUAGUCGU 5'
query=miR3712, target=RO3G_07104, score=0, range=558-578, strand=
target 5' UGGUCUUGAUCUUGAUCAGCA 3'
:::::::::::::::::::::
query 3' ACCAGAACUAGAACUAGUCGU 5'
query=miR3712, target=RO3G_03103, score=1, range=558-578, strand=
target 5' UGAUCUUGAUCUUGAUCAGCA 3'
:: ::::::::::::::::::
```

```
query 3' ACCAGAACUAGAACUAGUCGU 5'
query=miR3712, target=RO3G_12951, score=2, range=558-578, strand=
target 5' UGGUCUUGAUCUUGAUCAACA 3'
::::::::::::::::: ::
query 3' ACCAGAACUAGAACUAGUCGU 5'
query=miR3712, target=RO3G_07433, score=2, range=228-247, strand=
target 5' UGGUCUUGAUCUU-AUCAGCA 3'
::::::::::::: :::::::
query 3' ACCAGAACUAGAACUAGUCGU 5'
query=miR3712, target=RO3G_05884, score=3, range=2508-2528, stran
target 5' UCAUCUUCAUCUUGAUCAGCA 3'
: :::: :::::::::::::
query 3' ACCAGAACUAGAACUAGUCGU 5'
query=miR3712, target=RO3G_03260, score=3.5, range=150-171, stran
target 5' UUCUUUUGUAUCUUGAUCAGCA 3'
: :.::: ::::::::::::
query 3' ACCAGAAC-UAGAACUAGUCGU 5'
query=miR3712, target=RO3G_17220, score=4, range=516-536, strand=
target 5' UGGUCUUGAUCUUGAUCUGUC 3'
:::::::::::::::: :.
query 3' ACCAGAACUAGAACUAGUCGU 5'
query=miR3712, target=RO3G_05255, score=4, range=407-427, strand=
target 5' UGGCCUUGAUCUUUGUCAGCA 3'
::: ::::::::: .::::::
query 3' ACCAGAACUAGAACUAGUCGU 5'
query=miR3712, target=RO3G_01597, score=4, range=879-898, strand=
target 5' UGAUCUUGAUUUUGAU-AGCA 3'
:: :::::::.::::: ::::
query 3' ACCAGAACUAGAACUAGUCGU 5'
query=miR3712, target=RO3G_13054, score=4, range=291-311, strand=
target 5' AUGAGUUGAUCUUGAUCAGCA 3'
: :::::::::::::::
query 3' ACCAGAACUAGAACUAGUCGU 5'
query=miR3712, target=RO3G_08864, score=4, range=1172-1193, stran
target 5' UGGAACUGGAUCUUGGUCAGCA 3'
::: :: :::::::.::::::
query 3' ACC-AGAACUAGAACUAGUCGU 5'
query=miR158-5p, target=RO3G_14816, score=4, range=593-613, stran
target 5' UUUUGUGAACGAUAGGUAAAA 3'
:::: ..::::::::::.:::
query 3' AAAAGGUUUGCUAUCCGUUUC 5'
query=miR158-5p, target=RO3G_09479, score=4, range=588-608, stran
target 5' AUAUCCAAACGAUACGCAAAG 3'
: ::::::::::: ::::::
query 3' AAAAGGUUUGCUAUCCGUUUC 5'
```

query=miR163, target=RO3G_13072, score=0, range=755-777, strand=1
target 5' ACGAAUUCCAUGCUGCUCUUCAA 3'
:::::::::::::::::::::::
query 3' UGCUUAAGGUACGACGAGAAGUU 5'
query=miR163, target=RO3G_15256, score=1, range=851-873, strand=1
target 5' ACGAAUUCCAUGCUGUUCUUCAA 3'
:::::::::::::::.:::::::
query 3' UGCUUAAGGUACGACGAGAAGUU 5'
query=miR163, target=RO3G_10737, score=1, range=851-873, strand=1
target 5' ACGAAUUCCAUGCUGUUCUUCAA 3'
:::::::::::::::.:::::::
query 3' UGCUUAAGGUACGACGAGAAGUU 5'
query=miR163, target=RO3G_00259, score=1, range=851-873, strand=1
target 5' ACGAAUUCCAUGCUGUUCUUCAA 3'
:::::::::::::::.:::::::
query 3' UGCUUAAGGUACGACGAGAAGUU 5'
query=miR163, target=RO3G_10048, score=1, range=851-873, strand=1
target 5' ACGAAUUCCAUGCUGUUCUUCAA 3'
:::::::::::::::.:::::::
query 3' UGCUUAAGGUACGACGAGAAGUU 5'
query=miR163, target=RO3G_02231, score=1, range=851-873, strand=1
target 5' ACGAAUUCCAUGCUGUUCUUCAA 3'
:::::::::::::::.:::::::
query 3' UGCUUAAGGUACGACGAGAAGUU 5'
query=miR163, target=RO3G_16127, score=1, range=851-873, strand=1
target 5' ACGAAUUCCAUGCUGUUCUUCAA 3'
:::::::::::::::.:::::::
query 3' UGCUUAAGGUACGACGAGAAGUU 5'
query=miR163, target=RO3G_02827, score=1, range=851-873, strand=1
target 5' ACGAAUUCCAUGCUGUUCUUCAA 3'
:::::::::::::::.:::::::
query 3' UGCUUAAGGUACGACGAGAAGUU 5'
query=miR163, target=RO3G_13711, score=1, range=836-858, strand=1
target 5' ACGAAUUCCAUGCUGUUCUUCAA 3'
:::::::::::::::.:::::::
query 3' UGCUUAAGGUACGACGAGAAGUU 5'
query=miR163, target=RO3G_05910, score=1, range=851-873, strand=1
target 5' ACGAAUUCCAUGCUGUUCUUCAA 3'
:::::::::::::::.:::::::
query 3' UGCUUAAGGUACGACGAGAAGUU 5'
query=miR163, target=RO3G_07115, score=1, range=812-834, strand=1
target 5' ACGAAUUCCAUGCUGUUCUUCAA 3'
:::::::::::::::.:::::::
query 3' UGCUUAAGGUACGACGAGAAGUU 5'
query=miR163, target=RO3G_01198, score=1, range=785-807, strand=1

target 5' ACGAAUUCCAUGCUGUUCUUCAA 3'
::::::::::::::.:::::::
query 3' UGCUUAAGGUACGACGAGAAGUU 5'
query=miR163, target=RO3G_09184, score=1, range=563-585, strand=1
target 5' ACGAAUUCCAUGCUGUUCUUCAA 3'
::::::::::::::.:::::::
query 3' UGCUUAAGGUACGACGAGAAGUU 5'
query=miR163, target=RO3G_01723, score=1, range=272-294, strand=1
target 5' ACGAAUUCCAUGCUGUUCUUCAA 3'
::::::::::::::.:::::::
query 3' UGCUUAAGGUACGACGAGAAGUU 5'
query=miR163, target=RO3G_17241, score=1, range=170-192, strand=1
target 5' ACGAAUUCCAUGCUGUUCUUCAA 3'
::::::::::::::.:::::::
query 3' UGCUUAAGGUACGACGAGAAGUU 5'
query=miR163, target=RO3G_12195, score=1, range=44-66, strand=1
target 5' ACGAAUUCCAUGCUGUUCUUCAA 3'
::::::::::::::.:::::::
query 3' UGCUUAAGGUACGACGAGAAGUU 5'
query=miR163, target=RO3G_07933, score=3.5, range=1332-1353, stra
target 5' AC-AGAUGCAUGCUGCUCUUCAA 3'
:: :. : ::::::::::::::
query 3' UGCUUAAGGUACGACGAGAAGUU 5'
query=miR163, target=RO3G_12867, score=3.5, range=68-89, strand=-
target 5' AC-AAUUUCUUGCUGCUUUUCAA 3'
:: ::::.: :::::::.:::::
query 3' UGCUUAAGGUACGACGAGAAGUU 5'
query=miR163, target=RO3G_16486, score=4, range=2408-2430, strand
target 5' ACGGACUCCAGGCUGCUCUUCAG 3'
:::.: :::: :::::::::::.
query 3' UGCUUAAGGUACGACGAGAAGUU 5'
query=miR163, target=RO3G_16013, score=4, range=17-39, strand=-1
target 5' ACGGAUUCCGUCCUGUUCUUCAA 3'
:::.:::::.: :::.:::::::
query 3' UGCUUAAGGUACGACGAGAAGUU 5'
query=miR5648-5p, target=RO3G_14543, score=3, range=1452-1472,
strand=1
target 5' AGUCAAUCCUAAAUUUUUAAA 3'
:::::: :.:::::::::::
query 3' UCAGUUCCGGUUUAAAAAUUU 5'
query=miR5648-5p, target=RO3G_11833, score=4, range=2232-2252,
strand=-1
target 5' AUCCAAGCCCAAAUUUUUGAA 3'
: :::: ::::::::::.::
query 3' UCAGUUCCGGUUUAAAAAUUU 5'

```
query=miR5648-5p, target=RO3G_02881, score=4, range=1084-1105,
strand=-1
target 5' AACCAACUGCCAAAUUUUUAAA 3'
: ::: ::::::::::::::
query 3' UCAGUU-CCGGUUUAAAAAUUU 5'
query=miR1097, target=RO3G_16362, score=0, range=2704-2725, stran
target 5' CUUCCAACAACAAACAAUGGCA 3'
::::::::::::::::::::::
query 3' GAAGGUUGUUGUUUGUUACCGU 5'
query=miR1097, target=RO3G_03008, score=4, range=1781-1802, stran
target 5' AUAUUAACAACAAACAAUGGCU 3'
: ..::::::::::::::::
query 3' GAAGGUUGUUGUUUGUUACCGU 5'
query=miR536f, target=RO3G_02551, score=0, range=241-260, strand=
target 5' UCAACACAGCUUGGACGGAA 3'
::::::::::::::::::::
query 3' AGUUGUGUCGAACCUGCCUU 5'
query=miR536f, target=RO3G_01892, score=3.5, range=481-500, stran
target 5' UCGAAACAGUUUGGAUGGAA 3'
::.: ::::.:::::.::::
query 3' AGUUGUGUCGAACCUGCCUU 5'
query=miR536f, target=RO3G_10980, score=3.5, range=51-69, strand=
target 5' GUAG-ACAGCUUGGACGGAG 3'
.:. ::::::::::::::.
query 3' AGUUGUGUCGAACCUGCCUU 5'
query=miR536f, target=RO3G_08262, score=3.5, range=978-996, stran
target 5' GUAG-ACAGCUUGGACGGAG 3'
.:. ::::::::::::::.
query 3' AGUUGUGUCGAACCUGCCUU 5'
query=miR536f, target=RO3G_16396, score=3.5, range=978-996, stran
target 5' GUAG-ACAGCUUGGACGGAG 3'
.:. ::::::::::::::.
query 3' AGUUGUGUCGAACCUGCCUU 5'
query=miR536f, target=RO3G_01285, score=3.5, range=1008-1026,
strand=-1
target 5' GUAG-ACAGCUUGGACGGAG 3'
.:. ::::::::::::::.
query 3' AGUUGUGUCGAACCUGCCUU 5'
query=miR536f, target=RO3G_03381, score=4, range=1241-1259, stran
target 5' UCAACAC-GCUUGGAAGGAA 3'
::::::: ::::::: ::::
query 3' AGUUGUGUCGAACCUGCCUU 5'
query=miR444b, target=RO3G_11967, score=0, range=834-854, strand=
target 5' AACUUGGAAACAGCAACGGCA 3'
:::::::::::::::::::::
```

```
query 3' UUGAACCUUUGUCGUUGCCGU 5'
query=miR444b, target=RO3G_14350, score=0, range=834-854, strand=
target 5' AACUUGGAAACAGCAACGGCA 3'
:::::::::::::::::::::
query 3' UUGAACCUUUGUCGUUGCCGU 5'
query=miR444b, target=RO3G_13225, score=0, range=834-854, strand=
target 5' AACUUGGAAACAGCAACGGCA 3'
:::::::::::::::::::::
query 3' UUGAACCUUUGUCGUUGCCGU 5'
query=miR444b, target=RO3G_16799, score=0, range=834-854, strand=
target 5' AACUUGGAAACAGCAACGGCA 3'
:::::::::::::::::::::
query 3' UUGAACCUUUGUCGUUGCCGU 5'
query=miR444b, target=RO3G_05639, score=0, range=834-854, strand=
target 5' AACUUGGAAACAGCAACGGCA 3'
:::::::::::::::::::::
query 3' UUGAACCUUUGUCGUUGCCGU 5'
query=miR444b, target=RO3G_13222, score=0.5, range=786-806, stran
target 5' AACUUGGAAACAGCAACGGCG 3'
::::::::::::::::::::.
query 3' UUGAACCUUUGUCGUUGCCGU 5'
query=miR444b, target=RO3G_16818, score=0.5, range=564-584, stran
target 5' AACUUGGAAACAGCAACGGCG 3'
::::::::::::::::::::.
query 3' UUGAACCUUUGUCGUUGCCGU 5'
query=miR444b, target=RO3G_14778, score=0.5, range=825-845, stran
target 5' AACUUGGAAACAGCAACGGCG 3'
::::::::::::::::::::.
query 3' UUGAACCUUUGUCGUUGCCGU 5'
query=miR444b, target=RO3G_03991, score=0.5, range=834-854, stran
target 5' AACUUGGAAACAGCAACGGCG 3'
::::::::::::::::::::.
query 3' UUGAACCUUUGUCGUUGCCGU 5'
query=miR444b, target=RO3G_17015, score=2, range=834-854, strand=
target 5' AACUUGGAGACGGCAACGGCA 3'
::::::::.::.:::::::::
query 3' UUGAACCUUUGUCGUUGCCGU 5'
query=miR444b, target=RO3G_04845, score=3.5, range=63-83, strand=
target 5' AAUUUGGAAGCAGCAAUGGCU 3'
::.::::::.::::::.:::
query 3' UUGAACCUUUGUCGUUGCCGU 5'
query=miR444b, target=RO3G_03379, score=4, range=171-191, strand=
target 5' GGGUUAGAAAUAGCAACGGCA 3'
.. :: ::::.::::::::::
query 3' UUGAACCUUUGUCGUUGCCGU 5'
```

query=miR1886.2, target=RO3G_06442, score=0, range=3476-3494, strand=-1
target 5' CCAAUCACAAUAUCAUCCA 3'
:::::::::::::::::::
query 3' GGUUAGUGUUAUAGUAGGU 5'
query=miR1886.2, target=RO3G_02691, score=0, range=6308-6326, strand=-1
target 5' CCAAUCACAAUAUCAUCCA 3'
:::::::::::::::::::
query 3' GGUUAGUGUUAUAGUAGGU 5'
query=miR1886.2, target=RO3G_09965, score=0, range=2819-2837, strand=-1
target 5' CCAAUCACAAUAUCAUCCA 3'
:::::::::::::::::::
query 3' GGUUAGUGUUAUAGUAGGU 5'
query=miR1886.2, target=RO3G_13920, score=0, range=2819-2837, strand=-1
target 5' CCAAUCACAAUAUCAUCCA 3'
:::::::::::::::::::
query 3' GGUUAGUGUUAUAGUAGGU 5'
query=miR1886.2, target=RO3G_03547, score=0, range=2819-2837, strand=-1
target 5' CCAAUCACAAUAUCAUCCA 3'
:::::::::::::::::::
query 3' GGUUAGUGUUAUAGUAGGU 5'
query=miR1886.2, target=RO3G_02959, score=0, range=2819-2837, strand=-1
target 5' CCAAUCACAAUAUCAUCCA 3'
:::::::::::::::::::
query 3' GGUUAGUGUUAUAGUAGGU 5'
query=miR1886.2, target=RO3G_13867, score=0, range=2735-2753, strand=-1
target 5' CCAAUCACAAUAUCAUCCA 3'
:::::::::::::::::::
query 3' GGUUAGUGUUAUAGUAGGU 5'
query=miR1886.2, target=RO3G_02221, score=0, range=2741-2759, strand=-1
target 5' CCAAUCACAAUAUCAUCCA 3'
:::::::::::::::::::
query 3' GGUUAGUGUUAUAGUAGGU 5'
query=miR1886.2, target=RO3G_13749, score=0, range=1382-1400, strand=-1
target 5' CCAAUCACAAUAUCAUCCA 3'
:::::::::::::::::::
query 3' GGUUAGUGUUAUAGUAGGU 5'

query=miR1886.2, target=RO3G_01330, score=0, range=53-71, strand=
target 5' CCAAUCACAAUAUCAUCCA 3'
:::::::::::::::::::
query 3' GGUUAGUGUUAUAGUAGGU 5'
query=miR1886.2, target=RO3G_00306, score=0, range=1268-1286,
strand=-1
target 5' CCAAUCACAAUAUCAUCCA 3'
:::::::::::::::::::
query 3' GGUUAGUGUUAUAGUAGGU 5'
query=miR1886.2, target=RO3G_06428, score=0, range=1913-1931,
strand=-1
target 5' CCAAUCACAAUAUCAUCCA 3'
:::::::::::::::::::
query 3' GGUUAGUGUUAUAGUAGGU 5'
query=miR1886.2, target=RO3G_17353, score=0, range=53-71, strand=
target 5' CCAAUCACAAUAUCAUCCA 3'
:::::::::::::::::::
query 3' GGUUAGUGUUAUAGUAGGU 5'
query=miR1886.2, target=RO3G_05619, score=0, range=962-980, stran
target 5' CCAAUCACAAUAUCAUCCA 3'
:::::::::::::::::::
query 3' GGUUAGUGUUAUAGUAGGU 5'
query=miR1886.2, target=RO3G_06427, score=0, range=2411-2429,
strand=-1
target 5' CCAAUCACAAUAUCAUCCA 3'
:::::::::::::::::::
query 3' GGUUAGUGUUAUAGUAGGU 5'
query=miR1886.2, target=RO3G_17326, score=0, range=2651-2669,
strand=-1
target 5' CCAAUCACAAUAUCAUCCA 3'
:::::::::::::::::::
query 3' GGUUAGUGUUAUAGUAGGU 5'
query=miR1886.2, target=RO3G_10558, score=0, range=572-590, stran
target 5' CCAAUCACAAUAUCAUCCA 3'
:::::::::::::::::::
query 3' GGUUAGUGUUAUAGUAGGU 5'
query=miR1886.2, target=RO3G_04114, score=0, range=2819-2837,
strand=-1
target 5' CCAAUCACAAUAUCAUCCA 3'
:::::::::::::::::::
query 3' GGUUAGUGUUAUAGUAGGU 5'
query=miR1886.2, target=RO3G_15585, score=0, range=2774-2792,
strand=-1
target 5' CCAAUCACAAUAUCAUCCA 3'
:::::::::::::::::::

query 3' GGUUAGUGUUAUAGUAGGU 5'
query=miR1886.2, target=RO3G_15250, score=1, range=2774-2792, strand=-1
target 5' CCAACCACAAUAUCAUCCA 3'
:::: ::::::::::::::
query 3' GGUUAGUGUUAUAGUAGGU 5'
query=miR1886.2, target=RO3G_17371, score=1, range=2774-2792, strand=-1
target 5' CCAACCACAAUAUCAUCCA 3'
:::: ::::::::::::::
query 3' GGUUAGUGUUAUAGUAGGU 5'
query=miR1886.2, target=RO3G_14355, score=1, range=1025-1043, strand=-1
target 5' CCAACCACAAUAUCAUCCA 3'
:::: ::::::::::::::
query 3' GGUUAGUGUUAUAGUAGGU 5'
query=miR1886.2, target=RO3G_01613, score=2, range=1070-1088, strand=-1
target 5' CCAAUCAAAAUAUCAUCCA 3'
::::::: :::::::::::
query 3' GGUUAGUGUUAUAGUAGGU 5'
query=miR1886.2, target=RO3G_09845, score=2, range=197-215, stran
target 5' CCAAGCACAAUAUCAUUCA 3'
:::: ::::::::::::.::
query 3' GGUUAGUGUUAUAGUAGGU 5'
query=miR1886.2, target=RO3G_03427, score=2.5, range=422-440, strand=-1
target 5' CCAAGUACAAUAUCAUUCA 3'
:::: .::::::::::.::
query 3' GGUUAGUGUUAUAGUAGGU 5'
query=miR1886.2, target=RO3G_16330, score=3, range=128-145, stran
target 5' CCAAUCAUAAUAUC-UCCA 3'
:::::::.:::::: ::::
query 3' GGUUAGUGUUAUAGUAGGU 5'
query=miR1886.2, target=RO3G_12487, score=3, range=74-92, strand=
target 5' CCAAUAACAAUAUUAUCUA 3'
::::: ::::::::.::::.:
query 3' GGUUAGUGUUAUAGUAGGU 5'
query=miR1886.2, target=RO3G_11216, score=3, range=1751-1769, strand=-1
target 5' UCAGCCACGAUAUCAUCCA 3'
.::. :::.::::::::::
query 3' GGUUAGUGUUAUAGUAGGU 5'
query=miR1886.2, target=RO3G_00301, score=3, range=578-596, stran
target 5' UCGGCUACAAUAUCAUCCA 3'

.:.. .:::::::::::::
query 3' GGUUAGUGUUAUAGUAGGU 5'
query=miR1886.2, target=RO3G_04049, score=3, range=1415-1433, strand=-1
target 5' UCGGCUACAAUAUCAUCCA 3'
.:.. .:::::::::::::
query 3' GGUUAGUGUUAUAGUAGGU 5'
query=miR1886.2, target=RO3G_01379, score=3, range=1448-1466, strand=-1
target 5' ACAAUCUCAAUAUCAUCCA 3'
::::: ::::::::::::
query 3' GGUUAGUGUUAUAGUAGGU 5'
query=miR1886.2, target=RO3G_10472, score=3, range=1502-1519, strand=-1
target 5' CUGA-CACAAUAUCAUUCA 3'
:..: :::::::::::.::
query 3' GGUUAGUGUUAUAGUAGGU 5'
query=miR1886.2, target=RO3G_17029, score=3.5, range=812-830, strand=-1
target 5' CCAAUUACAGUAGCAUCCA 3'
:::::.:::.:: ::::::
query 3' GGUUAGUGUUAUAGUAGGU 5'
query=miR1886.2, target=RO3G_06657, score=3.5, range=159-176, strand=-1
target 5' UCAAUCACAAUAUUA-CCA 3'
.::::::::::::.: :::
query 3' GGUUAGUGUUAUAGUAGGU 5'
query=miR1886.2, target=RO3G_02781, score=3.5, range=260-277, strand=-1
target 5' CCAGU-AUGAUAUCAUCCA 3'
:::.: :..::::::::::
query 3' GGUUAGUGUUAUAGUAGGU 5'
query=miR1886.2, target=RO3G_08495, score=3.5, range=797-815, strand=-1
target 5' UCUAUCAAAAUAUCAUCCA 3'
.: :::: :::::::::::
query 3' GGUUAGUGUUAUAGUAGGU 5'
query=miR1886.2, target=RO3G_01120, score=3.5, range=1019-1037, strand=-1
target 5' UGAAUGACAAUAUCAUUCA 3'
. ::: ::::::::::.::
query 3' GGUUAGUGUUAUAGUAGGU 5'
query=miR1886.2, target=RO3G_15621, score=3.5, range=2804-2822, strand=-1
target 5' UGAAUAACAAUAUCAUCCU 3'

```
. ::: :::::::::::
query 3' GGUUAGUGUUAUAGUAGGU 5'
query=miR1886.2, target=RO3G_10408, score=4, range=1295-1313, str
target 5' CUAGUUACAAGAUCAUCCG 3'
:.:.:.:::: :::::::.
query 3' GGUUAGUGUUAUAGUAGGU 5'
query=miR1886.2, target=RO3G_04181, score=4, range=53-71, strand=
target 5' UCAAUAACAAUGUCAUUCG 3'
.:::: :::::.::::.:.
query 3' GGUUAGUGUUAUAGUAGGU 5'
query=miR1886.2, target=RO3G_04517, score=4, range=721-739, stran
target 5' GCAAGCGCGAUAUCAUCCA 3'
::: :.:.::::::::::
query 3' GGUUAGUGUUAUAGUAGGU 5'
query=miR1886.2, target=RO3G_14446, score=4, range=1000-1018, str
target 5' CCAAUCACAACACCAUCCA 3'
:::::::::: : ::::::
query 3' GGUUAGUGUUAUAGUAGGU 5'
query=miR1886.2, target=RO3G_00505, score=4, range=93-111, strand
target 5' CCCUUCACGAUAUCAUUCA 3'
:: ::::.:::::::.::
query 3' GGUUAGUGUUAUAGUAGGU 5'
query=miR1886.2, target=RO3G_03406, score=4, range=78-97, strand=
target 5' CUACAUUACAAUAUCACCCA 3'
:.: ::.:::::::: :::
query 3' GGU-UAGUGUUAUAGUAGGU 5'
query=miR1886.2, target=RO3G_10373, score=4, range=264-282, stran
target 5' UCAUCCACAAUAUCAUUCG 3'
.:: :::::::::::.:.
query 3' GGUUAGUGUUAUAGUAGGU 5'
query=miR1886.2, target=RO3G_16231, score=4, range=276-294, stran
target 5' UUAUGCACAAUAUUAUCCA 3'
..: ::::::::.:::::
query 3' GGUUAGUGUUAUAGUAGGU 5'
query=miR1886.2, target=RO3G_13557, score=4, range=3563-3581, str
target 5' CCAACGACAAUAUCAUCUU 3'
:::: :::::::::::.
query 3' GGUUAGUGUUAUAGUAGGU 5'
query=miR1886.2, target=RO3G_04659, score=4, range=35-52, strand=
target 5' CCAA-CAUAACAUCAUCCA 3'
:::: ::.:: ::::::::
query 3' GGUUAGUGUUAUAGUAGGU 5'
query=miR1886.2, target=RO3G_09238, score=4, range=379-397, stran
target 5' AAAAAGACAAUAUCAUCCA 3'
:: ::::::::::::
```

query 3' GGUUAGUGUUAUAGUAGGU 5'
query=miR1886.2, target=RO3G_10061, score=4, range=327-344, stran
target 5' UCAAU-ACAAUACCAUCCG 3'
.:::: :::::: :::::.
query 3' GGUUAGUGUUAUAGUAGGU 5'
query=miR1886.2, target=RO3G_10105, score=4, range=500-518, stran
target 5' CCAAUCUCGAUGUCAUCCA 3'
:::::: :.::.:::::::
query 3' GGUUAGUGUUAUAGUAGGU 5'
query=miR1886.2, target=RO3G_08529, score=4, range=215-233, stran
target 5' UCGAUCUCAAUGUCAUCCA 3'
.:.::: ::::.:::::::
query 3' GGUUAGUGUUAUAGUAGGU 5'
query=miR1886.2, target=RO3G_12492, score=4, range=470-488, stran
target 5' UUAAUCAUACUAUCAUCCA 3'
..:::::.: ::::::::
query 3' GGUUAGUGUUAUAGUAGGU 5'
query=miR1886.2, target=RO3G_09248, score=4, range=650-668, stran
target 5' UCAAAUACAAUAUUAUUCA 3'
.::: .::::::.::.::
query 3' GGUUAGUGUUAUAGUAGGU 5'
query=miR1886.2, target=RO3G_12484, score=4, range=2903-2921,
strand=-1
target 5' AAGGUCACAAUAUCGUCCA 3'
..:::::::::.::::
query 3' GGUUAGUGUUAUAGUAGGU 5'
query=miR1886.2, target=RO3G_10943, score=4, range=560-578, stran
target 5' GCAAUCACAAUAUCAUCAU 3'
:::::::::::::::
query 3' GGUUAGUGUUAUAGUAGGU 5'
query=miR1886.2, target=RO3G_07188, score=4, range=1355-1373,
strand=-1
target 5' AUAAUUACAAGAUCAUCCA 3'
.:::.:::: ::::::::
query 3' GGUUAGUGUUAUAGUAGGU 5'
query=miR1886.2, target=RO3G_11616, score=4, range=653-671, stran
target 5' CUGAGCAUAAUAUCAUCCU 3'
:..: ::.:::::::::
query 3' GGUUAGUGUUAUAGUAGGU 5'
query=miR1886.2, target=RO3G_11160, score=4, range=1973-1992,
strand=-1
target 5' CUGAUUCAUAAUAUCAUCCC 3'
:..: :::.:::::::::
query 3' GGUU-AGUGUUAUAGUAGGU 5'
query=miR1886.2, target=RO3G_15031, score=4, range=2153-2170,

strand=-1
target 5' CCAA-CAUGAUAUUAUCCA 3'
:::: ::..::::.:::::
query 3' GGUUAGUGUUAUAGUAGGU 5'
query=miR1886.2, target=RO3G_08439, score=4, range=737-755, stran
target 5' CCAAUCUCAAUAUCACCCA 3'
:::::: :::::::: :::
query 3' GGUUAGUGUUAUAGUAGGU 5'
query=miR1886.2, target=RO3G_16260, score=4, range=744-761, stran
target 5' CCAGUCACAAUAU-AUUCG 3'
:::.:::::::: ::.:.
query 3' GGUUAGUGUUAUAGUAGGU 5'
query=miR1886.2, target=RO3G_16032, score=4, range=228-245, stran
target 5' CUGAUCACAAUA-CGUCCA 3'
:..::::::::: :.:::
query 3' GGUUAGUGUUAUAGUAGGU 5'
query=miR1886.2, target=RO3G_03657, score=4, range=1505-1523,
strand=-1
target 5' CCCAUCACAACAUUAUCCA 3'
:: ::::::: ::.::::
query 3' GGUUAGUGUUAUAGUAGGU 5'
query=miR1886.2, target=RO3G_03862, score=4, range=861-879, stran
target 5' CAAAUCGCAAUAUCUUCCA 3'
: ::::.:::::: ::::
query 3' GGUUAGUGUUAUAGUAGGU 5'
query=miR1886.2, target=RO3G_16990, score=4, range=421-439, stran
target 5' CAAAUAAUGAUAUCAUCCA 3'
: ::: :..::::::::
query 3' GGUUAGUGUUAUAGUAGGU 5'
query=miR1886.2, target=RO3G_09557, score=4, range=2921-2939,
strand=-1
target 5' UCUGUCACAAUUUCAUCCA 3'
.: .::::::: :::::::
query 3' GGUUAGUGUUAUAGUAGGU 5'
query=miR1886.2, target=RO3G_00949, score=4, range=2768-2786,
strand=-1
target 5' UCCAUUACAAGAUCAUCCA 3'
.: ::.:::: ::::::::
query 3' GGUUAGUGUUAUAGUAGGU 5'
query=miR1886.2, target=RO3G_08277, score=4, range=1802-1820,
strand=-1
target 5' CAGGUCACAAUAUCUUCCA 3'
: ..:::::::: ::::
query 3' GGUUAGUGUUAUAGUAGGU 5'
query=miR1886.2, target=RO3G_14159, score=4, range=398-417, stran

target 5' CCAAUCCACAAUAUCAUCAU 3'
::::: ::::::::::::
query 3' GGUUA-GUGUUAUAGUAGGU 5'
query=miR1886.2, target=RO3G_04224, score=4, range=257-275, stran
target 5' UCAAACUCAAUAUCAUCCG 3'
.::: : :::::::::::.
query 3' GGUUAGUGUUAUAGUAGGU 5'
query=miR1886.2, target=RO3G_05711, score=4, range=131-149, stran
target 5' UCGAUGACAAUCUCAUCCA 3'
.:.:: ::::: :::::::
query 3' GGUUAGUGUUAUAGUAGGU 5'
query=miR1886.2, target=RO3G_01944, score=4, range=2552-2570,
strand=-1
target 5' AAAAUGAUAAUAUCAUCCA 3'
::: :.:::::::::::
query 3' GGUUAGUGUUAUAGUAGGU 5'
query=miR1886.2, target=RO3G_13849, score=4, range=2552-2570,
strand=-1
target 5' AAAAUGAUAAUAUCAUCCA 3'
::: :.:::::::::::
query 3' GGUUAGUGUUAUAGUAGGU 5'
query=miR1886.2, target=RO3G_17287, score=4, range=2552-2570,
strand=-1
target 5' AAAAUGAUAAUAUCAUCCA 3'
::: :.:::::::::::
query 3' GGUUAGUGUUAUAGUAGGU 5'
query=miR1886.2, target=RO3G_15573, score=4, range=2552-2570,
strand=-1
target 5' AAAAUGAUAAUAUCAUCCA 3'
::: :.:::::::::::
query 3' GGUUAGUGUUAUAGUAGGU 5'
query=miR1886.2, target=RO3G_10991, score=4, range=2552-2570,
strand=-1
target 5' AAAAUGAUAAUAUCAUCCA 3'
::: :.:::::::::::
query 3' GGUUAGUGUUAUAGUAGGU 5'
query=miR1886.2, target=RO3G_03749, score=4, range=2552-2570,
strand=-1
target 5' AAAAUGAUAAUAUCAUCCA 3'
::: :.:::::::::::
query 3' GGUUAGUGUUAUAGUAGGU 5'
query=miR1886.2, target=RO3G_06502, score=4, range=2429-2447,
strand=-1
target 5' AAAAUGAUAAUAUCAUCCA 3'
::: :.:::::::::::

query 3' GGUUAGUGUUAUAGUAGGU 5'
query=miR1886.2, target=RO3G_10420, score=4, range=2552-2570, strand=-1
target 5' AAAAUGAUAAUAUCAUCCA 3'
::: :.::::::::::
query 3' GGUUAGUGUUAUAGUAGGU 5'
query=miR1886.2, target=RO3G_01733, score=4, range=2354-2372, strand=-1
target 5' AAAAUGAUAAUAUCAUCCA 3'
::: :.::::::::::
query 3' GGUUAGUGUUAUAGUAGGU 5'
query=miR1886.2, target=RO3G_05865, score=4, range=2204-2222, strand=-1
target 5' AAAAUGAUAAUAUCAUCCA 3'
::: :.::::::::::
query 3' GGUUAGUGUUAUAGUAGGU 5'
query=miR1886.2, target=RO3G_02966, score=4, range=1823-1841, strand=-1
target 5' AAAAUGAUAAUAUCAUCCA 3'
::: :.::::::::::
query 3' GGUUAGUGUUAUAGUAGGU 5'
query=miR1886.2, target=RO3G_17446, score=4, range=2552-2570, strand=-1
target 5' AAAAUGAUAAUAUCAUCCA 3'
::: :.::::::::::
query 3' GGUUAGUGUUAUAGUAGGU 5'
query=miR1886.2, target=RO3G_02707, score=4, range=101-119, stran
target 5' AAAAUGAUAAUAUCAUCCA 3'
::: :.::::::::::
query 3' GGUUAGUGUUAUAGUAGGU 5'
query=miR1886.2, target=RO3G_09351, score=4, range=458-476, stran
target 5' GGAAUAAUAAUAUCAUCCA 3'
::: :.::::::::::
query 3' GGUUAGUGUUAUAGUAGGU 5'
query=miR1886.2, target=RO3G_02339, score=4, range=227-244, stran
target 5' AUGAU-ACGAUAUCAUCCA 3'
..:: ::.:::::::::
query 3' GGUUAGUGUUAUAGUAGGU 5'
query=miR1886.2, target=RO3G_14810, score=4, range=1454-1471, strand=-1
target 5' CCAA-CUCAAUAUCAUCUA 3'
:::: : :::::::::.:
query 3' GGUUAGUGUUAUAGUAGGU 5'
query=miR1886.2, target=RO3G_10974, score=4, range=1983-2000, strand=-1

```
target 5' CAAAUCACAAUAUC-UUCA 3'
: ::::::::::: :.::
query 3' GGUUAGUGUUAUAGUAGGU 5'
query=miR1886.2, target=RO3G_13031, score=4, range=41-58, strand=
target 5' CCAA-CACAAUAUCAGUCA 3'
:::: :::::::::: .::
query 3' GGUUAGUGUUAUAGUAGGU 5'
query=miR1886.2, target=RO3G_10294, score=4, range=407-424, stran
target 5' CUAG-CACAAUAUCACCCA 3'
:.:. ::::::::: :::
query 3' GGUUAGUGUUAUAGUAGGU 5'
query=miR1886.2, target=RO3G_01664, score=4, range=497-514, stran
target 5' CCCAU-GUAAUAUCAUCCA 3'
:: :: ..::::::::::
query 3' GGUUAGUGUUAUAGUAGGU 5'
query=miR1886.2, target=RO3G_13901, score=4, range=2-19, strand=-
target 5' CUAG-CACAAUAUCACCCA 3'
:.:. ::::::::: :::
query 3' GGUUAGUGUUAUAGUAGGU 5'
query=miR1886.2, target=RO3G_13930, score=4, range=24-41, strand=
target 5' GCAAACACAAUAUCA-CCA 3'
::: ::::::::: :::
query 3' GGUUAGUGUUAUAGUAGGU 5'
query=miR166b, target=RO3G_00261, score=0, range=129-149, strand=
target 5' UUGGAAGGAAUGCCUGGCCGA 3'
:::::::::::::::::::::
query 3' AACCUUCCUUACGGACCGGCU 5'
query=miR166b, target=RO3G_00248, score=0, range=129-149, strand=
target 5' UUGGAAGGAAUGCCUGGCCGA 3'
:::::::::::::::::::::
query 3' AACCUUCCUUACGGACCGGCU 5'
query=miR166b, target=RO3G_15127, score=1, range=141-161, strand=
target 5' UUGGAAGGAAUGCUUGGCCGA 3'
:::::::::::::.:::::::
query 3' AACCUUCCUUACGGACCGGCU 5'
query=miR166b, target=RO3G_01504, score=1, range=123-143, strand=
target 5' UUGGAAGGAAUGCUUGGCCGA 3'
:::::::::::::.:::::::
query 3' AACCUUCCUUACGGACCGGCU 5'
query=miR166b, target=RO3G_01650, score=1, range=123-143, strand=
target 5' UUGGAAGGAAUGCUUGGCCGA 3'
:::::::::::::.:::::::
query 3' AACCUUCCUUACGGACCGGCU 5'
query=miR166b, target=RO3G_00203, score=1.5, range=117-137, stran
target 5' UUGGAAGGAAUGCUUGGCCGG 3'
```

```
::::::::::::.::::::.
query 3' AACCUUCCUUACGGACCGGCU 5'
query=miR166b, target=RO3G_00466, score=3, range=129-149, strand=
target 5' UUGGAAGGAAUGCUUGACCGA 3'
:::::::::::::.:: ::::
query 3' AACCUUCCUUACGGACCGGCU 5'
query=miR166b, target=RO3G_01898, score=4, range=1111-1130, stran
target 5' UUGGCAGAAAUGCCU-GCCGA 3'
:::: :: ::::::: :::::
query 3' AACCUUCCUUACGGACCGGCU 5'
query=miR9677-5p, target=RO3G_14134, score=1, range=177-196, stra
target 5' CUUGGCCGUUGGAGAGGAAA 3'
:::::: :::::::::::::
query 3' GAACCGACAACCUCUCCUUU 5'
query=miR9677-5p, target=RO3G_10867, score=1, range=288-307, stra
target 5' CUUGGCCGUUGGAGAGGAAA 3'
:::::: :::::::::::::
query 3' GAACCGACAACCUCUCCUUU 5'
query=miR9677-5p, target=RO3G_05937, score=1, range=546-565, stra
target 5' CUUGGCCGUUGGAGAGGAAA 3'
:::::: :::::::::::::
query 3' GAACCGACAACCUCUCCUUU 5'
query=miR9677-5p, target=RO3G_08584, score=4, range=447-466, stra
target 5' AUUUACUGUUGGAGAGGAGA 3'
:: :::::::::::::.:
query 3' GAACCGACAACCUCUCCUUU 5'
query=miR833a-5p, target=RO3G_14793, score=0, range=1679-1702,
strand=-1
target 5' ACAGACCGGGUACAAACAAAGCCA 3'
::::::::::::::::::::::::
query 3' UGUCUGGCCCAUGUUUGUUUCGGU 5'
query=miR833a-5p, target=RO3G_14787, score=0, range=1679-1702,
strand=-1
target 5' ACAGACCGGGUACAAACAAAGCCA 3'
::::::::::::::::::::::::
query 3' UGUCUGGCCCAUGUUUGUUUCGGU 5'
query=miR833a-5p, target=RO3G_15875, score=0, range=1679-1702,
strand=-1
target 5' ACAGACCGGGUACAAACAAAGCCA 3'
::::::::::::::::::::::::
query 3' UGUCUGGCCCAUGUUUGUUUCGGU 5'
query=miR833a-5p, target=RO3G_14800, score=0, range=1679-1702,
strand=-1
target 5' ACAGACCGGGUACAAACAAAGCCA 3'
::::::::::::::::::::::::
```

```
query 3' UGUCUGGCCCAUGUUUGUUUCGGU 5'
query=miR833a-5p, target=RO3G_17411, score=0, range=1121-1144,
strand=-1
target 5' ACAGACCGGGUACAAACAAAGCCA 3'
::::::::::::::::::::::::
query 3' UGUCUGGCCCAUGUUUGUUUCGGU 5'
query=miR833a-5p, target=RO3G_17268, score=0, range=260-283, stra
target 5' ACAGACCGGGUACAAACAAAGCCA 3'
::::::::::::::::::::::::
query 3' UGUCUGGCCCAUGUUUGUUUCGGU 5'
query=miR833a-5p, target=RO3G_16711, score=0, range=2-25, strand=
target 5' ACAGACCGGGUACAAACAAAGCCA 3'
::::::::::::::::::::::::
query 3' UGUCUGGCCCAUGUUUGUUUCGGU 5'
query=miR833a-5p, target=RO3G_06431, score=0, range=2-25, strand=
target 5' ACAGACCGGGUACAAACAAAGCCA 3'
::::::::::::::::::::::::
query 3' UGUCUGGCCCAUGUUUGUUUCGGU 5'
query=miR833a-5p, target=RO3G_17267, score=0, range=2-25, strand=
target 5' ACAGACCGGGUACAAACAAAGCCA 3'
::::::::::::::::::::::::
query 3' UGUCUGGCCCAUGUUUGUUUCGGU 5'
query=miR833a-5p, target=RO3G_09186, score=0, range=2-25, strand=
target 5' ACAGACCGGGUACAAACAAAGCCA 3'
::::::::::::::::::::::::
query 3' UGUCUGGCCCAUGUUUGUUUCGGU 5'
query=miR833a-5p, target=RO3G_13288, score=1, range=1679-1702,
strand=-1
target 5' AUAGAUCGGGUACAAACAAAGCCA 3'
:.:::.::::::::::::::::::
query 3' UGUCUGGCCCAUGUUUGUUUCGGU 5'
query=miR833a-5p, target=RO3G_06734, score=1, range=980-1003,
strand=-1
target 5' AUAGAUCGGGUACAAACAAAGCCA 3'
:.:::.::::::::::::::::::
query 3' UGUCUGGCCCAUGUUUGUUUCGGU 5'
query=miR833a-5p, target=RO3G_17260, score=1.5, range=1544-1567,
strand=-1
target 5' AUAGAUUGGGUACAAACAAAGCCA 3'
:.:::..:::::::::::::::::
query 3' UGUCUGGCCCAUGUUUGUUUCGGU 5'
query=miR833a-5p, target=RO3G_17226, score=4, range=1679-1702,
strand=-1
target 5' AGAGGCUAGGUACGAACAAAGCCA 3'
: ::.:. ::::.:::::::::::
```

query 3' UGUCUGGCCCAUGUUUGUUUCGGU 5'
query=miR833a-5p, target=RO3G_14795, score=4, range=1679-1702, strand=-1
target 5' AGAGGCUAGGUACGAACAAAGCCA 3'
: ::.:. ::::.::::::::::
query 3' UGUCUGGCCCAUGUUUGUUUCGGU 5'
query=miR833a-5p, target=RO3G_16815, score=4, range=1679-1702, strand=-1
target 5' AGAGGCUAGGUACGAACAAAGCCA 3'
: ::.:. ::::.:::::::::
query 3' UGUCUGGCCCAUGUUUGUUUCGGU 5'
query=miR2678, target=RO3G_13722, score=0, range=1039-1059, stran
target 5' AAGUACAAUCCCAACAAUUCA 3'
:::::::::::::::::::::
query 3' UUCAUGUUAGGGUUGUUAAGU 5'
query=miR2678, target=RO3G_10856, score=3, range=190-210, strand=
target 5' AAGCACAAUUCCAACAAUUCC 3'
::: ::::.::::::::::
query 3' UUCAUGUUAGGGUUGUUAAGU 5'
query=miR2678, target=RO3G_13330, score=4, range=370-390, strand=
target 5' AAGUACAAUCCCAACGAGUCC 3'
:::::::::::::::.: ::
query 3' UUCAUGUUAGGGUUGUUAAGU 5'
query=miR2678, target=RO3G_02730, score=4, range=1308-1329, stran
target 5' AGGAAAUAAUCCCAACAAUUCU 3'
:.: :.::::::::::::
query 3' UUC-AUGUUAGGGUUGUUAAGU 5'
query=miR2678, target=RO3G_11051, score=4, range=951-972, strand=
target 5' CAAUAUCAUUCCCAACAAUUCA 3'
: :: :: ::::::::::::
query 3' UUCAU-GUUAGGGUUGUUAAGU 5'
query=miR2678, target=RO3G_12863, score=4, range=2097-2116, stran
target 5' AACUACAAU-CCAGCAAUUCA 3'
:: :::::: :::.:::::::
query 3' UUCAUGUUAGGGUUGUUAAGU 5'
query=miR2678, target=RO3G_14575, score=4, range=1722-1741, stran
target 5' CAGU-CAAUCCCAAUAAUUCU 3'
::: :::::::::.:::::
query 3' UUCAUGUUAGGGUUGUUAAGU 5'
query=miR2678, target=RO3G_02743, score=4, range=845-864, strand=
target 5' AAG-ACCUUUCCAACAAUUCA 3'
::: :: :.::::::::::
query 3' UUCAUGUUAGGGUUGUUAAGU 5'
query=miR157a-3p, target=RO3G_05078, score=3.5, range=49-68, stra
target 5' AAGGAAGAAGGCUGGAGAGC 3'

```
: :. ::::::::.::::::
query 3' UACUGUCUUCCGAUCUCUCG 5'
query=miR157a-3p, target=RO3G_12815, score=4, range=578-597, stra
target 5' AUUACAAAAGGCUAGAUAGC 3'
:: ::: ::::::::: :::
query 3' UACUGUCUUCCGAUCUCUCG 5'
query=miR157a-3p, target=RO3G_03369, score=4, range=829-847, stra
target 5' AAGAGA-AAGGCUAGAGAGA 3'
: :: : ::::::::::::
query 3' UACUGUCUUCCGAUCUCUCG 5'
query=miR9662a-3p, target=RO3G_09376, score=2, range=499-518, str
target 5' CCUGUCUCUGUGGAUGUUAA 3'
: :: ::::::::::::::
query 3' GAACCGAGACACCUACAAUU 5'
query=miR9662a-3p, target=RO3G_13152, score=2, range=499-518, str
target 5' CCUGUCUCUGUGGAUGUUAA 3'
: :: ::::::::::::::
query 3' GAACCGAGACACCUACAAUU 5'
query=miR9662a-3p, target=RO3G_03988, score=2, range=499-518, str
target 5' CCUGUCUCUGUGGAUGUUAA 3'
: :: ::::::::::::::
query 3' GAACCGAGACACCUACAAUU 5'
query=miR9662a-3p, target=RO3G_11970, score=2, range=499-518, str
target 5' CCUGUCUCUGUGGAUGUUAA 3'
: :: ::::::::::::::
query 3' GAACCGAGACACCUACAAUU 5'
query=miR9662a-3p, target=RO3G_05630, score=2, range=499-518, str
target 5' CCUGUCUCUGUGGAUGUUAA 3'
: :: ::::::::::::::
query 3' GAACCGAGACACCUACAAUU 5'
query=miR9662a-3p, target=RO3G_07104, score=2, range=499-518, str
target 5' CCUGUCUCUGUGGAUGUUAA 3'
: :: ::::::::::::::
query 3' GAACCGAGACACCUACAAUU 5'
query=miR9662a-3p, target=RO3G_13859, score=2, range=124-143, str
target 5' CCUGUCUCUGUGGAUGUUAA 3'
: :: ::::::::::::::
query 3' GAACCGAGACACCUACAAUU 5'
query=miR9662a-3p, target=RO3G_15869, score=2, range=499-518, str
target 5' CCUGUCUCUGUGGAUGUUAA 3'
: :: ::::::::::::::
query 3' GAACCGAGACACCUACAAUU 5'
query=miR9662a-3p, target=RO3G_01203, score=2, range=499-518, str
target 5' CCUGUCUCUGUGGAUGUUAA 3'
: :: ::::::::::::::
```

```
query 3' GAACCGAGACACCUACAAUU 5'
query=miR9662a-3p, target=RO3G_06416, score=2, range=499-518, str
target 5' CCUGUCUCUGUGGAUGUUAA 3'
: :: ::::::::::::::
query 3' GAACCGAGACACCUACAAUU 5'
query=miR9662a-3p, target=RO3G_01718, score=2, range=499-518, str
target 5' CCUGUCUCUGUGGAUGUUAA 3'
: :: ::::::::::::::
query 3' GAACCGAGACACCUACAAUU 5'
query=miR9662a-3p, target=RO3G_16744, score=2, range=499-518, str
target 5' CCUGUCUCUGUGGAUGUUAA 3'
: :: ::::::::::::::
query 3' GAACCGAGACACCUACAAUU 5'
query=miR9662a-3p, target=RO3G_11275, score=2, range=499-518, str
target 5' CCUGUCUCUGUGGAUGUUAA 3'
: :: ::::::::::::::
query 3' GAACCGAGACACCUACAAUU 5'
query=miR9662a-3p, target=RO3G_10560, score=2, range=499-518, str
target 5' CCUGUCUCUGUGGAUGUUAA 3'
: :: ::::::::::::::
query 3' GAACCGAGACACCUACAAUU 5'
query=miR9662a-3p, target=RO3G_11403, score=2, range=499-518, str
target 5' CCUGUCUCUGUGGAUGUUAA 3'
: :: ::::::::::::::
query 3' GAACCGAGACACCUACAAUU 5'
query=miR9662a-3p, target=RO3G_12951, score=2, range=499-518, str
target 5' CCUGUCUCUGUGGAUGUUAA 3'
: :: ::::::::::::::
query 3' GAACCGAGACACCUACAAUU 5'
query=miR9662a-3p, target=RO3G_13450, score=2, range=454-473, str
target 5' CCUGUCUCUGUGGAUGUUAA 3'
: :: ::::::::::::::
query 3' GAACCGAGACACCUACAAUU 5'
query=miR9662a-3p, target=RO3G_08612, score=2, range=499-518, str
target 5' CCUGUCUCUGUGGAUGUUAA 3'
: :: ::::::::::::::
query 3' GAACCGAGACACCUACAAUU 5'
query=miR9662a-3p, target=RO3G_07305, score=2, range=499-518, str
target 5' CCUGUCUCUGUGGAUGUUAA 3'
: :: ::::::::::::::
query 3' GAACCGAGACACCUACAAUU 5'
query=miR9662a-3p, target=RO3G_16686, score=2, range=499-518, str
target 5' CCUGUCUCUGUGGAUGUUAA 3'
: :: ::::::::::::::
query 3' GAACCGAGACACCUACAAUU 5'
```

query=miR9662a-3p, target=RO3G_12979, score=2, range=499-518, str
target 5' CCUGUCUCUGUGGAUGUUAA 3'
: :: ::::::::::::::
query 3' GAACCGAGACACCUACAAUU 5'
query=miR9662a-3p, target=RO3G_05280, score=2, range=499-518, str
target 5' CCUGUCUCUGUGGAUGUUAA 3'
: :: ::::::::::::::
query 3' GAACCGAGACACCUACAAUU 5'
query=miR9662a-3p, target=RO3G_03103, score=2, range=499-518, str
target 5' CCUGUCUCUGUGGAUGUUAA 3'
: :: ::::::::::::::
query 3' GAACCGAGACACCUACAAUU 5'
query=miR9662a-3p, target=RO3G_10555, score=2, range=499-518, str
target 5' CCUGUCUCUGUGGAUGUUAA 3'
: :: ::::::::::::::
query 3' GAACCGAGACACCUACAAUU 5'
query=miR9662a-3p, target=RO3G_12087, score=2, range=499-518, str
target 5' CCUGUCUCUGUGGAUGUUAA 3'
: :: ::::::::::::::
query 3' GAACCGAGACACCUACAAUU 5'
query=miR9662a-3p, target=RO3G_14704, score=2, range=499-518, str
target 5' CCUGUCUCUGUGGAUGUUAA 3'
: :: ::::::::::::::
query 3' GAACCGAGACACCUACAAUU 5'
query=miR9662a-3p, target=RO3G_12194, score=2, range=499-518, str
target 5' CCUGUCUCUGUGGAUGUUAA 3'
: :: ::::::::::::::
query 3' GAACCGAGACACCUACAAUU 5'
query=miR9662a-3p, target=RO3G_05389, score=3.5, range=1472-1490, strand=1
target 5' UUUGG-ACUGUGGAUGUUGA 3'
.:::: :::::::::::.:
query 3' GAACCGAGACACCUACAAUU 5'
query=miR9662a-3p, target=RO3G_07346, score=4, range=178-197, str
target 5' AUUGCCUCUGUGGCUGUUAA 3'
::: :::::::: ::::::
query 3' GAACCGAGACACCUACAAUU 5'
query=miR9662a-3p, target=RO3G_15934, score=4, range=150-170, str
target 5' UUUGGCUAUUGUGGGUGUUAG 3'
.:::::: .:::::.:::::.
query 3' GAACCGA-GACACCUACAAUU 5'
query=miR9662a-3p, target=RO3G_02065, score=4, range=885-905, str
target 5' CUUGGCACCUGUGGAUGAUAA 3'
:::::: ::::::::: :::
query 3' GAACCG-AGACACCUACAAUU 5'

```
query=miR6485, target=RO3G_17453, score=0, range=862-881, strand=
target 5' UUGAUGCUCUGGUACAUCCA 3'
::::::::::::::::::::
query 3' AACUACGAGACCAUGUAGGU 5'
query=miR6485, target=RO3G_07862, score=0, range=2959-2978, stran
target 5' UUGAUGCUCUGGUACAUCCA 3'
::::::::::::::::::::
query 3' AACUACGAGACCAUGUAGGU 5'
query=miR6485, target=RO3G_10543, score=0, range=2302-2321, stran
target 5' UUGAUGCUCUGGUACAUCCA 3'
::::::::::::::::::::
query 3' AACUACGAGACCAUGUAGGU 5'
query=miR6485, target=RO3G_10744, score=0, range=2848-2867, stran
target 5' UUGAUGCUCUGGUACAUCCA 3'
::::::::::::::::::::
query 3' AACUACGAGACCAUGUAGGU 5'
query=miR6485, target=RO3G_17255, score=0, range=2800-2819, stran
target 5' UUGAUGCUCUGGUACAUCCA 3'
::::::::::::::::::::
query 3' AACUACGAGACCAUGUAGGU 5'
query=miR6485, target=RO3G_16919, score=0, range=424-443, strand=
target 5' UUGAUGCUCUGGUACAUCCA 3'
::::::::::::::::::::
query 3' AACUACGAGACCAUGUAGGU 5'
query=miR6485, target=RO3G_10745, score=0, range=1816-1835, stran
target 5' UUGAUGCUCUGGUACAUCCA 3'
::::::::::::::::::::
query 3' AACUACGAGACCAUGUAGGU 5'
query=miR6485, target=RO3G_14563, score=0, range=412-431, strand=
target 5' UUGAUGCUCUGGUACAUCCA 3'
::::::::::::::::::::
query 3' AACUACGAGACCAUGUAGGU 5'
query=miR6485, target=RO3G_07735, score=1, range=2749-2768, stran
target 5' UCGAUGCUCUGGUACAUCCA 3'
: ::::::::::::::::::
query 3' AACUACGAGACCAUGUAGGU 5'
query=miR6485, target=RO3G_17156, score=3, range=1102-1121, stran
target 5' UUGAUGCUCUGGCAUAUCCA 3'
:::::::::::: :.:::::
query 3' AACUACGAGACCAUGUAGGU 5'
query=miR6485, target=RO3G_11333, score=3.5, range=1274-1294, str
target 5' AUGAUGAUUCUGGUACAUUCA 3'
::::: .::::::::::.::
query 3' AACUAC-GAGACCAUGUAGGU 5'
query=miR6485, target=RO3G_10064, score=4, range=662-682, strand=
```

```
target 5' UUGGUAGUUCUGGUACAUUUA 3'
:::.: :.::::::::::..:
query 3' AACUA-CGAGACCAUGUAGGU 5'
query=miR6485, target=RO3G_10827, score=4, range=409-427, strand=
target 5' AUCAU-CUCUGGUGCAUCCA 3'
: :: :::::::.::::::
query 3' AACUACGAGACCAUGUAGGU 5'
query=miR6485, target=RO3G_15326, score=4, range=1039-1057, stran
target 5' ACAAU-CUCUGGUACAUCCA 3'
:: ::::::::::::::
query 3' AACUACGAGACCAUGUAGGU 5'
query=miR6485, target=RO3G_11852, score=4, range=1105-1123, stran
target 5' ACCAU-CUCUGGUACAUCCA 3'
:: ::::::::::::::
query 3' AACUACGAGACCAUGUAGGU 5'
query=miR6485, target=RO3G_05346, score=4, range=151-169, strand=
target 5' UCGUUGC-CUGGUACAUCCA 3'
: : ::: ::::::::::::
query 3' AACUACGAGACCAUGUAGGU 5'
query=miR6485, target=RO3G_02318, score=4, range=344-363, strand=
target 5' UCGAUGCUCUGGUAAAUCUA 3'
: :::::::::::: :::.:
query 3' AACUACGAGACCAUGUAGGU 5'
query=miR6485, target=RO3G_13551, score=4, range=768-786, strand=
target 5' UUGAU-CUCCGGUAUAUCCA 3'
::::: ::: ::::.:::::
query 3' AACUACGAGACCAUGUAGGU 5'
query=miR6485, target=RO3G_11824, score=4, range=657-677, strand=
target 5' UUGAAGACUUUGGUACAUCCU 3'
:::: : ::.::::::::::
query 3' AACUAC-GAGACCAUGUAGGU 5'
query=miR6485, target=RO3G_03947, score=4, range=4286-4305, stran
target 5' UUCAUCCUCUGGUAAAUCCA 3'
:: :: :::::::: :::::
query 3' AACUACGAGACCAUGUAGGU 5'
query=miR6485, target=RO3G_04730, score=4, range=254-272, strand=
target 5' AUGAU-CUCUGGUAGAUCCA 3'
:::: :::::::: :::::
query 3' AACUACGAGACCAUGUAGGU 5'
query=miR6485, target=RO3G_08203, score=4, range=893-911, strand=
target 5' ACUAU-CUCUGGUACAUCCA 3'
:: ::::::::::::::
query 3' AACUACGAGACCAUGUAGGU 5'
query=miR6485, target=RO3G_02325, score=4, range=1385-1403, stran
target 5' UUCAU-CUCAGGUACAUCCA 3'
```

```
:: :: ::: ::::::::::
query 3' AACUACGAGACCAUGUAGGU 5'
query=miR6485, target=RO3G_14338, score=4, range=1385-1403, stran
target 5' UUCAU-CUCAGGUACAUCCA 3'
:: :: ::: ::::::::::
query 3' AACUACGAGACCAUGUAGGU 5'
query=miR6269, target=RO3G_13221, score=0, range=860-881, strand=
target 5' CCAUGGCUAAUCACUUGCAGCA 3'
::::::::::::::::::::::
query 3' GGUACCGAUUAGUGAACGUCGU 5'
query=miR6269, target=RO3G_04123, score=2, range=860-881, strand=
target 5' CCAUGGCUAACCACUUGCAGCA 3'
:::::::::: :::::::::::
query 3' GGUACCGAUUAGUGAACGUCGU 5'
query=miR6269, target=RO3G_13224, score=4, range=860-881, strand=
target 5' CCAUGGCUAACCACUUGCAACA 3'
:::::::::: :::::::: ::
query 3' GGUACCGAUUAGUGAACGUCGU 5'
query=miR6269, target=RO3G_04120, score=4, range=860-881, strand=
target 5' CCAUGGCUAACCACUUGCAACA 3'
:::::::::: :::::::: ::
query 3' GGUACCGAUUAGUGAACGUCGU 5'
query=miR6269, target=RO3G_17093, score=4, range=620-641, strand=
target 5' CCAUGGCUAACCACUUGCAACA 3'
:::::::::: :::::::: ::
query 3' GGUACCGAUUAGUGAACGUCGU 5'
query=miR6269, target=RO3G_16246, score=4, range=1704-1726, stran
target 5' CCAUGGUCAAAUCACUUGCACCA 3'
:::::: : :::::::::: ::
query 3' GGUACC-GAUUAGUGAACGUCGU 5'
query=miR6156, target=RO3G_05342, score=0, range=559-579, strand=
target 5' ACGAACAAAGAACUUCUUCAA 3'
:::::::::::::::::::::
query 3' UGCUUGUUUCUUGAAGAAGUU 5'
query=miR6156, target=RO3G_14552, score=0, range=559-579, strand=
target 5' ACGAACAAAGAACUUCUUCAA 3'
:::::::::::::::::::::
query 3' UGCUUGUUUCUUGAAGAAGUU 5'
query=miR6156, target=RO3G_16368, score=0, range=559-579, strand=
target 5' ACGAACAAAGAACUUCUUCAA 3'
:::::::::::::::::::::
query 3' UGCUUGUUUCUUGAAGAAGUU 5'
query=miR6156, target=RO3G_13451, score=0, range=394-414, strand=
target 5' ACGAACAAAGAACUUCUUCAA 3'
:::::::::::::::::::::
```

```
query 3' UGCUUGUUUCUUGAAGAAGUU 5'
query=miR6156, target=RO3G_13708, score=0, range=394-414, strand=
target 5' ACGAACAAAGAACUUCUUCAA 3'
:::::::::::::::::::::
query 3' UGCUUGUUUCUUGAAGAAGUU 5'
query=miR6156, target=RO3G_11542, score=0, range=394-414, strand=
target 5' ACGAACAAAGAACUUCUUCAA 3'
:::::::::::::::::::::
query 3' UGCUUGUUUCUUGAAGAAGUU 5'
query=miR6156, target=RO3G_12646, score=0, range=394-414, strand=
target 5' ACGAACAAAGAACUUCUUCAA 3'
:::::::::::::::::::::
query 3' UGCUUGUUUCUUGAAGAAGUU 5'
query=miR6156, target=RO3G_15886, score=0, range=394-414, strand=
target 5' ACGAACAAAGAACUUCUUCAA 3'
:::::::::::::::::::::
query 3' UGCUUGUUUCUUGAAGAAGUU 5'
query=miR6156, target=RO3G_05908, score=0, range=394-414, strand=
target 5' ACGAACAAAGAACUUCUUCAA 3'
:::::::::::::::::::::
query 3' UGCUUGUUUCUUGAAGAAGUU 5'
query=miR6156, target=RO3G_01193, score=0, range=259-279, strand=
target 5' ACGAACAAAGAACUUCUUCAA 3'
:::::::::::::::::::::
query 3' UGCUUGUUUCUUGAAGAAGUU 5'
query=miR6156, target=RO3G_12077, score=0, range=631-651, strand=
target 5' ACGAACAAAGAACUUCUUCAA 3'
:::::::::::::::::::::
query 3' UGCUUGUUUCUUGAAGAAGUU 5'
query=miR6156, target=RO3G_01716, score=0, range=394-414, strand=
target 5' ACGAACAAAGAACUUCUUCAA 3'
:::::::::::::::::::::
query 3' UGCUUGUUUCUUGAAGAAGUU 5'
query=miR6156, target=RO3G_17393, score=0, range=520-540, strand=
target 5' ACGAACAAAGAACUUCUUCAA 3'
:::::::::::::::::::::
query 3' UGCUUGUUUCUUGAAGAAGUU 5'
query=miR6156, target=RO3G_16918, score=0, range=394-414, strand=
target 5' ACGAACAAAGAACUUCUUCAA 3'
:::::::::::::::::::::
query 3' UGCUUGUUUCUUGAAGAAGUU 5'
query=miR6156, target=RO3G_07693, score=0, range=559-579, strand=
target 5' ACGAACAAAGAACUUCUUCAA 3'
:::::::::::::::::::::
query 3' UGCUUGUUUCUUGAAGAAGUU 5'
```

```
query=miR6156, target=RO3G_07978, score=0, range=559-579, strand=
target 5' ACGAACAAAGAACUUCUUCAA 3'
:::::::::::::::::::::
query 3' UGCUUGUUUCUUGAAGAAGUU 5'
query=miR6156, target=RO3G_04658, score=0, range=394-414, strand=
target 5' ACGAACAAAGAACUUCUUCAA 3'
:::::::::::::::::::::
query 3' UGCUUGUUUCUUGAAGAAGUU 5'
query=miR6156, target=RO3G_07113, score=0, range=406-426, strand=
target 5' ACGAACAAAGAACUUCUUCAA 3'
:::::::::::::::::::::
query 3' UGCUUGUUUCUUGAAGAAGUU 5'
query=miR6156, target=RO3G_17240, score=0, range=406-426, strand=
target 5' ACGAACAAAGAACUUCUUCAA 3'
:::::::::::::::::::::
query 3' UGCUUGUUUCUUGAAGAAGUU 5'
query=miR6156, target=RO3G_17450, score=0, range=394-414, strand=
target 5' ACGAACAAAGAACUUCUUCAA 3'
:::::::::::::::::::::
query 3' UGCUUGUUUCUUGAAGAAGUU 5'
query=miR6156, target=RO3G_13285, score=0, range=394-414, strand=
target 5' ACGAACAAAGAACUUCUUCAA 3'
:::::::::::::::::::::
query 3' UGCUUGUUUCUUGAAGAAGUU 5'
query=miR6156, target=RO3G_01323, score=0, range=394-414, strand=
target 5' ACGAACAAAGAACUUCUUCAA 3'
:::::::::::::::::::::
query 3' UGCUUGUUUCUUGAAGAAGUU 5'
query=miR6156, target=RO3G_17212, score=0, range=394-414, strand=
target 5' ACGAACAAAGAACUUCUUCAA 3'
:::::::::::::::::::::
query 3' UGCUUGUUUCUUGAAGAAGUU 5'
query=miR6156, target=RO3G_16740, score=0, range=394-414, strand=
target 5' ACGAACAAAGAACUUCUUCAA 3'
:::::::::::::::::::::
query 3' UGCUUGUUUCUUGAAGAAGUU 5'
query=miR6156, target=RO3G_10049, score=0, range=394-414, strand=
target 5' ACGAACAAAGAACUUCUUCAA 3'
:::::::::::::::::::::
query 3' UGCUUGUUUCUUGAAGAAGUU 5'
query=miR6156, target=RO3G_12012, score=0, range=559-579, strand=
target 5' ACGAACAAAGAACUUCUUCAA 3'
:::::::::::::::::::::
query 3' UGCUUGUUUCUUGAAGAAGUU 5'
query=miR6156, target=RO3G_09492, score=0, range=559-579, strand=
```

```
target 5' ACGAACAAAGAACUUCUUCAA 3'
:::::::::::::::::::::
query 3' UGCUUGUUUCUUGAAGAAGUU 5'
query=miR6156, target=RO3G_16559, score=0, range=394-414, strand=
target 5' ACGAACAAAGAACUUCUUCAA 3'
:::::::::::::::::::::
query 3' UGCUUGUUUCUUGAAGAAGUU 5'
query=miR6156, target=RO3G_12968, score=0.5, range=406-426, stran
target 5' ACGGACAAAGAACUUCUUCAA 3'
:::.:::::::::::::::::
query 3' UGCUUGUUUCUUGAAGAAGUU 5'
query=miR6156, target=RO3G_05954, score=0.5, range=406-426, stran
target 5' ACGGACAAAGAACUUCUUCAA 3'
:::.:::::::::::::::::
query 3' UGCUUGUUUCUUGAAGAAGUU 5'
query=miR6156, target=RO3G_12185, score=0.5, range=634-654, stran
target 5' ACGGACAAAGAACUUCUUCAA 3'
:::.:::::::::::::::::
query 3' UGCUUGUUUCUUGAAGAAGUU 5'
query=miR6156, target=RO3G_05911, score=0.5, range=259-279, stran
target 5' ACGGACAAAGAACUUCUUCAA 3'
:::.:::::::::::::::::
query 3' UGCUUGUUUCUUGAAGAAGUU 5'
query=miR6156, target=RO3G_14559, score=0.5, range=634-654, stran
target 5' ACGGACAAAGAACUUCUUCAA 3'
:::.:::::::::::::::::
query 3' UGCUUGUUUCUUGAAGAAGUU 5'
query=miR6156, target=RO3G_09185, score=0.5, range=634-654, stran
target 5' ACGGACAAAGAACUUCUUCAA 3'
:::.:::::::::::::::::
query 3' UGCUUGUUUCUUGAAGAAGUU 5'
query=miR6156, target=RO3G_00260, score=0.5, range=406-426, stran
target 5' ACGGACAAAGAACUUCUUCAA 3'
:::.:::::::::::::::::
query 3' UGCUUGUUUCUUGAAGAAGUU 5'
query=miR6156, target=RO3G_16128, score=0.5, range=406-426, stran
target 5' ACGGACAAAGAACUUCUUCAA 3'
:::.:::::::::::::::::
query 3' UGCUUGUUUCUUGAAGAAGUU 5'
query=miR6156, target=RO3G_10047, score=0.5, range=406-426, stran
target 5' ACGGACAAAGAACUUCUUCAA 3'
:::.:::::::::::::::::
query 3' UGCUUGUUUCUUGAAGAAGUU 5'
query=miR6156, target=RO3G_07691, score=0.5, range=406-426, stran
target 5' ACGGACAAAGAACUUCUUCAA 3'
```

```
:::.::::::::::::::::
query 3' UGCUUGUUUCUUGAAGAAGUU 5'
query=miR6156, target=RO3G_01724, score=0.5, range=406-426, stran
target 5' ACGGACAAAGAACUUCUUCAA 3'
:::.::::::::::::::::
query 3' UGCUUGUUUCUUGAAGAAGUU 5'
query=miR6156, target=RO3G_01199, score=0.5, range=406-426, stran
target 5' ACGGACAAAGAACUUCUUCAA 3'
:::.::::::::::::::::
query 3' UGCUUGUUUCUUGAAGAAGUU 5'
query=miR6156, target=RO3G_13712, score=0.5, range=406-426, stran
target 5' ACGGACAAAGAACUUCUUCAA 3'
:::.::::::::::::::::
query 3' UGCUUGUUUCUUGAAGAAGUU 5'
query=miR6156, target=RO3G_17434, score=0.5, range=406-426, stran
target 5' ACGGACAAAGAACUUCUUCAA 3'
:::.::::::::::::::::
query 3' UGCUUGUUUCUUGAAGAAGUU 5'
query=miR6156, target=RO3G_02232, score=0.5, range=361-381, stran
target 5' ACGGACAAAGAACUUCUUCAA 3'
:::.::::::::::::::::
query 3' UGCUUGUUUCUUGAAGAAGUU 5'
query=miR6156, target=RO3G_10518, score=0.5, range=559-579, stran
target 5' ACGAACGAAGAACUUCUUCAA 3'
::::::.:::::::::::::
query 3' UGCUUGUUUCUUGAAGAAGUU 5'
query=miR6156, target=RO3G_05426, score=0.5, range=406-426, stran
target 5' ACGGACAAAGAACUUCUUCAA 3'
:::.::::::::::::::::
query 3' UGCUUGUUUCUUGAAGAAGUU 5'
query=miR6156, target=RO3G_16899, score=1, range=559-579, strand=
target 5' ACGAACAAAGAACUUUUUCAA 3'
:::::::::::::::.:::::
query 3' UGCUUGUUUCUUGAAGAAGUU 5'
query=miR6156, target=RO3G_04683, score=3, range=478-498, strand=
target 5' ACGAACAAAAAACUUCUUUAA 3'
::::::::: ::::::::.::
query 3' UGCUUGUUUCUUGAAGAAGUU 5'
query=miR6156, target=RO3G_13273, score=3.5, range=478-498, stran
target 5' ACGAGCAAAAAACUUCUUUAA 3'
::::.:::: ::::::::.::
query 3' UGCUUGUUUCUUGAAGAAGUU 5'
query=miR6156, target=RO3G_13145, score=3.5, range=478-498, stran
target 5' ACGAGCAAAAAACUUCUUUAA 3'
::::.:::: ::::::::.::
```

```
query 3' UGCUUGUUUCUUGAAGAAGUU 5'
query=miR6156, target=RO3G_16749, score=3.5, range=478-498, stran
target 5' ACGAGCAAAAAACUUCUUUAA 3'
::::.:::: ::::::::.::
query 3' UGCUUGUUUCUUGAAGAAGUU 5'
query=miR6156, target=RO3G_10217, score=3.5, range=478-498, stran
target 5' ACGAGCAAAAAACUUCUUUAA 3'
::::.:::: ::::::::.::
query 3' UGCUUGUUUCUUGAAGAAGUU 5'
query=miR6156, target=RO3G_11969, score=3.5, range=478-498, stran
target 5' ACGAGCAAAAAACUUCUUUAA 3'
::::.:::: ::::::::.::
query 3' UGCUUGUUUCUUGAAGAAGUU 5'
query=miR6156, target=RO3G_07307, score=3.5, range=478-498, stran
target 5' ACGAGCAAAAAACUUCUUUAA 3'
::::.:::: ::::::::.::
query 3' UGCUUGUUUCUUGAAGAAGUU 5'
query=miR6156, target=RO3G_11409, score=3.5, range=478-498, stran
target 5' ACGAGCAAAAAACUUCUUUAA 3'
::::.:::: ::::::::.::
query 3' UGCUUGUUUCUUGAAGAAGUU 5'
query=miR6156, target=RO3G_09930, score=3.5, range=478-498, stran
target 5' ACGAGCAAAAAACUUCUUUAA 3'
::::.:::: ::::::::.::
query 3' UGCUUGUUUCUUGAAGAAGUU 5'
query=miR6156, target=RO3G_06731, score=3.5, range=478-498, stran
target 5' ACGAGCAAAAAACUUCUUUAA 3'
::::.:::: ::::::::.::
query 3' UGCUUGUUUCUUGAAGAAGUU 5'
query=miR6156, target=RO3G_04212, score=3.5, range=478-498, stran
target 5' ACGAGCAAAAAACUUCUUUAA 3'
::::.:::: ::::::::.::
query 3' UGCUUGUUUCUUGAAGAAGUU 5'
query=miR6156, target=RO3G_07698, score=3.5, range=478-498, stran
target 5' ACGAGCAAAAAACUUCUUUAA 3'
::::.:::: ::::::::.::
query 3' UGCUUGUUUCUUGAAGAAGUU 5'
query=miR6156, target=RO3G_09601, score=3.5, range=478-498, stran
target 5' ACGAGCAAAAAACUUCUUUAA 3'
::::.:::: ::::::::.::
query 3' UGCUUGUUUCUUGAAGAAGUU 5'
query=miR6156, target=RO3G_11538, score=3.5, range=478-498, stran
target 5' ACGAGCAAAAAACUUCUUUAA 3'
::::.:::: ::::::::.::
query 3' UGCUUGUUUCUUGAAGAAGUU 5'
```

```
query=miR6156, target=RO3G_14550, score=3.5, range=478-498, stran
target 5' ACGAGCAAAAAACUUCUUUAA 3'
::::.:::: ::::::::.::
query 3' UGCUUGUUUCUUGAAGAAGUU 5'
query=miR6156, target=RO3G_15898, score=3.5, range=478-498, stran
target 5' ACGAGCAAAAAACUUCUUUAA 3'
::::.:::: ::::::::.::
query 3' UGCUUGUUUCUUGAAGAAGUU 5'
query=miR6156, target=RO3G_07721, score=3.5, range=478-498, stran
target 5' ACGAGCAAAAAACUUCUUUAA 3'
::::.:::: ::::::::.::
query 3' UGCUUGUUUCUUGAAGAAGUU 5'
query=miR6156, target=RO3G_01728, score=3.5, range=436-456, stran
target 5' ACGAGCAAAAAACUUCUUUAA 3'
::::.:::: ::::::::.::
query 3' UGCUUGUUUCUUGAAGAAGUU 5'
query=miR6156, target=RO3G_16827, score=3.5, range=478-498, stran
target 5' ACGAGCAAAAAACUUCUUUAA 3'
::::.:::: ::::::::.::
query 3' UGCUUGUUUCUUGAAGAAGUU 5'
query=miR6156, target=RO3G_15150, score=3.5, range=418-438, stran
target 5' ACGAGCAAAAAACUUCUUUAA 3'
::::.:::: ::::::::.::
query 3' UGCUUGUUUCUUGAAGAAGUU 5'
query=miR6156, target=RO3G_02056, score=3.5, range=89-108, strand
target 5' ACG-ACGAAGAACUUCUUGAA 3'
::: ::.:::::::::: ::
query 3' UGCUUGUUUCUUGAAGAAGUU 5'
query=miR6156, target=RO3G_03999, score=3.5, range=89-108, strand
target 5' ACG-ACGAAGAACUUCUUGAA 3'
::: ::.:::::::::: ::
query 3' UGCUUGUUUCUUGAAGAAGUU 5'
query=miR6156, target=RO3G_10490, score=3.5, range=89-108, strand
target 5' ACG-ACGAAGAACUUCUUGAA 3'
::: ::.:::::::::: ::
query 3' UGCUUGUUUCUUGAAGAAGUU 5'
query=miR6156, target=RO3G_00809, score=4, range=974-993, strand=
target 5' AU-AAAGAAGAACUUCUUUAA 3'
:. :: .::::::::::.::
query 3' UGCUUGUUUCUUGAAGAAGUU 5'
query=miR6156, target=RO3G_15046, score=4, range=1322-1341, stran
target 5' UCGAAC-AAGAACUCCUUCAA 3'
::::: ::::::: ::::::
query 3' UGCUUGUUUCUUGAAGAAGUU 5'
query=miR6156, target=RO3G_14251, score=4, range=734-754, strand=
```

```
target 5' AUGAACAGAGCGCUUCUUCAA 3'
:.:::::.:: .::::::::
query 3' UGCUUGUUUCUUGAAGAAGUU 5'
query=miR6156, target=RO3G_10498, score=4, range=1223-1243, stran
target 5' ACUUGCGAAGAACUUCUUUAA 3'
:: .:.:::::::::::.::
query 3' UGCUUGUUUCUUGAAGAAGUU 5'
query=miR6156, target=RO3G_08677, score=4, range=668-687, strand=
target 5' ACGAGCCAAGAA-UUCUUCAG 3'
::::.: ::::: :::::::.
query 3' UGCUUGUUUCUUGAAGAAGUU 5'
query=miR6156, target=RO3G_12153, score=4, range=1571-1590, stran
target 5' ACAAUCAAAG-ACUUCUUCAA 3'
:: : ::::: :::::::::
query 3' UGCUUGUUUCUUGAAGAAGUU 5'
query=miR6156, target=RO3G_10466, score=4, range=214-233, strand=
target 5' GCUUGC-AAGAACUUCUUCAA 3'
.: .: :::::::::::::
query 3' UGCUUGUUUCUUGAAGAAGUU 5'
query=miR6156, target=RO3G_08561, score=4, range=284-303, strand=
target 5' ACGAAC-CAGAAUUUCUUUAA 3'
:::::: ::::.:::::.::
query 3' UGCUUGUUUCUUGAAGAAGUU 5'
query=miR6156, target=RO3G_16296, score=4, range=512-531, strand=
target 5' AGAAACAAAGAA-UUCUUCAA 3'
: ::::::::: ::::::::
query 3' UGCUUGUUUCUUGAAGAAGUU 5'
query=miR403-3p, target=RO3G_10567, score=4, range=5795-5813, str
target 5' CACUUU-UGCGUGAAUCUGA 3'
: ::: :::::::::::.:
query 3' CUCAAACACGCACUUAGAUU 5'
query=miR5244, target=RO3G_10142, score=0, range=338-359, strand=
target 5' ACCAACAACUACUUCAAGAGAA 3'
::::::::::::::::::::::
query 3' UGGUUGUUGAUGAAGUUCUCUU 5'
query=miR5244, target=RO3G_03107, score=0, range=338-359, strand=
target 5' ACCAACAACUACUUCAAGAGAA 3'
::::::::::::::::::::::
query 3' UGGUUGUUGAUGAAGUUCUCUU 5'
query=miR5244, target=RO3G_01078, score=4, range=207-228, strand=
target 5' ACAAACACCUGUUUCAAGAGAA 3'
:: :::: ::..::::::::::
query 3' UGGUUGUUGAUGAAGUUCUCUU 5'
query=miR2611, target=RO3G_15874, score=0, range=154-175, strand=
target 5' UUCUACCACAACACUGAAAAUA 3'
```

```
::::::::::::::::::::::
query 3' AAGAUGGUGUUGUGACUUUUAU 5'
query=miR2611, target=RO3G_11737, score=4, range=10-31, strand=1
target 5' UUGAACGAUAACACUGAAAAUG 3'
:: :: :.::::::::::::.
query 3' AAGAUGGUGUUGUGACUUUUAU 5'
query=miR2611, target=RO3G_03919, score=4, range=15-37, strand=1
target 5' AUCCACAACAAACACUGAAAAUA 3'
:: :: :: :::::::::::::
query 3' AAGAUGGUG-UUGUGACUUUUAU 5'
query=miR162-3p, target=RO3G_05801, score=3, range=644-664, stran
target 5' UGGCUGCCAGAGGUUUAUUGA 3'
::: :: :::::::::::.::
query 3' ACCUAC-GUCUCCAAAUAGCU 5'
query=miR162-3p, target=RO3G_10399, score=4, range=762-781, stran
target 5' AAGGCGUAGAGGUUUAUCGA 3'
:. :.:::::::::::::
query 3' ACCUACGUCUCCAAAUAGCU 5'
query=miR162-3p, target=RO3G_00548, score=4, range=105-124, stran
target 5' AGGAUGCAGAGGUUUGUUGU 3'
::::::::::::::.:.:
query 3' ACCUACGUCUCCAAAUAGCU 5'
query=miR5568e-3p, target=RO3G_02952, score=0, range=1502-1522,
strand=-1
target 5' CGUUUUGACCAUUUCUAGAUA 3'
:::::::::::::::::::::
query 3' GCAAAACUGGUAAAGAUCUAU 5'
query=miR5568e-3p, target=RO3G_01722, score=0, range=1502-1522,
strand=-1
target 5' CGUUUUGACCAUUUCUAGAUA 3'
:::::::::::::::::::::
query 3' GCAAAACUGGUAAAGAUCUAU 5'
query=miR5568e-3p, target=RO3G_17431, score=0, range=1469-1489,
strand=-1
target 5' CGUUUUGACCAUUUCUAGAUA 3'
:::::::::::::::::::::
query 3' GCAAAACUGGUAAAGAUCUAU 5'
query=miR5568e-3p, target=RO3G_09164, score=0, range=1469-1489,
strand=-1
target 5' CGUUUUGACCAUUUCUAGAUA 3'
:::::::::::::::::::::
query 3' GCAAAACUGGUAAAGAUCUAU 5'
query=miR5568e-3p, target=RO3G_17323, score=0, range=1469-1489,
strand=-1
target 5' CGUUUUGACCAUUUCUAGAUA 3'
```

```
:::::::::::::::::::::
query 3' GCAAAACUGGUAAAGAUCUAU 5'
query=miR5568e-3p, target=RO3G_17247, score=0, range=1469-1489,
strand=-1
target 5' CGUUUUGACCAUUUCUAGAUA 3'
:::::::::::::::::::::
query 3' GCAAAACUGGUAAAGAUCUAU 5'
query=miR5568e-3p, target=RO3G_07127, score=0, range=1469-1489,
strand=-1
target 5' CGUUUUGACCAUUUCUAGAUA 3'
:::::::::::::::::::::
query 3' GCAAAACUGGUAAAGAUCUAU 5'
query=miR5568e-3p, target=RO3G_10547, score=0, range=1469-1489,
strand=-1
target 5' CGUUUUGACCAUUUCUAGAUA 3'
:::::::::::::::::::::
query 3' GCAAAACUGGUAAAGAUCUAU 5'
query=miR5568e-3p, target=RO3G_12068, score=0, range=1328-1348,
strand=-1
target 5' CGUUUUGACCAUUUCUAGAUA 3'
:::::::::::::::::::::
query 3' GCAAAACUGGUAAAGAUCUAU 5'
query=miR5568e-3p, target=RO3G_17219, score=0, range=1328-1348,
strand=-1
target 5' CGUUUUGACCAUUUCUAGAUA 3'
:::::::::::::::::::::
query 3' GCAAAACUGGUAAAGAUCUAU 5'
query=miR5568e-3p, target=RO3G_15165, score=2, range=1469-1489,
strand=-1
target 5' CGUUUUGACCAUUGCUAGAUA 3'
::::::::::::: :::::::
query 3' GCAAAACUGGUAAAGAUCUAU 5'
query=miR6464, target=RO3G_17255, score=0, range=605-625, strand=
target 5' AUCAACAUCAACAAGCAAACA 3'
:::::::::::::::::::::
query 3' UAGUUGUAGUUGUUCGUUUGU 5'
query=miR6464, target=RO3G_10532, score=1, range=605-625, strand=
target 5' AUCAACAUCAACAAGCAAAUA 3'
:::::::::::::::::::.:
query 3' UAGUUGUAGUUGUUCGUUUGU 5'
query=miR6464, target=RO3G_10747, score=1, range=719-739, strand=
target 5' AUCAACAUCAACAAGCAAAUA 3'
:::::::::::::::::::.:
query 3' UAGUUGUAGUUGUUCGUUUGU 5'
query=miR6464, target=RO3G_16366, score=1.5, range=605-625, stran
```

```
target 5' AUCAACAUCAACAAGCAAAUG 3'
::::::::::::::::::..
query 3' UAGUUGUAGUUGUUCGUUUGU 5'
query=miR6464, target=RO3G_11979, score=1.5, range=589-609, stran
target 5' AUCAACAUCAACAAGCAAAUG 3'
::::::::::::::::::..
query 3' UAGUUGUAGUUGUUCGUUUGU 5'
query=miR6464, target=RO3G_13448, score=1.5, range=605-625, stran
target 5' AUCAACAUCAACAAGCAAAUG 3'
::::::::::::::::::..
query 3' UAGUUGUAGUUGUUCGUUUGU 5'
query=miR6464, target=RO3G_10543, score=1.5, range=77-97, strand=
target 5' AUCAACAUCAACAAGCAAAUG 3'
::::::::::::::::::..
query 3' UAGUUGUAGUUGUUCGUUUGU 5'
query=miR6464, target=RO3G_07862, score=2, range=719-739, strand=
target 5' AUCAACAUCAGCAAGCAAAUA 3'
:::::::::.:::::::.:
query 3' UAGUUGUAGUUGUUCGUUUGU 5'
query=miR6464, target=RO3G_10744, score=2, range=605-625, strand=
target 5' AUCAACAUCAGCAAGCAAAUA 3'
:::::::::.:::::::.:
query 3' UAGUUGUAGUUGUUCGUUUGU 5'
query=miR6464, target=RO3G_09173, score=2, range=719-739, strand=
target 5' AUCAACAUCAGCAAGCAAAUA 3'
:::::::::.:::::::.:
query 3' UAGUUGUAGUUGUUCGUUUGU 5'
query=miR6464, target=RO3G_17218, score=2, range=605-625, strand=
target 5' AUCAACAUCAACAAGCGAAUA 3'
:::::::::::::::.::.:
query 3' UAGUUGUAGUUGUUCGUUUGU 5'
query=miR6464, target=RO3G_17253, score=2, range=605-625, strand=
target 5' AUCAGCAUCAACAAGCAAAUG 3'
::::.:::::::::::::..
query 3' UAGUUGUAGUUGUUCGUUUGU 5'
query=miR6464, target=RO3G_10539, score=2, range=719-739, strand=
target 5' AUCAACACCAACAAGCAAAUA 3'
::::::: :::::::::::.:
query 3' UAGUUGUAGUUGUUCGUUUGU 5'
query=miR6464, target=RO3G_03861, score=2.5, range=72-92, strand=
target 5' AUCAUCAUCAACAAGUAAACG 3'
:::: ::::::::::.::::.
query 3' UAGUUGUAGUUGUUCGUUUGU 5'
query=miR6464, target=RO3G_01193, score=2.5, range=1655-1675, str
target 5' AACAUUAUCAACAAGCAAACA 3'
```

```
: :: .::::::::::::::
query 3' UAGUUGUAGUUGUUCGUUUGU 5'
query=miR6464, target=RO3G_02804, score=2.5, range=97-118, strand
target 5' AUCAAUCGGCAACAAGCAAACA 3'
::::: :. ::::::::::::
query 3' UAGUU-GUAGUUGUUCGUUUGU 5'
query=miR6464, target=RO3G_12315, score=3, range=2518-2538, stran
target 5' AUUUAUAUCAAUAAGCAAACA 3'
::. :.::::.::::::::
query 3' UAGUUGUAGUUGUUCGUUUGU 5'
query=miR6464, target=RO3G_07808, score=3, range=1162-1183, stran
target 5' AUCAAUCAGCAACAAGCAGACA 3'
::::: :: :::::::::.:::
query 3' UAGUU-GUAGUUGUUCGUUUGU 5'
query=miR6464, target=RO3G_10276, score=3, range=673-694, strand=
target 5' AUCAAUCAGCAACAAGCAGACA 3'
::::: :: :::::::::.:::
query 3' UAGUU-GUAGUUGUUCGUUUGU 5'
query=miR6464, target=RO3G_11178, score=3, range=676-697, strand=
target 5' AUCAAUCAGCAACAAGCAGACA 3'
::::: :: :::::::::.:::
query 3' UAGUU-GUAGUUGUUCGUUUGU 5'
query=miR6464, target=RO3G_01486, score=3, range=565-586, strand=
target 5' AUCAAUCAGCAACAAGCAGACA 3'
::::: :: :::::::::.:::
query 3' UAGUU-GUAGUUGUUCGUUUGU 5'
query=miR6464, target=RO3G_14655, score=3, range=676-697, strand=
target 5' AUCAAUCAACAACAAGCAGACA 3'
::::: :: :::::::::.:::
query 3' UAGUU-GUAGUUGUUCGUUUGU 5'
query=miR6464, target=RO3G_13646, score=3, range=289-310, strand=
target 5' AUCAAUCAGCAACAAGCAGACA 3'
::::: :: :::::::::.:::
query 3' UAGUU-GUAGUUGUUCGUUUGU 5'
query=miR6464, target=RO3G_14089, score=3, range=19-40, strand=1
target 5' AUCAAUCAACAACAAGCAGACA 3'
::::: :: :::::::::.:::
query 3' UAGUU-GUAGUUGUUCGUUUGU 5'
query=miR6464, target=RO3G_03317, score=3, range=829-849, strand=
target 5' AUCAAUAUCAACAGGUAAACG 3'
::::.:::::::.:.::::.
query 3' UAGUUGUAGUUGUUCGUUUGU 5'
query=miR6464, target=RO3G_09805, score=3.5, range=472-493, stran
target 5' AUUAAUCAGCAACAAGCAGACA 3'
::.:: :: :::::::::.:::
```

```
query 3' UAGUU-GUAGUUGUUCGUUUGU 5'
query=miR6464, target=RO3G_00530, score=3.5, range=97-118, strand
target 5' AUUAAUCAGCAACAAGCAGACA 3'
::.:: :: :::::::::.:::
query 3' UAGUU-GUAGUUGUUCGUUUGU 5'
query=miR6464, target=RO3G_07259, score=3.5, range=2026-2045, str
target 5' AUC-ACGUCCACAAGCAAACA 3'
::: ::.:: ::::::::::
query 3' UAGUUGUAGUUGUUCGUUUGU 5'
query=miR6464, target=RO3G_09333, score=3.5, range=331-350, stran
target 5' AUCAUUAUCAACAA-CAAACA 3'
:::: .:::::::: ::::::
query 3' UAGUUGUAGUUGUUCGUUUGU 5'
query=miR6464, target=RO3G_00639, score=3.5, range=562-581, stran
target 5' AUCAAU-UUGACAAGCAAACA 3'
:::::. :..::::::::::
query 3' UAGUUGUAGUUGUUCGUUUGU 5'
query=miR6464, target=RO3G_05074, score=4, range=1688-1708, stran
target 5' AUCAACAACAGCAAGUAAACU 3'
::::::: ::.::::.::::
query 3' UAGUUGUAGUUGUUCGUUUGU 5'
query=miR6464, target=RO3G_13401, score=4, range=686-706, strand=
target 5' AUCAACGCCAACAAGCACACG 3'
::::::. ::::::::: ::.
query 3' UAGUUGUAGUUGUUCGUUUGU 5'
query=miR6464, target=RO3G_14450, score=4, range=42-63, strand=1
target 5' AUCUUACAUCAAUGAGCAAACA 3'
::: :::::::..::::::::
query 3' UAG-UUGUAGUUGUUCGUUUGU 5'
query=miR6464, target=RO3G_17083, score=4, range=153-173, strand=
target 5' AGCAACAUCAACAAACAAACC 3'
: :::::::::::: :::::
query 3' UAGUUGUAGUUGUUCGUUUGU 5'
query=miR6464, target=RO3G_07291, score=4, range=1250-1270, stran
target 5' CUCAACAACAACAAGAAAACA 3'
:::::: ::::::: :::::
query 3' UAGUUGUAGUUGUUCGUUUGU 5'
query=miR6464, target=RO3G_04620, score=4, range=854-873, strand=
target 5' AUCAUCAUCAACAA-CAGACA 3'
:::: ::::::::: ::.:::
query 3' UAGUUGUAGUUGUUCGUUUGU 5'
query=miR6464, target=RO3G_04140, score=4, range=395-414, strand=
target 5' AUCAACAACAACAAG-AGACA 3'
::::::: ::::::: :.:::
query 3' UAGUUGUAGUUGUUCGUUUGU 5'
```

```
query=miR6464, target=RO3G_15991, score=4, range=959-980, strand=
target 5' AACAACAACAACAAGCAGAACA 3'
: ::::: ::::::::: ::::
query 3' UAGUUGUAGUUGUUCGU-UUGU 5'
query=miR6464, target=RO3G_17097, score=4, range=676-697, strand=
target 5' AUCAAUCAGCAACAAGCACACA 3'
::::: :: ::::::::: :::
query 3' UAGUU-GUAGUUGUUCGUUUGU 5'
query=miR6464, target=RO3G_00154, score=4, range=47-66, strand=1
target 5' AUAAUCAUCAACAAG-AAACA 3'
:: : :::::::::: :::::
query 3' UAGUUGUAGUUGUUCGUUUGU 5'
query=miR6464, target=RO3G_01487, score=4, range=18-37, strand=1
target 5' AACAACAACAACAA-CAAACA 3'
: ::::: :::::: ::::::
query 3' UAGUUGUAGUUGUUCGUUUGU 5'
query=miR6464, target=RO3G_00781, score=4, range=2404-2423, stran
target 5' GACAAUAUCAAC-AGCAAACA 3'
. :::.:::::: ::::::::
query 3' UAGUUGUAGUUGUUCGUUUGU 5'
query=miR6464, target=RO3G_01599, score=4, range=275-294, strand=
target 5' AGCAACAACAACAA-CAAACA 3'
: ::::: :::::: ::::::
query 3' UAGUUGUAGUUGUUCGUUUGU 5'
query=miR6464, target=RO3G_12687, score=4, range=232-252, strand=
target 5' AUUGACAACAACAAGAAAACA 3'
::..::: ::::::: :::::
query 3' UAGUUGUAGUUGUUCGUUUGU 5'
query=miR6464, target=RO3G_02376, score=4, range=3656-3675, stran
target 5' AUAAACACCAACAAGC-AACA 3'
:: :::: :::::::: ::::
query 3' UAGUUGUAGUUGUUCGUUUGU 5'
query=miR7541, target=RO3G_08829, score=0, range=170-192, strand=
target 5' GUGCCAGCAAAAGAGACAUUGCA 3'
:::::::::::::::::::::::
query 3' CACGGUCGUUUUCUCUGUAACGU 5'
query=miR5512a, target=RO3G_13852, score=1, range=462-484, strand
target 5' AUUUCAGCAUGACAAUGAUCCUA 3'
:::: ::::::::::::::::::
query 3' UAAAAUCGUACUGUUACUAGGAU 5'
query=miR5512a, target=RO3G_15271, score=2, range=711-733, strand
target 5' AUUUCAGCACGACAAUGAUCCUA 3'
:::: :::: :::::::::::::
query 3' UAAAAUCGUACUGUUACUAGGAU 5'
query=miR5512a, target=RO3G_10771, score=2, range=462-484, strand
```

```
target 5' AUUUCAACAUGACAAUGAUCCUA 3'
:::: : :::::::::::::::
query 3' UAAAAUCGUACUGUUACUAGGAU 5'
query=miR5512a, target=RO3G_16725, score=2, range=453-475, strand
target 5' AUUUCAGCACGACAAUGAUCCUA 3'
:::: :::: ::::::::::::
query 3' UAAAAUCGUACUGUUACUAGGAU 5'
query=miR5512a, target=RO3G_07739, score=3, range=462-484, strand
target 5' AUUUCAGCAUGAGAAUGAUCCUA 3'
:::: ::::::: :::::::::
query 3' UAAAAUCGUACUGUUACUAGGAU 5'
query=miR5512a, target=RO3G_03797, score=3, range=209-232, strand
target 5' AUUUUCAGCACGACAGUGAUCCUA 3'
::::: :::: ::::.::::::::
query 3' UAAAA-UCGUACUGUUACUAGGAU 5'
query=miR5512a, target=RO3G_00745, score=3, range=432-454, strand
target 5' AUUUCAACACGACAAUGAUCCUA 3'
:::: : :: ::::::::::::
query 3' UAAAAUCGUACUGUUACUAGGAU 5'
query=miR5512a, target=RO3G_12082, score=4, range=599-622, strand
target 5' AUUUUCAACACGACAGUGAUCCUA 3'
::::: : :: ::::.::::::::
query 3' UAAAA-UCGUACUGUUACUAGGAU 5'
query=miR5512a, target=RO3G_10226, score=4, range=521-544, strand
target 5' AUUUUCAACACGACAGUGAUCCUA 3'
::::: : :: ::::.::::::::
query 3' UAAAA-UCGUACUGUUACUAGGAU 5'
query=miR5512a, target=RO3G_14772, score=4, range=599-622, strand
target 5' AUUUUCAACACGACAGUGAUCCUA 3'
::::: : :: ::::.::::::::
query 3' UAAAA-UCGUACUGUUACUAGGAU 5'
query=miR5512a, target=RO3G_12670, score=4, range=521-544, strand
target 5' AUUUUCAACACGACAGUGAUCCUA 3'
::::: : :: ::::.::::::::
query 3' UAAAA-UCGUACUGUUACUAGGAU 5'
query=miR5512a, target=RO3G_17436, score=4, range=521-544, strand
target 5' AUUUUCAACACGACAGUGAUCCUA 3'
::::: : :: ::::.::::::::
query 3' UAAAA-UCGUACUGUUACUAGGAU 5'
query=miR5512a, target=RO3G_09167, score=4, range=521-544, strand
target 5' AUUUUCAACACGACAGUGAUCCUA 3'
::::: : :: ::::.::::::::
query 3' UAAAA-UCGUACUGUUACUAGGAU 5'
query=miR5512a, target=RO3G_07689, score=4, range=521-544, strand
target 5' AUUUUCAACACGACAGUGAUCCUA 3'
```

```
::::: : :: ::::.::::::::
query 3' UAAAA-UCGUACUGUUACUAGGAU 5'
query=miR5512a, target=RO3G_00247, score=4, range=521-544, strand
target 5' AUUUUCAACACGACAGUGAUCCUA 3'
::::: : :: ::::.::::::::
query 3' UAAAA-UCGUACUGUUACUAGGAU 5'
query=miR5512a, target=RO3G_04654, score=4, range=521-544, strand
target 5' AUUUUCAACACGACAGUGAUCCUA 3'
::::: : :: ::::.::::::::
query 3' UAAAA-UCGUACUGUUACUAGGAU 5'
query=miR5512a, target=RO3G_07302, score=4, range=521-544, strand
target 5' AUUUUCAACACGACAGUGAUCCUA 3'
::::: : :: ::::.::::::::
query 3' UAAAA-UCGUACUGUUACUAGGAU 5'
query=miR5512a, target=RO3G_04200, score=4, range=521-544, strand
target 5' AUUUUCAACACGACAGUGAUCCUA 3'
::::: : :: ::::.::::::::
query 3' UAAAA-UCGUACUGUUACUAGGAU 5'
query=miR5512a, target=RO3G_11405, score=4, range=521-544, strand
target 5' AUUUUCAACACGACAGUGAUCCUA 3'
::::: : :: ::::.::::::::
query 3' UAAAA-UCGUACUGUUACUAGGAU 5'
query=miR5512a, target=RO3G_09944, score=4, range=521-544, strand
target 5' AUUUUCAACACGACAGUGAUCCUA 3'
::::: : :: ::::.::::::::
query 3' UAAAA-UCGUACUGUUACUAGGAU 5'
query=miR5512a, target=RO3G_15672, score=4, range=521-544, strand
target 5' AUUUUCAACACGACAGUGAUCCUA 3'
::::: : :: ::::.::::::::
query 3' UAAAA-UCGUACUGUUACUAGGAU 5'
query=miR5512a, target=RO3G_06721, score=4, range=521-544, strand
target 5' AUUUUCAACACGACAGUGAUCCUA 3'
::::: : :: ::::.::::::::
query 3' UAAAA-UCGUACUGUUACUAGGAU 5'
query=miR5512a, target=RO3G_03785, score=4, range=326-349, strand
target 5' AUUUUCAACACGACAGUGAUCCUA 3'
::::: : :: ::::.::::::::
query 3' UAAAA-UCGUACUGUUACUAGGAU 5'
query=miR5512a, target=RO3G_14620, score=4, range=149-172, strand
target 5' AUUUUCAACACGACAGUGAUCCUA 3'
::::: : :: ::::.::::::::
query 3' UAAAA-UCGUACUGUUACUAGGAU 5'
query=miR5512a, target=RO3G_17349, score=4, range=13-35, strand=-
target 5' AUUUUAGCAUGACAAUGACCCAA 3'
:::::::::::::::::: :: :
```

```
query 3' UAAAAUCGUACUGUUACUAGGAU 5'
query=miR5512a, target=RO3G_16894, score=4, range=13-35, strand=-
target 5' AUUUUAGCAUGACAAUGACCCAA 3'
::::::::::::::::::: :: :
query 3' UAAAAUCGUACUGUUACUAGGAU 5'
query=miR5144-3p, target=RO3G_10780, score=0, range=350-373, stra
target 5' CUUUCUUGUGCUGUGCAGUGAGUA 3'
::::::::::::::::::::::::
query 3' GAAAGAACACGACACGUCACUCAU 5'
query=miR5144-3p, target=RO3G_10773, score=0, range=350-373, stra
target 5' CUUUCUUGUGCUGUGCAGUGAGUA 3'
::::::::::::::::::::::::
query 3' GAAAGAACACGACACGUCACUCAU 5'
query=miR5144-3p, target=RO3G_04210, score=0, range=350-373, stra
target 5' CUUUCUUGUGCUGUGCAGUGAGUA 3'
::::::::::::::::::::::::
query 3' GAAAGAACACGACACGUCACUCAU 5'
query=miR5144-3p, target=RO3G_09542, score=0, range=350-373, stra
target 5' CUUUCUUGUGCUGUGCAGUGAGUA 3'
::::::::::::::::::::::::
query 3' GAAAGAACACGACACGUCACUCAU 5'
query=miR5144-3p, target=RO3G_17449, score=0, range=350-373, stra
target 5' CUUUCUUGUGCUGUGCAGUGAGUA 3'
::::::::::::::::::::::::
query 3' GAAAGAACACGACACGUCACUCAU 5'
query=miR5144-3p, target=RO3G_09967, score=0, range=350-373, stra
target 5' CUUUCUUGUGCUGUGCAGUGAGUA 3'
::::::::::::::::::::::::
query 3' GAAAGAACACGACACGUCACUCAU 5'
query=miR5144-3p, target=RO3G_04207, score=0, range=350-373, stra
target 5' CUUUCUUGUGCUGUGCAGUGAGUA 3'
::::::::::::::::::::::::
query 3' GAAAGAACACGACACGUCACUCAU 5'
query=miR5144-3p, target=RO3G_05267, score=0, range=350-373, stra
target 5' CUUUCUUGUGCUGUGCAGUGAGUA 3'
::::::::::::::::::::::::
query 3' GAAAGAACACGACACGUCACUCAU 5'
query=miR5144-3p, target=RO3G_14361, score=0, range=350-373, stra
target 5' CUUUCUUGUGCUGUGCAGUGAGUA 3'
::::::::::::::::::::::::
query 3' GAAAGAACACGACACGUCACUCAU 5'
query=miR5144-3p, target=RO3G_00256, score=0, range=350-373, stra
target 5' CUUUCUUGUGCUGUGCAGUGAGUA 3'
::::::::::::::::::::::::
query 3' GAAAGAACACGACACGUCACUCAU 5'
```

```
query=miR5144-3p, target=RO3G_05921, score=0, range=350-373, stra
target 5' CUUUCUUGUGCUGUGCAGUGAGUA 3'
::::::::::::::::::::::::
query 3' GAAAGAACACGACACGUCACUCAU 5'
query=miR5144-3p, target=RO3G_01942, score=0, range=350-373, stra
target 5' CUUUCUUGUGCUGUGCAGUGAGUA 3'
::::::::::::::::::::::::
query 3' GAAAGAACACGACACGUCACUCAU 5'
query=miR5144-3p, target=RO3G_09194, score=0, range=350-373, stra
target 5' CUUUCUUGUGCUGUGCAGUGAGUA 3'
::::::::::::::::::::::::
query 3' GAAAGAACACGACACGUCACUCAU 5'
query=miR5144-3p, target=RO3G_10729, score=0, range=350-373, stra
target 5' CUUUCUUGUGCUGUGCAGUGAGUA 3'
::::::::::::::::::::::::
query 3' GAAAGAACACGACACGUCACUCAU 5'
query=miR5144-3p, target=RO3G_05939, score=0, range=350-373, stra
target 5' CUUUCUUGUGCUGUGCAGUGAGUA 3'
::::::::::::::::::::::::
query 3' GAAAGAACACGACACGUCACUCAU 5'
query=miR5144-3p, target=RO3G_07124, score=0, range=350-373, stra
target 5' CUUUCUUGUGCUGUGCAGUGAGUA 3'
::::::::::::::::::::::::
query 3' GAAAGAACACGACACGUCACUCAU 5'
query=miR5144-3p, target=RO3G_17069, score=0, range=215-238, stra
target 5' CUUUCUUGUGCUGUGCAGUGAGUA 3'
::::::::::::::::::::::::
query 3' GAAAGAACACGACACGUCACUCAU 5'
query=miR5144-3p, target=RO3G_09945, score=0, range=74-97, strand
target 5' CUUUCUUGUGCUGUGCAGUGAGUA 3'
::::::::::::::::::::::::
query 3' GAAAGAACACGACACGUCACUCAU 5'
query=miR5144-3p, target=RO3G_15664, score=0, range=350-373, stra
target 5' CUUUCUUGUGCUGUGCAGUGAGUA 3'
::::::::::::::::::::::::
query 3' GAAAGAACACGACACGUCACUCAU 5'
query=miR5144-3p, target=RO3G_11264, score=0, range=350-373, stra
target 5' CUUUCUUGUGCUGUGCAGUGAGUA 3'
::::::::::::::::::::::::
query 3' GAAAGAACACGACACGUCACUCAU 5'
query=miR5144-3p, target=RO3G_02956, score=0, range=350-373, stra
target 5' CUUUCUUGUGCUGUGCAGUGAGUA 3'
::::::::::::::::::::::::
query 3' GAAAGAACACGACACGUCACUCAU 5'
query=miR5144-3p, target=RO3G_05435, score=0, range=350-373, stra
```

```
target 5' CUUUCUUGUGCUGUGCAGUGAGUA 3'
::::::::::::::::::::::::
query 3' GAAAGAACACGACACGUCACUCAU 5'
query=miR5144-3p, target=RO3G_10743, score=0, range=350-373, stra
target 5' CUUUCUUGUGCUGUGCAGUGAGUA 3'
::::::::::::::::::::::::
query 3' GAAAGAACACGACACGUCACUCAU 5'
query=miR5144-3p, target=RO3G_07038, score=0, range=350-373, stra
target 5' CUUUCUUGUGCUGUGCAGUGAGUA 3'
::::::::::::::::::::::::
query 3' GAAAGAACACGACACGUCACUCAU 5'
query=miR5144-3p, target=RO3G_07040, score=0, range=350-373, stra
target 5' CUUUCUUGUGCUGUGCAGUGAGUA 3'
::::::::::::::::::::::::
query 3' GAAAGAACACGACACGUCACUCAU 5'
query=miR5144-3p, target=RO3G_07030, score=0, range=350-373, stra
target 5' CUUUCUUGUGCUGUGCAGUGAGUA 3'
::::::::::::::::::::::::
query 3' GAAAGAACACGACACGUCACUCAU 5'
query=miR5144-3p, target=RO3G_16142, score=0, range=353-376, stra
target 5' CUUUCUUGUGCUGUGCAGUGAGUA 3'
::::::::::::::::::::::::
query 3' GAAAGAACACGACACGUCACUCAU 5'
query=miR5144-3p, target=RO3G_05264, score=0, range=350-373, stra
target 5' CUUUCUUGUGCUGUGCAGUGAGUA 3'
::::::::::::::::::::::::
query 3' GAAAGAACACGACACGUCACUCAU 5'
query=miR5144-3p, target=RO3G_12070, score=0, range=350-373, stra
target 5' CUUUCUUGUGCUGUGCAGUGAGUA 3'
::::::::::::::::::::::::
query 3' GAAAGAACACGACACGUCACUCAU 5'
query=miR5144-3p, target=RO3G_16798, score=0, range=350-373, stra
target 5' CUUUCUUGUGCUGUGCAGUGAGUA 3'
::::::::::::::::::::::::
query 3' GAAAGAACACGACACGUCACUCAU 5'
query=miR5144-3p, target=RO3G_16796, score=0, range=350-373, stra
target 5' CUUUCUUGUGCUGUGCAGUGAGUA 3'
::::::::::::::::::::::::
query 3' GAAAGAACACGACACGUCACUCAU 5'
query=miR5144-3p, target=RO3G_04803, score=0, range=350-373, stra
target 5' CUUUCUUGUGCUGUGCAGUGAGUA 3'
::::::::::::::::::::::::
query 3' GAAAGAACACGACACGUCACUCAU 5'
query=miR5144-3p, target=RO3G_10516, score=0, range=350-373, stra
target 5' CUUUCUUGUGCUGUGCAGUGAGUA 3'
```

```
::::::::::::::::::::::::
query 3' GAAAGAACACGACACGUCACUCAU 5'
query=miR5144-3p, target=RO3G_05953, score=0.5, range=350-373,
strand=-1
target 5' CUUUUUUGUGCUGUGCAGUGAGUA 3'
::::.:::::::::::::::::::
query 3' GAAAGAACACGACACGUCACUCAU 5'
query=miR5144-3p, target=RO3G_11966, score=0.5, range=350-373,
strand=-1
target 5' CUUUUUUGUGCUGUGCAGUGAGUA 3'
::::.:::::::::::::::::::
query 3' GAAAGAACACGACACGUCACUCAU 5'
query=miR5144-3p, target=RO3G_17325, score=0.5, range=350-373,
strand=-1
target 5' CUUUUUUGUGCUGUGCAGUGAGUA 3'
::::.:::::::::::::::::::
query 3' GAAAGAACACGACACGUCACUCAU 5'
query=miR5144-3p, target=RO3G_08660, score=0.5, range=350-373,
strand=-1
target 5' UUUUCUUGUGCUGUGCAGUGAGUA 3'
.:::::::::::::::::::::::
query 3' GAAAGAACACGACACGUCACUCAU 5'
query=miR5144-3p, target=RO3G_07860, score=2, range=350-373, stra
target 5' CUUUCUUGUGCUCUGCAGUGAGUA 3'
:::::::::::: :::::::::::
query 3' GAAAGAACACGACACGUCACUCAU 5'
query=miR5144-3p, target=RO3G_16431, score=2, range=218-241, stra
target 5' CUUUCUUGUGCUGUACAGUGAGUA 3'
:::::::::::::: :::::::::
query 3' GAAAGAACACGACACGUCACUCAU 5'
query=miR5144-3p, target=RO3G_03557, score=2, range=176-199, stra
target 5' CUUUCUUGUGCUGCGCAGUGAGUA 3'
::::::::::::: ::::::::::
query 3' GAAAGAACACGACACGUCACUCAU 5'
query=miR5144-3p, target=RO3G_05959, score=2.5, range=350-373,
strand=-1
target 5' CUUUUUUGUGCUGUGCAGCGAGUA 3'
::::.::::::::::::: :::::
query 3' GAAAGAACACGACACGUCACUCAU 5'
query=miR5144-3p, target=RO3G_14568, score=2.5, range=350-373,
strand=-1
target 5' CUUUUUUGUGCUGUGCAGCGAGUA 3'
::::.::::::::::::: :::::
query 3' GAAAGAACACGACACGUCACUCAU 5'
query=miR5144-3p, target=RO3G_10798, score=2.5, range=350-373,
```

```
strand=-1
target 5' CUUUUUUGUGCUGUGCAGCGAGUA 3'
::::.:::::::::::::: :::::
query 3' GAAAGAACACGACACGUCACUCAU 5'
query=miR5144-3p, target=RO3G_16689, score=2.5, range=350-373,
strand=-1
target 5' CUUUUUUGUGCUGUGCAGCGAGUA 3'
::::.:::::::::::::: :::::
query 3' GAAAGAACACGACACGUCACUCAU 5'
query=miR5144-3p, target=RO3G_17308, score=2.5, range=350-373,
strand=-1
target 5' CUUUUUUGUGCUGUGCAGCGAGUA 3'
::::.:::::::::::::: :::::
query 3' GAAAGAACACGACACGUCACUCAU 5'
query=miR5144-3p, target=RO3G_05628, score=2.5, range=350-373,
strand=-1
target 5' CUUUUUUGUGCUGUGCAGCGAGUA 3'
::::.:::::::::::::: :::::
query 3' GAAAGAACACGACACGUCACUCAU 5'
query=miR5144-3p, target=RO3G_11238, score=2.5, range=218-241,
strand=-1
target 5' CUUUUUUGUGCUGUGCAGCGAGUA 3'
::::.:::::::::::::: :::::
query 3' GAAAGAACACGACACGUCACUCAU 5'
query=miR5144-3p, target=RO3G_07103, score=2.5, range=74-97, stra
target 5' CUUUUUUGUGCUGUGCAGCGAGUA 3'
::::.:::::::::::::: :::::
query 3' GAAAGAACACGACACGUCACUCAU 5'
query=miR894, target=RO3G_07465, score=3, range=343-362, strand=1
target 5' GAUGAACUCCGACGUGAAAC 3'
: ::::: :::::::::::
query 3' CCACUUG-GGCUGCACUUUG 5'
query=miR894, target=RO3G_04258, score=4, range=37-56, strand=1
target 5' GAUGAACUUCGACGUGAAAC 3'
: ::::: .::::::::::
query 3' CCACUUG-GGCUGCACUUUG 5'
query=miR894, target=RO3G_08009, score=4, range=208-227, strand=1
target 5' GAUGAACUUCGACGUGAAAC 3'
: ::::: .::::::::::
query 3' CCACUUG-GGCUGCACUUUG 5'
query=miR4385, target=RO3G_15671, score=0, range=1485-1506, stran
target 5' ACCAAUCCUUUUUACAACAUUA 3'
::::::::::::::::::::::
query 3' UGGUUAGGAAAAAUGUUGUAAU 5'
query=miR4385, target=RO3G_15169, score=0, range=1482-1503, stran
```

target 5' ACCAAUCCUUUUUACAACAUUA 3'
::::::::::::::::::::::
query 3' UGGUUAGGAAAAAUGUUGUAAU 5'
query=miR4385, target=RO3G_07740, score=0, range=1485-1506, stran
target 5' ACCAAUCCUUUUUACAACAUUA 3'
::::::::::::::::::::::
query 3' UGGUUAGGAAAAAUGUUGUAAU 5'
query=miR4385, target=RO3G_01208, score=0, range=1098-1119, stran
target 5' ACCAAUCCUUUUUACAACAUUA 3'
::::::::::::::::::::::
query 3' UGGUUAGGAAAAAUGUUGUAAU 5'
query=miR4385, target=RO3G_11965, score=0, range=1482-1503, stran
target 5' ACCAAUCCUUUUUACAACAUUA 3'
::::::::::::::::::::::
query 3' UGGUUAGGAAAAAUGUUGUAAU 5'
query=miR4385, target=RO3G_16746, score=0, range=1284-1305, stran
target 5' ACCAAUCCUUUUUACAACAUUA 3'
::::::::::::::::::::::
query 3' UGGUUAGGAAAAAUGUUGUAAU 5'
query=miR4385, target=RO3G_01616, score=0, range=1485-1506, stran
target 5' ACCAAUCCUUUUUACAACAUUA 3'
::::::::::::::::::::::
query 3' UGGUUAGGAAAAAUGUUGUAAU 5'
query=miR4385, target=RO3G_05411, score=0, range=57-78, strand=-1
target 5' ACCAAUCCUUUUUACAACAUUA 3'
::::::::::::::::::::::
query 3' UGGUUAGGAAAAAUGUUGUAAU 5'
query=miR4385, target=RO3G_10873, score=1, range=1485-1506, stran
target 5' ACCAAUCCUUUUUACAACGUUA 3'
::::::::::::::::::.:::
query 3' UGGUUAGGAAAAAUGUUGUAAU 5'
query=miR4385, target=RO3G_16691, score=1, range=1485-1506, stran
target 5' ACCAAUCCUUUUUACAACGUUA 3'
::::::::::::::::::.:::
query 3' UGGUUAGGAAAAAUGUUGUAAU 5'
query=miR4385, target=RO3G_16946, score=3.5, range=2304-2325, str
target 5' ACCAAUUCCUUCUACAACAUUA 3'
::::::.: :: ::::::::::
query 3' UGGUUAGGAAAAAUGUUGUAAU 5'
query=miR4385, target=RO3G_03357, score=4, range=1906-1927, stran
target 5' GAAAAUCCUUUUUAUAACAUUG 3'
. ::::::::::.::::::.
query 3' UGGUUAGGAAAAAUGUUGUAAU 5'
query=miR4385, target=RO3G_13355, score=4, range=416-438, strand=
target 5' AUCAAUCUUAUUUUACAAGAUUA 3'

```
:.:::::.: :::::::: ::::
query 3' UGGUUAGGA-AAAAUGUUGUAAU 5'
query=miR4385, target=RO3G_10498, score=4, range=1267-1289, stran
target 5' AUGCAAUCUUUUUUACAAUAUUU 3'
:. :::::.:::::::::.:::
query 3' UG-GUUAGGAAAAAUGUUGUAAU 5'
query=miR5239, target=RO3G_00944, score=1.5, range=652-670, stran
target 5' CAUCGUAUGUUUUCUCCCA 3'
::.: :::::::::::::
query 3' GUGG-AUACAAAAGAGGGU 5'
query=miR5239, target=RO3G_04853, score=2, range=923-939, strand=
target 5' CACC-AUGUUUUCUUCCA 3'
:::: :::::::::.:::
query 3' GUGGAUACAAAAGAGGGU 5'
query=miR5239, target=RO3G_04015, score=2, range=255-273, strand=
target 5' CACAAUAUGUUUUCUCCCA 3'
::: ::::::::::::::
query 3' GUG-GAUACAAAAGAGGGU 5'
query=miR5239, target=RO3G_16071, score=3, range=527-544, strand=
target 5' UAUAUAUGUUUUCUCCUA 3'
.:. ::::::::::::.:
query 3' GUGGAUACAAAAGAGGGU 5'
query=miR5239, target=RO3G_00647, score=3, range=1547-1564, stran
target 5' CACCCAUGUUGUCUCCCA 3'
:::: ::::: :::::::
query 3' GUGGAUACAAAAGAGGGU 5'
query=miR5239, target=RO3G_06416, score=3.5, range=2981-2998, str
target 5' UACCUAUGUUUUCUCGCU 3'
.:::::::::::::: :
query 3' GUGGAUACAAAAGAGGGU 5'
query=miR5239, target=RO3G_16744, score=3.5, range=2981-2998, str
target 5' UACCUAUGUUUUCUCGCU 3'
.:::::::::::::: :
query 3' GUGGAUACAAAAGAGGGU 5'
query=miR5239, target=RO3G_11275, score=3.5, range=2981-2998, str
target 5' UACCUAUGUUUUCUCGCU 3'
.:::::::::::::: :
query 3' GUGGAUACAAAAGAGGGU 5'
query=miR5239, target=RO3G_11403, score=3.5, range=2981-2998, str
target 5' UACCUAUGUUUUCUCGCU 3'
.:::::::::::::: :
query 3' GUGGAUACAAAAGAGGGU 5'
query=miR5239, target=RO3G_15869, score=3.5, range=2981-2998, str
target 5' UACCUAUGUUUUCUCGCU 3'
.:::::::::::::: :
```

```
query 3' GUGGAUACAAAAGAGGGU 5'
query=miR5239, target=RO3G_01718, score=3.5, range=2981-2998, str
target 5' UACCUAUGUUUUCUCGCU 3'
.:::::::::::::: :
query 3' GUGGAUACAAAAGAGGGU 5'
query=miR5239, target=RO3G_01203, score=3.5, range=2981-2998, str
target 5' UACCUAUGUUUUCUCGCU 3'
.:::::::::::::: :
query 3' GUGGAUACAAAAGAGGGU 5'
query=miR5239, target=RO3G_10560, score=3.5, range=2981-2998, str
target 5' UACCUAUGUUUUCUCGCU 3'
.:::::::::::::: :
query 3' GUGGAUACAAAAGAGGGU 5'
query=miR5239, target=RO3G_10555, score=3.5, range=2822-2839, str
target 5' UACCUAUGUUUUCUCGCU 3'
.:::::::::::::: :
query 3' GUGGAUACAAAAGAGGGU 5'
query=miR5239, target=RO3G_03103, score=3.5, range=2822-2839, str
target 5' UACCUAUGUUUUCUCGCU 3'
.:::::::::::::: :
query 3' GUGGAUACAAAAGAGGGU 5'
query=miR5239, target=RO3G_12979, score=3.5, range=2822-2839, str
target 5' UACCUAUGUUUUCUCGCU 3'
.:::::::::::::: :
query 3' GUGGAUACAAAAGAGGGU 5'
query=miR5239, target=RO3G_08612, score=3.5, range=2822-2839, str
target 5' UACCUAUGUUUUCUCGCU 3'
.:::::::::::::: :
query 3' GUGGAUACAAAAGAGGGU 5'
query=miR5239, target=RO3G_12087, score=3.5, range=2822-2839, str
target 5' UACCUAUGUUUUCUCGCU 3'
.:::::::::::::: :
query 3' GUGGAUACAAAAGAGGGU 5'
query=miR5239, target=RO3G_07305, score=3.5, range=2822-2839, str
target 5' UACCUAUGUUUUCUCGCU 3'
.:::::::::::::: :
query 3' GUGGAUACAAAAGAGGGU 5'
query=miR5239, target=RO3G_05280, score=3.5, range=2822-2839, str
target 5' UACCUAUGUUUUCUCGCU 3'
.:::::::::::::: :
query 3' GUGGAUACAAAAGAGGGU 5'
query=miR5239, target=RO3G_16686, score=3.5, range=2822-2839, str
target 5' UACCUAUGUUUUCUCGCU 3'
.:::::::::::::: :
query 3' GUGGAUACAAAAGAGGGU 5'
```

```
query=miR5239, target=RO3G_14704, score=3.5, range=2822-2839, str
target 5' UACCUAUGUUUUCUCGCU 3'
.::::::::::::::: :
query 3' GUGGAUACAAAAGAGGGU 5'
query=miR5239, target=RO3G_05630, score=3.5, range=2741-2758, str
target 5' UACCUAUGUUUUCUCGCU 3'
.::::::::::::::: :
query 3' GUGGAUACAAAAGAGGGU 5'
query=miR5239, target=RO3G_13450, score=3.5, range=2639-2656, str
target 5' UACCUAUGUUUUCUCGCU 3'
.::::::::::::::: :
query 3' GUGGAUACAAAAGAGGGU 5'
query=miR5239, target=RO3G_13859, score=3.5, range=2606-2623, str
target 5' UACCUAUGUUUUCUCGCU 3'
.::::::::::::::: :
query 3' GUGGAUACAAAAGAGGGU 5'
query=miR5239, target=RO3G_11970, score=3.5, range=2522-2539, str
target 5' UACCUAUGUUUUCUCGCU 3'
.::::::::::::::: :
query 3' GUGGAUACAAAAGAGGGU 5'
query=miR5239, target=RO3G_02235, score=3.5, range=2165-2182, str
target 5' UACCUAUGUUUUCUCGCU 3'
.::::::::::::::: :
query 3' GUGGAUACAAAAGAGGGU 5'
query=miR5239, target=RO3G_13152, score=3.5, range=2822-2839, str
target 5' UACCUAUGUUUUCUCGCU 3'
.::::::::::::::: :
query 3' GUGGAUACAAAAGAGGGU 5'
query=miR5239, target=RO3G_03988, score=3.5, range=2822-2839, str
target 5' UACCUAUGUUUUCUCGCU 3'
.::::::::::::::: :
query 3' GUGGAUACAAAAGAGGGU 5'
query=miR5239, target=RO3G_12951, score=3.5, range=2822-2839, str
target 5' UACCUAUGUUUUCUCGCU 3'
.::::::::::::::: :
query 3' GUGGAUACAAAAGAGGGU 5'
query=miR5239, target=RO3G_09376, score=3.5, range=2860-2877, str
target 5' UACCUAUGUUUUCUCGCU 3'
.::::::::::::::: :
query 3' GUGGAUACAAAAGAGGGU 5'
query=miR5239, target=RO3G_08968, score=3.5, range=536-553, stran
target 5' UACCUAUGUUUUCUCGCU 3'
.::::::::::::::: :
query 3' GUGGAUACAAAAGAGGGU 5'
query=miR5239, target=RO3G_11785, score=3.5, range=1142-1159, str
```

target 5' UACCUAUGUUUUCUCGCU 3'
.::::::::::::: :
query 3' GUGGAUACAAAAGAGGGU 5'
query=miR5239, target=RO3G_05561, score=3.5, range=60-76, strand=
target 5' UACC-GUGUUUUCUUCCA 3'
.::: .::::::::.:::
query 3' GUGGAUACAAAAGAGGGU 5'
query=miR5239, target=RO3G_01932, score=3.5, range=156-173, stran
target 5' UCCCAAUGUUUUCUCCUA 3'
. :: :::::::::::.:
query 3' GUGGAUACAAAAGAGGGU 5'
query=miR5239, target=RO3G_04844, score=3.5, range=599-616, stran
target 5' CAUCUAUGUUUUCUUCUU 3'
::.:::::::::::.:.
query 3' GUGGAUACAAAAGAGGGU 5'
query=miR5239, target=RO3G_15427, score=3.5, range=502-519, stran
target 5' CACUUGUGUUUUCUCCUC 3'
:::.:.::::::::::.
query 3' GUGGAUACAAAAGAGGGU 5'
query=miR5239, target=RO3G_02549, score=3.5, range=482-499, stran
target 5' AAUCUAUGUUUUCUGCCA 3'
:.::::::::::: :::
query 3' GUGGAUACAAAAGAGGGU 5'
query=miR5239, target=RO3G_09692, score=3.5, range=1322-1339,
strand=-1
target 5' CAUCCAUGUUUUCUUCCU 3'
::.: ::::::::::.::
query 3' GUGGAUACAAAAGAGGGU 5'
query=miR5239, target=RO3G_10337, score=3.5, range=287-303, stran
target 5' CAUCU-UGUUUUCUCUCA 3'
::.:: :::::::::.::
query 3' GUGGAUACAAAAGAGGGU 5'
query=miR5239, target=RO3G_08982, score=3.5, range=1151-1167,
strand=-1
target 5' UAUCU-UGUUUUCUCCCG 3'
.:.:: :::::::::::.
query 3' GUGGAUACAAAAGAGGGU 5'
query=miR5239, target=RO3G_07205, score=3.5, range=457-474, stran
target 5' CAUGUCUGUUUUCUCCCA 3'
::. : :::::::::::
query 3' GUGGAUACAAAAGAGGGU 5'
query=miR5239, target=RO3G_15947, score=4, range=887-904, strand=
target 5' UGCCUAUGUUUGCUCCUA 3'
..::::::::: ::::.:
query 3' GUGGAUACAAAAGAGGGU 5'

```
query=miR5239, target=RO3G_06471, score=4, range=186-203, strand=
target 5' CAUUGGUGUUUUCUCCCU 3'
::.. .:::::::::::
query 3' GUGGAUACAAAAGAGGGU 5'
query=miR5239, target=RO3G_08009, score=4, range=1931-1949, stran
target 5' CACAUAUGGUUUUUUCCCA 3'
::: ::: :::::.:::::
query 3' GUGGAUA-CAAAAGAGGGU 5'
query=miR5239, target=RO3G_13227, score=4, range=122-140, strand=
target 5' CAACUGUGUUUAUCUCCCG 3'
:: ::.::::: ::::::.
query 3' GUGGAUACAAA-AGAGGGU 5'
query=miR5239, target=RO3G_15763, score=4, range=1749-1765, stran
target 5' CAACUAUG-UUUCUUCCA 3'
:: ::::: :::::.:::
query 3' GUGGAUACAAAAGAGGGU 5'
query=miR5239, target=RO3G_12102, score=4, range=1386-1402, stran
target 5' CACCU-UGUUUGCUCCCA 3'
::::: ::::: ::::::
query 3' GUGGAUACAAAAGAGGGU 5'
query=miR5239, target=RO3G_13417, score=4, range=78-94, strand=1
target 5' CAAUUAU-UUUUCUCCCG 3'
:: .::: :::::::::.
query 3' GUGGAUACAAAAGAGGGU 5'
query=miR5239, target=RO3G_06179, score=4, range=149-166, strand=
target 5' UGCCUAUGUUUUCUUCUC 3'
..::::::::::::.:.
query 3' GUGGAUACAAAAGAGGGU 5'
query=miR5239, target=RO3G_08339, score=4, range=1322-1339, stran
target 5' AGUCCAUGUUUUCUUCCA 3'
..: :::::::::.:::
query 3' GUGGAUACAAAAGAGGGU 5'
query=miR5239, target=RO3G_08916, score=4, range=1991-2008, stran
target 5' CACCAAUGUCUUCUCUCA 3'
:::: :::: :::::.::
query 3' GUGGAUACAAAAGAGGGU 5'
query=miR5239, target=RO3G_07932, score=4, range=257-274, strand=
target 5' CACCAAUCUUUUCUUCCA 3'
:::: :: ::::::.:::
query 3' GUGGAUACAAAAGAGGGU 5'
query=miR5239, target=RO3G_12042, score=4, range=129-145, strand=
target 5' CGCC-AUGUUUUCUUUCG 3'
:.:: :::::::::..:.
query 3' GUGGAUACAAAAGAGGGU 5'
query=miR5239, target=RO3G_12083, score=4, range=407-424, strand=
```

```
target 5' CAUCGAUGUUUUCUCCAG 3'
::.: ::::::::::: .
query 3' GUGGAUACAAAAGAGGGU 5'
query=miR5239, target=RO3G_04976, score=4, range=491-509, strand=
target 5' CGCUUUUGUUUUCUCCACA 3'
:.:.: :::::::::: ::
query 3' GUGGAUACAAAAGAGG-GU 5'
query=miR5239, target=RO3G_14378, score=4, range=2288-2305, stran
target 5' CCCUCAUGUUUUCUUCCG 3'
: :. :::::::::.::.
query 3' GUGGAUACAAAAGAGGGU 5'
query=miR5239, target=RO3G_13112, score=4, range=1493-1510, stran
target 5' UUUCAAUGUUUUCUUCCA 3'
. .: :::::::::.:::
query 3' GUGGAUACAAAAGAGGGU 5'
query=miR5239, target=RO3G_06194, score=4, range=1364-1380, stran
target 5' CACC-AUGUAUUUUCCCA 3'
:::: :::: ::.:::::
query 3' GUGGAUACAAAAGAGGGU 5'
query=miR5239, target=RO3G_02877, score=4, range=105-121, strand=
target 5' CACC-AUGUUUACUCUCA 3'
:::: :::::: :::.::
query 3' GUGGAUACAAAAGAGGGU 5'
query=miR5239, target=RO3G_12241, score=4, range=376-394, strand=
target 5' CUCCAUAUGUUUUCUCUCC 3'
: :: :::::::::::.:
query 3' GUGG-AUACAAAAGAGGGU 5'
query=miR1076-5p, target=RO3G_14532, score=0, range=1807-1828,
strand=1
target 5' ACAAUUAGUCGCGCUUGUGAUA 3'
::::::::::::::::::::::
query 3' UGUUAAUCAGCGCGAACACUAU 5'
query=miR1076-5p, target=RO3G_14210, score=0, range=1200-1221,
strand=1
target 5' ACAAUUAGUCGCGCUUGUGAUA 3'
::::::::::::::::::::::
query 3' UGUUAAUCAGCGCGAACACUAU 5'
query=miR845a, target=RO3G_15031, score=4, range=2663-2682, stran
target 5' AUCAAUUGGUGUCAGGGCAG 3'
::::::::::.::::.:: :
query 3' UAGUUAACCAUAGUCUCGGC 5'
query=miR5252, target=RO3G_12976, score=0, range=220-241, strand=
target 5' GCAGACUUCAGUAGCUUUACAA 3'
::::::::::::::::::::::
query 3' CGUCUGAAGUCAUCGAAAUGUU 5'
```

```
query=miR5252, target=RO3G_01717, score=0.5, range=220-241, stran
target 5' GCAGGCUUCAGUAGCUUUACAA 3'
::::.:::::::::::::::::
query 3' CGUCUGAAGUCAUCGAAAUGUU 5'
query=miR5252, target=RO3G_12011, score=0.5, range=217-238, stran
target 5' GCAGGCUUCAGUAGCUUUACAA 3'
::::.:::::::::::::::::
query 3' CGUCUGAAGUCAUCGAAAUGUU 5'
query=miR5252, target=RO3G_02817, score=0.5, range=220-241, stran
target 5' GCAGGCUUCAGUAGCUUUACAA 3'
::::.:::::::::::::::::
query 3' CGUCUGAAGUCAUCGAAAUGUU 5'
query=miR5252, target=RO3G_17433, score=0.5, range=214-235, stran
target 5' GCAGGCUUCAGUAGCUUUACAA 3'
::::.:::::::::::::::::
query 3' CGUCUGAAGUCAUCGAAAUGUU 5'
query=miR5252, target=RO3G_16743, score=0.5, range=163-184, stran
target 5' GCAGGCUUCAGUAGCUUUACAA 3'
::::.:::::::::::::::::
query 3' CGUCUGAAGUCAUCGAAAUGUU 5'
query=miR5252, target=RO3G_07692, score=0.5, range=151-172, stran
target 5' GCAGGCUUCAGUAGCUUUACAA 3'
::::.:::::::::::::::::
query 3' CGUCUGAAGUCAUCGAAAUGUU 5'
query=miR5252, target=RO3G_04659, score=0.5, range=151-172, stran
target 5' GCAGGCUUCAGUAGCUUUACAA 3'
::::.:::::::::::::::::
query 3' CGUCUGAAGUCAUCGAAAUGUU 5'
query=miR5252, target=RO3G_10043, score=0.5, range=211-232, stran
target 5' GCAGGCUUCAGUAGCUUUACAA 3'
::::.:::::::::::::::::
query 3' CGUCUGAAGUCAUCGAAAUGUU 5'
query=miR5252, target=RO3G_17460, score=0.5, range=241-262, stran
target 5' GCAGGCUUCAGUAGCUUUACAA 3'
::::.:::::::::::::::::
query 3' CGUCUGAAGUCAUCGAAAUGUU 5'
query=miR5252, target=RO3G_13452, score=0.5, range=193-214, stran
target 5' GCAGGCUUCAGUAGCUUUACAA 3'
::::.:::::::::::::::::
query 3' CGUCUGAAGUCAUCGAAAUGUU 5'
query=miR5252, target=RO3G_16369, score=0.5, range=196-217, stran
target 5' GCAGGCUUCAGUAGCUUUACAA 3'
::::.:::::::::::::::::
query 3' CGUCUGAAGUCAUCGAAAUGUU 5'
query=miR5252, target=RO3G_11543, score=0.5, range=217-238, stran
```

target 5' GCAGGCUUCAGUAGCUUUACAA 3'
::::.:::::::::::::::::
query 3' CGUCUGAAGUCAUCGAAAUGUU 5'
query=miR5252, target=RO3G_14553, score=0.5, range=217-238, stran
target 5' GCAGGCUUCAGUAGCUUUACAA 3'
::::.:::::::::::::::::
query 3' CGUCUGAAGUCAUCGAAAUGUU 5'
query=miR5252, target=RO3G_15887, score=0.5, range=241-262, stran
target 5' GCAGGCUUCAGUAGCUUUACAA 3'
::::.:::::::::::::::::
query 3' CGUCUGAAGUCAUCGAAAUGUU 5'
query=miR5252, target=RO3G_01192, score=0.5, range=154-175, stran
target 5' GCAGGCUUCAGUAGCUUUACAA 3'
::::.:::::::::::::::::
query 3' CGUCUGAAGUCAUCGAAAUGUU 5'
query=miR5252, target=RO3G_17265, score=0.5, range=118-139, stran
target 5' GCAGGCUUCAGUAGCUUUACAA 3'
::::.:::::::::::::::::
query 3' CGUCUGAAGUCAUCGAAAUGUU 5'
query=miR5252, target=RO3G_05906, score=0.5, range=154-175, stran
target 5' GCAGGCUUCAGUAGCUUUACAA 3'
::::.:::::::::::::::::
query 3' CGUCUGAAGUCAUCGAAAUGUU 5'
query=miR5252, target=RO3G_05347, score=0.5, range=217-238, stran
target 5' GCAGGCUUCAGUAGCUUUACAA 3'
::::.:::::::::::::::::
query 3' CGUCUGAAGUCAUCGAAAUGUU 5'
query=miR5252, target=RO3G_17210, score=0.5, range=241-262, stran
target 5' GCAGGCUUCAGUAGCUUUACAA 3'
::::.:::::::::::::::::
query 3' CGUCUGAAGUCAUCGAAAUGUU 5'
query=miR5252, target=RO3G_15879, score=2.5, range=217-239, stran
target 5' GCAGGCUUCAGUAGGCUUUACAA 3'
::::.:::::::: :::::::::
query 3' CGUCUGAAGUCAU-CGAAAUGUU 5'
query=miR5252, target=RO3G_17244, score=2.5, range=214-235, stran
target 5' GCAGGCUUCAGUAACUUUACAA 3'
::::.:::::::: ::::::::
query 3' CGUCUGAAGUCAUCGAAAUGUU 5'
query=miR5252, target=RO3G_16901, score=3.5, range=211-232, stran
target 5' GCAGGAUUCAGUAGCUUCACAA 3'
::::. ::::::::::: ::::
query 3' CGUCUGAAGUCAUCGAAAUGUU 5'
query=miR5252, target=RO3G_16917, score=3.5, range=217-238, stran
target 5' GCAGGAUUCAGUAGCUUCACAA 3'

```
::::. :::::::::: ::::
query 3' CGUCUGAAGUCAUCGAAAUGUU 5'
query=miR1108, target=RO3G_10822, score=0, range=1093-1113, stran
target 5' GGGCGUUAUGUUUCCAGCUUA 3'
:::::::::::::::::::::
query 3' CCCGCAAUACAAAGGUCGAAU 5'
query=miR1108, target=RO3G_13723, score=1.5, range=1039-1059, str
target 5' GGAUGUUAUGUUUCCAGCUUA 3'
:: .:::::::::::::::::
query 3' CCCGCAAUACAAAGGUCGAAU 5'
query=miR1108, target=RO3G_16906, score=1.5, range=208-228, stran
target 5' GGAUGUUAUGUUUCCAGCUUA 3'
:: .:::::::::::::::::
query 3' CCCGCAAUACAAAGGUCGAAU 5'
query=miR1108, target=RO3G_01682, score=2.5, range=985-1005, stra
target 5' GGGUGUUAUGUUUCCAACUUA 3'
:::.::::::::::::: ::::
query 3' CCCGCAAUACAAAGGUCGAAU 5'
query=miR164b, target=RO3G_14967, score=3.5, range=915-934, stran
target 5' GUACGUCCUCUGCUUCUCCU 3'
:.:::: :.:::::::::
query 3' CGUGCACGGGACGAAGAGGU 5'
query=miR164b, target=RO3G_07776, score=4, range=264-282, strand=
target 5' GAACGU-CCCUGCUUCUCUU 3'
: :::: ::::::::::.
query 3' CGUGCACGGGACGAAGAGGU 5'
query=miR164b, target=RO3G_08297, score=4, range=537-555, strand=
target 5' GC-CAUCCCCUGCUUCUUCA 3'
:: : : ::::::::::.::
query 3' CGUGCACGGGACGAAGAGGU 5'
query=miR5658, target=RO3G_16744, score=0, range=3642-3663, stran
target 5' UUCUGCAACAUCAUCAUCAUCA 3'
::::::::::::::::::::::
query 3' AAGACGUUGUAGUAGUAGUAGU 5'
query=miR5658, target=RO3G_01203, score=0, range=3642-3663, stran
target 5' UUCUGCAACAUCAUCAUCAUCA 3'
::::::::::::::::::::::
query 3' AAGACGUUGUAGUAGUAGUAGU 5'
query=miR5658, target=RO3G_11403, score=0, range=3642-3663, stran
target 5' UUCUGCAACAUCAUCAUCAUCA 3'
::::::::::::::::::::::
query 3' AAGACGUUGUAGUAGUAGUAGU 5'
query=miR5658, target=RO3G_06416, score=0, range=3642-3663, stran
target 5' UUCUGCAACAUCAUCAUCAUCA 3'
::::::::::::::::::::::
```

```
query 3' AAGACGUUGUAGUAGUAGUAGU 5'
query=miR5658, target=RO3G_01718, score=0, range=3642-3663, stran
target 5' UUCUGCAACAUCAUCAUCAUCA 3'
::::::::::::::::::::::
query 3' AAGACGUUGUAGUAGUAGUAGU 5'
query=miR5658, target=RO3G_15869, score=0, range=3642-3663, stran
target 5' UUCUGCAACAUCAUCAUCAUCA 3'
::::::::::::::::::::::
query 3' AAGACGUUGUAGUAGUAGUAGU 5'
query=miR5658, target=RO3G_10560, score=0, range=3642-3663, stran
target 5' UUCUGCAACAUCAUCAUCAUCA 3'
::::::::::::::::::::::
query 3' AAGACGUUGUAGUAGUAGUAGU 5'
query=miR5658, target=RO3G_11275, score=0, range=3642-3663, stran
target 5' UUCUGCAACAUCAUCAUCAUCA 3'
::::::::::::::::::::::
query 3' AAGACGUUGUAGUAGUAGUAGU 5'
query=miR5658, target=RO3G_14704, score=0, range=3483-3504, stran
target 5' UUCUGCAACAUCAUCAUCAUCA 3'
::::::::::::::::::::::
query 3' AAGACGUUGUAGUAGUAGUAGU 5'
query=miR5658, target=RO3G_12979, score=0, range=3483-3504, stran
target 5' UUCUGCAACAUCAUCAUCAUCA 3'
::::::::::::::::::::::
query 3' AAGACGUUGUAGUAGUAGUAGU 5'
query=miR5658, target=RO3G_10555, score=0, range=3483-3504, stran
target 5' UUCUGCAACAUCAUCAUCAUCA 3'
::::::::::::::::::::::
query 3' AAGACGUUGUAGUAGUAGUAGU 5'
query=miR5658, target=RO3G_05280, score=0, range=3483-3504, stran
target 5' UUCUGCAACAUCAUCAUCAUCA 3'
::::::::::::::::::::::
query 3' AAGACGUUGUAGUAGUAGUAGU 5'
query=miR5658, target=RO3G_16686, score=0, range=3483-3504, stran
target 5' UUCUGCAACAUCAUCAUCAUCA 3'
::::::::::::::::::::::
query 3' AAGACGUUGUAGUAGUAGUAGU 5'
query=miR5658, target=RO3G_07305, score=0, range=3483-3504, stran
target 5' UUCUGCAACAUCAUCAUCAUCA 3'
::::::::::::::::::::::
query 3' AAGACGUUGUAGUAGUAGUAGU 5'
query=miR5658, target=RO3G_03103, score=0, range=3483-3504, stran
target 5' UUCUGCAACAUCAUCAUCAUCA 3'
::::::::::::::::::::::
query 3' AAGACGUUGUAGUAGUAGUAGU 5'
```

```
query=miR5658, target=RO3G_12087, score=0, range=3483-3504, stran
target 5' UUCUGCAACAUCAUCAUCAUCA 3'
::::::::::::::::::::::
query 3' AAGACGUUGUAGUAGUAGUAGU 5'
query=miR5658, target=RO3G_08612, score=0, range=3483-3504, stran
target 5' UUCUGCAACAUCAUCAUCAUCA 3'
::::::::::::::::::::::
query 3' AAGACGUUGUAGUAGUAGUAGU 5'
query=miR5658, target=RO3G_05630, score=0, range=3402-3423, stran
target 5' UUCUGCAACAUCAUCAUCAUCA 3'
::::::::::::::::::::::
query 3' AAGACGUUGUAGUAGUAGUAGU 5'
query=miR5658, target=RO3G_13450, score=0, range=3300-3321, stran
target 5' UUCUGCAACAUCAUCAUCAUCA 3'
::::::::::::::::::::::
query 3' AAGACGUUGUAGUAGUAGUAGU 5'
query=miR5658, target=RO3G_13859, score=0, range=3267-3288, stran
target 5' UUCUGCAACAUCAUCAUCAUCA 3'
::::::::::::::::::::::
query 3' AAGACGUUGUAGUAGUAGUAGU 5'
query=miR5658, target=RO3G_02235, score=0, range=2826-2847, stran
target 5' UUCUGCAACAUCAUCAUCAUCA 3'
::::::::::::::::::::::
query 3' AAGACGUUGUAGUAGUAGUAGU 5'
query=miR5658, target=RO3G_13152, score=0, range=3483-3504, stran
target 5' UUCUGCAACAUCAUCAUCAUCA 3'
::::::::::::::::::::::
query 3' AAGACGUUGUAGUAGUAGUAGU 5'
query=miR5658, target=RO3G_12193, score=0, range=489-510, strand=
target 5' UUCUGCAACAUCAUCAUCAUCA 3'
::::::::::::::::::::::
query 3' AAGACGUUGUAGUAGUAGUAGU 5'
query=miR5658, target=RO3G_03990, score=0, range=240-261, strand=
target 5' UUCUGCAACAUCAUCAUCAUCA 3'
::::::::::::::::::::::
query 3' AAGACGUUGUAGUAGUAGUAGU 5'
query=miR5658, target=RO3G_10467, score=2, range=180-201, strand=
target 5' UUCUUCAUCAUCAUCAUCAUCA 3'
:::: :: ::::::::::::::
query 3' AAGACGUUGUAGUAGUAGUAGU 5'
query=miR5658, target=RO3G_15427, score=2, range=633-654, strand=
target 5' UUCUUCAUCAUCAUCAUCAUCA 3'
:::: :: ::::::::::::::
query 3' AAGACGUUGUAGUAGUAGUAGU 5'
query=miR5658, target=RO3G_14198, score=2.5, range=29-50, strand=
```

```
target 5' UUUACCAACAUCAUCAUCAUCA 3'
::. :::::::::::::::::
query 3' AAGACGUUGUAGUAGUAGUAGU 5'
query=miR5658, target=RO3G_16068, score=2.5, range=1575-1596,
strand=-1
target 5' UUCUUCAAUAUCAUCGUCAUCA 3'
:::: :::.:::::::.::::::
query 3' AAGACGUUGUAGUAGUAGUAGU 5'
query=miR5658, target=RO3G_16449, score=2.5, range=381-402, stran
target 5' UUCGGCAGCAUUAUCAUCAUCA 3'
::: :::.:::.::::::::::
query 3' AAGACGUUGUAGUAGUAGUAGU 5'
query=miR5658, target=RO3G_03951, score=2.5, range=675-696, stran
target 5' UUCUUCAUUAUCAUCAUCAUCA 3'
:::: :: .::::::::::::
query 3' AAGACGUUGUAGUAGUAGUAGU 5'
query=miR5658, target=RO3G_15960, score=3, range=1604-1625, stran
target 5' AACAGCAACAUCAUCAUCAUCA 3'
: ::::::::::::::::::
query 3' AAGACGUUGUAGUAGUAGUAGU 5'
query=miR5658, target=RO3G_16962, score=3, range=389-410, strand=
target 5' UUGUCCAUCAUCAUCAUCAUCA 3'
:: : :: :::::::::::::
query 3' AAGACGUUGUAGUAGUAGUAGU 5'
query=miR5658, target=RO3G_14668, score=3, range=3063-3084, stran
target 5' UUCUACAUCAUCAUCGUCAUCA 3'
:::: :: :::::::.::::::
query 3' AAGACGUUGUAGUAGUAGUAGU 5'
query=miR5658, target=RO3G_03214, score=3, range=810-831, strand=
target 5' UUCUUCAUCGUCAUCAUCAUCA 3'
:::: :: :.:::::::::::
query 3' AAGACGUUGUAGUAGUAGUAGU 5'
query=miR5658, target=RO3G_08049, score=3, range=474-495, strand=
target 5' UUCUUCAUCAUCAUCAUCGUCA 3'
:::: :: ::::::::::.:::
query 3' AAGACGUUGUAGUAGUAGUAGU 5'
query=miR5658, target=RO3G_11363, score=3, range=981-1002, strand
target 5' UUCCAUAGCAUCAUCAUCAUCA 3'
::: .:.::::::::::::::
query 3' AAGACGUUGUAGUAGUAGUAGU 5'
query=miR5658, target=RO3G_02468, score=3, range=102-123, strand=
target 5' UUCUUCAAGAUCGUCAUCAUCA 3'
:::: ::: :::.:::::::::
query 3' AAGACGUUGUAGUAGUAGUAGU 5'
query=miR5658, target=RO3G_06266, score=3, range=1425-1446, stran
```

target 5' UUCUUCAUCAUCAUCAUCAUCU 3'
:::: :: :::::::::::::
query 3' AAGACGUUGUAGUAGUAGUAGU 5'
query=miR5658, target=RO3G_13668, score=3, range=216-237, strand=
target 5' AUCAGCAUCAUCAUCAUCAUCA 3'
:: ::: ::::::::::::::
query 3' AAGACGUUGUAGUAGUAGUAGU 5'
query=miR5658, target=RO3G_00068, score=3, range=933-954, strand=
target 5' UUCAUCAUCAUCAUCAUCAUCA 3'
::: :: ::::::::::::::
query 3' AAGACGUUGUAGUAGUAGUAGU 5'
query=miR5658, target=RO3G_05239, score=3, range=102-122, strand=
target 5' UUAU-CAUCAUCAUCAUCAUCA 3'
:: : :: ::::::::::::::
query 3' AAGACGUUGUAGUAGUAGUAGU 5'
query=miR5658, target=RO3G_01410, score=3.5, range=438-459, stran
target 5' AUCUUUAUCAUCAUCAUCAUCA 3'
::: .: ::::::::::::::
query 3' AAGACGUUGUAGUAGUAGUAGU 5'
query=miR5658, target=RO3G_12605, score=3.5, range=111-132, stran
target 5' UUCUUUAGCAUUAUCAUCAUCG 3'
:::: .:.:::.:::::::::.
query 3' AAGACGUUGUAGUAGUAGUAGU 5'
query=miR5658, target=RO3G_14141, score=3.5, range=528-549, stran
target 5' AUGUGUAACAUUAUCAUCAUCA 3'
: ::.::::.:::::::::::
query 3' AAGACGUUGUAGUAGUAGUAGU 5'
query=miR5658, target=RO3G_06694, score=3.5, range=645-666, stran
target 5' UUUUGCAUCAUCAUCACCAUCA 3'
::.:::: :::::::: :::::
query 3' AAGACGUUGUAGUAGUAGUAGU 5'
query=miR5658, target=RO3G_14413, score=3.5, range=1110-1131,
strand=-1
target 5' UUCUUCAUCAUCGUCAUCAUCG 3'
:::: :: ::::.::::::::.
query 3' AAGACGUUGUAGUAGUAGUAGU 5'
query=miR5658, target=RO3G_16322, score=3.5, range=1824-1845,
strand=-1
target 5' UUCAAUGAUAUCAUCAUCAUCA 3'
::: ..:.:::::::::::::
query 3' AAGACGUUGUAGUAGUAGUAGU 5'
query=miR5658, target=RO3G_12515, score=3.5, range=189-210, stran
target 5' CUCUUCAAUAUCAUCAUCAUCC 3'
::: :::.::::::::::::
query 3' AAGACGUUGUAGUAGUAGUAGU 5'

query=miR5658, target=RO3G_04817, score=3.5, range=2901-2922,
strand=-1
target 5' UUCGUCAGCAUCAUCAUCAUCU 3'
::: ::.:::::::::::::
query 3' AAGACGUUGUAGUAGUAGUAGU 5'
query=miR5658, target=RO3G_14543, score=3.5, range=1563-1584,
strand=-1
target 5' CUCUUUAUCAUCAUCAUCAUCA 3'
::: .: ::::::::::::::
query 3' AAGACGUUGUAGUAGUAGUAGU 5'
query=miR5658, target=RO3G_13781, score=3.5, range=210-232, stran
target 5' AUUUGUCAUCAUCAUCAUCAUCA 3'
:.:: :: ::::::::::::::
query 3' AAGAC-GUUGUAGUAGUAGUAGU 5'
query=miR5658, target=RO3G_03274, score=3.5, range=840-860, stran
target 5' AUCU-CAUCAUCAUCAUCAUCG 3'
::: :: :::::::::::::.
query 3' AAGACGUUGUAGUAGUAGUAGU 5'
query=miR5658, target=RO3G_08260, score=3.5, range=330-350, stran
target 5' UCCUGU-UCAUCAUCAUCAUCA 3'
: :::. ::::::::::::::
query 3' AAGACGUUGUAGUAGUAGUAGU 5'
query=miR5658, target=RO3G_16243, score=3.5, range=1164-1184,
strand=-1
target 5' AUUUGCAA-AUCAUCAUUAUCA 3'
:.:::: :::::::::.::::
query 3' AAGACGUUGUAGUAGUAGUAGU 5'
query=miR5658, target=RO3G_07768, score=4, range=861-881, strand=
target 5' UUCU-CAAAAUCAUUAUCAUUA 3'
:::: ::: :::::.:::::.:
query 3' AAGACGUUGUAGUAGUAGUAGU 5'
query=miR5658, target=RO3G_06536, score=4, range=395-416, strand=
target 5' AACAACAACAUCAUCAUCAUCA 3'
: :::::::::::::::::
query 3' AAGACGUUGUAGUAGUAGUAGU 5'
query=miR5658, target=RO3G_07545, score=4, range=444-465, strand=
target 5' ACCUGCUCCAUCAUCAUCAUCA 3'
:::: ::::::::::::::
query 3' AAGACGUUGUAGUAGUAGUAGU 5'
query=miR5658, target=RO3G_09577, score=4, range=261-282, strand=
target 5' CGCUGAAUCAUCAUCAUCAUCA 3'
::: : ::::::::::::::
query 3' AAGACGUUGUAGUAGUAGUAGU 5'
query=miR5658, target=RO3G_03075, score=4, range=1070-1091, stran
target 5' AUCAUCACCAUCAUCAUCAUCA 3'

```
:: :: ::::::::::::::
query 3' AAGACGUUGUAGUAGUAGUAGU 5'
query=miR5658, target=RO3G_03827, score=4, range=1064-1085, stran
target 5' AUCAUCAUCAUCAUCAUCAUCA 3'
:: :: ::::::::::::::
query 3' AAGACGUUGUAGUAGUAGUAGU 5'
query=miR5658, target=RO3G_13341, score=4, range=606-626, strand=
target 5' AUCUUC-ACGUCAUCAUCAUCA 3'
::: : ::.:::::::::::
query 3' AAGACGUUGUAGUAGUAGUAGU 5'
query=miR5658, target=RO3G_00729, score=4, range=1058-1079, stran
target 5' AUCAUCAUCAUCAUCAUCAUCA 3'
:: :: ::::::::::::::
query 3' AAGACGUUGUAGUAGUAGUAGU 5'
query=miR5658, target=RO3G_03011, score=4, range=380-401, strand=
target 5' GUCGUCAUCAUCAUCAUCAUCA 3'
:: :: ::::::::::::::
query 3' AAGACGUUGUAGUAGUAGUAGU 5'
query=miR5658, target=RO3G_06138, score=4, range=612-634, strand=
target 5' AUCAGCAUCUAUCAUCAUCAUCA 3'
:: ::: : :::::::::::::
query 3' AAGACGUUG-UAGUAGUAGUAGU 5'
query=miR5658, target=RO3G_00266, score=4, range=1206-1226, stran
target 5' CCCU-CAUCAUCAUCAUCAUCA 3'
:: :: ::::::::::::::
query 3' AAGACGUUGUAGUAGUAGUAGU 5'
query=miR5658, target=RO3G_09516, score=4, range=242-262, strand=
target 5' UUAU-UAUUAUCAUCAUCAUCA 3'
:: : .: .:::::::::::::
query 3' AAGACGUUGUAGUAGUAGUAGU 5'
query=miR5658, target=RO3G_09649, score=4, range=586-606, strand=
target 5' CUUUGUAA-AUCAUCAUCAUCC 3'
:.::.:: ::::::::::::
query 3' AAGACGUUGUAGUAGUAGUAGU 5'
query=miR5658, target=RO3G_14098, score=4, range=1632-1653, stran
target 5' UUCUGCAACGUCAUCUUCAUCU 3'
::::::::.::::: :::::
query 3' AAGACGUUGUAGUAGUAGUAGU 5'
query=miR5658, target=RO3G_06174, score=4, range=1053-1074, stran
target 5' UUCUGCAUCGUCAUCGUCAUCC 3'
::::::: :.:::::.:::::
query 3' AAGACGUUGUAGUAGUAGUAGU 5'
query=miR5658, target=RO3G_14654, score=4, range=543-564, strand=
target 5' AUCUGCAACAUCAUUAGCAUCA 3'
:::::::::::::.: :::::
```

query 3' AAGACGUUGUAGUAGUAGUAGU 5'
query=miR5658, target=RO3G_15062, score=4, range=420-441, strand=
target 5' UUCUUCUACAUUAUCGUCAUCA 3'
:::: : ::::.:::.::::::
query 3' AAGACGUUGUAGUAGUAGUAGU 5'
query=miR5658, target=RO3G_17136, score=4, range=909-930, strand=
target 5' UUGUGAGAUAUCAUCAUUAUCA 3'
:: :: .:.::::::::.::::
query 3' AAGACGUUGUAGUAGUAGUAGU 5'
query=miR5658, target=RO3G_04521, score=4, range=186-207, strand=
target 5' AACUGCAUCGUCAUCAUCAUCA 3'
::::: :.::::::::::::
query 3' AAGACGUUGUAGUAGUAGUAGU 5'
query=miR5658, target=RO3G_17058, score=4, range=402-423, strand=
target 5' UUUGGUAUCAUCAUCAUCAUUA 3'
::. :.: ::::::::::::.:
query 3' AAGACGUUGUAGUAGUAGUAGU 5'
query=miR5658, target=RO3G_05224, score=4, range=477-498, strand=
target 5' UUCAACAGCAUCAUCGUCAUCG 3'
::: ::.::::::::.:::::.
query 3' AAGACGUUGUAGUAGUAGUAGU 5'
query=miR5658, target=RO3G_10411, score=4, range=3885-3906, stran
target 5' CUCUUCAUCAUCAUCAUCGUCA 3'
::: :: ::::::::::.:::
query 3' AAGACGUUGUAGUAGUAGUAGU 5'
query=miR5658, target=RO3G_12721, score=4, range=1383-1404, stran
target 5' AUUUUCGUCAUCAUCAUCAUCA 3'
:.: :. ::::::::::::::
query 3' AAGACGUUGUAGUAGUAGUAGU 5'
query=miR5658, target=RO3G_02346, score=4, range=1554-1575, stran
target 5' UUCUUCUACAUCAUCAUCAUUC 3'
:::: : ::::::::::::::.
query 3' AAGACGUUGUAGUAGUAGUAGU 5'
query=miR5658, target=RO3G_07465, score=4, range=1080-1101, stran
target 5' GUCGGCAUCAUCGUCAUCAUCA 3'
:: ::: ::::.:::::::::
query 3' AAGACGUUGUAGUAGUAGUAGU 5'
query=miR5658, target=RO3G_04437, score=4, range=66-87, strand=-1
target 5' UUCUUCAACAUCUUCAUCAUCC 3'
:::: ::::::: ::::::::
query 3' AAGACGUUGUAGUAGUAGUAGU 5'
query=miR5658, target=RO3G_11688, score=4, range=1029-1050, stran
target 5' CUCUUCGUUAUCAUCAUCAUCA 3'
::: :. .:::::::::::::
query 3' AAGACGUUGUAGUAGUAGUAGU 5'

```
query=miR5658, target=RO3G_09578, score=4, range=2882-2902, stran
target 5' UUCU-UGACAUCAUCAUCAUGA 3'
:::: ..::::::::::::: :
query 3' AAGACGUUGUAGUAGUAGUAGU 5'
query=miR5658, target=RO3G_06187, score=4, range=423-444, strand=
target 5' UUCUUCAUCAUCAUCAUCGUCU 3'
:::: :: ::::::::::.::
query 3' AAGACGUUGUAGUAGUAGUAGU 5'
query=miR5658, target=RO3G_10170, score=4, range=3336-3356, stran
target 5' UUUUUUAA-AUCAUCGUCAUCA 3'
::.: .:: ::::::.::::::
query 3' AAGACGUUGUAGUAGUAGUAGU 5'
query=miR5658, target=RO3G_10853, score=4, range=138-158, strand=
target 5' UUUUG-AAUAUCUUCAUCAUCA 3'
::.:: ::.::: :::::::::
query 3' AAGACGUUGUAGUAGUAGUAGU 5'
query=miR5658, target=RO3G_05884, score=4, range=2109-2131, stran
target 5' UGCUGUCAUCAUCAUCGUCAUCA 3'
: ::: :: :::::::.::::::
query 3' AAGAC-GUUGUAGUAGUAGUAGU 5'
query=miR5658, target=RO3G_08246, score=4, range=207-227, strand=
target 5' UUC-GUAUCAUCAUCAUCAUUG 3'
::: :.: ::::::::::::..
query 3' AAGACGUUGUAGUAGUAGUAGU 5'
query=miR5658, target=RO3G_10143, score=4, range=252-273, strand=
target 5' UUCUUUUGCAUCAUCAUCAUCC 3'
:::: . .:::::::::::::
query 3' AAGACGUUGUAGUAGUAGUAGU 5'
query=miR5658, target=RO3G_02328, score=4, range=540-561, strand=
target 5' UUCUUCAUCAUCUUCAUCAUCA 3'
:::: :: :::: :::::::::
query 3' AAGACGUUGUAGUAGUAGUAGU 5'
query=miR5658, target=RO3G_10942, score=4, range=456-477, strand=
target 5' UUCCUCAUCAUCAUCGUCAUCA 3'
::: :: :::::::.::::::
query 3' AAGACGUUGUAGUAGUAGUAGU 5'
query=miR5658, target=RO3G_16404, score=4, range=1539-1560, stran
target 5' AUCACCAUCAUCAUCAUCAUCA 3'
:: :: ::::::::::::::
query 3' AAGACGUUGUAGUAGUAGUAGU 5'
query=miR5658, target=RO3G_03908, score=4, range=2556-2577, stran
target 5' UUCUUCUUCAUCAUCAUCAUCU 3'
:::: : :::::::::::::
query 3' AAGACGUUGUAGUAGUAGUAGU 5'
query=miR5658, target=RO3G_15942, score=4, range=177-198, strand=
```

```
target 5' GUCUACUUCAUCAUCAUCAUCA 3'
::: : :::::::::::::
query 3' AAGACGUUGUAGUAGUAGUAGU 5'
query=miR5658, target=RO3G_15993, score=4, range=723-744, strand=
target 5' GUCAUCAUCAUCAUCAUCAUCA 3'
:: :: :::::::::::::
query 3' AAGACGUUGUAGUAGUAGUAGU 5'
query=miR5658, target=RO3G_09445, score=4, range=513-534, strand=
target 5' AUCAUCAUCAUCAUCAUCAUCA 3'
:: :: :::::::::::::
query 3' AAGACGUUGUAGUAGUAGUAGU 5'
query=miR5658, target=RO3G_01836, score=4, range=243-264, strand=
target 5' AUCAUCAUCAUCAUCAUCAUCA 3'
:: :: :::::::::::::
query 3' AAGACGUUGUAGUAGUAGUAGU 5'
query=miR5658, target=RO3G_04948, score=4, range=426-447, strand=
target 5' GUCCUCAUCAUCAUCAUCAUCA 3'
:: :: :::::::::::::
query 3' AAGACGUUGUAGUAGUAGUAGU 5'
query=miR5658, target=RO3G_00749, score=4, range=972-994, strand=
target 5' CUCUGUAAUAUCUAUCAUCAUCA 3'
::::.::.::: :::::::::
query 3' AAGACGUUGUAG-UAGUAGUAGU 5'
query=miR5658, target=RO3G_03069, score=4, range=900-921, strand=
target 5' UUAUUCCUCAUCAUCAUCAUCA 3'
:: : : :::::::::::::
query 3' AAGACGUUGUAGUAGUAGUAGU 5'
query=miR5658, target=RO3G_05036, score=4, range=789-810, strand=
target 5' UUAUACUUCAUCAUCAUCAUCA 3'
:: : : :::::::::::::
query 3' AAGACGUUGUAGUAGUAGUAGU 5'
query=miR5658, target=RO3G_10266, score=4, range=726-747, strand=
target 5' UUAUUCAUAAUCAUCAUCAUCA 3'
:: : :: ::::::::::::
query 3' AAGACGUUGUAGUAGUAGUAGU 5'
query=miR5658, target=RO3G_13923, score=4, range=228-248, strand=
target 5' UUAU-UAUUAUCAUCAUCAUCA 3'
:: : .: .::::::::::::
query 3' AAGACGUUGUAGUAGUAGUAGU 5'
query=miR5658, target=RO3G_01085, score=4, range=945-965, strand=
target 5' AUCUGUAA-AUCAUCAUUAUCG 3'
::::.:: ::::::::.:::.
query 3' AAGACGUUGUAGUAGUAGUAGU 5'
query=miR5658, target=RO3G_02346, score=4, range=1734-1755, stran
target 5' UUCUUCUACAUCAUCAUCAUUC 3'
```

```
:::: : ::::::::::::.
query 3' AAGACGUUGUAGUAGUAGUAGU 5'
query=miR5658, target=RO3G_04948, score=4, range=393-414, strand=
target 5' AUCAUCACCAUCAUCAUCAUCA 3'
:: :: :::::::::::::
query 3' AAGACGUUGUAGUAGUAGUAGU 5'
query=miR5658, target=RO3G_02346, score=4, range=2379-2400, stran
target 5' UUCUUCUACAUCAUCAUCAUUC 3'
:::: : ::::::::::::.
query 3' AAGACGUUGUAGUAGUAGUAGU 5'
query=miR8034, target=RO3G_08042, score=0, range=1280-1300, stran
target 5' AGUUUGUGCAGGCUUGUCAUA 3'
:::::::::::::::::::::
query 3' UCAAACACGUCCGAACAGUAU 5'
query=miR8034, target=RO3G_07976, score=1, range=1745-1765, stran
target 5' AGUUUGUACAGGCUUGUCAUA 3'
::::::: :::::::::::::
query 3' UCAAACACGUCCGAACAGUAU 5'
query=miR8034, target=RO3G_03580, score=2, range=1673-1693, stran
target 5' AGUUUGUGCAGGCCUGUCAUA 3'
::::::::::::: :::::::
query 3' UCAAACACGUCCGAACAGUAU 5'
query=miR8034, target=RO3G_11531, score=2, range=1241-1261, stran
target 5' AGUUUGUGCAGGCCUGUCAUA 3'
::::::::::::: :::::::
query 3' UCAAACACGUCCGAACAGUAU 5'
query=miR8034, target=RO3G_03589, score=2, range=1673-1693, stran
target 5' AGUUUGUGCAGGCCUGUCAUA 3'
::::::::::::: :::::::
query 3' UCAAACACGUCCGAACAGUAU 5'
query=miR8034, target=RO3G_15580, score=2, range=1712-1732, stran
target 5' AGUUUGUGCAGGCCUGUCAUA 3'
::::::::::::: :::::::
query 3' UCAAACACGUCCGAACAGUAU 5'
query=miR8034, target=RO3G_07686, score=3, range=1748-1768, stran
target 5' AGUUUGUACAGGCCUGUCAUA 3'
::::::: ::::: :::::::
query 3' UCAAACACGUCCGAACAGUAU 5'
query=miR8034, target=RO3G_16138, score=3, range=1748-1768, stran
target 5' AGUUUGUACAGGCCUGUCAUA 3'
::::::: ::::: :::::::
query 3' UCAAACACGUCCGAACAGUAU 5'
query=miR8034, target=RO3G_11771, score=3, range=1748-1768, stran
target 5' AGUUUGUACAGGCCUGUCAUA 3'
::::::: ::::: :::::::
```

```
query 3' UCAAACACGUCCGAACAGUAU 5'
query=miR8034, target=RO3G_09940, score=3, range=1748-1768, stran
target 5' AGUUUGUACAGGCCUGUCAUA 3'
::::::: ::::: :::::::
query 3' UCAAACACGUCCGAACAGUAU 5'
query=miR8034, target=RO3G_14626, score=3, range=1748-1768, stran
target 5' AGUUUGUACAGGCCUGUCAUA 3'
::::::: ::::: :::::::
query 3' UCAAACACGUCCGAACAGUAU 5'
query=miR8034, target=RO3G_17385, score=3, range=1748-1768, stran
target 5' AGUUUGUACAGGCCUGUCAUA 3'
::::::: ::::: :::::::
query 3' UCAAACACGUCCGAACAGUAU 5'
query=miR8034, target=RO3G_12169, score=3, range=1748-1768, stran
target 5' AGUUUGUACAGGCCUGUCAUA 3'
::::::: ::::: :::::::
query 3' UCAAACACGUCCGAACAGUAU 5'
query=miR8034, target=RO3G_17358, score=3, range=1748-1768, stran
target 5' AGUUUGUACAGGCCUGUCAUA 3'
::::::: ::::: :::::::
query 3' UCAAACACGUCCGAACAGUAU 5'
query=miR8034, target=RO3G_14710, score=3, range=1748-1768, stran
target 5' AGUUUGUACAGGCCUGUCAUA 3'
::::::: ::::: :::::::
query 3' UCAAACACGUCCGAACAGUAU 5'
query=miR8034, target=RO3G_12072, score=3, range=1748-1768, stran
target 5' AGUUUGUACAGGCCUGUCAUA 3'
::::::: ::::: :::::::
query 3' UCAAACACGUCCGAACAGUAU 5'
query=miR8034, target=RO3G_01740, score=3, range=1712-1732, stran
target 5' AGUUUGUACAGGCCUGUCAUA 3'
::::::: ::::: :::::::
query 3' UCAAACACGUCCGAACAGUAU 5'
query=miR8034, target=RO3G_10725, score=3, range=1712-1732, stran
target 5' AGUUUGUACAGGCCUGUCAUA 3'
::::::: ::::: :::::::
query 3' UCAAACACGUCCGAACAGUAU 5'
query=miR8034, target=RO3G_05958, score=3, range=1712-1732, stran
target 5' AGUUUGUACAGGCCUGUCAUA 3'
::::::: ::::: :::::::
query 3' UCAAACACGUCCGAACAGUAU 5'
query=miR8034, target=RO3G_06430, score=3, range=1712-1732, stran
target 5' AGUUUGUACAGGCCUGUCAUA 3'
::::::: ::::: :::::::
query 3' UCAAACACGUCCGAACAGUAU 5'
```

```
query=miR8034, target=RO3G_01309, score=3, range=1697-1717, stran
target 5' AGUUUGUACAGGCCUGUCAUA 3'
::::::: ::::: :::::::
query 3' UCAAACACGUCCGAACAGUAU 5'
query=miR8034, target=RO3G_17441, score=3, range=1475-1495, stran
target 5' AGUUUGUACAGGCCUGUCAUA 3'
::::::: ::::: :::::::
query 3' UCAAACACGUCCGAACAGUAU 5'
query=miR8034, target=RO3G_10522, score=3, range=1712-1732, stran
target 5' AGUUUGUACAGGCCUGUCAUA 3'
::::::: ::::: :::::::
query 3' UCAAACACGUCCGAACAGUAU 5'
query=miR8034, target=RO3G_07294, score=3, range=1349-1369, stran
target 5' AGUUUGUACAGGCCUGUCAUA 3'
::::::: ::::: :::::::
query 3' UCAAACACGUCCGAACAGUAU 5'
query=miR8034, target=RO3G_04785, score=3, range=1712-1732, stran
target 5' AGUUUGUACAGGCCUGUCAUA 3'
::::::: ::::: :::::::
query 3' UCAAACACGUCCGAACAGUAU 5'
query=miR8034, target=RO3G_03555, score=3, range=1748-1768, stran
target 5' AGUUUGUACAGGCCUGUCAUA 3'
::::::: ::::: :::::::
query 3' UCAAACACGUCCGAACAGUAU 5'
query=miR8034, target=RO3G_01954, score=3, range=698-718, strand=
target 5' AGUUUGUACAGGCCUGUCAUA 3'
::::::: ::::: :::::::
query 3' UCAAACACGUCCGAACAGUAU 5'
query=miR8034, target=RO3G_13148, score=3, range=1481-1501, stran
target 5' AGUUUGUACAGGCCUGUCAUA 3'
::::::: ::::: :::::::
query 3' UCAAACACGUCCGAACAGUAU 5'
query=miR8034, target=RO3G_07128, score=3, range=266-286, strand=
target 5' AGUUUGUACAGGCCUGUCAUA 3'
::::::: ::::: :::::::
query 3' UCAAACACGUCCGAACAGUAU 5'
query=miR8034, target=RO3G_16738, score=3, range=665-685, strand=
target 5' AGUUUGUACAGGCCUGUCAUA 3'
::::::: ::::: :::::::
query 3' UCAAACACGUCCGAACAGUAU 5'
query=miR8034, target=RO3G_12009, score=3.5, range=1748-1768, str
target 5' GGUUUGUACAGGCCUGUCAUA 3'
.:::::: ::::: :::::::
query 3' UCAAACACGUCCGAACAGUAU 5'
query=miR8034, target=RO3G_13747, score=3.5, range=1748-1768, str
```

```
target 5' GGUUUGUACAGGCCUGUCAUA 3'
.:::::: ::::: :::::::
query 3' UCAAACACGUCCGAACAGUAU 5'
query=miR8034, target=RO3G_11404, score=3.5, range=1748-1768, str
target 5' GGUUUGUACAGGCCUGUCAUA 3'
.:::::: ::::: :::::::
query 3' UCAAACACGUCCGAACAGUAU 5'
query=miR8034, target=RO3G_05139, score=4, range=1390-1410, stran
target 5' AGUUUCAGCAGGCUUGUUAUU 3'
::::: ::::::::::.::
query 3' UCAAACACGUCCGAACAGUAU 5'
query=miR5506, target=RO3G_13745, score=0, range=660-677, strand=
target 5' CCUCAACGAAGCGAUCUA 3'
::::::::::::::::::
query 3' GGAGUUGCUUCGCUAGAU 5'
query=miR5506, target=RO3G_05524, score=0, range=660-677, strand=
target 5' CCUCAACGAAGCGAUCUA 3'
::::::::::::::::::
query 3' GGAGUUGCUUCGCUAGAU 5'
query=miR5506, target=RO3G_12964, score=0, range=660-677, strand=
target 5' CCUCAACGAAGCGAUCUA 3'
::::::::::::::::::
query 3' GGAGUUGCUUCGCUAGAU 5'
query=miR5506, target=RO3G_11952, score=0, range=1038-1055, stran
target 5' CCUCAACGAAGCGAUCUA 3'
::::::::::::::::::
query 3' GGAGUUGCUUCGCUAGAU 5'
query=miR5506, target=RO3G_12669, score=0, range=660-677, strand=
target 5' CCUCAACGAAGCGAUCUA 3'
::::::::::::::::::
query 3' GGAGUUGCUUCGCUAGAU 5'
query=miR5506, target=RO3G_16708, score=0, range=660-677, strand=
target 5' CCUCAACGAAGCGAUCUA 3'
::::::::::::::::::
query 3' GGAGUUGCUUCGCUAGAU 5'
query=miR5506, target=RO3G_12075, score=0, range=660-677, strand=
target 5' CCUCAACGAAGCGAUCUA 3'
::::::::::::::::::
query 3' GGAGUUGCUUCGCUAGAU 5'
query=miR5506, target=RO3G_05925, score=0.5, range=660-677, stran
target 5' UCUCAACGAAGCGAUCUA 3'
.:::::::::::::::::
query 3' GGAGUUGCUUCGCUAGAU 5'
query=miR5506, target=RO3G_12961, score=0.5, range=993-1010, stra
target 5' UCUCAACGAAGCGAUCUA 3'
```

```
.:::::::::::::::::
query 3' GGAGUUGCUUCGCUAGAU 5'
query=miR5506, target=RO3G_05927, score=0.5, range=660-677, stran
target 5' UCUCAACGAAGCGAUCUA 3'
.:::::::::::::::::
query 3' GGAGUUGCUUCGCUAGAU 5'
query=miR5506, target=RO3G_09927, score=1, range=660-677, strand=
target 5' CCUCAACGAGGCGAUCUA 3'
::::::::.::::::::
query 3' GGAGUUGCUUCGCUAGAU 5'
query=miR5506, target=RO3G_09951, score=2.5, range=918-935, stran
target 5' UCUGAAUGAAGCGAUCUA 3'
.:: ::.:::::::::::
query 3' GGAGUUGCUUCGCUAGAU 5'
query=miR5506, target=RO3G_00927, score=3, range=608-625, strand=
target 5' UUUCAAGGAAGCGAUCUA 3'
..:::: ::::::::::
query 3' GGAGUUGCUUCGCUAGAU 5'
query=miR5506, target=RO3G_08754, score=3, range=5278-5295, stran
target 5' GCUGAACGAAGUGAUCUA 3'
:: :::::::.::::::
query 3' GGAGUUGCUUCGCUAGAU 5'
query=miR5506, target=RO3G_02524, score=3, range=244-260, strand=
target 5' CCUC-ACGAAGCCAUCUA 3'
:::: ::::::: :::::
query 3' GGAGUUGCUUCGCUAGAU 5'
query=miR5506, target=RO3G_07756, score=3.5, range=309-326, stran
target 5' UCUUGACCAAGCGAUCUA 3'
.::..:: :::::::::
query 3' GGAGUUGCUUCGCUAGAU 5'
query=miR5506, target=RO3G_16652, score=4, range=181-199, strand=
target 5' CUUCAACGAAGUGAUGCUG 3'
:.:::::::::.::: ::.
query 3' GGAGUUGCUUCGCUA-GAU 5'
query=miR5506, target=RO3G_13173, score=4, range=1310-1328, stran
target 5' CCUCAGACGAAGUGAUCCA 3'
::::: ::::::.:::: :
query 3' GGAGU-UGCUUCGCUAGAU 5'
query=miR5506, target=RO3G_02307, score=4, range=696-713, strand=
target 5' CCUGAACGAAGCGAUCGC 3'
::: :::::::::::
query 3' GGAGUUGCUUCGCUAGAU 5'
query=miR5506, target=RO3G_07404, score=4, range=883-899, strand=
target 5' CCUCAACGAAG-GAUUUC 3'
:::::::::: ::::.:
```

```
query 3' GGAGUUGCUUCGCUAGAU 5'
query=miR5506, target=RO3G_12345, score=4, range=241-257, strand=
target 5' CAUC-GCGAAGCGAUCUU 3'
: :: .:::::::::::
query 3' GGAGUUGCUUCGCUAGAU 5'
query=miR5506, target=RO3G_12484, score=4, range=3550-3568, stran
target 5' GCUCAACGAAGACGAUCUC 3'
:::::::::: ::::::
query 3' GGAGUUGCUUC-GCUAGAU 5'
query=miR5506, target=RO3G_02322, score=4, range=1604-1621, stran
target 5' CCUCAGCAAAGUGAUCUA 3'
:::::.: :::.::::::
query 3' GGAGUUGCUUCGCUAGAU 5'
query=miR5506, target=RO3G_11393, score=4, range=254-271, strand=
target 5' CCUUGACGAAUUGAUCUA 3'
:::..::::: .::::::
query 3' GGAGUUGCUUCGCUAGAU 5'
query=miR5506, target=RO3G_09594, score=4, range=262-279, strand=
target 5' CUUGAAUGGAGCGAUCUG 3'
:.: ::.:.::::::::.
query 3' GGAGUUGCUUCGCUAGAU 5'
query=miR5506, target=RO3G_15771, score=4, range=254-271, strand=
target 5' CCUUGACGAAGCAAUCUU 3'
:::..::::::: ::::
query 3' GGAGUUGCUUCGCUAGAU 5'
query=miR9752, target=RO3G_02835, score=0, range=239-261, strand=
target 5' AAAUCAUGGAACAGCAAGAAGCA 3'
:::::::::::::::::::::::
query 3' UUUAGUACCUUGUCGUUCUUCGU 5'
query=miR9752, target=RO3G_07109, score=0, range=239-261, strand=
target 5' AAAUCAUGGAACAGCAAGAAGCA 3'
:::::::::::::::::::::::
query 3' UUUAGUACCUUGUCGUUCUUCGU 5'
query=miR9752, target=RO3G_07672, score=0, range=239-261, strand=
target 5' AAAUCAUGGAACAGCAAGAAGCA 3'
:::::::::::::::::::::::
query 3' UUUAGUACCUUGUCGUUCUUCGU 5'
query=miR9752, target=RO3G_09934, score=0, range=239-261, strand=
target 5' AAAUCAUGGAACAGCAAGAAGCA 3'
:::::::::::::::::::::::
query 3' UUUAGUACCUUGUCGUUCUUCGU 5'
query=miR9752, target=RO3G_09197, score=0, range=239-261, strand=
target 5' AAAUCAUGGAACAGCAAGAAGCA 3'
:::::::::::::::::::::::
query 3' UUUAGUACCUUGUCGUUCUUCGU 5'
```

```
query=miR9752, target=RO3G_15283, score=0, range=239-261, strand=
target 5' AAAUCAUGGAACAGCAAGAAGCA 3'
:::::::::::::::::::::::
query 3' UUUAGUACCUUGUCGUUCUUCGU 5'
query=miR9752, target=RO3G_10526, score=0, range=239-261, strand=
target 5' AAAUCAUGGAACAGCAAGAAGCA 3'
:::::::::::::::::::::::
query 3' UUUAGUACCUUGUCGUUCUUCGU 5'
query=miR9752, target=RO3G_01312, score=0, range=20-42, strand=1
target 5' AAAUCAUGGAACAGCAAGAAGCA 3'
:::::::::::::::::::::::
query 3' UUUAGUACCUUGUCGUUCUUCGU 5'
query=miR9752, target=RO3G_01935, score=0, range=239-261, strand=
target 5' AAAUCAUGGAACAGCAAGAAGCA 3'
:::::::::::::::::::::::
query 3' UUUAGUACCUUGUCGUUCUUCGU 5'
query=miR9752, target=RO3G_04795, score=3.5, range=289-312, stran
target 5' AAAUUAUUGGAAAAGCAAGAAGCA 3'
::::.: ::::: :::::::::::
query 3' UUUAGU-ACCUUGUCGUUCUUCGU 5'
query=miR9752, target=RO3G_06295, score=4, range=2715-2737, stran
target 5' AAAUCAGGGAAAAGCAAGAAGUA 3'
:::::: :::: :::::::::.:
query 3' UUUAGUACCUUGUCGUUCUUCGU 5'
query=miR9752, target=RO3G_00450, score=4, range=2424-2446, stran
target 5' AAAGCAAGGAAAAGCAAGAAGCA 3'
::: :: :::: :::::::::::
query 3' UUUAGUACCUUGUCGUUCUUCGU 5'
query=miR9752, target=RO3G_01819, score=4, range=410-431, strand=
target 5' AAAUGAAGGAACAGCAA-AAGCA 3'
:::: : :::::::::: :::::
query 3' UUUAGUACCUUGUCGUUCUUCGU 5'
query=miR5369, target=RO3G_16744, score=0, range=639-660, strand=
target 5' AUGGACAUCCUCCUUUUCUUGA 3'
::::::::::::::::::::::
query 3' UACCUGUAGGAGGAAAAGAACU 5'
query=miR5369, target=RO3G_01203, score=0, range=639-660, strand=
target 5' AUGGACAUCCUCCUUUUCUUGA 3'
::::::::::::::::::::::
query 3' UACCUGUAGGAGGAAAAGAACU 5'
query=miR5369, target=RO3G_11403, score=0, range=639-660, strand=
target 5' AUGGACAUCCUCCUUUUCUUGA 3'
::::::::::::::::::::::
query 3' UACCUGUAGGAGGAAAAGAACU 5'
query=miR5369, target=RO3G_06416, score=0, range=639-660, strand=
```

target 5' AUGGACAUCCUCCUUUUCUUGA 3'
::::::::::::::::::::::
query 3' UACCUGUAGGAGGAAAAGAACU 5'
query=miR5369, target=RO3G_01718, score=0, range=639-660, strand=
target 5' AUGGACAUCCUCCUUUUCUUGA 3'
::::::::::::::::::::::
query 3' UACCUGUAGGAGGAAAAGAACU 5'
query=miR5369, target=RO3G_15869, score=0, range=639-660, strand=
target 5' AUGGACAUCCUCCUUUUCUUGA 3'
::::::::::::::::::::::
query 3' UACCUGUAGGAGGAAAAGAACU 5'
query=miR5369, target=RO3G_10560, score=0, range=639-660, strand=
target 5' AUGGACAUCCUCCUUUUCUUGA 3'
::::::::::::::::::::::
query 3' UACCUGUAGGAGGAAAAGAACU 5'
query=miR5369, target=RO3G_11275, score=0, range=639-660, strand=
target 5' AUGGACAUCCUCCUUUUCUUGA 3'
::::::::::::::::::::::
query 3' UACCUGUAGGAGGAAAAGAACU 5'
query=miR5369, target=RO3G_14704, score=0, range=639-660, strand=
target 5' AUGGACAUCCUCCUUUUCUUGA 3'
::::::::::::::::::::::
query 3' UACCUGUAGGAGGAAAAGAACU 5'
query=miR5369, target=RO3G_12979, score=0, range=639-660, strand=
target 5' AUGGACAUCCUCCUUUUCUUGA 3'
::::::::::::::::::::::
query 3' UACCUGUAGGAGGAAAAGAACU 5'
query=miR5369, target=RO3G_10555, score=0, range=639-660, strand=
target 5' AUGGACAUCCUCCUUUUCUUGA 3'
::::::::::::::::::::::
query 3' UACCUGUAGGAGGAAAAGAACU 5'
query=miR5369, target=RO3G_05280, score=0, range=639-660, strand=
target 5' AUGGACAUCCUCCUUUUCUUGA 3'
::::::::::::::::::::::
query 3' UACCUGUAGGAGGAAAAGAACU 5'
query=miR5369, target=RO3G_16686, score=0, range=639-660, strand=
target 5' AUGGACAUCCUCCUUUUCUUGA 3'
::::::::::::::::::::::
query 3' UACCUGUAGGAGGAAAAGAACU 5'
query=miR5369, target=RO3G_07305, score=0, range=639-660, strand=
target 5' AUGGACAUCCUCCUUUUCUUGA 3'
::::::::::::::::::::::
query 3' UACCUGUAGGAGGAAAAGAACU 5'
query=miR5369, target=RO3G_03103, score=0, range=639-660, strand=
target 5' AUGGACAUCCUCCUUUUCUUGA 3'

```
::::::::::::::::::::::
query 3' UACCUGUAGGAGGAAAAGAACU 5'
query=miR5369, target=RO3G_12087, score=0, range=639-660, strand=
target 5' AUGGACAUCCUCCUUUUCUUGA 3'
::::::::::::::::::::::
query 3' UACCUGUAGGAGGAAAAGAACU 5'
query=miR5369, target=RO3G_08612, score=0, range=639-660, strand=
target 5' AUGGACAUCCUCCUUUUCUUGA 3'
::::::::::::::::::::::
query 3' UACCUGUAGGAGGAAAAGAACU 5'
query=miR5369, target=RO3G_05630, score=0, range=639-660, strand=
target 5' AUGGACAUCCUCCUUUUCUUGA 3'
::::::::::::::::::::::
query 3' UACCUGUAGGAGGAAAAGAACU 5'
query=miR5369, target=RO3G_13450, score=0, range=594-615, strand=
target 5' AUGGACAUCCUCCUUUUCUUGA 3'
::::::::::::::::::::::
query 3' UACCUGUAGGAGGAAAAGAACU 5'
query=miR5369, target=RO3G_13859, score=0, range=264-285, strand=
target 5' AUGGACAUCCUCCUUUUCUUGA 3'
::::::::::::::::::::::
query 3' UACCUGUAGGAGGAAAAGAACU 5'
query=miR5369, target=RO3G_11970, score=0, range=639-660, strand=
target 5' AUGGACAUCCUCCUUUUCUUGA 3'
::::::::::::::::::::::
query 3' UACCUGUAGGAGGAAAAGAACU 5'
query=miR5369, target=RO3G_13152, score=0, range=639-660, strand=
target 5' AUGGACAUCCUCCUUUUCUUGA 3'
::::::::::::::::::::::
query 3' UACCUGUAGGAGGAAAAGAACU 5'
query=miR5369, target=RO3G_03988, score=0, range=639-660, strand=
target 5' AUGGACAUCCUCCUUUUCUUGA 3'
::::::::::::::::::::::
query 3' UACCUGUAGGAGGAAAAGAACU 5'
query=miR5369, target=RO3G_12951, score=0, range=639-660, strand=
target 5' AUGGACAUCCUCCUUUUCUUGA 3'
::::::::::::::::::::::
query 3' UACCUGUAGGAGGAAAAGAACU 5'
query=miR5369, target=RO3G_09376, score=0, range=639-660, strand=
target 5' AUGGACAUCCUCCUUUUCUUGA 3'
::::::::::::::::::::::
query 3' UACCUGUAGGAGGAAAAGAACU 5'
query=miR5369, target=RO3G_12194, score=0, range=639-660, strand=
target 5' AUGGACAUCCUCCUUUUCUUGA 3'
::::::::::::::::::::::
```

```
query 3' UACCUGUAGGAGGAAAAGAACU 5'
query=miR5369, target=RO3G_07104, score=0, range=639-660, strand=
target 5' AUGGACAUCCUCCUUUUCUUGA 3'
::::::::::::::::::::::
query 3' UACCUGUAGGAGGAAAAGAACU 5'
query=miR5369, target=RO3G_05532, score=2.5, range=677-698, stran
target 5' AUAGAUAUCCUCCUUUUUUUGA 3'
:: ::.:::::::::::.::::
query 3' UACCUGUAGGAGGAAAAGAACU 5'
query=miR5369, target=RO3G_13954, score=3.5, range=300-321, stran
target 5' AUUGACAUAUUCCUUUUCUUGG 3'
:: ::::: .:::::::::::.
query 3' UACCUGUAGGAGGAAAAGAACU 5'
query=miR5369, target=RO3G_12264, score=3.5, range=133-154, stran
target 5' AUUGCCAUUCUCCUUUUCUUGC 3'
:: : :::.:::::::::::::
query 3' UACCUGUAGGAGGAAAAGAACU 5'
query=miR5369, target=RO3G_09088, score=4, range=243-263, strand=
target 5' AUCGA-AUCCUCCUUUUCUUAA 3'
:: :: ::::::::::::: :
query 3' UACCUGUAGGAGGAAAAGAACU 5'
query=miR5369, target=RO3G_16312, score=4, range=1137-1157, stran
target 5' AAGUACAU-CUUCUUUUCUUGA 3'
: : :::: ::.::::::::::
query 3' UACCUGUAGGAGGAAAAGAACU 5'
query=miR5369, target=RO3G_13188, score=4, range=60-81, strand=-1
target 5' AUCGACACCUUUCUUUUCUUGA 3'
:: :::: :.:.::::::::::
query 3' UACCUGUAGGAGGAAAAGAACU 5'
query=miR5369, target=RO3G_09005, score=4, range=759-780, strand=
target 5' AUUUACAUCCUCUUUUUCUUGC 3'
:: :::::::.::::::::
query 3' UACCUGUAGGAGGAAAAGAACU 5'
query=miR5369, target=RO3G_07455, score=4, range=90-111, strand=-
target 5' UUUGACACCUUCCUUUUCUUGA 3'
: :::: :.:::::::::::::
query 3' UACCUGUAGGAGGAAAAGAACU 5'
query=miR5369, target=RO3G_13619, score=4, range=153-174, strand=
target 5' UUGCUCGUCCUCCUUUUCUUGG 3'
:: :.::::::::::::::.
query 3' UACCUGUAGGAGGAAAAGAACU 5'
query=miR1072, target=RO3G_15574, score=0, range=1404-1425, stran
target 5' CCAAGCUCAAAUAACAACAAUA 3'
::::::::::::::::::::::
query 3' GGUUCGAGUUUAUUGUUGUUAU 5'
```

```
query=miR1072, target=RO3G_01425, score=2, range=1389-1410, stran
target 5' UCAAGUUCAGAUAACAACAAUA 3'
.::::.:::.:::::::::::::
query 3' GGUUCGAGUUUAUUGUUGUUAU 5'
query=miR1072, target=RO3G_13806, score=2, range=1389-1410, stran
target 5' UCAAGUUCAGAUAACAACAAUA 3'
.::::.:::.:::::::::::::
query 3' GGUUCGAGUUUAUUGUUGUUAU 5'
query=miR1072, target=RO3G_16566, score=2, range=1389-1410, stran
target 5' UCAAGUUCAGAUAACAACAAUA 3'
.::::.:::.:::::::::::::
query 3' GGUUCGAGUUUAUUGUUGUUAU 5'
query=miR1072, target=RO3G_03120, score=2, range=1389-1410, stran
target 5' UCAAGUUCAGAUAACAACAAUA 3'
.::::.:::.:::::::::::::
query 3' GGUUCGAGUUUAUUGUUGUUAU 5'
query=miR1072, target=RO3G_14533, score=2, range=1194-1215, stran
target 5' UCAAGUUCAGAUAACAACAAUA 3'
.::::.:::.:::::::::::::
query 3' GGUUCGAGUUUAUUGUUGUUAU 5'
query=miR1072, target=RO3G_03093, score=2.5, range=415-435, stran
target 5' CGAAG-UCAAAUAACAACAAUG 3'
: ::: :::::::::::::::.
query 3' GGUUCGAGUUUAUUGUUGUUAU 5'
query=miR1072, target=RO3G_15852, score=3.5, range=582-604, stran
target 5' UCAUAGUUCGAAUAACAGCAAUA 3'
.:: ::.::.:::::::.:::::
query 3' GGU-UCGAGUUUAUUGUUGUUAU 5'
query=miR1072, target=RO3G_12258, score=4, range=540-562, strand=
target 5' UCAACCUCAAAGUAACAACAAUG 3'
.::: :::::: ::::::::::.
query 3' GGUUCGAGUUU-AUUGUUGUUAU 5'
query=miR1072, target=RO3G_14612, score=4, range=49-70, strand=1
target 5' CCCGGCACAAAUAACAAUAAUG 3'
:: .:: ::::::::::.:::.
query 3' GGUUCGAGUUUAUUGUUGUUAU 5'
query=miR1072, target=RO3G_11195, score=4, range=1304-1324, stran
target 5' UUAAGC-CAAAUAACAAAAAUA 3'
..:::: :::::::::: ::::
query 3' GGUUCGAGUUUAUUGUUGUUAU 5'
query=miR1072, target=RO3G_16217, score=4, range=462-483, strand=
target 5' CUGUGAUCCAAUAACAACAAUA 3'
:.. : :: ::::::::::::
query 3' GGUUCGAGUUUAUUGUUGUUAU 5'
query=miR1072, target=RO3G_10289, score=4, range=948-968, strand=
```

```
target 5' CCAGGCUCUAAUAA-AACAAUG 3'
:::.:::: ::::: ::::::.
query 3' GGUUCGAGUUUAUUGUUGUUAU 5'
query=miR2105, target=RO3G_07019, score=2, range=124-144, strand=
target 5' GUCAGCC-UUCACAAAUCACAA 3'
.:::.:: ::::::::::::::
query 3' UAGUUGGUAAGUGUUUAGUGUU 5'
query=miR2105, target=RO3G_01808, score=3.5, range=2231-2252,
strand=-1
target 5' AGCAACCGAUCACAAAUUACAA 3'
: :::::. ::::::::.::::
query 3' UAGUUGGUAAGUGUUUAGUGUU 5'
query=miR2105, target=RO3G_13442, score=4, range=600-621, strand=
target 5' AAGAACCGUUCACAAGUCACAG 3'
: ::::.::::::::.::::.
query 3' UAGUUGGUAAGUGUUUAGUGUU 5'
query=miR2105, target=RO3G_13216, score=4, range=216-237, strand=
target 5' AAGAACCGUUCACAAGUCACAG 3'
: ::::.::::::::.::::.
query 3' UAGUUGGUAAGUGUUUAGUGUU 5'
query=miR2105, target=RO3G_15621, score=4, range=85-105, strand=1
target 5' GUUCAUU-UUCACAAAUCACAA 3'
.:. :.. ::::::::::::::
query 3' UAGUUGGUAAGUGUUUAGUGUU 5'
query=miR2105, target=RO3G_00180, score=4, range=2255-2277, stran
target 5' AUCAGACUGUUCAUGAAUCACAA 3'
:::: ::..::::..::::::::
query 3' UAGU-UGGUAAGUGUUUAGUGUU 5'
query=miR2105, target=RO3G_01213, score=4, range=98-118, strand=-
target 5' AUAAACCA-UGACAAAUCACAA 3'
:: ::::: : :::::::::::
query 3' UAGUUGGUAAGUGUUUAGUGUU 5'
query=miR9760, target=RO3G_12099, score=3, range=107-129, strand=
target 5' AUCAAUUAAUGACUGCAUCAUCA 3'
:.::::.:: ::::.::::::::
query 3' UGGUUAGUU-CUGAUGUAGUAGU 5'
query=miR9760, target=RO3G_14120, score=3.5, range=133-154, stran
target 5' AGCGAUGAGGACUACAUCAUCG 3'
: :.:: :.::::::::::::.
query 3' UGGUUAGUUCUGAUGUAGUAGU 5'
query=miR9760, target=RO3G_14105, score=3.5, range=174-195, stran
target 5' AGCGAUGAGGACUACAUCAUCG 3'
: :.:: :.::::::::::::.
query 3' UGGUUAGUUCUGAUGUAGUAGU 5'
query=miR9760, target=RO3G_11395, score=4, range=556-577, strand=
```

```
target 5' ACAAAUCAAGAUUACAACAUCA 3'
:: ::::::::.:::: :::::
query 3' UGGUUAGUUCUGAUGUAGUAGU 5'
query=miR9760, target=RO3G_03021, score=4, range=1882-1902, stran
target 5' AUCAAUCCAGACUACAUC-UCG 3'
:.:::: ::::::::: ::.
query 3' UGGUUAGUUCUGAUGUAGUAGU 5'
query=miR9760, target=RO3G_12520, score=4, range=1440-1461, stran
target 5' AGAGAUCAGGACUACAUCGUCA 3'
: .::::.::::::::.:::
query 3' UGGUUAGUUCUGAUGUAGUAGU 5'
query=miR9760, target=RO3G_00797, score=4, range=1440-1461, stran
target 5' AGAGAUCAGGACUACAUCGUCA 3'
: .::::.::::::::.:::
query 3' UGGUUAGUUCUGAUGUAGUAGU 5'
query=miR398b-3p, target=RO3G_15529, score=4, range=586-605, stra
target 5' AGUGGU-ACUUGAGAGCACAU 3'
:: ::: ::.:::::.:::::
query 3' UCCCCACUGGACUCUUGUGUA 5'
query=miR5260, target=RO3G_10729, score=0, range=781-802, strand=
target 5' AUAGCCAGUCAUCAAUACAACA 3'
::::::::::::::::::::::
query 3' UAUCGGUCAGUAGUUAUGUUGU 5'
query=miR5260, target=RO3G_05953, score=1.5, range=781-802, stran
target 5' AUGGCCAGUCAUCAAUAUAACA 3'
::.:::::::::::::::.::::
query 3' UAUCGGUCAGUAGUUAUGUUGU 5'
query=miR5260, target=RO3G_10780, score=1.5, range=781-802, stran
target 5' AUGGCCAGUCAUCAAUAUAACA 3'
::.:::::::::::::::.::::
query 3' UAUCGGUCAGUAGUUAUGUUGU 5'
query=miR5260, target=RO3G_10773, score=1.5, range=781-802, stran
target 5' AUGGCCAGUCAUCAAUAUAACA 3'
::.:::::::::::::::.::::
query 3' UAUCGGUCAGUAGUUAUGUUGU 5'
query=miR5260, target=RO3G_09542, score=1.5, range=781-802, stran
target 5' AUGGCCAGUCAUCAAUAUAACA 3'
::.:::::::::::::::.::::
query 3' UAUCGGUCAGUAGUUAUGUUGU 5'
query=miR5260, target=RO3G_17449, score=1.5, range=781-802, stran
target 5' AUGGCCAGUCAUCAAUAUAACA 3'
::.:::::::::::::::.::::
query 3' UAUCGGUCAGUAGUUAUGUUGU 5'
query=miR5260, target=RO3G_04210, score=1.5, range=781-802, stran
target 5' AUGGCCAGUCAUCAAUAUAACA 3'
```

```
::.:::::::::::::::.::::
query 3' UAUCGGUCAGUAGUUAUGUUGU 5'
query=miR5260, target=RO3G_07860, score=1.5, range=781-802, stran
target 5' AUGGCCAGUCAUCAAUAUAACA 3'
::.:::::::::::::::.::::
query 3' UAUCGGUCAGUAGUUAUGUUGU 5'
query=miR5260, target=RO3G_14361, score=1.5, range=781-802, stran
target 5' AUGGCCAGUCAUCAAUAUAACA 3'
::.:::::::::::::::.::::
query 3' UAUCGGUCAGUAGUUAUGUUGU 5'
query=miR5260, target=RO3G_05921, score=1.5, range=781-802, stran
target 5' AUGGCCAGUCAUCAAUAUAACA 3'
::.:::::::::::::::.::::
query 3' UAUCGGUCAGUAGUUAUGUUGU 5'
query=miR5260, target=RO3G_05959, score=1.5, range=781-802, stran
target 5' AUGGCCAGUCAUCAAUAUAACA 3'
::.:::::::::::::::.::::
query 3' UAUCGGUCAGUAGUUAUGUUGU 5'
query=miR5260, target=RO3G_14568, score=1.5, range=781-802, stran
target 5' AUGGCCAGUCAUCAAUAUAACA 3'
::.:::::::::::::::.::::
query 3' UAUCGGUCAGUAGUUAUGUUGU 5'
query=miR5260, target=RO3G_11966, score=1.5, range=781-802, stran
target 5' AUGGCCAGUCAUCAAUAUAACA 3'
::.:::::::::::::::.::::
query 3' UAUCGGUCAGUAGUUAUGUUGU 5'
query=miR5260, target=RO3G_09967, score=2, range=781-802, strand=
target 5' AUGGCCAGUCAUCAAUAUAACG 3'
::.:::::::::::::::.:::.
query 3' UAUCGGUCAGUAGUUAUGUUGU 5'
query=miR5260, target=RO3G_05267, score=2, range=781-802, strand=
target 5' AUGGCCAGUCAUCAAUAUAACG 3'
::.:::::::::::::::.:::.
query 3' UAUCGGUCAGUAGUUAUGUUGU 5'
query=miR5260, target=RO3G_00256, score=2, range=781-802, strand=
target 5' AUGGCCAGUCAUCAAUAUAACG 3'
::.:::::::::::::::.:::.
query 3' UAUCGGUCAGUAGUUAUGUUGU 5'
query=miR5260, target=RO3G_04207, score=2.5, range=781-802, stran
target 5' AUGGCCAGUCAUCAAUGUAACA 3'
::.::::::::::::::..::::
query 3' UAUCGGUCAGUAGUUAUGUUGU 5'
query=miR5260, target=RO3G_07124, score=2.5, range=781-802, stran
target 5' AUGGCCAGUCAUCAAUGUAACA 3'
::.::::::::::::::..::::
```

```
query 3' UAUCGGUCAGUAGUUAUGUUGU 5'
query=miR5260, target=RO3G_17069, score=3.5, range=646-667, stran
target 5' AUGGCCAGUCAUCAAUAUAAAA 3'
::.:::::::::::::::.:: :
query 3' UAUCGGUCAGUAGUUAUGUUGU 5'
query=miR5260, target=RO3G_01942, score=3.5, range=778-799, stran
target 5' AUGUCCAGUCAUCAAUGUAACA 3'
::. :::::::::::..::::
query 3' UAUCGGUCAGUAGUUAUGUUGU 5'
query=miR5260, target=RO3G_09945, score=3.5, range=505-526, stran
target 5' AUGUCCAGUCAUCAAUGUAACA 3'
::. :::::::::::..::::
query 3' UAUCGGUCAGUAGUUAUGUUGU 5'
query=miR5260, target=RO3G_06545, score=4, range=698-719, strand=
target 5' CUAGCCAAACAUUAAUACAACA 3'
:::::: :::.:::::::::
query 3' UAUCGGUCAGUAGUUAUGUUGU 5'
query=miR5260, target=RO3G_01220, score=4, range=3112-3132, stran
target 5' AUA-CCAAUCAUCAAUACAAUC 3'
::: ::: :::::::::::.
query 3' UAUCGGUCAGUAGUUAUGUUGU 5'
query=miR5260, target=RO3G_14415, score=4, range=571-591, strand=
target 5' AUA-CCAGGUAUCAAUACAACC 3'
::: :::: .::::::::::
query 3' UAUCGGUCAGUAGUUAUGUUGU 5'
query=miR5260, target=RO3G_11450, score=4, range=1-23, strand=1
target 5' AUGGCUACUACAUUAAUACAACA 3'
::.::.: : :::.:::::::::
query 3' UAUCGGUCA-GUAGUUAUGUUGU 5'
query=miR5260, target=RO3G_09194, score=4, range=781-802, strand=
target 5' AUGUCCAGUCAUCAAUGUAACG 3'
::. :::::::::::..:::.
query 3' UAUCGGUCAGUAGUUAUGUUGU 5'
query=miR169f-3p, target=RO3G_12009, score=0, range=2102-2122,
strand=-1
target 5' AGUAACCAAAGGAAGACAUCA 3'
:::::::::::::::::::::
query 3' UCAUUGGUUUCCUUCUGUAGU 5'
query=miR169f-3p, target=RO3G_16138, score=0, range=2102-2122,
strand=-1
target 5' AGUAACCAAAGGAAGACAUCA 3'
:::::::::::::::::::::
query 3' UCAUUGGUUUCCUUCUGUAGU 5'
query=miR169f-3p, target=RO3G_12072, score=0, range=2102-2122,
strand=-1
```

target 5' AGUAACCAAAGGAAGACAUCA 3'
:::::::::::::::::::::
query 3' UCAUUGGUUUCCUUCUGUAGU 5'
query=miR169f-3p, target=RO3G_14626, score=0, range=2102-2122, strand=-1
target 5' AGUAACCAAAGGAAGACAUCA 3'
:::::::::::::::::::::
query 3' UCAUUGGUUUCCUUCUGUAGU 5'
query=miR169f-3p, target=RO3G_05958, score=0, range=2066-2086, strand=-1
target 5' AGUAACCAAAGGAAGACAUCA 3'
:::::::::::::::::::::
query 3' UCAUUGGUUUCCUUCUGUAGU 5'
query=miR169f-3p, target=RO3G_01740, score=0, range=2066-2086, strand=-1
target 5' AGUAACCAAAGGAAGACAUCA 3'
:::::::::::::::::::::
query 3' UCAUUGGUUUCCUUCUGUAGU 5'
query=miR169f-3p, target=RO3G_03580, score=0, range=2027-2047, strand=-1
target 5' AGUAACCAAAGGAAGACAUCA 3'
:::::::::::::::::::::
query 3' UCAUUGGUUUCCUUCUGUAGU 5'
query=miR169f-3p, target=RO3G_10522, score=0, range=2066-2086, strand=-1
target 5' AGUAACCAAAGGAAGACAUCA 3'
:::::::::::::::::::::
query 3' UCAUUGGUUUCCUUCUGUAGU 5'
query=miR169f-3p, target=RO3G_11531, score=0, range=1595-1615, strand=-1
target 5' AGUAACCAAAGGAAGACAUCA 3'
:::::::::::::::::::::
query 3' UCAUUGGUUUCCUUCUGUAGU 5'
query=miR169f-3p, target=RO3G_07106, score=0, range=1430-1450, strand=-1
target 5' AGUAACCAAAGGAAGACAUCA 3'
:::::::::::::::::::::
query 3' UCAUUGGUUUCCUUCUGUAGU 5'
query=miR169f-3p, target=RO3G_16736, score=0, range=236-256, stra
target 5' AGUAACCAAAGGAAGACAUCA 3'
:::::::::::::::::::::
query 3' UCAUUGGUUUCCUUCUGUAGU 5'
query=miR169f-3p, target=RO3G_04784, score=0, range=152-172, stra
target 5' AGUAACCAAAGGAAGACAUCA 3'
:::::::::::::::::::::

query 3' UCAUUGGUUUCCUUCUGUAGU 5'
query=miR169f-3p, target=RO3G_15578, score=0, range=80-100, stran
target 5' AGUAACCAAAGGAAGACAUCA 3'
:::::::::::::::::::::
query 3' UCAUUGGUUUCCUUCUGUAGU 5'
query=miR169f-3p, target=RO3G_13149, score=0, range=299-319, stra
target 5' AGUAACCAAAGGAAGACAUCA 3'
:::::::::::::::::::::
query 3' UCAUUGGUUUCCUUCUGUAGU 5'
query=miR169f-3p, target=RO3G_08042, score=1, range=1634-1654, strand=-1
target 5' AAUAACCAAAGGAAGACAUCA 3'
: :::::::::::::::::::
query 3' UCAUUGGUUUCCUUCUGUAGU 5'
query=miR169f-3p, target=RO3G_07686, score=2.5, range=2102-2122, strand=-1
target 5' AAUAGACAAAGGAAGACAUCA 3'
: ::. :::::::::::::::
query 3' UCAUUGGUUUCCUUCUGUAGU 5'
query=miR169f-3p, target=RO3G_09940, score=2.5, range=2102-2122, strand=-1
target 5' AAUAGACAAAGGAAGACAUCA 3'
: ::. :::::::::::::::
query 3' UCAUUGGUUUCCUUCUGUAGU 5'
query=miR169f-3p, target=RO3G_17385, score=2.5, range=2102-2122, strand=-1
target 5' AAUAGACAAAGGAAGACAUCA 3'
: ::. :::::::::::::::
query 3' UCAUUGGUUUCCUUCUGUAGU 5'
query=miR169f-3p, target=RO3G_17358, score=2.5, range=2102-2122, strand=-1
target 5' AAUAGACAAAGGAAGACAUCA 3'
: ::. :::::::::::::::
query 3' UCAUUGGUUUCCUUCUGUAGU 5'
query=miR169f-3p, target=RO3G_12169, score=2.5, range=2102-2122, strand=-1
target 5' AAUAGACAAAGGAAGACAUCA 3'
: ::. :::::::::::::::
query 3' UCAUUGGUUUCCUUCUGUAGU 5'
query=miR169f-3p, target=RO3G_11771, score=2.5, range=2102-2122, strand=-1
target 5' AAUAGACAAAGGAAGACAUCA 3'
: ::. :::::::::::::::
query 3' UCAUUGGUUUCCUUCUGUAGU 5'
query=miR169f-3p, target=RO3G_14710, score=2.5, range=2102-2122,

strand=-1
target 5' AAUAGACAAAGGAAGACAUCA 3'
: ::. ::::::::::::::
query 3' UCAUUGGUUUCCUUCUGUAGU 5'
query=miR169f-3p, target=RO3G_07976, score=2.5, range=2099-2119, strand=-1
target 5' AAUAGACAAAGGAAGACAUCA 3'
: ::. ::::::::::::::
query 3' UCAUUGGUUUCCUUCUGUAGU 5'
query=miR169f-3p, target=RO3G_10725, score=2.5, range=2066-2086, strand=-1
target 5' AAUAGACAAAGGAAGACAUCA 3'
: ::. ::::::::::::::
query 3' UCAUUGGUUUCCUUCUGUAGU 5'
query=miR169f-3p, target=RO3G_06430, score=2.5, range=2066-2086, strand=-1
target 5' AAUAGACAAAGGAAGACAUCA 3'
: ::. ::::::::::::::
query 3' UCAUUGGUUUCCUUCUGUAGU 5'
query=miR169f-3p, target=RO3G_01309, score=2.5, range=2051-2071, strand=-1
target 5' AAUAGACAAAGGAAGACAUCA 3'
: ::. ::::::::::::::
query 3' UCAUUGGUUUCCUUCUGUAGU 5'
query=miR169f-3p, target=RO3G_01207, score=2.5, range=1919-1939, strand=-1
target 5' AAUAGACAAAGGAAGACAUCA 3'
: ::. ::::::::::::::
query 3' UCAUUGGUUUCCUUCUGUAGU 5'
query=miR169f-3p, target=RO3G_17441, score=2.5, range=1829-1849, strand=-1
target 5' AAUAGACAAAGGAAGACAUCA 3'
: ::. ::::::::::::::
query 3' UCAUUGGUUUCCUUCUGUAGU 5'
query=miR169f-3p, target=RO3G_13747, score=2.5, range=2102-2122, strand=-1
target 5' AAUAGACAAAGGAAGACAUCA 3'
: ::. ::::::::::::::
query 3' UCAUUGGUUUCCUUCUGUAGU 5'
query=miR169f-3p, target=RO3G_07294, score=2.5, range=1703-1723, strand=-1
target 5' AAUAGACAAAGGAAGACAUCA 3'
: ::. ::::::::::::::
query 3' UCAUUGGUUUCCUUCUGUAGU 5'
query=miR169f-3p, target=RO3G_01954, score=2.5, range=1052-1072,

```
strand=-1
target 5' AAUAGACAAAGGAAGACAUCA 3'
: ::. ::::::::::::::
query 3' UCAUUGGUUUCCUUCUGUAGU 5'
query=miR169f-3p, target=RO3G_03552, score=2.5, range=236-256,
strand=-1
target 5' AAUAGACAAAGGAAGACAUCA 3'
: ::. ::::::::::::::
query 3' UCAUUGGUUUCCUUCUGUAGU 5'
query=miR169f-3p, target=RO3G_07128, score=2.5, range=620-640,
strand=-1
target 5' AAUAGACAAAGGAAGACAUCA 3'
: ::. ::::::::::::::
query 3' UCAUUGGUUUCCUUCUGUAGU 5'
query=miR169f-3p, target=RO3G_06605, score=3, range=425-444, stra
target 5' AGUAA-CAAAGGCAGACAUCA 3'
::::: :::::: ::::::::
query 3' UCAUUGGUUUCCUUCUGUAGU 5'
query=miR169f-3p, target=RO3G_05462, score=4, range=973-994, stra
target 5' CGUAACUGUAAGGGAGACAUCA 3'
:::::.. ::::.::::::::
query 3' UCAUUGGU-UUCCUUCUGUAGU 5'
query=miR169f-3p, target=RO3G_13316, score=4, range=199-220, stra
target 5' AGUAGGUCAAAGGAAGAGAUCA 3'
:::: ..:::::::::: ::::
query 3' UCAU-UGGUUUCCUUCUGUAGU 5'
query=miR169f-3p, target=RO3G_15960, score=4, range=573-592, stra
target 5' AG-AACCAAAGGAAGAAAUUA 3'
:: :::::::::::: ::.:
query 3' UCAUUGGUUUCCUUCUGUAGU 5'
query=miR169f-3p, target=RO3G_13160, score=4, range=1325-1344,
strand=1
target 5' AGUUACUAAAGGAAGA-AUCG 3'
::: ::.::::::::: :::.
query 3' UCAUUGGUUUCCUUCUGUAGU 5'
query=miR169f-3p, target=RO3G_06547, score=4, range=22-43, strand
target 5' AGUAGUCAAAGAGAAGAUAUCA 3'
::::..::::: :::::.::::
query 3' UCAUUGGUUUC-CUUCUGUAGU 5'
query=miR169f-3p, target=RO3G_03246, score=4, range=791-810, stra
target 5' AGU-GUCAGAGGAAGGCAUCA 3'
::: ..::.:::::.:::::
query 3' UCAUUGGUUUCCUUCUGUAGU 5'
query=miR1857-3p, target=RO3G_10799, score=0, range=757-777, stra
target 5' CUUGUUUUGUUGGAGCCAUGA 3'
```

```
:::::::::::::::::::::
query 3' GAACAAAACAACCUCGGUACU 5'
query=miR1857-3p, target=RO3G_17239, score=0, range=757-777, stra
target 5' CUUGUUUUGUUGGAGCCAUGA 3'
:::::::::::::::::::::
query 3' GAACAAAACAACCUCGGUACU 5'
query=miR1857-3p, target=RO3G_04664, score=0, range=757-777, stra
target 5' CUUGUUUUGUUGGAGCCAUGA 3'
:::::::::::::::::::::
query 3' GAACAAAACAACCUCGGUACU 5'
query=miR1857-3p, target=RO3G_03573, score=1, range=757-777, stra
target 5' CGUGUUUUGUUGGAGCCAUGA 3'
: :::::::::::::::::::
query 3' GAACAAAACAACCUCGGUACU 5'
query=miR1857-3p, target=RO3G_15168, score=1, range=541-561, stra
target 5' CAUGUUUUGUUGGAGCCAUGA 3'
: :::::::::::::::::::
query 3' GAACAAAACAACCUCGGUACU 5'
query=miR1857-3p, target=RO3G_10749, score=3, range=4-24, strand=
target 5' CGUGUUUUUUUGGAGCCAUGA 3'
: :::::: ::::::::::::
query 3' GAACAAAACAACCUCGGUACU 5'
query=miR1857-3p, target=RO3G_12124, score=4, range=99-118, stran
target 5' CUUG-UCUGUUGGAGCCAAGA 3'
:::: : ::::::::::: ::
query 3' GAACAAAACAACCUCGGUACU 5'
query=miR168, target=RO3G_10879, score=3, range=487-505, strand=-
target 5' UCACG-CCUGCGCCAAGCGA 3'
:: :: :::::.::::::::
query 3' AGGGCUGGACGUGGUUCGCU 5'
query=miR9558-3p, target=RO3G_17440, score=0, range=297-318, stra
target 5' AGAGGAAAAGCUGGCCUUGCAA 3'
::::::::::::::::::::::
query 3' UCUCCUUUUCGACCGGAACGUU 5'
query=miR9558-3p, target=RO3G_03579, score=0, range=297-318, stra
target 5' AGAGGAAAAGCUGGCCUUGCAA 3'
::::::::::::::::::::::
query 3' UCUCCUUUUCGACCGGAACGUU 5'
query=miR9558-3p, target=RO3G_11530, score=0, range=297-318, stra
target 5' AGAGGAAAAGCUGGCCUUGCAA 3'
::::::::::::::::::::::
query 3' UCUCCUUUUCGACCGGAACGUU 5'
query=miR9558-3p, target=RO3G_15577, score=0, range=297-318, stra
target 5' AGAGGAAAAGCUGGCCUUGCAA 3'
::::::::::::::::::::::
```

query 3' UCUCCUUUUCGACCGGAACGUU 5'
query=miR9558-3p, target=RO3G_14625, score=0, range=297-318, stra
target 5' AGAGGAAAAGCUGGCCUUGCAA 3'
::::::::::::::::::::::
query 3' UCUCCUUUUCGACCGGAACGUU 5'
query=miR9558-3p, target=RO3G_07107, score=0, range=297-318, stra
target 5' AGAGGAAAAGCUGGCCUUGCAA 3'
::::::::::::::::::::::
query 3' UCUCCUUUUCGACCGGAACGUU 5'
query=miR9558-3p, target=RO3G_05957, score=0, range=297-318, stra
target 5' AGAGGAAAAGCUGGCCUUGCAA 3'
::::::::::::::::::::::
query 3' UCUCCUUUUCGACCGGAACGUU 5'
query=miR9558-3p, target=RO3G_13150, score=0, range=18-39, strand
target 5' AGAGGAAAAGCUGGCCUUGCAA 3'
::::::::::::::::::::::
query 3' UCUCCUUUUCGACCGGAACGUU 5'
query=miR9558-3p, target=RO3G_04783, score=0, range=159-180, stra
target 5' AGAGGAAAAGCUGGCCUUGCAA 3'
::::::::::::::::::::::
query 3' UCUCCUUUUCGACCGGAACGUU 5'
query=miR9558-3p, target=RO3G_03097, score=0, range=297-318, stra
target 5' AGAGGAAAAGCUGGCCUUGCAA 3'
::::::::::::::::::::::
query 3' UCUCCUUUUCGACCGGAACGUU 5'
query=miR9558-3p, target=RO3G_01734, score=0, range=297-318, stra
target 5' AGAGGAAAAGCUGGCCUUGCAA 3'
::::::::::::::::::::::
query 3' UCUCCUUUUCGACCGGAACGUU 5'
query=miR9558-3p, target=RO3G_10519, score=0, range=297-318, stra
target 5' AGAGGAAAAGCUGGCCUUGCAA 3'
::::::::::::::::::::::
query 3' UCUCCUUUUCGACCGGAACGUU 5'
query=miR9558-3p, target=RO3G_04093, score=3, range=1311-1332,
strand=1
target 5' UGAGAAGGAGCUGGCCUUGCAA 3'
::: :..::::::::::::::
query 3' UCUCCUUUUCGACCGGAACGUU 5'
query=miR9558-3p, target=RO3G_02685, score=4, range=18-39, strand
target 5' AGAGGAAAAACAGGCCUUGCAA 3'
::::::::: : ::::::::::
query 3' UCUCCUUUUCGACCGGAACGUU 5'
query=miR2633, target=RO3G_06442, score=0, range=4775-4795, stran
target 5' AGAAUCUGGUGCAAAUGUCGA 3'
:::::::::::::::::::::

```
query 3' UCUUAGACCACGUUUACAGCU 5'
query=miR2633, target=RO3G_02691, score=0, range=7607-7627, stran
target 5' AGAAUCUGGUGCAAAUGUCGA 3'
:::::::::::::::::::::
query 3' UCUUAGACCACGUUUACAGCU 5'
query=miR2633, target=RO3G_09965, score=0, range=4118-4138, stran
target 5' AGAAUCUGGUGCAAAUGUCGA 3'
:::::::::::::::::::::
query 3' UCUUAGACCACGUUUACAGCU 5'
query=miR2633, target=RO3G_13920, score=0, range=4118-4138, stran
target 5' AGAAUCUGGUGCAAAUGUCGA 3'
:::::::::::::::::::::
query 3' UCUUAGACCACGUUUACAGCU 5'
query=miR2633, target=RO3G_03547, score=0, range=4118-4138, stran
target 5' AGAAUCUGGUGCAAAUGUCGA 3'
:::::::::::::::::::::
query 3' UCUUAGACCACGUUUACAGCU 5'
query=miR2633, target=RO3G_13867, score=0, range=4034-4054, stran
target 5' AGAAUCUGGUGCAAAUGUCGA 3'
:::::::::::::::::::::
query 3' UCUUAGACCACGUUUACAGCU 5'
query=miR2633, target=RO3G_02959, score=0, range=4118-4138, stran
target 5' AGAAUCUGGUGCAAAUGUCGA 3'
:::::::::::::::::::::
query 3' UCUUAGACCACGUUUACAGCU 5'
query=miR2633, target=RO3G_15250, score=0, range=4073-4093, stran
target 5' AGAAUCUGGUGCAAAUGUCGA 3'
:::::::::::::::::::::
query 3' UCUUAGACCACGUUUACAGCU 5'
query=miR2633, target=RO3G_17371, score=0, range=4073-4093, stran
target 5' AGAAUCUGGUGCAAAUGUCGA 3'
:::::::::::::::::::::
query 3' UCUUAGACCACGUUUACAGCU 5'
query=miR2633, target=RO3G_06427, score=0, range=3710-3730, stran
target 5' AGAAUCUGGUGCAAAUGUCGA 3'
:::::::::::::::::::::
query 3' UCUUAGACCACGUUUACAGCU 5'
query=miR2633, target=RO3G_17326, score=0, range=3950-3970, stran
target 5' AGAAUCUGGUGCAAAUGUCGA 3'
:::::::::::::::::::::
query 3' UCUUAGACCACGUUUACAGCU 5'
query=miR2633, target=RO3G_06428, score=0, range=3212-3232, stran
target 5' AGAAUCUGGUGCAAAUGUCGA 3'
:::::::::::::::::::::
query 3' UCUUAGACCACGUUUACAGCU 5'
```

```
query=miR2633, target=RO3G_13749, score=0, range=2627-2647, stran
target 5' AGAAUCUGGUGCAAAUGUCGA 3'
:::::::::::::::::::::
query 3' UCUUAGACCACGUUUACAGCU 5'
query=miR2633, target=RO3G_05619, score=0, range=2261-2281, stran
target 5' AGAAUCUGGUGCAAAUGUCGA 3'
:::::::::::::::::::::
query 3' UCUUAGACCACGUUUACAGCU 5'
query=miR2633, target=RO3G_14355, score=0, range=2324-2344, stran
target 5' AGAAUCUGGUGCAAAUGUCGA 3'
:::::::::::::::::::::
query 3' UCUUAGACCACGUUUACAGCU 5'
query=miR2633, target=RO3G_01613, score=0, range=2315-2335, stran
target 5' AGAAUCUGGUGCAAAUGUCGA 3'
:::::::::::::::::::::
query 3' UCUUAGACCACGUUUACAGCU 5'
query=miR2633, target=RO3G_01330, score=0, range=1352-1372, stran
target 5' AGAAUCUGGUGCAAAUGUCGA 3'
:::::::::::::::::::::
query 3' UCUUAGACCACGUUUACAGCU 5'
query=miR2633, target=RO3G_11776, score=0, range=1208-1228, stran
target 5' AGAAUCUGGUGCAAAUGUCGA 3'
:::::::::::::::::::::
query 3' UCUUAGACCACGUUUACAGCU 5'
query=miR2633, target=RO3G_02222, score=0, range=1172-1192, stran
target 5' AGAAUCUGGUGCAAAUGUCGA 3'
:::::::::::::::::::::
query 3' UCUUAGACCACGUUUACAGCU 5'
query=miR2633, target=RO3G_10558, score=0, range=1871-1891, stran
target 5' AGAAUCUGGUGCAAAUGUCGA 3'
:::::::::::::::::::::
query 3' UCUUAGACCACGUUUACAGCU 5'
query=miR2633, target=RO3G_04115, score=0, range=1061-1081, stran
target 5' AGAAUCUGGUGCAAAUGUCGA 3'
:::::::::::::::::::::
query 3' UCUUAGACCACGUUUACAGCU 5'
query=miR2633, target=RO3G_17404, score=0, range=746-766, strand=
target 5' AGAAUCUGGUGCAAAUGUCGA 3'
:::::::::::::::::::::
query 3' UCUUAGACCACGUUUACAGCU 5'
query=miR2633, target=RO3G_08610, score=3, range=337-357, strand=
target 5' GGCAUUUGGUGCAGAUGUCGA 3'
.: ::.:::::::.:::::::
query 3' UCUUAGACCACGUUUACAGCU 5'
query=miR6155, target=RO3G_14837, score=0, range=19-38, strand=1
```

```
target 5' UGCAAGGCACAGGCAACUCA 3'
::::::::::::::::::::
query 3' ACGUUCCGUGUCCGUUGAGU 5'
query=miR6155, target=RO3G_00488, score=0, range=19-38, strand=1
target 5' UGCAAGGCACAGGCAACUCA 3'
::::::::::::::::::::
query 3' ACGUUCCGUGUCCGUUGAGU 5'
query=miR6155, target=RO3G_08656, score=0, range=241-260, strand=
target 5' UGCAAGGCACAGGCAACUCA 3'
::::::::::::::::::::
query 3' ACGUUCCGUGUCCGUUGAGU 5'
query=miR6155, target=RO3G_07068, score=3.5, range=272-292, stran
target 5' UGCAAAAGCGCAGGCAACUCG 3'
::: :: ::.::::::::::.
query 3' ACG-UUCCGUGUCCGUUGAGU 5'
query=miR6155, target=RO3G_03221, score=4, range=3592-3612, stran
target 5' CGCAGGCGCACAGGCAAUUCG 3'
:::.: ::::::::::.::.
query 3' ACGUUC-CGUGUCCGUUGAGU 5'
query=miR6155, target=RO3G_01298, score=4, range=93-113, strand=-
target 5' UGAAUAGGCAAAGGCAACUCA 3'
:: : ::::: ::::::::::
query 3' ACGU-UCCGUGUCCGUUGAGU 5'
query=miR6155, target=RO3G_14449, score=4, range=51-69, strand=-1
target 5' UCCAA-GCACAGGAAACUCA 3'
: ::: ::::::: ::::::
query 3' ACGUUCCGUGUCCGUUGAGU 5'
query=miR6155, target=RO3G_08954, score=4, range=54-72, strand=-1
target 5' UCCAA-GCACAGGAAACUCA 3'
: ::: ::::::: ::::::
query 3' ACGUUCCGUGUCCGUUGAGU 5'
query=miR6155, target=RO3G_06774, score=4, range=400-420, strand=
target 5' UGCAAGCUCAUAGGCAACUCU 3'
:::::: ::.:::::::::
query 3' ACGUUC-CGUGUCCGUUGAGU 5'
query=miR394a-3p, target=RO3G_03743, score=2.5, range=570-588,
strand=-1
target 5' UUG-AACAGAGAUGACAACA 3'
::. :: ::::::::::::
query 3' AAUGUUUCUCUACUGUUGU 5'
query=miR394a-3p, target=RO3G_01624, score=3.5, range=863-882,
strand=1
target 5' GUACAAGUGAGAUGACAACA 3'
:::::. :::::::::::
query 3' AAUGUUUCUCUACUGUUGU 5'
```

```
query=miR394a-3p, target=RO3G_03967, score=3.5, range=1040-1059,
strand=1
target 5' UGAAGAAAGAGGUGACAACA 3'
: : .:::::.::::::::
query 3' AAUGUUUUCUCUACUGUUGU 5'
query=miR394a-3p, target=RO3G_12023, score=3.5, range=2-22, stran
target 5' UGGCAGAAAGAGAUGACGACA 3'
: .:: :::::::::::.:::
query 3' AAUGU-UUUCUCUACUGUUGU 5'
query=miR394a-3p, target=RO3G_04896, score=3.5, range=2208-2226,
strand=1
target 5' GUACAAGAGAGAUGA-AACA 3'
::::.::::::::: ::::
query 3' AAUGUUUUCUCUACUGUUGU 5'
query=miR394a-3p, target=RO3G_13557, score=3.5, range=974-993,
strand=-1
target 5' UGGUAAGAGAGAUGAUAACA 3'
: ..::.:::::::.::::
query 3' AAUGUUUUCUCUACUGUUGU 5'
query=miR394a-3p, target=RO3G_16787, score=4, range=261-280, stra
target 5' UUACACAGGAGAUGAUGACA 3'
::::: :.:::::::..:::
query 3' AAUGUUUUCUCUACUGUUGU 5'
query=miR394a-3p, target=RO3G_12626, score=4, range=720-739, stra
target 5' UGACAAAGUAGAUGACAACA 3'
: :::::. ::::::::::
query 3' AAUGUUUUCUCUACUGUUGU 5'
query=miR394a-3p, target=RO3G_05768, score=4, range=135-154, stra
target 5' UUACUAUAGGGAUGAUAACA 3'
:::: : ::.:::::.::::
query 3' AAUGUUUUCUCUACUGUUGU 5'
query=miR394a-3p, target=RO3G_12883, score=4, range=176-194, stra
target 5' ACACAAAAGAGAUGAC-ACA 3'
:::::::::::::: :::
query 3' AAUGUUUUCUCUACUGUUGU 5'
query=miR394a-3p, target=RO3G_06457, score=4, range=439-459, stra
target 5' UUGCAAAAGAGAUGAGCGACG 3'
::.:::::::::::: :.::.
query 3' AAUGUUUUCUCUACU-GUUGU 5'
query=miR394a-3p, target=RO3G_03160, score=4, range=300-320, stra
target 5' UUAUAGAUAGAGGUGACAACU 3'
:::.:.: ::::.::::::::
query 3' AAUGUUU-UCUCUACUGUUGU 5'
query=miR394a-3p, target=RO3G_10070, score=4, range=362-381, stra
target 5' UGGCGUAAGGGAUGACAACA 3'
```

```
: .:. :::.::::::::::
query 3' AAUGUUUUCUCUACUGUUGU 5'
query=miR394a-3p, target=RO3G_11485, score=4, range=362-381, stra
target 5' UGGCGUAAGGGAUGACAACA 3'
: .:. :::.::::::::::
query 3' AAUGUUUUCUCUACUGUUGU 5'
query=miR394a-3p, target=RO3G_10359, score=4, range=362-381, stra
target 5' UGGCGUAAGGGAUGACAACA 3'
: .:. :::.::::::::::
query 3' AAUGUUUUCUCUACUGUUGU 5'
query=miR394a-3p, target=RO3G_17191, score=4, range=362-381, stra
target 5' UGGCGUAAGGGAUGACAACA 3'
: .:. :::.::::::::::
query 3' AAUGUUUUCUCUACUGUUGU 5'
query=miR394a-3p, target=RO3G_06230, score=4, range=13977-13995,
strand=-1
target 5' UUA-AAAGGAGAUGACCACA 3'
::: :::.:::::::: :::
query 3' AAUGUUUUCUCUACUGUUGU 5'
query=miR915, target=RO3G_09507, score=0, range=730-751, strand=1
target 5' GACGACGUUGUCCUUAUUGCCA 3'
::::::::::::::::::::::
query 3' CUGCUGCAACAGGAAUAACGGU 5'
query=miR915, target=RO3G_17033, score=0, range=676-697, strand=1
target 5' GACGACGUUGUCCUUAUUGCCA 3'
::::::::::::::::::::::
query 3' CUGCUGCAACAGGAAUAACGGU 5'
query=miR915, target=RO3G_12456, score=0, range=658-679, strand=1
target 5' GACGACGUUGUCCUUAUUGCCA 3'
::::::::::::::::::::::
query 3' CUGCUGCAACAGGAAUAACGGU 5'
query=miR915, target=RO3G_08181, score=0, range=433-454, strand=1
target 5' GACGACGUUGUCCUUAUUGCCA 3'
::::::::::::::::::::::
query 3' CUGCUGCAACAGGAAUAACGGU 5'
query=miR915, target=RO3G_03515, score=0, range=370-391, strand=1
target 5' GACGACGUUGUCCUUAUUGCCA 3'
::::::::::::::::::::::
query 3' CUGCUGCAACAGGAAUAACGGU 5'
query=miR915, target=RO3G_01653, score=0.5, range=598-619, strand
target 5' GACGAUGUUGUCCUUAUUGCCA 3'
:::::.::::::::::::::::
query 3' CUGCUGCAACAGGAAUAACGGU 5'
query=miR915, target=RO3G_14836, score=2, range=1495-1516, strand
target 5' GAUGACGUUGUCCUUAUUGCUG 3'
```

```
::.:::::::::::::::::..
query 3' CUGCUGCAACAGGAAUAACGGU 5'
query=miR915, target=RO3G_00484, score=3.5, range=45-66, strand=1
target 5' GAUGACAUUGUCCUUAUUGCUU 3'
::.::: :::::::::::::.
query 3' CUGCUGCAACAGGAAUAACGGU 5'
query=miR915, target=RO3G_00331, score=3.5, range=1284-1306, stra
target 5' CACGAAUGUUGUCCUUAUUGCCU 3'
::: :.:::::::::::::::
query 3' CUGC-UGCAACAGGAAUAACGGU 5'
query=miR414, target=RO3G_12063, score=0, range=1104-1125, strand
target 5' UGACGAUAUGAAGAAGAUGGUA 3'
::::::::::::::::::::::
query 3' ACUGCUAUACUUCUUCUACCAU 5'
query=miR414, target=RO3G_04206, score=0, range=657-678, strand=1
target 5' UGACGAUAUGAAGAAGAUGGUA 3'
::::::::::::::::::::::
query 3' ACUGCUAUACUUCUUCUACCAU 5'
query=miR414, target=RO3G_07712, score=0, range=108-129, strand=1
target 5' UGACGAUAUGAAGAAGAUGGUA 3'
::::::::::::::::::::::
query 3' ACUGCUAUACUUCUUCUACCAU 5'
query=miR414, target=RO3G_12187, score=0.5, range=1608-1629, stra
target 5' UGAUGAUAUGAAGAAGAUGGUA 3'
:::.::::::::::::::::::
query 3' ACUGCUAUACUUCUUCUACCAU 5'
query=miR414, target=RO3G_11395, score=3, range=108-129, strand=1
target 5' UGAAGAUAUGAAGACGAUGGUA 3'
::: :::::::::: :::::::
query 3' ACUGCUAUACUUCUUCUACCAU 5'
query=miR414, target=RO3G_02287, score=3.5, range=1829-1849, stra
target 5' UUUUGAU-UGAAGAAGAUGGUA 3'
: .::: ::::::::::::::
query 3' ACUGCUAUACUUCUUCUACCAU 5'
query=miR414, target=RO3G_14053, score=3.5, range=922-944, strand
target 5' UGCUGAUAUUGAAGAGGAUGGUA 3'
:: .:::: ::::::.:::::::
query 3' ACUGCUAU-ACUUCUUCUACCAU 5'
query=miR414, target=RO3G_12494, score=4, range=894-916, strand=1
target 5' UGAUGAAGAUGAAGGAGAUGGUG 3'
:::.: : ::::::.:::::::.
query 3' ACUGC-UAUACUUCUUCUACCAU 5'
query=miR414, target=RO3G_01516, score=4, range=2539-2560, strand
target 5' CGACGAAAAGAGGAAGAUGGUA 3'
::::: : ::.::::::::::
```

```
query 3' ACUGCUAUACUUCUUCUACCAU 5'
query=miR414, target=RO3G_15731, score=4, range=39-60, strand=1
target 5' UGACGAGAUGAAAAAGAUGGUU 3'
:::::: ::::: ::::::::
query 3' ACUGCUAUACUUCUUCUACCAU 5'
query=miR414, target=RO3G_03476, score=4, range=66-87, strand=1
target 5' UGUUGAAAAGAAGAAGAUGGUG 3'
:: .:: : :::::::::::.
query 3' ACUGCUAUACUUCUUCUACCAU 5'
query=miR414, target=RO3G_02379, score=4, range=3894-3916, strand
target 5' UGACGAUGUAGAAGAAGAUGAUG 3'
:::::::.: :::::::::: :.
query 3' ACUGCUAUA-CUUCUUCUACCAU 5'
query=miR414, target=RO3G_07465, score=4, range=158-180, strand=-
target 5' UGGAGUAUGAGAAGAAGAUGGUA 3'
::. : ::. ::::::::::::
query 3' ACUGC-UAUACUUCUUCUACCAU 5'
query=miR8043, target=RO3G_16392, score=0, range=381-402, strand=
target 5' GGCCAAAUCCAUAGUAUGAUCA 3'
::::::::::::::::::::::
query 3' CCGGUUUAGGUAUCAUACUAGU 5'
query=miR8043, target=RO3G_09180, score=1, range=1398-1419, stran
target 5' GGCCAAAUCCAUAGUAUGAUUA 3'
::::::::::::::::::::.:
query 3' CCGGUUUAGGUAUCAUACUAGU 5'
query=miR8043, target=RO3G_16812, score=1, range=1398-1419, stran
target 5' GGCCAAAUCCAUAGUAUGAUUA 3'
::::::::::::::::::::.:
query 3' CCGGUUUAGGUAUCAUACUAGU 5'
query=miR8043, target=RO3G_09189, score=1, range=1398-1419, stran
target 5' GGCCAAAUCCAUAGUAUGAUUA 3'
::::::::::::::::::::.:
query 3' CCGGUUUAGGUAUCAUACUAGU 5'
query=miR8043, target=RO3G_14798, score=1, range=1398-1419, stran
target 5' GGCCAAAUCCAUAGUAUGAUUA 3'
::::::::::::::::::::.:
query 3' CCGGUUUAGGUAUCAUACUAGU 5'
query=miR8043, target=RO3G_14791, score=1, range=1398-1419, stran
target 5' GGCCAAAUCCAUAGUAUGAUUA 3'
::::::::::::::::::::.:
query 3' CCGGUUUAGGUAUCAUACUAGU 5'
query=miR8043, target=RO3G_17260, score=1, range=1266-1287, stran
target 5' GGCCAAAUCCAUAGUAUGAUUA 3'
::::::::::::::::::::.:
query 3' CCGGUUUAGGUAUCAUACUAGU 5'
```

```
query=miR8043, target=RO3G_15270, score=1, range=447-468, strand=
target 5' GGCCAAAUCCAUAGUAUGAUUA 3'
::::::::::::::::::::.:
query 3' CCGGUUUAGGUAUCAUACUAGU 5'
query=miR8043, target=RO3G_06734, score=1, range=702-723, strand=
target 5' GGCCAAAUCCAUAGUAUGAUUA 3'
::::::::::::::::::::.:
query 3' CCGGUUUAGGUAUCAUACUAGU 5'
query=miR169d, target=RO3G_02691, score=0, range=335-356, strand=
target 5' CCGGUAAUCUCAUCCUUGCUAA 3'
::::::::::::::::::::::
query 3' GGCCAUUAGAGUAGGAACGAUU 5'
query=miR169d, target=RO3G_17318, score=0, range=1802-1823, stran
target 5' CCGGUAAUCUCAUCCUUGCUAA 3'
::::::::::::::::::::::
query 3' GGCCAUUAGAGUAGGAACGAUU 5'
query=miR169d, target=RO3G_04118, score=0, range=1802-1823, stran
target 5' CCGGUAAUCUCAUCCUUGCUAA 3'
::::::::::::::::::::::
query 3' GGCCAUUAGAGUAGGAACGAUU 5'
query=miR169d, target=RO3G_16143, score=0, range=1799-1820, stran
target 5' CCGGUAAUCUCAUCCUUGCUAA 3'
::::::::::::::::::::::
query 3' GGCCAUUAGAGUAGGAACGAUU 5'
query=miR169d, target=RO3G_17274, score=0, range=1802-1823, stran
target 5' CCGGUAAUCUCAUCCUUGCUAA 3'
::::::::::::::::::::::
query 3' GGCCAUUAGAGUAGGAACGAUU 5'
query=miR169d, target=RO3G_12533, score=0, range=1142-1163, stran
target 5' CCGGUAAUCUCAUCCUUGCUAA 3'
::::::::::::::::::::::
query 3' GGCCAUUAGAGUAGGAACGAUU 5'
query=miR169d, target=RO3G_17329, score=0, range=1139-1160, stran
target 5' CCGGUAAUCUCAUCCUUGCUAA 3'
::::::::::::::::::::::
query 3' GGCCAUUAGAGUAGGAACGAUU 5'
query=miR169d, target=RO3G_07028, score=0, range=872-893, strand=
target 5' CCGGUAAUCUCAUCCUUGCUAA 3'
::::::::::::::::::::::
query 3' GGCCAUUAGAGUAGGAACGAUU 5'
query=miR169d, target=RO3G_15135, score=0, range=1802-1823, stran
target 5' CCGGUAAUCUCAUCCUUGCUAA 3'
::::::::::::::::::::::
query 3' GGCCAUUAGAGUAGGAACGAUU 5'
query=miR169d, target=RO3G_17384, score=0, range=1802-1823, stran
```

```
target 5' CCGGUAAUCUCAUCCUUGCUAA 3'
::::::::::::::::::::::
query 3' GGCCAUUAGAGUAGGAACGAUU 5'
query=miR169d, target=RO3G_06727, score=0, range=1940-1961, stran
target 5' CCGGUAAUCUCAUCCUUGCUAA 3'
::::::::::::::::::::::
query 3' GGCCAUUAGAGUAGGAACGAUU 5'
query=miR169d, target=RO3G_02999, score=4, range=3362-3383, stran
target 5' UCUGUAAAAUCAUCCUUGCUAG 3'
.: :::: ::::::::::::.
query 3' GGCCAUUAGAGUAGGAACGAUU 5'
query=miR5499, target=RO3G_07972, score=0, range=428-447, strand=
target 5' UUCCAAACGAUCUUCCUGUA 3'
::::::::::::::::::::
query 3' AAGGUUUGCUAGAAGGACAU 5'
query=miR5499, target=RO3G_07991, score=0, range=428-447, strand=
target 5' UUCCAAACGAUCUUCCUGUA 3'
::::::::::::::::::::
query 3' AAGGUUUGCUAGAAGGACAU 5'
query=miR5499, target=RO3G_16015, score=3, range=950-969, strand=
target 5' UUUCAAGUGGUCUUCCUGUA 3'
::.:::..:.::::::::::
query 3' AAGGUUUGCUAGAAGGACAU 5'
query=miR5499, target=RO3G_11387, score=3, range=2596-2615, stran
target 5' UUACAAAUGAUCUUCUUGUA 3'
:: ::::.::::::::.::::
query 3' AAGGUUUGCUAGAAGGACAU 5'
query=miR5499, target=RO3G_12682, score=3.5, range=410-429, stran
target 5' CAAUAAACGAUCUUCCUGUA 3'
.:::::::::::::::::
query 3' AAGGUUUGCUAGAAGGACAU 5'
query=miR5499, target=RO3G_08132, score=4, range=161-179, strand=
target 5' UUCCCAACGAUUUUCCU-UA 3'
:::: ::::::.::::: ::
query 3' AAGGUUUGCUAGAAGGACAU 5'
query=miR5679, target=RO3G_16392, score=0, range=942-961, strand=
target 5' UCUAACUUCUUCUGCACCAA 3'
::::::::::::::::::::
query 3' AGAUUGAAGAAGACGUGGUU 5'
query=miR5679, target=RO3G_08382, score=1.5, range=1059-1078, str
target 5' UCCAGCUUCUUCUGCACCAA 3'
:: :.:::::::::::::::
query 3' AGAUUGAAGAAGACGUGGUU 5'
query=miR5679, target=RO3G_17260, score=2, range=1972-1991, stran
target 5' UCCAACUUCUUUUGCACCAA 3'
```

```
:: :::::::.::::::::
query 3' AGAUUGAAGAAGACGUGGUU 5'
query=miR5679, target=RO3G_15046, score=2.5, range=81-100, strand
target 5' UCUUCCUUCUUCUGCACCAG 3'
::: ::::::::::::::.
query 3' AGAUUGAAGAAGACGUGGUU 5'
query=miR5679, target=RO3G_15766, score=3, range=1717-1735, stran
target 5' UCU-ACUUCUUCUGUACUAA 3'
::: ::::::::::.::.::
query 3' AGAUUGAAGAAGACGUGGUU 5'
query=miR5679, target=RO3G_15382, score=3.5, range=563-582, stran
target 5' UUUGGGUUCUUCUGCACCGA 3'
:.:.. ::::::::::::.:
query 3' AGAUUGAAGAAGACGUGGUU 5'
query=miR5679, target=RO3G_16662, score=3.5, range=344-363, stran
target 5' UUUGGGUUCUUCUGCACCGA 3'
:.:.. ::::::::::::.:
query 3' AGAUUGAAGAAGACGUGGUU 5'
query=miR5679, target=RO3G_00611, score=3.5, range=4253-4272,
strand=-1
target 5' UUGAACUUCUUCUGGACCAA 3'
:. ::::::::::: :::::
query 3' AGAUUGAAGAAGACGUGGUU 5'
query=miR5679, target=RO3G_12393, score=3.5, range=167-186, stran
target 5' UCUGCCUUCUUCUUCACCAA 3'
:::. :::::::: ::::::
query 3' AGAUUGAAGAAGACGUGGUU 5'
query=miR5679, target=RO3G_01808, score=3.5, range=4676-4695,
strand=-1
target 5' UUCAGCAUCUUCUGCACCAG 3'
:. :.: ::::::::::::.
query 3' AGAUUGAAGAAGACGUGGUU 5'
query=miR5679, target=RO3G_06040, score=3.5, range=377-396, stran
target 5' GCUGUCUUCUUCUGCACCAC 3'
::. ::::::::::::::
query 3' AGAUUGAAGAAGACGUGGUU 5'
query=miR5679, target=RO3G_04287, score=4, range=489-508, strand=
target 5' UCUGAGUUAUUCUGCACCAG 3'
:::.: :: ::::::::::.
query 3' AGAUUGAAGAAGACGUGGUU 5'
query=miR5679, target=RO3G_10076, score=4, range=634-652, strand=
target 5' UCU-ACUUCUUCUGCCUCAA 3'
::: ::::::::::: .:::
query 3' AGAUUGAAGAAGACGUGGUU 5'
query=miR5679, target=RO3G_00105, score=4, range=6733-6751, stran
```

```
target 5' UCCAA-UUCUUCUGCAUCAC 3'
:: :: ::::::::::.::
query 3' AGAUUGAAGAAGACGUGGUU 5'
query=miR5679, target=RO3G_04826, score=4, range=502-521, strand=
target 5' UUUAAUUUUUUGUGCACCAA 3'
:.:::.::.:: ::::::::
query 3' AGAUUGAAGAAGACGUGGUU 5'
query=miR5679, target=RO3G_12932, score=4, range=1367-1386, stran
target 5' GUUACCUUCUUCUGUACCAG 3'
.:: :::::::::.::::.
query 3' AGAUUGAAGAAGACGUGGUU 5'
query=miR5679, target=RO3G_00509, score=4, range=1631-1650, stran
target 5' UCUAAAUUUUUCUGCACCCA 3'
::::: ::.::::::::: :
query 3' AGAUUGAAGAAGACGUGGUU 5'
query=miR5679, target=RO3G_12643, score=4, range=239-258, strand=
target 5' UCUACCUUUUUCUUCACCAA 3'
:::: :::.:::: ::::::
query 3' AGAUUGAAGAAGACGUGGUU 5'
query=miR5679, target=RO3G_05177, score=4, range=377-395, strand=
target 5' UUUGA-UUCUUCUGCGCUAA 3'
:.:.: :::::::::.:.::
query 3' AGAUUGAAGAAGACGUGGUU 5'
query=miR5679, target=RO3G_07463, score=4, range=4283-4302, stran
target 5' UCGACCUUCUUCUGUAUCAA 3'
:: : :::::::::.:.:::
query 3' AGAUUGAAGAAGACGUGGUU 5'
query=miR5679, target=RO3G_01798, score=4, range=2320-2339, stran
target 5' UCUAUAUUCUUUUGCAUCAA 3'
:::: :::::.::::.:::
query 3' AGAUUGAAGAAGACGUGGUU 5'
query=miR5679, target=RO3G_16149, score=4, range=161-179, strand=
target 5' ACUAA-UUCUUCUGCAUUAA 3'
:::: ::::::::::..::
query 3' AGAUUGAAGAAGACGUGGUU 5'
query=miR5679, target=RO3G_02261, score=4, range=590-608, strand=
target 5' UCU-ACUUCUUCUGUAGCAA 3'
::: ::::::::::.: :::
query 3' AGAUUGAAGAAGACGUGGUU 5'
query=miR5679, target=RO3G_15954, score=4, range=329-349, strand=
target 5' GCUCAUCUUCUUCUGCAUCAA 3'
:: : :::::::::::.:::
query 3' AGA-UUGAAGAAGACGUGGUU 5'
query=miR5679, target=RO3G_13384, score=4, range=1193-1211, stran
target 5' UC-AGCUUCUUCAGCACCAG 3'
```

```
:: :.::::::: ::::::.
query 3' AGAUUGAAGAAGACGUGGUU 5'
query=miR5679, target=RO3G_06572, score=4, range=179-197, strand=
target 5' ACUAA-UUCUUCUACACCAA 3'
:::: ::::::: ::::::
query 3' AGAUUGAAGAAGACGUGGUU 5'
query=miR5679, target=RO3G_15432, score=4, range=305-323, strand=
target 5' UCU-UCUUCUUCUGCACUAC 3'
::: ::::::::::::.:
query 3' AGAUUGAAGAAGACGUGGUU 5'
query=miR5679, target=RO3G_11023, score=4, range=1487-1505, stran
target 5' UCU-UCUUCUUCUUCACCAA 3'
::: :::::::: ::::::
query 3' AGAUUGAAGAAGACGUGGUU 5'
query=miR161.1, target=RO3G_13154, score=0, range=378-399, strand
target 5' ACCCCUUGAUGAGCCACUUUCA 3'
::::::::::::::::::::::
query 3' UGGGGAACUACUCGGUGAAAGU 5'
query=miR161.1, target=RO3G_02823, score=0, range=378-399, strand
target 5' ACCCCUUGAUGAGCCACUUUCA 3'
::::::::::::::::::::::
query 3' UGGGGAACUACUCGGUGAAAGU 5'
query=miR161.1, target=RO3G_03622, score=2, range=201-222, strand
target 5' ACCCCUUGAUGAACCACUUUCA 3'
:::::::::::: :::::::::
query 3' UGGGGAACUACUCGGUGAAAGU 5'
query=miR161.1, target=RO3G_12187, score=2, range=378-399, strand
target 5' ACCCCUUGAUGAACCACUUUCA 3'
:::::::::::: :::::::::
query 3' UGGGGAACUACUCGGUGAAAGU 5'
query=miR161.1, target=RO3G_14986, score=2, range=378-399, strand
target 5' ACCCCUUGAUGAACCACUUUCA 3'
:::::::::::: :::::::::
query 3' UGGGGAACUACUCGGUGAAAGU 5'
query=miR161.1, target=RO3G_07714, score=2, range=201-222, strand
target 5' ACCCCUUGAUGAACCACUUUCA 3'
:::::::::::: :::::::::
query 3' UGGGGAACUACUCGGUGAAAGU 5'
query=miR161.1, target=RO3G_05349, score=2, range=378-399, strand
target 5' ACCCCUUGAUGAACCACUUUCA 3'
:::::::::::: :::::::::
query 3' UGGGGAACUACUCGGUGAAAGU 5'
query=miR161.1, target=RO3G_04693, score=2, range=378-399, strand
target 5' ACCCCUUGAUGAACCACUUUCA 3'
:::::::::::: :::::::::
```

```
query 3' UGGGGAACUACUCGGUGAAAGU 5'
query=miR161.1, target=RO3G_03610, score=2, range=356-377, strand
target 5' ACCCCUUGAUGAACCACUUUCA 3'
:::::::::::: :::::::::
query 3' UGGGGAACUACUCGGUGAAAGU 5'
query=miR161.1, target=RO3G_04209, score=2.5, range=306-327, stra
target 5' GCCCCUUGAUGAACCACUUUCA 3'
.::::::::::: :::::::::
query 3' UGGGGAACUACUCGGUGAAAGU 5'
query=miR161.1, target=RO3G_03658, score=4, range=128-150, strand
target 5' UCUCCUUGGUUGAGCCACUUUCU 3'
:.:::::. :::::::::::
query 3' UGGGGAACU-ACUCGGUGAAAGU 5'
query=miR161.1, target=RO3G_11575, score=4, range=689-710, strand
target 5' AUACCUUGGUGAGCCACUAUCA 3'
:. ::::.::::::::: :::
query 3' UGGGGAACUACUCGGUGAAAGU 5'
query=miR3631a-3p, target=RO3G_03783, score=0, range=268-289, str
target 5' UUGUUAAGACAUAAUCCAAUUA 3'
::::::::::::::::::::::
query 3' AACAAUUCUGUAUUAGGUUAAU 5'
query=miR3631a-3p, target=RO3G_13292, score=0, range=268-289, str
target 5' UUGUUAAGACAUAAUCCAAUUA 3'
::::::::::::::::::::::
query 3' AACAAUUCUGUAUUAGGUUAAU 5'
query=miR3631a-3p, target=RO3G_17309, score=0, range=268-289, str
target 5' UUGUUAAGACAUAAUCCAAUUA 3'
::::::::::::::::::::::
query 3' AACAAUUCUGUAUUAGGUUAAU 5'
query=miR3631a-3p, target=RO3G_17372, score=0, range=268-289, str
target 5' UUGUUAAGACAUAAUCCAAUUA 3'
::::::::::::::::::::::
query 3' AACAAUUCUGUAUUAGGUUAAU 5'
query=miR3631a-3p, target=RO3G_06411, score=0, range=268-289, str
target 5' UUGUUAAGACAUAAUCCAAUUA 3'
::::::::::::::::::::::
query 3' AACAAUUCUGUAUUAGGUUAAU 5'
query=miR3631a-3p, target=RO3G_17311, score=0, range=268-289, str
target 5' UUGUUAAGACAUAAUCCAAUUA 3'
::::::::::::::::::::::
query 3' AACAAUUCUGUAUUAGGUUAAU 5'
query=miR3631a-3p, target=RO3G_14785, score=0, range=268-289, str
target 5' UUGUUAAGACAUAAUCCAAUUA 3'
::::::::::::::::::::::
query 3' AACAAUUCUGUAUUAGGUUAAU 5'
```

query=miR3631a-3p, target=RO3G_15743, score=2, range=268-289, str
target 5' CUAUUAAGACAUAAUCCAAUUA 3'
: :::::::::::::::::::
query 3' AACAAUUCUGUAUUAGGUUAAU 5'
query=miR3631a-3p, target=RO3G_04902, score=3, range=158-180, str
target 5' UUGUUAAGACCAGAAUCCAAUUA 3'
::::::::: :: ::::::::::
query 3' AACAAUUCU-GUAUUAGGUUAAU 5'
query=miR7697-3p, target=RO3G_15176, score=3.5, range=89-108, str
target 5' UAUCUGAUU-UUGUUAUUGGA 3'
::::.:::. ::::::::::
query 3' AUAGGCUAGUAACAAUAACCU 5'
query=miR7697-3p, target=RO3G_11623, score=4, range=1154-1173, strand=1
target 5' CAUGCGAU-AUUGUUAUUGGA 3'
:: :::: :::::::::::
query 3' AUAGGCUAGUAACAAUAACCU 5'
query=miR7697-3p, target=RO3G_16177, score=4, range=813-833, stra
target 5' CAUUUGAUCAUUGUUAAUGGA 3'
::..::::::::::: ::::
query 3' AUAGGCUAGUAACAAUAACCU 5'
query=miR164b-3p, target=RO3G_16322, score=3, range=984-1001, strand=-1
target 5' GGAGGAGAAGCUGGGGGC 3'
:: ::::::::::: ::
query 3' CCACCUCUUCGACCCGCG 5'
query=miR164b-3p, target=RO3G_14084, score=3, range=1524-1540, strand=-1
target 5' CGU-GAGAAGUUGGGCGC 3'
:: ::::::.:::::::
query 3' CCACCUCUUCGACCCGCG 5'
query=miR164b-3p, target=RO3G_08058, score=3.5, range=1028-1046, strand=-1
target 5' GGUGUGAGAAGCUGGGCUU 3'
:::: ::::::::::: .
query 3' CCAC-CUCUUCGACCCGCG 5'
query=miR164b-3p, target=RO3G_00245, score=4, range=190-208, stra
target 5' GUUGGAGGAGCUGGGGCGC 3'
: :::::.:::: ::::::
query 3' CCACCUCUUCGA-CCCGCG 5'
query=miR164b-3p, target=RO3G_07739, score=4, range=6-24, strand=
target 5' GUUGGAGGAGCUGGAGCGC 3'
: :::::.:::::: ::::
query 3' CCACCUCUUCGACC-CGCG 5'
query=miR164b-3p, target=RO3G_17278, score=4, range=190-208, stra

```
target 5' GUUGGAGGAGCUGGGGCGC 3'
: :::::.:::: ::::::
query 3' CCACCUCUUCGA-CCCGCG 5'
query=miR164b-3p, target=RO3G_09937, score=4, range=190-208, stra
target 5' GUUGGAGGAGCUGGGGCGC 3'
: :::::.:::: ::::::
query 3' CCACCUCUUCGA-CCCGCG 5'
query=miR164b-3p, target=RO3G_10795, score=4, range=255-273, stra
target 5' GUUGGAGGAGCUUGGGCGC 3'
: :::::.::: :::::::
query 3' CCACCUCUUCG-ACCCGCG 5'
query=miR164b-3p, target=RO3G_05521, score=4, range=255-273, stra
target 5' GUUGGAGGAGCUGGGGCGC 3'
: :::::.:::: ::::::
query 3' CCACCUCUUCGA-CCCGCG 5'
query=miR164b-3p, target=RO3G_10474, score=4, range=10-27, strand
target 5' AGUGGAGAAGCUGGUCGA 3'
::::::::::::: ::
query 3' CCACCUCUUCGACCCGCG 5'
query=miR164b-3p, target=RO3G_14969, score=4, range=606-623, stra
target 5' GGUGAAGAAGGUGGGCGG 3'
:::: ::::: ::::::
query 3' CCACCUCUUCGACCCGCG 5'
query=miR164b-3p, target=RO3G_01126, score=4, range=606-623, stra
target 5' GGUGAAGAAGGUGGGCGG 3'
:::: ::::: ::::::
query 3' CCACCUCUUCGACCCGCG 5'
query=miR164b-3p, target=RO3G_00050, score=4, range=768-786, stra
target 5' GGCGAGAGAUGCUGGGCGC 3'
:: : :::: :::::::::
query 3' CCAC-CUCUUCGACCCGCG 5'
query=miR164b-3p, target=RO3G_04977, score=4, range=1846-1863,
strand=-1
target 5' GGUGUAGAUGCUGGGUGC 3'
:::: ::: ::::::.::
query 3' CCACCUCUUCGACCCGCG 5'
query=miR164b-3p, target=RO3G_07640, score=4, range=70-86, strand
target 5' GGUGGAGAAG-UGGGCAC 3'
:::::::::: ::::: :
query 3' CCACCUCUUCGACCCGCG 5'
query=miR5818, target=RO3G_06866, score=3.5, range=973-990, stran
target 5' GACU-GCUCUUCUGUUGGA 3'
.: : ::.::::::::::
query 3' UUCACCGGGAAGACAACCU 5'
query=miR5818, target=RO3G_12153, score=3.5, range=1195-1213, str
```

```
target 5' AGUUAUCCCUUCUGUUGGA 3'
:. : ::::::::::::
query 3' UUCACCGGGAAGACAACCU 5'
query=miR5818, target=RO3G_06159, score=4, range=58-77, strand=1
target 5' AAAUGGCCCCUUCUGUUGAA 3'
:: ::: :::::::::: :
query 3' UUCACC-GGGAAGACAACCU 5'
query=miR5818, target=RO3G_03338, score=4, range=381-399, strand=
target 5' AAAUAUCCCUUCUGUUGGC 3'
:: : :::::::::::
query 3' UUCACCGGGAAGACAACCU 5'
query=miR5818, target=RO3G_06840, score=4, range=154-171, strand=
target 5' AAGUGUCCCUU-UGUUGGU 3'
::::: ::::: ::::::
query 3' UUCACCGGGAAGACAACCU 5'
query=miR5818, target=RO3G_13337, score=4, range=522-540, strand=
target 5' AAGUGUCUCUUCUGUUAGA 3'
::::: :.:::::::: ::
query 3' UUCACCGGGAAGACAACCU 5'
query=miR5818, target=RO3G_05500, score=4, range=169-187, strand=
target 5' AAGUUGCACUUCUGUUGGC 3'
:::: :: :::::::::
query 3' UUCACCGGGAAGACAACCU 5'
query=miR5818, target=RO3G_07064, score=4, range=989-1007, strand
target 5' AAGGCACCCUUCUGUUGGU 3'
::: :::::::::::
query 3' UUCACCGGGAAGACAACCU 5'
query=miR1211-3p, target=RO3G_01518, score=0, range=395-413, stra
target 5' GCAUGAAGAGACGGUGUGA 3'
:::::::::::::::::::
query 3' CGUACUUCUCUGCCACACU 5'
query=miR1211-3p, target=RO3G_01528, score=0, range=590-608, stra
target 5' GCAUGAAGAGACGGUGUGA 3'
:::::::::::::::::::
query 3' CGUACUUCUCUGCCACACU 5'
query=miR1211-3p, target=RO3G_05181, score=0.5, range=1022-1040,
strand=1
target 5' GUAUGAAGAGACGGUGUGA 3'
:.:::::::::::::::::
query 3' CGUACUUCUCUGCCACACU 5'
query=miR1211-3p, target=RO3G_13903, score=0.5, range=1022-1040,
strand=1
target 5' GUAUGAAGAGACGGUGUGA 3'
:.:::::::::::::::::
query 3' CGUACUUCUCUGCCACACU 5'
```

query=miR1211-3p, target=RO3G_13664, score=0.5, range=1022-1040, strand=1
target 5' GUAUGAAGAGACGGUGUGA 3'
:.:::::::::::::::::
query 3' CGUACUUCUCUGCCACACU 5'
query=miR1211-3p, target=RO3G_14722, score=0.5, range=1022-1040, strand=1
target 5' GUAUGAAGAGACGGUGUGA 3'
:.:::::::::::::::::
query 3' CGUACUUCUCUGCCACACU 5'
query=miR1211-3p, target=RO3G_08906, score=0.5, range=1022-1040, strand=1
target 5' GUAUGAAGAGACGGUGUGA 3'
:.:::::::::::::::::
query 3' CGUACUUCUCUGCCACACU 5'
query=miR1211-3p, target=RO3G_05303, score=0.5, range=1022-1040, strand=1
target 5' GUAUGAAGAGACGGUGUGA 3'
:.:::::::::::::::::
query 3' CGUACUUCUCUGCCACACU 5'
query=miR1211-3p, target=RO3G_02354, score=0.5, range=1022-1040, strand=1
target 5' GUAUGAAGAGACGGUGUGA 3'
:.:::::::::::::::::
query 3' CGUACUUCUCUGCCACACU 5'
query=miR1211-3p, target=RO3G_05194, score=0.5, range=1022-1040, strand=1
target 5' GUAUGAAGAGACGGUGUGA 3'
:.:::::::::::::::::
query 3' CGUACUUCUCUGCCACACU 5'
query=miR1211-3p, target=RO3G_13672, score=0.5, range=1022-1040, strand=1
target 5' GUAUGAAGAGACGGUGUGA 3'
:.:::::::::::::::::
query 3' CGUACUUCUCUGCCACACU 5'
query=miR1211-3p, target=RO3G_12139, score=0.5, range=395-413, strand=1
target 5' GUAUGAAGAGACGGUGUGA 3'
:.:::::::::::::::::
query 3' CGUACUUCUCUGCCACACU 5'
query=miR1211-3p, target=RO3G_11486, score=0.5, range=731-749, strand=1
target 5' GUAUGAAGAGACGGUGUGA 3'
:.:::::::::::::::::
query 3' CGUACUUCUCUGCCACACU 5'

query=miR1211-3p, target=RO3G_09419, score=0.5, range=842-860, strand=1
target 5' GUAUGAAGAGACGGUGUGA 3'
:.:::::::::::::::::
query 3' CGUACUUCUCUGCCACACU 5'
query=miR1211-3p, target=RO3G_05616, score=0.5, range=311-329, strand=1
target 5' GUAUGAAGAGACGGUGUGA 3'
:.:::::::::::::::::
query 3' CGUACUUCUCUGCCACACU 5'
query=miR1211-3p, target=RO3G_11567, score=0.5, range=1679-1697, strand=1
target 5' GUAUGAAGAGACGGUGUGA 3'
:.:::::::::::::::::
query 3' CGUACUUCUCUGCCACACU 5'
query=miR1211-3p, target=RO3G_10644, score=0.5, range=407-425, strand=1
target 5' GUAUGAAGAGACGGUGUGA 3'
:.:::::::::::::::::
query 3' CGUACUUCUCUGCCACACU 5'
query=miR1211-3p, target=RO3G_05603, score=0.5, range=281-299, strand=1
target 5' GUAUGAAGAGACGGUGUGA 3'
:.:::::::::::::::::
query 3' CGUACUUCUCUGCCACACU 5'
query=miR1211-3p, target=RO3G_01923, score=1.5, range=971-989, strand=1
target 5' GUAUGAAGAGAUGGUGUGA 3'
:.::::::::.::::::
query 3' CGUACUUCUCUGCCACACU 5'
query=miR1211-3p, target=RO3G_11757, score=2, range=407-425, stra
target 5' GCAUGAAGAGACGAUGUGA 3'
::::::::::::: :::::
query 3' CGUACUUCUCUGCCACACU 5'
query=miR1211-3p, target=RO3G_11765, score=2, range=407-425, stra
target 5' GCAUGAAGAGACGAUGUGA 3'
::::::::::::: :::::
query 3' CGUACUUCUCUGCCACACU 5'
query=miR1211-3p, target=RO3G_05535, score=2.5, range=407-425, strand=1
target 5' GUAUGAAGAGACGGCGUGA 3'
:.::::::::::: ::::
query 3' CGUACUUCUCUGCCACACU 5'
query=miR1211-3p, target=RO3G_04104, score=2.5, range=719-737, strand=1

```
target 5' GUAUGAAGAGACGGUAUGA 3'
:.:::::::::::: :::
query 3' CGUACUUCUCUGCCACACU 5'
query=miR1211-3p, target=RO3G_14467, score=3, range=551-569, stra
target 5' GCAUGAAGAGGCGAUGUGA 3'
::::::::::.:: :::::
query 3' CGUACUUCUCUGCCACACU 5'
query=miR1211-3p, target=RO3G_08684, score=3, range=440-458, stra
target 5' GCAUGAAGAGGCGAUGUGA 3'
::::::::::.:: :::::
query 3' CGUACUUCUCUGCCACACU 5'
query=miR1211-3p, target=RO3G_16873, score=3, range=649-666, stra
target 5' GCAU-CAGAGACGGUGUGU 3'
:::: :::::::::::
query 3' CGUACUUCUCUGCCACACU 5'
query=miR1211-3p, target=RO3G_14020, score=3.5, range=2867-2885,
strand=-1
target 5' GUAUGAAGAGGCGGUGGGA 3'
:.:::::::.::::: ::
query 3' CGUACUUCUCUGCCACACU 5'
query=miR1211-3p, target=RO3G_15811, score=4, range=377-395, stra
target 5' GCAUGAAGAGACGAUAUGA 3'
::::::::::::: : :::
query 3' CGUACUUCUCUGCCACACU 5'
query=miR1211-3p, target=RO3G_13558, score=4, range=444-462, stra
target 5' ACAAGAAGAGACGGUGUCA 3'
:: ::::::::::::: :
query 3' CGUACUUCUCUGCCACACU 5'
query=miR1211-3p, target=RO3G_08982, score=4, range=924-941, stra
target 5' GGAUGAGGAGACGG-GUGA 3'
: ::::.::::::: ::::
query 3' CGUACUUCUCUGCCACACU 5'
query=miR1211-3p, target=RO3G_03733, score=4, range=709-727, stra
target 5' GGAAGAAGAUACGGUGUGA 3'
: : ::::: :::::::::
query 3' CGUACUUCUCUGCCACACU 5'
query=miR1211-3p, target=RO3G_02626, score=4, range=310-327, stra
target 5' UCAAGAAGAGACGGUG-GA 3'
:: :::::::::::: ::
query 3' CGUACUUCUCUGCCACACU 5'
query=miR1211-3p, target=RO3G_01552, score=4, range=20-37, strand
target 5' GUGUCAAGA-ACGGUGUGA 3'
:..: :::: :::::::::
query 3' CGUACUUCUCUGCCACACU 5'
query=miR1211-3p, target=RO3G_15523, score=4, range=85-103, stran
```

```
target 5' GCAUGAAAAGACGAUGUGA 3'
::::::: ::::: :::::
query 3' CGUACUUCUCUGCCACACU 5'
query=miR1211-3p, target=RO3G_15009, score=4, range=602-619, stra
target 5' GCAGGAGGAGACGGUG-GA 3'
::: ::.:::::::: ::
query 3' CGUACUUCUCUGCCACACU 5'
query=miR1211-3p, target=RO3G_14020, score=4, range=2117-2134,
strand=-1
target 5' GGAU-AGGAGGCGGUGUGA 3'
: :: :.:::.:::::::
query 3' CGUACUUCUCUGCCACACU 5'
query=miR5246, target=RO3G_04671, score=0, range=727-744, strand=
target 5' AAGUCAAAGCUGUCUCAA 3'
::::::::::::::::::
query 3' UUCAGUUUCGACAGAGUU 5'
query=miR5246, target=RO3G_07029, score=0, range=226-243, strand=
target 5' AAGUCAAAGCUGUCUCAA 3'
::::::::::::::::::
query 3' UUCAGUUUCGACAGAGUU 5'
query=miR5246, target=RO3G_13139, score=0, range=226-243, strand=
target 5' AAGUCAAAGCUGUCUCAA 3'
::::::::::::::::::
query 3' UUCAGUUUCGACAGAGUU 5'
query=miR5246, target=RO3G_10036, score=0, range=226-243, strand=
target 5' AAGUCAAAGCUGUCUCAA 3'
::::::::::::::::::
query 3' UUCAGUUUCGACAGAGUU 5'
query=miR5246, target=RO3G_17463, score=0, range=226-243, strand=
target 5' AAGUCAAAGCUGUCUCAA 3'
::::::::::::::::::
query 3' UUCAGUUUCGACAGAGUU 5'
query=miR5246, target=RO3G_09487, score=0, range=226-243, strand=
target 5' AAGUCAAAGCUGUCUCAA 3'
::::::::::::::::::
query 3' UUCAGUUUCGACAGAGUU 5'
query=miR5246, target=RO3G_05933, score=0, range=226-243, strand=
target 5' AAGUCAAAGCUGUCUCAA 3'
::::::::::::::::::
query 3' UUCAGUUUCGACAGAGUU 5'
query=miR5246, target=RO3G_09485, score=0, range=226-243, strand=
target 5' AAGUCAAAGCUGUCUCAA 3'
::::::::::::::::::
query 3' UUCAGUUUCGACAGAGUU 5'
query=miR5246, target=RO3G_09942, score=0, range=226-243, strand=
```

```
target 5' AAGUCAAAGCUGUCUCAA 3'
::::::::::::::::::
query 3' UUCAGUUUCGACAGAGUU 5'
query=miR5246, target=RO3G_12672, score=0, range=655-672, strand=
target 5' AAGUCAAAGCUGUCUCAA 3'
::::::::::::::::::
query 3' UUCAGUUUCGACAGAGUU 5'
query=miR5246, target=RO3G_01738, score=0, range=781-798, strand=
target 5' AAGUCAAAGCUGUCUCAA 3'
::::::::::::::::::
query 3' UUCAGUUUCGACAGAGUU 5'
query=miR5246, target=RO3G_13702, score=0, range=781-798, strand=
target 5' AAGUCAAAGCUGUCUCAA 3'
::::::::::::::::::
query 3' UUCAGUUUCGACAGAGUU 5'
query=miR5246, target=RO3G_06719, score=0, range=781-798, strand=
target 5' AAGUCAAAGCUGUCUCAA 3'
::::::::::::::::::
query 3' UUCAGUUUCGACAGAGUU 5'
query=miR5246, target=RO3G_05934, score=0, range=781-798, strand=
target 5' AAGUCAAAGCUGUCUCAA 3'
::::::::::::::::::
query 3' UUCAGUUUCGACAGAGUU 5'
query=miR5246, target=RO3G_14972, score=0, range=781-798, strand=
target 5' AAGUCAAAGCUGUCUCAA 3'
::::::::::::::::::
query 3' UUCAGUUUCGACAGAGUU 5'
query=miR5246, target=RO3G_06740, score=0, range=781-798, strand=
target 5' AAGUCAAAGCUGUCUCAA 3'
::::::::::::::::::
query 3' UUCAGUUUCGACAGAGUU 5'
query=miR5246, target=RO3G_13000, score=0, range=781-798, strand=
target 5' AAGUCAAAGCUGUCUCAA 3'
::::::::::::::::::
query 3' UUCAGUUUCGACAGAGUU 5'
query=miR5246, target=RO3G_00252, score=0, range=904-921, strand=
target 5' AAGUCAAAGCUGUCUCAA 3'
::::::::::::::::::
query 3' UUCAGUUUCGACAGAGUU 5'
query=miR5246, target=RO3G_09598, score=0, range=781-798, strand=
target 5' AAGUCAAAGCUGUCUCAA 3'
::::::::::::::::::
query 3' UUCAGUUUCGACAGAGUU 5'
query=miR5246, target=RO3G_04694, score=0, range=313-330, strand=
target 5' AAGUCAAAGCUGUCUCAA 3'
```

::::::::::::::::::
query 3' UUCAGUUUCGACAGAGUU 5'
query=miR5246, target=RO3G_12189, score=0, range=313-330, strand=
target 5' AAGUCAAAGCUGUCUCAA 3'
::::::::::::::::::
query 3' UUCAGUUUCGACAGAGUU 5'
query=miR5246, target=RO3G_12062, score=0, range=313-330, strand=
target 5' AAGUCAAAGCUGUCUCAA 3'
::::::::::::::::::
query 3' UUCAGUUUCGACAGAGUU 5'
query=miR5246, target=RO3G_16540, score=0, range=313-330, strand=
target 5' AAGUCAAAGCUGUCUCAA 3'
::::::::::::::::::
query 3' UUCAGUUUCGACAGAGUU 5'
query=miR5246, target=RO3G_16120, score=0, range=313-330, strand=
target 5' AAGUCAAAGCUGUCUCAA 3'
::::::::::::::::::
query 3' UUCAGUUUCGACAGAGUU 5'
query=miR5246, target=RO3G_17461, score=0, range=313-330, strand=
target 5' AAGUCAAAGCUGUCUCAA 3'
::::::::::::::::::
query 3' UUCAGUUUCGACAGAGUU 5'
query=miR5246, target=RO3G_03608, score=0, range=313-330, strand=
target 5' AAGUCAAAGCUGUCUCAA 3'
::::::::::::::::::
query 3' UUCAGUUUCGACAGAGUU 5'
query=miR5246, target=RO3G_13295, score=0, range=586-603, strand=
target 5' AAGUCAAAGCUGUCUCAA 3'
::::::::::::::::::
query 3' UUCAGUUUCGACAGAGUU 5'
query=miR5246, target=RO3G_01310, score=0, range=586-603, strand=
target 5' AAGUCAAAGCUGUCUCAA 3'
::::::::::::::::::
query 3' UUCAGUUUCGACAGAGUU 5'
query=miR5246, target=RO3G_03989, score=0, range=586-603, strand=
target 5' AAGUCAAAGCUGUCUCAA 3'
::::::::::::::::::
query 3' UUCAGUUUCGACAGAGUU 5'
query=miR5246, target=RO3G_13754, score=0, range=850-867, strand=
target 5' AAGUCAAAGCUGUCUCAA 3'
::::::::::::::::::
query 3' UUCAGUUUCGACAGAGUU 5'
query=miR5246, target=RO3G_07673, score=0, range=850-867, strand=
target 5' AAGUCAAAGCUGUCUCAA 3'
::::::::::::::::::

```
query 3' UUCAGUUUCGACAGAGUU 5'
query=miR5246, target=RO3G_01938, score=0, range=850-867, strand=
target 5' AAGUCAAAGCUGUCUCAA 3'
::::::::::::::::::
query 3' UUCAGUUUCGACAGAGUU 5'
query=miR5246, target=RO3G_12173, score=0, range=646-663, strand=
target 5' AAGUCAAAGCUGUCUCAA 3'
::::::::::::::::::
query 3' UUCAGUUUCGACAGAGUU 5'
query=miR5246, target=RO3G_01710, score=0, range=238-255, strand=
target 5' AAGUCAAAGCUGUCUCAA 3'
::::::::::::::::::
query 3' UUCAGUUUCGACAGAGUU 5'
query=miR5246, target=RO3G_08455, score=1.5, range=762-779, stran
target 5' GAUUCAAAGCUGUCUCAA 3'
.: :::::::::::::::
query 3' UUCAGUUUCGACAGAGUU 5'
query=miR5246, target=RO3G_16144, score=2, range=850-867, strand=
target 5' AAGUCAAAACUGUCUCAA 3'
:::::::: :::::::::
query 3' UUCAGUUUCGACAGAGUU 5'
query=miR5246, target=RO3G_09215, score=2, range=477-493, strand=
target 5' AAG-CAAAGCUGUCUCGA 3'
::: ::::::::::::.:
query 3' UUCAGUUUCGACAGAGUU 5'
query=miR5246, target=RO3G_09763, score=3, range=628-645, strand=
target 5' GAAUUAAAGCUGUUUCAA 3'
.: :.:::::::::.::::
query 3' UUCAGUUUCGACAGAGUU 5'
query=miR5246, target=RO3G_16293, score=3, range=1171-1188, stran
target 5' AAUUCAAAGCUGUCUGAA 3'
:: :::::::::::: ::
query 3' UUCAGUUUCGACAGAGUU 5'
query=miR5246, target=RO3G_06799, score=3, range=2959-2977, stran
target 5' AUGUCAAAGGCUGUCUCAA 3'
: :::::: ::::::::::
query 3' UUCAGUUU-CGACAGAGUU 5'
query=miR5246, target=RO3G_01135, score=3, range=2618-2634, stran
target 5' AAGGCAAAGCUG-CUCAA 3'
::: :::::::: :::::
query 3' UUCAGUUUCGACAGAGUU 5'
query=miR5246, target=RO3G_00648, score=3, range=426-444, strand=
target 5' AAGUCCAAGCAUGUCUCAA 3'
::::: :::: ::::::::
query 3' UUCAGUUUCG-ACAGAGUU 5'
```

```
query=miR5246, target=RO3G_05035, score=3, range=1720-1738, stran
target 5' AAGUCAAAAGUUGUUUCAA 3'
::::: ::::.:::.::::
query 3' UUCAG-UUUCGACAGAGUU 5'
query=miR5246, target=RO3G_10647, score=3, range=914-931, strand=
target 5' UAGCCAAAGCUGUUUCAA 3'
:: ::::::::::.::::
query 3' UUCAGUUUCGACAGAGUU 5'
query=miR5246, target=RO3G_07588, score=3.5, range=106-122, stran
target 5' AAGUUGAAGCUGUC-CAA 3'
::::..:::::::: :::
query 3' UUCAGUUUCGACAGAGUU 5'
query=miR5246, target=RO3G_05836, score=3.5, range=767-785, stran
target 5' AAAUUAAAGCUGUCAUCAA 3'
:: :.::::::::: ::::
query 3' UUCAGUUUCGACAG-AGUU 5'
query=miR5246, target=RO3G_11399, score=3.5, range=830-847, stran
target 5' AAAAUAAAGCUGUCUUAA 3'
:: .::::::::::.::
query 3' UUCAGUUUCGACAGAGUU 5'
query=miR5246, target=RO3G_10474, score=3.5, range=1487-1503, str
target 5' GAG-CAAAGCUGUCACAA 3'
.:: ::::::::: :::
query 3' UUCAGUUUCGACAGAGUU 5'
query=miR5246, target=RO3G_01898, score=3.5, range=631-648, stran
target 5' AGGCCAAAGUUGUCUCAU 3'
:.: ::::.:::::::
query 3' UUCAGUUUCGACAGAGUU 5'
query=miR5246, target=RO3G_00739, score=3.5, range=793-810, stran
target 5' AAGGUAAAGCUGUUUCAU 3'
::: .::::::::.:::
query 3' UUCAGUUUCGACAGAGUU 5'
query=miR5246, target=RO3G_03011, score=3.5, range=587-604, stran
target 5' AUGCUAAAGUUGUCUCAA 3'
: : .::::.::::::::
query 3' UUCAGUUUCGACAGAGUU 5'
query=miR5246, target=RO3G_15244, score=4, range=605-622, strand=
target 5' AAGUCAAGGCUGUAUUAA 3'
:::::::.::::: :.::
query 3' UUCAGUUUCGACAGAGUU 5'
query=miR5246, target=RO3G_07201, score=4, range=883-901, strand=
target 5' AAGCUCAAAGCUGUUUUGA 3'
::: ::::::::::.:..:
query 3' UUC-AGUUUCGACAGAGUU 5'
query=miR5246, target=RO3G_04477, score=4, range=3244-3261, stran
```

```
target 5' AAGUCUCAGCUGUCUCAA 3'
::::: ::::::::::
query 3' UUCAGUUUCGACAGAGUU 5'
query=miR5246, target=RO3G_09560, score=4, range=239-257, strand=
target 5' AAAUCAAAGCUGUUUACAA 3'
:: :::::::::.: :::
query 3' UUCAGUUUCGACAGA-GUU 5'
query=miR5246, target=RO3G_16067, score=4, range=1127-1144, stran
target 5' UAGUCAAUGCUGUCUCAU 3'
:::::: ::::::::
query 3' UUCAGUUUCGACAGAGUU 5'
query=miR5246, target=RO3G_08131, score=4, range=1066-1083, stran
target 5' AAAUUAAAGCUCUCUCAG 3'
:: :.:::::: :::::.
query 3' UUCAGUUUCGACAGAGUU 5'
query=miR5246, target=RO3G_04841, score=4, range=407-424, strand=
target 5' AAGAAAAAGUUGUUUCAA 3'
::: ::::.:::.::::
query 3' UUCAGUUUCGACAGAGUU 5'
query=miR5246, target=RO3G_03464, score=4, range=244-262, strand=
target 5' AAUUUGAAGACUGUCUCAA 3'
:: :..::: ::::::::
query 3' UUCAGUUUC-GACAGAGUU 5'
query=miR5246, target=RO3G_09319, score=4, range=618-634, strand=
target 5' UAGU-AAGGCUGUUUCAA 3'
::: ::.::::.::::
query 3' UUCAGUUUCGACAGAGUU 5'
query=miR5246, target=RO3G_02881, score=4, range=1352-1370, stran
target 5' CAAUCGAGAGCUGUCUCAA 3'
: :: :.::::::::::
query 3' UUCAG-UUUCGACAGAGUU 5'
query=miR5246, target=RO3G_14233, score=4, range=1403-1419, stran
target 5' AAG-CAAAGCUGGUUCAA 3'
::: :::::::: .::::
query 3' UUCAGUUUCGACAGAGUU 5'
query=miR5246, target=RO3G_10027, score=4, range=719-735, strand=
target 5' AAGU-AAAGCUGUUUGAA 3'
:::: ::::::::.: ::
query 3' UUCAGUUUCGACAGAGUU 5'
query=miR5246, target=RO3G_16871, score=4, range=220-236, strand=
target 5' AGGCCAAAGCUGUC-CAG 3'
:.: ::::::::: ::.
query 3' UUCAGUUUCGACAGAGUU 5'
query=miR5246, target=RO3G_09863, score=4, range=308-325, strand=
target 5' AACAAAAGGCUGUCUCAA 3'
```

```
:: ::.::::::::::
query 3' UUCAGUUUCGACAGAGUU 5'
query=miR5246, target=RO3G_08520, score=4, range=245-261, strand=
target 5' AAG-CAAAGCUGUCUGAU 3'
::: ::::::::::: :
query 3' UUCAGUUUCGACAGAGUU 5'
query=miR5246, target=RO3G_09251, score=4, range=1394-1410, stran
target 5' AACUCAAGG-UGUCUCAA 3'
:: ::::.: ::::::::
query 3' UUCAGUUUCGACAGAGUU 5'
query=miR5246, target=RO3G_14233, score=4, range=773-789, strand=
target 5' AAG-AAAAUCUGUCUCAA 3'
::: ::: :::::::::
query 3' UUCAGUUUCGACAGAGUU 5'
query=miR5246, target=RO3G_04347, score=4, range=455-472, strand=
target 5' GAAUCGAAGCUGUUUCAG 3'
.: ::.::::::::.:::.
query 3' UUCAGUUUCGACAGAGUU 5'
query=miR5246, target=RO3G_05919, score=4, range=2729-2746, stran
target 5' CAGUAAAAGUUGUCUUAA 3'
::: ::::.:::::.::
query 3' UUCAGUUUCGACAGAGUU 5'
query=miR5246, target=RO3G_08711, score=4, range=857-874, strand=
target 5' AAGCCAAAGCUGUUUCGC 3'
::: :::::::::.::.
query 3' UUCAGUUUCGACAGAGUU 5'
query=miR5246, target=RO3G_06460, score=4, range=56-74, strand=-1
target 5' CAGUCUGAAGUUGUCUCAA 3'
:::: .:::.:::::::
query 3' UUCAG-UUUCGACAGAGUU 5'
query=miR5246, target=RO3G_06926, score=4, range=2503-2521, stran
target 5' AAGUCAAAGCUGUAGUCAA 3'
::::::::::::: ::::
query 3' UUCAGUUUCGACA-GAGUU 5'
query=miR5246, target=RO3G_16145, score=4, range=601-619, strand=
target 5' AAGUGAAAGCUGUUUUCAA 3'
:::: ::::::: :.::::
query 3' UUCAGUUUCGAC-AGAGUU 5'
query=miR5246, target=RO3G_14757, score=4, range=656-673, strand=
target 5' AAGGUAAAGCUGUAUCAG 3'
::: .:::::::: :::.
query 3' UUCAGUUUCGACAGAGUU 5'
query=miR5246, target=RO3G_02737, score=4, range=266-283, strand=
target 5' CAUUCAAAGCUUUCUCAA 3'
: :::::::: ::::::
```

```
query 3' UUCAGUUUCGACAGAGUU 5'
query=miR5246, target=RO3G_01341, score=4, range=413-430, strand=
target 5' UAUUCCAAGCUGUCUCAA 3'
: :: :::::::::::
query 3' UUCAGUUUCGACAGAGUU 5'
query=miR5246, target=RO3G_15299, score=4, range=347-364, strand=
target 5' AAGAAAGAGCUGUCUCAU 3'
::: :.:::::::::
query 3' UUCAGUUUCGACAGAGUU 5'
query=miR5246, target=RO3G_12118, score=4, range=235-251, strand=
target 5' AUGUC-AAGCUGUCUUAA 3'
: ::: ::::::::.::
query 3' UUCAGUUUCGACAGAGUU 5'
query=miR5246, target=RO3G_03045, score=4, range=254-270, strand=
target 5' GGGUAAAAGCUGU-UCAA 3'
..:: :::::::: ::::
query 3' UUCAGUUUCGACAGAGUU 5'
query=miR5246, target=RO3G_02075, score=4, range=1025-1042, stran
target 5' ACAUGAAGGCUGUCUCAA 3'
: : ::.::::::::::
query 3' UUCAGUUUCGACAGAGUU 5'
query=miR5246, target=RO3G_01514, score=4, range=1013-1030, stran
target 5' ACAUGAAGGCUGUCUCAA 3'
: : ::.::::::::::
query 3' UUCAGUUUCGACAGAGUU 5'
query=miR5246, target=RO3G_02021, score=4, range=58-74, strand=-1
target 5' AAAUAAAAG-UGUCUCAA 3'
:: : :::: ::::::::
query 3' UUCAGUUUCGACAGAGUU 5'
query=miR5246, target=RO3G_00655, score=4, range=76-92, strand=-1
target 5' AAGUAAAAG-UGUCUUAA 3'
:::: :::: :::::.::
query 3' UUCAGUUUCGACAGAGUU 5'
query=miR5246, target=RO3G_01126, score=4, range=466-484, strand=
target 5' AGGUGGAAGCUGCUCUCAA 3'
:.:: .:::::: ::::::
query 3' UUCAGUUUCGAC-AGAGUU 5'
query=miR7709-3p, target=RO3G_11131, score=4, range=657-674, stra
target 5' AUCAAAUAUGUCUA-GUCA 3'
:::::::::: ::: ::::
query 3' UAGUUUAUACUGAUCCAGU 5'
query=miR7709-3p, target=RO3G_00593, score=4, range=472-489, stra
target 5' AUGAAAUAUGACU-GGUUA 3'
:: :::::::::: :::.:
query 3' UAGUUUAUACUGAUCCAGU 5'
```

```
query=miR1098, target=RO3G_01119, score=3.5, range=889-908, stran
target 5' AUUAUAGAUAAAAACGCAUU 3'
::: .:::.::::::::::
query 3' UAAAGUCUGUUUUUGCGUAU 5'
query=miR1098, target=RO3G_04236, score=4, range=931-950, strand=
target 5' GUUUUUGACAAAAACGUAUC 3'
.:::. ::::::::::.::
query 3' UAAAGUCUGUUUUUGCGUAU 5'
query=miR1098, target=RO3G_11375, score=4, range=1422-1442, stran
target 5' AUUACAAGGUAAAAACGCAUA 3'
::: : ::..:::::::::::
query 3' UAAAG-UCUGUUUUUGCGUAU 5'
query=miR1098, target=RO3G_13496, score=4, range=252-271, strand=
target 5' GUUGCUGAUAAAAACGCAUG 3'
.:: : ::.::::::::::.
query 3' UAAAGUCUGUUUUUGCGUAU 5'
query=miR1098, target=RO3G_03094, score=4, range=1187-1205, stran
target 5' AUAUCA-ACAAAAAUGUAUA 3'
:: ::: :::::::.:.:::
query 3' UAAAGUCUGUUUUUGCGUAU 5'
query=miR1098, target=RO3G_07549, score=4, range=813-832, strand=
target 5' AGUUCCCACAAAGACGCAUA 3'
: ::: ::::.::::::::
query 3' UAAAGUCUGUUUUUGCGUAU 5'
query=miR1098, target=RO3G_15988, score=4, range=1420-1439, stran
target 5' GUUUGAGGCAAAAACGCGUG 3'
.::: ::.:::::::::.:.
query 3' UAAAGUCUGUUUUUGCGUAU 5'
query=miR1098, target=RO3G_06576, score=4, range=337-356, strand=
target 5' UUUUCAGACAAGAACCCAUA 3'
::::::::::.::: ::::
query 3' UAAAGUCUGUUUUUGCGUAU 5'
query=miR1098, target=RO3G_15436, score=4, range=1483-1502, stran
target 5' AUUUGAGGCAAGAACGCAUU 3'
:::: ::.:::.:::::::
query 3' UAAAGUCUGUUUUUGCGUAU 5'
query=miR1098, target=RO3G_14314, score=4, range=1009-1028, stran
target 5' AUUAAAGGCGAAAACGCAUA 3'
::: ::.:.::::::::::
query 3' UAAAGUCUGUUUUUGCGUAU 5'
query=miR867, target=RO3G_07788, score=0, range=202-222, strand=-
target 5' CUCUCUAAUAAUCCUGUUCAA 3'
:::::::::::::::::::::
query 3' GAGAGAUUAUUAGGACAAGUU 5'
query=miR867, target=RO3G_11567, score=0, range=1105-1125, strand
```

target 5' CUCUCUAAUAAUCCUGUUCAA 3'
:::::::::::::::::::::
query 3' GAGAGAUUAUUAGGACAAGUU 5'
query=miR867, target=RO3G_14722, score=0, range=253-273, strand=-
target 5' CUCUCUAAUAAUCCUGUUCAA 3'
:::::::::::::::::::::
query 3' GAGAGAUUAUUAGGACAAGUU 5'
query=miR867, target=RO3G_05181, score=0, range=253-273, strand=-
target 5' CUCUCUAAUAAUCCUGUUCAA 3'
:::::::::::::::::::::
query 3' GAGAGAUUAUUAGGACAAGUU 5'
query=miR867, target=RO3G_13664, score=0, range=253-273, strand=-
target 5' CUCUCUAAUAAUCCUGUUCAA 3'
:::::::::::::::::::::
query 3' GAGAGAUUAUUAGGACAAGUU 5'
query=miR867, target=RO3G_02354, score=0, range=253-273, strand=-
target 5' CUCUCUAAUAAUCCUGUUCAA 3'
:::::::::::::::::::::
query 3' GAGAGAUUAUUAGGACAAGUU 5'
query=miR867, target=RO3G_13903, score=0, range=253-273, strand=-
target 5' CUCUCUAAUAAUCCUGUUCAA 3'
:::::::::::::::::::::
query 3' GAGAGAUUAUUAGGACAAGUU 5'
query=miR867, target=RO3G_05303, score=0, range=253-273, strand=-
target 5' CUCUCUAAUAAUCCUGUUCAA 3'
:::::::::::::::::::::
query 3' GAGAGAUUAUUAGGACAAGUU 5'
query=miR867, target=RO3G_08906, score=0, range=253-273, strand=-
target 5' CUCUCUAAUAAUCCUGUUCAA 3'
:::::::::::::::::::::
query 3' GAGAGAUUAUUAGGACAAGUU 5'
query=miR867, target=RO3G_05194, score=0, range=253-273, strand=-
target 5' CUCUCUAAUAAUCCUGUUCAA 3'
:::::::::::::::::::::
query 3' GAGAGAUUAUUAGGACAAGUU 5'
query=miR867, target=RO3G_13672, score=0, range=253-273, strand=-
target 5' CUCUCUAAUAAUCCUGUUCAA 3'
:::::::::::::::::::::
query 3' GAGAGAUUAUUAGGACAAGUU 5'
query=miR867, target=RO3G_08301, score=0, range=178-198, strand=-
target 5' CUCUCUAAUAAUCCUGUUCAA 3'
:::::::::::::::::::::
query 3' GAGAGAUUAUUAGGACAAGUU 5'
query=miR867, target=RO3G_01923, score=2, range=202-222, strand=-
target 5' CUCUCUAAUUAUCCUGUUCAA 3'

```
::::::::: :::::::::::
query 3' GAGAGAUUAUUAGGACAAGUU 5'
query=miR867, target=RO3G_05133, score=2, range=202-222, strand=-
target 5' CUCUCUAAUUAUCCUGUUCAA 3'
::::::::: :::::::::::
query 3' GAGAGAUUAUUAGGACAAGUU 5'
query=miR867, target=RO3G_02168, score=2, range=202-222, strand=-
target 5' CUCUCUAAUUAUCCUGUUCAA 3'
::::::::: :::::::::::
query 3' GAGAGAUUAUUAGGACAAGUU 5'
query=miR867, target=RO3G_05402, score=3.5, range=527-548, strand
target 5' CUCUUUAAUAAUGUCUGUUCAA 3'
::::.:::::: .::::::::
query 3' GAGAGAUUAUUA-GGACAAGUU 5'
query=miR867, target=RO3G_06105, score=4, range=561-582, strand=1
target 5' ACCUCUAAAUAAUCCUGUUCAU 3'
:::: ::::::::::::::
query 3' GAGAGA-UUAUUAGGACAAGUU 5'
query=miR867, target=RO3G_14378, score=4, range=2905-2925, strand
target 5' CUCUCGAAUCAUCUUGUUCAA 3'
::::: ::: :::.:::::::
query 3' GAGAGAUUAUUAGGACAAGUU 5'
query=miR867, target=RO3G_01636, score=4, range=202-222, strand=-
target 5' CUCUCUAAUUAUCCUCUUCAA 3'
::::::::: ::::: :::::
query 3' GAGAGAUUAUUAGGACAAGUU 5'
query=miR867, target=RO3G_02936, score=4, range=253-273, strand=-
target 5' CUCUCUAAAUAUCCUGUUCAA 3'
:::::::: ::::::::::
query 3' GAGAGAUUAUUAGGACAAGUU 5'
query=miR867, target=RO3G_09465, score=4, range=1222-1242, strand
target 5' CUUUUUCACAAUCCUGUUCAA 3'
::.:.: : :::::::::::
query 3' GAGAGAUUAUUAGGACAAGUU 5'
query=miR867, target=RO3G_01015, score=4, range=1003-1022, strand
target 5' UUUUCUAAUAAU-CUGUUCAU 3'
.:.:::::::: :::::::
query 3' GAGAGAUUAUUAGGACAAGUU 5'
query=miR867, target=RO3G_08884, score=4, range=1339-1358, strand
target 5' AUAUC-AAUAAUCUUGUUCAA 3'
: :: :::::::.:::::::
query 3' GAGAGAUUAUUAGGACAAGUU 5'
query=miR8773, target=RO3G_16542, score=0, range=473-494, strand=
target 5' AUAAACCACCGAUUGCAUCCAA 3'
::::::::::::::::::::::
```

```
query 3' UAUUUGGUGGCUAACGUAGGUU 5'
query=miR8773, target=RO3G_07956, score=4, range=700-720, strand=
target 5' AUACACC-CUGAUUGUAUCCAA 3'
::: ::: :.:::::.:::::
query 3' UAUUUGGUGGCUAACGUAGGUU 5'
query=miR9738, target=RO3G_12193, score=0, range=1788-1807, stran
target 5' GACGAGCCCAAUAUGUUUCA 3'
::::::::::::::::::::
query 3' CUGCUCGGGUUAUACAAAGU 5'
query=miR9738, target=RO3G_12087, score=0, range=4782-4801, stran
target 5' GACGAGCCCAAUAUGUUUCA 3'
::::::::::::::::::::
query 3' CUGCUCGGGUUAUACAAAGU 5'
query=miR9738, target=RO3G_08612, score=0, range=4782-4801, stran
target 5' GACGAGCCCAAUAUGUUUCA 3'
::::::::::::::::::::
query 3' CUGCUCGGGUUAUACAAAGU 5'
query=miR9738, target=RO3G_14704, score=0, range=4782-4801, stran
target 5' GACGAGCCCAAUAUGUUUCA 3'
::::::::::::::::::::
query 3' CUGCUCGGGUUAUACAAAGU 5'
query=miR9738, target=RO3G_12979, score=0, range=4782-4801, stran
target 5' GACGAGCCCAAUAUGUUUCA 3'
::::::::::::::::::::
query 3' CUGCUCGGGUUAUACAAAGU 5'
query=miR9738, target=RO3G_16686, score=0, range=4782-4801, stran
target 5' GACGAGCCCAAUAUGUUUCA 3'
::::::::::::::::::::
query 3' CUGCUCGGGUUAUACAAAGU 5'
query=miR9738, target=RO3G_10555, score=0, range=4782-4801, stran
target 5' GACGAGCCCAAUAUGUUUCA 3'
::::::::::::::::::::
query 3' CUGCUCGGGUUAUACAAAGU 5'
query=miR9738, target=RO3G_05280, score=0, range=4782-4801, stran
target 5' GACGAGCCCAAUAUGUUUCA 3'
::::::::::::::::::::
query 3' CUGCUCGGGUUAUACAAAGU 5'
query=miR9738, target=RO3G_03103, score=0, range=4782-4801, stran
target 5' GACGAGCCCAAUAUGUUUCA 3'
::::::::::::::::::::
query 3' CUGCUCGGGUUAUACAAAGU 5'
query=miR9738, target=RO3G_07305, score=0, range=4782-4801, stran
target 5' GACGAGCCCAAUAUGUUUCA 3'
::::::::::::::::::::
query 3' CUGCUCGGGUUAUACAAAGU 5'
```

```
query=miR9738, target=RO3G_13859, score=0, range=4566-4585, stran
target 5' GACGAGCCCAAUAUGUUUCA 3'
::::::::::::::::::::
query 3' CUGCUCGGGUUAUACAAAGU 5'
query=miR9738, target=RO3G_05630, score=0, range=4632-4651, stran
target 5' GACGAGCCCAAUAUGUUUCA 3'
::::::::::::::::::::
query 3' CUGCUCGGGUUAUACAAAGU 5'
query=miR9738, target=RO3G_02235, score=0, range=4125-4144, stran
target 5' GACGAGCCCAAUAUGUUUCA 3'
::::::::::::::::::::
query 3' CUGCUCGGGUUAUACAAAGU 5'
query=miR9738, target=RO3G_03990, score=0, range=1539-1558, stran
target 5' GACGAGCCCAAUAUGUUUCA 3'
::::::::::::::::::::
query 3' CUGCUCGGGUUAUACAAAGU 5'
query=miR9738, target=RO3G_06416, score=0, range=4941-4960, stran
target 5' GACGAGCCCAAUAUGUUUCA 3'
::::::::::::::::::::
query 3' CUGCUCGGGUUAUACAAAGU 5'
query=miR9738, target=RO3G_16744, score=0, range=4941-4960, stran
target 5' GACGAGCCCAAUAUGUUUCA 3'
::::::::::::::::::::
query 3' CUGCUCGGGUUAUACAAAGU 5'
query=miR9738, target=RO3G_11275, score=0, range=4941-4960, stran
target 5' GACGAGCCCAAUAUGUUUCA 3'
::::::::::::::::::::
query 3' CUGCUCGGGUUAUACAAAGU 5'
query=miR9738, target=RO3G_01718, score=0, range=4941-4960, stran
target 5' GACGAGCCCAAUAUGUUUCA 3'
::::::::::::::::::::
query 3' CUGCUCGGGUUAUACAAAGU 5'
query=miR9738, target=RO3G_01203, score=0, range=4941-4960, stran
target 5' GACGAGCCCAAUAUGUUUCA 3'
::::::::::::::::::::
query 3' CUGCUCGGGUUAUACAAAGU 5'
query=miR9738, target=RO3G_10560, score=0, range=4941-4960, stran
target 5' GACGAGCCCAAUAUGUUUCA 3'
::::::::::::::::::::
query 3' CUGCUCGGGUUAUACAAAGU 5'
query=miR9738, target=RO3G_15869, score=0, range=4941-4960, stran
target 5' GACGAGCCCAAUAUGUUUCA 3'
::::::::::::::::::::
query 3' CUGCUCGGGUUAUACAAAGU 5'
query=miR9738, target=RO3G_11403, score=0, range=4941-4960, stran
```

```
target 5' GACGAGCCCAAUAUGUUUCA 3'
::::::::::::::::::::
query 3' CUGCUCGGGUUAUACAAAGU 5'
query=miR9738, target=RO3G_01967, score=0, range=1071-1090, stran
target 5' GACGAGCCCAAUAUGUUUCA 3'
::::::::::::::::::::
query 3' CUGCUCGGGUUAUACAAAGU 5'
query=miR9738, target=RO3G_16562, score=0, range=1071-1090, stran
target 5' GACGAGCCCAAUAUGUUUCA 3'
::::::::::::::::::::
query 3' CUGCUCGGGUUAUACAAAGU 5'
query=miR9738, target=RO3G_13450, score=0, range=4599-4618, stran
target 5' GACGAGCCCAAUAUGUUUCA 3'
::::::::::::::::::::
query 3' CUGCUCGGGUUAUACAAAGU 5'
query=miR9738, target=RO3G_12952, score=0, range=1218-1237, stran
target 5' GACGAGCCCAAUAUGUUUCA 3'
::::::::::::::::::::
query 3' CUGCUCGGGUUAUACAAAGU 5'
query=miR9738, target=RO3G_13294, score=0, range=51-70, strand=-1
target 5' GACGAGCCCAAUAUGUUUCA 3'
::::::::::::::::::::
query 3' CUGCUCGGGUUAUACAAAGU 5'
query=miR9738, target=RO3G_11970, score=0, range=4287-4306, stran
target 5' GACGAGCCCAAUAUGUUUCA 3'
::::::::::::::::::::
query 3' CUGCUCGGGUUAUACAAAGU 5'
query=miR9738, target=RO3G_13153, score=0, range=51-70, strand=-1
target 5' GACGAGCCCAAUAUGUUUCA 3'
::::::::::::::::::::
query 3' CUGCUCGGGUUAUACAAAGU 5'
query=miR9738, target=RO3G_03750, score=0, range=51-70, strand=-1
target 5' GACGAGCCCAAUAUGUUUCA 3'
::::::::::::::::::::
query 3' CUGCUCGGGUUAUACAAAGU 5'
query=miR9738, target=RO3G_14323, score=3.5, range=235-254, stran
target 5' GGCGUACUCAAUAUGUUUCA 3'
:.:: :.:::::::::::::
query 3' CUGCUCGGGUUAUACAAAGU 5'
query=miR9738, target=RO3G_03851, score=3.5, range=712-730, stran
target 5' GAC-AGGCCAAUAUGUUUUG 3'
::: :: ::::::::::..
query 3' CUGCUCGGGUUAUACAAAGU 5'
query=miR9738, target=RO3G_09281, score=4, range=2520-2538, stran
target 5' CACGAGCCCAGUAUG-UUCA 3'
```

```
:::::::::.:::: ::::
query 3' CUGCUCGGGUUAUACAAAGU 5'
query=miR9738, target=RO3G_01404, score=4, range=181-199, strand=
target 5' GAUG-GUCCAAUGUGUUUCC 3'
::.: :.:::::.::::::
query 3' CUGCUCGGGUUAUACAAAGU 5'
query=miR9738, target=RO3G_05389, score=4, range=2682-2700, stran
target 5' GAUGUGUCCAAUAU-UUUCA 3'
::.: :.::::::: :::::
query 3' CUGCUCGGGUUAUACAAAGU 5'
query=miR840-3p, target=RO3G_01367, score=0, range=916-935, stran
target 5' GAAACUAAGGACAUAAACCA 3'
::::::::::::::::::::
query 3' CUUUGAUUCCUGUAUUUGGU 5'
query=miR840-3p, target=RO3G_16979, score=0, range=796-815, stran
target 5' GAAACUAAGGACAUAAACCA 3'
::::::::::::::::::::
query 3' CUUUGAUUCCUGUAUUUGGU 5'
query=miR840-3p, target=RO3G_12953, score=1, range=802-821, stran
target 5' GAAACUAAGGACAUGAACCA 3'
::::::::::::::.:::::
query 3' CUUUGAUUCCUGUAUUUGGU 5'
query=miR840-3p, target=RO3G_05752, score=3, range=107-125, stran
target 5' GAAAC-AAGGAAAUAAACCA 3'
::::: ::::: ::::::::
query 3' CUUUGAUUCCUGUAUUUGGU 5'
query=miR840-3p, target=RO3G_03482, score=3.5, range=421-441, str
target 5' GAAAUUGAAGGAUAUAAACUA 3'
::::.: :::::::::::.:
query 3' CUUUGA-UUCCUGUAUUUGGU 5'
query=miR840-3p, target=RO3G_06511, score=4, range=2111-2129, str
target 5' AAAAC-AAGGAUGUAAACCA 3'
:::: :::::..:::::::
query 3' CUUUGAUUCCUGUAUUUGGU 5'
query=miR840-3p, target=RO3G_11502, score=4, range=432-450, stran
target 5' GGAGC-AAGUACAUAAACCA 3'
:.:.: ::: ::::::::::
query 3' CUUUGAUUCCUGUAUUUGGU 5'
query=miR840-3p, target=RO3G_02506, score=4, range=1563-1582,
strand=-1
target 5' GAAGUUCAGGACUUAAACCA 3'
:::..: ::::: :::::::
query 3' CUUUGAUUCCUGUAUUUGGU 5'
query=miR840-3p, target=RO3G_07057, score=4, range=689-709, stran
target 5' GAGGCAAGAGGACAUGAACCA 3'
```

```
::..: : :::::::.:::::
query 3' CUUUGAU-UCCUGUAUUUGGU 5'
query=miR840-3p, target=RO3G_00639, score=4, range=736-754, stran
target 5' CAAAC-AAGGUCAUAAACCA 3'
:::: :::: :::::::::
query 3' CUUUGAUUCCUGUAUUUGGU 5'
query=miR5015, target=RO3G_13225, score=0, range=1654-1673, stran
target 5' GUAGACUACUACAUCACCAA 3'
::::::::::::::::::::
query 3' CAUCUGAUGAUGUAGUGGUU 5'
query=miR5015, target=RO3G_13219, score=0, range=1642-1661, stran
target 5' GUAGACUACUACAUCACCAA 3'
::::::::::::::::::::
query 3' CAUCUGAUGAUGUAGUGGUU 5'
query=miR5015, target=RO3G_05952, score=0, range=1522-1541, stran
target 5' GUAGACUACUACAUCACCAA 3'
::::::::::::::::::::
query 3' CAUCUGAUGAUGUAGUGGUU 5'
query=miR5015, target=RO3G_11967, score=0, range=1654-1673, stran
target 5' GUAGACUACUACAUCACCAA 3'
::::::::::::::::::::
query 3' CAUCUGAUGAUGUAGUGGUU 5'
query=miR5015, target=RO3G_03992, score=0, range=373-392, strand=
target 5' GUAGACUACUACAUCACCAA 3'
::::::::::::::::::::
query 3' CAUCUGAUGAUGUAGUGGUU 5'
query=miR5015, target=RO3G_15266, score=0, range=1642-1661, stran
target 5' GUAGACUACUACAUCACCAA 3'
::::::::::::::::::::
query 3' CAUCUGAUGAUGUAGUGGUU 5'
query=miR5015, target=RO3G_16800, score=0, range=1642-1661, stran
target 5' GUAGACUACUACAUCACCAA 3'
::::::::::::::::::::
query 3' CAUCUGAUGAUGUAGUGGUU 5'
query=miR5015, target=RO3G_16818, score=0, range=1384-1403, stran
target 5' GUAGACUACUACAUCACCAA 3'
::::::::::::::::::::
query 3' CAUCUGAUGAUGUAGUGGUU 5'
query=miR5015, target=RO3G_04121, score=0, range=1642-1661, stran
target 5' GUAGACUACUACAUCACCAA 3'
::::::::::::::::::::
query 3' CAUCUGAUGAUGUAGUGGUU 5'
query=miR5015, target=RO3G_15265, score=0, range=1642-1661, stran
target 5' GUAGACUACUACAUCACCAA 3'
::::::::::::::::::::
```

```
query 3' CAUCUGAUGAUGUAGUGGUU 5'
query=miR5015, target=RO3G_13217, score=0, range=1642-1661, stran
target 5' GUAGACUACUACAUCACCAA 3'
::::::::::::::::::::
query 3' CAUCUGAUGAUGUAGUGGUU 5'
query=miR5015, target=RO3G_05640, score=0, range=1390-1409, stran
target 5' GUAGACUACUACAUCACCAA 3'
::::::::::::::::::::
query 3' CAUCUGAUGAUGUAGUGGUU 5'
query=miR5015, target=RO3G_15268, score=0, range=1642-1661, stran
target 5' GUAGACUACUACAUCACCAA 3'
::::::::::::::::::::
query 3' CAUCUGAUGAUGUAGUGGUU 5'
query=miR5015, target=RO3G_16797, score=0, range=643-662, strand=
target 5' GUAGACUACUACAUCACCAA 3'
::::::::::::::::::::
query 3' CAUCUGAUGAUGUAGUGGUU 5'
query=miR5015, target=RO3G_13222, score=0, range=1636-1655, stran
target 5' GUAGACUACUACAUCACCAA 3'
::::::::::::::::::::
query 3' CAUCUGAUGAUGUAGUGGUU 5'
query=miR5015, target=RO3G_14352, score=0, range=1642-1661, stran
target 5' GUAGACUACUACAUCACCAA 3'
::::::::::::::::::::
query 3' CAUCUGAUGAUGUAGUGGUU 5'
query=miR5015, target=RO3G_10870, score=3.5, range=373-392, stran
target 5' GUGGACAGCUAUAUCACCAA 3'
::.::: .:::.::::::::
query 3' CAUCUGAUGAUGUAGUGGUU 5'
query=miR5015, target=RO3G_15674, score=4, range=694-713, strand=
target 5' GUCGACUACUUUAUCACCAA 3'
:: ::::::: .::::::::
query 3' CAUCUGAUGAUGUAGUGGUU 5'
query=miR5015, target=RO3G_07869, score=4, range=1588-1607, stran
target 5' GUCGACUACUUUAUCACCAA 3'
:: ::::::: .::::::::
query 3' CAUCUGAUGAUGUAGUGGUU 5'
query=miR5015, target=RO3G_13865, score=4, range=1615-1634, stran
target 5' GUCGACUACUUUAUCACCAA 3'
:: ::::::: .::::::::
query 3' CAUCUGAUGAUGUAGUGGUU 5'
query=miR5015, target=RO3G_10777, score=4, range=1519-1538, stran
target 5' GUCGACUACUUUAUCACCAA 3'
:: ::::::: .::::::::
query 3' CAUCUGAUGAUGUAGUGGUU 5'
```

```
query=miR5015, target=RO3G_11278, score=4, range=1546-1565, stran
target 5' GUCGACUACUUUAUCACCAA 3'
:: ::::::: .::::::::
query 3' CAUCUGAUGAUGUAGUGGUU 5'
query=miR5015, target=RO3G_14624, score=4, range=1531-1550, stran
target 5' GUCGACUACUUUAUCACCAA 3'
:: ::::::: .::::::::
query 3' CAUCUGAUGAUGUAGUGGUU 5'
query=miR5015, target=RO3G_08609, score=4, range=328-347, strand=
target 5' GUCGACUACUUUAUCACCAA 3'
:: ::::::: .::::::::
query 3' CAUCUGAUGAUGUAGUGGUU 5'
query=miR5015, target=RO3G_01720, score=4, range=328-347, strand=
target 5' GUCGACUACUUUAUCACCAA 3'
:: ::::::: .::::::::
query 3' CAUCUGAUGAUGUAGUGGUU 5'
query=miR5015, target=RO3G_16171, score=4, range=11174-11192,
strand=-1
target 5' GUA-ACAGCUGCAUCACCAA 3'
::: :: .::.:::::::::
query 3' CAUCUGAUGAUGUAGUGGUU 5'
query=miR5015, target=RO3G_01332, score=4, range=851-869, strand=
target 5' GUA-ACAGCUGCAUCACCAA 3'
::: :: .::.:::::::::
query 3' CAUCUGAUGAUGUAGUGGUU 5'
query=miR4227, target=RO3G_14636, score=0, range=38-55, strand=1
target 5' UGAAAGAUUGGUACCAGA 3'
::::::::::::::::::
query 3' ACUUUCUAACCAUGGUCU 5'
query=miR4227, target=RO3G_13717, score=0, range=38-55, strand=1
target 5' UGAAAGAUUGGUACCAGA 3'
::::::::::::::::::
query 3' ACUUUCUAACCAUGGUCU 5'
query=miR4227, target=RO3G_12005, score=0, range=662-679, strand=
target 5' UGAAAGAUUGGUACCAGA 3'
::::::::::::::::::
query 3' ACUUUCUAACCAUGGUCU 5'
query=miR4227, target=RO3G_12080, score=0, range=245-262, strand=
target 5' UGAAAGAUUGGUACCAGA 3'
::::::::::::::::::
query 3' ACUUUCUAACCAUGGUCU 5'
query=miR4227, target=RO3G_11407, score=0, range=245-262, strand=
target 5' UGAAAGAUUGGUACCAGA 3'
::::::::::::::::::
query 3' ACUUUCUAACCAUGGUCU 5'
```

query=miR4227, target=RO3G_05928, score=0, range=434-451, strand=
target 5' UGAAAGAUUGGUACCAGA 3'
::::::::::::::::::
query 3' ACUUUCUAACCAUGGUCU 5'
query=miR4227, target=RO3G_15157, score=0, range=662-679, strand=
target 5' UGAAAGAUUGGUACCAGA 3'
::::::::::::::::::
query 3' ACUUUCUAACCAUGGUCU 5'
query=miR4227, target=RO3G_04676, score=1, range=662-679, strand=
target 5' UGAAUGAUUGGUACCAGA 3'
:::: :::::::::::::
query 3' ACUUUCUAACCAUGGUCU 5'
query=miR4227, target=RO3G_12687, score=2.5, range=38-55, strand=
target 5' UGAAAGAUUGGUACCAAG 3'
:::::::::::::::: .
query 3' ACUUUCUAACCAUGGUCU 5'
query=miR4227, target=RO3G_17182, score=3, range=593-610, strand=
target 5' ACAAGGAUUGGUACCAGG 3'
::.::::::::::::.
query 3' ACUUUCUAACCAUGGUCU 5'
query=miR4227, target=RO3G_06830, score=3, range=620-637, strand=
target 5' UUAAAGAUUUGUACCAGA 3'
: ::::::: ::::::::
query 3' ACUUUCUAACCAUGGUCU 5'
query=miR4227, target=RO3G_03064, score=3, range=836-853, strand=
target 5' UGAUAGGUUGGUACCAGU 3'
::: ::.::::::::::
query 3' ACUUUCUAACCAUGGUCU 5'
query=miR4227, target=RO3G_16810, score=3.5, range=305-322, stran
target 5' CGAAAGAUUGGUACCAAG 3'
::::::::::::::: .
query 3' ACUUUCUAACCAUGGUCU 5'
query=miR4227, target=RO3G_01968, score=3.5, range=662-679, stran
target 5' UAAAAGAUUGGUACCAAG 3'
: :::::::::::::: .
query 3' ACUUUCUAACCAUGGUCU 5'
query=miR4227, target=RO3G_10525, score=3.5, range=662-679, stran
target 5' UAAAAGAUUGGUACCAAG 3'
: :::::::::::::: .
query 3' ACUUUCUAACCAUGGUCU 5'
query=miR4227, target=RO3G_07678, score=3.5, range=662-679, stran
target 5' UAAAAGAUUGGUACCAAG 3'
: :::::::::::::: .
query 3' ACUUUCUAACCAUGGUCU 5'
query=miR4227, target=RO3G_17380, score=3.5, range=662-679, stran

```
target 5' UAAAAGAUUGGUACCAAG 3'
: ::::::::::::: .
query 3' ACUUUCUAACCAUGGUCU 5'
query=miR4227, target=RO3G_05523, score=3.5, range=662-679, stran
target 5' UAAAAGAUUGGUACCAAG 3'
: ::::::::::::: .
query 3' ACUUUCUAACCAUGGUCU 5'
query=miR4227, target=RO3G_10531, score=3.5, range=662-679, stran
target 5' UAAAAGAUUGGUACCAAG 3'
: ::::::::::::: .
query 3' ACUUUCUAACCAUGGUCU 5'
query=miR4227, target=RO3G_07110, score=3.5, range=662-679, stran
target 5' UAAAAGAUUGGUACCAAG 3'
: ::::::::::::: .
query 3' ACUUUCUAACCAUGGUCU 5'
query=miR4227, target=RO3G_03767, score=3.5, range=662-679, stran
target 5' UAAAAGAUUGGUACCAAG 3'
: ::::::::::::: .
query 3' ACUUUCUAACCAUGGUCU 5'
query=miR4227, target=RO3G_01949, score=3.5, range=662-679, stran
target 5' UAAAAGAUUGGUACCAAG 3'
: ::::::::::::: .
query 3' ACUUUCUAACCAUGGUCU 5'
query=miR4227, target=RO3G_07676, score=3.5, range=662-679, stran
target 5' UAAAAGAUUGGUACCAAG 3'
: ::::::::::::: .
query 3' ACUUUCUAACCAUGGUCU 5'
query=miR4227, target=RO3G_12960, score=3.5, range=323-340, stran
target 5' UAAAAGAUUGGUACCAAG 3'
: ::::::::::::: .
query 3' ACUUUCUAACCAUGGUCU 5'
query=miR4227, target=RO3G_12974, score=3.5, range=323-340, stran
target 5' UAAAAGAUUGGUACCAAG 3'
: ::::::::::::: .
query 3' ACUUUCUAACCAUGGUCU 5'
query=miR4227, target=RO3G_07866, score=3.5, range=38-55, strand=
target 5' UAAAAGAUUGGUACCAAG 3'
: ::::::::::::: .
query 3' ACUUUCUAACCAUGGUCU 5'
query=miR4227, target=RO3G_11953, score=3.5, range=539-556, stran
target 5' UAAAAGAUUGGUACCAAG 3'
: ::::::::::::: .
query 3' ACUUUCUAACCAUGGUCU 5'
query=miR4227, target=RO3G_04819, score=3.5, range=1030-1047,
strand=-1
```

```
target 5' AGGAAGAUUGGUACCCGA 3'
:.:::::::::::: ::
query 3' ACUUUCUAACCAUGGUCU 5'
query=miR4227, target=RO3G_03917, score=3.5, range=1756-1774,
strand=-1
target 5' UGUGAGAUUUGGUACCAGA 3'
:: .::: ::::::::::
query 3' ACUUUCU-AACCAUGGUCU 5'
query=miR4227, target=RO3G_16937, score=4, range=279-296, strand=
target 5' UGAAAAAUUGGUAUUAGA 3'
::::: :::::::..:::
query 3' ACUUUCUAACCAUGGUCU 5'
query=miR4227, target=RO3G_15726, score=4, range=1739-1756, stran
target 5' UGGACGAUUGGUGUCAGG 3'
::.: :::::::..:::.
query 3' ACUUUCUAACCAUGGUCU 5'
query=miR4227, target=RO3G_15739, score=4, range=292-309, strand=
target 5' CGAAAGAUUGAUAUCAGA 3'
::::::::: ::.::::
query 3' ACUUUCUAACCAUGGUCU 5'
query=miR4227, target=RO3G_01667, score=4, range=836-853, strand=
target 5' UGAAAGAUUUGUACCAAA 3'
::::::::: :::::: :
query 3' ACUUUCUAACCAUGGUCU 5'
query=miR4227, target=RO3G_02868, score=4, range=1106-1124, stran
target 5' UUGGAGAUUGGUACGCAGA 3'
: ..:::::::::: ::::
query 3' ACUUUCUAACCAUG-GUCU 5'
query=miR4227, target=RO3G_08086, score=4, range=184-201, strand=
target 5' UCAACCAUUGGUACCAGA 3'
: :: :::::::::::
query 3' ACUUUCUAACCAUGGUCU 5'
query=miR4227, target=RO3G_08153, score=4, range=880-897, strand=
target 5' GGAGGCAUUGGUACCAGA 3'
::.. :::::::::::
query 3' ACUUUCUAACCAUGGUCU 5'
query=miR4227, target=RO3G_13522, score=4, range=10-28, strand=-1
target 5' UGAAAGAAUUGGAACCAGA 3'
:::::: ::::: ::::::
query 3' ACUUUC-UAACCAUGGUCU 5'
query=miR4227, target=RO3G_01416, score=4, range=400-418, strand=
target 5' UGAAAAGGUUGAUACCAGA 3'
:: ::::.::: :::::::
query 3' AC-UUUCUAACCAUGGUCU 5'
query=miR4227, target=RO3G_02247, score=4, range=578-595, strand=
```

```
target 5' CAAAAGAUUUGUACCAGA 3'
::::::: ::::::::
query 3' ACUUUCUAACCAUGGUCU 5'
query=miR4227, target=RO3G_08233, score=4, range=69-87, strand=-1
target 5' UGGGUGAUUUGGUACCAGA 3'
::.. :: ::::::::::
query 3' ACUUUCU-AACCAUGGUCU 5'
query=miR4227, target=RO3G_14174, score=4, range=1067-1084, stran
target 5' CGAUAGAUUGGUCCCAGA 3'
:: :::::::: :::::
query 3' ACUUUCUAACCAUGGUCU 5'
query=miR4227, target=RO3G_02201, score=4, range=850-867, strand=
target 5' UAAAAGAGUGGUACCAGU 3'
: ::::: ::::::::
query 3' ACUUUCUAACCAUGGUCU 5'
query=miR8048-5p, target=RO3G_06427, score=0, range=4006-4024,
strand=-1
target 5' CAGAUGGAAUGUAAUGAUG 3'
:::::::::::::::::::
query 3' GUCUACCUUACAUUACUAC 5'
query=miR8048-5p, target=RO3G_02222, score=0, range=1468-1486,
strand=-1
target 5' CAGAUGGAAUGUAAUGAUG 3'
:::::::::::::::::::
query 3' GUCUACCUUACAUUACUAC 5'
query=miR8048-5p, target=RO3G_15250, score=0, range=4369-4387,
strand=-1
target 5' CAGAUGGAAUGUAAUGAUG 3'
:::::::::::::::::::
query 3' GUCUACCUUACAUUACUAC 5'
query=miR8048-5p, target=RO3G_17371, score=0, range=4369-4387,
strand=-1
target 5' CAGAUGGAAUGUAAUGAUG 3'
:::::::::::::::::::
query 3' GUCUACCUUACAUUACUAC 5'
query=miR8048-5p, target=RO3G_06442, score=0, range=5071-5089,
strand=-1
target 5' CAGAUGGAAUGUAAUGAUG 3'
:::::::::::::::::::
query 3' GUCUACCUUACAUUACUAC 5'
query=miR8048-5p, target=RO3G_11776, score=0, range=1504-1522,
strand=-1
target 5' CAGAUGGAAUGUAAUGAUG 3'
:::::::::::::::::::
query 3' GUCUACCUUACAUUACUAC 5'
```

query=miR8048-5p, target=RO3G_01330, score=0, range=1648-1666, strand=-1
target 5' CAGAUGGAAUGUAAUGAUG 3'
:::::::::::::::::::
query 3' GUCUACCUUACAUUACUAC 5'
query=miR8048-5p, target=RO3G_14355, score=0, range=2620-2638, strand=-1
target 5' CAGAUGGAAUGUAAUGAUG 3'
:::::::::::::::::::
query 3' GUCUACCUUACAUUACUAC 5'
query=miR8048-5p, target=RO3G_04115, score=0, range=1357-1375, strand=-1
target 5' CAGAUGGAAUGUAAUGAUG 3'
:::::::::::::::::::
query 3' GUCUACCUUACAUUACUAC 5'
query=miR8048-5p, target=RO3G_13867, score=0, range=4330-4348, strand=-1
target 5' CAGAUGGAAUGUAAUGAUG 3'
:::::::::::::::::::
query 3' GUCUACCUUACAUUACUAC 5'
query=miR8048-5p, target=RO3G_06428, score=0, range=3508-3526, strand=-1
target 5' CAGAUGGAAUGUAAUGAUG 3'
:::::::::::::::::::
query 3' GUCUACCUUACAUUACUAC 5'
query=miR8048-5p, target=RO3G_01613, score=0, range=2611-2629, strand=-1
target 5' CAGAUGGAAUGUAAUGAUG 3'
:::::::::::::::::::
query 3' GUCUACCUUACAUUACUAC 5'
query=miR8048-5p, target=RO3G_17326, score=0, range=4246-4264, strand=-1
target 5' CAGAUGGAAUGUAAUGAUG 3'
:::::::::::::::::::
query 3' GUCUACCUUACAUUACUAC 5'
query=miR8048-5p, target=RO3G_05903, score=0, range=226-244, stra
target 5' CAGAUGGAAUGUAAUGAUG 3'
:::::::::::::::::::
query 3' GUCUACCUUACAUUACUAC 5'
query=miR8048-5p, target=RO3G_03547, score=0, range=4414-4432, strand=-1
target 5' CAGAUGGAAUGUAAUGAUG 3'
:::::::::::::::::::
query 3' GUCUACCUUACAUUACUAC 5'
query=miR8048-5p, target=RO3G_09965, score=0, range=4414-4432,

```
strand=-1
target 5' CAGAUGGAAUGUAAUGAUG 3'
:::::::::::::::::::
query 3' GUCUACCUUACAUUACUAC 5'
query=miR8048-5p, target=RO3G_13920, score=0, range=4414-4432,
strand=-1
target 5' CAGAUGGAAUGUAAUGAUG 3'
:::::::::::::::::::
query 3' GUCUACCUUACAUUACUAC 5'
query=miR8048-5p, target=RO3G_02691, score=0, range=7903-7921,
strand=-1
target 5' CAGAUGGAAUGUAAUGAUG 3'
:::::::::::::::::::
query 3' GUCUACCUUACAUUACUAC 5'
query=miR8048-5p, target=RO3G_13749, score=0, range=2923-2941,
strand=-1
target 5' CAGAUGGAAUGUAAUGAUG 3'
:::::::::::::::::::
query 3' GUCUACCUUACAUUACUAC 5'
query=miR8048-5p, target=RO3G_05619, score=0, range=2557-2575,
strand=-1
target 5' CAGAUGGAAUGUAAUGAUG 3'
:::::::::::::::::::
query 3' GUCUACCUUACAUUACUAC 5'
query=miR8048-5p, target=RO3G_05265, score=0, range=226-244, stra
target 5' CAGAUGGAAUGUAAUGAUG 3'
:::::::::::::::::::
query 3' GUCUACCUUACAUUACUAC 5'
query=miR8048-5p, target=RO3G_07668, score=0, range=817-835, stra
target 5' CAGAUGGAAUGUAAUGAUG 3'
:::::::::::::::::::
query 3' GUCUACCUUACAUUACUAC 5'
query=miR8048-5p, target=RO3G_02959, score=0, range=4414-4432,
strand=-1
target 5' CAGAUGGAAUGUAAUGAUG 3'
:::::::::::::::::::
query 3' GUCUACCUUACAUUACUAC 5'
query=miR8048-5p, target=RO3G_17404, score=0, range=1042-1060,
strand=-1
target 5' CAGAUGGAAUGUAAUGAUG 3'
:::::::::::::::::::
query 3' GUCUACCUUACAUUACUAC 5'
query=miR8048-5p, target=RO3G_10181, score=2, range=368-385, stra
target 5' CAGA-AGAAUGUAAUGAUG 3'
:::: ::::::::::::
```

query 3' GUCUACCUUACAUUACUAC 5'
query=miR8048-5p, target=RO3G_15517, score=3, range=444-462, stra
target 5' CAGAAGGAAUGUGAUGGUG 3'
:::: :::::::.:::.::
query 3' GUCUACCUUACAUUACUAC 5'
query=miR8048-5p, target=RO3G_14818, score=3.5, range=778-795, strand=1
target 5' UGGGU-GAAUGUGAUGAUG 3'
..:.: ::::::.::::::
query 3' GUCUACCUUACAUUACUAC 5'
query=miR8048-5p, target=RO3G_11678, score=4, range=240-258, stra
target 5' AAGAUUGAAUGUGAUGAUA 3'
:::: ::::::.:::::
query 3' GUCUACCUUACAUUACUAC 5'
query=miR8048-5p, target=RO3G_06144, score=4, range=456-474, stra
target 5' AACAUGGAAUGUAAUGAAG 3'
: ::::::::::::: :
query 3' GUCUACCUUACAUUACUAC 5'
query=miR8048-5p, target=RO3G_01072, score=4, range=2965-2982, strand=1
target 5' CA-AUGUAAUGUGAUGAUG 3'
:: ::: :::::.::::::
query 3' GUCUACCUUACAUUACUAC 5'
query=miR8048-5p, target=RO3G_00502, score=4, range=563-580, stra
target 5' CAGA-GGAAUGUGAUGACG 3'
:::: ::::::.:::: :
query 3' GUCUACCUUACAUUACUAC 5'
query=miR8048-5p, target=RO3G_05121, score=4, range=905-922, stra
target 5' CAGA-GGAAUGUAAUGCUU 3'
:::: ::::::::::: :
query 3' GUCUACCUUACAUUACUAC 5'
query=miR8048-5p, target=RO3G_12982, score=4, range=281-298, stra
target 5' CAACUGGAAUGU-AUGAUG 3'
:: :::::::: ::::::
query 3' GUCUACCUUACAUUACUAC 5'
query=miR8048-5p, target=RO3G_06073, score=4, range=369-388, stra
target 5' CUGAUGGAGUGUAAUCGAUG 3'
: ::::::.:::::: ::::
query 3' GUCUACCUUACAUUA-CUAC 5'
query=miR8048-5p, target=RO3G_08757, score=4, range=941-958, stra
target 5' CAGCU-GAAUGAAAUGAUG 3'
::: : ::::: :::::::
query 3' GUCUACCUUACAUUACUAC 5'
query=miR869.1, target=RO3G_11401, score=0, range=811-831, strand
target 5' CAAUCACCAGACUUGAACCCA 3'

:::::::::::::::::::::
query 3' GUUAGUGGUCUGAACUUGGGU 5'
query=miR869.1, target=RO3G_16551, score=0, range=811-831, strand
target 5' CAAUCACCAGACUUGAACCCA 3'
:::::::::::::::::::::
query 3' GUUAGUGGUCUGAACUUGGGU 5'
query=miR869.1, target=RO3G_06441, score=0, range=811-831, strand
target 5' CAAUCACCAGACUUGAACCCA 3'
:::::::::::::::::::::
query 3' GUUAGUGGUCUGAACUUGGGU 5'
query=miR869.1, target=RO3G_08846, score=0, range=811-831, strand
target 5' CAAUCACCAGACUUGAACCCA 3'
:::::::::::::::::::::
query 3' GUUAGUGGUCUGAACUUGGGU 5'
query=miR869.1, target=RO3G_11773, score=0, range=811-831, strand
target 5' CAAUCACCAGACUUGAACCCA 3'
:::::::::::::::::::::
query 3' GUUAGUGGUCUGAACUUGGGU 5'
query=miR869.1, target=RO3G_05635, score=0, range=811-831, strand
target 5' CAAUCACCAGACUUGAACCCA 3'
:::::::::::::::::::::
query 3' GUUAGUGGUCUGAACUUGGGU 5'
query=miR869.1, target=RO3G_13848, score=0, range=811-831, strand
target 5' CAAUCACCAGACUUGAACCCA 3'
:::::::::::::::::::::
query 3' GUUAGUGGUCUGAACUUGGGU 5'
query=miR869.1, target=RO3G_01956, score=0, range=811-831, strand
target 5' CAAUCACCAGACUUGAACCCA 3'
:::::::::::::::::::::
query 3' GUUAGUGGUCUGAACUUGGGU 5'
query=miR869.1, target=RO3G_17285, score=0, range=811-831, strand
target 5' CAAUCACCAGACUUGAACCCA 3'
:::::::::::::::::::::
query 3' GUUAGUGGUCUGAACUUGGGU 5'
query=miR869.1, target=RO3G_08661, score=0, range=811-831, strand
target 5' CAAUCACCAGACUUGAACCCA 3'
:::::::::::::::::::::
query 3' GUUAGUGGUCUGAACUUGGGU 5'
query=miR869.1, target=RO3G_03571, score=0, range=811-831, strand
target 5' CAAUCACCAGACUUGAACCCA 3'
:::::::::::::::::::::
query 3' GUUAGUGGUCUGAACUUGGGU 5'
query=miR869.1, target=RO3G_16555, score=0, range=811-831, strand
target 5' CAAUCACCAGACUUGAACCCA 3'
:::::::::::::::::::::

```
query 3' GUUAGUGGUCUGAACUUGGGU 5'
query=miR869.1, target=RO3G_15675, score=0, range=811-831, strand
target 5' CAAUCACCAGACUUGAACCCA 3'
:::::::::::::::::::::
query 3' GUUAGUGGUCUGAACUUGGGU 5'
query=miR869.1, target=RO3G_02689, score=0, range=811-831, strand
target 5' CAAUCACCAGACUUGAACCCA 3'
:::::::::::::::::::::
query 3' GUUAGUGGUCUGAACUUGGGU 5'
query=miR869.1, target=RO3G_09607, score=0, range=811-831, strand
target 5' CAAUCACCAGACUUGAACCCA 3'
:::::::::::::::::::::
query 3' GUUAGUGGUCUGAACUUGGGU 5'
query=miR869.1, target=RO3G_13727, score=0, range=250-270, strand
target 5' CAAUCACCAGACUUGAACCCA 3'
:::::::::::::::::::::
query 3' GUUAGUGGUCUGAACUUGGGU 5'
query=miR869.1, target=RO3G_16822, score=0, range=250-270, strand
target 5' CAAUCACCAGACUUGAACCCA 3'
:::::::::::::::::::::
query 3' GUUAGUGGUCUGAACUUGGGU 5'
query=miR869.1, target=RO3G_13737, score=0, range=250-270, strand
target 5' CAAUCACCAGACUUGAACCCA 3'
:::::::::::::::::::::
query 3' GUUAGUGGUCUGAACUUGGGU 5'
query=miR869.1, target=RO3G_07706, score=0, range=148-168, strand
target 5' CAAUCACCAGACUUGAACCCA 3'
:::::::::::::::::::::
query 3' GUUAGUGGUCUGAACUUGGGU 5'
query=miR869.1, target=RO3G_06423, score=1, range=865-885, strand
target 5' CAAUCACCGGACUUGAACCCA 3'
::::::::.::::::::::::
query 3' GUUAGUGGUCUGAACUUGGGU 5'
query=miR869.1, target=RO3G_10235, score=1, range=811-831, strand
target 5' CAAUCACCGGACUUGAACCCA 3'
::::::::.::::::::::::
query 3' GUUAGUGGUCUGAACUUGGGU 5'
query=miR869.1, target=RO3G_00681, score=1, range=811-831, strand
target 5' CAAUCACCGGACUUGAACCCA 3'
::::::::.::::::::::::
query 3' GUUAGUGGUCUGAACUUGGGU 5'
query=miR869.1, target=RO3G_09483, score=1, range=811-831, strand
target 5' CAAUCACCGGACUUGAACCCA 3'
::::::::.::::::::::::
query 3' GUUAGUGGUCUGAACUUGGGU 5'
```

query=miR869.1, target=RO3G_07875, score=1, range=811-831, strand
target 5' CAAUCACCGGACUUGAACCCA 3'
::::::::.::::::::::::
query 3' GUUAGUGGUCUGAACUUGGGU 5'
query=miR869.1, target=RO3G_16807, score=1, range=811-831, strand
target 5' CAAUCACCGGACUUGAACCCA 3'
::::::::.::::::::::::
query 3' GUUAGUGGUCUGAACUUGGGU 5'
query=miR869.1, target=RO3G_05336, score=1, range=811-831, strand
target 5' CAAUCACCGGACUUGAACCCA 3'
::::::::.::::::::::::
query 3' GUUAGUGGUCUGAACUUGGGU 5'
query=miR869.1, target=RO3G_07303, score=1, range=811-831, strand
target 5' CAAUCACCGGACUUGAACCCA 3'
::::::::.::::::::::::
query 3' GUUAGUGGUCUGAACUUGGGU 5'
query=miR869.1, target=RO3G_10732, score=1, range=811-831, strand
target 5' CAAUCACCGGACUUGAACCCA 3'
::::::::.::::::::::::
query 3' GUUAGUGGUCUGAACUUGGGU 5'
query=miR869.1, target=RO3G_09499, score=1, range=811-831, strand
target 5' CAAUCACCGGACUUGAACCCA 3'
::::::::.::::::::::::
query 3' GUUAGUGGUCUGAACUUGGGU 5'
query=miR869.1, target=RO3G_01652, score=1, range=811-831, strand
target 5' CAAUCACCGGACUUGAACCCA 3'
::::::::.::::::::::::
query 3' GUUAGUGGUCUGAACUUGGGU 5'
query=miR869.1, target=RO3G_14359, score=1, range=811-831, strand
target 5' CAAUCACCGGACUUGAACCCA 3'
::::::::.::::::::::::
query 3' GUUAGUGGUCUGAACUUGGGU 5'
query=miR869.1, target=RO3G_17382, score=1, range=811-831, strand
target 5' CAAUCACCGGACUUGAACCCA 3'
::::::::.::::::::::::
query 3' GUUAGUGGUCUGAACUUGGGU 5'
query=miR869.1, target=RO3G_13151, score=1, range=811-831, strand
target 5' CAAUCACCGGACUUGAACCCA 3'
::::::::.::::::::::::
query 3' GUUAGUGGUCUGAACUUGGGU 5'
query=miR869.1, target=RO3G_17400, score=1, range=685-705, strand
target 5' CAAUCACCGGACUUGAACCCA 3'
::::::::.::::::::::::
query 3' GUUAGUGGUCUGAACUUGGGU 5'
query=miR869.1, target=RO3G_05915, score=1, range=811-831, strand

```
target 5' CAAUCACCGGACUUGAACCCA 3'
::::::::.::::::::::::
query 3' GUUAGUGGUCUGAACUUGGGU 5'
query=miR869.1, target=RO3G_01730, score=1, range=811-831, strand
target 5' CAAUCACCGGACUUGAACCCA 3'
::::::::.::::::::::::
query 3' GUUAGUGGUCUGAACUUGGGU 5'
query=miR869.1, target=RO3G_05622, score=1, range=811-831, strand
target 5' CAAUCACCGGACUUGAACCCA 3'
::::::::.::::::::::::
query 3' GUUAGUGGUCUGAACUUGGGU 5'
query=miR869.1, target=RO3G_13456, score=1, range=208-228, strand
target 5' CAAUCACCGGACUUGAACCCA 3'
::::::::.::::::::::::
query 3' GUUAGUGGUCUGAACUUGGGU 5'
query=miR869.1, target=RO3G_16824, score=1, range=250-270, strand
target 5' CAAUCACCGGACUUGAACCCA 3'
::::::::.::::::::::::
query 3' GUUAGUGGUCUGAACUUGGGU 5'
query=miR869.1, target=RO3G_09544, score=1.5, range=811-831, stra
target 5' CAAUUACCGGACUUGAACCCA 3'
::::.:::.::::::::::::
query 3' GUUAGUGGUCUGAACUUGGGU 5'
query=miR869.1, target=RO3G_01322, score=1.5, range=697-717, stra
target 5' CAAUCACUGGACUUGAACCCA 3'
:::::::..::::::::::::
query 3' GUUAGUGGUCUGAACUUGGGU 5'
query=miR869.1, target=RO3G_03616, score=2, range=250-270, strand
target 5' CAAUCACCAGACUUGAACCGA 3'
::::::::::::::::::: :
query 3' GUUAGUGGUCUGAACUUGGGU 5'
query=miR869.1, target=RO3G_15287, score=3.5, range=445-465, stra
target 5' CAGUCACCUGACUUGAAUCCA 3'
::.:::::: :::::::.:::
query 3' GUUAGUGGUCUGAACUUGGGU 5'
query=miR869.1, target=RO3G_03766, score=3.5, range=589-609, stra
target 5' CAGUCACCUGACUUGAAUCCA 3'
::.:::::: :::::::.:::
query 3' GUUAGUGGUCUGAACUUGGGU 5'
query=miR869.1, target=RO3G_02844, score=3.5, range=589-609, stra
target 5' CAGUCACCUGACUUGAAUCCA 3'
::.:::::: :::::::.:::
query 3' GUUAGUGGUCUGAACUUGGGU 5'
query=miR869.1, target=RO3G_12664, score=3.5, range=538-558, stra
target 5' CAGUCCCCGGACUUGAAUCCA 3'
```

```
::.:: ::.:::::::.:::
query 3' GUUAGUGGUCUGAACUUGGGU 5'
query=miR869.1, target=RO3G_06170, score=3.5, range=546-565, stra
target 5' CAAUCA-CAGACUUGAACUUG 3'
:::::: ::::::::::...
query 3' GUUAGUGGUCUGAACUUGGGU 5'
query=miR869.1, target=RO3G_00766, score=4, range=1800-1819, stra
target 5' CAAUCA-CAGGCUUGAACUUA 3'
:::::: :::.:::::::..:
query 3' GUUAGUGGUCUGAACUUGGGU 5'
query=miR869.1, target=RO3G_17260, score=4, range=1019-1039, stra
target 5' CGAUUCCCAUACUUGAACCCA 3'
:.::. ::: ::::::::::
query 3' GUUAGUGGUCUGAACUUGGGU 5'
query=miR447a-3p, target=RO3G_15872, score=0, range=111-130, stra
target 5' CACAAAAUUCUCGUCCGCAA 3'
::::::::::::::::::::
query 3' GUGUUUUAAGAGCAGGCGUU 5'
query=miR447a-3p, target=RO3G_09180, score=0, range=896-915, stra
target 5' CACAAAAUUCUCGUCCGCAA 3'
::::::::::::::::::::
query 3' GUGUUUUAAGAGCAGGCGUU 5'
query=miR447a-3p, target=RO3G_16812, score=0, range=896-915, stra
target 5' CACAAAAUUCUCGUCCGCAA 3'
::::::::::::::::::::
query 3' GUGUUUUAAGAGCAGGCGUU 5'
query=miR447a-3p, target=RO3G_09189, score=0, range=896-915, stra
target 5' CACAAAAUUCUCGUCCGCAA 3'
::::::::::::::::::::
query 3' GUGUUUUAAGAGCAGGCGUU 5'
query=miR447a-3p, target=RO3G_14798, score=0, range=896-915, stra
target 5' CACAAAAUUCUCGUCCGCAA 3'
::::::::::::::::::::
query 3' GUGUUUUAAGAGCAGGCGUU 5'
query=miR447a-3p, target=RO3G_14791, score=0, range=896-915, stra
target 5' CACAAAAUUCUCGUCCGCAA 3'
::::::::::::::::::::
query 3' GUGUUUUAAGAGCAGGCGUU 5'
query=miR447a-3p, target=RO3G_16733, score=0, range=896-915, stra
target 5' CACAAAAUUCUCGUCCGCAA 3'
::::::::::::::::::::
query 3' GUGUUUUAAGAGCAGGCGUU 5'
query=miR447a-3p, target=RO3G_13059, score=4, range=62-81, strand
target 5' GACAUAGUUCUCGUCUGCAG 3'
::: :.:::::::.:::.
```

```
query 3' GUGUUUUAAGAGCAGGCGUU 5'
query=miR4412-5p, target=RO3G_01190, score=0, range=208-229, stra
target 5' AGUGCAAAGAAUAUCCGCAACA 3'
::::::::::::::::::::::
query 3' UCACGUUUCUUAUAGGCGUUGU 5'
query=miR4412-5p, target=RO3G_16146, score=0, range=385-406, stra
target 5' AGUGCAAAGAAUAUCCGCAACA 3'
::::::::::::::::::::::
query 3' UCACGUUUCUUAUAGGCGUUGU 5'
query=miR4412-5p, target=RO3G_04677, score=0, range=355-376, stra
target 5' AGUGCAAAGAAUAUCCGCAACA 3'
::::::::::::::::::::::
query 3' UCACGUUUCUUAUAGGCGUUGU 5'
query=miR4412-5p, target=RO3G_17448, score=0, range=349-370, stra
target 5' AGUGCAAAGAAUAUCCGCAACA 3'
::::::::::::::::::::::
query 3' UCACGUUUCUUAUAGGCGUUGU 5'
query=miR4412-5p, target=RO3G_01209, score=0, range=349-370, stra
target 5' AGUGCAAAGAAUAUCCGCAACA 3'
::::::::::::::::::::::
query 3' UCACGUUUCUUAUAGGCGUUGU 5'
query=miR4412-5p, target=RO3G_10239, score=0, range=349-370, stra
target 5' AGUGCAAAGAAUAUCCGCAACA 3'
::::::::::::::::::::::
query 3' UCACGUUUCUUAUAGGCGUUGU 5'
query=miR4412-5p, target=RO3G_07037, score=0, range=295-316, stra
target 5' AGUGCAAAGAAUAUCCGCAACA 3'
::::::::::::::::::::::
query 3' UCACGUUUCUUAUAGGCGUUGU 5'
query=miR4412-5p, target=RO3G_07032, score=0, range=283-304, stra
target 5' AGUGCAAAGAAUAUCCGCAACA 3'
::::::::::::::::::::::
query 3' UCACGUUUCUUAUAGGCGUUGU 5'
query=miR4412-5p, target=RO3G_12466, score=0, range=208-229, stra
target 5' AGUGCAAAGAAUAUCCGCAACA 3'
::::::::::::::::::::::
query 3' UCACGUUUCUUAUAGGCGUUGU 5'
query=miR4412-5p, target=RO3G_02950, score=0, range=208-229, stra
target 5' AGUGCAAAGAAUAUCCGCAACA 3'
::::::::::::::::::::::
query 3' UCACGUUUCUUAUAGGCGUUGU 5'
query=miR4412-5p, target=RO3G_01732, score=0, range=208-229, stra
target 5' AGUGCAAAGAAUAUCCGCAACA 3'
::::::::::::::::::::::
query 3' UCACGUUUCUUAUAGGCGUUGU 5'
```

```
query=miR4412-5p, target=RO3G_07713, score=0, range=184-205, stra
target 5' AGUGCAAAGAAUAUCCGCAACA 3'
::::::::::::::::::::::
query 3' UCACGUUUCUUAUAGGCGUUGU 5'
query=miR4412-5p, target=RO3G_09616, score=1, range=349-370, stra
target 5' AGUGCAAAGAAUAUCCGCGACA 3'
::::::::::::::::::.:::
query 3' UCACGUUUCUUAUAGGCGUUGU 5'
query=miR4412-5p, target=RO3G_16716, score=1, range=334-355, stra
target 5' AGUGCAAAGGAUAUCCGCAACA 3'
:::::::::.::::::::::::
query 3' UCACGUUUCUUAUAGGCGUUGU 5'
query=miR4412-5p, target=RO3G_00243, score=1, range=334-355, stra
target 5' AGUGCAAAGAAUAUCCGUAACA 3'
:::::::::::::::::.::::
query 3' UCACGUUUCUUAUAGGCGUUGU 5'
query=miR4412-5p, target=RO3G_07031, score=1, range=355-376, stra
target 5' AGCGCAAAGAAUAUCCGCAACA 3'
:: :::::::::::::::::::
query 3' UCACGUUUCUUAUAGGCGUUGU 5'
query=miR4412-5p, target=RO3G_16554, score=1, range=355-376, stra
target 5' AGCGCAAAGAAUAUCCGCAACA 3'
:: :::::::::::::::::::
query 3' UCACGUUUCUUAUAGGCGUUGU 5'
query=miR4412-5p, target=RO3G_15146, score=1, range=235-256, stra
target 5' AGCGCAAAGAAUAUCCGCAACA 3'
:: :::::::::::::::::::
query 3' UCACGUUUCUUAUAGGCGUUGU 5'
query=miR4412-5p, target=RO3G_01932, score=1, range=559-580, stra
target 5' AGUACAAAGAAUAUCCGCAACA 3'
::: ::::::::::::::::::
query 3' UCACGUUUCUUAUAGGCGUUGU 5'
query=miR4412-5p, target=RO3G_15251, score=1, range=316-337, stra
target 5' AGUACAAAGAAUAUCCGCAACA 3'
::: ::::::::::::::::::
query 3' UCACGUUUCUUAUAGGCGUUGU 5'
query=miR4412-5p, target=RO3G_16373, score=1, range=208-229, stra
target 5' AGUACAAAGAAUAUCCGCAACA 3'
::: ::::::::::::::::::
query 3' UCACGUUUCUUAUAGGCGUUGU 5'
query=miR4412-5p, target=RO3G_03768, score=1, range=169-190, stra
target 5' AGUACAAAGAAUAUCCGCAACA 3'
::: ::::::::::::::::::
query 3' UCACGUUUCUUAUAGGCGUUGU 5'
query=miR4412-5p, target=RO3G_05212, score=3, range=425-446, stra
```

```
target 5' AAUUUGAAGAAUAUCCGCAACA 3'
: : ..::::::::::::::::
query 3' UCACGUUUCUUAUAGGCGUUGU 5'
query=miR4412-5p, target=RO3G_14895, score=4, range=118-139, stra
target 5' AGUCCAAAGAGUAUCUGCAGCA 3'
::: ::::::.::::.:::.::
query 3' UCACGUUUCUUAUAGGCGUUGU 5'
query=miR4412-5p, target=RO3G_11266, score=4, range=178-199, stra
target 5' AGUGCAAAAAAUAUCUGCAAGA 3'
:::::::: ::::::.:::: :
query 3' UCACGUUUCUUAUAGGCGUUGU 5'
query=miR4412-5p, target=RO3G_11240, score=4, range=178-199, stra
target 5' AGUGCAAAAAAUAUCUGCAAGA 3'
:::::::: ::::::.:::: :
query 3' UCACGUUUCUUAUAGGCGUUGU 5'
query=miR477, target=RO3G_15287, score=0, range=1696-1716, strand
target 5' GCUCCUCCAAAGAUACUCCAA 3'
:::::::::::::::::::::
query 3' CGAGGAGGUUUCUAUGAGGUU 5'
query=miR477, target=RO3G_13291, score=0, range=1141-1161, strand
target 5' GCUCCUCCAAAGAUACUCCAA 3'
:::::::::::::::::::::
query 3' CGAGGAGGUUUCUAUGAGGUU 5'
query=miR477, target=RO3G_17283, score=0, range=1006-1026, strand
target 5' GCUCCUCCAAAGAUACUCCAA 3'
:::::::::::::::::::::
query 3' CGAGGAGGUUUCUAUGAGGUU 5'
query=miR477, target=RO3G_07117, score=0, range=496-516, strand=-
target 5' GCUCCUCCAAAGAUACUCCAA 3'
:::::::::::::::::::::
query 3' CGAGGAGGUUUCUAUGAGGUU 5'
query=miR477, target=RO3G_16805, score=0, range=82-102, strand=-1
target 5' GCUCCUCCAAAGAUACUCCAA 3'
:::::::::::::::::::::
query 3' CGAGGAGGUUUCUAUGAGGUU 5'
query=miR477, target=RO3G_09166, score=0, range=82-102, strand=-1
target 5' GCUCCUCCAAAGAUACUCCAA 3'
:::::::::::::::::::::
query 3' CGAGGAGGUUUCUAUGAGGUU 5'
query=miR477, target=RO3G_07134, score=1.5, range=1141-1161, stra
target 5' GCUUCGCCAAAGAUACUCCAA 3'
:::.: :::::::::::::::
query 3' CGAGGAGGUUUCUAUGAGGUU 5'
query=miR477, target=RO3G_01722, score=1.5, range=1162-1182, stra
target 5' GCUUCGCCAAAGAUACUCCAA 3'
```

```
::::.: ::::::::::::::
query 3' CGAGGAGGUUUCUAUGAGGUU 5'
query=miR477, target=RO3G_15165, score=1.5, range=1129-1149, stra
target 5' GCUUCGCCAAAGAUACUCCAA 3'
::::.: ::::::::::::::
query 3' CGAGGAGGUUUCUAUGAGGUU 5'
query=miR477, target=RO3G_17431, score=1.5, range=1129-1149, stra
target 5' GCUUCGCCAAAGAUACUCCAA 3'
::::.: ::::::::::::::
query 3' CGAGGAGGUUUCUAUGAGGUU 5'
query=miR477, target=RO3G_10547, score=1.5, range=1129-1149, stra
target 5' GCUUCGCCAAAGAUACUCCAA 3'
::::.: ::::::::::::::
query 3' CGAGGAGGUUUCUAUGAGGUU 5'
query=miR477, target=RO3G_17323, score=1.5, range=1129-1149, stra
target 5' GCUUCGCCAAAGAUACUCCAA 3'
::::.: ::::::::::::::
query 3' CGAGGAGGUUUCUAUGAGGUU 5'
query=miR477, target=RO3G_17247, score=1.5, range=1129-1149, stra
target 5' GCUUCGCCAAAGAUACUCCAA 3'
::::.: ::::::::::::::
query 3' CGAGGAGGUUUCUAUGAGGUU 5'
query=miR477, target=RO3G_07127, score=1.5, range=1129-1149, stra
target 5' GCUUCGCCAAAGAUACUCCAA 3'
::::.: ::::::::::::::
query 3' CGAGGAGGUUUCUAUGAGGUU 5'
query=miR477, target=RO3G_12006, score=2, range=988-1008, strand=
target 5' GCUUCGCCAAAGAUACUCCAG 3'
::::.: :::::::::::::.
query 3' CGAGGAGGUUUCUAUGAGGUU 5'
query=miR477, target=RO3G_02952, score=2.5, range=1162-1182, stra
target 5' GCUUCGCCAAAGAUGCUCCAA 3'
::::.: :::::::.::::::
query 3' CGAGGAGGUUUCUAUGAGGUU 5'
query=miR477, target=RO3G_09164, score=2.5, range=1129-1149, stra
target 5' GCUUCGCCAAAGAUGCUCCAA 3'
::::.: :::::::.::::::
query 3' CGAGGAGGUUUCUAUGAGGUU 5'
query=miR477, target=RO3G_17219, score=2.5, range=988-1008, stran
target 5' GCUUCGCCAAAGAUGCUCCAA 3'
::::.: :::::::.::::::
query 3' CGAGGAGGUUUCUAUGAGGUU 5'
query=miR477, target=RO3G_12068, score=2.5, range=988-1008, stran
target 5' GCUUCGCCAAAGAUGCUCCAA 3'
::::.: :::::::.::::::
```

```
query 3' CGAGGAGGUUUCUAUGAGGUU 5'
query=miR477, target=RO3G_14707, score=3, range=988-1008, strand=
target 5' GCUUCGCCAAAGGUACUCCAG 3'
:::.: :::::.:::::::.
query 3' CGAGGAGGUUUCUAUGAGGUU 5'
query=miR477, target=RO3G_16910, score=3, range=988-1008, strand=
target 5' GCUUCGCCAAAGGUACUCCAG 3'
:::.: :::::.:::::::.
query 3' CGAGGAGGUUUCUAUGAGGUU 5'
query=miR477, target=RO3G_17245, score=3, range=988-1008, strand=
target 5' GCUUCGCCAAAGGUACUCCAG 3'
:::.: :::::.:::::::.
query 3' CGAGGAGGUUUCUAUGAGGUU 5'
query=miR477, target=RO3G_11285, score=3, range=988-1008, strand=
target 5' GCUUCGCCAAAGGUACUCCAG 3'
:::.: :::::.:::::::.
query 3' CGAGGAGGUUUCUAUGAGGUU 5'
query=miR477, target=RO3G_13710, score=3, range=988-1008, strand=
target 5' GCUUCGCCAAAGGUACUCCAG 3'
:::.: :::::.:::::::.
query 3' CGAGGAGGUUUCUAUGAGGUU 5'
query=miR477, target=RO3G_17379, score=3, range=82-102, strand=-1
target 5' GCUUCGCCAAAGGUACUCCAG 3'
:::.: :::::.:::::::.
query 3' CGAGGAGGUUUCUAUGAGGUU 5'
query=miR477, target=RO3G_01314, score=3, range=82-102, strand=-1
target 5' GCUUCGCCAAAGGUACUCCAG 3'
:::.: :::::.:::::::.
query 3' CGAGGAGGUUUCUAUGAGGUU 5'
query=miR477, target=RO3G_13739, score=3, range=82-102, strand=-1
target 5' GCUUCGCCAAAGGUACUCCAG 3'
:::.: :::::.:::::::.
query 3' CGAGGAGGUUUCUAUGAGGUU 5'
query=miR477, target=RO3G_16939, score=3.5, range=1040-1060, stra
target 5' GCUCUUUUAAAGAUAAUCCAA 3'
::::.:..::::::: :::::
query 3' CGAGGAGGUUUCUAUGAGGUU 5'
query=miR477, target=RO3G_10629, score=4, range=306-325, strand=1
target 5' GCU-CUCGAAAAAUACUCCAA 3'
::: ::: ::: :::::::::
query 3' CGAGGAGGUUUCUAUGAGGUU 5'
query=miR477, target=RO3G_13404, score=4, range=27-46, strand=1
target 5' GAUGCU-CAAAGAUAUUCCAA 3'
: : :: ::::::::.:::::
query 3' CGAGGAGGUUUCUAUGAGGUU 5'
```

```
query=miR477, target=RO3G_17365, score=4, range=988-1008, strand=
target 5' GCAUCGCCAAAGGUACUCCAG 3'
:: .: ::::::.:::::::.
query 3' CGAGGAGGUUUCUAUGAGGUU 5'
query=miR477, target=RO3G_09816, score=4, range=189-208, strand=-
target 5' GCUUCUCCAAA-AUAUUCCAG 3'
:::.::::::: :::.::::.
query 3' CGAGGAGGUUUCUAUGAGGUU 5'
query=miR398, target=RO3G_12259, score=3.5, range=1659-1677, stra
target 5' GGAGGU-ACCUGAGAGCACA 3'
.: ::: ::::::::.::::
query 3' UCCCCACUGGACUCUUGUGU 5'
query=miR398, target=RO3G_03646, score=4, range=665-684, strand=-
target 5' CAAGGUGACUUGAGAACACA 3'
::::::.:::::::::
query 3' UCCCCACUGGACUCUUGUGU 5'
query=miR398, target=RO3G_07257, score=4, range=824-843, strand=-
target 5' AUGGGUGGCCUGAGAACAAA 3'
: :::::.:::::::::: :
query 3' UCCCCACUGGACUCUUGUGU 5'
query=miR398, target=RO3G_16010, score=4, range=546-564, strand=-
target 5' AGGGGUGACCUCAGAAC-CA 3'
::::::::::: ::::: ::
query 3' UCCCCACUGGACUCUUGUGU 5'
query=miR398, target=RO3G_15529, score=4, range=587-605, strand=-
target 5' AGUGGU-ACUUGAGAGCACA 3'
:: ::: ::.:::::.::::
query 3' UCCCCACUGGACUCUUGUGU 5'
query=miR5632-5p, target=RO3G_08617, score=0, range=1689-1708,
strand=1
target 5' CAGUUGGAUAAAGAAUGACA 3'
::::::::::::::::::::
query 3' GUCAACCUAUUUCUUACUGU 5'
query=miR5632-5p, target=RO3G_05328, score=0, range=762-781, stra
target 5' CAGUUGGAUAAAGAAUGACA 3'
::::::::::::::::::::
query 3' GUCAACCUAUUUCUUACUGU 5'
query=miR5632-5p, target=RO3G_10952, score=3.5, range=36-55, stra
target 5' CGCUUGAAUAAAGAAUGGCA 3'
:. ::: ::::::::::.::
query 3' GUCAACCUAUUUCUUACUGU 5'
query=miR5632-5p, target=RO3G_09606, score=3.5, range=2085-2104,
strand=-1
target 5' UCUUUUGAUAAAGAAUGACA 3'
. :: :::::::::::::
```

query 3' GUCAACCUAUUUCUUACUGU 5'
query=miR5632-5p, target=RO3G_01857, score=4, range=649-667, stra
target 5' UAUUUGGAUAAAGAAU-ACG 3'
.: ::::::::::::: ::.
query 3' GUCAACCUAUUUCUUACUGU 5'
query=miR5632-5p, target=RO3G_07262, score=4, range=1394-1412,
strand=1
target 5' CAGUU-CAUAAAGAAUGGUA 3'
::::: ::::::::::..:
query 3' GUCAACCUAUUUCUUACUGU 5'
query=miR5632-5p, target=RO3G_16946, score=4, range=236-256, stra
target 5' CAGAUUGUAUAAAGAAUGAAA 3'
::: ::: :::::::::: :
query 3' GUC-AACCUAUUUCUUACUGU 5'
query=miR5632-5p, target=RO3G_06140, score=4, range=481-499, stra
target 5' CAUUUGGAUAAAG-AUGACC 3'
:: :::::::::: :::::
query 3' GUCAACCUAUUUCUUACUGU 5'
query=miR5632-5p, target=RO3G_12705, score=4, range=358-376, stra
target 5' AAUUUGGAUAAAGAAU-ACA 3'
: ::::::::::::: :::
query 3' GUCAACCUAUUUCUUACUGU 5'
query=miR5632-5p, target=RO3G_01483, score=4, range=457-475, stra
target 5' CAUUUGUAUAAAGAAU-ACA 3'
:: ::: ::::::::: :::
query 3' GUCAACCUAUUUCUUACUGU 5'
query=miR5632-5p, target=RO3G_10584, score=4, range=660-679, stra
target 5' CAGUUUGAUAAAGAUUGAUA 3'
::::: :::::::: :::.:
query 3' GUCAACCUAUUUCUUACUGU 5'
query=miR5632-5p, target=RO3G_16992, score=4, range=38-56, strand
target 5' UGGUU-GAUAAAGAAUGGUA 3'
..::: ::::::::::..:
query 3' GUCAACCUAUUUCUUACUGU 5'
query=miR5632-5p, target=RO3G_03359, score=4, range=498-516, stra
target 5' GAGGUGG-UAAAGAAUGACA 3'
:: ::: :::::::::::
query 3' GUCAACCUAUUUCUUACUGU 5'
query=miR5632-5p, target=RO3G_06155, score=4, range=1158-1176,
strand=-1
target 5' GAAUU-GAUAGAGAAUGACA 3'
: :: ::::.:::::::::
query 3' GUCAACCUAUUUCUUACUGU 5'
query=miR5632-5p, target=RO3G_09999, score=4, range=384-402, stra
target 5' CAG-UCCAUAAAGAAUGACC 3'

::: : :::::::::::
query 3' GUCAACCUAUUUCUUACUGU 5'
query=miR5632-5p, target=RO3G_13889, score=4, range=248-266, stra
target 5' UUGUUGGAUAAAG-AUGACG 3'
. ::::::::::: :::::.
query 3' GUCAACCUAUUUCUUACUGU 5'
query=miR6282, target=RO3G_03573, score=0, range=559-580, strand=
target 5' UUAUACAUCCCCACAUGAUCAA 3'
:::::::::::::::::::::
query 3' AAUAUGUAGGGGUGUACUAGUU 5'
query=miR6282, target=RO3G_10799, score=0, range=559-580, strand=
target 5' UUAUACAUCCCCACAUGAUCAA 3'
:::::::::::::::::::::
query 3' AAUAUGUAGGGGUGUACUAGUU 5'
query=miR6282, target=RO3G_17239, score=0, range=559-580, strand=
target 5' UUAUACAUCCCCACAUGAUCAA 3'
:::::::::::::::::::::
query 3' AAUAUGUAGGGGUGUACUAGUU 5'
query=miR6282, target=RO3G_04664, score=0, range=559-580, strand=
target 5' UUAUACAUCCCCACAUGAUCAA 3'
:::::::::::::::::::::
query 3' AAUAUGUAGGGGUGUACUAGUU 5'
query=miR6282, target=RO3G_15168, score=0, range=343-364, strand=
target 5' UUAUACAUCCCCACAUGAUCAA 3'
:::::::::::::::::::::
query 3' AAUAUGUAGGGGUGUACUAGUU 5'
query=miR6282, target=RO3G_01327, score=0, range=559-580, strand=
target 5' UUAUACAUCCCCACAUGAUCAA 3'
:::::::::::::::::::::
query 3' AAUAUGUAGGGGUGUACUAGUU 5'
query=miR6282, target=RO3G_05522, score=3, range=559-580, strand=
target 5' UUAUACAGCCCCACAUCAUCAA 3'
::::::: :::::::: :::::
query 3' AAUAUGUAGGGGUGUACUAGUU 5'
query=miR6282, target=RO3G_03112, score=3, range=559-580, strand=
target 5' UUAUACAGCCCCACAUCAUCAA 3'
::::::: :::::::: :::::
query 3' AAUAUGUAGGGGUGUACUAGUU 5'
query=miR6282, target=RO3G_00240, score=3, range=502-523, strand=
target 5' UUAUACAGCCCCACAUCAUCAA 3'
::::::: :::::::: :::::
query 3' AAUAUGUAGGGGUGUACUAGUU 5'
query=miR6282, target=RO3G_02227, score=3, range=559-580, strand=
target 5' UUAUACAGCCCCACAUCAUCAA 3'
::::::: :::::::: :::::

```
query 3' AAUAUGUAGGGGUGUACUAGUU 5'
query=miR6282, target=RO3G_04668, score=3, range=370-391, strand=
target 5' UUAUACAGCCCCACAUCAUCAA 3'
::::::: :::::::: :::::
query 3' AAUAUGUAGGGGUGUACUAGUU 5'
query=miR6282, target=RO3G_17423, score=3, range=193-214, strand=
target 5' UUAUACAGCCCCACAUCAUCAA 3'
::::::: :::::::: :::::
query 3' AAUAUGUAGGGGUGUACUAGUU 5'
query=miR6282, target=RO3G_10797, score=3, range=193-214, strand=
target 5' UUAUACAGCCCCACAUCAUCAA 3'
::::::: :::::::: :::::
query 3' AAUAUGUAGGGGUGUACUAGUU 5'
query=miR6282, target=RO3G_16737, score=3, range=193-214, strand=
target 5' UUAUACAGCCCCACAUCAUCAA 3'
::::::: :::::::: :::::
query 3' AAUAUGUAGGGGUGUACUAGUU 5'
query=miR6282, target=RO3G_14784, score=3, range=193-214, strand=
target 5' UUAUACAGCCCCACAUCAUCAA 3'
::::::: :::::::: :::::
query 3' AAUAUGUAGGGGUGUACUAGUU 5'
query=miR6282, target=RO3G_16121, score=3, range=148-169, strand=
target 5' UUAUACAGCCCCACAUCAUCAA 3'
::::::: :::::::: :::::
query 3' AAUAUGUAGGGGUGUACUAGUU 5'
query=miR6282, target=RO3G_00689, score=3, range=61-82, strand=-1
target 5' UUAUACAGCCCCACAUCAUCAA 3'
::::::: :::::::: :::::
query 3' AAUAUGUAGGGGUGUACUAGUU 5'
query=miR6282, target=RO3G_17092, score=3, range=193-214, strand=
target 5' UUAUACAGCCCCACAUCAUCAA 3'
::::::: :::::::: :::::
query 3' AAUAUGUAGGGGUGUACUAGUU 5'
query=miR6282, target=RO3G_15170, score=3, range=193-214, strand=
target 5' UUAUACAGCCCCACAUCAUCAA 3'
::::::: :::::::: :::::
query 3' AAUAUGUAGGGGUGUACUAGUU 5'
query=miR6282, target=RO3G_10050, score=3, range=193-214, strand=
target 5' UUAUACAGCCCCACAUCAUCAA 3'
::::::: :::::::: :::::
query 3' AAUAUGUAGGGGUGUACUAGUU 5'
query=miR6282, target=RO3G_14981, score=3, range=193-214, strand=
target 5' UUAUACAGCCCCACAUCAUCAA 3'
::::::: :::::::: :::::
query 3' AAUAUGUAGGGGUGUACUAGUU 5'
```

query=miR6282, target=RO3G_16141, score=3, range=193-214, strand=
target 5' UUAUACAGCCCCACAUCAUCAA 3'
::::::: :::::::: :::::
query 3' AAUAUGUAGGGGUGUACUAGUU 5'
query=miR6282, target=RO3G_17328, score=3, range=193-214, strand=
target 5' UUAUACAGCCCCACAUCAUCAA 3'
::::::: :::::::: :::::
query 3' AAUAUGUAGGGGUGUACUAGUU 5'
query=miR6282, target=RO3G_16541, score=3, range=193-214, strand=
target 5' UUAUACAGCCCCACAUCAUCAA 3'
::::::: :::::::: :::::
query 3' AAUAUGUAGGGGUGUACUAGUU 5'
query=miR6282, target=RO3G_07723, score=3, range=193-214, strand=
target 5' UUAUACAGCCCCACAUCAUCAA 3'
::::::: :::::::: :::::
query 3' AAUAUGUAGGGGUGUACUAGUU 5'
query=miR6282, target=RO3G_13752, score=3, range=193-214, strand=
target 5' UUAUACAGCCCCACAUCAUCAA 3'
::::::: :::::::: :::::
query 3' AAUAUGUAGGGGUGUACUAGUU 5'
query=miR6282, target=RO3G_03791, score=3, range=193-214, strand=
target 5' UUAUACAGCCCCACAUCAUCAA 3'
::::::: :::::::: :::::
query 3' AAUAUGUAGGGGUGUACUAGUU 5'
query=miR6282, target=RO3G_11536, score=3, range=193-214, strand=
target 5' UUAUACAGCCCCACAUCAUCAA 3'
::::::: :::::::: :::::
query 3' AAUAUGUAGGGGUGUACUAGUU 5'
query=miR6282, target=RO3G_01725, score=3, range=193-214, strand=
target 5' UUAUACAGCCCCACAUCAUCAA 3'
::::::: :::::::: :::::
query 3' AAUAUGUAGGGGUGUACUAGUU 5'
query=miR6282, target=RO3G_09926, score=3, range=193-214, strand=
target 5' UUAUACAGCCCCACAUCAUCAA 3'
::::::: :::::::: :::::
query 3' AAUAUGUAGGGGUGUACUAGUU 5'
query=miR6282, target=RO3G_16124, score=3, range=193-214, strand=
target 5' UUAUACAGCCCCACAUCAUCAA 3'
::::::: :::::::: :::::
query 3' AAUAUGUAGGGGUGUACUAGUU 5'
query=miR6282, target=RO3G_12950, score=3, range=193-214, strand=
target 5' UUAUACAGCCCCACAUCAUCAA 3'
::::::: :::::::: :::::
query 3' AAUAUGUAGGGGUGUACUAGUU 5'
query=miR6282, target=RO3G_15286, score=3, range=193-214, strand=

```
target 5' UUAUACAGCCCCACAUCAUCAA 3'
::::::: :::::::: :::::
query 3' AAUAUGUAGGGGUGUACUAGUU 5'
query=miR6282, target=RO3G_05516, score=3, range=193-214, strand=
target 5' UUAUACAGCCCCACAUCAUCAA 3'
::::::: :::::::: :::::
query 3' AAUAUGUAGGGGUGUACUAGUU 5'
query=miR6282, target=RO3G_15145, score=3, range=193-214, strand=
target 5' UUAUACAGCCCCACAUCAUCAA 3'
::::::: :::::::: :::::
query 3' AAUAUGUAGGGGUGUACUAGUU 5'
query=miR6282, target=RO3G_03560, score=3, range=193-214, strand=
target 5' UUAUACAGCCCCACAUCAUCAA 3'
::::::: :::::::: :::::
query 3' AAUAUGUAGGGGUGUACUAGUU 5'
query=miR6282, target=RO3G_00675, score=3, range=193-214, strand=
target 5' UUAUACAGCCCCACAUCAUCAA 3'
::::::: :::::::: :::::
query 3' AAUAUGUAGGGGUGUACUAGUU 5'
query=miR6282, target=RO3G_10237, score=3, range=133-154, strand=
target 5' UUAUACAGCCCCACAUCAUCAA 3'
::::::: :::::::: :::::
query 3' AAUAUGUAGGGGUGUACUAGUU 5'
query=miR6282, target=RO3G_07298, score=3, range=193-214, strand=
target 5' UUAUACAGCCCCACAUCAUCAA 3'
::::::: :::::::: :::::
query 3' AAUAUGUAGGGGUGUACUAGUU 5'
query=miR6282, target=RO3G_11533, score=3, range=193-214, strand=
target 5' UUAUACAGCCCCACAUCAUCAA 3'
::::::: :::::::: :::::
query 3' AAUAUGUAGGGGUGUACUAGUU 5'
query=miR6282, target=RO3G_01709, score=3, range=193-214, strand=
target 5' UUAUACAGCCCCACAUCAUCAA 3'
::::::: :::::::: :::::
query 3' AAUAUGUAGGGGUGUACUAGUU 5'
query=miR6282, target=RO3G_00250, score=3, range=193-214, strand=
target 5' UUAUACAGCCCCACAUCAUCAA 3'
::::::: :::::::: :::::
query 3' AAUAUGUAGGGGUGUACUAGUU 5'
query=miR6282, target=RO3G_10785, score=3, range=193-214, strand=
target 5' UUAUACAGCCCCACAUCAUCAA 3'
::::::: :::::::: :::::
query 3' AAUAUGUAGGGGUGUACUAGUU 5'
query=miR6282, target=RO3G_12084, score=3, range=193-214, strand=
target 5' UUAUACAGCCCCACAUCAUCAA 3'
```

::::::: :::::::: :::::
query 3' AAUAUGUAGGGGUGUACUAGUU 5'
query=miR6282, target=RO3G_13267, score=3, range=193-214, strand=
target 5' UUAUACAGCCCCACAUCAUCAA 3'
::::::: :::::::: :::::
query 3' AAUAUGUAGGGGUGUACUAGUU 5'
query=miR6282, target=RO3G_03777, score=3, range=193-214, strand=
target 5' UUAUACAGCCCCACAUCAUCAA 3'
::::::: :::::::: :::::
query 3' AAUAUGUAGGGGUGUACUAGUU 5'
query=miR6282, target=RO3G_17316, score=3, range=193-214, strand=
target 5' UUAUACAGCCCCACAUCAUCAA 3'
::::::: :::::::: :::::
query 3' AAUAUGUAGGGGUGUACUAGUU 5'
query=miR6282, target=RO3G_03617, score=3.5, range=559-580, stran
target 5' UCAUGCAUCCCCACAUCAUCAA 3'
: ::.:::::::::: :::::
query 3' AAUAUGUAGGGGUGUACUAGUU 5'
query=miR5163b-3p, target=RO3G_02942, score=0, range=207-226, str
target 5' UCCCACAACCAAAGUAUCUA 3'
::::::::::::::::::::
query 3' AGGGUGUUGGUUUCAUAGAU 5'
query=miR5163b-3p, target=RO3G_10657, score=0, range=207-226, str
target 5' UCCCACAACCAAAGUAUCUA 3'
::::::::::::::::::::
query 3' AGGGUGUUGGUUUCAUAGAU 5'
query=miR5163b-3p, target=RO3G_12243, score=0, range=207-226, str
target 5' UCCCACAACCAAAGUAUCUA 3'
::::::::::::::::::::
query 3' AGGGUGUUGGUUUCAUAGAU 5'
query=miR5163b-3p, target=RO3G_01530, score=1, range=207-226, str
target 5' UCCCACAACCAAGGUAUCUA 3'
::::::::::::.:::::::
query 3' AGGGUGUUGGUUUCAUAGAU 5'
query=miR5163b-3p, target=RO3G_04017, score=1, range=207-226, str
target 5' UCCCACAACCAAGGUAUCUA 3'
::::::::::::.:::::::
query 3' AGGGUGUUGGUUUCAUAGAU 5'
query=miR5163b-3p, target=RO3G_03523, score=1, range=207-226, str
target 5' UCCCACAACCAAGGUAUCUA 3'
::::::::::::.:::::::
query 3' AGGGUGUUGGUUUCAUAGAU 5'
query=miR5163b-3p, target=RO3G_04502, score=1, range=207-226, str
target 5' UCCCACAACCAAGGUAUCUA 3'
::::::::::::.:::::::

query 3' AGGGUGUUGGUUUCAUAGAU 5'
query=miR5163b-3p, target=RO3G_15497, score=1, range=135-154, str
target 5' UCCCACAACCAAGGUAUCUA 3'
:::::::::::.::::::::
query 3' AGGGUGUUGGUUUCAUAGAU 5'
query=miR5163b-3p, target=RO3G_09087, score=1, range=207-226, str
target 5' UCCCACAACCAAGGUAUCUA 3'
:::::::::::.::::::::
query 3' AGGGUGUUGGUUUCAUAGAU 5'
query=miR5163b-3p, target=RO3G_05724, score=1, range=168-187, str
target 5' UCCCACAACCAAGGUAUCUA 3'
:::::::::::.::::::::
query 3' AGGGUGUUGGUUUCAUAGAU 5'
query=miR5163b-3p, target=RO3G_12446, score=1, range=207-226, str
target 5' UCCCACAACCAAGGUAUCUA 3'
:::::::::::.::::::::
query 3' AGGGUGUUGGUUUCAUAGAU 5'
query=miR5163b-3p, target=RO3G_13022, score=1.5, range=207-226, strand=1
target 5' UCCUACAACCAAGGUAUCUA 3'
:::.:::::::.::::::::
query 3' AGGGUGUUGGUUUCAUAGAU 5'
query=miR5163b-3p, target=RO3G_08447, score=1.5, range=207-226, strand=1
target 5' UCCUACAACCAAGGUAUCUA 3'
:::.:::::::.::::::::
query 3' AGGGUGUUGGUUUCAUAGAU 5'
query=miR5163b-3p, target=RO3G_12685, score=3, range=189-208, str
target 5' UCCCACAACCAAGGUAUCCA 3'
:::::::::::.::::: :
query 3' AGGGUGUUGGUUUCAUAGAU 5'
query=miR5163b-3p, target=RO3G_15290, score=3, range=135-154, str
target 5' UCCCACAACCAAGAUAUCUA 3'
:::::::::::. ::::::
query 3' AGGGUGUUGGUUUCAUAGAU 5'
query=miR5163b-3p, target=RO3G_13307, score=3, range=168-187, str
target 5' UCCCACAACCAAGGUUUCUA 3'
:::::::::::.:: ::::
query 3' AGGGUGUUGGUUUCAUAGAU 5'
query=miR5163b-3p, target=RO3G_03564, score=3, range=620-639, str
target 5' UACCACAACUAAGGUAUCUA 3'
: :::::::.::.:::::::
query 3' AGGGUGUUGGUUUCAUAGAU 5'
query=miR5163b-3p, target=RO3G_03971, score=3.5, range=207-226, strand=1

```
target 5' UCCUACAACCAAGGAAUCUA 3'
:::.:::::::.: :::::
query 3' AGGGUGUUGGUUUCAUAGAU 5'
query=miR5163b-3p, target=RO3G_03492, score=3.5, range=1067-1087,
strand=-1
target 5' ACACACGAACCAAAGUAUCUG 3'
: ::: ::::::::::::.
query 3' AGGGUG-UUGGUUUCAUAGAU 5'
query=miR5163b-3p, target=RO3G_02418, score=4, range=2354-2373,
strand=-1
target 5' UUCUUCAACUAAAGUGUCUA 3'
:.:. ::::.:::::.::::
query 3' AGGGUGUUGGUUUCAUAGAU 5'
query=miR5163b-3p, target=RO3G_08626, score=4, range=1676-1695,
strand=-1
target 5' GCUUCCAACCAAAGUAUUUA 3'
:.. ::::::::::::.::
query 3' AGGGUGUUGGUUUCAUAGAU 5'
query=miR5163b-3p, target=RO3G_10409, score=4, range=131-151,
strand=-1
target 5' UGUCACAGCCAAAGGUAUCUA 3'
: .::::.::::: :::::::
query 3' AGGGUGUUGGUUU-CAUAGAU 5'
query=miR1522, target=RO3G_04747, score=3, range=40-59, strand=-1
target 5' UAUUUAUGCUUAACAAUAAU 3'
. ::.:::::::::::::
query 3' GAAAGUACGAAUUGUUAUUU 5'
query=miR1522, target=RO3G_13498, score=3.5, range=482-501, stran
target 5' CUUGUAUGUUUGACAAUAAA 3'
::: .:::.::.::::::::
query 3' GAAAGUACGAAUUGUUAUUU 5'
query=miR1522, target=RO3G_06415, score=4, range=440-458, strand=
target 5' CUGUCA-GUUUAACAGUAAA 3'
:: ::: :.:::::::.::::
query 3' GAAAGUACGAAUUGUUAUUU 5'
query=miR1522, target=RO3G_16475, score=4, range=590-609, strand=
target 5' UUUGCAUGCUUGACGAUAAG 3'
.:: :::::::.::.::::.
query 3' GAAAGUACGAAUUGUUAUUU 5'
query=miR1522, target=RO3G_10108, score=4, range=1803-1822, stran
target 5' CCUGCAUGCUUGAUAAUAAA 3'
: : :::::::.:.::::::
query 3' GAAAGUACGAAUUGUUAUUU 5'
query=miR1522, target=RO3G_08294, score=4, range=48-67, strand=-1
target 5' CAUUGAUGCUUAAUGAUAAA 3'
```

```
: :: ::::::::..:::::
query 3' GAAAGUACGAAUUGUUAUUU 5'
query=miR1522, target=RO3G_17276, score=4, range=4-22, strand=-1
target 5' CUGUCA-GUUUAACAGUAAA 3'
:: ::: :.::::::.::::
query 3' GAAAGUACGAAUUGUUAUUU 5'
query=miR1522, target=RO3G_01811, score=4, range=1122-1142, stran
target 5' GUAUCAAGGCUUAACAAUAAA 3'
: :: : :::::::::::::
query 3' GAAAG-UACGAAUUGUUAUUU 5'
query=miR1052, target=RO3G_00301, score=0, range=1185-1207, stran
target 5' UAACACAGUCAAUAAAAUAGGAA 3'
:::::::::::::::::::::::
query 3' AUUGUGUCAGUUAUUUUAUCCUU 5'
query=miR1052, target=RO3G_13432, score=2, range=579-601, strand=
target 5' UAACACAGUCAAUAAAACAGGAA 3'
::::::::::::::::: :::::
query 3' AUUGUGUCAGUUAUUUUAUCCUU 5'
query=miR1052, target=RO3G_05125, score=4, range=162-183, strand=
target 5' CAAGACAGUCAAUAAAA-AGGAA 3'
:: ::::::::::::: :::::
query 3' AUUGUGUCAGUUAUUUUAUCCUU 5'
query=miR1052, target=RO3G_01602, score=4, range=611-633, strand=
target 5' UGAGGCAGUAAAUAAAAUAGGAU 3'
:.: .:::: ::::::::::::
query 3' AUUGUGUCAGUUAUUUUAUCCUU 5'
query=miR8148, target=RO3G_16424, score=0.5, range=232-254, stran
target 5' ACCACGGUAUGCAGACCCAAUCA 3'
:::::::.:::::::::::::::
query 3' UGGUGCCGUACGUCUGGGUUAGU 5'
query=miR950, target=RO3G_10607, score=0, range=7023-7041, strand
target 5' AUCAAACCAGCUGACCAGA 3'
:::::::::::::::::::
query 3' UAGUUUGGUCGACUGGUCU 5'
query=miR950, target=RO3G_07540, score=2.5, range=2209-2228, stra
target 5' AUCAAGCCAGCUGACACAGA 3'
:::::.::::::::: ::::
query 3' UAGUUUGGUCGACUG-GUCU 5'
query=miR950, target=RO3G_03353, score=2.5, range=172-190, strand
target 5' ACCAAGCCAGCUGAUCAGA 3'
: :::.::::::::.::::
query 3' UAGUUUGGUCGACUGGUCU 5'
query=miR950, target=RO3G_01135, score=4, range=2821-2839, strand
target 5' AUCAAAUCAGCUUACCAGU 3'
::::::.::::: :::::
```

```
query 3' UAGUUUGGUCGACUGGUCU 5'
query=miR950, target=RO3G_06838, score=4, range=189-207, strand=-
target 5' AUGAAACCUGUUGACCAGA 3'
:: ::::: :.:::::::
query 3' UAGUUUGGUCGACUGGUCU 5'
query=miR950, target=RO3G_11022, score=4, range=953-971, strand=-
target 5' AACAUACCAGCUGAUCGGA 3'
: :: :::::::::.:.::
query 3' UAGUUUGGUCGACUGGUCU 5'
query=miR950, target=RO3G_09400, score=4, range=427-444, strand=-
target 5' AUC-AGCCAGCUGACCAUG 3'
::: :.:::::::::: .
query 3' UAGUUUGGUCGACUGGUCU 5'
query=miR950, target=RO3G_12482, score=4, range=572-590, strand=-
target 5' AUACAACCAGCAGACCAGA 3'
:: ::::::: :::::::
query 3' UAGUUUGGUCGACUGGUCU 5'
query=miR9486a, target=RO3G_06014, score=2.5, range=37-57, strand
target 5' CAAUUUUUAACUCUUGAAAGA 3'
::..:.:::::::::::::
query 3' CUUGGAGAUUGAGAACUUUCU 5'
query=miR9486a, target=RO3G_08391, score=3, range=1392-1412, stra
target 5' AAACCUAUAACUUUUGAAAGA 3'
::::: ::::.::::::::
query 3' CUUGGAGAUUGAGAACUUUCU 5'
query=miR9486a, target=RO3G_11550, score=3.5, range=546-566, stra
target 5' GGAUCUUUUACUCUUGAAAGA 3'
:.:.::.: :::::::::::
query 3' CUUGGAGAUUGAGAACUUUCU 5'
query=miR9486a, target=RO3G_13033, score=3.5, range=266-285, stra
target 5' GAA-UUGUAAUUCUUGAAAGA 3'
::: .: :::.::::::::::
query 3' CUUGGAGAUUGAGAACUUUCU 5'
query=miR9486a, target=RO3G_00830, score=4, range=582-602, strand
target 5' GAACAUCUCAUUCUUGAAAGA 3'
:::: ::: :.::::::::::
query 3' CUUGGAGAUUGAGAACUUUCU 5'
query=miR9486a, target=RO3G_12288, score=4, range=77-97, strand=-
target 5' CAACUUUUAACUCUUGAGAGC 3'
:::.:.:::::::::::.::
query 3' CUUGGAGAUUGAGAACUUUCU 5'
query=miR9486a, target=RO3G_02132, score=4, range=1305-1324, stra
target 5' GGA-UUAUAAUUCUUGAAAGA 3'
:.: .: :::.::::::::::
query 3' CUUGGAGAUUGAGAACUUUCU 5'
```

```
query=miR408-5p, target=RO3G_01481, score=3, range=131-150, stran
target 5' AUGUUUUGCUUGUUCCUUGA 3'
:::.:.:::::::::::.::
query 3' UACGAGACGAACAAGGGACA 5'
query=miR408-5p, target=RO3G_08864, score=4, range=1430-1448,
strand=-1
target 5' AU-CUCUGUUUGUUCCUUGA 3'
:: ::::.:::::::.::
query 3' UACGAGACGAACAAGGGACA 5'
query=miR408-5p, target=RO3G_13100, score=4, range=602-621, stran
target 5' AUUAUCAGCUUGUUCCUUGU 3'
:: :: :::::::::.:::
query 3' UACGAGACGAACAAGGGACA 5'
query=miR2927, target=RO3G_15737, score=4, range=661-682, strand=
target 5' UAUGGACGUAUCACGACAGACG 3'
.:::: : ::::::::::::.
query 3' GUACCCGACUAGUGCUGUCUGU 5'
query=miR2927, target=RO3G_12325, score=4, range=544-564, strand=
target 5' CAUGGACGGAUCACGACAG-CA 3'
::::: : ::::::::::: ::
query 3' GUACCCGACUAGUGCUGUCUGU 5'
query=miR847-3p, target=RO3G_16744, score=0, range=1038-1058,
strand=-1
target 5' CGACCAGAAGAAGAGGAGUCA 3'
:::::::::::::::::::::
query 3' GCUGGUCUUCUUCUCCUCAGU 5'
query=miR847-3p, target=RO3G_01203, score=0, range=1038-1058,
strand=-1
target 5' CGACCAGAAGAAGAGGAGUCA 3'
:::::::::::::::::::::
query 3' GCUGGUCUUCUUCUCCUCAGU 5'
query=miR847-3p, target=RO3G_11403, score=0, range=1038-1058,
strand=-1
target 5' CGACCAGAAGAAGAGGAGUCA 3'
:::::::::::::::::::::
query 3' GCUGGUCUUCUUCUCCUCAGU 5'
query=miR847-3p, target=RO3G_06416, score=0, range=1038-1058,
strand=-1
target 5' CGACCAGAAGAAGAGGAGUCA 3'
:::::::::::::::::::::
query 3' GCUGGUCUUCUUCUCCUCAGU 5'
query=miR847-3p, target=RO3G_01718, score=0, range=1038-1058,
strand=-1
target 5' CGACCAGAAGAAGAGGAGUCA 3'
:::::::::::::::::::::
```

query 3' GCUGGUCUUCUUCUCCUCAGU 5'
query=miR847-3p, target=RO3G_15869, score=0, range=1038-1058, strand=-1
target 5' CGACCAGAAGAAGAGGAGUCA 3'
:::::::::::::::::::::
query 3' GCUGGUCUUCUUCUCCUCAGU 5'
query=miR847-3p, target=RO3G_10560, score=0, range=1038-1058, strand=-1
target 5' CGACCAGAAGAAGAGGAGUCA 3'
:::::::::::::::::::::
query 3' GCUGGUCUUCUUCUCCUCAGU 5'
query=miR847-3p, target=RO3G_11275, score=0, range=1038-1058, strand=-1
target 5' CGACCAGAAGAAGAGGAGUCA 3'
:::::::::::::::::::::
query 3' GCUGGUCUUCUUCUCCUCAGU 5'
query=miR847-3p, target=RO3G_14704, score=0, range=1038-1058, strand=-1
target 5' CGACCAGAAGAAGAGGAGUCA 3'
:::::::::::::::::::::
query 3' GCUGGUCUUCUUCUCCUCAGU 5'
query=miR847-3p, target=RO3G_12979, score=0, range=1038-1058, strand=-1
target 5' CGACCAGAAGAAGAGGAGUCA 3'
:::::::::::::::::::::
query 3' GCUGGUCUUCUUCUCCUCAGU 5'
query=miR847-3p, target=RO3G_10555, score=0, range=1038-1058, strand=-1
target 5' CGACCAGAAGAAGAGGAGUCA 3'
:::::::::::::::::::::
query 3' GCUGGUCUUCUUCUCCUCAGU 5'
query=miR847-3p, target=RO3G_05280, score=0, range=1038-1058, strand=-1
target 5' CGACCAGAAGAAGAGGAGUCA 3'
:::::::::::::::::::::
query 3' GCUGGUCUUCUUCUCCUCAGU 5'
query=miR847-3p, target=RO3G_16686, score=0, range=1038-1058, strand=-1
target 5' CGACCAGAAGAAGAGGAGUCA 3'
:::::::::::::::::::::
query 3' GCUGGUCUUCUUCUCCUCAGU 5'
query=miR847-3p, target=RO3G_07305, score=0, range=1038-1058, strand=-1
target 5' CGACCAGAAGAAGAGGAGUCA 3'
:::::::::::::::::::::

query 3' GCUGGUCUUCUUCUCCUCAGU 5'
query=miR847-3p, target=RO3G_03103, score=0, range=1038-1058, strand=-1
target 5' CGACCAGAAGAAGAGGAGUCA 3'
:::::::::::::::::::::
query 3' GCUGGUCUUCUUCUCCUCAGU 5'
query=miR847-3p, target=RO3G_12087, score=0, range=1038-1058, strand=-1
target 5' CGACCAGAAGAAGAGGAGUCA 3'
:::::::::::::::::::::
query 3' GCUGGUCUUCUUCUCCUCAGU 5'
query=miR847-3p, target=RO3G_08612, score=0, range=1038-1058, strand=-1
target 5' CGACCAGAAGAAGAGGAGUCA 3'
:::::::::::::::::::::
query 3' GCUGGUCUUCUUCUCCUCAGU 5'
query=miR847-3p, target=RO3G_05630, score=0, range=1038-1058, strand=-1
target 5' CGACCAGAAGAAGAGGAGUCA 3'
:::::::::::::::::::::
query 3' GCUGGUCUUCUUCUCCUCAGU 5'
query=miR847-3p, target=RO3G_13450, score=0, range=918-938, stran
target 5' CGACCAGAAGAAGAGGAGUCA 3'
:::::::::::::::::::::
query 3' GCUGGUCUUCUUCUCCUCAGU 5'
query=miR847-3p, target=RO3G_13859, score=0, range=663-683, stran
target 5' CGACCAGAAGAAGAGGAGUCA 3'
:::::::::::::::::::::
query 3' GCUGGUCUUCUUCUCCUCAGU 5'
query=miR847-3p, target=RO3G_02235, score=0, range=381-401, stran
target 5' CGACCAGAAGAAGAGGAGUCA 3'
:::::::::::::::::::::
query 3' GCUGGUCUUCUUCUCCUCAGU 5'
query=miR847-3p, target=RO3G_13152, score=0, range=1038-1058, strand=-1
target 5' CGACCAGAAGAAGAGGAGUCA 3'
:::::::::::::::::::::
query 3' GCUGGUCUUCUUCUCCUCAGU 5'
query=miR847-3p, target=RO3G_03988, score=0, range=1038-1058, strand=-1
target 5' CGACCAGAAGAAGAGGAGUCA 3'
:::::::::::::::::::::
query 3' GCUGGUCUUCUUCUCCUCAGU 5'
query=miR847-3p, target=RO3G_12951, score=0, range=1038-1058, strand=-1

```
target 5' CGACCAGAAGAAGAGGAGUCA 3'
:::::::::::::::::::::
query 3' GCUGGUCUUCUUCUCCUCAGU 5'
query=miR847-3p, target=RO3G_09376, score=0, range=1038-1058,
strand=-1
target 5' CGACCAGAAGAAGAGGAGUCA 3'
:::::::::::::::::::::
query 3' GCUGGUCUUCUUCUCCUCAGU 5'
query=miR847-3p, target=RO3G_12194, score=0, range=1038-1058,
strand=-1
target 5' CGACCAGAAGAAGAGGAGUCA 3'
:::::::::::::::::::::
query 3' GCUGGUCUUCUUCUCCUCAGU 5'
query=miR847-3p, target=RO3G_02543, score=3.5, range=260-279, str
target 5' CGACCGCAAGAAGAGGAG-CA 3'
:::::. :::::::::: ::
query 3' GCUGGUCUUCUUCUCCUCAGU 5'
query=miR847-3p, target=RO3G_02368, score=4, range=223-243, stran
target 5' CCACCAGAAGGAGAGAAGUCA 3'
: ::::::::.:::: :::::
query 3' GCUGGUCUUCUUCUCCUCAGU 5'
query=miR847-3p, target=RO3G_02317, score=4, range=36-57, strand=
target 5' AAACCAAGAAGAAGAGGAGUCU 3'
::: ::::::::::::::
query 3' GCUGG-UCUUCUUCUCCUCAGU 5'
query=miR847-3p, target=RO3G_08213, score=4, range=54-73, strand=
target 5' CGACAAGAAGAAGA-GAGUCU 3'
:::: ::::::::: :::::
query 3' GCUGGUCUUCUUCUCCUCAGU 5'
query=miR847-3p, target=RO3G_08223, score=4, range=54-73, strand=
target 5' CGACAAGAAGAAGA-GAGUCU 3'
:::: ::::::::: :::::
query 3' GCUGGUCUUCUUCUCCUCAGU 5'
query=miR822-5p, target=RO3G_03379, score=2, range=2346-2364,
strand=-1
target 5' CUUGGCAAGUGCUUUUGCA 3'
:::::::.::::::.:::
query 3' GAACCGUUUACGAAAGCGU 5'
query=miR822-5p, target=RO3G_08314, score=3, range=135-153, stran
target 5' CUUGGCAAGUGCUUUAGCA 3'
:::::::.:::::: :::
query 3' GAACCGUUUACGAAAGCGU 5'
query=miR822-5p, target=RO3G_19004, score=3, range=939-957, stran
target 5' CGGGUCAAAUGCUUUCGCA 3'
: : :::::::::::::
```

query 3' GAACCGUUUACGAAAGCGU 5'
query=miR822-5p, target=RO3G_19001, score=3, range=643-661, stran
target 5' CGGGUCAAAUGCUUUCGCA 3'
: : :::::::::::::
query 3' GAACCGUUUACGAAAGCGU 5'
query=miR822-5p, target=RO3G_07478, score=3.5, range=1699-1716, strand=1
target 5' CUUGACAAAUGCUUU-GCG 3'
:::: :::::::::: ::.
query 3' GAACCGUUUACGAAAGCGU 5'
query=miR822-5p, target=RO3G_06931, score=3.5, range=1911-1928, strand=1
target 5' CUU-CCAAAUGCUUUUGCG 3'
::: ::::::::::.::.
query 3' GAACCGUUUACGAAAGCGU 5'
query=miR822-5p, target=RO3G_08161, score=3.5, range=42-60, stran
target 5' UUUGGCAAUUGCUUUUGCA 3'
.::::::: ::::::.:::
query 3' GAACCGUUUACGAAAGCGU 5'
query=miR822-5p, target=RO3G_12520, score=3.5, range=498-516, strand=-1
target 5' UUUGGCAAUUGCUUUUGCA 3'
.::::::: ::::::.:::
query 3' GAACCGUUUACGAAAGCGU 5'
query=miR822-5p, target=RO3G_06980, score=3.5, range=318-336, strand=-1
target 5' UUUGGCAAUUGCUUUUGCA 3'
.::::::: ::::::.:::
query 3' GAACCGUUUACGAAAGCGU 5'
query=miR822-5p, target=RO3G_00797, score=3.5, range=498-516, strand=-1
target 5' UUUGGCAAUUGCUUUUGCA 3'
.::::::: ::::::.:::
query 3' GAACCGUUUACGAAAGCGU 5'
query=miR822-5p, target=RO3G_02296, score=3.5, range=1104-1122, strand=-1
target 5' CUUUGCUAAUGCUUUCGCG 3'
::: :: :::::::::::.
query 3' GAACCGUUUACGAAAGCGU 5'
query=miR822-5p, target=RO3G_03451, score=3.5, range=1071-1089, strand=-1
target 5' UUUCACAAAUGCUUUUGCA 3'
.:: ::::::::::.:::
query 3' GAACCGUUUACGAAAGCGU 5'
query=miR822-5p, target=RO3G_13257, score=4, range=462-481, stran

```
target 5' CUUGGCCGAUGCCUUUCGCA 3'
:::::: .::: ::::::::
query 3' GAACCGUUUAC-GAAAGCGU 5'
query=miR822-5p, target=RO3G_12735, score=4, range=159-176, stran
target 5' AUUGG-AAAUGUUUUUGCA 3'
:::: ::::.:::.:::
query 3' GAACCGUUUACGAAAGCGU 5'
query=miR822-5p, target=RO3G_04232, score=4, range=1816-1833, str
target 5' CUUCG-AAAUGCUAUCGCA 3'
::: : ::::::: :::::
query 3' GAACCGUUUACGAAAGCGU 5'
query=miR822-5p, target=RO3G_01017, score=4, range=663-681, stran
target 5' AAUGGCAAAUGUUUUUGCA 3'
::::::::.:::.:::
query 3' GAACCGUUUACGAAAGCGU 5'
query=miR822-5p, target=RO3G_05717, score=4, range=825-843, stran
target 5' CUUGGGAAAGGCUUUUGCA 3'
::::: ::: :::::.:::
query 3' GAACCGUUUACGAAAGCGU 5'
query=miR822-5p, target=RO3G_09854, score=4, range=357-376, stran
target 5' CUUGUUCAAGUGCUUUUGCA 3'
:::: :::.:::::::.:::
query 3' GAAC-CGUUUACGAAAGCGU 5'
query=miR822-5p, target=RO3G_08886, score=4, range=1595-1612,
strand=-1
target 5' CUUGCCAAAU-CUUUUGCA 3'
:::: ::::: ::::.:::
query 3' GAACCGUUUACGAAAGCGU 5'
query=miR822-5p, target=RO3G_06862, score=4, range=713-730, stran
target 5' CUUGGAAAAUGUUUUC-CA 3'
::::: :::::.:::: ::
query 3' GAACCGUUUACGAAAGCGU 5'
query=miR822-5p, target=RO3G_00797, score=4, range=1690-1707,
strand=-1
target 5' CAUAGCAAAUGCUUU-GCA 3'
: : ::::::::::: :::
query 3' GAACCGUUUACGAAAGCGU 5'
query=miR7761-3p, target=RO3G_14801, score=3, range=238-259, stra
target 5' CCAGAGCGCCCUAGAUUCGAGA 3'
:::::::: ::: :::::::::
query 3' GGUCUCGCAGGAACUAAGCUCU 5'
query=miR7761-3p, target=RO3G_14788, score=3, range=238-259, stra
target 5' CCAGAGCGCCCUAGAUUCGAGA 3'
:::::::: ::: :::::::::
query 3' GGUCUCGCAGGAACUAAGCUCU 5'
```

```
query=miR3510, target=RO3G_06495, score=2, range=1063-1084, stran
target 5' UUUAGACCUGAAAGAUGGUUAA 3'
::.:: ::.:::::::::::::
query 3' AAGUCAGGGCUUUCUACCAAUU 5'
query=miR842, target=RO3G_11952, score=2, range=54-75, strand=-1
target 5' AGUUGACAGAUCUGGGACCAUA 3'
:::::: ::::::::::::::
query 3' CCAACUGCCUAGACCCUGGUAU 5'
query=miR842, target=RO3G_12444, score=4, range=808-830, strand=1
target 5' GGUAUGACGGAUUUGGGGCCAUU 3'
::: ::::::::.::::.::::
query 3' CCA-ACUGCCUAGACCCUGGUAU 5'
query=miR9667-5p, target=RO3G_15311, score=2.5, range=1160-1178,
strand=-1
target 5' GAAUCAUGUUUGUCAAUUU 3'
::.:::::::.::::::
query 3' GUUGGUACAAACGGUUAAA 5'
query=miR9667-5p, target=RO3G_00741, score=2.5, range=244-262,
strand=-1
target 5' UAACCAUGUUUGCCAACUU 3'
.:::::::::::::: ::
query 3' GUUGGUACAAACGGUUAAA 5'
query=miR9667-5p, target=RO3G_15715, score=2.5, range=518-535,
strand=-1
target 5' CAACU-UGUUUGCCAAUUG 3'
::::. :::::::::::
query 3' GUUGGUACAAACGGUUAAA 5'
query=miR9667-5p, target=RO3G_04417, score=3, range=1283-1301,
strand=1
target 5' CAAAGAUGUUUGCCGAUUU 3'
::: :::::::::.::::
query 3' GUUGGUACAAACGGUUAAA 5'
query=miR9667-5p, target=RO3G_10607, score=3, range=1722-1740,
strand=-1
target 5' GAGUUGUGUUUGCCAAUUU 3'
:....::::::::::::
query 3' GUUGGUACAAACGGUUAAA 5'
query=miR9667-5p, target=RO3G_12615, score=3.5, range=368-386,
strand=1
target 5' GUACUAUGUUUGCCAAUUG 3'
::.:::::::::::
query 3' GUUGGUACAAACGGUUAAA 5'
query=miR9667-5p, target=RO3G_11619, score=3.5, range=549-567,
strand=1
target 5' UAACCUUGUUGGCCAAUUU 3'
```

```
.:::: :::: ::::::::
query 3' GUUGGUACAAACGGUUAAA 5'
query=miR9667-5p, target=RO3G_14904, score=3.5, range=687-704,
strand=1
target 5' CGA-GAUGUUUGUCAAUUU 3'
:.: :::::::.::::::
query 3' GUUGGUACAAACGGUUAAA 5'
query=miR9667-5p, target=RO3G_15536, score=3.5, range=3429-3446,
strand=1
target 5' CAUCUAU-UUUGCCAAUUU 3'
:: :.:: ::::::::::
query 3' GUUGGUACAAACGGUUAAA 5'
query=miR9667-5p, target=RO3G_14199, score=3.5, range=569-586,
strand=-1
target 5' UGG-CAUGUUUGCCAAUUG 3'
... :::::::::::::
query 3' GUUGGUACAAACGGUUAAA 5'
query=miR9667-5p, target=RO3G_12350, score=3.5, range=1208-1225,
strand=-1
target 5' CAAAUAUGUUUG-CAAUUU 3'
::: .::::::: ::::::
query 3' GUUGGUACAAACGGUUAAA 5'
query=miR9667-5p, target=RO3G_04618, score=3.5, range=28-47, stra
target 5' UAACAAGUGUUUGCCAAUUG 3'
.::: : :::::::::::
query 3' GUUGGU-ACAAACGGUUAAA 5'
query=miR9667-5p, target=RO3G_09699, score=4, range=1058-1076,
strand=1
target 5' AAACCAUGUUUGCCAAUCC 3'
::::::::::::::::
query 3' GUUGGUACAAACGGUUAAA 5'
query=miR9667-5p, target=RO3G_11879, score=4, range=5495-5513,
strand=1
target 5' AAGCUAUGUUUGCCACUUU 3'
:.:.:::::::::: :::
query 3' GUUGGUACAAACGGUUAAA 5'
query=miR9667-5p, target=RO3G_14530, score=4, range=336-354, stra
target 5' UGCCCAUGUUUGUCAAUUG 3'
.. ::::::::.:::::
query 3' GUUGGUACAAACGGUUAAA 5'
query=miR9667-5p, target=RO3G_03060, score=4, range=1097-1115,
strand=1
target 5' CACCCAUUUUUGCCAGUUU 3'
:: :::: :::::::.:::
query 3' GUUGGUACAAACGGUUAAA 5'
```

query=miR9667-5p, target=RO3G_11200, score=4, range=1022-1040, strand=1
target 5' UAUCUAUGCUUGCCAAUUU 3'
.: :.::: :::::::::
query 3' GUUGGUACAAACGGUUAAA 5'
query=miR9667-5p, target=RO3G_03221, score=4, range=2489-2507, strand=1
target 5' CAAACUUGUAUGCCAAUUU 3'
::: : ::: ::::::::
query 3' GUUGGUACAAACGGUUAAA 5'
query=miR9667-5p, target=RO3G_11350, score=4, range=2374-2392, strand=1
target 5' CAUCCCUGUUUGCCAAGUU 3'
:: :: ::::::::: ::
query 3' GUUGGUACAAACGGUUAAA 5'
query=miR9667-5p, target=RO3G_02013, score=4, range=2596-2614, strand=-1
target 5' CAGCUAUGUUUGCUAAUCU 3'
::.:.:::::::.::: :
query 3' GUUGGUACAAACGGUUAAA 5'
query=miR9667-5p, target=RO3G_08508, score=4, range=515-533, stra
target 5' UGAACAUGUUUGCUGAUUU 3'
..: ::::::::..::::
query 3' GUUGGUACAAACGGUUAAA 5'
query=miR9667-5p, target=RO3G_06442, score=4, range=9290-9308, strand=-1
target 5' UAUCCGUGUUUGCCAAUAU 3'
.: ::.:::::::::: :
query 3' GUUGGUACAAACGGUUAAA 5'
query=miR9667-5p, target=RO3G_16557, score=4, range=3566-3584, strand=-1
target 5' UAUCCGUGUUUGCCAAUAU 3'
.: ::.:::::::::: :
query 3' GUUGGUACAAACGGUUAAA 5'
query=miR9667-5p, target=RO3G_01745, score=4, range=3566-3584, strand=-1
target 5' UAUCCGUGUUUGCCAAUAU 3'
.: ::.:::::::::: :
query 3' GUUGGUACAAACGGUUAAA 5'
query=miR9667-5p, target=RO3G_07098, score=4, range=3566-3584, strand=-1
target 5' UAUCCGUGUUUGCCAAUAU 3'
.: ::.:::::::::: :
query 3' GUUGGUACAAACGGUUAAA 5'
query=miR9667-5p, target=RO3G_17438, score=4, range=3098-3116,

strand=-1
target 5' UAUCCGUGUUUGCCAAUAU 3'
.: ::.:::::::::: :
query 3' GUUGGUACAAACGGUUAAA 5'
query=miR9667-5p, target=RO3G_14995, score=4, range=3584-3602,
strand=-1
target 5' UAUCCGUGUUUGCCAAUAU 3'
.: ::.:::::::::: :
query 3' GUUGGUACAAACGGUUAAA 5'
query=miR9667-5p, target=RO3G_05271, score=4, range=3584-3602,
strand=-1
target 5' UAUCCGUGUUUGCCAAUAU 3'
.: ::.:::::::::: :
query 3' GUUGGUACAAACGGUUAAA 5'
query=miR9667-5p, target=RO3G_03098, score=4, range=2792-2810,
strand=-1
target 5' UACCCGUGUUUGCCAAUAU 3'
.: ::.:::::::::: :
query 3' GUUGGUACAAACGGUUAAA 5'
query=miR9667-5p, target=RO3G_00290, score=4, range=3566-3584,
strand=-1
target 5' UAUCCGUGUUUGCCAAUAU 3'
.: ::.:::::::::: :
query 3' GUUGGUACAAACGGUUAAA 5'
query=miR9667-5p, target=RO3G_00486, score=4, range=2381-2399,
strand=-1
target 5' UACCCGUGUUUGCCAAUAU 3'
.: ::.:::::::::: :
query 3' GUUGGUACAAACGGUUAAA 5'
query=miR9667-5p, target=RO3G_16561, score=4, range=1328-1346,
strand=-1
target 5' UACCCGUGUUUGCCAAUAU 3'
.: ::.:::::::::: :
query 3' GUUGGUACAAACGGUUAAA 5'
query=miR9667-5p, target=RO3G_01927, score=4, range=1328-1346,
strand=-1
target 5' UAUCCGUGUUUGCCAAUAU 3'
.: ::.:::::::::: :
query 3' GUUGGUACAAACGGUUAAA 5'
query=miR9667-5p, target=RO3G_12471, score=4, range=1328-1346,
strand=-1
target 5' UAUCCGUGUUUGCCAAUAU 3'
.: ::.:::::::::: :
query 3' GUUGGUACAAACGGUUAAA 5'
query=miR9667-5p, target=RO3G_17023, score=4, range=173-191, stra

```
target 5' UACCCGUGUUUGCCAAUAU 3'
.: ::.:::::::::: :
query 3' GUUGGUACAAACGGUUAAA 5'
query=miR9667-5p, target=RO3G_09538, score=4, range=173-191, stra
target 5' UACCCGUGUUUGCCAAUAU 3'
.: ::.:::::::::: :
query 3' GUUGGUACAAACGGUUAAA 5'
query=miR9667-5p, target=RO3G_02705, score=4, range=173-191, stra
target 5' UACCCGUGUUUGCCAAUAU 3'
.: ::.:::::::::: :
query 3' GUUGGUACAAACGGUUAAA 5'
query=miR9667-5p, target=RO3G_03633, score=4, range=841-859, stra
target 5' CUGUCAUGUCUGCCAAUUU 3'
: ..::::: ::::::::
query 3' GUUGGUACAAACGGUUAAA 5'
query=miR9667-5p, target=RO3G_11520, score=4, range=173-191, stra
target 5' UAUCCGUGUUUGCCAAUAU 3'
.: ::.:::::::::: :
query 3' GUUGGUACAAACGGUUAAA 5'
query=miR9667-5p, target=RO3G_15133, score=4, range=173-191, stra
target 5' UAUCCGUGUUUGCCAAUAU 3'
.: ::.:::::::::: :
query 3' GUUGGUACAAACGGUUAAA 5'
query=miR9667-5p, target=RO3G_01347, score=4, range=485-504, stra
target 5' UAGCACAUCUUUGCCAAUUU 3'
.:.: ::: ::::::::::
query 3' GUUG-GUACAAACGGUUAAA 5'
query=miR9667-5p, target=RO3G_13340, score=4, range=604-621, stra
target 5' CAACCAUGUUU-CUAAUUG 3'
:::::::::: :.:::
query 3' GUUGGUACAAACGGUUAAA 5'
query=miR9667-5p, target=RO3G_10478, score=4, range=558-577, stra
target 5' CAACCACUGUUUCCCAGUUU 3'
:::::: ::::: :::.:::
query 3' GUUGGU-ACAAACGGUUAAA 5'
query=miR9667-5p, target=RO3G_01563, score=4, range=921-938, stra
target 5' UAACUAU-UUUGCCAGUUU 3'
.:::.:: :::::::.:::
query 3' GUUGGUACAAACGGUUAAA 5'
query=miR7807a-3p, target=RO3G_16691, score=0, range=266-284, str
target 5' GUAUGUAUGCAAAUCCUCA 3'
:::::::::::::::::::
query 3' CAUACAUACGUUUAGGAGU 5'
query=miR7807a-3p, target=RO3G_07740, score=0, range=266-284, str
target 5' GUAUGUAUGCAAAUCCUCA 3'
```

```
::::::::::::::::::
query 3' CAUACAUACGUUUAGGAGU 5'
query=miR7807a-3p, target=RO3G_10873, score=0, range=266-284, str
target 5' GUAUGUAUGCAAAUCCUCA 3'
::::::::::::::::::
query 3' CAUACAUACGUUUAGGAGU 5'
query=miR7807a-3p, target=RO3G_10052, score=0, range=266-284, str
target 5' GUAUGUAUGCAAAUCCUCA 3'
::::::::::::::::::
query 3' CAUACAUACGUUUAGGAGU 5'
query=miR7807a-3p, target=RO3G_11965, score=0, range=266-284, str
target 5' GUAUGUAUGCAAAUCCUCA 3'
::::::::::::::::::
query 3' CAUACAUACGUUUAGGAGU 5'
query=miR7807a-3p, target=RO3G_01616, score=0, range=266-284, str
target 5' GUAUGUAUGCAAAUCCUCA 3'
::::::::::::::::::
query 3' CAUACAUACGUUUAGGAGU 5'
query=miR7807a-3p, target=RO3G_15169, score=0, range=266-284, str
target 5' GUAUGUAUGCAAAUCCUCA 3'
::::::::::::::::::
query 3' CAUACAUACGUUUAGGAGU 5'
query=miR7807a-3p, target=RO3G_15671, score=0, range=266-284, str
target 5' GUAUGUAUGCAAAUCCUCA 3'
::::::::::::::::::
query 3' CAUACAUACGUUUAGGAGU 5'
query=miR7807a-3p, target=RO3G_01786, score=2.5, range=305-323,
strand=1
target 5' GUGUGGAUGCAAAUCUUCA 3'
::.:: :::::::::.:::
query 3' CAUACAUACGUUUAGGAGU 5'
query=miR7807a-3p, target=RO3G_05487, score=3.5, range=2871-2890,
strand=1
target 5' CAGUGCUAUGCAAAUCCUCA 3'
.:: ::::::::::::::
query 3' CAUAC-AUACGUUUAGGAGU 5'
query=miR7807a-3p, target=RO3G_08715, score=4, range=452-469, str
target 5' GUG-GUAUGCAAAUUUUCG 3'
::. :::::::::..::.
query 3' CAUACAUACGUUUAGGAGU 5'
query=miR7807a-3p, target=RO3G_12723, score=4, range=224-241, str
target 5' GUG-GUAUGCAAAUCUUUG 3'
::. ::::::::::.:..
query 3' CAUACAUACGUUUAGGAGU 5'
query=miR7807a-3p, target=RO3G_09457, score=4, range=661-680, str
```

target 5' GUGUUUGUUGCAAAUCCUCA 3'
::.: :. :::::::::::
query 3' CAUACAU-ACGUUUAGGAGU 5'
query=miR7807a-3p, target=RO3G_03919, score=4, range=371-389, str
target 5' GAAUCUAUGCAAAUGCUCA 3'
: :: ::::::::: ::::
query 3' CAUACAUACGUUUAGGAGU 5'
query=miR7807a-3p, target=RO3G_14504, score=4, range=364-382,
strand=-1
target 5' CAAUGUGUGCAAAUCCUCU 3'
::::.::::::::::
query 3' CAUACAUACGUUUAGGAGU 5'
query=miR7807a-3p, target=RO3G_15692, score=4, range=3492-3510,
strand=-1
target 5' GAAUGUAUGCACAUCCUUA 3'
: ::::::::: :::::.:
query 3' CAUACAUACGUUUAGGAGU 5'
query=miR7807a-3p, target=RO3G_13262, score=4, range=336-354,
strand=-1
target 5' AUAUGUAGGCAAAUCCUCC 3'
:::::: ::::::::::
query 3' CAUACAUACGUUUAGGAGU 5'
query=miR7807a-3p, target=RO3G_05776, score=4, range=333-350,
strand=-1
target 5' GUCU-UGUGCAAAUCUUCA 3'
:: : :.::::::::.:::
query 3' CAUACAUACGUUUAGGAGU 5'
query=miR7807a-3p, target=RO3G_08094, score=4, range=75-92, stran
target 5' UUAAG-AUGCAAAUUCUCA 3'
:: : ::::::::.::::
query 3' CAUACAUACGUUUAGGAGU 5'
query=miR3440, target=RO3G_15146, score=2, range=370-390, strand=
target 5' GCUUUCCCCUGAUCGAAUCCA 3'
:::::::: :::::::::::
query 3' CGAAAGGGAACUAGCUUAGGU 5'
query=miR3440, target=RO3G_01190, score=2, range=268-288, strand=
target 5' GCUUUCCCCUGAUCGAAUCCA 3'
:::::::: :::::::::::
query 3' CGAAAGGGAACUAGCUUAGGU 5'
query=miR3440, target=RO3G_14697, score=2.5, range=295-314, stran
target 5' GCUUUCUCUUGAUCG-AUCCA 3'
:::::.:::::::: :::::
query 3' CGAAAGGGAACUAGCUUAGGU 5'
query=miR3440, target=RO3G_00057, score=3, range=1046-1066, stran
target 5' ACUUUUUCUUGAUCGAAUUCA 3'

```
::::..:::::::::::.::
query 3' CGAAAGGGAACUAGCUUAGGU 5'
query=miR3440, target=RO3G_09612, score=3, range=490-510, strand=
target 5' GCUUUCCCCUGAUCGGAUCCA 3'
:::::::: ::::::.:::::
query 3' CGAAAGGGAACUAGCUUAGGU 5'
query=miR3440, target=RO3G_13271, score=3, range=490-510, strand=
target 5' GCUUUCCCCUGAUCGGAUCCA 3'
:::::::: ::::::.:::::
query 3' CGAAAGGGAACUAGCUUAGGU 5'
query=miR3440, target=RO3G_00255, score=3, range=490-510, strand=
target 5' GCUUUCCCCUGAUCGGAUCCA 3'
:::::::: ::::::.:::::
query 3' CGAAAGGGAACUAGCUUAGGU 5'
query=miR3440, target=RO3G_16545, score=3, range=490-510, strand=
target 5' GCUUUCCCCUGAUCGGAUCCA 3'
:::::::: ::::::.:::::
query 3' CGAAAGGGAACUAGCUUAGGU 5'
query=miR3440, target=RO3G_16359, score=3, range=490-510, strand=
target 5' GCUUUCCCCUGAUCGGAUCCA 3'
:::::::: ::::::.:::::
query 3' CGAAAGGGAACUAGCUUAGGU 5'
query=miR3440, target=RO3G_11266, score=3, range=313-333, strand=
target 5' GCUUUCCCUUGACCGGAUCCA 3'
:::::::::::: ::.:::::
query 3' CGAAAGGGAACUAGCUUAGGU 5'
query=miR3440, target=RO3G_14621, score=3, range=148-168, strand=
target 5' GCUUUCCCCUGAUCGGAUCCA 3'
:::::::: ::::::.:::::
query 3' CGAAAGGGAACUAGCUUAGGU 5'
query=miR3440, target=RO3G_10800, score=3, range=148-168, strand=
target 5' GCUUUCCCCUGAUCGGAUCCA 3'
:::::::: ::::::.:::::
query 3' CGAAAGGGAACUAGCUUAGGU 5'
query=miR3440, target=RO3G_14433, score=3.5, range=3575-3596,
strand=-1
target 5' GCAUUUCCUUGAUCGAUAUCCA 3'
:: ::.:::::::::: :::::
query 3' CGAAAGGGAACUAGCU-UAGGU 5'
query=miR3440, target=RO3G_09375, score=4, range=331-350, strand=
target 5' GCUUUUUCUUGGUC-AAUCCA 3'
::::..::::.:: ::::::
query 3' CGAAAGGGAACUAGCUUAGGU 5'
query=miR3440, target=RO3G_07031, score=4, range=490-510, strand=
target 5' GCUUUCCCCGGAUCGAAUCCA 3'
```

```
:::::::: :::::::::::
query 3' CGAAAGGGAACUAGCUUAGGU 5'
query=miR3440, target=RO3G_16554, score=4, range=490-510, strand=
target 5' GCUUUCCCCGGAUCGAAUCCA 3'
:::::::: :::::::::::
query 3' CGAAAGGGAACUAGCUUAGGU 5'
query=miR3440, target=RO3G_13277, score=4, range=490-510, strand=
target 5' GCUUUCCCCGGAUCGAAUCCA 3'
:::::::: :::::::::::
query 3' CGAAAGGGAACUAGCUUAGGU 5'
query=miR3440, target=RO3G_10930, score=4, range=761-782, strand=
target 5' GCUGUCUUUUUGAUUGAAUCCA 3'
::: :: ..:::::.:::::::
query 3' CGAAAG-GGAACUAGCUUAGGU 5'
query=miR3440, target=RO3G_10693, score=4, range=578-598, strand=
target 5' GCAUGUUAUUGAUCGAAUCCA 3'
:: : .. ::::::::::::
query 3' CGAAAGGGAACUAGCUUAGGU 5'
query=miR3440, target=RO3G_12584, score=4, range=1127-1147, stran
target 5' CCUUGACCUUGAUCGAAUCCU 3'
::: ::::::::::::::
query 3' CGAAAGGGAACUAGCUUAGGU 5'
query=miR6214, target=RO3G_01294, score=1, range=1752-1772, stran
target 5' UGAUCGUCGCUCUCGUCGUCA 3'
::::::: ::::::::::::
query 3' ACUAGCACCGAGAGCAGCAGU 5'
query=miR6214, target=RO3G_17251, score=1, range=387-407, strand=
target 5' UGAUCGUCGCUCUCGUCGUCA 3'
::::::: ::::::::::::
query 3' ACUAGCACCGAGAGCAGCAGU 5'
query=miR6214, target=RO3G_16366, score=3, range=2679-2699, stran
target 5' UGAUCGUCGCUCUCGCCGUCA 3'
::::::: ::::::: :::::
query 3' ACUAGCACCGAGAGCAGCAGU 5'
query=miR6214, target=RO3G_07735, score=3, range=2583-2603, stran
target 5' UGAUCGUCGCUCUCGCCGUCA 3'
::::::: ::::::: :::::
query 3' ACUAGCACCGAGAGCAGCAGU 5'
query=miR6214, target=RO3G_10543, score=3, range=2136-2156, stran
target 5' UGAUCGUCGCUCUCGCCGUCA 3'
::::::: ::::::: :::::
query 3' ACUAGCACCGAGAGCAGCAGU 5'
query=miR6214, target=RO3G_16919, score=3, range=258-278, strand=
target 5' UGAUCGUCGCUCUCGCCGUCA 3'
::::::: ::::::: :::::
```

```
query 3' ACUAGCACCGAGAGCAGCAGU 5'
query=miR6214, target=RO3G_14563, score=3, range=246-266, strand=
target 5' UGAUCGUCGCUCUCGCCGUCA 3'
::::::: ::::::: :::::
query 3' ACUAGCACCGAGAGCAGCAGU 5'
query=miR6214, target=RO3G_03008, score=3.5, range=1044-1064, str
target 5' UGGACGUGGCCCUCGUCGUCA 3'
::. :::::: ::::::::::
query 3' ACUAGCACCGAGAGCAGCAGU 5'
query=miR6214, target=RO3G_15931, score=3.5, range=1293-1313, str
target 5' CCAUUUUGGCUCUCGUCGUCA 3'
::. ::::::::::::::
query 3' ACUAGCACCGAGAGCAGCAGU 5'
query=miR6214, target=RO3G_17255, score=4, range=2634-2654, stran
target 5' CGAUCGUCGCUCUCGCCGUCA 3'
:::::: ::::::: :::::
query 3' ACUAGCACCGAGAGCAGCAGU 5'
query=miR6214, target=RO3G_13458, score=4, range=387-407, strand=
target 5' CGAUCGUCGCUCUCGUCGCCA 3'
:::::: ::::::::: ::
query 3' ACUAGCACCGAGAGCAGCAGU 5'
query=miR5788, target=RO3G_15250, score=0, range=2507-2529, stran
target 5' UACUAGAGUCUGAUAACUAUCCA 3'
:::::::::::::::::::::::
query 3' AUGAUCUCAGACUAUUGAUAGGU 5'
query=miR5788, target=RO3G_13749, score=0, range=1115-1137, stran
target 5' UACUAGAGUCUGAUAACUAUCCA 3'
:::::::::::::::::::::::
query 3' AUGAUCUCAGACUAUUGAUAGGU 5'
query=miR5788, target=RO3G_15585, score=0, range=2507-2529, stran
target 5' UACUAGAGUCUGAUAACUAUCCA 3'
:::::::::::::::::::::::
query 3' AUGAUCUCAGACUAUUGAUAGGU 5'
query=miR5788, target=RO3G_01613, score=0, range=803-825, strand=
target 5' UACUAGAGUCUGAUAACUAUCCA 3'
:::::::::::::::::::::::
query 3' AUGAUCUCAGACUAUUGAUAGGU 5'
query=miR5788, target=RO3G_00306, score=0, range=1001-1023, stran
target 5' UACUAGAGUCUGAUAACUAUCCA 3'
:::::::::::::::::::::::
query 3' AUGAUCUCAGACUAUUGAUAGGU 5'
query=miR5788, target=RO3G_17371, score=0.5, range=2507-2529, str
target 5' UACUGGAGUCUGAUAACUAUCCA 3'
::::.::::::::::::::::::
query 3' AUGAUCUCAGACUAUUGAUAGGU 5'
```

```
query=miR5788, target=RO3G_14355, score=0.5, range=758-780, stran
target 5' UACUGGAGUCUGAUAACUAUCCA 3'
::::.:::::::::::::::::
query 3' AUGAUCUCAGACUAUUGAUAGGU 5'
query=miR5788, target=RO3G_04114, score=0.5, range=2552-2574, str
target 5' UACUGGAGUCUGAUAACUAUCCA 3'
::::.:::::::::::::::::
query 3' AUGAUCUCAGACUAUUGAUAGGU 5'
query=miR5788, target=RO3G_05272, score=0.5, range=2552-2574, str
target 5' UACUGGAGUCUGAUAACUAUCCA 3'
::::.:::::::::::::::::
query 3' AUGAUCUCAGACUAUUGAUAGGU 5'
query=miR5788, target=RO3G_10558, score=0.5, range=305-327, stran
target 5' UACUGGAGUCUGAUAACUAUCCA 3'
::::.:::::::::::::::::
query 3' AUGAUCUCAGACUAUUGAUAGGU 5'
query=miR5788, target=RO3G_13867, score=1, range=2468-2490, stran
target 5' UAAUAGAGUCUGAUAACUAUCCA 3'
:: :::::::::::::::::::
query 3' AUGAUCUCAGACUAUUGAUAGGU 5'
query=miR5788, target=RO3G_06442, score=2, range=3209-3231, stran
target 5' UAUUGGAAUCUGAUAACUAUCCA 3'
::.:.:: ::::::::::::::
query 3' AUGAUCUCAGACUAUUGAUAGGU 5'
query=miR5788, target=RO3G_02691, score=2, range=6041-6063, stran
target 5' UAUUGGAAUCUGAUAACUAUCCA 3'
::.:.:: ::::::::::::::
query 3' AUGAUCUCAGACUAUUGAUAGGU 5'
query=miR5788, target=RO3G_09965, score=2, range=2552-2574, stran
target 5' UAUUGGAAUCUGAUAACUAUCCA 3'
::.:.:: ::::::::::::::
query 3' AUGAUCUCAGACUAUUGAUAGGU 5'
query=miR5788, target=RO3G_13920, score=2, range=2552-2574, stran
target 5' UAUUGGAAUCUGAUAACUAUCCA 3'
::.:.:: ::::::::::::::
query 3' AUGAUCUCAGACUAUUGAUAGGU 5'
query=miR5788, target=RO3G_03547, score=2, range=2552-2574, stran
target 5' UAUUGGAAUCUGAUAACUAUCCA 3'
::.:.:: ::::::::::::::
query 3' AUGAUCUCAGACUAUUGAUAGGU 5'
query=miR5788, target=RO3G_02959, score=2, range=2552-2574, stran
target 5' UAUUGGAAUCUGAUAACUAUCCA 3'
::.:.:: ::::::::::::::
query 3' AUGAUCUCAGACUAUUGAUAGGU 5'
query=miR5788, target=RO3G_06427, score=2, range=2144-2166, stran
```

```
target 5' UAUUGGAAUCUGAUAACUAUCCA 3'
::.:.:: ::::::::::::::
query 3' AUGAUCUCAGACUAUUGAUAGGU 5'
query=miR5788, target=RO3G_17326, score=2, range=2384-2406, stran
target 5' UAUUGGAAUCUGAUAACUAUCCA 3'
::.:.:: ::::::::::::::
query 3' AUGAUCUCAGACUAUUGAUAGGU 5'
query=miR5788, target=RO3G_06428, score=2, range=1646-1668, stran
target 5' UAUUGGAAUCUGAUAACUAUCCA 3'
::.:.:: ::::::::::::::
query 3' AUGAUCUCAGACUAUUGAUAGGU 5'
query=miR5788, target=RO3G_05619, score=2, range=695-717, strand=
target 5' UAUUGGAAUCUGAUAACUAUCCA 3'
::.:.:: ::::::::::::::
query 3' AUGAUCUCAGACUAUUGAUAGGU 5'
query=miR5788, target=RO3G_02221, score=2, range=2474-2496, stran
target 5' UAUUGGAAUCUGAUAACUAUCCA 3'
::.:.:: ::::::::::::::
query 3' AUGAUCUCAGACUAUUGAUAGGU 5'
query=miR5208d, target=RO3G_06502, score=2, range=1468-1489, stra
target 5' UUGCCUACAAACUAUGACUAUA 3'
:::::::::::::::::::: :
query 3' AACGGAUGUUUGAUACUGAUUU 5'
query=miR5208d, target=RO3G_01733, score=2, range=1392-1413, stra
target 5' UUGCCUACAAACUAUGACUAUA 3'
:::::::::::::::::::: :
query 3' AACGGAUGUUUGAUACUGAUUU 5'
query=miR5208d, target=RO3G_05865, score=2, range=1468-1489, stra
target 5' UUGCCUACAAACUAUGACUAUA 3'
:::::::::::::::::::: :
query 3' AACGGAUGUUUGAUACUGAUUU 5'
query=miR5208d, target=RO3G_04662, score=2, range=1210-1231, stra
target 5' UUGCCUACAAACUAUGACUAUA 3'
:::::::::::::::::::: :
query 3' AACGGAUGUUUGAUACUGAUUU 5'
query=miR5208d, target=RO3G_10991, score=3, range=1468-1489, stra
target 5' UUGCCUACAAACUAUGACUGUA 3'
:::::::::::::::::::. :
query 3' AACGGAUGUUUGAUACUGAUUU 5'
query=miR5208d, target=RO3G_17287, score=3, range=1468-1489, stra
target 5' UUGCCUACAAACUAUGACUGUA 3'
:::::::::::::::::::. :
query 3' AACGGAUGUUUGAUACUGAUUU 5'
query=miR5208d, target=RO3G_01944, score=3, range=1468-1489, stra
target 5' UUGCCUACAAACUAUGACUGUA 3'
```

```
:::::::::::::::::::. :
query 3' AACGGAUGUUUGAUACUGAUUU 5'
query=miR5208d, target=RO3G_13849, score=3, range=1468-1489, stra
target 5' UUGCCUACAAACUAUGACUGUA 3'
:::::::::::::::::::. :
query 3' AACGGAUGUUUGAUACUGAUUU 5'
query=miR5208d, target=RO3G_03749, score=3, range=1468-1489, stra
target 5' UUGCCUACAAACUAUGACUGUA 3'
:::::::::::::::::::. :
query 3' AACGGAUGUUUGAUACUGAUUU 5'
query=miR5208d, target=RO3G_15573, score=3, range=1468-1489, stra
target 5' UUGCCUACAAACUAUGACUGUA 3'
:::::::::::::::::::. :
query 3' AACGGAUGUUUGAUACUGAUUU 5'
query=miR5208d, target=RO3G_10420, score=3, range=1468-1489, stra
target 5' UUGCCUACAAACUAUGACUGUA 3'
:::::::::::::::::::. :
query 3' AACGGAUGUUUGAUACUGAUUU 5'
query=miR5208d, target=RO3G_02966, score=3, range=739-760, strand
target 5' UUGCCUACAAACUAUGACUGUA 3'
:::::::::::::::::::. :
query 3' AACGGAUGUUUGAUACUGAUUU 5'
query=miR5208d, target=RO3G_17446, score=3, range=1468-1489, stra
target 5' UUGCCUACAAACUAUGACUGUA 3'
:::::::::::::::::::. :
query 3' AACGGAUGUUUGAUACUGAUUU 5'
query=miR5208d, target=RO3G_02708, score=3, range=1201-1222, stra
target 5' UUGCCUACAAACUAUGACUGUA 3'
:::::::::::::::::::. :
query 3' AACGGAUGUUUGAUACUGAUUU 5'
query=miR5208d, target=RO3G_01056, score=4, range=1363-1384, stra
target 5' CUGCCUACAAACUAUGACUACU 3'
:::::::::::::::::::
query 3' AACGGAUGUUUGAUACUGAUUU 5'
query=miR3440b-3p, target=RO3G_11266, score=0, range=313-334,
strand=-1
target 5' AGCUUUCCCUUGACCGGAUCCA 3'
::::::::::::::::::::::
query 3' UCGAAAGGGAACUGGCCUAGGU 5'
query=miR3440b-3p, target=RO3G_10866, score=2, range=490-511,
strand=-1
target 5' AGCUUUCCCCUGACCGGAUCCA 3'
::::::::: ::::::::::::
query 3' UCGAAAGGGAACUGGCCUAGGU 5'
query=miR3440b-3p, target=RO3G_07688, score=2, range=490-511,
```

strand=-1
target 5' AGCUUUCCCCUGACCGGAUCCA 3'
:::::::: :::::::::::
query 3' UCGAAAGGGAACUGGCCUAGGU 5'
query=miR3440b-3p, target=RO3G_13266, score=2, range=268-289, strand=-1
target 5' AGCUUUCCCCUGACCGGAUCCA 3'
:::::::: :::::::::::
query 3' UCGAAAGGGAACUGGCCUAGGU 5'
query=miR3440b-3p, target=RO3G_13271, score=3, range=490-511, strand=-1
target 5' AGCUUUCCCCUGAUCGGAUCCA 3'
:::::::: :::.:::::::
query 3' UCGAAAGGGAACUGGCCUAGGU 5'
query=miR3440b-3p, target=RO3G_16359, score=3, range=490-511, strand=-1
target 5' AGCUUUCCCCUGAUCGGAUCCA 3'
:::::::: :::.:::::::
query 3' UCGAAAGGGAACUGGCCUAGGU 5'
query=miR3440b-3p, target=RO3G_16545, score=3, range=490-511, strand=-1
target 5' AGCUUUCCCCUGAUCGGAUCCA 3'
:::::::: :::.:::::::
query 3' UCGAAAGGGAACUGGCCUAGGU 5'
query=miR3440b-3p, target=RO3G_00255, score=3, range=490-511, strand=-1
target 5' AGCUUUCCCCUGAUCGGAUCCA 3'
:::::::: :::.:::::::
query 3' UCGAAAGGGAACUGGCCUAGGU 5'
query=miR3440b-3p, target=RO3G_09612, score=3, range=490-511, strand=-1
target 5' AGCUUUCCCCUGAUCGGAUCCA 3'
:::::::: :::.:::::::
query 3' UCGAAAGGGAACUGGCCUAGGU 5'
query=miR3440b-3p, target=RO3G_14621, score=3, range=148-169, strand=-1
target 5' AGCUUUCCCCUGAUCGGAUCCA 3'
:::::::: :::.:::::::
query 3' UCGAAAGGGAACUGGCCUAGGU 5'
query=miR3440b-3p, target=RO3G_10800, score=3, range=148-169, strand=-1
target 5' AGCUUUCCCCUGAUCGGAUCCA 3'
:::::::: :::.:::::::
query 3' UCGAAAGGGAACUGGCCUAGGU 5'
query=miR6209, target=RO3G_07716, score=0, range=214-233, strand=

```
target 5' CGCACAGCUUCUGAUGCUCA 3'
::::::::::::::::::::
query 3' GCGUGUCGAAGACUACGAGU 5'
query=miR6209, target=RO3G_17468, score=0, range=160-179, strand=
target 5' CGCACAGCUUCUGAUGCUCA 3'
::::::::::::::::::::
query 3' GCGUGUCGAAGACUACGAGU 5'
query=miR6209, target=RO3G_04671, score=0, range=229-248, strand=
target 5' CGCACAGCUUCUGAUGCUCA 3'
::::::::::::::::::::
query 3' GCGUGUCGAAGACUACGAGU 5'
query=miR6209, target=RO3G_17152, score=0, range=208-227, strand=
target 5' CGCACAGCUUCUGAUGCUCA 3'
::::::::::::::::::::
query 3' GCGUGUCGAAGACUACGAGU 5'
query=miR6209, target=RO3G_12665, score=0, range=208-227, strand=
target 5' CGCACAGCUUCUGAUGCUCA 3'
::::::::::::::::::::
query 3' GCGUGUCGAAGACUACGAGU 5'
query=miR6209, target=RO3G_00252, score=0, range=283-302, strand=
target 5' CGCACAGCUUCUGAUGCUCA 3'
::::::::::::::::::::
query 3' GCGUGUCGAAGACUACGAGU 5'
query=miR6209, target=RO3G_16144, score=0, range=229-248, strand=
target 5' CGCACAGCUUCUGAUGCUCA 3'
::::::::::::::::::::
query 3' GCGUGUCGAAGACUACGAGU 5'
query=miR6209, target=RO3G_13860, score=0, range=229-248, strand=
target 5' CGCACAGCUUCUGAUGCUCA 3'
::::::::::::::::::::
query 3' GCGUGUCGAAGACUACGAGU 5'
query=miR6209, target=RO3G_07111, score=0, range=229-248, strand=
target 5' CGCACAGCUUCUGAUGCUCA 3'
::::::::::::::::::::
query 3' GCGUGUCGAAGACUACGAGU 5'
query=miR6209, target=RO3G_09598, score=0, range=160-179, strand=
target 5' CGCACAGCUUCUGAUGCUCA 3'
::::::::::::::::::::
query 3' GCGUGUCGAAGACUACGAGU 5'
query=miR6209, target=RO3G_13000, score=0, range=160-179, strand=
target 5' CGCACAGCUUCUGAUGCUCA 3'
::::::::::::::::::::
query 3' GCGUGUCGAAGACUACGAGU 5'
query=miR6209, target=RO3G_14972, score=0, range=160-179, strand=
target 5' CGCACAGCUUCUGAUGCUCA 3'
```

```
::::::::::::::::::::
query 3' GCGUGUCGAAGACUACGAGU 5'
query=miR6209, target=RO3G_01310, score=0, range=160-179, strand=
target 5' CGCACAGCUUCUGAUGCUCA 3'
::::::::::::::::::::
query 3' GCGUGUCGAAGACUACGAGU 5'
query=miR6209, target=RO3G_01738, score=0, range=160-179, strand=
target 5' CGCACAGCUUCUGAUGCUCA 3'
::::::::::::::::::::
query 3' GCGUGUCGAAGACUACGAGU 5'
query=miR6209, target=RO3G_06740, score=0, range=160-179, strand=
target 5' CGCACAGCUUCUGAUGCUCA 3'
::::::::::::::::::::
query 3' GCGUGUCGAAGACUACGAGU 5'
query=miR6209, target=RO3G_13295, score=0, range=160-179, strand=
target 5' CGCACAGCUUCUGAUGCUCA 3'
::::::::::::::::::::
query 3' GCGUGUCGAAGACUACGAGU 5'
query=miR6209, target=RO3G_06719, score=0, range=160-179, strand=
target 5' CGCACAGCUUCUGAUGCUCA 3'
::::::::::::::::::::
query 3' GCGUGUCGAAGACUACGAGU 5'
query=miR6209, target=RO3G_03989, score=0, range=160-179, strand=
target 5' CGCACAGCUUCUGAUGCUCA 3'
::::::::::::::::::::
query 3' GCGUGUCGAAGACUACGAGU 5'
query=miR6209, target=RO3G_05934, score=0, range=160-179, strand=
target 5' CGCACAGCUUCUGAUGCUCA 3'
::::::::::::::::::::
query 3' GCGUGUCGAAGACUACGAGU 5'
query=miR6209, target=RO3G_13702, score=0, range=160-179, strand=
target 5' CGCACAGCUUCUGAUGCUCA 3'
::::::::::::::::::::
query 3' GCGUGUCGAAGACUACGAGU 5'
query=miR6209, target=RO3G_02701, score=0, range=193-212, strand=
target 5' CGCACAGCUUCUGAUGCUCA 3'
::::::::::::::::::::
query 3' GCGUGUCGAAGACUACGAGU 5'
query=miR6209, target=RO3G_10051, score=1, range=214-233, strand=
target 5' CGCACAGUUUCUGAUGCUCA 3'
:::::::.::::::::::::
query 3' GCGUGUCGAAGACUACGAGU 5'
query=miR6209, target=RO3G_10034, score=1, range=214-233, strand=
target 5' CGCACAGUUUCUGAUGCUCA 3'
:::::::.::::::::::::
```

```
query 3' GCGUGUCGAAGACUACGAGU 5'
query=miR6209, target=RO3G_03561, score=1, range=250-269, strand=
target 5' CGCACAGUUUCUGAUGCUCA 3'
:::::::.::::::::::::
query 3' GCGUGUCGAAGACUACGAGU 5'
query=miR6209, target=RO3G_15151, score=1, range=250-269, strand=
target 5' CGCACAGUUUCUGAUGCUCA 3'
:::::::.::::::::::::
query 3' GCGUGUCGAAGACUACGAGU 5'
query=miR6209, target=RO3G_17359, score=1, range=250-269, strand=
target 5' CGCACAGUUUCUGAUGCUCA 3'
:::::::.::::::::::::
query 3' GCGUGUCGAAGACUACGAGU 5'
query=miR6209, target=RO3G_09618, score=1, range=214-233, strand=
target 5' CACACAGCUUCUGAUGCUCA 3'
: ::::::::::::::::::
query 3' GCGUGUCGAAGACUACGAGU 5'
query=miR6209, target=RO3G_07719, score=1, range=157-176, strand=
target 5' CACACAGCUUCUGAUGCUCA 3'
: ::::::::::::::::::
query 3' GCGUGUCGAAGACUACGAGU 5'
query=miR6209, target=RO3G_13138, score=2, range=160-179, strand=
target 5' CGCACAGAUUCUGAUGCUCA 3'
::::::: ::::::::::::
query 3' GCGUGUCGAAGACUACGAGU 5'
query=miR6209, target=RO3G_05932, score=2, range=160-179, strand=
target 5' CGCACAGAUUCUGAUGCUCA 3'
::::::: ::::::::::::
query 3' GCGUGUCGAAGACUACGAGU 5'
query=miR6209, target=RO3G_17333, score=2, range=229-248, strand=
target 5' CGCACAGAUUCUGAUGCUCA 3'
::::::: ::::::::::::
query 3' GCGUGUCGAAGACUACGAGU 5'
query=miR6209, target=RO3G_06435, score=2, range=229-248, strand=
target 5' CGCACAGAUUCUGAUGCUCA 3'
::::::: ::::::::::::
query 3' GCGUGUCGAAGACUACGAGU 5'
query=miR6209, target=RO3G_12004, score=2, range=229-248, strand=
target 5' CGCACAGAUUCUGAUGCUCA 3'
::::::: ::::::::::::
query 3' GCGUGUCGAAGACUACGAGU 5'
query=miR6209, target=RO3G_09950, score=2, range=229-248, strand=
target 5' CGCACAGAUUCUGAUGCUCA 3'
::::::: ::::::::::::
query 3' GCGUGUCGAAGACUACGAGU 5'
```

```
query=miR6209, target=RO3G_10231, score=2, range=229-248, strand=
target 5' CGCACAGAUUCUGAUGCUCA 3'
::::::: :::::::::::
query 3' GCGUGUCGAAGACUACGAGU 5'
query=miR6209, target=RO3G_12173, score=2, range=229-248, strand=
target 5' CGCACAGAUUCUGAUGCUCA 3'
::::::: :::::::::::
query 3' GCGUGUCGAAGACUACGAGU 5'
query=miR6209, target=RO3G_16430, score=2, range=193-212, strand=
target 5' CGCACAGAUUCUGAUGCUCA 3'
::::::: :::::::::::
query 3' GCGUGUCGAAGACUACGAGU 5'
query=miR6209, target=RO3G_05351, score=2, range=214-233, strand=
target 5' CGCACAGAUUCUGAUGCUCA 3'
::::::: :::::::::::
query 3' GCGUGUCGAAGACUACGAGU 5'
query=miR6209, target=RO3G_01938, score=2, range=229-248, strand=
target 5' CGCACAGAUUCUGAUGCUCA 3'
::::::: :::::::::::
query 3' GCGUGUCGAAGACUACGAGU 5'
query=miR6209, target=RO3G_07673, score=2, range=229-248, strand=
target 5' CGCACAGCUUCUGACGCUCA 3'
::::::::::::: :::::
query 3' GCGUGUCGAAGACUACGAGU 5'
query=miR6209, target=RO3G_13754, score=2, range=229-248, strand=
target 5' CGCACAGAUUCUGAUGCUCA 3'
::::::: :::::::::::
query 3' GCGUGUCGAAGACUACGAGU 5'
query=miR6209, target=RO3G_12672, score=2, range=229-248, strand=
target 5' CGCACAGAUUCUGAUGCUCA 3'
::::::: :::::::::::
query 3' GCGUGUCGAAGACUACGAGU 5'
query=miR6209, target=RO3G_07859, score=2, range=124-143, strand=
target 5' CGCACAGAUUCUGAUGCUCA 3'
::::::: :::::::::::
query 3' GCGUGUCGAAGACUACGAGU 5'
query=miR6209, target=RO3G_04128, score=3, range=229-248, strand=
target 5' CGCACAACUCCUGAUGCUCA 3'
:::::: :: :::::::::
query 3' GCGUGUCGAAGACUACGAGU 5'
query=miR6209, target=RO3G_02745, score=3.5, range=595-614, stran
target 5' CGUACAGCUUCUGGUGCUAA 3'
::.:::::::::.:::: :
query 3' GCGUGUCGAAGACUACGAGU 5'
query=miR6209, target=RO3G_07424, score=4, range=1182-1201, stran
```

```
target 5' CUUAUCGCUUUUGAUGCUCA 3'
: .:. ::::.::::::::::
query 3' GCGUGUCGAAGACUACGAGU 5'
query=miR6209, target=RO3G_15447, score=4, range=554-572, strand=
target 5' GGUAU-GCUUCUGAUGCUUA 3'
:.:. ::::::::::::.:
query 3' GCGUGUCGAAGACUACGAGU 5'
query=miR6209, target=RO3G_05013, score=4, range=442-461, strand=
target 5' AGCGUUACUUCUGAUGCUCA 3'
::.. ::::::::::::
query 3' GCGUGUCGAAGACUACGAGU 5'
query=miR6209, target=RO3G_00766, score=4, range=1648-1667, stran
target 5' CCUUUAGCUUCUGAUGCUCC 3'
: . .:::::::::::::
query 3' GCGUGUCGAAGACUACGAGU 5'
query=miR6209, target=RO3G_15644, score=4, range=1338-1356, stran
target 5' UGCGC-GCUUUUGAUGUUCA 3'
.::.: ::::.:::::.:::
query 3' GCGUGUCGAAGACUACGAGU 5'
query=miR6232a-3p, target=RO3G_11980, score=0, range=851-873,
strand=-1
target 5' ACUUUCUUUUUUGCUAGCAUCCA 3'
:::::::::::::::::::::::
query 3' UGAAAGAAAAAACGAUCGUAGGU 5'
query=miR160b, target=RO3G_17051, score=2.5, range=295-314, stran
target 5' GGCAUCAAGGGAGCCAGGCG 3'
::::: ::::::::::::.
query 3' CCGUAUGUCCCUCGGUCCGU 5'
query=miR160b, target=RO3G_10617, score=3.5, range=776-794, stran
target 5' GGCAUG-AGGGAGCCAGUCA 3'
:::::. :::::::::: ::
query 3' CCGUAUGUCCCUCGGUCCGU 5'
query=miR1120c-5p, target=RO3G_05642, score=3.5, range=517-537,
strand=-1
target 5' UUCUAC-AAGCAUUUUAUAUUG 3'
:: :: ::::::::::::::.
query 3' CAGUUGUUUCGUAAAAUAUAAU 5'
query=miR4387a, target=RO3G_15629, score=0, range=863-885, strand
target 5' AGUGUCACCCACACCGGCUUGUU 3'
:::::::::::::::::::::::
query 3' UCACAGUGGGUGUGGCCGAACAA 5'
query=miR4387a, target=RO3G_01478, score=4, range=2360-2382, stra
target 5' AUUUUCACCGACACCGGCUUGUG 3'
: : ::::: ::::::::::::
query 3' UCACAGUGGGUGUGGCCGAACAA 5'
```

```
query=miR5718, target=RO3G_15268, score=0, range=2464-2486, stran
target 5' CUUGGCUCUGGUCUUGGUUCUGA 3'
:::::::::::::::::::::::
query 3' GAACCGAGACCAGAACCAAGACU 5'
query=miR5718, target=RO3G_14350, score=0.5, range=2329-2351, str
target 5' CUUGGCUCUGGUCUUGGUUCUGG 3'
::::::::::::::::::::::.
query 3' GAACCGAGACCAGAACCAAGACU 5'
query=miR5718, target=RO3G_14352, score=0.5, range=2467-2489, str
target 5' CUUGGCUCUGGUCUUGGUUCUGG 3'
::::::::::::::::::::::.
query 3' GAACCGAGACCAGAACCAAGACU 5'
query=miR5718, target=RO3G_13219, score=0.5, range=2464-2486, str
target 5' CUUGGCUCUGGUCUUGGUUCUGG 3'
::::::::::::::::::::::.
query 3' GAACCGAGACCAGAACCAAGACU 5'
query=miR5718, target=RO3G_05640, score=1, range=2221-2243, stran
target 5' CUUGGUUCUGGUCUUGGUUCUGG 3'
:::::.::::::::::::::::.
query 3' GAACCGAGACCAGAACCAAGACU 5'
query=miR5718, target=RO3G_03992, score=2.5, range=1033-1055, str
target 5' UCUGGUUCUGGUCUUGGUUCUGG 3'
. :::.::::::::::::::::.
query 3' GAACCGAGACCAGAACCAAGACU 5'
query=miR5718, target=RO3G_16818, score=3, range=2236-2258, stran
target 5' CUCGGCUCUGGUCUUGGUCCUGA 3'
:: :::::::::::::: ::::
query 3' GAACCGAGACCAGAACCAAGACU 5'
query=miR5718, target=RO3G_15265, score=3.5, range=2464-2486, str
target 5' CUUGGCUCUGGUCUUGGUCUUGG 3'
:::::::::::::::::: .::.
query 3' GAACCGAGACCAGAACCAAGACU 5'
query=miR5718, target=RO3G_04121, score=3.5, range=2464-2486, str
target 5' CUUGGCUCUGGUCUUGGUCUUGG 3'
:::::::::::::::::: .::.
query 3' GAACCGAGACCAGAACCAAGACU 5'
query=miR5718, target=RO3G_13217, score=3.5, range=2464-2486, str
target 5' CUUGGCUCUGGUCUUGGUCUUGG 3'
:::::::::::::::::: .::.
query 3' GAACCGAGACCAGAACCAAGACU 5'
query=miR5718, target=RO3G_16800, score=3.5, range=2464-2486, str
target 5' CUUGGCUCUGGUCUUGGUCUUGG 3'
:::::::::::::::::: .::.
query 3' GAACCGAGACCAGAACCAAGACU 5'
query=miR5718, target=RO3G_15267, score=3.5, range=226-248, stran
```

```
target 5' CUUGGCUCUGGUCUUGGUCUUGG 3'
::::::::::::::::: .::.
query 3' GAACCGAGACCAGAACCAAGACU 5'
query=miR5718, target=RO3G_14777, score=3.5, range=163-185, stran
target 5' CUUGGCUCUGGUCUUGGUCUUGG 3'
::::::::::::::::: .::.
query 3' GAACCGAGACCAGAACCAAGACU 5'
query=miR5718, target=RO3G_11967, score=3.5, range=2509-2531, str
target 5' CUCGGCUCUGGUCUUGGUCCUGG 3'
:: :::::::::::::: :::.
query 3' GAACCGAGACCAGAACCAAGACU 5'
query=miR5718, target=RO3G_13222, score=3.5, range=2491-2513, str
target 5' CUCGGCUCUGGUCUUGGUCCUGG 3'
:: :::::::::::::: :::.
query 3' GAACCGAGACCAGAACCAAGACU 5'
query=miR5718, target=RO3G_16171, score=3.5, range=7007-7028, str
target 5' CUU-CUUCUCGUCUUGGUUCUGA 3'
::: .::: ::::::::::::
query 3' GAACCGAGACCAGAACCAAGACU 5'
query=miR5718, target=RO3G_14350, score=3.5, range=2359-2381, str
target 5' CUCGGCUCUGGUCCUGGUUCUGG 3'
:: :::::::::: ::::::::.
query 3' GAACCGAGACCAGAACCAAGACU 5'
query=miR5718, target=RO3G_14352, score=3.5, range=2497-2519, str
target 5' CUCGGCUCUGGUCCUGGUUCUGG 3'
:: :::::::::: ::::::::.
query 3' GAACCGAGACCAGAACCAAGACU 5'
query=miR5718, target=RO3G_13219, score=3.5, range=2494-2516, str
target 5' CUCGGCUCUGGUCCUGGUUCUGG 3'
:: :::::::::: ::::::::.
query 3' GAACCGAGACCAGAACCAAGACU 5'
query=miR5718, target=RO3G_11967, score=4, range=2479-2501, stran
target 5' CUUAGUUCUGGUCUUGGCUCUGG 3'
::: :.:::::::::: ::::.
query 3' GAACCGAGACCAGAACCAAGACU 5'
query=miR5718, target=RO3G_16818, score=4, range=2206-2228, stran
target 5' CUUAGUUCUGGUCUUGGCUCUGG 3'
::: :.:::::::::: ::::.
query 3' GAACCGAGACCAGAACCAAGACU 5'
query=miR6182, target=RO3G_07088, score=2.5, range=250-269, stran
target 5' GACGCAUCCAUCACAGAGCA 3'
.:::::::::::::::: ::
query 3' UUGCGUAGGUAGUGUCUGGU 5'
query=miR6182, target=RO3G_15997, score=3, range=110-128, strand=
target 5' AACG-AACCAUCACAGACCU 3'
```

```
:::: : :::::::::::
query 3' UUGCGUAGGUAGUGUCUGGU 5'
query=miR6182, target=RO3G_15911, score=3.5, range=115-133, stran
target 5' AACGCGUCCAUCACA-ACCU 3'
::::.::::::::: :::
query 3' UUGCGUAGGUAGUGUCUGGU 5'
query=miR6182, target=RO3G_15722, score=3.5, range=398-416, stran
target 5' AGCUC-UCCAUCACGGACCA 3'
:.: : ::::::::.:::::
query 3' UUGCGUAGGUAGUGUCUGGU 5'
query=miR6182, target=RO3G_05899, score=4, range=1244-1262, stran
target 5' AAC-CAACCAUCACAGAACA 3'
::: :: ::::::::: ::
query 3' UUGCGUAGGUAGUGUCUGGU 5'
query=miR6182, target=RO3G_13262, score=4, range=832-851, strand=
target 5' AGCUCGUCCAUCAUAGACCC 3'
:.: :.:::::::.:::::
query 3' UUGCGUAGGUAGUGUCUGGU 5'
query=miR6182, target=RO3G_16215, score=4, range=1171-1189, stran
target 5' AACG-AUCCAUCAUAGACUC 3'
:::: ::::::::.::::.
query 3' UUGCGUAGGUAGUGUCUGGU 5'
query=miR8179, target=RO3G_13279, score=0, range=1928-1949, stran
target 5' ACGAUCAAGUGAAUGCAGAUCA 3'
::::::::::::::::::::::
query 3' UGCUAGUUCACUUACGUCUAGU 5'
query=miR8179, target=RO3G_17346, score=0, range=1835-1856, stran
target 5' ACGAUCAAGUGAAUGCAGAUCA 3'
::::::::::::::::::::::
query 3' UGCUAGUUCACUUACGUCUAGU 5'
query=miR8179, target=RO3G_17263, score=0, range=1085-1106, stran
target 5' ACGAUCAAGUGAAUGCAGAUCA 3'
::::::::::::::::::::::
query 3' UGCUAGUUCACUUACGUCUAGU 5'
query=miR8179, target=RO3G_10786, score=0, range=1076-1097, stran
target 5' ACGAUCAAGUGAAUGCAGAUCA 3'
::::::::::::::::::::::
query 3' UGCUAGUUCACUUACGUCUAGU 5'
query=miR8179, target=RO3G_14771, score=0, range=1028-1049, stran
target 5' ACGAUCAAGUGAAUGCAGAUCA 3'
::::::::::::::::::::::
query 3' UGCUAGUUCACUUACGUCUAGU 5'
query=miR8179, target=RO3G_15868, score=0, range=914-935, strand=
target 5' ACGAUCAAGUGAAUGCAGAUCA 3'
::::::::::::::::::::::
```

```
query 3' UGCUAGUUCACUUACGUCUAGU 5'
query=miR8179, target=RO3G_17419, score=0, range=914-935, strand=
target 5' ACGAUCAAGUGAAUGCAGAUCA 3'
::::::::::::::::::::::
query 3' UGCUAGUUCACUUACGUCUAGU 5'
query=miR8179, target=RO3G_03598, score=0, range=1076-1097, stran
target 5' ACGAUCAAGUGAAUGCAGAUCA 3'
::::::::::::::::::::::
query 3' UGCUAGUUCACUUACGUCUAGU 5'
query=miR8179, target=RO3G_09943, score=0, range=701-722, strand=
target 5' ACGAUCAAGUGAAUGCAGAUCA 3'
::::::::::::::::::::::
query 3' UGCUAGUUCACUUACGUCUAGU 5'
query=miR8179, target=RO3G_17312, score=0, range=515-536, strand=
target 5' ACGAUCAAGUGAAUGCAGAUCA 3'
::::::::::::::::::::::
query 3' UGCUAGUUCACUUACGUCUAGU 5'
query=miR8179, target=RO3G_12808, score=4, range=795-815, strand=
target 5' AAGA-CAAGUGAAUGCAGAUAA 3'
: :: :::::::::::::: :
query 3' UGCUAGUUCACUUACGUCUAGU 5'
query=miR8179, target=RO3G_12345, score=4, range=2510-2532, stran
target 5' CUUAUACAAGUGAAUGCAGAUCG 3'
. :: :::::::::::::::.
query 3' UGCUA-GUUCACUUACGUCUAGU 5'
query=miR847-5p, target=RO3G_03516, score=0, range=645-663, stran
target 5' UCCAUCCUCCUCAUCAAUA 3'
:::::::::::::::::::
query 3' AGGUAGGAGGAGUAGUUAU 5'
query=miR847-5p, target=RO3G_16309, score=1.5, range=174-192,
strand=-1
target 5' GUCAUCCUCCUCAUCAAUA 3'
.:::::::::::::::::
query 3' AGGUAGGAGGAGUAGUUAU 5'
query=miR847-5p, target=RO3G_00269, score=2, range=1221-1239,
strand=-1
target 5' UACAUCCUCUUCAUCAAUA 3'
: :::::::.:::::::::
query 3' AGGUAGGAGGAGUAGUUAU 5'
query=miR847-5p, target=RO3G_06561, score=2.5, range=432-449, str
target 5' AUCA-CCUCCUCAUCAAUA 3'
.:: ::::::::::::::
query 3' AGGUAGGAGGAGUAGUUAU 5'
query=miR847-5p, target=RO3G_03142, score=2.5, range=2607-2625,
strand=-1
```

target 5' UCCAUUCUUCUUAUCAAUA 3'
:::::.::.::.:::::::
query 3' AGGUAGGAGGAGUAGUUAU 5'
query=miR847-5p, target=RO3G_01295, score=2.5, range=168-186,
strand=-1
target 5' UUGAUCCUCUUCAUCAAUA 3'
:. :::::.::::::::
query 3' AGGUAGGAGGAGUAGUUAU 5'
query=miR847-5p, target=RO3G_08083, score=3, range=380-398, stran
target 5' UUCGUCCUCCUCGUCAAUU 3'
:.:.:::::::::.:::::
query 3' AGGUAGGAGGAGUAGUUAU 5'
query=miR847-5p, target=RO3G_13952, score=3, range=1370-1389, str
target 5' UUCAUUCUCCUCAUCAAGUA 3'
:.:::.:::::::::: ::
query 3' AGGUAGGAGGAGUAGUU-AU 5'
query=miR847-5p, target=RO3G_08484, score=3, range=135-152, stran
target 5' UCC-UCCUCCUCCUCAAUA 3'
::: :::::::: ::::::
query 3' AGGUAGGAGGAGUAGUUAU 5'
query=miR847-5p, target=RO3G_02989, score=3, range=453-470, stran
target 5' UCC-UCCUCCUCAACAAUA 3'
::: ::::::::: :::::
query 3' AGGUAGGAGGAGUAGUUAU 5'
query=miR847-5p, target=RO3G_08785, score=3, range=267-284, stran
target 5' UCC-UCCUCCUCCUCAAUA 3'
::: :::::::: ::::::
query 3' AGGUAGGAGGAGUAGUUAU 5'
query=miR847-5p, target=RO3G_07261, score=3, range=1853-1871,
strand=-1
target 5' UUCAUUUUCUUCAUCAAUA 3'
:.:::..::.:::::::::
query 3' AGGUAGGAGGAGUAGUUAU 5'
query=miR847-5p, target=RO3G_03755, score=3, range=72-90, strand=
target 5' UUCGACCUCUUCAUCAAUA 3'
:.:. ::::.:::::::::
query 3' AGGUAGGAGGAGUAGUUAU 5'
query=miR847-5p, target=RO3G_08201, score=3, range=837-855, stran
target 5' UCUUUUCUCUUCAUCAAUA 3'
::. :.:::.:::::::::
query 3' AGGUAGGAGGAGUAGUUAU 5'
query=miR847-5p, target=RO3G_14338, score=3, range=1503-1522,
strand=-1
target 5' ACCAUCCUUCCUCAUCAAUA 3'
:::::: :::::::::::

query 3' AGGUAGG-AGGAGUAGUUAU 5'
query=miR847-5p, target=RO3G_04646, score=3, range=2874-2891, strand=-1
target 5' UCC-UCUUCUUCAUCAAUA 3'
::: ::.::.:::::::::
query 3' AGGUAGGAGGAGUAGUUAU 5'
query=miR847-5p, target=RO3G_02104, score=3.5, range=719-737, str
target 5' GCUAUCCUCUUUAUCAAUA 3'
:.:::::.:.:::::::
query 3' AGGUAGGAGGAGUAGUUAU 5'
query=miR847-5p, target=RO3G_13827, score=3.5, range=1908-1926, strand=1
target 5' UUCAUCCUCCUCAUCCAUU 3'
:.::::::::::::: ::
query 3' AGGUAGGAGGAGUAGUUAU 5'
query=miR847-5p, target=RO3G_06547, score=3.5, range=506-523, str
target 5' UCUAUCCUCCUUA-CAAUA 3'
::.::::::::.: :::::
query 3' AGGUAGGAGGAGUAGUUAU 5'
query=miR847-5p, target=RO3G_15183, score=3.5, range=97-115, stra
target 5' UCCAUGCUCCUCAUCAACG 3'
::::: :::::::::: .
query 3' AGGUAGGAGGAGUAGUUAU 5'
query=miR847-5p, target=RO3G_00192, score=3.5, range=1179-1197, strand=-1
target 5' UUCGUUCUCCUCAUCAAAA 3'
:.:.:.:::::::::: :
query 3' AGGUAGGAGGAGUAGUUAU 5'
query=miR847-5p, target=RO3G_00121, score=3.5, range=2001-2019, strand=-1
target 5' UGUAUCUUCUUCAUCAAUA 3'
: .:::.::.::::::::
query 3' AGGUAGGAGGAGUAGUUAU 5'
query=miR847-5p, target=RO3G_03394, score=3.5, range=1875-1894, strand=-1
target 5' UCCAUUUUUCUUCAUCAAUA 3'
:::: :..::.:::::::::
query 3' AGGU-AGGAGGAGUAGUUAU 5'
query=miR847-5p, target=RO3G_07938, score=3.5, range=749-767, strand=-1
target 5' CCCAUGCUCUUCAUCAAUG 3'
:::: :::.:::::::.
query 3' AGGUAGGAGGAGUAGUUAU 5'
query=miR847-5p, target=RO3G_09251, score=4, range=878-896, stran
target 5' UUCAGUCUUUUCAUCAAUA 3'

```
:.:: .::..:::::::::
query 3' AGGUAGGAGGAGUAGUUAU 5'
query=miR847-5p, target=RO3G_11915, score=4, range=398-416, stran
target 5' AUCGUCCUCCUCGUCAAUU 3'
.:.::::::::.:::::
query 3' AGGUAGGAGGAGUAGUUAU 5'
query=miR847-5p, target=RO3G_08494, score=4, range=399-416, stran
target 5' UCCA-CCUCAUUAUCAAUA 3'
:::: :::: :.:::::::
query 3' AGGUAGGAGGAGUAGUUAU 5'
query=miR847-5p, target=RO3G_14883, score=4, range=93-110, strand
target 5' UCC-UCCUCCUGGUCAAUA 3'
::: ::::::: .::::::
query 3' AGGUAGGAGGAGUAGUUAU 5'
query=miR847-5p, target=RO3G_06535, score=4, range=12-29, strand=
target 5' UCC-UCCUCCUCAUCUAUU 3'
::: :::::::::: ::
query 3' AGGUAGGAGGAGUAGUUAU 5'
query=miR847-5p, target=RO3G_06879, score=4, range=342-360, stran
target 5' AUCAUCUUCUUCAUCAAUG 3'
.::::.::.::::::::.
query 3' AGGUAGGAGGAGUAGUUAU 5'
query=miR847-5p, target=RO3G_01696, score=4, range=993-1011, stra
target 5' UCCGUUCUCUUCUUCAAUA 3'
:::.:.:::.:: ::::::
query 3' AGGUAGGAGGAGUAGUUAU 5'
query=miR847-5p, target=RO3G_11807, score=4, range=89-108, strand
target 5' UCCAUUCUCUUCAUCAAAUG 3'
::::.:::.::::: :::.
query 3' AGGUAGGAGGAGUAG-UUAU 5'
query=miR847-5p, target=RO3G_12917, score=4, range=1002-1020,
strand=-1
target 5' UUCAUUAUCUUCAUCAAUA 3'
:.:::. ::.:::::::::
query 3' AGGUAGGAGGAGUAGUUAU 5'
query=miR847-5p, target=RO3G_00050, score=4, range=84-102, strand
target 5' UUCAUUCUCCUUAUCAACA 3'
:.:::.::::.::::: :
query 3' AGGUAGGAGGAGUAGUUAU 5'
query=miR847-5p, target=RO3G_15129, score=4, range=171-189, stran
target 5' UUCAUUCUCUUCAUCCAUA 3'
:.:::.:::.::::: :::
query 3' AGGUAGGAGGAGUAGUUAU 5'
query=miR847-5p, target=RO3G_12552, score=4, range=222-240, stran
target 5' UCCACCCUUUUUAUCAAUA 3'
```

```
:::: :::..:.::::::: 
query 3' AGGUAGGAGGAGUAGUUAU 5'
query=miR847-5p, target=RO3G_14552, score=4, range=147-165, stran
target 5' UCUUUCCUCUUCGUCAAUG 3'
::. :::::.::.:::::.
query 3' AGGUAGGAGGAGUAGUUAU 5'
query=miR847-5p, target=RO3G_05342, score=4, range=147-165, stran
target 5' UCUUUCCUCUUCGUCAAUG 3'
::. :::::.::.:::::.
query 3' AGGUAGGAGGAGUAGUUAU 5'
query=miR847-5p, target=RO3G_16368, score=4, range=147-165, stran
target 5' UCUUUCCUCUUCGUCAAUG 3'
::. :::::.::.:::::.
query 3' AGGUAGGAGGAGUAGUUAU 5'
query=miR847-5p, target=RO3G_12077, score=4, range=219-237, stran
target 5' UCUUUCCUCUUCGUCAAUG 3'
::. :::::.::.:::::.
query 3' AGGUAGGAGGAGUAGUUAU 5'
query=miR847-5p, target=RO3G_17393, score=4, range=219-237, stran
target 5' UCUUUCCUCUUCGUCAAUG 3'
::. :::::.::.:::::.
query 3' AGGUAGGAGGAGUAGUUAU 5'
query=miR847-5p, target=RO3G_07693, score=4, range=147-165, stran
target 5' UCUUUCCUCUUCGUCAAUG 3'
::. :::::.::.:::::.
query 3' AGGUAGGAGGAGUAGUUAU 5'
query=miR847-5p, target=RO3G_07978, score=4, range=147-165, stran
target 5' UCUUUCCUCUUCGUCAAUG 3'
::. :::::.::.:::::.
query 3' AGGUAGGAGGAGUAGUUAU 5'
query=miR847-5p, target=RO3G_16899, score=4, range=147-165, stran
target 5' UCUUUCCUCUUCGUCAAUG 3'
::. :::::.::.:::::.
query 3' AGGUAGGAGGAGUAGUUAU 5'
query=miR847-5p, target=RO3G_10518, score=4, range=147-165, stran
target 5' UCUUUCCUCUUCGUCAAUG 3'
::. :::::.::.:::::.
query 3' AGGUAGGAGGAGUAGUUAU 5'
query=miR847-5p, target=RO3G_12012, score=4, range=147-165, stran
target 5' UCUUUCCUCUUCGUCAAUG 3'
::. :::::.::.:::::.
query 3' AGGUAGGAGGAGUAGUUAU 5'
query=miR847-5p, target=RO3G_09492, score=4, range=147-165, stran
target 5' UCUUUCCUCUUCGUCAAUG 3'
::. :::::.::.:::::.
```

query 3' AGGUAGGAGGAGUAGUUAU 5'
query=miR847-5p, target=RO3G_09454, score=4, range=6057-6075, strand=-1
target 5' GCCAUCUUCAUCAUCAAUA 3'
:::::.:: :::::::::
query 3' AGGUAGGAGGAGUAGUUAU 5'
query=miR847-5p, target=RO3G_02202, score=4, range=804-822, stran
target 5' ACCAUCCUCUUCAUCACUA 3'
::::::::.:::::: ::
query 3' AGGUAGGAGGAGUAGUUAU 5'
query=miR847-5p, target=RO3G_06990, score=4, range=762-780, stran
target 5' GCCAUCCUCCUCAUCAGAA 3'
:::::::::::::::. :
query 3' AGGUAGGAGGAGUAGUUAU 5'
query=miR847-5p, target=RO3G_00272, score=4, range=1479-1497, strand=-1
target 5' GUCAUCCUCAUCAUCAAUG 3'
.::::::: ::::::::.
query 3' AGGUAGGAGGAGUAGUUAU 5'
query=miR847-5p, target=RO3G_11024, score=4, range=432-450, stran
target 5' GCCUUCUUCUUCAUCAAUA 3'
:: ::.::.:::::::::
query 3' AGGUAGGAGGAGUAGUUAU 5'
query=miR847-5p, target=RO3G_00079, score=4, range=2056-2073, strand=-1
target 5' UUCAUCCUCUUCAUC-AUG 3'
:.:::::::.::::: ::.
query 3' AGGUAGGAGGAGUAGUUAU 5'
query=miR847-5p, target=RO3G_08073, score=4, range=105-122, stran
target 5' UCUGU-CUUUUCAUCAAUA 3'
::..: ::..:::::::::
query 3' AGGUAGGAGGAGUAGUUAU 5'
query=miR847-5p, target=RO3G_01625, score=4, range=609-626, stran
target 5' UCUG-CUUCUUCAUCAAUA 3'
::.. :.::.:::::::::
query 3' AGGUAGGAGGAGUAGUUAU 5'
query=miR847-5p, target=RO3G_11685, score=4, range=141-159, stran
target 5' UCCUUCCUCCUCAUUAUUA 3'
::: ::::::::::.: ::
query 3' AGGUAGGAGGAGUAGUUAU 5'
query=miR847-5p, target=RO3G_04476, score=4, range=522-540, stran
target 5' UCCACCAUCCUCAUUAAUA 3'
:::: : ::::::::.::::
query 3' AGGUAGGAGGAGUAGUUAU 5'
query=miR847-5p, target=RO3G_07949, score=4, range=363-380, stran

target 5' UUUAU-UUCUUCAUCAAUA 3'
:..:: .::.:::::::::
query 3' AGGUAGGAGGAGUAGUUAU 5'
query=miR847-5p, target=RO3G_07589, score=4, range=1461-1480,
strand=-1
target 5' UGCAUCCUUCUCCAUCAAUA 3'
: ::::::.:: ::::::::
query 3' AGGUAGGAGGA-GUAGUUAU 5'
query=miR847-5p, target=RO3G_09974, score=4, range=768-787, stran
target 5' UCCAUCCCUUCUCAUCCAUA 3'
::::: :::.:::::: :::
query 3' AGGUA-GGAGGAGUAGUUAU 5'
query=miR847-5p, target=RO3G_12740, score=4, range=90-108, strand
target 5' UGUAUUCUCAUCAUCAAUA 3'
: .::.::: ::::::::
query 3' AGGUAGGAGGAGUAGUUAU 5'
query=miR847-5p, target=RO3G_03984, score=4, range=630-648, stran
target 5' UGCUUCCUUUUCAUCAAUA 3'
: : ::::..:::::::::
query 3' AGGUAGGAGGAGUAGUUAU 5'
query=miR847-5p, target=RO3G_15749, score=4, range=630-648, stran
target 5' UGCCUCCUUUUCAUCAAUA 3'
: : ::::..:::::::::
query 3' AGGUAGGAGGAGUAGUUAU 5'
query=miR847-5p, target=RO3G_14129, score=4, range=637-656, stran
target 5' UUCUUCUUCCUCAUCAGAUA 3'
:.: ::.::::::::: :::
query 3' AGGUAGGAGGAGUAGU-UAU 5'
query=miR847-5p, target=RO3G_06649, score=4, range=2271-2288,
strand=-1
target 5' UCCAU-CUUUUCAUCAAUC 3'
::::: ::..::::::::
query 3' AGGUAGGAGGAGUAGUUAU 5'
query=miR9559-5p, target=RO3G_03878, score=2.5, range=847-866,
strand=1
target 5' CAAGACGAGCCAAAAUCAAA 3'
:::.: ::.:::::::::::
query 3' GUUUUACUUGGUUUUAGUUU 5'
query=miR9559-5p, target=RO3G_16288, score=3, range=220-239, stra
target 5' CAAAAUGAAUUAAAAUCAAU 3'
::::::::..::::::::
query 3' GUUUUACUUGGUUUUAGUUU 5'
query=miR9559-5p, target=RO3G_04779, score=3, range=1075-1094,
strand=1
target 5' CAAAAUGAAAUAAAAUCAAA 3'

::::::::: .:::::::::
query 3' GUUUUACUUGGUUUUAGUUU 5'
query=miR9559-5p, target=RO3G_07643, score=3, range=254-272, stra
target 5' UAGAA-GAACCAAAAUCAGA 3'
.:.:: ::::::::::::.:
query 3' GUUUUACUUGGUUUUAGUUU 5'
query=miR9559-5p, target=RO3G_09425, score=3, range=1292-1311,
strand=1
target 5' CAACAUCAACCAAAGUCAAA 3'
::: :: :::::::.:::::
query 3' GUUUUACUUGGUUUUAGUUU 5'
query=miR9559-5p, target=RO3G_14948, score=3, range=64-83, strand
target 5' CAAAAGAAACCAAAAUCGAA 3'
::::: ::::::::::.::
query 3' GUUUUACUUGGUUUUAGUUU 5'
query=miR9559-5p, target=RO3G_07162, score=3, range=1104-1122,
strand=1
target 5' CAAAAU-AACCAAAAUGAAA 3'
:::::: ::::::::: :::
query 3' GUUUUACUUGGUUUUAGUUU 5'
query=miR9559-5p, target=RO3G_15268, score=3, range=2419-2438,
strand=-1
target 5' GAAACUGAACCAAGAUCAAA 3'
::: ::::::::.::::::
query 3' GUUUUACUUGGUUUUAGUUU 5'
query=miR9559-5p, target=RO3G_16797, score=3, range=1420-1439,
strand=-1
target 5' CAAACGGAACCAAGAUCAAA 3'
:::: :::::::.::::::
query 3' GUUUUACUUGGUUUUAGUUU 5'
query=miR9559-5p, target=RO3G_14347, score=3.5, range=576-595,
strand=1
target 5' CAAGAUGAACCGAGAUCAAC 3'
:::.:::::::.:.:::::
query 3' GUUUUACUUGGUUUUAGUUU 5'
query=miR9559-5p, target=RO3G_06425, score=3.5, range=576-595,
strand=1
target 5' CAAGAUGAACCGAGAUCAAC 3'
:::.:::::::.:.:::::
query 3' GUUUUACUUGGUUUUAGUUU 5'
query=miR9559-5p, target=RO3G_09547, score=3.5, range=576-595,
strand=1
target 5' CAAGAUGAACCGAGAUCAAC 3'
:::.:::::::.:.:::::
query 3' GUUUUACUUGGUUUUAGUUU 5'

query=miR9559-5p, target=RO3G_12677, score=3.5, range=576-595, strand=1
target 5' CAAGAUGAACCGAGAUCAAC 3'
:::.::::::.:.:::::
query 3' GUUUUACUUGGUUUUAGUUU 5'
query=miR9559-5p, target=RO3G_04933, score=3.5, range=576-595, strand=1
target 5' CAAGAUGAACCGAGAUCAAC 3'
:::.::::::.:.:::::
query 3' GUUUUACUUGGUUUUAGUUU 5'
query=miR9559-5p, target=RO3G_05330, score=3.5, range=576-595, strand=1
target 5' CAAGAUGAACCGAGAUCAAC 3'
:::.::::::.:.:::::
query 3' GUUUUACUUGGUUUUAGUUU 5'
query=miR9559-5p, target=RO3G_02704, score=3.5, range=576-595, strand=1
target 5' CAAGAUGAACCGAGAUCAAC 3'
:::.::::::.:.:::::
query 3' GUUUUACUUGGUUUUAGUUU 5'
query=miR9559-5p, target=RO3G_17021, score=3.5, range=576-595, strand=1
target 5' CAAGAUGAACCGAGAUCAAC 3'
:::.::::::.:.:::::
query 3' GUUUUACUUGGUUUUAGUUU 5'
query=miR9559-5p, target=RO3G_05344, score=3.5, range=576-595, strand=1
target 5' CAAGAUGAACCGAGAUCAAC 3'
:::.::::::.:.:::::
query 3' GUUUUACUUGGUUUUAGUUU 5'
query=miR9559-5p, target=RO3G_03778, score=3.5, range=576-595, strand=1
target 5' CAAGAUGAACCGAGAUCAAC 3'
:::.::::::.:.:::::
query 3' GUUUUACUUGGUUUUAGUUU 5'
query=miR9559-5p, target=RO3G_13722, score=3.5, range=573-592, strand=1
target 5' CAAGAUGAACCGAGAUCAAC 3'
:::.::::::.:.:::::
query 3' GUUUUACUUGGUUUUAGUUU 5'
query=miR9559-5p, target=RO3G_16939, score=3.5, range=1205-1225, strand=1
target 5' UGGAACUGAAUCAAAAUCAAA 3'
...:: ::::.::::::::::
query 3' GUUUU-ACUUGGUUUUAGUUU 5'

query=miR9559-5p, target=RO3G_03924, score=3.5, range=790-808, strand=1
target 5' CAAGAUGGACCAAAA-CAAA 3'
:::.:::.::::::: ::::
query 3' GUUUUACUUGGUUUUAGUUU 5'
query=miR9559-5p, target=RO3G_15766, score=3.5, range=389-407, strand=1
target 5' UGGAA-GAACCAAAAUCAGA 3'
...:: ::::::::::::.:
query 3' GUUUUACUUGGUUUUAGUUU 5'
query=miR9559-5p, target=RO3G_16050, score=3.5, range=230-248, strand=1
target 5' UGGAA-GAACCAAAAUCAGA 3'
...:: ::::::::::::.:
query 3' GUUUUACUUGGUUUUAGUUU 5'
query=miR9559-5p, target=RO3G_10152, score=3.5, range=2992-3011, strand=1
target 5' CAGAAAAAAUCAAAAUCAAA 3'
::.:: ::.::::::::::
query 3' GUUUUACUUGGUUUUAGUUU 5'
query=miR9559-5p, target=RO3G_02904, score=3.5, range=1124-1143, strand=1
target 5' CGAAAAUAACCGAAAUCAAA 3'
:.::: ::::.::::::::
query 3' GUUUUACUUGGUUUUAGUUU 5'
query=miR9559-5p, target=RO3G_08576, score=3.5, range=393-413, strand=1
target 5' CAAAAGGAAAUCAAAAUCAAG 3'
::::: : ::.:::::::::.
query 3' GUUUUAC-UUGGUUUUAGUUU 5'
query=miR9559-5p, target=RO3G_00193, score=3.5, range=682-700, strand=1
target 5' CAAGAUCAA-CAAAAUCAAA 3'
:::.:: :: ::::::::::
query 3' GUUUUACUUGGUUUUAGUUU 5'
query=miR9559-5p, target=RO3G_09727, score=3.5, range=1036-1055, strand=-1
target 5' CAAGAAGAACCAAAAUCGAU 3'
:::.: :::::::::::.:
query 3' GUUUUACUUGGUUUUAGUUU 5'
query=miR9559-5p, target=RO3G_14350, score=3.5, range=2284-2304, strand=-1
target 5' GGAAAUGGAACCAAGAUCAAA 3'
.:::: :::::::.::::::
query 3' GUUUUA-CUUGGUUUUAGUUU 5'

query=miR9559-5p, target=RO3G_14352, score=3.5, range=2422-2442, strand=-1
target 5' GGAAAUGGAACCAAGAUCAAA 3'
.:::: ::::::.::::::
query 3' GUUUUA-CUUGGUUUUAGUUU 5'
query=miR9559-5p, target=RO3G_13219, score=3.5, range=2419-2439, strand=-1
target 5' GGAAAUGGAACCAAGAUCAAA 3'
.:::: ::::::.::::::
query 3' GUUUUA-CUUGGUUUUAGUUU 5'
query=miR9559-5p, target=RO3G_14777, score=3.5, range=118-138, strand=-1
target 5' AGAAAUGGAACCAAGAUCAAA 3'
.:::: ::::::.::::::
query 3' GUUUUA-CUUGGUUUUAGUUU 5'
query=miR9559-5p, target=RO3G_05734, score=3.5, range=426-445, strand=-1
target 5' CAAGAUCAUCCAAAAUCAAA 3'
:::.:: : ::::::::::
query 3' GUUUUACUUGGUUUUAGUUU 5'
query=miR9559-5p, target=RO3G_01970, score=3.5, range=672-692, strand=-1
target 5' CAAGCUUGAAUCAAAAUCAAA 3'
:::. ::::.::::::::::
query 3' GUUU-UACUUGGUUUUAGUUU 5'
query=miR9559-5p, target=RO3G_11125, score=3.5, range=447-465, strand=-1
target 5' CUGAAU-AAUCAAAAUCAAA 3'
: .::: ::.::::::::::
query 3' GUUUUACUUGGUUUUAGUUU 5'
query=miR9559-5p, target=RO3G_14378, score=4, range=3034-3053, strand=1
target 5' CAAAAUGGAUCAGAAUUAAA 3'
::::::.:.::.:::.:::
query 3' GUUUUACUUGGUUUUAGUUU 5'
query=miR9559-5p, target=RO3G_09435, score=4, range=622-641, stra
target 5' GAAGAUGAACUAGAAUCAAG 3'
::.::::::.:.::::::.
query 3' GUUUUACUUGGUUUUAGUUU 5'
query=miR9559-5p, target=RO3G_09688, score=4, range=162-182, stra
target 5' UAGAAUCGAACCAAAGUCAGA 3'
.:.::: :::::::.:::.:
query 3' GUUUUA-CUUGGUUUUAGUUU 5'
query=miR9559-5p, target=RO3G_03163, score=4, range=726-745, stra
target 5' AGAAAUGAACCAAAAUGAAG 3'

```
.::::::::::::: ::.
query 3' GUUUUACUUGGUUUUAGUUU 5'
query=miR9559-5p, target=RO3G_13839, score=4, range=149-168, stra
target 5' CAAAACGAAUCAAAAUCGAC 3'
::::: :::.:::::::.:
query 3' GUUUUACUUGGUUUUAGUUU 5'
query=miR9559-5p, target=RO3G_00751, score=4, range=1132-1151,
strand=1
target 5' GGAGAUAAACCAGAAUCAAA 3'
.:.:: ::::.:::::::
query 3' GUUUUACUUGGUUUUAGUUU 5'
query=miR9559-5p, target=RO3G_02384, score=4, range=1033-1052,
strand=1
target 5' CAAAAGGAACCAAGAGCAAA 3'
::::: :::::::.: ::::
query 3' GUUUUACUUGGUUUUAGUUU 5'
query=miR9559-5p, target=RO3G_13423, score=4, range=1055-1073,
strand=1
target 5' UAAAG-GAACCAGAAUUAAA 3'
.:::. ::::::.:::.:::
query 3' GUUUUACUUGGUUUUAGUUU 5'
query=miR9559-5p, target=RO3G_08755, score=4, range=204-222, stra
target 5' CAGAA-GAGCCAAAGUCAAG 3'
::.:: ::.:::::.::::.
query 3' GUUUUACUUGGUUUUAGUUU 5'
query=miR9559-5p, target=RO3G_15310, score=4, range=220-239, stra
target 5' CAAAAUAAACCAAAAAUAAA 3'
:::::: :::::::: .:::
query 3' GUUUUACUUGGUUUUAGUUU 5'
query=miR9559-5p, target=RO3G_10410, score=4, range=415-434, stra
target 5' CACAAUGAACCAAAAUUAUA 3'
:: :::::::::::::.: :
query 3' GUUUUACUUGGUUUUAGUUU 5'
query=miR9559-5p, target=RO3G_05566, score=4, range=70-89, strand
target 5' CAAAAAGAAUCAAAUUCAAA 3'
::::: :::.:::: :::::
query 3' GUUUUACUUGGUUUUAGUUU 5'
query=miR9559-5p, target=RO3G_02908, score=4, range=43-62, strand
target 5' CAAAAGAAAUCAAAAUCAGA 3'
::::: ::.::::::::.:
query 3' GUUUUACUUGGUUUUAGUUU 5'
query=miR9559-5p, target=RO3G_02814, score=4, range=38-57, strand
target 5' CAAGUUGUACCAAAAUCAAG 3'
:::. :: ::::::::::.
query 3' GUUUUACUUGGUUUUAGUUU 5'
```

```
query=miR9559-5p, target=RO3G_00924, score=4, range=896-916, stra
target 5' CCAAAUGCAACCGAGAUCAAA 3'
: ::::: ::::.:.::::::
query 3' GUUUUAC-UUGGUUUUAGUUU 5'
query=miR9559-5p, target=RO3G_08017, score=4, range=687-707, stra
target 5' CAGGAUAGAACCAAAUUCAAA 3'
::..:: :::::::: :::::
query 3' GUUUUA-CUUGGUUUUAGUUU 5'
query=miR9559-5p, target=RO3G_15821, score=4, range=1344-1364,
strand=1
target 5' UAUAAACGAACUAAAAUCAAG 3'
.: ::: ::::.:::::::.
query 3' GU-UUUACUUGGUUUUAGUUU 5'
query=miR9559-5p, target=RO3G_11377, score=4, range=2386-2405,
strand=1
target 5' GACAAUGAAGCAAAAUCAAA 3'
: :::::: :::::::::
query 3' GUUUUACUUGGUUUUAGUUU 5'
query=miR9559-5p, target=RO3G_07551, score=4, range=373-393, stra
target 5' CCAACUGAACCAAAUAUCAAA 3'
: :: ::::::::: ::::::
query 3' GUUUUACUUGGUUU-UAGUUU 5'
query=miR9559-5p, target=RO3G_16525, score=4, range=739-758, stra
target 5' GGAAGUGAACCAAAAUUGAA 3'
.::.::::::::::..::
query 3' GUUUUACUUGGUUUUAGUUU 5'
query=miR9559-5p, target=RO3G_07501, score=4, range=210-229, stra
target 5' CAAAAUCAAUCAAAAACAAA 3'
:::::: ::.::::: ::::
query 3' GUUUUACUUGGUUUUAGUUU 5'
query=miR9559-5p, target=RO3G_01072, score=4, range=6006-6025,
strand=-1
target 5' CAUAAUCAGCCAAGAUCAAA 3'
:: ::: :.::::.::::::
query 3' GUUUUACUUGGUUUUAGUUU 5'
query=miR9559-5p, target=RO3G_06040, score=4, range=1027-1046,
strand=-1
target 5' AAAAAUGUACCAAAAUCAAU 3'
:::::: ::::::::::
query 3' GUUUUACUUGGUUUUAGUUU 5'
query=miR9559-5p, target=RO3G_16914, score=4, range=2771-2790,
strand=-1
target 5' CAAAAAAAACCAAAAUCGAC 3'
::::: ::::::::::.:
query 3' GUUUUACUUGGUUUUAGUUU 5'
```

query=miR9559-5p, target=RO3G_12627, score=4, range=693-711, stra
target 5' UAAAG-GAAGCAAAAUCAAA 3'
.:::. ::: ::::::::::
query 3' GUUUUACUUGGUUUUAGUUU 5'
query=miR9559-5p, target=RO3G_05212, score=4, range=307-326, stra
target 5' CCCAUUGAACUAAAAUCAAA 3'
: : :::::.:::::::::
query 3' GUUUUACUUGGUUUUAGUUU 5'
query=miR9559-5p, target=RO3G_11872, score=4, range=2328-2347,
strand=-1
target 5' CAACAUCACCCAAAAUCAAA 3'
::: :: : ::::::::::
query 3' GUUUUACUUGGUUUUAGUUU 5'
query=miR9559-5p, target=RO3G_01416, score=4, range=315-333, stra
target 5' UUGAAU-AAUCAAAAUCAAA 3'
. .::: ::.::::::::::
query 3' GUUUUACUUGGUUUUAGUUU 5'
query=miR5387a, target=RO3G_17217, score=1, range=142-163, strand
target 5' GAUACCGUAGACCGGCGUGUUA 3'
::::: :::::::::::::::
query 3' CUAUGACAUCUGGCCGCACAAU 5'
query=miR5387a, target=RO3G_07862, score=2, range=1558-1579, stra
target 5' GAUACCGUCGACCGGCGUGUUA 3'
::::: :: ::::::::::::
query 3' CUAUGACAUCUGGCCGCACAAU 5'
query=miR5387a, target=RO3G_10744, score=2, range=1444-1465, stra
target 5' GAUACCGUCGACCGGCGUGUUA 3'
::::: :: ::::::::::::
query 3' CUAUGACAUCUGGCCGCACAAU 5'
query=miR5387a, target=RO3G_16366, score=2, range=1444-1465, stra
target 5' GAUACCGUCGACCGGCGUGUUA 3'
::::: :: ::::::::::::
query 3' CUAUGACAUCUGGCCGCACAAU 5'
query=miR5387a, target=RO3G_17255, score=2, range=1414-1435, stra
target 5' GAUACCGUCGACCGGCGUGUUA 3'
::::: :: ::::::::::::
query 3' CUAUGACAUCUGGCCGCACAAU 5'
query=miR5387a, target=RO3G_07735, score=2, range=1363-1384, stra
target 5' GAUACCGUCGACCGGCGUGUUA 3'
::::: :: ::::::::::::
query 3' CUAUGACAUCUGGCCGCACAAU 5'
query=miR5387a, target=RO3G_11979, score=2, range=1417-1438, stra
target 5' GAUACCGUCGACCGGCGUGUUA 3'
::::: :: ::::::::::::
query 3' CUAUGACAUCUGGCCGCACAAU 5'

```
query=miR5387a, target=RO3G_13448, score=2, range=1429-1450, stra
target 5' GAUACCGUCGACCGGCGUGUUA 3'
::::: :: :::::::::::::
query 3' CUAUGACAUCUGGCCGCACAAU 5'
query=miR5387a, target=RO3G_10543, score=2, range=916-937, strand
target 5' GAUACCGUCGACCGGCGUGUUA 3'
::::: :: :::::::::::::
query 3' CUAUGACAUCUGGCCGCACAAU 5'
query=miR5387a, target=RO3G_10745, score=2, range=412-433, strand
target 5' GAUACCGUCGACCGGCGUGUUA 3'
::::: :: :::::::::::::
query 3' CUAUGACAUCUGGCCGCACAAU 5'
query=miR5387a, target=RO3G_10532, score=2, range=1429-1450, stra
target 5' GAUACCGUCGACCGGCGUGUUA 3'
::::: :: :::::::::::::
query 3' CUAUGACAUCUGGCCGCACAAU 5'
query=miR5387a, target=RO3G_10747, score=2, range=1558-1579, stra
target 5' GAUACCGUCGACCGGCGUGUUA 3'
::::: :: :::::::::::::
query 3' CUAUGACAUCUGGCCGCACAAU 5'
query=miR5387a, target=RO3G_17253, score=2, range=1414-1435, stra
target 5' GAUACCGUCGACCGGCGUGUUA 3'
::::: :: :::::::::::::
query 3' CUAUGACAUCUGGCCGCACAAU 5'
query=miR5256, target=RO3G_01070, score=2, range=1640-1660, stran
target 5' UUAGUGUCUUACAAGCCAUUG 3'
:::.: ::::::::::::::.
query 3' AAUUA-AGAAUGUUCGGUAAU 5'
query=miR5256, target=RO3G_16253, score=3, range=14-33, strand=-1
target 5' GUAGUUUUUACGAGCCAUUA 3'
::.::.:::.::::::::
query 3' AAUUAAGAAUGUUCGGUAAU 5'
query=miR5256, target=RO3G_15832, score=3, range=863-881, strand=
target 5' UUAAUU-UUACAAUCCAUUA 3'
:::::: :::::: ::::::
query 3' AAUUAAGAAUGUUCGGUAAU 5'
query=miR5256, target=RO3G_13101, score=4, range=477-495, strand=
target 5' UUCA-UCUUACAGGCUAUUA 3'
:: : :::::::.::.::::
query 3' AAUUAAGAAUGUUCGGUAAU 5'
query=miR5256, target=RO3G_14175, score=4, range=391-409, strand=
target 5' UUAG-AAUUACAAGCCAUUG 3'
:::. ::::::::::::.
query 3' AAUUAAGAAUGUUCGGUAAU 5'
query=miR5256, target=RO3G_12301, score=4, range=2043-2062, stran
```

```
target 5' UUAAAUCUUGCAAGUCAUUC 3'
:::: ::::.::::.::::
query 3' AAUUAAGAAUGUUCGGUAAU 5'
query=miR5256, target=RO3G_05898, score=4, range=292-311, strand=
target 5' UUAACUCUUACAAUCCAUUU 3'
:::: :::::::: :::::
query 3' AAUUAAGAAUGUUCGGUAAU 5'
query=miR775, target=RO3G_01528, score=0, range=133-152, strand=1
target 5' UUGACUGCAAGACAUCGCAA 3'
::::::::::::::::::::
query 3' AACUGACGUUCUGUAGCGUU 5'
query=miR775, target=RO3G_09419, score=0, range=226-245, strand=1
target 5' UUGACUGCAAGACAUCGCAA 3'
::::::::::::::::::::
query 3' AACUGACGUUCUGUAGCGUU 5'
query=miR775, target=RO3G_11486, score=0, range=178-197, strand=1
target 5' UUGACUGCAAGACAUCGCAA 3'
::::::::::::::::::::
query 3' AACUGACGUUCUGUAGCGUU 5'
query=miR775, target=RO3G_15496, score=1, range=49-68, strand=1
target 5' UUGACUGCAAGACAUUGCAA 3'
:::::::::::::::.::::
query 3' AACUGACGUUCUGUAGCGUU 5'
query=miR775, target=RO3G_09838, score=1, range=178-197, strand=1
target 5' UUGACUGCAAGACAUCGUAA 3'
:::::::::::::::::.::
query 3' AACUGACGUUCUGUAGCGUU 5'
query=miR775, target=RO3G_07273, score=1, range=103-122, strand=1
target 5' UUAACUGCAAGACAUCGCAA 3'
:: :::::::::::::::::
query 3' AACUGACGUUCUGUAGCGUU 5'
query=miR775, target=RO3G_09656, score=1.5, range=103-122, strand
target 5' UUGGCUGCAAGGCAUCGCAA 3'
:::.:::::::.::::::::
query 3' AACUGACGUUCUGUAGCGUU 5'
query=miR775, target=RO3G_04104, score=2, range=103-122, strand=1
target 5' UUGACUGCAAGGCAUCGUAA 3'
:::::::::::.:::::.::
query 3' AACUGACGUUCUGUAGCGUU 5'
query=miR775, target=RO3G_04110, score=2, range=103-122, strand=1
target 5' UUGACUGCAAGGCAUCGUAA 3'
:::::::::::.:::::.::
query 3' AACUGACGUUCUGUAGCGUU 5'
query=miR775, target=RO3G_08301, score=2, range=331-350, strand=1
target 5' UUGACUGCAAGACACCGCAA 3'
```

```
::::::::::::: :::::
query 3' AACUGACGUUCUGUAGCGUU 5'
query=miR775, target=RO3G_16429, score=2, range=118-137, strand=1
target 5' UUGACUGCAAGACACCGCAA 3'
::::::::::::: :::::
query 3' AACUGACGUUCUGUAGCGUU 5'
query=miR775, target=RO3G_02348, score=2, range=118-137, strand=1
target 5' UUGACUGCAAGACACCGCAA 3'
::::::::::::: :::::
query 3' AACUGACGUUCUGUAGCGUU 5'
query=miR775, target=RO3G_13903, score=2, range=406-425, strand=1
target 5' UUGACUGCAAGACACCGCAA 3'
::::::::::::: :::::
query 3' AACUGACGUUCUGUAGCGUU 5'
query=miR775, target=RO3G_14722, score=2, range=406-425, strand=1
target 5' UUGACUGCAAGACACCGCAA 3'
::::::::::::: :::::
query 3' AACUGACGUUCUGUAGCGUU 5'
query=miR775, target=RO3G_08906, score=2, range=406-425, strand=1
target 5' UUGACUGCAAGACACCGCAA 3'
::::::::::::: :::::
query 3' AACUGACGUUCUGUAGCGUU 5'
query=miR775, target=RO3G_13664, score=2, range=406-425, strand=1
target 5' UUGACUGCAAGACACCGCAA 3'
::::::::::::: :::::
query 3' AACUGACGUUCUGUAGCGUU 5'
query=miR775, target=RO3G_05133, score=2, range=355-374, strand=1
target 5' UUGACUGCAAGACACCGCAA 3'
::::::::::::: :::::
query 3' AACUGACGUUCUGUAGCGUU 5'
query=miR775, target=RO3G_05303, score=2, range=406-425, strand=1
target 5' UUGACUGCAAGACACCGCAA 3'
::::::::::::: :::::
query 3' AACUGACGUUCUGUAGCGUU 5'
query=miR775, target=RO3G_05181, score=2, range=406-425, strand=1
target 5' UUGACUGCAAGACACCGCAA 3'
::::::::::::: :::::
query 3' AACUGACGUUCUGUAGCGUU 5'
query=miR775, target=RO3G_05194, score=2, range=406-425, strand=1
target 5' UUGACUGCAAGACACCGCAA 3'
::::::::::::: :::::
query 3' AACUGACGUUCUGUAGCGUU 5'
query=miR775, target=RO3G_13672, score=2, range=406-425, strand=1
target 5' UUGACUGCAAGACACCGCAA 3'
::::::::::::: :::::
```

```
query 3' AACUGACGUUCUGUAGCGUU 5'
query=miR775, target=RO3G_02354, score=2, range=406-425, strand=1
target 5' UUGACUGCAAGACACCGCAA 3'
::::::::::::: :::::
query 3' AACUGACGUUCUGUAGCGUU 5'
query=miR775, target=RO3G_07580, score=2, range=130-149, strand=1
target 5' UUGACUGCAAGACACCGCAA 3'
::::::::::::: :::::
query 3' AACUGACGUUCUGUAGCGUU 5'
query=miR775, target=RO3G_11567, score=2, range=1258-1277, strand
target 5' UUGACUGCAAGACACCGCAA 3'
::::::::::::: :::::
query 3' AACUGACGUUCUGUAGCGUU 5'
query=miR775, target=RO3G_07788, score=2, range=355-374, strand=1
target 5' UUGACUGCAAGACACCGCAA 3'
::::::::::::: :::::
query 3' AACUGACGUUCUGUAGCGUU 5'
query=miR775, target=RO3G_01636, score=2, range=355-374, strand=1
target 5' UUGACUGCAAGACACCGCAA 3'
::::::::::::: :::::
query 3' AACUGACGUUCUGUAGCGUU 5'
query=miR775, target=RO3G_01545, score=2, range=370-389, strand=1
target 5' UUGACUGCAAGACACCGCAA 3'
::::::::::::: :::::
query 3' AACUGACGUUCUGUAGCGUU 5'
query=miR775, target=RO3G_14467, score=3, range=103-122, strand=1
target 5' UUGACUGCAAGGCACCGCAA 3'
::::::::::.:: :::::
query 3' AACUGACGUUCUGUAGCGUU 5'
query=miR775, target=RO3G_14806, score=3, range=313-332, strand=1
target 5' UUGACUGCAAGACGCCGCAA 3'
:::::::::::::. :::::
query 3' AACUGACGUUCUGUAGCGUU 5'
query=miR775, target=RO3G_07438, score=3, range=103-122, strand=1
target 5' UUGACUGCAAGGCACCGCAA 3'
::::::::::.:: :::::
query 3' AACUGACGUUCUGUAGCGUU 5'
query=miR775, target=RO3G_09279, score=3, range=172-191, strand=1
target 5' CUGACUGCAAGACACCGCAA 3'
:::::::::::: :::::
query 3' AACUGACGUUCUGUAGCGUU 5'
query=miR775, target=RO3G_14365, score=3, range=298-317, strand=1
target 5' UUGACCGCAAGACACCGCAA 3'
::::: :::::::: :::::
query 3' AACUGACGUUCUGUAGCGUU 5'
```

```
query=miR775, target=RO3G_01923, score=3, range=355-374, strand=1
target 5' UUGACUACAAGACACCGCAA 3'
:::::: ::::::: :::::
query 3' AACUGACGUUCUGUAGCGUU 5'
query=miR775, target=RO3G_02936, score=3.5, range=406-425, strand
target 5' UUGGCUGCAAGACACUGCAA 3'
:::.:::::::::: .::::
query 3' AACUGACGUUCUGUAGCGUU 5'
query=miR775, target=RO3G_06838, score=4, range=49-68, strand=1
target 5' GUGACUGCAAGGCACCGCAA 3'
::::::::::.:: :::::
query 3' AACUGACGUUCUGUAGCGUU 5'
query=miR775, target=RO3G_13574, score=4, range=184-203, strand=1
target 5' UUGACUGCAAUACACCGCAA 3'
:::::::::: ::: :::::
query 3' AACUGACGUUCUGUAGCGUU 5'
query=miR775, target=RO3G_08649, score=4, range=133-152, strand=1
target 5' UUGACUACAAGGCACCGCAA 3'
:::::: ::::.:: :::::
query 3' AACUGACGUUCUGUAGCGUU 5'
query=miR775, target=RO3G_09608, score=4, range=739-758, strand=1
target 5' UUUAAUGCAAGACAUCGUGA 3'
:: : ::::::::::::..:
query 3' AACUGACGUUCUGUAGCGUU 5'
query=miR775, target=RO3G_12158, score=4, range=29-47, strand=1
target 5' UUGACUACAAGAUAU-GCAA 3'
:::::: :::::.:: ::::
query 3' AACUGACGUUCUGUAGCGUU 5'
query=miR775, target=RO3G_05218, score=4, range=410-428, strand=1
target 5' UUAAC-GCAAGACAUCGUGA 3'
:: :: :::::::::::..:
query 3' AACUGACGUUCUGUAGCGUU 5'
query=miR775, target=RO3G_10000, score=4, range=103-122, strand=1
target 5' UUUACUACAAGACACCGCAA 3'
:: ::: ::::::: :::::
query 3' AACUGACGUUCUGUAGCGUU 5'
query=miR775, target=RO3G_07957, score=4, range=634-653, strand=-
target 5' UUGAUUCCAAGAUAUUGCAG 3'
::::.: :::::.::.:::.
query 3' AACUGACGUUCUGUAGCGUU 5'
query=miR775, target=RO3G_15244, score=4, range=439-457, strand=-
target 5' UUGGUUUCAAGACAUC-CAA 3'
:::..: ::::::::: :::
query 3' AACUGACGUUCUGUAGCGUU 5'
query=miR775, target=RO3G_06107, score=4, range=311-330, strand=-
```

target 5' UUGAACGCAUGACAUCGCAA 3'
:::: ::: ::::::::::
query 3' AACUGACGUUCUGUAGCGUU 5'
query=miR167d, target=RO3G_13498, score=3, range=394-414, strand=
target 5' GGAUCCGUGUUGGCAGCUUCA 3'
.::: :.::.:::::::::::
query 3' UCUA-GUACGACCGUCGAAGU 5'
query=miR167d, target=RO3G_02881, score=3, range=10143-10163,
strand=-1
target 5' AGAUAAUGGCUGGCAGCUUCC 3'
:::: :: ::::::::::::
query 3' UCUAGUA-CGACCGUCGAAGU 5'
query=miR167d, target=RO3G_16597, score=3.5, range=342-361, stran
target 5' GGGUCAUGCUGGCCGCUUCG 3'
.:.:::::::::: :::::.
query 3' UCUAGUACGACCGUCGAAGU 5'
query=miR167d, target=RO3G_01139, score=4, range=492-510, strand=
target 5' GGAU-AUGCUGGCUGCUUCG 3'
.::: :::::::: :::::.
query 3' UCUAGUACGACCGUCGAAGU 5'
query=miR167d, target=RO3G_02577, score=4, range=573-591, strand=
target 5' UGA-CUUGCUGGCAGUUUCA 3'
:: : :::::::::.::::
query 3' UCUAGUACGACCGUCGAAGU 5'
query=miR5678, target=RO3G_05622, score=0, range=475-494, strand=
target 5' GGAAAGUCUUAUGCAUGGAA 3'
::::::::::::::::::::
query 3' CCUUUCAGAAUACGUACCUU 5'
query=miR5678, target=RO3G_08846, score=0, range=475-494, strand=
target 5' GGAAAGUCUUAUGCAUGGAA 3'
::::::::::::::::::::
query 3' CCUUUCAGAAUACGUACCUU 5'
query=miR5678, target=RO3G_13848, score=0, range=475-494, strand=
target 5' GGAAAGUCUUAUGCAUGGAA 3'
::::::::::::::::::::
query 3' CCUUUCAGAAUACGUACCUU 5'
query=miR5678, target=RO3G_05635, score=0, range=475-494, strand=
target 5' GGAAAGUCUUAUGCAUGGAA 3'
::::::::::::::::::::
query 3' CCUUUCAGAAUACGUACCUU 5'
query=miR5678, target=RO3G_03571, score=0, range=475-494, strand=
target 5' GGAAAGUCUUAUGCAUGGAA 3'
::::::::::::::::::::
query 3' CCUUUCAGAAUACGUACCUU 5'
query=miR5678, target=RO3G_01652, score=0, range=475-494, strand=

```
target 5' GGAAAGUCUUAUGCAUGGAA 3'
::::::::::::::::::::
query 3' CCUUUCAGAAUACGUACCUU 5'
query=miR5678, target=RO3G_09607, score=0, range=475-494, strand=
target 5' GGAAAGUCUUAUGCAUGGAA 3'
::::::::::::::::::::
query 3' CCUUUCAGAAUACGUACCUU 5'
query=miR5678, target=RO3G_11401, score=0.5, range=475-494, stran
target 5' GGGAAGUCUUAUGCAUGGAA 3'
::.:::::::::::::::::
query 3' CCUUUCAGAAUACGUACCUU 5'
query=miR5678, target=RO3G_09483, score=0.5, range=475-494, stran
target 5' GGGAAGUCUUAUGCAUGGAA 3'
::.:::::::::::::::::
query 3' CCUUUCAGAAUACGUACCUU 5'
query=miR5678, target=RO3G_00681, score=0.5, range=475-494, stran
target 5' GGGAAGUCUUAUGCAUGGAA 3'
::.:::::::::::::::::
query 3' CCUUUCAGAAUACGUACCUU 5'
query=miR5678, target=RO3G_09499, score=0.5, range=475-494, stran
target 5' GGGAAGUCUUAUGCAUGGAA 3'
::.:::::::::::::::::
query 3' CCUUUCAGAAUACGUACCUU 5'
query=miR5678, target=RO3G_07303, score=0.5, range=475-494, stran
target 5' GGGAAGUCUUAUGCAUGGAA 3'
::.:::::::::::::::::
query 3' CCUUUCAGAAUACGUACCUU 5'
query=miR5678, target=RO3G_09544, score=0.5, range=475-494, stran
target 5' GGGAAGUCUUAUGCAUGGAA 3'
::.:::::::::::::::::
query 3' CCUUUCAGAAUACGUACCUU 5'
query=miR5678, target=RO3G_07875, score=0.5, range=475-494, stran
target 5' GGGAAGUCUUAUGCAUGGAA 3'
::.:::::::::::::::::
query 3' CCUUUCAGAAUACGUACCUU 5'
query=miR5678, target=RO3G_05336, score=0.5, range=475-494, stran
target 5' GGGAAGUCUUAUGCAUGGAA 3'
::.:::::::::::::::::
query 3' CCUUUCAGAAUACGUACCUU 5'
query=miR5678, target=RO3G_17382, score=0.5, range=475-494, stran
target 5' GGGAAGUCUUAUGCAUGGAA 3'
::.:::::::::::::::::
query 3' CCUUUCAGAAUACGUACCUU 5'
query=miR5678, target=RO3G_06423, score=0.5, range=529-548, stran
target 5' GGGAAGUCUUAUGCAUGGAA 3'
```

::.:::::::::::::::::
query 3' CCUUUCAGAAUACGUACCUU 5'
query=miR5678, target=RO3G_16807, score=0.5, range=475-494, stran
target 5' GGGAAGUCUUAUGCAUGGAA 3'
::.:::::::::::::::::
query 3' CCUUUCAGAAUACGUACCUU 5'
query=miR5678, target=RO3G_02689, score=0.5, range=475-494, stran
target 5' GGGAAGUCUUAUGCAUGGAA 3'
::.:::::::::::::::::
query 3' CCUUUCAGAAUACGUACCUU 5'
query=miR5678, target=RO3G_10732, score=0.5, range=475-494, stran
target 5' GGGAAGUCUUAUGCAUGGAA 3'
::.:::::::::::::::::
query 3' CCUUUCAGAAUACGUACCUU 5'
query=miR5678, target=RO3G_14359, score=0.5, range=475-494, stran
target 5' GGGAAGUCUUAUGCAUGGAA 3'
::.:::::::::::::::::
query 3' CCUUUCAGAAUACGUACCUU 5'
query=miR5678, target=RO3G_06441, score=0.5, range=475-494, stran
target 5' GGGAAGUCUUAUGCAUGGAA 3'
::.:::::::::::::::::
query 3' CCUUUCAGAAUACGUACCUU 5'
query=miR5678, target=RO3G_15675, score=0.5, range=475-494, stran
target 5' GGGAAGUCUUAUGCAUGGAA 3'
::.:::::::::::::::::
query 3' CCUUUCAGAAUACGUACCUU 5'
query=miR5678, target=RO3G_10235, score=0.5, range=475-494, stran
target 5' GGGAAGUCUUAUGCAUGGAA 3'
::.:::::::::::::::::
query 3' CCUUUCAGAAUACGUACCUU 5'
query=miR5678, target=RO3G_17400, score=0.5, range=349-368, stran
target 5' GGGAAGUCUUAUGCAUGGAA 3'
::.:::::::::::::::::
query 3' CCUUUCAGAAUACGUACCUU 5'
query=miR5678, target=RO3G_01730, score=0.5, range=475-494, stran
target 5' GGGAAGUCUUAUGCAUGGAA 3'
::.:::::::::::::::::
query 3' CCUUUCAGAAUACGUACCUU 5'
query=miR5678, target=RO3G_05915, score=0.5, range=475-494, stran
target 5' GGGAAGUCUUAUGCAUGGAA 3'
::.:::::::::::::::::
query 3' CCUUUCAGAAUACGUACCUU 5'
query=miR5678, target=RO3G_01322, score=0.5, range=361-380, stran
target 5' GGGAAGUCUUAUGCAUGGAA 3'
::.:::::::::::::::::

```
query 3' CCUUUCAGAAUACGUACCUU 5'
query=miR5678, target=RO3G_13720, score=0.5, range=352-371, stran
target 5' GGGAAGUCUUAUGCAUGGAA 3'
::.:::::::::::::::::
query 3' CCUUUCAGAAUACGUACCUU 5'
query=miR5678, target=RO3G_13742, score=1, range=475-494, strand=
target 5' GGAAAGUCUUAUGUAUGGAA 3'
:::::::::::::.::::::
query 3' CCUUUCAGAAUACGUACCUU 5'
query=miR5678, target=RO3G_12071, score=1, range=106-125, strand=
target 5' GGAAAGGCUUAUGCAUGGAA 3'
:::::: :::::::::::::
query 3' CCUUUCAGAAUACGUACCUU 5'
query=miR5678, target=RO3G_01188, score=1, range=343-362, strand=
target 5' GGAAAGGCUUAUGCAUGGAA 3'
:::::: :::::::::::::
query 3' CCUUUCAGAAUACGUACCUU 5'
query=miR5678, target=RO3G_07129, score=1, range=343-362, strand=
target 5' GGAAAGGCUUAUGCAUGGAA 3'
:::::: :::::::::::::
query 3' CCUUUCAGAAUACGUACCUU 5'
query=miR5678, target=RO3G_16555, score=1, range=475-494, strand=
target 5' GGAAAAUCUUAUGCAUGGAA 3'
::::: ::::::::::::::
query 3' CCUUUCAGAAUACGUACCUU 5'
query=miR5678, target=RO3G_16551, score=1, range=475-494, strand=
target 5' GGAAAAUCUUAUGCAUGGAA 3'
::::: ::::::::::::::
query 3' CCUUUCAGAAUACGUACCUU 5'
query=miR5678, target=RO3G_11773, score=1, range=475-494, strand=
target 5' GGAAAAUCUUAUGCAUGGAA 3'
::::: ::::::::::::::
query 3' CCUUUCAGAAUACGUACCUU 5'
query=miR5678, target=RO3G_04787, score=1, range=475-494, strand=
target 5' GGAAAGGCUUAUGCAUGGAA 3'
:::::: :::::::::::::
query 3' CCUUUCAGAAUACGUACCUU 5'
query=miR5678, target=RO3G_10770, score=1, range=475-494, strand=
target 5' GGAAAGGCUUAUGCAUGGAA 3'
:::::: :::::::::::::
query 3' CCUUUCAGAAUACGUACCUU 5'
query=miR5678, target=RO3G_02822, score=1, range=475-494, strand=
target 5' GGAAAGGCUUAUGCAUGGAA 3'
:::::: :::::::::::::
query 3' CCUUUCAGAAUACGUACCUU 5'
```

```
query=miR5678, target=RO3G_15141, score=1, range=475-494, strand=
target 5' GGAAAGGCUUAUGCAUGGAA 3'
:::::: :::::::::::::
query 3' CCUUUCAGAAUACGUACCUU 5'
query=miR5678, target=RO3G_09168, score=1, range=475-494, strand=
target 5' GGAAAGGCUUAUGCAUGGAA 3'
:::::: :::::::::::::
query 3' CCUUUCAGAAUACGUACCUU 5'
query=miR5678, target=RO3G_10762, score=1, range=475-494, strand=
target 5' GGAAAGGCUUAUGCAUGGAA 3'
:::::: :::::::::::::
query 3' CCUUUCAGAAUACGUACCUU 5'
query=miR5678, target=RO3G_13151, score=1.5, range=475-494, stran
target 5' AGGAAGUCUUAUGCAUGGAA 3'
:.:::::::::::::::::
query 3' CCUUUCAGAAUACGUACCUU 5'
query=miR5678, target=RO3G_01956, score=1.5, range=475-494, stran
target 5' GGGAAGCCUUAUGCAUGGAA 3'
::.::: :::::::::::::
query 3' CCUUUCAGAAUACGUACCUU 5'
query=miR5678, target=RO3G_17285, score=1.5, range=475-494, stran
target 5' GGGAAGCCUUAUGCAUGGAA 3'
::.::: :::::::::::::
query 3' CCUUUCAGAAUACGUACCUU 5'
query=miR5678, target=RO3G_03615, score=1.5, range=475-494, stran
target 5' GGGAAGACUUAUGCAUGGAA 3'
::.::: :::::::::::::
query 3' CCUUUCAGAAUACGUACCUU 5'
query=miR5678, target=RO3G_00304, score=2, range=475-494, strand=
target 5' AGAAAGGCUUAUGCAUGGAA 3'
::::: :::::::::::::
query 3' CCUUUCAGAAUACGUACCUU 5'
query=miR5678, target=RO3G_02713, score=2, range=205-224, strand=
target 5' AGAAAGGCUUAUGCAUGGAA 3'
::::: :::::::::::::
query 3' CCUUUCAGAAUACGUACCUU 5'
query=miR5678, target=RO3G_16729, score=2, range=343-362, strand=
target 5' AGAAAGGCUUAUGCAUGGAA 3'
::::: :::::::::::::
query 3' CCUUUCAGAAUACGUACCUU 5'
query=miR5678, target=RO3G_08661, score=2, range=475-494, strand=
target 5' GAAAAGCCUUAUGCAUGGAA 3'
: :::: :::::::::::::
query 3' CCUUUCAGAAUACGUACCUU 5'
query=miR5678, target=RO3G_14560, score=3, range=343-362, strand=
```

```
target 5' GGAAAGGCCUAUGCAUGGAA 3'
:::::: : ::::::::::: 
query 3' CCUUUCAGAAUACGUACCUU 5'
query=miR5678, target=RO3G_10529, score=3, range=106-125, strand=
target 5' GGAAAGGCCUAUGCAUGGAA 3'
:::::: : ::::::::::: 
query 3' CCUUUCAGAAUACGUACCUU 5'
query=miR5678, target=RO3G_12666, score=3, range=106-125, strand=
target 5' GGAAAGGCCUAUGCAUGGAA 3'
:::::: : ::::::::::: 
query 3' CCUUUCAGAAUACGUACCUU 5'
query=miR5678, target=RO3G_03764, score=3, range=106-125, strand=
target 5' GGAAAGGCCUAUGCAUGGAA 3'
:::::: : ::::::::::: 
query 3' CCUUUCAGAAUACGUACCUU 5'
query=miR5678, target=RO3G_05431, score=3, range=475-494, strand=
target 5' GGAAAGGCCUAUGCAUGGAA 3'
:::::: : ::::::::::: 
query 3' CCUUUCAGAAUACGUACCUU 5'
query=miR5678, target=RO3G_12954, score=3, range=475-494, strand=
target 5' GGAAAGGCCUAUGCAUGGAA 3'
:::::: : ::::::::::: 
query 3' CCUUUCAGAAUACGUACCUU 5'
query=miR5678, target=RO3G_13858, score=3, range=106-125, strand=
target 5' GGAAAGGCCUAUGCAUGGAA 3'
:::::: : ::::::::::: 
query 3' CCUUUCAGAAUACGUACCUU 5'
query=miR5678, target=RO3G_04690, score=3, range=475-494, strand=
target 5' GGAAAGGCCUAUGCAUGGAA 3'
:::::: : ::::::::::: 
query 3' CCUUUCAGAAUACGUACCUU 5'
query=miR5678, target=RO3G_05350, score=3, range=343-362, strand=
target 5' GGAAAGGCCUAUGCAUGGAA 3'
:::::: : ::::::::::: 
query 3' CCUUUCAGAAUACGUACCUU 5'
query=miR5678, target=RO3G_10787, score=3, range=475-494, strand=
target 5' GGUAAGCUUUAUGCAUGGAA 3'
:: ::: .:::::::::::: 
query 3' CCUUUCAGAAUACGUACCUU 5'
query=miR5678, target=RO3G_08847, score=3, range=475-494, strand=
target 5' GGUAAGCUUUAUGCAUGGAA 3'
:: ::: .:::::::::::: 
query 3' CCUUUCAGAAUACGUACCUU 5'
query=miR5678, target=RO3G_16751, score=3, range=64-83, strand=1
target 5' GGUAAGCUUUAUGCAUGGAA 3'
```

```
:: ::: .::::::::::::
query 3' CCUUUCAGAAUACGUACCUU 5'
query=miR5678, target=RO3G_00688, score=3, range=475-494, strand=
target 5' GGUAAGGUUUAUGCAUGGAA 3'
:: ::: .::::::::::::
query 3' CCUUUCAGAAUACGUACCUU 5'
query=miR5678, target=RO3G_12659, score=3, range=64-83, strand=1
target 5' GGUAAGCUUUAUGCAUGGAA 3'
:: ::: .::::::::::::
query 3' CCUUUCAGAAUACGUACCUU 5'
query=miR5678, target=RO3G_00679, score=3, range=667-686, strand=
target 5' GGUAAGGUUUAUGCAUGGAA 3'
:: ::: .::::::::::::
query 3' CCUUUCAGAAUACGUACCUU 5'
query=miR5678, target=RO3G_13919, score=3, range=667-686, strand=
target 5' GGUAAGCUUUAUGCAUGGAA 3'
:: ::: .::::::::::::
query 3' CCUUUCAGAAUACGUACCUU 5'
query=miR5678, target=RO3G_13286, score=3, range=667-686, strand=
target 5' GGUAAGGUUUAUGCAUGGAA 3'
:: ::: .::::::::::::
query 3' CCUUUCAGAAUACGUACCUU 5'
query=miR5678, target=RO3G_03624, score=3, range=475-494, strand=
target 5' GGUAAGCUUUAUGCAUGGAA 3'
:: ::: .::::::::::::
query 3' CCUUUCAGAAUACGUACCUU 5'
query=miR5678, target=RO3G_03602, score=3, range=616-635, strand=
target 5' GGUAAGGUUUAUGCAUGGAA 3'
:: ::: .::::::::::::
query 3' CCUUUCAGAAUACGUACCUU 5'
query=miR5678, target=RO3G_00284, score=3, range=484-503, strand=
target 5' GGUAAGCUUUAUGCAUGGAA 3'
:: ::: .::::::::::::
query 3' CCUUUCAGAAUACGUACCUU 5'
query=miR5678, target=RO3G_17408, score=3, range=475-494, strand=
target 5' GGUAAGGUUUAUGCAUGGAA 3'
:: ::: .::::::::::::
query 3' CCUUUCAGAAUACGUACCUU 5'
query=miR5678, target=RO3G_12678, score=3, range=667-686, strand=
target 5' GGUAAGGUUUAUGCAUGGAA 3'
:: ::: .::::::::::::
query 3' CCUUUCAGAAUACGUACCUU 5'
query=miR5678, target=RO3G_10763, score=3, range=475-494, strand=
target 5' GGUAAGCUUUAUGCAUGGAA 3'
:: ::: .::::::::::::
```

```
query 3' CCUUUCAGAAUACGUACCUU 5'
query=miR5678, target=RO3G_08115, score=3, range=179-197, strand=
target 5' CUAAAG-CUUAUGCAUGGAA 3'
:::: :::::::::::::
query 3' CCUUUCAGAAUACGUACCUU 5'
query=miR5678, target=RO3G_11281, score=4, range=429-449, strand=
target 5' UGAUAAGGUUUAUGCAUGGAA 3'
:: ::: .::::::::::::
query 3' CCU-UUCAGAAUACGUACCUU 5'
query=miR5678, target=RO3G_02063, score=4, range=352-370, strand=
target 5' GAAAAGUUUUAUG-AUGGAA 3'
: :::::.::::: ::::::
query 3' CCUUUCAGAAUACGUACCUU 5'
query=miR476, target=RO3G_17462, score=0, range=231-250, strand=-
target 5' GACCUUGAAAGAAGGAUCCA 3'
::::::::::::::::::::
query 3' CUGGAACUUUCUUCCUAGGU 5'
query=miR476, target=RO3G_13914, score=0, range=99-118, strand=-1
target 5' GACCUUGAAAGAAGGAUCCA 3'
::::::::::::::::::::
query 3' CUGGAACUUUCUUCCUAGGU 5'
query=miR476, target=RO3G_12058, score=0, range=99-118, strand=-1
target 5' GACCUUGAAAGAAGGAUCCA 3'
::::::::::::::::::::
query 3' CUGGAACUUUCUUCCUAGGU 5'
query=miR476, target=RO3G_03751, score=0, range=93-112, strand=-1
target 5' GACCUUGAAAGAAGGAUCCA 3'
::::::::::::::::::::
query 3' CUGGAACUUUCUUCCUAGGU 5'
query=miR476, target=RO3G_05618, score=0, range=93-112, strand=-1
target 5' GACCUUGAAAGAAGGAUCCA 3'
::::::::::::::::::::
query 3' CUGGAACUUUCUUCCUAGGU 5'
query=miR476, target=RO3G_17452, score=0, range=99-118, strand=-1
target 5' GACCUUGAAAGAAGGAUCCA 3'
::::::::::::::::::::
query 3' CUGGAACUUUCUUCCUAGGU 5'
query=miR476, target=RO3G_09195, score=0, range=99-118, strand=-1
target 5' GACCUUGAAAGAAGGAUCCA 3'
::::::::::::::::::::
query 3' CUGGAACUUUCUUCCUAGGU 5'
query=miR476, target=RO3G_13864, score=0, range=99-118, strand=-1
target 5' GACCUUGAAAGAAGGAUCCA 3'
::::::::::::::::::::
query 3' CUGGAACUUUCUUCCUAGGU 5'
```

query=miR476, target=RO3G_16821, score=0, range=99-118, strand=-1
target 5' GACCUUGAAAGAAGGAUCCA 3'
::::::::::::::::::::
query 3' CUGGAACUUUCUUCCUAGGU 5'
query=miR476, target=RO3G_01930, score=0, range=99-118, strand=-1
target 5' GACCUUGAAAGAAGGAUCCA 3'
::::::::::::::::::::
query 3' CUGGAACUUUCUUCCUAGGU 5'
query=miR476, target=RO3G_09921, score=0, range=99-118, strand=-1
target 5' GACCUUGAAAGAAGGAUCCA 3'
::::::::::::::::::::
query 3' CUGGAACUUUCUUCCUAGGU 5'
query=miR476, target=RO3G_16687, score=0, range=99-118, strand=-1
target 5' GACCUUGAAAGAAGGAUCCA 3'
::::::::::::::::::::
query 3' CUGGAACUUUCUUCCUAGGU 5'
query=miR476, target=RO3G_14357, score=0, range=99-118, strand=-1
target 5' GACCUUGAAAGAAGGAUCCA 3'
::::::::::::::::::::
query 3' CUGGAACUUUCUUCCUAGGU 5'
query=miR476, target=RO3G_13719, score=0, range=99-118, strand=-1
target 5' GACCUUGAAAGAAGGAUCCA 3'
::::::::::::::::::::
query 3' CUGGAACUUUCUUCCUAGGU 5'
query=miR476, target=RO3G_17169, score=0, range=99-118, strand=-1
target 5' GACCUUGAAAGAAGGAUCCA 3'
::::::::::::::::::::
query 3' CUGGAACUUUCUUCCUAGGU 5'
query=miR476, target=RO3G_04204, score=0, range=99-118, strand=-1
target 5' GACCUUGAAAGAAGGAUCCA 3'
::::::::::::::::::::
query 3' CUGGAACUUUCUUCCUAGGU 5'
query=miR476, target=RO3G_04699, score=0, range=99-118, strand=-1
target 5' GACCUUGAAAGAAGGAUCCA 3'
::::::::::::::::::::
query 3' CUGGAACUUUCUUCCUAGGU 5'
query=miR476, target=RO3G_10046, score=0, range=99-118, strand=-1
target 5' GACCUUGAAAGAAGGAUCCA 3'
::::::::::::::::::::
query 3' CUGGAACUUUCUUCCUAGGU 5'
query=miR476, target=RO3G_07674, score=0, range=99-118, strand=-1
target 5' GACCUUGAAAGAAGGAUCCA 3'
::::::::::::::::::::
query 3' CUGGAACUUUCUUCCUAGGU 5'
query=miR476, target=RO3G_02837, score=0, range=99-118, strand=-1

target 5' GACCUUGAAAGAAGGAUCCA 3'
::::::::::::::::::::
query 3' CUGGAACUUUCUUCCUAGGU 5'
query=miR476, target=RO3G_01311, score=0, range=99-118, strand=-1
target 5' GACCUUGAAAGAAGGAUCCA 3'
::::::::::::::::::::
query 3' CUGGAACUUUCUUCCUAGGU 5'
query=miR476, target=RO3G_16898, score=0, range=99-118, strand=-1
target 5' GACCUUGAAAGAAGGAUCCA 3'
::::::::::::::::::::
query 3' CUGGAACUUUCUUCCUAGGU 5'
query=miR476, target=RO3G_14623, score=0, range=99-118, strand=-1
target 5' GACCUUGAAAGAAGGAUCCA 3'
::::::::::::::::::::
query 3' CUGGAACUUUCUUCCUAGGU 5'
query=miR476, target=RO3G_13721, score=0, range=99-118, strand=-1
target 5' GACCUUGAAAGAAGGAUCCA 3'
::::::::::::::::::::
query 3' CUGGAACUUUCUUCCUAGGU 5'
query=miR476, target=RO3G_04125, score=0, range=99-118, strand=-1
target 5' GACCUUGAAAGAAGGAUCCA 3'
::::::::::::::::::::
query 3' CUGGAACUUUCUUCCUAGGU 5'
query=miR476, target=RO3G_03806, score=1, range=99-118, strand=-1
target 5' GACCUUGAAAGAAGGAUCUA 3'
::::::::::::::::::.:
query 3' CUGGAACUUUCUUCCUAGGU 5'
query=miR476, target=RO3G_03793, score=1, range=99-118, strand=-1
target 5' GACCUUGAAAGAAGGGUCCA 3'
:::::::::::::::.::::
query 3' CUGGAACUUUCUUCCUAGGU 5'
query=miR476, target=RO3G_09196, score=2, range=99-118, strand=-1
target 5' GACCUUGAAAGAAGGACCCA 3'
:::::::::::::::: :::
query 3' CUGGAACUUUCUUCCUAGGU 5'
query=miR476, target=RO3G_17373, score=2, range=99-118, strand=-1
target 5' GACCUUGAAAGAAGGACCCA 3'
:::::::::::::::: :::
query 3' CUGGAACUUUCUUCCUAGGU 5'
query=miR476, target=RO3G_03779, score=2, range=99-118, strand=-1
target 5' GACCUUGAAAGAAGGACCCA 3'
:::::::::::::::: :::
query 3' CUGGAACUUUCUUCCUAGGU 5'
query=miR476, target=RO3G_16802, score=2, range=99-118, strand=-1
target 5' GACCUUGAAAGAAGGACCCA 3'

```
:::::::::::::::: :::
query 3' CUGGAACUUUCUUCCUAGGU 5'
query=miR476, target=RO3G_08398, score=3, range=1228-1246, strand
target 5' GAACUUGAAAGAA-GAUCCA 3'
:: :::::::::: ::::::
query 3' CUGGAACUUUCUUCCUAGGU 5'
query=miR476, target=RO3G_05445, score=3.5, range=418-438, strand
target 5' GAUCAGUGGAAGAAGGAUCCA 3'
::.: ::.::::::::::::
query 3' CUGG-AACUUUCUUCCUAGGU 5'
query=miR476, target=RO3G_03482, score=3.5, range=265-284, strand
target 5' AACAUGGAAAGAAGGAUCCG 3'
:: : :::::::::::::.
query 3' CUGGAACUUUCUUCCUAGGU 5'
query=miR476, target=RO3G_11833, score=4, range=1109-1129, strand
target 5' UACUUGUGAAAGAAGGAUCUG 3'
::.: :::::::::::::..
query 3' CUGGA-ACUUUCUUCCUAGGU 5'
query=miR476, target=RO3G_08702, score=4, range=378-397, strand=1
target 5' AAACUUGAAAGAAGAAUCCA 3'
: :::::::::: :::::
query 3' CUGGAACUUUCUUCCUAGGU 5'
query=miR476, target=RO3G_15987, score=4, range=335-354, strand=1
target 5' AAUUGAGAAAGAAGGAUCCA 3'
:.. :::::::::::::
query 3' CUGGAACUUUCUUCCUAGGU 5'
query=miR476, target=RO3G_13408, score=4, range=1335-1355, strand
target 5' GGCUUUGGAAAGAUGGAUCCA 3'
:.:.:: :::::: :::::::
query 3' CUGGAA-CUUUCUUCCUAGGU 5'
query=miR476, target=RO3G_02901, score=4, range=1036-1054, strand
target 5' UACAUUGAAAGAAGG-UCCA 3'
:: :::::::::: ::::
query 3' CUGGAACUUUCUUCCUAGGU 5'
query=miR476, target=RO3G_15654, score=4, range=2181-2199, strand
target 5' GACCUUCAAA-AAGGAUUCA 3'
:::::: ::: ::::::.::
query 3' CUGGAACUUUCUUCCUAGGU 5'
query=miR8610.2, target=RO3G_02802, score=3.5, range=712-732,
strand=-1
target 5' ACUCAUCAAUUUGUUCGAGAA 3'
:.: :::::::::::.::::
query 3' CGGGAAGUUAAACAAGUUCUU 5'
query=miR8610.2, target=RO3G_09454, score=3.5, range=2708-2729,
strand=-1
```

```
target 5' GCCAUUACAAUUUGUUCAAGGG 3'
::: :: :::::::::::::..
query 3' CGGGAA-GUUAAACAAGUUCUU 5'
query=miR8610.2, target=RO3G_11579, score=4, range=5512-5532, str
target 5' AUUAUCCAAUUUGUUCAAGAA 3'
.. : ::::::::::::::
query 3' CGGGAAGUUAAACAAGUUCUU 5'
query=miR8610.2, target=RO3G_03165, score=4, range=1036-1056, str
target 5' GCACCUCAAUAUGUUCAAGAA 3'
:: : ::::: ::::::::::
query 3' CGGGAAGUUAAACAAGUUCUU 5'
query=miR8610.2, target=RO3G_03206, score=4, range=449-469, stran
target 5' UCUUUUCAAAUUGUUCAAGAA 3'
:..::::: ::::::::::
query 3' CGGGAAGUUAAACAAGUUCUU 5'
query=miR8610.2, target=RO3G_15328, score=4, range=173-192, stran
target 5' UUUCUUC-AUUUGUUCAAGGA 3'
..:::: :::::::::::.:
query 3' CGGGAAGUUAAACAAGUUCUU 5'
query=miR8610.2, target=RO3G_10956, score=4, range=1432-1451,
strand=-1
target 5' AUUCUU-AAUUUGUUCAAGAU 3'
..::: ::::::::::::
query 3' CGGGAAGUUAAACAAGUUCUU 5'
query=miR8610.2, target=RO3G_08417, score=4, range=107-126, stran
target 5' GCCCUUCAA-UUGUUCAUGAA 3'
::::::::: ::::::: :::
query 3' CGGGAAGUUAAACAAGUUCUU 5'
query=miR5261, target=RO3G_07863, score=0, range=932-952, strand=
target 5' AGACCAAAGCAUCUGACAUGA 3'
:::::::::::::::::::::
query 3' UCUGGUUUCGUAGACUGUACU 5'
query=miR5261, target=RO3G_17254, score=0, range=932-952, strand=
target 5' AGACCAAAGCAUCUGACAUGA 3'
:::::::::::::::::::::
query 3' UCUGGUUUCGUAGACUGUACU 5'
query=miR5261, target=RO3G_10748, score=0, range=932-952, strand=
target 5' AGACCAAAGCAUCUGACAUGA 3'
:::::::::::::::::::::
query 3' UCUGGUUUCGUAGACUGUACU 5'
query=miR5261, target=RO3G_07861, score=0, range=932-952, strand=
target 5' AGACCAAAGCAUCUGACAUGA 3'
:::::::::::::::::::::
query 3' UCUGGUUUCGUAGACUGUACU 5'
query=miR5261, target=RO3G_17201, score=0, range=929-949, strand=
```

target 5' AGACCAAAGCAUCUGACAUGA 3'
:::::::::::::::::::::
query 3' UCUGGUUUCGUAGACUGUACU 5'
query=miR5261, target=RO3G_16903, score=0, range=866-886, strand=
target 5' AGACCAAAGCAUCUGACAUGA 3'
:::::::::::::::::::::
query 3' UCUGGUUUCGUAGACUGUACU 5'
query=miR5261, target=RO3G_16358, score=0, range=761-781, strand=
target 5' AGACCAAAGCAUCUGACAUGA 3'
:::::::::::::::::::::
query 3' UCUGGUUUCGUAGACUGUACU 5'
query=miR5261, target=RO3G_11978, score=0.5, range=785-805, stran
target 5' AGACCGAAGCAUCUGACAUGA 3'
:::::.:::::::::::::::
query 3' UCUGGUUUCGUAGACUGUACU 5'
query=miR5261, target=RO3G_13449, score=2, range=932-952, strand=
target 5' AGACCAAAGCAUCUGACAUAA 3'
::::::::::::::::::: :
query 3' UCUGGUUUCGUAGACUGUACU 5'
query=miR5261, target=RO3G_16365, score=2, range=932-952, strand=
target 5' AGACCAAAGCAUCUAACAUGA 3'
:::::::::::::: ::::::
query 3' UCUGGUUUCGUAGACUGUACU 5'
query=miR5261, target=RO3G_07736, score=2, range=932-952, strand=
target 5' AGACCAAAGCAUCUGACAUAA 3'
::::::::::::::::::: :
query 3' UCUGGUUUCGUAGACUGUACU 5'
query=miR5261, target=RO3G_17250, score=2, range=932-952, strand=
target 5' AGACCAAAGCAUCUGACAUAA 3'
::::::::::::::::::: :
query 3' UCUGGUUUCGUAGACUGUACU 5'
query=miR5261, target=RO3G_10533, score=2, range=932-952, strand=
target 5' AGACCAAAGCAUCUGACAUAA 3'
::::::::::::::::::: :
query 3' UCUGGUUUCGUAGACUGUACU 5'
query=miR5261, target=RO3G_06414, score=2, range=929-949, strand=
target 5' AGACCAAAGCAUCUGACAUAA 3'
::::::::::::::::::: :
query 3' UCUGGUUUCGUAGACUGUACU 5'
query=miR5261, target=RO3G_09174, score=2, range=929-949, strand=
target 5' AGACCAAAGCAUCUGACAUAA 3'
::::::::::::::::::: :
query 3' UCUGGUUUCGUAGACUGUACU 5'
query=miR5261, target=RO3G_10538, score=2, range=890-910, strand=
target 5' AGACCAAAGCAUCUGACAUAA 3'

:::::::::::::::::: :
query 3' UCUGGUUUCGUAGACUGUACU 5'
query=miR5261, target=RO3G_17220, score=4, range=938-958, strand=
target 5' AGACCAAAGCAUCCGACAUAA 3'
::::::::::::: ::::: :
query 3' UCUGGUUUCGUAGACUGUACU 5'
query=miR5261, target=RO3G_12967, score=4, range=932-952, strand=
target 5' AGACCAAAGCAUCCGACAUAA 3'
::::::::::::: ::::: :
query 3' UCUGGUUUCGUAGACUGUACU 5'
query=miR5261, target=RO3G_14562, score=4, range=869-889, strand=
target 5' AGACCAAAGCAUCCGACAUAA 3'
::::::::::::: ::::: :
query 3' UCUGGUUUCGUAGACUGUACU 5'
query=miR5261, target=RO3G_16583, score=4, range=56-76, strand=-1
target 5' AGACAACAGUAUCUGACGUGA 3'
:::: : ::.:::::::.:::
query 3' UCUGGUUUCGUAGACUGUACU 5'
query=miR8658, target=RO3G_08999, score=3, range=415-435, strand=
target 5' AUGGUCUCUAUGCUUUGUCAA 3'
::::::::: :.:::::::::
query 3' UACCAGAGACAUGAAACAGUU 5'
query=miR8658, target=RO3G_17168, score=3, range=967-987, strand=
target 5' ACGGUCUCUGUUCUUUGUCAA 3'
: ::::::::: :::::::::
query 3' UACCAGAGACAUGAAACAGUU 5'
query=miR8658, target=RO3G_01279, score=3.5, range=52-72, strand=
target 5' AUGGUCCUUGUUCUUUGUCAA 3'
:::::: .::: :::::::::
query 3' UACCAGAGACAUGAAACAGUU 5'
query=miR8658, target=RO3G_12321, score=3.5, range=203-222, stran
target 5' AAGGU-UUUGUAUUUUGUCAA 3'
: ::: :.::::.::::::::
query 3' UACCAGAGACAUGAAACAGUU 5'
query=miR8658, target=RO3G_08552, score=4, range=1163-1183, stran
target 5' AUGCUCUCUGUGUUUUGUCAC 3'
::: :::::::..:::::::
query 3' UACCAGAGACAUGAAACAGUU 5'
query=miR8658, target=RO3G_03092, score=4, range=1163-1183, stran
target 5' AUGCUCUCUGUGUUUUGUCAC 3'
::: :::::::..:::::::
query 3' UACCAGAGACAUGAAACAGUU 5'
query=miR8658, target=RO3G_10756, score=4, range=32-51, strand=1
target 5' GUGG-CUUUGUACUUUGGCAA 3'
.::: ::.::::::::: :::

```
query 3' UACCAGAGACAUGAAACAGUU 5'
query=miR8658, target=RO3G_11224, score=4, range=319-339, strand=
target 5' AUGUUCACUGUAUUUUGUUAA 3'
::: :: :::::.:::::.::
query 3' UACCAGAGACAUGAAACAGUU 5'
query=miR8658, target=RO3G_16312, score=4, range=488-508, strand=
target 5' AAUCUCUAUGUACUUUGUCAA 3'
: ::: :::::::::::::
query 3' UACCAGAGACAUGAAACAGUU 5'
query=miR8658, target=RO3G_01139, score=4, range=721-741, strand=
target 5' AUCUUCACUGUACUUUGUCAC 3'
:: :: :::::::::::::
query 3' UACCAGAGACAUGAAACAGUU 5'
query=miR2111, target=RO3G_04502, score=0, range=239-258, strand=
target 5' CCAAACCCAAACACAGUUUA 3'
::::::::::::::::::::
query 3' GGUUUGGGUUUGUGUCAAAU 5'
query=miR2111, target=RO3G_02942, score=0, range=239-258, strand=
target 5' CCAAACCCAAACACAGUUUA 3'
::::::::::::::::::::
query 3' GGUUUGGGUUUGUGUCAAAU 5'
query=miR2111, target=RO3G_01530, score=0, range=239-258, strand=
target 5' CCAAACCCAAACACAGUUUA 3'
::::::::::::::::::::
query 3' GGUUUGGGUUUGUGUCAAAU 5'
query=miR2111, target=RO3G_04017, score=0, range=239-258, strand=
target 5' CCAAACCCAAACACAGUUUA 3'
::::::::::::::::::::
query 3' GGUUUGGGUUUGUGUCAAAU 5'
query=miR2111, target=RO3G_03523, score=0, range=239-258, strand=
target 5' CCAAACCCAAACACAGUUUA 3'
::::::::::::::::::::
query 3' GGUUUGGGUUUGUGUCAAAU 5'
query=miR2111, target=RO3G_12446, score=0, range=239-258, strand=
target 5' CCAAACCCAAACACAGUUUA 3'
::::::::::::::::::::
query 3' GGUUUGGGUUUGUGUCAAAU 5'
query=miR2111, target=RO3G_15497, score=0, range=167-186, strand=
target 5' CCAAACCCAAACACAGUUUA 3'
::::::::::::::::::::
query 3' GGUUUGGGUUUGUGUCAAAU 5'
query=miR2111, target=RO3G_05724, score=0, range=200-219, strand=
target 5' CCAAACCCAAACACAGUUUA 3'
::::::::::::::::::::
query 3' GGUUUGGGUUUGUGUCAAAU 5'
```

```
query=miR2111, target=RO3G_15290, score=0, range=167-186, strand=
target 5' CCAAACCCAAACACAGUUUA 3'
::::::::::::::::::::
query 3' GGUUUGGGUUUGUGUCAAAU 5'
query=miR2111, target=RO3G_09087, score=0.5, range=239-258, stran
target 5' UCAAACCCAAACACAGUUUA 3'
.:::::::::::::::::::
query 3' GGUUUGGGUUUGUGUCAAAU 5'
query=miR2111, target=RO3G_10657, score=0.5, range=239-258, stran
target 5' CCAAGCCCAAACACAGUUUA 3'
::::.:::::::::::::::
query 3' GGUUUGGGUUUGUGUCAAAU 5'
query=miR2111, target=RO3G_12685, score=1, range=221-240, strand=
target 5' CCAAACCCAAACAUAGUUUA 3'
:::::::::::::.::::::
query 3' GGUUUGGGUUUGUGUCAAAU 5'
query=miR2111, target=RO3G_12243, score=1, range=239-258, strand=
target 5' CCAAACCCAAACAUAGUUUA 3'
:::::::::::::.::::::
query 3' GGUUUGGGUUUGUGUCAAAU 5'
query=miR2111, target=RO3G_13307, score=1, range=200-219, strand=
target 5' CCCAACCCAAACACAGUUUA 3'
:: :::::::::::::::::
query 3' GGUUUGGGUUUGUGUCAAAU 5'
query=miR2111, target=RO3G_08447, score=1, range=239-258, strand=
target 5' CCAAAACCAAACACAGUUUA 3'
::::: ::::::::::::::
query 3' GGUUUGGGUUUGUGUCAAAU 5'
query=miR2111, target=RO3G_03971, score=1.5, range=239-258, stran
target 5' UCAAACCCAAGCACAGUUUA 3'
.:::::::::.:::::::::
query 3' GGUUUGGGUUUGUGUCAAAU 5'
query=miR2111, target=RO3G_13022, score=1.5, range=239-258, stran
target 5' UCAAACCCAAGCACAGUUUA 3'
.:::::::::.:::::::::
query 3' GGUUUGGGUUUGUGUCAAAU 5'
query=miR2111, target=RO3G_11292, score=2.5, range=3295-3314, str
target 5' ACAAAUACAAACACAGUUUA 3'
::::. :::::::::::::
query 3' GGUUUGGGUUUGUGUCAAAU 5'
query=miR2111, target=RO3G_10368, score=3, range=8704-8723, stran
target 5' GCAAACGCAAACACAGUUUU 3'
::::: ::::::::::::
query 3' GGUUUGGGUUUGUGUCAAAU 5'
query=miR2111, target=RO3G_05178, score=3.5, range=431-449, stran
```

```
target 5' UUAAA-UCAAACGCAGUUUA 3'
..::: .:::::.:::::::
query 3' GGUUUGGGUUUGUGUCAAAU 5'
query=miR2111, target=RO3G_04030, score=3.5, range=740-760, stran
target 5' CCAUACUUCAAACACAGUUUC 3'
::: :: .::::::::::::
query 3' GGUUUG-GGUUUGUGUCAAAU 5'
query=miR2111, target=RO3G_05582, score=3.5, range=271-289, stran
target 5' CUAAA-CCAAACAGAGUUUA 3'
:.::: ::::::: ::::::
query 3' GGUUUGGGUUUGUGUCAAAU 5'
query=miR2111, target=RO3G_00850, score=4, range=165-184, strand=
target 5' UCCAACUCAACCACAGUUUA 3'
.: :::.::: :::::::::
query 3' GGUUUGGGUUUGUGUCAAAU 5'
query=miR2111, target=RO3G_08461, score=4, range=423-443, strand=
target 5' UCAAAUCACAAACACAGGUUA 3'
.::::.: ::::::::: :::
query 3' GGUUUGG-GUUUGUGUCAAAU 5'
query=miR2111, target=RO3G_10271, score=4, range=910-929, strand=
target 5' AUUUAUCCAAACACAGUUUA 3'
. :.::::::::::::::
query 3' GGUUUGGGUUUGUGUCAAAU 5'
query=miR2111, target=RO3G_09085, score=4, range=1804-1822, stran
target 5' ACAGG-ACAAACACAGUUUA 3'
::.. :::::::::::::
query 3' GGUUUGGGUUUGUGUCAAAU 5'
query=miR2111, target=RO3G_00116, score=4, range=453-472, strand=
target 5' CCAUAGGCAAACACAGUUUC 3'
::: : ::::::::::::
query 3' GGUUUGGGUUUGUGUCAAAU 5'
query=miR7512, target=RO3G_16107, score=0, range=3015-3036, stran
target 5' AUGGCAUAAGCUACAAGUAGUA 3'
::::::::::::::::::::::
query 3' UACCGUAUUCGAUGUUCAUCAU 5'
query=miR7512, target=RO3G_03119, score=0, range=294-315, strand=
target 5' AUGGCAUAAGCUACAAGUAGUA 3'
::::::::::::::::::::::
query 3' UACCGUAUUCGAUGUUCAUCAU 5'
query=miR7817a, target=RO3G_12527, score=0, range=4341-4363, stra
target 5' UUCUCGAGACAAGUUCAACCAAA 3'
:::::::::::::::::::::::
query 3' AAGAGCUCUGUUCAAGUUGGUUU 5'
query=miR7817a, target=RO3G_13918, score=0, range=4341-4363, stra
target 5' UUCUCGAGACAAGUUCAACCAAA 3'
```

```
::::::::::::::::::::::
query 3' AAGAGCUCUGUUCAAGUUGGUUU 5'
query=miR7817a, target=RO3G_14347, score=0, range=4272-4294, stra
target 5' UUCUCGAGACAAGUUCAACCAAA 3'
::::::::::::::::::::::
query 3' AAGAGCUCUGUUCAAGUUGGUUU 5'
query=miR7817a, target=RO3G_01614, score=0, range=4182-4204, stra
target 5' UUCUCGAGACAAGUUCAACCAAA 3'
::::::::::::::::::::::
query 3' AAGAGCUCUGUUCAAGUUGGUUU 5'
query=miR7817a, target=RO3G_16361, score=0, range=4176-4198, stra
target 5' UUCUCGAGACAAGUUCAACCAAA 3'
::::::::::::::::::::::
query 3' AAGAGCUCUGUUCAAGUUGGUUU 5'
query=miR7817a, target=RO3G_04126, score=0, range=4335-4357, stra
target 5' UUCUCGAGACAAGUUCAACCAAA 3'
::::::::::::::::::::::
query 3' AAGAGCUCUGUUCAAGUUGGUUU 5'
query=miR7817a, target=RO3G_09493, score=0, range=2889-2911, stra
target 5' UUCUCGAGACAAGUUCAACCAAA 3'
::::::::::::::::::::::
query 3' AAGAGCUCUGUUCAAGUUGGUUU 5'
query=miR7817a, target=RO3G_17165, score=0, range=717-739, strand
target 5' UUCUCGAGACAAGUUCAACCAAA 3'
::::::::::::::::::::::
query 3' AAGAGCUCUGUUCAAGUUGGUUU 5'
query=miR7817a, target=RO3G_00298, score=0, range=696-718, strand
target 5' UUCUCGAGACAAGUUCAACCAAA 3'
::::::::::::::::::::::
query 3' AAGAGCUCUGUUCAAGUUGGUUU 5'
query=miR7817a, target=RO3G_12530, score=0, range=159-181, strand
target 5' UUCUCGAGACAAGUUCAACCAAA 3'
::::::::::::::::::::::
query 3' AAGAGCUCUGUUCAAGUUGGUUU 5'
query=miR7817a, target=RO3G_03618, score=0, range=159-181, strand
target 5' UUCUCGAGACAAGUUCAACCAAA 3'
::::::::::::::::::::::
query 3' AAGAGCUCUGUUCAAGUUGGUUU 5'
query=miR7817a, target=RO3G_08831, score=0, range=87-109, strand=
target 5' UUCUCGAGACAAGUUCAACCAAA 3'
::::::::::::::::::::::
query 3' AAGAGCUCUGUUCAAGUUGGUUU 5'
query=miR7817a, target=RO3G_03765, score=1, range=159-181, strand
target 5' UUCUCGAGACAAGUUCAAUCAAA 3'
::::::::::::::::::.::::
```

query 3' AAGAGCUCUGUUCAAGUUGGUUU 5'
query=miR7817a, target=RO3G_16693, score=1, range=4341-4363, stra
target 5' GUCUCGAGACAAGUUCAACCAAA 3'
::::::::::::::::::::::
query 3' AAGAGCUCUGUUCAAGUUGGUUU 5'
query=miR7817a, target=RO3G_16539, score=1, range=4233-4255, stra
target 5' GUCUCGAGACAAGUUCAACCAAA 3'
::::::::::::::::::::::
query 3' AAGAGCUCUGUUCAAGUUGGUUU 5'
query=miR7817a, target=RO3G_12538, score=1, range=4233-4255, stra
target 5' CUCUCGAGACAAGUUCAACCAAA 3'
::::::::::::::::::::::
query 3' AAGAGCUCUGUUCAAGUUGGUUU 5'
query=miR7817a, target=RO3G_07311, score=1, range=4182-4204, stra
target 5' CUCUCGAGACAAGUUCAACCAAA 3'
::::::::::::::::::::::
query 3' AAGAGCUCUGUUCAAGUUGGUUU 5'
query=miR7817a, target=RO3G_10804, score=1, range=4182-4204, stra
target 5' CUCUCGAGACAAGUUCAACCAAA 3'
::::::::::::::::::::::
query 3' AAGAGCUCUGUUCAAGUUGGUUU 5'
query=miR7817a, target=RO3G_09547, score=1, range=4176-4198, stra
target 5' CUCUCGAGACAAGUUCAACCAAA 3'
::::::::::::::::::::::
query 3' AAGAGCUCUGUUCAAGUUGGUUU 5'
query=miR7817a, target=RO3G_06425, score=1, range=4176-4198, stra
target 5' CUCUCGAGACAAGUUCAACCAAA 3'
::::::::::::::::::::::
query 3' AAGAGCUCUGUUCAAGUUGGUUU 5'
query=miR7817a, target=RO3G_17021, score=1, range=4176-4198, stra
target 5' CUCUCGAGACAAGUUCAACCAAA 3'
::::::::::::::::::::::
query 3' AAGAGCUCUGUUCAAGUUGGUUU 5'
query=miR7817a, target=RO3G_05330, score=1, range=4176-4198, stra
target 5' CUCUCGAGACAAGUUCAACCAAA 3'
::::::::::::::::::::::
query 3' AAGAGCUCUGUUCAAGUUGGUUU 5'
query=miR7817a, target=RO3G_02704, score=1, range=4176-4198, stra
target 5' CUCUCGAGACAAGUUCAACCAAA 3'
::::::::::::::::::::::
query 3' AAGAGCUCUGUUCAAGUUGGUUU 5'
query=miR7817a, target=RO3G_12677, score=1, range=4176-4198, stra
target 5' CUCUCGAGACAAGUUCAACCAAA 3'
::::::::::::::::::::::
query 3' AAGAGCUCUGUUCAAGUUGGUUU 5'

```
query=miR7817a, target=RO3G_04933, score=1, range=4176-4198, stra
target 5' CUCUCGAGACAAGUUCAACCAAA 3'
::::::::::::::::::::::
query 3' AAGAGCUCUGUUCAAGUUGGUUU 5'
query=miR7817a, target=RO3G_05344, score=1, range=4002-4024, stra
target 5' CUCUCGAGACAAGUUCAACCAAA 3'
::::::::::::::::::::::
query 3' AAGAGCUCUGUUCAAGUUGGUUU 5'
query=miR7817a, target=RO3G_03778, score=1, range=4134-4156, stra
target 5' CUCUCGAGACAAGUUCAACCAAA 3'
::::::::::::::::::::::
query 3' AAGAGCUCUGUUCAAGUUGGUUU 5'
query=miR7817a, target=RO3G_12673, score=1, range=3477-3499, stra
target 5' CUCUCGAGACAAGUUCAACCAAA 3'
::::::::::::::::::::::
query 3' AAGAGCUCUGUUCAAGUUGGUUU 5'
query=miR7817a, target=RO3G_17422, score=1, range=3477-3499, stra
target 5' CUCUCGAGACAAGUUCAACCAAA 3'
::::::::::::::::::::::
query 3' AAGAGCUCUGUUCAAGUUGGUUU 5'
query=miR7817a, target=RO3G_01966, score=1, range=1224-1246, stra
target 5' GUCUCGAGACAAGUUCAACCAAA 3'
::::::::::::::::::::::
query 3' AAGAGCUCUGUUCAAGUUGGUUU 5'
query=miR7817a, target=RO3G_08107, score=1, range=699-721, strand
target 5' GUCUCGAGACAAGUUCAACCAAA 3'
::::::::::::::::::::::
query 3' AAGAGCUCUGUUCAAGUUGGUUU 5'
query=miR7817a, target=RO3G_00239, score=1, range=696-718, strand
target 5' GUCUCGAGACAAGUUCAACCAAA 3'
::::::::::::::::::::::
query 3' AAGAGCUCUGUUCAAGUUGGUUU 5'
query=miR7817a, target=RO3G_05514, score=1, range=690-712, strand
target 5' CUCUCGAGACAAGUUCAACCAAA 3'
::::::::::::::::::::::
query 3' AAGAGCUCUGUUCAAGUUGGUUU 5'
query=miR7817a, target=RO3G_11473, score=1, range=159-181, strand
target 5' GUCUCGAGACAAGUUCAACCAAA 3'
::::::::::::::::::::::
query 3' AAGAGCUCUGUUCAAGUUGGUUU 5'
query=miR7817a, target=RO3G_02820, score=1, range=159-181, strand
target 5' GUCUCGAGACAAGUUCAACCAAA 3'
::::::::::::::::::::::
query 3' AAGAGCUCUGUUCAAGUUGGUUU 5'
query=miR7817a, target=RO3G_01937, score=1, range=159-181, strand
```

```
target 5' GUCUCGAGACAAGUUCAACCAAA 3'
::::::::::::::::::::::
query 3' AAGAGCUCUGUUCAAGUUGGUUU 5'
query=miR7817a, target=RO3G_00701, score=1, range=87-109, strand=
target 5' GUCUCGAGACAAGUUCAACCAAA 3'
::::::::::::::::::::::
query 3' AAGAGCUCUGUUCAAGUUGGUUU 5'
query=miR7817a, target=RO3G_04909, score=4, range=186-207, strand
target 5' UAUUCGA-CCAAGUUCAACCAAG 3'
: .:::: :::::::::::::.
query 3' AAGAGCUCUGUUCAAGUUGGUUU 5'
query=miR8581, target=RO3G_04697, score=0, range=944-965, strand=
target 5' CAGUUGUCUACAAAAUUCUCUA 3'
::::::::::::::::::::::
query 3' GUCAACAGAUGUUUUAAGAGAU 5'
query=miR8581, target=RO3G_00004, score=3.5, range=122-142, stran
target 5' UA-UUAUUUACAAAAUUCUCUG 3'
.: :: :.:::::::::::::.
query 3' GUCAACAGAUGUUUUAAGAGAU 5'
query=miR391, target=RO3G_00684, score=0, range=565-583, strand=1
target 5' CGACGUCUCCCUCUGCGUA 3'
:::::::::::::::::::
query 3' GCUGCAGAGGGAGACGCAU 5'
query=miR391, target=RO3G_15674, score=0, range=1537-1555, strand
target 5' CGACGUCUCCCUCUGCGUA 3'
:::::::::::::::::::
query 3' GCUGCAGAGGGAGACGCAU 5'
query=miR391, target=RO3G_12961, score=0, range=1519-1537, strand
target 5' CGACGUCUCCCUCUGCGUA 3'
:::::::::::::::::::
query 3' GCUGCAGAGGGAGACGCAU 5'
query=miR391, target=RO3G_12964, score=0, range=1186-1204, strand
target 5' CGACGUCUCCCUCUGCGUA 3'
:::::::::::::::::::
query 3' GCUGCAGAGGGAGACGCAU 5'
query=miR391, target=RO3G_11952, score=0, range=1564-1582, strand
target 5' CGACGUCUCCCUCUGCGUA 3'
:::::::::::::::::::
query 3' GCUGCAGAGGGAGACGCAU 5'
query=miR391, target=RO3G_13865, score=0, range=2458-2476, strand
target 5' CGACGUCUCCCUCUGCGUA 3'
:::::::::::::::::::
query 3' GCUGCAGAGGGAGACGCAU 5'
query=miR391, target=RO3G_09951, score=0, range=1444-1462, strand
target 5' CGACGUCUCCCUCUGCGUA 3'
```

```
:::::::::::::::::::
query 3' GCUGCAGAGGGAGACGCAU 5'
query=miR391, target=RO3G_12075, score=0, range=1186-1204, strand
target 5' CGACGUCUCCCUCUGCGUA 3'
:::::::::::::::::::
query 3' GCUGCAGAGGGAGACGCAU 5'
query=miR391, target=RO3G_09927, score=0, range=1186-1204, strand
target 5' CGACGUCUCCCUCUGCGUA 3'
:::::::::::::::::::
query 3' GCUGCAGAGGGAGACGCAU 5'
query=miR391, target=RO3G_14622, score=0, range=244-262, strand=1
target 5' CGACGUCUCCCUCUGCGUA 3'
:::::::::::::::::::
query 3' GCUGCAGAGGGAGACGCAU 5'
query=miR391, target=RO3G_05925, score=0, range=1186-1204, strand
target 5' CGACGUCUCCCUCUGCGUA 3'
:::::::::::::::::::
query 3' GCUGCAGAGGGAGACGCAU 5'
query=miR391, target=RO3G_01720, score=0, range=1171-1189, strand
target 5' CGACGUCUCCCUCUGCGUA 3'
:::::::::::::::::::
query 3' GCUGCAGAGGGAGACGCAU 5'
query=miR391, target=RO3G_10777, score=0, range=2362-2380, strand
target 5' CGACGUCUCCCUCUGCGUA 3'
:::::::::::::::::::
query 3' GCUGCAGAGGGAGACGCAU 5'
query=miR391, target=RO3G_08609, score=0, range=1171-1189, strand
target 5' CGACGUCUCCCUCUGCGUA 3'
:::::::::::::::::::
query 3' GCUGCAGAGGGAGACGCAU 5'
query=miR391, target=RO3G_11279, score=0, range=148-166, strand=1
target 5' CGACGUCUCCCUCUGCGUA 3'
:::::::::::::::::::
query 3' GCUGCAGAGGGAGACGCAU 5'
query=miR391, target=RO3G_13746, score=0, range=40-58, strand=1
target 5' CGACGUCUCCCUCUGCGUA 3'
:::::::::::::::::::
query 3' GCUGCAGAGGGAGACGCAU 5'
query=miR391, target=RO3G_12669, score=0, range=1186-1204, strand
target 5' CGACGUCUCCCUCUGCGUA 3'
:::::::::::::::::::
query 3' GCUGCAGAGGGAGACGCAU 5'
query=miR391, target=RO3G_16708, score=0, range=1186-1204, strand
target 5' CGACGUCUCCCUCUGCGUA 3'
:::::::::::::::::::
```

```
query 3' GCUGCAGAGGGAGACGCAU 5'
query=miR391, target=RO3G_05927, score=0, range=1186-1204, strand
target 5' CGACGUCUCCCUCUGCGUA 3'
:::::::::::::::::::
query 3' GCUGCAGAGGGAGACGCAU 5'
query=miR391, target=RO3G_07869, score=2, range=2431-2449, strand
target 5' CGACGUCUCCCUCUACGUA 3'
:::::::::::::: ::::
query 3' GCUGCAGAGGGAGACGCAU 5'
query=miR391, target=RO3G_05524, score=2, range=1186-1203, strand
target 5' CGACGUCUCCCUCUGC-UA 3'
:::::::::::::::: ::
query 3' GCUGCAGAGGGAGACGCAU 5'
query=miR391, target=RO3G_10122, score=4, range=774-793, strand=1
target 5' UGAUCUCUCCCUCAUGCGUA 3'
.::. :::::::: ::::::
query 3' GCUGCAGAGGGAG-ACGCAU 5'
query=miR391, target=RO3G_12669, score=4, range=1868-1885, strand
target 5' CGGUGUCUCCCUCUG-GUC 3'
::..:::::::::: ::
query 3' GCUGCAGAGGGAGACGCAU 5'
query=miR391, target=RO3G_02076, score=4, range=156-174, strand=-
target 5' UGAUUUCUUUCUCUGCGUA 3'
.::. :::..:::::::::
query 3' GCUGCAGAGGGAGACGCAU 5'
query=miR391, target=RO3G_12955, score=4, range=208-225, strand=-
target 5' CGGUGUCUCCCUCUG-GUC 3'
::..:::::::::: ::
query 3' GCUGCAGAGGGAGACGCAU 5'
query=miR399f, target=RO3G_11770, score=0, range=17-36, strand=1
target 5' CAGGACAAAUCGCGUUGGCA 3'
::::::::::::::::::::
query 3' GUCCUGUUUAGCGCAACCGU 5'
query=miR399f, target=RO3G_04217, score=0, range=17-36, strand=1
target 5' CAGGACAAAUCGCGUUGGCA 3'
::::::::::::::::::::
query 3' GUCCUGUUUAGCGCAACCGU 5'
query=miR399f, target=RO3G_16135, score=0, range=17-36, strand=1
target 5' CAGGACAAAUCGCGUUGGCA 3'
::::::::::::::::::::
query 3' GUCCUGUUUAGCGCAACCGU 5'
query=miR399f, target=RO3G_10523, score=0, range=17-36, strand=1
target 5' CAGGACAAAUCGCGUUGGCA 3'
::::::::::::::::::::
query 3' GUCCUGUUUAGCGCAACCGU 5'
```

```
query=miR399f, target=RO3G_01941, score=0, range=17-36, strand=1
target 5' CAGGACAAAUCGCGUUGGCA 3'
::::::::::::::::::::
query 3' GUCCUGUUUAGCGCAACCGU 5'
query=miR399f, target=RO3G_03105, score=0, range=17-36, strand=1
target 5' CAGGACAAAUCGCGUUGGCA 3'
::::::::::::::::::::
query 3' GUCCUGUUUAGCGCAACCGU 5'
query=miR399f, target=RO3G_15285, score=0, range=17-36, strand=1
target 5' CAGGACAAAUCGCGUUGGCA 3'
::::::::::::::::::::
query 3' GUCCUGUUUAGCGCAACCGU 5'
query=miR399f, target=RO3G_09599, score=0, range=17-36, strand=1
target 5' CAGGACAAAUCGCGUUGGCA 3'
::::::::::::::::::::
query 3' GUCCUGUUUAGCGCAACCGU 5'
query=miR399f, target=RO3G_09932, score=0, range=17-36, strand=1
target 5' CAGGACAAAUCGCGUUGGCA 3'
::::::::::::::::::::
query 3' GUCCUGUUUAGCGCAACCGU 5'
query=miR399f, target=RO3G_09956, score=2, range=17-36, strand=1
target 5' CAGGACAAAUCGCGUUAGCA 3'
:::::::::::::::: :::
query 3' GUCCUGUUUAGCGCAACCGU 5'
query=miR399f, target=RO3G_15161, score=2, range=17-36, strand=1
target 5' CAGAACAAAUCGUGUUGGCA 3'
::: ::::::::.:::::::
query 3' GUCCUGUUUAGCGCAACCGU 5'
query=miR399f, target=RO3G_07726, score=2, range=17-36, strand=1
target 5' CAGAACAAAUCGUGUUGGCA 3'
::: ::::::::.:::::::
query 3' GUCCUGUUUAGCGCAACCGU 5'
query=miR399f, target=RO3G_05912, score=3, range=11-30, strand=1
target 5' CAGGACAAAACGUGUUGGCA 3'
::::::::: ::.:::::::
query 3' GUCCUGUUUAGCGCAACCGU 5'
query=miR399f, target=RO3G_01715, score=4, range=17-36, strand=1
target 5' CAGGACAAACCGUGUUGGCU 3'
::::::::: ::.::::::
query 3' GUCCUGUUUAGCGCAACCGU 5'
query=miR399f, target=RO3G_15255, score=4, range=17-36, strand=1
target 5' CAGGACAAACCGUGUUGGCU 3'
::::::::: ::.::::::
query 3' GUCCUGUUUAGCGCAACCGU 5'
query=miR399f, target=RO3G_16425, score=4, range=17-36, strand=1
```

```
target 5' CAGGACAAACCGUGUUGGCU 3'
::::::::: ::.::::::
query 3' GUCCUGUUUAGCGCAACCGU 5'
query=miR399f, target=RO3G_12170, score=4, range=17-36, strand=1
target 5' CAGGACAAACCGUGUUGGCU 3'
::::::::: ::.::::::
query 3' GUCCUGUUUAGCGCAACCGU 5'
query=miR399f, target=RO3G_06412, score=4, range=17-36, strand=1
target 5' CCGGACAUAUCGCGUUGGCC 3'
: ::::: :::::::::::
query 3' GUCCUGUUUAGCGCAACCGU 5'
query=miR399f, target=RO3G_04214, score=4, range=17-36, strand=1
target 5' CCGGACAUAUCGCGUUGGCC 3'
: ::::: :::::::::::
query 3' GUCCUGUUUAGCGCAACCGU 5'
query=miR399f, target=RO3G_17339, score=4, range=17-36, strand=1
target 5' CCGGACAUAUCGCGUUGGCC 3'
: ::::: :::::::::::
query 3' GUCCUGUUUAGCGCAACCGU 5'
query=miR399f, target=RO3G_14494, score=4, range=17-36, strand=1
target 5' CCGGACAUAUCGCGUUGGCC 3'
: ::::: :::::::::::
query 3' GUCCUGUUUAGCGCAACCGU 5'
query=miR399f, target=RO3G_09935, score=4, range=17-36, strand=1
target 5' CCGGACAUAUCGCGUUGGCC 3'
: ::::: :::::::::::
query 3' GUCCUGUUUAGCGCAACCGU 5'
query=miR399f, target=RO3G_07843, score=4, range=17-36, strand=1
target 5' CCGGACAUAUCGCGUUGGCC 3'
: ::::: :::::::::::
query 3' GUCCUGUUUAGCGCAACCGU 5'
query=miR399f, target=RO3G_01885, score=4, range=17-36, strand=1
target 5' CCGGACAUAUCGCGUUGGCC 3'
: ::::: :::::::::::
query 3' GUCCUGUUUAGCGCAACCGU 5'
query=miR399f, target=RO3G_14628, score=4, range=17-36, strand=1
target 5' CCGGACAUAUCGCGUUGGCC 3'
: ::::: :::::::::::
query 3' GUCCUGUUUAGCGCAACCGU 5'
query=miR399f, target=RO3G_01882, score=4, range=17-36, strand=1
target 5' CCGGACAUAUCGCGUUGGCC 3'
: ::::: :::::::::::
query 3' GUCCUGUUUAGCGCAACCGU 5'
query=miR399f, target=RO3G_17290, score=4, range=17-36, strand=1
target 5' CCGGACAUAUCGCGUUGGCU 3'
```

```
: ::::: ::::::::::: 
query 3' GUCCUGUUUAGCGCAACCGU 5'
query=miR399f, target=RO3G_16556, score=4, range=17-36, strand=1
target 5' CCGGACAUAUCGCGUUGGCC 3'
: ::::: ::::::::::: 
query 3' GUCCUGUUUAGCGCAACCGU 5'
query=miR399f, target=RO3G_05543, score=4, range=606-624, strand=
target 5' CAUGUCAAAU-GCGUUGGCA 3'
:: : ::::: :::::::::
query 3' GUCCUGUUUAGCGCAACCGU 5'
query=miR824-5p, target=RO3G_09022, score=3.5, range=582-600,
strand=-1
target 5' CCCUUCU-AUAAAUGGUCUG 3'
::::::: :.:::::::::.
query 3' GGGAAGAGUGUUUACCAGAU 5'
query=miR824-5p, target=RO3G_11708, score=4, range=266-285, stran
target 5' CACGUAACACAAAUGGUCUA 3'
: : : ::::::::::::: 
query 3' GGGAAGAGUGUUUACCAGAU 5'
query=miR5298d, target=RO3G_02081, score=3, range=1650-1672, stra
target 5' CUCUUUCUGCUUCUUCAUCUCUU 3'
:::::: ::::::::::::::
query 3' AAGAAAGUCGAAGAAGUAGAGAU 5'
query=miR5298d, target=RO3G_03728, score=3, range=1353-1375, stra
target 5' CUCUUUCUGCUUCUUCAUCUCUU 3'
:::::: ::::::::::::::
query 3' AAGAAAGUCGAAGAAGUAGAGAU 5'
query=miR5298d, target=RO3G_03008, score=3.5, range=1713-1735,
strand=-1
target 5' UUGUUUCAGUUUCUUCAUCUCCA 3'
:: ::::::.:::::::::: :
query 3' AAGAAAGUCGAAGAAGUAGAGAU 5'
query=miR5298d, target=RO3G_05215, score=3.5, range=525-547, stra
target 5' UUUUUUCAUCUUCUUCAUCACUA 3'
::.::::: :::::::::: :::
query 3' AAGAAAGUCGAAGAAGUAGAGAU 5'
query=miR5298d, target=RO3G_05122, score=3.5, range=108-129, stra
target 5' UUCUUUCGG-UUCUUCAUCUUUU 3'
:::::::.: ::::::::::.:
query 3' AAGAAAGUCGAAGAAGUAGAGAU 5'
query=miR5298d, target=RO3G_11135, score=4, range=736-759, strand
target 5' UUAUUUCAACCUUCUUCAUCUUUA 3'
:: :::: : :::::::::::.::
query 3' AAGAAAG-UCGAAGAAGUAGAGAU 5'
query=miR5298d, target=RO3G_15171, score=4, range=123-144, strand
```

target 5' UUCAUU-AGCUUCUUCAUUUCUC 3'
::: :: :::::::::::.:::
query 3' AAGAAAGUCGAAGAAGUAGAGAU 5'
query=miR5298d, target=RO3G_11059, score=4, range=287-308, strand
target 5' UUCCUUC-UCUUCUUCAUCUCUU 3'
::: ::: :::::::::::::
query 3' AAGAAAGUCGAAGAAGUAGAGAU 5'
query=miR5632-3p, target=RO3G_07707, score=0, range=1004-1024, strand=1
target 5' AGUAUCCAGACUUAAAUCCAA 3'
:::::::::::::::::::::
query 3' UCAUAGGUCUGAAUUUAGGUU 5'
query=miR5632-3p, target=RO3G_13919, score=0, range=1004-1024, strand=1
target 5' AGUAUCCAGACUUAAAUCCAA 3'
:::::::::::::::::::::
query 3' UCAUAGGUCUGAAUUUAGGUU 5'
query=miR5632-3p, target=RO3G_07690, score=0, range=812-832, stra
target 5' AGUAUCCAGACUUAAAUCCAA 3'
:::::::::::::::::::::
query 3' UCAUAGGUCUGAAUUUAGGUU 5'
query=miR5632-3p, target=RO3G_00679, score=1, range=1004-1024, strand=1
target 5' AGUCUCCAGACUUAAAUCCAA 3'
::: :::::::::::::::::
query 3' UCAUAGGUCUGAAUUUAGGUU 5'
query=miR5632-3p, target=RO3G_10550, score=1, range=812-832, stra
target 5' AGUUUCCAGACUUAAAUCCAA 3'
::: :::::::::::::::::
query 3' UCAUAGGUCUGAAUUUAGGUU 5'
query=miR5632-3p, target=RO3G_17408, score=1, range=812-832, stra
target 5' AGUCUCCAGACUUAAAUCCAA 3'
::: :::::::::::::::::
query 3' UCAUAGGUCUGAAUUUAGGUU 5'
query=miR5632-3p, target=RO3G_11281, score=1, range=767-787, stra
target 5' AGUCUCCAGACUUAAAUCCAA 3'
::: :::::::::::::::::
query 3' UCAUAGGUCUGAAUUUAGGUU 5'
query=miR5632-3p, target=RO3G_12166, score=1, range=812-832, stra
target 5' AGUUUCCAGACUUAAAUCCAA 3'
::: :::::::::::::::::
query 3' UCAUAGGUCUGAAUUUAGGUU 5'
query=miR5632-3p, target=RO3G_10787, score=1, range=812-832, stra
target 5' AGUCUCCAGACUUAAAUCCAA 3'
::: :::::::::::::::::

query 3' UCAUAGGUCUGAAUUUAGGUU 5'
query=miR5632-3p, target=RO3G_08847, score=1, range=812-832, stra
target 5' AGUUUCCAGACUUAAAUCCAA 3'
::: ::::::::::::::::::
query 3' UCAUAGGUCUGAAUUUAGGUU 5'
query=miR5632-3p, target=RO3G_12659, score=1, range=401-421, stra
target 5' AGUCUCCAGACUUAAAUCCAA 3'
::: ::::::::::::::::::
query 3' UCAUAGGUCUGAAUUUAGGUU 5'
query=miR5632-3p, target=RO3G_00700, score=1, range=251-271, stra
target 5' AGUCUCCAGACUUAAAUCCAA 3'
::: ::::::::::::::::::
query 3' UCAUAGGUCUGAAUUUAGGUU 5'
query=miR5632-3p, target=RO3G_16140, score=1, range=251-271, stra
target 5' AGUUUCCAGACUUAAAUCCAA 3'
::: ::::::::::::::::::
query 3' UCAUAGGUCUGAAUUUAGGUU 5'
query=miR5632-3p, target=RO3G_15582, score=1, range=191-211, stra
target 5' AGUCUCCAGACUUAAAUCCAA 3'
::: ::::::::::::::::::
query 3' UCAUAGGUCUGAAUUUAGGUU 5'
query=miR5632-3p, target=RO3G_13453, score=1, range=191-211, stra
target 5' AGUCUCCAGACUUAAAUCCAA 3'
::: ::::::::::::::::::
query 3' UCAUAGGUCUGAAUUUAGGUU 5'
query=miR5632-3p, target=RO3G_10220, score=1, range=251-271, stra
target 5' AGUCUCCAGACUUAAAUCCAA 3'
::: ::::::::::::::::::
query 3' UCAUAGGUCUGAAUUUAGGUU 5'
query=miR5632-3p, target=RO3G_02846, score=1, range=17-37, strand
target 5' AGUCUCCAGACUUAAAUCCAA 3'
::: ::::::::::::::::::
query 3' UCAUAGGUCUGAAUUUAGGUU 5'
query=miR5632-3p, target=RO3G_03624, score=1.5, range=812-832,
strand=1
target 5' AGUCUUCAGACUUAAAUCCAA 3'
::: :.::::::::::::::::
query 3' UCAUAGGUCUGAAUUUAGGUU 5'
query=miR5632-3p, target=RO3G_03603, score=1.5, range=251-271,
strand=1
target 5' AGUUUUCAGACUUAAAUCCAA 3'
::: :.::::::::::::::::
query 3' UCAUAGGUCUGAAUUUAGGUU 5'
query=miR5632-3p, target=RO3G_03112, score=2, range=806-826, stra
target 5' AGAGUCCGGACUUAAAUCCAA 3'

```
:: .:::.:::::::::::::
query 3' UCAUAGGUCUGAAUUUAGGUU 5'
query=miR5632-3p, target=RO3G_05522, score=2, range=806-826, stra
target 5' AGAGUCCGGACUUAAAUCCAA 3'
:: .:::.:::::::::::::
query 3' UCAUAGGUCUGAAUUUAGGUU 5'
query=miR5632-3p, target=RO3G_00240, score=2, range=749-769, stra
target 5' AGAGUCCGGACUUAAAUCCAA 3'
:: .:::.:::::::::::::
query 3' UCAUAGGUCUGAAUUUAGGUU 5'
query=miR5632-3p, target=RO3G_02227, score=2, range=806-826, stra
target 5' AGAGUCCGGACUUAAAUCCAA 3'
:: .:::.:::::::::::::
query 3' UCAUAGGUCUGAAUUUAGGUU 5'
query=miR5632-3p, target=RO3G_14784, score=2, range=440-460, stra
target 5' AGAGUCCGGACUUAAAUCCAA 3'
:: .:::.:::::::::::::
query 3' UCAUAGGUCUGAAUUUAGGUU 5'
query=miR5632-3p, target=RO3G_17423, score=2, range=440-460, stra
target 5' AGAGUCCGGACUUAAAUCCAA 3'
:: .:::.:::::::::::::
query 3' UCAUAGGUCUGAAUUUAGGUU 5'
query=miR5632-3p, target=RO3G_16737, score=2, range=440-460, stra
target 5' AGAGUCCGGACUUAAAUCCAA 3'
:: .:::.:::::::::::::
query 3' UCAUAGGUCUGAAUUUAGGUU 5'
query=miR5632-3p, target=RO3G_10797, score=2, range=440-460, stra
target 5' AGAGUCCGGACUUAAAUCCAA 3'
:: .:::.:::::::::::::
query 3' UCAUAGGUCUGAAUUUAGGUU 5'
query=miR5632-3p, target=RO3G_03560, score=2, range=440-460, stra
target 5' AGAGUCCGGACUUAAAUCCAA 3'
:: .:::.:::::::::::::
query 3' UCAUAGGUCUGAAUUUAGGUU 5'
query=miR5632-3p, target=RO3G_17328, score=2, range=440-460, stra
target 5' AGAGUCCGGACUUAAAUCCAA 3'
:: .:::.:::::::::::::
query 3' UCAUAGGUCUGAAUUUAGGUU 5'
query=miR5632-3p, target=RO3G_12950, score=2, range=440-460, stra
target 5' AGAGUCCGGACUUAAAUCCAA 3'
:: .:::.:::::::::::::
query 3' UCAUAGGUCUGAAUUUAGGUU 5'
query=miR5632-3p, target=RO3G_14981, score=2, range=440-460, stra
target 5' AGAGUCCGGACUUAAAUCCAA 3'
:: .:::.:::::::::::::
```

```
query 3' UCAUAGGUCUGAAUUUAGGUU 5'
query=miR5632-3p, target=RO3G_16124, score=2, range=440-460, stra
target 5' AGAGUCCGGACUUAAAUCCAA 3'
:: .:::.:::::::::::::
query 3' UCAUAGGUCUGAAUUUAGGUU 5'
query=miR5632-3p, target=RO3G_15170, score=2, range=440-460, stra
target 5' AGAGUCCGGACUUAAAUCCAA 3'
:: .:::.:::::::::::::
query 3' UCAUAGGUCUGAAUUUAGGUU 5'
query=miR5632-3p, target=RO3G_10050, score=2, range=440-460, stra
target 5' AGAGUCCGGACUUAAAUCCAA 3'
:: .:::.:::::::::::::
query 3' UCAUAGGUCUGAAUUUAGGUU 5'
query=miR5632-3p, target=RO3G_15145, score=2, range=440-460, stra
target 5' AGAGUCCGGACUUAAAUCCAA 3'
:: .:::.:::::::::::::
query 3' UCAUAGGUCUGAAUUUAGGUU 5'
query=miR5632-3p, target=RO3G_13752, score=2, range=440-460, stra
target 5' AGAGUCCGGACUUAAAUCCAA 3'
:: .:::.:::::::::::::
query 3' UCAUAGGUCUGAAUUUAGGUU 5'
query=miR5632-3p, target=RO3G_03791, score=2, range=440-460, stra
target 5' AGAGUCCGGACUUAAAUCCAA 3'
:: .:::.:::::::::::::
query 3' UCAUAGGUCUGAAUUUAGGUU 5'
query=miR5632-3p, target=RO3G_07723, score=2, range=440-460, stra
target 5' AGAGUCCGGACUUAAAUCCAA 3'
:: .:::.:::::::::::::
query 3' UCAUAGGUCUGAAUUUAGGUU 5'
query=miR5632-3p, target=RO3G_17092, score=2, range=440-460, stra
target 5' AGAGUCCGGACUUAAAUCCAA 3'
:: .:::.:::::::::::::
query 3' UCAUAGGUCUGAAUUUAGGUU 5'
query=miR5632-3p, target=RO3G_16141, score=2, range=440-460, stra
target 5' AGAGUCCGGACUUAAAUCCAA 3'
:: .:::.:::::::::::::
query 3' UCAUAGGUCUGAAUUUAGGUU 5'
query=miR5632-3p, target=RO3G_16541, score=2, range=440-460, stra
target 5' AGAGUCCGGACUUAAAUCCAA 3'
:: .:::.:::::::::::::
query 3' UCAUAGGUCUGAAUUUAGGUU 5'
query=miR5632-3p, target=RO3G_11536, score=2, range=440-460, stra
target 5' AGAGUCCGGACUUAAAUCCAA 3'
:: .:::.:::::::::::::
query 3' UCAUAGGUCUGAAUUUAGGUU 5'
```

query=miR5632-3p, target=RO3G_15286, score=2, range=440-460, stra
target 5' AGAGUCCGGACUUAAAUCCAA 3'
:: .:::.::::::::::::::
query 3' UCAUAGGUCUGAAUUUAGGUU 5'
query=miR5632-3p, target=RO3G_09926, score=2, range=440-460, stra
target 5' AGAGUCCGGACUUAAAUCCAA 3'
:: .:::.::::::::::::::
query 3' UCAUAGGUCUGAAUUUAGGUU 5'
query=miR5632-3p, target=RO3G_05516, score=2, range=440-460, stra
target 5' AGAGUCCGGACUUAAAUCCAA 3'
:: .:::.::::::::::::::
query 3' UCAUAGGUCUGAAUUUAGGUU 5'
query=miR5632-3p, target=RO3G_01725, score=2, range=440-460, stra
target 5' AGAGUCCGGACUUAAAUCCAA 3'
:: .:::.::::::::::::::
query 3' UCAUAGGUCUGAAUUUAGGUU 5'
query=miR5632-3p, target=RO3G_07136, score=2, range=131-151, stra
target 5' AGAGUCCGGACUUAAAUCCAA 3'
:: .:::.::::::::::::::
query 3' UCAUAGGUCUGAAUUUAGGUU 5'
query=miR5632-3p, target=RO3G_11533, score=2, range=440-460, stra
target 5' AGAGUCCGGACUUAAAUCCAA 3'
:: .:::.::::::::::::::
query 3' UCAUAGGUCUGAAUUUAGGUU 5'
query=miR5632-3p, target=RO3G_13286, score=2, range=1004-1024,
strand=1
target 5' AGCCUCCAGACUUAAAUCCAA 3'
:: :::::::::::::::::
query 3' UCAUAGGUCUGAAUUUAGGUU 5'
query=miR5632-3p, target=RO3G_00284, score=2, range=821-841, stra
target 5' AGCCUCCAGACUUAAAUCCAA 3'
:: :::::::::::::::::
query 3' UCAUAGGUCUGAAUUUAGGUU 5'
query=miR5632-3p, target=RO3G_16727, score=2, range=812-832, stra
target 5' AGCCUCCAGACUUAAAUCCAA 3'
:: :::::::::::::::::
query 3' UCAUAGGUCUGAAUUUAGGUU 5'
query=miR5632-3p, target=RO3G_16719, score=2, range=812-832, stra
target 5' AGCCUCCAGACUUAAAUCCAA 3'
:: :::::::::::::::::
query 3' UCAUAGGUCUGAAUUUAGGUU 5'
query=miR5632-3p, target=RO3G_01959, score=2, range=251-271, stra
target 5' AGCCUCCAGACUUAAAUCCAA 3'
:: :::::::::::::::::
query 3' UCAUAGGUCUGAAUUUAGGUU 5'

query=miR5632-3p, target=RO3G_16751, score=2, range=401-421, stra
target 5' AGUUCCCAGACUUAAAUCCAA 3'
::: :::::::::::::::
query 3' UCAUAGGUCUGAAUUUAGGUU 5'
query=miR5632-3p, target=RO3G_03554, score=3, range=704-724, stra
target 5' AGUCUCCUGACUUGAAUCCAA 3'
::: ::: ::::.:::::::
query 3' UCAUAGGUCUGAAUUUAGGUU 5'
query=miR5632-3p, target=RO3G_08657, score=3, range=704-724, stra
target 5' AGUCUCCUGACUUGAAUCCAA 3'
::: ::: ::::.:::::::
query 3' UCAUAGGUCUGAAUUUAGGUU 5'
query=miR5632-3p, target=RO3G_10772, score=3, range=701-721, stra
target 5' AGUCUCCUGACUUGAAUCCAA 3'
::: ::: ::::.:::::::
query 3' UCAUAGGUCUGAAUUUAGGUU 5'
query=miR5632-3p, target=RO3G_10768, score=3, range=17-37, strand
target 5' AGUCUCCAGACUUAAAUGCAA 3'
::: :::::::::::: :::
query 3' UCAUAGGUCUGAAUUUAGGUU 5'
query=miR5632-3p, target=RO3G_04668, score=4, range=617-637, stra
target 5' AGAGUCCGGACUUAAAUCCCA 3'
:: .:::.:::::::::: :
query 3' UCAUAGGUCUGAAUUUAGGUU 5'
query=miR5632-3p, target=RO3G_16121, score=4, range=395-415, stra
target 5' AGAGUCCGGACUUAAAUCCCA 3'
:: .:::.:::::::::: :
query 3' UCAUAGGUCUGAAUUUAGGUU 5'
query=miR5632-3p, target=RO3G_00675, score=4, range=440-460, stra
target 5' AGAGUCCGGACUUAAAUCCCA 3'
:: .:::.:::::::::: :
query 3' UCAUAGGUCUGAAUUUAGGUU 5'
query=miR5632-3p, target=RO3G_10237, score=4, range=380-400, stra
target 5' AGAGUCCGGACUUAAAUCCCA 3'
:: .:::.:::::::::: :
query 3' UCAUAGGUCUGAAUUUAGGUU 5'
query=miR5632-3p, target=RO3G_07298, score=4, range=440-460, stra
target 5' AGAGUCCGGACUUAAAUCCCA 3'
:: .:::.:::::::::: :
query 3' UCAUAGGUCUGAAUUUAGGUU 5'
query=miR5632-3p, target=RO3G_12664, score=4, range=539-559, stra
target 5' AGUCCCCGGACUUGAAUCCAG 3'
::: ::.:::::.::::::.
query 3' UCAUAGGUCUGAAUUUAGGUU 5'
query=miR5632-3p, target=RO3G_15287, score=4, range=446-466, stra

```
target 5' AGUCACCUGACUUGAAUCCAA 3'
::: :: :::::.:::::::
query 3' UCAUAGGUCUGAAUUUAGGUU 5'
query=miR5632-3p, target=RO3G_16904, score=4, range=794-814, stra
target 5' ACUCUCCAGACUUAAAUCCUA 3'
: : :::::::::::::: :
query 3' UCAUAGGUCUGAAUUUAGGUU 5'
query=miR5632-3p, target=RO3G_16829, score=4, range=794-814, stra
target 5' ACUCUCCAGACUUAAAUCCUA 3'
: : :::::::::::::: :
query 3' UCAUAGGUCUGAAUUUAGGUU 5'
query=miR5632-3p, target=RO3G_13734, score=4, range=794-814, stra
target 5' ACUCUCCAGACUUAAAUCCUA 3'
: : :::::::::::::: :
query 3' UCAUAGGUCUGAAUUUAGGUU 5'
query=miR5632-3p, target=RO3G_17202, score=4, range=794-814, stra
target 5' ACUCUCCAGACUUAAAUCCUA 3'
: : :::::::::::::: :
query 3' UCAUAGGUCUGAAUUUAGGUU 5'
query=miR5632-3p, target=RO3G_16428, score=4, range=431-451, stra
target 5' ACUCUCCAGACUUAAAUCCUA 3'
: : :::::::::::::: :
query 3' UCAUAGGUCUGAAUUUAGGUU 5'
query=miR5632-3p, target=RO3G_03772, score=4, range=812-832, stra
target 5' AGUCCCCUGACUUGAAUCCAA 3'
::: :: :::::.:::::::
query 3' UCAUAGGUCUGAAUUUAGGUU 5'
query=miR5632-3p, target=RO3G_05945, score=4, range=431-451, stra
target 5' ACUCUCCAGACUUAAAUCCUA 3'
: : :::::::::::::: :
query 3' UCAUAGGUCUGAAUUUAGGUU 5'
query=miR5632-3p, target=RO3G_11411, score=4, range=431-451, stra
target 5' ACUCUCCAGACUUAAAUCCUA 3'
: : :::::::::::::: :
query 3' UCAUAGGUCUGAAUUUAGGUU 5'
query=miR5632-3p, target=RO3G_08842, score=4, range=812-832, stra
target 5' AGUCCCCUGACUUGAAUCCAA 3'
::: :: :::::.:::::::
query 3' UCAUAGGUCUGAAUUUAGGUU 5'
query=miR5632-3p, target=RO3G_15579, score=4, range=812-832, stra
target 5' AGUCCCCUGACUUGAAUCCAA 3'
::: :: :::::.:::::::
query 3' UCAUAGGUCUGAAUUUAGGUU 5'
query=miR5632-3p, target=RO3G_02844, score=4, range=590-610, stra
target 5' AGUCACCUGACUUGAAUCCAA 3'
```

```
::: :: :::::.:::::::
query 3' UCAUAGGUCUGAAUUUAGGUU 5'
query=miR5632-3p, target=RO3G_03766, score=4, range=590-610, stra
target 5' AGUCACCUGACUUGAAUCCAA 3'
::: :: :::::.:::::::
query 3' UCAUAGGUCUGAAUUUAGGUU 5'
query=miR5632-3p, target=RO3G_17154, score=4, range=431-451, stra
target 5' ACUCUCCAGACUUAAAUCCUA 3'
: : :::::::::::::: :
query 3' UCAUAGGUCUGAAUUUAGGUU 5'
query=miR5632-3p, target=RO3G_13137, score=4, range=710-730, stra
target 5' AGUCCCCUGACUUGAAUCCAA 3'
::: :: :::::.:::::::
query 3' UCAUAGGUCUGAAUUUAGGUU 5'
query=miR5632-3p, target=RO3G_04205, score=4, range=512-532, stra
target 5' AGUUCCCUGACUUGAAUCCAA 3'
::: :: :::::.:::::::
query 3' UCAUAGGUCUGAAUUUAGGUU 5'
query=miR5632-3p, target=RO3G_13733, score=4, range=383-403, stra
target 5' AGUCCCCUGACUUGAAUCCAA 3'
::: :: :::::.:::::::
query 3' UCAUAGGUCUGAAUUUAGGUU 5'
query=miR475a-3p, target=RO3G_16086, score=0, range=1825-1844,
strand=1
target 5' CUGAAGUCAAUGGGCAUUAA 3'
::::::::::::::::::::
query 3' GACUUCAGUUACCCGUAAUU 5'
query=miR475a-3p, target=RO3G_02364, score=0, range=3409-3428,
strand=1
target 5' CUGAAGUCAAUGGGCAUUAA 3'
::::::::::::::::::::
query 3' GACUUCAGUUACCCGUAAUU 5'
query=miR475a-3p, target=RO3G_15802, score=0, range=3409-3428,
strand=1
target 5' CUGAAGUCAAUGGGCAUUAA 3'
::::::::::::::::::::
query 3' GACUUCAGUUACCCGUAAUU 5'
query=miR475a-3p, target=RO3G_12567, score=0, range=3409-3428,
strand=1
target 5' CUGAAGUCAAUGGGCAUUAA 3'
::::::::::::::::::::
query 3' GACUUCAGUUACCCGUAAUU 5'
query=miR475a-3p, target=RO3G_04496, score=0, range=3031-3050,
strand=1
target 5' CUGAAGUCAAUGGGCAUUAA 3'
```

```
::::::::::::::::::::
query 3' GACUUCAGUUACCCGUAAUU 5'
query=miR475a-3p, target=RO3G_06775, score=0, range=3409-3428,
strand=1
target 5' CUGAAGUCAAUGGGCAUUAA 3'
::::::::::::::::::::
query 3' GACUUCAGUUACCCGUAAUU 5'
query=miR475a-3p, target=RO3G_04252, score=0, range=3409-3428,
strand=1
target 5' CUGAAGUCAAUGGGCAUUAA 3'
::::::::::::::::::::
query 3' GACUUCAGUUACCCGUAAUU 5'
query=miR475a-3p, target=RO3G_07854, score=0, range=3409-3428,
strand=1
target 5' CUGAAGUCAAUGGGCAUUAA 3'
::::::::::::::::::::
query 3' GACUUCAGUUACCCGUAAUU 5'
query=miR475a-3p, target=RO3G_14949, score=0, range=3031-3050,
strand=1
target 5' CUGAAGUCAAUGGGCAUUAA 3'
::::::::::::::::::::
query 3' GACUUCAGUUACCCGUAAUU 5'
query=miR475a-3p, target=RO3G_01156, score=3, range=778-797, stra
target 5' CUGAAGUCAAUUGGUAUUAA 3'
:::::::::: ::.:::::
query 3' GACUUCAGUUACCCGUAAUU 5'
query=miR475a-3p, target=RO3G_16981, score=3, range=304-323, stra
target 5' CUGAAUUCAAUUGGCAUUAA 3'
::::: ::::: ::::::::
query 3' GACUUCAGUUACCCGUAAUU 5'
query=miR475a-3p, target=RO3G_16987, score=3, range=286-305, stra
target 5' CUGAAUUCAAUUGGCAUUAA 3'
::::: ::::: ::::::::
query 3' GACUUCAGUUACCCGUAAUU 5'
query=miR475a-3p, target=RO3G_10320, score=4, range=449-467, stra
target 5' AUGAAG-AAAUGGGCAUUAA 3'
::::: ::::::::::::
query 3' GACUUCAGUUACCCGUAAUU 5'
query=miR475a-3p, target=RO3G_09513, score=4, range=421-439, stra
target 5' CUG-AGUCAAUGGGCAAUAU 3'
::: ::::::::::: ::
query 3' GACUUCAGUUACCCGUAAUU 5'
query=miR8044-5p, target=RO3G_17226, score=0, range=1843-1864,
strand=1
target 5' AAAUCUCCAACCAUUUUGACAA 3'
```

```
::::::::::::::::::::::
query 3' UUUAGAGGUUGGUAAAACUGUU 5'
query=miR8044-5p, target=RO3G_16815, score=0, range=1843-1864,
strand=1
target 5' AAAUCUCCAACCAUUUUGACAA 3'
::::::::::::::::::::::
query 3' UUUAGAGGUUGGUAAAACUGUU 5'
query=miR8044-5p, target=RO3G_14795, score=0, range=1843-1864,
strand=1
target 5' AAAUCUCCAACCAUUUUGACAA 3'
::::::::::::::::::::::
query 3' UUUAGAGGUUGGUAAAACUGUU 5'
query=miR8044-5p, target=RO3G_11557, score=4, range=363-383, stra
target 5' AAAUC-CCAAUCAUUUUGAUAU 3'
::::: ::::.::::::::.:
query 3' UUUAGAGGUUGGUAAAACUGUU 5'
query=miR7725a-3p, target=RO3G_02691, score=0, range=377-398,
strand=-1
target 5' UUGACGAAAUGAGUUGUUUGCA 3'
::::::::::::::::::::::
query 3' AACUGCUUUACUCAACAAACGU 5'
query=miR7725a-3p, target=RO3G_17318, score=0, range=1844-1865,
strand=-1
target 5' UUGACGAAAUGAGUUGUUUGCA 3'
::::::::::::::::::::::
query 3' AACUGCUUUACUCAACAAACGU 5'
query=miR7725a-3p, target=RO3G_04118, score=0, range=1844-1865,
strand=-1
target 5' UUGACGAAAUGAGUUGUUUGCA 3'
::::::::::::::::::::::
query 3' AACUGCUUUACUCAACAAACGU 5'
query=miR7725a-3p, target=RO3G_16143, score=0, range=1841-1862,
strand=-1
target 5' UUGACGAAAUGAGUUGUUUGCA 3'
::::::::::::::::::::::
query 3' AACUGCUUUACUCAACAAACGU 5'
query=miR7725a-3p, target=RO3G_17274, score=0, range=1844-1865,
strand=-1
target 5' UUGACGAAAUGAGUUGUUUGCA 3'
::::::::::::::::::::::
query 3' AACUGCUUUACUCAACAAACGU 5'
query=miR7725a-3p, target=RO3G_12533, score=0, range=1184-1205,
strand=-1
target 5' UUGACGAAAUGAGUUGUUUGCA 3'
::::::::::::::::::::::
```

query 3' AACUGCUUUACUCAACAAACGU 5'
query=miR7725a-3p, target=RO3G_17329, score=0, range=1181-1202,
strand=-1
target 5' UUGACGAAAUGAGUUGUUUGCA 3'
::::::::::::::::::::::
query 3' AACUGCUUUACUCAACAAACGU 5'
query=miR7725a-3p, target=RO3G_07028, score=0, range=914-935,
strand=-1
target 5' UUGACGAAAUGAGUUGUUUGCA 3'
::::::::::::::::::::::
query 3' AACUGCUUUACUCAACAAACGU 5'
query=miR7725a-3p, target=RO3G_15135, score=0, range=1844-1865,
strand=-1
target 5' UUGACGAAAUGAGUUGUUUGCA 3'
::::::::::::::::::::::
query 3' AACUGCUUUACUCAACAAACGU 5'
query=miR7725a-3p, target=RO3G_17384, score=0, range=1844-1865,
strand=-1
target 5' UUGACGAAAUGAGUUGUUUGCA 3'
::::::::::::::::::::::
query 3' AACUGCUUUACUCAACAAACGU 5'
query=miR7725a-3p, target=RO3G_05919, score=0, range=2018-2039,
strand=-1
target 5' UUGACGAAAUGAGUUGUUUGCA 3'
::::::::::::::::::::::
query 3' AACUGCUUUACUCAACAAACGU 5'
query=miR7725a-3p, target=RO3G_16914, score=0, range=1778-1799,
strand=-1
target 5' UUGACGAAAUGAGUUGUUUGCA 3'
::::::::::::::::::::::
query 3' AACUGCUUUACUCAACAAACGU 5'
query=miR7725a-3p, target=RO3G_06727, score=0, range=1982-2003,
strand=-1
target 5' UUGACGAAAUGAGUUGUUUGCA 3'
::::::::::::::::::::::
query 3' AACUGCUUUACUCAACAAACGU 5'
query=miR4240, target=RO3G_12541, score=0, range=429-449, strand=
target 5' GAAAUGUUACGAGUCAGUCGA 3'
:::::::::::::::::::::
query 3' CUUUACAAUGCUCAGUCAGCU 5'
query=miR4240, target=RO3G_09496, score=0, range=429-449, strand=
target 5' GAAAUGUUACGAGUCAGUCGA 3'
:::::::::::::::::::::
query 3' CUUUACAAUGCUCAGUCAGCU 5'
query=miR4240, target=RO3G_13916, score=0, range=429-449, strand=

```
target 5' GAAAUGUUACGAGUCAGUCGA 3'
:::::::::::::::::::::
query 3' CUUUACAAUGCUCAGUCAGCU 5'
query=miR4240, target=RO3G_11272, score=0, range=429-449, strand=
target 5' GAAAUGUUACGAGUCAGUCGA 3'
:::::::::::::::::::::
query 3' CUUUACAAUGCUCAGUCAGCU 5'
query=miR4240, target=RO3G_15274, score=0, range=429-449, strand=
target 5' GAAAUGUUACGAGUCAGUCGA 3'
:::::::::::::::::::::
query 3' CUUUACAAUGCUCAGUCAGCU 5'
query=miR4240, target=RO3G_11784, score=0, range=429-449, strand=
target 5' GAAAUGUUACGAGUCAGUCGA 3'
:::::::::::::::::::::
query 3' CUUUACAAUGCUCAGUCAGCU 5'
query=miR4240, target=RO3G_10860, score=4, range=429-449, strand=
target 5' GAAAUGCUAUGAAUCAGUCGA 3'
:::::: ::.:: ::::::::
query 3' CUUUACAAUGCUCAGUCAGCU 5'
query=miR4240, target=RO3G_17289, score=4, range=429-449, strand=
target 5' GAAAUGCUAUGAAUCAGUCGA 3'
:::::: ::.:: ::::::::
query 3' CUUUACAAUGCUCAGUCAGCU 5'
query=miR4240, target=RO3G_08111, score=4, range=429-449, strand=
target 5' GAAAUGCUAUGAAUCAGUCGA 3'
:::::: ::.:: ::::::::
query 3' CUUUACAAUGCUCAGUCAGCU 5'
query=miR4240, target=RO3G_02624, score=4, range=186-205, strand=
target 5' CGAGUGUUACGAGU-AGUCGA 3'
.:.:::::::::: ::::::
query 3' CUUUACAAUGCUCAGUCAGCU 5'
query=miR1222a, target=RO3G_10582, score=4, range=256-277, strand
target 5' UAUUAUCAG-GAACUUCUUCAUU 3'
:::.: ::. :::::::::::
query 3' AUAGUUGUUACUUGAAGAAGUAU 5'
query=miR6443, target=RO3G_02848, score=0, range=152-170, strand=
target 5' CUCAUCAUCUUGACCUUAC 3'
:::::::::::::::::::
query 3' GAGUAGUAGAACUGGAAUG 5'
query=miR6443, target=RO3G_03570, score=0, range=206-224, strand=
target 5' CUCAUCAUCUUGACCUUAC 3'
:::::::::::::::::::
query 3' GAGUAGUAGAACUGGAAUG 5'
query=miR6443, target=RO3G_02700, score=0, range=206-224, strand=
target 5' CUCAUCAUCUUGACCUUAC 3'
```

```
:::::::::::::::::::
query 3' GAGUAGUAGAACUGGAAUG 5'
query=miR6443, target=RO3G_09955, score=0, range=206-224, strand=
target 5' CUCAUCAUCUUGACCUUAC 3'
:::::::::::::::::::
query 3' GAGUAGUAGAACUGGAAUG 5'
query=miR6443, target=RO3G_16825, score=0, range=329-347, strand=
target 5' CUCAUCAUCUUGACCUUAC 3'
:::::::::::::::::::
query 3' GAGUAGUAGAACUGGAAUG 5'
query=miR6443, target=RO3G_04654, score=0, range=329-347, strand=
target 5' CUCAUCAUCUUGACCUUAC 3'
:::::::::::::::::::
query 3' GAGUAGUAGAACUGGAAUG 5'
query=miR6443, target=RO3G_09167, score=0, range=329-347, strand=
target 5' CUCAUCAUCUUGACCUUAC 3'
:::::::::::::::::::
query 3' GAGUAGUAGAACUGGAAUG 5'
query=miR6443, target=RO3G_12670, score=0, range=329-347, strand=
target 5' CUCAUCAUCUUGACCUUAC 3'
:::::::::::::::::::
query 3' GAGUAGUAGAACUGGAAUG 5'
query=miR6443, target=RO3G_00247, score=0, range=329-347, strand=
target 5' CUCAUCAUCUUGACCUUAC 3'
:::::::::::::::::::
query 3' GAGUAGUAGAACUGGAAUG 5'
query=miR6443, target=RO3G_16538, score=0, range=407-425, strand=
target 5' CUCAUCAUCUUGACCUUAC 3'
:::::::::::::::::::
query 3' GAGUAGUAGAACUGGAAUG 5'
query=miR6443, target=RO3G_07302, score=0, range=329-347, strand=
target 5' CUCAUCAUCUUGACCUUAC 3'
:::::::::::::::::::
query 3' GAGUAGUAGAACUGGAAUG 5'
query=miR6443, target=RO3G_09952, score=0, range=299-317, strand=
target 5' CUCAUCAUCUUGACCUUAC 3'
:::::::::::::::::::
query 3' GAGUAGUAGAACUGGAAUG 5'
query=miR6443, target=RO3G_10224, score=0, range=329-347, strand=
target 5' CUCAUCAUCUUGACCUUAC 3'
:::::::::::::::::::
query 3' GAGUAGUAGAACUGGAAUG 5'
query=miR6443, target=RO3G_17436, score=0, range=329-347, strand=
target 5' CUCAUCAUCUUGACCUUAC 3'
:::::::::::::::::::
```

```
query 3' GAGUAGUAGAACUGGAAUG 5'
query=miR6443, target=RO3G_17293, score=0, range=329-347, strand=
target 5' CUCAUCAUCUUGACCUUAC 3'
:::::::::::::::::::
query 3' GAGUAGUAGAACUGGAAUG 5'
query=miR6443, target=RO3G_09944, score=0, range=329-347, strand=
target 5' CUCAUCAUCUUGACCUUAC 3'
:::::::::::::::::::
query 3' GAGUAGUAGAACUGGAAUG 5'
query=miR6443, target=RO3G_15672, score=0, range=329-347, strand=
target 5' CUCAUCAUCUUGACCUUAC 3'
:::::::::::::::::::
query 3' GAGUAGUAGAACUGGAAUG 5'
query=miR6443, target=RO3G_04200, score=0, range=329-347, strand=
target 5' CUCAUCAUCUUGACCUUAC 3'
:::::::::::::::::::
query 3' GAGUAGUAGAACUGGAAUG 5'
query=miR6443, target=RO3G_11405, score=0, range=329-347, strand=
target 5' CUCAUCAUCUUGACCUUAC 3'
:::::::::::::::::::
query 3' GAGUAGUAGAACUGGAAUG 5'
query=miR6443, target=RO3G_07689, score=0, range=329-347, strand=
target 5' CUCAUCAUCUUGACCUUAC 3'
:::::::::::::::::::
query 3' GAGUAGUAGAACUGGAAUG 5'
query=miR6443, target=RO3G_04786, score=0, range=89-107, strand=1
target 5' CUCAUCAUCUUGACCUUAC 3'
:::::::::::::::::::
query 3' GAGUAGUAGAACUGGAAUG 5'
query=miR6443, target=RO3G_02850, score=0, range=521-539, strand=
target 5' CUCAUCAUCUUGACCUUAC 3'
:::::::::::::::::::
query 3' GAGUAGUAGAACUGGAAUG 5'
query=miR6443, target=RO3G_07121, score=0, range=521-539, strand=
target 5' CUCAUCAUCUUGACCUUAC 3'
:::::::::::::::::::
query 3' GAGUAGUAGAACUGGAAUG 5'
query=miR6443, target=RO3G_07024, score=0, range=521-539, strand=
target 5' CUCAUCAUCUUGACCUUAC 3'
:::::::::::::::::::
query 3' GAGUAGUAGAACUGGAAUG 5'
query=miR6443, target=RO3G_03599, score=0, range=521-539, strand=
target 5' CUCAUCAUCUUGACCUUAC 3'
:::::::::::::::::::
query 3' GAGUAGUAGAACUGGAAUG 5'
```

query=miR6443, target=RO3G_01925, score=0, range=521-539, strand=
target 5' CUCAUCAUCUUGACCUUAC 3'
:::::::::::::::::::
query 3' GAGUAGUAGAACUGGAAUG 5'
query=miR6443, target=RO3G_01587, score=0, range=338-356, strand=
target 5' CUCAUCAUCUUGACCUUAC 3'
:::::::::::::::::::
query 3' GAGUAGUAGAACUGGAAUG 5'
query=miR6443, target=RO3G_10515, score=0, range=443-461, strand=
target 5' CUCAUCAUCUUGACCUUAC 3'
:::::::::::::::::::
query 3' GAGUAGUAGAACUGGAAUG 5'
query=miR6443, target=RO3G_17361, score=0, range=89-107, strand=1
target 5' CUCAUCAUCUUGACCUUAC 3'
:::::::::::::::::::
query 3' GAGUAGUAGAACUGGAAUG 5'
query=miR6443, target=RO3G_06721, score=0, range=329-347, strand=
target 5' CUCAUCAUCUUGACCUUAC 3'
:::::::::::::::::::
query 3' GAGUAGUAGAACUGGAAUG 5'
query=miR6443, target=RO3G_14974, score=0, range=395-413, strand=
target 5' CUCAUCAUCUUGACCUUAC 3'
:::::::::::::::::::
query 3' GAGUAGUAGAACUGGAAUG 5'
query=miR6443, target=RO3G_13857, score=0, range=152-170, strand=
target 5' CUCAUCAUCUUGACCUUAC 3'
:::::::::::::::::::
query 3' GAGUAGUAGAACUGGAAUG 5'
query=miR6443, target=RO3G_14772, score=0, range=407-425, strand=
target 5' CUCAUCAUCUUGACCUUAC 3'
:::::::::::::::::::
query 3' GAGUAGUAGAACUGGAAUG 5'
query=miR6443, target=RO3G_10226, score=0, range=329-347, strand=
target 5' CUCAUCAUCUUGACCUUAC 3'
:::::::::::::::::::
query 3' GAGUAGUAGAACUGGAAUG 5'
query=miR6443, target=RO3G_12082, score=0, range=407-425, strand=
target 5' CUCAUCAUCUUGACCUUAC 3'
:::::::::::::::::::
query 3' GAGUAGUAGAACUGGAAUG 5'
query=miR6443, target=RO3G_16905, score=0, range=329-347, strand=
target 5' CUCAUCAUCUUGACCUUAC 3'
:::::::::::::::::::
query 3' GAGUAGUAGAACUGGAAUG 5'
query=miR6443, target=RO3G_17204, score=0, range=392-410, strand=

```
target 5' CUCAUCAUCUUGACCUUAC 3'
:::::::::::::::::::
query 3' GAGUAGUAGAACUGGAAUG 5'
query=miR6443, target=RO3G_12525, score=0, range=443-461, strand=
target 5' CUCAUCAUCUUGACCUUAC 3'
:::::::::::::::::::
query 3' GAGUAGUAGAACUGGAAUG 5'
query=miR6443, target=RO3G_03785, score=0, range=134-152, strand=
target 5' CUCAUCAUCUUGACCUUAC 3'
:::::::::::::::::::
query 3' GAGUAGUAGAACUGGAAUG 5'
query=miR6443, target=RO3G_08658, score=0, range=329-347, strand=
target 5' CUCAUCAUCUUGACCUUAC 3'
:::::::::::::::::::
query 3' GAGUAGUAGAACUGGAAUG 5'
query=miR6443, target=RO3G_03797, score=0, range=17-35, strand=1
target 5' CUCAUCAUCUUGACCUUAC 3'
:::::::::::::::::::
query 3' GAGUAGUAGAACUGGAAUG 5'
query=miR6443, target=RO3G_12196, score=0.5, range=443-461, stran
target 5' CUCGUCAUCUUGACCUUAC 3'
:::.:::::::::::::::
query 3' GAGUAGUAGAACUGGAAUG 5'
query=miR6443, target=RO3G_12186, score=0.5, range=521-539, stran
target 5' CUCGUCAUCUUGACCUUAC 3'
:::.:::::::::::::::
query 3' GAGUAGUAGAACUGGAAUG 5'
query=miR6443, target=RO3G_06424, score=1, range=122-140, strand=
target 5' CUCAUCAUCUUGACCUUGC 3'
:::::::::::::::::.:
query 3' GAGUAGUAGAACUGGAAUG 5'
query=miR6443, target=RO3G_04661, score=2, range=152-170, strand=
target 5' CUCAUCAUCUUGACCCUAC 3'
::::::::::::::: :::
query 3' GAGUAGUAGAACUGGAAUG 5'
query=miR6443, target=RO3G_02589, score=2, range=935-952, strand=
target 5' CUUGU-AUCUUGACCUUAC 3'
::..: :::::::::::::
query 3' GAGUAGUAGAACUGGAAUG 5'
query=miR6443, target=RO3G_05486, score=2.5, range=284-303, stran
target 5' CUCGUCAUCUUGACCUCUAC 3'
:::.::::::::::: :::
query 3' GAGUAGUAGAACUGGA-AUG 5'
query=miR6443, target=RO3G_17316, score=3, range=152-170, strand=
target 5' CUCAUCAUCUGGACUUUAC 3'
```

```
:::::::::: :::.::::
query 3' GAGUAGUAGAACUGGAAUG 5'
query=miR6443, target=RO3G_00689, score=3, range=20-38, strand=1
target 5' CUCAUCAUCUAGACUUUAC 3'
:::::::::: :::.::::
query 3' GAGUAGUAGAACUGGAAUG 5'
query=miR6443, target=RO3G_07298, score=3, range=152-170, strand=
target 5' CUCAUCAUCUAGACUUUAC 3'
:::::::::: :::.::::
query 3' GAGUAGUAGAACUGGAAUG 5'
query=miR6443, target=RO3G_03777, score=3, range=152-170, strand=
target 5' CUCAUCAUCUAGACUUUAC 3'
:::::::::: :::.::::
query 3' GAGUAGUAGAACUGGAAUG 5'
query=miR6443, target=RO3G_01709, score=3, range=152-170, strand=
target 5' CUCAUCAUCUAGACUUUAC 3'
:::::::::: :::.::::
query 3' GAGUAGUAGAACUGGAAUG 5'
query=miR6443, target=RO3G_00675, score=3, range=152-170, strand=
target 5' CUCAUCAUCUAGACUUUAC 3'
:::::::::: :::.::::
query 3' GAGUAGUAGAACUGGAAUG 5'
query=miR6443, target=RO3G_04668, score=3, range=329-347, strand=
target 5' CUCAUCAUCUAGACUUUAC 3'
:::::::::: :::.::::
query 3' GAGUAGUAGAACUGGAAUG 5'
query=miR6443, target=RO3G_16121, score=3, range=107-125, strand=
target 5' CUCAUCAUCUAGACUUUAC 3'
:::::::::: :::.::::
query 3' GAGUAGUAGAACUGGAAUG 5'
query=miR6443, target=RO3G_04710, score=3, range=339-356, strand=
target 5' CUC-UUAUCUUGAUCUUAU 3'
::: :.:::::::.::::.
query 3' GAGUAGUAGAACUGGAAUG 5'
query=miR6443, target=RO3G_09272, score=3, range=1222-1240, stran
target 5' CUCACCUUCUUGACCUUAC 3'
:::: : ::::::::::::
query 3' GAGUAGUAGAACUGGAAUG 5'
query=miR6443, target=RO3G_03106, score=3, range=74-92, strand=1
target 5' CUCCUCAUCUUGACCUGAC 3'
::: ::::::::::: ::
query 3' GAGUAGUAGAACUGGAAUG 5'
query=miR6443, target=RO3G_03096, score=3, range=326-344, strand=
target 5' CUCCUCAUCUUGACCUGAC 3'
::: ::::::::::: ::
```

```
query 3' GAGUAGUAGAACUGGAAUG 5'
query=miR6443, target=RO3G_03816, score=3, range=806-824, strand=
target 5' AUCAUCAUCUUGAUCUUAG 3'
:::::::::::.::::
query 3' GAGUAGUAGAACUGGAAUG 5'
query=miR6443, target=RO3G_04768, score=3, range=692-711, strand=
target 5' CACAUUCAUCUUGACCUUGC 3'
: :: ::::::::::::.:
query 3' GAGU-AGUAGAACUGGAAUG 5'
query=miR6443, target=RO3G_17059, score=3.5, range=1243-1260, str
target 5' CUUGUCAUCUU-ACCUUAU 3'
::..::::::: ::::::.
query 3' GAGUAGUAGAACUGGAAUG 5'
query=miR6443, target=RO3G_05791, score=3.5, range=520-538, stran
target 5' CGAAACAUCUUGACCUUAU 3'
: : ::::::::::::.
query 3' GAGUAGUAGAACUGGAAUG 5'
query=miR6443, target=RO3G_02810, score=3.5, range=320-338, stran
target 5' UUCAUCAUCUUUAUCUUAC 3'
.:::::::::: :.::::
query 3' GAGUAGUAGAACUGGAAUG 5'
query=miR6443, target=RO3G_06631, score=3.5, range=4769-4788,
strand=-1
target 5' UUCAUCAAUCUUGGCCUUAA 3'
.::::: :::::.::::
query 3' GAGUAG-UAGAACUGGAAUG 5'
query=miR6443, target=RO3G_04064, score=3.5, range=81-98, strand=
target 5' CUC-UUAUCUUGAUCUUGC 3'
::: :.:::::::.:::.:
query 3' GAGUAGUAGAACUGGAAUG 5'
query=miR6443, target=RO3G_00042, score=3.5, range=68-86, strand=
target 5' UUCACCUUCUUGACCUUAC 3'
.::: : :::::::::::
query 3' GAGUAGUAGAACUGGAAUG 5'
query=miR6443, target=RO3G_03345, score=3.5, range=659-677, stran
target 5' UUGAGGAUCUUGACCUUAC 3'
.: : ::::::::::::
query 3' GAGUAGUAGAACUGGAAUG 5'
query=miR6443, target=RO3G_12122, score=3.5, range=659-677, stran
target 5' UUGAGGAUCUUGACCUUAC 3'
.: : ::::::::::::
query 3' GAGUAGUAGAACUGGAAUG 5'
query=miR6443, target=RO3G_08799, score=3.5, range=551-569, stran
target 5' UUAAGGAUCUUGACCUUAC 3'
.: : ::::::::::::
```

```
query 3' GAGUAGUAGAACUGGAAUG 5'
query=miR6443, target=RO3G_15841, score=3.5, range=551-569, stran
target 5' UUGAGGAUCUUGACCUUAC 3'
.: : ::::::::::::
query 3' GAGUAGUAGAACUGGAAUG 5'
query=miR6443, target=RO3G_03965, score=3.5, range=551-569, stran
target 5' UUGAGGAUCUUGACCUUAC 3'
.: : ::::::::::::
query 3' GAGUAGUAGAACUGGAAUG 5'
query=miR6443, target=RO3G_11989, score=3.5, range=719-738, stran
target 5' CAUACCGAUCUUGACCUUAC 3'
: .: : ::::::::::::
query 3' GAGUAG-UAGAACUGGAAUG 5'
query=miR6443, target=RO3G_03450, score=4, range=1170-1188, stran
target 5' UUCAUCAUCUUCACCUUGU 3'
.:::::::::: :::::..
query 3' GAGUAGUAGAACUGGAAUG 5'
query=miR6443, target=RO3G_00272, score=4, range=997-1016, strand
target 5' CUCAUCAUCUUGACUUAUAG 3'
::::::::::::::.: ::
query 3' GAGUAGUAGAACUGGA-AUG 5'
query=miR6443, target=RO3G_05381, score=4, range=991-1009, strand
target 5' GGCAUCGUCUUGACCUUAA 3'
::::.:::::::::::
query 3' GAGUAGUAGAACUGGAAUG 5'
query=miR6443, target=RO3G_07041, score=4, range=443-461, strand=
target 5' CUCAUCAUCUCGACCUAAC 3'
:::::::::: ::::: ::
query 3' GAGUAGUAGAACUGGAAUG 5'
query=miR6443, target=RO3G_09349, score=4, range=767-785, strand=
target 5' CUCACCAUCCUGACUUUAC 3'
:::: :::: ::::.::::
query 3' GAGUAGUAGAACUGGAAUG 5'
query=miR6443, target=RO3G_14715, score=4, range=74-92, strand=1
target 5' CUCCUCAUCUUGAUCUGAC 3'
::: :::::::::.:: ::
query 3' GAGUAGUAGAACUGGAAUG 5'
query=miR6443, target=RO3G_05981, score=4, range=1126-1144, stran
target 5' CUGUUCAUCUUGACUUUGC 3'
:: ::::::::::.::.:
query 3' GAGUAGUAGAACUGGAAUG 5'
query=miR6443, target=RO3G_09638, score=4, range=514-532, strand=
target 5' AUCAUGAUCUUGGCCUUAA 3'
:::: ::::::.:::::
query 3' GAGUAGUAGAACUGGAAUG 5'
```

```
query=miR6443, target=RO3G_12785, score=4, range=557-575, strand=
target 5' GUCUUCGUCUUGACCUUAA 3'
:: ::.:::::::::::
query 3' GAGUAGUAGAACUGGAAUG 5'
query=miR6443, target=RO3G_05879, score=4, range=596-614, strand=
target 5' CUCAAGAUCGUGACCUUAC 3'
:::: ::: :::::::::
query 3' GAGUAGUAGAACUGGAAUG 5'
query=miR6443, target=RO3G_02040, score=4, range=1487-1505, stran
target 5' CUACUCAUCCUGACCUUAC 3'
:: ::::: :::::::::
query 3' GAGUAGUAGAACUGGAAUG 5'
query=miR6443, target=RO3G_03735, score=4, range=544-563, strand=
target 5' CUCCUCUUCUUGACCUCUAC 3'
::: :: ::::::::: :::
query 3' GAGUAGUAGAACUGGA-AUG 5'
query=miR6443, target=RO3G_09820, score=4, range=482-499, strand=
target 5' UUCCUCAUCUU-ACCUUAU 3'
.:: ::::::: ::::::.
query 3' GAGUAGUAGAACUGGAAUG 5'
query=miR6443, target=RO3G_08962, score=4, range=38-55, strand=1
target 5' UUCUUUAUCUU-ACCUUAC 3'
.:: :.::::: :::::::
query 3' GAGUAGUAGAACUGGAAUG 5'
query=miR6443, target=RO3G_03905, score=4, range=1995-2013, stran
target 5' UUCAUCUUCUUGACCUUGU 3'
.::::: ::::::::::..
query 3' GAGUAGUAGAACUGGAAUG 5'
query=miR6443, target=RO3G_16310, score=4, range=1464-1482, stran
target 5' UUCAUUAUCUUGACCUCGC 3'
.::::.:::::::::: .:
query 3' GAGUAGUAGAACUGGAAUG 5'
query=miR6443, target=RO3G_14746, score=4, range=2478-2496, stran
target 5' UUCCUCAUCUUGAUCUUGU 3'
.:: :::::::::.:::..
query 3' GAGUAGUAGAACUGGAAUG 5'
query=miR6443, target=RO3G_05993, score=4, range=1603-1622, stran
target 5' GUGAUCAUCUUGACCUGUAC 3'
: :::::::::::: :::
query 3' GAGUAGUAGAACUGGA-AUG 5'
query=miR6443, target=RO3G_03668, score=4, range=74-93, strand=-1
target 5' GUCAUCCUCUUGAUCCUUAC 3'
::::: :::::: ::::::
query 3' GAGUAGUAGAACU-GGAAUG 5'
query=miR6443, target=RO3G_01474, score=4, range=494-511, strand=
```

target 5' CUU-UCAUCUUGACCAUAU 3'
::. :::::::::: ::.
query 3' GAGUAGUAGAACUGGAAUG 5'
query=miR6443, target=RO3G_08748, score=4, range=279-296, strand=
target 5' AUC-UCAUCUUGACCUUGA 3'
:: :::::::::::::.
query 3' GAGUAGUAGAACUGGAAUG 5'
query=miR3441-5p.2, target=RO3G_03247, score=4, range=222-243, strand=-1
target 5' AAGAGCAAUUUCAUUUUCUUCA 3'
::.::: :.:::::::::: :
query 3' UUUUCGCUGAAGUAAAAGAACU 5'
query=miR3441-5p.2, target=RO3G_15367, score=4, range=27-48, stra
target 5' AACAGUGACUUCAAUUUCUUGG 3'
:: ::.::::::: ::::::.
query 3' UUUUCGCUGAAGUAAAAGAACU 5'
query=miR6029, target=RO3G_13006, score=4, range=1448-1468, stran
target 5' UAGCUG-AUAUUCACAACCUUG 3'
:::::: :.:::::::::...
query 3' AUCGACUUGUAAGUGUUGGGGU 5'
query=miR7508, target=RO3G_05769, score=3.5, range=87-105, strand
target 5' UUUACUGAUUUGUUUUUUA 3'
..: :.::.:::::::::
query 3' GGAGGGCUGAACAAAAAAU 5'
query=miR7508, target=RO3G_10957, score=3.5, range=1098-1115, str
target 5' UCU-UUGGCUUGUUUUUUA 3'
.:: ..:.:::::::::
query 3' GGAGGGCUGAACAAAAAAU 5'
query=miR7508, target=RO3G_04288, score=3.5, range=1924-1942, strand=-1
target 5' CCACCUGGCUUGUUUUUUU 3'
:: ::.:.:::::::::
query 3' GGAGGGCUGAACAAAAAAU 5'
query=miR7508, target=RO3G_09636, score=3.5, range=1014-1032, strand=-1
target 5' AUUAUCGACUUGUUUUUUG 3'
.: .:::::::::::::.
query 3' GGAGGGCUGAACAAAAAAU 5'
query=miR7508, target=RO3G_10151, score=3.5, range=5295-5312, strand=-1
target 5' UCUCUC-ACUUGUUUUUUG 3'
.:::.: ::::::::::.
query 3' GGAGGGCUGAACAAAAAAU 5'
query=miR7508, target=RO3G_13300, score=4, range=1750-1768, stran
target 5' UCUCCUGAUUUGUCUUUUA 3'

```
.::::.::.:::: :::::
query 3' GGAGGGCUGAACAAAAAAU 5'
query=miR7508, target=RO3G_09480, score=4, range=1750-1768, stran
target 5' UCUCCUGAUUUGUCUUUUA 3'
.::::.::.:::: :::::
query 3' GGAGGGCUGAACAAAAAAU 5'
query=miR7508, target=RO3G_10448, score=4, range=58-75, strand=1
target 5' CCU-UUGAAUUGUUUUUUA 3'
::: ..:: :::::::::
query 3' GGAGGGCUGAACAAAAAAU 5'
query=miR7508, target=RO3G_03982, score=4, range=120-139, strand=
target 5' CCUCUUGCAUUUGUUUUUUA 3'
::::..: :.:::::::::
query 3' GGAGGGC-UGAACAAAAAAU 5'
query=miR7508, target=RO3G_15626, score=4, range=105-123, strand=
target 5' UUUCUUGACUUGCUUUUUA 3'
..::..:::::: ::::::
query 3' GGAGGGCUGAACAAAAAAU 5'
query=miR7508, target=RO3G_04628, score=4, range=138-156, strand=
target 5' CUUUUUGACUUCUUUUUUA 3'
:.:...::::: :::::::
query 3' GGAGGGCUGAACAAAAAAU 5'
query=miR7508, target=RO3G_15226, score=4, range=28-47, strand=-1
target 5' UCUUUCGACUUGUGUUUUUG 3'
.::..::::::::: :::::.
query 3' GGAGGGCUGAACA-AAAAAU 5'
query=miR7508, target=RO3G_16275, score=4, range=290-309, strand=
target 5' UCUUUUGACUUUGUUUUUUA 3'
.::...::: :::::::::
query 3' GGAGGGCUG-AACAAAAAAU 5'
query=miR7508, target=RO3G_10108, score=4, range=1871-1890, stran
target 5' ACUCUUGACUUGCUUUUUUA 3'
:::..:::::: :::::::
query 3' GGAGGGCUGAAC-AAAAAAU 5'
query=miR7508, target=RO3G_09472, score=4, range=369-387, strand=
target 5' CUUUGCGAUUUGUUUUUUC 3'
:.:. :::.:::::::::
query 3' GGAGGGCUGAACAAAAAAU 5'
query=miR7508, target=RO3G_12789, score=4, range=822-839, strand=
target 5' UUUCUUGACUU-UUUUUUA 3'
..::..::::: :::::::
query 3' GGAGGGCUGAACAAAAAAU 5'
query=miR7508, target=RO3G_00476, score=4, range=183-202, strand=
target 5' CCUGUCGACUUGAUUUUUUG 3'
::: .::::::: ::::::.
```

```
query 3' GGAGGGCUGAAC-AAAAAAU 5'
query=miR7508, target=RO3G_09556, score=4, range=768-787, strand=
target 5' CUUCCAUGACUUGUUUUCUA 3'
:.::: .:::::::::: ::
query 3' GGAGG-GCUGAACAAAAAAU 5'
query=miR7508, target=RO3G_06450, score=4, range=678-697, strand=
target 5' CCUGUUCGACUUGUUUUUGA 3'
::: ..::::::::::: :
query 3' GGA-GGGCUGAACAAAAAAU 5'
query=miR7508, target=RO3G_01661, score=4, range=1738-1755, stran
target 5' CCUUCGGAC-UGUUUUUUG 3'
:::.: ::: ::::::::.
query 3' GGAGGGCUGAACAAAAAAU 5'
query=miR7508, target=RO3G_00129, score=4, range=176-194, strand=
target 5' CCUGGCGACCUGUUUUUUA 3'
::: :::: :::::::::
query 3' GGAGGGCUGAACAAAAAAU 5'
query=miR1061-3p, target=RO3G_19004, score=0, range=797-814, stra
target 5' CAUUAUUCCAUGCUAACA 3'
::::::::::::::::::
query 3' GUAAUAAGGUACGAUUGU 5'
query=miR1061-3p, target=RO3G_19001, score=0, range=501-518, stra
target 5' CAUUAUUCCAUGCUAACA 3'
::::::::::::::::::
query 3' GUAAUAAGGUACGAUUGU 5'
query=miR1061-3p, target=RO3G_10004, score=2, range=230-246, stra
target 5' CAUU-UUCCAUGCUAAUA 3'
:::: :::::::::::.:
query 3' GUAAUAAGGUACGAUUGU 5'
query=miR1061-3p, target=RO3G_07815, score=3, range=738-754, stra
target 5' CAUUAUUCCAUGC-AAUA 3'
:::::::::::::: ::.:
query 3' GUAAUAAGGUACGAUUGU 5'
query=miR1061-3p, target=RO3G_09588, score=3, range=663-679, stra
target 5' CAGU-UUCCAUGUUAACA 3'
:: : :::::::.:::::
query 3' GUAAUAAGGUACGAUUGU 5'
query=miR1061-3p, target=RO3G_08463, score=3, range=947-963, stra
target 5' CAGU-UUCCAUGCUAACU 3'
:: : ::::::::::::
query 3' GUAAUAAGGUACGAUUGU 5'
query=miR1061-3p, target=RO3G_13503, score=3.5, range=1240-1257,
strand=1
target 5' UAUUAUUUCAUGCUGAUA 3'
.::::::.::::::.:.:
```

query 3' GUAAUAAGGUACGAUUGU 5'
query=miR1061-3p, target=RO3G_07650, score=3.5, range=100-118, strand=1
target 5' UAUUAUUCCACUGCUGACA 3'
.::::::::: ::::.:::
query 3' GUAAUAAGGU-ACGAUUGU 5'
query=miR1061-3p, target=RO3G_12113, score=3.5, range=2253-2270, strand=-1
target 5' GAUUGUUUCAUGCUAAUA 3'
:::.::.::::::::.:
query 3' GUAAUAAGGUACGAUUGU 5'
query=miR1061-3p, target=RO3G_01111, score=3.5, range=273-289, strand=-1
target 5' UCUUAUUCCAUGCU-ACA 3'
. ::::::::::: :::
query 3' GUAAUAAGGUACGAUUGU 5'
query=miR1061-3p, target=RO3G_05753, score=4, range=110-127, stra
target 5' UGUUAUUCCUUGUUAACA 3'
..::::::: ::.:::::
query 3' GUAAUAAGGUACGAUUGU 5'
query=miR1061-3p, target=RO3G_00027, score=4, range=173-190, stra
target 5' AAUUAUUCUGUGCUAACU 3'
:::::::..:::::::
query 3' GUAAUAAGGUACGAUUGU 5'
query=miR1061-3p, target=RO3G_10806, score=4, range=1253-1270, strand=1
target 5' CAGGAUUCCAUGCUGGCA 3'
:: ::::::::::..::
query 3' GUAAUAAGGUACGAUUGU 5'
query=miR1061-3p, target=RO3G_07773, score=4, range=811-828, stra
target 5' CAUAUUUCUAUGCUAGCA 3'
::: :::.::::::.::
query 3' GUAAUAAGGUACGAUUGU 5'
query=miR1061-3p, target=RO3G_08438, score=4, range=2454-2470, strand=1
target 5' CA-UAUUCAAUGCUAACC 3'
:: ::::: ::::::::
query 3' GUAAUAAGGUACGAUUGU 5'
query=miR1061-3p, target=RO3G_00213, score=4, range=2244-2262, strand=-1
target 5' CAUUAUUCCAUAGCCAACA 3'
:::::::::: :: ::::
query 3' GUAAUAAGGUA-CGAUUGU 5'
query=miR1061-3p, target=RO3G_02280, score=4, range=372-389, stra
target 5' UAUUUUUCCAUGCUAUCG 3'

```
.::: ::::::::: :.
query 3' GUAAUAAGGUACGAUUGU 5'
query=miR1061-3p, target=RO3G_04817, score=4, range=650-667, stra
target 5' CCUUAUUCCCUGCUAACU 3'
: ::::::: :::::::
query 3' GUAAUAAGGUACGAUUGU 5'
query=miR1061-3p, target=RO3G_12684, score=4, range=336-352, stra
target 5' CAGU-UUCCAUGUUGACA 3'
:: : :::::::.:.:::
query 3' GUAAUAAGGUACGAUUGU 5'
query=miR1061-3p, target=RO3G_09476, score=4, range=315-331, stra
target 5' UGUUCUUCCAUGCU-ACA 3'
..:: ::::::::: :::
query 3' GUAAUAAGGUACGAUUGU 5'
query=miR1061-3p, target=RO3G_04299, score=4, range=258-276, stra
target 5' CACUUCUUCCUUGCUAACA 3'
:: :: :::: ::::::::
query 3' GU-AAUAAGGUACGAUUGU 5'
query=miR1061-3p, target=RO3G_08883, score=4, range=114-131, stra
target 5' CAGCCUUCCAUGCUGACA 3'
:: :::::::::.:::
query 3' GUAAUAAGGUACGAUUGU 5'
query=miR1061-3p, target=RO3G_05448, score=4, range=1535-1551,
strand=-1
target 5' CACU-UUCAAUGCUAACA 3'
:: : ::: :::::::::
query 3' GUAAUAAGGUACGAUUGU 5'
query=miR1061-3p, target=RO3G_08652, score=4, range=186-202, stra
target 5' CACU-UUCCAUGCCAACA 3'
:: : :::::::: ::::
query 3' GUAAUAAGGUACGAUUGU 5'
query=miR5821, target=RO3G_17015, score=0, range=1022-1042, stran
target 5' CUCUACCAUCGUCUCCGUCCA 3'
:::::::::::::::::::::
query 3' GAGAUGGUAGCAGAGGCAGGU 5'
query=miR5821, target=RO3G_10929, score=3.5, range=782-801, stran
target 5' CUCU-CCGUCGUCUCCGUUCU 3'
:::: ::.:::::::::.:
query 3' GAGAUGGUAGCAGAGGCAGGU 5'
query=miR5821, target=RO3G_01864, score=3.5, range=782-801, stran
target 5' CUCU-CCGUCGUCUCCGUUCC 3'
:::: ::.:::::::::.:
query 3' GAGAUGGUAGCAGAGGCAGGU 5'
query=miR5821, target=RO3G_11069, score=3.5, range=782-801, stran
target 5' CUCU-CCGUCGUCUCCGUUCC 3'
```

```
:::: ::.:::::::::.:
query 3' GAGAUGGUAGCAGAGGCAGGU 5'
query=miR5821, target=RO3G_05170, score=3.5, range=782-801, stran
target 5' CUCU-CCGUCGUCUCCGUUCU 3'
:::: ::.:::::::::.:
query 3' GAGAUGGUAGCAGAGGCAGGU 5'
query=miR5821, target=RO3G_04495, score=4, range=489-510, strand=
target 5' CCCUACAAUCGUCUCUCGUCCA 3'
: :::: :::::::: ::::::
query 3' GAGAUGGUAGCAGAG-GCAGGU 5'
query=miR5821, target=RO3G_10279, score=4, range=222-242, strand=
target 5' CGGUAGCAUCGUCUCUGUCCA 3'
: :: :::::::::.:::::
query 3' GAGAUGGUAGCAGAGGCAGGU 5'
query=miR5821, target=RO3G_14257, score=4, range=1135-1155, stran
target 5' UUCCACUAUUGUCUCUGUCCA 3'
.:: ::.::.:::::.:::::
query 3' GAGAUGGUAGCAGAGGCAGGU 5'
query=miR1048-3p, target=RO3G_06442, score=1, range=4680-4700,
strand=-1
target 5' CCAGGAACAUAAGCGUAGACA 3'
: ::::::::::::::::::
query 3' GAUCCUUGUAUUCGCAUCUGU 5'
query=miR1048-3p, target=RO3G_09965, score=1, range=4023-4043,
strand=-1
target 5' CCAGGAACAUAAGCGUAGACA 3'
: ::::::::::::::::::
query 3' GAUCCUUGUAUUCGCAUCUGU 5'
query=miR1048-3p, target=RO3G_13920, score=1, range=4023-4043,
strand=-1
target 5' CCAGGAACAUAAGCGUAGACA 3'
: ::::::::::::::::::
query 3' GAUCCUUGUAUUCGCAUCUGU 5'
query=miR1048-3p, target=RO3G_03547, score=1, range=4023-4043,
strand=-1
target 5' CCAGGAACAUAAGCGUAGACA 3'
: ::::::::::::::::::
query 3' GAUCCUUGUAUUCGCAUCUGU 5'
query=miR1048-3p, target=RO3G_13867, score=1, range=3939-3959,
strand=-1
target 5' CCAGGAACAUAAGCGUAGACA 3'
: ::::::::::::::::::
query 3' GAUCCUUGUAUUCGCAUCUGU 5'
query=miR1048-3p, target=RO3G_02959, score=1, range=4023-4043,
strand=-1
```

target 5' CCAGGAACAUAAGCGUAGACA 3'
: :::::::::::::::::::
query 3' GAUCCUUGUAUUCGCAUCUGU 5'
query=miR1048-3p, target=RO3G_15250, score=1, range=3978-3998, strand=-1
target 5' CCAGGAACAUAAGCGUAGACA 3'
: :::::::::::::::::::
query 3' GAUCCUUGUAUUCGCAUCUGU 5'
query=miR1048-3p, target=RO3G_17371, score=1, range=3978-3998, strand=-1
target 5' CCAGGAACAUAAGCGUAGACA 3'
: :::::::::::::::::::
query 3' GAUCCUUGUAUUCGCAUCUGU 5'
query=miR1048-3p, target=RO3G_06427, score=1, range=3615-3635, strand=-1
target 5' CCAGGAACAUAAGCGUAGACA 3'
: :::::::::::::::::::
query 3' GAUCCUUGUAUUCGCAUCUGU 5'
query=miR1048-3p, target=RO3G_17326, score=1, range=3855-3875, strand=-1
target 5' CCAGGAACAUAAGCGUAGACA 3'
: :::::::::::::::::::
query 3' GAUCCUUGUAUUCGCAUCUGU 5'
query=miR1048-3p, target=RO3G_06428, score=1, range=3117-3137, strand=-1
target 5' CCAGGAACAUAAGCGUAGACA 3'
: :::::::::::::::::::
query 3' GAUCCUUGUAUUCGCAUCUGU 5'
query=miR1048-3p, target=RO3G_13749, score=1, range=2532-2552, strand=-1
target 5' CCAGGAACAUAAGCGUAGACA 3'
: :::::::::::::::::::
query 3' GAUCCUUGUAUUCGCAUCUGU 5'
query=miR1048-3p, target=RO3G_05619, score=1, range=2166-2186, strand=-1
target 5' CCAGGAACAUAAGCGUAGACA 3'
: :::::::::::::::::::
query 3' GAUCCUUGUAUUCGCAUCUGU 5'
query=miR1048-3p, target=RO3G_14355, score=1, range=2229-2249, strand=-1
target 5' CCAGGAACAUAAGCGUAGACA 3'
: :::::::::::::::::::
query 3' GAUCCUUGUAUUCGCAUCUGU 5'
query=miR1048-3p, target=RO3G_01613, score=1, range=2220-2240, strand=-1

```
target 5' CCAGGAACAUAAGCGUAGACA 3'
: ::::::::::::::::::
query 3' GAUCCUUGUAUUCGCAUCUGU 5'
query=miR1048-3p, target=RO3G_01330, score=1, range=1257-1277,
strand=-1
target 5' CCAGGAACAUAAGCGUAGACA 3'
: ::::::::::::::::::
query 3' GAUCCUUGUAUUCGCAUCUGU 5'
query=miR1048-3p, target=RO3G_11776, score=1, range=1113-1133,
strand=-1
target 5' CCAGGAACAUAAGCGUAGACA 3'
: ::::::::::::::::::
query 3' GAUCCUUGUAUUCGCAUCUGU 5'
query=miR1048-3p, target=RO3G_02222, score=1, range=1077-1097,
strand=-1
target 5' CCAGGAACAUAAGCGUAGACA 3'
: ::::::::::::::::::
query 3' GAUCCUUGUAUUCGCAUCUGU 5'
query=miR1048-3p, target=RO3G_10558, score=1, range=1776-1796,
strand=-1
target 5' CCAGGAACAUAAGCGUAGACA 3'
: ::::::::::::::::::
query 3' GAUCCUUGUAUUCGCAUCUGU 5'
query=miR1048-3p, target=RO3G_04115, score=1, range=966-986, stra
target 5' CCAGGAACAUAAGCGUAGACA 3'
: ::::::::::::::::::
query 3' GAUCCUUGUAUUCGCAUCUGU 5'
query=miR1048-3p, target=RO3G_17404, score=1, range=651-671, stra
target 5' CCAGGAACAUAAGCGUAGACA 3'
: ::::::::::::::::::
query 3' GAUCCUUGUAUUCGCAUCUGU 5'
query=miR1048-3p, target=RO3G_07668, score=1, range=606-626, stra
target 5' CCAGGAACAUAAGCGUAGACA 3'
: ::::::::::::::::::
query 3' GAUCCUUGUAUUCGCAUCUGU 5'
query=miR1048-3p, target=RO3G_02691, score=2, range=7512-7532,
strand=-1
target 5' CCAGGAACAUAGGCGUAGACA 3'
: ::::::::.:::::::::
query 3' GAUCCUUGUAUUCGCAUCUGU 5'
query=miR482d-3p, target=RO3G_13222, score=0, range=676-697, stra
target 5' UGGCAUAGGUAGGGUCGGCCAA 3'
::::::::::::::::::::::
query 3' ACCGUAUCCAUCCCAGCCGGUU 5'
query=miR482d-3p, target=RO3G_14778, score=0, range=715-736, stra
```

target 5' UGGCAUAGGUAGGGUCGGCCAA 3'
::::::::::::::::::::::
query 3' ACCGUAUCCAUCCCAGCCGGUU 5'
query=miR482d-3p, target=RO3G_11967, score=1, range=724-745, stra
target 5' AGGCAUAGGUAGGGUCGGCCAA 3'
:::::::::::::::::::::
query 3' ACCGUAUCCAUCCCAGCCGGUU 5'
query=miR482d-3p, target=RO3G_14350, score=1, range=724-745, stra
target 5' AGGCAUAGGUAGGGUCGGCCAA 3'
:::::::::::::::::::::
query 3' ACCGUAUCCAUCCCAGCCGGUU 5'
query=miR482d-3p, target=RO3G_13225, score=1, range=724-745, stra
target 5' AGGCAUAGGUAGGGUCGGCCAA 3'
:::::::::::::::::::::
query 3' ACCGUAUCCAUCCCAGCCGGUU 5'
query=miR482d-3p, target=RO3G_16818, score=3.5, range=454-475,
strand=-1
target 5' AGGCGUAGGUUGGGUCGGCCAA 3'
:::.::::: :::::::::::
query 3' ACCGUAUCCAUCCCAGCCGGUU 5'
query=miR482d-3p, target=RO3G_16799, score=3.5, range=724-745,
strand=-1
target 5' AGGCAUAGGUAGGGUCGGCAAG 3'
:::::::::::::::::: :.
query 3' ACCGUAUCCAUCCCAGCCGGUU 5'
query=miR482d-3p, target=RO3G_05639, score=3.5, range=724-745,
strand=-1
target 5' AGGCAUAGGUAGGGUCGGCAAG 3'
:::::::::::::::::: :.
query 3' ACCGUAUCCAUCCCAGCCGGUU 5'
query=miR482d-3p, target=RO3G_03991, score=3.5, range=724-745,
strand=-1
target 5' AGGCAUAGGUAGGGUCGGCAAG 3'
:::::::::::::::::: :.
query 3' ACCGUAUCCAUCCCAGCCGGUU 5'
query=miR482d-3p, target=RO3G_17015, score=4, range=724-745, stra
target 5' AGGCGUAGGCAGGGUCGGCCAG 3'
:::.:::: :::::::::::.
query 3' ACCGUAUCCAUCCCAGCCGGUU 5'
query=miR6255, target=RO3G_07788, score=0, range=928-949, strand=
target 5' ACUCAUCGUCCAUUCUUUCCCA 3'
::::::::::::::::::::::
query 3' UGAGUAGCAGGUAAGAAAGGGU 5'
query=miR6255, target=RO3G_14722, score=0, range=979-1000, strand
target 5' ACUCAUCGUCCAUUCUUUCCCA 3'

```
:::::::::::::::::::::
query 3' UGAGUAGCAGGUAAGAAAGGGU 5'
query=miR6255, target=RO3G_05181, score=0, range=979-1000, strand
target 5' ACUCAUCGUCCAUUCUUUCCCA 3'
:::::::::::::::::::::
query 3' UGAGUAGCAGGUAAGAAAGGGU 5'
query=miR6255, target=RO3G_13664, score=0, range=979-1000, strand
target 5' ACUCAUCGUCCAUUCUUUCCCA 3'
:::::::::::::::::::::
query 3' UGAGUAGCAGGUAAGAAAGGGU 5'
query=miR6255, target=RO3G_02354, score=0, range=979-1000, strand
target 5' ACUCAUCGUCCAUUCUUUCCCA 3'
:::::::::::::::::::::
query 3' UGAGUAGCAGGUAAGAAAGGGU 5'
query=miR6255, target=RO3G_08906, score=0, range=979-1000, strand
target 5' ACUCAUCGUCCAUUCUUUCCCA 3'
:::::::::::::::::::::
query 3' UGAGUAGCAGGUAAGAAAGGGU 5'
query=miR6255, target=RO3G_13903, score=0, range=979-1000, strand
target 5' ACUCAUCGUCCAUUCUUUCCCA 3'
:::::::::::::::::::::
query 3' UGAGUAGCAGGUAAGAAAGGGU 5'
query=miR6255, target=RO3G_05303, score=0, range=979-1000, strand
target 5' ACUCAUCGUCCAUUCUUUCCCA 3'
:::::::::::::::::::::
query 3' UGAGUAGCAGGUAAGAAAGGGU 5'
query=miR6255, target=RO3G_05194, score=0, range=979-1000, strand
target 5' ACUCAUCGUCCAUUCUUUCCCA 3'
:::::::::::::::::::::
query 3' UGAGUAGCAGGUAAGAAAGGGU 5'
query=miR6255, target=RO3G_13672, score=0, range=979-1000, strand
target 5' ACUCAUCGUCCAUUCUUUCCCA 3'
:::::::::::::::::::::
query 3' UGAGUAGCAGGUAAGAAAGGGU 5'
query=miR6255, target=RO3G_11567, score=1, range=1636-1657, stran
target 5' ACUCAUCAUCCAUUCUUUCCCA 3'
::::::: :::::::::::::
query 3' UGAGUAGCAGGUAAGAAAGGGU 5'
query=miR6255, target=RO3G_01923, score=1, range=928-949, strand=
target 5' ACUCAUCAUCCAUUCUUUCCCA 3'
::::::: :::::::::::::
query 3' UGAGUAGCAGGUAAGAAAGGGU 5'
query=miR6255, target=RO3G_09419, score=1, range=799-820, strand=
target 5' ACUCAUCAUCCAUUCUUUCCCA 3'
::::::: :::::::::::::
```

```
query 3' UGAGUAGCAGGUAAGAAAGGGU 5'
query=miR6255, target=RO3G_11486, score=1, range=688-709, strand=
target 5' ACUCAUCAUCCAUUCUUUCCCA 3'
::::::: ::::::::::::::
query 3' UGAGUAGCAGGUAAGAAAGGGU 5'
query=miR6255, target=RO3G_04104, score=1, range=676-697, strand=
target 5' ACUCAUCAUCCAUUCUUUCCCA 3'
::::::: ::::::::::::::
query 3' UGAGUAGCAGGUAAGAAAGGGU 5'
query=miR6255, target=RO3G_04110, score=1, range=676-697, strand=
target 5' ACUCAUCAUCCAUUCUUUCCCA 3'
::::::: ::::::::::::::
query 3' UGAGUAGCAGGUAAGAAAGGGU 5'
query=miR6255, target=RO3G_05535, score=1, range=364-385, strand=
target 5' ACUCAUCAUCCAUUCUUUCCCA 3'
::::::: ::::::::::::::
query 3' UGAGUAGCAGGUAAGAAAGGGU 5'
query=miR6255, target=RO3G_11757, score=1, range=364-385, strand=
target 5' ACUCAUCAUCCAUUCUUUCCCA 3'
::::::: ::::::::::::::
query 3' UGAGUAGCAGGUAAGAAAGGGU 5'
query=miR6255, target=RO3G_11765, score=1, range=364-385, strand=
target 5' ACUCAUCAUCCAUUCUUUCCCA 3'
::::::: ::::::::::::::
query 3' UGAGUAGCAGGUAAGAAAGGGU 5'
query=miR6255, target=RO3G_10644, score=1, range=364-385, strand=
target 5' ACUCAUCAUCCAUUCUUUCCCA 3'
::::::: ::::::::::::::
query 3' UGAGUAGCAGGUAAGAAAGGGU 5'
query=miR6255, target=RO3G_12139, score=1, range=352-373, strand=
target 5' ACUCAUCAUCCAUUCUUUCCCA 3'
::::::: ::::::::::::::
query 3' UGAGUAGCAGGUAAGAAAGGGU 5'
query=miR6255, target=RO3G_05616, score=1, range=268-289, strand=
target 5' ACUCAUCAUCCAUUCUUUCCCA 3'
::::::: ::::::::::::::
query 3' UGAGUAGCAGGUAAGAAAGGGU 5'
query=miR6255, target=RO3G_01528, score=1.5, range=547-568, stran
target 5' ACUCAUCAUCCAUUCUUUCCCG 3'
::::::: :::::::::::::.
query 3' UGAGUAGCAGGUAAGAAAGGGU 5'
query=miR6255, target=RO3G_01518, score=1.5, range=352-373, stran
target 5' ACUCAUCAUCCAUUCUUUCCCG 3'
::::::: :::::::::::::.
query 3' UGAGUAGCAGGUAAGAAAGGGU 5'
```

```
query=miR6255, target=RO3G_14467, score=2, range=508-529, strand=
target 5' ACUCAUCAUCUAUUCUUUCCCA 3'
::::::: ::.:::::::::::
query 3' UGAGUAGCAGGUAAGAAAGGGU 5'
query=miR6255, target=RO3G_05603, score=2, range=238-259, strand=
target 5' ACUCAUCAUUCAUUCUUUCCCA 3'
::::::: :.::::::::::::
query 3' UGAGUAGCAGGUAAGAAAGGGU 5'
query=miR6255, target=RO3G_08684, score=3, range=397-418, strand=
target 5' AAACAUCAUCCAUUCUUUCCCA 3'
: :::: :::::::::::::
query 3' UGAGUAGCAGGUAAGAAAGGGU 5'
query=miR6255, target=RO3G_14050, score=4, range=815-835, strand=
target 5' ACUCAUC-UUCAUUCUUUACCA 3'
::::::: :.:::::::: :::
query 3' UGAGUAGCAGGUAAGAAAGGGU 5'
query=miR9409, target=RO3G_11521, score=0, range=585-604, strand=
target 5' GAAAAUGAACUAUGACAAAA 3'
::::::::::::::::::::
query 3' CUUUUACUUGAUACUGUUUU 5'
query=miR9409, target=RO3G_15711, score=0, range=1623-1642, stran
target 5' GAAAAUGAACUAUGACAAAA 3'
::::::::::::::::::::
query 3' CUUUUACUUGAUACUGUUUU 5'
query=miR9409, target=RO3G_09539, score=0, range=1623-1642, stran
target 5' GAAAAUGAACUAUGACAAAA 3'
::::::::::::::::::::
query 3' CUUUUACUUGAUACUGUUUU 5'
query=miR9409, target=RO3G_13796, score=0, range=1623-1642, stran
target 5' GAAAAUGAACUAUGACAAAA 3'
::::::::::::::::::::
query 3' CUUUUACUUGAUACUGUUUU 5'
query=miR9409, target=RO3G_01512, score=3, range=1611-1630, stran
target 5' GAAAAUGAAUUACGACAAAA 3'
:::::::::.:: :::::::
query 3' CUUUUACUUGAUACUGUUUU 5'
query=miR9409, target=RO3G_11616, score=3.5, range=2292-2311, str
target 5' AAAAGAAAACUAUGACAAAA 3'
:::. :::::::::::::
query 3' CUUUUACUUGAUACUGUUUU 5'
query=miR9409, target=RO3G_03121, score=4, range=1614-1633, stran
target 5' CAAAAUGAAUUAUGAUAAGA 3'
::::::::.:::::.::.:
query 3' CUUUUACUUGAUACUGUUUU 5'
query=miR9409, target=RO3G_14532, score=4, range=1614-1633, stran
```

```
target 5' CAAAAUGAAUUAUGAUAAGA 3'
::::::::.:::::.::.:
query 3' CUUUUACUUGAUACUGUUUU 5'
query=miR9409, target=RO3G_13807, score=4, range=876-895, strand=
target 5' CAAAAUGAAUUAUGAUAAGA 3'
::::::::.:::::.::.:
query 3' CUUUUACUUGAUACUGUUUU 5'
query=miR9409, target=RO3G_01426, score=4, range=876-895, strand=
target 5' CAAAAUGAAUUAUGAUAAGA 3'
::::::::.:::::.::.:
query 3' CUUUUACUUGAUACUGUUUU 5'
query=miR9409, target=RO3G_16107, score=4, range=1972-1991, stran
target 5' GAAAAUCAACUGUUACAAAA 3'
:::::: ::::.: ::::::
query 3' CUUUUACUUGAUACUGUUUU 5'
query=miR9409, target=RO3G_07873, score=4, range=829-848, strand=
target 5' GUUGAUGAACUAUGGCAAAG 3'
: .::::::::::.::::.
query 3' CUUUUACUUGAUACUGUUUU 5'
query=miR9409, target=RO3G_13728, score=4, range=817-836, strand=
target 5' GUUGAUGAACUAUGGCAAAG 3'
: .::::::::::.::::.
query 3' CUUUUACUUGAUACUGUUUU 5'
query=miR9409, target=RO3G_05922, score=4, range=328-347, strand=
target 5' GUUGAUGAACUAUGGCAAAG 3'
: .::::::::::.::::.
query 3' CUUUUACUUGAUACUGUUUU 5'
query=miR9409, target=RO3G_05273, score=4, range=313-332, strand=
target 5' GUUGAUGAACUAUGGCAAAG 3'
: .::::::::::.::::.
query 3' CUUUUACUUGAUACUGUUUU 5'
query=miR9409, target=RO3G_16521, score=4, range=640-659, strand=
target 5' GAAAAAGAACUAAGACAAAU 3'
::::: :::::: ::::::
query 3' CUUUUACUUGAUACUGUUUU 5'
query=miR9409, target=RO3G_06371, score=4, range=570-589, strand=
target 5' UGUAGCGAACUAUGACAAAA 3'
. :. :::::::::::::
query 3' CUUUUACUUGAUACUGUUUU 5'
query=miR9409, target=RO3G_03094, score=4, range=1883-1903, stran
target 5' GAAACACGAACUAUCACAAAA 3'
:::: : ::::::: ::::::
query 3' CUUU-UACUUGAUACUGUUUU 5'
query=miR9409, target=RO3G_17057, score=4, range=440-459, strand=
target 5' GCAAAUGAAUUGUGAUAAAA 3'
```

```
: :::::::.:.:::.::::
query 3' CUUUUACUUGAUACUGUUUU 5'
query=miR6194, target=RO3G_10233, score=0, range=2142-2164, stran
target 5' CUCGUGUGUCAAGAUCCCUACUA 3'
:::::::::::::::::::::::
query 3' GAGCACACAGUUCUAGGGAUGAU 5'
query=miR6194, target=RO3G_01744, score=0, range=2145-2167, stran
target 5' CUCGUGUGUCAAGAUCCCUACUA 3'
:::::::::::::::::::::::
query 3' GAGCACACAGUUCUAGGGAUGAU 5'
query=miR6194, target=RO3G_04025, score=0, range=2142-2164, stran
target 5' CUCGUGUGUCAAGAUCCCUACUA 3'
:::::::::::::::::::::::
query 3' GAGCACACAGUUCUAGGGAUGAU 5'
query=miR5059, target=RO3G_13235, score=4, range=1381-1399, stran
target 5' UGUAG-GCUGUCCAGGAUCG 3'
:: : ::::.:::::::::
query 3' CCACCACGACGGGUCCUAGC 5'
query=miR415, target=RO3G_07738, score=0, range=179-200, strand=1
target 5' CUGCUUCUGCUUCUGUUCCUUU 3'
::::::::::::::::::::::
query 3' GACGAAGACGAAGACAAGGAAA 5'
query=miR415, target=RO3G_11537, score=0, range=179-200, strand=1
target 5' CUGCUUCUGCUUCUGUUCCUUU 3'
::::::::::::::::::::::
query 3' GACGAAGACGAAGACAAGGAAA 5'
query=miR415, target=RO3G_11236, score=0, range=179-200, strand=1
target 5' CUGCUUCUGCUUCUGUUCCUUU 3'
::::::::::::::::::::::
query 3' GACGAAGACGAAGACAAGGAAA 5'
query=miR415, target=RO3G_17248, score=0, range=167-188, strand=1
target 5' CUGCUUCUGCUUCUGUUCCUUU 3'
::::::::::::::::::::::
query 3' GACGAAGACGAAGACAAGGAAA 5'
query=miR415, target=RO3G_10041, score=0, range=167-188, strand=1
target 5' CUGCUUCUGCUUCUGUUCCUUU 3'
::::::::::::::::::::::
query 3' GACGAAGACGAAGACAAGGAAA 5'
query=miR415, target=RO3G_06730, score=0, range=137-158, strand=1
target 5' CUGCUUCUGCUUCUGUUCCUUU 3'
::::::::::::::::::::::
query 3' GACGAAGACGAAGACAAGGAAA 5'
query=miR415, target=RO3G_07981, score=0, range=137-158, strand=1
target 5' CUGCUUCUGCUUCUGUUCCUUU 3'
::::::::::::::::::::::
```

```
query 3' GACGAAGACGAAGACAAGGAAA 5'
query=miR415, target=RO3G_15897, score=0, range=137-158, strand=1
target 5' CUGCUUCUGCUUCUGUUCCUUU 3'
::::::::::::::::::::::
query 3' GACGAAGACGAAGACAAGGAAA 5'
query=miR415, target=RO3G_07720, score=0, range=137-158, strand=1
target 5' CUGCUUCUGCUUCUGUUCCUUU 3'
::::::::::::::::::::::
query 3' GACGAAGACGAAGACAAGGAAA 5'
query=miR415, target=RO3G_09602, score=0, range=137-158, strand=1
target 5' CUGCUUCUGCUUCUGUUCCUUU 3'
::::::::::::::::::::::
query 3' GACGAAGACGAAGACAAGGAAA 5'
query=miR415, target=RO3G_07306, score=0, range=137-158, strand=1
target 5' CUGCUUCUGCUUCUGUUCCUUU 3'
::::::::::::::::::::::
query 3' GACGAAGACGAAGACAAGGAAA 5'
query=miR415, target=RO3G_04211, score=0, range=137-158, strand=1
target 5' CUGCUUCUGCUUCUGUUCCUUU 3'
::::::::::::::::::::::
query 3' GACGAAGACGAAGACAAGGAAA 5'
query=miR415, target=RO3G_07696, score=0, range=137-158, strand=1
target 5' CUGCUUCUGCUUCUGUUCCUUU 3'
::::::::::::::::::::::
query 3' GACGAAGACGAAGACAAGGAAA 5'
query=miR415, target=RO3G_14551, score=0, range=137-158, strand=1
target 5' CUGCUUCUGCUUCUGUUCCUUU 3'
::::::::::::::::::::::
query 3' GACGAAGACGAAGACAAGGAAA 5'
query=miR415, target=RO3G_09929, score=0, range=137-158, strand=1
target 5' CUGCUUCUGCUUCUGUUCCUUU 3'
::::::::::::::::::::::
query 3' GACGAAGACGAAGACAAGGAAA 5'
query=miR415, target=RO3G_15147, score=0, range=113-134, strand=1
target 5' CUGCUUCUGCUUCUGUUCCUUU 3'
::::::::::::::::::::::
query 3' GACGAAGACGAAGACAAGGAAA 5'
query=miR415, target=RO3G_10775, score=0, range=167-188, strand=1
target 5' CUGCUUCUGCUUCUGUUCCUUU 3'
::::::::::::::::::::::
query 3' GACGAAGACGAAGACAAGGAAA 5'
query=miR415, target=RO3G_03584, score=0, range=95-116, strand=1
target 5' CUGCUUCUGCUUCUGUUCCUUU 3'
::::::::::::::::::::::
query 3' GACGAAGACGAAGACAAGGAAA 5'
```

```
query=miR415, target=RO3G_09603, score=0, range=179-200, strand=1
target 5' CUGCUUCUGCUUCUGUUCCUUU 3'
::::::::::::::::::::::
query 3' GACGAAGACGAAGACAAGGAAA 5'
query=miR415, target=RO3G_15890, score=0, range=161-182, strand=1
target 5' CUGCUUCUGCUUCUGUUCCUUU 3'
::::::::::::::::::::::
query 3' GACGAAGACGAAGACAAGGAAA 5'
query=miR415, target=RO3G_13146, score=0, range=161-182, strand=1
target 5' CUGCUUCUGCUUCUGUUCCUUU 3'
::::::::::::::::::::::
query 3' GACGAAGACGAAGACAAGGAAA 5'
query=miR415, target=RO3G_05425, score=0, range=167-188, strand=1
target 5' CUGCUUCUGCUUCUGUUCCUUU 3'
::::::::::::::::::::::
query 3' GACGAAGACGAAGACAAGGAAA 5'
query=miR415, target=RO3G_17256, score=0, range=161-182, strand=1
target 5' CUGCUUCUGCUUCUGUUCCUUU 3'
::::::::::::::::::::::
query 3' GACGAAGACGAAGACAAGGAAA 5'
query=miR415, target=RO3G_16137, score=0, range=161-182, strand=1
target 5' CUGCUUCUGCUUCUGUUCCUUU 3'
::::::::::::::::::::::
query 3' GACGAAGACGAAGACAAGGAAA 5'
query=miR415, target=RO3G_17350, score=0, range=161-182, strand=1
target 5' CUGCUUCUGCUUCUGUUCCUUU 3'
::::::::::::::::::::::
query 3' GACGAAGACGAAGACAAGGAAA 5'
query=miR415, target=RO3G_10218, score=0, range=167-188, strand=1
target 5' CUGCUUCUGCUUCUGUUCCUUU 3'
::::::::::::::::::::::
query 3' GACGAAGACGAAGACAAGGAAA 5'
query=miR415, target=RO3G_09182, score=0, range=161-182, strand=1
target 5' CUGCUUCUGCUUCUGUUCCUUU 3'
::::::::::::::::::::::
query 3' GACGAAGACGAAGACAAGGAAA 5'
query=miR415, target=RO3G_16748, score=0, range=137-158, strand=1
target 5' CUGCUUCUGCUUCUGUUCCUUU 3'
::::::::::::::::::::::
query 3' GACGAAGACGAAGACAAGGAAA 5'
query=miR415, target=RO3G_02228, score=0, range=137-158, strand=1
target 5' CUGCUUCUGCUUCUGUUCCUUU 3'
::::::::::::::::::::::
query 3' GACGAAGACGAAGACAAGGAAA 5'
query=miR415, target=RO3G_13272, score=0, range=220-241, strand=1
```

```
target 5' CUGCUUCUGCUUCUGUUCCUUU 3'
::::::::::::::::::::::
query 3' GACGAAGACGAAGACAAGGAAA 5'
query=miR415, target=RO3G_01726, score=1, range=179-200, strand=1
target 5' CUGCUUCGGCUUCUGUUCCUUU 3'
::::::: ::::::::::::::
query 3' GACGAAGACGAAGACAAGGAAA 5'
query=miR415, target=RO3G_16901, score=2, range=188-209, strand=1
target 5' CUGCUUCUGCUUCUGUUCCUGU 3'
:::::::::::::::::::: :
query 3' GACGAAGACGAAGACAAGGAAA 5'
query=miR415, target=RO3G_16917, score=2, range=194-215, strand=1
target 5' CUGCUUCUGCUUCUGUUCCUGU 3'
:::::::::::::::::::: :
query 3' GACGAAGACGAAGACAAGGAAA 5'
query=miR415, target=RO3G_01717, score=2, range=197-218, strand=1
target 5' CUGCUUCUGCUUCUGUUCCUGU 3'
:::::::::::::::::::: :
query 3' GACGAAGACGAAGACAAGGAAA 5'
query=miR415, target=RO3G_02817, score=2, range=197-218, strand=1
target 5' CUGCUUCUGCUUCUGUUCCUGU 3'
:::::::::::::::::::: :
query 3' GACGAAGACGAAGACAAGGAAA 5'
query=miR415, target=RO3G_12011, score=2, range=194-215, strand=1
target 5' CUGCUUCUGCUUCUGUUCCUGU 3'
:::::::::::::::::::: :
query 3' GACGAAGACGAAGACAAGGAAA 5'
query=miR415, target=RO3G_17433, score=2, range=191-212, strand=1
target 5' CUGCUUCUGCUUCUGUUCCUGU 3'
:::::::::::::::::::: :
query 3' GACGAAGACGAAGACAAGGAAA 5'
query=miR415, target=RO3G_16743, score=2, range=140-161, strand=1
target 5' CUGCUUCUGCUUCUGUUCCUGU 3'
:::::::::::::::::::: :
query 3' GACGAAGACGAAGACAAGGAAA 5'
query=miR415, target=RO3G_04659, score=2, range=128-149, strand=1
target 5' CUGCUUCUGCUUCUGUUCCUGU 3'
:::::::::::::::::::: :
query 3' GACGAAGACGAAGACAAGGAAA 5'
query=miR415, target=RO3G_07692, score=2, range=128-149, strand=1
target 5' CUGCUUCUGCUUCUGUUCCUGU 3'
:::::::::::::::::::: :
query 3' GACGAAGACGAAGACAAGGAAA 5'
query=miR415, target=RO3G_15879, score=2, range=194-215, strand=1
target 5' CUGCUUCUGCUUCUGUUCCUGU 3'
```

```
:::::::::::::::::::: :
query 3' GACGAAGACGAAGACAAGGAAA 5'
query=miR415, target=RO3G_10043, score=2, range=188-209, strand=1
target 5' CUGCUUCUGCUUCUGUUCCUGU 3'
:::::::::::::::::::: :
query 3' GACGAAGACGAAGACAAGGAAA 5'
query=miR415, target=RO3G_17460, score=2, range=218-239, strand=1
target 5' CUGCUUCUGCUUCUGUUCCUGU 3'
:::::::::::::::::::: :
query 3' GACGAAGACGAAGACAAGGAAA 5'
query=miR415, target=RO3G_13452, score=2, range=170-191, strand=1
target 5' CUGCUUCUGCUUCUGUUCCUGU 3'
:::::::::::::::::::: :
query 3' GACGAAGACGAAGACAAGGAAA 5'
query=miR415, target=RO3G_16369, score=2, range=173-194, strand=1
target 5' CUGCUUCUGCUUCUGUUCCUGU 3'
:::::::::::::::::::: :
query 3' GACGAAGACGAAGACAAGGAAA 5'
query=miR415, target=RO3G_00288, score=2, range=161-182, strand=1
target 5' CUGCUUCUGCUUCUGUUCCUGU 3'
:::::::::::::::::::: :
query 3' GACGAAGACGAAGACAAGGAAA 5'
query=miR415, target=RO3G_14553, score=2, range=194-215, strand=1
target 5' CUGCUUCUGCUUCUGUUCCUGU 3'
:::::::::::::::::::: :
query 3' GACGAAGACGAAGACAAGGAAA 5'
query=miR415, target=RO3G_11543, score=2, range=194-215, strand=1
target 5' CUGCUUCUGCUUCUGUUCCUGU 3'
:::::::::::::::::::: :
query 3' GACGAAGACGAAGACAAGGAAA 5'
query=miR415, target=RO3G_15887, score=2, range=218-239, strand=1
target 5' CUGCUUCUGCUUCUGUUCCUGU 3'
:::::::::::::::::::: :
query 3' GACGAAGACGAAGACAAGGAAA 5'
query=miR415, target=RO3G_01192, score=2, range=131-152, strand=1
target 5' CUGCUUCUGCUUCUGUUCCUGU 3'
:::::::::::::::::::: :
query 3' GACGAAGACGAAGACAAGGAAA 5'
query=miR415, target=RO3G_17244, score=2, range=191-212, strand=1
target 5' CUGCUUCUGCUUCUGUUCCUGU 3'
:::::::::::::::::::: :
query 3' GACGAAGACGAAGACAAGGAAA 5'
query=miR415, target=RO3G_05347, score=2, range=194-215, strand=1
target 5' CUGCUUCUGCUUCUGUUCCUGU 3'
:::::::::::::::::::: :
```

```
query 3' GACGAAGACGAAGACAAGGAAA 5'
query=miR415, target=RO3G_17210, score=2, range=218-239, strand=1
target 5' CUGCUUCUGCUUCUGUUCCUGU 3'
:::::::::::::::::::: :
query 3' GACGAAGACGAAGACAAGGAAA 5'
query=miR415, target=RO3G_12976, score=2, range=197-218, strand=1
target 5' CUGCUUCUGCUUCUGUUCCUGU 3'
:::::::::::::::::::: :
query 3' GACGAAGACGAAGACAAGGAAA 5'
query=miR415, target=RO3G_15932, score=3, range=1440-1461, strand
target 5' CUUCUUCUUUUUCUGUUCCUUU 3'
:: ::::: .::::::::::::
query 3' GACGAAGACGAAGACAAGGAAA 5'
query=miR415, target=RO3G_10852, score=3, range=1687-1707, strand
target 5' CU-CAUCUUCUUCUGUUCCUUU 3'
:: : ::: :::::::::::::
query 3' GACGAAGACGAAGACAAGGAAA 5'
query=miR415, target=RO3G_02721, score=3.5, range=1208-1229, stra
target 5' CUGUUACAGCUUCUGUUCUUUU 3'
:::.: : ::::::::::.:::
query 3' GACGAAGACGAAGACAAGGAAA 5'
query=miR415, target=RO3G_05906, score=4, range=131-152, strand=1
target 5' CUGCUCCCGCUUCUGUUCCUGU 3'
::::: : :::::::::::: :
query 3' GACGAAGACGAAGACAAGGAAA 5'
query=miR415, target=RO3G_14812, score=4, range=1665-1687, strand
target 5' CUGCUUCUGCUGCUGCUUCCUUU 3'
::::::::::: ::: :::::::
query 3' GACGAAGACGAAGAC-AAGGAAA 5'
query=miR415, target=RO3G_12799, score=4, range=169-190, strand=-
target 5' CUGGUUCUGCUUUUGGUCCUUU 3'
::: ::::::::.:: ::::::
query 3' GACGAAGACGAAGACAAGGAAA 5'
query=miR415, target=RO3G_00853, score=4, range=31-51, strand=-1
target 5' UGGUUUCU-CUUCUGUUCUUUU 3'
. :.:::: :::::::::.:::
query 3' GACGAAGACGAAGACAAGGAAA 5'
query=miR415, target=RO3G_10466, score=4, range=1350-1371, strand
target 5' CUUCUUCUCCUUCUUUUCCUUU 3'
:: ::::: ::::: :::::::
query 3' GACGAAGACGAAGACAAGGAAA 5'
query=miR415, target=RO3G_03013, score=4, range=562-583, strand=-
target 5' CUUCCUCUUCUUCUGUUUCUUU 3'
:: : ::: ::::::::.::::
query 3' GACGAAGACGAAGACAAGGAAA 5'
```

```
query=miR415, target=RO3G_04112, score=4, range=46-67, strand=-1
target 5' CUUCUUCUUCUUCGGUUCCUUU 3'
:: ::::: :::: ::::::::
query 3' GACGAAGACGAAGACAAGGAAA 5'
query=miR415, target=RO3G_02603, score=4, range=1394-1414, strand
target 5' CUUCAUCU-CUUCUGUUUCUUU 3'
:: : ::: ::::::::.::::
query 3' GACGAAGACGAAGACAAGGAAA 5'
query=miR5185l-3p, target=RO3G_09199, score=0, range=740-759, str
target 5' GUUCUAAGUCAACCGUCCAA 3'
::::::::::::::::::::
query 3' CAAGAUUCAGUUGGCAGGUU 5'
query=miR5185l-3p, target=RO3G_01950, score=0, range=740-759, str
target 5' GUUCUAAGUCAACCGUCCAA 3'
::::::::::::::::::::
query 3' CAAGAUUCAGUUGGCAGGUU 5'
query=miR5185l-3p, target=RO3G_11948, score=0, range=740-759, str
target 5' GUUCUAAGUCAACCGUCCAA 3'
::::::::::::::::::::
query 3' CAAGAUUCAGUUGGCAGGUU 5'
query=miR5185l-3p, target=RO3G_09924, score=0, range=740-759, str
target 5' GUUCUAAGUCAACCGUCCAA 3'
::::::::::::::::::::
query 3' CAAGAUUCAGUUGGCAGGUU 5'
query=miR5185l-3p, target=RO3G_07295, score=0, range=572-591, str
target 5' GUUCUAAGUCAACCGUCCAA 3'
::::::::::::::::::::
query 3' CAAGAUUCAGUUGGCAGGUU 5'
query=miR5185l-3p, target=RO3G_10537, score=1, range=563-582, str
target 5' GUUCCAAGUCAACCGUCCAA 3'
:::: :::::::::::::::
query 3' CAAGAUUCAGUUGGCAGGUU 5'
query=miR5185l-3p, target=RO3G_12013, score=1, range=65-84, stran
target 5' GUUCCAAGUCAACCGUCCAA 3'
:::: :::::::::::::::
query 3' CAAGAUUCAGUUGGCAGGUU 5'
query=miR5185l-3p, target=RO3G_10528, score=1, range=65-84, stran
target 5' GUUCCAAGUCAACCGUCCAA 3'
:::: :::::::::::::::
query 3' CAAGAUUCAGUUGGCAGGUU 5'
query=miR5185l-3p, target=RO3G_07119, score=1, range=65-84, stran
target 5' GUUCCAAGUCAACCGUCCAA 3'
:::: :::::::::::::::
query 3' CAAGAUUCAGUUGGCAGGUU 5'
query=miR5185l-3p, target=RO3G_10742, score=1, range=65-84, stran
```

```
target 5' GUUCCAAGUCAACCGUCCAA 3'
:::: :::::::::::::::
query 3' CAAGAUUCAGUUGGCAGGUU 5'
query=miR5185l-3p, target=RO3G_08848, score=1, range=50-69, stran
target 5' GUUCCAAGUCAACCGUCCAA 3'
:::: :::::::::::::::
query 3' CAAGAUUCAGUUGGCAGGUU 5'
query=miR5185l-3p, target=RO3G_11528, score=1, range=65-84, stran
target 5' GUUCCAAGUCAACCGUCCAA 3'
:::: :::::::::::::::
query 3' CAAGAUUCAGUUGGCAGGUU 5'
query=miR5185l-3p, target=RO3G_01951, score=1, range=740-759, str
target 5' GUUCCAAGUCAACCGUCCAA 3'
:::: :::::::::::::::
query 3' CAAGAUUCAGUUGGCAGGUU 5'
query=miR5185l-3p, target=RO3G_11244, score=1, range=740-759, str
target 5' GUUCCAAGUCAACCGUCCAA 3'
:::: :::::::::::::::
query 3' CAAGAUUCAGUUGGCAGGUU 5'
query=miR5185l-3p, target=RO3G_01313, score=1, range=740-759, str
target 5' GUUCCAAGUCAACCGUCCAA 3'
:::: :::::::::::::::
query 3' CAAGAUUCAGUUGGCAGGUU 5'
query=miR5185l-3p, target=RO3G_01194, score=1, range=740-759, str
target 5' GUUCCAAGUCAACCGUCCAA 3'
:::: :::::::::::::::
query 3' CAAGAUUCAGUUGGCAGGUU 5'
query=miR5185l-3p, target=RO3G_10044, score=1, range=740-759, str
target 5' GUUCCAAGUCAACCGUCCAA 3'
:::: :::::::::::::::
query 3' CAAGAUUCAGUUGGCAGGUU 5'
query=miR5185l-3p, target=RO3G_11534, score=1, range=740-759, str
target 5' GUUCCAAGUCAACCGUCCAA 3'
:::: :::::::::::::::
query 3' CAAGAUUCAGUUGGCAGGUU 5'
query=miR5185l-3p, target=RO3G_03987, score=1, range=740-759, str
target 5' GUUCCAAGUCAACCGUCCAA 3'
:::: :::::::::::::::
query 3' CAAGAUUCAGUUGGCAGGUU 5'
query=miR5185l-3p, target=RO3G_15873, score=1, range=740-759, str
target 5' GUUCCAAGUCAACCGUCCAA 3'
:::: :::::::::::::::
query 3' CAAGAUUCAGUUGGCAGGUU 5'
query=miR5185l-3p, target=RO3G_10720, score=1, range=740-759, str
target 5' GUUCCAAGUCAACCGUCCAA 3'
```

```
:::: ::::::::::::::
query 3' CAAGAUUCAGUUGGCAGGUU 5'
query=miR5185l-3p, target=RO3G_05916, score=1, range=740-759, str
target 5' GUUCCAAGUCAACCGUCCAA 3'
:::: ::::::::::::::
query 3' CAAGAUUCAGUUGGCAGGUU 5'
query=miR5185l-3p, target=RO3G_07994, score=1, range=740-759, str
target 5' GUUCCAAGUCAACCGUCCAA 3'
:::: ::::::::::::::
query 3' CAAGAUUCAGUUGGCAGGUU 5'
query=miR5185l-3p, target=RO3G_07683, score=1, range=740-759, str
target 5' GUUCCAAGUCAACCGUCCAA 3'
:::: ::::::::::::::
query 3' CAAGAUUCAGUUGGCAGGUU 5'
query=miR5185l-3p, target=RO3G_16118, score=1, range=8-27, strand
target 5' GUUCCAAGUCAACCGUCCAA 3'
:::: ::::::::::::::
query 3' CAAGAUUCAGUUGGCAGGUU 5'
query=miR5185l-3p, target=RO3G_01735, score=1, range=740-759, str
target 5' GUUCCAAGUCAACCGUCCAA 3'
:::: ::::::::::::::
query 3' CAAGAUUCAGUUGGCAGGUU 5'
query=miR5185l-3p, target=RO3G_11263, score=2, range=65-84, stran
target 5' GUUCCAAGUCAGCCGUCCAA 3'
:::: ::::::.:::::::
query 3' CAAGAUUCAGUUGGCAGGUU 5'
query=miR5185l-3p, target=RO3G_13706, score=2, range=698-717, str
target 5' GUUCCAAGUCAGCCGUCCAA 3'
:::: ::::::.:::::::
query 3' CAAGAUUCAGUUGGCAGGUU 5'
query=miR5185l-3p, target=RO3G_12526, score=2, range=740-759, str
target 5' GUUCCAAGUCAGCCGUCCAA 3'
:::: ::::::.:::::::
query 3' CAAGAUUCAGUUGGCAGGUU 5'
query=miR5185l-3p, target=RO3G_03558, score=2, range=740-759, str
target 5' GUUCCAAGUCAACUGUCCAA 3'
:::: ::::::::.::::::
query 3' CAAGAUUCAGUUGGCAGGUU 5'
query=miR5185l-3p, target=RO3G_13447, score=2, range=740-759, str
target 5' GUUCCAAGUCAACUGUCCAA 3'
:::: ::::::::.::::::
query 3' CAAGAUUCAGUUGGCAGGUU 5'
query=miR5185l-3p, target=RO3G_17409, score=2, range=740-759, str
target 5' GUUCCAAGUCAGCCGUCCAA 3'
:::: ::::::.:::::::
```

```
query 3' CAAGAUUCAGUUGGCAGGUU 5'
query=miR5185l-3p, target=RO3G_15154, score=2, range=740-759, str
target 5' GUUCCAAGUCAGCCGUCCAA 3'
:::: ::::::.::::::::
query 3' CAAGAUUCAGUUGGCAGGUU 5'
query=miR5185l-3p, target=RO3G_12174, score=2, range=65-84, stran
target 5' GUUCCAAGUCAGCCGUCCAA 3'
:::: ::::::.::::::::
query 3' CAAGAUUCAGUUGGCAGGUU 5'
query=miR5185l-3p, target=RO3G_17213, score=2, range=65-84, stran
target 5' GUUCCAAGUCAGCCGUCCAA 3'
:::: ::::::.::::::::
query 3' CAAGAUUCAGUUGGCAGGUU 5'
query=miR5185l-3p, target=RO3G_03617, score=2, range=740-759, str
target 5' GUUCCAAGUCAACUGUCCAA 3'
:::: ::::::::.::::::
query 3' CAAGAUUCAGUUGGCAGGUU 5'
query=miR5185l-3p, target=RO3G_17211, score=2, range=740-759, str
target 5' GUUCCAAGUCAGCCGUCCAA 3'
:::: ::::::.::::::::
query 3' CAAGAUUCAGUUGGCAGGUU 5'
query=miR5185l-3p, target=RO3G_08835, score=3, range=50-69, stran
target 5' GUUCCAAGUCAACCAUCCAA 3'
:::: ::::::::: :::::
query 3' CAAGAUUCAGUUGGCAGGUU 5'
query=miR5185l-3p, target=RO3G_16377, score=4, range=65-84, stran
target 5' GUUCCAAGUCAACAGUUCAA 3'
:::: :::::::: ::.:::
query 3' CAAGAUUCAGUUGGCAGGUU 5'
query=miR5185l-3p, target=RO3G_07465, score=4, range=1455-1474,
strand=1
target 5' AUUCUAUGUCAACCAUCCAA 3'
::::: ::::::: :::::
query 3' CAAGAUUCAGUUGGCAGGUU 5'
query=miR5185l-3p, target=RO3G_00951, score=4, range=1834-1853,
strand=1
target 5' CUUCAACGUCAAUCGUCCAA 3'
::: : :::::.:::::::
query 3' CAAGAUUCAGUUGGCAGGUU 5'
query=miR5185l-3p, target=RO3G_12994, score=4, range=31-50, stran
target 5' GAUAAAAGUCAAUCGUCCAA 3'
: : :::::::.:::::::
query 3' CAAGAUUCAGUUGGCAGGUU 5'
query=miR6274b-3p, target=RO3G_13001, score=0, range=2234-2253,
strand=1
```

target 5' CUAUUUUCGGUCUACCACAA 3'
::::::::::::::::::::
query 3' GAUAAAAGCCAGAUGGUGUU 5'
query=miR6274b-3p, target=RO3G_06596, score=0, range=2234-2253, strand=1
target 5' CUAUUUUCGGUCUACCACAA 3'
::::::::::::::::::::
query 3' GAUAAAAGCCAGAUGGUGUU 5'
query=miR6274b-3p, target=RO3G_03002, score=0, range=2234-2253, strand=1
target 5' CUAUUUUCGGUCUACCACAA 3'
::::::::::::::::::::
query 3' GAUAAAAGCCAGAUGGUGUU 5'
query=miR6274b-3p, target=RO3G_15390, score=0, range=524-543, str
target 5' CUAUUUUCGGUCUACCACAA 3'
::::::::::::::::::::
query 3' GAUAAAAGCCAGAUGGUGUU 5'
query=miR6274b-3p, target=RO3G_10503, score=1, range=170-189, str
target 5' CUAUUUCCGGUCUACCACAA 3'
:::::: :::::::::::::
query 3' GAUAAAAGCCAGAUGGUGUU 5'
query=miR6274b-3p, target=RO3G_02145, score=4, range=116-135, str
target 5' GUAUUUCCGAUCUACCACAA 3'
::::: :: ::::::::::
query 3' GAUAAAAGCCAGAUGGUGUU 5'
query=miR6274b-3p, target=RO3G_11963, score=4, range=170-189, str
target 5' CUAUUCCCGAUCUACCACAA 3'
::::: :: ::::::::::
query 3' GAUAAAAGCCAGAUGGUGUU 5'
query=miR8126-3p, target=RO3G_15431, score=3.5, range=797-819, strand=1
target 5' CUCUGAUAGACAAGUAUAUUGAG 3'
::::::: :::::.::::.:::.
query 3' GAGACUA-CUGUUUAUAUGACUU 5'
query=miR8126-3p, target=RO3G_10563, score=4, range=3-24, strand=
target 5' GUCUGAUGACGAUUAUACUGAA 3'
:::::::::.: :::::::::
query 3' GAGACUACUGUUUAUAUGACUU 5'
query=miR8126-3p, target=RO3G_04045, score=4, range=269-290, stra
target 5' CUCUGAGGAGGAAUAUACUGAA 3'
:::::: :: .::::::::::
query 3' GAGACUACUGUUUAUAUGACUU 5'
query=miR8126-3p, target=RO3G_02673, score=4, range=773-794, stra
target 5' UUUUGCGGACAAAUAUACUGAU 3'
.:.:: :::::::::::::

query 3' GAGACUACUGUUUAUAUGACUU 5'
query=miR1428b, target=RO3G_03721, score=0, range=633-654, strand
target 5' AGAAUUCAUGGCAUUUCUUAUA 3'
:::::::::::::::::::::
query 3' UCUUAAGUACCGUAAAGAAUAU 5'
query=miR1428b, target=RO3G_05325, score=0.5, range=633-654, stra
target 5' AGAGUUCAUGGCAUUUCUUAUA 3'
:::.:::::::::::::::::
query 3' UCUUAAGUACCGUAAAGAAUAU 5'
query=miR1428b, target=RO3G_13882, score=0.5, range=633-654, stra
target 5' AGAGUUCAUGGCAUUUCUUAUA 3'
:::.:::::::::::::::::
query 3' UCUUAAGUACCGUAAAGAAUAU 5'
query=miR1428b, target=RO3G_00809, score=0.5, range=633-654, stra
target 5' AGAGUUCAUGGCAUUUCUUAUA 3'
:::.:::::::::::::::::
query 3' UCUUAAGUACCGUAAAGAAUAU 5'
query=miR1428b, target=RO3G_13730, score=0.5, range=396-417, stra
target 5' AGAGUUCAUGGCAUUUCUUAUA 3'
:::.:::::::::::::::::
query 3' UCUUAAGUACCGUAAAGAAUAU 5'
query=miR1428b, target=RO3G_00141, score=1, range=633-654, strand
target 5' AGAAUUCAUGGUAUUUCUUAUA 3'
::::::::::.::::::::::
query 3' UCUUAAGUACCGUAAAGAAUAU 5'
query=miR1428b, target=RO3G_02269, score=1, range=105-127, strand
target 5' AGAAAUUCAUGGCAUUUCUUAUA 3'
:: ::::::::::::::::::::
query 3' UC-UUAAGUACCGUAAAGAAUAU 5'
query=miR1428b, target=RO3G_12350, score=4, range=1371-1392, stra
target 5' UAAAUUCAUGGCAUUUCUAAUA 3'
:::::::::::::::: :::
query 3' UCUUAAGUACCGUAAAGAAUAU 5'
query=miR2111-3p, target=RO3G_19002, score=0, range=353-371, stra
target 5' GGUAAUCCCACCUGACUUC 3'
:::::::::::::::::::
query 3' CCAUUAGGGUGGACUGAAG 5'
query=miR2111-3p, target=RO3G_19000, score=0, range=353-371, stra
target 5' GGUAAUCCCACCUGACUUC 3'
:::::::::::::::::::
query 3' CCAUUAGGGUGGACUGAAG 5'
query=miR2111-3p, target=RO3G_04261, score=3, range=1575-1593,
strand=-1
target 5' AGAAAACCCACCUGACUUC 3'
: :: ::::::::::::

```
query 3' CCAUUAGGGUGGACUGAAG 5'
query=miR2111-3p, target=RO3G_06346, score=4, range=258-276, stra
target 5' GGUAAACCCAUCUGAGUUC 3'
::::: ::::.:::: :::
query 3' CCAUUAGGGUGGACUGAAG 5'
query=miR2111-3p, target=RO3G_16693, score=4, range=414-433, stra
target 5' UGUCAUCCCACCUGACCUUC 3'
:: ::::::::::: ::::
query 3' CCAUUAGGGUGGACU-GAAG 5'
query=miR2111-3p, target=RO3G_12527, score=4, range=414-433, stra
target 5' UGUUAUCCCACCUGACCUUC 3'
:: ::::::::::: ::::
query 3' CCAUUAGGGUGGACU-GAAG 5'
query=miR2111-3p, target=RO3G_07311, score=4, range=414-433, stra
target 5' UGUCAUCCCACCUGACCUUC 3'
:: ::::::::::: ::::
query 3' CCAUUAGGGUGGACU-GAAG 5'
query=miR2111-3p, target=RO3G_01614, score=4, range=414-433, stra
target 5' UGUCAUCCCACCUGACCUUC 3'
:: ::::::::::: ::::
query 3' CCAUUAGGGUGGACU-GAAG 5'
query=miR2111-3p, target=RO3G_10804, score=4, range=414-433, stra
target 5' UGUCAUCCCACCUGACCUUC 3'
:: ::::::::::: ::::
query 3' CCAUUAGGGUGGACU-GAAG 5'
query=miR2111-3p, target=RO3G_12532, score=4, range=414-433, stra
target 5' UGUCAUCCCACCUGACCUUC 3'
:: ::::::::::: ::::
query 3' CCAUUAGGGUGGACU-GAAG 5'
query=miR2111-3p, target=RO3G_01936, score=4, range=414-433, stra
target 5' UGUCAUCCCACCUGACCUUC 3'
:: ::::::::::: ::::
query 3' CCAUUAGGGUGGACU-GAAG 5'
query=miR2111-3p, target=RO3G_03619, score=4, range=414-433, stra
target 5' UGUCAUCCCACCUGACCUUC 3'
:: ::::::::::: ::::
query 3' CCAUUAGGGUGGACU-GAAG 5'
query=miR2111-3p, target=RO3G_02821, score=4, range=414-433, stra
target 5' UGUCAUCCCACCUGACCUUC 3'
:: ::::::::::: ::::
query 3' CCAUUAGGGUGGACU-GAAG 5'
query=miR2111-3p, target=RO3G_00242, score=4, range=414-433, stra
target 5' UGUCAUCCCACCUGACCUUC 3'
:: ::::::::::: ::::
query 3' CCAUUAGGGUGGACU-GAAG 5'
```

```
query=miR2111-3p, target=RO3G_00295, score=4, range=414-433, stra
target 5' UGUCAUCCCACCUGACCUUC 3'
:: ::::::::::: ::::
query 3' CCAUUAGGGUGGACU-GAAG 5'
query=miR2111-3p, target=RO3G_00687, score=4, range=414-433, stra
target 5' UGUCAUCCCACCUGACCUUC 3'
:: ::::::::::: ::::
query 3' CCAUUAGGGUGGACU-GAAG 5'
query=miR2111-3p, target=RO3G_05517, score=4, range=414-433, stra
target 5' UGUCAUCCCACCUGACCUUC 3'
:: ::::::::::: ::::
query 3' CCAUUAGGGUGGACU-GAAG 5'
query=miR2111-3p, target=RO3G_17170, score=4, range=414-433, stra
target 5' UGUUAUCCCACCUGACCUUC 3'
:: ::::::::::: ::::
query 3' CCAUUAGGGUGGACU-GAAG 5'
query=miR2111-3p, target=RO3G_12671, score=4, range=414-433, stra
target 5' UGUCAUCCCACCUGACCUUC 3'
:: ::::::::::: ::::
query 3' CCAUUAGGGUGGACU-GAAG 5'
query=miR825-3p, target=RO3G_14363, score=0, range=451-473, stran
target 5' GUUAAUGCACCUUAUCGAGACCA 3'
:::::::::::::::::::::::
query 3' CAAUUACGUGGAAUAGCUCUGGU 5'
query=miR825-3p, target=RO3G_13270, score=1, range=493-515, stran
target 5' GAUAAUGCACCUUAUCGAGACCA 3'
: :::::::::::::::::::::
query 3' CAAUUACGUGGAAUAGCUCUGGU 5'
query=miR825-3p, target=RO3G_10774, score=1, range=460-482, stran
target 5' GAUAAUGCACCUUAUCGAGACCA 3'
: :::::::::::::::::::::
query 3' CAAUUACGUGGAAUAGCUCUGGU 5'
query=miR825-3p, target=RO3G_13863, score=1, range=193-215, stran
target 5' GAUAAUGCACCUUAUCGAGACCA 3'
: :::::::::::::::::::::
query 3' CAAUUACGUGGAAUAGCUCUGGU 5'
query=miR825-3p, target=RO3G_13741, score=1, range=493-515, stran
target 5' GAUAAUGCACCUUAUCGAGACCA 3'
: :::::::::::::::::::::
query 3' CAAUUACGUGGAAUAGCUCUGGU 5'
query=miR825-3p, target=RO3G_08849, score=1, range=493-515, stran
target 5' GAUAAUGCACCUUAUCGAGACCA 3'
: :::::::::::::::::::::
query 3' CAAUUACGUGGAAUAGCUCUGGU 5'
query=miR825-3p, target=RO3G_05936, score=1, range=493-515, stran
```

```
target 5' GAUAAUGCACCUUAUCGAGACCA 3'
: :::::::::::::::::::::
query 3' CAAUUACGUGGAAUAGCUCUGGU 5'
query=miR825-3p, target=RO3G_14979, score=1, range=493-515, stran
target 5' GAUAAUGCACCUUAUCGAGACCA 3'
: :::::::::::::::::::::
query 3' CAAUUACGUGGAAUAGCUCUGGU 5'
query=miR825-3p, target=RO3G_17276, score=1.5, range=583-605, str
target 5' GAUAAUGCACCUUAUCGAGACCG 3'
: ::::::::::::::::::::.
query 3' CAAUUACGUGGAAUAGCUCUGGU 5'
query=miR825-3p, target=RO3G_05420, score=2, range=354-376, stran
target 5' GGAAAUGCACCUUAUCGAGACCA 3'
: ::::::::::::::::::::
query 3' CAAUUACGUGGAAUAGCUCUGGU 5'
query=miR825-3p, target=RO3G_13755, score=2, range=493-515, stran
target 5' GAUAAUGAACCUUAUCGAGACCA 3'
: ::::: :::::::::::::::
query 3' CAAUUACGUGGAAUAGCUCUGGU 5'
query=miR825-3p, target=RO3G_17249, score=3, range=493-515, stran
target 5' GAUAAUGCACCUUUUCGAGACCA 3'
: ::::::::::: :::::::::
query 3' CAAUUACGUGGAAUAGCUCUGGU 5'
query=miR5081, target=RO3G_10245, score=3.5, range=546-567, stran
target 5' AGAUAAAGAUUUUACAAAUUCA 3'
:: :: .::::::::::::::
query 3' UC-AUAGUUAAAAUGUUUAAGU 5'
query=miR5081, target=RO3G_13517, score=3.5, range=24-43, strand=
target 5' AGUAUU-CUUUUACAAGUUCA 3'
:::::. ::::::::.::::
query 3' UCAUAGUUAAAAUGUUUAAGU 5'
query=miR5081, target=RO3G_02815, score=3.5, range=780-799, stran
target 5' CGUAUU-CUUUUACAAAUUCA 3'
::::. :::::::::::::
query 3' UCAUAGUUAAAAUGUUUAAGU 5'
query=miR5081, target=RO3G_01688, score=4, range=1297-1317, stran
target 5' GGUAUCAAUUUUACACGUUCG 3'
.:::::::::::::: .:::.
query 3' UCAUAGUUAAAAUGUUUAAGU 5'
query=miR5081, target=RO3G_00403, score=4, range=4229-4249, stran
target 5' GCGGUCAAUUUUAUAAAUUCA 3'
. .:::::::::.:::::::
query 3' UCAUAGUUAAAAUGUUUAAGU 5'
query=miR5081, target=RO3G_15599, score=4, range=178-198, strand=
target 5' UGUAUUAAUUUUACUAAUUCG 3'
```

```
::::.:::::::: :::::.
query 3' UCAUAGUUAAAAUGUUUAAGU 5'
query=miR5081, target=RO3G_10909, score=4, range=66-87, strand=-1
target 5' AGAUAUCCAUUUUAUGAAUUCA 3'
:: :::: ::::::..::::::
query 3' UC-AUAGUUAAAAUGUUUAAGU 5'
query=miR5081, target=RO3G_01802, score=4, range=894-914, strand=
target 5' AAUAUCUCUUUUAUAAAUUCA 3'
: :::: :::::.:::::::
query 3' UCAUAGUUAAAAUGUUUAAGU 5'
query=miR5081, target=RO3G_10971, score=4, range=4119-4140, stran
target 5' AAUAGGAAUUUUUACAAAUUCA 3'
: :: :: :::::::::::::
query 3' UCAUAGUU-AAAAUGUUUAAGU 5'
query=miR6459a-3p, target=RO3G_10991, score=0, range=1101-1120,
strand=1
target 5' UCAAUUCAAGCCCAAAUCAA 3'
::::::::::::::::::::
query 3' AGUUAAGUUCGGGUUUAGUU 5'
query=miR6459a-3p, target=RO3G_15573, score=0, range=1101-1120,
strand=1
target 5' UCAAUUCAAGCCCAAAUCAA 3'
::::::::::::::::::::
query 3' AGUUAAGUUCGGGUUUAGUU 5'
query=miR6459a-3p, target=RO3G_02708, score=0, range=834-853, str
target 5' UCAAUUCAAGCCCAAAUCAA 3'
::::::::::::::::::::
query 3' AGUUAAGUUCGGGUUUAGUU 5'
query=miR6459a-3p, target=RO3G_06502, score=0, range=1101-1120,
strand=1
target 5' UCAAUUCAAGCCCAAAUCAA 3'
::::::::::::::::::::
query 3' AGUUAAGUUCGGGUUUAGUU 5'
query=miR6459a-3p, target=RO3G_02966, score=0, range=372-391, str
target 5' UCAAUUCAAGCCCAAAUCAA 3'
::::::::::::::::::::
query 3' AGUUAAGUUCGGGUUUAGUU 5'
query=miR6459a-3p, target=RO3G_16376, score=0, range=1026-1045,
strand=1
target 5' UCAAUUCAAGCCCAAAUCAA 3'
::::::::::::::::::::
query 3' AGUUAAGUUCGGGUUUAGUU 5'
query=miR6459a-3p, target=RO3G_01056, score=2, range=1008-1027,
strand=1
target 5' UCAAUUCAAGCCUAAGUCAA 3'
```

::::::::::::.::.::::
query 3' AGUUAAGUUCGGGUUUAGUU 5'
query=miR6459a-3p, target=RO3G_04662, score=2, range=953-972, str
target 5' UCAAUUCAAGCCUAAGUCAA 3'
::::::::::::.::.::::
query 3' AGUUAAGUUCGGGUUUAGUU 5'
query=miR6459a-3p, target=RO3G_03749, score=3, range=1101-1120, strand=1
target 5' UCAAUUCAAGCCGAAGUCAA 3'
:::::::::::: ::.::::
query 3' AGUUAAGUUCGGGUUUAGUU 5'
query=miR6459a-3p, target=RO3G_17287, score=3, range=1101-1120, strand=1
target 5' UCAAUUCAAGCCGAAGUCAA 3'
:::::::::::: ::.::::
query 3' AGUUAAGUUCGGGUUUAGUU 5'
query=miR6459a-3p, target=RO3G_01944, score=3, range=1101-1120, strand=1
target 5' UCAAUUCAAGCCGAAGUCAA 3'
:::::::::::: ::.::::
query 3' AGUUAAGUUCGGGUUUAGUU 5'
query=miR6459a-3p, target=RO3G_13849, score=3, range=1101-1120, strand=1
target 5' UCAAUUCAAGCCGAAGUCAA 3'
:::::::::::: ::.::::
query 3' AGUUAAGUUCGGGUUUAGUU 5'
query=miR6459a-3p, target=RO3G_10420, score=3, range=1101-1120, strand=1
target 5' UCAAUUCAAGCCGAAGUCAA 3'
:::::::::::: ::.::::
query 3' AGUUAAGUUCGGGUUUAGUU 5'
query=miR6459a-3p, target=RO3G_01733, score=3, range=1101-1120, strand=1
target 5' UCAAUUCAAGCCGAAGUCAA 3'
:::::::::::: ::.::::
query 3' AGUUAAGUUCGGGUUUAGUU 5'
query=miR6459a-3p, target=RO3G_05865, score=3, range=1101-1120, strand=1
target 5' UCAAUUCAAGCCGAAGUCAA 3'
:::::::::::: ::.::::
query 3' AGUUAAGUUCGGGUUUAGUU 5'
query=miR6459a-3p, target=RO3G_17446, score=3, range=1101-1120, strand=1
target 5' UCAAUUCAAGCCGAAGUCAA 3'
:::::::::::: ::.::::

query 3' AGUUAAGUUCGGGUUUAGUU 5'
query=miR6459a-3p, target=RO3G_00273, score=3, range=789-807, str
target 5' UC-AUUCAAGCCCAUAUCAA 3'
:: ::::::::::: :::::
query 3' AGUUAAGUUCGGGUUUAGUU 5'
query=miR6459a-3p, target=RO3G_01540, score=3.5, range=1012-1030, strand=1
target 5' UCGAUUCAAGCCC-AAUUAA 3'
::.:::::::::: :::.::
query 3' AGUUAAGUUCGGGUUUAGUU 5'
query=miR6459a-3p, target=RO3G_12262, score=3.5, range=258-278, strand=1
target 5' UCGCUGUCAAGCCCAAAUCAC 3'
::. : :::::::::::::
query 3' AGUUA-AGUUCGGGUUUAGUU 5'
query=miR6459a-3p, target=RO3G_00933, score=3.5, range=2737-2755, strand=1
target 5' UCAGAUCAAG-CCAAAUCAA 3'
:::. ::::: :::::::::
query 3' AGUUAAGUUCGGGUUUAGUU 5'
query=miR6459a-3p, target=RO3G_12505, score=4, range=548-567, str
target 5' UCAAUACAAGCUCCAAUCAA 3'
::::: :::::.: ::::::
query 3' AGUUAAGUUCGGGUUUAGUU 5'
query=miR6459a-3p, target=RO3G_12506, score=4, range=548-567, str
target 5' UCAAUACAAGCUCCAAUCAA 3'
::::: :::::.: ::::::
query 3' AGUUAAGUUCGGGUUUAGUU 5'
query=miR6459a-3p, target=RO3G_10202, score=4, range=801-820, str
target 5' UCAAUUAGAGCCCAAUUCAA 3'
:::::: .::::::: ::::
query 3' AGUUAAGUUCGGGUUUAGUU 5'
query=miR6459a-3p, target=RO3G_01464, score=4, range=1163-1182, strand=1
target 5' CCAAUUCAAGCCCAAAUAAU 3'
:::::::::::::::: :
query 3' AGUUAAGUUCGGGUUUAGUU 5'
query=miR6459a-3p, target=RO3G_15761, score=4, range=147-166, str
target 5' ACAAAUCAAGCCUAAAUCAU 3'
::: :::::::.::::::
query 3' AGUUAAGUUCGGGUUUAGUU 5'
query=miR6459a-3p, target=RO3G_06875, score=4, range=954-974, str
target 5' UCAAUUUCAAGCCCACAUCAU 3'
:::: ::::::::: ::::
query 3' AGUU-AAGUUCGGGUUUAGUU 5'

```
query=miR6459a-3p, target=RO3G_07372, score=4, range=757-777, str
target 5' UCAAAUACAAGCGCAAAUCAA 3'
:: ::: ::::: ::::::::
query 3' AG-UUAAGUUCGGGUUUAGUU 5'
query=miR6459a-3p, target=RO3G_09506, score=4, range=576-594, str
target 5' UCAACU-AGGCCCAAAUCAU 3'
:::: : :.:::::::::
query 3' AGUUAAGUUCGGGUUUAGUU 5'
query=miR6459a-3p, target=RO3G_05710, score=4, range=22-40, stran
target 5' UCCAUUCAA-CCCAAAUCAC 3'
:: :::::: :::::::::
query 3' AGUUAAGUUCGGGUUUAGUU 5'
query=miR6459a-3p, target=RO3G_04047, score=4, range=548-567,
strand=-1
target 5' UUCAUUCAAGCGCAAAUCAG 3'
:. :::::::: :::::::.
query 3' AGUUAAGUUCGGGUUUAGUU 5'
query=miR6459a-3p, target=RO3G_03236, score=4, range=1022-1041,
strand=-1
target 5' GCCCUUCAAGCCCAAGUCAA 3'
: :::::::::::.::::
query 3' AGUUAAGUUCGGGUUUAGUU 5'
query=miR6459a-3p, target=RO3G_02517, score=4, range=325-343,
strand=-1
target 5' UAAAUUCAAGUCC-AAUCAA 3'
: ::::::::.:: ::::::
query 3' AGUUAAGUUCGGGUUUAGUU 5'
query=miR6459a-3p, target=RO3G_09150, score=4, range=365-383,
strand=-1
target 5' UCAAUUCUA-CCCAAAUCAA 3'
::::::: : :::::::::
query 3' AGUUAAGUUCGGGUUUAGUU 5'
query=miR529a, target=RO3G_05953, score=0, range=2920-2939, stran
target 5' GAAGAAGAGAGCAGCGACAA 3'
::::::::::::::::::::
query 3' CUUCUUCUCUCGUCGCUGUU 5'
query=miR529a, target=RO3G_13226, score=0, range=73-92, strand=-1
target 5' GAAGAAGAGAGCAGCGACAA 3'
::::::::::::::::::::
query 3' CUUCUUCUCUCGUCGCUGUU 5'
query=miR529a, target=RO3G_16800, score=0, range=3961-3980, stran
target 5' GAAGAAGAGAGCAGCGACAA 3'
::::::::::::::::::::
query 3' CUUCUUCUCUCGUCGCUGUU 5'
query=miR529a, target=RO3G_13217, score=0, range=3961-3980, stran
```

```
target 5' GAAGAAGAGAGCAGCGACAA 3'
::::::::::::::::::::
query 3' CUUCUUCUCUCGUCGCUGUU 5'
query=miR529a, target=R03G_14350, score=0, range=3832-3851, stran
target 5' GAAGAAGAGAGCAGCGACAA 3'
::::::::::::::::::::
query 3' CUUCUUCUCUCGUCGCUGUU 5'
query=miR529a, target=R03G_15268, score=0, range=3967-3986, stran
target 5' GAAGAAGAGAGCAGCGACAA 3'
::::::::::::::::::::
query 3' CUUCUUCUCUCGUCGCUGUU 5'
query=miR529a, target=R03G_11967, score=0, range=3988-4007, stran
target 5' GAAGAAGAGAGCAGCGACAA 3'
::::::::::::::::::::
query 3' CUUCUUCUCUCGUCGCUGUU 5'
query=miR529a, target=R03G_14777, score=2, range=1660-1679, stran
target 5' GAAGAAGAGAGAAGCGACAA 3'
::::::::::: ::::::::
query 3' CUUCUUCUCUCGUCGCUGUU 5'
query=miR529a, target=R03G_15267, score=2, range=1723-1742, stran
target 5' GAAGAAGAGAGAAGCGACAA 3'
::::::::::: ::::::::
query 3' CUUCUUCUCUCGUCGCUGUU 5'
query=miR529a, target=R03G_04121, score=2, range=3961-3980, stran
target 5' GAAGAAGAGAGAAGCGACAA 3'
::::::::::: ::::::::
query 3' CUUCUUCUCUCGUCGCUGUU 5'
query=miR529a, target=R03G_15265, score=2, range=3961-3980, stran
target 5' GAAGAAGAGAGAAGCGACAA 3'
::::::::::: ::::::::
query 3' CUUCUUCUCUCGUCGCUGUU 5'
query=miR529a, target=R03G_15508, score=2.5, range=29-48, strand=
target 5' CAACAAGAGAGCAGCGACAG 3'
:: ::::::::::::::::.
query 3' CUUCUUCUCUCGUCGCUGUU 5'
query=miR529a, target=R03G_15510, score=2.5, range=29-48, strand=
target 5' CAACAAGAGAGCAGCGACAG 3'
:: ::::::::::::::::.
query 3' CUUCUUCUCUCGUCGCUGUU 5'
query=miR529a, target=R03G_15511, score=2.5, range=29-48, strand=
target 5' CAACAAGAGAGCAGCGACAG 3'
:: ::::::::::::::::.
query 3' CUUCUUCUCUCGUCGCUGUU 5'
query=miR529a, target=R03G_04591, score=3, range=647-666, strand=
target 5' GAAAAAGAGAGCAGCAACAA 3'
```

```
::: :::::::::: ::::
query 3' CUUCUUCUCUCGUCGCUGUU 5'
query=miR529a, target=RO3G_04603, score=3, range=73-93, strand=1
target 5' GAAGAAGAAGAGCAGCAACAA 3'
::::::: :::::::: ::::
query 3' CUUCUUC-UCUCGUCGCUGUU 5'
query=miR529a, target=RO3G_13647, score=3, range=11-30, strand=-1
target 5' GAUGAAAAGAGCAGCGAUAA 3'
:: ::: ::::::::::.::
query 3' CUUCUUCUCUCGUCGCUGUU 5'
query=miR529a, target=RO3G_15451, score=3.5, range=1558-1576, str
target 5' GAAG-AGAGAGCAGUGGCAG 3'
:::: :::::::::.:.::.
query 3' CUUCUUCUCUCGUCGCUGUU 5'
query=miR529a, target=RO3G_13394, score=4, range=150-169, strand=
target 5' GAAAAAGAGAGUAGUGACAC 3'
::: :::::::.::.::::
query 3' CUUCUUCUCUCGUCGCUGUU 5'
query=miR529a, target=RO3G_05374, score=4, range=181-199, strand=
target 5' GAAGAA-AGACCAGCGAUAA 3'
:::::: ::: ::::::.::
query 3' CUUCUUCUCUCGUCGCUGUU 5'
query=miR529a, target=RO3G_08150, score=4, range=73-93, strand=1
target 5' AAAGAAGCAGAGCAGCAACAA 3'
:::::: :::::::: ::::
query 3' CUUCUUC-UCUCGUCGCUGUU 5'
query=miR529a, target=RO3G_11952, score=4, range=2965-2984, stran
target 5' GAAGAAGACAGCAGAGACAA 3'
:::::::: ::::: :::::
query 3' CUUCUUCUCUCGUCGCUGUU 5'
query=miR529a, target=RO3G_12964, score=4, range=2587-2606, stran
target 5' GAAGAAGACAGCAGAGACAA 3'
:::::::: ::::: :::::
query 3' CUUCUUCUCUCGUCGCUGUU 5'
query=miR529a, target=RO3G_13746, score=4, range=1441-1460, stran
target 5' GAAGAAGACAGCAGAGACAA 3'
:::::::: ::::: :::::
query 3' CUUCUUCUCUCGUCGCUGUU 5'
query=miR529a, target=RO3G_00686, score=4, range=310-329, strand=
target 5' GAAGAAGACAGCAGAGACAA 3'
:::::::: ::::: :::::
query 3' CUUCUUCUCUCGUCGCUGUU 5'
query=miR529a, target=RO3G_07209, score=4, range=902-921, strand=
target 5' CAAGAAAGGAGCAGCGACAU 3'
::::: .::::::::::
```

```
query 3' CUUCUUCUCUCGUCGCUGUU 5'
query=miR529a, target=RO3G_08758, score=4, range=1754-1773, stran
target 5' CAUCAAGAGAGCAGCGACGA 3'
: ::::::::::::::.:
query 3' CUUCUUCUCUCGUCGCUGUU 5'
query=miR9492, target=RO3G_15743, score=0, range=940-958, strand=
target 5' AUACCAAGACAGAGAAGUA 3'
:::::::::::::::::::
query 3' UAUGGUUCUGUCUCUUCAU 5'
query=miR9492, target=RO3G_13691, score=3, range=1154-1172, stran
target 5' GAGCCAAGACAGAGAAGUU 3'
. .::::::::::::::
query 3' UAUGGUUCUGUCUCUUCAU 5'
query=miR9492, target=RO3G_14451, score=3, range=3207-3224, stran
target 5' AUA-CAAGGCAGGGAAGUA 3'
::: ::::.:::.::::::
query 3' UAUGGUUCUGUCUCUUCAU 5'
query=miR9492, target=RO3G_05900, score=3, range=311-328, strand=
target 5' AGACCAAGACAGAG-AGUA 3'
: ::::::::::: ::::
query 3' UAUGGUUCUGUCUCUUCAU 5'
query=miR9492, target=RO3G_14783, score=3.5, range=1-19, strand=1
target 5' AUGCCAAGGCAGAGAAAUA 3'
::.::::.::::::: ::
query 3' UAUGGUUCUGUCUCUUCAU 5'
query=miR9492, target=RO3G_17309, score=3.5, range=940-958, stran
target 5' AUGCCAAGGCAGAGAAAUA 3'
::.::::.::::::: ::
query 3' UAUGGUUCUGUCUCUUCAU 5'
query=miR9492, target=RO3G_17372, score=3.5, range=940-958, stran
target 5' AUGCCAAGGCAGAGAAAUA 3'
::.::::.::::::: ::
query 3' UAUGGUUCUGUCUCUUCAU 5'
query=miR9492, target=RO3G_06411, score=3.5, range=940-958, stran
target 5' AUGCCAAGGCAGAGAAAUA 3'
::.::::.::::::: ::
query 3' UAUGGUUCUGUCUCUUCAU 5'
query=miR9492, target=RO3G_03783, score=3.5, range=940-958, stran
target 5' AUGCCAAGGCAGAGAAAUA 3'
::.::::.::::::: ::
query 3' UAUGGUUCUGUCUCUUCAU 5'
query=miR9492, target=RO3G_17311, score=3.5, range=940-958, stran
target 5' AUGCCAAGGCAGAGAAAUA 3'
::.::::.::::::: ::
query 3' UAUGGUUCUGUCUCUUCAU 5'
```

```
query=miR9492, target=RO3G_13292, score=3.5, range=940-958, stran
target 5' AUGCCAAGGCAGAGAAAUA 3'
::.:::::.::::::: ::
query 3' UAUGGUUCUGUCUCUUCAU 5'
query=miR9492, target=RO3G_09359, score=3.5, range=1835-1853, str
target 5' AGACCAAGACAGAGAAGAG 3'
: ::::::::::::::: .
query 3' UAUGGUUCUGUCUCUUCAU 5'
query=miR9492, target=RO3G_09646, score=3.5, range=372-390, stran
target 5' UGAUGAAGACAGAGAAGUA 3'
:. ::::::::::::::
query 3' UAUGGUUCUGUCUCUUCAU 5'
query=miR9492, target=RO3G_02887, score=3.5, range=402-419, stran
target 5' GUACC-AGAAAGAGAAGUA 3'
.:::: ::: :::::::::
query 3' UAUGGUUCUGUCUCUUCAU 5'
query=miR9492, target=RO3G_15040, score=4, range=44-62, strand=1
target 5' AUGUCAAGACAGAGAAGGC 3'
::..:::::::::::::
query 3' UAUGGUUCUGUCUCUUCAU 5'
query=miR9492, target=RO3G_02575, score=4, range=482-500, strand=
target 5' AUAUCGAGACAGAAAAGUU 3'
:::.:.::::::: ::::
query 3' UAUGGUUCUGUCUCUUCAU 5'
query=miR9492, target=RO3G_06617, score=4, range=512-529, strand=
target 5' AUAUCAAGACAGAGGA-UG 3'
:::.::::::::::.: :.
query 3' UAUGGUUCUGUCUCUUCAU 5'
query=miR9492, target=RO3G_10607, score=4, range=3154-3173, stran
target 5' GUGUCCAAGACAGAGAAAUA 3'
.:. ::::::::::::: ::
query 3' UAU-GGUUCUGUCUCUUCAU 5'
query=miR9492, target=RO3G_00056, score=4, range=935-952, strand=
target 5' AUA-CAAGAUACAGAAGUA 3'
::: :::::.: :::::::
query 3' UAUGGUUCUGUCUCUUCAU 5'
query=miR9492, target=RO3G_13059, score=4, range=758-775, strand=
target 5' AUACAAAG-CAGAGAAGUC 3'
:::: ::: :::::::::
query 3' UAUGGUUCUGUCUCUUCAU 5'
query=miR9492, target=RO3G_13636, score=4, range=1515-1532, stran
target 5' AUACGAAGACA-AGAAGUU 3'
:::: :::::: ::::::
query 3' UAUGGUUCUGUCUCUUCAU 5'
query=miR9492, target=RO3G_11230, score=4, range=1515-1532, stran
```

```
target 5' AUACGAAGACA-AGAAGUU 3'
:::: :::::: ::::::
query 3' UAUGGUUCUGUCUCUUCAU 5'
query=miR9492, target=RO3G_07862, score=4, range=2058-2077, stran
target 5' CUACCAAAGGCAGAGGAGUA 3'
:::: :::.::::.::::
query 3' UAUGG-UUCUGUCUCUUCAU 5'
query=miR9492, target=RO3G_10744, score=4, range=1947-1966, stran
target 5' CUACCAAAGGCAGAGGAGUA 3'
:::: :::.::::.::::
query 3' UAUGG-UUCUGUCUCUUCAU 5'
query=miR9492, target=RO3G_10745, score=4, range=915-934, strand=
target 5' CUACCAAAGGCAGAGGAGUA 3'
:::: :::.::::.::::
query 3' UAUGG-UUCUGUCUCUUCAU 5'
query=miR9492, target=RO3G_16029, score=4, range=738-755, strand=
target 5' GUACCGAGAUAGA-AAGUA 3'
.::::.:::.::: :::::
query 3' UAUGGUUCUGUCUCUUCAU 5'
query=miR9492, target=RO3G_04232, score=4, range=698-715, strand=
target 5' AUA-CAUGACAGAGAGGUA 3'
::: :: ::::::::.:::
query 3' UAUGGUUCUGUCUCUUCAU 5'
query=miR7542, target=RO3G_01330, score=0, range=378-397, strand=
target 5' UACCAUCUAUGAACGCAACA 3'
::::::::::::::::::::
query 3' AUGGUAGAUACUUGCGUUGU 5'
query=miR7542, target=RO3G_06428, score=0.5, range=2238-2257,
strand=-1
target 5' UAUCAUCUAUGAACGCAACA 3'
::.:::::::::::::::::
query 3' AUGGUAGAUACUUGCGUUGU 5'
query=miR7542, target=RO3G_02222, score=0.5, range=198-217, stran
target 5' UAUCAUCUAUGAACGCAACA 3'
::.:::::::::::::::::
query 3' AUGGUAGAUACUUGCGUUGU 5'
query=miR7542, target=RO3G_02959, score=0.5, range=3144-3163,
strand=-1
target 5' UAUCAUCUAUGAACGCAACA 3'
::.:::::::::::::::::
query 3' AUGGUAGAUACUUGCGUUGU 5'
query=miR7542, target=RO3G_05619, score=0.5, range=1287-1306,
strand=-1
target 5' UAUCAUCUAUGAACGCAACA 3'
::.:::::::::::::::::
```

query 3' AUGGUAGAUACUUGCGUUGU 5'
query=miR7542, target=RO3G_06427, score=0.5, range=2736-2755, strand=-1
target 5' UAUCAUCUAUGAACGCAACA 3'
::.:::::::::::::::::
query 3' AUGGUAGAUACUUGCGUUGU 5'
query=miR7542, target=RO3G_09965, score=0.5, range=3144-3163, strand=-1
target 5' UAUCAUCUAUGAACGCAACA 3'
::.:::::::::::::::::
query 3' AUGGUAGAUACUUGCGUUGU 5'
query=miR7542, target=RO3G_13920, score=0.5, range=3144-3163, strand=-1
target 5' UAUCAUCUAUGAACGCAACA 3'
::.:::::::::::::::::
query 3' AUGGUAGAUACUUGCGUUGU 5'
query=miR7542, target=RO3G_03547, score=0.5, range=3144-3163, strand=-1
target 5' UAUCAUCUAUGAACGCAACA 3'
::.:::::::::::::::::
query 3' AUGGUAGAUACUUGCGUUGU 5'
query=miR7542, target=RO3G_02691, score=0.5, range=6633-6652, strand=-1
target 5' UAUCAUCUAUGAACGCAACA 3'
::.:::::::::::::::::
query 3' AUGGUAGAUACUUGCGUUGU 5'
query=miR7542, target=RO3G_06442, score=0.5, range=3801-3820, strand=-1
target 5' UAUCAUCUAUGAACGCAACA 3'
::.:::::::::::::::::
query 3' AUGGUAGAUACUUGCGUUGU 5'
query=miR7542, target=RO3G_11776, score=1.5, range=234-253, stran
target 5' UAUCAUCUAUGAAUGCAACA 3'
::.:::::::::.::::::
query 3' AUGGUAGAUACUUGCGUUGU 5'
query=miR7542, target=RO3G_13867, score=1.5, range=3060-3079, strand=-1
target 5' UAUCAUCUAUGAAUGCAACA 3'
::.:::::::::.::::::
query 3' AUGGUAGAUACUUGCGUUGU 5'
query=miR7542, target=RO3G_13749, score=1.5, range=1707-1726, strand=-1
target 5' UAUCAUCUAUGAAUGCAACA 3'
::.:::::::::.::::::
query 3' AUGGUAGAUACUUGCGUUGU 5'

query=miR7542, target=RO3G_04115, score=1.5, range=87-106, strand
target 5' UAUCAUCUAUGAAUGCAACA 3'
::.:::::::::.::::::
query 3' AUGGUAGAUACUUGCGUUGU 5'
query=miR7542, target=RO3G_01613, score=1.5, range=1395-1414, strand=-1
target 5' UAUCAUCUAUGAAUGCAACA 3'
::.:::::::::.::::::
query 3' AUGGUAGAUACUUGCGUUGU 5'
query=miR7542, target=RO3G_10558, score=1.5, range=897-916, stran
target 5' UAUCAUCUAUGAAUGCAACA 3'
::.:::::::::.::::::
query 3' AUGGUAGAUACUUGCGUUGU 5'
query=miR7542, target=RO3G_17326, score=1.5, range=2976-2995, strand=-1
target 5' UAUCAUCUAUGAAUGCAACA 3'
::.:::::::::.::::::
query 3' AUGGUAGAUACUUGCGUUGU 5'
query=miR7542, target=RO3G_14355, score=1.5, range=1350-1369, strand=-1
target 5' UAUCAUCUAUGAAUGCAACA 3'
::.:::::::::.::::::
query 3' AUGGUAGAUACUUGCGUUGU 5'
query=miR7542, target=RO3G_05279, score=1.5, range=90-109, strand
target 5' UAUCAUCUAUGAAUGCAACA 3'
::.:::::::::.::::::
query 3' AUGGUAGAUACUUGCGUUGU 5'
query=miR7542, target=RO3G_17371, score=1.5, range=3099-3118, strand=-1
target 5' UAUCAUCUAUGAAUGCAACA 3'
::.:::::::::.::::::
query 3' AUGGUAGAUACUUGCGUUGU 5'
query=miR7542, target=RO3G_15250, score=1.5, range=3099-3118, strand=-1
target 5' UAUCAUCUAUGAAUGCAACA 3'
::.:::::::::.::::::
query 3' AUGGUAGAUACUUGCGUUGU 5'
query=miR7542, target=RO3G_12788, score=3, range=348-366, strand=
target 5' UACGA-CUAUGAACGCAAUA 3'
::: : ::::::::::::.:
query 3' AUGGUAGAUACUUGCGUUGU 5'
query=miR7542, target=RO3G_13422, score=4, range=618-637, strand=
target 5' UGCUGUCUAUGAACUCAACG 3'
:.:..::::::::: ::::.
query 3' AUGGUAGAUACUUGCGUUGU 5'

```
query=miR7542, target=RO3G_12316, score=4, range=594-613, strand=
target 5' UGCUGUCUAUGAACUCAACG 3'
:.:..::::::::: ::::.
query 3' AUGGUAGAUACUUGCGUUGU 5'
query=miR7542, target=RO3G_13406, score=4, range=594-613, strand=
target 5' UGCUGUCUAUGAACUCAACG 3'
:.:..::::::::: ::::.
query 3' AUGGUAGAUACUUGCGUUGU 5'
query=miR7542, target=RO3G_01494, score=4, range=1754-1773, stran
target 5' AAUUGUUUAUGAACGCAACC 3'
:...:.::::::::::::
query 3' AUGGUAGAUACUUGCGUUGU 5'
query=miR7542, target=RO3G_11117, score=4, range=294-312, strand=
target 5' UACCAUCUAUGAA-GUAGCA 3'
::::::::::::: :.:.::
query 3' AUGGUAGAUACUUGCGUUGU 5'
query=miR7542, target=RO3G_02560, score=4, range=1093-1113, stran
target 5' UGUCAUCUUAUGAAUGCAACU 3'
:..:::: :::::.::::::
query 3' AUGGUAG-AUACUUGCGUUGU 5'
query=miR7542, target=RO3G_16285, score=4, range=53-72, strand=1
target 5' UUCGAUCUAUGAAUGCAGCA 3'
: : :::::::::.:::.::
query 3' AUGGUAGAUACUUGCGUUGU 5'
query=miR7542, target=RO3G_17390, score=4, range=594-614, strand=
target 5' UGCUACCUAUGAACGCAAGCA 3'
:.:.: :::::::::::: ::
query 3' AUGGUAGAUACUUGCGUU-GU 5'
query=miR7542, target=RO3G_02872, score=4, range=3755-3775, stran
target 5' CACCAGUCUAUGAACGCAAUC 3'
:::: :::::::::::::.
query 3' AUGGU-AGAUACUUGCGUUGU 5'
query=miR7542, target=RO3G_06268, score=4, range=140-160, strand=
target 5' AACCUUCUAUGAACAGCAACA 3'
::: ::::::::: ::::::
query 3' AUGGUAGAUACUUG-CGUUGU 5'
query=miR7542, target=RO3G_15995, score=4, range=375-393, strand=
target 5' UGCCAGCUAUGAA-GCAACG 3'
:.::: ::::::: :::::.
query 3' AUGGUAGAUACUUGCGUUGU 5'
query=miR159a, target=RO3G_01784, score=3, range=322-341, strand=
target 5' AGAAUUUCCUUCAAUUCAAA 3'
::: .:.::::::::.::::
query 3' UCUCGAGGGAAGUUAGGUUU 5'
query=miR159a, target=RO3G_12083, score=3.5, range=46-65, strand=
```

```
target 5' AGAAGUCCUUUCAAUCCAAG 3'
::: :::.:::::::::::.
query 3' UCUCGAGGGAAGUUAGGUUU 5'
query=miR159a, target=RO3G_14268, score=3.5, range=37-55, strand=
target 5' ACAGCU-UCUUCAAUCCAAG 3'
: :::: .:::::::::::.
query 3' UCUCGAGGGAAGUUAGGUUU 5'
query=miR159a, target=RO3G_02134, score=3.5, range=401-420, stran
target 5' GGAGCUACAUUCAAUCCAAA 3'
.::::: : ::::::::::
query 3' UCUCGAGGGAAGUUAGGUUU 5'
query=miR159a, target=RO3G_00458, score=4, range=48-66, strand=1
target 5' UGAGC-GCCUUCAAUCCGAA 3'
:::: ::::::::::.::
query 3' UCUCGAGGGAAGUUAGGUUU 5'
query=miR159a, target=RO3G_11680, score=4, range=801-819, strand=
target 5' AGAGAU-CAUUCAAUCCAAA 3'
:::: : : ::::::::::
query 3' UCUCGAGGGAAGUUAGGUUU 5'
query=miR159a, target=RO3G_06157, score=4, range=667-687, strand=
target 5' AGAGCUUUCUAUCGAUCCAAA 3'
::::::..:: ::.:::::::
query 3' UCUCGAGGGA-AGUUAGGUUU 5'
query=miR4244, target=RO3G_04497, score=1, range=221-240, strand=
target 5' CACAUCUCCAGGAACAAUUA 3'
:::::::::::::::::.::
query 3' GUGUAGAGGUCCUUGUUGAU 5'
query=miR4244, target=RO3G_02365, score=1, range=221-240, strand=
target 5' CACACCUCCAGGAACAACUA 3'
:::: :::::::::::::::
query 3' GUGUAGAGGUCCUUGUUGAU 5'
query=miR4244, target=RO3G_13029, score=1, range=155-174, strand=
target 5' CACAACUCCAGGAACAACUA 3'
:::: :::::::::::::::
query 3' GUGUAGAGGUCCUUGUUGAU 5'
query=miR4244, target=RO3G_16979, score=1, range=71-90, strand=1
target 5' CACACCUCCAGGAACAACUA 3'
:::: :::::::::::::::
query 3' GUGUAGAGGUCCUUGUUGAU 5'
query=miR4244, target=RO3G_11695, score=1.5, range=221-240, stran
target 5' CAUACCUCCAGGAACAACUA 3'
::.: :::::::::::::::
query 3' GUGUAGAGGUCCUUGUUGAU 5'
query=miR4244, target=RO3G_12568, score=2, range=464-483, strand=
target 5' CACACCUUCAGGAACAACUA 3'
```

```
:::: ::.::::::::::::
query 3' GUGUAGAGGUCCUUGUUGAU 5'
query=miR4244, target=RO3G_12482, score=4, range=617-637, strand=
target 5' CAUUAUUUCCAGGAACAACCA 3'
:: .::.::::::::::::: :
query 3' GU-GUAGAGGUCCUUGUUGAU 5'
query=miR4244, target=RO3G_02714, score=4, range=705-723, strand=
target 5' CACAU-UCAAGGAACAAUUA 3'
::::: :: ::::::::.::
query 3' GUGUAGAGGUCCUUGUUGAU 5'
query=miR4244, target=RO3G_03106, score=4, range=186-204, strand=
target 5' UA-AUCGUCAGGAACAACUG 3'
.: ::: .:::::::::::.
query 3' GUGUAGAGGUCCUUGUUGAU 5'
query=miR4244, target=RO3G_13937, score=4, range=184-202, strand=
target 5' CA-AACACCAGGAACAGCUA 3'
:: : : :::::::::.:::
query 3' GUGUAGAGGUCCUUGUUGAU 5'
query=miR4244, target=RO3G_11363, score=4, range=911-930, strand=
target 5' UACAGUUUCAGGAACAACUU 3'
.::: .:.::::::::::
query 3' GUGUAGAGGUCCUUGUUGAU 5'
query=miR4244, target=RO3G_04292, score=4, range=1586-1606, stran
target 5' UACAGCUUCCAGGAACAAUUG 3'
.::: : ::::::::::.:.
query 3' GUGUAG-AGGUCCUUGUUGAU 5'
query=miR2595, target=RO3G_15743, score=0, range=946-966, strand=
target 5' AGACAGAGAAGUAGAAAUGUA 3'
:::::::::::::::::::::
query 3' UCUGUCUCUUCAUCUUUACAU 5'
query=miR2595, target=RO3G_16060, score=4, range=18-38, strand=1
target 5' ACAAAAAGAAGUAGAAAUGUU 3'
: : : ::::::::::::::
query 3' UCUGUCUCUUCAUCUUUACAU 5'
query=miR2595, target=RO3G_07199, score=4, range=98-119, strand=1
target 5' AAAGAGAUGAAGUAGAAAUGUU 3'
: : ::: :::::::::::::
query 3' UCUGUCU-CUUCAUCUUUACAU 5'
query=miR2595, target=RO3G_08289, score=4, range=26-45, strand=1
target 5' AGAAAAAGAA-UAGAAAUGUA 3'
::: : :::: ::::::::::
query 3' UCUGUCUCUUCAUCUUUACAU 5'
query=miR2595, target=RO3G_11137, score=4, range=206-225, strand=
target 5' AGAUA-AGAAGUAGACAUGUG 3'
:::.: ::::::::: ::::.
```

query 3' UCUGUCUCUUCAUCUUUACAU 5'
query=miR6148b, target=RO3G_06693, score=2.5, range=66-83, strand
target 5' UGUACCAAACAUAACAGG 3'
:: : ::::::::::::.
query 3' ACCUUGUUUGUAUUGUCU 5'
query=miR6148b, target=RO3G_16582, score=2.5, range=256-273, stra
target 5' AGGAGCAAAUAUAACAGA 3'
:::.::::.:::::::
query 3' ACCUUGUUUGUAUUGUCU 5'
query=miR6148b, target=RO3G_02259, score=3, range=1633-1651, stra
target 5' UGGAACAAAUAUAAUCAGA 3'
:::::::.:::: ::::
query 3' ACCUUGUUUGUAUU-GUCU 5'
query=miR6148b, target=RO3G_13408, score=3, range=43-60, strand=1
target 5' AUGGGCAAACAUAACAGA 3'
:..:::::::::::
query 3' ACCUUGUUUGUAUUGUCU 5'
query=miR6148b, target=RO3G_16217, score=3, range=2122-2139, stra
target 5' UUGAAUAAACAUAACAGC 3'
: :::.::::::::::
query 3' ACCUUGUUUGUAUUGUCU 5'
query=miR6148b, target=RO3G_01753, score=3, range=749-765, strand
target 5' UAGAACAAACAU-ACAGA 3'
: ::::::::: :::::
query 3' ACCUUGUUUGUAUUGUCU 5'
query=miR6148b, target=RO3G_03392, score=3.5, range=208-225, stra
target 5' UGGGACAAACAUAUCGGA 3'
:::.:::::::: :.::
query 3' ACCUUGUUUGUAUUGUCU 5'
query=miR6148b, target=RO3G_14433, score=3.5, range=4312-4329,
strand=-1
target 5' UGUAGCAAACAUAGUAGA 3'
:: :.::::::::..:::
query 3' ACCUUGUUUGUAUUGUCU 5'
query=miR6148b, target=RO3G_12831, score=3.5, range=655-673, stra
target 5' UGGCAGCAAAUAUAGCAGA 3'
::: :.::::.:::.::::
query 3' ACC-UUGUUUGUAUUGUCU 5'
query=miR6148b, target=RO3G_07832, score=3.5, range=55-72, strand
target 5' AGAGACAAACAUAAUAGA 3'
: .::::::::::.:::
query 3' ACCUUGUUUGUAUUGUCU 5'
query=miR6148b, target=RO3G_08867, score=3.5, range=23-39, strand
target 5' UGCGACAAACA-AACAGA 3'
:: .::::::: ::::::

```
query 3' ACCUUGUUUGUAUUGUCU 5'
query=miR6148b, target=RO3G_09626, score=3.5, range=318-336, stra
target 5' UCGAGCAACACAUAACAGA 3'
: ::.::: :::::::::
query 3' ACCUUGUU-UGUAUUGUCU 5'
query=miR6148b, target=RO3G_05800, score=4, range=397-413, strand
target 5' UGGAACGAACA-AACGGA 3'
:::::.:::: :::.::
query 3' ACCUUGUUUGUAUUGUCU 5'
query=miR6148b, target=RO3G_09803, score=4, range=997-1014, stran
target 5' CUUAACAAAUAUAACAGA 3'
:::::.::::::::
query 3' ACCUUGUUUGUAUUGUCU 5'
query=miR6148b, target=RO3G_03474, score=4, range=450-467, strand
target 5' AUCAAUAAACAUAACAGA 3'
::.:::::::::::
query 3' ACCUUGUUUGUAUUGUCU 5'
query=miR6148b, target=RO3G_06487, score=4, range=814-830, strand
target 5' UGGGAUAAACAU-ACAGG 3'
:::.:.:::::: ::::.
query 3' ACCUUGUUUGUAUUGUCU 5'
query=miR6148b, target=RO3G_05297, score=4, range=2586-2603, stra
target 5' UAAAACAAGCAUGACAGA 3'
: :::::.:::.:::::
query 3' ACCUUGUUUGUAUUGUCU 5'
query=miR6148b, target=RO3G_03447, score=4, range=829-846, strand
target 5' UCAAACGAACAUAACGGA 3'
: :::.:::::::::.::
query 3' ACCUUGUUUGUAUUGUCU 5'
query=miR6148b, target=RO3G_03802, score=4, range=400-417, strand
target 5' UACAAUAGACAUAACAGA 3'
: ::.:.::::::::::
query 3' ACCUUGUUUGUAUUGUCU 5'
query=miR6148b, target=RO3G_12527, score=4, range=57-74, strand=1
target 5' UGAAACAAACAUAGAAGA 3'
:: ::::::::::. :::
query 3' ACCUUGUUUGUAUUGUCU 5'
query=miR6148b, target=RO3G_10494, score=4, range=237-254, strand
target 5' UGCAACAAAUAUAACAAA 3'
:: ::::::.:::::: :
query 3' ACCUUGUUUGUAUUGUCU 5'
query=miR6148b, target=RO3G_17170, score=4, range=57-74, strand=1
target 5' UGAAACAAACAUAGAAGA 3'
:: ::::::::::. :::
query 3' ACCUUGUUUGUAUUGUCU 5'
```

```
query=miR6148b, target=RO3G_09920, score=4, range=57-74, strand=1
target 5' UGAAACAAACAUAGAAGA 3'
:: ::::::::::. :::
query 3' ACCUUGUUUGUAUUGUCU 5'
query=miR6148b, target=RO3G_05519, score=4, range=57-74, strand=1
target 5' UGAAACAAACAUAGAAGA 3'
:: ::::::::::. :::
query 3' ACCUUGUUUGUAUUGUCU 5'
query=miR6148b, target=RO3G_05185, score=4, range=1021-1039, stra
target 5' UGUGAACAAGCAGAACAGA 3'
:: ::::::.:: ::::::
query 3' AC-CUUGUUUGUAUUGUCU 5'
query=miR6148b, target=RO3G_14029, score=4, range=249-266, strand
target 5' UUUGGCAAACAUGACAGA 3'
: ..::::::::.:::::
query 3' ACCUUGUUUGUAUUGUCU 5'
query=miR6148b, target=RO3G_08323, score=4, range=625-642, strand
target 5' AAGAACAAACAUAACACA 3'
:::::::::::::: :
query 3' ACCUUGUUUGUAUUGUCU 5'
query=miR6148b, target=RO3G_14174, score=4, range=3669-3686, stra
target 5' UGAUACAAACAUGAUAGA 3'
:: ::::::::.:.:::
query 3' ACCUUGUUUGUAUUGUCU 5'
query=miR6148b, target=RO3G_06253, score=4, range=185-201, strand
target 5' UGGAA-AAACAUAAUAGU 3'
::::: ::::::::.::
query 3' ACCUUGUUUGUAUUGUCU 5'
query=miR6148b, target=RO3G_12768, score=4, range=1347-1364, stra
target 5' UACAACAAACAUAACGGU 3'
: ::::::::::::.:
query 3' ACCUUGUUUGUAUUGUCU 5'
query=miR6148b, target=RO3G_11902, score=4, range=115-132, strand
target 5' UGGUACAAACAUCACAGU 3'
::: :::::::: ::::
query 3' ACCUUGUUUGUAUUGUCU 5'
query=miR6148b, target=RO3G_00837, score=4, range=1393-1410, stra
target 5' UUGAACAAACACAACAGC 3'
: ::::::::: :::::
query 3' ACCUUGUUUGUAUUGUCU 5'
query=miR6148b, target=RO3G_13559, score=4, range=132-149, strand
target 5' UUCAACAAACAUAACAAA 3'
: ::::::::::::: :
query 3' ACCUUGUUUGUAUUGUCU 5'
query=miR6148b, target=RO3G_17187, score=4, range=864-880, strand
```

target 5' UGCGACAAACAU-ACAGG 3'
:: .:::::::: ::::.
query 3' ACCUUGUUUGUAUUGUCU 5'
query=miR6148b, target=RO3G_06187, score=4, range=378-395, strand
target 5' UCAAACAAUCAUAACAGA 3'
: ::::: :::::::::
query 3' ACCUUGUUUGUAUUGUCU 5'
query=miR6148b, target=RO3G_01503, score=4, range=19-36, strand=1
target 5' UGCAUCAACCAUAACAGA 3'
:: : ::: :::::::::
query 3' ACCUUGUUUGUAUUGUCU 5'
query=miR6148b, target=RO3G_09701, score=4, range=115-131, strand
target 5' UUCAAC-AACAUAACAGA 3'
: ::: ::::::::::
query 3' ACCUUGUUUGUAUUGUCU 5'
query=miR6148b, target=RO3G_04288, score=4, range=473-489, strand
target 5' AGGAACAGACA-AACAGA 3'
::::::.::: ::::::
query 3' ACCUUGUUUGUAUUGUCU 5'
query=miR6148b, target=RO3G_13667, score=4, range=74-90, strand=1
target 5' UAAAACAAACAU-ACAGA 3'
: ::::::::: :::::
query 3' ACCUUGUUUGUAUUGUCU 5'
query=miR6148b, target=RO3G_08814, score=4, range=158-175, strand
target 5' UGGGGCAAGUAUAACAGU 3'
:::..:::..:::::::
query 3' ACCUUGUUUGUAUUGUCU 5'
query=miR6148b, target=RO3G_15457, score=4, range=15-32, strand=-
target 5' UGUAAUAAAUGUAACAGA 3'
:: ::.:::..:::::::
query 3' ACCUUGUUUGUAUUGUCU 5'
query=miR6148b, target=RO3G_01056, score=4, range=1462-1479, stra
target 5' UGUGGCAAAUAUAAUAGA 3'
:: ..::::.::::.:::
query 3' ACCUUGUUUGUAUUGUCU 5'
query=miR6148b, target=RO3G_08994, score=4, range=863-880, strand
target 5' UGGAAUAAACAUAAAAGU 3'
:::::.:::::::: ::
query 3' ACCUUGUUUGUAUUGUCU 5'
query=miR6148b, target=RO3G_00192, score=4, range=1738-1755, stra
target 5' UGCAACAGACAUAGCAGC 3'
:: ::::.:::::.:::
query 3' ACCUUGUUUGUAUUGUCU 5'
query=miR6148b, target=RO3G_09465, score=4, range=1399-1416, stra
target 5' AGGAUCAAAUGUAACAGA 3'

```
::: ::::..:::::::
query 3' ACCUUGUUUGUAUUGUCU 5'
query=miR6148b, target=RO3G_13835, score=4, range=10657-10674,
strand=-1
target 5' UGCAACAGAAAUAACAGA 3'
:: ::::.: ::::::::
query 3' ACCUUGUUUGUAUUGUCU 5'
query=miR6148b, target=RO3G_04094, score=4, range=1516-1533, stra
target 5' UGCAACAGAAAUAACAGA 3'
:: ::::.: ::::::::
query 3' ACCUUGUUUGUAUUGUCU 5'
query=miR6148b, target=RO3G_16608, score=4, range=880-897, strand
target 5' UGGACCAAACAAGACAGA 3'
:::: :::::: .:::::
query 3' ACCUUGUUUGUAUUGUCU 5'
query=miR6148b, target=RO3G_16604, score=4, range=880-897, strand
target 5' UGGACCAAACAAGACAGA 3'
:::: :::::: .:::::
query 3' ACCUUGUUUGUAUUGUCU 5'
query=miR6148b, target=RO3G_10476, score=4, range=1127-1144, stra
target 5' CCCAACAAACAUAACAGC 3'
::::::::::::::
query 3' ACCUUGUUUGUAUUGUCU 5'
query=miR6148b, target=RO3G_07479, score=4, range=379-396, strand
target 5' UUGACCAAGCAUAACAGU 3'
: :: :::.::::::::
query 3' ACCUUGUUUGUAUUGUCU 5'
query=miR6148b, target=RO3G_07765, score=4, range=737-753, strand
target 5' UGGCACAAAUAUAA-AGA 3'
::: :::::.:::: :::
query 3' ACCUUGUUUGUAUUGUCU 5'
query=miR6148b, target=RO3G_16036, score=4, range=1546-1563, stra
target 5' UUUACCAAACAUAGCAGA 3'
: : ::::::::.::::
query 3' ACCUUGUUUGUAUUGUCU 5'
query=miR6148b, target=RO3G_07543, score=4, range=265-282, strand
target 5' UGAAUCAAUCAUAACAGA 3'
:: : ::: :::::::::
query 3' ACCUUGUUUGUAUUGUCU 5'
query=miR1425-5p, target=RO3G_07690, score=0, range=175-194, stra
target 5' AGCAGCAAGGAUGAUCAGUA 3'
::::::::::::::::::::
query 3' UCGUCGUUCCUACUAGUCAU 5'
query=miR1425-5p, target=RO3G_00679, score=0, range=367-386, stra
target 5' AGCAGCAAGGAUGAUCAGUA 3'
```

```
::::::::::::::::::::
query 3' UCGUCGUUCCUACUAGUCAU 5'
query=miR1425-5p, target=RO3G_07707, score=0, range=367-386, stra
target 5' AGCAGCAAGGAUGAUCAGUA 3'
::::::::::::::::::::
query 3' UCGUCGUUCCUACUAGUCAU 5'
query=miR1425-5p, target=RO3G_11281, score=0, range=175-194, stra
target 5' AGCAGCAAGGAUGAUCAGUA 3'
::::::::::::::::::::
query 3' UCGUCGUUCCUACUAGUCAU 5'
query=miR1425-5p, target=RO3G_16719, score=0, range=175-194, stra
target 5' AGCAGCAAGGAUGAUCAGUA 3'
::::::::::::::::::::
query 3' UCGUCGUUCCUACUAGUCAU 5'
query=miR1425-5p, target=RO3G_16727, score=0, range=175-194, stra
target 5' AGCAGCAAGGAUGAUCAGUA 3'
::::::::::::::::::::
query 3' UCGUCGUUCCUACUAGUCAU 5'
query=miR1425-5p, target=RO3G_17408, score=0, range=175-194, stra
target 5' AGCAGCAAGGAUGAUCAGUA 3'
::::::::::::::::::::
query 3' UCGUCGUUCCUACUAGUCAU 5'
query=miR1425-5p, target=RO3G_03624, score=1, range=175-194, stra
target 5' AGCAGCAGGGAUGAUCAGUA 3'
:::::::.::::::::::::
query 3' UCGUCGUUCCUACUAGUCAU 5'
query=miR1425-5p, target=RO3G_01960, score=1, range=367-386, stra
target 5' AGCAGCAGGGAUGAUCAGUA 3'
:::::::.::::::::::::
query 3' UCGUCGUUCCUACUAGUCAU 5'
query=miR1425-5p, target=RO3G_16550, score=1, range=286-305, stra
target 5' AGCAGCAGGGAUGAUCAGUA 3'
:::::::.::::::::::::
query 3' UCGUCGUUCCUACUAGUCAU 5'
query=miR1425-5p, target=RO3G_02845, score=1, range=400-419, stra
target 5' AGCAGCAGGGAUGAUCAGUA 3'
:::::::.::::::::::::
query 3' UCGUCGUUCCUACUAGUCAU 5'
query=miR1425-5p, target=RO3G_03602, score=1, range=316-335, stra
target 5' AGCAGCAGGGAUGAUCAGUA 3'
:::::::.::::::::::::
query 3' UCGUCGUUCCUACUAGUCAU 5'
query=miR1425-5p, target=RO3G_02706, score=1, range=367-386, stra
target 5' AGCAGCAGGGAUGAUCAGUA 3'
:::::::.::::::::::::
```

```
query 3' UCGUCGUUCCUACUAGUCAU 5'
query=miR1425-5p, target=RO3G_13286, score=1, range=367-386, stra
target 5' AGCAGCAGGGAUGAUCAGUA 3'
:::::::.::::::::::::
query 3' UCGUCGUUCCUACUAGUCAU 5'
query=miR1425-5p, target=RO3G_13919, score=1, range=367-386, stra
target 5' AGCAGCAGGGAUGAUCAGUA 3'
:::::::.::::::::::::
query 3' UCGUCGUUCCUACUAGUCAU 5'
query=miR1425-5p, target=RO3G_00688, score=1, range=175-194, stra
target 5' AGCAGCAGGGAUGAUCAGUA 3'
:::::::.::::::::::::
query 3' UCGUCGUUCCUACUAGUCAU 5'
query=miR1425-5p, target=RO3G_10741, score=1, range=175-194, stra
target 5' AGCAGCAGGGAUGAUCAGUA 3'
:::::::.::::::::::::
query 3' UCGUCGUUCCUACUAGUCAU 5'
query=miR1425-5p, target=RO3G_13743, score=1, range=175-194, stra
target 5' AGCAGCAGGGAUGAUCAGUA 3'
:::::::.::::::::::::
query 3' UCGUCGUUCCUACUAGUCAU 5'
query=miR1425-5p, target=RO3G_10763, score=1, range=175-194, stra
target 5' AGCAGCAGGGAUGAUCAGUA 3'
:::::::.::::::::::::
query 3' UCGUCGUUCCUACUAGUCAU 5'
query=miR1425-5p, target=RO3G_10769, score=1, range=52-71, strand
target 5' AGCAGCAGGGAUGAUCAGUA 3'
:::::::.::::::::::::
query 3' UCGUCGUUCCUACUAGUCAU 5'
query=miR1425-5p, target=RO3G_10550, score=2, range=175-194, stra
target 5' AGCAACAGGGAUGAUCAGUA 3'
:::: ::.::::::::::::
query 3' UCGUCGUUCCUACUAGUCAU 5'
query=miR1425-5p, target=RO3G_17222, score=2, range=367-386, stra
target 5' AGCAGAAGGGAUGAUCAGUA 3'
::::: :.::::::::::::
query 3' UCGUCGUUCCUACUAGUCAU 5'
query=miR1425-5p, target=RO3G_12678, score=2, range=367-386, stra
target 5' AACAGCAGGGAUGAUCAGUA 3'
: :::::.::::::::::::
query 3' UCGUCGUUCCUACUAGUCAU 5'
query=miR1425-5p, target=RO3G_10787, score=2, range=175-194, stra
target 5' AGCAACAGGGAUGAUCAGUA 3'
:::: ::.::::::::::::
query 3' UCGUCGUUCCUACUAGUCAU 5'
```

```
query=miR1425-5p, target=RO3G_15583, score=3, range=40-59, strand
target 5' AGCAGCAGCGAUGAUCAGUA 3'
:::::::. ::::::::::: 
query 3' UCGUCGUUCCUACUAGUCAU 5'
query=miR1425-5p, target=RO3G_08847, score=3, range=175-194, stra
target 5' AGCAGCAGGAAUGAUCAGUA 3'
:::::::.: :::::::::: 
query 3' UCGUCGUUCCUACUAGUCAU 5'
query=miR1425-5p, target=RO3G_12166, score=3, range=175-194, stra
target 5' AGCAGCAGCGAUGAUCAGUA 3'
:::::::. ::::::::::: 
query 3' UCGUCGUUCCUACUAGUCAU 5'
query=miR1425-5p, target=RO3G_12658, score=3, range=175-194, stra
target 5' AGCAGCAGCGAUGAUCAGUA 3'
:::::::. ::::::::::: 
query 3' UCGUCGUUCCUACUAGUCAU 5'
query=miR1425-5p, target=RO3G_03188, score=3.5, range=2990-3008,
strand=1
target 5' GGCAGC-AGGAUGAUCGGUC 3'
.::::: :::::::::.::
query 3' UCGUCGUUCCUACUAGUCAU 5'
query=miR1425-5p, target=RO3G_00460, score=4, range=856-874, stra
target 5' AGUAGCAAGGAUGAU-GGUG 3'
::.::::::::::: .::.
query 3' UCGUCGUUCCUACUAGUCAU 5'
query=miR1425-5p, target=RO3G_15033, score=4, range=578-598, stra
target 5' AGCGACGAGGAUGAUCCAGUA 3'
:::. :.:::::::: :::::
query 3' UCGUCGUUCCUACUA-GUCAU 5'
query=miR1425-5p, target=RO3G_13880, score=4, range=968-988, stra
target 5' AGAAGCCAAGGAUGGUCAGUU 3'
:: :: ::::::::.:::::
query 3' UCGUC-GUUCCUACUAGUCAU 5'
query=miR1425-5p, target=RO3G_06641, score=4, range=282-300, stra
target 5' AGCA-CAAGGAUGAUUAUUA 3'
:::: ::::::::::.: ::
query 3' UCGUCGUUCCUACUAGUCAU 5'
query=miR1425-5p, target=RO3G_02515, score=4, range=360-378, stra
target 5' AGCA-UGAGGAUGAUCAGGA 3'
:::: ..:::::::::: :
query 3' UCGUCGUUCCUACUAGUCAU 5'
query=miR1425-5p, target=RO3G_07213, score=4, range=1079-1099,
strand=-1
target 5' AACAGGACAGGAUGAUCGGUA 3'
: ::: : :::::::::.:::
```

```
query 3' UCGUCGU-UCCUACUAGUCAU 5'
query=miR6270, target=RO3G_03556, score=0, range=702-723, strand=
target 5' AGAUGAAGAUUCCAAUAGCGAA 3'
::::::::::::::::::::::
query 3' UCUACUUCUAAGGUUAUCGCUU 5'
query=miR6270, target=RO3G_17281, score=0, range=138-159, strand=
target 5' AGAUGAAGAUUCCAAUAGCGAA 3'
::::::::::::::::::::::
query 3' UCUACUUCUAAGGUUAUCGCUU 5'
query=miR6270, target=RO3G_16803, score=0, range=138-159, strand=
target 5' AGAUGAAGAUUCCAAUAGCGAA 3'
::::::::::::::::::::::
query 3' UCUACUUCUAAGGUUAUCGCUU 5'
query=miR6270, target=RO3G_17374, score=0, range=138-159, strand=
target 5' AGAUGAAGAUUCCAAUAGCGAA 3'
::::::::::::::::::::::
query 3' UCUACUUCUAAGGUUAUCGCUU 5'
query=miR6270, target=RO3G_03794, score=0, range=138-159, strand=
target 5' AGAUGAAGAUUCCAAUAGCGAA 3'
::::::::::::::::::::::
query 3' UCUACUUCUAAGGUUAUCGCUU 5'
query=miR6270, target=RO3G_03780, score=0, range=138-159, strand=
target 5' AGAUGAAGAUUCCAAUAGCGAA 3'
::::::::::::::::::::::
query 3' UCUACUUCUAAGGUUAUCGCUU 5'
query=miR6270, target=RO3G_03583, score=1, range=702-723, strand=
target 5' AGAUGAAAAUUCCAAUAGCGAA 3'
::::::: ::::::::::::::
query 3' UCUACUUCUAAGGUUAUCGCUU 5'
query=miR6270, target=RO3G_05930, score=2, range=561-582, strand=
target 5' AGAUGAAGAUCCCAAUAGCGAA 3'
:::::::::: :::::::::::
query 3' UCUACUUCUAAGGUUAUCGCUU 5'
query=miR6270, target=RO3G_17462, score=2, range=1029-1050, stran
target 5' AGAUGACGAUUCCGAUAGCGAA 3'
:::::: ::::::.::::::::
query 3' UCUACUUCUAAGGUUAUCGCUU 5'
query=miR6270, target=RO3G_13914, score=2, range=897-918, strand=
target 5' AGAUGACGAUUCCGAUAGCGAA 3'
:::::: ::::::.::::::::
query 3' UCUACUUCUAAGGUUAUCGCUU 5'
query=miR6270, target=RO3G_12058, score=2, range=897-918, strand=
target 5' AGAUGACGAUUCCGAUAGCGAA 3'
:::::: ::::::.::::::::
query 3' UCUACUUCUAAGGUUAUCGCUU 5'
```

query=miR6270, target=RO3G_14983, score=2, range=702-723, strand=
target 5' AGAUGACGAUUCCGAUAGCGAA 3'
:::::: :::::.::::::::
query 3' UCUACUUCUAAGGUUAUCGCUU 5'
query=miR6270, target=RO3G_10801, score=2, range=702-723, strand=
target 5' AGAUGACGAUUCCGAUAGCGAA 3'
:::::: :::::.::::::::
query 3' UCUACUUCUAAGGUUAUCGCUU 5'
query=miR6270, target=RO3G_02233, score=2, range=426-447, strand=
target 5' AGAUGACGAUUCCGAUAGCGAA 3'
:::::: :::::.::::::::
query 3' UCUACUUCUAAGGUUAUCGCUU 5'
query=miR6270, target=RO3G_12667, score=2, range=267-288, strand=
target 5' AGAUGACGAUUCCGAUAGCGAA 3'
:::::: :::::.::::::::
query 3' UCUACUUCUAAGGUUAUCGCUU 5'
query=miR6270, target=RO3G_09614, score=2, range=228-249, strand=
target 5' AGAUGACGAUUCCGAUAGCGAA 3'
:::::: :::::.::::::::
query 3' UCUACUUCUAAGGUUAUCGCUU 5'
query=miR6270, target=RO3G_17451, score=2, range=138-159, strand=
target 5' AGAUGACGAUUCCGAUAGCGAA 3'
:::::: :::::.::::::::
query 3' UCUACUUCUAAGGUUAUCGCUU 5'
query=miR6270, target=RO3G_04831, score=2.5, range=1559-1580, str
target 5' AGAUGAGGAUUUCAAUAGCGAC 3'
::::::.:::.:::::::::
query 3' UCUACUUCUAAGGUUAUCGCUU 5'
query=miR6270, target=RO3G_04116, score=3, range=261-282, strand=
target 5' AGAAGAAGAUUCCAAUAUCGAA 3'
::: ::::::::::: ::::
query 3' UCUACUUCUAAGGUUAUCGCUU 5'
query=miR6270, target=RO3G_16714, score=4, range=255-276, strand=
target 5' AGACGAAGAUUCCAGUAUCGAA 3'
::: :::::::::.:: ::::
query 3' UCUACUUCUAAGGUUAUCGCUU 5'
query=miR827-3p, target=RO3G_06727, score=0, range=102-124, stran
target 5' CGUUUGUUGGAUGGAUCAUAUUA 3'
:::::::::::::::::::::::
query 3' GCAAACAACCUACCUAGUAUAAU 5'
query=miR5532, target=RO3G_03575, score=0, range=926-947, strand=
target 5' CCAACUUGUGUGAUAUAUUCCA 3'
::::::::::::::::::::::
query 3' GGUUGAACACACUAUAUAAGGU 5'
query=miR5532, target=RO3G_12067, score=0, range=926-947, strand=

```
target 5' CCAACUUGUGUGAUAUAUUCCA 3'
::::::::::::::::::::::
query 3' GGUUGAACACACUAUAUAAGGU 5'
query=miR5532, target=RO3G_13855, score=0, range=926-947, strand=
target 5' CCAACUUGUGUGAUAUAUUCCA 3'
::::::::::::::::::::::
query 3' GGUUGAACACACUAUAUAAGGU 5'
query=miR5532, target=RO3G_13701, score=0, range=926-947, strand=
target 5' CCAACUUGUGUGAUAUAUUCCA 3'
::::::::::::::::::::::
query 3' GGUUGAACACACUAUAUAAGGU 5'
query=miR5532, target=RO3G_10222, score=0, range=926-947, strand=
target 5' CCAACUUGUGUGAUAUAUUCCA 3'
::::::::::::::::::::::
query 3' GGUUGAACACACUAUAUAAGGU 5'
query=miR5532, target=RO3G_12462, score=0, range=791-812, strand=
target 5' CCAACUUGUGUGAUAUAUUCCA 3'
::::::::::::::::::::::
query 3' GGUUGAACACACUAUAUAAGGU 5'
query=miR5532, target=RO3G_03796, score=0, range=119-140, strand=
target 5' CCAACUUGUGUGAUAUAUUCCA 3'
::::::::::::::::::::::
query 3' GGUUGAACACACUAUAUAAGGU 5'
query=miR5532, target=RO3G_00303, score=0, range=926-947, strand=
target 5' CCAACUUGUGUGAUAUAUUCCA 3'
::::::::::::::::::::::
query 3' GGUUGAACACACUAUAUAAGGU 5'
query=miR5532, target=RO3G_07666, score=0, range=926-947, strand=
target 5' CCAACUUGUGUGAUAUAUUCCA 3'
::::::::::::::::::::::
query 3' GGUUGAACACACUAUAUAAGGU 5'
query=miR5532, target=RO3G_17370, score=0, range=926-947, strand=
target 5' CCAACUUGUGUGAUAUAUUCCA 3'
::::::::::::::::::::::
query 3' GGUUGAACACACUAUAUAAGGU 5'
query=miR5532, target=RO3G_05626, score=0, range=926-947, strand=
target 5' CCAACUUGUGUGAUAUAUUCCA 3'
::::::::::::::::::::::
query 3' GGUUGAACACACUAUAUAAGGU 5'
query=miR5532, target=RO3G_12014, score=1, range=926-947, strand=
target 5' CCAACCUGUGUGAUAUAUUCCA 3'
::::: ::::::::::::::::
query 3' GGUUGAACACACUAUAUAAGGU 5'
query=miR5532, target=RO3G_03752, score=1, range=926-947, strand=
target 5' CCAACCUGUGUGAUAUAUUCCA 3'
```

```
::::: :::::::::::::::
query 3' GGUUGAACACACUAUAUAAGGU 5'
query=miR5532, target=RO3G_01852, score=3.5, range=392-412, stran
target 5' CUAA-UUGUGUGAUAUAUUCUU 3'
:.:: ::::::::::::::.
query 3' GGUUGAACACACUAUAUAAGGU 5'
query=miR5532, target=RO3G_04039, score=4, range=374-395, strand=
target 5' UUGAUAUGUGUGAUAUAUUCCC 3'
...:. ::::::::::::::
query 3' GGUUGAACACACUAUAUAAGGU 5'
query=miR5532, target=RO3G_02087, score=4, range=360-380, strand=
target 5' CCAAGUU-UGUGAUAUAUUUCU 3'
:::: :: :::::::::::.:
query 3' GGUUGAACACACUAUAUAAGGU 5'
query=miR5144-5p, target=RO3G_09194, score=0, range=353-371, stra
target 5' UCACUGCACAGCACAAGAA 3'
:::::::::::::::::::
query 3' AGUGACGUGUCGUGUUCUU 5'
query=miR5144-5p, target=RO3G_16798, score=0, range=353-371, stra
target 5' UCACUGCACAGCACAAGAA 3'
:::::::::::::::::::
query 3' AGUGACGUGUCGUGUUCUU 5'
query=miR5144-5p, target=RO3G_07038, score=0, range=353-371, stra
target 5' UCACUGCACAGCACAAGAA 3'
:::::::::::::::::::
query 3' AGUGACGUGUCGUGUUCUU 5'
query=miR5144-5p, target=RO3G_04803, score=0, range=353-371, stra
target 5' UCACUGCACAGCACAAGAA 3'
:::::::::::::::::::
query 3' AGUGACGUGUCGUGUUCUU 5'
query=miR5144-5p, target=RO3G_16796, score=0, range=353-371, stra
target 5' UCACUGCACAGCACAAGAA 3'
:::::::::::::::::::
query 3' AGUGACGUGUCGUGUUCUU 5'
query=miR5144-5p, target=RO3G_10516, score=0, range=353-371, stra
target 5' UCACUGCACAGCACAAGAA 3'
:::::::::::::::::::
query 3' AGUGACGUGUCGUGUUCUU 5'
query=miR5144-5p, target=RO3G_07040, score=0, range=353-371, stra
target 5' UCACUGCACAGCACAAGAA 3'
:::::::::::::::::::
query 3' AGUGACGUGUCGUGUUCUU 5'
query=miR5144-5p, target=RO3G_07124, score=0, range=353-371, stra
target 5' UCACUGCACAGCACAAGAA 3'
:::::::::::::::::::
```

```
query 3' AGUGACGUGUCGUGUUCUU 5'
query=miR5144-5p, target=RO3G_15664, score=0, range=353-371, stra
target 5' UCACUGCACAGCACAAGAA 3'
:::::::::::::::::::
query 3' AGUGACGUGUCGUGUUCUU 5'
query=miR5144-5p, target=RO3G_10729, score=0, range=353-371, stra
target 5' UCACUGCACAGCACAAGAA 3'
:::::::::::::::::::
query 3' AGUGACGUGUCGUGUUCUU 5'
query=miR5144-5p, target=RO3G_10743, score=0, range=353-371, stra
target 5' UCACUGCACAGCACAAGAA 3'
:::::::::::::::::::
query 3' AGUGACGUGUCGUGUUCUU 5'
query=miR5144-5p, target=RO3G_09945, score=0, range=77-95, strand
target 5' UCACUGCACAGCACAAGAA 3'
:::::::::::::::::::
query 3' AGUGACGUGUCGUGUUCUU 5'
query=miR5144-5p, target=RO3G_01942, score=0, range=353-371, stra
target 5' UCACUGCACAGCACAAGAA 3'
:::::::::::::::::::
query 3' AGUGACGUGUCGUGUUCUU 5'
query=miR5144-5p, target=RO3G_11264, score=0, range=353-371, stra
target 5' UCACUGCACAGCACAAGAA 3'
:::::::::::::::::::
query 3' AGUGACGUGUCGUGUUCUU 5'
query=miR5144-5p, target=RO3G_02956, score=0, range=353-371, stra
target 5' UCACUGCACAGCACAAGAA 3'
:::::::::::::::::::
query 3' AGUGACGUGUCGUGUUCUU 5'
query=miR5144-5p, target=RO3G_05435, score=0, range=353-371, stra
target 5' UCACUGCACAGCACAAGAA 3'
:::::::::::::::::::
query 3' AGUGACGUGUCGUGUUCUU 5'
query=miR5144-5p, target=RO3G_07030, score=0, range=353-371, stra
target 5' UCACUGCACAGCACAAGAA 3'
:::::::::::::::::::
query 3' AGUGACGUGUCGUGUUCUU 5'
query=miR5144-5p, target=RO3G_00256, score=0, range=353-371, stra
target 5' UCACUGCACAGCACAAGAA 3'
:::::::::::::::::::
query 3' AGUGACGUGUCGUGUUCUU 5'
query=miR5144-5p, target=RO3G_10780, score=0, range=353-371, stra
target 5' UCACUGCACAGCACAAGAA 3'
:::::::::::::::::::
query 3' AGUGACGUGUCGUGUUCUU 5'
```

```
query=miR5144-5p, target=RO3G_12070, score=0, range=353-371, stra
target 5' UCACUGCACAGCACAAGAA 3'
:::::::::::::::::::
query 3' AGUGACGUGUCGUGUUCUU 5'
query=miR5144-5p, target=RO3G_09542, score=0, range=353-371, stra
target 5' UCACUGCACAGCACAAGAA 3'
:::::::::::::::::::
query 3' AGUGACGUGUCGUGUUCUU 5'
query=miR5144-5p, target=RO3G_17449, score=0, range=353-371, stra
target 5' UCACUGCACAGCACAAGAA 3'
:::::::::::::::::::
query 3' AGUGACGUGUCGUGUUCUU 5'
query=miR5144-5p, target=RO3G_05939, score=0, range=353-371, stra
target 5' UCACUGCACAGCACAAGAA 3'
:::::::::::::::::::
query 3' AGUGACGUGUCGUGUUCUU 5'
query=miR5144-5p, target=RO3G_14361, score=0, range=353-371, stra
target 5' UCACUGCACAGCACAAGAA 3'
:::::::::::::::::::
query 3' AGUGACGUGUCGUGUUCUU 5'
query=miR5144-5p, target=RO3G_09967, score=0, range=353-371, stra
target 5' UCACUGCACAGCACAAGAA 3'
:::::::::::::::::::
query 3' AGUGACGUGUCGUGUUCUU 5'
query=miR5144-5p, target=RO3G_05267, score=0, range=353-371, stra
target 5' UCACUGCACAGCACAAGAA 3'
:::::::::::::::::::
query 3' AGUGACGUGUCGUGUUCUU 5'
query=miR5144-5p, target=RO3G_05264, score=0, range=353-371, stra
target 5' UCACUGCACAGCACAAGAA 3'
:::::::::::::::::::
query 3' AGUGACGUGUCGUGUUCUU 5'
query=miR5144-5p, target=RO3G_04210, score=0, range=353-371, stra
target 5' UCACUGCACAGCACAAGAA 3'
:::::::::::::::::::
query 3' AGUGACGUGUCGUGUUCUU 5'
query=miR5144-5p, target=RO3G_16142, score=0, range=356-374, stra
target 5' UCACUGCACAGCACAAGAA 3'
:::::::::::::::::::
query 3' AGUGACGUGUCGUGUUCUU 5'
query=miR5144-5p, target=RO3G_08660, score=0, range=353-371, stra
target 5' UCACUGCACAGCACAAGAA 3'
:::::::::::::::::::
query 3' AGUGACGUGUCGUGUUCUU 5'
query=miR5144-5p, target=RO3G_05921, score=0, range=353-371, stra
```

```
target 5' UCACUGCACAGCACAAGAA 3'
:::::::::::::::::::
query 3' AGUGACGUGUCGUGUUCUU 5'
query=miR5144-5p, target=RO3G_17069, score=0, range=218-236, stra
target 5' UCACUGCACAGCACAAGAA 3'
:::::::::::::::::::
query 3' AGUGACGUGUCGUGUUCUU 5'
query=miR5144-5p, target=RO3G_04207, score=0, range=353-371, stra
target 5' UCACUGCACAGCACAAGAA 3'
:::::::::::::::::::
query 3' AGUGACGUGUCGUGUUCUU 5'
query=miR5144-5p, target=RO3G_10773, score=0, range=353-371, stra
target 5' UCACUGCACAGCACAAGAA 3'
:::::::::::::::::::
query 3' AGUGACGUGUCGUGUUCUU 5'
query=miR5144-5p, target=RO3G_16431, score=1, range=221-239, stra
target 5' UCACUGUACAGCACAAGAA 3'
::::::.::::::::::::
query 3' AGUGACGUGUCGUGUUCUU 5'
query=miR5144-5p, target=RO3G_03557, score=1, range=179-197, stra
target 5' UCACUGCGCAGCACAAGAA 3'
:::::::.:::::::::::
query 3' AGUGACGUGUCGUGUUCUU 5'
query=miR5144-5p, target=RO3G_17325, score=2, range=353-371, stra
target 5' UCACUGCACAGCACAAAAA 3'
:::::::::::::::: ::
query 3' AGUGACGUGUCGUGUUCUU 5'
query=miR5144-5p, target=RO3G_11966, score=2, range=353-371, stra
target 5' UCACUGCACAGCACAAAAA 3'
:::::::::::::::: ::
query 3' AGUGACGUGUCGUGUUCUU 5'
query=miR5144-5p, target=RO3G_07860, score=2, range=353-371, stra
target 5' UCACUGCAGAGCACAAGAA 3'
:::::::: ::::::::::
query 3' AGUGACGUGUCGUGUUCUU 5'
query=miR5144-5p, target=RO3G_05953, score=2, range=353-371, stra
target 5' UCACUGCACAGCACAAAAA 3'
:::::::::::::::: ::
query 3' AGUGACGUGUCGUGUUCUU 5'
query=miR5144-5p, target=RO3G_05959, score=2.5, range=353-371,
strand=1
target 5' UCGCUGCACAGCACAAAAA 3'
::.::::::::::::: ::
query 3' AGUGACGUGUCGUGUUCUU 5'
query=miR5144-5p, target=RO3G_11238, score=2.5, range=221-239,
```

strand=1
target 5' UCGCUGCACAGCACAAAAA 3'
::.:::::::::::: ::
query 3' AGUGACGUGUCGUGUUCUU 5'
query=miR5144-5p, target=RO3G_05628, score=2.5, range=353-371,
strand=1
target 5' UCGCUGCACAGCACAAAAA 3'
::.:::::::::::: ::
query 3' AGUGACGUGUCGUGUUCUU 5'
query=miR5144-5p, target=RO3G_07103, score=2.5, range=77-95, stra
target 5' UCGCUGCACAGCACAAAAA 3'
::.:::::::::::: ::
query 3' AGUGACGUGUCGUGUUCUU 5'
query=miR5144-5p, target=RO3G_14568, score=2.5, range=353-371,
strand=1
target 5' UCGCUGCACAGCACAAAAA 3'
::.:::::::::::: ::
query 3' AGUGACGUGUCGUGUUCUU 5'
query=miR5144-5p, target=RO3G_16689, score=2.5, range=353-371,
strand=1
target 5' UCGCUGCACAGCACAAAAA 3'
::.:::::::::::: ::
query 3' AGUGACGUGUCGUGUUCUU 5'
query=miR5144-5p, target=RO3G_17308, score=2.5, range=353-371,
strand=1
target 5' UCGCUGCACAGCACAAAAA 3'
::.:::::::::::: ::
query 3' AGUGACGUGUCGUGUUCUU 5'
query=miR5144-5p, target=RO3G_10798, score=2.5, range=353-371,
strand=1
target 5' UCGCUGCACAGCACAAAAA 3'
::.:::::::::::: ::
query 3' AGUGACGUGUCGUGUUCUU 5'
query=miR5144-5p, target=RO3G_03037, score=2.5, range=1836-1853,
strand=-1
target 5' UC-CUUCACAGCACAAGAG 3'
:: :: :::::::::::.
query 3' AGUGACGUGUCGUGUUCUU 5'
query=miR5144-5p, target=RO3G_17083, score=3, range=1703-1721,
strand=1
target 5' UUGCUGAACAGCACAAGAA 3'
:..::: :::::::::::
query 3' AGUGACGUGUCGUGUUCUU 5'
query=miR5144-5p, target=RO3G_07214, score=3, range=62-81, strand
target 5' UCACUUGCACAGCACGAGAU 3'

```
:::: ::::::::::.:::
query 3' AGUG-ACGUGUCGUGUUCUU 5'
query=miR5144-5p, target=RO3G_08312, score=3, range=216-234, stra
target 5' UCAGUGGACAGCACAAGAA 3'
::: :: ::::::::::::
query 3' AGUGACGUGUCGUGUUCUU 5'
query=miR5144-5p, target=RO3G_06678, score=3, range=843-860, stra
target 5' UC-CUGCUCAGCACAAGAA 3'
:: :::: :::::::::::
query 3' AGUGACGUGUCGUGUUCUU 5'
query=miR5144-5p, target=RO3G_05120, score=4, range=289-306, stra
target 5' UCACUGCACAGCGC-GGAA 3'
::::::::::::.: .:::
query 3' AGUGACGUGUCGUGUUCUU 5'
query=miR5144-5p, target=RO3G_14679, score=4, range=702-720, stra
target 5' UUAUUAUACAGCACAAGAU 3'
:.:.: .:::::::::::
query 3' AGUGACGUGUCGUGUUCUU 5'
query=miR5144-5p, target=RO3G_04155, score=4, range=161-180, stra
target 5' UGGCUGUACAGCACGAAGAA 3'
: .:::.:::::::: :::::
query 3' AGUGACGUGUCGUG-UUCUU 5'
query=miR5144-5p, target=RO3G_00637, score=4, range=3-20, strand=
target 5' GUGC-GCACAGCACAAGGA 3'
..: ::::::::::::.:
query 3' AGUGACGUGUCGUGUUCUU 5'
query=miR5144-5p, target=RO3G_08491, score=4, range=760-777, stra
target 5' ACAC-CCACAGUACAAGAA 3'
::: :::::.:::::::
query 3' AGUGACGUGUCGUGUUCUU 5'
query=miR5144-5p, target=RO3G_06174, score=4, range=2914-2931,
strand=1
target 5' UCACUGCACA-CACAAGGC 3'
:::::::::: :::::::.
query 3' AGUGACGUGUCGUGUUCUU 5'
query=miR5144-5p, target=RO3G_07934, score=4, range=325-342, stra
target 5' UUAUUUCA-AGCACAAGAA 3'
:.:.: :: ::::::::::
query 3' AGUGACGUGUCGUGUUCUU 5'
query=miR5144-5p, target=RO3G_17255, score=4, range=1093-1110,
strand=-1
target 5' CCACUG-ACAGCACAAGGA 3'
::::: ::::::::::.:
query 3' AGUGACGUGUCGUGUUCUU 5'
query=miR5144-5p, target=RO3G_11979, score=4, range=1096-1113,
```

strand=-1
target 5' CCACUG-ACAGCACAAGGA 3'
::::: ::::::::::.:
query 3' AGUGACGUGUCGUGUUCUU 5'
query=miR5144-5p, target=RO3G_10532, score=4, range=1108-1125,
strand=-1
target 5' CCACUG-ACAGCACAAGGA 3'
::::: ::::::::::.:
query 3' AGUGACGUGUCGUGUUCUU 5'
query=miR6462e, target=RO3G_06442, score=0, range=4812-4831, stra
target 5' AGUAGACAAAAAGGCAAAAA 3'
::::::::::::::::::::
query 3' UCAUCUGUUUUUCCGUUUUU 5'
query=miR6462e, target=RO3G_02691, score=0, range=7644-7663, stra
target 5' AGUAGACAAAAAGGCAAAAA 3'
::::::::::::::::::::
query 3' UCAUCUGUUUUUCCGUUUUU 5'
query=miR6462e, target=RO3G_09965, score=0, range=4155-4174, stra
target 5' AGUAGACAAAAAGGCAAAAA 3'
::::::::::::::::::::
query 3' UCAUCUGUUUUUCCGUUUUU 5'
query=miR6462e, target=RO3G_13920, score=0, range=4155-4174, stra
target 5' AGUAGACAAAAAGGCAAAAA 3'
::::::::::::::::::::
query 3' UCAUCUGUUUUUCCGUUUUU 5'
query=miR6462e, target=RO3G_03547, score=0, range=4155-4174, stra
target 5' AGUAGACAAAAAGGCAAAAA 3'
::::::::::::::::::::
query 3' UCAUCUGUUUUUCCGUUUUU 5'
query=miR6462e, target=RO3G_13867, score=0, range=4071-4090, stra
target 5' AGUAGACAAAAAGGCAAAAA 3'
::::::::::::::::::::
query 3' UCAUCUGUUUUUCCGUUUUU 5'
query=miR6462e, target=RO3G_02959, score=0, range=4155-4174, stra
target 5' AGUAGACAAAAAGGCAAAAA 3'
::::::::::::::::::::
query 3' UCAUCUGUUUUUCCGUUUUU 5'
query=miR6462e, target=RO3G_15250, score=0, range=4110-4129, stra
target 5' AGUAGACAAAAAGGCAAAAA 3'
::::::::::::::::::::
query 3' UCAUCUGUUUUUCCGUUUUU 5'
query=miR6462e, target=RO3G_17371, score=0, range=4110-4129, stra
target 5' AGUAGACAAAAAGGCAAAAA 3'
::::::::::::::::::::
query 3' UCAUCUGUUUUUCCGUUUUU 5'

```
query=miR6462e, target=RO3G_06427, score=0, range=3747-3766, stra
target 5' AGUAGACAAAAAGGCAAAAA 3'
::::::::::::::::::::
query 3' UCAUCUGUUUUUCCGUUUUU 5'
query=miR6462e, target=RO3G_02222, score=0, range=1209-1228, stra
target 5' AGUAGACAAAAAGGCAAAAA 3'
::::::::::::::::::::
query 3' UCAUCUGUUUUUCCGUUUUU 5'
query=miR6462e, target=RO3G_10558, score=0, range=1908-1927, stra
target 5' AGUAGACAAAAAGGCAAAAA 3'
::::::::::::::::::::
query 3' UCAUCUGUUUUUCCGUUUUU 5'
query=miR6462e, target=RO3G_04115, score=0, range=1098-1117, stra
target 5' AGUAGACAAAAAGGCAAAAA 3'
::::::::::::::::::::
query 3' UCAUCUGUUUUUCCGUUUUU 5'
query=miR6462e, target=RO3G_11776, score=0, range=1245-1264, stra
target 5' AGUAGACAAAAAGGCAAAAA 3'
::::::::::::::::::::
query 3' UCAUCUGUUUUUCCGUUUUU 5'
query=miR6462e, target=RO3G_14355, score=0, range=2361-2380, stra
target 5' AGUAGACAAAAAGGCAAAAA 3'
::::::::::::::::::::
query 3' UCAUCUGUUUUUCCGUUUUU 5'
query=miR6462e, target=RO3G_17404, score=0, range=783-802, strand
target 5' AGUAGACAAAAAGGCAAAAA 3'
::::::::::::::::::::
query 3' UCAUCUGUUUUUCCGUUUUU 5'
query=miR6462e, target=RO3G_13749, score=0, range=2664-2683, stra
target 5' AGUAGACAAAAAGGCAAAAA 3'
::::::::::::::::::::
query 3' UCAUCUGUUUUUCCGUUUUU 5'
query=miR6462e, target=RO3G_01330, score=0, range=1389-1408, stra
target 5' AGUAGACAAAAAGGCAAAAA 3'
::::::::::::::::::::
query 3' UCAUCUGUUUUUCCGUUUUU 5'
query=miR6462e, target=RO3G_06428, score=0, range=3249-3268, stra
target 5' AGUAGACAAAAAGGCAAAAA 3'
::::::::::::::::::::
query 3' UCAUCUGUUUUUCCGUUUUU 5'
query=miR6462e, target=RO3G_01613, score=0, range=2352-2371, stra
target 5' AGUAGACAAAAAGGCAAAAA 3'
::::::::::::::::::::
query 3' UCAUCUGUUUUUCCGUUUUU 5'
query=miR6462e, target=RO3G_05619, score=0, range=2298-2317, stra
```

target 5' AGUAGACAAAAAGGCAAAAA 3'
::::::::::::::::::::
query 3' UCAUCUGUUUUUCCGUUUUU 5'
query=miR6462e, target=RO3G_17326, score=1, range=3987-4006, stra
target 5' AGUACACAAAAAGGCAAAAA 3'
:::: :::::::::::::::
query 3' UCAUCUGUUUUUCCGUUUUU 5'
query=miR6462e, target=RO3G_16015, score=1.5, range=2154-2172,
strand=-1
target 5' AGU-GGCAAAAAGGCAAAAA 3'
::: :.::::::::::::::
query 3' UCAUCUGUUUUUCCGUUUUU 5'
query=miR6462e, target=RO3G_12588, score=3, range=861-880, strand
target 5' AGCAGACAAAAAGGCAAAGC 3'
:: ::::::::::::::::.
query 3' UCAUCUGUUUUUCCGUUUUU 5'
query=miR6462e, target=RO3G_14112, score=3, range=246-265, strand
target 5' AUUAGAGAAAAAGGCAAAGA 3'
: :::: :::::::::::.:
query 3' UCAUCUGUUUUUCCGUUUUU 5'
query=miR6462e, target=RO3G_01140, score=3, range=228-247, strand
target 5' AGAAGAAAAAAAGGCGAAAA 3'
:: ::: ::::::::.::::
query 3' UCAUCUGUUUUUCCGUUUUU 5'
query=miR6462e, target=RO3G_16912, score=3, range=634-652, strand
target 5' AUUA-AAAAAAAGGCAAAAA 3'
: :: : :::::::::::::
query 3' UCAUCUGUUUUUCCGUUUUU 5'
query=miR6462e, target=RO3G_13564, score=3.5, range=158-177, stra
target 5' AGUUGACAGAGAGGCAAAAG 3'
::: ::::.:.::::::::.
query 3' UCAUCUGUUUUUCCGUUUUU 5'
query=miR6462e, target=RO3G_15072, score=3.5, range=228-247, stra
target 5' AGUUGUUAAAAAGGCAGAAA 3'
::: : .:::::::::.:::
query 3' UCAUCUGUUUUUCCGUUUUU 5'
query=miR6462e, target=RO3G_15307, score=3.5, range=1110-1128,
strand=1
target 5' AG-AGAUAAAAAGGCAAAGU 3'
:: :::.:::::::::::.
query 3' UCAUCUGUUUUUCCGUUUUU 5'
query=miR6462e, target=RO3G_02652, score=3.5, range=48-66, strand
target 5' AGCAGAUAAAAA-GCAAAAA 3'
:: :::.::::: :::::::
query 3' UCAUCUGUUUUUCCGUUUUU 5'

```
query=miR6462e, target=RO3G_02732, score=3.5, range=295-313, stra
target 5' AGUA-UUAAAAAGGCAAAGA 3'
:::: .:::::::::::.:
query 3' UCAUCUGUUUUUCCGUUUUU 5'
query=miR6462e, target=RO3G_03214, score=3.5, range=229-247, stra
target 5' AGUCUG-AAAAAGGCAAAAA 3'
::: . ::::::::::::
query 3' UCAUCUGUUUUUCCGUUUUU 5'
query=miR6462e, target=RO3G_12330, score=4, range=573-592, strand
target 5' AGCAGACAAGAAGGCGAGAA 3'
:: :::::.:::::.:.::
query 3' UCAUCUGUUUUUCCGUUUUU 5'
query=miR6462e, target=RO3G_12274, score=4, range=375-394, strand
target 5' AGUAGAUAAAAAGGCAGUAG 3'
::::::.::::::::. :.
query 3' UCAUCUGUUUUUCCGUUUUU 5'
query=miR6462e, target=RO3G_06590, score=4, range=3001-3020, stra
target 5' AGUGCACAAAAAGGUGAAAG 3'
:::. :::::::::..:::.
query 3' UCAUCUGUUUUUCCGUUUUU 5'
query=miR6462e, target=RO3G_01430, score=4, range=6-25, strand=1
target 5' AGUAGAAAAGAAGGCAAAGU 3'
:::::: ::.::::::::.
query 3' UCAUCUGUUUUUCCGUUUUU 5'
query=miR6462e, target=RO3G_15381, score=4, range=823-842, strand
target 5' ACUAGACAAGAAGGCAAAGU 3'
: ::::::.::::::::.
query 3' UCAUCUGUUUUUCCGUUUUU 5'
query=miR6462e, target=RO3G_01423, score=4, range=6-25, strand=1
target 5' AGUAGAAAAGAAGGCAAAGU 3'
:::::: ::.::::::::.
query 3' UCAUCUGUUUUUCCGUUUUU 5'
query=miR6462e, target=RO3G_09727, score=4, range=446-465, strand
target 5' UGUUGGCAAAGAGGCAAAAG 3'
:: :.::::.::::::::.
query 3' UCAUCUGUUUUUCCGUUUUU 5'
query=miR6462e, target=RO3G_13128, score=4, range=474-492, strand
target 5' AGUAGAUGAAAA-GCAAAAG 3'
::::::..:::: ::::::.
query 3' UCAUCUGUUUUUCCGUUUUU 5'
query=miR6462e, target=RO3G_11598, score=4, range=654-673, strand
target 5' AGAAGACGAAAAGGAAAAAA 3'
:: ::::.:::::: :::::
query 3' UCAUCUGUUUUUCCGUUUUU 5'
query=miR6462e, target=RO3G_04477, score=4, range=2952-2971, stra
```

```
target 5' AAUAGAUAAAAAGGCCAAAG 3'
: ::::.:::::::: :::.
query 3' UCAUCUGUUUUUCCGUUUUU 5'
query=miR6462e, target=RO3G_02193, score=4, range=252-271, strand
target 5' AGAAGGCAAAAAAGCAAAAG 3'
:: ::.:::::: ::::::.
query 3' UCAUCUGUUUUUCCGUUUUU 5'
query=miR6462e, target=RO3G_04479, score=4, range=210-229, strand
target 5' AAUAGAAAAAGAGGCAAGAA 3'
: :::: :::.::::::.::
query 3' UCAUCUGUUUUUCCGUUUUU 5'
query=miR6462e, target=RO3G_02684, score=4, range=178-197, strand
target 5' AGUAAAAGAAAAGGUAAAAA 3'
:::: : .::::::.:::::
query 3' UCAUCUGUUUUUCCGUUUUU 5'
query=miR6462e, target=RO3G_07491, score=4, range=27-46, strand=1
target 5' GAUAGGCAAAAAGGGAAAAA 3'
. :::.:::::::: :::::
query 3' UCAUCUGUUUUUCCGUUUUU 5'
query=miR6462e, target=RO3G_17058, score=4, range=591-610, strand
target 5' AGAAAAUAAAAAGGCAAGAG 3'
:: : :.::::::::::.:.
query 3' UCAUCUGUUUUUCCGUUUUU 5'
query=miR6462e, target=RO3G_10924, score=4, range=536-554, strand
target 5' AGAAGACGAAAA-GCAAAAA 3'
:: ::::.:::: :::::::
query 3' UCAUCUGUUUUUCCGUUUUU 5'
query=miR6462e, target=RO3G_15382, score=4, range=2437-2455, stra
target 5' AG-AGAAAAGAAGGCAAAGA 3'
:: ::: ::.::::::::.:
query 3' UCAUCUGUUUUUCCGUUUUU 5'
query=miR6462e, target=RO3G_10256, score=4, range=569-587, strand
target 5' AGAAGA-AGAAAGGCAAGAA 3'
:: ::: :.::::::::.::
query 3' UCAUCUGUUUUUCCGUUUUU 5'
query=miR6462e, target=RO3G_09886, score=4, range=276-295, strand
target 5' AAUUGACAAAAAGUCAAAAA 3'
: : ::::::::: ::::::
query 3' UCAUCUGUUUUUCCGUUUUU 5'
query=miR6462e, target=RO3G_07436, score=4, range=1944-1963, stra
target 5' AGAAACCAAAAAGGCGAAAA 3'
:: : :::::::::.::::
query 3' UCAUCUGUUUUUCCGUUUUU 5'
query=miR6462e, target=RO3G_05744, score=4, range=864-883, strand
target 5' AGUUGAAAAAAAGCCAAAAA 3'
```

```
::: :: :::::: ::::::
query 3' UCAUCUGUUUUUCCGUUUUU 5'
query=miR6462e, target=RO3G_00717, score=4, range=198-217, strand
target 5' AGAAGAAAAAAAGGCCAAAA 3'
:: ::: :::::::: ::::
query 3' UCAUCUGUUUUUCCGUUUUU 5'
query=miR6462e, target=RO3G_10919, score=4, range=1349-1369, stra
target 5' AGUUUAUCAAGAAGGCAAAAA 3'
::: : :::.::::::::::
query 3' UCAUCU-GUUUUUCCGUUUUU 5'
query=miR6462e, target=RO3G_14082, score=4, range=700-718, strand
target 5' CCUACA-AAAAAGGCAAAAA 3'
:: : :::::::::::::
query 3' UCAUCUGUUUUUCCGUUUUU 5'
query=miR6462e, target=RO3G_03022, score=4, range=251-271, strand
target 5' AGUAAGACAAAAGGGUAAAGA 3'
::: ::::::::.::.:::.:
query 3' UCA-UCUGUUUUUCCGUUUUU 5'
query=miR6462e, target=RO3G_03656, score=4, range=661-681, strand
target 5' AGUAGAUGAAGAAGGCAAAGG 3'
::::::. ::.:::::::..
query 3' UCAUCUG-UUUUUCCGUUUUU 5'
query=miR6462e, target=RO3G_09847, score=4, range=2651-2669, stra
target 5' GGU-GGCAAAAAGGUGAAAA 3'
.:: :.::::::::..::::
query 3' UCAUCUGUUUUUCCGUUUUU 5'
query=miR6462e, target=RO3G_03960, score=4, range=59-77, strand=-
target 5' AAUA-AAAAAGAGGCAAAAA 3'
: :: : :::.:::::::::
query 3' UCAUCUGUUUUUCCGUUUUU 5'
query=miR9657a-3p, target=RO3G_05616, score=0, range=69-90, stran
target 5' ACCUGUACGACAGGAAGCACCA 3'
::::::::::::::::::::::
query 3' UGGACAUGCUGUCCUUCGUGGU 5'
query=miR9657a-3p, target=RO3G_06070, score=0.5, range=91-112,
strand=1
target 5' ACCUGUAUGACAGGAAGCACCA 3'
:::::::.::::::::::::::
query 3' UGGACAUGCUGUCCUUCGUGGU 5'
query=miR9657a-3p, target=RO3G_01923, score=0.5, range=728-749,
strand=-1
target 5' ACCUGUAUGACAGGAAGCACCA 3'
:::::::.::::::::::::::
query 3' UGGACAUGCUGUCCUUCGUGGU 5'
query=miR9657a-3p, target=RO3G_02936, score=0.5, range=777-798,
```

strand=-1
target 5' ACCUGUAUGACAGGAAGCACCA 3'
:::::::.:::::::::::::
query 3' UGGACAUGCUGUCCUUCGUGGU 5'
query=miR9657a-3p, target=RO3G_11486, score=0.5, range=489-510,
strand=-1
target 5' ACCUGUAUGACAGGAAGCACCA 3'
:::::::.:::::::::::::
query 3' UGGACAUGCUGUCCUUCGUGGU 5'
query=miR9657a-3p, target=RO3G_05133, score=0.5, range=543-564,
strand=-1
target 5' ACCUGUAUGACAGGAAGCACCA 3'
:::::::.:::::::::::::
query 3' UGGACAUGCUGUCCUUCGUGGU 5'
query=miR9657a-3p, target=RO3G_01528, score=0.5, range=348-369,
strand=-1
target 5' ACCUGUAUGACAGGAAGCACCA 3'
:::::::.:::::::::::::
query 3' UGGACAUGCUGUCCUUCGUGGU 5'
query=miR9657a-3p, target=RO3G_08649, score=0.5, range=485-506,
strand=-1
target 5' ACCUGUAUGACAGGAAGCACCA 3'
:::::::.:::::::::::::
query 3' UGGACAUGCUGUCCUUCGUGGU 5'
query=miR9657a-3p, target=RO3G_15496, score=0.5, range=423-444,
strand=-1
target 5' ACCUGUAUGACAGGAAGCACCA 3'
:::::::.:::::::::::::
query 3' UGGACAUGCUGUCCUUCGUGGU 5'
query=miR9657a-3p, target=RO3G_10644, score=0.5, range=165-186,
strand=-1
target 5' ACCUGUAUGACAGGAAGCACCA 3'
:::::::.:::::::::::::
query 3' UGGACAUGCUGUCCUUCGUGGU 5'
query=miR9657a-3p, target=RO3G_05535, score=0.5, range=165-186,
strand=-1
target 5' ACCUGUAUGACAGGAAGCACCA 3'
:::::::.:::::::::::::
query 3' UGGACAUGCUGUCCUUCGUGGU 5'
query=miR9657a-3p, target=RO3G_12139, score=0.5, range=153-174,
strand=-1
target 5' ACCUGUAUGACAGGAAGCACCA 3'
:::::::.:::::::::::::
query 3' UGGACAUGCUGUCCUUCGUGGU 5'
query=miR9657a-3p, target=RO3G_01518, score=0.5, range=153-174,

strand=-1
target 5' ACCUGUAUGACAGGAAGCACCA 3'
:::::::.::::::::::::::
query 3' UGGACAUGCUGUCCUUCGUGGU 5'
query=miR9657a-3p, target=RO3G_01013, score=0.5, range=222-243, strand=-1
target 5' ACCUGUAUGACAGGAAGCACCA 3'
:::::::.::::::::::::::
query 3' UGGACAUGCUGUCCUUCGUGGU 5'
query=miR9657a-3p, target=RO3G_16893, score=0.5, range=165-186, strand=-1
target 5' ACCUGUAUGACAGGAAGCACCA 3'
:::::::.::::::::::::::
query 3' UGGACAUGCUGUCCUUCGUGGU 5'
query=miR9657a-3p, target=RO3G_04866, score=0.5, range=165-186, strand=-1
target 5' ACCUGUAUGACAGGAAGCACCA 3'
:::::::.::::::::::::::
query 3' UGGACAUGCUGUCCUUCGUGGU 5'
query=miR9657a-3p, target=RO3G_11567, score=1.5, range=1437-1458, strand=-1
target 5' ACCUGUAUGAUAGGAAGCACCA 3'
:::::::.::.:::::::::::
query 3' UGGACAUGCUGUCCUUCGUGGU 5'
query=miR9657a-3p, target=RO3G_09419, score=1.5, range=600-621, strand=-1
target 5' ACCUGUAUGACAGGAGGCACCA 3'
:::::::.:::::::.::::::
query 3' UGGACAUGCUGUCCUUCGUGGU 5'
query=miR9657a-3p, target=RO3G_08301, score=1.5, range=675-696, strand=-1
target 5' ACCUGUAUGAUAGGAAGCACCA 3'
:::::::.::.:::::::::::
query 3' UGGACAUGCUGUCCUUCGUGGU 5'
query=miR9657a-3p, target=RO3G_06976, score=1.5, range=246-267, strand=-1
target 5' ACCUGUAUGACAGGAAGCACUA 3'
:::::::.::::::::::::.:
query 3' UGGACAUGCUGUCCUUCGUGGU 5'
query=miR9657a-3p, target=RO3G_15460, score=1.5, range=90-111, strand=-1
target 5' ACCUGUAUGAUAGGAAGCACCA 3'
:::::::.::.:::::::::::
query 3' UGGACAUGCUGUCCUUCGUGGU 5'
query=miR9657a-3p, target=RO3G_07788, score=1.5, range=729-750,

strand=-1
target 5' ACCAGUAUGACAGGAAGCACCA 3'
::: :::.::::::::::::::
query 3' UGGACAUGCUGUCCUUCGUGGU 5'
query=miR9657a-3p, target=RO3G_05603, score=1.5, range=39-60,
strand=-1
target 5' ACCUGUAUGACAGGAAGCACCC 3'
:::::::.:::::::::::::
query 3' UGGACAUGCUGUCCUUCGUGGU 5'
query=miR9657a-3p, target=RO3G_13903, score=1.5, range=780-801,
strand=-1
target 5' ACCAGUAUGACAGGAAGCACCA 3'
::: :::.::::::::::::::
query 3' UGGACAUGCUGUCCUUCGUGGU 5'
query=miR9657a-3p, target=RO3G_13672, score=1.5, range=780-801,
strand=-1
target 5' ACCAGUAUGACAGGAAGCACCA 3'
::: :::.::::::::::::::
query 3' UGGACAUGCUGUCCUUCGUGGU 5'
query=miR9657a-3p, target=RO3G_13664, score=1.5, range=780-801,
strand=-1
target 5' ACCAGUAUGACAGGAAGCACCA 3'
::: :::.::::::::::::::
query 3' UGGACAUGCUGUCCUUCGUGGU 5'
query=miR9657a-3p, target=RO3G_14722, score=1.5, range=780-801,
strand=-1
target 5' ACCAGUAUGACAGGAAGCACCA 3'
::: :::.::::::::::::::
query 3' UGGACAUGCUGUCCUUCGUGGU 5'
query=miR9657a-3p, target=RO3G_05303, score=1.5, range=780-801,
strand=-1
target 5' ACCAGUAUGACAGGAAGCACCA 3'
::: :::.::::::::::::::
query 3' UGGACAUGCUGUCCUUCGUGGU 5'
query=miR9657a-3p, target=RO3G_02354, score=1.5, range=780-801,
strand=-1
target 5' ACCAGUAUGACAGGAAGCACCA 3'
::: :::.::::::::::::::
query 3' UGGACAUGCUGUCCUUCGUGGU 5'
query=miR9657a-3p, target=RO3G_08906, score=1.5, range=780-801,
strand=-1
target 5' ACCAGUAUGACAGGAAGCACCA 3'
::: :::.::::::::::::::
query 3' UGGACAUGCUGUCCUUCGUGGU 5'
query=miR9657a-3p, target=RO3G_05194, score=1.5, range=780-801,

strand=-1
target 5' ACCAGUAUGACAGGAAGCACCA 3'
::: :::.::::::::::::::
query 3' UGGACAUGCUGUCCUUCGUGGU 5'
query=miR9657a-3p, target=RO3G_05181, score=1.5, range=780-801,
strand=-1
target 5' ACCAGUAUGACAGGAAGCACCA 3'
::: :::.::::::::::::::
query 3' UGGACAUGCUGUCCUUCGUGGU 5'
query=miR9657a-3p, target=RO3G_10707, score=2, range=267-288,
strand=-1
target 5' AUCUGUAUGACAGGAAGCGCCA 3'
:.:::::.::::::::::.:::
query 3' UGGACAUGCUGUCCUUCGUGGU 5'
query=miR9657a-3p, target=RO3G_00780, score=2, range=334-355,
strand=-1
target 5' ACCUGUACGACAGGAAGCUCCA 3'
:::::::::::::::::: :::
query 3' UGGACAUGCUGUCCUUCGUGGU 5'
query=miR9657a-3p, target=RO3G_04104, score=2, range=477-498,
strand=-1
target 5' ACCUAUGUGACAGGAAGCACCA 3'
:::: :..::::::::::::::
query 3' UGGACAUGCUGUCCUUCGUGGU 5'
query=miR9657a-3p, target=RO3G_04110, score=2, range=477-498,
strand=-1
target 5' ACCUAUGUGACAGGAAGCACCA 3'
:::: :..::::::::::::::
query 3' UGGACAUGCUGUCCUUCGUGGU 5'
query=miR9657a-3p, target=RO3G_15836, score=2.5, range=91-112,
strand=1
target 5' ACCUGUAUGACAGGAAGCUCCA 3'
:::::::.:::::::::: :::
query 3' UGGACAUGCUGUCCUUCGUGGU 5'
query=miR9657a-3p, target=RO3G_01545, score=2.5, range=576-597,
strand=-1
target 5' ACCUGUAUGACAGGAAGCUCCA 3'
:::::::.:::::::::: :::
query 3' UGGACAUGCUGUCCUUCGUGGU 5'
query=miR9657a-3p, target=RO3G_14365, score=2.5, range=522-543,
strand=-1
target 5' ACCUGUAUGACAGGAAGCUCCA 3'
:::::::.:::::::::: :::
query 3' UGGACAUGCUGUCCUUCGUGGU 5'
query=miR9657a-3p, target=RO3G_14123, score=2.5, range=264-285,

strand=-1
target 5' ACCUGUAUGACAGGAAGCUCCA 3'
:::::::.:::::::::: :::
query 3' UGGACAUGCUGUCCUUCGUGGU 5'
query=miR9657a-3p, target=RO3G_04776, score=2.5, range=726-746,
strand=-1
target 5' AUCUGUACG-CAGGAAGCACCA 3'
:.::::::: ::::::::::::
query 3' UGGACAUGCUGUCCUUCGUGGU 5'
query=miR9657a-3p, target=RO3G_11757, score=3.5, range=165-186,
strand=-1
target 5' ACCUGUAUGGCAGGAAGCUCCA 3'
:::::::.:.:::::::: :::
query 3' UGGACAUGCUGUCCUUCGUGGU 5'
query=miR9657a-3p, target=RO3G_11765, score=3.5, range=165-186,
strand=-1
target 5' ACCUGUAUGGCAGGAAGCUCCA 3'
:::::::.:.:::::::: :::
query 3' UGGACAUGCUGUCCUUCGUGGU 5'
query=miR9657a-3p, target=RO3G_06838, score=3.5, range=264-285,
strand=-1
target 5' ACCUGUAUGACAGGAAGCUCUA 3'
:::::::.:::::::::: :.:
query 3' UGGACAUGCUGUCCUUCGUGGU 5'
query=miR9657a-3p, target=RO3G_15336, score=3.5, range=267-287,
strand=-1
target 5' ACCUGUAUGACAGGAAGC-UCA 3'
:::::::.:::::::::: .::
query 3' UGGACAUGCUGUCCUUCGUGGU 5'
query=miR9657a-3p, target=RO3G_08684, score=3.5, range=198-219,
strand=-1
target 5' ACCUAUAUGACAGGAAGCUCCA 3'
:::: ::.:::::::::: :::
query 3' UGGACAUGCUGUCCUUCGUGGU 5'
query=miR9657a-3p, target=RO3G_09656, score=3.5, range=306-327,
strand=-1
target 5' ACCUGUAUAACAGGAAGCUCCA 3'
:::::::. ::::::::: :::
query 3' UGGACAUGCUGUCCUUCGUGGU 5'
query=miR9657a-3p, target=RO3G_13827, score=4, range=1203-1223,
strand=1
target 5' ACCUGU-CGACAGGAUGCAUCA 3'
:::::: :::::::: :::.::
query 3' UGGACAUGCUGUCCUUCGUGGU 5'
query=miR9657a-3p, target=RO3G_09838, score=4, range=552-573,

```
strand=-1
target 5' CUCUGUAUGACAGGAAGCUCCA 3'
.:::::.:::::::::: :::
query 3' UGGACAUGCUGUCCUUCGUGGU 5'
query=miR6202, target=RO3G_08114, score=0, range=16-35, strand=1
target 5' CUUCAAAGCUUAAAUCCUCA 3'
::::::::::::::::::::
query 3' GAAGUUUCGAAUUUAGGAGU 5'
query=miR6202, target=RO3G_00653, score=0, range=3964-3983, stran
target 5' CUUCAAAGCUUAAAUCCUCA 3'
::::::::::::::::::::
query 3' GAAGUUUCGAAUUUAGGAGU 5'
query=miR6202, target=RO3G_12753, score=0, range=16-35, strand=1
target 5' CUUCAAAGCUUAAAUCCUCA 3'
::::::::::::::::::::
query 3' GAAGUUUCGAAUUUAGGAGU 5'
query=miR6202, target=RO3G_04256, score=0, range=16-35, strand=1
target 5' CUUCAAAGCUUAAAUCCUCA 3'
::::::::::::::::::::
query 3' GAAGUUUCGAAUUUAGGAGU 5'
query=miR6202, target=RO3G_14345, score=0, range=16-35, strand=1
target 5' CUUCAAAGCUUAAAUCCUCA 3'
::::::::::::::::::::
query 3' GAAGUUUCGAAUUUAGGAGU 5'
query=miR6202, target=RO3G_08062, score=0, range=835-854, strand=
target 5' CUUCAAAGCUUAAAUCCUCA 3'
::::::::::::::::::::
query 3' GAAGUUUCGAAUUUAGGAGU 5'
query=miR6202, target=RO3G_01243, score=0, range=16-35, strand=1
target 5' CUUCAAAGCUUAAAUCCUCA 3'
::::::::::::::::::::
query 3' GAAGUUUCGAAUUUAGGAGU 5'
query=miR6202, target=RO3G_06977, score=0, range=619-638, strand=
target 5' CUUCAAAGCUUAAAUCCUCA 3'
::::::::::::::::::::
query 3' GAAGUUUCGAAUUUAGGAGU 5'
query=miR6202, target=RO3G_16319, score=0, range=4054-4073, stran
target 5' CUUCAAAGCUUAAAUCCUCA 3'
::::::::::::::::::::
query 3' GAAGUUUCGAAUUUAGGAGU 5'
query=miR6202, target=RO3G_12207, score=0, range=4054-4073, stran
target 5' CUUCAAAGCUUAAAUCCUCA 3'
::::::::::::::::::::
query 3' GAAGUUUCGAAUUUAGGAGU 5'
query=miR6202, target=RO3G_01242, score=0, range=16-35, strand=1
```

```
target 5' CUUCAAAGCUUAAAUCCUCA 3'
::::::::::::::::::::
query 3' GAAGUUUCGAAUUUAGGAGU 5'
query=miR6202, target=RO3G_02944, score=0, range=3373-3392, stran
target 5' CUUCAAAGCUUAAAUCCUCA 3'
::::::::::::::::::::
query 3' GAAGUUUCGAAUUUAGGAGU 5'
query=miR6202, target=RO3G_01026, score=0, range=16-35, strand=1
target 5' CUUCAAAGCUUAAAUCCUCA 3'
::::::::::::::::::::
query 3' GAAGUUUCGAAUUUAGGAGU 5'
query=miR6202, target=RO3G_09206, score=2, range=16-35, strand=1
target 5' CUUCAAACCUUAAAUCCUCA 3'
::::::: ::::::::::::
query 3' GAAGUUUCGAAUUUAGGAGU 5'
query=miR6202, target=RO3G_00493, score=2.5, range=358-377, stran
target 5' CUUCAAAGCUUAAAUCCUAG 3'
:::::::::::::::::: .
query 3' GAAGUUUCGAAUUUAGGAGU 5'
query=miR6202, target=RO3G_05992, score=3, range=806-825, strand=
target 5' CAUCUCAGCUUAAAUCCUCA 3'
: :: ::::::::::::::
query 3' GAAGUUUCGAAUUUAGGAGU 5'
query=miR6202, target=RO3G_11606, score=3, range=1047-1066, stran
target 5' UUUCCAAGCUUAAAUCUUCG 3'
.::: ::::::::::.::.
query 3' GAAGUUUCGAAUUUAGGAGU 5'
query=miR6202, target=RO3G_10954, score=4, range=16-35, strand=1
target 5' CUUCAAAGUUUAAGUCCUGA 3'
::::::::.::::.:::: :
query 3' GAAGUUUCGAAUUUAGGAGU 5'
query=miR6202, target=RO3G_15536, score=4, range=3628-3647, stran
target 5' GCUAAAAGCUUGAAUCCUCA 3'
: :::::::.::::::::
query 3' GAAGUUUCGAAUUUAGGAGU 5'
query=miR6202, target=RO3G_01906, score=4, range=882-900, strand=
target 5' CUUC-AAGCUAAAAUCCUCU 3'
:::: ::::: ::::::::
query 3' GAAGUUUCGAAUUUAGGAGU 5'
query=miR6202, target=RO3G_09575, score=4, range=957-976, strand=
target 5' CAUCAACGCUUAGAUCUUCA 3'
: :::: :::::.:::.:::
query 3' GAAGUUUCGAAUUUAGGAGU 5'
query=miR167c-3p, target=RO3G_10805, score=0, range=181-202, stra
target 5' GGUGAAACUACCAAGUCAUACA 3'
```

```
::::::::::::::::::::::
query 3' CCACUUUGAUGGUUCAGUAUGU 5'
query=miR167c-3p, target=RO3G_14561, score=0, range=181-202, stra
target 5' GGUGAAACUACCAAGUCAUACA 3'
::::::::::::::::::::::
query 3' CCACUUUGAUGGUUCAGUAUGU 5'
query=miR167c-3p, target=RO3G_12978, score=0, range=181-202, stra
target 5' GGUGAAACUACCAAGUCAUACA 3'
::::::::::::::::::::::
query 3' CCACUUUGAUGGUUCAGUAUGU 5'
query=miR167c-3p, target=RO3G_15140, score=0, range=34-55, strand
target 5' GGUGAAACUACCAAGUCAUACA 3'
::::::::::::::::::::::
query 3' CCACUUUGAUGGUUCAGUAUGU 5'
query=miR167c-3p, target=RO3G_03606, score=0, range=181-202, stra
target 5' GGUGAAACUACCAAGUCAUACA 3'
::::::::::::::::::::::
query 3' CCACUUUGAUGGUUCAGUAUGU 5'
query=miR167c-3p, target=RO3G_03620, score=0, range=181-202, stra
target 5' GGUGAAACUACCAAGUCAUACA 3'
::::::::::::::::::::::
query 3' CCACUUUGAUGGUUCAGUAUGU 5'
query=miR167c-3p, target=RO3G_08833, score=0, range=181-202, stra
target 5' GGUGAAACUACCAAGUCAUACA 3'
::::::::::::::::::::::
query 3' CCACUUUGAUGGUUCAGUAUGU 5'
query=miR167c-3p, target=RO3G_14546, score=0, range=130-151, stra
target 5' GGUGAAACUACCAAGUCAUACA 3'
::::::::::::::::::::::
query 3' CCACUUUGAUGGUUCAGUAUGU 5'
query=miR167c-3p, target=RO3G_09203, score=0, range=130-151, stra
target 5' GGUGAAACUACCAAGUCAUACA 3'
::::::::::::::::::::::
query 3' CCACUUUGAUGGUUCAGUAUGU 5'
query=miR167c-3p, target=RO3G_03613, score=0, range=130-151, stra
target 5' GGUGAAACUACCAAGUCAUACA 3'
::::::::::::::::::::::
query 3' CCACUUUGAUGGUUCAGUAUGU 5'
query=miR167c-3p, target=RO3G_13729, score=0, range=181-202, stra
target 5' GGUGAAACUACCAAGUCAUACA 3'
::::::::::::::::::::::
query 3' CCACUUUGAUGGUUCAGUAUGU 5'
query=miR167c-3p, target=RO3G_15666, score=0, range=181-202, stra
target 5' GGUGAAACUACCAAGUCAUACA 3'
::::::::::::::::::::::
```

```
query 3' CCACUUUGAUGGUUCAGUAUGU 5'
query=miR167c-3p, target=RO3G_05949, score=0, range=181-202, stra
target 5' GGUGAAACUACCAAGUCAUACA 3'
::::::::::::::::::::::
query 3' CCACUUUGAUGGUUCAGUAUGU 5'
query=miR167c-3p, target=RO3G_17238, score=0, range=181-202, stra
target 5' GGUGAAACUACCAAGUCAUACA 3'
::::::::::::::::::::::
query 3' CCACUUUGAUGGUUCAGUAUGU 5'
query=miR167c-3p, target=RO3G_03586, score=0, range=130-151, stra
target 5' GGUGAAACUACCAAGUCAUACA 3'
::::::::::::::::::::::
query 3' CCACUUUGAUGGUUCAGUAUGU 5'
query=miR167c-3p, target=RO3G_16718, score=0, range=34-55, strand
target 5' GGUGAAACUACCAAGUCAUACA 3'
::::::::::::::::::::::
query 3' CCACUUUGAUGGUUCAGUAUGU 5'
query=miR167c-3p, target=RO3G_17360, score=0, range=34-55, strand
target 5' GGUGAAACUACCAAGUCAUACA 3'
::::::::::::::::::::::
query 3' CCACUUUGAUGGUUCAGUAUGU 5'
query=miR167c-3p, target=RO3G_13713, score=0, range=34-55, strand
target 5' GGUGAAACUACCAAGUCAUACA 3'
::::::::::::::::::::::
query 3' CCACUUUGAUGGUUCAGUAUGU 5'
query=miR167c-3p, target=RO3G_13740, score=0, range=34-55, strand
target 5' GGUGAAACUACCAAGUCAUACA 3'
::::::::::::::::::::::
query 3' CCACUUUGAUGGUUCAGUAUGU 5'
query=miR167c-3p, target=RO3G_13851, score=0, range=34-55, strand
target 5' GGUGAAACUACCAAGUCAUACA 3'
::::::::::::::::::::::
query 3' CCACUUUGAUGGUUCAGUAUGU 5'
query=miR167c-3p, target=RO3G_15148, score=0, range=34-55, strand
target 5' GGUGAAACUACCAAGUCAUACA 3'
::::::::::::::::::::::
query 3' CCACUUUGAUGGUUCAGUAUGU 5'
query=miR167c-3p, target=RO3G_16690, score=0, range=34-55, strand
target 5' GGUGAAACUACCAAGUCAUACA 3'
::::::::::::::::::::::
query 3' CCACUUUGAUGGUUCAGUAUGU 5'
query=miR167c-3p, target=RO3G_15144, score=0, range=34-55, strand
target 5' GGUGAAACUACCAAGUCAUACA 3'
::::::::::::::::::::::
query 3' CCACUUUGAUGGUUCAGUAUGU 5'
```

```
query=miR167c-3p, target=RO3G_13282, score=0, range=181-202, stra
target 5' GGUGAAACUACCAAGUCAUACA 3'
::::::::::::::::::::::
query 3' CCACUUUGAUGGUUCAGUAUGU 5'
query=miR167c-3p, target=RO3G_14720, score=2, range=130-151, stra
target 5' GGUGAAACUACCAAUUCAUACA 3'
:::::::::::::: :::::::
query 3' CCACUUUGAUGGUUCAGUAUGU 5'
query=miR2082, target=RO3G_07738, score=0, range=93-114, strand=-
target 5' AAGAGAAGAAGAAGGAACAACA 3'
::::::::::::::::::::::
query 3' UUCUCUUCUUCUUCCUUGUUGU 5'
query=miR2082, target=RO3G_01726, score=0, range=93-114, strand=-
target 5' AAGAGAAGAAGAAGGAACAACA 3'
::::::::::::::::::::::
query 3' UUCUCUUCUUCUUCCUUGUUGU 5'
query=miR2082, target=RO3G_11537, score=0, range=93-114, strand=-
target 5' AAGAGAAGAAGAAGGAACAACA 3'
::::::::::::::::::::::
query 3' UUCUCUUCUUCUUCCUUGUUGU 5'
query=miR2082, target=RO3G_11236, score=0, range=93-114, strand=-
target 5' AAGAGAAGAAGAAGGAACAACA 3'
::::::::::::::::::::::
query 3' UUCUCUUCUUCUUCCUUGUUGU 5'
query=miR2082, target=RO3G_07981, score=0, range=93-114, strand=-
target 5' AAGAGAAGAAGAAGGAACAACA 3'
::::::::::::::::::::::
query 3' UUCUCUUCUUCUUCCUUGUUGU 5'
query=miR2082, target=RO3G_07696, score=0, range=93-114, strand=-
target 5' AAGAGAAGAAGAAGGAACAACA 3'
::::::::::::::::::::::
query 3' UUCUCUUCUUCUUCCUUGUUGU 5'
query=miR2082, target=RO3G_06730, score=0, range=93-114, strand=-
target 5' AAGAGAAGAAGAAGGAACAACA 3'
::::::::::::::::::::::
query 3' UUCUCUUCUUCUUCCUUGUUGU 5'
query=miR2082, target=RO3G_07720, score=0, range=93-114, strand=-
target 5' AAGAGAAGAAGAAGGAACAACA 3'
::::::::::::::::::::::
query 3' UUCUCUUCUUCUUCCUUGUUGU 5'
query=miR2082, target=RO3G_04211, score=0, range=93-114, strand=-
target 5' AAGAGAAGAAGAAGGAACAACA 3'
::::::::::::::::::::::
query 3' UUCUCUUCUUCUUCCUUGUUGU 5'
query=miR2082, target=RO3G_09602, score=0, range=93-114, strand=-
```

```
target 5' AAGAGAAGAAGAAGGAACAACA 3'
::::::::::::::::::::::
query 3' UUCUCUUCUUCUUCCUUGUUGU 5'
query=miR2082, target=RO3G_07306, score=0, range=93-114, strand=-
target 5' AAGAGAAGAAGAAGGAACAACA 3'
::::::::::::::::::::::
query 3' UUCUCUUCUUCUUCCUUGUUGU 5'
query=miR2082, target=RO3G_14551, score=0, range=93-114, strand=-
target 5' AAGAGAAGAAGAAGGAACAACA 3'
::::::::::::::::::::::
query 3' UUCUCUUCUUCUUCCUUGUUGU 5'
query=miR2082, target=RO3G_09929, score=0, range=93-114, strand=-
target 5' AAGAGAAGAAGAAGGAACAACA 3'
::::::::::::::::::::::
query 3' UUCUCUUCUUCUUCCUUGUUGU 5'
query=miR2082, target=RO3G_15897, score=0, range=93-114, strand=-
target 5' AAGAGAAGAAGAAGGAACAACA 3'
::::::::::::::::::::::
query 3' UUCUCUUCUUCUUCCUUGUUGU 5'
query=miR2082, target=RO3G_09603, score=0, range=93-114, strand=-
target 5' AAGAGAAGAAGAAGGAACAACA 3'
::::::::::::::::::::::
query 3' UUCUCUUCUUCUUCCUUGUUGU 5'
query=miR2082, target=RO3G_10218, score=0, range=93-114, strand=-
target 5' AAGAGAAGAAGAAGGAACAACA 3'
::::::::::::::::::::::
query 3' UUCUCUUCUUCUUCCUUGUUGU 5'
query=miR2082, target=RO3G_16748, score=0, range=93-114, strand=-
target 5' AAGAGAAGAAGAAGGAACAACA 3'
::::::::::::::::::::::
query 3' UUCUCUUCUUCUUCCUUGUUGU 5'
query=miR2082, target=RO3G_11977, score=0, range=93-114, strand=-
target 5' AAGAGAAGAAGAAGGAACAACA 3'
::::::::::::::::::::::
query 3' UUCUCUUCUUCUUCCUUGUUGU 5'
query=miR2082, target=RO3G_02228, score=0, range=93-114, strand=-
target 5' AAGAGAAGAAGAAGGAACAACA 3'
::::::::::::::::::::::
query 3' UUCUCUUCUUCUUCCUUGUUGU 5'
query=miR2082, target=RO3G_13272, score=0, range=93-114, strand=-
target 5' AAGAGAAGAAGAAGGAACAACA 3'
::::::::::::::::::::::
query 3' UUCUCUUCUUCUUCCUUGUUGU 5'
query=miR2082, target=RO3G_06990, score=1, range=93-114, strand=-
target 5' AAGAGAAGAAGAAGGAACAGCA 3'
```

```
:::::::::::::::::::.::
query 3' UUCUCUUCUUCUUCCUUGUUGU 5'
query=miR2082, target=RO3G_10041, score=1.5, range=93-115, strand
target 5' AAGAAGAAGGAGAAGGAACAACA 3'
::: :::::.::::::::::::::
query 3' UUC-UCUUCUUCUUCCUUGUUGU 5'
query=miR2082, target=RO3G_17248, score=1.5, range=93-115, strand
target 5' AAGAAGAAGGAGAAGGAACAACA 3'
::: :::::.::::::::::::::
query 3' UUC-UCUUCUUCUUCCUUGUUGU 5'
query=miR2082, target=RO3G_10775, score=1.5, range=93-115, strand
target 5' AAGAAGAAGGAGAAGGAACAACA 3'
::: :::::.::::::::::::::
query 3' UUC-UCUUCUUCUUCCUUGUUGU 5'
query=miR2082, target=RO3G_03584, score=1.5, range=27-49, strand=
target 5' AAGAAGAAGGAGAAGGAACAACA 3'
::: :::::.::::::::::::::
query 3' UUC-UCUUCUUCUUCCUUGUUGU 5'
query=miR2082, target=RO3G_13146, score=1.5, range=93-115, strand
target 5' AAGAAGAAGGAGAAGGAACAACA 3'
::: :::::.::::::::::::::
query 3' UUC-UCUUCUUCUUCCUUGUUGU 5'
query=miR2082, target=RO3G_15890, score=1.5, range=93-115, strand
target 5' AAGAAGAAGGAGAAGGAACAACA 3'
::: :::::.::::::::::::::
query 3' UUC-UCUUCUUCUUCCUUGUUGU 5'
query=miR2082, target=RO3G_17256, score=1.5, range=93-115, strand
target 5' AAGAAGAAGGAGAAGGAACAACA 3'
::: :::::.::::::::::::::
query 3' UUC-UCUUCUUCUUCCUUGUUGU 5'
query=miR2082, target=RO3G_05425, score=1.5, range=93-115, strand
target 5' AAGAAGAAGGAGAAGGAACAACA 3'
::: :::::.::::::::::::::
query 3' UUC-UCUUCUUCUUCCUUGUUGU 5'
query=miR2082, target=RO3G_17350, score=1.5, range=93-115, strand
target 5' AAGAAGAAGGAGAAGGAACAACA 3'
::: :::::.::::::::::::::
query 3' UUC-UCUUCUUCUUCCUUGUUGU 5'
query=miR2082, target=RO3G_16137, score=1.5, range=93-115, strand
target 5' AAGAAGAAGGAGAAGGAACAACA 3'
::: :::::.::::::::::::::
query 3' UUC-UCUUCUUCUUCCUUGUUGU 5'
query=miR2082, target=RO3G_00288, score=1.5, range=93-115, strand
target 5' AAGAAGAAGGAGAAGGAACAACA 3'
::: :::::.::::::::::::::
```

```
query 3' UUC-UCUUCUUCUUCCUUGUUGU 5'
query=miR2082, target=RO3G_09182, score=1.5, range=93-115, strand
target 5' AAGAAGAAGGAGAAGGAACAACA 3'
::: :::::.:::::::::::::
query 3' UUC-UCUUCUUCUUCCUUGUUGU 5'
query=miR2082, target=RO3G_04679, score=1.5, range=93-115, strand
target 5' AAGAAGAAGGAGAAGGAACAACA 3'
::: :::::.:::::::::::::
query 3' UUC-UCUUCUUCUUCCUUGUUGU 5'
query=miR2082, target=RO3G_10488, score=3, range=987-1008, strand
target 5' AAGAGUAGAAGAAGGAACACCA 3'
::::: :::::::::::: ::
query 3' UUCUCUUCUUCUUCCUUGUUGU 5'
query=miR2082, target=RO3G_01565, score=3, range=216-238, strand=
target 5' AAGAAGAAGCAGAGGGAACAACA 3'
::: ::::: :::.:::::::::
query 3' UUC-UCUUCUUCUUCCUUGUUGU 5'
query=miR2082, target=RO3G_13069, score=3.5, range=941-962, stran
target 5' AAGAAAGGAAGAAGAAACAACA 3'
:::: :.::::::: :::::::
query 3' UUCUCUUCUUCUUCCUUGUUGU 5'
query=miR2082, target=RO3G_06764, score=3.5, range=60-80, strand=
target 5' ACGGGAAGAAGAA-GAACAACA 3'
: :.::::::::: ::::::::
query 3' UUCUCUUCUUCUUCCUUGUUGU 5'
query=miR2082, target=RO3G_08916, score=3.5, range=9-30, strand=-
target 5' AAGAGAAGGAGGAGGAGCAACU 3'
::::::::.::.::::.::::
query 3' UUCUCUUCUUCUUCCUUGUUGU 5'
query=miR2082, target=RO3G_02306, score=3.5, range=498-519, stran
target 5' UGGAGCAGAAGAAGGAACAGCA 3'
.::: :::::::::::::.::
query 3' UUCUCUUCUUCUUCCUUGUUGU 5'
query=miR2082, target=RO3G_06282, score=3.5, range=216-238, stran
target 5' AAGAGGAAGCAGAGGGAACAACG 3'
:::: :::: :::.::::::::.
query 3' UUCU-CUUCUUCUUCCUUGUUGU 5'
query=miR2082, target=RO3G_10686, score=3.5, range=216-238, stran
target 5' AAGAGGAAGCAGAGGGAACAACG 3'
:::: :::: :::.::::::::.
query 3' UUCU-CUUCUUCUUCCUUGUUGU 5'
query=miR2082, target=RO3G_14846, score=3.5, range=216-238, stran
target 5' AAGAGGAAGCAGAGGGAACAACG 3'
:::: :::: :::.::::::::.
query 3' UUCU-CUUCUUCUUCCUUGUUGU 5'
```

```
query=miR2082, target=RO3G_03704, score=4, range=137-158, strand=
target 5' AAGAGGAGGAGGAGGAACAAGA 3'
:::::.::.::.:::::::: :
query 3' UUCUCUUCUUCUUCCUUGUUGU 5'
query=miR2082, target=RO3G_04239, score=4, range=1229-1249, stran
target 5' GGGAGAAGAAGAAGGAA-AAUA 3'
..::::::::::::::: ::.:
query 3' UUCUCUUCUUCUUCCUUGUUGU 5'
query=miR2082, target=RO3G_05980, score=4, range=210-231, strand=
target 5' UAGAGAAGAAGAAGGAAUAUCA 3'
::::::::::::::::.: ::
query 3' UUCUCUUCUUCUUCCUUGUUGU 5'
query=miR2082, target=RO3G_04776, score=4, range=510-530, strand=
target 5' AGGAGGAGAAGAAGG-ACAACU 3'
:.:::.::::::::: :::::
query 3' UUCUCUUCUUCUUCCUUGUUGU 5'
query=miR2082, target=RO3G_03818, score=4, range=3595-3617, stran
target 5' AAGCAAAAGAAGAAGGAACAAGA 3'
::: : ::::::::::::::: :
query 3' UUC-UCUUCUUCUUCCUUGUUGU 5'
query=miR2082, target=RO3G_14402, score=4, range=1206-1227, stran
target 5' AAGCGAUGAAGAAGAAACAACA 3'
::: :: ::::::: :::::::
query 3' UUCUCUUCUUCUUCCUUGUUGU 5'
query=miR2082, target=RO3G_06260, score=4, range=656-676, strand=
target 5' GGGAAAAGAAGAAGGAA-AACA 3'
..:: :::::::::::: ::::
query 3' UUCUCUUCUUCUUCCUUGUUGU 5'
query=miR2082, target=RO3G_08027, score=4, range=587-608, strand=
target 5' AAAAGAAAAAGAAGGAACAAGA 3'
:: :::: :::::::::::: :
query 3' UUCUCUUCUUCUUCCUUGUUGU 5'
query=miR2082, target=RO3G_05645, score=4, range=454-476, strand=
target 5' AAGAAGAACAAGAAGGAAAAACA 3'
::: :::: ::::::::: ::::
query 3' UUC-UCUUCUUCUUCCUUGUUGU 5'
query=miR2082, target=RO3G_08171, score=4, range=1410-1432, stran
target 5' AAGAUGGAGGAGAAGGAAUGACA 3'
:::: :.::.::::::::..:::
query 3' UUCU-CUUCUUCUUCCUUGUUGU 5'
query=miR2082, target=RO3G_15990, score=4, range=1192-1212, stran
target 5' AGGAGAAGGAGAAGGAG-AACA 3'
:.::::::.:::::::. ::::
query 3' UUCUCUUCUUCUUCCUUGUUGU 5'
query=miR2082, target=RO3G_12011, score=4, range=93-114, strand=-
```

```
target 5' GGGAGCAGAAGCAGGAACAACA 3'
..::: ::::: ::::::::::
query 3' UUCUCUUCUUCUUCCUUGUUGU 5'
query=miR2082, target=RO3G_17433, score=4, range=93-114, strand=-
target 5' GGGAGCAGAAGCAGGAACAACA 3'
..::: ::::: ::::::::::
query 3' UUCUCUUCUUCUUCCUUGUUGU 5'
query=miR2082, target=RO3G_16743, score=4, range=120-141, strand=
target 5' AGGAGCAGGAGCAGGAACAACA 3'
:.::: ::.:: ::::::::::
query 3' UUCUCUUCUUCUUCCUUGUUGU 5'
query=miR2082, target=RO3G_04659, score=4, range=54-75, strand=-1
target 5' GGGAGCAGAAGCAGGAACAACA 3'
..::: ::::: ::::::::::
query 3' UUCUCUUCUUCUUCCUUGUUGU 5'
query=miR2082, target=RO3G_07692, score=4, range=27-48, strand=-1
target 5' GGGAGCAGAAGCAGGAACAACA 3'
..::: ::::: ::::::::::
query 3' UUCUCUUCUUCUUCCUUGUUGU 5'
query=miR2082, target=RO3G_15879, score=4, range=93-114, strand=-
target 5' GGGAGCAGAAGCAGGAACAACA 3'
..::: ::::: ::::::::::
query 3' UUCUCUUCUUCUUCCUUGUUGU 5'
query=miR2082, target=RO3G_13452, score=4, range=93-114, strand=-
target 5' GGGAGCAGAAGCAGGAACAACA 3'
..::: ::::: ::::::::::
query 3' UUCUCUUCUUCUUCCUUGUUGU 5'
query=miR2082, target=RO3G_16369, score=4, range=93-114, strand=-
target 5' GGGAGCAGAAGCAGGAACAACA 3'
..::: ::::: ::::::::::
query 3' UUCUCUUCUUCUUCCUUGUUGU 5'
query=miR2082, target=RO3G_14553, score=4, range=93-114, strand=-
target 5' GGGAGCAGAAGCAGGAACAACA 3'
..::: ::::: ::::::::::
query 3' UUCUCUUCUUCUUCCUUGUUGU 5'
query=miR2082, target=RO3G_11543, score=4, range=93-114, strand=-
target 5' GGGAGCAGAAGCAGGAACAACA 3'
..::: ::::: ::::::::::
query 3' UUCUCUUCUUCUUCCUUGUUGU 5'
query=miR2082, target=RO3G_15887, score=4, range=93-114, strand=-
target 5' GGGAGCAGAAGCAGGAACAACA 3'
..::: ::::: ::::::::::
query 3' UUCUCUUCUUCUUCCUUGUUGU 5'
query=miR2082, target=RO3G_05347, score=4, range=93-114, strand=-
target 5' GGGAGCAGAAGCAGGAACAACA 3'
```

```
..::: ::::: ::::::::::
query 3' UUCUCUUCUUCUUCCUUGUUGU 5'
query=miR2082, target=RO3G_12976, score=4, range=93-114, strand=-
target 5' GGGAGCAGAAGCAGGAACAACA 3'
..::: ::::: ::::::::::
query 3' UUCUCUUCUUCUUCCUUGUUGU 5'
query=miR2082, target=RO3G_16743, score=4, range=93-114, strand=-
target 5' GGGAGCAGAAGCAGGAACAACA 3'
..::: ::::: ::::::::::
query 3' UUCUCUUCUUCUUCCUUGUUGU 5'
query=miR7803b-3p, target=RO3G_15271, score=1, range=411-433, str
target 5' AGUACAUGAUUGGCGACGUGUCA 3'
:::::::::::.::::::::::
query 3' UCAUGUACUAACUGCUGCACAGU 5'
query=miR7803b-3p, target=RO3G_07705, score=1, range=411-433, str
target 5' AGUACAUGAUUGGCGACGUGUCA 3'
:::::::::::.::::::::::
query 3' UCAUGUACUAACUGCUGCACAGU 5'
query=miR7803b-3p, target=RO3G_01328, score=1, range=411-433, str
target 5' AGUACAUGAUUGGCGACGUGUCA 3'
:::::::::::.::::::::::
query 3' UCAUGUACUAACUGCUGCACAGU 5'
query=miR7803b-3p, target=RO3G_17387, score=1, range=411-433, str
target 5' AGUACAUGAUUGGCGACGUGUCA 3'
:::::::::::.::::::::::
query 3' UCAUGUACUAACUGCUGCACAGU 5'
query=miR7803b-3p, target=RO3G_16123, score=1, range=411-433, str
target 5' AGUACAUGAUUGGCGACGUGUCA 3'
:::::::::::.::::::::::
query 3' UCAUGUACUAACUGCUGCACAGU 5'
query=miR7803b-3p, target=RO3G_17399, score=1, range=411-433, str
target 5' AGUACAUGAUUGGCGACGUGUCA 3'
:::::::::::.::::::::::
query 3' UCAUGUACUAACUGCUGCACAGU 5'
query=miR7803b-3p, target=RO3G_06717, score=1, range=411-433, str
target 5' AGUACAUGAUUGGCGACGUGUCA 3'
:::::::::::.::::::::::
query 3' UCAUGUACUAACUGCUGCACAGU 5'
query=miR7803b-3p, target=RO3G_07137, score=1, range=411-433, str
target 5' AGUACAUGAUUGGCGACGUGUCA 3'
:::::::::::.::::::::::
query 3' UCAUGUACUAACUGCUGCACAGU 5'
query=miR7803b-3p, target=RO3G_05268, score=1, range=411-433, str
target 5' AGUACAUGAUUGGCGACGUGUCA 3'
:::::::::::.::::::::::
```

```
query 3' UCAUGUACUAACUGCUGCACAGU 5'
query=miR7803b-3p, target=RO3G_04674, score=1, range=162-184, str
target 5' AGUACAUGAUUGGCGACGUGUCA 3'
:::::::::::.:::::::::::
query 3' UCAUGUACUAACUGCUGCACAGU 5'
query=miR7803b-3p, target=RO3G_17223, score=1, range=135-157, str
target 5' AGUACAUGAUUGGCGACGUGUCA 3'
:::::::::::.:::::::::::
query 3' UCAUGUACUAACUGCUGCACAGU 5'
query=miR7803b-3p, target=RO3G_07702, score=1, range=162-184, str
target 5' AGUACAUGAUUGGCGACGUGUCA 3'
:::::::::::.:::::::::::
query 3' UCAUGUACUAACUGCUGCACAGU 5'
query=miR7803b-3p, target=RO3G_16420, score=1, range=162-184, str
target 5' AGUACAUGAUUGGCGACGUGUCA 3'
:::::::::::.:::::::::::
query 3' UCAUGUACUAACUGCUGCACAGU 5'
query=miR7803b-3p, target=RO3G_15282, score=1, range=135-157, str
target 5' AGUACAUGAUUGGCGACGUGUCA 3'
:::::::::::.:::::::::::
query 3' UCAUGUACUAACUGCUGCACAGU 5'
query=miR7803b-3p, target=RO3G_10795, score=1, range=411-433, str
target 5' AGUACAUGAUUGGCGACGUGUCA 3'
:::::::::::.:::::::::::
query 3' UCAUGUACUAACUGCUGCACAGU 5'
query=miR7803b-3p, target=RO3G_15261, score=1, range=411-433, str
target 5' AGUACAUGAUUGGCGACGUGUCA 3'
:::::::::::.:::::::::::
query 3' UCAUGUACUAACUGCUGCACAGU 5'
query=miR7803b-3p, target=RO3G_17017, score=1, range=411-433, str
target 5' AGUACAUGAUUGGCGACGUGUCA 3'
:::::::::::.:::::::::::
query 3' UCAUGUACUAACUGCUGCACAGU 5'
query=miR7803b-3p, target=RO3G_04695, score=1, range=201-223, str
target 5' AGUACAUGAUUGGCGACGUGUCA 3'
:::::::::::.:::::::::::
query 3' UCAUGUACUAACUGCUGCACAGU 5'
query=miR7803b-3p, target=RO3G_07867, score=1, range=162-184, str
target 5' AGUACAUGAUUGGCGACGUGUCA 3'
:::::::::::.:::::::::::
query 3' UCAUGUACUAACUGCUGCACAGU 5'
query=miR7803b-3p, target=RO3G_16725, score=1, range=153-175, str
target 5' AGUACAUGAUUGGCGACGUGUCA 3'
:::::::::::.:::::::::::
query 3' UCAUGUACUAACUGCUGCACAGU 5'
```

query=miR7803b-3p, target=RO3G_13852, score=1, range=162-184, str
target 5' AGUACAUGAUUGGCGACGUGUCA 3'
::::::::::::.::::::::::
query 3' UCAUGUACUAACUGCUGCACAGU 5'
query=miR7803b-3p, target=RO3G_16383, score=1, range=411-433, str
target 5' AGUACAUGAUUGGCGACGUGUCA 3'
::::::::::::.::::::::::
query 3' UCAUGUACUAACUGCUGCACAGU 5'
query=miR7803b-3p, target=RO3G_06735, score=1, range=135-157, str
target 5' AGUACAUGAUUGGCGACGUGUCA 3'
::::::::::::.::::::::::
query 3' UCAUGUACUAACUGCUGCACAGU 5'
query=miR7803b-3p, target=RO3G_11962, score=1, range=135-157, str
target 5' AGUACAUGAUUGGCGACGUGUCA 3'
::::::::::::.::::::::::
query 3' UCAUGUACUAACUGCUGCACAGU 5'
query=miR7803b-3p, target=RO3G_06433, score=1, range=135-157, str
target 5' AGUACAUGAUUGGCGACGUGUCA 3'
::::::::::::.::::::::::
query 3' UCAUGUACUAACUGCUGCACAGU 5'
query=miR7803b-3p, target=RO3G_05521, score=1, range=290-312, str
target 5' CGUACAUGAUUGACGACGUGUCA 3'
::::::::::::::::::::::
query 3' UCAUGUACUAACUGCUGCACAGU 5'
query=miR7803b-3p, target=RO3G_01205, score=1.5, range=411-433, strand=1
target 5' AGUACAUGAUUGGCGACGUGUCG 3'
::::::::::::.:::::::::.
query 3' UCAUGUACUAACUGCUGCACAGU 5'
query=miR7803b-3p, target=RO3G_00238, score=1.5, range=411-433, strand=1
target 5' AGUACAUGAUUGGCGACGUGUCG 3'
::::::::::::.:::::::::.
query 3' UCAUGUACUAACUGCUGCACAGU 5'
query=miR7803b-3p, target=RO3G_16698, score=1.5, range=408-430, strand=1
target 5' AGUACAUGAUUGGCGACGUGUCG 3'
::::::::::::.:::::::::.
query 3' UCAUGUACUAACUGCUGCACAGU 5'
query=miR7803b-3p, target=RO3G_12537, score=1.5, range=162-184, strand=1
target 5' AGUACAUGAUUGGCGACGUGUCG 3'
::::::::::::.:::::::::.
query 3' UCAUGUACUAACUGCUGCACAGU 5'
query=miR7803b-3p, target=RO3G_17416, score=2, range=411-433, str

```
target 5' AGUACAUGAUUGGCGACGUGUUA 3'
::::::::::::.::::::::.:
query 3' UCAUGUACUAACUGCUGCACAGU 5'
query=miR7803b-3p, target=RO3G_17291, score=2, range=411-433, str
target 5' AGUACAUGAUUGGCGACGUGUUA 3'
::::::::::::.::::::::.:
query 3' UCAUGUACUAACUGCUGCACAGU 5'
query=miR7803b-3p, target=RO3G_16742, score=2, range=411-433, str
target 5' AGUACAUGAUUGGCGACGUGUUA 3'
::::::::::::.::::::::.:
query 3' UCAUGUACUAACUGCUGCACAGU 5'
query=miR7803b-3p, target=RO3G_02824, score=2, range=411-433, str
target 5' AGUACAUGAUUGGCGACGUGUUA 3'
::::::::::::.::::::::.:
query 3' UCAUGUACUAACUGCUGCACAGU 5'
query=miR7803b-3p, target=RO3G_13714, score=2, range=411-433, str
target 5' AGUACAUGAUUGGCGACGUGUUA 3'
::::::::::::.::::::::.:
query 3' UCAUGUACUAACUGCUGCACAGU 5'
query=miR7803b-3p, target=RO3G_16372, score=2, range=411-433, str
target 5' AGUACAUGAUUGGCGACGUGUUA 3'
::::::::::::.::::::::.:
query 3' UCAUGUACUAACUGCUGCACAGU 5'
query=miR7803b-3p, target=RO3G_12663, score=2, range=246-268, str
target 5' AGUACAUGAUUGGCGACGUGUUA 3'
::::::::::::.::::::::.:
query 3' UCAUGUACUAACUGCUGCACAGU 5'
query=miR7803b-3p, target=RO3G_17355, score=2, range=246-268, str
target 5' AGUACAUGAUUGGCGACGUGUUA 3'
::::::::::::.::::::::.:
query 3' UCAUGUACUAACUGCUGCACAGU 5'
query=miR7803b-3p, target=RO3G_01962, score=2, range=162-184, str
target 5' AGUACAUGAUUGGCGACGUGUUA 3'
::::::::::::.::::::::.:
query 3' UCAUGUACUAACUGCUGCACAGU 5'
query=miR7803b-3p, target=RO3G_16706, score=2, range=135-157, str
target 5' AGUACAUGAUUGGCGACGUGUUA 3'
::::::::::::.::::::::.:
query 3' UCAUGUACUAACUGCUGCACAGU 5'
query=miR7803b-3p, target=RO3G_09193, score=2, range=135-157, str
target 5' AGUACAUGAUUGGCGACGUGUUA 3'
::::::::::::.::::::::.:
query 3' UCAUGUACUAACUGCUGCACAGU 5'
query=miR7803b-3p, target=RO3G_04682, score=2, range=411-433, str
target 5' AGUACAUGAUUGGCGACGUGUUA 3'
```

```
:::::::::::.::::::::.:
query 3' UCAUGUACUAACUGCUGCACAGU 5'
query=miR7803b-3p, target=RO3G_10771, score=2, range=162-184, str
target 5' AGUACAUAAUUGGCGACGUGUCA 3'
::::::: ::::.:::::::::::
query 3' UCAUGUACUAACUGCUGCACAGU 5'
query=miR7803b-3p, target=RO3G_07739, score=3, range=162-184, str
target 5' AGUACAUGAUUGGCGUCGUGUCA 3'
::::::::::::.:: :::::::
query 3' UCAUGUACUAACUGCUGCACAGU 5'
query=miR7803b-3p, target=RO3G_14770, score=3.5, range=411-433,
strand=1
target 5' AGUACAUGAUUGGCGACGUGCCG 3'
:::::::::::.::::::: :.
query 3' UCAUGUACUAACUGCUGCACAGU 5'
query=miR7803b-3p, target=RO3G_07984, score=3.5, range=411-433,
strand=1
target 5' AGUACAUGAUUGGCGACGUGCCG 3'
:::::::::::.::::::: :.
query 3' UCAUGUACUAACUGCUGCACAGU 5'
query=miR5758, target=RO3G_11521, score=0, range=1471-1489, stran
target 5' CAAGGACAUUCUUCACUUA 3'
:::::::::::::::::::
query 3' GUUCCUGUAAGAAGUGAAU 5'
query=miR5758, target=RO3G_09539, score=0, range=2509-2527, stran
target 5' CAAGGACAUUCUUCACUUA 3'
:::::::::::::::::::
query 3' GUUCCUGUAAGAAGUGAAU 5'
query=miR5758, target=RO3G_13796, score=0, range=2509-2527, stran
target 5' CAAGGACAUUCUUCACUUA 3'
:::::::::::::::::::
query 3' GUUCCUGUAAGAAGUGAAU 5'
query=miR5758, target=RO3G_03627, score=2, range=526-544, strand=
target 5' CAAGGACAUUCUUCACUGA 3'
::::::::::::::::: :
query 3' GUUCCUGUAAGAAGUGAAU 5'
query=miR5758, target=RO3G_01708, score=2, range=526-544, strand=
target 5' CAAGGACAUUCUUCACUGA 3'
::::::::::::::::: :
query 3' GUUCCUGUAAGAAGUGAAU 5'
query=miR5758, target=RO3G_15437, score=3, range=798-816, strand=
target 5' CGGUGACAUUCUUCAUUUA 3'
:.. :::::::::::.:::
query 3' GUUCCUGUAAGAAGUGAAU 5'
query=miR5758, target=RO3G_01900, score=3, range=243-260, strand=
```

target 5' CAA-GAUAUUCUUCACUUU 3'
::: ::.:::::::::::
query 3' GUUCCUGUAAGAAGUGAAU 5'
query=miR5758, target=RO3G_14433, score=3.5, range=1861-1879,
strand=-1
target 5' CAUGGAUAUUCUUCAUUUG 3'
:: :::.::::::::.::.
query 3' GUUCCUGUAAGAAGUGAAU 5'
query=miR5758, target=RO3G_16926, score=3.5, range=236-254, stran
target 5' CGAGGCCAUUUUUCAUUUA 3'
:.::: ::::.::::.:::
query 3' GUUCCUGUAAGAAGUGAAU 5'
query=miR5758, target=RO3G_03353, score=3.5, range=1028-1045,
strand=-1
target 5' CAAGGGCAUU-UUCGCUUA 3'
:::::.:::: :::.::::
query 3' GUUCCUGUAAGAAGUGAAU 5'
query=miR5758, target=RO3G_04261, score=3.5, range=2581-2599,
strand=-1
target 5' UCAUGGCAUUCUUCACUUG 3'
. : :.::::::::::::.
query 3' GUUCCUGUAAGAAGUGAAU 5'
query=miR5758, target=RO3G_06098, score=3.5, range=68-86, strand=
target 5' GAAUUGCAUUCUUCACUUA 3'
:: .:::::::::::::
query 3' GUUCCUGUAAGAAGUGAAU 5'
query=miR5758, target=RO3G_16313, score=3.5, range=569-586, stran
target 5' CGA-GACAUUCUUCAAUUA 3'
:.: ::::::::::: :::
query 3' GUUCCUGUAAGAAGUGAAU 5'
query=miR5758, target=RO3G_09046, score=4, range=237-255, strand=
target 5' CAAGGGCAUCCUUCAUUUG 3'
:::::.::: :::::.::.
query 3' GUUCCUGUAAGAAGUGAAU 5'
query=miR5758, target=RO3G_08781, score=4, range=1421-1440, stran
target 5' UGAGAUAUAUUCUUCACUUA 3'
..:: :.::::::::::::
query 3' GUUC-CUGUAAGAAGUGAAU 5'
query=miR5758, target=RO3G_12248, score=4, range=879-897, strand=
target 5' CAACGUCAUUCUUCAUUUU 3'
::: : :::::::::.::
query 3' GUUCCUGUAAGAAGUGAAU 5'
query=miR5758, target=RO3G_03221, score=4, range=1203-1220, stran
target 5' CAA-GACAUUCUUCAUUCA 3'
::: :::::::::::.: :

```
query 3' GUUCCUGUAAGAAGUGAAU 5'
query=miR5758, target=RO3G_05240, score=4, range=215-232, strand=
target 5' CAAGGUUAUUCUU-ACUUA 3'
::::: .:::::: :::::
query 3' GUUCCUGUAAGAAGUGAAU 5'
query=miR5758, target=RO3G_02933, score=4, range=296-314, strand=
target 5' CAACUACAUCCUUCACUUA 3'
::: :::: :::::::::
query 3' GUUCCUGUAAGAAGUGAAU 5'
query=miR5758, target=RO3G_12572, score=4, range=387-405, strand=
target 5' CAUGAUCAUUCUUCGCUUA 3'
:: : ::::::::.::::
query 3' GUUCCUGUAAGAAGUGAAU 5'
query=miR5758, target=RO3G_01038, score=4, range=56-73, strand=1
target 5' CAAUG-CAUUCUUCACAUA 3'
::: : :::::::::: ::
query 3' GUUCCUGUAAGAAGUGAAU 5'
query=miR5758, target=RO3G_12835, score=4, range=1978-1997, stran
target 5' CAAGGCACAUUCUUCAAUUC 3'
::::: :::::::::: ::
query 3' GUUCC-UGUAAGAAGUGAAU 5'
query=miR5758, target=RO3G_00347, score=4, range=199-216, strand=
target 5' UAAAG-CAUUUUUCACUUG 3'
.:: : ::::.:::::::.
query 3' GUUCCUGUAAGAAGUGAAU 5'
query=miR5758, target=RO3G_07743, score=4, range=434-451, strand=
target 5' UAAAG-CAUUCUUUACUUG 3'
.:: : :::::::.::::.
query 3' GUUCCUGUAAGAAGUGAAU 5'
query=miR5758, target=RO3G_12515, score=4, range=223-240, strand=
target 5' CAA-GACAUUAUUCACUUC 3'
::: :::::: :::::::
query 3' GUUCCUGUAAGAAGUGAAU 5'
query=miR5038a, target=RO3G_17155, score=0, range=632-652, strand
target 5' UGGAAAAGGCCAAGAUUCCCA 3'
:::::::::::::::::::::
query 3' ACCUUUUCCGGUUCUAAGGGU 5'
query=miR5038a, target=RO3G_05926, score=0, range=632-652, strand
target 5' UGGAAAAGGCCAAGAUUCCCA 3'
:::::::::::::::::::::
query 3' ACCUUUUCCGGUUCUAAGGGU 5'
query=miR5038a, target=RO3G_09928, score=0, range=632-652, strand
target 5' UGGAAAAGGCCAAGAUUCCCA 3'
:::::::::::::::::::::
query 3' ACCUUUUCCGGUUCUAAGGGU 5'
```

```
query=miR5038a, target=RO3G_12668, score=0, range=545-565, strand
target 5' UGGAAAAGGCCAAGAUUCCCA 3'
:::::::::::::::::::::
query 3' ACCUUUUCCGGUUCUAAGGGU 5'
query=miR5038a, target=RO3G_12962, score=0, range=632-652, strand
target 5' UGGAAAAGGCCAAGAUUCCCA 3'
:::::::::::::::::::::
query 3' ACCUUUUCCGGUUCUAAGGGU 5'
query=miR5038a, target=RO3G_11954, score=2.5, range=632-652, stra
target 5' UCGAAAAGGCCAAGAUUCCUG 3'
: :::::::::::::::::..
query 3' ACCUUUUCCGGUUCUAAGGGU 5'
query=miR5038a, target=RO3G_16707, score=4, range=632-652, strand
target 5' UCGAAAAGGCCAAGAUCCCUA 3'
: ::::::::::::: ::.:
query 3' ACCUUUUCCGGUUCUAAGGGU 5'
query=miR5038a, target=RO3G_05924, score=4, range=632-652, strand
target 5' UCGAAAAGGCCAAGAUCCCUA 3'
: ::::::::::::: ::.:
query 3' ACCUUUUCCGGUUCUAAGGGU 5'
query=miR5038a, target=RO3G_12074, score=4, range=632-652, strand
target 5' UCGAAAAGGCCAAGAUCCCUA 3'
: ::::::::::::: ::.:
query 3' ACCUUUUCCGGUUCUAAGGGU 5'
query=miR5038a, target=RO3G_02229, score=4, range=632-652, strand
target 5' UCGAAAAGGCCAAGAUCCCUA 3'
: ::::::::::::: ::.:
query 3' ACCUUUUCCGGUUCUAAGGGU 5'
query=miR5038a, target=RO3G_13744, score=4, range=632-652, strand
target 5' UCGAAAAGGCCAAGAUCCCUA 3'
: ::::::::::::: ::.:
query 3' ACCUUUUCCGGUUCUAAGGGU 5'
query=miR5038a, target=RO3G_05525, score=4, range=632-652, strand
target 5' UCGAAAAGGCCAAGAUCCCUA 3'
: ::::::::::::: ::.:
query 3' ACCUUUUCCGGUUCUAAGGGU 5'
query=miR5038a, target=RO3G_12965, score=4, range=434-454, strand
target 5' UCGAAAAGGCCAAGAUCCCUA 3'
: ::::::::::::: ::.:
query 3' ACCUUUUCCGGUUCUAAGGGU 5'
query=miR5038a, target=RO3G_17443, score=4, range=632-652, strand
target 5' UCGAAAAGGCCAAGAUCCCUA 3'
: ::::::::::::: ::.:
query 3' ACCUUUUCCGGUUCUAAGGGU 5'
query=miR9674b-3p, target=RO3G_01294, score=0, range=1918-1937,
```

```
strand=1
target 5' CUGAUGCUCUGGCAUAUUCA 3'
::::::::::::::::::::
query 3' GACUACGAGACCGUAUAAGU 5'
query=miR9674b-3p, target=RO3G_13459, score=1, range=250-269, str
target 5' CCGAUGCUCUGGCAUAUUCA 3'
: ::::::::::::::::::
query 3' GACUACGAGACCGUAUAAGU 5'
query=miR9674b-3p, target=RO3G_17251, score=2, range=553-572, str
target 5' CUGAUGCUCUGGCAUAUACA 3'
::::::::::::::::: ::
query 3' GACUACGAGACCGUAUAAGU 5'
query=miR9674b-3p, target=RO3G_16366, score=2, range=2845-2864,
strand=1
target 5' CUGAUGCUCUGGCAUAUACA 3'
::::::::::::::::: ::
query 3' GACUACGAGACCGUAUAAGU 5'
query=miR9674b-3p, target=RO3G_17156, score=2.5, range=1102-1121,
strand=1
target 5' UUGAUGCUCUGGCAUAUCCA 3'
.:::::::::::::::: ::
query 3' GACUACGAGACCGUAUAAGU 5'
query=miR9674b-3p, target=RO3G_02475, score=3, range=1496-1514,
strand=1
target 5' AUUAU-CUCUGGCAUAUUCA 3'
: :: ::::::::::::::
query 3' GACUACGAGACCGUAUAAGU 5'
query=miR9674b-3p, target=RO3G_13580, score=3.5, range=332-351,
strand=-1
target 5' UAAAUGCUCUGGCAUGUUCA 3'
. ::::::::::::.::::
query 3' GACUACGAGACCGUAUAAGU 5'
query=miR9674b-3p, target=RO3G_12833, score=4, range=1483-1501,
strand=1
target 5' AUGAU-CUCUGGUAUAUUCC 3'
:::: ::::::.::::::
query 3' GACUACGAGACCGUAUAAGU 5'
query=miR9674b-3p, target=RO3G_07810, score=4, range=365-385,
strand=-1
target 5' CUGGUGUCUCUGGUAUAUUUG 3'
:::.:: ::::::.:::::..
query 3' GACUAC-GAGACCGUAUAAGU 5'
query=miR9674b-3p, target=RO3G_01002, score=4, range=1592-1611,
strand=-1
target 5' CUGUUGCUCUGGUAUAUUAA 3'
```

::: ::::::::.::::: :
query 3' GACUACGAGACCGUAUAAGU 5'
query=miR9674b-3p, target=RO3G_07279, score=4, range=3078-3097, strand=-1
target 5' CUGUUGCUUUGGCAGAUUCA 3'
::: ::::.::::: :::::
query 3' GACUACGAGACCGUAUAAGU 5'
query=miR9674b-3p, target=RO3G_16585, score=4, range=188-206, strand=-1
target 5' CUG-UGGUCUGGAAUAUUCA 3'
::: :: ::::: :::::::
query 3' GACUACGAGACCGUAUAAGU 5'
query=miR528-5p, target=RO3G_09951, score=0, range=29-49, strand=
target 5' CCUCCUCUCAUCCCCAUUCCA 3'
:::::::::::::::::::::
query 3' GGAGGAGAGUAGGGGUAAGGU 5'
query=miR528-5p, target=RO3G_05148, score=4, range=400-420, stran
target 5' CCACCUGUCAUCCCUAUUCCU 3'
:: ::: :::::::.:::::
query 3' GGAGGAGAGUAGGGGUAAGGU 5'
query=miR528-5p, target=RO3G_05364, score=4, range=1573-1593, strand=-1
target 5' UCUUUUUUCAUCCGCAUUCCA 3'
.::..:.::::::: :::::::
query 3' GGAGGAGAGUAGGGGUAAGGU 5'
query=miR528-5p, target=RO3G_05871, score=4, range=955-975, stran
target 5' UCUCCACUCAUCCCUAUUUCG 3'
.:::: :::::::::.:::.:.
query 3' GGAGGAGAGUAGGGGUAAGGU 5'
query=miR4397-3p, target=RO3G_07123, score=0, range=769-790, stra
target 5' AGUCUUUCUCCACAUCUUUGAA 3'
::::::::::::::::::::::
query 3' UCAGAAAGAGGUGUAGAAACUU 5'
query=miR4397-3p, target=RO3G_11284, score=0, range=1012-1033, strand=-1
target 5' AGUCUUUCUCCACAUCUUUGAA 3'
::::::::::::::::::::::
query 3' UCAGAAAGAGGUGUAGAAACUU 5'
query=miR4397-3p, target=RO3G_13853, score=0, range=58-79, strand
target 5' AGUCUUUCUCCACAUCUUUGAA 3'
::::::::::::::::::::::
query 3' UCAGAAAGAGGUGUAGAAACUU 5'
query=miR4397-3p, target=RO3G_00705, score=0, range=1012-1033, strand=-1
target 5' AGUCUUUCUCCACAUCUUUGAA 3'

```
:::::::::::::::::::::
query 3' UCAGAAAGAGGUGUAGAAACUU 5'
query=miR4397-3p, target=RO3G_03562, score=0, range=742-763, stra
target 5' AGUCUUUCUCCACAUCUUUGAA 3'
:::::::::::::::::::::
query 3' UCAGAAAGAGGUGUAGAAACUU 5'
query=miR4397-3p, target=RO3G_10802, score=0, range=130-151, stra
target 5' AGUCUUUCUCCACAUCUUUGAA 3'
:::::::::::::::::::::
query 3' UCAGAAAGAGGUGUAGAAACUU 5'
query=miR4397-3p, target=RO3G_13302, score=0, range=1009-1030,
strand=-1
target 5' AGUCUUUCUCCACAUCUUUGAA 3'
:::::::::::::::::::::
query 3' UCAGAAAGAGGUGUAGAAACUU 5'
query=miR4397-3p, target=RO3G_05372, score=4, range=1946-1967,
strand=-1
target 5' AGUGCAUCGCCACAUCUUUGAA 3'
::: :: :::::::::::::
query 3' UCAGAAAGAGGUGUAGAAACUU 5'
query=miR4413b, target=RO3G_14270, score=4, range=120-141, strand
target 5' GGGAGAGUUAUACUUGUUCUUG 3'
.:: :: :::::::::.::::.
query 3' UCCACUGAAUAUGAACGAGAAU 5'
query=miR7716-5p, target=RO3G_15587, score=1, range=212-234, stra
target 5' GUCUCGUUCUUACUUAUCAGCUA 3'
:::::::::::::::::::.:::
query 3' CAGAGCAAGAAUGAAUAGUUGAU 5'
query=miR7716-5p, target=RO3G_08791, score=3, range=128-150, stra
target 5' GUCUCGUUCUGGCUUAUCAACUA 3'
:::::::::: .:::::::::::
query 3' CAGAGCAAGAAUGAAUAGUUGAU 5'
query=miR482e, target=RO3G_02851, score=1, range=1072-1092, stran
target 5' CUUAGGCAGAGGAAUAGGCAA 3'
::: :::::::::::::::::
query 3' GAA-CCGUCUCCUUAUCCGUU 5'
query=miR482e, target=RO3G_12958, score=1, range=892-912, strand=
target 5' CUUAGGCAGAGGAAUAGGCAA 3'
::: :::::::::::::::::
query 3' GAA-CCGUCUCCUUAUCCGUU 5'
query=miR482e, target=RO3G_07982, score=1, range=1072-1092, stran
target 5' CUUAGGCAGAGGAAUAGGCAA 3'
::: :::::::::::::::::
query 3' GAA-CCGUCUCCUUAUCCGUU 5'
query=miR482e, target=RO3G_02849, score=1, range=1072-1092, stran
```

```
target 5' CUUAGGCAGAGGAAUAGGCAA 3'
::: :::::::::::::::::
query 3' GAA-CCGUCUCCUUAUCCGUU 5'
query=miR482e, target=RO3G_05217, score=1, range=1072-1092, stran
target 5' CUUAGGCAGAGGAAUAGGCAA 3'
::: :::::::::::::::::
query 3' GAA-CCGUCUCCUUAUCCGUU 5'
query=miR482e, target=RO3G_07035, score=1, range=1072-1092, stran
target 5' CUUAGGCAGAGGAAUAGGCAA 3'
::: :::::::::::::::::
query 3' GAA-CCGUCUCCUUAUCCGUU 5'
query=miR482e, target=RO3G_00697, score=1, range=1072-1092, stran
target 5' CUUAGGCAGAGGAAUAGGCAA 3'
::: :::::::::::::::::
query 3' GAA-CCGUCUCCUUAUCCGUU 5'
query=miR482e, target=RO3G_17425, score=1, range=1072-1092, stran
target 5' CUUAGGCAGAGGAAUAGGCAA 3'
::: :::::::::::::::::
query 3' GAA-CCGUCUCCUUAUCCGUU 5'
query=miR482e, target=RO3G_12650, score=1, range=1072-1092, stran
target 5' CUUAGGCAGAGGAAUAGGCAA 3'
::: :::::::::::::::::
query 3' GAA-CCGUCUCCUUAUCCGUU 5'
query=miR482e, target=RO3G_01965, score=1, range=1072-1092, stran
target 5' CUUAGGCAGAGGAAUAGGCAA 3'
::: :::::::::::::::::
query 3' GAA-CCGUCUCCUUAUCCGUU 5'
query=miR482e, target=RO3G_08655, score=1, range=1072-1092, stran
target 5' CUUAGGCAGAGGAAUAGGCAA 3'
::: :::::::::::::::::
query 3' GAA-CCGUCUCCUUAUCCGUU 5'
query=miR482e, target=RO3G_08650, score=1, range=1072-1092, stran
target 5' CUUAGGCAGAGGAAUAGGCAA 3'
::: :::::::::::::::::
query 3' GAA-CCGUCUCCUUAUCCGUU 5'
query=miR482e, target=RO3G_12973, score=1, range=1072-1092, stran
target 5' CUUAGGCAGAGGAAUAGGCAA 3'
::: :::::::::::::::::
query 3' GAA-CCGUCUCCUUAUCCGUU 5'
query=miR482e, target=RO3G_15259, score=2, range=1072-1092, stran
target 5' CUUAGGCAGAGGAAUAGGUAA 3'
::: ::::::::::::::.::
query 3' GAA-CCGUCUCCUUAUCCGUU 5'
query=miR482e, target=RO3G_11778, score=2, range=1072-1092, stran
target 5' CUUAGGCAGAGGAAUAGGUAA 3'
```

```
::: ::::::::::::::.::
query 3' GAA-CCGUCUCCUUAUCCGUU 5'
query=miR482e, target=RO3G_16408, score=2, range=1072-1092, stran
target 5' CUUGGGAAGAGGAAUAGGCAA 3'
::: :: ::::::::::::::
query 3' GAA-CCGUCUCCUUAUCCGUU 5'
query=miR482e, target=RO3G_08310, score=3, range=129-148, strand=
target 5' AUUGACCGAGGAAUAGGCAA 3'
::: : :::::::::::::
query 3' GAACCGUCUCCUUAUCCGUU 5'
query=miR482e, target=RO3G_00068, score=3.5, range=277-296, stran
target 5' GGUGGAGGAGGAAUAGGCAA 3'
::: .:::::::::::::
query 3' GAACCGUCUCCUUAUCCGUU 5'
query=miR482e, target=RO3G_05305, score=4, range=1161-1179, stran
target 5' CUUGGCUGAGGAA-GGGCAA 3'
:::::: :::::: .:::::
query 3' GAACCGUCUCCUUAUCCGUU 5'
query=miR482e, target=RO3G_08315, score=4, range=129-147, strand=
target 5' AUUAGCAGAGGAA-AGGCAA 3'
:: ::::::::: ::::::
query 3' GAACCGUCUCCUUAUCCGUU 5'
query=miR482e, target=RO3G_02737, score=4, range=2080-2100, stran
target 5' AUUAGCACGGGGAAUAGGCAA 3'
:: ::: :.:::::::::::
query 3' GAACCGU-CUCCUUAUCCGUU 5'
query=miR482e, target=RO3G_10728, score=4, range=991-1011, strand
target 5' CUUGGGAAGAGAAAUAGGCAA 3'
::: :: :::: :::::::::
query 3' GAA-CCGUCUCCUUAUCCGUU 5'
query=miR482e, target=RO3G_03247, score=4, range=508-526, strand=
target 5' CUUCACA-AGGAAUAGGCAA 3'
::: :: ::::::::::::
query 3' GAACCGUCUCCUUAUCCGUU 5'
query=miR5766, target=RO3G_05217, score=0, range=1071-1091, stran
target 5' CUUGCCUAUUCCUCUGCCUAA 3'
:::::::::::::::::::::
query 3' GAACGGAUAAGGAGACGGAUU 5'
query=miR5766, target=RO3G_07982, score=0, range=1071-1091, stran
target 5' CUUGCCUAUUCCUCUGCCUAA 3'
:::::::::::::::::::::
query 3' GAACGGAUAAGGAGACGGAUU 5'
query=miR5766, target=RO3G_02849, score=0, range=1071-1091, stran
target 5' CUUGCCUAUUCCUCUGCCUAA 3'
:::::::::::::::::::::
```

```
query 3' GAACGGAUAAGGAGACGGAUU 5'
query=miR5766, target=RO3G_07035, score=0, range=1071-1091, stran
target 5' CUUGCCUAUUCCUCUGCCUAA 3'
:::::::::::::::::::::
query 3' GAACGGAUAAGGAGACGGAUU 5'
query=miR5766, target=RO3G_00697, score=0, range=1071-1091, stran
target 5' CUUGCCUAUUCCUCUGCCUAA 3'
:::::::::::::::::::::
query 3' GAACGGAUAAGGAGACGGAUU 5'
query=miR5766, target=RO3G_01965, score=0, range=1071-1091, stran
target 5' CUUGCCUAUUCCUCUGCCUAA 3'
:::::::::::::::::::::
query 3' GAACGGAUAAGGAGACGGAUU 5'
query=miR5766, target=RO3G_08650, score=0, range=1071-1091, stran
target 5' CUUGCCUAUUCCUCUGCCUAA 3'
:::::::::::::::::::::
query 3' GAACGGAUAAGGAGACGGAUU 5'
query=miR5766, target=RO3G_08655, score=0, range=1071-1091, stran
target 5' CUUGCCUAUUCCUCUGCCUAA 3'
:::::::::::::::::::::
query 3' GAACGGAUAAGGAGACGGAUU 5'
query=miR5766, target=RO3G_12973, score=0, range=1071-1091, stran
target 5' CUUGCCUAUUCCUCUGCCUAA 3'
:::::::::::::::::::::
query 3' GAACGGAUAAGGAGACGGAUU 5'
query=miR5766, target=RO3G_02851, score=0, range=1071-1091, stran
target 5' CUUGCCUAUUCCUCUGCCUAA 3'
:::::::::::::::::::::
query 3' GAACGGAUAAGGAGACGGAUU 5'
query=miR5766, target=RO3G_12650, score=0, range=1071-1091, stran
target 5' CUUGCCUAUUCCUCUGCCUAA 3'
:::::::::::::::::::::
query 3' GAACGGAUAAGGAGACGGAUU 5'
query=miR5766, target=RO3G_12958, score=0, range=891-911, strand=
target 5' CUUGCCUAUUCCUCUGCCUAA 3'
:::::::::::::::::::::
query 3' GAACGGAUAAGGAGACGGAUU 5'
query=miR5766, target=RO3G_17425, score=0, range=1071-1091, stran
target 5' CUUGCCUAUUCCUCUGCCUAA 3'
:::::::::::::::::::::
query 3' GAACGGAUAAGGAGACGGAUU 5'
query=miR5766, target=RO3G_15259, score=1, range=1071-1091, stran
target 5' CUUACCUAUUCCUCUGCCUAA 3'
::: :::::::::::::::::
query 3' GAACGGAUAAGGAGACGGAUU 5'
```

query=miR5766, target=RO3G_11778, score=1, range=1071-1091, stran
target 5' CUUACCUAUUCCUCUGCCUAA 3'
::: :::::::::::::::::
query 3' GAACGGAUAAGGAGACGGAUU 5'
query=miR5766, target=RO3G_08741, score=3, range=1074-1094, stran
target 5' CUAGCAUAUUCCUCUGCCUGA 3'
:: :: :::::::::::::.:
query 3' GAACGGAUAAGGAGACGGAUU 5'
query=miR5766, target=RO3G_16408, score=4, range=1071-1091, stran
target 5' CUUGCCUAUUCCUCUUCCCAA 3'
::::::::::::::: :: ::
query 3' GAACGGAUAAGGAGACGGAUU 5'
query=miR7763-5p, target=RO3G_19004, score=0, range=1548-1569, strand=-1
target 5' CUAUCCCCAGCACGAUGAAGUU 3'
::::::::::::::::::::::
query 3' GAUAGGGGUCGUGCUACUUCAA 5'
query=miR7763-5p, target=RO3G_19001, score=0, range=1252-1273, strand=-1
target 5' CUAUCCCCAGCACGAUGAAGUU 3'
::::::::::::::::::::::
query 3' GAUAGGGGUCGUGCUACUUCAA 5'
query=miR7763-5p, target=RO3G_05488, score=4, range=1808-1829, strand=1
target 5' CUAACCCCAUCAUGAUGAAGUU 3'
::: ::::: ::.:::::::::
query 3' GAUAGGGGUCGUGCUACUUCAA 5'
query=miR7763-5p, target=RO3G_00708, score=4, range=1739-1760, strand=1
target 5' CUAACCCCAUCAUGAUGAAGUU 3'
::: ::::: ::.:::::::::
query 3' GAUAGGGGUCGUGCUACUUCAA 5'
query=miR7763-5p, target=RO3G_00340, score=4, range=1226-1246, strand=-1
target 5' CU-UCUUCAGCACGACGAAGUU 3'
:: ::..:::::::: ::::::
query 3' GAUAGGGGUCGUGCUACUUCAA 5'
query=miR5020c, target=RO3G_10281, score=3, range=947-964, strand
target 5' GUCGACCUCUUGC-UGCUA 3'
::::::::::::: :::.:
query 3' CAGCUGGAGAACGUACGGU 5'
query=miR5020c, target=RO3G_03849, score=3, range=258-275, strand
target 5' GUUG-UCUCUUGCAUGCCU 3'
::.: .::::::::::::
query 3' CAGCUGGAGAACGUACGGU 5'

```
query=miR5020c, target=RO3G_10250, score=3.5, range=457-475, stra
target 5' AUAGGCCUCUUGCAUGCCC 3'
: :.:::::::::::::
query 3' CAGCUGGAGAACGUACGGU 5'
query=miR5020c, target=RO3G_11325, score=4, range=330-348, strand
target 5' GUCGGUCUCUUCCAUGUCA 3'
::::..::::: ::::.::
query 3' CAGCUGGAGAACGUACGGU 5'
query=miR5020c, target=RO3G_05820, score=4, range=1348-1365, stra
target 5' UUCGACUUCUUGC-UGCCA 3'
:::::.:::::: :::::
query 3' CAGCUGGAGAACGUACGGU 5'
query=miR5020c, target=RO3G_14589, score=4, range=180-197, strand
target 5' GUC-UCUUCUUGCAUGUCA 3'
::: :.:::::::::.::
query 3' CAGCUGGAGAACGUACGGU 5'
query=miR5227, target=RO3G_04218, score=0, range=837-858, strand=
target 5' AUCCUCAACUUCUUCUUCUUCA 3'
::::::::::::::::::::::
query 3' UAGGAGUUGAAGAAGAAGAAGU 5'
query=miR5227, target=RO3G_02836, score=0, range=837-858, strand=
target 5' AUCCUCAACUUCUUCUUCUUCA 3'
::::::::::::::::::::::
query 3' UAGGAGUUGAAGAAGAAGAAGU 5'
query=miR5227, target=RO3G_09957, score=0, range=837-858, strand=
target 5' AUCCUCAACUUCUUCUUCUUCA 3'
::::::::::::::::::::::
query 3' UAGGAGUUGAAGAAGAAGAAGU 5'
query=miR5227, target=RO3G_16134, score=0, range=837-858, strand=
target 5' AUCCUCAACUUCUUCUUCUUCA 3'
::::::::::::::::::::::
query 3' UAGGAGUUGAAGAAGAAGAAGU 5'
query=miR5227, target=RO3G_15284, score=0, range=837-858, strand=
target 5' AUCCUCAACUUCUUCUUCUUCA 3'
::::::::::::::::::::::
query 3' UAGGAGUUGAAGAAGAAGAAGU 5'
query=miR5227, target=RO3G_03104, score=0, range=837-858, strand=
target 5' AUCCUCAACUUCUUCUUCUUCA 3'
::::::::::::::::::::::
query 3' UAGGAGUUGAAGAAGAAGAAGU 5'
query=miR5227, target=RO3G_09933, score=0, range=837-858, strand=
target 5' AUCCUCAACUUCUUCUUCUUCA 3'
::::::::::::::::::::::
query 3' UAGGAGUUGAAGAAGAAGAAGU 5'
query=miR5227, target=RO3G_01940, score=0, range=795-816, strand=
```

target 5' AUCCUCAACUUCUUCUUCUUCA 3'
::::::::::::::::::::::
query 3' UAGGAGUUGAAGAAGAAGAAGU 5'
query=miR5227, target=RO3G_10229, score=0, range=249-270, strand=
target 5' AUCCUCAACUUCUUCUUCUUCA 3'
::::::::::::::::::::::
query 3' UAGGAGUUGAAGAAGAAGAAGU 5'
query=miR5227, target=RO3G_07675, score=0, range=249-270, strand=
target 5' AUCCUCAACUUCUUCUUCUUCA 3'
::::::::::::::::::::::
query 3' UAGGAGUUGAAGAAGAAGAAGU 5'
query=miR5227, target=RO3G_10898, score=1.5, range=108-129, stran
target 5' UUCCUCAAUUUCUUCUUCUUCA 3'
:::::::.::::::::::::::
query 3' UAGGAGUUGAAGAAGAAGAAGU 5'
query=miR5227, target=RO3G_01819, score=1.5, range=1071-1092,
strand=-1
target 5' AUCCUCGUCUUCUUCUUCUUCA 3'
::::::. ::::::::::::::
query 3' UAGGAGUUGAAGAAGAAGAAGU 5'
query=miR5227, target=RO3G_08358, score=2, range=1089-1110, stran
target 5' AUCCUCUACUUCUUCUUCUUCU 3'
:::::: ::::::::::::::
query 3' UAGGAGUUGAAGAAGAAGAAGU 5'
query=miR5227, target=RO3G_04876, score=2, range=452-472, strand=
target 5' AUUC-CAAUUUCUUCUUCUUCA 3'
::.: :::.::::::::::::::
query 3' UAGGAGUUGAAGAAGAAGAAGU 5'
query=miR5227, target=RO3G_03732, score=2, range=510-530, strand=
target 5' AUCCUU-GCUUCUUCUUCUUCA 3'
:::::. .::::::::::::::
query 3' UAGGAGUUGAAGAAGAAGAAGU 5'
query=miR5227, target=RO3G_08993, score=2, range=144-165, strand=
target 5' AUCCUCUUCUUCUUCUUCUUCA 3'
:::::: ::::::::::::::
query 3' UAGGAGUUGAAGAAGAAGAAGU 5'
query=miR5227, target=RO3G_07832, score=2, range=630-651, strand=
target 5' AUCCUCCUCUUCUUCUUCUUCA 3'
:::::: ::::::::::::::
query 3' UAGGAGUUGAAGAAGAAGAAGU 5'
query=miR5227, target=RO3G_13189, score=2.5, range=216-237, stran
target 5' CUCCUCGACUUCUUCUUCUUCU 3'
:::::.::::::::::::::
query 3' UAGGAGUUGAAGAAGAAGAAGU 5'
query=miR5227, target=RO3G_16997, score=2.5, range=879-900, stran

target 5' AUCUUCGGAUUCUUCUUCUUCA 3'
:::.::.. :::::::::::::
query 3' UAGGAGUUGAAGAAGAAGAAGU 5'
query=miR5227, target=RO3G_09023, score=2.5, range=879-900, stran
target 5' AUCUUCGGAUUCUUCUUCUUCA 3'
:::.::.. :::::::::::::
query 3' UAGGAGUUGAAGAAGAAGAAGU 5'
query=miR5227, target=RO3G_00109, score=2.5, range=642-663, stran
target 5' AUCCUCACUUUCUUCUUCUUCC 3'
::::::: .::::::::::::
query 3' UAGGAGUUGAAGAAGAAGAAGU 5'
query=miR5227, target=RO3G_11355, score=2.5, range=1284-1305,
strand=-1
target 5' CUCUUCCACUUCUUCUUCUUCA 3'
::.:: :::::::::::::::
query 3' UAGGAGUUGAAGAAGAAGAAGU 5'
query=miR5227, target=RO3G_15530, score=2.5, range=552-573, stran
target 5' UUCUUCAUCUUCUUCUUCUUCA 3'
::.::: ::::::::::::::
query 3' UAGGAGUUGAAGAAGAAGAAGU 5'
query=miR5227, target=RO3G_07283, score=2.5, range=213-234, stran
target 5' ACCUUCAACUUCUUCUUCUUCU 3'
: :.:::::::::::::::::
query 3' UAGGAGUUGAAGAAGAAGAAGU 5'
query=miR5227, target=RO3G_04284, score=2.5, range=452-472, stran
target 5' AUUC-CAACUUCUUUUUCUUCA 3'
::.: :::::::::.:::::::
query 3' UAGGAGUUGAAGAAGAAGAAGU 5'
query=miR5227, target=RO3G_02035, score=2.5, range=2799-2820,
strand=-1
target 5' AUCUUCUUCUUCUUCUUCUUCA 3'
:::.:: ::::::::::::::
query 3' UAGGAGUUGAAGAAGAAGAAGU 5'
query=miR5227, target=RO3G_09029, score=2.5, range=450-471, stran
target 5' ACCUUCAUCUUCUUCUUCUUCA 3'
: :.::: ::::::::::::::
query 3' UAGGAGUUGAAGAAGAAGAAGU 5'
query=miR5227, target=RO3G_04071, score=2.5, range=987-1008, stra
target 5' AUCAUUAUCUUCUUCUUCUUCA 3'
::: :.: ::::::::::::::
query 3' UAGGAGUUGAAGAAGAAGAAGU 5'
query=miR5227, target=RO3G_08743, score=2.5, range=690-711, stran
target 5' AUCAUCAUCUUCUUCUUCUUCG 3'
::: ::: :::::::::::::.
query 3' UAGGAGUUGAAGAAGAAGAAGU 5'

```
query=miR5227, target=RO3G_03568, score=2.5, range=582-603, stran
target 5' AUCUUCUUCUUCUUCUUCUUCA 3'
:::.:: ::::::::::::::
query 3' UAGGAGUUGAAGAAGAAGAAGU 5'
query=miR5227, target=RO3G_14458, score=2.5, range=102-123, stran
target 5' AUGUUCACCUUCUUCUUCUUCA 3'
:: .::: ::::::::::::::
query 3' UAGGAGUUGAAGAAGAAGAAGU 5'
query=miR5227, target=RO3G_06538, score=3, range=1130-1151, stran
target 5' UCCCUCAACUUCUUCUUCUUCC 3'
:::::::::::::::::::
query 3' UAGGAGUUGAAGAAGAAGAAGU 5'
query=miR5227, target=RO3G_07001, score=3, range=171-192, strand=
target 5' AUCCUCUUCUUCUUCUUCUUCU 3'
:::::: :::::::::::::
query 3' UAGGAGUUGAAGAAGAAGAAGU 5'
query=miR5227, target=RO3G_07000, score=3, range=171-192, strand=
target 5' AUCCUCUUCUUCUUCUUCUUCU 3'
:::::: :::::::::::::
query 3' UAGGAGUUGAAGAAGAAGAAGU 5'
query=miR5227, target=RO3G_05565, score=3, range=663-684, strand=
target 5' CUCCACACCUUCUUCUUCUUCA 3'
::: :: ::::::::::::::
query 3' UAGGAGUUGAAGAAGAAGAAGU 5'
query=miR5227, target=RO3G_05857, score=3, range=27-48, strand=-1
target 5' CUUUUCAACUUUUUCUUCUUCA 3'
:..:::::::.:::::::::
query 3' UAGGAGUUGAAGAAGAAGAAGU 5'
query=miR5227, target=RO3G_07659, score=3, range=465-486, strand=
target 5' AUCUUCAAUCUCUUCUUCUUCA 3'
:::.::::. :::::::::::
query 3' UAGGAGUUGAAGAAGAAGAAGU 5'
query=miR5227, target=RO3G_07328, score=3, range=411-432, strand=
target 5' AUCCUCACUUUCUUUUUCUUCG 3'
::::::: .:::::.::::::.
query 3' UAGGAGUUGAAGAAGAAGAAGU 5'
query=miR5227, target=RO3G_06107, score=3, range=1311-1332, stran
target 5' CUCUUCGACUUCUUCUUCUUCU 3'
::.::.::::::::::::::
query 3' UAGGAGUUGAAGAAGAAGAAGU 5'
query=miR5227, target=RO3G_14397, score=3, range=1083-1104, stran
target 5' GGCUUCGAUUUCUUCUUCUUCA 3'
. :.::.:.:::::::::::::
query 3' UAGGAGUUGAAGAAGAAGAAGU 5'
query=miR5227, target=RO3G_14329, score=3, range=57-78, strand=-1
```

```
target 5' GUCUUCAUCUUCUUCUUCUUCC 3'
.::.::: :::::::::::::
query 3' UAGGAGUUGAAGAAGAAGAAGU 5'
query=miR5227, target=RO3G_01773, score=3, range=1197-1218, stran
target 5' AUCUUCGUCUUCUUCUUCUUCU 3'
:::.::. :::::::::::::
query 3' UAGGAGUUGAAGAAGAAGAAGU 5'
query=miR5227, target=RO3G_02077, score=3, range=396-417, strand=
target 5' UUCUUCCAUUUCUUCUUCUUCA 3'
::.:: :.:::::::::::::
query 3' UAGGAGUUGAAGAAGAAGAAGU 5'
query=miR5227, target=RO3G_05685, score=3, range=543-564, strand=
target 5' AUUCUCACUUUCUUCUUCUUCU 3'
::.:::: .::::::::::::
query 3' UAGGAGUUGAAGAAGAAGAAGU 5'
query=miR5227, target=RO3G_01903, score=3, range=1098-1119, stran
target 5' AUCCUCAUCCUCUUCUUCUUCA 3'
::::::: : :::::::::::
query 3' UAGGAGUUGAAGAAGAAGAAGU 5'
query=miR5227, target=RO3G_10088, score=3, range=651-672, strand=
target 5' AUCCUCAUCUUCAUCUUCUUCA 3'
::::::: :::: :::::::::
query 3' UAGGAGUUGAAGAAGAAGAAGU 5'
query=miR5227, target=RO3G_04729, score=3, range=363-384, strand=
target 5' UUCCUCUACUUCUUCUUCUUCC 3'
::::: ::::::::::::::
query 3' UAGGAGUUGAAGAAGAAGAAGU 5'
query=miR5227, target=RO3G_11868, score=3, range=1317-1338, stran
target 5' ACUCUCACUUUCUUCUUCUUCA 3'
: .:::: .:::::::::::::
query 3' UAGGAGUUGAAGAAGAAGAAGU 5'
query=miR5227, target=RO3G_07180, score=3, range=1338-1359, stran
target 5' GUCUUCCUCUUCUUCUUCUUCA 3'
.::.:: ::::::::::::::
query 3' UAGGAGUUGAAGAAGAAGAAGU 5'
query=miR5227, target=RO3G_16681, score=3, range=1413-1434, stran
target 5' GUCAUCGUCUUCUUCUUCUUCA 3'
.:: ::. ::::::::::::::
query 3' UAGGAGUUGAAGAAGAAGAAGU 5'
query=miR5227, target=RO3G_11086, score=3, range=411-432, strand=
target 5' AUUUUCUUCUUCUUCUUCUUCA 3'
::..:: :::::::::::::
query 3' UAGGAGUUGAAGAAGAAGAAGU 5'
query=miR5227, target=RO3G_04771, score=3, range=606-627, strand=
target 5' CUCCUCCUCUUCUUCUUCUUCA 3'
```

```
::::: :::::::::::::
query 3' UAGGAGUUGAAGAAGAAGAAGU 5'
query=miR5227, target=RO3G_14543, score=3, range=1545-1566, stran
target 5' AUCAUCAUCUUCUUCUUCUUCU 3'
::: ::: ::::::::::::
query 3' UAGGAGUUGAAGAAGAAGAAGU 5'
query=miR5227, target=RO3G_04550, score=3, range=993-1014, strand
target 5' AUCCUCCUCUUCUUCUUCUUCU 3'
:::::: ::::::::::::
query 3' UAGGAGUUGAAGAAGAAGAAGU 5'
query=miR5227, target=RO3G_13627, score=3, range=273-294, strand=
target 5' UUCCUCUUCUUCUUCUUCUUCA 3'
::::: :::::::::::::
query 3' UAGGAGUUGAAGAAGAAGAAGU 5'
query=miR5227, target=RO3G_16847, score=3, range=936-957, strand=
target 5' CUCCUCUUCUUCUUCUUCUUCA 3'
::::: :::::::::::::
query 3' UAGGAGUUGAAGAAGAAGAAGU 5'
query=miR5227, target=RO3G_08412, score=3, range=639-660, strand=
target 5' AUCCUCUUCUUCUUCUUCUUCU 3'
:::::: ::::::::::::
query 3' UAGGAGUUGAAGAAGAAGAAGU 5'
query=miR5227, target=RO3G_01052, score=3, range=879-900, strand=
target 5' CUCCUCUUCUUCUUCUUCUUCA 3'
::::: :::::::::::::
query 3' UAGGAGUUGAAGAAGAAGAAGU 5'
query=miR5227, target=RO3G_03638, score=3, range=162-183, strand=
target 5' CUCCUCCUCUUCUUCUUCUUCA 3'
::::: :::::::::::::
query 3' UAGGAGUUGAAGAAGAAGAAGU 5'
query=miR5227, target=RO3G_08508, score=3, range=540-561, strand=
target 5' AUCCUCUUCUUCUUCUUCUUCU 3'
:::::: ::::::::::::
query 3' UAGGAGUUGAAGAAGAAGAAGU 5'
query=miR5227, target=RO3G_01098, score=3, range=378-399, strand=
target 5' CUCCUCCUCUUCUUCUUCUUCA 3'
::::: :::::::::::::
query 3' UAGGAGUUGAAGAAGAAGAAGU 5'
query=miR5227, target=RO3G_15201, score=3, range=543-564, strand=
target 5' UUCCUCCUCUUCUUCUUCUUCA 3'
::::: :::::::::::::
query 3' UAGGAGUUGAAGAAGAAGAAGU 5'
query=miR5227, target=RO3G_11593, score=3, range=63-84, strand=-1
target 5' UUCAUCAUCUUCUUCUUCUUCA 3'
:: ::: :::::::::::::
```

```
query 3' UAGGAGUUGAAGAAGAAGAAGU 5'
query=miR5227, target=RO3G_08160, score=3, range=984-1004, strand
target 5' ACCUUUAA-UUCUUCUUCUUCA 3'
: :.:.:: :::::::::::::
query 3' UAGGAGUUGAAGAAGAAGAAGU 5'
query=miR5227, target=RO3G_09393, score=3, range=744-765, strand=
target 5' AUCAUCCUCUUCUUCUUCUUCA 3'
::: :: ::::::::::::::
query 3' UAGGAGUUGAAGAAGAAGAAGU 5'
query=miR5227, target=RO3G_08267, score=3, range=1272-1293, stran
target 5' AUCAUCCUCUUCUUCUUCUUCA 3'
::: :: ::::::::::::::
query 3' UAGGAGUUGAAGAAGAAGAAGU 5'
query=miR5227, target=RO3G_07283, score=3, range=105-126, strand=
target 5' CUCUUCGUCUUCUUCUUCUUCA 3'
::.::. ::::::::::::::
query 3' UAGGAGUUGAAGAAGAAGAAGU 5'
query=miR5227, target=RO3G_05857, score=3, range=174-195, strand=
target 5' CUCCUCUUCUUCUUCUUCUUCA 3'
::::: ::::::::::::::
query 3' UAGGAGUUGAAGAAGAAGAAGU 5'
query=miR5227, target=RO3G_10079, score=3, range=300-322, strand=
target 5' AUCCAUCACCUUCUUCUUCUUCC 3'
:::: ::: :::::::::::::
query 3' UAGG-AGUUGAAGAAGAAGAAGU 5'
query=miR5227, target=RO3G_11015, score=3.5, range=20-42, strand=
target 5' GUCUAUCGACUUCUUCUUCUUUA 3'
.::. ::.:::::::::::::.:
query 3' UAGG-AGUUGAAGAAGAAGAAGU 5'
query=miR5227, target=RO3G_07279, score=3.5, range=3573-3594, str
target 5' AUCCUCCGCUUCUUCUUCCUCA 3'
:::::: .:::::::::: :::
query 3' UAGGAGUUGAAGAAGAAGAAGU 5'
query=miR5227, target=RO3G_09917, score=3.5, range=684-705, stran
target 5' AUCUUCCACUUCUUCUUUUUCU 3'
:::.:: ::::::::::.:::
query 3' UAGGAGUUGAAGAAGAAGAAGU 5'
query=miR5227, target=RO3G_06244, score=3.5, range=1458-1479, str
target 5' AUCUUCCUCUUCUUCUUCUUUA 3'
:::.:: ::::::::::::.:
query 3' UAGGAGUUGAAGAAGAAGAAGU 5'
query=miR5227, target=RO3G_00057, score=3.5, range=1656-1677, str
target 5' AUCCAGAGCUUCUUCUUUUUCA 3'
:::: :.:::::::::.::::
query 3' UAGGAGUUGAAGAAGAAGAAGU 5'
```

```
query=miR5227, target=RO3G_03357, score=3.5, range=48-69, strand=
target 5' UUCUUCAAGUUCUUCUUCUUCU 3'
::.:::: ::::::::::::
query 3' UAGGAGUUGAAGAAGAAGAAGU 5'
query=miR5227, target=RO3G_01458, score=3.5, range=858-879, stran
target 5' CUCUUCUACUUCUUCUUCUUCU 3'
::.:: ::::::::::::::
query 3' UAGGAGUUGAAGAAGAAGAAGU 5'
query=miR5227, target=RO3G_12945, score=3.5, range=1503-1524, str
target 5' UUCUUCUUCUUCUUCUUCUUCA 3'
::.:: ::::::::::::::
query 3' UAGGAGUUGAAGAAGAAGAAGU 5'
query=miR5227, target=RO3G_00312, score=3.5, range=744-765, stran
target 5' UUCUUCUUCUUCUUCUUCUUCA 3'
::.:: ::::::::::::::
query 3' UAGGAGUUGAAGAAGAAGAAGU 5'
query=miR5227, target=RO3G_03738, score=3.5, range=1761-1782, str
target 5' AUCAGCUGCUUCUUCUUCUUCA 3'
::: : .::::::::::::::
query 3' UAGGAGUUGAAGAAGAAGAAGU 5'
query=miR5227, target=RO3G_15453, score=3.5, range=1580-1602, str
target 5' AAACUCAACAUUCUUCUUCUUCG 3'
: :::::: ::::::::::::.
query 3' UAGGAGUUG-AAGAAGAAGAAGU 5'
query=miR5227, target=RO3G_08622, score=3.5, range=1218-1239,
strand=-1
target 5' AUUCUUGACUUCUUCUUCUUUU 3'
::.::..::::::::::::::.
query 3' UAGGAGUUGAAGAAGAAGAAGU 5'
query=miR5227, target=RO3G_10411, score=3.5, range=1395-1416,
strand=-1
target 5' AAUUUCGACUUUUUCUUCUUCA 3'
: ..::.::::.::::::::::
query 3' UAGGAGUUGAAGAAGAAGAAGU 5'
query=miR5227, target=RO3G_10319, score=3.5, range=933-954, stran
target 5' AUCUUUAGGUUCUUUUUCUUCA 3'
:::.:.:. :::::.:::::::
query 3' UAGGAGUUGAAGAAGAAGAAGU 5'
query=miR5227, target=RO3G_02254, score=3.5, range=840-861, stran
target 5' GUCCACAAUUUUUUCUUCUUCG 3'
.::: :::.::.::::::::.
query 3' UAGGAGUUGAAGAAGAAGAAGU 5'
query=miR5227, target=RO3G_06538, score=3.5, range=195-216, stran
target 5' AUCUUCGUUUUCUUCUUUUUCA 3'
:::.::. .::::::::.::::
```

```
query 3' UAGGAGUUGAAGAAGAAGAAGU 5'
query=miR5227, target=RO3G_13396, score=3.5, range=177-198, stran
target 5' AUUUUCAAUUACUUCUUCUUCA 3'
::..::::.: ::::::::::
query 3' UAGGAGUUGAAGAAGAAGAAGU 5'
query=miR5227, target=RO3G_06685, score=3.5, range=1122-1143,
strand=-1
target 5' UUCUUCCACUUCUUCUUCUUUA 3'
::.:: :::::::::::::.:
query 3' UAGGAGUUGAAGAAGAAGAAGU 5'
query=miR5227, target=RO3G_11306, score=3.5, range=549-570, stran
target 5' AUUUUCCGCUUCUUCUUCUUCC 3'
::..:: .:::::::::::::
query 3' UAGGAGUUGAAGAAGAAGAAGU 5'
query=miR5227, target=RO3G_13926, score=3.5, range=1290-1311,
strand=-1
target 5' UUCCUUGUUUUCUUCUUCUUCA 3'
::::.. .:::::::::::::
query 3' UAGGAGUUGAAGAAGAAGAAGU 5'
query=miR5227, target=RO3G_05498, score=3.5, range=123-144, stran
target 5' UUCUUCAUCUUCUUCUUUUUCA 3'
::.::: :::::::::.::::
query 3' UAGGAGUUGAAGAAGAAGAAGU 5'
query=miR5227, target=RO3G_01827, score=3.5, range=99-120, strand
target 5' AUCUUCUACUUGUUCUUCUUCA 3'
:::.:: :::: :::::::::
query 3' UAGGAGUUGAAGAAGAAGAAGU 5'
query=miR5227, target=RO3G_03429, score=3.5, range=1113-1134,
strand=-1
target 5' AUCCUCAGAUUCUUCUUCUGCA 3'
:::::::. ::::::::: ::
query 3' UAGGAGUUGAAGAAGAAGAAGU 5'
query=miR5227, target=RO3G_06850, score=3.5, range=522-543, stran
target 5' AUCCUCAUCUUCUUCAUCUUCG 3'
::::::: ::::::: :::::.
query 3' UAGGAGUUGAAGAAGAAGAAGU 5'
query=miR5227, target=RO3G_05173, score=3.5, range=750-771, stran
target 5' AUCUUCAUCCUCUUCUUCUUCA 3'
:::.::: : :::::::::::
query 3' UAGGAGUUGAAGAAGAAGAAGU 5'
query=miR5227, target=RO3G_07261, score=3.5, range=810-831, stran
target 5' UUCCUCUAUUUCUUCUUCUUCU 3'
::::: :.:::::::::::
query 3' UAGGAGUUGAAGAAGAAGAAGU 5'
query=miR5227, target=RO3G_03381, score=3.5, range=507-528, stran
```

target 5' CUCUUCUACUUCUUCUUCUUCU 3'
::.:: :::::::::::::
query 3' UAGGAGUUGAAGAAGAAGAAGU 5'
query=miR5227, target=RO3G_11176, score=3.5, range=597-618, stran
target 5' CUCCUCGUCUUCUUCUUCUUCC 3'
:::::. ::::::::::::
query 3' UAGGAGUUGAAGAAGAAGAAGU 5'
query=miR5227, target=RO3G_15658, score=3.5, range=408-429, stran
target 5' UUCUUCUACUUCUUCUUCUUCU 3'
::.:: :::::::::::::
query 3' UAGGAGUUGAAGAAGAAGAAGU 5'
query=miR5227, target=RO3G_11924, score=3.5, range=252-273, stran
target 5' CUCAUCAGCUUCUUCUUCUUCU 3'
:: :::.:::::::::::::
query 3' UAGGAGUUGAAGAAGAAGAAGU 5'
query=miR5227, target=RO3G_09312, score=3.5, range=216-238, stran
target 5' GUUUUAAAGCUUCUUCUUCUUCA 3'
.:..: :: :::::::::::::
query 3' UAGGAGUU-GAAGAAGAAGAAGU 5'
query=miR5227, target=RO3G_09135, score=3.5, range=3882-3903,
strand=-1
target 5' UUCGGCAAUUUCUUCUUCUUCA 3'
:: :::.:::::::::::::
query 3' UAGGAGUUGAAGAAGAAGAAGU 5'
query=miR5227, target=RO3G_10467, score=3.5, range=195-216, stran
target 5' UUCUUCUUCUUCUUCUUCUUCA 3'
::.:: :::::::::::::
query 3' UAGGAGUUGAAGAAGAAGAAGU 5'
query=miR5227, target=RO3G_05884, score=3.5, range=1203-1224,
strand=-1
target 5' UUCUUCUUCUUCUUCUUCUUCA 3'
::.:: :::::::::::::
query 3' UAGGAGUUGAAGAAGAAGAAGU 5'
query=miR5227, target=RO3G_01472, score=3.5, range=1404-1425,
strand=-1
target 5' UUCUUCCUCUUCUUCUUCUUCA 3'
::.:: :::::::::::::
query 3' UAGGAGUUGAAGAAGAAGAAGU 5'
query=miR5227, target=RO3G_05552, score=3.5, range=99-120, strand
target 5' UUCAUCGUCUUCUUCUUCUUCA 3'
:: ::. :::::::::::::
query 3' UAGGAGUUGAAGAAGAAGAAGU 5'
query=miR5227, target=RO3G_07254, score=3.5, range=1494-1515,
strand=-1
target 5' CUCUUCCUCUUCUUCUUCUUCA 3'

```
::.:: :::::::::::::
query 3' UAGGAGUUGAAGAAGAAGAAGU 5'
query=miR5227, target=RO3G_07634, score=3.5, range=1629-1650,
strand=-1
target 5' UUCUUCUUCUUCUUCUUCUUCA 3'
::.:: :::::::::::::
query 3' UAGGAGUUGAAGAAGAAGAAGU 5'
query=miR5227, target=RO3G_00658, score=3.5, range=1200-1221,
strand=-1
target 5' UUCCUCUUCUUCUUCUUCUUCG 3'
::::: ::::::::::::.
query 3' UAGGAGUUGAAGAAGAAGAAGU 5'
query=miR5227, target=RO3G_12990, score=3.5, range=534-555, stran
target 5' UUCUUCUUCUUCUUCUUCUUCA 3'
::.:: :::::::::::::
query 3' UAGGAGUUGAAGAAGAAGAAGU 5'
query=miR5227, target=RO3G_06058, score=3.5, range=372-393, stran
target 5' UUCUUCUUCUUCUUCUUCUUCA 3'
::.:: :::::::::::::
query 3' UAGGAGUUGAAGAAGAAGAAGU 5'
query=miR5227, target=RO3G_03973, score=3.5, range=567-588, stran
target 5' AUCCGCACUUUCUUCUUCUUCC 3'
:::: :: .:::::::::::
query 3' UAGGAGUUGAAGAAGAAGAAGU 5'
query=miR5227, target=RO3G_14740, score=3.5, range=777-798, stran
target 5' UUCUUCUUCUUCUUCUUCUUCA 3'
::.:: :::::::::::::
query 3' UAGGAGUUGAAGAAGAAGAAGU 5'
query=miR5227, target=RO3G_00906, score=3.5, range=363-384, stran
target 5' UUCUUCUUCUUCUUCUUCUUCA 3'
::.:: :::::::::::::
query 3' UAGGAGUUGAAGAAGAAGAAGU 5'
query=miR5227, target=RO3G_15854, score=3.5, range=669-690, stran
target 5' AUCUUCUUCUUCUUCUUCUUCU 3'
:::.:: ::::::::::::
query 3' UAGGAGUUGAAGAAGAAGAAGU 5'
query=miR5227, target=RO3G_05540, score=3.5, range=180-201, stran
target 5' UUCUUCCUCUUCUUCUUCUUCA 3'
::.:: :::::::::::::
query 3' UAGGAGUUGAAGAAGAAGAAGU 5'
query=miR5227, target=RO3G_09134, score=3.5, range=555-576, stran
target 5' UUCUUCCUCUUCUUCUUCUUCA 3'
::.:: :::::::::::::
query 3' UAGGAGUUGAAGAAGAAGAAGU 5'
query=miR5227, target=RO3G_14175, score=3.5, range=135-156, stran
```

target 5' CUCUUCUUCUUCUUCUUCUUCA 3'
::.:: :::::::::::::
query 3' UAGGAGUUGAAGAAGAAGAAGU 5'
query=miR5227, target=RO3G_02089, score=3.5, range=315-336, stran
target 5' GGCGUCAACUUCUUCUUCUUCU 3'
. : ::::::::::::::::
query 3' UAGGAGUUGAAGAAGAAGAAGU 5'
query=miR5227, target=RO3G_03760, score=3.5, range=72-93, strand=
target 5' AAUAUCAACUUCUUCUUCUUCU 3'
: . ::::::::::::::::
query 3' UAGGAGUUGAAGAAGAAGAAGU 5'
query=miR5227, target=RO3G_08243, score=3.5, range=51-72, strand=
target 5' UUCUUCUUCUUCUUCUUCUUCA 3'
::.:: :::::::::::::
query 3' UAGGAGUUGAAGAAGAAGAAGU 5'
query=miR5227, target=RO3G_16184, score=3.5, range=3243-3265,
strand=-1
target 5' AUUCCAAAUCUUCUUCUUCUUCA 3'
::.: :: :::::::::::::
query 3' UAGGAGUU-GAAGAAGAAGAAGU 5'
query=miR5227, target=RO3G_13525, score=3.5, range=750-770, stran
target 5' AUCUUU-UUUUCUUCUUCUUCA 3'
:::.:. .:::::::::::::
query 3' UAGGAGUUGAAGAAGAAGAAGU 5'
query=miR5227, target=RO3G_09240, score=3.5, range=57-78, strand=
target 5' ACUCUCCUCUUCUUCUUCUUCA 3'
: .::: :::::::::::::
query 3' UAGGAGUUGAAGAAGAAGAAGU 5'
query=miR5227, target=RO3G_01410, score=3.5, range=306-327, stran
target 5' GUCAUCCUCUUCUUCUUCUUCA 3'
.:: :: :::::::::::::
query 3' UAGGAGUUGAAGAAGAAGAAGU 5'
query=miR5227, target=RO3G_13511, score=3.5, range=219-240, stran
target 5' AUCUCCUUCUUCUUCUUCUUCA 3'
:::. : :::::::::::::
query 3' UAGGAGUUGAAGAAGAAGAAGU 5'
query=miR5227, target=RO3G_13239, score=3.5, range=315-336, stran
target 5' AUCCAUUUCUUCUUCUUCUUCA 3'
:::: . :::::::::::::
query 3' UAGGAGUUGAAGAAGAAGAAGU 5'
query=miR5227, target=RO3G_12381, score=3.5, range=675-696, stran
target 5' AUCAUCCUCUUCUUCUUCUUCG 3'
::: :: ::::::::::::.
query 3' UAGGAGUUGAAGAAGAAGAAGU 5'
query=miR5227, target=RO3G_07422, score=3.5, range=321-341, stran

target 5' GUCGUCAAC-UCUUCUUCUUCA 3'
.:: ::::: ::::::::::::
query 3' UAGGAGUUGAAGAAGAAGAAGU 5'
query=miR5227, target=RO3G_04929, score=3.5, range=1083-1104, strand=-1
target 5' AUCUUCCUCUUCUUCUUCUUCU 3'
:::.:: :::::::::::::
query 3' UAGGAGUUGAAGAAGAAGAAGU 5'
query=miR5227, target=RO3G_15658, score=3.5, range=441-462, stran
target 5' UUCUUCUUCUUCUUCUUCUUCA 3'
::.:: ::::::::::::::
query 3' UAGGAGUUGAAGAAGAAGAAGU 5'
query=miR5227, target=RO3G_00049, score=3.5, range=252-272, stran
target 5' AUCUUU-UCUUCUUCUUCUUCG 3'
:::.:. :::::::::::::.
query 3' UAGGAGUUGAAGAAGAAGAAGU 5'
query=miR5227, target=RO3G_03381, score=3.5, range=924-946, stran
target 5' GUUCGUUAUCUUCUUCUUCUUCA 3'
.:.: :.: ::::::::::::::
query 3' UAGG-AGUUGAAGAAGAAGAAGU 5'
query=miR5227, target=RO3G_02972, score=4, range=201-222, strand=
target 5' UUCUUCUUCUUCUUCUUCUUCG 3'
::.:: :::::::::::::.
query 3' UAGGAGUUGAAGAAGAAGAAGU 5'
query=miR5227, target=RO3G_00345, score=4, range=3-24, strand=1
target 5' GACUUCCCCUUCUUCUUCUUCA 3'
. :.:: ::::::::::::::
query 3' UAGGAGUUGAAGAAGAAGAAGU 5'
query=miR5227, target=RO3G_14312, score=4, range=248-270, strand=
target 5' AUCGUACUACUUCUUCUUCUUCU 3'
::: : : ::::::::::::::
query 3' UAGGA-GUUGAAGAAGAAGAAGU 5'
query=miR5227, target=RO3G_03203, score=4, range=515-537, strand=
target 5' AUCCCGAAUCUUCUUCUUCUUCU 3'
:::: :: :::::::::::::
query 3' UAGGAGUU-GAAGAAGAAGAAGU 5'
query=miR5227, target=RO3G_11040, score=4, range=73-93, strand=1
target 5' AUCC-CAAGUUCAUCUUCUUCA 3'
:::: ::: ::: :::::::::
query 3' UAGGAGUUGAAGAAGAAGAAGU 5'
query=miR5227, target=RO3G_13835, score=4, range=13665-13686, strand=-1
target 5' CUUCUCAGCUUUUUCUUCUUUA 3'
:.::::.:::.:::::::.:
query 3' UAGGAGUUGAAGAAGAAGAAGU 5'

```
query=miR5227, target=RO3G_10982, score=4, range=1254-1275, stran
target 5' AUCAUUAAUUUCUUUUUCUUUA 3'
::: :.::.::::.:::::.:
query 3' UAGGAGUUGAAGAAGAAGAAGU 5'
query=miR5227, target=RO3G_09720, score=4, range=318-339, strand=
target 5' GUCCUUGAUUUCUUCUUCAUCA 3'
.::::..:.:::::::: :::
query 3' UAGGAGUUGAAGAAGAAGAAGU 5'
query=miR5227, target=RO3G_06767, score=4, range=3225-3246, stran
target 5' CCUUUUAGCUUCUUCUUCUUCA 3'
..:.:.:::::::::::::
query 3' UAGGAGUUGAAGAAGAAGAAGU 5'
query=miR5227, target=RO3G_07999, score=4, range=969-990, strand=
target 5' AUCUUCAGCUUCUUCUUCAUCU 3'
:::.:::.:::::::::: ::
query 3' UAGGAGUUGAAGAAGAAGAAGU 5'
query=miR5227, target=RO3G_08632, score=4, range=558-579, strand=
target 5' AUCCUCAACUUCUUCCUCAUCA 3'
:::::::::::::: :: :::
query 3' UAGGAGUUGAAGAAGAAGAAGU 5'
query=miR5227, target=RO3G_14961, score=4, range=3449-3470, stran
target 5' AUACUCAACAUCUUCUUCUUUA 3'
:: :::::: ::::::::::.:
query 3' UAGGAGUUGAAGAAGAAGAAGU 5'
query=miR5227, target=RO3G_00993, score=4, range=612-633, strand=
target 5' AUCCUCAUCGUUUUCUUCUUCA 3'
::::::: : :.:::::::::
query 3' UAGGAGUUGAAGAAGAAGAAGU 5'
query=miR5227, target=RO3G_13237, score=4, range=189-210, strand=
target 5' GUCCUCGUCUUCUUCCUCUUCA 3'
.:::::. ::::::: ::::::
query 3' UAGGAGUUGAAGAAGAAGAAGU 5'
query=miR5227, target=RO3G_14289, score=4, range=618-639, strand=
target 5' AUCGUCAUCUUUUUCUUCUUUA 3'
::: ::: :::.:::::::.:
query 3' UAGGAGUUGAAGAAGAAGAAGU 5'
query=miR5227, target=RO3G_07647, score=4, range=150-171, strand=
target 5' AUUUUGAACUUCUUCUUCUCCA 3'
::..: :::::::::::: ::
query 3' UAGGAGUUGAAGAAGAAGAAGU 5'
query=miR5227, target=RO3G_11504, score=4, range=270-291, strand=
target 5' AGCUUCAGCAUCUUCUUCUUCA 3'
: :.:::.: :::::::::::
query 3' UAGGAGUUGAAGAAGAAGAAGU 5'
query=miR5227, target=RO3G_04929, score=4, range=441-462, strand=
```

```
target 5' AUCUUCUUUUUCUUCUUCUUUA 3'
:::.:: .:::::::::::.:
query 3' UAGGAGUUGAAGAAGAAGAAGU 5'
query=miR5227, target=RO3G_04852, score=4, range=870-891, strand=
target 5' AUCUACAGAUUCUUCUUUUUCA 3'
:::. ::. ::::::::.::::
query 3' UAGGAGUUGAAGAAGAAGAAGU 5'
query=miR5227, target=RO3G_03409, score=4, range=414-435, strand=
target 5' AUCUUUCUCUUUUUCUUCUUCA 3'
:::.:. :::.::::::::::
query 3' UAGGAGUUGAAGAAGAAGAAGU 5'
query=miR5227, target=RO3G_02335, score=4, range=906-928, strand=
target 5' AUCUUUCAUUUUCUUCUUUUUCA 3'
::: .::: .::::::::.::::
query 3' UAG-GAGUUGAAGAAGAAGAAGU 5'
query=miR5227, target=RO3G_17101, score=4, range=665-686, strand=
target 5' UUCCAUAAUUUCUUCUUCUUCU 3'
::: .::.::::::::::::
query 3' UAGGAGUUGAAGAAGAAGAAGU 5'
query=miR5227, target=RO3G_10461, score=4, range=620-641, strand=
target 5' UUCCAUAGCUUCUUCUUCUUCU 3'
::: .:.:::::::::::::
query 3' UAGGAGUUGAAGAAGAAGAAGU 5'
query=miR5227, target=RO3G_16965, score=4, range=2358-2379, stran
target 5' AUCCUCAUCUUCUUCUUCAUCC 3'
::::::: :::::::::: ::
query 3' UAGGAGUUGAAGAAGAAGAAGU 5'
query=miR5227, target=RO3G_16470, score=4, range=426-448, strand=
target 5' GUUGUUCGUCUUCUUCUUCUUCA 3'
.:. .::. ::::::::::::::
query 3' UAG-GAGUUGAAGAAGAAGAAGU 5'
query=miR5227, target=RO3G_14853, score=4, range=246-267, strand=
target 5' UUCUUCGUCUUCUUCUUCUUCC 3'
::.::. :::::::::::::
query 3' UAGGAGUUGAAGAAGAAGAAGU 5'
query=miR5227, target=RO3G_04484, score=4, range=84-105, strand=-
target 5' UUCCUCAUCUUCCUCUUCUUCA 3'
:::::: :::: :::::::::
query 3' UAGGAGUUGAAGAAGAAGAAGU 5'
query=miR5227, target=RO3G_07020, score=4, range=735-756, strand=
target 5' AUCCUCAUCUUCUUCAUCUUCU 3'
::::::: ::::::: :::::
query 3' UAGGAGUUGAAGAAGAAGAAGU 5'
query=miR5227, target=RO3G_15353, score=4, range=657-678, strand=
target 5' CUCCUCAUCCUCUUCUUCUUCA 3'
```

```
:::::: : ::::::::::::
query 3' UAGGAGUUGAAGAAGAAGAAGU 5'
query=miR5227, target=RO3G_13505, score=4, range=381-402, strand=
target 5' UUCUUCCUCUUCUUCUUCUUCG 3'
::.:: :::::::::::::.
query 3' UAGGAGUUGAAGAAGAAGAAGU 5'
query=miR5227, target=RO3G_12517, score=4, range=27-48, strand=-1
target 5' UUCCUCAAGCUCUUCUUCUUCA 3'
::::::: ::::::::::::
query 3' UAGGAGUUGAAGAAGAAGAAGU 5'
query=miR5227, target=RO3G_01798, score=4, range=207-228, strand=
target 5' ACUCUCGCCUUCUUCUUCUUCU 3'
: .:::. :::::::::::::
query 3' UAGGAGUUGAAGAAGAAGAAGU 5'
query=miR5227, target=RO3G_14420, score=4, range=1773-1794, stran
target 5' UUCUUCUUCUUCUUCUUCUUCG 3'
::.:: :::::::::::::.
query 3' UAGGAGUUGAAGAAGAAGAAGU 5'
query=miR5227, target=RO3G_13513, score=4, range=1503-1524, stran
target 5' UUCUUCUUCUUCUUCUUCUUCG 3'
::.:: :::::::::::::.
query 3' UAGGAGUUGAAGAAGAAGAAGU 5'
query=miR5227, target=RO3G_00845, score=4, range=1437-1458, stran
target 5' GCCUUCCACUUCUUCUUCUUCC 3'
. :.:: ::::::::::::::
query 3' UAGGAGUUGAAGAAGAAGAAGU 5'
query=miR5227, target=RO3G_08989, score=4, range=1746-1767, stran
target 5' AAUUUCACCUUCUUCUUCUUCU 3'
: ..::: :::::::::::::
query 3' UAGGAGUUGAAGAAGAAGAAGU 5'
query=miR5227, target=RO3G_12945, score=4, range=72-93, strand=-1
target 5' UUCUUCUUCUUCUUCUUCUUCG 3'
::.:: :::::::::::::.
query 3' UAGGAGUUGAAGAAGAAGAAGU 5'
query=miR5227, target=RO3G_02556, score=4, range=3054-3075, stran
target 5' AUCAUCAAAUUCUUCUUCCUCA 3'
::: :::: ::::::::: :::
query 3' UAGGAGUUGAAGAAGAAGAAGU 5'
query=miR5227, target=RO3G_03969, score=4, range=600-621, strand=
target 5' AUCCGCGCUUUCUUCUUCUUCC 3'
:::: :. .::::::::::::
query 3' UAGGAGUUGAAGAAGAAGAAGU 5'
query=miR5227, target=RO3G_10712, score=4, range=1143-1164, stran
target 5' UUCUUCUUCUUCUUCUUCUUCG 3'
::.:: :::::::::::::.
```

```
query 3' UAGGAGUUGAAGAAGAAGAAGU 5'
query=miR5227, target=RO3G_00049, score=4, range=174-195, strand=
target 5' CUUUUCUUCUUCUUCUUCUUCA 3'
:..:: :::::::::::::
query 3' UAGGAGUUGAAGAAGAAGAAGU 5'
query=miR5227, target=RO3G_03544, score=4, range=735-756, strand=
target 5' UUCUUCUUCUUCUUCUUCUUCG 3'
::.:: ::::::::::::.
query 3' UAGGAGUUGAAGAAGAAGAAGU 5'
query=miR5227, target=RO3G_09400, score=4, range=612-633, strand=
target 5' UUCUUUUUCUUCUUCUUCUUCA 3'
::.:. :::::::::::::
query 3' UAGGAGUUGAAGAAGAAGAAGU 5'
query=miR5227, target=RO3G_11643, score=4, range=402-423, strand=
target 5' CUCUUCUUCUUCUUCUUCUUCG 3'
::.:: ::::::::::::.
query 3' UAGGAGUUGAAGAAGAAGAAGU 5'
query=miR5227, target=RO3G_10079, score=4, range=720-741, strand=
target 5' GUCUUCUUCUUCUUCUUCUUCU 3'
.::.:: ::::::::::::
query 3' UAGGAGUUGAAGAAGAAGAAGU 5'
query=miR5227, target=RO3G_16926, score=4, range=387-408, strand=
target 5' AUCUCUCACUUCUUCUUCUUCU 3'
:::. . :::::::::::::
query 3' UAGGAGUUGAAGAAGAAGAAGU 5'
query=miR5227, target=RO3G_04817, score=4, range=2955-2976, stran
target 5' AAAUACAAUUUCUUCUUCUUCA 3'
: . :::.:::::::::::::
query 3' UAGGAGUUGAAGAAGAAGAAGU 5'
query=miR5227, target=RO3G_01413, score=4, range=363-384, strand=
target 5' AUCUUCUGAUUCUUCUUCUUCC 3'
:::.:: . :::::::::::
query 3' UAGGAGUUGAAGAAGAAGAAGU 5'
query=miR5227, target=RO3G_11230, score=4, range=2004-2025, stran
target 5' AUCAACAACUUCUUCUUCAUCA 3'
::: ::::::::::::: :::
query 3' UAGGAGUUGAAGAAGAAGAAGU 5'
query=miR5227, target=RO3G_04278, score=4, range=1923-1945, stran
target 5' AUACCUCAUCAUCUUCUUCUUCA 3'
:: ::::: : :::::::::::
query 3' UA-GGAGUUGAAGAAGAAGAAGU 5'
query=miR5227, target=RO3G_10002, score=4, range=180-200, strand=
target 5' AUCUCUAACUU-UUCUUCUUCA 3'
:::. .::::: ::::::::::
query 3' UAGGAGUUGAAGAAGAAGAAGU 5'
```

```
query=miR5227, target=RO3G_12131, score=4, range=1437-1458, stran
target 5' AUCAUCAUCCUCUUCUUCUUCA 3'
::: ::: : ::::::::::::
query 3' UAGGAGUUGAAGAAGAAGAAGU 5'
query=miR5227, target=RO3G_03957, score=4, range=1284-1305, stran
target 5' AUCAUCAUCCUCUUCUUCUUCA 3'
::: ::: : ::::::::::::
query 3' UAGGAGUUGAAGAAGAAGAAGU 5'
query=miR5227, target=RO3G_15686, score=4, range=720-741, strand=
target 5' AUCAUCAUCUUCUUCUUCAUCA 3'
::: ::: :::::::::: :::
query 3' UAGGAGUUGAAGAAGAAGAAGU 5'
query=miR5227, target=RO3G_11890, score=4, range=111-132, strand=
target 5' AUCUUUUAAUUCUUCUUCUUCC 3'
:::.:. : :::::::::::
query 3' UAGGAGUUGAAGAAGAAGAAGU 5'
query=miR5227, target=RO3G_13255, score=4, range=195-217, strand=
target 5' CUCCUUGUUCUUCUUCUUCUUCA 3'
::::.. ::::::::::::::
query 3' UAGGAGU-UGAAGAAGAAGAAGU 5'
query=miR5227, target=RO3G_02114, score=4, range=459-480, strand=
target 5' AUCAUCAUCUUCUUCUUCCUCA 3'
::: ::: :::::::::: :::
query 3' UAGGAGUUGAAGAAGAAGAAGU 5'
query=miR5227, target=RO3G_01368, score=4, range=633-654, strand=
target 5' CUCAUCAAAUUCUUCUUCUUCC 3'
:: :::: :::::::::::
query 3' UAGGAGUUGAAGAAGAAGAAGU 5'
query=miR5227, target=RO3G_06704, score=4, range=171-192, strand=
target 5' AUCAUCAUCGUCUUCUUCUUCA 3'
::: ::: : ::::::::::::
query 3' UAGGAGUUGAAGAAGAAGAAGU 5'
query=miR5227, target=RO3G_09755, score=4, range=2232-2253, stran
target 5' UUCCUCUUCUUCUUCUUCUUCU 3'
::::: :::::::::::::
query 3' UAGGAGUUGAAGAAGAAGAAGU 5'
query=miR5227, target=RO3G_16240, score=4, range=390-411, strand=
target 5' AUCGUCACCUUCUUCUUCAUCA 3'
::: ::: :::::::::: :::
query 3' UAGGAGUUGAAGAAGAAGAAGU 5'
query=miR5227, target=RO3G_02991, score=4, range=135-156, strand=
target 5' UUCCUCUUCUUCUUCUUCUUCC 3'
::::: :::::::::::::
query 3' UAGGAGUUGAAGAAGAAGAAGU 5'
query=miR5227, target=RO3G_05313, score=4, range=1230-1251, stran
```

```
target 5' UUCCUCUUCUUCUUCUUCUUCU 3'
::::: :::::::::::::
query 3' UAGGAGUUGAAGAAGAAGAAGU 5'
query=miR5227, target=RO3G_02243, score=4, range=363-384, strand=
target 5' UUCCUCCUCUUCUUCUUCUUCC 3'
::::: :::::::::::::
query 3' UAGGAGUUGAAGAAGAAGAAGU 5'
query=miR5227, target=RO3G_12267, score=4, range=396-417, strand=
target 5' AUUUUCUUGUUCUUCUUCUUCA 3'
::..:: :::::::::::::
query 3' UAGGAGUUGAAGAAGAAGAAGU 5'
query=miR5227, target=RO3G_07091, score=4, range=357-378, strand=
target 5' CUCCUCCUCUUCUUCUUCUUCC 3'
::::: :::::::::::::
query 3' UAGGAGUUGAAGAAGAAGAAGU 5'
query=miR5227, target=RO3G_01019, score=4, range=72-93, strand=-1
target 5' CUCCUCCUCUUCUUCUUCUUCC 3'
::::: :::::::::::::
query 3' UAGGAGUUGAAGAAGAAGAAGU 5'
query=miR5227, target=RO3G_13923, score=4, range=105-126, strand=
target 5' UUCCUCUUCUUCUUCUUCUUCC 3'
::::: :::::::::::::
query 3' UAGGAGUUGAAGAAGAAGAAGU 5'
query=miR5227, target=RO3G_14653, score=4, range=318-339, strand=
target 5' AUCUCUUUCUUCUUCUUCUUCA 3'
:::. . ::::::::::::::
query 3' UAGGAGUUGAAGAAGAAGAAGU 5'
query=miR5227, target=RO3G_11915, score=4, range=804-826, strand=
target 5' GUCUUUCUAAUUCUUCUUCUUCA 3'
.:: .:: : :::::::::::::
query 3' UAG-GAGUUGAAGAAGAAGAAGU 5'
query=miR5227, target=RO3G_08247, score=4, range=678-700, strand=
target 5' GUACUAGUACUUCUUCUUCUUCA 3'
.: :: . :::::::::::::::
query 3' UAGGAGU-UGAAGAAGAAGAAGU 5'
query=miR5227, target=RO3G_13436, score=4, range=105-126, strand=
target 5' UUCCUCUUCUUCUUCUUCUUCU 3'
::::: :::::::::::::
query 3' UAGGAGUUGAAGAAGAAGAAGU 5'
query=miR5227, target=RO3G_04530, score=4, range=147-168, strand=
target 5' CACAUCAUCUUCUUCUUCUUCA 3'
: ::: ::::::::::::::
query 3' UAGGAGUUGAAGAAGAAGAAGU 5'
query=miR5227, target=RO3G_15929, score=4, range=56-78, strand=-1
target 5' AUUCAUUAUAUUCUUCUUCUUCA 3'
```

```
::.: :.: ::::::::::::
query 3' UAGG-AGUUGAAGAAGAAGAAGU 5'
query=miR5227, target=RO3G_01696, score=4, range=753-774, strand=
target 5' UUCUUUUUCUUCUUCUUCUUCA 3'
::.:. :::::::::::::
query 3' UAGGAGUUGAAGAAGAAGAAGU 5'
query=miR5227, target=RO3G_10492, score=4, range=129-150, strand=
target 5' AUCCUGCUCUUCUUCUUCUUCU 3'
::::: ::::::::::::
query 3' UAGGAGUUGAAGAAGAAGAAGU 5'
query=miR5227, target=RO3G_11587, score=4, range=1443-1465, stran
target 5' AUACUAAAUCUUCUUCUUCUUCU 3'
:: :: :: ::::::::::::
query 3' UAGGAGUU-GAAGAAGAAGAAGU 5'
query=miR5227, target=RO3G_05221, score=4, range=417-439, strand=
target 5' AUGCAUCUACUUCUUCUUCUUCU 3'
:: : :: ::::::::::::::
query 3' UA-GGAGUUGAAGAAGAAGAAGU 5'
query=miR5227, target=RO3G_03642, score=4, range=341-361, strand=
target 5' CUCCUCCA-UUCUUCUUUUUCA 3'
::::: : :::::::.::::
query 3' UAGGAGUUGAAGAAGAAGAAGU 5'
query=miR5227, target=RO3G_08632, score=4, range=369-390, strand=
target 5' UUCUUCAUUUUCUUCUUCUUCC 3'
::.::: .::::::::::::
query 3' UAGGAGUUGAAGAAGAAGAAGU 5'
query=miR5227, target=RO3G_03381, score=4, range=870-891, strand=
target 5' UUCUUCACUUUCUUCUUCUUCC 3'
::.::: .::::::::::::
query 3' UAGGAGUUGAAGAAGAAGAAGU 5'
query=miR5227, target=RO3G_14397, score=4, range=141-163, strand=
target 5' GUUCAUCUAUUUCUUCUUCUUCG 3'
.:.: :: :.:::::::::::.
query 3' UAGG-AGUUGAAGAAGAAGAAGU 5'
query=miR5227, target=RO3G_13923, score=4, range=296-318, strand=
target 5' AUCCUCUUCUUCUUCUUCGUUCA 3'
:::::: ::::::::: ::::
query 3' UAGGAGUUGAAGAAGAAG-AAGU 5'
query=miR5227, target=RO3G_08412, score=4, range=600-621, strand=
target 5' CUCCUCCUCUUCUUCUUCUUCC 3'
::::: ::::::::::::
query 3' UAGGAGUUGAAGAAGAAGAAGU 5'
query=miR5227, target=RO3G_03544, score=4, range=417-438, strand=
target 5' CUCCUCCUCUUCUUCUUCUUCC 3'
::::: ::::::::::::
```

query 3' UAGGAGUUGAAGAAGAAGAAGU 5'
query=miR823-5p, target=RO3G_06729, score=0, range=3316-3337, strand=-1
target 5' AAUGGUCAGUGAUCAAUACAAA 3'
::::::::::::::::::::::
query 3' UUACCAGUCACUAGUUAUGUUU 5'
query=miR823-5p, target=RO3G_15892, score=0, range=3316-3337, strand=-1
target 5' AAUGGUCAGUGAUCAAUACAAA 3'
::::::::::::::::::::::
query 3' UUACCAGUCACUAGUUAUGUUU 5'
query=miR823-5p, target=RO3G_16125, score=0, range=3316-3337, strand=-1
target 5' AAUGGUCAGUGAUCAAUACAAA 3'
::::::::::::::::::::::
query 3' UUACCAGUCACUAGUUAUGUUU 5'
query=miR823-5p, target=RO3G_04368, score=3.5, range=720-740, str
target 5' AGGGGUCA-UGAUCAAUACAGA 3'
:. ::::: :::::::::::.:
query 3' UUACCAGUCACUAGUUAUGUUU 5'
query=miR823-5p, target=RO3G_05538, score=3.5, range=717-737, str
target 5' AGGGGUCA-UGAUCAAUACAGA 3'
:. ::::: :::::::::::.:
query 3' UUACCAGUCACUAGUUAUGUUU 5'
query=miR823-5p, target=RO3G_02988, score=4, range=213-234, stran
target 5' UGUUGUCGGUGGUCAAUACAAA 3'
.: :::.:::.:::::::::
query 3' UUACCAGUCACUAGUUAUGUUU 5'
query=miR823-5p, target=RO3G_00371, score=4, range=576-597, stran
target 5' AAUGAUUGCUGAUCAAUAUAAA 3'
:::: :.. :::::::::.:::
query 3' UUACCAGUCACUAGUUAUGUUU 5'
query=miR823-5p, target=RO3G_02399, score=4, range=40-62, strand=
target 5' AAUGCUACAGUGAUCAAUGCAAC 3'
:::: : :::::::::::.:::
query 3' UUACCA-GUCACUAGUUAUGUUU 5'
query=miR823-5p, target=RO3G_09067, score=4, range=292-312, stran
target 5' GAU-GAGGGUGAUCAAUACAAA 3'
.:: : .:::::::::::::
query 3' UUACCAGUCACUAGUUAUGUUU 5'
query=miR823-5p, target=RO3G_12081, score=4, range=1492-1513, strand=-1
target 5' AAUGAUCAGUGAUCAGGACAAA 3'
:::: ::::::::::. :::::
query 3' UUACCAGUCACUAGUUAUGUUU 5'

```
query=miR823-5p, target=RO3G_11406, score=4, range=835-856, stran
target 5' AAUGAUCAGUGAUCAGGACAAA 3'
:::: ::::::::::. :::::
query 3' UUACCAGUCACUAGUUAUGUUU 5'
query=miR823-5p, target=RO3G_06563, score=4, range=2527-2547,
strand=-1
target 5' CAUGG-CAGUGAUUAAUGCAAA 3'
:::: :::::::.:::.::::
query 3' UUACCAGUCACUAGUUAUGUUU 5'
query=miR823-5p, target=RO3G_06887, score=4, range=1619-1639,
strand=-1
target 5' AAUGG-CAGUUAUCAAUACGAA 3'
::::: :::: ::::::::.::
query 3' UUACCAGUCACUAGUUAUGUUU 5'
query=miR2644, target=RO3G_06059, score=2.5, range=999-1018, stra
target 5' ACAAUCAUCAACUGAAGAAA 3'
::::.:::::::::::::: :
query 3' UGUUGGUAGUUGACUUCUCU 5'
query=miR2644, target=RO3G_13025, score=2.5, range=2013-2033, str
target 5' ACAGUCCAUCAACUGGAGAGA 3'
:::. ::::::::::.:::::
query 3' UGUU-GGUAGUUGACUUCUCU 5'
query=miR2644, target=RO3G_00272, score=3, range=5285-5305, stran
target 5' ACACACCAUCAACUGAAGGGC 3'
::: ::::::::::::::.:
query 3' UGU-UGGUAGUUGACUUCUCU 5'
query=miR2644, target=RO3G_10744, score=3.5, range=360-378, stran
target 5' GCUAUC-UCAACUGAAGAGG 3'
.: :.: :::::::::::.
query 3' UGUUGGUAGUUGACUUCUCU 5'
query=miR2644, target=RO3G_16366, score=3.5, range=360-378, stran
target 5' GCUAUC-UCAACUGAAGAGG 3'
.: :.: :::::::::::.
query 3' UGUUGGUAGUUGACUUCUCU 5'
query=miR2644, target=RO3G_13448, score=3.5, range=360-378, stran
target 5' GCUAUC-UCAACUGAAGAGG 3'
.: :.: :::::::::::.
query 3' UGUUGGUAGUUGACUUCUCU 5'
query=miR2644, target=RO3G_17253, score=3.5, range=360-378, stran
target 5' GCUAUC-UCAACUGAAGAGG 3'
.: :.: :::::::::::.
query 3' UGUUGGUAGUUGACUUCUCU 5'
query=miR2644, target=RO3G_17200, score=3.5, range=474-492, stran
target 5' GCUAUC-UCAACUGAAGAGG 3'
.: :.: :::::::::::.
```

```
query 3' UGUUGGUAGUUGACUUCUCU 5'
query=miR2644, target=RO3G_00074, score=4, range=456-475, strand=
target 5' ACAACAAUUAACUGUAGAGA 3'
::::: ::.::::: :::::
query 3' UGUUGGUAGUUGACUUCUCU 5'
query=miR2644, target=RO3G_01826, score=4, range=165-184, strand=
target 5' ACCUUUAUCAAUUGAAGAGA 3'
:: ..:::::.::::::::
query 3' UGUUGGUAGUUGACUUCUCU 5'
query=miR2644, target=RO3G_06628, score=4, range=242-262, strand=
target 5' AUAAUAAAUCAACUGAAGAGC 3'
:.::. :::::::::::::
query 3' UGUUG-GUAGUUGACUUCUCU 5'
query=miR863-5p, target=RO3G_14622, score=0, range=2022-2042,
strand=-1
target 5' AUGAUAUACAACAAGCCAUAA 3'
:::::::::::::::::::::
query 3' UACUAUAUGUUGUUCGGUAUU 5'
query=miR863-5p, target=RO3G_14810, score=2.5, range=488-508, str
target 5' AUGGAAUACAACAAGUCAUAA 3'
:::. ::::::::::.:::::
query 3' UACUAUAUGUUGUUCGGUAUU 5'
query=miR863-5p, target=RO3G_07492, score=3.5, range=116-135,
strand=-1
target 5' AAGAU-UGCGACAAGCCAUAA 3'
: ::: :.:.:::::::::::
query 3' UACUAUAUGUUGUUCGGUAUU 5'
query=miR863-5p, target=RO3G_14232, score=4, range=167-187, stran
target 5' UUGCUACACAACAAGCCAUAC 3'
:: :: :::::::::::::
query 3' UACUAUAUGUUGUUCGGUAUU 5'
query=miR863-5p, target=RO3G_11909, score=4, range=12-31, strand=
target 5' AUG-UUUCCAACAAGCCAUAC 3'
::: : : ::::::::::::
query 3' UACUAUAUGUUGUUCGGUAUU 5'
query=miR863-5p, target=RO3G_13469, score=4, range=39-59, strand=
target 5' AUCACAAACAACAAGCCAUGA 3'
:: : : ::::::::::::.:
query 3' UACUAUAUGUUGUUCGGUAUU 5'
query=miR863-5p, target=RO3G_08590, score=4, range=422-443, stran
target 5' ACGAUAUACAACAUGGCCAUAA 3'
: ::::::::::: .:::::::
query 3' UACUAUAUGUUGU-UCGGUAUU 5'
query=miR416, target=RO3G_04690, score=0, range=90-111, strand=-1
target 5' CUGAACAGUGGUACGACUAACA 3'
```

```
:::::::::::::::::::::
query 3' GACUUGUCACCAUGCUGAUUGU 5'
query=miR416, target=RO3G_05431, score=0, range=90-111, strand=-1
target 5' CUGAACAGUGGUACGACUAACA 3'
:::::::::::::::::::::
query 3' GACUUGUCACCAUGCUGAUUGU 5'
query=miR416, target=RO3G_02822, score=0, range=90-111, strand=-1
target 5' CUGAACAGUGGUACGACUAACA 3'
:::::::::::::::::::::
query 3' GACUUGUCACCAUGCUGAUUGU 5'
query=miR416, target=RO3G_15141, score=0, range=90-111, strand=-1
target 5' CUGAACAGUGGUACGACUAACA 3'
:::::::::::::::::::::
query 3' GACUUGUCACCAUGCUGAUUGU 5'
query=miR416, target=RO3G_09168, score=0, range=90-111, strand=-1
target 5' CUGAACAGUGGUACGACUAACA 3'
:::::::::::::::::::::
query 3' GACUUGUCACCAUGCUGAUUGU 5'
query=miR416, target=RO3G_04787, score=0, range=90-111, strand=-1
target 5' CUGAACAGUGGUACGACUAACA 3'
:::::::::::::::::::::
query 3' GACUUGUCACCAUGCUGAUUGU 5'
query=miR416, target=RO3G_00304, score=1, range=90-111, strand=-1
target 5' CUGAACAGUGGUACGACUAAUA 3'
:::::::::::::::::::.:
query 3' GACUUGUCACCAUGCUGAUUGU 5'
query=miR416, target=RO3G_12954, score=1, range=90-111, strand=-1
target 5' CUGGACAGUGGUACGACUAACG 3'
:::.:::::::::::::::::.
query 3' GACUUGUCACCAUGCUGAUUGU 5'
query=miR416, target=RO3G_10770, score=1, range=90-111, strand=-1
target 5' CUGGACAGUGGUACGACUAACG 3'
:::.:::::::::::::::::.
query 3' GACUUGUCACCAUGCUGAUUGU 5'
query=miR416, target=RO3G_10762, score=1, range=90-111, strand=-1
target 5' CUGGACAGUGGUACGACUAACG 3'
:::.:::::::::::::::::.
query 3' GACUUGUCACCAUGCUGAUUGU 5'
query=miR1917, target=RO3G_19001, score=0, range=672-690, strand=
target 5' ACUUUAGACCUUGAUCAAU 3'
:::::::::::::::::::
query 3' UGAAAUCUGGAACUAGUUA 5'
query=miR1917, target=RO3G_19004, score=0, range=968-986, strand=
target 5' ACUUUAGACCUUGAUCAAU 3'
:::::::::::::::::::
```

```
query 3' UGAAAUCUGGAACUAGUUA 5'
query=miR1917, target=RO3G_10315, score=2, range=1853-1870, stran
target 5' ACUUU-GACUUUGAUCAAU 3'
::::: :::.::::::::
query 3' UGAAAUCUGGAACUAGUUA 5'
query=miR1917, target=RO3G_08884, score=2, range=2014-2031, stran
target 5' ACUUU-GACUUUGAUCAAU 3'
::::: :::.::::::::
query 3' UGAAAUCUGGAACUAGUUA 5'
query=miR1917, target=RO3G_00502, score=2.5, range=244-261, stran
target 5' AC-UUGGACCUUGAUCGAU 3'
:: ::.:::::::::.::
query 3' UGAAAUCUGGAACUAGUUA 5'
query=miR1917, target=RO3G_05691, score=3, range=448-466, strand=
target 5' CUUUUGGACCUUGGUCAAU 3'
.:::.::::::::.:::::
query 3' UGAAAUCUGGAACUAGUUA 5'
query=miR1917, target=RO3G_10277, score=3, range=1653-1671, stran
target 5' ACCUUUGACCUUGAUCAAG 3'
:: :: :::::::::::
query 3' UGAAAUCUGGAACUAGUUA 5'
query=miR1917, target=RO3G_03637, score=3, range=525-542, strand=
target 5' CCUUU-GACCUUGAUCAAC 3'
:::: :::::::::::
query 3' UGAAAUCUGGAACUAGUUA 5'
query=miR1917, target=RO3G_01687, score=3.5, range=213-231, stran
target 5' GCUUUUGAUCUUGAUUAAU 3'
.:::: ::.::::::.:::
query 3' UGAAAUCUGGAACUAGUUA 5'
query=miR1917, target=RO3G_16595, score=3.5, range=792-810, stran
target 5' GCUUCAGACCUUGAUCGAG 3'
.::: :::::::::::.:
query 3' UGAAAUCUGGAACUAGUUA 5'
query=miR1917, target=RO3G_02854, score=3.5, range=871-889, stran
target 5' AUUGUAGACGUUGAUCAAU 3'
:.: ::::: :::::::::
query 3' UGAAAUCUGGAACUAGUUA 5'
query=miR1917, target=RO3G_12510, score=3.5, range=1126-1143, str
target 5' CUUUUAGACCUUGA-CAAU 3'
.:::::::::::: ::::
query 3' UGAAAUCUGGAACUAGUUA 5'
query=miR1917, target=RO3G_16647, score=3.5, range=265-283, stran
target 5' AUUUAUGACCUUGAUCAAC 3'
:.:: :::::::::::
query 3' UGAAAUCUGGAACUAGUUA 5'
```

```
query=miR1917, target=RO3G_15094, score=3.5, range=700-717, stran
target 5' GCUUCA-ACCUUGAUCAAU 3'
.::: : :::::::::::
query 3' UGAAAUCUGGAACUAGUUA 5'
query=miR1917, target=RO3G_16435, score=3.5, range=300-318, stran
target 5' AUUGUAGACCUUGAACAAU 3'
:.: :::::::::: ::::
query 3' UGAAAUCUGGAACUAGUUA 5'
query=miR1917, target=RO3G_14576, score=3.5, range=360-377, stran
target 5' GCUUUCG-CCUUGAUCAAU 3'
.:::: : ::::::::::
query 3' UGAAAUCUGGAACUAGUUA 5'
query=miR1917, target=RO3G_00267, score=3.5, range=1092-1110,
strand=-1
target 5' AUACAAGACCUUGAUCAAU 3'
:. :::::::::::::
query 3' UGAAAUCUGGAACUAGUUA 5'
query=miR1917, target=RO3G_10615, score=4, range=297-315, strand=
target 5' ACUUCACAUCUUGAUCAAU 3'
:::: : :.:::::::::
query 3' UGAAAUCUGGAACUAGUUA 5'
query=miR1917, target=RO3G_11623, score=4, range=309-327, strand=
target 5' ACCUUUGGCUUUGAUCAAU 3'
:: :: :.:.::::::::
query 3' UGAAAUCUGGAACUAGUUA 5'
query=miR1917, target=RO3G_11551, score=4, range=483-501, strand=
target 5' ACCUCAGACCUUGAUCGGU 3'
:: : :::::::::::..:
query 3' UGAAAUCUGGAACUAGUUA 5'
query=miR1917, target=RO3G_11168, score=4, range=732-750, strand=
target 5' CCUUUUGACCUGGAUCAAU 3'
:::: ::::: :::::::
query 3' UGAAAUCUGGAACUAGUUA 5'
query=miR1917, target=RO3G_11391, score=4, range=658-675, strand=
target 5' ACUUUAGUCCUUGAU-AAU 3'
::::::: ::::::: :::
query 3' UGAAAUCUGGAACUAGUUA 5'
query=miR1917, target=RO3G_04899, score=4, range=412-429, strand=
target 5' ACUUU-GACAUUGAUCGAU 3'
::::: ::: ::::::.::
query 3' UGAAAUCUGGAACUAGUUA 5'
query=miR1917, target=RO3G_03500, score=4, range=406-423, strand=
target 5' ACCUU-GACCUUGGUCGAU 3'
:: :: :::::::.::.::
query 3' UGAAAUCUGGAACUAGUUA 5'
```

```
query=miR1917, target=RO3G_09571, score=4, range=1101-1119, stran
target 5' ACUACAGACAUUGAUCAAU 3'
::: :::: :::::::::
query 3' UGAAAUCUGGAACUAGUUA 5'
query=miR1917, target=RO3G_05825, score=4, range=499-516, strand=
target 5' ACUCAAG-CCUUGAUCAAU 3'
::: :: :::::::::::
query 3' UGAAAUCUGGAACUAGUUA 5'
query=miR1917, target=RO3G_07626, score=4, range=106-123, strand=
target 5' ACCUU-GACCUUGACCAAU 3'
:: :: :::::::: ::::
query 3' UGAAAUCUGGAACUAGUUA 5'
query=miR1917, target=RO3G_17338, score=4, range=1652-1671, stran
target 5' GUUUUCGACCAUUGAUCAAU 3'
..:::: :::: :::::::::
query 3' UGAAAUCUGG-AACUAGUUA 5'
query=miR1917, target=RO3G_01807, score=4, range=6547-6566, stran
target 5' GUUUUAAGAUCUUGGUCAAU 3'
..:::: :::.::::.:::::
query 3' UGAAA-UCUGGAACUAGUUA 5'
query=miR1917, target=RO3G_10464, score=4, range=1720-1738, stran
target 5' AUUGUGGGCCUUGAUCAAC 3'
:.: :.:.::::::::::
query 3' UGAAAUCUGGAACUAGUUA 5'
query=miR1917, target=RO3G_14229, score=4, range=4072-4089, stran
target 5' AUUUUGGACUUUG-UCAAU 3'
:.:::.:::.::: :::::
query 3' UGAAAUCUGGAACUAGUUA 5'
query=miR1917, target=RO3G_06482, score=4, range=193-210, strand=
target 5' GUUUU-GACAUUGAUCAAU 3'
..:::: ::: :::::::::
query 3' UGAAAUCUGGAACUAGUUA 5'
query=miR1917, target=RO3G_11579, score=4, range=403-421, strand=
target 5' AGUACAGACCUUGAUCAAA 3'
: : :::::::::::::
query 3' UGAAAUCUGGAACUAGUUA 5'
query=miR1917, target=RO3G_01176, score=4, range=4905-4923, stran
target 5' ACCGAUGACCUUGAUCAAU 3'
:: :::::::::::::
query 3' UGAAAUCUGGAACUAGUUA 5'
query=miR1917, target=RO3G_05885, score=4, range=1773-1791, stran
target 5' ACCCUUGACCUUGAUCAAG 3'
:: : :::::::::::
query 3' UGAAAUCUGGAACUAGUUA 5'
query=miR1917, target=RO3G_04352, score=4, range=1453-1470, stran
```

target 5' ACCUU-CACCUUGAUCAAU 3'
:: :: :::::::::::
query 3' UGAAAUCUGGAACUAGUUA 5'
query=miR1917, target=RO3G_04057, score=4, range=633-650, strand=
target 5' UCUUUAGG-CUUGAUCAAU 3'
::::::. :::::::::
query 3' UGAAAUCUGGAACUAGUUA 5'
query=miR1857-5p, target=RO3G_17462, score=1, range=165-185, stra
target 5' CCGCAUCGCCAAAAGAAUCCA 3'
::::::: ::::::::::::
query 3' GGCGUAGAGGUUUUCUUAGGU 5'
query=miR1857-5p, target=RO3G_05618, score=1, range=27-47, strand
target 5' CCGCAUCGCCAAAAGAAUCCA 3'
::::::: ::::::::::::
query 3' GGCGUAGAGGUUUUCUUAGGU 5'
query=miR1857-5p, target=RO3G_03751, score=1, range=27-47, strand
target 5' CCGCAUCGCCAAAAGAAUCCA 3'
::::::: ::::::::::::
query 3' GGCGUAGAGGUUUUCUUAGGU 5'
query=miR1857-5p, target=RO3G_14280, score=3, range=29-49, strand
target 5' CUGCAAUUUCAAAAGAAUCCA 3'
:.::: .:.::::::::::::
query 3' GGCGUAGAGGUUUUCUUAGGU 5'
query=miR1857-5p, target=RO3G_07567, score=3, range=2240-2260,
strand=-1
target 5' CCGCAUAUCCAAAAGAAUCAA 3'
:::::: ::::::::::: :
query 3' GGCGUAGAGGUUUUCUUAGGU 5'
query=miR1857-5p, target=RO3G_00874, score=3, range=978-997, stra
target 5' CUUCGUC-CCAAAAGAAUCCA 3'
:. :.:: ::::::::::::
query 3' GGCGUAGAGGUUUUCUUAGGU 5'
query=miR1857-5p, target=RO3G_02342, score=4, range=2118-2137,
strand=1
target 5' UCGUAU-UCCAAAACAAUCCA 3'
.::.:: ::::::: ::::::
query 3' GGCGUAGAGGUUUUCUUAGGU 5'
query=miR1857-5p, target=RO3G_07329, score=4, range=387-406, stra
target 5' UCGC-UCCCUAAAAGAAUCCG 3'
.::: :: :.:::::::::.
query 3' GGCGUAGAGGUUUUCUUAGGU 5'
query=miR1857-5p, target=RO3G_11250, score=4, range=200-220, stra
target 5' GCGCAUCACCAAAGGAAUUCA 3'
:::::: ::::.::::.::
query 3' GGCGUAGAGGUUUUCUUAGGU 5'

```
query=miR1857-5p, target=RO3G_05595, score=4, range=783-802, stra
target 5' GUGAAUC-CCAAAAGAAUCCG 3'
.: ::: ::::::::::::.
query 3' GGCGUAGAGGUUUUCUUAGGU 5'
query=miR1857-5p, target=RO3G_06858, score=4, range=589-610, stra
target 5' CAGCAGCACCCAAAAGAAUCCA 3'
: ::: : :::::::::::::
query 3' GGCGUAG-AGGUUUUCUUAGGU 5'
query=miR5293, target=RO3G_16693, score=0, range=2989-3011, stran
target 5' UCUUCUGCGCCACUUCUUCAUCA 3'
:::::::::::::::::::::::
query 3' AGAAGACGCGGUGAAGAAGUAGU 5'
query=miR5293, target=RO3G_12527, score=0, range=2989-3011, stran
target 5' UCUUCUGCGCCACUUCUUCAUCA 3'
:::::::::::::::::::::::
query 3' AGAAGACGCGGUGAAGAAGUAGU 5'
query=miR5293, target=RO3G_13918, score=0, range=2989-3011, stran
target 5' UCUUCUGCGCCACUUCUUCAUCA 3'
:::::::::::::::::::::::
query 3' AGAAGACGCGGUGAAGAAGUAGU 5'
query=miR5293, target=RO3G_14347, score=0, range=2920-2942, stran
target 5' UCUUCUGCGCCACUUCUUCAUCA 3'
:::::::::::::::::::::::
query 3' AGAAGACGCGGUGAAGAAGUAGU 5'
query=miR5293, target=RO3G_16539, score=0, range=2881-2903, stran
target 5' UCUUCUGCGCCACUUCUUCAUCA 3'
:::::::::::::::::::::::
query 3' AGAAGACGCGGUGAAGAAGUAGU 5'
query=miR5293, target=RO3G_12538, score=0, range=2881-2903, stran
target 5' UCUUCUGCGCCACUUCUUCAUCA 3'
:::::::::::::::::::::::
query 3' AGAAGACGCGGUGAAGAAGUAGU 5'
query=miR5293, target=RO3G_10804, score=0, range=2830-2852, stran
target 5' UCUUCUGCGCCACUUCUUCAUCA 3'
:::::::::::::::::::::::
query 3' AGAAGACGCGGUGAAGAAGUAGU 5'
query=miR5293, target=RO3G_07311, score=0, range=2830-2852, stran
target 5' UCUUCUGCGCCACUUCUUCAUCA 3'
:::::::::::::::::::::::
query 3' AGAAGACGCGGUGAAGAAGUAGU 5'
query=miR5293, target=RO3G_01614, score=0, range=2830-2852, stran
target 5' UCUUCUGCGCCACUUCUUCAUCA 3'
:::::::::::::::::::::::
query 3' AGAAGACGCGGUGAAGAAGUAGU 5'
query=miR5293, target=RO3G_09547, score=0, range=2824-2846, stran
```

```
target 5' UCUUCUGCGCCACUUCUUCAUCA 3'
:::::::::::::::::::::::
query 3' AGAAGACGCGGUGAAGAAGUAGU 5'
query=miR5293, target=RO3G_16361, score=0, range=2824-2846, stran
target 5' UCUUCUGCGCCACUUCUUCAUCA 3'
:::::::::::::::::::::::
query 3' AGAAGACGCGGUGAAGAAGUAGU 5'
query=miR5293, target=RO3G_06425, score=0, range=2824-2846, stran
target 5' UCUUCUGCGCCACUUCUUCAUCA 3'
:::::::::::::::::::::::
query 3' AGAAGACGCGGUGAAGAAGUAGU 5'
query=miR5293, target=RO3G_05330, score=0, range=2824-2846, stran
target 5' UCUUCUGCGCCACUUCUUCAUCA 3'
:::::::::::::::::::::::
query 3' AGAAGACGCGGUGAAGAAGUAGU 5'
query=miR5293, target=RO3G_02704, score=0, range=2824-2846, stran
target 5' UCUUCUGCGCCACUUCUUCAUCA 3'
:::::::::::::::::::::::
query 3' AGAAGACGCGGUGAAGAAGUAGU 5'
query=miR5293, target=RO3G_12677, score=0, range=2824-2846, stran
target 5' UCUUCUGCGCCACUUCUUCAUCA 3'
:::::::::::::::::::::::
query 3' AGAAGACGCGGUGAAGAAGUAGU 5'
query=miR5293, target=RO3G_17021, score=0, range=2824-2846, stran
target 5' UCUUCUGCGCCACUUCUUCAUCA 3'
:::::::::::::::::::::::
query 3' AGAAGACGCGGUGAAGAAGUAGU 5'
query=miR5293, target=RO3G_04933, score=0, range=2824-2846, stran
target 5' UCUUCUGCGCCACUUCUUCAUCA 3'
:::::::::::::::::::::::
query 3' AGAAGACGCGGUGAAGAAGUAGU 5'
query=miR5293, target=RO3G_04126, score=0, range=2983-3005, stran
target 5' UCUUCUGCGCCACUUCUUCAUCA 3'
:::::::::::::::::::::::
query 3' AGAAGACGCGGUGAAGAAGUAGU 5'
query=miR5293, target=RO3G_05344, score=0, range=2650-2672, stran
target 5' UCUUCUGCGCCACUUCUUCAUCA 3'
:::::::::::::::::::::::
query 3' AGAAGACGCGGUGAAGAAGUAGU 5'
query=miR5293, target=RO3G_03778, score=0, range=2782-2804, stran
target 5' UCUUCUGCGCCACUUCUUCAUCA 3'
:::::::::::::::::::::::
query 3' AGAAGACGCGGUGAAGAAGUAGU 5'
query=miR5293, target=RO3G_12673, score=0, range=2125-2147, stran
target 5' UCUUCUGCGCCACUUCUUCAUCA 3'
```

```
::::::::::::::::::::::
query 3' AGAAGACGCGGUGAAGAAGUAGU 5'
query=miR5293, target=RO3G_17422, score=0, range=2125-2147, stran
target 5' UCUUCUGCGCCACUUCUUCAUCA 3'
::::::::::::::::::::::
query 3' AGAAGACGCGGUGAAGAAGUAGU 5'
query=miR5293, target=RO3G_12532, score=0, range=2830-2852, stran
target 5' UCUUCUGCGCCACUUCUUCAUCA 3'
::::::::::::::::::::::
query 3' AGAAGACGCGGUGAAGAAGUAGU 5'
query=miR5293, target=RO3G_01936, score=0, range=2830-2852, stran
target 5' UCUUCUGCGCCACUUCUUCAUCA 3'
::::::::::::::::::::::
query 3' AGAAGACGCGGUGAAGAAGUAGU 5'
query=miR5293, target=RO3G_03619, score=0, range=2716-2738, stran
target 5' UCUUCUGCGCCACUUCUUCAUCA 3'
::::::::::::::::::::::
query 3' AGAAGACGCGGUGAAGAAGUAGU 5'
query=miR5293, target=RO3G_09493, score=0, range=1648-1670, stran
target 5' UCUUCUGCGCCACUUCUUCAUCA 3'
::::::::::::::::::::::
query 3' AGAAGACGCGGUGAAGAAGUAGU 5'
query=miR5293, target=RO3G_02821, score=0, range=2989-3011, stran
target 5' UCUUCUGCGCCACUUCUUCAUCA 3'
::::::::::::::::::::::
query 3' AGAAGACGCGGUGAAGAAGUAGU 5'
query=miR5293, target=RO3G_05515, score=0, range=1819-1841, stran
target 5' UCUUCUGCGCCACUUCUUCAUCA 3'
::::::::::::::::::::::
query 3' AGAAGACGCGGUGAAGAAGUAGU 5'
query=miR5293, target=RO3G_00241, score=0, range=1114-1136, stran
target 5' UCUUCUGCGCCACUUCUUCAUCA 3'
::::::::::::::::::::::
query 3' AGAAGACGCGGUGAAGAAGUAGU 5'
query=miR5293, target=RO3G_00296, score=0, range=1114-1136, stran
target 5' UCUUCUGCGCCACUUCUUCAUCA 3'
::::::::::::::::::::::
query 3' AGAAGACGCGGUGAAGAAGUAGU 5'
query=miR5293, target=RO3G_03769, score=0, range=754-776, strand=
target 5' UCUUCUGCGCCACUUCUUCAUCA 3'
::::::::::::::::::::::
query 3' AGAAGACGCGGUGAAGAAGUAGU 5'
query=miR5293, target=RO3G_01964, score=0, range=1300-1322, stran
target 5' UCUUCUGCGCCACUUCUUCAUCA 3'
::::::::::::::::::::::
```

query 3' AGAAGACGCGGUGAAGAAGUAGU 5'
query=miR5293, target=RO3G_08830, score=1.5, range=103-125, stran
target 5' UCUUCUGCAUCACUUCUUCAUCA 3'
:::::::: .:::::::::::::
query 3' AGAAGACGCGGUGAAGAAGUAGU 5'
query=miR5293, target=RO3G_09036, score=3.5, range=429-452, stran
target 5' UCUUCUGCGUCACUUUCUUCAUCU 3'
:::::::::.::: :::::::::
query 3' AGAAGACGCGGUG-AAGAAGUAGU 5'
query=miR5293, target=RO3G_14008, score=3.5, range=729-751, stran
target 5' UCUUCUCUUCUACUUCUUCAUCA 3'
:::::: . :.::::::::::::
query 3' AGAAGACGCGGUGAAGAAGUAGU 5'
query=miR5293, target=RO3G_00589, score=4, range=418-441, strand=
target 5' UGUUCUUCAGCCACUUCUUCAUCU 3'
: :::: : :::::::::::::
query 3' AGAAGACG-CGGUGAAGAAGUAGU 5'
query=miR5293, target=RO3G_11977, score=4, range=738-761, strand=
target 5' UCUUCUGGGCCAUUCUCUUCAUCA 3'
::::::: ::::.: :::::::::
query 3' AGAAGACGCGGUGA-AGAAGUAGU 5'
query=miR3623-3p, target=RO3G_03300, score=3.5, range=435-456,
strand=-1
target 5' GUCAACUUCUUGCGCAGCACCA 3'
. ::: :::::::.::::::::
query 3' UCGUUUAAGAACGUGUCGUGGU 5'
query=miR9727, target=RO3G_10828, score=4, range=592-612, strand=
target 5' CUUAGUUUUCAGAGUU-AUACA 3'
::.::::.::::::: :.:::
query 3' GAGUCAAGAGUCUCAAUUGUGU 5'
query=miR535d, target=RO3G_02254, score=0, range=602-621, strand=
target 5' GUGCUCUCUAUCGUCGUUCA 3'
::::::::::::::::::::
query 3' CACGAGAGAUAGCAGCAAGU 5'
query=miR535d, target=RO3G_03535, score=3, range=20-39, strand=1
target 5' AUGAACUCUAUCGUCGUUCA 3'
:: :::::::::::::::
query 3' CACGAGAGAUAGCAGCAAGU 5'
query=miR535d, target=RO3G_08076, score=3.5, range=299-317, stran
target 5' GUAUUC-CUGUCGUCGUUCA 3'
:: .:: ::.::::::::::
query 3' CACGAGAGAUAGCAGCAAGU 5'
query=miR535d, target=RO3G_14153, score=4, range=614-633, strand=
target 5' GUGCUCUUUAUCGUCGAUCU 3'
:::::::.:::::::: ::

query 3' CACGAGAGAUAGCAGCAAGU 5'
query=miR535d, target=RO3G_13174, score=4, range=20-39, strand=1
target 5' AUGAACUUUAUCGUCGUUCA 3'
:: ::.:::::::::::::
query 3' CACGAGAGAUAGCAGCAAGU 5'
query=miR535d, target=RO3G_09252, score=4, range=443-462, strand=
target 5' AUUCUAUCUAUCGUCGUUCU 3'
: :: :::::::::::::
query 3' CACGAGAGAUAGCAGCAAGU 5'
query=miR535d, target=RO3G_14251, score=4, range=422-440, strand=
target 5' GU-CUAUCUAUCCUCGUUCA 3'
:: :: :::::: :::::::
query 3' CACGAGAGAUAGCAGCAAGU 5'
query=miR1513a-5p, target=RO3G_09180, score=0, range=1667-1687, strand=1
target 5' GCAAGUCCAUGGCUUUGUUCA 3'
:::::::::::::::::::::
query 3' CGUUCAGGUACCGAAACAAGU 5'
query=miR1513a-5p, target=RO3G_16812, score=0, range=1667-1687, strand=1
target 5' GCAAGUCCAUGGCUUUGUUCA 3'
:::::::::::::::::::::
query 3' CGUUCAGGUACCGAAACAAGU 5'
query=miR1513a-5p, target=RO3G_09189, score=0, range=1667-1687, strand=1
target 5' GCAAGUCCAUGGCUUUGUUCA 3'
:::::::::::::::::::::
query 3' CGUUCAGGUACCGAAACAAGU 5'
query=miR1513a-5p, target=RO3G_14798, score=0, range=1667-1687, strand=1
target 5' GCAAGUCCAUGGCUUUGUUCA 3'
:::::::::::::::::::::
query 3' CGUUCAGGUACCGAAACAAGU 5'
query=miR1513a-5p, target=RO3G_14791, score=0, range=1667-1687, strand=1
target 5' GCAAGUCCAUGGCUUUGUUCA 3'
:::::::::::::::::::::
query 3' CGUUCAGGUACCGAAACAAGU 5'
query=miR1513a-5p, target=RO3G_15270, score=0, range=716-736, str
target 5' GCAAGUCCAUGGCUUUGUUCA 3'
:::::::::::::::::::::
query 3' CGUUCAGGUACCGAAACAAGU 5'
query=miR1513a-5p, target=RO3G_16392, score=0, range=650-670, str
target 5' GCAAGUCCAUGGCUUUGUUCA 3'
:::::::::::::::::::::

query 3' CGUUCAGGUACCGAAACAAGU 5'
query=miR1513a-5p, target=RO3G_14795, score=1.5, range=1670-1690, strand=1
target 5' GCAAGUCAAUGGCUUUGUUCG 3'
::::::: ::::::::::::.
query 3' CGUUCAGGUACCGAAACAAGU 5'
query=miR1513a-5p, target=RO3G_16815, score=1.5, range=1670-1690, strand=1
target 5' GCAAGUCAAUGGCUUUGUUCG 3'
::::::: ::::::::::::.
query 3' CGUUCAGGUACCGAAACAAGU 5'
query=miR1513a-5p, target=RO3G_17226, score=1.5, range=1670-1690, strand=1
target 5' GCAAGUCAAUGGCUUUGUUCG 3'
::::::: ::::::::::::.
query 3' CGUUCAGGUACCGAAACAAGU 5'
query=miR1513a-5p, target=RO3G_13288, score=2, range=1670-1690, strand=1
target 5' GCAAGUCUAUGGCUUUGUUUG 3'
:::::::.:::::::::::..
query 3' CGUUCAGGUACCGAAACAAGU 5'
query=miR1513a-5p, target=RO3G_17260, score=2, range=1535-1555, strand=1
target 5' GCAAGUCUAUGGCUUUGUUUG 3'
:::::::.:::::::::::..
query 3' CGUUCAGGUACCGAAACAAGU 5'
query=miR1513a-5p, target=RO3G_06734, score=2, range=971-991, str
target 5' GCAAGUCUAUGGCUUUGUUUG 3'
:::::::.:::::::::::..
query 3' CGUUCAGGUACCGAAACAAGU 5'
query=miR1513a-5p, target=RO3G_14793, score=2.5, range=1670-1690, strand=1
target 5' GCAAGUCGAUGGCUUUGUUUG 3'
::::::: :::::::::::..
query 3' CGUUCAGGUACCGAAACAAGU 5'
query=miR1513a-5p, target=RO3G_14800, score=2.5, range=1670-1690, strand=1
target 5' GCAAGUCGAUGGCUUUGUUUG 3'
::::::: :::::::::::..
query 3' CGUUCAGGUACCGAAACAAGU 5'
query=miR1513a-5p, target=RO3G_17268, score=2.5, range=251-271, strand=1
target 5' GCAAGUCGAUGGCUUUGUUUG 3'
::::::: :::::::::::..
query 3' CGUUCAGGUACCGAAACAAGU 5'

query=miR1513a-5p, target=RO3G_15875, score=3.5, range=1670-1690,
strand=1
target 5' GCAAAUCGAUGGCUUUGUUUG 3'
:::: :: :::::::::::..
query 3' CGUUCAGGUACCGAAACAAGU 5'
query=miR1513a-5p, target=RO3G_14787, score=3.5, range=1670-1690,
strand=1
target 5' GCAAAUCGAUGGCUUUGUUUG 3'
:::: :: :::::::::::..
query 3' CGUUCAGGUACCGAAACAAGU 5'
query=miR1513a-5p, target=RO3G_17411, score=3.5, range=1112-1132,
strand=1
target 5' GCAAAUCGAUGGCUUUGUUUG 3'
:::: :: :::::::::::..
query 3' CGUUCAGGUACCGAAACAAGU 5'
query=miR1513a-5p, target=RO3G_03072, score=3.5, range=465-485,
strand=1
target 5' GAAAGGAUAUGGCUUUGUUCA 3'
: ::: .:::::::::::::
query 3' CGUUCAGGUACCGAAACAAGU 5'
query=miR1513a-5p, target=RO3G_06449, score=4, range=3435-3454,
strand=-1
target 5' GUGA-UCCAAGGCUUUGUUCA 3'
:..: :::: :::::::::::
query 3' CGUUCAGGUACCGAAACAAGU 5'
query=miR1513a-5p, target=RO3G_16135, score=4, range=267-286,
strand=-1
target 5' GCAAUUCAAUGGCUUUG-UCA 3'
:::: :: ::::::::: :::
query 3' CGUUCAGGUACCGAAACAAGU 5'
query=miR1513a-5p, target=RO3G_01941, score=4, range=267-286,
strand=-1
target 5' GCAAUUCAAUGGCUUUG-UCA 3'
:::: :: ::::::::: :::
query 3' CGUUCAGGUACCGAAACAAGU 5'
query=miR1513a-5p, target=RO3G_09956, score=4, range=267-286,
strand=-1
target 5' GCAAUUCAAUGGCUUUG-UCA 3'
:::: :: ::::::::: :::
query 3' CGUUCAGGUACCGAAACAAGU 5'
query=miR1513a-5p, target=RO3G_03105, score=4, range=267-286,
strand=-1
target 5' GCAAUUCAAUGGCUUUG-UCA 3'
:::: :: ::::::::: :::
query 3' CGUUCAGGUACCGAAACAAGU 5'

query=miR1513a-5p, target=RO3G_10523, score=4, range=267-286, strand=-1
target 5' GCAAUUCAAUGGCUUUG-UCA 3'
:::: :: ::::::::: :::
query 3' CGUUCAGGUACCGAAACAAGU 5'
query=miR1513a-5p, target=RO3G_15285, score=4, range=267-286, strand=-1
target 5' GCAAUUCAAUGGCUUUG-UCA 3'
:::: :: ::::::::: :::
query 3' CGUUCAGGUACCGAAACAAGU 5'
query=miR1513a-5p, target=RO3G_04217, score=4, range=267-286, strand=-1
target 5' GCAAUUCAAUGGCUUUG-UCA 3'
:::: :: ::::::::: :::
query 3' CGUUCAGGUACCGAAACAAGU 5'
query=miR1513a-5p, target=RO3G_07679, score=4, range=36-55, stran
target 5' GCAAUUCAAUGGCUUUG-UCA 3'
:::: :: ::::::::: :::
query 3' CGUUCAGGUACCGAAACAAGU 5'
query=miR1513a-5p, target=RO3G_09932, score=4, range=267-286, strand=-1
target 5' GCAAUUCAAUGGCUUUG-UCA 3'
:::: :: ::::::::: :::
query 3' CGUUCAGGUACCGAAACAAGU 5'
query=miR1513a-5p, target=RO3G_11770, score=4, range=267-286, strand=-1
target 5' GCAAUUCAAUGGCUUUG-UCA 3'
:::: :: ::::::::: :::
query 3' CGUUCAGGUACCGAAACAAGU 5'
query=miR1167, target=RO3G_04690, score=2, range=406-427, strand=
target 5' GUUUCCAGUCAUCCACAGUCCA 3'
::::: :::::::::::.:::
query 3' CAAAGUUCAGUAGGUGUCGGGU 5'
query=miR1167, target=RO3G_16729, score=2, range=274-295, strand=
target 5' GUUUCCAGUCAUCCACAGUCCA 3'
::::: :::::::::::.:::
query 3' CAAAGUUCAGUAGGUGUCGGGU 5'
query=miR1167, target=RO3G_14560, score=2, range=274-295, strand=
target 5' GUUUCCAGUCAUCCACAGUCCA 3'
::::: :::::::::::.:::
query 3' CAAAGUUCAGUAGGUGUCGGGU 5'
query=miR1167, target=RO3G_15141, score=2, range=406-427, strand=
target 5' GUUUCCAGUCAUCCACAGUCCA 3'
::::: :::::::::::.:::
query 3' CAAAGUUCAGUAGGUGUCGGGU 5'

query=miR1167, target=RO3G_02822, score=2, range=406-427, strand=
target 5' GUUUCCAGUCAUCCACAGUCCA 3'
::::: ::::::::::::.:::
query 3' CAAAGUUCAGUAGGUGUCGGGU 5'
query=miR1167, target=RO3G_02713, score=2, range=136-157, strand=
target 5' GUUUCCAGUCAUCCACAGUCCA 3'
::::: ::::::::::::.:::
query 3' CAAAGUUCAGUAGGUGUCGGGU 5'
query=miR1167, target=RO3G_08115, score=2, range=136-157, strand=
target 5' GUUUCCAGUCAUCCACAGUCCA 3'
::::: ::::::::::::.:::
query 3' CAAAGUUCAGUAGGUGUCGGGU 5'
query=miR1167, target=RO3G_05421, score=2, range=154-175, strand=
target 5' GUUUCCAGUCAUCCACAGUCCA 3'
::::: ::::::::::::.:::
query 3' CAAAGUUCAGUAGGUGUCGGGU 5'
query=miR1167, target=RO3G_00304, score=2, range=406-427, strand=
target 5' GUUUCCAGUCAUCCACAGUCCA 3'
::::: ::::::::::::.:::
query 3' CAAAGUUCAGUAGGUGUCGGGU 5'
query=miR1167, target=RO3G_05431, score=3, range=406-427, strand=
target 5' GUUUCCAGUCGUCCACAGUCCA 3'
::::: ::::.:::::::.:::
query 3' CAAAGUUCAGUAGGUGUCGGGU 5'
query=miR1167, target=RO3G_07112, score=3, range=229-250, strand=
target 5' GUUUCCAGUCAUCUACAGUCCA 3'
::::: :::::::.::::.:::
query 3' CAAAGUUCAGUAGGUGUCGGGU 5'
query=miR1167, target=RO3G_12666, score=3, range=37-58, strand=-1
target 5' GUUUCCAGUCGUCCACAGUCCA 3'
::::: ::::.:::::::.:::
query 3' CAAAGUUCAGUAGGUGUCGGGU 5'
query=miR1167, target=RO3G_09168, score=3, range=406-427, strand=
target 5' GUUUCCAGUCGUCCAUAGCCCA 3'
::::: ::::.::::.::::::
query 3' CAAAGUUCAGUAGGUGUCGGGU 5'
query=miR1167, target=RO3G_10762, score=3, range=406-427, strand=
target 5' GUUUCCAGUCGUCCAUAGCCCA 3'
::::: ::::.::::.::::::
query 3' CAAAGUUCAGUAGGUGUCGGGU 5'
query=miR1167, target=RO3G_10770, score=3, range=406-427, strand=
target 5' GUUUCCAGUCGUCCAUAGCCCA 3'
::::: ::::.::::.::::::
query 3' CAAAGUUCAGUAGGUGUCGGGU 5'
query=miR1167, target=RO3G_08663, score=3, range=406-427, strand=

```
target 5' GUUUCCAGUCAUCUACAGUCCA 3'
::::: :::::::.::::.:::
query 3' CAAAGUUCAGUAGGUGUCGGGU 5'
query=miR1167, target=RO3G_13858, score=3, range=37-58, strand=-1
target 5' GUUUCCAGUCGUCCACAGUCCA 3'
::::: ::::.:::::::.:::
query 3' CAAAGUUCAGUAGGUGUCGGGU 5'
query=miR1167, target=RO3G_07129, score=3, range=274-295, strand=
target 5' GUUUCCAGUCGUCCACAGUCCA 3'
::::: ::::.:::::::.:::
query 3' CAAAGUUCAGUAGGUGUCGGGU 5'
query=miR1167, target=RO3G_01188, score=3, range=274-295, strand=
target 5' GUUUCCAGUCGUCCACAGUCCA 3'
::::: ::::.:::::::.:::
query 3' CAAAGUUCAGUAGGUGUCGGGU 5'
query=miR1167, target=RO3G_12071, score=3, range=37-58, strand=-1
target 5' GUUUCCAGUCGUCCACAGUCCA 3'
::::: ::::.:::::::.:::
query 3' CAAAGUUCAGUAGGUGUCGGGU 5'
query=miR1167, target=RO3G_03764, score=3, range=37-58, strand=-1
target 5' GUUUCCAGUCGUCCACAGUCCA 3'
::::: ::::.:::::::.:::
query 3' CAAAGUUCAGUAGGUGUCGGGU 5'
query=miR1167, target=RO3G_04787, score=3, range=406-427, strand=
target 5' GUUUCCAGUCGUCCAUAGCCCA 3'
::::: ::::.::::.::::::
query 3' CAAAGUUCAGUAGGUGUCGGGU 5'
query=miR1167, target=RO3G_11266, score=3, range=229-250, strand=
target 5' GUUUCCAGUCAUCUACAGUCCA 3'
::::: :::::::.::::.:::
query 3' CAAAGUUCAGUAGGUGUCGGGU 5'
query=miR1167, target=RO3G_07120, score=3, range=229-250, strand=
target 5' GUUUCCAGUCAUCUACAGUCCA 3'
::::: :::::::.::::.:::
query 3' CAAAGUUCAGUAGGUGUCGGGU 5'
query=miR1167, target=RO3G_11240, score=3, range=229-250, strand=
target 5' GUUUCCAGUCAUCUACAGUCCA 3'
::::: :::::::.::::.:::
query 3' CAAAGUUCAGUAGGUGUCGGGU 5'
query=miR1167, target=RO3G_15001, score=3, range=229-250, strand=
target 5' GUUUCCAGUCAUCUACAGUCCA 3'
::::: :::::::.::::.:::
query 3' CAAAGUUCAGUAGGUGUCGGGU 5'
query=miR1167, target=RO3G_15575, score=3, range=229-250, strand=
target 5' GUUUCCAGUCAUCUACAGUCCA 3'
```

::::: :::::::.::::.:::
query 3' CAAAGUUCAGUAGGUGUCGGGU 5'
query=miR1167, target=RO3G_10529, score=3, range=37-58, strand=-1
target 5' GUUUCCAGUCGUCCACAGUCCA 3'
::::: ::::.::::::::.:::
query 3' CAAAGUUCAGUAGGUGUCGGGU 5'
query=miR1167, target=RO3G_01948, score=3, range=184-205, strand=
target 5' GUUUCCAGUCAUCUACAGUCCA 3'
::::: :::::::.::::.:::
query 3' CAAAGUUCAGUAGGUGUCGGGU 5'
query=miR1167, target=RO3G_05350, score=3.5, range=274-295, stran
target 5' GUUUCCAGUCGUCCACAGUCCG 3'
::::: ::::.::::::::.::.
query 3' CAAAGUUCAGUAGGUGUCGGGU 5'
query=miR1167, target=RO3G_12588, score=3.5, range=1185-1205, strand=-1
target 5' GAUUCAA-ACAUCCACAGCCCG 3'
: ::::: :::::::::::.
query 3' CAAAGUUCAGUAGGUGUCGGGU 5'
query=miR1167, target=RO3G_15287, score=4, range=184-205, strand=
target 5' GUUUCCAGUCAUCUAUAGUCCA 3'
::::: :::::::.:.::.:::
query 3' CAAAGUUCAGUAGGUGUCGGGU 5'
query=miR1167, target=RO3G_16359, score=4, range=406-427, strand=
target 5' GUUUCCAGUCAUCUAUAGUCCA 3'
::::: :::::::.:.::.:::
query 3' CAAAGUUCAGUAGGUGUCGGGU 5'
query=miR1167, target=RO3G_09612, score=4, range=406-427, strand=
target 5' GUUUCCAGUCAUCUAUAGUCCA 3'
::::: :::::::.:.::.:::
query 3' CAAAGUUCAGUAGGUGUCGGGU 5'
query=miR1167, target=RO3G_07688, score=4, range=406-427, strand=
target 5' GUUUCCAGUCAUCUAUAGUCCA 3'
::::: :::::::.:.::.:::
query 3' CAAAGUUCAGUAGGUGUCGGGU 5'
query=miR1167, target=RO3G_10866, score=4, range=406-427, strand=
target 5' GUUUCCAGUCAUCUAUAGUCCA 3'
::::: :::::::.:.::.:::
query 3' CAAAGUUCAGUAGGUGUCGGGU 5'
query=miR1167, target=RO3G_16545, score=4, range=406-427, strand=
target 5' GUUUCCAGUCAUCUAUAGUCCA 3'
::::: :::::::.:.::.:::
query 3' CAAAGUUCAGUAGGUGUCGGGU 5'
query=miR1167, target=RO3G_13271, score=4, range=406-427, strand=
target 5' GUUUCCAGUCAUCUAUAGUCCA 3'

```
::::: :::::::.:.::.:::
query 3' CAAAGUUCAGUAGGUGUCGGGU 5'
query=miR1167, target=RO3G_00255, score=4, range=406-427, strand=
target 5' GUUUCCAGUCAUCUAUAGUCCA 3'
::::: :::::::.:.::.:::
query 3' CAAAGUUCAGUAGGUGUCGGGU 5'
query=miR1167, target=RO3G_12954, score=4, range=406-427, strand=
target 5' GUUUCCAGUCGUUCACAGUCCA 3'
::::: ::::.:.:::::.:::
query 3' CAAAGUUCAGUAGGUGUCGGGU 5'
query=miR1167, target=RO3G_13266, score=4, range=184-205, strand=
target 5' GUUUCCAGUCAUCUAUAGUCCA 3'
::::: :::::::.:.::.:::
query 3' CAAAGUUCAGUAGGUGUCGGGU 5'
query=miR1167, target=RO3G_03766, score=4, range=184-205, strand=
target 5' GUUUCCAGUCAUCUAUAGUCCA 3'
::::: :::::::.:.::.:::
query 3' CAAAGUUCAGUAGGUGUCGGGU 5'
query=miR1167, target=RO3G_13147, score=4, range=184-205, strand=
target 5' GUUUCCAGUCAUCUAUAGUCCA 3'
::::: :::::::.:.::.:::
query 3' CAAAGUUCAGUAGGUGUCGGGU 5'
query=miR1167, target=RO3G_06722, score=4, range=406-427, strand=
target 5' GUUUCCAGUCAUCUAUAGUCCA 3'
::::: :::::::.:.::.:::
query 3' CAAAGUUCAGUAGGUGUCGGGU 5'
query=miR6114-5p, target=RO3G_00301, score=0, range=954-974, stra
target 5' UCACGUUCAUGGUGCUUUCGA 3'
:::::::::::::::::::::
query 3' AGUGCAAGUACCACGAAAGCU 5'
query=miR6114-5p, target=RO3G_13432, score=0, range=348-368, stra
target 5' UCACGUUCAUGGUGCUUUCGA 3'
:::::::::::::::::::::
query 3' AGUGCAAGUACCACGAAAGCU 5'
query=miR6478, target=RO3G_19017, score=0, range=1-22, strand=-1
target 5' CUACCAACUGAGCUAAGGCCGG 3'
::::::::::::::::::::::
query 3' GAUGGUUGACUCGAUUCCGGCC 5'
query=miR6478, target=RO3G_19074, score=0, range=1-22, strand=-1
target 5' CUACCAACUGAGCUAAGGCCGG 3'
::::::::::::::::::::::
query 3' GAUGGUUGACUCGAUUCCGGCC 5'
query=miR6478, target=RO3G_19106, score=0, range=1-22, strand=-1
target 5' CUACCAACUGAGCUAAGGCCGG 3'
::::::::::::::::::::::
```

```
query 3' GAUGGUUGACUCGAUUCCGGCC 5'
query=miR6478, target=RO3G_19164, score=0, range=1-22, strand=-1
target 5' CUACCAACUGAGCUAAGGCCGG 3'
::::::::::::::::::::::
query 3' GAUGGUUGACUCGAUUCCGGCC 5'
query=miR6478, target=RO3G_19144, score=0, range=1-22, strand=-1
target 5' CUACCAACUGAGCUAAGGCCGG 3'
::::::::::::::::::::::
query 3' GAUGGUUGACUCGAUUCCGGCC 5'
query=miR6478, target=RO3G_19194, score=0, range=1-22, strand=-1
target 5' CUACCAACUGAGCUAAGGCCGG 3'
::::::::::::::::::::::
query 3' GAUGGUUGACUCGAUUCCGGCC 5'
query=miR6478, target=RO3G_19112, score=0, range=1-22, strand=-1
target 5' CUACCAACUGAGCUAAGGCCGG 3'
::::::::::::::::::::::
query 3' GAUGGUUGACUCGAUUCCGGCC 5'
query=miR6478, target=RO3G_19210, score=0, range=1-22, strand=-1
target 5' CUACCAACUGAGCUAAGGCCGG 3'
::::::::::::::::::::::
query 3' GAUGGUUGACUCGAUUCCGGCC 5'
query=miR6478, target=RO3G_19195, score=0, range=1-22, strand=-1
target 5' CUACCAACUGAGCUAAGGCCGG 3'
::::::::::::::::::::::
query 3' GAUGGUUGACUCGAUUCCGGCC 5'
query=miR5809, target=RO3G_16804, score=0, range=1387-1404, stran
target 5' GCUUUGGUCGCCAGCCGA 3'
::::::::::::::::::
query 3' CGAAACCAGCGGUCGGCU 5'
query=miR5809, target=RO3G_10715, score=0, range=1387-1404, stran
target 5' GCUUUGGUCGCCAGCCGA 3'
::::::::::::::::::
query 3' CGAAACCAGCGGUCGGCU 5'
query=miR5809, target=RO3G_04309, score=0, range=1327-1344, stran
target 5' GCUUUGGUCGCCAGCCGA 3'
::::::::::::::::::
query 3' CGAAACCAGCGGUCGGCU 5'
query=miR5809, target=RO3G_16610, score=0, range=193-210, strand=
target 5' GCUUUGGUCGCCAGCCGA 3'
::::::::::::::::::
query 3' CGAAACCAGCGGUCGGCU 5'
query=miR5809, target=RO3G_16602, score=0, range=193-210, strand=
target 5' GCUUUGGUCGCCAGCCGA 3'
::::::::::::::::::
query 3' CGAAACCAGCGGUCGGCU 5'
```

```
query=miR5809, target=RO3G_15108, score=0, range=796-813, strand=
target 5' GCUUUGGUCGCCAGCCGA 3'
::::::::::::::::::
query 3' CGAAACCAGCGGUCGGCU 5'
query=miR5809, target=RO3G_13293, score=0, range=1387-1404, stran
target 5' GCUUUGGUCGCCAGCCGA 3'
::::::::::::::::::
query 3' CGAAACCAGCGGUCGGCU 5'
query=miR5809, target=RO3G_10177, score=0, range=1387-1404, stran
target 5' GCUUUGGUCGCCAGCCGA 3'
::::::::::::::::::
query 3' CGAAACCAGCGGUCGGCU 5'
query=miR5809, target=RO3G_12007, score=0, range=1387-1404, stran
target 5' GCUUUGGUCGCCAGCCGA 3'
::::::::::::::::::
query 3' CGAAACCAGCGGUCGGCU 5'
query=miR5809, target=RO3G_09718, score=0, range=1387-1404, stran
target 5' GCUUUGGUCGCCAGCCGA 3'
::::::::::::::::::
query 3' CGAAACCAGCGGUCGGCU 5'
query=miR5809, target=RO3G_03867, score=0, range=1384-1401, stran
target 5' GCUUUGGUCGCCAGCCGA 3'
::::::::::::::::::
query 3' CGAAACCAGCGGUCGGCU 5'
query=miR5809, target=RO3G_15131, score=0, range=1384-1401, stran
target 5' GCUUUGGUCGCCAGCCGA 3'
::::::::::::::::::
query 3' CGAAACCAGCGGUCGGCU 5'
query=miR5809, target=RO3G_13439, score=0, range=1384-1401, stran
target 5' GCUUUGGUCGCCAGCCGA 3'
::::::::::::::::::
query 3' CGAAACCAGCGGUCGGCU 5'
query=miR5809, target=RO3G_01475, score=0, range=1384-1401, stran
target 5' GCUUUGGUCGCCAGCCGA 3'
::::::::::::::::::
query 3' CGAAACCAGCGGUCGGCU 5'
query=miR5809, target=RO3G_15003, score=0, range=286-303, strand=
target 5' GCUUUGGUCGCCAGCCGA 3'
::::::::::::::::::
query 3' CGAAACCAGCGGUCGGCU 5'
query=miR5809, target=RO3G_01004, score=0, range=1384-1401, stran
target 5' GCUUUGGUCGCCAGCCGA 3'
::::::::::::::::::
query 3' CGAAACCAGCGGUCGGCU 5'
query=miR5809, target=RO3G_00977, score=0, range=646-663, strand=
```

```
target 5' GCUUUGGUCGCCAGCCGA 3'
::::::::::::::::::
query 3' CGAAACCAGCGGUCGGCU 5'
query=miR5809, target=RO3G_09043, score=0, range=658-675, strand=
target 5' GCUUUGGUCGCCAGCCGA 3'
::::::::::::::::::
query 3' CGAAACCAGCGGUCGGCU 5'
query=miR5809, target=RO3G_03851, score=3, range=820-838, strand=
target 5' GCUUACGGUCGCCAGCUGA 3'
:::: ::::::::::.::
query 3' CGAA-ACCAGCGGUCGGCU 5'
query=miR5809, target=RO3G_04551, score=3, range=2132-2148, stran
target 5' GCUUUGGUC-UCAGCCGA 3'
::::::::: .:::::::
query 3' CGAAACCAGCGGUCGGCU 5'
query=miR5809, target=RO3G_01282, score=4, range=712-729, strand=
target 5' GCUUUGGUCGACAGCCUA 3'
:::::::::: ::::: :
query 3' CGAAACCAGCGGUCGGCU 5'
query=miR5809, target=RO3G_02774, score=4, range=5454-5471, stran
target 5' CCUUUGGGCGCCAGCCGU 3'
:::::: :::::::::
query 3' CGAAACCAGCGGUCGGCU 5'
query=miR8578.2, target=RO3G_08806, score=0, range=1878-1896, str
target 5' CAUUGCUAAUACACUCAUA 3'
:::::::::::::::::::
query 3' GUAACGAUUAUGUGAGUAU 5'
query=miR8578.2, target=RO3G_14186, score=0, range=1878-1896, str
target 5' CAUUGCUAAUACACUCAUA 3'
:::::::::::::::::::
query 3' GUAACGAUUAUGUGAGUAU 5'
query=miR8578.2, target=RO3G_14870, score=0, range=1878-1896, str
target 5' CAUUGCUAAUACACUCAUA 3'
:::::::::::::::::::
query 3' GUAACGAUUAUGUGAGUAU 5'
query=miR8578.2, target=RO3G_14190, score=0, range=1878-1896, str
target 5' CAUUGCUAAUACACUCAUA 3'
:::::::::::::::::::
query 3' GUAACGAUUAUGUGAGUAU 5'
query=miR8578.2, target=RO3G_13228, score=0, range=1467-1485, str
target 5' CAUUGCUAAUACACUCAUA 3'
:::::::::::::::::::
query 3' GUAACGAUUAUGUGAGUAU 5'
query=miR8578.2, target=RO3G_01029, score=0, range=837-855, stran
target 5' CAUUGCUAAUACACUCAUA 3'
```

```
::::::::::::::::::
query 3' GUAACGAUUAUGUGAGUAU 5'
query=miR8578.2, target=RO3G_06628, score=3, range=1350-1368,
strand=-1
target 5' CUUUGCUAAUUCACUCAUA 3'
: :::::::: ::::::::
query 3' GUAACGAUUAUGUGAGUAU 5'
query=miR8578.2, target=RO3G_04839, score=3.5, range=293-312, str
target 5' CACUUGUUUAUACACUCAUA 3'
:: :::.: ::::::::::
query 3' GU-AACGAUUAUGUGAGUAU 5'
query=miR8578.2, target=RO3G_07324, score=3.5, range=1544-1563,
strand=1
target 5' UAUUACCUAAUACACUUAUA 3'
.::: ::::::::::.:::
query 3' GUAA-CGAUUAUGUGAGUAU 5'
query=miR8578.2, target=RO3G_07019, score=3.5, range=528-545,
strand=-1
target 5' UAAU-CUAAUACAUUCAUA 3'
.: : ::::::::.::::
query 3' GUAACGAUUAUGUGAGUAU 5'
query=miR8578.2, target=RO3G_15525, score=4, range=1326-1344, str
target 5' UGAUGCUAAUACACUCUUA 3'
.. :::::::::::: ::
query 3' GUAACGAUUAUGUGAGUAU 5'
query=miR8578.2, target=RO3G_11053, score=4, range=297-314, stran
target 5' CAUUGCUAAUA-AAUCAUA 3'
::::::::::: : :::::
query 3' GUAACGAUUAUGUGAGUAU 5'
query=miR8578.2, target=RO3G_02215, score=4, range=990-1008, stra
target 5' CAUUGCUAGUACAUUUAUU 3'
::::::::.::::.:.::
query 3' GUAACGAUUAUGUGAGUAU 5'
query=miR8578.2, target=RO3G_14686, score=4, range=823-841, stran
target 5' CAUUCUUGAUACGCUCAUG 3'
:::: .:.::::.::::.
query 3' GUAACGAUUAUGUGAGUAU 5'
query=miR1214, target=RO3G_17226, score=0, range=1271-1290, stran
target 5' GUGCCGCGAGACUCUAAGUA 3'
::::::::::::::::::::
query 3' CACGGCGCUCUGAGAUUCAU 5'
query=miR1214, target=RO3G_16815, score=0, range=1271-1290, stran
target 5' GUGCCGCGAGACUCUAAGUA 3'
::::::::::::::::::::
query 3' CACGGCGCUCUGAGAUUCAU 5'
```

```
query=miR1214, target=RO3G_14795, score=0, range=1271-1290, stran
target 5' GUGCCGCGAGACUCUAAGUA 3'
::::::::::::::::::::
query 3' CACGGCGCUCUGAGAUUCAU 5'
query=miR1214, target=RO3G_16392, score=2, range=251-270, strand=
target 5' GUGUGGUGAGACUCUAAGUA 3'
:::. :.::::::::::::::
query 3' CACGGCGCUCUGAGAUUCAU 5'
query=miR1214, target=RO3G_02091, score=3.5, range=112-130, stran
target 5' GUACC-CGAGACUUUAAGUG 3'
:: :: :::::::.:::::.
query 3' CACGGCGCUCUGAGAUUCAU 5'
query=miR1214, target=RO3G_16382, score=4, range=1123-1143, stran
target 5' GUAGCCGCGAGAUUCUACGUA 3'
:: ::::::::::.:::: :::
query 3' CA-CGGCGCUCUGAGAUUCAU 5'
query=miR7729a-5p, target=RO3G_17349, score=0, range=396-418, str
target 5' GCUCACUGUCCUACUUGAAAACA 3'
:::::::::::::::::::::::
query 3' CGAGUGACAGGAUGAACUUUUGU 5'
query=miR7729a-5p, target=RO3G_06738, score=0, range=399-421, str
target 5' GCUCACUGUCCUACUUGAAAACA 3'
:::::::::::::::::::::::
query 3' CGAGUGACAGGAUGAACUUUUGU 5'
query=miR7729a-5p, target=RO3G_12528, score=0, range=162-184, str
target 5' GCUCACUGUCCUACUUGAAAACA 3'
:::::::::::::::::::::::
query 3' CGAGUGACAGGAUGAACUUUUGU 5'
query=miR7729a-5p, target=RO3G_02826, score=0, range=162-184, str
target 5' GCUCACUGUCCUACUUGAAAACA 3'
:::::::::::::::::::::::
query 3' CGAGUGACAGGAUGAACUUUUGU 5'
query=miR7729a-5p, target=RO3G_15905, score=1, range=162-184, str
target 5' GCUCACUGUCCUGCUUGAAAACA 3'
:::::::::::.:::::::::::
query 3' CGAGUGACAGGAUGAACUUUUGU 5'
query=miR7729a-5p, target=RO3G_16371, score=1, range=162-184, str
target 5' GUUUACUGUCCUACUUGAAAACA 3'
:.:.:::::::::::::::::::
query 3' CGAGUGACAGGAUGAACUUUUGU 5'
query=miR7729a-5p, target=RO3G_07975, score=1, range=65-87, stran
target 5' GUUUACUGUCCUACUUGAAAACA 3'
:.:.:::::::::::::::::::
query 3' CGAGUGACAGGAUGAACUUUUGU 5'
query=miR7729a-5p, target=RO3G_17352, score=1, range=131-153,
```

```
strand=-1
target 5' GUUUACUGUCCUACUUGAAAACA 3'
:.:.:::::::::::::::::::
query 3' CGAGUGACAGGAUGAACUUUUGU 5'
query=miR7729a-5p, target=RO3G_16723, score=1.5, range=131-153,
strand=-1
target 5' GCUCAUUGUCCUAUUUGAAAACA 3'
:::::.:::::::.:::::::::
query 3' CGAGUGACAGGAUGAACUUUUGU 5'
query=miR7729a-5p, target=RO3G_04668, score=3, range=131-153,
strand=-1
target 5' GCUUAUUGUCCUACUUGAAAAAA 3'
:::.:.::::::::::::::: :
query 3' CGAGUGACAGGAUGAACUUUUGU 5'
query=miR7729a-5p, target=RO3G_03112, score=4, range=131-153,
strand=-1
target 5' GCUUUUUGUCCUAUUUGGAAACA 3'
:::. .:::::::.:::.:::::
query 3' CGAGUGACAGGAUGAACUUUUGU 5'
query=miR7729a-5p, target=RO3G_05522, score=4, range=131-153,
strand=-1
target 5' GCUUUUUGUCCUAUUUGGAAACA 3'
:::. .:::::::.:::.:::::
query 3' CGAGUGACAGGAUGAACUUUUGU 5'
query=miR7729a-5p, target=RO3G_02227, score=4, range=131-153,
strand=-1
target 5' GCUUUUUGUCCUAUUUGGAAACA 3'
:::. .:::::::.:::.:::::
query 3' CGAGUGACAGGAUGAACUUUUGU 5'
query=miR7729a-5p, target=RO3G_06406, score=4, range=131-153,
strand=-1
target 5' AUUUACUGUCCUACUCGAAAACA 3'
.:.::::::::::: :::::::
query 3' CGAGUGACAGGAUGAACUUUUGU 5'
query=miR7753-3p, target=RO3G_15889, score=0, range=434-453, stra
target 5' CCUUCUUCUCCAUUGCUCAA 3'
::::::::::::::::::::
query 3' GGAAGAAGAGGUAACGAGUU 5'
query=miR7753-3p, target=RO3G_17257, score=0, range=434-453, stra
target 5' CCUUCUUCUCCAUUGCUCAA 3'
::::::::::::::::::::
query 3' GGAAGAAGAGGUAACGAGUU 5'
query=miR7753-3p, target=RO3G_11951, score=0, range=434-453, stra
target 5' CCUUCUUCUCCAUUGCUCAA 3'
::::::::::::::::::::
```

query 3' GGAAGAAGAGGUAACGAGUU 5'
query=miR7753-3p, target=RO3G_16136, score=0, range=434-453, stra
target 5' CCUUCUUCUCCAUUGCUCAA 3'
::::::::::::::::::::
query 3' GGAAGAAGAGGUAACGAGUU 5'
query=miR7753-3p, target=RO3G_02828, score=0, range=434-453, stra
target 5' CCUUCUUCUCCAUUGCUCAA 3'
::::::::::::::::::::
query 3' GGAAGAAGAGGUAACGAGUU 5'
query=miR7753-3p, target=RO3G_00291, score=0, range=434-453, stra
target 5' CCUUCUUCUCCAUUGCUCAA 3'
::::::::::::::::::::
query 3' GGAAGAAGAGGUAACGAGUU 5'
query=miR7753-3p, target=RO3G_05424, score=0.5, range=434-453,
strand=-1
target 5' UCUUCUUCUCCAUUGCUCAA 3'
.:::::::::::::::::::
query 3' GGAAGAAGAGGUAACGAGUU 5'
query=miR7753-3p, target=RO3G_16427, score=0.5, range=434-453,
strand=-1
target 5' CCUUUUUCUCCAUUGCUCAA 3'
::::.:::::::::::::::
query 3' GGAAGAAGAGGUAACGAGUU 5'
query=miR7753-3p, target=RO3G_10039, score=0.5, range=434-453,
strand=-1
target 5' UCUUCUUCUCCAUUGCUCAA 3'
.:::::::::::::::::::
query 3' GGAAGAAGAGGUAACGAGUU 5'
query=miR7753-3p, target=RO3G_14994, score=2.5, range=434-453,
strand=-1
target 5' UCUUCUUCUGCAUUGCUCAA 3'
.:::::::: ::::::::::
query 3' GGAAGAAGAGGUAACGAGUU 5'
query=miR7753-3p, target=RO3G_09011, score=3.5, range=3563-3581,
strand=1
target 5' UCUU-UUCUCCUUUGCUCAA 3'
.::: :::::: ::::::::
query 3' GGAAGAAGAGGUAACGAGUU 5'
query=miR7753-3p, target=RO3G_00766, score=3.5, range=2407-2425,
strand=-1
target 5' UCUUCUUCUUCAUUGC-CAA 3'
.::::::::.:::::: :::
query 3' GGAAGAAGAGGUAACGAGUU 5'
query=miR7753-3p, target=RO3G_13145, score=3.5, range=518-537,
strand=-1

target 5' UCUACUUCUCCACUGCUCAA 3'
.:: :::::::: :::::::
query 3' GGAAGAAGAGGUAACGAGUU 5'
query=miR7753-3p, target=RO3G_13273, score=3.5, range=518-537, strand=-1
target 5' UCUACUUCUCCACUGCUCAA 3'
.:: :::::::: :::::::
query 3' GGAAGAAGAGGUAACGAGUU 5'
query=miR7753-3p, target=RO3G_10217, score=3.5, range=518-537, strand=-1
target 5' UCUACUUCUCCACUGCUCAA 3'
.:: :::::::: :::::::
query 3' GGAAGAAGAGGUAACGAGUU 5'
query=miR7753-3p, target=RO3G_11538, score=3.5, range=518-537, strand=-1
target 5' UCUACUUCUCCACUGCUCAA 3'
.:: :::::::: :::::::
query 3' GGAAGAAGAGGUAACGAGUU 5'
query=miR7753-3p, target=RO3G_11409, score=3.5, range=518-537, strand=-1
target 5' UCUACUUCUCCACUGCUCAA 3'
.:: :::::::: :::::::
query 3' GGAAGAAGAGGUAACGAGUU 5'
query=miR7753-3p, target=RO3G_07307, score=3.5, range=518-537, strand=-1
target 5' UCUACUUCUCCACUGCUCAA 3'
.:: :::::::: :::::::
query 3' GGAAGAAGAGGUAACGAGUU 5'
query=miR7753-3p, target=RO3G_09930, score=3.5, range=518-537, strand=-1
target 5' UCUACUUCUCCACUGCUCAA 3'
.:: :::::::: :::::::
query 3' GGAAGAAGAGGUAACGAGUU 5'
query=miR7753-3p, target=RO3G_14550, score=3.5, range=518-537, strand=-1
target 5' UCUACUUCUCCACUGCUCAA 3'
.:: :::::::: :::::::
query 3' GGAAGAAGAGGUAACGAGUU 5'
query=miR7753-3p, target=RO3G_15898, score=3.5, range=518-537, strand=-1
target 5' UCUACUUCUCCACUGCUCAA 3'
.:: :::::::: :::::::
query 3' GGAAGAAGAGGUAACGAGUU 5'
query=miR7753-3p, target=RO3G_11969, score=3.5, range=518-537, strand=-1

target 5' UCUACUUCUCCACUGCUCAA 3'
.:: :::::::: :::::::
query 3' GGAAGAAGAGGUAACGAGUU 5'
query=miR7753-3p, target=RO3G_09601, score=3.5, range=518-537, strand=-1
target 5' UCUACUUCUCCACUGCUCAA 3'
.:: :::::::: :::::::
query 3' GGAAGAAGAGGUAACGAGUU 5'
query=miR7753-3p, target=RO3G_11237, score=3.5, range=518-537, strand=-1
target 5' UCUACUUCUCCACUGCUCAA 3'
.:: :::::::: :::::::
query 3' GGAAGAAGAGGUAACGAGUU 5'
query=miR7753-3p, target=RO3G_04683, score=3.5, range=518-537, strand=-1
target 5' UCUACUUCUCCACUGCUCAA 3'
.:: :::::::: :::::::
query 3' GGAAGAAGAGGUAACGAGUU 5'
query=miR7753-3p, target=RO3G_04212, score=3.5, range=518-537, strand=-1
target 5' UCUACUUCUCCACUGCUCAA 3'
.:: :::::::: :::::::
query 3' GGAAGAAGAGGUAACGAGUU 5'
query=miR7753-3p, target=RO3G_07698, score=3.5, range=518-537, strand=-1
target 5' UCUACUUCUCCACUGCUCAA 3'
.:: :::::::: :::::::
query 3' GGAAGAAGAGGUAACGAGUU 5'
query=miR7753-3p, target=RO3G_07721, score=3.5, range=518-537, strand=-1
target 5' UCUACUUCUCCACUGCUCAA 3'
.:: :::::::: :::::::
query 3' GGAAGAAGAGGUAACGAGUU 5'
query=miR7753-3p, target=RO3G_01728, score=3.5, range=476-495, strand=-1
target 5' UCUACUUCUCCACUGCUCAA 3'
.:: :::::::: :::::::
query 3' GGAAGAAGAGGUAACGAGUU 5'
query=miR7753-3p, target=RO3G_16827, score=3.5, range=518-537, strand=-1
target 5' UCUACUUCUCCACUGCUCAA 3'
.:: :::::::: :::::::
query 3' GGAAGAAGAGGUAACGAGUU 5'
query=miR7753-3p, target=RO3G_15150, score=3.5, range=458-477, strand=-1

target 5' UCUACUUCUCCACUGCUCAA 3'
.:: :::::::: :::::::
query 3' GGAAGAAGAGGUAACGAGUU 5'
query=miR7753-3p, target=RO3G_07747, score=3.5, range=1382-1400, strand=-1
target 5' UCUUC-UCUCAAUUGCUCAA 3'
.:::: :::: :::::::::
query 3' GGAAGAAGAGGUAACGAGUU 5'
query=miR7753-3p, target=RO3G_04640, score=4, range=427-447, stra
target 5' UUUUCUUCUCUAUUGUCUCAA 3'
..::::::::.:::: :::::
query 3' GGAAGAAGAGGUAAC-GAGUU 5'
query=miR7753-3p, target=RO3G_16976, score=4, range=2018-2036, strand=1
target 5' UUUUCUUCUUCAUUGC-CAA 3'
..:::::::.:::::: :::
query 3' GGAAGAAGAGGUAACGAGUU 5'
query=miR7753-3p, target=RO3G_04562, score=4, range=1098-1117, strand=1
target 5' ACUUCAUCUCGAUUGCUCAA 3'
:::: :::: :::::::::
query 3' GGAAGAAGAGGUAACGAGUU 5'
query=miR7753-3p, target=RO3G_00546, score=4, range=298-317, stra
target 5' CCUUCUUCUUCAUCGCUUAA 3'
:::::::::.::: :::.::
query 3' GGAAGAAGAGGUAACGAGUU 5'
query=miR7753-3p, target=RO3G_15558, score=4, range=233-253, stra
target 5' CCUUCUUUUCCAAUUGUUCAG 3'
::::::::.::: ::::.:::.
query 3' GGAAGAAGAGG-UAACGAGUU 5'
query=miR7753-3p, target=RO3G_04458, score=4, range=703-722, stra
target 5' UCUCUUUCUUUAUUGCUCAA 3'
.:: .::::..:::::::::
query 3' GGAAGAAGAGGUAACGAGUU 5'
query=miR7753-3p, target=RO3G_02398, score=4, range=2630-2648, strand=-1
target 5' UCUUCUUCUUCAUU-CUCAG 3'
.::::::::.:::: ::::.
query 3' GGAAGAAGAGGUAACGAGUU 5'
query=miR7753-3p, target=RO3G_05246, score=4, range=197-217, stra
target 5' CCUGCUUCUUCAUUGCUACAA 3'
::: :::::.::::::: :::
query 3' GGAAGAAGAGGUAACGA-GUU 5'
query=miR7753-3p, target=RO3G_16749, score=4, range=518-537, stra
target 5' UCUACUUCUCCACUGCUCAG 3'

```
.:: :::::::: ::::::.
query 3' GGAAGAAGAGGUAACGAGUU 5'
query=miR7753-3p, target=RO3G_06731, score=4, range=518-537, stra
target 5' UCUACUUCUCCACUGCUCAG 3'
.:: :::::::: ::::::.
query 3' GGAAGAAGAGGUAACGAGUU 5'
query=miR7753-3p, target=RO3G_06286, score=4, range=634-653, stra
target 5' CCGUCGUCUUUAUUGCUCAA 3'
:: :: :::..:::::::::
query 3' GGAAGAAGAGGUAACGAGUU 5'
query=miR2100-5p, target=RO3G_05925, score=0, range=867-888, stra
target 5' UCGUUUGGCAGACUUGAGAGCA 3'
::::::::::::::::::::::
query 3' AGCAAACCGUCUGAACUCUCGU 5'
query=miR2100-5p, target=RO3G_12075, score=0, range=867-888, stra
target 5' UCGUUUGGCAGACUUGAGAGCA 3'
::::::::::::::::::::::
query 3' AGCAAACCGUCUGAACUCUCGU 5'
query=miR2100-5p, target=RO3G_16708, score=0, range=867-888, stra
target 5' UCGUUUGGCAGACUUGAGAGCA 3'
::::::::::::::::::::::
query 3' AGCAAACCGUCUGAACUCUCGU 5'
query=miR2100-5p, target=RO3G_09951, score=0, range=1125-1146,
strand=-1
target 5' UCGUUUGGCAGACUUGAGAGCA 3'
::::::::::::::::::::::
query 3' AGCAAACCGUCUGAACUCUCGU 5'
query=miR2100-5p, target=RO3G_00684, score=0, range=246-267, stra
target 5' UCGUUUGGCAGACUUGAGAGCA 3'
::::::::::::::::::::::
query 3' AGCAAACCGUCUGAACUCUCGU 5'
query=miR2100-5p, target=RO3G_11952, score=3, range=1245-1266,
strand=-1
target 5' ACGCUUAGCAGACUUGAGAGCA 3'
:: :: :::::::::::::::
query 3' AGCAAACCGUCUGAACUCUCGU 5'
query=miR2100-5p, target=RO3G_12964, score=3, range=867-888, stra
target 5' ACGCUUAGCAGACUUGAGAGCA 3'
:: :: :::::::::::::::
query 3' AGCAAACCGUCUGAACUCUCGU 5'
query=miR2100-5p, target=RO3G_09927, score=3, range=867-888, stra
target 5' ACGCUUAGCAGACUUGAGAGCA 3'
:: :: :::::::::::::::
query 3' AGCAAACCGUCUGAACUCUCGU 5'
query=miR2100-5p, target=RO3G_05927, score=3, range=867-888, stra
```

```
target 5' ACGCUUAGCAGACUUGAGAGCA 3'
:: :: ::::::::::::::
query 3' AGCAAACCGUCUGAACUCUCGU 5'
query=miR2100-5p, target=RO3G_12669, score=3, range=867-888, stra
target 5' ACGCUUAGCAGACUUGAGAGCA 3'
:: :: ::::::::::::::
query 3' AGCAAACCGUCUGAACUCUCGU 5'
query=miR2100-5p, target=RO3G_12961, score=3, range=1200-1221,
strand=-1
target 5' ACGCUUAGCAGACUUGAGAGCA 3'
:: :: ::::::::::::::
query 3' AGCAAACCGUCUGAACUCUCGU 5'
query=miR2100-5p, target=RO3G_05524, score=3, range=867-888, stra
target 5' ACGCUUAGCAGACUUGAGAGCA 3'
:: :: ::::::::::::::
query 3' AGCAAACCGUCUGAACUCUCGU 5'
query=miR6267a, target=RO3G_10719, score=0, range=901-921, strand
target 5' CACACUCUACCACCUCUCUCA 3'
:::::::::::::::::::::
query 3' GUGUGAGAUGGUGGAGAGAGU 5'
query=miR6267a, target=RO3G_01096, score=3, range=212-232, strand
target 5' CACACUCCACCACCUCCCUCA 3'
::::::: :::::::: ::::
query 3' GUGUGAGAUGGUGGAGAGAGU 5'
query=miR6267a, target=RO3G_04411, score=4, range=1135-1154, stra
target 5' CAGGC-CUAUCACCUCUCUCG 3'
:: .: :::.:::::::::.
query 3' GUGUGAGAUGGUGGAGAGAGU 5'
query=miR6267a, target=RO3G_08755, score=4, range=371-390, strand
target 5' CAC-CUCCACCACUUCUUUCA 3'
::: ::: :::::.:::.:::
query 3' GUGUGAGAUGGUGGAGAGAGU 5'
query=miR6267a, target=RO3G_05899, score=4, range=1329-1348, stra
target 5' CAC-CACUACCACCACUCUCA 3'
::: : :::::::: ::::::
query 3' GUGUGAGAUGGUGGAGAGAGU 5'
query=miR6267a, target=RO3G_16063, score=4, range=2-22, strand=-1
target 5' CAAUCUCUAUCAUCUCUCUCA 3'
:: :::::.::.::::::::
query 3' GUGUGAGAUGGUGGAGAGAGU 5'
query=miR6267a, target=RO3G_00821, score=4, range=539-559, strand
target 5' UACGCUCCUCCACCUCUCUCA 3'
.::.::: :::::::::::
query 3' GUGUGAGAUGGUGGAGAGAGU 5'
query=miR6267a, target=RO3G_11293, score=4, range=168-187, strand
```

```
target 5' CAC-CUCUAUCACCUCUGUCA 3'
::: :::::.::::::: :::
query 3' GUGUGAGAUGGUGGAGAGAGU 5'
query=miR6267a, target=RO3G_01095, score=4, range=317-336, strand
target 5' CACUCUUU-CCACCUCUCUCG 3'
::: ::.: :::::::::::.
query 3' GUGUGAGAUGGUGGAGAGAGU 5'
query=miR1044-3p, target=RO3G_16569, score=3.5, range=419-438,
strand=1
target 5' CAAACAGAUAUCUUACCACA 3'
::::::.:::: :.::::::
query 3' GUUUGUUUAUACAGUGGUGU 5'
query=miR1044-3p, target=RO3G_01127, score=3.5, range=738-756,
strand=1
target 5' UGAG-ACAUAUGUCACCACA 3'
..:. : ::::::::::::
query 3' GUUUGUUUAUACAGUGGUGU 5'
query=miR1044-3p, target=RO3G_15999, score=4, range=425-444, stra
target 5' GAAAAGAAUAUGUUACCACG 3'
::: .:::::::.:::::.
query 3' GUUUGUUUAUACAGUGGUGU 5'
query=miR1044-3p, target=RO3G_02003, score=4, range=1002-1021,
strand=-1
target 5' CAAGCGAGUCUGUCACCACA 3'
:::.:.:.: :::::::::
query 3' GUUUGUUUAUACAGUGGUGU 5'
query=miR1044-3p, target=RO3G_00409, score=4, range=220-240, stra
target 5' CAACACAAAUAUGUUAUCACC 3'
::: ::::::::::.:.:::
query 3' GUU-UGUUUAUACAGUGGUGU 5'
query=miR1044-3p, target=RO3G_04064, score=4, range=697-716, stra
target 5' UUAUCCGAUAUGUCACCACA 3'
. : : .:::::::::::::
query 3' GUUUGUUUAUACAGUGGUGU 5'
query=miR1044-3p, target=RO3G_07362, score=4, range=739-757, stra
target 5' CAAACAAA-AUGACACCACA 3'
:::::::: ::: :::::::
query 3' GUUUGUUUAUACAGUGGUGU 5'
query=miR5526, target=RO3G_17296, score=0, range=189-207, strand=
target 5' CUCAUACCUGACUCAACUC 3'
:::::::::::::::::::
query 3' GAGUAUGGACUGAGUUGAG 5'
query=miR5526, target=RO3G_04752, score=3, range=4864-4882, stran
target 5' UUAGUACCUGACUUAACUC 3'
.: .::::::::.:::::
```

query 3' GAGUAUGGACUGAGUUGAG 5'
query=miR5526, target=RO3G_15506, score=3, range=623-640, strand=
target 5' CCCA-ACCUGACUUAACUC 3'
: :: :::::::.:::::
query 3' GAGUAUGGACUGAGUUGAG 5'
query=miR5526, target=RO3G_02574, score=3.5, range=441-459, stran
target 5' CUCAUGUCUGGUUCAACUC 3'
:::::..:::..:::::::
query 3' GAGUAUGGACUGAGUUGAG 5'
query=miR5526, target=RO3G_10923, score=3.5, range=2912-2930,
strand=-1
target 5' UCCAUAUCUGACUCAACUA 3'
. ::::.:::::::::::
query 3' GAGUAUGGACUGAGUUGAG 5'
query=miR5526, target=RO3G_09283, score=3.5, range=1757-1774,
strand=-1
target 5' CUUCU-CUUGACUCAACUC 3'
::. : :.::::::::::
query 3' GAGUAUGGACUGAGUUGAG 5'
query=miR5526, target=RO3G_06462, score=4, range=1278-1296, stran
target 5' CUCCUGCUUGGCUCAACUU 3'
::: :.:.::.:::::::.
query 3' GAGUAUGGACUGAGUUGAG 5'
query=miR5526, target=RO3G_05580, score=4, range=40-57, strand=1
target 5' CUUA-ACUUGACUCAAUUU 3'
::.: ::.::::::::.:.
query 3' GAGUAUGGACUGAGUUGAG 5'
query=miR5526, target=RO3G_16064, score=4, range=233-250, strand=
target 5' CUCA-ACCUGACUCAAUCC 3'
:::: :::::::::::. :
query 3' GAGUAUGGACUGAGUUGAG 5'
query=miR5526, target=RO3G_06908, score=4, range=56-73, strand=1
target 5' CUC-UUCCUGAUUCAAUUC 3'
::: : :::::.::::.::
query 3' GAGUAUGGACUGAGUUGAG 5'
query=miR5526, target=RO3G_03913, score=4, range=141-159, strand=
target 5' CAGCUACCUGACUCAACUA 3'
: ::::::::::::::
query 3' GAGUAUGGACUGAGUUGAG 5'
query=miR5526, target=RO3G_12386, score=4, range=43-60, strand=1
target 5' AUCA-ACCUGAGUCAACUC 3'
::: :::::: :::::::
query 3' GAGUAUGGACUGAGUUGAG 5'
query=miR5526, target=RO3G_08080, score=4, range=13-30, strand=-1
target 5' CUU-UGACUGACUCAACUC 3'

```
::. :. :::::::::::
query 3' GAGUAUGGACUGAGUUGAG 5'
query=miR5526, target=RO3G_08626, score=4, range=562-579, strand=
target 5' AACA-ACCUGACUCAAUUC 3'
:: :::::::::::.::
query 3' GAGUAUGGACUGAGUUGAG 5'
query=miR2108b, target=RO3G_04592, score=0, range=1276-1295, stra
target 5' ACUACAAACACAUACAUUAA 3'
::::::::::::::::::::
query 3' UGAUGUUUGUGUAUGUAAUU 5'
query=miR2108b, target=RO3G_00289, score=1, range=832-851, strand
target 5' ACUACAAACACAUACAUUGA 3'
::::::::::::::::::.:
query 3' UGAUGUUUGUGUAUGUAAUU 5'
query=miR2108b, target=RO3G_09183, score=1, range=1237-1256, stra
target 5' ACUACAAACACAUACAUUGA 3'
::::::::::::::::::.:
query 3' UGAUGUUUGUGUAUGUAAUU 5'
query=miR2108b, target=RO3G_17351, score=1, range=832-851, strand
target 5' ACUACAAACACAUACAUUGA 3'
::::::::::::::::::.:
query 3' UGAUGUUUGUGUAUGUAAUU 5'
query=miR2108b, target=RO3G_10735, score=1, range=1549-1568, stra
target 5' ACUACAAACACAUACAUUGA 3'
::::::::::::::::::.:
query 3' UGAUGUUUGUGUAUGUAAUU 5'
query=miR2108b, target=RO3G_03585, score=1, range=694-713, strand
target 5' ACUACAAACACAUACAUUGA 3'
::::::::::::::::::.:
query 3' UGAUGUUUGUGUAUGUAAUU 5'
query=miR2108b, target=RO3G_10040, score=1, range=694-713, strand
target 5' ACUACAAACACAUACAUUGA 3'
::::::::::::::::::.:
query 3' UGAUGUUUGUGUAUGUAAUU 5'
query=miR2108b, target=RO3G_17257, score=1, range=2020-2039, stra
target 5' ACUACAAACACAUACAUUGA 3'
::::::::::::::::::.:
query 3' UGAUGUUUGUGUAUGUAAUU 5'
query=miR2108b, target=RO3G_15889, score=1, range=2020-2039, stra
target 5' ACUACAAACACAUACAUUGA 3'
::::::::::::::::::.:
query 3' UGAUGUUUGUGUAUGUAAUU 5'
query=miR2108b, target=RO3G_05424, score=1, range=2020-2039, stra
target 5' ACUACAAACACAUACAUUGA 3'
::::::::::::::::::.:
```

query 3' UGAUGUUUGUGUAUGUAAUU 5'
query=miR2108b, target=RO3G_00872, score=2.5, range=640-659, stra
target 5' ACAGAAAACACAUACAUUAA 3'
:: . ::::::::::::::
query 3' UGAUGUUUGUGUAUGUAAUU 5'
query=miR2108b, target=RO3G_09728, score=3.5, range=155-174, stra
target 5' AUUACAAACAUAUACAUAAA 3'
:.:::::::.:::::: ::
query 3' UGAUGUUUGUGUAUGUAAUU 5'
query=miR2108b, target=RO3G_07697, score=3.5, range=430-449, stra
target 5' AUAACAAAUACAUACAUUGA 3'
:. ::::.::::::::.:
query 3' UGAUGUUUGUGUAUGUAAUU 5'
query=miR2108b, target=RO3G_04681, score=3.5, range=694-713, stra
target 5' AUAACAAAUACAUACAUUGA 3'
:. ::::.::::::::.:
query 3' UGAUGUUUGUGUAUGUAAUU 5'
query=miR2108b, target=RO3G_16136, score=3.5, range=2020-2039,
strand=1
target 5' AUAACAAAUACAUACAUUGA 3'
:. ::::.::::::::.:
query 3' UGAUGUUUGUGUAUGUAAUU 5'
query=miR2108b, target=RO3G_10217, score=3.5, range=2104-2123,
strand=1
target 5' AUAACAAAUACAUACAUUGA 3'
:. ::::.::::::::.:
query 3' UGAUGUUUGUGUAUGUAAUU 5'
query=miR2108b, target=RO3G_16749, score=3.5, range=2104-2123,
strand=1
target 5' AUAACAAAUACAUACAUUGA 3'
:. ::::.::::::::.:
query 3' UGAUGUUUGUGUAUGUAAUU 5'
query=miR2108b, target=RO3G_13273, score=3.5, range=2104-2123,
strand=1
target 5' AUAACAAAUACAUACAUUGA 3'
:. ::::.::::::::.:
query 3' UGAUGUUUGUGUAUGUAAUU 5'
query=miR2108b, target=RO3G_13145, score=3.5, range=2104-2123,
strand=1
target 5' AUAACAAAUACAUACAUUGA 3'
:. ::::.::::::::.:
query 3' UGAUGUUUGUGUAUGUAAUU 5'
query=miR2108b, target=RO3G_09758, score=4, range=3946-3965, stra
target 5' AUUACAAAUACAAACAUUAG 3'
:.::::::.::: ::::::.

```
query 3' UGAUGUUUGUGUAUGUAAUU 5'
query=miR2108b, target=RO3G_04056, score=4, range=1187-1206, stra
target 5' AGUAUGAACACGUACAUUGA 3'
: ::..::::.::::::.:
query 3' UGAUGUUUGUGUAUGUAAUU 5'
query=miR2108b, target=RO3G_00433, score=4, range=739-758, strand
target 5' UCUUCAAACAUGUACAUUAA 3'
:: ::::::..::::::::
query 3' UGAUGUUUGUGUAUGUAAUU 5'
query=miR2108b, target=RO3G_15942, score=4, range=346-365, strand
target 5' ACGACAAACGCAUAGAUUAA 3'
:: ::::::.:::: :::::
query 3' UGAUGUUUGUGUAUGUAAUU 5'
query=miR2108b, target=RO3G_13950, score=4, range=695-713, strand
target 5' GCUG-AAGCACAUACAUUAU 3'
.::. ::.:::::::::::
query 3' UGAUGUUUGUGUAUGUAAUU 5'
query=miR2867-3p, target=RO3G_05953, score=0, range=796-815, stra
target 5' UGGCCAUCCCAAAGUCCUGA 3'
::::::::::::::::::::
query 3' ACCGGUAGGGUUUCAGGACU 5'
query=miR2867-3p, target=RO3G_17069, score=0, range=661-680, stra
target 5' UGGCCAUCCCAAAGUCCUGA 3'
::::::::::::::::::::
query 3' ACCGGUAGGGUUUCAGGACU 5'
query=miR2867-3p, target=RO3G_14568, score=0, range=796-815, stra
target 5' UGGCCAUCCCAAAGUCCUGA 3'
::::::::::::::::::::
query 3' ACCGGUAGGGUUUCAGGACU 5'
query=miR2867-3p, target=RO3G_09967, score=0, range=796-815, stra
target 5' UGGCCAUCCCAAAGUCCUGA 3'
::::::::::::::::::::
query 3' ACCGGUAGGGUUUCAGGACU 5'
query=miR2867-3p, target=RO3G_00256, score=0, range=796-815, stra
target 5' UGGCCAUCCCAAAGUCCUGA 3'
::::::::::::::::::::
query 3' ACCGGUAGGGUUUCAGGACU 5'
query=miR2867-3p, target=RO3G_17449, score=0, range=796-815, stra
target 5' UGGCCAUCCCAAAGUCCUGA 3'
::::::::::::::::::::
query 3' ACCGGUAGGGUUUCAGGACU 5'
query=miR2867-3p, target=RO3G_14361, score=0, range=796-815, stra
target 5' UGGCCAUCCCAAAGUCCUGA 3'
::::::::::::::::::::
query 3' ACCGGUAGGGUUUCAGGACU 5'
```

```
query=miR2867-3p, target=RO3G_07860, score=0, range=796-815, stra
target 5' UGGCCAUCCCAAAGUCCUGA 3'
::::::::::::::::::::
query 3' ACCGGUAGGGUUUCAGGACU 5'
query=miR2867-3p, target=RO3G_05267, score=0, range=796-815, stra
target 5' UGGCCAUCCCAAAGUCCUGA 3'
::::::::::::::::::::
query 3' ACCGGUAGGGUUUCAGGACU 5'
query=miR2867-3p, target=RO3G_05921, score=0, range=796-815, stra
target 5' UGGCCAUCCCAAAGUCCUGA 3'
::::::::::::::::::::
query 3' ACCGGUAGGGUUUCAGGACU 5'
query=miR2867-3p, target=RO3G_11966, score=0, range=796-815, stra
target 5' UGGCCAUCCCAAAGUCCUGA 3'
::::::::::::::::::::
query 3' ACCGGUAGGGUUUCAGGACU 5'
query=miR2867-3p, target=RO3G_05959, score=0, range=796-815, stra
target 5' UGGCCAUCCCAAAGUCCUGA 3'
::::::::::::::::::::
query 3' ACCGGUAGGGUUUCAGGACU 5'
query=miR2867-3p, target=RO3G_10729, score=0.5, range=796-815,
strand=1
target 5' UGGCUAUCCCAAAGUCCUGA 3'
::::.:::::::::::::::
query 3' ACCGGUAGGGUUUCAGGACU 5'
query=miR2867-3p, target=RO3G_04207, score=1, range=796-815, stra
target 5' UGGCCAUCCCAAAGUUCUGA 3'
:::::::::::::::.::::
query 3' ACCGGUAGGGUUUCAGGACU 5'
query=miR2867-3p, target=RO3G_09945, score=1, range=520-539, stra
target 5' UGGACAUCCCAAAGUCCUGA 3'
::: ::::::::::::::::
query 3' ACCGGUAGGGUUUCAGGACU 5'
query=miR2867-3p, target=RO3G_01942, score=1, range=793-812, stra
target 5' UGGACAUCCCAAAGUCCUGA 3'
::: ::::::::::::::::
query 3' ACCGGUAGGGUUUCAGGACU 5'
query=miR2867-3p, target=RO3G_09194, score=1, range=796-815, stra
target 5' UGGACAUCCCAAAGUCCUGA 3'
::: ::::::::::::::::
query 3' ACCGGUAGGGUUUCAGGACU 5'
query=miR2867-3p, target=RO3G_04210, score=2, range=796-815, stra
target 5' UGGCCAUCCCAAAGCCCUGA 3'
:::::::::::::: :::::
query 3' ACCGGUAGGGUUUCAGGACU 5'
```

query=miR2867-3p, target=RO3G_10780, score=2, range=796-815, stra
target 5' UGGCCAUCCCAAAGCCCUGA 3'
:::::::::::::: :::::
query 3' ACCGGUAGGGUUUCAGGACU 5'
query=miR2867-3p, target=RO3G_10773, score=2, range=796-815, stra
target 5' UGGCCAUCCCAAAGCCCUGA 3'
:::::::::::::: :::::
query 3' ACCGGUAGGGUUUCAGGACU 5'
query=miR2867-3p, target=RO3G_08835, score=2, range=106-125, stra
target 5' UGGCCAGCUCAAAGUCCUGA 3'
:::::: :.:::::::::::
query 3' ACCGGUAGGGUUUCAGGACU 5'
query=miR2867-3p, target=RO3G_11244, score=2, range=796-815, stra
target 5' UGGCCAGCUCAAAGUCCUGA 3'
:::::: :.:::::::::::
query 3' ACCGGUAGGGUUUCAGGACU 5'
query=miR2867-3p, target=RO3G_01313, score=2, range=796-815, stra
target 5' UGGCCAGCUCAAAGUCCUGA 3'
:::::: :.:::::::::::
query 3' ACCGGUAGGGUUUCAGGACU 5'
query=miR2867-3p, target=RO3G_13447, score=2, range=796-815, stra
target 5' UGGCCAGCUCAAAGUCCUGA 3'
:::::: :.:::::::::::
query 3' ACCGGUAGGGUUUCAGGACU 5'
query=miR2867-3p, target=RO3G_15154, score=2, range=796-815, stra
target 5' UGGCCAGCUCAAAGUCCUGA 3'
:::::: :.:::::::::::
query 3' ACCGGUAGGGUUUCAGGACU 5'
query=miR2867-3p, target=RO3G_10720, score=2, range=796-815, stra
target 5' UGGCCAGCUCAAAGUCCUGA 3'
:::::: :.:::::::::::
query 3' ACCGGUAGGGUUUCAGGACU 5'
query=miR2867-3p, target=RO3G_10044, score=2, range=796-815, stra
target 5' UGGCCAGCUCAAAGUCCUGA 3'
:::::: :.:::::::::::
query 3' ACCGGUAGGGUUUCAGGACU 5'
query=miR2867-3p, target=RO3G_03987, score=2, range=796-815, stra
target 5' UGGCCAGCUCAAAGUCCUGA 3'
:::::: :.:::::::::::
query 3' ACCGGUAGGGUUUCAGGACU 5'
query=miR2867-3p, target=RO3G_03558, score=2, range=796-815, stra
target 5' UGGCCAGCUCAAAGUCCUGA 3'
:::::: :.:::::::::::
query 3' ACCGGUAGGGUUUCAGGACU 5'
query=miR2867-3p, target=RO3G_01951, score=2, range=796-815, stra

```
target 5' UGGCCAGCUCAAAGUCCUGA 3'
:::::: :.:::::::::::
query 3' ACCGGUAGGGUUUCAGGACU 5'
query=miR2867-3p, target=RO3G_01194, score=2, range=796-815, stra
target 5' UGGCCAGCUCAAAGUCCUGA 3'
:::::: :.:::::::::::
query 3' ACCGGUAGGGUUUCAGGACU 5'
query=miR2867-3p, target=RO3G_09924, score=2, range=796-815, stra
target 5' UGGCCAGCUCAAAGUCCUGA 3'
:::::: :.:::::::::::
query 3' ACCGGUAGGGUUUCAGGACU 5'
query=miR2867-3p, target=RO3G_05916, score=2, range=796-815, stra
target 5' UGGCCAGCUCAAAGUCCUGA 3'
:::::: :.:::::::::::
query 3' ACCGGUAGGGUUUCAGGACU 5'
query=miR2867-3p, target=RO3G_11948, score=2, range=796-815, stra
target 5' UGGCCAGCUCAAAGUCCUGA 3'
:::::: :.:::::::::::
query 3' ACCGGUAGGGUUUCAGGACU 5'
query=miR2867-3p, target=RO3G_15873, score=2, range=796-815, stra
target 5' UGGCCAGCUCAAAGUCCUGA 3'
:::::: :.:::::::::::
query 3' ACCGGUAGGGUUUCAGGACU 5'
query=miR2867-3p, target=RO3G_17409, score=2, range=796-815, stra
target 5' UGGCCAGCUCAAAGUCCUGA 3'
:::::: :.:::::::::::
query 3' ACCGGUAGGGUUUCAGGACU 5'
query=miR2867-3p, target=RO3G_07683, score=2, range=796-815, stra
target 5' UGGCCAGCUCAAAGUCCUGA 3'
:::::: :.:::::::::::
query 3' ACCGGUAGGGUUUCAGGACU 5'
query=miR2867-3p, target=RO3G_07994, score=2, range=796-815, stra
target 5' UGGCCAGCUCAAAGUCCUGA 3'
:::::: :.:::::::::::
query 3' ACCGGUAGGGUUUCAGGACU 5'
query=miR2867-3p, target=RO3G_13706, score=2, range=754-773, stra
target 5' UGGCCAGCUCAAAGUCCUGA 3'
:::::: :.:::::::::::
query 3' ACCGGUAGGGUUUCAGGACU 5'
query=miR2867-3p, target=RO3G_12526, score=2, range=796-815, stra
target 5' UGGCCAGCUCAAAGUCCUGA 3'
:::::: :.:::::::::::
query 3' ACCGGUAGGGUUUCAGGACU 5'
query=miR2867-3p, target=RO3G_01950, score=2, range=796-815, stra
target 5' UGGCCAGCUCAAAGUCCUGA 3'
```

```
:::::: :.:::::::::::
query 3' ACCGGUAGGGUUUCAGGACU 5'
query=miR2867-3p, target=RO3G_09199, score=2, range=796-815, stra
target 5' UGGCCAGCUCAAAGUCCUGA 3'
:::::: :.:::::::::::
query 3' ACCGGUAGGGUUUCAGGACU 5'
query=miR2867-3p, target=RO3G_12174, score=2, range=121-140, stra
target 5' UGGCCAGCUCAAAGUCCUGA 3'
:::::: :.:::::::::::
query 3' ACCGGUAGGGUUUCAGGACU 5'
query=miR2867-3p, target=RO3G_17213, score=2, range=121-140, stra
target 5' UGGCCAGCUCAAAGUCCUGA 3'
:::::: :.:::::::::::
query 3' ACCGGUAGGGUUUCAGGACU 5'
query=miR2867-3p, target=RO3G_17211, score=2, range=796-815, stra
target 5' UGGCCAGCUCAAAGUCCUGA 3'
:::::: :.:::::::::::
query 3' ACCGGUAGGGUUUCAGGACU 5'
query=miR2867-3p, target=RO3G_12013, score=2, range=121-140, stra
target 5' UGGCCAGCUCAAAGUCCUGA 3'
:::::: :.:::::::::::
query 3' ACCGGUAGGGUUUCAGGACU 5'
query=miR2867-3p, target=RO3G_07119, score=2, range=121-140, stra
target 5' UGGCCAGCUCAAAGUCCUGA 3'
:::::: :.:::::::::::
query 3' ACCGGUAGGGUUUCAGGACU 5'
query=miR2867-3p, target=RO3G_10528, score=2, range=121-140, stra
target 5' UGGCCAGCUCAAAGUCCUGA 3'
:::::: :.:::::::::::
query 3' ACCGGUAGGGUUUCAGGACU 5'
query=miR2867-3p, target=RO3G_11263, score=2, range=121-140, stra
target 5' UGGCCAGCUCAAAGUCCUGA 3'
:::::: :.:::::::::::
query 3' ACCGGUAGGGUUUCAGGACU 5'
query=miR2867-3p, target=RO3G_10742, score=2, range=121-140, stra
target 5' UGGCCAGCUCAAAGUCCUGA 3'
:::::: :.:::::::::::
query 3' ACCGGUAGGGUUUCAGGACU 5'
query=miR2867-3p, target=RO3G_11528, score=2, range=121-140, stra
target 5' UGGCCAGCUCAAAGUCCUGA 3'
:::::: :.:::::::::::
query 3' ACCGGUAGGGUUUCAGGACU 5'
query=miR2867-3p, target=RO3G_07295, score=2, range=628-647, stra
target 5' UGGCCAGCUCAAAGUCCUGA 3'
:::::: :.:::::::::::
```

```
query 3' ACCGGUAGGGUUUCAGGACU 5'
query=miR2867-3p, target=RO3G_13276, score=2, range=178-197, stra
target 5' UGGCCAGCUCAAAGUCCUGA 3'
:::::: :.::::::::::
query 3' ACCGGUAGGGUUUCAGGACU 5'
query=miR2867-3p, target=RO3G_01735, score=2, range=796-815, stra
target 5' UGGCCAGCUCAAAGUCCUGA 3'
:::::: :.::::::::::
query 3' ACCGGUAGGGUUUCAGGACU 5'
query=miR2867-3p, target=RO3G_03617, score=2, range=796-815, stra
target 5' UGGCCAGCUCAAAGUCCUGA 3'
:::::: :.::::::::::
query 3' ACCGGUAGGGUUUCAGGACU 5'
query=miR2867-3p, target=RO3G_16118, score=2, range=64-83, strand
target 5' UGGCCAGCUCAAAGUCCUGA 3'
:::::: :.::::::::::
query 3' ACCGGUAGGGUUUCAGGACU 5'
query=miR2867-3p, target=RO3G_10537, score=2, range=619-638, stra
target 5' UGGCCAGCUCAAAGUCCUGA 3'
:::::: :.::::::::::
query 3' ACCGGUAGGGUUUCAGGACU 5'
query=miR2867-3p, target=RO3G_09542, score=2.5, range=796-815,
strand=1
target 5' UGGCCAUCCCAAAGCCCUGG 3'
:::::::::::::: ::::.
query 3' ACCGGUAGGGUUUCAGGACU 5'
query=miR2867-3p, target=RO3G_16377, score=3, range=121-140, stra
target 5' UGGCCAGCUCAAAGUUCUGA 3'
:::::: :.:::::::.::::
query 3' ACCGGUAGGGUUUCAGGACU 5'
query=miR2867-3p, target=RO3G_17406, score=3, range=157-176, stra
target 5' UGGCCAGCCCAAAGUCCCGA 3'
:::::: ::::::::: ::
query 3' ACCGGUAGGGUUUCAGGACU 5'
query=miR2867-3p, target=RO3G_05950, score=3, range=274-293, stra
target 5' UGGCCAGCCCAAAGUCCCGA 3'
:::::: ::::::::: ::
query 3' ACCGGUAGGGUUUCAGGACU 5'
query=miR2867-3p, target=RO3G_16715, score=3, range=256-275, stra
target 5' UGGCCAGCCCAAAGUCCCGA 3'
:::::: ::::::::: ::
query 3' ACCGGUAGGGUUUCAGGACU 5'
query=miR2867-3p, target=RO3G_03605, score=3, range=1258-1277,
strand=1
target 5' UGGCCAGCCCAAAGUCCCGA 3'
```

```
:::::: :::::::::: ::
query 3' ACCGGUAGGGUUUCAGGACU 5'
query=miR2867-3p, target=RO3G_00677, score=3, range=358-377, stra
target 5' UGGCCAGCCCAAAGUCCCGA 3'
:::::: :::::::::: ::
query 3' ACCGGUAGGGUUUCAGGACU 5'
query=miR2867-3p, target=RO3G_01588, score=3, range=136-155, stra
target 5' UGGCCAGCCCAAAGUCCCGA 3'
:::::: :::::::::: ::
query 3' ACCGGUAGGGUUUCAGGACU 5'
query=miR2867-3p, target=RO3G_14620, score=3, range=241-260, stra
target 5' UGGCCUGCUCAAAGUCCUGA 3'
::::: :.:::::::::::
query 3' ACCGGUAGGGUUUCAGGACU 5'
query=miR2867-3p, target=RO3G_03785, score=3, range=418-437, stra
target 5' UGGCCUGCUCAAAGUCCUGA 3'
::::: :.:::::::::::
query 3' ACCGGUAGGGUUUCAGGACU 5'
query=miR2867-3p, target=RO3G_10224, score=3, range=613-632, stra
target 5' UGGCCUGCUCAAAGUCCUGA 3'
::::: :.:::::::::::
query 3' ACCGGUAGGGUUUCAGGACU 5'
query=miR2867-3p, target=RO3G_07302, score=3, range=613-632, stra
target 5' UGGCCUGCUCAAAGUCCUGA 3'
::::: :.:::::::::::
query 3' ACCGGUAGGGUUUCAGGACU 5'
query=miR2867-3p, target=RO3G_09944, score=3, range=613-632, stra
target 5' UGGCCUGCUCAAAGUCCUGA 3'
::::: :.:::::::::::
query 3' ACCGGUAGGGUUUCAGGACU 5'
query=miR2867-3p, target=RO3G_00247, score=3, range=613-632, stra
target 5' UGGCCUGCUCAAAGUCCUGA 3'
::::: :.:::::::::::
query 3' ACCGGUAGGGUUUCAGGACU 5'
query=miR2867-3p, target=RO3G_17436, score=3, range=613-632, stra
target 5' UGGCCUGCUCAAAGUCCUGA 3'
::::: :.:::::::::::
query 3' ACCGGUAGGGUUUCAGGACU 5'
query=miR2867-3p, target=RO3G_07689, score=3, range=613-632, stra
target 5' UGGCCUGCUCAAAGUCCUGA 3'
::::: :.:::::::::::
query 3' ACCGGUAGGGUUUCAGGACU 5'
query=miR2867-3p, target=RO3G_17293, score=3, range=613-632, stra
target 5' UGGCCUGCUCAAAGUCCUGA 3'
::::: :.:::::::::::
```

```
query 3' ACCGGUAGGGUUUCAGGACU 5'
query=miR2867-3p, target=RO3G_11405, score=3, range=613-632, stra
target 5' UGGCCUGCUCAAAGUCCUGA 3'
::::: :.::::::::::
query 3' ACCGGUAGGGUUUCAGGACU 5'
query=miR2867-3p, target=RO3G_15672, score=3, range=613-632, stra
target 5' UGGCCUGCUCAAAGUCCUGA 3'
::::: :.::::::::::
query 3' ACCGGUAGGGUUUCAGGACU 5'
query=miR2867-3p, target=RO3G_12670, score=3, range=613-632, stra
target 5' UGGCCUGCUCAAAGUCCUGA 3'
::::: :.::::::::::
query 3' ACCGGUAGGGUUUCAGGACU 5'
query=miR2867-3p, target=RO3G_04200, score=3, range=613-632, stra
target 5' UGGCCUGCUCAAAGUCCUGA 3'
::::: :.::::::::::
query 3' ACCGGUAGGGUUUCAGGACU 5'
query=miR2867-3p, target=RO3G_04654, score=3, range=613-632, stra
target 5' UGGCCUGCUCAAAGUCCUGA 3'
::::: :.::::::::::
query 3' ACCGGUAGGGUUUCAGGACU 5'
query=miR2867-3p, target=RO3G_14772, score=3, range=691-710, stra
target 5' UGGCCUGCUCAAAGUCCUGA 3'
::::: :.::::::::::
query 3' ACCGGUAGGGUUUCAGGACU 5'
query=miR2867-3p, target=RO3G_10226, score=3, range=613-632, stra
target 5' UGGCCUGCUCAAAGUCCUGA 3'
::::: :.::::::::::
query 3' ACCGGUAGGGUUUCAGGACU 5'
query=miR2867-3p, target=RO3G_12082, score=3, range=691-710, stra
target 5' UGGCCUGCUCAAAGUCCUGA 3'
::::: :.::::::::::
query 3' ACCGGUAGGGUUUCAGGACU 5'
query=miR2867-3p, target=RO3G_03797, score=3, range=301-320, stra
target 5' UGGCCUGCUCAAAGUCCUGA 3'
::::: :.::::::::::
query 3' ACCGGUAGGGUUUCAGGACU 5'
query=miR2867-3p, target=RO3G_06721, score=3, range=610-629, stra
target 5' UGGCCUGCUCAAAGUCCUGA 3'
::::: :.::::::::::
query 3' ACCGGUAGGGUUUCAGGACU 5'
query=miR2867-3p, target=RO3G_16548, score=3, range=451-470, stra
target 5' UUGCCAGCUCAAAGUCCUGA 3'
: :::: :.::::::::::
query 3' ACCGGUAGGGUUUCAGGACU 5'
```

```
query=miR2867-3p, target=RO3G_14987, score=3, range=460-479, stra
target 5' UGGCCUGCUCAAAGUCCUGA 3'
::::: :.:::::::::::
query 3' ACCGGUAGGGUUUCAGGACU 5'
query=miR2867-3p, target=RO3G_17204, score=3, range=676-695, stra
target 5' UGGCCUGCUCAAAGUCCUGA 3'
::::: :.:::::::::::
query 3' ACCGGUAGGGUUUCAGGACU 5'
query=miR2867-3p, target=RO3G_08649, score=3, range=558-577, stra
target 5' UGGCCUGCUCAAAGUCCUGA 3'
::::: :.:::::::::::
query 3' ACCGGUAGGGUUUCAGGACU 5'
query=miR2867-3p, target=RO3G_11757, score=3.5, range=238-257,
strand=1
target 5' UGGCCUGUCCAAAGUCCUGG 3'
::::: .:::::::::::.
query 3' ACCGGUAGGGUUUCAGGACU 5'
query=miR2867-3p, target=RO3G_11765, score=3.5, range=238-257,
strand=1
target 5' UGGCCUGUCCAAAGUCCUGG 3'
::::: .:::::::::::.
query 3' ACCGGUAGGGUUUCAGGACU 5'
query=miR2867-3p, target=RO3G_12030, score=3.5, range=360-379,
strand=-1
target 5' UGAUCAUCCCAAAGUCUUGU 3'
:: .:::::::::::.::
query 3' ACCGGUAGGGUUUCAGGACU 5'
query=miR2867-3p, target=RO3G_15271, score=4, range=796-815, stra
target 5' UGGCCAUCACAAAGUCCAGA 3'
:::::::: :::::::: ::
query 3' ACCGGUAGGGUUUCAGGACU 5'
query=miR2867-3p, target=RO3G_01962, score=4, range=547-566, stra
target 5' UGGCCAUCACAAAGUCCAGA 3'
:::::::: :::::::: ::
query 3' ACCGGUAGGGUUUCAGGACU 5'
query=miR2867-3p, target=RO3G_16725, score=4, range=538-557, stra
target 5' UGGCCAUCACAAAGUCCAGA 3'
:::::::: :::::::: ::
query 3' ACCGGUAGGGUUUCAGGACU 5'
query=miR2867-3p, target=RO3G_13714, score=4, range=796-815, stra
target 5' UGGCCAUCACAAAGUCCAGA 3'
:::::::: :::::::: ::
query 3' ACCGGUAGGGUUUCAGGACU 5'
query=miR2867-3p, target=RO3G_16742, score=4, range=796-815, stra
target 5' UGGCCAUCACAAAGUCCAGA 3'
```

```
:::::::: :::::::: ::
query 3' ACCGGUAGGGUUUCAGGACU 5'
query=miR2867-3p, target=RO3G_04674, score=4, range=547-566, stra
target 5' UGGCCAUCACAAAGUCCAGA 3'
:::::::: :::::::: ::
query 3' ACCGGUAGGGUUUCAGGACU 5'
query=miR2867-3p, target=RO3G_17416, score=4, range=796-815, stra
target 5' UGGCCAUCACAAAGUCCAGA 3'
:::::::: :::::::: ::
query 3' ACCGGUAGGGUUUCAGGACU 5'
query=miR2867-3p, target=RO3G_02824, score=4, range=796-815, stra
target 5' UGGCCAUCACAAAGUCCAGA 3'
:::::::: :::::::: ::
query 3' ACCGGUAGGGUUUCAGGACU 5'
query=miR2867-3p, target=RO3G_17291, score=4, range=796-815, stra
target 5' UGGCCAUCACAAAGUCCAGA 3'
:::::::: :::::::: ::
query 3' ACCGGUAGGGUUUCAGGACU 5'
query=miR2867-3p, target=RO3G_07702, score=4, range=547-566, stra
target 5' UGGCCAUCGCAAAGUCCAGA 3'
:::::::: :::::::: ::
query 3' ACCGGUAGGGUUUCAGGACU 5'
query=miR2867-3p, target=RO3G_16420, score=4, range=547-566, stra
target 5' UGGCCAUCGCAAAGUCCAGA 3'
:::::::: :::::::: ::
query 3' ACCGGUAGGGUUUCAGGACU 5'
query=miR2867-3p, target=RO3G_07867, score=4, range=547-566, stra
target 5' UGGCCAUCACAAAGUCCAGA 3'
:::::::: :::::::: ::
query 3' ACCGGUAGGGUUUCAGGACU 5'
query=miR2867-3p, target=RO3G_16372, score=4, range=796-815, stra
target 5' UGGCCAUCACAAAGUCCAGA 3'
:::::::: :::::::: ::
query 3' ACCGGUAGGGUUUCAGGACU 5'
query=miR2867-3p, target=RO3G_07705, score=4, range=796-815, stra
target 5' UGGCCAUCGCAAAGUCCAGA 3'
:::::::: :::::::: ::
query 3' ACCGGUAGGGUUUCAGGACU 5'
query=miR2867-3p, target=RO3G_01328, score=4, range=796-815, stra
target 5' UGGCCAUCACAAAGUCCAGA 3'
:::::::: :::::::: ::
query 3' ACCGGUAGGGUUUCAGGACU 5'
query=miR2867-3p, target=RO3G_15282, score=4, range=520-539, stra
target 5' UGGCCAUCGCAAAGUCCAGA 3'
:::::::: :::::::: ::
```

query 3' ACCGGUAGGGUUUCAGGACU 5'
query=miR2867-3p, target=RO3G_17387, score=4, range=796-815, stra
target 5' UGGCCAUCACAAAGUCCAGA 3'
:::::::: :::::::: ::
query 3' ACCGGUAGGGUUUCAGGACU 5'
query=miR2867-3p, target=RO3G_17399, score=4, range=796-815, stra
target 5' UGGCCAUCACAAAGUCCAGA 3'
:::::::: :::::::: ::
query 3' ACCGGUAGGGUUUCAGGACU 5'
query=miR2867-3p, target=RO3G_06717, score=4, range=796-815, stra
target 5' UGGCCAUCACAAAGUCCAGA 3'
:::::::: :::::::: ::
query 3' ACCGGUAGGGUUUCAGGACU 5'
query=miR2867-3p, target=RO3G_16123, score=4, range=796-815, stra
target 5' UGGCCAUCACAAAGUCCAGA 3'
:::::::: :::::::: ::
query 3' ACCGGUAGGGUUUCAGGACU 5'
query=miR2867-3p, target=RO3G_07137, score=4, range=796-815, stra
target 5' UGGCCAUCACAAAGUCCAGA 3'
:::::::: :::::::: ::
query 3' ACCGGUAGGGUUUCAGGACU 5'
query=miR2867-3p, target=RO3G_05640, score=4, range=691-710, stra
target 5' UGGCCAUCACAAAGUCCAGA 3'
:::::::: :::::::: ::
query 3' ACCGGUAGGGUUUCAGGACU 5'
query=miR2867-3p, target=RO3G_11781, score=4, range=691-710, stra
target 5' UGGCCAUCACAAAGUCCAGA 3'
:::::::: :::::::: ::
query 3' ACCGGUAGGGUUUCAGGACU 5'
query=miR2867-3p, target=RO3G_16706, score=4, range=520-539, stra
target 5' UGGCCAUCACAAAGUCCAGA 3'
:::::::: :::::::: ::
query 3' ACCGGUAGGGUUUCAGGACU 5'
query=miR2867-3p, target=RO3G_05268, score=4, range=796-815, stra
target 5' UGGCCAUCACAAAGUCCAGA 3'
:::::::: :::::::: ::
query 3' ACCGGUAGGGUUUCAGGACU 5'
query=miR2867-3p, target=RO3G_09193, score=4, range=520-539, stra
target 5' UGGCCAUCACAAAGUCCAGA 3'
:::::::: :::::::: ::
query 3' ACCGGUAGGGUUUCAGGACU 5'
query=miR2867-3p, target=RO3G_17223, score=4, range=520-539, stra
target 5' UGGCCAUCACAAAGUCCAGA 3'
:::::::: :::::::: ::
query 3' ACCGGUAGGGUUUCAGGACU 5'

query=miR2867-3p, target=RO3G_12663, score=4, range=631-650, stra
target 5' UGGCCAUCACAAAGUCCAGA 3'
:::::::: :::::::: ::
query 3' ACCGGUAGGGUUUCAGGACU 5'
query=miR2867-3p, target=RO3G_17355, score=4, range=631-650, stra
target 5' UGGCCAUCACAAAGUCCAGA 3'
:::::::: :::::::: ::
query 3' ACCGGUAGGGUUUCAGGACU 5'
query=miR2867-3p, target=RO3G_00745, score=4, range=517-536, stra
target 5' UGGCCAUCACAAAGUCCAGA 3'
:::::::: :::::::: ::
query 3' ACCGGUAGGGUUUCAGGACU 5'
query=miR2867-3p, target=RO3G_09842, score=4, range=1450-1469,
strand=1
target 5' CCUUUAUCCCAAAGUCCUGA 3'
..::::::::::::::
query 3' ACCGGUAGGGUUUCAGGACU 5'
query=miR2867-3p, target=RO3G_11534, score=4, range=796-815, stra
target 5' UGGCCAGCUCAAAGCCCUGA 3'
:::::: :.::::: :::::
query 3' ACCGGUAGGGUUUCAGGACU 5'
query=miR2867-3p, target=RO3G_02700, score=4, range=490-509, stra
target 5' UGGCCAGCUCAAAGUCCCGA 3'
:::::: :.:::::::: ::
query 3' ACCGGUAGGGUUUCAGGACU 5'
query=miR2867-3p, target=RO3G_03570, score=4, range=490-509, stra
target 5' UGGCCAGCUCAAAGUCCCGA 3'
:::::: :.:::::::: ::
query 3' ACCGGUAGGGUUUCAGGACU 5'
query=miR2867-3p, target=RO3G_09955, score=4, range=490-509, stra
target 5' UGGCCAGCUCAAAGUCCCGA 3'
:::::: :.:::::::: ::
query 3' ACCGGUAGGGUUUCAGGACU 5'
query=miR2867-3p, target=RO3G_11273, score=4, range=136-155, stra
target 5' UGGCCAGCUCAAAGUCCCGA 3'
:::::: :.:::::::: ::
query 3' ACCGGUAGGGUUUCAGGACU 5'
query=miR2867-3p, target=RO3G_16905, score=4, range=613-632, stra
target 5' UGGCCAGCUCAAAGUCCCGA 3'
:::::: :.:::::::: ::
query 3' ACCGGUAGGGUUUCAGGACU 5'
query=miR2867-3p, target=RO3G_12196, score=4, range=727-746, stra
target 5' UGGCCAGCUCAAAGUCCAGA 3'
:::::: :.:::::::: ::
query 3' ACCGGUAGGGUUUCAGGACU 5'

```
query=miR2867-3p, target=RO3G_04786, score=4, range=373-392, stra
target 5' UGGCCAGCUCAAAGUCCCGA 3'
:::::: :.:::::::: ::
query 3' ACCGGUAGGGUUUCAGGACU 5'
query=miR2867-3p, target=RO3G_12525, score=4, range=727-746, stra
target 5' UGGCCAGCUCAAAGUCCCGA 3'
:::::: :.:::::::: ::
query 3' ACCGGUAGGGUUUCAGGACU 5'
query=miR2867-3p, target=RO3G_00690, score=4, range=745-764, stra
target 5' UGGCCAGCUCAAAGUCCCGA 3'
:::::: :.:::::::: ::
query 3' ACCGGUAGGGUUUCAGGACU 5'
query=miR2867-3p, target=RO3G_12186, score=4, range=805-824, stra
target 5' UGGCCAGCUCAAAGUCCAGA 3'
:::::: :.:::::::: ::
query 3' ACCGGUAGGGUUUCAGGACU 5'
query=miR2867-3p, target=RO3G_01925, score=4, range=805-824, stra
target 5' UGGCCAGCUCAAAGUCCCGA 3'
:::::: :.:::::::: ::
query 3' ACCGGUAGGGUUUCAGGACU 5'
query=miR2867-3p, target=RO3G_07024, score=4, range=805-824, stra
target 5' UGGCCAGCUCAAAGUCCCGA 3'
:::::: :.:::::::: ::
query 3' ACCGGUAGGGUUUCAGGACU 5'
query=miR2867-3p, target=RO3G_07121, score=4, range=805-824, stra
target 5' UGGCCAGCUCAAAGUCCCGA 3'
:::::: :.:::::::: ::
query 3' ACCGGUAGGGUUUCAGGACU 5'
query=miR2867-3p, target=RO3G_08848, score=4, range=106-125, stra
target 5' UGGCCAGCUCAAAGCCCUGA 3'
:::::: :.:::::: :::::
query 3' ACCGGUAGGGUUUCAGGACU 5'
query=miR2867-3p, target=RO3G_14467, score=4, range=382-401, stra
target 5' UGGCCUGCCCAAAGCCCUGA 3'
::::: ::::::: :::::
query 3' ACCGGUAGGGUUUCAGGACU 5'
query=miR2867-3p, target=RO3G_04661, score=4, range=436-455, stra
target 5' UGGCCUGCCCAAAGUCCCGA 3'
::::: ::::::::: ::
query 3' ACCGGUAGGGUUUCAGGACU 5'
query=miR2867-3p, target=RO3G_09167, score=4, range=614-632, stra
target 5' UGCCUG-CUCAAAGUCCUGA 3'
:: :.. :.:::::::::::
query 3' ACCGGUAGGGUUUCAGGACU 5'
query=miR5554a-5p, target=RO3G_07863, score=0, range=109-131, str
```

```
target 5' AACAUUGUUCAAGAUCUGCAACA 3'
:::::::::::::::::::::::
query 3' UUGUAACAAGUUCUAGACGUUGU 5'
query=miR5554a-5p, target=RO3G_14562, score=0, range=46-68, stran
target 5' AACAUUGUUCAAGAUCUGCAACA 3'
:::::::::::::::::::::::
query 3' UUGUAACAAGUUCUAGACGUUGU 5'
query=miR5554a-5p, target=RO3G_16903, score=0, range=46-68, stran
target 5' AACAUUGUUCAAGAUCUGCAACA 3'
:::::::::::::::::::::::
query 3' UUGUAACAAGUUCUAGACGUUGU 5'
query=miR5554a-5p, target=RO3G_17220, score=1, range=109-131, str
target 5' AACAUUGCUCAAGAUCUGCAACA 3'
::::::: :::::::::::::::
query 3' UUGUAACAAGUUCUAGACGUUGU 5'
query=miR5554a-5p, target=RO3G_12858, score=4, range=55-76, stran
target 5' AACAUUAUUCAAGAUCU-CAAUA 3'
:::::: :::::::::: :::.:
query 3' UUGUAACAAGUUCUAGACGUUGU 5'
query=miR5554a-5p, target=RO3G_08022, score=4, range=231-252, str
target 5' AAGAUU-UUCACGAUCUGCAACA 3'
:: ::: :::: ::::::::::
query 3' UUGUAACAAGUUCUAGACGUUGU 5'
query=miR5554a-5p, target=RO3G_02261, score=4, range=131-153,
strand=-1
target 5' AACAGGGAUCAAGAUCUGCAAUA 3'
:::: : :::::::::::::.:
query 3' UUGUAACAAGUUCUAGACGUUGU 5'
query=miR7539, target=RO3G_01611, score=0, range=303-323, strand=
target 5' CCUCAUAGCUCUUCUCUCUCA 3'
:::::::::::::::::::::
query 3' GGAGUAUCGAGAAGAGAGAGU 5'
query=miR7539, target=RO3G_05214, score=3.5, range=1791-1810,
strand=-1
target 5' GUUCA-AUCUCUUCUCUCUCA 3'
.::: : :::::::::::::
query 3' GGAGUAUCGAGAAGAGAGAGU 5'
query=miR7539, target=RO3G_06163, score=4, range=34-53, strand=1
target 5' CCUCAUUGCUCUUCU-UCUCC 3'
:::::: :::::::: ::::
query 3' GGAGUAUCGAGAAGAGAGAGU 5'
query=miR7539, target=RO3G_12884, score=4, range=510-529, strand=
target 5' UUUCA-AGCUCUUCUCUUUCU 3'
..::: :::::::::::.::
query 3' GGAGUAUCGAGAAGAGAGAGU 5'
```

```
query=miR7539, target=RO3G_02200, score=4, range=153-172, strand=
target 5' CAUCAU-GCUCUUCUUUCUUA 3'
: :::: ::::::::.::::.:
query 3' GGAGUAUCGAGAAGAGAGAGU 5'
query=miR7539, target=RO3G_08090, score=4, range=780-799, strand=
target 5' CUUCAUGUCUCUU-UCUCUCA 3'
:.::::. ::::: :::::::
query 3' GGAGUAUCGAGAAGAGAGAGU 5'
query=miR3703, target=RO3G_04592, score=0, range=495-514, strand=
target 5' GGCAAUGGCUGAUGAAUCUA 3'
::::::::::::::::::::
query 3' CCGUUACCGACUACUUAGAU 5'
query=miR3703, target=RO3G_07737, score=2, range=684-703, strand=
target 5' GGCAAUGGCUGAUGAAUCAA 3'
:::::::::::::::::: :
query 3' CCGUUACCGACUACUUAGAU 5'
query=miR3703, target=RO3G_11969, score=2, range=1323-1342, stran
target 5' GGCAAUGGCUGAUGAAUCAA 3'
:::::::::::::::::: :
query 3' CCGUUACCGACUACUUAGAU 5'
query=miR3703, target=RO3G_13273, score=2, range=1323-1342, stran
target 5' GGCAAUGGCUGAUGAAUCAA 3'
:::::::::::::::::: :
query 3' CCGUUACCGACUACUUAGAU 5'
query=miR3703, target=RO3G_15898, score=2, range=1323-1342, stran
target 5' GGCAAUGGCUGAUGAAUCAA 3'
:::::::::::::::::: :
query 3' CCGUUACCGACUACUUAGAU 5'
query=miR3703, target=RO3G_16749, score=2, range=1323-1342, stran
target 5' GGCAAUGGCUGAUGAAUCAA 3'
:::::::::::::::::: :
query 3' CCGUUACCGACUACUUAGAU 5'
query=miR3703, target=RO3G_11237, score=2, range=1323-1342, stran
target 5' GGCAAUGGCUGAUGAAUCAA 3'
:::::::::::::::::: :
query 3' CCGUUACCGACUACUUAGAU 5'
query=miR3703, target=RO3G_04683, score=2, range=1323-1342, stran
target 5' GGCAAUGGCUGAUGAAUCAA 3'
:::::::::::::::::: :
query 3' CCGUUACCGACUACUUAGAU 5'
query=miR3703, target=RO3G_11409, score=2, range=1323-1342, stran
target 5' GGCAAUGGCUGAUGAAUCAA 3'
:::::::::::::::::: :
query 3' CCGUUACCGACUACUUAGAU 5'
query=miR3703, target=RO3G_04212, score=2, range=1323-1342, stran
```

target 5' GGCAAUGGCUGAUGAAUCAA 3'
:::::::::::::::::: :
query 3' CCGUUACCGACUACUUAGAU 5'
query=miR3703, target=RO3G_13145, score=2, range=1323-1342, stran
target 5' GGCAAUGGCUGAUGAAUCAA 3'
:::::::::::::::::: :
query 3' CCGUUACCGACUACUUAGAU 5'
query=miR3703, target=RO3G_11538, score=2, range=1323-1342, stran
target 5' GGCAAUGGCUGAUGAAUCAA 3'
:::::::::::::::::: :
query 3' CCGUUACCGACUACUUAGAU 5'
query=miR3703, target=RO3G_09930, score=2, range=1323-1342, stran
target 5' GGCAAUGGCUGAUGAAUCAA 3'
:::::::::::::::::: :
query 3' CCGUUACCGACUACUUAGAU 5'
query=miR3703, target=RO3G_01727, score=2, range=408-427, strand=
target 5' GGCAAUGGCUGAUGAAUCAA 3'
:::::::::::::::::: :
query 3' CCGUUACCGACUACUUAGAU 5'
query=miR3703, target=RO3G_06731, score=2, range=1323-1342, stran
target 5' GGCAAUGGCUGAUGAAUCAA 3'
:::::::::::::::::: :
query 3' CCGUUACCGACUACUUAGAU 5'
query=miR3703, target=RO3G_07307, score=2, range=1323-1342, stran
target 5' GGCAAUGGCUGAUGAAUCAA 3'
:::::::::::::::::: :
query 3' CCGUUACCGACUACUUAGAU 5'
query=miR3703, target=RO3G_07698, score=2, range=1323-1342, stran
target 5' GGCAAUGGCUGAUGAAUCAA 3'
:::::::::::::::::: :
query 3' CCGUUACCGACUACUUAGAU 5'
query=miR3703, target=RO3G_09601, score=2, range=1323-1342, stran
target 5' GGCAAUGGCUGAUGAAUCAA 3'
:::::::::::::::::: :
query 3' CCGUUACCGACUACUUAGAU 5'
query=miR3703, target=RO3G_10217, score=2, range=1323-1342, stran
target 5' GGCAAUGGCUGAUGAAUCAA 3'
:::::::::::::::::: :
query 3' CCGUUACCGACUACUUAGAU 5'
query=miR3703, target=RO3G_14550, score=2, range=1323-1342, stran
target 5' GGCAAUGGCUGAUGAAUCAA 3'
:::::::::::::::::: :
query 3' CCGUUACCGACUACUUAGAU 5'
query=miR3703, target=RO3G_07980, score=2, range=711-730, strand=
target 5' GGCAAUGGCUGAUGAAUCAA 3'

```
:::::::::::::::::: :
query 3' CCGUUACCGACUACUUAGAU 5'
query=miR3703, target=RO3G_07721, score=2, range=1254-1273, stran
target 5' GGCAAUGGCUGAUGAAUCAA 3'
:::::::::::::::::: :
query 3' CCGUUACCGACUACUUAGAU 5'
query=miR3703, target=RO3G_00332, score=3.5, range=378-397, stran
target 5' GGCAAUGGCUGAAGAAUUUG 3'
:::::::::::: ::::.:.
query 3' CCGUUACCGACUACUUAGAU 5'
query=miR3703, target=RO3G_05557, score=3.5, range=969-988, stran
target 5' UUGGAUGGCUGAUGAAUCUA 3'
.::::::::::::::::
query 3' CCGUUACCGACUACUUAGAU 5'
query=miR3703, target=RO3G_10411, score=3.5, range=1855-1873, str
target 5' GAUAGU-GCUGAUGAAUCUG 3'
: .:.: ::::::::::::.
query 3' CCGUUACCGACUACUUAGAU 5'
query=miR3703, target=RO3G_08231, score=4, range=1242-1261, stran
target 5' GGCACUAGCUGAUAAAUCUA 3'
:::: : :::::: ::::::
query 3' CCGUUACCGACUACUUAGAU 5'
query=miR3703, target=RO3G_01507, score=4, range=1139-1158, stran
target 5' GGAAAAGGCAGAUGAAUCUA 3'
:: :: ::: ::::::::::
query 3' CCGUUACCGACUACUUAGAU 5'
query=miR3703, target=RO3G_09337, score=4, range=375-393, strand=
target 5' GAUAA-AGCUGAUGAAUCUG 3'
: .:: ::::::::::::.
query 3' CCGUUACCGACUACUUAGAU 5'
query=miR7543, target=RO3G_14371, score=0, range=626-645, strand=
target 5' UUGUCAAACAUGUAUAUGAA 3'
::::::::::::::::::::
query 3' AACAGUUUGUACAUAUACUU 5'
query=miR7543, target=RO3G_00716, score=0, range=611-630, strand=
target 5' UUGUCAAACAUGUAUAUGAA 3'
::::::::::::::::::::
query 3' AACAGUUUGUACAUAUACUU 5'
query=miR7543, target=RO3G_12320, score=1, range=578-597, strand=
target 5' UUGUCAAGCAUGUAUAUGAA 3'
:::::::.::::::::::::
query 3' AACAGUUUGUACAUAUACUU 5'
query=miR7543, target=RO3G_05704, score=1, range=578-597, strand=
target 5' UUGUCAAGCAUGUAUAUGAA 3'
:::::::.::::::::::::
```

```
query 3' AACAGUUUGUACAUAUACUU 5'
query=miR7543, target=RO3G_13908, score=1, range=461-480, strand=
target 5' UUGUCAAGCAUGUAUAUGAA 3'
:::::::.::::::::::::
query 3' AACAGUUUGUACAUAUACUU 5'
query=miR7543, target=RO3G_01392, score=1, range=89-108, strand=1
target 5' UUGUCAAGCAUGUAUAUGAA 3'
:::::::.::::::::::::
query 3' AACAGUUUGUACAUAUACUU 5'
query=miR7543, target=RO3G_10438, score=2, range=170-189, strand=
target 5' UUGACAAGCAUGUAUAUGAA 3'
::: :::.::::::::::::
query 3' AACAGUUUGUACAUAUACUU 5'
query=miR7543, target=RO3G_02935, score=2, range=191-210, strand=
target 5' UUGCCAAGCAUGUAUAUGAA 3'
::: :::.::::::::::::
query 3' AACAGUUUGUACAUAUACUU 5'
query=miR7543, target=RO3G_15644, score=3, range=1808-1826, stran
target 5' UUAUC-AACAUGUGUAUGAA 3'
:: :: :::::::.::::::
query 3' AACAGUUUGUACAUAUACUU 5'
query=miR7543, target=RO3G_15849, score=3, range=474-493, strand=
target 5' UUAUCAAACUUGUAUAUGAA 3'
:: :::::: ::::::::::
query 3' AACAGUUUGUACAUAUACUU 5'
query=miR7543, target=RO3G_15182, score=3, range=572-590, strand=
target 5' UUUUCAAACAUGUAU-UGAA 3'
:: :::::::::::: ::::
query 3' AACAGUUUGUACAUAUACUU 5'
query=miR7543, target=RO3G_08601, score=4, range=187-206, strand=
target 5' GUGUUAAACAUGUUUAUGAG 3'
:::.:::::::: :::::.
query 3' AACAGUUUGUACAUAUACUU 5'
query=miR7543, target=RO3G_13867, score=4, range=994-1013, strand
target 5' UACUUGAACAUGUAUGUGAA 3'
: :..::::::::::.::::
query 3' AACAGUUUGUACAUAUACUU 5'
query=miR7543, target=RO3G_04239, score=4, range=3170-3189, stran
target 5' UUGAAAAAUAUGUAUAUGAU 3'
::: :::.::::::::::
query 3' AACAGUUUGUACAUAUACUU 5'
query=miR7543, target=RO3G_00829, score=4, range=125-144, strand=
target 5' UUGGAAAACAAGUAUAUGAA 3'
::: ::::: :::::::::
query 3' AACAGUUUGUACAUAUACUU 5'
```

```
query=miR8554, target=RO3G_03575, score=0, range=2741-2762, stran
target 5' UCCCAAAUCAAAGUCAUAAGCA 3'
::::::::::::::::::::::
query 3' AGGGUUUAGUUUCAGUAUUCGU 5'
query=miR8554, target=RO3G_12067, score=0, range=2741-2762, stran
target 5' UCCCAAAUCAAAGUCAUAAGCA 3'
::::::::::::::::::::::
query 3' AGGGUUUAGUUUCAGUAUUCGU 5'
query=miR8554, target=RO3G_13855, score=0, range=2741-2762, stran
target 5' UCCCAAAUCAAAGUCAUAAGCA 3'
::::::::::::::::::::::
query 3' AGGGUUUAGUUUCAGUAUUCGU 5'
query=miR8554, target=RO3G_13701, score=0, range=2741-2762, stran
target 5' UCCCAAAUCAAAGUCAUAAGCA 3'
::::::::::::::::::::::
query 3' AGGGUUUAGUUUCAGUAUUCGU 5'
query=miR8554, target=RO3G_12014, score=0, range=2741-2762, stran
target 5' UCCCAAAUCAAAGUCAUAAGCA 3'
::::::::::::::::::::::
query 3' AGGGUUUAGUUUCAGUAUUCGU 5'
query=miR8554, target=RO3G_10222, score=0, range=2741-2762, stran
target 5' UCCCAAAUCAAAGUCAUAAGCA 3'
::::::::::::::::::::::
query 3' AGGGUUUAGUUUCAGUAUUCGU 5'
query=miR8554, target=RO3G_12462, score=0, range=2597-2618, stran
target 5' UCCCAAAUCAAAGUCAUAAGCA 3'
::::::::::::::::::::::
query 3' AGGGUUUAGUUUCAGUAUUCGU 5'
query=miR8554, target=RO3G_03796, score=0, range=1934-1955, stran
target 5' UCCCAAAUCAAAGUCAUAAGCA 3'
::::::::::::::::::::::
query 3' AGGGUUUAGUUUCAGUAUUCGU 5'
query=miR8554, target=RO3G_03752, score=0, range=2741-2762, stran
target 5' UCCCAAAUCAAAGUCAUAAGCA 3'
::::::::::::::::::::::
query 3' AGGGUUUAGUUUCAGUAUUCGU 5'
query=miR8554, target=RO3G_15845, score=3, range=750-770, strand=
target 5' UCCC-UUUCAAAGUCAUAAGCA 3'
:::: :::::::::::::::
query 3' AGGGUUUAGUUUCAGUAUUCGU 5'
query=miR8554, target=RO3G_01115, score=3.5, range=18-40, strand=
target 5' UCUCAGUAUUAAAGUCAUAAGCC 3'
::.::. ::.::::::::::::
query 3' AGGGUU-UAGUUUCAGUAUUCGU 5'
query=miR8554, target=RO3G_08541, score=4, range=192-214, strand=
```

target 5' UUUCAUAAUCAAAGUCAUAAACA 3'
:..:: ::::::::::::: ::
query 3' AGGGU-UUAGUUUCAGUAUUCGU 5'
query=miR1172.1, target=RO3G_00600, score=1, range=858-879, stran
target 5' CUCCUUUGCUGCUGCAAAUCCA 3'
::::::::::::::::::::
query 3' GAGGAAACGACGACGUUUAGGA 5'
query=miR1172.1, target=RO3G_16901, score=3.5, range=1053-1074, strand=1
target 5' UUCCUUUGCUGCUGCCAAUCCA 3'
.:::::::::::::: :::::
query 3' GAGGAAACGACGACGUUUAGGA 5'
query=miR1172.1, target=RO3G_16917, score=3.5, range=1059-1080, strand=1
target 5' UUCCUUUGCUGCUGCCAAUCCA 3'
.:::::::::::::: :::::
query 3' GAGGAAACGACGACGUUUAGGA 5'
query=miR1172.1, target=RO3G_01717, score=3.5, range=1050-1071, strand=1
target 5' UUCCUUUGCUGCUGCCAAUCCA 3'
.:::::::::::::: :::::
query 3' GAGGAAACGACGACGUUUAGGA 5'
query=miR1172.1, target=RO3G_12011, score=3.5, range=1047-1068, strand=1
target 5' UUCCUUUGCUGCUGCCAAUCCA 3'
.:::::::::::::: :::::
query 3' GAGGAAACGACGACGUUUAGGA 5'
query=miR1172.1, target=RO3G_02817, score=3.5, range=1047-1068, strand=1
target 5' UUCCUUUGCUGCUGCCAAUCCA 3'
.:::::::::::::: :::::
query 3' GAGGAAACGACGACGUUUAGGA 5'
query=miR1172.1, target=RO3G_17433, score=3.5, range=1044-1065, strand=1
target 5' UUCCUUUGCUGCUGCCAAUCCA 3'
.:::::::::::::: :::::
query 3' GAGGAAACGACGACGUUUAGGA 5'
query=miR1172.1, target=RO3G_07115, score=3.5, range=1002-1023, strand=1
target 5' CUCUUUUGCUGCUGCUAAUCCC 3'
:::.:::::::::: :::::
query 3' GAGGAAACGACGACGUUUAGGA 5'
query=miR1172.1, target=RO3G_16743, score=3.5, range=1005-1026, strand=1
target 5' UUCCUUUGCUGCUGCCAAUCCA 3'

.:::::::::::::: :::::
query 3' GAGGAAACGACGACGUUUAGGA 5'
query=miR1172.1, target=RO3G_07692, score=3.5, range=981-1002, strand=1
target 5' UUCCUUUGCUGCUGCCAAUCCA 3'
.:::::::::::::: :::::
query 3' GAGGAAACGACGACGUUUAGGA 5'
query=miR1172.1, target=RO3G_04659, score=3.5, range=981-1002, strand=1
target 5' UUCCUUUGCUGCUGCCAAUCCA 3'
.:::::::::::::: :::::
query 3' GAGGAAACGACGACGUUUAGGA 5'
query=miR1172.1, target=RO3G_15879, score=3.5, range=966-987, str
target 5' UUCCUUUGCUGCUGCCAAUCCA 3'
.:::::::::::::: :::::
query 3' GAGGAAACGACGACGUUUAGGA 5'
query=miR1172.1, target=RO3G_10043, score=3.5, range=954-975, str
target 5' UUCCUUUGCUGCUGCCAAUCCA 3'
.:::::::::::::: :::::
query 3' GAGGAAACGACGACGUUUAGGA 5'
query=miR1172.1, target=RO3G_12078, score=3.5, range=747-768, str
target 5' UUCCUUUGCUGCUGCCAAUCCA 3'
.:::::::::::::: :::::
query 3' GAGGAAACGACGACGUUUAGGA 5'
query=miR1172.1, target=RO3G_12975, score=3.5, range=345-366, str
target 5' UUCCUUUGCUGCUGCCAAUCCA 3'
.:::::::::::::: :::::
query 3' GAGGAAACGACGACGUUUAGGA 5'
query=miR1172.1, target=RO3G_05907, score=3.5, range=261-282, str
target 5' UUCCUUUGCUGCUGCCAAUCCA 3'
.:::::::::::::: :::::
query 3' GAGGAAACGACGACGUUUAGGA 5'
query=miR1172.1, target=RO3G_12645, score=3.5, range=219-240, str
target 5' UUCCUUUGCUGCUGCCAAUCCA 3'
.:::::::::::::: :::::
query 3' GAGGAAACGACGACGUUUAGGA 5'
query=miR1172.1, target=RO3G_15581, score=3.5, range=216-237, str
target 5' UUCCUUUGCUGCUGCCAAUCCA 3'
.:::::::::::::: :::::
query 3' GAGGAAACGACGACGUUUAGGA 5'
query=miR1172.1, target=RO3G_01325, score=3.5, range=156-177, str
target 5' UUCCUUUGCUGCUGCCAAUCCA 3'
.:::::::::::::: :::::
query 3' GAGGAAACGACGACGUUUAGGA 5'
query=miR5080, target=RO3G_09180, score=0, range=1098-1119, stran

```
target 5' CAUGUACCGUCAUGAUCCUUUU 3'
::::::::::::::::::::::
query 3' GUACAUGGCAGUACUAGGAAAA 5'
query=miR5080, target=RO3G_16812, score=0, range=1098-1119, stran
target 5' CAUGUACCGUCAUGAUCCUUUU 3'
::::::::::::::::::::::
query 3' GUACAUGGCAGUACUAGGAAAA 5'
query=miR5080, target=RO3G_09189, score=0, range=1098-1119, stran
target 5' CAUGUACCGUCAUGAUCCUUUU 3'
::::::::::::::::::::::
query 3' GUACAUGGCAGUACUAGGAAAA 5'
query=miR5080, target=RO3G_14798, score=0, range=1098-1119, stran
target 5' CAUGUACCGUCAUGAUCCUUUU 3'
::::::::::::::::::::::
query 3' GUACAUGGCAGUACUAGGAAAA 5'
query=miR5080, target=RO3G_14791, score=0, range=1098-1119, stran
target 5' CAUGUACCGUCAUGAUCCUUUU 3'
::::::::::::::::::::::
query 3' GUACAUGGCAGUACUAGGAAAA 5'
query=miR5080, target=RO3G_17260, score=0, range=977-998, strand=
target 5' CAUGUACCGUCAUGAUCCUUUU 3'
::::::::::::::::::::::
query 3' GUACAUGGCAGUACUAGGAAAA 5'
query=miR5080, target=RO3G_16733, score=0, range=1098-1119, stran
target 5' CAUGUACCGUCAUGAUCCUUUU 3'
::::::::::::::::::::::
query 3' GUACAUGGCAGUACUAGGAAAA 5'
query=miR5080, target=RO3G_15270, score=0, range=147-168, strand=
target 5' CAUGUACCGUCAUGAUCCUUUU 3'
::::::::::::::::::::::
query 3' GUACAUGGCAGUACUAGGAAAA 5'
query=miR5080, target=RO3G_06734, score=0, range=402-423, strand=
target 5' CAUGUACCGUCAUGAUCCUUUU 3'
::::::::::::::::::::::
query 3' GUACAUGGCAGUACUAGGAAAA 5'
query=miR5080, target=RO3G_08770, score=4, range=290-311, strand=
target 5' CUGGUACCGUCUUGAUCCUUUU 3'
: :::::::: ::::::::::
query 3' GUACAUGGCAGUACUAGGAAAA 5'
query=miR5022, target=RO3G_06594, score=3, range=1033-1050, stran
target 5' GUACAACAUACCCCAUUA 3'
: : :::::::::::::
query 3' GAAGCUGUAUGGGGUAAU 5'
query=miR5022, target=RO3G_00045, score=3.5, range=173-190, stran
target 5' GUUCGACAUACUCUAUUG 3'
```

```
:::::::::.:.:::.
query 3' GAAGCUGUAUGGGGUAAU 5'
query=miR5022, target=RO3G_12104, score=4, range=449-466, strand=
target 5' CUUCGACAGGCUCCAUUA 3'
:::::::: .:.::::::
query 3' GAAGCUGUAUGGGGUAAU 5'
query=miR5022, target=RO3G_10150, score=4, range=789-806, strand=
target 5' ACUCCACAUACCCCAUUC 3'
:: ::::::::::::
query 3' GAAGCUGUAUGGGGUAAU 5'
query=miR5022, target=RO3G_16088, score=4, range=854-870, strand=
target 5' UAUUGA-AUACCCCAUUA 3'
. :.:: ::::::::::
query 3' GAAGCUGUAUGGGGUAAU 5'
query=miR5022, target=RO3G_16316, score=4, range=87-104, strand=1
target 5' CUUGAACAUACCCGAUUA 3'
::: :::::::: ::::
query 3' GAAGCUGUAUGGGGUAAU 5'
query=miR5022, target=RO3G_03007, score=4, range=1043-1060, stran
target 5' CUUCCAUAUAUUCCAUUA 3'
:::: :.:::..::::::
query 3' GAAGCUGUAUGGGGUAAU 5'
query=miR5022, target=RO3G_03499, score=4, range=486-503, strand=
target 5' GUUGGACAUACUUCAUUA 3'
:: :::::::..:::::
query 3' GAAGCUGUAUGGGGUAAU 5'
query=miR5022, target=RO3G_16153, score=4, range=874-892, strand=
target 5' GUUUGACAUAGCCCCAUUG 3'
::.:::::: :::::::.
query 3' GAAGCUGUAU-GGGGUAAU 5'
query=miR5022, target=RO3G_04215, score=4, range=131-147, strand=
target 5' CUUC-ACGUAUUCCAUUA 3'
:::: ::.::..::::::
query 3' GAAGCUGUAUGGGGUAAU 5'
query=miR5022, target=RO3G_00439, score=4, range=41-57, strand=-1
target 5' CUUC-AUAUGCUCCAUUA 3'
:::: :.::.:.::::::
query 3' GAAGCUGUAUGGGGUAAU 5'
query=miR5022, target=RO3G_17299, score=4, range=1004-1021, stran
target 5' CCUCGUCAUACUCCAUUA 3'
: ::: ::::.::::::
query 3' GAAGCUGUAUGGGGUAAU 5'
query=miR5022, target=RO3G_05376, score=4, range=237-254, strand=
target 5' CUUCCACAUAUCGCAUUA 3'
:::: :::::.: :::::
```

```
query 3' GAAGCUGUAUGGGGUAAU 5'
query=miR5022, target=RO3G_10249, score=4, range=176-193, strand=
target 5' CUUGAACAUAUUCCAUUA 3'
::: :::::..::::::
query 3' GAAGCUGUAUGGGGUAAU 5'
query=miR5022, target=RO3G_00409, score=4, range=903-919, strand=
target 5' CUUC-ACAUAUCUCAUUU 3'
:::: :::::.:.::::
query 3' GAAGCUGUAUGGGGUAAU 5'
query=miR5022, target=RO3G_04533, score=4, range=3521-3538, stran
target 5' CUUGUACAUAUCCCAUUU 3'
::: :::::.::::::
query 3' GAAGCUGUAUGGGGUAAU 5'
query=miR5022, target=RO3G_15103, score=4, range=2208-2226, stran
target 5' CCUCGACAUGCCGCCAUUA 3'
: :::::::.:: ::::::
query 3' GAAGCUGUAUGG-GGUAAU 5'
query=miR5037c, target=RO3G_17063, score=3, range=731-747, strand
target 5' CGGG-CCAAACUUCAACU 3'
:::: ::::: :::::::
query 3' GCCCGGGUUUCAAGUUGA 5'
query=miR5037c, target=RO3G_17385, score=3.5, range=288-305, stra
target 5' UGUGCCCAAAGUUAAACU 3'
.: :::::::::: ::::
query 3' GCCCGGGUUUCAAGUUGA 5'
query=miR5037c, target=RO3G_11771, score=3.5, range=288-305, stra
target 5' UGUGCCCAAAGUUAAACU 3'
.: :::::::::: ::::
query 3' GCCCGGGUUUCAAGUUGA 5'
query=miR5037c, target=RO3G_12009, score=3.5, range=288-305, stra
target 5' UGUGCCCAAAGUUAAACU 3'
.: :::::::::: ::::
query 3' GCCCGGGUUUCAAGUUGA 5'
query=miR5037c, target=RO3G_09940, score=3.5, range=288-305, stra
target 5' UGUGCCCAAAGUUAAACU 3'
.: :::::::::: ::::
query 3' GCCCGGGUUUCAAGUUGA 5'
query=miR5037c, target=RO3G_17358, score=3.5, range=288-305, stra
target 5' UGUGCCCAAAGUUAAACU 3'
.: :::::::::: ::::
query 3' GCCCGGGUUUCAAGUUGA 5'
query=miR5037c, target=RO3G_12169, score=3.5, range=288-305, stra
target 5' UGUGCCCAAAGUUAAACU 3'
.: :::::::::: ::::
query 3' GCCCGGGUUUCAAGUUGA 5'
```

query=miR5037c, target=RO3G_12072, score=3.5, range=288-305, stra
target 5' UGUGCCCAAAGUUAAACU 3'
.: :::::::::: ::::
query 3' GCCCGGGUUUCAAGUUGA 5'
query=miR5037c, target=RO3G_16138, score=3.5, range=288-305, stra
target 5' UGUGCCCAAAGUUAAACU 3'
.: :::::::::: ::::
query 3' GCCCGGGUUUCAAGUUGA 5'
query=miR5037c, target=RO3G_07686, score=3.5, range=288-305, stra
target 5' UGUGCCCAAAGUUAAACU 3'
.: :::::::::: ::::
query 3' GCCCGGGUUUCAAGUUGA 5'
query=miR5037c, target=RO3G_07976, score=3.5, range=288-305, stra
target 5' UGUGCCCAAAGUUAAACU 3'
.: :::::::::: ::::
query 3' GCCCGGGUUUCAAGUUGA 5'
query=miR5037c, target=RO3G_10725, score=3.5, range=252-269, stra
target 5' UGUGCCCAAAGUUAAACU 3'
.: :::::::::: ::::
query 3' GCCCGGGUUUCAAGUUGA 5'
query=miR5037c, target=RO3G_01309, score=3.5, range=237-254, stra
target 5' UGUGCCCAAAGUUAAACU 3'
.: :::::::::: ::::
query 3' GCCCGGGUUUCAAGUUGA 5'
query=miR5037c, target=RO3G_01207, score=3.5, range=288-305, stra
target 5' UGUGCCCAAAGUUAAACU 3'
.: :::::::::: ::::
query 3' GCCCGGGUUUCAAGUUGA 5'
query=miR5037c, target=RO3G_13747, score=3.5, range=288-305, stra
target 5' UGUGCCCAAAGUUAAACU 3'
.: :::::::::: ::::
query 3' GCCCGGGUUUCAAGUUGA 5'
query=miR5037c, target=RO3G_11404, score=3.5, range=288-305, stra
target 5' UGUGCCCAAAGUUAAACU 3'
.: :::::::::: ::::
query 3' GCCCGGGUUUCAAGUUGA 5'
query=miR5037c, target=RO3G_05631, score=3.5, range=288-305, stra
target 5' UGUGCCCAAAGUUAAACU 3'
.: :::::::::: ::::
query 3' GCCCGGGUUUCAAGUUGA 5'
query=miR5037c, target=RO3G_03555, score=3.5, range=288-305, stra
target 5' UGUGCCCAAAGUUAAACU 3'
.: :::::::::: ::::
query 3' GCCCGGGUUUCAAGUUGA 5'
query=miR5037c, target=RO3G_09192, score=3.5, range=288-305, stra

```
target 5' UGUGCCCAAAGUUAAACU 3'
.: ::::::::: ::::
query 3' GCCCGGGUUUCAAGUUGA 5'
query=miR5037c, target=RO3G_17356, score=3.5, range=198-215, stra
target 5' UGUGCCCAAAGUUAAACU 3'
.: ::::::::: ::::
query 3' GCCCGGGUUUCAAGUUGA 5'
query=miR5037c, target=RO3G_07130, score=3.5, range=192-209, stra
target 5' UGUGCCCAAAGUUAAACU 3'
.: ::::::::: ::::
query 3' GCCCGGGUUUCAAGUUGA 5'
query=miR5037c, target=RO3G_03574, score=3.5, range=171-188, stra
target 5' UGUGCCCAAAGUUAAACU 3'
.: ::::::::: ::::
query 3' GCCCGGGUUUCAAGUUGA 5'
query=miR5037c, target=RO3G_16739, score=3.5, range=288-305, stra
target 5' UGUGCCCAAAGUUAAACU 3'
.: ::::::::: ::::
query 3' GCCCGGGUUUCAAGUUGA 5'
query=miR5037c, target=RO3G_13999, score=3.5, range=443-461, stra
target 5' UGGCACCCAAAGUUCAAUU 3'
.:: :::::::::::.:
query 3' GCC-CGGGUUUCAAGUUGA 5'
query=miR5037c, target=RO3G_07734, score=3.5, range=488-505, stra
target 5' UGAGCCCAAAGUUCAAGU 3'
.: ::::::::::::: :
query 3' GCCCGGGUUUCAAGUUGA 5'
query=miR5037c, target=RO3G_06072, score=3.5, range=635-652, stra
target 5' CUGCUUCAAAGUUCAACU 3'
: : ..::::::::::::
query 3' GCCCGGGUUUCAAGUUGA 5'
query=miR5037c, target=RO3G_13387, score=3.5, range=53-70, strand
target 5' CUGCUUCAAAGUUCAACU 3'
: : ..::::::::::::
query 3' GCCCGGGUUUCAAGUUGA 5'
query=miR5037c, target=RO3G_15223, score=3.5, range=210-226, stra
target 5' UGGG-CCAGAGUUCAACA 3'
.::: :::.::::::::
query 3' GCCCGGGUUUCAAGUUGA 5'
query=miR5037c, target=RO3G_02475, score=3.5, range=423-440, stra
target 5' AGAUUCCAAAGUUCAACU 3'
: .::::::::::::
query 3' GCCCGGGUUUCAAGUUGA 5'
query=miR5037c, target=RO3G_03632, score=4, range=336-353, strand
target 5' CGUGCCCAAAGGUCAAUU 3'
```

```
:: :::::::: ::::.:
query 3' GCCCGGGUUUCAAGUUGA 5'
query=miR5037c, target=RO3G_14710, score=4, range=288-305, strand
target 5' UGUGUCCAAAGUUAAACU 3'
.: :.:::::::: ::::
query 3' GCCCGGGUUUCAAGUUGA 5'
query=miR5037c, target=RO3G_04526, score=4, range=1389-1406, stra
target 5' UGGAUCCAAAGUUGAACU 3'
.:: .:::::::: ::::
query 3' GCCCGGGUUUCAAGUUGA 5'
query=miR5037c, target=RO3G_01349, score=4, range=690-707, strand
target 5' GGAACUCAAAGUUCAACU 3'
: :.:::::::::::
query 3' GCCCGGGUUUCAAGUUGA 5'
query=miR5037c, target=RO3G_09687, score=4, range=1060-1076, stra
target 5' CUGG-CCGAAGUUCAACG 3'
: :: ::.:::::::::
query 3' GCCCGGGUUUCAAGUUGA 5'
query=miR5037c, target=RO3G_13223, score=4, range=972-989, strand
target 5' CAAGGCCAAAGUUCAACC 3'
: : :::::::::::
query 3' GCCCGGGUUUCAAGUUGA 5'
query=miR5037c, target=RO3G_04124, score=4, range=972-989, strand
target 5' CAAGGCCAAAGUUCAACC 3'
: : :::::::::::
query 3' GCCCGGGUUUCAAGUUGA 5'
query=miR5037c, target=RO3G_13596, score=4, range=520-537, strand
target 5' CUGUCCCAAAGUUCGAUU 3'
: : ::::::::::.:.:
query 3' GCCCGGGUUUCAAGUUGA 5'
query=miR396a-3p, target=RO3G_09454, score=2, range=2269-2288,
strand=-1
target 5' UUUUCACAGCUUUGUUGAAC 3'
::..::::::::.:::::
query 3' AAGGGUGUCGAAAUAACUUG 5'
query=miR396a-3p, target=RO3G_15072, score=3, range=1209-1227,
strand=1
target 5' UGCCCA-AGCUUUGUUGAAC 3'
: :::: ::::::.::::::
query 3' AAGGGUGUCGAAAUAACUUG 5'
query=miR396a-3p, target=RO3G_02514, score=3.5, range=452-471,
strand=1
target 5' UUUUCACAGGUUUAUUGAAU 3'
::..::::: :::::::::.
query 3' AAGGGUGUCGAAAUAACUUG 5'
```

query=miR396a-3p, target=RO3G_04854, score=3.5, range=690-709, strand=-1
target 5' CAUCUAUAGCUUUAUUGAAC 3'
.:.:.:::::::::::::
query 3' AAGGGUGUCGAAAUAACUUG 5'
query=miR396a-3p, target=RO3G_11676, score=4, range=1413-1432, strand=1
target 5' UAAUCACAGUUUUAUUGAAU 3'
: .:::::.:::::::::.
query 3' AAGGGUGUCGAAAUAACUUG 5'
query=miR396a-3p, target=RO3G_06667, score=4, range=475-493, stra
target 5' AUACC-CAGUUUUAUUGAAC 3'
: :: :::.::::::::::
query 3' AAGGGUGUCGAAAUAACUUG 5'
query=miR4221, target=RO3G_04030, score=0, range=1211-1233, stran
target 5' UUUCCUACCUUUUGAACCUCUUA 3'
:::::::::::::::::::::::
query 3' AAAGGAUGGAAAACUUGGAGAAU 5'
query=miR4221, target=RO3G_05627, score=0, range=104-126, strand=
target 5' UUUCCUACCUUUUGAACCUCUUA 3'
:::::::::::::::::::::::
query 3' AAAGGAUGGAAAACUUGGAGAAU 5'
query=miR4221, target=RO3G_09361, score=0.5, range=890-912, stran
target 5' UUUCCUAUCUUUUGAACCUCUUA 3'
:::::::.:::::::::::::::
query 3' AAAGGAUGGAAAACUUGGAGAAU 5'
query=miR4221, target=RO3G_12138, score=0.5, range=845-867, stran
target 5' UUUCCUAUCUUUUGAACCUCUUA 3'
:::::::.:::::::::::::::
query 3' AAAGGAUGGAAAACUUGGAGAAU 5'
query=miR4221, target=RO3G_05034, score=4, range=158-179, strand=
target 5' UUUUCU-CUUUUUGAGCCUCUUU 3'
:::.:: :.::::::.::::::
query 3' AAAGGAUGGAAAACUUGGAGAAU 5'
query=miR156i, target=RO3G_11952, score=0, range=2971-2991, stran
target 5' CUGCUGUCUUCUUCUGUUCCA 3'
:::::::::::::::::::::
query 3' GACGACAGAAGAAGACAAGGU 5'
query=miR156i, target=RO3G_12964, score=0, range=2593-2613, stran
target 5' CUGCUGUCUUCUUCUGUUCCA 3'
:::::::::::::::::::::
query 3' GACGACAGAAGAAGACAAGGU 5'
query=miR156i, target=RO3G_13746, score=0, range=1447-1467, stran
target 5' CUGCUGUCUUCUUCUGUUCCA 3'
:::::::::::::::::::::

```
query 3' GACGACAGAAGAAGACAAGGU 5'
query=miR156i, target=RO3G_00686, score=0, range=316-336, strand=
target 5' CUGCUGUCUUCUUCUGUUCCA 3'
:::::::::::::::::::::
query 3' GACGACAGAAGAAGACAAGGU 5'
query=miR156i, target=RO3G_08024, score=3, range=2-22, strand=-1
target 5' AUUCUGUCUUCUUUUGUUCCA 3'
: ::::::::::.:::::::
query 3' GACGACAGAAGAAGACAAGGU 5'
query=miR156i, target=RO3G_02682, score=3.5, range=1016-1036, str
target 5' CUGGUGCUUUCUUUUGUUCCA 3'
::: :: .:::::.:::::::
query 3' GACGACAGAAGAAGACAAGGU 5'
query=miR156i, target=RO3G_15288, score=3.5, range=1016-1036, str
target 5' CUGGUGCUUUCUUUUGUUCCA 3'
::: :: .:::::.:::::::
query 3' GACGACAGAAGAAGACAAGGU 5'
query=miR156i, target=RO3G_07729, score=3.5, range=785-805, stran
target 5' CUGGUGCUUUCUUUUGUUCCA 3'
::: :: .:::::.:::::::
query 3' GACGACAGAAGAAGACAAGGU 5'
query=miR156i, target=RO3G_10263, score=3.5, range=21-41, strand=
target 5' UUGUCGUUUUCUUCUGUUUCA 3'
.::. ::.::::::::::.::
query 3' GACGACAGAAGAAGACAAGGU 5'
query=miR156i, target=RO3G_16684, score=3.5, range=578-599, stran
target 5' CUGUUAUCUUCUUCCUGUUCCA 3'
:::.: ::::::: ::::::::
query 3' GACGACAGAAGAA-GACAAGGU 5'
query=miR156i, target=RO3G_11120, score=3.5, range=879-899, stran
target 5' CUACCGUUUUCUUCUGUUUCA 3'
:: : ::.::::::::::.::
query 3' GACGACAGAAGAAGACAAGGU 5'
query=miR156i, target=RO3G_06158, score=3.5, range=2058-2077,
strand=-1
target 5' UGGCU-UCUUCUUCUGUUUCA 3'
. ::: ::::::::::::.::
query 3' GACGACAGAAGAAGACAAGGU 5'
query=miR156i, target=RO3G_11630, score=3.5, range=242-261, stran
target 5' CUGUUGU-UUCUUUUGUUCCU 3'
:::.::: :::::.::::::
query 3' GACGACAGAAGAAGACAAGGU 5'
query=miR156i, target=RO3G_16901, score=4, range=188-207, strand=
target 5' CUGCU-UCUGCUUCUGUUCCU 3'
::::: ::: ::::::::::
```

```
query 3' GACGACAGAAGAAGACAAGGU 5'
query=miR156i, target=RO3G_07738, score=4, range=179-198, strand=
target 5' CUGCU-UCUGCUUCUGUUCCU 3'
::::: ::: ::::::::::
query 3' GACGACAGAAGAAGACAAGGU 5'
query=miR156i, target=RO3G_11236, score=4, range=179-198, strand=
target 5' CUGCU-UCUGCUUCUGUUCCU 3'
::::: ::: ::::::::::
query 3' GACGACAGAAGAAGACAAGGU 5'
query=miR156i, target=RO3G_11537, score=4, range=179-198, strand=
target 5' CUGCU-UCUGCUUCUGUUCCU 3'
::::: ::: ::::::::::
query 3' GACGACAGAAGAAGACAAGGU 5'
query=miR156i, target=RO3G_17248, score=4, range=167-186, strand=
target 5' CUGCU-UCUGCUUCUGUUCCU 3'
::::: ::: ::::::::::
query 3' GACGACAGAAGAAGACAAGGU 5'
query=miR156i, target=RO3G_16917, score=4, range=194-213, strand=
target 5' CUGCU-UCUGCUUCUGUUCCU 3'
::::: ::: ::::::::::
query 3' GACGACAGAAGAAGACAAGGU 5'
query=miR156i, target=RO3G_01717, score=4, range=197-216, strand=
target 5' CUGCU-UCUGCUUCUGUUCCU 3'
::::: ::: ::::::::::
query 3' GACGACAGAAGAAGACAAGGU 5'
query=miR156i, target=RO3G_12011, score=4, range=194-213, strand=
target 5' CUGCU-UCUGCUUCUGUUCCU 3'
::::: ::: ::::::::::
query 3' GACGACAGAAGAAGACAAGGU 5'
query=miR156i, target=RO3G_02817, score=4, range=197-216, strand=
target 5' CUGCU-UCUGCUUCUGUUCCU 3'
::::: ::: ::::::::::
query 3' GACGACAGAAGAAGACAAGGU 5'
query=miR156i, target=RO3G_15897, score=4, range=137-156, strand=
target 5' CUGCU-UCUGCUUCUGUUCCU 3'
::::: ::: ::::::::::
query 3' GACGACAGAAGAAGACAAGGU 5'
query=miR156i, target=RO3G_04211, score=4, range=137-156, strand=
target 5' CUGCU-UCUGCUUCUGUUCCU 3'
::::: ::: ::::::::::
query 3' GACGACAGAAGAAGACAAGGU 5'
query=miR156i, target=RO3G_06730, score=4, range=137-156, strand=
target 5' CUGCU-UCUGCUUCUGUUCCU 3'
::::: ::: ::::::::::
query 3' GACGACAGAAGAAGACAAGGU 5'
```

```
query=miR156i, target=RO3G_09602, score=4, range=137-156, strand=
target 5' CUGCU-UCUGCUUCUGUUCCU 3'
::::: ::: ::::::::::
query 3' GACGACAGAAGAAGACAAGGU 5'
query=miR156i, target=RO3G_09929, score=4, range=137-156, strand=
target 5' CUGCU-UCUGCUUCUGUUCCU 3'
::::: ::: ::::::::::
query 3' GACGACAGAAGAAGACAAGGU 5'
query=miR156i, target=RO3G_07696, score=4, range=137-156, strand=
target 5' CUGCU-UCUGCUUCUGUUCCU 3'
::::: ::: ::::::::::
query 3' GACGACAGAAGAAGACAAGGU 5'
query=miR156i, target=RO3G_07306, score=4, range=137-156, strand=
target 5' CUGCU-UCUGCUUCUGUUCCU 3'
::::: ::: ::::::::::
query 3' GACGACAGAAGAAGACAAGGU 5'
query=miR156i, target=RO3G_14551, score=4, range=137-156, strand=
target 5' CUGCU-UCUGCUUCUGUUCCU 3'
::::: ::: ::::::::::
query 3' GACGACAGAAGAAGACAAGGU 5'
query=miR156i, target=RO3G_17433, score=4, range=191-210, strand=
target 5' CUGCU-UCUGCUUCUGUUCCU 3'
::::: ::: ::::::::::
query 3' GACGACAGAAGAAGACAAGGU 5'
query=miR156i, target=RO3G_07981, score=4, range=137-156, strand=
target 5' CUGCU-UCUGCUUCUGUUCCU 3'
::::: ::: ::::::::::
query 3' GACGACAGAAGAAGACAAGGU 5'
query=miR156i, target=RO3G_07720, score=4, range=137-156, strand=
target 5' CUGCU-UCUGCUUCUGUUCCU 3'
::::: ::: ::::::::::
query 3' GACGACAGAAGAAGACAAGGU 5'
query=miR156i, target=RO3G_10775, score=4, range=167-186, strand=
target 5' CUGCU-UCUGCUUCUGUUCCU 3'
::::: ::: ::::::::::
query 3' GACGACAGAAGAAGACAAGGU 5'
query=miR156i, target=RO3G_16743, score=4, range=140-159, strand=
target 5' CUGCU-UCUGCUUCUGUUCCU 3'
::::: ::: ::::::::::
query 3' GACGACAGAAGAAGACAAGGU 5'
query=miR156i, target=RO3G_03584, score=4, range=95-114, strand=1
target 5' CUGCU-UCUGCUUCUGUUCCU 3'
::::: ::: ::::::::::
query 3' GACGACAGAAGAAGACAAGGU 5'
query=miR156i, target=RO3G_07692, score=4, range=128-147, strand=
```

```
target 5' CUGCU-UCUGCUUCUGUUCCU 3'
::::: ::: ::::::::::
query 3' GACGACAGAAGAAGACAAGGU 5'
query=miR156i, target=RO3G_04659, score=4, range=86-105, strand=1
target 5' CUGCU-UCUUCUCCUGUUCCC 3'
::::: :::::: :::::::
query 3' GACGACAGAAGAAGACAAGGU 5'
query=miR156i, target=RO3G_15879, score=4, range=194-213, strand=
target 5' CUGCU-UCUGCUUCUGUUCCU 3'
::::: ::: ::::::::::
query 3' GACGACAGAAGAAGACAAGGU 5'
query=miR156i, target=RO3G_17155, score=4, range=290-309, strand=
target 5' CUGCU-UCUUCUUCUGCUCCU 3'
::::: :::::::::: :::
query 3' GACGACAGAAGAAGACAAGGU 5'
query=miR156i, target=RO3G_05926, score=4, range=290-309, strand=
target 5' CUGCU-UCUUCUUCUGCUCCU 3'
::::: :::::::::: :::
query 3' GACGACAGAAGAAGACAAGGU 5'
query=miR156i, target=RO3G_09928, score=4, range=290-309, strand=
target 5' CUGCU-UCUUCUUCUGCUCCU 3'
::::: :::::::::: :::
query 3' GACGACAGAAGAAGACAAGGU 5'
query=miR156i, target=RO3G_10043, score=4, range=188-207, strand=
target 5' CUGCU-UCUGCUUCUGUUCCU 3'
::::: ::: ::::::::::
query 3' GACGACAGAAGAAGACAAGGU 5'
query=miR156i, target=RO3G_17460, score=4, range=218-237, strand=
target 5' CUGCU-UCUGCUUCUGUUCCU 3'
::::: ::: ::::::::::
query 3' GACGACAGAAGAAGACAAGGU 5'
query=miR156i, target=RO3G_13452, score=4, range=170-189, strand=
target 5' CUGCU-UCUGCUUCUGUUCCU 3'
::::: ::: ::::::::::
query 3' GACGACAGAAGAAGACAAGGU 5'
query=miR156i, target=RO3G_16369, score=4, range=173-192, strand=
target 5' CUGCU-UCUGCUUCUGUUCCU 3'
::::: ::: ::::::::::
query 3' GACGACAGAAGAAGACAAGGU 5'
query=miR156i, target=RO3G_09603, score=4, range=179-198, strand=
target 5' CUGCU-UCUGCUUCUGUUCCU 3'
::::: ::: ::::::::::
query 3' GACGACAGAAGAAGACAAGGU 5'
query=miR156i, target=RO3G_13146, score=4, range=161-180, strand=
target 5' CUGCU-UCUGCUUCUGUUCCU 3'
```

```
::::: ::: ::::::::::
query 3' GACGACAGAAGAAGACAAGGU 5'
query=miR156i, target=RO3G_12668, score=4, range=203-222, strand=
target 5' CUGCU-UCUUCUUCUGCUCCU 3'
::::: :::::::::: :::
query 3' GACGACAGAAGAAGACAAGGU 5'
query=miR156i, target=RO3G_05425, score=4, range=167-186, strand=
target 5' CUGCU-UCUGCUUCUGUUCCU 3'
::::: ::: ::::::::::
query 3' GACGACAGAAGAAGACAAGGU 5'
query=miR156i, target=RO3G_16137, score=4, range=161-180, strand=
target 5' CUGCU-UCUGCUUCUGUUCCU 3'
::::: ::: ::::::::::
query 3' GACGACAGAAGAAGACAAGGU 5'
query=miR156i, target=RO3G_10218, score=4, range=167-186, strand=
target 5' CUGCU-UCUGCUUCUGUUCCU 3'
::::: ::: ::::::::::
query 3' GACGACAGAAGAAGACAAGGU 5'
query=miR156i, target=RO3G_11543, score=4, range=194-213, strand=
target 5' CUGCU-UCUGCUUCUGUUCCU 3'
::::: ::: ::::::::::
query 3' GACGACAGAAGAAGACAAGGU 5'
query=miR156i, target=RO3G_14553, score=4, range=194-213, strand=
target 5' CUGCU-UCUGCUUCUGUUCCU 3'
::::: ::: ::::::::::
query 3' GACGACAGAAGAAGACAAGGU 5'
query=miR156i, target=RO3G_12962, score=4, range=290-309, strand=
target 5' CUGCU-UCUUCUUCUGCUCCU 3'
::::: :::::::::: :::
query 3' GACGACAGAAGAAGACAAGGU 5'
query=miR156i, target=RO3G_16748, score=4, range=137-156, strand=
target 5' CUGCU-UCUGCUUCUGUUCCU 3'
::::: ::: ::::::::::
query 3' GACGACAGAAGAAGACAAGGU 5'
query=miR156i, target=RO3G_15887, score=4, range=218-237, strand=
target 5' CUGCU-UCUGCUUCUGUUCCU 3'
::::: ::: ::::::::::
query 3' GACGACAGAAGAAGACAAGGU 5'
query=miR156i, target=RO3G_02228, score=4, range=137-156, strand=
target 5' CUGCU-UCUGCUUCUGUUCCU 3'
::::: ::: ::::::::::
query 3' GACGACAGAAGAAGACAAGGU 5'
query=miR156i, target=RO3G_01192, score=4, range=131-150, strand=
target 5' CUGCU-UCUGCUUCUGUUCCU 3'
::::: ::: ::::::::::
```

query 3' GACGACAGAAGAAGACAAGGU 5'
query=miR156i, target=RO3G_17244, score=4, range=191-210, strand=
target 5' CUGCU-UCUGCUUCUGUUCCU 3'
::::: ::: ::::::::::
query 3' GACGACAGAAGAAGACAAGGU 5'
query=miR156i, target=RO3G_05347, score=4, range=194-213, strand=
target 5' CUGCU-UCUGCUUCUGUUCCU 3'
::::: ::: ::::::::::
query 3' GACGACAGAAGAAGACAAGGU 5'
query=miR156i, target=RO3G_17210, score=4, range=218-237, strand=
target 5' CUGCU-UCUGCUUCUGUUCCU 3'
::::: ::: ::::::::::
query 3' GACGACAGAAGAAGACAAGGU 5'
query=miR156i, target=RO3G_13272, score=4, range=220-239, strand=
target 5' CUGCU-UCUGCUUCUGUUCCU 3'
::::: ::: ::::::::::
query 3' GACGACAGAAGAAGACAAGGU 5'
query=miR156i, target=RO3G_12976, score=4, range=197-216, strand=
target 5' CUGCU-UCUGCUUCUGUUCCU 3'
::::: ::: ::::::::::
query 3' GACGACAGAAGAAGACAAGGU 5'
query=miR156i, target=RO3G_04659, score=4, range=128-147, strand=
target 5' CUGCU-UCUGCUUCUGUUCCU 3'
::::: ::: ::::::::::
query 3' GACGACAGAAGAAGACAAGGU 5'
query=miR156i, target=RO3G_13121, score=4, range=3829-3849, stran
target 5' UUCUUGUCUUCUUCUGCUCCA 3'
.: .:::::::::::: ::::
query 3' GACGACAGAAGAAGACAAGGU 5'
query=miR156i, target=RO3G_05595, score=4, range=513-534, strand=
target 5' CUGCUGUUCUUCUUCUGUUUGA 3'
:::::: ::::::::::::. :
query 3' GACGAC-AGAAGAAGACAAGGU 5'
query=miR156i, target=RO3G_05251, score=4, range=455-475, strand=
target 5' CUGAUGUCUUCUUCUUUUCCU 3'
::: ::::::::::: ::::
query 3' GACGACAGAAGAAGACAAGGU 5'
query=miR156i, target=RO3G_02549, score=4, range=213-232, strand=
target 5' CU-UUUUCUUCUUCUGUUUCG 3'
:: .: ::::::::::::.:.
query 3' GACGACAGAAGAAGACAAGGU 5'
query=miR156i, target=RO3G_06597, score=4, range=3060-3079, stran
target 5' CAUCUG-CUUCUUCUGUUUCA 3'
: ::: ::::::::::.::
query 3' GACGACAGAAGAAGACAAGGU 5'

```
query=miR156i, target=RO3G_15932, score=4, range=1442-1461, stran
target 5' CUUCU-UCUUUUUCUGUUCCU 3'
:: :: ::::.:::::::::
query 3' GACGACAGAAGAAGACAAGGU 5'
query=miR156i, target=RO3G_06191, score=4, range=937-956, strand=
target 5' CUUCUUUCUUCUUCUG-UCCA 3'
:: :: :::::::::: ::::
query 3' GACGACAGAAGAAGACAAGGU 5'
query=miR4374b, target=RO3G_17462, score=0, range=964-983, strand
target 5' CUCACAACCGUCUUUGAAGA 3'
::::::::::::::::::::
query 3' GAGUGUUGGCAGAAACUUCU 5'
query=miR4374b, target=RO3G_17451, score=0, range=73-92, strand=1
target 5' CUCACAACCGUCUUUGAAGA 3'
::::::::::::::::::::
query 3' GAGUGUUGGCAGAAACUUCU 5'
query=miR4374b, target=RO3G_13914, score=0, range=832-851, strand
target 5' CUCACAACCGUCUUUGAAGA 3'
::::::::::::::::::::
query 3' GAGUGUUGGCAGAAACUUCU 5'
query=miR4374b, target=RO3G_12058, score=0, range=832-851, strand
target 5' CUCACAACCGUCUUUGAAGA 3'
::::::::::::::::::::
query 3' GAGUGUUGGCAGAAACUUCU 5'
query=miR4374b, target=RO3G_02233, score=0, range=361-380, strand
target 5' CUCACAACCGUCUUUGAAGA 3'
::::::::::::::::::::
query 3' GAGUGUUGGCAGAAACUUCU 5'
query=miR4374b, target=RO3G_14983, score=0, range=637-656, strand
target 5' CUCACAACCGUCUUUGAAGA 3'
::::::::::::::::::::
query 3' GAGUGUUGGCAGAAACUUCU 5'
query=miR4374b, target=RO3G_10801, score=0, range=637-656, strand
target 5' CUCACAACCGUCUUUGAAGA 3'
::::::::::::::::::::
query 3' GAGUGUUGGCAGAAACUUCU 5'
query=miR4374b, target=RO3G_09614, score=0, range=163-182, strand
target 5' CUCACAACCGUCUUUGAAGA 3'
::::::::::::::::::::
query 3' GAGUGUUGGCAGAAACUUCU 5'
query=miR4374b, target=RO3G_03583, score=2, range=637-656, strand
target 5' CUCACAACAGUCUUUGAAGA 3'
:::::::: :::::::::::
query 3' GAGUGUUGGCAGAAACUUCU 5'
query=miR4374b, target=RO3G_12667, score=2, range=202-221, strand
```

```
target 5' CUCACAACCGUCUUGGAAGA 3'
::::::::::::: :::::
query 3' GAGUGUUGGCAGAAACUUCU 5'
query=miR4374b, target=RO3G_03116, score=3, range=256-276, strand
target 5' CUGCAUGACCGUUUUUGAAGA 3'
:: ::..::::.::::::::
query 3' GA-GUGUUGGCAGAAACUUCU 5'
query=miR4374b, target=RO3G_09836, score=3, range=780-798, strand
target 5' CUCAU-GCUGUCUUUGAAGA 3'
::::. .:.:::::::::::
query 3' GAGUGUUGGCAGAAACUUCU 5'
query=miR4374b, target=RO3G_11295, score=3.5, range=25-44, strand
target 5' ACCAUAACUGUCUUUGAAGA 3'
::.:::.::::::::::
query 3' GAGUGUUGGCAGAAACUUCU 5'
query=miR4374b, target=RO3G_16714, score=3.5, range=190-209, stra
target 5' UUGACAACCGUCUUAGAAGA 3'
.: :::::::::: :::::
query 3' GAGUGUUGGCAGAAACUUCU 5'
query=miR4374b, target=RO3G_09352, score=3.5, range=336-356, stra
target 5' CUCAAAAUCGUCUUAUGAAGA 3'
:::: ::.::::: ::::::
query 3' GAGUGUUGGCAGAA-ACUUCU 5'
query=miR4374b, target=RO3G_09578, score=3.5, range=2535-2553,
strand=-1
target 5' UUCA-AACUGUCUUUGAAGC 3'
.::: :::.:::::::::
query 3' GAGUGUUGGCAGAAACUUCU 5'
query=miR4374b, target=RO3G_15506, score=4, range=1832-1850, stra
target 5' CUU-CUGCUGUCUUUGAAGA 3'
::. : .:.:::::::::::
query 3' GAGUGUUGGCAGAAACUUCU 5'
query=miR4374b, target=RO3G_10348, score=4, range=1194-1213, stra
target 5' CUUGAAAUCGUCUUUGAGGA 3'
::.. ::.::::::::.::
query 3' GAGUGUUGGCAGAAACUUCU 5'
query=miR4374b, target=RO3G_11686, score=4, range=2820-2838, stra
target 5' CUCA-GGCCGUCUUUGAACA 3'
:::: ..:::::::::: :
query 3' GAGUGUUGGCAGAAACUUCU 5'
query=miR4374b, target=RO3G_13429, score=4, range=1953-1971, stra
target 5' CUCA-GGCCGUCUUUGAACA 3'
:::: ..:::::::::: :
query 3' GAGUGUUGGCAGAAACUUCU 5'
query=miR4374b, target=RO3G_07567, score=4, range=1122-1140, stra
```

```
target 5' CUCA-GGCCGUCUUUGAACA 3'
:::: ..:::::::::: :
query 3' GAGUGUUGGCAGAAACUUCU 5'
query=miR4374b, target=RO3G_09089, score=4, range=1122-1140, stra
target 5' CUCA-GGCCGUCUUUGAACA 3'
:::: ..:::::::::: :
query 3' GAGUGUUGGCAGAAACUUCU 5'
query=miR4374b, target=RO3G_15417, score=4, range=1371-1389, stra
target 5' CUCA-GGCCGUCUUUGAACA 3'
:::: ..:::::::::: :
query 3' GAGUGUUGGCAGAAACUUCU 5'
query=miR4374b, target=RO3G_15078, score=4, range=1122-1140, stra
target 5' CUCA-GGCCGUCUUUGAACA 3'
:::: ..:::::::::: :
query 3' GAGUGUUGGCAGAAACUUCU 5'
query=miR4374b, target=RO3G_09251, score=4, range=267-285, strand
target 5' CUCA-GGCCGUCUUUGAACA 3'
:::: ..:::::::::: :
query 3' GAGUGUUGGCAGAAACUUCU 5'
query=miR4374b, target=RO3G_13414, score=4, range=99-117, strand=
target 5' CUCA-GGCCGUCUUUGAACA 3'
:::: ..:::::::::: :
query 3' GAGUGUUGGCAGAAACUUCU 5'
query=miR4374b, target=RO3G_14856, score=4, range=924-942, strand
target 5' CUCA-GGCCGUCUUUGAACA 3'
:::: ..:::::::::: :
query 3' GAGUGUUGGCAGAAACUUCU 5'
query=miR4374b, target=RO3G_14875, score=4, range=924-942, strand
target 5' CUCA-GGCCGUCUUUGAACA 3'
:::: ..:::::::::: :
query 3' GAGUGUUGGCAGAAACUUCU 5'
query=miR4374b, target=RO3G_15709, score=4, range=264-282, strand
target 5' CUCA-GGCCGUCUUUGAACA 3'
:::: ..:::::::::: :
query 3' GAGUGUUGGCAGAAACUUCU 5'
query=miR4374b, target=RO3G_10584, score=4, range=747-765, strand
target 5' CUCA-GGCCGUCUUUGAACA 3'
:::: ..:::::::::: :
query 3' GAGUGUUGGCAGAAACUUCU 5'
query=miR4374b, target=RO3G_14154, score=4, range=99-117, strand=
target 5' CUCA-GGCCGUCUUUGAACA 3'
:::: ..:::::::::: :
query 3' GAGUGUUGGCAGAAACUUCU 5'
query=miR826b, target=RO3G_15287, score=0, range=828-849, strand=
target 5' AUUUUCAUCGAUGUCAAGACCA 3'
```

```
::::::::::::::::::::::
query 3' UAAAAGUAGCUACAGUUCUGGU 5'
query=miR826b, target=RO3G_07134, score=0, range=273-294, strand=
target 5' AUUUUCAUCGAUGUCAAGACCA 3'
::::::::::::::::::::::
query 3' UAAAAGUAGCUACAGUUCUGGU 5'
query=miR826b, target=RO3G_13291, score=0, range=273-294, strand=
target 5' AUUUUCAUCGAUGUCAAGACCA 3'
::::::::::::::::::::::
query 3' UAAAAGUAGCUACAGUUCUGGU 5'
query=miR826b, target=RO3G_01722, score=0, range=294-315, strand=
target 5' AUUUUCAUCGAUGUCAAGACCA 3'
::::::::::::::::::::::
query 3' UAAAAGUAGCUACAGUUCUGGU 5'
query=miR826b, target=RO3G_02952, score=0, range=294-315, strand=
target 5' AUUUUCAUCGAUGUCAAGACCA 3'
::::::::::::::::::::::
query 3' UAAAAGUAGCUACAGUUCUGGU 5'
query=miR826b, target=RO3G_17431, score=0, range=261-282, strand=
target 5' AUUUUCAUCGAUGUCAAGACCA 3'
::::::::::::::::::::::
query 3' UAAAAGUAGCUACAGUUCUGGU 5'
query=miR826b, target=RO3G_17247, score=0, range=261-282, strand=
target 5' AUUUUCAUCGAUGUCAAGACCA 3'
::::::::::::::::::::::
query 3' UAAAAGUAGCUACAGUUCUGGU 5'
query=miR826b, target=RO3G_15165, score=0, range=261-282, strand=
target 5' AUUUUCAUCGAUGUCAAGACCA 3'
::::::::::::::::::::::
query 3' UAAAAGUAGCUACAGUUCUGGU 5'
query=miR826b, target=RO3G_10547, score=0, range=261-282, strand=
target 5' AUUUUCAUCGAUGUCAAGACCA 3'
::::::::::::::::::::::
query 3' UAAAAGUAGCUACAGUUCUGGU 5'
query=miR826b, target=RO3G_07127, score=0, range=261-282, strand=
target 5' AUUUUCAUCGAUGUCAAGACCA 3'
::::::::::::::::::::::
query 3' UAAAAGUAGCUACAGUUCUGGU 5'
query=miR826b, target=RO3G_09164, score=0, range=261-282, strand=
target 5' AUUUUCAUCGAUGUCAAGACCA 3'
::::::::::::::::::::::
query 3' UAAAAGUAGCUACAGUUCUGGU 5'
query=miR826b, target=RO3G_17323, score=0, range=261-282, strand=
target 5' AUUUUCAUCGAUGUCAAGACCA 3'
::::::::::::::::::::::
```

```
query 3' UAAAAGUAGCUACAGUUCUGGU 5'
query=miR826b, target=RO3G_17283, score=0, range=216-237, strand=
target 5' AUUUUCAUCGAUGUCAAGACCA 3'
::::::::::::::::::::::
query 3' UAAAAGUAGCUACAGUUCUGGU 5'
query=miR826b, target=RO3G_12068, score=0, range=261-282, strand=
target 5' AUUUUCAUCGAUGUCAAGACCA 3'
::::::::::::::::::::::
query 3' UAAAAGUAGCUACAGUUCUGGU 5'
query=miR826b, target=RO3G_17219, score=0, range=261-282, strand=
target 5' AUUUUCAUCGAUGUCAAGACCA 3'
::::::::::::::::::::::
query 3' UAAAAGUAGCUACAGUUCUGGU 5'
query=miR826b, target=RO3G_14977, score=0, range=273-294, strand=
target 5' AUUUUCAUCGAUGUCAAGACCA 3'
::::::::::::::::::::::
query 3' UAAAAGUAGCUACAGUUCUGGU 5'
query=miR826b, target=RO3G_16806, score=0, range=273-294, strand=
target 5' AUUUUCAUCGAUGUCAAGACCA 3'
::::::::::::::::::::::
query 3' UAAAAGUAGCUACAGUUCUGGU 5'
query=miR826b, target=RO3G_14335, score=3, range=45-66, strand=-1
target 5' AUCUUUUUUGAUGUCAAGACCA 3'
:: ::. :.:::::::::::::
query 3' UAAAAGUAGCUACAGUUCUGGU 5'
query=miR826b, target=RO3G_14707, score=4, range=261-282, strand=
target 5' AUUUUCAUCAAUCUCAAGACCA 3'
::::::::: :: :::::::::
query 3' UAAAAGUAGCUACAGUUCUGGU 5'
query=miR826b, target=RO3G_12006, score=4, range=261-282, strand=
target 5' AUUUUCAUCAAUCUCAAGACCA 3'
::::::::: :: :::::::::
query 3' UAAAAGUAGCUACAGUUCUGGU 5'
query=miR826b, target=RO3G_16910, score=4, range=261-282, strand=
target 5' AUUUUCAUCAAUCUCAAGACCA 3'
::::::::: :: :::::::::
query 3' UAAAAGUAGCUACAGUUCUGGU 5'
query=miR826b, target=RO3G_17245, score=4, range=261-282, strand=
target 5' AUUUUCAUCAAUCUCAAGACCA 3'
::::::::: :: :::::::::
query 3' UAAAAGUAGCUACAGUUCUGGU 5'
query=miR826b, target=RO3G_11285, score=4, range=261-282, strand=
target 5' AUUUUCAUCAAUCUCAAGACCA 3'
::::::::: :: :::::::::
query 3' UAAAAGUAGCUACAGUUCUGGU 5'
```

```
query=miR826b, target=RO3G_13710, score=4, range=261-282, strand=
target 5' AUUUUCAUCAAUCUCAAGACCA 3'
::::::::: :: :::::::::
query 3' UAAAAGUAGCUACAGUUCUGGU 5'
query=miR826b, target=RO3G_02275, score=4, range=3357-3378, stran
target 5' AUUUUUUUUGAUGUCAAGAACA 3'
:::::. :.:::::::::: ::
query 3' UAAAAGUAGCUACAGUUCUGGU 5'
query=miR826b, target=RO3G_17365, score=4, range=261-282, strand=
target 5' AUUUUCAUCAAUCUCAAGACCA 3'
::::::::: :: :::::::::
query 3' UAAAAGUAGCUACAGUUCUGGU 5'
query=miR826b, target=RO3G_01196, score=4, range=261-282, strand=
target 5' AUUUUCAUCAAUCUCAAGACCA 3'
::::::::: :: :::::::::
query 3' UAAAAGUAGCUACAGUUCUGGU 5'
query=miR826b, target=RO3G_13738, score=4, range=261-282, strand=
target 5' AUUUUCAUCAAUCUCAAGACCA 3'
::::::::: :: :::::::::
query 3' UAAAAGUAGCUACAGUUCUGGU 5'
query=miR7800, target=RO3G_08663, score=2, range=437-456, strand=
target 5' GGUCAGACGAGACGAAAAUA 3'
:..:::::::::.::::::
query 3' CUGGUCUGCUCUGUUUUUAU 5'
query=miR7800, target=RO3G_06722, score=2, range=437-456, strand=
target 5' GGUCAGACGAGACGAAAAUA 3'
:..:::::::::.::::::
query 3' CUGGUCUGCUCUGUUUUUAU 5'
query=miR7800, target=RO3G_07112, score=2, range=260-279, strand=
target 5' GGUCAGACGAGACGAAAAUA 3'
:..:::::::::.::::::
query 3' CUGGUCUGCUCUGUUUUUAU 5'
query=miR7800, target=RO3G_16359, score=2, range=437-456, strand=
target 5' GGUCAGACGAGACGAAAAUA 3'
:..:::::::::.::::::
query 3' CUGGUCUGCUCUGUUUUUAU 5'
query=miR7800, target=RO3G_13271, score=2, range=437-456, strand=
target 5' GGUCAGACGAGACGAAAAUA 3'
:..:::::::::.::::::
query 3' CUGGUCUGCUCUGUUUUUAU 5'
query=miR7800, target=RO3G_09612, score=2, range=437-456, strand=
target 5' GGUCAGACGAGACGAAAAUA 3'
:..:::::::::.::::::
query 3' CUGGUCUGCUCUGUUUUUAU 5'
query=miR7800, target=RO3G_10866, score=2, range=437-456, strand=
```

target 5' GGUCAGACGAGACGAAAAUA 3'
:..::::::::::.::::::
query 3' CUGGUCUGCUCUGUUUUUAU 5'
query=miR7800, target=RO3G_16545, score=2, range=437-456, strand=
target 5' GGUCAGACGAGACGAAAAUA 3'
:..::::::::::.::::::
query 3' CUGGUCUGCUCUGUUUUUAU 5'
query=miR7800, target=RO3G_00255, score=2, range=437-456, strand=
target 5' GGUCAGACGAGACGAAAAUA 3'
:..::::::::::.::::::
query 3' CUGGUCUGCUCUGUUUUUAU 5'
query=miR7800, target=RO3G_13266, score=2, range=215-234, strand=
target 5' GGUCAGACGAGACGAAAAUA 3'
:..::::::::::.::::::
query 3' CUGGUCUGCUCUGUUUUUAU 5'
query=miR7800, target=RO3G_13277, score=2, range=437-456, strand=
target 5' GGUCAGACGAGACGAAAAUA 3'
:..::::::::::.::::::
query 3' CUGGUCUGCUCUGUUUUUAU 5'
query=miR7800, target=RO3G_11266, score=2, range=260-279, strand=
target 5' GGUCAGACGAGACGAAAAUA 3'
:..::::::::::.::::::
query 3' CUGGUCUGCUCUGUUUUUAU 5'
query=miR7800, target=RO3G_07120, score=2, range=260-279, strand=
target 5' GGUCAGACGAGACGAAAAUA 3'
:..::::::::::.::::::
query 3' CUGGUCUGCUCUGUUUUUAU 5'
query=miR7800, target=RO3G_15001, score=2, range=260-279, strand=
target 5' GGUCAGACGAGACGAAAAUA 3'
:..::::::::::.::::::
query 3' CUGGUCUGCUCUGUUUUUAU 5'
query=miR7800, target=RO3G_11240, score=2, range=260-279, strand=
target 5' GGUCAGACGAGACGAAAAUA 3'
:..::::::::::.::::::
query 3' CUGGUCUGCUCUGUUUUUAU 5'
query=miR7800, target=RO3G_07031, score=2, range=437-456, strand=
target 5' GGUCAGACGAGACAAAAAUC 3'
:..::::::::::::::::
query 3' CUGGUCUGCUCUGUUUUUAU 5'
query=miR7800, target=RO3G_16554, score=2, range=437-456, strand=
target 5' GGUCAGACGAGACAAAAAUC 3'
:..::::::::::::::::
query 3' CUGGUCUGCUCUGUUUUUAU 5'
query=miR7800, target=RO3G_15146, score=2, range=317-336, strand=
target 5' GGUCAGACGAGACAAAAAUC 3'

```
:..::::::::::::::::
query 3' CUGGUCUGCUCUGUUUUUAU 5'
query=miR7800, target=RO3G_08842, score=2.5, range=437-456, stran
target 5' GGUCGGACGAGACAAAAAUC 3'
:..:.::::::::::::::
query 3' CUGGUCUGCUCUGUUUUUAU 5'
query=miR7800, target=RO3G_16363, score=2.5, range=437-456, stran
target 5' GGUCGGACGAGACAAAAAUC 3'
:..:.::::::::::::::
query 3' CUGGUCUGCUCUGUUUUUAU 5'
query=miR7800, target=RO3G_07688, score=3, range=437-456, strand=
target 5' GGUCAGAUGAGACGAAAAUA 3'
:..::::.:::::.::::::
query 3' CUGGUCUGCUCUGUUUUUAU 5'
query=miR7800, target=RO3G_15575, score=3, range=260-279, strand=
target 5' GGUCAAACGAGACGAAAAUA 3'
:..:: :::::::.::::::
query 3' CUGGUCUGCUCUGUUUUUAU 5'
query=miR7800, target=RO3G_07877, score=3.5, range=215-234, stran
target 5' GGUCGGAUGAGACAAAAAUC 3'
:..:.::.:::::::::::
query 3' CUGGUCUGCUCUGUUUUUAU 5'
query=miR7800, target=RO3G_13718, score=3.5, range=215-234, stran
target 5' GGUCGGAUGAGACAAAAAUC 3'
:..:.::.:::::::::::
query 3' CUGGUCUGCUCUGUUUUUAU 5'
query=miR7800, target=RO3G_13147, score=3.5, range=215-234, stran
target 5' GGUCGGAUGAGACAAAAAUC 3'
:..:.::.:::::::::::
query 3' CUGGUCUGCUCUGUUUUUAU 5'
query=miR7800, target=RO3G_03772, score=3.5, range=437-456, stran
target 5' GGUCGGAUGAGACAAAAAUC 3'
:..:.::.:::::::::::
query 3' CUGGUCUGCUCUGUUUUUAU 5'
query=miR7800, target=RO3G_13733, score=3.5, range=215-234, stran
target 5' GGUCGGAUGAGACAAAAAUC 3'
:..:.::.:::::::::::
query 3' CUGGUCUGCUCUGUUUUUAU 5'
query=miR7800, target=RO3G_17363, score=3.5, range=179-198, stran
target 5' GGUCGGAUGAGACAAAAAUC 3'
:..:.::.:::::::::::
query 3' CUGGUCUGCUCUGUUUUUAU 5'
query=miR7800, target=RO3G_03113, score=3.5, range=179-198, stran
target 5' GGUCGGAUGAGACAAAAAUC 3'
:..:.::.:::::::::::
```

```
query 3' CUGGUCUGCUCUGUUUUUAU 5'
query=miR7800, target=RO3G_02844, score=3.5, range=215-234, stran
target 5' GGUCGGAUGAGACAAAAAUC 3'
:..:.::.::::::::::
query 3' CUGGUCUGCUCUGUUUUUAU 5'
query=miR7800, target=RO3G_03766, score=3.5, range=215-234, stran
target 5' GGUCGGAUGAGACAAAAAUC 3'
:..:.::.::::::::::
query 3' CUGGUCUGCUCUGUUUUUAU 5'
query=miR7800, target=RO3G_13137, score=3.5, range=437-456, stran
target 5' GGUCGGAUGAGACAAAAAUC 3'
:..:.::.::::::::::
query 3' CUGGUCUGCUCUGUUUUUAU 5'
query=miR7800, target=RO3G_01618, score=3.5, range=437-456, stran
target 5' GGUCGGAUGAGACAAAAAUC 3'
:..:.::.::::::::::
query 3' CUGGUCUGCUCUGUUUUUAU 5'
query=miR7800, target=RO3G_14150, score=4, range=118-137, strand=
target 5' GACGAGAUGAGGCAAAGAUA 3'
::: :::.:::.::::.:::
query 3' CUGGUCUGCUCUGUUUUUAU 5'
query=miR7800, target=RO3G_03803, score=4, range=260-279, strand=
target 5' GGUCAGAAGAGACGAAAAUA 3'
:..:::: ::::.::::::
query 3' CUGGUCUGCUCUGUUUUUAU 5'
query=miR7800, target=RO3G_13502, score=4, range=268-287, strand=
target 5' GACCAGUCGAGAAAAGAAUA 3'
:::::: ::::: ::.::::
query 3' CUGGUCUGCUCUGUUUUUAU 5'
query=miR7800, target=RO3G_00482, score=4, range=116-136, strand=
target 5' GAACAGACGAGAUGAAAAAUA 3'
:: :::::::::. :::::::
query 3' CUGGUCUGCUCUG-UUUUUAU 5'
query=miR7800, target=RO3G_15651, score=4, range=232-250, strand=
target 5' GA-CAGACGAUACAAAAAUC 3'
:: ::::::: ::::::::
query 3' CUGGUCUGCUCUGUUUUUAU 5'
query=miR7800, target=RO3G_06934, score=4, range=1364-1384, stran
target 5' GACCAGCAGAGACCAAAAAUA 3'
:::::: :::: ::::::::
query 3' CUGGUCUGCUCU-GUUUUUAU 5'
query=miR9759, target=RO3G_02835, score=0, range=188-209, strand=
target 5' AUAAACUCGUACUUGCUUACUA 3'
::::::::::::::::::::::
query 3' UAUUUGAGCAUGAACGAAUGAU 5'
```

```
query=miR9759, target=RO3G_07109, score=0, range=188-209, strand=
target 5' AUAAACUCGUACUUGCUUACUA 3'
::::::::::::::::::::::
query 3' UAUUUGAGCAUGAACGAAUGAU 5'
query=miR9759, target=RO3G_09197, score=0, range=188-209, strand=
target 5' AUAAACUCGUACUUGCUUACUA 3'
::::::::::::::::::::::
query 3' UAUUUGAGCAUGAACGAAUGAU 5'
query=miR9759, target=RO3G_09934, score=0, range=188-209, strand=
target 5' AUAAACUCGUACUUGCUUACUA 3'
::::::::::::::::::::::
query 3' UAUUUGAGCAUGAACGAAUGAU 5'
query=miR9759, target=RO3G_15283, score=0, range=188-209, strand=
target 5' AUAAACUCGUACUUGCUUACUA 3'
::::::::::::::::::::::
query 3' UAUUUGAGCAUGAACGAAUGAU 5'
query=miR9759, target=RO3G_10526, score=0, range=188-209, strand=
target 5' AUAAACUCGUACUUGCUUACUA 3'
::::::::::::::::::::::
query 3' UAUUUGAGCAUGAACGAAUGAU 5'
query=miR9759, target=RO3G_01935, score=0, range=188-209, strand=
target 5' AUAAACUCGUACUUGCUUACUA 3'
::::::::::::::::::::::
query 3' UAUUUGAGCAUGAACGAAUGAU 5'
query=miR9759, target=RO3G_02813, score=3.5, range=830-852, stran
target 5' AUGAAGAUCGUAUUUGCUUACUA 3'
:: ::. :::::.:::::::::
query 3' UA-UUUGAGCAUGAACGAAUGAU 5'
query=miR9759, target=RO3G_07672, score=4, range=188-209, strand=
target 5' AUAAACUCGUACUUGCCUACCA 3'
:::::::::::::::: ::: :
query 3' UAUUUGAGCAUGAACGAAUGAU 5'
query=miR857, target=RO3G_12721, score=1.5, range=81-100, strand=
target 5' CGCACCUUUAACAUACAAAA 3'
:.::::::.:::::::::::
query 3' GUGUGGAAGUUGUAUGUUUU 5'
query=miR857, target=RO3G_06407, score=3, range=306-325, strand=1
target 5' GACACCUUUAACAUACAAGA 3'
:::::::.:::::::::.:
query 3' GUGUGGAAGUUGUAUGUUUU 5'
query=miR857, target=RO3G_02274, score=3, range=2480-2499, strand
target 5' UGCCUUUUCAACAUACAAAA 3'
..: ..:::::::::::::
query 3' GUGUGGAAGUUGUAUGUUUU 5'
query=miR857, target=RO3G_13922, score=3, range=1522-1540, strand
```

target 5' CA-AAUUUCAACAUACAAAG 3'
:: : .:::::::::::::.
query 3' GUGUGGAAGUUGUAUGUUUU 5'
query=miR857, target=RO3G_16060, score=3.5, range=930-949, strand
target 5' CACAUCUUCAACAUGCAAGU 3'
::::.:::::::::.:::.
query 3' GUGUGGAAGUUGUAUGUUUU 5'
query=miR857, target=RO3G_07199, score=3.5, range=152-170, strand
target 5' UACG-UUUCGACAUACAAAA 3'
.::. .:::.::::::::::
query 3' GUGUGGAAGUUGUAUGUUUU 5'
query=miR857, target=RO3G_01274, score=3.5, range=378-397, strand
target 5' UAGCCCUUUAACAUACAAAA 3'
.: ::::.:::::::::::
query 3' GUGUGGAAGUUGUAUGUUUU 5'
query=miR857, target=RO3G_03519, score=3.5, range=1670-1689, stra
target 5' CACAUCAUCAGCAUAUAAAA 3'
::::.: :::.::::.::::
query 3' GUGUGGAAGUUGUAUGUUUU 5'
query=miR857, target=RO3G_03719, score=3.5, range=1586-1605, stra
target 5' CACAUCGUCAGCAUAUAAAA 3'
::::.: :::.::::.::::
query 3' GUGUGGAAGUUGUAUGUUUU 5'
query=miR857, target=RO3G_00794, score=3.5, range=1586-1605, stra
target 5' CACAUCGUCAGCAUAUAAAA 3'
::::.: :::.::::.::::
query 3' GUGUGGAAGUUGUAUGUUUU 5'
query=miR857, target=RO3G_08299, score=3.5, range=1232-1251, stra
target 5' CACAUCGUCAGCAUAUAAAA 3'
::::.: :::.::::.::::
query 3' GUGUGGAAGUUGUAUGUUUU 5'
query=miR857, target=RO3G_06780, score=3.5, range=761-780, strand
target 5' CACAUCAUCAGCAUACAAGA 3'
::::.: :::.:::::::.:
query 3' GUGUGGAAGUUGUAUGUUUU 5'
query=miR857, target=RO3G_01914, score=3.5, range=785-804, strand
target 5' AAUUUCUUCAACAUACAAAG 3'
:. .::::::::::::::.
query 3' GUGUGGAAGUUGUAUGUUUU 5'
query=miR857, target=RO3G_09731, score=3.5, range=1721-1740, stra
target 5' UUCACCUUCAACAUCCAAAA 3'
. ::::::::::: :::::
query 3' GUGUGGAAGUUGUAUGUUUU 5'
query=miR857, target=RO3G_10103, score=3.5, range=986-1005, stran
target 5' CAAAAUAUCAACAUACAAAA 3'

```
:: : . ::::::::::::
query 3' GUGUGGAAGUUGUAUGUUUU 5'
query=miR857, target=RO3G_00834, score=4, range=146-165, strand=1
target 5' CACAUUUACAAUAUACAAAA 3'
::::..: :::.:::::::
query 3' GUGUGGAAGUUGUAUGUUUU 5'
query=miR857, target=RO3G_14120, score=4, range=145-164, strand=1
target 5' UACAUCAUCGGCAUACAAAA 3'
.:::.: ::..::::::::
query 3' GUGUGGAAGUUGUAUGUUUU 5'
query=miR857, target=RO3G_12689, score=4, range=83-102, strand=1
target 5' CACAACUUCAAUAUACAGAC 3'
:::: :::::.:::::.:
query 3' GUGUGGAAGUUGUAUGUUUU 5'
query=miR857, target=RO3G_10706, score=4, range=30-48, strand=1
target 5' UAUACCUUUAACA-ACAAAA 3'
.:.:::::.:::: ::::::
query 3' GUGUGGAAGUUGUAUGUUUU 5'
query=miR857, target=RO3G_14364, score=4, range=146-165, strand=1
target 5' CACAGCUUCAACCUACAAAU 3'
:::: ::::::: ::::::
query 3' GUGUGGAAGUUGUAUGUUUU 5'
query=miR857, target=RO3G_06801, score=4, range=744-762, strand=1
target 5' CGCG-AUUCAACAUGCAAAA 3'
:.:. :::::::.:::::
query 3' GUGUGGAAGUUGUAUGUUUU 5'
query=miR857, target=RO3G_00772, score=4, range=445-465, strand=1
target 5' CACCACCGUCAACAUACCAAA 3'
:: :::: ::::::::: :::
query 3' GU-GUGGAAGUUGUAUGUUUU 5'
query=miR857, target=RO3G_17016, score=4, range=271-290, strand=1
target 5' CGAGCGGUCAACAUACAAAA 3'
:. .: ::::::::::::
query 3' GUGUGGAAGUUGUAUGUUUU 5'
query=miR857, target=RO3G_08895, score=4, range=354-372, strand=1
target 5' UUUACCUUCAACA-ACAAAA 3'
. .:::::::::: ::::::
query 3' GUGUGGAAGUUGUAUGUUUU 5'
query=miR857, target=RO3G_00356, score=4, range=536-555, strand=-
target 5' CACACCUUCAACAAAUGAAA 3'
::::::::::::: :..:::
query 3' GUGUGGAAGUUGUAUGUUUU 5'
query=miR857, target=RO3G_14105, score=4, range=164-183, strand=-
target 5' UACAUCAUCGGCAUACAAAA 3'
.:::.: ::..::::::::
```

```
query 3' GUGUGGAAGUUGUAUGUUUU 5'
query=miR6185, target=RO3G_13223, score=0, range=1206-1227, stran
target 5' UACUGUUACCCGUGCUGCCAGA 3'
::::::::::::::::::::::
query 3' AUGACAAUGGGCACGACGGUCU 5'
query=miR6185, target=RO3G_04122, score=0, range=1206-1227, stran
target 5' UACUGUUACCCGUGCUGCCAGA 3'
::::::::::::::::::::::
query 3' AUGACAAUGGGCACGACGGUCU 5'
query=miR6185, target=RO3G_13220, score=0, range=1206-1227, stran
target 5' UACUGUUACCCGUGCUGCCAGA 3'
::::::::::::::::::::::
query 3' AUGACAAUGGGCACGACGGUCU 5'
query=miR6185, target=RO3G_04119, score=0, range=1206-1227, stran
target 5' UACUGUUACCCGUGCUGCCAGA 3'
::::::::::::::::::::::
query 3' AUGACAAUGGGCACGACGGUCU 5'
query=miR1023e-3p, target=RO3G_17387, score=0, range=78-99, stran
target 5' AUGCACUCCAACCCGUUUCCCA 3'
::::::::::::::::::::::
query 3' UACGUGAGGUUGGGCAAAGGGU 5'
query=miR1023e-3p, target=RO3G_01205, score=0, range=78-99, stran
target 5' AUGCACUCCAACCCGUUUCCCA 3'
::::::::::::::::::::::
query 3' UACGUGAGGUUGGGCAAAGGGU 5'
query=miR1023e-3p, target=RO3G_00238, score=0, range=78-99, stran
target 5' AUGCACUCCAACCCGUUUCCCA 3'
::::::::::::::::::::::
query 3' UACGUGAGGUUGGGCAAAGGGU 5'
query=miR1023e-3p, target=RO3G_10795, score=0, range=78-99, stran
target 5' AUGCACUCCAACCCGUUUCCCA 3'
::::::::::::::::::::::
query 3' UACGUGAGGUUGGGCAAAGGGU 5'
query=miR1023e-3p, target=RO3G_16698, score=0, range=78-99, stran
target 5' AUGCACUCCAACCCGUUUCCCA 3'
::::::::::::::::::::::
query 3' UACGUGAGGUUGGGCAAAGGGU 5'
query=miR1023e-3p, target=RO3G_05521, score=0, range=78-99, stran
target 5' AUGCACUCCAACCCGUUUCCCA 3'
::::::::::::::::::::::
query 3' UACGUGAGGUUGGGCAAAGGGU 5'
query=miR1023e-3p, target=RO3G_12539, score=0.5, range=78-99,
strand=-1
target 5' AUGCACUUCAACCCGUUUCCCA 3'
:::::::.::::::::::::::
```

query 3' UACGUGAGGUUGGGCAAAGGGU 5'
query=miR1023e-3p, target=RO3G_09494, score=0.5, range=78-99, strand=-1
target 5' AUGCACUUCAACCCGUUUCCCA 3'
:::::::.::::::::::::::
query 3' UACGUGAGGUUGGGCAAAGGGU 5'
query=miR1023e-3p, target=RO3G_09498, score=0.5, range=78-99, strand=-1
target 5' AUGCACUUCAACCCGUUUCCCA 3'
:::::::.::::::::::::::
query 3' UACGUGAGGUUGGGCAAAGGGU 5'
query=miR1023e-3p, target=RO3G_12542, score=0.5, range=78-99, strand=-1
target 5' AUGCACUUCAACCCGUUUCCCA 3'
:::::::.::::::::::::::
query 3' UACGUGAGGUUGGGCAAAGGGU 5'
query=miR1023e-3p, target=RO3G_05640, score=1, range=78-99, stran
target 5' AUGCACUCCAACUCGUUUCCCA 3'
:::::::::::.::::::::::
query 3' UACGUGAGGUUGGGCAAAGGGU 5'
query=miR1023e-3p, target=RO3G_17416, score=1, range=78-99, stran
target 5' AUGCACUCCAACUCGUUUCCCA 3'
:::::::::::.::::::::::
query 3' UACGUGAGGUUGGGCAAAGGGU 5'
query=miR1023e-3p, target=RO3G_16742, score=1, range=78-99, stran
target 5' AUGCACUCCAACUCGUUUCCCA 3'
:::::::::::.::::::::::
query 3' UACGUGAGGUUGGGCAAAGGGU 5'
query=miR1023e-3p, target=RO3G_17291, score=1, range=78-99, stran
target 5' AUGCACUCCAACUCGUUUCCCA 3'
:::::::::::.::::::::::
query 3' UACGUGAGGUUGGGCAAAGGGU 5'
query=miR1023e-3p, target=RO3G_02824, score=1, range=78-99, stran
target 5' AUGCACUCCAACUCGUUUCCCA 3'
:::::::::::.::::::::::
query 3' UACGUGAGGUUGGGCAAAGGGU 5'
query=miR1023e-3p, target=RO3G_13714, score=1, range=78-99, stran
target 5' AUGCACUCCAACUCGUUUCCCA 3'
:::::::::::.::::::::::
query 3' UACGUGAGGUUGGGCAAAGGGU 5'
query=miR1023e-3p, target=RO3G_16372, score=1, range=78-99, stran
target 5' AUGCACUCCAACUCGUUUCCCA 3'
:::::::::::.::::::::::
query 3' UACGUGAGGUUGGGCAAAGGGU 5'
query=miR1023e-3p, target=RO3G_07705, score=1, range=78-99, stran

```
target 5' AUGCACUCCAACCCGUUUCUCA 3'
:::::::::::::::::::.::
query 3' UACGUGAGGUUGGGCAAAGGGU 5'
query=miR1023e-3p, target=RO3G_06717, score=1, range=78-99, stran
target 5' AUGCACUCCAACUCGUUUCCCA 3'
::::::::::::.:::::::::
query 3' UACGUGAGGUUGGGCAAAGGGU 5'
query=miR1023e-3p, target=RO3G_17399, score=1, range=78-99, stran
target 5' AUGCACUCCAACUCGUUUCCCA 3'
::::::::::::.:::::::::
query 3' UACGUGAGGUUGGGCAAAGGGU 5'
query=miR1023e-3p, target=RO3G_16123, score=1, range=78-99, stran
target 5' AUGCACUCCAACUCGUUUCCCA 3'
::::::::::::.:::::::::
query 3' UACGUGAGGUUGGGCAAAGGGU 5'
query=miR1023e-3p, target=RO3G_01328, score=1, range=78-99, stran
target 5' AUGCACUCCAACUCGUUUCCCA 3'
::::::::::::.:::::::::
query 3' UACGUGAGGUUGGGCAAAGGGU 5'
query=miR1023e-3p, target=RO3G_07137, score=1, range=78-99, stran
target 5' AUGCACUCCAACUCGUUUCCCA 3'
::::::::::::.:::::::::
query 3' UACGUGAGGUUGGGCAAAGGGU 5'
query=miR1023e-3p, target=RO3G_05268, score=1, range=78-99, stran
target 5' AUGCACUCCAACUCGUUUCCCA 3'
::::::::::::.:::::::::
query 3' UACGUGAGGUUGGGCAAAGGGU 5'
query=miR1023e-3p, target=RO3G_17355, score=1, range=78-99, stran
target 5' AUGCACUCCAACUCGUUUCCCA 3'
::::::::::::.:::::::::
query 3' UACGUGAGGUUGGGCAAAGGGU 5'
query=miR1023e-3p, target=RO3G_12663, score=1, range=78-99, stran
target 5' AUGCACUCCAACUCGUUUCCCA 3'
::::::::::::.:::::::::
query 3' UACGUGAGGUUGGGCAAAGGGU 5'
query=miR1023e-3p, target=RO3G_14770, score=1, range=78-99, stran
target 5' AUGCACUCCAACUCGUUUCCCA 3'
::::::::::::.:::::::::
query 3' UACGUGAGGUUGGGCAAAGGGU 5'
query=miR1023e-3p, target=RO3G_11781, score=1, range=78-99, stran
target 5' AUGCACUCCAACUCGUUUCCCA 3'
::::::::::::.:::::::::
query 3' UACGUGAGGUUGGGCAAAGGGU 5'
query=miR1023e-3p, target=RO3G_07984, score=1, range=78-99, stran
target 5' AUGCACUCCAACUCGUUUCCCA 3'
```

```
:::::::::::.:::::::::
query 3' UACGUGAGGUUGGGCAAAGGGU 5'
query=miR1023e-3p, target=RO3G_15261, score=1, range=78-99, stran
target 5' AUGCACUCCAACUCGUUUCCCA 3'
:::::::::::.:::::::::
query 3' UACGUGAGGUUGGGCAAAGGGU 5'
query=miR1023e-3p, target=RO3G_17017, score=1, range=78-99, stran
target 5' AUGCACUCCAACUCGUUUCCCA 3'
:::::::::::.:::::::::
query 3' UACGUGAGGUUGGGCAAAGGGU 5'
query=miR1023e-3p, target=RO3G_12653, score=1, range=516-537,
strand=-1
target 5' AUGCACUCCAACUCGUUUCCCA 3'
:::::::::::.:::::::::
query 3' UACGUGAGGUUGGGCAAAGGGU 5'
query=miR1023e-3p, target=RO3G_04682, score=1, range=78-99, stran
target 5' AUGCACUCCAACUCGUUUCCCA 3'
:::::::::::.:::::::::
query 3' UACGUGAGGUUGGGCAAAGGGU 5'
query=miR1023e-3p, target=RO3G_16383, score=1, range=78-99, stran
target 5' AUGCACUCCAACUCGUUUCCCA 3'
:::::::::::.:::::::::
query 3' UACGUGAGGUUGGGCAAAGGGU 5'
query=miR1023e-3p, target=RO3G_11270, score=1, range=78-99, stran
target 5' AUGCACUCCAACUCGUUUCCCA 3'
:::::::::::.:::::::::
query 3' UACGUGAGGUUGGGCAAAGGGU 5'
query=miR1023e-3p, target=RO3G_17466, score=1, range=78-99, stran
target 5' AUGCACUCCAACUCGUUUCCCA 3'
:::::::::::.:::::::::
query 3' UACGUGAGGUUGGGCAAAGGGU 5'
query=miR1023e-3p, target=RO3G_17278, score=1, range=78-99, stran
target 5' AUGCACUCCAACCCGUUUCUCA 3'
:::::::::::::::::::.::
query 3' UACGUGAGGUUGGGCAAAGGGU 5'
query=miR1023e-3p, target=RO3G_00245, score=1, range=78-99, stran
target 5' AUGCACUCCAACCCGUUUCUCA 3'
:::::::::::::::::::.::
query 3' UACGUGAGGUUGGGCAAAGGGU 5'
query=miR1023e-3p, target=RO3G_17344, score=1, range=78-99, stran
target 5' AUGCACUCCAACUCGUUUCCCA 3'
:::::::::::.:::::::::
query 3' UACGUGAGGUUGGGCAAAGGGU 5'
query=miR1023e-3p, target=RO3G_09937, score=1.5, range=78-99,
strand=-1
```

target 5' AUGCAUUCCAACCCGUUUCUCA 3'
:::::.:::::::::::::.::
query 3' UACGUGAGGUUGGGCAAAGGGU 5'
query=miR1023e-3p, target=RO3G_04117, score=2.5, range=78-99,
strand=-1
target 5' AUGCACUUCAACCCAUUUCCCA 3'
:::::::.:::::: :::::::
query 3' UACGUGAGGUUGGGCAAAGGGU 5'
query=miR1023e-3p, target=RO3G_15271, score=3, range=78-99, stran
target 5' AUGCACUCCGACACGUUUCCCA 3'
:::::::::.:: :::::::::
query 3' UACGUGAGGUUGGGCAAAGGGU 5'
query=miR1023e-3p, target=RO3G_11235, score=3, range=78-99, stran
target 5' AUGCACUCCGACACGUUUCCCA 3'
:::::::::.:: :::::::::
query 3' UACGUGAGGUUGGGCAAAGGGU 5'
query=miR1023e-3p, target=RO3G_04669, score=3, range=78-99, stran
target 5' AUGCACUCCAACUCGCUUCCCA 3'
::::::::::::.:: ::::::
query 3' UACGUGAGGUUGGGCAAAGGGU 5'
query=miR1023e-3p, target=RO3G_03115, score=3.5, range=78-99,
strand=-1
target 5' AUGCACUUCAACUCGCUUCCCA 3'
:::::::.::::.:: ::::::
query 3' UACGUGAGGUUGGGCAAAGGGU 5'
query=miR1023e-3p, target=RO3G_00745, score=4, range=159-180,
strand=-1
target 5' AUGCACUCCAACUCGUUCCUCA 3'
:::::::::::::.:::: :.::
query 3' UACGUGAGGUUGGGCAAAGGGU 5'
query=miR1535a, target=RO3G_17160, score=0, range=59-80, strand=1
target 5' AAGACAUCACGCACAAACAGAA 3'
::::::::::::::::::::::
query 3' UUCUGUAGUGCGUGUUUGUCUU 5'
query=miR1535a, target=RO3G_15884, score=0, range=59-80, strand=1
target 5' AAGACAUCACGCACAAACAGAA 3'
::::::::::::::::::::::
query 3' UUCUGUAGUGCGUGUUUGUCUU 5'
query=miR1535a, target=RO3G_09486, score=0, range=59-80, strand=1
target 5' AAGACAUCACGCACAAACAGAA 3'
::::::::::::::::::::::
query 3' UUCUGUAGUGCGUGUUUGUCUU 5'
query=miR1535a, target=RO3G_02698, score=0.5, range=59-80, strand
target 5' AAGACAUCACGCACAAACAGAG 3'
:::::::::::::::::::::.

```
query 3' UUCUGUAGUGCGUGUUUGUCUU 5'
query=miR1535a, target=RO3G_09380, score=4, range=254-274, strand
target 5' GAGG-AUCACGCACAAACACAA 3'
.::. ::::::::::::: ::
query 3' UUCUGUAGUGCGUGUUUGUCUU 5'
query=miR403a, target=RO3G_17156, score=0, range=327-348, strand=
target 5' CAAGAUUGGCGUGAAUCGUCAA 3'
::::::::::::::::::::::
query 3' GUUCUAACCGCACUUAGCAGUU 5'
query=miR403a, target=RO3G_17453, score=0, range=87-108, strand=1
target 5' CAAGAUUGGCGUGAAUCGUCAA 3'
::::::::::::::::::::::
query 3' GUUCUAACCGCACUUAGCAGUU 5'
query=miR403a, target=RO3G_17252, score=0, range=87-108, strand=1
target 5' CAAGAUUGGCGUGAAUCGUCAA 3'
::::::::::::::::::::::
query 3' GUUCUAACCGCACUUAGCAGUU 5'
query=miR403a, target=RO3G_07862, score=3, range=2184-2205, stran
target 5' CAAGAUUGGUGUCAAUCGUCAA 3'
:::::::::.:: :::::::::
query 3' GUUCUAACCGCACUUAGCAGUU 5'
query=miR403a, target=RO3G_10744, score=3, range=2073-2094, stran
target 5' CAAGAUUGGUGUCAAUCGUCAA 3'
:::::::::.:: :::::::::
query 3' GUUCUAACCGCACUUAGCAGUU 5'
query=miR403a, target=RO3G_16366, score=3, range=2070-2091, stran
target 5' CAAGAUUGGUGUCAAUCGUCAA 3'
:::::::::.:: :::::::::
query 3' GUUCUAACCGCACUUAGCAGUU 5'
query=miR403a, target=RO3G_10745, score=3, range=1041-1062, stran
target 5' CAAGAUUGGUGUCAAUCGUCAA 3'
:::::::::.:: :::::::::
query 3' GUUCUAACCGCACUUAGCAGUU 5'
query=miR5751, target=RO3G_15989, score=0, range=2918-2937, stran
target 5' AAAAGGCAUCUGCAAAUCAA 3'
::::::::::::::::::::
query 3' UUUUCCGUAGACGUUUAGUU 5'
query=miR5751, target=RO3G_05208, score=3, range=1070-1090, stran
target 5' AAAUGAGCAUCUGCAAGUCAA 3'
::: : ::::::::::.::::
query 3' UUUUC-CGUAGACGUUUAGUU 5'
query=miR5751, target=RO3G_09146, score=3.5, range=182-201, stran
target 5' GAAAGGCAUUAGCAAAUCAA 3'
.:::::::. :::::::::
query 3' UUUUCCGUAGACGUUUAGUU 5'
```

```
query=miR5751, target=RO3G_12400, score=3.5, range=701-720, stran
target 5' GCAAGGCAUCUUCAAAUCAA 3'
. ::::::::: ::::::::
query 3' UUUUCCGUAGACGUUUAGUU 5'
query=miR5751, target=RO3G_13699, score=3.5, range=965-985, stran
target 5' AAGAGUCAUCUGCAAAAUCAA 3'
::.:: ::::::: :::::::
query 3' UUUUCCGUAGACG-UUUAGUU 5'
query=miR5751, target=RO3G_05579, score=4, range=1529-1548, stran
target 5' AAAAGGAAUUUGAAAAUCAA 3'
:::::: ::.:: :::::::
query 3' UUUUCCGUAGACGUUUAGUU 5'
query=miR5751, target=RO3G_15989, score=4, range=2263-2282, stran
target 5' AAAAAGCGUCUGCAAAUCGU 3'
:::: ::.:::::::::::.
query 3' UUUUCCGUAGACGUUUAGUU 5'
query=miR5751, target=RO3G_13109, score=4, range=1910-1929, stran
target 5' AGUAGGUAUCUGCAAAUUAU 3'
:. :::.:::::::::::.:
query 3' UUUUCCGUAGACGUUUAGUU 5'
query=miR5751, target=RO3G_00105, score=4, range=2268-2287, stran
target 5' AGCGGGCAUCUGCAAAUAAA 3'
:. .:::::::::::: ::
query 3' UUUUCCGUAGACGUUUAGUU 5'
query=miR5751, target=RO3G_06132, score=4, range=590-610, strand=
target 5' AAAAGUUAUCUGCUAAAUCAG 3'
::::: .:::::: ::::::.
query 3' UUUUCCGUAGACG-UUUAGUU 5'
query=miR5751, target=RO3G_16407, score=4, range=383-403, strand=
target 5' AAAAGGAAAUCUGCAAAUUAU 3'
:::::: ::::::::::.:
query 3' UUUUCC-GUAGACGUUUAGUU 5'
query=miR5751, target=RO3G_02082, score=4, range=764-784, strand=
target 5' AAAAUCCAUCUGCCAAAUCAA 3'
:::: :::::: ::::::::
query 3' UUUUCCGUAGAC-GUUUAGUU 5'
query=miR530, target=RO3G_12973, score=0, range=4800-4819, strand
target 5' GAGAGCCAGGUGCAUAAUCA 3'
::::::::::::::::::::
query 3' CUCUCGGUCCACGUAUUAGU 5'
query=miR530, target=RO3G_15143, score=0, range=2298-2317, strand
target 5' GAGAGCCAGGUGCAUAAUCA 3'
::::::::::::::::::::
query 3' CUCUCGGUCCACGUAUUAGU 5'
query=miR530, target=RO3G_16143, score=0, range=4779-4798, strand
```

```
target 5' GAGAGCCAGGUGCAUAAUCA 3'
::::::::::::::::::::
query 3' CUCUCGGUCCACGUAUUAGU 5'
query=miR530, target=RO3G_08650, score=0, range=4641-4660, strand
target 5' GAGAGCCAGGUGCAUAAUCA 3'
::::::::::::::::::::
query 3' CUCUCGGUCCACGUAUUAGU 5'
query=miR530, target=RO3G_08655, score=0, range=4641-4660, strand
target 5' GAGAGCCAGGUGCAUAAUCA 3'
::::::::::::::::::::
query 3' CUCUCGGUCCACGUAUUAGU 5'
query=miR530, target=RO3G_07028, score=0, range=3765-3784, strand
target 5' GAGAGCCAGGUGCAUAAUCA 3'
::::::::::::::::::::
query 3' CUCUCGGUCCACGUAUUAGU 5'
query=miR530, target=RO3G_05520, score=0, range=1449-1468, strand
target 5' GAGAGCCAGGUGCAUAAUCA 3'
::::::::::::::::::::
query 3' CUCUCGGUCCACGUAUUAGU 5'
query=miR530, target=RO3G_10728, score=0, range=4560-4579, strand
target 5' GAGAGCCAGGUGCAUAAUCA 3'
::::::::::::::::::::
query 3' CUCUCGGUCCACGUAUUAGU 5'
query=miR530, target=RO3G_02816, score=0, range=579-598, strand=-
target 5' GAGAGCCAGGUGCAUAAUCA 3'
::::::::::::::::::::
query 3' CUCUCGGUCCACGUAUUAGU 5'
query=miR530, target=RO3G_12959, score=0, range=681-700, strand=-
target 5' GAGAGCCAGGUGCAUAAUCA 3'
::::::::::::::::::::
query 3' CUCUCGGUCCACGUAUUAGU 5'
query=miR530, target=RO3G_17383, score=0, range=1281-1300, strand
target 5' GAGAGCCAGGUGCAUAAUCA 3'
::::::::::::::::::::
query 3' CUCUCGGUCCACGUAUUAGU 5'
query=miR530, target=RO3G_12533, score=0, range=4122-4141, strand
target 5' GAGAGCCAGGUGCAUAAUCA 3'
::::::::::::::::::::
query 3' CUCUCGGUCCACGUAUUAGU 5'
query=miR530, target=RO3G_01965, score=0, range=4692-4711, strand
target 5' GAGAGCCAGGUGCAUAAUCA 3'
::::::::::::::::::::
query 3' CUCUCGGUCCACGUAUUAGU 5'
query=miR530, target=RO3G_15136, score=0, range=489-508, strand=-
target 5' GAGAGCCAGGUGCAUAAUCA 3'
```

```
::::::::::::::::::::
query 3' CUCUCGGUCCACGUAUUAGU 5'
query=miR530, target=RO3G_12651, score=0, range=489-508, strand=-
target 5' GAGAGCCAGGUGCAUAAUCA 3'
::::::::::::::::::::
query 3' CUCUCGGUCCACGUAUUAGU 5'
query=miR530, target=RO3G_02691, score=0, range=3228-3247, strand
target 5' GAGAGCCAGGUGCAUAAUCA 3'
::::::::::::::::::::
query 3' CUCUCGGUCCACGUAUUAGU 5'
query=miR530, target=RO3G_17318, score=0, range=4782-4801, strand
target 5' GAGAGCCAGGUGCAUAAUCA 3'
::::::::::::::::::::
query 3' CUCUCGGUCCACGUAUUAGU 5'
query=miR530, target=RO3G_04118, score=0, range=4782-4801, strand
target 5' GAGAGCCAGGUGCAUAAUCA 3'
::::::::::::::::::::
query 3' CUCUCGGUCCACGUAUUAGU 5'
query=miR530, target=RO3G_17329, score=0, range=4119-4138, strand
target 5' GAGAGCCAGGUGCAUAAUCA 3'
::::::::::::::::::::
query 3' CUCUCGGUCCACGUAUUAGU 5'
query=miR530, target=RO3G_07971, score=0, range=234-253, strand=-
target 5' GAGAGCCAGGUGCAUAAUCA 3'
::::::::::::::::::::
query 3' CUCUCGGUCCACGUAUUAGU 5'
query=miR530, target=RO3G_02849, score=0, range=4800-4819, strand
target 5' GAGAGCCAGGUGCAUAAUCA 3'
::::::::::::::::::::
query 3' CUCUCGGUCCACGUAUUAGU 5'
query=miR530, target=RO3G_15259, score=0, range=4800-4819, strand
target 5' GAGAGCCAGGUGCAUAAUCA 3'
::::::::::::::::::::
query 3' CUCUCGGUCCACGUAUUAGU 5'
query=miR530, target=RO3G_11778, score=0, range=4800-4819, strand
target 5' GAGAGCCAGGUGCAUAAUCA 3'
::::::::::::::::::::
query 3' CUCUCGGUCCACGUAUUAGU 5'
query=miR530, target=RO3G_05217, score=0, range=4800-4819, strand
target 5' GAGAGCCAGGUGCAUAAUCA 3'
::::::::::::::::::::
query 3' CUCUCGGUCCACGUAUUAGU 5'
query=miR530, target=RO3G_07982, score=0, range=4800-4819, strand
target 5' GAGAGCCAGGUGCAUAAUCA 3'
::::::::::::::::::::
```

```
query 3' CUCUCGGUCCACGUAUUAGU 5'
query=miR530, target=RO3G_00697, score=0, range=4800-4819, strand
target 5' GAGAGCCAGGUGCAUAAUCA 3'
::::::::::::::::::::
query 3' CUCUCGGUCCACGUAUUAGU 5'
query=miR530, target=RO3G_07035, score=0, range=4800-4819, strand
target 5' GAGAGCCAGGUGCAUAAUCA 3'
::::::::::::::::::::
query 3' CUCUCGGUCCACGUAUUAGU 5'
query=miR530, target=RO3G_10535, score=0, range=2427-2446, strand
target 5' GAGAGCCAGGUGCAUAAUCA 3'
::::::::::::::::::::
query 3' CUCUCGGUCCACGUAUUAGU 5'
query=miR530, target=RO3G_02851, score=0, range=4800-4819, strand
target 5' GAGAGCCAGGUGCAUAAUCA 3'
::::::::::::::::::::
query 3' CUCUCGGUCCACGUAUUAGU 5'
query=miR530, target=RO3G_17274, score=1, range=4605-4624, strand
target 5' GAGAACCAGGUGCAUAAUCA 3'
:::: :::::::::::::::
query 3' CUCUCGGUCCACGUAUUAGU 5'
query=miR530, target=RO3G_05852, score=4, range=517-536, strand=1
target 5' GAAAACCAGGUGCGUGAUCA 3'
:: : ::::::::.:.::::
query 3' CUCUCGGUCCACGUAUUAGU 5'
query=miR530, target=RO3G_12181, score=4, range=1596-1615, strand
target 5' GGGAGUCAGGAGCAUAAUCC 3'
:.:::.:::: ::::::::
query 3' CUCUCGGUCCACGUAUUAGU 5'
query=miR530, target=RO3G_05918, score=4, range=1302-1321, strand
target 5' GGGAGUCAGGAGCAUAAUCC 3'
:.:::.:::: ::::::::
query 3' CUCUCGGUCCACGUAUUAGU 5'
query=miR530, target=RO3G_01102, score=4, range=1863-1882, strand
target 5' CUGAGGCAGGUGCAUGAUCA 3'
::: ::::::::::.::::
query 3' CUCUCGGUCCACGUAUUAGU 5'
query=miR530, target=RO3G_05951, score=4, range=816-835, strand=-
target 5' GAAGUUCAGGUGCAUAGUCA 3'
:: . .::::::::::.:::
query 3' CUCUCGGUCCACGUAUUAGU 5'
query=miR530, target=RO3G_03890, score=4, range=396-416, strand=-
target 5' GGAAGUGCAGGUGUAUAAUCA 3'
:. ::. :::::.:::::::
query 3' CUCUCG-GUCCACGUAUUAGU 5'
```

query=miR6118-5p, target=RO3G_01147, score=0, range=212-231, stra
target 5' UUUCCCAAGCACCCAUUCCA 3'
::::::::::::::::::::
query 3' AAAGGGUUCGUGGGUAAGGU 5'
query=miR6118-5p, target=RO3G_01161, score=0, range=212-231, stra
target 5' UUUCCCAAGCACCCAUUCCA 3'
::::::::::::::::::::
query 3' AAAGGGUUCGUGGGUAAGGU 5'
query=miR6118-5p, target=RO3G_04428, score=1, range=1532-1551, strand=1
target 5' UCUCCCAAGCACCCAUUCCA 3'
: ::::::::::::::::::
query 3' AAAGGGUUCGUGGGUAAGGU 5'
query=miR6118-5p, target=RO3G_14109, score=2, range=65-84, strand
target 5' UCUCCCAAGCACCCAUUCUA 3'
: ::::::::::::::::.:
query 3' AAAGGGUUCGUGGGUAAGGU 5'
query=miR6118-5p, target=RO3G_11276, score=2, range=251-270, stra
target 5' UCUCCCAAGCACCUAUUCCA 3'
: :::::::::::.::::::
query 3' AAAGGGUUCGUGGGUAAGGU 5'
query=miR6118-5p, target=RO3G_07939, score=4, range=1538-1557, strand=1
target 5' CAUCCCAAGCAUCUAUUCCA 3'
:::::::::.:.::::::
query 3' AAAGGGUUCGUGGGUAAGGU 5'
query=miR6279, target=RO3G_13288, score=2, range=1245-1267, stran
target 5' CGUCUGGCUGGAAGUCUUGUCCA 3'
::::::::::::::::::::: :
query 3' GCAGACCGACCUUCAGAACAGAU 5'
query=miR6279, target=RO3G_14787, score=2, range=1245-1267, stran
target 5' CGUCUGGCUGGAAGUCUUGUCCA 3'
::::::::::::::::::::: :
query 3' GCAGACCGACCUUCAGAACAGAU 5'
query=miR6279, target=RO3G_14800, score=2, range=1245-1267, stran
target 5' CGUCUGGCUGGAAGUCUUGUCCA 3'
::::::::::::::::::::: :
query 3' GCAGACCGACCUUCAGAACAGAU 5'
query=miR6279, target=RO3G_15875, score=2, range=1245-1267, stran
target 5' CGUCUGGCUGGAAGUCUUGUCCA 3'
::::::::::::::::::::: :
query 3' GCAGACCGACCUUCAGAACAGAU 5'
query=miR6279, target=RO3G_14793, score=2, range=1245-1267, stran
target 5' CGUCUGGCUGGAAGUCUUGUCCA 3'
::::::::::::::::::::: :

```
query 3' GCAGACCGACCUUCAGAACAGAU 5'
query=miR6279, target=RO3G_17411, score=2, range=687-709, strand=
target 5' CGUCUGGCUGGAAGUCUUGUCCA 3'
:::::::::::::::::::: :
query 3' GCAGACCGACCUUCAGAACAGAU 5'
FGSC-9543 know miRNA targets (PsRobot)
#miRNA Score mRNA Pairing target and miRNA
>miR1160.1 Score: 0.5 RO3G_17249
Query: 1 TTCCCTCTTTTTAAGCATGGCC 22
|:||||||||||||||||||||
Sbjct: 774 AGGGGAGAAAAATTCGTACCGG 753
>miR1160.1 Score: 0.5 RO3G_14363
Query: 1 TTCCCTCTTTTTAAGCATGG-CC 22
||||||||||||||||||||*||
Sbjct: 807 AAGGGAGAAAAATTCGTACCAGG 785
>miR1160.1 Score: 0.5 RO3G_14547
Query: 1 TTCCCTCTTTTTAAGCATGG-CC 22
||||||||||||||||||||*||
Sbjct: 255 AAGGGAGAAAAATTCGTACCAGG 233
>miR1160.1 Score: 0.0 RO3G_13863
Query: 1 TTCCCTCTTTTTAAGCATGGCC 22
||||||||||||||||||||||
Sbjct: 549 AAGGGAGAAAAATTCGTACCGG 528
>miR1160.1 Score: 0.0 RO3G_13270
Query: 1 TTCCCTCTTTTTAAGCATGGCC 22
||||||||||||||||||||||
Sbjct: 849 AAGGGAGAAAAATTCGTACCGG 828
>miR1160.1 Score: 0.0 RO3G_11527
Query: 1 TTCCCTCTTTTTAAGCATGGCC 22
||||||||||||||||||||||
Sbjct: 255 AAGGGAGAAAAATTCGTACCGG 234
>miR1160.1 Score: 1.0 RO3G_10734
Query: 1 TTCCCTCTTTTTAAGCATGGCC 22
||||*|||||||||||||||||
Sbjct: 255 AAGGTAGAAAAATTCGTACCGG 234
>miR1160.1 Score: 0.0 RO3G_10774
Query: 1 TTCCCTCTTTTTAAGCATGGCC 22
||||||||||||||||||||||
Sbjct: 816 AAGGGAGAAAAATTCGTACCGG 795
>miR1160.1 Score: 0.0 RO3G_10869
Query: 1 TTCCCTCTTTTTAAGCATGGCC 22
||||||||||||||||||||||
Sbjct: 255 AAGGGAGAAAAATTCGTACCGG 234
>miR1160.1 Score: 0.0 RO3G_05935
Query: 1 TTCCCTCTTTTTAAGCATGGCC 22
```

||||||||||||||||||||||
Sbjct: 255 AAGGGAGAAAAATTCGTACCGG 234
>miR1160.1 Score: 0.0 RO3G_04790
Query: 1 TTCCCTCTTTTTAAGCATGGCC 22
||||||||||||||||||||||
Sbjct: 255 AAGGGAGAAAAATTCGTACCGG 234
>miR1160.1 Score: 0.0 RO3G_03593
Query: 1 TTCCCTCTTTTTAAGCATGGCC 22
||||||||||||||||||||||
Sbjct: 255 AAGGGAGAAAAATTCGTACCGG 234
>miR1160.1 Score: 0.0 RO3G_03626
Query: 1 TTCCCTCTTTTTAAGCATGGCC 22
||||||||||||||||||||||
Sbjct: 255 AAGGGAGAAAAATTCGTACCGG 234
>miR1160.1 Score: 0.0 RO3G_00678
Query: 1 TTCCCTCTTTTTAAGCATGGCC 22
||||||||||||||||||||||
Sbjct: 219 AAGGGAGAAAAATTCGTACCGG 198
>miR1160.1 Score: 0.2 RO3G_01589
Query: 1 TTCCCTCTTTTTAAGCATGGCC 22
|||||||||||||||||||:||
Sbjct: 255 AAGGGAGAAAAATTCGTACTGG 234
>miR1160.1 Score: 1.0 RO3G_17459
Query: 1 TTCCCTCTTTTTAAGCATGGCC 22
||||*|||||||||||||||||
Sbjct: 255 AAGGTAGAAAAATTCGTACCGG 234
>miR1160.1 Score: 0.0 RO3G_17276
Query: 1 TTCCCTCTTTTTAAGCATGGCC 22
||||||||||||||||||||||
Sbjct: 939 AAGGGAGAAAAATTCGTACCGG 918
>miR5281b Score: 2.5 RO3G_10068
Query: 1 TTTTAATAAAAGGAACGGAG 20
:*|||||||||||||*:||
Sbjct: 265 GTAATTATTTTCCTTGATTC 246
>miR5281b Score: 2.0 RO3G_08187
Query: 1 TTTTAATAAAAGGAACGG-AG 20
|||:||||||||||*|||*||
Sbjct: 1267 AAAGTTATTTTCCTAGCCCTC 1247
>miR5281b Score: 2.2 RO3G_06992
Query: 1 TTTTAATAAAAGGAACGGAG 20
:||:||||||||||||:*|*
Sbjct: 819 GAAGTTATTTTCCTTGT-TA 801
>miR5281b Score: 2.2 RO3G_17117
Query: 1 TTTTAATAAAAGGAACGGAG 20
||||||||||||||**|:||

Sbjct: 684 AAAATTATTTTCCTGACTTC 665
>miR5042-3p Score: 2.5 RO3G_07173
Query: 1 TGGGCTTGAGTACAAGAAGG 20
||||||||:||||||*||
Sbjct: 629 CCCCGAACGCGTGTTCT-CC 611
>miR5042-3p Score: 2.2 RO3G_00779
Query: 1 TGGGCTTGAGTACAAGAAGG 20
*||:|:||||:||||||||:
Sbjct: 3290 TCCTGGACTCGTGTTCTTCT 3271
>miR5565e Score: 0.0 RO3G_16107
Query: 1 TATGGTCTAGATGTTGTCGGA 21
|||||||||||||||||||||
Sbjct: 547 ATACCAGATCTACAACAGCCT 527
>miR3711 Score: 2.2 RO3G_13432
Query: 1 TAGCCAAGAAGGAGGTGCCT 20
:||||||||||:*|:|||||
Sbjct: 567 GTCGGTTCTTCTACTACGGA 548
>miR3711 Score: 2.2 RO3G_11375
Query: 1 TAGCCAAGAAGGAGGTGCCT 20
||:||||||||*||:||||:
Sbjct: 1413 ATTGGTTCTTCGTCTACGGG 1394
>miR3711 Score: 2.2 RO3G_00301
Query: 1 TAGCCAAGAAGGAGGTGCCT 20
:|:||||||||:*|||||||
Sbjct: 1173 GTTGGTTCTTCTACCACGGA 1154
>miR170-3p Score: 2.5 RO3G_15840
Query: 1 TATTGGCCGTGTAATATT 18
||||*|||:|:|||:|||
Sbjct: 1099 ATAAACGGTATATTGTAA 1082
>miR170-3p Score: 2.5 RO3G_10275
Query: 1 TATTGGCCGTGTAATATT 18
|||||||||:||||:|
Sbjct: 224 CTAACCGGCATATTACGA 207
>miR170-3p Score: 2.2 RO3G_11045
Query: 1 TATTGGCCGTGTAATATT 18
:|||||||:|:|||*|||
Sbjct: 319 GTAACCGGTATATTTTAA 302
>miR170-3p Score: 2.5 RO3G_10201
Query: 1 TATTGGCCGTGTAATATT 18
:|||*||||||:||:||:
Sbjct: 905 GTAAGCGGCACGTTGTAG 888
>miR170-3p Score: 2.5 RO3G_08494
Query: 1 TATTGGCCGTGTAATATT 18
:||||*||*||||||||:
Sbjct: 1032 GTAACGGGAACATTATAG 1015

>miR170-3p Score: 2.5 RO3G_08635
Query: 1 TATTGGCCGTGTAATATT 18
||||:||||||||||*
Sbjct: 805 CTAACTGGCACAGTATAC 788
>miR2275b-3p Score: 2.5 RO3G_16737
Query: 1 TTCAAGTTTCCATCGTAAATCT-A 23
||||*|||:||||||||||*|*|
Sbjct: 263 AAGTACAAGGGTAGCATTTAAACT 240
>miR2275b-3p Score: 2.5 RO3G_16541
Query: 1 TTCAAGTTTCCATCGTAAATCT-A 23
||||*|||:||||||||||*|*|
Sbjct: 263 AAGTACAAGGGTAGCATTTAAACT 240
>miR2275b-3p Score: 2.5 RO3G_16141
Query: 1 TTCAAGTTTCCATCGTAAATCT-A 23
||||*|||:||||||||||*|*|
Sbjct: 263 AAGTACAAGGGTAGCATTTAAACT 240
>miR2275b-3p Score: 2.5 RO3G_16124
Query: 1 TTCAAGTTTCCATCGTAAATCT-A 23
||||*|||:||||||||||*|*|
Sbjct: 263 AAGTACAAGGGTAGCATTTAAACT 240
>miR2275b-3p Score: 2.5 RO3G_15286
Query: 1 TTCAAGTTTCCATCGTAAATCT-A 23
||||*|||:||||||||||*|*|
Sbjct: 263 AAGTACAAGGGTAGCATTTAAACT 240
>miR2275b-3p Score: 2.5 RO3G_14981
Query: 1 TTCAAGTTTCCATCGTAAATCT-A 23
||||*|||:||||||||||*|*|
Sbjct: 263 AAGTACAAGGGTAGCATTTAAACT 240
>miR2275b-3p Score: 2.5 RO3G_15170
Query: 1 TTCAAGTTTCCATCGTAAATCT-A 23
||||*|||:||||||||||*|*|
Sbjct: 263 AAGTACAAGGGTAGCATTTAAACT 240
>miR2275b-3p Score: 2.5 RO3G_14784
Query: 1 TTCAAGTTTCCATCGTAAATCT-A 23
||||*|||:||||||||||*|*|
Sbjct: 263 AAGTACAAGGGTAGCATTTAAACT 240
>miR2275b-3p Score: 2.5 RO3G_13752
Query: 1 TTCAAGTTTCCATCGTAAATCT-A 23
||||*|||:||||||||||*|*|
Sbjct: 263 AAGTACAAGGGTAGCATTTAAACT 240
>miR2275b-3p Score: 2.5 RO3G_12950
Query: 1 TTCAAGTTTCCATCGTAAATCT-A 23
||||*|||:||||||||||*|*|
Sbjct: 263 AAGTACAAGGGTAGCATTTAAACT 240
>miR2275b-3p Score: 2.5 RO3G_13267

Query: 1 TTCAAGTTTCCATCGTAAATCT-A 23
||||*|||:|||||||||||*|*|
Sbjct: 263 AAGTACAAGGGTAGCATTTAAACT 240
>miR2275b-3p Score: 2.5 RO3G_12084
Query: 1 TTCAAGTTTCCATCGTAAATCT-A 23
||||*|||:|||||||||||*|*|
Sbjct: 263 AAGTACAAGGGTAGCATTTAAACT 240
>miR2275b-3p Score: 2.5 RO3G_11533
Query: 1 TTCAAGTTTCCATCGTAAATCT-A 23
||||*|||:|||||||||||*|*|
Sbjct: 263 AAGTACAAGGGTAGCATTTAAACT 240
>miR2275b-3p Score: 2.5 RO3G_11536
Query: 1 TTCAAGTTTCCATCGTAAATCT-A 23
||||*|||:|||||||||||*|*|
Sbjct: 263 AAGTACAAGGGTAGCATTTAAACT 240
>miR2275b-3p Score: 2.5 RO3G_10785
Query: 1 TTCAAGTTTCCATCGTAAATCT-A 23
||||*|||:|||||||||||*|*|
Sbjct: 263 AAGTACAAGGGTAGCATTTAAACT 240
>miR2275b-3p Score: 2.5 RO3G_09926
Query: 1 TTCAAGTTTCCATCGTAAATCT-A 23
||||*|||:|||||||||||*|*|
Sbjct: 263 AAGTACAAGGGTAGCATTTAAACT 240
>miR2275b-3p Score: 2.5 RO3G_10050
Query: 1 TTCAAGTTTCCATCGTAAATCT-A 23
||||*|||:|||||||||||*|*|
Sbjct: 263 AAGTACAAGGGTAGCATTTAAACT 240
>miR2275b-3p Score: 2.5 RO3G_07723
Query: 1 TTCAAGTTTCCATCGTAAATCT-A 23
||||*|||:|||||||||||*|*|
Sbjct: 263 AAGTACAAGGGTAGCATTTAAACT 240
>miR2275b-3p Score: 2.5 RO3G_05522
Query: 1 TTCAAGTTTCCATCGTAAATCT-A 23
||||*|||:|||||||||||*|*|
Sbjct: 629 AAGTACAAGGGTAGCATTTAAACT 606
>miR2275b-3p Score: 2.5 RO3G_05516
Query: 1 TTCAAGTTTCCATCGTAAATCT-A 23
||||*|||:|||||||||||*|*|
Sbjct: 263 AAGTACAAGGGTAGCATTTAAACT 240
>miR2275b-3p Score: 2.5 RO3G_03112
Query: 1 TTCAAGTTTCCATCGTAAATCT-A 23
||||*|||:|||||||||||*|*|
Sbjct: 629 AAGTACAAGGGTAGCATTTAAACT 606
>miR2275b-3p Score: 2.5 RO3G_03560
Query: 1 TTCAAGTTTCCATCGTAAATCT-A 23

```
||||*|||:|||||||||||*|*|
Sbjct: 263 AAGTACAAGGGTAGCATTTAAACT 240
>miR2275b-3p Score: 2.5 RO3G_03791
Query: 1 TTCAAGTTTCCATCGTAAATCT-A 23
||||*|||:|||||||||||*|*|
Sbjct: 263 AAGTACAAGGGTAGCATTTAAACT 240
>miR2275b-3p Score: 2.5 RO3G_00240
Query: 1 TTCAAGTTTCCATCGTAAATCT-A 23
||||*|||:|||||||||||*|*|
Sbjct: 572 AAGTACAAGGGTAGCATTTAAACT 549
>miR2275b-3p Score: 2.5 RO3G_00250
Query: 1 TTCAAGTTTCCATCGTAAATCT-A 23
||||*|||:|||||||||||*|*|
Sbjct: 263 AAGTACAAGGGTAGCATTTAAACT 240
>miR2275b-3p Score: 2.5 RO3G_01725
Query: 1 TTCAAGTTTCCATCGTAAATCT-A 23
||||*|||:|||||||||||*|*|
Sbjct: 263 AAGTACAAGGGTAGCATTTAAACT 240
>miR2275b-3p Score: 2.5 RO3G_02227
Query: 1 TTCAAGTTTCCATCGTAAATCT-A 23
||||*|||:|||||||||||*|*|
Sbjct: 629 AAGTACAAGGGTAGCATTTAAACT 606
>miR2275b-3p Score: 2.5 RO3G_17423
Query: 1 TTCAAGTTTCCATCGTAAATCT-A 23
||||*|||:|||||||||||*|*|
Sbjct: 263 AAGTACAAGGGTAGCATTTAAACT 240
>miR2275b-3p Score: 2.5 RO3G_17328
Query: 1 TTCAAGTTTCCATCGTAAATCT-A 23
||||*|||:|||||||||||*|*|
Sbjct: 263 AAGTACAAGGGTAGCATTTAAACT 240
>miR2275b-3p Score: 2.5 RO3G_17092
Query: 1 TTCAAGTTTCCATCGTAAATCT-A 23
||||*|||:|||||||||||*|*|
Sbjct: 263 AAGTACAAGGGTAGCATTTAAACT 240
>miR5743a Score: 0.0 RO3G_08617
Query: 1 TGAGAAGTTTTCCCACCATT 20
||||||||||||||||||||
Sbjct: 300 ACTCTTCAAAAGGGTGGTAA 281
>miR5743a Score: 0.5 RO3G_03516
Query: 1 TGAGAAGTTTTCCCACCATT 20
||||||:|||||||||||||
Sbjct: 1635 ACTCTTTAAAAGGGTGGTAA 1616
>miR159g-3p Score: 2.5 RO3G_15485
Query: 1 TTTGGAGTGAAGCGATTTGA 20
*|||:||||||||:||:*||
```

Sbjct: 3592 TAACTTCACTTCGTTAG-CT 3574
>miR159g-3p Score: 0.5 RO3G_10142
Query: 1 TTTGGAGTGAAGCGATTTGA 20
||||:|||||||||||||||
Sbjct: 598 AAACTTCACTTCGCTAAACT 579
>miR159g-3p Score: 2.5 RO3G_08255
Query: 1 TTTGGAGTGAAGCGATTTGA 20
|||||||||||:|||*||
Sbjct: 237 CAACCTCACTTAGTTAA-CT 219
>miR159g-3p Score: 2.5 RO3G_06080
Query: 1 TTTGGAGTGAAGCGATTTGA 20
||||||*|||||*||||*||
Sbjct: 855 AAACCTAACTTCCCTAA-CT 837
>miR159g-3p Score: 2.0 RO3G_02867
Query: 1 TTTGGAGTGAAGCGATTTGA 20
||||:|:*||||||||||||
Sbjct: 526 AAACTTTCCTTCGCTAAACT 507
>miR159g-3p Score: 2.0 RO3G_02881
Query: 1 TTTGGAGTGAAGCGATTTGA 20
||||||:|:|||*|||||||
Sbjct: 3834 AAACCTTATTTCTCTAAACT 3815
>miR159g-3p Score: 0.5 RO3G_03107
Query: 1 TTTGGAGTGAAGCGATTTGA 20
||||:|||||||||||||||
Sbjct: 598 AAACTTCACTTCGCTAAACT 579
>miR397a Score: 2.5 RO3G_11794
Query: 1 TCATTGAGTGCAGCGTTGATG 21
||||||||:||||||*|||
Sbjct: 2483 TGTAACTCATGCCGCAAGTAC 2463
>miR397a Score: 2.5 RO3G_03441
Query: 1 TCATTGAGTGCAGCGTTG-ATG 21
||||||::|||:||||||||
Sbjct: 477 TGTAACTTGCGTTGCAACCTAC 456
>miR8775 Score: 2.5 RO3G_09103
Query: 1 TTGCAGATTGAACCGATGG 19
||||**|||||||||||*|
Sbjct: 908 AACGAGTAACTTGGCTAAC 890
>miR8775 Score: 2.5 RO3G_04618
Query: 1 TTGCAGATTGAACCGATGG 19
||||*:||||||||:||*|
Sbjct: 37 AACGGTTAACTTGGTTAAC 19
>miR3946 Score: 1.0 RO3G_17023
Query: 1 TGTAGAGAAAAGAGAGAGCATG 22
||||||||||||||*|||||||
Sbjct: 1246 ACATCTCTTTTCTCCCTCGTAC 1225

>miR8748 Score: 1.0 RO3G_17257
Query: 1 TGATGGTGATGATAATAATGAAT 23
|||||||||||||*|||||||||
Sbjct: 2127 ACTACCACTACTACTATTACTTA 2105
>miR8748 Score: 1.0 RO3G_16749
Query: 1 TGATGGTGATGATAATAATGAAT 23
|||||||||||||*|||||||||
Sbjct: 2211 ACTACCACTACTACTATTACTTA 2189
>miR8748 Score: 2.5 RO3G_16764
Query: 1 TGATGGTGATGATAATAATGAAT 23
:||||||:|||||*||||:*|||
Sbjct: 722 GCTACCATTACTACTATTG-TTA 701
>miR8748 Score: 1.0 RO3G_16136
Query: 1 TGATGGTGATGATAATAATGAAT 23
|||||||||||||*|||||||||
Sbjct: 2127 ACTACCACTACTACTATTACTTA 2105
>miR8748 Score: 1.0 RO3G_15889
Query: 1 TGATGGTGATGATAATAATGAAT 23
|||||||||||||*|||||||||
Sbjct: 2127 ACTACCACTACTACTATTACTTA 2105
>miR8748 Score: 1.0 RO3G_13145
Query: 1 TGATGGTGATGATAATAATGAAT 23
|||||||||||||*|||||||||
Sbjct: 2211 ACTACCACTACTACTATTACTTA 2189
>miR8748 Score: 1.0 RO3G_13273
Query: 1 TGATGGTGATGATAATAATGAAT 23
|||||||||||||*|||||||||
Sbjct: 2211 ACTACCACTACTACTATTACTTA 2189
>miR8748 Score: 1.0 RO3G_10735
Query: 1 TGATGGTGATGATAATAATGAAT 23
|||||||||||||*|||||||||
Sbjct: 1656 ACTACCACTACTACTATTACTTA 1634
>miR8748 Score: 2.2 RO3G_11036
Query: 1 TGATGGTGATGATAATAATGAAT 23
::|||:||||||:|||||||*||
Sbjct: 1069 GTTACTACTACTGTTATTAC-TA 1048
>miR8748 Score: 1.0 RO3G_09516
Query: 1 TGATGGTGATGATAATAATGAAT 23
|||||:|||||||||||||*|||
Sbjct: 262 ACTACTACTACTATTATTA-TTA 241
>miR8748 Score: 1.0 RO3G_10040
Query: 1 TGATGGTGATGATAATAATGAAT 23
|||||||||||||*|||||||||
Sbjct: 801 ACTACCACTACTACTATTACTTA 779
>miR8748 Score: 1.0 RO3G_10217

Query: 1 TGATGGTGATGATAATAATGAAT 23
|||||||||||||*|||||||||
Sbjct: 2211 ACTACCACTACTACTATTACTTA 2189
>miR8748 Score: 2.0 RO3G_08929
Query: 1 TGATGGTGATGATAATAATGAAT 23
||||||*:|||||||||||*|||
Sbjct: 30 ACTACCTTTACTATTATTA-TTA 9
>miR8748 Score: 2.5 RO3G_08974
Query: 1 TGATGGTGATGATAATAATGAAT 23
:||||||:*|||||||||:||*|
Sbjct: 176 GCTACCATCACTATTATTGCTAA 154
>miR8748 Score: 1.0 RO3G_09183
Query: 1 TGATGGTGATGATAATAATGAAT 23
|||||||||||||*|||||||||
Sbjct: 1344 ACTACCACTACTACTATTACTTA 1322
>miR8748 Score: 2.5 RO3G_07191
Query: 1 TGATGGTGATGATAATAATGAAT 23
||||||||||||||||**|**||
Sbjct: 303 ACTACCACTACTATTACGAGATA 281
>miR8748 Score: 1.0 RO3G_07697
Query: 1 TGATGGTGATGATAATAATGAAT 23
|||||||||||||*|||||||||
Sbjct: 537 ACTACCACTACTACTATTACTTA 515
>miR8748 Score: 0.0 RO3G_04592
Query: 1 TGATGGTGATGATAATAATGAAT 23
|||||||||||||||||||||||
Sbjct: 1383 ACTACCACTACTATTATTACTTA 1361
>miR8748 Score: 1.0 RO3G_04681
Query: 1 TGATGGTGATGATAATAATGAAT 23
|||||||||||||*|||||||||
Sbjct: 801 ACTACCACTACTACTATTACTTA 779
>miR8748 Score: 2.5 RO3G_04843
Query: 1 TGATGGTGATGATAATAATGAAT 23
|:||:||:||:||||||||*|||
Sbjct: 368 ATTATCATTATTATTATTA-TTA 347
>miR8748 Score: 1.0 RO3G_05424
Query: 1 TGATGGTGATGATAATAATGAAT 23
|||||||||||||*|||||||||
Sbjct: 2127 ACTACCACTACTACTATTACTTA 2105
>miR8748 Score: 2.2 RO3G_02397
Query: 1 TGATGGTGATGATAATAATGAAT 23
|||||:||||||||*|||*:|||
Sbjct: 357 ACTACTACTACTATCATT-TTTA 336
>miR8748 Score: 2.0 RO3G_02763
Query: 1 TGATGGTGATGATAATAATGAAT 23

||||||::||:||||||||*|||
Sbjct: 865 ACTACCGTTATTATTATTA-TTA 844
>miR8748 Score: 1.5 RO3G_00289
Query: 1 TGATGGTGATGATAATAATGAAT 23
|||||||||||||*||||||*||
Sbjct: 945 ACTACCACTACTACTATTAC-TA 924
>miR8748 Score: 2.5 RO3G_01410
Query: 1 TGATGGTGATGATAATAATGAAT 23
|||||:|||||||*||||*|*||
Sbjct: 459 ACTACTACTACTACTATTTC-TA 438
>miR8748 Score: 2.2 RO3G_01780
Query: 1 TGATGGTGATGATAATAATGAAT 23
||||||:|||||:||||*|:*||
Sbjct: 1267 ACTACCGCTACTGTTAT-ATCTA 1246
>miR8748 Score: 1.0 RO3G_17351
Query: 1 TGATGGTGATGATAATAATGAAT 23
|||||||||||||*|||||||||
Sbjct: 939 ACTACCACTACTACTATTACTTA 917
>miR408-3p Score: 2.5 RO3G_12917
Query: 1 ATGCACTGCCTCTTCCCTGG 20
|||||::|||||||||::
Sbjct: 370 CACGTGGTGGAGAAGGGGTA 351
>miR408-3p Score: 2.5 RO3G_04352
Query: 1 ATGCACTGCCTCTTCCCTGG 20
:||||||||||||||*|
Sbjct: 706 GGCGTTACGGAGAAGGGAAC 687
>miR9663-5p Score: 0.0 RO3G_16744
Query: 1 AACGTAGTCGACCGATTCT 19
|||||||||||||||||||
Sbjct: 945 TTGCATCAGCTGGCTAAGA 927
>miR9663-5p Score: 0.0 RO3G_16686
Query: 1 AACGTAGTCGACCGATTCT 19
|||||||||||||||||||
Sbjct: 945 TTGCATCAGCTGGCTAAGA 927
>miR9663-5p Score: 2.5 RO3G_16171
Query: 1 AACGTAGTCGACCGATTCT 19
|||||||||||:|||*|
Sbjct: 7764 CTGCATCAGCCGGTTAA-A 7747
>miR9663-5p Score: 0.0 RO3G_15869
Query: 1 AACGTAGTCGACCGATTCT 19
|||||||||||||||||||
Sbjct: 945 TTGCATCAGCTGGCTAAGA 927
>miR9663-5p Score: 0.0 RO3G_14704
Query: 1 AACGTAGTCGACCGATTCT 19
|||||||||||||||||||

Sbjct: 945 TTGCATCAGCTGGCTAAGA 927
>miR9663-5p Score: 0.0 RO3G_13859
Query: 1 AACGTAGTCGACCGATTCT 19
|||||||||||||||||||
Sbjct: 570 TTGCATCAGCTGGCTAAGA 552
>miR9663-5p Score: 0.0 RO3G_12951
Query: 1 AACGTAGTCGACCGATTCT 19
|||||||||||||||||||
Sbjct: 945 TTGCATCAGCTGGCTAAGA 927
>miR9663-5p Score: 0.0 RO3G_12979
Query: 1 AACGTAGTCGACCGATTCT 19
|||||||||||||||||||
Sbjct: 945 TTGCATCAGCTGGCTAAGA 927
>miR9663-5p Score: 0.0 RO3G_13152
Query: 1 AACGTAGTCGACCGATTCT 19
|||||||||||||||||||
Sbjct: 945 TTGCATCAGCTGGCTAAGA 927
>miR9663-5p Score: 0.0 RO3G_11970
Query: 1 AACGTAGTCGACCGATTCT 19
|||||||||||||||||||
Sbjct: 945 TTGCATCAGCTGGCTAAGA 927
>miR9663-5p Score: 0.0 RO3G_12087
Query: 1 AACGTAGTCGACCGATTCT 19
|||||||||||||||||||
Sbjct: 945 TTGCATCAGCTGGCTAAGA 927
>miR9663-5p Score: 0.0 RO3G_12194
Query: 1 AACGTAGTCGACCGATTCT 19
|||||||||||||||||||
Sbjct: 945 TTGCATCAGCTGGCTAAGA 927
>miR9663-5p Score: 0.0 RO3G_11275
Query: 1 AACGTAGTCGACCGATTCT 19
|||||||||||||||||||
Sbjct: 945 TTGCATCAGCTGGCTAAGA 927
>miR9663-5p Score: 0.0 RO3G_11403
Query: 1 AACGTAGTCGACCGATTCT 19
|||||||||||||||||||
Sbjct: 945 TTGCATCAGCTGGCTAAGA 927
>miR9663-5p Score: 0.0 RO3G_10555
Query: 1 AACGTAGTCGACCGATTCT 19
|||||||||||||||||||
Sbjct: 945 TTGCATCAGCTGGCTAAGA 927
>miR9663-5p Score: 0.0 RO3G_10560
Query: 1 AACGTAGTCGACCGATTCT 19
|||||||||||||||||||
Sbjct: 945 TTGCATCAGCTGGCTAAGA 927

>miR9663-5p Score: 2.2 RO3G_10896
Query: 1 AACGTAGTCGACCGATTCT 19
|||:||||||||||||:
Sbjct: 431 ATGCGTAAGCTGGCTAAGG 413
>miR9663-5p Score: 0.0 RO3G_09376
Query: 1 AACGTAGTCGACCGATTCT 19
|||||||||||||||||||
Sbjct: 945 TTGCATCAGCTGGCTAAGA 927
>miR9663-5p Score: 0.0 RO3G_08612
Query: 1 AACGTAGTCGACCGATTCT 19
|||||||||||||||||||
Sbjct: 945 TTGCATCAGCTGGCTAAGA 927
>miR9663-5p Score: 0.0 RO3G_07104
Query: 1 AACGTAGTCGACCGATTCT 19
|||||||||||||||||||
Sbjct: 945 TTGCATCAGCTGGCTAAGA 927
>miR9663-5p Score: 0.0 RO3G_07305
Query: 1 AACGTAGTCGACCGATTCT 19
|||||||||||||||||||
Sbjct: 945 TTGCATCAGCTGGCTAAGA 927
>miR9663-5p Score: 0.0 RO3G_05630
Query: 1 AACGTAGTCGACCGATTCT 19
|||||||||||||||||||
Sbjct: 945 TTGCATCAGCTGGCTAAGA 927
>miR9663-5p Score: 0.0 RO3G_06416
Query: 1 AACGTAGTCGACCGATTCT 19
|||||||||||||||||||
Sbjct: 945 TTGCATCAGCTGGCTAAGA 927
>miR9663-5p Score: 0.0 RO3G_05280
Query: 1 AACGTAGTCGACCGATTCT 19
|||||||||||||||||||
Sbjct: 945 TTGCATCAGCTGGCTAAGA 927
>miR9663-5p Score: 0.0 RO3G_02235
Query: 1 AACGTAGTCGACCGATTCT 19
|||||||||||||||||||
Sbjct: 288 TTGCATCAGCTGGCTAAGA 270
>miR9663-5p Score: 0.0 RO3G_03103
Query: 1 AACGTAGTCGACCGATTCT 19
|||||||||||||||||||
Sbjct: 945 TTGCATCAGCTGGCTAAGA 927
>miR9663-5p Score: 0.0 RO3G_03988
Query: 1 AACGTAGTCGACCGATTCT 19
|||||||||||||||||||
Sbjct: 945 TTGCATCAGCTGGCTAAGA 927
>miR9663-5p Score: 0.0 RO3G_01203

```
Query: 1 AACGTAGTCGACCGATTCT 19
|||||||||||||||||||
Sbjct: 945 TTGCATCAGCTGGCTAAGA 927
>miR9663-5p Score: 0.0 RO3G_01718
Query: 1 AACGTAGTCGACCGATTCT 19
|||||||||||||||||||
Sbjct: 945 TTGCATCAGCTGGCTAAGA 927
>miR9554-3p Score: 2.5 RO3G_12339
Query: 1 TCATAGCTCAAGTCATCATTCAT 23
||||||*|||||||||||**|*|
Sbjct: 1340 AGTATCAAGTTCAGTAGT-CGAA 1319
>miR9554-3p Score: 0.0 RO3G_12520
Query: 1 TCATAGCTCAAGTCATCATTCAT 23
|||||||||||||||||||||||
Sbjct: 1583 AGTATCGAGTTCAGTAGTAAGTA 1561
>miR9554-3p Score: 2.5 RO3G_11933
Query: 1 TCATAGCTCAAGTCATCATTCAT 23
||*|||||*||||||||*|||||
Sbjct: 23 AGCATCGAATTCAGTAGCAAGTA 1
>miR9554-3p Score: 0.0 RO3G_00797
Query: 1 TCATAGCTCAAGTCATCATTCAT 23
|||||||||||||||||||||||
Sbjct: 1583 AGTATCGAGTTCAGTAGTAAGTA 1561
>miR9554-3p Score: 2.0 RO3G_01994
Query: 1 TCATAGCTCAAGTCATCATTCAT 23
|*|||:||||||||||||*||||
Sbjct: 443 AATATTGAGTTCAGTAGT-AGTA 422
>miR9554-3p Score: 1.5 RO3G_02103
Query: 1 TCATAGCTCAAGTCATCATTCAT 23
||||||||||||||*||*|||||
Sbjct: 110 AGTATCGAGTTCAGCAGCAAGTA 88
>miR858b Score: 2.5 RO3G_11693
Query: 1 TTCGTTCTGTTCGTCGCTTG 20
|||:||||*|||||||*|||
Sbjct: 153 AAGTAAGAAAAGCAGCAAAC 134
>miR827 Score: 2.5 RO3G_16161
Query: 1 TTAGATGACCATCAACAAAT 20
|||:||||||||||**||||
Sbjct: 694 AATTTACTGGTAGTAATTTA 675
>miR827 Score: 2.5 RO3G_15356
Query: 1 TTAGATGACCATCAACAAAT 20
|||||||||*|:||*|||||
Sbjct: 706 AATCTACTGTTGGTGGTTTA 687
>miR827 Score: 2.2 RO3G_05422
Query: 1 TTAGATGACCATCAACAAAT 20
```

||*||||||||:||||||*:
Sbjct: 352 AAGCTACTGGTGGTTGTTCG 333
>miR171a-3p Score: 2.5 RO3G_16320
Query: 1 TGATTGAGCCGCGCCAAT-AT 20
||||||||||:|||||*||
Sbjct: 366 CCTAACTCGACGTGGTTAGTA 346
>miR2656a Score: 1.5 RO3G_17222
Query: 1 TAAGTTGCATATTGGACGTTGG 22
||*|||||||||||||||*|||
Sbjct: 202 ATCCAACGTATAACCTGCTACC 181
>miR2656a Score: 1.0 RO3G_13919
Query: 1 TAAGTTGCATATTGGACGTTGG 22
||*|||||||||||||||||||
Sbjct: 202 ATCCAACGTATAACCTGCAACC 181
>miR2656a Score: 1.0 RO3G_13454
Query: 1 TAAGTTGCATATTGGACGTTGG 22
||*|||||||||||||||||||
Sbjct: 202 ATCCAACGTATAACCTGCAACC 181
>miR2656a Score: 1.0 RO3G_12678
Query: 1 TAAGTTGCATATTGGACGTTGG 22
||*|||||||||||||||||||
Sbjct: 202 ATCCAACGTATAACCTGCAACC 181
>miR2656a Score: 1.0 RO3G_13286
Query: 1 TAAGTTGCATATTGGACGTTGG 22
||*|||||||||||||||||||
Sbjct: 202 ATCCAACGTATAACCTGCAACC 181
>miR2656a Score: 2.0 RO3G_07707
Query: 1 TAAGTTGCATATTGGACGTTGG 22
||**||||||||||||||||||
Sbjct: 202 ATCAAACGTATAACCTGCAACC 181
>miR2656a Score: 1.0 RO3G_02706
Query: 1 TAAGTTGCATATTGGACGTTGG 22
||*|||||||||||||||||||
Sbjct: 202 ATCCAACGTATAACCTGCAACC 181
>miR2656a Score: 0.0 RO3G_02845
Query: 1 TAAGTTGCATATTGGACGTTGG 22
||||||||||||||||||||||
Sbjct: 235 ATTCAACGTATAACCTGCAACC 214
>miR2656a Score: 1.0 RO3G_03602
Query: 1 TAAGTTGCATATTGGACGTTGG 22
||*|||||||||||||||||||
Sbjct: 202 ATCCAACGTATAACCTGCAACC 181
>miR2656a Score: 1.0 RO3G_00284
Query: 1 TAAGTTGCATATTGGACGTTGG 22
||*|||||||||||||||||||

```
Sbjct: 202 ATCCAACGTATAACCTGCAACC 181
>miR2656a Score: 1.0 RO3G_00679
Query: 1 TAAGTTGCATATTGGACGTTGG 22
||*|||||||||||||||||||
Sbjct: 202 ATCCAACGTATAACCTGCAACC 181
>miR2656a Score: 2.2 RO3G_01914
Query: 1 TAAGTTGCATATTGGACGTTGG 22
|||:|||*|||||||||*|||:
Sbjct: 85 ATTTAACCTATAACCTG-AACT 65
>miR2656a Score: 1.0 RO3G_01960
Query: 1 TAAGTTGCATATTGGACGTTGG 22
||*|||||||||||||||||||
Sbjct: 202 ATCCAACGTATAACCTGCAACC 181
>miR8123-3p Score: 1.5 RO3G_17202
Query: 1 TTGTCGCCATTGGTCCAAGTC 21
||:|*||||||||||||||||
Sbjct: 503 AATAACGGTAACCAGGTTCAG 483
>miR8123-3p Score: 0.0 RO3G_16829
Query: 1 TTGTCGCCATTGGTCCAAGTC 21
|||||||||||||||||||||
Sbjct: 503 AACAGCGGTAACCAGGTTCAG 483
>miR8123-3p Score: 0.0 RO3G_16904
Query: 1 TTGTCGCCATTGGTCCAAGTC 21
|||||||||||||||||||||
Sbjct: 503 AACAGCGGTAACCAGGTTCAG 483
>miR8123-3p Score: 0.5 RO3G_16721
Query: 1 TTGTCGCCATTGGTCCAAGTC 21
||:||||||||||||||||||
Sbjct: 461 AATAGCGGTAACCAGGTTCAG 441
>miR8123-3p Score: 0.8 RO3G_16703
Query: 1 TTGTCGCCATTGGTCCAAGTC 21
||:||||||||||||||||:|
Sbjct: 503 AATAGCGGTAACCAGGTTCGG 483
>miR8123-3p Score: 0.0 RO3G_16428
Query: 1 TTGTCGCCATTGGTCCAAGTC 21
|||||||||||||||||||||
Sbjct: 140 AACAGCGGTAACCAGGTTCAG 120
>miR8123-3p Score: 1.0 RO3G_13734
Query: 1 TTGTCGCCATTGGTCCAAGTC 21
||:||||||||:|||||||||
Sbjct: 503 AATAGCGGTAATCAGGTTCAG 483
>miR8123-3p Score: 1.0 RO3G_12060
Query: 1 TTGTCGCCATTGGTCCAAGTC 21
||:||||||||:|||||||||
Sbjct: 140 AATAGCGGTAATCAGGTTCAG 120
```

>miR8123-3p Score: 0.5 RO3G_11411
Query: 1 TTGTCGCCATTGGTCCAAGTC 21
||:||||||||||||||||||
Sbjct: 140 AATAGCGGTAACCAGGTTCAG 120
>miR8123-3p Score: 0.5 RO3G_05945
Query: 1 TTGTCGCCATTGGTCCAAGTC 21
||:||||||||||||||||||
Sbjct: 140 AATAGCGGTAACCAGGTTCAG 120
>miR8123-3p Score: 1.5 RO3G_03594
Query: 1 TTGTCGCCATTGGTCCAAGTC 21
||:|*||||||||||||||||
Sbjct: 140 AATAACGGTAACCAGGTTCAG 120
>miR8123-3p Score: 1.5 RO3G_00676
Query: 1 TTGTCGCCATTGGTCCAAGTC 21
||:|||||||||*||||||||
Sbjct: 503 AATAGCGGTAACGAGGTTCAG 483
>miR8123-3p Score: 1.0 RO3G_02224
Query: 1 TTGTCGCCATTGGTCCAAGTC 21
||:||||||||:|||||||||
Sbjct: 140 AATAGCGGTAATCAGGTTCAG 120
>miR8123-3p Score: 1.0 RO3G_17154
Query: 1 TTGTCGCCATTGGTCCAAGTC 21
||:||||||||:|||||||||
Sbjct: 140 AATAGCGGTAATCAGGTTCAG 120
>miR2622 Score: 2.2 RO3G_14266
Query: 1 TTTGAGGCCATCGTGAAACAT-TA 23
||||||||||||:|:||*||:*||
Sbjct: 1032 AAACTCCGGTAGTATTTAGTGTAT 1009
>miR7495a Score: 2.5 RO3G_16104
Query: 1 TTCTTGTAGTGCCTCCTTCA 20
||||||:|:*:|||||||||
Sbjct: 67 AAGAACGTTTTGGAGGAAGT 48
>miR7495a Score: 2.5 RO3G_14467
Query: 1 TTCTTGTAGTGCCTCCTTCA 20
||||:||||*|||*||||||
Sbjct: 504 AAGAGCATCTCGGTGGAAGT 485
>miR7495a Score: 2.5 RO3G_14722
Query: 1 TTCTTGTAGTGCCTCCTTCA 20
||||:||||*|||*||||||
Sbjct: 975 AAGAGCATCTCGGTGGAAGT 956
>miR7495a Score: 2.5 RO3G_13903
Query: 1 TTCTTGTAGTGCCTCCTTCA 20
||||:||||*|||*||||||
Sbjct: 975 AAGAGCATCTCGGTGGAAGT 956
>miR7495a Score: 2.5 RO3G_13664

Query: 1 TTCTTGTAGTGCCTCCTTCA 20
||||:||||*|||*||||||
Sbjct: 975 AAGAGCATCTCGGTGGAAGT 956
>miR7495a Score: 2.5 RO3G_13672
Query: 1 TTCTTGTAGTGCCTCCTTCA 20
||||:||||*|||*||||||
Sbjct: 975 AAGAGCATCTCGGTGGAAGT 956
>miR7495a Score: 2.5 RO3G_12139
Query: 1 TTCTTGTAGTGCCTCCTTCA 20
||||:||||*|||*||||||
Sbjct: 348 AAGAGCATCTCGGTGGAAGT 329
>miR7495a Score: 2.5 RO3G_11171
Query: 1 TTCTTGTAGTGCCTCCTTCA 20
||||||||:::||||||*|*
Sbjct: 510 AAGAACATTGTGGAGGA-GA 492
>miR7495a Score: 2.5 RO3G_11486
Query: 1 TTCTTGTAGTGCCTCCTTCA 20
||||:||||*|||*||||||
Sbjct: 684 AAGAGCATCTCGGTGGAAGT 665
>miR7495a Score: 2.5 RO3G_11567
Query: 1 TTCTTGTAGTGCCTCCTTCA 20
||||:||||*|||*||||||
Sbjct: 1632 AAGAGCATCTCGGTGGAAGT 1613
>miR7495a Score: 2.5 RO3G_10644
Query: 1 TTCTTGTAGTGCCTCCTTCA 20
||||:||||*|||*||||||
Sbjct: 360 AAGAGCATCTCGGTGGAAGT 341
>miR7495a Score: 2.0 RO3G_09419
Query: 1 TTCTTGTAGTGCCTCCTTCA 20
|||||||||*|||*||||||
Sbjct: 795 AAGAACATCTCGGTGGAAGT 776
>miR7495a Score: 2.0 RO3G_08375
Query: 1 TTCTTGTAGTGCCTCCTTCA 20
|*||||||:|||||||||*|
Sbjct: 1014 ATGAACATTACGGAGGAAAT 995
>miR7495a Score: 2.5 RO3G_08906
Query: 1 TTCTTGTAGTGCCTCCTTCA 20
||||:||||*|||*||||||
Sbjct: 975 AAGAGCATCTCGGTGGAAGT 956
>miR7495a Score: 2.5 RO3G_07788
Query: 1 TTCTTGTAGTGCCTCCTTCA 20
||||:||||*|||*||||||
Sbjct: 924 AAGAGCATCTCGGTGGAAGT 905
>miR7495a Score: 2.5 RO3G_05535
Query: 1 TTCTTGTAGTGCCTCCTTCA 20

||||:||||*|||*||||||
Sbjct: 360 AAGAGCATCTCGGTGGAAGT 341
>miR7495a Score: 2.5 RO3G_04841
Query: 1 TTCTTGTAGTGCCTCCTTCA 20
::*|||||||||||||:|*
Sbjct: 922 GGAAACATCACGGAGGAGGA 903
>miR7495a Score: 2.0 RO3G_05161
Query: 1 TTCTTGTAGTGCCTCCTTCA 20
||||||||*:|||||||||*
Sbjct: 2095 AAGAACATAGCGGAGGAAGG 2076
>miR7495a Score: 2.5 RO3G_05181
Query: 1 TTCTTGTAGTGCCTCCTTCA 20
||||:||||*|||*||||||
Sbjct: 975 AAGAGCATCTCGGTGGAAGT 956
>miR7495a Score: 2.5 RO3G_05194
Query: 1 TTCTTGTAGTGCCTCCTTCA 20
||||:||||*|||*||||||
Sbjct: 975 AAGAGCATCTCGGTGGAAGT 956
>miR7495a Score: 2.5 RO3G_05303
Query: 1 TTCTTGTAGTGCCTCCTTCA 20
||||:||||*|||*||||||
Sbjct: 975 AAGAGCATCTCGGTGGAAGT 956
>miR7495a Score: 2.5 RO3G_02354
Query: 1 TTCTTGTAGTGCCTCCTTCA 20
||||:||||*|||*||||||
Sbjct: 975 AAGAGCATCTCGGTGGAAGT 956
>miR7495a Score: 2.5 RO3G_03896
Query: 1 TTCTTGTAGTGCCTCCTTCA 20
|||||*|*|||||||||*||
Sbjct: 1233 AAGAAAAACACGGAGGA-GT 1215
>miR7495a Score: 2.5 RO3G_04104
Query: 1 TTCTTGTAGTGCCTCCTTCA 20
||||:||||*|||*||||||
Sbjct: 672 AAGAGCATCTCGGCGGAAGT 653
>miR7495a Score: 2.5 RO3G_04110
Query: 1 TTCTTGTAGTGCCTCCTTCA 20
||||:||||*|||*||||||
Sbjct: 672 AAGAGCATCTCGGTGGAAGT 653
>miR7495a Score: 2.5 RO3G_00779
Query: 1 TTCTTGTAGTGCCTCCTTCA 20
|||||*||||*||||||*||
Sbjct: 2835 AAGAAGATCAGGGAGGA-GT 2817
>miR7495a Score: 2.5 RO3G_01518
Query: 1 TTCTTGTAGTGCCTCCTTCA 20
||||:||||*|||*||||||

```
Sbjct: 348 AAGAGCATCTCGGTGGAAGT 329
>miR7495a Score: 2.5 RO3G_01528
Query: 1 TTCTTGTAGTGCCTCCTTCA 20
||||:||||*|||*||||||
Sbjct: 543 AAGAGCATCTCGGTGGAAGT 524
>miR7495a Score: 2.5 RO3G_01838
Query: 1 TTCTTGTAGTGCCTCCTTCA 20
||||||*|||||||*|||*|
Sbjct: 486 AAGAACTTCACGGAAGAA-T 468
>miR7495a Score: 2.5 RO3G_01923
Query: 1 TTCTTGTAGTGCCTCCTTCA 20
||||:||||*|||*||||||
Sbjct: 924 AAGAGCATCTCGGTGGAAGT 905
>miR7495a Score: 2.0 RO3G_02132
Query: 1 TTCTTGTAGTGCCTCCTTCA 20
|||||*|*||||||||||||
Sbjct: 175 AAGAAAAGCACGGAGGAAGT 156
>miR7495a Score: 2.5 RO3G_02179
Query: 1 TTCTTGTAGTGCCTCCTTCA 20
||||*|||:*||||||||||
Sbjct: 3597 AAGACCATTCCGGAGGAAGT 3578
>miR9742 Score: 0.0 RO3G_15585
Query: 1 TGTATTGTTTGTTTCTGTGAGTA 23
|||||||||||||||||||||||
Sbjct: 2674 ACATAACAAACAAAGACACTCAT 2652
>miR9742 Score: 0.0 RO3G_15250
Query: 1 TGTATTGTTTGTTTCTGTGAGTA 23
|||||||||||||||||||||||
Sbjct: 2674 ACATAACAAACAAAGACACTCAT 2652
>miR9742 Score: 0.0 RO3G_14355
Query: 1 TGTATTGTTTGTTTCTGTGAGTA 23
|||||||||||||||||||||||
Sbjct: 925 ACATAACAAACAAAGACACTCAT 903
>miR9742 Score: 0.0 RO3G_13867
Query: 1 TGTATTGTTTGTTTCTGTGAGTA 23
|||||||||||||||||||||||
Sbjct: 2635 ACATAACAAACAAAGACACTCAT 2613
>miR9742 Score: 0.0 RO3G_13920
Query: 1 TGTATTGTTTGTTTCTGTGAGTA 23
|||||||||||||||||||||||
Sbjct: 2719 ACATAACAAACAAAGACACTCAT 2697
>miR9742 Score: 0.0 RO3G_13749
Query: 1 TGTATTGTTTGTTTCTGTGAGTA 23
|||||||||||||||||||||||
Sbjct: 1282 ACATAACAAACAAAGACACTCAT 1260
```

>miR9742 Score: 0.0 RO3G_10558
Query: 1 TGTATTGTTTGTTTCTGTGAGTA 23
|||||||||||||||||||||||
Sbjct: 472 ACATAACAAACAAAGACACTCAT 450
>miR9742 Score: 2.5 RO3G_09555
Query: 1 TGTATTGTTTGTTTCTGTGAGTA 23
|||||||*:|||:||||||||*|
Sbjct: 1308 ACATAACCGACAGAGACACTC-T 1287
>miR9742 Score: 0.0 RO3G_09965
Query: 1 TGTATTGTTTGTTTCTGTGAGTA 23
|||||||||||||||||||||||
Sbjct: 2719 ACATAACAAACAAAGACACTCAT 2697
>miR9742 Score: 2.2 RO3G_10158
Query: 1 TGTATTGTTTGTTTCTGTGAGTA 23
||||||*|||||:||||*:||||
Sbjct: 72 ACATAAAAAACAGAGAC-TTCAT 51
>miR9742 Score: 0.0 RO3G_05619
Query: 1 TGTATTGTTTGTTTCTGTGAGTA 23
|||||||||||||||||||||||
Sbjct: 862 ACATAACAAACAAAGACACTCAT 840
>miR9742 Score: 0.0 RO3G_06427
Query: 1 TGTATTGTTTGTTTCTGTGAGTA 23
|||||||||||||||||||||||
Sbjct: 2311 ACATAACAAACAAAGACACTCAT 2289
>miR9742 Score: 0.0 RO3G_06428
Query: 1 TGTATTGTTTGTTTCTGTGAGTA 23
|||||||||||||||||||||||
Sbjct: 1813 ACATAACAAACAAAGACACTCAT 1791
>miR9742 Score: 0.0 RO3G_06442
Query: 1 TGTATTGTTTGTTTCTGTGAGTA 23
|||||||||||||||||||||||
Sbjct: 3376 ACATAACAAACAAAGACACTCAT 3354
>miR9742 Score: 2.5 RO3G_06545
Query: 1 TGTATTGTTTGTTTCTGTGAGTA 23
:||||||:|||||||||*|||
Sbjct: 957 TTATAACAGACAAAGACTC-CAT 936
>miR9742 Score: 0.0 RO3G_05272
Query: 1 TGTATTGTTTGTTTCTGTGAGTA 23
|||||||||||||||||||||||
Sbjct: 2719 ACATAACAAACAAAGACACTCAT 2697
>miR9742 Score: 2.0 RO3G_05368
Query: 1 TGTATTGTTTGTTTCTGTGAGTA 23
||||||||||||||*|||*|*||
Sbjct: 1939 ACATAACAAACAAATACAATAAT 1917
>miR9742 Score: 2.5 RO3G_02393

```
Query: 1 TGTATTGTTTGTTTCTGTG-AGTA 23
||||:||||||||*|||||*|*||
Sbjct: 383 ACATGACAAACAACGACACGTAAT 360
>miR9742 Score: 0.0 RO3G_02691
Query: 1 TGTATTGTTTGTTTCTGTGAGTA 23
|||||||||||||||||||||||
Sbjct: 6208 ACATAACAAACAAAGACACTCAT 6186
>miR9742 Score: 0.0 RO3G_02959
Query: 1 TGTATTGTTTGTTTCTGTGAGTA 23
|||||||||||||||||||||||
Sbjct: 2719 ACATAACAAACAAAGACACTCAT 2697
>miR9742 Score: 0.0 RO3G_03547
Query: 1 TGTATTGTTTGTTTCTGTGAGTA 23
|||||||||||||||||||||||
Sbjct: 2719 ACATAACAAACAAAGACACTCAT 2697
>miR9742 Score: 0.0 RO3G_04114
Query: 1 TGTATTGTTTGTTTCTGTGAGTA 23
|||||||||||||||||||||||
Sbjct: 2719 ACATAACAAACAAAGACACTCAT 2697
>miR9742 Score: 0.0 RO3G_00306
Query: 1 TGTATTGTTTGTTTCTGTGAGTA 23
|||||||||||||||||||||||
Sbjct: 1168 ACATAACAAACAAAGACACTCAT 1146
>miR9742 Score: 0.0 RO3G_01613
Query: 1 TGTATTGTTTGTTTCTGTGAGTA 23
|||||||||||||||||||||||
Sbjct: 970 ACATAACAAACAAAGACACTCAT 948
>miR9742 Score: 2.0 RO3G_02099
Query: 1 TGTATTGTTTGTTTCTGTGA-GTA 23
|||||||:||:|||||||*|*|||
Sbjct: 1855 ACATAACGAATAAAGACAGTCCAT 1832
>miR9742 Score: 0.0 RO3G_02221
Query: 1 TGTATTGTTTGTTTCTGTGAGTA 23
|||||||||||||||||||||||
Sbjct: 2641 ACATAACAAACAAAGACACTCAT 2619
>miR9742 Score: 0.0 RO3G_17371
Query: 1 TGTATTGTTTGTTTCTGTGAGTA 23
|||||||||||||||||||||||
Sbjct: 2674 ACATAACAAACAAAGACACTCAT 2652
>miR9742 Score: 0.0 RO3G_17326
Query: 1 TGTATTGTTTGTTTCTGTGAGTA 23
|||||||||||||||||||||||
Sbjct: 2551 ACATAACAAACAAAGACACTCAT 2529
>miR3434-3p Score: 1.0 RO3G_17250
Query: 1 TCAGGATCAGCCAAGTTGA 19
```

```
||||||||*||||||||||
Sbjct: 686 AGTCCTAGGCGGTTCAACT 668
>miR3434-3p Score: 0.0 RO3G_17254
Query: 1 TCAGGATCAGCCAAGTTGA 19
|||||||||||||||||||
Sbjct: 686 AGTCCTAGTCGGTTCAACT 668
>miR3434-3p Score: 0.0 RO3G_17201
Query: 1 TCAGGATCAGCCAAGTTGA 19
|||||||||||||||||||
Sbjct: 683 AGTCCTAGTCGGTTCAACT 665
>miR3434-3p Score: 1.0 RO3G_17220
Query: 1 TCAGGATCAGCCAAGTTGA 19
||||||||*||||||||||
Sbjct: 692 AGTCCTAGGCGGTTCAACT 674
>miR3434-3p Score: 1.0 RO3G_16903
Query: 1 TCAGGATCAGCCAAGTTGA 19
||||||||*||||||||||
Sbjct: 620 AGTCCTAGGCGGTTCAACT 602
>miR3434-3p Score: 1.0 RO3G_16358
Query: 1 TCAGGATCAGCCAAGTTGA 19
|||||*|||||||||||||
Sbjct: 515 AGTCCCAGTCGGTTCAACT 497
>miR3434-3p Score: 1.0 RO3G_16365
Query: 1 TCAGGATCAGCCAAGTTGA 19
||||||||*||||||||||
Sbjct: 686 AGTCCTAGGCGGTTCAACT 668
>miR3434-3p Score: 2.0 RO3G_16217
Query: 1 TCAGGATCAGCCAAGTTGA 19
||||:|*||||||||||*|
Sbjct: 701 AGTCTTTGTCGGTTCAAGT 683
>miR3434-3p Score: 2.0 RO3G_15938
Query: 1 TCAGGATCAGCCAAGTTGA 19
||||:|:|||||||:|:||
Sbjct: 608 AGTCTTGGTCGGTTTAGCT 590
>miR3434-3p Score: 2.5 RO3G_15989
Query: 1 TCAGGATCAGCCAAGTTG-A 19
||||:|*||:||||||||*|
Sbjct: 1049 AGTCTTCGTTGGTTCAACAT 1030
>miR3434-3p Score: 2.5 RO3G_15543
Query: 1 TCAGGATCAGCCAAGTTGA 19
||||:||||:*||||||*|
Sbjct: 107 AGTCTTAGTTTGTTCAAGT 89
>miR3434-3p Score: 1.0 RO3G_14562
Query: 1 TCAGGATCAGCCAAGTTGA 19
||||||||*||||||||||
```

Sbjct: 623 AGTCCTAGGCGGTTCAACT 605
>miR3434-3p Score: 2.5 RO3G_14665
Query: 1 TCAGGATCAGCCAAGTTGA 19
|||||||||*|||||||
Sbjct: 983 CGTCGTAGTCGTTTCAACT 965
>miR3434-3p Score: 2.5 RO3G_14694
Query: 1 TCAGGATCAGCCAAGTTGA 19
||||||||||||*||*:||
Sbjct: 980 AGTCCTAGTCGGGTCTGCT 962
>miR3434-3p Score: 2.5 RO3G_14156
Query: 1 TCAGGATCAGCCAAGTTGA 19
|||:|||||||||||*|
Sbjct: 671 CGTCTTAGTGGGTTCAA-T 654
>miR3434-3p Score: 2.5 RO3G_14338
Query: 1 TCAGGATCAGCCAAGTTGA 19
|||::||||:*||||||||
Sbjct: 662 AGTTTTAGTTAGTTCAACT 644
>miR3434-3p Score: 0.0 RO3G_13449
Query: 1 TCAGGATCAGCCAAGTTGA 19
|||||||||||||||||||
Sbjct: 686 AGTCCTAGTCGGTTCAACT 668
>miR3434-3p Score: 0.0 RO3G_12967
Query: 1 TCAGGATCAGCCAAGTTGA 19
|||||||||||||||||||
Sbjct: 686 AGTCCTAGTCGGTTCAACT 668
>miR3434-3p Score: 2.0 RO3G_13067
Query: 1 TCAGGATCAGCCAAGTTGA 19
|||:||:|||||||||:*|
Sbjct: 683 AGTTCTGGTCGGTTCAG-T 666
>miR3434-3p Score: 0.0 RO3G_11978
Query: 1 TCAGGATCAGCCAAGTTGA 19
|||||||||||||||||||
Sbjct: 539 AGTCCTAGTCGGTTCAACT 521
>miR3434-3p Score: 2.5 RO3G_11115
Query: 1 TCAGGATCAGCCAAGTTGA 19
||||:||||:*|||||||*
Sbjct: 161 AGTCTTAGTTAGTTCAACA 143
>miR3434-3p Score: 1.5 RO3G_11790
Query: 1 TCAGGATCAGCCAAGTTGA 19
|||||||||||||||||
Sbjct: 1256 TGTCCTAGTCAGTTCAACT 1238
>miR3434-3p Score: 1.0 RO3G_11821
Query: 1 TCAGGATCAGCCAAGTTGA 19
|||:||:||||||||||||
Sbjct: 2222 AGTTCTGGTCGGTTCAACT 2204

```
>miR3434-3p Score: 0.0 RO3G_10533
Query: 1 TCAGGATCAGCCAAGTTGA 19
|||||||||||||||||||
Sbjct: 686 AGTCCTAGTCGGTTCAACT 668
>miR3434-3p Score: 1.0 RO3G_10538
Query: 1 TCAGGATCAGCCAAGTTGA 19
||||||||*||||||||||
Sbjct: 644 AGTCCTAGGCGGTTCAACT 626
>miR3434-3p Score: 2.5 RO3G_10672
Query: 1 TCAGGATCAGCCAAGTTGA 19
|||||||||:|||||*|**
Sbjct: 623 AGTCCTAGTTGGTTCTAAA 605
>miR3434-3p Score: 0.0 RO3G_10730
Query: 1 TCAGGATCAGCCAAGTTGA 19
|||||||||||||||||||
Sbjct: 686 AGTCCTAGTCGGTTCAACT 668
>miR3434-3p Score: 0.0 RO3G_10748
Query: 1 TCAGGATCAGCCAAGTTGA 19
|||||||||||||||||||
Sbjct: 686 AGTCCTAGTCGGTTCAACT 668
>miR3434-3p Score: 2.2 RO3G_09445
Query: 1 TCAGGATCAGCCAAGTTGA 19
||||*||||||*|||||:|
Sbjct: 104 AGTCATAGTCGTTTCAATT 86
>miR3434-3p Score: 2.5 RO3G_08291
Query: 1 TCAGGATCAGCCAAGTTGA 19
|||||*|||:*||||||||
Sbjct: 401 AGTCCAAGTTAGTTCAACT 383
>miR3434-3p Score: 0.0 RO3G_09174
Query: 1 TCAGGATCAGCCAAGTTGA 19
|||||||||||||||||||
Sbjct: 683 AGTCCTAGTCGGTTCAACT 665
>miR3434-3p Score: 1.8 RO3G_06992
Query: 1 TCAGGATCAGCCAAGTTGA 19
:||||||||:|*|||||||
Sbjct: 461 GGTCCTAGTTGTTTCAACT 443
>miR3434-3p Score: 0.0 RO3G_07736
Query: 1 TCAGGATCAGCCAAGTTGA 19
|||||||||||||||||||
Sbjct: 686 AGTCCTAGTCGGTTCAACT 668
>miR3434-3p Score: 0.0 RO3G_07861
Query: 1 TCAGGATCAGCCAAGTTGA 19
|||||||||||||||||||
Sbjct: 686 AGTCCTAGTCGGTTCAACT 668
>miR3434-3p Score: 0.0 RO3G_07863
```

Query: 1 TCAGGATCAGCCAAGTTGA 19
|||||||||||||||||||
Sbjct: 686 AGTCCTAGTCGGTTCAACT 668
>miR3434-3p Score: 2.5 RO3G_07931
Query: 1 TCAGGATCAGCCAAGTTGA 19
||||||*|*|||||:||||
Sbjct: 2729 AGTCCTCGCCGGTTTAACT 2711
>miR3434-3p Score: 2.2 RO3G_05895
Query: 1 TCAGGATCAGCCAAGTTGA 19
:||::|||||||||*||||
Sbjct: 311 GGTTTTAGTCGGTTAAACT 293
>miR3434-3p Score: 0.0 RO3G_06414
Query: 1 TCAGGATCAGCCAAGTTGA 19
|||||||||||||||||||
Sbjct: 683 AGTCCTAGTCGGTTCAACT 665
>miR3434-3p Score: 2.5 RO3G_06597
Query: 1 TCAGGATCAGCCAAGTTG-A 19
|||:||||||||||||*|
Sbjct: 6890 CGTCTTAATCGGTTCAACCT 6871
>miR3434-3p Score: 2.5 RO3G_06633
Query: 1 TCAGGATCAGCCAAGTTGA 19
||||:||||:||||:|*||
Sbjct: 224 AGTCTTAGTTGGTTTA-CT 207
>miR3434-3p Score: 2.5 RO3G_06674
Query: 1 TCAGGATCAGCCAAGTTGA 19
:||||||||:|||||*::|
Sbjct: 263 GGTCCTAGTTGGTTCCGTT 245
>miR3434-3p Score: 2.5 RO3G_04451
Query: 1 TCAGGATCAGCCAAGTTGA 19
||||||*|||*||||||*|
Sbjct: 863 AGTCCTTGTCTGTTCAA-T 846
>miR3434-3p Score: 2.5 RO3G_05324
Query: 1 TCAGGATCAGCCAAGTTGA 19
||||||||:||||:|||
Sbjct: 278 TGTCCTAGTTGGTTTA-CT 261
>miR3434-3p Score: 2.0 RO3G_03248
Query: 1 TCAGGATCAGCCAAGTTGA 19
*||||||||:|||||::||
Sbjct: 191 CGTCCTAGTTGGTTCGGCT 173
>miR3434-3p Score: 1.5 RO3G_04192
Query: 1 TCAGGATCAGCCAAGTTGA 19
||||:|:|||||||||:||
Sbjct: 629 AGTCTTGGTCGGTTCAGCT 611
>miR3434-3p Score: 2.5 RO3G_00405
Query: 1 TCAGGATCAGCCAAGTTGA 19

||||||*|||*||||||*|
Sbjct: 647 AGTCCTTGTCTGTTCAA-T 630
>miR3434-3p Score: 2.5 RO3G_00473
Query: 1 TCAGGATCAGCCAAGTTGA 19
|||:|||||*|||||||**
Sbjct: 986 AGTTCTAGTAGGTTCAAGA 968
>miR3434-3p Score: 2.5 RO3G_00996
Query: 1 TCAGGATCAGCCAAGTTGA 19
||||:*|||:|||||||*|
Sbjct: 584 AGTCTAAGTTGGTTCAAAT 566
>miR3434-3p Score: 2.5 RO3G_01084
Query: 1 TCAGGATCAGCCAAGTTGA 19
|||||*|||:*||||||||
Sbjct: 401 AGTCCAAGTTAGTTCAACT 383
>miR3434-3p Score: 2.5 RO3G_01089
Query: 1 TCAGGATCAGCCAAGTTGA 19
*||||||||:||||:||**
Sbjct: 6191 TGTCCTAGTTGGTTTAAGA 6173
>miR3434-3p Score: 2.5 RO3G_01571
Query: 1 TCAGGATCAGCCAAGTTGA 19
|||::||||||*||||:||
Sbjct: 1736 AGTTTTAGTCGTTTCAGCT 1718
>miR3434-3p Score: 1.5 RO3G_17108
Query: 1 TCAGGATCAGCCAAGTTG-A 19
||||:||||||||||||**|
Sbjct: 902 AGTCTTAGTCGGTTCAAGGT 883
>miR4348c Score: 2.5 RO3G_11064
Query: 1 TGTTAAACCTTGGAAGAGACCA 22
:||||||||||||||||*||
Sbjct: 1278 CTAATTTGGAACCTTCCCTCGT 1257
>miR4348c Score: 2.5 RO3G_09637
Query: 1 TGTTAAACCTTGGAAGAGACCA 22
|||:||*|||||:|||||*|||
Sbjct: 966 ACAGTTCGGAACTTTCTCCGGT 945
>miR1870-5p Score: 0.0 RO3G_16818
Query: 1 TTGCTGAATGGACCAGTGGCAT 22
||||||||||||||||||||||
Sbjct: 1426 AACGACTTACCTGGTCACCGTA 1405
>miR1870-5p Score: 0.0 RO3G_16797
Query: 1 TTGCTGAATGGACCAGTGGCAT 22
||||||||||||||||||||||
Sbjct: 685 AACGACTTACCTGGTCACCGTA 664
>miR1870-5p Score: 2.0 RO3G_16800
Query: 1 TTGCTGAATGGACCAGTGGCAT 22
|||||||*:|:|||||||||||

```
Sbjct: 1684 AACGACTCGCTTGGTCACCGTA 1663
>miR1870-5p Score: 0.0 RO3G_15265
Query: 1 TTGCTGAATGGACCAGTGGCAT 22
||||||||||||||||||||||
Sbjct: 1684 AACGACTTACCTGGTCACCGTA 1663
>miR1870-5p Score: 0.0 RO3G_15266
Query: 1 TTGCTGAATGGACCAGTGGCAT 22
||||||||||||||||||||||
Sbjct: 1684 AACGACTTACCTGGTCACCGTA 1663
>miR1870-5p Score: 0.0 RO3G_15268
Query: 1 TTGCTGAATGGACCAGTGGCAT 22
||||||||||||||||||||||
Sbjct: 1684 AACGACTTACCTGGTCACCGTA 1663
>miR1870-5p Score: 0.0 RO3G_14352
Query: 1 TTGCTGAATGGACCAGTGGCAT 22
||||||||||||||||||||||
Sbjct: 1684 AACGACTTACCTGGTCACCGTA 1663
>miR1870-5p Score: 0.0 RO3G_13217
Query: 1 TTGCTGAATGGACCAGTGGCAT 22
||||||||||||||||||||||
Sbjct: 1684 AACGACTTACCTGGTCACCGTA 1663
>miR1870-5p Score: 0.0 RO3G_13219
Query: 1 TTGCTGAATGGACCAGTGGCAT 22
||||||||||||||||||||||
Sbjct: 1684 AACGACTTACCTGGTCACCGTA 1663
>miR1870-5p Score: 0.0 RO3G_13222
Query: 1 TTGCTGAATGGACCAGTGGCAT 22
||||||||||||||||||||||
Sbjct: 1678 AACGACTTACCTGGTCACCGTA 1657
>miR1870-5p Score: 1.0 RO3G_13225
Query: 1 TTGCTGAATGGACCAGTGGCAT 22
||||||||:|:|||||||||||
Sbjct: 1696 AACGACTTGCTTGGTCACCGTA 1675
>miR1870-5p Score: 1.0 RO3G_11967
Query: 1 TTGCTGAATGGACCAGTGGCAT 22
||||||||:|:|||||||||||
Sbjct: 1696 AACGACTTGCTTGGTCACCGTA 1675
>miR1870-5p Score: 2.5 RO3G_08855
Query: 1 TTGCTGAATGGACCAGTGGCAT 22
|::|||||||||||*|||||*|
Sbjct: 935 AGTGACTTACCTGGACACCGCA 914
>miR1870-5p Score: 0.0 RO3G_05640
Query: 1 TTGCTGAATGGACCAGTGGCAT 22
||||||||||||||||||||||
Sbjct: 1432 AACGACTTACCTGGTCACCGTA 1411
```

>miR1870-5p Score: 2.5 RO3G_05952
Query: 1 TTGCTGAATGGACCAGTGGCAT 22
|||||||*:|:||||:||||||
Sbjct: 1564 AACGACTCGCTTGGTTACCGTA 1543
>miR1870-5p Score: 0.0 RO3G_03992
Query: 1 TTGCTGAATGGACCAGTGGCAT 22
||||||||||||||||||||||
Sbjct: 415 AACGACTTACCTGGTCACCGTA 394
>miR1870-5p Score: 0.0 RO3G_04121
Query: 1 TTGCTGAATGGACCAGTGGCAT 22
||||||||||||||||||||||
Sbjct: 1684 AACGACTTACCTGGTCACCGTA 1663
>miR2905 Score: 0.0 RO3G_15711
Query: 1 TACTGTAGCCGACAAAGGCA 20
||||||||||||||||||||
Sbjct: 2394 ATGACATCGGCTGTTTCCGT 2375
>miR2905 Score: 0.0 RO3G_13796
Query: 1 TACTGTAGCCGACAAAGGCA 20
||||||||||||||||||||
Sbjct: 2394 ATGACATCGGCTGTTTCCGT 2375
>miR2905 Score: 0.0 RO3G_11521
Query: 1 TACTGTAGCCGACAAAGGCA 20
||||||||||||||||||||
Sbjct: 1356 ATGACATCGGCTGTTTCCGT 1337
>miR2905 Score: 2.0 RO3G_11026
Query: 1 TACTGTAGCCGACAAAGGCA 20
|||::|||||:|||||:|||
Sbjct: 676 ATGGTATCGGTTGTTTTCGT 657
>miR2905 Score: 0.0 RO3G_09539
Query: 1 TACTGTAGCCGACAAAGGCA 20
||||||||||||||||||||
Sbjct: 2394 ATGACATCGGCTGTTTCCGT 2375
>miR2905 Score: 1.8 RO3G_03627
Query: 1 TACTGTAGCCGACAAAGGCA 20
:||||||||||||||*||*|
Sbjct: 411 GTGACATCGGCTGTTACCTT 392
>miR2905 Score: 1.8 RO3G_01708
Query: 1 TACTGTAGCCGACAAAGGCA 20
:||||||||||||||*||*|
Sbjct: 411 GTGACATCGGCTGTTACCTT 392
>miR5557-3p Score: 1.0 RO3G_17259
Query: 1 TGCTTGCATTATCTTGTTGA 20
|||||*||||||||||||||
Sbjct: 130 ACGAAAGTAATAGAACAACT 111
>miR5557-3p Score: 0.0 RO3G_17249

```
Query: 1 TGCTTGCATTATCTTGTTGA 20
||||||||||||||||||||
Sbjct: 649 ACGAACGTAATAGAACAACT 630
>miR5557-3p Score: 2.5 RO3G_16179
Query: 1 TGCTTGCATTATCTTGTTGA 20
|||||||*:||||||||*|*
Sbjct: 463 ACGAACGAGATAGAACA-CG 445
>miR5557-3p Score: 0.0 RO3G_14978
Query: 1 TGCTTGCATTATCTTGTTGA 20
||||||||||||||||||||
Sbjct: 130 ACGAACGTAATAGAACAACT 111
>miR5557-3p Score: 0.0 RO3G_14363
Query: 1 TGCTTGCATTATCTTGTTGA 20
||||||||||||||||||||
Sbjct: 682 ACGAACGTAATAGAACAACT 663
>miR5557-3p Score: 0.0 RO3G_14547
Query: 1 TGCTTGCATTATCTTGTTGA 20
||||||||||||||||||||
Sbjct: 130 ACGAACGTAATAGAACAACT 111
>miR5557-3p Score: 1.0 RO3G_13921
Query: 1 TGCTTGCATTATCTTGTTGA 20
|||||*||||||||||||||
Sbjct: 130 ACGAAGGTAATAGAACAACT 111
>miR5557-3p Score: 2.5 RO3G_13952
Query: 1 TGCTTGCATTATCTTGTTGA 20
|||||||||||*||||:*|*
Sbjct: 3724 ACGAACGTAATCGAACGTCA 3705
>miR5557-3p Score: 2.0 RO3G_13954
Query: 1 TGCTTGCATTATCTTGTTGA 20
|||||||||||||:|*|::|
Sbjct: 468 ACGAACGTAATAGGAGAGTT 449
>miR5557-3p Score: 0.0 RO3G_13863
Query: 1 TGCTTGCATTATCTTGTTGA 20
||||||||||||||||||||
Sbjct: 424 ACGAACGTAATAGAACAACT 405
>miR5557-3p Score: 0.0 RO3G_13270
Query: 1 TGCTTGCATTATCTTGTTGA 20
||||||||||||||||||||
Sbjct: 724 ACGAACGTAATAGAACAACT 705
>miR5557-3p Score: 0.0 RO3G_11527
Query: 1 TGCTTGCATTATCTTGTTGA 20
||||||||||||||||||||
Sbjct: 130 ACGAACGTAATAGAACAACT 111
>miR5557-3p Score: 2.5 RO3G_11925
Query: 1 TGCTTGCATTATCTTGTTGA 20
```

|||*||||||*|||||||*|
Sbjct: 160 ACGTACGTAAAAGAACAAAT 141
>miR5557-3p Score: 2.5 RO3G_10608
Query: 1 TGCTTGCATTATCTTGTTG-A 20
|||||||||:|:|||*|||*|
Sbjct: 8947 ACGAACGTAGTGGAAGAACGT 8927
>miR5557-3p Score: 1.0 RO3G_10734
Query: 1 TGCTTGCATTATCTTGTTGA 20
||||||||||||*|||||||
Sbjct: 130 ACGAACGTAATACAACAACT 111
>miR5557-3p Score: 0.0 RO3G_10774
Query: 1 TGCTTGCATTATCTTGTTGA 20
||||||||||||||||||||
Sbjct: 691 ACGAACGTAATAGAACAACT 672
>miR5557-3p Score: 1.0 RO3G_10869
Query: 1 TGCTTGCATTATCTTGTTGA 20
|||||||||||||*||||||
Sbjct: 130 ACGAACGTAATAGTACAACT 111
>miR5557-3p Score: 2.5 RO3G_05820
Query: 1 TGCTTGCATTATCTTGTTGA 20
||||||||:|||||||*|
Sbjct: 507 CCGAACGTAGTAGAAGAAGT 488
>miR5557-3p Score: 0.0 RO3G_05935
Query: 1 TGCTTGCATTATCTTGTTGA 20
||||||||||||||||||||
Sbjct: 130 ACGAACGTAATAGAACAACT 111
>miR5557-3p Score: 2.5 RO3G_04254
Query: 1 TGCTTGCATTATCTTGTTGA 20
||||*|*|||||||||||||*
Sbjct: 487 ACGATCTTAATAGAACAACC 468
>miR5557-3p Score: 0.0 RO3G_04790
Query: 1 TGCTTGCATTATCTTGTTGA 20
||||||||||||||||||||
Sbjct: 130 ACGAACGTAATAGAACAACT 111
>miR5557-3p Score: 1.5 RO3G_05423
Query: 1 TGCTTGCATTATCTTGTTGA 20
|||||:||||||||*|||||
Sbjct: 130 ACGAATGTAATAGATCAACT 111
>miR5557-3p Score: 1.0 RO3G_03593
Query: 1 TGCTTGCATTATCTTGTTGA 20
|||:||||:|||||||||||
Sbjct: 130 ACGGACGTGATAGAACAACT 111
>miR5557-3p Score: 0.0 RO3G_03626
Query: 1 TGCTTGCATTATCTTGTTGA 20
||||||||||||||||||||

```
Sbjct: 130 ACGAACGTAATAGAACAACT 111
>miR5557-3p Score: 2.5 RO3G_00114
Query: 1 TGCTTGCATTATCTTGTTGA 20
||*||||||||:*|||||||
Sbjct: 466 ACTAACGTAATGAAACAACT 447
>miR5557-3p Score: 0.0 RO3G_00678
Query: 1 TGCTTGCATTATCTTGTTGA 20
||||||||||||||||||||
Sbjct: 94 ACGAACGTAATAGAACAACT 75
>miR5557-3p Score: 0.0 RO3G_01589
Query: 1 TGCTTGCATTATCTTGTTGA 20
||||||||||||||||||||
Sbjct: 130 ACGAACGTAATAGAACAACT 111
>miR5557-3p Score: 1.0 RO3G_17459
Query: 1 TGCTTGCATTATCTTGTTGA 20
||||||||||||*|||||||
Sbjct: 130 ACGAACGTAATACAACAACT 111
>miR5557-3p Score: 0.0 RO3G_17276
Query: 1 TGCTTGCATTATCTTGTTGA 20
||||||||||||||||||||
Sbjct: 814 ACGAACGTAATAGAACAACT 795
>miR8135 Score: 2.5 RO3G_16054
Query: 1 AGGATTTATGCTTGGTGAT 19
||:||:||*||||||||*|
Sbjct: 2442 TCTTAGATTCGAACCAC-A 2425
>miR8135 Score: 2.5 RO3G_15106
Query: 1 AGGATTTATGCTTGGTGAT 19
|*||:|||||||||:|||*
Sbjct: 300 TACTGAATACGAACTACTT 282
>miR8135 Score: 2.0 RO3G_13681
Query: 1 AGGATTTATGCTTGGTGAT 19
*|||||||||||||*:|||
Sbjct: 280 GCCTAAATACGAACGGCTA 262
>miR8135 Score: 2.5 RO3G_12399
Query: 1 AGGATTTATGCTTGGTGAT 19
*|:|||||*||||||||*|
Sbjct: 495 ACTTAAATTCGAACCAC-A 478
>miR8135 Score: 2.5 RO3G_11749
Query: 1 AGGATTTATGCTTGGTGAT 19
*|:||||||||:||:|:||
Sbjct: 1590 ACTTAAATACGGACTATTA 1572
>miR8135 Score: 2.0 RO3G_05601
Query: 1 AGGATTTATGCTTGGTGA-T 19
|||*|||||||:||||||*|
Sbjct: 1131 TCCCAAATACGGACCACTTA 1112
```

>miR8135 Score: 2.0 RO3G_05297
Query: 1 AGGATTTATGCTTGGTGAT 19
|:|||||*|||||||||*|
Sbjct: 2621 TTCTAAAGACGAACCACAA 2603
>miR5241a Score: 2.0 RO3G_17221
Query: 1 TGATGATGGAAGAGGGAAT 19
|||||||||||*|:||||*
Sbjct: 857 ACTACTACCTTGTTCCTTT 839
>miR5241a Score: 2.0 RO3G_16861
Query: 1 TGATGATGGAAGAGGGAAT 19
|||||||::||:|||||*|
Sbjct: 421 ACTACTATTTTTTCCCT-A 404
>miR5241a Score: 2.5 RO3G_16170
Query: 1 TGATGATGGAAGAGGGAAT 19
||||||||:*|:|||||*|
Sbjct: 119 ACTACTACTCTTTCCCTGA 101
>miR5241a Score: 2.5 RO3G_16310
Query: 1 TGATGATGGAAGAGGGAAT 19
||||||||||||||::**|
Sbjct: 1248 ACTACTACCTTCTCTTGAA 1230
>miR5241a Score: 2.0 RO3G_16076
Query: 1 TGATGATGGAAGAGGGAAT 19
|||||||||||*|:||||*
Sbjct: 857 ACTACTACCTTGTTCCTTT 839
>miR5241a Score: 2.0 RO3G_14908
Query: 1 TGATGATGGAAGAGGGAAT 19
|||||||||||*|:||||*
Sbjct: 857 ACTACTACCTTGTTCCTTT 839
>miR5241a Score: 1.8 RO3G_15056
Query: 1 TGATGATGGAAGAGGGAAT 19
*||:||||:|||||||||:
Sbjct: 445 CCTGCTACTTTCTCCCTTG 427
>miR5241a Score: 2.0 RO3G_14748
Query: 1 TGATGATGGAAGAGGGAAT 19
|||||||||||*|:||||*
Sbjct: 857 ACTACTACCTTGTTCCTTT 839
>miR5241a Score: 2.0 RO3G_14037
Query: 1 TGATGATGGAAGAGGGAA-T 19
||||||||||*|||:|||*|
Sbjct: 213 ACTACTACCTGCTCTCTTTA 194
>miR5241a Score: 2.0 RO3G_13483
Query: 1 TGATGATGGAAGAGGGAAT 19
|||||||||||*|:||||*
Sbjct: 857 ACTACTACCTTGTTCCTTT 839
>miR5241a Score: 2.0 RO3G_13707

Query: 1 TGATGATGGAAGAGGGAAT 19
|||||||||||*|:||||*
Sbjct: 857 ACTACTACCTTGTTCCTTT 839
>miR5241a Score: 2.0 RO3G_12949
Query: 1 TGATGATGGAAGAGGGAAT 19
|||:|||:|||||:||||*
Sbjct: 721 ACTGCTATCTTCTTCCTTT 703
>miR5241a Score: 2.5 RO3G_13051
Query: 1 TGATGATGGAAGAGGGAAT 19
|*|||||||||||:|||**
Sbjct: 262 AGTACTACCTTCTTCCTCT 244
>miR5241a Score: 2.0 RO3G_13265
Query: 1 TGATGATGGAAGAGGGAAT 19
|||||||||||*|:||||*
Sbjct: 857 ACTACTACCTTGTTCCTTT 839
>miR5241a Score: 2.0 RO3G_11990
Query: 1 TGATGATGGAAGAGGGAAT 19
|||||||||||*|:||||*
Sbjct: 857 ACTACTACCTTGTTCCTTT 839
>miR5241a Score: 2.0 RO3G_12352
Query: 1 TGATGATGGAAGAGGGAAT 19
|||||||||||*|:||||*
Sbjct: 857 ACTACTACCTTGTTCCTTT 839
>miR5241a Score: 2.5 RO3G_10250
Query: 1 TGATGATGGAAGAGGGAAT 19
|||:*||||||||:||||*
Sbjct: 256 ACTGATACCTTCTTCCTTT 238
>miR5241a Score: 2.2 RO3G_09758
Query: 1 TGATGATGGAAGAGGGAAT 19
||||||::|||||:|:||:
Sbjct: 1978 ACTACTGTCTTCTTCTTTG 1960
>miR5241a Score: 2.0 RO3G_09951
Query: 1 TGATGATGGAAGAGGGAAT 19
||||||||:|*|||:||||
Sbjct: 126 ACTACTACTTCCTCTCTTA 108
>miR5241a Score: 2.0 RO3G_10082
Query: 1 TGATGATGGAAGAGGGAAT 19
|||||||||||*|:||||*
Sbjct: 857 ACTACTACCTTGTTCCTTT 839
>miR5241a Score: 2.0 RO3G_08729
Query: 1 TGATGATGGAAGAGGGAAT 19
|||||||||||*|:||||*
Sbjct: 857 ACTACTACCTTGTTCCTTT 839
>miR5241a Score: 2.0 RO3G_07318
Query: 1 TGATGATGGAAGAGGGAAT 19

```
|||||||||||*|:||||*
Sbjct: 857 ACTACTACCTTGTTCCTTT 839
>miR5241a Score: 2.5 RO3G_07775
Query: 1 TGATGATGGAAGAGGGAAT 19
*||||||||||||:|:|**
Sbjct: 1423 TCTACTACCTTCTTCTTCT 1405
>miR5241a Score: 2.0 RO3G_07919
Query: 1 TGATGATGGAAGAGGGAAT 19
|||||||||||*|:||||*
Sbjct: 857 ACTACTACCTTGTTCCTTT 839
>miR5241a Score: 2.5 RO3G_05571
Query: 1 TGATGATGGAAGAGGGAAT 19
|:|||||*|||||:|||*|
Sbjct: 343 ATTACTAGCTTCTTCCT-A 326
>miR5241a Score: 2.0 RO3G_05692
Query: 1 TGATGATGGAAGAGGGAAT 19
|||||||||||*|:||||*
Sbjct: 857 ACTACTACCTTGTTCCTTT 839
>miR5241a Score: 2.5 RO3G_05801
Query: 1 TGATGATGGAAGAGGGAAT 19
||||||*|||*|||:||||
Sbjct: 465 ACTACTTCCTGCTCTCTTA 447
>miR5241a Score: 2.2 RO3G_05858
Query: 1 TGATGATGGAAGAGGGAAT 19
||||:|||||||||:*||:
Sbjct: 694 ACTATTACCTTCTCTATTG 676
>miR5241a Score: 2.5 RO3G_06122
Query: 1 TGATGATGGAAGAGGGAAT 19
|||||||:||*|||*||||
Sbjct: 982 ACTACTATCTACTCACTTA 964
>miR5241a Score: 2.5 RO3G_06182
Query: 1 TGATGATGGAAGAGGGAA-T 19
|||||||:|||||||*|**|
Sbjct: 421 ACTACTATCTTCTCCATCCA 402
>miR5241a Score: 2.0 RO3G_06439
Query: 1 TGATGATGGAAGAGGGAAT 19
|||||||||||*|:||||*
Sbjct: 854 ACTACTACCTTGTTCCTTT 836
>miR5241a Score: 2.0 RO3G_05090
Query: 1 TGATGATGGAAGAGGGAAT 19
|||||||||||*|:||||*
Sbjct: 857 ACTACTACCTTGTTCCTTT 839
>miR5241a Score: 2.0 RO3G_05091
Query: 1 TGATGATGGAAGAGGGAAT 19
|||||||||||*|:||||*
```

Sbjct: 857 ACTACTACCTTGTTCCTTT 839
>miR5241a Score: 2.0 RO3G_05128
Query: 1 TGATGATGGAAGAGGGAAT 19
|||||||||||*|:||||*
Sbjct: 857 ACTACTACCTTGTTCCTTT 839
>miR5241a Score: 2.0 RO3G_05129
Query: 1 TGATGATGGAAGAGGGAAT 19
|||||||||||*|:||||*
Sbjct: 857 ACTACTACCTTGTTCCTTT 839
>miR5241a Score: 2.2 RO3G_02765
Query: 1 TGATGATGGAAGAGGGAAT 19
:|||:|||:|||||:||*|
Sbjct: 1376 GCTATTACTTTCTCTCT-A 1359
>miR5241a Score: 2.0 RO3G_02948
Query: 1 TGATGATGGAAGAGGGAAT 19
|||||||||||*|:||||*
Sbjct: 857 ACTACTACCTTGTTCCTTT 839
>miR5241a Score: 2.0 RO3G_03722
Query: 1 TGATGATGGAAGAGGGAAT 19
|||||||||||*|:||||*
Sbjct: 857 ACTACTACCTTGTTCCTTT 839
>miR5241a Score: 2.0 RO3G_03986
Query: 1 TGATGATGGAAGAGGGAAT 19
|||||||||||*|:||||*
Sbjct: 857 ACTACTACCTTGTTCCTTT 839
>miR5241a Score: 2.0 RO3G_04027
Query: 1 TGATGATGGAAGAGGGAAT 19
|||||||||||*|:||||*
Sbjct: 857 ACTACTACCTTGTTCCTTT 839
>miR5241a Score: 2.0 RO3G_04035
Query: 1 TGATGATGGAAGAGGGAAT 19
|||||||||||*|:||||*
Sbjct: 857 ACTACTACCTTGTTCCTTT 839
>miR5241a Score: 2.0 RO3G_04050
Query: 1 TGATGATGGAAGAGGGAAT 19
|||:|||:|||||:||||*
Sbjct: 1276 ACTGCTATCTTCTTCCTTT 1258
>miR5241a Score: 2.0 RO3G_00002
Query: 1 TGATGATGGAAGAGGGAAT 19
|||||||||||*|:||||*
Sbjct: 857 ACTACTACCTTGTTCCTTT 839
>miR5241a Score: 2.0 RO3G_00301
Query: 1 TGATGATGGAAGAGGGAAT 19
|||:|||:|||||:||||*
Sbjct: 2560 ACTGCTATCTTCTTCCTTT 2542

```
>miR5241a Score: 2.0 RO3G_00400
Query: 1 TGATGATGGAAGAGGGAAT 19
|||||||||||*|:||||*
Sbjct: 857 ACTACTACCTTGTTCCTTT 839
>miR5241a Score: 2.0 RO3G_01112
Query: 1 TGATGATGGAAGAGGGAAT 19
|||||||||||*|:||||*
Sbjct: 857 ACTACTACCTTGTTCCTTT 839
>miR5241a Score: 2.0 RO3G_01269
Query: 1 TGATGATGGAAGAGGGAAT 19
|||||||||||||:*||*|
Sbjct: 202 ACTACTACCTTCTTACT-A 185
>miR5241a Score: 2.5 RO3G_01630
Query: 1 TGATGATGGAAGAGGGAAT 19
*|||||*|||||||:||*|
Sbjct: 474 TCTACTTCCTTCTCTCTAA 456
>miR5241a Score: 1.8 RO3G_01868
Query: 1 TGATGATGGAAGAGGGAAT 19
:||||||||||:|||||**
Sbjct: 214 GCTACTACCTTTTCCCTCT 196
>miR5241a Score: 2.5 RO3G_02199
Query: 1 TGATGATGGAAGAGGGAAT 19
|||:||:|:||:|||:|||
Sbjct: 133 ACTGCTGCTTTTTCCTTTA 115
>miR5241a Score: 2.0 RO3G_17389
Query: 1 TGATGATGGAAGAGGGAAT 19
|||||||||||*|:||||*
Sbjct: 857 ACTACTACCTTGTTCCTTT 839
>miR5241a Score: 2.0 RO3G_17348
Query: 1 TGATGATGGAAGAGGGAAT 19
|||||||||||*|:||||*
Sbjct: 857 ACTACTACCTTGTTCCTTT 839
>miR5241a Score: 2.0 RO3G_17322
Query: 1 TGATGATGGAAGAGGGAAT 19
|||||||||||*|:||||*
Sbjct: 857 ACTACTACCTTGTTCCTTT 839
>miR5241a Score: 1.5 RO3G_17296
Query: 1 TGATGATGGAAGAGGGAAT 19
||||||||||||||||**|
Sbjct: 52 ACTACTACCTTCTCCC-AA 35
>miR1068 Score: 0.0 RO3G_16557
Query: 1 TAGCCAGTTTTGCTTGCAGGTC 22
||||||||||||||||||||||
Sbjct: 418 ATCGGTCAAAACGAACGTCCAG 397
>miR1068 Score: 0.0 RO3G_16132
```

```
Query: 1 TAGCCAGTTTTGCTTGCAGGTC 22
||||||||||||||||||||||
Sbjct: 418 ATCGGTCAAAACGAACGTCCAG 397
>miR1068 Score: 0.2 RO3G_14995
Query: 1 TAGCCAGTTTTGCTTGCAGGTC 22
||||||||||||||||||||:|
Sbjct: 418 ATCGGTCAAAACGAACGTCCGG 397
>miR1068 Score: 0.0 RO3G_15134
Query: 1 TAGCCAGTTTTGCTTGCAGGTC 22
||||||||||||||||||||||
Sbjct: 460 ATCGGTCAAAACGAACGTCCAG 439
>miR1068 Score: 0.0 RO3G_12470
Query: 1 TAGCCAGTTTTGCTTGCAGGTC 22
||||||||||||||||||||||
Sbjct: 418 ATCGGTCAAAACGAACGTCCAG 397
>miR1068 Score: 0.0 RO3G_11525
Query: 1 TAGCCAGTTTTGCTTGCAGGTC 22
||||||||||||||||||||||
Sbjct: 418 ATCGGTCAAAACGAACGTCCAG 397
>miR1068 Score: 0.0 RO3G_09479
Query: 1 TAGCCAGTTTTGCTTGCAGGTC 22
||||||||||||||||||||||
Sbjct: 418 ATCGGTCAAAACGAACGTCCAG 397
>miR1068 Score: 0.0 RO3G_09541
Query: 1 TAGCCAGTTTTGCTTGCAGGTC 22
||||||||||||||||||||||
Sbjct: 418 ATCGGTCAAAACGAACGTCCAG 397
>miR1068 Score: 2.0 RO3G_06842
Query: 1 TAGCCAGTTTTGCTTGCAGGTC 22
:|:*||||||||||||||:|||
Sbjct: 5311 GTTAGTCAAAACGAACGTTCAG 5290
>miR1068 Score: 0.0 RO3G_07098
Query: 1 TAGCCAGTTTTGCTTGCAGGTC 22
||||||||||||||||||||||
Sbjct: 418 ATCGGTCAAAACGAACGTCCAG 397
>miR1068 Score: 0.0 RO3G_06442
Query: 1 TAGCCAGTTTTGCTTGCAGGTC 22
||||||||||||||||||||||
Sbjct: 6142 ATCGGTCAAAACGAACGTCCAG 6121
>miR1068 Score: 2.5 RO3G_04655
Query: 1 TAGCCAGTTTTGCTTGCAGGTC 22
*||||||||||:|||*||*|||
Sbjct: 1811 TTCGGTCAAAATGAAAGTACAG 1790
>miR1068 Score: 0.0 RO3G_05271
Query: 1 TAGCCAGTTTTGCTTGCAGGTC 22
```

||||||||||||||||||||||
Sbjct: 418 ATCGGTCAAAACGAACGTCCAG 397
>miR1068 Score: 2.5 RO3G_02470
Query: 1 TAGCCAGTTTTGCTTGCAGGTC 22
:|||*|:|||||||||||*:||
Sbjct: 49 GTCGATTAAAACGAACGT-TAG 29
>miR1068 Score: 2.2 RO3G_02714
Query: 1 TAGCCAGTTTTGCTTGCAGGTC 22
||||*|||||||||||*|:|||
Sbjct: 418 ATCGATCAAAACGAACATTCAG 397
>miR1068 Score: 2.2 RO3G_02962
Query: 1 TAGCCAGTTTTGCTTGCAGGTC 22
||||||||||||||||:||*
Sbjct: 202 TTCGGTCAAAACGAAC-TTCAA 182
>miR1068 Score: 0.0 RO3G_00290
Query: 1 TAGCCAGTTTTGCTTGCAGGTC 22
||||||||||||||||||||||
Sbjct: 418 ATCGGTCAAAACGAACGTCCAG 397
>miR1068 Score: 0.0 RO3G_00485
Query: 1 TAGCCAGTTTTGCTTGCAGGTC 22
||||||||||||||||||||||
Sbjct: 418 ATCGGTCAAAACGAACGTCCAG 397
>miR1068 Score: 0.0 RO3G_01745
Query: 1 TAGCCAGTTTTGCTTGCAGGTC 22
||||||||||||||||||||||
Sbjct: 418 ATCGGTCAAAACGAACGTCCAG 397
>miR1068 Score: 0.0 RO3G_01928
Query: 1 TAGCCAGTTTTGCTTGCAGGTC 22
||||||||||||||||||||||
Sbjct: 418 ATCGGTCAAAACGAACGTCCAG 397
>miR9491 Score: 0.0 RO3G_16744
Query: 1 TGCTATCTTCCTCTGACTCAG 21
|||||||||||||||||||||
Sbjct: 298 ACGATAGAAGGAGACTGAGTC 278
>miR9491 Score: 0.0 RO3G_16686
Query: 1 TGCTATCTTCCTCTGACTCAG 21
|||||||||||||||||||||
Sbjct: 298 ACGATAGAAGGAGACTGAGTC 278
>miR9491 Score: 0.0 RO3G_15869
Query: 1 TGCTATCTTCCTCTGACTCAG 21
|||||||||||||||||||||
Sbjct: 298 ACGATAGAAGGAGACTGAGTC 278
>miR9491 Score: 0.0 RO3G_14704
Query: 1 TGCTATCTTCCTCTGACTCAG 21
|||||||||||||||||||||

```
Sbjct: 298 ACGATAGAAGGAGACTGAGTC 278
>miR9491 Score: 0.0 RO3G_13450
Query: 1 TGCTATCTTCCTCTGACTCAG 21
|||||||||||||||||||||
Sbjct: 298 ACGATAGAAGGAGACTGAGTC 278
>miR9491 Score: 0.0 RO3G_13861
Query: 1 TGCTATCTTCCTCTGACTCAG 21
|||||||||||||||||||||
Sbjct: 298 ACGATAGAAGGAGACTGAGTC 278
>miR9491 Score: 0.0 RO3G_12951
Query: 1 TGCTATCTTCCTCTGACTCAG 21
|||||||||||||||||||||
Sbjct: 298 ACGATAGAAGGAGACTGAGTC 278
>miR9491 Score: 0.0 RO3G_12979
Query: 1 TGCTATCTTCCTCTGACTCAG 21
|||||||||||||||||||||
Sbjct: 298 ACGATAGAAGGAGACTGAGTC 278
>miR9491 Score: 0.0 RO3G_13152
Query: 1 TGCTATCTTCCTCTGACTCAG 21
|||||||||||||||||||||
Sbjct: 298 ACGATAGAAGGAGACTGAGTC 278
>miR9491 Score: 0.0 RO3G_11970
Query: 1 TGCTATCTTCCTCTGACTCAG 21
|||||||||||||||||||||
Sbjct: 298 ACGATAGAAGGAGACTGAGTC 278
>miR9491 Score: 0.0 RO3G_12087
Query: 1 TGCTATCTTCCTCTGACTCAG 21
|||||||||||||||||||||
Sbjct: 298 ACGATAGAAGGAGACTGAGTC 278
>miR9491 Score: 0.0 RO3G_12194
Query: 1 TGCTATCTTCCTCTGACTCAG 21
|||||||||||||||||||||
Sbjct: 298 ACGATAGAAGGAGACTGAGTC 278
>miR9491 Score: 0.0 RO3G_11275
Query: 1 TGCTATCTTCCTCTGACTCAG 21
|||||||||||||||||||||
Sbjct: 298 ACGATAGAAGGAGACTGAGTC 278
>miR9491 Score: 0.0 RO3G_11403
Query: 1 TGCTATCTTCCTCTGACTCAG 21
|||||||||||||||||||||
Sbjct: 298 ACGATAGAAGGAGACTGAGTC 278
>miR9491 Score: 0.0 RO3G_10555
Query: 1 TGCTATCTTCCTCTGACTCAG 21
|||||||||||||||||||||
Sbjct: 298 ACGATAGAAGGAGACTGAGTC 278
```

```
>miR9491 Score: 0.0 RO3G_10560
Query: 1 TGCTATCTTCCTCTGACTCAG 21
|||||||||||||||||||||
Sbjct: 298 ACGATAGAAGGAGACTGAGTC 278
>miR9491 Score: 0.0 RO3G_09376
Query: 1 TGCTATCTTCCTCTGACTCAG 21
|||||||||||||||||||||
Sbjct: 298 ACGATAGAAGGAGACTGAGTC 278
>miR9491 Score: 0.0 RO3G_08612
Query: 1 TGCTATCTTCCTCTGACTCAG 21
|||||||||||||||||||||
Sbjct: 298 ACGATAGAAGGAGACTGAGTC 278
>miR9491 Score: 2.0 RO3G_08632
Query: 1 TGCTATCTTCCTCTGACTCAG 21
*|||||||||||||||*|||*
Sbjct: 523 TCGATAGAAGGAGACTAAGTG 503
>miR9491 Score: 0.0 RO3G_07104
Query: 1 TGCTATCTTCCTCTGACTCAG 21
|||||||||||||||||||||
Sbjct: 298 ACGATAGAAGGAGACTGAGTC 278
>miR9491 Score: 0.0 RO3G_07305
Query: 1 TGCTATCTTCCTCTGACTCAG 21
|||||||||||||||||||||
Sbjct: 298 ACGATAGAAGGAGACTGAGTC 278
>miR9491 Score: 0.0 RO3G_05630
Query: 1 TGCTATCTTCCTCTGACTCAG 21
|||||||||||||||||||||
Sbjct: 298 ACGATAGAAGGAGACTGAGTC 278
>miR9491 Score: 0.0 RO3G_06416
Query: 1 TGCTATCTTCCTCTGACTCAG 21
|||||||||||||||||||||
Sbjct: 298 ACGATAGAAGGAGACTGAGTC 278
>miR9491 Score: 0.0 RO3G_05280
Query: 1 TGCTATCTTCCTCTGACTCAG 21
|||||||||||||||||||||
Sbjct: 298 ACGATAGAAGGAGACTGAGTC 278
>miR9491 Score: 0.0 RO3G_03103
Query: 1 TGCTATCTTCCTCTGACTCAG 21
|||||||||||||||||||||
Sbjct: 298 ACGATAGAAGGAGACTGAGTC 278
>miR9491 Score: 0.0 RO3G_03988
Query: 1 TGCTATCTTCCTCTGACTCAG 21
|||||||||||||||||||||
Sbjct: 298 ACGATAGAAGGAGACTGAGTC 278
>miR9491 Score: 0.0 RO3G_01203
```

```
Query: 1 TGCTATCTTCCTCTGACTCAG 21
|||||||||||||||||||||
Sbjct: 298 ACGATAGAAGGAGACTGAGTC 278
>miR9491 Score: 0.0 RO3G_01718
Query: 1 TGCTATCTTCCTCTGACTCAG 21
|||||||||||||||||||||
Sbjct: 298 ACGATAGAAGGAGACTGAGTC 278
>miR2602a Score: 2.2 RO3G_17076
Query: 1 TTCCAGTGATTGCCAGCAT 19
::|||||:|||||||:|*|
Sbjct: 814 GGGGTCATTAACGGTTGCA 796
>miR2602a Score: 2.2 RO3G_16143
Query: 1 TTCCAGTGATTGCCAGCAT 19
|:|||||*|||||||:||:
Sbjct: 4493 AGGGTCAGTAACGGTTGTG 4475
>miR2602a Score: 0.5 RO3G_14983
Query: 1 TTCCAGTGATTGCCAGCAT 19
|||||||:|||||||||||
Sbjct: 560 AAGGTCATTAACGGTCGTA 542
>miR2602a Score: 2.2 RO3G_15136
Query: 1 TTCCAGTGATTGCCAGCAT 19
|:|||||*|||||||:||:
Sbjct: 203 AGGGTCAGTAACGGTTGTG 185
>miR2602a Score: 0.0 RO3G_13914
Query: 1 TTCCAGTGATTGCCAGCAT 19
|||||||||||||||||||
Sbjct: 755 AAGGTCACTAACGGTCGTA 737
>miR2602a Score: 2.0 RO3G_13096
Query: 1 TTCCAGTGATTGCCAGCAT 19
||||||:||:|||||*|||
Sbjct: 287 AAGGTCGCTGACGGTGGTA 269
>miR2602a Score: 0.0 RO3G_12058
Query: 1 TTCCAGTGATTGCCAGCAT 19
|||||||||||||||||||
Sbjct: 755 AAGGTCACTAACGGTCGTA 737
>miR2602a Score: 2.2 RO3G_12181
Query: 1 TTCCAGTGATTGCCAGCAT 19
|:|||||*|||||||:||:
Sbjct: 1310 AGGGTCAGTAACGGTTGTG 1292
>miR2602a Score: 2.2 RO3G_12533
Query: 1 TTCCAGTGATTGCCAGCAT 19
|:|||||*|||||||:||:
Sbjct: 3836 AGGGTCAGTAACGGTTGTG 3818
>miR2602a Score: 2.2 RO3G_10535
Query: 1 TTCCAGTGATTGCCAGCAT 19
```

```
|:|||||*|||||||:||:
Sbjct: 2141 AGGGTCAGTAACGGTTGTG 2123
>miR2602a Score: 0.0 RO3G_10801
Query: 1 TTCCAGTGATTGCCAGCAT 19
|||||||||||||||||||
Sbjct: 560 AAGGTCACTAACGGTCGTA 542
>miR2602a Score: 0.0 RO3G_09614
Query: 1 TTCCAGTGATTGCCAGCAT 19
|||||||||||||||||||
Sbjct: 86 AAGGTCACTAACGGTCGTA 68
>miR2602a Score: 2.2 RO3G_06752
Query: 1 TTCCAGTGATTGCCAGCAT 19
:||||||:|||:|||*|||
Sbjct: 197 GAGGTCATTAATGGTAGTA 179
>miR2602a Score: 2.2 RO3G_07028
Query: 1 TTCCAGTGATTGCCAGCAT 19
|:|||||*|||||||:||:
Sbjct: 3479 AGGGTCAGTAACGGTTGTG 3461
>miR2602a Score: 2.2 RO3G_05750
Query: 1 TTCCAGTGATTGCCAGCAT 19
:||*|||||:|||||||*|
Sbjct: 309 GAGATCACTGACGGTCGGA 291
>miR2602a Score: 2.2 RO3G_05918
Query: 1 TTCCAGTGATTGCCAGCAT 19
|:|||||*|||||||:||:
Sbjct: 1016 AGGGTCAGTAACGGTTGTG 998
>miR2602a Score: 2.2 RO3G_05520
Query: 1 TTCCAGTGATTGCCAGCAT 19
|:|||||*|||||||:||:
Sbjct: 1163 AGGGTCAGTAACGGTTGTG 1145
>miR2602a Score: 0.0 RO3G_02233
Query: 1 TTCCAGTGATTGCCAGCAT 19
|||||||||||||||||||
Sbjct: 284 AAGGTCACTAACGGTCGTA 266
>miR2602a Score: 2.2 RO3G_02691
Query: 1 TTCCAGTGATTGCCAGCAT 19
|:|||||*|||||||:||:
Sbjct: 2942 AGGGTCAGTAACGGTTGTG 2924
>miR2602a Score: 1.0 RO3G_03556
Query: 1 TTCCAGTGATTGCCAGCAT 19
||||||||||||*||||||
Sbjct: 560 AAGGTCACTAACAGTCGTA 542
>miR2602a Score: 0.5 RO3G_03583
Query: 1 TTCCAGTGATTGCCAGCAT 19
|||||||||||||||||*|
```

```
Sbjct: 560 AAGGTCACTAACGGTCGCA 542
>miR2602a Score: 2.0 RO3G_03950
Query: 1 TTCCAGTGATTGCCAGCAT 19
*||||:||*||||||||||
Sbjct: 220 CAGGTTACGAACGGTCGTA 202
>miR2602a Score: 2.5 RO3G_00540
Query: 1 TTCCAGTGATTGCCAGCAT 19
:|||*||||||||||:|*:
Sbjct: 346 GAGGACACTAACGGTTG-G 329
>miR2602a Score: 2.5 RO3G_00714
Query: 1 TTCCAGTGATTGCCAGCAT 19
*:||||||*:|||||||||
Sbjct: 1934 CGGGTCACAGACGGTCGTA 1916
>miR2602a Score: 2.5 RO3G_01041
Query: 1 TTCCAGTGATTGCCAGCAT 19
*||||:|*|||||||||*|
Sbjct: 452 TAGGTTAATAACGGTCG-A 435
>miR2602a Score: 0.0 RO3G_17462
Query: 1 TTCCAGTGATTGCCAGCAT 19
|||||||||||||||||||
Sbjct: 887 AAGGTCACTAACGGTCGTA 869
>miR2602a Score: 2.2 RO3G_17383
Query: 1 TTCCAGTGATTGCCAGCAT 19
|:|||||*|||||||:||:
Sbjct: 995 AGGGTCAGTAACGGTTGTG 977
>miR2602a Score: 2.2 RO3G_17329
Query: 1 TTCCAGTGATTGCCAGCAT 19
|:|||||*|||||||:||:
Sbjct: 3833 AGGGTCAGTAACGGTTGTG 3815
>miR2602a Score: 2.2 RO3G_17318
Query: 1 TTCCAGTGATTGCCAGCAT 19
|:|||||*|||||||:||:
Sbjct: 4496 AGGGTCAGTAACGGTTGTG 4478
>miR2602a Score: 2.2 RO3G_17274
Query: 1 TTCCAGTGATTGCCAGCAT 19
|:|||||*|||||||:||:
Sbjct: 4319 AGGGTCAGTAACGGTTGTG 4301
>miR396b Score: 2.5 RO3G_14017
Query: 1 TTCCACAGCTTTCTTGAACT 20
|||||*||||||*||||*||
Sbjct: 1794 AAGGTCTCGAAATAACT-GA 1776
>miR396b Score: 2.5 RO3G_09454
Query: 1 TTCCACAGCTTTCTTGAACT 20
||*|||||||||*||||||*
Sbjct: 2287 AAAGTGTCGAAACAACTTGC 2268
```

```
>miR396b Score: 2.5 RO3G_07651
Query: 1 TTCCACAGCTTTCTTGAACT 20
||*||||||||||||*||*|
Sbjct: 4225 AAAGTGTCGAAAGAAATTAA 4206
>miR396b Score: 2.0 RO3G_02790
Query: 1 TTCCACAGCTTTCTTGAAC-T 20
|||||*|||||||||||*|*|
Sbjct: 759 AAGGTATCGAAAGAACTCGCA 739
>miR396b Score: 2.5 RO3G_03278
Query: 1 TTCCACAGCTTTCTTGAACT 20
|||||||:||||*|:|||*|
Sbjct: 1794 AAGGTGTTGAAAAAGCTT-A 1776
>miR396b Score: 2.5 RO3G_03333
Query: 1 TTCCACAGCTTTCTTGAACT 20
|||||*||||:||||||**|
Sbjct: 912 AAGGTATCGAGAGAACT-CA 894
>miR396b Score: 2.5 RO3G_16940
Query: 1 TTCCACAGCTTTCTTGAACT 20
|||*||||*||||||||*||
Sbjct: 69 AAGCTGTCTAAAGAACTCGA 50
>miR7711-5p.1 Score: 2.0 RO3G_15141
Query: 1 TACTTAGCTGTTTGACTTCTTG 22
||*||*||||||||||||||||
Sbjct: 75 ATAAAACGACAAACTGAAGAAC 54
>miR7711-5p.1 Score: 1.0 RO3G_12954
Query: 1 TACTTAGCTGTTTGACTTCTTG 22
|||||*||||||||||||||||
Sbjct: 75 ATGAAACGACAAACTGAAGAAC 54
>miR7711-5p.1 Score: 1.0 RO3G_10762
Query: 1 TACTTAGCTGTTTGACTTCTTG 22
|||||*||||||||||||||||
Sbjct: 75 ATGAAACGACAAACTGAAGAAC 54
>miR7711-5p.1 Score: 1.0 RO3G_10770
Query: 1 TACTTAGCTGTTTGACTTCTTG 22
|||||*||||||||||||||||
Sbjct: 75 ATGAAACGACAAACTGAAGAAC 54
>miR7711-5p.1 Score: 2.0 RO3G_09168
Query: 1 TACTTAGCTGTTTGACTTCTTG 22
||*||*||||||||||||||||
Sbjct: 75 ATAAAACGACAAACTGAAGAAC 54
>miR7711-5p.1 Score: 2.0 RO3G_04690
Query: 1 TACTTAGCTGTTTGACTTCTTG 22
||*||*||||||||||||||||
Sbjct: 75 ATAAAGCGACAAACTGAAGAAC 54
>miR7711-5p.1 Score: 2.0 RO3G_04787
```

Query: 1 TACTTAGCTGTTTGACTTCTTG 22
||*||*||||||||||||||||
Sbjct: 75 ATAAAACGACAAACTGAAGAAC 54
>miR7711-5p.1 Score: 2.0 RO3G_05431
Query: 1 TACTTAGCTGTTTGACTTCTTG 22
||*||*||||||||||||||||
Sbjct: 75 ATAAAACGACAAACTGAAGAAC 54
>miR7711-5p.1 Score: 2.0 RO3G_02822
Query: 1 TACTTAGCTGTTTGACTTCTTG 22
||*||*||||||||||||||||
Sbjct: 75 ATAAAGCGACAAACTGAAGAAC 54
>miR7711-5p.1 Score: 1.0 RO3G_00304
Query: 1 TACTTAGCTGTTTGACTTCTTG 22
|||||*||||||||||||||||
Sbjct: 75 ATGAAACGACAAACTGAAGAAC 54
>miR5373 Score: 2.5 RO3G_15307
Query: 1 TCTCTTGTATGCTGATGATGT-T 22
|*||||||||||||*||||||*|
Sbjct: 1504 ACAGAACATACGACAACTACACA 1482
>miR5373 Score: 2.0 RO3G_14120
Query: 1 TCTCTTGTATGCTGATGATGTT 22
|||*||||||||:||||||||*
Sbjct: 166 AGAAAACATACGGCTACTACAT 145
>miR5373 Score: 2.5 RO3G_10168
Query: 1 TCTCTTGTATGCTGATGATGTT 22
|||||||||*:||||||*|*||
Sbjct: 281 AGAGAACATTTGACTACGA-AA 261
>miR832-3p Score: 1.8 RO3G_16359
Query: 1 TTGATCGAATCCAAGCCAAG 20
:||||||*|||||||||*||
Sbjct: 502 GACTAGCCTAGGTTCGGGTC 483
>miR832-3p Score: 1.8 RO3G_16545
Query: 1 TTGATCGAATCCAAGCCAAG 20
:||||||*|||||||||*||
Sbjct: 502 GACTAGCCTAGGTTCGGGTC 483
>miR832-3p Score: 1.8 RO3G_16554
Query: 1 TTGATCGAATCCAAGCCAAG 20
:*|||||||||||||||*||
Sbjct: 502 GCCTAGCTTAGGTTCGGGTC 483
>miR832-3p Score: 1.2 RO3G_15575
Query: 1 TTGATCGAATCCAAGCCAAG 20
::|||||||||||||||*||
Sbjct: 325 GGCTAGCTTAGGTTCGGGTC 306
>miR832-3p Score: 1.2 RO3G_15001
Query: 1 TTGATCGAATCCAAGCCAAG 20

```
::|||||||||||||||*||
Sbjct: 325 GGCTAGCTTAGGTTCGGGTC 306
>miR832-3p Score: 0.8 RO3G_15146
Query: 1 TTGATCGAATCCAAGCCAAG 20
:||||||||||||||||*||
Sbjct: 382 GACTAGCTTAGGTTCGGGTC 363
>miR832-3p Score: 1.8 RO3G_14621
Query: 1 TTGATCGAATCCAAGCCAAG 20
:||||||*|||||||||*||
Sbjct: 160 GACTAGCCTAGGTTCGGGTC 141
>miR832-3p Score: 1.2 RO3G_12664
Query: 1 TTGATCGAATCCAAGCCAAG 20
::|||||||||||||||*||
Sbjct: 229 GGCTAGCTTAGGTTCGGGTC 210
>miR832-3p Score: 2.5 RO3G_12847
Query: 1 TTGATCGAATCCAAGCCAAG 20
||||||||||||||*|*|*|
Sbjct: 312 AACTAGCTTAGGTTAGCTCC 293
>miR832-3p Score: 2.2 RO3G_13266
Query: 1 TTGATCGAATCCAAGCCAAG 20
:|||:||*|||||||||*||
Sbjct: 280 GACTGGCCTAGGTTCGGGTC 261
>miR832-3p Score: 1.8 RO3G_13271
Query: 1 TTGATCGAATCCAAGCCAAG 20
:||||||*|||||||||*||
Sbjct: 502 GACTAGCCTAGGTTCGGGTC 483
>miR832-3p Score: 1.8 RO3G_13277
Query: 1 TTGATCGAATCCAAGCCAAG 20
:*|||||||||||||||*||
Sbjct: 502 GCCTAGCTTAGGTTCGGGTC 483
>miR832-3p Score: 1.2 RO3G_11240
Query: 1 TTGATCGAATCCAAGCCAAG 20
::|||||||||||||||*||
Sbjct: 325 GGCTAGCTTAGGTTCGGGTC 306
>miR832-3p Score: 2.0 RO3G_11266
Query: 1 TTGATCGAATCCAAGCCAAG 20
||||:||*|||||||||*||
Sbjct: 325 AACTGGCCTAGGTTCGGGTC 306
>miR832-3p Score: 1.8 RO3G_10800
Query: 1 TTGATCGAATCCAAGCCAAG 20
:||||||*|||||||||*||
Sbjct: 160 GACTAGCCTAGGTTCGGGTC 141
>miR832-3p Score: 2.2 RO3G_10866
Query: 1 TTGATCGAATCCAAGCCAAG 20
:|||:||*|||||||||*||
```

Sbjct: 502 GACTGGCCTAGGTTCGGGTC 483
>miR832-3p Score: 1.8 RO3G_09612
Query: 1 TTGATCGAATCCAAGCCAAG 20
:||||||*|||||||||*||
Sbjct: 502 GACTAGCCTAGGTTCGGGTC 483
>miR832-3p Score: 1.2 RO3G_08663
Query: 1 TTGATCGAATCCAAGCCAAG 20
::||||||||||||||*||
Sbjct: 502 GGCTAGCTTAGGTTCGGGTC 483
>miR832-3p Score: 2.2 RO3G_06875
Query: 1 TTGATCGAATCCAAGCCAAG 20
|||*|||||||*|||||||:
Sbjct: 1739 AACGAGCTTAGTTTCGGTTT 1720
>miR832-3p Score: 1.8 RO3G_07031
Query: 1 TTGATCGAATCCAAGCCAAG 20
:*||||||||||||||*||
Sbjct: 502 GCCTAGCTTAGGTTCGGGTC 483
>miR832-3p Score: 1.2 RO3G_07112
Query: 1 TTGATCGAATCCAAGCCAAG 20
::||||||||||||||*||
Sbjct: 325 GGCTAGCTTAGGTTCGGGTC 306
>miR832-3p Score: 1.2 RO3G_07120
Query: 1 TTGATCGAATCCAAGCCAAG 20
::||||||||||||||*||
Sbjct: 325 GGCTAGCTTAGGTTCGGGTC 306
>miR832-3p Score: 2.2 RO3G_07688
Query: 1 TTGATCGAATCCAAGCCAAG 20
:|||:||*|||||||||*||
Sbjct: 502 GACTGGCCTAGGTTCGGGTC 483
>miR832-3p Score: 2.5 RO3G_05301
Query: 1 TTGATCGAATCCAAGCCAAG 20
:|||:|:|||||||*||||:
Sbjct: 454 GACTGGTTTAGGTTAGGTTT 435
>miR832-3p Score: 2.5 RO3G_03727
Query: 1 TTGATCGAATCCAAGCCAAG 20
|||||*||||||||:*||||
Sbjct: 1630 AACTAACTTAGGTTTAGTTC 1611
>miR832-3p Score: 2.2 RO3G_03803
Query: 1 TTGATCGAATCCAAGCCAAG 20
::|||||||||||||*|*||
Sbjct: 325 GGCTAGCTTAGGTTCAGGTC 306
>miR832-3p Score: 1.8 RO3G_00255
Query: 1 TTGATCGAATCCAAGCCAAG 20
:||||||*|||||||||*||
Sbjct: 502 GACTAGCCTAGGTTCGGGTC 483

>miR832-3p Score: 2.0 RO3G_01902
Query: 1 TTGATCGAATCCAAGCCA-AG 20
|||||*:|||||||||||*||
Sbjct: 3944 AACTACTTTAGGTTCGGTATC 3924
>miR172k Score: 0.0 RO3G_17256
Query: 1 TGAACTGGATGCTGCTGCT 19
|||||||||||||||||||
Sbjct: 374 ACTTGACCTACGACGACGA 356
>miR172k Score: 0.0 RO3G_17248
Query: 1 TGAACTGGATGCTGCTGCT 19
|||||||||||||||||||
Sbjct: 377 ACTTGACCTACGACGACGA 359
>miR172k Score: 2.2 RO3G_17215
Query: 1 TGAACTGGATGCTGCTGCT 19
:||||*:|||||||||*|
Sbjct: 203 GCTTGCTCTACGACGACAA 185
>miR172k Score: 2.2 RO3G_16897
Query: 1 TGAACTGGATGCTGCTGCT 19
:||||*:|||||||||*|
Sbjct: 203 GCTTGCTCTACGACGACAA 185
>miR172k Score: 1.0 RO3G_16748
Query: 1 TGAACTGGATGCTGCTGCT 19
||||||||*||||||||||
Sbjct: 347 ACTTGACCCACGACGACGA 329
>miR172k Score: 2.2 RO3G_16807
Query: 1 TGAACTGGATGCTGCTGCT 19
:||||*:|||||||||*|
Sbjct: 203 GCTTGCTCTACGACGACAA 185
>miR172k Score: 1.8 RO3G_16551
Query: 1 TGAACTGGATGCTGCTGCT 19
:||||*:||||||||||||
Sbjct: 203 GCTTGCTCTACGACGACGA 185
>miR172k Score: 1.8 RO3G_16555
Query: 1 TGAACTGGATGCTGCTGCT 19
:||||*:||||||||||||
Sbjct: 203 GCTTGCTCTACGACGACGA 185
>miR172k Score: 0.0 RO3G_16137
Query: 1 TGAACTGGATGCTGCTGCT 19
|||||||||||||||||||
Sbjct: 371 ACTTGACCTACGACGACGA 353
>miR172k Score: 0.0 RO3G_15890
Query: 1 TGAACTGGATGCTGCTGCT 19
|||||||||||||||||||
Sbjct: 377 ACTTGACCTACGACGACGA 359
>miR172k Score: 1.0 RO3G_15897

Query: 1 TGAACTGGATGCTGCTGCT 19
||||||||*||||||||||
Sbjct: 347 ACTTGACCCACGACGACGA 329
>miR172k Score: 2.5 RO3G_16070
Query: 1 TGAACTGGATGCTGCTGCT 19
||||||||*:|*|||||||
Sbjct: 522 ACTTGACCAGCCACGACGA 504
>miR172k Score: 2.2 RO3G_15675
Query: 1 TGAACTGGATGCTGCTGCT 19
:||||*:||||||||||*|
Sbjct: 203 GCTTGCTCTACGACGACAA 185
>miR172k Score: 2.5 RO3G_15446
Query: 1 TGAACTGGATGCTGCTGCT 19
**|||||:||||||||||*
Sbjct: 819 CATTGACTTACGACGACGC 801
>miR172k Score: 2.5 RO3G_15536
Query: 1 TGAACTGGATGCTGCTGCT 19
|||**||||||||||||*|
Sbjct: 660 ACTACACCTACGACGACTA 642
>miR172k Score: 0.0 RO3G_15147
Query: 1 TGAACTGGATGCTGCTGCT 19
|||||||||||||||||||
Sbjct: 341 ACTTGACCTACGACGACGA 323
>miR172k Score: 2.2 RO3G_14359
Query: 1 TGAACTGGATGCTGCTGCT 19
:||||*:||||||||||*|
Sbjct: 203 GCTTGCTCTACGACGACAA 185
>miR172k Score: 1.0 RO3G_14551
Query: 1 TGAACTGGATGCTGCTGCT 19
||||||||*||||||||||
Sbjct: 347 ACTTGACCCACGACGACGA 329
>miR172k Score: 1.8 RO3G_13726
Query: 1 TGAACTGGATGCTGCTGCT 19
:||||*:||||||||||||
Sbjct: 203 GCTTGCTCTACGACGACGA 185
>miR172k Score: 1.8 RO3G_13742
Query: 1 TGAACTGGATGCTGCTGCT 19
:||||*:||||||||||||
Sbjct: 203 GCTTGCTCTACGACGACGA 185
>miR172k Score: 2.5 RO3G_13827
Query: 1 TGAACTGGATGCTGCTGCT 19
|:|||||:||*||||||*|
Sbjct: 705 ATTTGACTTAGGACGACAA 687
>miR172k Score: 0.0 RO3G_13146
Query: 1 TGAACTGGATGCTGCTGCT 19

```
|||||||||||||||||||
Sbjct: 377 ACTTGACCTACGACGACGA 359
>miR172k Score: 2.0 RO3G_13150
Query: 1 TGAACTGGATGCTGCTGCT 19
*:||||||||||||||:*|
Sbjct: 766 CTTTGACCTACGACGATAA 748
>miR172k Score: 2.2 RO3G_13151
Query: 1 TGAACTGGATGCTGCTGCT 19
:||||*:||||||||||*|
Sbjct: 203 GCTTGCTCTACGACGACAA 185
>miR172k Score: 1.0 RO3G_11977
Query: 1 TGAACTGGATGCTGCTGCT 19
||||||||*||||||||||
Sbjct: 311 ACTTGACCCACGACGACGA 293
>miR172k Score: 1.0 RO3G_11236
Query: 1 TGAACTGGATGCTGCTGCT 19
||||||||*||||||||||
Sbjct: 389 ACTTGACCCACGACGACGA 371
>miR172k Score: 1.0 RO3G_11537
Query: 1 TGAACTGGATGCTGCTGCT 19
||||||||*||||||||||
Sbjct: 389 ACTTGACCCACGACGACGA 371
>miR172k Score: 1.8 RO3G_11773
Query: 1 TGAACTGGATGCTGCTGCT 19
:||||*:||||||||||||
Sbjct: 203 GCTTGCTCTACGACGACGA 185
>miR172k Score: 2.2 RO3G_10235
Query: 1 TGAACTGGATGCTGCTGCT 19
:||||*:||||||||||*|
Sbjct: 203 GCTTGCTCTACGACGACAA 185
>miR172k Score: 2.0 RO3G_10520
Query: 1 TGAACTGGATGCTGCTGCT 19
*:||||||||||||||:*|
Sbjct: 706 CTTTGACCTACGACGATAA 688
>miR172k Score: 2.5 RO3G_10706
Query: 1 TGAACTGGATGCTGCTGCT 19
|||**|||||||||||:||
Sbjct: 402 ACTACACCTACGACGATGA 384
>miR172k Score: 2.2 RO3G_10732
Query: 1 TGAACTGGATGCTGCTGCT 19
:||||*:||||||||||*|
Sbjct: 203 GCTTGCTCTACGACGACAA 185
>miR172k Score: 1.5 RO3G_10775
Query: 1 TGAACTGGATGCTGCTGCT 19
|||||||||||||*|||*|
```

Sbjct: 375 ACTTGACCTACGAAGACAA 357
>miR172k Score: 1.8 RO3G_09483
Query: 1 TGAACTGGATGCTGCTGCT 19
:||||*:||||||||||||
Sbjct: 203 GCTTGCTCTACGACGACGA 185
>miR172k Score: 0.5 RO3G_09497
Query: 1 TGAACTGGATGCTGCTGCT 19
|||||||||||||||||*|
Sbjct: 86 ACTTGACCTACGACGACAA 68
>miR172k Score: 2.2 RO3G_09499
Query: 1 TGAACTGGATGCTGCTGCT 19
:||||*:||||||||||*|
Sbjct: 203 GCTTGCTCTACGACGACAA 185
>miR172k Score: 2.2 RO3G_09544
Query: 1 TGAACTGGATGCTGCTGCT 19
:||||*:||||||||||*|
Sbjct: 203 GCTTGCTCTACGACGACAA 185
>miR172k Score: 1.0 RO3G_09602
Query: 1 TGAACTGGATGCTGCTGCT 19
||||||||*||||||||||
Sbjct: 347 ACTTGACCCACGACGACGA 329
>miR172k Score: 1.0 RO3G_09603
Query: 1 TGAACTGGATGCTGCTGCT 19
||||||||*||||||||||
Sbjct: 389 ACTTGACCCACGACGACGA 371
>miR172k Score: 1.8 RO3G_09607
Query: 1 TGAACTGGATGCTGCTGCT 19
:||||*:||||||||||||
Sbjct: 203 GCTTGCTCTACGACGACGA 185
>miR172k Score: 1.0 RO3G_09929
Query: 1 TGAACTGGATGCTGCTGCT 19
||||||||*||||||||||
Sbjct: 347 ACTTGACCCACGACGACGA 329
>miR172k Score: 0.0 RO3G_10041
Query: 1 TGAACTGGATGCTGCTGCT 19
|||||||||||||||||||
Sbjct: 377 ACTTGACCTACGACGACGA 359
>miR172k Score: 1.0 RO3G_10218
Query: 1 TGAACTGGATGCTGCTGCT 19
||||||||*||||||||||
Sbjct: 368 ACTTGACCCACGACGACGA 350
>miR172k Score: 1.0 RO3G_07981
Query: 1 TGAACTGGATGCTGCTGCT 19
||||||||*||||||||||
Sbjct: 347 ACTTGACCCACGACGACGA 329

>miR172k Score: 1.8 RO3G_08846
Query: 1 TGAACTGGATGCTGCTGCT 19
:||||*:||||||||||||
Sbjct: 203 GCTTGCTCTACGACGACGA 185
>miR172k Score: 0.0 RO3G_09182
Query: 1 TGAACTGGATGCTGCTGCT 19
|||||||||||||||||||
Sbjct: 353 ACTTGACCTACGACGACGA 335
>miR172k Score: 1.0 RO3G_06730
Query: 1 TGAACTGGATGCTGCTGCT 19
||||||||*||||||||||
Sbjct: 347 ACTTGACCCACGACGACGA 329
>miR172k Score: 2.5 RO3G_07051
Query: 1 TGAACTGGATGCTGCTGC-T 19
|:|*|||||||||||||**|
Sbjct: 1250 ATTAGACCTACGACGACCTA 1231
>miR172k Score: 2.2 RO3G_07303
Query: 1 TGAACTGGATGCTGCTGCT 19
:||||*:||||||||||*|
Sbjct: 203 GCTTGCTCTACGACGACAA 185
>miR172k Score: 1.0 RO3G_07306
Query: 1 TGAACTGGATGCTGCTGCT 19
||||||||*||||||||||
Sbjct: 347 ACTTGACCCACGACGACGA 329
>miR172k Score: 1.0 RO3G_07696
Query: 1 TGAACTGGATGCTGCTGCT 19
||||||||*||||||||||
Sbjct: 347 ACTTGACCCACGACGACGA 329
>miR172k Score: 1.8 RO3G_07704
Query: 1 TGAACTGGATGCTGCTGCT 19
:||||*:||||||||||||
Sbjct: 203 GCTTGCTCTACGACGACGA 185
>miR172k Score: 1.0 RO3G_07720
Query: 1 TGAACTGGATGCTGCTGCT 19
||||||||*||||||||||
Sbjct: 347 ACTTGACCCACGACGACGA 329
>miR172k Score: 1.0 RO3G_07738
Query: 1 TGAACTGGATGCTGCTGCT 19
||||||||*||||||||||
Sbjct: 398 ACTTGACCCACGACGACGA 380
>miR172k Score: 2.2 RO3G_07875
Query: 1 TGAACTGGATGCTGCTGCT 19
:||||*:||||||||||*|
Sbjct: 203 GCTTGCTCTACGACGACAA 185
>miR172k Score: 2.0 RO3G_05635

Query: 1 TGAACTGGATGCTGCTGCT 19
||||:||||||||||||
Sbjct: 203 CCTTGCTCTACGACGACGA 185
>miR172k Score: 2.5 RO3G_06335
Query: 1 TGAACTGGATGCTGCTGCT 19
|||||:|||||||||*|
Sbjct: 695 CCTTGATCAACGACGAC-A 678
>miR172k Score: 2.2 RO3G_06441
Query: 1 TGAACTGGATGCTGCTGCT 19
:||||*:||||||||||*|
Sbjct: 203 GCTTGCTCTACGACGACAA 185
>miR172k Score: 2.0 RO3G_06678
Query: 1 TGAACTGGATGCTGCTGCT 19
||*|||||||:||||||*|
Sbjct: 1136 ACATGACCTATGACGAC-A 1119
>miR172k Score: 2.0 RO3G_04559
Query: 1 TGAACTGGATGCTGCTGCT 19
||||||:|||||||||**|
Sbjct: 387 ACTTGATCTACGACGA-AA 370
>miR172k Score: 1.0 RO3G_04679
Query: 1 TGAACTGGATGCTGCTGCT 19
||||||||*||||||||||
Sbjct: 317 ACTTGACCCACGACGACGA 299
>miR172k Score: 2.5 RO3G_04867
Query: 1 TGAACTGGATGCTGCTGCT 19
||||*||||||*|||||*|
Sbjct: 565 ACTTCACCTACTACGACAA 547
>miR172k Score: 2.2 RO3G_05336
Query: 1 TGAACTGGATGCTGCTGCT 19
:||||*:||||||||||*|
Sbjct: 203 GCTTGCTCTACGACGACAA 185
>miR172k Score: 1.0 RO3G_05425
Query: 1 TGAACTGGATGCTGCTGCT 19
|||||||||||||*|||||
Sbjct: 374 ACTTGACCTACGAAGACGA 356
>miR172k Score: 1.0 RO3G_02228
Query: 1 TGAACTGGATGCTGCTGCT 19
||||||||*||||||||||
Sbjct: 347 ACTTGACCCACGACGACGA 329
>miR172k Score: 2.0 RO3G_02685
Query: 1 TGAACTGGATGCTGCTGCT 19
:||||||||||||||:|
Sbjct: 766 CTTTGACCTACGACGATAA 748
>miR172k Score: 2.2 RO3G_02689
Query: 1 TGAACTGGATGCTGCTGCT 19

:||||*:|||||||||*|
Sbjct: 203 GCTTGCTCTACGACGACAA 185
>miR172k Score: 2.5 RO3G_03439
Query: 1 TGAACTGGATGCTGCTGCT 19
||||||||*|:|||||*||
Sbjct: 537 ACTTGACCCATGACGAAGA 519
>miR172k Score: 0.0 RO3G_03584
Query: 1 TGAACTGGATGCTGCTGCT 19
|||||||||||||||||||
Sbjct: 305 ACTTGACCTACGACGACGA 287
>miR172k Score: 1.8 RO3G_03615
Query: 1 TGAACTGGATGCTGCTGCT 19
:||||*:||||||||||||
Sbjct: 203 GCTTGCTCTACGACGACGA 185
>miR172k Score: 1.0 RO3G_04211
Query: 1 TGAACTGGATGCTGCTGCT 19
||||||||*||||||||||
Sbjct: 347 ACTTGACCCACGACGACGA 329
>miR172k Score: 0.0 RO3G_00288
Query: 1 TGAACTGGATGCTGCTGCT 19
|||||||||||||||||||
Sbjct: 368 ACTTGACCTACGACGACGA 350
>miR172k Score: 2.2 RO3G_00681
Query: 1 TGAACTGGATGCTGCTGCT 19
:||||*:|||||||||*|
Sbjct: 203 GCTTGCTCTACGACGACAA 185
>miR172k Score: 2.5 RO3G_01016
Query: 1 TGAACTGGATGCTGCTGCT 19
|:|||:|||||||*|||*|
Sbjct: 33 ATTTGGCCTACGAGGACAA 15
>miR172k Score: 2.2 RO3G_01322
Query: 1 TGAACTGGATGCTGCTGCT 19
:||||*:|||||||||*|
Sbjct: 203 GCTTGCTCTACGACGACAA 185
>miR172k Score: 2.0 RO3G_01369
Query: 1 TGAACTGGATGCTGCTGCT 19
|||||:|||||||||||
Sbjct: 344 TCCTGACTTACGACGACGA 326
>miR172k Score: 1.8 RO3G_01612
Query: 1 TGAACTGGATGCTGCTGCT 19
:||||*:||||||||||||
Sbjct: 203 GCTTGCTCTACGACGACGA 185
>miR172k Score: 1.0 RO3G_01726
Query: 1 TGAACTGGATGCTGCTGCT 19
||||||||*||||||||||

Sbjct: 392 ACTTGACCCACGACGACGA 374
>miR172k Score: 2.0 RO3G_01737
Query: 1 TGAACTGGATGCTGCTGCT 19
:||||||||||||||:|
Sbjct: 559 CTTTGACCTACGACGATAA 541
>miR172k Score: 2.5 RO3G_01912
Query: 1 TGAACTGGATGCTGCTGCT 19
||||||:||::|*||||||
Sbjct: 33 ACTTGATCTGTGTCGACGA 15
>miR172k Score: 2.2 RO3G_01956
Query: 1 TGAACTGGATGCTGCTGCT 19
:||||*:||||||||||*|
Sbjct: 203 GCTTGCTCTACGACGACAA 185
>miR172k Score: 2.0 RO3G_17440
Query: 1 TGAACTGGATGCTGCTGCT 19
:||||||||||||||:|
Sbjct: 1045 CTTTGACCTACGACGATAA 1027
>miR172k Score: 2.2 RO3G_17400
Query: 1 TGAACTGGATGCTGCTGCT 19
:||||*:||||||||||*|
Sbjct: 77 GCTTGCTCTACGACGACAA 59
>miR172k Score: 2.2 RO3G_17382
Query: 1 TGAACTGGATGCTGCTGCT 19
:||||*:||||||||||*|
Sbjct: 203 GCTTGCTCTACGACGACAA 185
>miR172k Score: 0.0 RO3G_17350
Query: 1 TGAACTGGATGCTGCTGCT 19
|||||||||||||||||||
Sbjct: 371 ACTTGACCTACGACGACGA 353
>miR172k Score: 2.2 RO3G_17285
Query: 1 TGAACTGGATGCTGCTGCT 19
:||||*:||||||||||*|
Sbjct: 203 GCTTGCTCTACGACGACAA 185
>miR7837 Score: 0.0 RO3G_11243
Query: 1 TGGGTGTAGGAGTGGTAGCTG 21
|||||||||||||||||||||
Sbjct: 178 ACCCACATCCTCACCATCGAC 158
>miR7837 Score: 2.5 RO3G_08703
Query: 1 TGGGTGTAGGAGTGGTAGCTG 21
|||||||||:||*:||||*||
Sbjct: 34 ACCCACATCTTCTTCATCCAC 14
>miR7837 Score: 2.5 RO3G_06473
Query: 1 TGGGTGTAGGAGTGGTAGCTG 21
||||||||:|||:|:||||
Sbjct: 148 TCCCACATCTTCATCGT-GAC 129

>miR7837 Score: 2.5 RO3G_05294
Query: 1 TGGGTGTAGGAGTGGTAGCTG 21
::||:|||::||||||||||:
Sbjct: 247 GTCCGCATTTTCACCATCGAT 227
>miR7837 Score: 2.0 RO3G_00539
Query: 1 TGGGTGTAGGAGTGGTAGCTG 21
||||||*||||||||*|||||
Sbjct: 319 ACCCACTTCCTCACCCTCGAC 299
>miR7837 Score: 2.0 RO3G_01660
Query: 1 TGGGTGTAGGAGTGGTAGCTG 21
::||||:|||||||||||*|:
Sbjct: 853 GTCCACGTCCTCACCATC-AT 834
>miR7837 Score: 2.5 RO3G_02068
Query: 1 TGGGTGTAGGAGTGGTAGCTG 21
|||||||||||:*||||**||
Sbjct: 226 ACCCACATCCTTCCCAT-AAC 207
>miR8016 Score: 1.5 RO3G_16368
Query: 1 TTTTTGAATGAAGGGCCTGTGG 22
|||:||||||||||||*|||||
Sbjct: 1927 AAAGACTTACTTCCCGAACACC 1906
>miR8016 Score: 1.5 RO3G_15886
Query: 1 TTTTTGAATGAAGGGCCTGTGG 22
|||:||||||||||||*|||||
Sbjct: 1762 AAAGACTTACTTCCCGAACACC 1741
>miR8016 Score: 2.0 RO3G_15258
Query: 1 TTTTTGAATGAAGGGCCTGTGG 22
||||||||*|||||||*|||||
Sbjct: 613 AAAAACTTCCTTCCCGAACACC 592
>miR8016 Score: 1.5 RO3G_14552
Query: 1 TTTTTGAATGAAGGGCCTGTGG 22
|||:||||||||||||*|||||
Sbjct: 1927 AAAGACTTACTTCCCGAACACC 1906
>miR8016 Score: 1.0 RO3G_13451
Query: 1 TTTTTGAATGAAGGGCCTGTGG 22
||||||||||||||||*|||||
Sbjct: 1762 AAAAACTTACTTCCCGAACACC 1741
>miR8016 Score: 1.5 RO3G_13708
Query: 1 TTTTTGAATGAAGGGCCTGTGG 22
|||:||||||||||||*|||||
Sbjct: 1762 AAAGACTTACTTCCCGAACACC 1741
>miR8016 Score: 2.0 RO3G_12968
Query: 1 TTTTTGAATGAAGGGCCTGTGG 22
||||||||*|||||||*|||||
Sbjct: 1843 AAAAACTTCCTTCCCGAACACC 1822
>miR8016 Score: 1.5 RO3G_12646

```
Query: 1 TTTTTGAATGAAGGGCCTGTGG 22
|||:||||||||||||*|||||
Sbjct: 1762 AAAGACTTACTTCCCGAACACC 1741
>miR8016 Score: 1.5 RO3G_11542
Query: 1 TTTTTGAATGAAGGGCCTGTGG 22
|||:||||||||||||*|||||
Sbjct: 1762 AAAGACTTACTTCCCGAACACC 1741
>miR8016 Score: 1.5 RO3G_05908
Query: 1 TTTTTGAATGAAGGGCCTGTGG 22
|||:||||||||||||*|||||
Sbjct: 1762 AAAGACTTACTTCCCGAACACC 1741
>miR8016 Score: 2.0 RO3G_05911
Query: 1 TTTTTGAATGAAGGGCCTGTGG 22
||||||||*|||||||*|||||
Sbjct: 1696 AAAAACTTCCTTCCCGAACACC 1675
>miR8016 Score: 2.0 RO3G_05954
Query: 1 TTTTTGAATGAAGGGCCTGTGG 22
||||||||*|||||||*|||||
Sbjct: 1843 AAAAACTTCCTTCCCGAACACC 1822
>miR8016 Score: 1.5 RO3G_05342
Query: 1 TTTTTGAATGAAGGGCCTGTGG 22
|||:||||||||||||*|||||
Sbjct: 1927 AAAGACTTACTTCCCGAACACC 1906
>miR8016 Score: 2.0 RO3G_05427
Query: 1 TTTTTGAATGAAGGGCCTGTGG 22
||||||||*|||||||*|||||
Sbjct: 1123 AAAAACTTCCTTCCCGAACACC 1102
>miR8016 Score: 1.8 RO3G_02818
Query: 1 TTTTTGAATGAAGGGCCTGTGG 22
|||:||||||||||||*||:||
Sbjct: 262 AAAGACTTACTTCCCGAACGCC 241
>miR8016 Score: 2.5 RO3G_02910
Query: 1 TTTTTGAATGAAGGGCCTGTGG 22
|||||||||:||:|:|||*||*
Sbjct: 158 AAAAACTTATTTTCTGGA-ACA 138
>miR8016 Score: 2.5 RO3G_00696
Query: 1 TTTTTGAATGAAGGGCCTGTGG 22
||:|||*||:||:|||||||||
Sbjct: 621 AAGAACATATTTTCCGGACACC 600
>miR8016 Score: 1.0 RO3G_01193
Query: 1 TTTTTGAATGAAGGGCCTGTGG 22
||||||||||||||||*|||||
Sbjct: 1516 AAAAACTTACTTCCCGAACACC 1495
>miR8016 Score: 1.5 RO3G_01324
Query: 1 TTTTTGAATGAAGGGCCTGTGG 22
```

|||:||||||||||||*|||||
Sbjct: 613 AAAGACTTACTTCCCGAACACC 592
>miR1515 Score: 0.2 RO3G_16894
Query: 1 TATTTTAGCATGACAATGATCC 22
|||||||||||||||||||:||
Sbjct: 36 ATAAAATCGTACTGTTACTGGG 15
>miR1515 Score: 2.5 RO3G_16705
Query: 1 TATTTTAGCATGACAATGATCC 22
|*|||:||||*|||||||||||
Sbjct: 69 ACAAAGTCGTCCTGTTACTAGG 48
>miR1515 Score: 2.5 RO3G_16375
Query: 1 TATTTTAGCATGACAATGATCC 22
|*|||:||||*|||||||||||
Sbjct: 69 ACAAAGTCGTCCTGTTACTAGG 48
>miR1515 Score: 2.5 RO3G_16552
Query: 1 TATTTTAGCATGACAATGATCC 22
|*|||:||||*|||||||||||
Sbjct: 69 ACAAAGTCGTCCTGTTACTAGG 48
>miR1515 Score: 2.5 RO3G_15668
Query: 1 TATTTTAGCATGACAATGATCC 22
|*|||:||||*|||||||||||
Sbjct: 78 ACAAAGTCGTCCTGTTACTAGG 57
>miR1515 Score: 2.5 RO3G_14803
Query: 1 TATTTTAGCATGACAATGATCC 22
|*|||:||||*|||||||||||
Sbjct: 69 ACAAAGTCGTCCTGTTACTAGG 48
>miR1515 Score: 2.5 RO3G_15135
Query: 1 TATTTTAGCATGACAATGATCC 22
|*|||:||||*|||||||||||
Sbjct: 4260 ACAAAGTCGTCCTGTTACTAGG 4239
>miR1515 Score: 2.5 RO3G_14638
Query: 1 TATTTTAGCATGACAATGATCC 22
|*|||:||||*|||||||||||
Sbjct: 69 ACAAAGTCGTCCTGTTACTAGG 48
>miR1515 Score: 1.8 RO3G_13731
Query: 1 TATTTTAGCATGACAATGATCC 22
**|||||||||||||||||:||
Sbjct: 224 TGAAAATCGTACTGTTACTGGG 203
>miR1515 Score: 2.5 RO3G_12191
Query: 1 TATTTTAGCATGACAATGATCC 22
|*|||:||||*|||||||||||
Sbjct: 69 ACAAAGTCGTCCTGTTACTAGG 48
>miR1515 Score: 2.5 RO3G_12648
Query: 1 TATTTTAGCATGACAATGATCC 22
|*|||:||||*|||||||||||

Sbjct: 69 ACAAAGTCGTCCTGTTACTAGG 48
>miR1515 Score: 2.5 RO3G_11260
Query: 1 TATTTTAGCATGACAATGATCC 22
|*|||:||||*|||||||||||
Sbjct: 69 ACAAAGTCGTCCTGTTACTAGG 48
>miR1515 Score: 2.5 RO3G_11408
Query: 1 TATTTTAGCATGACAATGATCC 22
|*|||:||||*|||||||||||
Sbjct: 69 ACAAAGTCGTCCTGTTACTAGG 48
>miR1515 Score: 1.2 RO3G_10527
Query: 1 TATTTTAGCATGACAATGATCC 22
|*|||||||||||||||||:||
Sbjct: 210 ACAAAATCGTACTGTTACTGGG 189
>miR1515 Score: 2.5 RO3G_10534
Query: 1 TATTTTAGCATGACAATGATCC 22
|*|||:||||*|||||||||||
Sbjct: 69 ACAAAGTCGTCCTGTTACTAGG 48
>miR1515 Score: 2.5 RO3G_10536
Query: 1 TATTTTAGCATGACAATGATCC 22
|*|||:||||*|||||||||||
Sbjct: 69 ACAAAGTCGTCCTGTTACTAGG 48
>miR1515 Score: 1.8 RO3G_10553
Query: 1 TATTTTAGCATGACAATGATCC 22
**||||||||||||||||||:||
Sbjct: 210 TAAAAATCGTACTGTTACTGGG 189
>miR1515 Score: 2.5 RO3G_10554
Query: 1 TATTTTAGCATGACAATGATCC 22
|*|||:||||*|||||||||||
Sbjct: 69 ACAAAGTCGTCCTGTTACTAGG 48
>miR1515 Score: 1.8 RO3G_10559
Query: 1 TATTTTAGCATGACAATGATCC 22
**||||||||||||||||||:||
Sbjct: 210 TAAAAATCGTACTGTTACTGGG 189
>miR1515 Score: 2.5 RO3G_10872
Query: 1 TATTTTAGCATGACAATGATCC 22
|*|||:||||*|||||||||||
Sbjct: 69 ACAAAGTCGTCCTGTTACTAGG 48
>miR1515 Score: 1.2 RO3G_09962
Query: 1 TATTTTAGCATGACAATGATCC 22
|*|||||||||||||||||:||
Sbjct: 36 ACAAAATCGTACTGTTACTGGG 15
>miR1515 Score: 1.2 RO3G_09968
Query: 1 TATTTTAGCATGACAATGATCC 22
|*|||||||||||||||||:||
Sbjct: 36 ACAAAATCGTACTGTTACTGGG 15

>miR1515 Score: 2.5 RO3G_07986
Query: 1 TATTTTAGCATGACAATGATCC 22
|*|||:||||*|||||||||||
Sbjct: 69 ACAAAGTCGTCCTGTTACTAGG 48
>miR1515 Score: 2.5 RO3G_07987
Query: 1 TATTTTAGCATGACAATGATCC 22
|*|||:||||*|||||||||||
Sbjct: 69 ACAAAGTCGTCCTGTTACTAGG 48
>miR1515 Score: 2.5 RO3G_07724
Query: 1 TATTTTAGCATGACAATGATCC 22
|*|||:||||*|||||||||||
Sbjct: 69 ACAAAGTCGTCCTGTTACTAGG 48
>miR1515 Score: 2.5 RO3G_07731
Query: 1 TATTTTAGCATGACAATGATCC 22
|*|||:||||*|||||||||||
Sbjct: 69 ACAAAGTCGTCCTGTTACTAGG 48
>miR1515 Score: 2.5 RO3G_05917
Query: 1 TATTTTAGCATGACAATGATCC 22
|*|||:||||*|||||||||||
Sbjct: 69 ACAAAGTCGTCCTGTTACTAGG 48
>miR1515 Score: 2.5 RO3G_05931
Query: 1 TATTTTAGCATGACAATGATCC 22
|*|||:||||*|||||||||||
Sbjct: 69 ACAAAGTCGTCCTGTTACTAGG 48
>miR1515 Score: 2.5 RO3G_05948
Query: 1 TATTTTAGCATGACAATGATCC 22
|*|||:||||*|||||||||||
Sbjct: 69 ACAAAGTCGTCCTGTTACTAGG 48
>miR1515 Score: 2.5 RO3G_06419
Query: 1 TATTTTAGCATGACAATGATCC 22
|*|||:||||*|||||||||||
Sbjct: 69 ACAAAGTCGTCCTGTTACTAGG 48
>miR1515 Score: 2.5 RO3G_03592
Query: 1 TATTTTAGCATGACAATGATCC 22
|*|||:||||*|||||||||||
Sbjct: 69 ACAAAGTCGTCCTGTTACTAGG 48
>miR1515 Score: 2.5 RO3G_03596
Query: 1 TATTTTAGCATGACAATGATCC 22
|*|||:||||*|||||||||||
Sbjct: 69 ACAAAGTCGTCCTGTTACTAGG 48
>miR1515 Score: 2.5 RO3G_03788
Query: 1 TATTTTAGCATGACAATGATCC 22
|*|||:||||*|||||||||||
Sbjct: 69 ACAAAGTCGTCCTGTTACTAGG 48
>miR1515 Score: 2.5 RO3G_04026

Query: 1 TATTTTAGCATGACAATGATCC 22
|*|||:||||*|||||||||||
Sbjct: 69 ACAAAGTCGTCCTGTTACTAGG 48
>miR1515 Score: 2.5 RO3G_04199
Query: 1 TATTTTAGCATGACAATGATCC 22
|*|||:||||*|||||||||||
Sbjct: 69 ACAAAGTCGTCCTGTTACTAGG 48
>miR1515 Score: 2.5 RO3G_00246
Query: 1 TATTTTAGCATGACAATGATCC 22
|*|||:||||*|||||||||||
Sbjct: 69 ACAAAGTCGTCCTGTTACTAGG 48
>miR1515 Score: 2.5 RO3G_00685
Query: 1 TATTTTAGCATGACAATGATCC 22
|*|||:||||*|||||||||||
Sbjct: 69 ACAAAGTCGTCCTGTTACTAGG 48
>miR1515 Score: 2.5 RO3G_01200
Query: 1 TATTTTAGCATGACAATGATCC 22
|*|||:||||*|||||||||||
Sbjct: 69 ACAAAGTCGTCCTGTTACTAGG 48
>miR1515 Score: 2.5 RO3G_01316
Query: 1 TATTTTAGCATGACAATGATCC 22
|*|||:||||*|||||||||||
Sbjct: 69 ACAAAGTCGTCCTGTTACTAGG 48
>miR1515 Score: 2.5 RO3G_01736
Query: 1 TATTTTAGCATGACAATGATCC 22
|*|||:||||*|||||||||||
Sbjct: 69 ACAAAGTCGTCCTGTTACTAGG 48
>miR1515 Score: 2.5 RO3G_01957
Query: 1 TATTTTAGCATGACAATGATCC 22
|*|||:||||*|||||||||||
Sbjct: 69 ACAAAGTCGTCCTGTTACTAGG 48
>miR1515 Score: 2.5 RO3G_17368
Query: 1 TATTTTAGCATGACAATGATCC 22
|*|||:||||*|||||||||||
Sbjct: 69 ACAAAGTCGTCCTGTTACTAGG 48
>miR1515 Score: 0.2 RO3G_17349
Query: 1 TATTTTAGCATGACAATGATCC 22
|||||||||||||||||||:||
Sbjct: 36 ATAAAATCGTACTGTTACTGGG 15
>miR5561-5p Score: 2.5 RO3G_12299
Query: 1 CATTTGGTGAGAGACATG-GACAA 23
|||||:|:||||||||||*|||
Sbjct: 1374 TTAAACTATTCTCTGTACACAGTT 1351
>miR7502b Score: 2.5 RO3G_16530
Query: 1 TTGTTAATAAGTTCATGC-AT 20

||:|||||||:|||||*|*||
Sbjct: 1031 AATAATTATTTAAGTAAGTTA 1011
>miR7502b Score: 2.5 RO3G_14312
Query: 1 TTGTTAATAAGTTCATGCAT 20
|:||||||||||||*||
Sbjct: 962 CATATTTATTCAAGTACTTA 943
>miR7502b Score: 2.0 RO3G_12923
Query: 1 TTGTTAATAAGTTCATGCAT 20
|::|||||||:||||||||*
Sbjct: 602 AGTAATTATTTAAGTACGTT 583
>miR7502b Score: 2.5 RO3G_13261
Query: 1 TTGTTAATAAGTTCATGC-AT 20
|||:||||||:|||||:**||
Sbjct: 1246 AACGATTATTTAAGTATATTA 1226
>miR7502b Score: 2.2 RO3G_12569
Query: 1 TTGTTAATAAGTTCATGCAT 20
:||:||*:||||||||||||
Sbjct: 281 GACGATAGTTCAAGTACGTA 262
>miR7502b Score: 2.5 RO3G_08198
Query: 1 TTGTTAATAAGTTCATGCAT 20
|||::|||||||||*|||*|
Sbjct: 190 AACGGTTATTCAAGAACG-A 172
>miR7502b Score: 2.5 RO3G_08587
Query: 1 TTGTTAATAAGTTCATGCAT 20
|||:|||||||||||*||
Sbjct: 2039 TACAGTTATTCAAGAAC-TA 2021
>miR7502b Score: 2.5 RO3G_05880
Query: 1 TTGTTAATAAGTTCATGCAT 20
||||:||||||||*||*|||
Sbjct: 290 AACAGTTATTCAAATA-GTA 272
>miR7502b Score: 2.5 RO3G_03431
Query: 1 TTGTTAATAAGTTCATGCA-T 20
||:|:|||||||||||:*|*|
Sbjct: 3368 AATAGTTATTCAAGTATTTCA 3348
>miR7502b Score: 2.2 RO3G_01163
Query: 1 TTGTTAATAAGTTCATGCAT 20
:|||:||:|||:|||||*||
Sbjct: 335 GACAGTTGTTCGAGTACTTA 316
>miR6451 Score: 2.0 RO3G_16725
Query: 1 ATGTAGATCATGTTGATT 18
|||||:||||||:|:||*
Sbjct: 530 TACATTTAGTACGATTAT 513
>miR6451 Score: 2.5 RO3G_16741
Query: 1 ATGTAGATCATGTTGATT 18
||||||*|||||||||

Sbjct: 665 AACATCAACTACAACTAA 648
>miR6451 Score: 2.5 RO3G_16368
Query: 1 ATGTAGATCATGTTGATT 18
||||||*|||||||||
Sbjct: 1661 AACATCAACTACAACTAA 1644
>miR6451 Score: 2.5 RO3G_15886
Query: 1 ATGTAGATCATGTTGATT 18
||||||*|||||||||
Sbjct: 1496 AACATCAACTACAACTAA 1479
>miR6451 Score: 2.0 RO3G_15840
Query: 1 ATGTAGATCATGTTGATT 18
|||||:||||||||||
Sbjct: 7830 AACATGTGGTACAACTAA 7813
>miR6451 Score: 2.0 RO3G_15271
Query: 1 ATGTAGATCATGTTGATT 18
|||||:||||||:|:||*
Sbjct: 788 TACATTTAGTACGATTAT 771
>miR6451 Score: 2.5 RO3G_15359
Query: 1 ATGTAGATCATGTTGATT 18
||*|||||||||||||**
Sbjct: 123 TAGATCTAGTACAACTTC 106
>miR6451 Score: 2.5 RO3G_15507
Query: 1 ATGTAGATCATGTTGATT 18
||||||||||||||*|
Sbjct: 5124 AACACCTAGTACAACTTA 5107
>miR6451 Score: 2.5 RO3G_15560
Query: 1 ATGTAGATCATGTTGATT 18
|::|||||||*||||||*
Sbjct: 713 TGTATCTAGTCCAACTAT 696
>miR6451 Score: 2.5 RO3G_14552
Query: 1 ATGTAGATCATGTTGATT 18
||||||*|||||||||
Sbjct: 1661 AACATCAACTACAACTAA 1644
>miR6451 Score: 2.2 RO3G_14659
Query: 1 ATGTAGATCATGTTGATT 18
**:||||||||||||||:
Sbjct: 1080 ATTATCTAGTACAACTAG 1063
>miR6451 Score: 2.5 RO3G_13970
Query: 1 ATGTAGATCATGTTGATT 18
|||||||||||::|||
Sbjct: 362 GACATCTAGTACGGGTAA 345
>miR6451 Score: 2.0 RO3G_14199
Query: 1 ATGTAGATCATGTTGATT 18
*||:|:|:||||||||||
Sbjct: 3429 GACGTTTGGTACAACTAA 3412

>miR6451 Score: 2.5 RO3G_13451
Query: 1 ATGTAGATCATGTTGATT 18
||||||*|||||||||
Sbjct: 1496 AACATCAACTACAACTAA 1479
>miR6451 Score: 2.5 RO3G_13503
Query: 1 ATGTAGATCATGTTGATT 18
|||:||*|||*|||||||
Sbjct: 1125 TACGTCAAGTTCAACTAA 1108
>miR6451 Score: 2.5 RO3G_13708
Query: 1 ATGTAGATCATGTTGATT 18
||||||*|||||||||
Sbjct: 1496 AACATCAACTACAACTAA 1479
>miR6451 Score: 2.5 RO3G_12077
Query: 1 ATGTAGATCATGTTGATT 18
||||||*|||||||||
Sbjct: 1733 AACATCAACTACAACTAA 1716
>miR6451 Score: 2.5 RO3G_12646
Query: 1 ATGTAGATCATGTTGATT 18
||||||*|||||||||
Sbjct: 1496 AACATCAACTACAACTAA 1479
>miR6451 Score: 2.5 RO3G_11542
Query: 1 ATGTAGATCATGTTGATT 18
||||||*|||||||||
Sbjct: 1496 AACATCAACTACAACTAA 1479
>miR6451 Score: 2.5 RO3G_11799
Query: 1 ATGTAGATCATGTTGATT 18
||||*|||||*||||||*
Sbjct: 152 TACAACTAGTTCAACTAT 135
>miR6451 Score: 2.2 RO3G_10429
Query: 1 ATGTAGATCATGTTGATT 18
||||*||:|||:|||||:
Sbjct: 672 TACAGCTGGTATAACTAG 655
>miR6451 Score: 2.0 RO3G_10667
Query: 1 ATGTAGATCATGTTGATT 18
||||||||||||||*
Sbjct: 131 AACATCTAGTACAACAAT 114
>miR6451 Score: 2.0 RO3G_10669
Query: 1 ATGTAGATCATGTTGATT 18
||||||||||||||*
Sbjct: 1004 AACATCTAGTACAACAAT 987
>miR6451 Score: 2.0 RO3G_10673
Query: 1 ATGTAGATCATGTTGATT 18
||||||||||||||*
Sbjct: 1004 AACATCTAGTACAACAAT 987
>miR6451 Score: 2.0 RO3G_10684

Query: 1 ATGTAGATCATGTTGATT 18
||||||||||||||*
Sbjct: 1004 AACATCTAGTACAACAAT 987
>miR6451 Score: 2.2 RO3G_09462
Query: 1 ATGTAGATCATGTTGATT 18
|||:||||||||::||::
Sbjct: 842 TACGTCTAGTACGGCTGG 825
>miR6451 Score: 2.5 RO3G_09689
Query: 1 ATGTAGATCATGTTGATT 18
||||||*|||||||||
Sbjct: 297 AACTTCTATTACAACTAA 280
>miR6451 Score: 2.2 RO3G_08163
Query: 1 ATGTAGATCATGTTGATT 18
||||||||*||||||*|:
Sbjct: 126 TACATCTATTACAACAAG 109
>miR6451 Score: 2.5 RO3G_08513
Query: 1 ATGTAGATCATGTTGATT 18
||||*|||||*||||||*
Sbjct: 152 TACAACTAGTTCAACTAT 135
>miR6451 Score: 2.5 RO3G_08772
Query: 1 ATGTAGATCATGTTGATT 18
||||||:||||||||*
Sbjct: 3105 AACATCTGGTAAAACTAC 3088
>miR6451 Score: 2.5 RO3G_06746
Query: 1 ATGTAGATCATGTTGATT 18
|*|||*|||||:||||||
Sbjct: 546 TCCATGTAGTATAACTAA 529
>miR6451 Score: 2.0 RO3G_06872
Query: 1 ATGTAGATCATGTTGATT 18
|||||||||||:||||
Sbjct: 918 AACATCTAGTCCAGCTAA 901
>miR6451 Score: 2.5 RO3G_05908
Query: 1 ATGTAGATCATGTTGATT 18
||||||*|||||||||
Sbjct: 1496 AACATCAACTACAACTAA 1479
>miR6451 Score: 2.5 RO3G_06664
Query: 1 ATGTAGATCATGTTGATT 18
||||*|||||:*||||||
Sbjct: 677 TACACCTAGTGGAACTAA 660
>miR6451 Score: 2.5 RO3G_04899
Query: 1 ATGTAGATCATGTTGATT 18
::||||:|||||||||
Sbjct: 1319 AGTATCTGGTACAACTAC 1302
>miR6451 Score: 2.5 RO3G_04929
Query: 1 ATGTAGATCATGTTGATT 18

|||||:|||||:|*:|||
Sbjct: 279 TACATTTAGTATATTTAA 262
>miR6451 Score: 2.5 RO3G_05342
Query: 1 ATGTAGATCATGTTGATT 18
||||||*|||||||||
Sbjct: 1661 AACATCAACTACAACTAA 1644
>miR6451 Score: 2.0 RO3G_05460
Query: 1 ATGTAGATCATGTTGATT 18
|||*||||||:||||||*
Sbjct: 290 TACTTCTAGTGCAACTAC 273
>miR6451 Score: 2.5 RO3G_02238
Query: 1 ATGTAGATCATGTTGATT 18
||*||||:||||*|||||
Sbjct: 927 TAGATCTGGTACTACTAA 910
>miR6451 Score: 2.0 RO3G_02555
Query: 1 ATGTAGATCATGTTGATT 18
|||||||||||:||||
Sbjct: 97 CACATCTCGTACAGCTAA 80
>miR6451 Score: 2.2 RO3G_02771
Query: 1 ATGTAGATCATGTTGATT 18
*||||:||||||::|||:
Sbjct: 1110 AACATTTAGTACGGCTAG 1093
>miR6451 Score: 2.5 RO3G_02819
Query: 1 ATGTAGATCATGTTGATT 18
||||||*|||||||||
Sbjct: 941 AACATCAACTACAACTAA 924
>miR6451 Score: 2.5 RO3G_03205
Query: 1 ATGTAGATCATGTTGATT 18
||||*||:||:||||||*
Sbjct: 402 TACAGCTGGTGCAACTAC 385
>miR6451 Score: 2.5 RO3G_03967
Query: 1 ATGTAGATCATGTTGATT 18
||:||:|||*|||||||*
Sbjct: 33 TATATTTAGAACAACTAC 16
>miR6451 Score: 2.0 RO3G_00324
Query: 1 ATGTAGATCATGTTGATT 18
||||*|||*|||||||||
Sbjct: 834 TACAACTACTACAACTAA 817
>miR6451 Score: 2.0 RO3G_00403
Query: 1 ATGTAGATCATGTTGATT 18
||||:|||||||||||
Sbjct: 7842 CACATTTAGTCCAACTAA 7825
>miR6451 Score: 2.5 RO3G_00880
Query: 1 ATGTAGATCATGTTGATT 18
||||||||||||*::||*

Sbjct: 1097 TACATCTAGTACTGTTAT 1080
>miR6451 Score: 2.5 RO3G_01178
Query: 1 ATGTAGATCATGTTGATT 18
|||||||||:::||||
Sbjct: 92 AACATCTAGTGTGACTAT 75
>miR6451 Score: 2.5 RO3G_01324
Query: 1 ATGTAGATCATGTTGATT 18
||||||*|||||||||
Sbjct: 347 AACATCAACTACAACTAA 330
>miR6451 Score: 2.5 RO3G_01716
Query: 1 ATGTAGATCATGTTGATT 18
||||||*|||||||||
Sbjct: 1496 AACATCAACTACAACTAA 1479
>miR6451 Score: 2.5 RO3G_01915
Query: 1 ATGTAGATCATGTTGATT 18
||*||:||*|||||||||
Sbjct: 468 TAAATTTACTACAACTAA 451
>miR6451 Score: 2.2 RO3G_02047
Query: 1 ATGTAGATCATGTTGATT 18
|:||||||*|:||||||:
Sbjct: 81 TGCATCTAATGCAACTAG 64
>miR951 Score: 0.0 RO3G_13220
Query: 1 TGCTCTTGACGATCTGGCTACG 22
||||||||||||||||||||||
Sbjct: 217 ACGAGAACTGCTAGACCGATGC 196
>miR951 Score: 0.5 RO3G_13223
Query: 1 TGCTCTTGACGATCTGGCTACG 22
||||||||||||||||:|||||
Sbjct: 217 ACGAGAACTGCTAGACTGATGC 196
>miR951 Score: 2.5 RO3G_12464
Query: 1 TGCTCTTGACGATCTGGCTACG 22
||*|||||||*|||||:|||||
Sbjct: 217 ACAAGAACTGGTAGACTGATGC 196
>miR951 Score: 1.0 RO3G_04119
Query: 1 TGCTCTTGACGATCTGGCTACG 22
||||||||||*|||||||||||
Sbjct: 217 ACGAGAACTGGTAGACCGATGC 196
>miR951 Score: 0.0 RO3G_04122
Query: 1 TGCTCTTGACGATCTGGCTACG 22
||||||||||||||||||||||
Sbjct: 217 ACGAGAACTGCTAGACCGATGC 196
>miR951 Score: 0.0 RO3G_04124
Query: 1 TGCTCTTGACGATCTGGCTACG 22
||||||||||||||||||||||
Sbjct: 217 ACGAGAACTGCTAGACCGATGC 196

```
>miR7716-3p Score: 2.2 RO3G_12741
Query: 1 TTCTTCATCCTCCAGTAATGCC 22
:||||||||||||||:|*::*|
Sbjct: 1191 GAGAAGTAGGAGGTCGTGGTAG 1170
>miR7716-3p Score: 2.2 RO3G_07958
Query: 1 TTCTTCATCCTCCAGTAATGCC 22
||||||||||||||::|*:|*|
Sbjct: 3090 AAGAAGTAGGAGGTTGT-GCAG 3070
>miR7716-3p Score: 0.0 RO3G_17338
Query: 1 TTCTTCATCCTCCAGTAATGCC 22
||||||||||||||||||||||
Sbjct: 24 AAGAAGTAGGAGGTCATTACGG 3
>miR8741 Score: 0.0 RO3G_17257
Query: 1 TGGCCTGAATGAATGATGATGG 22
||||||||||||||||||||||
Sbjct: 2143 ACCGGACTTACTTACTACTACC 2122
>miR8741 Score: 0.0 RO3G_16749
Query: 1 TGGCCTGAATGAATGATGATGG 22
||||||||||||||||||||||
Sbjct: 2227 ACCGGACTTACTTACTACTACC 2206
>miR8741 Score: 0.0 RO3G_16136
Query: 1 TGGCCTGAATGAATGATGATGG 22
||||||||||||||||||||||
Sbjct: 2143 ACCGGACTTACTTACTACTACC 2122
>miR8741 Score: 0.0 RO3G_15889
Query: 1 TGGCCTGAATGAATGATGATGG 22
||||||||||||||||||||||
Sbjct: 2143 ACCGGACTTACTTACTACTACC 2122
>miR8741 Score: 0.0 RO3G_13145
Query: 1 TGGCCTGAATGAATGATGATGG 22
||||||||||||||||||||||
Sbjct: 2227 ACCGGACTTACTTACTACTACC 2206
>miR8741 Score: 0.0 RO3G_13273
Query: 1 TGGCCTGAATGAATGATGATGG 22
||||||||||||||||||||||
Sbjct: 2227 ACCGGACTTACTTACTACTACC 2206
>miR8741 Score: 1.2 RO3G_10735
Query: 1 TGGCCTGAATGAATGATGATGG 22
:*||||||||||||||||||||
Sbjct: 1672 GGCGGACTTACTTACTACTACC 1651
>miR8741 Score: 0.0 RO3G_10040
Query: 1 TGGCCTGAATGAATGATGATGG 22
||||||||||||||||||||||
Sbjct: 817 ACCGGACTTACTTACTACTACC 796
>miR8741 Score: 0.0 RO3G_10217
```

Query: 1 TGGCCTGAATGAATGATGATGG 22
||||||||||||||||||||||
Sbjct: 2227 ACCGGACTTACTTACTACTACC 2206
>miR8741 Score: 0.0 RO3G_09183
Query: 1 TGGCCTGAATGAATGATGATGG 22
||||||||||||||||||||||
Sbjct: 1360 ACCGGACTTACTTACTACTACC 1339
>miR8741 Score: 0.0 RO3G_07697
Query: 1 TGGCCTGAATGAATGATGATGG 22
||||||||||||||||||||||
Sbjct: 553 ACCGGACTTACTTACTACTACC 532
>miR8741 Score: 0.0 RO3G_04592
Query: 1 TGGCCTGAATGAATGATGATGG 22
||||||||||||||||||||||
Sbjct: 1399 ACCGGACTTACTTACTACTACC 1378
>miR8741 Score: 0.0 RO3G_04681
Query: 1 TGGCCTGAATGAATGATGATGG 22
||||||||||||||||||||||
Sbjct: 817 ACCGGACTTACTTACTACTACC 796
>miR8741 Score: 0.0 RO3G_05424
Query: 1 TGGCCTGAATGAATGATGATGG 22
||||||||||||||||||||||
Sbjct: 2143 ACCGGACTTACTTACTACTACC 2122
>miR8741 Score: 0.0 RO3G_00289
Query: 1 TGGCCTGAATGAATGATGATGG 22
||||||||||||||||||||||
Sbjct: 961 ACCGGACTTACTTACTACTACC 940
>miR8741 Score: 0.0 RO3G_17351
Query: 1 TGGCCTGAATGAATGATGATGG 22
||||||||||||||||||||||
Sbjct: 955 ACCGGACTTACTTACTACTACC 934
>miR913-5p Score: 0.0 RO3G_16800
Query: 1 TGACATCTTGCGAGACCGAGG 21
|||||||||||||||||||||
Sbjct: 492 ACTGTAGAACGCTCTGGCTCC 472
>miR913-5p Score: 0.0 RO3G_15265
Query: 1 TGACATCTTGCGAGACCGAGG 21
|||||||||||||||||||||
Sbjct: 492 ACTGTAGAACGCTCTGGCTCC 472
>miR913-5p Score: 2.0 RO3G_15266
Query: 1 TGACATCTTGCGAGACCGAGG 21
||||||||:||||||||||
Sbjct: 492 TCTGTAGAATGCCCTGGCTCC 472
>miR913-5p Score: 0.0 RO3G_15268
Query: 1 TGACATCTTGCGAGACCGAGG 21

|||||||||||||||||||||
Sbjct: 492 ACTGTAGAACGCTCTGGCTCC 472
>miR913-5p Score: 2.0 RO3G_14352
Query: 1 TGACATCTTGCGAGACCGAGG 21
||||||||:||||||||||
Sbjct: 492 TCTGTAGAATGCCCTGGCTCC 472
>miR913-5p Score: 2.0 RO3G_13217
Query: 1 TGACATCTTGCGAGACCGAGG 21
||||||||:||||||||||
Sbjct: 492 TCTGTAGAATGCCCTGGCTCC 472
>miR913-5p Score: 0.0 RO3G_13219
Query: 1 TGACATCTTGCGAGACCGAGG 21
|||||||||||||||||||||
Sbjct: 492 ACTGTAGAACGCTCTGGCTCC 472
>miR913-5p Score: 0.0 RO3G_04121
Query: 1 TGACATCTTGCGAGACCGAGG 21
|||||||||||||||||||||
Sbjct: 492 ACTGTAGAACGCTCTGGCTCC 472
>miR8603 Score: 2.5 RO3G_06113
Query: 1 TGAGCTTGTGGTGAGCATTGT 21
||||||:|:|*||||||*|||
Sbjct: 1285 ACTCGAGCGCGACTCGT-ACA 1266
>miR8603 Score: 2.5 RO3G_05236
Query: 1 TGAGCTTGTGGTGAGCA-TTGT 21
|:|:||||||:||||||*||||
Sbjct: 36 ATTTGAACACTACTCGTTAACA 15
>miR8603 Score: 2.5 RO3G_02600
Query: 1 TGAGCTTGTGGTGAGCATTGT 21
|||||||*||*||||||:|:|
Sbjct: 970 ACTCGAAAACGACTCGTGATA 950
>miR8603 Score: 2.5 RO3G_03300
Query: 1 TGAGCTTGTGGTGAGCATTGT 21
||||||||||:||*|||**||
Sbjct: 199 ACTCGAACACTACCCGT-TCA 180
>miR4401b Score: 2.5 RO3G_16714
Query: 1 TCAAAGACGGTTGTGAGCAA 20
||*|||||||||||*|:|||
Sbjct: 206 AGATTCTGCCAACAGTTGTT 187
>miR4401b Score: 0.0 RO3G_14983
Query: 1 TCAAAGACGGTTGTGAGCAA 20
||||||||||||||||||||
Sbjct: 653 AGTTTCTGCCAACACTCGTT 634
>miR4401b Score: 0.0 RO3G_13914
Query: 1 TCAAAGACGGTTGTGAGCAA 20
||||||||||||||||||||

Sbjct: 848 AGTTTCTGCCAACACTCGTT 829
>miR4401b Score: 1.0 RO3G_12667
Query: 1 TCAAAGACGGTTGTGAGCAA 20
||*|||||||||||||||||
Sbjct: 218 AGGTTCTGCCAACACTCGTT 199
>miR4401b Score: 0.0 RO3G_12058
Query: 1 TCAAAGACGGTTGTGAGCAA 20
||||||||||||||||||||
Sbjct: 848 AGTTTCTGCCAACACTCGTT 829
>miR4401b Score: 0.0 RO3G_10801
Query: 1 TCAAAGACGGTTGTGAGCAA 20
||||||||||||||||||||
Sbjct: 653 AGTTTCTGCCAACACTCGTT 634
>miR4401b Score: 0.0 RO3G_09614
Query: 1 TCAAAGACGGTTGTGAGCAA 20
||||||||||||||||||||
Sbjct: 179 AGTTTCTGCCAACACTCGTT 160
>miR4401b Score: 2.2 RO3G_08383
Query: 1 TCAAAGACGGTTGTGAGCAA 20
:|*|||||||:||:||||||
Sbjct: 300 GGGTTCTGCCGACGCTCGTT 281
>miR4401b Score: 0.0 RO3G_02233
Query: 1 TCAAAGACGGTTGTGAGCAA 20
||||||||||||||||||||
Sbjct: 377 AGTTTCTGCCAACACTCGTT 358
>miR4401b Score: 1.0 RO3G_03583
Query: 1 TCAAAGACGGTTGTGAGCAA 20
||||||||*|||||||||||
Sbjct: 653 AGTTTCTGACAACACTCGTT 634
>miR4401b Score: 2.5 RO3G_04116
Query: 1 TCAAAGACGGTTGTGAGCAA 20
||*|||||||||||*|:|||
Sbjct: 213 AGATTCTGCCAACAGTTGTT 194
>miR4401b Score: 0.0 RO3G_17462
Query: 1 TCAAAGACGGTTGTGAGCAA 20
||||||||||||||||||||
Sbjct: 980 AGTTTCTGCCAACACTCGTT 961
>miR4401b Score: 0.0 RO3G_17451
Query: 1 TCAAAGACGGTTGTGAGCAA 20
||||||||||||||||||||
Sbjct: 89 AGTTTCTGCCAACACTCGTT 70
>miR171n-5p Score: 2.2 RO3G_12220
Query: 1 TATTGGTGGGTTCAAGTACGG 21
**|||||:|||||||||||:|
Sbjct: 316 TCAACCATCCAAGTTCATGTC 296

>miR171n-5p Score: 2.2 RO3G_12505
Query: 1 TATTGGTGGGTTCAAGTACGG 21
|||||:|||||||||:||*:*
Sbjct: 28 ATAACTACCCAAGTTTAT-TA 9
>miR171n-5p Score: 2.2 RO3G_12506
Query: 1 TATTGGTGGGTTCAAGTACGG 21
|||||:|||||||||:||*:*
Sbjct: 28 ATAACTACCCAAGTTTAT-TA 9
>miR171n-5p Score: 2.5 RO3G_02538
Query: 1 TATTGGTGGGTTCAAGTACGG 21
:|:||:|:||||||||||*:|
Sbjct: 796 GTGACTATCCAAGTTCATTTC 776
>miR1431 Score: 2.5 RO3G_17253
Query: 1 TTTGGAGTTGGCCACCGCTTGAC 23
|||||*:||||||||*|||||||
Sbjct: 657 AAACCGTAACCGGTGTCGAACTG 635
>miR1431 Score: 2.5 RO3G_11979
Query: 1 TTTGGAGTTGGCCACCGCTTGAC 23
|||||*:||||||||*|||||||
Sbjct: 641 AAACCGTAACCGGTGTCGAACTG 619
>miR158-3p Score: 2.0 RO3G_15863
Query: 1 TTTCCAAATGTAGACAAAGC 20
|||*||*|||||||||||||
Sbjct: 29 AAAAGTCTACATCTGTTTCG 10
>miR158-3p Score: 2.0 RO3G_15573
Query: 1 TTTCCAAATGTAGACAAAGC 20
|||*||*|||||||||||||
Sbjct: 3101 AAAAGTCTACATCTGTTTCG 3082
>miR158-3p Score: 2.0 RO3G_13849
Query: 1 TTTCCAAATGTAGACAAAGC 20
|||*||*|||||||||||||
Sbjct: 3101 AAAAGTCTACATCTGTTTCG 3082
>miR158-3p Score: 2.0 RO3G_11945
Query: 1 TTTCCAAATGTAGACAAAGC 20
|||*||*|||||||||||||
Sbjct: 29 AAAAGTCTACATCTGTTTCG 10
>miR158-3p Score: 2.0 RO3G_10420
Query: 1 TTTCCAAATGTAGACAAAGC 20
|||*||*|||||||||||||
Sbjct: 3101 AAAAGTCTACATCTGTTTCG 3082
>miR158-3p Score: 2.5 RO3G_10923
Query: 1 TTTCCAAATGTAGACAAAGC 20
:||*|||||||||||||*:*
Sbjct: 3134 GAACGTTTACATCTGTTATA 3115
>miR158-3p Score: 2.0 RO3G_10991

Query: 1 TTTCCAAATGTAGACAAAGC 20
|||*||*|||||||||||||
Sbjct: 3101 AAAAGTCTACATCTGTTTCG 3082
>miR158-3p Score: 2.5 RO3G_09886
Query: 1 TTTCCAAATGTAGACAAAGC 20
|||||||||*||||||||
Sbjct: 1726 TAAGCTTTACAACTGTTTCG 1707
>miR158-3p Score: 2.5 RO3G_07325
Query: 1 TTTCCAAATGTAGACAAAGC 20
|||*||||||||:*||||||
Sbjct: 1325 AAAAGTTTACATTGGTTTCG 1306
>miR158-3p Score: 2.0 RO3G_05865
Query: 1 TTTCCAAATGTAGACAAAGC 20
|||*||*|||||||||||||
Sbjct: 2753 AAAAGTCTACATCTGTTTCG 2734
>miR158-3p Score: 2.0 RO3G_06502
Query: 1 TTTCCAAATGTAGACAAAGC 20
|||*||*|||||||||||||
Sbjct: 2978 AAAAGTCTACATCTGTTTCG 2959
>miR158-3p Score: 2.0 RO3G_02707
Query: 1 TTTCCAAATGTAGACAAAGC 20
|||*||*|||||||||||||
Sbjct: 650 AAAAGTCTACATCTGTTTCG 631
>miR158-3p Score: 2.0 RO3G_02966
Query: 1 TTTCCAAATGTAGACAAAGC 20
|||*||*|||||||||||||
Sbjct: 2372 AAAAGTCTACATCTGTTTCG 2353
>miR158-3p Score: 2.0 RO3G_03749
Query: 1 TTTCCAAATGTAGACAAAGC 20
|||*||*|||||||||||||
Sbjct: 3101 AAAAGTCTACATCTGTTTCG 3082
>miR158-3p Score: 2.5 RO3G_00647
Query: 1 TTTCCAAATGTAGACAAAGC 20
||||||||||:|||||*|
Sbjct: 4144 TAAGGTTTAGATTTGTTT-G 4126
>miR158-3p Score: 2.0 RO3G_01733
Query: 1 TTTCCAAATGTAGACAAAGC 20
|||*||*|||||||||||||
Sbjct: 2903 AAAAGTCTACATCTGTTTCG 2884
>miR158-3p Score: 2.0 RO3G_01944
Query: 1 TTTCCAAATGTAGACAAAGC 20
|||*||*|||||||||||||
Sbjct: 3101 AAAAGTCTACATCTGTTTCG 3082
>miR158-3p Score: 1.0 RO3G_02126
Query: 1 TTTCCAAATGTAGACAAA-GC 20

||||||||:|||||||||*||
Sbjct: 540 AAAGGTTTGCATCTGTTTACG 520
>miR158-3p Score: 2.0 RO3G_17446
Query: 1 TTTCCAAATGTAGACAAAGC 20
|||*||*|||||||||||||
Sbjct: 3101 AAAAGTCTACATCTGTTTCG 3082
>miR158-3p Score: 2.0 RO3G_17287
Query: 1 TTTCCAAATGTAGACAAAGC 20
|||*||*|||||||||||||
Sbjct: 3101 AAAAGTCTACATCTGTTTCG 3082
>miR1146 Score: 2.5 RO3G_05699
Query: 1 TTGGGTCCGACGGGAAGTCTT 21
||||||||:||::*|||||||
Sbjct: 101 AACCCAGGTTGTTGTTCAGAA 81
>miR4414b Score: 0.5 RO3G_11767
Query: 1 TGTGAATATGCGAGAGCCAAG 21
||||||||||||||||||*||
Sbjct: 559 ACACTTATACGCTCTCGG-TC 540
>miR4414b Score: 0.5 RO3G_17150
Query: 1 TGTGAATATGCGAGAGCCAAG 21
||||||||||||||||||*||
Sbjct: 1405 ACACTTATACGCTCTCGG-TC 1386
>miR5219 Score: 0.5 RO3G_15231
Query: 1 TCTGGACTCTCAGCTGCTGCGT 22
|||||||||||||||||||*||
Sbjct: 554 AGACCTGAGAGTCGACGACACA 533
>miR5219 Score: 0.5 RO3G_10585
Query: 1 TCTGGACTCTCAGCTGCTGCGT 22
|||||||||||||||||||*||
Sbjct: 155 AGACCTGAGAGTCGACGACACA 134
>miR5219 Score: 1.0 RO3G_10900
Query: 1 TCTGGACTCTCAGCTGCTGCGT 22
||||||*|||||||||||||||
Sbjct: 110 AGACCTAAGAGTCGACGACGCA 89
>miR8762d Score: 2.5 RO3G_17223
Query: 1 TTGTAAACTTTGATCGATGTCT 22
||||**||||||||||||||*|
Sbjct: 478 AACACCTGAAACTAGCTACAAA 457
>miR8762d Score: 2.5 RO3G_16725
Query: 1 TTGTAAACTTTGATCGATGTCT 22
||||**||||||||||||||*|
Sbjct: 496 AACACCTGAAACTAGCTACAAA 475
>miR8762d Score: 2.5 RO3G_16742
Query: 1 TTGTAAACTTTGATCGATGTCT 22
||||**||||||||||||||*|

Sbjct: 754 AACACCTGAAACTAGCTACAAA 733
>miR8762d Score: 2.5 RO3G_16706
Query: 1 TTGTAAACTTTGATCGATGTCT 22
||||**||||||||||||||*|
Sbjct: 478 AACACCTGAAACTAGCTACAAA 457
>miR8762d Score: 2.5 RO3G_16372
Query: 1 TTGTAAACTTTGATCGATGTCT 22
||||**||||||||||||||*|
Sbjct: 754 AACACCTGAAACTAGCTACAAA 733
>miR8762d Score: 2.5 RO3G_16420
Query: 1 TTGTAAACTTTGATCGATGTCT 22
||||**||||||||||||||*|
Sbjct: 505 AACACCTGAAACTAGCTACAAA 484
>miR8762d Score: 2.5 RO3G_16123
Query: 1 TTGTAAACTTTGATCGATGTCT 22
||||**||||||||||||||*|
Sbjct: 754 AACACCTGAAACTAGCTACAAA 733
>miR8762d Score: 2.5 RO3G_15271
Query: 1 TTGTAAACTTTGATCGATGTCT 22
||||**||||||||||||||*|
Sbjct: 754 AACACCTGAAACTAGCTACAAA 733
>miR8762d Score: 2.5 RO3G_15282
Query: 1 TTGTAAACTTTGATCGATGTCT 22
||||**||||||||||||||*|
Sbjct: 478 AACACCTGAAACTAGCTACAAA 457
>miR8762d Score: 2.5 RO3G_13714
Query: 1 TTGTAAACTTTGATCGATGTCT 22
||||**||||||||||||||*|
Sbjct: 754 AACACCTGAAACTAGCTACAAA 733
>miR8762d Score: 1.5 RO3G_13852
Query: 1 TTGTAAACTTTGATCGATGTCT 22
||||*|||||||||||||||*|
Sbjct: 505 AACACTTGAAACTAGCTACAAA 484
>miR8762d Score: 2.5 RO3G_12663
Query: 1 TTGTAAACTTTGATCGATGTCT 22
||||**||||||||||||||*|
Sbjct: 589 AACACCTGAAACTAGCTACAAA 568
>miR8762d Score: 2.5 RO3G_11966
Query: 1 TTGTAAACTTTGATCGATGTCT 22
|||||*||||||||||*|||*|
Sbjct: 754 AACATCTGAAACTAGCCACAAA 733
>miR8762d Score: 2.5 RO3G_11781
Query: 1 TTGTAAACTTTGATCGATGTCT 22
||||**||||||||||||||*|
Sbjct: 649 AACACCTGAAACTAGCTACAAA 628

>miR8762d Score: 2.5 RO3G_10729
Query: 1 TTGTAAACTTTGATCGATGTCT 22
|||||*||||||||||*|||*|
Sbjct: 754 AACATCTGAAACTAGCCACAAA 733
>miR8762d Score: 1.5 RO3G_10771
Query: 1 TTGTAAACTTTGATCGATGTCT 22
||||*|||||||||||||||*|
Sbjct: 505 AACACTTGAAACTAGCTACAAA 484
>miR8762d Score: 2.5 RO3G_09193
Query: 1 TTGTAAACTTTGATCGATGTCT 22
||||**||||||||||||||*|
Sbjct: 478 AACACCTGAAACTAGCTACAAA 457
>miR8762d Score: 2.5 RO3G_07137
Query: 1 TTGTAAACTTTGATCGATGTCT 22
||||**||||||||||||||*|
Sbjct: 754 AACACCTGAAACTAGCTACAAA 733
>miR8762d Score: 2.5 RO3G_07702
Query: 1 TTGTAAACTTTGATCGATGTCT 22
||||**||||||||||||||*|
Sbjct: 505 AACACCTGAAACTAGCTACAAA 484
>miR8762d Score: 2.5 RO3G_07705
Query: 1 TTGTAAACTTTGATCGATGTCT 22
||||**||||||||||||||*|
Sbjct: 754 AACACCTGAAACTAGCTACAAA 733
>miR8762d Score: 1.5 RO3G_07739
Query: 1 TTGTAAACTTTGATCGATGTCT 22
||||*|||||||||||||||*|
Sbjct: 505 AACACTTGAAACTAGCTACAAA 484
>miR8762d Score: 2.5 RO3G_07867
Query: 1 TTGTAAACTTTGATCGATGTCT 22
||||**||||||||||||||*|
Sbjct: 505 AACACCTGAAACTAGCTACAAA 484
>miR8762d Score: 2.5 RO3G_05640
Query: 1 TTGTAAACTTTGATCGATGTCT 22
||||**||||||||||||||*|
Sbjct: 649 AACACCTGAAACTAGCTACAAA 628
>miR8762d Score: 2.5 RO3G_05921
Query: 1 TTGTAAACTTTGATCGATGTCT 22
|||||*||||||||||*|||*|
Sbjct: 754 AACATCTGAAACTAGCCACAAA 733
>miR8762d Score: 2.5 RO3G_05939
Query: 1 TTGTAAACTTTGATCGATGTCT 22
|||||*||||||||||*|||*|
Sbjct: 754 AACATCTGAAACTAGCCACAAA 733
>miR8762d Score: 2.5 RO3G_05953

Query: 1 TTGTAAACTTTGATCGATGTCT 22
|||||*||||||||||*|||*|
Sbjct: 754 AACATCTGAAACTAGCCACAAA 733
>miR8762d Score: 2.5 RO3G_04327
Query: 1 TTGTAAACTTTGATCGATGTCT 22
|||||*|||*|||||||||*||
Sbjct: 864 AACATCTGACACTAGCTACCGA 843
>miR8762d Score: 2.5 RO3G_04674
Query: 1 TTGTAAACTTTGATCGATGTCT 22
||||**||||||||||||||*|
Sbjct: 505 AACACCTGAAACTAGCTACAAA 484
>miR8762d Score: 2.5 RO3G_04695
Query: 1 TTGTAAACTTTGATCGATGTCT 22
||||**||||||||||||||*|
Sbjct: 544 AACACCTGAAACTAGCTACAAA 523
>miR8762d Score: 2.5 RO3G_05268
Query: 1 TTGTAAACTTTGATCGATGTCT 22
||||**||||||||||||||*|
Sbjct: 754 AACACCTGAAACTAGCTACAAA 733
>miR8762d Score: 2.5 RO3G_02824
Query: 1 TTGTAAACTTTGATCGATGTCT 22
||||**||||||||||||||*|
Sbjct: 754 AACACCTGAAACTAGCTACAAA 733
>miR8762d Score: 2.5 RO3G_01328
Query: 1 TTGTAAACTTTGATCGATGTCT 22
||||**||||||||||||||*|
Sbjct: 754 AACACCTGAAACTAGCTACAAA 733
>miR8762d Score: 2.5 RO3G_01962
Query: 1 TTGTAAACTTTGATCGATGTCT 22
||||**||||||||||||||*|
Sbjct: 505 AACACCTGAAACTAGCTACAAA 484
>miR8762d Score: 2.5 RO3G_17449
Query: 1 TTGTAAACTTTGATCGATGTCT 22
|||||*||||||||||*|||*|
Sbjct: 754 AACATCTGAAACTAGCCACAAA 733
>miR8762d Score: 2.5 RO3G_17416
Query: 1 TTGTAAACTTTGATCGATGTCT 22
||||**||||||||||||||*|
Sbjct: 754 AACACCTGAAACTAGCTACAAA 733
>miR8762d Score: 2.5 RO3G_17399
Query: 1 TTGTAAACTTTGATCGATGTCT 22
||||**||||||||||||||*|
Sbjct: 754 AACACCTGAAACTAGCTACAAA 733
>miR8762d Score: 2.5 RO3G_17387
Query: 1 TTGTAAACTTTGATCGATGTCT 22

||||**|||||||||||||*|
Sbjct: 754 AACACCTGAAACTAGCTACAAA 733
>miR8762d Score: 2.5 RO3G_17355
Query: 1 TTGTAAACTTTGATCGATGTCT 22
||||**|||||||||||||*|
Sbjct: 589 AACACCTGAAACTAGCTACAAA 568
>miR8762d Score: 2.5 RO3G_17291
Query: 1 TTGTAAACTTTGATCGATGTCT 22
||||**|||||||||||||*|
Sbjct: 754 AACACCTGAAACTAGCTACAAA 733
>miR1216 Score: 0.0 RO3G_17261
Query: 1 TGATGGGTTAGCGCTTGTAC 20
||||||||||||||||||||
Sbjct: 183 ACTACCCAATCGCGAACATG 164
>miR1216 Score: 0.0 RO3G_16732
Query: 1 TGATGGGTTAGCGCTTGTAC 20
||||||||||||||||||||
Sbjct: 183 ACTACCCAATCGCGAACATG 164
>miR1216 Score: 0.0 RO3G_16813
Query: 1 TGATGGGTTAGCGCTTGTAC 20
||||||||||||||||||||
Sbjct: 183 ACTACCCAATCGCGAACATG 164
>miR1216 Score: 0.0 RO3G_14788
Query: 1 TGATGGGTTAGCGCTTGTAC 20
||||||||||||||||||||
Sbjct: 116 ACTACCCAATCGCGAACATG 97
>miR1216 Score: 0.0 RO3G_14792
Query: 1 TGATGGGTTAGCGCTTGTAC 20
||||||||||||||||||||
Sbjct: 183 ACTACCCAATCGCGAACATG 164
>miR1216 Score: 0.0 RO3G_14799
Query: 1 TGATGGGTTAGCGCTTGTAC 20
||||||||||||||||||||
Sbjct: 183 ACTACCCAATCGCGAACATG 164
>miR1216 Score: 0.0 RO3G_14801
Query: 1 TGATGGGTTAGCGCTTGTAC 20
||||||||||||||||||||
Sbjct: 116 ACTACCCAATCGCGAACATG 97
>miR1216 Score: 0.0 RO3G_09181
Query: 1 TGATGGGTTAGCGCTTGTAC 20
||||||||||||||||||||
Sbjct: 183 ACTACCCAATCGCGAACATG 164
>miR1216 Score: 0.0 RO3G_09190
Query: 1 TGATGGGTTAGCGCTTGTAC 20
||||||||||||||||||||

Sbjct: 183 ACTACCCAATCGCGAACATG 164
>miR2612 Score: 2.5 RO3G_13972
Query: 1 TGATAGTGGCAGTAGTATCAG 21
|:*||||:|||||||||*|||
Sbjct: 88 ATCATCATCGTCATCATCGTC 68
>miR2612 Score: 2.5 RO3G_13081
Query: 1 TGATAGTGGCAGTAGTATCAG 21
|:*||||:||||||||||*||
Sbjct: 574 ATCATCATCGTCATCATACTC 554
>miR2612 Score: 0.0 RO3G_13221
Query: 1 TGATAGTGGCAGTAGTATCAG 21
|||||||||||||||||||||
Sbjct: 787 ACTATCACCGTCATCATAGTC 767
>miR2612 Score: 1.0 RO3G_13224
Query: 1 TGATAGTGGCAGTAGTATCAG 21
|||||||||||||*|||||||
Sbjct: 787 ACTATCACCGTCACCATAGTC 767
>miR2612 Score: 2.5 RO3G_11742
Query: 1 TGATAGTGGCAGTAGTATCAG 21
|||:||:|||||*||||||*|
Sbjct: 823 ACTGTCGCCGTCTTCATAGAC 803
>miR2612 Score: 2.5 RO3G_10462
Query: 1 TGATAGTGGCAGTAGTATCAG 21
|:|||||:|||||||||*|**
Sbjct: 379 ATTATCATCGTCATCATCGAA 359
>miR2612 Score: 2.2 RO3G_11036
Query: 1 TGATAGTGGCAGTAGTATCAG 21
|||||:|:|||::||||:|||
Sbjct: 1294 ACTATTATCGTTGTCATGGTC 1274
>miR2612 Score: 2.0 RO3G_08172
Query: 1 TGATAGTGGCAGTAGTATCAG 21
||||||::|||:||||||||*
Sbjct: 625 ACTATCGTCGTTATCATAGTA 605
>miR2612 Score: 2.5 RO3G_09062
Query: 1 TGATAGTGGCAGTAGTATCAG 21
||||||::||||:||||**||
Sbjct: 64 ACTATCGTCGTCGTCATCATC 44
>miR2612 Score: 2.5 RO3G_04278
Query: 1 TGATAGTGGCAGTAGTATCAG 21
|:||||::|||||||:|*|||
Sbjct: 1357 ATTATCGTCGTCATCGTCGTC 1337
>miR2612 Score: 2.2 RO3G_02581
Query: 1 TGATAGTGGCAGTAGTATCAG 21
|||*||||||||:|||||*|:
Sbjct: 163 ACTCTCACCGTCGTCATATTT 143

>miR2612 Score: 2.5 RO3G_02911
Query: 1 TGATAGTGGCAGTAGTATCAG 21
||||||::||||||*:|||||
Sbjct: 133 ACTATCGTCGTCATAGTAGTC 113
>miR2612 Score: 2.0 RO3G_03005
Query: 1 TGATAGTGGCAGTAGTATCAG 21
::||||||||||||:||*||:
Sbjct: 127 GTTATCACCGTCATTATTGTT 107
>miR2612 Score: 2.5 RO3G_03352
Query: 1 TGATAGTGGCAGTAGTATCAG 21
|||||||:|*|||||||**||
Sbjct: 739 ACTATCATCATCATCATTATC 719
>miR2612 Score: 2.2 RO3G_03879
Query: 1 TGATAGTGGCAGTAGTATCAG 21
|:|||||:||||||::||||:
Sbjct: 136 ATTATCATCGTCATTGTAGTT 116
>miR2612 Score: 1.0 RO3G_04120
Query: 1 TGATAGTGGCAGTAGTATCAG 21
|||||||||||||*|||||||
Sbjct: 787 ACTATCACCGTCACCATAGTC 767
>miR2612 Score: 1.0 RO3G_04123
Query: 1 TGATAGTGGCAGTAGTATCAG 21
|||||||||||||*|||||||
Sbjct: 787 ACTATCACCGTCACCATAGTC 767
>miR2612 Score: 2.5 RO3G_00855
Query: 1 TGATAGTGGCAGTAGTATCAG 21
|||||:||:|||:||:||*||
Sbjct: 1111 ACTATTACTGTCGTCGTA-TC 1092
>miR2612 Score: 2.5 RO3G_00887
Query: 1 TGATAGTGGCAGTAGTATCAG 21
|:*||||||||||||||**||
Sbjct: 367 ATCATCACCGTCATCAT-CTC 348
>miR2612 Score: 2.5 RO3G_01770
Query: 1 TGATAGTGGCAGTAGTATCAG 21
|:|:|||:||||:||||*|||
Sbjct: 637 ATTGTCATCGTCGTCATTGTC 617
>miR2612 Score: 1.0 RO3G_17093
Query: 1 TGATAGTGGCAGTAGTATCAG 21
|||||||||||||*|||||||
Sbjct: 547 ACTATCACCGTCACCATAGTC 527
>miR1148.2 Score: 1.0 RO3G_17296
Query: 1 TGGAGATCCGCCTGTGCCGGG 21
||||||||||||*||||||||
Sbjct: 140 ACCTCTAGGCGGCCACGGCCC 120
>miR6476a Score: 2.5 RO3G_04527

Query: 1 TCGGTAGGTGATGAACATGA 20
||:||||:||||||*|||*|
Sbjct: 799 AGTCATCTACTACTAGTAGT 780
>miR5021 Score: 2.5 RO3G_14233
Query: 1 TGACGATAGAAGAAGGAAAA 20
:|:|||||||||||||||
Sbjct: 1123 CTAGTTATCTTCTTCCTTTT 1104
>miR5021 Score: 0.0 RO3G_12949
Query: 1 TGACGATAGAAGAAGGAAAA 20
||||||||||||||||||||
Sbjct: 721 ACTGCTATCTTCTTCCTTTT 702
>miR5021 Score: 2.0 RO3G_10250
Query: 1 TGACGATAGAAGAAGGAAAA 20
||||*||*||||||||||||
Sbjct: 256 ACTGATACCTTCTTCCTTTT 237
>miR5021 Score: 2.5 RO3G_09758
Query: 1 TGACGATAGAAGAAGGAA-AA 20
|||*||:||||||||:||*||
Sbjct: 1978 ACTACTGTCTTCTTCTTTGTT 1958
>miR5021 Score: 2.5 RO3G_08015
Query: 1 TGACGATAGAAGAAGGAAAA 20
||||:*|||||||||:||*|
Sbjct: 1231 ACTGTAATCTTCTTCTTTAT 1212
>miR5021 Score: 2.5 RO3G_04304
Query: 1 TGACGATAGAAGAAGGAAAA 20
||||||:*:|||||||||*|
Sbjct: 1141 ACTGCTGGTTTCTTCCTTGT 1122
>miR5021 Score: 0.0 RO3G_04050
Query: 1 TGACGATAGAAGAAGGAAAA 20
||||||||||||||||||||
Sbjct: 1276 ACTGCTATCTTCTTCCTTTT 1257
>miR5021 Score: 0.0 RO3G_00301
Query: 1 TGACGATAGAAGAAGGAAAA 20
||||||||||||||||||||
Sbjct: 2560 ACTGCTATCTTCTTCCTTTT 2541
>miR6025e Score: 2.0 RO3G_17214
Query: 1 TGCCATTATAGATCTGACATGC 22
||||||:|||*|:|||||||||
Sbjct: 622 ACGGTAGTATGTGGACTGTACG 601
>miR6025e Score: 0.0 RO3G_17223
Query: 1 TGCCATTATAGATCTGACATGC 22
||||||||||||||||||||||
Sbjct: 346 ACGGTAATATCTAGACTGTACG 325
>miR6025e Score: 0.5 RO3G_17017
Query: 1 TGCCATTATAGATCTGACATGC 22

|:||||||||||||||||||||
Sbjct: 622 ATGGTAATATCTAGACTGTACG 601
>miR6025e Score: 1.0 RO3G_16725
Query: 1 TGCCATTATAGATCTGACATGC 22
|:||||:|||||||||||||||
Sbjct: 364 ATGGTAGTATCTAGACTGTACG 343
>miR6025e Score: 0.0 RO3G_16742
Query: 1 TGCCATTATAGATCTGACATGC 22
||||||||||||||||||||||
Sbjct: 622 ACGGTAATATCTAGACTGTACG 601
>miR6025e Score: 1.5 RO3G_16689
Query: 1 TGCCATTATAGATCTGACATGC 22
||||||:|||*|||||||||||
Sbjct: 622 ACGGTAGTATATAGACTGTACG 601
>miR6025e Score: 2.5 RO3G_16698
Query: 1 TGCCATTATAGATCTGACATGC 22
|:||||:||||*|||||||||*
Sbjct: 619 ATGGTAGTATCCAGACTGTACA 598
>miR6025e Score: 0.0 RO3G_16706
Query: 1 TGCCATTATAGATCTGACATGC 22
||||||||||||||||||||||
Sbjct: 346 ACGGTAATATCTAGACTGTACG 325
>miR6025e Score: 0.0 RO3G_16372
Query: 1 TGCCATTATAGATCTGACATGC 22
||||||||||||||||||||||
Sbjct: 622 ACGGTAATATCTAGACTGTACG 601
>miR6025e Score: 0.5 RO3G_16420
Query: 1 TGCCATTATAGATCTGACATGC 22
|:||||||||||||||||||||
Sbjct: 373 ATGGTAATATCTAGACTGTACG 352
>miR6025e Score: 0.5 RO3G_16123
Query: 1 TGCCATTATAGATCTGACATGC 22
||||||:|||||||||||||||
Sbjct: 622 ACGGTAGTATCTAGACTGTACG 601
>miR6025e Score: 2.0 RO3G_15866
Query: 1 TGCCATTATAGATCTGACATGC 22
||||||:|||*|:|||||||||
Sbjct: 346 ACGGTAGTATGTGGACTGTACG 325
>miR6025e Score: 1.0 RO3G_15271
Query: 1 TGCCATTATAGATCTGACATGC 22
|:||||:|||||||||||||||
Sbjct: 622 ATGGTAGTATCTAGACTGTACG 601
>miR6025e Score: 0.5 RO3G_15282
Query: 1 TGCCATTATAGATCTGACATGC 22
|:||||||||||||||||||||

Sbjct: 346 ATGGTAATATCTAGACTGTACG 325
>miR6025e Score: 0.5 RO3G_15261
Query: 1 TGCCATTATAGATCTGACATGC 22
||||||:|||||||||||||||
Sbjct: 622 ACGGTAGTATCTAGACTGTACG 601
>miR6025e Score: 1.5 RO3G_14361
Query: 1 TGCCATTATAGATCTGACATGC 22
||||||:|||*|||||||||||
Sbjct: 622 ACGGTAGTATATAGACTGTACG 601
>miR6025e Score: 0.5 RO3G_14555
Query: 1 TGCCATTATAGATCTGACATGC 22
|:||||||||||||||||||||
Sbjct: 250 ATGGTAATATCTAGACTGTACG 229
>miR6025e Score: 2.0 RO3G_14568
Query: 1 TGCCATTATAGATCTGACATGC 22
||||||:|||*|:|||||||||
Sbjct: 622 ACGGTAGTATATGGACTGTACG 601
>miR6025e Score: 0.5 RO3G_14770
Query: 1 TGCCATTATAGATCTGACATGC 22
|:||||||||||||||||||||
Sbjct: 622 ATGGTAATATCTAGACTGTACG 601
>miR6025e Score: 0.5 RO3G_14786
Query: 1 TGCCATTATAGATCTGACATGC 22
|:||||||||||||||||||||
Sbjct: 250 ATGGTAATATCTAGACTGTACG 229
>miR6025e Score: 0.0 RO3G_13714
Query: 1 TGCCATTATAGATCTGACATGC 22
||||||||||||||||||||||
Sbjct: 622 ACGGTAATATCTAGACTGTACG 601
>miR6025e Score: 1.5 RO3G_13852
Query: 1 TGCCATTATAGATCTGACATGC 22
|:||||:||||||||||||||*
Sbjct: 373 ATGGTAGTATCTAGACTGTACA 352
>miR6025e Score: 2.0 RO3G_12663
Query: 1 TGCCATTATAGATCTGACATGC 22
||||||:|||:*||||||||||
Sbjct: 457 ACGGTAGTATTCAGACTGTACG 436
>miR6025e Score: 1.5 RO3G_11962
Query: 1 TGCCATTATAGATCTGACATGC 22
|:||||||*|||||||||||||
Sbjct: 346 ATGGTAATCTCTAGACTGTACG 325
>miR6025e Score: 2.0 RO3G_11966
Query: 1 TGCCATTATAGATCTGACATGC 22
||||||:|||*||||||||*||
Sbjct: 622 ACGGTAGTATATAGACTGTCCG 601

>miR6025e Score: 0.5 RO3G_11235
Query: 1 TGCCATTATAGATCTGACATGC 22
|:||||||||||||||||||||
Sbjct: 517 ATGGTAATATCTAGACTGTACG 496
>miR6025e Score: 1.5 RO3G_11264
Query: 1 TGCCATTATAGATCTGACATGC 22
||||||:|||*|||||||||||
Sbjct: 622 ACGGTAGTATATAGACTGTACG 601
>miR6025e Score: 0.5 RO3G_11781
Query: 1 TGCCATTATAGATCTGACATGC 22
|:||||||||||||||||||||
Sbjct: 517 ATGGTAATATCTAGACTGTACG 496
>miR6025e Score: 2.0 RO3G_10729
Query: 1 TGCCATTATAGATCTGACATGC 22
||||||:|||*|:|||||||||
Sbjct: 622 ACGGTAGTATATGGACTGTACG 601
>miR6025e Score: 1.5 RO3G_10771
Query: 1 TGCCATTATAGATCTGACATGC 22
|:||||:||||||||||||||*
Sbjct: 373 ATGGTAGTATCTAGACTGTACA 352
>miR6025e Score: 2.0 RO3G_10773
Query: 1 TGCCATTATAGATCTGACATGC 22
||||||:|||*|:|||||||||
Sbjct: 622 ACGGTAGTATGTGGACTGTACG 601
>miR6025e Score: 1.0 RO3G_10779
Query: 1 TGCCATTATAGATCTGACATGC 22
|:||||||||:|||||||||||
Sbjct: 238 ATGGTAATATTTAGACTGTACG 217
>miR6025e Score: 2.0 RO3G_10780
Query: 1 TGCCATTATAGATCTGACATGC 22
||||||:|||*|:|||||||||
Sbjct: 622 ACGGTAGTATGTGGACTGTACG 601
>miR6025e Score: 0.5 RO3G_10795
Query: 1 TGCCATTATAGATCTGACATGC 22
|:||||||||||||||||||||
Sbjct: 622 ATGGTAATATCTAGACTGTACG 601
>miR6025e Score: 2.0 RO3G_10798
Query: 1 TGCCATTATAGATCTGACATGC 22
||||||:|||*|:|||||||||
Sbjct: 622 ACGGTAGTATATGGACTGTACG 601
>miR6025e Score: 0.0 RO3G_09193
Query: 1 TGCCATTATAGATCTGACATGC 22
||||||||||||||||||||||
Sbjct: 346 ACGGTAATATCTAGACTGTACG 325
>miR6025e Score: 2.0 RO3G_09194

```
Query: 1 TGCCATTATAGATCTGACATGC 22
||||||:|||*||||||||*||
Sbjct: 622 ACGGTAGTATATAGACTGTCCG 601
>miR6025e Score: 2.0 RO3G_09542
Query: 1 TGCCATTATAGATCTGACATGC 22
||||||:|||*|:|||||||||
Sbjct: 622 ACGGTAGTATATGGACTGTACG 601
>miR6025e Score: 1.5 RO3G_09945
Query: 1 TGCCATTATAGATCTGACATGC 22
||||||:|||*|||||||||||
Sbjct: 346 ACGGTAGTATATAGACTGTACG 325
>miR6025e Score: 2.0 RO3G_09967
Query: 1 TGCCATTATAGATCTGACATGC 22
||||||:|||*||||||||*||
Sbjct: 622 ACGGTAGTATATAGACTGTCCG 601
>miR6025e Score: 2.5 RO3G_07984
Query: 1 TGCCATTATAGATCTGACATGC 22
|:||||:||||*|||||||||*
Sbjct: 622 ATGGTAGTATCCAGACTGTACA 601
>miR6025e Score: 0.5 RO3G_06735
Query: 1 TGCCATTATAGATCTGACATGC 22
|:||||||||||||||||||||
Sbjct: 346 ATGGTAATATCTAGACTGTACG 325
>miR6025e Score: 1.5 RO3G_07124
Query: 1 TGCCATTATAGATCTGACATGC 22
||||||:|||*|||||||||||
Sbjct: 622 ACGGTAGTATGTAGACTGTACG 601
>miR6025e Score: 0.5 RO3G_07137
Query: 1 TGCCATTATAGATCTGACATGC 22
||||||:|||||||||||||||
Sbjct: 622 ACGGTAGTATCTAGACTGTACG 601
>miR6025e Score: 0.5 RO3G_07702
Query: 1 TGCCATTATAGATCTGACATGC 22
|:||||||||||||||||||||
Sbjct: 373 ATGGTAATATCTAGACTGTACG 352
>miR6025e Score: 0.5 RO3G_07705
Query: 1 TGCCATTATAGATCTGACATGC 22
|:||||||||||||||||||||
Sbjct: 622 ATGGTAATATCTAGACTGTACG 601
>miR6025e Score: 1.0 RO3G_07739
Query: 1 TGCCATTATAGATCTGACATGC 22
|:||||:|||||||||||||||
Sbjct: 373 ATGGTAGTATCTAGACTGTACG 352
>miR6025e Score: 2.0 RO3G_07860
Query: 1 TGCCATTATAGATCTGACATGC 22
```

||||||:|||*||||||||*||
Sbjct: 622 ACGGTAGTATATAGACTGTCCG 601
>miR6025e Score: 0.5 RO3G_07867
Query: 1 TGCCATTATAGATCTGACATGC 22
||||||:|||||||||||||||
Sbjct: 373 ACGGTAGTATCTAGACTGTACG 352
>miR6025e Score: 2.5 RO3G_07876
Query: 1 TGCCATTATAGATCTGACATGC 22
|:||||:|||*|:|||||||||
Sbjct: 496 ATGGTAGTATATGGACTGTACG 475
>miR6025e Score: 1.0 RO3G_05632
Query: 1 TGCCATTATAGATCTGACATGC 22
|*||||||||||||||||||||
Sbjct: 307 AGGGTAATATCTAGACTGTACG 286
>miR6025e Score: 0.5 RO3G_05640
Query: 1 TGCCATTATAGATCTGACATGC 22
||||||:|||||||||||||||
Sbjct: 517 ACGGTAGTATCTAGACTGTACG 496
>miR6025e Score: 1.5 RO3G_05921
Query: 1 TGCCATTATAGATCTGACATGC 22
||||||:|||*|||||||||||
Sbjct: 622 ACGGTAGTATATAGACTGTACG 601
>miR6025e Score: 1.5 RO3G_05939
Query: 1 TGCCATTATAGATCTGACATGC 22
||||||:|||*|||||||||||
Sbjct: 622 ACGGTAGTATGTAGACTGTACG 601
>miR6025e Score: 2.5 RO3G_05953
Query: 1 TGCCATTATAGATCTGACATGC 22
|:||||:|||*||||||||*||
Sbjct: 622 ATGGTAGTATATAGACTGTCCG 601
>miR6025e Score: 2.0 RO3G_05959
Query: 1 TGCCATTATAGATCTGACATGC 22
||||||:|||*||||||||*||
Sbjct: 622 ACGGTAGTATATAGACTGTCCG 601
>miR6025e Score: 0.5 RO3G_06417
Query: 1 TGCCATTATAGATCTGACATGC 22
|:||||||||||||||||||||
Sbjct: 250 ATGGTAATATCTAGACTGTACG 229
>miR6025e Score: 1.0 RO3G_06433
Query: 1 TGCCATTATAGATCTGACATGC 22
|:||||||||:|||||||||||
Sbjct: 346 ATGGTAATATTTAGACTGTACG 325
>miR6025e Score: 0.5 RO3G_06717
Query: 1 TGCCATTATAGATCTGACATGC 22
||||||:|||||||||||||||

```
Sbjct: 622 ACGGTAGTATCTAGACTGTACG 601
>miR6025e Score: 0.5 RO3G_04674
Query: 1 TGCCATTATAGATCTGACATGC 22
||||||:|||||||||||||||
Sbjct: 373 ACGGTAGTATCTAGACTGTACG 352
>miR6025e Score: 0.0 RO3G_04682
Query: 1 TGCCATTATAGATCTGACATGC 22
||||||||||||||||||||||
Sbjct: 622 ACGGTAATATCTAGACTGTACG 601
>miR6025e Score: 0.5 RO3G_04695
Query: 1 TGCCATTATAGATCTGACATGC 22
||||||:|||||||||||||||
Sbjct: 412 ACGGTAGTATCTAGACTGTACG 391
>miR6025e Score: 2.0 RO3G_05267
Query: 1 TGCCATTATAGATCTGACATGC 22
||||||:|||*||||||||*||
Sbjct: 622 ACGGTAGTATATAGACTGTCCG 601
>miR6025e Score: 0.5 RO3G_05268
Query: 1 TGCCATTATAGATCTGACATGC 22
||||||:|||||||||||||||
Sbjct: 622 ACGGTAGTATCTAGACTGTACG 601
>miR6025e Score: 0.0 RO3G_02824
Query: 1 TGCCATTATAGATCTGACATGC 22
||||||||||||||||||||||
Sbjct: 622 ACGGTAATATCTAGACTGTACG 601
>miR6025e Score: 1.5 RO3G_03591
Query: 1 TGCCATTATAGATCTGACATGC 22
|:||||||||:|||:|||||||
Sbjct: 238 ATGGTAATATTTAGGCTGTACG 217
>miR6025e Score: 2.0 RO3G_04117
Query: 1 TGCCATTATAGATCTGACATGC 22
|:||||:||||*||||||||||
Sbjct: 517 ATGGTAGTATCCAGACTGTACG 496
>miR6025e Score: 1.5 RO3G_04207
Query: 1 TGCCATTATAGATCTGACATGC 22
||||||:|||*|||||||||||
Sbjct: 622 ACGGTAGTATATAGACTGTACG 601
>miR6025e Score: 2.0 RO3G_04210
Query: 1 TGCCATTATAGATCTGACATGC 22
||||||:|||*|:|||||||||
Sbjct: 622 ACGGTAGTATGTGGACTGTACG 601
>miR6025e Score: 2.5 RO3G_00238
Query: 1 TGCCATTATAGATCTGACATGC 22
|:||||:||||*|||||||||*
Sbjct: 622 ATGGTAGTATCCAGACTGTACA 601
```

```
>miR6025e Score: 2.0 RO3G_00256
Query: 1 TGCCATTATAGATCTGACATGC 22
||||||:|||*||||||||*||
Sbjct: 622 ACGGTAGTATATAGACTGTCCG 601
>miR6025e Score: 2.5 RO3G_01205
Query: 1 TGCCATTATAGATCTGACATGC 22
|:||||:||||*|||||||||*
Sbjct: 622 ATGGTAGTATCCAGACTGTACA 601
>miR6025e Score: 0.5 RO3G_01328
Query: 1 TGCCATTATAGATCTGACATGC 22
||||||:|||||||||||||||
Sbjct: 622 ACGGTAGTATCTAGACTGTACG 601
>miR6025e Score: 1.5 RO3G_01942
Query: 1 TGCCATTATAGATCTGACATGC 22
||||||:|||*|||||||||||
Sbjct: 619 ACGGTAGTATATAGACTGTACG 598
>miR6025e Score: 0.0 RO3G_01962
Query: 1 TGCCATTATAGATCTGACATGC 22
||||||||||||||||||||||
Sbjct: 373 ACGGTAATATCTAGACTGTACG 352
>miR6025e Score: 1.5 RO3G_17449
Query: 1 TGCCATTATAGATCTGACATGC 22
||||||:|||*|||||||||||
Sbjct: 622 ACGGTAGTATATAGACTGTACG 601
>miR6025e Score: 0.0 RO3G_17416
Query: 1 TGCCATTATAGATCTGACATGC 22
||||||||||||||||||||||
Sbjct: 622 ACGGTAATATCTAGACTGTACG 601
>miR6025e Score: 1.5 RO3G_17399
Query: 1 TGCCATTATAGATCTGACATGC 22
||*|||:|||||||||||||||
Sbjct: 622 ACAGTAGTATCTAGACTGTACG 601
>miR6025e Score: 0.5 RO3G_17387
Query: 1 TGCCATTATAGATCTGACATGC 22
||||||:|||||||||||||||
Sbjct: 622 ACGGTAGTATCTAGACTGTACG 601
>miR6025e Score: 0.0 RO3G_17355
Query: 1 TGCCATTATAGATCTGACATGC 22
||||||||||||||||||||||
Sbjct: 457 ACGGTAATATCTAGACTGTACG 436
>miR6025e Score: 0.0 RO3G_17291
Query: 1 TGCCATTATAGATCTGACATGC 22
||||||||||||||||||||||
Sbjct: 622 ACGGTAATATCTAGACTGTACG 601
>miR6025e Score: 1.0 RO3G_17277
```

Query: 1 TGCCATTATAGATCTGACATGC 22
|:||||||||:|||||||||||
Sbjct: 106 ATGGTAATATTTAGACTGTACG 85
>miR9553-3p Score: 2.5 RO3G_16362
Query: 1 TAATCATCCCAGCTATGAACAT 22
||*||||||||:|||||*||*|
Sbjct: 3050 ATAAGTAGGGTTGATACCTGGA 3029
>miR9553-3p Score: 0.0 RO3G_11541
Query: 1 TAATCATCCCAGCTATGAACAT 22
||||||||||||||||||||||
Sbjct: 1076 ATTAGTAGGGTCGATACTTGTA 1055
>miR9553-3p Score: 0.0 RO3G_09175
Query: 1 TAATCATCCCAGCTATGAACAT 22
||||||||||||||||||||||
Sbjct: 3686 ATTAGTAGGGTCGATACTTGTA 3665
>miR9553-3p Score: 0.0 RO3G_02695
Query: 1 TAATCATCCCAGCTATGAACAT 22
||||||||||||||||||||||
Sbjct: 3686 ATTAGTAGGGTCGATACTTGTA 3665
>miR9553-3p Score: 0.0 RO3G_03550
Query: 1 TAATCATCCCAGCTATGAACAT 22
||||||||||||||||||||||
Sbjct: 3686 ATTAGTAGGGTCGATACTTGTA 3665
>miR2868 Score: 2.5 RO3G_16417
Query: 1 TTGATTTTGTTGTAGTAGCA 20
||||||:|||||:|*|||*|
Sbjct: 747 AACTAAGACAACGTAATC-T 729
>miR2868 Score: 2.5 RO3G_15310
Query: 1 TTGATTTTGTTGTAGTAGCA 20
|||||:||*||:||||||*|
Sbjct: 670 AACTAGAAAAATATCATC-T 652
>miR2868 Score: 2.5 RO3G_14147
Query: 1 TTGATTTTGTTGTAGTAGCA 20
||||||||:||:|*|||*||
Sbjct: 1154 AACTAAAATAATAACAT-GT 1136
>miR2868 Score: 0.0 RO3G_14217
Query: 1 TTGATTTTGTTGTAGTAGCA 20
||||||||||||||||||||
Sbjct: 618 AACTAAAACAACATCATCGT 599
>miR2868 Score: 2.5 RO3G_13385
Query: 1 TTGATTTTGTTGTAGTAGCA 20
|||||||||||||*||*|*|
Sbjct: 111 AACTAAAACAACAACAAC-T 93
>miR2868 Score: 0.0 RO3G_13590
Query: 1 TTGATTTTGTTGTAGTAGCA 20

||||||||||||||||||||
Sbjct: 618 AACTAAAACAACATCATCGT 599
>miR2868 Score: 2.5 RO3G_12098
Query: 1 TTGATTTTGTTGTAGTAGCA 20
*||||:||||||||:|||**
Sbjct: 393 TACTAGAACAACATTATCAA 374
>miR2868 Score: 2.5 RO3G_11324
Query: 1 TTGATTTTGTTGTAGTAGCA 20
||||||||||||::||||
Sbjct: 151 CACCAAAACAACATTGTCGT 132
>miR2868 Score: 0.2 RO3G_11515
Query: 1 TTGATTTTGTTGTAGTAGCA 20
:|||||||||||||||||||
Sbjct: 351 GACTAAAACAACATCATCGT 332
>miR2868 Score: 2.5 RO3G_11675
Query: 1 TTGATTTTGTTGTAGTAGCA 20
|||*||:||:|||||:||||
Sbjct: 598 AACGAAGACGACATCGTCGT 579
>miR2868 Score: 2.2 RO3G_10461
Query: 1 TTGATTTTGTTGTAGTAGCA 20
|**||||||||||||||:||
Sbjct: 79 ATATAAAACAACATCATTGT 60
>miR2868 Score: 2.0 RO3G_10856
Query: 1 TTGATTTTGTTGTAGTAGCA 20
||*||||||:||||:|||||
Sbjct: 660 AAGTAAAACGACATTATCGT 641
>miR2868 Score: 2.0 RO3G_09230
Query: 1 TTGATTTTGTTGTAGTAGCA 20
||||||||||||||||*|
Sbjct: 214 CACGAAAACAACATCATCAT 195
>miR2868 Score: 2.5 RO3G_08621
Query: 1 TTGATTTTGTTGTAGTAGCA 20
|||*|*||||||||||||*|
Sbjct: 760 AACGACAACAACATCATCTT 741
>miR2868 Score: 2.5 RO3G_08992
Query: 1 TTGATTTTGTTGTAGTAGCA 20
|||*||||||||||:|*|||
Sbjct: 686 AACAAAAACAACATTA-CGT 668
>miR2868 Score: 2.0 RO3G_07279
Query: 1 TTGATTTTGTTGTAGTAGCA 20
|||*||:||||:||||||||
Sbjct: 3169 AACAAAGACAATATCATCGT 3150
>miR2868 Score: 2.5 RO3G_07846
Query: 1 TTGATTTTGTTGTAGTAGCA 20
||||||||||||||:|**|*

Sbjct: 857 AACTAAAACAACATTA-GGA 839
>miR2868 Score: 2.2 RO3G_06097
Query: 1 TTGATTTTGTTGTAGTAGCA 20
::||||:|*|||||||||||
Sbjct: 388 GGCTAAGAGAACATCATCGT 369
>miR2868 Score: 2.5 RO3G_06532
Query: 1 TTGATTTTGTTGTAGTAGCA 20
||||||||:||||||||*
Sbjct: 669 TACTAAAACGACATCAACGA 650
>miR2868 Score: 2.2 RO3G_04479
Query: 1 TTGATTTTGTTGTAGTAGCA 20
||||||:||*||||:||:||
Sbjct: 549 AACTAAGACCACATTATTGT 530
>miR2868 Score: 2.5 RO3G_02514
Query: 1 TTGATTTTGTTGTAGTAGCA 20
||||||||*|||:||||*|*
Sbjct: 561 AACTAAAAGAACGTCATAGA 542
>miR2868 Score: 2.5 RO3G_03026
Query: 1 TTGATTTTGTTGTAGTAGCA 20
||||||||||||||**||*|
Sbjct: 37 AACTAAAACAACATATTC-T 19
>miR2868 Score: 2.0 RO3G_03130
Query: 1 TTGATTTTGTTGTAGTAGCA 20
|||||||::||||||*||||
Sbjct: 928 AACTAAAGTAACATCTTCGT 909
>miR2868 Score: 2.5 RO3G_03352
Query: 1 TTGATTTTGTTGTAGTAGCA 20
|||*|*|||||||||||*|
Sbjct: 667 AACAACAACAACATCATCTT 648
>miR2868 Score: 0.5 RO3G_03617
Query: 1 TTGATTTTGTTGTAGTAGCA 20
|||||||||||||||:||||
Sbjct: 1587 AACTAAAACAACATCGTCGT 1568
>miR2868 Score: 2.2 RO3G_03720
Query: 1 TTGATTTTGTTGTAGTAGCA 20
|||*|*|||||||||||:||
Sbjct: 352 AACGACAACAACATCATTGT 333
>miR2868 Score: 2.5 RO3G_03818
Query: 1 TTGATTTTGTTGTAGTAGCA 20
|||*|*|||||||||:||||
Sbjct: 1756 AACGACAACAACATCGTCGT 1737
>miR2868 Score: 2.5 RO3G_03950
Query: 1 TTGATTTTGTTGTAGTAGCA 20
|||*|*||||||||||||*|
Sbjct: 373 AACGACAACAACATCATCAT 354

>miR2868 Score: 0.5 RO3G_04048
Query: 1 TTGATTTTGTTGTAGTAGCA 20
|||||||||||||||:||||
Sbjct: 1527 AACTAAAACAACATCGTCGT 1508
>miR2868 Score: 2.0 RO3G_04201
Query: 1 TTGATTTTGTTGTAGTAGCA 20
::||||||||||||:||:*|
Sbjct: 792 GGCTAAAACAACATTATT-T 774
>miR2868 Score: 2.5 RO3G_04209
Query: 1 TTGATTTTGTTGTAGTAGCA 20
||||||||*||:||:||*||
Sbjct: 85 AACTAAAAAAATATTATAGT 66
>miR2868 Score: 2.2 RO3G_00028
Query: 1 TTGATTTTGTTGTAGTAG-CA 20
|*|||||||||||||:|:*||
Sbjct: 791 ACCTAAAACAACATCGTTCGT 771
>miR2868 Score: 2.5 RO3G_00451
Query: 1 TTGATTTTGTTGTAGTAGCA 20
|||||:||:||||*|||||*
Sbjct: 298 AACTAGAATAACAACATCGA 279
>miR2868 Score: 2.0 RO3G_00460
Query: 1 TTGATTTTGTTGTAGTAGCA 20
||||:||||||*||||||*|
Sbjct: 310 AACTGAAACAAAATCATCCT 291
>miR2868 Score: 2.5 RO3G_00896
Query: 1 TTGATTTTGTTGTAGTAGCA 20
||||||||||||:|*||*|*
Sbjct: 378 AACTAAAACAACGTAATAGA 359
>miR2868 Score: 2.0 RO3G_01611
Query: 1 TTGATTTTGTTGTAGTAGCA 20
::||||||||||||:||:*|
Sbjct: 2832 GGCTAAAACAACATTATT-T 2814
>miR2868 Score: 2.5 RO3G_01651
Query: 1 TTGATTTTGTTGTAGTAGCA 20
||||||||||||||:*||**
Sbjct: 258 AACTAAAACAACATTCTCAA 239
>miR2868 Score: 1.8 RO3G_01909
Query: 1 TTGATTTTGTTGTAGTAGCA 20
:||||||||:|||||:|||*
Sbjct: 814 GACTAAAACGACATCGTCGA 795
>miR2868 Score: 2.5 RO3G_01918
Query: 1 TTGATTTTGTTGTAGTAGCA 20
|||||||||||||||*|*
Sbjct: 961 TACTAAAACAACACCAT-GA 943
>miR2868 Score: 2.5 RO3G_02044

```
Query: 1 TTGATTTTGTTGTAGTAGCA 20
||||||||||||*||*||*|
Sbjct: 502 AACTAAAACAACTTCTTC-T 484
>miR5639-5p Score: 0.0 RO3G_16544
Query: 1 TTGTTCCACGTGTCTAAGGC 20
||||||||||||||||||||
Sbjct: 582 AACAAGGTGCACAGATTCCG 563
>miR5639-5p Score: 2.2 RO3G_15468
Query: 1 TTGTTCCACGTGTCTAAGGC 20
:|||||||*|||:|:|||||
Sbjct: 1574 GACAAGGTACACGGGTTCCG 1555
>miR5639-5p Score: 2.5 RO3G_07160
Query: 1 TTGTTCCACGTGTCTAAGGC 20
|*|:|||||:|||||||*||
Sbjct: 674 ATCGAGGTGTACAGATT-CG 656
>miR2651 Score: 0.0 RO3G_16804
Query: 1 TGTTGATTGATATGCTGATT 20
||||||||||||||||||||
Sbjct: 178 ACAACTAACTATACGACTAA 159
>miR2651 Score: 2.5 RO3G_16681
Query: 1 TGTTGATTGATATGCTGATT 20
:::|||:|||||:||||||:
Sbjct: 161 GTGACTGACTATGCGACTAG 142
>miR2651 Score: 0.5 RO3G_15002
Query: 1 TGTTGATTGATATGCTGATT 20
||||||||||:|||||||||
Sbjct: 178 ACAACTAACTGTACGACTAA 159
>miR2651 Score: 0.5 RO3G_15131
Query: 1 TGTTGATTGATATGCTGATT 20
||||||||||:|||||||||
Sbjct: 178 ACAACTAACTGTACGACTAA 159
>miR2651 Score: 2.5 RO3G_15178
Query: 1 TGTTGATTGATATGCTGATT 20
:|||||||||||:|*|||:*
Sbjct: 1748 GCAACTAACTATGCAACTGC 1729
>miR2651 Score: 2.5 RO3G_14116
Query: 1 TGTTGATTGATATGCTGATT 20
|:|*||||||||:|||||::
Sbjct: 171 ATATCTAACTATGCGACTGG 152
>miR2651 Score: 2.5 RO3G_13370
Query: 1 TGTTGATTGATATGCTGATT 20
*:|:||||*|||||||||||
Sbjct: 348 CTAGCTAAGTATACGACTAA 329
>miR2651 Score: 0.5 RO3G_13439
Query: 1 TGTTGATTGATATGCTGATT 20
```

||||||||||:|||||||||
Sbjct: 178 ACAACTAACTGTACGACTAA 159
>miR2651 Score: 2.5 RO3G_13655
Query: 1 TGTTGATTGATATGCTGATT 20
|||||*|||||*||||||*|
Sbjct: 905 ACAACAAACTAAACGACTTA 886
>miR2651 Score: 2.5 RO3G_12971
Query: 1 TGTTGATTGATATGCTGATT 20
|||||||||*||||*||*||
Sbjct: 1130 ACAACTAACAATACAACCAA 1111
>miR2651 Score: 2.0 RO3G_13112
Query: 1 TGTTGATTGATATGCTGATT 20
:|:|||||||||:|||||:*
Sbjct: 1637 GCGACTAACTATGCGACTGC 1618
>miR2651 Score: 0.0 RO3G_13293
Query: 1 TGTTGATTGATATGCTGATT 20
||||||||||||||||||||
Sbjct: 178 ACAACTAACTATACGACTAA 159
>miR2651 Score: 0.5 RO3G_12007
Query: 1 TGTTGATTGATATGCTGATT 20
||||||||||:|||||||||
Sbjct: 178 ACAACTAACTGTACGACTAA 159
>miR2651 Score: 0.5 RO3G_12536
Query: 1 TGTTGATTGATATGCTGATT 20
||||||||||:|||||||||
Sbjct: 178 ACAACTAACTGTACGACTAA 159
>miR2651 Score: 2.5 RO3G_12637
Query: 1 TGTTGATTGATATGCTGATT 20
|||||||||*||:||::|||
Sbjct: 518 ACAACTAACAATGCGGTTAA 499
>miR2651 Score: 1.5 RO3G_11598
Query: 1 TGTTGATTGATATGCTGATT 20
||||||||||*||||||||*
Sbjct: 1091 ACAACTAACTTTACGACTAC 1072
>miR2651 Score: 0.5 RO3G_10715
Query: 1 TGTTGATTGATATGCTGATT 20
||||||||||:|||||||||
Sbjct: 178 ACAACTAACTGTACGACTAA 159
>miR2651 Score: 2.5 RO3G_09489
Query: 1 TGTTGATTGATATGCTGATT 20
|||||||||*||||*||*||
Sbjct: 1088 ACAACTAACAATACAACCAA 1069
>miR2651 Score: 0.5 RO3G_09718
Query: 1 TGTTGATTGATATGCTGATT 20
||||||||||:|||||||||

Sbjct: 178 ACAACTAACTGTACGACTAA 159
>miR2651 Score: 0.5 RO3G_10177
Query: 1 TGTTGATTGATATGCTGATT 20
||||||||||:|||||||||
Sbjct: 178 ACAACTAACTGTACGACTAA 159
>miR2651 Score: 2.5 RO3G_10225
Query: 1 TGTTGATTGATATGCTGATT 20
|||||||||*||||*||*||
Sbjct: 1088 ACAACTAACAATACAACCAA 1069
>miR2651 Score: 0.5 RO3G_09044
Query: 1 TGTTGATTGATATGCTGATT 20
||||||||||:|||||||||
Sbjct: 40 ACAACTAACTGTACGACTAA 21
>miR2651 Score: 2.0 RO3G_06877
Query: 1 TGTTGATTGATATGCTGATT 20
||||||||||||*||||*|*
Sbjct: 1160 ACAACTAACTATTCGAC-AC 1142
>miR2651 Score: 2.5 RO3G_07853
Query: 1 TGTTGATTGATATGCTGATT 20
|:||||||*|||||*|||||
Sbjct: 978 ATAACTAAATATACTACTAA 959
>miR2651 Score: 0.5 RO3G_04309
Query: 1 TGTTGATTGATATGCTGATT 20
||||||||||:|||||||||
Sbjct: 178 ACAACTAACTGTACGACTAA 159
>miR2651 Score: 2.5 RO3G_02696
Query: 1 TGTTGATTGATATGCTGATT 20
|||||||||*||||*||*||
Sbjct: 572 ACAACTAACAATACAACCAA 553
>miR2651 Score: 2.5 RO3G_03799
Query: 1 TGTTGATTGATATGCTGATT 20
|||||||||*||||*||*||
Sbjct: 1088 ACAACTAACAATACAACCAA 1069
>miR2651 Score: 2.5 RO3G_03855
Query: 1 TGTTGATTGATATGCTGATT 20
||||||||*||*|||||*||
Sbjct: 311 ACAACTAAATAGACGAC-AA 293
>miR2651 Score: 0.5 RO3G_03867
Query: 1 TGTTGATTGATATGCTGATT 20
||||||||||:|||||||||
Sbjct: 178 ACAACTAACTGTACGACTAA 159
>miR2651 Score: 0.5 RO3G_01004
Query: 1 TGTTGATTGATATGCTGATT 20
||||||||||:|||||||||
Sbjct: 178 ACAACTAACTGTACGACTAA 159

>miR2651 Score: 0.5 RO3G_01475
Query: 1 TGTTGATTGATATGCTGATT 20
||||||||||:|||||||||
Sbjct: 178 ACAACTAACTGTACGACTAA 159
>miR2651 Score: 2.5 RO3G_17129
Query: 1 TGTTGATTGATATGCTGATT 20
|||||||:||:||*|||*||
Sbjct: 2093 ACAACTAGCTGTAAGACGAA 2074
>miR8011a-5p Score: 0.0 RO3G_13920
Query: 1 TTGGGTTGAGCTTTCTTTTTTTC 23
|||||||||||||||||||||||
Sbjct: 5261 AACCCAACTCGAAAGAAAAAAAG 5239
>miR8011a-5p Score: 0.0 RO3G_09965
Query: 1 TTGGGTTGAGCTTTCTTTTTTTC 23
|||||||||||||||||||||||
Sbjct: 5261 AACCCAACTCGAAAGAAAAAAAG 5239
>miR8011a-5p Score: 1.5 RO3G_08253
Query: 1 TTGGGTTGAGCTTTCTTTTTTTC 23
|||:|||||||||||||||||
Sbjct: 367 CACCTAACTCGAAAGAAAAAAAT 345
>miR8011a-5p Score: 0.0 RO3G_05619
Query: 1 TTGGGTTGAGCTTTCTTTTTTTC 23
|||||||||||||||||||||||
Sbjct: 3404 AACCCAACTCGAAAGAAAAAAAG 3382
>miR8011a-5p Score: 0.0 RO3G_06427
Query: 1 TTGGGTTGAGCTTTCTTTTTTTC 23
|||||||||||||||||||||||
Sbjct: 4853 AACCCAACTCGAAAGAAAAAAAG 4831
>miR8011a-5p Score: 0.0 RO3G_06428
Query: 1 TTGGGTTGAGCTTTCTTTTTTTC 23
|||||||||||||||||||||||
Sbjct: 4355 AACCCAACTCGAAAGAAAAAAAG 4333
>miR8011a-5p Score: 0.0 RO3G_06442
Query: 1 TTGGGTTGAGCTTTCTTTTTTTC 23
|||||||||||||||||||||||
Sbjct: 5918 AACCCAACTCGAAAGAAAAAAAG 5896
>miR8011a-5p Score: 0.0 RO3G_02691
Query: 1 TTGGGTTGAGCTTTCTTTTTTTC 23
|||||||||||||||||||||||
Sbjct: 8750 AACCCAACTCGAAAGAAAAAAAG 8728
>miR8011a-5p Score: 0.0 RO3G_02959
Query: 1 TTGGGTTGAGCTTTCTTTTTTTC 23
|||||||||||||||||||||||
Sbjct: 5101 AACCCAACTCGAAAGAAAAAAAG 5079
>miR8011a-5p Score: 0.0 RO3G_03547

Query: 1 TTGGGTTGAGCTTTCTTTTTTTC 23
|||||||||||||||||||||||
Sbjct: 5261 AACCCAACTCGAAAGAAAAAAAG 5239
>miR8011a-5p Score: 1.0 RO3G_04115
Query: 1 TTGGGTTGAGCTTTCTTTTTTTC 23
||||:|||||||||||||||||*
Sbjct: 2053 AACCTAACTCGAAAGAAAAAAAA 2031
>miR8011a-5p Score: 0.5 RO3G_17404
Query: 1 TTGGGTTGAGCTTTCTTTTTTTC 23
||||||||||||||||||||||*
Sbjct: 1738 AACCCAACTCGAAAGAAAAAAAA 1716
>miR1152 Score: 0.0 RO3G_13297
Query: 1 TAAAAGGAGCGCGTCTTGAA 20
||||||||||||||||||||
Sbjct: 738 ATTTTCCTCGCGCAGAACTT 719
>miR1152 Score: 0.0 RO3G_10676
Query: 1 TAAAAGGAGCGCGTCTTGAA 20
||||||||||||||||||||
Sbjct: 4119 ATTTTCCTCGCGCAGAACTT 4100
>miR1152 Score: 0.0 RO3G_09480
Query: 1 TAAAAGGAGCGCGTCTTGAA 20
||||||||||||||||||||
Sbjct: 4977 ATTTTCCTCGCGCAGAACTT 4958
>miR854 Score: 2.5 RO3G_14229
Query: 1 TGATGAGGATATTGAGAAGGG 21
||||||||||:||||||::
Sbjct: 2660 CCTCCTCCTATAGCTCTTCTT 2640
>miR854 Score: 2.5 RO3G_13317
Query: 1 TGATGAGGATATTGAGAAGGG 21
|||||:||||||||||||*
Sbjct: 486 CCTACTTCTATAACTCCTCCA 466
>miR854 Score: 1.5 RO3G_11879
Query: 1 TGATGAGGATATTGAGAAGGG 21
||||:||||||||:|||||*|
Sbjct: 2158 ACTATTCCTATAATTCTTCAC 2138
>miR854 Score: 2.0 RO3G_03642
Query: 1 TGATGAGGATATTGAGAAGGG 21
::||:||:|||||||||||:|
Sbjct: 1195 GTTATTCTTATAACTCTTCTC 1175
>miR3449-5p Score: 0.0 RO3G_17222
Query: 1 TCCAATCGCTTTCGTCTGT 19
|||||||||||||||||||
Sbjct: 575 AGGTTAGCGAAAGCAGACA 557
>miR3449-5p Score: 0.0 RO3G_16719
Query: 1 TCCAATCGCTTTCGTCTGT 19

```
|||||||||||||||||||
Sbjct: 383 AGGTTAGCGAAAGCAGACA 365
>miR3449-5p Score: 0.0 RO3G_16727
Query: 1 TCCAATCGCTTTCGTCTGT 19
|||||||||||||||||||
Sbjct: 383 AGGTTAGCGAAAGCAGACA 365
>miR3449-5p Score: 2.5 RO3G_16465
Query: 1 TCCAATCGCTTTCGTCTGT 19
||||||**|||||||||*|
Sbjct: 2266 AGGTTATAGAAAGCAGAAA 2248
>miR3449-5p Score: 0.0 RO3G_16550
Query: 1 TCCAATCGCTTTCGTCTGT 19
|||||||||||||||||||
Sbjct: 494 AGGTTAGCGAAAGCAGACA 476
>miR3449-5p Score: 0.0 RO3G_13919
Query: 1 TCCAATCGCTTTCGTCTGT 19
|||||||||||||||||||
Sbjct: 575 AGGTTAGCGAAAGCAGACA 557
>miR3449-5p Score: 0.0 RO3G_13743
Query: 1 TCCAATCGCTTTCGTCTGT 19
|||||||||||||||||||
Sbjct: 383 AGGTTAGCGAAAGCAGACA 365
>miR3449-5p Score: 0.0 RO3G_12678
Query: 1 TCCAATCGCTTTCGTCTGT 19
|||||||||||||||||||
Sbjct: 575 AGGTTAGCGAAAGCAGACA 557
>miR3449-5p Score: 1.0 RO3G_13064
Query: 1 TCCAATCGCTTTCGTCTGT 19
||||||||||||*||||||
Sbjct: 41 AGGTTAGCGAAAACAGACA 23
>miR3449-5p Score: 0.0 RO3G_13286
Query: 1 TCCAATCGCTTTCGTCTGT 19
|||||||||||||||||||
Sbjct: 575 AGGTTAGCGAAAGCAGACA 557
>miR3449-5p Score: 0.2 RO3G_12166
Query: 1 TCCAATCGCTTTCGTCTGT 19
:||||||||||||||||||
Sbjct: 383 GGGTTAGCGAAAGCAGACA 365
>miR3449-5p Score: 0.2 RO3G_12658
Query: 1 TCCAATCGCTTTCGTCTGT 19
:||||||||||||||||||
Sbjct: 383 GGGTTAGCGAAAGCAGACA 365
>miR3449-5p Score: 1.0 RO3G_11281
Query: 1 TCCAATCGCTTTCGTCTGT 19
||||||*||||||||||||
```

Sbjct: 338 AGGTTAACGAAAGCAGACA 320
>miR3449-5p Score: 2.0 RO3G_11807
Query: 1 TCCAATCGCTTTCGTCTGT 19
||*||||||||||||*|||
Sbjct: 2510 AGTTTAGCGAAAGCATACA 2492
>miR3449-5p Score: 0.0 RO3G_10550
Query: 1 TCCAATCGCTTTCGTCTGT 19
|||||||||||||||||||
Sbjct: 383 AGGTTAGCGAAAGCAGACA 365
>miR3449-5p Score: 0.0 RO3G_10741
Query: 1 TCCAATCGCTTTCGTCTGT 19
|||||||||||||||||||
Sbjct: 383 AGGTTAGCGAAAGCAGACA 365
>miR3449-5p Score: 0.0 RO3G_10763
Query: 1 TCCAATCGCTTTCGTCTGT 19
|||||||||||||||||||
Sbjct: 383 AGGTTAGCGAAAGCAGACA 365
>miR3449-5p Score: 0.0 RO3G_10769
Query: 1 TCCAATCGCTTTCGTCTGT 19
|||||||||||||||||||
Sbjct: 260 AGGTTAGCGAAAGCAGACA 242
>miR3449-5p Score: 0.2 RO3G_10787
Query: 1 TCCAATCGCTTTCGTCTGT 19
:||||||||||||||||||
Sbjct: 383 GGGTTAGCGAAAGCAGACA 365
>miR3449-5p Score: 1.8 RO3G_10056
Query: 1 TCCAATCGCTTTCGTCTGT 19
||||||||*|:|||||||:
Sbjct: 2220 AGGTTAGCTAGAGCAGACG 2202
>miR3449-5p Score: 2.5 RO3G_08529
Query: 1 TCCAATCGCTTTCGTCTGT 19
|||||||*||:||:||:||
Sbjct: 793 AGGTTAGAGAGAGTAGGCA 775
>miR3449-5p Score: 0.0 RO3G_08847
Query: 1 TCCAATCGCTTTCGTCTGT 19
|||||||||||||||||||
Sbjct: 383 AGGTTAGCGAAAGCAGACA 365
>miR3449-5p Score: 2.2 RO3G_08994
Query: 1 TCCAATCGCTTTCGTCTGT 19
|**|||||||||||||||:
Sbjct: 85 AATTTAGCGAAAGCAGACG 67
>miR3449-5p Score: 0.0 RO3G_07690
Query: 1 TCCAATCGCTTTCGTCTGT 19
|||||||||||||||||||
Sbjct: 383 AGGTTAGCGAAAGCAGACA 365

>miR3449-5p Score: 0.0 RO3G_07707
Query: 1 TCCAATCGCTTTCGTCTGT 19
|||||||||||||||||||
Sbjct: 575 AGGTTAGCGAAAGCAGACA 557
>miR3449-5p Score: 2.5 RO3G_04768
Query: 1 TCCAATCGCTTTCGTCTGT 19
|||||||||:||:||:|
Sbjct: 163 CGGTTAGCGAGAGTAGG-A 146
>miR3449-5p Score: 2.5 RO3G_04776
Query: 1 TCCAATCGCTTTCGTCTGT 19
|||||||*|||||:|*|||
Sbjct: 144 AGGTTAGAGAAAGTAAACA 126
>miR3449-5p Score: 0.0 RO3G_02706
Query: 1 TCCAATCGCTTTCGTCTGT 19
|||||||||||||||||||
Sbjct: 575 AGGTTAGCGAAAGCAGACA 557
>miR3449-5p Score: 0.0 RO3G_02845
Query: 1 TCCAATCGCTTTCGTCTGT 19
|||||||||||||||||||
Sbjct: 608 AGGTTAGCGAAAGCAGACA 590
>miR3449-5p Score: 2.5 RO3G_03261
Query: 1 TCCAATCGCTTTCGTCTGT 19
||||||*||||||||*|*|
Sbjct: 1343 AGGTTACCGAAAGCACA-A 1326
>miR3449-5p Score: 1.0 RO3G_03588
Query: 1 TCCAATCGCTTTCGTCTGT 19
|||||||||||||||*|||
Sbjct: 260 AGGTTAGCGAAAGCATACA 242
>miR3449-5p Score: 1.0 RO3G_03602
Query: 1 TCCAATCGCTTTCGTCTGT 19
|||||||||||||||*|||
Sbjct: 524 AGGTTAGCGAAAGCATACA 506
>miR3449-5p Score: 0.0 RO3G_03624
Query: 1 TCCAATCGCTTTCGTCTGT 19
|||||||||||||||||||
Sbjct: 383 AGGTTAGCGAAAGCAGACA 365
>miR3449-5p Score: 2.5 RO3G_04064
Query: 1 TCCAATCGCTTTCGTCTGT 19
||||||||*||||:||*||
Sbjct: 813 AGGTTAGCTAAAGTAG-CA 796
>miR3449-5p Score: 2.5 RO3G_00079
Query: 1 TCCAATCGCTTTCGTCTGT 19
|||||||:||*||*|||||
Sbjct: 1135 AGGTTAGTGATAGGAGACA 1117
>miR3449-5p Score: 2.5 RO3G_00099

```
Query: 1 TCCAATCGCTTTCGTCTGT 19
|||||:||||||**|||||
Sbjct: 895 AGGTTGGCGAAAAGAGACA 877
>miR3449-5p Score: 0.0 RO3G_00284
Query: 1 TCCAATCGCTTTCGTCTGT 19
|||||||||||||||||||
Sbjct: 392 AGGTTAGCGAAAGCAGACA 374
>miR3449-5p Score: 2.5 RO3G_00413
Query: 1 TCCAATCGCTTTCGTCTGT 19
||||*||||||||*|||*|
Sbjct: 973 AGGTAAGCGAAAGGAGA-A 956
>miR3449-5p Score: 0.0 RO3G_00679
Query: 1 TCCAATCGCTTTCGTCTGT 19
|||||||||||||||||||
Sbjct: 575 AGGTTAGCGAAAGCAGACA 557
>miR3449-5p Score: 0.0 RO3G_00688
Query: 1 TCCAATCGCTTTCGTCTGT 19
|||||||||||||||||||
Sbjct: 383 AGGTTAGCGAAAGCAGACA 365
>miR3449-5p Score: 1.0 RO3G_01960
Query: 1 TCCAATCGCTTTCGTCTGT 19
|||||||||||||||*|||
Sbjct: 575 AGGTTAGCGAAAGCATACA 557
>miR3449-5p Score: 0.0 RO3G_17408
Query: 1 TCCAATCGCTTTCGTCTGT 19
|||||||||||||||||||
Sbjct: 383 AGGTTAGCGAAAGCAGACA 365
>miR1435a Score: 0.8 RO3G_17245
Query: 1 TTTCTTAAAGTGCAAGTTTTTC 22
|||||||||||||||:||:|||
Sbjct: 221 AAAGAATTTCACGTTTAAGAAG 200
>miR1435a Score: 0.8 RO3G_16910
Query: 1 TTTCTTAAAGTGCAAGTTTTTC 22
|||||||||||||||:||:|||
Sbjct: 221 AAAGAATTTCACGTTTAAGAAG 200
>miR1435a Score: 0.8 RO3G_14707
Query: 1 TTTCTTAAAGTGCAAGTTTTTC 22
|||||||||||||||:||:|||
Sbjct: 221 AAAGAATTTCACGTTTAAGAAG 200
>miR1435a Score: 0.0 RO3G_13710
Query: 1 TTTCTTAAAGTGCAAGTTTTTC 22
||||||||||||||||||||||
Sbjct: 221 AAAGAATTTCACGTTCAAAAAG 200
>miR1435a Score: 0.8 RO3G_13738
Query: 1 TTTCTTAAAGTGCAAGTTTTTC 22
```

||||||||||||||:||:|||
Sbjct: 221 AAAGAATTTCACGTTTAAGAAG 200
>miR1435a Score: 0.8 RO3G_12006
Query: 1 TTTCTTAAAGTGCAAGTTTTTC 22
||||||||||||||:||:|||
Sbjct: 221 AAAGAATTTCACGTTTAAGAAG 200
>miR1435a Score: 0.0 RO3G_11285
Query: 1 TTTCTTAAAGTGCAAGTTTTTC 22
|||||||||||||||||||||
Sbjct: 221 AAAGAATTTCACGTTCAAAAAG 200
>miR1435a Score: 2.5 RO3G_11681
Query: 1 TTTCTTAAAGTGCAAGTTTTTC 22
|||||||||||||||**|||
Sbjct: 1201 CAAGTATTTCACGTTCACTAAG 1180
>miR1435a Score: 0.8 RO3G_01196
Query: 1 TTTCTTAAAGTGCAAGTTTTTC 22
||||||||||||||:||:|||
Sbjct: 221 AAAGAATTTCACGTTTAAGAAG 200
>miR1435a Score: 0.8 RO3G_17365
Query: 1 TTTCTTAAAGTGCAAGTTTTTC 22
||||||||||||||:||:|||
Sbjct: 221 AAAGAATTTCACGTTTAAGAAG 200
>miR6023 Score: 2.0 RO3G_17214
Query: 1 TTGATGACAGTTTTTTGGAT 20
|||||:|||||*|||||*||
Sbjct: 116 AACTATTGTCACAAAAC-TA 98
>miR6023 Score: 2.0 RO3G_16689
Query: 1 TTGATGACAGTTTTTTGGAT 20
|||||:|||||*|||||*||
Sbjct: 116 AACTATTGTCACAAAAC-TA 98
>miR6023 Score: 2.0 RO3G_15664
Query: 1 TTGATGACAGTTTTTTGGAT 20
|||||:|||||*|||||*||
Sbjct: 116 AACTATTGTCACAAAAC-TA 98
>miR6023 Score: 2.5 RO3G_15800
Query: 1 TTGATGACAGTTTTTTGGAT 20
|::||*|||||:||||||||
Sbjct: 1541 AGTTAGTGTCAGAAAACCTA 1522
>miR6023 Score: 2.5 RO3G_15540
Query: 1 TTGATGACAGTTTTTTGGAT 20
|:||||||*||:|||||*||
Sbjct: 1385 AGCTACTGGCAGAAAAC-TA 1367
>miR6023 Score: 2.0 RO3G_14568
Query: 1 TTGATGACAGTTTTTTGGAT 20
|||||:|||||*|||||*||

```
Sbjct: 116 AACTATTGTCACAAAAC-TA 98
>miR6023 Score: 2.5 RO3G_13835
Query: 1 TTGATGACAGTTTTTTGGAT 20
:|||::|||:|||:|||:||
Sbjct: 10250 GACTGTTGTTAAAGAACTTA 10231
>miR6023 Score: 2.5 RO3G_13075
Query: 1 TTGATGACAGTTTTTTGGA-T 20
|||||||*||||:||||*|*|
Sbjct: 696 AACTACTTTCAAGAAACATCA 676
>miR6023 Score: 2.0 RO3G_10773
Query: 1 TTGATGACAGTTTTTTGGAT 20
|||||:|||||*|||||*||
Sbjct: 116 AACTATTGTCACAAAAC-TA 98
>miR6023 Score: 2.0 RO3G_10798
Query: 1 TTGATGACAGTTTTTTGGAT 20
|||||:|||||*|||||*||
Sbjct: 116 AACTATTGTCACAAAAC-TA 98
>miR6023 Score: 2.5 RO3G_10881
Query: 1 TTGATGACAGTTTTTTGGAT 20
|||||||||:*|||||:*||
Sbjct: 238 AACTACTGTTTAAAAAT-TA 220
>miR6023 Score: 2.5 RO3G_09261
Query: 1 TTGATGACAGTTTTTTGGAT 20
**|||||*||||||||||||
Sbjct: 72 CTCTACTTTCAAAAAAACCTA 53
>miR6023 Score: 2.0 RO3G_09542
Query: 1 TTGATGACAGTTTTTTGGAT 20
|||||:|||||*|||||*||
Sbjct: 116 AACTATTGTCACAAAAC-TA 98
>miR6023 Score: 2.5 RO3G_08626
Query: 1 TTGATGACAGTTTTTTGGAT 20
||||||*||||||||:||**|
Sbjct: 1601 AACTACAGTCAAAAGAC-AA 1583
>miR6023 Score: 2.5 RO3G_08783
Query: 1 TTGATGACAGTTTTTTGGAT 20
:|||*||||:||:||||:||
Sbjct: 244 GACTCCTGTTAAGAAACTTA 225
>miR6023 Score: 2.0 RO3G_07030
Query: 1 TTGATGACAGTTTTTTGGAT 20
|||||:|||||*|||||*||
Sbjct: 116 AACTATTGTCACAAAAC-TA 98
>miR6023 Score: 2.0 RO3G_07038
Query: 1 TTGATGACAGTTTTTTGGAT 20
|||||:|||||*|||||*||
Sbjct: 116 AACTATTGTCACAAAAC-TA 98
```

>miR6023 Score: 2.0 RO3G_07040
Query: 1 TTGATGACAGTTTTTTGGAT 20
|||||:|||||*|||||*||
Sbjct: 116 AACTATTGTCACAAAAC-TA 98
>miR6023 Score: 2.0 RO3G_07876
Query: 1 TTGATGACAGTTTTTTGGAT 20
|||||:|||||*|||||*||
Sbjct: 116 AACTATTGTCACAAAAC-TA 98
>miR6023 Score: 2.0 RO3G_05628
Query: 1 TTGATGACAGTTTTTTGGAT 20
|||||:|||||*|||||*||
Sbjct: 116 AACTATTGTCACAAAAC-TA 98
>miR6023 Score: 2.0 RO3G_05939
Query: 1 TTGATGACAGTTTTTTGGAT 20
|||||:|||||*|||||*||
Sbjct: 116 AACTATTGTCACAAAAC-TA 98
>miR6023 Score: 2.0 RO3G_05953
Query: 1 TTGATGACAGTTTTTTGGAT 20
|||||:|||||*|||||*||
Sbjct: 116 AACTATTGTCACAAAAC-TA 98
>miR6023 Score: 2.0 RO3G_05959
Query: 1 TTGATGACAGTTTTTTGGAT 20
|||||:|||||*|||||*||
Sbjct: 116 AACTATTGTCACAAAAC-TA 98
>miR6023 Score: 2.0 RO3G_04478
Query: 1 TTGATGACAGTTTTTTGGAT 20
|||||||||||||:||**||
Sbjct: 1684 AACTACTGTCAAAGAA-GTA 1666
>miR6023 Score: 2.0 RO3G_05264
Query: 1 TTGATGACAGTTTTTTGGAT 20
|||||:|||||*|||||*||
Sbjct: 116 AACTATTGTCACAAAAC-TA 98
>miR6023 Score: 2.0 RO3G_02956
Query: 1 TTGATGACAGTTTTTTGGAT 20
|||||:|||||*|||||*||
Sbjct: 116 AACTATTGTCACAAAAC-TA 98
>miR6023 Score: 2.5 RO3G_03267
Query: 1 TTGATGACAGTTTTTTGGAT 20
:||||||||*:||||||*|:
Sbjct: 842 GACTACTGTAGAAAAAC-TG 824
>miR6023 Score: 2.0 RO3G_03634
Query: 1 TTGATGACAGTTTTTTGGAT 20
|||||||||:||||||||
Sbjct: 68 TACAACTGTCAGAAAACCTA 49
>miR9722 Score: 2.5 RO3G_08462

```
Query: 1 TAAAGATGGGAAGATGAAGAA 21
*|||||||:|||||:||||**
Sbjct: 437 CTTTCTACTCTTCTGCTTCGA 417
>miR7492n Score: 2.5 RO3G_17204
Query: 1 TAAGATCAGGGCTTTGAGCTGG 22
|*|:||||||:||||||||*||
Sbjct: 700 AGTTTAGTCCTGAAACTCGTCC 679
>miR7492n Score: 0.5 RO3G_17211
Query: 1 TAAGATCAGGGCTTTGAGCTGG 22
||||||||||:|||||||||||
Sbjct: 820 ATTCTAGTCCTGAAACTCGACC 799
>miR7492n Score: 0.5 RO3G_17213
Query: 1 TAAGATCAGGGCTTTGAGCTGG 22
||||||||||:|||||||||||
Sbjct: 145 ATTCTAGTCCTGAAACTCGACC 124
>miR7492n Score: 2.0 RO3G_16893
Query: 1 TAAGATCAGGGCTTTGAGCTGG 22
|*|:|||||||||||||||*||
Sbjct: 262 AGTTTAGTCCCGAAACTCGTCC 241
>miR7492n Score: 2.0 RO3G_16377
Query: 1 TAAGATCAGGGCTTTGAGCTGG 22
||||*||||::|||||||||||
Sbjct: 145 ATTCCAGTCTTGAAACTCGACC 124
>miR7492n Score: 1.5 RO3G_16548
Query: 1 TAAGATCAGGGCTTTGAGCTGG 22
||||*|||||:|||||||||||
Sbjct: 475 ATTCCAGTCCTGAAACTCGACC 454
>miR7492n Score: 0.5 RO3G_16118
Query: 1 TAAGATCAGGGCTTTGAGCTGG 22
||||||||||:|||||||||||
Sbjct: 88 ATTCTAGTCCTGAAACTCGACC 67
>miR7492n Score: 2.5 RO3G_15672
Query: 1 TAAGATCAGGGCTTTGAGCTGG 22
|*|:||||||:||||||||*||
Sbjct: 637 AGTTTAGTCCTGAAACTCGTCC 616
>miR7492n Score: 0.5 RO3G_15873
Query: 1 TAAGATCAGGGCTTTGAGCTGG 22
||||||||||:|||||||||||
Sbjct: 820 ATTCTAGTCCTGAAACTCGACC 799
>miR7492n Score: 2.0 RO3G_15496
Query: 1 TAAGATCAGGGCTTTGAGCTGG 22
|*|:|||||||||||||||*||
Sbjct: 519 AGTTTAGTCCCGAAACTCGTCC 498
>miR7492n Score: 2.5 RO3G_14987
Query: 1 TAAGATCAGGGCTTTGAGCTGG 22
```

|*|:||||||:||||||||*||
Sbjct: 484 AGTTTAGTCCTGAAACTCGTCC 463
>miR7492n Score: 0.5 RO3G_15154
Query: 1 TAAGATCAGGGCTTTGAGCTGG 22
||||||||||:|||||||||||
Sbjct: 820 ATTCTAGTCCTGAAACTCGACC 799
>miR7492n Score: 2.2 RO3G_14596
Query: 1 TAAGATCAGGGCTTTGAGCTGG 22
|*|:|||||||||||||||*|:
Sbjct: 25 AGTTTAGTCCCGAAACTCGTCT 4
>miR7492n Score: 2.5 RO3G_14620
Query: 1 TAAGATCAGGGCTTTGAGCTGG 22
|*|:||||||:||||||||*||
Sbjct: 265 AGTTTAGTCCTGAAACTCGTCC 244
>miR7492n Score: 2.0 RO3G_14722
Query: 1 TAAGATCAGGGCTTTGAGCTGG 22
|*|:|||||||||||||||*||
Sbjct: 877 AGTTTAGTCCCGAAACTCGTCC 856
>miR7492n Score: 2.5 RO3G_14772
Query: 1 TAAGATCAGGGCTTTGAGCTGG 22
|*|:||||||:||||||||*||
Sbjct: 715 AGTTTAGTCCTGAAACTCGTCC 694
>miR7492n Score: 2.0 RO3G_13903
Query: 1 TAAGATCAGGGCTTTGAGCTGG 22
|*|:|||||||||||||||*||
Sbjct: 877 AGTTTAGTCCCGAAACTCGTCC 856
>miR7492n Score: 0.5 RO3G_13447
Query: 1 TAAGATCAGGGCTTTGAGCTGG 22
||||||||||:|||||||||||
Sbjct: 820 ATTCTAGTCCTGAAACTCGACC 799
>miR7492n Score: 2.0 RO3G_13664
Query: 1 TAAGATCAGGGCTTTGAGCTGG 22
|*|:|||||||||||||||*||
Sbjct: 877 AGTTTAGTCCCGAAACTCGTCC 856
>miR7492n Score: 2.0 RO3G_13672
Query: 1 TAAGATCAGGGCTTTGAGCTGG 22
|*|:|||||||||||||||*||
Sbjct: 877 AGTTTAGTCCCGAAACTCGTCC 856
>miR7492n Score: 0.5 RO3G_13706
Query: 1 TAAGATCAGGGCTTTGAGCTGG 22
||||||||||:|||||||||||
Sbjct: 778 ATTCTAGTCCTGAAACTCGACC 757
>miR7492n Score: 2.5 RO3G_12670
Query: 1 TAAGATCAGGGCTTTGAGCTGG 22
|*|:||||||:||||||||*||

Sbjct: 637 AGTTTAGTCCTGAAACTCGTCC 616
>miR7492n Score: 2.0 RO3G_13276
Query: 1 TAAGATCAGGGCTTTGAGCTGG 22
|*|:|||||:||||||||||||
Sbjct: 202 AGTTTAGTCCTGAAACTCGACC 181
>miR7492n Score: 0.5 RO3G_12013
Query: 1 TAAGATCAGGGCTTTGAGCTGG 22
||||||||||:|||||||||||
Sbjct: 145 ATTCTAGTCCTGAAACTCGACC 124
>miR7492n Score: 2.5 RO3G_12082
Query: 1 TAAGATCAGGGCTTTGAGCTGG 22
|*|:|||||:||||||||*||
Sbjct: 715 AGTTTAGTCCTGAAACTCGTCC 694
>miR7492n Score: 2.0 RO3G_12139
Query: 1 TAAGATCAGGGCTTTGAGCTGG 22
|*|:||||||||||||||*||
Sbjct: 250 AGTTTAGTCCCGAAACTCGTCC 229
>miR7492n Score: 0.5 RO3G_12174
Query: 1 TAAGATCAGGGCTTTGAGCTGG 22
||||||||||:|||||||||||
Sbjct: 145 ATTCTAGTCCTGAAACTCGACC 124
>miR7492n Score: 0.5 RO3G_12526
Query: 1 TAAGATCAGGGCTTTGAGCTGG 22
||||||||||:|||||||||||
Sbjct: 820 ATTCTAGTCCTGAAACTCGACC 799
>miR7492n Score: 0.5 RO3G_11244
Query: 1 TAAGATCAGGGCTTTGAGCTGG 22
||||||||||:|||||||||||
Sbjct: 820 ATTCTAGTCCTGAAACTCGACC 799
>miR7492n Score: 0.5 RO3G_11263
Query: 1 TAAGATCAGGGCTTTGAGCTGG 22
||||||||||:|||||||||||
Sbjct: 145 ATTCTAGTCCTGAAACTCGACC 124
>miR7492n Score: 2.0 RO3G_11402
Query: 1 TAAGATCAGGGCTTTGAGCTGG 22
|*|||||||||||||*|||||
Sbjct: 445 ACTCTAGTCCCGAAACACGACC 424
>miR7492n Score: 2.5 RO3G_11405
Query: 1 TAAGATCAGGGCTTTGAGCTGG 22
|*|:|||||:||||||||*||
Sbjct: 637 AGTTTAGTCCTGAAACTCGTCC 616
>miR7492n Score: 2.0 RO3G_11486
Query: 1 TAAGATCAGGGCTTTGAGCTGG 22
|*|:||||||||||||||*||
Sbjct: 586 AGTTTAGTCCCGAAACTCGTCC 565

>miR7492n Score: 1.5 RO3G_11528
Query: 1 TAAGATCAGGGCTTTGAGCTGG 22
||||*|||||:|||||||||||
Sbjct: 145 ATTCCAGTCCTGAAACTCGACC 124
>miR7492n Score: 0.0 RO3G_11534
Query: 1 TAAGATCAGGGCTTTGAGCTGG 22
||||||||||||||||||||||
Sbjct: 820 ATTCTAGTCCCGAAACTCGACC 799
>miR7492n Score: 2.0 RO3G_11567
Query: 1 TAAGATCAGGGCTTTGAGCTGG 22
|*|:|||||||||||||||*||
Sbjct: 1534 AGTTTAGTCCCGAAACTCGTCC 1513
>miR7492n Score: 2.2 RO3G_11827
Query: 1 TAAGATCAGGGCTTTGAGCTGG 22
|*|:||||||||||||||*|:|
Sbjct: 64 AGTTTAGTCCCGAAACTC-ATC 44
>miR7492n Score: 1.5 RO3G_11948
Query: 1 TAAGATCAGGGCTTTGAGCTGG 22
||||*|||||:|||||||||||
Sbjct: 820 ATTCCAGTCCTGAAACTCGACC 799
>miR7492n Score: 0.5 RO3G_10528
Query: 1 TAAGATCAGGGCTTTGAGCTGG 22
||||||||||:|||||||||||
Sbjct: 145 ATTCTAGTCCTGAAACTCGACC 124
>miR7492n Score: 1.5 RO3G_10537
Query: 1 TAAGATCAGGGCTTTGAGCTGG 22
||||*|||||:|||||||||||
Sbjct: 643 ATTCCAGTCCTGAAACTCGACC 622
>miR7492n Score: 2.0 RO3G_10644
Query: 1 TAAGATCAGGGCTTTGAGCTGG 22
|*|:|||||||||||||||*||
Sbjct: 262 AGTTTAGTCCCGAAACTCGTCC 241
>miR7492n Score: 0.5 RO3G_10720
Query: 1 TAAGATCAGGGCTTTGAGCTGG 22
||||||||||:|||||||||||
Sbjct: 820 ATTCTAGTCCTGAAACTCGACC 799
>miR7492n Score: 0.5 RO3G_10742
Query: 1 TAAGATCAGGGCTTTGAGCTGG 22
||||||||||:|||||||||||
Sbjct: 145 ATTCTAGTCCTGAAACTCGACC 124
>miR7492n Score: 1.5 RO3G_09199
Query: 1 TAAGATCAGGGCTTTGAGCTGG 22
||||*|||||:|||||||||||
Sbjct: 820 ATTCCAGTCCTGAAACTCGACC 799
>miR7492n Score: 2.2 RO3G_09279

```
Query: 1 TAAGATCAGGGCTTTGAGCTGG 22
|*|:|||||||||||||||*|:
Sbjct: 412 AGTTTAGTCCCGAAACTCGTCT 391
>miR7492n Score: 1.5 RO3G_09924
Query: 1 TAAGATCAGGGCTTTGAGCTGG 22
||||*|||||:|||||||||||
Sbjct: 820 ATTCCAGTCCTGAAACTCGACC 799
>miR7492n Score: 2.5 RO3G_09944
Query: 1 TAAGATCAGGGCTTTGAGCTGG 22
|*|:||||||:||||||||*||
Sbjct: 637 AGTTTAGTCCTGAAACTCGTCC 616
>miR7492n Score: 2.0 RO3G_09997
Query: 1 TAAGATCAGGGCTTTGAGCTGG 22
|||:||||||:||||||*||::
Sbjct: 1078 ATTTTAGTCCTGAAACTAGATT 1057
>miR7492n Score: 0.5 RO3G_10044
Query: 1 TAAGATCAGGGCTTTGAGCTGG 22
||||||||||:|||||||||||
Sbjct: 820 ATTCTAGTCCTGAAACTCGACC 799
>miR7492n Score: 2.5 RO3G_10224
Query: 1 TAAGATCAGGGCTTTGAGCTGG 22
|*|:||||||:||||||||*||
Sbjct: 637 AGTTTAGTCCTGAAACTCGTCC 616
>miR7492n Score: 2.5 RO3G_10226
Query: 1 TAAGATCAGGGCTTTGAGCTGG 22
|*|:||||||:||||||||*||
Sbjct: 637 AGTTTAGTCCTGAAACTCGTCC 616
>miR7492n Score: 0.5 RO3G_07994
Query: 1 TAAGATCAGGGCTTTGAGCTGG 22
||||||||||:|||||||||||
Sbjct: 820 ATTCTAGTCCTGAAACTCGACC 799
>miR7492n Score: 2.5 RO3G_08649
Query: 1 TAAGATCAGGGCTTTGAGCTGG 22
|*|:||||||:||||||||*||
Sbjct: 582 AGTTTAGTCCTGAAACTCGTCC 561
>miR7492n Score: 2.0 RO3G_08835
Query: 1 TAAGATCAGGGCTTTGAGCTGG 22
*|||*|||||:|||||||||||
Sbjct: 130 CTTCCAGTCCTGAAACTCGACC 109
>miR7492n Score: 0.0 RO3G_08848
Query: 1 TAAGATCAGGGCTTTGAGCTGG 22
||||||||||||||||||||||
Sbjct: 130 ATTCTAGTCCCGAAACTCGACC 109
>miR7492n Score: 2.0 RO3G_08906
Query: 1 TAAGATCAGGGCTTTGAGCTGG 22
```

|*|:|||||||||||||||*||
Sbjct: 877 AGTTTAGTCCCGAAACTCGTCC 856
>miR7492n Score: 2.5 RO3G_09167
Query: 1 TAAGATCAGGGCTTTGAGCTGG 22
|*|:||||||:||||||||*||
Sbjct: 637 AGTTTAGTCCTGAAACTCGTCC 616
>miR7492n Score: 0.5 RO3G_07119
Query: 1 TAAGATCAGGGCTTTGAGCTGG 22
||||||||||:|||||||||||
Sbjct: 145 ATTCTAGTCCTGAAACTCGACC 124
>miR7492n Score: 1.5 RO3G_07295
Query: 1 TAAGATCAGGGCTTTGAGCTGG 22
||||*|||||:|||||||||||
Sbjct: 652 ATTCCAGTCCTGAAACTCGACC 631
>miR7492n Score: 2.5 RO3G_07302
Query: 1 TAAGATCAGGGCTTTGAGCTGG 22
|*|:||||||:||||||||*||
Sbjct: 637 AGTTTAGTCCTGAAACTCGTCC 616
>miR7492n Score: 0.5 RO3G_07683
Query: 1 TAAGATCAGGGCTTTGAGCTGG 22
||||||||||:|||||||||||
Sbjct: 820 ATTCTAGTCCTGAAACTCGACC 799
>miR7492n Score: 2.5 RO3G_07689
Query: 1 TAAGATCAGGGCTTTGAGCTGG 22
|*|:||||||:||||||||*||
Sbjct: 637 AGTTTAGTCCTGAAACTCGTCC 616
>miR7492n Score: 2.0 RO3G_07788
Query: 1 TAAGATCAGGGCTTTGAGCTGG 22
|*|:|||||||||||||||*||
Sbjct: 826 AGTTTAGTCCCGAAACTCGTCC 805
>miR7492n Score: 2.0 RO3G_05535
Query: 1 TAAGATCAGGGCTTTGAGCTGG 22
|*|:|||||||||||||||*||
Sbjct: 262 AGTTTAGTCCCGAAACTCGTCC 241
>miR7492n Score: 2.0 RO3G_05603
Query: 1 TAAGATCAGGGCTTTGAGCTGG 22
|*|:|||||||||||||||*||
Sbjct: 136 AGTTTAGTCCCGAAACTCGTCC 115
>miR7492n Score: 2.0 RO3G_05616
Query: 1 TAAGATCAGGGCTTTGAGCTGG 22
|*|:|||||||||||||||*||
Sbjct: 166 AGTTTAGTCCCGAAACTCGTCC 145
>miR7492n Score: 1.5 RO3G_05916
Query: 1 TAAGATCAGGGCTTTGAGCTGG 22
||||*|||||:|||||||||||

```
Sbjct: 820 ATTCCAGTCCTGAAACTCGACC 799
>miR7492n Score: 2.5 RO3G_06721
Query: 1 TAAGATCAGGGCTTTGAGCTGG 22
|*|:||||||:||||||||*||
Sbjct: 634 AGTTTAGTCCTGAAACTCGTCC 613
>miR7492n Score: 2.5 RO3G_04654
Query: 1 TAAGATCAGGGCTTTGAGCTGG 22
|*|:||||||:||||||||*||
Sbjct: 637 AGTTTAGTCCTGAAACTCGTCC 616
>miR7492n Score: 2.0 RO3G_05181
Query: 1 TAAGATCAGGGCTTTGAGCTGG 22
|*|:|||||||||||||||*||
Sbjct: 877 AGTTTAGTCCCGAAACTCGTCC 856
>miR7492n Score: 2.0 RO3G_05194
Query: 1 TAAGATCAGGGCTTTGAGCTGG 22
|*|:|||||||||||||||*||
Sbjct: 877 AGTTTAGTCCCGAAACTCGTCC 856
>miR7492n Score: 2.0 RO3G_05303
Query: 1 TAAGATCAGGGCTTTGAGCTGG 22
|*|:|||||||||||||||*||
Sbjct: 877 AGTTTAGTCCCGAAACTCGTCC 856
>miR7492n Score: 2.0 RO3G_02354
Query: 1 TAAGATCAGGGCTTTGAGCTGG 22
|*|:|||||||||||||||*||
Sbjct: 877 AGTTTAGTCCCGAAACTCGTCC 856
>miR7492n Score: 0.5 RO3G_03558
Query: 1 TAAGATCAGGGCTTTGAGCTGG 22
||||||||||:|||||||||||
Sbjct: 820 ATTCTAGTCCTGAAACTCGACC 799
>miR7492n Score: 1.5 RO3G_03617
Query: 1 TAAGATCAGGGCTTTGAGCTGG 22
||||*|||||:|||||||||||
Sbjct: 820 ATTCCAGTCCTGAAACTCGACC 799
>miR7492n Score: 2.5 RO3G_03785
Query: 1 TAAGATCAGGGCTTTGAGCTGG 22
|*|:||||||:||||||||*||
Sbjct: 442 AGTTTAGTCCTGAAACTCGTCC 421
>miR7492n Score: 2.5 RO3G_03797
Query: 1 TAAGATCAGGGCTTTGAGCTGG 22
|*|:||||||:||||||||*||
Sbjct: 325 AGTTTAGTCCTGAAACTCGTCC 304
>miR7492n Score: 0.5 RO3G_03987
Query: 1 TAAGATCAGGGCTTTGAGCTGG 22
||||||||||:|||||||||||
Sbjct: 820 ATTCTAGTCCTGAAACTCGACC 799
```

>miR7492n Score: 2.0 RO3G_04110
Query: 1 TAAGATCAGGGCTTTGAGCTGG 22
|*|:|||||||||||||||*||
Sbjct: 574 AGTTTAGTCCCGAAACTCGTCC 553
>miR7492n Score: 2.5 RO3G_04200
Query: 1 TAAGATCAGGGCTTTGAGCTGG 22
|*|:||||||:||||||||*||
Sbjct: 637 AGTTTAGTCCTGAAACTCGTCC 616
>miR7492n Score: 2.5 RO3G_00247
Query: 1 TAAGATCAGGGCTTTGAGCTGG 22
|*|:||||||:||||||||*||
Sbjct: 637 AGTTTAGTCCTGAAACTCGTCC 616
>miR7492n Score: 0.5 RO3G_01194
Query: 1 TAAGATCAGGGCTTTGAGCTGG 22
||||||||||:|||||||||||
Sbjct: 820 ATTCTAGTCCTGAAACTCGACC 799
>miR7492n Score: 0.5 RO3G_01313
Query: 1 TAAGATCAGGGCTTTGAGCTGG 22
||||||||||:|||||||||||
Sbjct: 820 ATTCTAGTCCTGAAACTCGACC 799
>miR7492n Score: 2.0 RO3G_01518
Query: 1 TAAGATCAGGGCTTTGAGCTGG 22
|*|:|||||||||||||||*||
Sbjct: 250 AATTTAGTCCCGAAACTCGTCC 229
>miR7492n Score: 2.0 RO3G_01528
Query: 1 TAAGATCAGGGCTTTGAGCTGG 22
|*|:|||||||||||||||*||
Sbjct: 445 AATTTAGTCCCGAAACTCGTCC 424
>miR7492n Score: 0.5 RO3G_01735
Query: 1 TAAGATCAGGGCTTTGAGCTGG 22
||||||||||:|||||||||||
Sbjct: 820 ATTCTAGTCCTGAAACTCGACC 799
>miR7492n Score: 2.0 RO3G_01923
Query: 1 TAAGATCAGGGCTTTGAGCTGG 22
|*|:|||||||||||||||*||
Sbjct: 826 AGTTTAGTCCCGAAACTCGTCC 805
>miR7492n Score: 1.5 RO3G_01950
Query: 1 TAAGATCAGGGCTTTGAGCTGG 22
||||*|||||:|||||||||||
Sbjct: 820 ATTCCAGTCCTGAAACTCGACC 799
>miR7492n Score: 0.5 RO3G_01951
Query: 1 TAAGATCAGGGCTTTGAGCTGG 22
||||||||||:|||||||||||
Sbjct: 820 ATTCTAGTCCTGAAACTCGACC 799
>miR7492n Score: 2.5 RO3G_17436

Query: 1 TAAGATCAGGGCTTTGAGCTGG 22
|*|:||||||:||||||||*||
Sbjct: 637 AGTTTAGTCCTGAAACTCGTCC 616
>miR7492n Score: 0.5 RO3G_17409
Query: 1 TAAGATCAGGGCTTTGAGCTGG 22
||||||||||:|||||||||||
Sbjct: 820 ATTCTAGTCCTGAAACTCGACC 799
>miR7492n Score: 2.5 RO3G_17293
Query: 1 TAAGATCAGGGCTTTGAGCTGG 22
|*|:||||||:||||||||*||
Sbjct: 637 AGTTTAGTCCTGAAACTCGTCC 616
>miR319c Score: 2.5 RO3G_14171
Query: 1 TTGGATGAAAGGGGCATGCT 20
|:::|||||||:||||||*|
Sbjct: 989 AGTTTACTTTCTCCGTACTA 970
>miR319c Score: 2.5 RO3G_11493
Query: 1 TTGGATGAAAGGGGCATGCT 20
|||:|||||||::||||**|
Sbjct: 597 AACTTACTTTCTTCGTAACA 578
>miR319c Score: 2.5 RO3G_11610
Query: 1 TTGGATGAAAGGGGCATGCT 20
||:||||||||:||||||
Sbjct: 1755 CACTTACTTTCCGTGTACGA 1736
>miR319c Score: 2.5 RO3G_08477
Query: 1 TTGGATGAAAGGGGCATGCT 20
:*||||||||:|:||||:||
Sbjct: 954 GTCCTACTTTTCTCGTATGA 935
>miR319c Score: 0.5 RO3G_04030
Query: 1 TTGGATGAAAGGGGCATGCT 20
||||||||||:|||||||||
Sbjct: 1126 AACCTACTTTTCCCGTACGA 1107
>miR1169-3p Score: 2.5 RO3G_14434
Query: 1 TGTGGATGTTGCTTTCTGTGACC 23
||||||*:|||||||||*|||*|
Sbjct: 1375 ACACCTTTAACGAAAGA-ACTTG 1354
>miR1169-3p Score: 1.0 RO3G_13809
Query: 1 TGTGGATGTTGCTTTCTGTGACC 23
||||*||||||||||||||||||
Sbjct: 81 ACACATACAACGAAAGACACTGG 59
>miR1169-3p Score: 0.5 RO3G_13001
Query: 1 TGTGGATGTTGCTTTCTGTGACC 23
||||||||||:||||||||||||
Sbjct: 1603 ACACCTACAATGAAAGACACTGG 1581
>miR1169-3p Score: 0.0 RO3G_11964
Query: 1 TGTGGATGTTGCTTTCTGTGACC 23

||||||||||||||||||||||
Sbjct: 47 ACACCTACAACGAAAGACACTGG 25
>miR1169-3p Score: 2.5 RO3G_08182
Query: 1 TGTGGATGTTGCTTTCTGTGACC 23
|||:||:|:||||||||*|||*|
Sbjct: 237 ACATCTGCGACGAAAGA-ACTAG 216
>miR1169-3p Score: 2.0 RO3G_08215
Query: 1 TGTGGATGTTGCTTTCTGTGACC 23
|||:||:|:||||||||*|||||
Sbjct: 252 ACATCTGCGACGAAAGA-ACTGG 231
>miR1169-3p Score: 2.5 RO3G_08368
Query: 1 TGTGGATGTTGCTTTCTGTGACC 23
|||:||:|:||||||||*||*||
Sbjct: 109 ACATCTGCGACGAAAGA-ACCGG 88
>miR1169-3p Score: 0.5 RO3G_06596
Query: 1 TGTGGATGTTGCTTTCTGTGACC 23
||||||||||:||||||||||||
Sbjct: 1603 ACACCTACAATGAAAGACACTGG 1581
>miR1169-3p Score: 0.5 RO3G_03002
Query: 1 TGTGGATGTTGCTTTCTGTGACC 23
||||||||||:||||||||||||
Sbjct: 1603 ACACCTACAATGAAAGACACTGG 1581
>miR1169-3p Score: 1.0 RO3G_02146
Query: 1 TGTGGATGTTGCTTTCTGTGACC 23
||||||||||:|||||||||||*
Sbjct: 24 ACACCTACAATGAAAGACACTGT 2
>miR157a-5p Score: 2.5 RO3G_08552
Query: 1 TTGACAGAAGATAGAGAGCA 20
|||||:|||:||||||||
Sbjct: 1183 CACTGTTTTGTGTCTCTCGT 1164
>miR157a-5p Score: 2.5 RO3G_07943
Query: 1 TTGACAGAAGATAGAGAGCA 20
|||||||||||:||||*|
Sbjct: 434 CACTGTCTTCTACTTCTCCT 415
>miR157a-5p Score: 2.5 RO3G_05159
Query: 1 TTGACAGAAGATAGAGAGCA 20
:|:|*||||||||:|||:||
Sbjct: 98 GATTATCTTCTATTTCTTGT 79
>miR157a-5p Score: 2.5 RO3G_03092
Query: 1 TTGACAGAAGATAGAGAGCA 20
|||||:|||:||||||||
Sbjct: 1183 CACTGTTTTGTGTCTCTCGT 1164
>miR157a-5p Score: 2.0 RO3G_03674
Query: 1 TTGACAGAAGATAGAGAGCA 20
||||||*||||:|||||*||

```
Sbjct: 375 AACTGTGTTCTGTCTCTAGT 356
>miR1428e-3p Score: 0.0 RO3G_13882
Query: 1 TAAGAAATGCCATGAACTCTTT 22
||||||||||||||||||||||
Sbjct: 652 ATTCTTTACGGTACTTGAGAAA 631
>miR1428e-3p Score: 0.0 RO3G_13730
Query: 1 TAAGAAATGCCATGAACTCTTT 22
||||||||||||||||||||||
Sbjct: 415 ATTCTTTACGGTACTTGAGAAA 394
>miR1428e-3p Score: 0.0 RO3G_05325
Query: 1 TAAGAAATGCCATGAACTCTTT 22
||||||||||||||||||||||
Sbjct: 652 ATTCTTTACGGTACTTGAGAAA 631
>miR1428e-3p Score: 1.0 RO3G_03721
Query: 1 TAAGAAATGCCATGAACTCTTT 22
||||||||||||||||*|||||
Sbjct: 652 ATTCTTTACGGTACTTAAGAAA 631
>miR1428e-3p Score: 1.5 RO3G_00141
Query: 1 TAAGAAATGCCATGAACTCTTT 22
||||||||:|||||||*|||||
Sbjct: 652 ATTCTTTATGGTACTTAAGAAA 631
>miR1428e-3p Score: 0.0 RO3G_00809
Query: 1 TAAGAAATGCCATGAACTCTTT 22
||||||||||||||||||||||
Sbjct: 652 ATTCTTTACGGTACTTGAGAAA 631
>miR1428e-3p Score: 2.5 RO3G_02044
Query: 1 TAAGAAATGCCATGAACTCTTT 22
:||:||*||||||:|||||||:
Sbjct: 1542 GTTTTTGACGGTATTTGAGAAG 1521
>miR1850.1 Score: 2.0 RO3G_14387
Query: 1 TTGAAAGTTTGGGAGTTGGGA 21
||||||||:|||||*|||::|
Sbjct: 1248 AACTTTCAGACCCTAAACTTT 1228
>miR1850.1 Score: 0.5 RO3G_13985
Query: 1 TTGAAAGTTTGGGAGTTGGGA 21
*||||||||||||||||||||
Sbjct: 35 TACTTTCAAACCCTCAACCCT 15
>miR1850.1 Score: 1.0 RO3G_10032
Query: 1 TTGAAAGTTTGGGAGTTGGGA 21
|*|||||||||||||||||||
Sbjct: 29 ACCTTTCAAACCCTCAACCCT 9
>miR1850.1 Score: 2.5 RO3G_06362
Query: 1 TTGAAAGTTTGGGAGTTG-GGA 21
|:||||||:|||:||:||*|||
Sbjct: 2173 AGCTTTCAGACCTTCGACACCT 2152
```

>miR835-3p Score: 2.5 RO3G_17089
Query: 1 TGAAGAGATACGCCAGGAG 19
||*||||||*|||||:|||
Sbjct: 395 ACATCTCTAGGCGGTTCTC 377
>miR835-3p Score: 0.0 RO3G_14509
Query: 1 TGAAGAGATACGCCAGGAG 19
|||||||||||||||||||
Sbjct: 856 ACTTCTCTATGCGGTCCTC 838
>miR835-3p Score: 2.5 RO3G_05548
Query: 1 TGAAGAGATACGCCAGGAG 19
||||||||||||||*||
Sbjct: 972 CCTTCTCTATGCTGTC-TC 955
>miR835-3p Score: 2.5 RO3G_06167
Query: 1 TGAAGAGATACGCCAGGAG 19
|:|*||||||||*||||||
Sbjct: 338 ATTACTCTATGCAGTCCTC 320
>miR7536a Score: 2.5 RO3G_07618
Query: 1 TGAAGCATGCTCGAGACTG 19
||||:|||:*|||||||*|
Sbjct: 417 ACTTTGTATCAGCTCTG-C 400
>miR7536a Score: 2.0 RO3G_01272
Query: 1 TGAAGCATGCTCGAGACTG 19
:|||||||:|||*||||:|
Sbjct: 3269 GCTTCGTATGAGGTCTGGC 3251
>miR856-3p Score: 2.5 RO3G_16502
Query: 1 TGAATGTGATTGTGGTTA-AA 20
*||||:||||||||||:|**|
Sbjct: 326 CCTTATACTAACACCAGTAAT 306
>miR856-3p Score: 2.5 RO3G_09352
Query: 1 TGAATGTGATTGTGGTTAAA 20
*||||:||||||||||:|**
Sbjct: 500 CCTTATACTAACACCAGTAA 481
>miR856-3p Score: 2.5 RO3G_09388
Query: 1 TGAATGTGATTGTGGTTAAA 20
||*||||||||||:|||**|
Sbjct: 223 ACGTACACTAACATCAACAT 204
>miR856-3p Score: 2.5 RO3G_08586
Query: 1 TGAATGTGATTGTGGTTAAA 20
|||||||||||||**||*||
Sbjct: 225 ACTTACACTAACAAAAAATT 206
>miR856-3p Score: 2.5 RO3G_09191
Query: 1 TGAATGTGATTGTGGTTAAA 20
|||*||||*|||||||||*|
Sbjct: 301 ACTGACACCAACACCAATAT 282
>miR6030 Score: 2.5 RO3G_02123

```
Query: 1 TCCACCCATACCATACAGACC 21
*||||||||||||:*||*|||
Sbjct: 1728 TGGTGGGTATGGTGGGTATGG 1708
>miR773a Score: 1.5 RO3G_16853
Query: 1 TTTGCTGCCACTTTTGTCACG 21
||||||||||||||||*|||*
Sbjct: 670 AAACGACGGTGAAAAC-GTGG 651
>miR773a Score: 1.5 RO3G_16855
Query: 1 TTTGCTGCCACTTTTGTCACG 21
||||||||||||||||*|||*
Sbjct: 670 AAACGACGGTGAAAAC-GTGG 651
>miR773a Score: 1.5 RO3G_16780
Query: 1 TTTGCTGCCACTTTTGTCACG 21
||||||||||||||||*|||*
Sbjct: 670 AAACGACGGTGAAAAC-GTGG 651
>miR773a Score: 1.5 RO3G_16782
Query: 1 TTTGCTGCCACTTTTGTCACG 21
||||||||||||||||*|||*
Sbjct: 670 AAACGACGGTGAAAAC-GTGG 651
>miR773a Score: 1.5 RO3G_16361
Query: 1 TTTGCTGCCACTTTTGTCACG 21
|||||:||*||||||||||||
Sbjct: 920 AAACGGCGTTGAAAACAGTGC 900
>miR773a Score: 1.5 RO3G_15904
Query: 1 TTTGCTGCCACTTTTGTCACG 21
||||||||||||||||*|||*
Sbjct: 604 AAACGACGGTGAAAAC-GTGG 585
>miR773a Score: 2.0 RO3G_15475
Query: 1 TTTGCTGCCACTTTTGTCACG 21
||||||||||||||||**||*
Sbjct: 487 AAACGACGGTGAAAAC-CTGG 468
>miR773a Score: 2.0 RO3G_12985
Query: 1 TTTGCTGCCACTTTTGTCACG 21
||||||||||||||||**||*
Sbjct: 667 AAACGACGGTGAAAAC-CTGG 648
>miR773a Score: 2.0 RO3G_12986
Query: 1 TTTGCTGCCACTTTTGTCACG 21
||||||||||||||||**||*
Sbjct: 667 AAACGACGGTGAAAAC-CTGG 648
>miR773a Score: 2.0 RO3G_12228
Query: 1 TTTGCTGCCACTTTTGTCACG 21
||||||||||||||||**||*
Sbjct: 667 AAACGACGGTGAAAAC-CTGG 648
>miR773a Score: 2.0 RO3G_12229
Query: 1 TTTGCTGCCACTTTTGTCACG 21
```

```
||||||||||||||||**||*
Sbjct: 667 AAACGACGGTGAAAAC-CTGG 648
>miR773a Score: 2.5 RO3G_08059
Query: 1 TTTGCTGCCACTTTTGTCACG 21
*|||||||||*||||||*|*|
Sbjct: 427 TAACGACGGTAAAAACACTTC 407
>miR773a Score: 2.0 RO3G_06812
Query: 1 TTTGCTGCCACTTTTGTCACG 21
||||||||||||||||**||*
Sbjct: 667 AAACGACGGTGAAAAC-CTGG 648
>miR773a Score: 2.0 RO3G_06815
Query: 1 TTTGCTGCCACTTTTGTCACG 21
||||||||||||||||**||*
Sbjct: 601 AAACGACGGTGAAAAC-CTGG 582
>miR773a Score: 2.0 RO3G_07241
Query: 1 TTTGCTGCCACTTTTGTCACG 21
||||||||||||||||**||*
Sbjct: 601 AAACGACGGTGAAAAC-CTGG 582
>miR773a Score: 2.0 RO3G_07244
Query: 1 TTTGCTGCCACTTTTGTCACG 21
||||||||||||||||**||*
Sbjct: 694 AAACGACGGTGAAAAC-CTGG 675
>miR773a Score: 2.5 RO3G_04955
Query: 1 TTTGCTGCCACTTTTGTCA-CG 21
||||*||||||||:|||*|*||
Sbjct: 180 AAACCACGGTGAAGACACTAGC 159
>miR773a Score: 2.5 RO3G_03107
Query: 1 TTTGCTGCCACTTTTGTCACG 21
*|||||||||*||||||*|*|
Sbjct: 2734 TAACGACGGTAAAAACACTTC 2714
>miR773a Score: 0.0 RO3G_04126
Query: 1 TTTGCTGCCACTTTTGTCACG 21
|||||||||||||||||||||
Sbjct: 920 AAACGACGGTGAAAACAGTGC 900
>miR773a Score: 2.0 RO3G_00573
Query: 1 TTTGCTGCCACTTTTGTCACG 21
||||||||||||||||**||*
Sbjct: 439 AAACGACGGTGAAAAC-TTGG 420
>miR773a Score: 1.5 RO3G_17422
Query: 1 TTTGCTGCCACTTTTGTCACG 21
|||||:||*||||||||||||
Sbjct: 221 AAACGGCGTTGAAAACAGTGC 201
>miR6264 Score: 2.5 RO3G_17159
Query: 1 ATTCTTGGACCGTGTCAA 18
|||||||*|||:|*||||
```

Sbjct: 400 TAAGAACATGGTAAAGTT 383
>miR6264 Score: 2.5 RO3G_15603
Query: 1 ATTCTTGGACCGTGTCAA 18
||:||||:|||:*|||||
Sbjct: 382 TAGGAACTTGGTTCAGTT 365
>miR6264 Score: 2.5 RO3G_15562
Query: 1 ATTCTTGGACCGTGTCAA 18
||:|||||*|*|||||||
Sbjct: 603 TAGGAACCAGTCACAGTT 586
>miR6264 Score: 2.0 RO3G_14386
Query: 1 ATTCTTGGACCGTGTCAA 18
|:||||||||||||||
Sbjct: 303 GAGGAACCGGGCACAGTT 286
>miR6264 Score: 2.5 RO3G_14331
Query: 1 ATTCTTGGACCGTGTCAA 18
||||||:||||||||*
Sbjct: 4680 AAAGAACTTGGCAAAGTA 4663
>miR6264 Score: 2.5 RO3G_13789
Query: 1 ATTCTTGGACCGTGTCAA 18
:||:||||||||||||
Sbjct: 1594 GGAGGAACTGGCACAGTT 1577
>miR6264 Score: 2.5 RO3G_07205
Query: 1 ATTCTTGGACCGTGTCAA 18
|||||||||:|||||*
Sbjct: 555 GAAGAAACTGGTACAGTA 538
>miR6264 Score: 2.5 RO3G_07582
Query: 1 ATTCTTGGACCGTGTCAA 18
||||||:||:||||||
Sbjct: 1429 GAAGAACTTAGTACAGTT 1412
>miR6264 Score: 2.5 RO3G_04566
Query: 1 ATTCTTGGACCGTGTCAA 18
|||:|||||:||||||
Sbjct: 454 CACGAGCCTGGTACAGTT 437
>miR6264 Score: 2.5 RO3G_04729
Query: 1 ATTCTTGGACCGTGTCAA 18
**|||||||*||||||||
Sbjct: 972 GTAGAACCTAGCACAGTT 955
>miR6264 Score: 2.5 RO3G_02698
Query: 1 ATTCTTGGACCGTGTCAA 18
|||||||*|||:|*||||
Sbjct: 280 TAAGAACATGGTAAAGTT 263
>miR6264 Score: 2.0 RO3G_03474
Query: 1 ATTCTTGGACCGTGTCAA 18
|||||||||||:|||*|*
Sbjct: 555 TAAGAACCTGGTACAATG 538

>miR6264 Score: 2.5 RO3G_00321
Query: 1 ATTCTTGGACCGTGTCAA 18
||||||||*|||||||
Sbjct: 139 GAAGAACGTGACACAGTT 122
>miR9552b-3p Score: 2.5 RO3G_10258
Query: 1 TGGCGAGAGACCGATAGC 18
*|||||:|:|||:|:|||
Sbjct: 1399 CCCGCTTTTTGGTTGTCG 1382
>miR9552b-3p Score: 2.5 RO3G_09074
Query: 1 TGGCGAGAGACCGATAGC 18
||||:|||||||:|:|*|
Sbjct: 1074 ACCGTTCTCTGGTTGTGG 1057
>miR9552b-3p Score: 2.5 RO3G_09077
Query: 1 TGGCGAGAGACCGATAGC 18
||||:|||||||:|:|*|
Sbjct: 1074 ACCGTTCTCTGGTTGTGG 1057
>miR9552b-3p Score: 2.5 RO3G_07084
Query: 1 TGGCGAGAGACCGATAGC 18
:||:||||||||||||
Sbjct: 165 TTCGTTCTCTGGCCATCG 148
>miR9552b-3p Score: 2.5 RO3G_02584
Query: 1 TGGCGAGAGACCGATAGC 18
:||:||||||||||||
Sbjct: 165 CTCGTTCTCTGGCCATCG 148
>miR9552b-3p Score: 2.0 RO3G_03948
Query: 1 TGGCGAGAGACCGATAGC 18
|::*||||||||||||||
Sbjct: 84 ATTACTCTCTGGCTATCG 67
>miR9552b-3p Score: 2.0 RO3G_01749
Query: 1 TGGCGAGAGACCGATAGC 18
:|||||||||||||||
Sbjct: 204 CTCGCTCTCTGGCTACCG 187
>miR8141 Score: 1.0 RO3G_16729
Query: 1 TGCATACTAGTTGGTGGTGT 20
|||||||||*||||||||||
Sbjct: 758 ACGTATGATGAACCACCACA 739
>miR8141 Score: 0.2 RO3G_14560
Query: 1 TGCATACTAGTTGGTGGTGT 20
|||||||||||||||||||:
Sbjct: 758 ACGTATGATCAACCACCACG 739
>miR8141 Score: 0.2 RO3G_13858
Query: 1 TGCATACTAGTTGGTGGTGT 20
|||||||||||||||||||:
Sbjct: 521 ACGTATGATCAACCACCACG 502
>miR8141 Score: 0.2 RO3G_12666

```
Query: 1 TGCATACTAGTTGGTGGTGT 20
|||||||||||||||||||:
Sbjct: 521 ACGTATGATCAACCACCACG 502
>miR8141 Score: 0.2 RO3G_12954
Query: 1 TGCATACTAGTTGGTGGTGT 20
|||||||||||||||||||:
Sbjct: 890 ACGTATGATCAACCACCACG 871
>miR8141 Score: 0.2 RO3G_12071
Query: 1 TGCATACTAGTTGGTGGTGT 20
|||||||||||||||||||:
Sbjct: 521 ACGTATGATCAACCACCACG 502
>miR8141 Score: 1.2 RO3G_08115
Query: 1 TGCATACTAGTTGGTGGTGT 20
|||||||||*|||||||||:
Sbjct: 593 ACGTATGATGAACCACCACG 574
>miR8141 Score: 2.2 RO3G_07774
Query: 1 TGCATACTAGTTGGTGGTGT 20
*||||||:||:||:|||||:
Sbjct: 266 CCGTATGGTCGACTACCACG 247
>miR8141 Score: 1.2 RO3G_04690
Query: 1 TGCATACTAGTTGGTGGTGT 20
|||||||||*|||||||||:
Sbjct: 890 ACGTATGATGAACCACCACG 871
>miR8141 Score: 0.2 RO3G_05421
Query: 1 TGCATACTAGTTGGTGGTGT 20
|||||||||||||||||||:
Sbjct: 560 ACGTATGATCAACCACCACG 541
>miR8141 Score: 0.2 RO3G_05431
Query: 1 TGCATACTAGTTGGTGGTGT 20
|||||||||||||||||||:
Sbjct: 848 ACGTATGATCAACCACCACG 829
>miR8141 Score: 1.2 RO3G_02713
Query: 1 TGCATACTAGTTGGTGGTGT 20
|||||||||*|||||||||:
Sbjct: 620 ACGTATGATGAACCACCACG 601
>miR8141 Score: 0.2 RO3G_03764
Query: 1 TGCATACTAGTTGGTGGTGT 20
|||||||||||||||||||:
Sbjct: 521 ACGTATGATCAACCACCACG 502
>miR8141 Score: 0.2 RO3G_03776
Query: 1 TGCATACTAGTTGGTGGTGT 20
|||||||||||||||||||:
Sbjct: 350 ACGTATGATCAACCACCACG 331
>miR902a-5p Score: 1.0 RO3G_15157
Query: 1 TACTGCATGCACGCTTCTTCA 21
```

*|||||||:||||||||||||
Sbjct: 347 TTGACGTATGTGCGAAGAAGT 327
>miR902a-5p Score: 1.0 RO3G_14637
Query: 1 TACTGCATGCACGCTTCTTCA 21
*|||||||:||||||||||||
Sbjct: 347 TTGACGTATGTGCGAAGAAGT 327
>miR902a-5p Score: 0.5 RO3G_13735
Query: 1 TACTGCATGCACGCTTCTTCA 21
||||||||:||||||||||||
Sbjct: 347 ATGACGTATGTGCGAAGAAGT 327
>miR902a-5p Score: 0.2 RO3G_12957
Query: 1 TACTGCATGCACGCTTCTTCA 21
:||||||||||||||||||||
Sbjct: 347 GTGACGTACGTGCGAAGAAGT 327
>miR902a-5p Score: 1.0 RO3G_12005
Query: 1 TACTGCATGCACGCTTCTTCA 21
*|||||||:||||||||||||
Sbjct: 347 TTGACGTATGTGCGAAGAAGT 327
>miR902a-5p Score: 0.5 RO3G_12179
Query: 1 TACTGCATGCACGCTTCTTCA 21
||||||||:||||||||||||
Sbjct: 347 ATGACGTATGTGCGAAGAAGT 327
>miR902a-5p Score: 0.5 RO3G_11953
Query: 1 TACTGCATGCACGCTTCTTCA 21
*||||||||||||||||||||
Sbjct: 224 TTGACGTACGTGCGAAGAAGT 204
>miR902a-5p Score: 0.5 RO3G_10525
Query: 1 TACTGCATGCACGCTTCTTCA 21
*||||||||||||||||||||
Sbjct: 347 TTGACGTACGTGCGAAGAAGT 327
>miR902a-5p Score: 0.5 RO3G_10531
Query: 1 TACTGCATGCACGCTTCTTCA 21
*||||||||||||||||||||
Sbjct: 347 TTGACGTACGTGCGAAGAAGT 327
>miR902a-5p Score: 0.5 RO3G_10545
Query: 1 TACTGCATGCACGCTTCTTCA 21
*||||||||||||||||||||
Sbjct: 347 TTGACGTACGTGCGAAGAAGT 327
>miR902a-5p Score: 0.5 RO3G_07110
Query: 1 TACTGCATGCACGCTTCTTCA 21
*||||||||||||||||||||
Sbjct: 347 TTGACGTACGTGCGAAGAAGT 327
>miR902a-5p Score: 0.5 RO3G_07676
Query: 1 TACTGCATGCACGCTTCTTCA 21
*||||||||||||||||||||

```
Sbjct: 347 TTGACGTACGTGCGAAGAAGT 327
>miR902a-5p Score: 0.5 RO3G_07678
Query: 1 TACTGCATGCACGCTTCTTCA 21
*||||||||||||||||||||
Sbjct: 347 TTGACGTACGTGCGAAGAAGT 327
>miR902a-5p Score: 0.5 RO3G_07868
Query: 1 TACTGCATGCACGCTTCTTCA 21
*||||||||||||||||||||
Sbjct: 347 TTGACGTACGTGCGAAGAAGT 327
>miR902a-5p Score: 0.5 RO3G_05523
Query: 1 TACTGCATGCACGCTTCTTCA 21
*||||||||||||||||||||
Sbjct: 347 TTGACGTACGTGCGAAGAAGT 327
>miR902a-5p Score: 1.0 RO3G_04676
Query: 1 TACTGCATGCACGCTTCTTCA 21
*|||||||:||||||||||||
Sbjct: 347 TTGACGTATGTGCGAAGAAGT 327
>miR902a-5p Score: 0.5 RO3G_03767
Query: 1 TACTGCATGCACGCTTCTTCA 21
*||||||||||||||||||||
Sbjct: 347 TTGACGTACGTGCGAAGAAGT 327
>miR902a-5p Score: 0.5 RO3G_01949
Query: 1 TACTGCATGCACGCTTCTTCA 21
*||||||||||||||||||||
Sbjct: 347 TTGACGTACGTGCGAAGAAGT 327
>miR902a-5p Score: 0.5 RO3G_01968
Query: 1 TACTGCATGCACGCTTCTTCA 21
*||||||||||||||||||||
Sbjct: 347 TTGACGTACGTGCGAAGAAGT 327
>miR902a-5p Score: 0.5 RO3G_17380
Query: 1 TACTGCATGCACGCTTCTTCA 21
*||||||||||||||||||||
Sbjct: 347 TTGACGTACGTGCGAAGAAGT 327
>miR774b-5p Score: 0.2 RO3G_16736
Query: 1 TGAAGATGCATATTTGGAGTGAC 23
||||||||||||||||||||:||
Sbjct: 26 ACTTCTACGTATAAACCTCATTG 4
>miR774b-5p Score: 0.0 RO3G_16138
Query: 1 TGAAGATGCATATTTGGAGTGAC 23
|||||||||||||||||||||||
Sbjct: 1892 ACTTCTACGTATAAACCTCACTG 1870
>miR774b-5p Score: 0.5 RO3G_15580
Query: 1 TGAAGATGCATATTTGGAGTGA-C 23
||||||||||||||||||||||*|
Sbjct: 1856 ACTTCTACGTATAAACCTCACTAG 1833
```

>miR774b-5p Score: 0.0 RO3G_14626
Query: 1 TGAAGATGCATATTTGGAGTGAC 23
|||||||||||||||||||||||
Sbjct: 1892 ACTTCTACGTATAAACCTCACTG 1870
>miR774b-5p Score: 0.2 RO3G_14710
Query: 1 TGAAGATGCATATTTGGAGTGAC 23
||||||||||||||||||||:||
Sbjct: 1892 ACTTCTACGTATAAACCTCATTG 1870
>miR774b-5p Score: 0.2 RO3G_13747
Query: 1 TGAAGATGCATATTTGGAGTGAC 23
||||||||||||||||||||:||
Sbjct: 1892 ACTTCTACGTATAAACCTCATTG 1870
>miR774b-5p Score: 0.0 RO3G_13149
Query: 1 TGAAGATGCATATTTGGAGTGAC 23
|||||||||||||||||||||||
Sbjct: 89 ACTTCTACGTATAAACCTCACTG 67
>miR774b-5p Score: 0.2 RO3G_12009
Query: 1 TGAAGATGCATATTTGGAGTGAC 23
||||||||||||||||||||:||
Sbjct: 1892 ACTTCTACGTATAAACCTCATTG 1870
>miR774b-5p Score: 0.0 RO3G_12072
Query: 1 TGAAGATGCATATTTGGAGTGAC 23
|||||||||||||||||||||||
Sbjct: 1892 ACTTCTACGTATAAACCTCACTG 1870
>miR774b-5p Score: 0.2 RO3G_12169
Query: 1 TGAAGATGCATATTTGGAGTGAC 23
||||||||||||||||||||:||
Sbjct: 1892 ACTTCTACGTATAAACCTCATTG 1870
>miR774b-5p Score: 0.2 RO3G_11404
Query: 1 TGAAGATGCATATTTGGAGTGAC 23
||||||||||||||||||||:||
Sbjct: 1892 ACTTCTACGTATAAACCTCATTG 1870
>miR774b-5p Score: 0.5 RO3G_11531
Query: 1 TGAAGATGCATATTTGGAGTGA-C 23
||||||||||||||||||||||*|
Sbjct: 1385 ACTTCTACGTATAAACCTCACTAG 1362
>miR774b-5p Score: 0.2 RO3G_11771
Query: 1 TGAAGATGCATATTTGGAGTGAC 23
||||||||||||||||||||:||
Sbjct: 1892 ACTTCTACGTATAAACCTCATTG 1870
>miR774b-5p Score: 0.0 RO3G_10522
Query: 1 TGAAGATGCATATTTGGAGTGAC 23
|||||||||||||||||||||||
Sbjct: 1856 ACTTCTACGTATAAACCTCACTG 1834
>miR774b-5p Score: 0.2 RO3G_10725

```
Query: 1 TGAAGATGCATATTTGGAGTGAC 23
||||||||||||||||||||:||
Sbjct: 1856 ACTTCTACGTATAAACCTCATTG 1834
>miR774b-5p Score: 0.2 RO3G_09940
Query: 1 TGAAGATGCATATTTGGAGTGAC 23
||||||||||||||||||||:||
Sbjct: 1892 ACTTCTACGTATAAACCTCATTG 1870
>miR774b-5p Score: 0.2 RO3G_07128
Query: 1 TGAAGATGCATATTTGGAGTGAC 23
||||||||||||||||||||:||
Sbjct: 410 ACTTCTACGTATAAACCTCATTG 388
>miR774b-5p Score: 0.0 RO3G_07294
Query: 1 TGAAGATGCATATTTGGAGTGAC 23
|||||||||||||||||||||||
Sbjct: 1493 ACTTCTACGTATAAACCTCACTG 1471
>miR774b-5p Score: 0.2 RO3G_07686
Query: 1 TGAAGATGCATATTTGGAGTGAC 23
||||||||||||||||||||:||
Sbjct: 1892 ACTTCTACGTATAAACCTCATTG 1870
>miR774b-5p Score: 0.2 RO3G_07976
Query: 1 TGAAGATGCATATTTGGAGTGAC 23
||||||||||||||||||||:||
Sbjct: 1889 ACTTCTACGTATAAACCTCATTG 1867
>miR774b-5p Score: 0.0 RO3G_05958
Query: 1 TGAAGATGCATATTTGGAGTGAC 23
|||||||||||||||||||||||
Sbjct: 1856 ACTTCTACGTATAAACCTCACTG 1834
>miR774b-5p Score: 0.8 RO3G_06430
Query: 1 TGAAGATGCATATTTGGAGTGAC 23
||||:|||||||||||||||:||
Sbjct: 1856 ACTTTTACGTATAAACCTCATTG 1834
>miR774b-5p Score: 0.0 RO3G_04785
Query: 1 TGAAGATGCATATTTGGAGTGAC 23
|||||||||||||||||||||||
Sbjct: 1856 ACTTCTACGTATAAACCTCACTG 1834
>miR774b-5p Score: 0.2 RO3G_03552
Query: 1 TGAAGATGCATATTTGGAGTGAC 23
||||||||||||||||||||:||
Sbjct: 26 ACTTCTACGTATAAACCTCATTG 4
>miR774b-5p Score: 0.5 RO3G_03580
Query: 1 TGAAGATGCATATTTGGAGTGA-C 23
||||||||||||||||||||||*|
Sbjct: 1817 ACTTCTACGTATAAACCTCACTAG 1794
>miR774b-5p Score: 0.5 RO3G_03589
Query: 1 TGAAGATGCATATTTGGAGTGA-C 23
```

|||||||||||||||||||||*|
Sbjct: 1817 ACTTCTACGTATAAACCTCACTAG 1794
>miR774b-5p Score: 1.0 RO3G_01207
Query: 1 TGAAGATGCATATTTGGAGTGAC 23
||||:||||||||||||||*||
Sbjct: 1709 ACTTTTACGTATAAACCTCA-TG 1688
>miR774b-5p Score: 0.2 RO3G_01309
Query: 1 TGAAGATGCATATTTGGAGTGAC 23
|||||||||||||||||||:||
Sbjct: 1841 ACTTCTACGTATAAACCTCATTG 1819
>miR774b-5p Score: 0.0 RO3G_01740
Query: 1 TGAAGATGCATATTTGGAGTGAC 23
||||||||||||||||||||||
Sbjct: 1856 ACTTCTACGTATAAACCTCACTG 1834
>miR774b-5p Score: 0.0 RO3G_01954
Query: 1 TGAAGATGCATATTTGGAGTGAC 23
||||||||||||||||||||||
Sbjct: 842 ACTTCTACGTATAAACCTCACTG 820
>miR774b-5p Score: 0.0 RO3G_17441
Query: 1 TGAAGATGCATATTTGGAGTGAC 23
||||||||||||||||||||||
Sbjct: 1619 ACTTCTACGTATAAACCTCACTG 1597
>miR774b-5p Score: 0.2 RO3G_17385
Query: 1 TGAAGATGCATATTTGGAGTGAC 23
|||||||||||||||||||:||
Sbjct: 1892 ACTTCTACGTATAAACCTCATTG 1870
>miR774b-5p Score: 0.2 RO3G_17358
Query: 1 TGAAGATGCATATTTGGAGTGAC 23
|||||||||||||||||||:||
Sbjct: 1892 ACTTCTACGTATAAACCTCATTG 1870
>miR5741a Score: 2.5 RO3G_07788
Query: 1 TAGGAGCAAATTGATGTGTTT 21
|*:|||||||||||*||||||
Sbjct: 1540 ACTCTCGTTTAACTTCACAAA 1520
>miR9653a-3p Score: 2.5 RO3G_17204
Query: 1 TTGGGACTTTGGGCTGGCC 19
|::||||||||:||*||||
Sbjct: 695 AGTCCTGAAACTCGTCCGG 677
>miR9653a-3p Score: 1.5 RO3G_17211
Query: 1 TTGGGACTTTGGGCTGGCC 19
|::||||||||:|||||||
Sbjct: 815 AGTCCTGAAACTCGACCGG 797
>miR9653a-3p Score: 1.5 RO3G_17213
Query: 1 TTGGGACTTTGGGCTGGCC 19
|::||||||||:|||||||

Sbjct: 140 AGTCCTGAAACTCGACCGG 122
>miR9653a-3p Score: 2.0 RO3G_17069
Query: 1 TTGGGACTTTGGGCTGGCC 19
|::||||||||||*|||||
Sbjct: 680 AGTCCTGAAACCCTACCGG 662
>miR9653a-3p Score: 1.0 RO3G_16905
Query: 1 TTGGGACTTTGGGCTGGCC 19
|:|||||||||:|||||||
Sbjct: 632 AGCCCTGAAACTCGACCGG 614
>miR9653a-3p Score: 0.5 RO3G_16715
Query: 1 TTGGGACTTTGGGCTGGCC 19
|:|||||||||||||||||
Sbjct: 275 AGCCCTGAAACCCGACCGG 257
>miR9653a-3p Score: 2.5 RO3G_16737
Query: 1 TTGGGACTTTGGGCTGGCC 19
|:*|||||:||:|||||||
Sbjct: 449 AGGCCTGAGACTCGACCGG 431
>miR9653a-3p Score: 2.0 RO3G_16377
Query: 1 TTGGGACTTTGGGCTGGCC 19
|::|:||||||:|||||||
Sbjct: 140 AGTCTTGAAACTCGACCGG 122
>miR9653a-3p Score: 2.5 RO3G_16541
Query: 1 TTGGGACTTTGGGCTGGCC 19
|:*|||||:||:|||||||
Sbjct: 449 AGGCCTGAGACTCGACCGG 431
>miR9653a-3p Score: 2.0 RO3G_16548
Query: 1 TTGGGACTTTGGGCTGGCC 19
|::||||||||:||||||*
Sbjct: 470 AGTCCTGAAACTCGACCGT 452
>miR9653a-3p Score: 2.5 RO3G_16141
Query: 1 TTGGGACTTTGGGCTGGCC 19
|:*|||||:||:|||||||
Sbjct: 449 AGGCCTGAGACTCGACCGG 431
>miR9653a-3p Score: 1.5 RO3G_16118
Query: 1 TTGGGACTTTGGGCTGGCC 19
|::||||||||:|||||||
Sbjct: 83 AGTCCTGAAACTCGACCGG 65
>miR9653a-3p Score: 2.5 RO3G_16121
Query: 1 TTGGGACTTTGGGCTGGCC 19
|:*|||||:||:|||||||
Sbjct: 404 AGGCCTGAGACTCGACCGG 386
>miR9653a-3p Score: 2.5 RO3G_15672
Query: 1 TTGGGACTTTGGGCTGGCC 19
|::||||||||:||*||||
Sbjct: 632 AGTCCTGAAACTCGTCCGG 614

>miR9653a-3p Score: 1.5 RO3G_15873
Query: 1 TTGGGACTTTGGGCTGGCC 19
|::||||||||:|||||||
Sbjct: 815 AGTCCTGAAACTCGACCGG 797
>miR9653a-3p Score: 2.5 RO3G_15286
Query: 1 TTGGGACTTTGGGCTGGCC 19
|:*|||||:||:|||||||
Sbjct: 449 AGGCCTGAGACTCGACCGG 431
>miR9653a-3p Score: 2.5 RO3G_14972
Query: 1 TTGGGACTTTGGGCTGGCC 19
|:|||||||||*|*|||||
Sbjct: 746 AGCCCTGAAACACCACCGG 728
>miR9653a-3p Score: 2.5 RO3G_14981
Query: 1 TTGGGACTTTGGGCTGGCC 19
|:*|||||:||:|||||||
Sbjct: 449 AGGCCTGAGACTCGACCGG 431
>miR9653a-3p Score: 2.5 RO3G_14987
Query: 1 TTGGGACTTTGGGCTGGCC 19
|::||||||||:||*||||
Sbjct: 479 AGTCCTGAAACTCGTCCGG 461
>miR9653a-3p Score: 2.0 RO3G_14989
Query: 1 TTGGGACTTTGGGCTGGCC 19
|:|||||||||:||*||||
Sbjct: 671 AGCCCTGAAACTCGTCCGG 653
>miR9653a-3p Score: 2.5 RO3G_15145
Query: 1 TTGGGACTTTGGGCTGGCC 19
|:*|||||:||:|||||||
Sbjct: 449 AGGCCTGAGACTCGACCGG 431
>miR9653a-3p Score: 1.5 RO3G_15154
Query: 1 TTGGGACTTTGGGCTGGCC 19
|::||||||||:|||||||
Sbjct: 815 AGTCCTGAAACTCGACCGG 797
>miR9653a-3p Score: 2.5 RO3G_15170
Query: 1 TTGGGACTTTGGGCTGGCC 19
|:*|||||:||:|||||||
Sbjct: 449 AGGCCTGAGACTCGACCGG 431
>miR9653a-3p Score: 2.0 RO3G_14361
Query: 1 TTGGGACTTTGGGCTGGCC 19
|::||||||||||*|||||
Sbjct: 815 AGTCCTGAAACCCTACCGG 797
>miR9653a-3p Score: 2.0 RO3G_14568
Query: 1 TTGGGACTTTGGGCTGGCC 19
|::||||||||||*|||||
Sbjct: 815 AGTCCTGAAACCCTACCGG 797
>miR9653a-3p Score: 2.5 RO3G_14620

Query: 1 TTGGGACTTTGGGCTGGCC 19
|::||||||||:||*||||
Sbjct: 260 AGTCCTGAAACTCGTCCGG 242
>miR9653a-3p Score: 2.5 RO3G_14772
Query: 1 TTGGGACTTTGGGCTGGCC 19
|::||||||||:||*||||
Sbjct: 710 AGTCCTGAAACTCGTCCGG 692
>miR9653a-3p Score: 2.5 RO3G_14784
Query: 1 TTGGGACTTTGGGCTGGCC 19
|:*|||||:||:|||||||
Sbjct: 449 AGGCCTGAGACTCGACCGG 431
>miR9653a-3p Score: 1.5 RO3G_13447
Query: 1 TTGGGACTTTGGGCTGGCC 19
|::||||||||:|||||||
Sbjct: 815 AGTCCTGAAACTCGACCGG 797
>miR9653a-3p Score: 1.5 RO3G_13706
Query: 1 TTGGGACTTTGGGCTGGCC 19
|::||||||||:|||||||
Sbjct: 773 AGTCCTGAAACTCGACCGG 755
>miR9653a-3p Score: 2.5 RO3G_13752
Query: 1 TTGGGACTTTGGGCTGGCC 19
|:*|||||:||:|||||||
Sbjct: 449 AGGCCTGAGACTCGACCGG 431
>miR9653a-3p Score: 2.0 RO3G_13857
Query: 1 TTGGGACTTTGGGCTGGCC 19
|:|||||||||:||*||||
Sbjct: 455 AGCCCTGAAACTCGTCCGG 437
>miR9653a-3p Score: 2.5 RO3G_12670
Query: 1 TTGGGACTTTGGGCTGGCC 19
|::||||||||:||*||||
Sbjct: 632 AGTCCTGAAACTCGTCCGG 614
>miR9653a-3p Score: 2.5 RO3G_12950
Query: 1 TTGGGACTTTGGGCTGGCC 19
|:*|||||:||:|||||||
Sbjct: 449 AGGCCTGAGACTCGACCGG 431
>miR9653a-3p Score: 1.5 RO3G_13276
Query: 1 TTGGGACTTTGGGCTGGCC 19
|::||||||||:|||||||
Sbjct: 197 AGTCCTGAAACTCGACCGG 179
>miR9653a-3p Score: 2.0 RO3G_11966
Query: 1 TTGGGACTTTGGGCTGGCC 19
|::||||||||||*|||||
Sbjct: 815 AGTCCTGAAACCCTACCGG 797
>miR9653a-3p Score: 1.5 RO3G_12013
Query: 1 TTGGGACTTTGGGCTGGCC 19

|::||||||||:|||||||
Sbjct: 140 AGTCCTGAAACTCGACCGG 122
>miR9653a-3p Score: 2.5 RO3G_12082
Query: 1 TTGGGACTTTGGGCTGGCC 19
|::||||||||:||*||||
Sbjct: 710 AGTCCTGAAACTCGTCCGG 692
>miR9653a-3p Score: 1.5 RO3G_12174
Query: 1 TTGGGACTTTGGGCTGGCC 19
|::||||||||:|||||||
Sbjct: 140 AGTCCTGAAACTCGACCGG 122
>miR9653a-3p Score: 2.0 RO3G_12186
Query: 1 TTGGGACTTTGGGCTGGCC 19
|:*||||||||:|||||||
Sbjct: 824 AGACCTGAAACTCGACCGG 806
>miR9653a-3p Score: 2.0 RO3G_12196
Query: 1 TTGGGACTTTGGGCTGGCC 19
|:*||||||||:|||||||
Sbjct: 746 AGACCTGAAACTCGACCGG 728
>miR9653a-3p Score: 1.0 RO3G_12525
Query: 1 TTGGGACTTTGGGCTGGCC 19
|:|||||||||:|||||||
Sbjct: 746 AGCCCTGAAACTCGACCGG 728
>miR9653a-3p Score: 1.5 RO3G_12526
Query: 1 TTGGGACTTTGGGCTGGCC 19
|::||||||||:|||||||
Sbjct: 815 AGTCCTGAAACTCGACCGG 797
>miR9653a-3p Score: 2.5 RO3G_11158
Query: 1 TTGGGACTTTGGGCTGGCC 19
|||::|||*|||:||||||
Sbjct: 480 AACTTTGACACCTGACCGG 462
>miR9653a-3p Score: 1.5 RO3G_11244
Query: 1 TTGGGACTTTGGGCTGGCC 19
|::||||||||:|||||||
Sbjct: 815 AGTCCTGAAACTCGACCGG 797
>miR9653a-3p Score: 1.5 RO3G_11263
Query: 1 TTGGGACTTTGGGCTGGCC 19
|::||||||||:|||||||
Sbjct: 140 AGTCCTGAAACTCGACCGG 122
>miR9653a-3p Score: 1.0 RO3G_11273
Query: 1 TTGGGACTTTGGGCTGGCC 19
|:|||||||||:|||||||
Sbjct: 155 AGCCCTGAAACTCGACCGG 137
>miR9653a-3p Score: 2.5 RO3G_11405
Query: 1 TTGGGACTTTGGGCTGGCC 19
|::||||||||:||*||||

Sbjct: 632 AGTCCTGAAACTCGTCCGG 614
>miR9653a-3p Score: 1.5 RO3G_11528
Query: 1 TTGGGACTTTGGGCTGGCC 19
|::||||||||:|||||||
Sbjct: 140 AGTCCTGAAACTCGACCGG 122
>miR9653a-3p Score: 2.5 RO3G_11533
Query: 1 TTGGGACTTTGGGCTGGCC 19
|:*|||||:||:|||||||
Sbjct: 449 AGGCCTGAGACTCGACCGG 431
>miR9653a-3p Score: 2.5 RO3G_11534
Query: 1 TTGGGACTTTGGGCTGGCC 19
|::||*|||||:|||||||
Sbjct: 815 AGTCCCGAAACTCGACCGG 797
>miR9653a-3p Score: 2.5 RO3G_11536
Query: 1 TTGGGACTTTGGGCTGGCC 19
|:*|||||:||:|||||||
Sbjct: 449 AGGCCTGAGACTCGACCGG 431
>miR9653a-3p Score: 1.5 RO3G_11948
Query: 1 TTGGGACTTTGGGCTGGCC 19
|::||||||||:|||||||
Sbjct: 815 AGTCCTGAAACTCGACCGG 797
>miR9653a-3p Score: 1.5 RO3G_10528
Query: 1 TTGGGACTTTGGGCTGGCC 19
|::||||||||:|||||||
Sbjct: 140 AGTCCTGAAACTCGACCGG 122
>miR9653a-3p Score: 1.5 RO3G_10537
Query: 1 TTGGGACTTTGGGCTGGCC 19
|::||||||||:|||||||
Sbjct: 638 AGTCCTGAAACTCGACCGG 620
>miR9653a-3p Score: 1.5 RO3G_10720
Query: 1 TTGGGACTTTGGGCTGGCC 19
|::||||||||:|||||||
Sbjct: 815 AGTCCTGAAACTCGACCGG 797
>miR9653a-3p Score: 2.5 RO3G_10729
Query: 1 TTGGGACTTTGGGCTGGCC 19
|::||||||||||*|:|||
Sbjct: 815 AGTCCTGAAACCCTATCGG 797
>miR9653a-3p Score: 1.5 RO3G_10742
Query: 1 TTGGGACTTTGGGCTGGCC 19
|::||||||||:|||||||
Sbjct: 140 AGTCCTGAAACTCGACCGG 122
>miR9653a-3p Score: 1.5 RO3G_10805
Query: 1 TTGGGACTTTGGGCTGGCC 19
|:||||*||||||||||||
Sbjct: 824 AGCCCTTAAACCCGACCGG 806

>miR9653a-3p Score: 0.0 RO3G_10862
Query: 1 TTGGGACTTTGGGCTGGCC 19
|||||||||||||||||||
Sbjct: 347 AACCCTGAAACCCGACCGG 329
>miR9653a-3p Score: 1.5 RO3G_09199
Query: 1 TTGGGACTTTGGGCTGGCC 19
|::||||||||:|||||||
Sbjct: 815 AGTCCTGAAACTCGACCGG 797
>miR9653a-3p Score: 1.5 RO3G_09924
Query: 1 TTGGGACTTTGGGCTGGCC 19
|::||||||||:|||||||
Sbjct: 815 AGTCCTGAAACTCGACCGG 797
>miR9653a-3p Score: 2.5 RO3G_09926
Query: 1 TTGGGACTTTGGGCTGGCC 19
|:*|||||:||:|||||||
Sbjct: 449 AGGCCTGAGACTCGACCGG 431
>miR9653a-3p Score: 2.5 RO3G_09944
Query: 1 TTGGGACTTTGGGCTGGCC 19
|::||||||||:||*||||
Sbjct: 632 AGTCCTGAAACTCGTCCGG 614
>miR9653a-3p Score: 1.0 RO3G_09955
Query: 1 TTGGGACTTTGGGCTGGCC 19
|:|||||||||:|||||||
Sbjct: 509 AGCCCTGAAACTCGACCGG 491
>miR9653a-3p Score: 2.0 RO3G_09967
Query: 1 TTGGGACTTTGGGCTGGCC 19
|::||||||||||*|||||
Sbjct: 815 AGTCCTGAAACCCTACCGG 797
>miR9653a-3p Score: 1.5 RO3G_10044
Query: 1 TTGGGACTTTGGGCTGGCC 19
|::||||||||:|||||||
Sbjct: 815 AGTCCTGAAACTCGACCGG 797
>miR9653a-3p Score: 2.5 RO3G_10050
Query: 1 TTGGGACTTTGGGCTGGCC 19
|:*|||||:||:|||||||
Sbjct: 449 AGGCCTGAGACTCGACCGG 431
>miR9653a-3p Score: 2.5 RO3G_10224
Query: 1 TTGGGACTTTGGGCTGGCC 19
|::||||||||:||*||||
Sbjct: 632 AGTCCTGAAACTCGTCCGG 614
>miR9653a-3p Score: 2.5 RO3G_10226
Query: 1 TTGGGACTTTGGGCTGGCC 19
|::||||||||:||*||||
Sbjct: 632 AGTCCTGAAACTCGTCCGG 614
>miR9653a-3p Score: 1.5 RO3G_07994

```
Query: 1 TTGGGACTTTGGGCTGGCC 19
|::||||||||:|||||||
Sbjct: 815 AGTCCTGAAACTCGACCGG 797
>miR9653a-3p Score: 2.5 RO3G_08649
Query: 1 TTGGGACTTTGGGCTGGCC 19
|::||||||||:||*||||
Sbjct: 577 AGTCCTGAAACTCGTCCGG 559
>miR9653a-3p Score: 2.0 RO3G_08658
Query: 1 TTGGGACTTTGGGCTGGCC 19
|:|||||||||:||*||||
Sbjct: 632 AGCCCTGAAACTCGTCCGG 614
>miR9653a-3p Score: 1.5 RO3G_08835
Query: 1 TTGGGACTTTGGGCTGGCC 19
|::||||||||:|||||||
Sbjct: 125 AGTCCTGAAACTCGACCGG 107
>miR9653a-3p Score: 2.5 RO3G_08848
Query: 1 TTGGGACTTTGGGCTGGCC 19
|::||*|||||:|||||||
Sbjct: 125 AGTCCCGAAACTCGACCGG 107
>miR9653a-3p Score: 1.0 RO3G_07024
Query: 1 TTGGGACTTTGGGCTGGCC 19
|:|||||||||:|||||||
Sbjct: 824 AGCCCTGAAACTCGACCGG 806
>miR9653a-3p Score: 1.5 RO3G_07119
Query: 1 TTGGGACTTTGGGCTGGCC 19
|::||||||||:|||||||
Sbjct: 140 AGTCCTGAAACTCGACCGG 122
>miR9653a-3p Score: 1.0 RO3G_07121
Query: 1 TTGGGACTTTGGGCTGGCC 19
|:|||||||||:|||||||
Sbjct: 824 AGCCCTGAAACTCGACCGG 806
>miR9653a-3p Score: 1.5 RO3G_07295
Query: 1 TTGGGACTTTGGGCTGGCC 19
|::||||||||:|||||||
Sbjct: 647 AGTCCTGAAACTCGACCGG 629
>miR9653a-3p Score: 2.5 RO3G_07298
Query: 1 TTGGGACTTTGGGCTGGCC 19
|:*|||||:||:|||||||
Sbjct: 449 AGGCCTGAGACTCGACCGG 431
>miR9653a-3p Score: 2.5 RO3G_07302
Query: 1 TTGGGACTTTGGGCTGGCC 19
|::||||||||:||*||||
Sbjct: 632 AGTCCTGAAACTCGTCCGG 614
>miR9653a-3p Score: 2.0 RO3G_07651
Query: 1 TTGGGACTTTGGGCTGGCC 19
```

|||||||||||:|||:*||
Sbjct: 5211 AACCCTGAAACTCGAT-GG 5194
>miR9653a-3p Score: 1.5 RO3G_07683
Query: 1 TTGGGACTTTGGGCTGGCC 19
|::||||||||:|||||||
Sbjct: 815 AGTCCTGAAACTCGACCGG 797
>miR9653a-3p Score: 2.5 RO3G_07689
Query: 1 TTGGGACTTTGGGCTGGCC 19
|::||||||||:||*||||
Sbjct: 632 AGTCCTGAAACTCGTCCGG 614
>miR9653a-3p Score: 2.5 RO3G_07723
Query: 1 TTGGGACTTTGGGCTGGCC 19
|:*|||||:||:|||||||
Sbjct: 449 AGGCCTGAGACTCGACCGG 431
>miR9653a-3p Score: 2.0 RO3G_07860
Query: 1 TTGGGACTTTGGGCTGGCC 19
|::||||||||||*|||||
Sbjct: 815 AGTCCTGAAACCCTACCGG 797
>miR9653a-3p Score: 2.5 RO3G_05522
Query: 1 TTGGGACTTTGGGCTGGCC 19
|:*|||||:||:|||||||
Sbjct: 815 AGGCCTGAGACTCGACCGG 797
>miR9653a-3p Score: 1.5 RO3G_05916
Query: 1 TTGGGACTTTGGGCTGGCC 19
|::||||||||:|||||||
Sbjct: 815 AGTCCTGAAACTCGACCGG 797
>miR9653a-3p Score: 2.0 RO3G_05921
Query: 1 TTGGGACTTTGGGCTGGCC 19
|::||||||||||*|||||
Sbjct: 815 AGTCCTGAAACCCTACCGG 797
>miR9653a-3p Score: 0.5 RO3G_05950
Query: 1 TTGGGACTTTGGGCTGGCC 19
|:|||||||||||||||||
Sbjct: 293 AGCCCTGAAACCCGACCGG 275
>miR9653a-3p Score: 2.0 RO3G_05953
Query: 1 TTGGGACTTTGGGCTGGCC 19
|::||||||||||*|||||
Sbjct: 815 AGTCCTGAAACCCTACCGG 797
>miR9653a-3p Score: 2.0 RO3G_05959
Query: 1 TTGGGACTTTGGGCTGGCC 19
|::||||||||||*|||||
Sbjct: 815 AGTCCTGAAACCCTACCGG 797
>miR9653a-3p Score: 2.5 RO3G_06721
Query: 1 TTGGGACTTTGGGCTGGCC 19
|::||||||||:||*||||

```
Sbjct: 629 AGTCCTGAAACTCGTCCGG 611
>miR9653a-3p Score: 2.5 RO3G_04654
Query: 1 TTGGGACTTTGGGCTGGCC 19
|::||||||||:||*||||
Sbjct: 632 AGTCCTGAAACTCGTCCGG 614
>miR9653a-3p Score: 1.5 RO3G_04661
Query: 1 TTGGGACTTTGGGCTGGCC 19
|:||||||||||||*||||
Sbjct: 455 AGCCCTGAAACCCGTCCGG 437
>miR9653a-3p Score: 2.5 RO3G_04668
Query: 1 TTGGGACTTTGGGCTGGCC 19
|:*|||||:||:|||||||
Sbjct: 626 AGGCCTGAGACTCGACCGG 608
>miR9653a-3p Score: 1.0 RO3G_04786
Query: 1 TTGGGACTTTGGGCTGGCC 19
|:|||||||||:|||||||
Sbjct: 392 AGCCCTGAAACTCGACCGG 374
>miR9653a-3p Score: 2.0 RO3G_05267
Query: 1 TTGGGACTTTGGGCTGGCC 19
|::||||||||||*|||||
Sbjct: 815 AGTCCTGAAACCCTACCGG 797
>miR9653a-3p Score: 2.5 RO3G_05516
Query: 1 TTGGGACTTTGGGCTGGCC 19
|:*|||||:||:|||||||
Sbjct: 449 AGGCCTGAGACTCGACCGG 431
>miR9653a-3p Score: 1.0 RO3G_02700
Query: 1 TTGGGACTTTGGGCTGGCC 19
|:|||||||||:|||||||
Sbjct: 509 AGCCCTGAAACTCGACCGG 491
>miR9653a-3p Score: 1.5 RO3G_03558
Query: 1 TTGGGACTTTGGGCTGGCC 19
|::||||||||:|||||||
Sbjct: 815 AGTCCTGAAACTCGACCGG 797
>miR9653a-3p Score: 2.5 RO3G_03560
Query: 1 TTGGGACTTTGGGCTGGCC 19
|:*|||||:||:|||||||
Sbjct: 449 AGGCCTGAGACTCGACCGG 431
>miR9653a-3p Score: 1.0 RO3G_03570
Query: 1 TTGGGACTTTGGGCTGGCC 19
|:|||||||||:|||||||
Sbjct: 509 AGCCCTGAAACTCGACCGG 491
>miR9653a-3p Score: 2.0 RO3G_03599
Query: 1 TTGGGACTTTGGGCTGGCC 19
|:||||*||||:|||||||
Sbjct: 824 AGCCCTAAAACTCGACCGG 806
```

>miR9653a-3p Score: 0.5 RO3G_03605
Query: 1 TTGGGACTTTGGGCTGGCC 19
|:|||||||||||||||||
Sbjct: 1277 AGCCCTGAAACCCGACCGG 1259
>miR9653a-3p Score: 1.5 RO3G_03617
Query: 1 TTGGGACTTTGGGCTGGCC 19
|::||||||||:|||||||
Sbjct: 815 AGTCCTGAAACTCGACCGG 797
>miR9653a-3p Score: 2.5 RO3G_03785
Query: 1 TTGGGACTTTGGGCTGGCC 19
|::||||||||:||*||||
Sbjct: 437 AGTCCTGAAACTCGTCCGG 419
>miR9653a-3p Score: 2.5 RO3G_03791
Query: 1 TTGGGACTTTGGGCTGGCC 19
|:*|||||:||:|||||||
Sbjct: 449 AGGCCTGAGACTCGACCGG 431
>miR9653a-3p Score: 2.5 RO3G_03797
Query: 1 TTGGGACTTTGGGCTGGCC 19
|::||||||||:||*||||
Sbjct: 320 AGTCCTGAAACTCGTCCGG 302
>miR9653a-3p Score: 1.5 RO3G_03987
Query: 1 TTGGGACTTTGGGCTGGCC 19
|::||||||||:|||||||
Sbjct: 815 AGTCCTGAAACTCGACCGG 797
>miR9653a-3p Score: 2.5 RO3G_04200
Query: 1 TTGGGACTTTGGGCTGGCC 19
|::||||||||:||*||||
Sbjct: 632 AGTCCTGAAACTCGTCCGG 614
>miR9653a-3p Score: 2.5 RO3G_04207
Query: 1 TTGGGACTTTGGGCTGGCC 19
|::|:||||||||*|||||
Sbjct: 815 AGTCTTGAAACCCTACCGG 797
>miR9653a-3p Score: 2.5 RO3G_00240
Query: 1 TTGGGACTTTGGGCTGGCC 19
|:*|||||:||:|||||||
Sbjct: 758 AGGCCTGAGACTCGACCGG 740
>miR9653a-3p Score: 2.5 RO3G_00247
Query: 1 TTGGGACTTTGGGCTGGCC 19
|::||||||||:||*||||
Sbjct: 632 AGTCCTGAAACTCGTCCGG 614
>miR9653a-3p Score: 2.0 RO3G_00256
Query: 1 TTGGGACTTTGGGCTGGCC 19
|::||||||||||*|||||
Sbjct: 815 AGTCCTGAAACCCTACCGG 797
>miR9653a-3p Score: 2.5 RO3G_00675

Query: 1 TTGGGACTTTGGGCTGGCC 19
|:*|||||:||:|||||||
Sbjct: 449 AGGCCTGAGACTCGACCGG 431
>miR9653a-3p Score: 0.5 RO3G_00677
Query: 1 TTGGGACTTTGGGCTGGCC 19
|:|||||||||||||||||
Sbjct: 377 AGCCCTGAAACCCGACCGG 359
>miR9653a-3p Score: 2.5 RO3G_00689
Query: 1 TTGGGACTTTGGGCTGGCC 19
|:*|||||:||:|||||||
Sbjct: 317 AGGCCTGAGACTCGACCGG 299
>miR9653a-3p Score: 1.0 RO3G_00690
Query: 1 TTGGGACTTTGGGCTGGCC 19
|:||||||||:|||||||
Sbjct: 764 AGCCCTGAAACTCGACCGG 746
>miR9653a-3p Score: 1.5 RO3G_01194
Query: 1 TTGGGACTTTGGGCTGGCC 19
|::||||||||:|||||||
Sbjct: 815 AGTCCTGAAACTCGACCGG 797
>miR9653a-3p Score: 1.5 RO3G_01313
Query: 1 TTGGGACTTTGGGCTGGCC 19
|::||||||||:|||||||
Sbjct: 815 AGTCCTGAAACTCGACCGG 797
>miR9653a-3p Score: 2.0 RO3G_01587
Query: 1 TTGGGACTTTGGGCTGGCC 19
|:||||||||:||*||||
Sbjct: 641 AGCCCTGAAACTCGTCCGG 623
>miR9653a-3p Score: 0.5 RO3G_01588
Query: 1 TTGGGACTTTGGGCTGGCC 19
|:|||||||||||||||||
Sbjct: 155 AGCCCTGAAACCCGACCGG 137
>miR9653a-3p Score: 2.5 RO3G_01725
Query: 1 TTGGGACTTTGGGCTGGCC 19
|:*|||||:||:|||||||
Sbjct: 449 AGGCCTGAGACTCGACCGG 431
>miR9653a-3p Score: 1.5 RO3G_01735
Query: 1 TTGGGACTTTGGGCTGGCC 19
|::||||||||:|||||||
Sbjct: 815 AGTCCTGAAACTCGACCGG 797
>miR9653a-3p Score: 1.0 RO3G_01925
Query: 1 TTGGGACTTTGGGCTGGCC 19
|:||||||||:|||||||
Sbjct: 824 AGCCCTGAAACTCGACCGG 806
>miR9653a-3p Score: 1.5 RO3G_01950
Query: 1 TTGGGACTTTGGGCTGGCC 19

|::||||||||:|||||||
Sbjct: 815 AGTCCTGAAACTCGACCGG 797
>miR9653a-3p Score: 1.5 RO3G_01951
Query: 1 TTGGGACTTTGGGCTGGCC 19
|::||||||||:|||||||
Sbjct: 815 AGTCCTGAAACTCGACCGG 797
>miR9653a-3p Score: 2.5 RO3G_02227
Query: 1 TTGGGACTTTGGGCTGGCC 19
|:*|||||:||:|||||||
Sbjct: 815 AGGCCTGAGACTCGACCGG 797
>miR9653a-3p Score: 2.0 RO3G_17449
Query: 1 TTGGGACTTTGGGCTGGCC 19
|::||||||||||*|||||
Sbjct: 815 AGTCCTGAAACCCTACCGG 797
>miR9653a-3p Score: 2.5 RO3G_17436
Query: 1 TTGGGACTTTGGGCTGGCC 19
|::||||||||:||*||||
Sbjct: 632 AGTCCTGAAACTCGTCCGG 614
>miR9653a-3p Score: 2.5 RO3G_17423
Query: 1 TTGGGACTTTGGGCTGGCC 19
|:*|||||:||:|||||||
Sbjct: 449 AGGCCTGAGACTCGACCGG 431
>miR9653a-3p Score: 1.5 RO3G_17409
Query: 1 TTGGGACTTTGGGCTGGCC 19
|::||||||||:|||||||
Sbjct: 815 AGTCCTGAAACTCGACCGG 797
>miR9653a-3p Score: 0.5 RO3G_17406
Query: 1 TTGGGACTTTGGGCTGGCC 19
|:|||||||||||||||||
Sbjct: 176 AGCCCTGAAACCCGACCGG 158
>miR9653a-3p Score: 2.0 RO3G_17361
Query: 1 TTGGGACTTTGGGCTGGCC 19
|:|||||||||:||*||||
Sbjct: 392 AGCCCTGAAACTCGTCCGG 374
>miR9653a-3p Score: 2.5 RO3G_17328
Query: 1 TTGGGACTTTGGGCTGGCC 19
|:*|||||:||:|||||||
Sbjct: 449 AGGCCTGAGACTCGACCGG 431
>miR9653a-3p Score: 2.5 RO3G_17293
Query: 1 TTGGGACTTTGGGCTGGCC 19
|::||||||||:||*||||
Sbjct: 632 AGTCCTGAAACTCGTCCGG 614
>miR9653a-3p Score: 2.5 RO3G_17092
Query: 1 TTGGGACTTTGGGCTGGCC 19
|:*|||||:||:|||||||

```
Sbjct: 449 AGGCCTGAGACTCGACCGG 431
>miR419 Score: 2.0 RO3G_17196
Query: 1 TGAGGAATGATGACGTGA-G 19
|||:||||:||||||||**|
Sbjct: 518 ACTTCTTATTACTGCACAAC 499
>miR419 Score: 2.5 RO3G_14928
Query: 1 TGAGGAATGATGACGTGAG 19
*|||*|||||||||::|||
Sbjct: 47 TCTCATTACTACTGTGCTC 29
>miR419 Score: 2.5 RO3G_14934
Query: 1 TGAGGAATGATGACGTGAG 19
*|||*|||||||||::|||
Sbjct: 47 TCTCATTACTACTGTGCTC 29
>miR419 Score: 1.8 RO3G_13948
Query: 1 TGAGGAATGATGACGTGAG 19
:||:|||||||||*|||||
Sbjct: 1827 GCTTCTTACTACTCCACTC 1809
>miR419 Score: 2.5 RO3G_11316
Query: 1 TGAGGAATGATGACGTGAG 19
||*|||||:||:||||||*
Sbjct: 849 ACACCTTATTATTGCACTA 831
>miR419 Score: 2.5 RO3G_08802
Query: 1 TGAGGAATGATGACGTGAG 19
||||||||||||*|||:**
Sbjct: 626 ACTCCTTACTACAGCATAA 608
>miR419 Score: 2.0 RO3G_05065
Query: 1 TGAGGAATGATGACGTGA-G 19
|||:||||:||||||||**|
Sbjct: 518 ACTTCTTATTACTGCACAAC 499
>miR419 Score: 2.5 RO3G_02287
Query: 1 TGAGGAATGATGACGTGAG 19
|||:|||||||:*||||*|
Sbjct: 153 ACTTCTTACTATCGCAC-C 136
>miR419 Score: 2.2 RO3G_02333
Query: 1 TGAGGAATGATGACGTGAG 19
|||:|||:|||||*||||:
Sbjct: 138 ACTTCTTGCTACTACACTT 120
>miR419 Score: 2.5 RO3G_02459
Query: 1 TGAGGAATGATGACGTGAG 19
|||||||:||||*|:||*|
Sbjct: 656 ACTCCTTGCTACCGTACAC 638
>miR419 Score: 0.0 RO3G_17296
Query: 1 TGAGGAATGATGACGTGAG 19
|||||||||||||||||||
Sbjct: 192 ACTCCTTACTACTGCACTC 174
```

>miR419 Score: 2.5 RO3G_17114
Query: 1 TGAGGAATGATGACGTGAG 19
|||*||||||||||*|||*
Sbjct: 491 ACTACTTACTACTGAACTA 473
>miR6477 Score: 2.5 RO3G_17265
Query: 1 TGAACAGTAGAGGATGAT 18
|||||::|||||||||
Sbjct: 101 CCTTGTTGTCACCTACTA 84
>miR6477 Score: 2.5 RO3G_17244
Query: 1 TGAACAGTAGAGGATGAT 18
|||||::|||||||||
Sbjct: 101 CCTTGTTGTCACCTACTA 84
>miR6477 Score: 2.5 RO3G_17210
Query: 1 TGAACAGTAGAGGATGAT 18
|||||::|||||||||
Sbjct: 101 CCTTGTTGTCACCTACTA 84
>miR6477 Score: 0.0 RO3G_17215
Query: 1 TGAACAGTAGAGGATGAT 18
||||||||||||||||||
Sbjct: 110 ACTTGTCATCTCCTACTA 93
>miR6477 Score: 0.0 RO3G_16897
Query: 1 TGAACAGTAGAGGATGAT 18
||||||||||||||||||
Sbjct: 110 ACTTGTCATCTCCTACTA 93
>miR6477 Score: 2.5 RO3G_16743
Query: 1 TGAACAGTAGAGGATGAT 18
|||||::|||||||||
Sbjct: 101 CCTTGTTGTCACCTACTA 84
>miR6477 Score: 2.5 RO3G_16748
Query: 1 TGAACAGTAGAGGATGAT 18
|||||::|||||||||
Sbjct: 101 CCTTGTTGTCACCTACTA 84
>miR6477 Score: 0.0 RO3G_16807
Query: 1 TGAACAGTAGAGGATGAT 18
||||||||||||||||||
Sbjct: 110 ACTTGTCATCTCCTACTA 93
>miR6477 Score: 2.5 RO3G_16369
Query: 1 TGAACAGTAGAGGATGAT 18
|||||::|||||||||
Sbjct: 101 CCTTGTTGTCACCTACTA 84
>miR6477 Score: 2.2 RO3G_16436
Query: 1 TGAACAGTAGAGGATGAT 18
::|||||:||||:||:||
Sbjct: 444 GTTTGTCGTCTCTTATTA 427
>miR6477 Score: 2.0 RO3G_16482

Query: 1 TGAACAGTAGAGGATGAT 18
||*|*|||||||||||||
Sbjct: 122 ACATTTCATCTCCTACTA 105
>miR6477 Score: 1.0 RO3G_16551
Query: 1 TGAACAGTAGAGGATGAT 18
||||||||*|||||||||
Sbjct: 110 ACTTGTCACCTCCTACTA 93
>miR6477 Score: 1.0 RO3G_16555
Query: 1 TGAACAGTAGAGGATGAT 18
||||||||*|||||||||
Sbjct: 110 ACTTGTCACCTCCTACTA 93
>miR6477 Score: 2.5 RO3G_15879
Query: 1 TGAACAGTAGAGGATGAT 18
|||||::|||||||||
Sbjct: 101 CCTTGTTGTCACCTACTA 84
>miR6477 Score: 2.5 RO3G_15897
Query: 1 TGAACAGTAGAGGATGAT 18
|||||::|||||||||
Sbjct: 101 CCTTGTTGTCACCTACTA 84
>miR6477 Score: 1.5 RO3G_16099
Query: 1 TGAACAGTAGAGGATGAT 18
|||*|||||||:||||||
Sbjct: 764 ACTAGTCATCTTCTACTA 747
>miR6477 Score: 0.0 RO3G_15675
Query: 1 TGAACAGTAGAGGATGAT 18
||||||||||||||||||
Sbjct: 110 ACTTGTCATCTCCTACTA 93
>miR6477 Score: 2.5 RO3G_14963
Query: 1 TGAACAGTAGAGGATGAT 18
*|||||||||**||||||
Sbjct: 615 CCTTGTCATCAACTACTA 598
>miR6477 Score: 0.0 RO3G_14359
Query: 1 TGAACAGTAGAGGATGAT 18
||||||||||||||||||
Sbjct: 110 ACTTGTCATCTCCTACTA 93
>miR6477 Score: 2.5 RO3G_14551
Query: 1 TGAACAGTAGAGGATGAT 18
|||||::|||||||||
Sbjct: 101 CCTTGTTGTCACCTACTA 84
>miR6477 Score: 2.5 RO3G_14553
Query: 1 TGAACAGTAGAGGATGAT 18
|||||::|||||||||
Sbjct: 101 CCTTGTTGTCACCTACTA 84
>miR6477 Score: 2.5 RO3G_14690
Query: 1 TGAACAGTAGAGGATGAT 18

||||||||*|||||||
Sbjct: 122 CCATGTCATCACCTACTA 105
>miR6477 Score: 2.5 RO3G_14091
Query: 1 TGAACAGTAGAGGATGAT 18
||||||||:||||:||
Sbjct: 192 CCTTGTCATTTCCTGCCA 175
>miR6477 Score: 2.5 RO3G_13452
Query: 1 TGAACAGTAGAGGATGAT 18
|||||::|||||||||
Sbjct: 101 CCTTGTTGTCACCTACTA 84
>miR6477 Score: 1.0 RO3G_13726
Query: 1 TGAACAGTAGAGGATGAT 18
||||||||*|||||||||
Sbjct: 110 ACTTGTCACCTCCTACTA 93
>miR6477 Score: 1.0 RO3G_13742
Query: 1 TGAACAGTAGAGGATGAT 18
||||||||*|||||||||
Sbjct: 110 ACTTGTCACCTCCTACTA 93
>miR6477 Score: 1.0 RO3G_13848
Query: 1 TGAACAGTAGAGGATGAT 18
||||||||*|||||||||
Sbjct: 110 ACTTGTCACCTCCTACTA 93
>miR6477 Score: 2.5 RO3G_12976
Query: 1 TGAACAGTAGAGGATGAT 18
|||||::|||||||||
Sbjct: 101 CCTTGTTGTCACCTACTA 84
>miR6477 Score: 0.2 RO3G_13151
Query: 1 TGAACAGTAGAGGATGAT 18
|||||||||||||||||:
Sbjct: 110 ACTTGTCATCTCCTACTG 93
>miR6477 Score: 2.5 RO3G_13272
Query: 1 TGAACAGTAGAGGATGAT 18
|||||::|||||||||
Sbjct: 101 CCTTGTTGTCACCTACTA 84
>miR6477 Score: 2.5 RO3G_11977
Query: 1 TGAACAGTAGAGGATGAT 18
|||||::|||||||||
Sbjct: 101 CCTTGTTGTCACCTACTA 84
>miR6477 Score: 2.5 RO3G_12011
Query: 1 TGAACAGTAGAGGATGAT 18
|||||::|||||||||
Sbjct: 101 CCTTGTTGTCACCTACTA 84
>miR6477 Score: 2.5 RO3G_11236
Query: 1 TGAACAGTAGAGGATGAT 18
|||||::|||||||||

```
Sbjct: 101 CCTTGTTGTCACCTACTA 84
>miR6477 Score: 1.0 RO3G_11401
Query: 1 TGAACAGTAGAGGATGAT 18
||||||||*|||||||||
Sbjct: 110 ACTTGTCACCTCCTACTA 93
>miR6477 Score: 2.5 RO3G_11537
Query: 1 TGAACAGTAGAGGATGAT 18
*|||||::||*|||||||
Sbjct: 101 CCTTGTTGTCACCTACTA 84
>miR6477 Score: 2.5 RO3G_11543
Query: 1 TGAACAGTAGAGGATGAT 18
*|||||::||*|||||||
Sbjct: 101 CCTTGTTGTCACCTACTA 84
>miR6477 Score: 2.5 RO3G_11651
Query: 1 TGAACAGTAGAGGATGAT 18
:||||||*|||:||:||:
Sbjct: 3200 GCTTGTCTTCTTCTGCTG 3183
>miR6477 Score: 1.0 RO3G_11773
Query: 1 TGAACAGTAGAGGATGAT 18
||||||||*|||||||||
Sbjct: 110 ACTTGTCACCTCCTACTA 93
>miR6477 Score: 0.0 RO3G_10235
Query: 1 TGAACAGTAGAGGATGAT 18
||||||||||||||||||
Sbjct: 110 ACTTGTCATCTCCTACTA 93
>miR6477 Score: 0.0 RO3G_10732
Query: 1 TGAACAGTAGAGGATGAT 18
||||||||||||||||||
Sbjct: 110 ACTTGTCATCTCCTACTA 93
>miR6477 Score: 0.0 RO3G_09483
Query: 1 TGAACAGTAGAGGATGAT 18
||||||||||||||||||
Sbjct: 110 ACTTGTCATCTCCTACTA 93
>miR6477 Score: 0.0 RO3G_09499
Query: 1 TGAACAGTAGAGGATGAT 18
||||||||||||||||||
Sbjct: 110 ACTTGTCATCTCCTACTA 93
>miR6477 Score: 0.0 RO3G_09544
Query: 1 TGAACAGTAGAGGATGAT 18
||||||||||||||||||
Sbjct: 110 ACTTGTCATCTCCTACTA 93
>miR6477 Score: 2.5 RO3G_09602
Query: 1 TGAACAGTAGAGGATGAT 18
*|||||::||*|||||||
Sbjct: 101 CCTTGTTGTCACCTACTA 84
```

>miR6477 Score: 2.5 RO3G_09603
Query: 1 TGAACAGTAGAGGATGAT 18
|||||::|||||||||
Sbjct: 101 CCTTGTTGTCACCTACTA 84
>miR6477 Score: 1.0 RO3G_09607
Query: 1 TGAACAGTAGAGGATGAT 18
||||||||*|||||||||
Sbjct: 110 ACTTGTCACCTCCTACTA 93
>miR6477 Score: 2.5 RO3G_09842
Query: 1 TGAACAGTAGAGGATGAT 18
||||||:||||:|*|||*
Sbjct: 2078 ACTTGTTATCTTCAACTT 2061
>miR6477 Score: 2.5 RO3G_09929
Query: 1 TGAACAGTAGAGGATGAT 18
|||||::|||||||||
Sbjct: 101 CCTTGTTGTCACCTACTA 84
>miR6477 Score: 2.5 RO3G_10043
Query: 1 TGAACAGTAGAGGATGAT 18
|||||::|||||||||
Sbjct: 101 CCTTGTTGTCACCTACTA 84
>miR6477 Score: 2.5 RO3G_10218
Query: 1 TGAACAGTAGAGGATGAT 18
|||||::|||||||||
Sbjct: 101 CCTTGTTGTCACCTACTA 84
>miR6477 Score: 2.5 RO3G_07981
Query: 1 TGAACAGTAGAGGATGAT 18
|||||::|||||||||
Sbjct: 101 CCTTGTTGTCACCTACTA 84
>miR6477 Score: 2.2 RO3G_08245
Query: 1 TGAACAGTAGAGGATGAT 18
::|||||:|:||:|||||
Sbjct: 444 GTTTGTCGTTTCTTACTA 427
>miR6477 Score: 1.5 RO3G_08661
Query: 1 TGAACAGTAGAGGATGAT 18
|:||||||*|||||||||
Sbjct: 110 ATTTGTCACCTCCTACTA 93
>miR6477 Score: 1.0 RO3G_08846
Query: 1 TGAACAGTAGAGGATGAT 18
||||||||*|||||||||
Sbjct: 110 ACTTGTCACCTCCTACTA 93
>miR6477 Score: 2.0 RO3G_08852
Query: 1 TGAACAGTAGAGGATGAT 18
||||||:||||||:||
Sbjct: 578 CCTTGTCGTCTCCTGCTC 561
>miR6477 Score: 2.5 RO3G_06730

```
Query: 1 TGAACAGTAGAGGATGAT 18
*|||||::||*|||||||
Sbjct: 101 CCTTGTTGTCACCTACTA 84
>miR6477 Score: 2.5 RO3G_06990
Query: 1 TGAACAGTAGAGGATGAT 18
*||||||:||*|||:|||
Sbjct: 101 CCTTGTCGTCACCTGCTA 84
>miR6477 Score: 1.0 RO3G_07210
Query: 1 TGAACAGTAGAGGATGAT 18
||||||||||||||::||
Sbjct: 920 ACTTGTCATCTCCTGTTA 903
>miR6477 Score: 0.0 RO3G_07303
Query: 1 TGAACAGTAGAGGATGAT 18
||||||||||||||||||
Sbjct: 110 ACTTGTCATCTCCTACTA 93
>miR6477 Score: 2.5 RO3G_07306
Query: 1 TGAACAGTAGAGGATGAT 18
*|||||::||*|||||||
Sbjct: 101 CCTTGTTGTCACCTACTA 84
>miR6477 Score: 2.5 RO3G_07696
Query: 1 TGAACAGTAGAGGATGAT 18
*|||||::||*|||||||
Sbjct: 101 CCTTGTTGTCACCTACTA 84
>miR6477 Score: 1.0 RO3G_07704
Query: 1 TGAACAGTAGAGGATGAT 18
||||||||*|||||||||
Sbjct: 110 ACTTGTCACCTCCTACTA 93
>miR6477 Score: 2.5 RO3G_07720
Query: 1 TGAACAGTAGAGGATGAT 18
*|||||::||*|||||||
Sbjct: 101 CCTTGTTGTCACCTACTA 84
>miR6477 Score: 2.5 RO3G_07738
Query: 1 TGAACAGTAGAGGATGAT 18
*|||||::||*|||||||
Sbjct: 101 CCTTGTTGTCACCTACTA 84
>miR6477 Score: 0.0 RO3G_07875
Query: 1 TGAACAGTAGAGGATGAT 18
||||||||||||||||||
Sbjct: 110 ACTTGTCATCTCCTACTA 93
>miR6477 Score: 1.0 RO3G_05622
Query: 1 TGAACAGTAGAGGATGAT 18
||||||||*|||||||||
Sbjct: 110 ACTTGTCACCTCCTACTA 93
>miR6477 Score: 0.0 RO3G_05635
Query: 1 TGAACAGTAGAGGATGAT 18
```

```
||||||||||||||||||
Sbjct: 110 ACTTGTCATCTCCTACTA 93
>miR6477 Score: 2.5 RO3G_05906
Query: 1 TGAACAGTAGAGGATGAT 18
*|||||::||*|||||||
Sbjct: 101 CCTTGTTGTCACCTACTA 84
>miR6477 Score: 1.0 RO3G_05915
Query: 1 TGAACAGTAGAGGATGAT 18
||*|||||||||||||||
Sbjct: 110 ACGTGTCATCTCCTACTA 93
>miR6477 Score: 2.2 RO3G_06138
Query: 1 TGAACAGTAGAGGATGAT 18
|:||*|||||||:||||:
Sbjct: 531 ATTTTTCATCTCTTACTG 514
>miR6477 Score: 0.0 RO3G_06423
Query: 1 TGAACAGTAGAGGATGAT 18
||||||||||||||||||
Sbjct: 110 ACTTGTCATCTCCTACTA 93
>miR6477 Score: 0.0 RO3G_06441
Query: 1 TGAACAGTAGAGGATGAT 18
||||||||||||||||||
Sbjct: 110 ACTTGTCATCTCCTACTA 93
>miR6477 Score: 2.2 RO3G_06479
Query: 1 TGAACAGTAGAGGATGAT 18
:||||||:|||*||:|||
Sbjct: 4058 GCTTGTCGTCTGCTGCTA 4041
>miR6477 Score: 1.8 RO3G_04535
Query: 1 TGAACAGTAGAGGATGAT 18
:|||*||||||||||||*
Sbjct: 2036 GCTTATCATCTCCTACTC 2019
>miR6477 Score: 2.5 RO3G_04659
Query: 1 TGAACAGTAGAGGATGAT 18
*|||||::||*|||||||
Sbjct: 62 CCTTGTTGTCACCTACTA 45
>miR6477 Score: 2.5 RO3G_05171
Query: 1 TGAACAGTAGAGGATGAT 18
||||||:|||*||||:|*
Sbjct: 749 ACTTGTTATCGCCTATTC 732
>miR6477 Score: 0.0 RO3G_05336
Query: 1 TGAACAGTAGAGGATGAT 18
||||||||||||||||||
Sbjct: 110 ACTTGTCATCTCCTACTA 93
>miR6477 Score: 2.5 RO3G_05347
Query: 1 TGAACAGTAGAGGATGAT 18
*|||||::||*|||||||
```

```
Sbjct: 101 CCTTGTTGTCACCTACTA 84
>miR6477 Score: 2.5 RO3G_02228
Query: 1 TGAACAGTAGAGGATGAT 18
*|||||::||*|||||||
Sbjct: 101 CCTTGTTGTCACCTACTA 84
>miR6477 Score: 2.5 RO3G_02407
Query: 1 TGAACAGTAGAGGATGAT 18
|||||*|||:||||||*|
Sbjct: 417 ACTTGCCATTTCCTAC-A 401
>miR6477 Score: 0.0 RO3G_02689
Query: 1 TGAACAGTAGAGGATGAT 18
||||||||||||||||||
Sbjct: 110 ACTTGTCATCTCCTACTA 93
>miR6477 Score: 2.5 RO3G_02817
Query: 1 TGAACAGTAGAGGATGAT 18
*|||||::||*|||||||
Sbjct: 101 CCTTGTTGTCACCTACTA 84
>miR6477 Score: 0.0 RO3G_03109
Query: 1 TGAACAGTAGAGGATGAT 18
||||||||||||||||||
Sbjct: 110 ACTTGTCATCTCCTACTA 93
>miR6477 Score: 1.0 RO3G_03571
Query: 1 TGAACAGTAGAGGATGAT 18
||*|||||||||||||||
Sbjct: 110 ACATGTCATCTCCTACTA 93
>miR6477 Score: 0.0 RO3G_03615
Query: 1 TGAACAGTAGAGGATGAT 18
||||||||||||||||||
Sbjct: 110 ACTTGTCATCTCCTACTA 93
>miR6477 Score: 2.5 RO3G_04211
Query: 1 TGAACAGTAGAGGATGAT 18
*|||||::||*|||||||
Sbjct: 101 CCTTGTTGTCACCTACTA 84
>miR6477 Score: 2.5 RO3G_00513
Query: 1 TGAACAGTAGAGGATGAT 18
:||||||||:||||*:|:
Sbjct: 5609 GCTTGTCATTTCCTCTTG 5592
>miR6477 Score: 0.0 RO3G_00681
Query: 1 TGAACAGTAGAGGATGAT 18
||||||||||||||||||
Sbjct: 110 ACTTGTCATCTCCTACTA 93
>miR6477 Score: 1.0 RO3G_00999
Query: 1 TGAACAGTAGAGGATGAT 18
||||||||||||||::||
Sbjct: 1952 ACTTGTCATCTCCTGTTA 1935
```

>miR6477 Score: 2.5 RO3G_01108
Query: 1 TGAACAGTAGAGGATGAT 18
|||||:|||||||||*
Sbjct: 377 CCATGTCGTCTCCTACTT 360
>miR6477 Score: 2.5 RO3G_01192
Query: 1 TGAACAGTAGAGGATGAT 18
|||||::|||||||||
Sbjct: 101 CCTTGTTGTCACCTACTA 84
>miR6477 Score: 0.0 RO3G_01322
Query: 1 TGAACAGTAGAGGATGAT 18
||||||||||||||||||
Sbjct: 110 ACTTGTCATCTCCTACTA 93
>miR6477 Score: 1.0 RO3G_01612
Query: 1 TGAACAGTAGAGGATGAT 18
||||||||*|||||||||
Sbjct: 110 ACTTGTCACCTCCTACTA 93
>miR6477 Score: 0.0 RO3G_01652
Query: 1 TGAACAGTAGAGGATGAT 18
||||||||||||||||||
Sbjct: 110 ACTTGTCATCTCCTACTA 93
>miR6477 Score: 2.5 RO3G_01717
Query: 1 TGAACAGTAGAGGATGAT 18
|||||::|||||||||
Sbjct: 101 CCTTGTTGTCACCTACTA 84
>miR6477 Score: 2.5 RO3G_01726
Query: 1 TGAACAGTAGAGGATGAT 18
|||||::|||||||||
Sbjct: 101 CCTTGTTGTCACCTACTA 84
>miR6477 Score: 1.0 RO3G_01730
Query: 1 TGAACAGTAGAGGATGAT 18
||*|||||||||||||||
Sbjct: 110 ACGTGTCATCTCCTACTA 93
>miR6477 Score: 2.5 RO3G_01780
Query: 1 TGAACAGTAGAGGATGAT 18
|||||||:|||||||*
Sbjct: 696 CCTTTTCATTTCCTACTC 679
>miR6477 Score: 1.8 RO3G_01902
Query: 1 TGAACAGTAGAGGATGAT 18
|||||||||||:||*||:
Sbjct: 371 ACTTGTCATCTTCTTCTG 354
>miR6477 Score: 0.0 RO3G_01956
Query: 1 TGAACAGTAGAGGATGAT 18
||||||||||||||||||
Sbjct: 110 ACTTGTCATCTCCTACTA 93
>miR6477 Score: 2.5 RO3G_17460

Query: 1 TGAACAGTAGAGGATGAT 18
|||||::|||||||||
Sbjct: 101 CCTTGTTGTCACCTACTA 84
>miR6477 Score: 2.5 RO3G_17433
Query: 1 TGAACAGTAGAGGATGAT 18
|||||::|||||||||
Sbjct: 101 CCTTGTTGTCACCTACTA 84
>miR6477 Score: 0.0 RO3G_17382
Query: 1 TGAACAGTAGAGGATGAT 18
||||||||||||||||||
Sbjct: 110 ACTTGTCATCTCCTACTA 93
>miR6477 Score: 0.0 RO3G_17285
Query: 1 TGAACAGTAGAGGATGAT 18
||||||||||||||||||
Sbjct: 110 ACTTGTCATCTCCTACTA 93
>miR7734-3p Score: 1.0 RO3G_17257
Query: 1 TGGAACCAGTCCACATTCAACG 22
||||||||:|||||||||||*|
Sbjct: 584 ACCTTGGTTAGGTGTAAGTTAC 563
>miR7734-3p Score: 2.5 RO3G_17240
Query: 1 TGGAACCAGTCCACATTCAACG 22
||:|||||:||*||||||||*|
Sbjct: 596 ACTTTGGTTAGTTGTAAGTTAC 575
>miR7734-3p Score: 2.5 RO3G_17212
Query: 1 TGGAACCAGTCCACATTCAACG 22
||:|||||:||*||||||||*|
Sbjct: 584 ACTTTGGTTAGTTGTAAGTTAC 563
>miR7734-3p Score: 0.0 RO3G_16827
Query: 1 TGGAACCAGTCCACATTCAACG 22
||||||||||||||||||||||
Sbjct: 668 ACCTTGGTCAGGTGTAAGTTGC 647
>miR7734-3p Score: 2.0 RO3G_16739
Query: 1 TGGAACCAGTCCACATTCAACG 22
||:|||||:||*||||||||||
Sbjct: 887 ACTTTGGTTAGCTGTAAGTTGC 866
>miR7734-3p Score: 0.0 RO3G_16749
Query: 1 TGGAACCAGTCCACATTCAACG 22
||||||||||||||||||||||
Sbjct: 668 ACCTTGGTCAGGTGTAAGTTGC 647
>miR7734-3p Score: 1.0 RO3G_16136
Query: 1 TGGAACCAGTCCACATTCAACG 22
||||||||:|||||||||||*|
Sbjct: 584 ACCTTGGTTAGGTGTAAGTTAC 563
>miR7734-3p Score: 2.5 RO3G_16138
Query: 1 TGGAACCAGTCCACATTCAACG 22

||:|||||:||*|||||*||||
Sbjct: 887 ACTTTGGTTAGCTGTAAATTGC 866
>miR7734-3p Score: 1.0 RO3G_15889
Query: 1 TGGAACCAGTCCACATTCAACG 22
||||||||:|||||||||||*|
Sbjct: 584 ACCTTGGTTAGGTGTAAGTTAC 563
>miR7734-3p Score: 0.0 RO3G_15898
Query: 1 TGGAACCAGTCCACATTCAACG 22
||||||||||||||||||||||
Sbjct: 668 ACCTTGGTCAGGTGTAAGTTGC 647
>miR7734-3p Score: 2.5 RO3G_15580
Query: 1 TGGAACCAGTCCACATTCAACG 22
||:|||||:||*||||||||*|
Sbjct: 851 ACTTTGGTTAGCTGTAAGTTTC 830
>miR7734-3p Score: 1.0 RO3G_14991
Query: 1 TGGAACCAGTCCACATTCAACG 22
||||||||:|||||||||||*|
Sbjct: 29 ACCTTGGTTAGGTGTAAGTTAC 8
>miR7734-3p Score: 0.0 RO3G_14550
Query: 1 TGGAACCAGTCCACATTCAACG 22
||||||||||||||||||||||
Sbjct: 668 ACCTTGGTCAGGTGTAAGTTGC 647
>miR7734-3p Score: 2.5 RO3G_14626
Query: 1 TGGAACCAGTCCACATTCAACG 22
||:|||||:||*||||||||*|
Sbjct: 887 ACTTTGGTTAGCTGTAAGTTTC 866
>miR7734-3p Score: 2.0 RO3G_14710
Query: 1 TGGAACCAGTCCACATTCAACG 22
||:|||||:||*||||||||||
Sbjct: 887 ACTTTGGTTAGCTGTAAGTTGC 866
>miR7734-3p Score: 2.5 RO3G_13866
Query: 1 TGGAACCAGTCCACATTCAACG 22
||:|||||:||*||||||||*|
Sbjct: 719 ACTTTGGTTAGCTGTAAGTTTC 698
>miR7734-3p Score: 2.0 RO3G_13747
Query: 1 TGGAACCAGTCCACATTCAACG 22
||:|||||:||*||||||||||
Sbjct: 887 ACTTTGGTTAGCTGTAAGTTGC 866
>miR7734-3p Score: 0.0 RO3G_13145
Query: 1 TGGAACCAGTCCACATTCAACG 22
||||||||||||||||||||||
Sbjct: 668 ACCTTGGTCAGGTGTAAGTTGC 647
>miR7734-3p Score: 2.5 RO3G_13148
Query: 1 TGGAACCAGTCCACATTCAACG 22
||:|||||:||*||||||||*|

Sbjct: 620 ACTTTGGTTAGCTGTAAGTTTC 599
>miR7734-3p Score: 0.0 RO3G_13273
Query: 1 TGGAACCAGTCCACATTCAACG 22
||||||||||||||||||||||
Sbjct: 668 ACCTTGGTCAGGTGTAAGTTGC 647
>miR7734-3p Score: 0.0 RO3G_11969
Query: 1 TGGAACCAGTCCACATTCAACG 22
||||||||||||||||||||||
Sbjct: 668 ACCTTGGTCAGGTGTAAGTTGC 647
>miR7734-3p Score: 2.0 RO3G_12009
Query: 1 TGGAACCAGTCCACATTCAACG 22
||:|||||:||*||||||||||
Sbjct: 887 ACTTTGGTTAGCTGTAAGTTGC 866
>miR7734-3p Score: 2.0 RO3G_12072
Query: 1 TGGAACCAGTCCACATTCAACG 22
||:|||||:||*||||||||||
Sbjct: 887 ACTTTGGTTAGCTGTAAGTTGC 866
>miR7734-3p Score: 2.5 RO3G_12169
Query: 1 TGGAACCAGTCCACATTCAACG 22
||:|||||:||*||||||||*|
Sbjct: 887 ACTTTGGTTAGCTGTAAGTTTC 866
>miR7734-3p Score: 0.0 RO3G_11237
Query: 1 TGGAACCAGTCCACATTCAACG 22
||||||||||||||||||||||
Sbjct: 668 ACCTTGGTCAGGTGTAAGTTGC 647
>miR7734-3p Score: 2.5 RO3G_11404
Query: 1 TGGAACCAGTCCACATTCAACG 22
||:|||||:||*||||||||*|
Sbjct: 887 ACTTTGGTTAGCTGTAAGTTAC 866
>miR7734-3p Score: 0.0 RO3G_11409
Query: 1 TGGAACCAGTCCACATTCAACG 22
||||||||||||||||||||||
Sbjct: 668 ACCTTGGTCAGGTGTAAGTTGC 647
>miR7734-3p Score: 2.5 RO3G_11531
Query: 1 TGGAACCAGTCCACATTCAACG 22
||:|||||:||*||||||||*|
Sbjct: 380 ACTTTGGTTAGCTGTAAGTTTC 359
>miR7734-3p Score: 0.0 RO3G_11538
Query: 1 TGGAACCAGTCCACATTCAACG 22
||||||||||||||||||||||
Sbjct: 668 ACCTTGGTCAGGTGTAAGTTGC 647
>miR7734-3p Score: 2.5 RO3G_11542
Query: 1 TGGAACCAGTCCACATTCAACG 22
||:|||||:||*||||||||*|
Sbjct: 584 ACTTTGGTTAGTTGTAAGTTAC 563

>miR7734-3p Score: 2.5 RO3G_11771
Query: 1 TGGAACCAGTCCACATTCAACG 22
||:|||||:||*||||||||*|
Sbjct: 887 ACTTTGGTTAGCTGTAAGTTTC 866
>miR7734-3p Score: 1.0 RO3G_11949
Query: 1 TGGAACCAGTCCACATTCAACG 22
||||||||:|||||||||||*|
Sbjct: 29 ACCTTGGTTAGGTGTAAGTTAC 8
>miR7734-3p Score: 2.5 RO3G_10522
Query: 1 TGGAACCAGTCCACATTCAACG 22
||:|||||:||*||||||||*|
Sbjct: 851 ACTTTGGTTAGCTGTAAGTTTC 830
>miR7734-3p Score: 2.0 RO3G_10725
Query: 1 TGGAACCAGTCCACATTCAACG 22
||:|||||:||*||||||||||
Sbjct: 851 ACTTTGGTTAGCTGTAAGTTGC 830
>miR7734-3p Score: 1.0 RO3G_10735
Query: 1 TGGAACCAGTCCACATTCAACG 22
||||||||:|||||||||||*|
Sbjct: 113 ACCTTGGTTAGGTGTAAGTTAC 92
>miR7734-3p Score: 2.0 RO3G_09192
Query: 1 TGGAACCAGTCCACATTCAACG 22
||:|||||:||*||||||||||
Sbjct: 887 ACTTTGGTTAGCTGTAAGTTGC 866
>miR7734-3p Score: 0.0 RO3G_09601
Query: 1 TGGAACCAGTCCACATTCAACG 22
||||||||||||||||||||||
Sbjct: 668 ACCTTGGTCAGGTGTAAGTTGC 647
>miR7734-3p Score: 0.0 RO3G_09930
Query: 1 TGGAACCAGTCCACATTCAACG 22
||||||||||||||||||||||
Sbjct: 668 ACCTTGGTCAGGTGTAAGTTGC 647
>miR7734-3p Score: 2.0 RO3G_09940
Query: 1 TGGAACCAGTCCACATTCAACG 22
||:|||||:||*||||||||||
Sbjct: 887 ACTTTGGTTAGCTGTAAGTTGC 866
>miR7734-3p Score: 1.0 RO3G_10039
Query: 1 TGGAACCAGTCCACATTCAACG 22
||||||||:|||||||||||*|
Sbjct: 584 ACCTTGGTTAGGTGTAAGTTAC 563
>miR7734-3p Score: 0.0 RO3G_10217
Query: 1 TGGAACCAGTCCACATTCAACG 22
||||||||||||||||||||||
Sbjct: 668 ACCTTGGTCAGGTGTAAGTTGC 647
>miR7734-3p Score: 0.0 RO3G_07980

Query: 1 TGGAACCAGTCCACATTCAACG 22
||||||||||||||||||||||
Sbjct: 56 ACCTTGGTCAGGTGTAAGTTGC 35
>miR7734-3p Score: 2.0 RO3G_08042
Query: 1 TGGAACCAGTCCACATTCAACG 22
||:|||||:||*||||||||||
Sbjct: 419 ACTTTGGTTAGCTGTAAGTTGC 398
>miR7734-3p Score: 0.0 RO3G_06731
Query: 1 TGGAACCAGTCCACATTCAACG 22
||||||||||||||||||||||
Sbjct: 668 ACCTTGGTCAGGTGTAAGTTGC 647
>miR7734-3p Score: 2.0 RO3G_07130
Query: 1 TGGAACCAGTCCACATTCAACG 22
||:|||||:||*||||||||||
Sbjct: 791 ACTTTGGTTAGCTGTAAGTTGC 770
>miR7734-3p Score: 2.0 RO3G_07294
Query: 1 TGGAACCAGTCCACATTCAACG 22
||:|||||:||*||||||||||
Sbjct: 488 ACTTTGGTTAGCTGTAAGTTGC 467
>miR7734-3p Score: 0.0 RO3G_07307
Query: 1 TGGAACCAGTCCACATTCAACG 22
||||||||||||||||||||||
Sbjct: 668 ACCTTGGTCAGGTGTAAGTTGC 647
>miR7734-3p Score: 2.0 RO3G_07686
Query: 1 TGGAACCAGTCCACATTCAACG 22
||:|||||:||*||||||||||
Sbjct: 887 ACTTTGGTTAGCTGTAAGTTGC 866
>miR7734-3p Score: 2.5 RO3G_07693
Query: 1 TGGAACCAGTCCACATTCAACG 22
||:|||||:||*||||||||*|
Sbjct: 749 ACTTTGGTTAGTTGTAAGTTAC 728
>miR7734-3p Score: 0.0 RO3G_07698
Query: 1 TGGAACCAGTCCACATTCAACG 22
||||||||||||||||||||||
Sbjct: 668 ACCTTGGTCAGGTGTAAGTTGC 647
>miR7734-3p Score: 0.0 RO3G_07721
Query: 1 TGGAACCAGTCCACATTCAACG 22
||||||||||||||||||||||
Sbjct: 668 ACCTTGGTCAGGTGTAAGTTGC 647
>miR7734-3p Score: 0.0 RO3G_07737
Query: 1 TGGAACCAGTCCACATTCAACG 22
||||||||||||||||||||||
Sbjct: 29 ACCTTGGTCAGGTGTAAGTTGC 8
>miR7734-3p Score: 2.0 RO3G_07976
Query: 1 TGGAACCAGTCCACATTCAACG 22

||:|||||:||*||||||||||
Sbjct: 884 ACTTTGGTTAGCTGTAAGTTGC 863
>miR7734-3p Score: 2.0 RO3G_05631
Query: 1 TGGAACCAGTCCACATTCAACG 22
||:|||||:||*||||||||||
Sbjct: 887 ACTTTGGTTAGCTGTAAGTTGC 866
>miR7734-3p Score: 2.5 RO3G_05958
Query: 1 TGGAACCAGTCCACATTCAACG 22
||:|||||:||*||||||||*|
Sbjct: 851 ACTTTGGTTAGCTGTAAGTTTC 830
>miR7734-3p Score: 2.5 RO3G_06430
Query: 1 TGGAACCAGTCCACATTCAACG 22
||:|||||:||*||||||||*|
Sbjct: 851 ACTTTGGTTAGCTGTAAGTTTC 830
>miR7734-3p Score: 0.0 RO3G_04683
Query: 1 TGGAACCAGTCCACATTCAACG 22
||||||||||||||||||||||
Sbjct: 668 ACCTTGGTCAGGTGTAAGTTGC 647
>miR7734-3p Score: 2.5 RO3G_04785
Query: 1 TGGAACCAGTCCACATTCAACG 22
||:|||||:||*||||||||*|
Sbjct: 851 ACTTTGGTTAGCTGTAAGTTTC 830
>miR7734-3p Score: 1.0 RO3G_05424
Query: 1 TGGAACCAGTCCACATTCAACG 22
||||||||:|||||||||||*|
Sbjct: 584 ACCTTGGTTAGGTGTAAGTTAC 563
>miR7734-3p Score: 1.0 RO3G_02828
Query: 1 TGGAACCAGTCCACATTCAACG 22
||||||||:|||||||||||*|
Sbjct: 584 ACCTTGGTTAGGTGTAAGTTAC 563
>miR7734-3p Score: 2.5 RO3G_03555
Query: 1 TGGAACCAGTCCACATTCAACG 22
||:|||||:||*||||||||*|
Sbjct: 887 ACTTTGGTTAGCTGTAAGTTTC 866
>miR7734-3p Score: 2.0 RO3G_03580
Query: 1 TGGAACCAGTCCACATTCAACG 22
||:|||||:||*||||||||||
Sbjct: 812 ACTTTGGTTAGCTGTAAGTTGC 791
>miR7734-3p Score: 2.5 RO3G_03589
Query: 1 TGGAACCAGTCCACATTCAACG 22
||:|||||:||*||||||||*|
Sbjct: 812 ACTTTGGTTAGCTGTAAGTTTC 791
>miR7734-3p Score: 0.0 RO3G_04212
Query: 1 TGGAACCAGTCCACATTCAACG 22
||||||||||||||||||||||

```
Sbjct: 668 ACCTTGGTCAGGTGTAAGTTGC 647
>miR7734-3p Score: 1.0 RO3G_00291
Query: 1 TGGAACCAGTCCACATTCAACG 22
||||||||:|||||||||||*|
Sbjct: 584 ACCTTGGTTAGGTGTAAGTTAC 563
>miR7734-3p Score: 2.0 RO3G_00693
Query: 1 TGGAACCAGTCCACATTCAACG 22
||:|||||:||*||||||||||
Sbjct: 365 ACTTTGGTTAGCTGTAAGTTGC 344
>miR7734-3p Score: 2.5 RO3G_01207
Query: 1 TGGAACCAGTCCACATTCAACG 22
||:|||||:||*||||||||*|
Sbjct: 821 ACTTTGGTTAGCTGTAAGTTTC 800
>miR7734-3p Score: 2.5 RO3G_01309
Query: 1 TGGAACCAGTCCACATTCAACG 22
||:|||||:||*||||||||*|
Sbjct: 836 ACTTTGGTTAGCTGTAAGTTTC 815
>miR7734-3p Score: 0.0 RO3G_01728
Query: 1 TGGAACCAGTCCACATTCAACG 22
||||||||||||||||||||||
Sbjct: 626 ACCTTGGTCAGGTGTAAGTTGC 605
>miR7734-3p Score: 2.5 RO3G_01740
Query: 1 TGGAACCAGTCCACATTCAACG 22
||:|||||:||*||||||||*|
Sbjct: 851 ACTTTGGTTAGCTGTAAGTTTC 830
>miR7734-3p Score: 2.0 RO3G_17441
Query: 1 TGGAACCAGTCCACATTCAACG 22
||:|||||:||*||||||||||
Sbjct: 614 ACTTTGGTTAGCTGTAAGTTGC 593
>miR7734-3p Score: 2.0 RO3G_17385
Query: 1 TGGAACCAGTCCACATTCAACG 22
||:|||||:||*||||||||||
Sbjct: 887 ACTTTGGTTAGCTGTAAGTTGC 866
>miR7734-3p Score: 2.0 RO3G_17358
Query: 1 TGGAACCAGTCCACATTCAACG 22
||:|||||:||*||||||||||
Sbjct: 887 ACTTTGGTTAGCTGTAAGTTGC 866
>miR9476-5p Score: 2.2 RO3G_14596
Query: 1 TCTAGTCTTGCCTACTTTTTT-TT 23
||:|||||||||:||||||:|**|
Sbjct: 140 AGGTCAGAACGGGTGAAAAGATCA 117
>miR9476-5p Score: 2.5 RO3G_12740
Query: 1 TCTAGTCTTGCCTACTTTTTTTT 23
:|:*|||||||||||||||:|*
Sbjct: 444 GGGCCAGAACGGATGAAAAAGAC 422
```

>miR9476-5p Score: 2.0 RO3G_09279
Query: 1 TCTAGTCTTGCCTACTTTTTTTT 23
||:|||||||||:|||:|||||*
Sbjct: 527 AGGTCAGAACGGGTGAGAAAAAC 505
>miR3476-5p Score: 0.5 RO3G_17256
Query: 1 TGAACTGGGTGCTGCTGCTGC 21
||||||||:||||||||||||
Sbjct: 374 ACTTGACCTACGACGACGACG 354
>miR3476-5p Score: 0.5 RO3G_17248
Query: 1 TGAACTGGGTGCTGCTGCTGC 21
||||||||:||||||||||||
Sbjct: 377 ACTTGACCTACGACGACGACG 357
>miR3476-5p Score: 0.0 RO3G_16748
Query: 1 TGAACTGGGTGCTGCTGCTGC 21
|||||||||||||||||||||
Sbjct: 347 ACTTGACCCACGACGACGACG 327
>miR3476-5p Score: 0.5 RO3G_16137
Query: 1 TGAACTGGGTGCTGCTGCTGC 21
||||||||:||||||||||||
Sbjct: 371 ACTTGACCTACGACGACGACG 351
>miR3476-5p Score: 0.5 RO3G_15890
Query: 1 TGAACTGGGTGCTGCTGCTGC 21
||||||||:||||||||||||
Sbjct: 377 ACTTGACCTACGACGACGACG 357
>miR3476-5p Score: 0.0 RO3G_15897
Query: 1 TGAACTGGGTGCTGCTGCTGC 21
|||||||||||||||||||||
Sbjct: 347 ACTTGACCCACGACGACGACG 327
>miR3476-5p Score: 0.5 RO3G_15147
Query: 1 TGAACTGGGTGCTGCTGCTGC 21
||||||||:||||||||||||
Sbjct: 341 ACTTGACCTACGACGACGACG 321
>miR3476-5p Score: 0.0 RO3G_14551
Query: 1 TGAACTGGGTGCTGCTGCTGC 21
|||||||||||||||||||||
Sbjct: 347 ACTTGACCCACGACGACGACG 327
>miR3476-5p Score: 2.5 RO3G_13627
Query: 1 TGAACTGGGTGCTGCTGCTGC 21
:|*||||||||||:|||*:||
Sbjct: 393 GCCTGACCCACGATGACCGCG 373
>miR3476-5p Score: 0.5 RO3G_13146
Query: 1 TGAACTGGGTGCTGCTGCTGC 21
||||||||:||||||||||||
Sbjct: 377 ACTTGACCTACGACGACGACG 357
>miR3476-5p Score: 0.0 RO3G_11977

```
Query: 1 TGAACTGGGTGCTGCTGCTGC 21
|||||||||||||||||||||
Sbjct: 311 ACTTGACCCACGACGACGACG 291
>miR3476-5p Score: 0.0 RO3G_11236
Query: 1 TGAACTGGGTGCTGCTGCTGC 21
|||||||||||||||||||||
Sbjct: 389 ACTTGACCCACGACGACGACG 369
>miR3476-5p Score: 0.0 RO3G_11537
Query: 1 TGAACTGGGTGCTGCTGCTGC 21
|||||||||||||||||||||
Sbjct: 389 ACTTGACCCACGACGACGACG 369
>miR3476-5p Score: 2.5 RO3G_10502
Query: 1 TGAACTGGGTGCTGCTGCTGC 21
||||*:::|||||||||||||
Sbjct: 231 ACTTTGTTCACGACGACGACG 211
>miR3476-5p Score: 2.0 RO3G_10775
Query: 1 TGAACTGGGTGCTGCTGCTGC 21
||||||||:||||*|||*|||
Sbjct: 375 ACTTGACCTACGAAGACAACG 355
>miR3476-5p Score: 1.0 RO3G_09497
Query: 1 TGAACTGGGTGCTGCTGCTGC 21
||||||||:||||||||*|||
Sbjct: 86 ACTTGACCTACGACGACAACG 66
>miR3476-5p Score: 0.0 RO3G_09602
Query: 1 TGAACTGGGTGCTGCTGCTGC 21
|||||||||||||||||||||
Sbjct: 347 ACTTGACCCACGACGACGACG 327
>miR3476-5p Score: 0.0 RO3G_09603
Query: 1 TGAACTGGGTGCTGCTGCTGC 21
|||||||||||||||||||||
Sbjct: 389 ACTTGACCCACGACGACGACG 369
>miR3476-5p Score: 0.0 RO3G_09929
Query: 1 TGAACTGGGTGCTGCTGCTGC 21
|||||||||||||||||||||
Sbjct: 347 ACTTGACCCACGACGACGACG 327
>miR3476-5p Score: 0.5 RO3G_10041
Query: 1 TGAACTGGGTGCTGCTGCTGC 21
||||||||:||||||||||||
Sbjct: 377 ACTTGACCTACGACGACGACG 357
>miR3476-5p Score: 0.0 RO3G_10218
Query: 1 TGAACTGGGTGCTGCTGCTGC 21
|||||||||||||||||||||
Sbjct: 368 ACTTGACCCACGACGACGACG 348
>miR3476-5p Score: 0.0 RO3G_07981
Query: 1 TGAACTGGGTGCTGCTGCTGC 21
```

```
||||||||||||||||||||
Sbjct: 347 ACTTGACCCACGACGACGACG 327
>miR3476-5p Score: 0.5 RO3G_09182
Query: 1 TGAACTGGGTGCTGCTGCTGC 21
||||||||:||||||||||||
Sbjct: 353 ACTTGACCTACGACGACGACG 333
>miR3476-5p Score: 0.0 RO3G_06730
Query: 1 TGAACTGGGTGCTGCTGCTGC 21
||||||||||||||||||||
Sbjct: 347 ACTTGACCCACGACGACGACG 327
>miR3476-5p Score: 0.0 RO3G_07306
Query: 1 TGAACTGGGTGCTGCTGCTGC 21
||||||||||||||||||||
Sbjct: 347 ACTTGACCCACGACGACGACG 327
>miR3476-5p Score: 0.0 RO3G_07696
Query: 1 TGAACTGGGTGCTGCTGCTGC 21
||||||||||||||||||||
Sbjct: 347 ACTTGACCCACGACGACGACG 327
>miR3476-5p Score: 0.0 RO3G_07720
Query: 1 TGAACTGGGTGCTGCTGCTGC 21
||||||||||||||||||||
Sbjct: 347 ACTTGACCCACGACGACGACG 327
>miR3476-5p Score: 0.0 RO3G_07738
Query: 1 TGAACTGGGTGCTGCTGCTGC 21
||||||||||||||||||||
Sbjct: 398 ACTTGACCCACGACGACGACG 378
>miR3476-5p Score: 0.0 RO3G_04679
Query: 1 TGAACTGGGTGCTGCTGCTGC 21
||||||||||||||||||||
Sbjct: 317 ACTTGACCCACGACGACGACG 297
>miR3476-5p Score: 1.5 RO3G_05425
Query: 1 TGAACTGGGTGCTGCTGCTGC 21
||||||||:||||*|||||||
Sbjct: 374 ACTTGACCTACGAAGACGACG 354
>miR3476-5p Score: 0.0 RO3G_02228
Query: 1 TGAACTGGGTGCTGCTGCTGC 21
||||||||||||||||||||
Sbjct: 347 ACTTGACCCACGACGACGACG 327
>miR3476-5p Score: 2.5 RO3G_03439
Query: 1 TGAACTGGGTGCTGCTGCTGC 21
||||||||||:|||||*||**
Sbjct: 537 ACTTGACCCATGACGAAGAAA 517
>miR3476-5p Score: 0.5 RO3G_03584
Query: 1 TGAACTGGGTGCTGCTGCTGC 21
||||||||:||||||||||||
```

```
Sbjct: 305 ACTTGACCTACGACGACGACG 285
>miR3476-5p Score: 2.2 RO3G_03734
Query: 1 TGAACTGGGTGCTGCTGCTGC 21
|||*|||||||||:||||:*|
Sbjct: 286 ACTAGACCCACGATGACGGGG 266
>miR3476-5p Score: 0.0 RO3G_04211
Query: 1 TGAACTGGGTGCTGCTGCTGC 21
|||||||||||||||||||||
Sbjct: 347 ACTTGACCCACGACGACGACG 327
>miR3476-5p Score: 0.5 RO3G_00288
Query: 1 TGAACTGGGTGCTGCTGCTGC 21
||||||||:||||||||||||
Sbjct: 368 ACTTGACCTACGACGACGACG 348
>miR3476-5p Score: 0.0 RO3G_01726
Query: 1 TGAACTGGGTGCTGCTGCTGC 21
|||||||||||||||||||||
Sbjct: 392 ACTTGACCCACGACGACGACG 372
>miR3476-5p Score: 0.5 RO3G_17350
Query: 1 TGAACTGGGTGCTGCTGCTGC 21
||||||||:||||||||||||
Sbjct: 371 ACTTGACCTACGACGACGACG 351
>miR8137 Score: 0.5 RO3G_17257
Query: 1 TATCGCAACCAGTTCACAAAAT 22
||:|||||||||||||||||||
Sbjct: 1424 ATGGCGTTGGTCAAGTGTTTTA 1403
>miR8137 Score: 0.5 RO3G_16136
Query: 1 TATCGCAACCAGTTCACAAAAT 22
||:|||||||||||||||||||
Sbjct: 1424 ATGGCGTTGGTCAAGTGTTTTA 1403
>miR8137 Score: 0.5 RO3G_15889
Query: 1 TATCGCAACCAGTTCACAAAAT 22
||:|||||||||||||||||||
Sbjct: 1424 ATGGCGTTGGTCAAGTGTTTTA 1403
>miR8137 Score: 0.5 RO3G_11949
Query: 1 TATCGCAACCAGTTCACAAAAT 22
||:|||||||||||||||||||
Sbjct: 869 ATGGCGTTGGTCAAGTGTTTTA 848
>miR8137 Score: 0.5 RO3G_10735
Query: 1 TATCGCAACCAGTTCACAAAAT 22
||:|||||||||||||||||||
Sbjct: 953 ATGGCGTTGGTCAAGTGTTTTA 932
>miR8137 Score: 0.5 RO3G_10040
Query: 1 TATCGCAACCAGTTCACAAAAT 22
||:|||||||||||||||||||
Sbjct: 98 ATGGCGTTGGTCAAGTGTTTTA 77
```

```
>miR8137 Score: 0.5 RO3G_09183
Query: 1 TATCGCAACCAGTTCACAAAAT 22
||:|||||||||||||||||||
Sbjct: 641 ATGGCGTTGGTCAAGTGTTTTA 620
>miR8137 Score: 2.5 RO3G_06782
Query: 1 TATCGCAACCAGTTCACAAAAT 22
*|||||||||||||||*|*|*|
Sbjct: 143 TTAGCGTTGGTCAAGT-TGTGA 123
>miR8137 Score: 0.5 RO3G_05424
Query: 1 TATCGCAACCAGTTCACAAAAT 22
||:|||||||||||||||||||
Sbjct: 1424 ATGGCGTTGGTCAAGTGTTTTA 1403
>miR8137 Score: 0.0 RO3G_03585
Query: 1 TATCGCAACCAGTTCACAAAAT 22
||||||||||||||||||||||
Sbjct: 98 ATAGCGTTGGTCAAGTGTTTTA 77
>miR8137 Score: 0.5 RO3G_00289
Query: 1 TATCGCAACCAGTTCACAAAAT 22
||:|||||||||||||||||||
Sbjct: 236 ATGGCGTTGGTCAAGTGTTTTA 215
>miR8137 Score: 0.5 RO3G_17351
Query: 1 TATCGCAACCAGTTCACAAAAT 22
||:|||||||||||||||||||
Sbjct: 236 ATGGCGTTGGTCAAGTGTTTTA 215
>miR8571 Score: 0.0 RO3G_16560
Query: 1 TTCATCGTCAAGATCCTGTTCT 22
||||||||||||||||||||||
Sbjct: 534 AAGTAGCAGTTCTAGGACAAGA 513
>miR8571 Score: 0.0 RO3G_16557
Query: 1 TTCATCGTCAAGATCCTGTTCT 22
||||||||||||||||||||||
Sbjct: 1464 AAGTAGCAGTTCTAGGACAAGA 1443
>miR8571 Score: 0.0 RO3G_16132
Query: 1 TTCATCGTCAAGATCCTGTTCT 22
||||||||||||||||||||||
Sbjct: 1464 AAGTAGCAGTTCTAGGACAAGA 1443
>miR8571 Score: 2.5 RO3G_14835
Query: 1 TTCATCGTCAAGATCCTGTTCT 22
*||||||||||*||||||*|*|
Sbjct: 459 TAGTAGCAGTTGTAGGACTA-A 439
>miR8571 Score: 1.5 RO3G_14926
Query: 1 TTCATCGTCAAGATCCTGTTCT 22
||||||:||||:|||||*||||
Sbjct: 51 AAGTAGTAGTTTTAGGA-AAGA 31
>miR8571 Score: 0.0 RO3G_14995
```

Query: 1 TTCATCGTCAAGATCCTGTTCT 22
||||||||||||||||||||||
Sbjct: 1464 AAGTAGCAGTTCTAGGACAAGA 1443
>miR8571 Score: 0.0 RO3G_15134
Query: 1 TTCATCGTCAAGATCCTGTTCT 22
||||||||||||||||||||||
Sbjct: 1506 AAGTAGCAGTTCTAGGACAAGA 1485
>miR8571 Score: 2.5 RO3G_13437
Query: 1 TTCATCGTCAAGATCCTGTTCT 22
||||||*||||||:||||*|*|
Sbjct: 231 AAGTAGAAGTTCTGGGACTAAA 210
>miR8571 Score: 0.0 RO3G_12470
Query: 1 TTCATCGTCAAGATCCTGTTCT 22
||||||||||||||||||||||
Sbjct: 1464 AAGTAGCAGTTCTAGGACAAGA 1443
>miR8571 Score: 0.0 RO3G_11524
Query: 1 TTCATCGTCAAGATCCTGTTCT 22
||||||||||||||||||||||
Sbjct: 531 AAGTAGCAGTTCTAGGACAAGA 510
>miR8571 Score: 0.0 RO3G_09540
Query: 1 TTCATCGTCAAGATCCTGTTCT 22
||||||||||||||||||||||
Sbjct: 672 AAGTAGCAGTTCTAGGACAAGA 651
>miR8571 Score: 0.0 RO3G_07098
Query: 1 TTCATCGTCAAGATCCTGTTCT 22
||||||||||||||||||||||
Sbjct: 1464 AAGTAGCAGTTCTAGGACAAGA 1443
>miR8571 Score: 0.0 RO3G_06442
Query: 1 TTCATCGTCAAGATCCTGTTCT 22
||||||||||||||||||||||
Sbjct: 7188 AAGTAGCAGTTCTAGGACAAGA 7167
>miR8571 Score: 0.0 RO3G_05271
Query: 1 TTCATCGTCAAGATCCTGTTCT 22
||||||||||||||||||||||
Sbjct: 1464 AAGTAGCAGTTCTAGGACAAGA 1443
>miR8571 Score: 0.0 RO3G_02699
Query: 1 TTCATCGTCAAGATCCTGTTCT 22
||||||||||||||||||||||
Sbjct: 261 AAGTAGCAGTTCTAGGACAAGA 240
>miR8571 Score: 0.0 RO3G_03098
Query: 1 TTCATCGTCAAGATCCTGTTCT 22
||||||||||||||||||||||
Sbjct: 672 AAGTAGCAGTTCTAGGACAAGA 651
>miR8571 Score: 0.0 RO3G_00290
Query: 1 TTCATCGTCAAGATCCTGTTCT 22

```
||||||||||||||||||||||
Sbjct: 1464 AAGTAGCAGTTCTAGGACAAGA 1443
>miR8571 Score: 0.0 RO3G_00486
Query: 1 TTCATCGTCAAGATCCTGTTCT 22
||||||||||||||||||||||
Sbjct: 261 AAGTAGCAGTTCTAGGACAAGA 240
>miR8571 Score: 0.0 RO3G_01745
Query: 1 TTCATCGTCAAGATCCTGTTCT 22
||||||||||||||||||||||
Sbjct: 1464 AAGTAGCAGTTCTAGGACAAGA 1443
>miR8571 Score: 0.0 RO3G_01928
Query: 1 TTCATCGTCAAGATCCTGTTCT 22
||||||||||||||||||||||
Sbjct: 1464 AAGTAGCAGTTCTAGGACAAGA 1443
>miR8571 Score: 0.0 RO3G_17438
Query: 1 TTCATCGTCAAGATCCTGTTCT 22
||||||||||||||||||||||
Sbjct: 996 AAGTAGCAGTTCTAGGACAAGA 975
>miR7719-5p Score: 0.5 RO3G_17249
Query: 1 TCCTCCATAAGCATGTTTCTTC 22
|||||||||||||||||||||*
Sbjct: 546 AGGAGGTATTCGTACAAAGAAA 525
>miR7719-5p Score: 0.0 RO3G_14979
Query: 1 TCCTCCATAAGCATGTTTCTTC 22
||||||||||||||||||||||
Sbjct: 546 AGGAGGTATTCGTACAAAGAAG 525
>miR7719-5p Score: 0.0 RO3G_14363
Query: 1 TCCTCCATAAGCATGTTTCTTC 22
||||||||||||||||||||||
Sbjct: 504 AGGAGGTATTCGTACAAAGAAG 483
>miR7719-5p Score: 0.5 RO3G_13741
Query: 1 TCCTCCATAAGCATGTTTCTTC 22
|||||||||||||:||||||||
Sbjct: 546 AGGAGGTATTCGTGCAAAGAAG 525
>miR7719-5p Score: 0.0 RO3G_13755
Query: 1 TCCTCCATAAGCATGTTTCTTC 22
||||||||||||||||||||||
Sbjct: 546 AGGAGGTATTCGTACAAAGAAG 525
>miR7719-5p Score: 0.0 RO3G_13863
Query: 1 TCCTCCATAAGCATGTTTCTTC 22
||||||||||||||||||||||
Sbjct: 246 AGGAGGTATTCGTACAAAGAAG 225
>miR7719-5p Score: 0.0 RO3G_13270
Query: 1 TCCTCCATAAGCATGTTTCTTC 22
||||||||||||||||||||||
```

```
Sbjct: 546 AGGAGGTATTCGTACAAAGAAG 525
>miR7719-5p Score: 0.0 RO3G_10774
Query: 1 TCCTCCATAAGCATGTTTCTTC 22
||||||||||||||||||||||
Sbjct: 513 AGGAGGTATTCGTACAAAGAAG 492
>miR7719-5p Score: 0.0 RO3G_08849
Query: 1 TCCTCCATAAGCATGTTTCTTC 22
||||||||||||||||||||||
Sbjct: 546 AGGAGGTATTCGTACAAAGAAG 525
>miR7719-5p Score: 1.0 RO3G_05936
Query: 1 TCCTCCATAAGCATGTTTCTTC 22
|*||||||||||||||||||||
Sbjct: 546 ACGAGGTATTCGTACAAAGAAG 525
>miR7719-5p Score: 1.0 RO3G_05420
Query: 1 TCCTCCATAAGCATGTTTCTTC 22
|*||||||||||||||||||||
Sbjct: 407 ACGAGGTATTCGTACAAAGAAG 386
>miR7719-5p Score: 0.2 RO3G_17276
Query: 1 TCCTCCATAAGCATGTTTCTTC 22
|||||||||||||||||||:||
Sbjct: 636 AGGAGGTATTCGTACAAAGGAG 615
>miR5177 Score: 2.5 RO3G_16800
Query: 1 TGAGGTTGGAAAACGAGCAG 20
||||||:||||**|||||||
Sbjct: 1215 ACTCCAGCCTTCCGCTCGTC 1196
>miR5177 Score: 2.5 RO3G_15748
Query: 1 TGAGGTTGGAAAACGAGCAG 20
*||:||||||||||||:*|*
Sbjct: 69 TCTTCAACCTTTTGCTTATA 50
>miR5177 Score: 2.5 RO3G_15265
Query: 1 TGAGGTTGGAAAACGAGCAG 20
||||||:||||**|||||||
Sbjct: 1215 ACTCCAGCCTTCCGCTCGTC 1196
>miR5177 Score: 2.5 RO3G_15268
Query: 1 TGAGGTTGGAAAACGAGCAG 20
||||||:||||**|||||||
Sbjct: 1215 ACTCCAGCCTTCCGCTCGTC 1196
>miR5177 Score: 2.5 RO3G_13129
Query: 1 TGAGGTTGGAAAACGAGCAG 20
|:||||||||||*|||*|||
Sbjct: 365 ATTCCAACCTTTGGCTAGTC 346
>miR5177 Score: 2.5 RO3G_13219
Query: 1 TGAGGTTGGAAAACGAGCAG 20
||||||:||||**|||||||
Sbjct: 1215 ACTCCAGCCTTCCGCTCGTC 1196
```

>miR5177 Score: 2.5 RO3G_13222
Query: 1 TGAGGTTGGAAAACGAGCAG 20
||||||:||||**|||||||
Sbjct: 1209 ACTCCAGCCTTCCGCTCGTC 1190
>miR5177 Score: 1.5 RO3G_12627
Query: 1 TGAGGTTGGAAAACGAGCAG 20
||||||||||||||||||
Sbjct: 1137 CCTCCAACCTTCTGCTCGTC 1118
>miR5177 Score: 2.5 RO3G_05640
Query: 1 TGAGGTTGGAAAACGAGCAG 20
||||||:||||**|||||||
Sbjct: 1152 ACTCCAGCCTTCCGCTCGTC 1133
>miR5177 Score: 2.5 RO3G_05952
Query: 1 TGAGGTTGGAAAACGAGCAG 20
||||||:||||**|||||||
Sbjct: 1095 ACTCCAGCCTTCCGCTCGTC 1076
>miR5177 Score: 2.5 RO3G_05990
Query: 1 TGAGGTTGGAAAACGAGCAG 20
|||*|||||*|||||||*||
Sbjct: 1236 ACTACAACCCTTTGCTCTTC 1217
>miR5177 Score: 2.5 RO3G_06701
Query: 1 TGAGGTTGGAAAACGAGCAG 20
|||:*||||*||||||||||
Sbjct: 336 ACTTAAACCATTTGCTCGTC 317
>miR5177 Score: 2.5 RO3G_04121
Query: 1 TGAGGTTGGAAAACGAGCAG 20
||||||:||||**|||||||
Sbjct: 1215 ACTCCAGCCTTCCGCTCGTC 1196
>miR5177 Score: 2.5 RO3G_00075
Query: 1 TGAGGTTGGAAAACGAGCAG 20
||||||||*|||||||||
Sbjct: 1233 CCTACAACCTATTGCTCGTC 1214
>miR5177 Score: 2.0 RO3G_01141
Query: 1 TGAGGTTGGAAAACGAGCAG 20
||||:|||||||*||||*||
Sbjct: 517 ACTCTAACCTTTCGCTC-TC 499
>miR5177 Score: 0.0 RO3G_17015
Query: 1 TGAGGTTGGAAAACGAGCAG 20
||||||||||||||||||||
Sbjct: 1227 ACTCCAACCTTTTGCTCGTC 1208
>miR6248 Score: 2.5 RO3G_16143
Query: 1 TATTGAGGTGGAGGTTAGTG 20
||:||:||||||:|||||
Sbjct: 538 TTAGCTTCACCTCTAATAAC 519
>miR6248 Score: 2.5 RO3G_16292

Query: 1 TATTGAGGTGGAGGTTAGTG 20
:||:||||||||||*||:*|
Sbjct: 658 GTAGCTCCACCTCCCATT-C 640
>miR6248 Score: 2.5 RO3G_15135
Query: 1 TATTGAGGTGGAGGTTAGTG 20
||:||:||||||:|||||
Sbjct: 538 TTAGCTTCACCTCTAATAAC 519
>miR6248 Score: 0.0 RO3G_12949
Query: 1 TATTGAGGTGGAGGTTAGTG 20
||||||||||||||||||||
Sbjct: 766 ATAACTCCACCTCCAATCAC 747
>miR6248 Score: 2.5 RO3G_12534
Query: 1 TATTGAGGTGGAGGTTAGTG 20
||:||:||||||:|||||
Sbjct: 538 TTAGCTTCACCTCTAATAAC 519
>miR6248 Score: 2.5 RO3G_10206
Query: 1 TATTGAGGTGGAGGTTAG-TG 20
|||||||||||*||::||*||
Sbjct: 425 ATAACTCCACCACCGGTCTAC 405
>miR6248 Score: 2.5 RO3G_07027
Query: 1 TATTGAGGTGGAGGTTAGTG 20
||:||:||||||:|||||
Sbjct: 538 TTAGCTTCACCTCTAATAAC 519
>miR6248 Score: 2.5 RO3G_05683
Query: 1 TATTGAGGTGGAGGTTAGTG 20
|||||||||||*|||:*|||
Sbjct: 551 ATAACTCCACCACCAG-CAC 533
>miR6248 Score: 2.5 RO3G_06727
Query: 1 TATTGAGGTGGAGGTTAGTG 20
||:||:||||||:|||||
Sbjct: 676 TTAGCTTCACCTCTAATAAC 657
>miR6248 Score: 2.0 RO3G_04538
Query: 1 TATTGAGGTGGAGGTTAGTG 20
|||||||||||||||||*
Sbjct: 1595 CTAACTCCACCTCCAACCAA 1576
>miR6248 Score: 2.5 RO3G_02690
Query: 1 TATTGAGGTGGAGGTTAGTG 20
||:||:||||||:|||||
Sbjct: 118 TTAGCTTCACCTCTAATAAC 99
>miR6248 Score: 0.0 RO3G_04050
Query: 1 TATTGAGGTGGAGGTTAGTG 20
||||||||||||||||||||
Sbjct: 1321 ATAACTCCACCTCCAATCAC 1302
>miR6248 Score: 2.5 RO3G_04118
Query: 1 TATTGAGGTGGAGGTTAGTG 20

```
*||:||:||||||:|||*||
Sbjct: 538 TTAGCTTCACCTCTAATAAC 519
>miR6248 Score: 0.0 RO3G_00301
Query: 1 TATTGAGGTGGAGGTTAGTG 20
||||||||||||||||||||
Sbjct: 2605 ATAACTCCACCTCCAATCAC 2586
>miR6248 Score: 2.5 RO3G_00949
Query: 1 TATTGAGGTGGAGGTTAGTG 20
*||||*||||||||||*|||
Sbjct: 2045 CTAACACCACCTCCAA-CAC 2027
>miR6248 Score: 2.5 RO3G_17384
Query: 1 TATTGAGGTGGAGGTTAGTG 20
*||:||:||||||:|||*||
Sbjct: 538 TTAGCTTCACCTCTAATAAC 519
>miR6248 Score: 2.5 RO3G_17318
Query: 1 TATTGAGGTGGAGGTTAGTG 20
*||:||:||||||:|||*||
Sbjct: 538 TTAGCTTCACCTCTAATAAC 519
>miR6248 Score: 2.5 RO3G_17274
Query: 1 TATTGAGGTGGAGGTTAGTG 20
*||:||:||||||:|||*||
Sbjct: 538 TTAGCTTCACCTCTAATAAC 519
>miR1849 Score: 0.0 RO3G_16810
Query: 1 TAGCGTATCATAGGTTGGT 19
|||||||||||||||||||
Sbjct: 216 ATCGCATAGTATCCAACCA 198
>miR1849 Score: 0.0 RO3G_15157
Query: 1 TAGCGTATCATAGGTTGGT 19
|||||||||||||||||||
Sbjct: 573 ATCGCATAGTATCCAACCA 555
>miR1849 Score: 2.5 RO3G_14264
Query: 1 TAGCGTATCATAGGTTGGT 19
||*||:||||:||||||::
Sbjct: 290 ATAGCGTAGTGTCCAACTG 272
>miR1849 Score: 1.5 RO3G_12960
Query: 1 TAGCGTATCATAGGTTGGT 19
*||||:||||||||:||||
Sbjct: 234 CTCGCGTAGTATCCGACCA 216
>miR1849 Score: 1.5 RO3G_12974
Query: 1 TAGCGTATCATAGGTTGGT 19
*||||:||||||||:||||
Sbjct: 234 CTCGCGTAGTATCCGACCA 216
>miR1849 Score: 0.0 RO3G_12005
Query: 1 TAGCGTATCATAGGTTGGT 19
|||||||||||||||||||
```

```
Sbjct: 573 ATCGCATAGTATCCAACCA 555
>miR1849 Score: 0.0 RO3G_12080
Query: 1 TAGCGTATCATAGGTTGGT 19
|||||||||||||||||||
Sbjct: 156 ATCGCATAGTATCCAACCA 138
>miR1849 Score: 2.2 RO3G_12201
Query: 1 TAGCGTATCATAGGTTGGT 19
:|||*:||||||||||||*
Sbjct: 36 GTCGAGTAGTATCCAACCT 18
>miR1849 Score: 0.0 RO3G_11407
Query: 1 TAGCGTATCATAGGTTGGT 19
|||||||||||||||||||
Sbjct: 156 ATCGCATAGTATCCAACCA 138
>miR1849 Score: 1.5 RO3G_11953
Query: 1 TAGCGTATCATAGGTTGGT 19
*||||:||||||||:||||
Sbjct: 450 CTCGCGTAGTATCCGACCA 432
>miR1849 Score: 1.5 RO3G_10525
Query: 1 TAGCGTATCATAGGTTGGT 19
*||||:||||||||:||||
Sbjct: 573 CTCGCGTAGTATCCGACCA 555
>miR1849 Score: 1.5 RO3G_10531
Query: 1 TAGCGTATCATAGGTTGGT 19
*||||:||||||||:||||
Sbjct: 573 CTCGCGTAGTATCCGACCA 555
>miR1849 Score: 1.5 RO3G_10545
Query: 1 TAGCGTATCATAGGTTGGT 19
*||||:||||||||:||||
Sbjct: 573 CTCGCGTAGTATCCGACCA 555
>miR1849 Score: 2.5 RO3G_09022
Query: 1 TAGCGTATCATAGGTTGGT 19
**||:||||||||||||*|
Sbjct: 1337 CACGTATAGTATCCAAC-A 1320
>miR1849 Score: 1.5 RO3G_07110
Query: 1 TAGCGTATCATAGGTTGGT 19
*||||:||||||||:||||
Sbjct: 573 CTCGCGTAGTATCCGACCA 555
>miR1849 Score: 1.5 RO3G_07676
Query: 1 TAGCGTATCATAGGTTGGT 19
*||||:||||||||:||||
Sbjct: 573 CTCGCGTAGTATCCGACCA 555
>miR1849 Score: 1.5 RO3G_07678
Query: 1 TAGCGTATCATAGGTTGGT 19
*||||:||||||||:||||
Sbjct: 573 CTCGCGTAGTATCCGACCA 555
```

```
>miR1849 Score: 1.5 RO3G_05523
Query: 1 TAGCGTATCATAGGTTGGT 19
*||||:||||||||:||||
Sbjct: 573 CTCGCGTAGTATCCGACCA 555
>miR1849 Score: 0.0 RO3G_05928
Query: 1 TAGCGTATCATAGGTTGGT 19
|||||||||||||||||||
Sbjct: 345 ATCGCATAGTATCCAACCA 327
>miR1849 Score: 0.0 RO3G_04676
Query: 1 TAGCGTATCATAGGTTGGT 19
|||||||||||||||||||
Sbjct: 573 ATCGCATAGTATCCAACCA 555
>miR1849 Score: 2.5 RO3G_02881
Query: 1 TAGCGTATCATAGGTTGGT 19
|||||||||||*:|:||*|
Sbjct: 636 ATCGCATAGTACTCGAC-A 619
>miR1849 Score: 2.5 RO3G_03254
Query: 1 TAGCGTATCATAGGTTGGT 19
:|**|||||||||||||:|
Sbjct: 114 GTGACATAGTATCCAACTA 96
>miR1849 Score: 1.5 RO3G_03767
Query: 1 TAGCGTATCATAGGTTGGT 19
*||||:||||||||:||||
Sbjct: 573 CTCGCGTAGTATCCGACCA 555
>miR1849 Score: 2.5 RO3G_00382
Query: 1 TAGCGTATCATAGGTTGGT 19
*|||*|||||||:||||*|
Sbjct: 351 TTCGAATAGTATTCAAC-A 334
>miR1849 Score: 1.5 RO3G_01949
Query: 1 TAGCGTATCATAGGTTGGT 19
*||||:||||||||:||||
Sbjct: 573 CTCGCGTAGTATCCGACCA 555
>miR1849 Score: 1.5 RO3G_01968
Query: 1 TAGCGTATCATAGGTTGGT 19
*||||:||||||||:||||
Sbjct: 573 CTCGCGTAGTATCCGACCA 555
>miR1849 Score: 1.5 RO3G_17380
Query: 1 TAGCGTATCATAGGTTGGT 19
*||||:||||||||:||||
Sbjct: 573 CTCGCGTAGTATCCGACCA 555
>miR1047-3p Score: 0.0 RO3G_15127
Query: 1 TGATCAGCATACTTCAGTTG 20
||||||||||||||||||||
Sbjct: 1283 ACTAGTCGTATGAAGTCAAC 1264
>miR1047-3p Score: 2.5 RO3G_13898
```

```
Query: 1 TGATCAGCATACTTCAGTTG 20
||||||||||||::*||*||
Sbjct: 370 ACTAGTCGTATGGGTTCTAC 351
>miR1047-3p Score: 2.5 RO3G_13579
Query: 1 TGATCAGCATACTTCAGTTG 20
||||||||||||||**|*||
Sbjct: 1033 ACTAGTCGTATGAAAAC-AC 1015
>miR1047-3p Score: 2.5 RO3G_00203
Query: 1 TGATCAGCATACTTCAGTTG 20
*:||*||||||||||||*||
Sbjct: 1907 CTTAATCGTATGAAGTC-AC 1889
>miR1047-3p Score: 2.5 RO3G_00203
Query: 1 TGATCAGCATACTTCAGTTG 20
||||||||*|||*:||||||
Sbjct: 1208 ACTAGTCGCATGTGGTCAAC 1189
>miR1047-3p Score: 1.8 RO3G_00249
Query: 1 TGATCAGCATACTTCAGTTG 20
||||||||*||||:|||:||
Sbjct: 92 ACTAGTCGCATGAGGTCGAC 73
>miR1047-3p Score: 2.5 RO3G_01649
Query: 1 TGATCAGCATACTTCAGTT-G 20
|:||||||*||||:|||||*|
Sbjct: 476 ATTAGTCGCATGAGGTCAAGC 456
>miR1047-3p Score: 1.5 RO3G_02142
Query: 1 TGATCAGCATACTTCAGTTG 20
*||||||*||||||||||||
Sbjct: 1799 CCTAGTCCTATGAAGTCAAC 1780
>miR5176-5p Score: 0.2 RO3G_15862
Query: 1 TATTCCATGTGTCACATCTC 20
:|||||||||||||||||||
Sbjct: 595 GTAAGGTACACAGTGTAGAG 576
>miR5176-5p Score: 0.2 RO3G_15274
Query: 1 TATTCCATGTGTCACATCTC 20
:|||||||||||||||||||
Sbjct: 595 GTAAGGTACACAGTGTAGAG 576
>miR5176-5p Score: 0.2 RO3G_15288
Query: 1 TATTCCATGTGTCACATCTC 20
:|||||||||||||||||||
Sbjct: 595 GTAAGGTACACAGTGTAGAG 576
>miR5176-5p Score: 0.2 RO3G_13916
Query: 1 TATTCCATGTGTCACATCTC 20
:|||||||||||||||||||
Sbjct: 595 GTAAGGTACACAGTGTAGAG 576
>miR5176-5p Score: 0.0 RO3G_12182
Query: 1 TATTCCATGTGTCACATCTC 20
```

```
||||||||||||||||||||
Sbjct: 595 ATAAGGTACACAGTGTAGAG 576
>miR5176-5p Score: 0.2 RO3G_12541
Query: 1 TATTCCATGTGTCACATCTC 20
:|||||||||||||||||||
Sbjct: 595 GTAAGGTACACAGTGTAGAG 576
>miR5176-5p Score: 0.2 RO3G_11272
Query: 1 TATTCCATGTGTCACATCTC 20
:|||||||||||||||||||
Sbjct: 595 GTAAGGTACACAGTGTAGAG 576
>miR5176-5p Score: 0.2 RO3G_11784
Query: 1 TATTCCATGTGTCACATCTC 20
:|||||||||||||||||||
Sbjct: 595 GTAAGGTACACAGTGTAGAG 576
>miR5176-5p Score: 0.0 RO3G_10860
Query: 1 TATTCCATGTGTCACATCTC 20
||||||||||||||||||||
Sbjct: 595 ATAAGGTACACAGTGTAGAG 576
>miR5176-5p Score: 0.2 RO3G_09496
Query: 1 TATTCCATGTGTCACATCTC 20
:|||||||||||||||||||
Sbjct: 595 GTAAGGTACACAGTGTAGAG 576
>miR5176-5p Score: 0.0 RO3G_08111
Query: 1 TATTCCATGTGTCACATCTC 20
||||||||||||||||||||
Sbjct: 595 ATAAGGTACACAGTGTAGAG 576
>miR5176-5p Score: 0.0 RO3G_07729
Query: 1 TATTCCATGTGTCACATCTC 20
||||||||||||||||||||
Sbjct: 364 ATAAGGTACACAGTGTAGAG 345
>miR5176-5p Score: 2.5 RO3G_04287
Query: 1 TATTCCATGTGTCACATCTC 20
||:||*|:|||||||||*||
Sbjct: 1132 ATGAGCTGCACAGTGTA-AG 1114
>miR5176-5p Score: 2.5 RO3G_04878
Query: 1 TATTCCATGTGTCACATCTC 20
||:||*|:|||||||||*||
Sbjct: 943 ATGAGCTGCACAGTGTA-AG 925
>miR5176-5p Score: 0.0 RO3G_02682
Query: 1 TATTCCATGTGTCACATCTC 20
||||||||||||||||||||
Sbjct: 595 ATAAGGTACACAGTGTAGAG 576
>miR5176-5p Score: 2.0 RO3G_02988
Query: 1 TATTCCATGTGTCACATC-TC 20
*|||||||||*|||||||*||
```

```
Sbjct: 1347 TTAAGGTACAAAGTGTAGTAG 1327
>miR5176-5p Score: 0.0 RO3G_17289
Query: 1 TATTCCATGTGTCACATCTC 20
||||||||||||||||||||
Sbjct: 595 ATAAGGTACACAGTGTAGAG 576
>miR5726 Score: 0.0 RO3G_13448
Query: 1 TAAAGAGTTGCTTGAATCAGG 21
|||||||||||||||||||||
Sbjct: 975 ATTTCTCAACGAACTTAGTCC 955
>miR5726 Score: 2.2 RO3G_10510
Query: 1 TAAAGAGTTGCTTGAATCAGG 21
||||:|||:|*|||||||||:
Sbjct: 117 ATTTTTCAGCAAACTTAGTCT 97
>miR5243 Score: 2.5 RO3G_02034
Query: 1 TGGGCCAGAATCGTGGGC 18
**|||||:|||||||:||
Sbjct: 2027 TACCGGTTTTAGCACTCG 2010
>miR8602 Score: 0.0 RO3G_17259
Query: 1 TCAGGAGAGTATGCTGGCCA 20
||||||||||||||||||||
Sbjct: 209 AGTCCTCTCATACGACCGGT 190
>miR8602 Score: 0.0 RO3G_17249
Query: 1 TCAGGAGAGTATGCTGGCCA 20
||||||||||||||||||||
Sbjct: 728 AGTCCTCTCATACGACCGGT 709
>miR8602 Score: 2.0 RO3G_17207
Query: 1 TCAGGAGAGTATGCTGGCCA 20
|||||*||:|||:|||||||
Sbjct: 161 AGTCCACTTATATGACCGGT 142
>miR8602 Score: 1.5 RO3G_16536
Query: 1 TCAGGAGAGTATGCTGGCCA 20
|||||*||:|||||||||||
Sbjct: 122 AGTCCGCTTATACGACCGGT 103
>miR8602 Score: 2.0 RO3G_15895
Query: 1 TCAGGAGAGTATGCTGGCCA 20
|||||*||:|||:|||||||
Sbjct: 122 AGTCCACTTATATGACCGGT 103
>miR8602 Score: 1.5 RO3G_15166
Query: 1 TCAGGAGAGTATGCTGGCCA 20
|||||*||:|||||||||||
Sbjct: 809 AGTCCACTTATACGACCGGT 790
>miR8602 Score: 0.0 RO3G_14363
Query: 1 TCAGGAGAGTATGCTGGCCA 20
||||||||||||||||||||
Sbjct: 761 AGTCCTCTCATACGACCGGT 742
```

```
>miR8602 Score: 0.5 RO3G_14547
Query: 1 TCAGGAGAGTATGCTGGCCA 20
|||||||||||||||:||||
Sbjct: 209 AGTCCTCTCATACGATCGGT 190
>miR8602 Score: 0.5 RO3G_13921
Query: 1 TCAGGAGAGTATGCTGGCCA 20
|||:||||||||||||||||
Sbjct: 209 AGTTCTCTCATACGACCGGT 190
>miR8602 Score: 0.0 RO3G_13863
Query: 1 TCAGGAGAGTATGCTGGCCA 20
||||||||||||||||||||
Sbjct: 503 AGTCCTCTCATACGACCGGT 484
>miR8602 Score: 0.0 RO3G_13270
Query: 1 TCAGGAGAGTATGCTGGCCA 20
||||||||||||||||||||
Sbjct: 803 AGTCCTCTCATACGACCGGT 784
>miR8602 Score: 0.0 RO3G_11527
Query: 1 TCAGGAGAGTATGCTGGCCA 20
||||||||||||||||||||
Sbjct: 209 AGTCCTCTCATACGACCGGT 190
>miR8602 Score: 0.8 RO3G_10734
Query: 1 TCAGGAGAGTATGCTGGCCA 20
:|||||||||||||||:|||
Sbjct: 209 GGTCCTCTCATACGACTGGT 190
>miR8602 Score: 0.0 RO3G_10774
Query: 1 TCAGGAGAGTATGCTGGCCA 20
||||||||||||||||||||
Sbjct: 770 AGTCCTCTCATACGACCGGT 751
>miR8602 Score: 0.0 RO3G_10869
Query: 1 TCAGGAGAGTATGCTGGCCA 20
||||||||||||||||||||
Sbjct: 209 AGTCCTCTCATACGACCGGT 190
>miR8602 Score: 1.5 RO3G_07577
Query: 1 TCAGGAGAGTATGCTGGCCA 20
|||||*||:|||||||||||
Sbjct: 161 AGTCCACTTATACGACCGGT 142
>miR8602 Score: 2.5 RO3G_07783
Query: 1 TCAGGAGAGTATGCTGGCCA 20
|||||||||*||||||**||
Sbjct: 677 AGTCCTCTCTTACGACACGT 658
>miR8602 Score: 0.0 RO3G_05935
Query: 1 TCAGGAGAGTATGCTGGCCA 20
||||||||||||||||||||
Sbjct: 209 AGTCCTCTCATACGACCGGT 190
>miR8602 Score: 1.5 RO3G_05940
```

Query: 1 TCAGGAGAGTATGCTGGCCA 20
|||||*||:|||||||||||
Sbjct: 518 AGTCCACTTATACGACCGGT 499
>miR8602 Score: 2.0 RO3G_04692
Query: 1 TCAGGAGAGTATGCTGGCCA 20
|||||*||:|||:|||||||
Sbjct: 161 AGTCCACTTATATGACCGGT 142
>miR8602 Score: 0.0 RO3G_04790
Query: 1 TCAGGAGAGTATGCTGGCCA 20
||||||||||||||||||||
Sbjct: 209 AGTCCTCTCATACGACCGGT 190
>miR8602 Score: 0.0 RO3G_03593
Query: 1 TCAGGAGAGTATGCTGGCCA 20
||||||||||||||||||||
Sbjct: 209 AGTCCTCTCATACGACCGGT 190
>miR8602 Score: 0.0 RO3G_03626
Query: 1 TCAGGAGAGTATGCTGGCCA 20
||||||||||||||||||||
Sbjct: 209 AGTCCTCTCATACGACCGGT 190
>miR8602 Score: 2.5 RO3G_03629
Query: 1 TCAGGAGAGTATGCTGGCCA 20
||||||||:||||*|||**|
Sbjct: 1085 AGTCCTCTTATACCACC-TT 1067
>miR8602 Score: 1.5 RO3G_00237
Query: 1 TCAGGAGAGTATGCTGGCCA 20
|||||*||:|||||||||||
Sbjct: 278 AGTCCACTTATACGACCGGT 259
>miR8602 Score: 0.0 RO3G_00678
Query: 1 TCAGGAGAGTATGCTGGCCA 20
||||||||||||||||||||
Sbjct: 173 AGTCCTCTCATACGACCGGT 154
>miR8602 Score: 0.0 RO3G_01589
Query: 1 TCAGGAGAGTATGCTGGCCA 20
||||||||||||||||||||
Sbjct: 209 AGTCCTCTCATACGACCGGT 190
>miR8602 Score: 0.8 RO3G_17459
Query: 1 TCAGGAGAGTATGCTGGCCA 20
:|||||||||||||||:|||
Sbjct: 209 GGTCCTCTCATACGACTGGT 190
>miR8602 Score: 0.0 RO3G_17276
Query: 1 TCAGGAGAGTATGCTGGCCA 20
||||||||||||||||||||
Sbjct: 893 AGTCCTCTCATACGACCGGT 874
>miR7814 Score: 2.5 RO3G_16088
Query: 1 TAATATGTCTTTGATGCTT-TGA 22

:|||||||*||||||||*|*|:|
Sbjct: 553 GTTATACAAAAACTACGTATATT 531
>miR7814 Score: 0.0 RO3G_15585
Query: 1 TAATATGTCTTTGATGCTTTGA 22
||||||||||||||||||||||
Sbjct: 3018 ATTATACAGAAACTACGAAACT 2997
>miR7814 Score: 0.0 RO3G_15250
Query: 1 TAATATGTCTTTGATGCTTTGA 22
||||||||||||||||||||||
Sbjct: 3171 ATTATACAGAAACTACGAAACT 3150
>miR7814 Score: 0.0 RO3G_14355
Query: 1 TAATATGTCTTTGATGCTTTGA 22
||||||||||||||||||||||
Sbjct: 1422 ATTATACAGAAACTACGAAACT 1401
>miR7814 Score: 0.0 RO3G_13867
Query: 1 TAATATGTCTTTGATGCTTTGA 22
||||||||||||||||||||||
Sbjct: 3132 ATTATACAGAAACTACGAAACT 3111
>miR7814 Score: 0.5 RO3G_13920
Query: 1 TAATATGTCTTTGATGCTTTGA 22
||||||:|||||||||||||||
Sbjct: 3216 ATTATATAGAAACTACGAAACT 3195
>miR7814 Score: 0.0 RO3G_13749
Query: 1 TAATATGTCTTTGATGCTTTGA 22
||||||||||||||||||||||
Sbjct: 1779 ATTATACAGAAACTACGAAACT 1758
>miR7814 Score: 0.0 RO3G_11776
Query: 1 TAATATGTCTTTGATGCTTTGA 22
||||||||||||||||||||||
Sbjct: 306 ATTATACAGAAACTACGAAACT 285
>miR7814 Score: 0.0 RO3G_10558
Query: 1 TAATATGTCTTTGATGCTTTGA 22
||||||||||||||||||||||
Sbjct: 969 ATTATACAGAAACTACGAAACT 948
>miR7814 Score: 0.0 RO3G_09965
Query: 1 TAATATGTCTTTGATGCTTTGA 22
||||||||||||||||||||||
Sbjct: 3216 ATTATACAGAAACTACGAAACT 3195
>miR7814 Score: 0.0 RO3G_05619
Query: 1 TAATATGTCTTTGATGCTTTGA 22
||||||||||||||||||||||
Sbjct: 1359 ATTATACAGAAACTACGAAACT 1338
>miR7814 Score: 0.0 RO3G_06427
Query: 1 TAATATGTCTTTGATGCTTTGA 22
||||||||||||||||||||||

Sbjct: 2808 ATTATACAGAAACTACGAAACT 2787
>miR7814 Score: 0.0 RO3G_06428
Query: 1 TAATATGTCTTTGATGCTTTGA 22
||||||||||||||||||||||
Sbjct: 2310 ATTATACAGAAACTACGAAACT 2289
>miR7814 Score: 0.0 RO3G_06442
Query: 1 TAATATGTCTTTGATGCTTTGA 22
||||||||||||||||||||||
Sbjct: 3873 ATTATACAGAAACTACGAAACT 3852
>miR7814 Score: 0.0 RO3G_05279
Query: 1 TAATATGTCTTTGATGCTTTGA 22
||||||||||||||||||||||
Sbjct: 162 ATTATACAGAAACTACGAAACT 141
>miR7814 Score: 0.5 RO3G_02691
Query: 1 TAATATGTCTTTGATGCTTTGA 22
||||||:|||||||||||||||
Sbjct: 6705 ATTATATAGAAACTACGAAACT 6684
>miR7814 Score: 0.0 RO3G_02959
Query: 1 TAATATGTCTTTGATGCTTTGA 22
||||||||||||||||||||||
Sbjct: 3216 ATTATACAGAAACTACGAAACT 3195
>miR7814 Score: 0.5 RO3G_03547
Query: 1 TAATATGTCTTTGATGCTTTGA 22
||||||:|||||||||||||||
Sbjct: 3216 ATTATATAGAAACTACGAAACT 3195
>miR7814 Score: 0.0 RO3G_04115
Query: 1 TAATATGTCTTTGATGCTTTGA 22
||||||||||||||||||||||
Sbjct: 159 ATTATACAGAAACTACGAAACT 138
>miR7814 Score: 1.0 RO3G_00306
Query: 1 TAATATGTCTTTGATGCTTTGA 22
|*||||||||||||||||||||
Sbjct: 1512 ACTATACAGAAACTACGAAACT 1491
>miR7814 Score: 1.0 RO3G_01330
Query: 1 TAATATGTCTTTGATGCTTTGA 22
||*|||||||||||||||||||
Sbjct: 450 ATCATACAGAAACTACGAAACT 429
>miR7814 Score: 0.5 RO3G_01613
Query: 1 TAATATGTCTTTGATGCTTTGA 22
|||||||:||||||||||||||
Sbjct: 1467 ATTATACGGAAACTACGAAACT 1446
>miR7814 Score: 2.5 RO3G_02034
Query: 1 TAATATGTCTTTGATGCTTTGA 22
||||||:|||||:||:|::|*|
Sbjct: 1302 ATTATATAGAAATTATGGGAGT 1281

>miR7814 Score: 0.0 RO3G_02222
Query: 1 TAATATGTCTTTGATGCTTTGA 22
||||||||||||||||||||||
Sbjct: 270 ATTATACAGAAACTACGAAACT 249
>miR7814 Score: 0.0 RO3G_17371
Query: 1 TAATATGTCTTTGATGCTTTGA 22
||||||||||||||||||||||
Sbjct: 3171 ATTATACAGAAACTACGAAACT 3150
>miR7814 Score: 0.0 RO3G_17326
Query: 1 TAATATGTCTTTGATGCTTTGA 22
||||||||||||||||||||||
Sbjct: 3048 ATTATACAGAAACTACGAAACT 3027
>miR6116-3p Score: 0.0 RO3G_15681
Query: 1 TCATTTGTCATCCAAGCATTAG 22
||||||||||||||||||||||
Sbjct: 65 AGTAAACAGTAGGTTCGTAATC 44
>miR6116-3p Score: 0.0 RO3G_13488
Query: 1 TCATTTGTCATCCAAGCATTAG 22
||||||||||||||||||||||
Sbjct: 65 AGTAAACAGTAGGTTCGTAATC 44
>miR6116-3p Score: 0.0 RO3G_13491
Query: 1 TCATTTGTCATCCAAGCATTAG 22
||||||||||||||||||||||
Sbjct: 65 AGTAAACAGTAGGTTCGTAATC 44
>miR6116-3p Score: 0.0 RO3G_12001
Query: 1 TCATTTGTCATCCAAGCATTAG 22
||||||||||||||||||||||
Sbjct: 806 AGTAAACAGTAGGTTCGTAATC 785
>miR6116-3p Score: 0.0 RO3G_10556
Query: 1 TCATTTGTCATCCAAGCATTAG 22
||||||||||||||||||||||
Sbjct: 806 AGTAAACAGTAGGTTCGTAATC 785
>miR6116-3p Score: 0.0 RO3G_05824
Query: 1 TCATTTGTCATCCAAGCATTAG 22
||||||||||||||||||||||
Sbjct: 65 AGTAAACAGTAGGTTCGTAATC 44
>miR6116-3p Score: 0.0 RO3G_03517
Query: 1 TCATTTGTCATCCAAGCATTAG 22
||||||||||||||||||||||
Sbjct: 65 AGTAAACAGTAGGTTCGTAATC 44
>miR6116-3p Score: 0.0 RO3G_03518
Query: 1 TCATTTGTCATCCAAGCATTAG 22
||||||||||||||||||||||
Sbjct: 65 AGTAAACAGTAGGTTCGTAATC 44
>miR6116-3p Score: 0.0 RO3G_01302

Query: 1 TCATTTGTCATCCAAGCATTAG 22
||||||||||||||||||||||
Sbjct: 377 AGTAAACAGTAGGTTCGTAATC 356
>miR6116-3p Score: 0.0 RO3G_17369
Query: 1 TCATTTGTCATCCAAGCATTAG 22
||||||||||||||||||||||
Sbjct: 806 AGTAAACAGTAGGTTCGTAATC 785
>miR5065 Score: 2.5 RO3G_12990
Query: 1 TAGGCTTACTTATACATT 18
|||||||::|||||||
Sbjct: 188 TTCCGAAAGGGTATGTAA 171
>miR5065 Score: 2.5 RO3G_08665
Query: 1 TAGGCTTACTTATACATT 18
||:|||:|||||:|*|||
Sbjct: 1594 ATTCGAGTGAATGTTTAA 1577
>miR5065 Score: 2.5 RO3G_00543
Query: 1 TAGGCTTACTTATACATT 18
|*|:|||*||||||||||
Sbjct: 908 AACTGAAGGAATATGTAA 891
>miR5645a Score: 2.5 RO3G_05619
Query: 1 TTTGCAGTCACGGCGTTAAG 20
|||||||||||:||:||***
Sbjct: 3521 AAACGTCAGTGTCGTAAAAA 3502
>miR5645a Score: 2.5 RO3G_00812
Query: 1 TTTGCAGTCACGGCGTTAA-G 20
||||*|||||||||||:*|*|
Sbjct: 540 AAACTTCAGTGCCGCAGCTGC 520
>miR6473 Score: 2.2 RO3G_14532
Query: 1 TCCACAATGCCAAAGAGTT 19
|||||||::|||||:|::|
Sbjct: 3029 AGGTGTTGTGGTTTTTTGA 3011
>miR6473 Score: 2.5 RO3G_14295
Query: 1 TCCACAATGCCAAAGAGTT 19
||||||*|:|||||||:*|
Sbjct: 35 AGGTGTAATGGTTTCTTTA 17
>miR6473 Score: 2.2 RO3G_13808
Query: 1 TCCACAATGCCAAAGAGTT 19
|||||||::|||||:|::|
Sbjct: 329 AGGTGTTGTGGTTTTTTGA 311
>miR6473 Score: 2.5 RO3G_13244
Query: 1 TCCACAATGCCAAAGAGTT 19
|||**|||:||||||||||
Sbjct: 4040 AGGACTTATGGTTTCTCAA 4022
>miR6473 Score: 2.0 RO3G_05727
Query: 1 TCCACAATGCCAAAGAGTT 19

||||*|||||||||:||*|
Sbjct: 406 AGGTTTTACGGTTTTTCTA 388
>miR6473 Score: 2.0 RO3G_05738
Query: 1 TCCACAATGCCAAAGAGTT 19
||||*|||||||||:||*|
Sbjct: 406 AGGTTTTACGGTTTTTCTA 388
>miR6473 Score: 2.2 RO3G_03123
Query: 1 TCCACAATGCCAAAGAGTT 19
|||||||::|||||:|::|
Sbjct: 329 AGGTGTTGTGGTTTTTTGA 311
>miR6473 Score: 2.2 RO3G_01427
Query: 1 TCCACAATGCCAAAGAGTT 19
|||||||::|||||:|::|
Sbjct: 329 AGGTGTTGTGGTTTTTTGA 311
>miR3712 Score: 2.0 RO3G_17220
Query: 1 TGCTGATCAAGATCAAGACCA 21
:||||||||||||||||||
Sbjct: 536 CTGTCTAGTTCTAGTTCTGGT 516
>miR3712 Score: 0.0 RO3G_16744
Query: 1 TGCTGATCAAGATCAAGACCA 21
|||||||||||||||||||||
Sbjct: 578 ACGACTAGTTCTAGTTCTGGT 558
>miR3712 Score: 0.0 RO3G_16686
Query: 1 TGCTGATCAAGATCAAGACCA 21
|||||||||||||||||||||
Sbjct: 578 ACGACTAGTTCTAGTTCTGGT 558
>miR3712 Score: 0.0 RO3G_15869
Query: 1 TGCTGATCAAGATCAAGACCA 21
|||||||||||||||||||||
Sbjct: 578 ACGACTAGTTCTAGTTCTGGT 558
>miR3712 Score: 0.0 RO3G_14704
Query: 1 TGCTGATCAAGATCAAGACCA 21
|||||||||||||||||||||
Sbjct: 578 ACGACTAGTTCTAGTTCTGGT 558
>miR3712 Score: 0.0 RO3G_13450
Query: 1 TGCTGATCAAGATCAAGACCA 21
|||||||||||||||||||||
Sbjct: 533 ACGACTAGTTCTAGTTCTGGT 513
>miR3712 Score: 0.0 RO3G_13859
Query: 1 TGCTGATCAAGATCAAGACCA 21
|||||||||||||||||||||
Sbjct: 203 ACGACTAGTTCTAGTTCTGGT 183
>miR3712 Score: 1.0 RO3G_12951
Query: 1 TGCTGATCAAGATCAAGACCA 21
||*||||||||||||||||||

Sbjct: 578 ACAACTAGTTCTAGTTCTGGT 558
>miR3712 Score: 0.0 RO3G_12979
Query: 1 TGCTGATCAAGATCAAGACCA 21
|||||||||||||||||||||
Sbjct: 578 ACGACTAGTTCTAGTTCTGGT 558
>miR3712 Score: 0.0 RO3G_13152
Query: 1 TGCTGATCAAGATCAAGACCA 21
|||||||||||||||||||||
Sbjct: 578 ACGACTAGTTCTAGTTCTGGT 558
>miR3712 Score: 0.0 RO3G_11970
Query: 1 TGCTGATCAAGATCAAGACCA 21
|||||||||||||||||||||
Sbjct: 578 ACGACTAGTTCTAGTTCTGGT 558
>miR3712 Score: 0.0 RO3G_12087
Query: 1 TGCTGATCAAGATCAAGACCA 21
|||||||||||||||||||||
Sbjct: 578 ACGACTAGTTCTAGTTCTGGT 558
>miR3712 Score: 0.0 RO3G_12194
Query: 1 TGCTGATCAAGATCAAGACCA 21
|||||||||||||||||||||
Sbjct: 578 ACGACTAGTTCTAGTTCTGGT 558
>miR3712 Score: 0.0 RO3G_11275
Query: 1 TGCTGATCAAGATCAAGACCA 21
|||||||||||||||||||||
Sbjct: 578 ACGACTAGTTCTAGTTCTGGT 558
>miR3712 Score: 0.0 RO3G_11403
Query: 1 TGCTGATCAAGATCAAGACCA 21
|||||||||||||||||||||
Sbjct: 578 ACGACTAGTTCTAGTTCTGGT 558
>miR3712 Score: 0.0 RO3G_10555
Query: 1 TGCTGATCAAGATCAAGACCA 21
|||||||||||||||||||||
Sbjct: 578 ACGACTAGTTCTAGTTCTGGT 558
>miR3712 Score: 0.0 RO3G_10560
Query: 1 TGCTGATCAAGATCAAGACCA 21
|||||||||||||||||||||
Sbjct: 578 ACGACTAGTTCTAGTTCTGGT 558
>miR3712 Score: 2.5 RO3G_10759
Query: 1 TGCTGATCAAGATCAAGACCA 21
|||||*||||||||||:|**|
Sbjct: 518 ACGACAAGTTCTAGTTTT-TT 499
>miR3712 Score: 0.0 RO3G_09376
Query: 1 TGCTGATCAAGATCAAGACCA 21
|||||||||||||||||||||
Sbjct: 578 ACGACTAGTTCTAGTTCTGGT 558

>miR3712 Score: 0.0 RO3G_08612
Query: 1 TGCTGATCAAGATCAAGACCA 21
|||||||||||||||||||||
Sbjct: 578 ACGACTAGTTCTAGTTCTGGT 558
>miR3712 Score: 0.0 RO3G_07104
Query: 1 TGCTGATCAAGATCAAGACCA 21
|||||||||||||||||||||
Sbjct: 578 ACGACTAGTTCTAGTTCTGGT 558
>miR3712 Score: 0.0 RO3G_07305
Query: 1 TGCTGATCAAGATCAAGACCA 21
|||||||||||||||||||||
Sbjct: 578 ACGACTAGTTCTAGTTCTGGT 558
>miR3712 Score: 0.0 RO3G_05630
Query: 1 TGCTGATCAAGATCAAGACCA 21
|||||||||||||||||||||
Sbjct: 578 ACGACTAGTTCTAGTTCTGGT 558
>miR3712 Score: 0.0 RO3G_06416
Query: 1 TGCTGATCAAGATCAAGACCA 21
|||||||||||||||||||||
Sbjct: 578 ACGACTAGTTCTAGTTCTGGT 558
>miR3712 Score: 0.0 RO3G_05280
Query: 1 TGCTGATCAAGATCAAGACCA 21
|||||||||||||||||||||
Sbjct: 578 ACGACTAGTTCTAGTTCTGGT 558
>miR3712 Score: 2.5 RO3G_05369
Query: 1 TGCTGATCAAGATCAAGACCA 21
:||||||||||||||||**
Sbjct: 335 TTGACTAGTTCTAGTTC-GAA 316
>miR3712 Score: 0.5 RO3G_03103
Query: 1 TGCTGATCAAGATCAAGACCA 21
||||||||||||||||||*||
Sbjct: 578 ACGACTAGTTCTAGTTCTAGT 558
>miR3712 Score: 2.0 RO3G_03330
Query: 1 TGCTGATCAAGATCAAGACCA 21
|||||||||||||||||**
Sbjct: 1586 CCGACTAGTTCTAGTTC-GAA 1567
>miR3712 Score: 0.0 RO3G_03988
Query: 1 TGCTGATCAAGATCAAGACCA 21
|||||||||||||||||||||
Sbjct: 578 ACGACTAGTTCTAGTTCTGGT 558
>miR3712 Score: 0.0 RO3G_01203
Query: 1 TGCTGATCAAGATCAAGACCA 21
|||||||||||||||||||||
Sbjct: 578 ACGACTAGTTCTAGTTCTGGT 558
>miR3712 Score: 0.0 RO3G_01718

Query: 1 TGCTGATCAAGATCAAGACCA 21
|||||||||||||||||||||
Sbjct: 578 ACGACTAGTTCTAGTTCTGGT 558
>miR163 Score: 0.5 RO3G_17241
Query: 1 TTGAAGAGCAGCATGGAATTCGT 23
|||||||:|||||||||||||||
Sbjct: 192 AACTTCTTGTCGTACCTTAAGCA 170
>miR163 Score: 2.0 RO3G_16486
Query: 1 TTGAAGAGCAGCATGGAATTCGT 23
:|||||||||||*||||*|:|||
Sbjct: 2430 GACTTCTCGTCGGACCTCAGGCA 2408
>miR163 Score: 0.5 RO3G_16127
Query: 1 TTGAAGAGCAGCATGGAATTCGT 23
|||||||:|||||||||||||||
Sbjct: 873 AACTTCTTGTCGTACCTTAAGCA 851
>miR163 Score: 0.5 RO3G_15256
Query: 1 TTGAAGAGCAGCATGGAATTCGT 23
|||||||:|||||||||||||||
Sbjct: 873 AACTTCTTGTCGTACCTTAAGCA 851
>miR163 Score: 0.5 RO3G_13711
Query: 1 TTGAAGAGCAGCATGGAATTCGT 23
|||||||:|||||||||||||||
Sbjct: 858 AACTTCTTGTCGTACCTTAAGCA 836
>miR163 Score: 0.0 RO3G_13072
Query: 1 TTGAAGAGCAGCATGGAATTCGT 23
|||||||||||||||||||||||
Sbjct: 777 AACTTCTCGTCGTACCTTAAGCA 755
>miR163 Score: 0.5 RO3G_12195
Query: 1 TTGAAGAGCAGCATGGAATTCGT 23
|||||||:|||||||||||||||
Sbjct: 66 AACTTCTTGTCGTACCTTAAGCA 44
>miR163 Score: 0.5 RO3G_10737
Query: 1 TTGAAGAGCAGCATGGAATTCGT 23
|||||||:|||||||||||||||
Sbjct: 873 AACTTCTTGTCGTACCTTAAGCA 851
>miR163 Score: 0.5 RO3G_10048
Query: 1 TTGAAGAGCAGCATGGAATTCGT 23
|||||||:|||||||||||||||
Sbjct: 873 AACTTCTTGTCGTACCTTAAGCA 851
>miR163 Score: 0.5 RO3G_09184
Query: 1 TTGAAGAGCAGCATGGAATTCGT 23
|||||||:|||||||||||||||
Sbjct: 585 AACTTCTTGTCGTACCTTAAGCA 563
>miR163 Score: 0.5 RO3G_07115
Query: 1 TTGAAGAGCAGCATGGAATTCGT 23

|||||||:|||||||||||||||
Sbjct: 834 AACTTCTTGTCGTACCTTAAGCA 812
>miR163 Score: 2.2 RO3G_07933
Query: 1 TTGAAGAGCAGCATGGAATTCGT 23
|||||||||||||||*|*|:*||
Sbjct: 1353 AACTTCTCGTCGTACGT-AGACA 1332
>miR163 Score: 0.5 RO3G_05910
Query: 1 TTGAAGAGCAGCATGGAATTCGT 23
|||||||:|||||||||||||||
Sbjct: 873 AACTTCTTGTCGTACCTTAAGCA 851
>miR163 Score: 2.5 RO3G_04623
Query: 1 TTGAAGAGCAGCATGGAATTCGT 23
|:|||:|||||*||||||*||||
Sbjct: 141 AGCTTTTCGTCCTACCTT-AGCA 120
>miR163 Score: 0.5 RO3G_02231
Query: 1 TTGAAGAGCAGCATGGAATTCGT 23
|||||||:|||||||||||||||
Sbjct: 873 AACTTCTTGTCGTACCTTAAGCA 851
>miR163 Score: 0.5 RO3G_02827
Query: 1 TTGAAGAGCAGCATGGAATTCGT 23
|||||||:|||||||||||||||
Sbjct: 873 AACTTCTTGTCGTACCTTAAGCA 851
>miR163 Score: 0.5 RO3G_00259
Query: 1 TTGAAGAGCAGCATGGAATTCGT 23
|||||||:|||||||||||||||
Sbjct: 873 AACTTCTTGTCGTACCTTAAGCA 851
>miR163 Score: 0.5 RO3G_01198
Query: 1 TTGAAGAGCAGCATGGAATTCGT 23
|||||||:|||||||||||||||
Sbjct: 807 AACTTCTTGTCGTACCTTAAGCA 785
>miR163 Score: 0.5 RO3G_01723
Query: 1 TTGAAGAGCAGCATGGAATTCGT 23
|||||||:|||||||||||||||
Sbjct: 294 AACTTCTTGTCGTACCTTAAGCA 272
>miR5648-5p Score: 2.5 RO3G_14543
Query: 1 TTTAAAAATTTGGCCTTGACT 21
|||||||||||:|**||||||
Sbjct: 1472 AAATTTTTAAATCCTAACTGA 1452
>miR5648-5p Score: 2.2 RO3G_09804
Query: 1 TTTAAAAATTTGGCCTTGACT 21
:|:|||||:|||||||||**|
Sbjct: 239 GAGTTTTTGAACCGGAAC-CA 220
>miR1097 Score: 0.0 RO3G_16362
Query: 1 TGCCATTGTTTGTTGTTGGAAG 22
||||||||||||||||||||||

```
Sbjct: 2725 ACGGTAACAAACAACAACCTTC 2704
>miR1097 Score: 2.5 RO3G_15109
Query: 1 TGCCATTGTTTGTTGTTGGA-AG 22
||||||::||||:|||||||**|
Sbjct: 144 ACGGTAGTAAACGACAACCTAAC 122
>miR1097 Score: 2.5 RO3G_06371
Query: 1 TGCCATTGTTTGTTGTTGGAAG 22
||||||||||||:|||*||**|
Sbjct: 760 ACGGTAACAAACGACA-CCGAC 740
>miR1097 Score: 2.0 RO3G_03008
Query: 1 TGCCATTGTTTGTTGTTGGAAG 22
*|||||||||||||||::*|*
Sbjct: 1802 TCGGTAACAAACAACAATTATA 1781
>miR1097 Score: 2.5 RO3G_00127
Query: 1 TGCCATTGTTTGTTGTTGGAAG 22
||||||||*|:|:||||||*||
Sbjct: 1037 ACGGTAACTAGCGACAACCCTC 1016
>miR536f Score: 0.0 RO3G_02551
Query: 1 TTCCGTCCAAGCTGTGTTGA 20
||||||||||||||||||||
Sbjct: 260 AAGGCAGGTTCGACACAACT 241
>miR536f Score: 2.2 RO3G_01892
Query: 1 TTCCGTCCAAGCTGTGTTGA 20
||||:|||||:||||*|:||
Sbjct: 500 AAGGTAGGTTTGACAAAGCT 481
>miR444b Score: 2.5 RO3G_15616
Query: 1 TGCCGTTGCTGTTTCCAAGTT 21
*|*|||||||||:|||||:|:
Sbjct: 521 CCAGCAACGACAGAGGTTTAG 501
>miR444b Score: 2.5 RO3G_09068
Query: 1 TGCCGTTGCTGTTTCCAAGTT 21
|||*||||*||||||||*|||
Sbjct: 1139 ACGTCAACCACAAAGGT-CAA 1120
>miR444b Score: 2.5 RO3G_00707
Query: 1 TGCCGTTGCTGTTTCCAAGTT 21
*|||*|||||||||||||:*:
Sbjct: 2019 TCGGAAACGACAAAGGTTTCG 1999
>miR1886.2 Score: 2.5 RO3G_15363
Query: 1 TGGATGATATTGTGATTGG 19
|||*|||||:|:|||||*|
Sbjct: 112 ACCAACTATGATACTAAAC 94
>miR1886.2 Score: 2.0 RO3G_14446
Query: 1 TGGATGATATTGTGATTGG 19
||||||*|*||||||||||
Sbjct: 1018 ACCTACCACAACACTAACC 1000
```

```
>miR1886.2 Score: 2.5 RO3G_13371
Query: 1 TGGATGATATTGTGATTGG 19
||||||||||*:||||*||
Sbjct: 973 ACCTACTATACTACTA-CC 956
>miR1886.2 Score: 2.5 RO3G_12133
Query: 1 TGGATGATATTGTGATTGG 19
||||||||*|:|||||*||
Sbjct: 205 ACCTACTAAAGCACTA-CC 188
>miR1886.2 Score: 2.5 RO3G_12414
Query: 1 TGGATGATATTGTGATTGG 19
:||||||:||::|:||||:
Sbjct: 266 GCCTACTGTAGTATTAACT 248
>miR1886.2 Score: 2.5 RO3G_10408
Query: 1 TGGATGATATTGTGATTGG 19
:|||||||*||||:|:|:|
Sbjct: 1313 GCCTACTAGAACATTGATC 1295
>miR1886.2 Score: 2.5 RO3G_08298
Query: 1 TGGATGATATTGTGATTGG 19
*|||||*|||::|||||||
Sbjct: 512 TCCTACCATAGTACTAACC 494
>miR1886.2 Score: 2.5 RO3G_04517
Query: 1 TGGATGATATTGTGATTGG 19
||||||||||:|:|*|||*
Sbjct: 739 ACCTACTATAGCGCGAACG 721
>miR1886.2 Score: 2.5 RO3G_04181
Query: 1 TGGATGATATTGTGATTGG 19
:|:||||:|||||*||||:
Sbjct: 71 GCTTACTGTAACAATAACT 53
>miR1886.2 Score: 2.5 RO3G_00277
Query: 1 TGGATGATATTGTGATTGG 19
*||||:|*|||||||||*|
Sbjct: 2178 CCCTATTTTAACACTAAGC 2160
>miR166b Score: 0.5 RO3G_15127
Query: 1 TCGGCCAGGCATTCCTTCCAA 21
|||||||:|||||||||||||
Sbjct: 161 AGCCGGTTCGTAAGGAAGGTT 141
>miR166b Score: 0.8 RO3G_00203
Query: 1 TCGGCCAGGCATTCCTTCCAA 21
:||||||:|||||||||||||
Sbjct: 137 GGCCGGTTCGTAAGGAAGGTT 117
>miR166b Score: 0.0 RO3G_00248
Query: 1 TCGGCCAGGCATTCCTTCCAA 21
|||||||||||||||||||||
Sbjct: 149 AGCCGGTCCGTAAGGAAGGTT 129
>miR166b Score: 0.0 RO3G_00261
```

```
Query: 1 TCGGCCAGGCATTCCTTCCAA 21
|||||||||||||||||||||
Sbjct: 149 AGCCGGTCCGTAAGGAAGGTT 129
>miR166b Score: 1.5 RO3G_00466
Query: 1 TCGGCCAGGCATTCCTTCCAA 21
||||*||:|||||||||||||
Sbjct: 149 AGCCAGTTCGTAAGGAAGGTT 129
>miR166b Score: 0.5 RO3G_01504
Query: 1 TCGGCCAGGCATTCCTTCCAA 21
|||||||:|||||||||||||
Sbjct: 143 AGCCGGTTCGTAAGGAAGGTT 123
>miR166b Score: 0.5 RO3G_01650
Query: 1 TCGGCCAGGCATTCCTTCCAA 21
|||||||:|||||||||||||
Sbjct: 143 AGCCGGTTCGTAAGGAAGGTT 123
>miR9677-5p Score: 1.0 RO3G_14134
Query: 1 TTTCCTCTCCAACAGCCAAG 20
|||||||||||||*||||||
Sbjct: 196 AAAGGAGAGGTTGCCGGTTC 177
>miR9677-5p Score: 1.0 RO3G_10867
Query: 1 TTTCCTCTCCAACAGCCAAG 20
|||||||||||||*||||||
Sbjct: 307 AAAGGAGAGGTTGCCGGTTC 288
>miR9677-5p Score: 1.0 RO3G_05937
Query: 1 TTTCCTCTCCAACAGCCAAG 20
|||||||||||||*||||||
Sbjct: 565 AAAGGAGAGGTTGCCGGTTC 546
>miR2678 Score: 2.0 RO3G_13330
Query: 1 TGAATTGTTGGGATTGTACTT 21
*||*|:|||||||||||||||
Sbjct: 390 CCTGAGCAACCCTAACATGAA 370
>miR2678 Score: 0.0 RO3G_13722
Query: 1 TGAATTGTTGGGATTGTACTT 21
|||||||||||||||||||||
Sbjct: 1059 ACTTAACAACCCTAACATGAA 1039
>miR2678 Score: 2.2 RO3G_08781
Query: 1 TGAATTGTTGGGATTGTAC-TT 21
||||||||||::|||||*|*:|
Sbjct: 2794 ACTTAACAACTTTAACAAGTGA 2773
>miR2678 Score: 2.5 RO3G_08782
Query: 1 TGAATTGTTGGGATTGTACTT 21
*||||||||*|||||||**||
Sbjct: 208 TCTTAACAAACCTAACAAAAA 188
>miR2678 Score: 2.5 RO3G_06760
Query: 1 TGAATTGTTGGGATTGTACTT 21
```

```
||*||||||||||||:|**||
Sbjct: 162 ACATAACAACCCTAATAACAA 142
>miR2678 Score: 2.5 RO3G_05796
Query: 1 TGAATTGTTGGGATTGTACTT 21
||||||||||*:|||||*||*
Sbjct: 1485 ACTTAACAACATTAACA-GAT 1466
>miR2678 Score: 2.5 RO3G_03140
Query: 1 TGAATTGTTGGGATTGTACTT 21
||||||:||*|||||:||*||
Sbjct: 348 ACTTAATAAGCCTAATATTAA 328
>miR157a-3p Score: 2.5 RO3G_12815
Query: 1 GCTCTCTAGCCTTCTGTCAT 20
|||*|||||||||*|||*||
Sbjct: 597 CGATAGATCGGAAAACATTA 578
>miR157a-3p Score: 2.5 RO3G_05078
Query: 1 GCTCTCTAGCCTTCTGTCAT 20
||||||:||||||||*:|*|
Sbjct: 68 CGAGAGGTCGGAAGAAGGAA 49
>miR9662a-3p Score: 1.5 RO3G_16744
Query: 1 TTAACATCCACAGAGCCAAG 20
|||||||||||||||*||*|
Sbjct: 518 AATTGTAGGTGTCTCTGTCC 499
>miR9662a-3p Score: 1.5 RO3G_16686
Query: 1 TTAACATCCACAGAGCCAAG 20
|||||||||||||||*||*|
Sbjct: 518 AATTGTAGGTGTCTCTGTCC 499
>miR9662a-3p Score: 1.5 RO3G_15869
Query: 1 TTAACATCCACAGAGCCAAG 20
|||||||||||||||*||*|
Sbjct: 518 AATTGTAGGTGTCTCTGTCC 499
>miR9662a-3p Score: 1.5 RO3G_14704
Query: 1 TTAACATCCACAGAGCCAAG 20
|||||||||||||||*||*|
Sbjct: 518 AATTGTAGGTGTCTCTGTCC 499
>miR9662a-3p Score: 1.5 RO3G_13450
Query: 1 TTAACATCCACAGAGCCAAG 20
|||||||||||||||*||*|
Sbjct: 473 AATTGTAGGTGTCTCTGTCC 454
>miR9662a-3p Score: 1.5 RO3G_13859
Query: 1 TTAACATCCACAGAGCCAAG 20
|||||||||||||||*||*|
Sbjct: 143 AATTGTAGGTGTCTCTGTCC 124
>miR9662a-3p Score: 1.5 RO3G_12951
Query: 1 TTAACATCCACAGAGCCAAG 20
|||||||||||||||*||*|
```

Sbjct: 518 AATTGTAGGTGTCTCTGTCC 499
>miR9662a-3p Score: 1.5 RO3G_12979
Query: 1 TTAACATCCACAGAGCCAAG 20
||||||||||||||*||*|
Sbjct: 518 AATTGTAGGTGTCTCTGTCC 499
>miR9662a-3p Score: 1.5 RO3G_13152
Query: 1 TTAACATCCACAGAGCCAAG 20
||||||||||||||*||*|
Sbjct: 518 AATTGTAGGTGTCTCTGTCC 499
>miR9662a-3p Score: 1.5 RO3G_11970
Query: 1 TTAACATCCACAGAGCCAAG 20
||||||||||||||*||*|
Sbjct: 518 AATTGTAGGTGTCTCTGTCC 499
>miR9662a-3p Score: 1.5 RO3G_12087
Query: 1 TTAACATCCACAGAGCCAAG 20
||||||||||||||*||*|
Sbjct: 518 AATTGTAGGTGTCTCTGTCC 499
>miR9662a-3p Score: 1.5 RO3G_12194
Query: 1 TTAACATCCACAGAGCCAAG 20
||||||||||||||*||*|
Sbjct: 518 AATTGTAGGTGTCTCTGTCC 499
>miR9662a-3p Score: 1.5 RO3G_11275
Query: 1 TTAACATCCACAGAGCCAAG 20
||||||||||||||*||*|
Sbjct: 518 AATTGTAGGTGTCTCTGTCC 499
>miR9662a-3p Score: 1.5 RO3G_11403
Query: 1 TTAACATCCACAGAGCCAAG 20
||||||||||||||*||*|
Sbjct: 518 AATTGTAGGTGTCTCTGTCC 499
>miR9662a-3p Score: 1.5 RO3G_10555
Query: 1 TTAACATCCACAGAGCCAAG 20
||||||||||||||*||*|
Sbjct: 518 AATTGTAGGTGTCTCTGTCC 499
>miR9662a-3p Score: 1.5 RO3G_10560
Query: 1 TTAACATCCACAGAGCCAAG 20
||||||||||||||*||*|
Sbjct: 518 AATTGTAGGTGTCTCTGTCC 499
>miR9662a-3p Score: 1.5 RO3G_09376
Query: 1 TTAACATCCACAGAGCCAAG 20
||||||||||||||*||*|
Sbjct: 518 AATTGTAGGTGTCTCTGTCC 499
>miR9662a-3p Score: 1.5 RO3G_08612
Query: 1 TTAACATCCACAGAGCCAAG 20
||||||||||||||*||*|
Sbjct: 518 AATTGTAGGTGTCTCTGTCC 499

```
>miR9662a-3p Score: 1.5 RO3G_07104
Query: 1 TTAACATCCACAGAGCCAAG 20
||||||||||||||*||*|
Sbjct: 518 AATTGTAGGTGTCTCTGTCC 499
>miR9662a-3p Score: 1.5 RO3G_07305
Query: 1 TTAACATCCACAGAGCCAAG 20
||||||||||||||*||*|
Sbjct: 518 AATTGTAGGTGTCTCTGTCC 499
>miR9662a-3p Score: 2.5 RO3G_07346
Query: 1 TTAACATCCACAGAGCCAAG 20
||||||*||||||||*|||*
Sbjct: 197 AATTGTCGGTGTCTCCGTTA 178
>miR9662a-3p Score: 1.5 RO3G_05630
Query: 1 TTAACATCCACAGAGCCAAG 20
||||||||||||||*||*|
Sbjct: 518 AATTGTAGGTGTCTCTGTCC 499
>miR9662a-3p Score: 1.5 RO3G_06416
Query: 1 TTAACATCCACAGAGCCAAG 20
||||||||||||||*||*|
Sbjct: 518 AATTGTAGGTGTCTCTGTCC 499
>miR9662a-3p Score: 2.2 RO3G_06504
Query: 1 TTAACATCCACAGAGCCA-AG 20
||||||||*||||||||**|:
Sbjct: 509 AATTGTAGTTGTCTCGGGGTT 489
>miR9662a-3p Score: 2.2 RO3G_04960
Query: 1 TTAACATCCACAGAGCCAAG 20
:||||*||||||:||||*||
Sbjct: 383 GATTGCAGGTGTTTCGG-TC 365
>miR9662a-3p Score: 1.5 RO3G_05280
Query: 1 TTAACATCCACAGAGCCAAG 20
||||||||||||||*||*|
Sbjct: 518 AATTGTAGGTGTCTCTGTCC 499
>miR9662a-3p Score: 1.5 RO3G_03103
Query: 1 TTAACATCCACAGAGCCAAG 20
||||||||||||||*||*|
Sbjct: 518 AATTGTAGGTGTCTCTGTCC 499
>miR9662a-3p Score: 2.5 RO3G_03685
Query: 1 TTAACATCCACAGAGCCAAG 20
*|**||||||||||||||||
Sbjct: 827 TAAAGTAGGTGTCTCGGTTC 808
>miR9662a-3p Score: 1.5 RO3G_03988
Query: 1 TTAACATCCACAGAGCCAAG 20
||||||||||||||*||*|
Sbjct: 518 AATTGTAGGTGTCTCTGTCC 499
>miR9662a-3p Score: 1.5 RO3G_01203
```

Query: 1 TTAACATCCACAGAGCCAAG 20
||||||||||||||*||*|
Sbjct: 518 AATTGTAGGTGTCTCTGTCC 499
>miR9662a-3p Score: 1.5 RO3G_01718
Query: 1 TTAACATCCACAGAGCCAAG 20
||||||||||||||*||*|
Sbjct: 518 AATTGTAGGTGTCTCTGTCC 499
>miR6485 Score: 0.0 RO3G_17255
Query: 1 TGGATGTACCAGAGCATCAA 20
||||||||||||||||||||
Sbjct: 2819 ACCTACATGGTCTCGTAGTT 2800
>miR6485 Score: 1.5 RO3G_17156
Query: 1 TGGATGTACCAGAGCATCAA 20
|||||:|*||||||||||||
Sbjct: 1121 ACCTATACGGTCTCGTAGTT 1102
>miR6485 Score: 0.0 RO3G_16919
Query: 1 TGGATGTACCAGAGCATCAA 20
||||||||||||||||||||
Sbjct: 443 ACCTACATGGTCTCGTAGTT 424
>miR6485 Score: 2.5 RO3G_16519
Query: 1 TGGATGTACCAGAGCATCAA 20
|||||||*|*|:||||||||
Sbjct: 186 ACCTACAGGATTTCGTAGTT 167
>miR6485 Score: 0.0 RO3G_14563
Query: 1 TGGATGTACCAGAGCATCAA 20
||||||||||||||||||||
Sbjct: 431 ACCTACATGGTCTCGTAGTT 412
>miR6485 Score: 0.0 RO3G_10543
Query: 1 TGGATGTACCAGAGCATCAA 20
||||||||||||||||||||
Sbjct: 2321 ACCTACATGGTCTCGTAGTT 2302
>miR6485 Score: 0.0 RO3G_10744
Query: 1 TGGATGTACCAGAGCATCAA 20
||||||||||||||||||||
Sbjct: 2867 ACCTACATGGTCTCGTAGTT 2848
>miR6485 Score: 0.0 RO3G_10745
Query: 1 TGGATGTACCAGAGCATCAA 20
||||||||||||||||||||
Sbjct: 1835 ACCTACATGGTCTCGTAGTT 1816
>miR6485 Score: 0.5 RO3G_07735
Query: 1 TGGATGTACCAGAGCATCAA 20
||||||||||||||||||*|
Sbjct: 2768 ACCTACATGGTCTCGTAGCT 2749
>miR6485 Score: 0.0 RO3G_07862
Query: 1 TGGATGTACCAGAGCATCAA 20

||||||||||||||||||||
Sbjct: 2978 ACCTACATGGTCTCGTAGTT 2959
>miR6485 Score: 2.5 RO3G_00640
Query: 1 TGGATGTACCAGAGCATCAA 20
|::||:*|||||||||||||
Sbjct: 3146 ATTTATCTGGTCTCGTAGTT 3127
>miR6485 Score: 2.5 RO3G_01294
Query: 1 TGGATGTACCAGAGCATCA-A 20
||:||:|*|||||||||||*|
Sbjct: 1937 ACTTATACGGTCTCGTAGTCT 1917
>miR6485 Score: 0.0 RO3G_17453
Query: 1 TGGATGTACCAGAGCATCAA 20
||||||||||||||||||||
Sbjct: 881 ACCTACATGGTCTCGTAGTT 862
>miR6269 Score: 0.0 RO3G_13221
Query: 1 TGCTGCAAGTGATTAGCCATGG 22
||||||||||||||||||||||
Sbjct: 881 ACGACGTTCACTAATCGGTACC 860
>miR6269 Score: 2.0 RO3G_13224
Query: 1 TGCTGCAAGTGATTAGCCATGG 22
||*||||||||*||||||||||
Sbjct: 881 ACAACGTTCACCAATCGGTACC 860
>miR6269 Score: 2.0 RO3G_04120
Query: 1 TGCTGCAAGTGATTAGCCATGG 22
||*||||||||*||||||||||
Sbjct: 881 ACAACGTTCACCAATCGGTACC 860
>miR6269 Score: 1.0 RO3G_04123
Query: 1 TGCTGCAAGTGATTAGCCATGG 22
|||||||||||*||||||||||
Sbjct: 881 ACGACGTTCACCAATCGGTACC 860
>miR6269 Score: 2.0 RO3G_17093
Query: 1 TGCTGCAAGTGATTAGCCATGG 22
||*||||||||*||||||||||
Sbjct: 641 ACAACGTTCACCAATCGGTACC 620
>miR6156 Score: 2.5 RO3G_16582
Query: 1 TTGAAGAAGTTCTTTGTTCGT 21
||||||||||:|||||*||
Sbjct: 685 CACTTCTTCAACGAACAA-CA 666
>miR6156 Score: 2.0 RO3G_13795
Query: 1 TTGAAGAAGTTCTTTGTTCGT 21
|*|||||||||||||||*||*
Sbjct: 1087 ATCTTCTTCAAGAAACA-GCC 1068
>miR6156 Score: 2.5 RO3G_12847
Query: 1 TTGAAGAAGTTCTTTGTTCGT 21
|::||||*:||||||||||||

Sbjct: 589 AGTTTCTCTAAGAAACAAGCA 569
>miR403-3p Score: 2.5 RO3G_02749
Query: 1 TTAGATTCACGCACAAACTC 20
:|||||||||||||||*|
Sbjct: 536 CGTCTAAGTGGGTGTTTGTG 517
>miR403-3p Score: 2.5 RO3G_03878
Query: 1 TTAGATTCACGCACAAACTC 20
|||:|||||||*|||||**|
Sbjct: 275 AATTTAAGTGCTTGTTTATG 256
>miR5244 Score: 2.2 RO3G_12564
Query: 1 TTCTCTTGAAGTAGTTGTTGGT 22
||*|||||||:|||||||*|:|
Sbjct: 570 AAAAGAACTTTATCAACACCTA 549
>miR5244 Score: 0.0 RO3G_10142
Query: 1 TTCTCTTGAAGTAGTTGTTGGT 22
||||||||||||||||||||||
Sbjct: 359 AAGAGAACTTCATCAACAACCA 338
>miR5244 Score: 2.5 RO3G_02474
Query: 1 TTCTCTTGAAGTAGTTGTTGGT 22
||||*||||*||||||||*|||
Sbjct: 102 AAGAAAACTCCATCAACATCCA 81
>miR5244 Score: 0.0 RO3G_03107
Query: 1 TTCTCTTGAAGTAGTTGTTGGT 22
||||||||||||||||||||||
Sbjct: 359 AAGAGAACTTCATCAACAACCA 338
>miR5244 Score: 2.5 RO3G_01005
Query: 1 TTCTCTTGAAGTAGTTGTTGGT 22
||||||||*|||||||:|::*|
Sbjct: 193 AAGAGAACGTCATCAATAGTAA 172
>miR5244 Score: 2.5 RO3G_01078
Query: 1 TTCTCTTGAAGTAGTTGTTGGT 22
||||||||||::||*||||*||
Sbjct: 228 AAGAGAACTTTGTCCACAAACA 207
>miR2611 Score: 2.5 RO3G_15192
Query: 1 TATTTTCAGTGTTGTGGTAGAA 22
:|||||||:|:||||||:||**
Sbjct: 849 GTAAAAGTTATAACACCGTCGC 828
>miR6464 Score: 1.2 RO3G_17253
Query: 1 TGTTTGCTTGTTGATGTTGAT 21
::||||||||||||||:||||
Sbjct: 625 GTAAACGAACAACTACGACTA 605
>miR6464 Score: 0.0 RO3G_17255
Query: 1 TGTTTGCTTGTTGATGTTGAT 21
|||||||||||||||||||||
Sbjct: 625 ACAAACGAACAACTACAACTA 605

>miR6464 Score: 1.0 RO3G_17218
Query: 1 TGTTTGCTTGTTGATGTTGAT 21
|:||:||||||||||||||||
Sbjct: 625 ATAAGCGAACAACTACAACTA 605
>miR6464 Score: 2.0 RO3G_17083
Query: 1 TGTTTGCTTGTTGATGTTGAT 21
|||||||||||||||||*|
Sbjct: 173 CCAAACAAACAACTACAACGA 153
>miR6464 Score: 0.8 RO3G_16366
Query: 1 TGTTTGCTTGTTGATGTTGAT 21
::|||||||||||||||||||
Sbjct: 625 GTAAACGAACAACTACAACTA 605
>miR6464 Score: 2.5 RO3G_15299
Query: 1 TGTTTGCTTGTTGATGTTGAT 21
||**|||||||||||||||*|
Sbjct: 952 ACCTACGAACAACTACAAC-A 933
>miR6464 Score: 2.0 RO3G_14450
Query: 1 TGTTTGCTTGTTGATGTT-GAT 21
||||||||::|||||||**|||
Sbjct: 63 ACAAACGAGTAACTACATTCTA 42
>miR6464 Score: 0.8 RO3G_13448
Query: 1 TGTTTGCTTGTTGATGTTGAT 21
::|||||||||||||||||||
Sbjct: 625 GTAAACGAACAACTACAACTA 605
>miR6464 Score: 0.8 RO3G_11979
Query: 1 TGTTTGCTTGTTGATGTTGAT 21
::|||||||||||||||||||
Sbjct: 609 GTAAACGAACAACTACAACTA 589
>miR6464 Score: 1.8 RO3G_12315
Query: 1 TGTTTGCTTGTTGATGTTGAT 21
|||||||||:|||||:|*:||
Sbjct: 2538 ACAAACGAATAACTATATTTA 2518
>miR6464 Score: 0.5 RO3G_10532
Query: 1 TGTTTGCTTGTTGATGTTGAT 21
|:|||||||||||||||||||
Sbjct: 625 ATAAACGAACAACTACAACTA 605
>miR6464 Score: 1.5 RO3G_10539
Query: 1 TGTTTGCTTGTTGATGTTGAT 21
|:|||||||||||*|||||||
Sbjct: 739 ATAAACGAACAACCACAACTA 719
>miR6464 Score: 0.8 RO3G_10543
Query: 1 TGTTTGCTTGTTGATGTTGAT 21
::|||||||||||||||||||
Sbjct: 97 GTAAACGAACAACTACAACTA 77
>miR6464 Score: 1.0 RO3G_10744

Query: 1 TGTTTGCTTGTTGATGTTGAT 21
|:||||||||:||||||||||
Sbjct: 625 ATAAACGAACGACTACAACTA 605
>miR6464 Score: 0.5 RO3G_10747
Query: 1 TGTTTGCTTGTTGATGTTGAT 21
|:|||||||||||||||||||
Sbjct: 739 ATAAACGAACAACTACAACTA 719
>miR6464 Score: 2.2 RO3G_09582
Query: 1 TGTTTGCTTGTTGATGTTGAT 21
:*|||:|||||||||||||*|
Sbjct: 106 GAAAATGAACAACTACAACAA 86
>miR6464 Score: 2.5 RO3G_08583
Query: 1 TGTTTGCTTGTTGATGTTGAT 21
|||||*||*|:||||||||||
Sbjct: 3479 ACAAAAGATCGACTACAACTA 3459
>miR6464 Score: 2.5 RO3G_08623
Query: 1 TGTTTGCTTGTTGATGTTGAT 21
|||||||||||||:|||*|
Sbjct: 1195 CCTAACGAACAACTATAACAA 1175
>miR6464 Score: 1.0 RO3G_09173
Query: 1 TGTTTGCTTGTTGATGTTGAT 21
|:||||||||:||||||||||
Sbjct: 739 ATAAACGAACGACTACAACTA 719
>miR6464 Score: 2.0 RO3G_07259
Query: 1 TGTTTGCTTGTTGATGTTGAT 21
|||||||||||*||:||*|||
Sbjct: 2045 ACAAACGAACACCTGCA-CTA 2026
>miR6464 Score: 2.5 RO3G_07291
Query: 1 TGTTTGCTTGTTGATGTTGAT 21
|||||*|||||||*||||||*
Sbjct: 1270 ACAAAAGAACAACAACAACTC 1250
>miR6464 Score: 1.0 RO3G_07862
Query: 1 TGTTTGCTTGTTGATGTTGAT 21
|:||||||||:||||||||||
Sbjct: 739 ATAAACGAACGACTACAACTA 719
>miR6464 Score: 2.5 RO3G_05698
Query: 1 TGTTTGCTTGTTGATGTTGAT 21
|||||**|||||||||:||||
Sbjct: 826 ACAAAACAACAACTACGACTA 806
>miR6464 Score: 2.5 RO3G_06329
Query: 1 TGTTTGCTTGTTGATGTTGAT 21
||:||||||||||*||||**|
Sbjct: 355 ACGAACGAACAACAACAA-GA 336
>miR6464 Score: 2.5 RO3G_05074
Query: 1 TGTTTGCTTGTTGATGTTGAT 21

||||:||||:|||||||||
Sbjct: 1708 TCAAATGAACGACAACAACTA 1688
>miR6464 Score: 2.0 RO3G_05389
Query: 1 TGTTTGCTTGTTGATGTTGAT 21
|||||||||||||*|||*|*|
Sbjct: 679 ACAAACGAACAACAACA-CAA 660
>miR6464 Score: 1.8 RO3G_03861
Query: 1 TGTTTGCTTGTTGATGTTGAT 21
:||||:||||||||||*||||
Sbjct: 92 GCAAATGAACAACTACTACTA 72
>miR6464 Score: 2.0 RO3G_01193
Query: 1 TGTTTGCTTGTTGATGTTGAT 21
||||||||||||||:*||*|
Sbjct: 1675 ACAAACGAACAACTATTACAA 1655
>miR7541 Score: 2.5 RO3G_10208
Query: 1 TGCAATGTCTCTTTTGCTGGCAC 23
||||||||||||*|:|||*|*||
Sbjct: 629 ACGTTACAGAGATAGCGAAC-TG 608
>miR7541 Score: 0.0 RO3G_08829
Query: 1 TGCAATGTCTCTTTTGCTGGCAC 23
|||||||||||||||||||||||
Sbjct: 192 ACGTTACAGAGAAAACGACCGTG 170
>miR5512a Score: 1.5 RO3G_16725
Query: 1 TAGGATCATTGTCATGCTAAAAT 23
|||||||||||||*||||*||||
Sbjct: 475 ATCCTAGTAACAGCACGACTTTA 453
>miR5512a Score: 2.5 RO3G_16737
Query: 1 TAGGATCATTGTCATGCT-AAAAT 23
|*||||||||:|||||||*|||*|
Sbjct: 367 AACCTAGTAATAGTACGACTTTCA 344
>miR5512a Score: 2.0 RO3G_16359
Query: 1 TAGGATCATTGTCATGCT-AAAAT 23
||||||||||||||||*|*||||*
Sbjct: 742 ATCCTAGTAACAGTACAACTTTTT 719
>miR5512a Score: 2.0 RO3G_16378
Query: 1 TAGGATCATTGTCATGCT-AAAAT 23
||||||||||||||||*|*||||*
Sbjct: 211 ATCCTAGTAACAGTACAACTTTTT 188
>miR5512a Score: 2.5 RO3G_16541
Query: 1 TAGGATCATTGTCATGCT-AAAAT 23
|*||||||||:|||||||*|||*|
Sbjct: 367 AACCTAGTAATAGTACGACTTTCA 344
>miR5512a Score: 2.0 RO3G_16545
Query: 1 TAGGATCATTGTCATGCT-AAAAT 23
||||||||||||||||*|*||||*

Sbjct: 742 ATCCTAGTAACAGTACAACTTTTT 719
>miR5512a Score: 2.5 RO3G_16141
Query: 1 TAGGATCATTGTCATGCT-AAAAT 23
|*||||||||:|||||||*|||*|
Sbjct: 367 AACCTAGTAATAGTACGACTTTCA 344
>miR5512a Score: 2.5 RO3G_16124
Query: 1 TAGGATCATTGTCATGCT-AAAAT 23
|*||||||||:|||||||*|||*|
Sbjct: 367 AACCTAGTAATAGTACGACTTTCA 344
>miR5512a Score: 1.5 RO3G_15271
Query: 1 TAGGATCATTGTCATGCTAAAAT 23
|||||||||||||*||||*||||
Sbjct: 733 ATCCTAGTAACAGCACGACTTTA 711
>miR5512a Score: 2.5 RO3G_15286
Query: 1 TAGGATCATTGTCATGCT-AAAAT 23
|*||||||||:|||||||*|||*|
Sbjct: 367 AACCTAGTAATAGTACGACTTTCA 344
>miR5512a Score: 2.5 RO3G_14981
Query: 1 TAGGATCATTGTCATGCT-AAAAT 23
|*||||||||:|||||||*|||*|
Sbjct: 367 AACCTAGTAATAGTACGACTTTCA 344
>miR5512a Score: 2.5 RO3G_15145
Query: 1 TAGGATCATTGTCATGCT-AAAAT 23
|*||||||||:|||||||*|||*|
Sbjct: 367 AACCTAGTAATAGTACGACTTTCA 344
>miR5512a Score: 2.5 RO3G_15170
Query: 1 TAGGATCATTGTCATGCT-AAAAT 23
|*||||||||:|||||||*|||*|
Sbjct: 367 AACCTAGTAATAGTACGACTTTCA 344
>miR5512a Score: 2.5 RO3G_14784
Query: 1 TAGGATCATTGTCATGCT-AAAAT 23
|*||||||||:|||||||*|||*|
Sbjct: 367 AACCTAGTAATAGTACGACTTTCA 344
>miR5512a Score: 2.5 RO3G_13752
Query: 1 TAGGATCATTGTCATGCT-AAAAT 23
|*||||||||:|||||||*|||*|
Sbjct: 367 AACCTAGTAATAGTACGACTTTCA 344
>miR5512a Score: 0.5 RO3G_13852
Query: 1 TAGGATCATTGTCATGCTAAAAT 23
|||||||||||||||||*|||||
Sbjct: 484 ATCCTAGTAACAGTACGACTTTA 462
>miR5512a Score: 2.5 RO3G_12950
Query: 1 TAGGATCATTGTCATGCT-AAAAT 23
|*||||||||:|||||||*|||*|
Sbjct: 367 AACCTAGTAATAGTACGACTTTCA 344

>miR5512a Score: 2.5 RO3G_13267
Query: 1 TAGGATCATTGTCATGCT-AAAAT 23
|*||||||||:|||||||*|||*|
Sbjct: 367 AACCTAGTAATAGTACGACTTTCA 344
>miR5512a Score: 2.5 RO3G_12084
Query: 1 TAGGATCATTGTCATGCT-AAAAT 23
|*||||||||:|||||||*|||*|
Sbjct: 367 AACCTAGTAATAGTACGACTTTCA 344
>miR5512a Score: 2.5 RO3G_11533
Query: 1 TAGGATCATTGTCATGCT-AAAAT 23
|*||||||||:|||||||*|||*|
Sbjct: 367 AACCTAGTAATAGTACGACTTTCA 344
>miR5512a Score: 2.5 RO3G_11536
Query: 1 TAGGATCATTGTCATGCT-AAAAT 23
|*||||||||:|||||||*|||*|
Sbjct: 367 AACCTAGTAATAGTACGACTTTCA 344
>miR5512a Score: 1.5 RO3G_10771
Query: 1 TAGGATCATTGTCATGCTAAAAT 23
||||||||||||||||*|*||||
Sbjct: 484 ATCCTAGTAACAGTACAACTTTA 462
>miR5512a Score: 2.5 RO3G_10785
Query: 1 TAGGATCATTGTCATGCT-AAAAT 23
|*||||||||:|||||||*|||*|
Sbjct: 367 AACCTAGTAATAGTACGACTTTCA 344
>miR5512a Score: 2.5 RO3G_10797
Query: 1 TAGGATCATTGTCATGCT-AAAAT 23
|*||||||||:|||||||*|||*|
Sbjct: 367 AACCTAGTAATAGTACGACTTTCA 344
>miR5512a Score: 2.5 RO3G_09926
Query: 1 TAGGATCATTGTCATGCT-AAAAT 23
|*||||||||:|||||||*|||*|
Sbjct: 367 AACCTAGTAATAGTACGACTTTCA 344
>miR5512a Score: 2.5 RO3G_10050
Query: 1 TAGGATCATTGTCATGCT-AAAAT 23
|*||||||||:|||||||*|||*|
Sbjct: 367 AACCTAGTAATAGTACGACTTTCA 344
>miR5512a Score: 2.5 RO3G_07136
Query: 1 TAGGATCATTGTCATGCT-AAAAT 23
|*||||||||:|||||||*|||*|
Sbjct: 58 AACCTAGTAATAGTACGACTTTCA 35
>miR5512a Score: 2.5 RO3G_07723
Query: 1 TAGGATCATTGTCATGCT-AAAAT 23
|*||||||||:|||||||*|||*|
Sbjct: 367 AACCTAGTAATAGTACGACTTTCA 344
>miR5512a Score: 1.5 RO3G_07739

```
Query: 1 TAGGATCATTGTCATGCTAAAAT 23
||||||||||*|||||||*||||
Sbjct: 484 ATCCTAGTAAGAGTACGACTTTA 462
>miR5512a Score: 2.5 RO3G_05522
Query: 1 TAGGATCATTGTCATGCT-AAAAT 23
|*||||||||:|||||||*|||*|
Sbjct: 733 AACCTAGTAATAGTACGACTTTCA 710
>miR5512a Score: 2.5 RO3G_05516
Query: 1 TAGGATCATTGTCATGCT-AAAAT 23
|*||||||||:|||||||*|||*|
Sbjct: 367 AACCTAGTAATAGTACGACTTTCA 344
>miR5512a Score: 2.5 RO3G_02844
Query: 1 TAGGATCATTGTCATGCTAAAAT 23
|*|||||||||||||||**||*
Sbjct: 520 ACCCTAGTAACAGTACGACCTTC 498
>miR5512a Score: 2.5 RO3G_03112
Query: 1 TAGGATCATTGTCATGCT-AAAAT 23
|*||||||||:|||||||*|||*|
Sbjct: 733 AACCTAGTAATAGTACGACTTTCA 710
>miR5512a Score: 2.5 RO3G_03560
Query: 1 TAGGATCATTGTCATGCT-AAAAT 23
|*||||||||:|||||||*|||*|
Sbjct: 367 AACCTAGTAATAGTACGACTTTCA 344
>miR5512a Score: 2.5 RO3G_03791
Query: 1 TAGGATCATTGTCATGCT-AAAAT 23
|*||||||||:|||||||*|||*|
Sbjct: 367 AACCTAGTAATAGTACGACTTTCA 344
>miR5512a Score: 2.0 RO3G_03797
Query: 1 TAGGATCATTGTCATGCT-AAAAT 23
||||||||:||||*||||*|||||
Sbjct: 232 ATCCTAGTGACAGCACGACTTTTA 209
>miR5512a Score: 2.5 RO3G_00240
Query: 1 TAGGATCATTGTCATGCT-AAAAT 23
|*||||||||:|||||||*|||*|
Sbjct: 676 AACCTAGTAATAGTACGACTTTCA 653
>miR5512a Score: 2.5 RO3G_00250
Query: 1 TAGGATCATTGTCATGCT-AAAAT 23
|*||||||||:|||||||*|||*|
Sbjct: 367 AACCTAGTAATAGTACGACTTTCA 344
>miR5512a Score: 2.5 RO3G_00745
Query: 1 TAGGATCATTGTCATGCTAAAAT 23
|||||||||||||*||*|*||||
Sbjct: 454 ATCCTAGTAACAGCACAACTTTA 432
>miR5512a Score: 2.5 RO3G_01725
Query: 1 TAGGATCATTGTCATGCT-AAAAT 23
```

|*||||||||:|||||||*|||*|
Sbjct: 367 AACCTAGTAATAGTACGACTTTCA 344
>miR5512a Score: 2.5 RO3G_02227
Query: 1 TAGGATCATTGTCATGCT-AAAAT 23
|*||||||||:|||||||*|||*|
Sbjct: 733 AACCTAGTAATAGTACGACTTTCA 710
>miR5512a Score: 2.5 RO3G_17423
Query: 1 TAGGATCATTGTCATGCT-AAAAT 23
|*||||||||:|||||||*|||*|
Sbjct: 367 AACCTAGTAATAGTACGACTTTCA 344
>miR5512a Score: 2.5 RO3G_17328
Query: 1 TAGGATCATTGTCATGCT-AAAAT 23
|*||||||||:|||||||*|||*|
Sbjct: 367 AACCTAGTAATAGTACGACTTTCA 344
>miR5512a Score: 2.5 RO3G_17092
Query: 1 TAGGATCATTGTCATGCT-AAAAT 23
|*||||||||:|||||||*|||*|
Sbjct: 367 AACCTAGTAATAGTACGACTTTCA 344
>miR894 Score: 2.5 RO3G_15221
Query: 1 GTTTCACGTCGGGTTCACC 19
||||||*||||::||||*|
Sbjct: 910 CAAAGTTCAGCTTAAGTAG 892
>miR894 Score: 2.5 RO3G_11316
Query: 1 GTTTCACGTCGGGTTCACC 19
|||:|||::||||||||**
Sbjct: 1029 CAAGGTGTGGCCCAAGTCC 1011
>miR894 Score: 2.5 RO3G_10759
Query: 1 GTTTCACGTCGGGTTCACC 19
||||||||:|:||||||
Sbjct: 2182 GAAAGTGAAGTCTAAGTGG 2164
>miR4385 Score: 1.8 RO3G_03357
Query: 1 TAATGTTGTAAAAAGGATTGGT 22
:||||||:|||||||||||**|
Sbjct: 1927 GTTACAATATTTTTCCTAA-AA 1907
>miR4385 Score: 2.5 RO3G_00371
Query: 1 TAATGTTGTAAAAAGGATTGGT 22
|||||:|||||*|||:|*||||
Sbjct: 663 ATTACGACATTATTCTT-ACCA 643
>miR4385 Score: 2.5 RO3G_16946
Query: 1 TAATGTTGTAAAAAGGATTGGT 22
||||||||||*||*|:||||||
Sbjct: 2325 ATTACAACATCTTCCTTAACCA 2304
>miR5239 Score: 1.8 RO3G_16744
Query: 1 TGGGAGAAAACATAGGTG 18
|||||||||||||||:

Sbjct: 2998 TCGCTCTTTTGTATCCAT 2981
>miR5239 Score: 1.8 RO3G_16686
Query: 1 TGGGAGAAAACATAGGTG 18
|||||||||||||||:
Sbjct: 2839 TCGCTCTTTTGTATCCAT 2822
>miR5239 Score: 2.2 RO3G_15947
Query: 1 TGGGAGAAAACATAGGTG 18
|:||||*|||||||||::
Sbjct: 904 ATCCTCGTTTGTATCCGT 887
>miR5239 Score: 2.2 RO3G_15659
Query: 1 TGGGAGAAAACATAGGTG 18
|*||||*||||||||||:
Sbjct: 856 AACCTCGTTTGTATCCAT 839
>miR5239 Score: 1.8 RO3G_15869
Query: 1 TGGGAGAAAACATAGGTG 18
|||||||||||||||:
Sbjct: 2998 TCGCTCTTTTGTATCCAT 2981
>miR5239 Score: 1.8 RO3G_14704
Query: 1 TGGGAGAAAACATAGGTG 18
|||||||||||||||:
Sbjct: 2839 TCGCTCTTTTGTATCCAT 2822
>miR5239 Score: 1.8 RO3G_13450
Query: 1 TGGGAGAAAACATAGGTG 18
|||||||||||||||:
Sbjct: 2656 TCGCTCTTTTGTATCCAT 2639
>miR5239 Score: 1.8 RO3G_13859
Query: 1 TGGGAGAAAACATAGGTG 18
|||||||||||||||:
Sbjct: 2623 TCGCTCTTTTGTATCCAT 2606
>miR5239 Score: 1.8 RO3G_12951
Query: 1 TGGGAGAAAACATAGGTG 18
|||||||||||||||:
Sbjct: 2839 TCGCTCTTTTGTATCCAT 2822
>miR5239 Score: 1.8 RO3G_12979
Query: 1 TGGGAGAAAACATAGGTG 18
|||||||||||||||:
Sbjct: 2839 TCGCTCTTTTGTATCCAT 2822
>miR5239 Score: 1.8 RO3G_13152
Query: 1 TGGGAGAAAACATAGGTG 18
|||||||||||||||:
Sbjct: 2839 TCGCTCTTTTGTATCCAT 2822
>miR5239 Score: 1.8 RO3G_11970
Query: 1 TGGGAGAAAACATAGGTG 18
|||||||||||||||:
Sbjct: 2539 TCGCTCTTTTGTATCCAT 2522

>miR5239 Score: 2.5 RO3G_11987
Query: 1 TGGGAGAAAACATAGGTG 18
||:||||*||||:||||*
Sbjct: 428 ACTCTCTCTTGTGTCCAG 411
>miR5239 Score: 1.8 RO3G_12087
Query: 1 TGGGAGAAAACATAGGTG 18
|||||||||||||||:
Sbjct: 2839 TCGCTCTTTTGTATCCAT 2822
>miR5239 Score: 1.8 RO3G_11275
Query: 1 TGGGAGAAAACATAGGTG 18
|||||||||||||||:
Sbjct: 2998 TCGCTCTTTTGTATCCAT 2981
>miR5239 Score: 1.8 RO3G_11403
Query: 1 TGGGAGAAAACATAGGTG 18
|||||||||||||||:
Sbjct: 2998 TCGCTCTTTTGTATCCAT 2981
>miR5239 Score: 1.8 RO3G_11785
Query: 1 TGGGAGAAAACATAGGTG 18
|||||||||||||||:
Sbjct: 1159 TCGCTCTTTTGTATCCAT 1142
>miR5239 Score: 1.8 RO3G_10555
Query: 1 TGGGAGAAAACATAGGTG 18
|||||||||||||||:
Sbjct: 2839 TCGCTCTTTTGTATCCAT 2822
>miR5239 Score: 1.8 RO3G_10560
Query: 1 TGGGAGAAAACATAGGTG 18
|||||||||||||||:
Sbjct: 2998 TCGCTCTTTTGTATCCAT 2981
>miR5239 Score: 1.8 RO3G_09376
Query: 1 TGGGAGAAAACATAGGTG 18
|||||||||||||||:
Sbjct: 2877 TCGCTCTTTTGTATCCAT 2860
>miR5239 Score: 1.8 RO3G_08612
Query: 1 TGGGAGAAAACATAGGTG 18
|||||||||||||||:
Sbjct: 2839 TCGCTCTTTTGTATCCAT 2822
>miR5239 Score: 1.8 RO3G_08968
Query: 1 TGGGAGAAAACATAGGTG 18
|||||||||||||||:
Sbjct: 553 TCGCTCTTTTGTATCCAT 536
>miR5239 Score: 1.8 RO3G_07305
Query: 1 TGGGAGAAAACATAGGTG 18
|||||||||||||||:
Sbjct: 2839 TCGCTCTTTTGTATCCAT 2822
>miR5239 Score: 1.8 RO3G_05630

Query: 1 TGGGAGAAAACATAGGTG 18
|||||||||||||||:
Sbjct: 2758 TCGCTCTTTTGTATCCAT 2741
>miR5239 Score: 1.8 RO3G_06416
Query: 1 TGGGAGAAAACATAGGTG 18
|||||||||||||||:
Sbjct: 2998 TCGCTCTTTTGTATCCAT 2981
>miR5239 Score: 1.8 RO3G_05280
Query: 1 TGGGAGAAAACATAGGTG 18
|||||||||||||||:
Sbjct: 2839 TCGCTCTTTTGTATCCAT 2822
>miR5239 Score: 1.8 RO3G_02235
Query: 1 TGGGAGAAAACATAGGTG 18
|||||||||||||||:
Sbjct: 2182 TCGCTCTTTTGTATCCAT 2165
>miR5239 Score: 1.8 RO3G_03103
Query: 1 TGGGAGAAAACATAGGTG 18
|||||||||||||||:
Sbjct: 2839 TCGCTCTTTTGTATCCAT 2822
>miR5239 Score: 2.5 RO3G_03743
Query: 1 TGGGAGAAAACATAGGTG 18
||:||||*||||:||||*
Sbjct: 437 ACTCTCTCTTGTGTCCAG 420
>miR5239 Score: 1.8 RO3G_03988
Query: 1 TGGGAGAAAACATAGGTG 18
|||||||||||||||:
Sbjct: 2839 TCGCTCTTTTGTATCCAT 2822
>miR5239 Score: 2.2 RO3G_00944
Query: 1 TGGGAGAAAACATAGGTG 18
||||||||||||||*|*:
Sbjct: 670 ACCCTCTTTTGTATGC-T 654
>miR5239 Score: 1.8 RO3G_01203
Query: 1 TGGGAGAAAACATAGGTG 18
|||||||||||||||:
Sbjct: 2998 TCGCTCTTTTGTATCCAT 2981
>miR5239 Score: 1.8 RO3G_01718
Query: 1 TGGGAGAAAACATAGGTG 18
|||||||||||||||:
Sbjct: 2998 TCGCTCTTTTGTATCCAT 2981
>miR5239 Score: 2.5 RO3G_01932
Query: 1 TGGGAGAAAACATAGGTG 18
|:|||||||||||*||*|
Sbjct: 173 ATCCTCTTTTGTAACC-C 157
>miR1076-5p Score: 0.0 RO3G_14532
Query: 1 TATCACAAGCGCGACTAATTGT 22

||||||||||||||||||||||
Sbjct: 1828 ATAGTGTTCGCGCTGATTAACA 1807
>miR1076-5p Score: 0.0 RO3G_14210
Query: 1 TATCACAAGCGCGACTAATTGT 22
||||||||||||||||||||||
Sbjct: 1221 ATAGTGTTCGCGCTGATTAACA 1200
>miR845a Score: 2.5 RO3G_16081
Query: 1 CGGCTCTGATACCAATTGAT 20
:||||||||||||||*||
Sbjct: 309 TTCGAGACTATAGTTAAGTA 290
>miR845a Score: 2.2 RO3G_05232
Query: 1 CGGCTCTGATACCAATTGAT 20
:||||||||||||||||:
Sbjct: 873 ATCGAGCCTATGGTTAACTG 854
>miR5252 Score: 0.2 RO3G_17265
Query: 1 TTGTAAAGCTACTGAAGTCTGC 22
|||||||||||||||||:||||
Sbjct: 139 AACATTTCGATGACTTCGGACG 118
>miR5252 Score: 1.2 RO3G_17244
Query: 1 TTGTAAAGCTACTGAAGTCTGC 22
||||||||*||||||||:||||
Sbjct: 235 AACATTTCAATGACTTCGGACG 214
>miR5252 Score: 0.2 RO3G_17210
Query: 1 TTGTAAAGCTACTGAAGTCTGC 22
|||||||||||||||||:||||
Sbjct: 262 AACATTTCGATGACTTCGGACG 241
>miR5252 Score: 2.2 RO3G_16901
Query: 1 TTGTAAAGCTACTGAAGTCTGC 22
||||*|||||||||||*:||||
Sbjct: 232 AACACTTCGATGACTTAGGACG 211
>miR5252 Score: 2.2 RO3G_16917
Query: 1 TTGTAAAGCTACTGAAGTCTGC 22
||||*|||||||||||*:||||
Sbjct: 238 AACACTTCGATGACTTAGGACG 217
>miR5252 Score: 0.2 RO3G_16743
Query: 1 TTGTAAAGCTACTGAAGTCTGC 22
|||||||||||||||||:||||
Sbjct: 184 AACATTTCGATGACTTCGGACG 163
>miR5252 Score: 0.2 RO3G_16369
Query: 1 TTGTAAAGCTACTGAAGTCTGC 22
|||||||||||||||||:||||
Sbjct: 217 AACATTTCGATGACTTCGGACG 196
>miR5252 Score: 0.2 RO3G_15887
Query: 1 TTGTAAAGCTACTGAAGTCTGC 22
|||||||||||||||||:||||

```
Sbjct: 262 AACATTTCGATGACTTCGGACG 241
>miR5252 Score: 0.2 RO3G_14553
Query: 1 TTGTAAAGCTACTGAAGTCTGC 22
|||||||||||||||||:||||
Sbjct: 238 AACATTTCGATGACTTCGGACG 217
>miR5252 Score: 0.2 RO3G_13452
Query: 1 TTGTAAAGCTACTGAAGTCTGC 22
|||||||||||||||||:||||
Sbjct: 214 AACATTTCGATGACTTCGGACG 193
>miR5252 Score: 0.0 RO3G_12976
Query: 1 TTGTAAAGCTACTGAAGTCTGC 22
||||||||||||||||||||||
Sbjct: 241 AACATTTCGATGACTTCAGACG 220
>miR5252 Score: 0.2 RO3G_12011
Query: 1 TTGTAAAGCTACTGAAGTCTGC 22
|||||||||||||||||:||||
Sbjct: 238 AACATTTCGATGACTTCGGACG 217
>miR5252 Score: 0.2 RO3G_11543
Query: 1 TTGTAAAGCTACTGAAGTCTGC 22
|||||||||||||||||:||||
Sbjct: 238 AACATTTCGATGACTTCGGACG 217
>miR5252 Score: 0.2 RO3G_10043
Query: 1 TTGTAAAGCTACTGAAGTCTGC 22
|||||||||||||||||:||||
Sbjct: 232 AACATTTCGATGACTTCGGACG 211
>miR5252 Score: 0.2 RO3G_07692
Query: 1 TTGTAAAGCTACTGAAGTCTGC 22
|||||||||||||||||:||||
Sbjct: 172 AACATTTCGATGACTTCGGACG 151
>miR5252 Score: 0.2 RO3G_05906
Query: 1 TTGTAAAGCTACTGAAGTCTGC 22
|||||||||||||||||:||||
Sbjct: 175 AACATTTCGATGACTTCGGACG 154
>miR5252 Score: 0.2 RO3G_04659
Query: 1 TTGTAAAGCTACTGAAGTCTGC 22
|||||||||||||||||:||||
Sbjct: 172 AACATTTCGATGACTTCGGACG 151
>miR5252 Score: 0.2 RO3G_05347
Query: 1 TTGTAAAGCTACTGAAGTCTGC 22
|||||||||||||||||:||||
Sbjct: 238 AACATTTCGATGACTTCGGACG 217
>miR5252 Score: 0.2 RO3G_02817
Query: 1 TTGTAAAGCTACTGAAGTCTGC 22
|||||||||||||||||:||||
Sbjct: 241 AACATTTCGATGACTTCGGACG 220
```

>miR5252 Score: 0.2 RO3G_01192
Query: 1 TTGTAAAGCTACTGAAGTCTGC 22
|||||||||||||||||:||||
Sbjct: 175 AACATTTCGATGACTTCGGACG 154
>miR5252 Score: 0.2 RO3G_01717
Query: 1 TTGTAAAGCTACTGAAGTCTGC 22
|||||||||||||||||:||||
Sbjct: 241 AACATTTCGATGACTTCGGACG 220
>miR5252 Score: 0.2 RO3G_17460
Query: 1 TTGTAAAGCTACTGAAGTCTGC 22
|||||||||||||||||:||||
Sbjct: 262 AACATTTCGATGACTTCGGACG 241
>miR5252 Score: 0.2 RO3G_17433
Query: 1 TTGTAAAGCTACTGAAGTCTGC 22
|||||||||||||||||:||||
Sbjct: 235 AACATTTCGATGACTTCGGACG 214
>miR1108 Score: 0.8 RO3G_16906
Query: 1 TAAGCTGGAAACATAACG-CCC 21
|||||||||||||||||:*|||
Sbjct: 228 ATTCGACCTTTGTATTGTAGGG 207
>miR1108 Score: 0.8 RO3G_13723
Query: 1 TAAGCTGGAAACATAACG-CCC 21
|||||||||||||||||:*|||
Sbjct: 1059 ATTCGACCTTTGTATTGTAGGG 1038
>miR1108 Score: 0.0 RO3G_10822
Query: 1 TAAGCTGGAAACATAACGCCC 21
|||||||||||||||||||||
Sbjct: 1113 ATTCGACCTTTGTATTGCGGG 1093
>miR1108 Score: 1.2 RO3G_01682
Query: 1 TAAGCTGGAAACATAACGCCC 21
||||*||||||||||||:|||
Sbjct: 1005 ATTCAACCTTTGTATTGTGGG 985
>miR5658 Score: 0.0 RO3G_16744
Query: 1 TGATGATGATGATGTTGCAGAA 22
||||||||||||||||||||||
Sbjct: 3663 ACTACTACTACTACAACGTCTT 3642
>miR5658 Score: 0.0 RO3G_16686
Query: 1 TGATGATGATGATGTTGCAGAA 22
||||||||||||||||||||||
Sbjct: 3504 ACTACTACTACTACAACGTCTT 3483
>miR5658 Score: 1.5 RO3G_15960
Query: 1 TGATGATGATGATGTTGCAGAA 22
||||||||||||||||||*|**
Sbjct: 1625 ACTACTACTACTACAACGACAA 1604
>miR5658 Score: 0.0 RO3G_15869

Query: 1 TGATGATGATGATGTTGCAGAA 22
||||||||||||||||||||||
Sbjct: 3663 ACTACTACTACTACAACGTCTT 3642
>miR5658 Score: 0.0 RO3G_14704
Query: 1 TGATGATGATGATGTTGCAGAA 22
||||||||||||||||||||||
Sbjct: 3504 ACTACTACTACTACAACGTCTT 3483
>miR5658 Score: 1.2 RO3G_14198
Query: 1 TGATGATGATGATGTTGCAGAA 22
|||||||||||||||||**:||
Sbjct: 50 ACTACTACTACTACAACCATTT 29
>miR5658 Score: 2.5 RO3G_13341
Query: 1 TGATGATGATGATGTTGCAGAA 22
||||||||||||||*||**||*
Sbjct: 635 ACTACTACTACTACTACTACTG 614
>miR5658 Score: 0.0 RO3G_13450
Query: 1 TGATGATGATGATGTTGCAGAA 22
||||||||||||||||||||||
Sbjct: 3321 ACTACTACTACTACAACGTCTT 3300
>miR5658 Score: 0.0 RO3G_13859
Query: 1 TGATGATGATGATGTTGCAGAA 22
||||||||||||||||||||||
Sbjct: 3288 ACTACTACTACTACAACGTCTT 3267
>miR5658 Score: 0.0 RO3G_12979
Query: 1 TGATGATGATGATGTTGCAGAA 22
||||||||||||||||||||||
Sbjct: 3504 ACTACTACTACTACAACGTCTT 3483
>miR5658 Score: 0.0 RO3G_13152
Query: 1 TGATGATGATGATGTTGCAGAA 22
||||||||||||||||||||||
Sbjct: 3504 ACTACTACTACTACAACGTCTT 3483
>miR5658 Score: 0.0 RO3G_12087
Query: 1 TGATGATGATGATGTTGCAGAA 22
||||||||||||||||||||||
Sbjct: 3504 ACTACTACTACTACAACGTCTT 3483
>miR5658 Score: 0.0 RO3G_12193
Query: 1 TGATGATGATGATGTTGCAGAA 22
||||||||||||||||||||||
Sbjct: 510 ACTACTACTACTACAACGTCTT 489
>miR5658 Score: 0.0 RO3G_11275
Query: 1 TGATGATGATGATGTTGCAGAA 22
||||||||||||||||||||||
Sbjct: 3663 ACTACTACTACTACAACGTCTT 3642
>miR5658 Score: 0.0 RO3G_11403
Query: 1 TGATGATGATGATGTTGCAGAA 22

||||||||||||||||||||||
Sbjct: 3663 ACTACTACTACTACAACGTCTT 3642
>miR5658 Score: 0.0 RO3G_10555
Query: 1 TGATGATGATGATGTTGCAGAA 22
||||||||||||||||||||||
Sbjct: 3504 ACTACTACTACTACAACGTCTT 3483
>miR5658 Score: 0.0 RO3G_10560
Query: 1 TGATGATGATGATGTTGCAGAA 22
||||||||||||||||||||||
Sbjct: 3663 ACTACTACTACTACAACGTCTT 3642
>miR5658 Score: 0.0 RO3G_08612
Query: 1 TGATGATGATGATGTTGCAGAA 22
||||||||||||||||||||||
Sbjct: 3504 ACTACTACTACTACAACGTCTT 3483
>miR5658 Score: 0.0 RO3G_07305
Query: 1 TGATGATGATGATGTTGCAGAA 22
||||||||||||||||||||||
Sbjct: 3504 ACTACTACTACTACAACGTCTT 3483
>miR5658 Score: 2.5 RO3G_07545
Query: 1 TGATGATGATGATGTTGCAGAA 22
|||||||||||||||*||*|
Sbjct: 468 CCTACTACTACTACTACCTCGT 447
>miR5658 Score: 2.5 RO3G_07768
Query: 1 TGATGATGATGATGTTGCAGAA 22
|:|||||:|||||*|||*||||
Sbjct: 881 ATTACTATTACTAAAAC-TCTT 861
>miR5658 Score: 0.0 RO3G_05630
Query: 1 TGATGATGATGATGTTGCAGAA 22
||||||||||||||||||||||
Sbjct: 3423 ACTACTACTACTACAACGTCTT 3402
>miR5658 Score: 0.0 RO3G_06416
Query: 1 TGATGATGATGATGTTGCAGAA 22
||||||||||||||||||||||
Sbjct: 3663 ACTACTACTACTACAACGTCTT 3642
>miR5658 Score: 2.0 RO3G_06536
Query: 1 TGATGATGATGATGTTGCAGAA 22
|||||||||||||||||**|**
Sbjct: 416 ACTACTACTACTACAACAACAA 395
>miR5658 Score: 2.5 RO3G_06611
Query: 1 TGATGATGATGATGTTGCAGAA 22
||||||||||||||*||**|*|
Sbjct: 459 ACTACTACTACTACCACACCGT 438
>miR5658 Score: 2.5 RO3G_05207
Query: 1 TGATGATGATGATGTTGCAGAA 22
||||||||||||||*||**||*

Sbjct: 956 ACTACTACTACTACTACTACTG 935
>miR5658 Score: 0.0 RO3G_05280
Query: 1 TGATGATGATGATGTTGCAGAA 22
||||||||||||||||||||||
Sbjct: 3504 ACTACTACTACTACAACGTCTT 3483
>miR5658 Score: 0.0 RO3G_02235
Query: 1 TGATGATGATGATGTTGCAGAA 22
||||||||||||||||||||||
Sbjct: 2847 ACTACTACTACTACAACGTCTT 2826
>miR5658 Score: 2.5 RO3G_03011
Query: 1 TGATGATGATGATGTTGCAGAA 22
||||||||||||||*||**||*
Sbjct: 401 ACTACTACTACTACTACTGCTG 380
>miR5658 Score: 2.5 RO3G_03075
Query: 1 TGATGATGATGATGTTGCAGAA 22
||||||||||||||*||**||*
Sbjct: 1091 ACTACTACTACTACCACTACTA 1070
>miR5658 Score: 0.0 RO3G_03103
Query: 1 TGATGATGATGATGTTGCAGAA 22
||||||||||||||||||||||
Sbjct: 3504 ACTACTACTACTACAACGTCTT 3483
>miR5658 Score: 2.5 RO3G_03827
Query: 1 TGATGATGATGATGTTGCAGAA 22
||||||||||||||*||**||*
Sbjct: 1085 ACTACTACTACTACTACTACTA 1064
>miR5658 Score: 2.5 RO3G_03866
Query: 1 TGATGATGATGATGTTGCAGAA 22
||||||||||||||||*|***|
Sbjct: 110 ACTACTACTACTACAA-GAAGT 90
>miR5658 Score: 0.0 RO3G_03990
Query: 1 TGATGATGATGATGTTGCAGAA 22
||||||||||||||||||||||
Sbjct: 261 ACTACTACTACTACAACGTCTT 240
>miR5658 Score: 2.5 RO3G_04007
Query: 1 TGATGATGATGATGTTGCAGAA 22
|*||||||||||||:|||*||*
Sbjct: 1097 AATACTACTACTACGACGACTA 1076
>miR5658 Score: 2.5 RO3G_00266
Query: 1 TGATGATGATGATGTTGCAGAA 22
||||||||||||||*||*||**
Sbjct: 1226 ACTACTACTACTACTAC-TCCC 1206
>miR5658 Score: 2.5 RO3G_00272
Query: 1 TGATGATGATGATGTTGCAGAA 22
:||||||||||||:|||||*
Sbjct: 5961 TTTACTACTACTACGACCTCTA 5940

>miR5658 Score: 2.5 RO3G_00729
Query: 1 TGATGATGATGATGTTGCAGAA 22
|||||||||||||*||**||*
Sbjct: 1070 ACTACTACTACTACTACTACTA 1049
>miR5658 Score: 2.5 RO3G_00853
Query: 1 TGATGATGATGATGTTGCAGAA 22
|||||||||||||:||**|**
Sbjct: 1100 ACTACTACTACTACGACAACAA 1079
>miR5658 Score: 0.0 RO3G_01203
Query: 1 TGATGATGATGATGTTGCAGAA 22
||||||||||||||||||||||
Sbjct: 3663 ACTACTACTACTACAACGTCTT 3642
>miR5658 Score: 2.0 RO3G_01410
Query: 1 TGATGATGATGATGTTGCAGAA 22
|||||||||||||*||*|*||
Sbjct: 462 ACTACTACTACTACTAC-TATT 442
>miR5658 Score: 0.0 RO3G_01718
Query: 1 TGATGATGATGATGTTGCAGAA 22
||||||||||||||||||||||
Sbjct: 3663 ACTACTACTACTACAACGTCTT 3642
>miR5658 Score: 2.0 RO3G_16962
Query: 1 TGATGATGATGATGTTGCAGAA 22
|||||||||||||*||*|*||
Sbjct: 410 ACTACTACTACTACTACCTGTT 389
>miR8034 Score: 2.0 RO3G_16738
Query: 1 TATGACAAGCCTGCACAAACT 21
|||||||*|||||*|||||||
Sbjct: 685 ATACTGTCCGGACATGTTTGA 665
>miR8034 Score: 2.0 RO3G_16138
Query: 1 TATGACAAGCCTGCACAAACT 21
|||||||*|||||*|||||||
Sbjct: 1768 ATACTGTCCGGACATGTTTGA 1748
>miR8034 Score: 1.0 RO3G_15580
Query: 1 TATGACAAGCCTGCACAAACT 21
|||||||*|||||||||||||
Sbjct: 1732 ATACTGTCCGGACGTGTTTGA 1712
>miR8034 Score: 2.0 RO3G_14626
Query: 1 TATGACAAGCCTGCACAAACT 21
|||||||*|||||*|||||||
Sbjct: 1768 ATACTGTCCGGACATGTTTGA 1748
>miR8034 Score: 2.0 RO3G_14710
Query: 1 TATGACAAGCCTGCACAAACT 21
|||||||*|||||*|||||||
Sbjct: 1768 ATACTGTCCGGACATGTTTGA 1748
>miR8034 Score: 2.2 RO3G_13747

Query: 1 TATGACAAGCCTGCACAAACT 21
|||||||*|||||*||||||:
Sbjct: 1768 ATACTGTCCGGACATGTTTGG 1748
>miR8034 Score: 2.0 RO3G_13148
Query: 1 TATGACAAGCCTGCACAAACT 21
|||||||*|||||*|||||||
Sbjct: 1501 ATACTGTCCGGACATGTTTGA 1481
>miR8034 Score: 2.2 RO3G_12009
Query: 1 TATGACAAGCCTGCACAAACT 21
|||||||*|||||*||||||:
Sbjct: 1768 ATACTGTCCGGACATGTTTGG 1748
>miR8034 Score: 2.0 RO3G_12072
Query: 1 TATGACAAGCCTGCACAAACT 21
|||||||*|||||*|||||||
Sbjct: 1768 ATACTGTCCGGACATGTTTGA 1748
>miR8034 Score: 2.0 RO3G_12169
Query: 1 TATGACAAGCCTGCACAAACT 21
|||||||*|||||*|||||||
Sbjct: 1768 ATACTGTCCGGACATGTTTGA 1748
>miR8034 Score: 2.2 RO3G_11404
Query: 1 TATGACAAGCCTGCACAAACT 21
|||||||*|||||*||||||:
Sbjct: 1768 ATACTGTCCGGACATGTTTGG 1748
>miR8034 Score: 1.0 RO3G_11531
Query: 1 TATGACAAGCCTGCACAAACT 21
|||||||*|||||||||||||
Sbjct: 1261 ATACTGTCCGGACGTGTTTGA 1241
>miR8034 Score: 2.0 RO3G_11771
Query: 1 TATGACAAGCCTGCACAAACT 21
|||||||*|||||*|||||||
Sbjct: 1768 ATACTGTCCGGACATGTTTGA 1748
>miR8034 Score: 2.0 RO3G_10522
Query: 1 TATGACAAGCCTGCACAAACT 21
|||||||*|||||*|||||||
Sbjct: 1732 ATACTGTCCGGACATGTTTGA 1712
>miR8034 Score: 2.0 RO3G_10725
Query: 1 TATGACAAGCCTGCACAAACT 21
|||||||*|||||*|||||||
Sbjct: 1732 ATACTGTCCGGACATGTTTGA 1712
>miR8034 Score: 2.0 RO3G_09940
Query: 1 TATGACAAGCCTGCACAAACT 21
|||||||*|||||*|||||||
Sbjct: 1768 ATACTGTCCGGACATGTTTGA 1748
>miR8034 Score: 0.0 RO3G_08042
Query: 1 TATGACAAGCCTGCACAAACT 21

|||||||||||||||||||||
Sbjct: 1300 ATACTGTTCGGACGTGTTTGA 1280
>miR8034 Score: 2.0 RO3G_07128
Query: 1 TATGACAAGCCTGCACAAACT 21
|||||||*|||||*|||||||
Sbjct: 286 ATACTGTCCGGACATGTTTGA 266
>miR8034 Score: 2.0 RO3G_07294
Query: 1 TATGACAAGCCTGCACAAACT 21
|||||||*|||||*|||||||
Sbjct: 1369 ATACTGTCCGGACATGTTTGA 1349
>miR8034 Score: 2.0 RO3G_07686
Query: 1 TATGACAAGCCTGCACAAACT 21
|||||||*|||||*|||||||
Sbjct: 1768 ATACTGTCCGGACATGTTTGA 1748
>miR8034 Score: 1.0 RO3G_07976
Query: 1 TATGACAAGCCTGCACAAACT 21
|||||||||||||*|||||||
Sbjct: 1765 ATACTGTTCGGACATGTTTGA 1745
>miR8034 Score: 2.0 RO3G_05958
Query: 1 TATGACAAGCCTGCACAAACT 21
|||||||*|||||*|||||||
Sbjct: 1732 ATACTGTCCGGACATGTTTGA 1712
>miR8034 Score: 2.0 RO3G_06430
Query: 1 TATGACAAGCCTGCACAAACT 21
|||||||*|||||*|||||||
Sbjct: 1732 ATACTGTCCGGACATGTTTGA 1712
>miR8034 Score: 2.0 RO3G_04785
Query: 1 TATGACAAGCCTGCACAAACT 21
|||||||*|||||*|||||||
Sbjct: 1732 ATACTGTCCGGACATGTTTGA 1712
>miR8034 Score: 2.0 RO3G_03555
Query: 1 TATGACAAGCCTGCACAAACT 21
|||||||*|||||*|||||||
Sbjct: 1768 ATACTGTCCGGACATGTTTGA 1748
>miR8034 Score: 1.0 RO3G_03580
Query: 1 TATGACAAGCCTGCACAAACT 21
|||||||*|||||||||||||
Sbjct: 1693 ATACTGTCCGGACGTGTTTGA 1673
>miR8034 Score: 1.0 RO3G_03589
Query: 1 TATGACAAGCCTGCACAAACT 21
|||||||*|||||||||||||
Sbjct: 1693 ATACTGTCCGGACGTGTTTGA 1673
>miR8034 Score: 2.0 RO3G_01309
Query: 1 TATGACAAGCCTGCACAAACT 21
|||||||*|||||*|||||||

```
Sbjct: 1717 ATACTGTCCGGACATGTTTGA 1697
>miR8034 Score: 2.0 RO3G_01740
Query: 1 TATGACAAGCCTGCACAAACT 21
|||||||*|||||*|||||||
Sbjct: 1732 ATACTGTCCGGACATGTTTGA 1712
>miR8034 Score: 2.0 RO3G_01954
Query: 1 TATGACAAGCCTGCACAAACT 21
|||||||*|||||*|||||||
Sbjct: 718 ATACTGTCCGGACATGTTTGA 698
>miR8034 Score: 2.0 RO3G_17441
Query: 1 TATGACAAGCCTGCACAAACT 21
|||||||*|||||*|||||||
Sbjct: 1495 ATACTGTCCGGACATGTTTGA 1475
>miR8034 Score: 2.0 RO3G_17385
Query: 1 TATGACAAGCCTGCACAAACT 21
|||||||*|||||*|||||||
Sbjct: 1768 ATACTGTCCGGACATGTTTGA 1748
>miR8034 Score: 2.0 RO3G_17358
Query: 1 TATGACAAGCCTGCACAAACT 21
|||||||*|||||*|||||||
Sbjct: 1768 ATACTGTCCGGACATGTTTGA 1748
>miR5506 Score: 0.0 RO3G_16708
Query: 1 TAGATCGCTTCGTTGAGG 18
||||||||||||||||||
Sbjct: 677 ATCTAGCGAAGCAACTCC 660
>miR5506 Score: 0.0 RO3G_13745
Query: 1 TAGATCGCTTCGTTGAGG 18
||||||||||||||||||
Sbjct: 677 ATCTAGCGAAGCAACTCC 660
>miR5506 Score: 0.0 RO3G_12669
Query: 1 TAGATCGCTTCGTTGAGG 18
||||||||||||||||||
Sbjct: 677 ATCTAGCGAAGCAACTCC 660
>miR5506 Score: 0.2 RO3G_12961
Query: 1 TAGATCGCTTCGTTGAGG 18
|||||||||||||||||:
Sbjct: 1010 ATCTAGCGAAGCAACTCT 993
>miR5506 Score: 0.0 RO3G_12964
Query: 1 TAGATCGCTTCGTTGAGG 18
||||||||||||||||||
Sbjct: 677 ATCTAGCGAAGCAACTCC 660
>miR5506 Score: 0.0 RO3G_12075
Query: 1 TAGATCGCTTCGTTGAGG 18
||||||||||||||||||
Sbjct: 677 ATCTAGCGAAGCAACTCC 660
```

>miR5506 Score: 0.0 RO3G_11952
Query: 1 TAGATCGCTTCGTTGAGG 18
||||||||||||||||||
Sbjct: 1055 ATCTAGCGAAGCAACTCC 1038
>miR5506 Score: 0.5 RO3G_09927
Query: 1 TAGATCGCTTCGTTGAGG 18
||||||||:|||||||||
Sbjct: 677 ATCTAGCGGAGCAACTCC 660
>miR5506 Score: 1.8 RO3G_09951
Query: 1 TAGATCGCTTCGTTGAGG 18
|||||||||||:||*||:
Sbjct: 935 ATCTAGCGAAGTAAGTCT 918
>miR5506 Score: 2.5 RO3G_08587
Query: 1 TAGATCGCTTCGTTGAGG 18
||||||*|||*||||||*
Sbjct: 1023 ATCTAGAGAAACAACTCA 1006
>miR5506 Score: 2.0 RO3G_08754
Query: 1 TAGATCGCTTCGTTGAGG 18
||||||:|||||||*||*
Sbjct: 5295 ATCTAGTGAAGCAAGTCG 5278
>miR5506 Score: 0.0 RO3G_05524
Query: 1 TAGATCGCTTCGTTGAGG 18
||||||||||||||||||
Sbjct: 677 ATCTAGCGAAGCAACTCC 660
>miR5506 Score: 0.2 RO3G_05925
Query: 1 TAGATCGCTTCGTTGAGG 18
|||||||||||||||||:
Sbjct: 677 ATCTAGCGAAGCAACTCT 660
>miR5506 Score: 0.2 RO3G_05927
Query: 1 TAGATCGCTTCGTTGAGG 18
|||||||||||||||||:
Sbjct: 677 ATCTAGCGAAGCAACTCT 660
>miR5506 Score: 2.5 RO3G_02307
Query: 1 TAGATCGCTTCGTTGAGG 18
**||||||||||||*|||
Sbjct: 713 CGCTAGCGAAGCAAGTCC 696
>miR5506 Score: 1.8 RO3G_00927
Query: 1 TAGATCGCTTCGTTGAGG 18
|||||||||||*||||::
Sbjct: 625 ATCTAGCGAAGGAACTTT 608
>miR9752 Score: 0.0 RO3G_15283
Query: 1 TGCTTCTTGCTGTTCCATGATTT 23
|||||||||||||||||||||||
Sbjct: 261 ACGAAGAACGACAAGGTACTAAA 239
>miR9752 Score: 0.0 RO3G_10526

Query: 1 TGCTTCTTGCTGTTCCATGATTT 23
||||||||||||||||||||||
Sbjct: 261 ACGAAGAACGACAAGGTACTAAA 239
>miR9752 Score: 0.0 RO3G_09197
Query: 1 TGCTTCTTGCTGTTCCATGATTT 23
||||||||||||||||||||||
Sbjct: 261 ACGAAGAACGACAAGGTACTAAA 239
>miR9752 Score: 0.0 RO3G_09934
Query: 1 TGCTTCTTGCTGTTCCATGATTT 23
||||||||||||||||||||||
Sbjct: 261 ACGAAGAACGACAAGGTACTAAA 239
>miR9752 Score: 0.0 RO3G_07109
Query: 1 TGCTTCTTGCTGTTCCATGATTT 23
||||||||||||||||||||||
Sbjct: 261 ACGAAGAACGACAAGGTACTAAA 239
>miR9752 Score: 0.0 RO3G_07672
Query: 1 TGCTTCTTGCTGTTCCATGATTT 23
||||||||||||||||||||||
Sbjct: 261 ACGAAGAACGACAAGGTACTAAA 239
>miR9752 Score: 2.5 RO3G_06295
Query: 1 TGCTTCTTGCTGTTCCATGATTT 23
|:|||||||||*||||*||||||
Sbjct: 2737 ATGAAGAACGAAAAGGGACTAAA 2715
>miR9752 Score: 2.2 RO3G_04795
Query: 1 TGCTTCTTGCTGTTCCA-TGATTT 23
|||||||||||*|||||*|:||||
Sbjct: 312 ACGAAGAACGAAAAGGTTATTAAA 289
>miR9752 Score: 0.0 RO3G_02835
Query: 1 TGCTTCTTGCTGTTCCATGATTT 23
||||||||||||||||||||||
Sbjct: 261 ACGAAGAACGACAAGGTACTAAA 239
>miR9752 Score: 2.5 RO3G_00450
Query: 1 TGCTTCTTGCTGTTCCATGATTT 23
|||||||||||*||||*||*|||
Sbjct: 2446 ACGAAGAACGAAAAGGAACGAAA 2424
>miR9752 Score: 0.0 RO3G_01312
Query: 1 TGCTTCTTGCTGTTCCATGATTT 23
||||||||||||||||||||||
Sbjct: 42 ACGAAGAACGACAAGGTACTAAA 20
>miR9752 Score: 0.0 RO3G_01935
Query: 1 TGCTTCTTGCTGTTCCATGATTT 23
||||||||||||||||||||||
Sbjct: 261 ACGAAGAACGACAAGGTACTAAA 239
>miR5369 Score: 2.5 RO3G_15932
Query: 1 TCAAGAAAAGGAGGATGTCCAT 22

```
||||||||||*|||||:*|||*
Sbjct: 1706 AGTTCTTTTCATCCTATTGGTC 1685
>miR5369 Score: 2.5 RO3G_09088
Query: 1 TCAAGAAAAGGAGGATGTCCAT 22
|*|||||||||||||*||*||
Sbjct: 263 AATTCTTTTCCTCCTA-AGCTA 243
>miR5369 Score: 1.5 RO3G_05532
Query: 1 TCAAGAAAAGGAGGATGTCCAT 22
||||:|||||||||||:||*||
Sbjct: 698 AGTTTTTTTCCTCCTATAGATA 677
>miR5369 Score: 2.5 RO3G_04367
Query: 1 TCAAGAAAAGGAGGATGTCCAT 22
||||:*||||||||||:||||*
Sbjct: 824 AGTTTCTTTCCTCCTATAGGTC 803
>miR1072 Score: 1.2 RO3G_16566
Query: 1 TATTGTTGTTATTTGAGCTTGG 22
||||||||||||:|||:||||:
Sbjct: 1410 ATAACAACAATAGACTTGAACT 1389
>miR1072 Score: 2.2 RO3G_15852
Query: 1 TATTGTTGTTATTTGAGCT-TGG 22
|||||:|||||||:||:||*||:
Sbjct: 604 ATAACGACAATAAGCTTGATACT 582
>miR1072 Score: 2.5 RO3G_15543
Query: 1 TATTGTTGTTATTTGAGCTTGG 22
||||:||:||||||||:*||::
Sbjct: 1075 ATAATAATAATAAACTT-AATT 1055
>miR1072 Score: 0.0 RO3G_15574
Query: 1 TATTGTTGTTATTTGAGCTTGG 22
||||||||||||||||||||||
Sbjct: 1425 ATAACAACAATAAACTCGAACC 1404
>miR1072 Score: 1.2 RO3G_14533
Query: 1 TATTGTTGTTATTTGAGCTTGG 22
||||||||||||:|||:||||:
Sbjct: 1215 ATAACAACAATAGACTTGAACT 1194
>miR1072 Score: 2.5 RO3G_14612
Query: 1 TATTGTTGTTATTTGAGCTTGG 22
:|||:||||||||||*||:*||
Sbjct: 70 GTAATAACAATAAACACGGCCC 49
>miR1072 Score: 1.2 RO3G_13806
Query: 1 TATTGTTGTTATTTGAGCTTGG 22
||||||||||||:|||:||||:
Sbjct: 1410 ATAACAACAATAGACTTGAACT 1389
>miR1072 Score: 2.5 RO3G_06196
Query: 1 TATTGTTGTTATTTGAGCT-TGG 22
:||||||:|:|||||||||*:*|
```

```
Sbjct: 229 GTAACAATAGTAAACTCGATGGC 207
>miR1072 Score: 1.8 RO3G_03093
Query: 1 TATTGTTGTTATTTGAGCTTGG 22
:||||||||||||||*|||*|
Sbjct: 435 GTAACAACAATAAACT-GAAGC 415
>miR1072 Score: 1.2 RO3G_03120
Query: 1 TATTGTTGTTATTTGAGCTTGG 22
|||||||||||:|||:||||:
Sbjct: 1410 ATAACAACAATAGACTTGAACT 1389
>miR1072 Score: 1.2 RO3G_01425
Query: 1 TATTGTTGTTATTTGAGCTTGG 22
|||||||||||:|||:||||:
Sbjct: 1410 ATAACAACAATAGACTTGAACT 1389
>miR2105 Score: 2.2 RO3G_13442
Query: 1 TTGTGATTTGTGAATGGTTGAT 22
:|||||:|||||||:||||**|
Sbjct: 621 GACACTGAACACTTGCCAAGAA 600
>miR2105 Score: 2.2 RO3G_13216
Query: 1 TTGTGATTTGTGAATGGTTGAT 22
:|||||:|||||||:||||**|
Sbjct: 237 GACACTGAACACTTGCCAAGAA 216
>miR2105 Score: 2.5 RO3G_07019
Query: 1 TTGTGATTTGTGAATGGTTGAT 22
|||||||||||||*|*:|||:
Sbjct: 144 AACACTAAACACTTCC-GACTG 124
>miR2105 Score: 2.5 RO3G_00035
Query: 1 TTGTGATTTGTGAATGGTTGAT 22
:||||||||:||||||:*|:*|
Sbjct: 261 GACACTAAATACTTACTTATCA 240
>miR9760 Score: 2.5 RO3G_14120
Query: 1 TGATGATGTAGTCTTGATTGGT 22
:||||||||||||:|*||:|*|
Sbjct: 154 GCTACTACATCAGGAGTAGCGA 133
>miR9760 Score: 2.0 RO3G_11395
Query: 1 TGATGATGTAGTCTTGATTGGT 22
|||||*||||:||||||||*||
Sbjct: 577 ACTACAACATTAGAACTAAACA 556
>miR398b-3p Score: 2.5 RO3G_04368
Query: 1 ATGTGTTCTCAGGTCACCCCT 21
|||||||||||:||||***||
Sbjct: 2694 TACACAAGAGTTCAGT-CAGA 2675
>miR5260 Score: 2.5 RO3G_15050
Query: 1 TGTTGTATTGATGACTGGCTAT 22
|*||||||||*||||||*||||
Sbjct: 55 AAAACATAACAACTGAC-GATA 35
```

```
>miR5260 Score: 2.5 RO3G_14415
Query: 1 TGTTGTATTGATGACTGGCTAT 22
*|||||||||||:*||||*|||
Sbjct: 591 CCAACATAACTATGGACC-ATA 571
>miR5260 Score: 2.5 RO3G_01220
Query: 1 TGTTGTATTGATGACTGGCTAT 22
*:||||||||||||*|||*|||
Sbjct: 3132 CTAACATAACTACTAACC-ATA 3112
>miR169f-3p Score: 2.5 RO3G_16614
Query: 1 TGATGTCTTCCTTTGGTTACT 21
:|||*|||||||||||:*||:
Sbjct: 154 GCTAAAGAAGGAAACCG-TGG 135
>miR169f-3p Score: 2.0 RO3G_15960
Query: 1 TGATGTCTTCCTTTGGTTACT 21
|:||*|||||||||||||*||
Sbjct: 592 ATTAAAGAAGGAAACCAA-GA 573
>miR169f-3p Score: 2.5 RO3G_15382
Query: 1 TGATGTCTTCCTTTGGTTACT 21
|||||||:|*||||||||**|
Sbjct: 2320 ACTACAGGAAGAAACCAA-AA 2301
>miR169f-3p Score: 2.5 RO3G_14833
Query: 1 TGATGTCTTCCTTTGGTTACT 21
|:||||||||*||||||*|*|
Sbjct: 538 ATTACAGAAGTAAACCACT-A 519
>miR169f-3p Score: 2.2 RO3G_15222
Query: 1 TGATGTCTTCCTTTGGTTACT 21
|||||:|||||||||:|:*|*
Sbjct: 1801 ACTACGGAAGGAAACTAG-GT 1782
>miR169f-3p Score: 2.5 RO3G_09894
Query: 1 TGATGTCTTCCTTTGGTT-ACT 21
|:|:||||||*|||||||*|||
Sbjct: 1408 ATTGCAGAAGAAAACCAAGTGA 1387
>miR168 Score: 2.5 RO3G_16003
Query: 1 TCGCTTGGTGCAGGTCGGGA 20
|*|||||*|||||||||||*
Sbjct: 2980 ATCGAACAACGTCCAGCCCC 2961
>miR9558-3p Score: 0.0 RO3G_15577
Query: 1 TTGCAAGGCCAGCTTTTCCTCT 22
||||||||||||||||||||||
Sbjct: 318 AACGTTCCGGTCGAAAAGGAGA 297
>miR9558-3p Score: 0.0 RO3G_14625
Query: 1 TTGCAAGGCCAGCTTTTCCTCT 22
||||||||||||||||||||||
Sbjct: 318 AACGTTCCGGTCGAAAAGGAGA 297
>miR9558-3p Score: 0.0 RO3G_13150
```

```
Query: 1 TTGCAAGGCCAGCTTTTCCTCT 22
||||||||||||||||||||||
Sbjct: 39 AACGTTCCGGTCGAAAAGGAGA 18
>miR9558-3p Score: 0.0 RO3G_11530
Query: 1 TTGCAAGGCCAGCTTTTCCTCT 22
||||||||||||||||||||||
Sbjct: 318 AACGTTCCGGTCGAAAAGGAGA 297
>miR9558-3p Score: 0.0 RO3G_10519
Query: 1 TTGCAAGGCCAGCTTTTCCTCT 22
||||||||||||||||||||||
Sbjct: 318 AACGTTCCGGTCGAAAAGGAGA 297
>miR9558-3p Score: 2.5 RO3G_08428
Query: 1 TTGCAAGGCCAGCTTTTCCTCT 22
|||||||||**|||||||*|||
Sbjct: 318 AACGTTCCGAACGAAAAGAAGA 297
>miR9558-3p Score: 0.0 RO3G_07107
Query: 1 TTGCAAGGCCAGCTTTTCCTCT 22
||||||||||||||||||||||
Sbjct: 318 AACGTTCCGGTCGAAAAGGAGA 297
>miR9558-3p Score: 0.0 RO3G_05957
Query: 1 TTGCAAGGCCAGCTTTTCCTCT 22
||||||||||||||||||||||
Sbjct: 318 AACGTTCCGGTCGAAAAGGAGA 297
>miR9558-3p Score: 0.0 RO3G_04783
Query: 1 TTGCAAGGCCAGCTTTTCCTCT 22
||||||||||||||||||||||
Sbjct: 180 AACGTTCCGGTCGAAAAGGAGA 159
>miR9558-3p Score: 2.0 RO3G_02685
Query: 1 TTGCAAGGCCAGCTTTTCCTCT 22
||||||||||*|*|||||||||
Sbjct: 39 AACGTTCCGGACAAAAAGGAGA 18
>miR9558-3p Score: 0.0 RO3G_03097
Query: 1 TTGCAAGGCCAGCTTTTCCTCT 22
||||||||||||||||||||||
Sbjct: 318 AACGTTCCGGTCGAAAAGGAGA 297
>miR9558-3p Score: 0.0 RO3G_03579
Query: 1 TTGCAAGGCCAGCTTTTCCTCT 22
||||||||||||||||||||||
Sbjct: 318 AACGTTCCGGTCGAAAAGGAGA 297
>miR9558-3p Score: 2.0 RO3G_04093
Query: 1 TTGCAAGGCCAGCTTTTCCTCT 22
||||||||||||||::|*|||*
Sbjct: 1332 AACGTTCCGGTCGAGGAAGAGT 1311
>miR9558-3p Score: 2.5 RO3G_00117
Query: 1 TTGCAAGGCCAGCTTTTC-CTCT 22
```

||||||*||||||||||||***||
Sbjct: 115 AACGTTGCGGTCGAAAAGATCGA 93
>miR9558-3p Score: 0.0 RO3G_01734
Query: 1 TTGCAAGGCCAGCTTTTCCTCT 22
||||||||||||||||||||||
Sbjct: 318 AACGTTCCGGTCGAAAAGGAGA 297
>miR9558-3p Score: 0.0 RO3G_17440
Query: 1 TTGCAAGGCCAGCTTTTCCTCT 22
||||||||||||||||||||||
Sbjct: 318 AACGTTCCGGTCGAAAAGGAGA 297
>miR2633 Score: 2.5 RO3G_08201
Query: 1 TCGACATTTGCACCAGATTCT 21
|*||||||*||||||||*|||
Sbjct: 365 ATCTGTAACCGTGGTCTCAGA 345
>miR2633 Score: 2.2 RO3G_00832
Query: 1 TCGACATTTGCACCAGATTCT 21
|||||||:|||*|||||:|*|
Sbjct: 307 AGCTGTAGACGAGGTCTGA-A 288
>miR2633 Score: 2.5 RO3G_01808
Query: 1 TCGACATTTGCACCAGATTCT 21
**||||||*||||||||||||
Sbjct: 6977 TACTGTAATCGTGGTCTAAGA 6957
>miR6155 Score: 0.0 RO3G_14837
Query: 1 TGAGTTGCCTGTGCCTTGCA 20
||||||||||||||||||||
Sbjct: 38 ACTCAACGGACACGGAACGT 19
>miR6155 Score: 0.0 RO3G_08656
Query: 1 TGAGTTGCCTGTGCCTTGCA 20
||||||||||||||||||||
Sbjct: 260 ACTCAACGGACACGGAACGT 241
>miR6155 Score: 0.0 RO3G_00488
Query: 1 TGAGTTGCCTGTGCCTTGCA 20
||||||||||||||||||||
Sbjct: 38 ACTCAACGGACACGGAACGT 19
>miR394a-3p Score: 2.5 RO3G_16787
Query: 1 TGTTGTCATCTCTTTTGTAA 20
|||::|||||||:|*|||||
Sbjct: 280 ACAGTAGTAGAGGACACATT 261
>miR394a-3p Score: 2.5 RO3G_16640
Query: 1 TGTTGTCATCTCTTTTGTAA 20
|||||**|||||||||||||*
Sbjct: 83 ACAACTTTAGAGAAAACATG 64
>miR394a-3p Score: 2.5 RO3G_16302
Query: 1 TGTTGTCATCTCTTTTGTA-A 20
|:||||||*||||:|||||*|

```
Sbjct: 730 ATAACAGTCGAGAGAACATGT 710
>miR394a-3p Score: 2.5 RO3G_14609
Query: 1 TGTTGTCATCTCTTTTGTAA 20
|*:||||||||||:|||||*
Sbjct: 3405 AAGACAGTAGAGAGAACATC 3386
>miR394a-3p Score: 2.0 RO3G_12626
Query: 1 TGTTGTCATCTCTTTTGTAA 20
|||||||||||*:|||||*|
Sbjct: 739 ACAACAGTAGATGAAACAGT 720
>miR394a-3p Score: 2.5 RO3G_08077
Query: 1 TGTTGTCATCTCTTTTGT-AA 20
||||:*||||||||||||**|
Sbjct: 499 ACAATTGTAGAGAAAACAGAT 479
>miR394a-3p Score: 2.5 RO3G_08483
Query: 1 TGTTGTCATCTCTTTTGTAA 20
|||:|*||||||||:||||*
Sbjct: 897 ACAGCCGTAGAGAAGACATA 878
>miR394a-3p Score: 2.5 RO3G_07774
Query: 1 TGTTGTCATCTCTTTTGTAA 20
||||||**|||||||||*|
Sbjct: 762 ACAACATAAGAGAAAACAAT 743
>miR394a-3p Score: 2.5 RO3G_07818
Query: 1 TGTTGTCATCTCTTTTGTAA 20
|||||:*|||||:||||||*
Sbjct: 349 ACAACGTTAGAGGAAACATC 330
>miR394a-3p Score: 2.5 RO3G_06204
Query: 1 TGTTGTCATCTCTTTTGTAA 20
*|*|||||||||:||||||*
Sbjct: 3369 CCCACAGTAGAGGAAACATC 3350
>miR394a-3p Score: 2.5 RO3G_05214
Query: 1 TGTTGTCATCTCTTTTGTAA 20
|:|:|||||||*||||||*|
Sbjct: 1225 ATAGCAGTAGAAAAAACACT 1206
>miR394a-3p Score: 2.5 RO3G_03290
Query: 1 TGTTGTCATCTCTTTTGTAA 20
*|||||||||:*||||||*|
Sbjct: 54 CCAACAGTAGGTAAAACAAT 35
>miR394a-3p Score: 2.5 RO3G_03967
Query: 1 TGTTGTCATCTCTTTTGTAA 20
||||||||:||||||:*|*|
Sbjct: 1059 ACAACAGTGGAGAAAGAAGT 1040
>miR394a-3p Score: 2.5 RO3G_01453
Query: 1 TGTTGTCATCTCTTTTGTAA 20
|||||:||*|||||||||**
Sbjct: 1407 ACAACGGTCGAGAAAACAAA 1388
```

>miR394a-3p Score: 2.0 RO3G_01624
Query: 1 TGTTGTCATCTCTTTTGTA-A 20
||||||||||||*:|||||*|
Sbjct: 882 ACAACAGTAGAGTGAACATGT 862
>miR394a-3p Score: 2.5 RO3G_01975
Query: 1 TGTTGTCATCTCTTTTGTAA 20
||||:||||*||||||||**
Sbjct: 1617 ACAATAGTATAGAAAACACC 1598
>miR915 Score: 0.0 RO3G_17033
Query: 1 TGGCAATAAGGACAACGTCGTC 22
||||||||||||||||||||||
Sbjct: 697 ACCGTTATTCCTGTTGCAGCAG 676
>miR915 Score: 1.0 RO3G_14836
Query: 1 TGGCAATAAGGACAACGTCGTC 22
::|||||||||||||||||:||
Sbjct: 1516 GTCGTTATTCCTGTTGCAGTAG 1495
>miR915 Score: 0.0 RO3G_12456
Query: 1 TGGCAATAAGGACAACGTCGTC 22
||||||||||||||||||||||
Sbjct: 679 ACCGTTATTCCTGTTGCAGCAG 658
>miR915 Score: 0.0 RO3G_09507
Query: 1 TGGCAATAAGGACAACGTCGTC 22
||||||||||||||||||||||
Sbjct: 751 ACCGTTATTCCTGTTGCAGCAG 730
>miR915 Score: 0.0 RO3G_08181
Query: 1 TGGCAATAAGGACAACGTCGTC 22
||||||||||||||||||||||
Sbjct: 454 ACCGTTATTCCTGTTGCAGCAG 433
>miR915 Score: 2.5 RO3G_04583
Query: 1 TGGCAATAAGGACAACGTCGTC 22
||||*||*||||||||:|||||
Sbjct: 841 ACCGATACTCCTGTTGTAGCAG 820
>miR915 Score: 0.0 RO3G_03515
Query: 1 TGGCAATAAGGACAACGTCGTC 22
||||||||||||||||||||||
Sbjct: 391 ACCGTTATTCCTGTTGCAGCAG 370
>miR915 Score: 2.2 RO3G_00484
Query: 1 TGGCAATAAGGACAACGTCGTC 22
:||||||||||||||||:||
Sbjct: 66 TTCGTTATTCCTGTTACAGTAG 45
>miR915 Score: 2.5 RO3G_00570
Query: 1 TGGCAATAAGGACAACGTCGTC 22
|:||*||*||||||||||||||
Sbjct: 874 ATCGATACTCCTGTTGCAGCAG 853
>miR915 Score: 0.5 RO3G_01653

Query: 1 TGGCAATAAGGACAACGTCGTC 22
||||||||||||||||:|||||
Sbjct: 619 ACCGTTATTCCTGTTGTAGCAG 598
>miR414 Score: 2.5 RO3G_15731
Query: 1 TACCATCTTCTTCATATCGTCA 22
|||||||||||||*||||||
Sbjct: 60 TTGGTAGAAAAAGTAGAGCAGT 39
>miR414 Score: 2.5 RO3G_14147
Query: 1 TACCATCTTCTTCATATCGTCA 22
||||:||||||||:|||||*
Sbjct: 310 TTGGTGGAAGAAGTGTAG-AGA 290
>miR414 Score: 2.5 RO3G_12689
Query: 1 TACCATCTTCTTCATATCGTCA 22
|||||||||||||||*|**|*|
Sbjct: 1465 ATGGTAGAAGAAGTAGAAGA-T 1445
>miR414 Score: 0.0 RO3G_12063
Query: 1 TACCATCTTCTTCATATCGTCA 22
||||||||||||||||||||||
Sbjct: 1125 ATGGTAGAAGAAGTATAGCAGT 1104
>miR414 Score: 0.2 RO3G_12187
Query: 1 TACCATCTTCTTCATATCGTCA 22
||||||||||||||||||:|||
Sbjct: 1629 ATGGTAGAAGAAGTATAGTAGT 1608
>miR414 Score: 1.5 RO3G_11395
Query: 1 TACCATCTTCTTCATATCGTCA 22
|||||||*||||||||||*|||
Sbjct: 129 ATGGTAGCAGAAGTATAGAAGT 108
>miR414 Score: 0.0 RO3G_07712
Query: 1 TACCATCTTCTTCATATCGTCA 22
||||||||||||||||||||||
Sbjct: 129 ATGGTAGAAGAAGTATAGCAGT 108
>miR414 Score: 2.5 RO3G_04919
Query: 1 TACCATCTTCTTCATATCGTCA 22
|||||||||||||||||**|
Sbjct: 1452 CTGGTAGAAGAAGTAAAGC-CT 1432
>miR414 Score: 0.0 RO3G_04206
Query: 1 TACCATCTTCTTCATATCGTCA 22
||||||||||||||||||||||
Sbjct: 678 ATGGTAGAAGAAGTATAGCAGT 657
>miR169d Score: 0.0 RO3G_16143
Query: 1 TTAGCAAGGATGAGATTACCGG 22
||||||||||||||||||||||
Sbjct: 1820 AATCGTTCCTACTCTAATGGCC 1799
>miR169d Score: 0.0 RO3G_15135
Query: 1 TTAGCAAGGATGAGATTACCGG 22

```
||||||||||||||||||||||
Sbjct: 1823 AATCGTTCCTACTCTAATGGCC 1802
>miR169d Score: 0.0 RO3G_12533
Query: 1 TTAGCAAGGATGAGATTACCGG 22
||||||||||||||||||||||
Sbjct: 1163 AATCGTTCCTACTCTAATGGCC 1142
>miR169d Score: 0.0 RO3G_07028
Query: 1 TTAGCAAGGATGAGATTACCGG 22
||||||||||||||||||||||
Sbjct: 893 AATCGTTCCTACTCTAATGGCC 872
>miR169d Score: 0.0 RO3G_06727
Query: 1 TTAGCAAGGATGAGATTACCGG 22
||||||||||||||||||||||
Sbjct: 1961 AATCGTTCCTACTCTAATGGCC 1940
>miR169d Score: 0.0 RO3G_02691
Query: 1 TTAGCAAGGATGAGATTACCGG 22
||||||||||||||||||||||
Sbjct: 356 AATCGTTCCTACTCTAATGGCC 335
>miR169d Score: 0.0 RO3G_04118
Query: 1 TTAGCAAGGATGAGATTACCGG 22
||||||||||||||||||||||
Sbjct: 1823 AATCGTTCCTACTCTAATGGCC 1802
>miR169d Score: 0.0 RO3G_17384
Query: 1 TTAGCAAGGATGAGATTACCGG 22
||||||||||||||||||||||
Sbjct: 1823 AATCGTTCCTACTCTAATGGCC 1802
>miR169d Score: 0.0 RO3G_17329
Query: 1 TTAGCAAGGATGAGATTACCGG 22
||||||||||||||||||||||
Sbjct: 1160 AATCGTTCCTACTCTAATGGCC 1139
>miR169d Score: 0.0 RO3G_17318
Query: 1 TTAGCAAGGATGAGATTACCGG 22
||||||||||||||||||||||
Sbjct: 1823 AATCGTTCCTACTCTAATGGCC 1802
>miR169d Score: 0.0 RO3G_17274
Query: 1 TTAGCAAGGATGAGATTACCGG 22
||||||||||||||||||||||
Sbjct: 1823 AATCGTTCCTACTCTAATGGCC 1802
>miR5499 Score: 2.5 RO3G_13849
Query: 1 TACAGGAAGATCGTTTGGAA 20
|**|||||||||:|||||||
Sbjct: 438 ACATCCTTCTAGTAAACCTT 419
>miR5499 Score: 2.0 RO3G_12682
Query: 1 TACAGGAAGATCGTTTGGAA 20
||||||||||||||||:***
```

Sbjct: 429 ATGTCCTTCTAGCAAATAAC 410
>miR5499 Score: 2.5 RO3G_10420
Query: 1 TACAGGAAGATCGTTTGGAA 20
|**|||||||||:|||||||
Sbjct: 438 ACATCCTTCTAGTAAACCTT 419
>miR5499 Score: 2.5 RO3G_10965
Query: 1 TACAGGAAGATCGTTTGGAA 20
|**|||||||||:|||||||
Sbjct: 438 ACATCCTTCTAGTAAACCTT 419
>miR5499 Score: 0.0 RO3G_07991
Query: 1 TACAGGAAGATCGTTTGGAA 20
||||||||||||||||||||
Sbjct: 447 ATGTCCTTCTAGCAAACCTT 428
>miR5499 Score: 0.0 RO3G_07972
Query: 1 TACAGGAAGATCGTTTGGAA 20
||||||||||||||||||||
Sbjct: 447 ATGTCCTTCTAGCAAACCTT 428
>miR5499 Score: 2.5 RO3G_05865
Query: 1 TACAGGAAGATCGTTTGGAA 20
|**|||||||||:|||||||
Sbjct: 438 ACATCCTTCTAGTAAACCTT 419
>miR5499 Score: 2.2 RO3G_03352
Query: 1 TACAGGAAGATCGTTTGGAA 20
:|||:|||||||*|||||*|
Sbjct: 361 GTGTTCTTCTAGGAAACCCT 342
>miR5499 Score: 2.2 RO3G_03677
Query: 1 TACAGGAAGATCGTTTGGAA 20
:|||||||||:|:||||*|*
Sbjct: 381 GTGTCCTTCTGGTAAAC-TA 363
>miR5499 Score: 2.5 RO3G_01733
Query: 1 TACAGGAAGATCGTTTGGAA 20
|**|||||||||:|||||||
Sbjct: 438 ACATCCTTCTAGTAAACCTT 419
>miR5499 Score: 2.5 RO3G_01944
Query: 1 TACAGGAAGATCGTTTGGAA 20
|**|||||||||:|||||||
Sbjct: 438 ACATCCTTCTAGTAAACCTT 419
>miR5499 Score: 2.5 RO3G_17446
Query: 1 TACAGGAAGATCGTTTGGAA 20
|**|||||||||:|||||||
Sbjct: 438 ACATCCTTCTAGTAAACCTT 419
>miR5499 Score: 2.5 RO3G_17287
Query: 1 TACAGGAAGATCGTTTGGAA 20
|**|||||||||:|||||||
Sbjct: 438 ACATCCTTCTAGTAAACCTT 419

```
>miR5679 Score: 1.0 RO3G_17260
Query: 1 TTGGTGCAGAAGAAGTTAGA 20
||||||||:||||||||*||
Sbjct: 1991 AACCACGTTTTCTTCAACCT 1972
>miR5679 Score: 0.0 RO3G_16392
Query: 1 TTGGTGCAGAAGAAGTTAGA 20
||||||||||||||||||||
Sbjct: 961 AACCACGTCTTCTTCAATCT 942
>miR5679 Score: 2.0 RO3G_15766
Query: 1 TTGGTGCAGAAGAAGTTAGA 20
||:||:||||||||||*|||
Sbjct: 1735 AATCATGTCTTCTTCA-TCT 1717
>miR5679 Score: 2.2 RO3G_15046
Query: 1 TTGGTGCAGAAGAAGTTAGA 20
:||||||||||||||**|||
Sbjct: 100 GACCACGTCTTCTTCCTTCT 81
>miR5679 Score: 2.5 RO3G_13244
Query: 1 TTGGTGCAGAAGAAGTTAGA 20
|||:||*|:||||||||*||
Sbjct: 3965 AACTACCTTTTCTTCAA-CT 3947
>miR5679 Score: 2.5 RO3G_10076
Query: 1 TTGGTGCAGAAGAAGTTAGA 20
|||:*|||||||||||*|||
Sbjct: 652 AACTCCGTCTTCTTCA-TCT 634
>miR5679 Score: 2.5 RO3G_08276
Query: 1 TTGGTGCAGAAGAAGTTAGA 20
||||||**||||||||||*|
Sbjct: 409 AACCACTACTTCTTCAAT-T 391
>miR5679 Score: 1.0 RO3G_08382
Query: 1 TTGGTGCAGAAGAAGTTAGA 20
|||||||||||||||:|*||
Sbjct: 1078 AACCACGTCTTCTTCGACCT 1059
>miR161.1 Score: 1.0 RO3G_14986
Query: 1 TGAAAGTGGCTCATCAAGGGGT 22
|||||||||*||||||||||||
Sbjct: 399 ACTTTCACCAAGTAGTTCCCCA 378
>miR161.1 Score: 0.0 RO3G_13154
Query: 1 TGAAAGTGGCTCATCAAGGGGT 22
||||||||||||||||||||||
Sbjct: 399 ACTTTCACCGAGTAGTTCCCCA 378
>miR161.1 Score: 1.0 RO3G_12187
Query: 1 TGAAAGTGGCTCATCAAGGGGT 22
|||||||||*||||||||||||
Sbjct: 399 ACTTTCACCAAGTAGTTCCCCA 378
>miR161.1 Score: 1.0 RO3G_07714
```

Query: 1 TGAAAGTGGCTCATCAAGGGGT 22
|||||||||*||||||||||||
Sbjct: 222 ACTTTCACCAAGTAGTTCCCCA 201
>miR161.1 Score: 1.0 RO3G_04693
Query: 1 TGAAAGTGGCTCATCAAGGGGT 22
|||||||||*||||||||||||
Sbjct: 399 ACTTTCACCAAGTAGTTCCCCA 378
>miR161.1 Score: 1.0 RO3G_05349
Query: 1 TGAAAGTGGCTCATCAAGGGGT 22
|||||||||*||||||||||||
Sbjct: 399 ACTTTCACCAAGTAGTTCCCCA 378
>miR161.1 Score: 0.0 RO3G_02823
Query: 1 TGAAAGTGGCTCATCAAGGGGT 22
||||||||||||||||||||||
Sbjct: 399 ACTTTCACCGAGTAGTTCCCCA 378
>miR161.1 Score: 1.0 RO3G_03610
Query: 1 TGAAAGTGGCTCATCAAGGGGT 22
|||||||||*||||||||||||
Sbjct: 377 ACTTTCACCAAGTAGTTCCCCA 356
>miR161.1 Score: 1.0 RO3G_03622
Query: 1 TGAAAGTGGCTCATCAAGGGGT 22
|||||||||*||||||||||||
Sbjct: 222 ACTTTCACCAAGTAGTTCCCCA 201
>miR161.1 Score: 1.2 RO3G_04209
Query: 1 TGAAAGTGGCTCATCAAGGGGT 22
|||||||||*|||||||||||:
Sbjct: 327 ACTTTCACCAAGTAGTTCCCCG 306
>miR3631a-3p Score: 2.5 RO3G_16658
Query: 1 TAATTGGATTATGTCTTAACAA 22
|||||||||*|||||||*|**|
Sbjct: 197 ATTAACCTATTACAGAACTAAT 176
>miR3631a-3p Score: 1.0 RO3G_15743
Query: 1 TAATTGGATTATGTCTTAACAA 22
|||||||||||||||||||*|*
Sbjct: 289 ATTAACCTAATACAGAATTATC 268
>miR3631a-3p Score: 0.0 RO3G_14785
Query: 1 TAATTGGATTATGTCTTAACAA 22
||||||||||||||||||||||
Sbjct: 289 ATTAACCTAATACAGAATTGTT 268
>miR3631a-3p Score: 0.0 RO3G_13292
Query: 1 TAATTGGATTATGTCTTAACAA 22
||||||||||||||||||||||
Sbjct: 289 ATTAACCTAATACAGAATTGTT 268
>miR3631a-3p Score: 0.0 RO3G_06411
Query: 1 TAATTGGATTATGTCTTAACAA 22

||||||||||||||||||||||
Sbjct: 289 ATTAACCTAATACAGAATTGTT 268
>miR3631a-3p Score: 0.0 RO3G_03783
Query: 1 TAATTGGATTATGTCTTAACAA 22
||||||||||||||||||||||
Sbjct: 289 ATTAACCTAATACAGAATTGTT 268
>miR3631a-3p Score: 0.0 RO3G_17372
Query: 1 TAATTGGATTATGTCTTAACAA 22
||||||||||||||||||||||
Sbjct: 289 ATTAACCTAATACAGAATTGTT 268
>miR3631a-3p Score: 0.0 RO3G_17309
Query: 1 TAATTGGATTATGTCTTAACAA 22
||||||||||||||||||||||
Sbjct: 289 ATTAACCTAATACAGAATTGTT 268
>miR3631a-3p Score: 0.0 RO3G_17311
Query: 1 TAATTGGATTATGTCTTAACAA 22
||||||||||||||||||||||
Sbjct: 289 ATTAACCTAATACAGAATTGTT 268
>miR164b-3p Score: 2.5 RO3G_14969
Query: 1 GCGCCCAGCTTCTCCACC 18
|||||||||||*||||
Sbjct: 623 GGCGGGTGGAAGAAGTGG 606
>miR164b-3p Score: 2.0 RO3G_10474
Query: 1 GCGCCCAGCTTCTCCACC 18
|||||||||||||||*
Sbjct: 27 AGCTGGTCGAAGAGGTGA 10
>miR164b-3p Score: 2.5 RO3G_01126
Query: 1 GCGCCCAGCTTCTCCACC 18
|||||||||||*||||
Sbjct: 623 GGCGGGTGGAAGAAGTGG 606
>miR5818 Score: 2.5 RO3G_16292
Query: 1 TCCAACAGAAGGGCCACTT 19
|||||*|:||||*||||||
Sbjct: 435 AGGTTATTTTCCAGGTGAA 417
>miR5818 Score: 2.2 RO3G_12760
Query: 1 TCCAACAGAAGGGCCACTT 19
**||||||||:|||||||:
Sbjct: 102 TAGTTGTCTTTCCGGTGAG 84
>miR5818 Score: 2.5 RO3G_10403
Query: 1 TCCAACAGAAGGGCCACTT 19
|*||||||*|||:||||||
Sbjct: 1911 AAGTTGTCATCCTGGTGAA 1893
>miR5818 Score: 2.2 RO3G_08166
Query: 1 TCCAACAGAAGGGCCACT-T 19
|||||*|||||:|||||:*|

```
Sbjct: 654 AGGTTATCTTCTCGGTGGTA 635
>miR5818 Score: 2.5 RO3G_01784
Query: 1 TCCAACAGAAGGGCCACTT 19
||*|*||:|||||||||||
Sbjct: 125 AGTTCGTTTTCCCGGTGAA 107
>miR1211-3p Score: 2.0 RO3G_15811
Query: 1 TCACACCGTCTCTTCATGC 19
|||*|*|||||||||||||
Sbjct: 395 AGTATAGCAGAGAAGTACG 377
>miR1211-3p Score: 1.5 RO3G_14467
Query: 1 TCACACCGTCTCTTCATGC 19
|||||*||:||||||||||
Sbjct: 569 AGTGTAGCGGAGAAGTACG 551
>miR1211-3p Score: 0.2 RO3G_14722
Query: 1 TCACACCGTCTCTTCATGC 19
|||||||||||||||||:|
Sbjct: 1040 AGTGTGGCAGAGAAGTATG 1022
>miR1211-3p Score: 0.2 RO3G_13903
Query: 1 TCACACCGTCTCTTCATGC 19
|||||||||||||||||:|
Sbjct: 1040 AGTGTGGCAGAGAAGTATG 1022
>miR1211-3p Score: 2.5 RO3G_13957
Query: 1 TCACACCGTCTCTTCATGC 19
*||||||||*||:||||*|
Sbjct: 330 TGTGTGGCACAGGAGTA-G 313
>miR1211-3p Score: 2.5 RO3G_13558
Query: 1 TCACACCGTCTCTTCATGC 19
|*|||||||||||||*||*
Sbjct: 462 ACTGTGGCAGAGAAGAACA 444
>miR1211-3p Score: 0.2 RO3G_13664
Query: 1 TCACACCGTCTCTTCATGC 19
|||||||||||||||||:|
Sbjct: 1040 AGTGTGGCAGAGAAGTATG 1022
>miR1211-3p Score: 0.2 RO3G_13672
Query: 1 TCACACCGTCTCTTCATGC 19
|||||||||||||||||:|
Sbjct: 1040 AGTGTGGCAGAGAAGTATG 1022
>miR1211-3p Score: 0.2 RO3G_12139
Query: 1 TCACACCGTCTCTTCATGC 19
|||||||||||||||||:|
Sbjct: 413 AGTGTGGCAGAGAAGTATG 395
>miR1211-3p Score: 0.2 RO3G_11486
Query: 1 TCACACCGTCTCTTCATGC 19
|||||||||||||||||:|
Sbjct: 749 AGTGTGGCAGAGAAGTATG 731
```

>miR1211-3p Score: 0.2 RO3G_11567
Query: 1 TCACACCGTCTCTTCATGC 19
|||||||||||||||||:|
Sbjct: 1697 AGTGTGGCAGAGAAGTATG 1679
>miR1211-3p Score: 1.0 RO3G_11757
Query: 1 TCACACCGTCTCTTCATGC 19
|||||*|||||||||||||
Sbjct: 425 AGTGTAGCAGAGAAGTACG 407
>miR1211-3p Score: 1.0 RO3G_11765
Query: 1 TCACACCGTCTCTTCATGC 19
|||||*|||||||||||||
Sbjct: 425 AGTGTAGCAGAGAAGTACG 407
>miR1211-3p Score: 2.5 RO3G_11827
Query: 1 TCACACCGTCTCTTCATGC 19
|||||*||:*|||||||||
Sbjct: 227 AGTGTAGCGAAGAAGTACG 209
>miR1211-3p Score: 0.2 RO3G_10644
Query: 1 TCACACCGTCTCTTCATGC 19
|||||||||||||||||:|
Sbjct: 425 AGTGTGGCAGAGAAGTATG 407
>miR1211-3p Score: 2.5 RO3G_09279
Query: 1 TCACACCGTCTCTTCATGC 19
|||*|*||:||||||||||
Sbjct: 575 AGTATAGCGGAGAAGTACG 557
>miR1211-3p Score: 0.2 RO3G_09419
Query: 1 TCACACCGTCTCTTCATGC 19
|||||||||||||||||:|
Sbjct: 860 AGTGTGGCAGAGAAGTATG 842
>miR1211-3p Score: 1.5 RO3G_08684
Query: 1 TCACACCGTCTCTTCATGC 19
|||||*||:||||||||||
Sbjct: 458 AGTGTAGCGGAGAAGTACG 440
>miR1211-3p Score: 0.2 RO3G_08906
Query: 1 TCACACCGTCTCTTCATGC 19
|||||||||||||||||:|
Sbjct: 1040 AGTGTGGCAGAGAAGTATG 1022
>miR1211-3p Score: 1.2 RO3G_05535
Query: 1 TCACACCGTCTCTTCATGC 19
||||*||||||||||||:|
Sbjct: 425 AGTGCGGCAGAGAAGTATG 407
>miR1211-3p Score: 0.2 RO3G_05603
Query: 1 TCACACCGTCTCTTCATGC 19
|||||||||||||||||:|
Sbjct: 299 AGTGTGGCAGAGAAGTATG 281
>miR1211-3p Score: 0.2 RO3G_05616

Query: 1 TCACACCGTCTCTTCATGC 19
|||||||||||||||||:|
Sbjct: 329 AGTGTGGCAGAGAAGTATG 311
>miR1211-3p Score: 0.2 RO3G_05181
Query: 1 TCACACCGTCTCTTCATGC 19
|||||||||||||||||:|
Sbjct: 1040 AGTGTGGCAGAGAAGTATG 1022
>miR1211-3p Score: 0.2 RO3G_05194
Query: 1 TCACACCGTCTCTTCATGC 19
|||||||||||||||||:|
Sbjct: 1040 AGTGTGGCAGAGAAGTATG 1022
>miR1211-3p Score: 0.2 RO3G_05303
Query: 1 TCACACCGTCTCTTCATGC 19
|||||||||||||||||:|
Sbjct: 1040 AGTGTGGCAGAGAAGTATG 1022
>miR1211-3p Score: 0.2 RO3G_02354
Query: 1 TCACACCGTCTCTTCATGC 19
|||||||||||||||||:|
Sbjct: 1040 AGTGTGGCAGAGAAGTATG 1022
>miR1211-3p Score: 2.5 RO3G_02446
Query: 1 TCACACCGTCTCTTCATGC 19
||||||||:||||||*|
Sbjct: 225 TGTATGGCAGGGAAGTA-G 208
>miR1211-3p Score: 2.5 RO3G_03733
Query: 1 TCACACCGTCTCTTCATGC 19
|||||||||*|||||*|*|
Sbjct: 727 AGTGTGGCATAGAAGAAGG 709
>miR1211-3p Score: 1.2 RO3G_04104
Query: 1 TCACACCGTCTCTTCATGC 19
|||*|||||||||||||:|
Sbjct: 737 AGTATGGCAGAGAAGTATG 719
>miR1211-3p Score: 0.0 RO3G_01518
Query: 1 TCACACCGTCTCTTCATGC 19
|||||||||||||||||||
Sbjct: 413 AGTGTGGCAGAGAAGTACG 395
>miR1211-3p Score: 0.0 RO3G_01528
Query: 1 TCACACCGTCTCTTCATGC 19
|||||||||||||||||||
Sbjct: 608 AGTGTGGCAGAGAAGTACG 590
>miR1211-3p Score: 0.8 RO3G_01923
Query: 1 TCACACCGTCTCTTCATGC 19
|||||||:|||||||||:|
Sbjct: 989 AGTGTGGTAGAGAAGTATG 971
>miR5246 Score: 0.0 RO3G_16540
Query: 1 TTGAGACAGCTTTGACTT 18

||||||||||||||||||
Sbjct: 330 AACTCTGTCGAAACTGAA 313
>miR5246 Score: 1.0 RO3G_16144
Query: 1 TTGAGACAGCTTTGACTT 18
|||||||||*||||||||
Sbjct: 867 AACTCTGTCAAAACTGAA 850
>miR5246 Score: 2.0 RO3G_16293
Query: 1 TTGAGACAGCTTTGACTT 18
||*||||||||||||*||
Sbjct: 1188 AAGTCTGTCGAAACTTAA 1171
>miR5246 Score: 2.0 RO3G_16067
Query: 1 TTGAGACAGCTTTGACTT 18
|||||||||||||||*
Sbjct: 1144 TACTCTGTCGTAACTGAT 1127
>miR5246 Score: 0.0 RO3G_16120
Query: 1 TTGAGACAGCTTTGACTT 18
||||||||||||||||||
Sbjct: 330 AACTCTGTCGAAACTGAA 313
>miR5246 Score: 2.5 RO3G_14810
Query: 1 TTGAGACAGCTTTGACTT 18
||*|||||:*||||||||
Sbjct: 2541 AAATCTGTTAAAACTGAA 2524
>miR5246 Score: 0.0 RO3G_14972
Query: 1 TTGAGACAGCTTTGACTT 18
||||||||||||||||||
Sbjct: 798 AACTCTGTCGAAACTGAA 781
>miR5246 Score: 2.0 RO3G_15244
Query: 1 TTGAGACAGCTTTGACTT 18
||:|*|||||:|||||||
Sbjct: 622 AATTATGTCGGAACTGAA 605
>miR5246 Score: 0.0 RO3G_13295
Query: 1 TTGAGACAGCTTTGACTT 18
||||||||||||||||||
Sbjct: 603 AACTCTGTCGAAACTGAA 586
>miR5246 Score: 0.0 RO3G_13702
Query: 1 TTGAGACAGCTTTGACTT 18
||||||||||||||||||
Sbjct: 798 AACTCTGTCGAAACTGAA 781
>miR5246 Score: 0.0 RO3G_13754
Query: 1 TTGAGACAGCTTTGACTT 18
||||||||||||||||||
Sbjct: 867 AACTCTGTCGAAACTGAA 850
>miR5246 Score: 0.0 RO3G_12672
Query: 1 TTGAGACAGCTTTGACTT 18
||||||||||||||||||

Sbjct: 672 AACTCTGTCGAAACTGAA 655
>miR5246 Score: 0.0 RO3G_13000
Query: 1 TTGAGACAGCTTTGACTT 18
||||||||||||||||||
Sbjct: 798 AACTCTGTCGAAACTGAA 781
>miR5246 Score: 0.0 RO3G_13139
Query: 1 TTGAGACAGCTTTGACTT 18
||||||||||||||||||
Sbjct: 243 AACTCTGTCGAAACTGAA 226
>miR5246 Score: 0.0 RO3G_12062
Query: 1 TTGAGACAGCTTTGACTT 18
||||||||||||||||||
Sbjct: 330 AACTCTGTCGAAACTGAA 313
>miR5246 Score: 0.0 RO3G_12173
Query: 1 TTGAGACAGCTTTGACTT 18
||||||||||||||||||
Sbjct: 663 AACTCTGTCGAAACTGAA 646
>miR5246 Score: 0.0 RO3G_12189
Query: 1 TTGAGACAGCTTTGACTT 18
||||||||||||||||||
Sbjct: 330 AACTCTGTCGAAACTGAA 313
>miR5246 Score: 2.5 RO3G_11276
Query: 1 TTGAGACAGCTTTGACTT 18
||||||*|:|||::||||
Sbjct: 235 AACTCTCTTGAAGTTGAA 218
>miR5246 Score: 2.5 RO3G_09214
Query: 1 TTGAGACAGCTTTGACTT 18
||||||:||||:||||
Sbjct: 25 CACTATGTTGAAATTGAA 8
>miR5246 Score: 0.0 RO3G_09485
Query: 1 TTGAGACAGCTTTGACTT 18
||||||||||||||||||
Sbjct: 243 AACTCTGTCGAAACTGAA 226
>miR5246 Score: 0.0 RO3G_09487
Query: 1 TTGAGACAGCTTTGACTT 18
||||||||||||||||||
Sbjct: 243 AACTCTGTCGAAACTGAA 226
>miR5246 Score: 0.0 RO3G_09598
Query: 1 TTGAGACAGCTTTGACTT 18
||||||||||||||||||
Sbjct: 798 AACTCTGTCGAAACTGAA 781
>miR5246 Score: 2.2 RO3G_09763
Query: 1 TTGAGACAGCTTTGACTT 18
||||:||||||||:|*|:
Sbjct: 645 AACTTTGTCGAAATTAAG 628

```
>miR5246 Score: 0.0 RO3G_09942
Query: 1 TTGAGACAGCTTTGACTT 18
||||||||||||||||||
Sbjct: 243 AACTCTGTCGAAACTGAA 226
>miR5246 Score: 0.0 RO3G_10036
Query: 1 TTGAGACAGCTTTGACTT 18
||||||||||||||||||
Sbjct: 243 AACTCTGTCGAAACTGAA 226
>miR5246 Score: 1.2 RO3G_08455
Query: 1 TTGAGACAGCTTTGACTT 18
|||||||||||||||*|:
Sbjct: 779 AACTCTGTCGAAACTTAG 762
>miR5246 Score: 0.0 RO3G_06740
Query: 1 TTGAGACAGCTTTGACTT 18
||||||||||||||||||
Sbjct: 798 AACTCTGTCGAAACTGAA 781
>miR5246 Score: 0.0 RO3G_07029
Query: 1 TTGAGACAGCTTTGACTT 18
||||||||||||||||||
Sbjct: 243 AACTCTGTCGAAACTGAA 226
>miR5246 Score: 0.0 RO3G_07673
Query: 1 TTGAGACAGCTTTGACTT 18
||||||||||||||||||
Sbjct: 867 AACTCTGTCGAAACTGAA 850
>miR5246 Score: 0.0 RO3G_05933
Query: 1 TTGAGACAGCTTTGACTT 18
||||||||||||||||||
Sbjct: 243 AACTCTGTCGAAACTGAA 226
>miR5246 Score: 0.0 RO3G_05934
Query: 1 TTGAGACAGCTTTGACTT 18
||||||||||||||||||
Sbjct: 798 AACTCTGTCGAAACTGAA 781
>miR5246 Score: 0.0 RO3G_06719
Query: 1 TTGAGACAGCTTTGACTT 18
||||||||||||||||||
Sbjct: 798 AACTCTGTCGAAACTGAA 781
>miR5246 Score: 2.0 RO3G_04477
Query: 1 TTGAGACAGCTTTGACTT 18
|||||||||||**|||||
Sbjct: 3261 AACTCTGTCGACTCTGAA 3244
>miR5246 Score: 0.0 RO3G_04671
Query: 1 TTGAGACAGCTTTGACTT 18
||||||||||||||||||
Sbjct: 744 AACTCTGTCGAAACTGAA 727
>miR5246 Score: 0.0 RO3G_04694
```

Query: 1 TTGAGACAGCTTTGACTT 18
||||||||||||||||||
Sbjct: 330 AACTCTGTCGAAACTGAA 313
>miR5246 Score: 0.0 RO3G_03608
Query: 1 TTGAGACAGCTTTGACTT 18
||||||||||||||||||
Sbjct: 330 AACTCTGTCGAAACTGAA 313
>miR5246 Score: 0.0 RO3G_03989
Query: 1 TTGAGACAGCTTTGACTT 18
||||||||||||||||||
Sbjct: 603 AACTCTGTCGAAACTGAA 586
>miR5246 Score: 2.5 RO3G_04159
Query: 1 TTGAGACAGCTTTGACTT 18
||||*|||:*||||||||
Sbjct: 223 AACTATGTTTAAACTGAA 206
>miR5246 Score: 0.0 RO3G_00252
Query: 1 TTGAGACAGCTTTGACTT 18
||||||||||||||||||
Sbjct: 921 AACTCTGTCGAAACTGAA 904
>miR5246 Score: 0.0 RO3G_01310
Query: 1 TTGAGACAGCTTTGACTT 18
||||||||||||||||||
Sbjct: 603 AACTCTGTCGAAACTGAA 586
>miR5246 Score: 0.0 RO3G_01710
Query: 1 TTGAGACAGCTTTGACTT 18
||||||||||||||||||
Sbjct: 255 AACTCTGTCGAAACTGAA 238
>miR5246 Score: 0.0 RO3G_01738
Query: 1 TTGAGACAGCTTTGACTT 18
||||||||||||||||||
Sbjct: 798 AACTCTGTCGAAACTGAA 781
>miR5246 Score: 0.0 RO3G_01938
Query: 1 TTGAGACAGCTTTGACTT 18
||||||||||||||||||
Sbjct: 867 AACTCTGTCGAAACTGAA 850
>miR5246 Score: 0.0 RO3G_17463
Query: 1 TTGAGACAGCTTTGACTT 18
||||||||||||||||||
Sbjct: 243 AACTCTGTCGAAACTGAA 226
>miR5246 Score: 0.0 RO3G_17461
Query: 1 TTGAGACAGCTTTGACTT 18
||||||||||||||||||
Sbjct: 330 AACTCTGTCGAAACTGAA 313
>miR7709-3p Score: 2.5 RO3G_05793
Query: 1 TGACCTAGTCATATTTGAT 19

||||:||:|||||||||
Sbjct: 1363 TCTGGGTCGGTATAAA-TA 1346
>miR1098 Score: 2.5 RO3G_04797
Query: 1 TATGCGTTTTTGTCTGAAAT 20
||:|||||:|||||||||
Sbjct: 998 TTATGAAAAAGCAGACTTTA 979
>miR1098 Score: 2.5 RO3G_04907
Query: 1 TATGCGTTTTTGTCTGAAAT 20
|||||:|||||:||:|||
Sbjct: 45 CTACGCGAAAACGGATTTTT 26
>miR1098 Score: 2.5 RO3G_00337
Query: 1 TATGCGTTTTTGTCTGAAAT 20
||||:||||::|||||||
Sbjct: 593 CTACGTAAAAGTAGACTTCA 574
>miR1098 Score: 2.5 RO3G_01119
Query: 1 TATGCGTTTTTGTCTGAAAT 20
||||||||||:|||:|||
Sbjct: 908 TTACGCAAAAATAGATATTA 889
>miR867 Score: 2.5 RO3G_10282
Query: 1 TTGAACAGGATTATTAGAGAG 21
||*||||||||||||*||*||
Sbjct: 279 AAGTTGTCCTAATAAACT-TC 260
>miR867 Score: 2.5 RO3G_10164
Query: 1 TTGAACAGGATTATTAGAGAG 21
|||||||::|||||:|:|*||
Sbjct: 276 AACTTGTTTTAATAGTTT-TC 257
>miR867 Score: 2.5 RO3G_07276
Query: 1 TTGAACAGGATTATTAGAGAG 21
|||||||::|||||:|:|*||
Sbjct: 576 AACTTGTTTTAATAGTTT-TC 557
>miR867 Score: 2.5 RO3G_06105
Query: 1 TTGAACAGGATTATTAGAGAG 21
*||||||||||||||**||||
Sbjct: 582 TACTTGTCCTAATAAA-TCTC 563
>miR8773 Score: 0.0 RO3G_16542
Query: 1 TTGGATGCAATCGGTGGTTTAT 22
||||||||||||||||||||||
Sbjct: 494 AACCTACGTTAGCCACCAAATA 473
>miR9738 Score: 2.5 RO3G_08953
Query: 1 TGAAACATATTGGGCTCGTC 20
|||*|*|||||||||||*||
Sbjct: 212 ACTATTTATAACCCGAGAAG 193
>miR9738 Score: 2.5 RO3G_03642
Query: 1 TGAAACATATTGGGCTCGTC 20
|||||||:*:|||||||*||

Sbjct: 1938 ACTTTGTGAGACCCGAG-AG 1920
>miR840-3p Score: 0.5 RO3G_12953
Query: 1 TGGTTTATGTCCTTAGTTTC 20
|||||:||||||||||||||
Sbjct: 821 ACCAAGTACAGGAATCAAAG 802
>miR840-3p Score: 2.5 RO3G_09472
Query: 1 TGGTTTATGTCCTTAGTTTC 20
|*||||*||||||:||||||
Sbjct: 257 AACAAAAACAGGAGTCAAAG 238
>miR840-3p Score: 2.5 RO3G_04834
Query: 1 TGGTTTATGTCCTTAGTTTC 20
|||||*||:|||||||:||*
Sbjct: 283 ACCAATTATAGGAATCGAAA 264
>miR840-3p Score: 0.0 RO3G_01367
Query: 1 TGGTTTATGTCCTTAGTTTC 20
||||||||||||||||||||
Sbjct: 935 ACCAAATACAGGAATCAAAG 916
>miR840-3p Score: 0.0 RO3G_16979
Query: 1 TGGTTTATGTCCTTAGTTTC 20
||||||||||||||||||||
Sbjct: 815 ACCAAATACAGGAATCAAAG 796
>miR5015 Score: 0.0 RO3G_16818
Query: 1 TTGGTGATGTAGTAGTCTAC 20
||||||||||||||||||||
Sbjct: 1403 AACCACTACATCATCAGATG 1384
>miR5015 Score: 0.0 RO3G_16797
Query: 1 TTGGTGATGTAGTAGTCTAC 20
||||||||||||||||||||
Sbjct: 662 AACCACTACATCATCAGATG 643
>miR5015 Score: 0.0 RO3G_16800
Query: 1 TTGGTGATGTAGTAGTCTAC 20
||||||||||||||||||||
Sbjct: 1661 AACCACTACATCATCAGATG 1642
>miR5015 Score: 2.0 RO3G_16183
Query: 1 TTGGTGATGTAGTAGTCTAC 20
|*|||||||||||||||*|*
Sbjct: 19 ACCCACTACATCATCAG-TA 1
>miR5015 Score: 2.5 RO3G_16310
Query: 1 TTGGTGATGTAGTAGTCT-AC 20
|||:|||||*|||||||**||
Sbjct: 514 AACTACTACTTCATCAGTCTG 494
>miR5015 Score: 2.0 RO3G_15674
Query: 1 TTGGTGATGTAGTAGTCTAC 20
||||||||:*|||||||*||
Sbjct: 713 AACCACTATTTCATCAGCTG 694

```
>miR5015 Score: 0.0 RO3G_15265
Query: 1 TTGGTGATGTAGTAGTCTAC 20
||||||||||||||||||||
Sbjct: 1661 AACCACTACATCATCAGATG 1642
>miR5015 Score: 0.0 RO3G_15266
Query: 1 TTGGTGATGTAGTAGTCTAC 20
||||||||||||||||||||
Sbjct: 1661 AACCACTACATCATCAGATG 1642
>miR5015 Score: 0.0 RO3G_15268
Query: 1 TTGGTGATGTAGTAGTCTAC 20
||||||||||||||||||||
Sbjct: 1661 AACCACTACATCATCAGATG 1642
>miR5015 Score: 0.0 RO3G_14352
Query: 1 TTGGTGATGTAGTAGTCTAC 20
||||||||||||||||||||
Sbjct: 1661 AACCACTACATCATCAGATG 1642
>miR5015 Score: 2.0 RO3G_14624
Query: 1 TTGGTGATGTAGTAGTCTAC 20
||||||||:*|||||||*||
Sbjct: 1550 AACCACTATTTCATCAGCTG 1531
>miR5015 Score: 2.0 RO3G_13865
Query: 1 TTGGTGATGTAGTAGTCTAC 20
||||||||:*|||||||*||
Sbjct: 1634 AACCACTATTTCATCAGCTG 1615
>miR5015 Score: 0.0 RO3G_13217
Query: 1 TTGGTGATGTAGTAGTCTAC 20
||||||||||||||||||||
Sbjct: 1661 AACCACTACATCATCAGATG 1642
>miR5015 Score: 0.0 RO3G_13219
Query: 1 TTGGTGATGTAGTAGTCTAC 20
||||||||||||||||||||
Sbjct: 1661 AACCACTACATCATCAGATG 1642
>miR5015 Score: 0.0 RO3G_13222
Query: 1 TTGGTGATGTAGTAGTCTAC 20
||||||||||||||||||||
Sbjct: 1655 AACCACTACATCATCAGATG 1636
>miR5015 Score: 0.0 RO3G_13225
Query: 1 TTGGTGATGTAGTAGTCTAC 20
||||||||||||||||||||
Sbjct: 1673 AACCACTACATCATCAGATG 1654
>miR5015 Score: 0.0 RO3G_11967
Query: 1 TTGGTGATGTAGTAGTCTAC 20
||||||||||||||||||||
Sbjct: 1673 AACCACTACATCATCAGATG 1654
>miR5015 Score: 2.0 RO3G_11278
```

Query: 1 TTGGTGATGTAGTAGTCTAC 20
||||||||:*|||||||*||
Sbjct: 1565 AACCACTATTTCATCAGCTG 1546
>miR5015 Score: 2.0 RO3G_10777
Query: 1 TTGGTGATGTAGTAGTCTAC 20
||||||||:*|||||||*||
Sbjct: 1538 AACCACTATTTCATCAGCTG 1519
>miR5015 Score: 2.2 RO3G_10870
Query: 1 TTGGTGATGTAGTAGTCTAC 20
||||||||:|||:*|||:||
Sbjct: 392 AACCACTATATCGACAGGTG 373
>miR5015 Score: 2.0 RO3G_08609
Query: 1 TTGGTGATGTAGTAGTCTAC 20
||||||||:*|||||||*||
Sbjct: 347 AACCACTATTTCATCAGCTG 328
>miR5015 Score: 2.0 RO3G_07869
Query: 1 TTGGTGATGTAGTAGTCTAC 20
||||||||:*|||||||*||
Sbjct: 1607 AACCACTATTTCATCAGCTG 1588
>miR5015 Score: 0.0 RO3G_05640
Query: 1 TTGGTGATGTAGTAGTCTAC 20
||||||||||||||||||||
Sbjct: 1409 AACCACTACATCATCAGATG 1390
>miR5015 Score: 0.0 RO3G_05952
Query: 1 TTGGTGATGTAGTAGTCTAC 20
||||||||||||||||||||
Sbjct: 1541 AACCACTACATCATCAGATG 1522
>miR5015 Score: 0.0 RO3G_03992
Query: 1 TTGGTGATGTAGTAGTCTAC 20
||||||||||||||||||||
Sbjct: 392 AACCACTACATCATCAGATG 373
>miR5015 Score: 0.0 RO3G_04121
Query: 1 TTGGTGATGTAGTAGTCTAC 20
||||||||||||||||||||
Sbjct: 1661 AACCACTACATCATCAGATG 1642
>miR5015 Score: 2.0 RO3G_01720
Query: 1 TTGGTGATGTAGTAGTCTAC 20
||||||||:*|||||||*||
Sbjct: 347 AACCACTATTTCATCAGCTG 328
>miR4227 Score: 2.2 RO3G_17182
Query: 1 TCTGGTACCAATCTTTCA 18
:||||||||||||:||**
Sbjct: 610 GGACCATGGTTAGGAACA 593
>miR4227 Score: 1.8 RO3G_16810
Query: 1 TCTGGTACCAATCTTTCA 18

:*|||||||||||||||*
Sbjct: 322 GAACCATGGTTAGAAAGC 305
>miR4227 Score: 2.0 RO3G_15739
Query: 1 TCTGGTACCAATCTTTCA 18
||||:||*|||||||||*
Sbjct: 309 AGACTATAGTTAGAAAGC 292
>miR4227 Score: 0.0 RO3G_15157
Query: 1 TCTGGTACCAATCTTTCA 18
||||||||||||||||||
Sbjct: 679 AGACCATGGTTAGAAAGT 662
>miR4227 Score: 0.0 RO3G_14636
Query: 1 TCTGGTACCAATCTTTCA 18
||||||||||||||||||
Sbjct: 55 AGACCATGGTTAGAAAGT 38
>miR4227 Score: 2.5 RO3G_14648
Query: 1 TCTGGTACCAATCTTTCA 18
|||::|*||||||||||*
Sbjct: 1262 AGATTAAGGTTAGAAAGA 1245
>miR4227 Score: 2.5 RO3G_13870
Query: 1 TCTGGTACCAATCTTTCA 18
||||||*|||*||||||*
Sbjct: 237 AGACCACGGTAAGAAAGG 220
>miR4227 Score: 0.0 RO3G_13717
Query: 1 TCTGGTACCAATCTTTCA 18
||||||||||||||||||
Sbjct: 55 AGACCATGGTTAGAAAGT 38
>miR4227 Score: 1.2 RO3G_12687
Query: 1 TCTGGTACCAATCTTTCA 18
:*||||||||||||||||
Sbjct: 55 GAACCATGGTTAGAAAGT 38
>miR4227 Score: 2.2 RO3G_12960
Query: 1 TCTGGTACCAATCTTTCA 18
:*||||||||||||||*|
Sbjct: 340 GAACCATGGTTAGAAAAT 323
>miR4227 Score: 2.2 RO3G_12974
Query: 1 TCTGGTACCAATCTTTCA 18
:*||||||||||||||*|
Sbjct: 340 GAACCATGGTTAGAAAAT 323
>miR4227 Score: 0.0 RO3G_12005
Query: 1 TCTGGTACCAATCTTTCA 18
||||||||||||||||||
Sbjct: 679 AGACCATGGTTAGAAAGT 662
>miR4227 Score: 0.0 RO3G_12080
Query: 1 TCTGGTACCAATCTTTCA 18
||||||||||||||||||

```
Sbjct: 262 AGACCATGGTTAGAAAGT 245
>miR4227 Score: 2.2 RO3G_11343
Query: 1 TCTGGTACCAATCTTTCA 18
:|||||*|||*|||||||
Sbjct: 1051 GGACCACGGTAAGAAAGT 1034
>miR4227 Score: 0.0 RO3G_11407
Query: 1 TCTGGTACCAATCTTTCA 18
||||||||||||||||||
Sbjct: 262 AGACCATGGTTAGAAAGT 245
>miR4227 Score: 2.2 RO3G_11953
Query: 1 TCTGGTACCAATCTTTCA 18
:*||||||||||||||*|
Sbjct: 556 GAACCATGGTTAGAAAAT 539
>miR4227 Score: 2.2 RO3G_10525
Query: 1 TCTGGTACCAATCTTTCA 18
:*||||||||||||||*|
Sbjct: 679 GAACCATGGTTAGAAAAT 662
>miR4227 Score: 2.2 RO3G_10531
Query: 1 TCTGGTACCAATCTTTCA 18
:*||||||||||||||*|
Sbjct: 679 GAACCATGGTTAGAAAAT 662
>miR4227 Score: 2.5 RO3G_08073
Query: 1 TCTGGTACCAATCTTTCA 18
*||:|||||||*|||||*
Sbjct: 1253 CGATCATGGTTCGAAAGA 1236
>miR4227 Score: 2.0 RO3G_06830
Query: 1 TCTGGTACCAATCTTTCA 18
||||||||*|||||||*|
Sbjct: 637 AGACCATGTTTAGAAATT 620
>miR4227 Score: 2.2 RO3G_07110
Query: 1 TCTGGTACCAATCTTTCA 18
:*||||||||||||||*|
Sbjct: 679 GAACCATGGTTAGAAAAT 662
>miR4227 Score: 2.2 RO3G_07676
Query: 1 TCTGGTACCAATCTTTCA 18
:*||||||||||||||*|
Sbjct: 679 GAACCATGGTTAGAAAAT 662
>miR4227 Score: 2.2 RO3G_07678
Query: 1 TCTGGTACCAATCTTTCA 18
:*||||||||||||||*|
Sbjct: 679 GAACCATGGTTAGAAAAT 662
>miR4227 Score: 2.2 RO3G_07866
Query: 1 TCTGGTACCAATCTTTCA 18
:*||||||||||||||*|
Sbjct: 55 GAACCATGGTTAGAAAAT 38
```

```
>miR4227 Score: 2.2 RO3G_05523
Query: 1 TCTGGTACCAATCTTTCA 18
:*||||||||||||||*|
Sbjct: 679 GAACCATGGTTAGAAAAT 662
>miR4227 Score: 2.5 RO3G_05788
Query: 1 TCTGGTACCAATCTTTCA 18
|||**:||||||||||||
Sbjct: 1301 AGAAGGTGGTTAGAAAGT 1284
>miR4227 Score: 0.0 RO3G_05928
Query: 1 TCTGGTACCAATCTTTCA 18
||||||||||||||||||
Sbjct: 451 AGACCATGGTTAGAAAGT 434
>miR4227 Score: 1.0 RO3G_04676
Query: 1 TCTGGTACCAATCTTTCA 18
|||||||||||||*||||
Sbjct: 679 AGACCATGGTTAGTAAGT 662
>miR4227 Score: 2.5 RO3G_05187
Query: 1 TCTGGTACCAATCTTTCA 18
||||||*|||||*|:|||
Sbjct: 272 AGACCAAGGTTAAAGAGT 255
>miR4227 Score: 2.5 RO3G_03478
Query: 1 TCTGGTACCAATCTTTCA 18
|||||:|*|||||:|||*
Sbjct: 711 AGACCGTAGTTAGGAAGC 694
>miR4227 Score: 2.2 RO3G_03767
Query: 1 TCTGGTACCAATCTTTCA 18
:*||||||||||||||*|
Sbjct: 679 GAACCATGGTTAGAAAAT 662
>miR4227 Score: 2.5 RO3G_00192
Query: 1 TCTGGTACCAATCTTTCA 18
||||||||||*|||:|*|
Sbjct: 973 AGACCATGGTAAGAGA-T 957
>miR4227 Score: 2.0 RO3G_01667
Query: 1 TCTGGTACCAATCTTTCA 18
|*||||||*|||||||||
Sbjct: 853 AAACCATGTTTAGAAAGT 836
>miR4227 Score: 2.2 RO3G_01949
Query: 1 TCTGGTACCAATCTTTCA 18
:*||||||||||||||*|
Sbjct: 679 GAACCATGGTTAGAAAAT 662
>miR4227 Score: 2.2 RO3G_01968
Query: 1 TCTGGTACCAATCTTTCA 18
:*||||||||||||||*|
Sbjct: 679 GAACCATGGTTAGAAAAT 662
>miR4227 Score: 2.2 RO3G_17380
```

Query: 1 TCTGGTACCAATCTTTCA 18
:*||||||||||||||*|
Sbjct: 679 GAACCATGGTTAGAAAAT 662
>miR4227 Score: 2.0 RO3G_16937
Query: 1 TCTGGTACCAATCTTTCA 18
|||::|||||||*|||||
Sbjct: 296 AGATTATGGTTAAAAAGT 279
>miR8048-5p Score: 2.0 RO3G_15517
Query: 1 CATCATTACATTCCATCTG 19
||:|||:|||||||*||||
Sbjct: 462 GTGGTAGTGTAAGGAAGAC 444
>miR8048-5p Score: 2.5 RO3G_12453
Query: 1 CATCATTACATTCCATCTG 19
||||||||:|||||||*
Sbjct: 625 TTAGTAAGGTGAGGTAGAA 607
>miR8048-5p Score: 2.5 RO3G_11678
Query: 1 CATCATTACATTCCATCTG 19
|||||:||||||||||*
Sbjct: 258 ATAGTAGTGTAAGTTAGAA 240
>miR8048-5p Score: 2.5 RO3G_06144
Query: 1 CATCATTACATTCCATCTG 19
|*||||||||||||||*|*
Sbjct: 474 GAAGTAATGTAAGGTACAA 456
>miR8048-5p Score: 2.5 RO3G_01072
Query: 1 CATCATTACATTCCATCTG 19
||||||:|||||*|||*||
Sbjct: 2982 GTAGTAGTGTAATGTA-AC 2965
>miR1320-5p Score: 2.2 RO3G_04749
Query: 1 TGGAAGGAGTGTAAGTTTATAG 22
:|||||||:|:||||||*||*|
Sbjct: 88 GCCTTCCTTATATTCAATTACC 67
>miR1320-5p Score: 2.5 RO3G_04767
Query: 1 TGGAAGGAGTGTAAGTTTATAG 22
|:|||||:||||||||||||
Sbjct: 216 TCTTTCGTCGCATTCAAATATC 195
>miR869.1 Score: 0.0 RO3G_16822
Query: 1 TGGGTTCAAGTCTGGTGATTG 21
|||||||||||||||||||||
Sbjct: 270 ACCCAAGTTCAGACCACTAAC 250
>miR869.1 Score: 0.5 RO3G_16824
Query: 1 TGGGTTCAAGTCTGGTGATTG 21
||||||||||||:||||||||
Sbjct: 270 ACCCAAGTTCAGGCCACTAAC 250
>miR869.1 Score: 0.5 RO3G_16807
Query: 1 TGGGTTCAAGTCTGGTGATTG 21

```
||||||||||||:||||||||
Sbjct: 831 ACCCAAGTTCAGGCCACTAAC 811
>miR869.1 Score: 2.5 RO3G_16699
Query: 1 TGGGTTCAAGTCTGGTGATTG 21
||||||||*|||*||:|||||
Sbjct: 825 ACCCAAGTCCAGTCCGCTAAC 805
>miR869.1 Score: 0.0 RO3G_16551
Query: 1 TGGGTTCAAGTCTGGTGATTG 21
|||||||||||||||||||||
Sbjct: 831 ACCCAAGTTCAGACCACTAAC 811
>miR869.1 Score: 0.0 RO3G_16555
Query: 1 TGGGTTCAAGTCTGGTGATTG 21
|||||||||||||||||||||
Sbjct: 831 ACCCAAGTTCAGACCACTAAC 811
>miR869.1 Score: 0.0 RO3G_15675
Query: 1 TGGGTTCAAGTCTGGTGATTG 21
|||||||||||||||||||||
Sbjct: 831 ACCCAAGTTCAGACCACTAAC 811
>miR869.1 Score: 1.8 RO3G_15287
Query: 1 TGGGTTCAAGTCTGGTGATTG 21
|||:||||||||*|||||:||
Sbjct: 465 ACCTAAGTTCAGTCCACTGAC 445
>miR869.1 Score: 0.5 RO3G_14359
Query: 1 TGGGTTCAAGTCTGGTGATTG 21
||||||||||||:||||||||
Sbjct: 831 ACCCAAGTTCAGGCCACTAAC 811
>miR869.1 Score: 0.5 RO3G_13456
Query: 1 TGGGTTCAAGTCTGGTGATTG 21
||||||||||||:||||||||
Sbjct: 228 ACCCAAGTTCAGGCCACTAAC 208
>miR869.1 Score: 0.0 RO3G_13727
Query: 1 TGGGTTCAAGTCTGGTGATTG 21
|||||||||||||||||||||
Sbjct: 270 ACCCAAGTTCAGACCACTAAC 250
>miR869.1 Score: 0.0 RO3G_13737
Query: 1 TGGGTTCAAGTCTGGTGATTG 21
|||||||||||||||||||||
Sbjct: 270 ACCCAAGTTCAGACCACTAAC 250
>miR869.1 Score: 0.0 RO3G_13848
Query: 1 TGGGTTCAAGTCTGGTGATTG 21
|||||||||||||||||||||
Sbjct: 831 ACCCAAGTTCAGACCACTAAC 811
>miR869.1 Score: 2.2 RO3G_12664
Query: 1 TGGGTTCAAGTCTGGTGATTG 21
|||:||||||||:||*||:||
```

```
Sbjct: 558 ACCTAAGTTCAGGCCCCTGAC 538
>miR869.1 Score: 0.5 RO3G_13151
Query: 1 TGGGTTCAAGTCTGGTGATTG 21
||||||||||||:||||||||
Sbjct: 831 ACCCAAGTTCAGGCCACTAAC 811
>miR869.1 Score: 2.5 RO3G_12177
Query: 1 TGGGTTCAAGTCTGGTGATTG 21
||||||||*|||*||:|||||
Sbjct: 825 ACCCAAGTCCAGTCCGCTAAC 805
>miR869.1 Score: 2.5 RO3G_11267
Query: 1 TGGGTTCAAGTCTGGTGATTG 21
||||||||*|||*||:|||||
Sbjct: 825 ACCCAAGTCCAGTCCGCTAAC 805
>miR869.1 Score: 2.5 RO3G_11280
Query: 1 TGGGTTCAAGTCTGGTGATTG 21
||||||||*|||*||:|||||
Sbjct: 633 ACCCAAGTCCAGTCCGCTAAC 613
>miR869.1 Score: 0.0 RO3G_11401
Query: 1 TGGGTTCAAGTCTGGTGATTG 21
|||||||||||||||||||||
Sbjct: 831 ACCCAAGTTCAGACCACTAAC 811
>miR869.1 Score: 0.0 RO3G_11773
Query: 1 TGGGTTCAAGTCTGGTGATTG 21
|||||||||||||||||||||
Sbjct: 831 ACCCAAGTTCAGACCACTAAC 811
>miR869.1 Score: 0.5 RO3G_10235
Query: 1 TGGGTTCAAGTCTGGTGATTG 21
||||||||||||:||||||||
Sbjct: 831 ACCCAAGTTCAGGCCACTAAC 811
>miR869.1 Score: 2.5 RO3G_10549
Query: 1 TGGGTTCAAGTCTGGTGATTG 21
||||||||*|||*||:|||||
Sbjct: 825 ACCCAAGTCCAGTCCGCTAAC 805
>miR869.1 Score: 0.5 RO3G_10732
Query: 1 TGGGTTCAAGTCTGGTGATTG 21
||||||||||||:||||||||
Sbjct: 831 ACCCAAGTTCAGGCCACTAAC 811
>miR869.1 Score: 0.5 RO3G_09483
Query: 1 TGGGTTCAAGTCTGGTGATTG 21
||||||||||||:||||||||
Sbjct: 831 ACCCAAGTTCAGGCCACTAAC 811
>miR869.1 Score: 0.5 RO3G_09499
Query: 1 TGGGTTCAAGTCTGGTGATTG 21
||||||||||||:||||||||
Sbjct: 831 ACCCAAGTTCAGGCCACTAAC 811
```

>miR869.1 Score: 1.0 RO3G_09544
Query: 1 TGGGTTCAAGTCTGGTGATTG 21
||||||||||||:|||:||||
Sbjct: 831 ACCCAAGTTCAGGCCATTAAC 811
>miR869.1 Score: 0.0 RO3G_09607
Query: 1 TGGGTTCAAGTCTGGTGATTG 21
|||||||||||||||||||||
Sbjct: 831 ACCCAAGTTCAGACCACTAAC 811
>miR869.1 Score: 2.5 RO3G_07995
Query: 1 TGGGTTCAAGTCTGGTGATTG 21
||||||||*|||*||:|||||
Sbjct: 732 ACCCAAGTCCAGTCCGCTAAC 712
>miR869.1 Score: 0.0 RO3G_08661
Query: 1 TGGGTTCAAGTCTGGTGATTG 21
|||||||||||||||||||||
Sbjct: 831 ACCCAAGTTCAGACCACTAAC 811
>miR869.1 Score: 0.0 RO3G_08846
Query: 1 TGGGTTCAAGTCTGGTGATTG 21
|||||||||||||||||||||
Sbjct: 831 ACCCAAGTTCAGACCACTAAC 811
>miR869.1 Score: 0.5 RO3G_07303
Query: 1 TGGGTTCAAGTCTGGTGATTG 21
||||||||||||:||||||||
Sbjct: 831 ACCCAAGTTCAGGCCACTAAC 811
>miR869.1 Score: 2.2 RO3G_07688
Query: 1 TGGGTTCAAGTCTGGTGATTG 21
|||:||||||*|:|||||:||
Sbjct: 831 ACCTAAGTTCTGGCCACTGAC 811
>miR869.1 Score: 0.0 RO3G_07706
Query: 1 TGGGTTCAAGTCTGGTGATTG 21
|||||||||||||||||||||
Sbjct: 168 ACCCAAGTTCAGACCACTAAC 148
>miR869.1 Score: 0.5 RO3G_07875
Query: 1 TGGGTTCAAGTCTGGTGATTG 21
||||||||||||:||||||||
Sbjct: 831 ACCCAAGTTCAGGCCACTAAC 811
>miR869.1 Score: 0.5 RO3G_05622
Query: 1 TGGGTTCAAGTCTGGTGATTG 21
||||||||||||:||||||||
Sbjct: 831 ACCCAAGTTCAGGCCACTAAC 811
>miR869.1 Score: 0.0 RO3G_05635
Query: 1 TGGGTTCAAGTCTGGTGATTG 21
|||||||||||||||||||||
Sbjct: 831 ACCCAAGTTCAGACCACTAAC 811
>miR869.1 Score: 2.5 RO3G_05904

```
Query: 1 TGGGTTCAAGTCTGGTGATTG 21
||||||||*|||*||:|||||
Sbjct: 825 ACCCAAGTCCAGTCCGCTAAC 805
>miR869.1 Score: 0.5 RO3G_05915
Query: 1 TGGGTTCAAGTCTGGTGATTG 21
||||||||||||:||||||||
Sbjct: 831 ACCCAAGTTCAGGCCACTAAC 811
>miR869.1 Score: 0.5 RO3G_06423
Query: 1 TGGGTTCAAGTCTGGTGATTG 21
||||||||||||:||||||||
Sbjct: 885 ACCCAAGTTCAGGCCACTAAC 865
>miR869.1 Score: 0.0 RO3G_06441
Query: 1 TGGGTTCAAGTCTGGTGATTG 21
|||||||||||||||||||||
Sbjct: 831 ACCCAAGTTCAGACCACTAAC 811
>miR869.1 Score: 0.5 RO3G_05336
Query: 1 TGGGTTCAAGTCTGGTGATTG 21
||||||||||||:||||||||
Sbjct: 831 ACCCAAGTTCAGGCCACTAAC 811
>miR869.1 Score: 0.0 RO3G_02689
Query: 1 TGGGTTCAAGTCTGGTGATTG 21
|||||||||||||||||||||
Sbjct: 831 ACCCAAGTTCAGACCACTAAC 811
>miR869.1 Score: 1.8 RO3G_02844
Query: 1 TGGGTTCAAGTCTGGTGATTG 21
|||:||||||||*|||||:||
Sbjct: 609 ACCTAAGTTCAGTCCACTGAC 589
>miR869.1 Score: 0.0 RO3G_03571
Query: 1 TGGGTTCAAGTCTGGTGATTG 21
|||||||||||||||||||||
Sbjct: 831 ACCCAAGTTCAGACCACTAAC 811
>miR869.1 Score: 1.0 RO3G_03616
Query: 1 TGGGTTCAAGTCTGGTGATTG 21
|*|||||||||||||||||||
Sbjct: 270 AGCCAAGTTCAGACCACTAAC 250
>miR869.1 Score: 1.8 RO3G_03766
Query: 1 TGGGTTCAAGTCTGGTGATTG 21
|||:||||||||*|||||:||
Sbjct: 609 ACCTAAGTTCAGTCCACTGAC 589
>miR869.1 Score: 0.5 RO3G_00681
Query: 1 TGGGTTCAAGTCTGGTGATTG 21
||||||||||||:||||||||
Sbjct: 831 ACCCAAGTTCAGGCCACTAAC 811
>miR869.1 Score: 2.5 RO3G_01305
Query: 1 TGGGTTCAAGTCTGGTGATTG 21
```

```
||||||||*|||*||:|||||
Sbjct: 825 ACCCAAGTCCAGTCCGCTAAC 805
>miR869.1 Score: 1.0 RO3G_01322
Query: 1 TGGGTTCAAGTCTGGTGATTG 21
||||||||||||::|||||||
Sbjct: 717 ACCCAAGTTCAGGTCACTAAC 697
>miR869.1 Score: 0.5 RO3G_01652
Query: 1 TGGGTTCAAGTCTGGTGATTG 21
||||||||||||:||||||||
Sbjct: 831 ACCCAAGTTCAGGCCACTAAC 811
>miR869.1 Score: 0.5 RO3G_01730
Query: 1 TGGGTTCAAGTCTGGTGATTG 21
||||||||||||:||||||||
Sbjct: 831 ACCCAAGTTCAGGCCACTAAC 811
>miR869.1 Score: 0.0 RO3G_01956
Query: 1 TGGGTTCAAGTCTGGTGATTG 21
|||||||||||||||||||||
Sbjct: 831 ACCCAAGTTCAGACCACTAAC 811
>miR869.1 Score: 0.5 RO3G_17400
Query: 1 TGGGTTCAAGTCTGGTGATTG 21
||||||||||||:||||||||
Sbjct: 705 ACCCAAGTTCAGGCCACTAAC 685
>miR869.1 Score: 0.5 RO3G_17382
Query: 1 TGGGTTCAAGTCTGGTGATTG 21
||||||||||||:||||||||
Sbjct: 831 ACCCAAGTTCAGGCCACTAAC 811
>miR869.1 Score: 0.0 RO3G_17285
Query: 1 TGGGTTCAAGTCTGGTGATTG 21
|||||||||||||||||||||
Sbjct: 831 ACCCAAGTTCAGACCACTAAC 811
>miR447a-3p Score: 0.0 RO3G_15872
Query: 1 TTGCGGACGAGAATTTTGTG 20
||||||||||||||||||||
Sbjct: 130 AACGCCTGCTCTTAAAACAC 111
>miR447a-3p Score: 2.5 RO3G_11868
Query: 1 TTGCGGACGAGAATTTTGTG 20
|||*||||||*|||||||*|
Sbjct: 843 AACTCCTGCTATTAAAAC-C 825
>miR4412-5p Score: 0.5 RO3G_16716
Query: 1 TGTTGCGGATATTCTTTGCACT 22
||||||||||||:|||||||||
Sbjct: 355 ACAACGCCTATAGGAAACGTGA 334
>miR4412-5p Score: 0.5 RO3G_16373
Query: 1 TGTTGCGGATATTCTTTGCACT 22
||||||||||||||||||*|||
```

```
Sbjct: 229 ACAACGCCTATAAGAAACATGA 208
>miR4412-5p Score: 0.5 RO3G_16554
Query: 1 TGTTGCGGATATTCTTTGCACT 22
|||||||||||||||||||*||
Sbjct: 376 ACAACGCCTATAAGAAACGCGA 355
>miR4412-5p Score: 0.0 RO3G_16146
Query: 1 TGTTGCGGATATTCTTTGCACT 22
||||||||||||||||||||||
Sbjct: 406 ACAACGCCTATAAGAAACGTGA 385
>miR4412-5p Score: 2.0 RO3G_14895
Query: 1 TGTTGCGGATATTCTTTGCACT 22
||:|||:||||:||||||*|||
Sbjct: 139 ACGACGTCTATGAGAAACCTGA 118
>miR4412-5p Score: 0.5 RO3G_15146
Query: 1 TGTTGCGGATATTCTTTGCACT 22
|||||||||||||||||||*||
Sbjct: 256 ACAACGCCTATAAGAAACGCGA 235
>miR4412-5p Score: 0.5 RO3G_15251
Query: 1 TGTTGCGGATATTCTTTGCACT 22
||||||||||||||||||*|||
Sbjct: 337 ACAACGCCTATAAGAAACATGA 316
>miR4412-5p Score: 0.0 RO3G_12466
Query: 1 TGTTGCGGATATTCTTTGCACT 22
||||||||||||||||||||||
Sbjct: 229 ACAACGCCTATAAGAAACGTGA 208
>miR4412-5p Score: 2.5 RO3G_11240
Query: 1 TGTTGCGGATATTCTTTGCACT 22
|*||||:||||||*||||||||
Sbjct: 199 AGAACGTCTATAAAAAACGTGA 178
>miR4412-5p Score: 2.5 RO3G_11266
Query: 1 TGTTGCGGATATTCTTTGCACT 22
|*||||:||||||*||||||||
Sbjct: 199 AGAACGTCTATAAAAAACGTGA 178
>miR4412-5p Score: 0.0 RO3G_10239
Query: 1 TGTTGCGGATATTCTTTGCACT 22
||||||||||||||||||||||
Sbjct: 370 ACAACGCCTATAAGAAACGTGA 349
>miR4412-5p Score: 0.5 RO3G_09616
Query: 1 TGTTGCGGATATTCTTTGCACT 22
|||:||||||||||||||||||
Sbjct: 370 ACAGCGCCTATAAGAAACGTGA 349
>miR4412-5p Score: 0.5 RO3G_07031
Query: 1 TGTTGCGGATATTCTTTGCACT 22
|||||||||||||||||||*||
Sbjct: 376 ACAACGCCTATAAGAAACGCGA 355
```

>miR4412-5p Score: 0.0 RO3G_07032
Query: 1 TGTTGCGGATATTCTTTGCACT 22
||||||||||||||||||||||
Sbjct: 304 ACAACGCCTATAAGAAACGTGA 283
>miR4412-5p Score: 0.0 RO3G_07037
Query: 1 TGTTGCGGATATTCTTTGCACT 22
||||||||||||||||||||||
Sbjct: 316 ACAACGCCTATAAGAAACGTGA 295
>miR4412-5p Score: 0.0 RO3G_07713
Query: 1 TGTTGCGGATATTCTTTGCACT 22
||||||||||||||||||||||
Sbjct: 205 ACAACGCCTATAAGAAACGTGA 184
>miR4412-5p Score: 0.0 RO3G_04677
Query: 1 TGTTGCGGATATTCTTTGCACT 22
||||||||||||||||||||||
Sbjct: 376 ACAACGCCTATAAGAAACGTGA 355
>miR4412-5p Score: 1.8 RO3G_05212
Query: 1 TGTTGCGGATATTCTTTGCACT 22
||||||||||||||||::*|*|
Sbjct: 446 ACAACGCCTATAAGAAGTTTAA 425
>miR4412-5p Score: 0.0 RO3G_02950
Query: 1 TGTTGCGGATATTCTTTGCACT 22
||||||||||||||||||||||
Sbjct: 229 ACAACGCCTATAAGAAACGTGA 208
>miR4412-5p Score: 0.5 RO3G_03768
Query: 1 TGTTGCGGATATTCTTTGCACT 22
||||||||||||||||||*|||
Sbjct: 190 ACAACGCCTATAAGAAACATGA 169
>miR4412-5p Score: 0.5 RO3G_00243
Query: 1 TGTTGCGGATATTCTTTGCACT 22
||||:|||||||||||||||||
Sbjct: 355 ACAATGCCTATAAGAAACGTGA 334
>miR4412-5p Score: 0.0 RO3G_01190
Query: 1 TGTTGCGGATATTCTTTGCACT 22
||||||||||||||||||||||
Sbjct: 229 ACAACGCCTATAAGAAACGTGA 208
>miR4412-5p Score: 0.0 RO3G_01209
Query: 1 TGTTGCGGATATTCTTTGCACT 22
||||||||||||||||||||||
Sbjct: 370 ACAACGCCTATAAGAAACGTGA 349
>miR4412-5p Score: 0.0 RO3G_01732
Query: 1 TGTTGCGGATATTCTTTGCACT 22
||||||||||||||||||||||
Sbjct: 229 ACAACGCCTATAAGAAACGTGA 208
>miR4412-5p Score: 0.5 RO3G_01932

Query: 1 TGTTGCGGATATTCTTTGCACT 22
||||||||||||||||||*|||
Sbjct: 580 ACAACGCCTATAAGAAACATGA 559
>miR4412-5p Score: 0.0 RO3G_17448
Query: 1 TGTTGCGGATATTCTTTGCACT 22
||||||||||||||||||||||
Sbjct: 370 ACAACGCCTATAAGAAACGTGA 349
>miR477 Score: 2.5 RO3G_10629
Query: 1 TTGGAGTATCTTTGGAGGAGC 21
|||||||||*|||*|||*|||
Sbjct: 325 AACCTCATAAAAAGCTC-TCG 306
>miR5632-5p Score: 2.5 RO3G_16460
Query: 1 TGTCATTCTTTATCCAACTG 20
||**|||||||||||||*||
Sbjct: 2405 ACTATAAGAAATAGGTTAAC 2386
>miR5632-5p Score: 2.5 RO3G_10250
Query: 1 TGTCATTCTTTATCCAACTG 20
::||||||||||||||||
Sbjct: 1541 TTGTTAAGAAATAGGTTGAC 1522
>miR5632-5p Score: 2.2 RO3G_10750
Query: 1 TGTCATTCTTTATCCAACTG 20
||*|||:||||||||||*|:
Sbjct: 254 ACTGTAGGAAATAGGTTTAT 235
>miR5632-5p Score: 0.0 RO3G_08617
Query: 1 TGTCATTCTTTATCCAACTG 20
||||||||||||||||||||
Sbjct: 1708 ACAGTAAGAAATAGGTTGAC 1689
>miR5632-5p Score: 0.0 RO3G_05328
Query: 1 TGTCATTCTTTATCCAACTG 20
||||||||||||||||||||
Sbjct: 781 ACAGTAAGAAATAGGTTGAC 762
>miR5632-5p Score: 2.2 RO3G_17115
Query: 1 TGTCATTCTTTATCCAACTG 20
|:||*|||||||||||||*:
Sbjct: 961 ATAGAAAGAAATAGGTTGTT 942
>miR5163b-3p Score: 1.5 RO3G_15290
Query: 1 TAGATACTTTGGTTGTGGGA 20
||||||*:||||||||||||
Sbjct: 154 ATCTATAGAACCAACACCCT 135
>miR5163b-3p Score: 0.5 RO3G_15497
Query: 1 TAGATACTTTGGTTGTGGGA 20
|||||||:||||||||||||
Sbjct: 154 ATCTATGGAACCAACACCCT 135
>miR5163b-3p Score: 1.5 RO3G_13307
Query: 1 TAGATACTTTGGTTGTGGGA 20

||||*||:||||||||||||
Sbjct: 187 ATCTTTGGAACCAACACCCT 168
>miR5163b-3p Score: 1.5 RO3G_12685
Query: 1 TAGATACTTTGGTTGTGGGA 20
|*|||||:||||||||||||
Sbjct: 208 ACCTATGGAACCAACACCCT 189
>miR5163b-3p Score: 1.0 RO3G_13022
Query: 1 TAGATACTTTGGTTGTGGGA 20
|||||||:||||||||:|||
Sbjct: 226 ATCTATGGAACCAACATCCT 207
>miR5163b-3p Score: 0.0 RO3G_12243
Query: 1 TAGATACTTTGGTTGTGGGA 20
||||||||||||||||||||
Sbjct: 226 ATCTATGAAACCAACACCCT 207
>miR5163b-3p Score: 0.5 RO3G_12446
Query: 1 TAGATACTTTGGTTGTGGGA 20
|||||||:||||||||||||
Sbjct: 226 ATCTATGGAACCAACACCCT 207
>miR5163b-3p Score: 0.0 RO3G_10657
Query: 1 TAGATACTTTGGTTGTGGGA 20
||||||||||||||||||||
Sbjct: 226 ATCTATGAAACCAACACCCT 207
>miR5163b-3p Score: 2.5 RO3G_08353
Query: 1 TAGATACTTTGGTTGTGGGA 20
||:|||||||||||*||
Sbjct: 440 TTCAGTGAAACCAACAC-CT 422
>miR5163b-3p Score: 1.0 RO3G_08447
Query: 1 TAGATACTTTGGTTGTGGGA 20
|||||||:||||||||:|||
Sbjct: 226 ATCTATGGAACCAACATCCT 207
>miR5163b-3p Score: 0.5 RO3G_09087
Query: 1 TAGATACTTTGGTTGTGGGA 20
|||||||:||||||||||||
Sbjct: 226 ATCTATGGAACCAACACCCT 207
>miR5163b-3p Score: 2.5 RO3G_05552
Query: 1 TAGATACTTTGGTTGTGGGA 20
:|||*|||||::||||||:|
Sbjct: 208 GTCTCTGAAATTAACACCTT 189
>miR5163b-3p Score: 0.5 RO3G_05724
Query: 1 TAGATACTTTGGTTGTGGGA 20
|||||||:||||||||||||
Sbjct: 187 ATCTATGGAACCAACACCCT 168
>miR5163b-3p Score: 0.5 RO3G_04502
Query: 1 TAGATACTTTGGTTGTGGGA 20
|||||||:||||||||||||

Sbjct: 226 ATCTATGGAACCAACACCCT 207
>miR5163b-3p Score: 2.5 RO3G_05035
Query: 1 TAGATACTTTGGTTGTGGGA 20
:|||||||||:|||||**:|
Sbjct: 970 GTCTATGAAATCAACA-ATT 952
>miR5163b-3p Score: 0.0 RO3G_02942
Query: 1 TAGATACTTTGGTTGTGGGA 20
||||||||||||||||||||
Sbjct: 226 ATCTATGAAACCAACACCCT 207
>miR5163b-3p Score: 0.5 RO3G_03523
Query: 1 TAGATACTTTGGTTGTGGGA 20
|||||||:||||||||||||
Sbjct: 226 ATCTATGGAACCAACACCCT 207
>miR5163b-3p Score: 1.5 RO3G_03564
Query: 1 TAGATACTTTGGTTGTGGGA 20
|||||||:||:|||||||*|
Sbjct: 639 ATCTATGGAATCAACACCAT 620
>miR5163b-3p Score: 2.5 RO3G_03712
Query: 1 TAGATACTTTGGTTGTGGGA 20
|||||:|||||||:||||
Sbjct: 79 TTCTATTGAACCAACGCCCT 60
>miR5163b-3p Score: 2.0 RO3G_03971
Query: 1 TAGATACTTTGGTTGTGGGA 20
|||||*|:||||||||:|||
Sbjct: 226 ATCTAAGGAACCAACATCCT 207
>miR5163b-3p Score: 0.5 RO3G_04017
Query: 1 TAGATACTTTGGTTGTGGGA 20
|||||||:||||||||||||
Sbjct: 226 ATCTATGGAACCAACACCCT 207
>miR5163b-3p Score: 0.5 RO3G_01530
Query: 1 TAGATACTTTGGTTGTGGGA 20
|||||||:||||||||||||
Sbjct: 226 ATCTATGGAACCAACACCCT 207
>miR1522 Score: 2.5 RO3G_13498
Query: 1 TTTATTGTTAAGCATGAAAG 20
||||||||:||:|||:*|||
Sbjct: 501 AAATAACAGTTTGTATGTTC 482
>miR1052 Score: 2.2 RO3G_09342
Query: 1 TTCCTATTTTATTGACTGTGTTA 23
||*||*||||||||||||||:|
Sbjct: 24 AACGAAAAAATAACTGACACAGT 2
>miR8148 Score: 0.5 RO3G_16424
Query: 1 TGATTGGGTCTGCATGCCGTGGT 23
|||||||||||||||:|||||||
Sbjct: 254 ACTAACCCAGACGTATGGCACCA 232

```
>miR950 Score: 2.5 RO3G_14208
Query: 1 TCTGGTCAGCTGGTTTGAT 19
||||:|*|||||||||*||
Sbjct: 172 AGACTACTCGACCAAA-TA 155
>miR950 Score: 2.5 RO3G_13428
Query: 1 TCTGGTCAGCTGGTTTGAT 19
*|||::||||||*||||||
Sbjct: 1840 TGACTGGTCGACAAAACTA 1822
>miR950 Score: 2.2 RO3G_09575
Query: 1 TCTGGTCAGCTGGTTTGAT 19
*|||||||||*:||||||:
Sbjct: 310 CGACCAGTCGTTCAAACTG 292
>miR950 Score: 2.5 RO3G_08593
Query: 1 TCTGGTCAGCTGGTTTGAT 19
|*||:||||*|||||||||
Sbjct: 130 ATACTAGTCAACCAAACTA 112
>miR950 Score: 2.5 RO3G_05312
Query: 1 TCTGGTCAGCTGGTTTGAT 19
|||||*|||*|||||||*|
Sbjct: 506 AGACCTGTCTACCAAAC-A 489
>miR950 Score: 1.5 RO3G_03353
Query: 1 TCTGGTCAGCTGGTTTGAT 19
||||:||||||||:|||*|
Sbjct: 190 AGACTAGTCGACCGAACCA 172
>miR950 Score: 2.5 RO3G_03724
Query: 1 TCTGGTCAGCTGGTTTGAT 19
||||:|*|||||||||*||
Sbjct: 172 AGACTACTCGACCAAA-TA 155
>miR950 Score: 2.5 RO3G_04130
Query: 1 TCTGGTCAGCTGGTTTGAT 19
||:|||*|:||||:|||||
Sbjct: 566 AGGCCACTTGACCGAACTA 548
>miR950 Score: 2.0 RO3G_01135
Query: 1 TCTGGTCAGCTGGTTTGAT 19
*|||||*|||||:||||||
Sbjct: 2839 TGACCATTCGACTAAACTA 2821
>miR9486a Score: 2.0 RO3G_11550
Query: 1 TCTTTCAAGAGTTAGAGGTTC 21
||||||||||||*|:||:|:|
Sbjct: 566 AGAAAGTTCTCATTTTCTAGG 546
>miR9486a Score: 2.5 RO3G_07328
Query: 1 TCTTTCAAGAGTTAGAGGTTC 21
||||||*|||:|||:||*|||
Sbjct: 877 AGAAAGATCTTAATTTC-AAG 858
>miR9486a Score: 1.8 RO3G_06014
```

Query: 1 TCTTTCAAGAGTTAGAGGTTC 21
||||||||||||||:|::||*
Sbjct: 57 AGAAAGTTCTCAATTTTTAAC 37
>miR9486a Score: 2.5 RO3G_00830
Query: 1 TCTTTCAAGAGTTAGAGGTTC 21
||||||||||:|*|||*||||
Sbjct: 602 AGAAAGTTCTTACTCTACAAG 582
>miR6266a Score: 2.2 RO3G_13524
Query: 1 TATATGCAGGCGGCAAAT-AT 20
:||*||||||||:|||||*||
Sbjct: 2686 GTAGACGTCCGCTGTTTAATA 2666
>miR847-3p Score: 2.2 RO3G_12785
Query: 1 TGACTCCTCTTCTTCTGG-TCG 21
:||*|||||||||||||:*||:
Sbjct: 303 GCTTAGGAGAAGAAGACTAAGT 282
>miR847-3p Score: 2.0 RO3G_02368
Query: 1 TGACTCCTCTTCTTCTGGTCG 21
|||||*||||:||||||||*|
Sbjct: 243 ACTGAAGAGAGGAAGACCACC 223
>miR847-3p Score: 2.0 RO3G_03499
Query: 1 TGACTCCTCTTCTTCTGGTCG 21
||*||||||||||||||*|*|
Sbjct: 2118 ACAGAGGAGAAGAAGAC-AAC 2099
>miR822-5p Score: 2.5 RO3G_14440
Query: 1 TGCGAAAGCATTTGCCAAG 19
**|||||||||||:|||*|
Sbjct: 2182 TAGCTTTCGTAAATGGTCC 2164
>miR822-5p Score: 2.5 RO3G_13777
Query: 1 TGCGAAAGCATTTGCCAAG 19
||||||*:|||||:|||*|
Sbjct: 1033 ACGCTTATGTAAATGGT-C 1016
>miR822-5p Score: 2.5 RO3G_12088
Query: 1 TGCGAAAGCATTTGCCAAG 19
|||:*||||||:|||||*|
Sbjct: 329 ACGTATTCGTAGACGGT-C 312
>miR822-5p Score: 2.5 RO3G_12467
Query: 1 TGCGAAAGCATTTGCCAAG 19
|||:*||||||:|||||*|
Sbjct: 194 ACGTATTCGTAGACGGT-C 177
>miR822-5p Score: 2.2 RO3G_11521
Query: 1 TGCGAAAGCATTTGCCAAG 19
:||*||||||||:||||*|
Sbjct: 1552 GCGGTTTCGTAAGCGGT-C 1535
>miR822-5p Score: 2.5 RO3G_10792
Query: 1 TGCGAAAGCATTTGCCAAG 19

```
|||:*||||||:||||||*
Sbjct: 332 ACGTATTCGTAGACGGTTA 314
>miR822-5p Score: 2.5 RO3G_06858
Query: 1 TGCGAAAGCATTTGCCAAG 19
*||:||||*|||||||||*
Sbjct: 45 CCGTTTTCATAAACGGTTA 27
>miR822-5p Score: 2.5 RO3G_07964
Query: 1 TGCGAAAGCATTTGCCAAG 19
::*||||||||||:||||:
Sbjct: 766 GTTCTTTCGTAAATGGTTT 748
>miR822-5p Score: 2.5 RO3G_06260
Query: 1 TGCGAAAGCATTTGCCAAG 19
*:*||||||||||||||*|
Sbjct: 693 TTTCTTTCGTAAACGGTAC 675
>miR822-5p Score: 2.2 RO3G_06339
Query: 1 TGCGAAAGCATTTGCCAA-G 19
:||:|||:||:|||||||*|
Sbjct: 200 GCGTTTTTGTGAACGGTTAC 181
>miR822-5p Score: 2.5 RO3G_04523
Query: 1 TGCGAAAGCATTTGCCAAG 19
|*||*|||||:||||||||
Sbjct: 246 AAGCGTTCGTGAACGGTTC 228
>miR822-5p Score: 2.5 RO3G_03576
Query: 1 TGCGAAAGCATTTGCCAAG 19
|||:*||||||:|||||*|
Sbjct: 281 ACGTATTCGTAGACGGT-C 264
>miR822-5p Score: 2.5 RO3G_03795
Query: 1 TGCGAAAGCATTTGCCAAG 19
|||:*||||||:|||||*|
Sbjct: 281 ACGTATTCGTAGACGGT-C 264
>miR7761-3p Score: 2.0 RO3G_14788
Query: 1 TCTCGAATCAAGGACGCTCTGG 22
|||||||||*|||*||||||||
Sbjct: 259 AGAGCTTAGATCCCGCGAGACC 238
>miR7761-3p Score: 2.0 RO3G_14801
Query: 1 TCTCGAATCAAGGACGCTCTGG 22
|||||||||*|||*||||||||
Sbjct: 259 AGAGCTTAGATCCCGCGAGACC 238
>miR3510 Score: 2.5 RO3G_09902
Query: 1 TTAACCATCTTTCGGGACTGAA 22
*||||||||||:|:|||*|||*
Sbjct: 1153 CATTGGTAGAAGGTCCT-ACTA 1133
>miR3510 Score: 1.8 RO3G_06495
Query: 1 TTAACCATCTTTCGGGACTGAA 22
|||||||||||||:||*||:||
```

Sbjct: 1084 AATTGGTAGAAAGTCCAGATTT 1063
>miR842 Score: 2.0 RO3G_12444
Query: 1 TATGGTCCCAGATCCGTCA-ACC 22
||||:||||:|||||||||||
Sbjct: 830 TTACCGGGGTTTAGGCAGTATGG 808
>miR9667-5p Score: 2.5 RO3G_14530
Query: 1 AAATTGGCAAACATGGTTG 19
|||||:|||||||||::
Sbjct: 354 GTTAACTGTTTGTACCCGT 336
>miR9667-5p Score: 2.5 RO3G_14023
Query: 1 AAATTGGCAAACATGGTTG 19
|||||:|||||||:|||
Sbjct: 1643 CTTAACTGTTTCTACTAAC 1625
>miR9667-5p Score: 2.5 RO3G_13643
Query: 1 AAATTGGCAAACATGGTTG 19
|||||||||||**|||:||
Sbjct: 3365 TTTAACCGTTTAAACCGAC 3347
>miR9667-5p Score: 1.8 RO3G_12615
Query: 1 AAATTGGCAAACATGGTTG 19
|||||||||||||:||:
Sbjct: 386 GTTAACCGTTTGTATCA-T 369
>miR9667-5p Score: 2.2 RO3G_11619
Query: 1 AAATTGGCAAACATGGTTG 19
||||||||*||||*||||:
Sbjct: 567 TTTAACCGGTTGTTCCAAT 549
>miR9667-5p Score: 2.5 RO3G_11879
Query: 1 AAATTGGCAAACATGGTTG 19
|||*||||||||||:|:|*
Sbjct: 5513 TTTCACCGTTTGTATCGAA 5495
>miR9667-5p Score: 2.0 RO3G_09699
Query: 1 AAATTGGCAAACATGGTT-G 19
**||||||||||||||||*|
Sbjct: 1076 CCTAACCGTTTGTACCAAAC 1057
>miR9667-5p Score: 2.5 RO3G_08916
Query: 1 AAATTGGCAAACATGGTTG 19
|||||:||||*|||||:*|
Sbjct: 1328 TTTAATCGTTGGTACCGTC 1310
>miR9667-5p Score: 2.5 RO3G_04417
Query: 1 AAATTGGCAAACATGGTTG 19
||||:|||||||||**|||
Sbjct: 1301 TTTAGCCGTTTGTAGAAAC 1283
>miR9667-5p Score: 2.5 RO3G_03060
Query: 1 AAATTGGCAAACATGGTTG 19
|||:|||||||*||||*||
Sbjct: 1115 TTTGACCGTTTTTACCCAC 1097

>miR9667-5p Score: 2.5 RO3G_04061
Query: 1 AAATTGGCAAACATGGTTG 19
|||||:*||||||||:|*|
Sbjct: 56 TTTAATAGTTTGTACTACC 38
>miR9667-5p Score: 2.5 RO3G_01886
Query: 1 AAATTGGCAAACATGGTTG 19
|||||||*||||||:|:*|
Sbjct: 53 TTTAACCCTTTGTATCG-C 36
>miR7807a-3p Score: 0.0 RO3G_16691
Query: 1 TGAGGATTTGCATACATAC 19
|||||||||||||||||||
Sbjct: 284 ACTCCTAAACGTATGTATG 266
>miR7807a-3p Score: 0.0 RO3G_15671
Query: 1 TGAGGATTTGCATACATAC 19
|||||||||||||||||||
Sbjct: 284 ACTCCTAAACGTATGTATG 266
>miR7807a-3p Score: 0.0 RO3G_15169
Query: 1 TGAGGATTTGCATACATAC 19
|||||||||||||||||||
Sbjct: 284 ACTCCTAAACGTATGTATG 266
>miR7807a-3p Score: 0.0 RO3G_11965
Query: 1 TGAGGATTTGCATACATAC 19
|||||||||||||||||||
Sbjct: 284 ACTCCTAAACGTATGTATG 266
>miR7807a-3p Score: 2.5 RO3G_12385
Query: 1 TGAGGATTTGCATACATA-C 19
|||*||::||||||||||*|
Sbjct: 1403 ACTACTGGACGTATGTATTG 1384
>miR7807a-3p Score: 2.5 RO3G_11608
Query: 1 TGAGGATTTGCATACATAC 19
||||||:||:*||||||*|
Sbjct: 1971 ACTCCTGAATCTATGTA-G 1954
>miR7807a-3p Score: 2.5 RO3G_10760
Query: 1 TGAGGATTTGCATACATAC 19
||||*||:||||||||*||
Sbjct: 665 ACTCGTAGACGTATGT-TG 648
>miR7807a-3p Score: 0.0 RO3G_10873
Query: 1 TGAGGATTTGCATACATAC 19
|||||||||||||||||||
Sbjct: 284 ACTCCTAAACGTATGTATG 266
>miR7807a-3p Score: 0.0 RO3G_10052
Query: 1 TGAGGATTTGCATACATAC 19
|||||||||||||||||||
Sbjct: 284 ACTCCTAAACGTATGTATG 266
>miR7807a-3p Score: 0.0 RO3G_07740

Query: 1 TGAGGATTTGCATACATAC 19
|||||||||||||||||||
Sbjct: 284 ACTCCTAAACGTATGTATG 266
>miR7807a-3p Score: 2.5 RO3G_03206
Query: 1 TGAGGATTTGCATACATAC 19
|:||:||||*|||||||*|
Sbjct: 528 ATTCTTAAAGGTATGTA-G 511
>miR7807a-3p Score: 2.5 RO3G_03919
Query: 1 TGAGGATTTGCATACATAC 19
||||*|||||||||*||*|
Sbjct: 389 ACTCGTAAACGTATCTAAG 371
>miR7807a-3p Score: 2.5 RO3G_03940
Query: 1 TGAGGATTTGCATACATA-C 19
|:|||||*:|||||||||*|
Sbjct: 449 ATTCCTACGCGTATGTATCG 430
>miR7807a-3p Score: 0.0 RO3G_01616
Query: 1 TGAGGATTTGCATACATAC 19
|||||||||||||||||||
Sbjct: 284 ACTCCTAAACGTATGTATG 266
>miR7807a-3p Score: 2.0 RO3G_01786
Query: 1 TGAGGATTTGCATACATAC 19
|||:|||||||||*||:||
Sbjct: 323 ACTTCTAAACGTAGGTGTG 305
>miR3440 Score: 2.5 RO3G_02539
Query: 1 TGGATTCGATCAAGGGAAAGC 21
|:|||||:||||||||:|*||*
Sbjct: 865 ATCTAAGTTAGTTCCTTATCC 845
>miR6214 Score: 1.0 RO3G_17251
Query: 1 TGACGACGAGAGCCACGATCA 21
|||||||||||||*|||||||
Sbjct: 407 ACTGCTGCTCTCGCTGCTAGT 387
>miR6214 Score: 2.5 RO3G_17255
Query: 1 TGACGACGAGAGCCACGATCA 21
|||||*|||||||*||||||*
Sbjct: 2654 ACTGCCGCTCTCGCTGCTAGC 2634
>miR6214 Score: 2.0 RO3G_16919
Query: 1 TGACGACGAGAGCCACGATCA 21
|||||*|||||||*|||||||
Sbjct: 278 ACTGCCGCTCTCGCTGCTAGT 258
>miR6214 Score: 2.0 RO3G_16366
Query: 1 TGACGACGAGAGCCACGATCA 21
|||||*|||||||*|||||||
Sbjct: 2699 ACTGCCGCTCTCGCTGCTAGT 2679
>miR6214 Score: 2.5 RO3G_15931
Query: 1 TGACGACGAGAGCCACGATCA 21

```
|||||||||||||||*:||**
Sbjct: 1313 ACTGCTGCTCTCGGTTTTACC 1293
>miR6214 Score: 2.0 RO3G_14563
Query: 1 TGACGACGAGAGCCACGATCA 21
|||||*|||||||*|||||||
Sbjct: 266 ACTGCCGCTCTCGCTGCTAGT 246
>miR6214 Score: 2.5 RO3G_14273
Query: 1 TGACGACGAGAGCCACGA-TCA 21
||||||||||||||||***|*|
Sbjct: 215 ACTGCTGCTCTCGGTGGCCACT 194
>miR6214 Score: 2.5 RO3G_13458
Query: 1 TGACGACGAGAGCCACGATCA 21
||*||||||||||*||||||*
Sbjct: 407 ACCGCTGCTCTCGCTGCTAGC 387
>miR6214 Score: 2.2 RO3G_11579
Query: 1 TGACGACGAGAGCCACGATCA 21
:|||||||||||*||||*||*
Sbjct: 3530 GCTGCTGCTCTCTGTGCAAGA 3510
>miR6214 Score: 2.0 RO3G_10543
Query: 1 TGACGACGAGAGCCACGATCA 21
|||||*|||||||*|||||||
Sbjct: 2156 ACTGCCGCTCTCGCTGCTAGT 2136
>miR6214 Score: 2.0 RO3G_07735
Query: 1 TGACGACGAGAGCCACGATCA 21
|||||*|||||||*|||||||
Sbjct: 2603 ACTGCCGCTCTCGCTGCTAGT 2583
>miR6214 Score: 1.8 RO3G_03008
Query: 1 TGACGACGAGAGCCACGATCA 21
||||||||||*||||||*:||
Sbjct: 1064 ACTGCTGCTCCCGGTGCAGGT 1044
>miR6214 Score: 1.0 RO3G_01294
Query: 1 TGACGACGAGAGCCACGATCA 21
|||||||||||||*|||||||
Sbjct: 1772 ACTGCTGCTCTCGCTGCTAGT 1752
>miR5788 Score: 0.0 RO3G_15585
Query: 1 TGGATAGTTATCAGACTCTAGTA 23
|||||||||||||||||||||||
Sbjct: 2529 ACCTATCAATAGTCTGAGATCAT 2507
>miR5788 Score: 0.0 RO3G_15250
Query: 1 TGGATAGTTATCAGACTCTAGTA 23
|||||||||||||||||||||||
Sbjct: 2529 ACCTATCAATAGTCTGAGATCAT 2507
>miR5788 Score: 0.2 RO3G_14355
Query: 1 TGGATAGTTATCAGACTCTAGTA 23
||||||||||||||||||:||||
```

Sbjct: 780 ACCTATCAATAGTCTGAGGTCAT 758
>miR5788 Score: 0.5 RO3G_13867
Query: 1 TGGATAGTTATCAGACTCTAGTA 23
||||||||||||||||||||*||
Sbjct: 2490 ACCTATCAATAGTCTGAGATAAT 2468
>miR5788 Score: 1.5 RO3G_13920
Query: 1 TGGATAGTTATCAGACTCTAGTA 23
|||||||||||||||*||:|:||
Sbjct: 2574 ACCTATCAATAGTCTAAGGTTAT 2552
>miR5788 Score: 0.0 RO3G_13749
Query: 1 TGGATAGTTATCAGACTCTAGTA 23
|||||||||||||||||||||||
Sbjct: 1137 ACCTATCAATAGTCTGAGATCAT 1115
>miR5788 Score: 0.2 RO3G_10558
Query: 1 TGGATAGTTATCAGACTCTAGTA 23
||||||||||||||||||:||||
Sbjct: 327 ACCTATCAATAGTCTGAGGTCAT 305
>miR5788 Score: 1.5 RO3G_09965
Query: 1 TGGATAGTTATCAGACTCTAGTA 23
|||||||||||||||*||:|:||
Sbjct: 2574 ACCTATCAATAGTCTAAGGTTAT 2552
>miR5788 Score: 1.5 RO3G_05619
Query: 1 TGGATAGTTATCAGACTCTAGTA 23
|||||||||||||||*||:|:||
Sbjct: 717 ACCTATCAATAGTCTAAGGTTAT 695
>miR5788 Score: 1.5 RO3G_06427
Query: 1 TGGATAGTTATCAGACTCTAGTA 23
|||||||||||||||*||:|:||
Sbjct: 2166 ACCTATCAATAGTCTAAGGTTAT 2144
>miR5788 Score: 1.5 RO3G_06428
Query: 1 TGGATAGTTATCAGACTCTAGTA 23
|||||||||||||||*||:|:||
Sbjct: 1668 ACCTATCAATAGTCTAAGGTTAT 1646
>miR5788 Score: 1.5 RO3G_06442
Query: 1 TGGATAGTTATCAGACTCTAGTA 23
|||||||||||||||*||:|:||
Sbjct: 3231 ACCTATCAATAGTCTAAGGTTAT 3209
>miR5788 Score: 0.2 RO3G_05272
Query: 1 TGGATAGTTATCAGACTCTAGTA 23
||||||||||||||||||:||||
Sbjct: 2574 ACCTATCAATAGTCTGAGGTCAT 2552
>miR5788 Score: 1.5 RO3G_02691
Query: 1 TGGATAGTTATCAGACTCTAGTA 23
|||||||||||||||*||:|:||
Sbjct: 6063 ACCTATCAATAGTCTAAGGTTAT 6041

```
>miR5788 Score: 1.5 RO3G_02959
Query: 1 TGGATAGTTATCAGACTCTAGTA 23
|||||||||||||||*||:|:||
Sbjct: 2574 ACCTATCAATAGTCTAAGGTTAT 2552
>miR5788 Score: 1.5 RO3G_03547
Query: 1 TGGATAGTTATCAGACTCTAGTA 23
|||||||||||||||*||:|:||
Sbjct: 2574 ACCTATCAATAGTCTAAGGTTAT 2552
>miR5788 Score: 0.2 RO3G_04114
Query: 1 TGGATAGTTATCAGACTCTAGTA 23
||||||||||||||||||:||||
Sbjct: 2574 ACCTATCAATAGTCTGAGGTCAT 2552
>miR5788 Score: 0.0 RO3G_00306
Query: 1 TGGATAGTTATCAGACTCTAGTA 23
|||||||||||||||||||||||
Sbjct: 1023 ACCTATCAATAGTCTGAGATCAT 1001
>miR5788 Score: 0.0 RO3G_01613
Query: 1 TGGATAGTTATCAGACTCTAGTA 23
|||||||||||||||||||||||
Sbjct: 825 ACCTATCAATAGTCTGAGATCAT 803
>miR5788 Score: 1.5 RO3G_02221
Query: 1 TGGATAGTTATCAGACTCTAGTA 23
|||||||||||||||*||:|:||
Sbjct: 2496 ACCTATCAATAGTCTAAGGTTAT 2474
>miR5788 Score: 0.2 RO3G_17371
Query: 1 TGGATAGTTATCAGACTCTAGTA 23
||||||||||||||||||:||||
Sbjct: 2529 ACCTATCAATAGTCTGAGGTCAT 2507
>miR5788 Score: 1.5 RO3G_17326
Query: 1 TGGATAGTTATCAGACTCTAGTA 23
|||||||||||||||*||:|:||
Sbjct: 2406 ACCTATCAATAGTCTAAGGTTAT 2384
>miR5208d Score: 1.5 RO3G_15573
Query: 1 TTTAGTCATAGTTTGTAGGCAA 22
|*:|||||||||||||||||||
Sbjct: 1489 ATGTCAGTATCAAACATCCGTT 1468
>miR5208d Score: 1.5 RO3G_13849
Query: 1 TTTAGTCATAGTTTGTAGGCAA 22
|*:|||||||||||||||||||
Sbjct: 1489 ATGTCAGTATCAAACATCCGTT 1468
>miR5208d Score: 2.5 RO3G_12344
Query: 1 TTTAGTCATAGTTTGTAGGCAA 22
||:|*||||||||||||::*||
Sbjct: 2523 AAGTGAGTATCAAACATTTCTT 2502
>miR5208d Score: 1.5 RO3G_10420
```

Query: 1 TTTAGTCATAGTTTGTAGGCAA 22
|*:|||||||||||||||||||
Sbjct: 1489 ATGTCAGTATCAAACATCCGTT 1468
>miR5208d Score: 1.5 RO3G_10991
Query: 1 TTTAGTCATAGTTTGTAGGCAA 22
|*:|||||||||||||||||||
Sbjct: 1489 ATGTCAGTATCAAACATCCGTT 1468
>miR5208d Score: 1.0 RO3G_05865
Query: 1 TTTAGTCATAGTTTGTAGGCAA 22
|*||||||||||||||||||||
Sbjct: 1489 ATATCAGTATCAAACATCCGTT 1468
>miR5208d Score: 1.0 RO3G_06502
Query: 1 TTTAGTCATAGTTTGTAGGCAA 22
|*||||||||||||||||||||
Sbjct: 1489 ATATCAGTATCAAACATCCGTT 1468
>miR5208d Score: 1.0 RO3G_04662
Query: 1 TTTAGTCATAGTTTGTAGGCAA 22
|*||||||||||||||||||||
Sbjct: 1231 ATATCAGTATCAAACATCCGTT 1210
>miR5208d Score: 1.5 RO3G_02708
Query: 1 TTTAGTCATAGTTTGTAGGCAA 22
|*:|||||||||||||||||||
Sbjct: 1222 ATGTCAGTATCAAACATCCGTT 1201
>miR5208d Score: 1.5 RO3G_02966
Query: 1 TTTAGTCATAGTTTGTAGGCAA 22
|*:|||||||||||||||||||
Sbjct: 760 ATGTCAGTATCAAACATCCGTT 739
>miR5208d Score: 1.5 RO3G_03749
Query: 1 TTTAGTCATAGTTTGTAGGCAA 22
|*:|||||||||||||||||||
Sbjct: 1489 ATGTCAGTATCAAACATCCGTT 1468
>miR5208d Score: 2.0 RO3G_01056
Query: 1 TTTAGTCATAGTTTGTAGGCAA 22
**|||||||||||||||||||*
Sbjct: 1384 TCATCAGTATCAAACATCCGTC 1363
>miR5208d Score: 1.0 RO3G_01733
Query: 1 TTTAGTCATAGTTTGTAGGCAA 22
|*||||||||||||||||||||
Sbjct: 1413 ATATCAGTATCAAACATCCGTT 1392
>miR5208d Score: 1.5 RO3G_01944
Query: 1 TTTAGTCATAGTTTGTAGGCAA 22
|*:|||||||||||||||||||
Sbjct: 1489 ATGTCAGTATCAAACATCCGTT 1468
>miR5208d Score: 1.5 RO3G_17446
Query: 1 TTTAGTCATAGTTTGTAGGCAA 22

|*:|||||||||||||||||||
Sbjct: 1489 ATGTCAGTATCAAACATCCGTT 1468
>miR5208d Score: 1.5 RO3G_17287
Query: 1 TTTAGTCATAGTTTGTAGGCAA 22
|*:|||||||||||||||||||
Sbjct: 1489 ATGTCAGTATCAAACATCCGTT 1468
>miR6209 Score: 1.0 RO3G_16430
Query: 1 TGAGCATCAGAAGCTGTGCG 20
||||||||||||*|||||||
Sbjct: 212 ACTCGTAGTCTTAGACACGC 193
>miR6209 Score: 0.0 RO3G_16144
Query: 1 TGAGCATCAGAAGCTGTGCG 20
||||||||||||||||||||
Sbjct: 248 ACTCGTAGTCTTCGACACGC 229
>miR6209 Score: 0.0 RO3G_14972
Query: 1 TGAGCATCAGAAGCTGTGCG 20
||||||||||||||||||||
Sbjct: 179 ACTCGTAGTCTTCGACACGC 160
>miR6209 Score: 0.5 RO3G_15151
Query: 1 TGAGCATCAGAAGCTGTGCG 20
||||||||||||:|||||||
Sbjct: 269 ACTCGTAGTCTTTGACACGC 250
>miR6209 Score: 0.0 RO3G_13295
Query: 1 TGAGCATCAGAAGCTGTGCG 20
||||||||||||||||||||
Sbjct: 179 ACTCGTAGTCTTCGACACGC 160
>miR6209 Score: 0.0 RO3G_13702
Query: 1 TGAGCATCAGAAGCTGTGCG 20
||||||||||||||||||||
Sbjct: 179 ACTCGTAGTCTTCGACACGC 160
>miR6209 Score: 1.0 RO3G_13754
Query: 1 TGAGCATCAGAAGCTGTGCG 20
||||||||||||*|||||||
Sbjct: 248 ACTCGTAGTCTTAGACACGC 229
>miR6209 Score: 0.0 RO3G_13860
Query: 1 TGAGCATCAGAAGCTGTGCG 20
||||||||||||||||||||
Sbjct: 248 ACTCGTAGTCTTCGACACGC 229
>miR6209 Score: 0.0 RO3G_12665
Query: 1 TGAGCATCAGAAGCTGTGCG 20
||||||||||||||||||||
Sbjct: 227 ACTCGTAGTCTTCGACACGC 208
>miR6209 Score: 1.0 RO3G_12672
Query: 1 TGAGCATCAGAAGCTGTGCG 20
||||||||||||*|||||||

```
Sbjct: 248 ACTCGTAGTCTTAGACACGC 229
>miR6209 Score: 0.0 RO3G_13000
Query: 1 TGAGCATCAGAAGCTGTGCG 20
||||||||||||||||||||
Sbjct: 179 ACTCGTAGTCTTCGACACGC 160
>miR6209 Score: 1.0 RO3G_13138
Query: 1 TGAGCATCAGAAGCTGTGCG 20
||||||||||||*|||||||
Sbjct: 179 ACTCGTAGTCTTAGACACGC 160
>miR6209 Score: 1.0 RO3G_12004
Query: 1 TGAGCATCAGAAGCTGTGCG 20
||||||||||||*|||||||
Sbjct: 248 ACTCGTAGTCTTAGACACGC 229
>miR6209 Score: 1.0 RO3G_12173
Query: 1 TGAGCATCAGAAGCTGTGCG 20
||||||||||||*|||||||
Sbjct: 248 ACTCGTAGTCTTAGACACGC 229
>miR6209 Score: 2.5 RO3G_12181
Query: 1 TGAGCATCAGAAGCTGTGCG 20
*:||||||||||*||||*|
Sbjct: 722 TTTCGTAGTCTTCAACAC-C 704
>miR6209 Score: 2.0 RO3G_11253
Query: 1 TGAGCATCAGAAGCTGTGCG 20
||||||||*:||||||||*|
Sbjct: 794 ACTCGTAGGTTTCGACAC-C 776
>miR6209 Score: 2.5 RO3G_10340
Query: 1 TGAGCATCAGAAGCTGTGCG 20
|:||||*|||||||||||**
Sbjct: 467 ATTCGTTGTCTTCGACACAA 448
>miR6209 Score: 0.0 RO3G_09598
Query: 1 TGAGCATCAGAAGCTGTGCG 20
||||||||||||||||||||
Sbjct: 179 ACTCGTAGTCTTCGACACGC 160
>miR6209 Score: 0.5 RO3G_09618
Query: 1 TGAGCATCAGAAGCTGTGCG 20
||||||||||||||||||*|
Sbjct: 233 ACTCGTAGTCTTCGACACAC 214
>miR6209 Score: 1.0 RO3G_09950
Query: 1 TGAGCATCAGAAGCTGTGCG 20
||||||||||||*|||||||
Sbjct: 248 ACTCGTAGTCTTAGACACGC 229
>miR6209 Score: 0.5 RO3G_10034
Query: 1 TGAGCATCAGAAGCTGTGCG 20
||||||||||||:|||||||
Sbjct: 233 ACTCGTAGTCTTTGACACGC 214
```

```
>miR6209 Score: 0.5 RO3G_10051
Query: 1 TGAGCATCAGAAGCTGTGCG 20
||||||||||||:|||||||
Sbjct: 233 ACTCGTAGTCTTTGACACGC 214
>miR6209 Score: 1.0 RO3G_10231
Query: 1 TGAGCATCAGAAGCTGTGCG 20
||||||||||||*|||||||
Sbjct: 248 ACTCGTAGTCTTAGACACGC 229
>miR6209 Score: 0.0 RO3G_06740
Query: 1 TGAGCATCAGAAGCTGTGCG 20
||||||||||||||||||||
Sbjct: 179 ACTCGTAGTCTTCGACACGC 160
>miR6209 Score: 2.0 RO3G_07076
Query: 1 TGAGCATCAGAAGCTGTGCG 20
||||||||*:||||||||*|
Sbjct: 485 ACTCGTAGGTTTCGACAC-C 467
>miR6209 Score: 0.0 RO3G_07111
Query: 1 TGAGCATCAGAAGCTGTGCG 20
||||||||||||||||||||
Sbjct: 248 ACTCGTAGTCTTCGACACGC 229
>miR6209 Score: 1.0 RO3G_07673
Query: 1 TGAGCATCAGAAGCTGTGCG 20
|||||*||||||||||||||
Sbjct: 248 ACTCGCAGTCTTCGACACGC 229
>miR6209 Score: 0.0 RO3G_07716
Query: 1 TGAGCATCAGAAGCTGTGCG 20
||||||||||||||||||||
Sbjct: 233 ACTCGTAGTCTTCGACACGC 214
>miR6209 Score: 0.5 RO3G_07719
Query: 1 TGAGCATCAGAAGCTGTGCG 20
||||||||||||||||||*|
Sbjct: 176 ACTCGTAGTCTTCGACACAC 157
>miR6209 Score: 1.0 RO3G_07859
Query: 1 TGAGCATCAGAAGCTGTGCG 20
||||||||||||*|||||||
Sbjct: 143 ACTCGTAGTCTTAGACACGC 124
>miR6209 Score: 1.0 RO3G_05932
Query: 1 TGAGCATCAGAAGCTGTGCG 20
||||||||||||*|||||||
Sbjct: 179 ACTCGTAGTCTTAGACACGC 160
>miR6209 Score: 0.0 RO3G_05934
Query: 1 TGAGCATCAGAAGCTGTGCG 20
||||||||||||||||||||
Sbjct: 179 ACTCGTAGTCTTCGACACGC 160
>miR6209 Score: 1.0 RO3G_06435
```

```
Query: 1 TGAGCATCAGAAGCTGTGCG 20
||||||||||||*|||||||
Sbjct: 248 ACTCGTAGTCTTAGACACGC 229
>miR6209 Score: 0.0 RO3G_06719
Query: 1 TGAGCATCAGAAGCTGTGCG 20
||||||||||||||||||||
Sbjct: 179 ACTCGTAGTCTTCGACACGC 160
>miR6209 Score: 0.0 RO3G_04671
Query: 1 TGAGCATCAGAAGCTGTGCG 20
||||||||||||||||||||
Sbjct: 248 ACTCGTAGTCTTCGACACGC 229
>miR6209 Score: 2.5 RO3G_05007
Query: 1 TGAGCATCAGAAGCTGTGCG 20
:|||*|||||||||||*:||
Sbjct: 173 GCTCTTAGTCTTCGAC-TGC 155
>miR6209 Score: 1.0 RO3G_05351
Query: 1 TGAGCATCAGAAGCTGTGCG 20
||||||||||||*|||||||
Sbjct: 233 ACTCGTAGTCTTAGACACGC 214
>miR6209 Score: 0.0 RO3G_02701
Query: 1 TGAGCATCAGAAGCTGTGCG 20
||||||||||||||||||||
Sbjct: 212 ACTCGTAGTCTTCGACACGC 193
>miR6209 Score: 2.5 RO3G_02730
Query: 1 TGAGCATCAGAAGCTGTGCG 20
|||||||*||||||||*|*|
Sbjct: 1163 ACTCGTAATCTTCGAC-CAC 1145
>miR6209 Score: 1.8 RO3G_02745
Query: 1 TGAGCATCAGAAGCTGTGCG 20
|*||||:||||||||||:||
Sbjct: 614 AATCGTGGTCTTCGACATGC 595
>miR6209 Score: 0.5 RO3G_03561
Query: 1 TGAGCATCAGAAGCTGTGCG 20
||||||||||||:|||||||
Sbjct: 269 ACTCGTAGTCTTTGACACGC 250
>miR6209 Score: 0.0 RO3G_03989
Query: 1 TGAGCATCAGAAGCTGTGCG 20
||||||||||||||||||||
Sbjct: 179 ACTCGTAGTCTTCGACACGC 160
>miR6209 Score: 2.0 RO3G_04128
Query: 1 TGAGCATCAGAAGCTGTGCG 20
||||||||||*||*||||||
Sbjct: 248 ACTCGTAGTCCTCAACACGC 229
>miR6209 Score: 0.0 RO3G_00252
Query: 1 TGAGCATCAGAAGCTGTGCG 20
```

||||||||||||||||||||
Sbjct: 302 ACTCGTAGTCTTCGACACGC 283
>miR6209 Score: 2.5 RO3G_00502
Query: 1 TGAGCATCAGAAGCTGTGCG 20
||||||*||*|||||||*||
Sbjct: 529 ACTCGTCGTGTTCGACA-GC 511
>miR6209 Score: 0.0 RO3G_01310
Query: 1 TGAGCATCAGAAGCTGTGCG 20
||||||||||||||||||||
Sbjct: 179 ACTCGTAGTCTTCGACACGC 160
>miR6209 Score: 0.0 RO3G_01738
Query: 1 TGAGCATCAGAAGCTGTGCG 20
||||||||||||||||||||
Sbjct: 179 ACTCGTAGTCTTCGACACGC 160
>miR6209 Score: 1.0 RO3G_01938
Query: 1 TGAGCATCAGAAGCTGTGCG 20
||||||||||||*|||||||
Sbjct: 248 ACTCGTAGTCTTAGACACGC 229
>miR6209 Score: 0.0 RO3G_17468
Query: 1 TGAGCATCAGAAGCTGTGCG 20
||||||||||||||||||||
Sbjct: 179 ACTCGTAGTCTTCGACACGC 160
>miR6209 Score: 0.5 RO3G_17359
Query: 1 TGAGCATCAGAAGCTGTGCG 20
||||||||||||:|||||||
Sbjct: 269 ACTCGTAGTCTTTGACACGC 250
>miR6209 Score: 1.0 RO3G_17333
Query: 1 TGAGCATCAGAAGCTGTGCG 20
||||||||||||*|||||||
Sbjct: 248 ACTCGTAGTCTTAGACACGC 229
>miR6209 Score: 0.0 RO3G_17152
Query: 1 TGAGCATCAGAAGCTGTGCG 20
||||||||||||||||||||
Sbjct: 227 ACTCGTAGTCTTCGACACGC 208
>miR1120c-5p Score: 2.5 RO3G_02870
Query: 1 TAATATAAAATGCTTTGTTGAC 22
|||||||||||||||||**|
Sbjct: 688 CTTATACTTTACGAAACAAACG 667
>miR4387a Score: 2.5 RO3G_01478
Query: 1 AACAAGCCGGTGTGGGTGACACT 23
|||||||||||||||||*|*|
Sbjct: 2382 GTGTTCGGCCACAGCCACTTTTA 2360
>miR5718 Score: 1.5 RO3G_16818
Query: 1 TCAGAACCAAGACCAGAGCCAAG 23
||||*||||||||||||||*||

Sbjct: 2258 AGTCCTGGTTCTGGTCTCGGCTC 2236
>miR5718 Score: 2.0 RO3G_16818
Query: 1 TCAGAACCAAGACCAGAGCCAAG 23
:||||*|||||||||||:|*|||
Sbjct: 2228 GGTCTCGGTTCTGGTCTTGATTC 2206
>miR5718 Score: 1.8 RO3G_16800
Query: 1 TCAGAACCAAGACCAGAGCCAAG 23
:||:*||||||||||||||||||
Sbjct: 2486 GGTTCTGGTTCTGGTCTCGGTTC 2464
>miR5718 Score: 2.2 RO3G_16171
Query: 1 TCAGAACCAAGACCAGAGCCAAG 23
|||||||||||||*|||:**|||
Sbjct: 7028 AGTCTTGGTTCTGCTCTT-CTTC 7007
>miR5718 Score: 1.8 RO3G_15265
Query: 1 TCAGAACCAAGACCAGAGCCAAG 23
:||:*||||||||||||||||||
Sbjct: 2486 GGTTCTGGTTCTGGTCTCGGTTC 2464
>miR5718 Score: 1.8 RO3G_15267
Query: 1 TCAGAACCAAGACCAGAGCCAAG 23
:||:*||||||||||||||||||
Sbjct: 248 GGTTCTGGTTCTGGTCTCGGTTC 226
>miR5718 Score: 0.0 RO3G_15268
Query: 1 TCAGAACCAAGACCAGAGCCAAG 23
|||||||||||||||||||||||
Sbjct: 2486 AGTCTTGGTTCTGGTCTCGGTTC 2464
>miR5718 Score: 1.8 RO3G_14350
Query: 1 TCAGAACCAAGACCAGAGCCAAG 23
:||||||||*||||||||||*||
Sbjct: 2381 GGTCTTGGTCCTGGTCTCGGCTC 2359
>miR5718 Score: 0.2 RO3G_14350
Query: 1 TCAGAACCAAGACCAGAGCCAAG 23
:||||||||||||||||||||||
Sbjct: 2351 GGTCTTGGTTCTGGTCTCGGTTC 2329
>miR5718 Score: 1.8 RO3G_14352
Query: 1 TCAGAACCAAGACCAGAGCCAAG 23
:||||||||*||||||||||*||
Sbjct: 2519 GGTCTTGGTCCTGGTCTCGGCTC 2497
>miR5718 Score: 0.2 RO3G_14352
Query: 1 TCAGAACCAAGACCAGAGCCAAG 23
:||||||||||||||||||||||
Sbjct: 2489 GGTCTTGGTTCTGGTCTCGGTTC 2467
>miR5718 Score: 1.8 RO3G_14777
Query: 1 TCAGAACCAAGACCAGAGCCAAG 23
:||:*||||||||||||||||||
Sbjct: 185 GGTTCTGGTTCTGGTCTCGGTTC 163

```
>miR5718 Score: 1.8 RO3G_13217
Query: 1 TCAGAACCAAGACCAGAGCCAAG 23
:||:*||||||||||||||||||
Sbjct: 2486 GGTTCTGGTTCTGGTCTCGGTTC 2464
>miR5718 Score: 1.8 RO3G_13219
Query: 1 TCAGAACCAAGACCAGAGCCAAG 23
:||||||||*||||||||||*||
Sbjct: 2516 GGTCTTGGTCCTGGTCTCGGCTC 2494
>miR5718 Score: 0.2 RO3G_13219
Query: 1 TCAGAACCAAGACCAGAGCCAAG 23
:||||||||||||||||||||||
Sbjct: 2486 GGTCTTGGTTCTGGTCTCGGTTC 2464
>miR5718 Score: 1.8 RO3G_13222
Query: 1 TCAGAACCAAGACCAGAGCCAAG 23
:|||*|||||||||||||||*||
Sbjct: 2513 GGTCCTGGTTCTGGTCTCGGCTC 2491
>miR5718 Score: 1.8 RO3G_11967
Query: 1 TCAGAACCAAGACCAGAGCCAAG 23
:|||*|||||||||||||||*||
Sbjct: 2531 GGTCCTGGTTCTGGTCTCGGCTC 2509
>miR5718 Score: 2.0 RO3G_11967
Query: 1 TCAGAACCAAGACCAGAGCCAAG 23
:||||*|||||||||||:|*|||
Sbjct: 2501 GGTCTCGGTTCTGGTCTTGATTC 2479
>miR5718 Score: 0.5 RO3G_05640
Query: 1 TCAGAACCAAGACCAGAGCCAAG 23
:||||||||||||||||:|||||
Sbjct: 2243 GGTCTTGGTTCTGGTCTTGGTTC 2221
>miR5718 Score: 1.0 RO3G_03992
Query: 1 TCAGAACCAAGACCAGAGCCAAG 23
:||||||||||||||||:||*||
Sbjct: 1055 GGTCTTGGTTCTGGTCTTGG-TC 1034
>miR5718 Score: 1.8 RO3G_04121
Query: 1 TCAGAACCAAGACCAGAGCCAAG 23
:||:*||||||||||||||||||
Sbjct: 2486 GGTTCTGGTTCTGGTCTCGGTTC 2464
>miR6182 Score: 1.2 RO3G_07088
Query: 1 TGGTCTGTGATGGATGCGTT 20
||*||||||||||||||||:
Sbjct: 269 ACGAGACACTACCTACGCAG 250
>miR6182 Score: 2.2 RO3G_06253
Query: 1 TGGTCTGTGATGGATGCG-TT 20
|:|||||:|||||||||:**|
Sbjct: 1282 ATCAGACGCTACCTACGTTCA 1262
>miR6182 Score: 2.5 RO3G_04358
```

```
Query: 1 TGGTCTGTGATGGATGCGTT 20
|||**||||||||||||||*
Sbjct: 154 ACCCAACACTACCTACGCAC 135
>miR6182 Score: 2.5 RO3G_00234
Query: 1 TGGTCTGTGATGGATGCGTT 20
||||||*||||||||:|**|
Sbjct: 998 ACCAGAAACTACCTATGATA 979
>miR8179 Score: 0.0 RO3G_17263
Query: 1 TGATCTGCATTCACTTGATCGT 22
||||||||||||||||||||||
Sbjct: 1106 ACTAGACGTAAGTGAACTAGCA 1085
>miR8179 Score: 0.0 RO3G_15868
Query: 1 TGATCTGCATTCACTTGATCGT 22
||||||||||||||||||||||
Sbjct: 935 ACTAGACGTAAGTGAACTAGCA 914
>miR8179 Score: 2.5 RO3G_14486
Query: 1 TGATCTGCATTCACTTGATCGT 22
*|*||||||||||||||*||*|
Sbjct: 197 CCAAGACGTAAGTGAAC-AGGA 177
>miR8179 Score: 0.0 RO3G_14771
Query: 1 TGATCTGCATTCACTTGATCGT 22
||||||||||||||||||||||
Sbjct: 1049 ACTAGACGTAAGTGAACTAGCA 1028
>miR8179 Score: 2.0 RO3G_12808
Query: 1 TGATCTGCATTCACTTGATCGT 22
|*|||||||||||||||*||*|
Sbjct: 815 AATAGACGTAAGTGAAC-AGAA 795
>miR8179 Score: 2.2 RO3G_13234
Query: 1 TGATCTGCATTCACTTGATCGT 22
:|||||||*||||||||*:|:|
Sbjct: 293 GCTAGACGAAAGTGAAC-GGTA 273
>miR8179 Score: 0.0 RO3G_13279
Query: 1 TGATCTGCATTCACTTGATCGT 22
||||||||||||||||||||||
Sbjct: 1949 ACTAGACGTAAGTGAACTAGCA 1928
>miR8179 Score: 2.5 RO3G_11794
Query: 1 TGATCTGCATTCACTTGATC-GT 22
|||||||*||:|||||||*|*||
Sbjct: 68 ACTAGACTTAGGTGAACTTGACA 46
>miR8179 Score: 0.0 RO3G_10786
Query: 1 TGATCTGCATTCACTTGATCGT 22
||||||||||||||||||||||
Sbjct: 1097 ACTAGACGTAAGTGAACTAGCA 1076
>miR8179 Score: 0.0 RO3G_09943
Query: 1 TGATCTGCATTCACTTGATCGT 22
```

||||||||||||||||||||||
Sbjct: 722 ACTAGACGTAAGTGAACTAGCA 701
>miR8179 Score: 0.0 RO3G_03598
Query: 1 TGATCTGCATTCACTTGATCGT 22
||||||||||||||||||||||
Sbjct: 1097 ACTAGACGTAAGTGAACTAGCA 1076
>miR8179 Score: 0.0 RO3G_17419
Query: 1 TGATCTGCATTCACTTGATCGT 22
||||||||||||||||||||||
Sbjct: 935 ACTAGACGTAAGTGAACTAGCA 914
>miR8179 Score: 0.0 RO3G_17346
Query: 1 TGATCTGCATTCACTTGATCGT 22
||||||||||||||||||||||
Sbjct: 1856 ACTAGACGTAAGTGAACTAGCA 1835
>miR8179 Score: 0.0 RO3G_17312
Query: 1 TGATCTGCATTCACTTGATCGT 22
||||||||||||||||||||||
Sbjct: 536 ACTAGACGTAAGTGAACTAGCA 515
>miR847-5p Score: 2.5 RO3G_16366
Query: 1 TATTGATGAGGAGGATGGA 19
|*||||:|||||:||||*|
Sbjct: 1908 ACAACTGCTCCTTCTAC-T 1891
>miR847-5p Score: 2.5 RO3G_16534
Query: 1 TATTGATGAGGAGGATGGA 19
|||*|||||||||||::*|
Sbjct: 376 ATACCTACTCCTCCTGT-T 359
>miR847-5p Score: 2.5 RO3G_15958
Query: 1 TATTGATGAGGAGGATGGA 19
|:|||||||||::||||
Sbjct: 248 TTGACTACTCCTTTTAC-T 231
>miR847-5p Score: 2.2 RO3G_15183
Query: 1 TATTGATGAGGAGGATGGA 19
:*|||||||||||*|||||
Sbjct: 115 GCAACTACTCCTCGTACCT 97
>miR847-5p Score: 2.2 RO3G_13952
Query: 1 TATTGATGAGGAGGATGGA 19
**|||||||||||:|||:|
Sbjct: 1388 TGAACTACTCCTCTTACTT 1370
>miR847-5p Score: 2.2 RO3G_14140
Query: 1 TATTGATGAGGAGGATGGA 19
||||||*||*|||||||:|
Sbjct: 182 ATAACTCCTGCTCCTACTT 164
>miR847-5p Score: 2.2 RO3G_13674
Query: 1 TATTGATGAGGAGGATGGA 19
:||*|||||||||||||**

```
Sbjct: 223 GTACCTACTCCTCCTACGG 205
>miR847-5p Score: 1.8 RO3G_13827
Query: 1 TATTGATGAGGAGGATGGA 19
*||*|||||||||||||:|
Sbjct: 1926 TTACCTACTCCTCCTACTT 1908
>miR847-5p Score: 2.5 RO3G_12961
Query: 1 TATTGATGAGGAGGATGGA 19
***||||||||||||||||
Sbjct: 96 TCTACTACTCCTCCTACCT 78
>miR847-5p Score: 2.0 RO3G_11915
Query: 1 TATTGATGAGGAGGATGGA 19
*|||||:||||||||:|*|
Sbjct: 416 TTAACTGCTCCTCCTGC-T 399
>miR847-5p Score: 2.5 RO3G_11952
Query: 1 TATTGATGAGGAGGATGGA 19
***||||||||||||||||
Sbjct: 141 TCTACTACTCCTCCTACCT 123
>miR847-5p Score: 2.5 RO3G_10744
Query: 1 TATTGATGAGGAGGATGGA 19
|*||||:|||||:||||*|
Sbjct: 1911 ACAACTGCTCCTTCTAC-T 1894
>miR847-5p Score: 2.5 RO3G_10745
Query: 1 TATTGATGAGGAGGATGGA 19
|*||||:|||||:||||*|
Sbjct: 879 ACAACTGCTCCTTCTAC-T 862
>miR847-5p Score: 2.5 RO3G_10839
Query: 1 TATTGATGAGGAGGATGGA 19
:*:||||||:|||||||:|
Sbjct: 615 GGGACTACTTCTCCTACTT 597
>miR847-5p Score: 2.2 RO3G_11040
Query: 1 TATTGATGAGGAGGATGGA 19
|*|||||||:||:||||:|
Sbjct: 99 ACAACTACTTCTTCTACTT 81
>miR847-5p Score: 2.5 RO3G_09242
Query: 1 TATTGATGAGGAGGATGGA 19
|||*|||||||||||::*|
Sbjct: 526 ATACCTACTCCTCCTGT-T 509
>miR847-5p Score: 1.8 RO3G_08083
Query: 1 TATTGATGAGGAGGATGGA 19
*|||||:||||||||:|:|
Sbjct: 398 TTAACTGCTCCTCCTGCTT 380
>miR847-5p Score: 2.5 RO3G_08484
Query: 1 TATTGATGAGGAGGATGGA 19
||||||*||||||||*|*|
Sbjct: 152 ATAACTCCTCCTCCTCC-T 135
```

>miR847-5p Score: 2.5 RO3G_08785
Query: 1 TATTGATGAGGAGGATGGA 19
|||||*||||||||*|*|
Sbjct: 284 ATAACTCCTCCTCCTCC-T 267
>miR847-5p Score: 2.5 RO3G_07862
Query: 1 TATTGATGAGGAGGATGGA 19
|*||||:|||||:||||*|
Sbjct: 2022 ACAACTGCTCCTTCTAC-T 2005
>miR847-5p Score: 2.5 RO3G_06497
Query: 1 TATTGATGAGGAGGATGGA 19
|||||:|||||:||:||
Sbjct: 23 TTAACTGCTCCTTCTGCGT 5
>miR847-5p Score: 2.5 RO3G_04817
Query: 1 TATTGATGAGGAGGATGGA 19
**|||||||:||||||||*
Sbjct: 1335 TCAACTACTTCTCCTACCA 1317
>miR847-5p Score: 2.5 RO3G_05205
Query: 1 TATTGATGAGGAGGATGGA 19
|||*|||||||||||::*|
Sbjct: 382 ATACCTACTCCTCCTGT-T 365
>miR847-5p Score: 2.5 RO3G_02989
Query: 1 TATTGATGAGGAGGATGGA 19
|||||*|||||||||*|*|
Sbjct: 470 ATAACAACTCCTCCTCC-T 453
>miR847-5p Score: 0.0 RO3G_03516
Query: 1 TATTGATGAGGAGGATGGA 19
|||||||||||||||||||
Sbjct: 663 ATAACTACTCCTCCTACCT 645
>miR847-5p Score: 2.5 RO3G_00683
Query: 1 TATTGATGAGGAGGATGGA 19
***||||||||||||||||
Sbjct: 96 TCTACTACTCCTCCTACCT 78
>miR847-5p Score: 2.5 RO3G_01278
Query: 1 TATTGATGAGGAGGATGG-A 19
||:||||||*:|||||||*|
Sbjct: 211 ATGACTACTGTTCCTACCGT 192
>miR847-5p Score: 2.5 RO3G_01830
Query: 1 TATTGATGAGGAGGATGGA 19
|||:|*|:||||||||||*
Sbjct: 136 ATAGCAATTCCTCCTACCC 118
>miR847-5p Score: 2.5 RO3G_01875
Query: 1 TATTGATGAGGAGGATGGA 19
||||||||:||||||*|
Sbjct: 1136 TTAACTACTTCTCCAAC-T 1119
>miR847-5p Score: 2.0 RO3G_02104

Query: 1 TATTGATGAGGAGGATGG-A 19
|||||||:|:||||||:|*|
Sbjct: 737 ATAACTATTTCTCCTATCGT 718
>miR9559-5p Score: 2.0 RO3G_17021
Query: 1 TTTGATTTTGGTTCATTTTG 20
*|||||:|:|||||||:|||
Sbjct: 595 CAACTAGAGCCAAGTAGAAC 576
>miR9559-5p Score: 1.5 RO3G_16288
Query: 1 TTTGATTTTGGTTCATTTTG 20
*||||||||::|||||||||
Sbjct: 239 TAACTAAAATTAAGTAAAAC 220
>miR9559-5p Score: 2.5 RO3G_15821
Query: 1 TTTGATTTTGGTTCATTT-TG 20
:||||||||:||||*|||*|:
Sbjct: 1364 GAACTAAAATCAAGCAAATAT 1344
>miR9559-5p Score: 2.5 RO3G_15310
Query: 1 TTTGATTTTGGTTCATTTTG 20
|||:*||||||||*||||||
Sbjct: 239 AAATAAAAACCAAATAAAAC 220
>miR9559-5p Score: 2.5 RO3G_14948
Query: 1 TTTGATTTTGGTTCATTTTG 20
||:||||||||||**|||||
Sbjct: 83 AAGCTAAAACCAAAGAAAAC 64
>miR9559-5p Score: 2.0 RO3G_14378
Query: 1 TTTGATTTTGGTTCATTTTG 20
|||:|||:||:|:|||||||
Sbjct: 3053 AAATTAAGACTAGGTAAAAC 3034
>miR9559-5p Score: 2.5 RO3G_13883
Query: 1 TTTGATTTTGGTTCATTT-TG 20
|||||||:::|||||||||
Sbjct: 1622 TAACTAAAGTTAAGTAAAGAC 1602
>miR9559-5p Score: 2.0 RO3G_14347
Query: 1 TTTGATTTTGGTTCATTTTG 20
*|||||:|:|||||||:|||
Sbjct: 595 CAACTAGAGCCAAGTAGAAC 576
>miR9559-5p Score: 2.0 RO3G_13722
Query: 1 TTTGATTTTGGTTCATTTTG 20
*|||||:|:|||||||:|||
Sbjct: 592 CAACTAGAGCCAAGTAGAAC 573
>miR9559-5p Score: 2.5 RO3G_13839
Query: 1 TTTGATTTTGGTTCATTTTG 20
|:|||||||:||||||||
Sbjct: 168 CAGCTAAAACTAAGCAAAAC 149
>miR9559-5p Score: 2.0 RO3G_12677
Query: 1 TTTGATTTTGGTTCATTTTG 20

*|||||:|:|||||||:|||
Sbjct: 595 CAACTAGAGCCAAGTAGAAC 576
>miR9559-5p Score: 2.0 RO3G_11377
Query: 1 TTTGATTTTGGTTCATTTTG 20
||||||||||*||||||*|*
Sbjct: 2405 AAACTAAAACGAAGTAACAG 2386
>miR9559-5p Score: 2.5 RO3G_10354
Query: 1 TTTGATTTTGGTTCATTTTG 20
|||:*||||||*||||||||
Sbjct: 398 AAATAAAAACCTAGTAAAAC 379
>miR9559-5p Score: 2.0 RO3G_10410
Query: 1 TTTGATTTTGGTTCATTTTG 20
|*|:|||||||||||||*||
Sbjct: 434 ATATTAAAACCAAGTAACAC 415
>miR9559-5p Score: 2.5 RO3G_10487
Query: 1 TTTGATTTTGGTTCATTTTG 20
:|||*|||||||||||:|*:
Sbjct: 248 GAACGAAAACCAAGTAGATT 229
>miR9559-5p Score: 2.5 RO3G_10638
Query: 1 TTTGATTTTGGTTCATTTTG 20
||||||*:|||||||:|*||
Sbjct: 152 AAACTATGACCAAGTGA-AC 134
>miR9559-5p Score: 2.5 RO3G_09382
Query: 1 TTTGATTTTGGTTCATTTTG 20
|||:||:||||:|||||*::
Sbjct: 20 AAATTAGAACCGAGTAA-GT 2
>miR9559-5p Score: 2.5 RO3G_09425
Query: 1 TTTGATTTTGGTTCATTTTG 20
|||||:|||||||*||*|||
Sbjct: 1311 AAACTGAAACCAACTACAAC 1292
>miR9559-5p Score: 2.2 RO3G_09435
Query: 1 TTTGATTTTGGTTCATTTTG 20
:||||||:|:||||||:||*
Sbjct: 641 GAACTAAGATCAAGTAGAAG 622
>miR9559-5p Score: 2.2 RO3G_09506
Query: 1 TTTGATTTTGGTTCATTTTG 20
|||||:|||*||:||||||:
Sbjct: 733 AAACTGAAAGCAGGTAAAAT 714
>miR9559-5p Score: 2.0 RO3G_09547
Query: 1 TTTGATTTTGGTTCATTTTG 20
*|||||:|:|||||||:|||
Sbjct: 595 CAACTAGAGCCAAGTAGAAC 576
>miR9559-5p Score: 2.5 RO3G_09738
Query: 1 TTTGATTTTGGTTCATTTTG 20
||||||||:*|||||||**|

Sbjct: 95 AAACTAAAGACAAGTAA-CC 77
>miR9559-5p Score: 2.2 RO3G_09845
Query: 1 TTTGATTTTGGTTCATTTTG 20
||||||||||||||*:|
Sbjct: 1265 CAACTAAAACCAAGTTA-GC 1247
>miR9559-5p Score: 2.2 RO3G_08365
Query: 1 TTTGATTTTGGTTCATTTTG 20
*:|||||||::|||||||:|
Sbjct: 491 TGACTAAAATTAAGTAAAGC 472
>miR9559-5p Score: 2.5 RO3G_09064
Query: 1 TTTGATTTTGGTTCATTTTG 20
:|||*||:||||||||||*:
Sbjct: 47 GAACAAAGACCAAGTAAATT 28
>miR9559-5p Score: 2.5 RO3G_07376
Query: 1 TTTGATTTTGGTTCATTTTG 20
|:|:|||:||||||||:||*
Sbjct: 1580 AGATTAAGACCAAGTAGAAG 1561
>miR9559-5p Score: 2.5 RO3G_07643
Query: 1 TTTGATTTTGGTTCATTTTG 20
|:||||||||||||*|:|*|
Sbjct: 272 AGACTAAAACCAAGAAGATC 253
>miR9559-5p Score: 2.5 RO3G_05566
Query: 1 TTTGATTTTGGTTCATTTTG 20
|||||*||||:|||*|||||
Sbjct: 89 AAACTTAAACTAAGAAAAAC 70
>miR9559-5p Score: 2.2 RO3G_05706
Query: 1 TTTGATTTTGGTTCATTTTG 20
||||||||:::|:||||||:
Sbjct: 822 AAACTAAAGTTAGGTAAAAT 803
>miR9559-5p Score: 2.5 RO3G_05788
Query: 1 TTTGATTTTGGTTCATTTTG 20
||||*||:|:|||||||||*
Sbjct: 53 AAACAAAGATCAAGTAAAAA 34
>miR9559-5p Score: 2.2 RO3G_06401
Query: 1 TTTGATTTTGGTTCATTTTG 20
:||||||||*|*||||||||
Sbjct: 187 GAACTAAAAGCTAGTAAAAC 168
>miR9559-5p Score: 2.0 RO3G_06425
Query: 1 TTTGATTTTGGTTCATTTTG 20
*|||||:|:|||||||:|||
Sbjct: 595 CAACTAGAGCCAAGTAGAAC 576
>miR9559-5p Score: 1.5 RO3G_04779
Query: 1 TTTGATTTTGGTTCATTTTG 20
|||||||||:*|||||||||
Sbjct: 1094 AAACTAAAATAAAGTAAAAC 1075

>miR9559-5p Score: 2.0 RO3G_04933
Query: 1 TTTGATTTTGGTTCATTTTG 20
*|||||:|:|||||||:|||
Sbjct: 595 CAACTAGAGCCAAGTAGAAC 576
>miR9559-5p Score: 2.0 RO3G_04973
Query: 1 TTTGATTTTGGTTCATTTTG 20
||||||:|:|||:||||*||
Sbjct: 946 AAACTAGAGCCAGGTAA-AC 928
>miR9559-5p Score: 2.0 RO3G_05330
Query: 1 TTTGATTTTGGTTCATTTTG 20
*|||||:|:|||||||:|||
Sbjct: 595 CAACTAGAGCCAAGTAGAAC 576
>miR9559-5p Score: 2.0 RO3G_05344
Query: 1 TTTGATTTTGGTTCATTTTG 20
*|||||:|:|||||||:|||
Sbjct: 595 CAACTAGAGCCAAGTAGAAC 576
>miR9559-5p Score: 2.5 RO3G_02384
Query: 1 TTTGATTTTGGTTCATTTTG 20
||||*|:|||||||*|||||
Sbjct: 1052 AAACGAGAACCAAGGAAAAC 1033
>miR9559-5p Score: 2.5 RO3G_02426
Query: 1 TTTGATTTTGGTTCATTTTG 20
||||||||||*|||||:*|*
Sbjct: 4598 AAACTAAAACAAAGTAGCAG 4579
>miR9559-5p Score: 2.5 RO3G_02518
Query: 1 TTTGATTTTGGTTCATTTTG 20
|::|||:|||:||||||*||
Sbjct: 146 AGGCTAGAACTAAGTAA-AC 128
>miR9559-5p Score: 2.0 RO3G_02704
Query: 1 TTTGATTTTGGTTCATTTTG 20
*|||||:|:|||||||:|||
Sbjct: 595 CAACTAGAGCCAAGTAGAAC 576
>miR9559-5p Score: 2.2 RO3G_02770
Query: 1 TTTGATTTTGGTTCATTTTG 20
:|||*|||||:||||||*||
Sbjct: 232 GAACAAAAACTAAGTAA-AC 214
>miR9559-5p Score: 2.2 RO3G_03042
Query: 1 TTTGATTTTGGTTCATTTTG 20
|||*|||||||||||||*:*
Sbjct: 955 AAAATAAAACCAAGTAATGA 936
>miR9559-5p Score: 2.5 RO3G_03130
Query: 1 TTTGATTTTGGTTCATTTTG 20
|||||||||:*|||||:*||
Sbjct: 1001 AAACTAAAATAAAGTAG-AC 983
>miR9559-5p Score: 2.0 RO3G_03163

Query: 1 TTTGATTTTGGTTCATTTTG 20
:||*|||||||||||||:*
Sbjct: 745 GAAGTAAAACCAAGTAAAGA 726
>miR9559-5p Score: 2.0 RO3G_03778
Query: 1 TTTGATTTTGGTTCATTTTG 20
*|||||:|:|||||||:|||
Sbjct: 595 CAACTAGAGCCAAGTAGAAC 576
>miR9559-5p Score: 2.0 RO3G_03878
Query: 1 TTTGATTTTGGTTCATTTTG 20
|||||||||||:||*|:|||
Sbjct: 866 AAACTAAAACCGAGCAGAAC 847
>miR9559-5p Score: 2.5 RO3G_00162
Query: 1 TTTGATTTTGGTTCATTTTG 20
||||||:|||||||:|||
Sbjct: 632 TAACTAAGACCATGTAGAAC 613
>miR9559-5p Score: 2.2 RO3G_00170
Query: 1 TTTGATTTTGGTTCATTTTG 20
:|||||:||:|||||||:*:
Sbjct: 4763 GAACTAGAATCAAGTAAGTT 4744
>miR9559-5p Score: 2.2 RO3G_00366
Query: 1 TTTGATTTTGGTTCATTTTG 20
:||||*||||:|:|||||||
Sbjct: 553 GAACTCAAACTAGGTAAAAC 534
>miR9559-5p Score: 2.5 RO3G_02194
Query: 1 TTTGATTTTGGTTCATTTTG 20
|:|||:|||::|||||||*|
Sbjct: 704 AGACTGAAATTAAGTAAACC 685
>miR9559-5p Score: 2.2 RO3G_16939
Query: 1 TTTGATTTTGGTTCATTTTG 20
||||||||||:||||*||:*
Sbjct: 1225 AAACTAAAACTAAGTCAAGG 1206
>miR5387a Score: 2.0 RO3G_17253
Query: 1 TAACACGCCGGTCTACAGTATC 22
|||||||||||||*||*|||||
Sbjct: 1435 ATTGTGCGGCCAGCTGCCATAG 1414
>miR5387a Score: 2.0 RO3G_17255
Query: 1 TAACACGCCGGTCTACAGTATC 22
|||||||||||||*||*|||||
Sbjct: 1435 ATTGTGCGGCCAGCTGCCATAG 1414
>miR5387a Score: 1.0 RO3G_17217
Query: 1 TAACACGCCGGTCTACAGTATC 22
||||||||||||||||*|||||
Sbjct: 163 ATTGTGCGGCCAGATGCCATAG 142
>miR5387a Score: 2.0 RO3G_16366
Query: 1 TAACACGCCGGTCTACAGTATC 22

|||||||||||||*||*|||||
Sbjct: 1465 ATTGTGCGGCCAGCTGCCATAG 1444
>miR5387a Score: 2.0 RO3G_13448
Query: 1 TAACACGCCGGTCTACAGTATC 22
|||||||||||||*||*|||||
Sbjct: 1450 ATTGTGCGGCCAGCTGCCATAG 1429
>miR5387a Score: 2.0 RO3G_11979
Query: 1 TAACACGCCGGTCTACAGTATC 22
|||||||||||||*||*|||||
Sbjct: 1438 ATTGTGCGGCCAGCTGCCATAG 1417
>miR5387a Score: 2.0 RO3G_10532
Query: 1 TAACACGCCGGTCTACAGTATC 22
|||||||||||||*||*|||||
Sbjct: 1450 ATTGTGCGGCCAGCTGCCATAG 1429
>miR5387a Score: 2.0 RO3G_10543
Query: 1 TAACACGCCGGTCTACAGTATC 22
|||||||||||||*||*|||||
Sbjct: 937 ATTGTGCGGCCAGCTGCCATAG 916
>miR5387a Score: 2.0 RO3G_10744
Query: 1 TAACACGCCGGTCTACAGTATC 22
|||||||||||||*||*|||||
Sbjct: 1465 ATTGTGCGGCCAGCTGCCATAG 1444
>miR5387a Score: 2.0 RO3G_10745
Query: 1 TAACACGCCGGTCTACAGTATC 22
|||||||||||||*||*|||||
Sbjct: 433 ATTGTGCGGCCAGCTGCCATAG 412
>miR5387a Score: 2.0 RO3G_10747
Query: 1 TAACACGCCGGTCTACAGTATC 22
|||||||||||||*||*|||||
Sbjct: 1579 ATTGTGCGGCCAGCTGCCATAG 1558
>miR5387a Score: 2.0 RO3G_07735
Query: 1 TAACACGCCGGTCTACAGTATC 22
|||||||||||||*||*|||||
Sbjct: 1384 ATTGTGCGGCCAGCTGCCATAG 1363
>miR5387a Score: 2.0 RO3G_07862
Query: 1 TAACACGCCGGTCTACAGTATC 22
|||||||||||||*||*|||||
Sbjct: 1579 ATTGTGCGGCCAGCTGCCATAG 1558
>miR5256 Score: 2.2 RO3G_13428
Query: 1 TAATGGCTTGTAAGAATTAA 20
:|||:|||||*||||||*||
Sbjct: 2350 GTTATCGAACCTTCTTATTT 2331
>miR5256 Score: 2.2 RO3G_12931
Query: 1 TAATGGCTTGTAAGAATTAA 20
:|||:|||||*||||||*||

```
Sbjct: 328 GTTATCGAACCTTCTTATTT 309
>miR5256 Score: 2.5 RO3G_10947
Query: 1 TAATGGCTTGTAAGAATTAA 20
|*||*||||||||||||*||
Sbjct: 58 ACTAACGAACATTCTTACTT 39
>miR5256 Score: 2.2 RO3G_07278
Query: 1 TAATGGCTTGTAAGAATTAA 20
:|||||*|||||||||||**
Sbjct: 640 GTTACCTAACATTCTTAAAA 621
>miR5256 Score: 2.5 RO3G_05502
Query: 1 TAATGGCTTGTAAGAATTAA 20
|*||:||||||||:|||*||
Sbjct: 854 ACTATCGAACATTTTTA-TT 836
>miR5256 Score: 2.2 RO3G_01473
Query: 1 TAATGGCTTGTAAGAATTAA 20
:|||:|||||*||||||*||
Sbjct: 3724 GTTATCGAACCTTCTTATTT 3705
>miR775 Score: 1.0 RO3G_16429
Query: 1 TTGCGATGTCTTGCAGTCAA 20
|||||*||||||||||||||
Sbjct: 137 AACGCCACAGAACGTCAGTT 118
>miR775 Score: 2.5 RO3G_15745
Query: 1 TTGCGATGTCTTGCAGTCAA 20
||:*|*||||||||||||||
Sbjct: 122 AATACCACAGAACGTCAGTT 103
>miR775 Score: 0.5 RO3G_15496
Query: 1 TTGCGATGTCTTGCAGTCAA 20
||||:|||||||||||||||
Sbjct: 68 AACGTTACAGAACGTCAGTT 49
>miR775 Score: 1.5 RO3G_14806
Query: 1 TTGCGATGTCTTGCAGTCAA 20
|||||*:|||||||||||||
Sbjct: 332 AACGCCGCAGAACGTCAGTT 313
>miR775 Score: 2.0 RO3G_14365
Query: 1 TTGCGATGTCTTGCAGTCAA 20
|||||*||||||||*|||||
Sbjct: 317 AACGCCACAGAACGCCAGTT 298
>miR775 Score: 1.5 RO3G_14467
Query: 1 TTGCGATGTCTTGCAGTCAA 20
|||||*||:|||||||||||
Sbjct: 122 AACGCCACGGAACGTCAGTT 103
>miR775 Score: 1.0 RO3G_14722
Query: 1 TTGCGATGTCTTGCAGTCAA 20
|||||*||||||||||||||
Sbjct: 425 AACGCCACAGAACGTCAGTT 406
```

>miR775 Score: 1.0 RO3G_13903
Query: 1 TTGCGATGTCTTGCAGTCAA 20
|||||*||||||||||||||
Sbjct: 425 AACGCCACAGAACGTCAGTT 406
>miR775 Score: 2.0 RO3G_13574
Query: 1 TTGCGATGTCTTGCAGTCAA 20
|||||*|||*||||||||||
Sbjct: 203 AACGCCACATAACGTCAGTT 184
>miR775 Score: 1.0 RO3G_13664
Query: 1 TTGCGATGTCTTGCAGTCAA 20
|||||*||||||||||||||
Sbjct: 425 AACGCCACAGAACGTCAGTT 406
>miR775 Score: 1.0 RO3G_13672
Query: 1 TTGCGATGTCTTGCAGTCAA 20
|||||*||||||||||||||
Sbjct: 425 AACGCCACAGAACGTCAGTT 406
>miR775 Score: 0.0 RO3G_11486
Query: 1 TTGCGATGTCTTGCAGTCAA 20
||||||||||||||||||||
Sbjct: 197 AACGCTACAGAACGTCAGTT 178
>miR775 Score: 1.0 RO3G_11567
Query: 1 TTGCGATGTCTTGCAGTCAA 20
|||||*||||||||||||||
Sbjct: 1277 AACGCCACAGAACGTCAGTT 1258
>miR775 Score: 1.5 RO3G_09279
Query: 1 TTGCGATGTCTTGCAGTCAA 20
|||||*|||||||||||||*
Sbjct: 191 AACGCCACAGAACGTCAGTC 172
>miR775 Score: 0.0 RO3G_09419
Query: 1 TTGCGATGTCTTGCAGTCAA 20
||||||||||||||||||||
Sbjct: 245 AACGCTACAGAACGTCAGTT 226
>miR775 Score: 2.5 RO3G_09608
Query: 1 TTGCGATGTCTTGCAGTCAA 20
|::||||||||||||*|*||
Sbjct: 758 AGTGCTACAGAACGTAATTT 739
>miR775 Score: 1.0 RO3G_09656
Query: 1 TTGCGATGTCTTGCAGTCAA 20
||||||||:|||||||:|||
Sbjct: 122 AACGCTACGGAACGTCGGTT 103
>miR775 Score: 2.5 RO3G_09821
Query: 1 TTGCGATGTCTTGCAGTCAA 20
||||:*||||||*|||||||
Sbjct: 122 AACGTCACAGAAAGTCAGTT 103
>miR775 Score: 0.5 RO3G_09838

```
Query: 1 TTGCGATGTCTTGCAGTCAA 20
||:|||||||||||||||||
Sbjct: 197 AATGCTACAGAACGTCAGTT 178
>miR775 Score: 2.5 RO3G_10000
Query: 1 TTGCGATGTCTTGCAGTCAA 20
|||||*|||||||*|||*||
Sbjct: 122 AACGCCACAGAACATCATTT 103
>miR775 Score: 1.0 RO3G_08301
Query: 1 TTGCGATGTCTTGCAGTCAA 20
|||||*||||||||||||||
Sbjct: 350 AACGCCACAGAACGTCAGTT 331
>miR775 Score: 2.5 RO3G_08649
Query: 1 TTGCGATGTCTTGCAGTCAA 20
|||||*||:||||*||||||
Sbjct: 152 AACGCCACGGAACATCAGTT 133
>miR775 Score: 1.0 RO3G_08906
Query: 1 TTGCGATGTCTTGCAGTCAA 20
|||||*||||||||||||||
Sbjct: 425 AACGCCACAGAACGTCAGTT 406
>miR775 Score: 2.5 RO3G_09032
Query: 1 TTGCGATGTCTTGCAGTCAA 20
|||||*|||||::||||||*
Sbjct: 117 AACGCCACAGAGTGTCAGTA 98
>miR775 Score: 2.0 RO3G_06838
Query: 1 TTGCGATGTCTTGCAGTCAA 20
|||||*||:||||||||||*
Sbjct: 68 AACGCCACGGAACGTCAGTG 49
>miR775 Score: 0.5 RO3G_07273
Query: 1 TTGCGATGTCTTGCAGTCAA 20
|||||||||||||||||*||
Sbjct: 122 AACGCTACAGAACGTCAATT 103
>miR775 Score: 1.5 RO3G_07438
Query: 1 TTGCGATGTCTTGCAGTCAA 20
|||||*||:|||||||||||
Sbjct: 122 AACGCCACGGAACGTCAGTT 103
>miR775 Score: 1.0 RO3G_07580
Query: 1 TTGCGATGTCTTGCAGTCAA 20
|||||*||||||||||||||
Sbjct: 149 AACGCCACAGAACGTCAGTT 130
>miR775 Score: 1.0 RO3G_07788
Query: 1 TTGCGATGTCTTGCAGTCAA 20
|||||*||||||||||||||
Sbjct: 374 AACGCCACAGAACGTCAGTT 355
>miR775 Score: 1.0 RO3G_05133
Query: 1 TTGCGATGTCTTGCAGTCAA 20
```

|||||*||||||||||||||
Sbjct: 374 AACGCCACAGAACGTCAGTT 355
>miR775 Score: 1.0 RO3G_05181
Query: 1 TTGCGATGTCTTGCAGTCAA 20
|||||*||||||||||||||
Sbjct: 425 AACGCCACAGAACGTCAGTT 406
>miR775 Score: 1.0 RO3G_05194
Query: 1 TTGCGATGTCTTGCAGTCAA 20
|||||*||||||||||||||
Sbjct: 425 AACGCCACAGAACGTCAGTT 406
>miR775 Score: 1.0 RO3G_05303
Query: 1 TTGCGATGTCTTGCAGTCAA 20
|||||*||||||||||||||
Sbjct: 425 AACGCCACAGAACGTCAGTT 406
>miR775 Score: 1.0 RO3G_02348
Query: 1 TTGCGATGTCTTGCAGTCAA 20
|||||*||||||||||||||
Sbjct: 137 AACGCCACAGAACGTCAGTT 118
>miR775 Score: 1.0 RO3G_02354
Query: 1 TTGCGATGTCTTGCAGTCAA 20
|||||*||||||||||||||
Sbjct: 425 AACGCCACAGAACGTCAGTT 406
>miR775 Score: 2.0 RO3G_02936
Query: 1 TTGCGATGTCTTGCAGTCAA 20
||||:*||||||||||:|||
Sbjct: 425 AACGTCACAGAACGTCGGTT 406
>miR775 Score: 1.0 RO3G_04104
Query: 1 TTGCGATGTCTTGCAGTCAA 20
||:|||||:|||||||||||
Sbjct: 122 AATGCTACGGAACGTCAGTT 103
>miR775 Score: 1.0 RO3G_04110
Query: 1 TTGCGATGTCTTGCAGTCAA 20
||:|||||:|||||||||||
Sbjct: 122 AATGCTACGGAACGTCAGTT 103
>miR775 Score: 2.5 RO3G_00450
Query: 1 TTGCGATGTCTTGCAGTCAA 20
|||**|||||||||||:|||
Sbjct: 1859 AACAATACAGAACGTCGGTT 1840
>miR775 Score: 2.5 RO3G_01013
Query: 1 TTGCGATGTCTTGCAGTCAA 20
|||||*|||*|||||:||||
Sbjct: 122 AACGCCACAAAACGTTAGTT 103
>miR775 Score: 0.0 RO3G_01528
Query: 1 TTGCGATGTCTTGCAGTCAA 20
||||||||||||||||||||

```
Sbjct: 152 AACGCTACAGAACGTCAGTT 133
>miR775 Score: 1.0 RO3G_01545
Query: 1 TTGCGATGTCTTGCAGTCAA 20
|||||*||||||||||||||
Sbjct: 389 AACGCCACAGAACGTCAGTT 370
>miR775 Score: 1.0 RO3G_01636
Query: 1 TTGCGATGTCTTGCAGTCAA 20
|||||*||||||||||||||
Sbjct: 374 AACGCCACAGAACGTCAGTT 355
>miR775 Score: 2.0 RO3G_01923
Query: 1 TTGCGATGTCTTGCAGTCAA 20
|||||*|||||||*||||||
Sbjct: 374 AACGCCACAGAACATCAGTT 355
>miR7120a Score: 2.5 RO3G_10474
Query: 1 TGTTATTTGTCAGATTGATTCT 22
:|||*||||||||||||*:|*|
Sbjct: 2030 GCAAGAAACAGTCTAAC-GACA 2010
>miR5678 Score: 1.5 RO3G_16729
Query: 1 TTCCATGCATAAGACTTTCC 20
|||||||||||||*|||||*
Sbjct: 362 AAGGTACGTATTCGGAAAGA 343
>miR5678 Score: 2.0 RO3G_16751
Query: 1 TTCCATGCATAAGACTTTCC 20
||||||||||||:*|||*||
Sbjct: 83 AAGGTACGTATTTCGAATGG 64
>miR5678 Score: 0.2 RO3G_16807
Query: 1 TTCCATGCATAAGACTTTCC 20
|||||||||||||||||:||
Sbjct: 494 AAGGTACGTATTCTGAAGGG 475
>miR5678 Score: 1.0 RO3G_16551
Query: 1 TTCCATGCATAAGACTTTCC 20
||||||||||||||*|||||
Sbjct: 494 AAGGTACGTATTCTAAAAGG 475
>miR5678 Score: 1.0 RO3G_16555
Query: 1 TTCCATGCATAAGACTTTCC 20
||||||||||||||*|||||
Sbjct: 494 AAGGTACGTATTCTAAAAGG 475
>miR5678 Score: 0.2 RO3G_15675
Query: 1 TTCCATGCATAAGACTTTCC 20
|||||||||||||||||:||
Sbjct: 494 AAGGTACGTATTCTGAAGGG 475
>miR5678 Score: 1.0 RO3G_15141
Query: 1 TTCCATGCATAAGACTTTCC 20
|||||||||||||*||||||
Sbjct: 494 AAGGTACGTATTCGGAAAGG 475
```

```
>miR5678 Score: 0.2 RO3G_14359
Query: 1 TTCCATGCATAAGACTTTCC 20
|||||||||||||||||:||
Sbjct: 494 AAGGTACGTATTCTGAAGGG 475
>miR5678 Score: 2.0 RO3G_14560
Query: 1 TTCCATGCATAAGACTTTCC 20
|||||||||||*|*||||||
Sbjct: 362 AAGGTACGTATCCGGAAAGG 343
>miR5678 Score: 2.0 RO3G_13919
Query: 1 TTCCATGCATAAGACTTTCC 20
||||||||||||:*|||*||
Sbjct: 686 AAGGTACGTATTTCGAATGG 667
>miR5678 Score: 0.2 RO3G_13720
Query: 1 TTCCATGCATAAGACTTTCC 20
|||||||||||||||||:||
Sbjct: 371 AAGGTACGTATTCTGAAGGG 352
>miR5678 Score: 0.5 RO3G_13742
Query: 1 TTCCATGCATAAGACTTTCC 20
||||||:|||||||||||||
Sbjct: 494 AAGGTATGTATTCTGAAAGG 475
>miR5678 Score: 0.0 RO3G_13848
Query: 1 TTCCATGCATAAGACTTTCC 20
||||||||||||||||||||
Sbjct: 494 AAGGTACGTATTCTGAAAGG 475
>miR5678 Score: 2.0 RO3G_13858
Query: 1 TTCCATGCATAAGACTTTCC 20
|||||||||||*|*||||||
Sbjct: 125 AAGGTACGTATCCGGAAAGG 106
>miR5678 Score: 2.0 RO3G_12666
Query: 1 TTCCATGCATAAGACTTTCC 20
|||||||||||*|*||||||
Sbjct: 125 AAGGTACGTATCCGGAAAGG 106
>miR5678 Score: 2.0 RO3G_12678
Query: 1 TTCCATGCATAAGACTTTCC 20
||||||||||||:*|||*||
Sbjct: 686 AAGGTACGTATTTGGAATGG 667
>miR5678 Score: 2.0 RO3G_12954
Query: 1 TTCCATGCATAAGACTTTCC 20
|||||||||||*|*||||||
Sbjct: 494 AAGGTACGTATCCGGAAAGG 475
>miR5678 Score: 0.5 RO3G_13151
Query: 1 TTCCATGCATAAGACTTTCC 20
|||||||||||||||||*||
Sbjct: 494 AAGGTACGTATTCTGAA-GG 476
>miR5678 Score: 2.0 RO3G_13286
```

```
Query: 1 TTCCATGCATAAGACTTTCC 20
||||||||||||:*|||*||
Sbjct: 686 AAGGTACGTATTTGGAATGG 667
>miR5678 Score: 1.0 RO3G_12071
Query: 1 TTCCATGCATAAGACTTTCC 20
|||||||||||||*||||||
Sbjct: 125 AAGGTACGTATTCGGAAAGG 106
>miR5678 Score: 2.0 RO3G_12659
Query: 1 TTCCATGCATAAGACTTTCC 20
||||||||||||:*|||*||
Sbjct: 83 AAGGTACGTATTTCGAATGG 64
>miR5678 Score: 2.5 RO3G_11281
Query: 1 TTCCATGCATAAGACTTTCC 20
||||||||||||:*|||**|
Sbjct: 449 AAGGTACGTATTTGGAATAG 430
>miR5678 Score: 0.2 RO3G_11401
Query: 1 TTCCATGCATAAGACTTTCC 20
|||||||||||||||||:||
Sbjct: 494 AAGGTACGTATTCTGAAGGG 475
>miR5678 Score: 1.0 RO3G_11773
Query: 1 TTCCATGCATAAGACTTTCC 20
||||||||||||||*|||||
Sbjct: 494 AAGGTACGTATTCTAAAAGG 475
>miR5678 Score: 0.2 RO3G_10235
Query: 1 TTCCATGCATAAGACTTTCC 20
|||||||||||||||||:||
Sbjct: 494 AAGGTACGTATTCTGAAGGG 475
>miR5678 Score: 2.0 RO3G_10529
Query: 1 TTCCATGCATAAGACTTTCC 20
|||||||||||*|*||||||
Sbjct: 125 AAGGTACGTATCCGGAAAGG 106
>miR5678 Score: 0.2 RO3G_10732
Query: 1 TTCCATGCATAAGACTTTCC 20
|||||||||||||||||:||
Sbjct: 494 AAGGTACGTATTCTGAAGGG 475
>miR5678 Score: 1.0 RO3G_10762
Query: 1 TTCCATGCATAAGACTTTCC 20
|||||||||||||*||||||
Sbjct: 494 AAGGTACGTATTCGGAAAGG 475
>miR5678 Score: 2.0 RO3G_10763
Query: 1 TTCCATGCATAAGACTTTCC 20
||||||||||||:*|||*||
Sbjct: 494 AAGGTACGTATTTCGAATGG 475
>miR5678 Score: 1.0 RO3G_10770
Query: 1 TTCCATGCATAAGACTTTCC 20
```

|||||||||||||*||||||
Sbjct: 494 AAGGTACGTATTCGGAAAGG 475
>miR5678 Score: 2.0 RO3G_10787
Query: 1 TTCCATGCATAAGACTTTCC 20
||||||||||||:*|||*||
Sbjct: 494 AAGGTACGTATTTCGAATGG 475
>miR5678 Score: 0.2 RO3G_09483
Query: 1 TTCCATGCATAAGACTTTCC 20
|||||||||||||||||:||
Sbjct: 494 AAGGTACGTATTCTGAAGGG 475
>miR5678 Score: 0.2 RO3G_09499
Query: 1 TTCCATGCATAAGACTTTCC 20
|||||||||||||||||:||
Sbjct: 494 AAGGTACGTATTCTGAAGGG 475
>miR5678 Score: 0.2 RO3G_09544
Query: 1 TTCCATGCATAAGACTTTCC 20
|||||||||||||||||:||
Sbjct: 494 AAGGTACGTATTCTGAAGGG 475
>miR5678 Score: 0.0 RO3G_09607
Query: 1 TTCCATGCATAAGACTTTCC 20
||||||||||||||||||||
Sbjct: 494 AAGGTACGTATTCTGAAAGG 475
>miR5678 Score: 1.5 RO3G_08661
Query: 1 TTCCATGCATAAGACTTTCC 20
|||||||||||||*||||*|
Sbjct: 494 AAGGTACGTATTCCGAAAAG 475
>miR5678 Score: 0.0 RO3G_08846
Query: 1 TTCCATGCATAAGACTTTCC 20
||||||||||||||||||||
Sbjct: 494 AAGGTACGTATTCTGAAAGG 475
>miR5678 Score: 2.0 RO3G_08847
Query: 1 TTCCATGCATAAGACTTTCC 20
||||||||||||:*|||*||
Sbjct: 494 AAGGTACGTATTTCGAATGG 475
>miR5678 Score: 1.0 RO3G_09168
Query: 1 TTCCATGCATAAGACTTTCC 20
|||||||||||||*||||||
Sbjct: 494 AAGGTACGTATTCGGAAAGG 475
>miR5678 Score: 1.0 RO3G_07129
Query: 1 TTCCATGCATAAGACTTTCC 20
|||||||||||||*||||||
Sbjct: 362 AAGGTACGTATTCGGAAAGG 343
>miR5678 Score: 0.2 RO3G_07303
Query: 1 TTCCATGCATAAGACTTTCC 20
|||||||||||||||||:||

```
Sbjct: 494 AAGGTACGTATTCTGAAGGG 475
>miR5678 Score: 2.5 RO3G_07704
Query: 3 CCATGCATAAGACTTTCC 20
|*||||||||||||||||
Sbjct: 492 GATACGTATTCTGAAAGG 475
>miR5678 Score: 0.2 RO3G_07875
Query: 1 TTCCATGCATAAGACTTTCC 20
|||||||||||||||||:||
Sbjct: 494 AAGGTACGTATTCTGAAGGG 475
>miR5678 Score: 0.0 RO3G_05622
Query: 1 TTCCATGCATAAGACTTTCC 20
||||||||||||||||||||
Sbjct: 494 AAGGTACGTATTCTGAAAGG 475
>miR5678 Score: 0.0 RO3G_05635
Query: 1 TTCCATGCATAAGACTTTCC 20
||||||||||||||||||||
Sbjct: 494 AAGGTACGTATTCTGAAAGG 475
>miR5678 Score: 0.2 RO3G_05915
Query: 1 TTCCATGCATAAGACTTTCC 20
|||||||||||||||||:||
Sbjct: 494 AAGGTACGTATTCTGAAGGG 475
>miR5678 Score: 0.2 RO3G_06423
Query: 1 TTCCATGCATAAGACTTTCC 20
|||||||||||||||||:||
Sbjct: 548 AAGGTACGTATTCTGAAGGG 529
>miR5678 Score: 0.2 RO3G_06441
Query: 1 TTCCATGCATAAGACTTTCC 20
|||||||||||||||||:||
Sbjct: 494 AAGGTACGTATTCTGAAGGG 475
>miR5678 Score: 2.0 RO3G_04690
Query: 1 TTCCATGCATAAGACTTTCC 20
|||||||||||*|*||||||
Sbjct: 494 AAGGTACGTATCCGGAAAGG 475
>miR5678 Score: 1.0 RO3G_04787
Query: 1 TTCCATGCATAAGACTTTCC 20
|||||||||||||*||||||
Sbjct: 494 AAGGTACGTATTCGGAAAGG 475
>miR5678 Score: 0.2 RO3G_05336
Query: 1 TTCCATGCATAAGACTTTCC 20
|||||||||||||||||:||
Sbjct: 494 AAGGTACGTATTCTGAAGGG 475
>miR5678 Score: 2.0 RO3G_05350
Query: 1 TTCCATGCATAAGACTTTCC 20
|||||||||||*|*||||||
Sbjct: 362 AAGGTACGTATCCGGAAAGG 343
```

>miR5678 Score: 2.0 RO3G_05431
Query: 1 TTCCATGCATAAGACTTTCC 20
|||||||||||*|*||||||
Sbjct: 494 AAGGTACGTATCCGGAAAGG 475
>miR5678 Score: 0.2 RO3G_02689
Query: 1 TTCCATGCATAAGACTTTCC 20
|||||||||||||||||:||
Sbjct: 494 AAGGTACGTATTCTGAAGGG 475
>miR5678 Score: 1.5 RO3G_02713
Query: 1 TTCCATGCATAAGACTTTCC 20
|||||||||||||*|||||*
Sbjct: 224 AAGGTACGTATTCGGAAAGA 205
>miR5678 Score: 1.0 RO3G_02822
Query: 1 TTCCATGCATAAGACTTTCC 20
|||||||||||||*||||||
Sbjct: 494 AAGGTACGTATTCGGAAAGG 475
>miR5678 Score: 0.0 RO3G_03571
Query: 1 TTCCATGCATAAGACTTTCC 20
||||||||||||||||||||
Sbjct: 494 AAGGTACGTATTCTGAAAGG 475
>miR5678 Score: 2.0 RO3G_03602
Query: 1 TTCCATGCATAAGACTTTCC 20
||||||||||||:*|||*||
Sbjct: 635 AAGGTACGTATTTGGAATGG 616
>miR5678 Score: 1.2 RO3G_03615
Query: 1 TTCCATGCATAAGACTTTCC 20
|||||||||||||*|||:||
Sbjct: 494 AAGGTACGTATTCAGAAGGG 475
>miR5678 Score: 2.0 RO3G_03624
Query: 1 TTCCATGCATAAGACTTTCC 20
||||||||||||:*|||*||
Sbjct: 494 AAGGTACGTATTTCGAATGG 475
>miR5678 Score: 2.0 RO3G_03764
Query: 1 TTCCATGCATAAGACTTTCC 20
|||||||||||*|*||||||
Sbjct: 125 AAGGTACGTATCCGGAAAGG 106
>miR5678 Score: 2.0 RO3G_00284
Query: 1 TTCCATGCATAAGACTTTCC 20
||||||||||||:*|||*||
Sbjct: 503 AAGGTACGTATTTCGAATGG 484
>miR5678 Score: 1.5 RO3G_00304
Query: 1 TTCCATGCATAAGACTTTCC 20
|||||||||||||*|||||*
Sbjct: 494 AAGGTACGTATTCGGAAAGA 475
>miR5678 Score: 2.0 RO3G_00679

Query: 1 TTCCATGCATAAGACTTTCC 20
||||||||||||:*|||*||
Sbjct: 686 AAGGTACGTATTTGGAATGG 667
>miR5678 Score: 0.2 RO3G_00681
Query: 1 TTCCATGCATAAGACTTTCC 20
|||||||||||||||||:||
Sbjct: 494 AAGGTACGTATTCTGAAGGG 475
>miR5678 Score: 2.0 RO3G_00688
Query: 1 TTCCATGCATAAGACTTTCC 20
||||||||||||:*|||*||
Sbjct: 494 AAGGTACGTATTTGGAATGG 475
>miR5678 Score: 1.0 RO3G_01188
Query: 1 TTCCATGCATAAGACTTTCC 20
|||||||||||||*||||||
Sbjct: 362 AAGGTACGTATTCGGAAAGG 343
>miR5678 Score: 0.2 RO3G_01322
Query: 1 TTCCATGCATAAGACTTTCC 20
|||||||||||||||||:||
Sbjct: 380 AAGGTACGTATTCTGAAGGG 361
>miR5678 Score: 0.0 RO3G_01652
Query: 1 TTCCATGCATAAGACTTTCC 20
||||||||||||||||||||
Sbjct: 494 AAGGTACGTATTCTGAAAGG 475
>miR5678 Score: 0.2 RO3G_01730
Query: 1 TTCCATGCATAAGACTTTCC 20
|||||||||||||||||:||
Sbjct: 494 AAGGTACGTATTCTGAAGGG 475
>miR5678 Score: 1.2 RO3G_01956
Query: 1 TTCCATGCATAAGACTTTCC 20
|||||||||||||*|||:||
Sbjct: 494 AAGGTACGTATTCCGAAGGG 475
>miR5678 Score: 2.0 RO3G_17408
Query: 1 TTCCATGCATAAGACTTTCC 20
||||||||||||:*|||*||
Sbjct: 494 AAGGTACGTATTTGGAATGG 475
>miR5678 Score: 0.2 RO3G_17400
Query: 1 TTCCATGCATAAGACTTTCC 20
|||||||||||||||||:||
Sbjct: 368 AAGGTACGTATTCTGAAGGG 349
>miR5678 Score: 0.2 RO3G_17382
Query: 1 TTCCATGCATAAGACTTTCC 20
|||||||||||||||||:||
Sbjct: 494 AAGGTACGTATTCTGAAGGG 475
>miR5678 Score: 1.2 RO3G_17285
Query: 1 TTCCATGCATAAGACTTTCC 20

|||||||||||||*|||:||
Sbjct: 494 AAGGTACGTATTCCGAAGGG 475
>miR476 Score: 2.2 RO3G_15416
Query: 1 TGGATCCTTCTTTCAAGGTC 20
||||||||||:*:||||:||
Sbjct: 1012 ACCTAGGAAGGTGGTTCTAG 993
>miR476 Score: 2.5 RO3G_10494
Query: 1 TGGATCCTTCTTTCAAGGTC 20
|:*||||||||||||||**|
Sbjct: 1345 ATATAGGAAGAAAGTTCATG 1326
>miR476 Score: 2.5 RO3G_10759
Query: 1 TGGATCCTTCTTTCAAGGTC 20
|||||||||||:*|||:||*
Sbjct: 1835 ACCTAGGAAGAGTGTTTCAA 1816
>miR476 Score: 2.0 RO3G_08702
Query: 1 TGGATCCTTCTTTCAAGGTC 20
|||||*|||||||||||*|*
Sbjct: 397 ACCTAAGAAGAAAGTTCAAA 378
>miR476 Score: 2.5 RO3G_01272
Query: 1 TGGATCCTTCTTTCAAGGTC 20
||:||*|||||:|||||*||
Sbjct: 2428 ACTTAAGAAGAGAGTTCAAG 2409
>miR476 Score: 2.5 RO3G_02060
Query: 1 TGGATCCTTCTTTCAAGGTC 20
||||||||**||||||||*|
Sbjct: 235 ACCTAGGACCAAAGTTCC-G 217
>miR8610.2 Score: 2.5 RO3G_16381
Query: 1 TTCTTGAACAAATTGAAGGGC 21
|||||*|*|||||||||*|||
Sbjct: 420 AAGAAGTGGTTTAACTTACCG 400
>miR8610.2 Score: 2.5 RO3G_11579
Query: 1 TTCTTGAACAAATTGAAGGG-C 21
|||||||||||||||*|*::*|
Sbjct: 5532 AAGAACTTGTTTAACCTATTAG 5511
>miR8610.2 Score: 2.5 RO3G_05735
Query: 1 TTCTTGAACAAATTGAAGGGC 21
||||||||*|||:||||**||
Sbjct: 1590 AAGAACTTATTTGACTT-ACG 1571
>miR8610.2 Score: 2.5 RO3G_06550
Query: 1 TTCTTGAACAAATTGAAGGGC 21
||||||||||||||||*|*
Sbjct: 1184 TAGAACATGTTTAACTTCGCT 1164
>miR8610.2 Score: 2.5 RO3G_03165
Query: 1 TTCTTGAACAAATTGAAGGGC 21
||||||||||*|||||*|*||

Sbjct: 1056 AAGAACTTGTATAACTCCACG 1036
>miR8610.2 Score: 2.5 RO3G_01654
Query: 1 TTCTTGAACAAATTGAAG-GGC 21
||||||||||||||||*::|
Sbjct: 104 CAGAACTTCTTTAACTTCTTTG 83
>miR8610.2 Score: 2.5 RO3G_01917
Query: 1 TTCTTGAACAAATTGAAGGG-C 21
**|||||||||||||||*||*|
Sbjct: 78 TCGAACTTGTTTAACTTGCCAG 57
>miR5261 Score: 2.5 RO3G_07791
Query: 1 TCATGTCAGATGCTTTGGTCT 21
||||||||:||*|||:|||*|
Sbjct: 517 AGTACAGTTTAGGAAGCCA-A 498
>miR8658 Score: 2.0 RO3G_08552
Query: 1 TTGACAAAGTACAGAGACCAT 21
|||||||::||||||||||
Sbjct: 1183 CACTGTTTTGTGTCTCTCGTA 1163
>miR8658 Score: 1.5 RO3G_08999
Query: 1 TTGACAAAGTACAGAGACCAT 21
|||||||||:|*|||||||||
Sbjct: 435 AACTGTTTCGTATCTCTGGTA 415
>miR8658 Score: 2.0 RO3G_03092
Query: 1 TTGACAAAGTACAGAGACCAT 21
|||||||::||||||||||
Sbjct: 1183 CACTGTTTTGTGTCTCTCGTA 1163
>miR2111 Score: 0.0 RO3G_15290
Query: 1 TAAACTGTGTTTGGGTTTGG 20
||||||||||||||||||||
Sbjct: 186 ATTTGACACAAACCCAAACC 167
>miR2111 Score: 0.0 RO3G_15497
Query: 1 TAAACTGTGTTTGGGTTTGG 20
||||||||||||||||||||
Sbjct: 186 ATTTGACACAAACCCAAACC 167
>miR2111 Score: 0.5 RO3G_13307
Query: 1 TAAACTGTGTTTGGGTTTGG 20
|||||||||||||||||*||
Sbjct: 219 ATTTGACACAAACCCAACCC 200
>miR2111 Score: 0.5 RO3G_12685
Query: 1 TAAACTGTGTTTGGGTTTGG 20
||||||:|||||||||||||
Sbjct: 240 ATTTGATACAAACCCAAACC 221
>miR2111 Score: 0.8 RO3G_13022
Query: 1 TAAACTGTGTTTGGGTTTGG 20
|||||||||:|||||||||:
Sbjct: 258 ATTTGACACGAACCCAAACT 239

```
>miR2111 Score: 0.5 RO3G_12243
Query: 1 TAAACTGTGTTTGGGTTTGG 20
||||||:|||||||||||||
Sbjct: 258 ATTTGATACAAACCCAAACC 239
>miR2111 Score: 0.0 RO3G_12446
Query: 1 TAAACTGTGTTTGGGTTTGG 20
||||||||||||||||||||
Sbjct: 258 ATTTGACACAAACCCAAACC 239
>miR2111 Score: 2.0 RO3G_11292
Query: 1 TAAACTGTGTTTGGGTTTGG 20
|||||||||||||*:||||*
Sbjct: 3314 ATTTGACACAAACATAAACA 3295
>miR2111 Score: 2.5 RO3G_10271
Query: 1 TAAACTGTGTTTGGGTTTGG 20
||||||||||||||:|**::
Sbjct: 929 ATTTGACACAAACCTA-TTT 911
>miR2111 Score: 2.0 RO3G_10368
Query: 1 TAAACTGTGTTTGGGTTTGG 20
*||||||||||||*|||||*
Sbjct: 8723 TTTTGACACAAACGCAAACG 8704
>miR2111 Score: 0.5 RO3G_10657
Query: 1 TAAACTGTGTTTGGGTTTGG 20
|||||||||||||||:||||
Sbjct: 258 ATTTGACACAAACCCGAACC 239
>miR2111 Score: 2.5 RO3G_10979
Query: 1 TAAACTGTGTTTGGGTTT-GG 20
|||||*:||:||||||||*||
Sbjct: 24 ATTTGCTACGAACCCAAAGCC 4
>miR2111 Score: 1.0 RO3G_08447
Query: 1 TAAACTGTGTTTGGGTTTGG 20
||||||||||||||*|||||
Sbjct: 258 ATTTGACACAAACCAAAACC 239
>miR2111 Score: 0.2 RO3G_09087
Query: 1 TAAACTGTGTTTGGGTTTGG 20
|||||||||||||||||||:
Sbjct: 258 ATTTGACACAAACCCAAACT 239
>miR2111 Score: 0.0 RO3G_05724
Query: 1 TAAACTGTGTTTGGGTTTGG 20
||||||||||||||||||||
Sbjct: 219 ATTTGACACAAACCCAAACC 200
>miR2111 Score: 2.5 RO3G_06640
Query: 1 TAAACTGTGTTTGGGTTTGG 20
|||||*|||*||||||||*|
Sbjct: 884 ATTTGTCACTAACCCAAAAC 865
>miR2111 Score: 0.0 RO3G_04502
```

Query: 1 TAAACTGTGTTTGGGTTTGG 20
||||||||||||||||||||
Sbjct: 258 ATTTGACACAAACCCAAACC 239
>miR2111 Score: 0.0 RO3G_02942
Query: 1 TAAACTGTGTTTGGGTTTGG 20
||||||||||||||||||||
Sbjct: 258 ATTTGACACAAACCCAAACC 239
>miR2111 Score: 0.0 RO3G_03523
Query: 1 TAAACTGTGTTTGGGTTTGG 20
||||||||||||||||||||
Sbjct: 258 ATTTGACACAAACCCAAACC 239
>miR2111 Score: 0.8 RO3G_03971
Query: 1 TAAACTGTGTTTGGGTTTGG 20
|||||||||:|||||||||:
Sbjct: 258 ATTTGACACGAACCCAAACT 239
>miR2111 Score: 0.0 RO3G_04017
Query: 1 TAAACTGTGTTTGGGTTTGG 20
||||||||||||||||||||
Sbjct: 258 ATTTGACACAAACCCAAACC 239
>miR2111 Score: 2.0 RO3G_00850
Query: 1 TAAACTGTGTTTGGGTTTGG 20
|||||||||*|||:|||*||
Sbjct: 184 ATTTGACACCAACTCAA-CC 166
>miR2111 Score: 0.0 RO3G_01530
Query: 1 TAAACTGTGTTTGGGTTTGG 20
||||||||||||||||||||
Sbjct: 258 ATTTGACACAAACCCAAACC 239
>miR7817a Score: 0.0 RO3G_17165
Query: 1 TTTGGTTGAACTTGTCTCGAGAA 23
|||||||||||||||||||||||
Sbjct: 739 AAACCAACTTGAACAGAGCTCTT 717
>miR7817a Score: 0.5 RO3G_17021
Query: 1 TTTGGTTGAACTTGTCTCGAGAA 23
||||||||||||||||||||||*
Sbjct: 4198 AAACCAACTTGAACAGAGCTCTC 4176
>miR7817a Score: 0.5 RO3G_16693
Query: 1 TTTGGTTGAACTTGTCTCGAGAA 23
||||||||||||||||||||||*
Sbjct: 4363 AAACCAACTTGAACAGAGCTCTG 4341
>miR7817a Score: 0.0 RO3G_16361
Query: 1 TTTGGTTGAACTTGTCTCGAGAA 23
|||||||||||||||||||||||
Sbjct: 4198 AAACCAACTTGAACAGAGCTCTT 4176
>miR7817a Score: 0.5 RO3G_16539
Query: 1 TTTGGTTGAACTTGTCTCGAGAA 23

||||||||||||||||||||||*
Sbjct: 4255 AAACCAACTTGAACAGAGCTCTG 4233
>miR7817a Score: 0.0 RO3G_13918
Query: 1 TTTGGTTGAACTTGTCTCGAGAA 23
|||||||||||||||||||||||
Sbjct: 4363 AAACCAACTTGAACAGAGCTCTT 4341
>miR7817a Score: 0.0 RO3G_14347
Query: 1 TTTGGTTGAACTTGTCTCGAGAA 23
|||||||||||||||||||||||
Sbjct: 4294 AAACCAACTTGAACAGAGCTCTT 4272
>miR7817a Score: 0.5 RO3G_12673
Query: 1 TTTGGTTGAACTTGTCTCGAGAA 23
||||||||||||||||||||||*
Sbjct: 3499 AAACCAACTTGAACAGAGCTCTC 3477
>miR7817a Score: 0.5 RO3G_12677
Query: 1 TTTGGTTGAACTTGTCTCGAGAA 23
||||||||||||||||||||||*
Sbjct: 4198 AAACCAACTTGAACAGAGCTCTC 4176
>miR7817a Score: 0.0 RO3G_12527
Query: 1 TTTGGTTGAACTTGTCTCGAGAA 23
|||||||||||||||||||||||
Sbjct: 4363 AAACCAACTTGAACAGAGCTCTT 4341
>miR7817a Score: 0.0 RO3G_12530
Query: 1 TTTGGTTGAACTTGTCTCGAGAA 23
|||||||||||||||||||||||
Sbjct: 181 AAACCAACTTGAACAGAGCTCTT 159
>miR7817a Score: 0.5 RO3G_12538
Query: 1 TTTGGTTGAACTTGTCTCGAGAA 23
||||||||||||||||||||||*
Sbjct: 4255 AAACCAACTTGAACAGAGCTCTC 4233
>miR7817a Score: 2.2 RO3G_11232
Query: 1 TTTGGTTGAACTTGTCTCGAGAA 23
||||*||:||||||||||*|:||
Sbjct: 28 AAACGAATTTGAACAGAGATTTT 6
>miR7817a Score: 0.5 RO3G_11473
Query: 1 TTTGGTTGAACTTGTCTCGAGAA 23
||||||||||||||||||||||*
Sbjct: 181 AAACCAACTTGAACAGAGCTCTG 159
>miR7817a Score: 0.5 RO3G_10804
Query: 1 TTTGGTTGAACTTGTCTCGAGAA 23
||||||||||||||||||||||*
Sbjct: 4204 AAACCAACTTGAACAGAGCTCTC 4182
>miR7817a Score: 0.0 RO3G_09493
Query: 1 TTTGGTTGAACTTGTCTCGAGAA 23
|||||||||||||||||||||||

Sbjct: 2911 AAACCAACTTGAACAGAGCTCTT 2889
>miR7817a Score: 0.5 RO3G_09547
Query: 1 TTTGGTTGAACTTGTCTCGAGAA 23
||||||||||||||||||||||*
Sbjct: 4198 AAACCAACTTGAACAGAGCTCTC 4176
>miR7817a Score: 0.5 RO3G_08107
Query: 1 TTTGGTTGAACTTGTCTCGAGAA 23
||||||||||||||||||||||*
Sbjct: 721 AAACCAACTTGAACAGAGCTCTG 699
>miR7817a Score: 0.0 RO3G_08831
Query: 1 TTTGGTTGAACTTGTCTCGAGAA 23
|||||||||||||||||||||||
Sbjct: 109 AAACCAACTTGAACAGAGCTCTT 87
>miR7817a Score: 0.5 RO3G_07311
Query: 1 TTTGGTTGAACTTGTCTCGAGAA 23
||||||||||||||||||||||*
Sbjct: 4204 AAACCAACTTGAACAGAGCTCTC 4182
>miR7817a Score: 0.5 RO3G_06425
Query: 1 TTTGGTTGAACTTGTCTCGAGAA 23
||||||||||||||||||||||*
Sbjct: 4198 AAACCAACTTGAACAGAGCTCTC 4176
>miR7817a Score: 0.5 RO3G_04933
Query: 1 TTTGGTTGAACTTGTCTCGAGAA 23
||||||||||||||||||||||*
Sbjct: 4198 AAACCAACTTGAACAGAGCTCTC 4176
>miR7817a Score: 0.5 RO3G_05330
Query: 1 TTTGGTTGAACTTGTCTCGAGAA 23
||||||||||||||||||||||*
Sbjct: 4198 AAACCAACTTGAACAGAGCTCTC 4176
>miR7817a Score: 0.5 RO3G_05344
Query: 1 TTTGGTTGAACTTGTCTCGAGAA 23
||||||||||||||||||||||*
Sbjct: 4024 AAACCAACTTGAACAGAGCTCTC 4002
>miR7817a Score: 0.5 RO3G_05514
Query: 1 TTTGGTTGAACTTGTCTCGAGAA 23
||||||||||||||||||||||*
Sbjct: 712 AAACCAACTTGAACAGAGCTCTC 690
>miR7817a Score: 0.5 RO3G_02704
Query: 1 TTTGGTTGAACTTGTCTCGAGAA 23
||||||||||||||||||||||*
Sbjct: 4198 AAACCAACTTGAACAGAGCTCTC 4176
>miR7817a Score: 0.5 RO3G_02820
Query: 1 TTTGGTTGAACTTGTCTCGAGAA 23
||||||||||||||||||||||*
Sbjct: 181 AAACCAACTTGAACAGAGCTCTG 159

>miR7817a Score: 0.0 RO3G_03618
Query: 1 TTTGGTTGAACTTGTCTCGAGAA 23
|||||||||||||||||||||||
Sbjct: 181 AAACCAACTTGAACAGAGCTCTT 159
>miR7817a Score: 0.5 RO3G_03765
Query: 1 TTTGGTTGAACTTGTCTCGAGAA 23
||||:||||||||||||||||||
Sbjct: 181 AAACTAACTTGAACAGAGCTCTT 159
>miR7817a Score: 0.5 RO3G_03778
Query: 1 TTTGGTTGAACTTGTCTCGAGAA 23
||||||||||||||||||||||*
Sbjct: 4156 AAACCAACTTGAACAGAGCTCTC 4134
>miR7817a Score: 0.0 RO3G_04126
Query: 1 TTTGGTTGAACTTGTCTCGAGAA 23
|||||||||||||||||||||||
Sbjct: 4357 AAACCAACTTGAACAGAGCTCTT 4335
>miR7817a Score: 0.5 RO3G_00239
Query: 1 TTTGGTTGAACTTGTCTCGAGAA 23
||||||||||||||||||||||*
Sbjct: 718 AAACCAACTTGAACAGAGCTCTG 696
>miR7817a Score: 0.0 RO3G_00298
Query: 1 TTTGGTTGAACTTGTCTCGAGAA 23
|||||||||||||||||||||||
Sbjct: 718 AAACCAACTTGAACAGAGCTCTT 696
>miR7817a Score: 0.5 RO3G_00701
Query: 1 TTTGGTTGAACTTGTCTCGAGAA 23
||||||||||||||||||||||*
Sbjct: 109 AAACCAACTTGAACAGAGCTCTG 87
>miR7817a Score: 2.5 RO3G_00973
Query: 1 TTTGGTTGAACTTGTCTCGAGAA 23
||||||||*||||||||*|*||*
Sbjct: 219 AAACCAACATGAACAGAAC-CTA 198
>miR7817a Score: 0.0 RO3G_01614
Query: 1 TTTGGTTGAACTTGTCTCGAGAA 23
|||||||||||||||||||||||
Sbjct: 4204 AAACCAACTTGAACAGAGCTCTT 4182
>miR7817a Score: 0.5 RO3G_01937
Query: 1 TTTGGTTGAACTTGTCTCGAGAA 23
||||||||||||||||||||||*
Sbjct: 181 AAACCAACTTGAACAGAGCTCTG 159
>miR7817a Score: 0.5 RO3G_01966
Query: 1 TTTGGTTGAACTTGTCTCGAGAA 23
||||||||||||||||||||||*
Sbjct: 1246 AAACCAACTTGAACAGAGCTCTG 1224
>miR7817a Score: 0.5 RO3G_17422

```
Query: 1 TTTGGTTGAACTTGTCTCGAGAA 23
||||||||||||||||||||||*
Sbjct: 3499 AAACCAACTTGAACAGAGCTCTC 3477
>miR8581 Score: 2.5 RO3G_00004
Query: 1 TAGAGAATTTTGTAGACAACTG 22
:|||||||||||||:|*||*|:
Sbjct: 142 GTCTCTTAAAACATTTATT-AT 122
>miR391 Score: 0.0 RO3G_16708
Query: 1 TACGCAGAGGGAGACGTCG 19
|||||||||||||||||||
Sbjct: 1204 ATGCGTCTCCCTCTGCAGC 1186
>miR391 Score: 0.0 RO3G_15674
Query: 1 TACGCAGAGGGAGACGTCG 19
|||||||||||||||||||
Sbjct: 1555 ATGCGTCTCCCTCTGCAGC 1537
>miR391 Score: 0.0 RO3G_14622
Query: 1 TACGCAGAGGGAGACGTCG 19
|||||||||||||||||||
Sbjct: 262 ATGCGTCTCCCTCTGCAGC 244
>miR391 Score: 0.0 RO3G_13746
Query: 1 TACGCAGAGGGAGACGTCG 19
|||||||||||||||||||
Sbjct: 58 ATGCGTCTCCCTCTGCAGC 40
>miR391 Score: 0.0 RO3G_13865
Query: 1 TACGCAGAGGGAGACGTCG 19
|||||||||||||||||||
Sbjct: 2476 ATGCGTCTCCCTCTGCAGC 2458
>miR391 Score: 0.0 RO3G_12669
Query: 1 TACGCAGAGGGAGACGTCG 19
|||||||||||||||||||
Sbjct: 1204 ATGCGTCTCCCTCTGCAGC 1186
>miR391 Score: 0.0 RO3G_12961
Query: 1 TACGCAGAGGGAGACGTCG 19
|||||||||||||||||||
Sbjct: 1537 ATGCGTCTCCCTCTGCAGC 1519
>miR391 Score: 0.0 RO3G_12964
Query: 1 TACGCAGAGGGAGACGTCG 19
|||||||||||||||||||
Sbjct: 1204 ATGCGTCTCCCTCTGCAGC 1186
>miR391 Score: 0.0 RO3G_12075
Query: 1 TACGCAGAGGGAGACGTCG 19
|||||||||||||||||||
Sbjct: 1204 ATGCGTCTCCCTCTGCAGC 1186
>miR391 Score: 0.0 RO3G_11279
Query: 1 TACGCAGAGGGAGACGTCG 19
```

|||||||||||||||||||
Sbjct: 166 ATGCGTCTCCCTCTGCAGC 148
>miR391 Score: 0.0 RO3G_11952
Query: 1 TACGCAGAGGGAGACGTCG 19
|||||||||||||||||||
Sbjct: 1582 ATGCGTCTCCCTCTGCAGC 1564
>miR391 Score: 0.0 RO3G_10777
Query: 1 TACGCAGAGGGAGACGTCG 19
|||||||||||||||||||
Sbjct: 2380 ATGCGTCTCCCTCTGCAGC 2362
>miR391 Score: 0.0 RO3G_09927
Query: 1 TACGCAGAGGGAGACGTCG 19
|||||||||||||||||||
Sbjct: 1204 ATGCGTCTCCCTCTGCAGC 1186
>miR391 Score: 0.0 RO3G_09951
Query: 1 TACGCAGAGGGAGACGTCG 19
|||||||||||||||||||
Sbjct: 1462 ATGCGTCTCCCTCTGCAGC 1444
>miR391 Score: 0.0 RO3G_08609
Query: 1 TACGCAGAGGGAGACGTCG 19
|||||||||||||||||||
Sbjct: 1189 ATGCGTCTCCCTCTGCAGC 1171
>miR391 Score: 1.0 RO3G_07869
Query: 1 TACGCAGAGGGAGACGTCG 19
||||*||||||||||||||
Sbjct: 2449 ATGCATCTCCCTCTGCAGC 2431
>miR391 Score: 2.5 RO3G_05524
Query: 1 TACGCAGAGGGAGACGTCG 19
***||||||||||||||||
Sbjct: 1204 TATCGTCTCCCTCTGCAGC 1186
>miR391 Score: 0.0 RO3G_05925
Query: 1 TACGCAGAGGGAGACGTCG 19
|||||||||||||||||||
Sbjct: 1204 ATGCGTCTCCCTCTGCAGC 1186
>miR391 Score: 0.0 RO3G_05927
Query: 1 TACGCAGAGGGAGACGTCG 19
|||||||||||||||||||
Sbjct: 1204 ATGCGTCTCCCTCTGCAGC 1186
>miR391 Score: 0.0 RO3G_00684
Query: 1 TACGCAGAGGGAGACGTCG 19
|||||||||||||||||||
Sbjct: 583 ATGCGTCTCCCTCTGCAGC 565
>miR391 Score: 0.0 RO3G_01720
Query: 1 TACGCAGAGGGAGACGTCG 19
|||||||||||||||||||

Sbjct: 1189 ATGCGTCTCCCTCTGCAGC 1171
>miR399f Score: 2.0 RO3G_16425
Query: 1 TGCCAACGCGATTTGTCCTG 20
||||||:|||||||||||
Sbjct: 36 TCGGTTGTGCCAAACAGGAC 17
>miR399f Score: 2.0 RO3G_16556
Query: 1 TGCCAACGCGATTTGTCCTG 20
||||||||||||||||*|
Sbjct: 36 CCGGTTGCGCTATACAGGCC 17
>miR399f Score: 0.0 RO3G_16135
Query: 1 TGCCAACGCGATTTGTCCTG 20
||||||||||||||||||||
Sbjct: 36 ACGGTTGCGCTAAACAGGAC 17
>miR399f Score: 0.0 RO3G_15285
Query: 1 TGCCAACGCGATTTGTCCTG 20
||||||||||||||||||||
Sbjct: 36 ACGGTTGCGCTAAACAGGAC 17
>miR399f Score: 1.5 RO3G_15161
Query: 1 TGCCAACGCGATTTGTCCTG 20
|||||||:||||||||*|||
Sbjct: 36 ACGGTTGTGCTAAACAAGAC 17
>miR399f Score: 2.0 RO3G_15255
Query: 1 TGCCAACGCGATTTGTCCTG 20
||||||:|||||||||||
Sbjct: 36 TCGGTTGTGCCAAACAGGAC 17
>miR399f Score: 2.0 RO3G_14494
Query: 1 TGCCAACGCGATTTGTCCTG 20
||||||||||||||||*|
Sbjct: 36 CCGGTTGCGCTATACAGGCC 17
>miR399f Score: 2.0 RO3G_14628
Query: 1 TGCCAACGCGATTTGTCCTG 20
||||||||||||||||*|
Sbjct: 36 CCGGTTGCGCTATACAGGCC 17
>miR399f Score: 2.0 RO3G_12170
Query: 1 TGCCAACGCGATTTGTCCTG 20
||||||:|||||||||||
Sbjct: 36 TCGGTTGTGCCAAACAGGAC 17
>miR399f Score: 0.0 RO3G_11770
Query: 1 TGCCAACGCGATTTGTCCTG 20
||||||||||||||||||||
Sbjct: 36 ACGGTTGCGCTAAACAGGAC 17
>miR399f Score: 0.0 RO3G_10523
Query: 1 TGCCAACGCGATTTGTCCTG 20
||||||||||||||||||||
Sbjct: 36 ACGGTTGCGCTAAACAGGAC 17

>miR399f Score: 0.0 RO3G_09599
Query: 1 TGCCAACGCGATTTGTCCTG 20
||||||||||||||||||||
Sbjct: 36 ACGGTTGCGCTAAACAGGAC 17
>miR399f Score: 0.0 RO3G_09932
Query: 1 TGCCAACGCGATTTGTCCTG 20
||||||||||||||||||||
Sbjct: 36 ACGGTTGCGCTAAACAGGAC 17
>miR399f Score: 2.0 RO3G_09935
Query: 1 TGCCAACGCGATTTGTCCTG 20
||||||||||||||||*|
Sbjct: 36 CCGGTTGCGCTATACAGGCC 17
>miR399f Score: 1.0 RO3G_09956
Query: 1 TGCCAACGCGATTTGTCCTG 20
|||*||||||||||||||||
Sbjct: 36 ACGATTGCGCTAAACAGGAC 17
>miR399f Score: 1.5 RO3G_07726
Query: 1 TGCCAACGCGATTTGTCCTG 20
|||||||:||||||||*|||
Sbjct: 36 ACGGTTGTGCTAAACAAGAC 17
>miR399f Score: 2.0 RO3G_07843
Query: 1 TGCCAACGCGATTTGTCCTG 20
||||||||||||||||*|
Sbjct: 36 CCGGTTGCGCTATACAGGCC 17
>miR399f Score: 1.5 RO3G_05912
Query: 1 TGCCAACGCGATTTGTCCTG 20
|||||||:||*|||||||||
Sbjct: 30 ACGGTTGTGCAAAACAGGAC 11
>miR399f Score: 2.0 RO3G_06412
Query: 1 TGCCAACGCGATTTGTCCTG 20
||||||||||||||||*|
Sbjct: 36 CCGGTTGCGCTATACAGGCC 17
>miR399f Score: 2.5 RO3G_04734
Query: 1 TGCCAACGCGATTTGTCCTG 20
||||||||||:||||||*
Sbjct: 516 CCAGTTGCGCTAGACAGGAA 497
>miR399f Score: 0.0 RO3G_03105
Query: 1 TGCCAACGCGATTTGTCCTG 20
||||||||||||||||||||
Sbjct: 36 ACGGTTGCGCTAAACAGGAC 17
>miR399f Score: 2.0 RO3G_04214
Query: 1 TGCCAACGCGATTTGTCCTG 20
||||||||||||||||*|
Sbjct: 36 CCGGTTGCGCTATACAGGCC 17
>miR399f Score: 0.0 RO3G_04217

Query: 1 TGCCAACGCGATTTGTCCTG 20
||||||||||||||||||||
Sbjct: 36 ACGGTTGCGCTAAACAGGAC 17
>miR399f Score: 2.0 RO3G_01715
Query: 1 TGCCAACGCGATTTGTCCTG 20
||||||:|||||||||||
Sbjct: 36 TCGGTTGTGCCAAACAGGAC 17
>miR399f Score: 2.0 RO3G_01882
Query: 1 TGCCAACGCGATTTGTCCTG 20
||||||||||||||||*|
Sbjct: 36 CCGGTTGCGCTATACAGGCC 17
>miR399f Score: 2.0 RO3G_01885
Query: 1 TGCCAACGCGATTTGTCCTG 20
||||||||||||||||*|
Sbjct: 36 CCGGTTGCGCTATACAGGCC 17
>miR399f Score: 0.0 RO3G_01941
Query: 1 TGCCAACGCGATTTGTCCTG 20
||||||||||||||||||||
Sbjct: 36 ACGGTTGCGCTAAACAGGAC 17
>miR399f Score: 2.0 RO3G_17339
Query: 1 TGCCAACGCGATTTGTCCTG 20
||||||||||||||||*|
Sbjct: 36 CCGGTTGCGCTATACAGGCC 17
>miR399f Score: 2.0 RO3G_17290
Query: 1 TGCCAACGCGATTTGTCCTG 20
||||||||||||||||*|
Sbjct: 36 TCGGTTGCGCTATACAGGCC 17
>miR824-5p Score: 2.5 RO3G_05999
Query: 1 TAGACCATTTGTGAGAAG-GG 20
|||||||||||::|:||:*:|
Sbjct: 48 ATCTGGTAAACGTTTTTTGTC 28
>miR824-5p Score: 2.5 RO3G_06174
Query: 1 TAGACCATTTGTGAGAAGGG 20
:|:|*||||:|||||||:||
Sbjct: 178 GTTTTGTAAGCACTCTTTCC 159
>miR824-5p Score: 2.5 RO3G_01069
Query: 1 TAGACCATTTGTGAGAAGGG 20
*|:|||||||||||:||**|
Sbjct: 2009 CTTTGGTAAACACTTTTAGC 1990
>miR824-5p Score: 2.2 RO3G_01701
Query: 1 TAGACCATTTGTGAGAAGG-G 20
|||||||||:*|||||||:*|
Sbjct: 136 ATCTGGTAAGAACTCTTCTGC 116
>miR5632-3p Score: 2.0 RO3G_17202
Query: 1 TTGGATTTAAGTCTGGATACT 21

|*||||||||||||||*|*|
Sbjct: 814 ATCCTAAATTCAGACCTCTCA 794
>miR5632-3p Score: 2.0 RO3G_16829
Query: 1 TTGGATTTAAGTCTGGATACT 21
|*||||||||||||||*|*|
Sbjct: 814 ATCCTAAATTCAGACCTCTCA 794
>miR5632-3p Score: 2.0 RO3G_16904
Query: 1 TTGGATTTAAGTCTGGATACT 21
|*||||||||||||||*|*|
Sbjct: 814 ATCCTAAATTCAGACCTCTCA 794
>miR5632-3p Score: 1.0 RO3G_16719
Query: 1 TTGGATTTAAGTCTGGATACT 21
||||||||||||||||**||
Sbjct: 832 AACCTAAATTCAGACCTCCGA 812
>miR5632-3p Score: 1.0 RO3G_16727
Query: 1 TTGGATTTAAGTCTGGATACT 21
||||||||||||||||**||
Sbjct: 832 AACCTAAATTCAGACCTCCGA 812
>miR5632-3p Score: 1.2 RO3G_16737
Query: 1 TTGGATTTAAGTCTGGATACT 21
||||||||||||:|||:*||
Sbjct: 460 AACCTAAATTCAGGCCTGAGA 440
>miR5632-3p Score: 1.5 RO3G_16751
Query: 1 TTGGATTTAAGTCTGGATACT 21
|||||||||||||||**|||
Sbjct: 421 AACCTAAATTCAGACCCTTGA 401
>miR5632-3p Score: 2.0 RO3G_16428
Query: 1 TTGGATTTAAGTCTGGATACT 21
|*||||||||||||||*|*|
Sbjct: 451 ATCCTAAATTCAGACCTCTCA 431
>miR5632-3p Score: 1.2 RO3G_16541
Query: 1 TTGGATTTAAGTCTGGATACT 21
||||||||||||:|||:*||
Sbjct: 460 AACCTAAATTCAGGCCTGAGA 440
>miR5632-3p Score: 0.5 RO3G_16140
Query: 1 TTGGATTTAAGTCTGGATACT 21
||||||||||||||||*|||
Sbjct: 271 AACCTAAATTCAGACCTTTGA 251
>miR5632-3p Score: 1.2 RO3G_16141
Query: 1 TTGGATTTAAGTCTGGATACT 21
||||||||||||:|||:*||
Sbjct: 460 AACCTAAATTCAGGCCTGAGA 440
>miR5632-3p Score: 2.2 RO3G_16121
Query: 1 TTGGATTTAAGTCTGGATACT 21
|*||||||||||:|||:*||

Sbjct: 415 ACCCTAAATTCAGGCCTGAGA 395
>miR5632-3p Score: 1.2 RO3G_16124
Query: 1 TTGGATTTAAGTCTGGATACT 21
|||||||||||||:|||:*||
Sbjct: 460 AACCTAAATTCAGGCCTGAGA 440
>miR5632-3p Score: 1.2 RO3G_15286
Query: 1 TTGGATTTAAGTCTGGATACT 21
|||||||||||||:|||:*||
Sbjct: 460 AACCTAAATTCAGGCCTGAGA 440
>miR5632-3p Score: 0.5 RO3G_15582
Query: 1 TTGGATTTAAGTCTGGATACT 21
|||||||||||||||||*|||
Sbjct: 211 AACCTAAATTCAGACCTCTGA 191
>miR5632-3p Score: 1.2 RO3G_14981
Query: 1 TTGGATTTAAGTCTGGATACT 21
|||||||||||||:|||:*||
Sbjct: 460 AACCTAAATTCAGGCCTGAGA 440
>miR5632-3p Score: 1.2 RO3G_15145
Query: 1 TTGGATTTAAGTCTGGATACT 21
|||||||||||||:|||:*||
Sbjct: 460 AACCTAAATTCAGGCCTGAGA 440
>miR5632-3p Score: 1.2 RO3G_15170
Query: 1 TTGGATTTAAGTCTGGATACT 21
|||||||||||||:|||:*||
Sbjct: 460 AACCTAAATTCAGGCCTGAGA 440
>miR5632-3p Score: 1.2 RO3G_14784
Query: 1 TTGGATTTAAGTCTGGATACT 21
|||||||||||||:|||:*||
Sbjct: 460 AACCTAAATTCAGGCCTGAGA 440
>miR5632-3p Score: 0.0 RO3G_13919
Query: 1 TTGGATTTAAGTCTGGATACT 21
|||||||||||||||||||||
Sbjct: 1024 AACCTAAATTCAGACCTATGA 1004
>miR5632-3p Score: 0.5 RO3G_13453
Query: 1 TTGGATTTAAGTCTGGATACT 21
|||||||||||||||||*|||
Sbjct: 211 AACCTAAATTCAGACCTCTGA 191
>miR5632-3p Score: 2.0 RO3G_13734
Query: 1 TTGGATTTAAGTCTGGATACT 21
|*|||||||||||||||*|*|
Sbjct: 814 ATCCTAAATTCAGACCTCTCA 794
>miR5632-3p Score: 1.2 RO3G_13752
Query: 1 TTGGATTTAAGTCTGGATACT 21
|||||||||||||:|||:*||
Sbjct: 460 AACCTAAATTCAGGCCTGAGA 440

```
>miR5632-3p Score: 2.5 RO3G_13855
Query: 1 TTGGATTTAAGTCTGGATACT 21
||**|||||||||||||||*|
Sbjct: 2962 AAAATAAATTCAGACCTATTA 2942
>miR5632-3p Score: 1.2 RO3G_12950
Query: 1 TTGGATTTAAGTCTGGATACT 21
|||||||||||||:|||:*||
Sbjct: 460 AACCTAAATTCAGGCCTGAGA 440
>miR5632-3p Score: 1.0 RO3G_13286
Query: 1 TTGGATTTAAGTCTGGATACT 21
|||||||||||||||||**||
Sbjct: 1024 AACCTAAATTCAGACCTCCGA 1004
>miR5632-3p Score: 2.5 RO3G_12067
Query: 1 TTGGATTTAAGTCTGGATACT 21
||**|||||||||||||||*|
Sbjct: 2962 AAAATAAATTCAGACCTATTA 2942
>miR5632-3p Score: 0.5 RO3G_12166
Query: 1 TTGGATTTAAGTCTGGATACT 21
|||||||||||||||||*|||
Sbjct: 832 AACCTAAATTCAGACCTTTGA 812
>miR5632-3p Score: 0.5 RO3G_12659
Query: 1 TTGGATTTAAGTCTGGATACT 21
|||||||||||||||||*|||
Sbjct: 421 AACCTAAATTCAGACCTCTGA 401
>miR5632-3p Score: 0.5 RO3G_11281
Query: 1 TTGGATTTAAGTCTGGATACT 21
|||||||||||||||||*|||
Sbjct: 787 AACCTAAATTCAGACCTCTGA 767
>miR5632-3p Score: 2.0 RO3G_11411
Query: 1 TTGGATTTAAGTCTGGATACT 21
|*|||||||||||||||*|*|
Sbjct: 451 ATCCTAAATTCAGACCTCTCA 431
>miR5632-3p Score: 1.2 RO3G_11533
Query: 1 TTGGATTTAAGTCTGGATACT 21
|||||||||||||:|||:*||
Sbjct: 460 AACCTAAATTCAGGCCTGAGA 440
>miR5632-3p Score: 1.2 RO3G_11536
Query: 1 TTGGATTTAAGTCTGGATACT 21
|||||||||||||:|||:*||
Sbjct: 460 AACCTAAATTCAGGCCTGAGA 440
>miR5632-3p Score: 2.2 RO3G_10237
Query: 1 TTGGATTTAAGTCTGGATACT 21
|*|||||||||||:|||:*||
Sbjct: 400 ACCCTAAATTCAGGCCTGAGA 380
>miR5632-3p Score: 0.5 RO3G_10550
```

```
Query: 1 TTGGATTTAAGTCTGGATACT 21
|||||||||||||||||*|||
Sbjct: 832 AACCTAAATTCAGACCTTTGA 812
>miR5632-3p Score: 1.5 RO3G_10768
Query: 1 TTGGATTTAAGTCTGGATACT 21
|||*|||||||||||||*|||
Sbjct: 37 AACGTAAATTCAGACCTCTGA 17
>miR5632-3p Score: 2.0 RO3G_10772
Query: 1 TTGGATTTAAGTCTGGATACT 21
|||||||:|||||*|||*|||
Sbjct: 721 AACCTAAGTTCAGTCCTCTGA 701
>miR5632-3p Score: 0.5 RO3G_10787
Query: 1 TTGGATTTAAGTCTGGATACT 21
|||||||||||||||||*|||
Sbjct: 832 AACCTAAATTCAGACCTCTGA 812
>miR5632-3p Score: 1.2 RO3G_10797
Query: 1 TTGGATTTAAGTCTGGATACT 21
|||||||||||||:|||:*||
Sbjct: 460 AACCTAAATTCAGGCCTGAGA 440
>miR5632-3p Score: 1.2 RO3G_09926
Query: 1 TTGGATTTAAGTCTGGATACT 21
|||||||||||||:|||:*||
Sbjct: 460 AACCTAAATTCAGGCCTGAGA 440
>miR5632-3p Score: 1.2 RO3G_10050
Query: 1 TTGGATTTAAGTCTGGATACT 21
|||||||||||||:|||:*||
Sbjct: 460 AACCTAAATTCAGGCCTGAGA 440
>miR5632-3p Score: 0.5 RO3G_10220
Query: 1 TTGGATTTAAGTCTGGATACT 21
|||||||||||||||||*|||
Sbjct: 271 AACCTAAATTCAGACCTCTGA 251
>miR5632-3p Score: 2.0 RO3G_08657
Query: 1 TTGGATTTAAGTCTGGATACT 21
|||||||:|||||*|||*|||
Sbjct: 724 AACCTAAGTTCAGTCCTCTGA 704
>miR5632-3p Score: 0.5 RO3G_08847
Query: 1 TTGGATTTAAGTCTGGATACT 21
|||||||||||||||||*|||
Sbjct: 832 AACCTAAATTCAGACCTTTGA 812
>miR5632-3p Score: 1.2 RO3G_07136
Query: 1 TTGGATTTAAGTCTGGATACT 21
|||||||||||||:|||:*||
Sbjct: 151 AACCTAAATTCAGGCCTGAGA 131
>miR5632-3p Score: 2.2 RO3G_07298
Query: 1 TTGGATTTAAGTCTGGATACT 21
```

|*|||||||||||:|||:*||
Sbjct: 460 ACCCTAAATTCAGGCCTGAGA 440
>miR5632-3p Score: 0.0 RO3G_07690
Query: 1 TTGGATTTAAGTCTGGATACT 21
|||||||||||||||||||||
Sbjct: 832 AACCTAAATTCAGACCTATGA 812
>miR5632-3p Score: 0.0 RO3G_07707
Query: 1 TTGGATTTAAGTCTGGATACT 21
|||||||||||||||||||||
Sbjct: 1024 AACCTAAATTCAGACCTATGA 1004
>miR5632-3p Score: 1.2 RO3G_07723
Query: 1 TTGGATTTAAGTCTGGATACT 21
|||||||||||||:|||:*||
Sbjct: 460 AACCTAAATTCAGGCCTGAGA 440
>miR5632-3p Score: 1.2 RO3G_05522
Query: 1 TTGGATTTAAGTCTGGATACT 21
|||||||||||||:|||:*||
Sbjct: 826 AACCTAAATTCAGGCCTGAGA 806
>miR5632-3p Score: 2.0 RO3G_05945
Query: 1 TTGGATTTAAGTCTGGATACT 21
|*|||||||||||||||*|*|
Sbjct: 451 ATCCTAAATTCAGACCTCTCA 431
>miR5632-3p Score: 2.5 RO3G_06322
Query: 1 TTGGATTTAAGTCTGGATACT 21
|||||||*||||*|||||*||
Sbjct: 331 AACCTAACTTCAAACCTACGA 311
>miR5632-3p Score: 2.2 RO3G_04668
Query: 1 TTGGATTTAAGTCTGGATACT 21
|*|||||||||||:|||:*||
Sbjct: 637 ACCCTAAATTCAGGCCTGAGA 617
>miR5632-3p Score: 1.2 RO3G_05516
Query: 1 TTGGATTTAAGTCTGGATACT 21
|||||||||||||:|||:*||
Sbjct: 460 AACCTAAATTCAGGCCTGAGA 440
>miR5632-3p Score: 0.5 RO3G_02846
Query: 1 TTGGATTTAAGTCTGGATACT 21
|||||||||||||||||*|||
Sbjct: 37 AACCTAAATTCAGACCTCTGA 17
>miR5632-3p Score: 1.2 RO3G_03112
Query: 1 TTGGATTTAAGTCTGGATACT 21
|||||||||||||:|||:*||
Sbjct: 826 AACCTAAATTCAGGCCTGAGA 806
>miR5632-3p Score: 2.0 RO3G_03554
Query: 1 TTGGATTTAAGTCTGGATACT 21
|||||||:|||||*|||*|||

```
Sbjct: 724 AACCTAAGTTCAGTCCTCTGA 704
>miR5632-3p Score: 1.2 RO3G_03560
Query: 1 TTGGATTTAAGTCTGGATACT 21
|||||||||||||:|||:*||
Sbjct: 460 AACCTAAATTCAGGCCTGAGA 440
>miR5632-3p Score: 2.5 RO3G_03575
Query: 1 TTGGATTTAAGTCTGGATACT 21
||**|||||||||||||||*|
Sbjct: 2962 AAAATAAATTCAGACCTATTA 2942
>miR5632-3p Score: 1.0 RO3G_03603
Query: 1 TTGGATTTAAGTCTGGATACT 21
|||||||||||||||:|*|||
Sbjct: 271 AACCTAAATTCAGACTTTTGA 251
>miR5632-3p Score: 1.0 RO3G_03624
Query: 1 TTGGATTTAAGTCTGGATACT 21
|||||||||||||||:|*|||
Sbjct: 832 AACCTAAATTCAGACTTCTGA 812
>miR5632-3p Score: 1.2 RO3G_03791
Query: 1 TTGGATTTAAGTCTGGATACT 21
|||||||||||||:|||:*||
Sbjct: 460 AACCTAAATTCAGGCCTGAGA 440
>miR5632-3p Score: 2.5 RO3G_03796
Query: 1 TTGGATTTAAGTCTGGATACT 21
||**|||||||||||||||*|
Sbjct: 2155 AAAATAAATTCAGACCTATTA 2135
>miR5632-3p Score: 1.2 RO3G_00240
Query: 1 TTGGATTTAAGTCTGGATACT 21
|||||||||||||:|||:*||
Sbjct: 769 AACCTAAATTCAGGCCTGAGA 749
>miR5632-3p Score: 1.0 RO3G_00284
Query: 1 TTGGATTTAAGTCTGGATACT 21
|||||||||||||||||**||
Sbjct: 841 AACCTAAATTCAGACCTCCGA 821
>miR5632-3p Score: 2.2 RO3G_00675
Query: 1 TTGGATTTAAGTCTGGATACT 21
|*|||||||||||:|||:*||
Sbjct: 460 ACCCTAAATTCAGGCCTGAGA 440
>miR5632-3p Score: 0.5 RO3G_00679
Query: 1 TTGGATTTAAGTCTGGATACT 21
|||||||||||||||||*|||
Sbjct: 1024 AACCTAAATTCAGACCTCTGA 1004
>miR5632-3p Score: 0.5 RO3G_00700
Query: 1 TTGGATTTAAGTCTGGATACT 21
|||||||||||||||||*|||
Sbjct: 271 AACCTAAATTCAGACCTCTGA 251
```

>miR5632-3p Score: 1.2 RO3G_01725
Query: 1 TTGGATTTAAGTCTGGATACT 21
|||||||||||||:|||:*||
Sbjct: 460 AACCTAAATTCAGGCCTGAGA 440
>miR5632-3p Score: 1.0 RO3G_01959
Query: 1 TTGGATTTAAGTCTGGATACT 21
|||||||||||||||||**||
Sbjct: 271 AACCTAAATTCAGACCTCCGA 251
>miR5632-3p Score: 2.5 RO3G_02224
Query: 1 TTGGATTTAAGTCTGGATACT 21
|*|||||||||:|||||*|*|
Sbjct: 451 ATCCTAAATTCGGACCTCTCA 431
>miR5632-3p Score: 1.2 RO3G_02227
Query: 1 TTGGATTTAAGTCTGGATACT 21
|||||||||||||:|||:*||
Sbjct: 826 AACCTAAATTCAGGCCTGAGA 806
>miR5632-3p Score: 1.2 RO3G_17423
Query: 1 TTGGATTTAAGTCTGGATACT 21
|||||||||||||:|||:*||
Sbjct: 460 AACCTAAATTCAGGCCTGAGA 440
>miR5632-3p Score: 0.5 RO3G_17408
Query: 1 TTGGATTTAAGTCTGGATACT 21
|||||||||||||||||*|||
Sbjct: 832 AACCTAAATTCAGACCTCTGA 812
>miR5632-3p Score: 1.2 RO3G_17328
Query: 1 TTGGATTTAAGTCTGGATACT 21
|||||||||||||:|||:*||
Sbjct: 460 AACCTAAATTCAGGCCTGAGA 440
>miR5632-3p Score: 1.2 RO3G_17092
Query: 1 TTGGATTTAAGTCTGGATACT 21
|||||||||||||:|||:*||
Sbjct: 460 AACCTAAATTCAGGCCTGAGA 440
>miR5632-3p Score: 2.0 RO3G_17154
Query: 1 TTGGATTTAAGTCTGGATACT 21
|*|||||||||||||||*|*|
Sbjct: 451 ATCCTAAATTCAGACCTCTCA 431
>miR475a-3p Score: 0.0 RO3G_16086
Query: 1 TTAATGCCCATTGACTTCAG 20
||||||||||||||||||||
Sbjct: 1844 AATTACGGGTAACTGAAGTC 1825
>miR475a-3p Score: 0.0 RO3G_15802
Query: 1 TTAATGCCCATTGACTTCAG 20
||||||||||||||||||||
Sbjct: 3428 AATTACGGGTAACTGAAGTC 3409
>miR475a-3p Score: 0.0 RO3G_14949

Query: 1 TTAATGCCCATTGACTTCAG 20
||||||||||||||||||||
Sbjct: 3050 AATTACGGGTAACTGAAGTC 3031
>miR475a-3p Score: 0.0 RO3G_12567
Query: 1 TTAATGCCCATTGACTTCAG 20
||||||||||||||||||||
Sbjct: 3428 AATTACGGGTAACTGAAGTC 3409
>miR475a-3p Score: 0.0 RO3G_06775
Query: 1 TTAATGCCCATTGACTTCAG 20
||||||||||||||||||||
Sbjct: 3428 AATTACGGGTAACTGAAGTC 3409
>miR475a-3p Score: 0.0 RO3G_07854
Query: 1 TTAATGCCCATTGACTTCAG 20
||||||||||||||||||||
Sbjct: 3428 AATTACGGGTAACTGAAGTC 3409
>miR475a-3p Score: 0.0 RO3G_04252
Query: 1 TTAATGCCCATTGACTTCAG 20
||||||||||||||||||||
Sbjct: 3428 AATTACGGGTAACTGAAGTC 3409
>miR475a-3p Score: 0.0 RO3G_04496
Query: 1 TTAATGCCCATTGACTTCAG 20
||||||||||||||||||||
Sbjct: 3050 AATTACGGGTAACTGAAGTC 3031
>miR475a-3p Score: 0.0 RO3G_02364
Query: 1 TTAATGCCCATTGACTTCAG 20
||||||||||||||||||||
Sbjct: 3428 AATTACGGGTAACTGAAGTC 3409
>miR475a-3p Score: 1.5 RO3G_01156
Query: 1 TTAATGCCCATTGACTTCAG 20
|||||:||*|||||||||||
Sbjct: 797 AATTATGGTTAACTGAAGTC 778
>miR475a-3p Score: 2.0 RO3G_16981
Query: 1 TTAATGCCCATTGACTTCAG 20
||||||||*|||||*|||||
Sbjct: 323 AATTACGGTTAACTTAAGTC 304
>miR475a-3p Score: 2.0 RO3G_16987
Query: 1 TTAATGCCCATTGACTTCAG 20
||||||||*|||||*|||||
Sbjct: 305 AATTACGGTTAACTTAAGTC 286
>miR8044-5p Score: 0.0 RO3G_17226
Query: 1 TTGTCAAAATGGTTGGAGATTT 22
||||||||||||||||||||||
Sbjct: 1864 AACAGTTTTACCAACCTCTAAA 1843
>miR8044-5p Score: 0.0 RO3G_16815
Query: 1 TTGTCAAAATGGTTGGAGATTT 22

||||||||||||||||||||||
Sbjct: 1864 AACAGTTTTACCAACCTCTAAA 1843
>miR8044-5p Score: 0.0 RO3G_14795
Query: 1 TTGTCAAAATGGTTGGAGATTT 22
||||||||||||||||||||||
Sbjct: 1864 AACAGTTTTACCAACCTCTAAA 1843
>miR8044-5p Score: 2.5 RO3G_11557
Query: 1 TTGTCAAAATGGTTGGAGATTT 22
|:||||||||:|||||||||
Sbjct: 383 TATAGTTTTACTAACC-CTAAA 363
>miR8044-5p Score: 2.2 RO3G_07485
Query: 1 TTGTCAAAATGGTTGGAGATTT 22
:||||||*||:||||||*||||
Sbjct: 977 GACAGTTCTATCAACCTGTAAA 956
>miR8044-5p Score: 2.2 RO3G_01429
Query: 1 TTGTCAAAATGGTTGGAGATTT 22
:||||||*||:||||||*||||
Sbjct: 1565 GACAGTTCTATCAACCTGTAAA 1544
>miR4240 Score: 2.5 RO3G_16915
Query: 1 TCGACTGACTCGTAACATTTC 21
|||||*||*||:|||||||||
Sbjct: 218 AGCTGTCTAAGTATTGTAAAG 198
>miR4240 Score: 2.5 RO3G_16752
Query: 1 TCGACTGACTCGTAACATTTC 21
|||||*||*||:|||||||||
Sbjct: 419 AGCTGTCTAAGTATTGTAAAG 399
>miR4240 Score: 0.0 RO3G_15274
Query: 1 TCGACTGACTCGTAACATTTC 21
|||||||||||||||||||||
Sbjct: 449 AGCTGACTGAGCATTGTAAAG 429
>miR4240 Score: 0.0 RO3G_13916
Query: 1 TCGACTGACTCGTAACATTTC 21
|||||||||||||||||||||
Sbjct: 449 AGCTGACTGAGCATTGTAAAG 429
>miR4240 Score: 2.5 RO3G_14292
Query: 1 TCGACTGACTCGTAACATTTC 21
|||*|:|||||||||||*|*|
Sbjct: 706 AGCCGGCTGAGCATTGT-ACG 687
>miR4240 Score: 2.5 RO3G_13144
Query: 1 TCGACTGACTCGTAACATTTC 21
|||||*||*||:|||||||||
Sbjct: 419 AGCTGTCTAAGTATTGTAAAG 399
>miR4240 Score: 0.0 RO3G_12541
Query: 1 TCGACTGACTCGTAACATTTC 21
|||||||||||||||||||||

```
Sbjct: 449 AGCTGACTGAGCATTGTAAAG 429
>miR4240 Score: 0.0 RO3G_11272
Query: 1 TCGACTGACTCGTAACATTTC 21
|||||||||||||||||||||
Sbjct: 449 AGCTGACTGAGCATTGTAAAG 429
>miR4240 Score: 0.0 RO3G_11784
Query: 1 TCGACTGACTCGTAACATTTC 21
|||||||||||||||||||||
Sbjct: 449 AGCTGACTGAGCATTGTAAAG 429
>miR4240 Score: 2.5 RO3G_10860
Query: 1 TCGACTGACTCGTAACATTTC 21
||||||||*||:||*||||||
Sbjct: 449 AGCTGACTAAGTATCGTAAAG 429
>miR4240 Score: 0.0 RO3G_09496
Query: 1 TCGACTGACTCGTAACATTTC 21
|||||||||||||||||||||
Sbjct: 449 AGCTGACTGAGCATTGTAAAG 429
>miR4240 Score: 2.5 RO3G_08111
Query: 1 TCGACTGACTCGTAACATTTC 21
||||||||*||:||*||||||
Sbjct: 449 AGCTGACTAAGTATCGTAAAG 429
>miR4240 Score: 2.5 RO3G_05353
Query: 1 TCGACTGACTCGTAACATTTC 21
|||||*||*||:|||||||||
Sbjct: 419 AGCTGTCTAAGTATTGTAAAG 399
>miR4240 Score: 2.5 RO3G_02834
Query: 1 TCGACTGACTCGTAACATTTC 21
|||||*||*||:|||||||||
Sbjct: 218 AGCTGTCTAAGTATTGTAAAG 198
>miR4240 Score: 2.5 RO3G_17289
Query: 1 TCGACTGACTCGTAACATTTC 21
||||||||*||:||*||||||
Sbjct: 449 AGCTGACTAAGTATCGTAAAG 429
>miR6443 Score: 0.0 RO3G_17204
Query: 1 GTAAGGTCAAGATGATGAG 19
|||||||||||||||||||
Sbjct: 410 CATTCCAGTTCTACTACTC 392
>miR6443 Score: 0.0 RO3G_16825
Query: 1 GTAAGGTCAAGATGATGAG 19
|||||||||||||||||||
Sbjct: 347 CATTCCAGTTCTACTACTC 329
>miR6443 Score: 0.0 RO3G_16905
Query: 1 GTAAGGTCAAGATGATGAG 19
|||||||||||||||||||
Sbjct: 347 CATTCCAGTTCTACTACTC 329
```

>miR6443 Score: 2.5 RO3G_16737
Query: 1 GTAAGGTCAAGATGATGAG 19
|||||*||*||||||||*|
Sbjct: 170 CATTCAAGATCTACTACGC 152
>miR6443 Score: 0.0 RO3G_16538
Query: 1 GTAAGGTCAAGATGATGAG 19
|||||||||||||||||||
Sbjct: 425 CATTCCAGTTCTACTACTC 407
>miR6443 Score: 2.5 RO3G_16541
Query: 1 GTAAGGTCAAGATGATGAG 19
|||||*||*||||||||*|
Sbjct: 170 CATTCAAGATCTACTACGC 152
>miR6443 Score: 2.5 RO3G_16141
Query: 1 GTAAGGTCAAGATGATGAG 19
|||||*||*||||||||*|
Sbjct: 170 CATTCAAGATCTACTACGC 152
>miR6443 Score: 1.5 RO3G_16121
Query: 1 GTAAGGTCAAGATGATGAG 19
||||:|||*||||||||||
Sbjct: 125 CATTTCAGATCTACTACTC 107
>miR6443 Score: 0.0 RO3G_15672
Query: 1 GTAAGGTCAAGATGATGAG 19
|||||||||||||||||||
Sbjct: 347 CATTCCAGTTCTACTACTC 329
>miR6443 Score: 2.5 RO3G_15286
Query: 1 GTAAGGTCAAGATGATGAG 19
|||||*||*||||||||*|
Sbjct: 170 CATTCAAGATCTACTACGC 152
>miR6443 Score: 2.5 RO3G_15432
Query: 1 GTAAGGTCAAGATGATGAG 19
|:|||||||*||:||:|||
Sbjct: 908 CGTTCCAGTGCTGCTGCTC 890
>miR6443 Score: 0.0 RO3G_14974
Query: 1 GTAAGGTCAAGATGATGAG 19
|||||||||||||||||||
Sbjct: 413 CATTCCAGTTCTACTACTC 395
>miR6443 Score: 2.5 RO3G_14981
Query: 1 GTAAGGTCAAGATGATGAG 19
|||||*||*||||||||*|
Sbjct: 170 CATTCAAGATCTACTACGC 152
>miR6443 Score: 2.5 RO3G_15145
Query: 1 GTAAGGTCAAGATGATGAG 19
|||||*||*||||||||*|
Sbjct: 170 CATTCAAGATCTACTACGC 152
>miR6443 Score: 2.5 RO3G_15170

```
Query: 1 GTAAGGTCAAGATGATGAG 19
|||||*||*||||||||*|
Sbjct: 170 CATTCAAGATCTACTACGC 152
>miR6443 Score: 2.5 RO3G_14715
Query: 1 GTAAGGTCAAGATGATGAG 19
||*||:|||||||||*|||
Sbjct: 92 CAGTCTAGTTCTACTCCTC 74
>miR6443 Score: 0.0 RO3G_14772
Query: 1 GTAAGGTCAAGATGATGAG 19
|||||||||||||||||||
Sbjct: 425 CATTCCAGTTCTACTACTC 407
>miR6443 Score: 2.5 RO3G_14784
Query: 1 GTAAGGTCAAGATGATGAG 19
|||||*||*||||||||*|
Sbjct: 170 CATTCAAGATCTACTACGC 152
>miR6443 Score: 2.5 RO3G_13752
Query: 1 GTAAGGTCAAGATGATGAG 19
|||||*||*||||||||*|
Sbjct: 170 CATTCAAGATCTACTACGC 152
>miR6443 Score: 0.0 RO3G_13857
Query: 1 GTAAGGTCAAGATGATGAG 19
|||||||||||||||||||
Sbjct: 170 CATTCCAGTTCTACTACTC 152
>miR6443 Score: 0.0 RO3G_12670
Query: 1 GTAAGGTCAAGATGATGAG 19
|||||||||||||||||||
Sbjct: 347 CATTCCAGTTCTACTACTC 329
>miR6443 Score: 2.5 RO3G_12785
Query: 1 GTAAGGTCAAGATGATGAG 19
*|||||||||||:||*||*
Sbjct: 575 AATTCCAGTTCTGCTTCTG 557
>miR6443 Score: 2.5 RO3G_12950
Query: 1 GTAAGGTCAAGATGATGAG 19
|||||*||*||||||||*|
Sbjct: 170 CATTCAAGATCTACTACGC 152
>miR6443 Score: 2.5 RO3G_13267
Query: 1 GTAAGGTCAAGATGATGAG 19
|||||*||*||||||||*|
Sbjct: 170 CATTCAAGATCTACTACGC 152
>miR6443 Score: 0.0 RO3G_12082
Query: 1 GTAAGGTCAAGATGATGAG 19
|||||||||||||||||||
Sbjct: 425 CATTCCAGTTCTACTACTC 407
>miR6443 Score: 2.5 RO3G_12084
Query: 1 GTAAGGTCAAGATGATGAG 19
```

|||||*||*||||||||*|
Sbjct: 170 CATTCAAGATCTACTACGC 152
>miR6443 Score: 0.5 RO3G_12186
Query: 1 GTAAGGTCAAGATGATGAG 19
|||||||||||||||:|||
Sbjct: 539 CATTCCAGTTCTACTGCTC 521
>miR6443 Score: 0.5 RO3G_12196
Query: 1 GTAAGGTCAAGATGATGAG 19
|||||||||||||||:|||
Sbjct: 461 CATTCCAGTTCTACTGCTC 443
>miR6443 Score: 0.0 RO3G_12525
Query: 1 GTAAGGTCAAGATGATGAG 19
|||||||||||||||||||
Sbjct: 461 CATTCCAGTTCTACTACTC 443
>miR6443 Score: 0.0 RO3G_11405
Query: 1 GTAAGGTCAAGATGATGAG 19
|||||||||||||||||||
Sbjct: 347 CATTCCAGTTCTACTACTC 329
>miR6443 Score: 2.5 RO3G_11533
Query: 1 GTAAGGTCAAGATGATGAG 19
|||||*||*||||||||*|
Sbjct: 170 CATTCAAGATCTACTACGC 152
>miR6443 Score: 2.5 RO3G_11857
Query: 1 GTAAGGTCAAGATGATGAG 19
|||*|:|||||||:|||*|
Sbjct: 1310 CATCCTAGTTCTATTAC-C 1293
>miR6443 Score: 0.0 RO3G_10515
Query: 1 GTAAGGTCAAGATGATGAG 19
|||||||||||||||||||
Sbjct: 461 CATTCCAGTTCTACTACTC 443
>miR6443 Score: 2.5 RO3G_10797
Query: 1 GTAAGGTCAAGATGATGAG 19
|||||*||*||||||||*|
Sbjct: 170 CATTCAAGATCTACTACGC 152
>miR6443 Score: 2.0 RO3G_09272
Query: 1 GTAAGGTCAAGATGATGAG 19
||||||||||||*|*||||
Sbjct: 1240 CATTCCAGTTCTTCCACTC 1222
>miR6443 Score: 2.5 RO3G_09349
Query: 1 GTAAGGTCAAGATGATGAG 19
||||:||||*||||*||||
Sbjct: 785 CATTTCAGTCCTACCACTC 767
>miR6443 Score: 2.5 RO3G_09638
Query: 1 GTAAGGTCAAGATGATGAG 19
|||||:||||||||||*

Sbjct: 532 AATTCCGGTTCTAGTACTA 514
>miR6443 Score: 2.5 RO3G_09926
Query: 1 GTAAGGTCAAGATGATGAG 19
|||||*||*||||||||*|
Sbjct: 170 CATTCAAGATCTACTACGC 152
>miR6443 Score: 0.0 RO3G_09944
Query: 1 GTAAGGTCAAGATGATGAG 19
|||||||||||||||||||
Sbjct: 347 CATTCCAGTTCTACTACTC 329
>miR6443 Score: 0.0 RO3G_09952
Query: 1 GTAAGGTCAAGATGATGAG 19
|||||||||||||||||||
Sbjct: 317 CATTCCAGTTCTACTACTC 299
>miR6443 Score: 0.0 RO3G_09955
Query: 1 GTAAGGTCAAGATGATGAG 19
|||||||||||||||||||
Sbjct: 224 CATTCCAGTTCTACTACTC 206
>miR6443 Score: 2.5 RO3G_10050
Query: 1 GTAAGGTCAAGATGATGAG 19
|||||*||*||||||||*|
Sbjct: 170 CATTCAAGATCTACTACGC 152
>miR6443 Score: 0.0 RO3G_10224
Query: 1 GTAAGGTCAAGATGATGAG 19
|||||||||||||||||||
Sbjct: 347 CATTCCAGTTCTACTACTC 329
>miR6443 Score: 0.0 RO3G_10226
Query: 1 GTAAGGTCAAGATGATGAG 19
|||||||||||||||||||
Sbjct: 347 CATTCCAGTTCTACTACTC 329
>miR6443 Score: 0.0 RO3G_08658
Query: 1 GTAAGGTCAAGATGATGAG 19
|||||||||||||||||||
Sbjct: 347 CATTCCAGTTCTACTACTC 329
>miR6443 Score: 2.2 RO3G_09080
Query: 1 GTAAGGTCAAGATGATGAG 19
|||||:|*||||:|||||:
Sbjct: 1435 CATTCTATTTCTGCTACTT 1417
>miR6443 Score: 0.0 RO3G_09167
Query: 1 GTAAGGTCAAGATGATGAG 19
|||||||||||||||||||
Sbjct: 347 CATTCCAGTTCTACTACTC 329
>miR6443 Score: 0.0 RO3G_07024
Query: 1 GTAAGGTCAAGATGATGAG 19
|||||||||||||||||||
Sbjct: 539 CATTCCAGTTCTACTACTC 521

```
>miR6443 Score: 2.0 RO3G_07041
Query: 1 GTAAGGTCAAGATGATGAG 19
||*|||||*||||||||||
Sbjct: 461 CAATCCAGCTCTACTACTC 443
>miR6443 Score: 0.0 RO3G_07121
Query: 1 GTAAGGTCAAGATGATGAG 19
|||||||||||||||||||
Sbjct: 539 CATTCCAGTTCTACTACTC 521
>miR6443 Score: 1.5 RO3G_07298
Query: 1 GTAAGGTCAAGATGATGAG 19
||||:|||*||||||||||
Sbjct: 170 CATTTCAGATCTACTACTC 152
>miR6443 Score: 0.0 RO3G_07302
Query: 1 GTAAGGTCAAGATGATGAG 19
|||||||||||||||||||
Sbjct: 347 CATTCCAGTTCTACTACTC 329
>miR6443 Score: 0.0 RO3G_07689
Query: 1 GTAAGGTCAAGATGATGAG 19
|||||||||||||||||||
Sbjct: 347 CATTCCAGTTCTACTACTC 329
>miR6443 Score: 2.5 RO3G_07723
Query: 1 GTAAGGTCAAGATGATGAG 19
|||||*||*||||||||*|
Sbjct: 170 CATTCAAGATCTACTACGC 152
>miR6443 Score: 2.5 RO3G_05522
Query: 1 GTAAGGTCAAGATGATGAG 19
|||||*||*||||||||*|
Sbjct: 536 CATTCAAGATCTACTACGC 518
>miR6443 Score: 2.5 RO3G_06384
Query: 1 GTAAGGTCAAGATGATGAG 19
||||:||*|||||:||||*
Sbjct: 668 CATTTCAATTCTATTACTA 650
>miR6443 Score: 0.5 RO3G_06424
Query: 1 GTAAGGTCAAGATGATGAG 19
|:|||||||||||||||||
Sbjct: 140 CGTTCCAGTTCTACTACTC 122
>miR6443 Score: 0.0 RO3G_06721
Query: 1 GTAAGGTCAAGATGATGAG 19
|||||||||||||||||||
Sbjct: 347 CATTCCAGTTCTACTACTC 329
>miR6443 Score: 0.0 RO3G_04654
Query: 1 GTAAGGTCAAGATGATGAG 19
|||||||||||||||||||
Sbjct: 347 CATTCCAGTTCTACTACTC 329
>miR6443 Score: 1.0 RO3G_04661
```

Query: 1 GTAAGGTCAAGATGATGAG 19
|||*|||||||||||||||
Sbjct: 170 CATCCCAGTTCTACTACTC 152
>miR6443 Score: 1.5 RO3G_04668
Query: 1 GTAAGGTCAAGATGATGAG 19
||||:|||*||||||||||
Sbjct: 347 CATTTCAGATCTACTACTC 329
>miR6443 Score: 0.0 RO3G_04786
Query: 1 GTAAGGTCAAGATGATGAG 19
|||||||||||||||||||
Sbjct: 107 CATTCCAGTTCTACTACTC 89
>miR6443 Score: 2.0 RO3G_05381
Query: 1 GTAAGGTCAAGATGATGAG 19
*|||||||||||:||||**
Sbjct: 1009 AATTCCAGTTCTGCTACGG 991
>miR6443 Score: 2.5 RO3G_05516
Query: 1 GTAAGGTCAAGATGATGAG 19
|||||*||*||||||||*|
Sbjct: 170 CATTCAAGATCTACTACGC 152
>miR6443 Score: 0.0 RO3G_02700
Query: 1 GTAAGGTCAAGATGATGAG 19
|||||||||||||||||||
Sbjct: 224 CATTCCAGTTCTACTACTC 206
>miR6443 Score: 0.0 RO3G_02848
Query: 1 GTAAGGTCAAGATGATGAG 19
|||||||||||||||||||
Sbjct: 170 CATTCCAGTTCTACTACTC 152
>miR6443 Score: 0.0 RO3G_02850
Query: 1 GTAAGGTCAAGATGATGAG 19
|||||||||||||||||||
Sbjct: 539 CATTCCAGTTCTACTACTC 521
>miR6443 Score: 2.0 RO3G_03096
Query: 1 GTAAGGTCAAGATGATGAG 19
||*||||||||||||*|||
Sbjct: 344 CAGTCCAGTTCTACTCCTC 326
>miR6443 Score: 2.0 RO3G_03106
Query: 1 GTAAGGTCAAGATGATGAG 19
||*||||||||||||*|||
Sbjct: 92 CAGTCCAGTTCTACTCCTC 74
>miR6443 Score: 2.5 RO3G_03112
Query: 1 GTAAGGTCAAGATGATGAG 19
|||||*||*||||||||*|
Sbjct: 536 CATTCAAGATCTACTACGC 518
>miR6443 Score: 2.0 RO3G_03450
Query: 1 GTAAGGTCAAGATGATGAG 19

::|||||*||||||||||:
Sbjct: 1188 TGTTCCACTTCTACTACTT 1170
>miR6443 Score: 2.5 RO3G_03560
Query: 1 GTAAGGTCAAGATGATGAG 19
|||||*||*||||||||*|
Sbjct: 170 CATTCAAGATCTACTACGC 152
>miR6443 Score: 0.0 RO3G_03570
Query: 1 GTAAGGTCAAGATGATGAG 19
|||||||||||||||||||
Sbjct: 224 CATTCCAGTTCTACTACTC 206
>miR6443 Score: 0.0 RO3G_03599
Query: 1 GTAAGGTCAAGATGATGAG 19
|||||||||||||||||||
Sbjct: 539 CATTCCAGTTCTACTACTC 521
>miR6443 Score: 1.5 RO3G_03777
Query: 1 GTAAGGTCAAGATGATGAG 19
||||:|||*||||||||||
Sbjct: 170 CATTTCAGATCTACTACTC 152
>miR6443 Score: 0.0 RO3G_03785
Query: 1 GTAAGGTCAAGATGATGAG 19
|||||||||||||||||||
Sbjct: 152 CATTCCAGTTCTACTACTC 134
>miR6443 Score: 2.5 RO3G_03791
Query: 1 GTAAGGTCAAGATGATGAG 19
|||||*||*||||||||*|
Sbjct: 170 CATTCAAGATCTACTACGC 152
>miR6443 Score: 0.0 RO3G_03797
Query: 1 GTAAGGTCAAGATGATGAG 19
|||||||||||||||||||
Sbjct: 35 CATTCCAGTTCTACTACTC 17
>miR6443 Score: 0.0 RO3G_04200
Query: 1 GTAAGGTCAAGATGATGAG 19
|||||||||||||||||||
Sbjct: 347 CATTCCAGTTCTACTACTC 329
>miR6443 Score: 2.5 RO3G_00240
Query: 1 GTAAGGTCAAGATGATGAG 19
|||||*||*||||||||*|
Sbjct: 479 CATTCAAGATCTACTACGC 461
>miR6443 Score: 0.0 RO3G_00247
Query: 1 GTAAGGTCAAGATGATGAG 19
|||||||||||||||||||
Sbjct: 347 CATTCCAGTTCTACTACTC 329
>miR6443 Score: 2.5 RO3G_00250
Query: 1 GTAAGGTCAAGATGATGAG 19
|||||*||*||||||||*|

```
Sbjct: 170 CATTCAAGATCTACTACGC 152
>miR6443 Score: 1.5 RO3G_00675
Query: 1 GTAAGGTCAAGATGATGAG 19
||||:|||*||||||||||
Sbjct: 170 CATTTCAGATCTACTACTC 152
>miR6443 Score: 1.5 RO3G_00689
Query: 1 GTAAGGTCAAGATGATGAG 19
||||:|||*||||||||||
Sbjct: 38 CATTTCAGATCTACTACTC 20
>miR6443 Score: 2.5 RO3G_01185
Query: 1 GTAAGGTCAAGATGATGAG 19
::|||::||||||||||*:
Sbjct: 900 TGTTCTGGTTCTACTACAT 882
>miR6443 Score: 0.0 RO3G_01587
Query: 1 GTAAGGTCAAGATGATGAG 19
|||||||||||||||||||
Sbjct: 356 CATTCCAGTTCTACTACTC 338
>miR6443 Score: 1.5 RO3G_01709
Query: 1 GTAAGGTCAAGATGATGAG 19
||||:|||*||||||||||
Sbjct: 170 CATTTCAGATCTACTACTC 152
>miR6443 Score: 2.5 RO3G_01725
Query: 1 GTAAGGTCAAGATGATGAG 19
|||||*||*||||||||*|
Sbjct: 170 CATTCAAGATCTACTACGC 152
>miR6443 Score: 0.0 RO3G_01925
Query: 1 GTAAGGTCAAGATGATGAG 19
|||||||||||||||||||
Sbjct: 539 CATTCCAGTTCTACTACTC 521
>miR6443 Score: 2.5 RO3G_02227
Query: 1 GTAAGGTCAAGATGATGAG 19
|||||*||*||||||||*|
Sbjct: 536 CATTCAAGATCTACTACGC 518
>miR6443 Score: 0.0 RO3G_17436
Query: 1 GTAAGGTCAAGATGATGAG 19
|||||||||||||||||||
Sbjct: 347 CATTCCAGTTCTACTACTC 329
>miR6443 Score: 2.5 RO3G_17423
Query: 1 GTAAGGTCAAGATGATGAG 19
|||||*||*||||||||*|
Sbjct: 170 CATTCAAGATCTACTACGC 152
>miR6443 Score: 0.0 RO3G_17361
Query: 1 GTAAGGTCAAGATGATGAG 19
|||||||||||||||||||
Sbjct: 107 CATTCCAGTTCTACTACTC 89
```

>miR6443 Score: 2.5 RO3G_17328
Query: 1 GTAAGGTCAAGATGATGAG 19
|||||*||*||||||||*|
Sbjct: 170 CATTCAAGATCTACTACGC 152
>miR6443 Score: 1.5 RO3G_17316
Query: 1 GTAAGGTCAAGATGATGAG 19
||||:|||*||||||||||
Sbjct: 170 CATTTCAGGTCTACTACTC 152
>miR6443 Score: 0.0 RO3G_17293
Query: 1 GTAAGGTCAAGATGATGAG 19
|||||||||||||||||||
Sbjct: 347 CATTCCAGTTCTACTACTC 329
>miR6443 Score: 2.5 RO3G_17092
Query: 1 GTAAGGTCAAGATGATGAG 19
|||||*||*||||||||*|
Sbjct: 170 CATTCAAGATCTACTACGC 152
>miR7508 Score: 2.2 RO3G_13300
Query: 1 TAAAAAACAAGTCGGGAGG 19
|||||*||||:||:||||:
Sbjct: 1768 ATTTTCTGTTTAGTCCTCT 1750
>miR7508 Score: 2.5 RO3G_09384
Query: 1 TAAAAAACAAGTCGGGAGG 19
|||||||*|||||:|||**
Sbjct: 1201 ATTTTTTTTCAGTCCTAA 1183
>miR7508 Score: 2.2 RO3G_09480
Query: 1 TAAAAAACAAGTCGGGAGG 19
|||||*||||:||:||||:
Sbjct: 1768 ATTTTCTGTTTAGTCCTCT 1750
>miR7508 Score: 2.5 RO3G_05685
Query: 1 TAAAAAACAAGTCGGGAGG 19
|*|||||||||:|||||**
Sbjct: 124 AATTTTTGTTCGGCCCTAG 106
>miR7508 Score: 2.5 RO3G_05769
Query: 1 TAAAAAACAAGTCGGGAGG 19
||||||||||:||:|*|::
Sbjct: 105 ATTTTTTGTTTAGTCATTT 87
>miR1061-3p Score: 1.8 RO3G_13503
Query: 1 TGTTAGCATGGAATAATG 18
|:|:||||||:||||||:
Sbjct: 1257 ATAGTCGTACTTTATTAT 1240
>miR1061-3p Score: 2.5 RO3G_11420
Query: 1 TGTTAGCATGGAATAATG 18
|||:|||*|||*||||||
Sbjct: 684 ACAGTCGCACCCTATTAC 667
>miR1061-3p Score: 2.5 RO3G_09891

Query: 1 TGTTAGCATGGAATAATG 18
*|::|:|||||||:||||
Sbjct: 345 TCGGTTGTACCTTGTTAC 328
>miR1061-3p Score: 2.2 RO3G_05753
Query: 1 TGTTAGCATGGAATAATG 18
|||||:||*|||||||::
Sbjct: 127 ACAATTGTTCCTTATTGT 110
>miR1061-3p Score: 2.0 RO3G_00027
Query: 1 TGTTAGCATGGAATAATG 18
|||||||::|||||||
Sbjct: 190 TCAATCGTGTCTTATTAA 173
>miR1061-3p Score: 2.5 RO3G_00751
Query: 1 TGTTAGCATGGAATAATG 18
|:|||||||::*||||||
Sbjct: 1123 ATAATCGTATTATATTAC 1106
>miR1061-3p Score: 2.2 RO3G_01853
Query: 1 TGTTAGCATGGAATAATG 18
|||:||*|||:||||||:
Sbjct: 364 ACAGTCCTACTTTATTAT 347
>miR5821 Score: 2.5 RO3G_10279
Query: 1 TGGACGGAGACGATGGTAGAG 21
|||||:|||||||||*||**|
Sbjct: 242 ACCTGTCTCTGCTACGATGGC 222
>miR5821 Score: 2.5 RO3G_10929
Query: 1 TGGACGGAGACGATGGTAGAG 21
|:||||||||||:||||||
Sbjct: 801 TCTTGCCTCTGCTGCC-TCTC 782
>miR5821 Score: 2.5 RO3G_11069
Query: 1 TGGACGGAGACGATGGTAGAG 21
|:||||||||||:||||||
Sbjct: 801 CCTTGCCTCTGCTGCC-TCTC 782
>miR5821 Score: 2.5 RO3G_05170
Query: 1 TGGACGGAGACGATGGTAGAG 21
|:||||||||||:||||||
Sbjct: 801 TCTTGCCTCTGCTGCC-TCTC 782
>miR5821 Score: 2.5 RO3G_01864
Query: 1 TGGACGGAGACGATGGTAGAG 21
|:||||||||||:||||||
Sbjct: 801 CCTTGCCTCTGCTGCC-TCTC 782
>miR5821 Score: 0.0 RO3G_17015
Query: 1 TGGACGGAGACGATGGTAGAG 21
|||||||||||||||||||||
Sbjct: 1042 ACCTGCCTCTGCTACCATCTC 1022
>miR9409 Score: 2.2 RO3G_16817
Query: 1 TTTTGTCATAGTTCATTTTC 20

:|||:|*||||||||||||*
Sbjct: 46 GAAATATTATCAAGTAAAAA 27
>miR9409 Score: 2.5 RO3G_16521
Query: 1 TTTTGTCATAGTTCATTTTC 20
||||||||||||*|||||
Sbjct: 659 TAAACAGAATCAAGAAAAAG 640
>miR9409 Score: 2.5 RO3G_16107
Query: 1 TTTTGTCATAGTTCATTTTC 20
||||||*|:||||*||||||
Sbjct: 1991 AAAACATTGTCAACTAAAAG 1972
>miR9409 Score: 0.0 RO3G_15711
Query: 1 TTTTGTCATAGTTCATTTTC 20
||||||||||||||||||||
Sbjct: 1642 AAAACAGTATCAAGTAAAAG 1623
>miR9409 Score: 2.0 RO3G_14532
Query: 1 TTTTGTCATAGTTCATTTTC 20
|:||:|||||:||||||||*
Sbjct: 1633 AGAATAGTATTAAGTAAAAC 1614
>miR9409 Score: 2.5 RO3G_14210
Query: 1 TTTTGTCATAGTTCATTTTC 20
|:|::|||||:||||||||*
Sbjct: 1027 AGAGTAGTATTAAGTAAAAC 1008
>miR9409 Score: 2.5 RO3G_13589
Query: 1 TTTTGTCATAGTTCATTTTC 20
|:||||||*|:||||||||*
Sbjct: 556 AGAACAGTTTTAAGTAAAAC 537
>miR9409 Score: 2.5 RO3G_13624
Query: 1 TTTTGTCATAGTTCATTTTC 20
|:||||||*|:||||||||*
Sbjct: 343 AGAACAGTTTTAAGTAAAAC 324
>miR9409 Score: 2.2 RO3G_13728
Query: 1 TTTTGTCATAGTTCATTTTC 20
:||||:||||||||||:**|
Sbjct: 836 GAAACGGTATCAAGTAGTTG 817
>miR9409 Score: 0.0 RO3G_13796
Query: 1 TTTTGTCATAGTTCATTTTC 20
||||||||||||||||||||
Sbjct: 1642 AAAACAGTATCAAGTAAAAG 1623
>miR9409 Score: 2.0 RO3G_13807
Query: 1 TTTTGTCATAGTTCATTTTC 20
|:||:|||||:||||||||*
Sbjct: 895 AGAATAGTATTAAGTAAAAC 876
>miR9409 Score: 0.0 RO3G_11521
Query: 1 TTTTGTCATAGTTCATTTTC 20
||||||||||||||||||||

```
Sbjct: 604 AAAACAGTATCAAGTAAAAG 585
>miR9409 Score: 0.0 RO3G_09539
Query: 1 TTTTGTCATAGTTCATTTTC 20
||||||||||||||||||||
Sbjct: 1642 AAAACAGTATCAAGTAAAAG 1623
>miR9409 Score: 2.2 RO3G_07873
Query: 1 TTTTGTCATAGTTCATTTTC 20
:||||:||||||||||:**|
Sbjct: 848 GAAACGGTATCAAGTAGTTG 829
>miR9409 Score: 2.2 RO3G_05922
Query: 1 TTTTGTCATAGTTCATTTTC 20
:||||:||||||||||:**|
Sbjct: 347 GAAACGGTATCAAGTAGTTG 328
>miR9409 Score: 2.5 RO3G_06371
Query: 1 TTTTGTCATAGTTCATTTTC 20
||||||||||||||*:|**|
Sbjct: 589 AAAACAGTATCAAGCGA-TG 571
>miR9409 Score: 2.2 RO3G_04478
Query: 1 TTTTGTCATAGTTCATTTTC 20
|:||||||||*||||||:|*
Sbjct: 1663 AGAACAGTATGAAGTAAGAC 1644
>miR9409 Score: 2.2 RO3G_05273
Query: 1 TTTTGTCATAGTTCATTTTC 20
:||||:||||||||||:**|
Sbjct: 332 GAAACGGTATCAAGTAGTTG 313
>miR9409 Score: 2.5 RO3G_02302
Query: 1 TTTTGTCATAGTTCATTTTC 20
:||:*|||||||||||*:|
Sbjct: 70 GAAGAAGTATCAAGTAA-GG 52
>miR9409 Score: 2.0 RO3G_03121
Query: 1 TTTTGTCATAGTTCATTTTC 20
|:||:|||||:||||||||*
Sbjct: 1633 AGAATAGTATTAAGTAAAAC 1614
>miR9409 Score: 2.5 RO3G_00521
Query: 1 TTTTGTCATAGTTCATTT-TC 20
||||*|*||||||||||*||
Sbjct: 301 AAAAAACTATCAAGTAAACAG 281
>miR9409 Score: 2.5 RO3G_01204
Query: 1 TTTTGTCATAGTTCATTTTC 20
*|||||||*|:||||||*||
Sbjct: 106 TAAACAGTTTTAAGTAATAG 87
>miR9409 Score: 2.0 RO3G_01426
Query: 1 TTTTGTCATAGTTCATTTTC 20
|:||:|||||:||||||||*
Sbjct: 895 AGAATAGTATTAAGTAAAAC 876
```

>miR9409 Score: 1.5 RO3G_01512
Query: 1 TTTTGTCATAGTTCATTTTC 20
|||||||*||:|||||||||
Sbjct: 1630 AAAACAGCATTAAGTAAAAG 1611
>miR9409 Score: 2.5 RO3G_01924
Query: 1 TTTTGTCATAGTTCATTTTC 20
|:||||||*|:||||||||*
Sbjct: 1027 AGAACAGTTTAAGTAAAAC 1008
>miR6194 Score: 0.0 RO3G_10233
Query: 1 TAGTAGGGATCTTGACACACGAG 23
|||||||||||||||||||||||
Sbjct: 2164 ATCATCCCTAGAACTGTGTGCTC 2142
>miR6194 Score: 0.0 RO3G_04025
Query: 1 TAGTAGGGATCTTGACACACGAG 23
|||||||||||||||||||||||
Sbjct: 2164 ATCATCCCTAGAACTGTGTGCTC 2142
>miR6194 Score: 0.0 RO3G_01744
Query: 1 TAGTAGGGATCTTGACACACGAG 23
|||||||||||||||||||||||
Sbjct: 2167 ATCATCCCTAGAACTGTGTGCTC 2145
>miR5059 Score: 2.5 RO3G_00398
Query: 1 CGATCCTGGGCAGCACCACC 20
|*|||*||||||||||||*|
Sbjct: 1583 GATAGTACCCGTCGTGGTCG 1564
>miR415 Score: 0.0 RO3G_17256
Query: 1 AAAGGAACAGAAGCAGAAGCAG 22
||||||||||||||||||||||
Sbjct: 182 TTTCCTTGTCTTCGTCTTCGTC 161
>miR415 Score: 1.0 RO3G_17244
Query: 1 AAAGGAACAGAAGCAGAAGCAG 22
|*||||||||||||||||||||
Sbjct: 212 TGTCCTTGTCTTCGTCTTCGTC 191
>miR415 Score: 2.5 RO3G_17245
Query: 1 AAAGGAACAGAAGCAGAAGCAG 22
|||||||||||||||*|*||
Sbjct: 35 CTTACTTGTCTTCGTCTAC-TC 15
>miR415 Score: 0.0 RO3G_17248
Query: 1 AAAGGAACAGAAGCAGAAGCAG 22
||||||||||||||||||||||
Sbjct: 188 TTTCCTTGTCTTCGTCTTCGTC 167
>miR415 Score: 1.0 RO3G_17210
Query: 1 AAAGGAACAGAAGCAGAAGCAG 22
|*||||||||||||||||||||
Sbjct: 239 TGTCCTTGTCTTCGTCTTCGTC 218
>miR415 Score: 1.0 RO3G_16901

```
Query: 1 AAAGGAACAGAAGCAGAAGCAG 22
|*||||||||||||||||||||
Sbjct: 209 TGTCCTTGTCTTCGTCTTCGTC 188
>miR415 Score: 2.5 RO3G_16910
Query: 1 AAAGGAACAGAAGCAGAAGCAG 22
*||*|||||||||||||*|*||
Sbjct: 35 CTTACTTGTCTTCGTCTAC-TC 15
>miR415 Score: 1.0 RO3G_16917
Query: 1 AAAGGAACAGAAGCAGAAGCAG 22
|*||||||||||||||||||||
Sbjct: 215 TGTCCTTGTCTTCGTCTTCGTC 194
>miR415 Score: 1.0 RO3G_16743
Query: 1 AAAGGAACAGAAGCAGAAGCAG 22
|*||||||||||||||||||||
Sbjct: 161 TGTCCTTGTCTTCGTCTTCGTC 140
>miR415 Score: 0.0 RO3G_16748
Query: 1 AAAGGAACAGAAGCAGAAGCAG 22
||||||||||||||||||||||
Sbjct: 158 TTTCCTTGTCTTCGTCTTCGTC 137
>miR415 Score: 1.0 RO3G_16369
Query: 1 AAAGGAACAGAAGCAGAAGCAG 22
|*||||||||||||||||||||
Sbjct: 194 TGTCCTTGTCTTCGTCTTCGTC 173
>miR415 Score: 0.0 RO3G_16137
Query: 1 AAAGGAACAGAAGCAGAAGCAG 22
||||||||||||||||||||||
Sbjct: 182 TTTCCTTGTCTTCGTCTTCGTC 161
>miR415 Score: 1.0 RO3G_15879
Query: 1 AAAGGAACAGAAGCAGAAGCAG 22
|*||||||||||||||||||||
Sbjct: 215 TGTCCTTGTCTTCGTCTTCGTC 194
>miR415 Score: 1.0 RO3G_15887
Query: 1 AAAGGAACAGAAGCAGAAGCAG 22
|*||||||||||||||||||||
Sbjct: 239 TGTCCTTGTCTTCGTCTTCGTC 218
>miR415 Score: 0.0 RO3G_15890
Query: 1 AAAGGAACAGAAGCAGAAGCAG 22
||||||||||||||||||||||
Sbjct: 182 TTTCCTTGTCTTCGTCTTCGTC 161
>miR415 Score: 0.0 RO3G_15897
Query: 1 AAAGGAACAGAAGCAGAAGCAG 22
||||||||||||||||||||||
Sbjct: 158 TTTCCTTGTCTTCGTCTTCGTC 137
>miR415 Score: 2.5 RO3G_15509
Query: 1 AAAGGAACAGAAGCAGAAGCAG 22
```

||||*||*|:||||||||||||
Sbjct: 26 TTTCATTATTTTCGTCTTCGTC 5
>miR415 Score: 0.0 RO3G_15147
Query: 1 AAAGGAACAGAAGCAGAAGCAG 22
||||||||||||||||||||||
Sbjct: 134 TTTCCTTGTCTTCGTCTTCGTC 113
>miR415 Score: 0.0 RO3G_14551
Query: 1 AAAGGAACAGAAGCAGAAGCAG 22
||||||||||||||||||||||
Sbjct: 158 TTTCCTTGTCTTCGTCTTCGTC 137
>miR415 Score: 1.0 RO3G_14553
Query: 1 AAAGGAACAGAAGCAGAAGCAG 22
|*||||||||||||||||||||
Sbjct: 215 TGTCCTTGTCTTCGTCTTCGTC 194
>miR415 Score: 2.5 RO3G_14707
Query: 1 AAAGGAACAGAAGCAGAAGCAG 22
|||||||||||||||*|*||
Sbjct: 35 CTTACTTGTCTTCGTCTAC-TC 15
>miR415 Score: 1.0 RO3G_13452
Query: 1 AAAGGAACAGAAGCAGAAGCAG 22
|*||||||||||||||||||||
Sbjct: 191 TGTCCTTGTCTTCGTCTTCGTC 170
>miR415 Score: 2.5 RO3G_13710
Query: 1 AAAGGAACAGAAGCAGAAGCAG 22
|||||||||||||||*|*||
Sbjct: 35 CTTACTTGTCTTCGTCTAC-TC 15
>miR415 Score: 2.5 RO3G_13738
Query: 1 AAAGGAACAGAAGCAGAAGCAG 22
|||||||||||||||*|*||
Sbjct: 35 CTTACTTGTCTTCGTCTAC-TC 15
>miR415 Score: 1.0 RO3G_12976
Query: 1 AAAGGAACAGAAGCAGAAGCAG 22
|*||||||||||||||||||||
Sbjct: 218 TGTCCTTGTCTTCGTCTTCGTC 197
>miR415 Score: 0.0 RO3G_13146
Query: 1 AAAGGAACAGAAGCAGAAGCAG 22
||||||||||||||||||||||
Sbjct: 182 TTTCCTTGTCTTCGTCTTCGTC 161
>miR415 Score: 0.0 RO3G_13272
Query: 1 AAAGGAACAGAAGCAGAAGCAG 22
||||||||||||||||||||||
Sbjct: 241 TTTCCTTGTCTTCGTCTTCGTC 220
>miR415 Score: 2.5 RO3G_12006
Query: 1 AAAGGAACAGAAGCAGAAGCAG 22
|||||||||||||||*|*||

```
Sbjct: 35 CTTACTTGTCTTCGTCTAC-TC 15
>miR415 Score: 1.0 RO3G_12011
Query: 1 AAAGGAACAGAAGCAGAAGCAG 22
|*||||||||||||||||||||
Sbjct: 215 TGTCCTTGTCTTCGTCTTCGTC 194
>miR415 Score: 0.0 RO3G_11236
Query: 1 AAAGGAACAGAAGCAGAAGCAG 22
||||||||||||||||||||||
Sbjct: 200 TTTCCTTGTCTTCGTCTTCGTC 179
>miR415 Score: 0.0 RO3G_11537
Query: 1 AAAGGAACAGAAGCAGAAGCAG 22
||||||||||||||||||||||
Sbjct: 200 TTTCCTTGTCTTCGTCTTCGTC 179
>miR415 Score: 1.0 RO3G_11543
Query: 1 AAAGGAACAGAAGCAGAAGCAG 22
|*||||||||||||||||||||
Sbjct: 215 TGTCCTTGTCTTCGTCTTCGTC 194
>miR415 Score: 0.0 RO3G_10775
Query: 1 AAAGGAACAGAAGCAGAAGCAG 22
||||||||||||||||||||||
Sbjct: 188 TTTCCTTGTCTTCGTCTTCGTC 167
>miR415 Score: 0.0 RO3G_09602
Query: 1 AAAGGAACAGAAGCAGAAGCAG 22
||||||||||||||||||||||
Sbjct: 158 TTTCCTTGTCTTCGTCTTCGTC 137
>miR415 Score: 0.0 RO3G_09603
Query: 1 AAAGGAACAGAAGCAGAAGCAG 22
||||||||||||||||||||||
Sbjct: 200 TTTCCTTGTCTTCGTCTTCGTC 179
>miR415 Score: 0.0 RO3G_09929
Query: 1 AAAGGAACAGAAGCAGAAGCAG 22
||||||||||||||||||||||
Sbjct: 158 TTTCCTTGTCTTCGTCTTCGTC 137
>miR415 Score: 0.0 RO3G_10041
Query: 1 AAAGGAACAGAAGCAGAAGCAG 22
||||||||||||||||||||||
Sbjct: 188 TTTCCTTGTCTTCGTCTTCGTC 167
>miR415 Score: 1.0 RO3G_10043
Query: 1 AAAGGAACAGAAGCAGAAGCAG 22
|*||||||||||||||||||||
Sbjct: 209 TGTCCTTGTCTTCGTCTTCGTC 188
>miR415 Score: 2.5 RO3G_10043
Query: 1 AAAGGAACAGAAGCAGAAGCAG 22
|*||||*|||||||||||||*|
Sbjct: 161 TGTCCTCGTCTTCGTCTTCGCC 140
```

>miR415 Score: 2.5 RO3G_10076
Query: 1 AAAGGAACAGAAGCAGAAGCAG 22
||:||||||||||||||*||
Sbjct: 512 CTGCTTTGTCTTCGTCTTCTTC 491
>miR415 Score: 0.0 RO3G_10218
Query: 1 AAAGGAACAGAAGCAGAAGCAG 22
||||||||||||||||||||||
Sbjct: 188 TTTCCTTGTCTTCGTCTTCGTC 167
>miR415 Score: 0.0 RO3G_07981
Query: 1 AAAGGAACAGAAGCAGAAGCAG 22
||||||||||||||||||||||
Sbjct: 158 TTTCCTTGTCTTCGTCTTCGTC 137
>miR415 Score: 0.0 RO3G_09182
Query: 1 AAAGGAACAGAAGCAGAAGCAG 22
||||||||||||||||||||||
Sbjct: 182 TTTCCTTGTCTTCGTCTTCGTC 161
>miR415 Score: 0.0 RO3G_06730
Query: 1 AAAGGAACAGAAGCAGAAGCAG 22
||||||||||||||||||||||
Sbjct: 158 TTTCCTTGTCTTCGTCTTCGTC 137
>miR415 Score: 0.0 RO3G_07306
Query: 1 AAAGGAACAGAAGCAGAAGCAG 22
||||||||||||||||||||||
Sbjct: 158 TTTCCTTGTCTTCGTCTTCGTC 137
>miR415 Score: 1.0 RO3G_07692
Query: 1 AAAGGAACAGAAGCAGAAGCAG 22
|*||||||||||||||||||||
Sbjct: 149 TGTCCTTGTCTTCGTCTTCGTC 128
>miR415 Score: 0.0 RO3G_07696
Query: 1 AAAGGAACAGAAGCAGAAGCAG 22
||||||||||||||||||||||
Sbjct: 158 TTTCCTTGTCTTCGTCTTCGTC 137
>miR415 Score: 0.0 RO3G_07720
Query: 1 AAAGGAACAGAAGCAGAAGCAG 22
||||||||||||||||||||||
Sbjct: 158 TTTCCTTGTCTTCGTCTTCGTC 137
>miR415 Score: 0.0 RO3G_07738
Query: 1 AAAGGAACAGAAGCAGAAGCAG 22
||||||||||||||||||||||
Sbjct: 200 TTTCCTTGTCTTCGTCTTCGTC 179
>miR415 Score: 1.0 RO3G_04659
Query: 1 AAAGGAACAGAAGCAGAAGCAG 22
|*||||||||||||||||||||
Sbjct: 149 TGTCCTTGTCTTCGTCTTCGTC 128
>miR415 Score: 2.5 RO3G_04776

Query: 1 AAAGGAACAGAAGCAGAAGCAG 22
||||||**|||||||||||*||
Sbjct: 1427 TTTCCTCTTCTTCGTCTTCCTC 1406
>miR415 Score: 1.0 RO3G_05347
Query: 1 AAAGGAACAGAAGCAGAAGCAG 22
|*||||||||||||||||||||
Sbjct: 215 TGTCCTTGTCTTCGTCTTCGTC 194
>miR415 Score: 0.0 RO3G_05425
Query: 1 AAAGGAACAGAAGCAGAAGCAG 22
||||||||||||||||||||||
Sbjct: 188 TTTCCTTGTCTTCGTCTTCGTC 167
>miR415 Score: 0.0 RO3G_02228
Query: 1 AAAGGAACAGAAGCAGAAGCAG 22
||||||||||||||||||||||
Sbjct: 158 TTTCCTTGTCTTCGTCTTCGTC 137
>miR415 Score: 1.0 RO3G_02817
Query: 1 AAAGGAACAGAAGCAGAAGCAG 22
|*||||||||||||||||||||
Sbjct: 218 TGTCCTTGTCTTCGTCTTCGTC 197
>miR415 Score: 0.0 RO3G_03584
Query: 1 AAAGGAACAGAAGCAGAAGCAG 22
||||||||||||||||||||||
Sbjct: 116 TTTCCTTGTCTTCGTCTTCGTC 95
>miR415 Score: 0.0 RO3G_04211
Query: 1 AAAGGAACAGAAGCAGAAGCAG 22
||||||||||||||||||||||
Sbjct: 158 TTTCCTTGTCTTCGTCTTCGTC 137
>miR415 Score: 1.0 RO3G_00288
Query: 1 AAAGGAACAGAAGCAGAAGCAG 22
|*||||||||||||||||||||
Sbjct: 182 TGTCCTTGTCTTCGTCTTCGTC 161
>miR415 Score: 2.2 RO3G_00874
Query: 1 AAAGGAACAGAAGCAGAAGCAG 22
||||||*||||||||||*:*||
Sbjct: 1889 TTTCCTAGTCTTCGTCTAT-TC 1869
>miR415 Score: 1.0 RO3G_01192
Query: 1 AAAGGAACAGAAGCAGAAGCAG 22
|*||||||||||||||||||||
Sbjct: 152 TGTCCTTGTCTTCGTCTTCGTC 131
>miR415 Score: 2.5 RO3G_01196
Query: 1 AAAGGAACAGAAGCAGAAGCAG 22
|||||||||||||||*|*||
Sbjct: 35 CTTACTTGTCTTCGTCTAC-TC 15
>miR415 Score: 1.0 RO3G_01717
Query: 1 AAAGGAACAGAAGCAGAAGCAG 22

```
|*||||||||||||||||||||
Sbjct: 218 TGTCCTTGTCTTCGTCTTCGTC 197
>miR415 Score: 1.0 RO3G_01726
Query: 1 AAAGGAACAGAAGCAGAAGCAG 22
||||||||||||||*|||||||
Sbjct: 200 TTTCCTTGTCTTCGGCTTCGTC 179
>miR415 Score: 1.0 RO3G_17460
Query: 1 AAAGGAACAGAAGCAGAAGCAG 22
|*||||||||||||||||||||
Sbjct: 239 TGTCCTTGTCTTCGTCTTCGTC 218
>miR415 Score: 1.0 RO3G_17433
Query: 1 AAAGGAACAGAAGCAGAAGCAG 22
|*||||||||||||||||||||
Sbjct: 212 TGTCCTTGTCTTCGTCTTCGTC 191
>miR415 Score: 2.5 RO3G_17365
Query: 1 AAAGGAACAGAAGCAGAAGCAG 22
*||*|||||||||||||*|*||
Sbjct: 35 CTTACTTGTCTTCGTCTAC-TC 15
>miR415 Score: 0.0 RO3G_17350
Query: 1 AAAGGAACAGAAGCAGAAGCAG 22
||||||||||||||||||||||
Sbjct: 182 TTTCCTTGTCTTCGTCTTCGTC 161
>miR5185l-3p Score: 1.5 RO3G_17211
Query: 1 TTGGACGGTTGACTTAGAAC 20
||||||||:||||||*||||
Sbjct: 759 AACCTGCCGACTGAACCTTG 740
>miR5185l-3p Score: 1.5 RO3G_17213
Query: 1 TTGGACGGTTGACTTAGAAC 20
||||||||:||||||*||||
Sbjct: 84 AACCTGCCGACTGAACCTTG 65
>miR5185l-3p Score: 2.5 RO3G_16377
Query: 1 TTGGACGGTTGACTTAGAAC 20
|||:||*||||||||*||||
Sbjct: 84 AACTTGACAACTGAACCTTG 65
>miR5185l-3p Score: 1.0 RO3G_16118
Query: 1 TTGGACGGTTGACTTAGAAC 20
|||||||||||||||*||||
Sbjct: 27 AACCTGCCAACTGAACCTTG 8
>miR5185l-3p Score: 1.0 RO3G_15873
Query: 1 TTGGACGGTTGACTTAGAAC 20
|||||||||||||||*||||
Sbjct: 759 AACCTGCCAACTGAACCTTG 740
>miR5185l-3p Score: 1.5 RO3G_15154
Query: 1 TTGGACGGTTGACTTAGAAC 20
||||||||:||||||*||||
```

```
Sbjct: 759 AACCTGCCGACTGAACCTTG 740
>miR5185l-3p Score: 1.5 RO3G_13447
Query: 1 TTGGACGGTTGACTTAGAAC 20
||||||:||||||||*||||
Sbjct: 759 AACCTGTCAACTGAACCTTG 740
>miR5185l-3p Score: 1.5 RO3G_13706
Query: 1 TTGGACGGTTGACTTAGAAC 20
||||||||:||||||*||||
Sbjct: 717 AACCTGCCGACTGAACCTTG 698
>miR5185l-3p Score: 1.0 RO3G_12013
Query: 1 TTGGACGGTTGACTTAGAAC 20
|||||||||||||||*||||
Sbjct: 84 AACCTGCCAACTGAACCTTG 65
>miR5185l-3p Score: 1.5 RO3G_12174
Query: 1 TTGGACGGTTGACTTAGAAC 20
||||||||:||||||*||||
Sbjct: 84 AACCTGCCGACTGAACCTTG 65
>miR5185l-3p Score: 1.5 RO3G_12526
Query: 1 TTGGACGGTTGACTTAGAAC 20
||||||||:||||||*||||
Sbjct: 759 AACCTGCCGACTGAACCTTG 740
>miR5185l-3p Score: 1.0 RO3G_11244
Query: 1 TTGGACGGTTGACTTAGAAC 20
|||||||||||||||*||||
Sbjct: 759 AACCTGCCAACTGAACCTTG 740
>miR5185l-3p Score: 1.5 RO3G_11263
Query: 1 TTGGACGGTTGACTTAGAAC 20
||||||||:||||||*||||
Sbjct: 84 AACCTGCCGACTGAACCTTG 65
>miR5185l-3p Score: 1.0 RO3G_11528
Query: 1 TTGGACGGTTGACTTAGAAC 20
|||||||||||||||*||||
Sbjct: 84 AACCTGCCAACTGAACCTTG 65
>miR5185l-3p Score: 1.0 RO3G_11534
Query: 1 TTGGACGGTTGACTTAGAAC 20
|||||||||||||||*||||
Sbjct: 759 AACCTGCCAACTGAACCTTG 740
>miR5185l-3p Score: 0.0 RO3G_11948
Query: 1 TTGGACGGTTGACTTAGAAC 20
||||||||||||||||||||
Sbjct: 759 AACCTGCCAACTGAATCTTG 740
>miR5185l-3p Score: 1.0 RO3G_10528
Query: 1 TTGGACGGTTGACTTAGAAC 20
|||||||||||||||*||||
Sbjct: 84 AACCTGCCAACTGAACCTTG 65
```

>miR5185l-3p Score: 1.0 RO3G_10537
Query: 1 TTGGACGGTTGACTTAGAAC 20
|||||||||||||||*||||
Sbjct: 582 AACCTGCCAACTGAACCTTG 563
>miR5185l-3p Score: 1.0 RO3G_10720
Query: 1 TTGGACGGTTGACTTAGAAC 20
|||||||||||||||*||||
Sbjct: 759 AACCTGCCAACTGAACCTTG 740
>miR5185l-3p Score: 1.0 RO3G_10742
Query: 1 TTGGACGGTTGACTTAGAAC 20
|||||||||||||||*||||
Sbjct: 84 AACCTGCCAACTGAACCTTG 65
>miR5185l-3p Score: 0.0 RO3G_09199
Query: 1 TTGGACGGTTGACTTAGAAC 20
||||||||||||||||||||
Sbjct: 759 AACCTGCCAACTGAATCTTG 740
>miR5185l-3p Score: 0.0 RO3G_09924
Query: 1 TTGGACGGTTGACTTAGAAC 20
||||||||||||||||||||
Sbjct: 759 AACCTGCCAACTGAATCTTG 740
>miR5185l-3p Score: 1.0 RO3G_10044
Query: 1 TTGGACGGTTGACTTAGAAC 20
|||||||||||||||*||||
Sbjct: 759 AACCTGCCAACTGAACCTTG 740
>miR5185l-3p Score: 1.0 RO3G_07994
Query: 1 TTGGACGGTTGACTTAGAAC 20
|||||||||||||||*||||
Sbjct: 759 AACCTGCCAACTGAACCTTG 740
>miR5185l-3p Score: 2.0 RO3G_08835
Query: 1 TTGGACGGTTGACTTAGAAC 20
|||||*|||||||||*||||
Sbjct: 69 AACCTACCAACTGAACCTTG 50
>miR5185l-3p Score: 1.0 RO3G_08848
Query: 1 TTGGACGGTTGACTTAGAAC 20
|||||||||||||||*||||
Sbjct: 69 AACCTGCCAACTGAACCTTG 50
>miR5185l-3p Score: 1.0 RO3G_07119
Query: 1 TTGGACGGTTGACTTAGAAC 20
|||||||||||||||*||||
Sbjct: 84 AACCTGCCAACTGAACCTTG 65
>miR5185l-3p Score: 0.0 RO3G_07295
Query: 1 TTGGACGGTTGACTTAGAAC 20
||||||||||||||||||||
Sbjct: 591 AACCTGCCAACTGAATCTTG 572
>miR5185l-3p Score: 2.5 RO3G_07465

```
Query: 1 TTGGACGGTTGACTTAGAA-C 20
|||||*|||||||*|||||*|
Sbjct: 1474 AACCTACCAACTGTATCTTAG 1454
>miR5185l-3p Score: 1.0 RO3G_07683
Query: 1 TTGGACGGTTGACTTAGAAC 20
||||||||||||||*||||
Sbjct: 759 AACCTGCCAACTGAACCTTG 740
>miR5185l-3p Score: 1.0 RO3G_05916
Query: 1 TTGGACGGTTGACTTAGAAC 20
||||||||||||||*||||
Sbjct: 759 AACCTGCCAACTGAACCTTG 740
>miR5185l-3p Score: 1.5 RO3G_03558
Query: 1 TTGGACGGTTGACTTAGAAC 20
||||||:||||||||*||||
Sbjct: 759 AACCTGTCAACTGAACCTTG 740
>miR5185l-3p Score: 1.5 RO3G_03617
Query: 1 TTGGACGGTTGACTTAGAAC 20
||||||:||||||||*||||
Sbjct: 759 AACCTGTCAACTGAACCTTG 740
>miR5185l-3p Score: 1.0 RO3G_03987
Query: 1 TTGGACGGTTGACTTAGAAC 20
||||||||||||||*||||
Sbjct: 759 AACCTGCCAACTGAACCTTG 740
>miR5185l-3p Score: 1.0 RO3G_01194
Query: 1 TTGGACGGTTGACTTAGAAC 20
||||||||||||||*||||
Sbjct: 759 AACCTGCCAACTGAACCTTG 740
>miR5185l-3p Score: 1.0 RO3G_01313
Query: 1 TTGGACGGTTGACTTAGAAC 20
||||||||||||||*||||
Sbjct: 759 AACCTGCCAACTGAACCTTG 740
>miR5185l-3p Score: 1.0 RO3G_01735
Query: 1 TTGGACGGTTGACTTAGAAC 20
||||||||||||||*||||
Sbjct: 759 AACCTGCCAACTGAACCTTG 740
>miR5185l-3p Score: 0.0 RO3G_01950
Query: 1 TTGGACGGTTGACTTAGAAC 20
|||||||||||||||||||
Sbjct: 759 AACCTGCCAACTGAATCTTG 740
>miR5185l-3p Score: 1.0 RO3G_01951
Query: 1 TTGGACGGTTGACTTAGAAC 20
||||||||||||||*||||
Sbjct: 759 AACCTGCCAACTGAACCTTG 740
>miR5185l-3p Score: 1.5 RO3G_17409
Query: 1 TTGGACGGTTGACTTAGAAC 20
```

||||||||:||||||*||||
Sbjct: 759 AACCTGCCGACTGAACCTTG 740
>miR6274b-3p Score: 0.0 RO3G_15390
Query: 1 TTGTGGTAGACCGAAAATAG 20
||||||||||||||||||||
Sbjct: 543 AACACCATCTGGCTTTTATC 524
>miR6274b-3p Score: 0.0 RO3G_13001
Query: 1 TTGTGGTAGACCGAAAATAG 20
||||||||||||||||||||
Sbjct: 2253 AACACCATCTGGCTTTTATC 2234
>miR6274b-3p Score: 1.0 RO3G_10503
Query: 1 TTGTGGTAGACCGAAAATAG 20
|||||||||||||*||||||
Sbjct: 189 AACACCATCTGGCCTTTATC 170
>miR6274b-3p Score: 0.0 RO3G_06596
Query: 1 TTGTGGTAGACCGAAAATAG 20
||||||||||||||||||||
Sbjct: 2253 AACACCATCTGGCTTTTATC 2234
>miR6274b-3p Score: 0.0 RO3G_03002
Query: 1 TTGTGGTAGACCGAAAATAG 20
||||||||||||||||||||
Sbjct: 2253 AACACCATCTGGCTTTTATC 2234
>miR6274b-3p Score: 2.5 RO3G_02145
Query: 1 TTGTGGTAGACCGAAAATAG 20
||||||||||*||*|||||*
Sbjct: 135 AACACCATCTAGCCTTTATG 116
>miR8126-3p Score: 2.5 RO3G_16842
Query: 1 TTCAGTATATTTGTCATCAGAG 22
|||||*|||||::||||||:|:
Sbjct: 258 AAGTCTTATAAGTAGTAGTTTT 237
>miR8126-3p Score: 2.0 RO3G_10563
Query: 1 TTCAGTATATTTGTCATCAGAG 22
|||||||||*|:|||||||||*
Sbjct: 24 AAGTCATATTAGCAGTAGTCTG 3
>miR8126-3p Score: 2.5 RO3G_10119
Query: 1 TTCAGTATATTTGTCATCAGAG 22
|||||:||*|:|:|||||||||
Sbjct: 84 AAGTCGTACAGATAGTAGTCTC 63
>miR8126-3p Score: 2.5 RO3G_06496
Query: 1 TTCAGTATATTTGTCATCAGAG 22
|||||||||:||*||||||**|
Sbjct: 1602 AAGTCATATGAAAAGTAGT-AC 1582
>miR1428b Score: 0.2 RO3G_13882
Query: 1 TATAAGAAATGCCATGAATTCT 22
||||||||||||||||||:|||

```
Sbjct: 654 ATATTCTTTACGGTACTTGAGA 633
>miR1428b Score: 0.2 RO3G_13730
Query: 1 TATAAGAAATGCCATGAATTCT 22
||||||||||||||||||:|||
Sbjct: 417 ATATTCTTTACGGTACTTGAGA 396
>miR1428b Score: 2.2 RO3G_08084
Query: 1 TATAAGAAATGCCATGAATTCT 22
||||||*||||*|||||||||:
Sbjct: 180 ATATTCCTTACTGTACTTAAGG 159
>miR1428b Score: 0.2 RO3G_05325
Query: 1 TATAAGAAATGCCATGAATTCT 22
||||||||||||||||||:|||
Sbjct: 654 ATATTCTTTACGGTACTTGAGA 633
>miR1428b Score: 0.0 RO3G_03721
Query: 1 TATAAGAAATGCCATGAATTCT 22
||||||||||||||||||||||
Sbjct: 654 ATATTCTTTACGGTACTTAAGA 633
>miR1428b Score: 0.5 RO3G_00141
Query: 1 TATAAGAAATGCCATGAATTCT 22
||||||||||:|||||||||||
Sbjct: 654 ATATTCTTTATGGTACTTAAGA 633
>miR1428b Score: 0.2 RO3G_00809
Query: 1 TATAAGAAATGCCATGAATTCT 22
||||||||||||||||||:|||
Sbjct: 654 ATATTCTTTACGGTACTTGAGA 633
>miR2111-3p Score: 2.5 RO3G_06346
Query: 1 GAAGTCAGGTGGGATTACC 19
|||*||||:||||*|||||
Sbjct: 276 CTTGAGTCTACCCAAATGG 258
>miR825-3p Score: 1.5 RO3G_17249
Query: 1 TGGTCTCGATAAGGTGCATTAAC 23
|||||||||*|||||||||||*|
Sbjct: 515 ACCAGAGCTTTTCCACGTAATAG 493
>miR825-3p Score: 0.5 RO3G_14979
Query: 1 TGGTCTCGATAAGGTGCATTAAC 23
|||||||||||||||||||||*|
Sbjct: 515 ACCAGAGCTATTCCACGTAATAG 493
>miR825-3p Score: 0.0 RO3G_14363
Query: 1 TGGTCTCGATAAGGTGCATTAAC 23
|||||||||||||||||||||||
Sbjct: 473 ACCAGAGCTATTCCACGTAATTG 451
>miR825-3p Score: 0.5 RO3G_13741
Query: 1 TGGTCTCGATAAGGTGCATTAAC 23
|||||||||||||||||||||*|
Sbjct: 515 ACCAGAGCTATTCCACGTAATAG 493
```

```
>miR825-3p Score: 1.5 RO3G_13755
Query: 1 TGGTCTCGATAAGGTGCATTAAC 23
|||||||||||||||*|||||*|
Sbjct: 515 ACCAGAGCTATTCCAAGTAATAG 493
>miR825-3p Score: 0.5 RO3G_13863
Query: 1 TGGTCTCGATAAGGTGCATTAAC 23
|||||||||||||||||||||*|
Sbjct: 215 ACCAGAGCTATTCCACGTAATAG 193
>miR825-3p Score: 0.5 RO3G_13270
Query: 1 TGGTCTCGATAAGGTGCATTAAC 23
|||||||||||||||||||||*|
Sbjct: 515 ACCAGAGCTATTCCACGTAATAG 493
>miR825-3p Score: 0.5 RO3G_10774
Query: 1 TGGTCTCGATAAGGTGCATTAAC 23
|||||||||||||||||||||*|
Sbjct: 482 ACCAGAGCTATTCCACGTAATAG 460
>miR825-3p Score: 0.5 RO3G_08849
Query: 1 TGGTCTCGATAAGGTGCATTAAC 23
|||||||||||||||||||||*|
Sbjct: 515 ACCAGAGCTATTCCACGTAATAG 493
>miR825-3p Score: 0.5 RO3G_05936
Query: 1 TGGTCTCGATAAGGTGCATTAAC 23
|||||||||||||||||||||*|
Sbjct: 515 ACCAGAGCTATTCCACGTAATAG 493
>miR825-3p Score: 1.0 RO3G_05420
Query: 1 TGGTCTCGATAAGGTGCATTAAC 23
||||||||||||||||||||**|
Sbjct: 376 ACCAGAGCTATTCCACGTAAAGG 354
>miR825-3p Score: 0.8 RO3G_17276
Query: 1 TGGTCTCGATAAGGTGCATTAAC 23
:||||||||||||||||||||*|
Sbjct: 605 GCCAGAGCTATTCCACGTAATAG 583
>miR5566 Score: 2.5 RO3G_07176
Query: 1 TACATCACCTCCCTGTCTGT 20
*|||:|||||||*|||||*|
Sbjct: 192 TTGTGGTGGAGGTACAGA-A 174
>miR5081 Score: 1.8 RO3G_00403
Query: 1 TGAATTTGTAAAATTGATACT 21
|||||||:|||||||||:*|*
Sbjct: 4249 ACTTAAATATTTTAACTG-GC 4230
>miR5081 Score: 2.0 RO3G_01688
Query: 1 TGAATTTGTAAAATTGATACT 21
:|||:*||||||||||||||:
Sbjct: 1317 GCTTGCACATTTTAACTATGG 1297
>miR6459a-3p Score: 0.0 RO3G_16376
```

Query: 1 TTGATTTGGGCTTGAATTGA 20
||||||||||||||||||||
Sbjct: 1045 AACTAAACCCGAACTTAACT 1026
>miR6459a-3p Score: 2.5 RO3G_15761
Query: 1 TTGATTTGGGCTTGAATTGA 20
||||||:||||||||||*
Sbjct: 166 TACTAAATCCGAACTAAACA 147
>miR6459a-3p Score: 0.0 RO3G_15573
Query: 1 TTGATTTGGGCTTGAATTGA 20
||||||||||||||||||||
Sbjct: 1120 AACTAAACCCGAACTTAACT 1101
>miR6459a-3p Score: 1.5 RO3G_13849
Query: 1 TTGATTTGGGCTTGAATTGA 20
||||:||*||||||||||||
Sbjct: 1120 AACTGAAGCCGAACTTAACT 1101
>miR6459a-3p Score: 2.5 RO3G_12505
Query: 1 TTGATTTGGGCTTGAATTGA 20
||||||*|:|||||*|||||
Sbjct: 567 AACTAACCTCGAACATAACT 548
>miR6459a-3p Score: 2.5 RO3G_12506
Query: 1 TTGATTTGGGCTTGAATTGA 20
||||||*|:|||||*|||||
Sbjct: 567 AACTAACCTCGAACATAACT 548
>miR6459a-3p Score: 1.5 RO3G_10420
Query: 1 TTGATTTGGGCTTGAATTGA 20
||||:||*||||||||||||
Sbjct: 1120 AACTGAAGCCGAACTTAACT 1101
>miR6459a-3p Score: 0.0 RO3G_10991
Query: 1 TTGATTTGGGCTTGAATTGA 20
||||||||||||||||||||
Sbjct: 1120 AACTAAACCCGAACTTAACT 1101
>miR6459a-3p Score: 2.5 RO3G_09502
Query: 1 TTGATTTGGGCTTGAATTGA 20
|||||*||||*||||||*||
Sbjct: 311 AACTATACCCCAACTTACCT 292
>miR6459a-3p Score: 2.5 RO3G_10202
Query: 1 TTGATTTGGGCTTGAATTGA 20
||||*|||||||:*||||||
Sbjct: 820 AACTTAACCCGAGATTAACT 801
>miR6459a-3p Score: 2.5 RO3G_07372
Query: 1 TTGATTTGGGCTTGAATT-GA 20
||||||||*|||||*|||*||
Sbjct: 777 AACTAAACGCGAACATAAACT 757
>miR6459a-3p Score: 1.5 RO3G_05865
Query: 1 TTGATTTGGGCTTGAATTGA 20

||||:||*||||||||||||
Sbjct: 1120 AACTGAAGCCGAACTTAACT 1101
>miR6459a-3p Score: 2.5 RO3G_05991
Query: 1 TTGATTTGGGCTTGAATTGA 20
|**|||||||||||||*||
Sbjct: 980 ACGTAAACCCGAACTTA-CT 962
>miR6459a-3p Score: 0.0 RO3G_06502
Query: 1 TTGATTTGGGCTTGAATTGA 20
||||||||||||||||||||
Sbjct: 1120 AACTAAACCCGAACTTAACT 1101
>miR6459a-3p Score: 1.0 RO3G_04662
Query: 1 TTGATTTGGGCTTGAATTGA 20
||||:||:||||||||||||
Sbjct: 972 AACTGAATCCGAACTTAACT 953
>miR6459a-3p Score: 2.2 RO3G_04718
Query: 1 TTGATTTGGGCTTGAATTGA 20
|||||*||||*||||||:||
Sbjct: 611 AACTACACCCTAACTTAGCT 592
>miR6459a-3p Score: 0.0 RO3G_02708
Query: 1 TTGATTTGGGCTTGAATTGA 20
||||||||||||||||||||
Sbjct: 853 AACTAAACCCGAACTTAACT 834
>miR6459a-3p Score: 0.0 RO3G_02966
Query: 1 TTGATTTGGGCTTGAATTGA 20
||||||||||||||||||||
Sbjct: 391 AACTAAACCCGAACTTAACT 372
>miR6459a-3p Score: 1.5 RO3G_03749
Query: 1 TTGATTTGGGCTTGAATTGA 20
||||:||*||||||||||||
Sbjct: 1120 AACTGAAGCCGAACTTAACT 1101
>miR6459a-3p Score: 1.5 RO3G_00273
Query: 1 TTGATTTGGGCTTGAATTGA 20
|||||*|||||||||||*||
Sbjct: 807 AACTATACCCGAACTTA-CT 789
>miR6459a-3p Score: 1.0 RO3G_01056
Query: 1 TTGATTTGGGCTTGAATTGA 20
||||:||:||||||||||||
Sbjct: 1027 AACTGAATCCGAACTTAACT 1008
>miR6459a-3p Score: 2.0 RO3G_01464
Query: 1 TTGATTTGGGCTTGAATTGA 20
|||||||||||||||||*
Sbjct: 1182 TAATAAACCCGAACTTAACC 1163
>miR6459a-3p Score: 2.2 RO3G_01540
Query: 1 TTGATTTGGGCTTGAATTGA 20
||*|*||||||||||||:||

```
Sbjct: 1031 AAATTAACCCGAACTTAGCT 1012
>miR6459a-3p Score: 1.5 RO3G_01733
Query: 1 TTGATTTGGGCTTGAATTGA 20
||||:||*||||||||||||
Sbjct: 1120 AACTGAAGCCGAACTTAACT 1101
>miR6459a-3p Score: 1.5 RO3G_01944
Query: 1 TTGATTTGGGCTTGAATTGA 20
||||:||*||||||||||||
Sbjct: 1120 AACTGAAGCCGAACTTAACT 1101
>miR6459a-3p Score: 1.5 RO3G_17446
Query: 1 TTGATTTGGGCTTGAATTGA 20
||||:||*||||||||||||
Sbjct: 1120 AACTGAAGCCGAACTTAACT 1101
>miR6459a-3p Score: 1.5 RO3G_17287
Query: 1 TTGATTTGGGCTTGAATTGA 20
||||:||*||||||||||||
Sbjct: 1120 AACTGAAGCCGAACTTAACT 1101
>miR529a Score: 2.5 RO3G_16449
Query: 1 TTGTCGCTGCTCTCTTCTTC 20
|::||:|||||*||||||||
Sbjct: 362 AGTAGTGACGAAAGAAGAAG 343
>miR529a Score: 2.5 RO3G_16256
Query: 1 TTGTCGCTGCTCTCTTCTTC 20
|:*||||||||||||||*|*
Sbjct: 1265 AGAAGCGACGAGAGAAGTAA 1246
>miR529a Score: 2.5 RO3G_13394
Query: 1 TTGTCGCTGCTCTCTTCTTC 20
*||||:||:|||||||*|||
Sbjct: 169 CACAGTGATGAGAGAAAAAG 150
>miR529a Score: 2.5 RO3G_12731
Query: 1 TTGTCGCTGCTCTCTTCTTC 20
|||||:||:*:|||||||||
Sbjct: 683 AACAGTGATAGGAGAAGAAG 664
>miR529a Score: 2.5 RO3G_05533
Query: 1 TTGTCGCTGCTCTCTTCTTC 20
|:|||:*|:|||||||||||
Sbjct: 617 AGCAGTAATGAGAGAAGAAG 598
>miR529a Score: 2.0 RO3G_04591
Query: 1 TTGTCGCTGCTCTCTTCTTC 20
||||*|||||||||||*|||
Sbjct: 666 AACAACGACGAGAGAAAAAG 647
>miR529a Score: 2.5 RO3G_02598
Query: 1 TTGTCGCTGCTCTCTTCTTC 20
*|*||*||||||||||||||
Sbjct: 685 TAGAGAGACGAGAGAAGAAG 666
```

```
>miR529a Score: 2.5 RO3G_00635
Query: 1 TTGTCGCTGCTCTCTTCTTC 20
|:|||:||:||*||||||||
Sbjct: 1898 AGCAGTGATGAAAGAAGAAG 1879
>miR9492 Score: 2.5 RO3G_16691
Query: 1 TACTTCTCTGTCTTGGTAT 19
|||:|||||*:||||||*|
Sbjct: 321 ATGGAGAGAAGGAACCA-A 304
>miR9492 Score: 2.5 RO3G_15671
Query: 1 TACTTCTCTGTCTTGGTAT 19
|||:|||||*:||||||*|
Sbjct: 321 ATGGAGAGAAGGAACCA-A 304
>miR9492 Score: 0.0 RO3G_15743
Query: 1 TACTTCTCTGTCTTGGTAT 19
|||||||||||||||||||
Sbjct: 958 ATGAAGAGACAGAACCATA 940
>miR9492 Score: 2.5 RO3G_15559
Query: 1 TACTTCTCTGTCTTGGTAT 19
*||||*||||||||||:*|
Sbjct: 1172 TTGAAAAGACAGAACCG-A 1155
>miR9492 Score: 2.5 RO3G_15040
Query: 1 TACTTCTCTGTCTTGGTAT 19
**|||||||||||||::||
Sbjct: 62 CGGAAGAGACAGAACTGTA 44
>miR9492 Score: 2.5 RO3G_15169
Query: 1 TACTTCTCTGTCTTGGTAT 19
|||:|||||*:||||||*|
Sbjct: 321 ATGGAGAGAAGGAACCA-A 304
>miR9492 Score: 2.0 RO3G_14783
Query: 1 TACTTCTCTGTCTTGGTAT 19
||*|||||||:|||||:||
Sbjct: 19 ATAAAGAGACGGAACCGTA 1
>miR9492 Score: 1.5 RO3G_13691
Query: 1 TACTTCTCTGTCTTGGTAT 19
*|||||||||||||||:*|
Sbjct: 1172 TTGAAGAGACAGAACCG-A 1155
>miR9492 Score: 2.0 RO3G_13292
Query: 1 TACTTCTCTGTCTTGGTAT 19
||*|||||||:|||||:||
Sbjct: 958 ATAAAGAGACGGAACCGTA 940
>miR9492 Score: 2.5 RO3G_11965
Query: 1 TACTTCTCTGTCTTGGTAT 19
|||:|||||*:||||||*|
Sbjct: 321 ATGGAGAGAAGGAACCA-A 304
>miR9492 Score: 2.5 RO3G_12112
```

Query: 1 TACTTCTCTGTCTTGGTAT 19
**|||||||*|||||||||
Sbjct: 721 TAGAAGAGAAAGAACCATA 703
>miR9492 Score: 2.2 RO3G_12555
Query: 1 TACTTCTCTGTCTTGGTAT 19
||||||||*||*||||||:
Sbjct: 1464 ATGAAGAGCCACAACCATG 1446
>miR9492 Score: 2.2 RO3G_10607
Query: 1 TACTTCTCTGTCTTGGTAT 19
||*|||||||||||||*|:
Sbjct: 3173 ATAAAGAGACAGAACC-TG 3156
>miR9492 Score: 2.5 RO3G_10873
Query: 1 TACTTCTCTGTCTTGGTAT 19
|||:|||||*:||||||*|
Sbjct: 321 ATGGAGAGAAGGAACCA-A 304
>miR9492 Score: 1.8 RO3G_09359
Query: 1 TACTTCTCTGTCTTGGTAT 19
:*|||||||||||||||*|
Sbjct: 1853 GAGAAGAGACAGAACCAGA 1835
>miR9492 Score: 2.5 RO3G_10052
Query: 1 TACTTCTCTGTCTTGGTAT 19
|||:|||||*:||||||*|
Sbjct: 321 ATGGAGAGAAGGAACCA-A 304
>miR9492 Score: 2.5 RO3G_07740
Query: 1 TACTTCTCTGTCTTGGTAT 19
|||:|||||*:||||||*|
Sbjct: 321 ATGGAGAGAAGGAACCA-A 304
>miR9492 Score: 2.0 RO3G_06411
Query: 1 TACTTCTCTGTCTTGGTAT 19
||*|||||||:|||||:||
Sbjct: 958 ATAAAGAGACGGAACCGTA 940
>miR9492 Score: 2.5 RO3G_04538
Query: 1 TACTTCTCTGTCTTGGTAT 19
|||||*||:||||||:|*|
Sbjct: 1548 ATGAACAGGCAGAACTACA 1530
>miR9492 Score: 2.5 RO3G_02575
Query: 1 TACTTCTCTGTCTTGGTAT 19
|||||||||||:|:|||
Sbjct: 500 TTGAAAAGACAGAGCTATA 482
>miR9492 Score: 2.5 RO3G_03336
Query: 1 TACTTCTCTGTCTTGGTAT 19
||||*||*||||||||:||
Sbjct: 973 ATGACGACACAGAACCGTA 955
>miR9492 Score: 2.0 RO3G_03783
Query: 1 TACTTCTCTGTCTTGGTAT 19

||*|||||||:|||||:||
Sbjct: 958 ATAAAGAGACGGAACCGTA 940
>miR9492 Score: 2.5 RO3G_00668
Query: 1 TACTTCTCTGTCTTGGTAT 19
||||||||||:||:|||
Sbjct: 189 CTAAAGAGACAGGACTATA 171
>miR9492 Score: 2.5 RO3G_01616
Query: 1 TACTTCTCTGTCTTGGTAT 19
|||:|||||*:||||||*|
Sbjct: 321 ATGGAGAGAAGGAACCA-A 304
>miR9492 Score: 2.0 RO3G_17372
Query: 1 TACTTCTCTGTCTTGGTAT 19
||*|||||||:|||||:||
Sbjct: 958 ATAAAGAGACGGAACCGTA 940
>miR9492 Score: 2.0 RO3G_17309
Query: 1 TACTTCTCTGTCTTGGTAT 19
||*|||||||:|||||:||
Sbjct: 958 ATAAAGAGACGGAACCGTA 940
>miR9492 Score: 2.0 RO3G_17311
Query: 1 TACTTCTCTGTCTTGGTAT 19
||*|||||||:|||||:||
Sbjct: 958 ATAAAGAGACGGAACCGTA 940
>miR7542 Score: 2.5 RO3G_16171
Query: 1 TGTTGCGTTCATAGATGGTA 20
|||||||||||*||*||*||
Sbjct: 4270 ACAACGCAAGTTTCCAC-AT 4252
>miR7542 Score: 2.5 RO3G_16285
Query: 1 TGTTGCGTTCATAGATGGTA 20
||:|||:|||||||||*|*|
Sbjct: 72 ACGACGTAAGTATCTAGCTT 53
>miR7542 Score: 2.5 RO3G_16050
Query: 1 TGTTGCGTTCATAGATGGTA 20
||*||*||||||||||||*|
Sbjct: 1669 ACTACCCAAGTATCTACCTT 1650
>miR7542 Score: 2.5 RO3G_13406
Query: 1 TGTTGCGTTCATAGATGGTA 20
:||||*|||||||||::|:|
Sbjct: 613 GCAACTCAAGTATCTGTCGT 594
>miR7542 Score: 2.5 RO3G_13422
Query: 1 TGTTGCGTTCATAGATGGTA 20
:||||*|||||||||::|:|
Sbjct: 637 GCAACTCAAGTATCTGTCGT 618
>miR7542 Score: 2.5 RO3G_12721
Query: 1 TGTTGCGTTCATAGATGGTA 20
||:|||:|||||||||:*|*

Sbjct: 57 ACGACGTAAGTATCTATAAA 38
>miR7542 Score: 2.5 RO3G_12788
Query: 1 TGTTGCGTTCATAGATGGTA 20
|:||||||||||||*:|*||
Sbjct: 366 ATAACGCAAGTATCAGC-AT 348
>miR7542 Score: 2.5 RO3G_12316
Query: 1 TGTTGCGTTCATAGATGGTA 20
:||||*|||||||||::|:|
Sbjct: 613 GCAACTCAAGTATCTGTCGT 594
>miR7542 Score: 2.5 RO3G_05240
Query: 1 TGTTGCGTTCATAGATGGTA 20
:|||:*|||||||||:||:|
Sbjct: 613 GCAATTCAAGTATCTGCCGT 594
>miR166h-3p Score: 2.5 RO3G_12495
Query: 1 TCGGACCAGGCTTCATTC-CC 20
||||*|*|||||||||||*||
Sbjct: 644 AGCCGGTTCCGAAGTAAGTGG 624
>miR159a Score: 2.5 RO3G_16775
Query: 1 TTTGGATTGAAGGGAGCTCT 20
||||||||:*|||||:||*|
Sbjct: 844 AAACCTAATGTCCCTTGA-A 826
>miR159a Score: 2.5 RO3G_14108
Query: 1 TTTGGATTGAAGGGAGCTCT 20
|||::||:|||||:|||*||
Sbjct: 185 AAATTTAGCTTCCTTCG-GA 167
>miR159a Score: 2.5 RO3G_13093
Query: 1 TTTGGATTGAAGGGAGCTCT 20
**||||||:|||||||||*|
Sbjct: 311 TTACCTAATTTCCCTCGA-A 293
>miR159a Score: 2.5 RO3G_10905
Query: 1 TTTGGATTGAAGGGAGCTCT 20
|||||*|||||:||||||**
Sbjct: 569 AAACCAAACTTTCCTCGATT 550
>miR159a Score: 2.5 RO3G_08331
Query: 1 TTTGGATTGAAGGGAGCTCT 20
|||:||||||:|||||||
Sbjct: 123 TAACTAAACTTCTCTCGAGA 104
>miR159a Score: 2.5 RO3G_02808
Query: 1 TTTGGATTGAAGGGAGCTCT 20
|||||||::||:|||||**|
Sbjct: 41 AAACCTAGTTTTCCTCGTCA 22
>miR4244 Score: 1.0 RO3G_13029
Query: 1 TAGTTGTTCCTGGAGATGTG 20
|||||||||||||||*||||
Sbjct: 174 ATCAACAAGGACCTCAACAC 155

>miR4244 Score: 2.2 RO3G_12482
Query: 1 TAGTTGTTCCTGGAGATG-TG 20
|*||||||||||||:||:*||
Sbjct: 637 ACCAACAAGGACCTTTATTAC 617
>miR4244 Score: 1.5 RO3G_12568
Query: 1 TAGTTGTTCCTGGAGATGTG 20
||||||||||||:||*||||
Sbjct: 483 ATCAACAAGGACTTCCACAC 464
>miR4244 Score: 1.2 RO3G_11695
Query: 1 TAGTTGTTCCTGGAGATGTG 20
|||||||||||||||*|:||
Sbjct: 240 ATCAACAAGGACCTCCATAC 221
>miR4244 Score: 2.5 RO3G_05564
Query: 1 TAGTTGTTCCTGGAGATGTG 20
|||*|:||||||*|||||||
Sbjct: 721 ATCCATAAGGACATCTACAC 702
>miR4244 Score: 2.2 RO3G_04377
Query: 1 TAGTTGTTCCTGGAGATGTG 20
:||||*||||||:||||||*
Sbjct: 570 GTCAAGAAGGACTTCTACAA 551
>miR4244 Score: 0.5 RO3G_04497
Query: 1 TAGTTGTTCCTGGAGATGTG 20
||:|||||||||||||||||
Sbjct: 240 ATTAACAAGGACCTCTACAC 221
>miR4244 Score: 2.5 RO3G_05311
Query: 1 TAGTTGTTCCTGGAGATGTG 20
||*|:|||||||:||||*||
Sbjct: 306 ATAAGCAAGGACTTCTA-AC 288
>miR4244 Score: 1.0 RO3G_02365
Query: 1 TAGTTGTTCCTGGAGATGTG 20
|||||||||||||||*||||
Sbjct: 240 ATCAACAAGGACCTCCACAC 221
>miR4244 Score: 2.5 RO3G_03106
Query: 1 TAGTTGTTCCTGGAGATGTG 20
:|||||||||||:*|||*|:
Sbjct: 204 GTCAACAAGGACTGCTA-AT 186
>miR4244 Score: 1.0 RO3G_16979
Query: 1 TAGTTGTTCCTGGAGATGTG 20
|||||||||||||||*||||
Sbjct: 90 ATCAACAAGGACCTCCACAC 71
>miR2595 Score: 2.5 RO3G_16060
Query: 1 TACATTTCTACTTCTCTGTCT 21
|||||||||||||||*|*|
Sbjct: 38 TTGTAAAGATGAAGAAAAACA 18
>miR2595 Score: 0.0 RO3G_15743

```
Query: 1 TACATTTCTACTTCTCTGTCT 21
|||||||||||||||||||||
Sbjct: 966 ATGTAAAGATGAAGAGACAGA 946
>miR6148b Score: 2.5 RO3G_17170
Query: 1 TCTGTTATGTTTGTTCCA 18
|||*:||||||||||*||
Sbjct: 74 AGAAGATACAAACAAAGT 57
>miR6148b Score: 2.0 RO3G_13408
Query: 1 TCTGTTATGTTTGTTCCA 18
|||||||||||||::|*|
Sbjct: 60 AGACAATACAAACGGG-T 44
>miR6148b Score: 2.5 RO3G_12835
Query: 1 TCTGTTATGTTTGTTCCA 18
*|||*|||*|||||||||
Sbjct: 1844 TGACCATAGAAACAAGGT 1827
>miR6148b Score: 2.5 RO3G_12527
Query: 1 TCTGTTATGTTTGTTCCA 18
|||*:||||||||||*||
Sbjct: 74 AGAAGATACAAACAAAGT 57
>miR6148b Score: 2.5 RO3G_11902
Query: 1 TCTGTTATGTTTGTTCCA 18
*||||*||||||||*|||
Sbjct: 132 TGACACTACAAACATGGT 115
>miR6148b Score: 2.5 RO3G_10494
Query: 1 TCTGTTATGTTTGTTCCA 18
|*||||||:||||||*||
Sbjct: 254 AAACAATATAAACAACGT 237
>miR6148b Score: 2.5 RO3G_09803
Query: 1 TCTGTTATGTTTGTTCCA 18
||||||||:||||||**|
Sbjct: 1014 AGACAATATAAACAAT-T 998
>miR6148b Score: 2.5 RO3G_09920
Query: 1 TCTGTTATGTTTGTTCCA 18
|||*:||||||||||*||
Sbjct: 74 AGAAGATACAAACAAAGT 57
>miR6148b Score: 2.2 RO3G_10057
Query: 1 TCTGTTATGTTTGTTCCA 18
:|||||*|*|||||||||
Sbjct: 1255 GGACAAAAAAAACAAGGT 1238
>miR6148b Score: 2.5 RO3G_08323
Query: 1 TCTGTTATGTTTGTTCCA 18
|*||||||||||||||**
Sbjct: 642 ACACAATACAAACAAGAA 625
>miR6148b Score: 2.5 RO3G_06478
Query: 1 TCTGTTATGTTTGTTCCA 18
```

|||||||:|||||||*
Sbjct: 262 TGACAAAACGAACAAGGA 245
>miR6148b Score: 2.5 RO3G_06500
Query: 1 TCTGTTATGTTTGTTCCA 18
|||:|*|:||||:|||||
Sbjct: 32 AGATATTGCAAATAAGGT 15
>miR6148b Score: 2.2 RO3G_06693
Query: 1 TCTGTTATGTTTGTTCCA 18
:||||||||||||*|*||
Sbjct: 83 GGACAATACAAACCATGT 66
>miR6148b Score: 2.5 RO3G_05185
Query: 1 TCTGTTATGTTTGTTCCA 18
||||||*||:||||||*|
Sbjct: 1039 AGACAAGACGAACAAG-T 1023
>miR6148b Score: 2.5 RO3G_05406
Query: 1 TCTGTTATGTTTGTTCCA 18
|||||:||*||::|||||
Sbjct: 234 AGACAGTAGAAGTAAGGT 217
>miR6148b Score: 2.5 RO3G_05519
Query: 1 TCTGTTATGTTTGTTCCA 18
|||*:||||||||||*||
Sbjct: 74 AGAAGATACAAACAAAGT 57
>miR6148b Score: 2.5 RO3G_03032
Query: 1 TCTGTTATGTTTGTTCCA 18
|||*:|||:||||||||*
Sbjct: 1988 AGAAGATATAAACAAGGA 1971
>miR6148b Score: 2.0 RO3G_03392
Query: 1 TCTGTTATGTTTGTTCCA 18
||:|*|||||||||:|||
Sbjct: 225 AGGCTATACAAACAGGGT 208
>miR6148b Score: 2.5 RO3G_03474
Query: 1 TCTGTTATGTTTGTTCCA 18
||||||||||||:||**|
Sbjct: 467 AGACAATACAAATAAC-T 451
>miR6148b Score: 2.5 RO3G_00076
Query: 1 TCTGTTATGTTTGTTCCA 18
**||||||||*|||||||
Sbjct: 348 TAACAATACACACAAGGT 331
>miR6148b Score: 2.5 RO3G_00104
Query: 1 TCTGTTATGTTTGTTCCA 18
**||||||||*|||||||
Sbjct: 348 TAACAATACACACAAGGT 331
>miR6148b Score: 2.5 RO3G_00837
Query: 1 TCTGTTATGTTTGTTCCA 18
||||||||||||||*|

Sbjct: 1410 CGACAACACAAACAAGTT 1393
>miR6148b Score: 2.5 RO3G_00940
Query: 1 TCTGTTATGTTTGTTCCA 18
||:||:|||*||||:|||
Sbjct: 699 AGGCAGTACTAACAGGGT 682
>miR6148b Score: 2.5 RO3G_01252
Query: 1 TCTGTTATGTTTGTTCCA 18
||:|*|||:||||||||*
Sbjct: 540 AGGCCATATAAACAAGGA 523
>miR6148b Score: 2.5 RO3G_01450
Query: 1 TCTGTTATGTTTGTTCCA 18
||||||||:::|*|||||
Sbjct: 788 AGACAATATGGAGAAGGT 771
>miR6148b Score: 2.5 RO3G_02202
Query: 1 TCTGTTATGTTTGTTCCA 18
||||*|||||||||||
Sbjct: 2089 CGAGAACACAAACAAGGT 2072
>miR1425-5p Score: 1.5 RO3G_17222
Query: 1 TACTGATCATCCTTGCTGCT 20
||||||||||||:|*|||||
Sbjct: 386 ATGACTAGTAGGGAAGACGA 367
>miR1425-5p Score: 0.0 RO3G_16719
Query: 1 TACTGATCATCCTTGCTGCT 20
||||||||||||||||||||
Sbjct: 194 ATGACTAGTAGGAACGACGA 175
>miR1425-5p Score: 0.0 RO3G_16727
Query: 1 TACTGATCATCCTTGCTGCT 20
||||||||||||||||||||
Sbjct: 194 ATGACTAGTAGGAACGACGA 175
>miR1425-5p Score: 0.5 RO3G_16550
Query: 1 TACTGATCATCCTTGCTGCT 20
||||||||||||:|||||||
Sbjct: 305 ATGACTAGTAGGGACGACGA 286
>miR1425-5p Score: 1.5 RO3G_15583
Query: 1 TACTGATCATCCTTGCTGCT 20
|||||||||||*:|||||||
Sbjct: 59 ATGACTAGTAGCGACGACGA 40
>miR1425-5p Score: 0.5 RO3G_13919
Query: 1 TACTGATCATCCTTGCTGCT 20
||||||||||||:|||||||
Sbjct: 386 ATGACTAGTAGGGACGACGA 367
>miR1425-5p Score: 0.5 RO3G_13743
Query: 1 TACTGATCATCCTTGCTGCT 20
||||||||||||:|||||||
Sbjct: 194 ATGACTAGTAGGGACGACGA 175

```
>miR1425-5p Score: 1.0 RO3G_12678
Query: 1 TACTGATCATCCTTGCTGCT 20
||||||||||||:|||||*|
Sbjct: 386 ATGACTAGTAGGGACGACAA 367
>miR1425-5p Score: 0.5 RO3G_13286
Query: 1 TACTGATCATCCTTGCTGCT 20
||||||||||||:|||||||
Sbjct: 386 ATGACTAGTAGGGACGACGA 367
>miR1425-5p Score: 1.5 RO3G_12166
Query: 1 TACTGATCATCCTTGCTGCT 20
|||||||||||*:|||||||
Sbjct: 194 ATGACTAGTAGCGACGACGA 175
>miR1425-5p Score: 1.5 RO3G_12658
Query: 1 TACTGATCATCCTTGCTGCT 20
|||||||||||*:|||||||
Sbjct: 194 ATGACTAGTAGCGACGACGA 175
>miR1425-5p Score: 0.0 RO3G_11281
Query: 1 TACTGATCATCCTTGCTGCT 20
||||||||||||||||||||
Sbjct: 194 ATGACTAGTAGGAACGACGA 175
>miR1425-5p Score: 1.5 RO3G_10550
Query: 1 TACTGATCATCCTTGCTGCT 20
||||||||||||:||*||||
Sbjct: 194 ATGACTAGTAGGGACAACGA 175
>miR1425-5p Score: 0.5 RO3G_10741
Query: 1 TACTGATCATCCTTGCTGCT 20
||||||||||||:|||||||
Sbjct: 194 ATGACTAGTAGGGACGACGA 175
>miR1425-5p Score: 0.5 RO3G_10763
Query: 1 TACTGATCATCCTTGCTGCT 20
||||||||||||:|||||||
Sbjct: 194 ATGACTAGTAGGGACGACGA 175
>miR1425-5p Score: 0.5 RO3G_10769
Query: 1 TACTGATCATCCTTGCTGCT 20
||||||||||||:|||||||
Sbjct: 71 ATGACTAGTAGGGACGACGA 52
>miR1425-5p Score: 1.5 RO3G_10787
Query: 1 TACTGATCATCCTTGCTGCT 20
||||||||||||:||*||||
Sbjct: 194 ATGACTAGTAGGGACAACGA 175
>miR1425-5p Score: 1.5 RO3G_08847
Query: 1 TACTGATCATCCTTGCTGCT 20
||||||||||*|:|||||||
Sbjct: 194 ATGACTAGTAAGGACGACGA 175
>miR1425-5p Score: 0.0 RO3G_07690
```

Query: 1 TACTGATCATCCTTGCTGCT 20
||||||||||||||||||||
Sbjct: 194 ATGACTAGTAGGAACGACGA 175
>miR1425-5p Score: 0.0 RO3G_07707
Query: 1 TACTGATCATCCTTGCTGCT 20
||||||||||||||||||||
Sbjct: 386 ATGACTAGTAGGAACGACGA 367
>miR1425-5p Score: 0.5 RO3G_02706
Query: 1 TACTGATCATCCTTGCTGCT 20
||||||||||||:|||||||
Sbjct: 386 ATGACTAGTAGGGACGACGA 367
>miR1425-5p Score: 0.5 RO3G_02845
Query: 1 TACTGATCATCCTTGCTGCT 20
||||||||||||:|||||||
Sbjct: 419 ATGACTAGTAGGGACGACGA 400
>miR1425-5p Score: 0.5 RO3G_03602
Query: 1 TACTGATCATCCTTGCTGCT 20
||||||||||||:|||||||
Sbjct: 335 ATGACTAGTAGGGACGACGA 316
>miR1425-5p Score: 0.5 RO3G_03624
Query: 1 TACTGATCATCCTTGCTGCT 20
||||||||||||:|||||||
Sbjct: 194 ATGACTAGTAGGGACGACGA 175
>miR1425-5p Score: 0.0 RO3G_00679
Query: 1 TACTGATCATCCTTGCTGCT 20
||||||||||||||||||||
Sbjct: 386 ATGACTAGTAGGAACGACGA 367
>miR1425-5p Score: 0.5 RO3G_00688
Query: 1 TACTGATCATCCTTGCTGCT 20
||||||||||||:|||||||
Sbjct: 194 ATGACTAGTAGGGACGACGA 175
>miR1425-5p Score: 2.5 RO3G_01212
Query: 1 TACTGATCATCCTTGCTGCT 20
|||||||||||||||*|*
Sbjct: 1508 TTGACTAGTCGGAACGA-GT 1490
>miR1425-5p Score: 0.5 RO3G_01960
Query: 1 TACTGATCATCCTTGCTGCT 20
||||||||||||:|||||||
Sbjct: 386 ATGACTAGTAGGGACGACGA 367
>miR1425-5p Score: 0.0 RO3G_17408
Query: 1 TACTGATCATCCTTGCTGCT 20
||||||||||||||||||||
Sbjct: 194 ATGACTAGTAGGAACGACGA 175
>miR6270 Score: 2.0 RO3G_16714
Query: 1 TTCGCTATTGGAATCTTCATCT 22

||||*||:||||||||||*|||
Sbjct: 276 AAGCTATGACCTTAGAAGCAGA 255
>miR6270 Score: 0.0 RO3G_16803
Query: 1 TTCGCTATTGGAATCTTCATCT 22
||||||||||||||||||||||
Sbjct: 159 AAGCGATAACCTTAGAAGTAGA 138
>miR6270 Score: 1.5 RO3G_14983
Query: 1 TTCGCTATTGGAATCTTCATCT 22
||||||||:||||||*||||||
Sbjct: 723 AAGCGATAGCCTTAGCAGTAGA 702
>miR6270 Score: 1.5 RO3G_13914
Query: 1 TTCGCTATTGGAATCTTCATCT 22
||||||||:||||||*||||||
Sbjct: 918 AAGCGATAGCCTTAGCAGTAGA 897
>miR6270 Score: 1.5 RO3G_12667
Query: 1 TTCGCTATTGGAATCTTCATCT 22
||||||||:||||||*||||||
Sbjct: 288 AAGCGATAGCCTTAGCAGTAGA 267
>miR6270 Score: 1.5 RO3G_12058
Query: 1 TTCGCTATTGGAATCTTCATCT 22
||||||||:||||||*||||||
Sbjct: 918 AAGCGATAGCCTTAGCAGTAGA 897
>miR6270 Score: 1.5 RO3G_10801
Query: 1 TTCGCTATTGGAATCTTCATCT 22
||||||||:||||||*||||||
Sbjct: 723 AAGCGATAGCCTTAGCAGTAGA 702
>miR6270 Score: 1.5 RO3G_09614
Query: 1 TTCGCTATTGGAATCTTCATCT 22
||||||||:||||||*||||||
Sbjct: 249 AAGCGATAGCCTTAGCAGTAGA 228
>miR6270 Score: 1.0 RO3G_05930
Query: 1 TTCGCTATTGGAATCTTCATCT 22
|||||||||||*||||||||||
Sbjct: 582 AAGCGATAACCCTAGAAGTAGA 561
>miR6270 Score: 1.5 RO3G_04831
Query: 1 TTCGCTATTGGAATCTTCATCT 22
*|||||||||:||||:||||||
Sbjct: 1580 CAGCGATAACTTTAGGAGTAGA 1559
>miR6270 Score: 1.5 RO3G_02233
Query: 1 TTCGCTATTGGAATCTTCATCT 22
||||||||:||||||*||||||
Sbjct: 447 AAGCGATAGCCTTAGCAGTAGA 426
>miR6270 Score: 0.0 RO3G_03556
Query: 1 TTCGCTATTGGAATCTTCATCT 22
||||||||||||||||||||||

```
Sbjct: 723 AAGCGATAACCTTAGAAGTAGA 702
>miR6270 Score: 1.0 RO3G_03583
Query: 1 TTCGCTATTGGAATCTTCATCT 22
||||||||||||||*|||||||
Sbjct: 723 AAGCGATAACCTTAAAAGTAGA 702
>miR6270 Score: 0.0 RO3G_03780
Query: 1 TTCGCTATTGGAATCTTCATCT 22
||||||||||||||||||||||
Sbjct: 159 AAGCGATAACCTTAGAAGTAGA 138
>miR6270 Score: 0.0 RO3G_03794
Query: 1 TTCGCTATTGGAATCTTCATCT 22
||||||||||||||||||||||
Sbjct: 159 AAGCGATAACCTTAGAAGTAGA 138
>miR6270 Score: 1.5 RO3G_04116
Query: 1 TTCGCTATTGGAATCTTCATCT 22
||||*|||||||||||||*|||
Sbjct: 282 AAGCTATAACCTTAGAAGAAGA 261
>miR6270 Score: 1.5 RO3G_17462
Query: 1 TTCGCTATTGGAATCTTCATCT 22
||||||||:||||||*||||||
Sbjct: 1050 AAGCGATAGCCTTAGCAGTAGA 1029
>miR6270 Score: 1.5 RO3G_17451
Query: 1 TTCGCTATTGGAATCTTCATCT 22
||||||||:||||||*||||||
Sbjct: 159 AAGCGATAGCCTTAGCAGTAGA 138
>miR6270 Score: 0.0 RO3G_17374
Query: 1 TTCGCTATTGGAATCTTCATCT 22
||||||||||||||||||||||
Sbjct: 159 AAGCGATAACCTTAGAAGTAGA 138
>miR6270 Score: 0.0 RO3G_17281
Query: 1 TTCGCTATTGGAATCTTCATCT 22
||||||||||||||||||||||
Sbjct: 159 AAGCGATAACCTTAGAAGTAGA 138
>miR5532 Score: 2.5 RO3G_04039
Query: 1 TGGAATATATCACACAAGTTGG 22
*|||||||||||||||*:|:::
Sbjct: 395 CCCTTATATAGTGTGTATAGTT 374
>miR5144-5p Score: 2.5 RO3G_17223
Query: 1 TTCTTGTGCTGTGCAGTGA 19
||||||||||*|*||||*|
Sbjct: 95 AAGAACACGAAAAGTCAGT 77
>miR5144-5p Score: 2.5 RO3G_17017
Query: 1 TTCTTGTGCTGTGCAGTGA 19
||||||||||*|*||||*|
Sbjct: 371 AAGAACACGAAAAGTCAGT 353
```

>miR5144-5p Score: 0.0 RO3G_17069
Query: 1 TTCTTGTGCTGTGCAGTGA 19
|||||||||||||||||||
Sbjct: 236 AAGAACACGACACGTCACT 218
>miR5144-5p Score: 1.8 RO3G_17083
Query: 1 TTCTTGTGCTGTGCAGTGA 19
||||||||||||*|||::|
Sbjct: 1721 AAGAACACGACAAGTCGTT 1703
>miR5144-5p Score: 2.5 RO3G_16725
Query: 1 TTCTTGTGCTGTGCAGTGA 19
||||||||||*|*||||*|
Sbjct: 113 AAGAACACGAAAAGTCAGT 95
>miR5144-5p Score: 2.5 RO3G_16742
Query: 1 TTCTTGTGCTGTGCAGTGA 19
||||||||||*|*||||*|
Sbjct: 371 AAGAACACGAAAAGTCAGT 353
>miR5144-5p Score: 0.0 RO3G_16796
Query: 1 TTCTTGTGCTGTGCAGTGA 19
|||||||||||||||||||
Sbjct: 371 AAGAACACGACACGTCACT 353
>miR5144-5p Score: 0.0 RO3G_16798
Query: 1 TTCTTGTGCTGTGCAGTGA 19
|||||||||||||||||||
Sbjct: 371 AAGAACACGACACGTCACT 353
>miR5144-5p Score: 1.5 RO3G_16689
Query: 1 TTCTTGTGCTGTGCAGTGA 19
||*|||||||||||||:||
Sbjct: 371 AAAAACACGACACGTCGCT 353
>miR5144-5p Score: 2.5 RO3G_16698
Query: 1 TTCTTGTGCTGTGCAGTGA 19
||||||||||*|*||||*|
Sbjct: 368 AAGAACACGAAAAGTCAGT 350
>miR5144-5p Score: 2.5 RO3G_16706
Query: 1 TTCTTGTGCTGTGCAGTGA 19
||||||||||*|*||||*|
Sbjct: 95 AAGAACACGAAAAGTCAGT 77
>miR5144-5p Score: 2.5 RO3G_16372
Query: 1 TTCTTGTGCTGTGCAGTGA 19
||||||||||*|*||||*|
Sbjct: 371 AAGAACACGAAAAGTCAGT 353
>miR5144-5p Score: 2.5 RO3G_16383
Query: 1 TTCTTGTGCTGTGCAGTGA 19
||||||||||*|*||||*|
Sbjct: 371 AAGAACACGAAAAGTCAGT 353
>miR5144-5p Score: 2.5 RO3G_16420

```
Query: 1 TTCTTGTGCTGTGCAGTGA 19
||||||||||*|*||||*|
Sbjct: 122 AAGAACACGAAAAGTCAGT 104
>miR5144-5p Score: 0.5 RO3G_16431
Query: 1 TTCTTGTGCTGTGCAGTGA 19
||||||||||||:||||||
Sbjct: 239 AAGAACACGACATGTCACT 221
>miR5144-5p Score: 0.0 RO3G_16142
Query: 1 TTCTTGTGCTGTGCAGTGA 19
|||||||||||||||||||
Sbjct: 374 AAGAACACGACACGTCACT 356
>miR5144-5p Score: 0.0 RO3G_15664
Query: 1 TTCTTGTGCTGTGCAGTGA 19
|||||||||||||||||||
Sbjct: 371 AAGAACACGACACGTCACT 353
>miR5144-5p Score: 2.5 RO3G_15271
Query: 1 TTCTTGTGCTGTGCAGTGA 19
||||||||||*|*||||*|
Sbjct: 371 AAGAACACGAAAAGTCAGT 353
>miR5144-5p Score: 2.5 RO3G_15282
Query: 1 TTCTTGTGCTGTGCAGTGA 19
||||||||||*|*||||*|
Sbjct: 95 AAGAACACGAAAAGTCAGT 77
>miR5144-5p Score: 2.5 RO3G_15261
Query: 1 TTCTTGTGCTGTGCAGTGA 19
||||||||||*|*||||*|
Sbjct: 371 AAGAACACGAAAAGTCAGT 353
>miR5144-5p Score: 0.0 RO3G_14361
Query: 1 TTCTTGTGCTGTGCAGTGA 19
|||||||||||||||||||
Sbjct: 371 AAGAACACGACACGTCACT 353
>miR5144-5p Score: 1.5 RO3G_14568
Query: 1 TTCTTGTGCTGTGCAGTGA 19
||*|||||||||||||:||
Sbjct: 371 AAAAACACGACACGTCGCT 353
>miR5144-5p Score: 2.5 RO3G_14770
Query: 1 TTCTTGTGCTGTGCAGTGA 19
||||||||||*|*||||*|
Sbjct: 371 AAGAACACGAAAAGTCAGT 353
>miR5144-5p Score: 2.5 RO3G_14284
Query: 1 TTCTTGTGCTGTGCAGTGA 19
*||||||||||:*||||*|
Sbjct: 447 TAGAACACGACGGGTCA-T 430
>miR5144-5p Score: 2.5 RO3G_13714
Query: 1 TTCTTGTGCTGTGCAGTGA 19
```

```
||||||||||*|*||||*|
Sbjct: 371 AAGAACACGAAAAGTCAGT 353
>miR5144-5p Score: 2.5 RO3G_13852
Query: 1 TTCTTGTGCTGTGCAGTGA 19
||||||||||*|*||||*|
Sbjct: 122 AAGAACACGAAAAGTCAGT 104
>miR5144-5p Score: 2.5 RO3G_12663
Query: 1 TTCTTGTGCTGTGCAGTGA 19
||||||||||*|*||||*|
Sbjct: 206 AAGAACACGAAAAGTCAGT 188
>miR5144-5p Score: 1.0 RO3G_11966
Query: 1 TTCTTGTGCTGTGCAGTGA 19
||*||||||||||||||||
Sbjct: 371 AAAAACACGACACGTCACT 353
>miR5144-5p Score: 0.0 RO3G_12070
Query: 1 TTCTTGTGCTGTGCAGTGA 19
|||||||||||||||||||
Sbjct: 371 AAGAACACGACACGTCACT 353
>miR5144-5p Score: 2.5 RO3G_12537
Query: 1 TTCTTGTGCTGTGCAGTGA 19
||||||||||*|*||||*|
Sbjct: 122 AAGAACACGAAAAGTCAGT 104
>miR5144-5p Score: 2.5 RO3G_12653
Query: 1 TTCTTGTGCTGTGCAGTGA 19
||||||||||*|*||||*|
Sbjct: 644 AAGAACACGAAAAGTCAGT 626
>miR5144-5p Score: 1.5 RO3G_11238
Query: 1 TTCTTGTGCTGTGCAGTGA 19
||*|||||||||||||:||
Sbjct: 239 AAAAACACGACACGTCGCT 221
>miR5144-5p Score: 0.0 RO3G_11264
Query: 1 TTCTTGTGCTGTGCAGTGA 19
|||||||||||||||||||
Sbjct: 371 AAGAACACGACACGTCACT 353
>miR5144-5p Score: 2.5 RO3G_11781
Query: 1 TTCTTGTGCTGTGCAGTGA 19
||||||||||*|*||||*|
Sbjct: 371 AAGAACACGAAAAGTCAGT 353
>miR5144-5p Score: 0.0 RO3G_10516
Query: 1 TTCTTGTGCTGTGCAGTGA 19
|||||||||||||||||||
Sbjct: 371 AAGAACACGACACGTCACT 353
>miR5144-5p Score: 0.0 RO3G_10729
Query: 1 TTCTTGTGCTGTGCAGTGA 19
|||||||||||||||||||
```

Sbjct: 371 AAGAACACGACACGTCACT 353
>miR5144-5p Score: 0.0 RO3G_10743
Query: 1 TTCTTGTGCTGTGCAGTGA 19
|||||||||||||||||||
Sbjct: 371 AAGAACACGACACGTCACT 353
>miR5144-5p Score: 0.0 RO3G_10773
Query: 1 TTCTTGTGCTGTGCAGTGA 19
|||||||||||||||||||
Sbjct: 371 AAGAACACGACACGTCACT 353
>miR5144-5p Score: 0.0 RO3G_10780
Query: 1 TTCTTGTGCTGTGCAGTGA 19
|||||||||||||||||||
Sbjct: 371 AAGAACACGACACGTCACT 353
>miR5144-5p Score: 1.5 RO3G_10798
Query: 1 TTCTTGTGCTGTGCAGTGA 19
||*|||||||||||||:||
Sbjct: 371 AAAAACACGACACGTCGCT 353
>miR5144-5p Score: 2.5 RO3G_09193
Query: 1 TTCTTGTGCTGTGCAGTGA 19
||||||||||*|*||||*|
Sbjct: 95 AAGAACACGAAAAGTCAGT 77
>miR5144-5p Score: 0.0 RO3G_09194
Query: 1 TTCTTGTGCTGTGCAGTGA 19
|||||||||||||||||||
Sbjct: 371 AAGAACACGACACGTCACT 353
>miR5144-5p Score: 0.0 RO3G_09542
Query: 1 TTCTTGTGCTGTGCAGTGA 19
|||||||||||||||||||
Sbjct: 371 AAGAACACGACACGTCACT 353
>miR5144-5p Score: 0.0 RO3G_09945
Query: 1 TTCTTGTGCTGTGCAGTGA 19
|||||||||||||||||||
Sbjct: 95 AAGAACACGACACGTCACT 77
>miR5144-5p Score: 0.0 RO3G_09967
Query: 1 TTCTTGTGCTGTGCAGTGA 19
|||||||||||||||||||
Sbjct: 371 AAGAACACGACACGTCACT 353
>miR5144-5p Score: 2.5 RO3G_07984
Query: 1 TTCTTGTGCTGTGCAGTGA 19
||||||||||*|*||||*|
Sbjct: 371 AAGAACACGAAAAGTCAGT 353
>miR5144-5p Score: 2.0 RO3G_08312
Query: 1 TTCTTGTGCTGTGCAGTGA 19
||||||||||||*||*|||
Sbjct: 234 AAGAACACGACAGGTGACT 216

>miR5144-5p Score: 0.0 RO3G_08660
Query: 1 TTCTTGTGCTGTGCAGTGA 19
|||||||||||||||||||
Sbjct: 371 AAGAACACGACACGTCACT 353
>miR5144-5p Score: 0.0 RO3G_07030
Query: 1 TTCTTGTGCTGTGCAGTGA 19
|||||||||||||||||||
Sbjct: 371 AAGAACACGACACGTCACT 353
>miR5144-5p Score: 0.0 RO3G_07038
Query: 1 TTCTTGTGCTGTGCAGTGA 19
|||||||||||||||||||
Sbjct: 371 AAGAACACGACACGTCACT 353
>miR5144-5p Score: 0.0 RO3G_07040
Query: 1 TTCTTGTGCTGTGCAGTGA 19
|||||||||||||||||||
Sbjct: 371 AAGAACACGACACGTCACT 353
>miR5144-5p Score: 1.5 RO3G_07103
Query: 1 TTCTTGTGCTGTGCAGTGA 19
||*|||||||||||||:||
Sbjct: 95 AAAAACACGACACGTCGCT 77
>miR5144-5p Score: 0.0 RO3G_07124
Query: 1 TTCTTGTGCTGTGCAGTGA 19
|||||||||||||||||||
Sbjct: 371 AAGAACACGACACGTCACT 353
>miR5144-5p Score: 2.5 RO3G_07137
Query: 1 TTCTTGTGCTGTGCAGTGA 19
||||||||||*|*||||*|
Sbjct: 371 AAGAACACGAAAAGTCAGT 353
>miR5144-5p Score: 2.5 RO3G_07702
Query: 1 TTCTTGTGCTGTGCAGTGA 19
||||||||||*|*||||*|
Sbjct: 122 AAGAACACGAAAAGTCAGT 104
>miR5144-5p Score: 2.5 RO3G_07705
Query: 1 TTCTTGTGCTGTGCAGTGA 19
||||||||||*|*||||*|
Sbjct: 371 AAGAACACGAAAAGTCAGT 353
>miR5144-5p Score: 1.0 RO3G_07860
Query: 1 TTCTTGTGCTGTGCAGTGA 19
||||||||||*||||||||
Sbjct: 371 AAGAACACGAGACGTCACT 353
>miR5144-5p Score: 2.5 RO3G_07867
Query: 1 TTCTTGTGCTGTGCAGTGA 19
||||||||||*|*||||*|
Sbjct: 122 AAGAACACGAAAAGTCAGT 104
>miR5144-5p Score: 1.5 RO3G_05628

```
Query: 1 TTCTTGTGCTGTGCAGTGA 19
||*|||||||||||||:||
Sbjct: 371 AAAAACACGACACGTCGCT 353
>miR5144-5p Score: 0.0 RO3G_05921
Query: 1 TTCTTGTGCTGTGCAGTGA 19
|||||||||||||||||||
Sbjct: 371 AAGAACACGACACGTCACT 353
>miR5144-5p Score: 0.0 RO3G_05939
Query: 1 TTCTTGTGCTGTGCAGTGA 19
|||||||||||||||||||
Sbjct: 371 AAGAACACGACACGTCACT 353
>miR5144-5p Score: 1.0 RO3G_05953
Query: 1 TTCTTGTGCTGTGCAGTGA 19
||*||||||||||||||||
Sbjct: 371 AAAAACACGACACGTCACT 353
>miR5144-5p Score: 1.5 RO3G_05959
Query: 1 TTCTTGTGCTGTGCAGTGA 19
||*|||||||||||||:||
Sbjct: 371 AAAAACACGACACGTCGCT 353
>miR5144-5p Score: 2.5 RO3G_06008
Query: 1 TTCTTGTGCTGTGCAGTGA 19
|||:|||*||||||||:*|
Sbjct: 136 AAGGACAAGACACGTCG-T 119
>miR5144-5p Score: 2.0 RO3G_06678
Query: 1 TTCTTGTGCTGTGCAGTGA 19
|||||||||||*||||*||
Sbjct: 860 AAGAACACGACTCGTC-CT 843
>miR5144-5p Score: 2.5 RO3G_06717
Query: 1 TTCTTGTGCTGTGCAGTGA 19
||||||||||*|*||||*|
Sbjct: 371 AAGAACACGAAAAGTCAGT 353
>miR5144-5p Score: 2.5 RO3G_04674
Query: 1 TTCTTGTGCTGTGCAGTGA 19
||||||||||*|*||||*|
Sbjct: 122 AAGAACACGAAAAGTCAGT 104
>miR5144-5p Score: 2.5 RO3G_04682
Query: 1 TTCTTGTGCTGTGCAGTGA 19
||||||||||*|*||||*|
Sbjct: 371 AAGAACACGAAAAGTCAGT 353
>miR5144-5p Score: 2.5 RO3G_04695
Query: 1 TTCTTGTGCTGTGCAGTGA 19
||||||||||*|*||||*|
Sbjct: 161 AAGAACACGAAAAGTCAGT 143
>miR5144-5p Score: 2.5 RO3G_04759
Query: 1 TTCTTGTGCTGTGCAGTGA 19
```

```
*|||||||*|||*||||||
Sbjct: 285 TAGAACACCACAGGTCACT 267
>miR5144-5p Score: 0.0 RO3G_04803
Query: 1 TTCTTGTGCTGTGCAGTGA 19
|||||||||||||||||||
Sbjct: 371 AAGAACACGACACGTCACT 353
>miR5144-5p Score: 0.0 RO3G_05264
Query: 1 TTCTTGTGCTGTGCAGTGA 19
|||||||||||||||||||
Sbjct: 371 AAGAACACGACACGTCACT 353
>miR5144-5p Score: 0.0 RO3G_05267
Query: 1 TTCTTGTGCTGTGCAGTGA 19
|||||||||||||||||||
Sbjct: 371 AAGAACACGACACGTCACT 353
>miR5144-5p Score: 2.5 RO3G_05268
Query: 1 TTCTTGTGCTGTGCAGTGA 19
||||||||||*|*||||*|
Sbjct: 371 AAGAACACGAAAAGTCAGT 353
>miR5144-5p Score: 0.0 RO3G_05435
Query: 1 TTCTTGTGCTGTGCAGTGA 19
|||||||||||||||||||
Sbjct: 371 AAGAACACGACACGTCACT 353
>miR5144-5p Score: 2.5 RO3G_02824
Query: 1 TTCTTGTGCTGTGCAGTGA 19
||||||||||*|*||||*|
Sbjct: 371 AAGAACACGAAAAGTCAGT 353
>miR5144-5p Score: 0.0 RO3G_02956
Query: 1 TTCTTGTGCTGTGCAGTGA 19
|||||||||||||||||||
Sbjct: 371 AAGAACACGACACGTCACT 353
>miR5144-5p Score: 0.5 RO3G_03557
Query: 1 TTCTTGTGCTGTGCAGTGA 19
|||||||||||:|||||||
Sbjct: 197 AAGAACACGACGCGTCACT 179
>miR5144-5p Score: 0.0 RO3G_04207
Query: 1 TTCTTGTGCTGTGCAGTGA 19
|||||||||||||||||||
Sbjct: 371 AAGAACACGACACGTCACT 353
>miR5144-5p Score: 0.0 RO3G_04210
Query: 1 TTCTTGTGCTGTGCAGTGA 19
|||||||||||||||||||
Sbjct: 371 AAGAACACGACACGTCACT 353
>miR5144-5p Score: 2.5 RO3G_00238
Query: 1 TTCTTGTGCTGTGCAGTGA 19
||||||||||*|*||||*|
```

Sbjct: 371 AAGAACACGAAAAGTCAGT 353
>miR5144-5p Score: 0.0 RO3G_00256
Query: 1 TTCTTGTGCTGTGCAGTGA 19
|||||||||||||||||||
Sbjct: 371 AAGAACACGACACGTCACT 353
>miR5144-5p Score: 2.5 RO3G_00446
Query: 1 TTCTTGTGCTGTGCAGTGA 19
|||||||*||||*||||*|
Sbjct: 804 AAGAACAAGACAGGTCAGT 786
>miR5144-5p Score: 2.5 RO3G_01205
Query: 1 TTCTTGTGCTGTGCAGTGA 19
||||||||||*|*||||*|
Sbjct: 371 AAGAACACGAAAAGTCAGT 353
>miR5144-5p Score: 2.5 RO3G_01328
Query: 1 TTCTTGTGCTGTGCAGTGA 19
||||||||||*|*||||*|
Sbjct: 371 AAGAACACGAAAAGTCAGT 353
>miR5144-5p Score: 0.0 RO3G_01942
Query: 1 TTCTTGTGCTGTGCAGTGA 19
|||||||||||||||||||
Sbjct: 371 AAGAACACGACACGTCACT 353
>miR5144-5p Score: 2.5 RO3G_01962
Query: 1 TTCTTGTGCTGTGCAGTGA 19
||||||||||*|*||||*|
Sbjct: 122 AAGAACACGAAAAGTCAGT 104
>miR5144-5p Score: 0.0 RO3G_17449
Query: 1 TTCTTGTGCTGTGCAGTGA 19
|||||||||||||||||||
Sbjct: 371 AAGAACACGACACGTCACT 353
>miR5144-5p Score: 2.5 RO3G_17416
Query: 1 TTCTTGTGCTGTGCAGTGA 19
||||||||||*|*||||*|
Sbjct: 371 AAGAACACGAAAAGTCAGT 353
>miR5144-5p Score: 2.5 RO3G_17399
Query: 1 TTCTTGTGCTGTGCAGTGA 19
||||||||||*|*||||*|
Sbjct: 371 AAGAACACGAAAAGTCAGT 353
>miR5144-5p Score: 2.5 RO3G_17387
Query: 1 TTCTTGTGCTGTGCAGTGA 19
||||||||||*|*||||*|
Sbjct: 371 AAGAACACGAAAAGTCAGT 353
>miR5144-5p Score: 2.5 RO3G_17355
Query: 1 TTCTTGTGCTGTGCAGTGA 19
||||||||||*|*||||*|
Sbjct: 206 AAGAACACGAAAAGTCAGT 188

```
>miR5144-5p Score: 1.0 RO3G_17325
Query: 1 TTCTTGTGCTGTGCAGTGA 19
||*||||||||||||||||
Sbjct: 371 AAAAACACGACACGTCACT 353
>miR5144-5p Score: 1.5 RO3G_17308
Query: 1 TTCTTGTGCTGTGCAGTGA 19
||*|||||||||||||:||
Sbjct: 371 AAAAACACGACACGTCGCT 353
>miR5144-5p Score: 2.5 RO3G_17291
Query: 1 TTCTTGTGCTGTGCAGTGA 19
||||||||||*|*||||*|
Sbjct: 371 AAGAACACGAAAAGTCAGT 353
>miR6462e Score: 2.5 RO3G_16134
Query: 1 TTTTTGCCTTTTTGTCTACT 20
|||:|||||||||||*|**|
Sbjct: 646 AAAGACGGAAAAACAAA-AA 628
>miR6462e Score: 2.5 RO3G_16191
Query: 1 TTTTTGCCTTTTTGTCTA-CT 20
*|||||*|||||||||:|*||
Sbjct: 3674 CAAAACTGAAAAACAGGTAGA 3654
>miR6462e Score: 2.5 RO3G_16217
Query: 1 TTTTTGCCTTTTTGTCTACT 20
||||||||:||:|||*|*||
Sbjct: 339 AAAAACGGGAAGACAAA-GA 321
>miR6462e Score: 1.8 RO3G_16043
Query: 1 TTTTTGCCTTTTTGTCTACT 20
::||||||||||:|||||*|
Sbjct: 991 GGAAACGGAAAAGCAGAT-A 973
>miR6462e Score: 2.5 RO3G_15793
Query: 1 TTTTTGCCTTTTTGTCTACT 20
|||||:|||:||||:||**|
Sbjct: 865 AAAAATGGAGAAACGGA-AA 847
>miR6462e Score: 2.5 RO3G_15284
Query: 1 TTTTTGCCTTTTTGTCTACT 20
|||:|||||||||||*|**|
Sbjct: 646 AAAGACGGAAAAACAAA-AA 628
>miR6462e Score: 2.0 RO3G_15307
Query: 1 TTTTTGCCTTTTTGTCTACT 20
*:|||||||||||:|||*||
Sbjct: 1128 TGAAACGGAAAAATAGA-GA 1110
>miR6462e Score: 2.0 RO3G_15381
Query: 1 TTTTTGCCTTTTTGTCTACT 20
*:||||||||:|||||||*|
Sbjct: 842 TGAAACGGAAGAACAGATCA 823
>miR6462e Score: 2.5 RO3G_15382
```

```
Query: 1 TTTTTGCCTTTTTGTCTACT 20
|:||||||||:||*|||*||
Sbjct: 2455 AGAAACGGAAGAAAAGA-GA 2437
>miR6462e Score: 0.0 RO3G_15250
Query: 1 TTTTTGCCTTTTTGTCTACT 20
||||||||||||||||||||
Sbjct: 4129 AAAAACGGAAAAACAGATGA 4110
>miR6462e Score: 0.0 RO3G_14355
Query: 1 TTTTTGCCTTTTTGTCTACT 20
||||||||||||||||||||
Sbjct: 2380 AAAAACGGAAAAACAGATGA 2361
>miR6462e Score: 2.5 RO3G_14686
Query: 1 TTTTTGCCTTTTTGTCTACT 20
||||::|||||||:|||**|
Sbjct: 577 AAAAGTGGAAAAATAGA-AA 559
>miR6462e Score: 0.0 RO3G_13867
Query: 1 TTTTTGCCTTTTTGTCTACT 20
||||||||||||||||||||
Sbjct: 4090 AAAAACGGAAAAACAGATGA 4071
>miR6462e Score: 0.0 RO3G_13920
Query: 1 TTTTTGCCTTTTTGTCTACT 20
||||||||||||||||||||
Sbjct: 4174 AAAAACGGAAAAACAGATGA 4155
>miR6462e Score: 2.0 RO3G_14112
Query: 1 TTTTTGCCTTTTTGTCTACT 20
|:|||||||||||*||||*|
Sbjct: 265 AGAAACGGAAAAAGAGATTA 246
>miR6462e Score: 2.2 RO3G_13564
Query: 1 TTTTTGCCTTTTTGTCTACT 20
:||||||||:|:||||*|||
Sbjct: 177 GAAAACGGAGAGACAGTTGA 158
>miR6462e Score: 0.0 RO3G_13749
Query: 1 TTTTTGCCTTTTTGTCTACT 20
||||||||||||||||||||
Sbjct: 2683 AAAAACGGAAAAACAGATGA 2664
>miR6462e Score: 2.5 RO3G_12946
Query: 1 TTTTTGCCTTTTTGTCTACT 20
|:|||||||||||:|*|*||
Sbjct: 232 AGAAACGGAAAAATAAA-GA 214
>miR6462e Score: 2.5 RO3G_12236
Query: 1 TTTTTGCCTTTTTGTCTAC-T 20
|||||||*|||||:|||*|*|
Sbjct: 727 AAAAACGAAAAAATAGACGTA 707
>miR6462e Score: 2.2 RO3G_12274
Query: 1 TTTTTGCCTTTTTGTCTACT 20
```

```
:|*:|||||||||:||||||
Sbjct: 394 GATGACGGAAAAATAGATGA 375
>miR6462e Score: 2.0 RO3G_12330
Query: 1 TTTTTGCCTTTTTGTCTACT 20
||:|:|||||:||||||*||
Sbjct: 592 AAGAGCGGAAGAACAGACGA 573
>miR6462e Score: 1.5 RO3G_12588
Query: 1 TTTTTGCCTTTTTGTCTACT 20
*:|||||||||||||||*||
Sbjct: 880 CGAAACGGAAAAACAGACGA 861
>miR6462e Score: 2.0 RO3G_11598
Query: 1 TTTTTGCCTTTTTGTCTACT 20
|||||*||||||:||||*||
Sbjct: 673 AAAAAAGGAAAAGCAGAAGA 654
>miR6462e Score: 0.0 RO3G_11776
Query: 1 TTTTTGCCTTTTTGTCTACT 20
||||||||||||||||||||
Sbjct: 1264 AAAAACGGAAAAACAGATGA 1245
>miR6462e Score: 2.5 RO3G_10466
Query: 1 TTTTTGCCTTTTTGTCTA-CT 20
|:|||*|||||||||||**||
Sbjct: 1360 AGAAAAGGAAAAACAGAAAGA 1340
>miR6462e Score: 0.0 RO3G_10558
Query: 1 TTTTTGCCTTTTTGTCTACT 20
||||||||||||||||||||
Sbjct: 1927 AAAAACGGAAAAACAGATGA 1908
>miR6462e Score: 2.5 RO3G_11011
Query: 1 TTTTTGCCTTTTTGTCTAC-T 20
|||||||*|:|||||||*|*|
Sbjct: 525 AAAAACGAAGAAACAGAAGCA 505
>miR6462e Score: 2.5 RO3G_09886
Query: 1 TTTTTGCCTTTTTGTCTACT 20
||||||*|||||||||*|*|
Sbjct: 295 AAAAACTGAAAAACAGTTAA 276
>miR6462e Score: 2.5 RO3G_09933
Query: 1 TTTTTGCCTTTTTGTCTACT 20
|||:|||||||||||*|**|
Sbjct: 646 AAAGACGGAAAAACAAA-AA 628
>miR6462e Score: 2.5 RO3G_09957
Query: 1 TTTTTGCCTTTTTGTCTACT 20
|||:|||||||||||*|**|
Sbjct: 646 AAAGACGGAAAAACAAA-AA 628
>miR6462e Score: 0.0 RO3G_09965
Query: 1 TTTTTGCCTTTTTGTCTACT 20
||||||||||||||||||||
```

```
Sbjct: 4174 AAAAACGGAAAAACAGATGA 4155
>miR6462e Score: 2.5 RO3G_10229
Query: 1 TTTTTGCCTTTTTGTCTACT 20
|||:|||||||||||*|**|
Sbjct: 58 AAAGACGGAAAAACAAA-AA 40
>miR6462e Score: 2.2 RO3G_08307
Query: 1 TTTTTGCCTTTTTGTCTACT 20
:||||||||*||:|||||*|
Sbjct: 2137 GAAAACGGACAAGCAGAT-A 2119
>miR6462e Score: 2.0 RO3G_07491
Query: 1 TTTTTGCCTTTTTGTCTACT 20
|||||*||||||||:|||*|
Sbjct: 46 AAAAAGGGAAAAACGGAT-A 28
>miR6462e Score: 2.5 RO3G_07675
Query: 1 TTTTTGCCTTTTTGTCTACT 20
|||:|||||||||||*|**|
Sbjct: 58 AAAGACGGAAAAACAAA-AA 40
>miR6462e Score: 0.0 RO3G_05619
Query: 1 TTTTTGCCTTTTTGTCTACT 20
||||||||||||||||||||
Sbjct: 2317 AAAAACGGAAAAACAGATGA 2298
>miR6462e Score: 0.0 RO3G_06427
Query: 1 TTTTTGCCTTTTTGTCTACT 20
||||||||||||||||||||
Sbjct: 3766 AAAAACGGAAAAACAGATGA 3747
>miR6462e Score: 0.0 RO3G_06428
Query: 1 TTTTTGCCTTTTTGTCTACT 20
||||||||||||||||||||
Sbjct: 3268 AAAAACGGAAAAACAGATGA 3249
>miR6462e Score: 0.0 RO3G_06442
Query: 1 TTTTTGCCTTTTTGTCTACT 20
||||||||||||||||||||
Sbjct: 4831 AAAAACGGAAAAACAGATGA 4812
>miR6462e Score: 2.2 RO3G_06452
Query: 1 TTTTTGCCTTTTTGTCTACT 20
:||||*|||||||||||**|
Sbjct: 565 GAAAAAGGAAAAACAGAACA 546
>miR6462e Score: 2.2 RO3G_04477
Query: 1 TTTTTGCCTTTTTGTCTACT 20
:|||*||||||||:||||*|
Sbjct: 2971 GAAACCGGAAAAATAGATAA 2952
>miR6462e Score: 2.5 RO3G_04479
Query: 1 TTTTTGCCTTTTTGTCTACT 20
||:||||||:|||*||||*|
Sbjct: 229 AAGAACGGAGAAAAAGATAA 210
```

>miR6462e Score: 2.5 RO3G_02685
Query: 1 TTTTTGCCTTTTTGTCTACT 20
|:|||||||:||||:||**|
Sbjct: 741 AGAAACGGAGAAACGGAACA 722
>miR6462e Score: 0.0 RO3G_02691
Query: 1 TTTTTGCCTTTTTGTCTACT 20
||||||||||||||||||||
Sbjct: 7663 AAAAACGGAAAAACAGATGA 7644
>miR6462e Score: 2.5 RO3G_02760
Query: 1 TTTTTGCCTTTTTGTCTACT 20
|||:|*|||:|||||||*||
Sbjct: 412 AAAGAAGGAGAAACAGAAGA 393
>miR6462e Score: 2.5 RO3G_02836
Query: 1 TTTTTGCCTTTTTGTCTACT 20
|||:|||||||||||*|**|
Sbjct: 646 AAAGACGGAAAAACAAA-AA 628
>miR6462e Score: 0.0 RO3G_02959
Query: 1 TTTTTGCCTTTTTGTCTACT 20
||||||||||||||||||||
Sbjct: 4174 AAAAACGGAAAAACAGATGA 4155
>miR6462e Score: 2.5 RO3G_03104
Query: 1 TTTTTGCCTTTTTGTCTACT 20
|||:|||||||||||*|**|
Sbjct: 646 AAAGACGGAAAAACAAA-AA 628
>miR6462e Score: 0.0 RO3G_03547
Query: 1 TTTTTGCCTTTTTGTCTACT 20
||||||||||||||||||||
Sbjct: 4174 AAAAACGGAAAAACAGATGA 4155
>miR6462e Score: 0.0 RO3G_04115
Query: 1 TTTTTGCCTTTTTGTCTACT 20
||||||||||||||||||||
Sbjct: 1117 AAAAACGGAAAAACAGATGA 1098
>miR6462e Score: 2.5 RO3G_04218
Query: 1 TTTTTGCCTTTTTGTCTACT 20
|||:|||||||||||*|**|
Sbjct: 646 AAAGACGGAAAAACAAA-AA 628
>miR6462e Score: 2.5 RO3G_00105
Query: 1 TTTTTGCCTTTTTGTCTACT 20
||||||||*:||:||||*||
Sbjct: 2170 AAAAACGGTGAAGCAGAAGA 2151
>miR6462e Score: 2.5 RO3G_00458
Query: 1 TTTTTGCCTTTTTGTCTA-CT 20
|:|||*|||||||||||**||
Sbjct: 1447 AGAAAAGGAAAAACAGAAAGA 1427
>miR6462e Score: 2.5 RO3G_00717

```
Query: 1 TTTTTGCCTTTTTGTCTACT 20
||||*||||||||*|||*||
Sbjct: 217 AAAACCGGAAAAAAAGAAGA 198
>miR6462e Score: 2.0 RO3G_01140
Query: 1 TTTTTGCCTTTTTGTCTACT 20
||||:||||||||*|||*||
Sbjct: 247 AAAAGCGGAAAAAAAGAAGA 228
>miR6462e Score: 0.0 RO3G_01330
Query: 1 TTTTTGCCTTTTTGTCTACT 20
||||||||||||||||||||
Sbjct: 1408 AAAAACGGAAAAACAGATGA 1389
>miR6462e Score: 2.5 RO3G_01423
Query: 1 TTTTTGCCTTTTTGTCTACT 20
*:||||||||:||*||||||
Sbjct: 25 TGAAACGGAAGAAAAGATGA 6
>miR6462e Score: 2.5 RO3G_01430
Query: 1 TTTTTGCCTTTTTGTCTACT 20
*:||||||||:||*||||||
Sbjct: 25 TGAAACGGAAGAAAAGATGA 6
>miR6462e Score: 0.0 RO3G_01613
Query: 1 TTTTTGCCTTTTTGTCTACT 20
||||||||||||||||||||
Sbjct: 2371 AAAAACGGAAAAACAGATGA 2352
>miR6462e Score: 2.5 RO3G_01940
Query: 1 TTTTTGCCTTTTTGTCTACT 20
|||:|||||||||||*|**|
Sbjct: 646 AAAGACGGAAAAACAAA-AA 628
>miR6462e Score: 2.5 RO3G_02035
Query: 1 TTTTTGCCTTTTTGTCTACT 20
*|||||||||*||||||**|
Sbjct: 2454 TAAAACGGAACAACAGA-AA 2436
>miR6462e Score: 2.5 RO3G_02179
Query: 1 TTTTTGCCTTTTTGTCTACT 20
*|||||||*||:|||||*||
Sbjct: 2214 CAAAACGGTAAGACAGACGA 2195
>miR6462e Score: 2.2 RO3G_02193
Query: 1 TTTTTGCCTTTTTGTCTACT 20
:||||||*||||||:||*||
Sbjct: 271 GAAAACGAAAAAACGGAAGA 252
>miR6462e Score: 0.0 RO3G_02222
Query: 1 TTTTTGCCTTTTTGTCTACT 20
||||||||||||||||||||
Sbjct: 1228 AAAAACGGAAAAACAGATGA 1209
>miR6462e Score: 0.0 RO3G_17404
Query: 1 TTTTTGCCTTTTTGTCTACT 20
```

```
||||||||||||||||||||
Sbjct: 802 AAAAACGGAAAAACAGATGA 783
>miR6462e Score: 0.0 RO3G_17371
Query: 1 TTTTTGCCTTTTTGTCTACT 20
||||||||||||||||||||
Sbjct: 4129 AAAAACGGAAAAACAGATGA 4110
>miR6462e Score: 1.0 RO3G_17326
Query: 1 TTTTTGCCTTTTTGTCTACT 20
|||||||||||||||*||||
Sbjct: 4006 AAAAACGGAAAAACACATGA 3987
>miR9657a-3p Score: 1.5 RO3G_15836
Query: 1 TGGTGCTTCCTGTCGTACAGGT 22
|||*||||||||||:|||||||
Sbjct: 112 ACCTCGAAGGACAGTATGTCCA 91
>miR9657a-3p Score: 0.5 RO3G_06070
Query: 1 TGGTGCTTCCTGTCGTACAGGT 22
||||||||||||||:|||||||
Sbjct: 112 ACCACGAAGGACAGTATGTCCA 91
>miR6202 Score: 0.0 RO3G_16319
Query: 1 TGAGGATTTAAGCTTTGAAG 20
||||||||||||||||||||
Sbjct: 4073 ACTCCTAAATTCGAAACTTC 4054
>miR6202 Score: 2.0 RO3G_15536
Query: 1 TGAGGATTTAAGCTTTGAAG 20
||||||||:|||||||**||
Sbjct: 3647 ACTCCTAAGTTCGAAA-ATC 3629
>miR6202 Score: 0.0 RO3G_14345
Query: 1 TGAGGATTTAAGCTTTGAAG 20
||||||||||||||||||||
Sbjct: 35 ACTCCTAAATTCGAAACTTC 16
>miR6202 Score: 0.0 RO3G_12753
Query: 1 TGAGGATTTAAGCTTTGAAG 20
||||||||||||||||||||
Sbjct: 35 ACTCCTAAATTCGAAACTTC 16
>miR6202 Score: 0.0 RO3G_12207
Query: 1 TGAGGATTTAAGCTTTGAAG 20
||||||||||||||||||||
Sbjct: 4073 ACTCCTAAATTCGAAACTTC 4054
>miR6202 Score: 2.0 RO3G_10954
Query: 1 TGAGGATTTAAGCTTTGAAG 20
|*||||:||||:||||||||
Sbjct: 35 AGTCCTGAATTTGAAACTTC 16
>miR6202 Score: 1.0 RO3G_09206
Query: 1 TGAGGATTTAAGCTTTGAAG 20
||||||||||||*|||||||
```

Sbjct: 35 ACTCCTAAATTCCAAACTTC 16
>miR6202 Score: 2.5 RO3G_09360
Query: 1 TGAGGATTTAAGCTTTGAAG 20
||**||||||||||||:|||
Sbjct: 309 ACAACTAAATTCGAAATTTC 290
>miR6202 Score: 2.5 RO3G_09423
Query: 1 TGAGGATTTAAGCTTTGAAG 20
|||||:|||:||||||||
Sbjct: 351 TCTGCTAGATTTGAAACTTC 332
>miR6202 Score: 0.0 RO3G_08062
Query: 1 TGAGGATTTAAGCTTTGAAG 20
||||||||||||||||||||
Sbjct: 854 ACTCCTAAATTCGAAACTTC 835
>miR6202 Score: 0.0 RO3G_08114
Query: 1 TGAGGATTTAAGCTTTGAAG 20
||||||||||||||||||||
Sbjct: 35 ACTCCTAAATTCGAAACTTC 16
>miR6202 Score: 0.0 RO3G_06977
Query: 1 TGAGGATTTAAGCTTTGAAG 20
||||||||||||||||||||
Sbjct: 638 ACTCCTAAATTCGAAACTTC 619
>miR6202 Score: 2.5 RO3G_05992
Query: 1 TGAGGATTTAAGCTTTGAAG 20
||||||||||||||**||*|
Sbjct: 825 ACTCCTAAATTCGACTCTAC 806
>miR6202 Score: 0.0 RO3G_04256
Query: 1 TGAGGATTTAAGCTTTGAAG 20
||||||||||||||||||||
Sbjct: 35 ACTCCTAAATTCGAAACTTC 16
>miR6202 Score: 0.0 RO3G_02944
Query: 1 TGAGGATTTAAGCTTTGAAG 20
||||||||||||||||||||
Sbjct: 3392 ACTCCTAAATTCGAAACTTC 3373
>miR6202 Score: 1.2 RO3G_00493
Query: 1 TGAGGATTTAAGCTTTGAAG 20
:*||||||||||||||||||
Sbjct: 377 GATCCTAAATTCGAAACTTC 358
>miR6202 Score: 0.0 RO3G_00653
Query: 1 TGAGGATTTAAGCTTTGAAG 20
||||||||||||||||||||
Sbjct: 3983 ACTCCTAAATTCGAAACTTC 3964
>miR6202 Score: 0.0 RO3G_01026
Query: 1 TGAGGATTTAAGCTTTGAAG 20
||||||||||||||||||||
Sbjct: 35 ACTCCTAAATTCGAAACTTC 16

>miR6202 Score: 0.0 RO3G_01242
Query: 1 TGAGGATTTAAGCTTTGAAG 20
||||||||||||||||||||
Sbjct: 35 ACTCCTAAATTCGAAACTTC 16
>miR6202 Score: 0.0 RO3G_01243
Query: 1 TGAGGATTTAAGCTTTGAAG 20
||||||||||||||||||||
Sbjct: 35 ACTCCTAAATTCGAAACTTC 16
>miR6202 Score: 2.5 RO3G_01379
Query: 1 TGAGGATTTAAGCTTTGAAG 20
|||*:|||*|||||||||||
Sbjct: 324 ACTATTAACTTCGAAACTTC 305
>miR6202 Score: 2.2 RO3G_17137
Query: 1 TGAGGATTTAAGCTTTGAAG 20
|*|||*|||||||||||||:
Sbjct: 1523 AGTCCCAAATTCGAAACTTT 1504
>miR167c-3p Score: 0.0 RO3G_17238
Query: 1 TGTATGACTTGGTAGTTTCACC 22
||||||||||||||||||||||
Sbjct: 202 ACATACTGAACCATCAAAGTGG 181
>miR167c-3p Score: 0.0 RO3G_16718
Query: 1 TGTATGACTTGGTAGTTTCACC 22
||||||||||||||||||||||
Sbjct: 55 ACATACTGAACCATCAAAGTGG 34
>miR167c-3p Score: 0.0 RO3G_16690
Query: 1 TGTATGACTTGGTAGTTTCACC 22
||||||||||||||||||||||
Sbjct: 55 ACATACTGAACCATCAAAGTGG 34
>miR167c-3p Score: 0.0 RO3G_15666
Query: 1 TGTATGACTTGGTAGTTTCACC 22
||||||||||||||||||||||
Sbjct: 202 ACATACTGAACCATCAAAGTGG 181
>miR167c-3p Score: 0.0 RO3G_15140
Query: 1 TGTATGACTTGGTAGTTTCACC 22
||||||||||||||||||||||
Sbjct: 55 ACATACTGAACCATCAAAGTGG 34
>miR167c-3p Score: 0.0 RO3G_15144
Query: 1 TGTATGACTTGGTAGTTTCACC 22
||||||||||||||||||||||
Sbjct: 55 ACATACTGAACCATCAAAGTGG 34
>miR167c-3p Score: 0.0 RO3G_15148
Query: 1 TGTATGACTTGGTAGTTTCACC 22
||||||||||||||||||||||
Sbjct: 55 ACATACTGAACCATCAAAGTGG 34
>miR167c-3p Score: 0.0 RO3G_14546

```
Query: 1 TGTATGACTTGGTAGTTTCACC 22
||||||||||||||||||||||
Sbjct: 151 ACATACTGAACCATCAAAGTGG 130
>miR167c-3p Score: 0.0 RO3G_14561
Query: 1 TGTATGACTTGGTAGTTTCACC 22
||||||||||||||||||||||
Sbjct: 202 ACATACTGAACCATCAAAGTGG 181
>miR167c-3p Score: 1.0 RO3G_14720
Query: 1 TGTATGACTTGGTAGTTTCACC 22
|||||||*||||||||||||||
Sbjct: 151 ACATACTTAACCATCAAAGTGG 130
>miR167c-3p Score: 0.0 RO3G_13713
Query: 1 TGTATGACTTGGTAGTTTCACC 22
||||||||||||||||||||||
Sbjct: 55 ACATACTGAACCATCAAAGTGG 34
>miR167c-3p Score: 0.0 RO3G_13729
Query: 1 TGTATGACTTGGTAGTTTCACC 22
||||||||||||||||||||||
Sbjct: 202 ACATACTGAACCATCAAAGTGG 181
>miR167c-3p Score: 0.0 RO3G_13740
Query: 1 TGTATGACTTGGTAGTTTCACC 22
||||||||||||||||||||||
Sbjct: 55 ACATACTGAACCATCAAAGTGG 34
>miR167c-3p Score: 0.0 RO3G_13851
Query: 1 TGTATGACTTGGTAGTTTCACC 22
||||||||||||||||||||||
Sbjct: 55 ACATACTGAACCATCAAAGTGG 34
>miR167c-3p Score: 0.0 RO3G_12978
Query: 1 TGTATGACTTGGTAGTTTCACC 22
||||||||||||||||||||||
Sbjct: 202 ACATACTGAACCATCAAAGTGG 181
>miR167c-3p Score: 0.0 RO3G_13282
Query: 1 TGTATGACTTGGTAGTTTCACC 22
||||||||||||||||||||||
Sbjct: 202 ACATACTGAACCATCAAAGTGG 181
>miR167c-3p Score: 0.0 RO3G_10805
Query: 1 TGTATGACTTGGTAGTTTCACC 22
||||||||||||||||||||||
Sbjct: 202 ACATACTGAACCATCAAAGTGG 181
>miR167c-3p Score: 0.0 RO3G_09203
Query: 1 TGTATGACTTGGTAGTTTCACC 22
||||||||||||||||||||||
Sbjct: 151 ACATACTGAACCATCAAAGTGG 130
>miR167c-3p Score: 0.0 RO3G_08833
Query: 1 TGTATGACTTGGTAGTTTCACC 22
```

```
||||||||||||||||||||||
Sbjct: 202 ACATACTGAACCATCAAAGTGG 181
>miR167c-3p Score: 0.0 RO3G_05949
Query: 1 TGTATGACTTGGTAGTTTCACC 22
||||||||||||||||||||||
Sbjct: 202 ACATACTGAACCATCAAAGTGG 181
>miR167c-3p Score: 0.0 RO3G_03586
Query: 1 TGTATGACTTGGTAGTTTCACC 22
||||||||||||||||||||||
Sbjct: 151 ACATACTGAACCATCAAAGTGG 130
>miR167c-3p Score: 0.0 RO3G_03606
Query: 1 TGTATGACTTGGTAGTTTCACC 22
||||||||||||||||||||||
Sbjct: 202 ACATACTGAACCATCAAAGTGG 181
>miR167c-3p Score: 0.0 RO3G_03613
Query: 1 TGTATGACTTGGTAGTTTCACC 22
||||||||||||||||||||||
Sbjct: 151 ACATACTGAACCATCAAAGTGG 130
>miR167c-3p Score: 0.0 RO3G_03620
Query: 1 TGTATGACTTGGTAGTTTCACC 22
||||||||||||||||||||||
Sbjct: 202 ACATACTGAACCATCAAAGTGG 181
>miR167c-3p Score: 0.0 RO3G_17360
Query: 1 TGTATGACTTGGTAGTTTCACC 22
||||||||||||||||||||||
Sbjct: 55 ACATACTGAACCATCAAAGTGG 34
>miR2082 Score: 2.0 RO3G_16753
Query: 1 TGTTGTTCCTTCTTCTTCTCTT 22
|:||:|:||||||||||||*||
Sbjct: 421 ATAATAGGGAAGAAGAAGA-AA 401
>miR2082 Score: 2.5 RO3G_16006
Query: 1 TGTTGTTCCTTCTTCTTCTCTT 22
|*:|*|||||||||||||||||
Sbjct: 1460 AAGAAAAGGAAGAAGAAGAGAA 1439
>miR2082 Score: 2.5 RO3G_15307
Query: 1 TGTTGTTCCTTCTTCTTCTCTT 22
||:||||*|||||||||*|*||
Sbjct: 1017 ACGACAAAGAAGAAGAA-ATAA 997
>miR2082 Score: 2.5 RO3G_15547
Query: 1 TGTTGTTCCTTCTTCTTCT-CTT 22
:||:|||||:||||||||:*|*|
Sbjct: 606 GCAGCAAGGGAGAAGAAGGTGCA 584
>miR2082 Score: 2.5 RO3G_15565
Query: 1 TGTTGTTCCTTCTTCTTC-TCTT 22
|*|||:|||:||||||||*||||
```

Sbjct: 713 AGAACGAGGGAGAAGAAGAAGAA 691
>miR2082 Score: 2.5 RO3G_15097
Query: 1 TGTTGTTCCTTCTTCTTCTCTT 22
||:|*||||||||||||*|||*
Sbjct: 1256 ACGAGAAGGAAGAAGAAAAGAC 1235
>miR2082 Score: 2.5 RO3G_14402
Query: 1 TGTTGTTCCTTCTTCTTCTCTT 22
|||||||*|||||||*||*|||
Sbjct: 1227 ACAACAAAGAAGAAGTAGCGAA 1206
>miR2082 Score: 2.5 RO3G_13891
Query: 1 TGTTGTTCCTTCTTCTTC-TCTT 22
||*|||*|||||||||||*||||
Sbjct: 1140 ACCACACGGAAGAAGAAGAAGAA 1118
>miR2082 Score: 2.5 RO3G_13935
Query: 1 TGTTGTTCCTTCTTCTTCT-CTT 22
||:|||||||||*||||||**||
Sbjct: 1005 ACGACAAGGAAGTAGAAGATTAA 983
>miR2082 Score: 2.5 RO3G_12789
Query: 1 TGTTGTTCCTTCTTCTTCTCTT 22
|||||||||*||||*||*||||
Sbjct: 80 ACAACAAGGTAGAAAAA-AGAA 60
>miR2082 Score: 2.0 RO3G_13069
Query: 1 TGTTGTTCCTTCTTCTTCTCTT 22
|||||||*|||||||:|*||||
Sbjct: 962 ACAACAAAGAAGAAGGAAAGAA 941
>miR2082 Score: 2.2 RO3G_13123
Query: 1 TGTTGTTCCTTCTTCTTCTCTT 22
|:||||:||:||||||||*||:
Sbjct: 834 ATAACAGGGGAGAAGAAGTGAG 813
>miR2082 Score: 2.5 RO3G_12266
Query: 1 TGTTGTTCCTTCTTCTTCTCTT 22
|*|||||*|||||||||||*||
Sbjct: 599 AAAACAAAGAAGAAGAAGAAAA 578
>miR2082 Score: 2.5 RO3G_11023
Query: 1 TGTTGTTCCTTCTTCTTC-TCTT 22
|||::||*||||||||||*||||
Sbjct: 1515 ACAGTAAAGAAGAAGAAGAAGAA 1493
>miR2082 Score: 2.5 RO3G_09811
Query: 1 TGTTGTTCCTTCTTCTTCTCTT 22
||:|:||*|||||||||||*||
Sbjct: 135 ACGATAAAGAAGAAGAAGAAAA 114
>miR2082 Score: 2.5 RO3G_08027
Query: 1 TGTTGTTCCTTCTTCTTCTCTT 22
|*||||||||||||*||||*||
Sbjct: 608 AGAACAAGGAAGAAAAAGAAAA 587

```
>miR2082 Score: 2.5 RO3G_09036
Query: 1 TGTTGTTCCTTCTTCTTC-TCTT 22
|*|||||*||||||||||*||||
Sbjct: 477 AAAACAAAGAAGAAGAAGGAGAA 455
>miR2082 Score: 2.5 RO3G_06853
Query: 1 TGTTGTTCCTTCTTCTTCTCTT 22
|*||*||||||||||||*||||
Sbjct: 1131 AAAAGAAGGAAGAAGAAAAGAA 1110
>miR2082 Score: 2.5 RO3G_06856
Query: 1 TGTTGTTCCTTCTTCTTCTCTT 22
|*||*||||||||||||*||||
Sbjct: 552 AAAAGAAGGAAGAAGAAAAGAA 531
>miR2082 Score: 2.5 RO3G_07217
Query: 1 TGTTGTTCCTTCTTCTTC-TCTT 22
|*||*|||||||||||||*||||
Sbjct: 449 AGAAGAAGGAAGAAGAAGAAGAA 427
>miR2082 Score: 2.5 RO3G_07516
Query: 1 TGTTGTTCCTTCTTCTTCTCTT 22
*|:|*|||||:|||||||||||
Sbjct: 1553 CCGAGAAGGAGGAAGAAGAGAA 1532
>miR2082 Score: 2.5 RO3G_05645
Query: 1 TGTTGTTCCTTCTTCTTC-TCTT 22
||||*|||||||||*|||*||||
Sbjct: 476 ACAAAAAGGAAGAACAAGAAGAA 454
>miR2082 Score: 2.0 RO3G_05980
Query: 1 TGTTGTTCCTTCTTCTTCTCTT 22
||*|:||||||||||||||||*
Sbjct: 231 ACTATAAGGAAGAAGAAGAGAT 210
>miR2082 Score: 2.5 RO3G_06032
Query: 1 TGTTGTTCCTTCTTCTTCTCTT 22
|::::||||||||||||*||||
Sbjct: 405 ATGGTAAGGAAGAAGAA-AGAA 385
>miR2082 Score: 2.5 RO3G_06076
Query: 1 TGTTGTTCCTTCTTCTTCTCTT 22
|:||||||||:*||||:|||||
Sbjct: 50 ATAACAAGGAGAAAGAGGAGAA 29
>miR2082 Score: 2.5 RO3G_06212
Query: 1 TGTTGTTCCTTCTTCTTCTCTT 22
*||||::|||||||||:*||||
Sbjct: 379 TCAACGGGGAAGAAGAGAAGAA 358
>miR2082 Score: 2.0 RO3G_06567
Query: 1 TGTTGTTCCTTCTTCTTCTCTT 22
|||||:|*|||||||||||*||
Sbjct: 641 ACAACGAAGAAGAAGAAGA-AA 621
>miR2082 Score: 2.5 RO3G_02630
```

Query: 1 TGTTGTTCCTTCTTCTTCTCTT 22
|*:|*|||||||||||||||||
Sbjct: 327 AAGAGAAGGAAGAAGAAGAGAA 306
>miR2082 Score: 2.5 RO3G_03261
Query: 1 TGTTGTTCCTTCTTCTTCTCTT 22
||||*|||||||::|||*||||
Sbjct: 36 ACAAAAAGGAAGGGGAACAGAA 15
>miR2082 Score: 2.5 RO3G_03385
Query: 1 TGTTGTTCCTTCTTCTTCTCTT 22
|*||||||||||||*||||*||
Sbjct: 1148 AAAACAAGGAAGAAAAAGA-AA 1128
>miR2082 Score: 2.5 RO3G_03704
Query: 1 TGTTGTTCCTTCTTCTTCTCTT 22
|*||||||||:||:||:|||||
Sbjct: 158 AGAACAAGGAGGAGGAGGAGAA 137
>miR2082 Score: 2.0 RO3G_03818
Query: 1 TGTTGTTCCTTCTTCTTCT-CTT 22
|*|||||||||||||||*|*|||
Sbjct: 3617 AGAACAAGGAAGAAGAAAACGAA 3595
>miR2082 Score: 2.5 RO3G_03958
Query: 1 TGTTGTTCCTTCTTCTTCTCTT 22
||:||||*|||||||||*|*||
Sbjct: 188 ACGACAAAGAAGAAGAAAAAAA 167
>miR7803b-3p Score: 0.5 RO3G_17223
Query: 1 TGACACGTCGTCAATCATGTACT 23
||||||||||:||||||||||||
Sbjct: 157 ACTGTGCAGCGGTTAGTACATGA 135
>miR7803b-3p Score: 0.5 RO3G_17017
Query: 1 TGACACGTCGTCAATCATGTACT 23
||||||||||:||||||||||||
Sbjct: 433 ACTGTGCAGCGGTTAGTACATGA 411
>miR7803b-3p Score: 0.5 RO3G_16725
Query: 1 TGACACGTCGTCAATCATGTACT 23
||||||||||:||||||||||||
Sbjct: 175 ACTGTGCAGCGGTTAGTACATGA 153
>miR7803b-3p Score: 1.0 RO3G_16742
Query: 1 TGACACGTCGTCAATCATGTACT 23
|:||||||||:||||||||||||
Sbjct: 433 ATTGTGCAGCGGTTAGTACATGA 411
>miR7803b-3p Score: 0.8 RO3G_16698
Query: 1 TGACACGTCGTCAATCATGTACT 23
:|||||||||:||||||||||||
Sbjct: 430 GCTGTGCAGCGGTTAGTACATGA 408
>miR7803b-3p Score: 1.0 RO3G_16706
Query: 1 TGACACGTCGTCAATCATGTACT 23

|:||||||||:||||||||||||
Sbjct: 157 ATTGTGCAGCGGTTAGTACATGA 135
>miR7803b-3p Score: 1.0 RO3G_16372
Query: 1 TGACACGTCGTCAATCATGTACT 23
|:||||||||:||||||||||||
Sbjct: 433 ATTGTGCAGCGGTTAGTACATGA 411
>miR7803b-3p Score: 0.5 RO3G_16383
Query: 1 TGACACGTCGTCAATCATGTACT 23
||||||||||:||||||||||||
Sbjct: 433 ACTGTGCAGCGGTTAGTACATGA 411
>miR7803b-3p Score: 0.5 RO3G_16420
Query: 1 TGACACGTCGTCAATCATGTACT 23
||||||||||:||||||||||||
Sbjct: 184 ACTGTGCAGCGGTTAGTACATGA 162
>miR7803b-3p Score: 0.5 RO3G_16123
Query: 1 TGACACGTCGTCAATCATGTACT 23
||||||||||:||||||||||||
Sbjct: 433 ACTGTGCAGCGGTTAGTACATGA 411
>miR7803b-3p Score: 0.5 RO3G_15271
Query: 1 TGACACGTCGTCAATCATGTACT 23
||||||||||:||||||||||||
Sbjct: 433 ACTGTGCAGCGGTTAGTACATGA 411
>miR7803b-3p Score: 0.5 RO3G_15282
Query: 1 TGACACGTCGTCAATCATGTACT 23
||||||||||:||||||||||||
Sbjct: 157 ACTGTGCAGCGGTTAGTACATGA 135
>miR7803b-3p Score: 0.5 RO3G_15261
Query: 1 TGACACGTCGTCAATCATGTACT 23
||||||||||:||||||||||||
Sbjct: 433 ACTGTGCAGCGGTTAGTACATGA 411
>miR7803b-3p Score: 1.8 RO3G_14770
Query: 1 TGACACGTCGTCAATCATGTACT 23
:|*|||||||:||||||||||||
Sbjct: 433 GCCGTGCAGCGGTTAGTACATGA 411
>miR7803b-3p Score: 1.0 RO3G_13714
Query: 1 TGACACGTCGTCAATCATGTACT 23
|:||||||||:||||||||||||
Sbjct: 433 ATTGTGCAGCGGTTAGTACATGA 411
>miR7803b-3p Score: 0.5 RO3G_13852
Query: 1 TGACACGTCGTCAATCATGTACT 23
||||||||||:||||||||||||
Sbjct: 184 ACTGTGCAGCGGTTAGTACATGA 162
>miR7803b-3p Score: 1.0 RO3G_12663
Query: 1 TGACACGTCGTCAATCATGTACT 23
|:||||||||:||||||||||||

Sbjct: 268 ATTGTGCAGCGGTTAGTACATGA 246
>miR7803b-3p Score: 0.5 RO3G_11962
Query: 1 TGACACGTCGTCAATCATGTACT 23
||||||||||:||||||||||||
Sbjct: 157 ACTGTGCAGCGGTTAGTACATGA 135
>miR7803b-3p Score: 0.8 RO3G_12537
Query: 1 TGACACGTCGTCAATCATGTACT 23
:|||||||||:||||||||||||
Sbjct: 184 GCTGTGCAGCGGTTAGTACATGA 162
>miR7803b-3p Score: 1.5 RO3G_10771
Query: 1 TGACACGTCGTCAATCATGTACT 23
||||||||||:||||*|||||||
Sbjct: 184 ACTGTGCAGCGGTTAATACATGA 162
>miR7803b-3p Score: 0.5 RO3G_10795
Query: 1 TGACACGTCGTCAATCATGTACT 23
||||||||||:||||||||||||
Sbjct: 433 ACTGTGCAGCGGTTAGTACATGA 411
>miR7803b-3p Score: 1.0 RO3G_09193
Query: 1 TGACACGTCGTCAATCATGTACT 23
|:||||||||:||||||||||||
Sbjct: 157 ATTGTGCAGCGGTTAGTACATGA 135
>miR7803b-3p Score: 1.8 RO3G_07984
Query: 1 TGACACGTCGTCAATCATGTACT 23
:|*|||||||:||||||||||||
Sbjct: 433 GCCGTGCAGCGGTTAGTACATGA 411
>miR7803b-3p Score: 0.5 RO3G_06735
Query: 1 TGACACGTCGTCAATCATGTACT 23
||||||||||:||||||||||||
Sbjct: 157 ACTGTGCAGCGGTTAGTACATGA 135
>miR7803b-3p Score: 0.5 RO3G_07137
Query: 1 TGACACGTCGTCAATCATGTACT 23
||||||||||:||||||||||||
Sbjct: 433 ACTGTGCAGCGGTTAGTACATGA 411
>miR7803b-3p Score: 0.5 RO3G_07702
Query: 1 TGACACGTCGTCAATCATGTACT 23
||||||||||:||||||||||||
Sbjct: 184 ACTGTGCAGCGGTTAGTACATGA 162
>miR7803b-3p Score: 0.5 RO3G_07705
Query: 1 TGACACGTCGTCAATCATGTACT 23
||||||||||:||||||||||||
Sbjct: 433 ACTGTGCAGCGGTTAGTACATGA 411
>miR7803b-3p Score: 1.5 RO3G_07739
Query: 1 TGACACGTCGTCAATCATGTACT 23
|||||||*||:||||||||||||
Sbjct: 184 ACTGTGCTGCGGTTAGTACATGA 162

>miR7803b-3p Score: 0.5 RO3G_07867
Query: 1 TGACACGTCGTCAATCATGTACT 23
||||||||||:||||||||||||
Sbjct: 184 ACTGTGCAGCGGTTAGTACATGA 162
>miR7803b-3p Score: 0.5 RO3G_05521
Query: 1 TGACACGTCGTCAATCATGTACT 23
||||||||||||||||||||||*
Sbjct: 312 ACTGTGCAGCAGTTAGTACATGC 290
>miR7803b-3p Score: 0.5 RO3G_06433
Query: 1 TGACACGTCGTCAATCATGTACT 23
||||||||||:||||||||||||
Sbjct: 157 ACTGTGCAGCGGTTAGTACATGA 135
>miR7803b-3p Score: 0.5 RO3G_06717
Query: 1 TGACACGTCGTCAATCATGTACT 23
||||||||||:||||||||||||
Sbjct: 433 ACTGTGCAGCGGTTAGTACATGA 411
>miR7803b-3p Score: 0.5 RO3G_04674
Query: 1 TGACACGTCGTCAATCATGTACT 23
||||||||||:||||||||||||
Sbjct: 184 ACTGTGCAGCGGTTAGTACATGA 162
>miR7803b-3p Score: 1.0 RO3G_04682
Query: 1 TGACACGTCGTCAATCATGTACT 23
|:||||||||:||||||||||||
Sbjct: 433 ATTGTGCAGCGGTTAGTACATGA 411
>miR7803b-3p Score: 0.5 RO3G_04695
Query: 1 TGACACGTCGTCAATCATGTACT 23
||||||||||:||||||||||||
Sbjct: 223 ACTGTGCAGCGGTTAGTACATGA 201
>miR7803b-3p Score: 0.5 RO3G_05268
Query: 1 TGACACGTCGTCAATCATGTACT 23
||||||||||:||||||||||||
Sbjct: 433 ACTGTGCAGCGGTTAGTACATGA 411
>miR7803b-3p Score: 1.0 RO3G_02824
Query: 1 TGACACGTCGTCAATCATGTACT 23
|:||||||||:||||||||||||
Sbjct: 433 ATTGTGCAGCGGTTAGTACATGA 411
>miR7803b-3p Score: 0.8 RO3G_00238
Query: 1 TGACACGTCGTCAATCATGTACT 23
:|||||||||:||||||||||||
Sbjct: 433 GCTGTGCAGCGGTTAGTACATGA 411
>miR7803b-3p Score: 0.8 RO3G_01205
Query: 1 TGACACGTCGTCAATCATGTACT 23
:|||||||||:||||||||||||
Sbjct: 433 GCTGTGCAGCGGTTAGTACATGA 411
>miR7803b-3p Score: 0.5 RO3G_01328

```
Query: 1 TGACACGTCGTCAATCATGTACT 23
||||||||||:||||||||||||
Sbjct: 433 ACTGTGCAGCGGTTAGTACATGA 411
>miR7803b-3p Score: 1.0 RO3G_01962
Query: 1 TGACACGTCGTCAATCATGTACT 23
|:||||||||:||||||||||||
Sbjct: 184 ATTGTGCAGCGGTTAGTACATGA 162
>miR7803b-3p Score: 1.0 RO3G_17416
Query: 1 TGACACGTCGTCAATCATGTACT 23
|:||||||||:||||||||||||
Sbjct: 433 ATTGTGCAGCGGTTAGTACATGA 411
>miR7803b-3p Score: 0.5 RO3G_17399
Query: 1 TGACACGTCGTCAATCATGTACT 23
||||||||||:||||||||||||
Sbjct: 433 ACTGTGCAGCGGTTAGTACATGA 411
>miR7803b-3p Score: 0.5 RO3G_17387
Query: 1 TGACACGTCGTCAATCATGTACT 23
||||||||||:||||||||||||
Sbjct: 433 ACTGTGCAGCGGTTAGTACATGA 411
>miR7803b-3p Score: 1.0 RO3G_17355
Query: 1 TGACACGTCGTCAATCATGTACT 23
|:||||||||:||||||||||||
Sbjct: 268 ATTGTGCAGCGGTTAGTACATGA 246
>miR7803b-3p Score: 1.0 RO3G_17291
Query: 1 TGACACGTCGTCAATCATGTACT 23
|:||||||||:||||||||||||
Sbjct: 433 ATTGTGCAGCGGTTAGTACATGA 411
>miR5758 Score: 2.2 RO3G_15437
Query: 1 TAAGTGAAGAATGTCCTTG 19
|||:|||||||||||*::|
Sbjct: 816 ATTTACTTCTTACAGTGGC 798
>miR5758 Score: 0.0 RO3G_13796
Query: 1 TAAGTGAAGAATGTCCTTG 19
|||||||||||||||||||
Sbjct: 2527 ATTCACTTCTTACAGGAAC 2509
>miR5758 Score: 2.2 RO3G_13165
Query: 1 TAAGTGAAGAATGTCCTTG 19
:||:||*||||||||||*|
Sbjct: 70 GTTTACATCTTACAGGA-C 53
>miR5758 Score: 0.0 RO3G_11521
Query: 1 TAAGTGAAGAATGTCCTTG 19
|||||||||||||||||||
Sbjct: 1489 ATTCACTTCTTACAGGAAC 1471
>miR5758 Score: 2.5 RO3G_11550
Query: 1 TAAGTGAAGAATGTCCTTG 19
```

||||:|||||||||||*
Sbjct: 1732 TTTCATTTCCTACAGGAAG 1714
>miR5758 Score: 0.0 RO3G_09539
Query: 1 TAAGTGAAGAATGTCCTTG 19
|||||||||||||||||||
Sbjct: 2527 ATTCACTTCTTACAGGAAC 2509
>miR5758 Score: 2.2 RO3G_09046
Query: 1 TAAGTGAAGAATGTCCTTG 19
:||:|||||*|||:|||||
Sbjct: 255 GTTTACTTCCTACGGGAAC 237
>miR5758 Score: 2.5 RO3G_07962
Query: 1 TAAGTGAAGAATGTCCTTG 19
|||:||*|||*||||||||
Sbjct: 34 ATTTACATCTAACAGGAAC 16
>miR5758 Score: 2.0 RO3G_03267
Query: 1 TAAGTGAAGAATGTCCTTG 19
:|||||||:|||:||||*:
Sbjct: 811 GTTCACTTTTTATAGGA-T 794
>miR5758 Score: 1.0 RO3G_03627
Query: 1 TAAGTGAAGAATGTCCTTG 19
|*|||||||||||||||||
Sbjct: 544 AGTCACTTCTTACAGGAAC 526
>miR5758 Score: 1.0 RO3G_01708
Query: 1 TAAGTGAAGAATGTCCTTG 19
|*|||||||||||||||||
Sbjct: 544 AGTCACTTCTTACAGGAAC 526
>miR5758 Score: 2.5 RO3G_01900
Query: 1 TAAGTGAAGAATGTCCTTG 19
||||||||||:|||*|
Sbjct: 260 TTTCACTTCTTATAGAA-C 243
>miR5758 Score: 2.5 RO3G_02110
Query: 1 TAAGTGAAGAATGTCCTTG 19
||*||||||*||||||||*
Sbjct: 781 ATCCACTTCCTACAGGAAG 763
>miR5038a Score: 2.0 RO3G_16707
Query: 1 TGGGAATCTTGGCCTTTTCCA 21
|:||*||||||||||||||*|
Sbjct: 652 ATCCCTAGAACCGGAAAAGCT 632
>miR5038a Score: 2.5 RO3G_14583
Query: 1 TGGGAATCTTGGCCTTTT-CCA 21
||||*|||||||||||:|**||
Sbjct: 697 ACCCATAGAACCGGAAGAACGT 676
>miR5038a Score: 2.0 RO3G_13744
Query: 1 TGGGAATCTTGGCCTTTTCCA 21
|:||*||||||||||||||*|

```
Sbjct: 652 ATCCCTAGAACCGGAAAAGCT 632
>miR5038a Score: 0.0 RO3G_12668
Query: 1 TGGGAATCTTGGCCTTTTCCA 21
|||||||||||||||||||||
Sbjct: 565 ACCCTTAGAACCGGAAAAGGT 545
>miR5038a Score: 0.0 RO3G_12962
Query: 1 TGGGAATCTTGGCCTTTTCCA 21
|||||||||||||||||||||
Sbjct: 652 ACCCTTAGAACCGGAAAAGGT 632
>miR5038a Score: 2.0 RO3G_12965
Query: 1 TGGGAATCTTGGCCTTTTCCA 21
|:||*||||||||||||||*|
Sbjct: 454 ATCCCTAGAACCGGAAAAGCT 434
>miR5038a Score: 2.0 RO3G_12074
Query: 1 TGGGAATCTTGGCCTTTTCCA 21
|:||*||||||||||||||*|
Sbjct: 652 ATCCCTAGAACCGGAAAAGCT 632
>miR5038a Score: 1.2 RO3G_11954
Query: 1 TGGGAATCTTGGCCTTTTCCA 21
::|||||||||||||||||*|
Sbjct: 652 GTCCTTAGAACCGGAAAAGCT 632
>miR5038a Score: 2.5 RO3G_10341
Query: 1 TGGGAATCTTGGCCTTTTCCA 21
*:||||:||||||||:||*||
Sbjct: 1261 TTCCTTGGAACCGGAGAAAGT 1241
>miR5038a Score: 0.0 RO3G_09928
Query: 1 TGGGAATCTTGGCCTTTTCCA 21
|||||||||||||||||||||
Sbjct: 652 ACCCTTAGAACCGGAAAAGGT 632
>miR5038a Score: 2.5 RO3G_08982
Query: 1 TGGGAATCTTGGCCTTTTCCA 21
|||:||||||*:||||||||*
Sbjct: 1094 ACCTTTAGAAGTGGAAAAGGC 1074
>miR5038a Score: 2.0 RO3G_05525
Query: 1 TGGGAATCTTGGCCTTTTCCA 21
|:||*||||||||||||||*|
Sbjct: 652 ATCCCTAGAACCGGAAAAGCT 632
>miR5038a Score: 2.0 RO3G_05924
Query: 1 TGGGAATCTTGGCCTTTTCCA 21
|:||*||||||||||||||*|
Sbjct: 652 ATCCCTAGAACCGGAAAAGCT 632
>miR5038a Score: 0.0 RO3G_05926
Query: 1 TGGGAATCTTGGCCTTTTCCA 21
|||||||||||||||||||||
Sbjct: 652 ACCCTTAGAACCGGAAAAGGT 632
```

>miR5038a Score: 2.0 RO3G_02229
Query: 1 TGGGAATCTTGGCCTTTTCCA 21
|:||*||||||||||||||*|
Sbjct: 652 ATCCCTAGAACCGGAAAAGCT 632
>miR5038a Score: 2.0 RO3G_17443
Query: 1 TGGGAATCTTGGCCTTTTCCA 21
|:||*||||||||||||||*|
Sbjct: 652 ATCCCTAGAACCGGAAAAGCT 632
>miR5038a Score: 0.0 RO3G_17155
Query: 1 TGGGAATCTTGGCCTTTTCCA 21
|||||||||||||||||||||
Sbjct: 652 ACCCTTAGAACCGGAAAAGGT 632
>miR9674b-3p Score: 1.0 RO3G_17251
Query: 1 TGAATATGCCAGAGCATCAG 20
||*|||||||||||||||||
Sbjct: 572 ACATATACGGTCTCGTAGTC 553
>miR9674b-3p Score: 1.2 RO3G_17156
Query: 1 TGAATATGCCAGAGCATCAG 20
||*||||||||||||||||:
Sbjct: 1121 ACCTATACGGTCTCGTAGTT 1102
>miR9674b-3p Score: 1.0 RO3G_16366
Query: 1 TGAATATGCCAGAGCATCAG 20
||*|||||||||||||||||
Sbjct: 2864 ACATATACGGTCTCGTAGTC 2845
>miR9674b-3p Score: 0.5 RO3G_13459
Query: 1 TGAATATGCCAGAGCATCAG 20
||||||||||||||||||*|
Sbjct: 269 ACTTATACGGTCTCGTAGCC 250
>miR9674b-3p Score: 2.2 RO3G_00640
Query: 1 TGAATATGCCAGAGCATCAG 20
|:||||*:|||||||||||:
Sbjct: 3146 ATTTATCTGGTCTCGTAGTT 3127
>miR9674b-3p Score: 0.0 RO3G_01294
Query: 1 TGAATATGCCAGAGCATCAG 20
||||||||||||||||||||
Sbjct: 1937 ACTTATACGGTCTCGTAGTC 1918
>miR528-5p Score: 2.5 RO3G_13887
Query: 1 TGGAATGGGGATGAGAGG-AGG 21
||||||:|||||||||*|||
Sbjct: 481 CCCTTACTACTACTCTCCATCC 460
>miR528-5p Score: 0.0 RO3G_09951
Query: 1 TGGAATGGGGATGAGAGGAGG 21
|||||||||||||||||||||
Sbjct: 49 ACCTTACCCCTACTCTCCTCC 29
>miR528-5p Score: 2.2 RO3G_06616

```
Query: 1 TGGAATGGGGATGAGAGGAGG 21
||:|||:|||||||:|:||:|
Sbjct: 298 ACTTTATCCCTACTTTTCTTC 278
>miR528-5p Score: 2.5 RO3G_05148
Query: 1 TGGAATGGGGATGAGAGGAGG 21
*|||||:|||||||*|||*||
Sbjct: 420 TCCTTATCCCTACTGTCCACC 400
>miR7716-5p Score: 0.5 RO3G_15587
Query: 1 TAGTTGATAAGTAAGAACGAGAC 23
|||:|||||||||||||||||||
Sbjct: 234 ATCGACTATTCATTCTTGCTCTG 212
>miR7716-5p Score: 2.2 RO3G_13422
Query: 1 TAGTTGATAAGTAAGAACGAGAC 23
|*||||||||||||||||*|:*|
Sbjct: 263 AGCAACTATTCATTCTTG-TTAG 242
>miR7716-5p Score: 1.5 RO3G_08791
Query: 1 TAGTTGATAAGTAAGAACGAGAC 23
|||||||||||:*||||||||||
Sbjct: 150 ATCAACTATTCGGTCTTGCTCTG 128
>miR482e Score: 2.5 RO3G_00068
Query: 1 TTGCCTATTCCTCTGCCAAG 20
|||||||||||||:*|||**
Sbjct: 296 AACGGATAAGGAGGAGGTGG 277
>miR5766 Score: 2.0 RO3G_16408
Query: 1 TTAGGCAGAGGAATAGGCAAG 21
||*||*|||||||||||||||
Sbjct: 1091 AACCCTTCTCCTTATCCGTTC 1071
>miR5766 Score: 0.5 RO3G_15259
Query: 1 TTAGGCAGAGGAATAGGCAAG 21
|||||||||||||||||*|||
Sbjct: 1091 AATCCGTCTCCTTATCCATTC 1071
>miR5766 Score: 0.0 RO3G_12958
Query: 1 TTAGGCAGAGGAATAGGCAAG 21
|||||||||||||||||||||
Sbjct: 911 AATCCGTCTCCTTATCCGTTC 891
>miR5766 Score: 0.0 RO3G_12973
Query: 1 TTAGGCAGAGGAATAGGCAAG 21
|||||||||||||||||||||
Sbjct: 1091 AATCCGTCTCCTTATCCGTTC 1071
>miR5766 Score: 0.0 RO3G_12650
Query: 1 TTAGGCAGAGGAATAGGCAAG 21
|||||||||||||||||||||
Sbjct: 1091 AATCCGTCTCCTTATCCGTTC 1071
>miR5766 Score: 0.5 RO3G_11778
Query: 1 TTAGGCAGAGGAATAGGCAAG 21
```

```
|||||||||||||||||*|||
Sbjct: 1091 AATCCGTCTCCTTATCCATTC 1071
>miR5766 Score: 0.0 RO3G_07982
Query: 1 TTAGGCAGAGGAATAGGCAAG 21
|||||||||||||||||||||
Sbjct: 1091 AATCCGTCTCCTTATCCGTTC 1071
>miR5766 Score: 0.0 RO3G_08650
Query: 1 TTAGGCAGAGGAATAGGCAAG 21
|||||||||||||||||||||
Sbjct: 1091 AATCCGTCTCCTTATCCGTTC 1071
>miR5766 Score: 0.0 RO3G_08655
Query: 1 TTAGGCAGAGGAATAGGCAAG 21
|||||||||||||||||||||
Sbjct: 1091 AATCCGTCTCCTTATCCGTTC 1071
>miR5766 Score: 0.0 RO3G_07035
Query: 1 TTAGGCAGAGGAATAGGCAAG 21
|||||||||||||||||||||
Sbjct: 1091 AATCCGTCTCCTTATCCGTTC 1071
>miR5766 Score: 0.0 RO3G_05217
Query: 1 TTAGGCAGAGGAATAGGCAAG 21
|||||||||||||||||||||
Sbjct: 1091 AATCCGTCTCCTTATCCGTTC 1071
>miR5766 Score: 0.0 RO3G_02849
Query: 1 TTAGGCAGAGGAATAGGCAAG 21
|||||||||||||||||||||
Sbjct: 1091 AATCCGTCTCCTTATCCGTTC 1071
>miR5766 Score: 0.0 RO3G_02851
Query: 1 TTAGGCAGAGGAATAGGCAAG 21
|||||||||||||||||||||
Sbjct: 1091 AATCCGTCTCCTTATCCGTTC 1071
>miR5766 Score: 2.5 RO3G_03289
Query: 1 TTAGGCAGAGGAATAGGCAAG 21
*:|||||:|:|||:|||||||
Sbjct: 998 CGTCCGTTTTCTTGTCCGTTC 978
>miR5766 Score: 0.0 RO3G_00697
Query: 1 TTAGGCAGAGGAATAGGCAAG 21
|||||||||||||||||||||
Sbjct: 1091 AATCCGTCTCCTTATCCGTTC 1071
>miR5766 Score: 0.0 RO3G_01965
Query: 1 TTAGGCAGAGGAATAGGCAAG 21
|||||||||||||||||||||
Sbjct: 1091 AATCCGTCTCCTTATCCGTTC 1071
>miR5766 Score: 0.0 RO3G_17425
Query: 1 TTAGGCAGAGGAATAGGCAAG 21
|||||||||||||||||||||
```

Sbjct: 1091 AATCCGTCTCCTTATCCGTTC 1071
>miR7763-5p Score: 2.0 RO3G_05488
Query: 1 AACTTCATCGTGCTGGGGATAG 22
|||||||||:||*|||||*|||
Sbjct: 1829 TTGAAGTAGTACTACCCCAATC 1808
>miR7763-5p Score: 2.0 RO3G_00708
Query: 1 AACTTCATCGTGCTGGGGATAG 22
|||||||||:||*|||||*|||
Sbjct: 1760 TTGAAGTAGTACTACCCCAATC 1739
>miR5227 Score: 2.5 RO3G_17229
Query: 1 TGAAGAAGAAGAAGTTGAGGAT 22
:||||||||||||||||*|
Sbjct: 504 CTTTCTTCTTCTTCCACTCC-A 484
>miR5227 Score: 2.5 RO3G_12224
Query: 1 TGAAGAAGAAGAAGTTGA-GGAT 22
||||||*||||||||||***|||
Sbjct: 1289 ACTTCTGCTTCTTCAACAAACTA 1267
>miR5227 Score: 2.0 RO3G_10883
Query: 1 TGAAGAAGAAGAAGTTGAGGAT 22
|:|*|||||||||||||||*|
Sbjct: 759 ATTCCTTCTTCTTCAACTCC-A 739
>miR5227 Score: 1.0 RO3G_10898
Query: 1 TGAAGAAGAAGAAGTTGAGG-AT 22
|||||||||||||:||||||*||
Sbjct: 129 ACTTCTTCTTCTTTAACTCCTTA 107
>miR5227 Score: 2.0 RO3G_11015
Query: 1 TGAAGAAGAAGAAGTTGA-GGAT 22
|:|||||||||||||:||*:||:
Sbjct: 42 ATTTCTTCTTCTTCAGCTATCTG 20
>miR5227 Score: 2.5 RO3G_11040
Query: 1 TGAAGAAGAAGAAGTTGAGGAT 22
|||||||||*|||*|||*||||
Sbjct: 93 ACTTCTTCTACTTGAAC-CCTA 73
>miR5227 Score: 2.2 RO3G_09917
Query: 1 TGAAGAAGAAGAAGTTGAGGAT 22
|||:||||||||||||:|||
Sbjct: 705 TCTTTTTCTTCTTCACCTTCTA 684
>miR5227 Score: 2.0 RO3G_10043
Query: 1 TGAAGAAGAAGAAGTTGAGGAT 22
|||||||||||||:|||:|:
Sbjct: 957 CCTTCTTCTTCTTCGACT-TTG 937
>miR5227 Score: 2.5 RO3G_07000
Query: 1 TGAAGAAGAAGAAGTTGAGGAT 22
*|||||||||||||**||||||
Sbjct: 192 TCTTCTTCTTCTTCTTCTCCTA 171

```
>miR5227 Score: 2.5 RO3G_07001
Query: 1 TGAAGAAGAAGAAGTTGAGGAT 22
*|||||||||||||**||||||
Sbjct: 192 TCTTCTTCTTCTTCTTCTCCTA 171
>miR5227 Score: 2.5 RO3G_07279
Query: 1 TGAAGAAGAAGAAGTTGAGGAT 22
|||*||||||||||:*||||||
Sbjct: 3594 ACTCCTTCTTCTTCGCCTCCTA 3573
>miR5227 Score: 2.0 RO3G_05565
Query: 1 TGAAGAAGAAGAAGTTGAGGAT 22
||||||||||||||*||*|||*
Sbjct: 684 ACTTCTTCTTCTTCCACACCTC 663
>miR5227 Score: 2.2 RO3G_06171
Query: 1 TGAAGAAGAAGAAGTTGA-GGAT 22
*|||*|||||||||||||*:|||
Sbjct: 216 TCTTGTTCTTCTTCAACTTTCTA 194
>miR5227 Score: 1.5 RO3G_06538
Query: 1 TGAAGAAGAAGAAGTTGA-GGAT 22
*|||||||||||||||||*|||*
Sbjct: 1151 CCTTCTTCTTCTTCAACTCCCTC 1129
>miR5227 Score: 2.2 RO3G_03357
Query: 1 TGAAGAAGAAGAAGTTGAGGAT 22
*||||||||||||*||||:||*
Sbjct: 69 TCTTCTTCTTCTTGAACTTCTT 48
>miR5227 Score: 2.5 RO3G_03738
Query: 1 TGAAGAAGAAGAAGTTGAGGAT 22
||||||||||||||:*|**|||
Sbjct: 1782 ACTTCTTCTTCTTCGTCGACTA 1761
>miR5227 Score: 2.5 RO3G_00057
Query: 1 TGAAGAAGAAGAAGTTGAGGAT 22
||||:|||||||||:|**||||
Sbjct: 1677 ACTTTTTCTTCTTCGAGACCTA 1656
>miR5227 Score: 2.5 RO3G_01232
Query: 1 TGAAGAAGAAGAAGTTGA-GGAT 22
*||||||||||||||:||**|*|
Sbjct: 189 CCTTCTTCTTCTTCAGCTAGCCA 167
>miR5227 Score: 2.5 RO3G_01236
Query: 1 TGAAGAAGAAGAAGTTGA-GGAT 22
*||||||||||||||:||**|*|
Sbjct: 189 CCTTCTTCTTCTTCAGCTAGCCA 167
>miR5227 Score: 2.2 RO3G_01458
Query: 1 TGAAGAAGAAGAAGTTGAGGAT 22
*||||||||||||||*||:||*
Sbjct: 879 TCTTCTTCTTCTTCATCTTCTC 858
>miR823-5p Score: 2.2 RO3G_02988
```

Query: 1 TTTGTATTGATCACTGACCATT 22
||||||||||:|||:|||*|:*
Sbjct: 234 AAACATAACTGGTGGCTGTTGT 213
>miR823-5p Score: 2.5 RO3G_01483
Query: 1 TTTGTATTGATCACTGACCATT 22
|*|::|||||||||||||*|||
Sbjct: 570 ATATGTAACTAGTGACTGTTAA 549
>miR2644 Score: 2.2 RO3G_13025
Query: 1 TCTCTTCAGTTGATGGTT-GT 20
|||||:||||||||||*:*||
Sbjct: 2033 AGAGAGGTCAACTACCTGACA 2013
>miR2644 Score: 2.2 RO3G_12411
Query: 1 TCTCTTCAGTTGATGGTTGT 20
:*||||||:|||||||||*|
Sbjct: 979 GAAGAAGTTAACTACCAA-A 961
>miR2644 Score: 2.5 RO3G_11845
Query: 1 TCTCTTCAGTTGATGGTTGT 20
||*|||||*||||||||*||
Sbjct: 472 AGTGAAGTGAACTACCA-CA 454
>miR2644 Score: 2.5 RO3G_09501
Query: 1 TCTCTTCAGTTGATGGTTGT 20
|||||||::|:|||||||
Sbjct: 175 TGAGAAGTTGATTACCAA-A 157
>miR2644 Score: 2.5 RO3G_07648
Query: 1 TCTCTTCAGTTGATGGTT-GT 20
|||||||:||||||||*||
Sbjct: 661 CGTGAAGTCGACTACCAAACA 641
>miR2644 Score: 1.5 RO3G_06059
Query: 1 TCTCTTCAGTTGATGGTTGT 20
|*|||||||||||||:||||
Sbjct: 1018 AAAGAAGTCAACTACTAACA 999
>miR2644 Score: 2.5 RO3G_04985
Query: 1 TCTCTTCAGTTGATGGTTGT 20
|||||||||:||||:|||
Sbjct: 322 TGAGAAGTCATTTACCGACA 303
>miR2644 Score: 2.5 RO3G_00074
Query: 1 TCTCTTCAGTTGATGGTTGT 20
|||||*|||||:||*|||||
Sbjct: 475 AGAGATGTCAATTAACAACA 456
>miR2644 Score: 1.5 RO3G_00272
Query: 1 TCTCTTCAGTTGATGGTTGT 20
|:||||||||||||||||
Sbjct: 5305 CGGGAAGTCAACTACCA-CA 5287
>miR2644 Score: 2.5 RO3G_01873
Query: 1 TCTCTTCAGTTGATGGTTGT 20

|||||:||:||||:|||::*
Sbjct: 872 AGAGAGGTTAACTGCCAGTC 853
>miR863-5p Score: 1.8 RO3G_14810
Query: 1 TTATGGCTTGTTGTATATCAT 21
|||||:||||||||||*:|||
Sbjct: 508 AATACTGAACAACATAAGGTA 488
>miR863-5p Score: 2.5 RO3G_14232
Query: 1 TTATGGCTTGTTGTATATCAT 21
|||||||||||||||*||*
Sbjct: 187 CATACCGAACAACACATCGTT 167
>miR863-5p Score: 2.2 RO3G_08164
Query: 1 TTATGGCTTGTTGTATATCAT 21
:||||||||*|||||||*||*
Sbjct: 772 GATACCGAAGAACATATCGTC 752
>miR863-5p Score: 2.5 RO3G_00542
Query: 1 TTATGGCTTGTTGTATATC-AT 21
|:|||:||||||*||||||*||
Sbjct: 730 AGTACTGAACAAAATATAGTTA 709
>miR863-5p Score: 2.5 RO3G_17110
Query: 1 TTATGGCTTGTTGTATATCAT 21
|||:||||||||||*|||
Sbjct: 265 TATATGGAACAACATATTGTA 245
>miR1917 Score: 2.2 RO3G_16595
Query: 1 ATTGATCAAGGTCTAAAGT 19
|:||||||||||||||:
Sbjct: 810 GAGCTAGTTCCAGACTTCG 792
>miR1917 Score: 2.5 RO3G_16678
Query: 1 ATTGATCAAGGTCTAAAGT 19
|:||||||*|:||:|||||
Sbjct: 276 TGACTAGTACTAGGTTTCA 258
>miR1917 Score: 2.5 RO3G_11168
Query: 1 ATTGATCAAGGTCTAAAGT 19
|||||||*|||||*||||*
Sbjct: 750 TAACTAGGTCCAGTTTTCC 732
>miR1917 Score: 2.5 RO3G_10315
Query: 1 ATTGATCAAGGTCTAAAGT 19
|||||||||:|||*||*||
Sbjct: 1870 TAACTAGTTTCAGTTT-CA 1853
>miR1917 Score: 2.5 RO3G_10565
Query: 1 ATTGATCAAGGTCTAAAGT 19
|||||||||:|:|||||
Sbjct: 1026 CAACAAGTTCCGGGTTTCA 1008
>miR1917 Score: 2.5 RO3G_10615
Query: 1 ATTGATCAAGGTCTAAAGT 19
||||||||||:|*|*||||

Sbjct: 315 TAACTAGTTCTACACTTCA 297
>miR1917 Score: 2.5 RO3G_10660
Query: 1 ATTGATCAAGGTCTAAAGT 19
:|||||||||||||||*
Sbjct: 1204 GGTCTAGTTCCAGATTTCT 1186
>miR1917 Score: 2.2 RO3G_08230
Query: 1 ATTGATCAAGGTCTAAAGT 19
||*|||*||||||||||:|
Sbjct: 260 TATCTACTTCCAGATTTTA 242
>miR1917 Score: 2.5 RO3G_06399
Query: 1 ATTGATCAAGGTCTAAAGT 19
|||||:||||:||||*|*|
Sbjct: 3103 TAACTGGTTCTAGATGT-A 3086
>miR1917 Score: 2.0 RO3G_02427
Query: 1 ATTGATCAAGGTCTAAAGT 19
|||||||||||||||*|
Sbjct: 683 AAACTACTTCCAGATTT-A 666
>miR1917 Score: 2.2 RO3G_02854
Query: 1 ATTGATCAAGGTCTAAAGT 19
|||||||||*|||||*|:|
Sbjct: 889 TAACTAGTTGCAGATGTTA 871
>miR1917 Score: 2.5 RO3G_03973
Query: 1 ATTGATCAAGGTCTAAAGT 19
||*|||||||:|*||||||
Sbjct: 684 TATCTAGTTCTATATTTCA 666
>miR1917 Score: 2.5 RO3G_00170
Query: 1 ATTGATCAAGGTCTAAAGT 19
|||||||||||:|||*|
Sbjct: 4480 GAACTAGTTACAGGTTT-A 4463
>miR1917 Score: 2.0 RO3G_00502
Query: 1 ATTGATCAAGGTCTAAAGT 19
||:||||||||||:||*||
Sbjct: 261 TAGCTAGTTCCAGGTT-CA 244
>miR1917 Score: 2.2 RO3G_01687
Query: 1 ATTGATCAAGGTCTAAAGT 19
|||:||||||:||*||||:
Sbjct: 231 TAATTAGTTCTAGTTTTCG 213
>miR1857-5p Score: 2.2 RO3G_14280
Query: 1 TGGATTCTTTTGGAGATGCGG 21
||||||||||||:|:*|||:|
Sbjct: 49 ACCTAAGAAAACTTTAACGTC 29
>miR1857-5p Score: 2.2 RO3G_12733
Query: 1 TGGATTCTTTTGGAGATGCGG 21
::||||||||||||:||*|::
Sbjct: 426 GTCTAAGAAAACCTTTA-GTT 407

>miR1857-5p Score: 2.5 RO3G_10745
Query: 1 TGGATTCTTTTGGAGATGCGG 21
:||||||||||*:|||||*|:
Sbjct: 1398 GCCTAAGAAAAGTTCTAC-CT 1379
>miR1857-5p Score: 2.5 RO3G_07862
Query: 1 TGGATTCTTTTGGAGATGCGG 21
:||||||||||*:|||||*|:
Sbjct: 2541 GCCTAAGAAAAGTTCTAC-CT 2522
>miR1857-5p Score: 2.5 RO3G_05880
Query: 1 TGGATTCTTTTGGAGATGCGG 21
|||||||*|||:|||||:|:*
Sbjct: 217 ACCTAAGTAAATCTCTATGTG 197
>miR1857-5p Score: 2.2 RO3G_03713
Query: 1 TGGATTCTTTTGGAGATGCGG 21
|||||||:||||:||||**:|
Sbjct: 469 ACCTAAGGAAACTTCTAAATC 449
>miR1857-5p Score: 2.5 RO3G_04137
Query: 1 TGGATTCTTTTGGAGATGCGG 21
|||||:||||||:|||::*:|
Sbjct: 1104 ACCTAGGAAAACTTCTGT-TC 1085
>miR5293 Score: 0.0 RO3G_17021
Query: 1 TGATGAAGAAGTGGCGCAGAAGA 23
|||||||||||||||||||||||
Sbjct: 2846 ACTACTTCTTCACCGCGTCTTCT 2824
>miR5293 Score: 0.0 RO3G_16693
Query: 1 TGATGAAGAAGTGGCGCAGAAGA 23
|||||||||||||||||||||||
Sbjct: 3011 ACTACTTCTTCACCGCGTCTTCT 2989
>miR5293 Score: 0.0 RO3G_16361
Query: 1 TGATGAAGAAGTGGCGCAGAAGA 23
|||||||||||||||||||||||
Sbjct: 2846 ACTACTTCTTCACCGCGTCTTCT 2824
>miR5293 Score: 0.0 RO3G_16539
Query: 1 TGATGAAGAAGTGGCGCAGAAGA 23
|||||||||||||||||||||||
Sbjct: 2903 ACTACTTCTTCACCGCGTCTTCT 2881
>miR5293 Score: 0.0 RO3G_13918
Query: 1 TGATGAAGAAGTGGCGCAGAAGA 23
|||||||||||||||||||||||
Sbjct: 3011 ACTACTTCTTCACCGCGTCTTCT 2989
>miR5293 Score: 0.0 RO3G_14347
Query: 1 TGATGAAGAAGTGGCGCAGAAGA 23
|||||||||||||||||||||||
Sbjct: 2942 ACTACTTCTTCACCGCGTCTTCT 2920
>miR5293 Score: 0.0 RO3G_12673

Query: 1 TGATGAAGAAGTGGCGCAGAAGA 23
|||||||||||||||||||||||
Sbjct: 2147 ACTACTTCTTCACCGCGTCTTCT 2125
>miR5293 Score: 0.0 RO3G_12677
Query: 1 TGATGAAGAAGTGGCGCAGAAGA 23
|||||||||||||||||||||||
Sbjct: 2846 ACTACTTCTTCACCGCGTCTTCT 2824
>miR5293 Score: 0.0 RO3G_12527
Query: 1 TGATGAAGAAGTGGCGCAGAAGA 23
|||||||||||||||||||||||
Sbjct: 3011 ACTACTTCTTCACCGCGTCTTCT 2989
>miR5293 Score: 0.0 RO3G_12532
Query: 1 TGATGAAGAAGTGGCGCAGAAGA 23
|||||||||||||||||||||||
Sbjct: 2852 ACTACTTCTTCACCGCGTCTTCT 2830
>miR5293 Score: 0.0 RO3G_12538
Query: 1 TGATGAAGAAGTGGCGCAGAAGA 23
|||||||||||||||||||||||
Sbjct: 2903 ACTACTTCTTCACCGCGTCTTCT 2881
>miR5293 Score: 0.0 RO3G_10804
Query: 1 TGATGAAGAAGTGGCGCAGAAGA 23
|||||||||||||||||||||||
Sbjct: 2852 ACTACTTCTTCACCGCGTCTTCT 2830
>miR5293 Score: 0.0 RO3G_09493
Query: 1 TGATGAAGAAGTGGCGCAGAAGA 23
|||||||||||||||||||||||
Sbjct: 1670 ACTACTTCTTCACCGCGTCTTCT 1648
>miR5293 Score: 0.0 RO3G_09547
Query: 1 TGATGAAGAAGTGGCGCAGAAGA 23
|||||||||||||||||||||||
Sbjct: 2846 ACTACTTCTTCACCGCGTCTTCT 2824
>miR5293 Score: 1.5 RO3G_08830
Query: 1 TGATGAAGAAGTGGCGCAGAAGA 23
|||||||||||||:*||||||||
Sbjct: 125 ACTACTTCTTCACTACGTCTTCT 103
>miR5293 Score: 0.0 RO3G_07311
Query: 1 TGATGAAGAAGTGGCGCAGAAGA 23
|||||||||||||||||||||||
Sbjct: 2852 ACTACTTCTTCACCGCGTCTTCT 2830
>miR5293 Score: 0.0 RO3G_06425
Query: 1 TGATGAAGAAGTGGCGCAGAAGA 23
|||||||||||||||||||||||
Sbjct: 2846 ACTACTTCTTCACCGCGTCTTCT 2824
>miR5293 Score: 0.0 RO3G_04933
Query: 1 TGATGAAGAAGTGGCGCAGAAGA 23

||||||||||||||||||||||
Sbjct: 2846 ACTACTTCTTCACCGCGTCTTCT 2824
>miR5293 Score: 0.0 RO3G_05330
Query: 1 TGATGAAGAAGTGGCGCAGAAGA 23
||||||||||||||||||||||
Sbjct: 2846 ACTACTTCTTCACCGCGTCTTCT 2824
>miR5293 Score: 0.0 RO3G_05344
Query: 1 TGATGAAGAAGTGGCGCAGAAGA 23
||||||||||||||||||||||
Sbjct: 2672 ACTACTTCTTCACCGCGTCTTCT 2650
>miR5293 Score: 0.0 RO3G_05515
Query: 1 TGATGAAGAAGTGGCGCAGAAGA 23
||||||||||||||||||||||
Sbjct: 1841 ACTACTTCTTCACCGCGTCTTCT 1819
>miR5293 Score: 0.0 RO3G_02704
Query: 1 TGATGAAGAAGTGGCGCAGAAGA 23
||||||||||||||||||||||
Sbjct: 2846 ACTACTTCTTCACCGCGTCTTCT 2824
>miR5293 Score: 0.0 RO3G_02821
Query: 1 TGATGAAGAAGTGGCGCAGAAGA 23
||||||||||||||||||||||
Sbjct: 3011 ACTACTTCTTCACCGCGTCTTCT 2989
>miR5293 Score: 0.0 RO3G_03619
Query: 1 TGATGAAGAAGTGGCGCAGAAGA 23
||||||||||||||||||||||
Sbjct: 2738 ACTACTTCTTCACCGCGTCTTCT 2716
>miR5293 Score: 0.0 RO3G_03769
Query: 1 TGATGAAGAAGTGGCGCAGAAGA 23
||||||||||||||||||||||
Sbjct: 776 ACTACTTCTTCACCGCGTCTTCT 754
>miR5293 Score: 0.0 RO3G_03778
Query: 1 TGATGAAGAAGTGGCGCAGAAGA 23
||||||||||||||||||||||
Sbjct: 2804 ACTACTTCTTCACCGCGTCTTCT 2782
>miR5293 Score: 0.0 RO3G_04126
Query: 1 TGATGAAGAAGTGGCGCAGAAGA 23
||||||||||||||||||||||
Sbjct: 3005 ACTACTTCTTCACCGCGTCTTCT 2983
>miR5293 Score: 0.0 RO3G_00241
Query: 1 TGATGAAGAAGTGGCGCAGAAGA 23
||||||||||||||||||||||
Sbjct: 1136 ACTACTTCTTCACCGCGTCTTCT 1114
>miR5293 Score: 0.0 RO3G_00296
Query: 1 TGATGAAGAAGTGGCGCAGAAGA 23
||||||||||||||||||||||

```
Sbjct: 1136 ACTACTTCTTCACCGCGTCTTCT 1114
>miR5293 Score: 0.0 RO3G_01614
Query: 1 TGATGAAGAAGTGGCGCAGAAGA 23
|||||||||||||||||||||||
Sbjct: 2852 ACTACTTCTTCACCGCGTCTTCT 2830
>miR5293 Score: 0.0 RO3G_01936
Query: 1 TGATGAAGAAGTGGCGCAGAAGA 23
|||||||||||||||||||||||
Sbjct: 2852 ACTACTTCTTCACCGCGTCTTCT 2830
>miR5293 Score: 0.0 RO3G_01964
Query: 1 TGATGAAGAAGTGGCGCAGAAGA 23
|||||||||||||||||||||||
Sbjct: 1322 ACTACTTCTTCACCGCGTCTTCT 1300
>miR5293 Score: 0.0 RO3G_17422
Query: 1 TGATGAAGAAGTGGCGCAGAAGA 23
|||||||||||||||||||||||
Sbjct: 2147 ACTACTTCTTCACCGCGTCTTCT 2125
>miR535d Score: 2.5 RO3G_15968
Query: 1 TGAACGACGATAGAGAGCAC 20
||||*|||||||:|||||**
Sbjct: 1710 ACTTCCTGCTATTTCTCGCA 1691
>miR535d Score: 2.0 RO3G_14153
Query: 1 TGAACGACGATAGAGAGCAC 20
*||*||||||||:|||||||
Sbjct: 633 TCTAGCTGCTATTTCTCGTG 614
>miR535d Score: 2.5 RO3G_14251
Query: 1 TGAACGACGATAGAGAGCAC 20
|||||||*||||||*||*||
Sbjct: 440 ACTTGCTCCTATCTATC-TG 422
>miR535d Score: 2.5 RO3G_09252
Query: 1 TGAACGACGATAGAGAGCAC 20
*|||||||||||||*||*|*
Sbjct: 462 TCTTGCTGCTATCTATCTTA 443
>miR535d Score: 2.2 RO3G_06941
Query: 1 TGAACGACGATAGAGAGCAC 20
::||||||||||:||||**|
Sbjct: 819 GTTTGCTGCTATTTCTCCAG 800
>miR535d Score: 2.5 RO3G_04512
Query: 1 TGAACGACGATAGAGAGCAC 20
*|||||||||||||*||*|*
Sbjct: 462 TCTTGCTGCTATCTATC-TC 444
>miR535d Score: 0.0 RO3G_02254
Query: 1 TGAACGACGATAGAGAGCAC 20
||||||||||||||||||||
Sbjct: 621 ACTTGCTGCTATCTCTCGTG 602
```

```
>miR535d Score: 2.5 RO3G_03471
Query: 1 TGAACGACGATAGAGAGCAC 20
*|||||||:*||||||:|||
Sbjct: 635 TCTTGCTGTCATCTCTTGTG 616
>miR535d Score: 2.5 RO3G_03535
Query: 1 TGAACGACGATAGAGAGCAC 20
|||||||||||||||**||*
Sbjct: 39 ACTTGCTGCTATCTCAAGTA 20
>miR535d Score: 2.5 RO3G_03945
Query: 1 TGAACGACGATAGAGAGCAC 20
|:||||||*|||||:||||*
Sbjct: 1071 ATTTGCTGGTATCTTTCGTA 1052
>miR1513a-5p Score: 1.2 RO3G_17260
Query: 1 TGAACAAAGCCATGGACTTGC 21
::|||||||||||:|||||||
Sbjct: 1555 GTTTGTTTCGGTATCTGAACG 1535
>miR1513a-5p Score: 1.8 RO3G_17268
Query: 1 TGAACAAAGCCATGGACTTGC 21
::|||||||||||*|||||||
Sbjct: 271 GTTTGTTTCGGTAGCTGAACG 251
>miR1513a-5p Score: 1.2 RO3G_17226
Query: 1 TGAACAAAGCCATGGACTTGC 21
:||||||||||||*|||||||
Sbjct: 1690 GCTTGTTTCGGTAACTGAACG 1670
>miR1513a-5p Score: 0.0 RO3G_16812
Query: 1 TGAACAAAGCCATGGACTTGC 21
|||||||||||||||||||||
Sbjct: 1687 ACTTGTTTCGGTACCTGAACG 1667
>miR1513a-5p Score: 1.2 RO3G_16815
Query: 1 TGAACAAAGCCATGGACTTGC 21
:||||||||||||*|||||||
Sbjct: 1690 GCTTGTTTCGGTAACTGAACG 1670
>miR1513a-5p Score: 0.0 RO3G_16392
Query: 1 TGAACAAAGCCATGGACTTGC 21
|||||||||||||||||||||
Sbjct: 670 ACTTGTTTCGGTACCTGAACG 650
>miR1513a-5p Score: 0.0 RO3G_15270
Query: 1 TGAACAAAGCCATGGACTTGC 21
|||||||||||||||||||||
Sbjct: 736 ACTTGTTTCGGTACCTGAACG 716
>miR1513a-5p Score: 0.0 RO3G_14791
Query: 1 TGAACAAAGCCATGGACTTGC 21
|||||||||||||||||||||
Sbjct: 1687 ACTTGTTTCGGTACCTGAACG 1667
>miR1513a-5p Score: 1.8 RO3G_14793
```

```
Query: 1 TGAACAAAGCCATGGACTTGC 21
::|||||||||||*|||||||
Sbjct: 1690 GTTTGTTTCGGTAGCTGAACG 1670
>miR1513a-5p Score: 1.2 RO3G_14795
Query: 1 TGAACAAAGCCATGGACTTGC 21
:||||||||||||*|||||||
Sbjct: 1690 GCTTGTTTCGGTAACTGAACG 1670
>miR1513a-5p Score: 0.0 RO3G_14798
Query: 1 TGAACAAAGCCATGGACTTGC 21
|||||||||||||||||||||
Sbjct: 1687 ACTTGTTTCGGTACCTGAACG 1667
>miR1513a-5p Score: 1.8 RO3G_14800
Query: 1 TGAACAAAGCCATGGACTTGC 21
::|||||||||||*|||||||
Sbjct: 1690 GTTTGTTTCGGTAGCTGAACG 1670
>miR1513a-5p Score: 1.2 RO3G_13288
Query: 1 TGAACAAAGCCATGGACTTGC 21
::|||||||||||:|||||||
Sbjct: 1690 GTTTGTTTCGGTATCTGAACG 1670
>miR1513a-5p Score: 0.0 RO3G_09180
Query: 1 TGAACAAAGCCATGGACTTGC 21
|||||||||||||||||||||
Sbjct: 1687 ACTTGTTTCGGTACCTGAACG 1667
>miR1513a-5p Score: 0.0 RO3G_09189
Query: 1 TGAACAAAGCCATGGACTTGC 21
|||||||||||||||||||||
Sbjct: 1687 ACTTGTTTCGGTACCTGAACG 1667
>miR1513a-5p Score: 1.2 RO3G_06734
Query: 1 TGAACAAAGCCATGGACTTGC 21
::|||||||||||:|||||||
Sbjct: 991 GTTTGTTTCGGTATCTGAACG 971
>miR1167 Score: 2.5 RO3G_15066
Query: 1 TGGGCTGTGGATGACTTGAAAC 22
||::|||||||*|||||||*||
Sbjct: 565 ACTTGACACCTCCTGAACTATG 544
>miR6114-5p Score: 0.0 RO3G_13432
Query: 1 TCGAAAGCACCATGAACGTGA 21
|||||||||||||||||||||
Sbjct: 368 AGCTTTCGTGGTACTTGCACT 348
>miR6114-5p Score: 0.0 RO3G_00301
Query: 1 TCGAAAGCACCATGAACGTGA 21
|||||||||||||||||||||
Sbjct: 974 AGCTTTCGTGGTACTTGCACT 954
>miR5809 Score: 0.0 RO3G_16804
Query: 1 TCGGCTGGCGACCAAAGC 18
```

||||||||||||||||||
Sbjct: 1404 AGCCGACCGCTGGTTTCG 1387
>miR5809 Score: 0.0 RO3G_16602
Query: 1 TCGGCTGGCGACCAAAGC 18
||||||||||||||||||
Sbjct: 210 AGCCGACCGCTGGTTTCG 193
>miR5809 Score: 0.0 RO3G_16610
Query: 1 TCGGCTGGCGACCAAAGC 18
||||||||||||||||||
Sbjct: 210 AGCCGACCGCTGGTTTCG 193
>miR5809 Score: 0.0 RO3G_15003
Query: 1 TCGGCTGGCGACCAAAGC 18
||||||||||||||||||
Sbjct: 303 AGCCGACCGCTGGTTTCG 286
>miR5809 Score: 0.0 RO3G_15108
Query: 1 TCGGCTGGCGACCAAAGC 18
||||||||||||||||||
Sbjct: 813 AGCCGACCGCTGGTTTCG 796
>miR5809 Score: 0.0 RO3G_15131
Query: 1 TCGGCTGGCGACCAAAGC 18
||||||||||||||||||
Sbjct: 1401 AGCCGACCGCTGGTTTCG 1384
>miR5809 Score: 0.0 RO3G_13439
Query: 1 TCGGCTGGCGACCAAAGC 18
||||||||||||||||||
Sbjct: 1401 AGCCGACCGCTGGTTTCG 1384
>miR5809 Score: 0.0 RO3G_13293
Query: 1 TCGGCTGGCGACCAAAGC 18
||||||||||||||||||
Sbjct: 1404 AGCCGACCGCTGGTTTCG 1387
>miR5809 Score: 0.0 RO3G_12007
Query: 1 TCGGCTGGCGACCAAAGC 18
||||||||||||||||||
Sbjct: 1404 AGCCGACCGCTGGTTTCG 1387
>miR5809 Score: 2.5 RO3G_12638
Query: 1 TCGGCTGGCGACCAAAGC 18
||||*||:||||||||:*
Sbjct: 1590 AGCCTACTGCTGGTTTTT 1573
>miR5809 Score: 0.0 RO3G_10715
Query: 1 TCGGCTGGCGACCAAAGC 18
||||||||||||||||||
Sbjct: 1404 AGCCGACCGCTGGTTTCG 1387
>miR5809 Score: 0.0 RO3G_09718
Query: 1 TCGGCTGGCGACCAAAGC 18
||||||||||||||||||

```
Sbjct: 1404 AGCCGACCGCTGGTTTCG 1387
>miR5809 Score: 0.0 RO3G_10177
Query: 1 TCGGCTGGCGACCAAAGC 18
||||||||||||||||||
Sbjct: 1404 AGCCGACCGCTGGTTTCG 1387
>miR5809 Score: 0.0 RO3G_09043
Query: 1 TCGGCTGGCGACCAAAGC 18
||||||||||||||||||
Sbjct: 675 AGCCGACCGCTGGTTTCG 658
>miR5809 Score: 0.0 RO3G_04309
Query: 1 TCGGCTGGCGACCAAAGC 18
||||||||||||||||||
Sbjct: 1344 AGCCGACCGCTGGTTTCG 1327
>miR5809 Score: 0.0 RO3G_03867
Query: 1 TCGGCTGGCGACCAAAGC 18
||||||||||||||||||
Sbjct: 1401 AGCCGACCGCTGGTTTCG 1384
>miR5809 Score: 2.2 RO3G_00519
Query: 1 TCGGCTGGCGACCAAAGC 18
:|:||*|||:||||||||
Sbjct: 179 GGTCGTCCGTTGGTTTCG 162
>miR5809 Score: 0.0 RO3G_00977
Query: 1 TCGGCTGGCGACCAAAGC 18
||||||||||||||||||
Sbjct: 663 AGCCGACCGCTGGTTTCG 646
>miR5809 Score: 0.0 RO3G_01004
Query: 1 TCGGCTGGCGACCAAAGC 18
||||||||||||||||||
Sbjct: 1401 AGCCGACCGCTGGTTTCG 1384
>miR5809 Score: 2.0 RO3G_01282
Query: 1 TCGGCTGGCGACCAAAGC 18
|*|||||*||||||||||
Sbjct: 729 ATCCGACAGCTGGTTTCG 712
>miR5809 Score: 2.2 RO3G_01428
Query: 1 TCGGCTGGCGACCAAAGC 18
:||||||*||||||||*|
Sbjct: 19 GGCCGACAGCTGGTTT-G 3
>miR5809 Score: 0.0 RO3G_01475
Query: 1 TCGGCTGGCGACCAAAGC 18
||||||||||||||||||
Sbjct: 1401 AGCCGACCGCTGGTTTCG 1384
>miR8578.2 Score: 2.5 RO3G_16055
Query: 1 TATGAGTGTATTAGCAATG 19
*||||:||:||:|||||*|
Sbjct: 1542 TTACTTACGTAGTCGTT-C 1525
```

>miR8578.2 Score: 2.2 RO3G_15696
Query: 1 TATGAGTGTATTAGCAATG 19
:|||||||*|||||||*||
Sbjct: 1095 GTACTCACCTAATCGT-AC 1078
>miR8578.2 Score: 2.5 RO3G_15525
Query: 1 TATGAGTGTATTAGCAATG 19
||*|||||||||||||*::
Sbjct: 1344 ATTCTCACATAATCGTAGT 1326
>miR8578.2 Score: 0.0 RO3G_14870
Query: 1 TATGAGTGTATTAGCAATG 19
|||||||||||||||||||
Sbjct: 1896 ATACTCACATAATCGTTAC 1878
>miR8578.2 Score: 0.0 RO3G_14186
Query: 1 TATGAGTGTATTAGCAATG 19
|||||||||||||||||||
Sbjct: 1896 ATACTCACATAATCGTTAC 1878
>miR8578.2 Score: 0.0 RO3G_14190
Query: 1 TATGAGTGTATTAGCAATG 19
|||||||||||||||||||
Sbjct: 1896 ATACTCACATAATCGTTAC 1878
>miR8578.2 Score: 0.0 RO3G_13228
Query: 1 TATGAGTGTATTAGCAATG 19
|||||||||||||||||||
Sbjct: 1485 ATACTCACATAATCGTTAC 1467
>miR8578.2 Score: 2.2 RO3G_08552
Query: 1 TATGAGTGTATTAGCAATG 19
:|||*:||||||||||||*
Sbjct: 720 GTACCTACATAATCGTTAG 702
>miR8578.2 Score: 0.0 RO3G_08806
Query: 1 TATGAGTGTATTAGCAATG 19
|||||||||||||||||||
Sbjct: 1896 ATACTCACATAATCGTTAC 1878
>miR8578.2 Score: 2.5 RO3G_07900
Query: 1 TATGAGTGTATTAGCAATG 19
|*|:|:|||||||:|||||
Sbjct: 261 ACATTTACATAATTGTTAC 243
>miR8578.2 Score: 2.0 RO3G_04839
Query: 1 TATGAGTGTATTAGCAATG 19
|||||||||||*|:|||*|
Sbjct: 312 ATACTCACATATTTGTT-C 295
>miR8578.2 Score: 0.0 RO3G_01029
Query: 1 TATGAGTGTATTAGCAATG 19
|||||||||||||||||||
Sbjct: 855 ATACTCACATAATCGTTAC 837
>miR7729a-5p Score: 0.5 RO3G_16371

```
Query: 1 TGTTTTCAAGTAGGACAGTGAGC 23
|||||||||||||||||||:|:|
Sbjct: 184 ACAAAAGTTCATCCTGTCATTTG 162
>miR7729a-5p Score: 0.5 RO3G_15905
Query: 1 TGTTTTCAAGTAGGACAGTGAGC 23
||||||||||:||||||||||||
Sbjct: 184 ACAAAAGTTCGTCCTGTCACTCG 162
>miR7729a-5p Score: 2.5 RO3G_15142
Query: 1 TGTTTTCAAGTAGGACAGTGAGC 23
||||||*|||||||||||**|*|
Sbjct: 190 ACAAAACTTCATCCTGTCTGTGG 168
>miR7729a-5p Score: 0.0 RO3G_12528
Query: 1 TGTTTTCAAGTAGGACAGTGAGC 23
|||||||||||||||||||||||
Sbjct: 184 ACAAAAGTTCATCCTGTCACTCG 162
>miR7729a-5p Score: 0.0 RO3G_06738
Query: 1 TGTTTTCAAGTAGGACAGTGAGC 23
|||||||||||||||||||||||
Sbjct: 421 ACAAAAGTTCATCCTGTCACTCG 399
>miR7729a-5p Score: 0.0 RO3G_02826
Query: 1 TGTTTTCAAGTAGGACAGTGAGC 23
|||||||||||||||||||||||
Sbjct: 184 ACAAAAGTTCATCCTGTCACTCG 162
>miR7729a-5p Score: 0.0 RO3G_17349
Query: 1 TGTTTTCAAGTAGGACAGTGAGC 23
|||||||||||||||||||||||
Sbjct: 418 ACAAAAGTTCATCCTGTCACTCG 396
>miR7753-3p Score: 2.5 RO3G_15882
Query: 1 TTGAGCAATGGAGAAGAAGG 20
:|:||*||||:||||||||:
Sbjct: 874 GATTCTTTACTTCTTCTTCT 855
>miR7753-3p Score: 2.5 RO3G_15183
Query: 1 TTGAGCAATGGAGAAGAAGG 20
*|||||||*|||:|||||*|
Sbjct: 2019 TACTCGTTTCCTTTTCTTGC 2000
>miR7753-3p Score: 2.5 RO3G_08425
Query: 1 TTGAGCAATGGAGAAGAAGG 20
:|||:||||:|||||:|*:|
Sbjct: 594 GACTTGTTATCTCTTTTATC 575
>miR7753-3p Score: 2.2 RO3G_09011
Query: 1 TTGAGCAATGGAGAAGAAGG 20
||||||||*||||||:|*|:
Sbjct: 3581 AACTCGTTTCCTCTTTT-CT 3563
>miR7753-3p Score: 2.2 RO3G_07292
Query: 1 TTGAGCAATGGAGAAGAAGG 20
```

|:|*||||||:||||||||:
Sbjct: 341 AGCACGTTACTTCTTCTTCT 322
>miR7753-3p Score: 2.5 RO3G_04562
Query: 1 TTGAGCAATGGAGAAGAAGG 20
|||||||||*||||*||||*
Sbjct: 1117 AACTCGTTAGCTCTACTTCA 1098
>miR7753-3p Score: 2.5 RO3G_04995
Query: 1 TTGAGCAATGGAGAAGAAGG 20
||||*||||:|||||:|||*
Sbjct: 813 AACTGGTTATCTCTTTTTCA 794
>miR7753-3p Score: 2.5 RO3G_03787
Query: 1 TTGAGCAATGGAGAAGAAGG 20
:|:||*||||:||||||||:
Sbjct: 478 GATTCTTTACTTCTTCTTCT 459
>miR7753-3p Score: 2.5 RO3G_00031
Query: 1 TTGAGCAATGGAGAAGAAGG 20
||*|:|||||:|:|||||||
Sbjct: 519 AAGTTGTTACTTTTTCTTCC 500
>miR7753-3p Score: 2.5 RO3G_00684
Query: 1 TTGAGCAATGGAGAAGAAGG 20
:|:||*||||:||||||||:
Sbjct: 1649 GATTCTTTACTTCTTCTTCT 1630
>miR7753-3p Score: 2.5 RO3G_01315
Query: 1 TTGAGCAATGGAGAAGAAGG 20
:|:||*||||:||||||||:
Sbjct: 869 GATTCTTTACTTCTTCTTCT 850
>miR6267a Score: 2.5 RO3G_13299
Query: 1 TGAGAGAGGTGGTAGAGTGTG 21
|||||||||:||||||*||
Sbjct: 1387 TCTCTCTCGACTATCTCA-AC 1368
>miR6267a Score: 2.5 RO3G_13412
Query: 1 TGAGAGAGGTGGTAGAGTGTG 21
|||||:|||:||:|:||||*|
Sbjct: 355 ACTCTTTCCGCCGTTTCACTC 335
>miR6267a Score: 0.0 RO3G_10719
Query: 1 TGAGAGAGGTGGTAGAGTGTG 21
|||||||||||||||||||||
Sbjct: 921 ACTCTCTCCACCATCTCACAC 901
>miR6267a Score: 2.5 RO3G_08755
Query: 1 TGAGAGAGGTGGTAGAGTGTG 21
|||:|||:|||||*|||*|||
Sbjct: 390 ACTTTCTTCACCACCTC-CAC 371
>miR6267a Score: 2.5 RO3G_05899
Query: 1 TGAGAGAGGTGGTAGAGTGTG 21
||||||*||||||||*|*|||

Sbjct: 1348 ACTCTCACCACCATCAC-CAC 1329
>miR6267a Score: 2.5 RO3G_00488
Query: 1 TGAGAGAGGTGGTAGAGTGTG 21
:||||:|||::||||||:|*|
Sbjct: 289 GCTCTTTCCGTCATCTCGCCC 269
>miR6267a Score: 2.0 RO3G_01096
Query: 1 TGAGAGAGGTGGTAGAGTGTG 21
||||*||||||||*|||||||
Sbjct: 232 ACTCCCTCCACCACCTCACAC 212
>miR1044-3p Score: 2.0 RO3G_16569
Query: 1 TGTGGTGACATATTTGTTTG 20
||||||:|*||||:||||||
Sbjct: 438 ACACCATTCTATAGACAAAC 419
>miR1044-3p Score: 2.5 RO3G_16223
Query: 1 TGTGGTGACATATTTGTTTG 20
||*|||||||||:|:|||*|
Sbjct: 1184 ACCCCACTGTATGAGCAACC 1165
>miR1044-3p Score: 2.5 RO3G_09753
Query: 1 TGTGGTGACATATTTGTTTG 20
|*|*|||||||||||||||*|
Sbjct: 593 AGAACACTGTATAAACAATC 574
>miR5526 Score: 2.5 RO3G_15634
Query: 1 GAGTTGAGTCAGGTATGAG 19
||:|||||:||::||||*|
Sbjct: 918 CTTAACTCGGTTTATAC-C 901
>miR5526 Score: 2.5 RO3G_11851
Query: 1 GAGTTGAGTCAGGTATGAG 19
|||||||:|||:|*|||*|
Sbjct: 591 CTCAACTTAGTTCTTAC-C 574
>miR5526 Score: 2.2 RO3G_04752
Query: 1 GAGTTGAGTCAGGTATGAG 19
|||||:|||||||||:*|:
Sbjct: 4882 CTCAATTCAGTCCATGATT 4864
>miR5526 Score: 2.0 RO3G_02574
Query: 1 GAGTTGAGTCAGGTATGAG 19
|||||||::|||::|||||
Sbjct: 459 CTCAACTTGGTCTGTACTC 441
>miR5526 Score: 2.5 RO3G_00272
Query: 1 GAGTTGAGTCAGGTATGAG 19
|||*||||||||*|||||*
Sbjct: 4609 CTCTACTCAGTCGATACTG 4591
>miR5526 Score: 0.0 RO3G_17296
Query: 1 GAGTTGAGTCAGGTATGAG 19
|||||||||||||||||||
Sbjct: 207 CTCAACTCAGTCCATACTC 189

>miR2108b Score: 0.5 RO3G_17257
Query: 1 TTAATGTATGTGTTTGTAGT 20
|:||||||||||||||||||
Sbjct: 2039 AGTTACATACACAAACATCA 2020
>miR2108b Score: 1.8 RO3G_16749
Query: 1 TTAATGTATGTGTTTGTAGT 20
|:|||||||||:|||||*:|
Sbjct: 2123 AGTTACATACATAAACAATA 2104
>miR2108b Score: 1.8 RO3G_16136
Query: 1 TTAATGTATGTGTTTGTAGT 20
|:|||||||||:|||||*:|
Sbjct: 2039 AGTTACATACATAAACAATA 2020
>miR2108b Score: 0.5 RO3G_15889
Query: 1 TTAATGTATGTGTTTGTAGT 20
|:||||||||||||||||||
Sbjct: 2039 AGTTACATACACAAACATCA 2020
>miR2108b Score: 2.5 RO3G_14674
Query: 1 TTAATGTATGTGTTTGTAGT 20
|*||||||||||||*|||*|
Sbjct: 517 ACTTACATACACAACCAT-A 499
>miR2108b Score: 2.2 RO3G_13523
Query: 1 TTAATGTATGTGTTTGTAGT 20
|:||||||:*|||||||||:
Sbjct: 410 AGTTACATGAACAAACATCG 391
>miR2108b Score: 1.8 RO3G_13145
Query: 1 TTAATGTATGTGTTTGTAGT 20
|:|||||||||:|||||*:|
Sbjct: 2123 AGTTACATACATAAACAATA 2104
>miR2108b Score: 1.8 RO3G_13273
Query: 1 TTAATGTATGTGTTTGTAGT 20
|:|||||||||:|||||*:|
Sbjct: 2123 AGTTACATACATAAACAATA 2104
>miR2108b Score: 0.5 RO3G_10735
Query: 1 TTAATGTATGTGTTTGTAGT 20
|:||||||||||||||||||
Sbjct: 1568 AGTTACATACACAAACATCA 1549
>miR2108b Score: 1.8 RO3G_09728
Query: 1 TTAATGTATGTGTTTGTAGT 20
||*||||||:||||||||:|
Sbjct: 174 AAATACATATACAAACATTA 155
>miR2108b Score: 0.5 RO3G_10040
Query: 1 TTAATGTATGTGTTTGTAGT 20
|:||||||||||||||||||
Sbjct: 713 AGTTACATACACAAACATCA 694
>miR2108b Score: 1.8 RO3G_10217

```
Query: 1 TTAATGTATGTGTTTGTAGT 20
|:|||||||||:|||||*:|
Sbjct: 2123 AGTTACATACATAAACAATA 2104
>miR2108b Score: 0.5 RO3G_09183
Query: 1 TTAATGTATGTGTTTGTAGT 20
|:||||||||||||||||||
Sbjct: 1256 AGTTACATACACAAACATCA 1237
>miR2108b Score: 2.2 RO3G_07555
Query: 1 TTAATGTATGTGTTTGTAGT 20
*:||||||||||||||:|*:
Sbjct: 446 CGTTACATACACAAACGT-G 428
>miR2108b Score: 1.8 RO3G_07697
Query: 1 TTAATGTATGTGTTTGTAGT 20
|:|||||||||:|||||*:|
Sbjct: 449 AGTTACATACATAAACAATA 430
>miR2108b Score: 2.5 RO3G_04258
Query: 1 TTAATGTATGTGTTTGTAGT 20
|||||:|||:|*|||||*||
Sbjct: 1408 AATTATATATAAAAACA-CA 1390
>miR2108b Score: 0.0 RO3G_04592
Query: 1 TTAATGTATGTGTTTGTAGT 20
||||||||||||||||||||
Sbjct: 1295 AATTACATACACAAACATCA 1276
>miR2108b Score: 1.8 RO3G_04681
Query: 1 TTAATGTATGTGTTTGTAGT 20
|:|||||||||:|||||*:|
Sbjct: 713 AGTTACATACATAAACAATA 694
>miR2108b Score: 0.5 RO3G_05424
Query: 1 TTAATGTATGTGTTTGTAGT 20
|:||||||||||||||||||
Sbjct: 2039 AGTTACATACACAAACATCA 2020
>miR2108b Score: 0.5 RO3G_03585
Query: 1 TTAATGTATGTGTTTGTAGT 20
|:||||||||||||||||||
Sbjct: 713 AGTTACATACACAAACATCA 694
>miR2108b Score: 0.5 RO3G_00289
Query: 1 TTAATGTATGTGTTTGTAGT 20
|:||||||||||||||||||
Sbjct: 851 AGTTACATACACAAACATCA 832
>miR2108b Score: 0.5 RO3G_17351
Query: 1 TTAATGTATGTGTTTGTAGT 20
|:||||||||||||||||||
Sbjct: 851 AGTTACATACACAAACATCA 832
>miR2867-3p Score: 2.5 RO3G_17204
Query: 1 TCAGGACTTTGGGATGGCCA 20
```

```
|||||||||||:|**|||||
Sbjct: 695 AGTCCTGAAACTCGTCCGGT 676
>miR2867-3p Score: 1.5 RO3G_17211
Query: 1 TCAGGACTTTGGGATGGCCA 20
|||||||||||:|*||||||
Sbjct: 815 AGTCCTGAAACTCGACCGGT 796
>miR2867-3p Score: 1.5 RO3G_17213
Query: 1 TCAGGACTTTGGGATGGCCA 20
|||||||||||:|*||||||
Sbjct: 140 AGTCCTGAAACTCGACCGGT 121
>miR2867-3p Score: 2.0 RO3G_17223
Query: 1 TCAGGACTTTGGGATGGCCA 20
||*||||||||*||||||||
Sbjct: 539 AGACCTGAAACACTACCGGT 520
>miR2867-3p Score: 0.0 RO3G_17069
Query: 1 TCAGGACTTTGGGATGGCCA 20
||||||||||||||||||||
Sbjct: 680 AGTCCTGAAACCCTACCGGT 661
>miR2867-3p Score: 2.5 RO3G_16905
Query: 1 TCAGGACTTTGGGATGGCCA 20
||*||||||||:|*||||||
Sbjct: 632 AGCCCTGAAACTCGACCGGT 613
>miR2867-3p Score: 2.0 RO3G_16715
Query: 1 TCAGGACTTTGGGATGGCCA 20
||*||||||||||*||||||
Sbjct: 275 AGCCCTGAAACCCGACCGGT 256
>miR2867-3p Score: 2.0 RO3G_16725
Query: 1 TCAGGACTTTGGGATGGCCA 20
||*||||||||*||||||||
Sbjct: 557 AGACCTGAAACACTACCGGT 538
>miR2867-3p Score: 2.0 RO3G_16742
Query: 1 TCAGGACTTTGGGATGGCCA 20
||*||||||||*||||||||
Sbjct: 815 AGACCTGAAACACTACCGGT 796
>miR2867-3p Score: 2.0 RO3G_16706
Query: 1 TCAGGACTTTGGGATGGCCA 20
||*||||||||*||||||||
Sbjct: 539 AGACCTGAAACACTACCGGT 520
>miR2867-3p Score: 2.0 RO3G_16372
Query: 1 TCAGGACTTTGGGATGGCCA 20
||*||||||||*||||||||
Sbjct: 815 AGACCTGAAACACTACCGGT 796
>miR2867-3p Score: 2.0 RO3G_16377
Query: 1 TCAGGACTTTGGGATGGCCA 20
||||:||||||:|*||||||
```

```
Sbjct: 140 AGTCTTGAAACTCGACCGGT 121
>miR2867-3p Score: 2.0 RO3G_16420
Query: 1 TCAGGACTTTGGGATGGCCA 20
||*||||||||*||||||||
Sbjct: 566 AGACCTGAAACGCTACCGGT 547
>miR2867-3p Score: 2.2 RO3G_16533
Query: 1 TCAGGACTTTGGGATGGCCA 20
:||:|||:|||||||||**|
Sbjct: 1411 GGTTCTGGAACCCTACCACT 1392
>miR2867-3p Score: 2.0 RO3G_16548
Query: 1 TCAGGACTTTGGGATGGCCA 20
|||||||||||:|*||||*|
Sbjct: 470 AGTCCTGAAACTCGACCGTT 451
>miR2867-3p Score: 1.5 RO3G_16118
Query: 1 TCAGGACTTTGGGATGGCCA 20
|||||||||||:|*||||||
Sbjct: 83 AGTCCTGAAACTCGACCGGT 64
>miR2867-3p Score: 2.0 RO3G_16123
Query: 1 TCAGGACTTTGGGATGGCCA 20
||*||||||||*||||||||
Sbjct: 815 AGACCTGAAACACTACCGGT 796
>miR2867-3p Score: 2.5 RO3G_15672
Query: 1 TCAGGACTTTGGGATGGCCA 20
|||||||||||:|**|||||
Sbjct: 632 AGTCCTGAAACTCGTCCGGT 613
>miR2867-3p Score: 1.5 RO3G_15873
Query: 1 TCAGGACTTTGGGATGGCCA 20
|||||||||||:|*||||||
Sbjct: 815 AGTCCTGAAACTCGACCGGT 796
>miR2867-3p Score: 2.0 RO3G_15271
Query: 1 TCAGGACTTTGGGATGGCCA 20
||*||||||||*||||||||
Sbjct: 815 AGACCTGAAACACTACCGGT 796
>miR2867-3p Score: 2.0 RO3G_15282
Query: 1 TCAGGACTTTGGGATGGCCA 20
||*||||||||*||||||||
Sbjct: 539 AGACCTGAAACGCTACCGGT 520
>miR2867-3p Score: 2.5 RO3G_14987
Query: 1 TCAGGACTTTGGGATGGCCA 20
|||||||||||:|**|||||
Sbjct: 479 AGTCCTGAAACTCGTCCGGT 460
>miR2867-3p Score: 1.5 RO3G_15154
Query: 1 TCAGGACTTTGGGATGGCCA 20
|||||||||||:|*||||||
Sbjct: 815 AGTCCTGAAACTCGACCGGT 796
```

```
>miR2867-3p Score: 0.0 RO3G_14361
Query: 1 TCAGGACTTTGGGATGGCCA 20
||||||||||||||||||||
Sbjct: 815 AGTCCTGAAACCCTACCGGT 796
>miR2867-3p Score: 0.0 RO3G_14568
Query: 1 TCAGGACTTTGGGATGGCCA 20
||||||||||||||||||||
Sbjct: 815 AGTCCTGAAACCCTACCGGT 796
>miR2867-3p Score: 2.5 RO3G_14620
Query: 1 TCAGGACTTTGGGATGGCCA 20
|||||||||||:|**|||||
Sbjct: 260 AGTCCTGAAACTCGTCCGGT 241
>miR2867-3p Score: 2.5 RO3G_14772
Query: 1 TCAGGACTTTGGGATGGCCA 20
|||||||||||:|**|||||
Sbjct: 710 AGTCCTGAAACTCGTCCGGT 691
>miR2867-3p Score: 2.5 RO3G_14050
Query: 1 TCAGGACTTTGGGATGGC-CA 20
|||||||:||||||||***||
Sbjct: 1504 AGTCCTGGAACCCTACAACGT 1484
>miR2867-3p Score: 1.5 RO3G_13447
Query: 1 TCAGGACTTTGGGATGGCCA 20
|||||||||||:|*||||||
Sbjct: 815 AGTCCTGAAACTCGACCGGT 796
>miR2867-3p Score: 1.5 RO3G_13706
Query: 1 TCAGGACTTTGGGATGGCCA 20
|||||||||||:|*||||||
Sbjct: 773 AGTCCTGAAACTCGACCGGT 754
>miR2867-3p Score: 2.0 RO3G_13714
Query: 1 TCAGGACTTTGGGATGGCCA 20
||*||||||||*||||||||
Sbjct: 815 AGACCTGAAACACTACCGGT 796
>miR2867-3p Score: 2.5 RO3G_13852
Query: 1 TCAGGACTTTGGGATGGCCA 20
||*||||||||*||||:|||
Sbjct: 566 AGACCTGAAACACTACTGGT 547
>miR2867-3p Score: 2.0 RO3G_12663
Query: 1 TCAGGACTTTGGGATGGCCA 20
||*||||||||*||||||||
Sbjct: 650 AGACCTGAAACACTACCGGT 631
>miR2867-3p Score: 2.5 RO3G_12670
Query: 1 TCAGGACTTTGGGATGGCCA 20
|||||||||||:|**|||||
Sbjct: 632 AGTCCTGAAACTCGTCCGGT 613
>miR2867-3p Score: 1.5 RO3G_13276
```

Query: 1 TCAGGACTTTGGGATGGCCA 20
|||||||||||:|*||||||
Sbjct: 197 AGTCCTGAAACTCGACCGGT 178
>miR2867-3p Score: 0.0 RO3G_11966
Query: 1 TCAGGACTTTGGGATGGCCA 20
||||||||||||||||||||
Sbjct: 815 AGTCCTGAAACCCTACCGGT 796
>miR2867-3p Score: 1.5 RO3G_12013
Query: 1 TCAGGACTTTGGGATGGCCA 20
|||||||||||:|*||||||
Sbjct: 140 AGTCCTGAAACTCGACCGGT 121
>miR2867-3p Score: 2.5 RO3G_12082
Query: 1 TCAGGACTTTGGGATGGCCA 20
|||||||||||:|**|||||
Sbjct: 710 AGTCCTGAAACTCGTCCGGT 691
>miR2867-3p Score: 1.5 RO3G_12174
Query: 1 TCAGGACTTTGGGATGGCCA 20
|||||||||||:|*||||||
Sbjct: 140 AGTCCTGAAACTCGACCGGT 121
>miR2867-3p Score: 2.5 RO3G_12186
Query: 1 TCAGGACTTTGGGATGGCCA 20
||*||||||||:|*||||||
Sbjct: 824 AGACCTGAAACTCGACCGGT 805
>miR2867-3p Score: 2.5 RO3G_12196
Query: 1 TCAGGACTTTGGGATGGCCA 20
||*||||||||:|*||||||
Sbjct: 746 AGACCTGAAACTCGACCGGT 727
>miR2867-3p Score: 2.5 RO3G_12525
Query: 1 TCAGGACTTTGGGATGGCCA 20
||*||||||||:|*||||||
Sbjct: 746 AGCCCTGAAACTCGACCGGT 727
>miR2867-3p Score: 1.5 RO3G_12526
Query: 1 TCAGGACTTTGGGATGGCCA 20
|||||||||||:|*||||||
Sbjct: 815 AGTCCTGAAACTCGACCGGT 796
>miR2867-3p Score: 1.5 RO3G_11244
Query: 1 TCAGGACTTTGGGATGGCCA 20
|||||||||||:|*||||||
Sbjct: 815 AGTCCTGAAACTCGACCGGT 796
>miR2867-3p Score: 1.5 RO3G_11263
Query: 1 TCAGGACTTTGGGATGGCCA 20
|||||||||||:|*||||||
Sbjct: 140 AGTCCTGAAACTCGACCGGT 121
>miR2867-3p Score: 2.5 RO3G_11273
Query: 1 TCAGGACTTTGGGATGGCCA 20

||*||||||||:|*||||||
Sbjct: 155 AGCCCTGAAACTCGACCGGT 136
>miR2867-3p Score: 2.5 RO3G_11405
Query: 1 TCAGGACTTTGGGATGGCCA 20
|||||||||||:|**|||||
Sbjct: 632 AGTCCTGAAACTCGTCCGGT 613
>miR2867-3p Score: 1.5 RO3G_11528
Query: 1 TCAGGACTTTGGGATGGCCA 20
|||||||||||:|*||||||
Sbjct: 140 AGTCCTGAAACTCGACCGGT 121
>miR2867-3p Score: 2.5 RO3G_11534
Query: 1 TCAGGACTTTGGGATGGCCA 20
|||||*|||||:|*||||||
Sbjct: 815 AGTCCCGAAACTCGACCGGT 796
>miR2867-3p Score: 2.0 RO3G_11781
Query: 1 TCAGGACTTTGGGATGGCCA 20
||*||||||||*||||||||
Sbjct: 710 AGACCTGAAACACTACCGGT 691
>miR2867-3p Score: 1.5 RO3G_11948
Query: 1 TCAGGACTTTGGGATGGCCA 20
|||||||||||:|*||||||
Sbjct: 815 AGTCCTGAAACTCGACCGGT 796
>miR2867-3p Score: 1.5 RO3G_10528
Query: 1 TCAGGACTTTGGGATGGCCA 20
|||||||||||:|*||||||
Sbjct: 140 AGTCCTGAAACTCGACCGGT 121
>miR2867-3p Score: 1.5 RO3G_10537
Query: 1 TCAGGACTTTGGGATGGCCA 20
|||||||||||:|*||||||
Sbjct: 638 AGTCCTGAAACTCGACCGGT 619
>miR2867-3p Score: 1.5 RO3G_10720
Query: 1 TCAGGACTTTGGGATGGCCA 20
|||||||||||:|*||||||
Sbjct: 815 AGTCCTGAAACTCGACCGGT 796
>miR2867-3p Score: 0.5 RO3G_10729
Query: 1 TCAGGACTTTGGGATGGCCA 20
|||||||||||||||:||||
Sbjct: 815 AGTCCTGAAACCCTATCGGT 796
>miR2867-3p Score: 1.5 RO3G_10742
Query: 1 TCAGGACTTTGGGATGGCCA 20
|||||||||||:|*||||||
Sbjct: 140 AGTCCTGAAACTCGACCGGT 121
>miR2867-3p Score: 1.0 RO3G_10773
Query: 1 TCAGGACTTTGGGATGGCCA 20
|||||*||||||||||||||

```
Sbjct: 815 AGTCCCGAAACCCTACCGGT 796
>miR2867-3p Score: 1.0 RO3G_10780
Query: 1 TCAGGACTTTGGGATGGCCA 20
|||||*||||||||||||||
Sbjct: 815 AGTCCCGAAACCCTACCGGT 796
>miR2867-3p Score: 2.0 RO3G_09193
Query: 1 TCAGGACTTTGGGATGGCCA 20
||*||||||||*||||||||
Sbjct: 539 AGACCTGAAACACTACCGGT 520
>miR2867-3p Score: 1.0 RO3G_09194
Query: 1 TCAGGACTTTGGGATGGCCA 20
||||||||||||||||*|||
Sbjct: 815 AGTCCTGAAACCCTACAGGT 796
>miR2867-3p Score: 1.5 RO3G_09199
Query: 1 TCAGGACTTTGGGATGGCCA 20
|||||||||||:|*||||||
Sbjct: 815 AGTCCTGAAACTCGACCGGT 796
>miR2867-3p Score: 1.2 RO3G_09542
Query: 1 TCAGGACTTTGGGATGGCCA 20
:||||*||||||||||||||
Sbjct: 815 GGTCCCGAAACCCTACCGGT 796
>miR2867-3p Score: 1.5 RO3G_09924
Query: 1 TCAGGACTTTGGGATGGCCA 20
|||||||||||:|*||||||
Sbjct: 815 AGTCCTGAAACTCGACCGGT 796
>miR2867-3p Score: 2.5 RO3G_09944
Query: 1 TCAGGACTTTGGGATGGCCA 20
|||||||||||:|**|||||
Sbjct: 632 AGTCCTGAAACTCGTCCGGT 613
>miR2867-3p Score: 1.0 RO3G_09945
Query: 1 TCAGGACTTTGGGATGGCCA 20
||||||||||||||||*|||
Sbjct: 539 AGTCCTGAAACCCTACAGGT 520
>miR2867-3p Score: 2.5 RO3G_09955
Query: 1 TCAGGACTTTGGGATGGCCA 20
||*||||||||:|*||||||
Sbjct: 509 AGCCCTGAAACTCGACCGGT 490
>miR2867-3p Score: 0.0 RO3G_09967
Query: 1 TCAGGACTTTGGGATGGCCA 20
||||||||||||||||||||
Sbjct: 815 AGTCCTGAAACCCTACCGGT 796
>miR2867-3p Score: 1.5 RO3G_10044
Query: 1 TCAGGACTTTGGGATGGCCA 20
|||||||||||:|*||||||
Sbjct: 815 AGTCCTGAAACTCGACCGGT 796
```

>miR2867-3p Score: 2.5 RO3G_10224
Query: 1 TCAGGACTTTGGGATGGCCA 20
|||||||||||:|**|||||
Sbjct: 632 AGTCCTGAAACTCGTCCGGT 613
>miR2867-3p Score: 2.5 RO3G_10226
Query: 1 TCAGGACTTTGGGATGGCCA 20
|||||||||||:|**|||||
Sbjct: 632 AGTCCTGAAACTCGTCCGGT 613
>miR2867-3p Score: 1.5 RO3G_07994
Query: 1 TCAGGACTTTGGGATGGCCA 20
|||||||||||:|*||||||
Sbjct: 815 AGTCCTGAAACTCGACCGGT 796
>miR2867-3p Score: 2.5 RO3G_08649
Query: 1 TCAGGACTTTGGGATGGCCA 20
|||||||||||:|**|||||
Sbjct: 577 AGTCCTGAAACTCGTCCGGT 558
>miR2867-3p Score: 1.5 RO3G_08835
Query: 1 TCAGGACTTTGGGATGGCCA 20
|||||||||||:|*||||||
Sbjct: 125 AGTCCTGAAACTCGACCGGT 106
>miR2867-3p Score: 2.5 RO3G_08848
Query: 1 TCAGGACTTTGGGATGGCCA 20
|||||*|||||:|*||||||
Sbjct: 125 AGTCCCGAAACTCGACCGGT 106
>miR2867-3p Score: 2.5 RO3G_07024
Query: 1 TCAGGACTTTGGGATGGCCA 20
||*||||||||:|*||||||
Sbjct: 824 AGCCCTGAAACTCGACCGGT 805
>miR2867-3p Score: 1.5 RO3G_07119
Query: 1 TCAGGACTTTGGGATGGCCA 20
|||||||||||:|*||||||
Sbjct: 140 AGTCCTGAAACTCGACCGGT 121
>miR2867-3p Score: 2.5 RO3G_07121
Query: 1 TCAGGACTTTGGGATGGCCA 20
||*||||||||:|*||||||
Sbjct: 824 AGCCCTGAAACTCGACCGGT 805
>miR2867-3p Score: 2.0 RO3G_07137
Query: 1 TCAGGACTTTGGGATGGCCA 20
||*||||||||*||||||||
Sbjct: 815 AGACCTGAAACACTACCGGT 796
>miR2867-3p Score: 1.5 RO3G_07295
Query: 1 TCAGGACTTTGGGATGGCCA 20
|||||||||||:|*||||||
Sbjct: 647 AGTCCTGAAACTCGACCGGT 628
>miR2867-3p Score: 2.5 RO3G_07302

```
Query: 1 TCAGGACTTTGGGATGGCCA 20
|||||||||||:|**|||||
Sbjct: 632 AGTCCTGAAACTCGTCCGGT 613
>miR2867-3p Score: 2.5 RO3G_07673
Query: 1 TCAGGACTTTGGGATGGCCA 20
||*||||||||*||:|||||
Sbjct: 815 AGCCCTGAAACACTGCCGGT 796
>miR2867-3p Score: 1.5 RO3G_07683
Query: 1 TCAGGACTTTGGGATGGCCA 20
|||||||||||:|*||||||
Sbjct: 815 AGTCCTGAAACTCGACCGGT 796
>miR2867-3p Score: 2.5 RO3G_07689
Query: 1 TCAGGACTTTGGGATGGCCA 20
|||||||||||:|**|||||
Sbjct: 632 AGTCCTGAAACTCGTCCGGT 613
>miR2867-3p Score: 2.0 RO3G_07702
Query: 1 TCAGGACTTTGGGATGGCCA 20
||*||||||||*||||||||
Sbjct: 566 AGACCTGAAACGCTACCGGT 547
>miR2867-3p Score: 2.0 RO3G_07705
Query: 1 TCAGGACTTTGGGATGGCCA 20
||*||||||||*||||||||
Sbjct: 815 AGACCTGAAACGCTACCGGT 796
>miR2867-3p Score: 2.5 RO3G_07739
Query: 1 TCAGGACTTTGGGATGGCCA 20
||*||||||||*||||:|||
Sbjct: 566 AGACCTGAAACACTACTGGT 547
>miR2867-3p Score: 0.0 RO3G_07860
Query: 1 TCAGGACTTTGGGATGGCCA 20
||||||||||||||||||||
Sbjct: 815 AGTCCTGAAACCCTACCGGT 796
>miR2867-3p Score: 2.0 RO3G_07867
Query: 1 TCAGGACTTTGGGATGGCCA 20
||*||||||||*||||||||
Sbjct: 566 AGACCTGAAACACTACCGGT 547
>miR2867-3p Score: 2.0 RO3G_05640
Query: 1 TCAGGACTTTGGGATGGCCA 20
||*||||||||*||||||||
Sbjct: 710 AGACCTGAAACACTACCGGT 691
>miR2867-3p Score: 1.5 RO3G_05916
Query: 1 TCAGGACTTTGGGATGGCCA 20
|||||||||||:|*||||||
Sbjct: 815 AGTCCTGAAACTCGACCGGT 796
>miR2867-3p Score: 0.0 RO3G_05921
Query: 1 TCAGGACTTTGGGATGGCCA 20
```

||||||||||||||||||||
Sbjct: 815 AGTCCTGAAACCCTACCGGT 796
>miR2867-3p Score: 2.0 RO3G_05950
Query: 1 TCAGGACTTTGGGATGGCCA 20
||*||||||||||*||||||
Sbjct: 293 AGCCCTGAAACCCGACCGGT 274
>miR2867-3p Score: 0.0 RO3G_05953
Query: 1 TCAGGACTTTGGGATGGCCA 20
||||||||||||||||||||
Sbjct: 815 AGTCCTGAAACCCTACCGGT 796
>miR2867-3p Score: 0.0 RO3G_05959
Query: 1 TCAGGACTTTGGGATGGCCA 20
||||||||||||||||||||
Sbjct: 815 AGTCCTGAAACCCTACCGGT 796
>miR2867-3p Score: 2.0 RO3G_06717
Query: 1 TCAGGACTTTGGGATGGCCA 20
||*||||||||*||||||||
Sbjct: 815 AGACCTGAAACACTACCGGT 796
>miR2867-3p Score: 2.5 RO3G_06721
Query: 1 TCAGGACTTTGGGATGGCCA 20
|||||||||||:|**|||||
Sbjct: 629 AGTCCTGAAACTCGTCCGGT 610
>miR2867-3p Score: 2.5 RO3G_04654
Query: 1 TCAGGACTTTGGGATGGCCA 20
|||||||||||:|**|||||
Sbjct: 632 AGTCCTGAAACTCGTCCGGT 613
>miR2867-3p Score: 2.0 RO3G_04674
Query: 1 TCAGGACTTTGGGATGGCCA 20
||*||||||||*||||||||
Sbjct: 566 AGACCTGAAACACTACCGGT 547
>miR2867-3p Score: 2.5 RO3G_04695
Query: 1 TCAGGACTTTGGGATGGCCA 20
||*|:||||||*||||||||
Sbjct: 605 AGACTTGAAACACTACCGGT 586
>miR2867-3p Score: 2.5 RO3G_04786
Query: 1 TCAGGACTTTGGGATGGCCA 20
||*||||||||:|*||||||
Sbjct: 392 AGCCCTGAAACTCGACCGGT 373
>miR2867-3p Score: 0.0 RO3G_05267
Query: 1 TCAGGACTTTGGGATGGCCA 20
||||||||||||||||||||
Sbjct: 815 AGTCCTGAAACCCTACCGGT 796
>miR2867-3p Score: 2.0 RO3G_05268
Query: 1 TCAGGACTTTGGGATGGCCA 20
||*||||||||*||||||||

Sbjct: 815 AGACCTGAAACACTACCGGT 796
>miR2867-3p Score: 2.5 RO3G_02700
Query: 1 TCAGGACTTTGGGATGGCCA 20
||*||||||||:|*||||||
Sbjct: 509 AGCCCTGAAACTCGACCGGT 490
>miR2867-3p Score: 2.0 RO3G_02824
Query: 1 TCAGGACTTTGGGATGGCCA 20
||*||||||||*||||||||
Sbjct: 815 AGACCTGAAACACTACCGGT 796
>miR2867-3p Score: 1.5 RO3G_03558
Query: 1 TCAGGACTTTGGGATGGCCA 20
|||||||||||:|*||||||
Sbjct: 815 AGTCCTGAAACTCGACCGGT 796
>miR2867-3p Score: 2.5 RO3G_03570
Query: 1 TCAGGACTTTGGGATGGCCA 20
||*||||||||:|*||||||
Sbjct: 509 AGCCCTGAAACTCGACCGGT 490
>miR2867-3p Score: 2.0 RO3G_03605
Query: 1 TCAGGACTTTGGGATGGCCA 20
||*||||||||||*||||||
Sbjct: 1277 AGCCCTGAAACCCGACCGGT 1258
>miR2867-3p Score: 1.5 RO3G_03617
Query: 1 TCAGGACTTTGGGATGGCCA 20
|||||||||||:|*||||||
Sbjct: 815 AGTCCTGAAACTCGACCGGT 796
>miR2867-3p Score: 2.5 RO3G_03785
Query: 1 TCAGGACTTTGGGATGGCCA 20
|||||||||||:|**|||||
Sbjct: 437 AGTCCTGAAACTCGTCCGGT 418
>miR2867-3p Score: 2.5 RO3G_03797
Query: 1 TCAGGACTTTGGGATGGCCA 20
|||||||||||:|**|||||
Sbjct: 320 AGTCCTGAAACTCGTCCGGT 301
>miR2867-3p Score: 1.5 RO3G_03987
Query: 1 TCAGGACTTTGGGATGGCCA 20
|||||||||||:|*||||||
Sbjct: 815 AGTCCTGAAACTCGACCGGT 796
>miR2867-3p Score: 2.5 RO3G_04200
Query: 1 TCAGGACTTTGGGATGGCCA 20
|||||||||||:|**|||||
Sbjct: 632 AGTCCTGAAACTCGTCCGGT 613
>miR2867-3p Score: 0.5 RO3G_04207
Query: 1 TCAGGACTTTGGGATGGCCA 20
||||:|||||||||||||||
Sbjct: 815 AGTCTTGAAACCCTACCGGT 796

>miR2867-3p Score: 1.0 RO3G_04210
Query: 1 TCAGGACTTTGGGATGGCCA 20
|||||*||||||||||||||
Sbjct: 815 AGTCCCGAAACCCTACCGGT 796
>miR2867-3p Score: 2.5 RO3G_00247
Query: 1 TCAGGACTTTGGGATGGCCA 20
|||||||||||:|**|||||
Sbjct: 632 AGTCCTGAAACTCGTCCGGT 613
>miR2867-3p Score: 0.0 RO3G_00256
Query: 1 TCAGGACTTTGGGATGGCCA 20
||||||||||||||||||||
Sbjct: 815 AGTCCTGAAACCCTACCGGT 796
>miR2867-3p Score: 2.0 RO3G_00677
Query: 1 TCAGGACTTTGGGATGGCCA 20
||*||||||||||*||||||
Sbjct: 377 AGCCCTGAAACCCGACCGGT 358
>miR2867-3p Score: 2.5 RO3G_00690
Query: 1 TCAGGACTTTGGGATGGCCA 20
||*||||||||:|*||||||
Sbjct: 764 AGCCCTGAAACTCGACCGGT 745
>miR2867-3p Score: 2.0 RO3G_00745
Query: 1 TCAGGACTTTGGGATGGCCA 20
||*||||||||*||||||||
Sbjct: 536 AGACCTGAAACACTACCGGT 517
>miR2867-3p Score: 1.5 RO3G_01194
Query: 1 TCAGGACTTTGGGATGGCCA 20
|||||||||||:|*||||||
Sbjct: 815 AGTCCTGAAACTCGACCGGT 796
>miR2867-3p Score: 1.5 RO3G_01313
Query: 1 TCAGGACTTTGGGATGGCCA 20
|||||||||||:|*||||||
Sbjct: 815 AGTCCTGAAACTCGACCGGT 796
>miR2867-3p Score: 2.0 RO3G_01328
Query: 1 TCAGGACTTTGGGATGGCCA 20
||*||||||||*||||||||
Sbjct: 815 AGACCTGAAACACTACCGGT 796
>miR2867-3p Score: 2.5 RO3G_01468
Query: 1 TCAGGACTTTGGGATGGCCA 20
||:||||||||:|:||||
Sbjct: 469 CGTTCTGAAACCTTGCCCGT 450
>miR2867-3p Score: 2.0 RO3G_01588
Query: 1 TCAGGACTTTGGGATGGCCA 20
||*||||||||||*||||||
Sbjct: 155 AGCCCTGAAACCCGACCGGT 136
>miR2867-3p Score: 1.5 RO3G_01735

```
Query: 1 TCAGGACTTTGGGATGGCCA 20
|||||||||||:|*||||||
Sbjct: 815 AGTCCTGAAACTCGACCGGT 796
>miR2867-3p Score: 2.5 RO3G_01925
Query: 1 TCAGGACTTTGGGATGGCCA 20
||*||||||||:|*||||||
Sbjct: 824 AGCCCTGAAACTCGACCGGT 805
>miR2867-3p Score: 1.0 RO3G_01942
Query: 1 TCAGGACTTTGGGATGGCCA 20
||||||||||||||||*|||
Sbjct: 812 AGTCCTGAAACCCTACAGGT 793
>miR2867-3p Score: 1.5 RO3G_01950
Query: 1 TCAGGACTTTGGGATGGCCA 20
|||||||||||:|*||||||
Sbjct: 815 AGTCCTGAAACTCGACCGGT 796
>miR2867-3p Score: 1.5 RO3G_01951
Query: 1 TCAGGACTTTGGGATGGCCA 20
|||||||||||:|*||||||
Sbjct: 815 AGTCCTGAAACTCGACCGGT 796
>miR2867-3p Score: 2.0 RO3G_01962
Query: 1 TCAGGACTTTGGGATGGCCA 20
||*||||||||*||||||||
Sbjct: 566 AGACCTGAAACACTACCGGT 547
>miR2867-3p Score: 0.0 RO3G_17449
Query: 1 TCAGGACTTTGGGATGGCCA 20
||||||||||||||||||||
Sbjct: 815 AGTCCTGAAACCCTACCGGT 796
>miR2867-3p Score: 2.5 RO3G_17436
Query: 1 TCAGGACTTTGGGATGGCCA 20
|||||||||||:|**|||||
Sbjct: 632 AGTCCTGAAACTCGTCCGGT 613
>miR2867-3p Score: 2.0 RO3G_17416
Query: 1 TCAGGACTTTGGGATGGCCA 20
||*||||||||*||||||||
Sbjct: 815 AGACCTGAAACACTACCGGT 796
>miR2867-3p Score: 1.5 RO3G_17409
Query: 1 TCAGGACTTTGGGATGGCCA 20
|||||||||||:|*||||||
Sbjct: 815 AGTCCTGAAACTCGACCGGT 796
>miR2867-3p Score: 2.0 RO3G_17406
Query: 1 TCAGGACTTTGGGATGGCCA 20
||*||||||||||*||||||
Sbjct: 176 AGCCCTGAAACCCGACCGGT 157
>miR2867-3p Score: 2.0 RO3G_17399
Query: 1 TCAGGACTTTGGGATGGCCA 20
```

||*||||||||*||||||||
Sbjct: 815 AGACCTGAAACACTACCGGT 796
>miR2867-3p Score: 2.0 RO3G_17387
Query: 1 TCAGGACTTTGGGATGGCCA 20
||*||||||||*||||||||
Sbjct: 815 AGACCTGAAACACTACCGGT 796
>miR2867-3p Score: 2.0 RO3G_17355
Query: 1 TCAGGACTTTGGGATGGCCA 20
||*||||||||*||||||||
Sbjct: 650 AGACCTGAAACACTACCGGT 631
>miR2867-3p Score: 2.0 RO3G_17291
Query: 1 TCAGGACTTTGGGATGGCCA 20
||*||||||||*||||||||
Sbjct: 815 AGACCTGAAACACTACCGGT 796
>miR2867-3p Score: 2.5 RO3G_17293
Query: 1 TCAGGACTTTGGGATGGCCA 20
|||||||||||:|**|||||
Sbjct: 632 AGTCCTGAAACTCGTCCGGT 613
>miR5554a-5p Score: 1.0 RO3G_17220
Query: 1 TGTTGCAGATCTTGAACAATGTT 23
||||||||||||||*||||||||
Sbjct: 131 ACAACGTCTAGAACTCGTTACAA 109
>miR5554a-5p Score: 0.0 RO3G_16903
Query: 1 TGTTGCAGATCTTGAACAATGTT 23
|||||||||||||||||||||||
Sbjct: 68 ACAACGTCTAGAACTTGTTACAA 46
>miR5554a-5p Score: 0.0 RO3G_14562
Query: 1 TGTTGCAGATCTTGAACAATGTT 23
|||||||||||||||||||||||
Sbjct: 68 ACAACGTCTAGAACTTGTTACAA 46
>miR5554a-5p Score: 2.5 RO3G_12583
Query: 1 TGTTGCAGATCTTGAACAATGTT 23
|*||||||||*|||||||||*||
Sbjct: 443 AAAACGTCTAAAACTTGTTA-AA 422
>miR5554a-5p Score: 2.5 RO3G_08022
Query: 1 TGTTGCAGATCTTGAACAATGTT 23
|||||||||||*||||*|||*||
Sbjct: 252 ACAACGTCTAGCACTT-TTAGAA 231
>miR5554a-5p Score: 0.0 RO3G_07863
Query: 1 TGTTGCAGATCTTGAACAATGTT 23
|||||||||||||||||||||||
Sbjct: 131 ACAACGTCTAGAACTTGTTACAA 109
>miR7539 Score: 0.0 RO3G_01611
Query: 1 TGAGAGAGAAGAGCTATGAGG 21
|||||||||||||||||||||

Sbjct: 323 ACTCTCTCTTCTCGATACTCC 303
>miR3703 Score: 1.0 RO3G_16749
Query: 1 TAGATTCATCAGCCATTGCC 20
|*||||||||||||||||||
Sbjct: 1342 AACTAAGTAGTCGGTAACGG 1323
>miR3703 Score: 2.5 RO3G_16457
Query: 1 TAGATTCATCAGCCATTGCC 20
|||*||||||||||||*|*|
Sbjct: 1162 ATCGAAGTAGTCGGTA-CTG 1144
>miR3703 Score: 1.0 RO3G_15898
Query: 1 TAGATTCATCAGCCATTGCC 20
|*||||||||||||||||||
Sbjct: 1342 AACTAAGTAGTCGGTAACGG 1323
>miR3703 Score: 1.0 RO3G_14550
Query: 1 TAGATTCATCAGCCATTGCC 20
|*||||||||||||||||||
Sbjct: 1342 AACTAAGTAGTCGGTAACGG 1323
>miR3703 Score: 1.0 RO3G_13145
Query: 1 TAGATTCATCAGCCATTGCC 20
|*||||||||||||||||||
Sbjct: 1342 AACTAAGTAGTCGGTAACGG 1323
>miR3703 Score: 1.0 RO3G_13273
Query: 1 TAGATTCATCAGCCATTGCC 20
|*||||||||||||||||||
Sbjct: 1342 AACTAAGTAGTCGGTAACGG 1323
>miR3703 Score: 1.0 RO3G_11969
Query: 1 TAGATTCATCAGCCATTGCC 20
|*||||||||||||||||||
Sbjct: 1342 AACTAAGTAGTCGGTAACGG 1323
>miR3703 Score: 2.2 RO3G_12602
Query: 1 TAGATTCATCAGCCATTGCC 20
:|||||||:||||||||***
Sbjct: 871 GTCTAAGTGGTCGGTAAAAT 852
>miR3703 Score: 1.0 RO3G_11237
Query: 1 TAGATTCATCAGCCATTGCC 20
|*||||||||||||||||||
Sbjct: 1342 AACTAAGTAGTCGGTAACGG 1323
>miR3703 Score: 1.0 RO3G_11409
Query: 1 TAGATTCATCAGCCATTGCC 20
|*||||||||||||||||||
Sbjct: 1342 AACTAAGTAGTCGGTAACGG 1323
>miR3703 Score: 1.0 RO3G_11538
Query: 1 TAGATTCATCAGCCATTGCC 20
|*||||||||||||||||||
Sbjct: 1342 AACTAAGTAGTCGGTAACGG 1323

```
>miR3703 Score: 2.5 RO3G_11638
Query: 1 TAGATTCATCAGCCATTGCC 20
||*||*|||||||||||*||
Sbjct: 583 ATATATGTAGTCGGTAA-GG 565
>miR3703 Score: 2.5 RO3G_11706
Query: 1 TAGATTCATCAGCCATTGCC 20
|*:||||||||||||||*|*
Sbjct: 610 ACTTAAGTAGTCGGTAAAGA 591
>miR3703 Score: 1.0 RO3G_09601
Query: 1 TAGATTCATCAGCCATTGCC 20
|*||||||||||||||||||
Sbjct: 1342 AACTAAGTAGTCGGTAACGG 1323
>miR3703 Score: 1.0 RO3G_09930
Query: 1 TAGATTCATCAGCCATTGCC 20
|*||||||||||||||||||
Sbjct: 1342 AACTAAGTAGTCGGTAACGG 1323
>miR3703 Score: 1.0 RO3G_10217
Query: 1 TAGATTCATCAGCCATTGCC 20
|*||||||||||||||||||
Sbjct: 1342 AACTAAGTAGTCGGTAACGG 1323
>miR3703 Score: 1.0 RO3G_07980
Query: 1 TAGATTCATCAGCCATTGCC 20
|*||||||||||||||||||
Sbjct: 730 AACTAAGTAGTCGGTAACGG 711
>miR3703 Score: 2.2 RO3G_08415
Query: 1 TAGATTCATCAGCCATTGCC 20
:|:|:||*||||||||||||
Sbjct: 397 GTTTGAGAAGTCGGTAACGG 378
>miR3703 Score: 1.0 RO3G_06731
Query: 1 TAGATTCATCAGCCATTGCC 20
|*||||||||||||||||||
Sbjct: 1342 AACTAAGTAGTCGGTAACGG 1323
>miR3703 Score: 1.0 RO3G_07307
Query: 1 TAGATTCATCAGCCATTGCC 20
|*||||||||||||||||||
Sbjct: 1342 AACTAAGTAGTCGGTAACGG 1323
>miR3703 Score: 1.0 RO3G_07698
Query: 1 TAGATTCATCAGCCATTGCC 20
|*||||||||||||||||||
Sbjct: 1342 AACTAAGTAGTCGGTAACGG 1323
>miR3703 Score: 1.0 RO3G_07721
Query: 1 TAGATTCATCAGCCATTGCC 20
|*||||||||||||||||||
Sbjct: 1273 AACTAAGTAGTCGGTAACGG 1254
>miR3703 Score: 1.0 RO3G_07737
```

Query: 1 TAGATTCATCAGCCATTGCC 20
|*||||||||||||||||||
Sbjct: 703 AACTAAGTAGTCGGTAACGG 684
>miR3703 Score: 2.2 RO3G_07895
Query: 1 TAGATTCATCAGCCATTGCC 20
:|||||||:||||||||***
Sbjct: 892 GTCTAAGTGGTCGGTAAAAT 873
>miR3703 Score: 1.5 RO3G_05557
Query: 1 TAGATTCATCAGCCATTGCC 20
||||||||||||||||:*|*
Sbjct: 988 ATCTAAGTAGTCGGTAG-GT 970
>miR3703 Score: 0.0 RO3G_04592
Query: 1 TAGATTCATCAGCCATTGCC 20
||||||||||||||||||||
Sbjct: 514 ATCTAAGTAGTCGGTAACGG 495
>miR3703 Score: 1.0 RO3G_04683
Query: 1 TAGATTCATCAGCCATTGCC 20
|*||||||||||||||||||
Sbjct: 1342 AACTAAGTAGTCGGTAACGG 1323
>miR3703 Score: 1.0 RO3G_04212
Query: 1 TAGATTCATCAGCCATTGCC 20
|*||||||||||||||||||
Sbjct: 1342 AACTAAGTAGTCGGTAACGG 1323
>miR3703 Score: 1.8 RO3G_00332
Query: 1 TAGATTCATCAGCCATTGCC 20
:|:||||*||||||||||||
Sbjct: 397 GTTTAAGAAGTCGGTAACGG 378
>miR3703 Score: 1.0 RO3G_01727
Query: 1 TAGATTCATCAGCCATTGCC 20
|*||||||||||||||||||
Sbjct: 427 AACTAAGTAGTCGGTAACGG 408
>miR7543 Score: 2.2 RO3G_16327
Query: 1 TTCATATACATGTTTGACAA 20
:||||*:|||||:|||||||
Sbjct: 198 GAGTACGTGTACGAACTGTT 179
>miR7543 Score: 2.5 RO3G_15734
Query: 1 TTCATATACATGTTTGAC-AA 20
||||||:||*||:|||||*||
Sbjct: 1176 AAGTATGTGAACGAACTGCTT 1156
>miR7543 Score: 0.0 RO3G_14371
Query: 1 TTCATATACATGTTTGACAA 20
||||||||||||||||||||
Sbjct: 645 AAGTATATGTACAAACTGTT 626
>miR7543 Score: 2.5 RO3G_13867
Query: 1 TTCATATACATGTTTGACAA 20

||||:|||||||||::|**|
Sbjct: 1013 AAGTGTATGTACAAGTTCAT 994
>miR7543 Score: 0.5 RO3G_13908
Query: 1 TTCATATACATGTTTGACAA 20
||||||||||||:|||||||
Sbjct: 480 AAGTATATGTACGAACTGTT 461
>miR7543 Score: 0.5 RO3G_12320
Query: 1 TTCATATACATGTTTGACAA 20
||||||||||||:|||||||
Sbjct: 597 AAGTATATGTACGAACTGTT 578
>miR7543 Score: 2.5 RO3G_11852
Query: 1 TTCATATACATGTTTGACAA 20
||||||*||||||||:|*|*
Sbjct: 288 AAGTATTTGTACAAATTATG 269
>miR7543 Score: 1.5 RO3G_10438
Query: 1 TTCATATACATGTTTGACAA 20
||||||||||||:|||*|||
Sbjct: 189 AAGTATATGTACGAACAGTT 170
>miR7543 Score: 2.5 RO3G_08239
Query: 1 TTCATATACATGTTTGACAA 20
||||||*||||||||*|*||
Sbjct: 333 AAGTATTTGTACAAAAT-TT 315
>miR7543 Score: 2.2 RO3G_08601
Query: 1 TTCATATACATGTTTGACAA 20
:|||||*||||||||:|||*
Sbjct: 206 GAGTATTTGTACAAATTGTG 187
>miR7543 Score: 0.5 RO3G_05704
Query: 1 TTCATATACATGTTTGACAA 20
||||||||||||:|||||||
Sbjct: 597 AAGTATATGTACGAACTGTT 578
>miR7543 Score: 2.5 RO3G_04493
Query: 1 TTCATATACATGTTTGACAA 20
|||||||**|||:|||||||
Sbjct: 108 AAGTATACATACGAACTGTT 89
>miR7543 Score: 1.5 RO3G_02935
Query: 1 TTCATATACATGTTTGACAA 20
||||||||||||:|||*|||
Sbjct: 210 AAGTATATGTACGAACCGTT 191
>miR7543 Score: 2.5 RO3G_03353
Query: 1 TTCATATACATGTTTGACAA 20
||||||**||:|||||||||
Sbjct: 540 AAGTATTCGTGCAAACTGTT 521
>miR7543 Score: 2.5 RO3G_04007
Query: 1 TTCATATACATGTTTGACAA 20
||||||*||*|||||:||||

```
Sbjct: 1158 AAGTATCTGAACAAATTGTT 1139
>miR7543 Score: 2.5 RO3G_00576
Query: 1 TTCATATACATGTTTGAC-AA 20
|:|||||||||*|||:||*||
Sbjct: 59 AGGTATATGTAGAAATTGGTT 39
>miR7543 Score: 2.5 RO3G_00599
Query: 1 TTCATATACATGTTTGAC-AA 20
|:|||||||||*|||:||*||
Sbjct: 1698 AGGTATATGTAGAAATTGGTT 1678
>miR7543 Score: 0.0 RO3G_00716
Query: 1 TTCATATACATGTTTGACAA 20
||||||||||||||||||||
Sbjct: 630 AAGTATATGTACAAACTGTT 611
>miR7543 Score: 0.5 RO3G_01392
Query: 1 TTCATATACATGTTTGACAA 20
||||||||||||:|||||||
Sbjct: 108 AAGTATATGTACGAACTGTT 89
>miR7543 Score: 2.5 RO3G_01429
Query: 1 TTCATATACATGTTTGAC-AA 20
|:|||||||||*|||:||*||
Sbjct: 1872 AGGTATATGTAGAAATTGGTT 1852
>miR8554 Score: 0.0 RO3G_13701
Query: 1 TGCTTATGACTTTGATTTGGGA 22
||||||||||||||||||||||
Sbjct: 2762 ACGAATACTGAAACTAAACCCT 2741
>miR8554 Score: 0.0 RO3G_13855
Query: 1 TGCTTATGACTTTGATTTGGGA 22
||||||||||||||||||||||
Sbjct: 2762 ACGAATACTGAAACTAAACCCT 2741
>miR8554 Score: 0.0 RO3G_12014
Query: 1 TGCTTATGACTTTGATTTGGGA 22
||||||||||||||||||||||
Sbjct: 2762 ACGAATACTGAAACTAAACCCT 2741
>miR8554 Score: 0.0 RO3G_12067
Query: 1 TGCTTATGACTTTGATTTGGGA 22
||||||||||||||||||||||
Sbjct: 2762 ACGAATACTGAAACTAAACCCT 2741
>miR8554 Score: 0.0 RO3G_12462
Query: 1 TGCTTATGACTTTGATTTGGGA 22
||||||||||||||||||||||
Sbjct: 2618 ACGAATACTGAAACTAAACCCT 2597
>miR8554 Score: 0.0 RO3G_10222
Query: 1 TGCTTATGACTTTGATTTGGGA 22
||||||||||||||||||||||
Sbjct: 2762 ACGAATACTGAAACTAAACCCT 2741
```

```
>miR8554 Score: 0.0 RO3G_03575
Query: 1 TGCTTATGACTTTGATTTGGGA 22
||||||||||||||||||||||
Sbjct: 2762 ACGAATACTGAAACTAAACCCT 2741
>miR8554 Score: 0.0 RO3G_03752
Query: 1 TGCTTATGACTTTGATTTGGGA 22
||||||||||||||||||||||
Sbjct: 2762 ACGAATACTGAAACTAAACCCT 2741
>miR8554 Score: 0.0 RO3G_03796
Query: 1 TGCTTATGACTTTGATTTGGGA 22
||||||||||||||||||||||
Sbjct: 1955 ACGAATACTGAAACTAAACCCT 1934
>miR1172.1 Score: 1.8 RO3G_16901
Query: 1 AGGATTTGCAGCAGCAAAGGAG 22
*|||||*||||||||||||||:
Sbjct: 1074 ACCTAACCGTCGTCGTTTCCTT 1053
>miR1172.1 Score: 1.8 RO3G_16917
Query: 1 AGGATTTGCAGCAGCAAAGGAG 22
*|||||*||||||||||||||:
Sbjct: 1080 ACCTAACCGTCGTCGTTTCCTT 1059
>miR1172.1 Score: 1.8 RO3G_16743
Query: 1 AGGATTTGCAGCAGCAAAGGAG 22
*|||||*||||||||||||||:
Sbjct: 1026 ACCTAACCGTCGTCGTTTCCTT 1005
>miR1172.1 Score: 1.8 RO3G_15879
Query: 1 AGGATTTGCAGCAGCAAAGGAG 22
*|||||*||||||||||||||:
Sbjct: 987 ACCTAACCGTCGTCGTTTCCTT 966
>miR1172.1 Score: 1.8 RO3G_15581
Query: 1 AGGATTTGCAGCAGCAAAGGAG 22
*|||||*||||||||||||||:
Sbjct: 237 ACCTAACCGTCGTCGTTTCCTT 216
>miR1172.1 Score: 1.8 RO3G_12975
Query: 1 AGGATTTGCAGCAGCAAAGGAG 22
*|||||*||||||||||||||:
Sbjct: 366 ACCTAACCGTCGTCGTTTCCTT 345
>miR1172.1 Score: 1.8 RO3G_12011
Query: 1 AGGATTTGCAGCAGCAAAGGAG 22
*|||||*||||||||||||||:
Sbjct: 1068 ACCTAACCGTCGTCGTTTCCTT 1047
>miR1172.1 Score: 1.8 RO3G_12078
Query: 1 AGGATTTGCAGCAGCAAAGGAG 22
*|||||*||||||||||||||:
Sbjct: 768 ACCTAACCGTCGTCGTTTCCTT 747
>miR1172.1 Score: 1.8 RO3G_12645
```

Query: 1 AGGATTTGCAGCAGCAAAGGAG 22
|||||||||||||||||||:
Sbjct: 240 ACCTAACCGTCGTCGTTTCCTT 219
>miR1172.1 Score: 1.8 RO3G_10043
Query: 1 AGGATTTGCAGCAGCAAAGGAG 22
|||||||||||||||||||:
Sbjct: 975 ACCTAACCGTCGTCGTTTCCTT 954
>miR1172.1 Score: 1.8 RO3G_07115
Query: 1 AGGATTTGCAGCAGCAAAGGAG 22
||||||||||||||||:|||
Sbjct: 1023 CCCTAATCGTCGTCGTTTTCTC 1002
>miR1172.1 Score: 1.8 RO3G_07692
Query: 1 AGGATTTGCAGCAGCAAAGGAG 22
|||||||||||||||||||:
Sbjct: 1002 ACCTAACCGTCGTCGTTTCCTT 981
>miR1172.1 Score: 1.8 RO3G_05907
Query: 1 AGGATTTGCAGCAGCAAAGGAG 22
|||||||||||||||||||:
Sbjct: 282 ACCTAACCGTCGTCGTTTCCTT 261
>miR1172.1 Score: 1.8 RO3G_04659
Query: 1 AGGATTTGCAGCAGCAAAGGAG 22
|||||||||||||||||||:
Sbjct: 1002 ACCTAACCGTCGTCGTTTCCTT 981
>miR1172.1 Score: 1.8 RO3G_02817
Query: 1 AGGATTTGCAGCAGCAAAGGAG 22
|||||||||||||||||||:
Sbjct: 1068 ACCTAACCGTCGTCGTTTCCTT 1047
>miR1172.1 Score: 0.5 RO3G_00600
Query: 1 AGGATTTGCAGCAGCAAAGGAG 22
*|||||||||||||||||||||
Sbjct: 879 ACCTAAACGTCGTCGTTTCCTC 858
>miR1172.1 Score: 1.8 RO3G_01325
Query: 1 AGGATTTGCAGCAGCAAAGGAG 22
|||||||||||||||||||:
Sbjct: 177 ACCTAACCGTCGTCGTTTCCTT 156
>miR1172.1 Score: 1.8 RO3G_01717
Query: 1 AGGATTTGCAGCAGCAAAGGAG 22
|||||||||||||||||||:
Sbjct: 1071 ACCTAACCGTCGTCGTTTCCTT 1050
>miR1172.1 Score: 1.8 RO3G_17433
Query: 1 AGGATTTGCAGCAGCAAAGGAG 22
|||||||||||||||||||:
Sbjct: 1065 ACCTAACCGTCGTCGTTTCCTT 1044
>miR5080 Score: 0.0 RO3G_17260
Query: 1 AAAAGGATCATGACGGTACATG 22

```
||||||||||||||||||||||
Sbjct: 998 TTTTCCTAGTACTGCCATGTAC 977
>miR5080 Score: 2.5 RO3G_17226
Query: 1 AAAAGGATCATGACGGTACATG 22
|||||||||*|||||:|||*|*
Sbjct: 1122 TTTTCCTAGCACTGCTATGAAA 1101
>miR5080 Score: 0.0 RO3G_16733
Query: 1 AAAAGGATCATGACGGTACATG 22
||||||||||||||||||||||
Sbjct: 1119 TTTTCCTAGTACTGCCATGTAC 1098
>miR5080 Score: 0.0 RO3G_16812
Query: 1 AAAAGGATCATGACGGTACATG 22
||||||||||||||||||||||
Sbjct: 1119 TTTTCCTAGTACTGCCATGTAC 1098
>miR5080 Score: 2.5 RO3G_16815
Query: 1 AAAAGGATCATGACGGTACATG 22
|||||||||*|||||:|||*|*
Sbjct: 1122 TTTTCCTAGCACTGCTATGAAA 1101
>miR5080 Score: 2.5 RO3G_16392
Query: 1 AAAAGGATCATGACGGTACATG 22
|||||||||*|||||:|||*|*
Sbjct: 102 TTTTCCTAGCACTGCTATGAAA 81
>miR5080 Score: 0.0 RO3G_15270
Query: 1 AAAAGGATCATGACGGTACATG 22
||||||||||||||||||||||
Sbjct: 168 TTTTCCTAGTACTGCCATGTAC 147
>miR5080 Score: 0.0 RO3G_14791
Query: 1 AAAAGGATCATGACGGTACATG 22
||||||||||||||||||||||
Sbjct: 1119 TTTTCCTAGTACTGCCATGTAC 1098
>miR5080 Score: 2.5 RO3G_14795
Query: 1 AAAAGGATCATGACGGTACATG 22
|||||||||*|||||:|||*|*
Sbjct: 1122 TTTTCCTAGCACTGCTATGAAA 1101
>miR5080 Score: 0.0 RO3G_14798
Query: 1 AAAAGGATCATGACGGTACATG 22
||||||||||||||||||||||
Sbjct: 1119 TTTTCCTAGTACTGCCATGTAC 1098
>miR5080 Score: 2.0 RO3G_08770
Query: 1 AAAAGGATCATGACGGTA-CATG 22
||||||||||*|||||||*||*|
Sbjct: 311 TTTTCCTAGTTCTGCCATGGTCC 289
>miR5080 Score: 0.0 RO3G_09180
Query: 1 AAAAGGATCATGACGGTACATG 22
||||||||||||||||||||||
```

Sbjct: 1119 TTTTCCTAGTACTGCCATGTAC 1098
>miR5080 Score: 0.0 RO3G_09189
Query: 1 AAAAGGATCATGACGGTACATG 22
||||||||||||||||||||||
Sbjct: 1119 TTTTCCTAGTACTGCCATGTAC 1098
>miR5080 Score: 0.0 RO3G_06734
Query: 1 AAAAGGATCATGACGGTACATG 22
||||||||||||||||||||||
Sbjct: 423 TTTTCCTAGTACTGCCATGTAC 402
>miR5022 Score: 2.0 RO3G_12104
Query: 1 TAATGGGGTATGTCGAAG 18
||||||:|:*||||||||
Sbjct: 466 ATTACCTCGGACAGCTTC 449
>miR5022 Score: 2.5 RO3G_11898
Query: 1 TAATGGGGTATGTCGAAG 18
||||:||:||:|:||||*
Sbjct: 229 ATTATCCTATGCGGCTTG 212
>miR5022 Score: 2.5 RO3G_10150
Query: 1 TAATGGGGTATGTCGAAG 18
||||||||||||||*|
Sbjct: 806 CTTACCCCATACACCT-C 790
>miR5022 Score: 2.5 RO3G_06594
Query: 1 TAATGGGGTATGTCGAAG 18
|||||||||||||*|*|*
Sbjct: 1050 ATTACCCCATACAACATG 1033
>miR5037c Score: 2.2 RO3G_16739
Query: 1 AGTTGAACTTTGGGCCCG 18
||||*||||||||||*|:
Sbjct: 305 TCAAATTGAAACCCGTGT 288
>miR5037c Score: 2.2 RO3G_16138
Query: 1 AGTTGAACTTTGGGCCCG 18
||||*||||||||||*|:
Sbjct: 305 TCAAATTGAAACCCGTGT 288
>miR5037c Score: 2.2 RO3G_13747
Query: 1 AGTTGAACTTTGGGCCCG 18
||||*||||||||||*|:
Sbjct: 305 TCAAATTGAAACCCGTGT 288
>miR5037c Score: 2.2 RO3G_12009
Query: 1 AGTTGAACTTTGGGCCCG 18
||||*||||||||||*|:
Sbjct: 305 TCAAATTGAAACCCGTGT 288
>miR5037c Score: 2.2 RO3G_12072
Query: 1 AGTTGAACTTTGGGCCCG 18
||||*||||||||||*|:
Sbjct: 305 TCAAATTGAAACCCGTGT 288

>miR5037c Score: 2.2 RO3G_12169
Query: 1 AGTTGAACTTTGGGCCCG 18
||||*||||||||||*|:
Sbjct: 305 TCAAATTGAAACCCGTGT 288
>miR5037c Score: 2.2 RO3G_11404
Query: 1 AGTTGAACTTTGGGCCCG 18
||||*||||||||||*|:
Sbjct: 305 TCAAATTGAAACCCGTGT 288
>miR5037c Score: 2.2 RO3G_11771
Query: 1 AGTTGAACTTTGGGCCCG 18
||||*||||||||||*|:
Sbjct: 305 TCAAATTGAAACCCGTGT 288
>miR5037c Score: 2.2 RO3G_10725
Query: 1 AGTTGAACTTTGGGCCCG 18
||||*||||||||||*|:
Sbjct: 269 TCAAATTGAAACCCGTGT 252
>miR5037c Score: 2.2 RO3G_09192
Query: 1 AGTTGAACTTTGGGCCCG 18
||||*||||||||||*|:
Sbjct: 305 TCAAATTGAAACCCGTGT 288
>miR5037c Score: 2.2 RO3G_09940
Query: 1 AGTTGAACTTTGGGCCCG 18
||||*||||||||||*|:
Sbjct: 305 TCAAATTGAAACCCGTGT 288
>miR5037c Score: 2.2 RO3G_07130
Query: 1 AGTTGAACTTTGGGCCCG 18
||||*||||||||||*|:
Sbjct: 209 TCAAATTGAAACCCGTGT 192
>miR5037c Score: 2.2 RO3G_07686
Query: 1 AGTTGAACTTTGGGCCCG 18
||||*||||||||||*|:
Sbjct: 305 TCAAATTGAAACCCGTGT 288
>miR5037c Score: 2.2 RO3G_07976
Query: 1 AGTTGAACTTTGGGCCCG 18
||||*||||||||||*|:
Sbjct: 305 TCAAATTGAAACCCGTGT 288
>miR5037c Score: 2.2 RO3G_05631
Query: 1 AGTTGAACTTTGGGCCCG 18
||||*||||||||||*|:
Sbjct: 305 TCAAATTGAAACCCGTGT 288
>miR5037c Score: 2.2 RO3G_03555
Query: 1 AGTTGAACTTTGGGCCCG 18
||||*||||||||||*|:
Sbjct: 305 TCAAATTGAAACCCGTGT 288
>miR5037c Score: 2.2 RO3G_03574

Query: 1 AGTTGAACTTTGGGCCCG 18
||||*||||||||||*|:
Sbjct: 188 TCAAATTGAAACCCGTGT 171
>miR5037c Score: 2.5 RO3G_03632
Query: 1 AGTTGAACTTTGGGCCCG 18
|:||||*||||||||*||
Sbjct: 353 TTAACTGGAAACCCGTGC 336
>miR5037c Score: 2.2 RO3G_01207
Query: 1 AGTTGAACTTTGGGCCCG 18
||||*||||||||||*|:
Sbjct: 305 TCAAATTGAAACCCGTGT 288
>miR5037c Score: 2.2 RO3G_01309
Query: 1 AGTTGAACTTTGGGCCCG 18
||||*||||||||||*|:
Sbjct: 254 TCAAATTGAAACCCGTGT 237
>miR5037c Score: 2.5 RO3G_01807
Query: 1 AGTTGAACTTTGGGCCCG 18
|||||||||*::|||||*
Sbjct: 590 TCAACTTGATGTCCGGGG 573
>miR5037c Score: 2.2 RO3G_17385
Query: 1 AGTTGAACTTTGGGCCCG 18
||||*||||||||||*|:
Sbjct: 305 TCAAATTGAAACCCGTGT 288
>miR5037c Score: 2.2 RO3G_17358
Query: 1 AGTTGAACTTTGGGCCCG 18
||||*||||||||||*|:
Sbjct: 305 TCAAATTGAAACCCGTGT 288
>miR5037c Score: 2.2 RO3G_17356
Query: 1 AGTTGAACTTTGGGCCCG 18
||||*||||||||||*|:
Sbjct: 215 TCAAATTGAAACCCGTGT 198
>miR396a-3p Score: 2.2 RO3G_11676
Query: 1 GTTCAATAAAGCTGTGGGAA 20
:|||||||||:|||||:**|
Sbjct: 1432 TAAGTTATTTGACACTAAT 1413
>miR396a-3p Score: 2.5 RO3G_08313
Query: 1 GTTCAATAAAGCTGTGGGAA 20
||:||||||||||*|||*|*
Sbjct: 1089 CAGGTTATTTCGAAACC-TA 1071
>miR396a-3p Score: 2.0 RO3G_02514
Query: 1 GTTCAATAAAGCTGTGGGAA 20
:|||||||||*|||||::||
Sbjct: 471 TAAGTTATTTGGACACTTTT 452
>miR4221 Score: 0.5 RO3G_12138
Query: 1 TAAGAGGTTCAAAAGGTAGGAAA 23

```
|||||||||||||||:|||||||
Sbjct: 867 ATTCTCCAAGTTTTCTATCCTTT 845
>miR4221 Score: 0.5 RO3G_09361
Query: 1 TAAGAGGTTCAAAAGGTAGGAAA 23
|||||||||||||||:|||||||
Sbjct: 912 ATTCTCCAAGTTTTCTATCCTTT 890
>miR4221 Score: 0.0 RO3G_05627
Query: 1 TAAGAGGTTCAAAAGGTAGGAAA 23
|||||||||||||||||||||||
Sbjct: 126 ATTCTCCAAGTTTTCCATCCTTT 104
>miR4221 Score: 0.0 RO3G_04030
Query: 1 TAAGAGGTTCAAAAGGTAGGAAA 23
|||||||||||||||||||||||
Sbjct: 1233 ATTCTCCAAGTTTTCCATCCTTT 1211
>miR156i Score: 2.5 RO3G_15674
Query: 1 TGGAACAGAAGAAGACAGCAG 21
*|:|*||||||||||||||*|
Sbjct: 3384 TCTTCGTCTTCTTCTGTCGCC 3364
>miR156i Score: 2.5 RO3G_15766
Query: 1 TGGAACAGAAGAAGACAGCAG 21
|||||*|||||||||*|||*|
Sbjct: 840 ACCTTATCTTCTTCTTTCG-C 821
>miR156i Score: 2.5 RO3G_15288
Query: 1 TGGAACAGAAGAAGACAGCAG 21
|||||||:|||||:*||*|||
Sbjct: 1036 ACCTTGTTTTCTTTCGTGGTC 1016
>miR156i Score: 2.2 RO3G_13465
Query: 1 TGGAACAGAAGAAGACAGCAG 21
||:|||||||||||||*:*||
Sbjct: 477 ACTTTGTCTTCTTCTGGT-TC 458
>miR156i Score: 2.5 RO3G_13732
Query: 1 TGGAACAGAAGAAGACAGCAG 21
*|:|*||||||||||||||*|
Sbjct: 528 TCTTCGTCTTCTTCTGTCGCC 508
>miR156i Score: 0.0 RO3G_13746
Query: 1 TGGAACAGAAGAAGACAGCAG 21
|||||||||||||||||||||
Sbjct: 1467 ACCTTGTCTTCTTCTGTCGTC 1447
>miR156i Score: 2.2 RO3G_12669
Query: 1 TGGAACAGAAGAAGACAGCAG 21
*|:||||||*|||||||:|||
Sbjct: 2613 CCTTTGTCTACTTCTGTTGTC 2593
>miR156i Score: 2.5 RO3G_12769
Query: 1 TGGAACAGAAGAAGACAGCAG 21
*|||*||||||*|||||||||
```

```
Sbjct: 273 TCCTAGTCTTCCTCTGTCGTC 253
>miR156i Score: 0.0 RO3G_12964
Query: 1 TGGAACAGAAGAAGACAGCAG 21
|||||||||||||||||||||
Sbjct: 2613 ACCTTGTCTTCTTCTGTCGTC 2593
>miR156i Score: 2.5 RO3G_11279
Query: 1 TGGAACAGAAGAAGACAGCAG 21
*|:|*||||||||||||||*|
Sbjct: 1995 TCTTCGTCTTCTTCTGTCGCC 1975
>miR156i Score: 0.0 RO3G_11952
Query: 1 TGGAACAGAAGAAGACAGCAG 21
|||||||||||||||||||||
Sbjct: 2991 ACCTTGTCTTCTTCTGTCGTC 2971
>miR156i Score: 2.5 RO3G_07729
Query: 1 TGGAACAGAAGAAGACAGCAG 21
|||||||:|||||:*||*|||
Sbjct: 805 ACCTTGTTTTCTTTCGTGGTC 785
>miR156i Score: 2.5 RO3G_07865
Query: 1 TGGAACAGAAGAAGACAGCAG 21
*|:|*||||||||||||||*|
Sbjct: 765 TCTTCGTCTTCTTCTGTCGCC 745
>miR156i Score: 2.5 RO3G_02682
Query: 1 TGGAACAGAAGAAGACAGCAG 21
|||||||:|||||:*||*|||
Sbjct: 1036 ACCTTGTTTTCTTTCGTGGTC 1016
>miR156i Score: 0.0 RO3G_00686
Query: 1 TGGAACAGAAGAAGACAGCAG 21
|||||||||||||||||||||
Sbjct: 336 ACCTTGTCTTCTTCTGTCGTC 316
>miR156i Score: 2.5 RO3G_01721
Query: 1 TGGAACAGAAGAAGACAGCAG 21
*|:|*||||||||||||||*|
Sbjct: 891 TCTTCGTCTTCTTCTGTCGCC 871
>miR4374b Score: 1.8 RO3G_16714
Query: 1 TCTTCAAAGACGGTTGTGAG 20
|||||*|||||||||||*|:
Sbjct: 209 AGAAGATTCTGCCAACAGTT 190
>miR4374b Score: 2.5 RO3G_16803
Query: 1 TCTTCAAAGACGGTTGTGAG 20
|||||*|*|||||||||*||
Sbjct: 92 AGAAGATCCTGCCAACAGTC 73
>miR4374b Score: 0.0 RO3G_14983
Query: 1 TCTTCAAAGACGGTTGTGAG 20
||||||||||||||||||||
Sbjct: 656 AGAAGTTTCTGCCAACACTC 637
```

```
>miR4374b Score: 0.0 RO3G_13914
Query: 1 TCTTCAAAGACGGTTGTGAG 20
||||||||||||||||||||
Sbjct: 851 AGAAGTTTCTGCCAACACTC 832
>miR4374b Score: 1.0 RO3G_12667
Query: 1 TCTTCAAAGACGGTTGTGAG 20
|||||*||||||||||||||
Sbjct: 221 AGAAGGTTCTGCCAACACTC 202
>miR4374b Score: 0.0 RO3G_12058
Query: 1 TCTTCAAAGACGGTTGTGAG 20
||||||||||||||||||||
Sbjct: 851 AGAAGTTTCTGCCAACACTC 832
>miR4374b Score: 1.5 RO3G_11295
Query: 1 TCTTCAAAGACGGTTGTGAG 20
|||||||||||:|||:||*|
Sbjct: 44 AGAAGTTTCTGTCAATAC-C 26
>miR4374b Score: 0.0 RO3G_10801
Query: 1 TCTTCAAAGACGGTTGTGAG 20
||||||||||||||||||||
Sbjct: 656 AGAAGTTTCTGCCAACACTC 637
>miR4374b Score: 2.5 RO3G_09552
Query: 1 TCTTCAAAGACGGTTGTGAG 20
||::||||||||:||||**|
Sbjct: 490 AGGGGTTTCTGCTAACAACC 471
>miR4374b Score: 0.0 RO3G_09614
Query: 1 TCTTCAAAGACGGTTGTGAG 20
||||||||||||||||||||
Sbjct: 182 AGAAGTTTCTGCCAACACTC 163
>miR4374b Score: 2.5 RO3G_05930
Query: 1 TCTTCAAAGACGGTTGTGAG 20
|||||*|*|||||||||*||
Sbjct: 515 AGAAGATCCTGCCAACAGTC 496
>miR4374b Score: 0.0 RO3G_02233
Query: 1 TCTTCAAAGACGGTTGTGAG 20
||||||||||||||||||||
Sbjct: 380 AGAAGTTTCTGCCAACACTC 361
>miR4374b Score: 2.5 RO3G_03556
Query: 1 TCTTCAAAGACGGTTGTGAG 20
|||||*|*|||||||||*||
Sbjct: 656 AGAAGATCCTGCCAACAGTC 637
>miR4374b Score: 1.0 RO3G_03583
Query: 1 TCTTCAAAGACGGTTGTGAG 20
|||||||||||*||||||||
Sbjct: 656 AGAAGTTTCTGACAACACTC 637
>miR4374b Score: 2.5 RO3G_03794
```

```
Query: 1 TCTTCAAAGACGGTTGTGAG 20
|||||*|*|||||||||*||
Sbjct: 92 AGAAGATCCTGCCAACAGTC 73
>miR4374b Score: 0.0 RO3G_17462
Query: 1 TCTTCAAAGACGGTTGTGAG 20
||||||||||||||||||||
Sbjct: 983 AGAAGTTTCTGCCAACACTC 964
>miR4374b Score: 0.0 RO3G_17451
Query: 1 TCTTCAAAGACGGTTGTGAG 20
||||||||||||||||||||
Sbjct: 92 AGAAGTTTCTGCCAACACTC 73
>miR4374b Score: 2.5 RO3G_17374
Query: 1 TCTTCAAAGACGGTTGTGAG 20
|||||*|*|||||||||*||
Sbjct: 92 AGAAGATCCTGCCAACAGTC 73
>miR4374b Score: 2.5 RO3G_17281
Query: 1 TCTTCAAAGACGGTTGTGAG 20
|||||*|*|||||||||*||
Sbjct: 92 AGAAGATCCTGCCAACAGTC 73
>miR826b Score: 2.5 RO3G_16423
Query: 1 TGGTCTTGACATCGATGAAAAT 22
|:|*||||||||||||||*|*|
Sbjct: 53 ATCTGAACTGTAGCTACTGTAA 32
>miR826b Score: 2.5 RO3G_15253
Query: 1 TGGTCTTGACATCGATGAAAAT 22
|:|*||||||||||||||*|*|
Sbjct: 53 ATCTGAACTGTAGCTACTGTAA 32
>miR826b Score: 2.5 RO3G_12175
Query: 1 TGGTCTTGACATCGATGAAAAT 22
|:|*||||||||||||||*|*|
Sbjct: 53 ATCTGAACTGTAGCTACTGTAA 32
>miR826b Score: 2.5 RO3G_01713
Query: 1 TGGTCTTGACATCGATGAAAAT 22
|:|*||||||||||||||*|*|
Sbjct: 53 ATCTGAACTGTAGCTACTGTAA 32
>miR7800 Score: 2.5 RO3G_16617
Query: 1 TATTTTTGTCTCGTCTGGTC 20
*|:|||::|||||||||*||
Sbjct: 115 CTGAAAGTAGAGCAGAC-AG 97
>miR7800 Score: 1.0 RO3G_16359
Query: 1 TATTTTTGTCTCGTCTGGTC 20
||||||:||||||||||::|
Sbjct: 456 ATAAAAGCAGAGCAGACTGG 437
>miR7800 Score: 1.5 RO3G_16363
Query: 1 TATTTTTGTCTCGTCTGGTC 20
```

*||||||||||||||:|::|
Sbjct: 456 CTAAAAACAGAGCAGGCTGG 437
>miR7800 Score: 1.0 RO3G_16545
Query: 1 TATTTTTGTCTCGTCTGGTC 20
||||||:||||||||||::|
Sbjct: 456 ATAAAAGCAGAGCAGACTGG 437
>miR7800 Score: 1.0 RO3G_16554
Query: 1 TATTTTTGTCTCGTCTGGTC 20
*||||||||||||||||::|
Sbjct: 456 CTAAAAACAGAGCAGACTGG 437
>miR7800 Score: 2.2 RO3G_16206
Query: 1 TATTTTTGTCTCGTCTGGTC 20
|||:||:||||*||||||:|
Sbjct: 2221 ATAGAAGCAGAACAGACCGG 2202
>miR7800 Score: 2.0 RO3G_15651
Query: 1 TATTTTTGTCTCGTCTGGTC 20
|||||||||||||||*||
Sbjct: 250 CTAAAAACATAGCAGAC-AG 232
>miR7800 Score: 2.0 RO3G_15575
Query: 1 TATTTTTGTCTCGTCTGGTC 20
||||||:|||||||*||::|
Sbjct: 279 ATAAAAGCAGAGCAAACTGG 260
>miR7800 Score: 2.5 RO3G_15579
Query: 1 TATTTTTGTCTCGTCTGGTC 20
*||:||||||||:||:|::|
Sbjct: 456 CTAGAAACAGAGTAGGCTGG 437
>miR7800 Score: 1.0 RO3G_15001
Query: 1 TATTTTTGTCTCGTCTGGTC 20
||||||:||||||||||::|
Sbjct: 279 ATAAAAGCAGAGCAGACTGG 260
>miR7800 Score: 1.0 RO3G_15146
Query: 1 TATTTTTGTCTCGTCTGGTC 20
*||||||||||||||||::|
Sbjct: 336 CTAAAAACAGAGCAGACTGG 317
>miR7800 Score: 2.5 RO3G_14567
Query: 1 TATTTTTGTCTCGTCTGGTC 20
*||:||||||||:||:|::|
Sbjct: 234 CTAGAAACAGAGTAGGCTGG 215
>miR7800 Score: 2.5 RO3G_14150
Query: 1 TATTTTTGTCTCGTCTGGTC 20
|||:||||:|||:|||*|||
Sbjct: 137 ATAGAAACGGAGTAGAGCAG 118
>miR7800 Score: 2.5 RO3G_14349
Query: 1 TATTTTTGTCTCGTCTGGTC 20
*|:|||||||||:||:|::|

Sbjct: 456 CTGAAAACAGAGTAGGCTGG 437
>miR7800 Score: 2.5 RO3G_13502
Query: 1 TATTTTTGTCTCGTCTGGTC 20
||||:||*|||||*||||||
Sbjct: 287 ATAAGAAAAGAGCTGACCAG 268
>miR7800 Score: 2.0 RO3G_13718
Query: 1 TATTTTTGTCTCGTCTGGTC 20
*||||||||||:||:|::|
Sbjct: 234 CTAAAAACAGAGTAGGCTGG 215
>miR7800 Score: 2.0 RO3G_13733
Query: 1 TATTTTTGTCTCGTCTGGTC 20
*||||||||||:||:|::|
Sbjct: 234 CTAAAAACAGAGTAGGCTGG 215
>miR7800 Score: 2.0 RO3G_13137
Query: 1 TATTTTTGTCTCGTCTGGTC 20
*||||||||||:||:|::|
Sbjct: 456 CTAAAAACAGAGTAGGCTGG 437
>miR7800 Score: 2.0 RO3G_13147
Query: 1 TATTTTTGTCTCGTCTGGTC 20
*||||||||||:||:|::|
Sbjct: 234 CTAAAAACAGAGTAGGCTGG 215
>miR7800 Score: 1.0 RO3G_13266
Query: 1 TATTTTTGTCTCGTCTGGTC 20
||||||:||||||||||::|
Sbjct: 234 ATAAAAGCAGAGCAGACTGG 215
>miR7800 Score: 1.0 RO3G_13271
Query: 1 TATTTTTGTCTCGTCTGGTC 20
||||||:||||||||||::|
Sbjct: 456 ATAAAAGCAGAGCAGACTGG 437
>miR7800 Score: 1.0 RO3G_13277
Query: 1 TATTTTTGTCTCGTCTGGTC 20
||||||:||||||||||::|
Sbjct: 456 ATAAAAGCAGAGCAGACTGG 437
>miR7800 Score: 1.0 RO3G_11240
Query: 1 TATTTTTGTCTCGTCTGGTC 20
||||||:||||||||||::|
Sbjct: 279 ATAAAAGCAGAGCAGACTGG 260
>miR7800 Score: 2.5 RO3G_11254
Query: 1 TATTTTTGTCTCGTCTGGTC 20
|*|||||||||*|||||*||
Sbjct: 21 AAAAAAACAGAACAGAC-AG 3
>miR7800 Score: 1.0 RO3G_11266
Query: 1 TATTTTTGTCTCGTCTGGTC 20
||||||:||||||||||::|
Sbjct: 279 ATAAAAGCAGAGCAGACTGG 260

>miR7800 Score: 2.5 RO3G_10837
Query: 1 TATTTTTGTCTCGTCTGGTC 20
|||::|||||||:||:|||*
Sbjct: 1170 ATAGGAACAGAGTAGGCCAC 1151
>miR7800 Score: 2.5 RO3G_10846
Query: 1 TATTTTTGTCTCGTCTGGTC 20
|||::|||||||:||:|||*
Sbjct: 1170 ATAGGAACAGAGTAGGCCAC 1151
>miR7800 Score: 1.0 RO3G_10866
Query: 1 TATTTTTGTCTCGTCTGGTC 20
||||||:||||||||||::|
Sbjct: 456 ATAAAAGCAGAGCAGACTGG 437
>miR7800 Score: 1.0 RO3G_09612
Query: 1 TATTTTTGTCTCGTCTGGTC 20
||||||:||||||||||::|
Sbjct: 456 ATAAAAGCAGAGCAGACTGG 437
>miR7800 Score: 1.0 RO3G_08663
Query: 1 TATTTTTGTCTCGTCTGGTC 20
||||||:||||||||||::|
Sbjct: 456 ATAAAAGCAGAGCAGACTGG 437
>miR7800 Score: 1.5 RO3G_08842
Query: 1 TATTTTTGTCTCGTCTGGTC 20
*||||||||||||||:|::|
Sbjct: 456 CTAAAAACAGAGCAGGCTGG 437
>miR7800 Score: 2.2 RO3G_08964
Query: 1 TATTTTTGTCTCGTCTGGTC 20
|||*|:||||||:|||||:|
Sbjct: 921 ATATAGACAGAGTAGACCGG 902
>miR7800 Score: 1.0 RO3G_07031
Query: 1 TATTTTTGTCTCGTCTGGTC 20
*||||||||||||||||::|
Sbjct: 456 CTAAAAACAGAGCAGACTGG 437
>miR7800 Score: 1.0 RO3G_07112
Query: 1 TATTTTTGTCTCGTCTGGTC 20
||||||:||||||||||::|
Sbjct: 279 ATAAAAGCAGAGCAGACTGG 260
>miR7800 Score: 1.0 RO3G_07120
Query: 1 TATTTTTGTCTCGTCTGGTC 20
||||||:||||||||||::|
Sbjct: 279 ATAAAAGCAGAGCAGACTGG 260
>miR7800 Score: 1.5 RO3G_07688
Query: 1 TATTTTTGTCTCGTCTGGTC 20
||||||:|||||:||||::|
Sbjct: 456 ATAAAAGCAGAGTAGACTGG 437
>miR7800 Score: 2.0 RO3G_07877

Query: 1 TATTTTTGTCTCGTCTGGTC 20
*||||||||||:||:|::|
Sbjct: 234 CTAAAAACAGAGTAGGCTGG 215
>miR7800 Score: 1.0 RO3G_06722
Query: 1 TATTTTTGTCTCGTCTGGTC 20
||||||:||||||||||::|
Sbjct: 456 ATAAAAGCAGAGCAGACTGG 437
>miR7800 Score: 2.0 RO3G_02844
Query: 1 TATTTTTGTCTCGTCTGGTC 20
*||||||||||:||:|::|
Sbjct: 234 CTAAAAACAGAGTAGGCTGG 215
>miR7800 Score: 2.0 RO3G_03017
Query: 1 TATTTTTGTCTCGTCTGGTC 20
|||||||:|||||||||*
Sbjct: 163 TTAAAAACGGAGCAGAC-AC 145
>miR7800 Score: 2.0 RO3G_03113
Query: 1 TATTTTTGTCTCGTCTGGTC 20
*||||||||||:||:|::|
Sbjct: 198 CTAAAAACAGAGTAGGCTGG 179
>miR7800 Score: 2.0 RO3G_03766
Query: 1 TATTTTTGTCTCGTCTGGTC 20
*||||||||||:||:|::|
Sbjct: 234 CTAAAAACAGAGTAGGCTGG 215
>miR7800 Score: 2.0 RO3G_03772
Query: 1 TATTTTTGTCTCGTCTGGTC 20
*||||||||||:||:|::|
Sbjct: 456 CTAAAAACAGAGTAGGCTGG 437
>miR7800 Score: 2.0 RO3G_03803
Query: 1 TATTTTTGTCTCGTCTGGTC 20
||||||:|||||*||||::|
Sbjct: 279 ATAAAAGCAGAGAAGACTGG 260
>miR7800 Score: 2.5 RO3G_03862
Query: 1 TATTTTTGTCTCGTCTGGTC 20
|||||||||:|||||*||
Sbjct: 752 TTAAAAACAGGACAGACGAG 733
>miR7800 Score: 2.5 RO3G_04205
Query: 1 TATTTTTGTCTCGTCTGGTC 20
*||:||||||||:||:|::|
Sbjct: 234 CTAGAAACAGAGTAGGCTGG 215
>miR7800 Score: 1.0 RO3G_00255
Query: 1 TATTTTTGTCTCGTCTGGTC 20
||||||:||||||||||::|
Sbjct: 456 ATAAAAGCAGAGCAGACTGG 437
>miR7800 Score: 2.0 RO3G_01618
Query: 1 TATTTTTGTCTCGTCTGGTC 20

*|||||||||||:||:|::|
Sbjct: 456 CTAAAAACAGAGTAGGCTGG 437
>miR7800 Score: 2.0 RO3G_17363
Query: 1 TATTTTTGTCTCGTCTGGTC 20
*|||||||||||:||:|::|
Sbjct: 198 CTAAAAACAGAGTAGGCTGG 179
>miR9759 Score: 2.2 RO3G_02813
Query: 1 TAGTAAGCAAGTACGAGTTT-AT 22
||||||||||:|||||*:||*||
Sbjct: 852 ATCATTCGTTTATGCTAGAAGTA 830
>miR857 Score: 2.0 RO3G_16060
Query: 1 TTTTGTATGTTGAAGGTGTG 20
*:|||:|||||||||:||||
Sbjct: 949 TGAACGTACAACTTCTACAC 930
>miR857 Score: 2.5 RO3G_14364
Query: 1 TTTTGTATGTTGAAGGTGTG 20
|||||||||||||*||||
Sbjct: 165 TAAACATCCAACTTCGACAC 146
>miR857 Score: 2.2 RO3G_13922
Query: 1 TTTTGTATGTTGAAGGTGTG 20
:|||||||||||||:*|*||
Sbjct: 1540 GAAACATACAACTTTAA-AC 1522
>miR857 Score: 2.5 RO3G_12689
Query: 1 TTTTGTATGTTGAAGGTGTG 20
|:|||||:||||||||||
Sbjct: 102 CAGACATATAACTTCAACAC 83
>miR857 Score: 0.8 RO3G_12721
Query: 1 TTTTGTATGTTGAAGGTGTG 20
|||||||||||:||||||:|
Sbjct: 100 AAAACATACAATTTCCACGC 81
>miR857 Score: 2.2 RO3G_12281
Query: 1 TTTTGTATGTTGAAGGTGTG 20
||:|||*|||||||||||:*
Sbjct: 765 AAGACACACAACTTCCACGG 746
>miR857 Score: 2.5 RO3G_10291
Query: 1 TTTTGTATGTTGAAGGTGTG 20
||||||*|||||*|||||*|
Sbjct: 4699 AAAACAAACAACATCCAC-C 4681
>miR857 Score: 2.2 RO3G_10294
Query: 1 TTTTGTATGTTGAAGGTGTG 20
:||:||||||||*||||||*
Sbjct: 346 GAAGCATACAACCTCCACAA 327
>miR857 Score: 2.5 RO3G_07876
Query: 1 TTTTGTATGTTGAAGGTGTG 20
|||:|||||:|:|||:|*||

```
Sbjct: 25 AAAGCATACGATTTCTAAAC 6
>miR857 Score: 2.5 RO3G_05896
Query: 1 TTTTGTATGTTGAAGGTGTG 20
*::|||||||||||||:*||
Sbjct: 102 TGGACATACAACTTCCGGAC 83
>miR857 Score: 2.5 RO3G_06365
Query: 1 TTTTGTATGTTGAAGGTGTG 20
|||||:||*:|||||||*||
Sbjct: 1203 AAAACGTAAGACTTCCAGAC 1184
>miR857 Score: 1.5 RO3G_06407
Query: 1 TTTTGTATGTTGAAGGTGT-G 20
|:|||||||||:|||||||*|
Sbjct: 325 AGAACATACAATTTCCACAGC 305
>miR857 Score: 2.5 RO3G_04379
Query: 1 TTTTGTATGTTGAAGGTGTG 20
|||||*|||*|||||||*||
Sbjct: 188 AAAACCTACTACTTCCA-AC 170
>miR857 Score: 2.5 RO3G_02261
Query: 1 TTTTGTATGTTGAAGGTGTG 20
||||||||:||||*|||*::
Sbjct: 102 AAAACATATAACTACCA-GT 84
>miR857 Score: 2.5 RO3G_02274
Query: 1 TTTTGTATGTTGAAGGTGTG 20
||||||||||||||::*|::
Sbjct: 2499 AAAACATACAACTTTTCCGT 2480
>miR857 Score: 2.2 RO3G_02414
Query: 1 TTTTGTATGTTGAAGGTGTG 20
||:|||*|||||||||||:*
Sbjct: 765 AAGACACACAACTTCCACGG 746
>miR857 Score: 2.5 RO3G_02897
Query: 1 TTTTGTATGTTGAAGGTGTG 20
||||||||*|||||||*|*|
Sbjct: 2737 AAAACATAAAACTTCCTC-C 2719
>miR857 Score: 2.2 RO3G_03544
Query: 1 TTTTGTATGTTGAAGGTGTG 20
:|||:*|||||||||||:|:
Sbjct: 51 GAAATTTACAACTTCCATAT 32
>miR857 Score: 2.5 RO3G_00772
Query: 1 TTTTGTATGTTGAAGGTG-TG 20
|||*|||||||||*||||*||
Sbjct: 465 AAACCATACAACTGCCACCAC 445
>miR857 Score: 2.5 RO3G_00834
Query: 1 TTTTGTATGTTGAAGGTGTG 20
||||||||:|||*|::||||
Sbjct: 165 AAAACATATAACATTTACAC 146
```

>miR857 Score: 2.2 RO3G_01274
Query: 1 TTTTGTATGTTGAAGGTGTG 20
|||||||||||:||||**|:
Sbjct: 397 AAAACATACAATTTCCCGAT 378
>miR857 Score: 2.5 RO3G_17402
Query: 1 TTTTGTATGTTGAAGGTGTG 20
:|||||*||||:|||||*|:
Sbjct: 208 GAAACACACAATTTCCAAAT 189
>miR6185 Score: 0.0 RO3G_13220
Query: 1 TCTGGCAGCACGGGTAACAGTA 22
||||||||||||||||||||||
Sbjct: 1227 AGACCGTCGTGCCCATTGTCAT 1206
>miR6185 Score: 0.0 RO3G_13223
Query: 1 TCTGGCAGCACGGGTAACAGTA 22
||||||||||||||||||||||
Sbjct: 1227 AGACCGTCGTGCCCATTGTCAT 1206
>miR6185 Score: 0.0 RO3G_04119
Query: 1 TCTGGCAGCACGGGTAACAGTA 22
||||||||||||||||||||||
Sbjct: 1227 AGACCGTCGTGCCCATTGTCAT 1206
>miR6185 Score: 0.0 RO3G_04122
Query: 1 TCTGGCAGCACGGGTAACAGTA 22
||||||||||||||||||||||
Sbjct: 1227 AGACCGTCGTGCCCATTGTCAT 1206
>miR1535a Score: 0.0 RO3G_17160
Query: 1 TTCTGTTTGTGCGTGATGTCTT 22
||||||||||||||||||||||
Sbjct: 80 AAGACAAACACGCACTACAGAA 59
>miR1535a Score: 0.0 RO3G_15884
Query: 1 TTCTGTTTGTGCGTGATGTCTT 22
||||||||||||||||||||||
Sbjct: 80 AAGACAAACACGCACTACAGAA 59
>miR1535a Score: 2.0 RO3G_09380
Query: 1 TTCTGTTTGTGCGTGATGTCTT 22
||*||||||||||||||*:||:
Sbjct: 274 AACACAAACACGCACTA-GGAG 254
>miR1535a Score: 0.0 RO3G_09486
Query: 1 TTCTGTTTGTGCGTGATGTCTT 22
||||||||||||||||||||||
Sbjct: 80 AAGACAAACACGCACTACAGAA 59
>miR1535a Score: 2.5 RO3G_09571
Query: 1 TTCTGTTTGTGCGTGATGTCTT 22
||||*|||||||:||||*:||:
Sbjct: 960 AAGAGAAACACGTACTA-GGAG 940
>miR1535a Score: 0.2 RO3G_02698

Query: 1 TTCTGTTTGTGCGTGATGTCTT 22
:|||||||||||||||||||||
Sbjct: 80 GAGACAAACACGCACTACAGAA 59
>miR403a Score: 0.0 RO3G_17252
Query: 1 TTGACGATTCACGCCAATCTTG 22
||||||||||||||||||||||
Sbjct: 108 AACTGCTAAGTGCGGTTAGAAC 87
>miR403a Score: 0.0 RO3G_17156
Query: 1 TTGACGATTCACGCCAATCTTG 22
||||||||||||||||||||||
Sbjct: 348 AACTGCTAAGTGCGGTTAGAAC 327
>miR403a Score: 1.5 RO3G_16366
Query: 1 TTGACGATTCACGCCAATCTTG 22
|||||||||*||:|||||||||
Sbjct: 2091 AACTGCTAACTGTGGTTAGAAC 2070
>miR403a Score: 1.5 RO3G_10744
Query: 1 TTGACGATTCACGCCAATCTTG 22
|||||||||*||:|||||||||
Sbjct: 2094 AACTGCTAACTGTGGTTAGAAC 2073
>miR403a Score: 1.5 RO3G_10745
Query: 1 TTGACGATTCACGCCAATCTTG 22
|||||||||*||:|||||||||
Sbjct: 1062 AACTGCTAACTGTGGTTAGAAC 1041
>miR403a Score: 1.5 RO3G_07862
Query: 1 TTGACGATTCACGCCAATCTTG 22
|||||||||*||:|||||||||
Sbjct: 2205 AACTGCTAACTGTGGTTAGAAC 2184
>miR403a Score: 2.5 RO3G_01294
Query: 1 TTGACGATTCACGCCAATCTTG 22
||||||*||*||:|||||||||
Sbjct: 1164 AACTGCCAACTGTGGTTAGAAC 1143
>miR403a Score: 0.0 RO3G_17453
Query: 1 TTGACGATTCACGCCAATCTTG 22
||||||||||||||||||||||
Sbjct: 108 AACTGCTAAGTGCGGTTAGAAC 87
>miR5751 Score: 0.0 RO3G_15989
Query: 1 TTGATTTGCAGATGCCTTTT 20
||||||||||||||||||||
Sbjct: 2937 AACTAAACGTCTACGGAAAA 2918
>miR5751 Score: 2.5 RO3G_15989
Query: 1 TTGATTTGCAGATGCCTTTT 20
:||||||||||:||||||
Sbjct: 2282 TGCTAAACGTCTGCGAAAAA 2263
>miR5751 Score: 1.8 RO3G_12400
Query: 1 TTGATTTGCAGATGCCTTTT 20

||||||||*|||||||||*:
Sbjct: 720 AACTAAACTTCTACGGAACG 701
>miR5751 Score: 1.8 RO3G_09146
Query: 1 TTGATTTGCAGATGCCTTTT 20
|||||||||*:||||||||:
Sbjct: 201 AACTAAACGATTACGGAAAG 182
>miR5751 Score: 2.5 RO3G_05579
Query: 1 TTGATTTGCAGATGCCTTTT 20
|||||||*||:||*||||||
Sbjct: 1548 AACTAAAAGTTTAAGGAAAA 1529
>miR5751 Score: 2.5 RO3G_05085
Query: 1 TTGATTTGCAGATGCCTT-TT 20
||||||*:||:|||||||*||
Sbjct: 192 AACTAATTGTTTACGGAACAA 172
>miR5751 Score: 2.5 RO3G_02776
Query: 1 TTGATTTGCAGATGCCTTTT 20
|:||||||:|||||||||
Sbjct: 177 CATCAAACGTTTACGGAAAA 158
>miR530 Score: 2.5 RO3G_05852
Query: 1 TGATTATGCACCTGGCTCTC 20
||||:|:||||||||*|*||
Sbjct: 536 ACTAGTGCGTGGACCAAAAG 517
>miR6118-5p Score: 1.0 RO3G_14109
Query: 1 TGGAATGGGTGCTTGGGAAA 20
|:||||||||||||||||*|
Sbjct: 84 ATCTTACCCACGAACCCTCT 65
>miR6118-5p Score: 1.0 RO3G_11276
Query: 1 TGGAATGGGTGCTTGGGAAA 20
||||||:|||||||||||*|
Sbjct: 270 ACCTTATCCACGAACCCTCT 251
>miR6118-5p Score: 2.5 RO3G_09531
Query: 1 TGGAATGGGTGCTTGGGA-AA 20
||||||||||||||:|*||
Sbjct: 618 TCCGTACCCACGAACCTTATT 598
>miR6118-5p Score: 0.5 RO3G_04428
Query: 1 TGGAATGGGTGCTTGGGAAA 20
||||||||||||||||||*|
Sbjct: 1551 ACCTTACCCACGAACCCTCT 1532
>miR6118-5p Score: 0.0 RO3G_01147
Query: 1 TGGAATGGGTGCTTGGGAAA 20
||||||||||||||||||||
Sbjct: 231 ACCTTACCCACGAACCCTTT 212
>miR6118-5p Score: 0.0 RO3G_01161
Query: 1 TGGAATGGGTGCTTGGGAAA 20
||||||||||||||||||||

Sbjct: 231 ACCTTACCCACGAACCCTTT 212

Supplemental Table 4: CDC-8219 know miRNA target (TF)
#miRNA mRNA Score Match_pos Strand Pairing target and
miRNA
query=miR1160.1, target=RO3G_17276, score=0, range=918-939, stran
target 5' GGCCAUGCUUAAAAAGAGGGAA 3'
::::::::::::::::::::::
query 3' CCGGUACGAAUUUUUCUCCCUU 5'
query=miR1160.1, target=RO3G_13270, score=0, range=828-849, stran
target 5' GGCCAUGCUUAAAAAGAGGGAA 3'
::::::::::::::::::::::
query 3' CCGGUACGAAUUUUUCUCCCUU 5'
query=miR1160.1, target=RO3G_10774, score=0, range=795-816, stran
target 5' GGCCAUGCUUAAAAAGAGGGAA 3'
::::::::::::::::::::::
query 3' CCGGUACGAAUUUUUCUCCCUU 5'
query=miR1160.1, target=RO3G_13863, score=0, range=528-549, stran
target 5' GGCCAUGCUUAAAAAGAGGGAA 3'
::::::::::::::::::::::
query 3' CCGGUACGAAUUUUUCUCCCUU 5'
query=miR1160.1, target=RO3G_05935, score=0, range=234-255, stran
target 5' GGCCAUGCUUAAAAAGAGGGAA 3'
::::::::::::::::::::::
query 3' CCGGUACGAAUUUUUCUCCCUU 5'
query=miR1160.1, target=RO3G_03626, score=0, range=234-255, stran
target 5' GGCCAUGCUUAAAAAGAGGGAA 3'
::::::::::::::::::::::
query 3' CCGGUACGAAUUUUUCUCCCUU 5'
query=miR1160.1, target=RO3G_03593, score=0, range=234-255, stran
target 5' GGCCAUGCUUAAAAAGAGGGAA 3'
::::::::::::::::::::::
query 3' CCGGUACGAAUUUUUCUCCCUU 5'
query=miR1160.1, target=RO3G_04790, score=0, range=234-255, stran
target 5' GGCCAUGCUUAAAAAGAGGGAA 3'
::::::::::::::::::::::
query 3' CCGGUACGAAUUUUUCUCCCUU 5'
query=miR1160.1, target=RO3G_00678, score=0, range=198-219, stran
target 5' GGCCAUGCUUAAAAAGAGGGAA 3'
::::::::::::::::::::::
query 3' CCGGUACGAAUUUUUCUCCCUU 5'
query=miR1160.1, target=RO3G_11527, score=0, range=234-255, stran
target 5' GGCCAUGCUUAAAAAGAGGGAA 3'
::::::::::::::::::::::
query 3' CCGGUACGAAUUUUUCUCCCUU 5'
query=miR1160.1, target=RO3G_10869, score=0, range=234-255, stran
target 5' GGCCAUGCUUAAAAAGAGGGAA 3'

```
::::::::::::::::::::::
query 3' CCGGUACGAAUUUUUCUCCCUU 5'
query=miR1160.1, target=RO3G_01589, score=0.5, range=234-255, str
target 5' GGUCAUGCUUAAAAAGAGGGAA 3'
::.:::::::::::::::::::
query 3' CCGGUACGAAUUUUUCUCCCUU 5'
query=miR1160.1, target=RO3G_17249, score=1, range=753-774, stran
target 5' GGCCAUGCUUAAAAAGAGGGGA 3'
::::::::::::::::::::.:
query 3' CCGGUACGAAUUUUUCUCCCUU 5'
query=miR1160.1, target=RO3G_14363, score=1, range=786-807, stran
target 5' GACCAUGCUUAAAAAGAGGGAA 3'
: ::::::::::::::::::::
query 3' CCGGUACGAAUUUUUCUCCCUU 5'
query=miR1160.1, target=RO3G_14547, score=1, range=234-255, stran
target 5' GACCAUGCUUAAAAAGAGGGAA 3'
: ::::::::::::::::::::
query 3' CCGGUACGAAUUUUUCUCCCUU 5'
query=miR1160.1, target=RO3G_10734, score=2, range=234-255, stran
target 5' GGCCAUGCUUAAAAAGAUGGAA 3'
:::::::::::::::::: ::::
query 3' CCGGUACGAAUUUUUCUCCCUU 5'
query=miR1160.1, target=RO3G_17459, score=2, range=234-255, stran
target 5' GGCCAUGCUUAAAAAGAUGGAA 3'
:::::::::::::::::: ::::
query 3' CCGGUACGAAUUUUUCUCCCUU 5'
query=miR1160.1, target=RO3G_05134, score=3.5, range=1120-1141,
strand=-1
target 5' UGCCAUCCUUAAAGAGAGGGAG 3'
::::: ::::::.:::::::.
query 3' CCGGUACGAAUUUUUCUCCCUU 5'
query=miR1160.1, target=RO3G_05127, score=3.5, range=1120-1141,
strand=-1
target 5' UGCCAUCCUUAAAGAGAGGGAG 3'
::::: ::::::.:::::::.
query 3' CCGGUACGAAUUUUUCUCCCUU 5'
query=miR8632, target=RO3G_19000, score=0, range=450-468, strand=
target 5' GGUUCCAACUUUGAGCUCU 3'
:::::::::::::::::::
query 3' CCAAGGUUGAAACUCGAGA 5'
query=miR8632, target=RO3G_19002, score=0, range=450-468, strand=
target 5' GGUUCCAACUUUGAGCUCU 3'
:::::::::::::::::::
query 3' CCAAGGUUGAAACUCGAGA 5'
query=miR8632, target=RO3G_15016, score=3, range=2706-2723, stran
```

```
target 5' GGUUCCAA-UUUGAGUUCU 3'
:::::::: :::::::.:::
query 3' CCAAGGUUGAAACUCGAGA 5'
query=miR8632, target=RO3G_04352, score=3, range=1925-1943, stran
target 5' GAUUCAAACUUUGAGCUCG 3'
: ::: ::::::::::::
query 3' CCAAGGUUGAAACUCGAGA 5'
query=miR8632, target=RO3G_15392, score=3.5, range=2639-2657,
strand=-1
target 5' GGUUCUAACUUUAAGCUCA 3'
:::::.::::::: :::::
query 3' CCAAGGUUGAAACUCGAGA 5'
query=miR8632, target=RO3G_03452, score=4, range=757-774, strand=
target 5' GAUUCCAACUUUGA-CUUU 3'
: :::::::::::: ::.:
query 3' CCAAGGUUGAAACUCGAGA 5'
query=miR8632, target=RO3G_12046, score=4, range=775-792, strand=
target 5' GGUUCCAG-UUUGAGUUCU 3'
:::::::. :::::::.:::
query 3' CCAAGGUUGAAACUCGAGA 5'
query=miR8632, target=RO3G_00895, score=4, range=1416-1433, stran
target 5' AGUUCCAA-UUUGAGCUUU 3'
::::::: :::::::::.:
query 3' CCAAGGUUGAAACUCGAGA 5'
query=miR8632, target=RO3G_06934, score=4, range=1429-1448, stran
target 5' UGUACACGACUUUGAGCUCU 3'
:: : :.:::::::::::
query 3' CCAAG-GUUGAAACUCGAGA 5'
query=miR5565e, target=RO3G_16107, score=0, range=527-547, strand
target 5' UCCGACAACAUCUAGACCAUA 3'
:::::::::::::::::::::
query 3' AGGCUGUUGUAGAUCUGGUAU 5'
query=miR5565e, target=RO3G_09773, score=4, range=571-590, strand
target 5' UCCGAUAAC-UCUGGACCAUG 3'
:::::.::: :::.::::::.
query 3' AGGCUGUUGUAGAUCUGGUAU 5'
query=miR5565e, target=RO3G_14812, score=4, range=115-134, strand
target 5' UCC-ACAACAUCUAAAUCAUA 3'
::: :::::::::: :.::::
query 3' AGGCUGUUGUAGAUCUGGUAU 5'
query=miR3711, target=RO3G_09910, score=3, range=704-724, strand=
target 5' GGGCAGCUUCCUUCUUGGCUU 3'
.:::: :.::::::::::::
query 3' UCCGU-GGAGGAAGAACCGAU 5'
query=miR3711, target=RO3G_01286, score=3.5, range=121-140, stran
```

```
target 5' AUGCGCAUCCUUCUUGGCUU 3'
: ::.: :::::::::::
query 3' UCCGUGGAGGAAGAACCGAU 5'
query=miR3711, target=RO3G_13432, score=4, range=548-567, strand=
target 5' AGGCAUCAUCUUCUUGGCUG 3'
:::::.: .::::::::::.
query 3' UCCGUGGAGGAAGAACCGAU 5'
query=miR3711, target=RO3G_11375, score=4, range=1394-1413, stran
target 5' GGGCAUCUGCUUCUUGGUUA 3'
.::::.:: ::::::::.::
query 3' UCCGUGGAGGAAGAACCGAU 5'
query=miR3711, target=RO3G_11895, score=4, range=983-1003, strand
target 5' AUGCAACUUCCUUUUUGGCUG 3'
: :: ::.:::::.:::::.
query 3' UCCG-UGGAGGAAGAACCGAU 5'
query=miR3711, target=RO3G_03385, score=4, range=1172-1192, stran
target 5' AGGCGUCUUCUUCUUGAGCUA 3'
::::..::.::::::: ::::
query 3' UCCGUGGAGGAAGAAC-CGAU 5'
query=miR3711, target=RO3G_11900, score=4, range=1400-1420, stran
target 5' AGGCGGCUUCCUUCUUGGCAA 3'
:::: .:.:::::::::: :
query 3' UCCG-UGGAGGAAGAACCGAU 5'
query=miR3711, target=RO3G_15319, score=4, range=1145-1165, stran
target 5' AGUUAUCUCCUUCUUUGGCUA 3'
:: .:.::::::: :::::::
query 3' UCCGUGGAGGAAG-AACCGAU 5'
query=miR3711, target=RO3G_04341, score=4, range=643-661, strand=
target 5' AUGU-CCUCCUUCUUGGUUG 3'
: :. ::::::::::::.:.
query 3' UCCGUGGAGGAAGAACCGAU 5'
query=miR1057, target=RO3G_15507, score=4, range=5822-5841, stran
target 5' AUCAAUCA-CUAAACCUAGAA 3'
:.:: .:: :::::::::::
query 3' UGGUGGGUCGAUUUGGAUCUU 5'
query=miR397a, target=RO3G_06442, score=4, range=5174-5194, stran
target 5' CAUCAUCCCUGUAUUCAAUGA 3'
::::: : :::.:.:::::::
query 3' GUAGUUGCGACGUGAGUUACU 5'
query=miR397a, target=RO3G_02691, score=4, range=8006-8026, stran
target 5' CAUCAUCCCUGUAUUCAAUGA 3'
::::: : :::.:.:::::::
query 3' GUAGUUGCGACGUGAGUUACU 5'
query=miR397a, target=RO3G_09965, score=4, range=4517-4537, stran
target 5' CAUCAUCCCUGUAUUCAAUGA 3'
```

```
::::: : :::.:.::::::
query 3' GUAGUUGCGACGUGAGUUACU 5'
query=miR397a, target=RO3G_13920, score=4, range=4517-4537, stran
target 5' CAUCAUCCCUGUAUUCAAUGA 3'
::::: : :::.:.::::::
query 3' GUAGUUGCGACGUGAGUUACU 5'
query=miR397a, target=RO3G_03547, score=4, range=4517-4537, stran
target 5' CAUCAUCCCUGUAUUCAAUGA 3'
::::: : :::.:.::::::
query 3' GUAGUUGCGACGUGAGUUACU 5'
query=miR397a, target=RO3G_13867, score=4, range=4433-4453, stran
target 5' CAUCAUCCCUGUAUUCAAUGA 3'
::::: : :::.:.::::::
query 3' GUAGUUGCGACGUGAGUUACU 5'
query=miR397a, target=RO3G_02959, score=4, range=4517-4537, stran
target 5' CAUCAUCCCUGUAUUCAAUGA 3'
::::: : :::.:.::::::
query 3' GUAGUUGCGACGUGAGUUACU 5'
query=miR397a, target=RO3G_15250, score=4, range=4472-4492, stran
target 5' CAUCAUCCCUGUAUUCAAUGA 3'
::::: : :::.:.::::::
query 3' GUAGUUGCGACGUGAGUUACU 5'
query=miR397a, target=RO3G_17371, score=4, range=4472-4492, stran
target 5' CAUCAUCCCUGUAUUCAAUGA 3'
::::: : :::.:.::::::
query 3' GUAGUUGCGACGUGAGUUACU 5'
query=miR397a, target=RO3G_06427, score=4, range=4109-4129, stran
target 5' CAUCAUCCCUGUAUUCAAUGA 3'
::::: : :::.:.::::::
query 3' GUAGUUGCGACGUGAGUUACU 5'
query=miR397a, target=RO3G_17326, score=4, range=4349-4369, stran
target 5' CAUCAUCCCUGUAUUCAAUGA 3'
::::: : :::.:.::::::
query 3' GUAGUUGCGACGUGAGUUACU 5'
query=miR397a, target=RO3G_06428, score=4, range=3611-3631, stran
target 5' CAUCAUCCCUGUAUUCAAUGA 3'
::::: : :::.:.::::::
query 3' GUAGUUGCGACGUGAGUUACU 5'
query=miR397a, target=RO3G_13749, score=4, range=3026-3046, stran
target 5' CAUCAUCCCUGUAUUCAAUGA 3'
::::: : :::.:.::::::
query 3' GUAGUUGCGACGUGAGUUACU 5'
query=miR397a, target=RO3G_05619, score=4, range=2660-2680, stran
target 5' CAUCAUCCCUGUAUUCAAUGA 3'
::::: : :::.:.::::::
```

```
query 3' GUAGUUGCGACGUGAGUUACU 5'
query=miR397a, target=RO3G_14355, score=4, range=2723-2743, stran
target 5' CAUCAUCCCUGUAUUCAAUGA 3'
::::: : :::.:.::::::
query 3' GUAGUUGCGACGUGAGUUACU 5'
query=miR397a, target=RO3G_01330, score=4, range=1751-1771, stran
target 5' CAUCAUCCCUGUAUUCAAUGA 3'
::::: : :::.:.::::::
query 3' GUAGUUGCGACGUGAGUUACU 5'
query=miR397a, target=RO3G_11776, score=4, range=1607-1627, stran
target 5' CAUCAUCCCUGUAUUCAAUGA 3'
::::: : :::.:.::::::
query 3' GUAGUUGCGACGUGAGUUACU 5'
query=miR397a, target=RO3G_02222, score=4, range=1571-1591, stran
target 5' CAUCAUCCCUGUAUUCAAUGA 3'
::::: : :::.:.::::::
query 3' GUAGUUGCGACGUGAGUUACU 5'
query=miR397a, target=RO3G_04115, score=4, range=1460-1480, stran
target 5' CAUCAUCCCUGUAUUCAAUGA 3'
::::: : :::.:.::::::
query 3' GUAGUUGCGACGUGAGUUACU 5'
query=miR397a, target=RO3G_17404, score=4, range=1145-1165, stran
target 5' CAUCAUCCCUGUAUUCAAUGA 3'
::::: : :::.:.::::::
query 3' GUAGUUGCGACGUGAGUUACU 5'
query=miR397a, target=RO3G_07668, score=4, range=920-940, strand=
target 5' CAUCAUCCCUGUAUUCAAUGA 3'
::::: : :::.:.::::::
query 3' GUAGUUGCGACGUGAGUUACU 5'
query=miR397a, target=RO3G_05265, score=4, range=329-349, strand=
target 5' CAUCAUCCCUGUAUUCAAUGA 3'
::::: : :::.:.::::::
query 3' GUAGUUGCGACGUGAGUUACU 5'
query=miR397a, target=RO3G_05903, score=4, range=329-349, strand=
target 5' CAUCAUCCCUGUAUUCAAUGA 3'
::::: : :::.:.::::::
query 3' GUAGUUGCGACGUGAGUUACU 5'
query=miR5786, target=RO3G_05613, score=4, range=678-698, strand=
target 5' AGCUUUACGU-UAUCCUGCGAA 3'
::::: :: : ::::::::::
query 3' UCGAACUGGAGAUAGGACGCUU 5'
query=miR2645, target=RO3G_14778, score=0, range=185-204, strand=
target 5' AAAAUGCUCAUCUCAAGAAA 3'
::::::::::::::::::::
query 3' UUUUACGAGUAGAGUUCUUU 5'
```

```
query=miR2645, target=RO3G_11967, score=0, range=185-204, strand=
target 5' AAAAUGCUCAUCUCAAGAAA 3'
::::::::::::::::::::
query 3' UUUUACGAGUAGAGUUCUUU 5'
query=miR2645, target=RO3G_13225, score=0, range=185-204, strand=
target 5' AAAAUGCUCAUCUCAAGAAA 3'
::::::::::::::::::::
query 3' UUUUACGAGUAGAGUUCUUU 5'
query=miR2645, target=RO3G_17015, score=0, range=185-204, strand=
target 5' AAAAUGCUCAUCUCAAGAAA 3'
::::::::::::::::::::
query 3' UUUUACGAGUAGAGUUCUUU 5'
query=miR2645, target=RO3G_14350, score=0, range=185-204, strand=
target 5' AAAAUGCUCAUCUCAAGAAA 3'
::::::::::::::::::::
query 3' UUUUACGAGUAGAGUUCUUU 5'
query=miR2645, target=RO3G_03991, score=0, range=185-204, strand=
target 5' AAAAUGCUCAUCUCAAGAAA 3'
::::::::::::::::::::
query 3' UUUUACGAGUAGAGUUCUUU 5'
query=miR2645, target=RO3G_13222, score=0, range=137-156, strand=
target 5' AAAAUGCUCAUCUCAAGAAA 3'
::::::::::::::::::::
query 3' UUUUACGAGUAGAGUUCUUU 5'
query=miR2645, target=RO3G_16799, score=2, range=185-204, strand=
target 5' AAAAUGCUCAUCUCAAAAAA 3'
:::::::::::::::: :::
query 3' UUUUACGAGUAGAGUUCUUU 5'
query=miR2645, target=RO3G_11650, score=2.5, range=521-541, stran
target 5' AGAAUGCUUUAUCUCAAGAAA 3'
:.::::: :.::::::::::
query 3' UUUUACG-AGUAGAGUUCUUU 5'
query=miR2645, target=RO3G_07237, score=3, range=129-148, strand=
target 5' AAUACGCUCAUUUCAAGAAA 3'
:: : ::::::.::::::::
query 3' UUUUACGAGUAGAGUUCUUU 5'
query=miR2645, target=RO3G_02261, score=3, range=1717-1735, stran
target 5' AAAGU-UUCAUCUCAAGAAU 3'
:::.: .:::::::::::
query 3' UUUUACGAGUAGAGUUCUUU 5'
query=miR2645, target=RO3G_02809, score=3.5, range=446-466, stran
target 5' AGAAUGCGUUAUCUCAAGAGA 3'
:.::::: :.::::::::.:
query 3' UUUUACG-AGUAGAGUUCUUU 5'
query=miR2645, target=RO3G_16003, score=3.5, range=212-231, stran
```

```
target 5' GAGGAGCUCAUCUCAAGAAC 3'
.:.. :::::::::::::
query 3' UUUUACGAGUAGAGUUCUUU 5'
query=miR2645, target=RO3G_13460, score=3.5, range=472-491, stran
target 5' AACUUUUUCAUCUCAAGAAA 3'
:: : .:::::::::::
query 3' UUUUACGAGUAGAGUUCUUU 5'
query=miR2645, target=RO3G_02728, score=3.5, range=515-535, stran
target 5' AAUUUGCUUCAUCUCAAGAAG 3'
:: ::: :::::::::::.
query 3' UUUUACG-AGUAGAGUUCUUU 5'
query=miR2645, target=RO3G_13885, score=3.5, range=76-95, strand=
target 5' CAGUUGCUCAUUUCAAGAAA 3'
:. :::::::.::::::::
query 3' UUUUACGAGUAGAGUUCUUU 5'
query=miR2645, target=RO3G_14053, score=4, range=119-138, strand=
target 5' UAACUGCUUAUUUCAAGAAA 3'
:: ::::.::.::::::::
query 3' UUUUACGAGUAGAGUUCUUU 5'
query=miR2645, target=RO3G_05639, score=4, range=185-204, strand=
target 5' AAAAUGCUCAUCUCAAACAA 3'
:::::::::::::::: ::
query 3' UUUUACGAGUAGAGUUCUUU 5'
query=miR2645, target=RO3G_06767, score=4, range=312-331, strand=
target 5' AAAAGGCUCAACUCGAGAAA 3'
:::: ::::: :::.:::::
query 3' UUUUACGAGUAGAGUUCUUU 5'
query=miR2645, target=RO3G_05469, score=4, range=785-804, strand=
target 5' AACGUGCUCAGCUCAAGAAG 3'
:: .:::::: ::::::::.
query 3' UUUUACGAGUAGAGUUCUUU 5'
query=miR2645, target=RO3G_11691, score=4, range=687-705, strand=
target 5' AAAAU-CUCAGCUCAGGAAA 3'
::::: :::: ::::.::::
query 3' UUUUACGAGUAGAGUUCUUU 5'
query=miR2645, target=RO3G_05056, score=4, range=275-294, strand=
target 5' AAUCUGCUCAUCACAAGAAA 3'
:: :::::::: :::::::
query 3' UUUUACGAGUAGAGUUCUUU 5'
query=miR2645, target=RO3G_11668, score=4, range=298-317, strand=
target 5' GAUACGGUCAUCUCAAGAAG 3'
.: : : :::::::::::.
query 3' UUUUACGAGUAGAGUUCUUU 5'
query=miR2645, target=RO3G_01374, score=4, range=250-268, strand=
target 5' CAAAAG-UUAUCUCAAGAAA 3'
```

```
::: : :.:::::::::::
query 3' UUUUACGAGUAGAGUUCUUU 5'
query=miR2645, target=RO3G_06106, score=4, range=1295-1313, stran
target 5' AUUAUG-UUAUCUCAAGAAA 3'
: ::: :.:::::::::::
query 3' UUUUACGAGUAGAGUUCUUU 5'
query=miR2645, target=RO3G_09549, score=4, range=106-124, strand=
target 5' AAAAAG-UCAUCUAAAGAAA 3'
:::: : :::::: ::::::
query 3' UUUUACGAGUAGAGUUCUUU 5'
query=miR2645, target=RO3G_09863, score=4, range=1198-1216, stran
target 5' AAAAAGUUCAUC-CAAGAAG 3'
:::: :.::::: ::::::.
query 3' UUUUACGAGUAGAGUUCUUU 5'
query=miR2645, target=RO3G_06697, score=4, range=274-292, strand=
target 5' AGAAGGUUCAU-UCAAGAAA 3'
:.:: :.:::: ::::::::
query 3' UUUUACGAGUAGAGUUCUUU 5'
query=miR2645, target=RO3G_05056, score=4, range=593-612, strand=
target 5' AGGCUGCUCAUCACAAGAAA 3'
:.. :::::::: :::::::
query 3' UUUUACGAGUAGAGUUCUUU 5'
query=miR2645, target=RO3G_11502, score=4, range=175-195, strand=
target 5' AGAGUGCUCAUGCUCAAGAAC 3'
:.:.::::::: ::::::::
query 3' UUUUACGAGUA-GAGUUCUUU 5'
query=miR3946, target=RO3G_17023, score=1, range=1225-1246, stran
target 5' CAUGCUCCCUCUUUUCUCUACA 3'
::::::: :::::::::::::
query 3' GUACGAGAGAGAAAAGAGAUGU 5'
query=miR3946, target=RO3G_10379, score=3, range=319-340, strand=
target 5' CUUGUUCUUUCUUUUCUUUACA 3'
: ::.:::.::::::::.::::
query 3' GUACGAGAGAGAAAAGAGAUGU 5'
query=miR3946, target=RO3G_02013, score=4, range=1467-1489, stran
target 5' CAUCUUCUUUCUUUUCUUCUACA 3'
::: .:::.:::::::: ::::::
query 3' GUACGAGAGAGAAAAG-AGAUGU 5'
query=miR8748, target=RO3G_04592, score=0, range=1361-1383, stran
target 5' AUUCAUUAUUAUCAUCACCAUCA 3'
:::::::::::::::::::::::
query 3' UAAGUAAUAAUAGUAGUGGUAGU 5'
query=miR8748, target=RO3G_10217, score=1, range=2189-2211, stran
target 5' AUUCAUUAUCAUCAUCACCAUCA 3'
::::::::: ::::::::::::
```

```
query 3' UAAGUAAUAAUAGUAGUGGUAGU 5'
query=miR8748, target=RO3G_16749, score=1, range=2189-2211, stran
target 5' AUUCAUUAUCAUCAUCACCAUCA 3'
::::::::: ::::::::::::
query 3' UAAGUAAUAAUAGUAGUGGUAGU 5'
query=miR8748, target=RO3G_13273, score=1, range=2189-2211, stran
target 5' AUUCAUUAUCAUCAUCACCAUCA 3'
::::::::: ::::::::::::
query 3' UAAGUAAUAAUAGUAGUGGUAGU 5'
query=miR8748, target=RO3G_13145, score=1, range=2189-2211, stran
target 5' AUUCAUUAUCAUCAUCACCAUCA 3'
::::::::: ::::::::::::
query 3' UAAGUAAUAAUAGUAGUGGUAGU 5'
query=miR8748, target=RO3G_05424, score=1, range=2105-2127, stran
target 5' AUUCAUUAUCAUCAUCACCAUCA 3'
::::::::: ::::::::::::
query 3' UAAGUAAUAAUAGUAGUGGUAGU 5'
query=miR8748, target=RO3G_17257, score=1, range=2105-2127, stran
target 5' AUUCAUUAUCAUCAUCACCAUCA 3'
::::::::: ::::::::::::
query 3' UAAGUAAUAAUAGUAGUGGUAGU 5'
query=miR8748, target=RO3G_16136, score=1, range=2105-2127, stran
target 5' AUUCAUUAUCAUCAUCACCAUCA 3'
::::::::: ::::::::::::
query 3' UAAGUAAUAAUAGUAGUGGUAGU 5'
query=miR8748, target=RO3G_15889, score=1, range=2105-2127, stran
target 5' AUUCAUUAUCAUCAUCACCAUCA 3'
::::::::: ::::::::::::
query 3' UAAGUAAUAAUAGUAGUGGUAGU 5'
query=miR8748, target=RO3G_10735, score=1, range=1634-1656, stran
target 5' AUUCAUUAUCAUCAUCACCAUCA 3'
::::::::: ::::::::::::
query 3' UAAGUAAUAAUAGUAGUGGUAGU 5'
query=miR8748, target=RO3G_09183, score=1, range=1322-1344, stran
target 5' AUUCAUUAUCAUCAUCACCAUCA 3'
::::::::: ::::::::::::
query 3' UAAGUAAUAAUAGUAGUGGUAGU 5'
query=miR8748, target=RO3G_17351, score=1, range=917-939, strand=
target 5' AUUCAUUAUCAUCAUCACCAUCA 3'
::::::::: ::::::::::::
query 3' UAAGUAAUAAUAGUAGUGGUAGU 5'
query=miR8748, target=RO3G_04681, score=1, range=779-801, strand=
target 5' AUUCAUUAUCAUCAUCACCAUCA 3'
::::::::: ::::::::::::
query 3' UAAGUAAUAAUAGUAGUGGUAGU 5'
```

```
query=miR8748, target=RO3G_07697, score=1, range=515-537, strand=
target 5' AUUCAUUAUCAUCAUCACCAUCA 3'
::::::::: ::::::::::::: 
query 3' UAAGUAAUAAUAGUAGUGGUAGU 5'
query=miR8748, target=RO3G_10040, score=1, range=779-801, strand=
target 5' AUUCAUUAUCAUCAUCACCAUCA 3'
::::::::: ::::::::::::: 
query 3' UAAGUAAUAAUAGUAGUGGUAGU 5'
query=miR8748, target=RO3G_00422, score=2.5, range=36-57, strand=
target 5' AUUC-UUGUUAUCAUCACUAUCA 3'
:::: ::.::::::::::.::::
query 3' UAAGUAAUAAUAGUAGUGGUAGU 5'
query=miR8748, target=RO3G_00289, score=3, range=917-939, strand=
target 5' AUUCAUUAUCAUUAUCAUCAUCA 3'
::::::::: ::.::::.:::::
query 3' UAAGUAAUAAUAGUAGUGGUAGU 5'
query=miR8748, target=RO3G_13923, score=3, range=228-250, strand=
target 5' GCUUAUUAUUAUCAUCAUCAUCA 3'
. :.:::::::::::::.:::::
query 3' UAAGUAAUAAUAGUAGUGGUAGU 5'
query=miR8748, target=RO3G_01836, score=3, range=258-280, strand=
target 5' AUUCAUCAUAAUCAUCAUCAUCA 3'
:::::: :: :::::::.:::::
query 3' UAAGUAAUAAUAGUAGUGGUAGU 5'
query=miR8748, target=RO3G_09516, score=3.5, range=240-262, stran
target 5' UAUUAUUAUUAUCAUCAUCAUCA 3'
:.:::::::::::::.:::::
query 3' UAAGUAAUAAUAGUAGUGGUAGU 5'
query=miR8748, target=RO3G_03951, score=3.5, range=672-694, stran
target 5' CUUCAUUAUCAUCAUCAUCAUCG 3'
:::::::: ::::::::.::::.
query 3' UAAGUAAUAAUAGUAGUGGUAGU 5'
query=miR8748, target=RO3G_10591, score=3.5, range=999-1021, stra
target 5' AAUCGUCAUUAUCAUCAUCAUCA 3'
: ::.: ::::::::::.:::::
query 3' UAAGUAAUAAUAGUAGUGGUAGU 5'
query=miR8748, target=RO3G_14401, score=4, range=776-799, strand=
target 5' AAUUGUUAUUAUCAUCACCCAUCA 3'
: :..:::::::::::: ::::::
query 3' UAAGUAAUAAUAGUAGU-GGUAGU 5'
query=miR8748, target=RO3G_03827, score=4, range=1063-1085, stran
target 5' AAUCAUCAUCAUCAUCAUCAUCA 3'
: :::: :: :::::::.:::::
query 3' UAAGUAAUAAUAGUAGUGGUAGU 5'
query=miR8748, target=RO3G_00729, score=4, range=1048-1070, stran
```

```
target 5' AAUCAUCAUCAUCAUCAUCAUCA 3'
: :::: :: :::::::.:::::
query 3' UAAGUAAUAAUAGUAGUGGUAGU 5'
query=miR8748, target=RO3G_07191, score=4, range=281-303, strand=
target 5' AUAGAGCAUUAUCAUCACCAUCA 3'
:: : ::::::::::::::::
query 3' UAAGUAAUAAUAGUAGUGGUAGU 5'
query=miR8748, target=RO3G_10973, score=4, range=576-598, strand=
target 5' GGUCGUUAUUAUCAUCGUCAUCA 3'
. ::.:::::::::::..:::::
query 3' UAAGUAAUAAUAGUAGUGGUAGU 5'
query=miR8748, target=RO3G_14835, score=4, range=450-473, strand=
target 5' GUUCUGUUAUUAUCAUCAUCGUCA 3'
.::: .::::::::::::.:.:::
query 3' UAAG-UAAUAAUAGUAGUGGUAGU 5'
query=miR8748, target=RO3G_09517, score=4, range=507-529, strand=
target 5' GUUCAUUGUUAUCAUCAACAUCC 3'
.:::::.:::::::::: ::::
query 3' UAAGUAAUAAUAGUAGUGGUAGU 5'
query=miR8748, target=RO3G_13781, score=4, range=210-232, strand=
target 5' AUUUGUCAUCAUCAUCAUCAUCA 3'
:::..: :: :::::::.:::::
query 3' UAAGUAAUAAUAGUAGUGGUAGU 5'
query=miR8748, target=RO3G_11361, score=4, range=192-214, strand=
target 5' AUUCAUCGAUAUCAUCGCCAUCG 3'
::::: . :::::::.:::::.
query 3' UAAGUAAUAAUAGUAGUGGUAGU 5'
query=miR8748, target=RO3G_00068, score=4, range=933-955, strand=
target 5' CUUCAUCAUCAUCAUCAUCAUCA 3'
::::: :: :::::::.:::::
query 3' UAAGUAAUAAUAGUAGUGGUAGU 5'
query=miR8748, target=RO3G_15427, score=4, range=630-652, strand=
target 5' CUUCAUCAUCAUCAUCAUCAUCA 3'
::::: :: :::::::.:::::
query 3' UAAGUAAUAAUAGUAGUGGUAGU 5'
query=miR8748, target=RO3G_10691, score=4, range=522-544, strand=
target 5' AGUCAUCAUCAUCAUCAUCAUCA 3'
: :::: :: :::::::.:::::
query 3' UAAGUAAUAAUAGUAGUGGUAGU 5'
query=miR8748, target=RO3G_15993, score=4, range=723-746, strand=
target 5' AUGUCAUCAUCAUCAUCAUCAUCA 3'
:: :::: :: :::::::.:::::
query 3' UA-AGUAAUAAUAGUAGUGGUAGU 5'
query=miR8748, target=RO3G_15201, score=4, range=492-514, strand=
target 5' AAUCAUCAUCAUCAUCAUCAUCA 3'
```

```
: :::: :: :::::::.:::::
query 3' UAAGUAAUAAUAGUAGUGGUAGU 5'
query=miR8748, target=RO3G_04948, score=4, range=402-424, strand=
target 5' CAUCAUCAUCAUCAUCACCAUCA 3'
:::: :: ::::::::::::
query 3' UAAGUAAUAAUAGUAGUGGUAGU 5'
query=miR8748, target=RO3G_06092, score=4, range=4407-4428, stran
target 5' CUUCA-ACUUAUCAUCACCAUCA 3'
:::: ::::::::::::::
query 3' UAAGUAAUAAUAGUAGUGGUAGU 5'
query=miR408-3p, target=RO3G_11207, score=4, range=2798-2816, str
target 5' CGAUGGAAGAGGCAG-GCAU 3'
: : ::::::::::: ::::
query 3' GGUCCCUUCUCCGUCACGUA 5'
query=miR408-3p, target=RO3G_04583, score=4, range=378-396, stran
target 5' CCAAGGAAGAGG-AGUGUAU 3'
::: :::::::: ::::.::
query 3' GGUCCCUUCUCCGUCACGUA 5'
query=miR395b, target=RO3G_07684, score=3.5, range=372-391, stran
target 5' AGACCUCCCAAAUAUUUCAG 3'
:: ::.:::::.:.:::::
query 3' UCAGGGGGGUUUGUGAAGUC 5'
query=miR395b, target=RO3G_11383, score=4, range=50-69, strand=1
target 5' GGUCUCACCAAACACUUUAU 3'
.:::.: ::::::::::.:
query 3' UCAGGGGGGUUUGUGAAGUC 5'
query=miR395b, target=RO3G_05297, score=4, range=470-489, strand=
target 5' AGUCCCCCCAAACUCCUCAG 3'
:::::::::::: : ::::
query 3' UCAGGGGGGUUUGUGAAGUC 5'
query=miR395b, target=RO3G_07160, score=4, range=173-192, strand=
target 5' GAUGCCUUCAAACACUUCAG 3'
. : ::..::::::::::::
query 3' UCAGGGGGGUUUGUGAAGUC 5'
query=miR7767-5p, target=RO3G_14587, score=4, range=1821-1840,
strand=-1
target 5' GCAUUACACUCAUCUUGGGG 3'
: : ::::::::::::::
query 3' CCUCUCGUGAGUAGAACCCC 5'
query=miR5769, target=RO3G_08042, score=0, range=1243-1262, stran
target 5' UCGCGUCUUCCAGUUCCCCA 3'
::::::::::::::::::::
query 3' AGCGCAGAAGGUCAAGGGGU 5'
query=miR5769, target=RO3G_01807, score=3, range=1078-1097, stran
target 5' GCGCCUCUUCCAGUUCUCCA 3'
```

```
::: :::::::::::.:::
query 3' AGCGCAGAAGGUCAAGGGGU 5'
query=miR5769, target=RO3G_15707, score=3, range=472-492, strand=
target 5' UCGACGACUUCCAGUUCUCCA 3'
::: :: ::::::::::.:::
query 3' AGC-GCAGAAGGUCAAGGGGU 5'
query=miR5769, target=RO3G_05115, score=3.5, range=492-510, stran
target 5' UUG-GUCUUCCAGUUCCUUA 3'
:.: ::::::::::::..:
query 3' AGCGCAGAAGGUCAAGGGGU 5'
query=miR5769, target=RO3G_09242, score=4, range=351-369, strand=
target 5' UCGC-ACUUUCAGUUCCCCU 3'
:::: :::.:::::::::
query 3' AGCGCAGAAGGUCAAGGGGU 5'
query=miR5769, target=RO3G_09290, score=4, range=903-922, strand=
target 5' UAGCGGCUUCUAGUUCCUCA 3'
: ::: ::::.::::::.::
query 3' AGCGCAGAAGGUCAAGGGGU 5'
query=miR5769, target=RO3G_03922, score=4, range=472-492, strand=
target 5' GCGACGACUUCCAGUUCUCCA 3'
:: :: ::::::::::.:::
query 3' AGC-GCAGAAGGUCAAGGGGU 5'
query=miR827, target=RO3G_16161, score=3, range=675-694, strand=1
target 5' AUUUAAUGAUGGUCAUUUAA 3'
:::: ::::::::::.:::
query 3' UAAACAACUACCAGUAGAUU 5'
query=miR827, target=RO3G_15356, score=4, range=687-706, strand=1
target 5' AUUUGGUGGUUGUCAUCUAA 3'
::::: ::.: :::::::::
query 3' UAAACAACUACCAGUAGAUU 5'
query=miR827, target=RO3G_00714, score=4, range=616-635, strand=-
target 5' AUUUGUUGAUGGUUUUUUAA 3'
:::::::::::::. :.:::
query 3' UAAACAACUACCAGUAGAUU 5'
query=miR827, target=RO3G_16787, score=4, range=2140-2160, strand
target 5' CCUUGUUGAUGGUUCAUCUAA 3'
:::::::::: ::::::::
query 3' UAAACAACUACC-AGUAGAUU 5'
query=miR171a-3p, target=RO3G_16852, score=4, range=398-416, stra
target 5' AUAUUUUCG-GGCUCAAUCA 3'
::::: :: ::::::::::
query 3' UAUAACCGCGCCGAGUUAGU 5'
query=miR8123-3p, target=RO3G_16904, score=0, range=483-503, stra
target 5' GACUUGGACCAAUGGCGACAA 3'
::::::::::::::::::::
```

```
query 3' CUGAACCUGGUUACCGCUGUU 5'
query=miR8123-3p, target=RO3G_16829, score=0, range=483-503, stra
target 5' GACUUGGACCAAUGGCGACAA 3'
:::::::::::::::::::::
query 3' CUGAACCUGGUUACCGCUGUU 5'
query=miR8123-3p, target=RO3G_16428, score=0, range=120-140, stra
target 5' GACUUGGACCAAUGGCGACAA 3'
:::::::::::::::::::::
query 3' CUGAACCUGGUUACCGCUGUU 5'
query=miR8123-3p, target=RO3G_16721, score=1, range=441-461, stra
target 5' GACUUGGACCAAUGGCGAUAA 3'
::::::::::::::::::.::
query 3' CUGAACCUGGUUACCGCUGUU 5'
query=miR8123-3p, target=RO3G_11411, score=1, range=120-140, stra
target 5' GACUUGGACCAAUGGCGAUAA 3'
::::::::::::::::::.::
query 3' CUGAACCUGGUUACCGCUGUU 5'
query=miR8123-3p, target=RO3G_05945, score=1, range=120-140, stra
target 5' GACUUGGACCAAUGGCGAUAA 3'
::::::::::::::::::.::
query 3' CUGAACCUGGUUACCGCUGUU 5'
query=miR8123-3p, target=RO3G_16703, score=1.5, range=483-503,
strand=1
target 5' GGCUUGGACCAAUGGCGAUAA 3'
:.::::::::::::::::.::
query 3' CUGAACCUGGUUACCGCUGUU 5'
query=miR8123-3p, target=RO3G_13734, score=2, range=483-503, stra
target 5' GACUUGGACUAAUGGCGAUAA 3'
:::::::::.::::::::.::
query 3' CUGAACCUGGUUACCGCUGUU 5'
query=miR8123-3p, target=RO3G_17154, score=2, range=120-140, stra
target 5' GACUUGGACUAAUGGCGAUAA 3'
:::::::::.::::::::.::
query 3' CUGAACCUGGUUACCGCUGUU 5'
query=miR8123-3p, target=RO3G_02224, score=2, range=120-140, stra
target 5' GACUUGGACUAAUGGCGAUAA 3'
:::::::::.::::::::.::
query 3' CUGAACCUGGUUACCGCUGUU 5'
query=miR8123-3p, target=RO3G_12060, score=2, range=120-140, stra
target 5' GACUUGGACUAAUGGCGAUAA 3'
:::::::::.::::::::.::
query 3' CUGAACCUGGUUACCGCUGUU 5'
query=miR8123-3p, target=RO3G_17202, score=3, range=483-503, stra
target 5' GACUUGGACCAAUGGCAAUAA 3'
:::::::::::::::: :.::
```

```
query 3' CUGAACCUGGUUACCGCUGUU 5'
query=miR8123-3p, target=RO3G_00676, score=3, range=483-503, stra
target 5' GACUUGGAGCAAUGGCGAUAA 3'
:::::::: :::::::::.::
query 3' CUGAACCUGGUUACCGCUGUU 5'
query=miR8123-3p, target=RO3G_03594, score=3, range=120-140, stra
target 5' GACUUGGACCAAUGGCAAUAA 3'
:::::::::::::::: :.::
query 3' CUGAACCUGGUUACCGCUGUU 5'
query=miR8123-3p, target=RO3G_14688, score=3, range=1604-1624,
strand=-1
target 5' UAUUUUGGCCAAUGGCGACAA 3'
:.:: :.:::::::::::::
query 3' CUGAACCUGGUUACCGCUGUU 5'
query=miR7823, target=RO3G_04655, score=3, range=802-820, strand=
target 5' AUCAAAGAUCAGCAUGUAU 3'
::: ::::::::::::.:
query 3' UAGAUUCUAGUCGUACGUU 5'
query=miR7823, target=RO3G_01867, score=3, range=259-277, strand=
target 5' AUGUACGAUCAGCAUGCAC 3'
:: :: :::::::::::
query 3' UAGAUUCUAGUCGUACGUU 5'
query=miR7823, target=RO3G_03536, score=3, range=117-134, strand=
target 5' AUCUACGAUCAGCA-GCAA 3'
::::: :::::::: ::::
query 3' UAGAUUCUAGUCGUACGUU 5'
query=miR7823, target=RO3G_00505, score=3.5, range=8-26, strand=1
target 5' GUUUGAGAUCAGCAUGUAC 3'
.:.:.:::::::::::.:
query 3' UAGAUUCUAGUCGUACGUU 5'
query=miR7823, target=RO3G_05698, score=3.5, range=799-816, stran
target 5' AUUAAAGAUCAGCAU-CAA 3'
::. :::::::::: :::
query 3' UAGAUUCUAGUCGUACGUU 5'
query=miR7823, target=RO3G_11434, score=3.5, range=261-279, stran
target 5' AUCAAAGGUCAGCAUGUAG 3'
::: :::.::::::::.:.
query 3' UAGAUUCUAGUCGUACGUU 5'
query=miR7823, target=RO3G_03241, score=3.5, range=122-139, stran
target 5' GACUAAGAUCAGCA-GCAA 3'
. ::::::::::: ::::
query 3' UAGAUUCUAGUCGUACGUU 5'
query=miR7823, target=RO3G_15964, score=3.5, range=84-101, strand
target 5' AUC-AAGAUCAGCAGGCAG 3'
::: ::::::::: :::.
```

```
query 3' UAGAUUCUAGUCGUACGUU 5'
query=miR7823, target=RO3G_07541, score=4, range=210-228, strand=
target 5' AUCCAAGGUCAGCAAGCAA 3'
::: :::.:::::: ::::
query 3' UAGAUUCUAGUCGUACGUU 5'
query=miR7823, target=RO3G_00264, score=4, range=378-396, strand=
target 5' AUCCAAGGUCAGCAAGCAA 3'
::: :::.:::::: ::::
query 3' UAGAUUCUAGUCGUACGUU 5'
query=miR7823, target=RO3G_06546, score=4, range=207-225, strand=
target 5' ACCUAAGGUCAGCAAGCAA 3'
: ::::.:::::: ::::
query 3' UAGAUUCUAGUCGUACGUU 5'
query=miR7823, target=RO3G_04372, score=4, range=648-666, strand=
target 5' CUCUACGAUCAACAUGCAA 3'
:::: ::::: :::::::
query 3' UAGAUUCUAGUCGUACGUU 5'
query=miR7823, target=RO3G_05963, score=4, range=2524-2542, stran
target 5' AGCUAUGAACAGCAUGCAA 3'
: ::: :: :::::::::
query 3' UAGAUUCUAGUCGUACGUU 5'
query=miR7823, target=RO3G_08106, score=4, range=2458-2476, stran
target 5' AGCUAUGAACAGCAUGCAA 3'
: ::: :: :::::::::
query 3' UAGAUUCUAGUCGUACGUU 5'
query=miR7823, target=RO3G_17163, score=4, range=2338-2356, stran
target 5' AGCUAUGAACAGCAUGCAA 3'
: ::: :: :::::::::
query 3' UAGAUUCUAGUCGUACGUU 5'
query=miR7823, target=RO3G_15896, score=4, range=2524-2542, stran
target 5' AGCUAUGAACAGCAUGCAA 3'
: ::: :: :::::::::
query 3' UAGAUUCUAGUCGUACGUU 5'
query=miR7823, target=RO3G_02711, score=4, range=2458-2476, stran
target 5' AACUAUGAACAGCAUGCAA 3'
: ::: :: :::::::::
query 3' UAGAUUCUAGUCGUACGUU 5'
query=miR7823, target=RO3G_14242, score=4, range=622-640, strand=
target 5' AUCCAUGAUCAGCAUGCUA 3'
::: : :::::::::: :
query 3' UAGAUUCUAGUCGUACGUU 5'
query=miR7823, target=RO3G_11906, score=4, range=170-189, strand=
target 5' AUCUAAGUAUCAGCAUGCGC 3'
::::::: :::::::::.
query 3' UAGAUUC-UAGUCGUACGUU 5'
```

```
query=miR7823, target=RO3G_04189, score=4, range=1370-1387, stran
target 5' AUUUGAGAUCAG-GUGCAA 3'
::.:.:::::::: .:::::
query 3' UAGAUUCUAGUCGUACGUU 5'
query=miR7823, target=RO3G_01347, score=4, range=518-535, strand=
target 5' GUCUAGGAUCA-CAUGUAA 3'
.::::.::::: ::::.::
query 3' UAGAUUCUAGUCGUACGUU 5'
query=miR7495a, target=RO3G_15523, score=0, range=150-169, strand
target 5' UGAAGGAGGCACUACAAGAA 3'
::::::::::::::::::::
query 3' ACUUCCUCCGUGAUGUUCUU 5'
query=miR7495a, target=RO3G_09419, score=3, range=776-795, strand
target 5' UGAAGGUGGCUCUACAAGAA 3'
:::::: ::: :::::::::
query 3' ACUUCCUCCGUGAUGUUCUU 5'
query=miR7495a, target=RO3G_04309, score=3.5, range=1169-1189,
strand=1
target 5' UCAAGGCAGGCACUGCAAGAG 3'
: :::: :::::::.:::::.
query 3' ACUUCC-UCCGUGAUGUUCUU 5'
query=miR7495a, target=RO3G_00977, score=3.5, range=488-508, stra
target 5' UCAAGGCAGGCACUGCAAGAG 3'
: :::: :::::::.:::::.
query 3' ACUUCC-UCCGUGAUGUUCUU 5'
query=miR7495a, target=RO3G_05161, score=4, range=2076-2095, stra
target 5' GGAAGGAGGCGAUACAAGAA 3'
:::::::::. ::::::::
query 3' ACUUCCUCCGUGAUGUUCUU 5'
query=miR7495a, target=RO3G_02132, score=4, range=156-175, strand
target 5' UGAAGGAGGCACGAAAAGAA 3'
:::::::::::: : :::::
query 3' ACUUCCUCCGUGAUGUUCUU 5'
query=miR7495a, target=RO3G_07788, score=4, range=905-924, strand
target 5' UGAAGGUGGCUCUACGAGAA 3'
:::::: ::: ::::.::::
query 3' ACUUCCUCCGUGAUGUUCUU 5'
query=miR7495a, target=RO3G_01923, score=4, range=905-924, strand
target 5' UGAAGGUGGCUCUACGAGAA 3'
:::::: ::: ::::.::::
query 3' ACUUCCUCCGUGAUGUUCUU 5'
query=miR7495a, target=RO3G_14467, score=4, range=485-504, strand
target 5' UGAAGGUGGCUCUACGAGAA 3'
:::::: ::: ::::.::::
query 3' ACUUCCUCCGUGAUGUUCUU 5'
```

query=miR7495a, target=RO3G_11567, score=4, range=1613-1632, stra
target 5' UGAAGGUGGCUCUACGAGAA 3'
:::::: ::: ::::.::::
query 3' ACUUCCUCCGUGAUGUUCUU 5'
query=miR7495a, target=RO3G_11486, score=4, range=665-684, strand
target 5' UGAAGGUGGCUCUACGAGAA 3'
:::::: ::: ::::.::::
query 3' ACUUCCUCCGUGAUGUUCUU 5'
query=miR7495a, target=RO3G_02354, score=4, range=956-975, strand
target 5' UGAAGGUGGCUCUACGAGAA 3'
:::::: ::: ::::.::::
query 3' ACUUCCUCCGUGAUGUUCUU 5'
query=miR7495a, target=RO3G_08906, score=4, range=956-975, strand
target 5' UGAAGGUGGCUCUACGAGAA 3'
:::::: ::: ::::.::::
query 3' ACUUCCUCCGUGAUGUUCUU 5'
query=miR7495a, target=RO3G_05303, score=4, range=956-975, strand
target 5' UGAAGGUGGCUCUACGAGAA 3'
:::::: ::: ::::.::::
query 3' ACUUCCUCCGUGAUGUUCUU 5'
query=miR7495a, target=RO3G_13903, score=4, range=956-975, strand
target 5' UGAAGGUGGCUCUACGAGAA 3'
:::::: ::: ::::.::::
query 3' ACUUCCUCCGUGAUGUUCUU 5'
query=miR7495a, target=RO3G_13672, score=4, range=956-975, strand
target 5' UGAAGGUGGCUCUACGAGAA 3'
:::::: ::: ::::.::::
query 3' ACUUCCUCCGUGAUGUUCUU 5'
query=miR7495a, target=RO3G_14722, score=4, range=956-975, strand
target 5' UGAAGGUGGCUCUACGAGAA 3'
:::::: ::: ::::.::::
query 3' ACUUCCUCCGUGAUGUUCUU 5'
query=miR7495a, target=RO3G_13664, score=4, range=956-975, strand
target 5' UGAAGGUGGCUCUACGAGAA 3'
:::::: ::: ::::.::::
query 3' ACUUCCUCCGUGAUGUUCUU 5'
query=miR7495a, target=RO3G_05181, score=4, range=956-975, strand
target 5' UGAAGGUGGCUCUACGAGAA 3'
:::::: ::: ::::.::::
query 3' ACUUCCUCCGUGAUGUUCUU 5'
query=miR7495a, target=RO3G_05194, score=4, range=956-975, strand
target 5' UGAAGGUGGCUCUACGAGAA 3'
:::::: ::: ::::.::::
query 3' ACUUCCUCCGUGAUGUUCUU 5'
query=miR7495a, target=RO3G_01528, score=4, range=524-543, strand

```
target 5' UGAAGGUGGCUCUACGAGAA 3'
:::::: ::: ::::.::::
query 3' ACUUCCUCCGUGAUGUUCUU 5'
query=miR7495a, target=RO3G_08375, score=4, range=995-1014, stran
target 5' UAAAGGAGGCAUUACAAGUA 3'
: ::::::::.:::::: :
query 3' ACUUCCUCCGUGAUGUUCUU 5'
query=miR7495a, target=RO3G_04110, score=4, range=653-672, strand
target 5' UGAAGGUGGCUCUACGAGAA 3'
:::::: ::: ::::.::::
query 3' ACUUCCUCCGUGAUGUUCUU 5'
query=miR7495a, target=RO3G_04104, score=4, range=653-672, strand
target 5' UGAAGGCGGCUCUACGAGAA 3'
:::::: ::: ::::.::::
query 3' ACUUCCUCCGUGAUGUUCUU 5'
query=miR7495a, target=RO3G_12139, score=4, range=329-348, strand
target 5' UGAAGGUGGCUCUACGAGAA 3'
:::::: ::: ::::.::::
query 3' ACUUCCUCCGUGAUGUUCUU 5'
query=miR7495a, target=RO3G_01518, score=4, range=329-348, strand
target 5' UGAAGGUGGCUCUACGAGAA 3'
:::::: ::: ::::.::::
query 3' ACUUCCUCCGUGAUGUUCUU 5'
query=miR7495a, target=RO3G_05535, score=4, range=341-360, strand
target 5' UGAAGGUGGCUCUACGAGAA 3'
:::::: ::: ::::.::::
query 3' ACUUCCUCCGUGAUGUUCUU 5'
query=miR7495a, target=RO3G_10644, score=4, range=341-360, strand
target 5' UGAAGGUGGCUCUACGAGAA 3'
:::::: ::: ::::.::::
query 3' ACUUCCUCCGUGAUGUUCUU 5'
query=miR7495a, target=RO3G_02412, score=4, range=342-360, strand
target 5' AGAA-GAGGCACUACAAGCA 3'
::: ::::::::::::: :
query 3' ACUUCCUCCGUGAUGUUCUU 5'
query=miR7495a, target=RO3G_02795, score=4, range=555-575, strand
target 5' UGAAGAAGGCUAUUACAAGAA 3'
::::: :::: :.::::::::
query 3' ACUUCCUCCG-UGAUGUUCUU 5'
query=miR7495a, target=RO3G_06278, score=4, range=165-183, strand
target 5' UGAAGGAGGC-UUACGAGAA 3'
:::::::::: .:::.::::
query 3' ACUUCCUCCGUGAUGUUCUU 5'
query=miR9742, target=RO3G_06442, score=0, range=3354-3376, stran
target 5' UACUCACAGAAACAAACAAUACA 3'
```

```
::::::::::::::::::::::
query 3' AUGAGUGUCUUUGUUUGUUAUGU 5'
query=miR9742, target=RO3G_02691, score=0, range=6186-6208, stran
target 5' UACUCACAGAAACAAACAAUACA 3'
::::::::::::::::::::::
query 3' AUGAGUGUCUUUGUUUGUUAUGU 5'
query=miR9742, target=RO3G_09965, score=0, range=2697-2719, stran
target 5' UACUCACAGAAACAAACAAUACA 3'
::::::::::::::::::::::
query 3' AUGAGUGUCUUUGUUUGUUAUGU 5'
query=miR9742, target=RO3G_13920, score=0, range=2697-2719, stran
target 5' UACUCACAGAAACAAACAAUACA 3'
::::::::::::::::::::::
query 3' AUGAGUGUCUUUGUUUGUUAUGU 5'
query=miR9742, target=RO3G_03547, score=0, range=2697-2719, stran
target 5' UACUCACAGAAACAAACAAUACA 3'
::::::::::::::::::::::
query 3' AUGAGUGUCUUUGUUUGUUAUGU 5'
query=miR9742, target=RO3G_13867, score=0, range=2613-2635, stran
target 5' UACUCACAGAAACAAACAAUACA 3'
::::::::::::::::::::::
query 3' AUGAGUGUCUUUGUUUGUUAUGU 5'
query=miR9742, target=RO3G_02959, score=0, range=2697-2719, stran
target 5' UACUCACAGAAACAAACAAUACA 3'
::::::::::::::::::::::
query 3' AUGAGUGUCUUUGUUUGUUAUGU 5'
query=miR9742, target=RO3G_15250, score=0, range=2652-2674, stran
target 5' UACUCACAGAAACAAACAAUACA 3'
::::::::::::::::::::::
query 3' AUGAGUGUCUUUGUUUGUUAUGU 5'
query=miR9742, target=RO3G_17371, score=0, range=2652-2674, stran
target 5' UACUCACAGAAACAAACAAUACA 3'
::::::::::::::::::::::
query 3' AUGAGUGUCUUUGUUUGUUAUGU 5'
query=miR9742, target=RO3G_06427, score=0, range=2289-2311, stran
target 5' UACUCACAGAAACAAACAAUACA 3'
::::::::::::::::::::::
query 3' AUGAGUGUCUUUGUUUGUUAUGU 5'
query=miR9742, target=RO3G_17326, score=0, range=2529-2551, stran
target 5' UACUCACAGAAACAAACAAUACA 3'
::::::::::::::::::::::
query 3' AUGAGUGUCUUUGUUUGUUAUGU 5'
query=miR9742, target=RO3G_06428, score=0, range=1791-1813, stran
target 5' UACUCACAGAAACAAACAAUACA 3'
::::::::::::::::::::::
```

```
query 3' AUGAGUGUCUUUGUUUGUUAUGU 5'
query=miR9742, target=RO3G_13749, score=0, range=1260-1282, stran
target 5' UACUCACAGAAACAAACAAUACA 3'
:::::::::::::::::::::::
query 3' AUGAGUGUCUUUGUUUGUUAUGU 5'
query=miR9742, target=RO3G_05619, score=0, range=840-862, strand=
target 5' UACUCACAGAAACAAACAAUACA 3'
:::::::::::::::::::::::
query 3' AUGAGUGUCUUUGUUUGUUAUGU 5'
query=miR9742, target=RO3G_14355, score=0, range=903-925, strand=
target 5' UACUCACAGAAACAAACAAUACA 3'
:::::::::::::::::::::::
query 3' AUGAGUGUCUUUGUUUGUUAUGU 5'
query=miR9742, target=RO3G_15585, score=0, range=2652-2674, stran
target 5' UACUCACAGAAACAAACAAUACA 3'
:::::::::::::::::::::::
query 3' AUGAGUGUCUUUGUUUGUUAUGU 5'
query=miR9742, target=RO3G_04114, score=0, range=2697-2719, stran
target 5' UACUCACAGAAACAAACAAUACA 3'
:::::::::::::::::::::::
query 3' AUGAGUGUCUUUGUUUGUUAUGU 5'
query=miR9742, target=RO3G_02221, score=0, range=2619-2641, stran
target 5' UACUCACAGAAACAAACAAUACA 3'
:::::::::::::::::::::::
query 3' AUGAGUGUCUUUGUUUGUUAUGU 5'
query=miR9742, target=RO3G_05272, score=0, range=2697-2719, stran
target 5' UACUCACAGAAACAAACAAUACA 3'
:::::::::::::::::::::::
query 3' AUGAGUGUCUUUGUUUGUUAUGU 5'
query=miR9742, target=RO3G_01613, score=0, range=948-970, strand=
target 5' UACUCACAGAAACAAACAAUACA 3'
:::::::::::::::::::::::
query 3' AUGAGUGUCUUUGUUUGUUAUGU 5'
query=miR9742, target=RO3G_00306, score=0, range=1146-1168, stran
target 5' UACUCACAGAAACAAACAAUACA 3'
:::::::::::::::::::::::
query 3' AUGAGUGUCUUUGUUUGUUAUGU 5'
query=miR9742, target=RO3G_10558, score=0, range=450-472, strand=
target 5' UACUCACAGAAACAAACAAUACA 3'
:::::::::::::::::::::::
query 3' AUGAGUGUCUUUGUUUGUUAUGU 5'
query=miR9742, target=RO3G_05368, score=3, range=1917-1939, stran
target 5' UAAUAACAUAAACAAACAAUACA 3'
:: : ::: :::::::::::::
query 3' AUGAGUGUCUUUGUUUGUUAUGU 5'
```

```
query=miR9742, target=RO3G_02099, score=4, range=1832-1855, stran
target 5' UACCUGACAGAAAUAAGCAAUACA 3'
:: :: :::::::.::.:::::::
query 3' AU-GAGUGUCUUUGUUUGUUAUGU 5'
query=miR823, target=RO3G_15228, score=0, range=138-157, strand=1
target 5' AUCUUCAUUUGAUCACCACA 3'
::::::::::::::::::::
query 3' UAGAAGUAAACUAGUGGUGU 5'
query=miR823, target=RO3G_02750, score=3, range=1018-1036, strand
target 5' AUCUU-AUUUGAUCACCAAA 3'
::::: :::::::::::: :
query 3' UAGAAGUAAACUAGUGGUGU 5'
query=miR823, target=RO3G_06586, score=3, range=250-270, strand=-
target 5' AUCUUCAGUUUGAUCAUCACC 3'
::::::: ::::::::.:::
query 3' UAGAAGU-AAACUAGUGGUGU 5'
query=miR823, target=RO3G_16990, score=3, range=970-989, strand=-
target 5' ACCUUCUUUUGAUCACCACU 3'
: :::: ::::::::::::
query 3' UAGAAGUAAACUAGUGGUGU 5'
query=miR823, target=RO3G_12691, score=3.5, range=354-373, strand
target 5' GUCUUCAUUCGAUCACCAUA 3'
.:::::::: ::::::::.:
query 3' UAGAAGUAAACUAGUGGUGU 5'
query=miR823, target=RO3G_01418, score=3.5, range=235-253, strand
target 5' AUC-UCGUUUGAUCAUCAUA 3'
::: ::.::::::::.::.:
query 3' UAGAAGUAAACUAGUGGUGU 5'
query=miR823, target=RO3G_14897, score=3.5, range=138-157, strand
target 5' AGCAUCGUUUGAUCACCGCA 3'
: : ::.::::::::::.::
query 3' UAGAAGUAAACUAGUGGUGU 5'
query=miR823, target=RO3G_08367, score=3.5, range=6352-6370, stra
target 5' GCCUUC-UUUGAUCAUCACA 3'
. :::: ::::::::.::::
query 3' UAGAAGUAAACUAGUGGUGU 5'
query=miR823, target=RO3G_06012, score=3.5, range=636-654, strand
target 5' GUCUUC-UUUGAUUGCCACA 3'
.::::: ::::::..:::::
query 3' UAGAAGUAAACUAGUGGUGU 5'
query=miR823, target=RO3G_06365, score=4, range=350-370, strand=1
target 5' AUUUCUCGUUUGGUCACUACA 3'
::.: ::.::::.::::.:::
query 3' UAGA-AGUAAACUAGUGGUGU 5'
query=miR823, target=RO3G_13123, score=4, range=1653-1672, strand
```

```
target 5' AUCUUCUUUUGAUUACAACA 3'
:::::: ::::::.:: :::
query 3' UAGAAGUAAACUAGUGGUGU 5'
query=miR823, target=RO3G_00332, score=4, range=527-546, strand=1
target 5' AUCUUUCUUUGAUCACCAAG 3'
:::::. ::::::::::: .
query 3' UAGAAGUAAACUAGUGGUGU 5'
query=miR823, target=RO3G_08415, score=4, range=527-546, strand=1
target 5' AUCUUUCUUUGAUCACCAAG 3'
:::::. ::::::::::: .
query 3' UAGAAGUAAACUAGUGGUGU 5'
query=miR823, target=RO3G_04590, score=4, range=51-70, strand=1
target 5' AUCCUGGUUUGAUCAUCACG 3'
::: : .::::::::.:::.
query 3' UAGAAGUAAACUAGUGGUGU 5'
query=miR823, target=RO3G_05246, score=4, range=342-361, strand=1
target 5' AAGCUCAUUUGAUCAUCACA 3'
: :::::::::::.::::
query 3' UAGAAGUAAACUAGUGGUGU 5'
query=miR823, target=RO3G_14309, score=4, range=60-78, strand=1
target 5' AGCUUCAUUUGGUC-CCACA 3'
: :::::::::.:: :::::
query 3' UAGAAGUAAACUAGUGGUGU 5'
query=miR823, target=RO3G_09981, score=4, range=135-154, strand=1
target 5' AAGUUCCUUUGAUCAUCACA 3'
: ::: ::::::::.::::
query 3' UAGAAGUAAACUAGUGGUGU 5'
query=miR823, target=RO3G_09470, score=4, range=723-742, strand=1
target 5' AGCUUCCUUUGAUGACCACA 3'
: :::: :::::: ::::::
query 3' UAGAAGUAAACUAGUGGUGU 5'
query=miR823, target=RO3G_07622, score=4, range=150-169, strand=1
target 5' GAUUGCCUUUGAUCACCACA 3'
. .: : :::::::::::::
query 3' UAGAAGUAAACUAGUGGUGU 5'
query=miR823, target=RO3G_10140, score=4, range=429-448, strand=1
target 5' AGCAGGAUUUGAUCACCACA 3'
: : ::::::::::::::
query 3' UAGAAGUAAACUAGUGGUGU 5'
query=miR823, target=RO3G_14735, score=4, range=384-403, strand=1
target 5' AGCAGGAUUUGAUCACCACA 3'
: : ::::::::::::::
query 3' UAGAAGUAAACUAGUGGUGU 5'
query=miR823, target=RO3G_14693, score=4, range=1285-1303, strand
target 5' AUCCU-AUUUUAUCACCACA 3'
```

```
::: : :::: :::::::::
query 3' UAGAAGUAAACUAGUGGUGU 5'
query=miR823, target=RO3G_11943, score=4, range=484-502, strand=1
target 5' AUAUGC-UUUGAUCACCAUA 3'
:: : : :::::::::::.:
query 3' UAGAAGUAAACUAGUGGUGU 5'
query=miR823, target=RO3G_03819, score=4, range=858-876, strand=1
target 5' AUCCUC-UUUGAUCUCCACA 3'
::: :: ::::::: :::::
query 3' UAGAAGUAAACUAGUGGUGU 5'
query=miR823, target=RO3G_15097, score=4, range=3039-3058, strand
target 5' GUAUUUCUUUGGUCACCACA 3'
.: ::. ::::.::::::::
query 3' UAGAAGUAAACUAGUGGUGU 5'
query=miR823, target=RO3G_00657, score=4, range=286-306, strand=-
target 5' AUCUUGCUUUUGAUCAACACA 3'
::::: : :::::::: ::::
query 3' UAGAA-GUAAACUAGUGGUGU 5'
query=miR823, target=RO3G_03238, score=4, range=850-868, strand=-
target 5' GUUUCCAUUUGA-CACCACA 3'
.:.: ::::::: :::::::
query 3' UAGAAGUAAACUAGUGGUGU 5'
query=miR7699-3p, target=RO3G_17377, score=0, range=37-58, strand
target 5' GCAUUUAAUGAUGGUAUUUACA 3'
::::::::::::::::::::::
query 3' CGUAAAUUACUACCAUAAAUGU 5'
query=miR4348c, target=RO3G_16361, score=0, range=869-890, strand
target 5' UGGUCUCUUCCAAGGUUUAACA 3'
::::::::::::::::::::::
query 3' ACCAGAGAAGGUUCCAAAUUGU 5'
query=miR4348c, target=RO3G_04126, score=0, range=869-890, strand
target 5' UGGUCUCUUCCAAGGUUUAACA 3'
::::::::::::::::::::::
query 3' ACCAGAGAAGGUUCCAAAUUGU 5'
query=miR4348c, target=RO3G_17422, score=0, range=170-191, strand
target 5' UGGUCUCUUCCAAGGUUUAACA 3'
::::::::::::::::::::::
query 3' ACCAGAGAAGGUUCCAAAUUGU 5'
query=miR4348c, target=RO3G_13722, score=3, range=872-893, strand
target 5' UGAUCUCGUCCAAGGUUUAGCA 3'
:: :::: :::::::::::.::
query 3' ACCAGAGAAGGUUCCAAAUUGU 5'
query=miR4348c, target=RO3G_11064, score=4, range=1257-1278, stra
target 5' UGCUCCCUUCCAAGGUUUAAUC 3'
:: :: ::::::::::::::.
```

query 3' ACCAGAGAAGGUUCCAAAUUGU 5'
query=miR4348c, target=RO3G_14347, score=4, range=875-896, strand
target 5' UGAUCUCGACCAAGGUUUAGCA 3'
:: :::: ::::::::::.::
query 3' ACCAGAGAAGGUUCCAAAUUGU 5'
query=miR4348c, target=RO3G_09547, score=4, range=875-896, strand
target 5' UGAUCUCGACCAAGGUUUAGCA 3'
:: :::: ::::::::::.::
query 3' ACCAGAGAAGGUUCCAAAUUGU 5'
query=miR4348c, target=RO3G_06425, score=4, range=875-896, strand
target 5' UGAUCUCGACCAAGGUUUAGCA 3'
:: :::: ::::::::::.::
query 3' ACCAGAGAAGGUUCCAAAUUGU 5'
query=miR4348c, target=RO3G_17021, score=4, range=875-896, strand
target 5' UGAUCUCGACCAAGGUUUAGCA 3'
:: :::: ::::::::::.::
query 3' ACCAGAGAAGGUUCCAAAUUGU 5'
query=miR4348c, target=RO3G_12677, score=4, range=875-896, strand
target 5' UGAUCUCGACCAAGGUUUAGCA 3'
:: :::: ::::::::::.::
query 3' ACCAGAGAAGGUUCCAAAUUGU 5'
query=miR4348c, target=RO3G_04933, score=4, range=875-896, strand
target 5' UGAUCUCGACCAAGGUUUAGCA 3'
:: :::: ::::::::::.::
query 3' ACCAGAGAAGGUUCCAAAUUGU 5'
query=miR4348c, target=RO3G_05330, score=4, range=875-896, strand
target 5' UGAUCUCGACCAAGGUUUAGCA 3'
:: :::: ::::::::::.::
query 3' ACCAGAGAAGGUUCCAAAUUGU 5'
query=miR4348c, target=RO3G_02704, score=4, range=875-896, strand
target 5' UGAUCUCGACCAAGGUUUAGCA 3'
:: :::: ::::::::::.::
query 3' ACCAGAGAAGGUUCCAAAUUGU 5'
query=miR4348c, target=RO3G_05344, score=4, range=875-896, strand
target 5' UGAUCUCGACCAAGGUUUAGCA 3'
:: :::: ::::::::::.::
query 3' ACCAGAGAAGGUUCCAAAUUGU 5'
query=miR1870-5p, target=RO3G_15268, score=0, range=1663-1684, strand=1
target 5' AUGCCACUGGUCCAUUCAGCAA 3'
::::::::::::::::::::::
query 3' UACGGUGACCAGGUAAGUCGUU 5'
query=miR1870-5p, target=RO3G_04121, score=0, range=1663-1684, strand=1
target 5' AUGCCACUGGUCCAUUCAGCAA 3'

```
::::::::::::::::::::::
query 3' UACGGUGACCAGGUAAGUCGUU 5'
query=miR1870-5p, target=RO3G_13217, score=0, range=1663-1684,
strand=1
target 5' AUGCCACUGGUCCAUUCAGCAA 3'
::::::::::::::::::::::
query 3' UACGGUGACCAGGUAAGUCGUU 5'
query=miR1870-5p, target=RO3G_15265, score=0, range=1663-1684,
strand=1
target 5' AUGCCACUGGUCCAUUCAGCAA 3'
::::::::::::::::::::::
query 3' UACGGUGACCAGGUAAGUCGUU 5'
query=miR1870-5p, target=RO3G_14352, score=0, range=1663-1684,
strand=1
target 5' AUGCCACUGGUCCAUUCAGCAA 3'
::::::::::::::::::::::
query 3' UACGGUGACCAGGUAAGUCGUU 5'
query=miR1870-5p, target=RO3G_13222, score=0, range=1657-1678,
strand=1
target 5' AUGCCACUGGUCCAUUCAGCAA 3'
::::::::::::::::::::::
query 3' UACGGUGACCAGGUAAGUCGUU 5'
query=miR1870-5p, target=RO3G_13219, score=0, range=1663-1684,
strand=1
target 5' AUGCCACUGGUCCAUUCAGCAA 3'
::::::::::::::::::::::
query 3' UACGGUGACCAGGUAAGUCGUU 5'
query=miR1870-5p, target=RO3G_16818, score=0, range=1405-1426,
strand=1
target 5' AUGCCACUGGUCCAUUCAGCAA 3'
::::::::::::::::::::::
query 3' UACGGUGACCAGGUAAGUCGUU 5'
query=miR1870-5p, target=RO3G_05640, score=0, range=1411-1432,
strand=1
target 5' AUGCCACUGGUCCAUUCAGCAA 3'
::::::::::::::::::::::
query 3' UACGGUGACCAGGUAAGUCGUU 5'
query=miR1870-5p, target=RO3G_15266, score=0, range=1663-1684,
strand=1
target 5' AUGCCACUGGUCCAUUCAGCAA 3'
::::::::::::::::::::::
query 3' UACGGUGACCAGGUAAGUCGUU 5'
query=miR1870-5p, target=RO3G_03992, score=0, range=394-415, stra
target 5' AUGCCACUGGUCCAUUCAGCAA 3'
::::::::::::::::::::::
```

```
query 3' UACGGUGACCAGGUAAGUCGUU 5'
query=miR1870-5p, target=RO3G_16797, score=0, range=664-685, stra
target 5' AUGCCACUGGUCCAUUCAGCAA 3'
::::::::::::::::::::::
query 3' UACGGUGACCAGGUAAGUCGUU 5'
query=miR1870-5p, target=RO3G_11967, score=2, range=1675-1696,
strand=1
target 5' AUGCCACUGGUUCGUUCAGCAA 3'
:::::::::::.:.::::::::
query 3' UACGGUGACCAGGUAAGUCGUU 5'
query=miR1870-5p, target=RO3G_13225, score=2, range=1675-1696,
strand=1
target 5' AUGCCACUGGUUCGUUCAGCAA 3'
:::::::::::.:.::::::::
query 3' UACGGUGACCAGGUAAGUCGUU 5'
query=miR1870-5p, target=RO3G_16800, score=4, range=1663-1684,
strand=1
target 5' AUGCCACUGGUUCGCUCAGCAA 3'
:::::::::::.:. :::::::
query 3' UACGGUGACCAGGUAAGUCGUU 5'
query=miR1870-5p, target=RO3G_08855, score=4, range=914-935, stra
target 5' ACGCCACAGGUCCAUUCAGUGA 3'
: ::::: :::::::::::..:
query 3' UACGGUGACCAGGUAAGUCGUU 5'
query=miR2905, target=RO3G_15711, score=0, range=2375-2394, stran
target 5' UGCCUUUGUCGGCUACAGUA 3'
::::::::::::::::::::
query 3' ACGGAAACAGCCGAUGUCAU 5'
query=miR2905, target=RO3G_13796, score=0, range=2375-2394, stran
target 5' UGCCUUUGUCGGCUACAGUA 3'
::::::::::::::::::::
query 3' ACGGAAACAGCCGAUGUCAU 5'
query=miR2905, target=RO3G_09539, score=0, range=2375-2394, stran
target 5' UGCCUUUGUCGGCUACAGUA 3'
::::::::::::::::::::
query 3' ACGGAAACAGCCGAUGUCAU 5'
query=miR2905, target=RO3G_11521, score=0, range=1337-1356, stran
target 5' UGCCUUUGUCGGCUACAGUA 3'
::::::::::::::::::::
query 3' ACGGAAACAGCCGAUGUCAU 5'
query=miR2905, target=RO3G_03627, score=2.5, range=392-411, stran
target 5' UUCCAUUGUCGGCUACAGUG 3'
: :: ::::::::::::::.
query 3' ACGGAAACAGCCGAUGUCAU 5'
query=miR2905, target=RO3G_01708, score=2.5, range=392-411, stran
```

```
target 5' UUCCAUUGUCGGCUACAGUG 3'
: :: ::::::::::::::.
query 3' ACGGAAACAGCCGAUGUCAU 5'
query=miR2905, target=RO3G_11026, score=3.5, range=657-676, stran
target 5' UGCUUUUGUUGGCUAUGGUA 3'
:::.:::::.:::::..:::
query 3' ACGGAAACAGCCGAUGUCAU 5'
query=miR5557-3p, target=RO3G_14978, score=0, range=111-130, stra
target 5' UCAACAAGAUAAUGCAAGCA 3'
::::::::::::::::::::
query 3' AGUUGUUCUAUUACGUUCGU 5'
query=miR5557-3p, target=RO3G_17249, score=0, range=630-649, stra
target 5' UCAACAAGAUAAUGCAAGCA 3'
::::::::::::::::::::
query 3' AGUUGUUCUAUUACGUUCGU 5'
query=miR5557-3p, target=RO3G_11527, score=0, range=111-130, stra
target 5' UCAACAAGAUAAUGCAAGCA 3'
::::::::::::::::::::
query 3' AGUUGUUCUAUUACGUUCGU 5'
query=miR5557-3p, target=RO3G_10774, score=0, range=672-691, stra
target 5' UCAACAAGAUAAUGCAAGCA 3'
::::::::::::::::::::
query 3' AGUUGUUCUAUUACGUUCGU 5'
query=miR5557-3p, target=RO3G_13270, score=0, range=705-724, stra
target 5' UCAACAAGAUAAUGCAAGCA 3'
::::::::::::::::::::
query 3' AGUUGUUCUAUUACGUUCGU 5'
query=miR5557-3p, target=RO3G_17276, score=0, range=795-814, stra
target 5' UCAACAAGAUAAUGCAAGCA 3'
::::::::::::::::::::
query 3' AGUUGUUCUAUUACGUUCGU 5'
query=miR5557-3p, target=RO3G_03626, score=0, range=111-130, stra
target 5' UCAACAAGAUAAUGCAAGCA 3'
::::::::::::::::::::
query 3' AGUUGUUCUAUUACGUUCGU 5'
query=miR5557-3p, target=RO3G_05935, score=0, range=111-130, stra
target 5' UCAACAAGAUAAUGCAAGCA 3'
::::::::::::::::::::
query 3' AGUUGUUCUAUUACGUUCGU 5'
query=miR5557-3p, target=RO3G_01589, score=0, range=111-130, stra
target 5' UCAACAAGAUAAUGCAAGCA 3'
::::::::::::::::::::
query 3' AGUUGUUCUAUUACGUUCGU 5'
query=miR5557-3p, target=RO3G_14547, score=0, range=111-130, stra
target 5' UCAACAAGAUAAUGCAAGCA 3'
```

```
::::::::::::::::::::
query 3' AGUUGUUCUAUUACGUUCGU 5'
query=miR5557-3p, target=RO3G_00678, score=0, range=75-94, strand
target 5' UCAACAAGAUAAUGCAAGCA 3'
::::::::::::::::::::
query 3' AGUUGUUCUAUUACGUUCGU 5'
query=miR5557-3p, target=RO3G_04790, score=0, range=111-130, stra
target 5' UCAACAAGAUAAUGCAAGCA 3'
::::::::::::::::::::
query 3' AGUUGUUCUAUUACGUUCGU 5'
query=miR5557-3p, target=RO3G_13863, score=0, range=405-424, stra
target 5' UCAACAAGAUAAUGCAAGCA 3'
::::::::::::::::::::
query 3' AGUUGUUCUAUUACGUUCGU 5'
query=miR5557-3p, target=RO3G_14363, score=0, range=663-682, stra
target 5' UCAACAAGAUAAUGCAAGCA 3'
::::::::::::::::::::
query 3' AGUUGUUCUAUUACGUUCGU 5'
query=miR5557-3p, target=RO3G_10869, score=1, range=111-130, stra
target 5' UCAACAUGAUAAUGCAAGCA 3'
:::::: :::::::::::::
query 3' AGUUGUUCUAUUACGUUCGU 5'
query=miR5557-3p, target=RO3G_03593, score=2, range=111-130, stra
target 5' UCAACAAGAUAGUGCAGGCA 3'
::::::::::.::::.:::
query 3' AGUUGUUCUAUUACGUUCGU 5'
query=miR5557-3p, target=RO3G_17259, score=2, range=111-130, stra
target 5' UCAACAAGAUAAUGAAAGCA 3'
:::::::::::::: :::::
query 3' AGUUGUUCUAUUACGUUCGU 5'
query=miR5557-3p, target=RO3G_13921, score=2, range=111-130, stra
target 5' UCAACAAGAUAAUGGAAGCA 3'
:::::::::::::: :::::
query 3' AGUUGUUCUAUUACGUUCGU 5'
query=miR5557-3p, target=RO3G_10734, score=2, range=111-130, stra
target 5' UCAACAACAUAAUGCAAGCA 3'
::::::: ::::::::::::
query 3' AGUUGUUCUAUUACGUUCGU 5'
query=miR5557-3p, target=RO3G_17459, score=2, range=111-130, stra
target 5' UCAACAACAUAAUGCAAGCA 3'
::::::: ::::::::::::
query 3' AGUUGUUCUAUUACGUUCGU 5'
query=miR5557-3p, target=RO3G_05423, score=2, range=111-130, stra
target 5' UCAACUAGAUAAUGUAAGCA 3'
::::: ::::::::.:::::
```

```
query 3' AGUUGUUCUAUUACGUUCGU 5'
query=miR5557-3p, target=RO3G_10645, score=2, range=2-21, strand=
target 5' ACAAAAAGAUAAUGCAAGCA 3'
::: ::::::::::::::
query 3' AGUUGUUCUAUUACGUUCGU 5'
query=miR5557-3p, target=RO3G_13954, score=2.5, range=449-468,
strand=1
target 5' UUGAGAGGAUAAUGCAAGCA 3'
:..: :.:::::::::::::
query 3' AGUUGUUCUAUUACGUUCGU 5'
query=miR5557-3p, target=RO3G_06186, score=3, range=10-29, strand
target 5' UCAAUGAGGUAAUGCAAGUA 3'
::::..::.:::::::::.:
query 3' AGUUGUUCUAUUACGUUCGU 5'
query=miR5557-3p, target=RO3G_01707, score=3.5, range=158-177,
strand=-1
target 5' ACAACAGGAUAAUGCAAGGA 3'
::::.::::::::::: :
query 3' AGUUGUUCUAUUACGUUCGU 5'
query=miR5557-3p, target=RO3G_10608, score=4, range=8928-8947,
strand=1
target 5' GCAAGAAGGUGAUGCAAGCA 3'
::: :::.:.:::::::::
query 3' AGUUGUUCUAUUACGUUCGU 5'
query=miR5557-3p, target=RO3G_06444, score=4, range=326-344, stra
target 5' UCAAUAAGGUAAUG-AAGCG 3'
::::.:::.:::: ::::.
query 3' AGUUGUUCUAUUACGUUCGU 5'
query=miR5557-3p, target=RO3G_05820, score=4, range=488-507, stra
target 5' UGAAGAAGAUGAUGCAAGCC 3'
: :: :::::.::::::::
query 3' AGUUGUUCUAUUACGUUCGU 5'
query=miR5557-3p, target=RO3G_00743, score=4, range=2349-2367,
strand=1
target 5' UCAACAUGAUAAUGC-AGUA 3'
:::::: :::::::: ::.:
query 3' AGUUGUUCUAUUACGUUCGU 5'
query=miR5557-3p, target=RO3G_03883, score=4, range=586-604, stra
target 5' UCAGC-AGAUAAUGCAAGAG 3'
:::.: ::::::::::: .
query 3' AGUUGUUCUAUUACGUUCGU 5'
query=miR5557-3p, target=RO3G_04024, score=4, range=209-227, stra
target 5' UGAACAAGAUGAU-CAAGCA 3'
: ::::::::.:: ::::::
query 3' AGUUGUUCUAUUACGUUCGU 5'
```

```
query=miR5557-3p, target=RO3G_12622, score=4, range=1143-1162,
strand=-1
target 5' UGAACAAGAUGAUGUAAGUA 3'
: :::::::.:::.:::.:
query 3' AGUUGUUCUAUUACGUUCGU 5'
query=miR5557-3p, target=RO3G_02506, score=4, range=2209-2228,
strand=-1
target 5' UCAACAAGAGAAUGCAAGUU 3'
::::::::: ::::::::.
query 3' AGUUGUUCUAUUACGUUCGU 5'
query=miR5557-3p, target=RO3G_01224, score=4, range=369-388, stra
target 5' UCUGCUGGAUAAUGCAGGCA 3'
:: .: .:::::::::.:::
query 3' AGUUGUUCUAUUACGUUCGU 5'
query=miR5557-3p, target=RO3G_01221, score=4, range=369-388, stra
target 5' UCUGCUGGAUAAUGCAGGCA 3'
:: .: .:::::::::.:::
query 3' AGUUGUUCUAUUACGUUCGU 5'
query=miR5557-3p, target=RO3G_02795, score=4, range=491-509, stra
target 5' CGAACAAGAUAAUG-AAGCA 3'
:::::::::::: :::::
query 3' AGUUGUUCUAUUACGUUCGU 5'
query=miR5557-3p, target=RO3G_02641, score=4, range=1145-1163,
strand=-1
target 5' UCUGC-AGAUAGUGCAAGCG 3'
:: .: :::::.:::::::.
query 3' AGUUGUUCUAUUACGUUCGU 5'
query=miR5232, target=RO3G_15134, score=0, range=2073-2091, stran
target 5' UUCAGGUAGAGGACAUGCA 3'
:::::::::::::::::::
query 3' AAGUCCAUCUCCUGUACGU 5'
query=miR5232, target=RO3G_16132, score=0, range=2031-2049, stran
target 5' UUCAGGUAGAGGACAUGCA 3'
:::::::::::::::::::
query 3' AAGUCCAUCUCCUGUACGU 5'
query=miR5232, target=RO3G_16560, score=0, range=1101-1119, stran
target 5' UUCAGGUAGAGGACAUGCA 3'
:::::::::::::::::::
query 3' AAGUCCAUCUCCUGUACGU 5'
query=miR5232, target=RO3G_09540, score=0, range=1239-1257, stran
target 5' UUCAGGUAGAGGACAUGCA 3'
:::::::::::::::::::
query 3' AAGUCCAUCUCCUGUACGU 5'
query=miR5232, target=RO3G_00486, score=0, range=828-846, strand=
target 5' UUCAGGUAGAGGACAUGCA 3'
```

```
:::::::::::::::::::
query 3' AAGUCCAUCUCCUGUACGU 5'
query=miR5232, target=RO3G_05271, score=0, range=2031-2049, stran
target 5' UUCAGGUAGAGGACAUGCA 3'
:::::::::::::::::::
query 3' AAGUCCAUCUCCUGUACGU 5'
query=miR5232, target=RO3G_03098, score=0, range=1239-1257, stran
target 5' UUCAGGUAGAGGACAUGCA 3'
:::::::::::::::::::
query 3' AAGUCCAUCUCCUGUACGU 5'
query=miR5232, target=RO3G_00290, score=0, range=2031-2049, stran
target 5' UUCAGGUAGAGGACAUGCA 3'
:::::::::::::::::::
query 3' AAGUCCAUCUCCUGUACGU 5'
query=miR5232, target=RO3G_02703, score=0, range=198-216, strand=
target 5' UUCAGGUAGAGGACAUGCA 3'
:::::::::::::::::::
query 3' AAGUCCAUCUCCUGUACGU 5'
query=miR5232, target=RO3G_06442, score=0, range=7755-7773, stran
target 5' UUCAGGUAGAGGACAUGCA 3'
:::::::::::::::::::
query 3' AAGUCCAUCUCCUGUACGU 5'
query=miR5232, target=RO3G_12470, score=0, range=2031-2049, stran
target 5' UUCAGGUAGAGGACAUGCA 3'
:::::::::::::::::::
query 3' AAGUCCAUCUCCUGUACGU 5'
query=miR5232, target=RO3G_14995, score=0, range=2031-2049, stran
target 5' UUCAGGUAGAGGACAUGCA 3'
:::::::::::::::::::
query 3' AAGUCCAUCUCCUGUACGU 5'
query=miR5232, target=RO3G_17438, score=0, range=1563-1581, stran
target 5' UUCAGGUAGAGGACAUGCA 3'
:::::::::::::::::::
query 3' AAGUCCAUCUCCUGUACGU 5'
query=miR5232, target=RO3G_11524, score=0, range=1098-1116, stran
target 5' UUCAGGUAGAGGACAUGCA 3'
:::::::::::::::::::
query 3' AAGUCCAUCUCCUGUACGU 5'
query=miR5232, target=RO3G_07098, score=0, range=2031-2049, stran
target 5' UUCAGGUAGAGGACAUGCA 3'
:::::::::::::::::::
query 3' AAGUCCAUCUCCUGUACGU 5'
query=miR5232, target=RO3G_16557, score=0, range=2031-2049, stran
target 5' UUCAGGUAGAGGACAUGCA 3'
:::::::::::::::::::
```

```
query 3' AAGUCCAUCUCCUGUACGU 5'
query=miR5232, target=RO3G_01745, score=0, range=2031-2049, stran
target 5' UUCAGGUAGAGGACAUGCA 3'
:::::::::::::::::::
query 3' AAGUCCAUCUCCUGUACGU 5'
query=miR5232, target=RO3G_10258, score=3.5, range=2750-2768, str
target 5' UUUAUGUAGAGGGUAUGCA 3'
::.: :::::::..:::::
query 3' AAGUCCAUCUCCUGUACGU 5'
query=miR5232, target=RO3G_03614, score=4, range=271-289, strand=
target 5' UUCAGGUCGAGGAUAUGCU 3'
::::::: :::::.::::
query 3' AAGUCCAUCUCCUGUACGU 5'
query=miR5232, target=RO3G_16099, score=4, range=510-528, strand=
target 5' UGCUGGUAGAGGAUAUGUA 3'
: : :::::::::.:::.:
query 3' AAGUCCAUCUCCUGUACGU 5'
query=miR5232, target=RO3G_09272, score=4, range=426-443, strand=
target 5' UUCA-GUAGAGGGUAUGCU 3'
:::: :::::::..::::
query 3' AAGUCCAUCUCCUGUACGU 5'
query=miR5232, target=RO3G_06489, score=4, range=1488-1506, stran
target 5' UGCUGGUGGAGGACAUGCU 3'
: : :::.::::::::::
query 3' AAGUCCAUCUCCUGUACGU 5'
query=miR5232, target=RO3G_12749, score=4, range=889-906, strand=
target 5' UGCA-GUAGAGGAUAUGCU 3'
: :: :::::::::.::::
query 3' AAGUCCAUCUCCUGUACGU 5'
query=miR9491, target=RO3G_16744, score=0, range=278-298, strand=
target 5' CUGAGUCAGAGGAAGAUAGCA 3'
:::::::::::::::::::::
query 3' GACUCAGUCUCCUUCUAUCGU 5'
query=miR9491, target=RO3G_01203, score=0, range=278-298, strand=
target 5' CUGAGUCAGAGGAAGAUAGCA 3'
:::::::::::::::::::::
query 3' GACUCAGUCUCCUUCUAUCGU 5'
query=miR9491, target=RO3G_11403, score=0, range=278-298, strand=
target 5' CUGAGUCAGAGGAAGAUAGCA 3'
:::::::::::::::::::::
query 3' GACUCAGUCUCCUUCUAUCGU 5'
query=miR9491, target=RO3G_06416, score=0, range=278-298, strand=
target 5' CUGAGUCAGAGGAAGAUAGCA 3'
:::::::::::::::::::::
query 3' GACUCAGUCUCCUUCUAUCGU 5'
```

```
query=miR9491, target=RO3G_01718, score=0, range=278-298, strand=
target 5' CUGAGUCAGAGGAAGAUAGCA 3'
:::::::::::::::::::::
query 3' GACUCAGUCUCCUUCUAUCGU 5'
query=miR9491, target=RO3G_15869, score=0, range=278-298, strand=
target 5' CUGAGUCAGAGGAAGAUAGCA 3'
:::::::::::::::::::::
query 3' GACUCAGUCUCCUUCUAUCGU 5'
query=miR9491, target=RO3G_10560, score=0, range=278-298, strand=
target 5' CUGAGUCAGAGGAAGAUAGCA 3'
:::::::::::::::::::::
query 3' GACUCAGUCUCCUUCUAUCGU 5'
query=miR9491, target=RO3G_11275, score=0, range=278-298, strand=
target 5' CUGAGUCAGAGGAAGAUAGCA 3'
:::::::::::::::::::::
query 3' GACUCAGUCUCCUUCUAUCGU 5'
query=miR9491, target=RO3G_14704, score=0, range=278-298, strand=
target 5' CUGAGUCAGAGGAAGAUAGCA 3'
:::::::::::::::::::::
query 3' GACUCAGUCUCCUUCUAUCGU 5'
query=miR9491, target=RO3G_12979, score=0, range=278-298, strand=
target 5' CUGAGUCAGAGGAAGAUAGCA 3'
:::::::::::::::::::::
query 3' GACUCAGUCUCCUUCUAUCGU 5'
query=miR9491, target=RO3G_10555, score=0, range=278-298, strand=
target 5' CUGAGUCAGAGGAAGAUAGCA 3'
:::::::::::::::::::::
query 3' GACUCAGUCUCCUUCUAUCGU 5'
query=miR9491, target=RO3G_05280, score=0, range=278-298, strand=
target 5' CUGAGUCAGAGGAAGAUAGCA 3'
:::::::::::::::::::::
query 3' GACUCAGUCUCCUUCUAUCGU 5'
query=miR9491, target=RO3G_16686, score=0, range=278-298, strand=
target 5' CUGAGUCAGAGGAAGAUAGCA 3'
:::::::::::::::::::::
query 3' GACUCAGUCUCCUUCUAUCGU 5'
query=miR9491, target=RO3G_07305, score=0, range=278-298, strand=
target 5' CUGAGUCAGAGGAAGAUAGCA 3'
:::::::::::::::::::::
query 3' GACUCAGUCUCCUUCUAUCGU 5'
query=miR9491, target=RO3G_03103, score=0, range=278-298, strand=
target 5' CUGAGUCAGAGGAAGAUAGCA 3'
:::::::::::::::::::::
query 3' GACUCAGUCUCCUUCUAUCGU 5'
query=miR9491, target=RO3G_12087, score=0, range=278-298, strand=
```

```
target 5' CUGAGUCAGAGGAAGAUAGCA 3'
:::::::::::::::::::::
query 3' GACUCAGUCUCCUUCUAUCGU 5'
query=miR9491, target=RO3G_08612, score=0, range=278-298, strand=
target 5' CUGAGUCAGAGGAAGAUAGCA 3'
:::::::::::::::::::::
query 3' GACUCAGUCUCCUUCUAUCGU 5'
query=miR9491, target=RO3G_05630, score=0, range=278-298, strand=
target 5' CUGAGUCAGAGGAAGAUAGCA 3'
:::::::::::::::::::::
query 3' GACUCAGUCUCCUUCUAUCGU 5'
query=miR9491, target=RO3G_13450, score=0, range=278-298, strand=
target 5' CUGAGUCAGAGGAAGAUAGCA 3'
:::::::::::::::::::::
query 3' GACUCAGUCUCCUUCUAUCGU 5'
query=miR9491, target=RO3G_11970, score=0, range=278-298, strand=
target 5' CUGAGUCAGAGGAAGAUAGCA 3'
:::::::::::::::::::::
query 3' GACUCAGUCUCCUUCUAUCGU 5'
query=miR9491, target=RO3G_13152, score=0, range=278-298, strand=
target 5' CUGAGUCAGAGGAAGAUAGCA 3'
:::::::::::::::::::::
query 3' GACUCAGUCUCCUUCUAUCGU 5'
query=miR9491, target=RO3G_03988, score=0, range=278-298, strand=
target 5' CUGAGUCAGAGGAAGAUAGCA 3'
:::::::::::::::::::::
query 3' GACUCAGUCUCCUUCUAUCGU 5'
query=miR9491, target=RO3G_12951, score=0, range=278-298, strand=
target 5' CUGAGUCAGAGGAAGAUAGCA 3'
:::::::::::::::::::::
query 3' GACUCAGUCUCCUUCUAUCGU 5'
query=miR9491, target=RO3G_09376, score=0, range=278-298, strand=
target 5' CUGAGUCAGAGGAAGAUAGCA 3'
:::::::::::::::::::::
query 3' GACUCAGUCUCCUUCUAUCGU 5'
query=miR9491, target=RO3G_12194, score=0, range=278-298, strand=
target 5' CUGAGUCAGAGGAAGAUAGCA 3'
:::::::::::::::::::::
query 3' GACUCAGUCUCCUUCUAUCGU 5'
query=miR9491, target=RO3G_07104, score=0, range=278-298, strand=
target 5' CUGAGUCAGAGGAAGAUAGCA 3'
:::::::::::::::::::::
query 3' GACUCAGUCUCCUUCUAUCGU 5'
query=miR9491, target=RO3G_13861, score=0, range=278-298, strand=
target 5' CUGAGUCAGAGGAAGAUAGCA 3'
```

```
:::::::::::::::::::::
query 3' GACUCAGUCUCCUUCUAUCGU 5'
query=miR9491, target=RO3G_08632, score=3, range=503-523, strand=
target 5' GUGAAUCAGAGGAAGAUAGCU 3'
::: :::::::::::::::
query 3' GACUCAGUCUCCUUCUAUCGU 5'
query=miR9491, target=RO3G_12301, score=3.5, range=3578-3598, str
target 5' UUGACUUGGAGGAAGAUAGUA 3'
.::: :..:::::::::::.:
query 3' GACUCAGUCUCCUUCUAUCGU 5'
query=miR1068, target=RO3G_06442, score=0, range=6121-6142, stran
target 5' GACCUGCAAGCAAAACUGGCUA 3'
::::::::::::::::::::::
query 3' CUGGACGUUCGUUUUGACCGAU 5'
query=miR1068, target=RO3G_16557, score=0, range=397-418, strand=
target 5' GACCUGCAAGCAAAACUGGCUA 3'
::::::::::::::::::::::
query 3' CUGGACGUUCGUUUUGACCGAU 5'
query=miR1068, target=RO3G_07098, score=0, range=397-418, strand=
target 5' GACCUGCAAGCAAAACUGGCUA 3'
::::::::::::::::::::::
query 3' CUGGACGUUCGUUUUGACCGAU 5'
query=miR1068, target=RO3G_01745, score=0, range=397-418, strand=
target 5' GACCUGCAAGCAAAACUGGCUA 3'
::::::::::::::::::::::
query 3' CUGGACGUUCGUUUUGACCGAU 5'
query=miR1068, target=RO3G_05271, score=0, range=397-418, strand=
target 5' GACCUGCAAGCAAAACUGGCUA 3'
::::::::::::::::::::::
query 3' CUGGACGUUCGUUUUGACCGAU 5'
query=miR1068, target=RO3G_00290, score=0, range=397-418, strand=
target 5' GACCUGCAAGCAAAACUGGCUA 3'
::::::::::::::::::::::
query 3' CUGGACGUUCGUUUUGACCGAU 5'
query=miR1068, target=RO3G_15134, score=0, range=439-460, strand=
target 5' GACCUGCAAGCAAAACUGGCUA 3'
::::::::::::::::::::::
query 3' CUGGACGUUCGUUUUGACCGAU 5'
query=miR1068, target=RO3G_12470, score=0, range=397-418, strand=
target 5' GACCUGCAAGCAAAACUGGCUA 3'
::::::::::::::::::::::
query 3' CUGGACGUUCGUUUUGACCGAU 5'
query=miR1068, target=RO3G_16132, score=0, range=397-418, strand=
target 5' GACCUGCAAGCAAAACUGGCUA 3'
::::::::::::::::::::::
```

```
query 3' CUGGACGUUCGUUUUGACCGAU 5'
query=miR1068, target=RO3G_01928, score=0, range=397-418, strand=
target 5' GACCUGCAAGCAAAACUGGCUA 3'
::::::::::::::::::::::
query 3' CUGGACGUUCGUUUUGACCGAU 5'
query=miR1068, target=RO3G_11525, score=0, range=397-418, strand=
target 5' GACCUGCAAGCAAAACUGGCUA 3'
::::::::::::::::::::::
query 3' CUGGACGUUCGUUUUGACCGAU 5'
query=miR1068, target=RO3G_00485, score=0, range=397-418, strand=
target 5' GACCUGCAAGCAAAACUGGCUA 3'
::::::::::::::::::::::
query 3' CUGGACGUUCGUUUUGACCGAU 5'
query=miR1068, target=RO3G_09479, score=0, range=397-418, strand=
target 5' GACCUGCAAGCAAAACUGGCUA 3'
::::::::::::::::::::::
query 3' CUGGACGUUCGUUUUGACCGAU 5'
query=miR1068, target=RO3G_09541, score=0, range=397-418, strand=
target 5' GACCUGCAAGCAAAACUGGCUA 3'
::::::::::::::::::::::
query 3' CUGGACGUUCGUUUUGACCGAU 5'
query=miR1068, target=RO3G_14995, score=0.5, range=397-418, stran
target 5' GGCCUGCAAGCAAAACUGGCUA 3'
:.::::::::::::::::::::
query 3' CUGGACGUUCGUUUUGACCGAU 5'
query=miR1068, target=RO3G_02714, score=3.5, range=397-418, stran
target 5' GACUUACAAGCAAAACUAGCUA 3'
:::.: ::::::::::: ::::
query 3' CUGGACGUUCGUUUUGACCGAU 5'
query=miR1068, target=RO3G_02962, score=3.5, range=182-202, stran
target 5' AACUU-CAAGCAAAACUGGCUU 3'
::.: :::::::::::::::
query 3' CUGGACGUUCGUUUUGACCGAU 5'
query=miR1068, target=RO3G_06842, score=4, range=5290-5311, stran
target 5' GACUUGCAAGCAAAACUGAUUG 3'
:::.:::::::::::::: .:.
query 3' CUGGACGUUCGUUUUGACCGAU 5'
query=miR1068, target=RO3G_04655, score=4, range=1790-1811, stran
target 5' GACAUGAAAGUAAAACUGGCUU 3'
::: :: :::.::::::::::
query 3' CUGGACGUUCGUUUUGACCGAU 5'
query=miR1068, target=RO3G_14692, score=4, range=524-546, strand=
target 5' GACCUGCUGGGCAAAACUCGCUA 3'
::::::: ..:::::::: ::::
query 3' CUGGACG-UUCGUUUUGACCGAU 5'
```

query=miR6300, target=RO3G_01979, score=3, range=828-845, strand=
target 5' UCGCUAUACUACA-CCACC 3'
.:.:::::::::: :::::
query 3' GGUGAUAUGAUGUCGGUGG 5'
query=miR6300, target=RO3G_12567, score=3, range=656-674, strand=
target 5' CCAGAAUAUUACAGCCACC 3'
::: :::.::::::::::
query 3' GGUGAUAUGAUGUCGGUGG 5'
query=miR6300, target=RO3G_04252, score=3, range=656-674, strand=
target 5' CCAGAAUAUUACAGCCACC 3'
::: :::.::::::::::
query 3' GGUGAUAUGAUGUCGGUGG 5'
query=miR6300, target=RO3G_07854, score=3, range=656-674, strand=
target 5' CCAGAAUAUUACAGCCACC 3'
::: :::.::::::::::
query 3' GGUGAUAUGAUGUCGGUGG 5'
query=miR6300, target=RO3G_06775, score=3, range=656-674, strand=
target 5' CCAGAAUAUUACAGCCACC 3'
::: :::.::::::::::
query 3' GGUGAUAUGAUGUCGGUGG 5'
query=miR6300, target=RO3G_15802, score=3, range=656-674, strand=
target 5' CCAGAAUAUUACAGCCACC 3'
::: :::.::::::::::
query 3' GGUGAUAUGAUGUCGGUGG 5'
query=miR6300, target=RO3G_02364, score=3, range=656-674, strand=
target 5' CCAGAAUAUUACAGCCACC 3'
::: :::.::::::::::
query 3' GGUGAUAUGAUGUCGGUGG 5'
query=miR6300, target=RO3G_16779, score=3, range=656-674, strand=
target 5' CCAGAAUAUUACAGCCACC 3'
::: :::.::::::::::
query 3' GGUGAUAUGAUGUCGGUGG 5'
query=miR6300, target=RO3G_14949, score=3, range=278-296, strand=
target 5' CCAGAAUAUUACAGCCACC 3'
::: :::.::::::::::
query 3' GGUGAUAUGAUGUCGGUGG 5'
query=miR6300, target=RO3G_04496, score=3, range=278-296, strand=
target 5' CCAGAAUAUUACAGCCACC 3'
::: :::.::::::::::
query 3' GGUGAUAUGAUGUCGGUGG 5'
query=miR6300, target=RO3G_01157, score=3, range=278-296, strand=
target 5' CCAGAAUAUUACAGCCACC 3'
::: :::.::::::::::
query 3' GGUGAUAUGAUGUCGGUGG 5'
query=miR6300, target=RO3G_16085, score=3, range=656-674, strand=

```
target 5' CCAGAAUAUUACAGCCACC 3'
::: :::.::::::::::
query 3' GGUGAUAUGAUGUCGGUGG 5'
query=miR6300, target=RO3G_14215, score=3, range=656-674, strand=
target 5' CCAGAAUAUUACAGCCACC 3'
::: :::.::::::::::
query 3' GGUGAUAUGAUGUCGGUGG 5'
query=miR6300, target=RO3G_17397, score=3, range=278-296, strand=
target 5' CCAGAAUAUUACAGCCACC 3'
::: :::.::::::::::
query 3' GGUGAUAUGAUGUCGGUGG 5'
query=miR6300, target=RO3G_16776, score=3, range=278-296, strand=
target 5' CCAGAAUAUUACAGCCACC 3'
::: :::.::::::::::
query 3' GGUGAUAUGAUGUCGGUGG 5'
query=miR6300, target=RO3G_06350, score=3, range=278-296, strand=
target 5' CCAGAAUAUUACAGCCACC 3'
::: :::.::::::::::
query 3' GGUGAUAUGAUGUCGGUGG 5'
query=miR6300, target=RO3G_01591, score=3, range=142-160, strand=
target 5' CCAUUAUACAACAGCCACU 3'
:::.::::: ::::::::.
query 3' GGUGAUAUGAUGUCGGUGG 5'
query=miR6300, target=RO3G_09749, score=3, range=462-479, strand=
target 5' CCGUU-UGCUACAGCCACC 3'
::..: :.::::::::::
query 3' GGUGAUAUGAUGUCGGUGG 5'
query=miR6300, target=RO3G_14229, score=3, range=483-500, strand=
target 5' CCA-GAUACUACAGCCACA 3'
::: ::::::::::::
query 3' GGUGAUAUGAUGUCGGUGG 5'
query=miR6300, target=RO3G_03914, score=3.5, range=629-648, stran
target 5' CCACUCAUACUACAACCACU 3'
::::: :::::::: ::::.
query 3' GGUGA-UAUGAUGUCGGUGG 5'
query=miR6300, target=RO3G_08344, score=3.5, range=516-533, stran
target 5' UAACU-UAUUACAGCCACC 3'
. ::: ::.::::::::::
query 3' GGUGAUAUGAUGUCGGUGG 5'
query=miR6300, target=RO3G_06083, score=3.5, range=1456-1474,
strand=-1
target 5' CUACAAUACUAUAGCCAUC 3'
:.:: :::::.:::::.:
query 3' GGUGAUAUGAUGUCGGUGG 5'
query=miR6300, target=RO3G_08103, score=4, range=75-93, strand=1
```

```
target 5' CCAUUAUACAACAGCUACU 3'
:::.::::: :::::.::.
query 3' GGUGAUAUGAUGUCGGUGG 5'
query=miR6300, target=RO3G_08634, score=4, range=278-296, strand=
target 5' CCAGAAUAUUACAGUCACC 3'
::: :::.:::::.::::
query 3' GGUGAUAUGAUGUCGGUGG 5'
query=miR6300, target=RO3G_10181, score=4, range=904-921, strand=
target 5' CCA-UAUACUAUAGCCAAC 3'
::: ::::::.::::: :
query 3' GGUGAUAUGAUGUCGGUGG 5'
query=miR6300, target=RO3G_09282, score=4, range=24-41, strand=1
target 5' CCA-UAUACUUCAGCCGCC 3'
::: :::::: :::::.::
query 3' GGUGAUAUGAUGUCGGUGG 5'
query=miR6300, target=RO3G_08200, score=4, range=7-24, strand=1
target 5' CCCCU-UACUACAGCCAUG 3'
:: :: ::::::::::.
query 3' GGUGAUAUGAUGUCGGUGG 5'
query=miR6300, target=RO3G_13391, score=4, range=638-657, strand=
target 5' CUGCUACUACUACUGCCACC 3'
:..::: :::::: ::::::
query 3' GGUGAU-AUGAUGUCGGUGG 5'
query=miR6300, target=RO3G_02303, score=4, range=445-464, strand=
target 5' CCACCAUUAUUAUAGCCACC 3'
:::: : ::.::.:::::::
query 3' GGUGAU-AUGAUGUCGGUGG 5'
query=miR6300, target=RO3G_06641, score=4, range=982-1000, strand
target 5' CAACAUUACUACAGCCGCC 3'
: :: ::::::::::.::
query 3' GGUGAUAUGAUGUCGGUGG 5'
query=miR6300, target=RO3G_07030, score=4, range=472-489, strand=
target 5' UCACUGUA-UACAGCCAUC 3'
.::::.:: ::::::::.:
query 3' GGUGAUAUGAUGUCGGUGG 5'
query=miR2602a, target=RO3G_13914, score=0, range=737-755, strand
target 5' AUGCUGGCAAUCACUGGAA 3'
:::::::::::::::::::
query 3' UACGACCGUUAGUGACCUU 5'
query=miR2602a, target=RO3G_12058, score=0, range=737-755, strand
target 5' AUGCUGGCAAUCACUGGAA 3'
:::::::::::::::::::
query 3' UACGACCGUUAGUGACCUU 5'
query=miR2602a, target=RO3G_10801, score=0, range=542-560, strand
target 5' AUGCUGGCAAUCACUGGAA 3'
```

```
::::::::::::::::::::
query 3' UACGACCGUUAGUGACCUU 5'
query=miR2602a, target=RO3G_09614, score=0, range=68-86, strand=1
target 5' AUGCUGGCAAUCACUGGAA 3'
::::::::::::::::::::
query 3' UACGACCGUUAGUGACCUU 5'
query=miR2602a, target=RO3G_17462, score=0, range=869-887, strand
target 5' AUGCUGGCAAUCACUGGAA 3'
::::::::::::::::::::
query 3' UACGACCGUUAGUGACCUU 5'
query=miR2602a, target=RO3G_02233, score=0, range=266-284, strand
target 5' AUGCUGGCAAUCACUGGAA 3'
::::::::::::::::::::
query 3' UACGACCGUUAGUGACCUU 5'
query=miR2602a, target=RO3G_14983, score=1, range=542-560, strand
target 5' AUGCUGGCAAUUACUGGAA 3'
::::::::::.:::::::
query 3' UACGACCGUUAGUGACCUU 5'
query=miR2602a, target=RO3G_03583, score=1, range=542-560, strand
target 5' ACGCUGGCAAUCACUGGAA 3'
: ::::::::::::::::::
query 3' UACGACCGUUAGUGACCUU 5'
query=miR2602a, target=RO3G_03556, score=2, range=542-560, strand
target 5' AUGCUGACAAUCACUGGAA 3'
:::::: ::::::::::::
query 3' UACGACCGUUAGUGACCUU 5'
query=miR2602a, target=RO3G_13096, score=3, range=269-287, strand
target 5' AUGGUGGCAGUCGCUGGAA 3'
::: :::::.::.::::::
query 3' UACGACCGUUAGUGACCUU 5'
query=miR2602a, target=RO3G_12495, score=3, range=320-339, strand
target 5' AUGCUUAGCAAUCACUGGAU 3'
:::: : ::::::::::::
query 3' UACG-ACCGUUAGUGACCUU 5'
query=miR2602a, target=RO3G_06752, score=3.5, range=179-197, stra
target 5' AUGAUGGUAAUUACUGGAG 3'
::: :::.:::.::::::.
query 3' UACGACCGUUAGUGACCUU 5'
query=miR2602a, target=RO3G_10535, score=4, range=2123-2141, stra
target 5' GUGUUGGCAAUGACUGGGA 3'
.::.:::::: :::::.:
query 3' UACGACCGUUAGUGACCUU 5'
query=miR2602a, target=RO3G_12181, score=4, range=1292-1310, stra
target 5' GUGUUGGCAAUGACUGGGA 3'
.::.:::::: :::::.:
```

```
query 3' UACGACCGUUAGUGACCUU 5'
query=miR2602a, target=RO3G_15136, score=4, range=185-203, strand
target 5' GUGUUGGCAAUGACUGGGA 3'
.::.:::::: :::::.:
query 3' UACGACCGUUAGUGACCUU 5'
query=miR2602a, target=RO3G_16143, score=4, range=4475-4493, stra
target 5' GUGUUGGCAAUGACUGGGA 3'
.::.:::::: :::::.:
query 3' UACGACCGUUAGUGACCUU 5'
query=miR2602a, target=RO3G_02691, score=4, range=2924-2942, stra
target 5' GUGUUGGCAAUGACUGGGA 3'
.::.:::::: :::::.:
query 3' UACGACCGUUAGUGACCUU 5'
query=miR2602a, target=RO3G_17318, score=4, range=4478-4496, stra
target 5' GUGUUGGCAAUGACUGGGA 3'
.::.:::::: :::::.:
query 3' UACGACCGUUAGUGACCUU 5'
query=miR2602a, target=RO3G_05918, score=4, range=998-1016, stran
target 5' GUGUUGGCAAUGACUGGGA 3'
.::.:::::: :::::.:
query 3' UACGACCGUUAGUGACCUU 5'
query=miR2602a, target=RO3G_12533, score=4, range=3818-3836, stra
target 5' GUGUUGGCAAUGACUGGGA 3'
.::.:::::: :::::.:
query 3' UACGACCGUUAGUGACCUU 5'
query=miR2602a, target=RO3G_07028, score=4, range=3461-3479, stra
target 5' GUGUUGGCAAUGACUGGGA 3'
.::.:::::: :::::.:
query 3' UACGACCGUUAGUGACCUU 5'
query=miR2602a, target=RO3G_05520, score=4, range=1145-1163, stra
target 5' GUGUUGGCAAUGACUGGGA 3'
.::.:::::: :::::.:
query 3' UACGACCGUUAGUGACCUU 5'
query=miR2602a, target=RO3G_17383, score=4, range=977-995, strand
target 5' GUGUUGGCAAUGACUGGGA 3'
.::.:::::: :::::.:
query 3' UACGACCGUUAGUGACCUU 5'
query=miR2602a, target=RO3G_17329, score=4, range=3815-3833, stra
target 5' GUGUUGGCAAUGACUGGGA 3'
.::.:::::: :::::.:
query 3' UACGACCGUUAGUGACCUU 5'
query=miR2602a, target=RO3G_17274, score=4, range=4301-4319, stra
target 5' GUGUUGGCAAUGACUGGGA 3'
.::.:::::: :::::.:
query 3' UACGACCGUUAGUGACCUU 5'
```

```
query=miR2602a, target=RO3G_17076, score=4, range=796-814, strand
target 5' ACGUUGGCAAUUACUGGGG 3'
: :.::::::::.:::::..
query 3' UACGACCGUUAGUGACCUU 5'
query=miR2602a, target=RO3G_03950, score=4, range=202-220, strand
target 5' AUGCUGGCAAGCAUUGGAC 3'
:::::::::: ::.::::
query 3' UACGACCGUUAGUGACCUU 5'
query=miR2602a, target=RO3G_14739, score=4, range=749-766, strand
target 5' AUGCUGGCGAU-AUUGGAA 3'
::::::::.:: :.:::::
query 3' UACGACCGUUAGUGACCUU 5'
query=miR2602a, target=RO3G_15498, score=4, range=1311-1328, stra
target 5' GU-UUGGUAAUUACUGGAA 3'
.: .:::.:::.:::::::
query 3' UACGACCGUUAGUGACCUU 5'
query=miR2602a, target=RO3G_08685, score=4, range=503-521, strand
target 5' AUACUUGCAAUUAUUGGAA 3'
:: :: :::::.:.:::::
query 3' UACGACCGUUAGUGACCUU 5'
query=miR2602a, target=RO3G_12519, score=4, range=285-302, strand
target 5' AUGGU-GCAAUCACUGGGC 3'
::: : :::::::::::.
query 3' UACGACCGUUAGUGACCUU 5'
query=miR2602a, target=RO3G_06658, score=4, range=1991-2009, stra
target 5' AAGAUGGCAAUCGCUGGGA 3'
: : ::::::::.::::.:
query 3' UACGACCGUUAGUGACCUU 5'
query=miR2602a, target=RO3G_16999, score=4, range=4591-4608, stra
target 5' AU-CUAGCAAUUGCUGGAA 3'
:: :: :::::..::::::
query 3' UACGACCGUUAGUGACCUU 5'
query=miR2602a, target=RO3G_00024, score=4, range=470-488, strand
target 5' GCUUUAGCAAUCACUGGAA 3'
. .: ::::::::::::
query 3' UACGACCGUUAGUGACCUU 5'
query=miR2602a, target=RO3G_02639, score=4, range=416-435, strand
target 5' AUCCGGAGCAAUCACUGGAU 3'
:: : : :::::::::::
query 3' UACGAC-CGUUAGUGACCUU 5'
query=miR2602a, target=RO3G_10099, score=4, range=137-154, strand
target 5' CUGCUGGCAAU-GCUGGAA 3'
:::::::::: .::::::
query 3' UACGACCGUUAGUGACCUU 5'
query=miR8138, target=RO3G_00553, score=1.5, range=489-508, stran
```

```
target 5' UUUGCUUUGUUGCCUCUUUA 3'
.:: ::::::::::::::::
query 3' GAAGGAAACAACGGAGAAAU 5'
query=miR8138, target=RO3G_08778, score=2, range=629-648, strand=
target 5' CUGCCUUUGUUGCCUCUUUC 3'
:: ::::::::::::::::
query 3' GAAGGAAACAACGGAGAAAU 5'
query=miR8138, target=RO3G_04385, score=2.5, range=407-426, stran
target 5' CUGCCCUUGUUGCCUCUUUG 3'
:: :: :::::::::::::.
query 3' GAAGGAAACAACGGAGAAAU 5'
query=miR8138, target=RO3G_04628, score=2.5, range=206-225, stran
target 5' UUUCCUUUGUUGUUUCUUUA 3'
.:::::::::::..::::::
query 3' GAAGGAAACAACGGAGAAAU 5'
query=miR8138, target=RO3G_03926, score=2.5, range=148-166, stran
target 5' CUUCCUUUG-UGCCUCUUUG 3'
::::::::: :::::::::.
query 3' GAAGGAAACAACGGAGAAAU 5'
query=miR8138, target=RO3G_16000, score=3, range=794-813, strand=
target 5' GUUCCUUUGUAGCCUCUUUA 3'
::::::::: :::::::::
query 3' GAAGGAAACAACGGAGAAAU 5'
query=miR8138, target=RO3G_02293, score=3, range=956-975, strand=
target 5' UUUCUUUUGUUGUUUCUUUA 3'
.:::.:::::::..::::::
query 3' GAAGGAAACAACGGAGAAAU 5'
query=miR8138, target=RO3G_01819, score=3, range=1316-1336, stran
target 5' CUUCCUCUUGUUGUUUCUUUA 3'
:::::: ::::::..::::::
query 3' GAAGGA-AACAACGGAGAAAU 5'
query=miR8138, target=RO3G_14329, score=3.5, range=1205-1224, str
target 5' CUUCAGUUGUUGUCUCUUUG 3'
:::: ::::::.::::::.
query 3' GAAGGAAACAACGGAGAAAU 5'
query=miR8138, target=RO3G_11173, score=3.5, range=2106-2124, str
target 5' CUACC-UUGUUGCCUUUUUG 3'
:: :: :::::::::.:::.
query 3' GAAGGAAACAACGGAGAAAU 5'
query=miR8138, target=RO3G_08998, score=3.5, range=1179-1198,
strand=-1
target 5' GUGUCUUUGUUGCCUUUUUA 3'
: .:::::::::::.::::
query 3' GAAGGAAACAACGGAGAAAU 5'
query=miR8138, target=RO3G_04233, score=3.5, range=966-984, stran
```

```
target 5' CUUCUUUU-UUGCUUCUUUA 3'
::::.::: ::::.::::::
query 3' GAAGGAAACAACGGAGAAAU 5'
query=miR8138, target=RO3G_15081, score=3.5, range=177-196, stran
target 5' CCUUCUUAGUUGCCUCUUUA 3'
: :.::: ::::::::::::
query 3' GAAGGAAACAACGGAGAAAU 5'
query=miR8138, target=RO3G_05122, score=3.5, range=150-169, stran
target 5' CUUUAUUUGUUUCCUCUUUA 3'
:::. :::::: ::::::::
query 3' GAAGGAAACAACGGAGAAAU 5'
query=miR8138, target=RO3G_03896, score=3.5, range=1426-1446,
strand=-1
target 5' CUGCCUUUGUUGCUCUCUUUG 3'
:: :::::::::: ::::::.
query 3' GAAGGAAACAACG-GAGAAAU 5'
query=miR8138, target=RO3G_12214, score=3.5, range=300-320, stran
target 5' UUUGCUUCUGUUGCUUCUUUA 3'
.:: ::: ::::::.::::::
query 3' GAAGGAA-ACAACGGAGAAAU 5'
query=miR8138, target=RO3G_13059, score=3.5, range=357-375, stran
target 5' UUUACUUUGUU-CCUCUUUA 3'
.:: ::::::: ::::::::
query 3' GAAGGAAACAACGGAGAAAU 5'
query=miR8138, target=RO3G_06184, score=4, range=221-240, strand=
target 5' CUUCUCGUGUUGUCUCUUUG 3'
::::. :::::.::::::.
query 3' GAAGGAAACAACGGAGAAAU 5'
query=miR8138, target=RO3G_16495, score=4, range=497-516, strand=
target 5' CAACCUUUGUUGCUUCUUUU 3'
: ::::::::::.:::::
query 3' GAAGGAAACAACGGAGAAAU 5'
query=miR8138, target=RO3G_16489, score=4, range=497-516, strand=
target 5' CAACCUUUGUUGCUUCUUUU 3'
: ::::::::::.:::::
query 3' GAAGGAAACAACGGAGAAAU 5'
query=miR8138, target=RO3G_04748, score=4, range=4034-4053, stran
target 5' CUGCCUCUGUUUCCUCUUUA 3'
:: ::: :::: ::::::::
query 3' GAAGGAAACAACGGAGAAAU 5'
query=miR8138, target=RO3G_13822, score=4, range=477-496, strand=
target 5' CUUCCUUUGUUGUCUCUUCU 3'
::::::::::::.:::::
query 3' GAAGGAAACAACGGAGAAAU 5'
query=miR8138, target=RO3G_06085, score=4, range=177-196, strand=
```

target 5' CUUUUUUAGUUGCUUCUUUA 3'
:::..:: :::::.::::::
query 3' GAAGGAAACAACGGAGAAAU 5'
query=miR8138, target=RO3G_04369, score=4, range=212-231, strand=
target 5' CUUUUUUGGUUGCUUCUUUA 3'
:::..:: :::::.::::::
query 3' GAAGGAAACAACGGAGAAAU 5'
query=miR8138, target=RO3G_11011, score=4, range=512-531, strand=
target 5' CUUUCUUUUUUGCUUCUUUG 3'
:::.:::: ::::.:::::.
query 3' GAAGGAAACAACGGAGAAAU 5'
query=miR8138, target=RO3G_10851, score=4, range=767-787, strand=
target 5' CUUGUUUUUGUUGCUUUUUUA 3'
::: ..:::::::.:.:::::
query 3' GAA-GGAAACAACGGAGAAAU 5'
query=miR8138, target=RO3G_15381, score=4, range=231-251, strand=
target 5' CUUCCUUUGUAUGCUUCUUUU 3'
:::::::::: :::.:::::
query 3' GAAGGAAACA-ACGGAGAAAU 5'
query=miR8138, target=RO3G_00386, score=4, range=27-47, strand=-1
target 5' CUUUUUUUGUUGCCUUCUUUU 3'
:::..::::::::: :::::
query 3' GAAGGAAACAACGG-AGAAAU 5'
query=miR8138, target=RO3G_07955, score=4, range=282-300, strand=
target 5' UUUCCUAUGUUG-CUCUUUG 3'
.::::: ::::: ::::::.
query 3' GAAGGAAACAACGGAGAAAU 5'
query=miR6147, target=RO3G_05963, score=0, range=387-408, strand=
target 5' AGUGGGGUUUUGAUGCAUGUCA 3'
::::::::::::::::::::::
query 3' UCACCCCAAAACUACGUACAGU 5'
query=miR6147, target=RO3G_08106, score=0, range=387-408, strand=
target 5' AGUGGGGUUUUGAUGCAUGUCA 3'
::::::::::::::::::::::
query 3' UCACCCCAAAACUACGUACAGU 5'
query=miR6147, target=RO3G_17163, score=0, range=387-408, strand=
target 5' AGUGGGGUUUUGAUGCAUGUCA 3'
::::::::::::::::::::::
query 3' UCACCCCAAAACUACGUACAGU 5'
query=miR6147, target=RO3G_15896, score=0, range=387-408, strand=
target 5' AGUGGGGUUUUGAUGCAUGUCA 3'
::::::::::::::::::::::
query 3' UCACCCCAAAACUACGUACAGU 5'
query=miR6147, target=RO3G_14346, score=0, range=387-408, strand=
target 5' AGUGGGGUUUUGAUGCAUGUCA 3'

```
:::::::::::::::::::::
query 3' UCACCCCAAAACUACGUACAGU 5'
query=miR6147, target=RO3G_02711, score=0, range=387-408, strand=
target 5' AGUGGGGUUUUGAUGCAUGUCA 3'
:::::::::::::::::::::
query 3' UCACCCCAAAACUACGUACAGU 5'
query=miR396b, target=RO3G_03508, score=2, range=780-800, strand=
target 5' UGUUCAAAGAAAGCUGUGGAA 3'
:::: :::::::::::::::
query 3' UCAAG-UUCUUUCGACACCUU 5'
query=miR396b, target=RO3G_02790, score=4, range=740-759, strand=
target 5' CGCUCAAGAAAGCUAUGGAA 3'
: ::::::::::: :::::
query 3' UCAAGUUCUUUCGACACCUU 5'
query=miR396b, target=RO3G_16340, score=4, range=605-624, strand=
target 5' AGUUGAUGGAAGCUGUGGAU 3'
:::: : :.:::::::::
query 3' UCAAGUUCUUUCGACACCUU 5'
query=miR396b, target=RO3G_07651, score=4, range=4206-4225, stran
target 5' AAUUAAAGAAAGCUGUGAAA 3'
: :: ::::::::::: ::
query 3' UCAAGUUCUUUCGACACCUU 5'
query=miR396b, target=RO3G_08307, score=4, range=804-822, strand=
target 5' AGUUGAAGAAAGCU-UGGAU 3'
:::: ::::::::: ::::
query 3' UCAAGUUCUUUCGACACCUU 5'
query=miR396b, target=RO3G_06158, score=4, range=1190-1209, stran
target 5' UGCGCAUGAAAGCUGUGGAA 3'
: :: :::::::::::::
query 3' UCAAGUUCUUUCGACACCUU 5'
query=miR7711-5p.1, target=RO3G_12954, score=2, range=54-75, stra
target 5' CAAGAAGUCAAACAGCAAAGUA 3'
:::::::::::::::: :::::
query 3' GUUCUUCAGUUUGUCGAUUCAU 5'
query=miR7711-5p.1, target=RO3G_10762, score=2, range=54-75, stra
target 5' CAAGAAGUCAAACAGCAAAGUA 3'
:::::::::::::::: :::::
query 3' GUUCUUCAGUUUGUCGAUUCAU 5'
query=miR7711-5p.1, target=RO3G_10770, score=2, range=54-75, stra
target 5' CAAGAAGUCAAACAGCAAAGUA 3'
:::::::::::::::: :::::
query 3' GUUCUUCAGUUUGUCGAUUCAU 5'
query=miR7711-5p.1, target=RO3G_00304, score=2, range=54-75, stra
target 5' CAAGAAGUCAAACAGCAAAGUA 3'
:::::::::::::::: :::::
```

```
query 3' GUUCUUCAGUUUGUCGAUUCAU 5'
query=miR7711-5p.1, target=RO3G_04690, score=4, range=54-75, stra
target 5' CAAGAAGUCAAACAGCGAAAUA 3'
:::::::::::::::: :: ::
query 3' GUUCUUCAGUUUGUCGAUUCAU 5'
query=miR7711-5p.1, target=RO3G_05431, score=4, range=54-75, stra
target 5' CAAGAAGUCAAACAGCAAAAUA 3'
:::::::::::::::: :: ::
query 3' GUUCUUCAGUUUGUCGAUUCAU 5'
query=miR7711-5p.1, target=RO3G_02822, score=4, range=54-75, stra
target 5' CAAGAAGUCAAACAGCGAAAUA 3'
:::::::::::::::: :: ::
query 3' GUUCUUCAGUUUGUCGAUUCAU 5'
query=miR7711-5p.1, target=RO3G_09168, score=4, range=54-75, stra
target 5' CAAGAAGUCAAACAGCAAAAUA 3'
:::::::::::::::: :: ::
query 3' GUUCUUCAGUUUGUCGAUUCAU 5'
query=miR7711-5p.1, target=RO3G_15141, score=4, range=54-75, stra
target 5' CAAGAAGUCAAACAGCAAAAUA 3'
:::::::::::::::: :: ::
query 3' GUUCUUCAGUUUGUCGAUUCAU 5'
query=miR7711-5p.1, target=RO3G_04787, score=4, range=54-75, stra
target 5' CAAGAAGUCAAACAGCAAAAUA 3'
:::::::::::::::: :: ::
query 3' GUUCUUCAGUUUGUCGAUUCAU 5'
query=miR5373, target=RO3G_13221, score=0, range=1731-1752, stran
target 5' AACAUCAUCAGCAUACAAGAGA 3'
::::::::::::::::::::::
query 3' UUGUAGUAGUCGUAUGUUCUCU 5'
query=miR5373, target=RO3G_13224, score=0, range=1731-1752, stran
target 5' AACAUCAUCAGCAUACAAGAGA 3'
::::::::::::::::::::::
query 3' UUGUAGUAGUCGUAUGUUCUCU 5'
query=miR5373, target=RO3G_04123, score=0, range=1731-1752, stran
target 5' AACAUCAUCAGCAUACAAGAGA 3'
::::::::::::::::::::::
query 3' UUGUAGUAGUCGUAUGUUCUCU 5'
query=miR5373, target=RO3G_04120, score=0, range=1731-1752, stran
target 5' AACAUCAUCAGCAUACAAGAGA 3'
::::::::::::::::::::::
query 3' UUGUAGUAGUCGUAUGUUCUCU 5'
query=miR5373, target=RO3G_04203, score=0, range=675-696, strand=
target 5' AACAUCAUCAGCAUACAAGAGA 3'
::::::::::::::::::::::
query 3' UUGUAGUAGUCGUAUGUUCUCU 5'
```

```
query=miR5373, target=RO3G_13218, score=0, range=144-165, strand=
target 5' AACAUCAUCAGCAUACAAGAGA 3'
::::::::::::::::::::::
query 3' UUGUAGUAGUCGUAUGUUCUCU 5'
query=miR5373, target=RO3G_16122, score=0, range=144-165, strand=
target 5' AACAUCAUCAGCAUACAAGAGA 3'
::::::::::::::::::::::
query 3' UUGUAGUAGUCGUAUGUUCUCU 5'
query=miR5373, target=RO3G_14836, score=1, range=1482-1503, stran
target 5' AACGUCAUCAGCAUACAAGAGG 3'
:::.:::::::::::::::::.
query 3' UUGUAGUAGUCGUAUGUUCUCU 5'
query=miR5373, target=RO3G_06780, score=1, range=759-780, strand=
target 5' CACAUCAUCAGCAUACAAGAGA 3'
:::::::::::::::::::::
query 3' UUGUAGUAGUCGUAUGUUCUCU 5'
query=miR5373, target=RO3G_00484, score=1.5, range=32-53, strand=
target 5' AAUGUCAUCAGCAUACAAGAGG 3'
::..:::::::::::::::::.
query 3' UUGUAGUAGUCGUAUGUUCUCU 5'
query=miR5373, target=RO3G_16800, score=2, range=3363-3384, stran
target 5' CACAUCAUCAGCAUACAGGAGA 3'
::::::::::::::::.::::
query 3' UUGUAGUAGUCGUAUGUUCUCU 5'
query=miR5373, target=RO3G_15267, score=2, range=1125-1146, stran
target 5' CACAUCAUCAGCAUACAGGAGA 3'
::::::::::::::::.::::
query 3' UUGUAGUAGUCGUAUGUUCUCU 5'
query=miR5373, target=RO3G_17246, score=2, range=564-585, strand=
target 5' CACAUCAUCAGCAUACAGGAGA 3'
::::::::::::::::.::::
query 3' UUGUAGUAGUCGUAUGUUCUCU 5'
query=miR5373, target=RO3G_16819, score=2, range=135-156, strand=
target 5' CACAUCAUCAGCAUACAGGAGA 3'
::::::::::::::::.::::
query 3' UUGUAGUAGUCGUAUGUUCUCU 5'
query=miR5373, target=RO3G_12339, score=3.5, range=1656-1677,
strand=-1
target 5' GACUUCAUCAGCAUUCAAGAGA 3'
.:: :::::::::: :::::::
query 3' UUGUAGUAGUCGUAUGUUCUCU 5'
query=miR5373, target=RO3G_14120, score=4, range=145-166, strand=
target 5' UACAUCAUCGGCAUACAAAAGA 3'
::::::::.:::::::: :::
query 3' UUGUAGUAGUCGUAUGUUCUCU 5'
```

```
query=miR5373, target=RO3G_15307, score=4, range=1483-1504, stran
target 5' CACAUCAACAGCAUACAAGACA 3'
:::::: ::::::::::: :
query 3' UUGUAGUAGUCGUAUGUUCUCU 5'
query=miR5373, target=RO3G_14105, score=4, range=162-183, strand=
target 5' UACAUCAUCGGCAUACAAAAGA 3'
::::::::.::::::: :::
query 3' UUGUAGUAGUCGUAUGUUCUCU 5'
query=miR5373, target=RO3G_13384, score=4, range=1656-1677, stran
target 5' GACUUCGUCAGCAUUCAAGAGA 3'
.:: ::.::::::: :::::::
query 3' UUGUAGUAGUCGUAUGUUCUCU 5'
query=miR7837, target=RO3G_11243, score=0, range=158-178, strand=
target 5' CAGCUACCACUCCUACACCCA 3'
:::::::::::::::::::::
query 3' GUCGAUGGUGAGGAUGUGGGU 5'
query=miR7837, target=RO3G_00539, score=3, range=299-319, strand=
target 5' CAGCUCCCACUCCUUCACCCA 3'
::::: :::::::: ::::::
query 3' GUCGAUGGUGAGGAUGUGGGU 5'
query=miR7837, target=RO3G_01660, score=4, range=834-853, strand=
target 5' UA-CUACCACUCCUGCACCUG 3'
.: ::::::::::.::::..
query 3' GUCGAUGGUGAGGAUGUGGGU 5'
query=miR7837, target=RO3G_14336, score=4, range=1193-1213, stran
target 5' CAGUAUCUACUCCUACAUCCA 3'
:::. :.:::::::::.:::
query 3' GUCGAUGGUGAGGAUGUGGGU 5'
query=miR1515, target=RO3G_17349, score=0.5, range=15-36, strand=
target 5' GGGUCAUUGUCAUGCUAAAAUA 3'
::.:::::::::::::::::::
query 3' CCUAGUAACAGUACGAUUUUAU 5'
query=miR1515, target=RO3G_16894, score=0.5, range=15-36, strand=
target 5' GGGUCAUUGUCAUGCUAAAAUA 3'
::.:::::::::::::::::::
query 3' CCUAGUAACAGUACGAUUUUAU 5'
query=miR1515, target=RO3G_13852, score=1, range=461-482, strand=
target 5' GGAUCAUUGUCAUGCUGAAAUA 3'
::::::::::::::::.:::::
query 3' CCUAGUAACAGUACGAUUUUAU 5'
query=miR1515, target=RO3G_15271, score=2, range=710-731, strand=
target 5' GGAUCAUUGUCGUGCUGAAAUA 3'
:::::::::::.::::.:::::
query 3' CCUAGUAACAGUACGAUUUUAU 5'
query=miR1515, target=RO3G_10771, score=2, range=461-482, strand=
```

```
target 5' GGAUCAUUGUCAUGUUGAAAUA 3'
::::::::::::::.:.:::::
query 3' CCUAGUAACAGUACGAUUUUAU 5'
query=miR1515, target=RO3G_16725, score=2, range=452-473, strand=
target 5' GGAUCAUUGUCGUGCUGAAAUA 3'
:::::::::::.::::.:::::
query 3' CCUAGUAACAGUACGAUUUUAU 5'
query=miR1515, target=RO3G_07739, score=2, range=461-482, strand=
target 5' GGAUCAUUCUCAUGCUGAAAUA 3'
:::::::: :::::::.:::::
query 3' CCUAGUAACAGUACGAUUUUAU 5'
query=miR1515, target=RO3G_13731, score=2.5, range=203-225, stran
target 5' GGGUCAUUGUCAUGCUAAAAGUA 3'
::.::::::::::::::::: ::
query 3' CCUAGUAACAGUACGAUUUU-AU 5'
query=miR1515, target=RO3G_10527, score=2.5, range=189-210, stran
target 5' GGGUCAUUGUCAUGCUAAAACA 3'
::.::::::::::::::::: :
query 3' CCUAGUAACAGUACGAUUUUAU 5'
query=miR1515, target=RO3G_09968, score=2.5, range=15-36, strand=
target 5' GGGUCAUUGUCAUGCUAAAACA 3'
::.::::::::::::::::: :
query 3' CCUAGUAACAGUACGAUUUUAU 5'
query=miR1515, target=RO3G_09962, score=2.5, range=15-36, strand=
target 5' GGGUCAUUGUCAUGCUAAAACA 3'
::.::::::::::::::::: :
query 3' CCUAGUAACAGUACGAUUUUAU 5'
query=miR1515, target=RO3G_00745, score=3, range=431-452, strand=
target 5' GGAUCAUUGUCGUGUUGAAAUA 3'
:::::::::::.::.:.:::::
query 3' CCUAGUAACAGUACGAUUUUAU 5'
query=miR1515, target=RO3G_05640, score=3, range=605-626, strand=
target 5' GGAUCAUUAUCGUGCUGAAAUA 3'
:::::::: ::.::::.:::::
query 3' CCUAGUAACAGUACGAUUUUAU 5'
query=miR1515, target=RO3G_10866, score=3, range=719-740, strand=
target 5' GGAUCAUUGUCAUGCUGAAAAA 3'
::::::::::::::::.::: :
query 3' CCUAGUAACAGUACGAUUUUAU 5'
query=miR1515, target=RO3G_00255, score=3, range=719-740, strand=
target 5' GGAUCAUUGUCAUGCUGAAAAA 3'
::::::::::::::::.::: :
query 3' CCUAGUAACAGUACGAUUUUAU 5'
query=miR1515, target=RO3G_09612, score=3, range=719-740, strand=
target 5' GGAUCAUUGUCAUGCUGAAAAA 3'
```

```
::::::::::::::::.::: :
query 3' CCUAGUAACAGUACGAUUUUAU 5'
query=miR1515, target=RO3G_13271, score=3, range=719-740, strand=
target 5' GGAUCAUUGUCAUGCUGAAAAA 3'
::::::::::::::::.::: :
query 3' CCUAGUAACAGUACGAUUUUAU 5'
query=miR1515, target=RO3G_07112, score=3, range=542-563, strand=
target 5' GGAUCAUUGUCAUGCUGAAAAA 3'
::::::::::::::::.::: :
query 3' CCUAGUAACAGUACGAUUUUAU 5'
query=miR1515, target=RO3G_13266, score=3, range=497-518, strand=
target 5' GGAUCAUUGUCAUGCUGAAAAA 3'
::::::::::::::::.::: :
query 3' CCUAGUAACAGUACGAUUUUAU 5'
query=miR1515, target=RO3G_15166, score=3, range=707-728, strand=
target 5' GGAUCAUUAUCAUGUUGAAAUA 3'
:::::::: :::::.:.:::::
query 3' CCUAGUAACAGUACGAUUUUAU 5'
query=miR1515, target=RO3G_13714, score=3, range=710-731, strand=
target 5' GGAUCAUUAUCGUGCUGAAAUA 3'
:::::::: ::.::::.:::::
query 3' CCUAGUAACAGUACGAUUUUAU 5'
query=miR1515, target=RO3G_16742, score=3, range=710-731, strand=
target 5' GGAUCAUUAUCGUGCUGAAAUA 3'
:::::::: ::.::::.:::::
query 3' CCUAGUAACAGUACGAUUUUAU 5'
query=miR1515, target=RO3G_17416, score=3, range=710-731, strand=
target 5' GGAUCAUUAUCGUGCUGAAAUA 3'
:::::::: ::.::::.:::::
query 3' CCUAGUAACAGUACGAUUUUAU 5'
query=miR1515, target=RO3G_17291, score=3, range=710-731, strand=
target 5' GGAUCAUUAUCGUGCUGAAAUA 3'
:::::::: ::.::::.:::::
query 3' CCUAGUAACAGUACGAUUUUAU 5'
query=miR1515, target=RO3G_02824, score=3, range=710-731, strand=
target 5' GGAUCAUUAUCGUGCUGAAAUA 3'
:::::::: ::.::::.:::::
query 3' CCUAGUAACAGUACGAUUUUAU 5'
query=miR1515, target=RO3G_16372, score=3, range=710-731, strand=
target 5' GGAUCAUUAUCGUGCUGAAAUA 3'
:::::::: ::.::::.:::::
query 3' CCUAGUAACAGUACGAUUUUAU 5'
query=miR1515, target=RO3G_07705, score=3, range=710-731, strand=
target 5' GGAUCAUUAUCGUGCUGAAAUA 3'
:::::::: ::.::::.:::::
```

```
query 3' CCUAGUAACAGUACGAUUUUAU 5'
query=miR1515, target=RO3G_16123, score=3, range=710-731, strand=
target 5' GGAUCAUUAUCGUGCUGAAAUA 3'
:::::::: ::.::::.::::
query 3' CCUAGUAACAGUACGAUUUUAU 5'
query=miR1515, target=RO3G_17387, score=3, range=710-731, strand=
target 5' GGAUCAUUAUCGUGCUGAAAUA 3'
:::::::: ::.::::.::::
query 3' CCUAGUAACAGUACGAUUUUAU 5'
query=miR1515, target=RO3G_06717, score=3, range=710-731, strand=
target 5' GGAUCAUUAUCGUGCUGAAAUA 3'
:::::::: ::.::::.::::
query 3' CCUAGUAACAGUACGAUUUUAU 5'
query=miR1515, target=RO3G_17399, score=3, range=710-731, strand=
target 5' GGAUCAUUAUCGUGCUGAAAUA 3'
:::::::: ::.::::.::::
query 3' CCUAGUAACAGUACGAUUUUAU 5'
query=miR1515, target=RO3G_01328, score=3, range=710-731, strand=
target 5' GGAUCAUUAUCGUGCUGAAAUA 3'
:::::::: ::.::::.::::
query 3' CCUAGUAACAGUACGAUUUUAU 5'
query=miR1515, target=RO3G_07137, score=3, range=710-731, strand=
target 5' GGAUCAUUAUCGUGCUGAAAUA 3'
:::::::: ::.::::.::::
query 3' CCUAGUAACAGUACGAUUUUAU 5'
query=miR1515, target=RO3G_05268, score=3, range=710-731, strand=
target 5' GGAUCAUUAUCGUGCUGAAAUA 3'
:::::::: ::.::::.::::
query 3' CCUAGUAACAGUACGAUUUUAU 5'
query=miR1515, target=RO3G_16911, score=3, range=305-326, strand=
target 5' GGAUCAUUGUCAUGCUGAAAAA 3'
:::::::::::::::.::: :
query 3' CCUAGUAACAGUACGAUUUUAU 5'
query=miR1515, target=RO3G_12663, score=3, range=545-566, strand=
target 5' GGAUCAUUAUCGUGCUGAAAUA 3'
:::::::: ::.::::.::::
query 3' CCUAGUAACAGUACGAUUUUAU 5'
query=miR1515, target=RO3G_17355, score=3, range=545-566, strand=
target 5' GGAUCAUUAUCGUGCUGAAAUA 3'
:::::::: ::.::::.::::
query 3' CCUAGUAACAGUACGAUUUUAU 5'
query=miR1515, target=RO3G_04674, score=3, range=461-482, strand=
target 5' GGAUCAUUAUCGUGCUGAAAUA 3'
:::::::: ::.::::.::::
query 3' CCUAGUAACAGUACGAUUUUAU 5'
```

```
query=miR1515, target=RO3G_01962, score=3, range=461-482, strand=
target 5' GGAUCAUUAUCGUGCUGAAAUA 3'
:::::::: ::.::::.::::
query 3' CCUAGUAACAGUACGAUUUUAU 5'
query=miR1515, target=RO3G_16706, score=3, range=434-455, strand=
target 5' GGAUCAUUAUCGUGCUGAAAUA 3'
:::::::: ::.::::.::::
query 3' CCUAGUAACAGUACGAUUUUAU 5'
query=miR1515, target=RO3G_17223, score=3, range=434-455, strand=
target 5' GGAUCAUUAUCGUGCUGAAAUA 3'
:::::::: ::.::::.::::
query 3' CCUAGUAACAGUACGAUUUUAU 5'
query=miR1515, target=RO3G_09193, score=3, range=434-455, strand=
target 5' GGAUCAUUAUCGUGCUGAAAUA 3'
:::::::: ::.::::.::::
query 3' CCUAGUAACAGUACGAUUUUAU 5'
query=miR1515, target=RO3G_14770, score=3, range=710-731, strand=
target 5' GGAUCAUUAUCGUGCUGAAAUA 3'
:::::::: ::.::::.::::
query 3' CCUAGUAACAGUACGAUUUUAU 5'
query=miR1515, target=RO3G_11781, score=3, range=605-626, strand=
target 5' GGAUCAUUAUCGUGCUGAAAUA 3'
:::::::: ::.::::.::::
query 3' CCUAGUAACAGUACGAUUUUAU 5'
query=miR1515, target=RO3G_16420, score=3, range=461-482, strand=
target 5' GGAUCAUUAUCGUGCUGAAAUA 3'
:::::::: ::.::::.::::
query 3' CCUAGUAACAGUACGAUUUUAU 5'
query=miR1515, target=RO3G_07702, score=3, range=461-482, strand=
target 5' GGAUCAUUAUCGUGCUGAAAUA 3'
:::::::: ::.::::.::::
query 3' CCUAGUAACAGUACGAUUUUAU 5'
query=miR1515, target=RO3G_10800, score=3, range=377-398, strand=
target 5' GGAUCAUUGUCAUGCUGAAAAA 3'
::::::::::::::::.::: :
query 3' CCUAGUAACAGUACGAUUUUAU 5'
query=miR1515, target=RO3G_14621, score=3, range=377-398, strand=
target 5' GGAUCAUUGUCAUGCUGAAAAA 3'
::::::::::::::::.::: :
query 3' CCUAGUAACAGUACGAUUUUAU 5'
query=miR1515, target=RO3G_15282, score=3, range=434-455, strand=
target 5' GGAUCAUUAUCGUGCUGAAAUA 3'
:::::::: ::.::::.::::
query 3' CCUAGUAACAGUACGAUUUUAU 5'
query=miR1515, target=RO3G_05634, score=3, range=188-209, strand=
```

```
target 5' GGAUCAUUGUCAUGCUGAAAAA 3'
::::::::::::::::.::: :
query 3' CCUAGUAACAGUACGAUUUUAU 5'
query=miR1515, target=RO3G_12069, score=3, range=188-209, strand=
target 5' GGAUCAUUGUCAUGCUGAAAAA 3'
::::::::::::::::.::: :
query 3' CCUAGUAACAGUACGAUUUUAU 5'
query=miR1515, target=RO3G_04695, score=3, range=500-521, strand=
target 5' GGAUCAUUAUCGUGCUGAAAUA 3'
:::::::: ::.::::.:::::
query 3' CCUAGUAACAGUACGAUUUUAU 5'
query=miR1515, target=RO3G_07867, score=3, range=461-482, strand=
target 5' GGAUCAUUAUCGUGCUGAAAUA 3'
:::::::: ::.::::.:::::
query 3' CCUAGUAACAGUACGAUUUUAU 5'
query=miR1515, target=RO3G_10793, score=3, range=188-209, strand=
target 5' GGAUCAUUGUCAUGCUGAAAAA 3'
::::::::::::::::.::: :
query 3' CCUAGUAACAGUACGAUUUUAU 5'
query=miR1515, target=RO3G_10553, score=3.5, range=189-210, stran
target 5' GGGUCAUUGUCAUGCUAAAAAU 3'
::.:::::::::::::::::
query 3' CCUAGUAACAGUACGAUUUUAU 5'
query=miR1515, target=RO3G_10559, score=3.5, range=189-210, stran
target 5' GGGUCAUUGUCAUGCUAAAAAU 3'
::.:::::::::::::::::
query 3' CCUAGUAACAGUACGAUUUUAU 5'
query=miR1515, target=RO3G_07031, score=3.5, range=719-740, stran
target 5' GGGUCAUUGUCAUGCUGAAAAA 3'
::.:::::::::::::.::: :
query 3' CCUAGUAACAGUACGAUUUUAU 5'
query=miR1515, target=RO3G_16554, score=3.5, range=719-740, stran
target 5' GGGUCAUUGUCAUGCUGAAAAA 3'
::.:::::::::::::.::: :
query 3' CCUAGUAACAGUACGAUUUUAU 5'
query=miR1515, target=RO3G_00237, score=3.5, range=176-197, stran
target 5' GGAUCGUUAUCAUGUUGAAAUA 3'
:::::.:: :::::.:.:::::
query 3' CCUAGUAACAGUACGAUUUUAU 5'
query=miR1515, target=RO3G_04692, score=3.5, range=59-80, strand=
target 5' GGAUCGUUAUCAUGUUGAAAUA 3'
:::::.:: :::::.:.:::::
query 3' CCUAGUAACAGUACGAUUUUAU 5'
query=miR1515, target=RO3G_17207, score=3.5, range=59-80, strand=
target 5' GGAUCGUUAUCAUGUUGAAAUA 3'
```

```
:::::.:: :::::.:.:::::
query 3' CCUAGUAACAGUACGAUUUUAU 5'
query=miR1515, target=RO3G_15895, score=3.5, range=20-41, strand=
target 5' GGAUCGUUAUCAUGUUGAAAUA 3'
:::::.:: :::::.:.:::::
query 3' CCUAGUAACAGUACGAUUUUAU 5'
query=miR1515, target=RO3G_16536, score=3.5, range=20-41, strand=
target 5' GGAUCGUUAUCAUGUUGAAAUA 3'
:::::.:: :::::.:.:::::
query 3' CCUAGUAACAGUACGAUUUUAU 5'
query=miR1515, target=RO3G_05953, score=4, range=710-731, strand=
target 5' GGGUCGUUGUCAUGCUGAAAAA 3'
::.::.::::::::::.::: :
query 3' CCUAGUAACAGUACGAUUUUAU 5'
query=miR1515, target=RO3G_07688, score=4, range=719-740, strand=
target 5' GGAUCAUUGUCAUGUUGAAAAA 3'
::::::::::::::.:.::: :
query 3' CCUAGUAACAGUACGAUUUUAU 5'
query=miR1515, target=RO3G_16359, score=4, range=719-740, strand=
target 5' GGAUCAUUGUCAUGUUGAAAAA 3'
::::::::::::::.:.::: :
query 3' CCUAGUAACAGUACGAUUUUAU 5'
query=miR1515, target=RO3G_16545, score=4, range=719-740, strand=
target 5' GGAUCAUUGUCAUGUUGAAAAA 3'
::::::::::::::.:.::: :
query 3' CCUAGUAACAGUACGAUUUUAU 5'
query=miR1515, target=RO3G_03772, score=4, range=719-740, strand=
target 5' GGAUCAUUGUCAUGCUGGAAGA 3'
::::::::::::::::..:: :
query 3' CCUAGUAACAGUACGAUUUUAU 5'
query=miR1515, target=RO3G_01618, score=4, range=719-740, strand=
target 5' GGAUCAUUGUCAUGCUGGAAGA 3'
::::::::::::::::..:: :
query 3' CCUAGUAACAGUACGAUUUUAU 5'
query=miR1515, target=RO3G_08842, score=4, range=719-740, strand=
target 5' GGAUCAUUGUCAUGCUGGAAGA 3'
::::::::::::::::..:: :
query 3' CCUAGUAACAGUACGAUUUUAU 5'
query=miR1515, target=RO3G_02844, score=4, range=497-518, strand=
target 5' GGAUCAUUGUCAUGCUGGAAGA 3'
::::::::::::::::..:: :
query 3' CCUAGUAACAGUACGAUUUUAU 5'
query=miR1515, target=RO3G_03766, score=4, range=497-518, strand=
target 5' GGAUCAUUGUCAUGCUGGAAGA 3'
::::::::::::::::..:: :
```

```
query 3' CCUAGUAACAGUACGAUUUUAU 5'
query=miR1515, target=RO3G_13137, score=4, range=617-638, strand=
target 5' GGAUCAUUGUCAUGCUGGAAGA 3'
::::::::::::::::..:: :
query 3' CCUAGUAACAGUACGAUUUUAU 5'
query=miR1515, target=RO3G_03554, score=4, range=611-632, strand=
target 5' GGAUCAUUGUCAUGCUGGAAGA 3'
::::::::::::::::..:: :
query 3' CCUAGUAACAGUACGAUUUUAU 5'
query=miR1515, target=RO3G_08657, score=4, range=611-632, strand=
target 5' GGAUCAUUGUCAUGCUGGAAGA 3'
::::::::::::::::..:: :
query 3' CCUAGUAACAGUACGAUUUUAU 5'
query=miR1515, target=RO3G_10772, score=4, range=608-629, strand=
target 5' GGAUCAUUGUCAUGCUGGAAGA 3'
::::::::::::::::..:: :
query 3' CCUAGUAACAGUACGAUUUUAU 5'
query=miR1515, target=RO3G_10780, score=4, range=710-731, strand=
target 5' GGGUCGUUGUCAUGCUGAAAAA 3'
::.::.::::::::::.::: :
query 3' CCUAGUAACAGUACGAUUUUAU 5'
query=miR1515, target=RO3G_00256, score=4, range=710-731, strand=
target 5' GGGUCGUUGUCAUGCUGAAAAA 3'
::.::.::::::::::.::: :
query 3' CCUAGUAACAGUACGAUUUUAU 5'
query=miR1515, target=RO3G_14361, score=4, range=710-731, strand=
target 5' GGGUCGUUGUCAUGCUGAAAAA 3'
::.::.::::::::::.::: :
query 3' CCUAGUAACAGUACGAUUUUAU 5'
query=miR1515, target=RO3G_05267, score=4, range=710-731, strand=
target 5' GGGUCGUUGUCAUGCUGAAAAA 3'
::.::.::::::::::.::: :
query 3' CCUAGUAACAGUACGAUUUUAU 5'
query=miR1515, target=RO3G_09967, score=4, range=710-731, strand=
target 5' GGGUCGUUGUCAUGCUGAAAAA 3'
::.::.::::::::::.::: :
query 3' CCUAGUAACAGUACGAUUUUAU 5'
query=miR1515, target=RO3G_04207, score=4, range=710-731, strand=
target 5' GGGUCGUUGUCAUGCUGAAAAA 3'
::.::.::::::::::.::: :
query 3' CCUAGUAACAGUACGAUUUUAU 5'
query=miR1515, target=RO3G_04210, score=4, range=710-731, strand=
target 5' GGGUCGUUGUCAUGCUGAAAAA 3'
::.::.::::::::::.::: :
query 3' CCUAGUAACAGUACGAUUUUAU 5'
```

```
query=miR1515, target=RO3G_07860, score=4, range=710-731, strand=
target 5' GGGUCGUUGUCAUGCUGAAAAA 3'
::.::.::::::::::.::: :
query 3' CCUAGUAACAGUACGAUUUUAU 5'
query=miR1515, target=RO3G_09542, score=4, range=710-731, strand=
target 5' GGGUCGUUGUCAUGCUGAAAAA 3'
::.::.::::::::::.::: :
query 3' CCUAGUAACAGUACGAUUUUAU 5'
query=miR1515, target=RO3G_10773, score=4, range=710-731, strand=
target 5' GGGUCGUUGUCAUGCUGAAAAA 3'
::.::.::::::::::.::: :
query 3' CCUAGUAACAGUACGAUUUUAU 5'
query=miR1515, target=RO3G_04127, score=4, range=671-692, strand=
target 5' GGAUCAUUGUCAUGCUGGAAGA 3'
::::::::::::::::..:: :
query 3' CCUAGUAACAGUACGAUUUUAU 5'
query=miR1515, target=RO3G_00244, score=4, range=671-692, strand=
target 5' GGAUCAUUGUCAUGCUGGAAGA 3'
::::::::::::::::..:: :
query 3' CCUAGUAACAGUACGAUUUUAU 5'
query=miR1515, target=RO3G_05959, score=4, range=710-731, strand=
target 5' GGGUCGUUGUCAUGCUGAAAAA 3'
::.::.::::::::::.::: :
query 3' CCUAGUAACAGUACGAUUUUAU 5'
query=miR1515, target=RO3G_13147, score=4, range=497-518, strand=
target 5' GGAUCAUUGUCAUGCUGGAAGA 3'
::::::::::::::::..:: :
query 3' CCUAGUAACAGUACGAUUUUAU 5'
query=miR1515, target=RO3G_09194, score=4, range=710-731, strand=
target 5' GGGUCGUUGUCAUGCUGAAAAA 3'
::.::.::::::::::.::: :
query 3' CCUAGUAACAGUACGAUUUUAU 5'
query=miR1515, target=RO3G_11966, score=4, range=710-731, strand=
target 5' GGGUCGUUGUCAUGCUGAAAAA 3'
::.::.::::::::::.::: :
query 3' CCUAGUAACAGUACGAUUUUAU 5'
query=miR1515, target=RO3G_07124, score=4, range=710-731, strand=
target 5' GGGUCGUUGUCAUGCUGAAAAA 3'
::.::.::::::::::.::: :
query 3' CCUAGUAACAGUACGAUUUUAU 5'
query=miR1515, target=RO3G_04205, score=4, range=419-440, strand=
target 5' GGAUCAUUGUCAUGCUGGAAGA 3'
::::::::::::::::..:: :
query 3' CCUAGUAACAGUACGAUUUUAU 5'
query=miR1515, target=RO3G_09945, score=4, range=434-455, strand=
```

```
target 5' GGGUCGUUGUCAUGCUGAAAAA 3'
::.::.::::::::::.::: :
query 3' CCUAGUAACAGUACGAUUUUAU 5'
query=miR1515, target=RO3G_00287, score=4, range=599-620, strand=
target 5' GGAUUGUUAUCAUGUUGAAAUA 3'
::::..:: :::::.:.:::::
query 3' CCUAGUAACAGUACGAUUUUAU 5'
query=miR1515, target=RO3G_16378, score=4, range=188-209, strand=
target 5' GGAUCAUUGUCAUGUUGAAAAA 3'
::::::::::::::.:.::: :
query 3' CCUAGUAACAGUACGAUUUUAU 5'
query=miR1515, target=RO3G_12649, score=4, range=98-119, strand=-
target 5' GGAUCAUUGUCAUGCUGGAAGA 3'
::::::::::::::::..:: :
query 3' CCUAGUAACAGUACGAUUUUAU 5'
query=miR1515, target=RO3G_16704, score=4, range=128-149, strand=
target 5' GGAUCAUUGUCAUGCUGGAAGA 3'
::::::::::::::::..:: :
query 3' CCUAGUAACAGUACGAUUUUAU 5'
query=miR1515, target=RO3G_12198, score=4, range=107-128, strand=
target 5' GGAUUGUUAUCAUGUUGAAAUA 3'
::::..:: :::::.:.:::::
query 3' CCUAGUAACAGUACGAUUUUAU 5'
query=miR1515, target=RO3G_07725, score=4, range=98-119, strand=-
target 5' GGAUUGUUAUCAUGUUGAAAUA 3'
::::..:: :::::.:.:::::
query 3' CCUAGUAACAGUACGAUUUUAU 5'
query=miR1515, target=RO3G_16747, score=4, range=98-119, strand=-
target 5' GGAUUGUUAUCAUGUUGAAAUA 3'
::::..:: :::::.:.:::::
query 3' CCUAGUAACAGUACGAUUUUAU 5'
query=miR1515, target=RO3G_07577, score=4, range=59-80, strand=-1
target 5' GGAUUGUUAUCAUGUUGAAAUA 3'
::::..:: :::::.:.:::::
query 3' CCUAGUAACAGUACGAUUUUAU 5'
query=miR1515, target=RO3G_03553, score=4, range=59-80, strand=-1
target 5' GGAUUGUUAUCAUGUUGAAAUA 3'
::::..:: :::::.:.:::::
query 3' CCUAGUAACAGUACGAUUUUAU 5'
query=miR1515, target=RO3G_10562, score=4, range=59-80, strand=-1
target 5' GGAUUGUUAUCAUGUUGAAAUA 3'
::::..:: :::::.:.:::::
query 3' CCUAGUAACAGUACGAUUUUAU 5'
query=miR1515, target=RO3G_16809, score=4, range=59-80, strand=-1
target 5' GGAUUGUUAUCAUGUUGAAAUA 3'
```

```
::::..:: ::::.:.::::
query 3' CCUAGUAACAGUACGAUUUUAU 5'
query=miR1515, target=RO3G_17167, score=4, range=59-80, strand=-1
target 5' GGAUUGUUAUCAUGUUGAAAUA 3'
::::..:: ::::.:.::::
query 3' CCUAGUAACAGUACGAUUUUAU 5'
query=miR1515, target=RO3G_01952, score=4, range=59-80, strand=-1
target 5' GGAUUGUUAUCAUGUUGAAAUA 3'
::::..:: ::::.:.::::
query 3' CCUAGUAACAGUACGAUUUUAU 5'
query=miR1515, target=RO3G_07301, score=4, range=59-80, strand=-1
target 5' GGAUUGUUAUCAUGUUGAAAUA 3'
::::..:: ::::.:.::::
query 3' CCUAGUAACAGUACGAUUUUAU 5'
query=miR1515, target=RO3G_04678, score=4, range=59-80, strand=-1
target 5' GGAUUGUUAUCAUGUUGAAAUA 3'
::::..:: ::::.:.::::
query 3' CCUAGUAACAGUACGAUUUUAU 5'
query=miR1515, target=RO3G_07681, score=4, range=59-80, strand=-1
target 5' GGAUUGUUAUCAUGUUGAAAUA 3'
::::..:: ::::.:.::::
query 3' CCUAGUAACAGUACGAUUUUAU 5'
query=miR1515, target=RO3G_06723, score=4, range=59-80, strand=-1
target 5' GGAUUGUUAUCAUGUUGAAAUA 3'
::::..:: ::::.:.::::
query 3' CCUAGUAACAGUACGAUUUUAU 5'
query=miR1515, target=RO3G_02720, score=4, range=59-80, strand=-1
target 5' GGAUUGUUAUCAUGUUGAAAUA 3'
::::..:: ::::.:.::::
query 3' CCUAGUAACAGUACGAUUUUAU 5'
query=miR1515, target=RO3G_12676, score=4, range=20-41, strand=-1
target 5' GGAUUGUUAUCAUGUUGAAAUA 3'
::::..:: ::::.:.::::
query 3' CCUAGUAACAGUACGAUUUUAU 5'
query=miR1515, target=RO3G_03112, score=4, range=709-731, strand=
target 5' GGAUCAUUAUCAUGCUGAAAGUA 3'
:::::::: ::::::: :::.::
query 3' CCUAGUAACAGUACGA-UUUUAU 5'
query=miR1515, target=RO3G_05522, score=4, range=709-731, strand=
target 5' GGAUCAUUAUCAUGCUGAAAGUA 3'
:::::::: ::::::: :::.::
query 3' CCUAGUAACAGUACGA-UUUUAU 5'
query=miR1515, target=RO3G_00240, score=4, range=652-674, strand=
target 5' GGAUCAUUAUCAUGCUGAAAGUA 3'
:::::::: ::::::: :::.::
```

```
query 3' CCUAGUAACAGUACGA-UUUUAU 5'
query=miR1515, target=RO3G_02227, score=4, range=709-731, strand=
target 5' GGAUCAUUAUCAUGCUGAAAGUA 3'
:::::::: ::::::: :::.::
query 3' CCUAGUAACAGUACGA-UUUUAU 5'
query=miR1515, target=RO3G_10797, score=4, range=343-365, strand=
target 5' GGAUCAUUAUCAUGCUGAAAGUA 3'
:::::::: ::::::: :::.::
query 3' CCUAGUAACAGUACGA-UUUUAU 5'
query=miR1515, target=RO3G_17423, score=4, range=343-365, strand=
target 5' GGAUCAUUAUCAUGCUGAAAGUA 3'
:::::::: ::::::: :::.::
query 3' CCUAGUAACAGUACGA-UUUUAU 5'
query=miR1515, target=RO3G_14784, score=4, range=343-365, strand=
target 5' GGAUCAUUAUCAUGCUGAAAGUA 3'
:::::::: ::::::: :::.::
query 3' CCUAGUAACAGUACGA-UUUUAU 5'
query=miR1515, target=RO3G_16737, score=4, range=343-365, strand=
target 5' GGAUCAUUAUCAUGCUGAAAGUA 3'
:::::::: ::::::: :::.::
query 3' CCUAGUAACAGUACGA-UUUUAU 5'
query=miR1515, target=RO3G_01725, score=4, range=343-365, strand=
target 5' GGAUCAUUAUCAUGCUGAAAGUA 3'
:::::::: ::::::: :::.::
query 3' CCUAGUAACAGUACGA-UUUUAU 5'
query=miR1515, target=RO3G_07723, score=4, range=343-365, strand=
target 5' GGAUCAUUAUCAUGCUGAAAGUA 3'
:::::::: ::::::: :::.::
query 3' CCUAGUAACAGUACGA-UUUUAU 5'
query=miR1515, target=RO3G_17092, score=4, range=343-365, strand=
target 5' GGAUCAUUAUCAUGCUGAAAGUA 3'
:::::::: ::::::: :::.::
query 3' CCUAGUAACAGUACGA-UUUUAU 5'
query=miR1515, target=RO3G_10050, score=4, range=343-365, strand=
target 5' GGAUCAUUAUCAUGCUGAAAGUA 3'
:::::::: ::::::: :::.::
query 3' CCUAGUAACAGUACGA-UUUUAU 5'
query=miR1515, target=RO3G_14981, score=4, range=343-365, strand=
target 5' GGAUCAUUAUCAUGCUGAAAGUA 3'
:::::::: ::::::: :::.::
query 3' CCUAGUAACAGUACGA-UUUUAU 5'
query=miR1515, target=RO3G_12950, score=4, range=343-365, strand=
target 5' GGAUCAUUAUCAUGCUGAAAGUA 3'
:::::::: ::::::: :::.::
query 3' CCUAGUAACAGUACGA-UUUUAU 5'
```

query=miR1515, target=RO3G_15145, score=4, range=343-365, strand=
target 5' GGAUCAUUAUCAUGCUGAAAGUA 3'
:::::::: ::::::: :::.::
query 3' CCUAGUAACAGUACGA-UUUUAU 5'
query=miR1515, target=RO3G_03791, score=4, range=343-365, strand=
target 5' GGAUCAUUAUCAUGCUGAAAGUA 3'
:::::::: ::::::: :::.::
query 3' CCUAGUAACAGUACGA-UUUUAU 5'
query=miR1515, target=RO3G_09926, score=4, range=343-365, strand=
target 5' GGAUCAUUAUCAUGCUGAAAGUA 3'
:::::::: ::::::: :::.::
query 3' CCUAGUAACAGUACGA-UUUUAU 5'
query=miR1515, target=RO3G_11536, score=4, range=343-365, strand=
target 5' GGAUCAUUAUCAUGCUGAAAGUA 3'
:::::::: ::::::: :::.::
query 3' CCUAGUAACAGUACGA-UUUUAU 5'
query=miR1515, target=RO3G_05516, score=4, range=343-365, strand=
target 5' GGAUCAUUAUCAUGCUGAAAGUA 3'
:::::::: ::::::: :::.::
query 3' CCUAGUAACAGUACGA-UUUUAU 5'
query=miR1515, target=RO3G_17328, score=4, range=343-365, strand=
target 5' GGAUCAUUAUCAUGCUGAAAGUA 3'
:::::::: ::::::: :::.::
query 3' CCUAGUAACAGUACGA-UUUUAU 5'
query=miR1515, target=RO3G_15170, score=4, range=343-365, strand=
target 5' GGAUCAUUAUCAUGCUGAAAGUA 3'
:::::::: ::::::: :::.::
query 3' CCUAGUAACAGUACGA-UUUUAU 5'
query=miR1515, target=RO3G_16541, score=4, range=343-365, strand=
target 5' GGAUCAUUAUCAUGCUGAAAGUA 3'
:::::::: ::::::: :::.::
query 3' CCUAGUAACAGUACGA-UUUUAU 5'
query=miR1515, target=RO3G_16124, score=4, range=343-365, strand=
target 5' GGAUCAUUAUCAUGCUGAAAGUA 3'
:::::::: ::::::: :::.::
query 3' CCUAGUAACAGUACGA-UUUUAU 5'
query=miR1515, target=RO3G_15286, score=4, range=343-365, strand=
target 5' GGAUCAUUAUCAUGCUGAAAGUA 3'
:::::::: ::::::: :::.::
query 3' CCUAGUAACAGUACGA-UUUUAU 5'
query=miR1515, target=RO3G_13752, score=4, range=343-365, strand=
target 5' GGAUCAUUAUCAUGCUGAAAGUA 3'
:::::::: ::::::: :::.::
query 3' CCUAGUAACAGUACGA-UUUUAU 5'
query=miR1515, target=RO3G_03560, score=4, range=343-365, strand=

```
target 5' GGAUCAUUAUCAUGCUGAAAGUA 3'
:::::::: ::::::: :::.::
query 3' CCUAGUAACAGUACGA-UUUUAU 5'
query=miR1515, target=RO3G_16141, score=4, range=343-365, strand=
target 5' GGAUCAUUAUCAUGCUGAAAGUA 3'
:::::::: ::::::: :::.::
query 3' CCUAGUAACAGUACGA-UUUUAU 5'
query=miR1515, target=RO3G_07136, score=4, range=34-56, strand=-1
target 5' GGAUCAUUAUCAUGCUGAAAGUA 3'
:::::::: ::::::: :::.::
query 3' CCUAGUAACAGUACGA-UUUUAU 5'
query=miR1515, target=RO3G_11533, score=4, range=343-365, strand=
target 5' GGAUCAUUAUCAUGCUGAAAGUA 3'
:::::::: ::::::: :::.::
query 3' CCUAGUAACAGUACGA-UUUUAU 5'
query=miR1515, target=RO3G_10785, score=4, range=343-365, strand=
target 5' GGAUCAUUAUCAUGCUGAAAGUA 3'
:::::::: ::::::: :::.::
query 3' CCUAGUAACAGUACGA-UUUUAU 5'
query=miR1515, target=RO3G_00250, score=4, range=343-365, strand=
target 5' GGAUCAUUAUCAUGCUGAAAGUA 3'
:::::::: ::::::: :::.::
query 3' CCUAGUAACAGUACGA-UUUUAU 5'
query=miR1515, target=RO3G_13267, score=4, range=343-365, strand=
target 5' GGAUCAUUAUCAUGCUGAAAGUA 3'
:::::::: ::::::: :::.::
query 3' CCUAGUAACAGUACGA-UUUUAU 5'
query=miR1515, target=RO3G_12084, score=4, range=343-365, strand=
target 5' GGAUCAUUAUCAUGCUGAAAGUA 3'
:::::::: ::::::: :::.::
query 3' CCUAGUAACAGUACGA-UUUUAU 5'
query=miR1515, target=RO3G_03797, score=4, range=208-230, strand=
target 5' GGAUCACUGUCGUGCUGAAAAUA 3'
:::::: ::::.:::: ::::::
query 3' CCUAGUAACAGUACGA-UUUUAU 5'
query=miR7502b, target=RO3G_05880, score=3, range=272-290, strand
target 5' AUG-AUAAACUUAUUGACAA 3'
::: :: ::::::::.::::
query 3' UACGUACUUGAAUAAUUGUU 5'
query=miR7502b, target=RO3G_10183, score=3.5, range=260-278, stra
target 5' UUGC-UGAACUUAUUGACAG 3'
::: ::::::::::.:::.
query 3' UACGUACUUGAAUAAUUGUU 5'
query=miR7502b, target=RO3G_16944, score=3.5, range=191-210, stra
target 5' AUCUGUCAACUUAUUAACAG 3'
```

```
:: ..: :::::::::::.
query 3' UACGUACUUGAAUAAUUGUU 5'
query=miR7502b, target=RO3G_12923, score=4, range=583-602, strand
target 5' UUGCAUGAAUUUAUUAAUGA 3'
:::::::.:::::::..:
query 3' UACGUACUUGAAUAAUUGUU 5'
query=miR7502b, target=RO3G_16530, score=4, range=1012-1031, stra
target 5' UUGAAUGAAUUUAUUAAUAA 3'
:: ::::.:::::::.::
query 3' UACGUACUUGAAUAAUUGUU 5'
query=miR7502b, target=RO3G_02695, score=4, range=3805-3825, stra
target 5' AUGAAUGAAUUUAUUGAACAA 3'
::: ::::.::::: :::::
query 3' UACGUACUUGAAUAA-UUGUU 5'
query=miR7502b, target=RO3G_03550, score=4, range=3805-3825, stra
target 5' AUGAAUGAAUUUAUUGAACAA 3'
::: ::::.::::: :::::
query 3' UACGUACUUGAAUAA-UUGUU 5'
query=miR7502b, target=RO3G_09175, score=4, range=3805-3825, stra
target 5' AUGAAUGAAUUUAUUGAACAA 3'
::: ::::.::::: :::::
query 3' UACGUACUUGAAUAA-UUGUU 5'
query=miR7502b, target=RO3G_11541, score=4, range=1195-1215, stra
target 5' AUGAAUGAAUUUAUUGAACAA 3'
::: ::::.::::: :::::
query 3' UACGUACUUGAAUAA-UUGUU 5'
query=miR7502b, target=RO3G_08532, score=4, range=1304-1322, stra
target 5' AUGCA-GAACUUAUUAAAGA 3'
::::: :::::::::: .:
query 3' UACGUACUUGAAUAAUUGUU 5'
query=miR7502b, target=RO3G_11910, score=4, range=24-43, strand=1
target 5' AUCCUUAAAUUUAUUAACAA 3'
:: : : ::.:::::::::
query 3' UACGUACUUGAAUAAUUGUU 5'
query=miR7502b, target=RO3G_08587, score=4, range=2021-2039, stra
target 5' AU-CAAGAACUUAUUGACAU 3'
:: :: :::::::::.:::
query 3' UACGUACUUGAAUAAUUGUU 5'
query=miR7502b, target=RO3G_02564, score=4, range=443-461, strand
target 5' UUACA-GAAUUUAUUAACAA 3'
: :: :::.::::::::::
query 3' UACGUACUUGAAUAAUUGUU 5'
query=miR7502b, target=RO3G_09426, score=4, range=1132-1151, stra
target 5' AAGCACAAACUUAUUAACAU 3'
: ::: :::::::::::
```

```
query 3' UACGUACUUGAAUAAUUGUU 5'
query=miR7502b, target=RO3G_08891, score=4, range=109-127, strand
target 5' AUGCUCGAA-UUAUUAACAA 3'
:::: ::: ::::::::::
query 3' UACGUACUUGAAUAAUUGUU 5'
query=miR7502b, target=RO3G_06589, score=4, range=316-335, strand
target 5' GUGCUUGAGCUUAUUAAUAG 3'
.::: :::.::::::::.:.
query 3' UACGUACUUGAAUAAUUGUU 5'
query=miR7502b, target=RO3G_09234, score=4, range=83-101, strand=
target 5' AU-CAUGGGCUUAUUAGCAA 3'
:: ::::..:::::::.:::
query 3' UACGUACUUGAAUAAUUGUU 5'
query=miR7502b, target=RO3G_02236, score=4, range=308-327, strand
target 5' AUUCUUGAACUUAAUAACAA 3'
:: : :::::::: ::::::
query 3' UACGUACUUGAAUAAUUGUU 5'
query=miR7502b, target=RO3G_08139, score=4, range=407-427, strand
target 5' AUGACAUUAACUUAUUAUCAA 3'
::: ::: ::::::::: :::
query 3' UAC-GUACUUGAAUAAUUGUU 5'
query=miR7502b, target=RO3G_08587, score=4, range=620-639, strand
target 5' UUGCUUUAACUUAUUAACAU 3'
::: : :::::::::::
query 3' UACGUACUUGAAUAAUUGUU 5'
query=miR951, target=RO3G_04122, score=0, range=196-217, strand=1
target 5' CGUAGCCAGAUCGUCAAGAGCA 3'
::::::::::::::::::::::
query 3' GCAUCGGUCUAGCAGUUCUCGU 5'
query=miR951, target=RO3G_13220, score=0, range=196-217, strand=1
target 5' CGUAGCCAGAUCGUCAAGAGCA 3'
::::::::::::::::::::::
query 3' GCAUCGGUCUAGCAGUUCUCGU 5'
query=miR951, target=RO3G_04124, score=0, range=196-217, strand=1
target 5' CGUAGCCAGAUCGUCAAGAGCA 3'
::::::::::::::::::::::
query 3' GCAUCGGUCUAGCAGUUCUCGU 5'
query=miR951, target=RO3G_13223, score=0.5, range=196-217, strand
target 5' CGUAGUCAGAUCGUCAAGAGCA 3'
:::::.::::::::::::::::
query 3' GCAUCGGUCUAGCAGUUCUCGU 5'
query=miR951, target=RO3G_04119, score=2, range=196-217, strand=1
target 5' CGUAGCCAGAUGGUCAAGAGCA 3'
::::::::::: ::::::::::
query 3' GCAUCGGUCUAGCAGUUCUCGU 5'
```

```
query=miR8741, target=RO3G_13273, score=0, range=2206-2227, stran
target 5' CCAUCAUCAUUCAUUCAGGCCA 3'
::::::::::::::::::::::
query 3' GGUAGUAGUAAGUAAGUCCGGU 5'
query=miR8741, target=RO3G_10217, score=0, range=2206-2227, stran
target 5' CCAUCAUCAUUCAUUCAGGCCA 3'
::::::::::::::::::::::
query 3' GGUAGUAGUAAGUAAGUCCGGU 5'
query=miR8741, target=RO3G_16749, score=0, range=2206-2227, stran
target 5' CCAUCAUCAUUCAUUCAGGCCA 3'
::::::::::::::::::::::
query 3' GGUAGUAGUAAGUAAGUCCGGU 5'
query=miR8741, target=RO3G_13145, score=0, range=2206-2227, stran
target 5' CCAUCAUCAUUCAUUCAGGCCA 3'
::::::::::::::::::::::
query 3' GGUAGUAGUAAGUAAGUCCGGU 5'
query=miR8741, target=RO3G_17257, score=0, range=2122-2143, stran
target 5' CCAUCAUCAUUCAUUCAGGCCA 3'
::::::::::::::::::::::
query 3' GGUAGUAGUAAGUAAGUCCGGU 5'
query=miR8741, target=RO3G_05424, score=0, range=2122-2143, stran
target 5' CCAUCAUCAUUCAUUCAGGCCA 3'
::::::::::::::::::::::
query 3' GGUAGUAGUAAGUAAGUCCGGU 5'
query=miR8741, target=RO3G_15889, score=0, range=2122-2143, stran
target 5' CCAUCAUCAUUCAUUCAGGCCA 3'
::::::::::::::::::::::
query 3' GGUAGUAGUAAGUAAGUCCGGU 5'
query=miR8741, target=RO3G_16136, score=0, range=2122-2143, stran
target 5' CCAUCAUCAUUCAUUCAGGCCA 3'
::::::::::::::::::::::
query 3' GGUAGUAGUAAGUAAGUCCGGU 5'
query=miR8741, target=RO3G_04592, score=0, range=1378-1399, stran
target 5' CCAUCAUCAUUCAUUCAGGCCA 3'
::::::::::::::::::::::
query 3' GGUAGUAGUAAGUAAGUCCGGU 5'
query=miR8741, target=RO3G_09183, score=0, range=1339-1360, stran
target 5' CCAUCAUCAUUCAUUCAGGCCA 3'
::::::::::::::::::::::
query 3' GGUAGUAGUAAGUAAGUCCGGU 5'
query=miR8741, target=RO3G_00289, score=0, range=940-961, strand=
target 5' CCAUCAUCAUUCAUUCAGGCCA 3'
::::::::::::::::::::::
query 3' GGUAGUAGUAAGUAAGUCCGGU 5'
query=miR8741, target=RO3G_17351, score=0, range=934-955, strand=
```

target 5' CCAUCAUCAUUCAUUCAGGCCA 3'
::::::::::::::::::::::
query 3' GGUAGUAGUAAGUAAGUCCGGU 5'
query=miR8741, target=RO3G_04681, score=0, range=796-817, strand=
target 5' CCAUCAUCAUUCAUUCAGGCCA 3'
::::::::::::::::::::::
query 3' GGUAGUAGUAAGUAAGUCCGGU 5'
query=miR8741, target=RO3G_07697, score=0, range=532-553, strand=
target 5' CCAUCAUCAUUCAUUCAGGCCA 3'
::::::::::::::::::::::
query 3' GGUAGUAGUAAGUAAGUCCGGU 5'
query=miR8741, target=RO3G_10040, score=0, range=796-817, strand=
target 5' CCAUCAUCAUUCAUUCAGGCCA 3'
::::::::::::::::::::::
query 3' GGUAGUAGUAAGUAAGUCCGGU 5'
query=miR8741, target=RO3G_10735, score=2.5, range=1651-1672, str
target 5' CCAUCAUCAUUCAUUCAGGCGG 3'
:::::::::::::::::::: .
query 3' GGUAGUAGUAAGUAAGUCCGGU 5'
query=miR7716-3p, target=RO3G_17338, score=0, range=3-24, strand=
target 5' GGCAUUACUGGAGGAUGAAGAA 3'
::::::::::::::::::::::
query 3' CCGUAAUGACCUCCUACUUCUU 5'
query=miR7716-3p, target=RO3G_07958, score=3.5, range=3070-3090,
strand=1
target 5' GACG-UGUUGGAGGAUGAAGAA 3'
: :. :..::::::::::::::
query 3' CCGUAAUGACCUCCUACUUCUU 5'
query=miR7716-3p, target=RO3G_12741, score=4, range=1170-1191,
strand=1
target 5' GAUGGUGCUGGAGGAUGAAGAG 3'
: .. :.:::::::::::::::.
query 3' CCGUAAUGACCUCCUACUUCUU 5'
query=miR7716-3p, target=RO3G_14666, score=4, range=840-860, stra
target 5' AGCAUUAAUGGA-GAUGAAGAA 3'
:::::: :::: :::::::::
query 3' CCGUAAUGACCUCCUACUUCUU 5'
query=miR8603, target=RO3G_05069, score=3.5, range=1125-1145,
strand=-1
target 5' ACUAUAUUCAUCACAAGCUCA 3'
:: :: .:::.:::::::::
query 3' UGUUACGAGUGGUGUUCGAGU 5'
query=miR8603, target=RO3G_05236, score=4, range=15-36, strand=1
target 5' ACAAUUGCUCAUCACAAGUUUA 3'
:::: ::::::.::::::.:.:

query 3' UGUU-ACGAGUGGUGUUCGAGU 5'
query=miR5641, target=RO3G_17273, score=2.5, range=1754-1776, strand=-1
target 5' UAGUUCUUUACUGUCUUCUUCCA 3'
::.::::::::.:::::.::::
query 3' AUUAAGAAAUGAUAGAAGGAGGU 5'
query=miR5641, target=RO3G_15160, score=2.5, range=1652-1674, strand=-1
target 5' UAGUUCUUUACUGUCUUCUUCCA 3'
::.::::::::.:::::.::::
query 3' AUUAAGAAAUGAUAGAAGGAGGU 5'
query=miR5641, target=RO3G_03100, score=4, range=3206-3228, stran
target 5' UAGUUCUUUGCUAUCCUCUUCCA 3'
::.::::::.::::: ::.::::
query 3' AUUAAGAAAUGAUAGAAGGAGGU 5'
query=miR5641, target=RO3G_05340, score=4, range=1589-1611, stran
target 5' UAGUUCUUUGCUAUCCUCUUCCA 3'
::.::::::.::::: ::.::::
query 3' AUUAAGAAAUGAUAGAAGGAGGU 5'
query=miR4401b, target=RO3G_17451, score=0, range=70-89, strand=1
target 5' UUGCUCACAACCGUCUUUGA 3'
::::::::::::::::::::
query 3' AACGAGUGUUGGCAGAAACU 5'
query=miR4401b, target=RO3G_13914, score=0, range=829-848, strand
target 5' UUGCUCACAACCGUCUUUGA 3'
::::::::::::::::::::
query 3' AACGAGUGUUGGCAGAAACU 5'
query=miR4401b, target=RO3G_12058, score=0, range=829-848, strand
target 5' UUGCUCACAACCGUCUUUGA 3'
::::::::::::::::::::
query 3' AACGAGUGUUGGCAGAAACU 5'
query=miR4401b, target=RO3G_14983, score=0, range=634-653, strand
target 5' UUGCUCACAACCGUCUUUGA 3'
::::::::::::::::::::
query 3' AACGAGUGUUGGCAGAAACU 5'
query=miR4401b, target=RO3G_10801, score=0, range=634-653, strand
target 5' UUGCUCACAACCGUCUUUGA 3'
::::::::::::::::::::
query 3' AACGAGUGUUGGCAGAAACU 5'
query=miR4401b, target=RO3G_09614, score=0, range=160-179, strand
target 5' UUGCUCACAACCGUCUUUGA 3'
::::::::::::::::::::
query 3' AACGAGUGUUGGCAGAAACU 5'
query=miR4401b, target=RO3G_17462, score=0, range=961-980, strand
target 5' UUGCUCACAACCGUCUUUGA 3'

```
::::::::::::::::::::
query 3' AACGAGUGUUGGCAGAAACU 5'
query=miR4401b, target=RO3G_02233, score=0, range=358-377, strand
target 5' UUGCUCACAACCGUCUUUGA 3'
::::::::::::::::::::
query 3' AACGAGUGUUGGCAGAAACU 5'
query=miR4401b, target=RO3G_12667, score=2, range=199-218, strand
target 5' UUGCUCACAACCGUCUUGGA 3'
::::::::::::::::: ::
query 3' AACGAGUGUUGGCAGAAACU 5'
query=miR4401b, target=RO3G_03583, score=2, range=634-653, strand
target 5' UUGCUCACAACAGUCUUUGA 3'
::::::::::: ::::::::
query 3' AACGAGUGUUGGCAGAAACU 5'
query=miR4401b, target=RO3G_16714, score=3.5, range=187-206, stra
target 5' UUGUUGACAACCGUCUUAGA 3'
:::.: ::::::::::: ::
query 3' AACGAGUGUUGGCAGAAACU 5'
query=miR4401b, target=RO3G_04116, score=3.5, range=194-213, stra
target 5' UUGUUGACAACCGUCUUAGA 3'
:::.: ::::::::::: ::
query 3' AACGAGUGUUGGCAGAAACU 5'
query=miR4401b, target=RO3G_01079, score=3.5, range=466-486, stra
target 5' UGGCUCACCAACCGUUUUUGG 3'
: ::::: ::::::.::::.
query 3' AACGAGU-GUUGGCAGAAACU 5'
query=miR4401b, target=RO3G_01425, score=3.5, range=1419-1437,
strand=-1
target 5' UUUCUU-CAACCGUUUUUGA 3'
:: ::. ::::::.:::::
query 3' AACGAGUGUUGGCAGAAACU 5'
query=miR4401b, target=RO3G_03120, score=3.5, range=1419-1437,
strand=-1
target 5' UUUCUU-CAACCGUUUUUGA 3'
:: ::. ::::::.:::::
query 3' AACGAGUGUUGGCAGAAACU 5'
query=miR4401b, target=RO3G_16566, score=3.5, range=1419-1437,
strand=-1
target 5' UUUCUU-CAACCGUUUUUGA 3'
:: ::. ::::::.:::::
query 3' AACGAGUGUUGGCAGAAACU 5'
query=miR4401b, target=RO3G_13806, score=3.5, range=1419-1437,
strand=-1
target 5' UUUCUU-CAACCGUUUUUGA 3'
:: ::. ::::::.:::::
```

query 3' AACGAGUGUUGGCAGAAACU 5'
query=miR4401b, target=RO3G_14533, score=3.5, range=1224-1242, strand=-1
target 5' UUUCUU-CAACCGUUUUUGA 3'
:: ::. :::::::.:::::
query 3' AACGAGUGUUGGCAGAAACU 5'
query=miR4401b, target=RO3G_08383, score=4, range=281-300, strand
target 5' UUGCUCGCAGCCGUCUUGGG 3'
::::::.::.::::::: :.
query 3' AACGAGUGUUGGCAGAAACU 5'
query=miR4401b, target=RO3G_12286, score=4, range=242-260, strand
target 5' UUUCUGA-AACCGUCUUUGA 3'
:: :: : :::::::::::
query 3' AACGAGUGUUGGCAGAAACU 5'
query=miR4401b, target=RO3G_07324, score=4, range=567-586, strand
target 5' UGGCUGCCAAUCGUCUUUGA 3'
: ::: :::.:::::::::
query 3' AACGAGUGUUGGCAGAAACU 5'
query=miR4401b, target=RO3G_08428, score=4, range=401-419, strand
target 5' CUUCUCAC-ACCGUCUUUGA 3'
: ::::: ::::::::::
query 3' AACGAGUGUUGGCAGAAACU 5'
query=miR4401b, target=RO3G_11133, score=4, range=95-113, strand=
target 5' UUGCUC-CAAUAGUCUUUGA 3'
:::::: :::. ::::::::
query 3' AACGAGUGUUGGCAGAAACU 5'
query=miR158-3p, target=RO3G_02126, score=2, range=520-540, stran
target 5' GCAUUUGUCUACGUUUGGAAA 3'
:: :::::::::.::::::::
query 3' CG-AAACAGAUGUAAACCUUU 5'
query=miR158-3p, target=RO3G_05865, score=4, range=2734-2753, str
target 5' GCUUUGUCUACAUCUGAAAA 3'
::::::::::::: :: :::
query 3' CGAAACAGAUGUAAACCUUU 5'
query=miR158-3p, target=RO3G_02707, score=4, range=631-650, stran
target 5' GCUUUGUCUACAUCUGAAAA 3'
::::::::::::: :: :::
query 3' CGAAACAGAUGUAAACCUUU 5'
query=miR158-3p, target=RO3G_17446, score=4, range=3082-3101, str
target 5' GCUUUGUCUACAUCUGAAAA 3'
::::::::::::: :: :::
query 3' CGAAACAGAUGUAAACCUUU 5'
query=miR158-3p, target=RO3G_15863, score=4, range=10-29, strand=
target 5' GCUUUGUCUACAUCUGAAAA 3'
::::::::::::: :: :::

```
query 3' CGAAACAGAUGUAAACCUUU 5'
query=miR158-3p, target=RO3G_01733, score=4, range=2884-2903, str
target 5' GCUUUGUCUACAUCUGAAAA 3'
:::::::::::: :: :::
query 3' CGAAACAGAUGUAAACCUUU 5'
query=miR158-3p, target=RO3G_11945, score=4, range=10-29, strand=
target 5' GCUUUGUCUACAUCUGAAAA 3'
:::::::::::: :: :::
query 3' CGAAACAGAUGUAAACCUUU 5'
query=miR158-3p, target=RO3G_10420, score=4, range=3082-3101, str
target 5' GCUUUGUCUACAUCUGAAAA 3'
:::::::::::: :: :::
query 3' CGAAACAGAUGUAAACCUUU 5'
query=miR158-3p, target=RO3G_02966, score=4, range=2353-2372, str
target 5' GCUUUGUCUACAUCUGAAAA 3'
:::::::::::: :: :::
query 3' CGAAACAGAUGUAAACCUUU 5'
query=miR158-3p, target=RO3G_10991, score=4, range=3082-3101, str
target 5' GCUUUGUCUACAUCUGAAAA 3'
:::::::::::: :: :::
query 3' CGAAACAGAUGUAAACCUUU 5'
query=miR158-3p, target=RO3G_01944, score=4, range=3082-3101, str
target 5' GCUUUGUCUACAUCUGAAAA 3'
:::::::::::: :: :::
query 3' CGAAACAGAUGUAAACCUUU 5'
query=miR158-3p, target=RO3G_13849, score=4, range=3082-3101, str
target 5' GCUUUGUCUACAUCUGAAAA 3'
:::::::::::: :: :::
query 3' CGAAACAGAUGUAAACCUUU 5'
query=miR158-3p, target=RO3G_06502, score=4, range=2959-2978, str
target 5' GCUUUGUCUACAUCUGAAAA 3'
:::::::::::: :: :::
query 3' CGAAACAGAUGUAAACCUUU 5'
query=miR158-3p, target=RO3G_17287, score=4, range=3082-3101, str
target 5' GCUUUGUCUACAUCUGAAAA 3'
:::::::::::: :: :::
query 3' CGAAACAGAUGUAAACCUUU 5'
query=miR158-3p, target=RO3G_03749, score=4, range=3082-3101, str
target 5' GCUUUGUCUACAUCUGAAAA 3'
:::::::::::: :: :::
query 3' CGAAACAGAUGUAAACCUUU 5'
query=miR158-3p, target=RO3G_15573, score=4, range=3082-3101, str
target 5' GCUUUGUCUACAUCUGAAAA 3'
:::::::::::: :: :::
query 3' CGAAACAGAUGUAAACCUUU 5'
```

```
query=miR158-3p, target=RO3G_07325, score=4, range=1306-1325, str
target 5' GCUUUGGUUACAUUUGAAAA 3'
:::::: .:::::::: :::
query 3' CGAAACAGAUGUAAACCUUU 5'
query=miR158-3p, target=RO3G_09758, score=4, range=3112-3131, str
target 5' GCUUUUGCUACAUUAGGAAA 3'
::::: ::::::: :::::
query 3' CGAAACAGAUGUAAACCUUU 5'
query=miR158-3p, target=RO3G_06983, score=4, range=895-914, stran
target 5' GCUUGGUCUACAUCUGGAAU 3'
:::: :::::::: :::::
query 3' CGAAACAGAUGUAAACCUUU 5'
query=miR158-3p, target=RO3G_08644, score=4, range=829-847, stran
target 5' GUGUUG-CUAUAUUUGGAAG 3'
:. ::: :::.::::::::.
query 3' CGAAACAGAUGUAAACCUUU 5'
query=miR158-3p, target=RO3G_17196, score=4, range=215-233, stran
target 5' GCAUUG-CUACAUUUGAAAA 3'
:: ::: ::::::::: :::
query 3' CGAAACAGAUGUAAACCUUU 5'
query=miR1146, target=RO3G_06423, score=0, range=518-538, strand=
target 5' AAGACUUCCCGUCGGACCCAA 3'
:::::::::::::::::::::
query 3' UUCUGAAGGGCAGCCUGGGUU 5'
query=miR1146, target=RO3G_10235, score=0, range=464-484, strand=
target 5' AAGACUUCCCGUCGGACCCAA 3'
:::::::::::::::::::::
query 3' UUCUGAAGGGCAGCCUGGGUU 5'
query=miR1146, target=RO3G_09483, score=0, range=464-484, strand=
target 5' AAGACUUCCCGUCGGACCCAA 3'
:::::::::::::::::::::
query 3' UUCUGAAGGGCAGCCUGGGUU 5'
query=miR1146, target=RO3G_06441, score=0, range=464-484, strand=
target 5' AAGACUUCCCGUCGGACCCAA 3'
:::::::::::::::::::::
query 3' UUCUGAAGGGCAGCCUGGGUU 5'
query=miR1146, target=RO3G_09499, score=0, range=464-484, strand=
target 5' AAGACUUCCCGUCGGACCCAA 3'
:::::::::::::::::::::
query 3' UUCUGAAGGGCAGCCUGGGUU 5'
query=miR1146, target=RO3G_15675, score=0, range=464-484, strand=
target 5' AAGACUUCCCGUCGGACCCAA 3'
:::::::::::::::::::::
query 3' UUCUGAAGGGCAGCCUGGGUU 5'
query=miR1146, target=RO3G_11401, score=0, range=464-484, strand=
```

```
target 5' AAGACUUCCCGUCGGACCCAA 3'
:::::::::::::::::::::
query 3' UUCUGAAGGGCAGCCUGGGUU 5'
query=miR1146, target=RO3G_07875, score=0, range=464-484, strand=
target 5' AAGACUUCCCGUCGGACCCAA 3'
:::::::::::::::::::::
query 3' UUCUGAAGGGCAGCCUGGGUU 5'
query=miR1146, target=RO3G_07303, score=0, range=464-484, strand=
target 5' AAGACUUCCCGUCGGACCCAA 3'
:::::::::::::::::::::
query 3' UUCUGAAGGGCAGCCUGGGUU 5'
query=miR1146, target=RO3G_14359, score=0, range=464-484, strand=
target 5' AAGACUUCCCGUCGGACCCAA 3'
:::::::::::::::::::::
query 3' UUCUGAAGGGCAGCCUGGGUU 5'
query=miR1146, target=RO3G_00681, score=0, range=464-484, strand=
target 5' AAGACUUCCCGUCGGACCCAA 3'
:::::::::::::::::::::
query 3' UUCUGAAGGGCAGCCUGGGUU 5'
query=miR1146, target=RO3G_05336, score=0, range=464-484, strand=
target 5' AAGACUUCCCGUCGGACCCAA 3'
:::::::::::::::::::::
query 3' UUCUGAAGGGCAGCCUGGGUU 5'
query=miR1146, target=RO3G_09544, score=0, range=464-484, strand=
target 5' AAGACUUCCCGUCGGACCCAA 3'
:::::::::::::::::::::
query 3' UUCUGAAGGGCAGCCUGGGUU 5'
query=miR1146, target=RO3G_10732, score=0, range=464-484, strand=
target 5' AAGACUUCCCGUCGGACCCAA 3'
:::::::::::::::::::::
query 3' UUCUGAAGGGCAGCCUGGGUU 5'
query=miR1146, target=RO3G_16807, score=0, range=464-484, strand=
target 5' AAGACUUCCCGUCGGACCCAA 3'
:::::::::::::::::::::
query 3' UUCUGAAGGGCAGCCUGGGUU 5'
query=miR1146, target=RO3G_17382, score=0, range=464-484, strand=
target 5' AAGACUUCCCGUCGGACCCAA 3'
:::::::::::::::::::::
query 3' UUCUGAAGGGCAGCCUGGGUU 5'
query=miR1146, target=RO3G_02689, score=0, range=464-484, strand=
target 5' AAGACUUCCCGUCGGACCCAA 3'
:::::::::::::::::::::
query 3' UUCUGAAGGGCAGCCUGGGUU 5'
query=miR1146, target=RO3G_01322, score=0, range=350-370, strand=
target 5' AAGACUUCCCGUCGGACCCAA 3'
```

```
:::::::::::::::::::::
query 3' UUCUGAAGGGCAGCCUGGGUU 5'
query=miR1146, target=RO3G_17400, score=0, range=338-358, strand=
target 5' AAGACUUCCCGUCGGACCCAA 3'
:::::::::::::::::::::
query 3' UUCUGAAGGGCAGCCUGGGUU 5'
query=miR1146, target=RO3G_05915, score=0, range=464-484, strand=
target 5' AAGACUUCCCGUCGGACCCAA 3'
:::::::::::::::::::::
query 3' UUCUGAAGGGCAGCCUGGGUU 5'
query=miR1146, target=RO3G_01730, score=0, range=464-484, strand=
target 5' AAGACUUCCCGUCGGACCCAA 3'
:::::::::::::::::::::
query 3' UUCUGAAGGGCAGCCUGGGUU 5'
query=miR1146, target=RO3G_13720, score=0, range=341-361, strand=
target 5' AAGACUUCCCGUCGGACCCAA 3'
:::::::::::::::::::::
query 3' UUCUGAAGGGCAGCCUGGGUU 5'
query=miR1146, target=RO3G_16897, score=0, range=464-484, strand=
target 5' AAGACUUCCCGUCGGACCCAA 3'
:::::::::::::::::::::
query 3' UUCUGAAGGGCAGCCUGGGUU 5'
query=miR1146, target=RO3G_17285, score=0.5, range=464-484, stran
target 5' AAGGCUUCCCGUCGGACCCAA 3'
:::.:::::::::::::::::
query 3' UUCUGAAGGGCAGCCUGGGUU 5'
query=miR1146, target=RO3G_01652, score=0.5, range=464-484, stran
target 5' AAGACUUUCCGUCGGACCCAA 3'
:::::::.:::::::::::::
query 3' UUCUGAAGGGCAGCCUGGGUU 5'
query=miR1146, target=RO3G_01956, score=0.5, range=464-484, stran
target 5' AAGGCUUCCCGUCGGACCCAA 3'
:::.:::::::::::::::::
query 3' UUCUGAAGGGCAGCCUGGGUU 5'
query=miR1146, target=RO3G_08846, score=0.5, range=464-484, stran
target 5' AAGACUUUCCGUCGGACCCAA 3'
:::::::.:::::::::::::
query 3' UUCUGAAGGGCAGCCUGGGUU 5'
query=miR1146, target=RO3G_03571, score=0.5, range=464-484, stran
target 5' AAGACUUUCCGUCGGACCCAA 3'
:::::::.:::::::::::::
query 3' UUCUGAAGGGCAGCCUGGGUU 5'
query=miR1146, target=RO3G_05635, score=0.5, range=464-484, stran
target 5' AAGACUUUCCGUCGGACCCAA 3'
:::::::.:::::::::::::
```

```
query 3' UUCUGAAGGGCAGCCUGGGUU 5'
query=miR1146, target=RO3G_13848, score=0.5, range=464-484, stran
target 5' AAGACUUUCCGUCGGACCCAA 3'
:::::::.:::::::::::::
query 3' UUCUGAAGGGCAGCCUGGGUU 5'
query=miR1146, target=RO3G_09607, score=0.5, range=464-484, stran
target 5' AAGACUUUCCGUCGGACCCAA 3'
:::::::.:::::::::::::
query 3' UUCUGAAGGGCAGCCUGGGUU 5'
query=miR1146, target=RO3G_05622, score=0.5, range=464-484, stran
target 5' AAGACUUUCCGUCGGACCCAA 3'
:::::::.:::::::::::::
query 3' UUCUGAAGGGCAGCCUGGGUU 5'
query=miR1146, target=RO3G_13742, score=0.5, range=464-484, stran
target 5' AAGACUUUCCGUCGGACCCAA 3'
:::::::.:::::::::::::
query 3' UUCUGAAGGGCAGCCUGGGUU 5'
query=miR1146, target=RO3G_07704, score=0.5, range=464-484, stran
target 5' AAGACUUUCCGUCGGACCCAA 3'
:::::::.:::::::::::::
query 3' UUCUGAAGGGCAGCCUGGGUU 5'
query=miR1146, target=RO3G_13151, score=1, range=464-484, strand=
target 5' AAGACUUCCUGUCGGACCCAA 3'
:::::::::.:::::::::::
query 3' UUCUGAAGGGCAGCCUGGGUU 5'
query=miR1146, target=RO3G_11773, score=1, range=464-484, strand=
target 5' AAGAUUUUCCGUCGGACCCAA 3'
::::.::.:::::::::::::
query 3' UUCUGAAGGGCAGCCUGGGUU 5'
query=miR1146, target=RO3G_16551, score=1, range=464-484, strand=
target 5' AAGAUUUUCCGUCGGACCCAA 3'
::::.::.:::::::::::::
query 3' UUCUGAAGGGCAGCCUGGGUU 5'
query=miR1146, target=RO3G_16555, score=1, range=464-484, strand=
target 5' AAGAUUUUCCGUCGGACCCAA 3'
::::.::.:::::::::::::
query 3' UUCUGAAGGGCAGCCUGGGUU 5'
query=miR1146, target=RO3G_03615, score=1, range=464-484, strand=
target 5' AAGUCUUCCCGUCGGACCCAA 3'
::: :::::::::::::::::
query 3' UUCUGAAGGGCAGCCUGGGUU 5'
query=miR1146, target=RO3G_13726, score=1.5, range=464-484, stran
target 5' UAGACUUUCCGUCGGACCCAA 3'
::::::.:::::::::::::
query 3' UUCUGAAGGGCAGCCUGGGUU 5'
```

```
query=miR1146, target=RO3G_08661, score=2, range=464-484, strand=
target 5' AAGGCUUUUCGUCGGACCCAA 3'
:::.:::..::::::::::::
query 3' UUCUGAAGGGCAGCCUGGGUU 5'
query=miR1146, target=RO3G_05699, score=4, range=81-101, strand=1
target 5' AAGACUUGUUGUUGGACCCAA 3'
::::::: ..::.:::::::
query 3' UUCUGAAGGGCAGCCUGGGUU 5'
query=miR5219, target=RO3G_04102, score=0, range=292-313, strand=
target 5' ACGCAGCAGCUGAGAGUCCAGA 3'
::::::::::::::::::::::
query 3' UGCGUCGUCGACUCUCAGGUCU 5'
query=miR5219, target=RO3G_17126, score=0, range=320-341, strand=
target 5' ACGCAGCAGCUGAGAGUCCAGA 3'
::::::::::::::::::::::
query 3' UGCGUCGUCGACUCUCAGGUCU 5'
query=miR5219, target=RO3G_09090, score=0, range=290-311, strand=
target 5' ACGCAGCAGCUGAGAGUCCAGA 3'
::::::::::::::::::::::
query 3' UGCGUCGUCGACUCUCAGGUCU 5'
query=miR5219, target=RO3G_07568, score=0, range=193-214, strand=
target 5' ACGCAGCAGCUGAGAGUCCAGA 3'
::::::::::::::::::::::
query 3' UGCGUCGUCGACUCUCAGGUCU 5'
query=miR5219, target=RO3G_02387, score=0.5, range=235-256, stran
target 5' AUGCAGCAGCUGAGAGUCCAGA 3'
:.::::::::::::::::::::
query 3' UGCGUCGUCGACUCUCAGGUCU 5'
query=miR5219, target=RO3G_15231, score=1, range=533-554, strand=
target 5' ACACAGCAGCUGAGAGUCCAGA 3'
:: :::::::::::::::::::
query 3' UGCGUCGUCGACUCUCAGGUCU 5'
query=miR5219, target=RO3G_10585, score=1, range=134-155, strand=
target 5' ACACAGCAGCUGAGAGUCCAGA 3'
:: :::::::::::::::::::
query 3' UGCGUCGUCGACUCUCAGGUCU 5'
query=miR5219, target=RO3G_08723, score=1, range=197-218, strand=
target 5' ACGCAGCAGCUGAGAGUCCGGA 3'
:::::::::::::::::::.::
query 3' UGCGUCGUCGACUCUCAGGUCU 5'
query=miR5219, target=RO3G_08544, score=1.5, range=139-160, stran
target 5' ACGUAUCAGCUGAGAGUCCAGA 3'
:::.: ::::::::::::::::
query 3' UGCGUCGUCGACUCUCAGGUCU 5'
query=miR5219, target=RO3G_10900, score=2, range=89-110, strand=1
```

```
target 5' ACGCAGCAGCUGAGAAUCCAGA 3'
::::::::::::::: ::::::
query 3' UGCGUCGUCGACUCUCAGGUCU 5'
query=miR5219, target=RO3G_12762, score=2, range=290-311, strand=
target 5' ACGCAGCAGCUGAGAGUCGAGA 3'
:::::::::::::::::: :::
query 3' UGCGUCGUCGACUCUCAGGUCU 5'
query=miR5219, target=RO3G_10892, score=2, range=131-152, strand=
target 5' ACGCAGCAGCUGAGAAUCCAGA 3'
::::::::::::::: ::::::
query 3' UGCGUCGUCGACUCUCAGGUCU 5'
query=miR5219, target=RO3G_09875, score=2, range=193-214, strand=
target 5' ACACAGCAGUUGAGAGUCCAGA 3'
:: ::::::.::::::::::::
query 3' UGCGUCGUCGACUCUCAGGUCU 5'
query=miR5219, target=RO3G_03449, score=2, range=116-137, strand=
target 5' GCACAGCAGCUGAGAGUCCAGG 3'
.: ::::::::::::::::::.
query 3' UGCGUCGUCGACUCUCAGGUCU 5'
query=miR5219, target=RO3G_06453, score=2.5, range=136-157, stran
target 5' ACAUAGCAGCUGAGGGUCCAGA 3'
:: .::::::::::.:::::::
query 3' UGCGUCGUCGACUCUCAGGUCU 5'
query=miR5219, target=RO3G_01907, score=3.5, range=103-124, stran
target 5' GCAUAGUAGCUGAGAGUUCAGA 3'
.: .::.::::::::::.::::
query 3' UGCGUCGUCGACUCUCAGGUCU 5'
query=miR7729a-3p, target=RO3G_14637, score=0, range=73-94, stran
target 5' UCCUCCAACAACCCCAUUGCUA 3'
::::::::::::::::::::::
query 3' AGGAGGUUGUUGGGGUAACGAU 5'
query=miR7729a-3p, target=RO3G_01201, score=0, range=73-94, stran
target 5' UCCUCCAACAACCCCAUUGCUA 3'
::::::::::::::::::::::
query 3' AGGAGGUUGUUGGGGUAACGAU 5'
query=miR7729a-3p, target=RO3G_12005, score=1, range=73-94, stran
target 5' UCCUCCAACAACCCCAUUGUUA 3'
:::::::::::::::::::.::
query 3' AGGAGGUUGUUGGGGUAACGAU 5'
query=miR7729a-3p, target=RO3G_15157, score=1, range=73-94, stran
target 5' UCCUCCAACAACCCCAUUGUUA 3'
:::::::::::::::::::.::
query 3' AGGAGGUUGUUGGGGUAACGAU 5'
query=miR7729a-3p, target=RO3G_05928, score=1, range=73-94, stran
target 5' UCCUCCAACAACCCCAUUGUUA 3'
```

```
:::::::::::::::::::.::
query 3' AGGAGGUUGUUGGGGUAACGAU 5'
query=miR7729a-3p, target=RO3G_12079, score=1, range=73-94, stran
target 5' UCCUCCAACAACCCCAUUGUUA 3'
:::::::::::::::::::.::
query 3' AGGAGGUUGUUGGGGUAACGAU 5'
query=miR7729a-3p, target=RO3G_12179, score=1, range=73-94, stran
target 5' UCCUCCAACAACCCCAUUGUUA 3'
:::::::::::::::::::.::
query 3' AGGAGGUUGUUGGGGUAACGAU 5'
query=miR7729a-3p, target=RO3G_13735, score=1, range=73-94, stran
target 5' UCCUCCAACAACCCCAUUGUUA 3'
:::::::::::::::::::.::
query 3' AGGAGGUUGUUGGGGUAACGAU 5'
query=miR7729a-3p, target=RO3G_04676, score=3, range=73-94, stran
target 5' UCCUCCAACAACCCAAUUGUUA 3'
:::::::::::::: ::::.::
query 3' AGGAGGUUGUUGGGGUAACGAU 5'
query=miR7729a-3p, target=RO3G_07676, score=4, range=73-94, stran
target 5' UCCCCCAACAGCCCUAUUGUUA 3'
::: ::::::.:::.::::.::
query 3' AGGAGGUUGUUGGGGUAACGAU 5'
query=miR7729a-3p, target=RO3G_01968, score=4, range=73-94, stran
target 5' UCCCCCAACAGCCCUAUUGUUA 3'
::: ::::::.:::.::::.::
query 3' AGGAGGUUGUUGGGGUAACGAU 5'
query=miR7729a-3p, target=RO3G_07678, score=4, range=73-94, stran
target 5' UCCCCCAACAGCCCUAUUGUUA 3'
::: ::::::.:::.::::.::
query 3' AGGAGGUUGUUGGGGUAACGAU 5'
query=miR7729a-3p, target=RO3G_07110, score=4, range=73-94, stran
target 5' UCCCCCAACAGCCCUAUUGUUA 3'
::: ::::::.:::.::::.::
query 3' AGGAGGUUGUUGGGGUAACGAU 5'
query=miR7729a-3p, target=RO3G_17380, score=4, range=73-94, stran
target 5' UCCCCCAACAGCCCUAUUGUUA 3'
::: ::::::.:::.::::.::
query 3' AGGAGGUUGUUGGGGUAACGAU 5'
query=miR7729a-3p, target=RO3G_01949, score=4, range=73-94, stran
target 5' UCCCCCAACAGCCCUAUUGUUA 3'
::: ::::::.:::.::::.::
query 3' AGGAGGUUGUUGGGGUAACGAU 5'
query=miR7729a-3p, target=RO3G_03767, score=4, range=73-94, stran
target 5' UCCCCCAACAGCCCUAUUGUUA 3'
::: ::::::.:::.::::.::
```

```
query 3' AGGAGGUUGUUGGGGUAACGAU 5'
query=miR7729a-3p, target=RO3G_05523, score=4, range=73-94, stran
target 5' UCCCCCAACAGCCCUAUUGUUA 3'
::: ::::::.:::.::::.::
query 3' AGGAGGUUGUUGGGGUAACGAU 5'
query=miR7729a-3p, target=RO3G_10531, score=4, range=73-94, stran
target 5' UCCCCCAACAGCCCUAUUGUUA 3'
::: ::::::.:::.::::.::
query 3' AGGAGGUUGUUGGGGUAACGAU 5'
query=miR7729a-3p, target=RO3G_10525, score=4, range=73-94, stran
target 5' UCCCCCAACAGCCCUAUUGUUA 3'
::: ::::::.:::.::::.::
query 3' AGGAGGUUGUUGGGGUAACGAU 5'
query=miR7729a-3p, target=RO3G_07868, score=4, range=73-94, stran
target 5' UCCCCCAACAGCCCUAUUGUUA 3'
::: ::::::.:::.::::.::
query 3' AGGAGGUUGUUGGGGUAACGAU 5'
query=miR7729a-3p, target=RO3G_10545, score=4, range=73-94, stran
target 5' UCCCCCAACAGCCCUAUUGUUA 3'
::: ::::::.:::.::::.::
query 3' AGGAGGUUGUUGGGGUAACGAU 5'
query=miR7729a-3p, target=RO3G_00430, score=4, range=52-73, stran
target 5' UCUUCCAACACCUCCAUUGCUG 3'
::.::::::: :.::::::::.
query 3' AGGAGGUUGUUGGGGUAACGAU 5'
query=miR7729a-3p, target=RO3G_12957, score=4, range=73-94, stran
target 5' UCCCCCAACAGCCCUAUUGUUA 3'
::: ::::::.:::.::::.::
query 3' AGGAGGUUGUUGGGGUAACGAU 5'
query=miR7729a-3p, target=RO3G_16712, score=4, range=73-94, stran
target 5' UCCCCCAACAGCCCUAUUGUUA 3'
::: ::::::.:::.::::.::
query 3' AGGAGGUUGUUGGGGUAACGAU 5'
query=miR7729a-3p, target=RO3G_16734, score=4, range=73-94, stran
target 5' UCCCCCAACAGCCCUAUUGUUA 3'
::: ::::::.:::.::::.::
query 3' AGGAGGUUGUUGGGGUAACGAU 5'
query=miR7729a-3p, target=RO3G_12972, score=4, range=73-94, stran
target 5' UCCCCCAACAGCCCUAUUGUUA 3'
::: ::::::.:::.::::.::
query 3' AGGAGGUUGUUGGGGUAACGAU 5'
query=miR8762d, target=RO3G_10771, score=3, range=484-505, strand
target 5' AAACAUCGAUCAAAGUUCACAA 3'
: :::::::::::::: ::::
query 3' UCUGUAGCUAGUUUCAAAUGUU 5'
```

```
query=miR8762d, target=RO3G_07739, score=3, range=484-505, strand
target 5' AAACAUCGAUCAAAGUUCACAA 3'
: :::::::::::::: ::::
query 3' UCUGUAGCUAGUUUCAAAUGUU 5'
query=miR8762d, target=RO3G_13852, score=3, range=484-505, strand
target 5' AAACAUCGAUCAAAGUUCACAA 3'
: :::::::::::::: ::::
query 3' UCUGUAGCUAGUUUCAAAUGUU 5'
query=miR8762d, target=RO3G_05953, score=4, range=733-754, strand
target 5' AAACACCGAUCAAAGUCUACAA 3'
: ::: ::::::::: :::::
query 3' UCUGUAGCUAGUUUCAAAUGUU 5'
query=miR8762d, target=RO3G_17449, score=4, range=733-754, strand
target 5' AAACACCGAUCAAAGUCUACAA 3'
: ::: ::::::::: :::::
query 3' UCUGUAGCUAGUUUCAAAUGUU 5'
query=miR8762d, target=RO3G_05921, score=4, range=733-754, strand
target 5' AAACACCGAUCAAAGUCUACAA 3'
: ::: ::::::::: :::::
query 3' UCUGUAGCUAGUUUCAAAUGUU 5'
query=miR8762d, target=RO3G_10729, score=4, range=733-754, strand
target 5' AAACACCGAUCAAAGUCUACAA 3'
: ::: ::::::::: :::::
query 3' UCUGUAGCUAGUUUCAAAUGUU 5'
query=miR8762d, target=RO3G_11966, score=4, range=733-754, strand
target 5' AAACACCGAUCAAAGUCUACAA 3'
: ::: ::::::::: :::::
query 3' UCUGUAGCUAGUUUCAAAUGUU 5'
query=miR8762d, target=RO3G_05939, score=4, range=733-754, strand
target 5' AAACACCGAUCAAAGUCUACAA 3'
: ::: ::::::::: :::::
query 3' UCUGUAGCUAGUUUCAAAUGUU 5'
query=miR1051-3p, target=RO3G_06734, score=0, range=443-461, stra
target 5' GUGGGUUCAAGUACGGGAA 3'
:::::::::::::::::::
query 3' CACCCAAGUUCAUGCCCUU 5'
query=miR1051-3p, target=RO3G_16733, score=0, range=1139-1157,
strand=1
target 5' GUGGGUUCAAGUACGGGAA 3'
:::::::::::::::::::
query 3' CACCCAAGUUCAUGCCCUU 5'
query=miR1051-3p, target=RO3G_15270, score=0, range=188-206, stra
target 5' GUGGGUUCAAGUACGGGAA 3'
:::::::::::::::::::
query 3' CACCCAAGUUCAUGCCCUU 5'
```

```
query=miR1051-3p, target=RO3G_09180, score=0, range=1139-1157,
strand=1
target 5' GUGGGUUCAAGUACGGGAA 3'
:::::::::::::::::::
query 3' CACCCAAGUUCAUGCCCUU 5'
query=miR1051-3p, target=RO3G_16812, score=0, range=1139-1157,
strand=1
target 5' GUGGGUUCAAGUACGGGAA 3'
:::::::::::::::::::
query 3' CACCCAAGUUCAUGCCCUU 5'
query=miR1051-3p, target=RO3G_09189, score=0, range=1139-1157,
strand=1
target 5' GUGGGUUCAAGUACGGGAA 3'
:::::::::::::::::::
query 3' CACCCAAGUUCAUGCCCUU 5'
query=miR1051-3p, target=RO3G_14798, score=0, range=1139-1157,
strand=1
target 5' GUGGGUUCAAGUACGGGAA 3'
:::::::::::::::::::
query 3' CACCCAAGUUCAUGCCCUU 5'
query=miR1051-3p, target=RO3G_14791, score=0, range=1139-1157,
strand=1
target 5' GUGGGUUCAAGUACGGGAA 3'
:::::::::::::::::::
query 3' CACCCAAGUUCAUGCCCUU 5'
query=miR1051-3p, target=RO3G_17260, score=1, range=1018-1036,
strand=1
target 5' GUGGGUUCAAGUAUGGGAA 3'
:::::::::::::.:::::
query 3' CACCCAAGUUCAUGCCCUU 5'
query=miR1051-3p, target=RO3G_13789, score=4, range=2531-2548,
strand=1
target 5' GUGGG-GCAAGUACGGGAC 3'
::::: :::::::::::
query 3' CACCCAAGUUCAUGCCCUU 5'
query=miR1051-3p, target=RO3G_09447, score=4, range=110-127, stra
target 5' GU-GGUUCAAGUAUUGGAA 3'
:: ::::::::::. ::::
query 3' CACCCAAGUUCAUGCCCUU 5'
query=miR1051-3p, target=RO3G_01211, score=4, range=810-827, stra
target 5' GUGGGAUCAA-UACGGGAU 3'
::::: :::: :::::::
query 3' CACCCAAGUUCAUGCCCUU 5'
query=miR1216, target=RO3G_14801, score=0, range=97-116, strand=1
target 5' GUACAAGCGCUAACCCAUCA 3'
```

```
::::::::::::::::::::
query 3' CAUGUUCGCGAUUGGGUAGU 5'
query=miR1216, target=RO3G_14788, score=0, range=97-116, strand=1
target 5' GUACAAGCGCUAACCCAUCA 3'
::::::::::::::::::::
query 3' CAUGUUCGCGAUUGGGUAGU 5'
query=miR1216, target=RO3G_17261, score=0, range=164-183, strand=
target 5' GUACAAGCGCUAACCCAUCA 3'
::::::::::::::::::::
query 3' CAUGUUCGCGAUUGGGUAGU 5'
query=miR1216, target=RO3G_09190, score=0, range=164-183, strand=
target 5' GUACAAGCGCUAACCCAUCA 3'
::::::::::::::::::::
query 3' CAUGUUCGCGAUUGGGUAGU 5'
query=miR1216, target=RO3G_09181, score=0, range=164-183, strand=
target 5' GUACAAGCGCUAACCCAUCA 3'
::::::::::::::::::::
query 3' CAUGUUCGCGAUUGGGUAGU 5'
query=miR1216, target=RO3G_16732, score=0, range=164-183, strand=
target 5' GUACAAGCGCUAACCCAUCA 3'
::::::::::::::::::::
query 3' CAUGUUCGCGAUUGGGUAGU 5'
query=miR1216, target=RO3G_14792, score=0, range=164-183, strand=
target 5' GUACAAGCGCUAACCCAUCA 3'
::::::::::::::::::::
query 3' CAUGUUCGCGAUUGGGUAGU 5'
query=miR1216, target=RO3G_14799, score=0, range=164-183, strand=
target 5' GUACAAGCGCUAACCCAUCA 3'
::::::::::::::::::::
query 3' CAUGUUCGCGAUUGGGUAGU 5'
query=miR1216, target=RO3G_16813, score=0, range=164-183, strand=
target 5' GUACAAGCGCUAACCCAUCA 3'
::::::::::::::::::::
query 3' CAUGUUCGCGAUUGGGUAGU 5'
query=miR1216, target=RO3G_09818, score=4, range=432-452, strand=
target 5' GUACGAGUGGCUAACCUAUCA 3'
::::.::. :::::::.::::
query 3' CAUGUUCG-CGAUUGGGUAGU 5'
query=miR1216, target=RO3G_13574, score=4, range=219-237, strand=
target 5' GUAAAAGCGC-AACUCAUCA 3'
::: :::::: :::.:::::
query 3' CAUGUUCGCGAUUGGGUAGU 5'
query=miR2612, target=RO3G_13221, score=0, range=767-787, strand=
target 5' CUGAUACUACUGCCACUAUCA 3'
:::::::::::::::::::::
```

```
query 3' GACUAUGAUGACGGUGAUAGU 5'
query=miR2612, target=RO3G_04123, score=1, range=767-787, strand=
target 5' CUGAUACCACUGCCACUAUCA 3'
::::::: :::::::::::::
query 3' GACUAUGAUGACGGUGAUAGU 5'
query=miR2612, target=RO3G_04120, score=1, range=767-787, strand=
target 5' CUGAUACCACUGCCACUAUCA 3'
::::::: :::::::::::::
query 3' GACUAUGAUGACGGUGAUAGU 5'
query=miR2612, target=RO3G_13224, score=1, range=767-787, strand=
target 5' CUGAUACCACUGCCACUAUCA 3'
::::::: :::::::::::::
query 3' GACUAUGAUGACGGUGAUAGU 5'
query=miR2612, target=RO3G_17093, score=1, range=527-547, strand=
target 5' CUGAUACCACUGCCACUAUCA 3'
::::::: :::::::::::::
query 3' GACUAUGAUGACGGUGAUAGU 5'
query=miR2612, target=RO3G_11597, score=2.5, range=895-916, stran
target 5' UUGAUCACUACUGCCAUUAUCA 3'
.:::: :::::::::.:::::
query 3' GACUA-UGAUGACGGUGAUAGU 5'
query=miR2612, target=RO3G_01516, score=3, range=2025-2045, stran
target 5' UUGAUGCUGCUGCUACUAUCA 3'
.::::.::.:::.::::::::
query 3' GACUAUGAUGACGGUGAUAGU 5'
query=miR2612, target=RO3G_02911, score=3.5, range=113-133, stran
target 5' CUGAUGAUACUGCUGCUAUCA 3'
:::::. ::::::..::::::
query 3' GACUAUGAUGACGGUGAUAGU 5'
query=miR2612, target=RO3G_03005, score=3.5, range=107-127, stran
target 5' UUGUUAUUACUGCCACUAUUG 3'
.:: ::.:::::::::::..
query 3' GACUAUGAUGACGGUGAUAGU 5'
query=miR2612, target=RO3G_08542, score=3.5, range=237-256, stran
target 5' CUG-UGCUACUUCCACUAUCA 3'
::: :.::::: :::::::::
query 3' GACUAUGAUGACGGUGAUAGU 5'
query=miR2612, target=RO3G_02624, score=3.5, range=315-334, stran
target 5' UUGAUAC-ACUGCCGUUAUCA 3'
.:::::: ::::::..:::::
query 3' GACUAUGAUGACGGUGAUAGU 5'
query=miR2612, target=RO3G_08172, score=4, range=605-625, strand=
target 5' AUGAUACUAUUGCUGCUAUCA 3'
::::::::.:::..::::::
query 3' GACUAUGAUGACGGUGAUAGU 5'
```

```
query=miR2612, target=RO3G_02915, score=4, range=901-922, strand=
target 5' GUGAUAACUACUGCCAUUGUCA 3'
:::: :::::::::.:.:::
query 3' GACUA-UGAUGACGGUGAUAGU 5'
query=miR2612, target=RO3G_01852, score=4, range=164-183, strand=
target 5' CUGCUACUACUGCUACU-UCA 3'
::: :::::::::.::: :::
query 3' GACUAUGAUGACGGUGAUAGU 5'
query=miR2612, target=RO3G_04617, score=4, range=675-694, strand=
target 5' CUUUUACUACUGCCAC-AUCA 3'
:: ::::::::::: ::::
query 3' GACUAUGAUGACGGUGAUAGU 5'
query=miR6476a, target=RO3G_03666, score=4, range=110-128, strand
target 5' UCAU-UUCAUUACUUGCCGA 3'
:::: ::::.::.:.::::
query 3' AGUACAAGUAGUGGAUGGCU 5'
query=miR6476a, target=RO3G_04527, score=4, range=780-799, strand
target 5' UGAUGAUCAUCAUCUACUGA 3'
: ::: :::::.::::.::
query 3' AGUACAAGUAGUGGAUGGCU 5'
query=miR6476a, target=RO3G_05448, score=4, range=1076-1094, stra
target 5' UCAUGGUCAUCACCU-CUGA 3'
::::: ::::::::: :.::
query 3' AGUACAAGUAGUGGAUGGCU 5'
query=miR6476a, target=RO3G_10979, score=4, range=962-980, strand
target 5' UCAAG-GCAUCACCUACUGA 3'
::: : ::::::::::.::
query 3' AGUACAAGUAGUGGAUGGCU 5'
query=miR6476a, target=RO3G_00932, score=4, range=124-142, strand
target 5' UCGUG-UCAUCAUCUACUGG 3'
::.:: :::::.::::.:.
query 3' AGUACAAGUAGUGGAUGGCU 5'
query=miR1446, target=RO3G_12063, score=0, range=814-835, strand=
target 5' AUGAUGCGCAGAGAUUUCAGAA 3'
::::::::::::::::::::::
query 3' UACUACGCGUCUCUAAAGUCUU 5'
query=miR1446, target=RO3G_03622, score=0, range=1048-1069, stran
target 5' AUGAUGCGCAGAGAUUUCAGAA 3'
::::::::::::::::::::::
query 3' UACUACGCGUCUCUAAAGUCUU 5'
query=miR1446, target=RO3G_12187, score=0, range=1318-1339, stran
target 5' AUGAUGCGCAGAGAUUUCAGAA 3'
::::::::::::::::::::::
query 3' UACUACGCGUCUCUAAAGUCUU 5'
query=miR1446, target=RO3G_14986, score=0, range=1318-1339, stran
```

```
target 5' AUGAUGCGCAGAGAUUUCAGAA 3'
::::::::::::::::::::::
query 3' UACUACGCGUCUCUAAAGUCUU 5'
query=miR1446, target=RO3G_07714, score=0, range=1120-1141, stran
target 5' AUGAUGCGCAGAGAUUUCAGAA 3'
::::::::::::::::::::::
query 3' UACUACGCGUCUCUAAAGUCUU 5'
query=miR1446, target=RO3G_04206, score=0, range=367-388, strand=
target 5' AUGAUGCGCAGAGAUUUCAGAA 3'
::::::::::::::::::::::
query 3' UACUACGCGUCUCUAAAGUCUU 5'
query=miR1446, target=RO3G_13835, score=4, range=13844-13866, str
target 5' AUGCUGAACAAGAGAUUUCAGAA 3'
::: :: : ::::::::::::
query 3' UACUACGCG-UCUCUAAAGUCUU 5'
query=miR1446, target=RO3G_02903, score=4, range=318-339, strand=
target 5' UUGAAGCGCAGAGAGUUCAGAA 3'
::: ::::::::: :::::::
query 3' UACUACGCGUCUCUAAAGUCUU 5'
query=miR1446, target=RO3G_01098, score=4, range=108-128, strand=
target 5' GAAAUG-GUAGAGAUUUCAGAA 3'
. ::: :.:::::::::::::
query 3' UACUACGCGUCUCUAAAGUCUU 5'
query=miR5021, target=RO3G_00301, score=0, range=2541-2560, stran
target 5' UUUUCCUUCUUCUAUCGUCA 3'
::::::::::::::::::::
query 3' AAAAGGAAGAAGAUAGCAGU 5'
query=miR5021, target=RO3G_04050, score=0, range=1257-1276, stran
target 5' UUUUCCUUCUUCUAUCGUCA 3'
::::::::::::::::::::
query 3' AAAAGGAAGAAGAUAGCAGU 5'
query=miR5021, target=RO3G_12949, score=0, range=702-721, strand=
target 5' UUUUCCUUCUUCUAUCGUCA 3'
::::::::::::::::::::
query 3' AAAAGGAAGAAGAUAGCAGU 5'
query=miR5021, target=RO3G_06744, score=3, range=972-992, strand=
target 5' UUUUCCUUCUUUUCAUCGUCA 3'
:::::::::::.: :::::::
query 3' AAAAGGAAGAAGA-UAGCAGU 5'
query=miR5021, target=RO3G_14233, score=3.5, range=1104-1124, str
target 5' UUUUCCUUCUUCUAUUGAUCG 3'
::::::::::::::.: ::.
query 3' AAAAGGAAGAAGAUAGC-AGU 5'
query=miR5021, target=RO3G_00801, score=3.5, range=857-876, stran
target 5' UUUUCUUUCUUCAGUCGUCA 3'
```

```
::::.:::::: .::::::
query 3' AAAAGGAAGAAGAUAGCAGU 5'
query=miR5021, target=RO3G_09998, score=3.5, range=132-151, stran
target 5' UUUUUCUUCUUCUGUCUUCA 3'
::::.:::::::.:: :::
query 3' AAAAGGAAGAAGAUAGCAGU 5'
query=miR5021, target=RO3G_15993, score=3.5, range=162-181, stran
target 5' UAUUCUUUCUUUUAUUGUCA 3'
: :::.::::.:::.::::
query 3' AAAAGGAAGAAGAUAGCAGU 5'
query=miR5021, target=RO3G_06767, score=3.5, range=2738-2757,
strand=-1
target 5' UUCUUCUUCUUCUAUUGUCU 3'
:: :.:::::::::.:::
query 3' AAAAGGAAGAAGAUAGCAGU 5'
query=miR5021, target=RO3G_16410, score=3.5, range=890-909, stran
target 5' UUCUUCUUCUUUUAUCGUCU 3'
:: :.::::::.:::::::
query 3' AAAAGGAAGAAGAUAGCAGU 5'
query=miR5021, target=RO3G_03413, score=3.5, range=590-609, stran
target 5' UCUUUCUUCUUCUAUCGUUU 3'
: ::.:::::::::::::.
query 3' AAAAGGAAGAAGAUAGCAGU 5'
query=miR5021, target=RO3G_04712, score=3.5, range=74-93, strand=
target 5' UAUAUCUUCUUCUGUCGUCA 3'
: : .::::::::.::::::
query 3' AAAAGGAAGAAGAUAGCAGU 5'
query=miR5021, target=RO3G_08238, score=3.5, range=992-1011, stra
target 5' UUGCUCUUCUUCUGUCGUCA 3'
:: .::::::::.::::::
query 3' AAAAGGAAGAAGAUAGCAGU 5'
query=miR5021, target=RO3G_14008, score=3.5, range=792-810, stran
target 5' CUUUUCUUCUUC-AUCGUCA 3'
:::.::::::: :::::::
query 3' AAAAGGAAGAAGAUAGCAGU 5'
query=miR5021, target=RO3G_13112, score=3.5, range=1851-1869,
strand=-1
target 5' UUCUUCUUCUUCUA-CGUCA 3'
:: :.::::::::: :::::
query 3' AAAAGGAAGAAGAUAGCAGU 5'
query=miR5021, target=RO3G_10608, score=3.5, range=9639-9659,
strand=-1
target 5' AUUUCUUUCUUCUUAUCGUCA 3'
::::.:::::: ::::::::
query 3' AAAAGGAAGAAG-AUAGCAGU 5'
```

```
query=miR5021, target=RO3G_06189, score=3.5, range=87-105, strand
target 5' UUGUUCUUCUUC-AUCGUCA 3'
:: :.::::::: :::::::
query 3' AAAAGGAAGAAGAUAGCAGU 5'
query=miR5021, target=RO3G_10250, score=4, range=237-256, strand=
target 5' UUUUCCUUCUUCCAUAGUCA 3'
:::::::::::: :: ::::
query 3' AAAAGGAAGAAGAUAGCAGU 5'
query=miR5021, target=RO3G_03019, score=4, range=209-228, strand=
target 5' AUUUUUUUCUUCUGUUGUCA 3'
:::..:::::::.:.::::
query 3' AAAAGGAAGAAGAUAGCAGU 5'
query=miR5021, target=RO3G_12821, score=4, range=812-831, strand=
target 5' UCUUACUUUUUCUGUCGUCA 3'
: :: :::.::::.::::::
query 3' AAAAGGAAGAAGAUAGCAGU 5'
query=miR5021, target=RO3G_09002, score=4, range=881-900, strand=
target 5' AUUACCUUCUUCAAUCGUCA 3'
:: :::::::: :::::::
query 3' AAAAGGAAGAAGAUAGCAGU 5'
query=miR5021, target=RO3G_13930, score=4, range=338-356, strand=
target 5' UCUUCUUUCUU-UAUCGUCG 3'
: :::.::::: :::::::.
query 3' AAAAGGAAGAAGAUAGCAGU 5'
query=miR5021, target=RO3G_07645, score=4, range=494-513, strand=
target 5' CUUGACUUCUUCUAUCGUCU 3'
:: ::::::::::::::
query 3' AAAAGGAAGAAGAUAGCAGU 5'
query=miR5021, target=RO3G_03189, score=4, range=365-385, strand=
target 5' AAUUCACAUCUUCUAUCGUCA 3'
::: : :::::::::::::
query 3' AAAAG-GAAGAAGAUAGCAGU 5'
query=miR5021, target=RO3G_09319, score=4, range=558-576, strand=
target 5' UUCUACUUCUUCUAU-GUCA 3'
:: : :::::::::: ::::
query 3' AAAAGGAAGAAGAUAGCAGU 5'
query=miR6025e, target=RO3G_16742, score=0, range=601-622, strand
target 5' GCAUGUCAGAUCUAUAAUGGCA 3'
::::::::::::::::::::::
query 3' CGUACAGUCUAGAUAUUACCGU 5'
query=miR6025e, target=RO3G_02824, score=0, range=601-622, strand
target 5' GCAUGUCAGAUCUAUAAUGGCA 3'
::::::::::::::::::::::
query 3' CGUACAGUCUAGAUAUUACCGU 5'
query=miR6025e, target=RO3G_17416, score=0, range=601-622, strand
```

```
target 5' GCAUGUCAGAUCUAUAAUGGCA 3'
::::::::::::::::::::::
query 3' CGUACAGUCUAGAUAUUACCGU 5'
query=miR6025e, target=RO3G_17291, score=0, range=601-622, strand
target 5' GCAUGUCAGAUCUAUAAUGGCA 3'
::::::::::::::::::::::
query 3' CGUACAGUCUAGAUAUUACCGU 5'
query=miR6025e, target=RO3G_13714, score=0, range=601-622, strand
target 5' GCAUGUCAGAUCUAUAAUGGCA 3'
::::::::::::::::::::::
query 3' CGUACAGUCUAGAUAUUACCGU 5'
query=miR6025e, target=RO3G_16372, score=0, range=601-622, strand
target 5' GCAUGUCAGAUCUAUAAUGGCA 3'
::::::::::::::::::::::
query 3' CGUACAGUCUAGAUAUUACCGU 5'
query=miR6025e, target=RO3G_17355, score=0, range=436-457, strand
target 5' GCAUGUCAGAUCUAUAAUGGCA 3'
::::::::::::::::::::::
query 3' CGUACAGUCUAGAUAUUACCGU 5'
query=miR6025e, target=RO3G_01962, score=0, range=352-373, strand
target 5' GCAUGUCAGAUCUAUAAUGGCA 3'
::::::::::::::::::::::
query 3' CGUACAGUCUAGAUAUUACCGU 5'
query=miR6025e, target=RO3G_16706, score=0, range=325-346, strand
target 5' GCAUGUCAGAUCUAUAAUGGCA 3'
::::::::::::::::::::::
query 3' CGUACAGUCUAGAUAUUACCGU 5'
query=miR6025e, target=RO3G_09193, score=0, range=325-346, strand
target 5' GCAUGUCAGAUCUAUAAUGGCA 3'
::::::::::::::::::::::
query 3' CGUACAGUCUAGAUAUUACCGU 5'
query=miR6025e, target=RO3G_17223, score=0, range=325-346, strand
target 5' GCAUGUCAGAUCUAUAAUGGCA 3'
::::::::::::::::::::::
query 3' CGUACAGUCUAGAUAUUACCGU 5'
query=miR6025e, target=RO3G_04682, score=0, range=601-622, strand
target 5' GCAUGUCAGAUCUAUAAUGGCA 3'
::::::::::::::::::::::
query 3' CGUACAGUCUAGAUAUUACCGU 5'
query=miR6025e, target=RO3G_05640, score=1, range=496-517, strand
target 5' GCAUGUCAGAUCUAUGAUGGCA 3'
::::::::::::::::.:::::
query 3' CGUACAGUCUAGAUAUUACCGU 5'
query=miR6025e, target=RO3G_07705, score=1, range=601-622, strand
target 5' GCAUGUCAGAUCUAUAAUGGUA 3'
```

```
::::::::::::::::::::.:
query 3' CGUACAGUCUAGAUAUUACCGU 5'
query=miR6025e, target=RO3G_17387, score=1, range=601-622, strand
target 5' GCAUGUCAGAUCUAUGAUGGCA 3'
:::::::::::::::.::::::
query 3' CGUACAGUCUAGAUAUUACCGU 5'
query=miR6025e, target=RO3G_16123, score=1, range=601-622, strand
target 5' GCAUGUCAGAUCUAUGAUGGCA 3'
:::::::::::::::.::::::
query 3' CGUACAGUCUAGAUAUUACCGU 5'
query=miR6025e, target=RO3G_06717, score=1, range=601-622, strand
target 5' GCAUGUCAGAUCUAUGAUGGCA 3'
:::::::::::::::.::::::
query 3' CGUACAGUCUAGAUAUUACCGU 5'
query=miR6025e, target=RO3G_07137, score=1, range=601-622, strand
target 5' GCAUGUCAGAUCUAUGAUGGCA 3'
:::::::::::::::.::::::
query 3' CGUACAGUCUAGAUAUUACCGU 5'
query=miR6025e, target=RO3G_01328, score=1, range=601-622, strand
target 5' GCAUGUCAGAUCUAUGAUGGCA 3'
:::::::::::::::.::::::
query 3' CGUACAGUCUAGAUAUUACCGU 5'
query=miR6025e, target=RO3G_05268, score=1, range=601-622, strand
target 5' GCAUGUCAGAUCUAUGAUGGCA 3'
:::::::::::::::.::::::
query 3' CGUACAGUCUAGAUAUUACCGU 5'
query=miR6025e, target=RO3G_04674, score=1, range=352-373, strand
target 5' GCAUGUCAGAUCUAUGAUGGCA 3'
:::::::::::::::.::::::
query 3' CGUACAGUCUAGAUAUUACCGU 5'
query=miR6025e, target=RO3G_14770, score=1, range=601-622, strand
target 5' GCAUGUCAGAUCUAUAAUGGUA 3'
::::::::::::::::::::.:
query 3' CGUACAGUCUAGAUAUUACCGU 5'
query=miR6025e, target=RO3G_11781, score=1, range=496-517, strand
target 5' GCAUGUCAGAUCUAUAAUGGUA 3'
::::::::::::::::::::.:
query 3' CGUACAGUCUAGAUAUUACCGU 5'
query=miR6025e, target=RO3G_07702, score=1, range=352-373, strand
target 5' GCAUGUCAGAUCUAUAAUGGUA 3'
::::::::::::::::::::.:
query 3' CGUACAGUCUAGAUAUUACCGU 5'
query=miR6025e, target=RO3G_16420, score=1, range=352-373, strand
target 5' GCAUGUCAGAUCUAUAAUGGUA 3'
::::::::::::::::::::.:
```

query 3' CGUACAGUCUAGAUAUUACCGU 5'
query=miR6025e, target=RO3G_15282, score=1, range=325-346, strand
target 5' GCAUGUCAGAUCUAUAAUGGUA 3'
::::::::::::::::::::.:
query 3' CGUACAGUCUAGAUAUUACCGU 5'
query=miR6025e, target=RO3G_15261, score=1, range=601-622, strand
target 5' GCAUGUCAGAUCUAUGAUGGCA 3'
:::::::::::::::.::::::
query 3' CGUACAGUCUAGAUAUUACCGU 5'
query=miR6025e, target=RO3G_10795, score=1, range=601-622, strand
target 5' GCAUGUCAGAUCUAUAAUGGUA 3'
::::::::::::::::::::.:
query 3' CGUACAGUCUAGAUAUUACCGU 5'
query=miR6025e, target=RO3G_17017, score=1, range=601-622, strand
target 5' GCAUGUCAGAUCUAUAAUGGUA 3'
::::::::::::::::::::.:
query 3' CGUACAGUCUAGAUAUUACCGU 5'
query=miR6025e, target=RO3G_04695, score=1, range=391-412, strand
target 5' GCAUGUCAGAUCUAUGAUGGCA 3'
:::::::::::::::.::::::
query 3' CGUACAGUCUAGAUAUUACCGU 5'
query=miR6025e, target=RO3G_07867, score=1, range=352-373, strand
target 5' GCAUGUCAGAUCUAUGAUGGCA 3'
:::::::::::::::.::::::
query 3' CGUACAGUCUAGAUAUUACCGU 5'
query=miR6025e, target=RO3G_11235, score=1, range=496-517, strand
target 5' GCAUGUCAGAUCUAUAAUGGUA 3'
::::::::::::::::::::.:
query 3' CGUACAGUCUAGAUAUUACCGU 5'
query=miR6025e, target=RO3G_06735, score=1, range=325-346, strand
target 5' GCAUGUCAGAUCUAUAAUGGUA 3'
::::::::::::::::::::.:
query 3' CGUACAGUCUAGAUAUUACCGU 5'
query=miR6025e, target=RO3G_14555, score=1, range=229-250, strand
target 5' GCAUGUCAGAUCUAUAAUGGUA 3'
::::::::::::::::::::.:
query 3' CGUACAGUCUAGAUAUUACCGU 5'
query=miR6025e, target=RO3G_06417, score=1, range=229-250, strand
target 5' GCAUGUCAGAUCUAUAAUGGUA 3'
::::::::::::::::::::.:
query 3' CGUACAGUCUAGAUAUUACCGU 5'
query=miR6025e, target=RO3G_14786, score=1, range=229-250, strand
target 5' GCAUGUCAGAUCUAUAAUGGUA 3'
::::::::::::::::::::.:
query 3' CGUACAGUCUAGAUAUUACCGU 5'

```
query=miR6025e, target=RO3G_15271, score=2, range=601-622, strand
target 5' GCAUGUCAGAUCUAUGAUGGUA 3'
:::::::::::::::.::::.:
query 3' CGUACAGUCUAGAUAUUACCGU 5'
query=miR6025e, target=RO3G_07739, score=2, range=352-373, strand
target 5' GCAUGUCAGAUCUAUGAUGGUA 3'
:::::::::::::::.::::.:
query 3' CGUACAGUCUAGAUAUUACCGU 5'
query=miR6025e, target=RO3G_16725, score=2, range=343-364, strand
target 5' GCAUGUCAGAUCUAUGAUGGUA 3'
:::::::::::::::.::::.:
query 3' CGUACAGUCUAGAUAUUACCGU 5'
query=miR6025e, target=RO3G_06433, score=2, range=325-346, strand
target 5' GCAUGUCAGAUUUAUAAUGGUA 3'
:::::::::::.::::::::.:
query 3' CGUACAGUCUAGAUAUUACCGU 5'
query=miR6025e, target=RO3G_10779, score=2, range=217-238, strand
target 5' GCAUGUCAGAUUUAUAAUGGUA 3'
:::::::::::.::::::::.:
query 3' CGUACAGUCUAGAUAUUACCGU 5'
query=miR6025e, target=RO3G_17277, score=2, range=85-106, strand=
target 5' GCAUGUCAGAUUUAUAAUGGUA 3'
:::::::::::.::::::::.:
query 3' CGUACAGUCUAGAUAUUACCGU 5'
query=miR6025e, target=RO3G_05632, score=2, range=286-307, strand
target 5' GCAUGUCAGAUCUAUAAUGGGA 3'
:::::::::::::::::::: :
query 3' CGUACAGUCUAGAUAUUACCGU 5'
query=miR6025e, target=RO3G_03591, score=2.5, range=217-238, stra
target 5' GCAUGUCGGAUUUAUAAUGGUA 3'
:::::::.:::.::::::::.:
query 3' CGUACAGUCUAGAUAUUACCGU 5'
query=miR6025e, target=RO3G_10771, score=3, range=352-373, strand
target 5' ACAUGUCAGAUCUAUGAUGGUA 3'
::::::::::::::.::::.:
query 3' CGUACAGUCUAGAUAUUACCGU 5'
query=miR6025e, target=RO3G_13852, score=3, range=352-373, strand
target 5' ACAUGUCAGAUCUAUGAUGGUA 3'
::::::::::::::.::::.:
query 3' CGUACAGUCUAGAUAUUACCGU 5'
query=miR6025e, target=RO3G_04207, score=3, range=601-622, strand
target 5' GCAUGUCAGAUAUAUGAUGGCA 3'
::::::::::: :::.::::::
query 3' CGUACAGUCUAGAUAUUACCGU 5'
query=miR6025e, target=RO3G_17449, score=3, range=601-622, strand
```

```
target 5' GCAUGUCAGAUAUAUGAUGGCA 3'
:::::::::: :::.::::::
query 3' CGUACAGUCUAGAUAUUACCGU 5'
query=miR6025e, target=RO3G_05921, score=3, range=601-622, strand
target 5' GCAUGUCAGAUAUAUGAUGGCA 3'
:::::::::: :::.::::::
query 3' CGUACAGUCUAGAUAUUACCGU 5'
query=miR6025e, target=RO3G_14361, score=3, range=601-622, strand
target 5' GCAUGUCAGAUAUAUGAUGGCA 3'
:::::::::: :::.::::::
query 3' CGUACAGUCUAGAUAUUACCGU 5'
query=miR6025e, target=RO3G_01942, score=3, range=598-619, strand
target 5' GCAUGUCAGAUAUAUGAUGGCA 3'
:::::::::: :::.::::::
query 3' CGUACAGUCUAGAUAUUACCGU 5'
query=miR6025e, target=RO3G_17399, score=3, range=601-622, strand
target 5' GCAUGUCAGAUCUAUGAUGACA 3'
::::::::::::::.::: ::
query 3' CGUACAGUCUAGAUAUUACCGU 5'
query=miR6025e, target=RO3G_05939, score=3, range=601-622, strand
target 5' GCAUGUCAGAUGUAUGAUGGCA 3'
:::::::::: :::.::::::
query 3' CGUACAGUCUAGAUAUUACCGU 5'
query=miR6025e, target=RO3G_07124, score=3, range=601-622, strand
target 5' GCAUGUCAGAUGUAUGAUGGCA 3'
:::::::::: :::.::::::
query 3' CGUACAGUCUAGAUAUUACCGU 5'
query=miR6025e, target=RO3G_09945, score=3, range=325-346, strand
target 5' GCAUGUCAGAUAUAUGAUGGCA 3'
:::::::::: :::.::::::
query 3' CGUACAGUCUAGAUAUUACCGU 5'
query=miR6025e, target=RO3G_11264, score=3, range=601-622, strand
target 5' GCAUGUCAGAUAUAUGAUGGCA 3'
:::::::::: :::.::::::
query 3' CGUACAGUCUAGAUAUUACCGU 5'
query=miR6025e, target=RO3G_16689, score=3, range=601-622, strand
target 5' GCAUGUCAGAUAUAUGAUGGCA 3'
:::::::::: :::.::::::
query 3' CGUACAGUCUAGAUAUUACCGU 5'
query=miR6025e, target=RO3G_11962, score=3, range=325-346, strand
target 5' GCAUGUCAGAUCUCUAAUGGUA 3'
:::::::::::: ::::::.:
query 3' CGUACAGUCUAGAUAUUACCGU 5'
query=miR6025e, target=RO3G_09542, score=4, range=601-622, strand
target 5' GCAUGUCAGGUAUAUGAUGGCA 3'
```

```
:::::::::.: :::.::::::
query 3' CGUACAGUCUAGAUAUUACCGU 5'
query=miR6025e, target=RO3G_04210, score=4, range=601-622, strand
target 5' GCAUGUCAGGUGUAUGAUGGCA 3'
:::::::::.: :::.::::::
query 3' CGUACAGUCUAGAUAUUACCGU 5'
query=miR6025e, target=RO3G_10773, score=4, range=601-622, strand
target 5' GCAUGUCAGGUGUAUGAUGGCA 3'
:::::::::.: :::.::::::
query 3' CGUACAGUCUAGAUAUUACCGU 5'
query=miR6025e, target=RO3G_10780, score=4, range=601-622, strand
target 5' GCAUGUCAGGUGUAUGAUGGCA 3'
:::::::::.: :::.::::::
query 3' CGUACAGUCUAGAUAUUACCGU 5'
query=miR6025e, target=RO3G_14568, score=4, range=601-622, strand
target 5' GCAUGUCAGGUAUAUGAUGGCA 3'
:::::::::.: :::.::::::
query 3' CGUACAGUCUAGAUAUUACCGU 5'
query=miR6025e, target=RO3G_10729, score=4, range=601-622, strand
target 5' GCAUGUCAGGUAUAUGAUGGCA 3'
:::::::::.: :::.::::::
query 3' CGUACAGUCUAGAUAUUACCGU 5'
query=miR6025e, target=RO3G_12663, score=4, range=436-457, strand
target 5' GCAUGUCAGACUUAUGAUGGCA 3'
:::::::::: .:::.::::::
query 3' CGUACAGUCUAGAUAUUACCGU 5'
query=miR6025e, target=RO3G_10798, score=4, range=601-622, strand
target 5' GCAUGUCAGGUAUAUGAUGGCA 3'
:::::::::.: :::.::::::
query 3' CGUACAGUCUAGAUAUUACCGU 5'
query=miR6025e, target=RO3G_17214, score=4, range=601-622, strand
target 5' GCAUGUCAGGUGUAUGAUGGCA 3'
:::::::::.: :::.::::::
query 3' CGUACAGUCUAGAUAUUACCGU 5'
query=miR6025e, target=RO3G_04117, score=4, range=496-517, strand
target 5' GCAUGUCAGACCUAUGAUGGUA 3'
:::::::::: ::::.::::.:
query 3' CGUACAGUCUAGAUAUUACCGU 5'
query=miR6025e, target=RO3G_15866, score=4, range=325-346, strand
target 5' GCAUGUCAGGUGUAUGAUGGCA 3'
:::::::::.: :::.::::::
query 3' CGUACAGUCUAGAUAUUACCGU 5'
query=miR6025e, target=RO3G_05267, score=4, range=601-622, strand
target 5' GCCUGUCAGAUAUAUGAUGGCA 3'
:: :::::::: :::.::::::
```

```
query 3' CGUACAGUCUAGAUAUUACCGU 5'
query=miR6025e, target=RO3G_09967, score=4, range=601-622, strand
target 5' GCCUGUCAGAUAUAUGAUGGCA 3'
:: :::::::: :::.::::::
query 3' CGUACAGUCUAGAUAUUACCGU 5'
query=miR6025e, target=RO3G_07860, score=4, range=601-622, strand
target 5' GCCUGUCAGAUAUAUGAUGGCA 3'
:: :::::::: :::.::::::
query 3' CGUACAGUCUAGAUAUUACCGU 5'
query=miR6025e, target=RO3G_00256, score=4, range=601-622, strand
target 5' GCCUGUCAGAUAUAUGAUGGCA 3'
:: :::::::: :::.::::::
query 3' CGUACAGUCUAGAUAUUACCGU 5'
query=miR6025e, target=RO3G_05959, score=4, range=601-622, strand
target 5' GCCUGUCAGAUAUAUGAUGGCA 3'
:: :::::::: :::.::::::
query 3' CGUACAGUCUAGAUAUUACCGU 5'
query=miR6025e, target=RO3G_09194, score=4, range=601-622, strand
target 5' GCCUGUCAGAUAUAUGAUGGCA 3'
:: :::::::: :::.::::::
query 3' CGUACAGUCUAGAUAUUACCGU 5'
query=miR6025e, target=RO3G_11966, score=4, range=601-622, strand
target 5' GCCUGUCAGAUAUAUGAUGGCA 3'
:: :::::::: :::.::::::
query 3' CGUACAGUCUAGAUAUUACCGU 5'
query=miR319f, target=RO3G_04030, score=1, range=1106-1126, stran
target 5' AAGCAUGCCCUUUUCAUCCAA 3'
:::::::::.::::::::::
query 3' UUCGUACGGGGAAAGUAGGUU 5'
query=miR319f, target=RO3G_12114, score=2.5, range=169-190, stran
target 5' GAGCAUGUCCUCUUUCAUCCAA 3'
.:::::: ::.:::::::::::
query 3' UUCGUAC-GGGGAAAGUAGGUU 5'
query=miR319f, target=RO3G_13957, score=3.5, range=394-415, stran
target 5' AAGCAAUCUCUCUUUCAUCCAA 3'
:::: :: .:.:::::::::::
query 3' UUCG-UACGGGGAAAGUAGGUU 5'
query=miR319f, target=RO3G_01412, score=4, range=79-98, strand=1
target 5' AAGGAU-CCCUUUUCAUCCGA 3'
::: :: :::.::::::::.:
query 3' UUCGUACGGGGAAAGUAGGUU 5'
query=miR319f, target=RO3G_12773, score=4, range=259-279, strand=
target 5' AGGCCUGUCCUUUUCAUCCAC 3'
:.:: ::.::.:::::::::
query 3' UUCGUACGGGGAAAGUAGGUU 5'
```

query=miR9553-3p, target=RO3G_02695, score=0, range=3665-3686, strand=1
target 5' AUGUUCAUAGCUGGGAUGAUUA 3'
::::::::::::::::::::::
query 3' UACAAGUAUCGACCCUACUAAU 5'
[truncated: 315,419 more chars]
